# Counting animal species with DNA barcodes: Canadian insects

**DOI:** 10.1098/rstb.2015.0333

**Published:** 2016-09-05

**Authors:** Paul D. N. Hebert, Sujeevan Ratnasingham, Evgeny V. Zakharov, Angela C. Telfer, Valerie Levesque-Beaudin, Megan A. Milton, Stephanie Pedersen, Paul Jannetta, Jeremy R. deWaard

**Affiliations:** Centre for Biodiversity Genomics, Biodiversity Institute of Ontario, University of Guelph, Guelph, Ontario, Canada N1G 2W1

**Keywords:** biodiversity, Cecidomyiidae, breeding systems, mitochondrial DNA

## Abstract

Recent estimates suggest that the global insect fauna includes fewer than six million species, but this projection is very uncertain because taxonomic work has been limited on some highly diverse groups. Validation of current estimates minimally requires the investigation of all lineages that are diverse enough to have a substantial impact on the final species count. This study represents a first step in this direction; it employs DNA barcoding to evaluate patterns of species richness in 27 orders of Canadian insects. The analysis of over one million specimens revealed species counts congruent with earlier results for most orders. However, Diptera and Hymenoptera were unexpectedly diverse, representing two-thirds of the 46 937 barcode index numbers (=species) detected. Correspondence checks between known species and barcoded taxa showed that sampling was incomplete, a result confirmed by extrapolations from the barcode results which suggest the occurrence of at least 94 000 species of insects in Canada, a near doubling from the prior estimate of 54 000 species. One dipteran family, the Cecidomyiidae, was extraordinarily diverse with an estimated 16 000 species, a 10-fold increase from its predicted diversity. If Canada possesses about 1% of the global fauna, as it does for known taxa, the results of this study suggest the presence of 10 million insect species with about 1.8 million of these taxa in the Cecidomyiidae. If so, the global species count for this fly family may exceed the combined total for all 142 beetle families. If extended to more geographical regions and to all hyperdiverse groups, DNA barcoding can rapidly resolve the current uncertainty surrounding a species count for the animal kingdom. A newly detailed understanding of species diversity may illuminate processes important in speciation, as suggested by the discovery that the most diverse insect lineages in Canada employ an unusual mode of reproduction, haplodiploidy.

This article is part of the themed issue ‘From DNA barcodes to biomes’.

## 1. Introduction

Although most animal species are undescribed, it is presumed that the final count will not exceed 10 million [1]. In practice, any upward revision of this estimate is only likely to result from studies on hyperdiverse groups. The six animal lineages that may include more than a million species represent an obvious priority for analysis. Among the 28 phyla, the Nematoda is a strong prospect [2], while the Arthropoda undoubtedly qualifies, despite uncertainty concerning the species count for some of its component lineages. The Harpacticoida, a diverse order of copepod crustaceans, is the sole group of marine arthropods that may possess more than a million species [3], while the four terrestrial prospects include a superorder of mites (Acariformes) [4] and three orders of insects (beetles: Coleoptera; flies: Diptera; ants, bees and wasps: Hymenoptera). Estimates of global species richness, based upon varying presumptions of host plant specificity, led to an initial prediction of 28 million insect species [5], which was subsequently reduced to 7 million [6]. A recent investigation tightened the estimate to 5.6 million species [7]; it employed beetles as a proxy for all insects, presuming that current species counts for other orders provide a good indication of their relative diversities. There is reason to question this presumption because two of the most diverse orders (Diptera, Hymenoptera) possess families that have received little taxonomic attention despite signs of very high species richness.

The need for further biological inventories is evidenced by the fact that some researchers believe species registration has barely begun [1], while others suggest it is well advanced [8]. Until recently, the prospects for resolving uncertainty in species numbers appeared remote. Carbayo & Marque [9] estimated that a comprehensive inventory of animal species using morphology would require $250 billion and another 600 years. DNA barcoding represents an alternate approach, one allowing an expedited assessment of species richness for all animal lineages at far lower cost. Its capacity to accelerate progress rests on the fact that members of most species form a distinct barcode cluster [10–12], a relationship that has now been operationalized for large-scale surveys by the barcode index number (BIN) system [13]. The strong correspondence between BIN and species counts has been established through studies on groups with well-validated taxonomy [13–15]. Consequently, it is now clear that DNA barcode surveys can rapidly quantify species diversity, enabling analysis on geographical and taxonomic scales that were formerly impossible.

Although implementation of BIN-based biodiversity assessments for the entire animal kingdom will require substantial resources, less expansive work is likely to preview global patterns. This study employs DNA barcodes to evaluate insect diversity in Canada. Covering 7% of the planet's land surface, the only comprehensive checklist for its fauna [16] recorded 29 100 known insect species and proposed that another 25 000 awaited discovery. Updated checklists for Coleoptera [17], for Hemiptera, true bugs, [18] and for Lepidoptera, butterflies and moths, [19] have established species counts for these orders close to earlier estimates [16], and have raised the known species count for Canada to 33 572 species, suggesting the fauna is well understood. Viewed as a whole, the number of insect species in Canada is thought to represent 0.5–1.0% of the estimated global species count for each order (e.g. 5432 species of Lepidoptera in Canada versus 500 000 globally [20], 8107 species of Coleoptera in Canada versus 1.5 million globally [21]). Viewed across all orders, the predicted size of the Canadian insect fauna (54 000 species) is a close approximation to 1% of the latest planetary estimate of 5.6 million species [7].

This study employs DNA barcoding to obtain a fresh estimate for the number of insect species in Canada. Because all barcoded specimens, even those belonging to undescribed species, are assigned to a BIN, the present analysis represents the first taxonomically comprehensive survey of the Canadian insect fauna. It asks first if any insect orders have unexpectedly high diversity. As such groups are likely to show the same pattern in other regions, the present analysis provides a preliminary opportunity to check the accuracy of current estimates for global insect diversity [7]. If, for example, barcode analysis suggests the presence of 100 000 insect species in Canada, this would require an upward revision either in the global insect fauna to 10 million species or in the fraction of global biodiversity that resides in Canada to 2%. Because this investigation reveals a particularly high species count for Diptera, this order was investigated in more detail to determine if diversity was elevated across all or a fraction of its families. This additional taxonomic resolution provided a basis for considering the relationship between patterns of species richness and breeding system.

## 2. Experimental

### (a) Material

**Figure 1.**
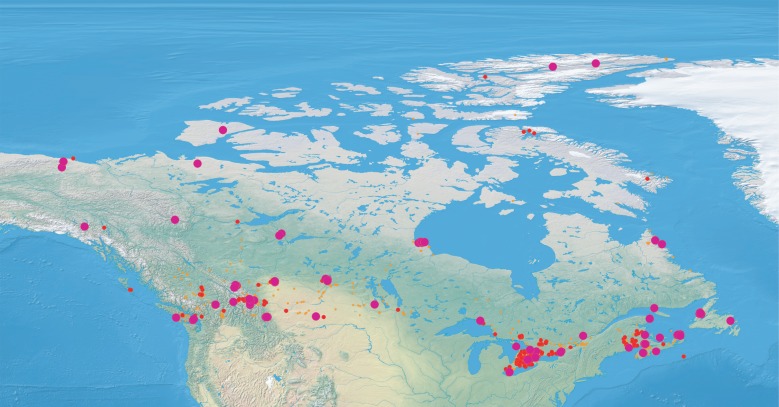
Heat map of sites in Canada where the one million insects analysed in this study were collected. Sites with 1–100 specimens in orange dots, 100–1000 are shown in small red dots, and 1000–10 000 are shown in larger pink dots.

Specimens were collected at sites across Canada from 2004 to 2014, but most (90%) were obtained over the last 4 years (figure 1). The sampling effort during this interval involved approximately three person-years devoted to collecting and another five person-years to processing the specimens for submission to DNA sequencing. The sampling programme employed Malaise traps, pitfall traps, sweep nets, intercept traps and light traps. Several million specimens were collected, but resource constraints limited analysis to approximately one million, enough to allow good geographical and seasonal coverage. Most samples were preserved in 95% EtOH and held at −20°C until they were sorted. Larger specimens were pinned, while smaller ones were retained in EtOH. When subsampling was required, specimens were selected haphazardly with respect to size, taxonomic group and life stage. All specimens were identified to an order before sequence analysis and more detailed taxonomic assignments were made later. Collection data, voucher information and taxonomy for each specimen are available in the Barcode of Life Data Systems [22] (BOLD, www.boldsystems.org) within 102 public datasets (electronic supplementary material, table S1).

### (b) Methods

#### (i) DNA extraction

Genomic DNA was extracted from 1 085 146 specimens. DNA extracts were prepared from a single leg from each large specimen and from the whole body of smaller taxa; the latter specimens were recovered as vouchers after DNA extraction. Tissue lysis, DNA extraction, PCR amplification, cycle sequencing and sequence analysis were performed at the Canadian Centre for DNA Barcoding employing standard protocols [23–25]. For most samples, the primer cocktail of C_LepFolF and C_LepFolR [26,27] was used for PCR amplification of the barcode region, while unidirectional sequence analysis of the amplicon employed C_LepFolR. Sequences, electropherograms and primer details for each specimen were uploaded to BOLD and GenBank (see electronic supplementary material, table S1, for DOIs). All voucher specimens and residual DNA extracts are archived at the Centre for Biodiversity Genomics.

#### (ii) Sequence analysis

Data were initially analysed using the workbench and tools on BOLD [22]. Owing to the large size of the dataset, conventional multiple sequence alignment was intractable so the sequences were aligned using a profile HMM [28] of the cytochrome *c* oxidase 1 (COI) protein [29]. All sequences were reviewed to ensure their high quality; those matching contaminants (e.g. human, proteobacteria) or with exceptionally low Hidden Markov Model (HMM) alignment scores, indicative of reading frame shifts, were excised.

#### (iii) Barcode index numbers

Most of the specimens delivered a sequence and 939 868 met the quality criteria (more than 500 bp, less than 1% uncertain base calls (Ns)) required to allow their assignment to a BIN. The refined single linkage (RESL) algorithm runs weekly on all qualifying barcode sequences in BOLD. Records were available for 4.6 million specimens representing 435 000 BINs on 1 December 2015, with nearly one-quarter of these specimens derived from this study. Every BIN has a publicly accessible web page that summarizes specimen and sequence data for its members (e.g. *Danaus plexippus*, http://dx.doi.org/10.5883/BOLD:AAA9566). Three representatives of each BIN, when available, were photographed and the resultant images are displayed on the corresponding BIN page.

#### (iv) Taxonomic assignment and validation

Following BIN assignment, each record received at least a family placement based on one of three methods. First, if the record's BIN contained specimens identified to a family, genus or species by a taxonomic expert, the record received this identification so long as all taxonomic assignments for the BIN were consistent. Second, sequences assigned to a new BIN were queried through the BOLD Identification Engine (http://www.boldsystems.org/index.php/IDS_OpenIdEngine). If this query yielded a close match (less than 10% for family, less than 5% for genus) and the query sequence fell within a monophyletic cluster of BINs in this genus or family, the record was assigned to this taxon. If these two approaches failed to deliver a placement, specimens were identified to a family (or better) through morphology. In every case where a taxonomic assignment conflicted with the ordinal identification made prior to sequence analysis, the specimen was inspected to confirm or correct its placement. Certain conflicts reflected sequence recovery from a non-target organism. For example, some larval Lepidoptera delivered a sequence from a tachinid fly or braconid wasp, reflecting cases where DNA was amplified from a parasitoid rather than its host. Other cases lacked an obvious explanation, but were assumed to reflect the amplification of DNA extracted from a non-target tissue fragment on the specimen that was analysed.

To further validate each record, two neighbour-joining (NJ) trees were generated in BOLD using the Kimura-2-Parameter distance model. The first tree included a representative from each of the 23 591 BINs of Diptera (electronic supplementary material, figure S1), while the other tree included a representative from each of the 23 346 BINs belonging to the other 26 orders (electronic supplementary material, figure S2). Both trees were inspected for unexpected placements, which might indicate contamination or analytical error. For example, the detection of a BIN assigned to Coleoptera within many BINs in another order would lead to examination of the voucher specimen and either validation or correction of its identification.

#### (v) Barcode index number analysis and species estimates

The overall dataset was initially analysed to ascertain the number of BINs in each order and the ratio of the BIN count to the known species count for Canada was determined, a value hereafter termed BIN/SP. The Diptera records were subsequently analysed in more detail by calculating the BIN/SP for each of the 98 families represented in the study. Lognormal abundance plots were also generated for the seven dipteran families with more than 20 000 barcode records and for the other 91 families combined. All statistical analyses were conducted using Python 2.7 (Python Software Foundation, http://www.python.org) or R, v. 3.1.1 (R Development Core Team, http://www.r-project.org) and the ‘vegan0’ 3 package [30]. Estimation of species numbers was based on the *prestonfit* function.

BIN/SP values more than 1.0 occur when there are more sequence clusters than recognized species, a situation that generally indicates the presence of species overlooked by the current taxonomic system. By contrast, BIN/SP values less than 1.0 arise when certain known species are not recovered by a sampling programme or when BIN sharing by different species is common. Past studies have indicated that BIN sharing is infrequent in insects [13–15], so low BIN/SP values typically reflect undersampling. The species in 25 families from three of the orders (Coleoptera, Hemiptera and Lepidoptera) with a low BIN/SP ratio were compared with Canadian checklists to verify that undersampling had excluded some known species from our collections.

## 3. Results

The single primer set employed in this study recovered an amplicon from 89.7% of the specimens that were analysed and 86.6% of all specimens delivered a barcode-compliant record although amplicons were only sequenced in one direction. Despite this high success, there was significant variation in sequence recovery among the orders, being highest in Diptera and Lepidoptera, lowest in Hemiptera and Hymenoptera, with Coleoptera and the other orders intermediate (table 1). The recovery of contaminated sequences varied little among orders, but there was substantial variation in the frequency of short sequences, reflecting premature termination of sequencing reactions. In fact, they composed 10% of the records for Hymenoptera versus less than 1% for Diptera. Also, there were clear differences among orders in the incidence of PCR failure, situations that probably reflect compromised primer binding.

**Table 1.** The number of specimens sampled in thousands (k) and percentage of specimens in five major insect orders and 22 other orders that delivered a BIN-compliant sequence (more than 487 bp, less than 1% Ns), a short sequence, a contaminated sequence or no sequence for the COI barcode region.

order	*n* (k)	BIN-compliant (%)	short (%)	contaminated (%)	none (%)
Coleoptera	47.6	83.9	1.7	0.6	13.7
Diptera	654.3	93.9	0.9	0.1	4.9
Hemiptera	67.9	67.7	5.2	1.4	25.4
Hymenoptera	192.7	65.2	9.9	1.2	23.6
Lepidoptera	81.9	96.0	0.8	0.3	2.7
other orders	40.8	86.1	3.6	0.8	9.4

Considering all orders, 0.94 million specimens received a BIN assignment and they included representatives of 46 937 BINs belonging to 478 insect families. This study did not encounter any specimens from 111 of the 589 insect families known from Canada (electronic supplementary material, table S2), all represented in the fauna by fewer than 10 species excepting two families of lice (Menoponidae, 100 species; Philopteridae, 200 species) and one of fleas (Leptopsyllidae, 19 species) whose vertebrate hosts were not sampled. Two-thirds of the missing families belonged to five major orders (20 Coleoptera, 10 Diptera, 22 Hemiptera, 12 Hymenoptera and 13 Lepidoptera), while the others were scattered across other orders.

**Figure 2.**
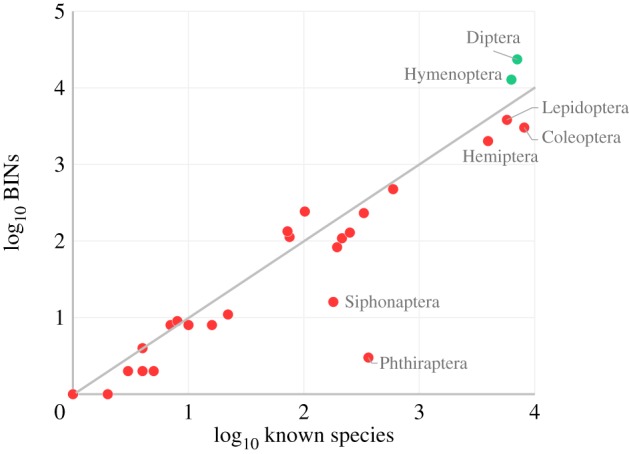
BIN count versus known species count for 27 orders of Canadian insects. Points above the line represent orders with more BINs than known species.

**Figure 3.**
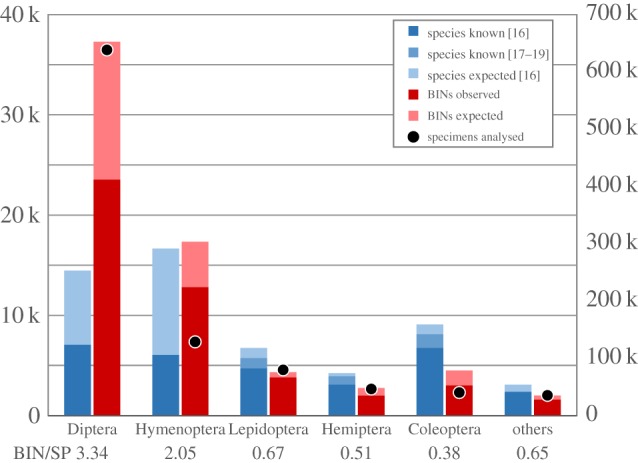
The number of observed/estimated BINs versus the numbers of known/estimated species for the five largest orders of Canadian insects and the other 22 orders. The known and estimated species counts for each order are based on Danks [16], but more recent counts are available for three orders [17–19]. Blue bars indicate species counts, while red bars indicate BIN counts (both in thousands). Circles indicate the number of BIN records for each order. BIN/SP is the ratio of observed BINs to known species.

BIN counts were compared with known species counts for the 27 orders with barcode data (figure 2). BIN/SP was less than 1.0 in 20 of these orders and more than 1.0 in seven. The Diptera and Hymenoptera represented the most striking examples of high BIN/SP as they possessed far more BINs than known species. Subsequent analysis examined the five most diverse orders and pooled results for the other 22 (figure 3). All values for known and estimated species counts in figure 3 are based on Danks [16] to employ a standard point of reference. Newer checklists are available for three of the five major orders (Coleoptera [17], Hemiptera [18], Lepidoptera [19]), but none of these updates has raised the known species count above the estimated values in Danks [16]. In three of the large orders, BIN/SP was substantially less than 1.0, ranging from 0.38 in Coleoptera, to 0.51 in Hemiptera and to 0.67 in Lepidoptera, suggesting that the sampling programme missed certain known species. This possibility was tested by identifying the BINs in 25 families from these three orders to a species level; this analysis confirmed the absence of many known taxa as our collections included just 23% of the species in nine beetle families, 32% of those in six hemipteran families, and 54% of the known species in 10 lepidopteran families (table 2). Despite this evidence for undersampling, BIN/SP was more than 1.0 for Hymenoptera (2.05) and Diptera (3.34). The estimated BIN counts, based upon the lognormal abundance model [31], strongly suggest that Diptera is the most diverse insect order in Canada, with more than 37 000 species estimated to occur at the sites examined in this study.

**Table 2.** Number of species known from Canada (*K*) versus the number of species captured (*C*) in the current sampling programme for each of 25 families in three insect orders. The capture fraction is *C*/*K*. Analysis examined families with more than 15 Canadian species known and with more than 85% BINs identified to a species level.

order	family	*K*	*C*	*C*/*K*	no. BINs
Coleoptera	Carabidae	945	223	0.24	263
	Cerambycidae	358	90	0.25	125
	Dermestidae	48	8	0.17	8
	Heteroceridae	28	6	0.21	4
	Scarabaeidae	219	43	0.20	60
	Silphidae	26	12	0.46	14
	Silvanidae	15	3	0.20	3
	Trogidae	15	4	0.27	4
	Trogossitidae	22	1	0.05	1
Hemiptera	Cicadidae	21	2	0.10	3
	Diaspididae	29	2	0.07	2
	Gerridae	23	7	0.30	8
	Nabidae	20	10	0.50	13
	Pentatomidae	69	27	0.39	36
	Rhopalidae	16	9	0.56	10
Lepidoptera	Erebidae	328	191	0.58	216
	Hesperiidae	81	27	0.33	26
	Lycaenidae	77	36	0.47	33
	Noctuidae	1145	652	0.57	641
	Notodontidae	57	49	0.86	54
	Nymphalidae	110	69	0.63	66
	Papilionidae	18	10	0.56	9
	Pieridae	43	21	0.49	11
	Saturniidae	26	11	0.42	9
	Sphingidae	65	30	0.46	34

**Figure 4.**
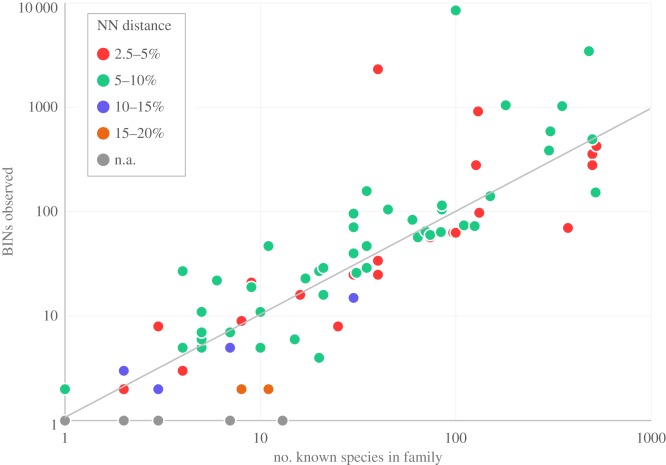
BIN count versus known species count for 87 families of Canadian Diptera. Points above the line represent families with more BINs than known species. Different colours indicate variation in mean nearest-neighbour (NN) distances (K2P) for the BINs in each family based on sequence divergences in the COI barcode region. n.a., data unavailable because only one BIN was analysed in this family.

To better understand the origins of the high diversity in Diptera, BIN/SP was examined on a family-by-family basis (electronic supplementary material, table S4). This calculation revealed that most families possessed a ratio close to unity (figure 4). However, the Cecidomyiidae and Sciaridae showed BIN counts far higher than their known species count: over 85× higher for the Cecidomyiidae and nearly 75× higher for the Sciaridae. In fact, the Cecidomyiidae composed 18.0% of all BINs (8467/46 937) collected in the present survey, making them, by far, the most diverse insect family in Canada. Although variation in the mean nearest-neighbour (NN) distance among the species composing each family did occur, the differences were generally small. As expected, NN distances were highest (more than 15%) in families with few species, reflecting the fact that their component taxa were usually members of different genera. By comparison, divergences in NN distance among families represented by more species were muted. For example, the 38 families of Diptera represented by more than 50 BINs had NN divergences (mean ± s.d.) averaging 5.44 ± 0.26%, with the Cecidomyiidae (5.06 ± 0.04%) and Sciaridae (4.82 ± 0.07%) showing no evidence of unusually low sequence divergences among their BINs (figure 4).

**Figure 5.**
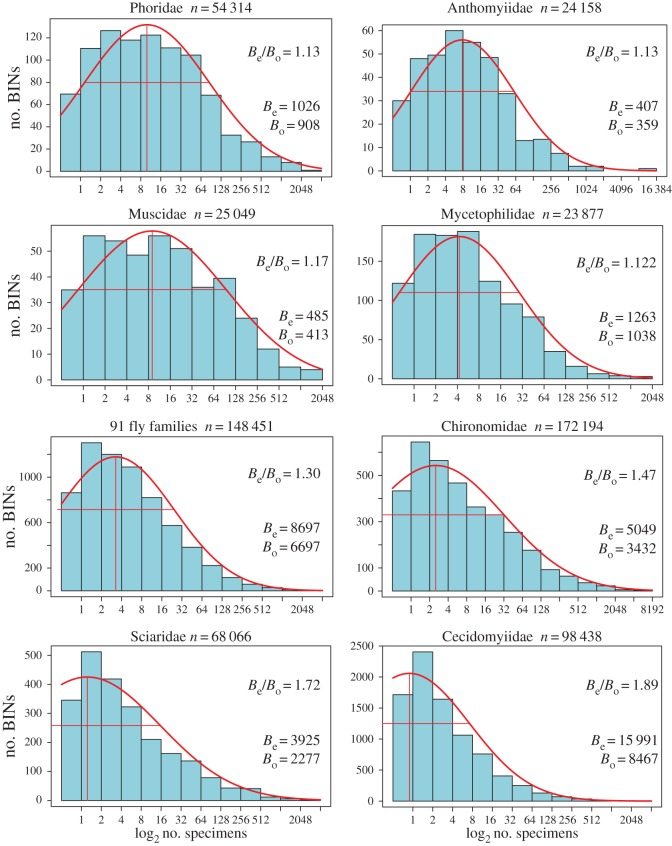
Lognormal abundance plots for seven families of Diptera with more than 20 000 barcode records and a composite curve for the other 91 families. *B*_e_, expected BIN count; *B*_o_, observed count.

The lognormal abundance model [31] was used to estimate total BIN counts for the seven fly families with the most specimens and for the composite of all others (figure 5). Four families (Anthomyiidae, Muscidae, Mycetophilidae and Phoridae) were well sampled, as BIN estimates were just 13–22% higher than current values. The Chironomidae and the composite families were less well sampled, with BIN estimates 30–47% higher than the current count, while the Sciaridae and Cecidomyiidae were severely under-sampled as BIN estimates were 72% and 89% higher than current counts. Lognormal projections suggest these two families include at least 3900 and 15 900 BINs, respectively, more species than the other 96 families of Canadian Diptera combined.

## 4. Discussion

This study represents, by far, the largest effort to recover DNA barcode records from a diverse assemblage of arthropods. Although representatives of 478 insect families were analysed with a regimented protocol, success in sequence recovery was high. This is an important result because it indicates that very simple analytical protocols can provide an effective assessment of species diversity across all insect lineages. Despite the high success, there was variation among taxonomic groups, with the lower recovery from Hymenoptera and Hemiptera probably reflecting two factors. Firstly, the very high AT composition of the mitochondrial genomes of some lineages in these orders produces difficulties in sequencing amplicons because of long homopolymer runs. This effect was particularly clear in the Hymenoptera, where 10% of all records experienced premature termination of the sequencing reaction versus less than 1% in Diptera. In addition, sequence recovery was lowered in these orders by difficulties in primer binding, a pattern almost certainly linked to the strong rate acceleration in Hymenoptera [32] and certain groups of Hemiptera, which leads to mismatches between standard primer sets and their target regions. Although specimens that failed to amplify could have been ‘rescued’ through reanalysis with an additional primer set, this approach was not employed in this study because of cost implications. As a consequence, it is likely that about one-third of the species of Hemiptera and Hymenoptera analysed in this study did not deliver a barcode record, meaning that species richness in these orders has been substantially underestimated.

BIN analysis represents a major advance for biodiversity science because it overcomes one of the key elements of the taxonomic impediment [13] by permitting comprehensive assessments of species richness, even for groups that have received very little taxonomic investigation. Work on the well-studied European insect fauna has shown strong correspondence between species and BIN boundaries in the three major orders (Coleoptera [12,15], Hymenoptera [11], Lepidoptera [14]) that have been examined. The Canadian fauna has seen less comprehensive analysis, but results suggest a similar pattern. For example, a study that examined nearly all (1541/1555) species of noctuoid moths revealed a BIN/SP of 0.98 [33]. As these studies on groups with an advanced taxonomic system have confirmed that BIN counts are a strong surrogate for species counts, BIN/SP values less than 1.0 generally reflect undersampling, while those more than 1.0 indicate overlooked species, a conclusion that is gaining confirmation as taxonomic work proceeds. Landry *et al*. [34] found 30 overlooked species of Canadian Lepidoptera based on DNA barcode results, while Fernandez-Triana (personal communication 2016) concluded that the morphological inspection of lineages assigned to different BINs supports the presence of at least 200 undescribed species of microgastrine Braconidae. In a similar fashion, barcode studies added 68 species to the Canadian spider fauna and revealed the possible presence of several hundred more cryptic species [35].

The present analysis of one million specimens from Canada revealed patterns of species richness that were generally congruent with expectations, as evidenced by the strong correlation between BIN counts and known species counts for most orders and families. However, BIN counts averaged 50% less than the number of known species for 18 orders, a difference that reflected the failure of the current sampling programme to collect some known species. This shortfall was expected because sampling examined relatively few sites for relatively brief intervals. Undersampling was further indicated by the failure to collect representatives of 111 insect families known from Canada and 63% of the species in 25 families targeted for detailed taxonomic analysis. Despite this undersampling, BIN counts for Diptera and Hymenoptera were far higher than their numbers of known species and substantially higher than past predictions of their total diversity, especially for Diptera. Because BIN counts averaged 50% less than the known species count for orders with strong taxonomy, the current BIN projections for Diptera and Hymenoptera are probably substantial underestimates of their true diversity. As a consequence, Canada may host more than 50 000 species of Diptera and perhaps 30 000 species of Hymenoptera, suggesting that its entire insect fauna may exceed 100 000 species. If Canada hosts 1% of the global fauna, this suggests the presence of some 10 million insect species. If implemented on a global scale, BIN analysis can quickly resolve the uncertainty in species numbers across the animal kingdom, but what then? The ‘big sky survey’ led astronomers to abandon their practice of naming stars when it revealed ‘there were simply too many to name’. The presence of millions of species-in-waiting of dipterans (this study), harpacticoids [3], mites [4] and nematodes [2] might stimulate a similar movement in taxonomy [36].

Extrapolating from species richness values based on morphological study, Stork *et al*. [7] recently concluded that the global species count for Coleoptera is unlikely to exceed 1.5 million species. Might molecular analysis lead to a radical increase in the species count for this order? It seems not; DNA barcoding studies on nearly 5000 species of European coleopterans revealed few overlooked species [12,15], suggesting that the current taxonomic system for beetles is robust. By contrast, DNA barcode analysis has indicated many overlooked species in groups of Diptera and Hymenoptera whose taxonomy was thought to be well understood [37,38]. This study has extended this earlier work by conducting a Canada-wide analysis of species richness in all families of Diptera and Hymenoptera. This work revealed BIN counts that were in close congruence with known species numbers for several families with well-established taxonomy in these two orders. However, when analysis extended to families where taxonomic work has been less intense, there was general evidence for the underestimation of species numbers, especially for Canadian Cecidomyiidae. Although it has been estimated that 1600 species of this family occur in Canada, just 100 species have been documented [16]. By contrast, this study established the occurrence of nearly 9000 species and further suggests that as many as 16 000 species occur at the sites examined in this study, implying the presence of considerably more than 20 000 species in Canada. If this count represents 1% of its global diversity, there could be two million species of cecidomyiids. While sometimes proposed as the most diverse dipteran family [39], quantification of its species richness has been viewed as an insurmountable task, leaving the species count for the family as ‘inestimable’ [40]. The results of this study suggest that DNA barcoding can resolve this uncertainty, although more geographical regions need to be analysed. Interestingly, ongoing barcode projects confirm that cecidomyiids are also a dominant component of Malaise trap catches in the tropics, comprising 23–35% of the BINs at several sites in Argentina and Costa Rica (P.H. 2016, personal observation).

This study provides a first sense of the impact of the comprehensive biodiversity assessments enabled by DNA barcoding on our understanding of both global species richness values and the contributions of particular taxonomic lineages to it. What are some of the implications of this work? The revelation of extremely high diversity within certain lineages of dipterans suggests that the long-standing recognition of Coleoptera as holding first place in the diversification race reflects a rush to judgement in a world of taxonomic uncertainty. If further work confirms the extraordinary diversity of Cecidomyiidae, Haldane's quip [41–43] needs revision to reflect an inordinate fondness for midges rather than beetles. By enabling the determination of species richness in all insect groups, DNA barcoding may also help to resolve the long-standing uncertainty concerning the factors that limit species diversity. Hutchinson [42] argued that competition was the primary force, but this conclusion has been challenged [44]. Felsenstein [45] proposed that the primary constraint was genetic, suggesting that the species count would be far higher (‘a species under every bush’) if speciation was easier to accomplish. The fact that the key drivers of animal diversity remain uncertain [46–48] reveals the need to extend knowledge of biodiversity patterns and the variation in genetic systems that might influence them. By documenting the spectacular diversity of the Cecidomyiidae, this study suggests the importance of genetic constraints on species diversity because members of this family employ a mode of reproduction, haplodiploidy, that is otherwise almost unknown among Diptera [49]. Is it simply by chance that this breeding system is also shared by Hymenoptera and by at least some Sciaridae? Might the linkage between haplodiploidy and extraordinary species numbers indicate that this mode of reproduction accelerates speciation enough to be an important driver of biodiversity? In order to critically evaluate this possibility, there is a need for more detailed information on breeding system variation, particularly in groups such as the Cecidomyiidae and Sciaridae. Do all species in these families reproduce by haplodiploidy or just some? Past cytogenetic studies have only examined a small fraction of their component species and interpretational complexities arise because they employ a form of haplodiploidy, pseudoarrhenotoky, where males are diploid, but the paternally derived genome is inactivated early in embryogenesis [49]. Transcriptomics offers a pathway to rapid, large-scale assessments of breeding systems in these groups as males of haplodiploid species will lack heterozygosity, while females will possess it at many loci. Beyond a more detailed understanding of breeding system variation, a mechanistic explanation for the impact of this breeding system on speciation is required. If the rate acceleration characteristic of the mitochondrial genomes in Hymenoptera [32] extends to other haplodiploids, this could be an important agent in their accelerated speciation as it could lead to an increased rate in the acquisition of nuclear–mitochondrial divergences [50], leading population isolates to rapidly gain reproductive incompatibility.

This study has revealed the scale of sampling and sequencing efforts required to assess insect diversity on a hemi-continent. Eight person-years of sampling and sorting, coupled with similar effort for the sequence analysis of one million specimens, led to the detection of 46 937 BINs. This total probably includes about half the 30 000 insect species known from Canada and another 30 000 species that are either undescribed or newly reported. Because lognormal projections suggest the presence of an additional 50 000 species, another one million or more specimens will need analysis to develop strong barcode coverage for Canadian insects. Because Canada occupies about half of the continent and its fauna includes half of its known insect species, it should be possible to gain a detailed perspective of insect diversity in North America by sequencing an additional three to four million specimens. As the Canadian Centre for DNA Barcoding now processes one million specimens annually, it could analyse the insect fauna of this continent in a few years. If this approach was applied globally, by establishing more analytical facilities with a similar capacity, ten million insect species could easily be registered within two decades, presuming a target of 10× coverage per species. Although this research programme might cost $500 million, this represents just 1% of the amount required to complete the same task using morphological approaches [9]. By providing new details on the patterning and extent of their diversity, this census of the insects would also advance understanding of evolutionary processes in the most diverse of animal lineages and provide the DNA extracts needed for their genomic characterization.

## Supplementary Material

Supplementary materials

## Data accessibility

The datasets supporting this article are accessible through the electronic supplementary material. All sequence records and specimen data can be accessed via 102 datasets (each with an individual DOI) listed in the electronic supplementary material, table S1. All sequences have been deposited in GenBank and accession numbers are listed in the corresponding dataset.
