# Supplementary material for: Counting animal species with DNA barcodes: Canadian insects
Source: Philos Trans R Soc Lond B Biol Sci. 2016 Sep 5;371(1702):20150333. doi: 10.1098/rstb.2015.0333 (PMC4971185; doi:10.1098/rstb.2015.0333)
Supplement: Supplementary materials [file rstb20150333supp1.pdf]

Phil. Trans. R. Soc. B **371** doi: 10.1098/rstb.2015.0333

Counting Animal Species with DNA Barcodes: Canadian Insects

Paul D. N. Hebert, Sujeevan Ratnasingham, Evgeny V. Zakharov, Angela C. Telfer, Valerie Levesque-Beaudin, Megan A. Milton, Stephanie Pedersen, Paul Jannetta, Jeremy R. deWaard

Supplementary material

Supplementary Table S1: Summary of datasets archived in the Barcode of Life Data Systems (BOLD, www.boldsystems.org).

Supplementary Table S2: The number of species known for each of 589 Canadian insect families and the number of BINs assigned to it in this study. Numeric superscripts indicate the reference for the species counts for the families in each order. When more than one reference is cited, the numeric superscript indicates it provided the species count for a particular family. Subfamilies in ten families have been raised to family status since publication of the source reference. These cases are shown in colour and are designated as a 'group' with the species total for the families in each group based on the value in the source. Families designated with † are a composite of two or more families in the original source, while those marked with * have undergone a name change.

Supplementary Table S3: The number of species known for each of 27 Canadian insect orders and the number of BINs assigned to it based on specimens collected in this study. The species totals derive from Table S2.

Supplementary Table S4: Nearest-Neighbor distances and BIN counts for the 98 families of Canadian Diptera encountered in this study. Species counts are only available for the 87 families included in [16].

Supplementary Table S5: Staff, researchers and students who contributed to the processing of specimens, to sequence analysis or to the development of the informatics platform required for data analysis. * Personnel who worked for more than one unit within the Centre for Biodiversity Genomics.

Supplementary Figure S1: Neighbor-Joining tree based on sequence divergences (K2P) at COI for the 23,591 BINs of Diptera from Canada. Entries in the tree are colorized by family.

Supplementary Figure S2: Neighbor-Joining tree based on sequence divergences (K2P) at COI for the 23,346 BINs of non-dipteran insects from Canada. Entries in the tree are colorized by family.

Electronic Supplementary Material for:
Counting Species with DNA Barcodes: Canadian Insects. 2016. PDN Hebert, S Ratnasingham, EV Zakharov, AC Telfer, V Levesque-Beaudin, MA Milton, S Pedersen, P Jannetta, JR deWaard. Philosophical Transactions of the Royal Society B. doi: 10.1098/rstb.2015.0333.

S1 Table. Summary of datasets archived in the Barcode of Life Data Systems (BOLD, www.boldsystems.org).

Dataset	Taxonomic composition	DOI for BOLD Data Access	Records	BINs
DS-FOND001	Coleoptera, Curculionidae	dx.doi.org/10.5883/DS-FOND001	5879	228
DS-FOND002	Coleoptera, Staphylinidae	dx.doi.org/10.5883/DS-FOND002	4829	618
DS-FOND003	Coleoptera, Cantharidae	dx.doi.org/10.5883/DS-FOND003	3974	86
DS-FOND004	Coleoptera, Latridiidae	dx.doi.org/10.5883/DS-FOND004	2606	69
DS-FOND005	Coleoptera, Elateridae	dx.doi.org/10.5883/DS-FOND005	2466	191
DS-FOND006	Coleoptera, Chrysomelidae	dx.doi.org/10.5883/DS-FOND006	2444	211
DS-FOND007	Coleoptera, Coccinellidae	dx.doi.org/10.5883/DS-FOND007	2103	84
DS-FOND008	Coleoptera, Scirtidae	dx.doi.org/10.5883/DS-FOND008	1616	37
DS-FOND009	Coleoptera, Carabidae	dx.doi.org/10.5883/DS-FOND009	1576	263
DS-FOND010	Coleoptera, Throscidae	dx.doi.org/10.5883/DS-FOND010	1369	16
DS-FOND011	Coleoptera, Scraptiidae	dx.doi.org/10.5883/DS-FOND011	1325	28
DS-FOND012	Coleoptera, Dytiscidae	dx.doi.org/10.5883/DS-FOND012	1199	90
DS-FOND013	Coleoptera, Remainder	dx.doi.org/10.5883/DS-FOND013	8516	1121
DS-FOND014	Diptera, Chironomidae	dx.doi.org/10.5883/DS-FOND014	172194	3432
DS-FOND015	Diptera, Cecidomyiidae	dx.doi.org/10.5883/DS-FOND015	98438	8467
DS-FOND016	Diptera, Sciaridae	dx.doi.org/10.5883/DS-FOND016	68066	2277
DS-FOND017	Diptera, Phoridae	dx.doi.org/10.5883/DS-FOND017	54314	908
DS-FOND018	Diptera, Muscidae	dx.doi.org/10.5883/DS-FOND018	25049	413
DS-FOND019	Diptera, Anthomyiidae	dx.doi.org/10.5883/DS-FOND019	24158	359
DS-FOND020	Diptera, Mycetophilidae	dx.doi.org/10.5883/DS-FOND020	23877	1038
DS-FOND021	Diptera, Ceratopogonidae	dx.doi.org/10.5883/DS-FOND021	16018	1054
DS-FOND022	Diptera, Dolichopodidae	dx.doi.org/10.5883/DS-FOND022	15024	499
DS-FOND023	Diptera, Hybotidae	dx.doi.org/10.5883/DS-FOND023	8027	256
DS-FOND024	Diptera, Psychodidae	dx.doi.org/10.5883/DS-FOND024	7688	99
DS-FOND025	Diptera, Chloropidae	dx.doi.org/10.5883/DS-FOND025	7428	280
DS-FOND026	Diptera, Culicidae	dx.doi.org/10.5883/DS-FOND026	7117	63
DS-FOND027	Diptera, Syrphidae	dx.doi.org/10.5883/DS-FOND027	6753	277
DS-FOND028	Diptera, Empididae	dx.doi.org/10.5883/DS-FOND028	5624	399
DS-FOND029	Diptera, Agromyzidae	dx.doi.org/10.5883/DS-FOND029	5425	589
DS-FOND030	Diptera, Scathophagidae	dx.doi.org/10.5883/DS-FOND030	5332	96
DS-FOND031	Diptera, Tachinidae	dx.doi.org/10.5883/DS-FOND031	4813	434
DS-FOND032	Diptera, Sphaeroceridae	dx.doi.org/10.5883/DS-FOND032	4558	158
DS-FOND033	Diptera, Drosophilidae	dx.doi.org/10.5883/DS-FOND033	4449	86
DS-FOND034	Diptera, Limoniidae	dx.doi.org/10.5883/DS-FOND034	4283	259
DS-FOND035	Diptera, Tabanidae	dx.doi.org/10.5883/DS-FOND035	4211	66
DS-FOND036	Diptera, Calliphoridae	dx.doi.org/10.5883/DS-FOND036	3834	33
DS-FOND037	Diptera, Simuliidae	dx.doi.org/10.5883/DS-FOND037	3612	74
DS-FOND038	Diptera, Heleomyzidae	dx.doi.org/10.5883/DS-FOND038	3570	61
DS-FOND039	Diptera, Fanniidae	dx.doi.org/10.5883/DS-FOND039	3032	70
DS-FOND040	Diptera, Ephydridae	dx.doi.org/10.5883/DS-FOND040	2335	143
DS-FOND041	Diptera, Sarcophagidae	dx.doi.org/10.5883/DS-FOND041	2147	107
DS-FOND042	Diptera, Sciomyzidae	dx.doi.org/10.5883/DS-FOND042	1908	118

DS-FOND043	Diptera, Anisopodidae	dx.doi.org/10.5883/DS-FOND043	1717	13
DS-FOND044	Diptera, Rhagionidae	dx.doi.org/10.5883/DS-FOND044	1506	47
DS-FOND045	Diptera, Tipulidae	dx.doi.org/10.5883/DS-FOND045	1481	151
DS-FOND046	Diptera, Pipunculidae	dx.doi.org/10.5883/DS-FOND046	1305	108
DS-FOND047	Diptera, Keroplatidae	dx.doi.org/10.5883/DS-FOND047	1282	71
DS-FOND048	Diptera, Lauxaniidae	dx.doi.org/10.5883/DS-FOND048	1113	72
DS-FOND049	Diptera, Sepsidae	dx.doi.org/10.5883/DS-FOND049	1085	23
DS-FOND050	Diptera, Bibionidae	dx.doi.org/10.5883/DS-FOND050	1071	27
DS-FOND051	Diptera, Remainder	dx.doi.org/10.5883/DS-FOND051	10703	964
DS-FOND052	Ephemeroptera, Baetidae	dx.doi.org/10.5883/DS-FOND052	992	67
DS-FOND053	Ephemeroptera, Remainder	dx.doi.org/10.5883/DS-FOND053	1987	165
DS-FOND054	Hemiptera, Cicadellidae	dx.doi.org/10.5883/DS-FOND054	21955	731
DS-FOND055	Hemiptera, Aphididae	dx.doi.org/10.5883/DS-FOND055	10823	462
DS-FOND056	Hemiptera, Miridae	dx.doi.org/10.5883/DS-FOND056	4746	287
DS-FOND057	Hemiptera, Aleyrodidae	dx.doi.org/10.5883/DS-FOND057	1357	31
DS-FOND058	Hemiptera, Remainder	dx.doi.org/10.5883/DS-FOND058	7084	511
DS-FOND059	Hymenoptera, Ichneumonidae	dx.doi.org/10.5883/DS-FOND059	37382	3851
DS-FOND060	Hymenoptera, Platygastridae	dx.doi.org/10.5883/DS-FOND060	20028	1654
DS-FOND061	Hymenoptera, Braconidae	dx.doi.org/10.5883/DS-FOND061	19309	2444
DS-FOND062	Hymenoptera, Formicidae	dx.doi.org/10.5883/DS-FOND062	12893	178
DS-FOND063	Hymenoptera, Diapriidae	dx.doi.org/10.5883/DS-FOND063	8031	526
DS-FOND064	Hymenoptera, Tenthredinidae	dx.doi.org/10.5883/DS-FOND064	3772	397
DS-FOND065	Hymenoptera, Mymaridae	dx.doi.org/10.5883/DS-FOND065	3210	238
DS-FOND066	Hymenoptera, Eulophidae	dx.doi.org/10.5883/DS-FOND066	2798	772
DS-FOND067	Hymenoptera, Megaspilidae	dx.doi.org/10.5883/DS-FOND067	2568	265
DS-FOND068	Hymenoptera, Cynipidae	dx.doi.org/10.5883/DS-FOND068	460	56
DS-FOND069	Hymenoptera, Pteromalidae	dx.doi.org/10.5883/DS-FOND069	1569	397
DS-FOND070	Hymenoptera, Apidae	dx.doi.org/10.5883/DS-FOND070	1314	76
DS-FOND071	Hymenoptera, Vespidae	dx.doi.org/10.5883/DS-FOND071	1158	67
DS-FOND072	Hymenoptera, Halictidae	dx.doi.org/10.5883/DS-FOND072	1130	94
DS-FOND073	Hymenoptera, Crabronidae	dx.doi.org/10.5883/DS-FOND073	1129	203
DS-FOND074	Hymenoptera, Trichogrammatidae	dx.doi.org/10.5883/DS-FOND074	1028	81
DS-FOND075	Hymenoptera, Remainder	dx.doi.org/10.5883/DS-FOND075	7926	1561
DS-FOND076	Lepidoptera, Noctuidae	dx.doi.org/10.5883/DS-FOND076	13516	641
DS-FOND077	Lepidoptera, Geometridae	dx.doi.org/10.5883/DS-FOND077	13253	438
DS-FOND078	Lepidoptera, Tortricidae	dx.doi.org/10.5883/DS-FOND078	8959	524
DS-FOND079	Lepidoptera, Gelechiidae	dx.doi.org/10.5883/DS-FOND079	8364	351
DS-FOND080	Lepidoptera, Crambidae	dx.doi.org/10.5883/DS-FOND080	6602	208
DS-FOND081	Lepidoptera, Erebidae	dx.doi.org/10.5883/DS-FOND081	4938	216
DS-FOND082	Lepidoptera, Gracillariidae	dx.doi.org/10.5883/DS-FOND082	4259	164
DS-FOND083	Lepidoptera, Elachistidae	dx.doi.org/10.5883/DS-FOND083	1221	77
DS-FOND084	Lepidoptera, Notodontidae	dx.doi.org/10.5883/DS-FOND084	1363	54
DS-FOND085	Lepidoptera, Pyralidae	dx.doi.org/10.5883/DS-FOND085	1345	123
DS-FOND086	Lepidoptera, Coleophoridae	dx.doi.org/10.5883/DS-FOND086	1196	111
DS-FOND087	Lepidoptera, Nepticulidae	dx.doi.org/10.5883/DS-FOND087	1176	75

DS-FOND088	Lepidoptera, Blastobasidae	dx.doi.org/10.5883/DS-FOND088	1176	33
DS-FOND089	Lepidoptera, Nymphalidae	dx.doi.org/10.5883/DS-FOND089	1122	66
DS-FOND090	Lepidoptera, Remainder	dx.doi.org/10.5883/DS-FOND090	10159	749
DS-FOND091	Orthoptera, Acrididae	dx.doi.org/10.5883/DS-FOND091	1243	59
DS-FOND092	Orthoptera, Remainder	dx.doi.org/10.5883/DS-FOND092	421	50
DS-FOND093	Psocoptera, Caeciliusidae	dx.doi.org/10.5883/DS-FOND093	2987	17
DS-FOND094	Psocoptera, Remainder	dx.doi.org/10.5883/DS-FOND094	4530	117
DS-FOND095	Thysanoptera, Thripidae	dx.doi.org/10.5883/DS-FOND095	5058	137
DS-FOND096	Thysanoptera, Remainder	dx.doi.org/10.5883/DS-FOND096	1393	105
DS-FOND097	Trichoptera, Limnephilidae	dx.doi.org/10.5883/DS-FOND097	3316	101
DS-FOND098	Trichoptera, Hydropsychidae	dx.doi.org/10.5883/DS-FOND098	1872	54
DS-FOND099	Trichoptera, Leptoceridae	dx.doi.org/10.5883/DS-FOND099	1228	95
DS-FOND100	Trichoptera, Hydroptilidae	dx.doi.org/10.5883/DS-FOND100	1054	60
DS-FOND101	Trichoptera, Remainder	dx.doi.org/10.5883/DS-FOND101	3642	166
DS-FOND102	Other Orders, Remainder *	dx.doi.org/10.5883/DS-FOND102	5377	400
		**Totals:**	**939868**	**46938**

*17 orders include Archaeognatha, Blattodea, Dermaptera, Grylloblattodea, Isoptera, Mantodea, Mecoptera, Megaloptera, Neuroptera, Odonata, Phasmatodea, Phthiraptera, Plecoptera, Psocoptera, Raphidioptera, Siphonaptera, Strepsiptera, and Thysanura

Supplementary Table S2: The number of species known for each of 589 Canadian insect families and the number of BINs assigned to it in this study. Numeric superscripts indicate the reference for the species counts for the families in each order. When more than one reference is cited, the numeric superscript indicates it provided the species count for a particular family. Subfamilies in ten families have been raised to family status since publication of the source reference. These cases are shown in colour and are designated as a 'group' with the species total for the families in each group based on the value in the source. Families designated with † are a composite of two or more families in the original source, while those marked with * have undergone a name change.

Order	Family	Species	BINs
**Archaeognatha**[2]	Machilidae	7	9
	Meinertellidae	1	0
**Blattodea**[1]	Blaberidae	1	2
	Blattidae	4	2
	Ectobiidae*	5	3
**Coleoptera**[3]	Aderidae	10	3
	Agyrtidae	8	0
	Amphizoidae	3	0
	Anthicidae	65	18
	Anthribidae	20	11
	Artematopodidae	5	3
	Attelabidae	14	6
	Biphyllidae	1	0
	Boridae	2	0
	Bostrichidae	24	2
	Bothrideridae	4	0
	Brachyceridae	19	5
	Brentidae	49	16
	Buprestidae	176	33
	Byrrhidae	26	10
	Byturidae	1	1
	Cantharidae	152	86
	Carabidae	945	263
	Cerambycidae	358	125
	Cerylonidae	8	3
	Chrysomelidae	579	211
	Ciidae	29	6
	Clambidae	7	4
	Cleridae	50	26
	Coccinellidae	151	84
	Corylophidae	16	15
	Cryptophagidae	73	52
	Cucujidae	7	5
	Cupedidae	3	0
	Curculionidae	812	228
	Dermestidae	48	8
	Derodontidae	8	1
	Dryophthoridae	27	3
	Dryopidae	6	0
	Dytiscidae	282	90
	Elateridae	382	191
	Elmidae	31	8
	Endecatomidae	1	0
	Endomychidae	16	5

Order	Family	Species	BINs
**Coleoptera**[3]	Erotylidae	27	14
	Eucinetidae	7	5
	Eucnemidae	38	17
	Geotrupidae	12	3
	Glaphyridae	1	0
	Glaresidae	2	0
	Gyrinidae	34	18
	Haliplidae	34	7
	Heteroceridae	28	4
	Histeridae	136	3
	Hybosoridae	1	1
	Hydraenidae	27	3
	Hydrophilidae	148	48
	Ischaliidae	2	1
	Kateretidae	7	4
	Laemophloeidae	13	4
	Lampyridae	32	19
	Latridiidae	64	69
	Leiodidae	182	95
	Limnichidae	3	0
	Lucanidae	14	6
	Lutrochidae	1	0
	Lycidae	29	19
	Lymexylidae	1	1
	Megalopodidae	7	1
	Melandryidae	43	32
	Meloidae	46	15
	Melyridae	53	29
	Micromalthidae	1	0
	Monotomidae	26	4
	Mordellidae	73	66
	Mycetophagidae	16	4
	Mycteridae	4	1
	Nemonychidae	8	2
	Nitidulidae	101	47
	Nosodendridae	2	0
	Noteridae	2	1
	Ochodaeidae	4	1
	Oedemeridae	13	4
	Orsodacnidae	1	1
	Passalidae	1	0
	Passandridae	1	1
	Phalacridae	8	13
	Phengodidae	1	1
	Prostomidae	1	0
	Psephenidae	4	3
	Ptiliidae	49	41
	Ptilodactylidae	4	4
	Ptinidae	98	45
	Pyrochroidae	22	12
	Pythidae	6	4
	Rhipiceridae	1	0

Order	Family	Species	BINs	
**Coleoptera**[3]	Rhysodidae	2	0	
	Ripiphoridae	11	2	
	Salpingidae	15	7	
	Scarabaeidae	219	60	
	Scirtidae	25	37	
	Scraptiidae	20	28	
	Silphidae	26	14	
	Silvanidae	15	3	
	Sphaeritidae	1	0	
	Sphindidae	6	3	
	Staphylinidae	1674	618	
	Stenotrachelidae	9	5	
	Synchroidae	2	2	
	Tenebrionidae	139	40	
	Tetratomidae	20	6	
	Throscidae	8	16	
	Trachypachidae	2	0	
	Trogidae	15	4	
	Trogossitidae	22	1	
	Zopheridae	19	1	
**Dermaptera**[1]	Forficulidae	5	2	
**Diptera**[1]	Acartophthalmidae	2	1	
	Acroceridae	20	4	
	Agromyzidae	305	589	
	Anisopodidae	5	13	
	Anthomyiidae	375	359	
	Anthomyzidae	4	30	
	Apioceridae	1	0	
	Asilidae	125	75	
	Asteiidae	5	6	
	Athericidae	3	0	
	Aulacigastridae	3	3	
	Axymyiidae	1	1	
	Bibionidae	40	27	
	Hesperinidae		1	**Bibionidae Group**
	**Total**	**40**	**28**	
	Blephariceridae	11	2	
	Bombyliidae	70	66	
	Braulidae	1	0	
	Calliphoridae	40	33	
	Camillidae	1	1	
	Canacidae†	5	9	
	Canthyloscelidae*	1	1	
	Carnidae	8	19	
	Cecidomyiidae	100	8467	
	Ceratopogonidae	180	1054	
	Chamaemyiidae	30	71	
	Chaoboridae	9	19	
	Chironomidae	480	3432	
	Chloropidae	100	280	
	Chyromyidae	5	5	
	Clusiidae	6	19	

Order	Family	Species	BINs	
**Diptera**[1]	Coelopidae	4	3	
	Conopidae	30	16	
	Culicidae	74	63	
	Curtonotidae	1	0	
	Deuterophlebiidae	1	0	
	Diastatidae	5	8	
	Diopsidae	1	2	
	Dixidae	21	15	
	Dolichopodidae	500	499	
	Drosophilidae	60	86	
	Dryomyzidae	7	7	
	Empididae	300	399	
	Brachystomatidae		5	**Empididae Group**
	Hybotidae		256	
	**Total**	**300**	**660**	
	Ephydridae	150	143	
	Heleomyzidae†	74	61	
	Hilarimorphidae	7	1	
	Hippoboscidae†	13	1	
	Lauxaniidae	64	72	
	Lonchaeidae	97	64	
	Lonchopteridae	4	5	
	Micropezidae	16	8	
	Milichiidae	11	46	
	Muscidae	525	413	
	Fanniidae		70	**Muscidae Group**
	**Total**	**525**	**483**	
	Mycetophilidae	350	1038	
	Bolitophilidae		21	
	Diadocidiidae		5	**Mycetophilidae Group**
	Ditomyiidae		7	
	Keroplatidae		71	
	**Total**	**350**	**1142**	
	Mydidae	2	1	
	Nemestrinidae	2	0	
	Nymphomyiidae	2	0	
	Odiniidae	5	5	
	Oestridae	15	6	
	Opomyzidae	10	11	
	Pachyneuridae	1	0	
	Pallopteridae	9	5	
	Pelecorhynchidae	4	0	
	Periscelididae	2	7	
	Phoridae	127	908	
	Piophilidae	31	26	
	Pipunculidae	45	108	
	Platypezidae	21	27	
	Platystomatidae	10	6	
	Psilidae	25	26	
	Psychodidae	30	99	
	Ptychopteridae	7	5	
	Pyrgotidae	3	1	

Order	Family	Species	BINs	
**Diptera**[1]	Rhagionidae	35	47	
	Rhinophoridae	2	3	
	Sarcophagidae	85	107	
	Scathophagidae	130	96	
	Scatopsidae	30	40	
	Scenopinidae	8	2	
	Sciaridae	30	2277	
	Sciomyzidae	85	118	
	Sepsidae	17	23	
	Simuliidae	110	74	
	Sphaeroceridae	35	159	
	Stratiomyidae	84	55	
	Strongylophthalmyiidae	1	2	
	Syrphidae	500	277	
	Tabanidae	132	66	
	Tachinidae	500	434	
	Tanyderidae	2	0	
	Tanypezidae	1	1	
	Tephritidae	40	53	
	Tipulidae	520	151	
	Cylindrotomidae		5	
	Limoniidae		259	**Tipulidae Group**
	Pediciidae		29	
	**Total**	**520**	**444**	
	Thaumaleidae	3	2	
	Therevidae	30	25	
	Trichoceridae	20	27	
	Ulidiidae*	35	29	
	Xylomyidae	4	5	
	Xylophagidae	15	12	
**Ephemeroptera**[1]	Ameletidae[4]	21	5	
	Ametropodidae	2	1	
	Baetidae	71	67	
	Baetiscidae	7	2	
	Caenidae	10	12	
	Ephemerellidae	44	34	
	Ephemeridae	12	8	
	Heptageniidae	75	51	
	Isonychiidae[4]	4	4	
	Leptohyphidae[4]	5	6	
	Leptophlebiidae	24	24	
	Metretopodidae	3	3	
	Neoephemeridae	1	0	
	Oligoneuriidae	1	0	
	Polymitarcyidae*	3	4	
	Potamanthidae	3	0	
	Siphlonuridae	41	11	
	Tricorythidae	4	0	
**Grylloblattodea**[1]	Grylloblattidae	2	1	
**Hemiptera**[5]	Acanthosomatidae	4	2	
	Achilidae	19	10	
	Aclerdidae	1	0	

Order	Family	Species	BINs	
**Hemiptera**[5]	Adelgidae	18	6	
	Aleyrodidae	11	31	
	Alydidae	11	7	
	Anthocoridae	38	22	
	Aphididae	836	462	
	Aradidae	54	5	
	Artheneidae	1	1	
	Asterolecaniidae	2	0	
	Belostomatidae	4	2	
	Berytidae	5	5	
	Blissidae	6	1	
	Caliscelidae	11	3	
	Calophyidae	1	0	
	Ceratocombidae	1	5	
	Cercopidae	24	12	
	Cicadellidae	1092	731	
	Cicadidae	21	3	
	Cimicidae	7	0	
	Cixiidae	35	13	
	Clastopteridae	12	8	
	Coccidae	24	4	
	Coreidae	13	6	
	Corixidae	81	31	
	Cryptococcidae	2	0	
	Cydnidae	10	5	
	Cymidae	5	4	
	Dactylopiidae	1	0	
	Delphacidae	125	58	
	Derbidae	14	7	
	Diaspididae	29	2	
	Enicocephalidae	1	1	
	Eriococcidae	3	1	
	Flatidae	1	2	
	Fulgoridae	8	2	
	Gelastocoridae	1	0	
	Geocoridae	10	4	
	Gerridae	23	8	
	Hebridae	6	0	
	Heterogastridae	1	0	
	Hydrometridae	1	0	
	Issidae	3	0	
	Acanaloniidae		2	**Issidae Group**
	**Total**	**3**	**2**	
	Kermesidae	1	0	
	Lasiochilidae	1	0	
	Lyctocoridae	6	1	
	Lygaeidae	29	13	
	Margarodidae	5	0	
	Membracidae	105	27	
	Mesoveliidae	2	1	
	Microphysidae	4	0	
	Miridae	698	287	

Order	Family	Species	BINs
**Hemiptera**[5]	Nabidae	20	13
	Naucoridae	1	0
	Nepidae	4	0
	Notonectidae	12	6
	Ochteridae	1	0
	Ortheziidae	5	0
	Oxycarenidae	4	0
	Pachygronthidae	3	2
	Pentatomidae	69	36
	Phylloxeridae	8	2
	Phymatidae	4	1
	Piesmatidae	4	1
	Pleidae	1	0
	Pseudococcidae	23	6
	Psyllidae	105	71
	Reduviidae	26	8
	Rhopalidae	16	10
	Rhyparochromidae	65	28
	Saldidae	40	11
	Scutelleridae	14	8
	Spondyliaspididae	1	0
	Thyreocoridae	11	2
	Tingidae	47	16
	Triozidae	25	4
	Veliidae	7	1
**Hymenoptera**[1]	Andrenidae	250	55
	Aphelinidae	30	94
	Apidae[†]	189	76
	Argidae	14	9
	Aulacidae	17	3
	Bethylidae	35	54
	Braconidae	830	2444
	Cephidae	16	5
	Ceraphronidae	35	1
	Chalcididae	22	5
	Chrysididae[†]	33	60
	Cimbicidae	4	10
	Colletidae	45	27
	Cynipidae	110	56
	Diapriidae	150	526
	Diprionidae	22	10
	Dryinidae	35	103
	Embolemidae	2	0
	Encyrtidae	65	120
	Eucharitidae	10	5
	Eulophidae[†]	173	772
	Eupelmidae	14	12
	Eurytomidae[6]	248	53
	Evaniidae	4	0
	Figitidae[†]	36	409
	Formicidae	139	178
	Gasteruptiidae	10	9

Order	Family		Species	BINs	
**Hymenoptera**[1]	Halictidae		110	94	
	Heloridae		2	2	
	Ibaliidae		4	0	
	Ichneumonidae†		2001	3851	
	Leucospidae		1	1	
	Megachilidae		150	81	
	Megaspilidae		35	265	
	Melittidae		2	1	
	Mutillidae		30	5	
	Myrmosidae			7	**Mutillidae Group**
		**Total**	**30**	**12**	
	Mymaridae		15	238	
	Ormyridae		5	1	
	Orussidae		3	0	
	Pamphiliidae		47	10	
	Pelecinidae		1	0	
	Pergidae		5	2	
	Perilampidae		11	13	
	Platygastridae†		250	1654	
	Pompilidae		150	72	
	Proctotrupidae		60	52	
	Pteromalidae		110	397	
	Rhopalosomatidae		2	0	
	Roproniidae		2	0	
	Sapygidae		6	4	
	Scoliidae		2	1	
	Sierolomorphidae		1	0	
	Signiphoridae		4	0	
	Siricidae		14	6	
	Sphecidae		225	33	
	Crabronidae			203	**Sphecidae Group**
		**Total**	**225**	**234**	
	Stephanidae		2	0	
	Tenthredinidae		300	397	
	Tetracampidae		2	0	
	Tiphiidae		25	5	
	Torymidae		32	96	
	Trichogrammatidae		6	81	
	Trigonalidae		4	3	
	Vanhorniidae		1	0	
	Vespidae		100	67	
	Xiphydriidae		5	2	
	Xyelidae		13	9	
**Isoptera**[1]	Rhinotermitidae		2	3	
	Termopsidae		2	1	
**Lepidoptera**[7]	Acanthopteroctetidae		4	0	
	Adelidae		10	10	
	Alucitidae		3	3	
	Apatelodidae		2	0	
	Argyresthiidae		33	24	
	Attevidae		1	0	
	Autostichidae†		10	4	

Order	Family		Species	BINs	
**Lepidoptera**[7]	Batrachedridae		6	5	
	Bedelliidae		1	1	
	Blastobasidae		22	33	
	Bombycidae		1	0	
	Bucculatricidae		38	39	
	Carposinidae		4	7	
	Choreutidae		20	13	
	Coelopoetidae		1	0	
	Coleophoridae		118	111	
	Copromorphidae		2	2	
	Cosmopterigidae		32	32	
	Cossidae		6	3	
	Crambidae		313	208	
	Douglasiidae		5	3	
	Drepanidae		14	12	
	Dryadaulidae		1	0	
	Elachistidae		167	77	**Elachistidae Group**
	Depressariidae†			69	
		**Total**	**167**	**146**	
	Epermeniidae		8	10	
	Erebidae[8]		328	216	
	Eriocraniidae		5	2	
	Euteliidae[8]		8	5	
	Galacticidae		1	0	
	Gelechiidae		395	351	
	Geometridae		596	438	
	Glyphipterigidae		14	8	
	Gracillariidae		177	164	
	Heliodinidae		4	2	
	Heliozelidae		17	19	
	Hepialidae		16	8	
	Hesperiidae		81	26	
	Incurvariidae		4	6	
	Lasiocampidae		12	12	
	Limacodidae		23	9	
	Lycaenidae		77	33	
	Lyonetiidae		11	11	
	Lypusidae†*		1	0	
	Meesiidae		2	0	
	Micropterigidae		3	4	
	Mimallonidae		2	0	
	Momphidae		24	39	
	Nepticulidae		63	75	
	Noctuidae[8]		1145	641	
	Nolidae[8]		17	15	
	Notodontidae[8]		57	54	
	Nymphalidae		110	66	
	Oecophoridae		22	18	
	Opostegidae		4	8	
	Papilionidae		18	9	
	Pieridae		43	11	

Order	Family	Species	BINs
**Lepidoptera**[7]	Plutellidae	10	8
	Praydidae	1	0
	Prodoxidae	22	10
	Psychidae	12	6
	Pterolonchidae	1	0
	Pterophoridae	92	61
	Pyralidae	271	123
	Riodinidae	1	0
	Saturniidae	26	9
	Schreckensteiniidae	3	4
	Scythrididae	15	14
	Sesiidae	69	11
	Sphingidae	65	34
	Stathmopodidae	1	1
	Thyrididae	3	1
	Tineidae	71	67
	Tischeriidae	13	11
	Tortricidae	906	524
	Uraniidae†	2	3
	Urodidae	1	1
	Yponomeutidae	26	12
	Ypsolophidae	18	12
	Zygaenidae	3	2
**Mantodea**[1]	Mantidae	3	2
**Mecoptera**[1]	Bittacidae	4	1
	Boreidae	7	1
	Meropeidae	1	1
	Panorpidae	10	8
**Megaloptera**[1]	Corydalidae	6	4
	Sialidae	10	4
**Neuroptera**[1]	Berothidae	1	0
	Chrysopidae	25	13
	Coniopterygidae	7	51
	Hemerobiidae†	28	38
	Ithonidae*	1	0
	Mantispidae	3	0
	Myrmeleontidae	7	7
	Sisyridae	3	3
**Odonata**[1]	Aeshnidae	24	17
	Calopterygidae	4	2
	Coenagrionidae	38	19
	Cordulegastridae	4	2
	Corduliidae	28	11
	Gomphidae	36	8
	Lestidae	9	6
	Libellulidae	45	17
	Macromiidae	4	1
	Petaluridae	2	0
**Orthoptera**[1]	Acrididae	124	59
	Gryllidae†	17	17
	Gryllotalpidae	1	0
	Myrmecophilidae	1	0

Order	Family	Species	BINs	
**Orthoptera**[1]	Haglidae	2	0	
	Prophalangopsidae		2	**Haglidae Group**
	**Total**	**2**	**2**	
	Rhaphidophoridae	21	9	
	Stenopelmatidae	2	0	
	Tetrigidae	7	6	
	Tettigoniidae†	36	16	
	Tridactylidae	2	0	
	Trigonidiidae	1	0	
**Phasmatodea**[1]	Heteronemiidae	1	1	
**Phthiraptera**[1]	Boopidae	1	0	
	Echinophthiriidae	3	0	
	Gyropidae	2	0	
	Haematopinidae	3	0	
	Hoplopleuridae	19	1	
	Laemobothriidae	2	0	
	Linognathidae	6	0	
	Menoponidae	100	0	
	Pediculidae	2	1	
	Philopteridae	200	0	
	Ricinidae	3	0	
	Trichodectidae	20	1	
	Trimenoponidae	1	0	
**Plecoptera**[1]	Capniidae	49	10	
	Chloroperlidae	44	28	
	Leuctridae	21	18	
	Nemouridae	32	37	
	Peltoperlidae	4	1	
	Perlidae	15	9	
	Perlodidae	61	18	
	Pteronarcyidae*	9	2	
	Taeniopterygidae	15	6	
**Psocoptera**[1]	Amphipsocidae	2	2	
	Caeciliusidae*	13	17	
	Ectopsocidae	2	7	
	Elipsocidae	3	3	
	Epipsocidae	2	3	
	Lachesillidae	11	30	
	Lepidopsocidae	2	2	
	Liposcelidae	2	1	
	Mesopsocidae	2	3	
	Myopsocidae	1	1	
	Peripsocidae	5	7	
	Philotarsidae	2	2	
	Psocidae	22	40	
	Stenopsocidae[9]	0	6	
	Trogiidae	3	3	
**Raphidioptera**[1]	Inocelliidae	1	1	
	Raphidiidae	6	7	
**Siphonaptera**[1]	Ceratophyllidae	80	12	
	Hystrichopsyllidae	62	3	
	Ischnopsyllidae	4	0	

Order	Family	Species	BINs
**Siphonaptera**[1]	Leptopsyllidae	19	0
	Pulicidae	10	1
	Vermipsyllidae	5	0
**Strepsiptera**[10]	Corioxenidae	1	0
	Elenchidae	0	2
	Halictophagidae	2	0
	Stylopidae	1	0
**Thysanoptera**[1]	Aeolothripidae	13	32
	Phlaeothripidae	30	72
	Thripidae	59	137
**Thysanura**[1]	Lepismatidae	2	1
**Trichoptera**[1]	Apataniidae[11]	16	4
	Beraeidae	1	0
	Brachycentridae	15	11
	Calamoceratidae	2	1
	Dipseudopsidae[11]	3	3
	Ecnomidae[11]	0	1
	Glossosomatidae	21	13
	Goeridae[11]	5	3
	Helicopsychidae	1	4
	Hydropsychidae	48	54
	Hydroptilidae	52	61
	Lepidostomatidae	26	23
	Leptoceridae	56	95
	Limnephilidae	179	101
	Molannidae	7	3
	Odontoceridae	4	3
	Philopotamidae	13	15
	Phryganeidae	24	25
	Polycentropodidae	36	24
	Psychomyiidae	2	2
	Ptilocolepidae[11]	3	0
	Rhyacophilidae	57	20
	Rossianidae[11]	1	0
	Sericostomatidae	2	1
	Uenoidae†[11]	18	9

References

1. Danks, H.V., editor. 1979. Canada and its Insect Fauna. *Memoirs Entomological Society of Canada*, **108**, 1–573.

2. Bowser, M.L. 2012 [Internet]. Key and checklist of the bristletails (*Microcoryphia*) of America north of Mexico. Fairbanks (AK): Alaska Entomological Society [cited 2016 Feb 12]. Available from: http://www.akentsoc.org/doc/Archaeognatha/Archaeognatha.pdf.

3. Bousquet, Y., Bouchard, P., Davies, A.E. & Sikes, D.S. 2013. Checklist of Beetles (Coleoptera) of Canada and Alaska. 2nd ed. Sofia (Bulgaria). Pensoft Publishers.

4. McCafferty, W. P. & Randolph, R. P. 1998. Canada mayflies: a faunistic compendium. *Proceedings of the Entomological Society of Ontario*, **129**, 47-97.

5. Maw, H.E.L., Foottit, R. G., Hamilton, K.G.A. & Scudder, G.G.E. 2000. Checklist for the Hemiptera of Canada and Alaska. NRC Press. Ottawa. 220pp

6. Grissell, E. E. & Schauff, M. E. 1990. A handbook of the families of Nearctic Chalcidoidea (Hymenoptera) (No. 1). Washington (DC). Entomological Society of Washington. 85 pp.

7. Pohl, G.R. et al. 2016 Annotated checklist of the Lepidoptera of Canada, Alaska and Greenland. *Zootaxa*, in review.

8. Zahiri, R., Lafontaine, D., Schmidt, C.D. Zakharov, E. & Hebert, P.D.N. 2014. A transcontinental challenge – A test of DNA barcode performance for 1541 species of Canadian Noctuoidea. *PLOS ONE* **9**: e92797.

9. Mockford, E.L., 2012 [Internet]. Aspects of the Biogeography of North American Psocoptera (Insecta), Global Advances in Biogeography, Dr. Lawrence Stevens (Ed.). Rijeka (Croatia): INTECH Open Access Publisher. (doi: 10.5772/33413).

10. Kathirithamby, J. & Taylor, S.J. 2005. A new species of *Halictophagus* (Insecta: Strepsiptera: Halictophagidae) from Texas, and a checklist of Strepsiptera from the United States and Canada. *Zootaxa*, **1056**, 1-18.

11. Rasmussen, A.K. & Morse, J.C. 2014 [Internet]. Distributional Checklist of Nearctic Trichoptera (Summer 2014 Revision). Tallahassee (FL): Unpublished, Florida A&M University. [cited 2016 Feb 10]. 487 pp. Available from: http://www.Trichoptera.org.

Supplementary Table S3: The number of species known for each of 27 Canadian insect orders and the number of BINs assigned to it based on specimens collected in this study. The species totals derive from Table S2.

Order	Species	BINs	BIN/SP
Archaeognatha	8	9	1.13
Blattodea	10	8	0.80
Coleoptera	8107	3042	0.38
Dermaptera	5	2	0.40
Diptera	7056	23591	3.34
Ephemeroptera	331	232	0.70
Grylloblattodea	2	1	0.50
Hemiptera	3943	2022	0.51
Hymenoptera	6276	12859	2.05
Isoptera	4	4	1.00
Lepidoptera	5734	3830	0.67
Mantodea	3	2	0.67
Mecoptera	22	11	0.50
Megaloptera	16	8	0.50
Neuroptera	75	112	1.49
Odonata	194	83	0.43
Orthoptera	214	109	0.51
Phasmatodea	1	1	1.00
Phthiraptera	362	3	0.01
Plecoptera	250	129	0.52
Psocoptera	72	134	1.86
Raphidioptera	7	8	1.14
Siphonaptera	180	16	0.09
Strepsiptera	4	2	0.50
Thysanoptera	102	242	2.37
Thysanura	2	1	0.50
Trichoptera	592	476	0.80

Electronic Supplementary Material for:
Counting Species with DNA Barcodes: Canadian Insects. 2016. PDN Hebert, S Ratnasingham, EV Zakharov, AC Telfer, V Levesque-Beaudin, MA Milton, S Pedersen, P Jannetta, JR deWaard. Philosophical Transactions of the Royal Society B. doi: 10.1098/rstb.2015.0333.

Supplementary Table S4: Nearest-Neighbor distances and BIN counts for the 98 families of Canadian Diptera that were encountered in this study. Species counts are only available for the 87 families included in [16].

Family	BINs	Known Species	Average Nearest Neighbour (NN) Distance
Acartophthalmidae	1	2	NA
Acroceridae	4	20	9.47
Agromyzidae	588	305	5.59
Anisopodidae	11	5	8.25
Anthomyiidae	357	375	3.47
Anthomyzidae	27	4	5.63
Asilidae	73	125	6.68
Asteiidae	6	5	9.36
Aulacigastridae	3	3	3.72
Axymyiidae	1	1	NA
Bibionidae	25	40	4.64
Hesperinidae	1	NA	NA
Blephariceridae	2	11	18.67
Bombyliidae	65	70	6.53
Calliphoridae	34	40	3.59
Camillidae	1	1	NA
Canacidae	9	5	4.83
Canthyloscelidae	1	1	NA
Carnidae	21	8	3.63
Cecidomyiidae	8484	100	5.01
Ceratopogonidae	1046	180	5.43
Chamaemyiidae	71	30	6.20
Chaoboridae	19	9	6.74
Chironomidae	3445	480	5.37
Chloropidae	280	100	3.42
Chyromyidae	5	5	5.11
Clusiidae	22	6	7.46
Coelopidae	3	4	4.50
Conopidae	15	30	11.92
Culicidae	63	74	4.40
Diastatidae	7	5	5.04
Diopsidae	2	1	2.61
Dixidae	16	21	8.27
Dolichopodidae	495	500	5.60
Drosophilidae	84	60	5.86
Dryomyzidae	7	7	8.30
Empididae	387	300	6.24
Brachystomatidae	5	NA	6.13
Hybotidae	259	NA	6.59
Ephydridae	141	150	5.71
Heleomyzidae	60	74	6.06
Hilarimorphidae	1	7	NA
Hippoboscidae	1	13	NA
Lauxaniidae	57	64	5.94
Lonchaeidae	63	97	4.53
Lonchopteridae	5	4	7.72
Micropezidae	8	16	4.28
Milichiidae	47	11	5.67
Muscidae	414	525	4.04
Fanniidae	72	NA	4.88

Family	BINs	Known Species	Average Nearest Neighbour (NN) Distance
Mycetophilidae	1027	350	5.82
Bolitophilidae	21	NA	5.70
Diadocidiidae	5	NA	14.55
Ditomyiidae	7	NA	9.23
Keroplatidae	71	NA	8.54
Mydidae	1	2	NA
Odiniidae	5	5	9.05
Oestridae	6	15	7.88
Opomyzidae	11	10	5.29
Pallopteridae	6	9	4.06
Periscelididae	8	2	2.74
Phoridae	915	127	4.68
Piophilidae	26	31	6.34
Pipunculidae	105	45	5.78
Platypezidae	29	21	7.79
Platystomatidae	5	10	5.60
Psilidae	26	25	4.25
Psychodidae	96	30	6.25
Ptychopteridae	5	7	10.11
Pyrgotidae	1	3	NA
Rhagionidae	47	35	6.11
Rhinophoridae	3	2	10.30
Sarcophagidae	105	85	5.42
Scathophagidae	98	130	3.50
Scatopsidae	40	30	8.43
Scenopinidae	2	8	15.50
Sciaridae	2317	30	4.82
Sciomyzidae	115	85	6.81
Sepsidae	23	17	5.38
Simuliidae	74	110	6.87
Sphaeroceridae	158	35	5.34
Stratiomyidae	64	84	6.19
Strongylophthalmyiidae	2	1	5.67
Syrphidae	280	500	4.00
Tabanidae	70	132	2.77
Tachinidae	426	500	4.83
Tanypezidae	1	1	NA
Tephritidae	57	40	4.89
Tipulidae	153	520	5.00
Cylindrotomidae	5	NA	7.08
Limoniidae	264	NA	7.46
Pediciidae	29	NA	7.97
Thaumaleidae	2	3	12.44
Therevidae	25	30	4.94
Trichoceridae	27	20	7.46
Ulidiidae	29	35	8.32
Xylomyidae	5	4	3.84
Xylophagidae	16	15	4.43

Supplementary Table S5: Students, staff and researchers who contributed to the processing of specimens, to sequence analysis or to the development of the informatics platform required for data analysis.
* Personnel who worked for more than one unit within the Centre for Biodiversity Genomics.

Collections

Laboratory Technicians

Addesi, Joseph	Hall, Mitchell John	St. Jacques, Brianne
Ansell, Graham	Layton, Kara*	Sukumar, Smera
Breton, Vanessa	McClenaghan, Beverley	Turner, Rick
Cossey, Jay	Pawlowski, Agata	Walker, Charles
Derbyshire, Rachael	Sills-Gilligan, Jesse	Young, Andrew
Eagalle, Thanushi	Smith, JDL	Zunder, Adam

Postdoctoral Fellows and Research Associates

Ashfaq, Muhammad	Gwiazdowski, Rodger	Steinke, Dirk
Berzitis, Emily	Hernandez Triana, Luis	Webb, Jeffrey
Blagoev, Gergin	Kekkonen, Mari	Zahiri, Reza
Borisenko, Alex	Porco, David	Zhou, Xin
Dinca, Vlad	Rougerie, Rodolphe	
Fernandez Triana, Jose	Stahlhut, Julie	

Undergraduate Students

Amin, Mahmoud	Engelking, Daniel	Morra, Michael
Ammendolia, Justine	Fife, Danielle	Nagabaskaran, Gokulan
Anderson, Hannah	Forde, Alison	Noel, Emily
Arora, Drupad	Gibson, Daniel	Persi, Joshua
Arroyo Gerez, Maria	Hammerton, Stephanie	Ronnenberg, Kylee*
Bannon, Grace	Hermack, Jessica	Sones, Jillian
Barron, Hannah	Huissen, Haedar	Swan, Megan
Belluz, Vincent	Jeffery, Nicholas	Telfer, Zachariah
Bergstrome, Jill	Jones, Nathaniel	Tsao, Natalie
Boskovic, Adrian	Junea, Vadim	Ul-Huda, Mohammad
Boyle, Elizabeth	Laforest, Brandon	Van Patter, Lauren
Braschel, Melissa	Lutes, Katelyn	Vandermeer, Caitlin
Burke, Grace	Lyttle, Hilary	Vender, Reid
Cardarelli-Goddard, Liam	Mancuso, Kristin	Wajmer, Nicole
Collins, Colina	McClenaghan, Beverly	Walker, Michael
Dickson, Liam	McCubbin, Sandra	Wallace, Lauren
D'Souza, Kareina	McLister, Corey	Williams, Jonathan
Dykeman, Sarah	Miles, Jizel	Wright, Danielle
Eagle, Shannon	Miranda Romo, Susanna	Zanki, Jenna
Eberl, Emma	Monaghan, Dominique	Zlatkin, Martin

Informatics

Informatics Staff

Abraham, Lily	Gonzales Ramos, Karina	Pierossi, Ola*
Berkers, T	Ho, Chris	Roes, Daniel
Bertrand, Claudia*	Jackson, Gregory	Rosco, Filip
Chan, Dean	Johansson, Jessica	Russell, John
Cicchello, Orlando	Kwiatkowski, Andy	Singh, Harman
De Ridder, Michael	Li, Jeff	Steinke, Claudia
Dobosz, Rafal	Ma, Eddie	van Konynenurg, Steven
Duquette, Karen	Manjunath, Ramya	Van Wyngaarden, Mallory

Informatics Staff

El-Bastrami, Mohammed
Esmaeilsabzali, Shahram
Fernandes, Rebecca
Gazo, Viktor
Gervais, Sapphyre
Mannan, Riadul
Marquhardt, Pia
Mohamed, Younis
Morawski, Mike
Papp, Andras
Von Konigslow, Taika
Will, Elise
Zepeda, Raul

Postdoctoral Fellows and Research Associates

Radulovici, Adriana*
Schonfeld, Justin
Zemlak, Tyler

Undergraduate Students

Andrews, Brittany*
Bai, Bryan
Bond, Kaitlin
Chabot, Chad
Chan, Irenaeus
Chen, Alexander
Dussault, Forest*
Lam, Derrick
Li, Xin
Nolet, Luke Joel
Poblete, Tashana
Pomezanski, Jennifer*
Saeed, Abiya
Sauk, Alexandra
Shamshoom, Dina
Silva, Anjali
St. Jacques, Jason*
Tukhareli, Tamara
Turgeon, Justin

Sequencing

Laboratory Technicians

Abrahamyan, Arusyak
Castillo, Anibal
Hortie, Jared
Iskandar, Marianne
Johnson, Laura
Kodos, Neveen
Macke, Katrena
Malo, Catherine
McKenna, Charly
Monkhouse, Norman
Morgan, Thomas
Nikolova, Nadya
Prosser, Sean
Seidle, Kristy
Shireen, Tasnina
Tabone, Robert
Whitmell, Chad
Yasmin, Munira

Postdoctoral Fellows and Research Associates

Braukmann, Thomas
Cao, H
Ivanova, Natalia
Meusnier, Isabelle
Remigio, Ed
Rodrigues, Anibal

Undergraduate Students

Arias, Joshua
Burcea, Katherine
Cote, Jordan
Fuhrman, Tyler
Knapp, Alexandra*
Kuzmin, Peter
Lau, Vincent
Lim, Jan Edward
MacDonald, Shannon*
McDonald, Jessica
Mouaaz, Samar
Parker, Colleen
Sebastian, Manuel
Shostak, Kristina
Thevanayagam, Abinah
Tonin, Attilio

Electronic Supplementary Material for:
Counting Species with DNA Barcodes: Canadian Insects. 2016. PDN Hebert, S Ratnasingham, EV Zakharov, AC Telfer, V Levesque-Beaudin, MA Milton, S Pedersen, P Jannetta, JR deWaard. Philosophical Transactions of the Royal Society B. doi: 10.1098/rstb.2015.0333.

Supplementary Figure S1: Neighbor-Joining tree based on sequence divergences (K2P) at COI for the 23,591 BINs of Diptera from Canada. Entries in the tree are colorized by family.

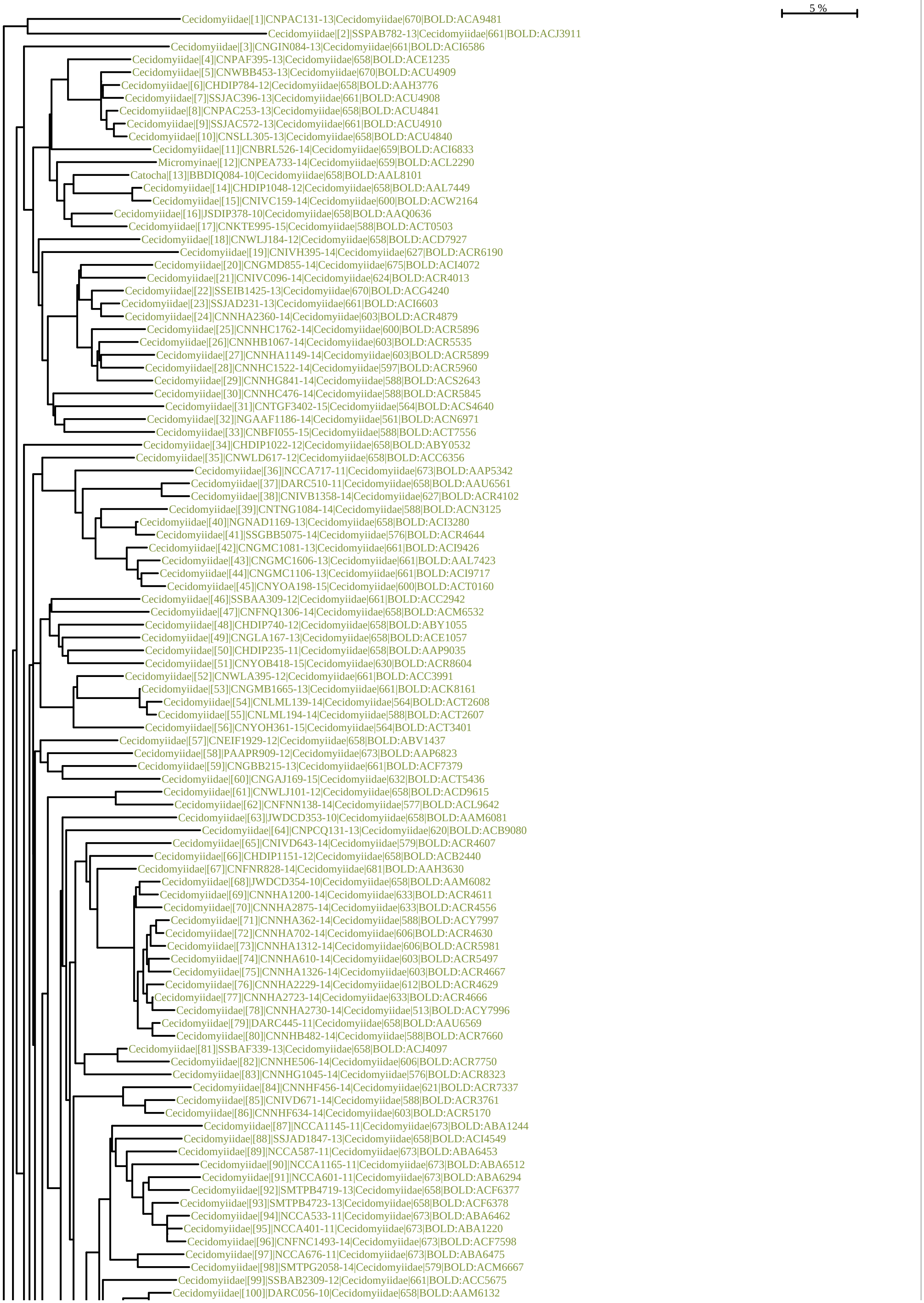

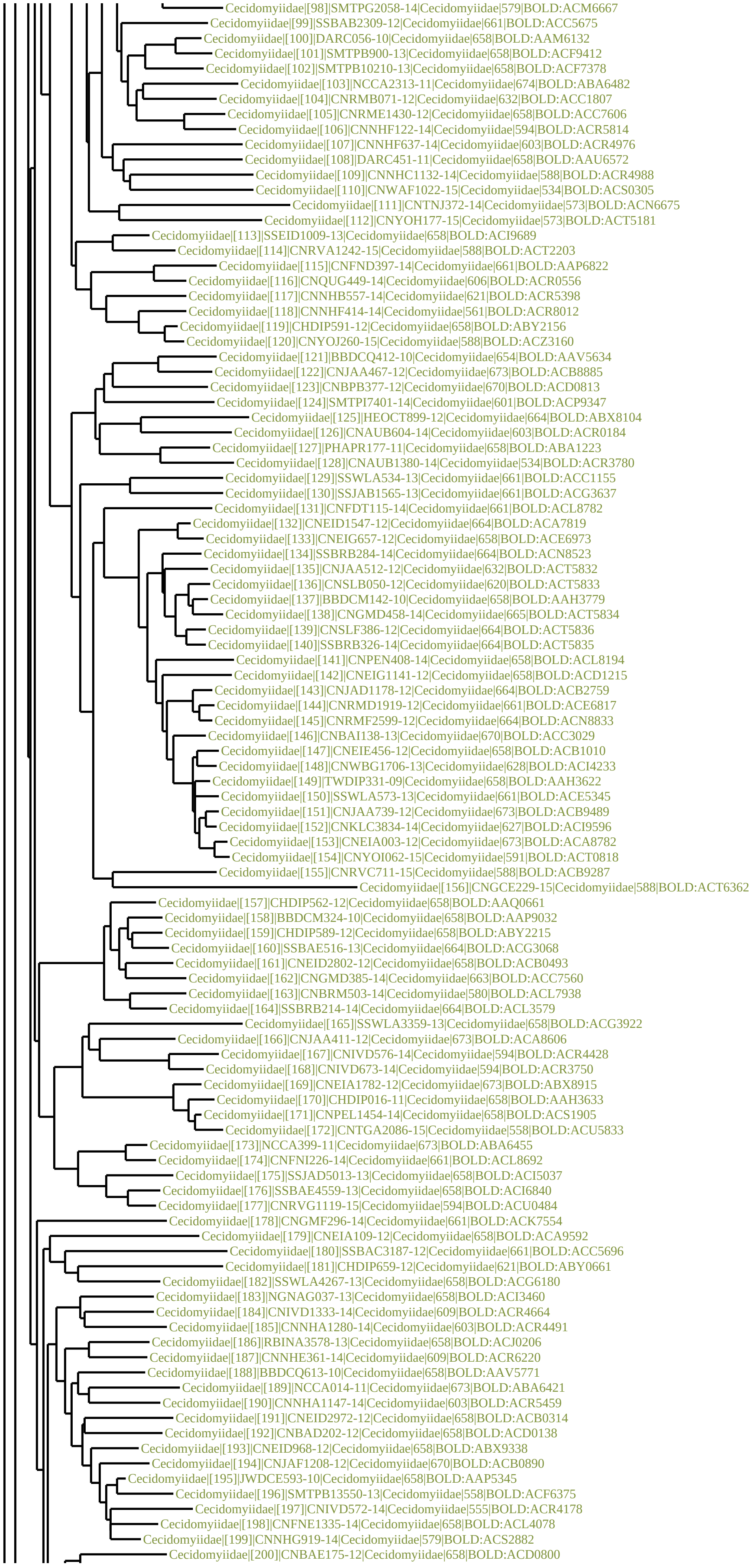
Cecidomyiidae|[98]|SMTPG2058-14|Cecidomyiidae|579|BOLD:ACM6667
Cecidomyiidae|[99]|SSBAB2309-12|Cecidomyiidae|661|BOLD:ACC5675
Cecidomyiidae|[100]|DARC056-10|Cecidomyiidae|658|BOLD:AAM6132
Cecidomyiidae|[101]|SMTPB900-13|Cecidomyiidae|658|BOLD:ACF9412
Cecidomyiidae|[102]|SMTPB10210-13|Cecidomyiidae|658|BOLD:ACF7378
Cecidomyiidae|[103]|NCCA2313-11|Cecidomyiidae|674|BOLD:ABA6482
Cecidomyiidae|[104]|CNRMB071-12|Cecidomyiidae|632|BOLD:ACC1807
Cecidomyiidae|[105]|CNRME1430-12|Cecidomyiidae|658|BOLD:ACC7606
Cecidomyiidae|[106]|CNNHF122-14|Cecidomyiidae|594|BOLD:ACR5814
Cecidomyiidae|[107]|CNNHF637-14|Cecidomyiidae|603|BOLD:ACR4976
Cecidomyiidae|[108]|DARC451-11|Cecidomyiidae|658|BOLD:AAU6572
Cecidomyiidae|[109]|CNNHC1132-14|Cecidomyiidae|588|BOLD:ACR4988
Cecidomyiidae|[110]|CNWAF1022-15|Cecidomyiidae|534|BOLD:ACS0305
Cecidomyiidae|[111]|CNTNJ372-14|Cecidomyiidae|573|BOLD:ACN6675
Cecidomyiidae|[112]|CNYOH177-15|Cecidomyiidae|573|BOLD:ACT5181
Cecidomyiidae|[113]|SSEID1009-13|Cecidomyiidae|658|BOLD:ACI9689
Cecidomyiidae|[114]|CNRVA1242-15|Cecidomyiidae|588|BOLD:ACT2203
Cecidomyiidae|[115]|CNFND397-14|Cecidomyiidae|661|BOLD:AAP6822
Cecidomyiidae|[116]|CNQUG449-14|Cecidomyiidae|606|BOLD:ACR0556
Cecidomyiidae|[117]|CNNHB557-14|Cecidomyiidae|621|BOLD:ACR5398
Cecidomyiidae|[118]|CNNHF414-14|Cecidomyiidae|561|BOLD:ACR8012
Cecidomyiidae|[119]|CHDIP591-12|Cecidomyiidae|658|BOLD:ABY2156
Cecidomyiidae|[120]|CNYOJ260-15|Cecidomyiidae|588|BOLD:ACZ3160
Cecidomyiidae|[121]|BBDCQ412-10|Cecidomyiidae|654|BOLD:AAV5634
Cecidomyiidae|[122]|CNJAA467-12|Cecidomyiidae|673|BOLD:ACB8885
Cecidomyiidae|[123]|CNBPB377-12|Cecidomyiidae|670|BOLD:ACD0813
Cecidomyiidae|[124]|SMTPI7401-14|Cecidomyiidae|601|BOLD:ACP9347
Cecidomyiidae|[125]|HEOCT899-12|Cecidomyiidae|664|BOLD:ABX8104
Cecidomyiidae|[126]|CNAUB604-14|Cecidomyiidae|603|BOLD:ACR0184
Cecidomyiidae|[127]|PHAPR177-11|Cecidomyiidae|658|BOLD:ABA1223
Cecidomyiidae|[128]|CNAUB1380-14|Cecidomyiidae|534|BOLD:ACR3780
Cecidomyiidae|[129]|SSWLA534-13|Cecidomyiidae|661|BOLD:ACC1155
Cecidomyiidae|[130]|SSJAB1565-13|Cecidomyiidae|661|BOLD:ACG3637
Cecidomyiidae|[131]|CNFDT115-14|Cecidomyiidae|661|BOLD:ACL8782
Cecidomyiidae|[132]|CNEID1547-12|Cecidomyiidae|664|BOLD:ACA7819
Cecidomyiidae|[133]|CNEIG657-12|Cecidomyiidae|658|BOLD:ACE6973
Cecidomyiidae|[134]|SSBRB284-14|Cecidomyiidae|664|BOLD:ACN8523
Cecidomyiidae|[135]|CNJAA512-12|Cecidomyiidae|632|BOLD:ACT5832
Cecidomyiidae|[136]|CNSLB050-12|Cecidomyiidae|620|BOLD:ACT5833
Cecidomyiidae|[137]|BBDCM142-10|Cecidomyiidae|658|BOLD:AAH3779
Cecidomyiidae|[138]|CNGMD458-14|Cecidomyiidae|665|BOLD:ACT5834
Cecidomyiidae|[139]|CNSLF386-12|Cecidomyiidae|664|BOLD:ACT5836
Cecidomyiidae|[140]|SSBRB326-14|Cecidomyiidae|664|BOLD:ACT5835
Cecidomyiidae|[141]|CNPEN408-14|Cecidomyiidae|658|BOLD:ACL8194
Cecidomyiidae|[142]|CNEIG1141-12|Cecidomyiidae|658|BOLD:ACD1215
Cecidomyiidae|[143]|CNJAD1178-12|Cecidomyiidae|664|BOLD:ACB2759
Cecidomyiidae|[144]|CNRMD1919-12|Cecidomyiidae|661|BOLD:ACE6817
Cecidomyiidae|[145]|CNRMF2599-12|Cecidomyiidae|664|BOLD:ACN8833
Cecidomyiidae|[146]|CNBAI138-13|Cecidomyiidae|670|BOLD:ACC3029
Cecidomyiidae|[147]|CNEIE456-12|Cecidomyiidae|658|BOLD:ACB1010
Cecidomyiidae|[148]|CNWBG1706-13|Cecidomyiidae|628|BOLD:ACI4233
Cecidomyiidae|[149]|TWDIP331-09|Cecidomyiidae|658|BOLD:AAH3622
Cecidomyiidae|[150]|SSWLA573-13|Cecidomyiidae|661|BOLD:ACE5345
Cecidomyiidae|[151]|CNJAA739-12|Cecidomyiidae|673|BOLD:ACB9489
Cecidomyiidae|[152]|CNKLC3834-14|Cecidomyiidae|627|BOLD:ACI9596
Cecidomyiidae|[153]|CNEIA003-12|Cecidomyiidae|673|BOLD:ACA8782
Cecidomyiidae|[154]|CNYOI062-15|Cecidomyiidae|591|BOLD:ACT0818
Cecidomyiidae|[155]|CNRVC711-15|Cecidomyiidae|588|BOLD:ACB9287
Cecidomyiidae|[156]|CNGCE229-15|Cecidomyiidae|588|BOLD:ACT6362
Cecidomyiidae|[157]|CHDIP562-12|Cecidomyiidae|658|BOLD:AAQ0661
Cecidomyiidae|[158]|BBDCM324-10|Cecidomyiidae|658|BOLD:AAP9032
Cecidomyiidae|[159]|CHDIP589-12|Cecidomyiidae|658|BOLD:ABY2215
Cecidomyiidae|[160]|SSBAE516-13|Cecidomyiidae|664|BOLD:ACG3068
Cecidomyiidae|[161]|CNEID2802-12|Cecidomyiidae|658|BOLD:ACB0493
Cecidomyiidae|[162]|CNGMD385-14|Cecidomyiidae|663|BOLD:ACC7560
Cecidomyiidae|[163]|CNBRM503-14|Cecidomyiidae|580|BOLD:ACL7938
Cecidomyiidae|[164]|SSBRB214-14|Cecidomyiidae|664|BOLD:ACL3579
Cecidomyiidae|[165]|SSWLA3359-13|Cecidomyiidae|658|BOLD:ACG3922
Cecidomyiidae|[166]|CNJAA411-12|Cecidomyiidae|673|BOLD:ACA8606
Cecidomyiidae|[167]|CNIVD576-14|Cecidomyiidae|594|BOLD:ACR4428
Cecidomyiidae|[168]|CNIVD673-14|Cecidomyiidae|594|BOLD:ACR3750
Cecidomyiidae|[169]|CNEIA1782-12|Cecidomyiidae|673|BOLD:ABX8915
Cecidomyiidae|[170]|CHDIP016-11|Cecidomyiidae|658|BOLD:AAH3633
Cecidomyiidae|[171]|CNPEL1454-14|Cecidomyiidae|658|BOLD:ACS1905
Cecidomyiidae|[172]|CNTGA2086-15|Cecidomyiidae|558|BOLD:ACU5833
Cecidomyiidae|[173]|NCCA399-11|Cecidomyiidae|673|BOLD:ABA6455
Cecidomyiidae|[174]|CNFNI226-14|Cecidomyiidae|661|BOLD:ACL8692
Cecidomyiidae|[175]|SSJAD5013-13|Cecidomyiidae|658|BOLD:ACI5037
Cecidomyiidae|[176]|SSBAE4559-13|Cecidomyiidae|658|BOLD:ACI6840
Cecidomyiidae|[177]|CNRVG1119-15|Cecidomyiidae|594|BOLD:ACU0484
Cecidomyiidae|[178]|CNGMF296-14|Cecidomyiidae|661|BOLD:ACK7554
Cecidomyiidae|[179]|CNEIA109-12|Cecidomyiidae|658|BOLD:ACA9592
Cecidomyiidae|[180]|SSBAC3187-12|Cecidomyiidae|661|BOLD:ACC5696
Cecidomyiidae|[181]|CHDIP659-12|Cecidomyiidae|621|BOLD:ABY0661
Cecidomyiidae|[182]|SSWLA4267-13|Cecidomyiidae|658|BOLD:ACG6180
Cecidomyiidae|[183]|NGNAG037-13|Cecidomyiidae|658|BOLD:ACI3460
Cecidomyiidae|[184]|CNIVD1333-14|Cecidomyiidae|609|BOLD:ACR4664
Cecidomyiidae|[185]|CNNHA1280-14|Cecidomyiidae|603|BOLD:ACR4491
Cecidomyiidae|[186]|RBINA3578-13|Cecidomyiidae|658|BOLD:ACJ0206
Cecidomyiidae|[187]|CNNHE361-14|Cecidomyiidae|609|BOLD:ACR6220
Cecidomyiidae|[188]|BBDCQ613-10|Cecidomyiidae|658|BOLD:AAV5771
Cecidomyiidae|[189]|NCCA014-11|Cecidomyiidae|673|BOLD:ABA6421
Cecidomyiidae|[190]|CNNHA1147-14|Cecidomyiidae|603|BOLD:ACR5459
Cecidomyiidae|[191]|CNEID2972-12|Cecidomyiidae|658|BOLD:ACB0314
Cecidomyiidae|[192]|CNBAD202-12|Cecidomyiidae|658|BOLD:ACD0138
Cecidomyiidae|[193]|CNEID968-12|Cecidomyiidae|658|BOLD:ABX9338
Cecidomyiidae|[194]|CNJAF1208-12|Cecidomyiidae|670|BOLD:ACB0890
Cecidomyiidae|[195]|JWDCE593-10|Cecidomyiidae|658|BOLD:AAP5345
Cecidomyiidae|[196]|SMTPB13550-13|Cecidomyiidae|558|BOLD:ACF6375
Cecidomyiidae|[197]|CNIVD572-14|Cecidomyiidae|555|BOLD:ACR4178
Cecidomyiidae|[198]|CNFNE1335-14|Cecidomyiidae|658|BOLD:ACL4078
Cecidomyiidae|[199]|CNNHG919-14|Cecidomyiidae|579|BOLD:ACS2882
Cecidomyiidae|[200]|CNBAE175-12|Cecidomyiidae|658|BOLD:ACD0800

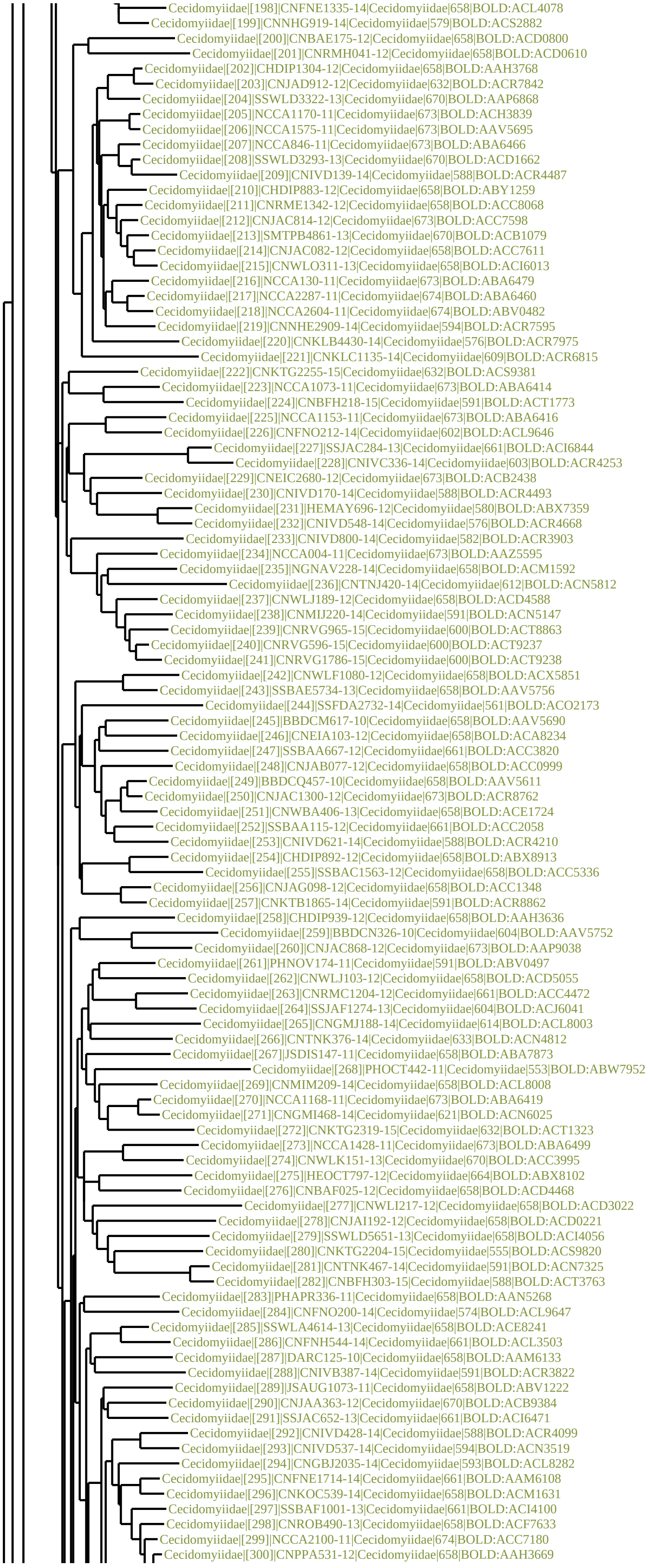
Cecidomyiidae|[198]|CNFNE1335-14|Cecidomyiidae|658|BOLD:ACL4078
Cecidomyiidae|[199]|CNNHG919-14|Cecidomyiidae|579|BOLD:ACS2882
Cecidomyiidae|[200]|CNBAE175-12|Cecidomyiidae|658|BOLD:ACD0800
Cecidomyiidae|[201]|CNRMH041-12|Cecidomyiidae|658|BOLD:ACD0610
Cecidomyiidae|[202]|CHDIP1304-12|Cecidomyiidae|658|BOLD:AAH3768
Cecidomyiidae|[203]|CNJAD912-12|Cecidomyiidae|632|BOLD:ACR7842
Cecidomyiidae|[204]|SSWLD3322-13|Cecidomyiidae|670|BOLD:AAP6868
Cecidomyiidae|[205]|NCCA1170-11|Cecidomyiidae|673|BOLD:ACH3839
Cecidomyiidae|[206]|NCCA1575-11|Cecidomyiidae|673|BOLD:AAV5695
Cecidomyiidae|[207]|NCCA846-11|Cecidomyiidae|673|BOLD:ABA6466
Cecidomyiidae|[208]|SSWLD3293-13|Cecidomyiidae|670|BOLD:ACD1662
Cecidomyiidae|[209]|CNIVD139-14|Cecidomyiidae|588|BOLD:ACR4487
Cecidomyiidae|[210]|CHDIP883-12|Cecidomyiidae|658|BOLD:ABY1259
Cecidomyiidae|[211]|CNRME1342-12|Cecidomyiidae|658|BOLD:ACC8068
Cecidomyiidae|[212]|CNJAC814-12|Cecidomyiidae|673|BOLD:ACC7598
Cecidomyiidae|[213]|SMTPB4861-13|Cecidomyiidae|670|BOLD:ACB1079
Cecidomyiidae|[214]|CNJAC082-12|Cecidomyiidae|658|BOLD:ACC7611
Cecidomyiidae|[215]|CNWLO311-13|Cecidomyiidae|658|BOLD:ACI6013
Cecidomyiidae|[216]|NCCA130-11|Cecidomyiidae|673|BOLD:ABA6479
Cecidomyiidae|[217]|NCCA2287-11|Cecidomyiidae|674|BOLD:ABA6460
Cecidomyiidae|[218]|NCCA2604-11|Cecidomyiidae|674|BOLD:ABV0482
Cecidomyiidae|[219]|CNNHE2909-14|Cecidomyiidae|594|BOLD:ACR7595
Cecidomyiidae|[220]|CNKLB4430-14|Cecidomyiidae|576|BOLD:ACR7975
Cecidomyiidae|[221]|CNKLC1135-14|Cecidomyiidae|609|BOLD:ACR6815
Cecidomyiidae|[222]|CNKTG2255-15|Cecidomyiidae|632|BOLD:ACS9381
Cecidomyiidae|[223]|NCCA1073-11|Cecidomyiidae|673|BOLD:ABA6414
Cecidomyiidae|[224]|CNBFH218-15|Cecidomyiidae|591|BOLD:ACT1773
Cecidomyiidae|[225]|NCCA1153-11|Cecidomyiidae|673|BOLD:ABA6416
Cecidomyiidae|[226]|CNFNO212-14|Cecidomyiidae|602|BOLD:ACL9646
Cecidomyiidae|[227]|SSJAC284-13|Cecidomyiidae|661|BOLD:ACI6844
Cecidomyiidae|[228]|CNIVC336-14|Cecidomyiidae|603|BOLD:ACR4253
Cecidomyiidae|[229]|CNEIC2680-12|Cecidomyiidae|673|BOLD:ACB2438
Cecidomyiidae|[230]|CNIVD170-14|Cecidomyiidae|588|BOLD:ACR4493
Cecidomyiidae|[231]|HEMAY696-12|Cecidomyiidae|580|BOLD:ABX7359
Cecidomyiidae|[232]|CNIVD548-14|Cecidomyiidae|576|BOLD:ACR4668
Cecidomyiidae|[233]|CNIVD800-14|Cecidomyiidae|582|BOLD:ACR3903
Cecidomyiidae|[234]|NCCA004-11|Cecidomyiidae|673|BOLD:AAZ5595
Cecidomyiidae|[235]|NGNAV228-14|Cecidomyiidae|658|BOLD:ACM1592
Cecidomyiidae|[236]|CNTNJ420-14|Cecidomyiidae|612|BOLD:ACN5812
Cecidomyiidae|[237]|CNWLJ189-12|Cecidomyiidae|658|BOLD:ACD4588
Cecidomyiidae|[238]|CNMIJ220-14|Cecidomyiidae|591|BOLD:ACN5147
Cecidomyiidae|[239]|CNRVG965-15|Cecidomyiidae|600|BOLD:ACT8863
Cecidomyiidae|[240]|CNRVG596-15|Cecidomyiidae|600|BOLD:ACT9237
Cecidomyiidae|[241]|CNRVG1786-15|Cecidomyiidae|600|BOLD:ACT9238
Cecidomyiidae|[242]|CNWLF1080-12|Cecidomyiidae|658|BOLD:ACX5851
Cecidomyiidae|[243]|SSBAE5734-13|Cecidomyiidae|658|BOLD:AAV5756
Cecidomyiidae|[244]|SSFDA2732-14|Cecidomyiidae|561|BOLD:ACO2173
Cecidomyiidae|[245]|BBDCM617-10|Cecidomyiidae|658|BOLD:AAV5690
Cecidomyiidae|[246]|CNEIA103-12|Cecidomyiidae|658|BOLD:ACA8234
Cecidomyiidae|[247]|SSBAA667-12|Cecidomyiidae|661|BOLD:ACC3820
Cecidomyiidae|[248]|CNJAB077-12|Cecidomyiidae|658|BOLD:ACC0999
Cecidomyiidae|[249]|BBDCQ457-10|Cecidomyiidae|658|BOLD:AAV5611
Cecidomyiidae|[250]|CNJAC1300-12|Cecidomyiidae|673|BOLD:ACR8762
Cecidomyiidae|[251]|CNWBA406-13|Cecidomyiidae|658|BOLD:ACE1724
Cecidomyiidae|[252]|SSBAA115-12|Cecidomyiidae|661|BOLD:ACC2058
Cecidomyiidae|[253]|CNIVD621-14|Cecidomyiidae|588|BOLD:ACR4210
Cecidomyiidae|[254]|CHDIP892-12|Cecidomyiidae|658|BOLD:ABX8913
Cecidomyiidae|[255]|SSBAC1563-12|Cecidomyiidae|658|BOLD:ACC5336
Cecidomyiidae|[256]|CNJAG098-12|Cecidomyiidae|658|BOLD:ACC1348
Cecidomyiidae|[257]|CNKTB1865-14|Cecidomyiidae|591|BOLD:ACR8862
Cecidomyiidae|[258]|CHDIP939-12|Cecidomyiidae|658|BOLD:AAH3636
Cecidomyiidae|[259]|BBDCN326-10|Cecidomyiidae|604|BOLD:AAV5752
Cecidomyiidae|[260]|CNJAC868-12|Cecidomyiidae|673|BOLD:AAP9038
Cecidomyiidae|[261]|PHNOV174-11|Cecidomyiidae|591|BOLD:ABV0497
Cecidomyiidae|[262]|CNWLJ103-12|Cecidomyiidae|658|BOLD:ACD5055
Cecidomyiidae|[263]|CNRMC1204-12|Cecidomyiidae|661|BOLD:ACC4472
Cecidomyiidae|[264]|SSJAF1274-13|Cecidomyiidae|604|BOLD:ACJ6041
Cecidomyiidae|[265]|CNGMJ188-14|Cecidomyiidae|614|BOLD:ACL8003
Cecidomyiidae|[266]|CNTNK376-14|Cecidomyiidae|633|BOLD:ACN4812
Cecidomyiidae|[267]|JSDIS147-11|Cecidomyiidae|658|BOLD:ABA7873
Cecidomyiidae|[268]|PHOCT442-11|Cecidomyiidae|553|BOLD:ABW7952
Cecidomyiidae|[269]|CNMIM209-14|Cecidomyiidae|658|BOLD:ACL8008
Cecidomyiidae|[270]|NCCA1168-11|Cecidomyiidae|673|BOLD:ABA6419
Cecidomyiidae|[271]|CNGMI468-14|Cecidomyiidae|621|BOLD:ACN6025
Cecidomyiidae|[272]|CNKTG2319-15|Cecidomyiidae|632|BOLD:ACT1323
Cecidomyiidae|[273]|NCCA1428-11|Cecidomyiidae|673|BOLD:ABA6499
Cecidomyiidae|[274]|CNWLK151-13|Cecidomyiidae|670|BOLD:ACC3995
Cecidomyiidae|[275]|HEOCT797-12|Cecidomyiidae|664|BOLD:ABX8102
Cecidomyiidae|[276]|CNBAF025-12|Cecidomyiidae|658|BOLD:ACD4468
Cecidomyiidae|[277]|CNWLI217-12|Cecidomyiidae|658|BOLD:ACD3022
Cecidomyiidae|[278]|CNJAI192-12|Cecidomyiidae|658|BOLD:ACD0221
Cecidomyiidae|[279]|SSWLD5651-13|Cecidomyiidae|658|BOLD:ACI4056
Cecidomyiidae|[280]|CNKTG2204-15|Cecidomyiidae|555|BOLD:ACS9820
Cecidomyiidae|[281]|CNTNK467-14|Cecidomyiidae|591|BOLD:ACN7325
Cecidomyiidae|[282]|CNBFH303-15|Cecidomyiidae|588|BOLD:ACT3763
Cecidomyiidae|[283]|PHAPR336-11|Cecidomyiidae|658|BOLD:AAN5268
Cecidomyiidae|[284]|CNFNO200-14|Cecidomyiidae|574|BOLD:ACL9647
Cecidomyiidae|[285]|SSWLA4614-13|Cecidomyiidae|658|BOLD:ACE8241
Cecidomyiidae|[286]|CNFNH544-14|Cecidomyiidae|661|BOLD:ACL3503
Cecidomyiidae|[287]|DARC125-10|Cecidomyiidae|658|BOLD:AAM6133
Cecidomyiidae|[288]|CNIVB387-14|Cecidomyiidae|591|BOLD:ACR3822
Cecidomyiidae|[289]|JSAUG1073-11|Cecidomyiidae|658|BOLD:ABV1222
Cecidomyiidae|[290]|CNJAA363-12|Cecidomyiidae|670|BOLD:ACB9384
Cecidomyiidae|[291]|SSJAC652-13|Cecidomyiidae|661|BOLD:ACI6471
Cecidomyiidae|[292]|CNIVD428-14|Cecidomyiidae|588|BOLD:ACR4099
Cecidomyiidae|[293]|CNIVD537-14|Cecidomyiidae|594|BOLD:ACN3519
Cecidomyiidae|[294]|CNGBJ2035-14|Cecidomyiidae|593|BOLD:ACL8282
Cecidomyiidae|[295]|CNFNE1714-14|Cecidomyiidae|661|BOLD:AAM6108
Cecidomyiidae|[296]|CNKOC539-14|Cecidomyiidae|658|BOLD:ACM1631
Cecidomyiidae|[297]|SSBAF1001-13|Cecidomyiidae|661|BOLD:ACI4100
Cecidomyiidae|[298]|CNROB490-13|Cecidomyiidae|658|BOLD:ACF7633
Cecidomyiidae|[299]|NCCA2100-11|Cecidomyiidae|674|BOLD:ACC7180
Cecidomyiidae|[300]|CNPPA531-12|Cecidomyiidae|658|BOLD:AAH3669

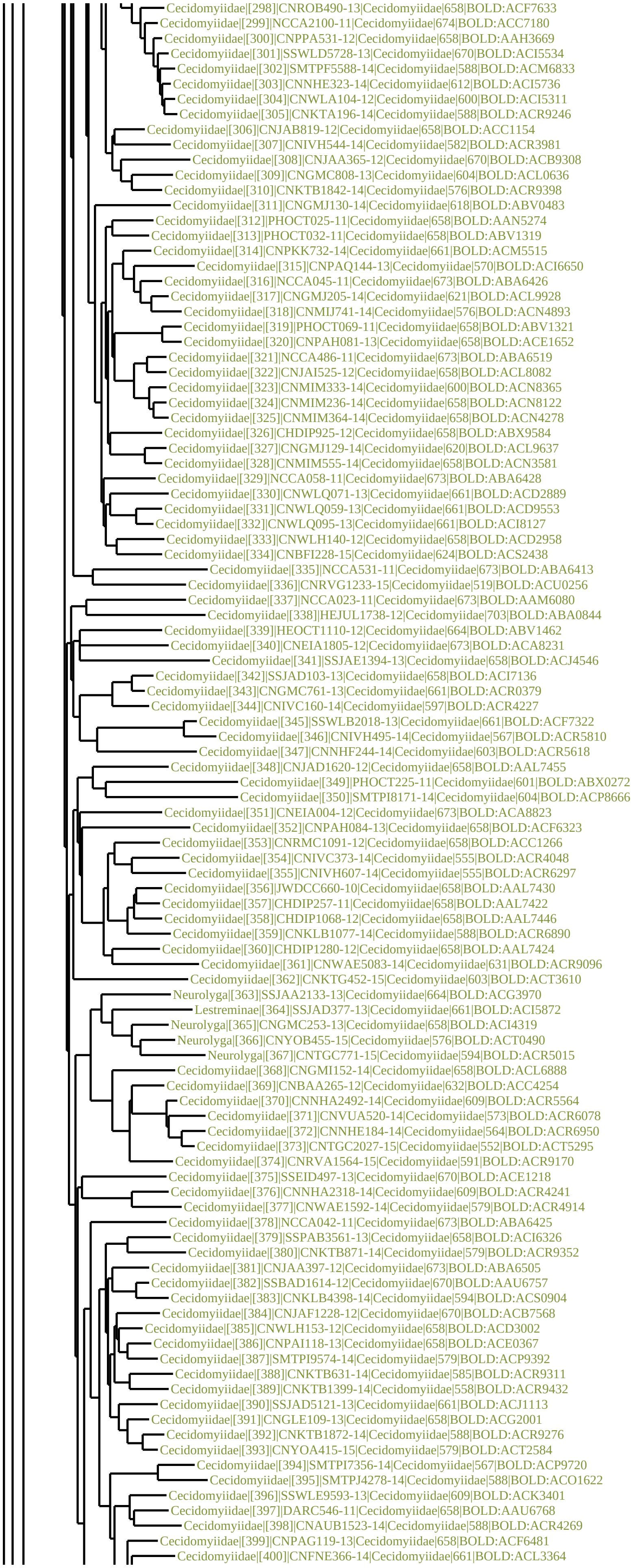
Cecidomyiidae|[298]|CNROB490-13|Cecidomyiidae|658|BOLD:ACF7633
Cecidomyiidae|[299]|NCCA2100-11|Cecidomyiidae|674|BOLD:ACC7180
Cecidomyiidae|[300]|CNPPA531-12|Cecidomyiidae|658|BOLD:AAH3669
Cecidomyiidae|[301]|SSWLD5728-13|Cecidomyiidae|670|BOLD:ACI5534
Cecidomyiidae|[302]|SMTPF5588-14|Cecidomyiidae|588|BOLD:ACM6833
Cecidomyiidae|[303]|CNNHE323-14|Cecidomyiidae|612|BOLD:ACI5736
Cecidomyiidae|[304]|CNWLA104-12|Cecidomyiidae|600|BOLD:ACI5311
Cecidomyiidae|[305]|CNKTA196-14|Cecidomyiidae|588|BOLD:ACR9246
Cecidomyiidae|[306]|CNJAB819-12|Cecidomyiidae|658|BOLD:ACC1154
Cecidomyiidae|[307]|CNIVH544-14|Cecidomyiidae|582|BOLD:ACR3981
Cecidomyiidae|[308]|CNJAA365-12|Cecidomyiidae|670|BOLD:ACB9308
Cecidomyiidae|[309]|CNGMC808-13|Cecidomyiidae|604|BOLD:ACL0636
Cecidomyiidae|[310]|CNKTB1842-14|Cecidomyiidae|576|BOLD:ACR9398
Cecidomyiidae|[311]|CNGMJ130-14|Cecidomyiidae|618|BOLD:ABV0483
Cecidomyiidae|[312]|PHOCT025-11|Cecidomyiidae|658|BOLD:AAN5274
Cecidomyiidae|[313]|PHOCT032-11|Cecidomyiidae|658|BOLD:ABV1319
Cecidomyiidae|[314]|CNPKK732-14|Cecidomyiidae|661|BOLD:ACM5515
Cecidomyiidae|[315]|CNPAQ144-13|Cecidomyiidae|570|BOLD:ACI6650
Cecidomyiidae|[316]|NCCA045-11|Cecidomyiidae|673|BOLD:ABA6426
Cecidomyiidae|[317]|CNGMJ205-14|Cecidomyiidae|621|BOLD:ACL9928
Cecidomyiidae|[318]|CNMIJ741-14|Cecidomyiidae|576|BOLD:ACN4893
Cecidomyiidae|[319]|PHOCT069-11|Cecidomyiidae|658|BOLD:ABV1321
Cecidomyiidae|[320]|CNPAH081-13|Cecidomyiidae|658|BOLD:ACE1652
Cecidomyiidae|[321]|NCCA486-11|Cecidomyiidae|673|BOLD:ABA6519
Cecidomyiidae|[322]|CNJAI525-12|Cecidomyiidae|658|BOLD:ACL8082
Cecidomyiidae|[323]|CNMIM333-14|Cecidomyiidae|600|BOLD:ACN8365
Cecidomyiidae|[324]|CNMIM236-14|Cecidomyiidae|658|BOLD:ACN8122
Cecidomyiidae|[325]|CNMIM364-14|Cecidomyiidae|658|BOLD:ACN4278
Cecidomyiidae|[326]|CHDIP925-12|Cecidomyiidae|658|BOLD:ABX9584
Cecidomyiidae|[327]|CNGMJ129-14|Cecidomyiidae|620|BOLD:ACL9637
Cecidomyiidae|[328]|CNMIM555-14|Cecidomyiidae|658|BOLD:ACN3581
Cecidomyiidae|[329]|NCCA058-11|Cecidomyiidae|673|BOLD:ABA6428
Cecidomyiidae|[330]|CNWLQ071-13|Cecidomyiidae|661|BOLD:ACD2889
Cecidomyiidae|[331]|CNWLQ059-13|Cecidomyiidae|661|BOLD:ACD9553
Cecidomyiidae|[332]|CNWLQ095-13|Cecidomyiidae|661|BOLD:ACI8127
Cecidomyiidae|[333]|CNWLH140-12|Cecidomyiidae|658|BOLD:ACD2958
Cecidomyiidae|[334]|CNBFI228-15|Cecidomyiidae|624|BOLD:ACS2438
Cecidomyiidae|[335]|NCCA531-11|Cecidomyiidae|673|BOLD:ABA6413
Cecidomyiidae|[336]|CNRVG1233-15|Cecidomyiidae|519|BOLD:ACU0256
Cecidomyiidae|[337]|NCCA023-11|Cecidomyiidae|673|BOLD:AAM6080
Cecidomyiidae|[338]|HEJUL1738-12|Cecidomyiidae|703|BOLD:ABA0844
Cecidomyiidae|[339]|HEOCT1110-12|Cecidomyiidae|664|BOLD:ABV1462
Cecidomyiidae|[340]|CNEIA1805-12|Cecidomyiidae|673|BOLD:ACA8231
Cecidomyiidae|[341]|SSJAE1394-13|Cecidomyiidae|658|BOLD:ACJ4546
Cecidomyiidae|[342]|SSJAD103-13|Cecidomyiidae|658|BOLD:ACI7136
Cecidomyiidae|[343]|CNGMC761-13|Cecidomyiidae|661|BOLD:ACR0379
Cecidomyiidae|[344]|CNIVC160-14|Cecidomyiidae|597|BOLD:ACR4227
Cecidomyiidae|[345]|SSWLB2018-13|Cecidomyiidae|661|BOLD:ACF7322
Cecidomyiidae|[346]|CNIVH495-14|Cecidomyiidae|567|BOLD:ACR5810
Cecidomyiidae|[347]|CNNHF244-14|Cecidomyiidae|603|BOLD:ACR5618
Cecidomyiidae|[348]|CNJAD1620-12|Cecidomyiidae|658|BOLD:AAL7455
Cecidomyiidae|[349]|PHOCT225-11|Cecidomyiidae|601|BOLD:ABX0272
Cecidomyiidae|[350]|SMTPI8171-14|Cecidomyiidae|604|BOLD:ACP8666
Cecidomyiidae|[351]|CNEIA004-12|Cecidomyiidae|673|BOLD:ACA8823
Cecidomyiidae|[352]|CNPAH084-13|Cecidomyiidae|658|BOLD:ACF6323
Cecidomyiidae|[353]|CNRMC1091-12|Cecidomyiidae|658|BOLD:ACC1266
Cecidomyiidae|[354]|CNIVC373-14|Cecidomyiidae|555|BOLD:ACR4048
Cecidomyiidae|[355]|CNIVH607-14|Cecidomyiidae|555|BOLD:ACR6297
Cecidomyiidae|[356]|JWDCC660-10|Cecidomyiidae|658|BOLD:AAL7430
Cecidomyiidae|[357]|CHDIP257-11|Cecidomyiidae|658|BOLD:AAL7422
Cecidomyiidae|[358]|CHDIP1068-12|Cecidomyiidae|658|BOLD:AAL7446
Cecidomyiidae|[359]|CNKLB1077-14|Cecidomyiidae|588|BOLD:ACR6890
Cecidomyiidae|[360]|CHDIP1280-12|Cecidomyiidae|658|BOLD:AAL7424
Cecidomyiidae|[361]|CNWAE5083-14|Cecidomyiidae|631|BOLD:ACR9096
Cecidomyiidae|[362]|CNKTG452-15|Cecidomyiidae|603|BOLD:ACT3610
Neurolyga|[363]|SSJAA2133-13|Cecidomyiidae|664|BOLD:ACG3970
Lestreminae|[364]|SSJAD377-13|Cecidomyiidae|661|BOLD:ACI5872
Neurolyga|[365]|CNGMC253-13|Cecidomyiidae|658|BOLD:ACI4319
Neurolyga|[366]|CNYOB455-15|Cecidomyiidae|576|BOLD:ACT0490
Neurolyga|[367]|CNTGC771-15|Cecidomyiidae|594|BOLD:ACR5015
Cecidomyiidae|[368]|CNGMI152-14|Cecidomyiidae|658|BOLD:ACL6888
Cecidomyiidae|[369]|CNBAA265-12|Cecidomyiidae|632|BOLD:ACC4254
Cecidomyiidae|[370]|CNNHA2492-14|Cecidomyiidae|609|BOLD:ACR5564
Cecidomyiidae|[371]|CNVUA520-14|Cecidomyiidae|573|BOLD:ACR6078
Cecidomyiidae|[372]|CNNHE184-14|Cecidomyiidae|564|BOLD:ACR6950
Cecidomyiidae|[373]|CNTGC2027-15|Cecidomyiidae|552|BOLD:ACT5295
Cecidomyiidae|[374]|CNRVA1564-15|Cecidomyiidae|591|BOLD:ACR9170
Cecidomyiidae|[375]|SSEID497-13|Cecidomyiidae|670|BOLD:ACE1218
Cecidomyiidae|[376]|CNNHA2318-14|Cecidomyiidae|609|BOLD:ACR4241
Cecidomyiidae|[377]|CNWAE1592-14|Cecidomyiidae|579|BOLD:ACR4914
Cecidomyiidae|[378]|NCCA042-11|Cecidomyiidae|673|BOLD:ABA6425
Cecidomyiidae|[379]|SSPAB3561-13|Cecidomyiidae|658|BOLD:ACI6326
Cecidomyiidae|[380]|CNKTB871-14|Cecidomyiidae|579|BOLD:ACR9352
Cecidomyiidae|[381]|CNJAA397-12|Cecidomyiidae|673|BOLD:ABA6505
Cecidomyiidae|[382]|SSBAD1614-12|Cecidomyiidae|670|BOLD:AAU6757
Cecidomyiidae|[383]|CNKLB4398-14|Cecidomyiidae|594|BOLD:ACS0904
Cecidomyiidae|[384]|CNJAF1228-12|Cecidomyiidae|670|BOLD:ACB7568
Cecidomyiidae|[385]|CNWLH153-12|Cecidomyiidae|658|BOLD:ACD3002
Cecidomyiidae|[386]|CNPAI118-13|Cecidomyiidae|658|BOLD:ACE0367
Cecidomyiidae|[387]|SMTPI9574-14|Cecidomyiidae|579|BOLD:ACP9392
Cecidomyiidae|[388]|CNKTB631-14|Cecidomyiidae|585|BOLD:ACR9311
Cecidomyiidae|[389]|CNKTB1399-14|Cecidomyiidae|558|BOLD:ACR9432
Cecidomyiidae|[390]|SSJAD5121-13|Cecidomyiidae|661|BOLD:ACJ1113
Cecidomyiidae|[391]|CNGLE109-13|Cecidomyiidae|658|BOLD:ACG2001
Cecidomyiidae|[392]|CNKTB1872-14|Cecidomyiidae|588|BOLD:ACR9276
Cecidomyiidae|[393]|CNYOA415-15|Cecidomyiidae|579|BOLD:ACT2584
Cecidomyiidae|[394]|SMTPI7356-14|Cecidomyiidae|567|BOLD:ACP9720
Cecidomyiidae|[395]|SMTPJ4278-14|Cecidomyiidae|588|BOLD:ACO1622
Cecidomyiidae|[396]|SSWLE9593-13|Cecidomyiidae|609|BOLD:ACK3401
Cecidomyiidae|[397]|DARC546-11|Cecidomyiidae|658|BOLD:AAU6768
Cecidomyiidae|[398]|CNAUB1523-14|Cecidomyiidae|588|BOLD:ACR4269
Cecidomyiidae|[399]|CNPAG119-13|Cecidomyiidae|658|BOLD:ACF6481
Cecidomyiidae|[400]|CNFNE366-14|Cecidomyiidae|661|BOLD:ACL3364

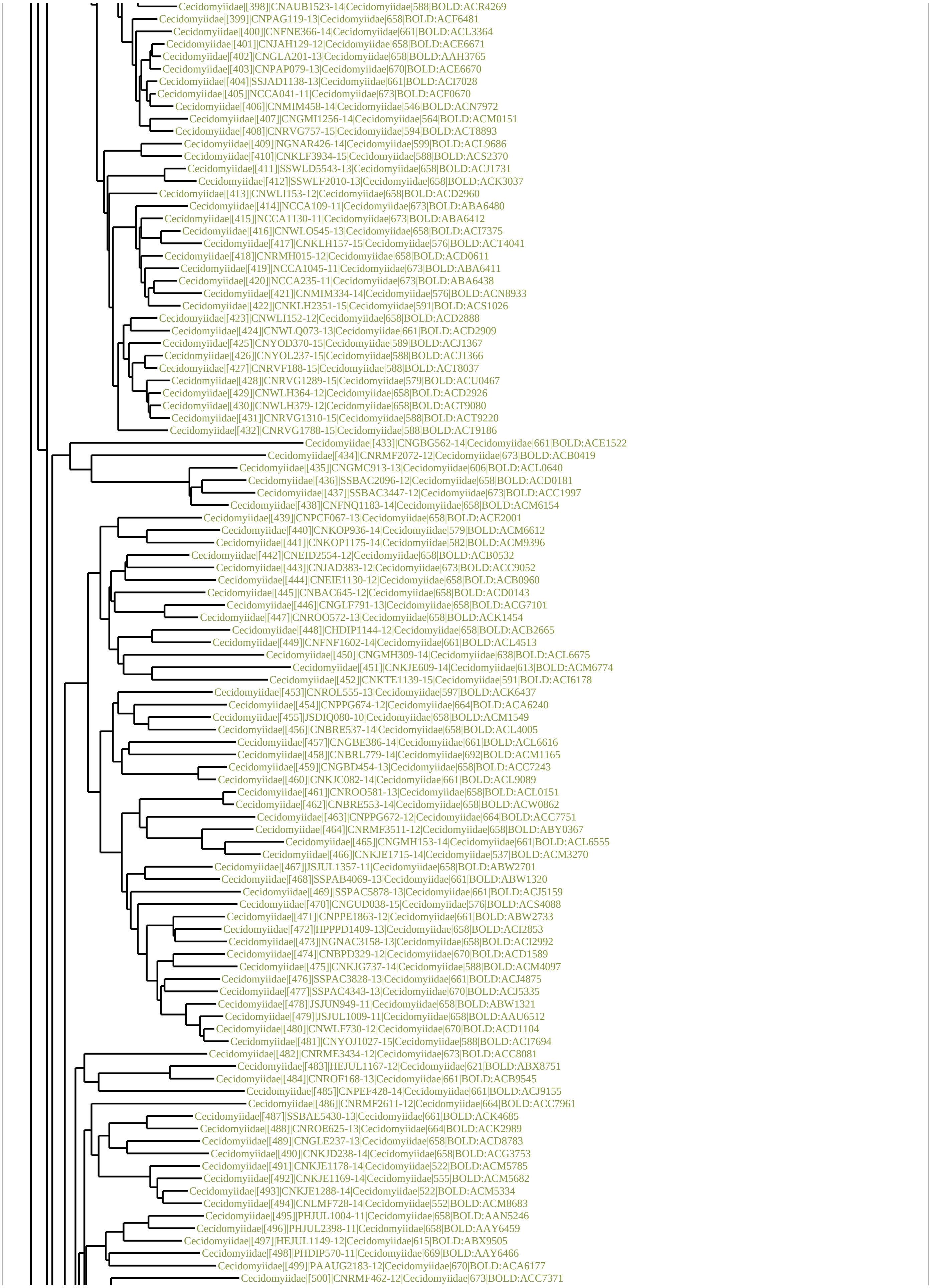
Cecidomyiidae|[398]|CNAUB1523-14|Cecidomyiidae|588|BOLD:ACR4269
Cecidomyiidae|[399]|CNPAG119-13|Cecidomyiidae|658|BOLD:ACF6481
Cecidomyiidae|[400]|CNFNE366-14|Cecidomyiidae|661|BOLD:ACL3364
Cecidomyiidae|[401]|CNJAH129-12|Cecidomyiidae|658|BOLD:ACE6671
Cecidomyiidae|[402]|CNGLA201-13|Cecidomyiidae|658|BOLD:AAH3765
Cecidomyiidae|[403]|CNPAP079-13|Cecidomyiidae|670|BOLD:ACE6670
Cecidomyiidae|[404]|SSJAD1138-13|Cecidomyiidae|661|BOLD:ACI7028
Cecidomyiidae|[405]|NCCA041-11|Cecidomyiidae|673|BOLD:ACF0670
Cecidomyiidae|[406]|CNMIM458-14|Cecidomyiidae|546|BOLD:ACN7972
Cecidomyiidae|[407]|CNGMI1256-14|Cecidomyiidae|564|BOLD:ACM0151
Cecidomyiidae|[408]|CNRVG757-15|Cecidomyiidae|594|BOLD:ACT8893
Cecidomyiidae|[409]|NGNAR426-14|Cecidomyiidae|599|BOLD:ACL9686
Cecidomyiidae|[410]|CNKLF3934-15|Cecidomyiidae|588|BOLD:ACS2370
Cecidomyiidae|[411]|SSWLD5543-13|Cecidomyiidae|658|BOLD:ACJ1731
Cecidomyiidae|[412]|SSWLF2010-13|Cecidomyiidae|658|BOLD:ACK3037
Cecidomyiidae|[413]|CNWLI153-12|Cecidomyiidae|658|BOLD:ACD2960
Cecidomyiidae|[414]|NCCA109-11|Cecidomyiidae|673|BOLD:ABA6480
Cecidomyiidae|[415]|NCCA1130-11|Cecidomyiidae|673|BOLD:ABA6412
Cecidomyiidae|[416]|CNWLO545-13|Cecidomyiidae|658|BOLD:ACI7375
Cecidomyiidae|[417]|CNKLH157-15|Cecidomyiidae|576|BOLD:ACT4041
Cecidomyiidae|[418]|CNRMH015-12|Cecidomyiidae|658|BOLD:ACD0611
Cecidomyiidae|[419]|NCCA1045-11|Cecidomyiidae|673|BOLD:ABA6411
Cecidomyiidae|[420]|NCCA235-11|Cecidomyiidae|673|BOLD:ABA6438
Cecidomyiidae|[421]|CNMIM334-14|Cecidomyiidae|576|BOLD:ACN8933
Cecidomyiidae|[422]|CNKLH2351-15|Cecidomyiidae|591|BOLD:ACS1026
Cecidomyiidae|[423]|CNWLI152-12|Cecidomyiidae|658|BOLD:ACD2888
Cecidomyiidae|[424]|CNWLQ073-13|Cecidomyiidae|661|BOLD:ACD2909
Cecidomyiidae|[425]|CNYOD370-15|Cecidomyiidae|589|BOLD:ACJ1367
Cecidomyiidae|[426]|CNYOL237-15|Cecidomyiidae|588|BOLD:ACJ1366
Cecidomyiidae|[427]|CNRVF188-15|Cecidomyiidae|588|BOLD:ACT8037
Cecidomyiidae|[428]|CNRVG1289-15|Cecidomyiidae|579|BOLD:ACU0467
Cecidomyiidae|[429]|CNWLH364-12|Cecidomyiidae|658|BOLD:ACD2926
Cecidomyiidae|[430]|CNWLH379-12|Cecidomyiidae|658|BOLD:ACT9080
Cecidomyiidae|[431]|CNRVG1310-15|Cecidomyiidae|588|BOLD:ACT9220
Cecidomyiidae|[432]|CNRVG1788-15|Cecidomyiidae|588|BOLD:ACT9186
Cecidomyiidae|[433]|CNGBG562-14|Cecidomyiidae|661|BOLD:ACE1522
Cecidomyiidae|[434]|CNRMF2072-12|Cecidomyiidae|673|BOLD:ACB0419
Cecidomyiidae|[435]|CNGMC913-13|Cecidomyiidae|606|BOLD:ACL0640
Cecidomyiidae|[436]|SSBAC2096-12|Cecidomyiidae|658|BOLD:ACD0181
Cecidomyiidae|[437]|SSBAC3447-12|Cecidomyiidae|673|BOLD:ACC1997
Cecidomyiidae|[438]|CNFNQ1183-14|Cecidomyiidae|658|BOLD:ACM6154
Cecidomyiidae|[439]|CNPCF067-13|Cecidomyiidae|658|BOLD:ACE2001
Cecidomyiidae|[440]|CNKOP936-14|Cecidomyiidae|579|BOLD:ACM6612
Cecidomyiidae|[441]|CNKOP1175-14|Cecidomyiidae|582|BOLD:ACM9396
Cecidomyiidae|[442]|CNEID2554-12|Cecidomyiidae|658|BOLD:ACB0532
Cecidomyiidae|[443]|CNJAD383-12|Cecidomyiidae|673|BOLD:ACC9052
Cecidomyiidae|[444]|CNEIE1130-12|Cecidomyiidae|658|BOLD:ACB0960
Cecidomyiidae|[445]|CNBAC645-12|Cecidomyiidae|658|BOLD:ACD0143
Cecidomyiidae|[446]|CNGLF791-13|Cecidomyiidae|658|BOLD:ACG7101
Cecidomyiidae|[447]|CNROO572-13|Cecidomyiidae|658|BOLD:ACK1454
Cecidomyiidae|[448]|CHDIP1144-12|Cecidomyiidae|658|BOLD:ACB2665
Cecidomyiidae|[449]|CNFNF1602-14|Cecidomyiidae|661|BOLD:ACL4513
Cecidomyiidae|[450]|CNGMH309-14|Cecidomyiidae|638|BOLD:ACL6675
Cecidomyiidae|[451]|CNKJE609-14|Cecidomyiidae|613|BOLD:ACM6774
Cecidomyiidae|[452]|CNKTE1139-15|Cecidomyiidae|591|BOLD:ACI6178
Cecidomyiidae|[453]|CNROL555-13|Cecidomyiidae|597|BOLD:ACK6437
Cecidomyiidae|[454]|CNPPG674-12|Cecidomyiidae|664|BOLD:ACA6240
Cecidomyiidae|[455]|JSDIQ080-10|Cecidomyiidae|658|BOLD:ACM1549
Cecidomyiidae|[456]|CNBRE537-14|Cecidomyiidae|658|BOLD:ACL4005
Cecidomyiidae|[457]|CNGBE386-14|Cecidomyiidae|661|BOLD:ACL6616
Cecidomyiidae|[458]|CNBRL779-14|Cecidomyiidae|692|BOLD:ACM1165
Cecidomyiidae|[459]|CNGBD454-13|Cecidomyiidae|658|BOLD:ACC7243
Cecidomyiidae|[460]|CNKJC082-14|Cecidomyiidae|661|BOLD:ACL9089
Cecidomyiidae|[461]|CNROO581-13|Cecidomyiidae|658|BOLD:ACL0151
Cecidomyiidae|[462]|CNBRE553-14|Cecidomyiidae|658|BOLD:ACW0862
Cecidomyiidae|[463]|CNPPG672-12|Cecidomyiidae|664|BOLD:ACC7751
Cecidomyiidae|[464]|CNRMF3511-12|Cecidomyiidae|658|BOLD:ABY0367
Cecidomyiidae|[465]|CNGMH153-14|Cecidomyiidae|661|BOLD:ACL6555
Cecidomyiidae|[466]|CNKJE1715-14|Cecidomyiidae|537|BOLD:ACM3270
Cecidomyiidae|[467]|JSJUL1357-11|Cecidomyiidae|658|BOLD:ABW2701
Cecidomyiidae|[468]|SSPAB4069-13|Cecidomyiidae|661|BOLD:ABW1320
Cecidomyiidae|[469]|SSPAC5878-13|Cecidomyiidae|661|BOLD:ACJ5159
Cecidomyiidae|[470]|CNGUD038-15|Cecidomyiidae|576|BOLD:ACS4088
Cecidomyiidae|[471]|CNPPE1863-12|Cecidomyiidae|661|BOLD:ABW2733
Cecidomyiidae|[472]|HPPPD1409-13|Cecidomyiidae|658|BOLD:ACI2853
Cecidomyiidae|[473]|NGNAC3158-13|Cecidomyiidae|658|BOLD:ACI2992
Cecidomyiidae|[474]|CNBPD329-12|Cecidomyiidae|670|BOLD:ACD1589
Cecidomyiidae|[475]|CNKJG737-14|Cecidomyiidae|588|BOLD:ACM4097
Cecidomyiidae|[476]|SSPAC3828-13|Cecidomyiidae|661|BOLD:ACJ4875
Cecidomyiidae|[477]|SSPAC4343-13|Cecidomyiidae|670|BOLD:ACJ5335
Cecidomyiidae|[478]|JSJUN949-11|Cecidomyiidae|658|BOLD:ABW1321
Cecidomyiidae|[479]|JSJUL1009-11|Cecidomyiidae|658|BOLD:AAU6512
Cecidomyiidae|[480]|CNWLF730-12|Cecidomyiidae|670|BOLD:ACD1104
Cecidomyiidae|[481]|CNYOJ1027-15|Cecidomyiidae|588|BOLD:ACI7694
Cecidomyiidae|[482]|CNRME3434-12|Cecidomyiidae|673|BOLD:ACC8081
Cecidomyiidae|[483]|HEJUL1167-12|Cecidomyiidae|621|BOLD:ABX8751
Cecidomyiidae|[484]|CNROF168-13|Cecidomyiidae|661|BOLD:ACB9545
Cecidomyiidae|[485]|CNPEF428-14|Cecidomyiidae|661|BOLD:ACJ9155
Cecidomyiidae|[486]|CNRMF2611-12|Cecidomyiidae|664|BOLD:ACC7961
Cecidomyiidae|[487]|SSBAE5430-13|Cecidomyiidae|661|BOLD:ACK4685
Cecidomyiidae|[488]|CNROE625-13|Cecidomyiidae|664|BOLD:ACK2989
Cecidomyiidae|[489]|CNGLE237-13|Cecidomyiidae|658|BOLD:ACD8783
Cecidomyiidae|[490]|CNKJD238-14|Cecidomyiidae|658|BOLD:ACG3753
Cecidomyiidae|[491]|CNKJE1178-14|Cecidomyiidae|522|BOLD:ACM5785
Cecidomyiidae|[492]|CNKJE1169-14|Cecidomyiidae|555|BOLD:ACM5682
Cecidomyiidae|[493]|CNKJE1288-14|Cecidomyiidae|522|BOLD:ACM5334
Cecidomyiidae|[494]|CNLMF728-14|Cecidomyiidae|552|BOLD:ACM8683
Cecidomyiidae|[495]|PHJUL1004-11|Cecidomyiidae|658|BOLD:AAN5246
Cecidomyiidae|[496]|PHJUL2398-11|Cecidomyiidae|658|BOLD:AAY6459
Cecidomyiidae|[497]|HEJUL1149-12|Cecidomyiidae|615|BOLD:ABX9505
Cecidomyiidae|[498]|PHDIP570-11|Cecidomyiidae|669|BOLD:AAY6466
Cecidomyiidae|[499]|PAAUG2183-12|Cecidomyiidae|670|BOLD:ACA6177
Cecidomyiidae|[500]|CNRMF462-12|Cecidomyiidae|673|BOLD:ACC7371

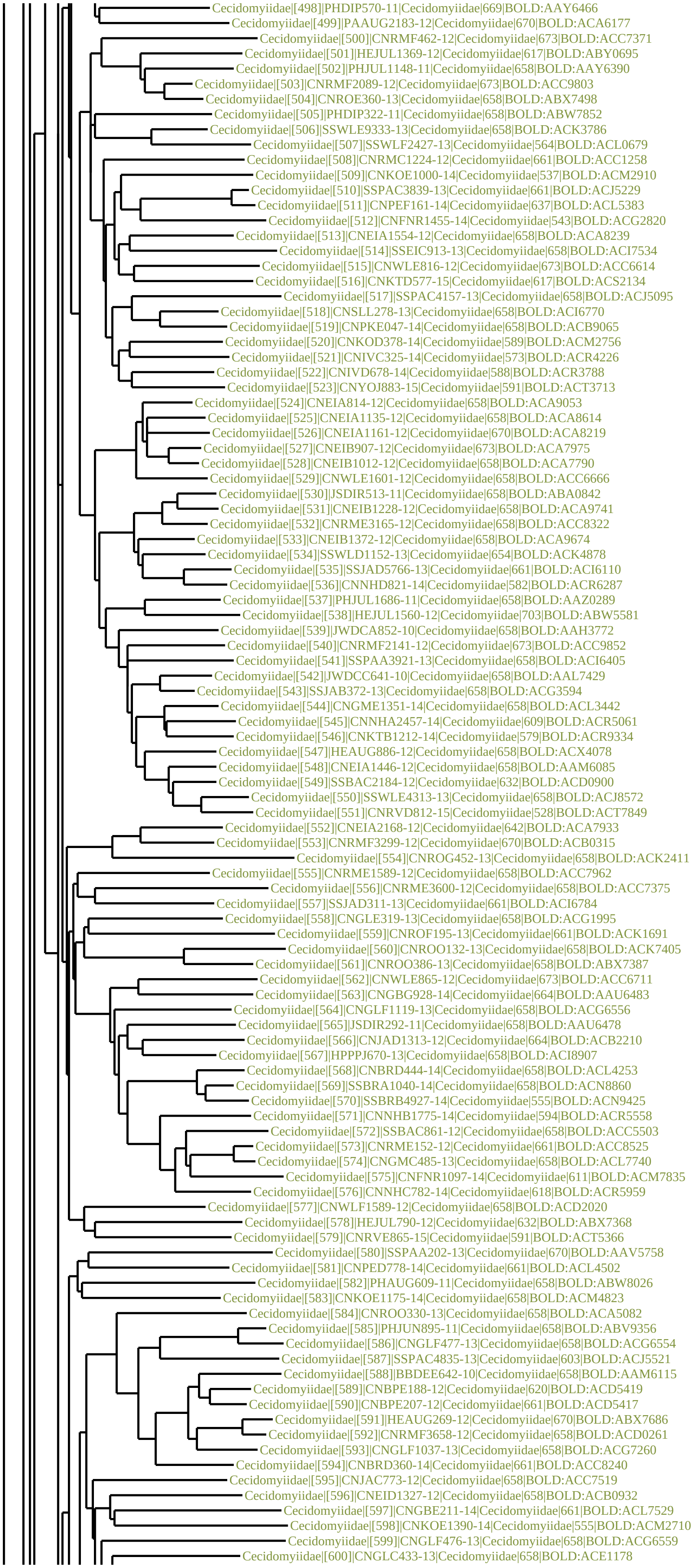

Cecidomyiidae|[498]|PHDIP570-11|Cecidomyiidae|669|BOLD:AAY6466
Cecidomyiidae|[499]|PAAUG2183-12|Cecidomyiidae|670|BOLD:ACA6177
Cecidomyiidae|[500]|CNRMF462-12|Cecidomyiidae|673|BOLD:ACC7371
Cecidomyiidae|[501]|HEJUL1369-12|Cecidomyiidae|617|BOLD:ABY0695
Cecidomyiidae|[502]|PHJUL1148-11|Cecidomyiidae|658|BOLD:AAY6390
Cecidomyiidae|[503]|CNRMF2089-12|Cecidomyiidae|673|BOLD:ACC9803
Cecidomyiidae|[504]|CNROE360-13|Cecidomyiidae|658|BOLD:ABX7498
Cecidomyiidae|[505]|PHDIP322-11|Cecidomyiidae|658|BOLD:ABW7852
Cecidomyiidae|[506]|SSWLE9333-13|Cecidomyiidae|658|BOLD:ACK3786
Cecidomyiidae|[507]|SSWLF2427-13|Cecidomyiidae|564|BOLD:ACL0679
Cecidomyiidae|[508]|CNRMC1224-12|Cecidomyiidae|661|BOLD:ACC1258
Cecidomyiidae|[509]|CNKOE1000-14|Cecidomyiidae|537|BOLD:ACM2910
Cecidomyiidae|[510]|SSPAC3839-13|Cecidomyiidae|661|BOLD:ACJ5229
Cecidomyiidae|[511]|CNPEF161-14|Cecidomyiidae|637|BOLD:ACL5383
Cecidomyiidae|[512]|CNFNR1455-14|Cecidomyiidae|543|BOLD:ACG2820
Cecidomyiidae|[513]|CNEIA1554-12|Cecidomyiidae|658|BOLD:ACA8239
Cecidomyiidae|[514]|SSEIC913-13|Cecidomyiidae|658|BOLD:ACI7534
Cecidomyiidae|[515]|CNWLE816-12|Cecidomyiidae|673|BOLD:ACC6614
Cecidomyiidae|[516]|CNKTD577-15|Cecidomyiidae|617|BOLD:ACS2134
Cecidomyiidae|[517]|SSPAC4157-13|Cecidomyiidae|658|BOLD:ACJ5095
Cecidomyiidae|[518]|CNSLL278-13|Cecidomyiidae|658|BOLD:ACI6770
Cecidomyiidae|[519]|CNPKE047-14|Cecidomyiidae|658|BOLD:ACB9065
Cecidomyiidae|[520]|CNKOD378-14|Cecidomyiidae|589|BOLD:ACM2756
Cecidomyiidae|[521]|CNIVC325-14|Cecidomyiidae|573|BOLD:ACR4226
Cecidomyiidae|[522]|CNIVD678-14|Cecidomyiidae|588|BOLD:ACR3788
Cecidomyiidae|[523]|CNYOJ883-15|Cecidomyiidae|591|BOLD:ACT3713
Cecidomyiidae|[524]|CNEIA814-12|Cecidomyiidae|658|BOLD:ACA9053
Cecidomyiidae|[525]|CNEIA1135-12|Cecidomyiidae|658|BOLD:ACA8614
Cecidomyiidae|[526]|CNEIA1161-12|Cecidomyiidae|670|BOLD:ACA8219
Cecidomyiidae|[527]|CNEIB907-12|Cecidomyiidae|673|BOLD:ACA7975
Cecidomyiidae|[528]|CNEIB1012-12|Cecidomyiidae|658|BOLD:ACA7790
Cecidomyiidae|[529]|CNWLE1601-12|Cecidomyiidae|658|BOLD:ACC6666
Cecidomyiidae|[530]|JSDIR513-11|Cecidomyiidae|658|BOLD:ABA0842
Cecidomyiidae|[531]|CNEIB1228-12|Cecidomyiidae|658|BOLD:ACA9741
Cecidomyiidae|[532]|CNRME3165-12|Cecidomyiidae|658|BOLD:ACC8322
Cecidomyiidae|[533]|CNEIB1372-12|Cecidomyiidae|658|BOLD:ACA9674
Cecidomyiidae|[534]|SSWLD1152-13|Cecidomyiidae|654|BOLD:ACK4878
Cecidomyiidae|[535]|SSJAD5766-13|Cecidomyiidae|661|BOLD:ACI6110
Cecidomyiidae|[536]|CNNHD821-14|Cecidomyiidae|582|BOLD:ACR6287
Cecidomyiidae|[537]|PHJUL1686-11|Cecidomyiidae|658|BOLD:AAZ0289
Cecidomyiidae|[538]|HEJUL1560-12|Cecidomyiidae|703|BOLD:ABW5581
Cecidomyiidae|[539]|JWDCA852-10|Cecidomyiidae|658|BOLD:AAH3772
Cecidomyiidae|[540]|CNRMF2141-12|Cecidomyiidae|673|BOLD:ACC9852
Cecidomyiidae|[541]|SSPAA3921-13|Cecidomyiidae|658|BOLD:ACI6405
Cecidomyiidae|[542]|JWDCC641-10|Cecidomyiidae|658|BOLD:AAL7429
Cecidomyiidae|[543]|SSJAB372-13|Cecidomyiidae|658|BOLD:ACG3594
Cecidomyiidae|[544]|CNGME1351-14|Cecidomyiidae|658|BOLD:ACL3442
Cecidomyiidae|[545]|CNNHA2457-14|Cecidomyiidae|609|BOLD:ACR5061
Cecidomyiidae|[546]|CNKTB1212-14|Cecidomyiidae|579|BOLD:ACR9334
Cecidomyiidae|[547]|HEAUG886-12|Cecidomyiidae|658|BOLD:ACX4078
Cecidomyiidae|[548]|CNEIA1446-12|Cecidomyiidae|658|BOLD:AAM6085
Cecidomyiidae|[549]|SSBAC2184-12|Cecidomyiidae|632|BOLD:ACD0900
Cecidomyiidae|[550]|SSWLE4313-13|Cecidomyiidae|658|BOLD:ACJ8572
Cecidomyiidae|[551]|CNRVD812-15|Cecidomyiidae|528|BOLD:ACT7849
Cecidomyiidae|[552]|CNEIA2168-12|Cecidomyiidae|642|BOLD:ACA7933
Cecidomyiidae|[553]|CNRMF3299-12|Cecidomyiidae|670|BOLD:ACB0315
Cecidomyiidae|[554]|CNROG452-13|Cecidomyiidae|658|BOLD:ACK2411
Cecidomyiidae|[555]|CNRME1589-12|Cecidomyiidae|658|BOLD:ACC7962
Cecidomyiidae|[556]|CNRME3600-12|Cecidomyiidae|658|BOLD:ACC7375
Cecidomyiidae|[557]|SSJAD311-13|Cecidomyiidae|661|BOLD:ACI6784
Cecidomyiidae|[558]|CNGLE319-13|Cecidomyiidae|658|BOLD:ACG1995
Cecidomyiidae|[559]|CNROF195-13|Cecidomyiidae|661|BOLD:ACK1691
Cecidomyiidae|[560]|CNROO132-13|Cecidomyiidae|658|BOLD:ACK7405
Cecidomyiidae|[561]|CNROO386-13|Cecidomyiidae|658|BOLD:ABX7387
Cecidomyiidae|[562]|CNWLE865-12|Cecidomyiidae|673|BOLD:ACC6711
Cecidomyiidae|[563]|CNGBG928-14|Cecidomyiidae|664|BOLD:AAU6483
Cecidomyiidae|[564]|CNGLF1119-13|Cecidomyiidae|658|BOLD:ACG6556
Cecidomyiidae|[565]|JSDIR292-11|Cecidomyiidae|658|BOLD:AAU6478
Cecidomyiidae|[566]|CNJAD1313-12|Cecidomyiidae|664|BOLD:ACB2210
Cecidomyiidae|[567]|HPPPJ670-13|Cecidomyiidae|658|BOLD:ACI8907
Cecidomyiidae|[568]|CNBRD444-14|Cecidomyiidae|658|BOLD:ACL4253
Cecidomyiidae|[569]|SSBRA1040-14|Cecidomyiidae|658|BOLD:ACN8860
Cecidomyiidae|[570]|SSBRB4927-14|Cecidomyiidae|555|BOLD:ACN9425
Cecidomyiidae|[571]|CNNHB1775-14|Cecidomyiidae|594|BOLD:ACR5558
Cecidomyiidae|[572]|SSBAC861-12|Cecidomyiidae|658|BOLD:ACC5503
Cecidomyiidae|[573]|CNRME152-12|Cecidomyiidae|661|BOLD:ACC8525
Cecidomyiidae|[574]|CNGMC485-13|Cecidomyiidae|658|BOLD:ACL7740
Cecidomyiidae|[575]|CNFNR1097-14|Cecidomyiidae|611|BOLD:ACM7835
Cecidomyiidae|[576]|CNNHC782-14|Cecidomyiidae|618|BOLD:ACR5959
Cecidomyiidae|[577]|CNWLF1589-12|Cecidomyiidae|658|BOLD:ACD2020
Cecidomyiidae|[578]|HEJUL790-12|Cecidomyiidae|632|BOLD:ABX7368
Cecidomyiidae|[579]|CNRVE865-15|Cecidomyiidae|591|BOLD:ACT5366
Cecidomyiidae|[580]|SSPAA202-13|Cecidomyiidae|670|BOLD:AAV5758
Cecidomyiidae|[581]|CNPED778-14|Cecidomyiidae|661|BOLD:ACL4502
Cecidomyiidae|[582]|PHAUG609-11|Cecidomyiidae|658|BOLD:ABW8026
Cecidomyiidae|[583]|CNKOE1175-14|Cecidomyiidae|658|BOLD:ACM4823
Cecidomyiidae|[584]|CNROO330-13|Cecidomyiidae|658|BOLD:ACA5082
Cecidomyiidae|[585]|PHJUN895-11|Cecidomyiidae|658|BOLD:ABV9356
Cecidomyiidae|[586]|CNGLF477-13|Cecidomyiidae|658|BOLD:ACG6554
Cecidomyiidae|[587]|SSPAC4835-13|Cecidomyiidae|603|BOLD:ACJ5521
Cecidomyiidae|[588]|BBDEE642-10|Cecidomyiidae|658|BOLD:AAM6115
Cecidomyiidae|[589]|CNBPE188-12|Cecidomyiidae|620|BOLD:ACD5419
Cecidomyiidae|[590]|CNBPE207-12|Cecidomyiidae|661|BOLD:ACD5417
Cecidomyiidae|[591]|HEAUG269-12|Cecidomyiidae|670|BOLD:ABX7686
Cecidomyiidae|[592]|CNRMF3658-12|Cecidomyiidae|658|BOLD:ACD0261
Cecidomyiidae|[593]|CNGLF1037-13|Cecidomyiidae|658|BOLD:ACG7260
Cecidomyiidae|[594]|CNBRD360-14|Cecidomyiidae|661|BOLD:ACC8240
Cecidomyiidae|[595]|CNJAC773-12|Cecidomyiidae|658|BOLD:ACC7519
Cecidomyiidae|[596]|CNEID1327-12|Cecidomyiidae|658|BOLD:ACB0932
Cecidomyiidae|[597]|CNGBE211-14|Cecidomyiidae|661|BOLD:ACL7529
Cecidomyiidae|[598]|CNKOE1390-14|Cecidomyiidae|555|BOLD:ACM2710
Cecidomyiidae|[599]|CNGLF476-13|Cecidomyiidae|658|BOLD:ACG6559
Cecidomyiidae|[600]|CNGLC433-13|Cecidomyiidae|658|BOLD:ACE1178

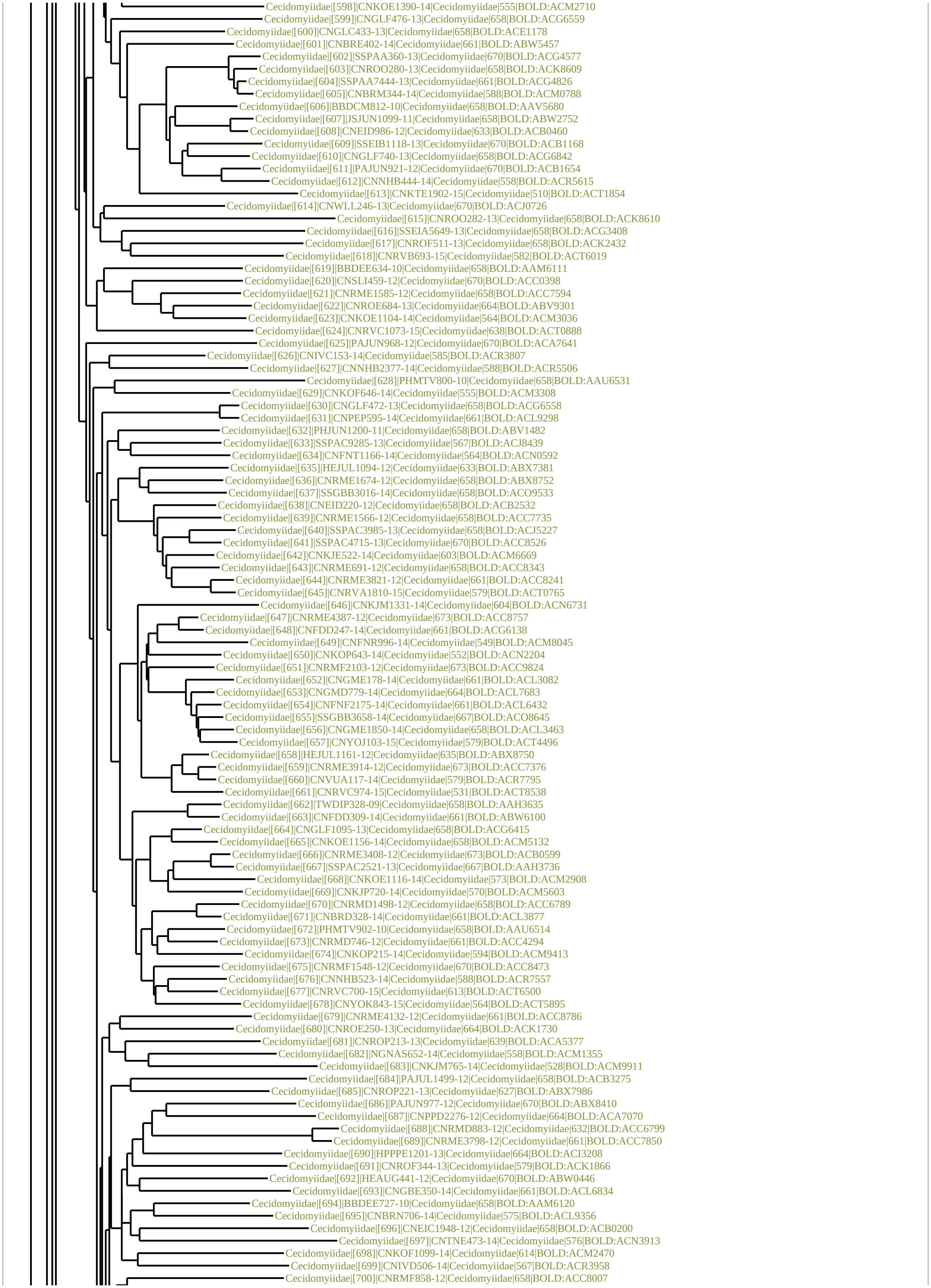
Cecidomyiidae|[598]|CNKOE1390-14|Cecidomyiidae|555|BOLD:ACM2710
Cecidomyiidae|[599]|CNGLF476-13|Cecidomyiidae|658|BOLD:ACG6559
Cecidomyiidae|[600]|CNGLC433-13|Cecidomyiidae|658|BOLD:ACE1178
Cecidomyiidae|[601]|CNBRE402-14|Cecidomyiidae|661|BOLD:ABW5457
Cecidomyiidae|[602]|SSPAA360-13|Cecidomyiidae|670|BOLD:ACG4577
Cecidomyiidae|[603]|CNROO280-13|Cecidomyiidae|658|BOLD:ACK8609
Cecidomyiidae|[604]|SSPAA7444-13|Cecidomyiidae|661|BOLD:ACG4826
Cecidomyiidae|[605]|CNBRM344-14|Cecidomyiidae|588|BOLD:ACM0788
Cecidomyiidae|[606]|BBDCM812-10|Cecidomyiidae|658|BOLD:AAV5680
Cecidomyiidae|[607]|JSJUN1099-11|Cecidomyiidae|658|BOLD:ABW2752
Cecidomyiidae|[608]|CNEID986-12|Cecidomyiidae|633|BOLD:ACB0460
Cecidomyiidae|[609]|SSEIB1118-13|Cecidomyiidae|670|BOLD:ACB1168
Cecidomyiidae|[610]|CNGLF740-13|Cecidomyiidae|658|BOLD:ACG6842
Cecidomyiidae|[611]|PAJUN921-12|Cecidomyiidae|670|BOLD:ACB1654
Cecidomyiidae|[612]|CNNHB444-14|Cecidomyiidae|558|BOLD:ACR5615
Cecidomyiidae|[613]|CNKTE1902-15|Cecidomyiidae|510|BOLD:ACT1854
Cecidomyiidae|[614]|CNWLL246-13|Cecidomyiidae|670|BOLD:ACJ0726
Cecidomyiidae|[615]|CNROO282-13|Cecidomyiidae|658|BOLD:ACK8610
Cecidomyiidae|[616]|SSEIA5649-13|Cecidomyiidae|658|BOLD:ACG3408
Cecidomyiidae|[617]|CNROF511-13|Cecidomyiidae|658|BOLD:ACK2432
Cecidomyiidae|[618]|CNRVB693-15|Cecidomyiidae|582|BOLD:ACT6019
Cecidomyiidae|[619]|BBDEE634-10|Cecidomyiidae|658|BOLD:AAM6111
Cecidomyiidae|[620]|CNSLI459-12|Cecidomyiidae|670|BOLD:ACC0398
Cecidomyiidae|[621]|CNRME1585-12|Cecidomyiidae|658|BOLD:ACC7594
Cecidomyiidae|[622]|CNROE684-13|Cecidomyiidae|664|BOLD:ABV9301
Cecidomyiidae|[623]|CNKOE1104-14|Cecidomyiidae|564|BOLD:ACM3036
Cecidomyiidae|[624]|CNRVC1073-15|Cecidomyiidae|638|BOLD:ACT0888
Cecidomyiidae|[625]|PAJUN968-12|Cecidomyiidae|670|BOLD:ACA7641
Cecidomyiidae|[626]|CNIVC153-14|Cecidomyiidae|585|BOLD:ACR3807
Cecidomyiidae|[627]|CNNHB2377-14|Cecidomyiidae|588|BOLD:ACR5506
Cecidomyiidae|[628]|PHMTV800-10|Cecidomyiidae|658|BOLD:AAU6531
Cecidomyiidae|[629]|CNKOF646-14|Cecidomyiidae|555|BOLD:ACM3308
Cecidomyiidae|[630]|CNGLF472-13|Cecidomyiidae|658|BOLD:ACG6558
Cecidomyiidae|[631]|CNPEP595-14|Cecidomyiidae|661|BOLD:ACL9298
Cecidomyiidae|[632]|PHJUN1200-11|Cecidomyiidae|658|BOLD:ABV1482
Cecidomyiidae|[633]|SSPAC9285-13|Cecidomyiidae|567|BOLD:ACJ8439
Cecidomyiidae|[634]|CNFNT1166-14|Cecidomyiidae|564|BOLD:ACN0592
Cecidomyiidae|[635]|HEJUL1094-12|Cecidomyiidae|633|BOLD:ABX7381
Cecidomyiidae|[636]|CNRME1674-12|Cecidomyiidae|658|BOLD:ABX8752
Cecidomyiidae|[637]|SSGBB3016-14|Cecidomyiidae|658|BOLD:ACO9533
Cecidomyiidae|[638]|CNEID220-12|Cecidomyiidae|658|BOLD:ACB2532
Cecidomyiidae|[639]|CNRME1566-12|Cecidomyiidae|658|BOLD:ACC7735
Cecidomyiidae|[640]|SSPAC3985-13|Cecidomyiidae|658|BOLD:ACJ5227
Cecidomyiidae|[641]|SSPAC4715-13|Cecidomyiidae|670|BOLD:ACC8526
Cecidomyiidae|[642]|CNKJE522-14|Cecidomyiidae|603|BOLD:ACM6669
Cecidomyiidae|[643]|CNRME691-12|Cecidomyiidae|658|BOLD:ACC8343
Cecidomyiidae|[644]|CNRME3821-12|Cecidomyiidae|661|BOLD:ACC8241
Cecidomyiidae|[645]|CNRVA1810-15|Cecidomyiidae|579|BOLD:ACT0765
Cecidomyiidae|[646]|CNKJM1331-14|Cecidomyiidae|604|BOLD:ACN6731
Cecidomyiidae|[647]|CNRME4387-12|Cecidomyiidae|673|BOLD:ACC8757
Cecidomyiidae|[648]|CNFDD247-14|Cecidomyiidae|661|BOLD:ACG6138
Cecidomyiidae|[649]|CNFNR996-14|Cecidomyiidae|549|BOLD:ACM8045
Cecidomyiidae|[650]|CNKOP643-14|Cecidomyiidae|552|BOLD:ACN2204
Cecidomyiidae|[651]|CNRMF2103-12|Cecidomyiidae|673|BOLD:ACC9824
Cecidomyiidae|[652]|CNGME178-14|Cecidomyiidae|661|BOLD:ACL3082
Cecidomyiidae|[653]|CNGMD779-14|Cecidomyiidae|664|BOLD:ACL7683
Cecidomyiidae|[654]|CNFNF2175-14|Cecidomyiidae|661|BOLD:ACL6432
Cecidomyiidae|[655]|SSGBB3658-14|Cecidomyiidae|667|BOLD:ACO8645
Cecidomyiidae|[656]|CNGME1850-14|Cecidomyiidae|658|BOLD:ACL3463
Cecidomyiidae|[657]|CNYOJ103-15|Cecidomyiidae|579|BOLD:ACT4496
Cecidomyiidae|[658]|HEJUL1161-12|Cecidomyiidae|635|BOLD:ABX8750
Cecidomyiidae|[659]|CNRME3914-12|Cecidomyiidae|673|BOLD:ACC7376
Cecidomyiidae|[660]|CNVUA117-14|Cecidomyiidae|579|BOLD:ACR7795
Cecidomyiidae|[661]|CNRVC974-15|Cecidomyiidae|531|BOLD:ACT8538
Cecidomyiidae|[662]|TWDIP328-09|Cecidomyiidae|658|BOLD:AAH3635
Cecidomyiidae|[663]|CNFDD309-14|Cecidomyiidae|661|BOLD:ABW6100
Cecidomyiidae|[664]|CNGLF1095-13|Cecidomyiidae|658|BOLD:ACG6415
Cecidomyiidae|[665]|CNKOE1156-14|Cecidomyiidae|658|BOLD:ACM5132
Cecidomyiidae|[666]|CNRME3408-12|Cecidomyiidae|673|BOLD:ACB0599
Cecidomyiidae|[667]|SSPAC2521-13|Cecidomyiidae|667|BOLD:AAH3736
Cecidomyiidae|[668]|CNKOE1116-14|Cecidomyiidae|573|BOLD:ACM2908
Cecidomyiidae|[669]|CNKJP720-14|Cecidomyiidae|570|BOLD:ACM5603
Cecidomyiidae|[670]|CNRMD1498-12|Cecidomyiidae|658|BOLD:ACC6789
Cecidomyiidae|[671]|CNBRD328-14|Cecidomyiidae|661|BOLD:ACL3877
Cecidomyiidae|[672]|PHMTV902-10|Cecidomyiidae|658|BOLD:AAU6514
Cecidomyiidae|[673]|CNRMD746-12|Cecidomyiidae|661|BOLD:ACC4294
Cecidomyiidae|[674]|CNKOP215-14|Cecidomyiidae|594|BOLD:ACM9413
Cecidomyiidae|[675]|CNRMF1548-12|Cecidomyiidae|670|BOLD:ACC8473
Cecidomyiidae|[676]|CNNHB523-14|Cecidomyiidae|588|BOLD:ACR7557
Cecidomyiidae|[677]|CNRVC700-15|Cecidomyiidae|613|BOLD:ACT6500
Cecidomyiidae|[678]|CNYOK843-15|Cecidomyiidae|564|BOLD:ACT5895
Cecidomyiidae|[679]|CNRME4132-12|Cecidomyiidae|661|BOLD:ACC8786
Cecidomyiidae|[680]|CNROE250-13|Cecidomyiidae|664|BOLD:ACK1730
Cecidomyiidae|[681]|CNROP213-13|Cecidomyiidae|639|BOLD:ACA5377
Cecidomyiidae|[682]|NGNAS652-14|Cecidomyiidae|558|BOLD:ACM1355
Cecidomyiidae|[683]|CNKJM765-14|Cecidomyiidae|528|BOLD:ACM9911
Cecidomyiidae|[684]|PAJUL1499-12|Cecidomyiidae|658|BOLD:ACB3275
Cecidomyiidae|[685]|CNROP221-13|Cecidomyiidae|627|BOLD:ABX7986
Cecidomyiidae|[686]|PAJUN977-12|Cecidomyiidae|670|BOLD:ABX8410
Cecidomyiidae|[687]|CNPPD2276-12|Cecidomyiidae|664|BOLD:ACA7070
Cecidomyiidae|[688]|CNRMD883-12|Cecidomyiidae|632|BOLD:ACC6799
Cecidomyiidae|[689]|CNRME3798-12|Cecidomyiidae|661|BOLD:ACC7850
Cecidomyiidae|[690]|HPPPE1201-13|Cecidomyiidae|664|BOLD:ACI3208
Cecidomyiidae|[691]|CNROF344-13|Cecidomyiidae|579|BOLD:ACK1866
Cecidomyiidae|[692]|HEAUG441-12|Cecidomyiidae|670|BOLD:ABW0446
Cecidomyiidae|[693]|CNGBE350-14|Cecidomyiidae|661|BOLD:ACL6834
Cecidomyiidae|[694]|BBDEE727-10|Cecidomyiidae|658|BOLD:AAM6120
Cecidomyiidae|[695]|CNBRN706-14|Cecidomyiidae|575|BOLD:ACL9356
Cecidomyiidae|[696]|CNEIC1948-12|Cecidomyiidae|658|BOLD:ACB0200
Cecidomyiidae|[697]|CNTNE473-14|Cecidomyiidae|576|BOLD:ACN3913
Cecidomyiidae|[698]|CNKOF1099-14|Cecidomyiidae|614|BOLD:ACM2470
Cecidomyiidae|[699]|CNIVD506-14|Cecidomyiidae|567|BOLD:ACR3958
Cecidomyiidae|[700]|CNRMF858-12|Cecidomyiidae|658|BOLD:ACC8007

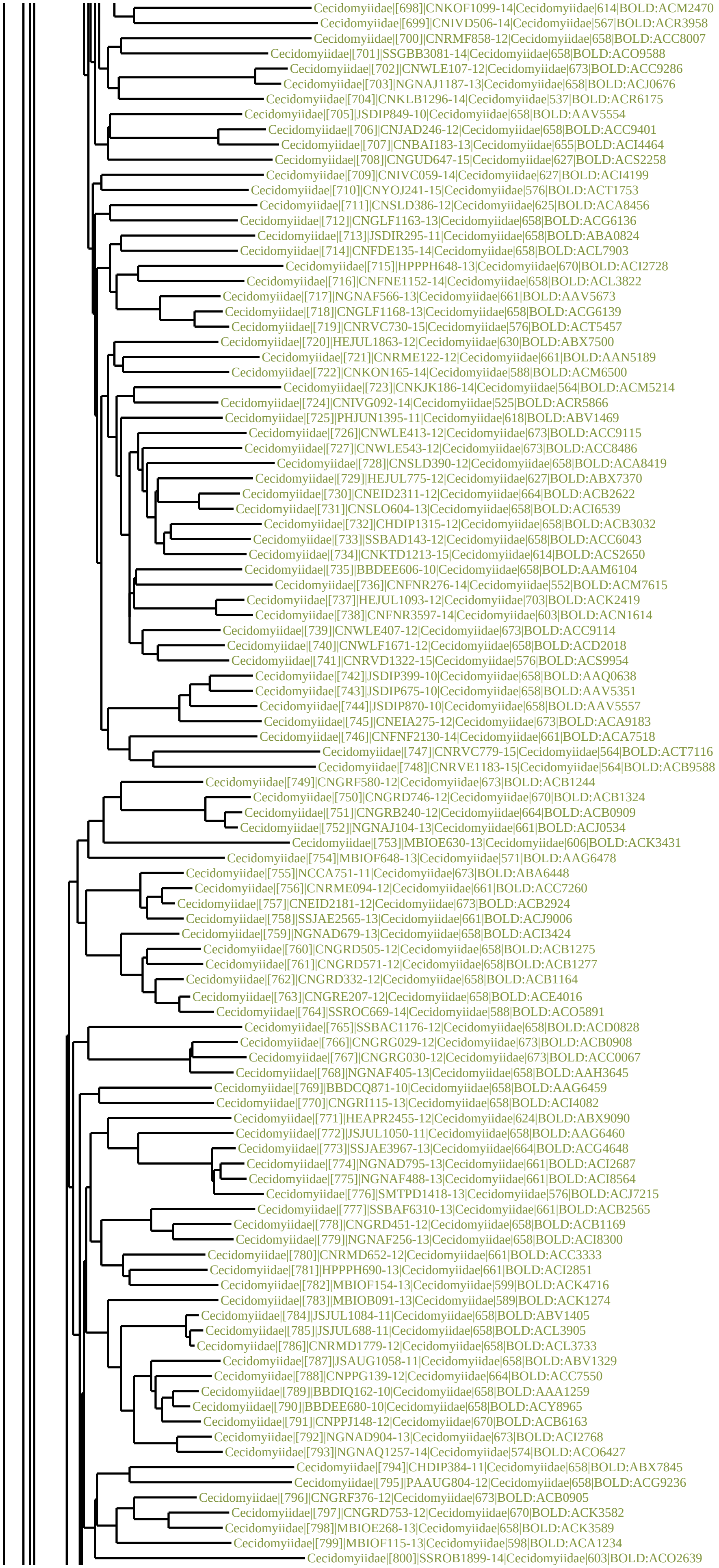
Cecidomyiidae|[698]|CNKOF1099-14|Cecidomyiidae|614|BOLD:ACM2470
Cecidomyiidae|[699]|CNIVD506-14|Cecidomyiidae|567|BOLD:ACR3958
Cecidomyiidae|[700]|CNRMF858-12|Cecidomyiidae|658|BOLD:ACC8007
Cecidomyiidae|[701]|SSGBB3081-14|Cecidomyiidae|658|BOLD:ACO9588
Cecidomyiidae|[702]|CNWLE107-12|Cecidomyiidae|673|BOLD:ACC9286
Cecidomyiidae|[703]|NGNAJ1187-13|Cecidomyiidae|658|BOLD:ACJ0676
Cecidomyiidae|[704]|CNKLB1296-14|Cecidomyiidae|537|BOLD:ACR6175
Cecidomyiidae|[705]|JSDIP849-10|Cecidomyiidae|658|BOLD:AAV5554
Cecidomyiidae|[706]|CNJAD246-12|Cecidomyiidae|658|BOLD:ACC9401
Cecidomyiidae|[707]|CNBAI183-13|Cecidomyiidae|655|BOLD:ACI4464
Cecidomyiidae|[708]|CNGUD647-15|Cecidomyiidae|627|BOLD:ACS2258
Cecidomyiidae|[709]|CNIVC059-14|Cecidomyiidae|627|BOLD:ACI4199
Cecidomyiidae|[710]|CNYOJ241-15|Cecidomyiidae|576|BOLD:ACT1753
Cecidomyiidae|[711]|CNSLD386-12|Cecidomyiidae|625|BOLD:ACA8456
Cecidomyiidae|[712]|CNGLF1163-13|Cecidomyiidae|658|BOLD:ACG6136
Cecidomyiidae|[713]|JSDIR295-11|Cecidomyiidae|658|BOLD:ABA0824
Cecidomyiidae|[714]|CNFDE135-14|Cecidomyiidae|658|BOLD:ACL7903
Cecidomyiidae|[715]|HPPPH648-13|Cecidomyiidae|670|BOLD:ACI2728
Cecidomyiidae|[716]|CNFNE1152-14|Cecidomyiidae|658|BOLD:ACL3822
Cecidomyiidae|[717]|NGNAF566-13|Cecidomyiidae|661|BOLD:AAV5673
Cecidomyiidae|[718]|CNGLF1168-13|Cecidomyiidae|658|BOLD:ACG6139
Cecidomyiidae|[719]|CNRVC730-15|Cecidomyiidae|576|BOLD:ACT5457
Cecidomyiidae|[720]|HEJUL1863-12|Cecidomyiidae|630|BOLD:ABX7500
Cecidomyiidae|[721]|CNRME122-12|Cecidomyiidae|661|BOLD:AAN5189
Cecidomyiidae|[722]|CNKON165-14|Cecidomyiidae|588|BOLD:ACM6500
Cecidomyiidae|[723]|CNKJK186-14|Cecidomyiidae|564|BOLD:ACM5214
Cecidomyiidae|[724]|CNIVG092-14|Cecidomyiidae|525|BOLD:ACR5866
Cecidomyiidae|[725]|PHJUN1395-11|Cecidomyiidae|618|BOLD:ABV1469
Cecidomyiidae|[726]|CNWLE413-12|Cecidomyiidae|673|BOLD:ACC9115
Cecidomyiidae|[727]|CNWLE543-12|Cecidomyiidae|673|BOLD:ACC8486
Cecidomyiidae|[728]|CNSLD390-12|Cecidomyiidae|658|BOLD:ACA8419
Cecidomyiidae|[729]|HEJUL775-12|Cecidomyiidae|627|BOLD:ABX7370
Cecidomyiidae|[730]|CNEID2311-12|Cecidomyiidae|664|BOLD:ACB2622
Cecidomyiidae|[731]|CNSLO604-13|Cecidomyiidae|658|BOLD:ACI6539
Cecidomyiidae|[732]|CHDIP1315-12|Cecidomyiidae|658|BOLD:ACB3032
Cecidomyiidae|[733]|SSBAD143-12|Cecidomyiidae|658|BOLD:ACC6043
Cecidomyiidae|[734]|CNKTD1213-15|Cecidomyiidae|614|BOLD:ACS2650
Cecidomyiidae|[735]|BBDEE606-10|Cecidomyiidae|658|BOLD:AAM6104
Cecidomyiidae|[736]|CNFNR276-14|Cecidomyiidae|552|BOLD:ACM7615
Cecidomyiidae|[737]|HEJUL1093-12|Cecidomyiidae|703|BOLD:ACK2419
Cecidomyiidae|[738]|CNFNR3597-14|Cecidomyiidae|603|BOLD:ACN1614
Cecidomyiidae|[739]|CNWLE407-12|Cecidomyiidae|673|BOLD:ACC9114
Cecidomyiidae|[740]|CNWLF1671-12|Cecidomyiidae|658|BOLD:ACD2018
Cecidomyiidae|[741]|CNRVD1322-15|Cecidomyiidae|576|BOLD:ACS9954
Cecidomyiidae|[742]|JSDIP399-10|Cecidomyiidae|658|BOLD:AAQ0638
Cecidomyiidae|[743]|JSDIP675-10|Cecidomyiidae|658|BOLD:AAV5351
Cecidomyiidae|[744]|JSDIP870-10|Cecidomyiidae|658|BOLD:AAV5557
Cecidomyiidae|[745]|CNEIA275-12|Cecidomyiidae|673|BOLD:ACA9183
Cecidomyiidae|[746]|CNFNF2130-14|Cecidomyiidae|661|BOLD:ACA7518
Cecidomyiidae|[747]|CNRVC779-15|Cecidomyiidae|564|BOLD:ACT7116
Cecidomyiidae|[748]|CNRVE1183-15|Cecidomyiidae|564|BOLD:ACB9588
Cecidomyiidae|[749]|CNGRF580-12|Cecidomyiidae|673|BOLD:ACB1244
Cecidomyiidae|[750]|CNGRD746-12|Cecidomyiidae|670|BOLD:ACB1324
Cecidomyiidae|[751]|CNGRB240-12|Cecidomyiidae|664|BOLD:ACB0909
Cecidomyiidae|[752]|NGNAJ104-13|Cecidomyiidae|661|BOLD:ACJ0534
Cecidomyiidae|[753]|MBIOE630-13|Cecidomyiidae|606|BOLD:ACK3431
Cecidomyiidae|[754]|MBIOF648-13|Cecidomyiidae|571|BOLD:AAG6478
Cecidomyiidae|[755]|NCCA751-11|Cecidomyiidae|673|BOLD:ABA6448
Cecidomyiidae|[756]|CNRME094-12|Cecidomyiidae|661|BOLD:ACC7260
Cecidomyiidae|[757]|CNEID2181-12|Cecidomyiidae|673|BOLD:ACB2924
Cecidomyiidae|[758]|SSJAE2565-13|Cecidomyiidae|661|BOLD:ACJ9006
Cecidomyiidae|[759]|NGNAD679-13|Cecidomyiidae|658|BOLD:ACI3424
Cecidomyiidae|[760]|CNGRD505-12|Cecidomyiidae|658|BOLD:ACB1275
Cecidomyiidae|[761]|CNGRD571-12|Cecidomyiidae|658|BOLD:ACB1277
Cecidomyiidae|[762]|CNGRD332-12|Cecidomyiidae|658|BOLD:ACB1164
Cecidomyiidae|[763]|CNGRE207-12|Cecidomyiidae|658|BOLD:ACE4016
Cecidomyiidae|[764]|SSROC669-14|Cecidomyiidae|588|BOLD:ACO5891
Cecidomyiidae|[765]|SSBAC1176-12|Cecidomyiidae|658|BOLD:ACD0828
Cecidomyiidae|[766]|CNGRG029-12|Cecidomyiidae|673|BOLD:ACB0908
Cecidomyiidae|[767]|CNGRG030-12|Cecidomyiidae|673|BOLD:ACC0067
Cecidomyiidae|[768]|NGNAF405-13|Cecidomyiidae|658|BOLD:AAH3645
Cecidomyiidae|[769]|BBDCQ871-10|Cecidomyiidae|658|BOLD:AAG6459
Cecidomyiidae|[770]|CNGRI115-13|Cecidomyiidae|658|BOLD:ACI4082
Cecidomyiidae|[771]|HEAPR2455-12|Cecidomyiidae|624|BOLD:ABX9090
Cecidomyiidae|[772]|JSJUL1050-11|Cecidomyiidae|658|BOLD:AAG6460
Cecidomyiidae|[773]|SSJAE3967-13|Cecidomyiidae|664|BOLD:ACG4648
Cecidomyiidae|[774]|NGNAD795-13|Cecidomyiidae|661|BOLD:ACI2687
Cecidomyiidae|[775]|NGNAF488-13|Cecidomyiidae|661|BOLD:ACI8564
Cecidomyiidae|[776]|SMTPD1418-13|Cecidomyiidae|576|BOLD:ACJ7215
Cecidomyiidae|[777]|SSBAF6310-13|Cecidomyiidae|661|BOLD:ACB2565
Cecidomyiidae|[778]|CNGRD451-12|Cecidomyiidae|658|BOLD:ACB1169
Cecidomyiidae|[779]|NGNAF256-13|Cecidomyiidae|658|BOLD:ACI8300
Cecidomyiidae|[780]|CNRMD652-12|Cecidomyiidae|661|BOLD:ACC3333
Cecidomyiidae|[781]|HPPPH690-13|Cecidomyiidae|661|BOLD:ACI2851
Cecidomyiidae|[782]|MBIOF154-13|Cecidomyiidae|599|BOLD:ACK4716
Cecidomyiidae|[783]|MBIOB091-13|Cecidomyiidae|589|BOLD:ACK1274
Cecidomyiidae|[784]|JSJUL1084-11|Cecidomyiidae|658|BOLD:ABV1405
Cecidomyiidae|[785]|JSJUL688-11|Cecidomyiidae|658|BOLD:ACL3905
Cecidomyiidae|[786]|CNRMD1779-12|Cecidomyiidae|658|BOLD:ACL3733
Cecidomyiidae|[787]|JSAUG1058-11|Cecidomyiidae|658|BOLD:ABV1329
Cecidomyiidae|[788]|CNPPG139-12|Cecidomyiidae|664|BOLD:ACC7550
Cecidomyiidae|[789]|BBDIQ162-10|Cecidomyiidae|658|BOLD:AAA1259
Cecidomyiidae|[790]|BBDEE680-10|Cecidomyiidae|658|BOLD:ACY8965
Cecidomyiidae|[791]|CNPPJ148-12|Cecidomyiidae|670|BOLD:ACB6163
Cecidomyiidae|[792]|NGNAD904-13|Cecidomyiidae|673|BOLD:ACI2768
Cecidomyiidae|[793]|NGNAQ1257-14|Cecidomyiidae|574|BOLD:ACO6427
Cecidomyiidae|[794]|CHDIP384-11|Cecidomyiidae|658|BOLD:ABX7845
Cecidomyiidae|[795]|PAAUG804-12|Cecidomyiidae|658|BOLD:ACG9236
Cecidomyiidae|[796]|CNGRF376-12|Cecidomyiidae|673|BOLD:ACB0905
Cecidomyiidae|[797]|CNGRD753-12|Cecidomyiidae|670|BOLD:ACK3582
Cecidomyiidae|[798]|MBIOE268-13|Cecidomyiidae|658|BOLD:ACK3589
Cecidomyiidae|[799]|MBIOF115-13|Cecidomyiidae|598|BOLD:ACA1234
Cecidomyiidae|[800]|SSROB1899-14|Cecidomyiidae|603|BOLD:ACO2639

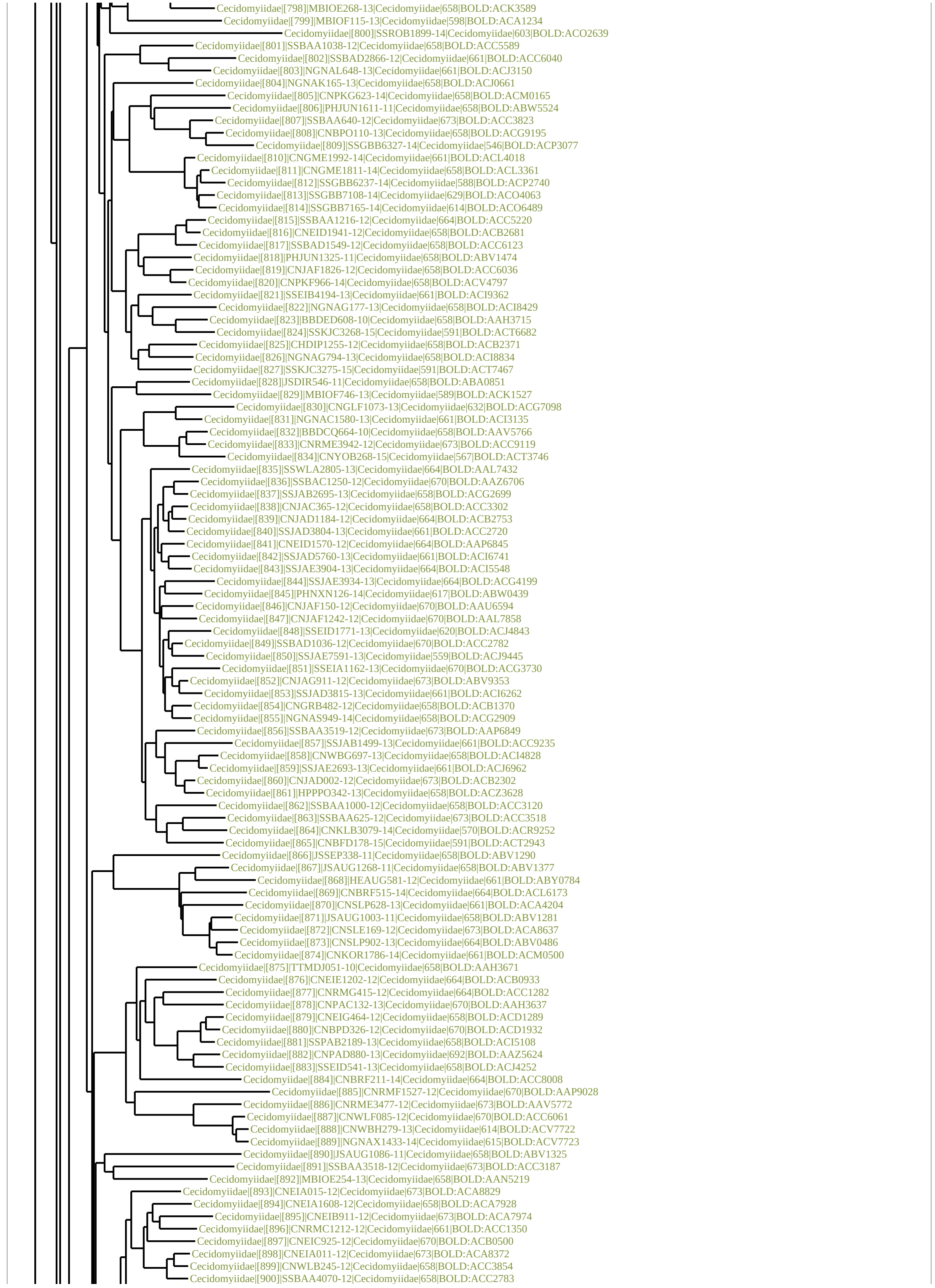
Cecidomyiidae|[798]|MBIOE268-13|Cecidomyiidae|658|BOLD:ACK3589
Cecidomyiidae|[799]|MBIOF115-13|Cecidomyiidae|598|BOLD:ACA1234
Cecidomyiidae|[800]|SSROB1899-14|Cecidomyiidae|603|BOLD:ACO2639
Cecidomyiidae|[801]|SSBAA1038-12|Cecidomyiidae|658|BOLD:ACC5589
Cecidomyiidae|[802]|SSBAD2866-12|Cecidomyiidae|661|BOLD:ACC6040
Cecidomyiidae|[803]|NGNAL648-13|Cecidomyiidae|661|BOLD:ACJ3150
Cecidomyiidae|[804]|NGNAK165-13|Cecidomyiidae|658|BOLD:ACJ0661
Cecidomyiidae|[805]|CNPKG623-14|Cecidomyiidae|658|BOLD:ACM0165
Cecidomyiidae|[806]|PHJUN1611-11|Cecidomyiidae|658|BOLD:ABW5524
Cecidomyiidae|[807]|SSBAA640-12|Cecidomyiidae|673|BOLD:ACC3823
Cecidomyiidae|[808]|CNBPO110-13|Cecidomyiidae|658|BOLD:ACG9195
Cecidomyiidae|[809]|SSGBB6327-14|Cecidomyiidae|546|BOLD:ACP3077
Cecidomyiidae|[810]|CNGME1992-14|Cecidomyiidae|661|BOLD:ACL4018
Cecidomyiidae|[811]|CNGME1811-14|Cecidomyiidae|658|BOLD:ACL3361
Cecidomyiidae|[812]|SSGBB6237-14|Cecidomyiidae|588|BOLD:ACP2740
Cecidomyiidae|[813]|SSGBB7108-14|Cecidomyiidae|629|BOLD:ACO4063
Cecidomyiidae|[814]|SSGBB7165-14|Cecidomyiidae|614|BOLD:ACO6489
Cecidomyiidae|[815]|SSBAA1216-12|Cecidomyiidae|664|BOLD:ACC5220
Cecidomyiidae|[816]|CNEID1941-12|Cecidomyiidae|658|BOLD:ACB2681
Cecidomyiidae|[817]|SSBAD1549-12|Cecidomyiidae|658|BOLD:ACC6123
Cecidomyiidae|[818]|PHJUN1325-11|Cecidomyiidae|658|BOLD:ABV1474
Cecidomyiidae|[819]|CNJAF1826-12|Cecidomyiidae|658|BOLD:ACC6036
Cecidomyiidae|[820]|CNPKF966-14|Cecidomyiidae|658|BOLD:ACV4797
Cecidomyiidae|[821]|SSEIB4194-13|Cecidomyiidae|661|BOLD:ACI9362
Cecidomyiidae|[822]|NGNAG177-13|Cecidomyiidae|658|BOLD:ACI8429
Cecidomyiidae|[823]|BBDED608-10|Cecidomyiidae|658|BOLD:AAH3715
Cecidomyiidae|[824]|SSKJC3268-15|Cecidomyiidae|591|BOLD:ACT6682
Cecidomyiidae|[825]|CHDIP1255-12|Cecidomyiidae|658|BOLD:ACB2371
Cecidomyiidae|[826]|NGNAG794-13|Cecidomyiidae|658|BOLD:ACI8834
Cecidomyiidae|[827]|SSKJC3275-15|Cecidomyiidae|591|BOLD:ACT7467
Cecidomyiidae|[828]|JSDIR546-11|Cecidomyiidae|658|BOLD:ABA0851
Cecidomyiidae|[829]|MBIOF746-13|Cecidomyiidae|589|BOLD:ACK1527
Cecidomyiidae|[830]|CNGLF1073-13|Cecidomyiidae|632|BOLD:ACG7098
Cecidomyiidae|[831]|NGNAC1580-13|Cecidomyiidae|661|BOLD:ACI3135
Cecidomyiidae|[832]|BBDCQ664-10|Cecidomyiidae|658|BOLD:AAV5766
Cecidomyiidae|[833]|CNRME3942-12|Cecidomyiidae|673|BOLD:ACC9119
Cecidomyiidae|[834]|CNYOB268-15|Cecidomyiidae|567|BOLD:ACT3746
Cecidomyiidae|[835]|SSWLA2805-13|Cecidomyiidae|664|BOLD:AAL7432
Cecidomyiidae|[836]|SSBAC1250-12|Cecidomyiidae|670|BOLD:AAZ6706
Cecidomyiidae|[837]|SSJAB2695-13|Cecidomyiidae|658|BOLD:ACG2699
Cecidomyiidae|[838]|CNJAC365-12|Cecidomyiidae|658|BOLD:ACC3302
Cecidomyiidae|[839]|CNJAD1184-12|Cecidomyiidae|664|BOLD:ACB2753
Cecidomyiidae|[840]|SSJAD3804-13|Cecidomyiidae|661|BOLD:ACC2720
Cecidomyiidae|[841]|CNEID1570-12|Cecidomyiidae|664|BOLD:AAP6845
Cecidomyiidae|[842]|SSJAD5760-13|Cecidomyiidae|661|BOLD:ACI6741
Cecidomyiidae|[843]|SSJAE3904-13|Cecidomyiidae|664|BOLD:ACI5548
Cecidomyiidae|[844]|SSJAE3934-13|Cecidomyiidae|664|BOLD:ACG4199
Cecidomyiidae|[845]|PHNXN126-14|Cecidomyiidae|617|BOLD:ABW0439
Cecidomyiidae|[846]|CNJAF150-12|Cecidomyiidae|670|BOLD:AAU6594
Cecidomyiidae|[847]|CNJAF1242-12|Cecidomyiidae|670|BOLD:AAL7858
Cecidomyiidae|[848]|SSEID1771-13|Cecidomyiidae|620|BOLD:ACJ4843
Cecidomyiidae|[849]|SSBAD1036-12|Cecidomyiidae|670|BOLD:ACC2782
Cecidomyiidae|[850]|SSJAE7591-13|Cecidomyiidae|559|BOLD:ACJ9445
Cecidomyiidae|[851]|SSEIA1162-13|Cecidomyiidae|670|BOLD:ACG3730
Cecidomyiidae|[852]|CNJAG911-12|Cecidomyiidae|673|BOLD:ABV9353
Cecidomyiidae|[853]|SSJAD3815-13|Cecidomyiidae|661|BOLD:ACI6262
Cecidomyiidae|[854]|CNGRB482-12|Cecidomyiidae|658|BOLD:ACB1370
Cecidomyiidae|[855]|NGNAS949-14|Cecidomyiidae|658|BOLD:ACG2909
Cecidomyiidae|[856]|SSBAA3519-12|Cecidomyiidae|673|BOLD:AAP6849
Cecidomyiidae|[857]|SSJAB1499-13|Cecidomyiidae|661|BOLD:ACC9235
Cecidomyiidae|[858]|CNWBG697-13|Cecidomyiidae|658|BOLD:ACI4828
Cecidomyiidae|[859]|SSJAE2693-13|Cecidomyiidae|661|BOLD:ACJ6962
Cecidomyiidae|[860]|CNJAD002-12|Cecidomyiidae|673|BOLD:ACB2302
Cecidomyiidae|[861]|HPPPO342-13|Cecidomyiidae|658|BOLD:ACZ3628
Cecidomyiidae|[862]|SSBAA1000-12|Cecidomyiidae|658|BOLD:ACC3120
Cecidomyiidae|[863]|SSBAA625-12|Cecidomyiidae|673|BOLD:ACC3518
Cecidomyiidae|[864]|CNKLB3079-14|Cecidomyiidae|570|BOLD:ACR9252
Cecidomyiidae|[865]|CNBFD178-15|Cecidomyiidae|591|BOLD:ACT2943
Cecidomyiidae|[866]|JSSEP338-11|Cecidomyiidae|658|BOLD:ABV1290
Cecidomyiidae|[867]|JSAUG1268-11|Cecidomyiidae|658|BOLD:ABV1377
Cecidomyiidae|[868]|HEAUG581-12|Cecidomyiidae|661|BOLD:ABY0784
Cecidomyiidae|[869]|CNBRF515-14|Cecidomyiidae|664|BOLD:ACL6173
Cecidomyiidae|[870]|CNSLP628-13|Cecidomyiidae|661|BOLD:ACA4204
Cecidomyiidae|[871]|JSAUG1003-11|Cecidomyiidae|658|BOLD:ABV1281
Cecidomyiidae|[872]|CNSLE169-12|Cecidomyiidae|673|BOLD:ACA8637
Cecidomyiidae|[873]|CNSLP902-13|Cecidomyiidae|664|BOLD:ABV0486
Cecidomyiidae|[874]|CNKOR1786-14|Cecidomyiidae|661|BOLD:ACM0500
Cecidomyiidae|[875]|TTMDJ051-10|Cecidomyiidae|658|BOLD:AAH3671
Cecidomyiidae|[876]|CNEIE1202-12|Cecidomyiidae|664|BOLD:ACB0933
Cecidomyiidae|[877]|CNRMG415-12|Cecidomyiidae|664|BOLD:ACC1282
Cecidomyiidae|[878]|CNPAC132-13|Cecidomyiidae|670|BOLD:AAH3637
Cecidomyiidae|[879]|CNEIG464-12|Cecidomyiidae|658|BOLD:ACD1289
Cecidomyiidae|[880]|CNBPD326-12|Cecidomyiidae|670|BOLD:ACD1932
Cecidomyiidae|[881]|SSPAB2189-13|Cecidomyiidae|658|BOLD:ACI5108
Cecidomyiidae|[882]|CNPAD880-13|Cecidomyiidae|692|BOLD:AAZ5624
Cecidomyiidae|[883]|SSEID541-13|Cecidomyiidae|658|BOLD:ACJ4252
Cecidomyiidae|[884]|CNBRF211-14|Cecidomyiidae|664|BOLD:ACC8008
Cecidomyiidae|[885]|CNRMF1527-12|Cecidomyiidae|670|BOLD:AAP9028
Cecidomyiidae|[886]|CNRME3477-12|Cecidomyiidae|673|BOLD:AAV5772
Cecidomyiidae|[887]|CNWLF085-12|Cecidomyiidae|670|BOLD:ACC6061
Cecidomyiidae|[888]|CNWBH279-13|Cecidomyiidae|614|BOLD:ACV7722
Cecidomyiidae|[889]|NGNAX1433-14|Cecidomyiidae|615|BOLD:ACV7723
Cecidomyiidae|[890]|JSAUG1086-11|Cecidomyiidae|658|BOLD:ABV1325
Cecidomyiidae|[891]|SSBAA3518-12|Cecidomyiidae|673|BOLD:ACC3187
Cecidomyiidae|[892]|MBIOE254-13|Cecidomyiidae|658|BOLD:AAN5219
Cecidomyiidae|[893]|CNEIA015-12|Cecidomyiidae|673|BOLD:ACA8829
Cecidomyiidae|[894]|CNEIA1608-12|Cecidomyiidae|658|BOLD:ACA7928
Cecidomyiidae|[895]|CNEIB911-12|Cecidomyiidae|673|BOLD:ACA7974
Cecidomyiidae|[896]|CNRMC1212-12|Cecidomyiidae|661|BOLD:ACC1350
Cecidomyiidae|[897]|CNEIC925-12|Cecidomyiidae|670|BOLD:ACB0500
Cecidomyiidae|[898]|CNEIA011-12|Cecidomyiidae|673|BOLD:ACA8372
Cecidomyiidae|[899]|CNWLB245-12|Cecidomyiidae|658|BOLD:ACC3854
Cecidomyiidae|[900]|SSBAA4070-12|Cecidomyiidae|658|BOLD:ACC2783

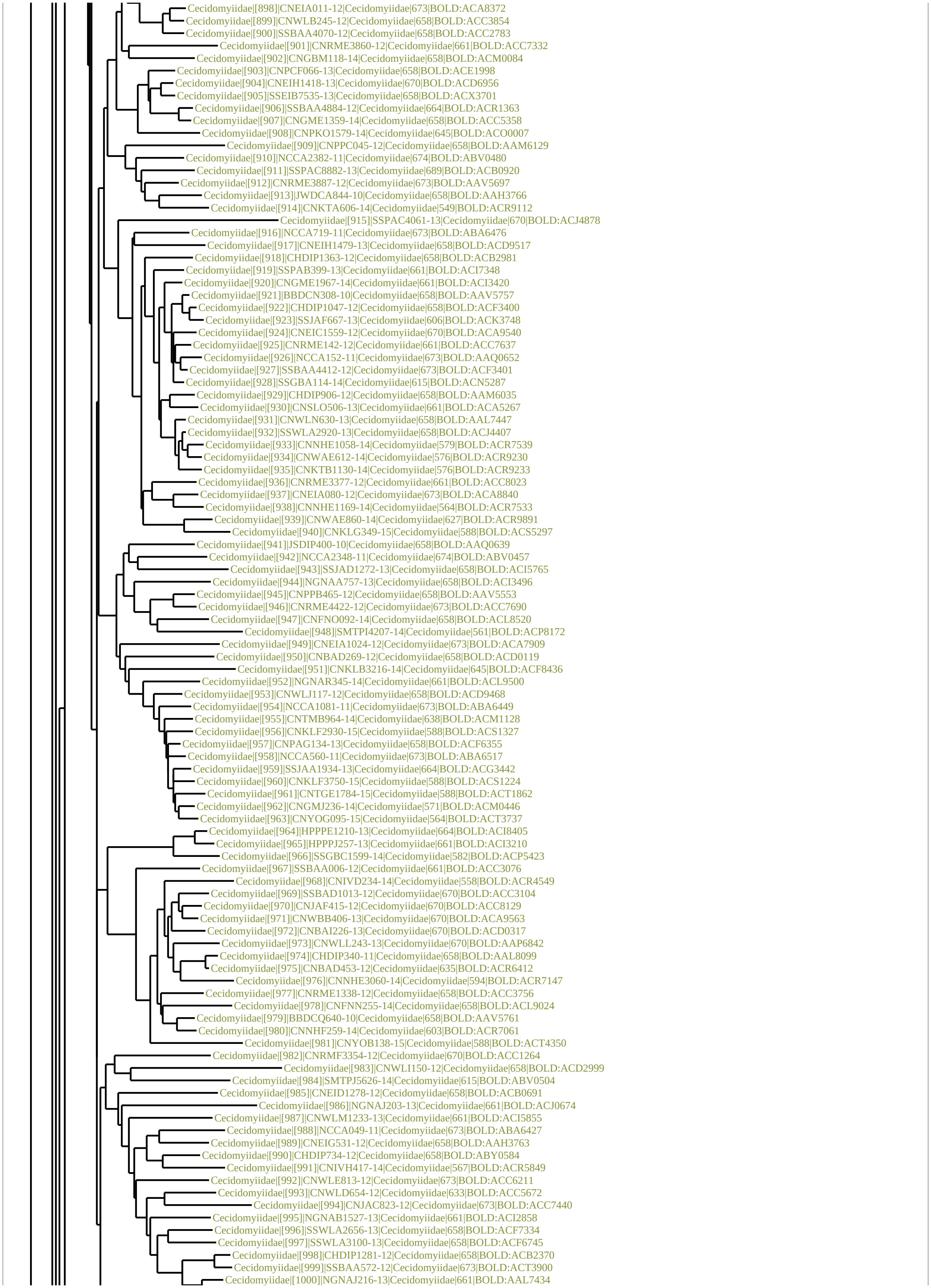
Cecidomyiidae|[898]|CNEIA011-12|Cecidomyiidae|673|BOLD:ACA8372
Cecidomyiidae|[899]|CNWLB245-12|Cecidomyiidae|658|BOLD:ACC3854
Cecidomyiidae|[900]|SSBAA4070-12|Cecidomyiidae|658|BOLD:ACC2783
Cecidomyiidae|[901]|CNRME3860-12|Cecidomyiidae|661|BOLD:ACC7332
Cecidomyiidae|[902]|CNGBM118-14|Cecidomyiidae|658|BOLD:ACM0084
Cecidomyiidae|[903]|CNPCF066-13|Cecidomyiidae|658|BOLD:ACE1998
Cecidomyiidae|[904]|CNEIH1418-13|Cecidomyiidae|670|BOLD:ACD6956
Cecidomyiidae|[905]|SSEIB7535-13|Cecidomyiidae|658|BOLD:ACX3701
Cecidomyiidae|[906]|SSBAA4884-12|Cecidomyiidae|664|BOLD:ACR1363
Cecidomyiidae|[907]|CNGME1359-14|Cecidomyiidae|658|BOLD:ACC5358
Cecidomyiidae|[908]|CNPKO1579-14|Cecidomyiidae|645|BOLD:ACO0007
Cecidomyiidae|[909]|CNPPC045-12|Cecidomyiidae|658|BOLD:AAM6129
Cecidomyiidae|[910]|NCCA2382-11|Cecidomyiidae|674|BOLD:ABV0480
Cecidomyiidae|[911]|SSPAC8882-13|Cecidomyiidae|689|BOLD:ACB0920
Cecidomyiidae|[912]|CNRME3887-12|Cecidomyiidae|673|BOLD:AAV5697
Cecidomyiidae|[913]|JWDCA844-10|Cecidomyiidae|658|BOLD:AAH3766
Cecidomyiidae|[914]|CNKTA606-14|Cecidomyiidae|549|BOLD:ACR9112
Cecidomyiidae|[915]|SSPAC4061-13|Cecidomyiidae|670|BOLD:ACJ4878
Cecidomyiidae|[916]|NCCA719-11|Cecidomyiidae|673|BOLD:ABA6476
Cecidomyiidae|[917]|CNEIH1479-13|Cecidomyiidae|658|BOLD:ACD9517
Cecidomyiidae|[918]|CHDIP1363-12|Cecidomyiidae|658|BOLD:ACB2981
Cecidomyiidae|[919]|SSPAB399-13|Cecidomyiidae|661|BOLD:ACI7348
Cecidomyiidae|[920]|CNGME1967-14|Cecidomyiidae|661|BOLD:ACI3420
Cecidomyiidae|[921]|BBDCN308-10|Cecidomyiidae|658|BOLD:AAV5757
Cecidomyiidae|[922]|CHDIP1047-12|Cecidomyiidae|658|BOLD:ACF3400
Cecidomyiidae|[923]|SSJAF667-13|Cecidomyiidae|606|BOLD:ACK3748
Cecidomyiidae|[924]|CNEIC1559-12|Cecidomyiidae|670|BOLD:ACA9540
Cecidomyiidae|[925]|CNRME142-12|Cecidomyiidae|661|BOLD:ACC7637
Cecidomyiidae|[926]|NCCA152-11|Cecidomyiidae|673|BOLD:AAQ0652
Cecidomyiidae|[927]|SSBAA4412-12|Cecidomyiidae|673|BOLD:ACF3401
Cecidomyiidae|[928]|SSGBA114-14|Cecidomyiidae|615|BOLD:ACN5287
Cecidomyiidae|[929]|CHDIP906-12|Cecidomyiidae|658|BOLD:AAM6035
Cecidomyiidae|[930]|CNSLO506-13|Cecidomyiidae|661|BOLD:ACA5267
Cecidomyiidae|[931]|CNWLN630-13|Cecidomyiidae|658|BOLD:AAL7447
Cecidomyiidae|[932]|SSWLA2920-13|Cecidomyiidae|658|BOLD:ACJ4407
Cecidomyiidae|[933]|CNNHE1058-14|Cecidomyiidae|579|BOLD:ACR7539
Cecidomyiidae|[934]|CNWAE612-14|Cecidomyiidae|576|BOLD:ACR9230
Cecidomyiidae|[935]|CNKTB1130-14|Cecidomyiidae|576|BOLD:ACR9233
Cecidomyiidae|[936]|CNRME3377-12|Cecidomyiidae|661|BOLD:ACC8023
Cecidomyiidae|[937]|CNEIA080-12|Cecidomyiidae|673|BOLD:ACA8840
Cecidomyiidae|[938]|CNNHE1169-14|Cecidomyiidae|564|BOLD:ACR7533
Cecidomyiidae|[939]|CNWAE860-14|Cecidomyiidae|627|BOLD:ACR9891
Cecidomyiidae|[940]|CNKLG349-15|Cecidomyiidae|588|BOLD:ACS5297
Cecidomyiidae|[941]|JSDIP400-10|Cecidomyiidae|658|BOLD:AAQ0639
Cecidomyiidae|[942]|NCCA2348-11|Cecidomyiidae|674|BOLD:ABV0457
Cecidomyiidae|[943]|SSJAD1272-13|Cecidomyiidae|658|BOLD:ACI5765
Cecidomyiidae|[944]|NGNAA757-13|Cecidomyiidae|658|BOLD:ACI3496
Cecidomyiidae|[945]|CNPPB465-12|Cecidomyiidae|658|BOLD:AAV5553
Cecidomyiidae|[946]|CNRME4422-12|Cecidomyiidae|673|BOLD:ACC7690
Cecidomyiidae|[947]|CNFNO092-14|Cecidomyiidae|658|BOLD:ACL8520
Cecidomyiidae|[948]|SMTPI4207-14|Cecidomyiidae|561|BOLD:ACP8172
Cecidomyiidae|[949]|CNEIA1024-12|Cecidomyiidae|673|BOLD:ACA7909
Cecidomyiidae|[950]|CNBAD269-12|Cecidomyiidae|658|BOLD:ACD0119
Cecidomyiidae|[951]|CNKLB3216-14|Cecidomyiidae|645|BOLD:ACF8436
Cecidomyiidae|[952]|NGNAR345-14|Cecidomyiidae|661|BOLD:ACL9500
Cecidomyiidae|[953]|CNWLJ117-12|Cecidomyiidae|658|BOLD:ACD9468
Cecidomyiidae|[954]|NCCA1081-11|Cecidomyiidae|673|BOLD:ABA6449
Cecidomyiidae|[955]|CNTMB964-14|Cecidomyiidae|638|BOLD:ACM1128
Cecidomyiidae|[956]|CNKLF2930-15|Cecidomyiidae|588|BOLD:ACS1327
Cecidomyiidae|[957]|CNPAG134-13|Cecidomyiidae|658|BOLD:ACF6355
Cecidomyiidae|[958]|NCCA560-11|Cecidomyiidae|673|BOLD:ABA6517
Cecidomyiidae|[959]|SSJAA1934-13|Cecidomyiidae|664|BOLD:ACG3442
Cecidomyiidae|[960]|CNKLF3750-15|Cecidomyiidae|588|BOLD:ACS1224
Cecidomyiidae|[961]|CNTGE1784-15|Cecidomyiidae|588|BOLD:ACT1862
Cecidomyiidae|[962]|CNGMJ236-14|Cecidomyiidae|571|BOLD:ACM0446
Cecidomyiidae|[963]|CNYOG095-15|Cecidomyiidae|564|BOLD:ACT3737
Cecidomyiidae|[964]|HPPPE1210-13|Cecidomyiidae|664|BOLD:ACI8405
Cecidomyiidae|[965]|HPPPJ257-13|Cecidomyiidae|661|BOLD:ACI3210
Cecidomyiidae|[966]|SSGBC1599-14|Cecidomyiidae|582|BOLD:ACP5423
Cecidomyiidae|[967]|SSBAA006-12|Cecidomyiidae|661|BOLD:ACC3076
Cecidomyiidae|[968]|CNIVD234-14|Cecidomyiidae|558|BOLD:ACR4549
Cecidomyiidae|[969]|SSBAD1013-12|Cecidomyiidae|670|BOLD:ACC3104
Cecidomyiidae|[970]|CNJAF415-12|Cecidomyiidae|670|BOLD:ACC8129
Cecidomyiidae|[971]|CNWBB406-13|Cecidomyiidae|670|BOLD:ACA9563
Cecidomyiidae|[972]|CNBAI226-13|Cecidomyiidae|670|BOLD:ACD0317
Cecidomyiidae|[973]|CNWLL243-13|Cecidomyiidae|670|BOLD:AAP6842
Cecidomyiidae|[974]|CHDIP340-11|Cecidomyiidae|658|BOLD:AAL8099
Cecidomyiidae|[975]|CNBAD453-12|Cecidomyiidae|635|BOLD:ACR6412
Cecidomyiidae|[976]|CNNHE3060-14|Cecidomyiidae|594|BOLD:ACR7147
Cecidomyiidae|[977]|CNRME1338-12|Cecidomyiidae|658|BOLD:ACC3756
Cecidomyiidae|[978]|CNFNN255-14|Cecidomyiidae|658|BOLD:ACL9024
Cecidomyiidae|[979]|BBDCQ640-10|Cecidomyiidae|658|BOLD:AAV5761
Cecidomyiidae|[980]|CNNHF259-14|Cecidomyiidae|603|BOLD:ACR7061
Cecidomyiidae|[981]|CNYOB138-15|Cecidomyiidae|588|BOLD:ACT4350
Cecidomyiidae|[982]|CNRMF3354-12|Cecidomyiidae|670|BOLD:ACC1264
Cecidomyiidae|[983]|CNWLI150-12|Cecidomyiidae|658|BOLD:ACD2999
Cecidomyiidae|[984]|SMTPJ5626-14|Cecidomyiidae|615|BOLD:ABV0504
Cecidomyiidae|[985]|CNEID1278-12|Cecidomyiidae|658|BOLD:ACB0691
Cecidomyiidae|[986]|NGNAJ203-13|Cecidomyiidae|661|BOLD:ACJ0674
Cecidomyiidae|[987]|CNWLM1233-13|Cecidomyiidae|661|BOLD:ACI5855
Cecidomyiidae|[988]|NCCA049-11|Cecidomyiidae|673|BOLD:ABA6427
Cecidomyiidae|[989]|CNEIG531-12|Cecidomyiidae|658|BOLD:AAH3763
Cecidomyiidae|[990]|CHDIP734-12|Cecidomyiidae|658|BOLD:ABY0584
Cecidomyiidae|[991]|CNIVH417-14|Cecidomyiidae|567|BOLD:ACR5849
Cecidomyiidae|[992]|CNWLE813-12|Cecidomyiidae|673|BOLD:ACC6211
Cecidomyiidae|[993]|CNWLD654-12|Cecidomyiidae|633|BOLD:ACC5672
Cecidomyiidae|[994]|CNJAC823-12|Cecidomyiidae|673|BOLD:ACC7440
Cecidomyiidae|[995]|NGNAB1527-13|Cecidomyiidae|661|BOLD:ACI2858
Cecidomyiidae|[996]|SSWLA2656-13|Cecidomyiidae|658|BOLD:ACF7334
Cecidomyiidae|[997]|SSWLA3100-13|Cecidomyiidae|658|BOLD:ACF6745
Cecidomyiidae|[998]|CHDIP1281-12|Cecidomyiidae|658|BOLD:ACB2370
Cecidomyiidae|[999]|SSBAA572-12|Cecidomyiidae|673|BOLD:ACT3900
Cecidomyiidae|[1000]|NGNAJ216-13|Cecidomyiidae|661|BOLD:AAL7434

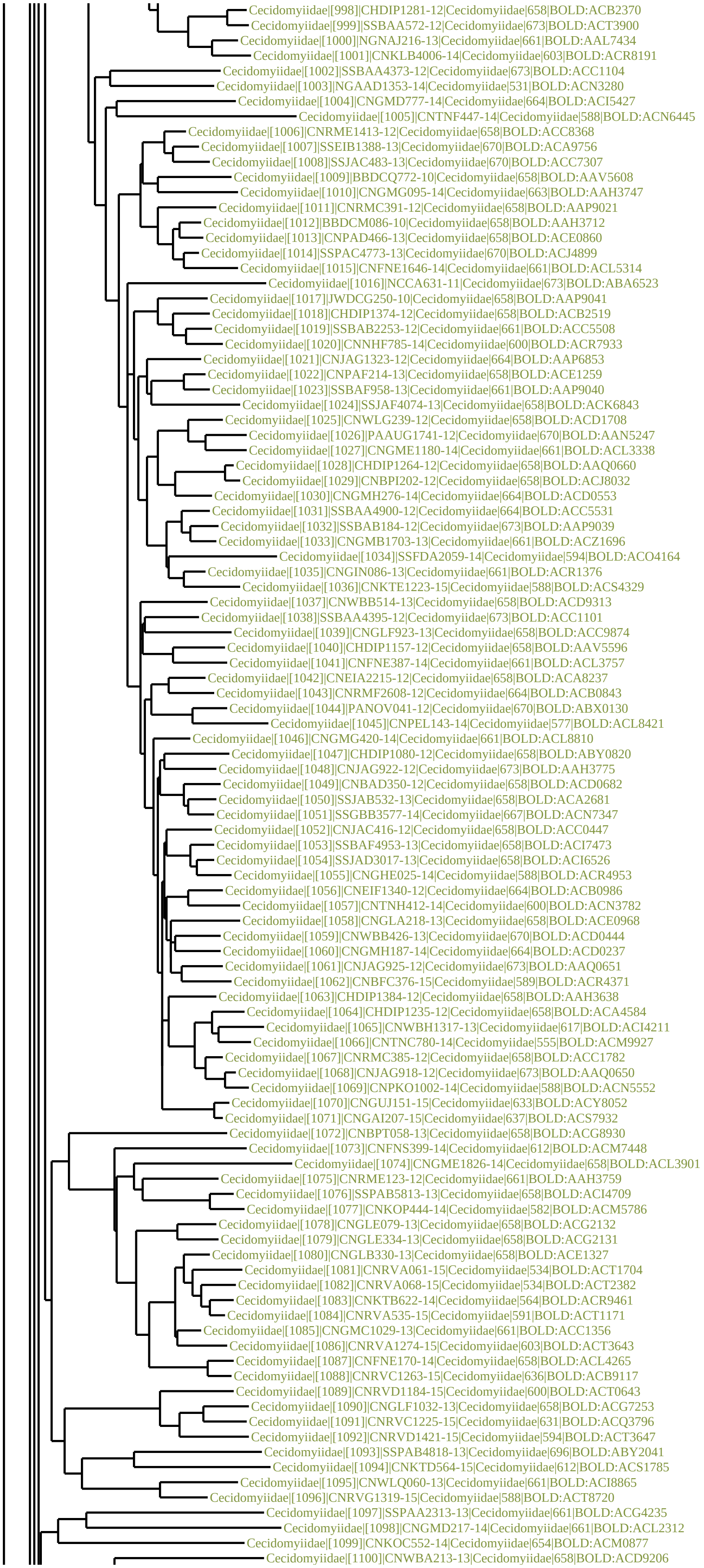
Cecidomyiidae|[998]|CHDIP1281-12|Cecidomyiidae|658|BOLD:ACB2370
Cecidomyiidae|[999]|SSBAA572-12|Cecidomyiidae|673|BOLD:ACT3900
Cecidomyiidae|[1000]|NGNAJ216-13|Cecidomyiidae|661|BOLD:AAL7434
Cecidomyiidae|[1001]|CNKLB4006-14|Cecidomyiidae|603|BOLD:ACR8191
Cecidomyiidae|[1002]|SSBAA4373-12|Cecidomyiidae|673|BOLD:ACC1104
Cecidomyiidae|[1003]|NGAAD1353-14|Cecidomyiidae|531|BOLD:ACN3280
Cecidomyiidae|[1004]|CNGMD777-14|Cecidomyiidae|664|BOLD:ACI5427
Cecidomyiidae|[1005]|CNTNF447-14|Cecidomyiidae|588|BOLD:ACN6445
Cecidomyiidae|[1006]|CNRME1413-12|Cecidomyiidae|658|BOLD:ACC8368
Cecidomyiidae|[1007]|SSEIB1388-13|Cecidomyiidae|670|BOLD:ACA9756
Cecidomyiidae|[1008]|SSJAC483-13|Cecidomyiidae|670|BOLD:ACC7307
Cecidomyiidae|[1009]|BBDCQ772-10|Cecidomyiidae|658|BOLD:AAV5608
Cecidomyiidae|[1010]|CNGMG095-14|Cecidomyiidae|663|BOLD:AAH3747
Cecidomyiidae|[1011]|CNRMC391-12|Cecidomyiidae|658|BOLD:AAP9021
Cecidomyiidae|[1012]|BBDCM086-10|Cecidomyiidae|658|BOLD:AAH3712
Cecidomyiidae|[1013]|CNPAD466-13|Cecidomyiidae|658|BOLD:ACE0860
Cecidomyiidae|[1014]|SSPAC4773-13|Cecidomyiidae|670|BOLD:ACJ4899
Cecidomyiidae|[1015]|CNFNE1646-14|Cecidomyiidae|661|BOLD:ACL5314
Cecidomyiidae|[1016]|NCCA631-11|Cecidomyiidae|673|BOLD:ABA6523
Cecidomyiidae|[1017]|JWDCG250-10|Cecidomyiidae|658|BOLD:AAP9041
Cecidomyiidae|[1018]|CHDIP1374-12|Cecidomyiidae|658|BOLD:ACB2519
Cecidomyiidae|[1019]|SSBAB2253-12|Cecidomyiidae|661|BOLD:ACC5508
Cecidomyiidae|[1020]|CNNHF785-14|Cecidomyiidae|600|BOLD:ACR7933
Cecidomyiidae|[1021]|CNJAG1323-12|Cecidomyiidae|664|BOLD:AAP6853
Cecidomyiidae|[1022]|CNPAF214-13|Cecidomyiidae|658|BOLD:ACE1259
Cecidomyiidae|[1023]|SSBAF958-13|Cecidomyiidae|661|BOLD:AAP9040
Cecidomyiidae|[1024]|SSJAF4074-13|Cecidomyiidae|658|BOLD:ACK6843
Cecidomyiidae|[1025]|CNWLG239-12|Cecidomyiidae|658|BOLD:ACD1708
Cecidomyiidae|[1026]|PAAUG1741-12|Cecidomyiidae|670|BOLD:AAN5247
Cecidomyiidae|[1027]|CNGME1180-14|Cecidomyiidae|661|BOLD:ACL3338
Cecidomyiidae|[1028]|CHDIP1264-12|Cecidomyiidae|658|BOLD:AAQ0660
Cecidomyiidae|[1029]|CNBPI202-12|Cecidomyiidae|658|BOLD:ACJ8032
Cecidomyiidae|[1030]|CNGMH276-14|Cecidomyiidae|664|BOLD:ACD0553
Cecidomyiidae|[1031]|SSBAA4900-12|Cecidomyiidae|664|BOLD:ACC5531
Cecidomyiidae|[1032]|SSBAB184-12|Cecidomyiidae|673|BOLD:AAP9039
Cecidomyiidae|[1033]|CNGMB1703-13|Cecidomyiidae|661|BOLD:ACZ1696
Cecidomyiidae|[1034]|SSFDA2059-14|Cecidomyiidae|594|BOLD:ACO4164
Cecidomyiidae|[1035]|CNGIN086-13|Cecidomyiidae|661|BOLD:ACR1376
Cecidomyiidae|[1036]|CNKTE1223-15|Cecidomyiidae|588|BOLD:ACS4329
Cecidomyiidae|[1037]|CNWBB514-13|Cecidomyiidae|658|BOLD:ACD9313
Cecidomyiidae|[1038]|SSBAA4395-12|Cecidomyiidae|673|BOLD:ACC1101
Cecidomyiidae|[1039]|CNGLF923-13|Cecidomyiidae|658|BOLD:ACC9874
Cecidomyiidae|[1040]|CHDIP1157-12|Cecidomyiidae|658|BOLD:AAV5596
Cecidomyiidae|[1041]|CNFNE387-14|Cecidomyiidae|661|BOLD:ACL3757
Cecidomyiidae|[1042]|CNEIA2215-12|Cecidomyiidae|658|BOLD:ACA8237
Cecidomyiidae|[1043]|CNRMF2608-12|Cecidomyiidae|664|BOLD:ACB0843
Cecidomyiidae|[1044]|PANOV041-12|Cecidomyiidae|670|BOLD:ABX0130
Cecidomyiidae|[1045]|CNPEL143-14|Cecidomyiidae|577|BOLD:ACL8421
Cecidomyiidae|[1046]|CNGMG420-14|Cecidomyiidae|661|BOLD:ACL8810
Cecidomyiidae|[1047]|CHDIP1080-12|Cecidomyiidae|658|BOLD:ABY0820
Cecidomyiidae|[1048]|CNJAG922-12|Cecidomyiidae|673|BOLD:AAH3775
Cecidomyiidae|[1049]|CNBAD350-12|Cecidomyiidae|658|BOLD:ACD0682
Cecidomyiidae|[1050]|SSJAB532-13|Cecidomyiidae|658|BOLD:ACA2681
Cecidomyiidae|[1051]|SSGBB3577-14|Cecidomyiidae|667|BOLD:ACN7347
Cecidomyiidae|[1052]|CNJAC416-12|Cecidomyiidae|658|BOLD:ACC0447
Cecidomyiidae|[1053]|SSBAF4953-13|Cecidomyiidae|658|BOLD:ACI7473
Cecidomyiidae|[1054]|SSJAD3017-13|Cecidomyiidae|658|BOLD:ACI6526
Cecidomyiidae|[1055]|CNGHE025-14|Cecidomyiidae|588|BOLD:ACR4953
Cecidomyiidae|[1056]|CNEIF1340-12|Cecidomyiidae|664|BOLD:ACB0986
Cecidomyiidae|[1057]|CNTNH412-14|Cecidomyiidae|600|BOLD:ACN3782
Cecidomyiidae|[1058]|CNGLA218-13|Cecidomyiidae|658|BOLD:ACE0968
Cecidomyiidae|[1059]|CNWBB426-13|Cecidomyiidae|670|BOLD:ACD0444
Cecidomyiidae|[1060]|CNGMH187-14|Cecidomyiidae|664|BOLD:ACD0237
Cecidomyiidae|[1061]|CNJAG925-12|Cecidomyiidae|673|BOLD:AAQ0651
Cecidomyiidae|[1062]|CNBFC376-15|Cecidomyiidae|589|BOLD:ACR4371
Cecidomyiidae|[1063]|CHDIP1384-12|Cecidomyiidae|658|BOLD:AAH3638
Cecidomyiidae|[1064]|CHDIP1235-12|Cecidomyiidae|658|BOLD:ACA4584
Cecidomyiidae|[1065]|CNWBH1317-13|Cecidomyiidae|617|BOLD:ACI4211
Cecidomyiidae|[1066]|CNTNC780-14|Cecidomyiidae|555|BOLD:ACM9927
Cecidomyiidae|[1067]|CNRMC385-12|Cecidomyiidae|658|BOLD:ACC1782
Cecidomyiidae|[1068]|CNJAG918-12|Cecidomyiidae|673|BOLD:AAQ0650
Cecidomyiidae|[1069]|CNPKO1002-14|Cecidomyiidae|588|BOLD:ACN5552
Cecidomyiidae|[1070]|CNGUJ151-15|Cecidomyiidae|633|BOLD:ACY8052
Cecidomyiidae|[1071]|CNGAI207-15|Cecidomyiidae|637|BOLD:ACS7932
Cecidomyiidae|[1072]|CNBPT058-13|Cecidomyiidae|658|BOLD:ACG8930
Cecidomyiidae|[1073]|CNFNS399-14|Cecidomyiidae|612|BOLD:ACM7448
Cecidomyiidae|[1074]|CNGME1826-14|Cecidomyiidae|658|BOLD:ACL3901
Cecidomyiidae|[1075]|CNRME123-12|Cecidomyiidae|661|BOLD:AAH3759
Cecidomyiidae|[1076]|SSPAB5813-13|Cecidomyiidae|658|BOLD:ACI4709
Cecidomyiidae|[1077]|CNKOP444-14|Cecidomyiidae|582|BOLD:ACM5786
Cecidomyiidae|[1078]|CNGLE079-13|Cecidomyiidae|658|BOLD:ACG2132
Cecidomyiidae|[1079]|CNGLE334-13|Cecidomyiidae|658|BOLD:ACG2131
Cecidomyiidae|[1080]|CNGLB330-13|Cecidomyiidae|658|BOLD:ACE1327
Cecidomyiidae|[1081]|CNRVA061-15|Cecidomyiidae|534|BOLD:ACT1704
Cecidomyiidae|[1082]|CNRVA068-15|Cecidomyiidae|534|BOLD:ACT2382
Cecidomyiidae|[1083]|CNKTB622-14|Cecidomyiidae|564|BOLD:ACR9461
Cecidomyiidae|[1084]|CNRVA535-15|Cecidomyiidae|591|BOLD:ACT1171
Cecidomyiidae|[1085]|CNGMC1029-13|Cecidomyiidae|661|BOLD:ACC1356
Cecidomyiidae|[1086]|CNRVA1274-15|Cecidomyiidae|603|BOLD:ACT3643
Cecidomyiidae|[1087]|CNFNE170-14|Cecidomyiidae|658|BOLD:ACL4265
Cecidomyiidae|[1088]|CNRVC1263-15|Cecidomyiidae|636|BOLD:ACB9117
Cecidomyiidae|[1089]|CNRVD1184-15|Cecidomyiidae|600|BOLD:ACT0643
Cecidomyiidae|[1090]|CNGLF1032-13|Cecidomyiidae|658|BOLD:ACG7253
Cecidomyiidae|[1091]|CNRVC1225-15|Cecidomyiidae|631|BOLD:ACQ3796
Cecidomyiidae|[1092]|CNRVD1421-15|Cecidomyiidae|594|BOLD:ACT3647
Cecidomyiidae|[1093]|SSPAB4818-13|Cecidomyiidae|696|BOLD:ABY2041
Cecidomyiidae|[1094]|CNKTD564-15|Cecidomyiidae|612|BOLD:ACS1785
Cecidomyiidae|[1095]|CNWLQ060-13|Cecidomyiidae|661|BOLD:ACI8865
Cecidomyiidae|[1096]|CNRVG1319-15|Cecidomyiidae|588|BOLD:ACT8720
Cecidomyiidae|[1097]|SSPAA2313-13|Cecidomyiidae|661|BOLD:ACG4235
Cecidomyiidae|[1098]|CNGMD217-14|Cecidomyiidae|661|BOLD:ACL2312
Cecidomyiidae|[1099]|CNKOC552-14|Cecidomyiidae|654|BOLD:ACM0877
Cecidomyiidae|[1100]|CNWBA213-13|Cecidomyiidae|658|BOLD:ACD9206

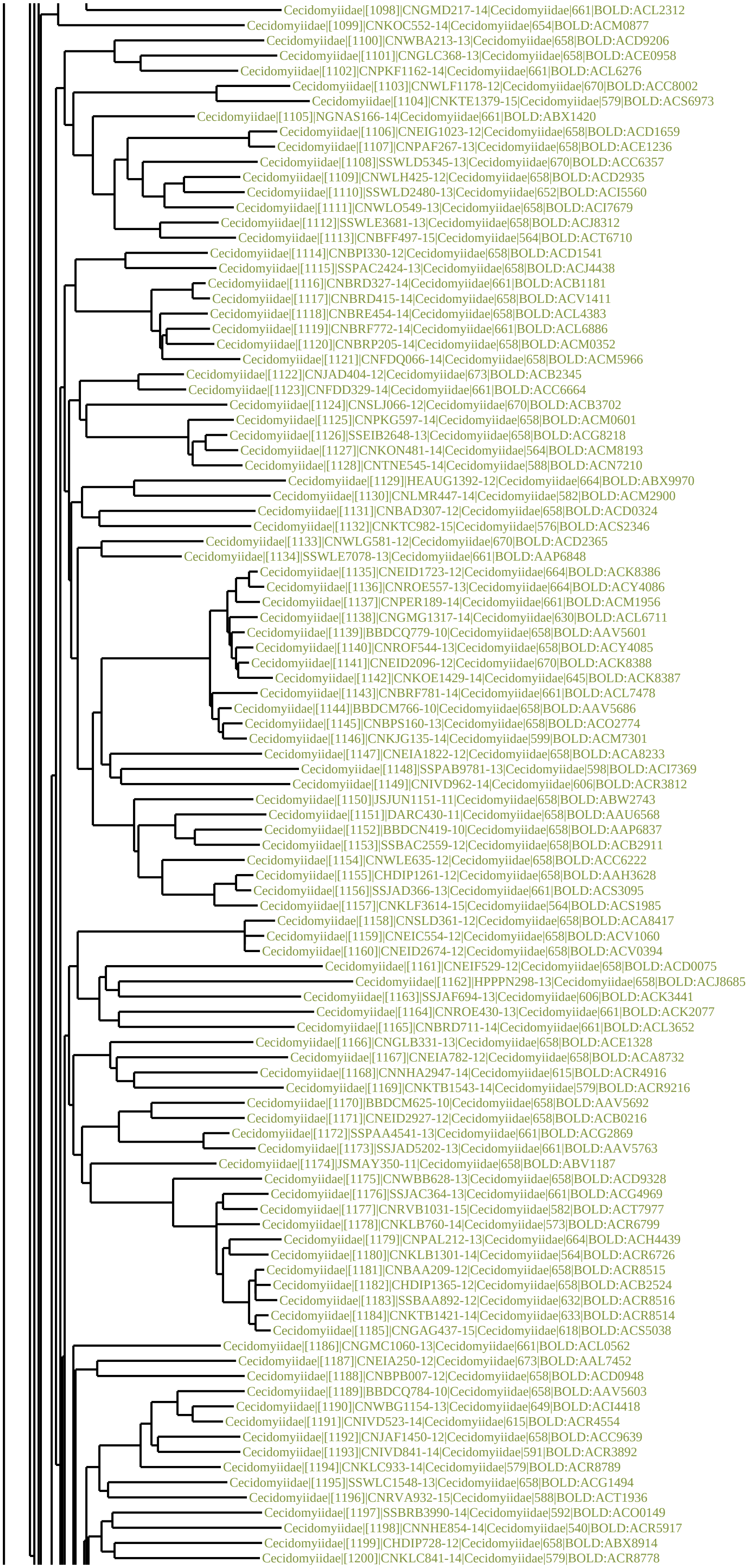
Cecidomyiidae|[1098]|CNGMD217-14|Cecidomyiidae|661|BOLD:ACL2312
Cecidomyiidae|[1099]|CNKOC552-14|Cecidomyiidae|654|BOLD:ACM0877
Cecidomyiidae|[1100]|CNWBA213-13|Cecidomyiidae|658|BOLD:ACD9206
Cecidomyiidae|[1101]|CNGLC368-13|Cecidomyiidae|658|BOLD:ACE0958
Cecidomyiidae|[1102]|CNPKF1162-14|Cecidomyiidae|661|BOLD:ACL6276
Cecidomyiidae|[1103]|CNWLF1178-12|Cecidomyiidae|670|BOLD:ACC8002
Cecidomyiidae|[1104]|CNKTE1379-15|Cecidomyiidae|579|BOLD:ACS6973
Cecidomyiidae|[1105]|NGNAS166-14|Cecidomyiidae|661|BOLD:ABX1420
Cecidomyiidae|[1106]|CNEIG1023-12|Cecidomyiidae|658|BOLD:ACD1659
Cecidomyiidae|[1107]|CNPAF267-13|Cecidomyiidae|658|BOLD:ACE1236
Cecidomyiidae|[1108]|SSWLD5345-13|Cecidomyiidae|670|BOLD:ACC6357
Cecidomyiidae|[1109]|CNWLH425-12|Cecidomyiidae|658|BOLD:ACD2935
Cecidomyiidae|[1110]|SSWLD2480-13|Cecidomyiidae|652|BOLD:ACI5560
Cecidomyiidae|[1111]|CNWLO549-13|Cecidomyiidae|658|BOLD:ACI7679
Cecidomyiidae|[1112]|SSWLE3681-13|Cecidomyiidae|658|BOLD:ACJ8312
Cecidomyiidae|[1113]|CNBFF497-15|Cecidomyiidae|564|BOLD:ACT6710
Cecidomyiidae|[1114]|CNBPI330-12|Cecidomyiidae|658|BOLD:ACD1541
Cecidomyiidae|[1115]|SSPAC2424-13|Cecidomyiidae|658|BOLD:ACJ4438
Cecidomyiidae|[1116]|CNBRD327-14|Cecidomyiidae|661|BOLD:ACB1181
Cecidomyiidae|[1117]|CNBRD415-14|Cecidomyiidae|658|BOLD:ACV1411
Cecidomyiidae|[1118]|CNBRE454-14|Cecidomyiidae|658|BOLD:ACL4383
Cecidomyiidae|[1119]|CNBRF772-14|Cecidomyiidae|661|BOLD:ACL6886
Cecidomyiidae|[1120]|CNBRP205-14|Cecidomyiidae|658|BOLD:ACM0352
Cecidomyiidae|[1121]|CNFDQ066-14|Cecidomyiidae|658|BOLD:ACM5966
Cecidomyiidae|[1122]|CNJAD404-12|Cecidomyiidae|673|BOLD:ACB2345
Cecidomyiidae|[1123]|CNFDD329-14|Cecidomyiidae|661|BOLD:ACC6664
Cecidomyiidae|[1124]|CNSLJ066-12|Cecidomyiidae|670|BOLD:ACB3702
Cecidomyiidae|[1125]|CNPKG597-14|Cecidomyiidae|658|BOLD:ACM0601
Cecidomyiidae|[1126]|SSEIB2648-13|Cecidomyiidae|658|BOLD:ACG8218
Cecidomyiidae|[1127]|CNKON481-14|Cecidomyiidae|564|BOLD:ACM8193
Cecidomyiidae|[1128]|CNTNE545-14|Cecidomyiidae|588|BOLD:ACN7210
Cecidomyiidae|[1129]|HEAUG1392-12|Cecidomyiidae|664|BOLD:ABX9970
Cecidomyiidae|[1130]|CNLMR447-14|Cecidomyiidae|582|BOLD:ACM2900
Cecidomyiidae|[1131]|CNBAD307-12|Cecidomyiidae|658|BOLD:ACD0324
Cecidomyiidae|[1132]|CNKTC982-15|Cecidomyiidae|576|BOLD:ACS2346
Cecidomyiidae|[1133]|CNWLG581-12|Cecidomyiidae|670|BOLD:ACD2365
Cecidomyiidae|[1134]|SSWLE7078-13|Cecidomyiidae|661|BOLD:AAP6848
Cecidomyiidae|[1135]|CNEID1723-12|Cecidomyiidae|664|BOLD:ACK8386
Cecidomyiidae|[1136]|CNROE557-13|Cecidomyiidae|664|BOLD:ACY4086
Cecidomyiidae|[1137]|CNPER189-14|Cecidomyiidae|661|BOLD:ACM1956
Cecidomyiidae|[1138]|CNGMG1317-14|Cecidomyiidae|630|BOLD:ACL6711
Cecidomyiidae|[1139]|BBDCQ779-10|Cecidomyiidae|658|BOLD:AAV5601
Cecidomyiidae|[1140]|CNROF544-13|Cecidomyiidae|658|BOLD:ACY4085
Cecidomyiidae|[1141]|CNEID2096-12|Cecidomyiidae|670|BOLD:ACK8388
Cecidomyiidae|[1142]|CNKOE1429-14|Cecidomyiidae|645|BOLD:ACK8387
Cecidomyiidae|[1143]|CNBRF781-14|Cecidomyiidae|661|BOLD:ACL7478
Cecidomyiidae|[1144]|BBDCM766-10|Cecidomyiidae|658|BOLD:AAV5686
Cecidomyiidae|[1145]|CNBPS160-13|Cecidomyiidae|658|BOLD:ACO2774
Cecidomyiidae|[1146]|CNKJG135-14|Cecidomyiidae|599|BOLD:ACM7301
Cecidomyiidae|[1147]|CNEIA1822-12|Cecidomyiidae|658|BOLD:ACA8233
Cecidomyiidae|[1148]|SSPAB9781-13|Cecidomyiidae|598|BOLD:ACI7369
Cecidomyiidae|[1149]|CNIVD962-14|Cecidomyiidae|606|BOLD:ACR3812
Cecidomyiidae|[1150]|JSJUN1151-11|Cecidomyiidae|658|BOLD:ABW2743
Cecidomyiidae|[1151]|DARC430-11|Cecidomyiidae|658|BOLD:AAU6568
Cecidomyiidae|[1152]|BBDCN419-10|Cecidomyiidae|658|BOLD:AAP6837
Cecidomyiidae|[1153]|SSBAC2559-12|Cecidomyiidae|658|BOLD:ACB2911
Cecidomyiidae|[1154]|CNWLE635-12|Cecidomyiidae|658|BOLD:ACC6222
Cecidomyiidae|[1155]|CHDIP1261-12|Cecidomyiidae|658|BOLD:AAH3628
Cecidomyiidae|[1156]|SSJAD366-13|Cecidomyiidae|661|BOLD:ACS3095
Cecidomyiidae|[1157]|CNKLF3614-15|Cecidomyiidae|564|BOLD:ACS1985
Cecidomyiidae|[1158]|CNSLD361-12|Cecidomyiidae|658|BOLD:ACA8417
Cecidomyiidae|[1159]|CNEIC554-12|Cecidomyiidae|658|BOLD:ACV1060
Cecidomyiidae|[1160]|CNEID2674-12|Cecidomyiidae|658|BOLD:ACV0394
Cecidomyiidae|[1161]|CNEIF529-12|Cecidomyiidae|658|BOLD:ACD0075
Cecidomyiidae|[1162]|HPPPN298-13|Cecidomyiidae|658|BOLD:ACJ8685
Cecidomyiidae|[1163]|SSJAF694-13|Cecidomyiidae|606|BOLD:ACK3441
Cecidomyiidae|[1164]|CNROE430-13|Cecidomyiidae|661|BOLD:ACK2077
Cecidomyiidae|[1165]|CNBRD711-14|Cecidomyiidae|661|BOLD:ACL3652
Cecidomyiidae|[1166]|CNGLB331-13|Cecidomyiidae|658|BOLD:ACE1328
Cecidomyiidae|[1167]|CNEIA782-12|Cecidomyiidae|658|BOLD:ACA8732
Cecidomyiidae|[1168]|CNNHA2947-14|Cecidomyiidae|615|BOLD:ACR4916
Cecidomyiidae|[1169]|CNKTB1543-14|Cecidomyiidae|579|BOLD:ACR9216
Cecidomyiidae|[1170]|BBDCM625-10|Cecidomyiidae|658|BOLD:AAV5692
Cecidomyiidae|[1171]|CNEID2927-12|Cecidomyiidae|658|BOLD:ACB0216
Cecidomyiidae|[1172]|SSPAA4541-13|Cecidomyiidae|661|BOLD:ACG2869
Cecidomyiidae|[1173]|SSJAD5202-13|Cecidomyiidae|661|BOLD:AAV5763
Cecidomyiidae|[1174]|JSMAY350-11|Cecidomyiidae|658|BOLD:ABV1187
Cecidomyiidae|[1175]|CNWBB628-13|Cecidomyiidae|658|BOLD:ACD9328
Cecidomyiidae|[1176]|SSJAC364-13|Cecidomyiidae|661|BOLD:ACG4969
Cecidomyiidae|[1177]|CNRVB1031-15|Cecidomyiidae|582|BOLD:ACT7977
Cecidomyiidae|[1178]|CNKLB760-14|Cecidomyiidae|573|BOLD:ACR6799
Cecidomyiidae|[1179]|CNPAL212-13|Cecidomyiidae|664|BOLD:ACH4439
Cecidomyiidae|[1180]|CNKLB1301-14|Cecidomyiidae|564|BOLD:ACR6726
Cecidomyiidae|[1181]|CNBAA209-12|Cecidomyiidae|658|BOLD:ACR8515
Cecidomyiidae|[1182]|CHDIP1365-12|Cecidomyiidae|658|BOLD:ACB2524
Cecidomyiidae|[1183]|SSBAA892-12|Cecidomyiidae|632|BOLD:ACR8516
Cecidomyiidae|[1184]|CNKTB1421-14|Cecidomyiidae|633|BOLD:ACR8514
Cecidomyiidae|[1185]|CNGAG437-15|Cecidomyiidae|618|BOLD:ACS5038
Cecidomyiidae|[1186]|CNGMC1060-13|Cecidomyiidae|661|BOLD:ACL0562
Cecidomyiidae|[1187]|CNEIA250-12|Cecidomyiidae|673|BOLD:AAL7452
Cecidomyiidae|[1188]|CNBPB007-12|Cecidomyiidae|658|BOLD:ACD0948
Cecidomyiidae|[1189]|BBDCQ784-10|Cecidomyiidae|658|BOLD:AAV5603
Cecidomyiidae|[1190]|CNWBG1154-13|Cecidomyiidae|649|BOLD:ACI4418
Cecidomyiidae|[1191]|CNIVD523-14|Cecidomyiidae|615|BOLD:ACR4554
Cecidomyiidae|[1192]|CNJAF1450-12|Cecidomyiidae|658|BOLD:ACC9639
Cecidomyiidae|[1193]|CNIVD841-14|Cecidomyiidae|591|BOLD:ACR3892
Cecidomyiidae|[1194]|CNKLC933-14|Cecidomyiidae|579|BOLD:ACR8789
Cecidomyiidae|[1195]|SSWLC1548-13|Cecidomyiidae|658|BOLD:ACG1494
Cecidomyiidae|[1196]|CNRVA932-15|Cecidomyiidae|588|BOLD:ACT1936
Cecidomyiidae|[1197]|SSBRB3990-14|Cecidomyiidae|592|BOLD:ACO0149
Cecidomyiidae|[1198]|CNNHE854-14|Cecidomyiidae|540|BOLD:ACR5917
Cecidomyiidae|[1199]|CHDIP728-12|Cecidomyiidae|658|BOLD:ABX8914
Cecidomyiidae|[1200]|CNKLC841-14|Cecidomyiidae|579|BOLD:ACR8778

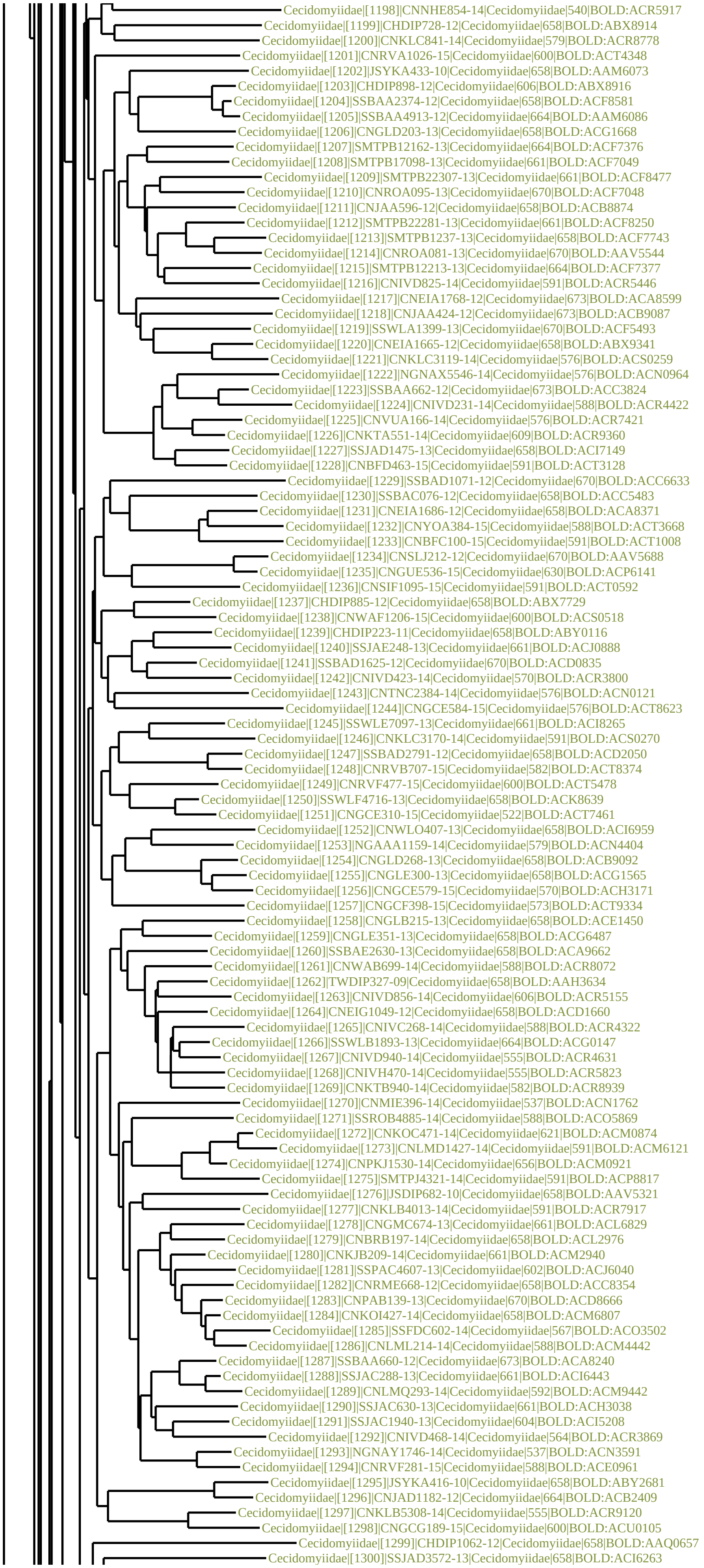
Cecidomyiidae|[1198]|CNNHE854-14|Cecidomyiidae|540|BOLD:ACR5917
Cecidomyiidae|[1199]|CHDIP728-12|Cecidomyiidae|658|BOLD:ABX8914
Cecidomyiidae|[1200]|CNKLC841-14|Cecidomyiidae|579|BOLD:ACR8778
Cecidomyiidae|[1201]|CNRVA1026-15|Cecidomyiidae|600|BOLD:ACT4348
Cecidomyiidae|[1202]|JSYKA433-10|Cecidomyiidae|658|BOLD:AAM6073
Cecidomyiidae|[1203]|CHDIP898-12|Cecidomyiidae|606|BOLD:ABX8916
Cecidomyiidae|[1204]|SSBAA2374-12|Cecidomyiidae|658|BOLD:ACF8581
Cecidomyiidae|[1205]|SSBAA4913-12|Cecidomyiidae|664|BOLD:AAM6086
Cecidomyiidae|[1206]|CNGLD203-13|Cecidomyiidae|658|BOLD:ACG1668
Cecidomyiidae|[1207]|SMTPB12162-13|Cecidomyiidae|664|BOLD:ACF7376
Cecidomyiidae|[1208]|SMTPB17098-13|Cecidomyiidae|661|BOLD:ACF7049
Cecidomyiidae|[1209]|SMTPB22307-13|Cecidomyiidae|661|BOLD:ACF8477
Cecidomyiidae|[1210]|CNROA095-13|Cecidomyiidae|670|BOLD:ACF7048
Cecidomyiidae|[1211]|CNJAA596-12|Cecidomyiidae|658|BOLD:ACB8874
Cecidomyiidae|[1212]|SMTPB22281-13|Cecidomyiidae|661|BOLD:ACF8250
Cecidomyiidae|[1213]|SMTPB1237-13|Cecidomyiidae|658|BOLD:ACF7743
Cecidomyiidae|[1214]|CNROA081-13|Cecidomyiidae|670|BOLD:AAV5544
Cecidomyiidae|[1215]|SMTPB12213-13|Cecidomyiidae|664|BOLD:ACF7377
Cecidomyiidae|[1216]|CNIVD825-14|Cecidomyiidae|591|BOLD:ACR5446
Cecidomyiidae|[1217]|CNEIA1768-12|Cecidomyiidae|673|BOLD:ACA8599
Cecidomyiidae|[1218]|CNJAA424-12|Cecidomyiidae|673|BOLD:ACB9087
Cecidomyiidae|[1219]|SSWLA1399-13|Cecidomyiidae|670|BOLD:ACF5493
Cecidomyiidae|[1220]|CNEIA1665-12|Cecidomyiidae|658|BOLD:ABX9341
Cecidomyiidae|[1221]|CNKLC3119-14|Cecidomyiidae|576|BOLD:ACS0259
Cecidomyiidae|[1222]|NGNAX5546-14|Cecidomyiidae|576|BOLD:ACN0964
Cecidomyiidae|[1223]|SSBAA662-12|Cecidomyiidae|673|BOLD:ACC3824
Cecidomyiidae|[1224]|CNIVD231-14|Cecidomyiidae|588|BOLD:ACR4422
Cecidomyiidae|[1225]|CNVUA166-14|Cecidomyiidae|576|BOLD:ACR7421
Cecidomyiidae|[1226]|CNKTA551-14|Cecidomyiidae|609|BOLD:ACR9360
Cecidomyiidae|[1227]|SSJAD1475-13|Cecidomyiidae|658|BOLD:ACI7149
Cecidomyiidae|[1228]|CNBFD463-15|Cecidomyiidae|591|BOLD:ACT3128
Cecidomyiidae|[1229]|SSBAD1071-12|Cecidomyiidae|670|BOLD:ACC6633
Cecidomyiidae|[1230]|SSBAC076-12|Cecidomyiidae|658|BOLD:ACC5483
Cecidomyiidae|[1231]|CNEIA1686-12|Cecidomyiidae|658|BOLD:ACA8371
Cecidomyiidae|[1232]|CNYOA384-15|Cecidomyiidae|588|BOLD:ACT3668
Cecidomyiidae|[1233]|CNBFC100-15|Cecidomyiidae|591|BOLD:ACT1008
Cecidomyiidae|[1234]|CNSLJ212-12|Cecidomyiidae|670|BOLD:AAV5688
Cecidomyiidae|[1235]|CNGUE536-15|Cecidomyiidae|630|BOLD:ACP6141
Cecidomyiidae|[1236]|CNSIF1095-15|Cecidomyiidae|591|BOLD:ACT0592
Cecidomyiidae|[1237]|CHDIP885-12|Cecidomyiidae|658|BOLD:ABX7729
Cecidomyiidae|[1238]|CNWAF1206-15|Cecidomyiidae|600|BOLD:ACS0518
Cecidomyiidae|[1239]|CHDIP223-11|Cecidomyiidae|658|BOLD:ABY0116
Cecidomyiidae|[1240]|SSJAE248-13|Cecidomyiidae|661|BOLD:ACJ0888
Cecidomyiidae|[1241]|SSBAD1625-12|Cecidomyiidae|670|BOLD:ACD0835
Cecidomyiidae|[1242]|CNIVD423-14|Cecidomyiidae|570|BOLD:ACR3800
Cecidomyiidae|[1243]|CNTNC2384-14|Cecidomyiidae|576|BOLD:ACN0121
Cecidomyiidae|[1244]|CNGCE584-15|Cecidomyiidae|576|BOLD:ACT8623
Cecidomyiidae|[1245]|SSWLE7097-13|Cecidomyiidae|661|BOLD:ACI8265
Cecidomyiidae|[1246]|CNKLC3170-14|Cecidomyiidae|591|BOLD:ACS0270
Cecidomyiidae|[1247]|SSBAD2791-12|Cecidomyiidae|658|BOLD:ACD2050
Cecidomyiidae|[1248]|CNRVB707-15|Cecidomyiidae|582|BOLD:ACT8374
Cecidomyiidae|[1249]|CNRVF477-15|Cecidomyiidae|600|BOLD:ACT5478
Cecidomyiidae|[1250]|SSWLF4716-13|Cecidomyiidae|658|BOLD:ACK8639
Cecidomyiidae|[1251]|CNGCE310-15|Cecidomyiidae|522|BOLD:ACT7461
Cecidomyiidae|[1252]|CNWLO407-13|Cecidomyiidae|658|BOLD:ACI6959
Cecidomyiidae|[1253]|NGAAA1159-14|Cecidomyiidae|579|BOLD:ACN4404
Cecidomyiidae|[1254]|CNGLD268-13|Cecidomyiidae|658|BOLD:ACB9092
Cecidomyiidae|[1255]|CNGLE300-13|Cecidomyiidae|658|BOLD:ACG1565
Cecidomyiidae|[1256]|CNGCE579-15|Cecidomyiidae|570|BOLD:ACH3171
Cecidomyiidae|[1257]|CNGCF398-15|Cecidomyiidae|573|BOLD:ACT9334
Cecidomyiidae|[1258]|CNGLB215-13|Cecidomyiidae|658|BOLD:ACE1450
Cecidomyiidae|[1259]|CNGLE351-13|Cecidomyiidae|658|BOLD:ACG6487
Cecidomyiidae|[1260]|SSBAE2630-13|Cecidomyiidae|658|BOLD:ACA9662
Cecidomyiidae|[1261]|CNWAB699-14|Cecidomyiidae|588|BOLD:ACR8072
Cecidomyiidae|[1262]|TWDIP327-09|Cecidomyiidae|658|BOLD:AAH3634
Cecidomyiidae|[1263]|CNIVD856-14|Cecidomyiidae|606|BOLD:ACR5155
Cecidomyiidae|[1264]|CNEIG1049-12|Cecidomyiidae|658|BOLD:ACD1660
Cecidomyiidae|[1265]|CNIVC268-14|Cecidomyiidae|588|BOLD:ACR4322
Cecidomyiidae|[1266]|SSWLB1893-13|Cecidomyiidae|664|BOLD:ACG0147
Cecidomyiidae|[1267]|CNIVD940-14|Cecidomyiidae|555|BOLD:ACR4631
Cecidomyiidae|[1268]|CNIVH470-14|Cecidomyiidae|555|BOLD:ACR5823
Cecidomyiidae|[1269]|CNKTB940-14|Cecidomyiidae|582|BOLD:ACR8939
Cecidomyiidae|[1270]|CNMIE396-14|Cecidomyiidae|537|BOLD:ACN1762
Cecidomyiidae|[1271]|SSROB4885-14|Cecidomyiidae|588|BOLD:ACO5869
Cecidomyiidae|[1272]|CNKOC471-14|Cecidomyiidae|621|BOLD:ACM0874
Cecidomyiidae|[1273]|CNLMD1427-14|Cecidomyiidae|591|BOLD:ACM6121
Cecidomyiidae|[1274]|CNPKJ1530-14|Cecidomyiidae|656|BOLD:ACM0921
Cecidomyiidae|[1275]|SMTPJ4321-14|Cecidomyiidae|591|BOLD:ACP8817
Cecidomyiidae|[1276]|JSDIP682-10|Cecidomyiidae|658|BOLD:AAV5321
Cecidomyiidae|[1277]|CNKLB4013-14|Cecidomyiidae|591|BOLD:ACR7917
Cecidomyiidae|[1278]|CNGMC674-13|Cecidomyiidae|661|BOLD:ACL6829
Cecidomyiidae|[1279]|CNBRB197-14|Cecidomyiidae|658|BOLD:ACL2976
Cecidomyiidae|[1280]|CNKJB209-14|Cecidomyiidae|661|BOLD:ACM2940
Cecidomyiidae|[1281]|SSPAC4607-13|Cecidomyiidae|602|BOLD:ACJ6040
Cecidomyiidae|[1282]|CNRME668-12|Cecidomyiidae|658|BOLD:ACC8354
Cecidomyiidae|[1283]|CNPAB139-13|Cecidomyiidae|670|BOLD:ACD8666
Cecidomyiidae|[1284]|CNKOI427-14|Cecidomyiidae|658|BOLD:ACM6807
Cecidomyiidae|[1285]|SSFDC602-14|Cecidomyiidae|567|BOLD:ACO3502
Cecidomyiidae|[1286]|CNLML214-14|Cecidomyiidae|588|BOLD:ACM4442
Cecidomyiidae|[1287]|SSBAA660-12|Cecidomyiidae|673|BOLD:ACA8240
Cecidomyiidae|[1288]|SSJAC288-13|Cecidomyiidae|661|BOLD:ACI6443
Cecidomyiidae|[1289]|CNLMQ293-14|Cecidomyiidae|592|BOLD:ACM9442
Cecidomyiidae|[1290]|SSJAC630-13|Cecidomyiidae|661|BOLD:ACH3038
Cecidomyiidae|[1291]|SSJAC1940-13|Cecidomyiidae|604|BOLD:ACI5208
Cecidomyiidae|[1292]|CNIVD468-14|Cecidomyiidae|564|BOLD:ACR3869
Cecidomyiidae|[1293]|NGNAY1746-14|Cecidomyiidae|537|BOLD:ACN3591
Cecidomyiidae|[1294]|CNRVF281-15|Cecidomyiidae|588|BOLD:ACE0961
Cecidomyiidae|[1295]|JSYKA416-10|Cecidomyiidae|658|BOLD:ABY2681
Cecidomyiidae|[1296]|CNJAD1182-12|Cecidomyiidae|664|BOLD:ACB2409
Cecidomyiidae|[1297]|CNKLB5308-14|Cecidomyiidae|555|BOLD:ACR9120
Cecidomyiidae|[1298]|CNGCG189-15|Cecidomyiidae|600|BOLD:ACU0105
Cecidomyiidae|[1299]|CHDIP1062-12|Cecidomyiidae|658|BOLD:AAQ0657
Cecidomyiidae|[1300]|SSJAD3572-13|Cecidomyiidae|658|BOLD:ACI6263

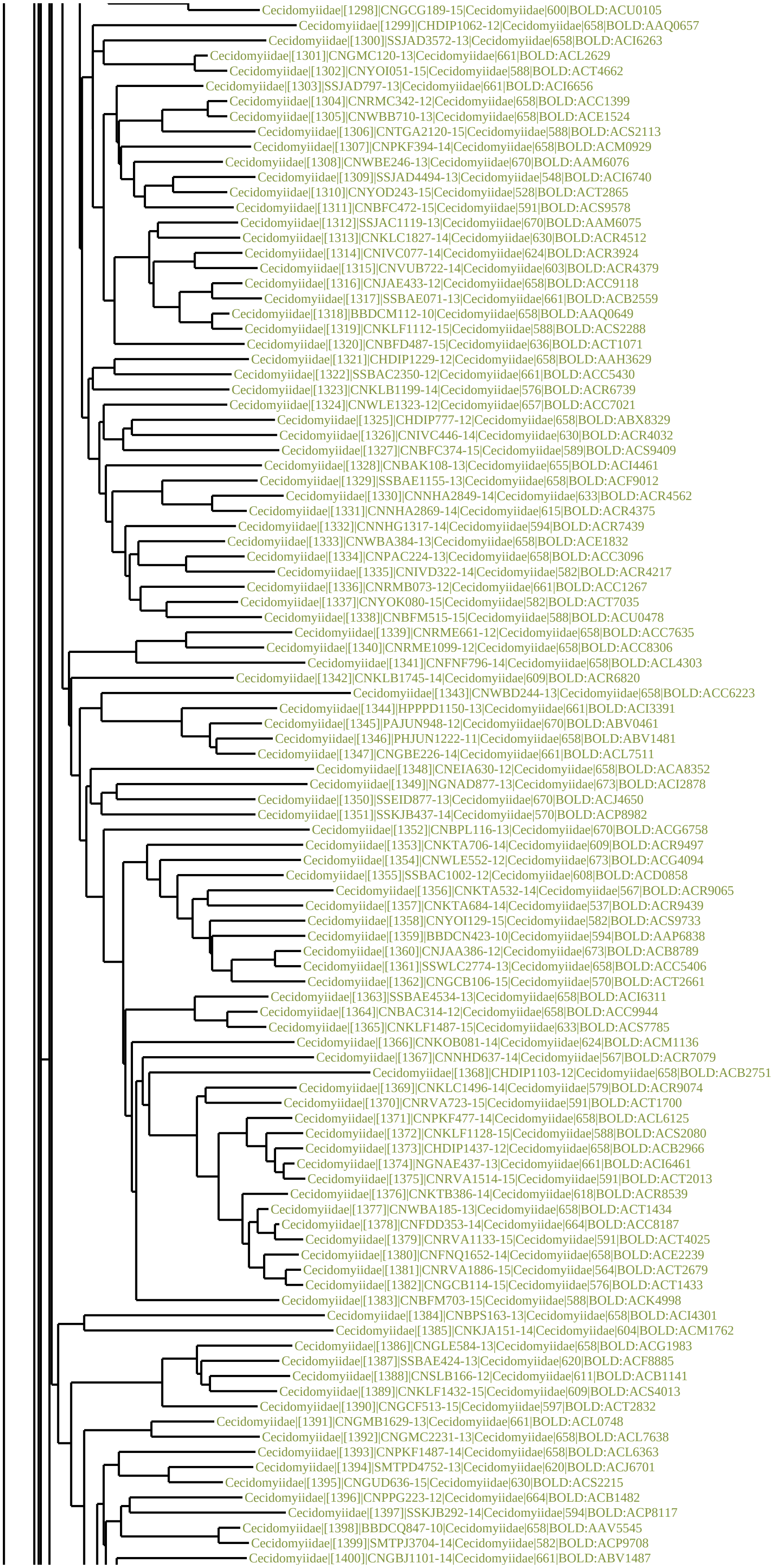

Cecidomyiidae|[1298]|CNGCG189-15|Cecidomyiidae|600|BOLD:ACU0105
Cecidomyiidae|[1299]|CHDIP1062-12|Cecidomyiidae|658|BOLD:AAQ0657
Cecidomyiidae|[1300]|SSJAD3572-13|Cecidomyiidae|658|BOLD:ACI6263
Cecidomyiidae|[1301]|CNGMC120-13|Cecidomyiidae|661|BOLD:ACL2629
Cecidomyiidae|[1302]|CNYOI051-15|Cecidomyiidae|588|BOLD:ACT4662
Cecidomyiidae|[1303]|SSJAD797-13|Cecidomyiidae|661|BOLD:ACI6656
Cecidomyiidae|[1304]|CNRMC342-12|Cecidomyiidae|658|BOLD:ACC1399
Cecidomyiidae|[1305]|CNWBB710-13|Cecidomyiidae|658|BOLD:ACE1524
Cecidomyiidae|[1306]|CNTGA2120-15|Cecidomyiidae|588|BOLD:ACS2113
Cecidomyiidae|[1307]|CNPKF394-14|Cecidomyiidae|658|BOLD:ACM0929
Cecidomyiidae|[1308]|CNWBE246-13|Cecidomyiidae|670|BOLD:AAM6076
Cecidomyiidae|[1309]|SSJAD4494-13|Cecidomyiidae|548|BOLD:ACI6740
Cecidomyiidae|[1310]|CNYOD243-15|Cecidomyiidae|528|BOLD:ACT2865
Cecidomyiidae|[1311]|CNBFC472-15|Cecidomyiidae|591|BOLD:ACS9578
Cecidomyiidae|[1312]|SSJAC1119-13|Cecidomyiidae|670|BOLD:AAM6075
Cecidomyiidae|[1313]|CNKLC1827-14|Cecidomyiidae|630|BOLD:ACR4512
Cecidomyiidae|[1314]|CNIVC077-14|Cecidomyiidae|624|BOLD:ACR3924
Cecidomyiidae|[1315]|CNVUB722-14|Cecidomyiidae|603|BOLD:ACR4379
Cecidomyiidae|[1316]|CNJAE433-12|Cecidomyiidae|658|BOLD:ACC9118
Cecidomyiidae|[1317]|SSBAE071-13|Cecidomyiidae|661|BOLD:ACB2559
Cecidomyiidae|[1318]|BBDCM112-10|Cecidomyiidae|658|BOLD:AAQ0649
Cecidomyiidae|[1319]|CNKLF1112-15|Cecidomyiidae|588|BOLD:ACS2288
Cecidomyiidae|[1320]|CNBFD487-15|Cecidomyiidae|636|BOLD:ACT1071
Cecidomyiidae|[1321]|CHDIP1229-12|Cecidomyiidae|658|BOLD:AAH3629
Cecidomyiidae|[1322]|SSBAC2350-12|Cecidomyiidae|661|BOLD:ACC5430
Cecidomyiidae|[1323]|CNKLB1199-14|Cecidomyiidae|576|BOLD:ACR6739
Cecidomyiidae|[1324]|CNWLE1323-12|Cecidomyiidae|657|BOLD:ACC7021
Cecidomyiidae|[1325]|CHDIP777-12|Cecidomyiidae|658|BOLD:ABX8329
Cecidomyiidae|[1326]|CNIVC446-14|Cecidomyiidae|630|BOLD:ACR4032
Cecidomyiidae|[1327]|CNBFC374-15|Cecidomyiidae|589|BOLD:ACS9409
Cecidomyiidae|[1328]|CNBAK108-13|Cecidomyiidae|655|BOLD:ACI4461
Cecidomyiidae|[1329]|SSBAE1155-13|Cecidomyiidae|658|BOLD:ACF9012
Cecidomyiidae|[1330]|CNNHA2849-14|Cecidomyiidae|633|BOLD:ACR4562
Cecidomyiidae|[1331]|CNNHA2869-14|Cecidomyiidae|615|BOLD:ACR4375
Cecidomyiidae|[1332]|CNNHG1317-14|Cecidomyiidae|594|BOLD:ACR7439
Cecidomyiidae|[1333]|CNWBA384-13|Cecidomyiidae|658|BOLD:ACE1832
Cecidomyiidae|[1334]|CNPAC224-13|Cecidomyiidae|658|BOLD:ACC3096
Cecidomyiidae|[1335]|CNIVD322-14|Cecidomyiidae|582|BOLD:ACR4217
Cecidomyiidae|[1336]|CNRMB073-12|Cecidomyiidae|661|BOLD:ACC1267
Cecidomyiidae|[1337]|CNYOK080-15|Cecidomyiidae|582|BOLD:ACT7035
Cecidomyiidae|[1338]|CNBFM515-15|Cecidomyiidae|588|BOLD:ACU0478
Cecidomyiidae|[1339]|CNRME661-12|Cecidomyiidae|658|BOLD:ACC7635
Cecidomyiidae|[1340]|CNRME1099-12|Cecidomyiidae|658|BOLD:ACC8306
Cecidomyiidae|[1341]|CNFNF796-14|Cecidomyiidae|658|BOLD:ACL4303
Cecidomyiidae|[1342]|CNKLB1745-14|Cecidomyiidae|609|BOLD:ACR6820
Cecidomyiidae|[1343]|CNWBD244-13|Cecidomyiidae|658|BOLD:ACC6223
Cecidomyiidae|[1344]|HPPPD1150-13|Cecidomyiidae|661|BOLD:ACI3391
Cecidomyiidae|[1345]|PAJUN948-12|Cecidomyiidae|670|BOLD:ABV0461
Cecidomyiidae|[1346]|PHJUN1222-11|Cecidomyiidae|658|BOLD:ABV1481
Cecidomyiidae|[1347]|CNGBE226-14|Cecidomyiidae|661|BOLD:ACL7511
Cecidomyiidae|[1348]|CNEIA630-12|Cecidomyiidae|658|BOLD:ACA8352
Cecidomyiidae|[1349]|NGNAD877-13|Cecidomyiidae|673|BOLD:ACI2878
Cecidomyiidae|[1350]|SSEID877-13|Cecidomyiidae|670|BOLD:ACJ4650
Cecidomyiidae|[1351]|SSKJB437-14|Cecidomyiidae|570|BOLD:ACP8982
Cecidomyiidae|[1352]|CNBPL116-13|Cecidomyiidae|670|BOLD:ACG6758
Cecidomyiidae|[1353]|CNKTA706-14|Cecidomyiidae|609|BOLD:ACR9497
Cecidomyiidae|[1354]|CNWLE552-12|Cecidomyiidae|673|BOLD:ACG4094
Cecidomyiidae|[1355]|SSBAC1002-12|Cecidomyiidae|608|BOLD:ACD0858
Cecidomyiidae|[1356]|CNKTA532-14|Cecidomyiidae|567|BOLD:ACR9065
Cecidomyiidae|[1357]|CNKTA684-14|Cecidomyiidae|537|BOLD:ACR9439
Cecidomyiidae|[1358]|CNYOI129-15|Cecidomyiidae|582|BOLD:ACS9733
Cecidomyiidae|[1359]|BBDCN423-10|Cecidomyiidae|594|BOLD:AAP6838
Cecidomyiidae|[1360]|CNJAA386-12|Cecidomyiidae|673|BOLD:ACB8789
Cecidomyiidae|[1361]|SSWLC2774-13|Cecidomyiidae|658|BOLD:ACC5406
Cecidomyiidae|[1362]|CNGCB106-15|Cecidomyiidae|570|BOLD:ACT2661
Cecidomyiidae|[1363]|SSBAE4534-13|Cecidomyiidae|658|BOLD:ACI6311
Cecidomyiidae|[1364]|CNBAC314-12|Cecidomyiidae|658|BOLD:ACC9944
Cecidomyiidae|[1365]|CNKLF1487-15|Cecidomyiidae|633|BOLD:ACS7785
Cecidomyiidae|[1366]|CNKOB081-14|Cecidomyiidae|624|BOLD:ACM1136
Cecidomyiidae|[1367]|CNNHD637-14|Cecidomyiidae|567|BOLD:ACR7079
Cecidomyiidae|[1368]|CHDIP1103-12|Cecidomyiidae|658|BOLD:ACB2751
Cecidomyiidae|[1369]|CNKLC1496-14|Cecidomyiidae|579|BOLD:ACR9074
Cecidomyiidae|[1370]|CNRVA723-15|Cecidomyiidae|591|BOLD:ACT1700
Cecidomyiidae|[1371]|CNPKF477-14|Cecidomyiidae|658|BOLD:ACL6125
Cecidomyiidae|[1372]|CNKLF1128-15|Cecidomyiidae|588|BOLD:ACS2080
Cecidomyiidae|[1373]|CHDIP1437-12|Cecidomyiidae|658|BOLD:ACB2966
Cecidomyiidae|[1374]|NGNAE437-13|Cecidomyiidae|661|BOLD:ACI6461
Cecidomyiidae|[1375]|CNRVA1514-15|Cecidomyiidae|591|BOLD:ACT2013
Cecidomyiidae|[1376]|CNKTB386-14|Cecidomyiidae|618|BOLD:ACR8539
Cecidomyiidae|[1377]|CNWBA185-13|Cecidomyiidae|658|BOLD:ACT1434
Cecidomyiidae|[1378]|CNFDD353-14|Cecidomyiidae|664|BOLD:ACC8187
Cecidomyiidae|[1379]|CNRVA1133-15|Cecidomyiidae|591|BOLD:ACT4025
Cecidomyiidae|[1380]|CNFNQ1652-14|Cecidomyiidae|658|BOLD:ACE2239
Cecidomyiidae|[1381]|CNRVA1886-15|Cecidomyiidae|564|BOLD:ACT2679
Cecidomyiidae|[1382]|CNGCB114-15|Cecidomyiidae|576|BOLD:ACT1433
Cecidomyiidae|[1383]|CNBFM703-15|Cecidomyiidae|588|BOLD:ACK4998
Cecidomyiidae|[1384]|CNBPS163-13|Cecidomyiidae|658|BOLD:ACI4301
Cecidomyiidae|[1385]|CNKJA151-14|Cecidomyiidae|604|BOLD:ACM1762
Cecidomyiidae|[1386]|CNGLE584-13|Cecidomyiidae|658|BOLD:ACG1983
Cecidomyiidae|[1387]|SSBAE424-13|Cecidomyiidae|620|BOLD:ACF8885
Cecidomyiidae|[1388]|CNSLB166-12|Cecidomyiidae|611|BOLD:ACB1141
Cecidomyiidae|[1389]|CNKLF1432-15|Cecidomyiidae|609|BOLD:ACS4013
Cecidomyiidae|[1390]|CNGCF513-15|Cecidomyiidae|597|BOLD:ACT2832
Cecidomyiidae|[1391]|CNGMB1629-13|Cecidomyiidae|661|BOLD:ACL0748
Cecidomyiidae|[1392]|CNGMC2231-13|Cecidomyiidae|658|BOLD:ACL7638
Cecidomyiidae|[1393]|CNPKF1487-14|Cecidomyiidae|658|BOLD:ACL6363
Cecidomyiidae|[1394]|SMTPD4752-13|Cecidomyiidae|620|BOLD:ACJ6701
Cecidomyiidae|[1395]|CNGUD636-15|Cecidomyiidae|630|BOLD:ACS2215
Cecidomyiidae|[1396]|CNPPG223-12|Cecidomyiidae|664|BOLD:ACB1482
Cecidomyiidae|[1397]|SSKJB292-14|Cecidomyiidae|594|BOLD:ACP8117
Cecidomyiidae|[1398]|BBDCQ847-10|Cecidomyiidae|658|BOLD:AAV5545
Cecidomyiidae|[1399]|SMTPJ3704-14|Cecidomyiidae|582|BOLD:ACP9708
Cecidomyiidae|[1400]|CNGBJ1101-14|Cecidomyiidae|661|BOLD:ABV1487

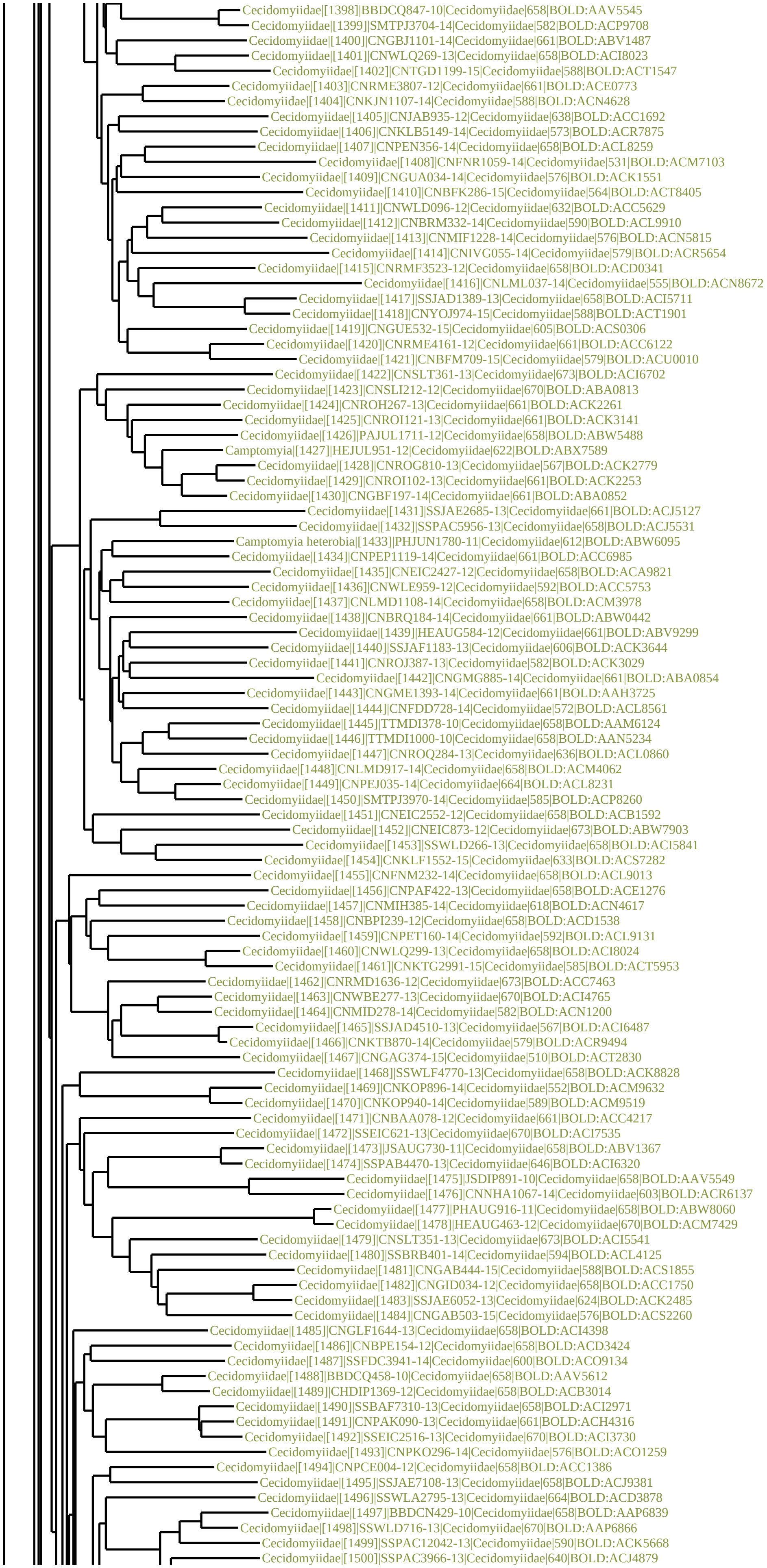
Cecidomyiidae|[1398]|BBDCQ847-10|Cecidomyiidae|658|BOLD:AAV5545
Cecidomyiidae|[1399]|SMTPJ3704-14|Cecidomyiidae|582|BOLD:ACP9708
Cecidomyiidae|[1400]|CNGBJ1101-14|Cecidomyiidae|661|BOLD:ABV1487
Cecidomyiidae|[1401]|CNWLQ269-13|Cecidomyiidae|658|BOLD:ACI8023
Cecidomyiidae|[1402]|CNTGD1199-15|Cecidomyiidae|588|BOLD:ACT1547
Cecidomyiidae|[1403]|CNRME3807-12|Cecidomyiidae|661|BOLD:ACE0773
Cecidomyiidae|[1404]|CNKJN1107-14|Cecidomyiidae|588|BOLD:ACN4628
Cecidomyiidae|[1405]|CNJAB935-12|Cecidomyiidae|638|BOLD:ACC1692
Cecidomyiidae|[1406]|CNKLB5149-14|Cecidomyiidae|573|BOLD:ACR7875
Cecidomyiidae|[1407]|CNPEN356-14|Cecidomyiidae|658|BOLD:ACL8259
Cecidomyiidae|[1408]|CNFNR1059-14|Cecidomyiidae|531|BOLD:ACM7103
Cecidomyiidae|[1409]|CNGUA034-14|Cecidomyiidae|576|BOLD:ACK1551
Cecidomyiidae|[1410]|CNBFK286-15|Cecidomyiidae|564|BOLD:ACT8405
Cecidomyiidae|[1411]|CNWLD096-12|Cecidomyiidae|632|BOLD:ACC5629
Cecidomyiidae|[1412]|CNBRM332-14|Cecidomyiidae|590|BOLD:ACL9910
Cecidomyiidae|[1413]|CNMIF1228-14|Cecidomyiidae|576|BOLD:ACN5815
Cecidomyiidae|[1414]|CNIVG055-14|Cecidomyiidae|579|BOLD:ACR5654
Cecidomyiidae|[1415]|CNRMF3523-12|Cecidomyiidae|658|BOLD:ACD0341
Cecidomyiidae|[1416]|CNLML037-14|Cecidomyiidae|555|BOLD:ACN8672
Cecidomyiidae|[1417]|SSJAD1389-13|Cecidomyiidae|658|BOLD:ACI5711
Cecidomyiidae|[1418]|CNYOJ974-15|Cecidomyiidae|588|BOLD:ACT1901
Cecidomyiidae|[1419]|CNGUE532-15|Cecidomyiidae|605|BOLD:ACS0306
Cecidomyiidae|[1420]|CNRME4161-12|Cecidomyiidae|661|BOLD:ACC6122
Cecidomyiidae|[1421]|CNBFM709-15|Cecidomyiidae|579|BOLD:ACU0010
Cecidomyiidae|[1422]|CNSLT361-13|Cecidomyiidae|673|BOLD:ACI6702
Cecidomyiidae|[1423]|CNSLI212-12|Cecidomyiidae|670|BOLD:ABA0813
Cecidomyiidae|[1424]|CNROH267-13|Cecidomyiidae|661|BOLD:ACK2261
Cecidomyiidae|[1425]|CNROI121-13|Cecidomyiidae|661|BOLD:ACK3141
Cecidomyiidae|[1426]|PAJUL1711-12|Cecidomyiidae|658|BOLD:ABW5488
Camptomyia|[1427]|HEJUL951-12|Cecidomyiidae|622|BOLD:ABX7589
Cecidomyiidae|[1428]|CNROG810-13|Cecidomyiidae|567|BOLD:ACK2779
Cecidomyiidae|[1429]|CNROI102-13|Cecidomyiidae|661|BOLD:ACK2253
Cecidomyiidae|[1430]|CNGBF197-14|Cecidomyiidae|661|BOLD:ABA0852
Cecidomyiidae|[1431]|SSJAE2685-13|Cecidomyiidae|661|BOLD:ACJ5127
Cecidomyiidae|[1432]|SSPAC5956-13|Cecidomyiidae|658|BOLD:ACJ5531
Camptomyia heterobia|[1433]|PHJUN1780-11|Cecidomyiidae|612|BOLD:ABW6095
Cecidomyiidae|[1434]|CNPEP1119-14|Cecidomyiidae|661|BOLD:ACC6985
Cecidomyiidae|[1435]|CNEIC2427-12|Cecidomyiidae|658|BOLD:ACA9821
Cecidomyiidae|[1436]|CNWLE959-12|Cecidomyiidae|592|BOLD:ACC5753
Cecidomyiidae|[1437]|CNLMD1108-14|Cecidomyiidae|658|BOLD:ACM3978
Cecidomyiidae|[1438]|CNBRQ184-14|Cecidomyiidae|661|BOLD:ABW0442
Cecidomyiidae|[1439]|HEAUG584-12|Cecidomyiidae|661|BOLD:ABV9299
Cecidomyiidae|[1440]|SSJAF1183-13|Cecidomyiidae|606|BOLD:ACK3644
Cecidomyiidae|[1441]|CNROJ387-13|Cecidomyiidae|582|BOLD:ACK3029
Cecidomyiidae|[1442]|CNGMG885-14|Cecidomyiidae|661|BOLD:ABA0854
Cecidomyiidae|[1443]|CNGME1393-14|Cecidomyiidae|661|BOLD:AAH3725
Cecidomyiidae|[1444]|CNFDD728-14|Cecidomyiidae|572|BOLD:ACL8561
Cecidomyiidae|[1445]|TTMDI378-10|Cecidomyiidae|658|BOLD:AAM6124
Cecidomyiidae|[1446]|TTMDI1000-10|Cecidomyiidae|658|BOLD:AAN5234
Cecidomyiidae|[1447]|CNROQ284-13|Cecidomyiidae|636|BOLD:ACL0860
Cecidomyiidae|[1448]|CNLMD917-14|Cecidomyiidae|658|BOLD:ACM4062
Cecidomyiidae|[1449]|CNPEJ035-14|Cecidomyiidae|664|BOLD:ACL8231
Cecidomyiidae|[1450]|SMTPJ3970-14|Cecidomyiidae|585|BOLD:ACP8260
Cecidomyiidae|[1451]|CNEIC2552-12|Cecidomyiidae|658|BOLD:ACB1592
Cecidomyiidae|[1452]|CNEIC873-12|Cecidomyiidae|673|BOLD:ABW7903
Cecidomyiidae|[1453]|SSWLD266-13|Cecidomyiidae|658|BOLD:ACI5841
Cecidomyiidae|[1454]|CNKLF1552-15|Cecidomyiidae|633|BOLD:ACS7282
Cecidomyiidae|[1455]|CNFNM232-14|Cecidomyiidae|658|BOLD:ACL9013
Cecidomyiidae|[1456]|CNPAF422-13|Cecidomyiidae|658|BOLD:ACE1276
Cecidomyiidae|[1457]|CNMIH385-14|Cecidomyiidae|618|BOLD:ACN4617
Cecidomyiidae|[1458]|CNBPI239-12|Cecidomyiidae|658|BOLD:ACD1538
Cecidomyiidae|[1459]|CNPET160-14|Cecidomyiidae|592|BOLD:ACL9131
Cecidomyiidae|[1460]|CNWLQ299-13|Cecidomyiidae|658|BOLD:ACI8024
Cecidomyiidae|[1461]|CNKTG2991-15|Cecidomyiidae|585|BOLD:ACT5953
Cecidomyiidae|[1462]|CNRMD1636-12|Cecidomyiidae|673|BOLD:ACC7463
Cecidomyiidae|[1463]|CNWBE277-13|Cecidomyiidae|670|BOLD:ACI4765
Cecidomyiidae|[1464]|CNMID278-14|Cecidomyiidae|582|BOLD:ACN1200
Cecidomyiidae|[1465]|SSJAD4510-13|Cecidomyiidae|567|BOLD:ACI6487
Cecidomyiidae|[1466]|CNKTB870-14|Cecidomyiidae|579|BOLD:ACR9494
Cecidomyiidae|[1467]|CNGAG374-15|Cecidomyiidae|510|BOLD:ACT2830
Cecidomyiidae|[1468]|SSWLF4770-13|Cecidomyiidae|658|BOLD:ACK8828
Cecidomyiidae|[1469]|CNKOP896-14|Cecidomyiidae|552|BOLD:ACM9632
Cecidomyiidae|[1470]|CNKOP940-14|Cecidomyiidae|589|BOLD:ACM9519
Cecidomyiidae|[1471]|CNBAA078-12|Cecidomyiidae|661|BOLD:ACC4217
Cecidomyiidae|[1472]|SSEIC621-13|Cecidomyiidae|670|BOLD:ACI7535
Cecidomyiidae|[1473]|JSAUG730-11|Cecidomyiidae|658|BOLD:ABV1367
Cecidomyiidae|[1474]|SSPAB4470-13|Cecidomyiidae|646|BOLD:ACI6320
Cecidomyiidae|[1475]|JSDIP891-10|Cecidomyiidae|658|BOLD:AAV5549
Cecidomyiidae|[1476]|CNNHA1067-14|Cecidomyiidae|603|BOLD:ACR6137
Cecidomyiidae|[1477]|PHAUG916-11|Cecidomyiidae|658|BOLD:ABW8060
Cecidomyiidae|[1478]|HEAUG463-12|Cecidomyiidae|670|BOLD:ACM7429
Cecidomyiidae|[1479]|CNSLT351-13|Cecidomyiidae|673|BOLD:ACI5541
Cecidomyiidae|[1480]|SSBRB401-14|Cecidomyiidae|594|BOLD:ACL4125
Cecidomyiidae|[1481]|CNGAB444-15|Cecidomyiidae|588|BOLD:ACS1855
Cecidomyiidae|[1482]|CNGID034-12|Cecidomyiidae|658|BOLD:ACC1750
Cecidomyiidae|[1483]|SSJAE6052-13|Cecidomyiidae|624|BOLD:ACK2485
Cecidomyiidae|[1484]|CNGAB503-15|Cecidomyiidae|576|BOLD:ACS2260
Cecidomyiidae|[1485]|CNGLF1644-13|Cecidomyiidae|658|BOLD:ACI4398
Cecidomyiidae|[1486]|CNBPE154-12|Cecidomyiidae|658|BOLD:ACD3424
Cecidomyiidae|[1487]|SSFDC3941-14|Cecidomyiidae|600|BOLD:ACO9134
Cecidomyiidae|[1488]|BBDCQ458-10|Cecidomyiidae|658|BOLD:AAV5612
Cecidomyiidae|[1489]|CHDIP1369-12|Cecidomyiidae|658|BOLD:ACB3014
Cecidomyiidae|[1490]|SSBAF7310-13|Cecidomyiidae|658|BOLD:ACI2971
Cecidomyiidae|[1491]|CNPAK090-13|Cecidomyiidae|661|BOLD:ACH4316
Cecidomyiidae|[1492]|SSEIC2516-13|Cecidomyiidae|670|BOLD:ACI3730
Cecidomyiidae|[1493]|CNPKO296-14|Cecidomyiidae|576|BOLD:ACO1259
Cecidomyiidae|[1494]|CNPCE004-12|Cecidomyiidae|658|BOLD:ACC1386
Cecidomyiidae|[1495]|SSJAE7108-13|Cecidomyiidae|658|BOLD:ACJ9381
Cecidomyiidae|[1496]|SSWLA2795-13|Cecidomyiidae|664|BOLD:ACD3878
Cecidomyiidae|[1497]|BBDCN429-10|Cecidomyiidae|658|BOLD:AAP6839
Cecidomyiidae|[1498]|SSWLD716-13|Cecidomyiidae|670|BOLD:AAP6866
Cecidomyiidae|[1499]|SSPAC12042-13|Cecidomyiidae|590|BOLD:ACK5668
Cecidomyiidae|[1500]|SSPAC3966-13|Cecidomyiidae|640|BOLD:ACJ4879

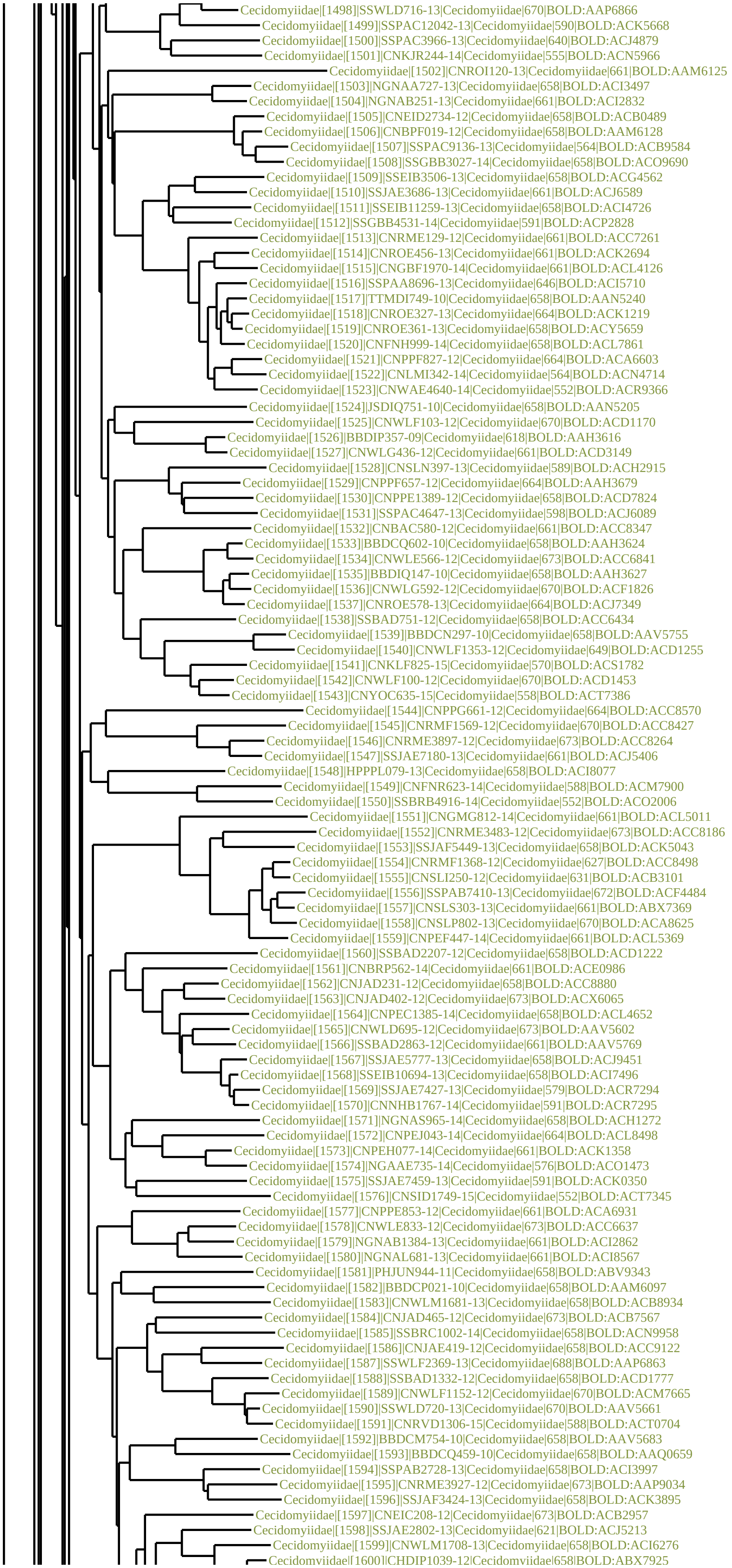
Cecidomyiidae|[1498]|SSWLD716-13|Cecidomyiidae|670|BOLD:AAP6866
Cecidomyiidae|[1499]|SSPAC12042-13|Cecidomyiidae|590|BOLD:ACK5668
Cecidomyiidae|[1500]|SSPAC3966-13|Cecidomyiidae|640|BOLD:ACJ4879
Cecidomyiidae|[1501]|CNKJR244-14|Cecidomyiidae|555|BOLD:ACN5966
Cecidomyiidae|[1502]|CNROI120-13|Cecidomyiidae|661|BOLD:AAM6125
Cecidomyiidae|[1503]|NGNAA727-13|Cecidomyiidae|658|BOLD:ACI3497
Cecidomyiidae|[1504]|NGNAB251-13|Cecidomyiidae|661|BOLD:ACI2832
Cecidomyiidae|[1505]|CNEID2734-12|Cecidomyiidae|658|BOLD:ACB0489
Cecidomyiidae|[1506]|CNBPF019-12|Cecidomyiidae|658|BOLD:AAM6128
Cecidomyiidae|[1507]|SSPAC9136-13|Cecidomyiidae|564|BOLD:ACB9584
Cecidomyiidae|[1508]|SSGBB3027-14|Cecidomyiidae|658|BOLD:ACO9690
Cecidomyiidae|[1509]|SSEIB3506-13|Cecidomyiidae|658|BOLD:ACG4562
Cecidomyiidae|[1510]|SSJAE3686-13|Cecidomyiidae|661|BOLD:ACJ6589
Cecidomyiidae|[1511]|SSEIB11259-13|Cecidomyiidae|658|BOLD:ACI4726
Cecidomyiidae|[1512]|SSGBB4531-14|Cecidomyiidae|591|BOLD:ACP2828
Cecidomyiidae|[1513]|CNRME129-12|Cecidomyiidae|661|BOLD:ACC7261
Cecidomyiidae|[1514]|CNROE456-13|Cecidomyiidae|661|BOLD:ACK2694
Cecidomyiidae|[1515]|CNGBF1970-14|Cecidomyiidae|661|BOLD:ACL4126
Cecidomyiidae|[1516]|SSPAA8696-13|Cecidomyiidae|646|BOLD:ACI5710
Cecidomyiidae|[1517]|TTMDI749-10|Cecidomyiidae|658|BOLD:AAN5240
Cecidomyiidae|[1518]|CNROE327-13|Cecidomyiidae|664|BOLD:ACK1219
Cecidomyiidae|[1519]|CNROE361-13|Cecidomyiidae|658|BOLD:ACY5659
Cecidomyiidae|[1520]|CNFNH999-14|Cecidomyiidae|658|BOLD:ACL7861
Cecidomyiidae|[1521]|CNPPF827-12|Cecidomyiidae|664|BOLD:ACA6603
Cecidomyiidae|[1522]|CNLMI342-14|Cecidomyiidae|564|BOLD:ACN4714
Cecidomyiidae|[1523]|CNWAE4640-14|Cecidomyiidae|552|BOLD:ACR9366
Cecidomyiidae|[1524]|JSDIQ751-10|Cecidomyiidae|658|BOLD:AAN5205
Cecidomyiidae|[1525]|CNWLF103-12|Cecidomyiidae|670|BOLD:ACD1170
Cecidomyiidae|[1526]|BBDIP357-09|Cecidomyiidae|618|BOLD:AAH3616
Cecidomyiidae|[1527]|CNWLG436-12|Cecidomyiidae|661|BOLD:ACD3149
Cecidomyiidae|[1528]|CNSLN397-13|Cecidomyiidae|589|BOLD:ACH2915
Cecidomyiidae|[1529]|CNPPF657-12|Cecidomyiidae|664|BOLD:AAH3679
Cecidomyiidae|[1530]|CNPPE1389-12|Cecidomyiidae|658|BOLD:ACD7824
Cecidomyiidae|[1531]|SSPAC4647-13|Cecidomyiidae|598|BOLD:ACJ6089
Cecidomyiidae|[1532]|CNBAC580-12|Cecidomyiidae|661|BOLD:ACC8347
Cecidomyiidae|[1533]|BBDCQ602-10|Cecidomyiidae|658|BOLD:AAH3624
Cecidomyiidae|[1534]|CNWLE566-12|Cecidomyiidae|673|BOLD:ACC6841
Cecidomyiidae|[1535]|BBDIQ147-10|Cecidomyiidae|658|BOLD:AAH3627
Cecidomyiidae|[1536]|CNWLG592-12|Cecidomyiidae|670|BOLD:ACF1826
Cecidomyiidae|[1537]|CNROE578-13|Cecidomyiidae|664|BOLD:ACJ7349
Cecidomyiidae|[1538]|SSBAD751-12|Cecidomyiidae|658|BOLD:ACC6434
Cecidomyiidae|[1539]|BBDCN297-10|Cecidomyiidae|658|BOLD:AAV5755
Cecidomyiidae|[1540]|CNWLF1353-12|Cecidomyiidae|649|BOLD:ACD1255
Cecidomyiidae|[1541]|CNKLF825-15|Cecidomyiidae|570|BOLD:ACS1782
Cecidomyiidae|[1542]|CNWLF100-12|Cecidomyiidae|670|BOLD:ACD1453
Cecidomyiidae|[1543]|CNYOC635-15|Cecidomyiidae|558|BOLD:ACT7386
Cecidomyiidae|[1544]|CNPPG661-12|Cecidomyiidae|664|BOLD:ACC8570
Cecidomyiidae|[1545]|CNRMF1569-12|Cecidomyiidae|670|BOLD:ACC8427
Cecidomyiidae|[1546]|CNRME3897-12|Cecidomyiidae|673|BOLD:ACC8264
Cecidomyiidae|[1547]|SSJAE7180-13|Cecidomyiidae|661|BOLD:ACJ5406
Cecidomyiidae|[1548]|HPPPL079-13|Cecidomyiidae|658|BOLD:ACI8077
Cecidomyiidae|[1549]|CNFNR623-14|Cecidomyiidae|588|BOLD:ACM7900
Cecidomyiidae|[1550]|SSBRB4916-14|Cecidomyiidae|552|BOLD:ACO2006
Cecidomyiidae|[1551]|CNGMG812-14|Cecidomyiidae|661|BOLD:ACL5011
Cecidomyiidae|[1552]|CNRME3483-12|Cecidomyiidae|673|BOLD:ACC8186
Cecidomyiidae|[1553]|SSJAF5449-13|Cecidomyiidae|658|BOLD:ACK5043
Cecidomyiidae|[1554]|CNRMF1368-12|Cecidomyiidae|627|BOLD:ACC8498
Cecidomyiidae|[1555]|CNSLI250-12|Cecidomyiidae|631|BOLD:ACB3101
Cecidomyiidae|[1556]|SSPAB7410-13|Cecidomyiidae|672|BOLD:ACF4484
Cecidomyiidae|[1557]|CNSLS303-13|Cecidomyiidae|661|BOLD:ABX7369
Cecidomyiidae|[1558]|CNSLP802-13|Cecidomyiidae|670|BOLD:ACA8625
Cecidomyiidae|[1559]|CNPEF447-14|Cecidomyiidae|661|BOLD:ACL5369
Cecidomyiidae|[1560]|SSBAD2207-12|Cecidomyiidae|658|BOLD:ACD1222
Cecidomyiidae|[1561]|CNBRP562-14|Cecidomyiidae|661|BOLD:ACE0986
Cecidomyiidae|[1562]|CNJAD231-12|Cecidomyiidae|658|BOLD:ACC8880
Cecidomyiidae|[1563]|CNJAD402-12|Cecidomyiidae|673|BOLD:ACX6065
Cecidomyiidae|[1564]|CNPEC1385-14|Cecidomyiidae|658|BOLD:ACL4652
Cecidomyiidae|[1565]|CNWLD695-12|Cecidomyiidae|673|BOLD:AAV5602
Cecidomyiidae|[1566]|SSBAD2863-12|Cecidomyiidae|661|BOLD:AAV5769
Cecidomyiidae|[1567]|SSJAE5777-13|Cecidomyiidae|658|BOLD:ACJ9451
Cecidomyiidae|[1568]|SSEIB10694-13|Cecidomyiidae|658|BOLD:ACI7496
Cecidomyiidae|[1569]|SSJAE7427-13|Cecidomyiidae|579|BOLD:ACR7294
Cecidomyiidae|[1570]|CNNHB1767-14|Cecidomyiidae|591|BOLD:ACR7295
Cecidomyiidae|[1571]|NGNAS965-14|Cecidomyiidae|658|BOLD:ACH1272
Cecidomyiidae|[1572]|CNPEJ043-14|Cecidomyiidae|664|BOLD:ACL8498
Cecidomyiidae|[1573]|CNPEH077-14|Cecidomyiidae|661|BOLD:ACK1358
Cecidomyiidae|[1574]|NGAAE735-14|Cecidomyiidae|576|BOLD:ACO1473
Cecidomyiidae|[1575]|SSJAE7459-13|Cecidomyiidae|591|BOLD:ACK0350
Cecidomyiidae|[1576]|CNSID1749-15|Cecidomyiidae|552|BOLD:ACT7345
Cecidomyiidae|[1577]|CNPPE853-12|Cecidomyiidae|661|BOLD:ACA6931
Cecidomyiidae|[1578]|CNWLE833-12|Cecidomyiidae|673|BOLD:ACC6637
Cecidomyiidae|[1579]|NGNAB1384-13|Cecidomyiidae|661|BOLD:ACI2862
Cecidomyiidae|[1580]|NGNAL681-13|Cecidomyiidae|661|BOLD:ACI8567
Cecidomyiidae|[1581]|PHJUN944-11|Cecidomyiidae|658|BOLD:ABV9343
Cecidomyiidae|[1582]|BBDCP021-10|Cecidomyiidae|658|BOLD:AAM6097
Cecidomyiidae|[1583]|CNWLM1681-13|Cecidomyiidae|658|BOLD:ACB8934
Cecidomyiidae|[1584]|CNJAD465-12|Cecidomyiidae|673|BOLD:ACB7567
Cecidomyiidae|[1585]|SSBRC1002-14|Cecidomyiidae|658|BOLD:ACN9958
Cecidomyiidae|[1586]|CNJAE419-12|Cecidomyiidae|658|BOLD:ACC9122
Cecidomyiidae|[1587]|SSWLF2369-13|Cecidomyiidae|688|BOLD:AAP6863
Cecidomyiidae|[1588]|SSBAD1332-12|Cecidomyiidae|658|BOLD:ACD1777
Cecidomyiidae|[1589]|CNWLF1152-12|Cecidomyiidae|670|BOLD:ACM7665
Cecidomyiidae|[1590]|SSWLD720-13|Cecidomyiidae|670|BOLD:AAV5661
Cecidomyiidae|[1591]|CNRVD1306-15|Cecidomyiidae|588|BOLD:ACT0704
Cecidomyiidae|[1592]|BBDCM754-10|Cecidomyiidae|658|BOLD:AAV5683
Cecidomyiidae|[1593]|BBDCQ459-10|Cecidomyiidae|658|BOLD:AAQ0659
Cecidomyiidae|[1594]|SSPAB2728-13|Cecidomyiidae|658|BOLD:ACI3997
Cecidomyiidae|[1595]|CNRME3927-12|Cecidomyiidae|673|BOLD:AAP9034
Cecidomyiidae|[1596]|SSJAF3424-13|Cecidomyiidae|658|BOLD:ACK3895
Cecidomyiidae|[1597]|CNEIC208-12|Cecidomyiidae|673|BOLD:ACB2957
Cecidomyiidae|[1598]|SSJAE2802-13|Cecidomyiidae|621|BOLD:ACJ5213
Cecidomyiidae|[1599]|CNWLM1708-13|Cecidomyiidae|658|BOLD:ACI6276
Cecidomyiidae|[1600]|CHDIP1039-12|Cecidomyiidae|658|BOLD:ABX7925

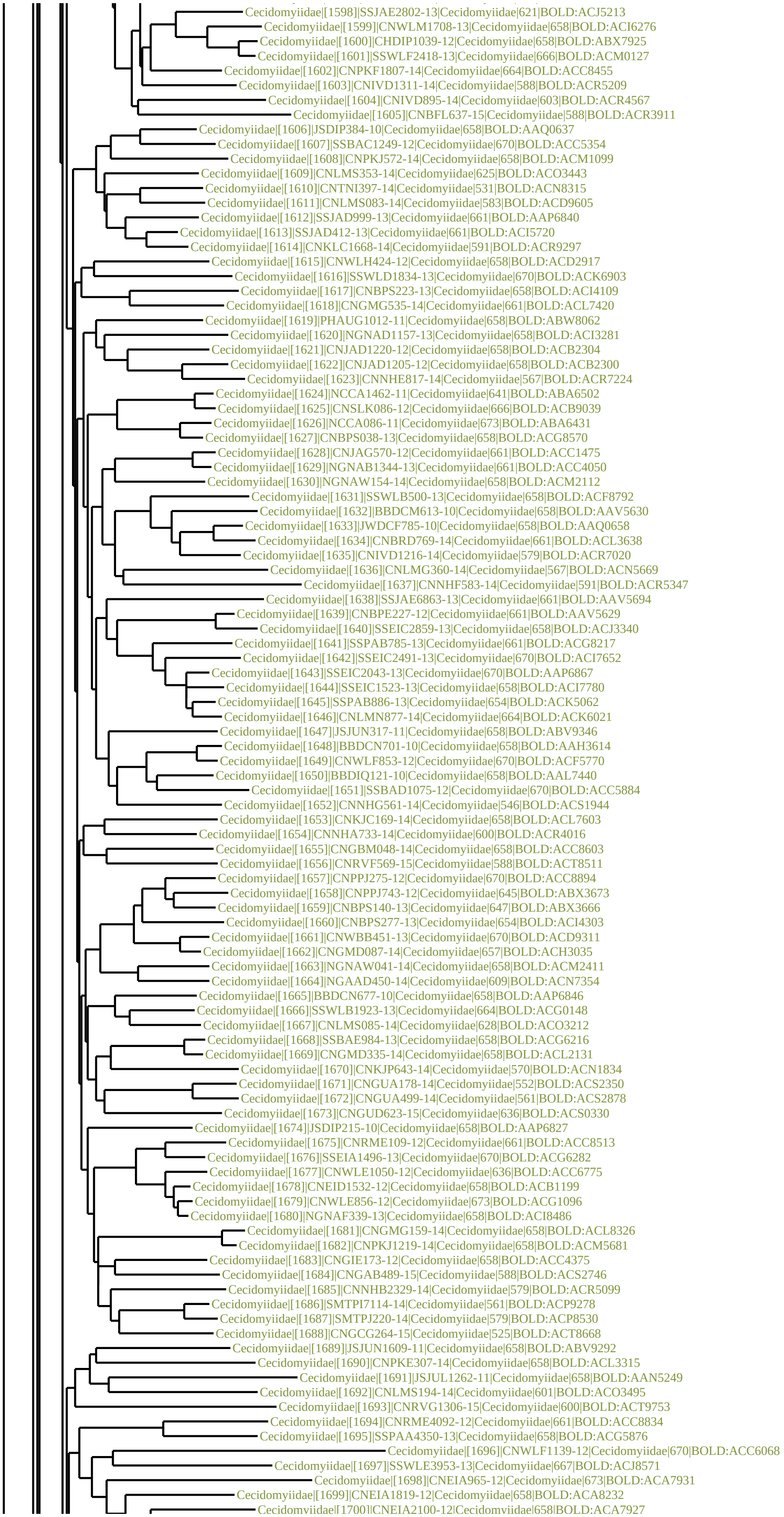
Cecidomyiidae|[1598]|SSJAE2802-13|Cecidomyiidae|621|BOLD:ACJ5213
Cecidomyiidae|[1599]|CNWLM1708-13|Cecidomyiidae|658|BOLD:ACI6276
Cecidomyiidae|[1600]|CHDIP1039-12|Cecidomyiidae|658|BOLD:ABX7925
Cecidomyiidae|[1601]|SSWLF2418-13|Cecidomyiidae|666|BOLD:ACM0127
Cecidomyiidae|[1602]|CNPKF1807-14|Cecidomyiidae|664|BOLD:ACC8455
Cecidomyiidae|[1603]|CNIVD1311-14|Cecidomyiidae|588|BOLD:ACR5209
Cecidomyiidae|[1604]|CNIVD895-14|Cecidomyiidae|603|BOLD:ACR4567
Cecidomyiidae|[1605]|CNBFL637-15|Cecidomyiidae|588|BOLD:ACR3911
Cecidomyiidae|[1606]|JSDIP384-10|Cecidomyiidae|658|BOLD:AAQ0637
Cecidomyiidae|[1607]|SSBAC1249-12|Cecidomyiidae|670|BOLD:ACC5354
Cecidomyiidae|[1608]|CNPKJ572-14|Cecidomyiidae|658|BOLD:ACM1099
Cecidomyiidae|[1609]|CNLMS353-14|Cecidomyiidae|625|BOLD:ACO3443
Cecidomyiidae|[1610]|CNTNI397-14|Cecidomyiidae|531|BOLD:ACN8315
Cecidomyiidae|[1611]|CNLMS083-14|Cecidomyiidae|583|BOLD:ACD9605
Cecidomyiidae|[1612]|SSJAD999-13|Cecidomyiidae|661|BOLD:AAP6840
Cecidomyiidae|[1613]|SSJAD412-13|Cecidomyiidae|661|BOLD:ACI5720
Cecidomyiidae|[1614]|CNKLC1668-14|Cecidomyiidae|591|BOLD:ACR9297
Cecidomyiidae|[1615]|CNWLH424-12|Cecidomyiidae|658|BOLD:ACD2917
Cecidomyiidae|[1616]|SSWLD1834-13|Cecidomyiidae|670|BOLD:ACK6903
Cecidomyiidae|[1617]|CNBPS223-13|Cecidomyiidae|658|BOLD:ACI4109
Cecidomyiidae|[1618]|CNGMG535-14|Cecidomyiidae|661|BOLD:ACL7420
Cecidomyiidae|[1619]|PHAUG1012-11|Cecidomyiidae|658|BOLD:ABW8062
Cecidomyiidae|[1620]|NGNAD1157-13|Cecidomyiidae|658|BOLD:ACI3281
Cecidomyiidae|[1621]|CNJAD1220-12|Cecidomyiidae|658|BOLD:ACB2304
Cecidomyiidae|[1622]|CNJAD1205-12|Cecidomyiidae|658|BOLD:ACB2300
Cecidomyiidae|[1623]|CNNHE817-14|Cecidomyiidae|567|BOLD:ACR7224
Cecidomyiidae|[1624]|NCCA1462-11|Cecidomyiidae|641|BOLD:ABA6502
Cecidomyiidae|[1625]|CNSLK086-12|Cecidomyiidae|666|BOLD:ACB9039
Cecidomyiidae|[1626]|NCCA086-11|Cecidomyiidae|673|BOLD:ABA6431
Cecidomyiidae|[1627]|CNBPS038-13|Cecidomyiidae|658|BOLD:ACG8570
Cecidomyiidae|[1628]|CNJAG570-12|Cecidomyiidae|661|BOLD:ACC1475
Cecidomyiidae|[1629]|NGNAB1344-13|Cecidomyiidae|661|BOLD:ACC4050
Cecidomyiidae|[1630]|NGNAW154-14|Cecidomyiidae|658|BOLD:ACM2112
Cecidomyiidae|[1631]|SSWLB500-13|Cecidomyiidae|658|BOLD:ACF8792
Cecidomyiidae|[1632]|BBDCM613-10|Cecidomyiidae|658|BOLD:AAV5630
Cecidomyiidae|[1633]|JWDCF785-10|Cecidomyiidae|658|BOLD:AAQ0658
Cecidomyiidae|[1634]|CNBRD769-14|Cecidomyiidae|661|BOLD:ACL3638
Cecidomyiidae|[1635]|CNIVD1216-14|Cecidomyiidae|579|BOLD:ACR7020
Cecidomyiidae|[1636]|CNLMG360-14|Cecidomyiidae|567|BOLD:ACN5669
Cecidomyiidae|[1637]|CNNHF583-14|Cecidomyiidae|591|BOLD:ACR5347
Cecidomyiidae|[1638]|SSJAE6863-13|Cecidomyiidae|661|BOLD:AAV5694
Cecidomyiidae|[1639]|CNBPE227-12|Cecidomyiidae|661|BOLD:AAV5629
Cecidomyiidae|[1640]|SSEIC2859-13|Cecidomyiidae|658|BOLD:ACJ3340
Cecidomyiidae|[1641]|SSPAB785-13|Cecidomyiidae|661|BOLD:ACG8217
Cecidomyiidae|[1642]|SSEIC2491-13|Cecidomyiidae|670|BOLD:ACI7652
Cecidomyiidae|[1643]|SSEIC2043-13|Cecidomyiidae|670|BOLD:AAP6867
Cecidomyiidae|[1644]|SSEIC1523-13|Cecidomyiidae|658|BOLD:ACI7780
Cecidomyiidae|[1645]|SSPAB886-13|Cecidomyiidae|654|BOLD:ACK5062
Cecidomyiidae|[1646]|CNLMN877-14|Cecidomyiidae|664|BOLD:ACK6021
Cecidomyiidae|[1647]|JSJUN317-11|Cecidomyiidae|658|BOLD:ABV9346
Cecidomyiidae|[1648]|BBDCN701-10|Cecidomyiidae|658|BOLD:AAH3614
Cecidomyiidae|[1649]|CNWLF853-12|Cecidomyiidae|670|BOLD:ACF5770
Cecidomyiidae|[1650]|BBDIQ121-10|Cecidomyiidae|658|BOLD:AAL7440
Cecidomyiidae|[1651]|SSBAD1075-12|Cecidomyiidae|670|BOLD:ACC5884
Cecidomyiidae|[1652]|CNNHG561-14|Cecidomyiidae|546|BOLD:ACS1944
Cecidomyiidae|[1653]|CNKJC169-14|Cecidomyiidae|658|BOLD:ACL7603
Cecidomyiidae|[1654]|CNNHA733-14|Cecidomyiidae|600|BOLD:ACR4016
Cecidomyiidae|[1655]|CNGBM048-14|Cecidomyiidae|658|BOLD:ACC8603
Cecidomyiidae|[1656]|CNRVF569-15|Cecidomyiidae|588|BOLD:ACT8511
Cecidomyiidae|[1657]|CNPPJ275-12|Cecidomyiidae|670|BOLD:ACC8894
Cecidomyiidae|[1658]|CNPPJ743-12|Cecidomyiidae|645|BOLD:ABX3673
Cecidomyiidae|[1659]|CNBPS140-13|Cecidomyiidae|647|BOLD:ABX3666
Cecidomyiidae|[1660]|CNBPS277-13|Cecidomyiidae|654|BOLD:ACI4303
Cecidomyiidae|[1661]|CNWBB451-13|Cecidomyiidae|670|BOLD:ACD9311
Cecidomyiidae|[1662]|CNGMD087-14|Cecidomyiidae|657|BOLD:ACH3035
Cecidomyiidae|[1663]|NGNAW041-14|Cecidomyiidae|658|BOLD:ACM2411
Cecidomyiidae|[1664]|NGAAD450-14|Cecidomyiidae|609|BOLD:ACN7354
Cecidomyiidae|[1665]|BBDCN677-10|Cecidomyiidae|658|BOLD:AAP6846
Cecidomyiidae|[1666]|SSWLB1923-13|Cecidomyiidae|664|BOLD:ACG0148
Cecidomyiidae|[1667]|CNLMS085-14|Cecidomyiidae|628|BOLD:ACO3212
Cecidomyiidae|[1668]|SSBAE984-13|Cecidomyiidae|658|BOLD:ACG6216
Cecidomyiidae|[1669]|CNGMD335-14|Cecidomyiidae|658|BOLD:ACL2131
Cecidomyiidae|[1670]|CNKJP643-14|Cecidomyiidae|570|BOLD:ACN1834
Cecidomyiidae|[1671]|CNGUA178-14|Cecidomyiidae|552|BOLD:ACS2350
Cecidomyiidae|[1672]|CNGUA499-14|Cecidomyiidae|561|BOLD:ACS2878
Cecidomyiidae|[1673]|CNGUD623-15|Cecidomyiidae|636|BOLD:ACS0330
Cecidomyiidae|[1674]|JSDIP215-10|Cecidomyiidae|658|BOLD:AAP6827
Cecidomyiidae|[1675]|CNRME109-12|Cecidomyiidae|661|BOLD:ACC8513
Cecidomyiidae|[1676]|SSEIA1496-13|Cecidomyiidae|670|BOLD:ACG6282
Cecidomyiidae|[1677]|CNWLE1050-12|Cecidomyiidae|636|BOLD:ACC6775
Cecidomyiidae|[1678]|CNEID1532-12|Cecidomyiidae|658|BOLD:ACB1199
Cecidomyiidae|[1679]|CNWLE856-12|Cecidomyiidae|673|BOLD:ACG1096
Cecidomyiidae|[1680]|NGNAF339-13|Cecidomyiidae|658|BOLD:ACI8486
Cecidomyiidae|[1681]|CNGMG159-14|Cecidomyiidae|658|BOLD:ACL8326
Cecidomyiidae|[1682]|CNPKJ1219-14|Cecidomyiidae|658|BOLD:ACM5681
Cecidomyiidae|[1683]|CNGIE173-12|Cecidomyiidae|658|BOLD:ACC4375
Cecidomyiidae|[1684]|CNGAB489-15|Cecidomyiidae|588|BOLD:ACS2746
Cecidomyiidae|[1685]|CNNHB2329-14|Cecidomyiidae|579|BOLD:ACR5099
Cecidomyiidae|[1686]|SMTPI7114-14|Cecidomyiidae|561|BOLD:ACP9278
Cecidomyiidae|[1687]|SMTPJ220-14|Cecidomyiidae|579|BOLD:ACP8530
Cecidomyiidae|[1688]|CNGCG264-15|Cecidomyiidae|525|BOLD:ACT8668
Cecidomyiidae|[1689]|JSJUN1609-11|Cecidomyiidae|658|BOLD:ABV9292
Cecidomyiidae|[1690]|CNPKE307-14|Cecidomyiidae|658|BOLD:ACL3315
Cecidomyiidae|[1691]|JSJUL1262-11|Cecidomyiidae|658|BOLD:AAN5249
Cecidomyiidae|[1692]|CNLMS194-14|Cecidomyiidae|601|BOLD:ACO3495
Cecidomyiidae|[1693]|CNRVG1306-15|Cecidomyiidae|600|BOLD:ACT9753
Cecidomyiidae|[1694]|CNRME4092-12|Cecidomyiidae|661|BOLD:ACC8834
Cecidomyiidae|[1695]|SSPAA4350-13|Cecidomyiidae|658|BOLD:ACG5876
Cecidomyiidae|[1696]|CNWLF1139-12|Cecidomyiidae|670|BOLD:ACC6068
Cecidomyiidae|[1697]|SSWLE3953-13|Cecidomyiidae|667|BOLD:ACJ8571
Cecidomyiidae|[1698]|CNEIA965-12|Cecidomyiidae|673|BOLD:ACA7931
Cecidomyiidae|[1699]|CNEIA1819-12|Cecidomyiidae|658|BOLD:ACA8232
Cecidomyiidae|[1700]|CNEIA2100-12|Cecidomyiidae|658|BOLD:ACA7927

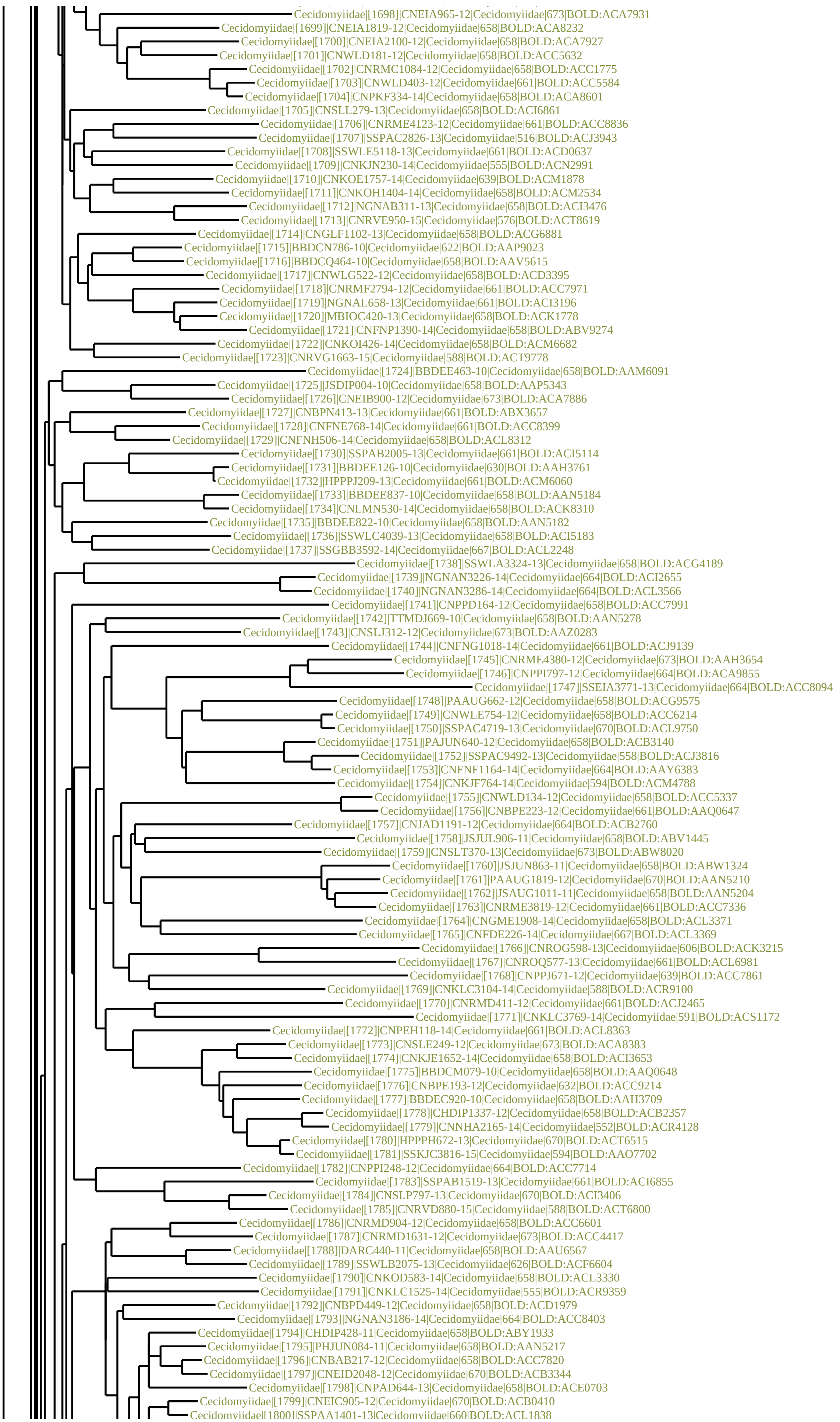
Cecidomyiidae|[1698]|CNEIA965-12|Cecidomyiidae|673|BOLD:ACA7931
Cecidomyiidae|[1699]|CNEIA1819-12|Cecidomyiidae|658|BOLD:ACA8232
Cecidomyiidae|[1700]|CNEIA2100-12|Cecidomyiidae|658|BOLD:ACA7927
Cecidomyiidae|[1701]|CNWLD181-12|Cecidomyiidae|658|BOLD:ACC5632
Cecidomyiidae|[1702]|CNRMC1084-12|Cecidomyiidae|658|BOLD:ACC1775
Cecidomyiidae|[1703]|CNWLD403-12|Cecidomyiidae|661|BOLD:ACC5584
Cecidomyiidae|[1704]|CNPKF334-14|Cecidomyiidae|658|BOLD:ACA8601
Cecidomyiidae|[1705]|CNSLL279-13|Cecidomyiidae|658|BOLD:ACI6861
Cecidomyiidae|[1706]|CNRME4123-12|Cecidomyiidae|661|BOLD:ACC8836
Cecidomyiidae|[1707]|SSPAC2826-13|Cecidomyiidae|516|BOLD:ACJ3943
Cecidomyiidae|[1708]|SSWLE5118-13|Cecidomyiidae|661|BOLD:ACD0637
Cecidomyiidae|[1709]|CNKJN230-14|Cecidomyiidae|555|BOLD:ACN2991
Cecidomyiidae|[1710]|CNKOE1757-14|Cecidomyiidae|639|BOLD:ACM1878
Cecidomyiidae|[1711]|CNKOH1404-14|Cecidomyiidae|658|BOLD:ACM2534
Cecidomyiidae|[1712]|NGNAB311-13|Cecidomyiidae|658|BOLD:ACI3476
Cecidomyiidae|[1713]|CNRVE950-15|Cecidomyiidae|576|BOLD:ACT8619
Cecidomyiidae|[1714]|CNGLF1102-13|Cecidomyiidae|658|BOLD:ACG6881
Cecidomyiidae|[1715]|BBDCN786-10|Cecidomyiidae|622|BOLD:AAP9023
Cecidomyiidae|[1716]|BBDCQ464-10|Cecidomyiidae|658|BOLD:AAV5615
Cecidomyiidae|[1717]|CNWLG522-12|Cecidomyiidae|658|BOLD:ACD3395
Cecidomyiidae|[1718]|CNRMF2794-12|Cecidomyiidae|661|BOLD:ACC7971
Cecidomyiidae|[1719]|NGNAL658-13|Cecidomyiidae|661|BOLD:ACI3196
Cecidomyiidae|[1720]|MBIOC420-13|Cecidomyiidae|658|BOLD:ACK1778
Cecidomyiidae|[1721]|CNFNP1390-14|Cecidomyiidae|658|BOLD:ABV9274
Cecidomyiidae|[1722]|CNKOI426-14|Cecidomyiidae|658|BOLD:ACM6682
Cecidomyiidae|[1723]|CNRVG1663-15|Cecidomyiidae|588|BOLD:ACT9778
Cecidomyiidae|[1724]|BBDEE463-10|Cecidomyiidae|658|BOLD:AAM6091
Cecidomyiidae|[1725]|JSDIP004-10|Cecidomyiidae|658|BOLD:AAP5343
Cecidomyiidae|[1726]|CNEIB900-12|Cecidomyiidae|673|BOLD:ACA7886
Cecidomyiidae|[1727]|CNBPN413-13|Cecidomyiidae|661|BOLD:ABX3657
Cecidomyiidae|[1728]|CNFNE768-14|Cecidomyiidae|661|BOLD:ACC8399
Cecidomyiidae|[1729]|CNFNH506-14|Cecidomyiidae|658|BOLD:ACL8312
Cecidomyiidae|[1730]|SSPAB2005-13|Cecidomyiidae|661|BOLD:ACI5114
Cecidomyiidae|[1731]|BBDEE126-10|Cecidomyiidae|630|BOLD:AAH3761
Cecidomyiidae|[1732]|HPPPJ209-13|Cecidomyiidae|661|BOLD:ACM6060
Cecidomyiidae|[1733]|BBDEE837-10|Cecidomyiidae|658|BOLD:AAN5184
Cecidomyiidae|[1734]|CNLMN530-14|Cecidomyiidae|658|BOLD:ACK8310
Cecidomyiidae|[1735]|BBDEE822-10|Cecidomyiidae|658|BOLD:AAN5182
Cecidomyiidae|[1736]|SSWLC4039-13|Cecidomyiidae|658|BOLD:ACI5183
Cecidomyiidae|[1737]|SSGBB3592-14|Cecidomyiidae|667|BOLD:ACL2248
Cecidomyiidae|[1738]|SSWLA3324-13|Cecidomyiidae|658|BOLD:ACG4189
Cecidomyiidae|[1739]|NGNAN3226-14|Cecidomyiidae|664|BOLD:ACI2655
Cecidomyiidae|[1740]|NGNAN3286-14|Cecidomyiidae|664|BOLD:ACL3566
Cecidomyiidae|[1741]|CNPPD164-12|Cecidomyiidae|658|BOLD:ACC7991
Cecidomyiidae|[1742]|TTMDJ669-10|Cecidomyiidae|658|BOLD:AAN5278
Cecidomyiidae|[1743]|CNSLJ312-12|Cecidomyiidae|673|BOLD:AAZ0283
Cecidomyiidae|[1744]|CNFNG1018-14|Cecidomyiidae|661|BOLD:ACJ9139
Cecidomyiidae|[1745]|CNRME4380-12|Cecidomyiidae|673|BOLD:AAH3654
Cecidomyiidae|[1746]|CNPPI797-12|Cecidomyiidae|664|BOLD:ACA9855
Cecidomyiidae|[1747]|SSEIA3771-13|Cecidomyiidae|664|BOLD:ACC8094
Cecidomyiidae|[1748]|PAAUG662-12|Cecidomyiidae|658|BOLD:ACG9575
Cecidomyiidae|[1749]|CNWLE754-12|Cecidomyiidae|658|BOLD:ACC6214
Cecidomyiidae|[1750]|SSPAC4719-13|Cecidomyiidae|670|BOLD:ACL9750
Cecidomyiidae|[1751]|PAJUN640-12|Cecidomyiidae|658|BOLD:ACB3140
Cecidomyiidae|[1752]|SSPAC9492-13|Cecidomyiidae|558|BOLD:ACJ3816
Cecidomyiidae|[1753]|CNFNF1164-14|Cecidomyiidae|664|BOLD:AAY6383
Cecidomyiidae|[1754]|CNKJF764-14|Cecidomyiidae|594|BOLD:ACM4788
Cecidomyiidae|[1755]|CNWLD134-12|Cecidomyiidae|658|BOLD:ACC5337
Cecidomyiidae|[1756]|CNBPE223-12|Cecidomyiidae|661|BOLD:AAQ0647
Cecidomyiidae|[1757]|CNJAD1191-12|Cecidomyiidae|664|BOLD:ACB2760
Cecidomyiidae|[1758]|JSJUL906-11|Cecidomyiidae|658|BOLD:ABV1445
Cecidomyiidae|[1759]|CNSLT370-13|Cecidomyiidae|673|BOLD:ABW8020
Cecidomyiidae|[1760]|JSJUN863-11|Cecidomyiidae|658|BOLD:ABW1324
Cecidomyiidae|[1761]|PAAUG1819-12|Cecidomyiidae|670|BOLD:AAN5210
Cecidomyiidae|[1762]|JSAUG1011-11|Cecidomyiidae|658|BOLD:AAN5204
Cecidomyiidae|[1763]|CNRME3819-12|Cecidomyiidae|661|BOLD:ACC7336
Cecidomyiidae|[1764]|CNGME1908-14|Cecidomyiidae|658|BOLD:ACL3371
Cecidomyiidae|[1765]|CNFDE226-14|Cecidomyiidae|667|BOLD:ACL3369
Cecidomyiidae|[1766]|CNROG598-13|Cecidomyiidae|606|BOLD:ACK3215
Cecidomyiidae|[1767]|CNROQ577-13|Cecidomyiidae|661|BOLD:ACL6981
Cecidomyiidae|[1768]|CNPPJ671-12|Cecidomyiidae|639|BOLD:ACC7861
Cecidomyiidae|[1769]|CNKLC3104-14|Cecidomyiidae|588|BOLD:ACR9100
Cecidomyiidae|[1770]|CNRMD411-12|Cecidomyiidae|661|BOLD:ACJ2465
Cecidomyiidae|[1771]|CNKLC3769-14|Cecidomyiidae|591|BOLD:ACS1172
Cecidomyiidae|[1772]|CNPEH118-14|Cecidomyiidae|661|BOLD:ACL8363
Cecidomyiidae|[1773]|CNSLE249-12|Cecidomyiidae|673|BOLD:ACA8383
Cecidomyiidae|[1774]|CNKJE1652-14|Cecidomyiidae|658|BOLD:ACI3653
Cecidomyiidae|[1775]|BBDCM079-10|Cecidomyiidae|658|BOLD:AAQ0648
Cecidomyiidae|[1776]|CNBPE193-12|Cecidomyiidae|632|BOLD:ACC9214
Cecidomyiidae|[1777]|BBDEC920-10|Cecidomyiidae|658|BOLD:AAH3709
Cecidomyiidae|[1778]|CHDIP1337-12|Cecidomyiidae|658|BOLD:ACB2357
Cecidomyiidae|[1779]|CNNHA2165-14|Cecidomyiidae|552|BOLD:ACR4128
Cecidomyiidae|[1780]|HPPPH672-13|Cecidomyiidae|670|BOLD:ACT6515
Cecidomyiidae|[1781]|SSKJC3816-15|Cecidomyiidae|594|BOLD:AAO7702
Cecidomyiidae|[1782]|CNPPI248-12|Cecidomyiidae|664|BOLD:ACC7714
Cecidomyiidae|[1783]|SSPAB1519-13|Cecidomyiidae|661|BOLD:ACI6855
Cecidomyiidae|[1784]|CNSLP797-13|Cecidomyiidae|670|BOLD:ACI3406
Cecidomyiidae|[1785]|CNRVD880-15|Cecidomyiidae|588|BOLD:ACT6800
Cecidomyiidae|[1786]|CNRMD904-12|Cecidomyiidae|658|BOLD:ACC6601
Cecidomyiidae|[1787]|CNRMD1631-12|Cecidomyiidae|673|BOLD:ACC4417
Cecidomyiidae|[1788]|DARC440-11|Cecidomyiidae|658|BOLD:AAU6567
Cecidomyiidae|[1789]|SSWLB2075-13|Cecidomyiidae|626|BOLD:ACF6604
Cecidomyiidae|[1790]|CNKOD583-14|Cecidomyiidae|658|BOLD:ACL3330
Cecidomyiidae|[1791]|CNKLC1525-14|Cecidomyiidae|555|BOLD:ACR9359
Cecidomyiidae|[1792]|CNBPD449-12|Cecidomyiidae|658|BOLD:ACD1979
Cecidomyiidae|[1793]|NGNAN3186-14|Cecidomyiidae|664|BOLD:ACC8403
Cecidomyiidae|[1794]|CHDIP428-11|Cecidomyiidae|658|BOLD:ABY1933
Cecidomyiidae|[1795]|PHJUN084-11|Cecidomyiidae|658|BOLD:AAN5217
Cecidomyiidae|[1796]|CNBAB217-12|Cecidomyiidae|658|BOLD:ACC7820
Cecidomyiidae|[1797]|CNEID2048-12|Cecidomyiidae|670|BOLD:ACB3344
Cecidomyiidae|[1798]|CNPAD644-13|Cecidomyiidae|658|BOLD:ACE0703
Cecidomyiidae|[1799]|CNEIC905-12|Cecidomyiidae|670|BOLD:ACB0410
Cecidomyiidae|[1800]|SSPAA1401-13|Cecidomyiidae|660|BOLD:ACL1838

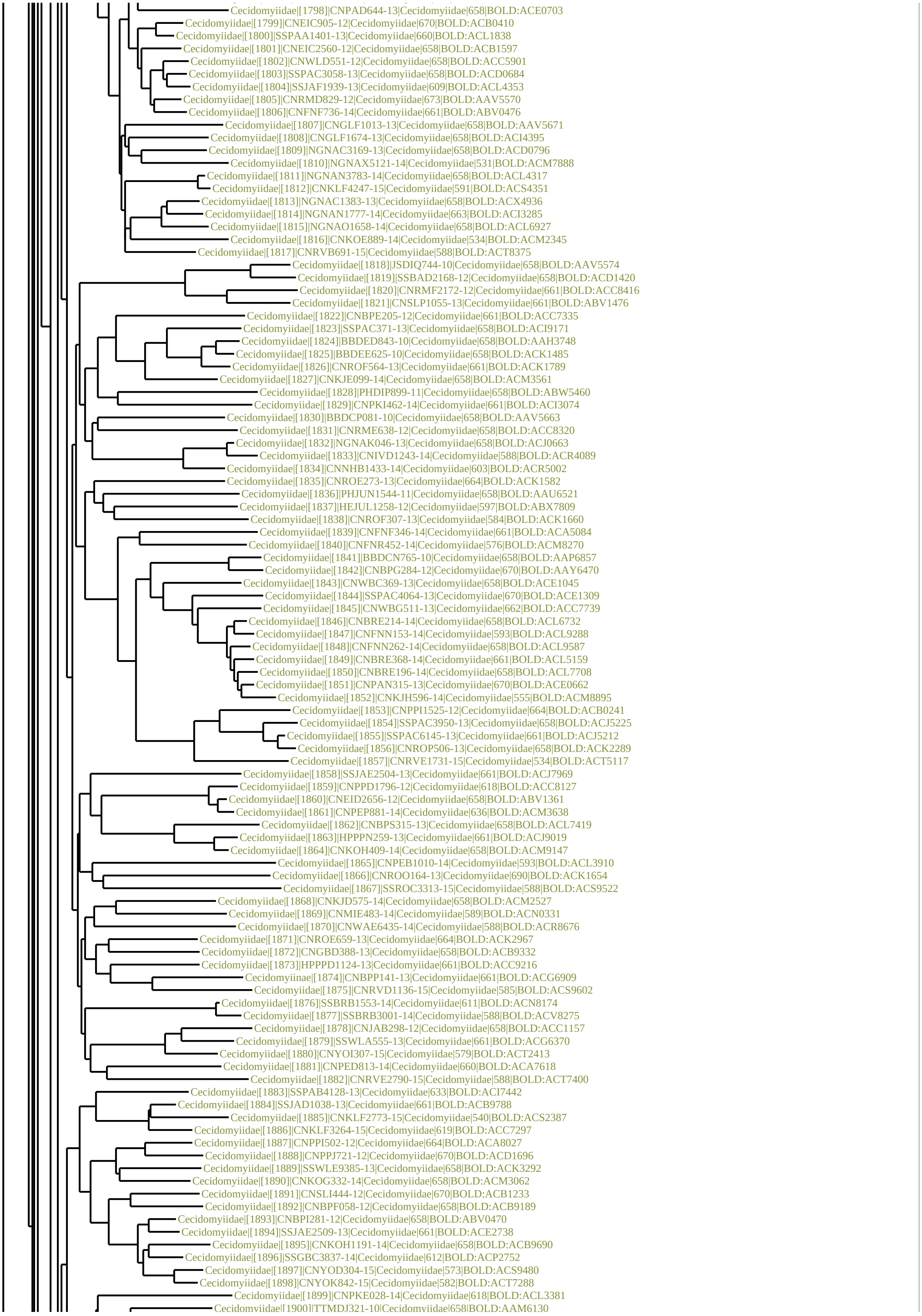

Cecidomyiidae|[1798]|CNPAD644-13|Cecidomyiidae|658|BOLD:ACE0703
Cecidomyiidae|[1799]|CNEIC905-12|Cecidomyiidae|670|BOLD:ACB0410
Cecidomyiidae|[1800]|SSPAA1401-13|Cecidomyiidae|660|BOLD:ACL1838
Cecidomyiidae|[1801]|CNEIC2560-12|Cecidomyiidae|658|BOLD:ACB1597
Cecidomyiidae|[1802]|CNWLD551-12|Cecidomyiidae|658|BOLD:ACC5901
Cecidomyiidae|[1803]|SSPAC3058-13|Cecidomyiidae|658|BOLD:ACD0684
Cecidomyiidae|[1804]|SSJAF1939-13|Cecidomyiidae|609|BOLD:ACL4353
Cecidomyiidae|[1805]|CNRMD829-12|Cecidomyiidae|673|BOLD:AAV5570
Cecidomyiidae|[1806]|CNFNF736-14|Cecidomyiidae|661|BOLD:ABV0476
Cecidomyiidae|[1807]|CNGLF1013-13|Cecidomyiidae|658|BOLD:AAV5671
Cecidomyiidae|[1808]|CNGLF1674-13|Cecidomyiidae|658|BOLD:ACI4395
Cecidomyiidae|[1809]|NGNAC3169-13|Cecidomyiidae|658|BOLD:ACD0796
Cecidomyiidae|[1810]|NGNAX5121-14|Cecidomyiidae|531|BOLD:ACM7888
Cecidomyiidae|[1811]|NGNAN3783-14|Cecidomyiidae|658|BOLD:ACL4317
Cecidomyiidae|[1812]|CNKLF4247-15|Cecidomyiidae|591|BOLD:ACS4351
Cecidomyiidae|[1813]|NGNAC1383-13|Cecidomyiidae|658|BOLD:ACX4936
Cecidomyiidae|[1814]|NGNAN1777-14|Cecidomyiidae|663|BOLD:ACI3285
Cecidomyiidae|[1815]|NGNAO1658-14|Cecidomyiidae|658|BOLD:ACL6927
Cecidomyiidae|[1816]|CNKOE889-14|Cecidomyiidae|534|BOLD:ACM2345
Cecidomyiidae|[1817]|CNRVB691-15|Cecidomyiidae|588|BOLD:ACT8375
Cecidomyiidae|[1818]|JSDIQ744-10|Cecidomyiidae|658|BOLD:AAV5574
Cecidomyiidae|[1819]|SSBAD2168-12|Cecidomyiidae|658|BOLD:ACD1420
Cecidomyiidae|[1820]|CNRMF2172-12|Cecidomyiidae|661|BOLD:ACC8416
Cecidomyiidae|[1821]|CNSLP1055-13|Cecidomyiidae|661|BOLD:ABV1476
Cecidomyiidae|[1822]|CNBPE205-12|Cecidomyiidae|661|BOLD:ACC7335
Cecidomyiidae|[1823]|SSPAC371-13|Cecidomyiidae|658|BOLD:ACI9171
Cecidomyiidae|[1824]|BBDED843-10|Cecidomyiidae|658|BOLD:AAH3748
Cecidomyiidae|[1825]|BBDEE625-10|Cecidomyiidae|658|BOLD:ACK1485
Cecidomyiidae|[1826]|CNROF564-13|Cecidomyiidae|661|BOLD:ACK1789
Cecidomyiidae|[1827]|CNKJE099-14|Cecidomyiidae|658|BOLD:ACM3561
Cecidomyiidae|[1828]|PHDIP899-11|Cecidomyiidae|658|BOLD:ABW5460
Cecidomyiidae|[1829]|CNPKI462-14|Cecidomyiidae|661|BOLD:ACI3074
Cecidomyiidae|[1830]|BBDCP081-10|Cecidomyiidae|658|BOLD:AAV5663
Cecidomyiidae|[1831]|CNRME638-12|Cecidomyiidae|658|BOLD:ACC8320
Cecidomyiidae|[1832]|NGNAK046-13|Cecidomyiidae|658|BOLD:ACJ0663
Cecidomyiidae|[1833]|CNIVD1243-14|Cecidomyiidae|588|BOLD:ACR4089
Cecidomyiidae|[1834]|CNNHB1433-14|Cecidomyiidae|603|BOLD:ACR5002
Cecidomyiidae|[1835]|CNROE273-13|Cecidomyiidae|664|BOLD:ACK1582
Cecidomyiidae|[1836]|PHJUN1544-11|Cecidomyiidae|658|BOLD:AAU6521
Cecidomyiidae|[1837]|HEJUL1258-12|Cecidomyiidae|597|BOLD:ABX7809
Cecidomyiidae|[1838]|CNROF307-13|Cecidomyiidae|584|BOLD:ACK1660
Cecidomyiidae|[1839]|CNFNF346-14|Cecidomyiidae|661|BOLD:ACA5084
Cecidomyiidae|[1840]|CNFNR452-14|Cecidomyiidae|576|BOLD:ACM8270
Cecidomyiidae|[1841]|BBDCN765-10|Cecidomyiidae|658|BOLD:AAP6857
Cecidomyiidae|[1842]|CNBPG284-12|Cecidomyiidae|670|BOLD:AAY6470
Cecidomyiidae|[1843]|CNWBC369-13|Cecidomyiidae|658|BOLD:ACE1045
Cecidomyiidae|[1844]|SSPAC4064-13|Cecidomyiidae|670|BOLD:ACE1309
Cecidomyiidae|[1845]|CNWBG511-13|Cecidomyiidae|662|BOLD:ACC7739
Cecidomyiidae|[1846]|CNBRE214-14|Cecidomyiidae|658|BOLD:ACL6732
Cecidomyiidae|[1847]|CNFNN153-14|Cecidomyiidae|593|BOLD:ACL9288
Cecidomyiidae|[1848]|CNFNN262-14|Cecidomyiidae|658|BOLD:ACL9587
Cecidomyiidae|[1849]|CNBRE368-14|Cecidomyiidae|661|BOLD:ACL5159
Cecidomyiidae|[1850]|CNBRE196-14|Cecidomyiidae|658|BOLD:ACL7708
Cecidomyiidae|[1851]|CNPAN315-13|Cecidomyiidae|670|BOLD:ACE0662
Cecidomyiidae|[1852]|CNKJH596-14|Cecidomyiidae|555|BOLD:ACM8895
Cecidomyiidae|[1853]|CNPPI1525-12|Cecidomyiidae|664|BOLD:ACB0241
Cecidomyiidae|[1854]|SSPAC3950-13|Cecidomyiidae|658|BOLD:ACJ5225
Cecidomyiidae|[1855]|SSPAC6145-13|Cecidomyiidae|661|BOLD:ACJ5212
Cecidomyiidae|[1856]|CNROP506-13|Cecidomyiidae|658|BOLD:ACK2289
Cecidomyiidae|[1857]|CNRVE1731-15|Cecidomyiidae|534|BOLD:ACT5117
Cecidomyiidae|[1858]|SSJAE2504-13|Cecidomyiidae|661|BOLD:ACJ7969
Cecidomyiidae|[1859]|CNPPD1796-12|Cecidomyiidae|618|BOLD:ACC8127
Cecidomyiidae|[1860]|CNEID2656-12|Cecidomyiidae|658|BOLD:ABV1361
Cecidomyiidae|[1861]|CNPEP881-14|Cecidomyiidae|636|BOLD:ACM3638
Cecidomyiidae|[1862]|CNBPS315-13|Cecidomyiidae|658|BOLD:ACL7419
Cecidomyiidae|[1863]|HPPPN259-13|Cecidomyiidae|661|BOLD:ACJ9019
Cecidomyiidae|[1864]|CNKOH409-14|Cecidomyiidae|658|BOLD:ACM9147
Cecidomyiidae|[1865]|CNPEB1010-14|Cecidomyiidae|593|BOLD:ACL3910
Cecidomyiidae|[1866]|CNROO164-13|Cecidomyiidae|690|BOLD:ACK1654
Cecidomyiidae|[1867]|SSROC3313-15|Cecidomyiidae|588|BOLD:ACS9522
Cecidomyiidae|[1868]|CNKJD575-14|Cecidomyiidae|658|BOLD:ACM2527
Cecidomyiidae|[1869]|CNMIE483-14|Cecidomyiidae|589|BOLD:ACN0331
Cecidomyiidae|[1870]|CNWAE6435-14|Cecidomyiidae|588|BOLD:ACR8676
Cecidomyiidae|[1871]|CNROE659-13|Cecidomyiidae|664|BOLD:ACK2967
Cecidomyiidae|[1872]|CNGBD388-13|Cecidomyiidae|658|BOLD:ACB9332
Cecidomyiidae|[1873]|HPPPD1124-13|Cecidomyiidae|661|BOLD:ACC9216
Cecidomyiinae|[1874]|CNBPP141-13|Cecidomyiidae|661|BOLD:ACG6909
Cecidomyiidae|[1875]|CNRVD1136-15|Cecidomyiidae|585|BOLD:ACS9602
Cecidomyiidae|[1876]|SSBRB1553-14|Cecidomyiidae|611|BOLD:ACN8174
Cecidomyiidae|[1877]|SSBRB3001-14|Cecidomyiidae|588|BOLD:ACV8275
Cecidomyiidae|[1878]|CNJAB298-12|Cecidomyiidae|658|BOLD:ACC1157
Cecidomyiidae|[1879]|SSWLA555-13|Cecidomyiidae|661|BOLD:ACG6370
Cecidomyiidae|[1880]|CNYOI307-15|Cecidomyiidae|579|BOLD:ACT2413
Cecidomyiidae|[1881]|CNPED813-14|Cecidomyiidae|660|BOLD:ACA7618
Cecidomyiidae|[1882]|CNRVE2790-15|Cecidomyiidae|588|BOLD:ACT7400
Cecidomyiidae|[1883]|SSPAB4128-13|Cecidomyiidae|633|BOLD:ACI7442
Cecidomyiidae|[1884]|SSJAD1038-13|Cecidomyiidae|661|BOLD:ACB9788
Cecidomyiidae|[1885]|CNKLF2773-15|Cecidomyiidae|540|BOLD:ACS2387
Cecidomyiidae|[1886]|CNKLF3264-15|Cecidomyiidae|619|BOLD:ACC7297
Cecidomyiidae|[1887]|CNPPI502-12|Cecidomyiidae|664|BOLD:ACA8027
Cecidomyiidae|[1888]|CNPPJ721-12|Cecidomyiidae|670|BOLD:ACD1696
Cecidomyiidae|[1889]|SSWLE9385-13|Cecidomyiidae|658|BOLD:ACK3292
Cecidomyiidae|[1890]|CNKOG332-14|Cecidomyiidae|658|BOLD:ACM3062
Cecidomyiidae|[1891]|CNSLI444-12|Cecidomyiidae|670|BOLD:ACB1233
Cecidomyiidae|[1892]|CNBPF058-12|Cecidomyiidae|658|BOLD:ACB9189
Cecidomyiidae|[1893]|CNBPI281-12|Cecidomyiidae|658|BOLD:ABV0470
Cecidomyiidae|[1894]|SSJAE2509-13|Cecidomyiidae|661|BOLD:ACE2738
Cecidomyiidae|[1895]|CNKOH1191-14|Cecidomyiidae|658|BOLD:ACB9690
Cecidomyiidae|[1896]|SSGBC3837-14|Cecidomyiidae|612|BOLD:ACP2752
Cecidomyiidae|[1897]|CNYOD304-15|Cecidomyiidae|573|BOLD:ACS9480
Cecidomyiidae|[1898]|CNYOK842-15|Cecidomyiidae|582|BOLD:ACT7288
Cecidomyiidae|[1899]|CNPKE028-14|Cecidomyiidae|618|BOLD:ACL3381
Cecidomyiidae|[1900]|TTMDJ321-10|Cecidomyiidae|658|BOLD:AAM6130

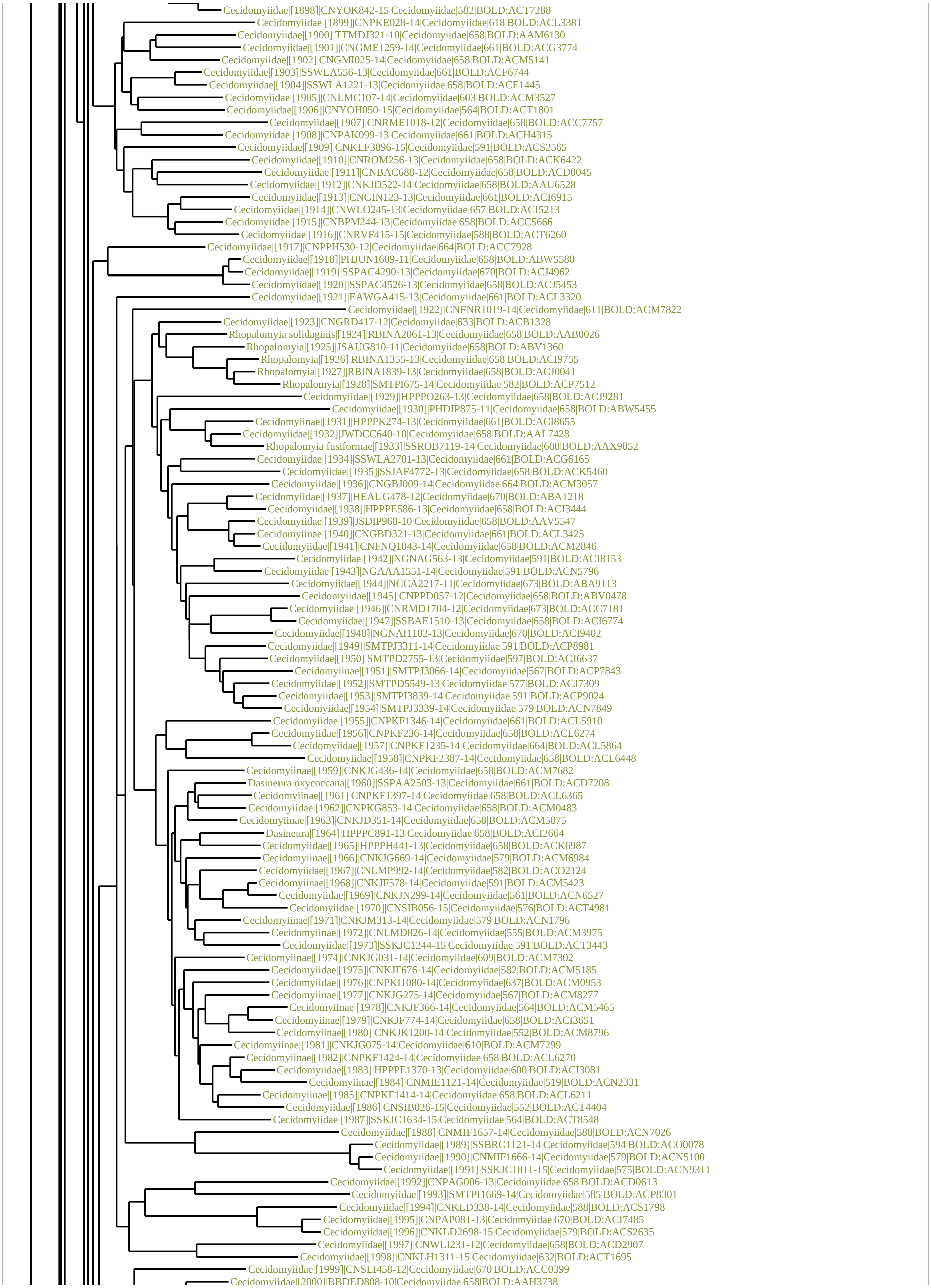
Cecidomyiidae|[1898]|CNYOK842-15|Cecidomyiidae|582|BOLD:ACT7288
Cecidomyiidae|[1899]|CNPKE028-14|Cecidomyiidae|618|BOLD:ACL3381
Cecidomyiidae|[1900]|TTMDJ321-10|Cecidomyiidae|658|BOLD:AAM6130
Cecidomyiidae|[1901]|CNGME1259-14|Cecidomyiidae|661|BOLD:ACG3774
Cecidomyiidae|[1902]|CNGMI025-14|Cecidomyiidae|658|BOLD:ACM5141
Cecidomyiidae|[1903]|SSWLA556-13|Cecidomyiidae|661|BOLD:ACF6744
Cecidomyiidae|[1904]|SSWLA1221-13|Cecidomyiidae|658|BOLD:ACE1445
Cecidomyiidae|[1905]|CNLMC107-14|Cecidomyiidae|603|BOLD:ACM3527
Cecidomyiidae|[1906]|CNYOH050-15|Cecidomyiidae|564|BOLD:ACT1801
Cecidomyiidae|[1907]|CNRME1018-12|Cecidomyiidae|658|BOLD:ACC7757
Cecidomyiidae|[1908]|CNPAK099-13|Cecidomyiidae|661|BOLD:ACH4315
Cecidomyiidae|[1909]|CNKLF3896-15|Cecidomyiidae|591|BOLD:ACS2565
Cecidomyiidae|[1910]|CNROM256-13|Cecidomyiidae|658|BOLD:ACK6422
Cecidomyiidae|[1911]|CNBAC688-12|Cecidomyiidae|658|BOLD:ACD0045
Cecidomyiidae|[1912]|CNKJD522-14|Cecidomyiidae|658|BOLD:AAU6528
Cecidomyiidae|[1913]|CNGIN123-13|Cecidomyiidae|661|BOLD:ACI6915
Cecidomyiidae|[1914]|CNWLO245-13|Cecidomyiidae|657|BOLD:ACI5213
Cecidomyiidae|[1915]|CNBPM244-13|Cecidomyiidae|658|BOLD:ACC5666
Cecidomyiidae|[1916]|CNRVF415-15|Cecidomyiidae|588|BOLD:ACT6260
Cecidomyiidae|[1917]|CNPPH530-12|Cecidomyiidae|664|BOLD:ACC7928
Cecidomyiidae|[1918]|PHJUN1609-11|Cecidomyiidae|658|BOLD:ABW5580
Cecidomyiidae|[1919]|SSPAC4290-13|Cecidomyiidae|670|BOLD:ACJ4962
Cecidomyiidae|[1920]|SSPAC4526-13|Cecidomyiidae|658|BOLD:ACJ5453
Cecidomyiidae|[1921]|EAWGA415-13|Cecidomyiidae|661|BOLD:ACL3320
Cecidomyiidae|[1922]|CNFNR1019-14|Cecidomyiidae|611|BOLD:ACM7822
Cecidomyiidae|[1923]|CNGRD417-12|Cecidomyiidae|633|BOLD:ACB1328
Rhopalomyia solidaginis|[1924]|RBINA2061-13|Cecidomyiidae|658|BOLD:AAB0026
Rhopalomyia|[1925]|JSAUG810-11|Cecidomyiidae|658|BOLD:ABV1360
Rhopalomyia|[1926]|RBINA1355-13|Cecidomyiidae|658|BOLD:ACI9755
Rhopalomyia|[1927]|RBINA1839-13|Cecidomyiidae|658|BOLD:ACJ0041
Rhopalomyia|[1928]|SMTPI675-14|Cecidomyiidae|582|BOLD:ACP7512
Cecidomyiidae|[1929]|HPPPO263-13|Cecidomyiidae|658|BOLD:ACJ9281
Cecidomyiidae|[1930]|PHDIP875-11|Cecidomyiidae|658|BOLD:ABW5455
Cecidomyiinae|[1931]|HPPPK274-13|Cecidomyiidae|661|BOLD:ACI8655
Cecidomyiidae|[1932]|JWDCC640-10|Cecidomyiidae|658|BOLD:AAL7428
Rhopalomyia fusiformae|[1933]|SSROB7119-14|Cecidomyiidae|600|BOLD:AAX9052
Cecidomyiidae|[1934]|SSWLA2701-13|Cecidomyiidae|661|BOLD:ACG6165
Cecidomyiidae|[1935]|SSJAF4772-13|Cecidomyiidae|658|BOLD:ACK5460
Cecidomyiidae|[1936]|CNGBJ009-14|Cecidomyiidae|664|BOLD:ACM3057
Cecidomyiidae|[1937]|HEAUG478-12|Cecidomyiidae|670|BOLD:ABA1218
Cecidomyiidae|[1938]|HPPPE586-13|Cecidomyiidae|658|BOLD:ACI3444
Cecidomyiidae|[1939]|JSDIP968-10|Cecidomyiidae|658|BOLD:AAV5547
Cecidomyiinae|[1940]|CNGBD321-13|Cecidomyiidae|661|BOLD:ACL3425
Cecidomyiidae|[1941]|CNFNQ1043-14|Cecidomyiidae|658|BOLD:ACM2846
Cecidomyiidae|[1942]|NGNAG563-13|Cecidomyiidae|591|BOLD:ACI8153
Cecidomyiidae|[1943]|NGAAA1551-14|Cecidomyiidae|591|BOLD:ACN5796
Cecidomyiidae|[1944]|NCCA2217-11|Cecidomyiidae|673|BOLD:ABA9113
Cecidomyiidae|[1945]|CNPPD057-12|Cecidomyiidae|658|BOLD:ABV0478
Cecidomyiidae|[1946]|CNRMD1704-12|Cecidomyiidae|673|BOLD:ACC7181
Cecidomyiidae|[1947]|SSBAE1510-13|Cecidomyiidae|658|BOLD:ACI6774
Cecidomyiidae|[1948]|NGNAI1102-13|Cecidomyiidae|670|BOLD:ACI9402
Cecidomyiidae|[1949]|SMTPJ3311-14|Cecidomyiidae|591|BOLD:ACP8981
Cecidomyiidae|[1950]|SMTPD2755-13|Cecidomyiidae|597|BOLD:ACJ6637
Cecidomyiinae|[1951]|SMTPJ3066-14|Cecidomyiidae|567|BOLD:ACP7843
Cecidomyiidae|[1952]|SMTPD5549-13|Cecidomyiidae|577|BOLD:ACJ7309
Cecidomyiidae|[1953]|SMTPI3839-14|Cecidomyiidae|591|BOLD:ACP9024
Cecidomyiidae|[1954]|SMTPJ3339-14|Cecidomyiidae|579|BOLD:ACN7849
Cecidomyiidae|[1955]|CNPKF1346-14|Cecidomyiidae|661|BOLD:ACL5910
Cecidomyiidae|[1956]|CNPKF236-14|Cecidomyiidae|658|BOLD:ACL6274
Cecidomyiidae|[1957]|CNPKF1235-14|Cecidomyiidae|664|BOLD:ACL5864
Cecidomyiidae|[1958]|CNPKF2387-14|Cecidomyiidae|658|BOLD:ACL6448
Cecidomyiinae|[1959]|CNKJG436-14|Cecidomyiidae|658|BOLD:ACM7682
Dasineura oxycoccana|[1960]|SSPAA2503-13|Cecidomyiidae|661|BOLD:ACD7208
Cecidomyiinae|[1961]|CNPKF1397-14|Cecidomyiidae|658|BOLD:ACL6365
Cecidomyiidae|[1962]|CNPKG853-14|Cecidomyiidae|658|BOLD:ACM0483
Cecidomyiinae|[1963]|CNKJD351-14|Cecidomyiidae|658|BOLD:ACM5875
Dasineura|[1964]|HPPPC891-13|Cecidomyiidae|658|BOLD:ACI2664
Cecidomyiidae|[1965]|HPPPH441-13|Cecidomyiidae|658|BOLD:ACK6987
Cecidomyiinae|[1966]|CNKJG669-14|Cecidomyiidae|579|BOLD:ACM6984
Cecidomyiidae|[1967]|CNLMP992-14|Cecidomyiidae|582|BOLD:ACO2124
Cecidomyiinae|[1968]|CNKJF578-14|Cecidomyiidae|591|BOLD:ACM5423
Cecidomyiidae|[1969]|CNKJN299-14|Cecidomyiidae|561|BOLD:ACN6527
Cecidomyiidae|[1970]|CNSIB056-15|Cecidomyiidae|576|BOLD:ACT4981
Cecidomyiinae|[1971]|CNKJM313-14|Cecidomyiidae|579|BOLD:ACN1796
Cecidomyiinae|[1972]|CNLMD826-14|Cecidomyiidae|555|BOLD:ACM3975
Cecidomyiidae|[1973]|SSKJC1244-15|Cecidomyiidae|591|BOLD:ACT3443
Cecidomyiinae|[1974]|CNKJG031-14|Cecidomyiidae|609|BOLD:ACM7302
Cecidomyiidae|[1975]|CNKJF676-14|Cecidomyiidae|582|BOLD:ACM5185
Cecidomyiidae|[1976]|CNPKI1080-14|Cecidomyiidae|637|BOLD:ACM0953
Cecidomyiinae|[1977]|CNKJG275-14|Cecidomyiidae|567|BOLD:ACM8277
Cecidomyiinae|[1978]|CNKJF366-14|Cecidomyiidae|564|BOLD:ACM5465
Cecidomyiinae|[1979]|CNKJF774-14|Cecidomyiidae|658|BOLD:ACI3651
Cecidomyiinae|[1980]|CNKJK1200-14|Cecidomyiidae|552|BOLD:ACM8796
Cecidomyiinae|[1981]|CNKJG075-14|Cecidomyiidae|610|BOLD:ACM7299
Cecidomyiinae|[1982]|CNPKF1424-14|Cecidomyiidae|658|BOLD:ACL6270
Cecidomyiidae|[1983]|HPPPE1370-13|Cecidomyiidae|600|BOLD:ACI3081
Cecidomyiinae|[1984]|CNMIE1121-14|Cecidomyiidae|519|BOLD:ACN2331
Cecidomyiinae|[1985]|CNPKF1414-14|Cecidomyiidae|658|BOLD:ACL6211
Cecidomyiidae|[1986]|CNSIB026-15|Cecidomyiidae|552|BOLD:ACT4404
Cecidomyiidae|[1987]|SSKJC1634-15|Cecidomyiidae|564|BOLD:ACT8548
Cecidomyiidae|[1988]|CNMIF1657-14|Cecidomyiidae|588|BOLD:ACN7026
Cecidomyiidae|[1989]|SSBRC1121-14|Cecidomyiidae|594|BOLD:ACO0078
Cecidomyiidae|[1990]|CNMIF1666-14|Cecidomyiidae|579|BOLD:ACN5100
Cecidomyiidae|[1991]|SSKJC1811-15|Cecidomyiidae|575|BOLD:ACN9311
Cecidomyiidae|[1992]|CNPAG006-13|Cecidomyiidae|658|BOLD:ACD0613
Cecidomyiidae|[1993]|SMTPI1669-14|Cecidomyiidae|585|BOLD:ACP8301
Cecidomyiidae|[1994]|CNKLD338-14|Cecidomyiidae|588|BOLD:ACS1798
Cecidomyiidae|[1995]|CNPAP081-13|Cecidomyiidae|670|BOLD:ACI7485
Cecidomyiidae|[1996]|CNKLD2698-15|Cecidomyiidae|579|BOLD:ACS2635
Cecidomyiidae|[1997]|CNWLI231-12|Cecidomyiidae|658|BOLD:ACD2907
Cecidomyiidae|[1998]|CNKLH1311-15|Cecidomyiidae|632|BOLD:ACT1695
Cecidomyiidae|[1999]|CNSLI458-12|Cecidomyiidae|670|BOLD:ACC0399
Cecidomyiidae|[2000]|BBDED808-10|Cecidomyiidae|658|BOLD:AAH3738

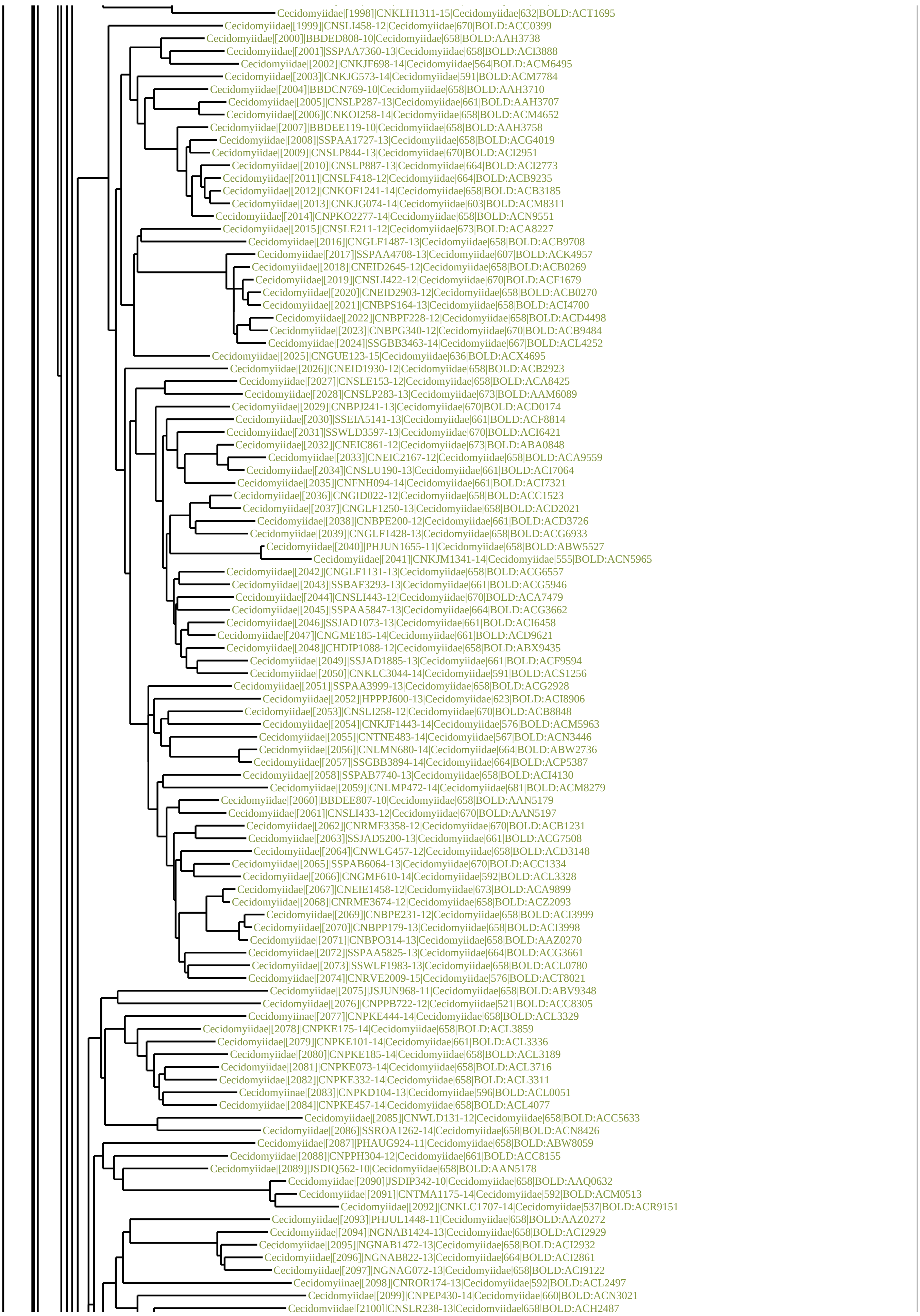
Cecidomyiidae|[1998]|CNKLH1311-15|Cecidomyiidae|632|BOLD:ACT1695
Cecidomyiidae|[1999]|CNSLI458-12|Cecidomyiidae|670|BOLD:ACC0399
Cecidomyiidae|[2000]|BBDED808-10|Cecidomyiidae|658|BOLD:AAH3738
Cecidomyiidae|[2001]|SSPAA7360-13|Cecidomyiidae|658|BOLD:ACI3888
Cecidomyiidae|[2002]|CNKJF698-14|Cecidomyiidae|564|BOLD:ACM6495
Cecidomyiidae|[2003]|CNKJG573-14|Cecidomyiidae|591|BOLD:ACM7784
Cecidomyiidae|[2004]|BBDCN769-10|Cecidomyiidae|658|BOLD:AAH3710
Cecidomyiidae|[2005]|CNSLP287-13|Cecidomyiidae|661|BOLD:AAH3707
Cecidomyiidae|[2006]|CNKOI258-14|Cecidomyiidae|658|BOLD:ACM4652
Cecidomyiidae|[2007]|BBDEE119-10|Cecidomyiidae|658|BOLD:AAH3758
Cecidomyiidae|[2008]|SSPAA1727-13|Cecidomyiidae|658|BOLD:ACG4019
Cecidomyiidae|[2009]|CNSLP844-13|Cecidomyiidae|670|BOLD:ACI2951
Cecidomyiidae|[2010]|CNSLP887-13|Cecidomyiidae|664|BOLD:ACI2773
Cecidomyiidae|[2011]|CNSLF418-12|Cecidomyiidae|664|BOLD:ACB9235
Cecidomyiidae|[2012]|CNKOF1241-14|Cecidomyiidae|658|BOLD:ACB3185
Cecidomyiidae|[2013]|CNKJG074-14|Cecidomyiidae|603|BOLD:ACM8311
Cecidomyiidae|[2014]|CNPKO2277-14|Cecidomyiidae|658|BOLD:ACN9551
Cecidomyiidae|[2015]|CNSLE211-12|Cecidomyiidae|673|BOLD:ACA8227
Cecidomyiidae|[2016]|CNGLF1487-13|Cecidomyiidae|658|BOLD:ACB9708
Cecidomyiidae|[2017]|SSPAA4708-13|Cecidomyiidae|607|BOLD:ACK4957
Cecidomyiidae|[2018]|CNEID2645-12|Cecidomyiidae|658|BOLD:ACB0269
Cecidomyiidae|[2019]|CNSLI422-12|Cecidomyiidae|670|BOLD:ACF1679
Cecidomyiidae|[2020]|CNEID2903-12|Cecidomyiidae|658|BOLD:ACB0270
Cecidomyiidae|[2021]|CNBPS164-13|Cecidomyiidae|658|BOLD:ACI4700
Cecidomyiidae|[2022]|CNBPF228-12|Cecidomyiidae|658|BOLD:ACD4498
Cecidomyiidae|[2023]|CNBPG340-12|Cecidomyiidae|670|BOLD:ACB9484
Cecidomyiidae|[2024]|SSGBB3463-14|Cecidomyiidae|667|BOLD:ACL4252
Cecidomyiidae|[2025]|CNGUE123-15|Cecidomyiidae|636|BOLD:ACX4695
Cecidomyiidae|[2026]|CNEID1930-12|Cecidomyiidae|658|BOLD:ACB2923
Cecidomyiidae|[2027]|CNSLE153-12|Cecidomyiidae|658|BOLD:ACA8425
Cecidomyiidae|[2028]|CNSLP283-13|Cecidomyiidae|673|BOLD:AAM6089
Cecidomyiidae|[2029]|CNBPJ241-13|Cecidomyiidae|670|BOLD:ACD0174
Cecidomyiidae|[2030]|SSEIA5141-13|Cecidomyiidae|661|BOLD:ACF8814
Cecidomyiidae|[2031]|SSWLD3597-13|Cecidomyiidae|670|BOLD:ACI6421
Cecidomyiidae|[2032]|CNEIC861-12|Cecidomyiidae|673|BOLD:ABA0848
Cecidomyiidae|[2033]|CNEIC2167-12|Cecidomyiidae|658|BOLD:ACA9559
Cecidomyiidae|[2034]|CNSLU190-13|Cecidomyiidae|661|BOLD:ACI7064
Cecidomyiidae|[2035]|CNFNH094-14|Cecidomyiidae|661|BOLD:ACI7321
Cecidomyiidae|[2036]|CNGID022-12|Cecidomyiidae|658|BOLD:ACC1523
Cecidomyiidae|[2037]|CNGLF1250-13|Cecidomyiidae|658|BOLD:ACD2021
Cecidomyiidae|[2038]|CNBPE200-12|Cecidomyiidae|661|BOLD:ACD3726
Cecidomyiidae|[2039]|CNGLF1428-13|Cecidomyiidae|658|BOLD:ACG6933
Cecidomyiidae|[2040]|PHJUN1655-11|Cecidomyiidae|658|BOLD:ABW5527
Cecidomyiidae|[2041]|CNKJM1341-14|Cecidomyiidae|555|BOLD:ACN5965
Cecidomyiidae|[2042]|CNGLF1131-13|Cecidomyiidae|658|BOLD:ACG6557
Cecidomyiidae|[2043]|SSBAF3293-13|Cecidomyiidae|661|BOLD:ACG5946
Cecidomyiidae|[2044]|CNSLI443-12|Cecidomyiidae|670|BOLD:ACA7479
Cecidomyiidae|[2045]|SSPAA5847-13|Cecidomyiidae|664|BOLD:ACG3662
Cecidomyiidae|[2046]|SSJAD1073-13|Cecidomyiidae|661|BOLD:ACI6458
Cecidomyiidae|[2047]|CNGME185-14|Cecidomyiidae|661|BOLD:ACD9621
Cecidomyiidae|[2048]|CHDIP1088-12|Cecidomyiidae|658|BOLD:ABX9435
Cecidomyiidae|[2049]|SSJAD1885-13|Cecidomyiidae|661|BOLD:ACF9594
Cecidomyiidae|[2050]|CNKLC3044-14|Cecidomyiidae|591|BOLD:ACS1256
Cecidomyiidae|[2051]|SSPAA3999-13|Cecidomyiidae|658|BOLD:ACG2928
Cecidomyiidae|[2052]|HPPPJ600-13|Cecidomyiidae|623|BOLD:ACI8906
Cecidomyiidae|[2053]|CNSLI258-12|Cecidomyiidae|670|BOLD:ACB8848
Cecidomyiidae|[2054]|CNKJF1443-14|Cecidomyiidae|576|BOLD:ACM5963
Cecidomyiidae|[2055]|CNTNE483-14|Cecidomyiidae|567|BOLD:ACN3446
Cecidomyiidae|[2056]|CNLMN680-14|Cecidomyiidae|664|BOLD:ABW2736
Cecidomyiidae|[2057]|SSGBB3894-14|Cecidomyiidae|664|BOLD:ACP5387
Cecidomyiidae|[2058]|SSPAB7740-13|Cecidomyiidae|658|BOLD:ACI4130
Cecidomyiidae|[2059]|CNLMP472-14|Cecidomyiidae|681|BOLD:ACM8279
Cecidomyiidae|[2060]|BBDEE807-10|Cecidomyiidae|658|BOLD:AAN5179
Cecidomyiidae|[2061]|CNSLI433-12|Cecidomyiidae|670|BOLD:AAN5197
Cecidomyiidae|[2062]|CNRMF3358-12|Cecidomyiidae|670|BOLD:ACB1231
Cecidomyiidae|[2063]|SSJAD5200-13|Cecidomyiidae|661|BOLD:ACG7508
Cecidomyiidae|[2064]|CNWLG457-12|Cecidomyiidae|658|BOLD:ACD3148
Cecidomyiidae|[2065]|SSPAB6064-13|Cecidomyiidae|670|BOLD:ACC1334
Cecidomyiidae|[2066]|CNGMF610-14|Cecidomyiidae|592|BOLD:ACL3328
Cecidomyiidae|[2067]|CNEIE1458-12|Cecidomyiidae|673|BOLD:ACA9899
Cecidomyiidae|[2068]|CNRME3674-12|Cecidomyiidae|658|BOLD:ACZ2093
Cecidomyiidae|[2069]|CNBPE231-12|Cecidomyiidae|658|BOLD:ACI3999
Cecidomyiidae|[2070]|CNBPP179-13|Cecidomyiidae|658|BOLD:ACI3998
Cecidomyiidae|[2071]|CNBPO314-13|Cecidomyiidae|658|BOLD:AAZ0270
Cecidomyiidae|[2072]|SSPAA5825-13|Cecidomyiidae|664|BOLD:ACG3661
Cecidomyiidae|[2073]|SSWLF1983-13|Cecidomyiidae|658|BOLD:ACL0780
Cecidomyiidae|[2074]|CNRVE2009-15|Cecidomyiidae|576|BOLD:ACT8021
Cecidomyiidae|[2075]|JSJUN968-11|Cecidomyiidae|658|BOLD:ABV9348
Cecidomyiidae|[2076]|CNPPB722-12|Cecidomyiidae|521|BOLD:ACC8305
Cecidomyiinae|[2077]|CNPKE444-14|Cecidomyiidae|658|BOLD:ACL3329
Cecidomyiidae|[2078]|CNPKE175-14|Cecidomyiidae|658|BOLD:ACL3859
Cecidomyiidae|[2079]|CNPKE101-14|Cecidomyiidae|661|BOLD:ACL3336
Cecidomyiidae|[2080]|CNPKE185-14|Cecidomyiidae|658|BOLD:ACL3189
Cecidomyiidae|[2081]|CNPKE073-14|Cecidomyiidae|658|BOLD:ACL3716
Cecidomyiidae|[2082]|CNPKE332-14|Cecidomyiidae|658|BOLD:ACL3311
Cecidomyiinae|[2083]|CNPKD104-13|Cecidomyiidae|596|BOLD:ACL0051
Cecidomyiidae|[2084]|CNPKE457-14|Cecidomyiidae|658|BOLD:ACL4077
Cecidomyiidae|[2085]|CNWLD131-12|Cecidomyiidae|658|BOLD:ACC5633
Cecidomyiidae|[2086]|SSROA1262-14|Cecidomyiidae|658|BOLD:ACN8426
Cecidomyiidae|[2087]|PHAUG924-11|Cecidomyiidae|658|BOLD:ABW8059
Cecidomyiidae|[2088]|CNPPH304-12|Cecidomyiidae|661|BOLD:ACC8155
Cecidomyiidae|[2089]|JSDIQ562-10|Cecidomyiidae|658|BOLD:AAN5178
Cecidomyiidae|[2090]|JSDIP342-10|Cecidomyiidae|658|BOLD:AAQ0632
Cecidomyiidae|[2091]|CNTMA1175-14|Cecidomyiidae|592|BOLD:ACM0513
Cecidomyiidae|[2092]|CNKLC1707-14|Cecidomyiidae|537|BOLD:ACR9151
Cecidomyiidae|[2093]|PHJUL1448-11|Cecidomyiidae|658|BOLD:AAZ0272
Cecidomyiidae|[2094]|NGNAB1424-13|Cecidomyiidae|658|BOLD:ACI2929
Cecidomyiidae|[2095]|NGNAB1472-13|Cecidomyiidae|658|BOLD:ACI2932
Cecidomyiidae|[2096]|NGNAB822-13|Cecidomyiidae|664|BOLD:ACI2861
Cecidomyiidae|[2097]|NGNAG072-13|Cecidomyiidae|658|BOLD:ACI9122
Cecidomyiinae|[2098]|CNROR174-13|Cecidomyiidae|592|BOLD:ACL2497
Cecidomyiidae|[2099]|CNPEP430-14|Cecidomyiidae|660|BOLD:ACN3021
Cecidomyiidae|[2100]|CNSLR238-13|Cecidomyiidae|658|BOLD:ACH2487

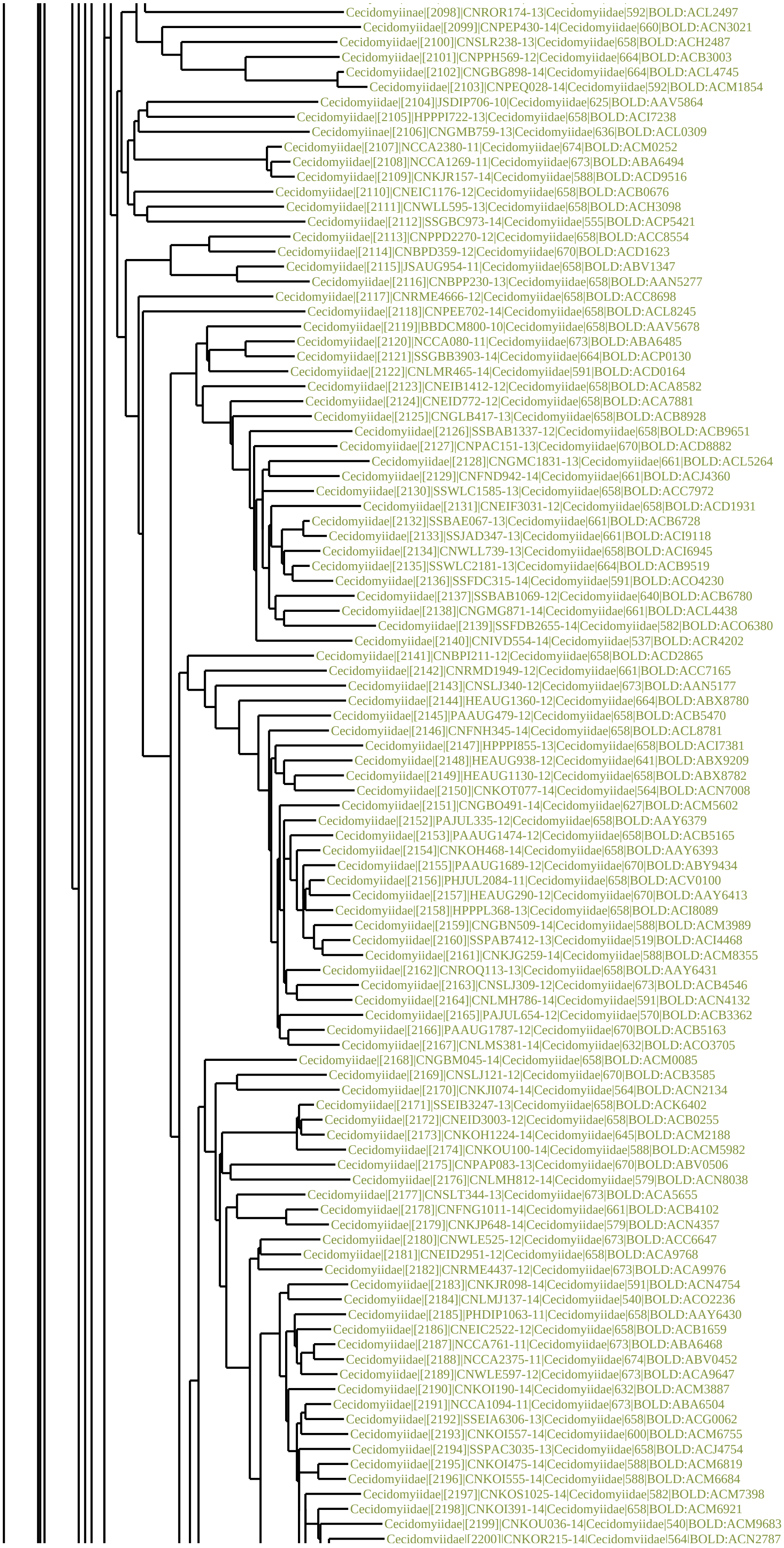
Cecidomyiinae|[2098]|CNROR174-13|Cecidomyiidae|592|BOLD:ACL2497
Cecidomyiidae|[2099]|CNPEP430-14|Cecidomyiidae|660|BOLD:ACN3021
Cecidomyiidae|[2100]|CNSLR238-13|Cecidomyiidae|658|BOLD:ACH2487
Cecidomyiidae|[2101]|CNPPH569-12|Cecidomyiidae|664|BOLD:ACB3003
Cecidomyiidae|[2102]|CNGBG898-14|Cecidomyiidae|664|BOLD:ACL4745
Cecidomyiidae|[2103]|CNPEQ028-14|Cecidomyiidae|592|BOLD:ACM1854
Cecidomyiidae|[2104]|JSDIP706-10|Cecidomyiidae|625|BOLD:AAV5864
Cecidomyiidae|[2105]|HPPPI722-13|Cecidomyiidae|658|BOLD:ACI7238
Cecidomyiinae|[2106]|CNGMB759-13|Cecidomyiidae|636|BOLD:ACL0309
Cecidomyiidae|[2107]|NCCA2380-11|Cecidomyiidae|674|BOLD:ACM0252
Cecidomyiidae|[2108]|NCCA1269-11|Cecidomyiidae|673|BOLD:ABA6494
Cecidomyiidae|[2109]|CNKJR157-14|Cecidomyiidae|588|BOLD:ACD9516
Cecidomyiidae|[2110]|CNEIC1176-12|Cecidomyiidae|658|BOLD:ACB0676
Cecidomyiidae|[2111]|CNWLL595-13|Cecidomyiidae|658|BOLD:ACH3098
Cecidomyiidae|[2112]|SSGBC973-14|Cecidomyiidae|555|BOLD:ACP5421
Cecidomyiidae|[2113]|CNPPD2270-12|Cecidomyiidae|658|BOLD:ACC8554
Cecidomyiidae|[2114]|CNBPD359-12|Cecidomyiidae|670|BOLD:ACD1623
Cecidomyiidae|[2115]|JSAUG954-11|Cecidomyiidae|658|BOLD:ABV1347
Cecidomyiidae|[2116]|CNBPP230-13|Cecidomyiidae|658|BOLD:AAN5277
Cecidomyiidae|[2117]|CNRME4666-12|Cecidomyiidae|658|BOLD:ACC8698
Cecidomyiidae|[2118]|CNPEE702-14|Cecidomyiidae|658|BOLD:ACL8245
Cecidomyiidae|[2119]|BBDCM800-10|Cecidomyiidae|658|BOLD:AAV5678
Cecidomyiidae|[2120]|NCCA080-11|Cecidomyiidae|673|BOLD:ABA6485
Cecidomyiidae|[2121]|SSGBB3903-14|Cecidomyiidae|664|BOLD:ACP0130
Cecidomyiidae|[2122]|CNLMR465-14|Cecidomyiidae|591|BOLD:ACD0164
Cecidomyiidae|[2123]|CNEIB1412-12|Cecidomyiidae|658|BOLD:ACA8582
Cecidomyiidae|[2124]|CNEID772-12|Cecidomyiidae|658|BOLD:ACA7881
Cecidomyiidae|[2125]|CNGLB417-13|Cecidomyiidae|658|BOLD:ACB8928
Cecidomyiidae|[2126]|SSBAB1337-12|Cecidomyiidae|658|BOLD:ACB9651
Cecidomyiidae|[2127]|CNPAC151-13|Cecidomyiidae|670|BOLD:ACD8882
Cecidomyiidae|[2128]|CNGMC1831-13|Cecidomyiidae|661|BOLD:ACL5264
Cecidomyiidae|[2129]|CNFND942-14|Cecidomyiidae|661|BOLD:ACJ4360
Cecidomyiidae|[2130]|SSWLC1585-13|Cecidomyiidae|658|BOLD:ACC7972
Cecidomyiidae|[2131]|CNEIF3031-12|Cecidomyiidae|658|BOLD:ACD1931
Cecidomyiidae|[2132]|SSBAE067-13|Cecidomyiidae|661|BOLD:ACB6728
Cecidomyiidae|[2133]|SSJAD347-13|Cecidomyiidae|661|BOLD:ACI9118
Cecidomyiidae|[2134]|CNWLL739-13|Cecidomyiidae|658|BOLD:ACI6945
Cecidomyiidae|[2135]|SSWLC2181-13|Cecidomyiidae|664|BOLD:ACB9519
Cecidomyiidae|[2136]|SSFDC315-14|Cecidomyiidae|591|BOLD:ACO4230
Cecidomyiidae|[2137]|SSBAB1069-12|Cecidomyiidae|640|BOLD:ACB6780
Cecidomyiidae|[2138]|CNGMG871-14|Cecidomyiidae|661|BOLD:ACL4438
Cecidomyiidae|[2139]|SSFDB2655-14|Cecidomyiidae|582|BOLD:ACO6380
Cecidomyiidae|[2140]|CNIVD554-14|Cecidomyiidae|537|BOLD:ACR4202
Cecidomyiidae|[2141]|CNBPI211-12|Cecidomyiidae|658|BOLD:ACD2865
Cecidomyiidae|[2142]|CNRMD1949-12|Cecidomyiidae|661|BOLD:ACC7165
Cecidomyiidae|[2143]|CNSLJ340-12|Cecidomyiidae|673|BOLD:AAN5177
Cecidomyiidae|[2144]|HEAUG1360-12|Cecidomyiidae|664|BOLD:ABX8780
Cecidomyiidae|[2145]|PAAUG479-12|Cecidomyiidae|658|BOLD:ACB5470
Cecidomyiidae|[2146]|CNFNH345-14|Cecidomyiidae|658|BOLD:ACL8781
Cecidomyiidae|[2147]|HPPPI855-13|Cecidomyiidae|658|BOLD:ACI7381
Cecidomyiidae|[2148]|HEAUG938-12|Cecidomyiidae|641|BOLD:ABX9209
Cecidomyiidae|[2149]|HEAUG1130-12|Cecidomyiidae|658|BOLD:ABX8782
Cecidomyiidae|[2150]|CNKOT077-14|Cecidomyiidae|564|BOLD:ACN7008
Cecidomyiidae|[2151]|CNGBO491-14|Cecidomyiidae|627|BOLD:ACM5602
Cecidomyiidae|[2152]|PAJUL335-12|Cecidomyiidae|658|BOLD:AAY6379
Cecidomyiidae|[2153]|PAAUG1474-12|Cecidomyiidae|658|BOLD:ACB5165
Cecidomyiidae|[2154]|CNKOH468-14|Cecidomyiidae|658|BOLD:AAY6393
Cecidomyiidae|[2155]|PAAUG1689-12|Cecidomyiidae|670|BOLD:ABY9434
Cecidomyiidae|[2156]|PHJUL2084-11|Cecidomyiidae|658|BOLD:ACV0100
Cecidomyiidae|[2157]|HEAUG290-12|Cecidomyiidae|670|BOLD:AAY6413
Cecidomyiidae|[2158]|HPPPL368-13|Cecidomyiidae|658|BOLD:ACI8089
Cecidomyiidae|[2159]|CNGBN509-14|Cecidomyiidae|588|BOLD:ACM3989
Cecidomyiidae|[2160]|SSPAB7412-13|Cecidomyiidae|519|BOLD:ACI4468
Cecidomyiidae|[2161]|CNKJG259-14|Cecidomyiidae|588|BOLD:ACM8355
Cecidomyiidae|[2162]|CNROQ113-13|Cecidomyiidae|658|BOLD:AAY6431
Cecidomyiidae|[2163]|CNSLJ309-12|Cecidomyiidae|673|BOLD:ACB4546
Cecidomyiidae|[2164]|CNLMH786-14|Cecidomyiidae|591|BOLD:ACN4132
Cecidomyiidae|[2165]|PAJUL654-12|Cecidomyiidae|570|BOLD:ACB3362
Cecidomyiidae|[2166]|PAAUG1787-12|Cecidomyiidae|670|BOLD:ACB5163
Cecidomyiidae|[2167]|CNLMS381-14|Cecidomyiidae|632|BOLD:ACO3705
Cecidomyiidae|[2168]|CNGBM045-14|Cecidomyiidae|658|BOLD:ACM0085
Cecidomyiidae|[2169]|CNSLJ121-12|Cecidomyiidae|670|BOLD:ACB3585
Cecidomyiidae|[2170]|CNKJI074-14|Cecidomyiidae|564|BOLD:ACN2134
Cecidomyiidae|[2171]|SSEIB3247-13|Cecidomyiidae|658|BOLD:ACK6402
Cecidomyiidae|[2172]|CNEID3003-12|Cecidomyiidae|658|BOLD:ACB0255
Cecidomyiidae|[2173]|CNKOH1224-14|Cecidomyiidae|645|BOLD:ACM2188
Cecidomyiidae|[2174]|CNKOU100-14|Cecidomyiidae|588|BOLD:ACM5982
Cecidomyiidae|[2175]|CNPAP083-13|Cecidomyiidae|670|BOLD:ABV0506
Cecidomyiidae|[2176]|CNLMH812-14|Cecidomyiidae|579|BOLD:ACN8038
Cecidomyiidae|[2177]|CNSLT344-13|Cecidomyiidae|673|BOLD:ACA5655
Cecidomyiidae|[2178]|CNFNG1011-14|Cecidomyiidae|661|BOLD:ACB4102
Cecidomyiidae|[2179]|CNKJP648-14|Cecidomyiidae|579|BOLD:ACN4357
Cecidomyiidae|[2180]|CNWLE525-12|Cecidomyiidae|673|BOLD:ACC6647
Cecidomyiidae|[2181]|CNEID2951-12|Cecidomyiidae|658|BOLD:ACA9768
Cecidomyiidae|[2182]|CNRME4437-12|Cecidomyiidae|673|BOLD:ACA9976
Cecidomyiidae|[2183]|CNKJR098-14|Cecidomyiidae|591|BOLD:ACN4754
Cecidomyiidae|[2184]|CNLMJ137-14|Cecidomyiidae|540|BOLD:ACO2236
Cecidomyiidae|[2185]|PHDIP1063-11|Cecidomyiidae|658|BOLD:AAY6430
Cecidomyiidae|[2186]|CNEIC2522-12|Cecidomyiidae|658|BOLD:ACB1659
Cecidomyiidae|[2187]|NCCA761-11|Cecidomyiidae|673|BOLD:ABA6468
Cecidomyiidae|[2188]|NCCA2375-11|Cecidomyiidae|674|BOLD:ABV0452
Cecidomyiidae|[2189]|CNWLE597-12|Cecidomyiidae|673|BOLD:ACA9647
Cecidomyiidae|[2190]|CNKOI190-14|Cecidomyiidae|632|BOLD:ACM3887
Cecidomyiidae|[2191]|NCCA1094-11|Cecidomyiidae|673|BOLD:ABA6504
Cecidomyiidae|[2192]|SSEIA6306-13|Cecidomyiidae|658|BOLD:ACG0062
Cecidomyiidae|[2193]|CNKOI557-14|Cecidomyiidae|600|BOLD:ACM6755
Cecidomyiidae|[2194]|SSPAC3035-13|Cecidomyiidae|658|BOLD:ACJ4754
Cecidomyiidae|[2195]|CNKOI475-14|Cecidomyiidae|588|BOLD:ACM6819
Cecidomyiidae|[2196]|CNKOI555-14|Cecidomyiidae|588|BOLD:ACM6684
Cecidomyiidae|[2197]|CNKOS1025-14|Cecidomyiidae|582|BOLD:ACM7398
Cecidomyiidae|[2198]|CNKOI391-14|Cecidomyiidae|658|BOLD:ACM6921
Cecidomyiidae|[2199]|CNKOU036-14|Cecidomyiidae|540|BOLD:ACM9683
Cecidomyiidae|[2200]|CNKOR215-14|Cecidomyiidae|564|BOLD:ACN2787

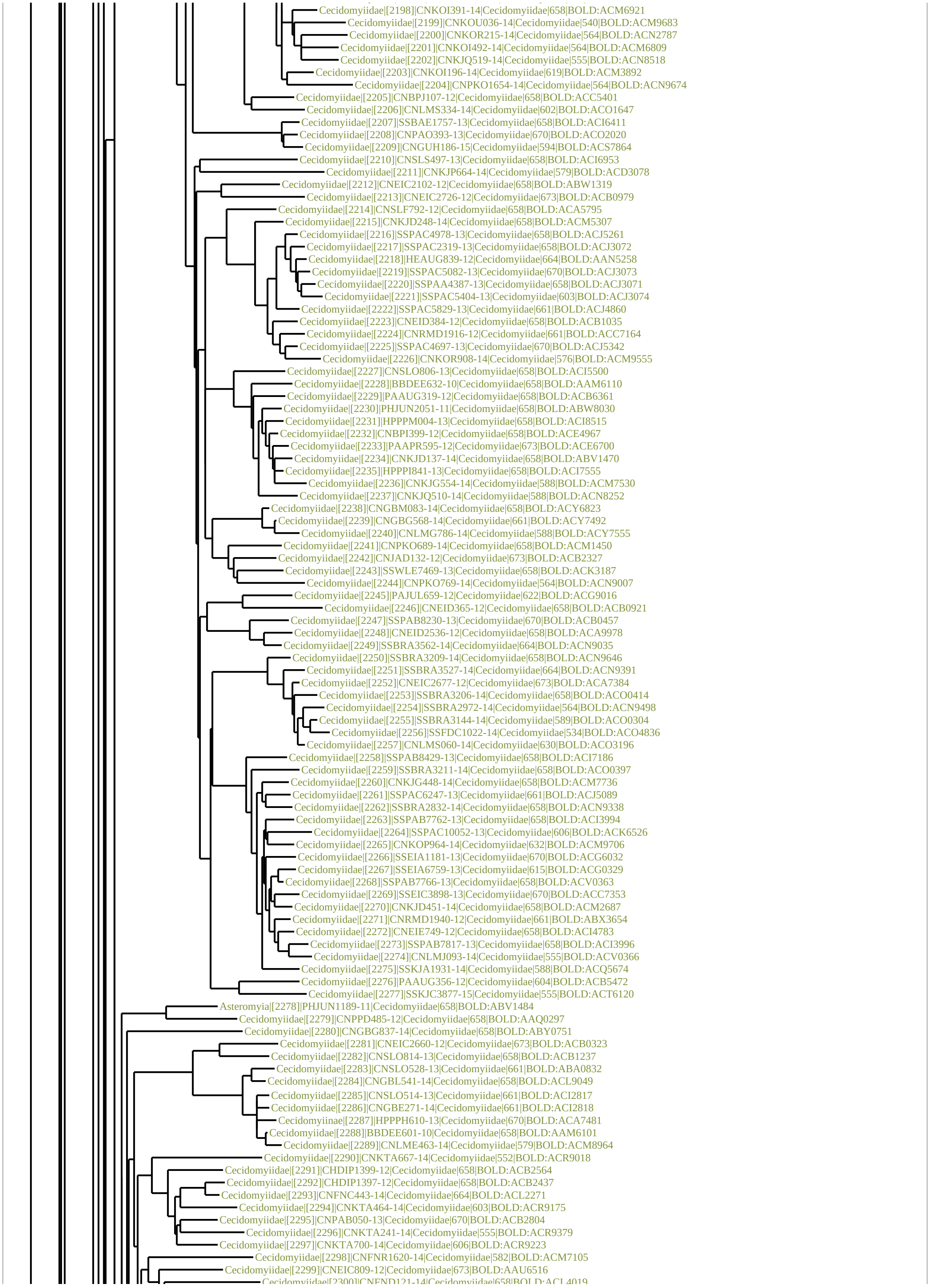
Cecidomyiidae|[2198]|CNKOI391-14|Cecidomyiidae|658|BOLD:ACM6921
Cecidomyiidae|[2199]|CNKOU036-14|Cecidomyiidae|540|BOLD:ACM9683
Cecidomyiidae|[2200]|CNKOR215-14|Cecidomyiidae|564|BOLD:ACN2787
Cecidomyiidae|[2201]|CNKOI492-14|Cecidomyiidae|564|BOLD:ACM6809
Cecidomyiidae|[2202]|CNKJQ519-14|Cecidomyiidae|555|BOLD:ACN8518
Cecidomyiidae|[2203]|CNKOI196-14|Cecidomyiidae|619|BOLD:ACM3892
Cecidomyiidae|[2204]|CNPKO1654-14|Cecidomyiidae|564|BOLD:ACN9674
Cecidomyiidae|[2205]|CNBPJ107-12|Cecidomyiidae|658|BOLD:ACC5401
Cecidomyiidae|[2206]|CNLMS334-14|Cecidomyiidae|602|BOLD:ACO1647
Cecidomyiidae|[2207]|SSBAE1757-13|Cecidomyiidae|658|BOLD:ACI6411
Cecidomyiidae|[2208]|CNPAO393-13|Cecidomyiidae|670|BOLD:ACO2020
Cecidomyiidae|[2209]|CNGUH186-15|Cecidomyiidae|594|BOLD:ACS7864
Cecidomyiidae|[2210]|CNSLS497-13|Cecidomyiidae|658|BOLD:ACI6953
Cecidomyiidae|[2211]|CNKJP664-14|Cecidomyiidae|579|BOLD:ACD3078
Cecidomyiidae|[2212]|CNEIC2102-12|Cecidomyiidae|658|BOLD:ABW1319
Cecidomyiidae|[2213]|CNEIC2726-12|Cecidomyiidae|673|BOLD:ACB0979
Cecidomyiidae|[2214]|CNSLF792-12|Cecidomyiidae|658|BOLD:ACA5795
Cecidomyiidae|[2215]|CNKJD248-14|Cecidomyiidae|658|BOLD:ACM5307
Cecidomyiidae|[2216]|SSPAC4978-13|Cecidomyiidae|658|BOLD:ACJ5261
Cecidomyiidae|[2217]|SSPAC2319-13|Cecidomyiidae|658|BOLD:ACJ3072
Cecidomyiidae|[2218]|HEAUG839-12|Cecidomyiidae|664|BOLD:AAN5258
Cecidomyiidae|[2219]|SSPAC5082-13|Cecidomyiidae|670|BOLD:ACJ3073
Cecidomyiidae|[2220]|SSPAA4387-13|Cecidomyiidae|658|BOLD:ACJ3071
Cecidomyiidae|[2221]|SSPAC5404-13|Cecidomyiidae|603|BOLD:ACJ3074
Cecidomyiidae|[2222]|SSPAC5829-13|Cecidomyiidae|661|BOLD:ACJ4860
Cecidomyiidae|[2223]|CNEID384-12|Cecidomyiidae|658|BOLD:ACB1035
Cecidomyiidae|[2224]|CNRMD1916-12|Cecidomyiidae|661|BOLD:ACC7164
Cecidomyiidae|[2225]|SSPAC4697-13|Cecidomyiidae|670|BOLD:ACJ5342
Cecidomyiidae|[2226]|CNKOR908-14|Cecidomyiidae|576|BOLD:ACM9555
Cecidomyiidae|[2227]|CNSLO806-13|Cecidomyiidae|658|BOLD:ACI5500
Cecidomyiidae|[2228]|BBDEE632-10|Cecidomyiidae|658|BOLD:AAM6110
Cecidomyiidae|[2229]|PAAUG319-12|Cecidomyiidae|658|BOLD:ACB6361
Cecidomyiidae|[2230]|PHJUN2051-11|Cecidomyiidae|658|BOLD:ABW8030
Cecidomyiidae|[2231]|HPPPM004-13|Cecidomyiidae|658|BOLD:ACI8515
Cecidomyiidae|[2232]|CNBPI399-12|Cecidomyiidae|658|BOLD:ACE4967
Cecidomyiidae|[2233]|PAAPR595-12|Cecidomyiidae|673|BOLD:ACE6700
Cecidomyiidae|[2234]|CNKJD137-14|Cecidomyiidae|658|BOLD:ABV1470
Cecidomyiidae|[2235]|HPPPI841-13|Cecidomyiidae|658|BOLD:ACI7555
Cecidomyiidae|[2236]|CNKJG554-14|Cecidomyiidae|588|BOLD:ACM7530
Cecidomyiidae|[2237]|CNKJQ510-14|Cecidomyiidae|588|BOLD:ACN8252
Cecidomyiidae|[2238]|CNGBM083-14|Cecidomyiidae|658|BOLD:ACY6823
Cecidomyiidae|[2239]|CNGBG568-14|Cecidomyiidae|661|BOLD:ACY7492
Cecidomyiidae|[2240]|CNLMG786-14|Cecidomyiidae|588|BOLD:ACY7555
Cecidomyiidae|[2241]|CNPKO689-14|Cecidomyiidae|658|BOLD:ACM1450
Cecidomyiidae|[2242]|CNJAD132-12|Cecidomyiidae|673|BOLD:ACB2327
Cecidomyiidae|[2243]|SSWLE7469-13|Cecidomyiidae|658|BOLD:ACK3187
Cecidomyiidae|[2244]|CNPKO769-14|Cecidomyiidae|564|BOLD:ACN9007
Cecidomyiidae|[2245]|PAJUL659-12|Cecidomyiidae|622|BOLD:ACG9016
Cecidomyiidae|[2246]|CNEID365-12|Cecidomyiidae|658|BOLD:ACB0921
Cecidomyiidae|[2247]|SSPAB8230-13|Cecidomyiidae|670|BOLD:ACB0457
Cecidomyiidae|[2248]|CNEID2536-12|Cecidomyiidae|658|BOLD:ACA9978
Cecidomyiidae|[2249]|SSBRA3562-14|Cecidomyiidae|664|BOLD:ACN9035
Cecidomyiidae|[2250]|SSBRA3209-14|Cecidomyiidae|658|BOLD:ACN9646
Cecidomyiidae|[2251]|SSBRA3527-14|Cecidomyiidae|664|BOLD:ACN9391
Cecidomyiidae|[2252]|CNEIC2677-12|Cecidomyiidae|673|BOLD:ACA7384
Cecidomyiidae|[2253]|SSBRA3206-14|Cecidomyiidae|658|BOLD:ACO0414
Cecidomyiidae|[2254]|SSBRA2972-14|Cecidomyiidae|564|BOLD:ACN9498
Cecidomyiidae|[2255]|SSBRA3144-14|Cecidomyiidae|589|BOLD:ACO0304
Cecidomyiidae|[2256]|SSFDC1022-14|Cecidomyiidae|534|BOLD:ACO4836
Cecidomyiidae|[2257]|CNLMS060-14|Cecidomyiidae|630|BOLD:ACO3196
Cecidomyiidae|[2258]|SSPAB8429-13|Cecidomyiidae|658|BOLD:ACI7186
Cecidomyiidae|[2259]|SSBRA3211-14|Cecidomyiidae|658|BOLD:ACO0397
Cecidomyiidae|[2260]|CNKJG448-14|Cecidomyiidae|658|BOLD:ACM7736
Cecidomyiidae|[2261]|SSPAC6247-13|Cecidomyiidae|661|BOLD:ACJ5089
Cecidomyiidae|[2262]|SSBRA2832-14|Cecidomyiidae|658|BOLD:ACN9338
Cecidomyiidae|[2263]|SSPAB7762-13|Cecidomyiidae|658|BOLD:ACI3994
Cecidomyiidae|[2264]|SSPAC10052-13|Cecidomyiidae|606|BOLD:ACK6526
Cecidomyiidae|[2265]|CNKOP964-14|Cecidomyiidae|632|BOLD:ACM9706
Cecidomyiidae|[2266]|SSEIA1181-13|Cecidomyiidae|670|BOLD:ACG6032
Cecidomyiidae|[2267]|SSEIA6759-13|Cecidomyiidae|615|BOLD:ACG0329
Cecidomyiidae|[2268]|SSPAB7766-13|Cecidomyiidae|658|BOLD:ACV0363
Cecidomyiidae|[2269]|SSEIC3898-13|Cecidomyiidae|670|BOLD:ACC7353
Cecidomyiidae|[2270]|CNKJD451-14|Cecidomyiidae|658|BOLD:ACM2687
Cecidomyiidae|[2271]|CNRMD1940-12|Cecidomyiidae|661|BOLD:ABX3654
Cecidomyiidae|[2272]|CNEIE749-12|Cecidomyiidae|658|BOLD:ACI4783
Cecidomyiidae|[2273]|SSPAB7817-13|Cecidomyiidae|658|BOLD:ACI3996
Cecidomyiidae|[2274]|CNLMJ093-14|Cecidomyiidae|555|BOLD:ACV0366
Cecidomyiidae|[2275]|SSKJA1931-14|Cecidomyiidae|588|BOLD:ACQ5674
Cecidomyiidae|[2276]|PAAUG356-12|Cecidomyiidae|604|BOLD:ACB5472
Cecidomyiidae|[2277]|SSKJC3877-15|Cecidomyiidae|555|BOLD:ACT6120
Asteromyia|[2278]|PHJUN1189-11|Cecidomyiidae|658|BOLD:ABV1484
Cecidomyiidae|[2279]|CNPPD485-12|Cecidomyiidae|658|BOLD:AAQ0297
Cecidomyiidae|[2280]|CNGBG837-14|Cecidomyiidae|658|BOLD:ABY0751
Cecidomyiidae|[2281]|CNEIC2660-12|Cecidomyiidae|673|BOLD:ACB0323
Cecidomyiidae|[2282]|CNSLO814-13|Cecidomyiidae|658|BOLD:ACB1237
Cecidomyiidae|[2283]|CNSLO528-13|Cecidomyiidae|661|BOLD:ABA0832
Cecidomyiidae|[2284]|CNGBL541-14|Cecidomyiidae|658|BOLD:ACL9049
Cecidomyiidae|[2285]|CNSLO514-13|Cecidomyiidae|661|BOLD:ACI2817
Cecidomyiidae|[2286]|CNGBE271-14|Cecidomyiidae|661|BOLD:ACI2818
Cecidomyiinae|[2287]|HPPPH610-13|Cecidomyiidae|670|BOLD:ACA7481
Cecidomyiidae|[2288]|BBDEE601-10|Cecidomyiidae|658|BOLD:AAM6101
Cecidomyiidae|[2289]|CNLME463-14|Cecidomyiidae|579|BOLD:ACM8964
Cecidomyiidae|[2290]|CNKTA667-14|Cecidomyiidae|552|BOLD:ACR9018
Cecidomyiidae|[2291]|CHDIP1399-12|Cecidomyiidae|658|BOLD:ACB2564
Cecidomyiidae|[2292]|CHDIP1397-12|Cecidomyiidae|658|BOLD:ACB2437
Cecidomyiidae|[2293]|CNFNC443-14|Cecidomyiidae|664|BOLD:ACL2271
Cecidomyiidae|[2294]|CNKTA464-14|Cecidomyiidae|603|BOLD:ACR9175
Cecidomyiidae|[2295]|CNPAB050-13|Cecidomyiidae|670|BOLD:ACB2804
Cecidomyiidae|[2296]|CNKTA241-14|Cecidomyiidae|555|BOLD:ACR9379
Cecidomyiidae|[2297]|CNKTA700-14|Cecidomyiidae|606|BOLD:ACR9223
Cecidomyiidae|[2298]|CNFNR1620-14|Cecidomyiidae|582|BOLD:ACM7105
Cecidomyiidae|[2299]|CNEIC809-12|Cecidomyiidae|673|BOLD:AAU6516
Cecidomyiidae|[2300]|CNFND121-14|Cecidomyiidae|658|BOLD:ACL4019

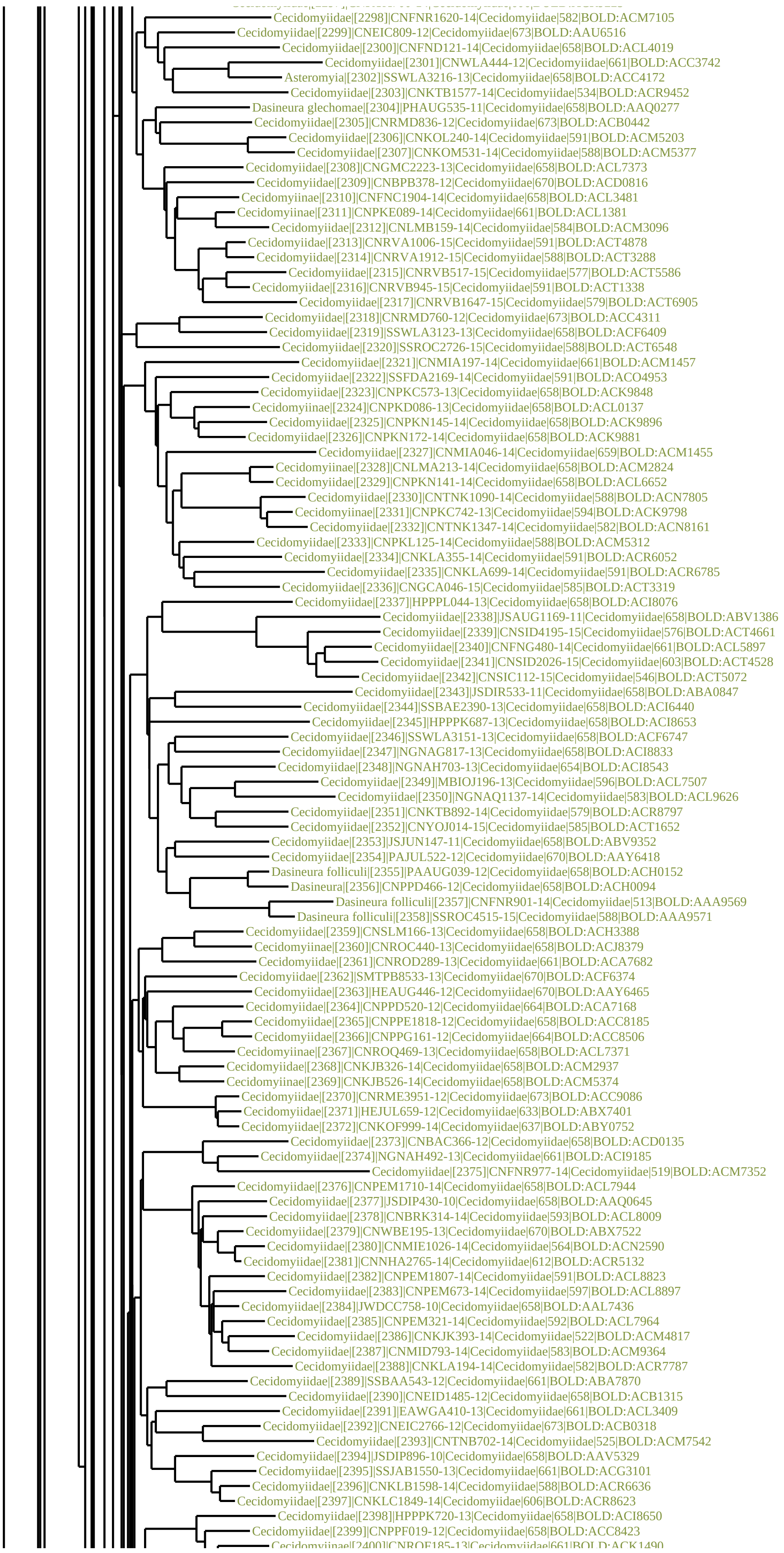
Cecidomyiidae|[2298]|CNFNR1620-14|Cecidomyiidae|582|BOLD:ACM7105
Cecidomyiidae|[2299]|CNEIC809-12|Cecidomyiidae|673|BOLD:AAU6516
Cecidomyiidae|[2300]|CNFND121-14|Cecidomyiidae|658|BOLD:ACL4019
Cecidomyiidae|[2301]|CNWLA444-12|Cecidomyiidae|661|BOLD:ACC3742
Asteromyia|[2302]|SSWLA3216-13|Cecidomyiidae|658|BOLD:ACC4172
Cecidomyiidae|[2303]|CNKTB1577-14|Cecidomyiidae|534|BOLD:ACR9452
Dasineura glechomae|[2304]|PHAUG535-11|Cecidomyiidae|658|BOLD:AAQ0277
Cecidomyiidae|[2305]|CNRMD836-12|Cecidomyiidae|673|BOLD:ACB0442
Cecidomyiidae|[2306]|CNKOL240-14|Cecidomyiidae|591|BOLD:ACM5203
Cecidomyiidae|[2307]|CNKOM531-14|Cecidomyiidae|588|BOLD:ACM5377
Cecidomyiidae|[2308]|CNGMC2223-13|Cecidomyiidae|658|BOLD:ACL7373
Cecidomyiidae|[2309]|CNBPB378-12|Cecidomyiidae|670|BOLD:ACD0816
Cecidomyiinae|[2310]|CNFNC1904-14|Cecidomyiidae|658|BOLD:ACL3481
Cecidomyiinae|[2311]|CNPKE089-14|Cecidomyiidae|661|BOLD:ACL1381
Cecidomyiidae|[2312]|CNLMB159-14|Cecidomyiidae|584|BOLD:ACM3096
Cecidomyiidae|[2313]|CNRVA1006-15|Cecidomyiidae|591|BOLD:ACT4878
Cecidomyiidae|[2314]|CNRVA1912-15|Cecidomyiidae|588|BOLD:ACT3288
Cecidomyiidae|[2315]|CNRVB517-15|Cecidomyiidae|577|BOLD:ACT5586
Cecidomyiidae|[2316]|CNRVB945-15|Cecidomyiidae|591|BOLD:ACT1338
Cecidomyiidae|[2317]|CNRVB1647-15|Cecidomyiidae|579|BOLD:ACT6905
Cecidomyiidae|[2318]|CNRMD760-12|Cecidomyiidae|673|BOLD:ACC4311
Cecidomyiidae|[2319]|SSWLA3123-13|Cecidomyiidae|658|BOLD:ACF6409
Cecidomyiidae|[2320]|SSROC2726-15|Cecidomyiidae|588|BOLD:ACT6548
Cecidomyiidae|[2321]|CNMIA197-14|Cecidomyiidae|661|BOLD:ACM1457
Cecidomyiidae|[2322]|SSFDA2169-14|Cecidomyiidae|591|BOLD:ACO4953
Cecidomyiidae|[2323]|CNPKC573-13|Cecidomyiidae|658|BOLD:ACK9848
Cecidomyiinae|[2324]|CNPKD086-13|Cecidomyiidae|658|BOLD:ACL0137
Cecidomyiidae|[2325]|CNPKN145-14|Cecidomyiidae|658|BOLD:ACK9896
Cecidomyiidae|[2326]|CNPKN172-14|Cecidomyiidae|658|BOLD:ACK9881
Cecidomyiidae|[2327]|CNMIA046-14|Cecidomyiidae|659|BOLD:ACM1455
Cecidomyiinae|[2328]|CNLMA213-14|Cecidomyiidae|658|BOLD:ACM2824
Cecidomyiidae|[2329]|CNPKN141-14|Cecidomyiidae|658|BOLD:ACL6652
Cecidomyiidae|[2330]|CNTNK1090-14|Cecidomyiidae|588|BOLD:ACN7805
Cecidomyiinae|[2331]|CNPKC742-13|Cecidomyiidae|594|BOLD:ACK9798
Cecidomyiidae|[2332]|CNTNK1347-14|Cecidomyiidae|582|BOLD:ACN8161
Cecidomyiidae|[2333]|CNPKL125-14|Cecidomyiidae|588|BOLD:ACM5312
Cecidomyiidae|[2334]|CNKLA355-14|Cecidomyiidae|591|BOLD:ACR6052
Cecidomyiidae|[2335]|CNKLA699-14|Cecidomyiidae|591|BOLD:ACR6785
Cecidomyiidae|[2336]|CNGCA046-15|Cecidomyiidae|585|BOLD:ACT3319
Cecidomyiidae|[2337]|HPPPL044-13|Cecidomyiidae|658|BOLD:ACI8076
Cecidomyiidae|[2338]|JSAUG1169-11|Cecidomyiidae|658|BOLD:ABV1386
Cecidomyiidae|[2339]|CNSID4195-15|Cecidomyiidae|576|BOLD:ACT4661
Cecidomyiidae|[2340]|CNFNG480-14|Cecidomyiidae|661|BOLD:ACL5897
Cecidomyiidae|[2341]|CNSID2026-15|Cecidomyiidae|603|BOLD:ACT4528
Cecidomyiidae|[2342]|CNSIC112-15|Cecidomyiidae|546|BOLD:ACT5072
Cecidomyiidae|[2343]|JSDIR533-11|Cecidomyiidae|658|BOLD:ABA0847
Cecidomyiidae|[2344]|SSBAE2390-13|Cecidomyiidae|658|BOLD:ACI6440
Cecidomyiidae|[2345]|HPPPK687-13|Cecidomyiidae|658|BOLD:ACI8653
Cecidomyiidae|[2346]|SSWLA3151-13|Cecidomyiidae|658|BOLD:ACF6747
Cecidomyiidae|[2347]|NGNAG817-13|Cecidomyiidae|658|BOLD:ACI8833
Cecidomyiidae|[2348]|NGNAH703-13|Cecidomyiidae|654|BOLD:ACI8543
Cecidomyiidae|[2349]|MBIOJ196-13|Cecidomyiidae|596|BOLD:ACL7507
Cecidomyiidae|[2350]|NGNAQ1137-14|Cecidomyiidae|583|BOLD:ACL9626
Cecidomyiidae|[2351]|CNKTB892-14|Cecidomyiidae|579|BOLD:ACR8797
Cecidomyiidae|[2352]|CNYOJ014-15|Cecidomyiidae|585|BOLD:ACT1652
Cecidomyiidae|[2353]|JSJUN147-11|Cecidomyiidae|658|BOLD:ABV9352
Cecidomyiidae|[2354]|PAJUL522-12|Cecidomyiidae|670|BOLD:AAY6418
Dasineura folliculi|[2355]|PAAUG039-12|Cecidomyiidae|658|BOLD:ACH0152
Dasineura|[2356]|CNPPD466-12|Cecidomyiidae|658|BOLD:ACH0094
Dasineura folliculi|[2357]|CNFNR901-14|Cecidomyiidae|513|BOLD:AAA9569
Dasineura folliculi|[2358]|SSROC4515-15|Cecidomyiidae|588|BOLD:AAA9571
Cecidomyiidae|[2359]|CNSLM166-13|Cecidomyiidae|658|BOLD:ACH3388
Cecidomyiinae|[2360]|CNROC440-13|Cecidomyiidae|658|BOLD:ACJ8379
Cecidomyiidae|[2361]|CNROD289-13|Cecidomyiidae|661|BOLD:ACA7682
Cecidomyiidae|[2362]|SMTPB8533-13|Cecidomyiidae|670|BOLD:ACF6374
Cecidomyiidae|[2363]|HEAUG446-12|Cecidomyiidae|670|BOLD:AAY6465
Cecidomyiidae|[2364]|CNPPD520-12|Cecidomyiidae|664|BOLD:ACA7168
Cecidomyiidae|[2365]|CNPPE1818-12|Cecidomyiidae|658|BOLD:ACC8185
Cecidomyiidae|[2366]|CNPPG161-12|Cecidomyiidae|664|BOLD:ACC8506
Cecidomyiinae|[2367]|CNROQ469-13|Cecidomyiidae|658|BOLD:ACL7371
Cecidomyiidae|[2368]|CNKJB326-14|Cecidomyiidae|658|BOLD:ACM2937
Cecidomyiinae|[2369]|CNKJB526-14|Cecidomyiidae|658|BOLD:ACM5374
Cecidomyiidae|[2370]|CNRME3951-12|Cecidomyiidae|673|BOLD:ACC9086
Cecidomyiidae|[2371]|HEJUL659-12|Cecidomyiidae|633|BOLD:ABX7401
Cecidomyiidae|[2372]|CNKOF999-14|Cecidomyiidae|637|BOLD:ABY0752
Cecidomyiidae|[2373]|CNBAC366-12|Cecidomyiidae|658|BOLD:ACD0135
Cecidomyiidae|[2374]|NGNAH492-13|Cecidomyiidae|661|BOLD:ACI9185
Cecidomyiidae|[2375]|CNFNR977-14|Cecidomyiidae|519|BOLD:ACM7352
Cecidomyiidae|[2376]|CNPEM1710-14|Cecidomyiidae|658|BOLD:ACL7944
Cecidomyiidae|[2377]|JSDIP430-10|Cecidomyiidae|658|BOLD:AAQ0645
Cecidomyiidae|[2378]|CNBRK314-14|Cecidomyiidae|593|BOLD:ACL8009
Cecidomyiidae|[2379]|CNWBE195-13|Cecidomyiidae|670|BOLD:ABX7522
Cecidomyiidae|[2380]|CNMIE1026-14|Cecidomyiidae|564|BOLD:ACN2590
Cecidomyiidae|[2381]|CNNHA2765-14|Cecidomyiidae|612|BOLD:ACR5132
Cecidomyiidae|[2382]|CNPEM1807-14|Cecidomyiidae|591|BOLD:ACL8823
Cecidomyiidae|[2383]|CNPEM673-14|Cecidomyiidae|597|BOLD:ACL8897
Cecidomyiidae|[2384]|JWDCC758-10|Cecidomyiidae|658|BOLD:AAL7436
Cecidomyiidae|[2385]|CNPEM321-14|Cecidomyiidae|592|BOLD:ACL7964
Cecidomyiidae|[2386]|CNKJK393-14|Cecidomyiidae|522|BOLD:ACM4817
Cecidomyiidae|[2387]|CNMID793-14|Cecidomyiidae|583|BOLD:ACM9364
Cecidomyiidae|[2388]|CNKLA194-14|Cecidomyiidae|582|BOLD:ACR7787
Cecidomyiidae|[2389]|SSBAA543-12|Cecidomyiidae|661|BOLD:ABA7870
Cecidomyiidae|[2390]|CNEID1485-12|Cecidomyiidae|658|BOLD:ACB1315
Cecidomyiidae|[2391]|EAWGA410-13|Cecidomyiidae|661|BOLD:ACL3409
Cecidomyiidae|[2392]|CNEIC2766-12|Cecidomyiidae|673|BOLD:ACB0318
Cecidomyiidae|[2393]|CNTNB702-14|Cecidomyiidae|525|BOLD:ACM7542
Cecidomyiidae|[2394]|JSDIP896-10|Cecidomyiidae|658|BOLD:AAV5329
Cecidomyiidae|[2395]|SSJAB1550-13|Cecidomyiidae|661|BOLD:ACG3101
Cecidomyiidae|[2396]|CNKLB1598-14|Cecidomyiidae|588|BOLD:ACR6636
Cecidomyiidae|[2397]|CNKLC1849-14|Cecidomyiidae|606|BOLD:ACR8623
Cecidomyiidae|[2398]|HPPPK720-13|Cecidomyiidae|658|BOLD:ACI8650
Cecidomyiidae|[2399]|CNPPF019-12|Cecidomyiidae|658|BOLD:ACC8423
Cecidomyiinae|[2400]|CNROF185-13|Cecidomyiidae|661|BOLD:ACK1490

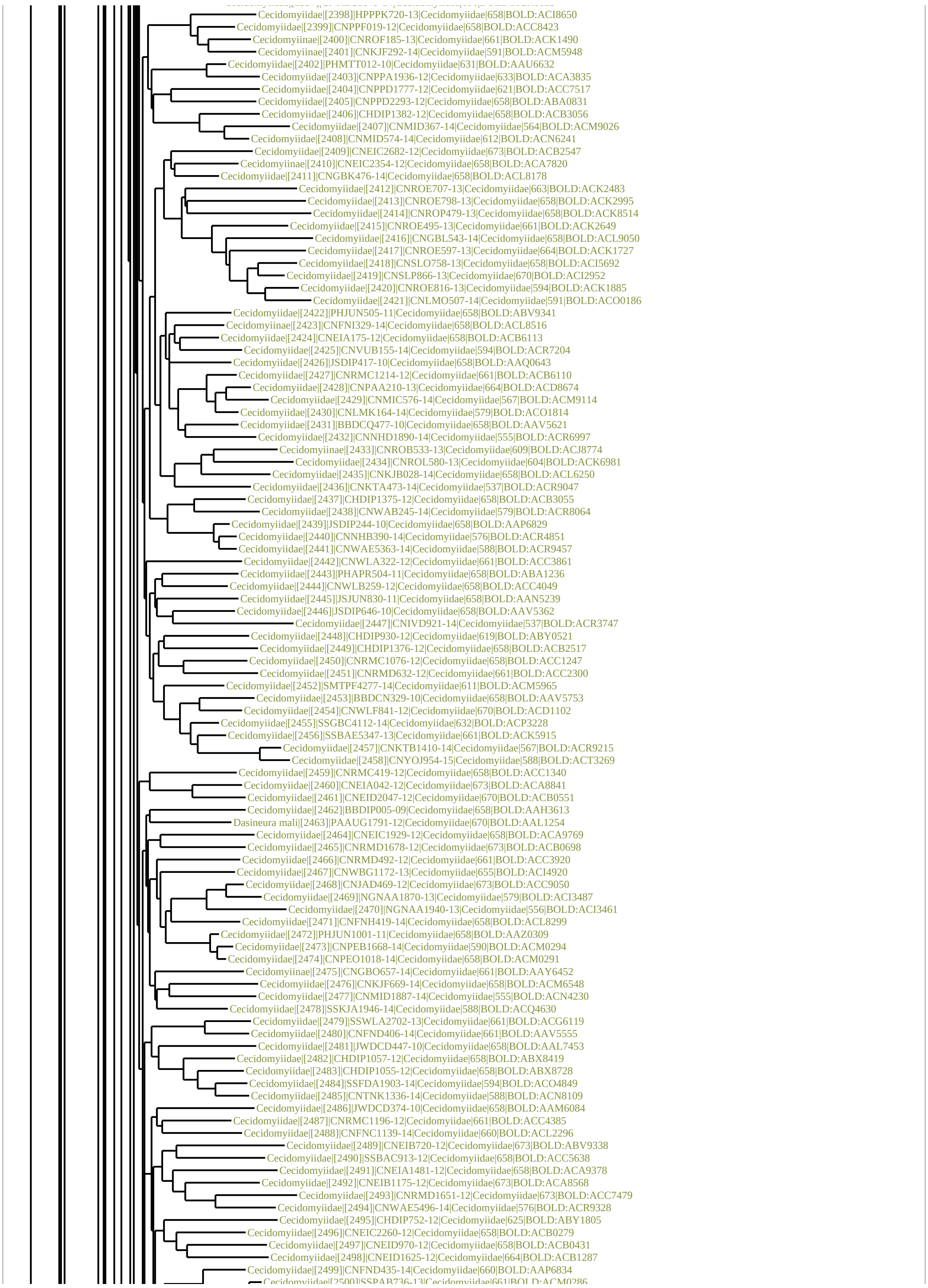
Cecidomyiidae|[2398]|HPPPK720-13|Cecidomyiidae|658|BOLD:ACI8650
Cecidomyiidae|[2399]|CNPPF019-12|Cecidomyiidae|658|BOLD:ACC8423
Cecidomyiinae|[2400]|CNROF185-13|Cecidomyiidae|661|BOLD:ACK1490
Cecidomyiinae|[2401]|CNKJF292-14|Cecidomyiidae|591|BOLD:ACM5948
Cecidomyiidae|[2402]|PHMTT012-10|Cecidomyiidae|631|BOLD:AAU6632
Cecidomyiidae|[2403]|CNPPA1936-12|Cecidomyiidae|633|BOLD:ACA3835
Cecidomyiidae|[2404]|CNPPD1777-12|Cecidomyiidae|621|BOLD:ACC7517
Cecidomyiidae|[2405]|CNPPD2293-12|Cecidomyiidae|658|BOLD:ABA0831
Cecidomyiidae|[2406]|CHDIP1382-12|Cecidomyiidae|658|BOLD:ACB3056
Cecidomyiidae|[2407]|CNMID367-14|Cecidomyiidae|564|BOLD:ACM9026
Cecidomyiidae|[2408]|CNMID574-14|Cecidomyiidae|612|BOLD:ACN6241
Cecidomyiidae|[2409]|CNEIC2682-12|Cecidomyiidae|673|BOLD:ACB2547
Cecidomyiinae|[2410]|CNEIC2354-12|Cecidomyiidae|658|BOLD:ACA7820
Cecidomyiidae|[2411]|CNGBK476-14|Cecidomyiidae|658|BOLD:ACL8178
Cecidomyiidae|[2412]|CNROE707-13|Cecidomyiidae|663|BOLD:ACK2483
Cecidomyiidae|[2413]|CNROE798-13|Cecidomyiidae|658|BOLD:ACK2995
Cecidomyiidae|[2414]|CNROP479-13|Cecidomyiidae|658|BOLD:ACK8514
Cecidomyiidae|[2415]|CNROE495-13|Cecidomyiidae|661|BOLD:ACK2649
Cecidomyiidae|[2416]|CNGBL543-14|Cecidomyiidae|658|BOLD:ACL9050
Cecidomyiidae|[2417]|CNROE597-13|Cecidomyiidae|664|BOLD:ACK1727
Cecidomyiidae|[2418]|CNSLO758-13|Cecidomyiidae|658|BOLD:ACI5692
Cecidomyiidae|[2419]|CNSLP866-13|Cecidomyiidae|670|BOLD:ACI2952
Cecidomyiidae|[2420]|CNROE816-13|Cecidomyiidae|594|BOLD:ACK1885
Cecidomyiidae|[2421]|CNLMO507-14|Cecidomyiidae|591|BOLD:ACO0186
Cecidomyiidae|[2422]|PHJUN505-11|Cecidomyiidae|658|BOLD:ABV9341
Cecidomyiinae|[2423]|CNFNI329-14|Cecidomyiidae|658|BOLD:ACL8516
Cecidomyiidae|[2424]|CNEIA175-12|Cecidomyiidae|658|BOLD:ACB6113
Cecidomyiidae|[2425]|CNVUB155-14|Cecidomyiidae|594|BOLD:ACR7204
Cecidomyiidae|[2426]|JSDIP417-10|Cecidomyiidae|658|BOLD:AAQ0643
Cecidomyiidae|[2427]|CNRMC1214-12|Cecidomyiidae|661|BOLD:ACB6110
Cecidomyiidae|[2428]|CNPAA210-13|Cecidomyiidae|664|BOLD:ACD8674
Cecidomyiidae|[2429]|CNMIC576-14|Cecidomyiidae|567|BOLD:ACM9114
Cecidomyiidae|[2430]|CNLMK164-14|Cecidomyiidae|579|BOLD:ACO1814
Cecidomyiidae|[2431]|BBDCQ477-10|Cecidomyiidae|658|BOLD:AAV5621
Cecidomyiidae|[2432]|CNNHD1890-14|Cecidomyiidae|555|BOLD:ACR6997
Cecidomyiinae|[2433]|CNROB533-13|Cecidomyiidae|609|BOLD:ACJ8774
Cecidomyiidae|[2434]|CNROL580-13|Cecidomyiidae|604|BOLD:ACK6981
Cecidomyiidae|[2435]|CNKJB028-14|Cecidomyiidae|658|BOLD:ACL6250
Cecidomyiidae|[2436]|CNKTA473-14|Cecidomyiidae|537|BOLD:ACR9047
Cecidomyiidae|[2437]|CHDIP1375-12|Cecidomyiidae|658|BOLD:ACB3055
Cecidomyiidae|[2438]|CNWAB245-14|Cecidomyiidae|579|BOLD:ACR8064
Cecidomyiidae|[2439]|JSDIP244-10|Cecidomyiidae|658|BOLD:AAP6829
Cecidomyiidae|[2440]|CNNHB390-14|Cecidomyiidae|576|BOLD:ACR4851
Cecidomyiidae|[2441]|CNWAE5363-14|Cecidomyiidae|588|BOLD:ACR9457
Cecidomyiidae|[2442]|CNWLA322-12|Cecidomyiidae|661|BOLD:ACC3861
Cecidomyiidae|[2443]|PHAPR504-11|Cecidomyiidae|658|BOLD:ABA1236
Cecidomyiidae|[2444]|CNWLB259-12|Cecidomyiidae|658|BOLD:ACC4049
Cecidomyiidae|[2445]|JSJUN830-11|Cecidomyiidae|658|BOLD:AAN5239
Cecidomyiidae|[2446]|JSDIP646-10|Cecidomyiidae|658|BOLD:AAV5362
Cecidomyiidae|[2447]|CNIVD921-14|Cecidomyiidae|537|BOLD:ACR3747
Cecidomyiidae|[2448]|CHDIP930-12|Cecidomyiidae|619|BOLD:ABY0521
Cecidomyiidae|[2449]|CHDIP1376-12|Cecidomyiidae|658|BOLD:ACB2517
Cecidomyiidae|[2450]|CNRMC1076-12|Cecidomyiidae|658|BOLD:ACC1247
Cecidomyiidae|[2451]|CNRMD632-12|Cecidomyiidae|661|BOLD:ACC2300
Cecidomyiidae|[2452]|SMTPF4277-14|Cecidomyiidae|611|BOLD:ACM5965
Cecidomyiidae|[2453]|BBDCN329-10|Cecidomyiidae|658|BOLD:AAV5753
Cecidomyiidae|[2454]|CNWLF841-12|Cecidomyiidae|670|BOLD:ACD1102
Cecidomyiidae|[2455]|SSGBC4112-14|Cecidomyiidae|632|BOLD:ACP3228
Cecidomyiidae|[2456]|SSBAE5347-13|Cecidomyiidae|661|BOLD:ACK5915
Cecidomyiidae|[2457]|CNKTB1410-14|Cecidomyiidae|567|BOLD:ACR9215
Cecidomyiidae|[2458]|CNYOJ954-15|Cecidomyiidae|588|BOLD:ACT3269
Cecidomyiidae|[2459]|CNRMC419-12|Cecidomyiidae|658|BOLD:ACC1340
Cecidomyiidae|[2460]|CNEIA042-12|Cecidomyiidae|673|BOLD:ACA8841
Cecidomyiidae|[2461]|CNEID2047-12|Cecidomyiidae|670|BOLD:ACB0551
Cecidomyiidae|[2462]|BBDIP005-09|Cecidomyiidae|658|BOLD:AAH3613
Dasineura mali|[2463]|PAAUG1791-12|Cecidomyiidae|670|BOLD:AAL1254
Cecidomyiidae|[2464]|CNEIC1929-12|Cecidomyiidae|658|BOLD:ACA9769
Cecidomyiidae|[2465]|CNRMD1678-12|Cecidomyiidae|673|BOLD:ACB0698
Cecidomyiidae|[2466]|CNRMD492-12|Cecidomyiidae|661|BOLD:ACC3920
Cecidomyiidae|[2467]|CNWBG1172-13|Cecidomyiidae|655|BOLD:ACI4920
Cecidomyiidae|[2468]|CNJAD469-12|Cecidomyiidae|673|BOLD:ACC9050
Cecidomyiidae|[2469]|NGNAA1870-13|Cecidomyiidae|579|BOLD:ACI3487
Cecidomyiidae|[2470]|NGNAA1940-13|Cecidomyiidae|556|BOLD:ACI3461
Cecidomyiidae|[2471]|CNFNH419-14|Cecidomyiidae|658|BOLD:ACL8299
Cecidomyiidae|[2472]|PHJUN1001-11|Cecidomyiidae|658|BOLD:AAZ0309
Cecidomyiidae|[2473]|CNPEB1668-14|Cecidomyiidae|590|BOLD:ACM0294
Cecidomyiidae|[2474]|CNPEO1018-14|Cecidomyiidae|658|BOLD:ACM0291
Cecidomyiinae|[2475]|CNGBO657-14|Cecidomyiidae|661|BOLD:AAY6452
Cecidomyiidae|[2476]|CNKJF669-14|Cecidomyiidae|658|BOLD:ACM6548
Cecidomyiidae|[2477]|CNMID1887-14|Cecidomyiidae|555|BOLD:ACN4230
Cecidomyiidae|[2478]|SSKJA1946-14|Cecidomyiidae|588|BOLD:ACQ4630
Cecidomyiidae|[2479]|SSWLA2702-13|Cecidomyiidae|661|BOLD:ACG6119
Cecidomyiidae|[2480]|CNFND406-14|Cecidomyiidae|661|BOLD:AAV5555
Cecidomyiidae|[2481]|JWDCD447-10|Cecidomyiidae|658|BOLD:AAL7453
Cecidomyiidae|[2482]|CHDIP1057-12|Cecidomyiidae|658|BOLD:ABX8419
Cecidomyiidae|[2483]|CHDIP1055-12|Cecidomyiidae|658|BOLD:ABX8728
Cecidomyiidae|[2484]|SSFDA1903-14|Cecidomyiidae|594|BOLD:ACO4849
Cecidomyiidae|[2485]|CNTNK1336-14|Cecidomyiidae|588|BOLD:ACN8109
Cecidomyiidae|[2486]|JWDCD374-10|Cecidomyiidae|658|BOLD:AAM6084
Cecidomyiidae|[2487]|CNRMC1196-12|Cecidomyiidae|661|BOLD:ACC4385
Cecidomyiidae|[2488]|CNFNC1139-14|Cecidomyiidae|660|BOLD:ACL2296
Cecidomyiidae|[2489]|CNEIB720-12|Cecidomyiidae|673|BOLD:ABV9338
Cecidomyiidae|[2490]|SSBAC913-12|Cecidomyiidae|658|BOLD:ACC5638
Cecidomyiidae|[2491]|CNEIA1481-12|Cecidomyiidae|658|BOLD:ACA9378
Cecidomyiidae|[2492]|CNEIB1175-12|Cecidomyiidae|673|BOLD:ACA8568
Cecidomyiidae|[2493]|CNRMD1651-12|Cecidomyiidae|673|BOLD:ACC7479
Cecidomyiidae|[2494]|CNWAE5496-14|Cecidomyiidae|576|BOLD:ACR9328
Cecidomyiidae|[2495]|CHDIP752-12|Cecidomyiidae|625|BOLD:ABY1805
Cecidomyiidae|[2496]|CNEIC2260-12|Cecidomyiidae|658|BOLD:ACB0279
Cecidomyiidae|[2497]|CNEID970-12|Cecidomyiidae|658|BOLD:ACB0431
Cecidomyiidae|[2498]|CNEID1625-12|Cecidomyiidae|664|BOLD:ACB1287
Cecidomyiidae|[2499]|CNFND435-14|Cecidomyiidae|660|BOLD:AAP6834
Cecidomyiidae|[2500]|SSPAB736-13|Cecidomyiidae|661|BOLD:ACM0286

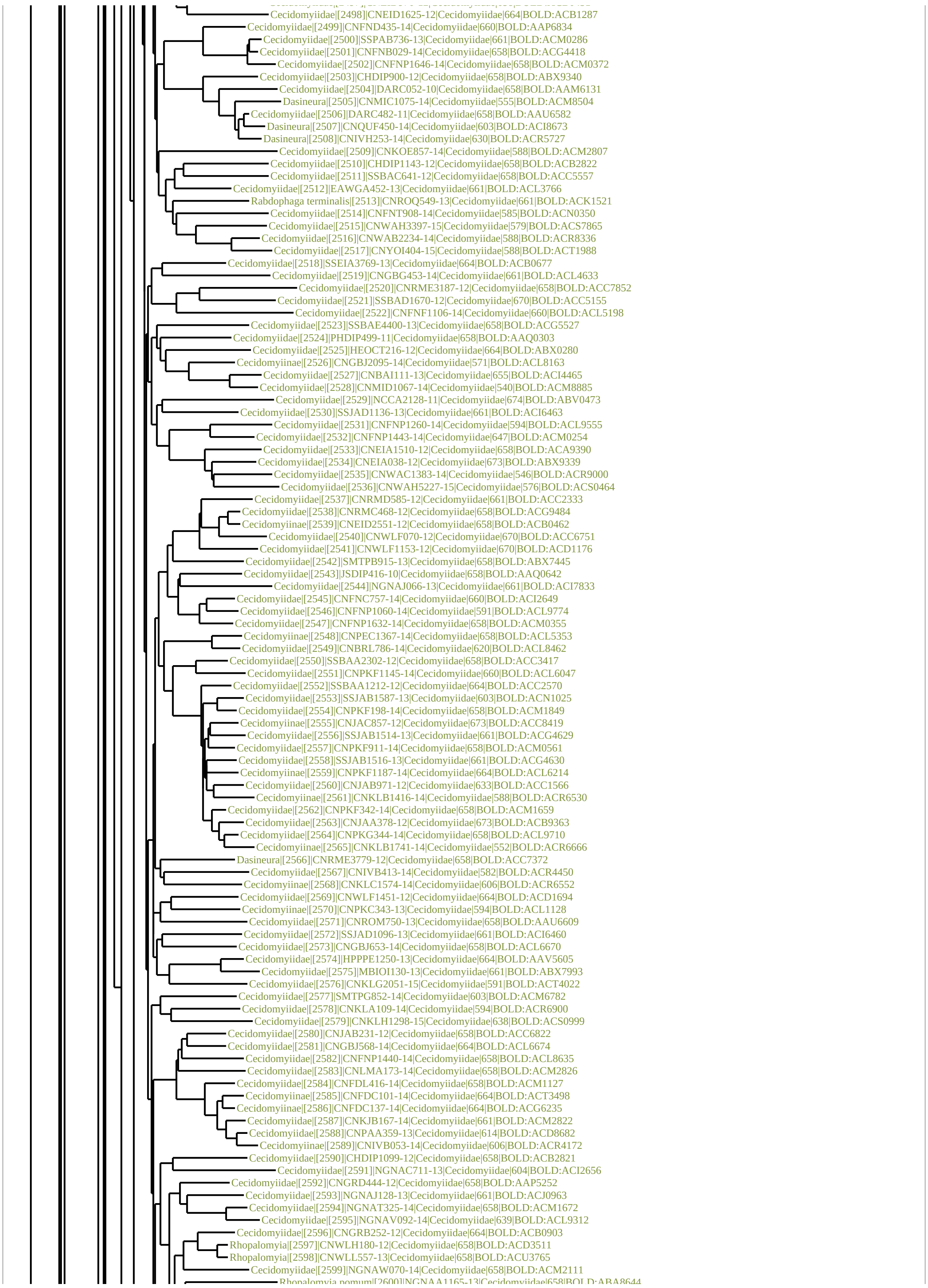

Cecidomyiidae|[2498]|CNEID1625-12|Cecidomyiidae|664|BOLD:ACB1287
Cecidomyiidae|[2499]|CNFND435-14|Cecidomyiidae|660|BOLD:AAP6834
Cecidomyiidae|[2500]|SSPAB736-13|Cecidomyiidae|661|BOLD:ACM0286
Cecidomyiidae|[2501]|CNFNB029-14|Cecidomyiidae|658|BOLD:ACG4418
Cecidomyiidae|[2502]|CNFNP1646-14|Cecidomyiidae|658|BOLD:ACM0372
Cecidomyiidae|[2503]|CHDIP900-12|Cecidomyiidae|658|BOLD:ABX9340
Cecidomyiidae|[2504]|DARC052-10|Cecidomyiidae|658|BOLD:AAM6131
Dasineura|[2505]|CNMIC1075-14|Cecidomyiidae|555|BOLD:ACM8504
Cecidomyiidae|[2506]|DARC482-11|Cecidomyiidae|658|BOLD:AAU6582
Dasineura|[2507]|CNQUF450-14|Cecidomyiidae|603|BOLD:ACI8673
Dasineura|[2508]|CNIVH253-14|Cecidomyiidae|630|BOLD:ACR5727
Cecidomyiidae|[2509]|CNKOE857-14|Cecidomyiidae|588|BOLD:ACM2807
Cecidomyiidae|[2510]|CHDIP1143-12|Cecidomyiidae|658|BOLD:ACB2822
Cecidomyiidae|[2511]|SSBAC641-12|Cecidomyiidae|658|BOLD:ACC5557
Cecidomyiidae|[2512]|EAWGA452-13|Cecidomyiidae|661|BOLD:ACL3766
Rabdophaga terminalis|[2513]|CNROQ549-13|Cecidomyiidae|661|BOLD:ACK1521
Cecidomyiidae|[2514]|CNFNT908-14|Cecidomyiidae|585|BOLD:ACN0350
Cecidomyiidae|[2515]|CNWAH3397-15|Cecidomyiidae|579|BOLD:ACS7865
Cecidomyiidae|[2516]|CNWAB2234-14|Cecidomyiidae|588|BOLD:ACR8336
Cecidomyiidae|[2517]|CNYOI404-15|Cecidomyiidae|588|BOLD:ACT1988
Cecidomyiidae|[2518]|SSEIA3769-13|Cecidomyiidae|664|BOLD:ACB0677
Cecidomyiidae|[2519]|CNGBG453-14|Cecidomyiidae|661|BOLD:ACL4633
Cecidomyiidae|[2520]|CNRME3187-12|Cecidomyiidae|658|BOLD:ACC7852
Cecidomyiidae|[2521]|SSBAD1670-12|Cecidomyiidae|670|BOLD:ACC5155
Cecidomyiidae|[2522]|CNFNF1106-14|Cecidomyiidae|660|BOLD:ACL5198
Cecidomyiidae|[2523]|SSBAE4400-13|Cecidomyiidae|658|BOLD:ACG5527
Cecidomyiidae|[2524]|PHDIP499-11|Cecidomyiidae|658|BOLD:AAQ0303
Cecidomyiidae|[2525]|HEOCT216-12|Cecidomyiidae|664|BOLD:ABX0280
Cecidomyiinae|[2526]|CNGBJ2095-14|Cecidomyiidae|571|BOLD:ACL8163
Cecidomyiidae|[2527]|CNBAI111-13|Cecidomyiidae|655|BOLD:ACI4465
Cecidomyiidae|[2528]|CNMID1067-14|Cecidomyiidae|540|BOLD:ACM8885
Cecidomyiidae|[2529]|NCCA2128-11|Cecidomyiidae|674|BOLD:ABV0473
Cecidomyiidae|[2530]|SSJAD1136-13|Cecidomyiidae|661|BOLD:ACI6463
Cecidomyiidae|[2531]|CNFNP1260-14|Cecidomyiidae|594|BOLD:ACL9555
Cecidomyiidae|[2532]|CNFNP1443-14|Cecidomyiidae|647|BOLD:ACM0254
Cecidomyiidae|[2533]|CNEIA1510-12|Cecidomyiidae|658|BOLD:ACA9390
Cecidomyiidae|[2534]|CNEIA038-12|Cecidomyiidae|673|BOLD:ABX9339
Cecidomyiidae|[2535]|CNWAC1383-14|Cecidomyiidae|546|BOLD:ACR9000
Cecidomyiidae|[2536]|CNWAH5227-15|Cecidomyiidae|576|BOLD:ACS0464
Cecidomyiidae|[2537]|CNRMD585-12|Cecidomyiidae|661|BOLD:ACC2333
Cecidomyiidae|[2538]|CNRMC468-12|Cecidomyiidae|658|BOLD:ACG9484
Cecidomyiinae|[2539]|CNEID2551-12|Cecidomyiidae|658|BOLD:ACB0462
Cecidomyiidae|[2540]|CNWLF070-12|Cecidomyiidae|670|BOLD:ACC6751
Cecidomyiidae|[2541]|CNWLF1153-12|Cecidomyiidae|670|BOLD:ACD1176
Cecidomyiidae|[2542]|SMTPB915-13|Cecidomyiidae|658|BOLD:ABX7445
Cecidomyiidae|[2543]|JSDIP416-10|Cecidomyiidae|658|BOLD:AAQ0642
Cecidomyiidae|[2544]|NGNAJ066-13|Cecidomyiidae|661|BOLD:ACI7833
Cecidomyiidae|[2545]|CNFNC757-14|Cecidomyiidae|660|BOLD:ACI2649
Cecidomyiidae|[2546]|CNFNP1060-14|Cecidomyiidae|591|BOLD:ACL9774
Cecidomyiidae|[2547]|CNFNP1632-14|Cecidomyiidae|658|BOLD:ACM0355
Cecidomyiinae|[2548]|CNPEC1367-14|Cecidomyiidae|658|BOLD:ACL5353
Cecidomyiidae|[2549]|CNBRL786-14|Cecidomyiidae|620|BOLD:ACL8462
Cecidomyiidae|[2550]|SSBAA2302-12|Cecidomyiidae|658|BOLD:ACC3417
Cecidomyiidae|[2551]|CNPKF1145-14|Cecidomyiidae|660|BOLD:ACL6047
Cecidomyiidae|[2552]|SSBAA1212-12|Cecidomyiidae|664|BOLD:ACC2570
Cecidomyiidae|[2553]|SSJAB1587-13|Cecidomyiidae|603|BOLD:ACN1025
Cecidomyiidae|[2554]|CNPKF198-14|Cecidomyiidae|658|BOLD:ACM1849
Cecidomyiinae|[2555]|CNJAC857-12|Cecidomyiidae|673|BOLD:ACC8419
Cecidomyiidae|[2556]|SSJAB1514-13|Cecidomyiidae|661|BOLD:ACG4629
Cecidomyiidae|[2557]|CNPKF911-14|Cecidomyiidae|658|BOLD:ACM0561
Cecidomyiidae|[2558]|SSJAB1516-13|Cecidomyiidae|661|BOLD:ACG4630
Cecidomyiinae|[2559]|CNPKF1187-14|Cecidomyiidae|664|BOLD:ACL6214
Cecidomyiidae|[2560]|CNJAB971-12|Cecidomyiidae|633|BOLD:ACC1566
Cecidomyiinae|[2561]|CNKLB1416-14|Cecidomyiidae|588|BOLD:ACR6530
Cecidomyiidae|[2562]|CNPKF342-14|Cecidomyiidae|658|BOLD:ACM1659
Cecidomyiidae|[2563]|CNJAA378-12|Cecidomyiidae|673|BOLD:ACB9363
Cecidomyiidae|[2564]|CNPKG344-14|Cecidomyiidae|658|BOLD:ACL9710
Cecidomyiinae|[2565]|CNKLB1741-14|Cecidomyiidae|552|BOLD:ACR6666
Dasineura|[2566]|CNRME3779-12|Cecidomyiidae|658|BOLD:ACC7372
Cecidomyiidae|[2567]|CNIVB413-14|Cecidomyiidae|582|BOLD:ACR4450
Cecidomyiinae|[2568]|CNKLC1574-14|Cecidomyiidae|606|BOLD:ACR6552
Cecidomyiidae|[2569]|CNWLF1451-12|Cecidomyiidae|664|BOLD:ACD1694
Cecidomyiinae|[2570]|CNPKC343-13|Cecidomyiidae|594|BOLD:ACL1128
Cecidomyiidae|[2571]|CNROM750-13|Cecidomyiidae|658|BOLD:AAU6609
Cecidomyiidae|[2572]|SSJAD1096-13|Cecidomyiidae|661|BOLD:ACI6460
Cecidomyiidae|[2573]|CNGBJ653-14|Cecidomyiidae|658|BOLD:ACL6670
Cecidomyiidae|[2574]|HPPPE1250-13|Cecidomyiidae|664|BOLD:AAV5605
Cecidomyiidae|[2575]|MBIOI130-13|Cecidomyiidae|661|BOLD:ABX7993
Cecidomyiidae|[2576]|CNKLG2051-15|Cecidomyiidae|591|BOLD:ACT4022
Cecidomyiidae|[2577]|SMTPG852-14|Cecidomyiidae|603|BOLD:ACM6782
Cecidomyiidae|[2578]|CNKLA109-14|Cecidomyiidae|594|BOLD:ACR6900
Cecidomyiidae|[2579]|CNKLH1298-15|Cecidomyiidae|638|BOLD:ACS0999
Cecidomyiidae|[2580]|CNJAB231-12|Cecidomyiidae|658|BOLD:ACC6822
Cecidomyiidae|[2581]|CNGBJ568-14|Cecidomyiidae|664|BOLD:ACL6674
Cecidomyiidae|[2582]|CNFNP1440-14|Cecidomyiidae|658|BOLD:ACL8635
Cecidomyiidae|[2583]|CNLMA173-14|Cecidomyiidae|658|BOLD:ACM2826
Cecidomyiidae|[2584]|CNFDL416-14|Cecidomyiidae|658|BOLD:ACM1127
Cecidomyiinae|[2585]|CNFDC101-14|Cecidomyiidae|664|BOLD:ACT3498
Cecidomyiinae|[2586]|CNFDC137-14|Cecidomyiidae|664|BOLD:ACG6235
Cecidomyiidae|[2587]|CNKJB167-14|Cecidomyiidae|661|BOLD:ACM2822
Cecidomyiidae|[2588]|CNPAA359-13|Cecidomyiidae|614|BOLD:ACD8682
Cecidomyiinae|[2589]|CNIVB053-14|Cecidomyiidae|606|BOLD:ACR4172
Cecidomyiidae|[2590]|CHDIP1099-12|Cecidomyiidae|658|BOLD:ACB2821
Cecidomyiidae|[2591]|NGNAC711-13|Cecidomyiidae|604|BOLD:ACI2656
Cecidomyiidae|[2592]|CNGRD444-12|Cecidomyiidae|658|BOLD:AAP5252
Cecidomyiidae|[2593]|NGNAJ128-13|Cecidomyiidae|661|BOLD:ACJ0963
Cecidomyiidae|[2594]|NGNAT325-14|Cecidomyiidae|658|BOLD:ACM1672
Cecidomyiidae|[2595]|NGNAV092-14|Cecidomyiidae|639|BOLD:ACL9312
Cecidomyiidae|[2596]|CNGRB252-12|Cecidomyiidae|664|BOLD:ACB0903
Rhopalomyia|[2597]|CNWLH180-12|Cecidomyiidae|658|BOLD:ACD3511
Rhopalomyia|[2598]|CNWLL557-13|Cecidomyiidae|658|BOLD:ACU3765
Cecidomyiidae|[2599]|NGNAW070-14|Cecidomyiidae|658|BOLD:ACM2111
Rhopalomyia pomum|[2600]|NGNAA1165-13|Cecidomyiidae|658|BOLD:ABA8644

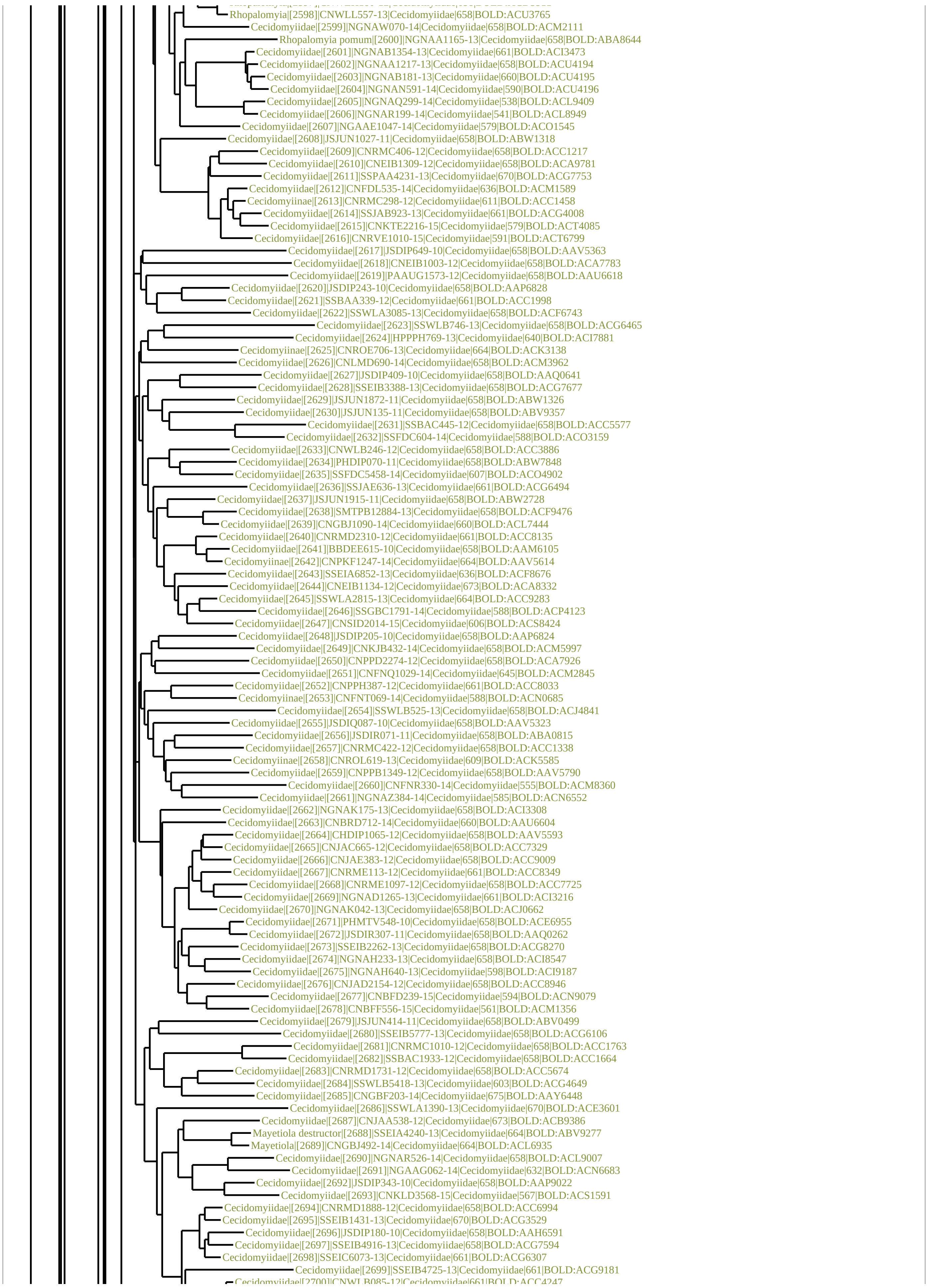
Rhopalomyia|[2598]|CNWLL557-13|Cecidomyiidae|658|BOLD:ACU3765
Cecidomyiidae|[2599]|NGNAW070-14|Cecidomyiidae|658|BOLD:ACM2111
Rhopalomyia pomum|[2600]|NGNAA1165-13|Cecidomyiidae|658|BOLD:ABA8644
Cecidomyiidae|[2601]|NGNAB1354-13|Cecidomyiidae|661|BOLD:ACI3473
Cecidomyiidae|[2602]|NGNAA1217-13|Cecidomyiidae|658|BOLD:ACU4194
Cecidomyiidae|[2603]|NGNAB181-13|Cecidomyiidae|660|BOLD:ACU4195
Cecidomyiidae|[2604]|NGNAN591-14|Cecidomyiidae|590|BOLD:ACU4196
Cecidomyiidae|[2605]|NGNAQ299-14|Cecidomyiidae|538|BOLD:ACL9409
Cecidomyiidae|[2606]|NGNAR199-14|Cecidomyiidae|541|BOLD:ACL8949
Cecidomyiidae|[2607]|NGAAE1047-14|Cecidomyiidae|579|BOLD:ACO1545
Cecidomyiidae|[2608]|JSJUN1027-11|Cecidomyiidae|658|BOLD:ABW1318
Cecidomyiidae|[2609]|CNRMC406-12|Cecidomyiidae|658|BOLD:ACC1217
Cecidomyiidae|[2610]|CNEIB1309-12|Cecidomyiidae|658|BOLD:ACA9781
Cecidomyiidae|[2611]|SSPAA4231-13|Cecidomyiidae|670|BOLD:ACG7753
Cecidomyiidae|[2612]|CNFDL535-14|Cecidomyiidae|636|BOLD:ACM1589
Cecidomyiinae|[2613]|CNRMC298-12|Cecidomyiidae|611|BOLD:ACC1458
Cecidomyiidae|[2614]|SSJAB923-13|Cecidomyiidae|661|BOLD:ACG4008
Cecidomyiidae|[2615]|CNKTE2216-15|Cecidomyiidae|579|BOLD:ACT4085
Cecidomyiidae|[2616]|CNRVE1010-15|Cecidomyiidae|591|BOLD:ACT6799
Cecidomyiidae|[2617]|JSDIP649-10|Cecidomyiidae|658|BOLD:AAV5363
Cecidomyiidae|[2618]|CNEIB1003-12|Cecidomyiidae|658|BOLD:ACA7783
Cecidomyiidae|[2619]|PAAUG1573-12|Cecidomyiidae|658|BOLD:AAU6618
Cecidomyiidae|[2620]|JSDIP243-10|Cecidomyiidae|658|BOLD:AAP6828
Cecidomyiidae|[2621]|SSBAA339-12|Cecidomyiidae|661|BOLD:ACC1998
Cecidomyiidae|[2622]|SSWLA3085-13|Cecidomyiidae|658|BOLD:ACF6743
Cecidomyiidae|[2623]|SSWLB746-13|Cecidomyiidae|658|BOLD:ACG6465
Cecidomyiidae|[2624]|HPPPH769-13|Cecidomyiidae|640|BOLD:ACI7881
Cecidomyiinae|[2625]|CNROE706-13|Cecidomyiidae|664|BOLD:ACK3138
Cecidomyiidae|[2626]|CNLMD690-14|Cecidomyiidae|658|BOLD:ACM3962
Cecidomyiidae|[2627]|JSDIP409-10|Cecidomyiidae|658|BOLD:AAQ0641
Cecidomyiidae|[2628]|SSEIB3388-13|Cecidomyiidae|658|BOLD:ACG7677
Cecidomyiidae|[2629]|JSJUN1872-11|Cecidomyiidae|658|BOLD:ABW1326
Cecidomyiidae|[2630]|JSJUN135-11|Cecidomyiidae|658|BOLD:ABV9357
Cecidomyiidae|[2631]|SSBAC445-12|Cecidomyiidae|658|BOLD:ACC5577
Cecidomyiidae|[2632]|SSFDC604-14|Cecidomyiidae|588|BOLD:ACO3159
Cecidomyiidae|[2633]|CNWLB246-12|Cecidomyiidae|658|BOLD:ACC3886
Cecidomyiidae|[2634]|PHDIP070-11|Cecidomyiidae|658|BOLD:ABW7848
Cecidomyiidae|[2635]|SSFDC5458-14|Cecidomyiidae|607|BOLD:ACO4902
Cecidomyiidae|[2636]|SSJAE636-13|Cecidomyiidae|661|BOLD:ACG6494
Cecidomyiidae|[2637]|JSJUN1915-11|Cecidomyiidae|658|BOLD:ABW2728
Cecidomyiidae|[2638]|SMTPB12884-13|Cecidomyiidae|658|BOLD:ACF9476
Cecidomyiidae|[2639]|CNGBJ1090-14|Cecidomyiidae|660|BOLD:ACL7444
Cecidomyiidae|[2640]|CNRMD2310-12|Cecidomyiidae|661|BOLD:ACC8135
Cecidomyiidae|[2641]|BBDEE615-10|Cecidomyiidae|658|BOLD:AAM6105
Cecidomyiinae|[2642]|CNPKF1247-14|Cecidomyiidae|664|BOLD:AAV5614
Cecidomyiidae|[2643]|SSEIA6852-13|Cecidomyiidae|636|BOLD:ACF8676
Cecidomyiidae|[2644]|CNEIB1134-12|Cecidomyiidae|673|BOLD:ACA8332
Cecidomyiidae|[2645]|SSWLA2815-13|Cecidomyiidae|664|BOLD:ACC9283
Cecidomyiidae|[2646]|SSGBC1791-14|Cecidomyiidae|588|BOLD:ACP4123
Cecidomyiidae|[2647]|CNSID2014-15|Cecidomyiidae|606|BOLD:ACS8424
Cecidomyiidae|[2648]|JSDIP205-10|Cecidomyiidae|658|BOLD:AAP6824
Cecidomyiidae|[2649]|CNKJB432-14|Cecidomyiidae|658|BOLD:ACM5997
Cecidomyiidae|[2650]|CNPPD2274-12|Cecidomyiidae|658|BOLD:ACA7926
Cecidomyiidae|[2651]|CNFNQ1029-14|Cecidomyiidae|645|BOLD:ACM2845
Cecidomyiidae|[2652]|CNPPH387-12|Cecidomyiidae|661|BOLD:ACC8033
Cecidomyiinae|[2653]|CNFNT069-14|Cecidomyiidae|588|BOLD:ACN0685
Cecidomyiidae|[2654]|SSWLB525-13|Cecidomyiidae|658|BOLD:ACJ4841
Cecidomyiidae|[2655]|JSDIQ087-10|Cecidomyiidae|658|BOLD:AAV5323
Cecidomyiidae|[2656]|JSDIR071-11|Cecidomyiidae|658|BOLD:ABA0815
Cecidomyiidae|[2657]|CNRMC422-12|Cecidomyiidae|658|BOLD:ACC1338
Cecidomyiinae|[2658]|CNROL619-13|Cecidomyiidae|609|BOLD:ACK5585
Cecidomyiidae|[2659]|CNPPB1349-12|Cecidomyiidae|658|BOLD:AAV5790
Cecidomyiidae|[2660]|CNFNR330-14|Cecidomyiidae|555|BOLD:ACM8360
Cecidomyiidae|[2661]|NGNAZ384-14|Cecidomyiidae|585|BOLD:ACN6552
Cecidomyiidae|[2662]|NGNAK175-13|Cecidomyiidae|658|BOLD:ACI3308
Cecidomyiidae|[2663]|CNBRD712-14|Cecidomyiidae|660|BOLD:AAU6604
Cecidomyiidae|[2664]|CHDIP1065-12|Cecidomyiidae|658|BOLD:AAV5593
Cecidomyiidae|[2665]|CNJAC665-12|Cecidomyiidae|658|BOLD:ACC7329
Cecidomyiidae|[2666]|CNJAE383-12|Cecidomyiidae|658|BOLD:ACC9009
Cecidomyiidae|[2667]|CNRME113-12|Cecidomyiidae|661|BOLD:ACC8349
Cecidomyiidae|[2668]|CNRME1097-12|Cecidomyiidae|658|BOLD:ACC7725
Cecidomyiidae|[2669]|NGNAD1265-13|Cecidomyiidae|661|BOLD:ACI3216
Cecidomyiidae|[2670]|NGNAK042-13|Cecidomyiidae|658|BOLD:ACJ0662
Cecidomyiidae|[2671]|PHMTV548-10|Cecidomyiidae|658|BOLD:ACE6955
Cecidomyiidae|[2672]|JSDIR307-11|Cecidomyiidae|658|BOLD:AAQ0262
Cecidomyiidae|[2673]|SSEIB2262-13|Cecidomyiidae|658|BOLD:ACG8270
Cecidomyiidae|[2674]|NGNAH233-13|Cecidomyiidae|658|BOLD:ACI8547
Cecidomyiidae|[2675]|NGNAH640-13|Cecidomyiidae|598|BOLD:ACI9187
Cecidomyiidae|[2676]|CNJAD2154-12|Cecidomyiidae|658|BOLD:ACC8946
Cecidomyiidae|[2677]|CNBFD239-15|Cecidomyiidae|594|BOLD:ACN9079
Cecidomyiidae|[2678]|CNBFF556-15|Cecidomyiidae|561|BOLD:ACM1356
Cecidomyiidae|[2679]|JSJUN414-11|Cecidomyiidae|658|BOLD:ABV0499
Cecidomyiidae|[2680]|SSEIB5777-13|Cecidomyiidae|658|BOLD:ACG6106
Cecidomyiidae|[2681]|CNRMC1010-12|Cecidomyiidae|658|BOLD:ACC1763
Cecidomyiidae|[2682]|SSBAC1933-12|Cecidomyiidae|658|BOLD:ACC1664
Cecidomyiidae|[2683]|CNRMD1731-12|Cecidomyiidae|658|BOLD:ACC5674
Cecidomyiidae|[2684]|SSWLB5418-13|Cecidomyiidae|603|BOLD:ACG4649
Cecidomyiidae|[2685]|CNGBF203-14|Cecidomyiidae|675|BOLD:AAY6448
Cecidomyiidae|[2686]|SSWLA1390-13|Cecidomyiidae|670|BOLD:ACE3601
Cecidomyiidae|[2687]|CNJAA538-12|Cecidomyiidae|673|BOLD:ACB9386
Mayetiola destructor|[2688]|SSEIA4240-13|Cecidomyiidae|664|BOLD:ABV9277
Mayetiola|[2689]|CNGBJ492-14|Cecidomyiidae|664|BOLD:ACL6935
Cecidomyiidae|[2690]|NGNAR526-14|Cecidomyiidae|658|BOLD:ACL9007
Cecidomyiidae|[2691]|NGAAG062-14|Cecidomyiidae|632|BOLD:ACN6683
Cecidomyiidae|[2692]|JSDIP343-10|Cecidomyiidae|658|BOLD:AAP9022
Cecidomyiidae|[2693]|CNKLD3568-15|Cecidomyiidae|567|BOLD:ACS1591
Cecidomyiidae|[2694]|CNRMD1888-12|Cecidomyiidae|658|BOLD:ACC6994
Cecidomyiidae|[2695]|SSEIB1431-13|Cecidomyiidae|670|BOLD:ACG3529
Cecidomyiidae|[2696]|JSDIP180-10|Cecidomyiidae|658|BOLD:AAH6591
Cecidomyiidae|[2697]|SSEIB4916-13|Cecidomyiidae|658|BOLD:ACG7594
Cecidomyiidae|[2698]|SSEIC6073-13|Cecidomyiidae|661|BOLD:ACG6307
Cecidomyiidae|[2699]|SSEIB4725-13|Cecidomyiidae|661|BOLD:ACG9181
Cecidomyiidae|[2700]|CNWLB085-12|Cecidomyiidae|661|BOLD:ACC4247

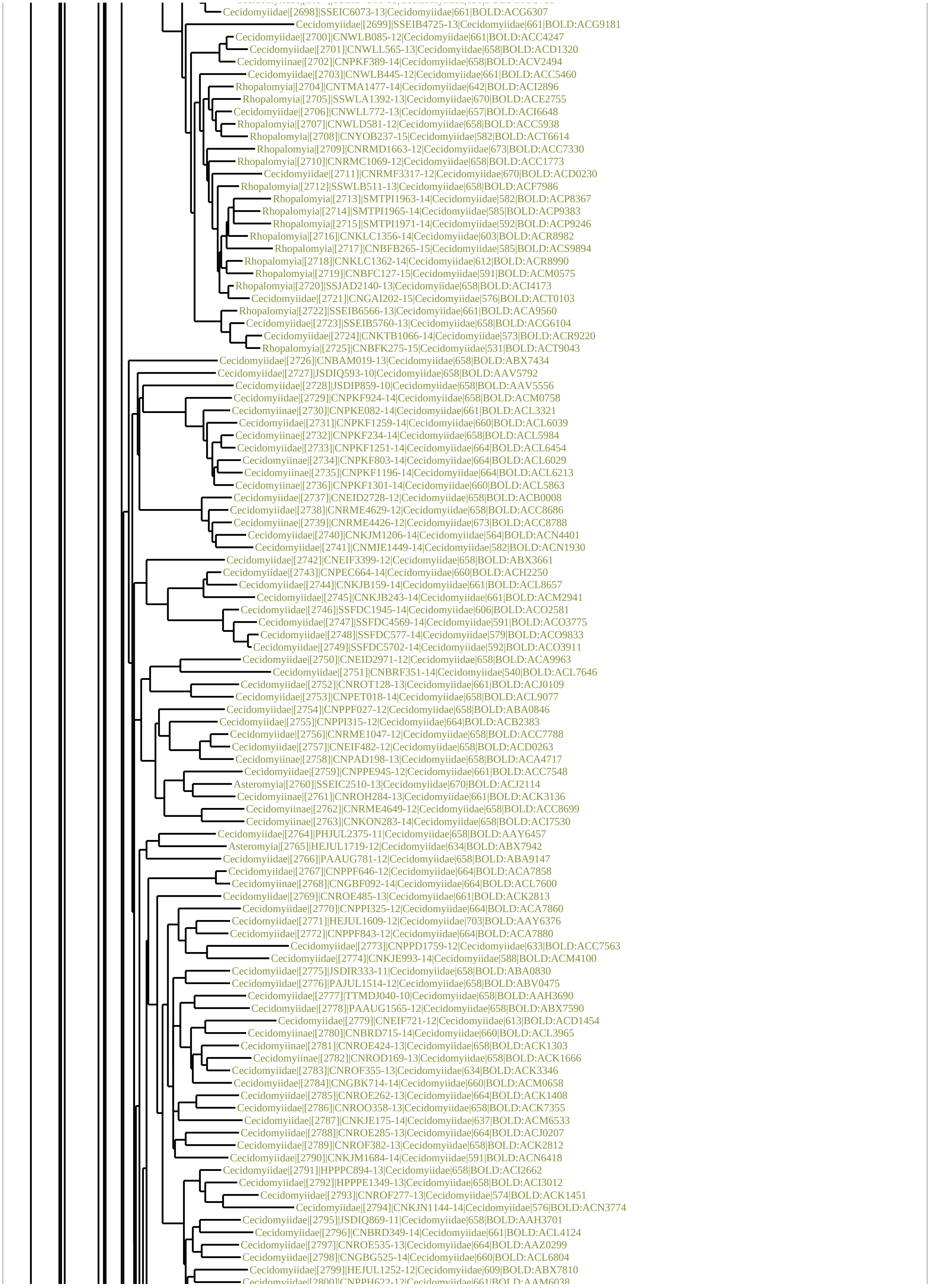
Cecidomyiidae|[2698]|SSEIC6073-13|Cecidomyiidae|661|BOLD:ACG6307
Cecidomyiidae|[2699]|SSEIB4725-13|Cecidomyiidae|661|BOLD:ACG9181
Cecidomyiidae|[2700]|CNWLB085-12|Cecidomyiidae|661|BOLD:ACC4247
Cecidomyiidae|[2701]|CNWLL565-13|Cecidomyiidae|658|BOLD:ACD1320
Cecidomyiinae|[2702]|CNPKF389-14|Cecidomyiidae|658|BOLD:ACV2494
Cecidomyiidae|[2703]|CNWLB445-12|Cecidomyiidae|661|BOLD:ACC5460
Rhopalomyia|[2704]|CNTMA1477-14|Cecidomyiidae|642|BOLD:ACI2896
Rhopalomyia|[2705]|SSWLA1392-13|Cecidomyiidae|670|BOLD:ACE2755
Cecidomyiidae|[2706]|CNWLL772-13|Cecidomyiidae|657|BOLD:ACI6648
Rhopalomyia|[2707]|CNWLD581-12|Cecidomyiidae|658|BOLD:ACC5938
Rhopalomyia|[2708]|CNYOB237-15|Cecidomyiidae|582|BOLD:ACT6614
Rhopalomyia|[2709]|CNRMD1663-12|Cecidomyiidae|673|BOLD:ACC7330
Rhopalomyia|[2710]|CNRMC1069-12|Cecidomyiidae|658|BOLD:ACC1773
Cecidomyiidae|[2711]|CNRMF3317-12|Cecidomyiidae|670|BOLD:ACD0230
Rhopalomyia|[2712]|SSWLB511-13|Cecidomyiidae|658|BOLD:ACF7986
Rhopalomyia|[2713]|SMTPI1963-14|Cecidomyiidae|582|BOLD:ACP8367
Rhopalomyia|[2714]|SMTPI1965-14|Cecidomyiidae|585|BOLD:ACP9383
Rhopalomyia|[2715]|SMTPI1971-14|Cecidomyiidae|592|BOLD:ACP9246
Rhopalomyia|[2716]|CNKLC1356-14|Cecidomyiidae|603|BOLD:ACR8982
Rhopalomyia|[2717]|CNBFB265-15|Cecidomyiidae|585|BOLD:ACS9894
Rhopalomyia|[2718]|CNKLC1362-14|Cecidomyiidae|612|BOLD:ACR8990
Rhopalomyia|[2719]|CNBFC127-15|Cecidomyiidae|591|BOLD:ACM0575
Rhopalomyia|[2720]|SSJAD2140-13|Cecidomyiidae|658|BOLD:ACI4173
Cecidomyiidae|[2721]|CNGAI202-15|Cecidomyiidae|576|BOLD:ACT0103
Rhopalomyia|[2722]|SSEIB6566-13|Cecidomyiidae|661|BOLD:ACA9560
Cecidomyiidae|[2723]|SSEIB5760-13|Cecidomyiidae|658|BOLD:ACG6104
Cecidomyiidae|[2724]|CNKTB1066-14|Cecidomyiidae|573|BOLD:ACR9220
Rhopalomyia|[2725]|CNBFK275-15|Cecidomyiidae|531|BOLD:ACT9043
Cecidomyiidae|[2726]|CNBAM019-13|Cecidomyiidae|658|BOLD:ABX7434
Cecidomyiidae|[2727]|JSDIQ593-10|Cecidomyiidae|658|BOLD:AAV5792
Cecidomyiidae|[2728]|JSDIP859-10|Cecidomyiidae|658|BOLD:AAV5556
Cecidomyiidae|[2729]|CNPKF924-14|Cecidomyiidae|658|BOLD:ACM0758
Cecidomyiinae|[2730]|CNPKE082-14|Cecidomyiidae|661|BOLD:ACL3321
Cecidomyiidae|[2731]|CNPKF1259-14|Cecidomyiidae|660|BOLD:ACL6039
Cecidomyiinae|[2732]|CNPKF234-14|Cecidomyiidae|658|BOLD:ACL5984
Cecidomyiidae|[2733]|CNPKF1251-14|Cecidomyiidae|664|BOLD:ACL6454
Cecidomyiinae|[2734]|CNPKF803-14|Cecidomyiidae|664|BOLD:ACL6029
Cecidomyiinae|[2735]|CNPKF1196-14|Cecidomyiidae|664|BOLD:ACL6213
Cecidomyiinae|[2736]|CNPKF1301-14|Cecidomyiidae|660|BOLD:ACL5863
Cecidomyiidae|[2737]|CNEID2728-12|Cecidomyiidae|658|BOLD:ACB0008
Cecidomyiidae|[2738]|CNRME4629-12|Cecidomyiidae|658|BOLD:ACC8686
Cecidomyiinae|[2739]|CNRME4426-12|Cecidomyiidae|673|BOLD:ACC8788
Cecidomyiidae|[2740]|CNKJM1206-14|Cecidomyiidae|564|BOLD:ACN4401
Cecidomyiidae|[2741]|CNMIE1449-14|Cecidomyiidae|582|BOLD:ACN1930
Cecidomyiidae|[2742]|CNEIF3399-12|Cecidomyiidae|658|BOLD:ABX3661
Cecidomyiidae|[2743]|CNPEC664-14|Cecidomyiidae|660|BOLD:ACH2250
Cecidomyiidae|[2744]|CNKJB159-14|Cecidomyiidae|661|BOLD:ACL8657
Cecidomyiidae|[2745]|CNKJB243-14|Cecidomyiidae|661|BOLD:ACM2941
Cecidomyiidae|[2746]|SSFDC1945-14|Cecidomyiidae|606|BOLD:ACO2581
Cecidomyiidae|[2747]|SSFDC4569-14|Cecidomyiidae|591|BOLD:ACO3775
Cecidomyiidae|[2748]|SSFDC577-14|Cecidomyiidae|579|BOLD:ACO9833
Cecidomyiidae|[2749]|SSFDC5702-14|Cecidomyiidae|592|BOLD:ACO3911
Cecidomyiidae|[2750]|CNEID2971-12|Cecidomyiidae|658|BOLD:ACA9963
Cecidomyiidae|[2751]|CNBRF351-14|Cecidomyiidae|540|BOLD:ACL7646
Cecidomyiidae|[2752]|CNROT128-13|Cecidomyiidae|661|BOLD:ACJ0109
Cecidomyiidae|[2753]|CNPET018-14|Cecidomyiidae|658|BOLD:ACL9077
Cecidomyiidae|[2754]|CNPPF027-12|Cecidomyiidae|658|BOLD:ABA0846
Cecidomyiidae|[2755]|CNPPI315-12|Cecidomyiidae|664|BOLD:ACB2383
Cecidomyiidae|[2756]|CNRME1047-12|Cecidomyiidae|658|BOLD:ACC7788
Cecidomyiidae|[2757]|CNEIF482-12|Cecidomyiidae|658|BOLD:ACD0263
Cecidomyiinae|[2758]|CNPAD198-13|Cecidomyiidae|658|BOLD:ACA4717
Cecidomyiidae|[2759]|CNPPE945-12|Cecidomyiidae|661|BOLD:ACC7548
Asteromyia|[2760]|SSEIC2510-13|Cecidomyiidae|670|BOLD:ACJ2114
Cecidomyiinae|[2761]|CNROH284-13|Cecidomyiidae|661|BOLD:ACK3136
Cecidomyiinae|[2762]|CNRME4649-12|Cecidomyiidae|658|BOLD:ACC8699
Cecidomyiinae|[2763]|CNKON283-14|Cecidomyiidae|658|BOLD:ACI7530
Cecidomyiidae|[2764]|PHJUL2375-11|Cecidomyiidae|658|BOLD:AAY6457
Asteromyia|[2765]|HEJUL1719-12|Cecidomyiidae|634|BOLD:ABX7942
Cecidomyiidae|[2766]|PAAUG781-12|Cecidomyiidae|658|BOLD:ABA9147
Cecidomyiidae|[2767]|CNPPF646-12|Cecidomyiidae|664|BOLD:ACA7858
Cecidomyiinae|[2768]|CNGBF092-14|Cecidomyiidae|664|BOLD:ACL7600
Cecidomyiidae|[2769]|CNROE485-13|Cecidomyiidae|661|BOLD:ACK2813
Cecidomyiidae|[2770]|CNPPI325-12|Cecidomyiidae|664|BOLD:ACA7860
Cecidomyiidae|[2771]|HEJUL1609-12|Cecidomyiidae|703|BOLD:AAY6376
Cecidomyiidae|[2772]|CNPPF843-12|Cecidomyiidae|664|BOLD:ACA7880
Cecidomyiidae|[2773]|CNPPD1759-12|Cecidomyiidae|633|BOLD:ACC7563
Cecidomyiidae|[2774]|CNKJE993-14|Cecidomyiidae|588|BOLD:ACM4100
Cecidomyiidae|[2775]|JSDIR333-11|Cecidomyiidae|658|BOLD:ABA0830
Cecidomyiidae|[2776]|PAJUL1514-12|Cecidomyiidae|658|BOLD:ABV0475
Cecidomyiidae|[2777]|TTMDJ040-10|Cecidomyiidae|658|BOLD:AAH3690
Cecidomyiidae|[2778]|PAAUG1565-12|Cecidomyiidae|658|BOLD:ABX7590
Cecidomyiidae|[2779]|CNEIF721-12|Cecidomyiidae|613|BOLD:ACD1454
Cecidomyiinae|[2780]|CNBRD715-14|Cecidomyiidae|660|BOLD:ACL3965
Cecidomyiinae|[2781]|CNROE424-13|Cecidomyiidae|658|BOLD:ACK1303
Cecidomyiinae|[2782]|CNROD169-13|Cecidomyiidae|658|BOLD:ACK1666
Cecidomyiidae|[2783]|CNROF355-13|Cecidomyiidae|634|BOLD:ACK3346
Cecidomyiidae|[2784]|CNGBK714-14|Cecidomyiidae|660|BOLD:ACM0658
Cecidomyiidae|[2785]|CNROE262-13|Cecidomyiidae|664|BOLD:ACK1408
Cecidomyiidae|[2786]|CNROO358-13|Cecidomyiidae|658|BOLD:ACK7355
Cecidomyiidae|[2787]|CNKJE175-14|Cecidomyiidae|637|BOLD:ACM6533
Cecidomyiidae|[2788]|CNROE285-13|Cecidomyiidae|664|BOLD:ACJ0207
Cecidomyiidae|[2789]|CNROF382-13|Cecidomyiidae|658|BOLD:ACK2812
Cecidomyiidae|[2790]|CNKJM1684-14|Cecidomyiidae|591|BOLD:ACN6418
Cecidomyiidae|[2791]|HPPPC894-13|Cecidomyiidae|658|BOLD:ACI2662
Cecidomyiidae|[2792]|HPPPE1349-13|Cecidomyiidae|658|BOLD:ACI3012
Cecidomyiidae|[2793]|CNROF277-13|Cecidomyiidae|574|BOLD:ACK1451
Cecidomyiidae|[2794]|CNKJN1144-14|Cecidomyiidae|576|BOLD:ACN3774
Cecidomyiidae|[2795]|JSDIQ869-11|Cecidomyiidae|658|BOLD:AAH3701
Cecidomyiidae|[2796]|CNBRD349-14|Cecidomyiidae|661|BOLD:ACL4124
Cecidomyiidae|[2797]|CNROE535-13|Cecidomyiidae|664|BOLD:AAZ0299
Cecidomyiidae|[2798]|CNGBG525-14|Cecidomyiidae|660|BOLD:ACL6804
Cecidomyiidae|[2799]|HEJUL1252-12|Cecidomyiidae|609|BOLD:ABX7810
Cecidomyiidae|[2800]|CNPPH622-12|Cecidomyiidae|661|BOLD:AAM6038

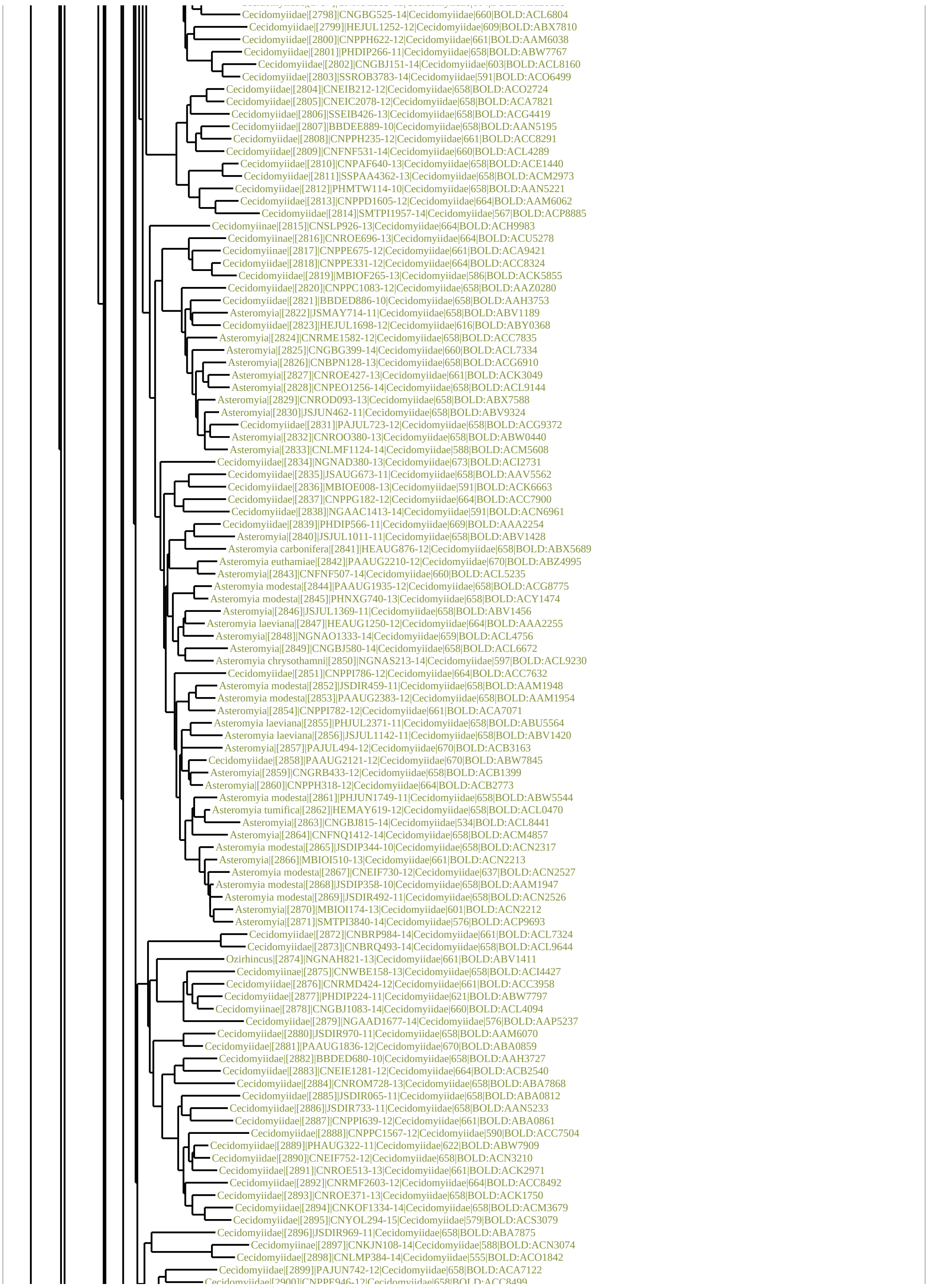
Cecidomyiidae|[2798]|CNGBG525-14|Cecidomyiidae|660|BOLD:ACL6804
Cecidomyiidae|[2799]|HEJUL1252-12|Cecidomyiidae|609|BOLD:ABX7810
Cecidomyiidae|[2800]|CNPPH622-12|Cecidomyiidae|661|BOLD:AAM6038
Cecidomyiidae|[2801]|PHDIP266-11|Cecidomyiidae|658|BOLD:ABW7767
Cecidomyiidae|[2802]|CNGBJ151-14|Cecidomyiidae|603|BOLD:ACL8160
Cecidomyiidae|[2803]|SSROB3783-14|Cecidomyiidae|591|BOLD:ACO6499
Cecidomyiidae|[2804]|CNEIB212-12|Cecidomyiidae|658|BOLD:ACO2724
Cecidomyiidae|[2805]|CNEIC2078-12|Cecidomyiidae|658|BOLD:ACA7821
Cecidomyiidae|[2806]|SSEIB426-13|Cecidomyiidae|658|BOLD:ACG4419
Cecidomyiidae|[2807]|BBDEE889-10|Cecidomyiidae|658|BOLD:AAN5195
Cecidomyiidae|[2808]|CNPPH235-12|Cecidomyiidae|661|BOLD:ACC8291
Cecidomyiidae|[2809]|CNFNF531-14|Cecidomyiidae|660|BOLD:ACL4289
Cecidomyiidae|[2810]|CNPAF640-13|Cecidomyiidae|658|BOLD:ACE1440
Cecidomyiidae|[2811]|SSPAA4362-13|Cecidomyiidae|658|BOLD:ACM2973
Cecidomyiidae|[2812]|PHMTW114-10|Cecidomyiidae|658|BOLD:AAN5221
Cecidomyiidae|[2813]|CNPPD1605-12|Cecidomyiidae|664|BOLD:AAM6062
Cecidomyiidae|[2814]|SMTPI1957-14|Cecidomyiidae|567|BOLD:ACP8885
Cecidomyiinae|[2815]|CNSLP926-13|Cecidomyiidae|664|BOLD:ACH9983
Cecidomyiinae|[2816]|CNROE696-13|Cecidomyiidae|664|BOLD:ACU5278
Cecidomyiinae|[2817]|CNPPE675-12|Cecidomyiidae|661|BOLD:ACA9421
Cecidomyiidae|[2818]|CNPPE331-12|Cecidomyiidae|664|BOLD:ACC8324
Cecidomyiidae|[2819]|MBIOF265-13|Cecidomyiidae|586|BOLD:ACK5855
Cecidomyiidae|[2820]|CNPPC1083-12|Cecidomyiidae|658|BOLD:AAZ0280
Cecidomyiidae|[2821]|BBDED886-10|Cecidomyiidae|658|BOLD:AAH3753
Asteromyia|[2822]|JSMAY714-11|Cecidomyiidae|658|BOLD:ABV1189
Cecidomyiidae|[2823]|HEJUL1698-12|Cecidomyiidae|616|BOLD:ABY0368
Asteromyia|[2824]|CNRME1582-12|Cecidomyiidae|658|BOLD:ACC7835
Asteromyia|[2825]|CNGBG399-14|Cecidomyiidae|660|BOLD:ACL7334
Asteromyia|[2826]|CNBPN128-13|Cecidomyiidae|658|BOLD:ACG6910
Asteromyia|[2827]|CNROE427-13|Cecidomyiidae|661|BOLD:ACK3049
Asteromyia|[2828]|CNPEO1256-14|Cecidomyiidae|658|BOLD:ACL9144
Asteromyia|[2829]|CNROD093-13|Cecidomyiidae|658|BOLD:ABX7588
Asteromyia|[2830]|JSJUN462-11|Cecidomyiidae|658|BOLD:ABV9324
Cecidomyiidae|[2831]|PAJUL723-12|Cecidomyiidae|658|BOLD:ACG9372
Asteromyia|[2832]|CNROO380-13|Cecidomyiidae|658|BOLD:ABW0440
Asteromyia|[2833]|CNLMF1124-14|Cecidomyiidae|588|BOLD:ACM5608
Cecidomyiidae|[2834]|NGNAD380-13|Cecidomyiidae|673|BOLD:ACI2731
Cecidomyiidae|[2835]|JSAUG673-11|Cecidomyiidae|658|BOLD:AAV5562
Cecidomyiidae|[2836]|MBIOE008-13|Cecidomyiidae|591|BOLD:ACK6663
Cecidomyiidae|[2837]|CNPPG182-12|Cecidomyiidae|664|BOLD:ACC7900
Cecidomyiidae|[2838]|NGAAC1413-14|Cecidomyiidae|591|BOLD:ACN6961
Cecidomyiidae|[2839]|PHDIP566-11|Cecidomyiidae|669|BOLD:AAA2254
Asteromyia|[2840]|JSJUL1011-11|Cecidomyiidae|658|BOLD:ABV1428
Asteromyia carbonifera|[2841]|HEAUG876-12|Cecidomyiidae|658|BOLD:ABX5689
Asteromyia euthamiae|[2842]|PAAUG2210-12|Cecidomyiidae|670|BOLD:ABZ4995
Asteromyia|[2843]|CNFNF507-14|Cecidomyiidae|660|BOLD:ACL5235
Asteromyia modesta|[2844]|PAAUG1935-12|Cecidomyiidae|658|BOLD:ACG8775
Asteromyia modesta|[2845]|PHNXG740-13|Cecidomyiidae|658|BOLD:ACY1474
Asteromyia|[2846]|JSJUL1369-11|Cecidomyiidae|658|BOLD:ABV1456
Asteromyia laeviana|[2847]|HEAUG1250-12|Cecidomyiidae|664|BOLD:AAA2255
Asteromyia|[2848]|NGNAO1333-14|Cecidomyiidae|659|BOLD:ACL4756
Asteromyia|[2849]|CNGBJ580-14|Cecidomyiidae|658|BOLD:ACL6672
Asteromyia chrysothamni|[2850]|NGNAS213-14|Cecidomyiidae|597|BOLD:ACL9230
Cecidomyiidae|[2851]|CNPPI786-12|Cecidomyiidae|664|BOLD:ACC7632
Asteromyia modesta|[2852]|JSDIR459-11|Cecidomyiidae|658|BOLD:AAM1948
Asteromyia modesta|[2853]|PAAUG2383-12|Cecidomyiidae|658|BOLD:AAM1954
Asteromyia|[2854]|CNPPI782-12|Cecidomyiidae|661|BOLD:ACA7071
Asteromyia laeviana|[2855]|PHJUL2371-11|Cecidomyiidae|658|BOLD:ABU5564
Asteromyia laeviana|[2856]|JSJUL1142-11|Cecidomyiidae|658|BOLD:ABV1420
Asteromyia|[2857]|PAJUL494-12|Cecidomyiidae|670|BOLD:ACB3163
Cecidomyiidae|[2858]|PAAUG2121-12|Cecidomyiidae|670|BOLD:ABW7845
Asteromyia|[2859]|CNGRB433-12|Cecidomyiidae|658|BOLD:ACB1399
Asteromyia|[2860]|CNPPH318-12|Cecidomyiidae|664|BOLD:ACB2773
Asteromyia modesta|[2861]|PHJUN1749-11|Cecidomyiidae|658|BOLD:ABW5544
Asteromyia tumifica|[2862]|HEMAY619-12|Cecidomyiidae|658|BOLD:ACL0470
Asteromyia|[2863]|CNGBJ815-14|Cecidomyiidae|534|BOLD:ACL8441
Asteromyia|[2864]|CNFNQ1412-14|Cecidomyiidae|658|BOLD:ACM4857
Asteromyia modesta|[2865]|JSDIP344-10|Cecidomyiidae|658|BOLD:ACN2317
Asteromyia|[2866]|MBIOI510-13|Cecidomyiidae|661|BOLD:ACN2213
Asteromyia modesta|[2867]|CNEIF730-12|Cecidomyiidae|637|BOLD:ACN2527
Asteromyia modesta|[2868]|JSDIP358-10|Cecidomyiidae|658|BOLD:AAM1947
Asteromyia modesta|[2869]|JSDIR492-11|Cecidomyiidae|658|BOLD:ACN2526
Asteromyia|[2870]|MBIOI174-13|Cecidomyiidae|601|BOLD:ACN2212
Asteromyia|[2871]|SMTPI3840-14|Cecidomyiidae|576|BOLD:ACP9693
Cecidomyiidae|[2872]|CNBRP984-14|Cecidomyiidae|661|BOLD:ACL7324
Cecidomyiidae|[2873]|CNBRQ493-14|Cecidomyiidae|658|BOLD:ACL9644
Ozirhincus|[2874]|NGNAH821-13|Cecidomyiidae|661|BOLD:ABV1411
Cecidomyiinae|[2875]|CNWBE158-13|Cecidomyiidae|658|BOLD:ACI4427
Cecidomyiidae|[2876]|CNRMD424-12|Cecidomyiidae|661|BOLD:ACC3958
Cecidomyiidae|[2877]|PHDIP224-11|Cecidomyiidae|621|BOLD:ABW7797
Cecidomyiinae|[2878]|CNGBJ1083-14|Cecidomyiidae|660|BOLD:ACL4094
Cecidomyiidae|[2879]|NGAAD1677-14|Cecidomyiidae|576|BOLD:AAP5237
Cecidomyiidae|[2880]|JSDIR970-11|Cecidomyiidae|658|BOLD:AAM6070
Cecidomyiidae|[2881]|PAAUG1836-12|Cecidomyiidae|670|BOLD:ABA0859
Cecidomyiidae|[2882]|BBDED680-10|Cecidomyiidae|658|BOLD:AAH3727
Cecidomyiidae|[2883]|CNEIE1281-12|Cecidomyiidae|664|BOLD:ACB2540
Cecidomyiidae|[2884]|CNROM728-13|Cecidomyiidae|658|BOLD:ABA7868
Cecidomyiidae|[2885]|JSDIR065-11|Cecidomyiidae|658|BOLD:ABA0812
Cecidomyiidae|[2886]|JSDIR733-11|Cecidomyiidae|658|BOLD:AAN5233
Cecidomyiidae|[2887]|CNPPI639-12|Cecidomyiidae|661|BOLD:ABA0861
Cecidomyiidae|[2888]|CNPPC1567-12|Cecidomyiidae|590|BOLD:ACC7504
Cecidomyiidae|[2889]|PHAUG322-11|Cecidomyiidae|622|BOLD:ABW7909
Cecidomyiidae|[2890]|CNEIF752-12|Cecidomyiidae|658|BOLD:ACN3210
Cecidomyiidae|[2891]|CNROE513-13|Cecidomyiidae|661|BOLD:ACK2971
Cecidomyiidae|[2892]|CNRMF2603-12|Cecidomyiidae|664|BOLD:ACC8492
Cecidomyiidae|[2893]|CNROE371-13|Cecidomyiidae|658|BOLD:ACK1750
Cecidomyiidae|[2894]|CNKOF1334-14|Cecidomyiidae|658|BOLD:ACM3679
Cecidomyiidae|[2895]|CNYOL294-15|Cecidomyiidae|579|BOLD:ACS3079
Cecidomyiidae|[2896]|JSDIR969-11|Cecidomyiidae|658|BOLD:ABA7875
Cecidomyiinae|[2897]|CNKJN108-14|Cecidomyiidae|588|BOLD:ACN3074
Cecidomyiidae|[2898]|CNLMP384-14|Cecidomyiidae|555|BOLD:ACO1842
Cecidomyiidae|[2899]|PAJUN742-12|Cecidomyiidae|658|BOLD:ACA7122
Cecidomyiidae|[2900]|CNPPF946-12|Cecidomyiidae|658|BOLD:ACC8499

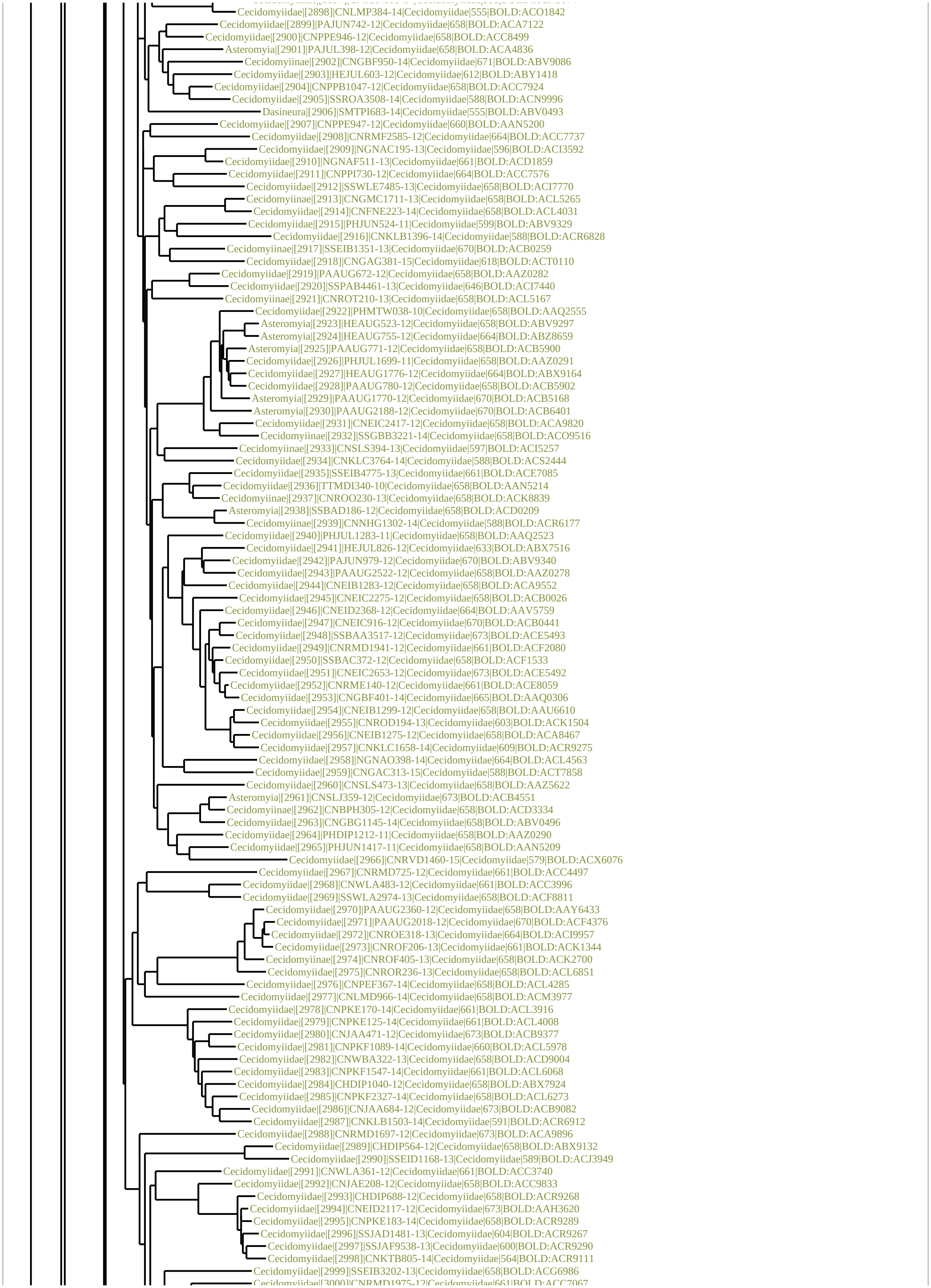

Cecidomyiidae|[2898]|CNLMP384-14|Cecidomyiidae|555|BOLD:ACO1842
Cecidomyiidae|[2899]|PAJUN742-12|Cecidomyiidae|658|BOLD:ACA7122
Cecidomyiidae|[2900]|CNPPE946-12|Cecidomyiidae|658|BOLD:ACC8499
Asteromyia|[2901]|PAJUL398-12|Cecidomyiidae|658|BOLD:ACA4836
Cecidomyiinae|[2902]|CNGBF950-14|Cecidomyiidae|671|BOLD:ABV9086
Cecidomyiidae|[2903]|HEJUL603-12|Cecidomyiidae|612|BOLD:ABY1418
Cecidomyiidae|[2904]|CNPPB1047-12|Cecidomyiidae|658|BOLD:ACC7924
Cecidomyiidae|[2905]|SSROA3508-14|Cecidomyiidae|588|BOLD:ACN9996
Dasineura|[2906]|SMTPI683-14|Cecidomyiidae|555|BOLD:ABV0493
Cecidomyiidae|[2907]|CNPPE947-12|Cecidomyiidae|660|BOLD:AAN5200
Cecidomyiidae|[2908]|CNRMF2585-12|Cecidomyiidae|664|BOLD:ACC7737
Cecidomyiidae|[2909]|NGNAC195-13|Cecidomyiidae|596|BOLD:ACI3592
Cecidomyiidae|[2910]|NGNAF511-13|Cecidomyiidae|661|BOLD:ACD1859
Cecidomyiidae|[2911]|CNPPI730-12|Cecidomyiidae|664|BOLD:ACC7576
Cecidomyiidae|[2912]|SSWLE7485-13|Cecidomyiidae|658|BOLD:ACI7770
Cecidomyiinae|[2913]|CNGMC1711-13|Cecidomyiidae|658|BOLD:ACL5265
Cecidomyiidae|[2914]|CNFNE223-14|Cecidomyiidae|658|BOLD:ACL4031
Cecidomyiidae|[2915]|PHJUN524-11|Cecidomyiidae|599|BOLD:ABV9329
Cecidomyiidae|[2916]|CNKLB1396-14|Cecidomyiidae|588|BOLD:ACR6828
Cecidomyiinae|[2917]|SSEIB1351-13|Cecidomyiidae|670|BOLD:ACB0259
Cecidomyiidae|[2918]|CNGAG381-15|Cecidomyiidae|618|BOLD:ACT0110
Cecidomyiidae|[2919]|PAAUG672-12|Cecidomyiidae|658|BOLD:AAZ0282
Cecidomyiidae|[2920]|SSPAB4461-13|Cecidomyiidae|646|BOLD:ACI7440
Cecidomyiinae|[2921]|CNROT210-13|Cecidomyiidae|658|BOLD:ACL5167
Cecidomyiidae|[2922]|PHMTW038-10|Cecidomyiidae|658|BOLD:AAQ2555
Asteromyia|[2923]|HEAUG523-12|Cecidomyiidae|658|BOLD:ABV9297
Asteromyia|[2924]|HEAUG755-12|Cecidomyiidae|664|BOLD:ABZ8659
Asteromyia|[2925]|PAAUG771-12|Cecidomyiidae|658|BOLD:ACB5900
Cecidomyiidae|[2926]|PHJUL1699-11|Cecidomyiidae|658|BOLD:AAZ0291
Cecidomyiidae|[2927]|HEAUG1776-12|Cecidomyiidae|664|BOLD:ABX9164
Cecidomyiidae|[2928]|PAAUG780-12|Cecidomyiidae|658|BOLD:ACB5902
Asteromyia|[2929]|PAAUG1770-12|Cecidomyiidae|670|BOLD:ACB5168
Asteromyia|[2930]|PAAUG2188-12|Cecidomyiidae|670|BOLD:ACB6401
Cecidomyiidae|[2931]|CNEIC2417-12|Cecidomyiidae|658|BOLD:ACA9820
Cecidomyiinae|[2932]|SSGBB3221-14|Cecidomyiidae|658|BOLD:ACO9516
Cecidomyiinae|[2933]|CNSLS394-13|Cecidomyiidae|597|BOLD:ACI5257
Cecidomyiidae|[2934]|CNKLC3764-14|Cecidomyiidae|588|BOLD:ACS2444
Cecidomyiidae|[2935]|SSEIB4775-13|Cecidomyiidae|661|BOLD:ACE7085
Cecidomyiidae|[2936]|TTMDI340-10|Cecidomyiidae|658|BOLD:AAN5214
Cecidomyiinae|[2937]|CNROO230-13|Cecidomyiidae|658|BOLD:ACK8839
Asteromyia|[2938]|SSBAD186-12|Cecidomyiidae|658|BOLD:ACD0209
Cecidomyiinae|[2939]|CNNHG1302-14|Cecidomyiidae|588|BOLD:ACR6177
Cecidomyiidae|[2940]|PHJUL1283-11|Cecidomyiidae|658|BOLD:AAQ2523
Cecidomyiidae|[2941]|HEJUL826-12|Cecidomyiidae|633|BOLD:ABX7516
Cecidomyiidae|[2942]|PAJUN979-12|Cecidomyiidae|670|BOLD:ABV9340
Cecidomyiidae|[2943]|PAAUG2522-12|Cecidomyiidae|658|BOLD:AAZ0278
Cecidomyiidae|[2944]|CNEIB1283-12|Cecidomyiidae|658|BOLD:ACA9552
Cecidomyiidae|[2945]|CNEIC2275-12|Cecidomyiidae|658|BOLD:ACB0026
Cecidomyiidae|[2946]|CNEID2368-12|Cecidomyiidae|664|BOLD:AAV5759
Cecidomyiidae|[2947]|CNEIC916-12|Cecidomyiidae|670|BOLD:ACB0441
Cecidomyiidae|[2948]|SSBAA3517-12|Cecidomyiidae|673|BOLD:ACE5493
Cecidomyiidae|[2949]|CNRMD1941-12|Cecidomyiidae|661|BOLD:ACF2080
Cecidomyiidae|[2950]|SSBAC372-12|Cecidomyiidae|658|BOLD:ACF1533
Cecidomyiidae|[2951]|CNEIC2653-12|Cecidomyiidae|673|BOLD:ACE5492
Cecidomyiidae|[2952]|CNRME140-12|Cecidomyiidae|661|BOLD:ACE8059
Cecidomyiidae|[2953]|CNGBF401-14|Cecidomyiidae|665|BOLD:AAQ0306
Cecidomyiidae|[2954]|CNEIB1299-12|Cecidomyiidae|658|BOLD:AAU6610
Cecidomyiidae|[2955]|CNROD194-13|Cecidomyiidae|603|BOLD:ACK1504
Cecidomyiidae|[2956]|CNEIB1275-12|Cecidomyiidae|658|BOLD:ACA8467
Cecidomyiidae|[2957]|CNKLC1658-14|Cecidomyiidae|609|BOLD:ACR9275
Cecidomyiidae|[2958]|NGNAO398-14|Cecidomyiidae|664|BOLD:ACL4563
Cecidomyiidae|[2959]|CNGAC313-15|Cecidomyiidae|588|BOLD:ACT7858
Cecidomyiidae|[2960]|CNSLS473-13|Cecidomyiidae|658|BOLD:AAZ5622
Asteromyia|[2961]|CNSLJ359-12|Cecidomyiidae|673|BOLD:ACB4551
Cecidomyiinae|[2962]|CNBPH305-12|Cecidomyiidae|658|BOLD:ACD3334
Cecidomyiidae|[2963]|CNGBG1145-14|Cecidomyiidae|658|BOLD:ABV0496
Cecidomyiidae|[2964]|PHDIP1212-11|Cecidomyiidae|658|BOLD:AAZ0290
Cecidomyiidae|[2965]|PHJUN1417-11|Cecidomyiidae|658|BOLD:AAN5209
Cecidomyiidae|[2966]|CNRVD1460-15|Cecidomyiidae|579|BOLD:ACX6076
Cecidomyiidae|[2967]|CNRMD725-12|Cecidomyiidae|661|BOLD:ACC4497
Cecidomyiidae|[2968]|CNWLA483-12|Cecidomyiidae|661|BOLD:ACC3996
Cecidomyiidae|[2969]|SSWLA2974-13|Cecidomyiidae|658|BOLD:ACF8811
Cecidomyiidae|[2970]|PAAUG2360-12|Cecidomyiidae|658|BOLD:AAY6433
Cecidomyiidae|[2971]|PAAUG2018-12|Cecidomyiidae|670|BOLD:ACF4376
Cecidomyiidae|[2972]|CNROE318-13|Cecidomyiidae|664|BOLD:ACI9957
Cecidomyiidae|[2973]|CNROF206-13|Cecidomyiidae|661|BOLD:ACK1344
Cecidomyiinae|[2974]|CNROF405-13|Cecidomyiidae|658|BOLD:ACK2700
Cecidomyiidae|[2975]|CNROR236-13|Cecidomyiidae|658|BOLD:ACL6851
Cecidomyiidae|[2976]|CNPEF367-14|Cecidomyiidae|658|BOLD:ACL4285
Cecidomyiidae|[2977]|CNLMD966-14|Cecidomyiidae|658|BOLD:ACM3977
Cecidomyiidae|[2978]|CNPKE170-14|Cecidomyiidae|661|BOLD:ACL3916
Cecidomyiidae|[2979]|CNPKE125-14|Cecidomyiidae|661|BOLD:ACL4008
Cecidomyiidae|[2980]|CNJAA471-12|Cecidomyiidae|673|BOLD:ACB9377
Cecidomyiidae|[2981]|CNPKF1089-14|Cecidomyiidae|660|BOLD:ACL5978
Cecidomyiidae|[2982]|CNWBA322-13|Cecidomyiidae|658|BOLD:ACD9004
Cecidomyiidae|[2983]|CNPKF1547-14|Cecidomyiidae|661|BOLD:ACL6068
Cecidomyiidae|[2984]|CHDIP1040-12|Cecidomyiidae|658|BOLD:ABX7924
Cecidomyiidae|[2985]|CNPKF2327-14|Cecidomyiidae|658|BOLD:ACL6273
Cecidomyiidae|[2986]|CNJAA684-12|Cecidomyiidae|673|BOLD:ACB9082
Cecidomyiidae|[2987]|CNKLB1503-14|Cecidomyiidae|591|BOLD:ACR6912
Cecidomyiidae|[2988]|CNRMD1697-12|Cecidomyiidae|673|BOLD:ACA9896
Cecidomyiidae|[2989]|CHDIP564-12|Cecidomyiidae|658|BOLD:ABX9132
Cecidomyiidae|[2990]|SSEID1168-13|Cecidomyiidae|589|BOLD:ACJ3949
Cecidomyiidae|[2991]|CNWLA361-12|Cecidomyiidae|661|BOLD:ACC3740
Cecidomyiidae|[2992]|CNJAE208-12|Cecidomyiidae|658|BOLD:ACC9833
Cecidomyiidae|[2993]|CHDIP688-12|Cecidomyiidae|658|BOLD:ACR9268
Cecidomyiidae|[2994]|CNEID2117-12|Cecidomyiidae|673|BOLD:AAH3620
Cecidomyiidae|[2995]|CNPKE183-14|Cecidomyiidae|658|BOLD:ACR9289
Cecidomyiidae|[2996]|SSJAD1481-13|Cecidomyiidae|604|BOLD:ACR9267
Cecidomyiidae|[2997]|SSJAF9538-13|Cecidomyiidae|600|BOLD:ACR9290
Cecidomyiidae|[2998]|CNKTB805-14|Cecidomyiidae|564|BOLD:ACR9111
Cecidomyiidae|[2999]|SSEIB3202-13|Cecidomyiidae|658|BOLD:ACG6986
Cecidomyiidae|[3000]|CNRMD1975-12|Cecidomyiidae|661|BOLD:ACC7067

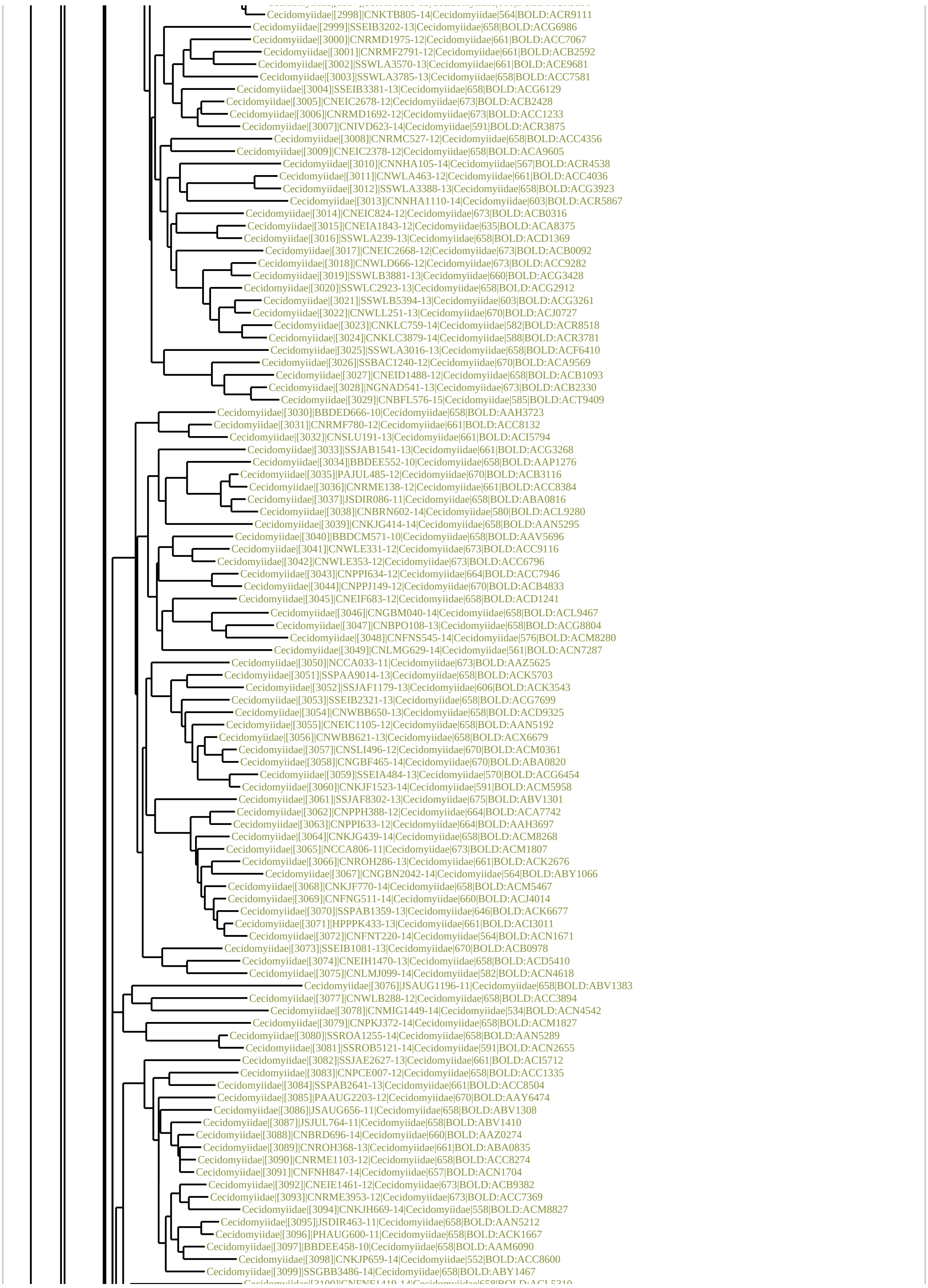
Cecidomyiidae|[2998]|CNKTB805-14|Cecidomyiidae|564|BOLD:ACR9111
Cecidomyiidae|[2999]|SSEIB3202-13|Cecidomyiidae|658|BOLD:ACG6986
Cecidomyiidae|[3000]|CNRMD1975-12|Cecidomyiidae|661|BOLD:ACC7067
Cecidomyiidae|[3001]|CNRMF2791-12|Cecidomyiidae|661|BOLD:ACB2592
Cecidomyiidae|[3002]|SSWLA3570-13|Cecidomyiidae|661|BOLD:ACE9681
Cecidomyiidae|[3003]|SSWLA3785-13|Cecidomyiidae|658|BOLD:ACC7581
Cecidomyiidae|[3004]|SSEIB3381-13|Cecidomyiidae|658|BOLD:ACG6129
Cecidomyiidae|[3005]|CNEIC2678-12|Cecidomyiidae|673|BOLD:ACB2428
Cecidomyiidae|[3006]|CNRMD1692-12|Cecidomyiidae|673|BOLD:ACC1233
Cecidomyiidae|[3007]|CNIVD623-14|Cecidomyiidae|591|BOLD:ACR3875
Cecidomyiidae|[3008]|CNRMC527-12|Cecidomyiidae|658|BOLD:ACC4356
Cecidomyiidae|[3009]|CNEIC2378-12|Cecidomyiidae|658|BOLD:ACA9605
Cecidomyiidae|[3010]|CNNHA105-14|Cecidomyiidae|567|BOLD:ACR4538
Cecidomyiidae|[3011]|CNWLA463-12|Cecidomyiidae|661|BOLD:ACC4036
Cecidomyiidae|[3012]|SSWLA3388-13|Cecidomyiidae|658|BOLD:ACG3923
Cecidomyiidae|[3013]|CNNHA1110-14|Cecidomyiidae|603|BOLD:ACR5867
Cecidomyiidae|[3014]|CNEIC824-12|Cecidomyiidae|673|BOLD:ACB0316
Cecidomyiidae|[3015]|CNEIA1843-12|Cecidomyiidae|635|BOLD:ACA8375
Cecidomyiidae|[3016]|SSWLA239-13|Cecidomyiidae|658|BOLD:ACD1369
Cecidomyiidae|[3017]|CNEIC2668-12|Cecidomyiidae|673|BOLD:ACB0092
Cecidomyiidae|[3018]|CNWLD666-12|Cecidomyiidae|673|BOLD:ACC9282
Cecidomyiidae|[3019]|SSWLB3881-13|Cecidomyiidae|660|BOLD:ACG3428
Cecidomyiidae|[3020]|SSWLC2923-13|Cecidomyiidae|658|BOLD:ACG2912
Cecidomyiidae|[3021]|SSWLB5394-13|Cecidomyiidae|603|BOLD:ACG3261
Cecidomyiidae|[3022]|CNWLL251-13|Cecidomyiidae|670|BOLD:ACJ0727
Cecidomyiidae|[3023]|CNKLC759-14|Cecidomyiidae|582|BOLD:ACR8518
Cecidomyiidae|[3024]|CNKLC3879-14|Cecidomyiidae|588|BOLD:ACR3781
Cecidomyiidae|[3025]|SSWLA3016-13|Cecidomyiidae|658|BOLD:ACF6410
Cecidomyiidae|[3026]|SSBAC1240-12|Cecidomyiidae|670|BOLD:ACA9569
Cecidomyiidae|[3027]|CNEID1488-12|Cecidomyiidae|658|BOLD:ACB1093
Cecidomyiidae|[3028]|NGNAD541-13|Cecidomyiidae|673|BOLD:ACB2330
Cecidomyiidae|[3029]|CNBFL576-15|Cecidomyiidae|585|BOLD:ACT9409
Cecidomyiidae|[3030]|BBDED666-10|Cecidomyiidae|658|BOLD:AAH3723
Cecidomyiidae|[3031]|CNRMF780-12|Cecidomyiidae|661|BOLD:ACC8132
Cecidomyiidae|[3032]|CNSLU191-13|Cecidomyiidae|661|BOLD:ACI5794
Cecidomyiidae|[3033]|SSJAB1541-13|Cecidomyiidae|661|BOLD:ACG3268
Cecidomyiidae|[3034]|BBDEE552-10|Cecidomyiidae|658|BOLD:AAP1276
Cecidomyiidae|[3035]|PAJUL485-12|Cecidomyiidae|670|BOLD:ACB3116
Cecidomyiidae|[3036]|CNRME138-12|Cecidomyiidae|661|BOLD:ACC8384
Cecidomyiidae|[3037]|JSDIR086-11|Cecidomyiidae|658|BOLD:ABA0816
Cecidomyiidae|[3038]|CNBRN602-14|Cecidomyiidae|580|BOLD:ACL9280
Cecidomyiidae|[3039]|CNKJG414-14|Cecidomyiidae|658|BOLD:AAN5295
Cecidomyiidae|[3040]|BBDCM571-10|Cecidomyiidae|658|BOLD:AAV5696
Cecidomyiidae|[3041]|CNWLE331-12|Cecidomyiidae|673|BOLD:ACC9116
Cecidomyiidae|[3042]|CNWLE353-12|Cecidomyiidae|673|BOLD:ACC6796
Cecidomyiidae|[3043]|CNPPI634-12|Cecidomyiidae|664|BOLD:ACC7946
Cecidomyiidae|[3044]|CNPPJ149-12|Cecidomyiidae|670|BOLD:ACB4833
Cecidomyiidae|[3045]|CNEIF683-12|Cecidomyiidae|658|BOLD:ACD1241
Cecidomyiidae|[3046]|CNGBM040-14|Cecidomyiidae|658|BOLD:ACL9467
Cecidomyiidae|[3047]|CNBPO108-13|Cecidomyiidae|658|BOLD:ACG8804
Cecidomyiidae|[3048]|CNFNS545-14|Cecidomyiidae|576|BOLD:ACM8280
Cecidomyiidae|[3049]|CNLMG629-14|Cecidomyiidae|561|BOLD:ACN7287
Cecidomyiidae|[3050]|NCCA033-11|Cecidomyiidae|673|BOLD:AAZ5625
Cecidomyiidae|[3051]|SSPAA9014-13|Cecidomyiidae|658|BOLD:ACK5703
Cecidomyiidae|[3052]|SSJAF1179-13|Cecidomyiidae|606|BOLD:ACK3543
Cecidomyiidae|[3053]|SSEIB2321-13|Cecidomyiidae|658|BOLD:ACG7699
Cecidomyiidae|[3054]|CNWBB650-13|Cecidomyiidae|658|BOLD:ACD9325
Cecidomyiidae|[3055]|CNEIC1105-12|Cecidomyiidae|658|BOLD:AAN5192
Cecidomyiidae|[3056]|CNWBB621-13|Cecidomyiidae|658|BOLD:ACX6679
Cecidomyiidae|[3057]|CNSLI496-12|Cecidomyiidae|670|BOLD:ACM0361
Cecidomyiidae|[3058]|CNGBF465-14|Cecidomyiidae|670|BOLD:ABA0820
Cecidomyiidae|[3059]|SSEIA484-13|Cecidomyiidae|570|BOLD:ACG6454
Cecidomyiidae|[3060]|CNKJF1523-14|Cecidomyiidae|591|BOLD:ACM5958
Cecidomyiidae|[3061]|SSJAF8302-13|Cecidomyiidae|675|BOLD:ABV1301
Cecidomyiidae|[3062]|CNPPH388-12|Cecidomyiidae|664|BOLD:ACA7742
Cecidomyiidae|[3063]|CNPPI633-12|Cecidomyiidae|664|BOLD:AAH3697
Cecidomyiidae|[3064]|CNKJG439-14|Cecidomyiidae|658|BOLD:ACM8268
Cecidomyiidae|[3065]|NCCA806-11|Cecidomyiidae|673|BOLD:ACM1807
Cecidomyiidae|[3066]|CNROH286-13|Cecidomyiidae|661|BOLD:ACK2676
Cecidomyiidae|[3067]|CNGBN2042-14|Cecidomyiidae|564|BOLD:ABY1066
Cecidomyiidae|[3068]|CNKJF770-14|Cecidomyiidae|658|BOLD:ACM5467
Cecidomyiidae|[3069]|CNFNG511-14|Cecidomyiidae|660|BOLD:ACJ4014
Cecidomyiidae|[3070]|SSPAB1359-13|Cecidomyiidae|646|BOLD:ACK6677
Cecidomyiidae|[3071]|HPPPK433-13|Cecidomyiidae|661|BOLD:ACI3011
Cecidomyiidae|[3072]|CNFNT220-14|Cecidomyiidae|564|BOLD:ACN1671
Cecidomyiidae|[3073]|SSEIB1081-13|Cecidomyiidae|670|BOLD:ACB0978
Cecidomyiidae|[3074]|CNEIH1470-13|Cecidomyiidae|658|BOLD:ACD5410
Cecidomyiidae|[3075]|CNLMJ099-14|Cecidomyiidae|582|BOLD:ACN4618
Cecidomyiidae|[3076]|JSAUG1196-11|Cecidomyiidae|658|BOLD:ABV1383
Cecidomyiidae|[3077]|CNWLB288-12|Cecidomyiidae|658|BOLD:ACC3894
Cecidomyiidae|[3078]|CNMIG1449-14|Cecidomyiidae|534|BOLD:ACN4542
Cecidomyiidae|[3079]|CNPKJ372-14|Cecidomyiidae|658|BOLD:ACM1827
Cecidomyiidae|[3080]|SSROA1255-14|Cecidomyiidae|658|BOLD:AAN5289
Cecidomyiidae|[3081]|SSROB5121-14|Cecidomyiidae|591|BOLD:ACN2655
Cecidomyiidae|[3082]|SSJAE2627-13|Cecidomyiidae|661|BOLD:ACI5712
Cecidomyiidae|[3083]|CNPCE007-12|Cecidomyiidae|658|BOLD:ACC1335
Cecidomyiidae|[3084]|SSPAB2641-13|Cecidomyiidae|661|BOLD:ACC8504
Cecidomyiidae|[3085]|PAAUG2203-12|Cecidomyiidae|670|BOLD:AAY6474
Cecidomyiidae|[3086]|JSAUG656-11|Cecidomyiidae|658|BOLD:ABV1308
Cecidomyiidae|[3087]|JSJUL764-11|Cecidomyiidae|658|BOLD:ABV1410
Cecidomyiidae|[3088]|CNBRD696-14|Cecidomyiidae|660|BOLD:AAZ0274
Cecidomyiidae|[3089]|CNROH368-13|Cecidomyiidae|661|BOLD:ABA0835
Cecidomyiidae|[3090]|CNRME1103-12|Cecidomyiidae|658|BOLD:ACC8274
Cecidomyiidae|[3091]|CNFNH847-14|Cecidomyiidae|657|BOLD:ACN1704
Cecidomyiidae|[3092]|CNEIE1461-12|Cecidomyiidae|673|BOLD:ACB9382
Cecidomyiidae|[3093]|CNRME3953-12|Cecidomyiidae|673|BOLD:ACC7369
Cecidomyiidae|[3094]|CNKJH669-14|Cecidomyiidae|558|BOLD:ACM8827
Cecidomyiidae|[3095]|JSDIR463-11|Cecidomyiidae|658|BOLD:AAN5212
Cecidomyiidae|[3096]|PHAUG600-11|Cecidomyiidae|658|BOLD:ACK1667
Cecidomyiidae|[3097]|BBDEE458-10|Cecidomyiidae|658|BOLD:AAM6090
Cecidomyiidae|[3098]|CNKJP659-14|Cecidomyiidae|552|BOLD:ACC8600
Cecidomyiidae|[3099]|SSGBB3486-14|Cecidomyiidae|658|BOLD:ABY1467

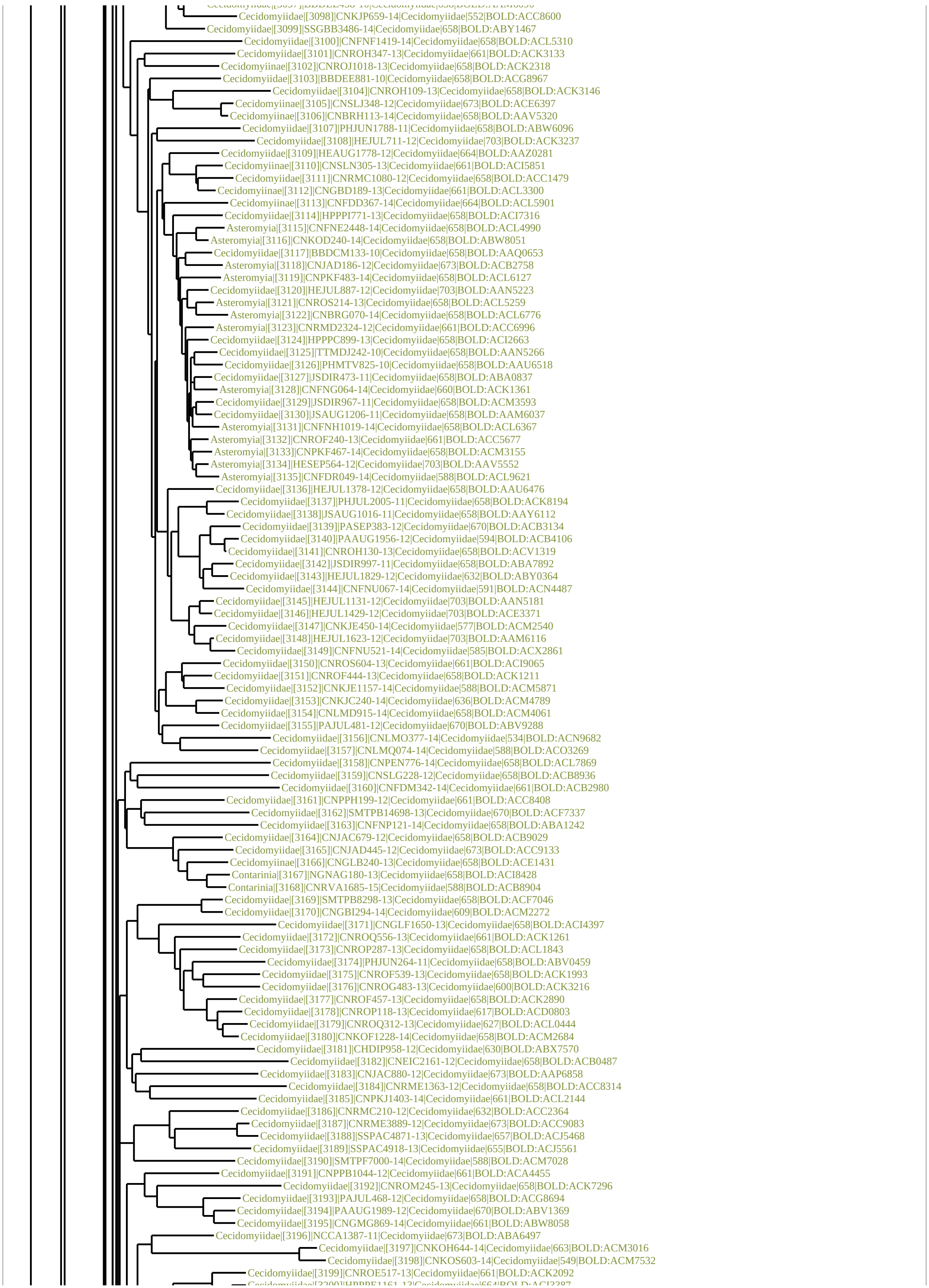
Cecidomyiidae|[3098]|CNKJP659-14|Cecidomyiidae|552|BOLD:ACC8600
Cecidomyiidae|[3099]|SSGBB3486-14|Cecidomyiidae|658|BOLD:ABY1467
Cecidomyiidae|[3100]|CNFNF1419-14|Cecidomyiidae|658|BOLD:ACL5310
Cecidomyiidae|[3101]|CNROH347-13|Cecidomyiidae|661|BOLD:ACK3133
Cecidomyiinae|[3102]|CNROJ1018-13|Cecidomyiidae|658|BOLD:ACK2318
Cecidomyiidae|[3103]|BBDEE881-10|Cecidomyiidae|658|BOLD:ACG8967
Cecidomyiidae|[3104]|CNROH109-13|Cecidomyiidae|658|BOLD:ACK3146
Cecidomyiinae|[3105]|CNSLJ348-12|Cecidomyiidae|673|BOLD:ACE6397
Cecidomyiinae|[3106]|CNBRH113-14|Cecidomyiidae|658|BOLD:AAV5320
Cecidomyiidae|[3107]|PHJUN1788-11|Cecidomyiidae|658|BOLD:ABW6096
Cecidomyiidae|[3108]|HEJUL711-12|Cecidomyiidae|703|BOLD:ACK3237
Cecidomyiidae|[3109]|HEAUG1778-12|Cecidomyiidae|664|BOLD:AAZ0281
Cecidomyiinae|[3110]|CNSLN305-13|Cecidomyiidae|661|BOLD:ACI5851
Cecidomyiidae|[3111]|CNRMC1080-12|Cecidomyiidae|658|BOLD:ACC1479
Cecidomyiinae|[3112]|CNGBD189-13|Cecidomyiidae|661|BOLD:ACL3300
Cecidomyiinae|[3113]|CNFDD367-14|Cecidomyiidae|664|BOLD:ACL5901
Cecidomyiidae|[3114]|HPPPI771-13|Cecidomyiidae|658|BOLD:ACI7316
Asteromyia|[3115]|CNFNE2448-14|Cecidomyiidae|658|BOLD:ACL4990
Asteromyia|[3116]|CNKOD240-14|Cecidomyiidae|658|BOLD:ABW8051
Cecidomyiidae|[3117]|BBDCM133-10|Cecidomyiidae|658|BOLD:AAQ0653
Asteromyia|[3118]|CNJAD186-12|Cecidomyiidae|673|BOLD:ACB2758
Asteromyia|[3119]|CNPKF483-14|Cecidomyiidae|658|BOLD:ACL6127
Cecidomyiidae|[3120]|HEJUL887-12|Cecidomyiidae|703|BOLD:AAN5223
Asteromyia|[3121]|CNROS214-13|Cecidomyiidae|658|BOLD:ACL5259
Asteromyia|[3122]|CNBRG070-14|Cecidomyiidae|658|BOLD:ACL6776
Asteromyia|[3123]|CNRMD2324-12|Cecidomyiidae|661|BOLD:ACC6996
Cecidomyiidae|[3124]|HPPPC899-13|Cecidomyiidae|658|BOLD:ACI2663
Cecidomyiidae|[3125]|TTMDJ242-10|Cecidomyiidae|658|BOLD:AAN5266
Cecidomyiidae|[3126]|PHMTV825-10|Cecidomyiidae|658|BOLD:AAU6518
Cecidomyiidae|[3127]|JSDIR473-11|Cecidomyiidae|658|BOLD:ABA0837
Asteromyia|[3128]|CNFNG064-14|Cecidomyiidae|660|BOLD:ACK1361
Cecidomyiidae|[3129]|JSDIR967-11|Cecidomyiidae|658|BOLD:ACM3593
Cecidomyiidae|[3130]|JSAUG1206-11|Cecidomyiidae|658|BOLD:AAM6037
Asteromyia|[3131]|CNFNH1019-14|Cecidomyiidae|658|BOLD:ACL6367
Asteromyia|[3132]|CNROF240-13|Cecidomyiidae|661|BOLD:ACC5677
Asteromyia|[3133]|CNPKF467-14|Cecidomyiidae|658|BOLD:ACM3155
Asteromyia|[3134]|HESEP564-12|Cecidomyiidae|703|BOLD:AAV5552
Asteromyia|[3135]|CNFDR049-14|Cecidomyiidae|588|BOLD:ACL9621
Cecidomyiidae|[3136]|HEJUL1378-12|Cecidomyiidae|658|BOLD:AAU6476
Cecidomyiidae|[3137]|PHJUL2005-11|Cecidomyiidae|658|BOLD:ACK8194
Cecidomyiidae|[3138]|JSAUG1016-11|Cecidomyiidae|658|BOLD:AAY6112
Cecidomyiidae|[3139]|PASEP383-12|Cecidomyiidae|670|BOLD:ACB3134
Cecidomyiidae|[3140]|PAAUG1956-12|Cecidomyiidae|594|BOLD:ACB4106
Cecidomyiidae|[3141]|CNROH130-13|Cecidomyiidae|658|BOLD:ACV1319
Cecidomyiidae|[3142]|JSDIR997-11|Cecidomyiidae|658|BOLD:ABA7892
Cecidomyiidae|[3143]|HEJUL1829-12|Cecidomyiidae|632|BOLD:ABY0364
Cecidomyiidae|[3144]|CNFNU067-14|Cecidomyiidae|591|BOLD:ACN4487
Cecidomyiidae|[3145]|HEJUL1131-12|Cecidomyiidae|703|BOLD:AAN5181
Cecidomyiidae|[3146]|HEJUL1429-12|Cecidomyiidae|703|BOLD:ACE3371
Cecidomyiidae|[3147]|CNKJE450-14|Cecidomyiidae|577|BOLD:ACM2540
Cecidomyiidae|[3148]|HEJUL1623-12|Cecidomyiidae|703|BOLD:AAM6116
Cecidomyiidae|[3149]|CNFNU521-14|Cecidomyiidae|585|BOLD:ACX2861
Cecidomyiidae|[3150]|CNROS604-13|Cecidomyiidae|661|BOLD:ACI9065
Cecidomyiidae|[3151]|CNROF444-13|Cecidomyiidae|658|BOLD:ACK1211
Cecidomyiidae|[3152]|CNKJE1157-14|Cecidomyiidae|588|BOLD:ACM5871
Cecidomyiidae|[3153]|CNKJC240-14|Cecidomyiidae|636|BOLD:ACM4789
Cecidomyiidae|[3154]|CNLMD915-14|Cecidomyiidae|658|BOLD:ACM4061
Cecidomyiidae|[3155]|PAJUL481-12|Cecidomyiidae|670|BOLD:ABV9288
Cecidomyiidae|[3156]|CNLMO377-14|Cecidomyiidae|534|BOLD:ACN9682
Cecidomyiidae|[3157]|CNLMQ074-14|Cecidomyiidae|588|BOLD:ACO3269
Cecidomyiidae|[3158]|CNPEN776-14|Cecidomyiidae|658|BOLD:ACL7869
Cecidomyiidae|[3159]|CNSLG228-12|Cecidomyiidae|658|BOLD:ACB8936
Cecidomyiidae|[3160]|CNFDM342-14|Cecidomyiidae|661|BOLD:ACB2980
Cecidomyiidae|[3161]|CNPPH199-12|Cecidomyiidae|661|BOLD:ACC8408
Cecidomyiidae|[3162]|SMTPB14698-13|Cecidomyiidae|670|BOLD:ACF7337
Cecidomyiidae|[3163]|CNFNP121-14|Cecidomyiidae|658|BOLD:ABA1242
Cecidomyiidae|[3164]|CNJAC679-12|Cecidomyiidae|658|BOLD:ACB9029
Cecidomyiidae|[3165]|CNJAD445-12|Cecidomyiidae|673|BOLD:ACC9133
Cecidomyiinae|[3166]|CNGLB240-13|Cecidomyiidae|658|BOLD:ACE1431
Contarinia|[3167]|NGNAG180-13|Cecidomyiidae|658|BOLD:ACI8428
Contarinia|[3168]|CNRVA1685-15|Cecidomyiidae|588|BOLD:ACB8904
Cecidomyiidae|[3169]|SMTPB8298-13|Cecidomyiidae|658|BOLD:ACF7046
Cecidomyiidae|[3170]|CNGBI294-14|Cecidomyiidae|609|BOLD:ACM2272
Cecidomyiidae|[3171]|CNGLF1650-13|Cecidomyiidae|658|BOLD:ACI4397
Cecidomyiidae|[3172]|CNROQ556-13|Cecidomyiidae|661|BOLD:ACK1261
Cecidomyiidae|[3173]|CNROP287-13|Cecidomyiidae|658|BOLD:ACL1843
Cecidomyiidae|[3174]|PHJUN264-11|Cecidomyiidae|658|BOLD:ABV0459
Cecidomyiidae|[3175]|CNROF539-13|Cecidomyiidae|658|BOLD:ACK1993
Cecidomyiidae|[3176]|CNROG483-13|Cecidomyiidae|600|BOLD:ACK3216
Cecidomyiidae|[3177]|CNROF457-13|Cecidomyiidae|658|BOLD:ACK2890
Cecidomyiidae|[3178]|CNROP118-13|Cecidomyiidae|617|BOLD:ACD0803
Cecidomyiidae|[3179]|CNROQ312-13|Cecidomyiidae|627|BOLD:ACL0444
Cecidomyiidae|[3180]|CNKOF1228-14|Cecidomyiidae|658|BOLD:ACM2684
Cecidomyiidae|[3181]|CHDIP958-12|Cecidomyiidae|630|BOLD:ABX7570
Cecidomyiidae|[3182]|CNEIC2161-12|Cecidomyiidae|658|BOLD:ACB0487
Cecidomyiidae|[3183]|CNJAC880-12|Cecidomyiidae|673|BOLD:AAP6858
Cecidomyiidae|[3184]|CNRME1363-12|Cecidomyiidae|658|BOLD:ACC8314
Cecidomyiidae|[3185]|CNPKJ1403-14|Cecidomyiidae|661|BOLD:ACL2144
Cecidomyiidae|[3186]|CNRMC210-12|Cecidomyiidae|632|BOLD:ACC2364
Cecidomyiidae|[3187]|CNRME3889-12|Cecidomyiidae|673|BOLD:ACC9083
Cecidomyiidae|[3188]|SSPAC4871-13|Cecidomyiidae|657|BOLD:ACJ5468
Cecidomyiidae|[3189]|SSPAC4918-13|Cecidomyiidae|655|BOLD:ACJ5561
Cecidomyiidae|[3190]|SMTPF7000-14|Cecidomyiidae|588|BOLD:ACM7028
Cecidomyiidae|[3191]|CNPPB1044-12|Cecidomyiidae|661|BOLD:ACA4455
Cecidomyiidae|[3192]|CNROM245-13|Cecidomyiidae|658|BOLD:ACK7296
Cecidomyiidae|[3193]|PAJUL468-12|Cecidomyiidae|658|BOLD:ACG8694
Cecidomyiidae|[3194]|PAAUG1989-12|Cecidomyiidae|670|BOLD:ABV1369
Cecidomyiidae|[3195]|CNGMG869-14|Cecidomyiidae|661|BOLD:ABW8058
Cecidomyiidae|[3196]|NCCA1387-11|Cecidomyiidae|673|BOLD:ABA6497
Cecidomyiidae|[3197]|CNKOH644-14|Cecidomyiidae|663|BOLD:ACM3016
Cecidomyiidae|[3198]|CNKOS603-14|Cecidomyiidae|549|BOLD:ACM7532
Cecidomyiidae|[3199]|CNROE517-13|Cecidomyiidae|661|BOLD:ACK2092

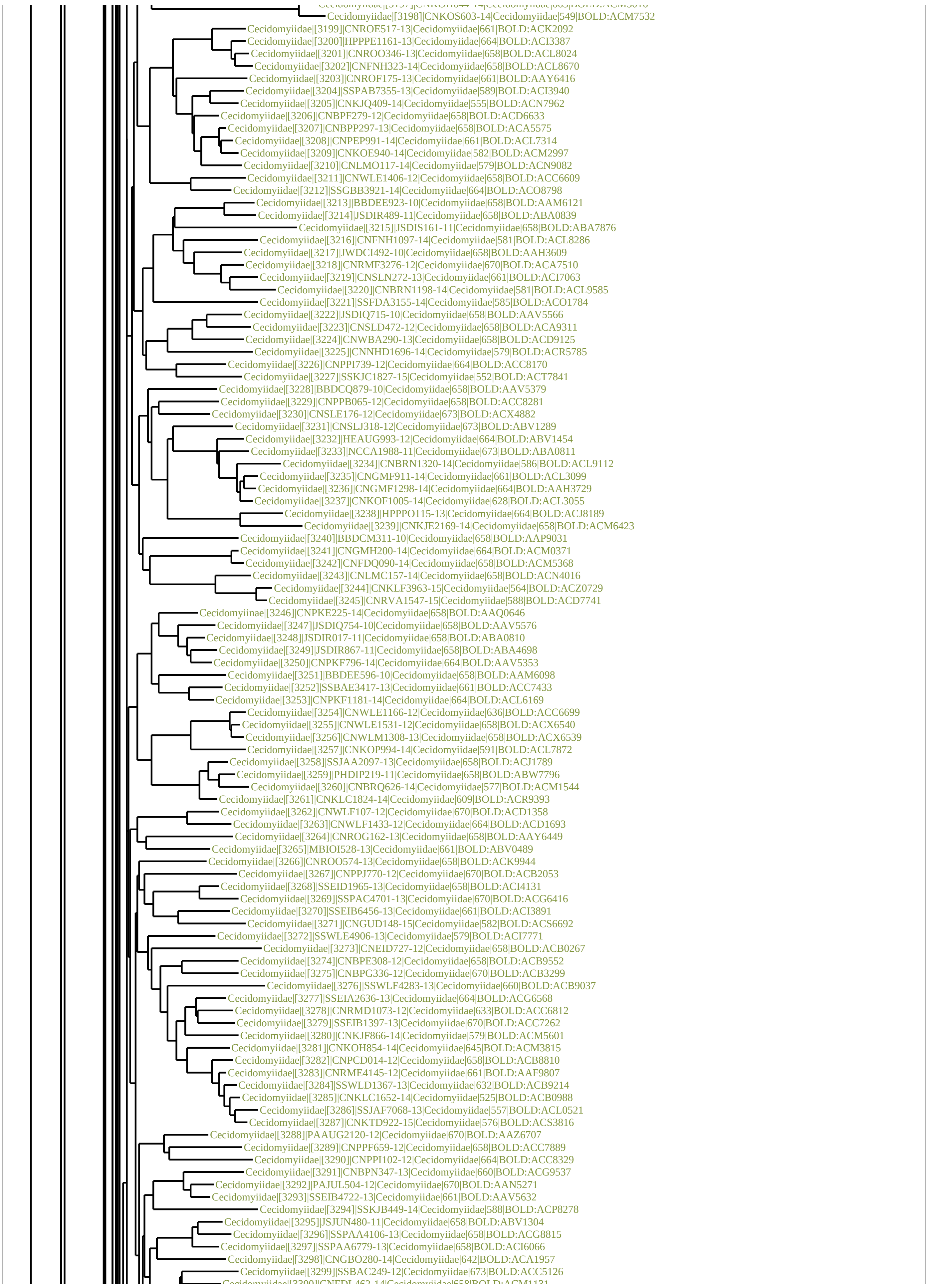
Cecidomyiidae|[3198]|CNKOS603-14|Cecidomyiidae|549|BOLD:ACM7532
Cecidomyiidae|[3199]|CNROE517-13|Cecidomyiidae|661|BOLD:ACK2092
Cecidomyiidae|[3200]|HPPPE1161-13|Cecidomyiidae|664|BOLD:ACI3387
Cecidomyiidae|[3201]|CNROO346-13|Cecidomyiidae|658|BOLD:ACL8024
Cecidomyiidae|[3202]|CNFNH323-14|Cecidomyiidae|658|BOLD:ACL8670
Cecidomyiidae|[3203]|CNROF175-13|Cecidomyiidae|661|BOLD:AAY6416
Cecidomyiidae|[3204]|SSPAB7355-13|Cecidomyiidae|589|BOLD:ACI3940
Cecidomyiidae|[3205]|CNKJQ409-14|Cecidomyiidae|555|BOLD:ACN7962
Cecidomyiidae|[3206]|CNBPF279-12|Cecidomyiidae|658|BOLD:ACD6633
Cecidomyiidae|[3207]|CNBPP297-13|Cecidomyiidae|658|BOLD:ACA5575
Cecidomyiidae|[3208]|CNPEP991-14|Cecidomyiidae|661|BOLD:ACL7314
Cecidomyiidae|[3209]|CNKOE940-14|Cecidomyiidae|582|BOLD:ACM2997
Cecidomyiidae|[3210]|CNLMO117-14|Cecidomyiidae|579|BOLD:ACN9082
Cecidomyiidae|[3211]|CNWLE1406-12|Cecidomyiidae|658|BOLD:ACC6609
Cecidomyiidae|[3212]|SSGBB3921-14|Cecidomyiidae|664|BOLD:ACO8798
Cecidomyiidae|[3213]|BBDEE923-10|Cecidomyiidae|658|BOLD:AAM6121
Cecidomyiidae|[3214]|JSDIR489-11|Cecidomyiidae|658|BOLD:ABA0839
Cecidomyiidae|[3215]|JSDIS161-11|Cecidomyiidae|658|BOLD:ABA7876
Cecidomyiidae|[3216]|CNFNH1097-14|Cecidomyiidae|581|BOLD:ACL8286
Cecidomyiidae|[3217]|JWDCI492-10|Cecidomyiidae|658|BOLD:AAH3609
Cecidomyiidae|[3218]|CNRMF3276-12|Cecidomyiidae|670|BOLD:ACA7510
Cecidomyiidae|[3219]|CNSLN272-13|Cecidomyiidae|661|BOLD:ACI7063
Cecidomyiidae|[3220]|CNBRN1198-14|Cecidomyiidae|581|BOLD:ACL9585
Cecidomyiidae|[3221]|SSFDA3155-14|Cecidomyiidae|585|BOLD:ACO1784
Cecidomyiidae|[3222]|JSDIQ715-10|Cecidomyiidae|658|BOLD:AAV5566
Cecidomyiidae|[3223]|CNSLD472-12|Cecidomyiidae|658|BOLD:ACA9311
Cecidomyiidae|[3224]|CNWBA290-13|Cecidomyiidae|658|BOLD:ACD9125
Cecidomyiidae|[3225]|CNNHD1696-14|Cecidomyiidae|579|BOLD:ACR5785
Cecidomyiidae|[3226]|CNPPI739-12|Cecidomyiidae|664|BOLD:ACC8170
Cecidomyiidae|[3227]|SSKJC1827-15|Cecidomyiidae|552|BOLD:ACT7841
Cecidomyiidae|[3228]|BBDCQ879-10|Cecidomyiidae|658|BOLD:AAV5379
Cecidomyiidae|[3229]|CNPPB065-12|Cecidomyiidae|658|BOLD:ACC8281
Cecidomyiidae|[3230]|CNSLE176-12|Cecidomyiidae|673|BOLD:ACX4882
Cecidomyiidae|[3231]|CNSLJ318-12|Cecidomyiidae|673|BOLD:ABV1289
Cecidomyiidae|[3232]|HEAUG993-12|Cecidomyiidae|664|BOLD:ABV1454
Cecidomyiidae|[3233]|NCCA1988-11|Cecidomyiidae|673|BOLD:ABA0811
Cecidomyiidae|[3234]|CNBRN1320-14|Cecidomyiidae|586|BOLD:ACL9112
Cecidomyiidae|[3235]|CNGMF911-14|Cecidomyiidae|661|BOLD:ACL3099
Cecidomyiidae|[3236]|CNGMF1298-14|Cecidomyiidae|664|BOLD:AAH3729
Cecidomyiidae|[3237]|CNKOF1005-14|Cecidomyiidae|628|BOLD:ACL3055
Cecidomyiidae|[3238]|HPPPO115-13|Cecidomyiidae|664|BOLD:ACJ8189
Cecidomyiidae|[3239]|CNKJE2169-14|Cecidomyiidae|658|BOLD:ACM6423
Cecidomyiidae|[3240]|BBDCM311-10|Cecidomyiidae|658|BOLD:AAP9031
Cecidomyiidae|[3241]|CNGMH200-14|Cecidomyiidae|664|BOLD:ACM0371
Cecidomyiidae|[3242]|CNFDQ090-14|Cecidomyiidae|658|BOLD:ACM5368
Cecidomyiidae|[3243]|CNLMC157-14|Cecidomyiidae|658|BOLD:ACN4016
Cecidomyiidae|[3244]|CNKLF3963-15|Cecidomyiidae|564|BOLD:ACZ0729
Cecidomyiidae|[3245]|CNRVA1547-15|Cecidomyiidae|588|BOLD:ACD7741
Cecidomyiinae|[3246]|CNPKE225-14|Cecidomyiidae|658|BOLD:AAQ0646
Cecidomyiidae|[3247]|JSDIQ754-10|Cecidomyiidae|658|BOLD:AAV5576
Cecidomyiidae|[3248]|JSDIR017-11|Cecidomyiidae|658|BOLD:ABA0810
Cecidomyiidae|[3249]|JSDIR867-11|Cecidomyiidae|658|BOLD:ABA4698
Cecidomyiidae|[3250]|CNPKF796-14|Cecidomyiidae|664|BOLD:AAV5353
Cecidomyiidae|[3251]|BBDEE596-10|Cecidomyiidae|658|BOLD:AAM6098
Cecidomyiidae|[3252]|SSBAE3417-13|Cecidomyiidae|661|BOLD:ACC7433
Cecidomyiidae|[3253]|CNPKF1181-14|Cecidomyiidae|664|BOLD:ACL6169
Cecidomyiidae|[3254]|CNWLE1166-12|Cecidomyiidae|636|BOLD:ACC6699
Cecidomyiidae|[3255]|CNWLE1531-12|Cecidomyiidae|658|BOLD:ACX6540
Cecidomyiidae|[3256]|CNWLM1308-13|Cecidomyiidae|658|BOLD:ACX6539
Cecidomyiidae|[3257]|CNKOP994-14|Cecidomyiidae|591|BOLD:ACL7872
Cecidomyiidae|[3258]|SSJAA2097-13|Cecidomyiidae|658|BOLD:ACJ1789
Cecidomyiidae|[3259]|PHDIP219-11|Cecidomyiidae|658|BOLD:ABW7796
Cecidomyiidae|[3260]|CNBRQ626-14|Cecidomyiidae|577|BOLD:ACM1544
Cecidomyiidae|[3261]|CNKLC1824-14|Cecidomyiidae|609|BOLD:ACR9393
Cecidomyiidae|[3262]|CNWLF107-12|Cecidomyiidae|670|BOLD:ACD1358
Cecidomyiidae|[3263]|CNWLF1433-12|Cecidomyiidae|664|BOLD:ACD1693
Cecidomyiidae|[3264]|CNROG162-13|Cecidomyiidae|658|BOLD:AAY6449
Cecidomyiidae|[3265]|MBIOI528-13|Cecidomyiidae|661|BOLD:ABV0489
Cecidomyiidae|[3266]|CNROO574-13|Cecidomyiidae|658|BOLD:ACK9944
Cecidomyiidae|[3267]|CNPPJ770-12|Cecidomyiidae|670|BOLD:ACB2053
Cecidomyiidae|[3268]|SSEID1965-13|Cecidomyiidae|658|BOLD:ACI4131
Cecidomyiidae|[3269]|SSPAC4701-13|Cecidomyiidae|670|BOLD:ACG6416
Cecidomyiidae|[3270]|SSEIB6456-13|Cecidomyiidae|661|BOLD:ACI3891
Cecidomyiidae|[3271]|CNGUD148-15|Cecidomyiidae|582|BOLD:ACS6692
Cecidomyiidae|[3272]|SSWLE4906-13|Cecidomyiidae|579|BOLD:ACI7771
Cecidomyiidae|[3273]|CNEID727-12|Cecidomyiidae|658|BOLD:ACB0267
Cecidomyiidae|[3274]|CNBPE308-12|Cecidomyiidae|658|BOLD:ACB9552
Cecidomyiidae|[3275]|CNBPG336-12|Cecidomyiidae|670|BOLD:ACB3299
Cecidomyiidae|[3276]|SSWLF4283-13|Cecidomyiidae|660|BOLD:ACB9037
Cecidomyiidae|[3277]|SSEIA2636-13|Cecidomyiidae|664|BOLD:ACG6568
Cecidomyiidae|[3278]|CNRMD1073-12|Cecidomyiidae|633|BOLD:ACC6812
Cecidomyiidae|[3279]|SSEIB1397-13|Cecidomyiidae|670|BOLD:ACC7262
Cecidomyiidae|[3280]|CNKJF866-14|Cecidomyiidae|579|BOLD:ACM5601
Cecidomyiidae|[3281]|CNKOH854-14|Cecidomyiidae|645|BOLD:ACM3815
Cecidomyiidae|[3282]|CNPCD014-12|Cecidomyiidae|658|BOLD:ACB8810
Cecidomyiidae|[3283]|CNRME4145-12|Cecidomyiidae|661|BOLD:AAF9807
Cecidomyiidae|[3284]|SSWLD1367-13|Cecidomyiidae|632|BOLD:ACB9214
Cecidomyiidae|[3285]|CNKLC1652-14|Cecidomyiidae|525|BOLD:ACB0988
Cecidomyiidae|[3286]|SSJAF7068-13|Cecidomyiidae|557|BOLD:ACL0521
Cecidomyiidae|[3287]|CNKTD922-15|Cecidomyiidae|576|BOLD:ACS3816
Cecidomyiidae|[3288]|PAAUG2120-12|Cecidomyiidae|670|BOLD:AAZ6707
Cecidomyiidae|[3289]|CNPPF659-12|Cecidomyiidae|658|BOLD:ACC7889
Cecidomyiidae|[3290]|CNPPI102-12|Cecidomyiidae|664|BOLD:ACC8329
Cecidomyiidae|[3291]|CNBPN347-13|Cecidomyiidae|660|BOLD:ACG9537
Cecidomyiidae|[3292]|PAJUL504-12|Cecidomyiidae|670|BOLD:AAN5271
Cecidomyiidae|[3293]|SSEIB4722-13|Cecidomyiidae|661|BOLD:AAV5632
Cecidomyiidae|[3294]|SSKJB449-14|Cecidomyiidae|588|BOLD:ACP8278
Cecidomyiidae|[3295]|JSJUN480-11|Cecidomyiidae|658|BOLD:ABV1304
Cecidomyiidae|[3296]|SSPAA4106-13|Cecidomyiidae|658|BOLD:ACG8815
Cecidomyiidae|[3297]|SSPAA6779-13|Cecidomyiidae|658|BOLD:ACI6066
Cecidomyiidae|[3298]|CNGBO280-14|Cecidomyiidae|642|BOLD:ACA1957
Cecidomyiidae|[3299]|SSBAC249-12|Cecidomyiidae|673|BOLD:ACC5126

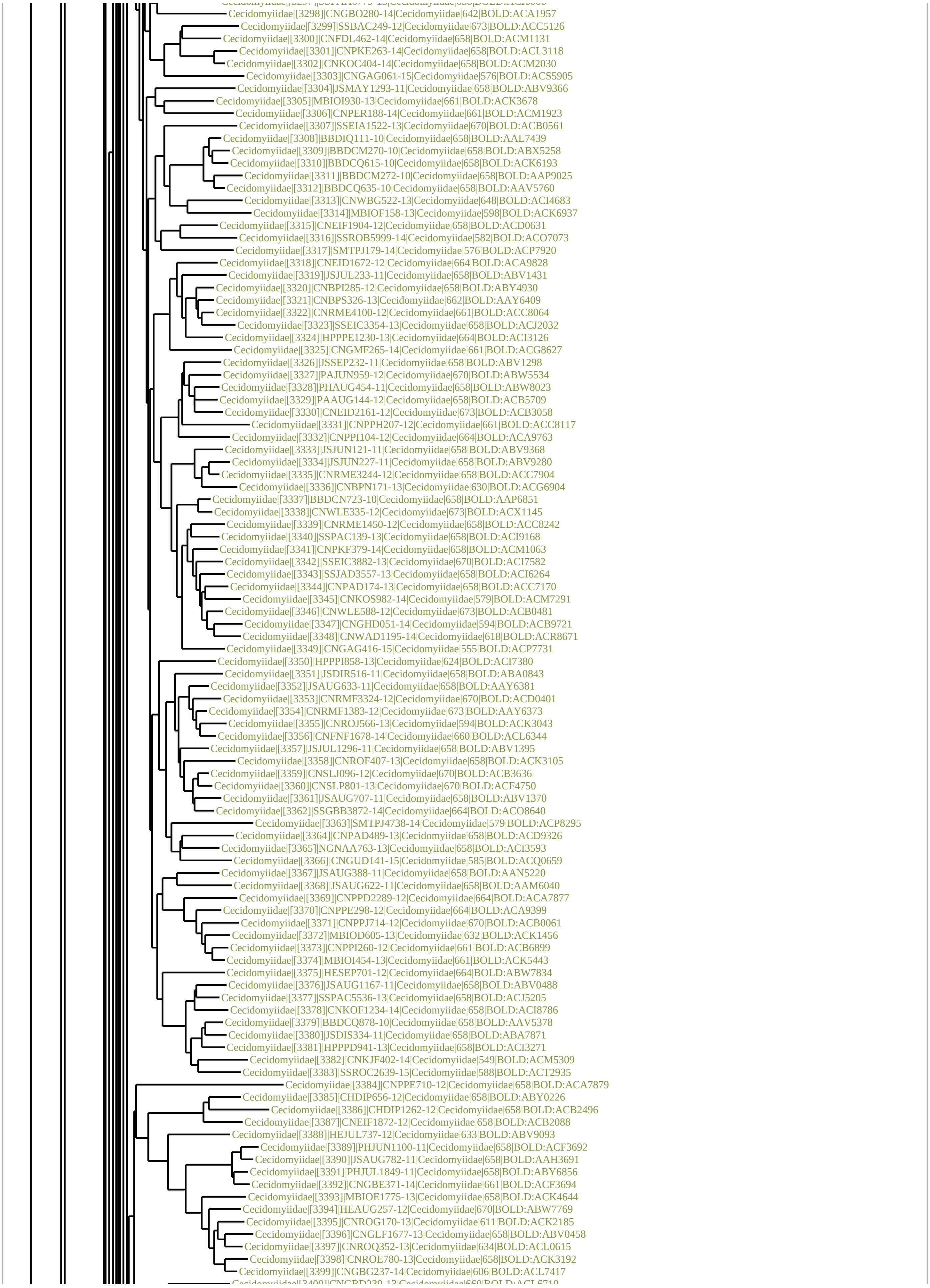
Cecidomyiidae|[3298]|CNGBO280-14|Cecidomyiidae|642|BOLD:ACA1957
Cecidomyiidae|[3299]|SSBAC249-12|Cecidomyiidae|673|BOLD:ACC5126
Cecidomyiidae|[3300]|CNFDL462-14|Cecidomyiidae|658|BOLD:ACM1131
Cecidomyiidae|[3301]|CNPKE263-14|Cecidomyiidae|658|BOLD:ACL3118
Cecidomyiidae|[3302]|CNKOC404-14|Cecidomyiidae|658|BOLD:ACM2030
Cecidomyiidae|[3303]|CNGAG061-15|Cecidomyiidae|576|BOLD:ACS5905
Cecidomyiidae|[3304]|JSMAY1293-11|Cecidomyiidae|658|BOLD:ABV9366
Cecidomyiidae|[3305]|MBIOI930-13|Cecidomyiidae|661|BOLD:ACK3678
Cecidomyiidae|[3306]|CNPER188-14|Cecidomyiidae|661|BOLD:ACM1923
Cecidomyiidae|[3307]|SSEIA1522-13|Cecidomyiidae|670|BOLD:ACB0561
Cecidomyiidae|[3308]|BBDIQ111-10|Cecidomyiidae|658|BOLD:AAL7439
Cecidomyiidae|[3309]|BBDCM270-10|Cecidomyiidae|658|BOLD:ABX5258
Cecidomyiidae|[3310]|BBDCQ615-10|Cecidomyiidae|658|BOLD:ACK6193
Cecidomyiidae|[3311]|BBDCM272-10|Cecidomyiidae|658|BOLD:AAP9025
Cecidomyiidae|[3312]|BBDCQ635-10|Cecidomyiidae|658|BOLD:AAV5760
Cecidomyiidae|[3313]|CNWBG522-13|Cecidomyiidae|648|BOLD:ACI4683
Cecidomyiidae|[3314]|MBIOF158-13|Cecidomyiidae|598|BOLD:ACK6937
Cecidomyiidae|[3315]|CNEIF1904-12|Cecidomyiidae|658|BOLD:ACD0631
Cecidomyiidae|[3316]|SSROB5999-14|Cecidomyiidae|582|BOLD:ACO7073
Cecidomyiidae|[3317]|SMTPJ179-14|Cecidomyiidae|576|BOLD:ACP7920
Cecidomyiidae|[3318]|CNEID1672-12|Cecidomyiidae|664|BOLD:ACA9828
Cecidomyiidae|[3319]|JSJUL233-11|Cecidomyiidae|658|BOLD:ABV1431
Cecidomyiidae|[3320]|CNBPI285-12|Cecidomyiidae|658|BOLD:ABY4930
Cecidomyiidae|[3321]|CNBPS326-13|Cecidomyiidae|662|BOLD:AAY6409
Cecidomyiidae|[3322]|CNRME4100-12|Cecidomyiidae|661|BOLD:ACC8064
Cecidomyiidae|[3323]|SSEIC3354-13|Cecidomyiidae|658|BOLD:ACJ2032
Cecidomyiidae|[3324]|HPPPE1230-13|Cecidomyiidae|664|BOLD:ACI3126
Cecidomyiidae|[3325]|CNGMF265-14|Cecidomyiidae|661|BOLD:ACG8627
Cecidomyiidae|[3326]|JSSEP232-11|Cecidomyiidae|658|BOLD:ABV1298
Cecidomyiidae|[3327]|PAJUN959-12|Cecidomyiidae|670|BOLD:ABW5534
Cecidomyiidae|[3328]|PHAUG454-11|Cecidomyiidae|658|BOLD:ABW8023
Cecidomyiidae|[3329]|PAAUG144-12|Cecidomyiidae|658|BOLD:ACB5709
Cecidomyiidae|[3330]|CNEID2161-12|Cecidomyiidae|673|BOLD:ACB3058
Cecidomyiidae|[3331]|CNPPH207-12|Cecidomyiidae|661|BOLD:ACC8117
Cecidomyiidae|[3332]|CNPPI104-12|Cecidomyiidae|664|BOLD:ACA9763
Cecidomyiidae|[3333]|JSJUN121-11|Cecidomyiidae|658|BOLD:ABV9368
Cecidomyiidae|[3334]|JSJUN227-11|Cecidomyiidae|658|BOLD:ABV9280
Cecidomyiidae|[3335]|CNRME3244-12|Cecidomyiidae|658|BOLD:ACC7904
Cecidomyiidae|[3336]|CNBPN171-13|Cecidomyiidae|630|BOLD:ACG6904
Cecidomyiidae|[3337]|BBDCN723-10|Cecidomyiidae|658|BOLD:AAP6851
Cecidomyiidae|[3338]|CNWLE335-12|Cecidomyiidae|673|BOLD:ACX1145
Cecidomyiidae|[3339]|CNRME1450-12|Cecidomyiidae|658|BOLD:ACC8242
Cecidomyiidae|[3340]|SSPAC139-13|Cecidomyiidae|658|BOLD:ACI9168
Cecidomyiidae|[3341]|CNPKF379-14|Cecidomyiidae|658|BOLD:ACM1063
Cecidomyiidae|[3342]|SSEIC3882-13|Cecidomyiidae|670|BOLD:ACI7582
Cecidomyiidae|[3343]|SSJAD3557-13|Cecidomyiidae|658|BOLD:ACI6264
Cecidomyiidae|[3344]|CNPAD174-13|Cecidomyiidae|658|BOLD:ACC7170
Cecidomyiidae|[3345]|CNKOS982-14|Cecidomyiidae|579|BOLD:ACM7291
Cecidomyiidae|[3346]|CNWLE588-12|Cecidomyiidae|673|BOLD:ACB0481
Cecidomyiidae|[3347]|CNGHD051-14|Cecidomyiidae|594|BOLD:ACB9721
Cecidomyiidae|[3348]|CNWAD1195-14|Cecidomyiidae|618|BOLD:ACR8671
Cecidomyiidae|[3349]|CNGAG416-15|Cecidomyiidae|555|BOLD:ACP7731
Cecidomyiidae|[3350]|HPPPI858-13|Cecidomyiidae|624|BOLD:ACI7380
Cecidomyiidae|[3351]|JSDIR516-11|Cecidomyiidae|658|BOLD:ABA0843
Cecidomyiidae|[3352]|JSAUG633-11|Cecidomyiidae|658|BOLD:AAY6381
Cecidomyiidae|[3353]|CNRMF3324-12|Cecidomyiidae|670|BOLD:ACD0401
Cecidomyiidae|[3354]|CNRMF1383-12|Cecidomyiidae|673|BOLD:AAY6373
Cecidomyiidae|[3355]|CNROJ566-13|Cecidomyiidae|594|BOLD:ACK3043
Cecidomyiidae|[3356]|CNFNF1678-14|Cecidomyiidae|660|BOLD:ACL6344
Cecidomyiidae|[3357]|JSJUL1296-11|Cecidomyiidae|658|BOLD:ABV1395
Cecidomyiidae|[3358]|CNROF407-13|Cecidomyiidae|658|BOLD:ACK3105
Cecidomyiidae|[3359]|CNSLJ096-12|Cecidomyiidae|670|BOLD:ACB3636
Cecidomyiidae|[3360]|CNSLP801-13|Cecidomyiidae|670|BOLD:ACF4750
Cecidomyiidae|[3361]|JSAUG707-11|Cecidomyiidae|658|BOLD:ABV1370
Cecidomyiidae|[3362]|SSGBB3872-14|Cecidomyiidae|664|BOLD:ACO8640
Cecidomyiidae|[3363]|SMTPJ4738-14|Cecidomyiidae|579|BOLD:ACP8295
Cecidomyiidae|[3364]|CNPAD489-13|Cecidomyiidae|658|BOLD:ACD9326
Cecidomyiidae|[3365]|NGNAA763-13|Cecidomyiidae|658|BOLD:ACI3593
Cecidomyiidae|[3366]|CNGUD141-15|Cecidomyiidae|585|BOLD:ACQ0659
Cecidomyiidae|[3367]|JSAUG388-11|Cecidomyiidae|658|BOLD:AAN5220
Cecidomyiidae|[3368]|JSAUG622-11|Cecidomyiidae|658|BOLD:AAM6040
Cecidomyiidae|[3369]|CNPPD2289-12|Cecidomyiidae|664|BOLD:ACA7877
Cecidomyiidae|[3370]|CNPPE298-12|Cecidomyiidae|664|BOLD:ACA9399
Cecidomyiidae|[3371]|CNPPJ714-12|Cecidomyiidae|670|BOLD:ACB0061
Cecidomyiidae|[3372]|MBIOD605-13|Cecidomyiidae|632|BOLD:ACK1456
Cecidomyiidae|[3373]|CNPPI260-12|Cecidomyiidae|661|BOLD:ACB6899
Cecidomyiidae|[3374]|MBIOI454-13|Cecidomyiidae|661|BOLD:ACK5443
Cecidomyiidae|[3375]|HESEP701-12|Cecidomyiidae|664|BOLD:ABW7834
Cecidomyiidae|[3376]|JSAUG1167-11|Cecidomyiidae|658|BOLD:ABV0488
Cecidomyiidae|[3377]|SSPAC5536-13|Cecidomyiidae|658|BOLD:ACJ5205
Cecidomyiidae|[3378]|CNKOF1234-14|Cecidomyiidae|658|BOLD:ACI8786
Cecidomyiidae|[3379]|BBDCQ878-10|Cecidomyiidae|658|BOLD:AAV5378
Cecidomyiidae|[3380]|JSDIS334-11|Cecidomyiidae|658|BOLD:ABA7871
Cecidomyiidae|[3381]|HPPPD941-13|Cecidomyiidae|658|BOLD:ACI3271
Cecidomyiidae|[3382]|CNKJF402-14|Cecidomyiidae|549|BOLD:ACM5309
Cecidomyiidae|[3383]|SSROC2639-15|Cecidomyiidae|588|BOLD:ACT2935
Cecidomyiidae|[3384]|CNPPE710-12|Cecidomyiidae|658|BOLD:ACA7879
Cecidomyiidae|[3385]|CHDIP656-12|Cecidomyiidae|658|BOLD:ABY0226
Cecidomyiidae|[3386]|CHDIP1262-12|Cecidomyiidae|658|BOLD:ACB2496
Cecidomyiidae|[3387]|CNEIF1872-12|Cecidomyiidae|658|BOLD:ACB2088
Cecidomyiidae|[3388]|HEJUL737-12|Cecidomyiidae|633|BOLD:ABV9093
Cecidomyiidae|[3389]|PHJUN1100-11|Cecidomyiidae|658|BOLD:ACF3692
Cecidomyiidae|[3390]|JSAUG782-11|Cecidomyiidae|658|BOLD:AAH3691
Cecidomyiidae|[3391]|PHJUL1849-11|Cecidomyiidae|658|BOLD:ABY6856
Cecidomyiidae|[3392]|CNGBE371-14|Cecidomyiidae|661|BOLD:ACF3694
Cecidomyiidae|[3393]|MBIOE1775-13|Cecidomyiidae|658|BOLD:ACK4644
Cecidomyiidae|[3394]|HEAUG257-12|Cecidomyiidae|670|BOLD:ABW7769
Cecidomyiidae|[3395]|CNROG170-13|Cecidomyiidae|611|BOLD:ACK2185
Cecidomyiidae|[3396]|CNGLF1677-13|Cecidomyiidae|658|BOLD:ABV0458
Cecidomyiidae|[3397]|CNROQ352-13|Cecidomyiidae|634|BOLD:ACL0615
Cecidomyiidae|[3398]|CNROE780-13|Cecidomyiidae|658|BOLD:ACK3192
Cecidomyiidae|[3399]|CNGBG237-14|Cecidomyiidae|606|BOLD:ACL7417

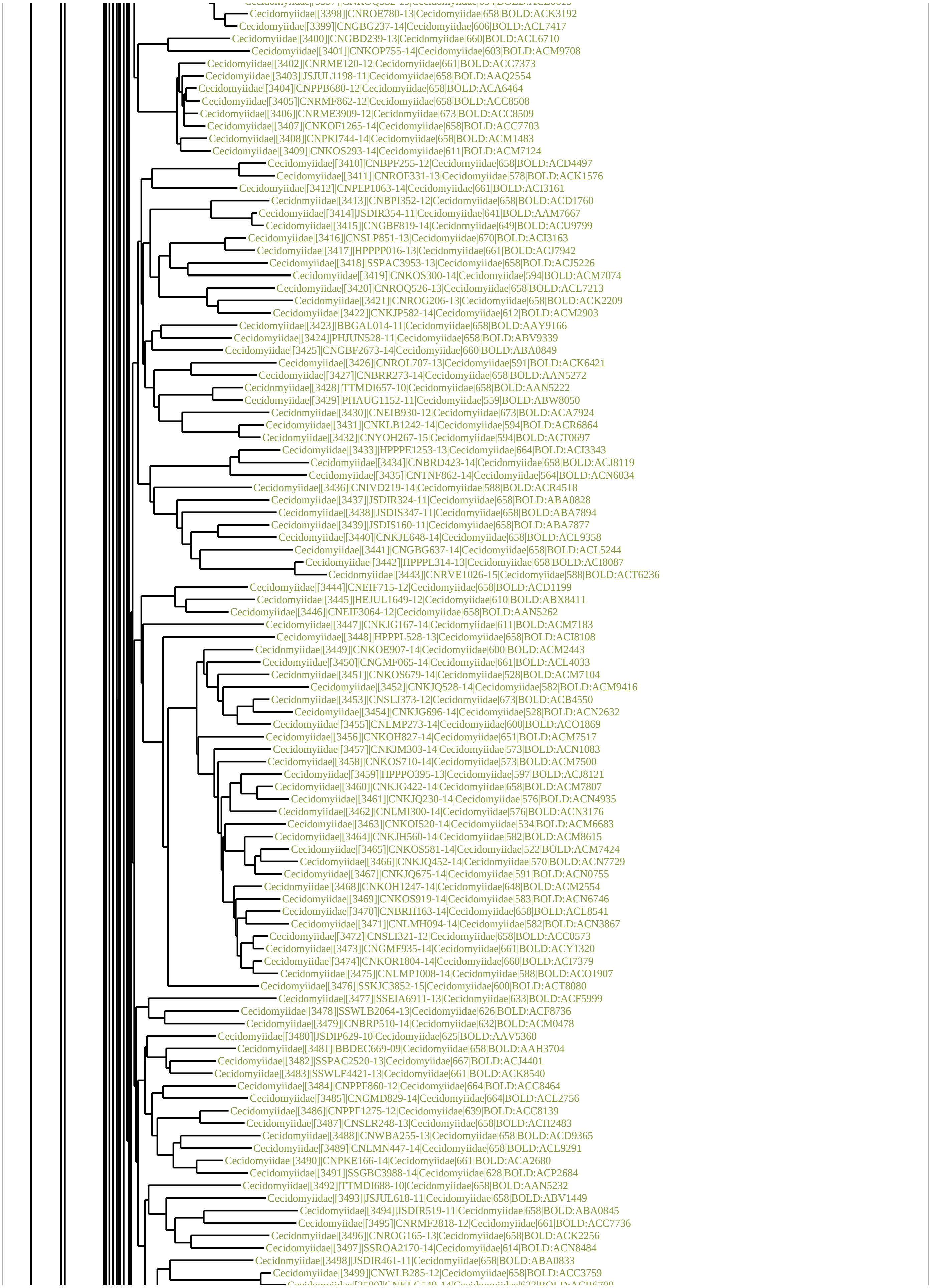
Cecidomyiidae|[3398]|CNROE780-13|Cecidomyiidae|658|BOLD:ACK3192
Cecidomyiidae|[3399]|CNGBG237-14|Cecidomyiidae|606|BOLD:ACL7417
Cecidomyiidae|[3400]|CNGBD239-13|Cecidomyiidae|660|BOLD:ACL6710
Cecidomyiidae|[3401]|CNKOP755-14|Cecidomyiidae|603|BOLD:ACM9708
Cecidomyiidae|[3402]|CNRME120-12|Cecidomyiidae|661|BOLD:ACC7373
Cecidomyiidae|[3403]|JSJUL1198-11|Cecidomyiidae|658|BOLD:AAQ2554
Cecidomyiidae|[3404]|CNPPB680-12|Cecidomyiidae|658|BOLD:ACA6464
Cecidomyiidae|[3405]|CNRMF862-12|Cecidomyiidae|658|BOLD:ACC8508
Cecidomyiidae|[3406]|CNRME3909-12|Cecidomyiidae|673|BOLD:ACC8509
Cecidomyiidae|[3407]|CNKOF1265-14|Cecidomyiidae|658|BOLD:ACC7703
Cecidomyiidae|[3408]|CNPKI744-14|Cecidomyiidae|658|BOLD:ACM1483
Cecidomyiidae|[3409]|CNKOS293-14|Cecidomyiidae|611|BOLD:ACM7124
Cecidomyiidae|[3410]|CNBPF255-12|Cecidomyiidae|658|BOLD:ACD4497
Cecidomyiidae|[3411]|CNROF331-13|Cecidomyiidae|578|BOLD:ACK1576
Cecidomyiidae|[3412]|CNPEP1063-14|Cecidomyiidae|661|BOLD:ACI3161
Cecidomyiidae|[3413]|CNBPI352-12|Cecidomyiidae|658|BOLD:ACD1760
Cecidomyiidae|[3414]|JSDIR354-11|Cecidomyiidae|641|BOLD:AAM7667
Cecidomyiidae|[3415]|CNGBF819-14|Cecidomyiidae|649|BOLD:ACU9799
Cecidomyiidae|[3416]|CNSLP851-13|Cecidomyiidae|670|BOLD:ACI3163
Cecidomyiidae|[3417]|HPPPP016-13|Cecidomyiidae|661|BOLD:ACJ7942
Cecidomyiidae|[3418]|SSPAC3953-13|Cecidomyiidae|658|BOLD:ACJ5226
Cecidomyiidae|[3419]|CNKOS300-14|Cecidomyiidae|594|BOLD:ACM7074
Cecidomyiidae|[3420]|CNROQ526-13|Cecidomyiidae|658|BOLD:ACL7213
Cecidomyiidae|[3421]|CNROG206-13|Cecidomyiidae|658|BOLD:ACK2209
Cecidomyiidae|[3422]|CNKJP582-14|Cecidomyiidae|612|BOLD:ACM2903
Cecidomyiidae|[3423]|BBGAL014-11|Cecidomyiidae|658|BOLD:AAY9166
Cecidomyiidae|[3424]|PHJUN528-11|Cecidomyiidae|658|BOLD:ABV9339
Cecidomyiidae|[3425]|CNGBF2673-14|Cecidomyiidae|660|BOLD:ABA0849
Cecidomyiidae|[3426]|CNROL707-13|Cecidomyiidae|591|BOLD:ACK6421
Cecidomyiidae|[3427]|CNBRR273-14|Cecidomyiidae|658|BOLD:AAN5272
Cecidomyiidae|[3428]|TTMDI657-10|Cecidomyiidae|658|BOLD:AAN5222
Cecidomyiidae|[3429]|PHAUG1152-11|Cecidomyiidae|559|BOLD:ABW8050
Cecidomyiidae|[3430]|CNEIB930-12|Cecidomyiidae|673|BOLD:ACA7924
Cecidomyiidae|[3431]|CNKLB1242-14|Cecidomyiidae|594|BOLD:ACR6864
Cecidomyiidae|[3432]|CNYOH267-15|Cecidomyiidae|594|BOLD:ACT0697
Cecidomyiidae|[3433]|HPPPE1253-13|Cecidomyiidae|664|BOLD:ACI3343
Cecidomyiidae|[3434]|CNBRD423-14|Cecidomyiidae|658|BOLD:ACJ8119
Cecidomyiidae|[3435]|CNTNF862-14|Cecidomyiidae|564|BOLD:ACN6034
Cecidomyiidae|[3436]|CNIVD219-14|Cecidomyiidae|588|BOLD:ACR4518
Cecidomyiidae|[3437]|JSDIR324-11|Cecidomyiidae|658|BOLD:ABA0828
Cecidomyiidae|[3438]|JSDIS347-11|Cecidomyiidae|658|BOLD:ABA7894
Cecidomyiidae|[3439]|JSDIS160-11|Cecidomyiidae|658|BOLD:ABA7877
Cecidomyiidae|[3440]|CNKJE648-14|Cecidomyiidae|658|BOLD:ACL9358
Cecidomyiidae|[3441]|CNGBG637-14|Cecidomyiidae|658|BOLD:ACL5244
Cecidomyiidae|[3442]|HPPPL314-13|Cecidomyiidae|658|BOLD:ACI8087
Cecidomyiidae|[3443]|CNRVE1026-15|Cecidomyiidae|588|BOLD:ACT6236
Cecidomyiidae|[3444]|CNEIF715-12|Cecidomyiidae|658|BOLD:ACD1199
Cecidomyiidae|[3445]|HEJUL1649-12|Cecidomyiidae|610|BOLD:ABX8411
Cecidomyiidae|[3446]|CNEIF3064-12|Cecidomyiidae|658|BOLD:AAN5262
Cecidomyiidae|[3447]|CNKJG167-14|Cecidomyiidae|611|BOLD:ACM7183
Cecidomyiidae|[3448]|HPPPL528-13|Cecidomyiidae|658|BOLD:ACI8108
Cecidomyiidae|[3449]|CNKOE907-14|Cecidomyiidae|600|BOLD:ACM2443
Cecidomyiidae|[3450]|CNGMF065-14|Cecidomyiidae|661|BOLD:ACL4033
Cecidomyiidae|[3451]|CNKOS679-14|Cecidomyiidae|528|BOLD:ACM7104
Cecidomyiidae|[3452]|CNKJQ528-14|Cecidomyiidae|582|BOLD:ACM9416
Cecidomyiidae|[3453]|CNSLJ373-12|Cecidomyiidae|673|BOLD:ACB4550
Cecidomyiidae|[3454]|CNKJG696-14|Cecidomyiidae|528|BOLD:ACN2632
Cecidomyiidae|[3455]|CNLMP273-14|Cecidomyiidae|600|BOLD:ACO1869
Cecidomyiidae|[3456]|CNKOH827-14|Cecidomyiidae|651|BOLD:ACM7517
Cecidomyiidae|[3457]|CNKJM303-14|Cecidomyiidae|573|BOLD:ACN1083
Cecidomyiidae|[3458]|CNKOS710-14|Cecidomyiidae|573|BOLD:ACM7500
Cecidomyiidae|[3459]|HPPPO395-13|Cecidomyiidae|597|BOLD:ACJ8121
Cecidomyiidae|[3460]|CNKJG422-14|Cecidomyiidae|658|BOLD:ACM7807
Cecidomyiidae|[3461]|CNKJQ230-14|Cecidomyiidae|576|BOLD:ACN4935
Cecidomyiidae|[3462]|CNLMI300-14|Cecidomyiidae|576|BOLD:ACN3176
Cecidomyiidae|[3463]|CNKOI520-14|Cecidomyiidae|534|BOLD:ACM6683
Cecidomyiidae|[3464]|CNKJH560-14|Cecidomyiidae|582|BOLD:ACM8615
Cecidomyiidae|[3465]|CNKOS581-14|Cecidomyiidae|522|BOLD:ACM7424
Cecidomyiidae|[3466]|CNKJQ452-14|Cecidomyiidae|570|BOLD:ACN7729
Cecidomyiidae|[3467]|CNKJQ675-14|Cecidomyiidae|591|BOLD:ACN0755
Cecidomyiidae|[3468]|CNKOH1247-14|Cecidomyiidae|648|BOLD:ACM2554
Cecidomyiidae|[3469]|CNKOS919-14|Cecidomyiidae|583|BOLD:ACN6746
Cecidomyiidae|[3470]|CNBRH163-14|Cecidomyiidae|658|BOLD:ACL8541
Cecidomyiidae|[3471]|CNLMH094-14|Cecidomyiidae|582|BOLD:ACN3867
Cecidomyiidae|[3472]|CNSLI321-12|Cecidomyiidae|658|BOLD:ACC0573
Cecidomyiidae|[3473]|CNGMF935-14|Cecidomyiidae|661|BOLD:ACY1320
Cecidomyiidae|[3474]|CNKOR1804-14|Cecidomyiidae|660|BOLD:ACI7379
Cecidomyiidae|[3475]|CNLMP1008-14|Cecidomyiidae|588|BOLD:ACO1907
Cecidomyiidae|[3476]|SSKJC3852-15|Cecidomyiidae|600|BOLD:ACT8080
Cecidomyiidae|[3477]|SSEIA6911-13|Cecidomyiidae|633|BOLD:ACF5999
Cecidomyiidae|[3478]|SSWLB2064-13|Cecidomyiidae|626|BOLD:ACF8736
Cecidomyiidae|[3479]|CNBRP510-14|Cecidomyiidae|632|BOLD:ACM0478
Cecidomyiidae|[3480]|JSDIP629-10|Cecidomyiidae|625|BOLD:AAV5360
Cecidomyiidae|[3481]|BBDEC669-09|Cecidomyiidae|658|BOLD:AAH3704
Cecidomyiidae|[3482]|SSPAC2520-13|Cecidomyiidae|667|BOLD:ACJ4401
Cecidomyiidae|[3483]|SSWLF4421-13|Cecidomyiidae|661|BOLD:ACK8540
Cecidomyiidae|[3484]|CNPPF860-12|Cecidomyiidae|664|BOLD:ACC8464
Cecidomyiidae|[3485]|CNGMD829-14|Cecidomyiidae|664|BOLD:ACL2756
Cecidomyiidae|[3486]|CNPPF1275-12|Cecidomyiidae|639|BOLD:ACC8139
Cecidomyiidae|[3487]|CNSLR248-13|Cecidomyiidae|658|BOLD:ACH2483
Cecidomyiidae|[3488]|CNWBA255-13|Cecidomyiidae|658|BOLD:ACD9365
Cecidomyiidae|[3489]|CNLMN447-14|Cecidomyiidae|658|BOLD:ACL9291
Cecidomyiidae|[3490]|CNPKE166-14|Cecidomyiidae|661|BOLD:ACA2680
Cecidomyiidae|[3491]|SSGBC3988-14|Cecidomyiidae|628|BOLD:ACP2684
Cecidomyiidae|[3492]|TTMDI688-10|Cecidomyiidae|658|BOLD:AAN5232
Cecidomyiidae|[3493]|JSJUL618-11|Cecidomyiidae|658|BOLD:ABV1449
Cecidomyiidae|[3494]|JSDIR519-11|Cecidomyiidae|658|BOLD:ABA0845
Cecidomyiidae|[3495]|CNRMF2818-12|Cecidomyiidae|661|BOLD:ACC7736
Cecidomyiidae|[3496]|CNROG165-13|Cecidomyiidae|658|BOLD:ACK2256
Cecidomyiidae|[3497]|SSROA2170-14|Cecidomyiidae|614|BOLD:ACN8484
Cecidomyiidae|[3498]|JSDIR461-11|Cecidomyiidae|658|BOLD:ABA0833
Cecidomyiidae|[3499]|CNWLB285-12|Cecidomyiidae|658|BOLD:ACC3759

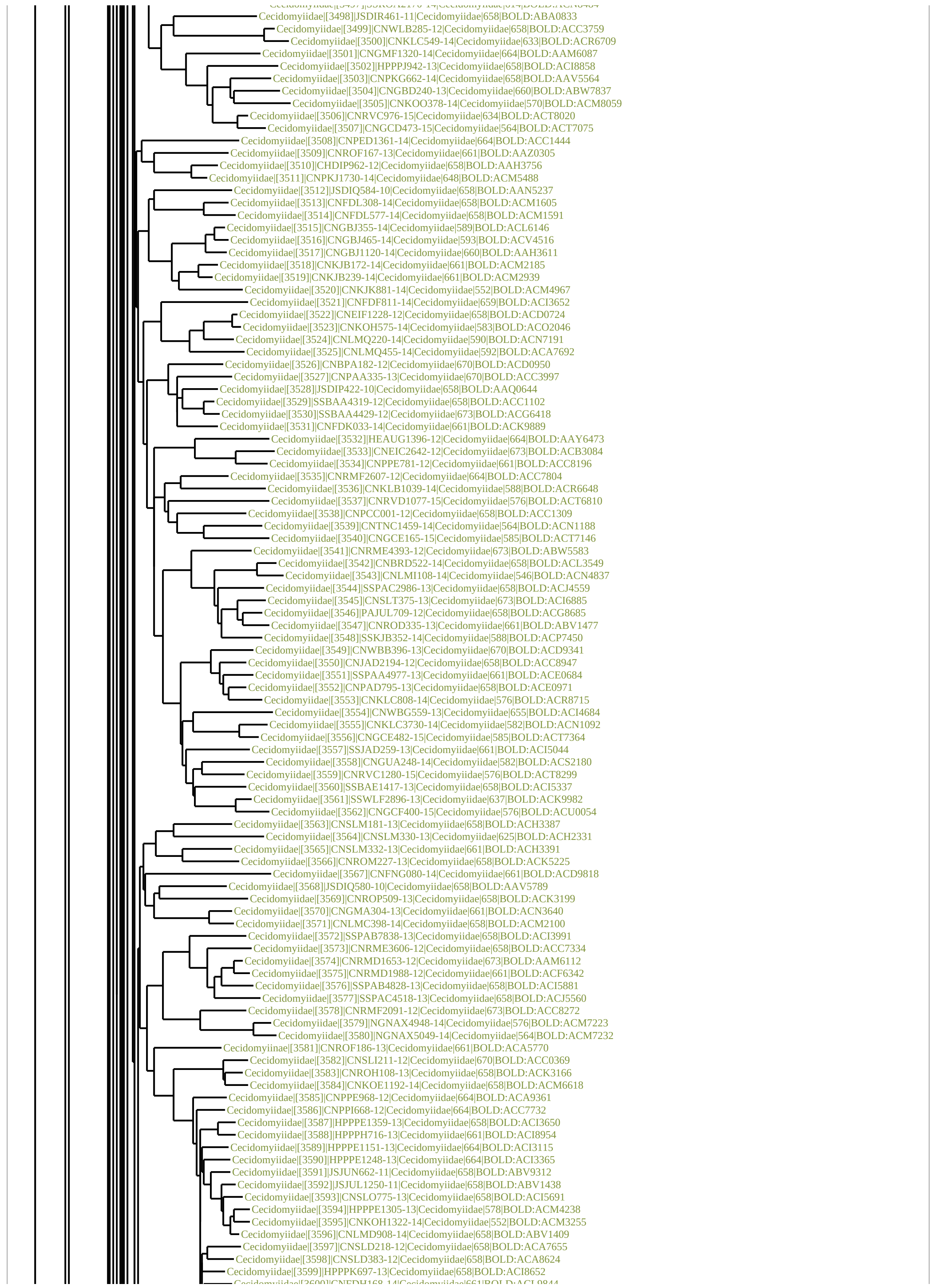
Cecidomyiidae|[3498]|JSDIR461-11|Cecidomyiidae|658|BOLD:ABA0833
Cecidomyiidae|[3499]|CNWLB285-12|Cecidomyiidae|658|BOLD:ACC3759
Cecidomyiidae|[3500]|CNKLC549-14|Cecidomyiidae|633|BOLD:ACR6709
Cecidomyiidae|[3501]|CNGMF1320-14|Cecidomyiidae|664|BOLD:AAM6087
Cecidomyiidae|[3502]|HPPPJ942-13|Cecidomyiidae|658|BOLD:ACI8858
Cecidomyiidae|[3503]|CNPKG662-14|Cecidomyiidae|658|BOLD:AAV5564
Cecidomyiidae|[3504]|CNGBD240-13|Cecidomyiidae|660|BOLD:ABW7837
Cecidomyiidae|[3505]|CNKOO378-14|Cecidomyiidae|570|BOLD:ACM8059
Cecidomyiidae|[3506]|CNRVC976-15|Cecidomyiidae|634|BOLD:ACT8020
Cecidomyiidae|[3507]|CNGCD473-15|Cecidomyiidae|564|BOLD:ACT7075
Cecidomyiidae|[3508]|CNPED1361-14|Cecidomyiidae|664|BOLD:ACC1444
Cecidomyiidae|[3509]|CNROF167-13|Cecidomyiidae|661|BOLD:AAZ0305
Cecidomyiidae|[3510]|CHDIP962-12|Cecidomyiidae|658|BOLD:AAH3756
Cecidomyiidae|[3511]|CNPKJ1730-14|Cecidomyiidae|648|BOLD:ACM5488
Cecidomyiidae|[3512]|JSDIQ584-10|Cecidomyiidae|658|BOLD:AAN5237
Cecidomyiidae|[3513]|CNFDL308-14|Cecidomyiidae|658|BOLD:ACM1605
Cecidomyiidae|[3514]|CNFDL577-14|Cecidomyiidae|658|BOLD:ACM1591
Cecidomyiidae|[3515]|CNGBJ355-14|Cecidomyiidae|589|BOLD:ACL6146
Cecidomyiidae|[3516]|CNGBJ465-14|Cecidomyiidae|593|BOLD:ACV4516
Cecidomyiidae|[3517]|CNGBJ1120-14|Cecidomyiidae|660|BOLD:AAH3611
Cecidomyiidae|[3518]|CNKJB172-14|Cecidomyiidae|661|BOLD:ACM2185
Cecidomyiidae|[3519]|CNKJB239-14|Cecidomyiidae|661|BOLD:ACM2939
Cecidomyiidae|[3520]|CNKJK881-14|Cecidomyiidae|552|BOLD:ACM4967
Cecidomyiidae|[3521]|CNFDF811-14|Cecidomyiidae|659|BOLD:ACI3652
Cecidomyiidae|[3522]|CNEIF1228-12|Cecidomyiidae|658|BOLD:ACD0724
Cecidomyiidae|[3523]|CNKOH575-14|Cecidomyiidae|583|BOLD:ACO2046
Cecidomyiidae|[3524]|CNLMQ220-14|Cecidomyiidae|590|BOLD:ACN7191
Cecidomyiidae|[3525]|CNLMQ455-14|Cecidomyiidae|592|BOLD:ACA7692
Cecidomyiidae|[3526]|CNBPA182-12|Cecidomyiidae|670|BOLD:ACD0950
Cecidomyiidae|[3527]|CNPAA335-13|Cecidomyiidae|670|BOLD:ACC3997
Cecidomyiidae|[3528]|JSDIP422-10|Cecidomyiidae|658|BOLD:AAQ0644
Cecidomyiidae|[3529]|SSBAA4319-12|Cecidomyiidae|658|BOLD:ACC1102
Cecidomyiidae|[3530]|SSBAA4429-12|Cecidomyiidae|673|BOLD:ACG6418
Cecidomyiidae|[3531]|CNFDK033-14|Cecidomyiidae|661|BOLD:ACK9889
Cecidomyiidae|[3532]|HEAUG1396-12|Cecidomyiidae|664|BOLD:AAY6473
Cecidomyiidae|[3533]|CNEIC2642-12|Cecidomyiidae|673|BOLD:ACB3084
Cecidomyiidae|[3534]|CNPPE781-12|Cecidomyiidae|661|BOLD:ACC8196
Cecidomyiidae|[3535]|CNRMF2607-12|Cecidomyiidae|664|BOLD:ACC7804
Cecidomyiidae|[3536]|CNKLB1039-14|Cecidomyiidae|588|BOLD:ACR6648
Cecidomyiidae|[3537]|CNRVD1077-15|Cecidomyiidae|576|BOLD:ACT6810
Cecidomyiidae|[3538]|CNPCC001-12|Cecidomyiidae|658|BOLD:ACC1309
Cecidomyiidae|[3539]|CNTNC1459-14|Cecidomyiidae|564|BOLD:ACN1188
Cecidomyiidae|[3540]|CNGCE165-15|Cecidomyiidae|585|BOLD:ACT7146
Cecidomyiidae|[3541]|CNRME4393-12|Cecidomyiidae|673|BOLD:ABW5583
Cecidomyiidae|[3542]|CNBRD522-14|Cecidomyiidae|658|BOLD:ACL3549
Cecidomyiidae|[3543]|CNLMI108-14|Cecidomyiidae|546|BOLD:ACN4837
Cecidomyiidae|[3544]|SSPAC2986-13|Cecidomyiidae|658|BOLD:ACJ4559
Cecidomyiidae|[3545]|CNSLT375-13|Cecidomyiidae|673|BOLD:ACI6885
Cecidomyiidae|[3546]|PAJUL709-12|Cecidomyiidae|658|BOLD:ACG8685
Cecidomyiidae|[3547]|CNROD335-13|Cecidomyiidae|661|BOLD:ABV1477
Cecidomyiidae|[3548]|SSKJB352-14|Cecidomyiidae|588|BOLD:ACP7450
Cecidomyiidae|[3549]|CNWBB396-13|Cecidomyiidae|670|BOLD:ACD9341
Cecidomyiidae|[3550]|CNJAD2194-12|Cecidomyiidae|658|BOLD:ACC8947
Cecidomyiidae|[3551]|SSPAA4977-13|Cecidomyiidae|661|BOLD:ACE0684
Cecidomyiidae|[3552]|CNPAD795-13|Cecidomyiidae|658|BOLD:ACE0971
Cecidomyiidae|[3553]|CNKLC808-14|Cecidomyiidae|576|BOLD:ACR8715
Cecidomyiidae|[3554]|CNWBG559-13|Cecidomyiidae|655|BOLD:ACI4684
Cecidomyiidae|[3555]|CNKLC3730-14|Cecidomyiidae|582|BOLD:ACN1092
Cecidomyiidae|[3556]|CNGCE482-15|Cecidomyiidae|585|BOLD:ACT7364
Cecidomyiidae|[3557]|SSJAD259-13|Cecidomyiidae|661|BOLD:ACI5044
Cecidomyiidae|[3558]|CNGUA248-14|Cecidomyiidae|582|BOLD:ACS2180
Cecidomyiidae|[3559]|CNRVC1280-15|Cecidomyiidae|576|BOLD:ACT8299
Cecidomyiidae|[3560]|SSBAE1417-13|Cecidomyiidae|658|BOLD:ACI5337
Cecidomyiidae|[3561]|SSWLF2896-13|Cecidomyiidae|637|BOLD:ACK9982
Cecidomyiidae|[3562]|CNGCF400-15|Cecidomyiidae|576|BOLD:ACU0054
Cecidomyiidae|[3563]|CNSLM181-13|Cecidomyiidae|658|BOLD:ACH3387
Cecidomyiidae|[3564]|CNSLM330-13|Cecidomyiidae|625|BOLD:ACH2331
Cecidomyiidae|[3565]|CNSLM332-13|Cecidomyiidae|661|BOLD:ACH3391
Cecidomyiidae|[3566]|CNROM227-13|Cecidomyiidae|658|BOLD:ACK5225
Cecidomyiidae|[3567]|CNFNG080-14|Cecidomyiidae|661|BOLD:ACD9818
Cecidomyiidae|[3568]|JSDIQ580-10|Cecidomyiidae|658|BOLD:AAV5789
Cecidomyiidae|[3569]|CNROP509-13|Cecidomyiidae|658|BOLD:ACK3199
Cecidomyiidae|[3570]|CNGMA304-13|Cecidomyiidae|661|BOLD:ACN3640
Cecidomyiidae|[3571]|CNLMC398-14|Cecidomyiidae|658|BOLD:ACM2100
Cecidomyiidae|[3572]|SSPAB7838-13|Cecidomyiidae|658|BOLD:ACI3991
Cecidomyiidae|[3573]|CNRME3606-12|Cecidomyiidae|658|BOLD:ACC7334
Cecidomyiidae|[3574]|CNRMD1653-12|Cecidomyiidae|673|BOLD:AAM6112
Cecidomyiidae|[3575]|CNRMD1988-12|Cecidomyiidae|661|BOLD:ACF6342
Cecidomyiidae|[3576]|SSPAB4828-13|Cecidomyiidae|658|BOLD:ACI5881
Cecidomyiidae|[3577]|SSPAC4518-13|Cecidomyiidae|658|BOLD:ACJ5560
Cecidomyiidae|[3578]|CNRMF2091-12|Cecidomyiidae|673|BOLD:ACC8272
Cecidomyiidae|[3579]|NGNAX4948-14|Cecidomyiidae|576|BOLD:ACM7223
Cecidomyiidae|[3580]|NGNAX5049-14|Cecidomyiidae|564|BOLD:ACM7232
Cecidomyiinae|[3581]|CNROF186-13|Cecidomyiidae|661|BOLD:ACA5770
Cecidomyiidae|[3582]|CNSLI211-12|Cecidomyiidae|670|BOLD:ACC0369
Cecidomyiidae|[3583]|CNROH108-13|Cecidomyiidae|658|BOLD:ACK3166
Cecidomyiidae|[3584]|CNKOE1192-14|Cecidomyiidae|658|BOLD:ACM6618
Cecidomyiidae|[3585]|CNPPE968-12|Cecidomyiidae|664|BOLD:ACA9361
Cecidomyiidae|[3586]|CNPPI668-12|Cecidomyiidae|664|BOLD:ACC7732
Cecidomyiidae|[3587]|HPPPE1359-13|Cecidomyiidae|658|BOLD:ACI3650
Cecidomyiidae|[3588]|HPPPH716-13|Cecidomyiidae|661|BOLD:ACI8954
Cecidomyiidae|[3589]|HPPPE1151-13|Cecidomyiidae|664|BOLD:ACI3115
Cecidomyiidae|[3590]|HPPPE1248-13|Cecidomyiidae|664|BOLD:ACI3365
Cecidomyiidae|[3591]|JSJUN662-11|Cecidomyiidae|658|BOLD:ABV9312
Cecidomyiidae|[3592]|JSJUL1250-11|Cecidomyiidae|658|BOLD:ABV1438
Cecidomyiidae|[3593]|CNSLO775-13|Cecidomyiidae|658|BOLD:ACI5691
Cecidomyiidae|[3594]|HPPPE1305-13|Cecidomyiidae|578|BOLD:ACM4238
Cecidomyiidae|[3595]|CNKOH1322-14|Cecidomyiidae|552|BOLD:ACM3255
Cecidomyiidae|[3596]|CNLMD908-14|Cecidomyiidae|658|BOLD:ABV1409
Cecidomyiidae|[3597]|CNSLD218-12|Cecidomyiidae|658|BOLD:ACA7655
Cecidomyiidae|[3598]|CNSLD383-12|Cecidomyiidae|658|BOLD:ACA8624
Cecidomyiidae|[3599]|HPPPK697-13|Cecidomyiidae|658|BOLD:ACI8652

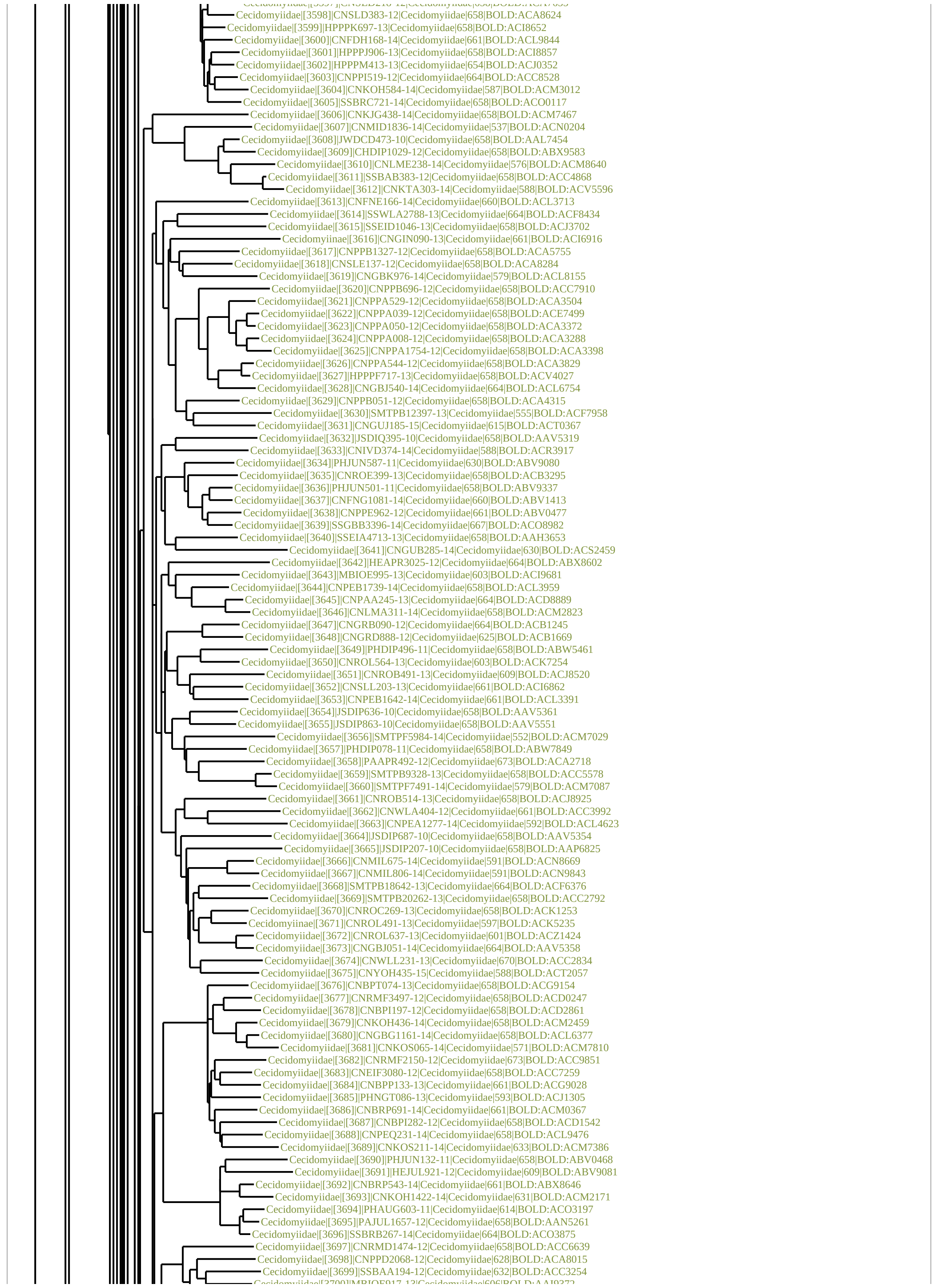

Cecidomyiidae|[3598]|CNSLD383-12|Cecidomyiidae|658|BOLD:ACA8624
Cecidomyiidae|[3599]|HPPPK697-13|Cecidomyiidae|658|BOLD:ACI8652
Cecidomyiidae|[3600]|CNFDH168-14|Cecidomyiidae|661|BOLD:ACL9844
Cecidomyiidae|[3601]|HPPPJ906-13|Cecidomyiidae|658|BOLD:ACI8857
Cecidomyiidae|[3602]|HPPPM413-13|Cecidomyiidae|654|BOLD:ACJ0352
Cecidomyiidae|[3603]|CNPPI519-12|Cecidomyiidae|664|BOLD:ACC8528
Cecidomyiidae|[3604]|CNKOH584-14|Cecidomyiidae|587|BOLD:ACM3012
Cecidomyiidae|[3605]|SSBRC721-14|Cecidomyiidae|658|BOLD:ACO0117
Cecidomyiidae|[3606]|CNKJG438-14|Cecidomyiidae|658|BOLD:ACM7467
Cecidomyiidae|[3607]|CNMID1836-14|Cecidomyiidae|537|BOLD:ACN0204
Cecidomyiidae|[3608]|JWDCD473-10|Cecidomyiidae|658|BOLD:AAL7454
Cecidomyiidae|[3609]|CHDIP1029-12|Cecidomyiidae|658|BOLD:ABX9583
Cecidomyiidae|[3610]|CNLME238-14|Cecidomyiidae|576|BOLD:ACM8640
Cecidomyiidae|[3611]|SSBAB383-12|Cecidomyiidae|658|BOLD:ACC4868
Cecidomyiidae|[3612]|CNKTA303-14|Cecidomyiidae|588|BOLD:ACV5596
Cecidomyiidae|[3613]|CNFNE166-14|Cecidomyiidae|660|BOLD:ACL3713
Cecidomyiidae|[3614]|SSWLA2788-13|Cecidomyiidae|664|BOLD:ACF8434
Cecidomyiidae|[3615]|SSEID1046-13|Cecidomyiidae|658|BOLD:ACJ3702
Cecidomyiinae|[3616]|CNGIN090-13|Cecidomyiidae|661|BOLD:ACI6916
Cecidomyiidae|[3617]|CNPPB1327-12|Cecidomyiidae|658|BOLD:ACA5755
Cecidomyiidae|[3618]|CNSLE137-12|Cecidomyiidae|658|BOLD:ACA8284
Cecidomyiidae|[3619]|CNGBK976-14|Cecidomyiidae|579|BOLD:ACL8155
Cecidomyiidae|[3620]|CNPPB696-12|Cecidomyiidae|658|BOLD:ACC7910
Cecidomyiidae|[3621]|CNPPA529-12|Cecidomyiidae|658|BOLD:ACA3504
Cecidomyiidae|[3622]|CNPPA039-12|Cecidomyiidae|658|BOLD:ACE7499
Cecidomyiidae|[3623]|CNPPA050-12|Cecidomyiidae|658|BOLD:ACA3372
Cecidomyiidae|[3624]|CNPPA008-12|Cecidomyiidae|658|BOLD:ACA3288
Cecidomyiidae|[3625]|CNPPA1754-12|Cecidomyiidae|658|BOLD:ACA3398
Cecidomyiidae|[3626]|CNPPA544-12|Cecidomyiidae|658|BOLD:ACA3829
Cecidomyiidae|[3627]|HPPPF717-13|Cecidomyiidae|658|BOLD:ACV4027
Cecidomyiidae|[3628]|CNGBJ540-14|Cecidomyiidae|664|BOLD:ACL6754
Cecidomyiidae|[3629]|CNPPB051-12|Cecidomyiidae|658|BOLD:ACA4315
Cecidomyiidae|[3630]|SMTPB12397-13|Cecidomyiidae|555|BOLD:ACF7958
Cecidomyiidae|[3631]|CNGUJ185-15|Cecidomyiidae|615|BOLD:ACT0367
Cecidomyiidae|[3632]|JSDIQ395-10|Cecidomyiidae|658|BOLD:AAV5319
Cecidomyiidae|[3633]|CNIVD374-14|Cecidomyiidae|588|BOLD:ACR3917
Cecidomyiidae|[3634]|PHJUN587-11|Cecidomyiidae|630|BOLD:ABV9080
Cecidomyiidae|[3635]|CNROE399-13|Cecidomyiidae|658|BOLD:ACB3295
Cecidomyiidae|[3636]|PHJUN501-11|Cecidomyiidae|658|BOLD:ABV9337
Cecidomyiidae|[3637]|CNFNG1081-14|Cecidomyiidae|660|BOLD:ABV1413
Cecidomyiidae|[3638]|CNPPE962-12|Cecidomyiidae|661|BOLD:ABV0477
Cecidomyiidae|[3639]|SSGBB3396-14|Cecidomyiidae|667|BOLD:ACO8982
Cecidomyiidae|[3640]|SSEIA4713-13|Cecidomyiidae|658|BOLD:AAH3653
Cecidomyiidae|[3641]|CNGUB285-14|Cecidomyiidae|630|BOLD:ACS2459
Cecidomyiidae|[3642]|HEAPR3025-12|Cecidomyiidae|664|BOLD:ABX8602
Cecidomyiidae|[3643]|MBIOE995-13|Cecidomyiidae|603|BOLD:ACI9681
Cecidomyiidae|[3644]|CNPEB1739-14|Cecidomyiidae|658|BOLD:ACL3959
Cecidomyiidae|[3645]|CNPAA245-13|Cecidomyiidae|664|BOLD:ACD8889
Cecidomyiidae|[3646]|CNLMA311-14|Cecidomyiidae|658|BOLD:ACM2823
Cecidomyiidae|[3647]|CNGRB090-12|Cecidomyiidae|664|BOLD:ACB1245
Cecidomyiidae|[3648]|CNGRD888-12|Cecidomyiidae|625|BOLD:ACB1669
Cecidomyiidae|[3649]|PHDIP496-11|Cecidomyiidae|658|BOLD:ABW5461
Cecidomyiidae|[3650]|CNROL564-13|Cecidomyiidae|603|BOLD:ACK7254
Cecidomyiidae|[3651]|CNROB491-13|Cecidomyiidae|609|BOLD:ACJ8520
Cecidomyiidae|[3652]|CNSLL203-13|Cecidomyiidae|661|BOLD:ACI6862
Cecidomyiidae|[3653]|CNPEB1642-14|Cecidomyiidae|661|BOLD:ACL3391
Cecidomyiidae|[3654]|JSDIP636-10|Cecidomyiidae|658|BOLD:AAV5361
Cecidomyiidae|[3655]|JSDIP863-10|Cecidomyiidae|658|BOLD:AAV5551
Cecidomyiidae|[3656]|SMTPF5984-14|Cecidomyiidae|552|BOLD:ACM7029
Cecidomyiidae|[3657]|PHDIP078-11|Cecidomyiidae|658|BOLD:ABW7849
Cecidomyiidae|[3658]|PAAPR492-12|Cecidomyiidae|673|BOLD:ACA2718
Cecidomyiidae|[3659]|SMTPB9328-13|Cecidomyiidae|658|BOLD:ACC5578
Cecidomyiidae|[3660]|SMTPF7491-14|Cecidomyiidae|579|BOLD:ACM7087
Cecidomyiidae|[3661]|CNROB514-13|Cecidomyiidae|658|BOLD:ACJ8925
Cecidomyiidae|[3662]|CNWLA404-12|Cecidomyiidae|661|BOLD:ACC3992
Cecidomyiidae|[3663]|CNPEA1277-14|Cecidomyiidae|592|BOLD:ACL4623
Cecidomyiidae|[3664]|JSDIP687-10|Cecidomyiidae|658|BOLD:AAV5354
Cecidomyiidae|[3665]|JSDIP207-10|Cecidomyiidae|658|BOLD:AAP6825
Cecidomyiidae|[3666]|CNMIL675-14|Cecidomyiidae|591|BOLD:ACN8669
Cecidomyiidae|[3667]|CNMIL806-14|Cecidomyiidae|591|BOLD:ACN9843
Cecidomyiidae|[3668]|SMTPB18642-13|Cecidomyiidae|664|BOLD:ACF6376
Cecidomyiidae|[3669]|SMTPB20262-13|Cecidomyiidae|658|BOLD:ACC2792
Cecidomyiidae|[3670]|CNROC269-13|Cecidomyiidae|658|BOLD:ACK1253
Cecidomyiinae|[3671]|CNROL491-13|Cecidomyiidae|597|BOLD:ACK5235
Cecidomyiidae|[3672]|CNROL637-13|Cecidomyiidae|601|BOLD:ACZ1424
Cecidomyiidae|[3673]|CNGBJ051-14|Cecidomyiidae|664|BOLD:AAV5358
Cecidomyiidae|[3674]|CNWLL231-13|Cecidomyiidae|670|BOLD:ACC2834
Cecidomyiidae|[3675]|CNYOH435-15|Cecidomyiidae|588|BOLD:ACT2057
Cecidomyiidae|[3676]|CNBPT074-13|Cecidomyiidae|658|BOLD:ACG9154
Cecidomyiidae|[3677]|CNRMF3497-12|Cecidomyiidae|658|BOLD:ACD0247
Cecidomyiidae|[3678]|CNBPI197-12|Cecidomyiidae|658|BOLD:ACD2861
Cecidomyiidae|[3679]|CNKOH436-14|Cecidomyiidae|658|BOLD:ACM2459
Cecidomyiidae|[3680]|CNGBG1161-14|Cecidomyiidae|658|BOLD:ACL6377
Cecidomyiidae|[3681]|CNKOS065-14|Cecidomyiidae|571|BOLD:ACM7810
Cecidomyiidae|[3682]|CNRMF2150-12|Cecidomyiidae|673|BOLD:ACC9851
Cecidomyiidae|[3683]|CNEIF3080-12|Cecidomyiidae|658|BOLD:ACC7259
Cecidomyiidae|[3684]|CNBPP133-13|Cecidomyiidae|661|BOLD:ACG9028
Cecidomyiidae|[3685]|PHNGT086-13|Cecidomyiidae|593|BOLD:ACJ1305
Cecidomyiidae|[3686]|CNBRP691-14|Cecidomyiidae|661|BOLD:ACM0367
Cecidomyiidae|[3687]|CNBPI282-12|Cecidomyiidae|658|BOLD:ACD1542
Cecidomyiidae|[3688]|CNPEQ231-14|Cecidomyiidae|658|BOLD:ACL9476
Cecidomyiidae|[3689]|CNKOS211-14|Cecidomyiidae|633|BOLD:ACM7386
Cecidomyiidae|[3690]|PHJUN132-11|Cecidomyiidae|658|BOLD:ABV0468
Cecidomyiidae|[3691]|HEJUL921-12|Cecidomyiidae|609|BOLD:ABV9081
Cecidomyiidae|[3692]|CNBRP543-14|Cecidomyiidae|661|BOLD:ABX8646
Cecidomyiidae|[3693]|CNKOH1422-14|Cecidomyiidae|631|BOLD:ACM2171
Cecidomyiidae|[3694]|PHAUG603-11|Cecidomyiidae|614|BOLD:ACO3197
Cecidomyiidae|[3695]|PAJUL1657-12|Cecidomyiidae|658|BOLD:AAN5261
Cecidomyiidae|[3696]|SSBRB267-14|Cecidomyiidae|664|BOLD:ACO3875
Cecidomyiidae|[3697]|CNRMD1474-12|Cecidomyiidae|658|BOLD:ACC6639
Cecidomyiidae|[3698]|CNPPD2068-12|Cecidomyiidae|628|BOLD:ACA8015
Cecidomyiidae|[3699]|SSBAA194-12|Cecidomyiidae|632|BOLD:ACC3254

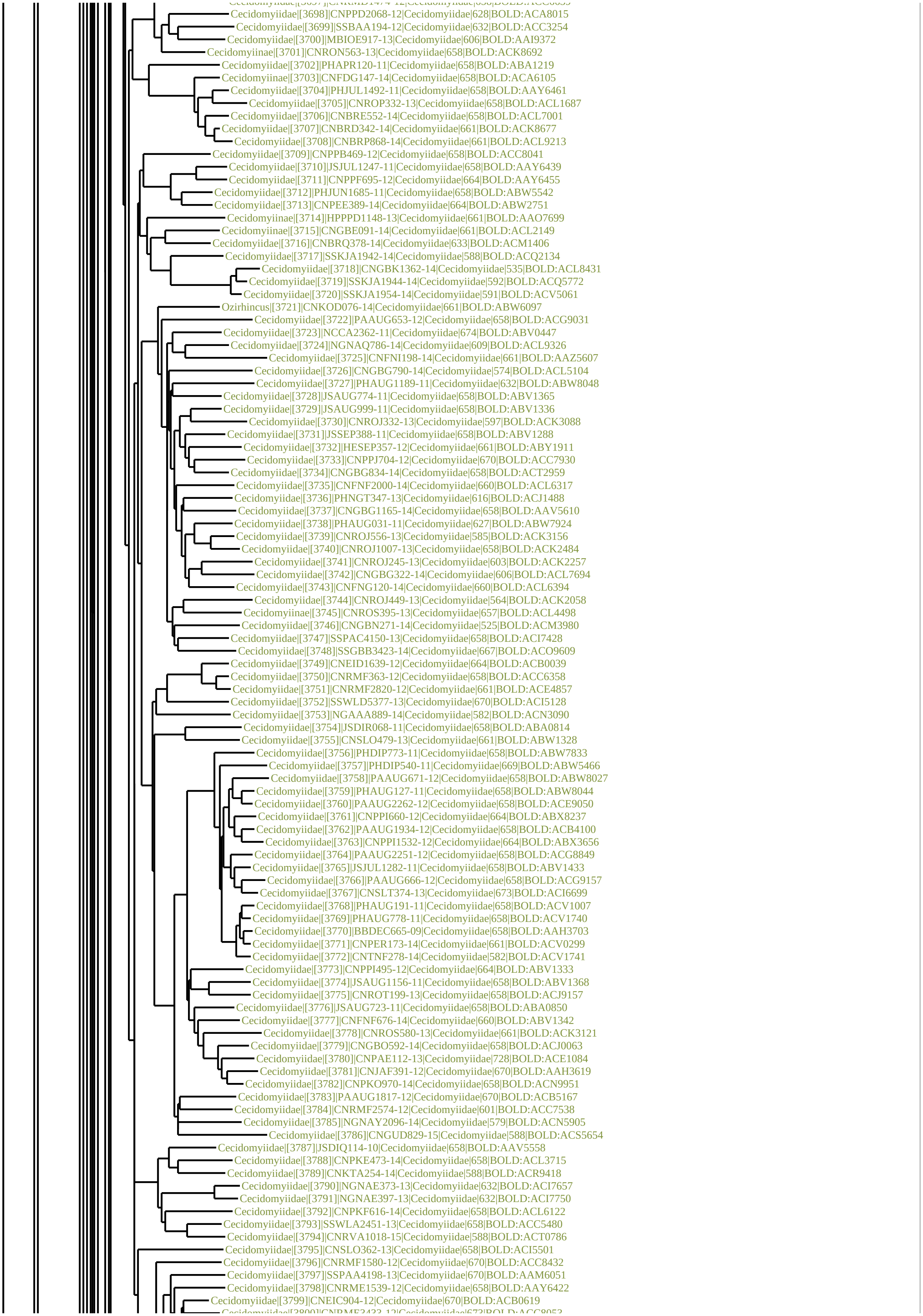
Cecidomyiidae|[3698]|CNPPD2068-12|Cecidomyiidae|628|BOLD:ACA8015
Cecidomyiidae|[3699]|SSBAA194-12|Cecidomyiidae|632|BOLD:ACC3254
Cecidomyiidae|[3700]|MBIOE917-13|Cecidomyiidae|606|BOLD:AAI9372
Cecidomyiinae|[3701]|CNRON563-13|Cecidomyiidae|658|BOLD:ACK8692
Cecidomyiidae|[3702]|PHAPR120-11|Cecidomyiidae|658|BOLD:ABA1219
Cecidomyiinae|[3703]|CNFDG147-14|Cecidomyiidae|658|BOLD:ACA6105
Cecidomyiidae|[3704]|PHJUL1492-11|Cecidomyiidae|658|BOLD:AAY6461
Cecidomyiidae|[3705]|CNROP332-13|Cecidomyiidae|658|BOLD:ACL1687
Cecidomyiidae|[3706]|CNBRE552-14|Cecidomyiidae|658|BOLD:ACL7001
Cecidomyiidae|[3707]|CNBRD342-14|Cecidomyiidae|661|BOLD:ACK8677
Cecidomyiidae|[3708]|CNBRP868-14|Cecidomyiidae|661|BOLD:ACL9213
Cecidomyiidae|[3709]|CNPPB469-12|Cecidomyiidae|658|BOLD:ACC8041
Cecidomyiidae|[3710]|JSJUL1247-11|Cecidomyiidae|658|BOLD:AAY6439
Cecidomyiidae|[3711]|CNPPF695-12|Cecidomyiidae|664|BOLD:AAY6455
Cecidomyiidae|[3712]|PHJUN1685-11|Cecidomyiidae|658|BOLD:ABW5542
Cecidomyiidae|[3713]|CNPEE389-14|Cecidomyiidae|664|BOLD:ABW2751
Cecidomyiinae|[3714]|HPPPD1148-13|Cecidomyiidae|661|BOLD:AAO7699
Cecidomyiinae|[3715]|CNGBE091-14|Cecidomyiidae|661|BOLD:ACL2149
Cecidomyiidae|[3716]|CNBRQ378-14|Cecidomyiidae|633|BOLD:ACM1406
Cecidomyiidae|[3717]|SSKJA1942-14|Cecidomyiidae|588|BOLD:ACQ2134
Cecidomyiidae|[3718]|CNGBK1362-14|Cecidomyiidae|535|BOLD:ACL8431
Cecidomyiidae|[3719]|SSKJA1944-14|Cecidomyiidae|592|BOLD:ACQ5772
Cecidomyiidae|[3720]|SSKJA1954-14|Cecidomyiidae|591|BOLD:ACV5061
Ozirhincus|[3721]|CNKOD076-14|Cecidomyiidae|661|BOLD:ABW6097
Cecidomyiidae|[3722]|PAAUG653-12|Cecidomyiidae|658|BOLD:ACG9031
Cecidomyiidae|[3723]|NCCA2362-11|Cecidomyiidae|674|BOLD:ABV0447
Cecidomyiidae|[3724]|NGNAQ786-14|Cecidomyiidae|609|BOLD:ACL9326
Cecidomyiidae|[3725]|CNFNI198-14|Cecidomyiidae|661|BOLD:AAZ5607
Cecidomyiidae|[3726]|CNGBG790-14|Cecidomyiidae|574|BOLD:ACL5104
Cecidomyiidae|[3727]|PHAUG1189-11|Cecidomyiidae|632|BOLD:ABW8048
Cecidomyiidae|[3728]|JSAUG774-11|Cecidomyiidae|658|BOLD:ABV1365
Cecidomyiidae|[3729]|JSAUG999-11|Cecidomyiidae|658|BOLD:ABV1336
Cecidomyiidae|[3730]|CNROJ332-13|Cecidomyiidae|597|BOLD:ACK3088
Cecidomyiidae|[3731]|JSSEP388-11|Cecidomyiidae|658|BOLD:ABV1288
Cecidomyiidae|[3732]|HESEP357-12|Cecidomyiidae|661|BOLD:ABY1911
Cecidomyiidae|[3733]|CNPPJ704-12|Cecidomyiidae|670|BOLD:ACC7930
Cecidomyiidae|[3734]|CNGBG834-14|Cecidomyiidae|658|BOLD:ACT2959
Cecidomyiidae|[3735]|CNFNF2000-14|Cecidomyiidae|660|BOLD:ACL6317
Cecidomyiidae|[3736]|PHNGT347-13|Cecidomyiidae|616|BOLD:ACJ1488
Cecidomyiidae|[3737]|CNGBG1165-14|Cecidomyiidae|658|BOLD:AAV5610
Cecidomyiidae|[3738]|PHAUG031-11|Cecidomyiidae|627|BOLD:ABW7924
Cecidomyiidae|[3739]|CNROJ556-13|Cecidomyiidae|585|BOLD:ACK3156
Cecidomyiidae|[3740]|CNROJ1007-13|Cecidomyiidae|658|BOLD:ACK2484
Cecidomyiidae|[3741]|CNROJ245-13|Cecidomyiidae|603|BOLD:ACK2257
Cecidomyiidae|[3742]|CNGBG322-14|Cecidomyiidae|606|BOLD:ACL7694
Cecidomyiidae|[3743]|CNFNG120-14|Cecidomyiidae|660|BOLD:ACL6394
Cecidomyiidae|[3744]|CNROJ449-13|Cecidomyiidae|564|BOLD:ACK2058
Cecidomyiinae|[3745]|CNROS395-13|Cecidomyiidae|657|BOLD:ACL4498
Cecidomyiidae|[3746]|CNGBN271-14|Cecidomyiidae|525|BOLD:ACM3980
Cecidomyiidae|[3747]|SSPAC4150-13|Cecidomyiidae|658|BOLD:ACI7428
Cecidomyiidae|[3748]|SSGBB3423-14|Cecidomyiidae|667|BOLD:ACO9609
Cecidomyiidae|[3749]|CNEID1639-12|Cecidomyiidae|664|BOLD:ACB0039
Cecidomyiidae|[3750]|CNRMF363-12|Cecidomyiidae|658|BOLD:ACC6358
Cecidomyiidae|[3751]|CNRMF2820-12|Cecidomyiidae|661|BOLD:ACE4857
Cecidomyiidae|[3752]|SSWLD5377-13|Cecidomyiidae|670|BOLD:ACI5128
Cecidomyiidae|[3753]|NGAAA889-14|Cecidomyiidae|582|BOLD:ACN3090
Cecidomyiidae|[3754]|JSDIR068-11|Cecidomyiidae|658|BOLD:ABA0814
Cecidomyiidae|[3755]|CNSLO479-13|Cecidomyiidae|661|BOLD:ABW1328
Cecidomyiidae|[3756]|PHDIP773-11|Cecidomyiidae|658|BOLD:ABW7833
Cecidomyiidae|[3757]|PHDIP540-11|Cecidomyiidae|669|BOLD:ABW5466
Cecidomyiidae|[3758]|PAAUG671-12|Cecidomyiidae|658|BOLD:ABW8027
Cecidomyiidae|[3759]|PHAUG127-11|Cecidomyiidae|658|BOLD:ABW8044
Cecidomyiidae|[3760]|PAAUG2262-12|Cecidomyiidae|658|BOLD:ACE9050
Cecidomyiidae|[3761]|CNPPI660-12|Cecidomyiidae|664|BOLD:ABX8237
Cecidomyiidae|[3762]|PAAUG1934-12|Cecidomyiidae|658|BOLD:ACB4100
Cecidomyiidae|[3763]|CNPPI1532-12|Cecidomyiidae|664|BOLD:ABX3656
Cecidomyiidae|[3764]|PAAUG2251-12|Cecidomyiidae|658|BOLD:ACG8849
Cecidomyiidae|[3765]|JSJUL1282-11|Cecidomyiidae|658|BOLD:ABV1433
Cecidomyiidae|[3766]|PAAUG666-12|Cecidomyiidae|658|BOLD:ACG9157
Cecidomyiidae|[3767]|CNSLT374-13|Cecidomyiidae|673|BOLD:ACI6699
Cecidomyiidae|[3768]|PHAUG191-11|Cecidomyiidae|658|BOLD:ACV1007
Cecidomyiidae|[3769]|PHAUG778-11|Cecidomyiidae|658|BOLD:ACV1740
Cecidomyiidae|[3770]|BBDEC665-09|Cecidomyiidae|658|BOLD:AAH3703
Cecidomyiidae|[3771]|CNPER173-14|Cecidomyiidae|661|BOLD:ACV0299
Cecidomyiidae|[3772]|CNTNF278-14|Cecidomyiidae|582|BOLD:ACV1741
Cecidomyiidae|[3773]|CNPPI495-12|Cecidomyiidae|664|BOLD:ABV1333
Cecidomyiidae|[3774]|JSAUG1156-11|Cecidomyiidae|658|BOLD:ABV1368
Cecidomyiidae|[3775]|CNROT199-13|Cecidomyiidae|658|BOLD:ACJ9157
Cecidomyiidae|[3776]|JSAUG723-11|Cecidomyiidae|658|BOLD:ABA0850
Cecidomyiidae|[3777]|CNFNF676-14|Cecidomyiidae|660|BOLD:ABV1342
Cecidomyiidae|[3778]|CNROS580-13|Cecidomyiidae|661|BOLD:ACK3121
Cecidomyiidae|[3779]|CNGBO592-14|Cecidomyiidae|658|BOLD:ACJ0063
Cecidomyiidae|[3780]|CNPAE112-13|Cecidomyiidae|728|BOLD:ACE1084
Cecidomyiidae|[3781]|CNJAF391-12|Cecidomyiidae|670|BOLD:AAH3619
Cecidomyiidae|[3782]|CNPKO970-14|Cecidomyiidae|658|BOLD:ACN9951
Cecidomyiidae|[3783]|PAAUG1817-12|Cecidomyiidae|670|BOLD:ACB5167
Cecidomyiidae|[3784]|CNRMF2574-12|Cecidomyiidae|601|BOLD:ACC7538
Cecidomyiidae|[3785]|NGNAY2096-14|Cecidomyiidae|579|BOLD:ACN5905
Cecidomyiidae|[3786]|CNGUD829-15|Cecidomyiidae|588|BOLD:ACS5654
Cecidomyiidae|[3787]|JSDIQ114-10|Cecidomyiidae|658|BOLD:AAV5558
Cecidomyiidae|[3788]|CNPKE473-14|Cecidomyiidae|658|BOLD:ACL3715
Cecidomyiidae|[3789]|CNKTA254-14|Cecidomyiidae|588|BOLD:ACR9418
Cecidomyiidae|[3790]|NGNAE373-13|Cecidomyiidae|632|BOLD:ACI7657
Cecidomyiidae|[3791]|NGNAE397-13|Cecidomyiidae|632|BOLD:ACI7750
Cecidomyiidae|[3792]|CNPKF616-14|Cecidomyiidae|658|BOLD:ACL6122
Cecidomyiidae|[3793]|SSWLA2451-13|Cecidomyiidae|658|BOLD:ACC5480
Cecidomyiidae|[3794]|CNRVA1018-15|Cecidomyiidae|588|BOLD:ACT0786
Cecidomyiidae|[3795]|CNSLO362-13|Cecidomyiidae|658|BOLD:ACI5501
Cecidomyiidae|[3796]|CNRMF1580-12|Cecidomyiidae|670|BOLD:ACC8432
Cecidomyiidae|[3797]|SSPAA4198-13|Cecidomyiidae|670|BOLD:AAM6051
Cecidomyiidae|[3798]|CNRME1539-12|Cecidomyiidae|658|BOLD:AAY6422
Cecidomyiidae|[3799]|CNEIC904-12|Cecidomyiidae|670|BOLD:ACB0619

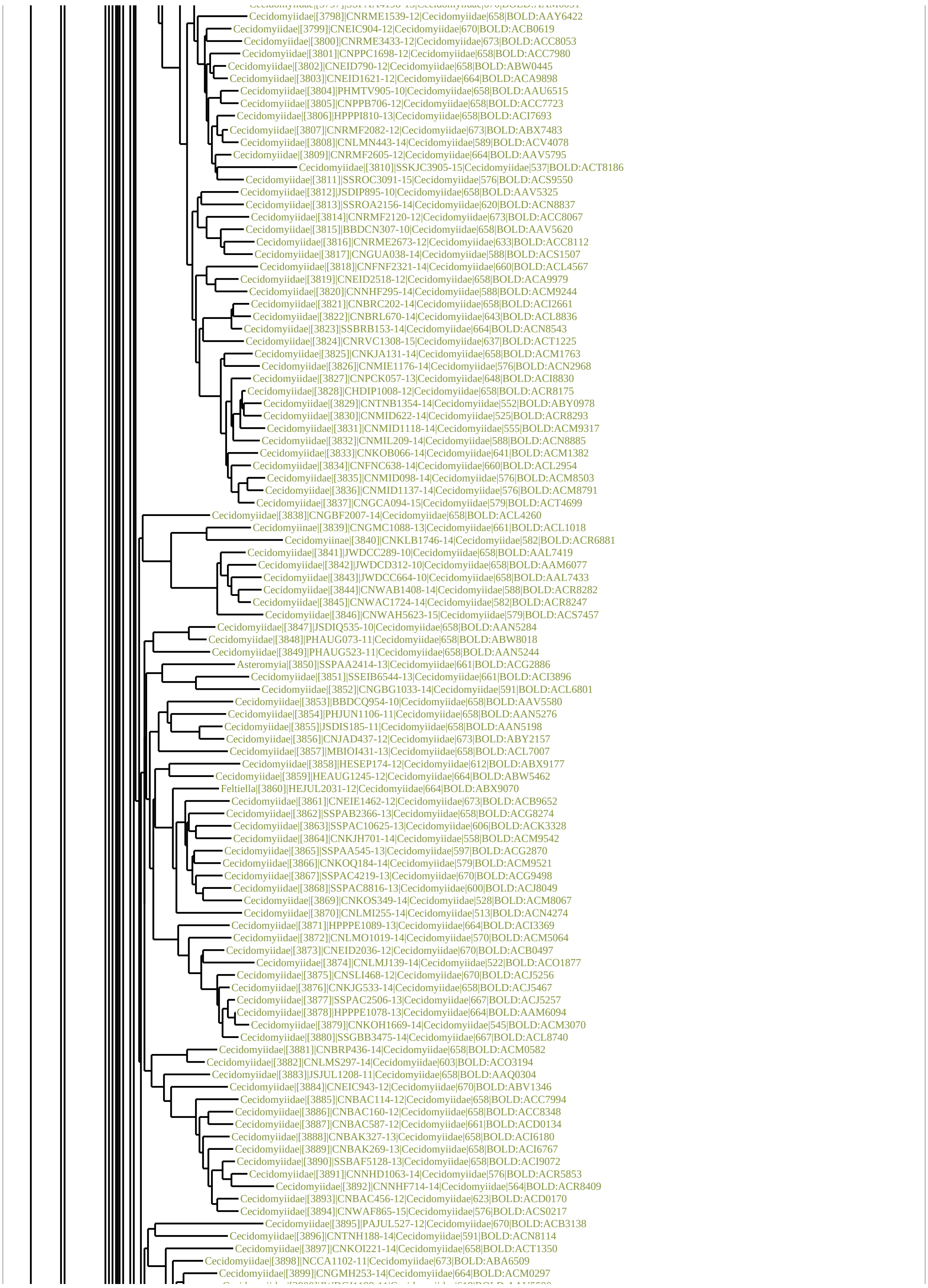
Cecidomyiidae|[3798]|CNRME1539-12|Cecidomyiidae|658|BOLD:AAY6422
Cecidomyiidae|[3799]|CNEIC904-12|Cecidomyiidae|670|BOLD:ACB0619
Cecidomyiidae|[3800]|CNRME3433-12|Cecidomyiidae|673|BOLD:ACC8053
Cecidomyiidae|[3801]|CNPPC1698-12|Cecidomyiidae|658|BOLD:ACC7980
Cecidomyiidae|[3802]|CNEID790-12|Cecidomyiidae|658|BOLD:ABW0445
Cecidomyiidae|[3803]|CNEID1621-12|Cecidomyiidae|664|BOLD:ACA9898
Cecidomyiidae|[3804]|PHMTV905-10|Cecidomyiidae|658|BOLD:AAU6515
Cecidomyiidae|[3805]|CNPPB706-12|Cecidomyiidae|658|BOLD:ACC7723
Cecidomyiidae|[3806]|HPPPI810-13|Cecidomyiidae|658|BOLD:ACI7693
Cecidomyiidae|[3807]|CNRMF2082-12|Cecidomyiidae|673|BOLD:ABX7483
Cecidomyiidae|[3808]|CNLMN443-14|Cecidomyiidae|589|BOLD:ACV4078
Cecidomyiidae|[3809]|CNRMF2605-12|Cecidomyiidae|664|BOLD:AAV5795
Cecidomyiidae|[3810]|SSKJC3905-15|Cecidomyiidae|537|BOLD:ACT8186
Cecidomyiidae|[3811]|SSROC3091-15|Cecidomyiidae|576|BOLD:ACS9550
Cecidomyiidae|[3812]|JSDIP895-10|Cecidomyiidae|658|BOLD:AAV5325
Cecidomyiidae|[3813]|SSROA2156-14|Cecidomyiidae|620|BOLD:ACN8837
Cecidomyiidae|[3814]|CNRMF2120-12|Cecidomyiidae|673|BOLD:ACC8067
Cecidomyiidae|[3815]|BBDCN307-10|Cecidomyiidae|658|BOLD:AAV5620
Cecidomyiidae|[3816]|CNRME2673-12|Cecidomyiidae|633|BOLD:ACC8112
Cecidomyiidae|[3817]|CNGUA038-14|Cecidomyiidae|588|BOLD:ACS1507
Cecidomyiidae|[3818]|CNFNF2321-14|Cecidomyiidae|660|BOLD:ACL4567
Cecidomyiidae|[3819]|CNEID2518-12|Cecidomyiidae|658|BOLD:ACA9979
Cecidomyiidae|[3820]|CNNHF295-14|Cecidomyiidae|588|BOLD:ACM9244
Cecidomyiidae|[3821]|CNBRC202-14|Cecidomyiidae|658|BOLD:ACI2661
Cecidomyiidae|[3822]|CNBRL670-14|Cecidomyiidae|643|BOLD:ACL8836
Cecidomyiidae|[3823]|SSBRB153-14|Cecidomyiidae|664|BOLD:ACN8543
Cecidomyiidae|[3824]|CNRVC1308-15|Cecidomyiidae|637|BOLD:ACT1225
Cecidomyiidae|[3825]|CNKJA131-14|Cecidomyiidae|658|BOLD:ACM1763
Cecidomyiidae|[3826]|CNMIE1176-14|Cecidomyiidae|576|BOLD:ACN2968
Cecidomyiidae|[3827]|CNPCK057-13|Cecidomyiidae|648|BOLD:ACI8830
Cecidomyiidae|[3828]|CHDIP1008-12|Cecidomyiidae|658|BOLD:ACR8175
Cecidomyiidae|[3829]|CNTNB1354-14|Cecidomyiidae|552|BOLD:ABY0978
Cecidomyiidae|[3830]|CNMID622-14|Cecidomyiidae|525|BOLD:ACR8293
Cecidomyiidae|[3831]|CNMID1118-14|Cecidomyiidae|555|BOLD:ACM9317
Cecidomyiidae|[3832]|CNMIL209-14|Cecidomyiidae|588|BOLD:ACN8885
Cecidomyiidae|[3833]|CNKOB066-14|Cecidomyiidae|641|BOLD:ACM1382
Cecidomyiidae|[3834]|CNFNC638-14|Cecidomyiidae|660|BOLD:ACL2954
Cecidomyiidae|[3835]|CNMID098-14|Cecidomyiidae|576|BOLD:ACM8503
Cecidomyiidae|[3836]|CNMID1137-14|Cecidomyiidae|576|BOLD:ACM8791
Cecidomyiidae|[3837]|CNGCA094-15|Cecidomyiidae|579|BOLD:ACT4699
Cecidomyiidae|[3838]|CNGBF2007-14|Cecidomyiidae|658|BOLD:ACL4260
Cecidomyiinae|[3839]|CNGMC1088-13|Cecidomyiidae|661|BOLD:ACL1018
Cecidomyiinae|[3840]|CNKLB1746-14|Cecidomyiidae|582|BOLD:ACR6881
Cecidomyiidae|[3841]|JWDCC289-10|Cecidomyiidae|658|BOLD:AAL7419
Cecidomyiidae|[3842]|JWDCD312-10|Cecidomyiidae|658|BOLD:AAM6077
Cecidomyiidae|[3843]|JWDCC664-10|Cecidomyiidae|658|BOLD:AAL7433
Cecidomyiidae|[3844]|CNWAB1408-14|Cecidomyiidae|588|BOLD:ACR8282
Cecidomyiidae|[3845]|CNWAC1724-14|Cecidomyiidae|582|BOLD:ACR8247
Cecidomyiidae|[3846]|CNWAH5623-15|Cecidomyiidae|579|BOLD:ACS7457
Cecidomyiidae|[3847]|JSDIQ535-10|Cecidomyiidae|658|BOLD:AAN5284
Cecidomyiidae|[3848]|PHAUG073-11|Cecidomyiidae|658|BOLD:ABW8018
Cecidomyiidae|[3849]|PHAUG523-11|Cecidomyiidae|658|BOLD:AAN5244
Asteromyia|[3850]|SSPAA2414-13|Cecidomyiidae|661|BOLD:ACG2886
Cecidomyiidae|[3851]|SSEIB6544-13|Cecidomyiidae|661|BOLD:ACI3896
Cecidomyiidae|[3852]|CNGBG1033-14|Cecidomyiidae|591|BOLD:ACL6801
Cecidomyiidae|[3853]|BBDCQ954-10|Cecidomyiidae|658|BOLD:AAV5580
Cecidomyiidae|[3854]|PHJUN1106-11|Cecidomyiidae|658|BOLD:AAN5276
Cecidomyiidae|[3855]|JSDIS185-11|Cecidomyiidae|658|BOLD:AAN5198
Cecidomyiidae|[3856]|CNJAD437-12|Cecidomyiidae|673|BOLD:ABY2157
Cecidomyiidae|[3857]|MBIOI431-13|Cecidomyiidae|658|BOLD:ACL7007
Cecidomyiidae|[3858]|HESEP174-12|Cecidomyiidae|612|BOLD:ABX9177
Cecidomyiidae|[3859]|HEAUG1245-12|Cecidomyiidae|664|BOLD:ABW5462
Feltiella|[3860]|HEJUL2031-12|Cecidomyiidae|664|BOLD:ABX9070
Cecidomyiidae|[3861]|CNEIE1462-12|Cecidomyiidae|673|BOLD:ACB9652
Cecidomyiidae|[3862]|SSPAB2366-13|Cecidomyiidae|658|BOLD:ACG8274
Cecidomyiidae|[3863]|SSPAC10625-13|Cecidomyiidae|606|BOLD:ACK3328
Cecidomyiidae|[3864]|CNKJH701-14|Cecidomyiidae|558|BOLD:ACM9542
Cecidomyiidae|[3865]|SSPAA545-13|Cecidomyiidae|597|BOLD:ACG2870
Cecidomyiidae|[3866]|CNKOQ184-14|Cecidomyiidae|579|BOLD:ACM9521
Cecidomyiidae|[3867]|SSPAC4219-13|Cecidomyiidae|670|BOLD:ACG9498
Cecidomyiidae|[3868]|SSPAC8816-13|Cecidomyiidae|600|BOLD:ACJ8049
Cecidomyiidae|[3869]|CNKOS349-14|Cecidomyiidae|528|BOLD:ACM8067
Cecidomyiidae|[3870]|CNLMI255-14|Cecidomyiidae|513|BOLD:ACN4274
Cecidomyiidae|[3871]|HPPPE1089-13|Cecidomyiidae|664|BOLD:ACI3369
Cecidomyiidae|[3872]|CNLMO1019-14|Cecidomyiidae|570|BOLD:ACM5064
Cecidomyiidae|[3873]|CNEID2036-12|Cecidomyiidae|670|BOLD:ACB0497
Cecidomyiidae|[3874]|CNLMJ139-14|Cecidomyiidae|522|BOLD:ACO1877
Cecidomyiidae|[3875]|CNSLI468-12|Cecidomyiidae|670|BOLD:ACJ5256
Cecidomyiidae|[3876]|CNKJG533-14|Cecidomyiidae|658|BOLD:ACJ5467
Cecidomyiidae|[3877]|SSPAC2506-13|Cecidomyiidae|667|BOLD:ACJ5257
Cecidomyiidae|[3878]|HPPPE1078-13|Cecidomyiidae|664|BOLD:AAM6094
Cecidomyiidae|[3879]|CNKOH1669-14|Cecidomyiidae|545|BOLD:ACM3070
Cecidomyiidae|[3880]|SSGBB3475-14|Cecidomyiidae|667|BOLD:ACL8740
Cecidomyiidae|[3881]|CNBRP436-14|Cecidomyiidae|658|BOLD:ACM0582
Cecidomyiidae|[3882]|CNLMS297-14|Cecidomyiidae|603|BOLD:ACO3194
Cecidomyiidae|[3883]|JSJUL1208-11|Cecidomyiidae|658|BOLD:AAQ0304
Cecidomyiidae|[3884]|CNEIC943-12|Cecidomyiidae|670|BOLD:ABV1346
Cecidomyiidae|[3885]|CNBAC114-12|Cecidomyiidae|658|BOLD:ACC7994
Cecidomyiidae|[3886]|CNBAC160-12|Cecidomyiidae|658|BOLD:ACC8348
Cecidomyiidae|[3887]|CNBAC587-12|Cecidomyiidae|661|BOLD:ACD0134
Cecidomyiidae|[3888]|CNBAK327-13|Cecidomyiidae|658|BOLD:ACI6180
Cecidomyiidae|[3889]|CNBAK269-13|Cecidomyiidae|658|BOLD:ACI6767
Cecidomyiidae|[3890]|SSBAF5128-13|Cecidomyiidae|658|BOLD:ACI9072
Cecidomyiidae|[3891]|CNNHD1063-14|Cecidomyiidae|576|BOLD:ACR5853
Cecidomyiidae|[3892]|CNNHF714-14|Cecidomyiidae|564|BOLD:ACR8409
Cecidomyiidae|[3893]|CNBAC456-12|Cecidomyiidae|623|BOLD:ACD0170
Cecidomyiidae|[3894]|CNWAF865-15|Cecidomyiidae|576|BOLD:ACS0217
Cecidomyiidae|[3895]|PAJUL527-12|Cecidomyiidae|670|BOLD:ACB3138
Cecidomyiidae|[3896]|CNTNH188-14|Cecidomyiidae|591|BOLD:ACN8114
Cecidomyiidae|[3897]|CNKOI221-14|Cecidomyiidae|658|BOLD:ACT1350
Cecidomyiidae|[3898]|NCCA1102-11|Cecidomyiidae|673|BOLD:ABA6509
Cecidomyiidae|[3899]|CNGMH253-14|Cecidomyiidae|664|BOLD:ACM0297

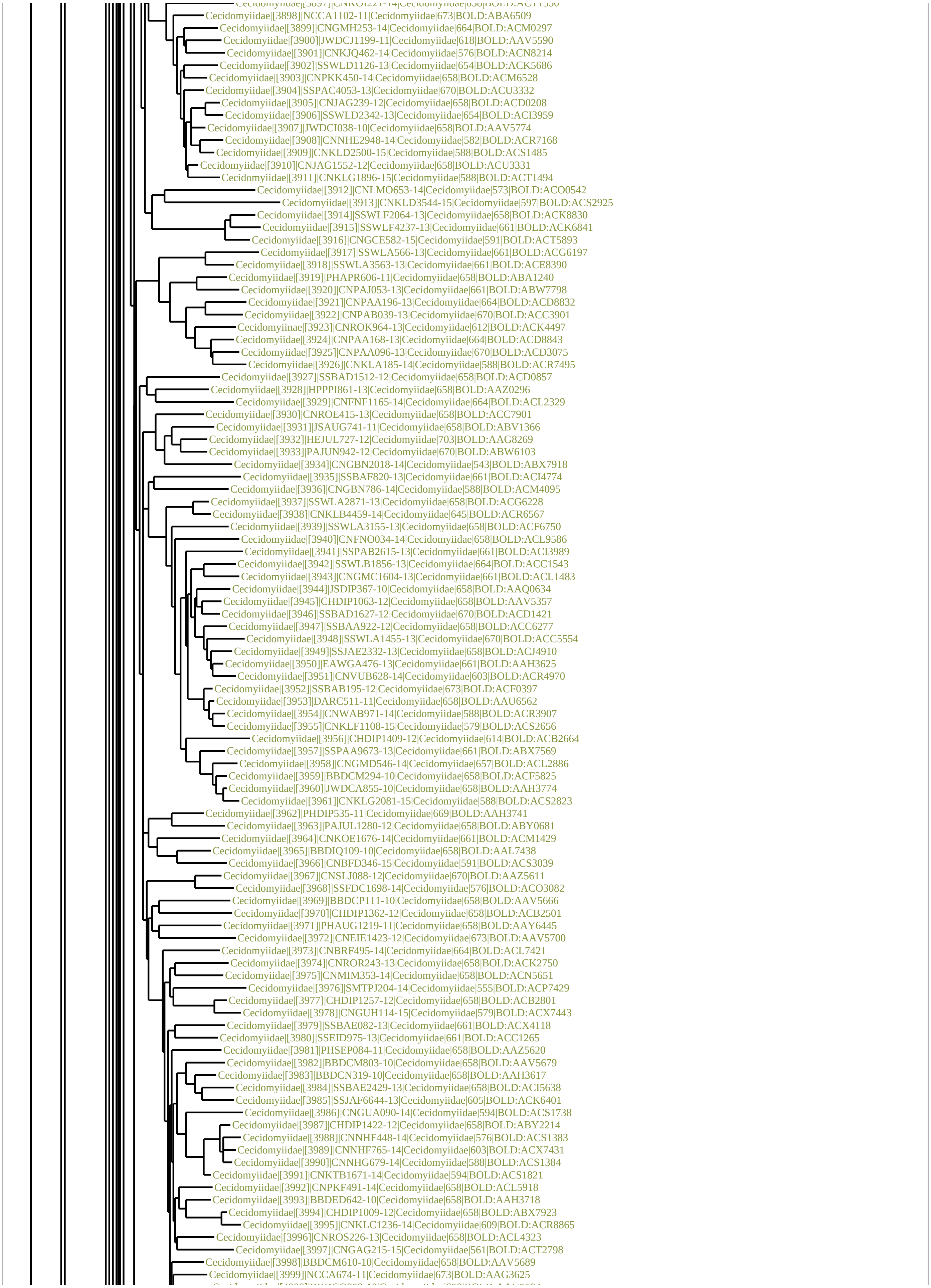

Cecidomyiidae|[3898]|NCCA1102-11|Cecidomyiidae|673|BOLD:ABA6509
Cecidomyiidae|[3899]|CNGMH253-14|Cecidomyiidae|664|BOLD:ACM0297
Cecidomyiidae|[3900]|JWDCJ1199-11|Cecidomyiidae|618|BOLD:AAV5590
Cecidomyiidae|[3901]|CNKJQ462-14|Cecidomyiidae|576|BOLD:ACN8214
Cecidomyiidae|[3902]|SSWLD1126-13|Cecidomyiidae|654|BOLD:ACK5686
Cecidomyiidae|[3903]|CNPKK450-14|Cecidomyiidae|658|BOLD:ACM6528
Cecidomyiidae|[3904]|SSPAC4053-13|Cecidomyiidae|670|BOLD:ACU3332
Cecidomyiidae|[3905]|CNJAG239-12|Cecidomyiidae|658|BOLD:ACD0208
Cecidomyiidae|[3906]|SSWLD2342-13|Cecidomyiidae|654|BOLD:ACI3959
Cecidomyiidae|[3907]|JWDCI038-10|Cecidomyiidae|658|BOLD:AAV5774
Cecidomyiidae|[3908]|CNNHE2948-14|Cecidomyiidae|582|BOLD:ACR7168
Cecidomyiidae|[3909]|CNKLD2500-15|Cecidomyiidae|588|BOLD:ACS1485
Cecidomyiidae|[3910]|CNJAG1552-12|Cecidomyiidae|658|BOLD:ACU3331
Cecidomyiidae|[3911]|CNKLG1896-15|Cecidomyiidae|588|BOLD:ACT1494
Cecidomyiidae|[3912]|CNLMO653-14|Cecidomyiidae|573|BOLD:ACO0542
Cecidomyiidae|[3913]|CNKLD3544-15|Cecidomyiidae|597|BOLD:ACS2925
Cecidomyiidae|[3914]|SSWLF2064-13|Cecidomyiidae|658|BOLD:ACK8830
Cecidomyiidae|[3915]|SSWLF4237-13|Cecidomyiidae|661|BOLD:ACK6841
Cecidomyiidae|[3916]|CNGCE582-15|Cecidomyiidae|591|BOLD:ACT5893
Cecidomyiidae|[3917]|SSWLA566-13|Cecidomyiidae|661|BOLD:ACG6197
Cecidomyiidae|[3918]|SSWLA3563-13|Cecidomyiidae|661|BOLD:ACE8390
Cecidomyiidae|[3919]|PHAPR606-11|Cecidomyiidae|658|BOLD:ABA1240
Cecidomyiidae|[3920]|CNPAJ053-13|Cecidomyiidae|661|BOLD:ABW7798
Cecidomyiidae|[3921]|CNPAA196-13|Cecidomyiidae|664|BOLD:ACD8832
Cecidomyiidae|[3922]|CNPAB039-13|Cecidomyiidae|670|BOLD:ACC3901
Cecidomyiinae|[3923]|CNROK964-13|Cecidomyiidae|612|BOLD:ACK4497
Cecidomyiidae|[3924]|CNPAA168-13|Cecidomyiidae|664|BOLD:ACD8843
Cecidomyiidae|[3925]|CNPAA096-13|Cecidomyiidae|670|BOLD:ACD3075
Cecidomyiidae|[3926]|CNKLA185-14|Cecidomyiidae|588|BOLD:ACR7495
Cecidomyiidae|[3927]|SSBAD1512-12|Cecidomyiidae|658|BOLD:ACD0857
Cecidomyiidae|[3928]|HPPPI861-13|Cecidomyiidae|658|BOLD:AAZ0296
Cecidomyiidae|[3929]|CNFNF1165-14|Cecidomyiidae|664|BOLD:ACL2329
Cecidomyiidae|[3930]|CNROE415-13|Cecidomyiidae|658|BOLD:ACC7901
Cecidomyiidae|[3931]|JSAUG741-11|Cecidomyiidae|658|BOLD:ABV1366
Cecidomyiidae|[3932]|HEJUL727-12|Cecidomyiidae|703|BOLD:AAG8269
Cecidomyiidae|[3933]|PAJUN942-12|Cecidomyiidae|670|BOLD:ABW6103
Cecidomyiidae|[3934]|CNGBN2018-14|Cecidomyiidae|543|BOLD:ABX7918
Cecidomyiidae|[3935]|SSBAF820-13|Cecidomyiidae|661|BOLD:ACI4774
Cecidomyiidae|[3936]|CNGBN786-14|Cecidomyiidae|588|BOLD:ACM4095
Cecidomyiidae|[3937]|SSWLA2871-13|Cecidomyiidae|658|BOLD:ACG6228
Cecidomyiidae|[3938]|CNKLB4459-14|Cecidomyiidae|645|BOLD:ACR6567
Cecidomyiidae|[3939]|SSWLA3155-13|Cecidomyiidae|658|BOLD:ACF6750
Cecidomyiidae|[3940]|CNFNO034-14|Cecidomyiidae|658|BOLD:ACL9586
Cecidomyiidae|[3941]|SSPAB2615-13|Cecidomyiidae|661|BOLD:ACI3989
Cecidomyiidae|[3942]|SSWLB1856-13|Cecidomyiidae|664|BOLD:ACC1543
Cecidomyiidae|[3943]|CNGMC1604-13|Cecidomyiidae|661|BOLD:ACL1483
Cecidomyiidae|[3944]|JSDIP367-10|Cecidomyiidae|658|BOLD:AAQ0634
Cecidomyiidae|[3945]|CHDIP1063-12|Cecidomyiidae|658|BOLD:AAV5357
Cecidomyiidae|[3946]|SSBAD1627-12|Cecidomyiidae|670|BOLD:ACD1421
Cecidomyiidae|[3947]|SSBAA922-12|Cecidomyiidae|658|BOLD:ACC6277
Cecidomyiidae|[3948]|SSWLA1455-13|Cecidomyiidae|670|BOLD:ACC5554
Cecidomyiidae|[3949]|SSJAE2332-13|Cecidomyiidae|658|BOLD:ACJ4910
Cecidomyiidae|[3950]|EAWGA476-13|Cecidomyiidae|661|BOLD:AAH3625
Cecidomyiidae|[3951]|CNVUB628-14|Cecidomyiidae|603|BOLD:ACR4970
Cecidomyiidae|[3952]|SSBAB195-12|Cecidomyiidae|673|BOLD:ACF0397
Cecidomyiidae|[3953]|DARC511-11|Cecidomyiidae|658|BOLD:AAU6562
Cecidomyiidae|[3954]|CNWAB971-14|Cecidomyiidae|588|BOLD:ACR3907
Cecidomyiidae|[3955]|CNKLF1108-15|Cecidomyiidae|579|BOLD:ACS2656
Cecidomyiidae|[3956]|CHDIP1409-12|Cecidomyiidae|614|BOLD:ACB2664
Cecidomyiidae|[3957]|SSPAA9673-13|Cecidomyiidae|661|BOLD:ABX7569
Cecidomyiidae|[3958]|CNGMD546-14|Cecidomyiidae|657|BOLD:ACL2886
Cecidomyiidae|[3959]|BBDCM294-10|Cecidomyiidae|658|BOLD:ACF5825
Cecidomyiidae|[3960]|JWDCA855-10|Cecidomyiidae|658|BOLD:AAH3774
Cecidomyiidae|[3961]|CNKLG2081-15|Cecidomyiidae|588|BOLD:ACS2823
Cecidomyiidae|[3962]|PHDIP535-11|Cecidomyiidae|669|BOLD:AAH3741
Cecidomyiidae|[3963]|PAJUL1280-12|Cecidomyiidae|658|BOLD:ABY0681
Cecidomyiidae|[3964]|CNKOE1676-14|Cecidomyiidae|661|BOLD:ACM1429
Cecidomyiidae|[3965]|BBDIQ109-10|Cecidomyiidae|658|BOLD:AAL7438
Cecidomyiidae|[3966]|CNBFD346-15|Cecidomyiidae|591|BOLD:ACS3039
Cecidomyiidae|[3967]|CNSLJ088-12|Cecidomyiidae|670|BOLD:AAZ5611
Cecidomyiidae|[3968]|SSFDC1698-14|Cecidomyiidae|576|BOLD:ACO3082
Cecidomyiidae|[3969]|BBDCP111-10|Cecidomyiidae|658|BOLD:AAV5666
Cecidomyiidae|[3970]|CHDIP1362-12|Cecidomyiidae|658|BOLD:ACB2501
Cecidomyiidae|[3971]|PHAUG1219-11|Cecidomyiidae|658|BOLD:AAY6445
Cecidomyiidae|[3972]|CNEIE1423-12|Cecidomyiidae|673|BOLD:AAV5700
Cecidomyiidae|[3973]|CNBRF495-14|Cecidomyiidae|664|BOLD:ACL7421
Cecidomyiidae|[3974]|CNROR243-13|Cecidomyiidae|658|BOLD:ACK2750
Cecidomyiidae|[3975]|CNMIM353-14|Cecidomyiidae|658|BOLD:ACN5651
Cecidomyiidae|[3976]|SMTPJ204-14|Cecidomyiidae|555|BOLD:ACP7429
Cecidomyiidae|[3977]|CHDIP1257-12|Cecidomyiidae|658|BOLD:ACB2801
Cecidomyiidae|[3978]|CNGUH114-15|Cecidomyiidae|579|BOLD:ACX7443
Cecidomyiidae|[3979]|SSBAE082-13|Cecidomyiidae|661|BOLD:ACX4118
Cecidomyiidae|[3980]|SSEID975-13|Cecidomyiidae|661|BOLD:ACC1265
Cecidomyiidae|[3981]|PHSEP084-11|Cecidomyiidae|658|BOLD:AAZ5620
Cecidomyiidae|[3982]|BBDCM803-10|Cecidomyiidae|658|BOLD:AAV5679
Cecidomyiidae|[3983]|BBDCN319-10|Cecidomyiidae|658|BOLD:AAH3617
Cecidomyiidae|[3984]|SSBAE2429-13|Cecidomyiidae|658|BOLD:ACI5638
Cecidomyiidae|[3985]|SSJAF6644-13|Cecidomyiidae|605|BOLD:ACK6401
Cecidomyiidae|[3986]|CNGUA090-14|Cecidomyiidae|594|BOLD:ACS1738
Cecidomyiidae|[3987]|CHDIP1422-12|Cecidomyiidae|658|BOLD:ABY2214
Cecidomyiidae|[3988]|CNNHF448-14|Cecidomyiidae|576|BOLD:ACS1383
Cecidomyiidae|[3989]|CNNHF765-14|Cecidomyiidae|603|BOLD:ACX7431
Cecidomyiidae|[3990]|CNNHG679-14|Cecidomyiidae|588|BOLD:ACS1384
Cecidomyiidae|[3991]|CNKTB1671-14|Cecidomyiidae|594|BOLD:ACS1821
Cecidomyiidae|[3992]|CNPKF491-14|Cecidomyiidae|658|BOLD:ACL5918
Cecidomyiidae|[3993]|BBDED642-10|Cecidomyiidae|658|BOLD:AAH3718
Cecidomyiidae|[3994]|CHDIP1009-12|Cecidomyiidae|658|BOLD:ABX7923
Cecidomyiidae|[3995]|CNKLC1236-14|Cecidomyiidae|609|BOLD:ACR8865
Cecidomyiidae|[3996]|CNROS226-13|Cecidomyiidae|658|BOLD:ACL4323
Cecidomyiidae|[3997]|CNGAG215-15|Cecidomyiidae|561|BOLD:ACT2798
Cecidomyiidae|[3998]|BBDCM610-10|Cecidomyiidae|658|BOLD:AAV5689
Cecidomyiidae|[3999]|NCCA674-11|Cecidomyiidae|673|BOLD:AAG3625

Cecidomyiidae|[3997]|CNGAG[illegible]|Cecidomyiidae|561|BOLD:ACT2798
Cecidomyiidae|[3998]|BBDCM610-10|Cecidomyiidae|658|BOLD:AAV5689
Cecidomyiidae|[3999]|NCCA674-11|Cecidomyiidae|673|BOLD:AAG3625
Cecidomyiidae|[4000]|BBDCQ858-10|Cecidomyiidae|658|BOLD:AAV5594
Cecidomyiidae|[4001]|SSBAE091-13|Cecidomyiidae|661|BOLD:ACE4217
Cecidomyiidae|[4002]|HPPPJ960-13|Cecidomyiidae|658|BOLD:ABV9290
Cecidomyiidae|[4003]|BBDED695-10|Cecidomyiidae|658|BOLD:AAH3731
Cecidomyiidae|[4004]|JSDIP681-10|Cecidomyiidae|658|BOLD:AAV5352
Cecidomyiidae|[4005]|SSBAF466-13|Cecidomyiidae|661|BOLD:AAP6847
Cecidomyiidae|[4006]|CNROQ204-13|Cecidomyiidae|636|BOLD:ACL0899
Cecidomyiidae|[4007]|CNGMG1495-14|Cecidomyiidae|664|BOLD:AAZ5617
Cecidomyiidae|[4008]|CHDIP1279-12|Cecidomyiidae|658|BOLD:AAH3778
Cecidomyiidae|[4009]|SSBAA412-12|Cecidomyiidae|661|BOLD:ACC1610
Cecidomyiidae|[4010]|CHDIP951-12|Cecidomyiidae|658|BOLD:ABX7961
Cecidomyiidae|[4011]|EAWGA477-13|Cecidomyiidae|661|BOLD:ACL3499
Cecidomyiidae|[4012]|SSEID905-13|Cecidomyiidae|661|BOLD:ACJ3795
Cecidomyiidae|[4013]|CNMIH707-14|Cecidomyiidae|588|BOLD:ACN7399
Cecidomyiidae|[4014]|PHAUG1451-11|Cecidomyiidae|657|BOLD:ABV1466
Cecidomyiidae|[4015]|SSBAA3525-12|Cecidomyiidae|673|BOLD:ACC2638
Cecidomyiidae|[4016]|CNEIF3365-12|Cecidomyiidae|658|BOLD:ACD2293
Cecidomyiidae|[4017]|CNGBF1828-14|Cecidomyiidae|658|BOLD:ACD4561
Cecidomyiidae|[4018]|PHNXN368-14|Cecidomyiidae|604|BOLD:ACX3842
Cecidomyiidae|[4019]|CNKTB100-14|Cecidomyiidae|576|BOLD:ACX3843
Cecidomyiidae|[4020]|SSBAA663-12|Cecidomyiidae|673|BOLD:AAL7437
Cecidomyiidae|[4021]|JSDIR318-11|Cecidomyiidae|658|BOLD:ABA0827
Cecidomyiidae|[4022]|CNEID2778-12|Cecidomyiidae|658|BOLD:ACB0663
Cecidomyiidae|[4023]|SSBAE579-13|Cecidomyiidae|664|BOLD:ACG1226
Cecidomyiidae|[4024]|SSWLA1626-13|Cecidomyiidae|658|BOLD:ACA2015
Cecidomyiidae|[4025]|CNWBG581-13|Cecidomyiidae|645|BOLD:ACI4561
Cecidomyiidae|[4026]|JSAUG1084-11|Cecidomyiidae|658|BOLD:ABV1326
Cecidomyiidae|[4027]|RBINA464-13|Cecidomyiidae|632|BOLD:ACI9839
Cecidomyiidae|[4028]|CNPKF369-14|Cecidomyiidae|658|BOLD:AAM6074
Cecidomyiidae|[4029]|BBDCQ851-10|Cecidomyiidae|658|BOLD:AAH3662
Cecidomyiidae|[4030]|CNPPE214-12|Cecidomyiidae|664|BOLD:ABW8055
Cecidomyiidae|[4031]|CNGAB375-15|Cecidomyiidae|591|BOLD:ACX4327
Cecidomyiidae|[4032]|CNGAD188-15|Cecidomyiidae|487|BOLD:ACN4979
Cecidomyiidae|[4033]|CNGAG346-15|Cecidomyiidae|618|BOLD:ACS1818
Cecidomyiidae|[4034]|CNROQ231-13|Cecidomyiidae|636|BOLD:ACL0900
Cecidomyiidae|[4035]|CNROR229-13|Cecidomyiidae|658|BOLD:ACL7149
Cecidomyiidae|[4036]|CNROF473-13|Cecidomyiidae|658|BOLD:ACK3071
Cecidomyiidae|[4037]|CNROG481-13|Cecidomyiidae|603|BOLD:ACK2316
Cecidomyiidae|[4038]|CNROQ252-13|Cecidomyiidae|658|BOLD:ACL1473
Cecidomyiidae|[4039]|CNROH348-13|Cecidomyiidae|661|BOLD:ACK3011
Cecidomyiidae|[4040]|CNROQ543-13|Cecidomyiidae|661|BOLD:ACK2119
Cecidomyiidae|[4041]|CNROH204-13|Cecidomyiidae|661|BOLD:ABA0853
Cecidomyiidae|[4042]|CNROT123-13|Cecidomyiidae|661|BOLD:ACL5038
Cecidomyiidae|[4043]|CNROG358-13|Cecidomyiidae|658|BOLD:ACK2823
Cecidomyiidae|[4044]|CNROH211-13|Cecidomyiidae|643|BOLD:ACK2789
Cecidomyiidae|[4045]|SSPAA4137-13|Cecidomyiidae|658|BOLD:ACG9000
Cecidomyiidae|[4046]|JSAUG1227-11|Cecidomyiidae|658|BOLD:ABV1313
Cecidomyiidae|[4047]|JSAUG1184-11|Cecidomyiidae|658|BOLD:ABV1384
Cecidomyiidae|[4048]|JSSEP257-11|Cecidomyiidae|658|BOLD:ABV1273
Cecidomyiidae|[4049]|CNFNI132-14|Cecidomyiidae|658|BOLD:ACF4932
Cecidomyiidae|[4050]|JSAUG619-11|Cecidomyiidae|658|BOLD:ABV1375
Cecidomyiidae|[4051]|CNPPE1851-12|Cecidomyiidae|661|BOLD:ACA6970
Cecidomyiidae|[4052]|JSAUG1014-11|Cecidomyiidae|658|BOLD:AAH3655
Cecidomyiidae|[4053]|PHAUG282-11|Cecidomyiidae|658|BOLD:ACU7593
Cecidomyiidae|[4054]|SSKJC1311-15|Cecidomyiidae|549|BOLD:ACT1582
Cecidomyiidae|[4055]|RBINA2726-13|Cecidomyiidae|658|BOLD:ACJ0108
Cecidomyiidae|[4056]|CNFNJ139-14|Cecidomyiidae|658|BOLD:ACK2997
Cecidomyiidae|[4057]|SSPAC8843-13|Cecidomyiidae|600|BOLD:ACJ8441
Cecidomyiidae|[4058]|CNKLG715-15|Cecidomyiidae|611|BOLD:ACR5199
Cecidomyiidae|[4059]|CNGCF301-15|Cecidomyiidae|534|BOLD:ACU0310
Cecidomyiidae|[4060]|JSJUL1432-11|Cecidomyiidae|658|BOLD:ABW2741
Cecidomyiidae|[4061]|JSJUL1034-11|Cecidomyiidae|658|BOLD:ABV1427
Cecidomyiidae|[4062]|JSAUG794-11|Cecidomyiidae|658|BOLD:ABV1196
Cecidomyiidae|[4063]|CNRMC1025-12|Cecidomyiidae|658|BOLD:ACC1235
Cecidomyiidae|[4064]|CNPPJ755-12|Cecidomyiidae|670|BOLD:ACC8369
Cecidomyiidae|[4065]|CNPPI253-12|Cecidomyiidae|664|BOLD:ACB3408
Cecidomyiidae|[4066]|PASEP370-12|Cecidomyiidae|670|BOLD:AAV5569
Cecidomyiidae|[4067]|MBIOI463-13|Cecidomyiidae|661|BOLD:AAP8816
Cecidomyiidae|[4068]|JSJUL1408-11|Cecidomyiidae|658|BOLD:AAZ0311
Cecidomyiidae|[4069]|CNGBF531-14|Cecidomyiidae|658|BOLD:ACI9539
Cecidomyiidae|[4070]|JSJUL2175-11|Cecidomyiidae|658|BOLD:ABV1397
Cecidomyiidae|[4071]|PHAUG759-11|Cecidomyiidae|658|BOLD:ABW8038
Cecidomyiidae|[4072]|BBDCN477-10|Cecidomyiidae|658|BOLD:AAP6843
Cecidomyiidae|[4073]|CNFNE152-14|Cecidomyiidae|660|BOLD:ACL3515
Cecidomyiidae|[4074]|CNGBH443-14|Cecidomyiidae|660|BOLD:ACC8303
Cecidomyiidae|[4075]|CNGBE353-14|Cecidomyiidae|661|BOLD:ACL7126
Cecidomyiidae|[4076]|CNGBC1048-13|Cecidomyiidae|658|BOLD:ACK9858
Cecidomyiidae|[4077]|PHAUG797-11|Cecidomyiidae|658|BOLD:ABV9350
Cecidomyiidae|[4078]|CNPPD071-12|Cecidomyiidae|658|BOLD:ACC7800
Cecidomyiidae|[4079]|PHDIP530-11|Cecidomyiidae|669|BOLD:AAY6403
Cecidomyiidae|[4080]|HEAUG689-12|Cecidomyiidae|664|BOLD:AAY6432
Cecidomyiidae|[4081]|HPPPD1138-13|Cecidomyiidae|661|BOLD:ACM1820
Cecidomyiidae|[4082]|SSGBA4253-14|Cecidomyiidae|630|BOLD:ACO5325
Cecidomyiidae|[4083]|JSJUL2192-11|Cecidomyiidae|658|BOLD:ABV1393
Cecidomyiidae|[4084]|HPPPE616-13|Cecidomyiidae|658|BOLD:ACM1821
Cecidomyiidae|[4085]|CNGBO624-14|Cecidomyiidae|649|BOLD:ACM2054
Cecidomyiidae|[4086]|NCCA576-11|Cecidomyiidae|673|BOLD:ABA6530
Cecidomyiidae|[4087]|CNBRO320-14|Cecidomyiidae|562|BOLD:ACL9399
Cecidomyiidae|[4088]|HPPPM001-13|Cecidomyiidae|658|BOLD:ACY0893
Cecidomyiidae|[4089]|SSBRC2120-14|Cecidomyiidae|543|BOLD:ACO1730
Cecidomyiidae|[4090]|SSGBA3926-14|Cecidomyiidae|564|BOLD:ACP3253
Cecidomyiidae|[4091]|SSGBA5510-14|Cecidomyiidae|591|BOLD:ACO5324
Cecidomyiidae|[4092]|HPPPJ242-13|Cecidomyiidae|661|BOLD:ACI8791
Cecidomyiidae|[4093]|CNSID1955-15|Cecidomyiidae|609|BOLD:ACT1511
Cecidomyiidae|[4094]|CNSID2165-15|Cecidomyiidae|591|BOLD:ACT4709
Cecidomyiidae|[4095]|CNSID2169-15|Cecidomyiidae|585|BOLD:ACT4710
Cecidomyiidae|[4096]|CNSID2173-15|Cecidomyiidae|558|BOLD:ACT3253
Cecidomyiidae|[4097]|JSJUL1275-11|Cecidomyiidae|658|BOLD:ABV1435
Cecidomyiidae|[4098]|CNPPI557-12|Cecidomyiidae|664|BOLD:ACA6549
Cecidomyiidae|[4099]|SSBAD1567-12|Cecidomyiidae|658|BOLD:ACD2007
[illegible]

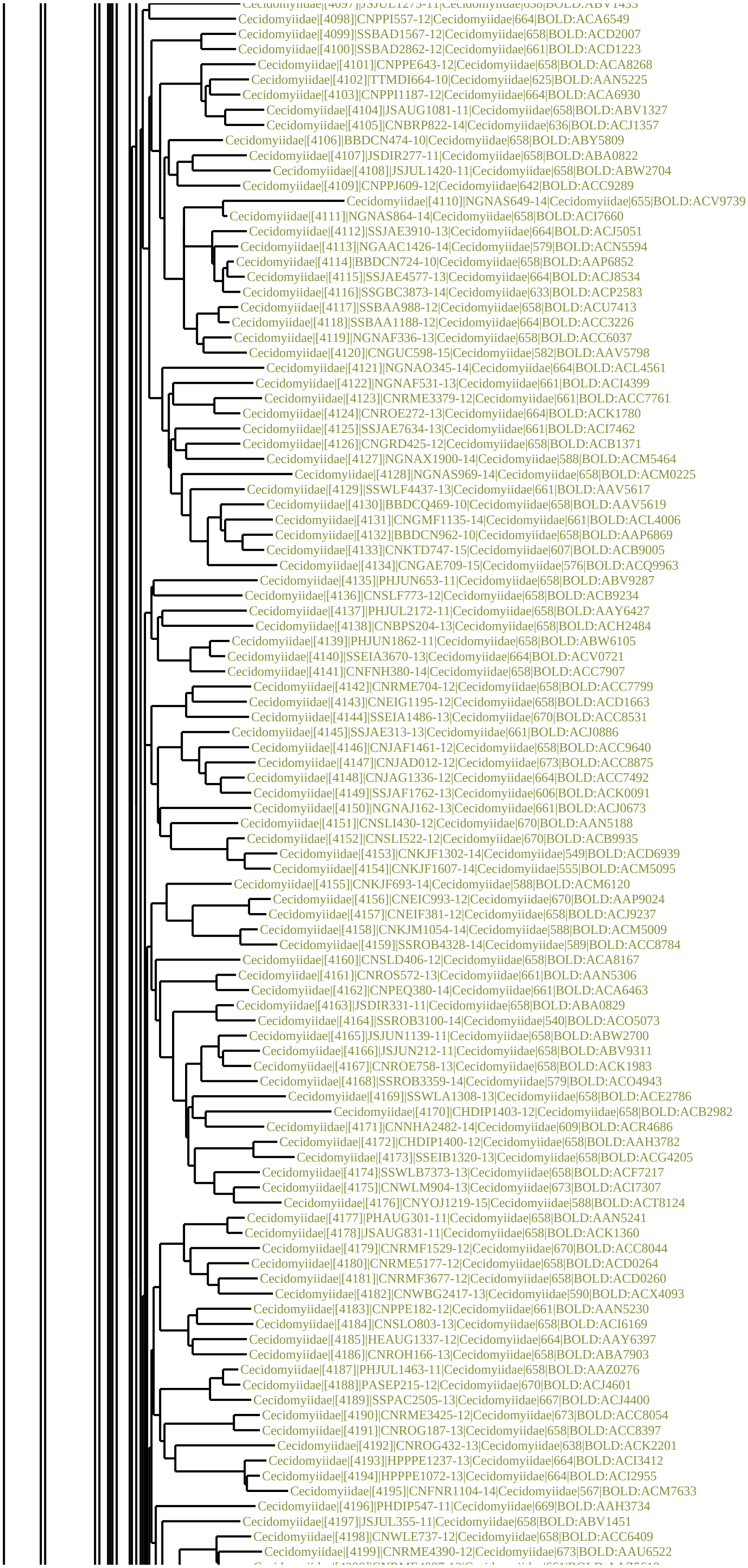
Cecidomyiidae|[4098]|CNPPI557-12|Cecidomyiidae|664|BOLD:ACA6549
Cecidomyiidae|[4099]|SSBAD1567-12|Cecidomyiidae|658|BOLD:ACD2007
Cecidomyiidae|[4100]|SSBAD2862-12|Cecidomyiidae|661|BOLD:ACD1223
Cecidomyiidae|[4101]|CNPPE643-12|Cecidomyiidae|658|BOLD:ACA8268
Cecidomyiidae|[4102]|TTMDI664-10|Cecidomyiidae|625|BOLD:AAN5225
Cecidomyiidae|[4103]|CNPPI1187-12|Cecidomyiidae|664|BOLD:ACA6930
Cecidomyiidae|[4104]|JSAUG1081-11|Cecidomyiidae|658|BOLD:ABV1327
Cecidomyiidae|[4105]|CNBRP822-14|Cecidomyiidae|636|BOLD:ACJ1357
Cecidomyiidae|[4106]|BBDCN474-10|Cecidomyiidae|658|BOLD:ABY5809
Cecidomyiidae|[4107]|JSDIR277-11|Cecidomyiidae|658|BOLD:ABA0822
Cecidomyiidae|[4108]|JSJUL1420-11|Cecidomyiidae|658|BOLD:ABW2704
Cecidomyiidae|[4109]|CNPPJ609-12|Cecidomyiidae|642|BOLD:ACC9289
Cecidomyiidae|[4110]|NGNAS649-14|Cecidomyiidae|655|BOLD:ACV9739
Cecidomyiidae|[4111]|NGNAS864-14|Cecidomyiidae|658|BOLD:ACI7660
Cecidomyiidae|[4112]|SSJAE3910-13|Cecidomyiidae|664|BOLD:ACJ5051
Cecidomyiidae|[4113]|NGAAC1426-14|Cecidomyiidae|579|BOLD:ACN5594
Cecidomyiidae|[4114]|BBDCN724-10|Cecidomyiidae|658|BOLD:AAP6852
Cecidomyiidae|[4115]|SSJAE4577-13|Cecidomyiidae|664|BOLD:ACJ8534
Cecidomyiidae|[4116]|SSGBC3873-14|Cecidomyiidae|633|BOLD:ACP2583
Cecidomyiidae|[4117]|SSBAA988-12|Cecidomyiidae|658|BOLD:ACU7413
Cecidomyiidae|[4118]|SSBAA1188-12|Cecidomyiidae|664|BOLD:ACC3226
Cecidomyiidae|[4119]|NGNAF336-13|Cecidomyiidae|658|BOLD:ACC6037
Cecidomyiidae|[4120]|CNGUC598-15|Cecidomyiidae|582|BOLD:AAV5798
Cecidomyiidae|[4121]|NGNAO345-14|Cecidomyiidae|664|BOLD:ACL4561
Cecidomyiidae|[4122]|NGNAF531-13|Cecidomyiidae|661|BOLD:ACI4399
Cecidomyiidae|[4123]|CNRME3379-12|Cecidomyiidae|661|BOLD:ACC7761
Cecidomyiidae|[4124]|CNROE272-13|Cecidomyiidae|664|BOLD:ACK1780
Cecidomyiidae|[4125]|SSJAE7634-13|Cecidomyiidae|661|BOLD:ACI7462
Cecidomyiidae|[4126]|CNGRD425-12|Cecidomyiidae|658|BOLD:ACB1371
Cecidomyiidae|[4127]|NGNAX1900-14|Cecidomyiidae|588|BOLD:ACM5464
Cecidomyiidae|[4128]|NGNAS969-14|Cecidomyiidae|658|BOLD:ACM0225
Cecidomyiidae|[4129]|SSWLF4437-13|Cecidomyiidae|661|BOLD:AAV5617
Cecidomyiidae|[4130]|BBDCQ469-10|Cecidomyiidae|658|BOLD:AAV5619
Cecidomyiidae|[4131]|CNGMF1135-14|Cecidomyiidae|661|BOLD:ACL4006
Cecidomyiidae|[4132]|BBDCN962-10|Cecidomyiidae|658|BOLD:AAP6869
Cecidomyiidae|[4133]|CNKTD747-15|Cecidomyiidae|607|BOLD:ACB9005
Cecidomyiidae|[4134]|CNGAE709-15|Cecidomyiidae|576|BOLD:ACQ9963
Cecidomyiidae|[4135]|PHJUN653-11|Cecidomyiidae|658|BOLD:ABV9287
Cecidomyiidae|[4136]|CNSLF773-12|Cecidomyiidae|658|BOLD:ACB9234
Cecidomyiidae|[4137]|PHJUL2172-11|Cecidomyiidae|658|BOLD:AAY6427
Cecidomyiidae|[4138]|CNBPS204-13|Cecidomyiidae|658|BOLD:ACH2484
Cecidomyiidae|[4139]|PHJUN1862-11|Cecidomyiidae|658|BOLD:ABW6105
Cecidomyiidae|[4140]|SSEIA3670-13|Cecidomyiidae|664|BOLD:ACV0721
Cecidomyiidae|[4141]|CNFNH380-14|Cecidomyiidae|658|BOLD:ACC7907
Cecidomyiidae|[4142]|CNRME704-12|Cecidomyiidae|658|BOLD:ACC7799
Cecidomyiidae|[4143]|CNEIG1195-12|Cecidomyiidae|658|BOLD:ACD1663
Cecidomyiidae|[4144]|SSEIA1486-13|Cecidomyiidae|670|BOLD:ACC8531
Cecidomyiidae|[4145]|SSJAE313-13|Cecidomyiidae|661|BOLD:ACJ0886
Cecidomyiidae|[4146]|CNJAF1461-12|Cecidomyiidae|658|BOLD:ACC9640
Cecidomyiidae|[4147]|CNJAD012-12|Cecidomyiidae|673|BOLD:ACC8875
Cecidomyiidae|[4148]|CNJAG1336-12|Cecidomyiidae|664|BOLD:ACC7492
Cecidomyiidae|[4149]|SSJAF1762-13|Cecidomyiidae|606|BOLD:ACK0091
Cecidomyiidae|[4150]|NGNAJ162-13|Cecidomyiidae|661|BOLD:ACJ0673
Cecidomyiidae|[4151]|CNSLI430-12|Cecidomyiidae|670|BOLD:AAN5188
Cecidomyiidae|[4152]|CNSLI522-12|Cecidomyiidae|670|BOLD:ACB9935
Cecidomyiidae|[4153]|CNKJF1302-14|Cecidomyiidae|549|BOLD:ACD6939
Cecidomyiidae|[4154]|CNKJF1607-14|Cecidomyiidae|555|BOLD:ACM5095
Cecidomyiidae|[4155]|CNKJF693-14|Cecidomyiidae|588|BOLD:ACM6120
Cecidomyiidae|[4156]|CNEIC993-12|Cecidomyiidae|670|BOLD:AAP9024
Cecidomyiidae|[4157]|CNEIF381-12|Cecidomyiidae|658|BOLD:ACJ9237
Cecidomyiidae|[4158]|CNKJM1054-14|Cecidomyiidae|588|BOLD:ACM5009
Cecidomyiidae|[4159]|SSROB4328-14|Cecidomyiidae|589|BOLD:ACC8784
Cecidomyiidae|[4160]|CNSLD406-12|Cecidomyiidae|658|BOLD:ACA8167
Cecidomyiidae|[4161]|CNROS572-13|Cecidomyiidae|661|BOLD:AAN5306
Cecidomyiidae|[4162]|CNPEQ380-14|Cecidomyiidae|661|BOLD:ACA6463
Cecidomyiidae|[4163]|JSDIR331-11|Cecidomyiidae|658|BOLD:ABA0829
Cecidomyiidae|[4164]|SSROB3100-14|Cecidomyiidae|540|BOLD:ACO5073
Cecidomyiidae|[4165]|JSJUN1139-11|Cecidomyiidae|658|BOLD:ABW2700
Cecidomyiidae|[4166]|JSJUN212-11|Cecidomyiidae|658|BOLD:ABV9311
Cecidomyiidae|[4167]|CNROE758-13|Cecidomyiidae|658|BOLD:ACK1983
Cecidomyiidae|[4168]|SSROB3359-14|Cecidomyiidae|579|BOLD:ACO4943
Cecidomyiidae|[4169]|SSWLA1308-13|Cecidomyiidae|658|BOLD:ACE2786
Cecidomyiidae|[4170]|CHDIP1403-12|Cecidomyiidae|658|BOLD:ACB2982
Cecidomyiidae|[4171]|CNNHA2482-14|Cecidomyiidae|609|BOLD:ACR4686
Cecidomyiidae|[4172]|CHDIP1400-12|Cecidomyiidae|658|BOLD:AAH3782
Cecidomyiidae|[4173]|SSEIB1320-13|Cecidomyiidae|658|BOLD:ACG4205
Cecidomyiidae|[4174]|SSWLB7373-13|Cecidomyiidae|658|BOLD:ACF7217
Cecidomyiidae|[4175]|CNWLM904-13|Cecidomyiidae|673|BOLD:ACI7307
Cecidomyiidae|[4176]|CNYOJ1219-15|Cecidomyiidae|588|BOLD:ACT8124
Cecidomyiidae|[4177]|PHAUG301-11|Cecidomyiidae|658|BOLD:AAN5241
Cecidomyiidae|[4178]|JSAUG831-11|Cecidomyiidae|658|BOLD:ACK1360
Cecidomyiidae|[4179]|CNRMF1529-12|Cecidomyiidae|670|BOLD:ACC8044
Cecidomyiidae|[4180]|CNRME5177-12|Cecidomyiidae|658|BOLD:ACD0264
Cecidomyiidae|[4181]|CNRMF3677-12|Cecidomyiidae|658|BOLD:ACD0260
Cecidomyiidae|[4182]|CNWBG2417-13|Cecidomyiidae|590|BOLD:ACX4093
Cecidomyiidae|[4183]|CNPPE182-12|Cecidomyiidae|661|BOLD:AAN5230
Cecidomyiidae|[4184]|CNSLO803-13|Cecidomyiidae|658|BOLD:ACI6169
Cecidomyiidae|[4185]|HEAUG1337-12|Cecidomyiidae|664|BOLD:AAY6397
Cecidomyiidae|[4186]|CNROH166-13|Cecidomyiidae|658|BOLD:ABA7903
Cecidomyiidae|[4187]|PHJUL1463-11|Cecidomyiidae|658|BOLD:AAZ0276
Cecidomyiidae|[4188]|PASEP215-12|Cecidomyiidae|670|BOLD:ACJ4601
Cecidomyiidae|[4189]|SSPAC2505-13|Cecidomyiidae|667|BOLD:ACJ4400
Cecidomyiidae|[4190]|CNRME3425-12|Cecidomyiidae|673|BOLD:ACC8054
Cecidomyiidae|[4191]|CNROG187-13|Cecidomyiidae|658|BOLD:ACC8397
Cecidomyiidae|[4192]|CNROG432-13|Cecidomyiidae|638|BOLD:ACK2201
Cecidomyiidae|[4193]|HPPPE1237-13|Cecidomyiidae|664|BOLD:ACI3412
Cecidomyiidae|[4194]|HPPPE1072-13|Cecidomyiidae|664|BOLD:ACI2955
Cecidomyiidae|[4195]|CNFNR1104-14|Cecidomyiidae|567|BOLD:ACM7633
Cecidomyiidae|[4196]|PHDIP547-11|Cecidomyiidae|669|BOLD:AAH3734
Cecidomyiidae|[4197]|JSJUL355-11|Cecidomyiidae|658|BOLD:ABV1451
Cecidomyiidae|[4198]|CNWLE737-12|Cecidomyiidae|658|BOLD:ACC6409
Cecidomyiidae|[4199]|CNRME4390-12|Cecidomyiidae|673|BOLD:AAU6522

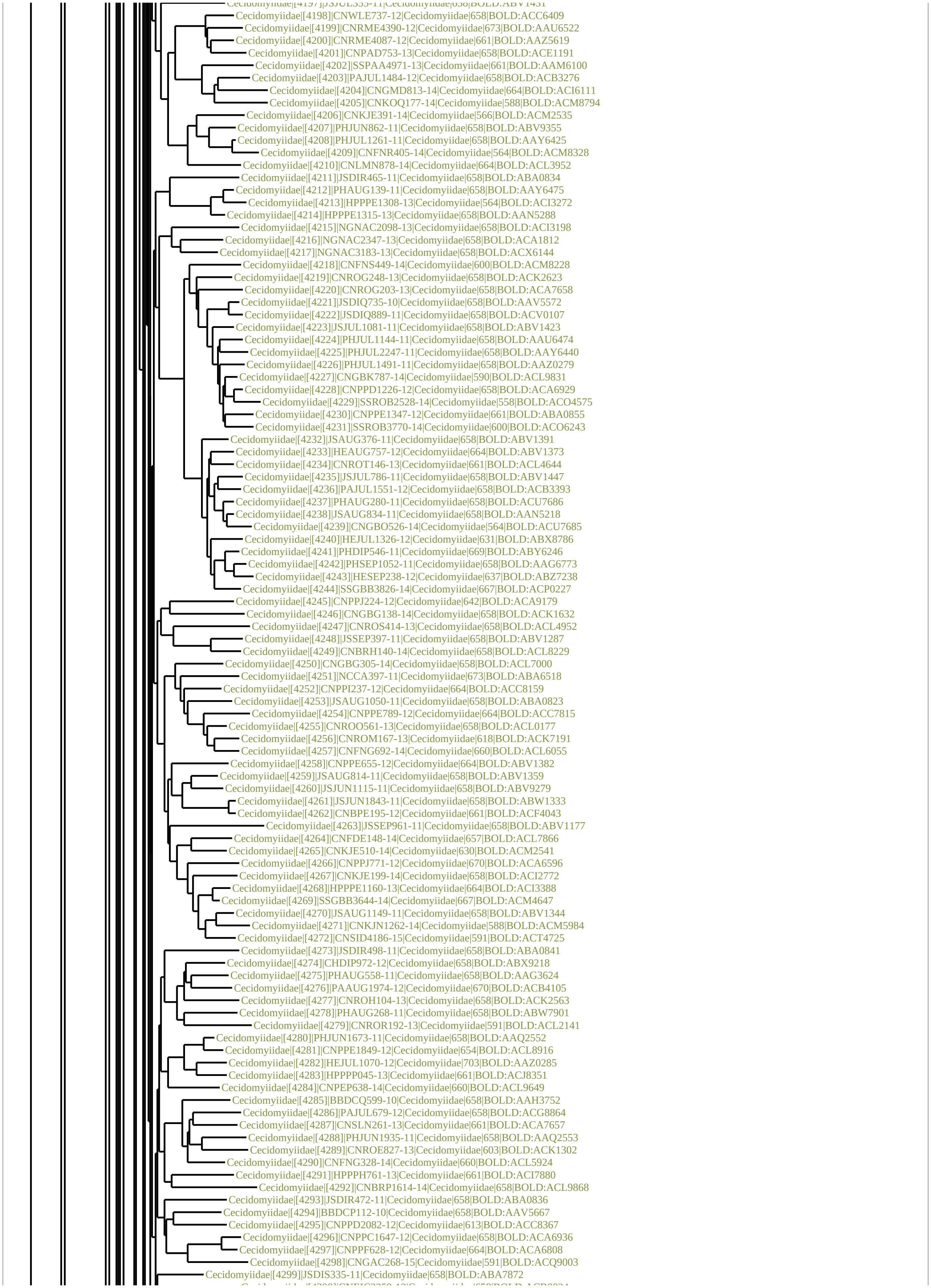

Cecidomyiidae|[4198]|CNWLE737-12|Cecidomyiidae|658|BOLD:ACC6409
Cecidomyiidae|[4199]|CNRME4390-12|Cecidomyiidae|673|BOLD:AAU6522
Cecidomyiidae|[4200]|CNRME4087-12|Cecidomyiidae|661|BOLD:AAZ5619
Cecidomyiidae|[4201]|CNPAD753-13|Cecidomyiidae|658|BOLD:ACE1191
Cecidomyiidae|[4202]|SSPAA4971-13|Cecidomyiidae|661|BOLD:AAM6100
Cecidomyiidae|[4203]|PAJUL1484-12|Cecidomyiidae|658|BOLD:ACB3276
Cecidomyiidae|[4204]|CNGMD813-14|Cecidomyiidae|664|BOLD:ACI6111
Cecidomyiidae|[4205]|CNKOQ177-14|Cecidomyiidae|588|BOLD:ACM8794
Cecidomyiidae|[4206]|CNKJE391-14|Cecidomyiidae|566|BOLD:ACM2535
Cecidomyiidae|[4207]|PHJUN862-11|Cecidomyiidae|658|BOLD:ABV9355
Cecidomyiidae|[4208]|PHJUL1261-11|Cecidomyiidae|658|BOLD:AAY6425
Cecidomyiidae|[4209]|CNFNR405-14|Cecidomyiidae|564|BOLD:ACM8328
Cecidomyiidae|[4210]|CNLMN878-14|Cecidomyiidae|664|BOLD:ACL3952
Cecidomyiidae|[4211]|JSDIR465-11|Cecidomyiidae|658|BOLD:ABA0834
Cecidomyiidae|[4212]|PHAUG139-11|Cecidomyiidae|658|BOLD:AAY6475
Cecidomyiidae|[4213]|HPPPE1308-13|Cecidomyiidae|564|BOLD:ACI3272
Cecidomyiidae|[4214]|HPPPE1315-13|Cecidomyiidae|658|BOLD:AAN5288
Cecidomyiidae|[4215]|NGNAC2098-13|Cecidomyiidae|658|BOLD:ACI3198
Cecidomyiidae|[4216]|NGNAC2347-13|Cecidomyiidae|658|BOLD:ACA1812
Cecidomyiidae|[4217]|NGNAC3183-13|Cecidomyiidae|658|BOLD:ACX6144
Cecidomyiidae|[4218]|CNFNS449-14|Cecidomyiidae|600|BOLD:ACM8228
Cecidomyiidae|[4219]|CNROG248-13|Cecidomyiidae|658|BOLD:ACK2623
Cecidomyiidae|[4220]|CNROG203-13|Cecidomyiidae|658|BOLD:ACA7658
Cecidomyiidae|[4221]|JSDIQ735-10|Cecidomyiidae|658|BOLD:AAV5572
Cecidomyiidae|[4222]|JSDIQ889-11|Cecidomyiidae|658|BOLD:ACV0107
Cecidomyiidae|[4223]|JSJUL1081-11|Cecidomyiidae|658|BOLD:ABV1423
Cecidomyiidae|[4224]|PHJUL1144-11|Cecidomyiidae|658|BOLD:AAU6474
Cecidomyiidae|[4225]|PHJUL2247-11|Cecidomyiidae|658|BOLD:AAY6440
Cecidomyiidae|[4226]|PHJUL1491-11|Cecidomyiidae|658|BOLD:AAZ0279
Cecidomyiidae|[4227]|CNGBK787-14|Cecidomyiidae|590|BOLD:ACL9831
Cecidomyiidae|[4228]|CNPPD1226-12|Cecidomyiidae|658|BOLD:ACA6929
Cecidomyiidae|[4229]|SSROB2528-14|Cecidomyiidae|558|BOLD:ACO4575
Cecidomyiidae|[4230]|CNPPE1347-12|Cecidomyiidae|661|BOLD:ABA0855
Cecidomyiidae|[4231]|SSROB3770-14|Cecidomyiidae|600|BOLD:ACO6243
Cecidomyiidae|[4232]|JSAUG376-11|Cecidomyiidae|658|BOLD:ABV1391
Cecidomyiidae|[4233]|HEAUG757-12|Cecidomyiidae|664|BOLD:ABV1373
Cecidomyiidae|[4234]|CNROT146-13|Cecidomyiidae|661|BOLD:ACL4644
Cecidomyiidae|[4235]|JSJUL786-11|Cecidomyiidae|658|BOLD:ABV1447
Cecidomyiidae|[4236]|PAJUL1551-12|Cecidomyiidae|658|BOLD:ACB3393
Cecidomyiidae|[4237]|PHAUG280-11|Cecidomyiidae|658|BOLD:ACU7686
Cecidomyiidae|[4238]|JSAUG834-11|Cecidomyiidae|658|BOLD:AAN5218
Cecidomyiidae|[4239]|CNGBO526-14|Cecidomyiidae|564|BOLD:ACU7685
Cecidomyiidae|[4240]|HEJUL1326-12|Cecidomyiidae|631|BOLD:ABX8786
Cecidomyiidae|[4241]|PHDIP546-11|Cecidomyiidae|669|BOLD:ABY6246
Cecidomyiidae|[4242]|PHSEP1052-11|Cecidomyiidae|658|BOLD:AAG6773
Cecidomyiidae|[4243]|HESEP238-12|Cecidomyiidae|637|BOLD:ABZ7238
Cecidomyiidae|[4244]|SSGBB3826-14|Cecidomyiidae|667|BOLD:ACP0227
Cecidomyiidae|[4245]|CNPPJ224-12|Cecidomyiidae|642|BOLD:ACA9179
Cecidomyiidae|[4246]|CNGBG138-14|Cecidomyiidae|658|BOLD:ACK1632
Cecidomyiidae|[4247]|CNROS414-13|Cecidomyiidae|658|BOLD:ACL4952
Cecidomyiidae|[4248]|JSSEP397-11|Cecidomyiidae|658|BOLD:ABV1287
Cecidomyiidae|[4249]|CNBRH140-14|Cecidomyiidae|658|BOLD:ACL8229
Cecidomyiidae|[4250]|CNGBG305-14|Cecidomyiidae|658|BOLD:ACL7000
Cecidomyiidae|[4251]|NCCA397-11|Cecidomyiidae|673|BOLD:ABA6518
Cecidomyiidae|[4252]|CNPPI237-12|Cecidomyiidae|664|BOLD:ACC8159
Cecidomyiidae|[4253]|JSAUG1050-11|Cecidomyiidae|658|BOLD:ABA0823
Cecidomyiidae|[4254]|CNPPE789-12|Cecidomyiidae|664|BOLD:ACC7815
Cecidomyiidae|[4255]|CNROO561-13|Cecidomyiidae|658|BOLD:ACL0177
Cecidomyiidae|[4256]|CNROM167-13|Cecidomyiidae|618|BOLD:ACK7191
Cecidomyiidae|[4257]|CNFNG692-14|Cecidomyiidae|660|BOLD:ACL6055
Cecidomyiidae|[4258]|CNPPE655-12|Cecidomyiidae|664|BOLD:ABV1382
Cecidomyiidae|[4259]|JSAUG814-11|Cecidomyiidae|658|BOLD:ABV1359
Cecidomyiidae|[4260]|JSJUN1115-11|Cecidomyiidae|658|BOLD:ABV9279
Cecidomyiidae|[4261]|JSJUN1843-11|Cecidomyiidae|658|BOLD:ABW1333
Cecidomyiidae|[4262]|CNBPE195-12|Cecidomyiidae|661|BOLD:ACF4043
Cecidomyiidae|[4263]|JSSEP961-11|Cecidomyiidae|658|BOLD:ABV1177
Cecidomyiidae|[4264]|CNFDE148-14|Cecidomyiidae|657|BOLD:ACL7866
Cecidomyiidae|[4265]|CNKJE510-14|Cecidomyiidae|630|BOLD:ACM2541
Cecidomyiidae|[4266]|CNPPJ771-12|Cecidomyiidae|670|BOLD:ACA6596
Cecidomyiidae|[4267]|CNKJE199-14|Cecidomyiidae|658|BOLD:ACI2772
Cecidomyiidae|[4268]|HPPPE1160-13|Cecidomyiidae|664|BOLD:ACI3388
Cecidomyiidae|[4269]|SSGBB3644-14|Cecidomyiidae|667|BOLD:ACM4647
Cecidomyiidae|[4270]|JSAUG1149-11|Cecidomyiidae|658|BOLD:ABV1344
Cecidomyiidae|[4271]|CNKJN1262-14|Cecidomyiidae|588|BOLD:ACM5984
Cecidomyiidae|[4272]|CNSID4186-15|Cecidomyiidae|591|BOLD:ACT4725
Cecidomyiidae|[4273]|JSDIR498-11|Cecidomyiidae|658|BOLD:ABA0841
Cecidomyiidae|[4274]|CHDIP972-12|Cecidomyiidae|658|BOLD:ABX9218
Cecidomyiidae|[4275]|PHAUG558-11|Cecidomyiidae|658|BOLD:AAG3624
Cecidomyiidae|[4276]|PAAUG1974-12|Cecidomyiidae|670|BOLD:ACB4105
Cecidomyiidae|[4277]|CNROH104-13|Cecidomyiidae|658|BOLD:ACK2563
Cecidomyiidae|[4278]|PHAUG268-11|Cecidomyiidae|658|BOLD:ABW7901
Cecidomyiidae|[4279]|CNROR192-13|Cecidomyiidae|591|BOLD:ACL2141
Cecidomyiidae|[4280]|PHJUN1673-11|Cecidomyiidae|658|BOLD:AAQ2552
Cecidomyiidae|[4281]|CNPPE1849-12|Cecidomyiidae|654|BOLD:ACL8916
Cecidomyiidae|[4282]|HEJUL1070-12|Cecidomyiidae|703|BOLD:AAZ0285
Cecidomyiidae|[4283]|HPPPP045-13|Cecidomyiidae|661|BOLD:ACJ8351
Cecidomyiidae|[4284]|CNPEP638-14|Cecidomyiidae|660|BOLD:ACL9649
Cecidomyiidae|[4285]|BBDCQ599-10|Cecidomyiidae|658|BOLD:AAH3752
Cecidomyiidae|[4286]|PAJUL679-12|Cecidomyiidae|658|BOLD:ACG8864
Cecidomyiidae|[4287]|CNSLN261-13|Cecidomyiidae|661|BOLD:ACA7657
Cecidomyiidae|[4288]|PHJUN1935-11|Cecidomyiidae|658|BOLD:AAQ2553
Cecidomyiidae|[4289]|CNROE827-13|Cecidomyiidae|603|BOLD:ACK1302
Cecidomyiidae|[4290]|CNFNG328-14|Cecidomyiidae|660|BOLD:ACL5924
Cecidomyiidae|[4291]|HPPPH761-13|Cecidomyiidae|661|BOLD:ACI7880
Cecidomyiidae|[4292]|CNBRP1614-14|Cecidomyiidae|658|BOLD:ACL9868
Cecidomyiidae|[4293]|JSDIR472-11|Cecidomyiidae|658|BOLD:ABA0836
Cecidomyiidae|[4294]|BBDCP112-10|Cecidomyiidae|658|BOLD:AAV5667
Cecidomyiidae|[4295]|CNPPD2082-12|Cecidomyiidae|613|BOLD:ACC8367
Cecidomyiidae|[4296]|CNPPC1647-12|Cecidomyiidae|658|BOLD:ACA6936
Cecidomyiidae|[4297]|CNPPF628-12|Cecidomyiidae|664|BOLD:ACA6808
Cecidomyiidae|[4298]|CNGAC268-15|Cecidomyiidae|591|BOLD:ACQ9003
Cecidomyiidae|[4299]|JSDIS335-11|Cecidomyiidae|658|BOLD:ABA7872

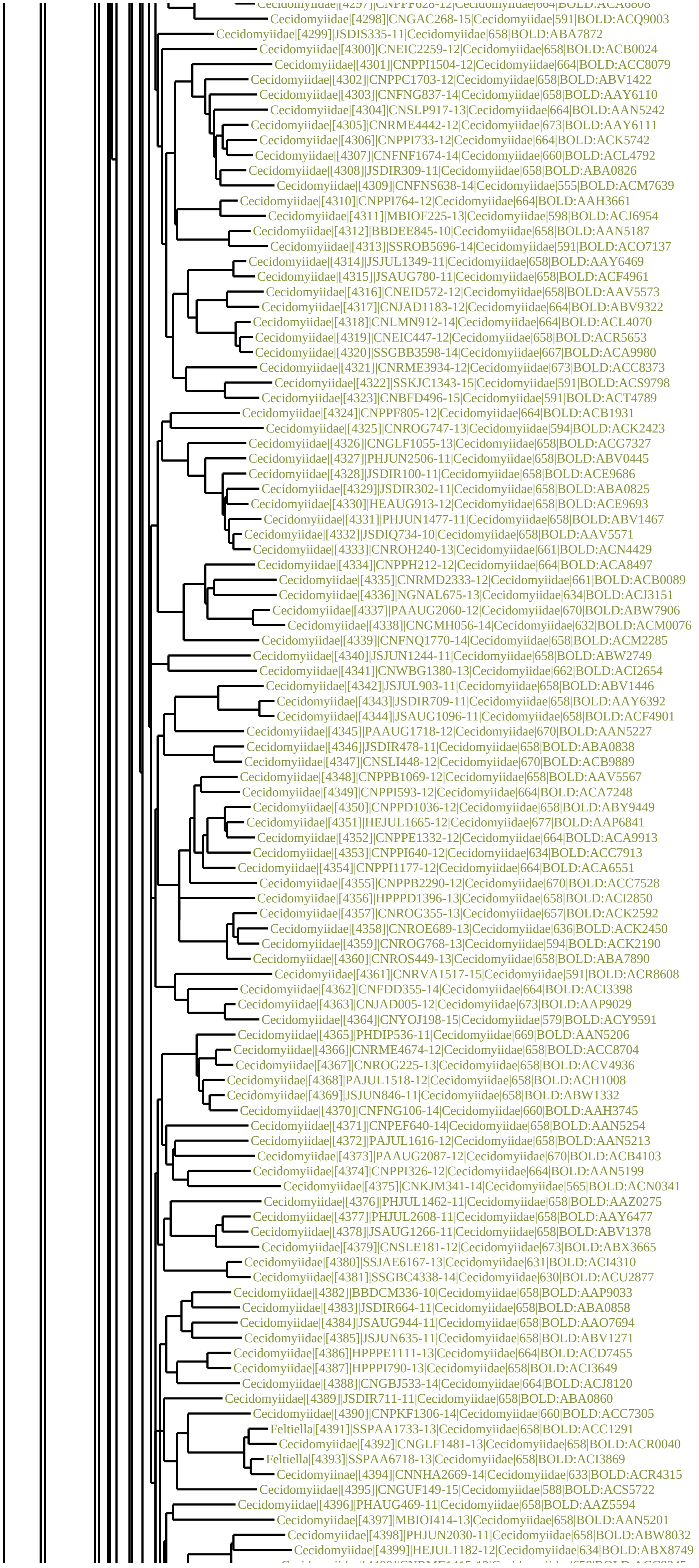
Cecidomyiidae|[4297]|CNPPF628-12|Cecidomyiidae|664|BOLD:ACA6808
Cecidomyiidae|[4298]|CNGAC268-15|Cecidomyiidae|591|BOLD:ACQ9003
Cecidomyiidae|[4299]|JSDIS335-11|Cecidomyiidae|658|BOLD:ABA7872
Cecidomyiidae|[4300]|CNEIC2259-12|Cecidomyiidae|658|BOLD:ACB0024
Cecidomyiidae|[4301]|CNPPI1504-12|Cecidomyiidae|664|BOLD:ACC8079
Cecidomyiidae|[4302]|CNPPC1703-12|Cecidomyiidae|658|BOLD:ABV1422
Cecidomyiidae|[4303]|CNFNG837-14|Cecidomyiidae|658|BOLD:AAY6110
Cecidomyiidae|[4304]|CNSLP917-13|Cecidomyiidae|664|BOLD:AAN5242
Cecidomyiidae|[4305]|CNRME4442-12|Cecidomyiidae|673|BOLD:AAY6111
Cecidomyiidae|[4306]|CNPPI733-12|Cecidomyiidae|664|BOLD:ACK5742
Cecidomyiidae|[4307]|CNFNF1674-14|Cecidomyiidae|660|BOLD:ACL4792
Cecidomyiidae|[4308]|JSDIR309-11|Cecidomyiidae|658|BOLD:ABA0826
Cecidomyiidae|[4309]|CNFNS638-14|Cecidomyiidae|555|BOLD:ACM7639
Cecidomyiidae|[4310]|CNPPI764-12|Cecidomyiidae|664|BOLD:AAH3661
Cecidomyiidae|[4311]|MBIOF225-13|Cecidomyiidae|598|BOLD:ACJ6954
Cecidomyiidae|[4312]|BBDEE845-10|Cecidomyiidae|658|BOLD:AAN5187
Cecidomyiidae|[4313]|SSROB5696-14|Cecidomyiidae|591|BOLD:ACO7137
Cecidomyiidae|[4314]|JSJUL1349-11|Cecidomyiidae|658|BOLD:AAY6469
Cecidomyiidae|[4315]|JSAUG780-11|Cecidomyiidae|658|BOLD:ACF4961
Cecidomyiidae|[4316]|CNEID572-12|Cecidomyiidae|658|BOLD:AAV5573
Cecidomyiidae|[4317]|CNJAD1183-12|Cecidomyiidae|664|BOLD:ABV9322
Cecidomyiidae|[4318]|CNLMN912-14|Cecidomyiidae|664|BOLD:ACL4070
Cecidomyiidae|[4319]|CNEIC447-12|Cecidomyiidae|658|BOLD:ACR5653
Cecidomyiidae|[4320]|SSGBB3598-14|Cecidomyiidae|667|BOLD:ACA9980
Cecidomyiidae|[4321]|CNRME3934-12|Cecidomyiidae|673|BOLD:ACC8373
Cecidomyiidae|[4322]|SSKJC1343-15|Cecidomyiidae|591|BOLD:ACS9798
Cecidomyiidae|[4323]|CNBFD496-15|Cecidomyiidae|591|BOLD:ACT4789
Cecidomyiidae|[4324]|CNPPF805-12|Cecidomyiidae|664|BOLD:ACB1931
Cecidomyiidae|[4325]|CNROG747-13|Cecidomyiidae|594|BOLD:ACK2423
Cecidomyiidae|[4326]|CNGLF1055-13|Cecidomyiidae|658|BOLD:ACG7327
Cecidomyiidae|[4327]|PHJUN2506-11|Cecidomyiidae|658|BOLD:ABV0445
Cecidomyiidae|[4328]|JSDIR100-11|Cecidomyiidae|658|BOLD:ACE9686
Cecidomyiidae|[4329]|JSDIR302-11|Cecidomyiidae|658|BOLD:ABA0825
Cecidomyiidae|[4330]|HEAUG913-12|Cecidomyiidae|658|BOLD:ACE9693
Cecidomyiidae|[4331]|PHJUN1477-11|Cecidomyiidae|658|BOLD:ABV1467
Cecidomyiidae|[4332]|JSDIQ734-10|Cecidomyiidae|658|BOLD:AAV5571
Cecidomyiidae|[4333]|CNROH240-13|Cecidomyiidae|661|BOLD:ACN4429
Cecidomyiidae|[4334]|CNPPH212-12|Cecidomyiidae|664|BOLD:ACA8497
Cecidomyiidae|[4335]|CNRMD2333-12|Cecidomyiidae|661|BOLD:ACB0089
Cecidomyiidae|[4336]|NGNAL675-13|Cecidomyiidae|634|BOLD:ACJ3151
Cecidomyiidae|[4337]|PAAUG2060-12|Cecidomyiidae|670|BOLD:ABW7906
Cecidomyiidae|[4338]|CNGMH056-14|Cecidomyiidae|632|BOLD:ACM0076
Cecidomyiidae|[4339]|CNFNQ1770-14|Cecidomyiidae|658|BOLD:ACM2285
Cecidomyiidae|[4340]|JSJUN1244-11|Cecidomyiidae|658|BOLD:ABW2749
Cecidomyiidae|[4341]|CNWBG1380-13|Cecidomyiidae|662|BOLD:ACI2654
Cecidomyiidae|[4342]|JSJUL903-11|Cecidomyiidae|658|BOLD:ABV1446
Cecidomyiidae|[4343]|JSDIR709-11|Cecidomyiidae|658|BOLD:AAY6392
Cecidomyiidae|[4344]|JSAUG1096-11|Cecidomyiidae|658|BOLD:ACF4901
Cecidomyiidae|[4345]|PAAUG1718-12|Cecidomyiidae|670|BOLD:AAN5227
Cecidomyiidae|[4346]|JSDIR478-11|Cecidomyiidae|658|BOLD:ABA0838
Cecidomyiidae|[4347]|CNSLI448-12|Cecidomyiidae|670|BOLD:ACB9889
Cecidomyiidae|[4348]|CNPPB1069-12|Cecidomyiidae|658|BOLD:AAV5567
Cecidomyiidae|[4349]|CNPPI593-12|Cecidomyiidae|664|BOLD:ACA7248
Cecidomyiidae|[4350]|CNPPD1036-12|Cecidomyiidae|658|BOLD:ABY9449
Cecidomyiidae|[4351]|HEJUL1665-12|Cecidomyiidae|677|BOLD:AAP6841
Cecidomyiidae|[4352]|CNPPE1332-12|Cecidomyiidae|664|BOLD:ACA9913
Cecidomyiidae|[4353]|CNPPI640-12|Cecidomyiidae|634|BOLD:ACC7913
Cecidomyiidae|[4354]|CNPPI1177-12|Cecidomyiidae|664|BOLD:ACA6551
Cecidomyiidae|[4355]|CNPPB2290-12|Cecidomyiidae|670|BOLD:ACC7528
Cecidomyiidae|[4356]|HPPPD1396-13|Cecidomyiidae|658|BOLD:ACI2850
Cecidomyiidae|[4357]|CNROG355-13|Cecidomyiidae|657|BOLD:ACK2592
Cecidomyiidae|[4358]|CNROE689-13|Cecidomyiidae|636|BOLD:ACK2450
Cecidomyiidae|[4359]|CNROG768-13|Cecidomyiidae|594|BOLD:ACK2190
Cecidomyiidae|[4360]|CNROS449-13|Cecidomyiidae|658|BOLD:ABA7890
Cecidomyiidae|[4361]|CNRVA1517-15|Cecidomyiidae|591|BOLD:ACR8608
Cecidomyiidae|[4362]|CNFDD355-14|Cecidomyiidae|664|BOLD:ACI3398
Cecidomyiidae|[4363]|CNJAD005-12|Cecidomyiidae|673|BOLD:AAP9029
Cecidomyiidae|[4364]|CNYOJ198-15|Cecidomyiidae|579|BOLD:ACY9591
Cecidomyiidae|[4365]|PHDIP536-11|Cecidomyiidae|669|BOLD:AAN5206
Cecidomyiidae|[4366]|CNRME4674-12|Cecidomyiidae|658|BOLD:ACC8704
Cecidomyiidae|[4367]|CNROG225-13|Cecidomyiidae|658|BOLD:ACV4936
Cecidomyiidae|[4368]|PAJUL1518-12|Cecidomyiidae|658|BOLD:ACH1008
Cecidomyiidae|[4369]|JSJUN846-11|Cecidomyiidae|658|BOLD:ABW1332
Cecidomyiidae|[4370]|CNFNG106-14|Cecidomyiidae|660|BOLD:AAH3745
Cecidomyiidae|[4371]|CNPEF640-14|Cecidomyiidae|658|BOLD:AAN5254
Cecidomyiidae|[4372]|PAJUL1616-12|Cecidomyiidae|658|BOLD:AAN5213
Cecidomyiidae|[4373]|PAAUG2087-12|Cecidomyiidae|670|BOLD:ACB4103
Cecidomyiidae|[4374]|CNPPI326-12|Cecidomyiidae|664|BOLD:AAN5199
Cecidomyiidae|[4375]|CNKJM341-14|Cecidomyiidae|565|BOLD:ACN0341
Cecidomyiidae|[4376]|PHJUL1462-11|Cecidomyiidae|658|BOLD:AAZ0275
Cecidomyiidae|[4377]|PHJUL2608-11|Cecidomyiidae|658|BOLD:AAY6477
Cecidomyiidae|[4378]|JSAUG1266-11|Cecidomyiidae|658|BOLD:ABV1378
Cecidomyiidae|[4379]|CNSLE181-12|Cecidomyiidae|673|BOLD:ABX3665
Cecidomyiidae|[4380]|SSJAE6167-13|Cecidomyiidae|631|BOLD:ACI4310
Cecidomyiidae|[4381]|SSGBC4338-14|Cecidomyiidae|630|BOLD:ACU2877
Cecidomyiidae|[4382]|BBDCM336-10|Cecidomyiidae|658|BOLD:AAP9033
Cecidomyiidae|[4383]|JSDIR664-11|Cecidomyiidae|658|BOLD:ABA0858
Cecidomyiidae|[4384]|JSAUG944-11|Cecidomyiidae|658|BOLD:AAO7694
Cecidomyiidae|[4385]|JSJUN635-11|Cecidomyiidae|658|BOLD:ABV1271
Cecidomyiidae|[4386]|HPPPE1111-13|Cecidomyiidae|664|BOLD:ACD7455
Cecidomyiidae|[4387]|HPPPI790-13|Cecidomyiidae|658|BOLD:ACI3649
Cecidomyiidae|[4388]|CNGBJ533-14|Cecidomyiidae|664|BOLD:ACJ8120
Cecidomyiidae|[4389]|JSDIR711-11|Cecidomyiidae|658|BOLD:ABA0860
Cecidomyiidae|[4390]|CNPKF1306-14|Cecidomyiidae|660|BOLD:ACC7305
Feltiella|[4391]|SSPAA1733-13|Cecidomyiidae|658|BOLD:ACC1291
Cecidomyiidae|[4392]|CNGLF1481-13|Cecidomyiidae|658|BOLD:ACR0040
Feltiella|[4393]|SSPAA6718-13|Cecidomyiidae|658|BOLD:ACI3869
Cecidomyiinae|[4394]|CNNHA2669-14|Cecidomyiidae|633|BOLD:ACR4315
Cecidomyiidae|[4395]|CNGUF149-15|Cecidomyiidae|588|BOLD:ACS5722
Cecidomyiidae|[4396]|PHAUG469-11|Cecidomyiidae|658|BOLD:AAZ5594
Cecidomyiidae|[4397]|MBIOI414-13|Cecidomyiidae|658|BOLD:AAN5201
Cecidomyiidae|[4398]|PHJUN2030-11|Cecidomyiidae|658|BOLD:ABW8032
Cecidomyiidae|[4399]|HEJUL1182-12|Cecidomyiidae|634|BOLD:ABX8749

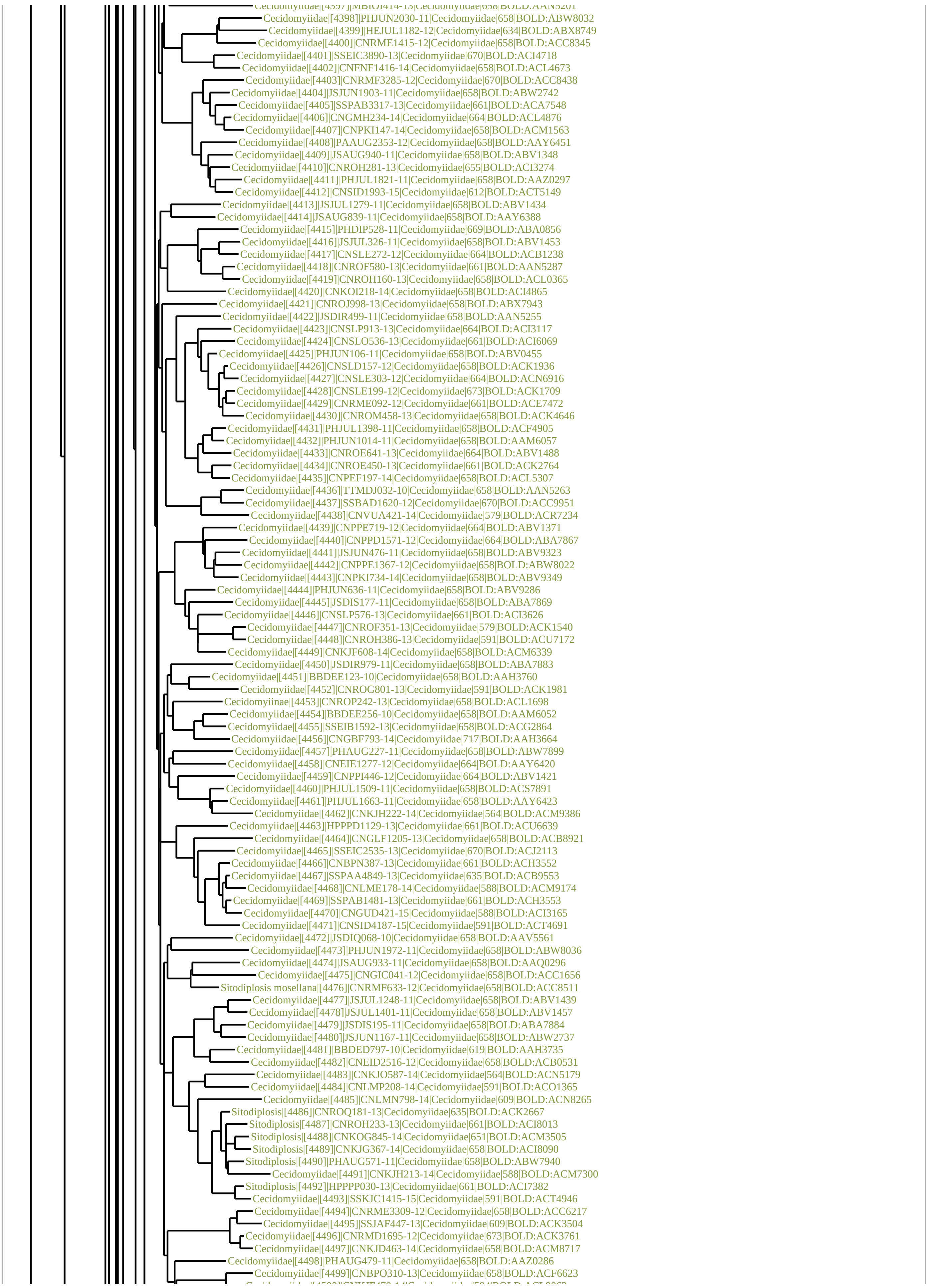
Cecidomyiidae|[4398]|PHJUN2030-11|Cecidomyiidae|658|BOLD:ABW8032
Cecidomyiidae|[4399]|HEJUL1182-12|Cecidomyiidae|634|BOLD:ABX8749
Cecidomyiidae|[4400]|CNRME1415-12|Cecidomyiidae|658|BOLD:ACC8345
Cecidomyiidae|[4401]|SSEIC3890-13|Cecidomyiidae|670|BOLD:ACI4718
Cecidomyiidae|[4402]|CNFNF1416-14|Cecidomyiidae|658|BOLD:ACL4673
Cecidomyiidae|[4403]|CNRMF3285-12|Cecidomyiidae|670|BOLD:ACC8438
Cecidomyiidae|[4404]|JSJUN1903-11|Cecidomyiidae|658|BOLD:ABW2742
Cecidomyiidae|[4405]|SSPAB3317-13|Cecidomyiidae|661|BOLD:ACA7548
Cecidomyiidae|[4406]|CNGMH234-14|Cecidomyiidae|664|BOLD:ACL4876
Cecidomyiidae|[4407]|CNPKI147-14|Cecidomyiidae|658|BOLD:ACM1563
Cecidomyiidae|[4408]|PAAUG2353-12|Cecidomyiidae|658|BOLD:AAY6451
Cecidomyiidae|[4409]|JSAUG940-11|Cecidomyiidae|658|BOLD:ABV1348
Cecidomyiidae|[4410]|CNROH281-13|Cecidomyiidae|655|BOLD:ACI3274
Cecidomyiidae|[4411]|PHJUL1821-11|Cecidomyiidae|658|BOLD:AAZ0297
Cecidomyiidae|[4412]|CNSID1993-15|Cecidomyiidae|612|BOLD:ACT5149
Cecidomyiidae|[4413]|JSJUL1279-11|Cecidomyiidae|658|BOLD:ABV1434
Cecidomyiidae|[4414]|JSAUG839-11|Cecidomyiidae|658|BOLD:AAY6388
Cecidomyiidae|[4415]|PHDIP528-11|Cecidomyiidae|669|BOLD:ABA0856
Cecidomyiidae|[4416]|JSJUL326-11|Cecidomyiidae|658|BOLD:ABV1453
Cecidomyiidae|[4417]|CNSLE272-12|Cecidomyiidae|664|BOLD:ACB1238
Cecidomyiidae|[4418]|CNROF580-13|Cecidomyiidae|661|BOLD:AAN5287
Cecidomyiidae|[4419]|CNROH160-13|Cecidomyiidae|658|BOLD:ACL0365
Cecidomyiidae|[4420]|CNKOI218-14|Cecidomyiidae|658|BOLD:ACI4865
Cecidomyiidae|[4421]|CNROJ998-13|Cecidomyiidae|658|BOLD:ABX7943
Cecidomyiidae|[4422]|JSDIR499-11|Cecidomyiidae|658|BOLD:AAN5255
Cecidomyiidae|[4423]|CNSLP913-13|Cecidomyiidae|664|BOLD:ACI3117
Cecidomyiidae|[4424]|CNSLO536-13|Cecidomyiidae|661|BOLD:ACI6069
Cecidomyiidae|[4425]|PHJUN106-11|Cecidomyiidae|658|BOLD:ABV0455
Cecidomyiidae|[4426]|CNSLD157-12|Cecidomyiidae|658|BOLD:ACK1936
Cecidomyiidae|[4427]|CNSLE303-12|Cecidomyiidae|664|BOLD:ACN6916
Cecidomyiidae|[4428]|CNSLE199-12|Cecidomyiidae|673|BOLD:ACK1709
Cecidomyiidae|[4429]|CNRME092-12|Cecidomyiidae|661|BOLD:ACE7472
Cecidomyiidae|[4430]|CNROM458-13|Cecidomyiidae|658|BOLD:ACK4646
Cecidomyiidae|[4431]|PHJUL1398-11|Cecidomyiidae|658|BOLD:ACF4905
Cecidomyiidae|[4432]|PHJUN1014-11|Cecidomyiidae|658|BOLD:AAM6057
Cecidomyiidae|[4433]|CNROE641-13|Cecidomyiidae|664|BOLD:ABV1488
Cecidomyiidae|[4434]|CNROE450-13|Cecidomyiidae|661|BOLD:ACK2764
Cecidomyiidae|[4435]|CNPEF197-14|Cecidomyiidae|658|BOLD:ACL5307
Cecidomyiidae|[4436]|TTMDJ032-10|Cecidomyiidae|658|BOLD:AAN5263
Cecidomyiidae|[4437]|SSBAD1620-12|Cecidomyiidae|670|BOLD:ACC9951
Cecidomyiidae|[4438]|CNVUA421-14|Cecidomyiidae|579|BOLD:ACR7234
Cecidomyiidae|[4439]|CNPPE719-12|Cecidomyiidae|664|BOLD:ABV1371
Cecidomyiidae|[4440]|CNPPD1571-12|Cecidomyiidae|664|BOLD:ABA7867
Cecidomyiidae|[4441]|JSJUN476-11|Cecidomyiidae|658|BOLD:ABV9323
Cecidomyiidae|[4442]|CNPPE1367-12|Cecidomyiidae|658|BOLD:ABW8022
Cecidomyiidae|[4443]|CNPKI734-14|Cecidomyiidae|658|BOLD:ABV9349
Cecidomyiidae|[4444]|PHJUN636-11|Cecidomyiidae|658|BOLD:ABV9286
Cecidomyiidae|[4445]|JSDIS177-11|Cecidomyiidae|658|BOLD:ABA7869
Cecidomyiidae|[4446]|CNSLP576-13|Cecidomyiidae|661|BOLD:ACI3626
Cecidomyiidae|[4447]|CNROF351-13|Cecidomyiidae|579|BOLD:ACK1540
Cecidomyiidae|[4448]|CNROH386-13|Cecidomyiidae|591|BOLD:ACU7172
Cecidomyiidae|[4449]|CNKJF608-14|Cecidomyiidae|658|BOLD:ACM6339
Cecidomyiidae|[4450]|JSDIR979-11|Cecidomyiidae|658|BOLD:ABA7883
Cecidomyiidae|[4451]|BBDEE123-10|Cecidomyiidae|658|BOLD:AAH3760
Cecidomyiidae|[4452]|CNROG801-13|Cecidomyiidae|591|BOLD:ACK1981
Cecidomyiinae|[4453]|CNROP242-13|Cecidomyiidae|658|BOLD:ACL1698
Cecidomyiidae|[4454]|BBDEE256-10|Cecidomyiidae|658|BOLD:AAM6052
Cecidomyiidae|[4455]|SSEIB1592-13|Cecidomyiidae|658|BOLD:ACG2864
Cecidomyiidae|[4456]|CNGBF793-14|Cecidomyiidae|717|BOLD:AAH3664
Cecidomyiidae|[4457]|PHAUG227-11|Cecidomyiidae|658|BOLD:ABW7899
Cecidomyiidae|[4458]|CNEIE1277-12|Cecidomyiidae|664|BOLD:AAY6420
Cecidomyiidae|[4459]|CNPPI446-12|Cecidomyiidae|664|BOLD:ABV1421
Cecidomyiidae|[4460]|PHJUL1509-11|Cecidomyiidae|658|BOLD:ACS7891
Cecidomyiidae|[4461]|PHJUL1663-11|Cecidomyiidae|658|BOLD:AAY6423
Cecidomyiidae|[4462]|CNKJH222-14|Cecidomyiidae|564|BOLD:ACM9386
Cecidomyiidae|[4463]|HPPPD1129-13|Cecidomyiidae|661|BOLD:ACU6639
Cecidomyiidae|[4464]|CNGLF1205-13|Cecidomyiidae|658|BOLD:ACB8921
Cecidomyiidae|[4465]|SSEIC2535-13|Cecidomyiidae|670|BOLD:ACJ2113
Cecidomyiidae|[4466]|CNBPN387-13|Cecidomyiidae|661|BOLD:ACH3552
Cecidomyiidae|[4467]|SSPAA4849-13|Cecidomyiidae|635|BOLD:ACB9553
Cecidomyiidae|[4468]|CNLME178-14|Cecidomyiidae|588|BOLD:ACM9174
Cecidomyiidae|[4469]|SSPAB1481-13|Cecidomyiidae|661|BOLD:ACH3553
Cecidomyiidae|[4470]|CNGUD421-15|Cecidomyiidae|588|BOLD:ACI3165
Cecidomyiidae|[4471]|CNSID4187-15|Cecidomyiidae|591|BOLD:ACT4691
Cecidomyiidae|[4472]|JSDIQ068-10|Cecidomyiidae|658|BOLD:AAV5561
Cecidomyiidae|[4473]|PHJUN1972-11|Cecidomyiidae|658|BOLD:ABW8036
Cecidomyiidae|[4474]|JSAUG933-11|Cecidomyiidae|658|BOLD:AAQ0296
Cecidomyiidae|[4475]|CNGIC041-12|Cecidomyiidae|658|BOLD:ACC1656
Sitodiplosis mosellana|[4476]|CNRMF633-12|Cecidomyiidae|658|BOLD:ACC8511
Cecidomyiidae|[4477]|JSJUL1248-11|Cecidomyiidae|658|BOLD:ABV1439
Cecidomyiidae|[4478]|JSJUL1401-11|Cecidomyiidae|658|BOLD:ABV1457
Cecidomyiidae|[4479]|JSDIS195-11|Cecidomyiidae|658|BOLD:ABA7884
Cecidomyiidae|[4480]|JSJUN1167-11|Cecidomyiidae|658|BOLD:ABW2737
Cecidomyiidae|[4481]|BBDED797-10|Cecidomyiidae|619|BOLD:AAH3735
Cecidomyiidae|[4482]|CNEID2516-12|Cecidomyiidae|658|BOLD:ACB0531
Cecidomyiidae|[4483]|CNKJO587-14|Cecidomyiidae|564|BOLD:ACN5179
Cecidomyiidae|[4484]|CNLMP208-14|Cecidomyiidae|591|BOLD:ACO1365
Cecidomyiidae|[4485]|CNLMN798-14|Cecidomyiidae|609|BOLD:ACN8265
Sitodiplosis|[4486]|CNROQ181-13|Cecidomyiidae|635|BOLD:ACK2667
Sitodiplosis|[4487]|CNROH233-13|Cecidomyiidae|661|BOLD:ACI8013
Sitodiplosis|[4488]|CNKOG845-14|Cecidomyiidae|651|BOLD:ACM3505
Sitodiplosis|[4489]|CNKJG367-14|Cecidomyiidae|658|BOLD:ACI8090
Sitodiplosis|[4490]|PHAUG571-11|Cecidomyiidae|658|BOLD:ABW7940
Cecidomyiidae|[4491]|CNKJH213-14|Cecidomyiidae|588|BOLD:ACM7300
Sitodiplosis|[4492]|HPPPP030-13|Cecidomyiidae|661|BOLD:ACI7382
Cecidomyiidae|[4493]|SSKJC1415-15|Cecidomyiidae|591|BOLD:ACT4946
Cecidomyiidae|[4494]|CNRME3309-12|Cecidomyiidae|658|BOLD:ACC6217
Cecidomyiidae|[4495]|SSJAF447-13|Cecidomyiidae|609|BOLD:ACK3504
Cecidomyiidae|[4496]|CNRMD1695-12|Cecidomyiidae|673|BOLD:ACK3761
Cecidomyiidae|[4497]|CNKJD463-14|Cecidomyiidae|658|BOLD:ACM8717
Cecidomyiidae|[4498]|PHAUG479-11|Cecidomyiidae|658|BOLD:AAZ0286
Cecidomyiidae|[4499]|CNBPO310-13|Cecidomyiidae|658|BOLD:ACF6623

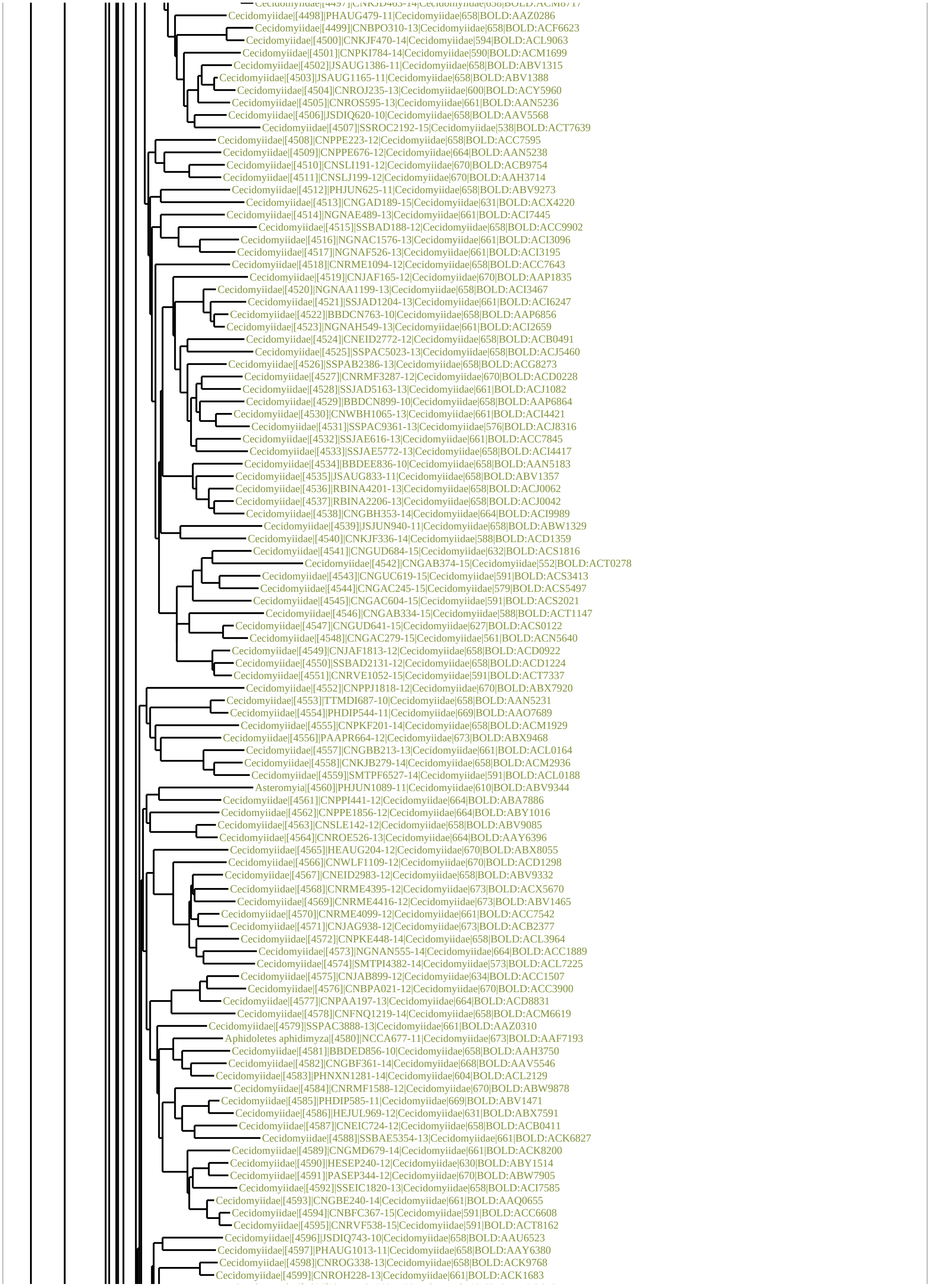

Cecidomyiidae|[4497]|CNKJD463-14|Cecidomyiidae|658|BOLD:ACM8717
Cecidomyiidae|[4498]|PHAUG479-11|Cecidomyiidae|658|BOLD:AAZ0286
Cecidomyiidae|[4499]|CNBPO310-13|Cecidomyiidae|658|BOLD:ACF6623
Cecidomyiidae|[4500]|CNKJF470-14|Cecidomyiidae|594|BOLD:ACL9063
Cecidomyiidae|[4501]|CNPKI784-14|Cecidomyiidae|590|BOLD:ACM1699
Cecidomyiidae|[4502]|JSAUG1386-11|Cecidomyiidae|658|BOLD:ABV1315
Cecidomyiidae|[4503]|JSAUG1165-11|Cecidomyiidae|658|BOLD:ABV1388
Cecidomyiidae|[4504]|CNROJ235-13|Cecidomyiidae|600|BOLD:ACY5960
Cecidomyiidae|[4505]|CNROS595-13|Cecidomyiidae|661|BOLD:AAN5236
Cecidomyiidae|[4506]|JSDIQ620-10|Cecidomyiidae|658|BOLD:AAV5568
Cecidomyiidae|[4507]|SSROC2192-15|Cecidomyiidae|538|BOLD:ACT7639
Cecidomyiidae|[4508]|CNPPE223-12|Cecidomyiidae|658|BOLD:ACC7595
Cecidomyiidae|[4509]|CNPPE676-12|Cecidomyiidae|664|BOLD:AAN5238
Cecidomyiidae|[4510]|CNSLI191-12|Cecidomyiidae|670|BOLD:ACB9754
Cecidomyiidae|[4511]|CNSLJ199-12|Cecidomyiidae|670|BOLD:AAH3714
Cecidomyiidae|[4512]|PHJUN625-11|Cecidomyiidae|658|BOLD:ABV9273
Cecidomyiidae|[4513]|CNGAD189-15|Cecidomyiidae|631|BOLD:ACX4220
Cecidomyiidae|[4514]|NGNAE489-13|Cecidomyiidae|661|BOLD:ACI7445
Cecidomyiidae|[4515]|SSBAD188-12|Cecidomyiidae|658|BOLD:ACC9902
Cecidomyiidae|[4516]|NGNAC1576-13|Cecidomyiidae|661|BOLD:ACI3096
Cecidomyiidae|[4517]|NGNAF526-13|Cecidomyiidae|661|BOLD:ACI3195
Cecidomyiidae|[4518]|CNRME1094-12|Cecidomyiidae|658|BOLD:ACC7643
Cecidomyiidae|[4519]|CNJAF165-12|Cecidomyiidae|670|BOLD:AAP1835
Cecidomyiidae|[4520]|NGNAA1199-13|Cecidomyiidae|658|BOLD:ACI3467
Cecidomyiidae|[4521]|SSJAD1204-13|Cecidomyiidae|661|BOLD:ACI6247
Cecidomyiidae|[4522]|BBDCN763-10|Cecidomyiidae|658|BOLD:AAP6856
Cecidomyiidae|[4523]|NGNAH549-13|Cecidomyiidae|661|BOLD:ACI2659
Cecidomyiidae|[4524]|CNEID2772-12|Cecidomyiidae|658|BOLD:ACB0491
Cecidomyiidae|[4525]|SSPAC5023-13|Cecidomyiidae|658|BOLD:ACJ5460
Cecidomyiidae|[4526]|SSPAB2386-13|Cecidomyiidae|658|BOLD:ACG8273
Cecidomyiidae|[4527]|CNRMF3287-12|Cecidomyiidae|670|BOLD:ACD0228
Cecidomyiidae|[4528]|SSJAD5163-13|Cecidomyiidae|661|BOLD:ACJ1082
Cecidomyiidae|[4529]|BBDCN899-10|Cecidomyiidae|658|BOLD:AAP6864
Cecidomyiidae|[4530]|CNWBH1065-13|Cecidomyiidae|661|BOLD:ACI4421
Cecidomyiidae|[4531]|SSPAC9361-13|Cecidomyiidae|576|BOLD:ACJ8316
Cecidomyiidae|[4532]|SSJAE616-13|Cecidomyiidae|661|BOLD:ACC7845
Cecidomyiidae|[4533]|SSJAE5772-13|Cecidomyiidae|658|BOLD:ACI4417
Cecidomyiidae|[4534]|BBDEE836-10|Cecidomyiidae|658|BOLD:AAN5183
Cecidomyiidae|[4535]|JSAUG833-11|Cecidomyiidae|658|BOLD:ABV1357
Cecidomyiidae|[4536]|RBINA4201-13|Cecidomyiidae|658|BOLD:ACJ0062
Cecidomyiidae|[4537]|RBINA2206-13|Cecidomyiidae|658|BOLD:ACJ0042
Cecidomyiidae|[4538]|CNGBH353-14|Cecidomyiidae|664|BOLD:ACI9989
Cecidomyiidae|[4539]|JSJUN940-11|Cecidomyiidae|658|BOLD:ABW1329
Cecidomyiidae|[4540]|CNKJF336-14|Cecidomyiidae|588|BOLD:ACD1359
Cecidomyiidae|[4541]|CNGUD684-15|Cecidomyiidae|632|BOLD:ACS1816
Cecidomyiidae|[4542]|CNGAB374-15|Cecidomyiidae|552|BOLD:ACT0278
Cecidomyiidae|[4543]|CNGUC619-15|Cecidomyiidae|591|BOLD:ACS3413
Cecidomyiidae|[4544]|CNGAC245-15|Cecidomyiidae|579|BOLD:ACS5497
Cecidomyiidae|[4545]|CNGAC604-15|Cecidomyiidae|591|BOLD:ACS2021
Cecidomyiidae|[4546]|CNGAB334-15|Cecidomyiidae|588|BOLD:ACT1147
Cecidomyiidae|[4547]|CNGUD641-15|Cecidomyiidae|627|BOLD:ACS0122
Cecidomyiidae|[4548]|CNGAC279-15|Cecidomyiidae|561|BOLD:ACN5640
Cecidomyiidae|[4549]|CNJAF1813-12|Cecidomyiidae|658|BOLD:ACD0922
Cecidomyiidae|[4550]|SSBAD2131-12|Cecidomyiidae|658|BOLD:ACD1224
Cecidomyiidae|[4551]|CNRVE1052-15|Cecidomyiidae|591|BOLD:ACT7337
Cecidomyiidae|[4552]|CNPPJ1818-12|Cecidomyiidae|670|BOLD:ABX7920
Cecidomyiidae|[4553]|TTMDI687-10|Cecidomyiidae|658|BOLD:AAN5231
Cecidomyiidae|[4554]|PHDIP544-11|Cecidomyiidae|669|BOLD:AAO7689
Cecidomyiidae|[4555]|CNPKF201-14|Cecidomyiidae|658|BOLD:ACM1929
Cecidomyiidae|[4556]|PAAPR664-12|Cecidomyiidae|673|BOLD:ABX9468
Cecidomyiidae|[4557]|CNGBB213-13|Cecidomyiidae|661|BOLD:ACL0164
Cecidomyiidae|[4558]|CNKJB279-14|Cecidomyiidae|658|BOLD:ACM2936
Cecidomyiidae|[4559]|SMTPF6527-14|Cecidomyiidae|591|BOLD:ACL0188
Asteromyia|[4560]|PHJUN1089-11|Cecidomyiidae|610|BOLD:ABV9344
Cecidomyiidae|[4561]|CNPPI441-12|Cecidomyiidae|664|BOLD:ABA7886
Cecidomyiidae|[4562]|CNPPE1856-12|Cecidomyiidae|664|BOLD:ABY1016
Cecidomyiidae|[4563]|CNSLE142-12|Cecidomyiidae|658|BOLD:ABV9085
Cecidomyiidae|[4564]|CNROE526-13|Cecidomyiidae|664|BOLD:AAY6396
Cecidomyiidae|[4565]|HEAUG204-12|Cecidomyiidae|670|BOLD:ABX8055
Cecidomyiidae|[4566]|CNWLF1109-12|Cecidomyiidae|670|BOLD:ACD1298
Cecidomyiidae|[4567]|CNEID2983-12|Cecidomyiidae|658|BOLD:ABV9332
Cecidomyiidae|[4568]|CNRME4395-12|Cecidomyiidae|673|BOLD:ACX5670
Cecidomyiidae|[4569]|CNRME4416-12|Cecidomyiidae|673|BOLD:ABV1465
Cecidomyiidae|[4570]|CNRME4099-12|Cecidomyiidae|661|BOLD:ACC7542
Cecidomyiidae|[4571]|CNJAG938-12|Cecidomyiidae|673|BOLD:ACB2377
Cecidomyiidae|[4572]|CNPKE448-14|Cecidomyiidae|658|BOLD:ACL3964
Cecidomyiidae|[4573]|NGNAN555-14|Cecidomyiidae|664|BOLD:ACC1889
Cecidomyiidae|[4574]|SMTPI4382-14|Cecidomyiidae|573|BOLD:ACL7225
Cecidomyiidae|[4575]|CNJAB899-12|Cecidomyiidae|634|BOLD:ACC1507
Cecidomyiidae|[4576]|CNBPA021-12|Cecidomyiidae|670|BOLD:ACC3900
Cecidomyiidae|[4577]|CNPAA197-13|Cecidomyiidae|664|BOLD:ACD8831
Cecidomyiidae|[4578]|CNFNQ1219-14|Cecidomyiidae|658|BOLD:ACM6619
Cecidomyiidae|[4579]|SSPAC3888-13|Cecidomyiidae|661|BOLD:AAZ0310
Aphidoletes aphidimyza|[4580]|NCCA677-11|Cecidomyiidae|673|BOLD:AAF7193
Cecidomyiidae|[4581]|BBDED856-10|Cecidomyiidae|658|BOLD:AAH3750
Cecidomyiidae|[4582]|CNGBF361-14|Cecidomyiidae|668|BOLD:AAV5546
Cecidomyiidae|[4583]|PHNXN1281-14|Cecidomyiidae|604|BOLD:ACL2129
Cecidomyiidae|[4584]|CNRMF1588-12|Cecidomyiidae|670|BOLD:ABW9878
Cecidomyiidae|[4585]|PHDIP585-11|Cecidomyiidae|669|BOLD:ABV1471
Cecidomyiidae|[4586]|HEJUL969-12|Cecidomyiidae|631|BOLD:ABX7591
Cecidomyiidae|[4587]|CNEIC724-12|Cecidomyiidae|658|BOLD:ACB0411
Cecidomyiidae|[4588]|SSBAE5354-13|Cecidomyiidae|661|BOLD:ACK6827
Cecidomyiidae|[4589]|CNGMD679-14|Cecidomyiidae|661|BOLD:ACK8200
Cecidomyiidae|[4590]|HESEP240-12|Cecidomyiidae|630|BOLD:ABY1514
Cecidomyiidae|[4591]|PASEP344-12|Cecidomyiidae|670|BOLD:ABW7905
Cecidomyiidae|[4592]|SSEIC1820-13|Cecidomyiidae|658|BOLD:ACI7585
Cecidomyiidae|[4593]|CNGBE240-14|Cecidomyiidae|661|BOLD:AAQ0655
Cecidomyiidae|[4594]|CNBFC367-15|Cecidomyiidae|591|BOLD:ACC6608
Cecidomyiidae|[4595]|CNRVF538-15|Cecidomyiidae|591|BOLD:ACT8162
Cecidomyiidae|[4596]|JSDIQ743-10|Cecidomyiidae|658|BOLD:AAU6523
Cecidomyiidae|[4597]|PHAUG1013-11|Cecidomyiidae|658|BOLD:AAY6380
Cecidomyiidae|[4598]|CNROG338-13|Cecidomyiidae|658|BOLD:ACK9768
Cecidomyiidae|[4599]|CNROH228-13|Cecidomyiidae|661|BOLD:ACK1683

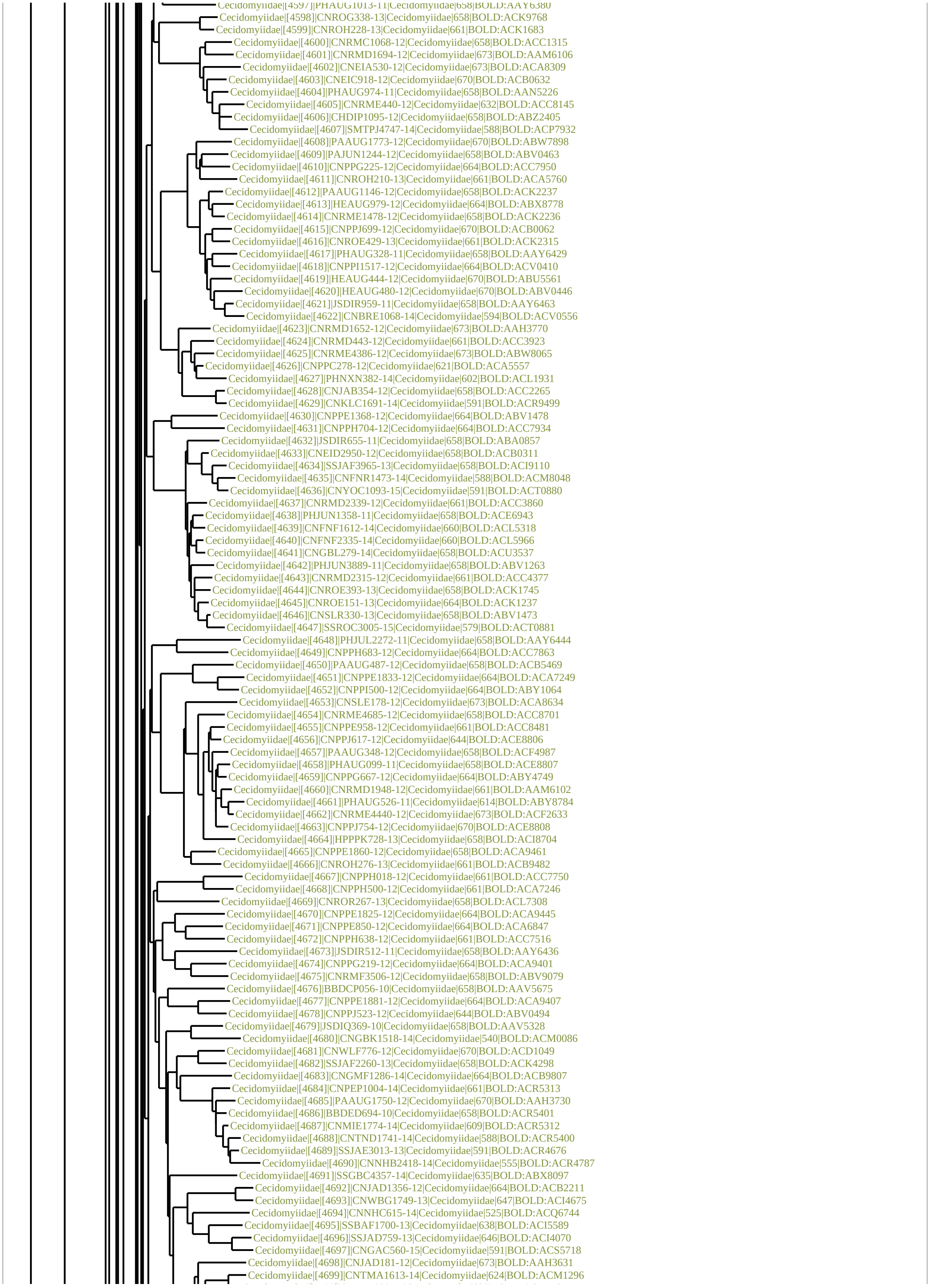

Cecidomyiidae|[4597]|PHAUG1013-11|Cecidomyiidae|658|BOLD:AAY6380
Cecidomyiidae|[4598]|CNROG338-13|Cecidomyiidae|658|BOLD:ACK9768
Cecidomyiidae|[4599]|CNROH228-13|Cecidomyiidae|661|BOLD:ACK1683
Cecidomyiidae|[4600]|CNRMC1068-12|Cecidomyiidae|658|BOLD:ACC1315
Cecidomyiidae|[4601]|CNRMD1694-12|Cecidomyiidae|673|BOLD:AAM6106
Cecidomyiidae|[4602]|CNEIA530-12|Cecidomyiidae|673|BOLD:ACA8309
Cecidomyiidae|[4603]|CNEIC918-12|Cecidomyiidae|670|BOLD:ACB0632
Cecidomyiidae|[4604]|PHAUG974-11|Cecidomyiidae|658|BOLD:AAN5226
Cecidomyiidae|[4605]|CNRME440-12|Cecidomyiidae|632|BOLD:ACC8145
Cecidomyiidae|[4606]|CHDIP1095-12|Cecidomyiidae|658|BOLD:ABZ2405
Cecidomyiidae|[4607]|SMTPJ4747-14|Cecidomyiidae|588|BOLD:ACP7932
Cecidomyiidae|[4608]|PAAUG1773-12|Cecidomyiidae|670|BOLD:ABW7898
Cecidomyiidae|[4609]|PAJUN1244-12|Cecidomyiidae|658|BOLD:ABV0463
Cecidomyiidae|[4610]|CNPPG225-12|Cecidomyiidae|664|BOLD:ACC7950
Cecidomyiidae|[4611]|CNROH210-13|Cecidomyiidae|661|BOLD:ACA5760
Cecidomyiidae|[4612]|PAAUG1146-12|Cecidomyiidae|658|BOLD:ACK2237
Cecidomyiidae|[4613]|HEAUG979-12|Cecidomyiidae|664|BOLD:ABX8778
Cecidomyiidae|[4614]|CNRME1478-12|Cecidomyiidae|658|BOLD:ACK2236
Cecidomyiidae|[4615]|CNPPJ699-12|Cecidomyiidae|670|BOLD:ACB0062
Cecidomyiidae|[4616]|CNROE429-13|Cecidomyiidae|661|BOLD:ACK2315
Cecidomyiidae|[4617]|PHAUG328-11|Cecidomyiidae|658|BOLD:AAY6429
Cecidomyiidae|[4618]|CNPPI1517-12|Cecidomyiidae|664|BOLD:ACV0410
Cecidomyiidae|[4619]|HEAUG444-12|Cecidomyiidae|670|BOLD:ABU5561
Cecidomyiidae|[4620]|HEAUG480-12|Cecidomyiidae|670|BOLD:ABV0446
Cecidomyiidae|[4621]|JSDIR959-11|Cecidomyiidae|658|BOLD:AAY6463
Cecidomyiidae|[4622]|CNBRE1068-14|Cecidomyiidae|594|BOLD:ACV0556
Cecidomyiidae|[4623]|CNRMD1652-12|Cecidomyiidae|673|BOLD:AAH3770
Cecidomyiidae|[4624]|CNRMD443-12|Cecidomyiidae|661|BOLD:ACC3923
Cecidomyiidae|[4625]|CNRME4386-12|Cecidomyiidae|673|BOLD:ABW8065
Cecidomyiidae|[4626]|CNPPC278-12|Cecidomyiidae|621|BOLD:ACA5557
Cecidomyiidae|[4627]|PHNXN382-14|Cecidomyiidae|602|BOLD:ACL1931
Cecidomyiidae|[4628]|CNJAB354-12|Cecidomyiidae|658|BOLD:ACC2265
Cecidomyiidae|[4629]|CNKLC1691-14|Cecidomyiidae|591|BOLD:ACR9499
Cecidomyiidae|[4630]|CNPPE1368-12|Cecidomyiidae|664|BOLD:ABV1478
Cecidomyiidae|[4631]|CNPPH704-12|Cecidomyiidae|664|BOLD:ACC7934
Cecidomyiidae|[4632]|JSDIR655-11|Cecidomyiidae|658|BOLD:ABA0857
Cecidomyiidae|[4633]|CNEID2950-12|Cecidomyiidae|658|BOLD:ACB0311
Cecidomyiidae|[4634]|SSJAF3965-13|Cecidomyiidae|658|BOLD:ACI9110
Cecidomyiidae|[4635]|CNFNR1473-14|Cecidomyiidae|588|BOLD:ACM8048
Cecidomyiidae|[4636]|CNYOC1093-15|Cecidomyiidae|591|BOLD:ACT0880
Cecidomyiidae|[4637]|CNRMD2339-12|Cecidomyiidae|661|BOLD:ACC3860
Cecidomyiidae|[4638]|PHJUN1358-11|Cecidomyiidae|658|BOLD:ACE6943
Cecidomyiidae|[4639]|CNFNF1612-14|Cecidomyiidae|660|BOLD:ACL5318
Cecidomyiidae|[4640]|CNFNF2335-14|Cecidomyiidae|660|BOLD:ACL5966
Cecidomyiidae|[4641]|CNGBL279-14|Cecidomyiidae|658|BOLD:ACU3537
Cecidomyiidae|[4642]|PHJUN3889-11|Cecidomyiidae|658|BOLD:ABV1263
Cecidomyiidae|[4643]|CNRMD2315-12|Cecidomyiidae|661|BOLD:ACC4377
Cecidomyiidae|[4644]|CNROE393-13|Cecidomyiidae|658|BOLD:ACK1745
Cecidomyiidae|[4645]|CNROE151-13|Cecidomyiidae|664|BOLD:ACK1237
Cecidomyiidae|[4646]|CNSLR330-13|Cecidomyiidae|658|BOLD:ABV1473
Cecidomyiidae|[4647]|SSROC3005-15|Cecidomyiidae|579|BOLD:ACT0881
Cecidomyiidae|[4648]|PHJUL2272-11|Cecidomyiidae|658|BOLD:AAY6444
Cecidomyiidae|[4649]|CNPPH683-12|Cecidomyiidae|664|BOLD:ACC7863
Cecidomyiidae|[4650]|PAAUG487-12|Cecidomyiidae|658|BOLD:ACB5469
Cecidomyiidae|[4651]|CNPPE1833-12|Cecidomyiidae|664|BOLD:ACA7249
Cecidomyiidae|[4652]|CNPPI500-12|Cecidomyiidae|664|BOLD:ABY1064
Cecidomyiidae|[4653]|CNSLE178-12|Cecidomyiidae|673|BOLD:ACA8634
Cecidomyiidae|[4654]|CNRME4685-12|Cecidomyiidae|658|BOLD:ACC8701
Cecidomyiidae|[4655]|CNPPE958-12|Cecidomyiidae|661|BOLD:ACC8481
Cecidomyiidae|[4656]|CNPPJ617-12|Cecidomyiidae|644|BOLD:ACE8806
Cecidomyiidae|[4657]|PAAUG348-12|Cecidomyiidae|658|BOLD:ACF4987
Cecidomyiidae|[4658]|PHAUG099-11|Cecidomyiidae|658|BOLD:ACE8807
Cecidomyiidae|[4659]|CNPPG667-12|Cecidomyiidae|664|BOLD:ABY4749
Cecidomyiidae|[4660]|CNRMD1948-12|Cecidomyiidae|661|BOLD:AAM6102
Cecidomyiidae|[4661]|PHAUG526-11|Cecidomyiidae|614|BOLD:ABY8784
Cecidomyiidae|[4662]|CNRME4440-12|Cecidomyiidae|673|BOLD:ACF2633
Cecidomyiidae|[4663]|CNPPJ754-12|Cecidomyiidae|670|BOLD:ACE8808
Cecidomyiidae|[4664]|HPPPK728-13|Cecidomyiidae|658|BOLD:ACI8704
Cecidomyiidae|[4665]|CNPPE1860-12|Cecidomyiidae|658|BOLD:ACA9461
Cecidomyiidae|[4666]|CNROH276-13|Cecidomyiidae|661|BOLD:ACB9482
Cecidomyiidae|[4667]|CNPPH018-12|Cecidomyiidae|661|BOLD:ACC7750
Cecidomyiidae|[4668]|CNPPH500-12|Cecidomyiidae|661|BOLD:ACA7246
Cecidomyiidae|[4669]|CNROR267-13|Cecidomyiidae|658|BOLD:ACL7308
Cecidomyiidae|[4670]|CNPPE1825-12|Cecidomyiidae|664|BOLD:ACA9445
Cecidomyiidae|[4671]|CNPPE850-12|Cecidomyiidae|664|BOLD:ACA6847
Cecidomyiidae|[4672]|CNPPH638-12|Cecidomyiidae|661|BOLD:ACC7516
Cecidomyiidae|[4673]|JSDIR512-11|Cecidomyiidae|658|BOLD:AAY6436
Cecidomyiidae|[4674]|CNPPG219-12|Cecidomyiidae|664|BOLD:ACA9401
Cecidomyiidae|[4675]|CNRMF3506-12|Cecidomyiidae|658|BOLD:ABV9079
Cecidomyiidae|[4676]|BBDCP056-10|Cecidomyiidae|658|BOLD:AAV5675
Cecidomyiidae|[4677]|CNPPE1881-12|Cecidomyiidae|664|BOLD:ACA9407
Cecidomyiidae|[4678]|CNPPJ523-12|Cecidomyiidae|644|BOLD:ABV0494
Cecidomyiidae|[4679]|JSDIQ369-10|Cecidomyiidae|658|BOLD:AAV5328
Cecidomyiidae|[4680]|CNGBK1518-14|Cecidomyiidae|540|BOLD:ACM0086
Cecidomyiidae|[4681]|CNWLF776-12|Cecidomyiidae|670|BOLD:ACD1049
Cecidomyiidae|[4682]|SSJAF2260-13|Cecidomyiidae|658|BOLD:ACK4298
Cecidomyiidae|[4683]|CNGMF1286-14|Cecidomyiidae|664|BOLD:ACB9807
Cecidomyiidae|[4684]|CNPEP1004-14|Cecidomyiidae|661|BOLD:ACR5313
Cecidomyiidae|[4685]|PAAUG1750-12|Cecidomyiidae|670|BOLD:AAH3730
Cecidomyiidae|[4686]|BBDED694-10|Cecidomyiidae|658|BOLD:ACR5401
Cecidomyiidae|[4687]|CNMIE1774-14|Cecidomyiidae|609|BOLD:ACR5312
Cecidomyiidae|[4688]|CNTND1741-14|Cecidomyiidae|588|BOLD:ACR5400
Cecidomyiidae|[4689]|SSJAE3013-13|Cecidomyiidae|591|BOLD:ACR4676
Cecidomyiidae|[4690]|CNNHB2418-14|Cecidomyiidae|555|BOLD:ACR4787
Cecidomyiidae|[4691]|SSGBC4357-14|Cecidomyiidae|635|BOLD:ABX8097
Cecidomyiidae|[4692]|CNJAD1356-12|Cecidomyiidae|664|BOLD:ACB2211
Cecidomyiidae|[4693]|CNWBG1749-13|Cecidomyiidae|647|BOLD:ACI4675
Cecidomyiidae|[4694]|CNNHC615-14|Cecidomyiidae|525|BOLD:ACQ6744
Cecidomyiidae|[4695]|SSBAF1700-13|Cecidomyiidae|638|BOLD:ACI5589
Cecidomyiidae|[4696]|SSJAD759-13|Cecidomyiidae|646|BOLD:ACI4070
Cecidomyiidae|[4697]|CNGAC560-15|Cecidomyiidae|591|BOLD:ACS5718
Cecidomyiidae|[4698]|CNJAD181-12|Cecidomyiidae|673|BOLD:AAH3631
Cecidomyiidae|[4699]|CNTMA1613-14|Cecidomyiidae|624|BOLD:ACM1296

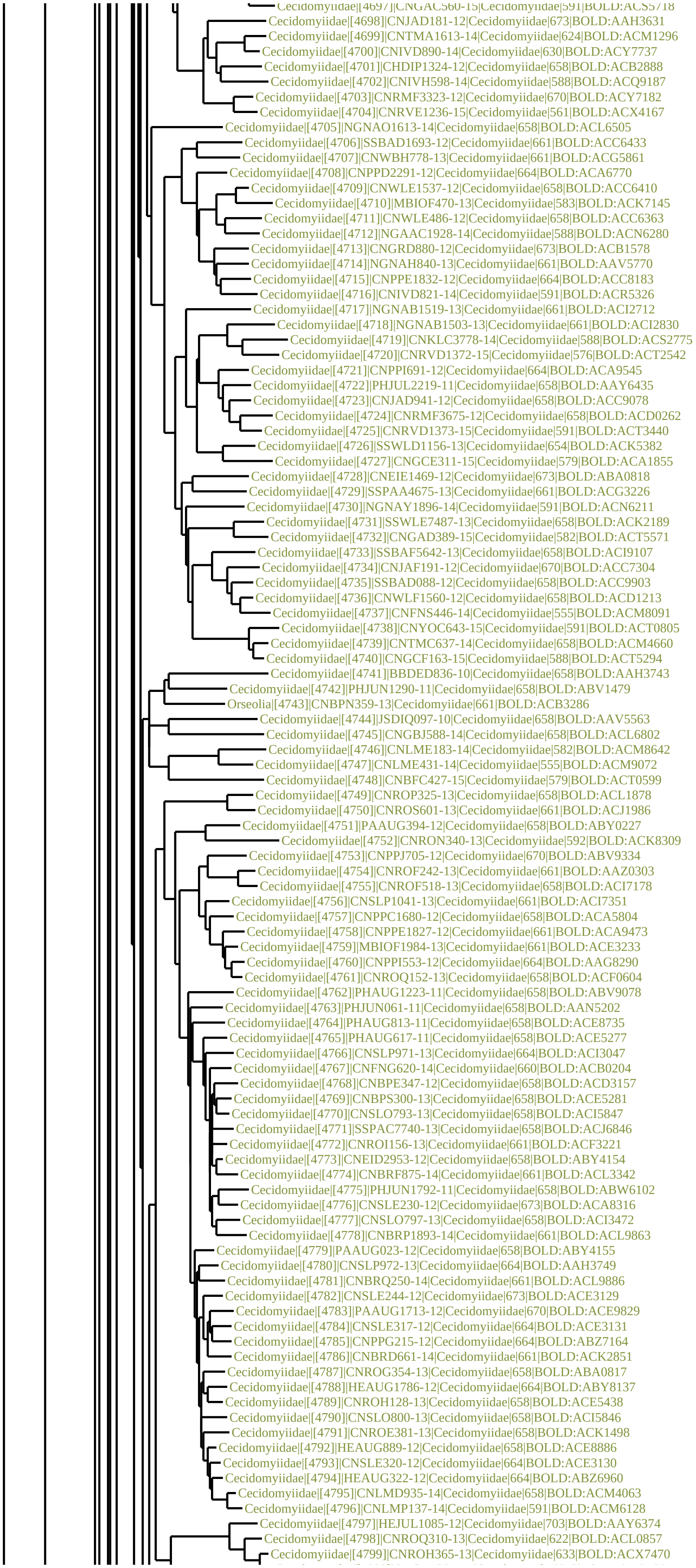

Cecidomyiidae|[4697]|CNGAC560-15|Cecidomyiidae|591|BOLD:ACS5718
Cecidomyiidae|[4698]|CNJAD181-12|Cecidomyiidae|673|BOLD:AAH3631
Cecidomyiidae|[4699]|CNTMA1613-14|Cecidomyiidae|624|BOLD:ACM1296
Cecidomyiidae|[4700]|CNIVD890-14|Cecidomyiidae|630|BOLD:ACY7737
Cecidomyiidae|[4701]|CHDIP1324-12|Cecidomyiidae|658|BOLD:ACB2888
Cecidomyiidae|[4702]|CNIVH598-14|Cecidomyiidae|588|BOLD:ACQ9187
Cecidomyiidae|[4703]|CNRMF3323-12|Cecidomyiidae|670|BOLD:ACY7182
Cecidomyiidae|[4704]|CNRVE1236-15|Cecidomyiidae|561|BOLD:ACX4167
Cecidomyiidae|[4705]|NGNAO1613-14|Cecidomyiidae|658|BOLD:ACL6505
Cecidomyiidae|[4706]|SSBAD1693-12|Cecidomyiidae|661|BOLD:ACC6433
Cecidomyiidae|[4707]|CNWBH778-13|Cecidomyiidae|661|BOLD:ACG5861
Cecidomyiidae|[4708]|CNPPD2291-12|Cecidomyiidae|664|BOLD:ACA6770
Cecidomyiidae|[4709]|CNWLE1537-12|Cecidomyiidae|658|BOLD:ACC6410
Cecidomyiidae|[4710]|MBIOF470-13|Cecidomyiidae|583|BOLD:ACK7145
Cecidomyiidae|[4711]|CNWLE486-12|Cecidomyiidae|658|BOLD:ACC6363
Cecidomyiidae|[4712]|NGAAC1928-14|Cecidomyiidae|588|BOLD:ACN6280
Cecidomyiidae|[4713]|CNGRD880-12|Cecidomyiidae|673|BOLD:ACB1578
Cecidomyiidae|[4714]|NGNAH840-13|Cecidomyiidae|661|BOLD:AAV5770
Cecidomyiidae|[4715]|CNPPE1832-12|Cecidomyiidae|664|BOLD:ACC8183
Cecidomyiidae|[4716]|CNIVD821-14|Cecidomyiidae|591|BOLD:ACR5326
Cecidomyiidae|[4717]|NGNAB1519-13|Cecidomyiidae|661|BOLD:ACI2712
Cecidomyiidae|[4718]|NGNAB1503-13|Cecidomyiidae|661|BOLD:ACI2830
Cecidomyiidae|[4719]|CNKLC3778-14|Cecidomyiidae|588|BOLD:ACS2775
Cecidomyiidae|[4720]|CNRVD1372-15|Cecidomyiidae|576|BOLD:ACT2542
Cecidomyiidae|[4721]|CNPPI691-12|Cecidomyiidae|664|BOLD:ACA9545
Cecidomyiidae|[4722]|PHJUL2219-11|Cecidomyiidae|658|BOLD:AAY6435
Cecidomyiidae|[4723]|CNJAD941-12|Cecidomyiidae|658|BOLD:ACC9078
Cecidomyiidae|[4724]|CNRMF3675-12|Cecidomyiidae|658|BOLD:ACD0262
Cecidomyiidae|[4725]|CNRVD1373-15|Cecidomyiidae|591|BOLD:ACT3440
Cecidomyiidae|[4726]|SSWLD1156-13|Cecidomyiidae|654|BOLD:ACK5382
Cecidomyiidae|[4727]|CNGCE311-15|Cecidomyiidae|579|BOLD:ACA1855
Cecidomyiidae|[4728]|CNEIE1469-12|Cecidomyiidae|673|BOLD:ABA0818
Cecidomyiidae|[4729]|SSPAA4675-13|Cecidomyiidae|661|BOLD:ACG3226
Cecidomyiidae|[4730]|NGNAY1896-14|Cecidomyiidae|591|BOLD:ACN6211
Cecidomyiidae|[4731]|SSWLE7487-13|Cecidomyiidae|658|BOLD:ACK2189
Cecidomyiidae|[4732]|CNGAD389-15|Cecidomyiidae|582|BOLD:ACT5571
Cecidomyiidae|[4733]|SSBAF5642-13|Cecidomyiidae|658|BOLD:ACI9107
Cecidomyiidae|[4734]|CNJAF191-12|Cecidomyiidae|670|BOLD:ACC7304
Cecidomyiidae|[4735]|SSBAD088-12|Cecidomyiidae|658|BOLD:ACC9903
Cecidomyiidae|[4736]|CNWLF1560-12|Cecidomyiidae|658|BOLD:ACD1213
Cecidomyiidae|[4737]|CNFNS446-14|Cecidomyiidae|555|BOLD:ACM8091
Cecidomyiidae|[4738]|CNYOC643-15|Cecidomyiidae|591|BOLD:ACT0805
Cecidomyiidae|[4739]|CNTMC637-14|Cecidomyiidae|658|BOLD:ACM4660
Cecidomyiidae|[4740]|CNGCF163-15|Cecidomyiidae|588|BOLD:ACT5294
Cecidomyiidae|[4741]|BBDED836-10|Cecidomyiidae|658|BOLD:AAH3743
Cecidomyiidae|[4742]|PHJUN1290-11|Cecidomyiidae|658|BOLD:ABV1479
Orseolia|[4743]|CNBPN359-13|Cecidomyiidae|661|BOLD:ACB3286
Cecidomyiidae|[4744]|JSDIQ097-10|Cecidomyiidae|658|BOLD:AAV5563
Cecidomyiidae|[4745]|CNGBJ588-14|Cecidomyiidae|658|BOLD:ACL6802
Cecidomyiidae|[4746]|CNLME183-14|Cecidomyiidae|582|BOLD:ACM8642
Cecidomyiidae|[4747]|CNLME431-14|Cecidomyiidae|555|BOLD:ACM9072
Cecidomyiidae|[4748]|CNBFC427-15|Cecidomyiidae|579|BOLD:ACT0599
Cecidomyiidae|[4749]|CNROP325-13|Cecidomyiidae|658|BOLD:ACL1878
Cecidomyiidae|[4750]|CNROS601-13|Cecidomyiidae|661|BOLD:ACJ1986
Cecidomyiidae|[4751]|PAAUG394-12|Cecidomyiidae|658|BOLD:ABY0227
Cecidomyiidae|[4752]|CNRON340-13|Cecidomyiidae|592|BOLD:ACK8309
Cecidomyiidae|[4753]|CNPPJ705-12|Cecidomyiidae|670|BOLD:ABV9334
Cecidomyiidae|[4754]|CNROF242-13|Cecidomyiidae|661|BOLD:AAZ0303
Cecidomyiidae|[4755]|CNROF518-13|Cecidomyiidae|658|BOLD:ACI7178
Cecidomyiidae|[4756]|CNSLP1041-13|Cecidomyiidae|661|BOLD:ACI7351
Cecidomyiidae|[4757]|CNPPC1680-12|Cecidomyiidae|658|BOLD:ACA5804
Cecidomyiidae|[4758]|CNPPE1827-12|Cecidomyiidae|661|BOLD:ACA9473
Cecidomyiidae|[4759]|MBIOF1984-13|Cecidomyiidae|661|BOLD:ACE3233
Cecidomyiidae|[4760]|CNPPI553-12|Cecidomyiidae|664|BOLD:AAG8290
Cecidomyiidae|[4761]|CNROQ152-13|Cecidomyiidae|658|BOLD:ACF0604
Cecidomyiidae|[4762]|PHAUG1223-11|Cecidomyiidae|658|BOLD:ABV9078
Cecidomyiidae|[4763]|PHJUN061-11|Cecidomyiidae|658|BOLD:AAN5202
Cecidomyiidae|[4764]|PHAUG813-11|Cecidomyiidae|658|BOLD:ACE8735
Cecidomyiidae|[4765]|PHAUG617-11|Cecidomyiidae|658|BOLD:ACE5277
Cecidomyiidae|[4766]|CNSLP971-13|Cecidomyiidae|664|BOLD:ACI3047
Cecidomyiidae|[4767]|CNFNG620-14|Cecidomyiidae|660|BOLD:ACB0204
Cecidomyiidae|[4768]|CNBPE347-12|Cecidomyiidae|658|BOLD:ACD3157
Cecidomyiidae|[4769]|CNBPS300-13|Cecidomyiidae|658|BOLD:ACE5281
Cecidomyiidae|[4770]|CNSLO793-13|Cecidomyiidae|658|BOLD:ACI5847
Cecidomyiidae|[4771]|SSPAC7740-13|Cecidomyiidae|658|BOLD:ACJ6846
Cecidomyiidae|[4772]|CNROI156-13|Cecidomyiidae|661|BOLD:ACF3221
Cecidomyiidae|[4773]|CNEID2953-12|Cecidomyiidae|658|BOLD:ABY4154
Cecidomyiidae|[4774]|CNBRF875-14|Cecidomyiidae|661|BOLD:ACL3342
Cecidomyiidae|[4775]|PHJUN1792-11|Cecidomyiidae|658|BOLD:ABW6102
Cecidomyiidae|[4776]|CNSLE230-12|Cecidomyiidae|673|BOLD:ACA8316
Cecidomyiidae|[4777]|CNSLO797-13|Cecidomyiidae|658|BOLD:ACI3472
Cecidomyiidae|[4778]|CNBRP1893-14|Cecidomyiidae|661|BOLD:ACL9863
Cecidomyiidae|[4779]|PAAUG023-12|Cecidomyiidae|658|BOLD:ABY4155
Cecidomyiidae|[4780]|CNSLP972-13|Cecidomyiidae|664|BOLD:AAH3749
Cecidomyiidae|[4781]|CNBRQ250-14|Cecidomyiidae|661|BOLD:ACL9886
Cecidomyiidae|[4782]|CNSLE244-12|Cecidomyiidae|673|BOLD:ACE3129
Cecidomyiidae|[4783]|PAAUG1713-12|Cecidomyiidae|670|BOLD:ACE9829
Cecidomyiidae|[4784]|CNSLE317-12|Cecidomyiidae|664|BOLD:ACE3131
Cecidomyiidae|[4785]|CNPPG215-12|Cecidomyiidae|664|BOLD:ABZ7164
Cecidomyiidae|[4786]|CNBRD661-14|Cecidomyiidae|661|BOLD:ACK2851
Cecidomyiidae|[4787]|CNROG354-13|Cecidomyiidae|658|BOLD:ABA0817
Cecidomyiidae|[4788]|HEAUG1786-12|Cecidomyiidae|664|BOLD:ABY8137
Cecidomyiidae|[4789]|CNROH128-13|Cecidomyiidae|658|BOLD:ACE5438
Cecidomyiidae|[4790]|CNSLO800-13|Cecidomyiidae|658|BOLD:ACI5846
Cecidomyiidae|[4791]|CNROE381-13|Cecidomyiidae|658|BOLD:ACK1498
Cecidomyiidae|[4792]|HEAUG889-12|Cecidomyiidae|658|BOLD:ACE8886
Cecidomyiidae|[4793]|CNSLE320-12|Cecidomyiidae|664|BOLD:ACE3130
Cecidomyiidae|[4794]|HEAUG322-12|Cecidomyiidae|664|BOLD:ABZ6960
Cecidomyiidae|[4795]|CNLMD935-14|Cecidomyiidae|658|BOLD:ACM4063
Cecidomyiidae|[4796]|CNLMP137-14|Cecidomyiidae|591|BOLD:ACM6128
Cecidomyiidae|[4797]|HEJUL1085-12|Cecidomyiidae|703|BOLD:AAY6374
Cecidomyiidae|[4798]|CNROQ310-13|Cecidomyiidae|622|BOLD:ACL0857
Cecidomyiidae|[4799]|CNROH365-13|Cecidomyiidae|633|BOLD:ACX7470

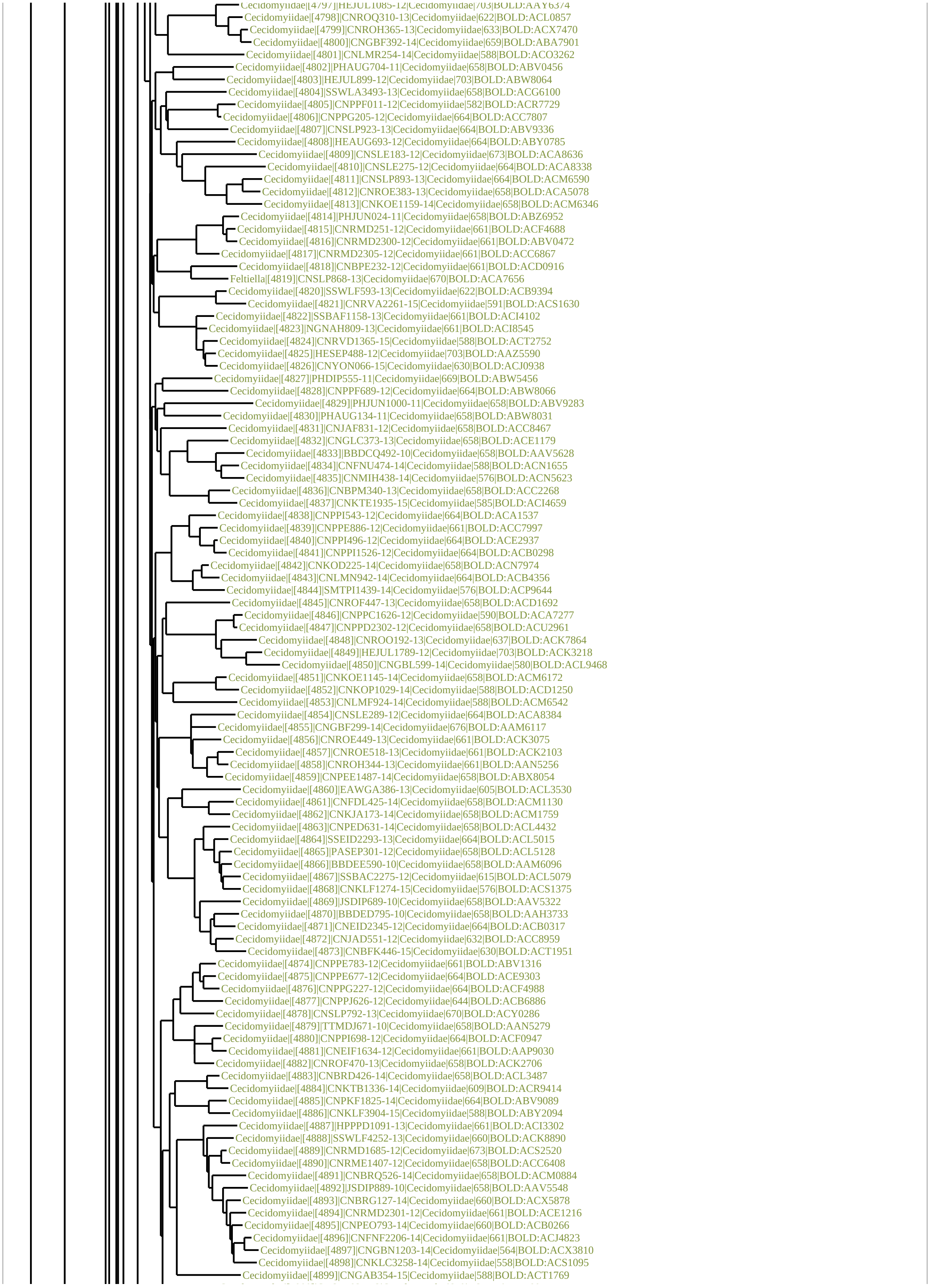
Cecidomyiidae|[4797]|HEJUL1085-12|Cecidomyiidae|703|BOLD:AAY6374
Cecidomyiidae|[4798]|CNROQ310-13|Cecidomyiidae|622|BOLD:ACL0857
Cecidomyiidae|[4799]|CNROH365-13|Cecidomyiidae|633|BOLD:ACX7470
Cecidomyiidae|[4800]|CNGBF392-14|Cecidomyiidae|659|BOLD:ABA7901
Cecidomyiidae|[4801]|CNLMR254-14|Cecidomyiidae|588|BOLD:ACO3262
Cecidomyiidae|[4802]|PHAUG704-11|Cecidomyiidae|658|BOLD:ABV0456
Cecidomyiidae|[4803]|HEJUL899-12|Cecidomyiidae|703|BOLD:ABW8064
Cecidomyiidae|[4804]|SSWLA3493-13|Cecidomyiidae|658|BOLD:ACG6100
Cecidomyiidae|[4805]|CNPPF011-12|Cecidomyiidae|582|BOLD:ACR7729
Cecidomyiidae|[4806]|CNPPG205-12|Cecidomyiidae|664|BOLD:ACC7807
Cecidomyiidae|[4807]|CNSLP923-13|Cecidomyiidae|664|BOLD:ABV9336
Cecidomyiidae|[4808]|HEAUG693-12|Cecidomyiidae|664|BOLD:ABY0785
Cecidomyiidae|[4809]|CNSLE183-12|Cecidomyiidae|673|BOLD:ACA8636
Cecidomyiidae|[4810]|CNSLE275-12|Cecidomyiidae|664|BOLD:ACA8338
Cecidomyiidae|[4811]|CNSLP893-13|Cecidomyiidae|664|BOLD:ACM6590
Cecidomyiidae|[4812]|CNROE383-13|Cecidomyiidae|658|BOLD:ACA5078
Cecidomyiidae|[4813]|CNKOE1159-14|Cecidomyiidae|658|BOLD:ACM6346
Cecidomyiidae|[4814]|PHJUN024-11|Cecidomyiidae|658|BOLD:ABZ6952
Cecidomyiidae|[4815]|CNRMD251-12|Cecidomyiidae|661|BOLD:ACF4688
Cecidomyiidae|[4816]|CNRMD2300-12|Cecidomyiidae|661|BOLD:ABV0472
Cecidomyiidae|[4817]|CNRMD2305-12|Cecidomyiidae|661|BOLD:ACC6867
Cecidomyiidae|[4818]|CNBPE232-12|Cecidomyiidae|661|BOLD:ACD0916
Feltiella|[4819]|CNSLP868-13|Cecidomyiidae|670|BOLD:ACA7656
Cecidomyiidae|[4820]|SSWLF593-13|Cecidomyiidae|622|BOLD:ACB9394
Cecidomyiidae|[4821]|CNRVA2261-15|Cecidomyiidae|591|BOLD:ACS1630
Cecidomyiidae|[4822]|SSBAF1158-13|Cecidomyiidae|661|BOLD:ACI4102
Cecidomyiidae|[4823]|NGNAH809-13|Cecidomyiidae|661|BOLD:ACI8545
Cecidomyiidae|[4824]|CNRVD1365-15|Cecidomyiidae|588|BOLD:ACT2752
Cecidomyiidae|[4825]|HESEP488-12|Cecidomyiidae|703|BOLD:AAZ5590
Cecidomyiidae|[4826]|CNYON066-15|Cecidomyiidae|630|BOLD:ACJ0938
Cecidomyiidae|[4827]|PHDIP555-11|Cecidomyiidae|669|BOLD:ABW5456
Cecidomyiidae|[4828]|CNPPF689-12|Cecidomyiidae|664|BOLD:ABW8066
Cecidomyiidae|[4829]|PHJUN1000-11|Cecidomyiidae|658|BOLD:ABV9283
Cecidomyiidae|[4830]|PHAUG134-11|Cecidomyiidae|658|BOLD:ABW8031
Cecidomyiidae|[4831]|CNJAF831-12|Cecidomyiidae|658|BOLD:ACC8467
Cecidomyiidae|[4832]|CNGLC373-13|Cecidomyiidae|658|BOLD:ACE1179
Cecidomyiidae|[4833]|BBDCQ492-10|Cecidomyiidae|658|BOLD:AAV5628
Cecidomyiidae|[4834]|CNFNU474-14|Cecidomyiidae|588|BOLD:ACN1655
Cecidomyiidae|[4835]|CNMIH438-14|Cecidomyiidae|576|BOLD:ACN5623
Cecidomyiidae|[4836]|CNBPM340-13|Cecidomyiidae|658|BOLD:ACC2268
Cecidomyiidae|[4837]|CNKTE1935-15|Cecidomyiidae|585|BOLD:ACI4659
Cecidomyiidae|[4838]|CNPPI543-12|Cecidomyiidae|664|BOLD:ACA1537
Cecidomyiidae|[4839]|CNPPE886-12|Cecidomyiidae|661|BOLD:ACC7997
Cecidomyiidae|[4840]|CNPPI496-12|Cecidomyiidae|664|BOLD:ACE2937
Cecidomyiidae|[4841]|CNPPI1526-12|Cecidomyiidae|664|BOLD:ACB0298
Cecidomyiidae|[4842]|CNKOD225-14|Cecidomyiidae|658|BOLD:ACN7974
Cecidomyiidae|[4843]|CNLMN942-14|Cecidomyiidae|664|BOLD:ACB4356
Cecidomyiidae|[4844]|SMTPI1439-14|Cecidomyiidae|576|BOLD:ACP9644
Cecidomyiidae|[4845]|CNROF447-13|Cecidomyiidae|658|BOLD:ACD1692
Cecidomyiidae|[4846]|CNPPC1626-12|Cecidomyiidae|590|BOLD:ACA7277
Cecidomyiidae|[4847]|CNPPD2302-12|Cecidomyiidae|658|BOLD:ACU2961
Cecidomyiidae|[4848]|CNROO192-13|Cecidomyiidae|637|BOLD:ACK7864
Cecidomyiidae|[4849]|HEJUL1789-12|Cecidomyiidae|703|BOLD:ACK3218
Cecidomyiidae|[4850]|CNGBL599-14|Cecidomyiidae|580|BOLD:ACL9468
Cecidomyiidae|[4851]|CNKOE1145-14|Cecidomyiidae|658|BOLD:ACM6172
Cecidomyiidae|[4852]|CNKOP1029-14|Cecidomyiidae|588|BOLD:ACD1250
Cecidomyiidae|[4853]|CNLMF924-14|Cecidomyiidae|588|BOLD:ACM6542
Cecidomyiidae|[4854]|CNSLE289-12|Cecidomyiidae|664|BOLD:ACA8384
Cecidomyiidae|[4855]|CNGBF299-14|Cecidomyiidae|676|BOLD:AAM6117
Cecidomyiidae|[4856]|CNROE449-13|Cecidomyiidae|661|BOLD:ACK3075
Cecidomyiidae|[4857]|CNROE518-13|Cecidomyiidae|661|BOLD:ACK2103
Cecidomyiidae|[4858]|CNROH344-13|Cecidomyiidae|661|BOLD:AAN5256
Cecidomyiidae|[4859]|CNPEE1487-14|Cecidomyiidae|658|BOLD:ABX8054
Cecidomyiidae|[4860]|EAWGA386-13|Cecidomyiidae|605|BOLD:ACL3530
Cecidomyiidae|[4861]|CNFDL425-14|Cecidomyiidae|658|BOLD:ACM1130
Cecidomyiidae|[4862]|CNKJA173-14|Cecidomyiidae|658|BOLD:ACM1759
Cecidomyiidae|[4863]|CNPED631-14|Cecidomyiidae|658|BOLD:ACL4432
Cecidomyiidae|[4864]|SSEID2293-13|Cecidomyiidae|664|BOLD:ACL5015
Cecidomyiidae|[4865]|PASEP301-12|Cecidomyiidae|658|BOLD:ACL5128
Cecidomyiidae|[4866]|BBDEE590-10|Cecidomyiidae|658|BOLD:AAM6096
Cecidomyiidae|[4867]|SSBAC2275-12|Cecidomyiidae|615|BOLD:ACL5079
Cecidomyiidae|[4868]|CNKLF1274-15|Cecidomyiidae|576|BOLD:ACS1375
Cecidomyiidae|[4869]|JSDIP689-10|Cecidomyiidae|658|BOLD:AAV5322
Cecidomyiidae|[4870]|BBDED795-10|Cecidomyiidae|658|BOLD:AAH3733
Cecidomyiidae|[4871]|CNEID2345-12|Cecidomyiidae|664|BOLD:ACB0317
Cecidomyiidae|[4872]|CNJAD551-12|Cecidomyiidae|632|BOLD:ACC8959
Cecidomyiidae|[4873]|CNBFK446-15|Cecidomyiidae|630|BOLD:ACT1951
Cecidomyiidae|[4874]|CNPPE783-12|Cecidomyiidae|661|BOLD:ABV1316
Cecidomyiidae|[4875]|CNPPE677-12|Cecidomyiidae|664|BOLD:ACE9303
Cecidomyiidae|[4876]|CNPPG227-12|Cecidomyiidae|664|BOLD:ACF4988
Cecidomyiidae|[4877]|CNPPJ626-12|Cecidomyiidae|644|BOLD:ACB6886
Cecidomyiidae|[4878]|CNSLP792-13|Cecidomyiidae|670|BOLD:ACY0286
Cecidomyiidae|[4879]|TTMDJ671-10|Cecidomyiidae|658|BOLD:AAN5279
Cecidomyiidae|[4880]|CNPPI698-12|Cecidomyiidae|664|BOLD:ACF0947
Cecidomyiidae|[4881]|CNEIF1634-12|Cecidomyiidae|661|BOLD:AAP9030
Cecidomyiidae|[4882]|CNROF470-13|Cecidomyiidae|658|BOLD:ACK2706
Cecidomyiidae|[4883]|CNBRD426-14|Cecidomyiidae|658|BOLD:ACL3487
Cecidomyiidae|[4884]|CNKTB1336-14|Cecidomyiidae|609|BOLD:ACR9414
Cecidomyiidae|[4885]|CNPKF1825-14|Cecidomyiidae|664|BOLD:ABV9089
Cecidomyiidae|[4886]|CNKLF3904-15|Cecidomyiidae|588|BOLD:ABY2094
Cecidomyiidae|[4887]|HPPPD1091-13|Cecidomyiidae|661|BOLD:ACI3302
Cecidomyiidae|[4888]|SSWLF4252-13|Cecidomyiidae|660|BOLD:ACK8890
Cecidomyiidae|[4889]|CNRMD1685-12|Cecidomyiidae|673|BOLD:ACS2520
Cecidomyiidae|[4890]|CNRME1407-12|Cecidomyiidae|658|BOLD:ACC6408
Cecidomyiidae|[4891]|CNBRQ526-14|Cecidomyiidae|658|BOLD:ACM0884
Cecidomyiidae|[4892]|JSDIP889-10|Cecidomyiidae|658|BOLD:AAV5548
Cecidomyiidae|[4893]|CNBRG127-14|Cecidomyiidae|660|BOLD:ACX5878
Cecidomyiidae|[4894]|CNRMD2301-12|Cecidomyiidae|661|BOLD:ACE1216
Cecidomyiidae|[4895]|CNPEO793-14|Cecidomyiidae|660|BOLD:ACB0266
Cecidomyiidae|[4896]|CNFNF2206-14|Cecidomyiidae|661|BOLD:ACJ4823
Cecidomyiidae|[4897]|CNGBN1203-14|Cecidomyiidae|564|BOLD:ACX3810
Cecidomyiidae|[4898]|CNKLC3258-14|Cecidomyiidae|558|BOLD:ACS1095
Cecidomyiidae|[4899]|CNGAB354-15|Cecidomyiidae|588|BOLD:ACT1769

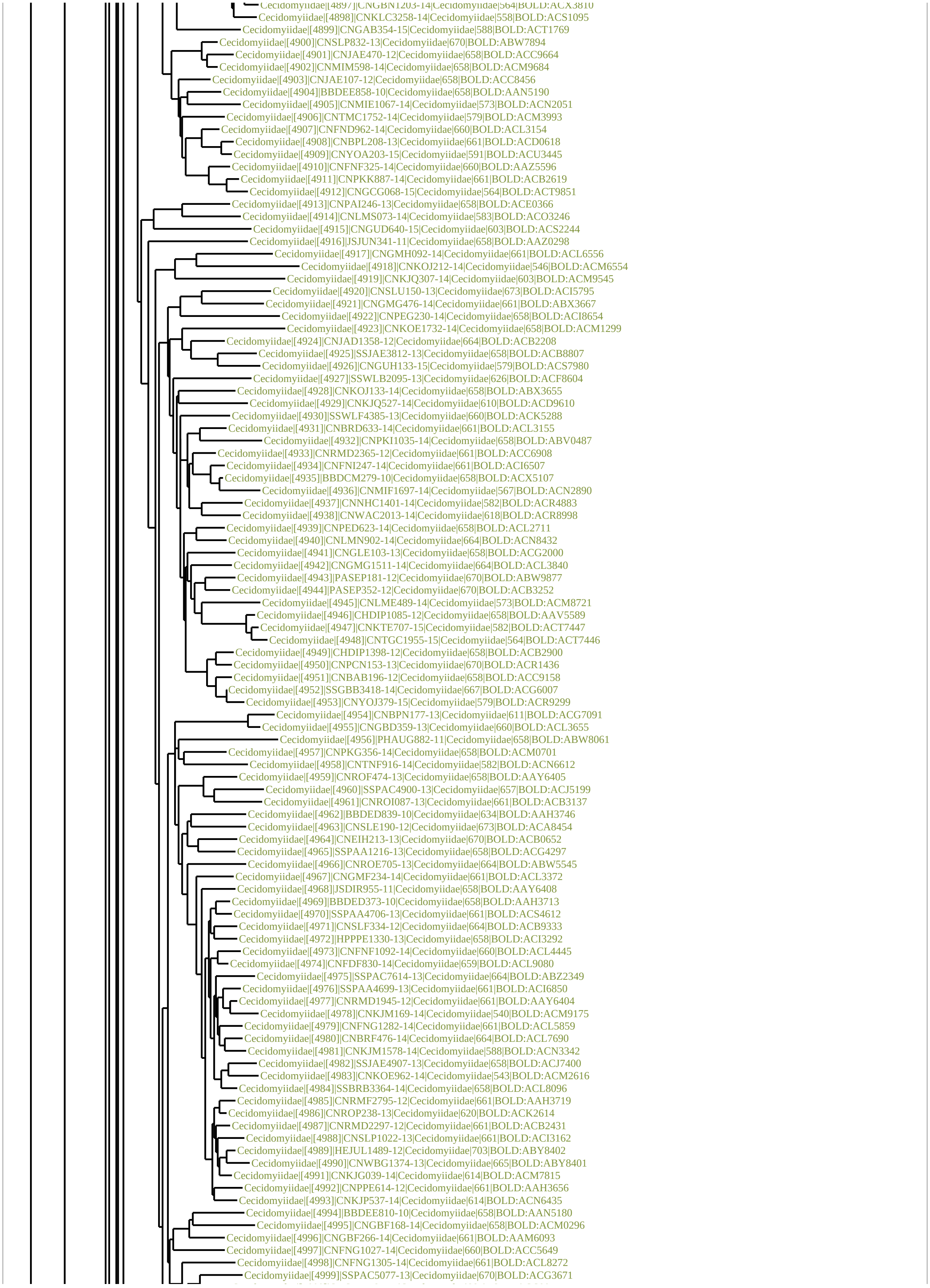
Cecidomyiidae|[4897]|CNGBN1203-14|Cecidomyiidae|564|BOLD:ACX3810
Cecidomyiidae|[4898]|CNKLC3258-14|Cecidomyiidae|558|BOLD:ACS1095
Cecidomyiidae|[4899]|CNGAB354-15|Cecidomyiidae|588|BOLD:ACT1769
Cecidomyiidae|[4900]|CNSLP832-13|Cecidomyiidae|670|BOLD:ABW7894
Cecidomyiidae|[4901]|CNJAE470-12|Cecidomyiidae|658|BOLD:ACC9664
Cecidomyiidae|[4902]|CNMIM598-14|Cecidomyiidae|658|BOLD:ACM9684
Cecidomyiidae|[4903]|CNJAE107-12|Cecidomyiidae|658|BOLD:ACC8456
Cecidomyiidae|[4904]|BBDEE858-10|Cecidomyiidae|658|BOLD:AAN5190
Cecidomyiidae|[4905]|CNMIE1067-14|Cecidomyiidae|573|BOLD:ACN2051
Cecidomyiidae|[4906]|CNTMC1752-14|Cecidomyiidae|579|BOLD:ACM3993
Cecidomyiidae|[4907]|CNFND962-14|Cecidomyiidae|660|BOLD:ACL3154
Cecidomyiidae|[4908]|CNBPL208-13|Cecidomyiidae|661|BOLD:ACD0618
Cecidomyiidae|[4909]|CNYOA203-15|Cecidomyiidae|591|BOLD:ACU3445
Cecidomyiidae|[4910]|CNFNF325-14|Cecidomyiidae|660|BOLD:AAZ5596
Cecidomyiidae|[4911]|CNPKK887-14|Cecidomyiidae|661|BOLD:ACB2619
Cecidomyiidae|[4912]|CNGCG068-15|Cecidomyiidae|564|BOLD:ACT9851
Cecidomyiidae|[4913]|CNPAI246-13|Cecidomyiidae|658|BOLD:ACE0366
Cecidomyiidae|[4914]|CNLMS073-14|Cecidomyiidae|583|BOLD:ACO3246
Cecidomyiidae|[4915]|CNGUD640-15|Cecidomyiidae|603|BOLD:ACS2244
Cecidomyiidae|[4916]|JSJUN341-11|Cecidomyiidae|658|BOLD:AAZ0298
Cecidomyiidae|[4917]|CNGMH092-14|Cecidomyiidae|661|BOLD:ACL6556
Cecidomyiidae|[4918]|CNKOJ212-14|Cecidomyiidae|546|BOLD:ACM6554
Cecidomyiidae|[4919]|CNKJQ307-14|Cecidomyiidae|603|BOLD:ACM9545
Cecidomyiidae|[4920]|CNSLU150-13|Cecidomyiidae|673|BOLD:ACI5795
Cecidomyiidae|[4921]|CNGMG476-14|Cecidomyiidae|661|BOLD:ABX3667
Cecidomyiidae|[4922]|CNPEG230-14|Cecidomyiidae|658|BOLD:ACI8654
Cecidomyiidae|[4923]|CNKOE1732-14|Cecidomyiidae|658|BOLD:ACM1299
Cecidomyiidae|[4924]|CNJAD1358-12|Cecidomyiidae|664|BOLD:ACB2208
Cecidomyiidae|[4925]|SSJAE3812-13|Cecidomyiidae|658|BOLD:ACB8807
Cecidomyiidae|[4926]|CNGUH133-15|Cecidomyiidae|579|BOLD:ACS7980
Cecidomyiidae|[4927]|SSWLB2095-13|Cecidomyiidae|626|BOLD:ACF8604
Cecidomyiidae|[4928]|CNKOJ133-14|Cecidomyiidae|658|BOLD:ABX3655
Cecidomyiidae|[4929]|CNKJQ527-14|Cecidomyiidae|610|BOLD:ACD9610
Cecidomyiidae|[4930]|SSWLF4385-13|Cecidomyiidae|660|BOLD:ACK5288
Cecidomyiidae|[4931]|CNBRD633-14|Cecidomyiidae|661|BOLD:ACL3155
Cecidomyiidae|[4932]|CNPKI1035-14|Cecidomyiidae|658|BOLD:ABV0487
Cecidomyiidae|[4933]|CNRMD2365-12|Cecidomyiidae|661|BOLD:ACC6908
Cecidomyiidae|[4934]|CNFNI247-14|Cecidomyiidae|661|BOLD:ACI6507
Cecidomyiidae|[4935]|BBDCM279-10|Cecidomyiidae|658|BOLD:ACX5107
Cecidomyiidae|[4936]|CNMIF1697-14|Cecidomyiidae|567|BOLD:ACN2890
Cecidomyiidae|[4937]|CNNHC1401-14|Cecidomyiidae|582|BOLD:ACR4883
Cecidomyiidae|[4938]|CNWAC2013-14|Cecidomyiidae|618|BOLD:ACR8998
Cecidomyiidae|[4939]|CNPED623-14|Cecidomyiidae|658|BOLD:ACL2711
Cecidomyiidae|[4940]|CNLMN902-14|Cecidomyiidae|664|BOLD:ACN8432
Cecidomyiidae|[4941]|CNGLE103-13|Cecidomyiidae|658|BOLD:ACG2000
Cecidomyiidae|[4942]|CNGMG1511-14|Cecidomyiidae|664|BOLD:ACL3840
Cecidomyiidae|[4943]|PASEP181-12|Cecidomyiidae|670|BOLD:ABW9877
Cecidomyiidae|[4944]|PASEP352-12|Cecidomyiidae|670|BOLD:ACB3252
Cecidomyiidae|[4945]|CNLME489-14|Cecidomyiidae|573|BOLD:ACM8721
Cecidomyiidae|[4946]|CHDIP1085-12|Cecidomyiidae|658|BOLD:AAV5589
Cecidomyiidae|[4947]|CNKTE707-15|Cecidomyiidae|582|BOLD:ACT7447
Cecidomyiidae|[4948]|CNTGC1955-15|Cecidomyiidae|564|BOLD:ACT7446
Cecidomyiidae|[4949]|CHDIP1398-12|Cecidomyiidae|658|BOLD:ACB2900
Cecidomyiidae|[4950]|CNPCN153-13|Cecidomyiidae|670|BOLD:ACR1436
Cecidomyiidae|[4951]|CNBAB196-12|Cecidomyiidae|658|BOLD:ACC9158
Cecidomyiidae|[4952]|SSGBB3418-14|Cecidomyiidae|667|BOLD:ACG6007
Cecidomyiidae|[4953]|CNYOJ379-15|Cecidomyiidae|579|BOLD:ACR9299
Cecidomyiidae|[4954]|CNBPN177-13|Cecidomyiidae|611|BOLD:ACG7091
Cecidomyiidae|[4955]|CNGBD359-13|Cecidomyiidae|660|BOLD:ACL3655
Cecidomyiidae|[4956]|PHAUG882-11|Cecidomyiidae|658|BOLD:ABW8061
Cecidomyiidae|[4957]|CNPKG356-14|Cecidomyiidae|658|BOLD:ACM0701
Cecidomyiidae|[4958]|CNTNF916-14|Cecidomyiidae|582|BOLD:ACN6612
Cecidomyiidae|[4959]|CNROF474-13|Cecidomyiidae|658|BOLD:AAY6405
Cecidomyiidae|[4960]|SSPAC4900-13|Cecidomyiidae|657|BOLD:ACJ5199
Cecidomyiidae|[4961]|CNROI087-13|Cecidomyiidae|661|BOLD:ACB3137
Cecidomyiidae|[4962]|BBDED839-10|Cecidomyiidae|634|BOLD:AAH3746
Cecidomyiidae|[4963]|CNSLE190-12|Cecidomyiidae|673|BOLD:ACA8454
Cecidomyiidae|[4964]|CNEIH213-13|Cecidomyiidae|670|BOLD:ACB0652
Cecidomyiidae|[4965]|SSPAA1216-13|Cecidomyiidae|658|BOLD:ACG4297
Cecidomyiidae|[4966]|CNROE705-13|Cecidomyiidae|664|BOLD:ABW5545
Cecidomyiidae|[4967]|CNGMF234-14|Cecidomyiidae|661|BOLD:ACL3372
Cecidomyiidae|[4968]|JSDIR955-11|Cecidomyiidae|658|BOLD:AAY6408
Cecidomyiidae|[4969]|BBDED373-10|Cecidomyiidae|658|BOLD:AAH3713
Cecidomyiidae|[4970]|SSPAA4706-13|Cecidomyiidae|661|BOLD:ACS4612
Cecidomyiidae|[4971]|CNSLF334-12|Cecidomyiidae|664|BOLD:ACB9333
Cecidomyiidae|[4972]|HPPPE1330-13|Cecidomyiidae|658|BOLD:ACI3292
Cecidomyiidae|[4973]|CNFNF1092-14|Cecidomyiidae|660|BOLD:ACL4445
Cecidomyiidae|[4974]|CNFDF830-14|Cecidomyiidae|659|BOLD:ACL9080
Cecidomyiidae|[4975]|SSPAC7614-13|Cecidomyiidae|664|BOLD:ABZ2349
Cecidomyiidae|[4976]|SSPAA4699-13|Cecidomyiidae|661|BOLD:ACI6850
Cecidomyiidae|[4977]|CNRMD1945-12|Cecidomyiidae|661|BOLD:AAY6404
Cecidomyiidae|[4978]|CNKJM169-14|Cecidomyiidae|540|BOLD:ACM9175
Cecidomyiidae|[4979]|CNFNG1282-14|Cecidomyiidae|661|BOLD:ACL5859
Cecidomyiidae|[4980]|CNBRF476-14|Cecidomyiidae|664|BOLD:ACL7690
Cecidomyiidae|[4981]|CNKJM1578-14|Cecidomyiidae|588|BOLD:ACN3342
Cecidomyiidae|[4982]|SSJAE4907-13|Cecidomyiidae|658|BOLD:ACJ7400
Cecidomyiidae|[4983]|CNKOE962-14|Cecidomyiidae|543|BOLD:ACM2616
Cecidomyiidae|[4984]|SSBRB3364-14|Cecidomyiidae|658|BOLD:ACL8096
Cecidomyiidae|[4985]|CNRMF2795-12|Cecidomyiidae|661|BOLD:AAH3719
Cecidomyiidae|[4986]|CNROP238-13|Cecidomyiidae|620|BOLD:ACK2614
Cecidomyiidae|[4987]|CNRMD2297-12|Cecidomyiidae|661|BOLD:ACB2431
Cecidomyiidae|[4988]|CNSLP1022-13|Cecidomyiidae|661|BOLD:ACI3162
Cecidomyiidae|[4989]|HEJUL1489-12|Cecidomyiidae|703|BOLD:ABY8402
Cecidomyiidae|[4990]|CNWBG1374-13|Cecidomyiidae|665|BOLD:ABY8401
Cecidomyiidae|[4991]|CNKJG039-14|Cecidomyiidae|614|BOLD:ACM7815
Cecidomyiidae|[4992]|CNPPE614-12|Cecidomyiidae|661|BOLD:AAH3656
Cecidomyiidae|[4993]|CNKJP537-14|Cecidomyiidae|614|BOLD:ACN6435
Cecidomyiidae|[4994]|BBDEE810-10|Cecidomyiidae|658|BOLD:AAN5180
Cecidomyiidae|[4995]|CNGBF168-14|Cecidomyiidae|658|BOLD:ACM0296
Cecidomyiidae|[4996]|CNGBF266-14|Cecidomyiidae|661|BOLD:AAM6093
Cecidomyiidae|[4997]|CNFNG1027-14|Cecidomyiidae|660|BOLD:ACC5649
Cecidomyiidae|[4998]|CNFNG1305-14|Cecidomyiidae|661|BOLD:ACL8272
Cecidomyiidae|[4999]|SSPAC5077-13|Cecidomyiidae|670|BOLD:ACG3671

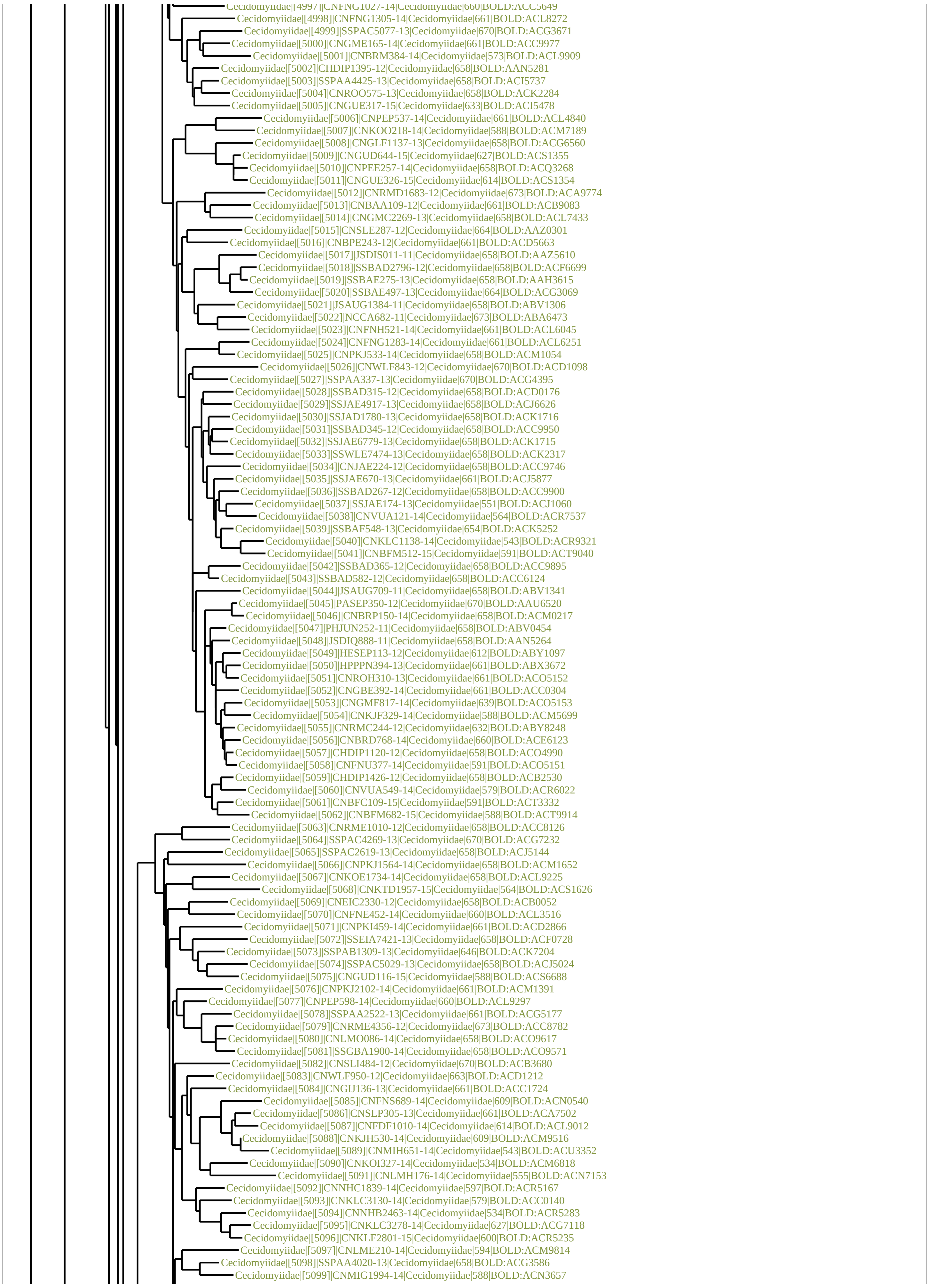
Cecidomyiidae|[4997]|CNFNG1027-14|Cecidomyiidae|660|BOLD:ACC5649
Cecidomyiidae|[4998]|CNFNG1305-14|Cecidomyiidae|661|BOLD:ACL8272
Cecidomyiidae|[4999]|SSPAC5077-13|Cecidomyiidae|670|BOLD:ACG3671
Cecidomyiidae|[5000]|CNGME165-14|Cecidomyiidae|661|BOLD:ACC9977
Cecidomyiidae|[5001]|CNBRM384-14|Cecidomyiidae|573|BOLD:ACL9909
Cecidomyiidae|[5002]|CHDIP1395-12|Cecidomyiidae|658|BOLD:AAN5281
Cecidomyiidae|[5003]|SSPAA4425-13|Cecidomyiidae|658|BOLD:ACI5737
Cecidomyiidae|[5004]|CNROO575-13|Cecidomyiidae|658|BOLD:ACK2284
Cecidomyiidae|[5005]|CNGUE317-15|Cecidomyiidae|633|BOLD:ACI5478
Cecidomyiidae|[5006]|CNPEP537-14|Cecidomyiidae|661|BOLD:ACL4840
Cecidomyiidae|[5007]|CNKOO218-14|Cecidomyiidae|588|BOLD:ACM7189
Cecidomyiidae|[5008]|CNGLF1137-13|Cecidomyiidae|658|BOLD:ACG6560
Cecidomyiidae|[5009]|CNGUD644-15|Cecidomyiidae|627|BOLD:ACS1355
Cecidomyiidae|[5010]|CNPEE257-14|Cecidomyiidae|658|BOLD:ACQ3268
Cecidomyiidae|[5011]|CNGUE326-15|Cecidomyiidae|614|BOLD:ACS1354
Cecidomyiidae|[5012]|CNRMD1683-12|Cecidomyiidae|673|BOLD:ACA9774
Cecidomyiidae|[5013]|CNBAA109-12|Cecidomyiidae|661|BOLD:ACB9083
Cecidomyiidae|[5014]|CNGMC2269-13|Cecidomyiidae|658|BOLD:ACL7433
Cecidomyiidae|[5015]|CNSLE287-12|Cecidomyiidae|664|BOLD:AAZ0301
Cecidomyiidae|[5016]|CNBPE243-12|Cecidomyiidae|661|BOLD:ACD5663
Cecidomyiidae|[5017]|JSDIS011-11|Cecidomyiidae|658|BOLD:AAZ5610
Cecidomyiidae|[5018]|SSBAD2796-12|Cecidomyiidae|658|BOLD:ACF6699
Cecidomyiidae|[5019]|SSBAE275-13|Cecidomyiidae|658|BOLD:AAH3615
Cecidomyiidae|[5020]|SSBAE497-13|Cecidomyiidae|664|BOLD:ACG3069
Cecidomyiidae|[5021]|JSAUG1384-11|Cecidomyiidae|658|BOLD:ABV1306
Cecidomyiidae|[5022]|NCCA682-11|Cecidomyiidae|673|BOLD:ABA6473
Cecidomyiidae|[5023]|CNFNH521-14|Cecidomyiidae|661|BOLD:ACL6045
Cecidomyiidae|[5024]|CNFNG1283-14|Cecidomyiidae|661|BOLD:ACL6251
Cecidomyiidae|[5025]|CNPKJ533-14|Cecidomyiidae|658|BOLD:ACM1054
Cecidomyiidae|[5026]|CNWLF843-12|Cecidomyiidae|670|BOLD:ACD1098
Cecidomyiidae|[5027]|SSPAA337-13|Cecidomyiidae|670|BOLD:ACG4395
Cecidomyiidae|[5028]|SSBAD315-12|Cecidomyiidae|658|BOLD:ACD0176
Cecidomyiidae|[5029]|SSJAE4917-13|Cecidomyiidae|658|BOLD:ACJ6626
Cecidomyiidae|[5030]|SSJAD1780-13|Cecidomyiidae|658|BOLD:ACK1716
Cecidomyiidae|[5031]|SSBAD345-12|Cecidomyiidae|658|BOLD:ACC9950
Cecidomyiidae|[5032]|SSJAE6779-13|Cecidomyiidae|658|BOLD:ACK1715
Cecidomyiidae|[5033]|SSWLE7474-13|Cecidomyiidae|658|BOLD:ACK2317
Cecidomyiidae|[5034]|CNJAE224-12|Cecidomyiidae|658|BOLD:ACC9746
Cecidomyiidae|[5035]|SSJAE670-13|Cecidomyiidae|661|BOLD:ACJ5877
Cecidomyiidae|[5036]|SSBAD267-12|Cecidomyiidae|658|BOLD:ACC9900
Cecidomyiidae|[5037]|SSJAE174-13|Cecidomyiidae|551|BOLD:ACJ1060
Cecidomyiidae|[5038]|CNVUA121-14|Cecidomyiidae|564|BOLD:ACR7537
Cecidomyiidae|[5039]|SSBAF548-13|Cecidomyiidae|654|BOLD:ACK5252
Cecidomyiidae|[5040]|CNKLC1138-14|Cecidomyiidae|543|BOLD:ACR9321
Cecidomyiidae|[5041]|CNBFM512-15|Cecidomyiidae|591|BOLD:ACT9040
Cecidomyiidae|[5042]|SSBAD365-12|Cecidomyiidae|658|BOLD:ACC9895
Cecidomyiidae|[5043]|SSBAD582-12|Cecidomyiidae|658|BOLD:ACC6124
Cecidomyiidae|[5044]|JSAUG709-11|Cecidomyiidae|658|BOLD:ABV1341
Cecidomyiidae|[5045]|PASEP350-12|Cecidomyiidae|670|BOLD:AAU6520
Cecidomyiidae|[5046]|CNBRP150-14|Cecidomyiidae|658|BOLD:ACM0217
Cecidomyiidae|[5047]|PHJUN252-11|Cecidomyiidae|658|BOLD:ABV0454
Cecidomyiidae|[5048]|JSDIQ888-11|Cecidomyiidae|658|BOLD:AAN5264
Cecidomyiidae|[5049]|HESEP113-12|Cecidomyiidae|612|BOLD:ABY1097
Cecidomyiidae|[5050]|HPPPN394-13|Cecidomyiidae|661|BOLD:ABX3672
Cecidomyiidae|[5051]|CNROH310-13|Cecidomyiidae|661|BOLD:ACO5152
Cecidomyiidae|[5052]|CNGBE392-14|Cecidomyiidae|661|BOLD:ACC0304
Cecidomyiidae|[5053]|CNGMF817-14|Cecidomyiidae|639|BOLD:ACO5153
Cecidomyiidae|[5054]|CNKJF329-14|Cecidomyiidae|588|BOLD:ACM5699
Cecidomyiidae|[5055]|CNRMC244-12|Cecidomyiidae|632|BOLD:ABY8248
Cecidomyiidae|[5056]|CNBRD768-14|Cecidomyiidae|660|BOLD:ACE6123
Cecidomyiidae|[5057]|CHDIP1120-12|Cecidomyiidae|658|BOLD:ACO4990
Cecidomyiidae|[5058]|CNFNU377-14|Cecidomyiidae|591|BOLD:ACO5151
Cecidomyiidae|[5059]|CHDIP1426-12|Cecidomyiidae|658|BOLD:ACB2530
Cecidomyiidae|[5060]|CNVUA549-14|Cecidomyiidae|579|BOLD:ACR6022
Cecidomyiidae|[5061]|CNBFC109-15|Cecidomyiidae|591|BOLD:ACT3332
Cecidomyiidae|[5062]|CNBFM682-15|Cecidomyiidae|588|BOLD:ACT9914
Cecidomyiidae|[5063]|CNRME1010-12|Cecidomyiidae|658|BOLD:ACC8126
Cecidomyiidae|[5064]|SSPAC4269-13|Cecidomyiidae|670|BOLD:ACG7232
Cecidomyiidae|[5065]|SSPAC2619-13|Cecidomyiidae|658|BOLD:ACJ5144
Cecidomyiidae|[5066]|CNPKJ1564-14|Cecidomyiidae|658|BOLD:ACM1652
Cecidomyiidae|[5067]|CNKOE1734-14|Cecidomyiidae|658|BOLD:ACL9225
Cecidomyiidae|[5068]|CNKTD1957-15|Cecidomyiidae|564|BOLD:ACS1626
Cecidomyiidae|[5069]|CNEIC2330-12|Cecidomyiidae|658|BOLD:ACB0052
Cecidomyiidae|[5070]|CNFNE452-14|Cecidomyiidae|660|BOLD:ACL3516
Cecidomyiidae|[5071]|CNPKI459-14|Cecidomyiidae|661|BOLD:ACD2866
Cecidomyiidae|[5072]|SSEIA7421-13|Cecidomyiidae|658|BOLD:ACF0728
Cecidomyiidae|[5073]|SSPAB1309-13|Cecidomyiidae|646|BOLD:ACK7204
Cecidomyiidae|[5074]|SSPAC5029-13|Cecidomyiidae|658|BOLD:ACJ5024
Cecidomyiidae|[5075]|CNGUD116-15|Cecidomyiidae|588|BOLD:ACS6688
Cecidomyiidae|[5076]|CNPKJ2102-14|Cecidomyiidae|661|BOLD:ACM1391
Cecidomyiidae|[5077]|CNPEP598-14|Cecidomyiidae|660|BOLD:ACL9297
Cecidomyiidae|[5078]|SSPAA2522-13|Cecidomyiidae|661|BOLD:ACG5177
Cecidomyiidae|[5079]|CNRME4356-12|Cecidomyiidae|673|BOLD:ACC8782
Cecidomyiidae|[5080]|CNLMO086-14|Cecidomyiidae|658|BOLD:ACO9617
Cecidomyiidae|[5081]|SSGBA1900-14|Cecidomyiidae|658|BOLD:ACO9571
Cecidomyiidae|[5082]|CNSLI484-12|Cecidomyiidae|670|BOLD:ACB3680
Cecidomyiidae|[5083]|CNWLF950-12|Cecidomyiidae|663|BOLD:ACD1212
Cecidomyiidae|[5084]|CNGIJ136-13|Cecidomyiidae|661|BOLD:ACC1724
Cecidomyiidae|[5085]|CNFNS689-14|Cecidomyiidae|609|BOLD:ACN0540
Cecidomyiidae|[5086]|CNSLP305-13|Cecidomyiidae|661|BOLD:ACA7502
Cecidomyiidae|[5087]|CNFDF1010-14|Cecidomyiidae|614|BOLD:ACL9012
Cecidomyiidae|[5088]|CNKJH530-14|Cecidomyiidae|609|BOLD:ACM9516
Cecidomyiidae|[5089]|CNMIH651-14|Cecidomyiidae|543|BOLD:ACU3352
Cecidomyiidae|[5090]|CNKOI327-14|Cecidomyiidae|534|BOLD:ACM6818
Cecidomyiidae|[5091]|CNLMH176-14|Cecidomyiidae|555|BOLD:ACN7153
Cecidomyiidae|[5092]|CNNHC1839-14|Cecidomyiidae|597|BOLD:ACR5167
Cecidomyiidae|[5093]|CNKLC3130-14|Cecidomyiidae|579|BOLD:ACC0140
Cecidomyiidae|[5094]|CNNHB2463-14|Cecidomyiidae|534|BOLD:ACR5283
Cecidomyiidae|[5095]|CNKLC3278-14|Cecidomyiidae|627|BOLD:ACG7118
Cecidomyiidae|[5096]|CNKLF2801-15|Cecidomyiidae|600|BOLD:ACR5235
Cecidomyiidae|[5097]|CNLME210-14|Cecidomyiidae|594|BOLD:ACM9814
Cecidomyiidae|[5098]|SSPAA4020-13|Cecidomyiidae|658|BOLD:ACG3586
Cecidomyiidae|[5099]|CNMIG1994-14|Cecidomyiidae|588|BOLD:ACN3657

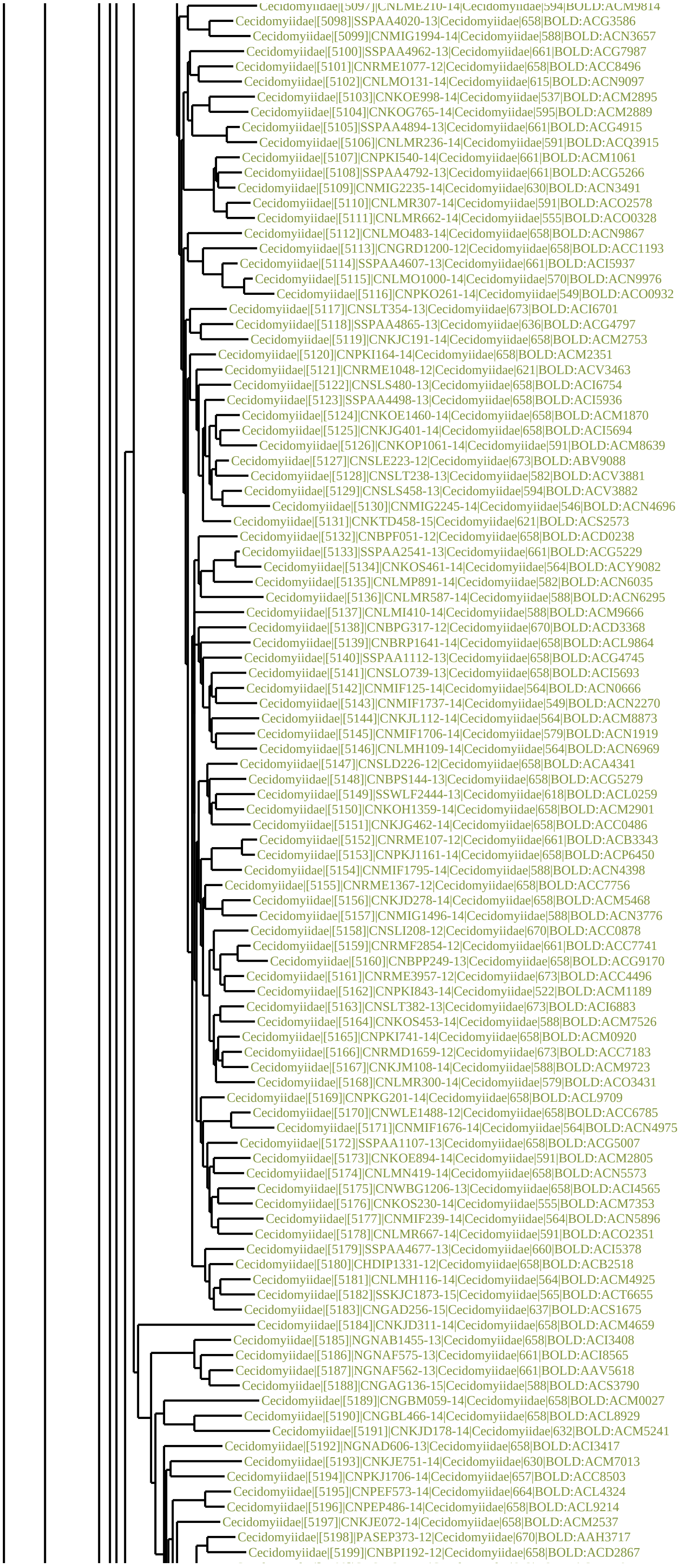

Cecidomyiidae|[5097]|CNLME210-14|Cecidomyiidae|594|BOLD:ACM9814
Cecidomyiidae|[5098]|SSPAA4020-13|Cecidomyiidae|658|BOLD:ACG3586
Cecidomyiidae|[5099]|CNMIG1994-14|Cecidomyiidae|588|BOLD:ACN3657
Cecidomyiidae|[5100]|SSPAA4962-13|Cecidomyiidae|661|BOLD:ACG7987
Cecidomyiidae|[5101]|CNRME1077-12|Cecidomyiidae|658|BOLD:ACC8496
Cecidomyiidae|[5102]|CNLMO131-14|Cecidomyiidae|615|BOLD:ACN9097
Cecidomyiidae|[5103]|CNKOE998-14|Cecidomyiidae|537|BOLD:ACM2895
Cecidomyiidae|[5104]|CNKOG765-14|Cecidomyiidae|595|BOLD:ACM2889
Cecidomyiidae|[5105]|SSPAA4894-13|Cecidomyiidae|661|BOLD:ACG4915
Cecidomyiidae|[5106]|CNLMR236-14|Cecidomyiidae|591|BOLD:ACQ3915
Cecidomyiidae|[5107]|CNPKI540-14|Cecidomyiidae|661|BOLD:ACM1061
Cecidomyiidae|[5108]|SSPAA4792-13|Cecidomyiidae|661|BOLD:ACG5266
Cecidomyiidae|[5109]|CNMIG2235-14|Cecidomyiidae|630|BOLD:ACN3491
Cecidomyiidae|[5110]|CNLMR307-14|Cecidomyiidae|591|BOLD:ACO2578
Cecidomyiidae|[5111]|CNLMR662-14|Cecidomyiidae|555|BOLD:ACO0328
Cecidomyiidae|[5112]|CNLMO483-14|Cecidomyiidae|658|BOLD:ACN9867
Cecidomyiidae|[5113]|CNGRD1200-12|Cecidomyiidae|658|BOLD:ACC1193
Cecidomyiidae|[5114]|SSPAA4607-13|Cecidomyiidae|661|BOLD:ACI5937
Cecidomyiidae|[5115]|CNLMO1000-14|Cecidomyiidae|570|BOLD:ACN9976
Cecidomyiidae|[5116]|CNPKO261-14|Cecidomyiidae|549|BOLD:ACO0932
Cecidomyiidae|[5117]|CNSLT354-13|Cecidomyiidae|673|BOLD:ACI6701
Cecidomyiidae|[5118]|SSPAA4865-13|Cecidomyiidae|636|BOLD:ACG4797
Cecidomyiidae|[5119]|CNKJC191-14|Cecidomyiidae|658|BOLD:ACM2753
Cecidomyiidae|[5120]|CNPKI164-14|Cecidomyiidae|658|BOLD:ACM2351
Cecidomyiidae|[5121]|CNRME1048-12|Cecidomyiidae|621|BOLD:ACV3463
Cecidomyiidae|[5122]|CNSLS480-13|Cecidomyiidae|658|BOLD:ACI6754
Cecidomyiidae|[5123]|SSPAA4498-13|Cecidomyiidae|658|BOLD:ACI5936
Cecidomyiidae|[5124]|CNKOE1460-14|Cecidomyiidae|658|BOLD:ACM1870
Cecidomyiidae|[5125]|CNKJG401-14|Cecidomyiidae|658|BOLD:ACI5694
Cecidomyiidae|[5126]|CNKOP1061-14|Cecidomyiidae|591|BOLD:ACM8639
Cecidomyiidae|[5127]|CNSLE223-12|Cecidomyiidae|673|BOLD:ABV9088
Cecidomyiidae|[5128]|CNSLT238-13|Cecidomyiidae|582|BOLD:ACV3881
Cecidomyiidae|[5129]|CNSLS458-13|Cecidomyiidae|594|BOLD:ACV3882
Cecidomyiidae|[5130]|CNMIG2245-14|Cecidomyiidae|546|BOLD:ACN4696
Cecidomyiidae|[5131]|CNKTD458-15|Cecidomyiidae|621|BOLD:ACS2573
Cecidomyiidae|[5132]|CNBPF051-12|Cecidomyiidae|658|BOLD:ACD0238
Cecidomyiidae|[5133]|SSPAA2541-13|Cecidomyiidae|661|BOLD:ACG5229
Cecidomyiidae|[5134]|CNKOS461-14|Cecidomyiidae|564|BOLD:ACY9082
Cecidomyiidae|[5135]|CNLMP891-14|Cecidomyiidae|582|BOLD:ACN6035
Cecidomyiidae|[5136]|CNLMR587-14|Cecidomyiidae|588|BOLD:ACN6295
Cecidomyiidae|[5137]|CNLMI410-14|Cecidomyiidae|588|BOLD:ACM9666
Cecidomyiidae|[5138]|CNBPG317-12|Cecidomyiidae|670|BOLD:ACD3368
Cecidomyiidae|[5139]|CNBRP1641-14|Cecidomyiidae|658|BOLD:ACL9864
Cecidomyiidae|[5140]|SSPAA1112-13|Cecidomyiidae|658|BOLD:ACG4745
Cecidomyiidae|[5141]|CNSLO739-13|Cecidomyiidae|658|BOLD:ACI5693
Cecidomyiidae|[5142]|CNMIF125-14|Cecidomyiidae|564|BOLD:ACN0666
Cecidomyiidae|[5143]|CNMIF1737-14|Cecidomyiidae|549|BOLD:ACN2270
Cecidomyiidae|[5144]|CNKJL112-14|Cecidomyiidae|564|BOLD:ACM8873
Cecidomyiidae|[5145]|CNMIF1706-14|Cecidomyiidae|579|BOLD:ACN1919
Cecidomyiidae|[5146]|CNLMH109-14|Cecidomyiidae|564|BOLD:ACN6969
Cecidomyiidae|[5147]|CNSLD226-12|Cecidomyiidae|658|BOLD:ACA4341
Cecidomyiidae|[5148]|CNBPS144-13|Cecidomyiidae|658|BOLD:ACG5279
Cecidomyiidae|[5149]|SSWLF2444-13|Cecidomyiidae|618|BOLD:ACL0259
Cecidomyiidae|[5150]|CNKOH1359-14|Cecidomyiidae|658|BOLD:ACM2901
Cecidomyiidae|[5151]|CNKJG462-14|Cecidomyiidae|658|BOLD:ACC0486
Cecidomyiidae|[5152]|CNRME107-12|Cecidomyiidae|661|BOLD:ACB3343
Cecidomyiidae|[5153]|CNPKJ1161-14|Cecidomyiidae|658|BOLD:ACP6450
Cecidomyiidae|[5154]|CNMIF1795-14|Cecidomyiidae|588|BOLD:ACN4398
Cecidomyiidae|[5155]|CNRME1367-12|Cecidomyiidae|658|BOLD:ACC7756
Cecidomyiidae|[5156]|CNKJD278-14|Cecidomyiidae|658|BOLD:ACM5468
Cecidomyiidae|[5157]|CNMIG1496-14|Cecidomyiidae|588|BOLD:ACN3776
Cecidomyiidae|[5158]|CNSLI208-12|Cecidomyiidae|670|BOLD:ACC0878
Cecidomyiidae|[5159]|CNRMF2854-12|Cecidomyiidae|661|BOLD:ACC7741
Cecidomyiidae|[5160]|CNBPP249-13|Cecidomyiidae|658|BOLD:ACG9170
Cecidomyiidae|[5161]|CNRME3957-12|Cecidomyiidae|673|BOLD:ACC4496
Cecidomyiidae|[5162]|CNPKI843-14|Cecidomyiidae|522|BOLD:ACM1189
Cecidomyiidae|[5163]|CNSLT382-13|Cecidomyiidae|673|BOLD:ACI6883
Cecidomyiidae|[5164]|CNKOS453-14|Cecidomyiidae|588|BOLD:ACM7526
Cecidomyiidae|[5165]|CNPKI741-14|Cecidomyiidae|658|BOLD:ACM0920
Cecidomyiidae|[5166]|CNRMD1659-12|Cecidomyiidae|673|BOLD:ACC7183
Cecidomyiidae|[5167]|CNKJM108-14|Cecidomyiidae|588|BOLD:ACM9723
Cecidomyiidae|[5168]|CNLMR300-14|Cecidomyiidae|579|BOLD:ACO3431
Cecidomyiidae|[5169]|CNPKG201-14|Cecidomyiidae|658|BOLD:ACL9709
Cecidomyiidae|[5170]|CNWLE1488-12|Cecidomyiidae|658|BOLD:ACC6785
Cecidomyiidae|[5171]|CNMIF1676-14|Cecidomyiidae|564|BOLD:ACN4975
Cecidomyiidae|[5172]|SSPAA1107-13|Cecidomyiidae|658|BOLD:ACG5007
Cecidomyiidae|[5173]|CNKOE894-14|Cecidomyiidae|591|BOLD:ACM2805
Cecidomyiidae|[5174]|CNLMN419-14|Cecidomyiidae|658|BOLD:ACN5573
Cecidomyiidae|[5175]|CNWBG1206-13|Cecidomyiidae|658|BOLD:ACI4565
Cecidomyiidae|[5176]|CNKOS230-14|Cecidomyiidae|555|BOLD:ACM7353
Cecidomyiidae|[5177]|CNMIF239-14|Cecidomyiidae|564|BOLD:ACN5896
Cecidomyiidae|[5178]|CNLMR667-14|Cecidomyiidae|591|BOLD:ACO2351
Cecidomyiidae|[5179]|SSPAA4677-13|Cecidomyiidae|660|BOLD:ACI5378
Cecidomyiidae|[5180]|CHDIP1331-12|Cecidomyiidae|658|BOLD:ACB2518
Cecidomyiidae|[5181]|CNLMH116-14|Cecidomyiidae|564|BOLD:ACM4925
Cecidomyiidae|[5182]|SSKJC1873-15|Cecidomyiidae|565|BOLD:ACT6655
Cecidomyiidae|[5183]|CNGAD256-15|Cecidomyiidae|637|BOLD:ACS1675
Cecidomyiidae|[5184]|CNKJD311-14|Cecidomyiidae|658|BOLD:ACM4659
Cecidomyiidae|[5185]|NGNAB1455-13|Cecidomyiidae|658|BOLD:ACI3408
Cecidomyiidae|[5186]|NGNAF575-13|Cecidomyiidae|661|BOLD:ACI8565
Cecidomyiidae|[5187]|NGNAF562-13|Cecidomyiidae|661|BOLD:AAV5618
Cecidomyiidae|[5188]|CNGAG136-15|Cecidomyiidae|588|BOLD:ACS3790
Cecidomyiidae|[5189]|CNGBM059-14|Cecidomyiidae|658|BOLD:ACM0027
Cecidomyiidae|[5190]|CNGBL466-14|Cecidomyiidae|658|BOLD:ACL8929
Cecidomyiidae|[5191]|CNKJD178-14|Cecidomyiidae|632|BOLD:ACM5241
Cecidomyiidae|[5192]|NGNAD606-13|Cecidomyiidae|658|BOLD:ACI3417
Cecidomyiidae|[5193]|CNKJE751-14|Cecidomyiidae|630|BOLD:ACM7013
Cecidomyiidae|[5194]|CNPKJ1706-14|Cecidomyiidae|657|BOLD:ACC8503
Cecidomyiidae|[5195]|CNPEF573-14|Cecidomyiidae|664|BOLD:ACL4324
Cecidomyiidae|[5196]|CNPEP486-14|Cecidomyiidae|658|BOLD:ACL9214
Cecidomyiidae|[5197]|CNKJE072-14|Cecidomyiidae|658|BOLD:ACM2537
Cecidomyiidae|[5198]|PASEP373-12|Cecidomyiidae|670|BOLD:AAH3717
Cecidomyiidae|[5199]|CNBPI192-12|Cecidomyiidae|658|BOLD:ACD2867

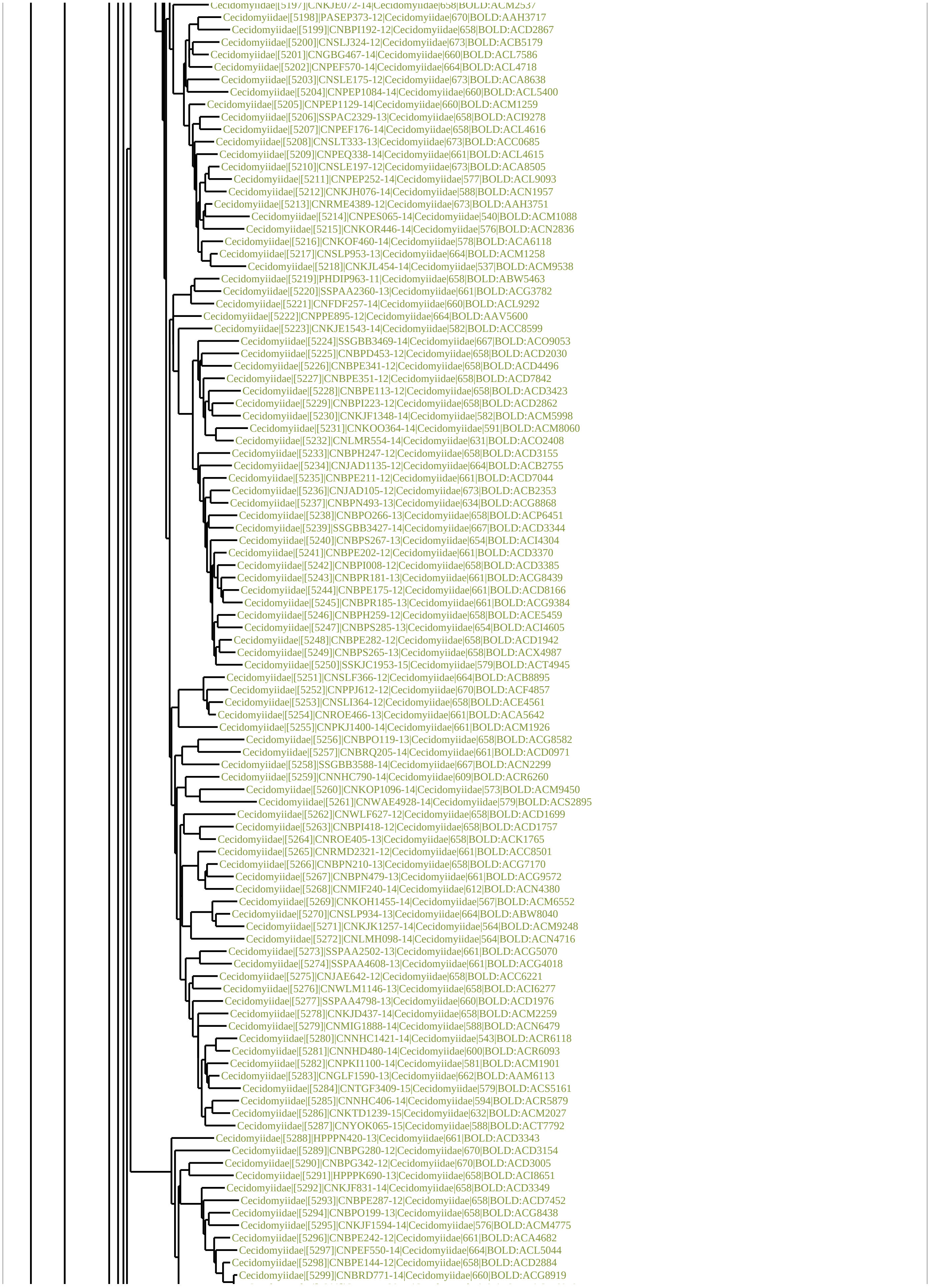

Cecidomyiidae|[5197]|CNKJE072-14|Cecidomyiidae|658|BOLD:ACM2537
Cecidomyiidae|[5198]|PASEP373-12|Cecidomyiidae|670|BOLD:AAH3717
Cecidomyiidae|[5199]|CNBPI192-12|Cecidomyiidae|658|BOLD:ACD2867
Cecidomyiidae|[5200]|CNSLJ324-12|Cecidomyiidae|673|BOLD:ACB5179
Cecidomyiidae|[5201]|CNGBG467-14|Cecidomyiidae|660|BOLD:ACL7586
Cecidomyiidae|[5202]|CNPEF570-14|Cecidomyiidae|664|BOLD:ACL4718
Cecidomyiidae|[5203]|CNSLE175-12|Cecidomyiidae|673|BOLD:ACA8638
Cecidomyiidae|[5204]|CNPEP1084-14|Cecidomyiidae|660|BOLD:ACL5400
Cecidomyiidae|[5205]|CNPEP1129-14|Cecidomyiidae|660|BOLD:ACM1259
Cecidomyiidae|[5206]|SSPAC2329-13|Cecidomyiidae|658|BOLD:ACI9278
Cecidomyiidae|[5207]|CNPEF176-14|Cecidomyiidae|658|BOLD:ACL4616
Cecidomyiidae|[5208]|CNSLT333-13|Cecidomyiidae|673|BOLD:ACC0685
Cecidomyiidae|[5209]|CNPEQ338-14|Cecidomyiidae|661|BOLD:ACL4615
Cecidomyiidae|[5210]|CNSLE197-12|Cecidomyiidae|673|BOLD:ACA8505
Cecidomyiidae|[5211]|CNPEP252-14|Cecidomyiidae|577|BOLD:ACL9093
Cecidomyiidae|[5212]|CNKJH076-14|Cecidomyiidae|588|BOLD:ACN1957
Cecidomyiidae|[5213]|CNRME4389-12|Cecidomyiidae|673|BOLD:AAH3751
Cecidomyiidae|[5214]|CNPES065-14|Cecidomyiidae|540|BOLD:ACM1088
Cecidomyiidae|[5215]|CNKOR446-14|Cecidomyiidae|576|BOLD:ACN2836
Cecidomyiidae|[5216]|CNKOF460-14|Cecidomyiidae|578|BOLD:ACA6118
Cecidomyiidae|[5217]|CNSLP953-13|Cecidomyiidae|664|BOLD:ACM1258
Cecidomyiidae|[5218]|CNKJL454-14|Cecidomyiidae|537|BOLD:ACM9538
Cecidomyiidae|[5219]|PHDIP963-11|Cecidomyiidae|658|BOLD:ABW5463
Cecidomyiidae|[5220]|SSPAA2360-13|Cecidomyiidae|661|BOLD:ACG3782
Cecidomyiidae|[5221]|CNFDF257-14|Cecidomyiidae|660|BOLD:ACL9292
Cecidomyiidae|[5222]|CNPPE895-12|Cecidomyiidae|664|BOLD:AAV5600
Cecidomyiidae|[5223]|CNKJE1543-14|Cecidomyiidae|582|BOLD:ACC8599
Cecidomyiidae|[5224]|SSGBB3469-14|Cecidomyiidae|667|BOLD:ACO9053
Cecidomyiidae|[5225]|CNBPD453-12|Cecidomyiidae|658|BOLD:ACD2030
Cecidomyiidae|[5226]|CNBPE341-12|Cecidomyiidae|658|BOLD:ACD4496
Cecidomyiidae|[5227]|CNBPE351-12|Cecidomyiidae|658|BOLD:ACD7842
Cecidomyiidae|[5228]|CNBPE113-12|Cecidomyiidae|658|BOLD:ACD3423
Cecidomyiidae|[5229]|CNBPI223-12|Cecidomyiidae|658|BOLD:ACD2862
Cecidomyiidae|[5230]|CNKJF1348-14|Cecidomyiidae|582|BOLD:ACM5998
Cecidomyiidae|[5231]|CNKOO364-14|Cecidomyiidae|591|BOLD:ACM8060
Cecidomyiidae|[5232]|CNLMR554-14|Cecidomyiidae|631|BOLD:ACO2408
Cecidomyiidae|[5233]|CNBPH247-12|Cecidomyiidae|658|BOLD:ACD3155
Cecidomyiidae|[5234]|CNJAD1135-12|Cecidomyiidae|664|BOLD:ACB2755
Cecidomyiidae|[5235]|CNBPE211-12|Cecidomyiidae|661|BOLD:ACD7044
Cecidomyiidae|[5236]|CNJAD105-12|Cecidomyiidae|673|BOLD:ACB2353
Cecidomyiidae|[5237]|CNBPN493-13|Cecidomyiidae|634|BOLD:ACG8868
Cecidomyiidae|[5238]|CNBPO266-13|Cecidomyiidae|658|BOLD:ACP6451
Cecidomyiidae|[5239]|SSGBB3427-14|Cecidomyiidae|667|BOLD:ACD3344
Cecidomyiidae|[5240]|CNBPS267-13|Cecidomyiidae|654|BOLD:ACI4304
Cecidomyiidae|[5241]|CNBPE202-12|Cecidomyiidae|661|BOLD:ACD3370
Cecidomyiidae|[5242]|CNBPI008-12|Cecidomyiidae|658|BOLD:ACD3385
Cecidomyiidae|[5243]|CNBPR181-13|Cecidomyiidae|661|BOLD:ACG8439
Cecidomyiidae|[5244]|CNBPE175-12|Cecidomyiidae|661|BOLD:ACD8166
Cecidomyiidae|[5245]|CNBPR185-13|Cecidomyiidae|661|BOLD:ACG9384
Cecidomyiidae|[5246]|CNBPH259-12|Cecidomyiidae|658|BOLD:ACE5459
Cecidomyiidae|[5247]|CNBPS285-13|Cecidomyiidae|654|BOLD:ACI4605
Cecidomyiidae|[5248]|CNBPE282-12|Cecidomyiidae|658|BOLD:ACD1942
Cecidomyiidae|[5249]|CNBPS265-13|Cecidomyiidae|658|BOLD:ACX4987
Cecidomyiidae|[5250]|SSKJC1953-15|Cecidomyiidae|579|BOLD:ACT4945
Cecidomyiidae|[5251]|CNSLF366-12|Cecidomyiidae|664|BOLD:ACB8895
Cecidomyiidae|[5252]|CNPPJ612-12|Cecidomyiidae|670|BOLD:ACF4857
Cecidomyiidae|[5253]|CNSLI364-12|Cecidomyiidae|658|BOLD:ACE4561
Cecidomyiidae|[5254]|CNROE466-13|Cecidomyiidae|661|BOLD:ACA5642
Cecidomyiidae|[5255]|CNPKJ1400-14|Cecidomyiidae|661|BOLD:ACM1926
Cecidomyiidae|[5256]|CNBPO119-13|Cecidomyiidae|658|BOLD:ACG8582
Cecidomyiidae|[5257]|CNBRQ205-14|Cecidomyiidae|661|BOLD:ACD0971
Cecidomyiidae|[5258]|SSGBB3588-14|Cecidomyiidae|667|BOLD:ACN2299
Cecidomyiidae|[5259]|CNNHC790-14|Cecidomyiidae|609|BOLD:ACR6260
Cecidomyiidae|[5260]|CNKOP1096-14|Cecidomyiidae|573|BOLD:ACM9450
Cecidomyiidae|[5261]|CNWAE4928-14|Cecidomyiidae|579|BOLD:ACS2895
Cecidomyiidae|[5262]|CNWLF627-12|Cecidomyiidae|658|BOLD:ACD1699
Cecidomyiidae|[5263]|CNBPI418-12|Cecidomyiidae|658|BOLD:ACD1757
Cecidomyiidae|[5264]|CNROE405-13|Cecidomyiidae|658|BOLD:ACK1765
Cecidomyiidae|[5265]|CNRMD2321-12|Cecidomyiidae|661|BOLD:ACC8501
Cecidomyiidae|[5266]|CNBPN210-13|Cecidomyiidae|658|BOLD:ACG7170
Cecidomyiidae|[5267]|CNBPN479-13|Cecidomyiidae|661|BOLD:ACG9572
Cecidomyiidae|[5268]|CNMIF240-14|Cecidomyiidae|612|BOLD:ACN4380
Cecidomyiidae|[5269]|CNKOH1455-14|Cecidomyiidae|567|BOLD:ACM6552
Cecidomyiidae|[5270]|CNSLP934-13|Cecidomyiidae|664|BOLD:ABW8040
Cecidomyiidae|[5271]|CNKJK1257-14|Cecidomyiidae|564|BOLD:ACM9248
Cecidomyiidae|[5272]|CNLMH098-14|Cecidomyiidae|564|BOLD:ACN4716
Cecidomyiidae|[5273]|SSPAA2502-13|Cecidomyiidae|661|BOLD:ACG5070
Cecidomyiidae|[5274]|SSPAA4608-13|Cecidomyiidae|661|BOLD:ACG4018
Cecidomyiidae|[5275]|CNJAE642-12|Cecidomyiidae|658|BOLD:ACC6221
Cecidomyiidae|[5276]|CNWLM1146-13|Cecidomyiidae|658|BOLD:ACI6277
Cecidomyiidae|[5277]|SSPAA4798-13|Cecidomyiidae|660|BOLD:ACD1976
Cecidomyiidae|[5278]|CNKJD437-14|Cecidomyiidae|658|BOLD:ACM2259
Cecidomyiidae|[5279]|CNMIG1888-14|Cecidomyiidae|588|BOLD:ACN6479
Cecidomyiidae|[5280]|CNNHC1421-14|Cecidomyiidae|543|BOLD:ACR6118
Cecidomyiidae|[5281]|CNNHD480-14|Cecidomyiidae|600|BOLD:ACR6093
Cecidomyiidae|[5282]|CNPKI1100-14|Cecidomyiidae|581|BOLD:ACM1901
Cecidomyiidae|[5283]|CNGLF1590-13|Cecidomyiidae|662|BOLD:AAM6113
Cecidomyiidae|[5284]|CNTGF3409-15|Cecidomyiidae|579|BOLD:ACS5161
Cecidomyiidae|[5285]|CNNHC406-14|Cecidomyiidae|594|BOLD:ACR5879
Cecidomyiidae|[5286]|CNKTD1239-15|Cecidomyiidae|632|BOLD:ACM2027
Cecidomyiidae|[5287]|CNYOK065-15|Cecidomyiidae|588|BOLD:ACT7792
Cecidomyiidae|[5288]|HPPPN420-13|Cecidomyiidae|661|BOLD:ACD3343
Cecidomyiidae|[5289]|CNBPG280-12|Cecidomyiidae|670|BOLD:ACD3154
Cecidomyiidae|[5290]|CNBPG342-12|Cecidomyiidae|670|BOLD:ACD3005
Cecidomyiidae|[5291]|HPPPK690-13|Cecidomyiidae|658|BOLD:ACI8651
Cecidomyiidae|[5292]|CNKJF831-14|Cecidomyiidae|658|BOLD:ACD3349
Cecidomyiidae|[5293]|CNBPE287-12|Cecidomyiidae|658|BOLD:ACD7452
Cecidomyiidae|[5294]|CNBPO199-13|Cecidomyiidae|658|BOLD:ACG8438
Cecidomyiidae|[5295]|CNKJF1594-14|Cecidomyiidae|576|BOLD:ACM4775
Cecidomyiidae|[5296]|CNBPE242-12|Cecidomyiidae|661|BOLD:ACA4682
Cecidomyiidae|[5297]|CNPEF550-14|Cecidomyiidae|664|BOLD:ACL5044
Cecidomyiidae|[5298]|CNBPE144-12|Cecidomyiidae|658|BOLD:ACD2884
Cecidomyiidae|[5299]|CNBRD771-14|Cecidomyiidae|660|BOLD:ACG8919

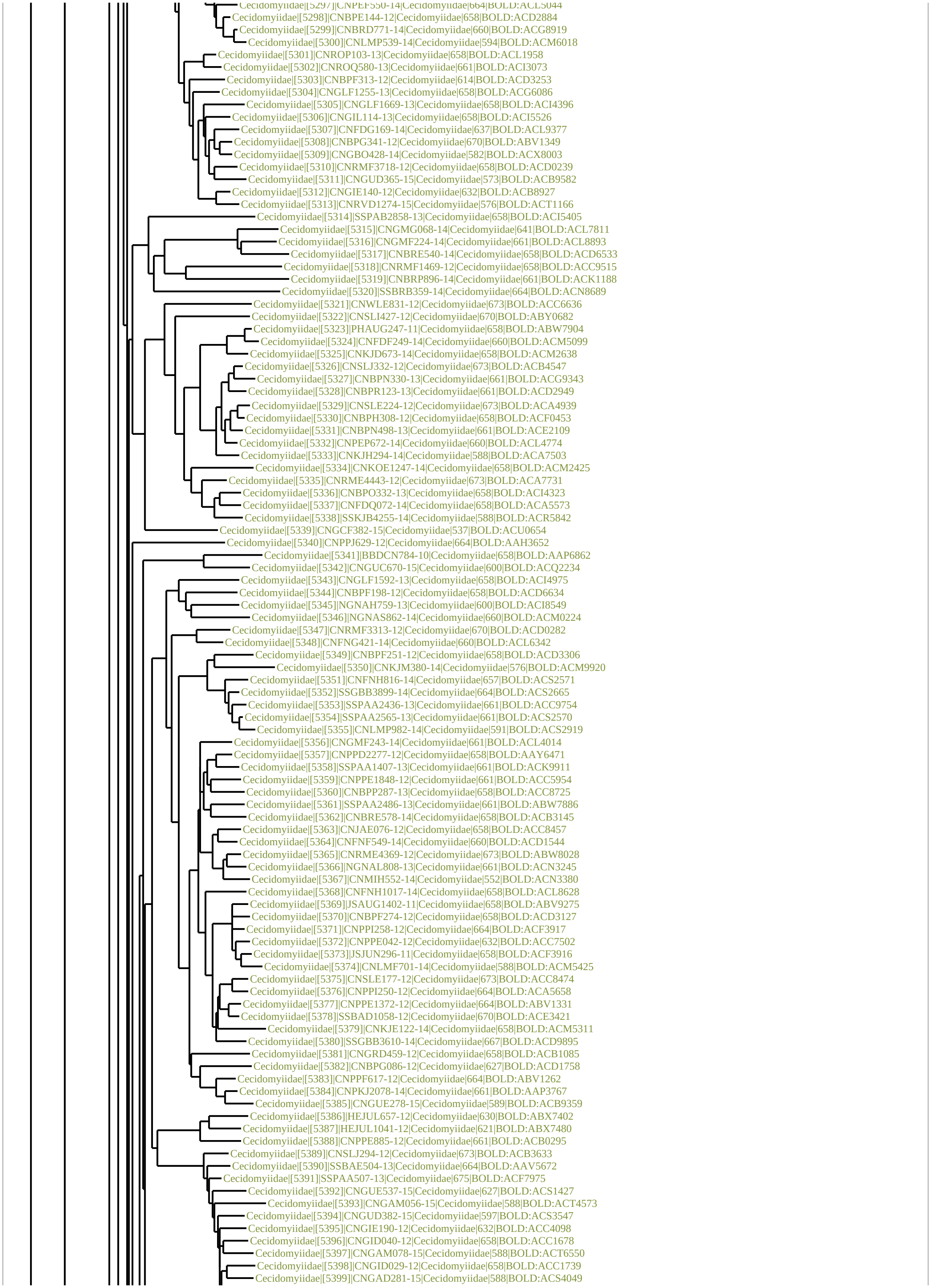
Cecidomyiidae|[5297]|CNPEF550-14|Cecidomyiidae|664|BOLD:ACL5044
Cecidomyiidae|[5298]|CNBPE144-12|Cecidomyiidae|658|BOLD:ACD2884
Cecidomyiidae|[5299]|CNBRD771-14|Cecidomyiidae|660|BOLD:ACG8919
Cecidomyiidae|[5300]|CNLMP539-14|Cecidomyiidae|594|BOLD:ACM6018
Cecidomyiidae|[5301]|CNROP103-13|Cecidomyiidae|658|BOLD:ACL1958
Cecidomyiidae|[5302]|CNROQ580-13|Cecidomyiidae|661|BOLD:ACI3073
Cecidomyiidae|[5303]|CNBPF313-12|Cecidomyiidae|614|BOLD:ACD3253
Cecidomyiidae|[5304]|CNGLF1255-13|Cecidomyiidae|658|BOLD:ACG6086
Cecidomyiidae|[5305]|CNGLF1669-13|Cecidomyiidae|658|BOLD:ACI4396
Cecidomyiidae|[5306]|CNGIL114-13|Cecidomyiidae|658|BOLD:ACI5526
Cecidomyiidae|[5307]|CNFDG169-14|Cecidomyiidae|637|BOLD:ACL9377
Cecidomyiidae|[5308]|CNBPG341-12|Cecidomyiidae|670|BOLD:ABV1349
Cecidomyiidae|[5309]|CNGBO428-14|Cecidomyiidae|582|BOLD:ACX8003
Cecidomyiidae|[5310]|CNRMF3718-12|Cecidomyiidae|658|BOLD:ACD0239
Cecidomyiidae|[5311]|CNGUD365-15|Cecidomyiidae|573|BOLD:ACB9582
Cecidomyiidae|[5312]|CNGIE140-12|Cecidomyiidae|632|BOLD:ACB8927
Cecidomyiidae|[5313]|CNRVD1274-15|Cecidomyiidae|576|BOLD:ACT1166
Cecidomyiidae|[5314]|SSPAB2858-13|Cecidomyiidae|658|BOLD:ACI5405
Cecidomyiidae|[5315]|CNGMG068-14|Cecidomyiidae|641|BOLD:ACL7811
Cecidomyiidae|[5316]|CNGMF224-14|Cecidomyiidae|661|BOLD:ACL8893
Cecidomyiidae|[5317]|CNBRE540-14|Cecidomyiidae|658|BOLD:ACD6533
Cecidomyiidae|[5318]|CNRMF1469-12|Cecidomyiidae|658|BOLD:ACC9515
Cecidomyiidae|[5319]|CNBRP896-14|Cecidomyiidae|661|BOLD:ACK1188
Cecidomyiidae|[5320]|SSBRB359-14|Cecidomyiidae|664|BOLD:ACN8689
Cecidomyiidae|[5321]|CNWLE831-12|Cecidomyiidae|673|BOLD:ACC6636
Cecidomyiidae|[5322]|CNSLI427-12|Cecidomyiidae|670|BOLD:ABY0682
Cecidomyiidae|[5323]|PHAUG247-11|Cecidomyiidae|658|BOLD:ABW7904
Cecidomyiidae|[5324]|CNFDF249-14|Cecidomyiidae|660|BOLD:ACM5099
Cecidomyiidae|[5325]|CNKJD673-14|Cecidomyiidae|658|BOLD:ACM2638
Cecidomyiidae|[5326]|CNSLJ332-12|Cecidomyiidae|673|BOLD:ACB4547
Cecidomyiidae|[5327]|CNBPN330-13|Cecidomyiidae|661|BOLD:ACG9343
Cecidomyiidae|[5328]|CNBPR123-13|Cecidomyiidae|661|BOLD:ACD2949
Cecidomyiidae|[5329]|CNSLE224-12|Cecidomyiidae|673|BOLD:ACA4939
Cecidomyiidae|[5330]|CNBPH308-12|Cecidomyiidae|658|BOLD:ACF0453
Cecidomyiidae|[5331]|CNBPN498-13|Cecidomyiidae|661|BOLD:ACE2109
Cecidomyiidae|[5332]|CNPEP672-14|Cecidomyiidae|660|BOLD:ACL4774
Cecidomyiidae|[5333]|CNKJH294-14|Cecidomyiidae|588|BOLD:ACA7503
Cecidomyiidae|[5334]|CNKOE1247-14|Cecidomyiidae|658|BOLD:ACM2425
Cecidomyiidae|[5335]|CNRME4443-12|Cecidomyiidae|673|BOLD:ACA7731
Cecidomyiidae|[5336]|CNBPO332-13|Cecidomyiidae|658|BOLD:ACI4323
Cecidomyiidae|[5337]|CNFDQ072-14|Cecidomyiidae|658|BOLD:ACA5573
Cecidomyiidae|[5338]|SSKJB4255-14|Cecidomyiidae|588|BOLD:ACR5842
Cecidomyiidae|[5339]|CNGCF382-15|Cecidomyiidae|537|BOLD:ACU0654
Cecidomyiidae|[5340]|CNPPJ629-12|Cecidomyiidae|664|BOLD:AAH3652
Cecidomyiidae|[5341]|BBDCN784-10|Cecidomyiidae|658|BOLD:AAP6862
Cecidomyiidae|[5342]|CNGUC670-15|Cecidomyiidae|600|BOLD:ACQ2234
Cecidomyiidae|[5343]|CNGLF1592-13|Cecidomyiidae|658|BOLD:ACI4975
Cecidomyiidae|[5344]|CNBPF198-12|Cecidomyiidae|658|BOLD:ACD6634
Cecidomyiidae|[5345]|NGNAH759-13|Cecidomyiidae|600|BOLD:ACI8549
Cecidomyiidae|[5346]|NGNAS862-14|Cecidomyiidae|660|BOLD:ACM0224
Cecidomyiidae|[5347]|CNRMF3313-12|Cecidomyiidae|670|BOLD:ACD0282
Cecidomyiidae|[5348]|CNFNG421-14|Cecidomyiidae|660|BOLD:ACL6342
Cecidomyiidae|[5349]|CNBPF251-12|Cecidomyiidae|658|BOLD:ACD3306
Cecidomyiidae|[5350]|CNKJM380-14|Cecidomyiidae|576|BOLD:ACM9920
Cecidomyiidae|[5351]|CNFNH816-14|Cecidomyiidae|657|BOLD:ACS2571
Cecidomyiidae|[5352]|SSGBB3899-14|Cecidomyiidae|664|BOLD:ACS2665
Cecidomyiidae|[5353]|SSPAA2436-13|Cecidomyiidae|661|BOLD:ACC9754
Cecidomyiidae|[5354]|SSPAA2565-13|Cecidomyiidae|661|BOLD:ACS2570
Cecidomyiidae|[5355]|CNLMP982-14|Cecidomyiidae|591|BOLD:ACS2919
Cecidomyiidae|[5356]|CNGMF243-14|Cecidomyiidae|661|BOLD:ACL4014
Cecidomyiidae|[5357]|CNPPD2277-12|Cecidomyiidae|658|BOLD:AAY6471
Cecidomyiidae|[5358]|SSPAA1407-13|Cecidomyiidae|661|BOLD:ACK9911
Cecidomyiidae|[5359]|CNPPE1848-12|Cecidomyiidae|661|BOLD:ACC5954
Cecidomyiidae|[5360]|CNBPP287-13|Cecidomyiidae|658|BOLD:ACC8725
Cecidomyiidae|[5361]|SSPAA2486-13|Cecidomyiidae|661|BOLD:ABW7886
Cecidomyiidae|[5362]|CNBRE578-14|Cecidomyiidae|658|BOLD:ACB3145
Cecidomyiidae|[5363]|CNJAE076-12|Cecidomyiidae|658|BOLD:ACC8457
Cecidomyiidae|[5364]|CNFNF549-14|Cecidomyiidae|660|BOLD:ACD1544
Cecidomyiidae|[5365]|CNRME4369-12|Cecidomyiidae|673|BOLD:ABW8028
Cecidomyiidae|[5366]|NGNAL808-13|Cecidomyiidae|661|BOLD:ACN3245
Cecidomyiidae|[5367]|CNMIH552-14|Cecidomyiidae|552|BOLD:ACN3380
Cecidomyiidae|[5368]|CNFNH1017-14|Cecidomyiidae|658|BOLD:ACL8628
Cecidomyiidae|[5369]|JSAUG1402-11|Cecidomyiidae|658|BOLD:ABV9275
Cecidomyiidae|[5370]|CNBPF274-12|Cecidomyiidae|658|BOLD:ACD3127
Cecidomyiidae|[5371]|CNPPI258-12|Cecidomyiidae|664|BOLD:ACF3917
Cecidomyiidae|[5372]|CNPPE042-12|Cecidomyiidae|632|BOLD:ACC7502
Cecidomyiidae|[5373]|JSJUN296-11|Cecidomyiidae|658|BOLD:ACF3916
Cecidomyiidae|[5374]|CNLMF701-14|Cecidomyiidae|588|BOLD:ACM5425
Cecidomyiidae|[5375]|CNSLE177-12|Cecidomyiidae|673|BOLD:ACC8474
Cecidomyiidae|[5376]|CNPPI250-12|Cecidomyiidae|664|BOLD:ACA5658
Cecidomyiidae|[5377]|CNPPE1372-12|Cecidomyiidae|664|BOLD:ABV1331
Cecidomyiidae|[5378]|SSBAD1058-12|Cecidomyiidae|670|BOLD:ACE3421
Cecidomyiidae|[5379]|CNKJE122-14|Cecidomyiidae|658|BOLD:ACM5311
Cecidomyiidae|[5380]|SSGBB3610-14|Cecidomyiidae|667|BOLD:ACD9895
Cecidomyiidae|[5381]|CNGRD459-12|Cecidomyiidae|658|BOLD:ACB1085
Cecidomyiidae|[5382]|CNBPG086-12|Cecidomyiidae|627|BOLD:ACD1758
Cecidomyiidae|[5383]|CNPPF617-12|Cecidomyiidae|664|BOLD:ABV1262
Cecidomyiidae|[5384]|CNPKJ2078-14|Cecidomyiidae|661|BOLD:AAP3767
Cecidomyiidae|[5385]|CNGUE278-15|Cecidomyiidae|589|BOLD:ACB9359
Cecidomyiidae|[5386]|HEJUL657-12|Cecidomyiidae|630|BOLD:ABX7402
Cecidomyiidae|[5387]|HEJUL1041-12|Cecidomyiidae|621|BOLD:ABX7480
Cecidomyiidae|[5388]|CNPPE885-12|Cecidomyiidae|661|BOLD:ACB0295
Cecidomyiidae|[5389]|CNSLJ294-12|Cecidomyiidae|673|BOLD:ACB3633
Cecidomyiidae|[5390]|SSBAE504-13|Cecidomyiidae|664|BOLD:AAV5672
Cecidomyiidae|[5391]|SSPAA507-13|Cecidomyiidae|675|BOLD:ACF7975
Cecidomyiidae|[5392]|CNGUE537-15|Cecidomyiidae|627|BOLD:ACS1427
Cecidomyiidae|[5393]|CNGAM056-15|Cecidomyiidae|588|BOLD:ACT4573
Cecidomyiidae|[5394]|CNGUD382-15|Cecidomyiidae|597|BOLD:ACS3547
Cecidomyiidae|[5395]|CNGIE190-12|Cecidomyiidae|632|BOLD:ACC4098
Cecidomyiidae|[5396]|CNGID040-12|Cecidomyiidae|658|BOLD:ACC1678
Cecidomyiidae|[5397]|CNGAM078-15|Cecidomyiidae|588|BOLD:ACT6550
Cecidomyiidae|[5398]|CNGID029-12|Cecidomyiidae|658|BOLD:ACC1739
Cecidomyiidae|[5399]|CNGAD281-15|Cecidomyiidae|588|BOLD:ACS4049

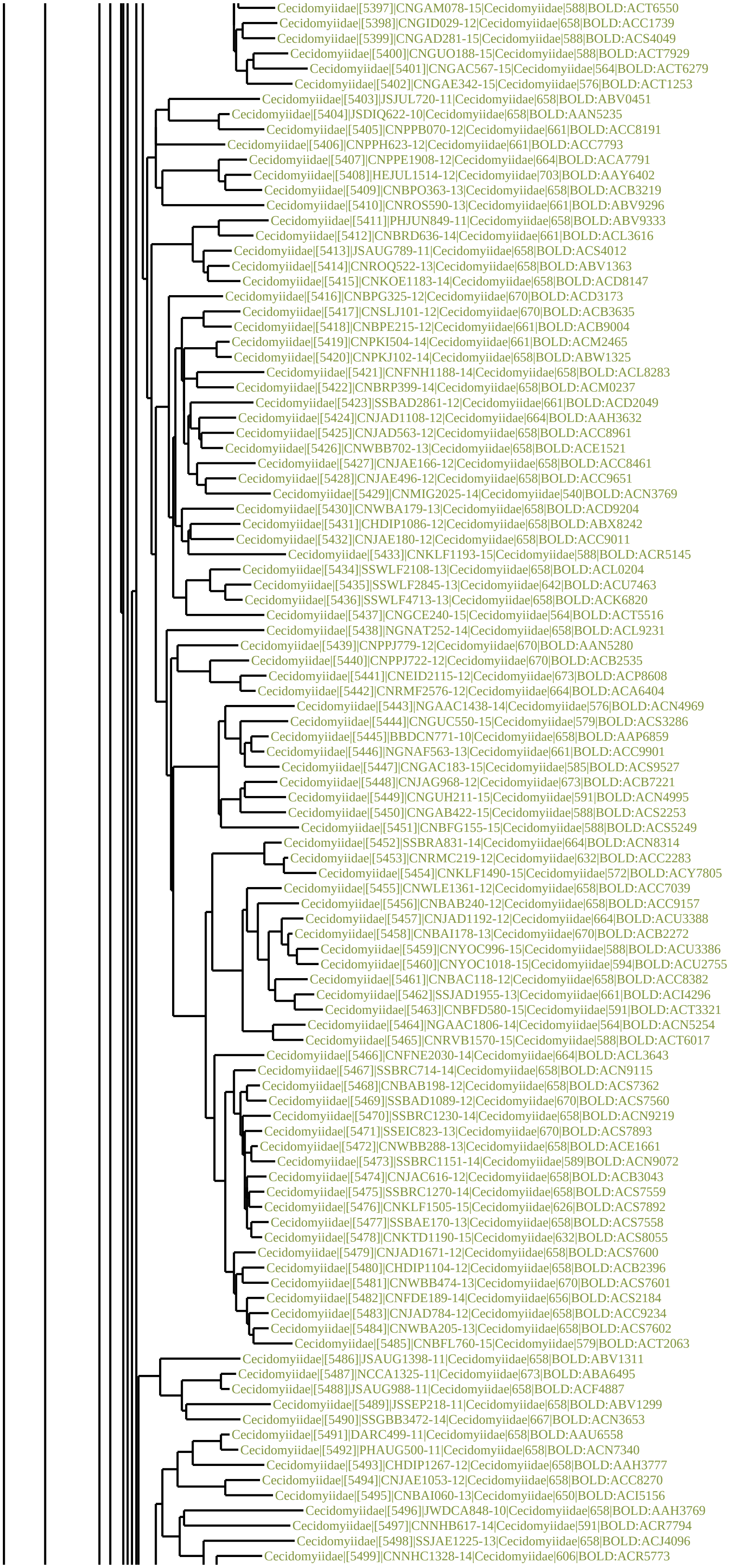
Cecidomyiidae|[5397]|CNGAM078-15|Cecidomyiidae|588|BOLD:ACT6550
Cecidomyiidae|[5398]|CNGID029-12|Cecidomyiidae|658|BOLD:ACC1739
Cecidomyiidae|[5399]|CNGAD281-15|Cecidomyiidae|588|BOLD:ACS4049
Cecidomyiidae|[5400]|CNGUO188-15|Cecidomyiidae|588|BOLD:ACT7929
Cecidomyiidae|[5401]|CNGAC567-15|Cecidomyiidae|564|BOLD:ACT6279
Cecidomyiidae|[5402]|CNGAE342-15|Cecidomyiidae|576|BOLD:ACT1253
Cecidomyiidae|[5403]|JSJUL720-11|Cecidomyiidae|658|BOLD:ABV0451
Cecidomyiidae|[5404]|JSDIQ622-10|Cecidomyiidae|658|BOLD:AAN5235
Cecidomyiidae|[5405]|CNPPB070-12|Cecidomyiidae|661|BOLD:ACC8191
Cecidomyiidae|[5406]|CNPPH623-12|Cecidomyiidae|661|BOLD:ACC7793
Cecidomyiidae|[5407]|CNPPE1908-12|Cecidomyiidae|664|BOLD:ACA7791
Cecidomyiidae|[5408]|HEJUL1514-12|Cecidomyiidae|703|BOLD:AAY6402
Cecidomyiidae|[5409]|CNBPO363-13|Cecidomyiidae|658|BOLD:ACB3219
Cecidomyiidae|[5410]|CNROS590-13|Cecidomyiidae|661|BOLD:ABV9296
Cecidomyiidae|[5411]|PHJUN849-11|Cecidomyiidae|658|BOLD:ABV9333
Cecidomyiidae|[5412]|CNBRD636-14|Cecidomyiidae|661|BOLD:ACL3616
Cecidomyiidae|[5413]|JSAUG789-11|Cecidomyiidae|658|BOLD:ACS4012
Cecidomyiidae|[5414]|CNROQ522-13|Cecidomyiidae|658|BOLD:ABV1363
Cecidomyiidae|[5415]|CNKOE1183-14|Cecidomyiidae|658|BOLD:ACD8147
Cecidomyiidae|[5416]|CNBPG325-12|Cecidomyiidae|670|BOLD:ACD3173
Cecidomyiidae|[5417]|CNSLJ101-12|Cecidomyiidae|670|BOLD:ACB3635
Cecidomyiidae|[5418]|CNBPE215-12|Cecidomyiidae|661|BOLD:ACB9004
Cecidomyiidae|[5419]|CNPKI504-14|Cecidomyiidae|661|BOLD:ACM2465
Cecidomyiidae|[5420]|CNPKJ102-14|Cecidomyiidae|658|BOLD:ABW1325
Cecidomyiidae|[5421]|CNFNH1188-14|Cecidomyiidae|658|BOLD:ACL8283
Cecidomyiidae|[5422]|CNBRP399-14|Cecidomyiidae|658|BOLD:ACM0237
Cecidomyiidae|[5423]|SSBAD2861-12|Cecidomyiidae|661|BOLD:ACD2049
Cecidomyiidae|[5424]|CNJAD1108-12|Cecidomyiidae|664|BOLD:AAH3632
Cecidomyiidae|[5425]|CNJAD563-12|Cecidomyiidae|658|BOLD:ACC8961
Cecidomyiidae|[5426]|CNWBB702-13|Cecidomyiidae|658|BOLD:ACE1521
Cecidomyiidae|[5427]|CNJAE166-12|Cecidomyiidae|658|BOLD:ACC8461
Cecidomyiidae|[5428]|CNJAE496-12|Cecidomyiidae|658|BOLD:ACC9651
Cecidomyiidae|[5429]|CNMIG2025-14|Cecidomyiidae|540|BOLD:ACN3769
Cecidomyiidae|[5430]|CNWBA179-13|Cecidomyiidae|658|BOLD:ACD9204
Cecidomyiidae|[5431]|CHDIP1086-12|Cecidomyiidae|658|BOLD:ABX8242
Cecidomyiidae|[5432]|CNJAE180-12|Cecidomyiidae|658|BOLD:ACC9011
Cecidomyiidae|[5433]|CNKLF1193-15|Cecidomyiidae|588|BOLD:ACR5145
Cecidomyiidae|[5434]|SSWLF2108-13|Cecidomyiidae|658|BOLD:ACL0204
Cecidomyiidae|[5435]|SSWLF2845-13|Cecidomyiidae|642|BOLD:ACU7463
Cecidomyiidae|[5436]|SSWLF4713-13|Cecidomyiidae|658|BOLD:ACK6820
Cecidomyiidae|[5437]|CNGCE240-15|Cecidomyiidae|564|BOLD:ACT5516
Cecidomyiidae|[5438]|NGNAT252-14|Cecidomyiidae|658|BOLD:ACL9231
Cecidomyiidae|[5439]|CNPPJ779-12|Cecidomyiidae|670|BOLD:AAN5280
Cecidomyiidae|[5440]|CNPPJ722-12|Cecidomyiidae|670|BOLD:ACB2535
Cecidomyiidae|[5441]|CNEID2115-12|Cecidomyiidae|673|BOLD:ACP8608
Cecidomyiidae|[5442]|CNRMF2576-12|Cecidomyiidae|664|BOLD:ACA6404
Cecidomyiidae|[5443]|NGAAC1438-14|Cecidomyiidae|576|BOLD:ACN4969
Cecidomyiidae|[5444]|CNGUC550-15|Cecidomyiidae|579|BOLD:ACS3286
Cecidomyiidae|[5445]|BBDCN771-10|Cecidomyiidae|658|BOLD:AAP6859
Cecidomyiidae|[5446]|NGNAF563-13|Cecidomyiidae|661|BOLD:ACC9901
Cecidomyiidae|[5447]|CNGAC183-15|Cecidomyiidae|585|BOLD:ACS9527
Cecidomyiidae|[5448]|CNJAG968-12|Cecidomyiidae|673|BOLD:ACB7221
Cecidomyiidae|[5449]|CNGUH211-15|Cecidomyiidae|591|BOLD:ACN4995
Cecidomyiidae|[5450]|CNGAB422-15|Cecidomyiidae|588|BOLD:ACS2253
Cecidomyiidae|[5451]|CNBFG155-15|Cecidomyiidae|588|BOLD:ACS5249
Cecidomyiidae|[5452]|SSBRA831-14|Cecidomyiidae|664|BOLD:ACN8314
Cecidomyiidae|[5453]|CNRMC219-12|Cecidomyiidae|632|BOLD:ACC2283
Cecidomyiidae|[5454]|CNKLF1490-15|Cecidomyiidae|572|BOLD:ACY7805
Cecidomyiidae|[5455]|CNWLE1361-12|Cecidomyiidae|658|BOLD:ACC7039
Cecidomyiidae|[5456]|CNBAB240-12|Cecidomyiidae|658|BOLD:ACC9157
Cecidomyiidae|[5457]|CNJAD1192-12|Cecidomyiidae|664|BOLD:ACU3388
Cecidomyiidae|[5458]|CNBAI178-13|Cecidomyiidae|670|BOLD:ACB2272
Cecidomyiidae|[5459]|CNYOC996-15|Cecidomyiidae|588|BOLD:ACU3386
Cecidomyiidae|[5460]|CNYOC1018-15|Cecidomyiidae|594|BOLD:ACU2755
Cecidomyiidae|[5461]|CNBAC118-12|Cecidomyiidae|658|BOLD:ACC8382
Cecidomyiidae|[5462]|SSJAD1955-13|Cecidomyiidae|661|BOLD:ACI4296
Cecidomyiidae|[5463]|CNBFD580-15|Cecidomyiidae|591|BOLD:ACT3321
Cecidomyiidae|[5464]|NGAAC1806-14|Cecidomyiidae|564|BOLD:ACN5254
Cecidomyiidae|[5465]|CNRVB1570-15|Cecidomyiidae|588|BOLD:ACT6017
Cecidomyiidae|[5466]|CNFNE2030-14|Cecidomyiidae|664|BOLD:ACL3643
Cecidomyiidae|[5467]|SSBRC714-14|Cecidomyiidae|658|BOLD:ACN9115
Cecidomyiidae|[5468]|CNBAB198-12|Cecidomyiidae|658|BOLD:ACS7362
Cecidomyiidae|[5469]|SSBAD1089-12|Cecidomyiidae|670|BOLD:ACS7560
Cecidomyiidae|[5470]|SSBRC1230-14|Cecidomyiidae|658|BOLD:ACN9219
Cecidomyiidae|[5471]|SSEIC823-13|Cecidomyiidae|670|BOLD:ACS7893
Cecidomyiidae|[5472]|CNWBB288-13|Cecidomyiidae|658|BOLD:ACE1661
Cecidomyiidae|[5473]|SSBRC1151-14|Cecidomyiidae|589|BOLD:ACN9072
Cecidomyiidae|[5474]|CNJAC616-12|Cecidomyiidae|658|BOLD:ACB3043
Cecidomyiidae|[5475]|SSBRC1270-14|Cecidomyiidae|658|BOLD:ACS7559
Cecidomyiidae|[5476]|CNKLF1505-15|Cecidomyiidae|626|BOLD:ACS7892
Cecidomyiidae|[5477]|SSBAE170-13|Cecidomyiidae|658|BOLD:ACS7558
Cecidomyiidae|[5478]|CNKTD1190-15|Cecidomyiidae|632|BOLD:ACS8055
Cecidomyiidae|[5479]|CNJAD1671-12|Cecidomyiidae|658|BOLD:ACS7600
Cecidomyiidae|[5480]|CHDIP1104-12|Cecidomyiidae|658|BOLD:ACB2396
Cecidomyiidae|[5481]|CNWBB474-13|Cecidomyiidae|670|BOLD:ACS7601
Cecidomyiidae|[5482]|CNFDE189-14|Cecidomyiidae|656|BOLD:ACS2184
Cecidomyiidae|[5483]|CNJAD784-12|Cecidomyiidae|658|BOLD:ACC9234
Cecidomyiidae|[5484]|CNWBA205-13|Cecidomyiidae|658|BOLD:ACS7602
Cecidomyiidae|[5485]|CNBFL760-15|Cecidomyiidae|579|BOLD:ACT2063
Cecidomyiidae|[5486]|JSAUG1398-11|Cecidomyiidae|658|BOLD:ABV1311
Cecidomyiidae|[5487]|NCCA1325-11|Cecidomyiidae|673|BOLD:ABA6495
Cecidomyiidae|[5488]|JSAUG988-11|Cecidomyiidae|658|BOLD:ACF4887
Cecidomyiidae|[5489]|JSSEP218-11|Cecidomyiidae|658|BOLD:ABV1299
Cecidomyiidae|[5490]|SSGBB3472-14|Cecidomyiidae|667|BOLD:ACN3653
Cecidomyiidae|[5491]|DARC499-11|Cecidomyiidae|658|BOLD:AAU6558
Cecidomyiidae|[5492]|PHAUG500-11|Cecidomyiidae|658|BOLD:ACN7340
Cecidomyiidae|[5493]|CHDIP1267-12|Cecidomyiidae|658|BOLD:AAH3777
Cecidomyiidae|[5494]|CNJAE1053-12|Cecidomyiidae|658|BOLD:ACC8270
Cecidomyiidae|[5495]|CNBAI060-13|Cecidomyiidae|650|BOLD:ACI5156
Cecidomyiidae|[5496]|JWDCA848-10|Cecidomyiidae|658|BOLD:AAH3769
Cecidomyiidae|[5497]|CNNHB617-14|Cecidomyiidae|591|BOLD:ACR7794
Cecidomyiidae|[5498]|SSJAE1225-13|Cecidomyiidae|658|BOLD:ACJ4096
Cecidomyiidae|[5499]|CNNHC1328-14|Cecidomyiidae|606|BOLD:ACR5773

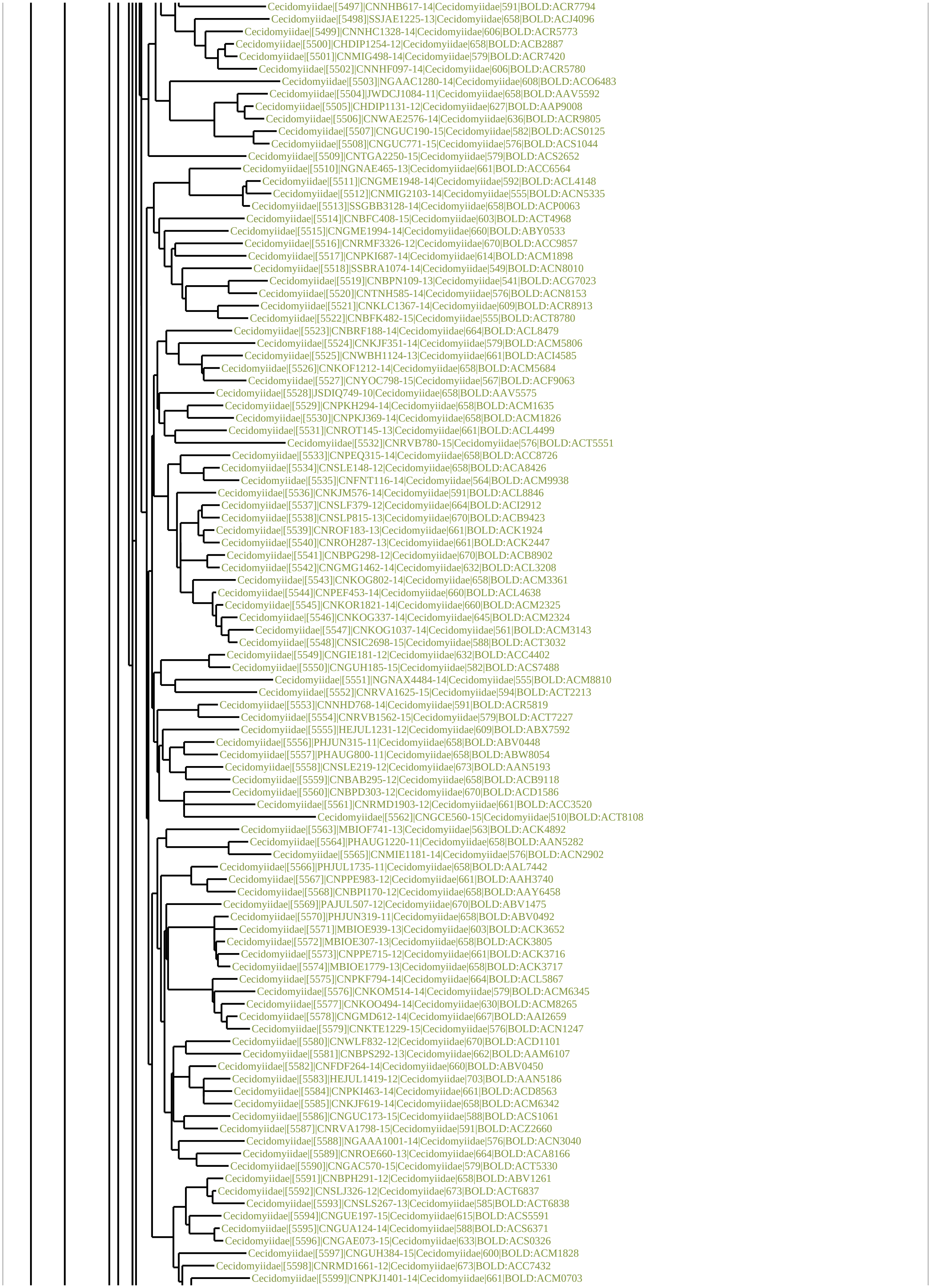

Cecidomyiidae|[5497]|CNNHB617-14|Cecidomyiidae|591|BOLD:ACR7794
Cecidomyiidae|[5498]|SSJAE1225-13|Cecidomyiidae|658|BOLD:ACJ4096
Cecidomyiidae|[5499]|CNNHC1328-14|Cecidomyiidae|606|BOLD:ACR5773
Cecidomyiidae|[5500]|CHDIP1254-12|Cecidomyiidae|658|BOLD:ACB2887
Cecidomyiidae|[5501]|CNMIG498-14|Cecidomyiidae|579|BOLD:ACR7420
Cecidomyiidae|[5502]|CNNHF097-14|Cecidomyiidae|606|BOLD:ACR5780
Cecidomyiidae|[5503]|NGAAC1280-14|Cecidomyiidae|608|BOLD:ACO6483
Cecidomyiidae|[5504]|JWDCJ1084-11|Cecidomyiidae|658|BOLD:AAV5592
Cecidomyiidae|[5505]|CHDIP1131-12|Cecidomyiidae|627|BOLD:AAP9008
Cecidomyiidae|[5506]|CNWAE2576-14|Cecidomyiidae|636|BOLD:ACR9805
Cecidomyiidae|[5507]|CNGUC190-15|Cecidomyiidae|582|BOLD:ACS0125
Cecidomyiidae|[5508]|CNGUC771-15|Cecidomyiidae|576|BOLD:ACS1044
Cecidomyiidae|[5509]|CNTGA2250-15|Cecidomyiidae|579|BOLD:ACS2652
Cecidomyiidae|[5510]|NGNAE465-13|Cecidomyiidae|661|BOLD:ACC6564
Cecidomyiidae|[5511]|CNGME1948-14|Cecidomyiidae|592|BOLD:ACL4148
Cecidomyiidae|[5512]|CNMIG2103-14|Cecidomyiidae|555|BOLD:ACN5335
Cecidomyiidae|[5513]|SSGBB3128-14|Cecidomyiidae|658|BOLD:ACP0063
Cecidomyiidae|[5514]|CNBFC408-15|Cecidomyiidae|603|BOLD:ACT4968
Cecidomyiidae|[5515]|CNGME1994-14|Cecidomyiidae|660|BOLD:ABY0533
Cecidomyiidae|[5516]|CNRMF3326-12|Cecidomyiidae|670|BOLD:ACC9857
Cecidomyiidae|[5517]|CNPKI687-14|Cecidomyiidae|614|BOLD:ACM1898
Cecidomyiidae|[5518]|SSBRA1074-14|Cecidomyiidae|549|BOLD:ACN8010
Cecidomyiidae|[5519]|CNBPN109-13|Cecidomyiidae|541|BOLD:ACG7023
Cecidomyiidae|[5520]|CNTNH585-14|Cecidomyiidae|576|BOLD:ACN8153
Cecidomyiidae|[5521]|CNKLC1367-14|Cecidomyiidae|609|BOLD:ACR8913
Cecidomyiidae|[5522]|CNBFK482-15|Cecidomyiidae|555|BOLD:ACT8780
Cecidomyiidae|[5523]|CNBRF188-14|Cecidomyiidae|664|BOLD:ACL8479
Cecidomyiidae|[5524]|CNKJF351-14|Cecidomyiidae|579|BOLD:ACM5806
Cecidomyiidae|[5525]|CNWBH1124-13|Cecidomyiidae|661|BOLD:ACI4585
Cecidomyiidae|[5526]|CNKOF1212-14|Cecidomyiidae|658|BOLD:ACM5684
Cecidomyiidae|[5527]|CNYOC798-15|Cecidomyiidae|567|BOLD:ACF9063
Cecidomyiidae|[5528]|JSDIQ749-10|Cecidomyiidae|658|BOLD:AAV5575
Cecidomyiidae|[5529]|CNPKH294-14|Cecidomyiidae|658|BOLD:ACM1635
Cecidomyiidae|[5530]|CNPKJ369-14|Cecidomyiidae|658|BOLD:ACM1826
Cecidomyiidae|[5531]|CNROT145-13|Cecidomyiidae|661|BOLD:ACL4499
Cecidomyiidae|[5532]|CNRVB780-15|Cecidomyiidae|576|BOLD:ACT5551
Cecidomyiidae|[5533]|CNPEQ315-14|Cecidomyiidae|658|BOLD:ACC8726
Cecidomyiidae|[5534]|CNSLE148-12|Cecidomyiidae|658|BOLD:ACA8426
Cecidomyiidae|[5535]|CNFNT116-14|Cecidomyiidae|564|BOLD:ACM9938
Cecidomyiidae|[5536]|CNKJM576-14|Cecidomyiidae|591|BOLD:ACL8846
Cecidomyiidae|[5537]|CNSLF379-12|Cecidomyiidae|664|BOLD:ACI2912
Cecidomyiidae|[5538]|CNSLP815-13|Cecidomyiidae|670|BOLD:ACB9423
Cecidomyiidae|[5539]|CNROF183-13|Cecidomyiidae|661|BOLD:ACK1924
Cecidomyiidae|[5540]|CNROH287-13|Cecidomyiidae|661|BOLD:ACK2447
Cecidomyiidae|[5541]|CNBPG298-12|Cecidomyiidae|670|BOLD:ACB8902
Cecidomyiidae|[5542]|CNGMG1462-14|Cecidomyiidae|632|BOLD:ACL3208
Cecidomyiidae|[5543]|CNKOG802-14|Cecidomyiidae|658|BOLD:ACM3361
Cecidomyiidae|[5544]|CNPEF453-14|Cecidomyiidae|660|BOLD:ACL4638
Cecidomyiidae|[5545]|CNKOR1821-14|Cecidomyiidae|660|BOLD:ACM2325
Cecidomyiidae|[5546]|CNKOG337-14|Cecidomyiidae|645|BOLD:ACM2324
Cecidomyiidae|[5547]|CNKOG1037-14|Cecidomyiidae|561|BOLD:ACM3143
Cecidomyiidae|[5548]|CNSIC2698-15|Cecidomyiidae|588|BOLD:ACT3032
Cecidomyiidae|[5549]|CNGIE181-12|Cecidomyiidae|632|BOLD:ACC4402
Cecidomyiidae|[5550]|CNGUH185-15|Cecidomyiidae|582|BOLD:ACS7488
Cecidomyiidae|[5551]|NGNAX4484-14|Cecidomyiidae|555|BOLD:ACM8810
Cecidomyiidae|[5552]|CNRVA1625-15|Cecidomyiidae|594|BOLD:ACT2213
Cecidomyiidae|[5553]|CNNHD768-14|Cecidomyiidae|591|BOLD:ACR5819
Cecidomyiidae|[5554]|CNRVB1562-15|Cecidomyiidae|579|BOLD:ACT7227
Cecidomyiidae|[5555]|HEJUL1231-12|Cecidomyiidae|609|BOLD:ABX7592
Cecidomyiidae|[5556]|PHJUN315-11|Cecidomyiidae|658|BOLD:ABV0448
Cecidomyiidae|[5557]|PHAUG800-11|Cecidomyiidae|658|BOLD:ABW8054
Cecidomyiidae|[5558]|CNSLE219-12|Cecidomyiidae|673|BOLD:AAN5193
Cecidomyiidae|[5559]|CNBAB295-12|Cecidomyiidae|658|BOLD:ACB9118
Cecidomyiidae|[5560]|CNBPD303-12|Cecidomyiidae|670|BOLD:ACD1586
Cecidomyiidae|[5561]|CNRMD1903-12|Cecidomyiidae|661|BOLD:ACC3520
Cecidomyiidae|[5562]|CNGCE560-15|Cecidomyiidae|510|BOLD:ACT8108
Cecidomyiidae|[5563]|MBIOF741-13|Cecidomyiidae|563|BOLD:ACK4892
Cecidomyiidae|[5564]|PHAUG1220-11|Cecidomyiidae|658|BOLD:AAN5282
Cecidomyiidae|[5565]|CNMIE1181-14|Cecidomyiidae|576|BOLD:ACN2902
Cecidomyiidae|[5566]|PHJUL1735-11|Cecidomyiidae|658|BOLD:AAL7442
Cecidomyiidae|[5567]|CNPPE983-12|Cecidomyiidae|661|BOLD:AAH3740
Cecidomyiidae|[5568]|CNBPI170-12|Cecidomyiidae|658|BOLD:AAY6458
Cecidomyiidae|[5569]|PAJUL507-12|Cecidomyiidae|670|BOLD:ABV1475
Cecidomyiidae|[5570]|PHJUN319-11|Cecidomyiidae|658|BOLD:ABV0492
Cecidomyiidae|[5571]|MBIOE939-13|Cecidomyiidae|603|BOLD:ACK3652
Cecidomyiidae|[5572]|MBIOE307-13|Cecidomyiidae|658|BOLD:ACK3805
Cecidomyiidae|[5573]|CNPPE715-12|Cecidomyiidae|661|BOLD:ACK3716
Cecidomyiidae|[5574]|MBIOE1779-13|Cecidomyiidae|658|BOLD:ACK3717
Cecidomyiidae|[5575]|CNPKF794-14|Cecidomyiidae|664|BOLD:ACL5867
Cecidomyiidae|[5576]|CNKOM514-14|Cecidomyiidae|579|BOLD:ACM6345
Cecidomyiidae|[5577]|CNKOO494-14|Cecidomyiidae|630|BOLD:ACM8265
Cecidomyiidae|[5578]|CNGMD612-14|Cecidomyiidae|667|BOLD:AAI2659
Cecidomyiidae|[5579]|CNKTE1229-15|Cecidomyiidae|576|BOLD:ACN1247
Cecidomyiidae|[5580]|CNWLF832-12|Cecidomyiidae|670|BOLD:ACD1101
Cecidomyiidae|[5581]|CNBPS292-13|Cecidomyiidae|662|BOLD:AAM6107
Cecidomyiidae|[5582]|CNFDF264-14|Cecidomyiidae|660|BOLD:ABV0450
Cecidomyiidae|[5583]|HEJUL1419-12|Cecidomyiidae|703|BOLD:AAN5186
Cecidomyiidae|[5584]|CNPKI463-14|Cecidomyiidae|661|BOLD:ACD8563
Cecidomyiidae|[5585]|CNKJF619-14|Cecidomyiidae|658|BOLD:ACM6342
Cecidomyiidae|[5586]|CNGUC173-15|Cecidomyiidae|588|BOLD:ACS1061
Cecidomyiidae|[5587]|CNRVA1798-15|Cecidomyiidae|591|BOLD:ACZ2660
Cecidomyiidae|[5588]|NGAAA1001-14|Cecidomyiidae|576|BOLD:ACN3040
Cecidomyiidae|[5589]|CNROE660-13|Cecidomyiidae|664|BOLD:ACA8166
Cecidomyiidae|[5590]|CNGAC570-15|Cecidomyiidae|579|BOLD:ACT5330
Cecidomyiidae|[5591]|CNBPH291-12|Cecidomyiidae|658|BOLD:ABV1261
Cecidomyiidae|[5592]|CNSLJ326-12|Cecidomyiidae|673|BOLD:ACT6837
Cecidomyiidae|[5593]|CNSLS267-13|Cecidomyiidae|585|BOLD:ACT6838
Cecidomyiidae|[5594]|CNGUE197-15|Cecidomyiidae|615|BOLD:ACS5591
Cecidomyiidae|[5595]|CNGUA124-14|Cecidomyiidae|588|BOLD:ACS6371
Cecidomyiidae|[5596]|CNGAE073-15|Cecidomyiidae|633|BOLD:ACS0326
Cecidomyiidae|[5597]|CNGUH384-15|Cecidomyiidae|600|BOLD:ACM1828
Cecidomyiidae|[5598]|CNRMD1661-12|Cecidomyiidae|673|BOLD:ACC7432
Cecidomyiidae|[5599]|CNPKJ1401-14|Cecidomyiidae|661|BOLD:ACM0703

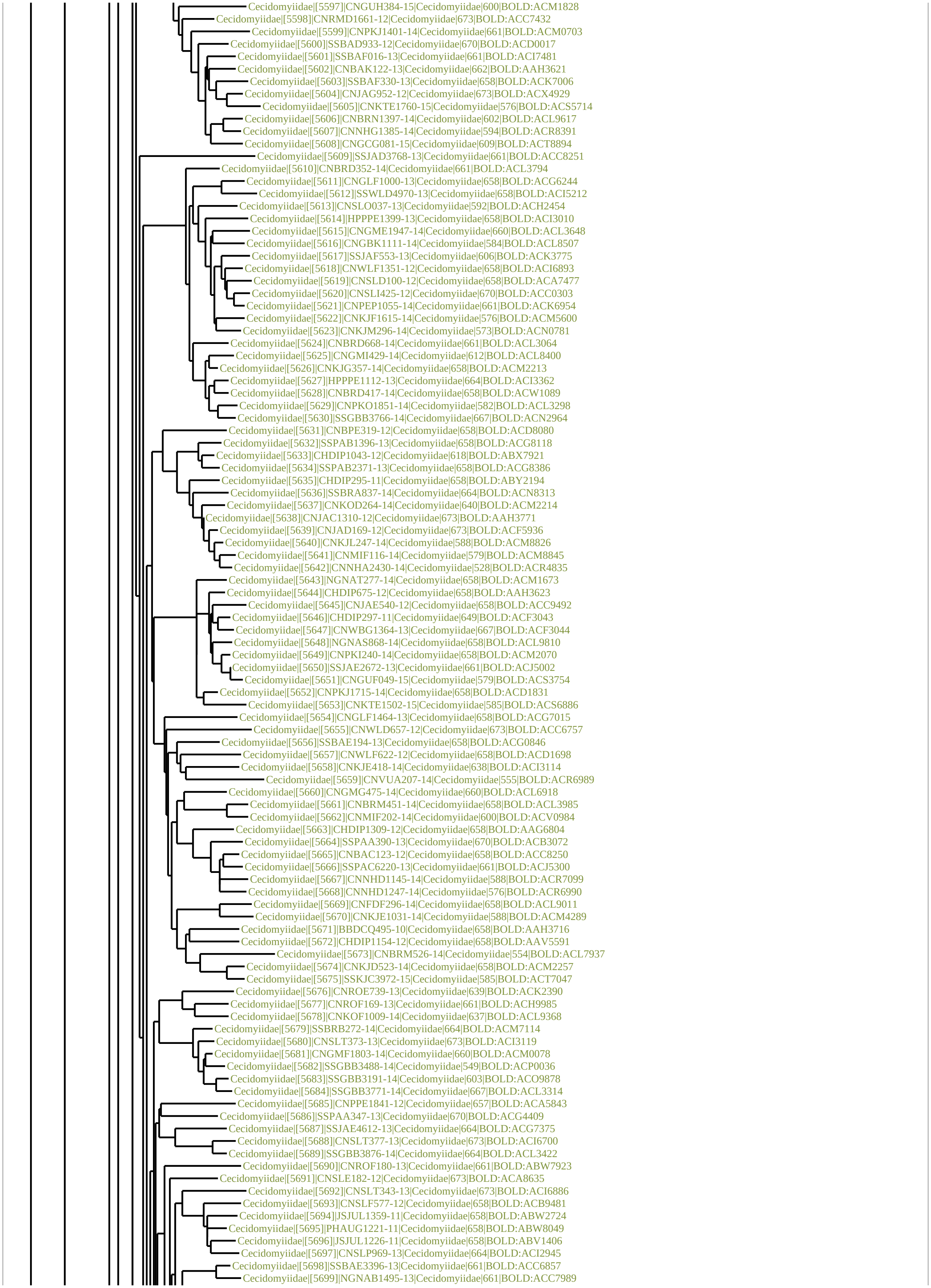
Cecidomyiidae|[5597]|CNGUH384-15|Cecidomyiidae|600|BOLD:ACM1828
Cecidomyiidae|[5598]|CNRMD1661-12|Cecidomyiidae|673|BOLD:ACC7432
Cecidomyiidae|[5599]|CNPKJ1401-14|Cecidomyiidae|661|BOLD:ACM0703
Cecidomyiidae|[5600]|SSBAD933-12|Cecidomyiidae|670|BOLD:ACD0017
Cecidomyiidae|[5601]|SSBAF016-13|Cecidomyiidae|661|BOLD:ACI7481
Cecidomyiidae|[5602]|CNBAK122-13|Cecidomyiidae|662|BOLD:AAH3621
Cecidomyiidae|[5603]|SSBAF330-13|Cecidomyiidae|658|BOLD:ACK7006
Cecidomyiidae|[5604]|CNJAG952-12|Cecidomyiidae|673|BOLD:ACX4929
Cecidomyiidae|[5605]|CNKTE1760-15|Cecidomyiidae|576|BOLD:ACS5714
Cecidomyiidae|[5606]|CNBRN1397-14|Cecidomyiidae|602|BOLD:ACL9617
Cecidomyiidae|[5607]|CNNHG1385-14|Cecidomyiidae|594|BOLD:ACR8391
Cecidomyiidae|[5608]|CNGCG081-15|Cecidomyiidae|609|BOLD:ACT8894
Cecidomyiidae|[5609]|SSJAD3768-13|Cecidomyiidae|661|BOLD:ACC8251
Cecidomyiidae|[5610]|CNBRD352-14|Cecidomyiidae|661|BOLD:ACL3794
Cecidomyiidae|[5611]|CNGLF1000-13|Cecidomyiidae|658|BOLD:ACG6244
Cecidomyiidae|[5612]|SSWLD4970-13|Cecidomyiidae|658|BOLD:ACI5212
Cecidomyiidae|[5613]|CNSLO037-13|Cecidomyiidae|592|BOLD:ACH2454
Cecidomyiidae|[5614]|HPPPE1399-13|Cecidomyiidae|658|BOLD:ACI3010
Cecidomyiidae|[5615]|CNGME1947-14|Cecidomyiidae|660|BOLD:ACL3648
Cecidomyiidae|[5616]|CNGBK1111-14|Cecidomyiidae|584|BOLD:ACL8507
Cecidomyiidae|[5617]|SSJAF553-13|Cecidomyiidae|606|BOLD:ACK3775
Cecidomyiidae|[5618]|CNWLF1351-12|Cecidomyiidae|658|BOLD:ACI6893
Cecidomyiidae|[5619]|CNSLD100-12|Cecidomyiidae|658|BOLD:ACA7477
Cecidomyiidae|[5620]|CNSLI425-12|Cecidomyiidae|670|BOLD:ACC0303
Cecidomyiidae|[5621]|CNPEP1055-14|Cecidomyiidae|661|BOLD:ACK6954
Cecidomyiidae|[5622]|CNKJF1615-14|Cecidomyiidae|576|BOLD:ACM5600
Cecidomyiidae|[5623]|CNKJM296-14|Cecidomyiidae|573|BOLD:ACN0781
Cecidomyiidae|[5624]|CNBRD668-14|Cecidomyiidae|661|BOLD:ACL3064
Cecidomyiidae|[5625]|CNGMI429-14|Cecidomyiidae|612|BOLD:ACL8400
Cecidomyiidae|[5626]|CNKJG357-14|Cecidomyiidae|658|BOLD:ACM2213
Cecidomyiidae|[5627]|HPPPE1112-13|Cecidomyiidae|664|BOLD:ACI3362
Cecidomyiidae|[5628]|CNBRD417-14|Cecidomyiidae|658|BOLD:ACW1089
Cecidomyiidae|[5629]|CNPKO1851-14|Cecidomyiidae|582|BOLD:ACL3298
Cecidomyiidae|[5630]|SSGBB3766-14|Cecidomyiidae|667|BOLD:ACN2964
Cecidomyiidae|[5631]|CNBPE319-12|Cecidomyiidae|658|BOLD:ACD8080
Cecidomyiidae|[5632]|SSPAB1396-13|Cecidomyiidae|658|BOLD:ACG8118
Cecidomyiidae|[5633]|CHDIP1043-12|Cecidomyiidae|618|BOLD:ABX7921
Cecidomyiidae|[5634]|SSPAB2371-13|Cecidomyiidae|658|BOLD:ACG8386
Cecidomyiidae|[5635]|CHDIP295-11|Cecidomyiidae|658|BOLD:ABY2194
Cecidomyiidae|[5636]|SSBRA837-14|Cecidomyiidae|664|BOLD:ACN8313
Cecidomyiidae|[5637]|CNKOD264-14|Cecidomyiidae|640|BOLD:ACM2214
Cecidomyiidae|[5638]|CNJAC1310-12|Cecidomyiidae|673|BOLD:AAH3771
Cecidomyiidae|[5639]|CNJAD169-12|Cecidomyiidae|673|BOLD:ACF5936
Cecidomyiidae|[5640]|CNKJL247-14|Cecidomyiidae|588|BOLD:ACM8826
Cecidomyiidae|[5641]|CNMIF116-14|Cecidomyiidae|579|BOLD:ACM8845
Cecidomyiidae|[5642]|CNNHA2430-14|Cecidomyiidae|528|BOLD:ACR4835
Cecidomyiidae|[5643]|NGNAT277-14|Cecidomyiidae|658|BOLD:ACM1673
Cecidomyiidae|[5644]|CHDIP675-12|Cecidomyiidae|658|BOLD:AAH3623
Cecidomyiidae|[5645]|CNJAE540-12|Cecidomyiidae|658|BOLD:ACC9492
Cecidomyiidae|[5646]|CHDIP297-11|Cecidomyiidae|649|BOLD:ACF3043
Cecidomyiidae|[5647]|CNWBG1364-13|Cecidomyiidae|667|BOLD:ACF3044
Cecidomyiidae|[5648]|NGNAS868-14|Cecidomyiidae|658|BOLD:ACL9810
Cecidomyiidae|[5649]|CNPKI240-14|Cecidomyiidae|658|BOLD:ACM2070
Cecidomyiidae|[5650]|SSJAE2672-13|Cecidomyiidae|661|BOLD:ACJ5002
Cecidomyiidae|[5651]|CNGUF049-15|Cecidomyiidae|579|BOLD:ACS3754
Cecidomyiidae|[5652]|CNPKJ1715-14|Cecidomyiidae|658|BOLD:ACD1831
Cecidomyiidae|[5653]|CNKTE1502-15|Cecidomyiidae|585|BOLD:ACS6886
Cecidomyiidae|[5654]|CNGLF1464-13|Cecidomyiidae|658|BOLD:ACG7015
Cecidomyiidae|[5655]|CNWLD657-12|Cecidomyiidae|673|BOLD:ACC6757
Cecidomyiidae|[5656]|SSBAE194-13|Cecidomyiidae|658|BOLD:ACG0846
Cecidomyiidae|[5657]|CNWLF622-12|Cecidomyiidae|658|BOLD:ACD1698
Cecidomyiidae|[5658]|CNKJE418-14|Cecidomyiidae|638|BOLD:ACI3114
Cecidomyiidae|[5659]|CNVUA207-14|Cecidomyiidae|555|BOLD:ACR6989
Cecidomyiidae|[5660]|CNGMG475-14|Cecidomyiidae|660|BOLD:ACL6918
Cecidomyiidae|[5661]|CNBRM451-14|Cecidomyiidae|658|BOLD:ACL3985
Cecidomyiidae|[5662]|CNMIF202-14|Cecidomyiidae|600|BOLD:ACV0984
Cecidomyiidae|[5663]|CHDIP1309-12|Cecidomyiidae|658|BOLD:AAG6804
Cecidomyiidae|[5664]|SSPAA390-13|Cecidomyiidae|670|BOLD:ACB3072
Cecidomyiidae|[5665]|CNBAC123-12|Cecidomyiidae|658|BOLD:ACC8250
Cecidomyiidae|[5666]|SSPAC6220-13|Cecidomyiidae|661|BOLD:ACJ5300
Cecidomyiidae|[5667]|CNNHD1145-14|Cecidomyiidae|588|BOLD:ACR7099
Cecidomyiidae|[5668]|CNNHD1247-14|Cecidomyiidae|576|BOLD:ACR6990
Cecidomyiidae|[5669]|CNFDF296-14|Cecidomyiidae|658|BOLD:ACL9011
Cecidomyiidae|[5670]|CNKJE1031-14|Cecidomyiidae|588|BOLD:ACM4289
Cecidomyiidae|[5671]|BBDCQ495-10|Cecidomyiidae|658|BOLD:AAH3716
Cecidomyiidae|[5672]|CHDIP1154-12|Cecidomyiidae|658|BOLD:AAV5591
Cecidomyiidae|[5673]|CNBRM526-14|Cecidomyiidae|554|BOLD:ACL7937
Cecidomyiidae|[5674]|CNKJD523-14|Cecidomyiidae|658|BOLD:ACM2257
Cecidomyiidae|[5675]|SSKJC3972-15|Cecidomyiidae|585|BOLD:ACT7047
Cecidomyiidae|[5676]|CNROE739-13|Cecidomyiidae|639|BOLD:ACK2390
Cecidomyiidae|[5677]|CNROF169-13|Cecidomyiidae|661|BOLD:ACH9985
Cecidomyiidae|[5678]|CNKOF1009-14|Cecidomyiidae|637|BOLD:ACL9368
Cecidomyiidae|[5679]|SSBRB272-14|Cecidomyiidae|664|BOLD:ACM7114
Cecidomyiidae|[5680]|CNSLT373-13|Cecidomyiidae|673|BOLD:ACI3119
Cecidomyiidae|[5681]|CNGMF1803-14|Cecidomyiidae|660|BOLD:ACM0078
Cecidomyiidae|[5682]|SSGBB3488-14|Cecidomyiidae|549|BOLD:ACP0036
Cecidomyiidae|[5683]|SSGBB3191-14|Cecidomyiidae|603|BOLD:ACO9878
Cecidomyiidae|[5684]|SSGBB3771-14|Cecidomyiidae|667|BOLD:ACL3314
Cecidomyiidae|[5685]|CNPPE1841-12|Cecidomyiidae|657|BOLD:ACA5843
Cecidomyiidae|[5686]|SSPAA347-13|Cecidomyiidae|670|BOLD:ACG4409
Cecidomyiidae|[5687]|SSJAE4612-13|Cecidomyiidae|664|BOLD:ACG7375
Cecidomyiidae|[5688]|CNSLT377-13|Cecidomyiidae|673|BOLD:ACI6700
Cecidomyiidae|[5689]|SSGBB3876-14|Cecidomyiidae|664|BOLD:ACL3422
Cecidomyiidae|[5690]|CNROF180-13|Cecidomyiidae|661|BOLD:ABW7923
Cecidomyiidae|[5691]|CNSLE182-12|Cecidomyiidae|673|BOLD:ACA8635
Cecidomyiidae|[5692]|CNSLT343-13|Cecidomyiidae|673|BOLD:ACI6886
Cecidomyiidae|[5693]|CNSLF577-12|Cecidomyiidae|658|BOLD:ACB9481
Cecidomyiidae|[5694]|JSJUL1359-11|Cecidomyiidae|658|BOLD:ABW2724
Cecidomyiidae|[5695]|PHAUG1221-11|Cecidomyiidae|658|BOLD:ABW8049
Cecidomyiidae|[5696]|JSJUL1226-11|Cecidomyiidae|658|BOLD:ABV1406
Cecidomyiidae|[5697]|CNSLP969-13|Cecidomyiidae|664|BOLD:ACI2945
Cecidomyiidae|[5698]|SSBAE3396-13|Cecidomyiidae|661|BOLD:ACC6857
Cecidomyiidae|[5699]|NGNAB1495-13|Cecidomyiidae|661|BOLD:ACC7989

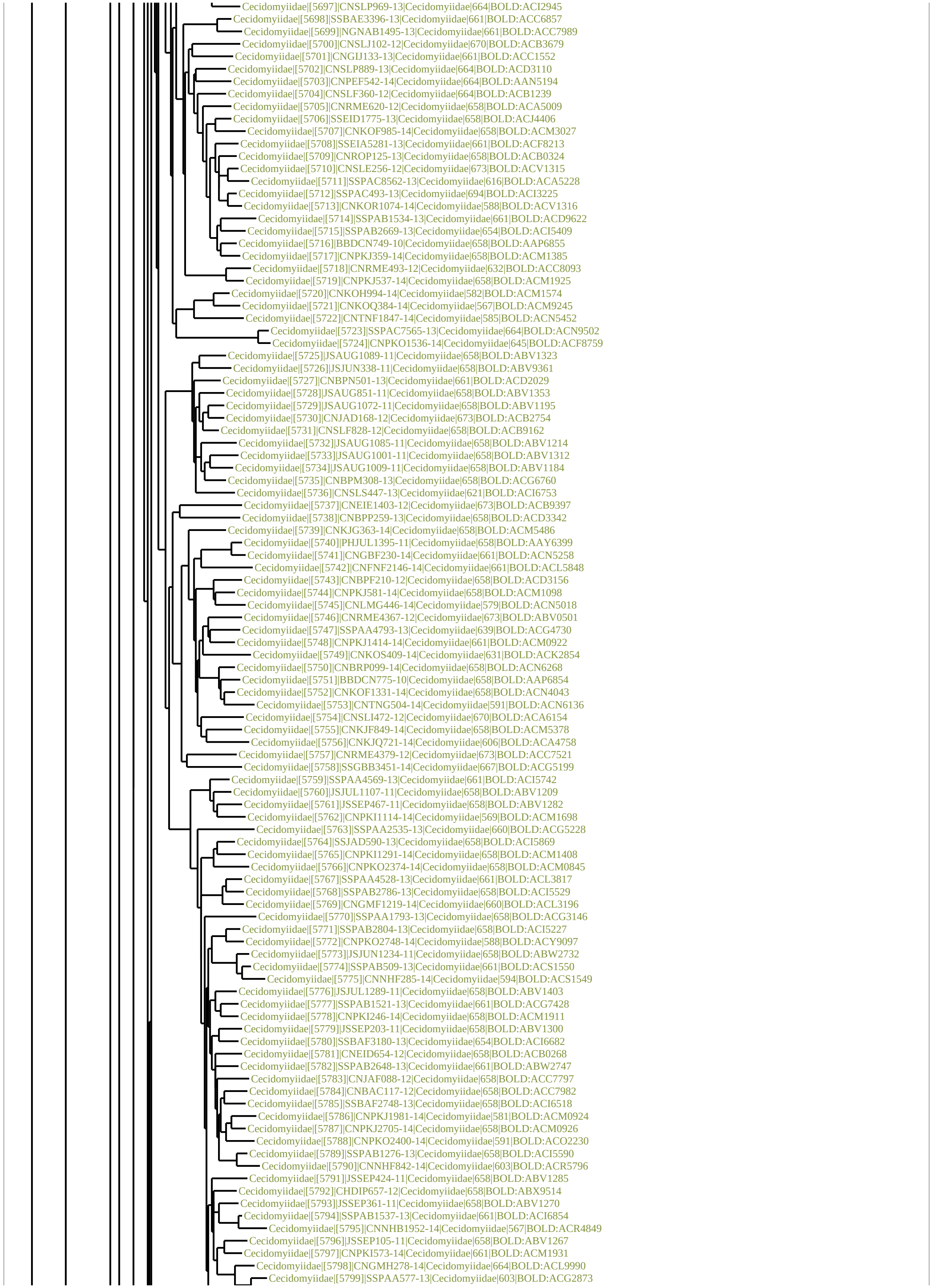
Cecidomyiidae|[5697]|CNSLP969-13|Cecidomyiidae|664|BOLD:ACI2945
Cecidomyiidae|[5698]|SSBAE3396-13|Cecidomyiidae|661|BOLD:ACC6857
Cecidomyiidae|[5699]|NGNAB1495-13|Cecidomyiidae|661|BOLD:ACC7989
Cecidomyiidae|[5700]|CNSLJ102-12|Cecidomyiidae|670|BOLD:ACB3679
Cecidomyiidae|[5701]|CNGIJ133-13|Cecidomyiidae|661|BOLD:ACC1552
Cecidomyiidae|[5702]|CNSLP889-13|Cecidomyiidae|664|BOLD:ACD3110
Cecidomyiidae|[5703]|CNPEF542-14|Cecidomyiidae|664|BOLD:AAN5194
Cecidomyiidae|[5704]|CNSLF360-12|Cecidomyiidae|664|BOLD:ACB1239
Cecidomyiidae|[5705]|CNRME620-12|Cecidomyiidae|658|BOLD:ACA5009
Cecidomyiidae|[5706]|SSEID1775-13|Cecidomyiidae|658|BOLD:ACJ4406
Cecidomyiidae|[5707]|CNKOF985-14|Cecidomyiidae|658|BOLD:ACM3027
Cecidomyiidae|[5708]|SSEIA5281-13|Cecidomyiidae|661|BOLD:ACF8213
Cecidomyiidae|[5709]|CNROP125-13|Cecidomyiidae|658|BOLD:ACB0324
Cecidomyiidae|[5710]|CNSLE256-12|Cecidomyiidae|673|BOLD:ACV1315
Cecidomyiidae|[5711]|SSPAC8562-13|Cecidomyiidae|616|BOLD:ACA5228
Cecidomyiidae|[5712]|SSPAC493-13|Cecidomyiidae|694|BOLD:ACI3225
Cecidomyiidae|[5713]|CNKOR1074-14|Cecidomyiidae|588|BOLD:ACV1316
Cecidomyiidae|[5714]|SSPAB1534-13|Cecidomyiidae|661|BOLD:ACD9622
Cecidomyiidae|[5715]|SSPAB2669-13|Cecidomyiidae|654|BOLD:ACI5409
Cecidomyiidae|[5716]|BBDCN749-10|Cecidomyiidae|658|BOLD:AAP6855
Cecidomyiidae|[5717]|CNPKJ359-14|Cecidomyiidae|658|BOLD:ACM1385
Cecidomyiidae|[5718]|CNRME493-12|Cecidomyiidae|632|BOLD:ACC8093
Cecidomyiidae|[5719]|CNPKJ537-14|Cecidomyiidae|658|BOLD:ACM1925
Cecidomyiidae|[5720]|CNKOH994-14|Cecidomyiidae|582|BOLD:ACM1574
Cecidomyiidae|[5721]|CNKOQ384-14|Cecidomyiidae|567|BOLD:ACM9245
Cecidomyiidae|[5722]|CNTNF1847-14|Cecidomyiidae|585|BOLD:ACN5452
Cecidomyiidae|[5723]|SSPAC7565-13|Cecidomyiidae|664|BOLD:ACN9502
Cecidomyiidae|[5724]|CNPKO1536-14|Cecidomyiidae|645|BOLD:ACF8759
Cecidomyiidae|[5725]|JSAUG1089-11|Cecidomyiidae|658|BOLD:ABV1323
Cecidomyiidae|[5726]|JSJUN338-11|Cecidomyiidae|658|BOLD:ABV9361
Cecidomyiidae|[5727]|CNBPN501-13|Cecidomyiidae|661|BOLD:ACD2029
Cecidomyiidae|[5728]|JSAUG851-11|Cecidomyiidae|658|BOLD:ABV1353
Cecidomyiidae|[5729]|JSAUG1072-11|Cecidomyiidae|658|BOLD:ABV1195
Cecidomyiidae|[5730]|CNJAD168-12|Cecidomyiidae|673|BOLD:ACB2754
Cecidomyiidae|[5731]|CNSLF828-12|Cecidomyiidae|658|BOLD:ACB9162
Cecidomyiidae|[5732]|JSAUG1085-11|Cecidomyiidae|658|BOLD:ABV1214
Cecidomyiidae|[5733]|JSAUG1001-11|Cecidomyiidae|658|BOLD:ABV1312
Cecidomyiidae|[5734]|JSAUG1009-11|Cecidomyiidae|658|BOLD:ABV1184
Cecidomyiidae|[5735]|CNBPM308-13|Cecidomyiidae|658|BOLD:ACG6760
Cecidomyiidae|[5736]|CNSLS447-13|Cecidomyiidae|621|BOLD:ACI6753
Cecidomyiidae|[5737]|CNEIE1403-12|Cecidomyiidae|673|BOLD:ACB9397
Cecidomyiidae|[5738]|CNBPP259-13|Cecidomyiidae|658|BOLD:ACD3342
Cecidomyiidae|[5739]|CNKJG363-14|Cecidomyiidae|658|BOLD:ACM5486
Cecidomyiidae|[5740]|PHJUL1395-11|Cecidomyiidae|658|BOLD:AAY6399
Cecidomyiidae|[5741]|CNGBF230-14|Cecidomyiidae|661|BOLD:ACN5258
Cecidomyiidae|[5742]|CNFNF2146-14|Cecidomyiidae|661|BOLD:ACL5848
Cecidomyiidae|[5743]|CNBPF210-12|Cecidomyiidae|658|BOLD:ACD3156
Cecidomyiidae|[5744]|CNPKJ581-14|Cecidomyiidae|658|BOLD:ACM1098
Cecidomyiidae|[5745]|CNLMG446-14|Cecidomyiidae|579|BOLD:ACN5018
Cecidomyiidae|[5746]|CNRME4367-12|Cecidomyiidae|673|BOLD:ABV0501
Cecidomyiidae|[5747]|SSPAA4793-13|Cecidomyiidae|639|BOLD:ACG4730
Cecidomyiidae|[5748]|CNPKJ1414-14|Cecidomyiidae|661|BOLD:ACM0922
Cecidomyiidae|[5749]|CNKOS409-14|Cecidomyiidae|631|BOLD:ACK2854
Cecidomyiidae|[5750]|CNBRP099-14|Cecidomyiidae|658|BOLD:ACN6268
Cecidomyiidae|[5751]|BBDCN775-10|Cecidomyiidae|658|BOLD:AAP6854
Cecidomyiidae|[5752]|CNKOF1331-14|Cecidomyiidae|658|BOLD:ACN4043
Cecidomyiidae|[5753]|CNTNG504-14|Cecidomyiidae|591|BOLD:ACN6136
Cecidomyiidae|[5754]|CNSLI472-12|Cecidomyiidae|670|BOLD:ACA6154
Cecidomyiidae|[5755]|CNKJF849-14|Cecidomyiidae|658|BOLD:ACM5378
Cecidomyiidae|[5756]|CNKJQ721-14|Cecidomyiidae|606|BOLD:ACA4758
Cecidomyiidae|[5757]|CNRME4379-12|Cecidomyiidae|673|BOLD:ACC7521
Cecidomyiidae|[5758]|SSGBB3451-14|Cecidomyiidae|667|BOLD:ACG5199
Cecidomyiidae|[5759]|SSPAA4569-13|Cecidomyiidae|661|BOLD:ACI5742
Cecidomyiidae|[5760]|JSJUL1107-11|Cecidomyiidae|658|BOLD:ABV1209
Cecidomyiidae|[5761]|JSSEP467-11|Cecidomyiidae|658|BOLD:ABV1282
Cecidomyiidae|[5762]|CNPKI1114-14|Cecidomyiidae|569|BOLD:ACM1698
Cecidomyiidae|[5763]|SSPAA2535-13|Cecidomyiidae|660|BOLD:ACG5228
Cecidomyiidae|[5764]|SSJAD590-13|Cecidomyiidae|658|BOLD:ACI5869
Cecidomyiidae|[5765]|CNPKI1291-14|Cecidomyiidae|658|BOLD:ACM1408
Cecidomyiidae|[5766]|CNPKO2374-14|Cecidomyiidae|658|BOLD:ACM0845
Cecidomyiidae|[5767]|SSPAA4528-13|Cecidomyiidae|661|BOLD:ACL3817
Cecidomyiidae|[5768]|SSPAB2786-13|Cecidomyiidae|658|BOLD:ACI5529
Cecidomyiidae|[5769]|CNGMF1219-14|Cecidomyiidae|660|BOLD:ACL3196
Cecidomyiidae|[5770]|SSPAA1793-13|Cecidomyiidae|658|BOLD:ACG3146
Cecidomyiidae|[5771]|SSPAB2804-13|Cecidomyiidae|658|BOLD:ACI5227
Cecidomyiidae|[5772]|CNPKO2748-14|Cecidomyiidae|588|BOLD:ACY9097
Cecidomyiidae|[5773]|JSJUN1234-11|Cecidomyiidae|658|BOLD:ABW2732
Cecidomyiidae|[5774]|SSPAB509-13|Cecidomyiidae|661|BOLD:ACS1550
Cecidomyiidae|[5775]|CNNHF285-14|Cecidomyiidae|594|BOLD:ACS1549
Cecidomyiidae|[5776]|JSJUL1289-11|Cecidomyiidae|658|BOLD:ABV1403
Cecidomyiidae|[5777]|SSPAB1521-13|Cecidomyiidae|661|BOLD:ACG7428
Cecidomyiidae|[5778]|CNPKI246-14|Cecidomyiidae|658|BOLD:ACM1911
Cecidomyiidae|[5779]|JSSEP203-11|Cecidomyiidae|658|BOLD:ABV1300
Cecidomyiidae|[5780]|SSBAF3180-13|Cecidomyiidae|654|BOLD:ACI6682
Cecidomyiidae|[5781]|CNEID654-12|Cecidomyiidae|658|BOLD:ACB0268
Cecidomyiidae|[5782]|SSPAB2648-13|Cecidomyiidae|661|BOLD:ABW2747
Cecidomyiidae|[5783]|CNJAF088-12|Cecidomyiidae|658|BOLD:ACC7797
Cecidomyiidae|[5784]|CNBAC117-12|Cecidomyiidae|658|BOLD:ACC7982
Cecidomyiidae|[5785]|SSBAF2748-13|Cecidomyiidae|658|BOLD:ACI6518
Cecidomyiidae|[5786]|CNPKJ1981-14|Cecidomyiidae|581|BOLD:ACM0924
Cecidomyiidae|[5787]|CNPKJ2705-14|Cecidomyiidae|658|BOLD:ACM0926
Cecidomyiidae|[5788]|CNPKO2400-14|Cecidomyiidae|591|BOLD:ACO2230
Cecidomyiidae|[5789]|SSPAB1276-13|Cecidomyiidae|658|BOLD:ACI5590
Cecidomyiidae|[5790]|CNNHF842-14|Cecidomyiidae|603|BOLD:ACR5796
Cecidomyiidae|[5791]|JSSEP424-11|Cecidomyiidae|658|BOLD:ABV1285
Cecidomyiidae|[5792]|CHDIP657-12|Cecidomyiidae|658|BOLD:ABX9514
Cecidomyiidae|[5793]|JSSEP361-11|Cecidomyiidae|658|BOLD:ABV1270
Cecidomyiidae|[5794]|SSPAB1537-13|Cecidomyiidae|661|BOLD:ACI6854
Cecidomyiidae|[5795]|CNNHB1952-14|Cecidomyiidae|567|BOLD:ACR4849
Cecidomyiidae|[5796]|JSSEP105-11|Cecidomyiidae|658|BOLD:ABV1267
Cecidomyiidae|[5797]|CNPKI573-14|Cecidomyiidae|661|BOLD:ACM1931
Cecidomyiidae|[5798]|CNGMH278-14|Cecidomyiidae|664|BOLD:ACL9990
Cecidomyiidae|[5799]|SSPAA577-13|Cecidomyiidae|603|BOLD:ACG2873

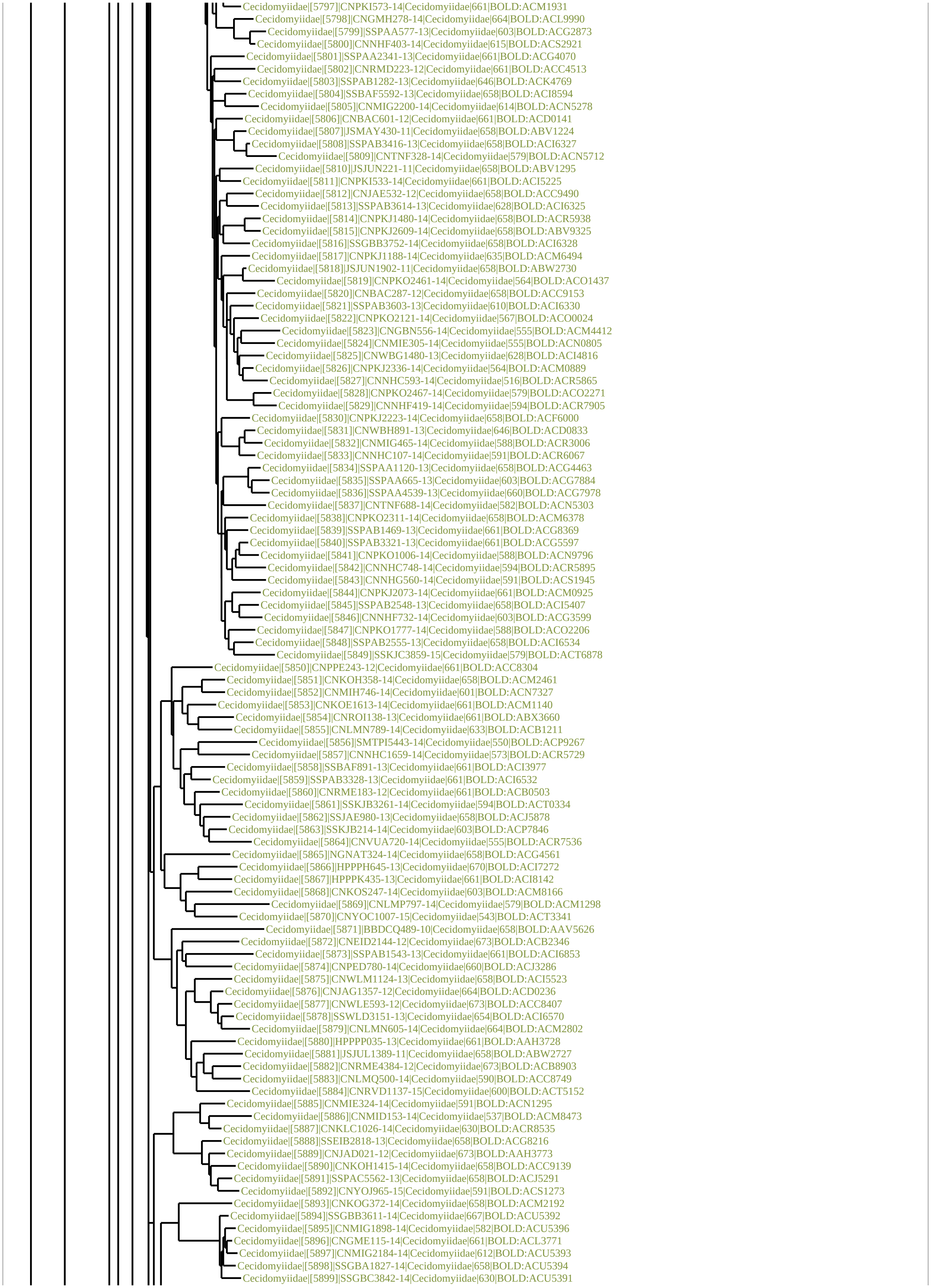
Cecidomyiidae|[5797]|CNPKI573-14|Cecidomyiidae|661|BOLD:ACM1931
Cecidomyiidae|[5798]|CNGMH278-14|Cecidomyiidae|664|BOLD:ACL9990
Cecidomyiidae|[5799]|SSPAA577-13|Cecidomyiidae|603|BOLD:ACG2873
Cecidomyiidae|[5800]|CNNHF403-14|Cecidomyiidae|615|BOLD:ACS2921
Cecidomyiidae|[5801]|SSPAA2341-13|Cecidomyiidae|661|BOLD:ACG4070
Cecidomyiidae|[5802]|CNRMD223-12|Cecidomyiidae|661|BOLD:ACC4513
Cecidomyiidae|[5803]|SSPAB1282-13|Cecidomyiidae|646|BOLD:ACK4769
Cecidomyiidae|[5804]|SSBAF5592-13|Cecidomyiidae|658|BOLD:ACI8594
Cecidomyiidae|[5805]|CNMIG2200-14|Cecidomyiidae|614|BOLD:ACN5278
Cecidomyiidae|[5806]|CNBAC601-12|Cecidomyiidae|661|BOLD:ACD0141
Cecidomyiidae|[5807]|JSMAY430-11|Cecidomyiidae|658|BOLD:ABV1224
Cecidomyiidae|[5808]|SSPAB3416-13|Cecidomyiidae|658|BOLD:ACI6327
Cecidomyiidae|[5809]|CNTNF328-14|Cecidomyiidae|579|BOLD:ACN5712
Cecidomyiidae|[5810]|JSJUN221-11|Cecidomyiidae|658|BOLD:ABV1295
Cecidomyiidae|[5811]|CNPKI533-14|Cecidomyiidae|661|BOLD:ACI5225
Cecidomyiidae|[5812]|CNJAE532-12|Cecidomyiidae|658|BOLD:ACC9490
Cecidomyiidae|[5813]|SSPAB3614-13|Cecidomyiidae|628|BOLD:ACI6325
Cecidomyiidae|[5814]|CNPKJ1480-14|Cecidomyiidae|658|BOLD:ACR5938
Cecidomyiidae|[5815]|CNPKJ2609-14|Cecidomyiidae|658|BOLD:ABV9325
Cecidomyiidae|[5816]|SSGBB3752-14|Cecidomyiidae|658|BOLD:ACI6328
Cecidomyiidae|[5817]|CNPKJ1188-14|Cecidomyiidae|635|BOLD:ACM6494
Cecidomyiidae|[5818]|JSJUN1902-11|Cecidomyiidae|658|BOLD:ABW2730
Cecidomyiidae|[5819]|CNPKO2461-14|Cecidomyiidae|564|BOLD:ACO1437
Cecidomyiidae|[5820]|CNBAC287-12|Cecidomyiidae|658|BOLD:ACC9153
Cecidomyiidae|[5821]|SSPAB3603-13|Cecidomyiidae|610|BOLD:ACI6330
Cecidomyiidae|[5822]|CNPKO2121-14|Cecidomyiidae|567|BOLD:ACO0024
Cecidomyiidae|[5823]|CNGBN556-14|Cecidomyiidae|555|BOLD:ACM4412
Cecidomyiidae|[5824]|CNMIE305-14|Cecidomyiidae|555|BOLD:ACN0805
Cecidomyiidae|[5825]|CNWBG1480-13|Cecidomyiidae|628|BOLD:ACI4816
Cecidomyiidae|[5826]|CNPKJ2336-14|Cecidomyiidae|564|BOLD:ACM0889
Cecidomyiidae|[5827]|CNNHC593-14|Cecidomyiidae|516|BOLD:ACR5865
Cecidomyiidae|[5828]|CNPKO2467-14|Cecidomyiidae|579|BOLD:ACO2271
Cecidomyiidae|[5829]|CNNHF419-14|Cecidomyiidae|594|BOLD:ACR7905
Cecidomyiidae|[5830]|CNPKJ2223-14|Cecidomyiidae|658|BOLD:ACF6000
Cecidomyiidae|[5831]|CNWBH891-13|Cecidomyiidae|646|BOLD:ACD0833
Cecidomyiidae|[5832]|CNMIG465-14|Cecidomyiidae|588|BOLD:ACR3006
Cecidomyiidae|[5833]|CNNHC107-14|Cecidomyiidae|591|BOLD:ACR6067
Cecidomyiidae|[5834]|SSPAA1120-13|Cecidomyiidae|658|BOLD:ACG4463
Cecidomyiidae|[5835]|SSPAA665-13|Cecidomyiidae|603|BOLD:ACG7884
Cecidomyiidae|[5836]|SSPAA4539-13|Cecidomyiidae|660|BOLD:ACG7978
Cecidomyiidae|[5837]|CNTNF688-14|Cecidomyiidae|582|BOLD:ACN5303
Cecidomyiidae|[5838]|CNPKO2311-14|Cecidomyiidae|658|BOLD:ACM6378
Cecidomyiidae|[5839]|SSPAB1469-13|Cecidomyiidae|661|BOLD:ACG8369
Cecidomyiidae|[5840]|SSPAB3321-13|Cecidomyiidae|661|BOLD:ACG5597
Cecidomyiidae|[5841]|CNPKO1006-14|Cecidomyiidae|588|BOLD:ACN9796
Cecidomyiidae|[5842]|CNNHC748-14|Cecidomyiidae|594|BOLD:ACR5895
Cecidomyiidae|[5843]|CNNHG560-14|Cecidomyiidae|591|BOLD:ACS1945
Cecidomyiidae|[5844]|CNPKJ2073-14|Cecidomyiidae|661|BOLD:ACM0925
Cecidomyiidae|[5845]|SSPAB2548-13|Cecidomyiidae|658|BOLD:ACI5407
Cecidomyiidae|[5846]|CNNHF732-14|Cecidomyiidae|603|BOLD:ACG3599
Cecidomyiidae|[5847]|CNPKO1777-14|Cecidomyiidae|588|BOLD:ACO2206
Cecidomyiidae|[5848]|SSPAB2555-13|Cecidomyiidae|658|BOLD:ACI6534
Cecidomyiidae|[5849]|SSKJC3859-15|Cecidomyiidae|579|BOLD:ACT6878
Cecidomyiidae|[5850]|CNPPE243-12|Cecidomyiidae|661|BOLD:ACC8304
Cecidomyiidae|[5851]|CNKOH358-14|Cecidomyiidae|658|BOLD:ACM2461
Cecidomyiidae|[5852]|CNMIH746-14|Cecidomyiidae|601|BOLD:ACN7327
Cecidomyiidae|[5853]|CNKOE1613-14|Cecidomyiidae|661|BOLD:ACM1140
Cecidomyiidae|[5854]|CNROI138-13|Cecidomyiidae|661|BOLD:ABX3660
Cecidomyiidae|[5855]|CNLMN789-14|Cecidomyiidae|633|BOLD:ACB1211
Cecidomyiidae|[5856]|SMTPI5443-14|Cecidomyiidae|550|BOLD:ACP9267
Cecidomyiidae|[5857]|CNNHC1659-14|Cecidomyiidae|573|BOLD:ACR5729
Cecidomyiidae|[5858]|SSBAF891-13|Cecidomyiidae|661|BOLD:ACI3977
Cecidomyiidae|[5859]|SSPAB3328-13|Cecidomyiidae|661|BOLD:ACI6532
Cecidomyiidae|[5860]|CNRME183-12|Cecidomyiidae|661|BOLD:ACB0503
Cecidomyiidae|[5861]|SSKJB3261-14|Cecidomyiidae|594|BOLD:ACT0334
Cecidomyiidae|[5862]|SSJAE980-13|Cecidomyiidae|658|BOLD:ACJ5878
Cecidomyiidae|[5863]|SSKJB214-14|Cecidomyiidae|603|BOLD:ACP7846
Cecidomyiidae|[5864]|CNVUA720-14|Cecidomyiidae|555|BOLD:ACR7536
Cecidomyiidae|[5865]|NGNAT324-14|Cecidomyiidae|658|BOLD:ACG4561
Cecidomyiidae|[5866]|HPPPH645-13|Cecidomyiidae|670|BOLD:ACI7272
Cecidomyiidae|[5867]|HPPPK435-13|Cecidomyiidae|661|BOLD:ACI8142
Cecidomyiidae|[5868]|CNKOS247-14|Cecidomyiidae|603|BOLD:ACM8166
Cecidomyiidae|[5869]|CNLMP797-14|Cecidomyiidae|579|BOLD:ACM1298
Cecidomyiidae|[5870]|CNYOC1007-15|Cecidomyiidae|543|BOLD:ACT3341
Cecidomyiidae|[5871]|BBDCQ489-10|Cecidomyiidae|658|BOLD:AAV5626
Cecidomyiidae|[5872]|CNEID2144-12|Cecidomyiidae|673|BOLD:ACB2346
Cecidomyiidae|[5873]|SSPAB1543-13|Cecidomyiidae|661|BOLD:ACI6853
Cecidomyiidae|[5874]|CNPED780-14|Cecidomyiidae|660|BOLD:ACJ3286
Cecidomyiidae|[5875]|CNWLM1124-13|Cecidomyiidae|658|BOLD:ACI5523
Cecidomyiidae|[5876]|CNJAG1357-12|Cecidomyiidae|664|BOLD:ACD0236
Cecidomyiidae|[5877]|CNWLE593-12|Cecidomyiidae|673|BOLD:ACC8407
Cecidomyiidae|[5878]|SSWLD3151-13|Cecidomyiidae|654|BOLD:ACI6570
Cecidomyiidae|[5879]|CNLMN605-14|Cecidomyiidae|664|BOLD:ACM2802
Cecidomyiidae|[5880]|HPPPO035-13|Cecidomyiidae|661|BOLD:AAH3728
Cecidomyiidae|[5881]|JSJUL1389-11|Cecidomyiidae|658|BOLD:ABW2727
Cecidomyiidae|[5882]|CNRME4384-12|Cecidomyiidae|673|BOLD:ACB8903
Cecidomyiidae|[5883]|CNLMQ500-14|Cecidomyiidae|590|BOLD:ACC8749
Cecidomyiidae|[5884]|CNRVD1137-15|Cecidomyiidae|600|BOLD:ACT5152
Cecidomyiidae|[5885]|CNMIE324-14|Cecidomyiidae|591|BOLD:ACN1295
Cecidomyiidae|[5886]|CNMID153-14|Cecidomyiidae|537|BOLD:ACM8473
Cecidomyiidae|[5887]|CNKLC1026-14|Cecidomyiidae|630|BOLD:ACR8535
Cecidomyiidae|[5888]|SSEIB2818-13|Cecidomyiidae|658|BOLD:ACG8216
Cecidomyiidae|[5889]|CNJAD021-12|Cecidomyiidae|673|BOLD:AAH3773
Cecidomyiidae|[5890]|CNKOH1415-14|Cecidomyiidae|658|BOLD:ACC9139
Cecidomyiidae|[5891]|SSPAC5562-13|Cecidomyiidae|658|BOLD:ACJ5291
Cecidomyiidae|[5892]|CNYOJ965-15|Cecidomyiidae|591|BOLD:ACS1273
Cecidomyiidae|[5893]|CNKOG372-14|Cecidomyiidae|658|BOLD:ACM2192
Cecidomyiidae|[5894]|SSGBB3611-14|Cecidomyiidae|667|BOLD:ACU5392
Cecidomyiidae|[5895]|CNMIG1898-14|Cecidomyiidae|582|BOLD:ACU5396
Cecidomyiidae|[5896]|CNGME115-14|Cecidomyiidae|661|BOLD:ACL3771
Cecidomyiidae|[5897]|CNMIG2184-14|Cecidomyiidae|612|BOLD:ACU5393
Cecidomyiidae|[5898]|SSGBA1827-14|Cecidomyiidae|658|BOLD:ACU5394
Cecidomyiidae|[5899]|SSGBC3842-14|Cecidomyiidae|630|BOLD:ACU5391

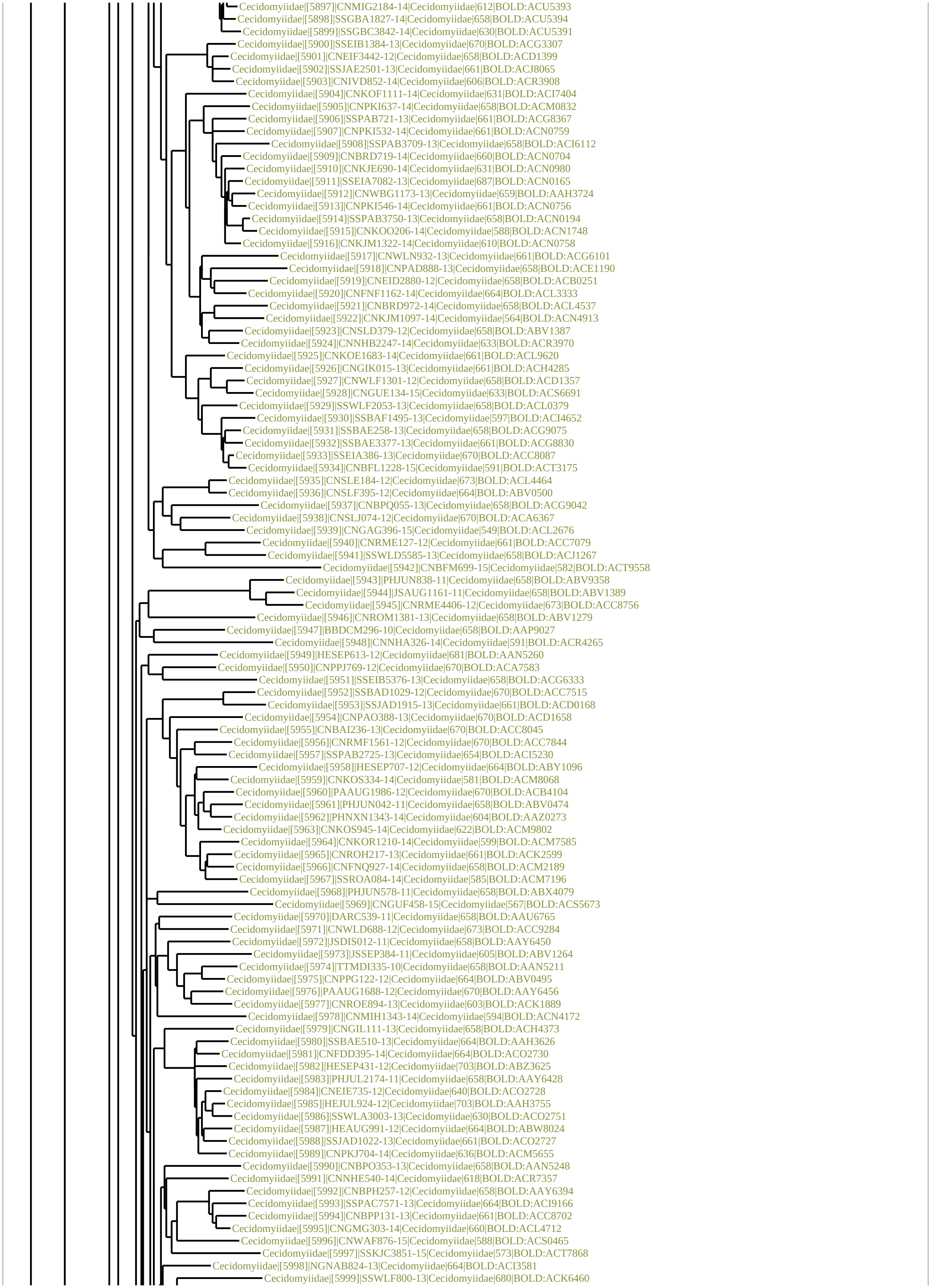

Cecidomyiidae|[5897]|CNMIG2184-14|Cecidomyiidae|612|BOLD:ACU5393
Cecidomyiidae|[5898]|SSGBA1827-14|Cecidomyiidae|658|BOLD:ACU5394
Cecidomyiidae|[5899]|SSGBC3842-14|Cecidomyiidae|630|BOLD:ACU5391
Cecidomyiidae|[5900]|SSEIB1384-13|Cecidomyiidae|670|BOLD:ACG3307
Cecidomyiidae|[5901]|CNEIF3442-12|Cecidomyiidae|658|BOLD:ACD1399
Cecidomyiidae|[5902]|SSJAE2501-13|Cecidomyiidae|661|BOLD:ACJ8065
Cecidomyiidae|[5903]|CNIVD852-14|Cecidomyiidae|606|BOLD:ACR3908
Cecidomyiidae|[5904]|CNKOF1111-14|Cecidomyiidae|631|BOLD:ACI7404
Cecidomyiidae|[5905]|CNPKI637-14|Cecidomyiidae|658|BOLD:ACM0832
Cecidomyiidae|[5906]|SSPAB721-13|Cecidomyiidae|661|BOLD:ACG8367
Cecidomyiidae|[5907]|CNPKI532-14|Cecidomyiidae|661|BOLD:ACN0759
Cecidomyiidae|[5908]|SSPAB3709-13|Cecidomyiidae|658|BOLD:ACI6112
Cecidomyiidae|[5909]|CNBRD719-14|Cecidomyiidae|660|BOLD:ACN0704
Cecidomyiidae|[5910]|CNKJE690-14|Cecidomyiidae|631|BOLD:ACN0980
Cecidomyiidae|[5911]|SSEIA7082-13|Cecidomyiidae|687|BOLD:ACN0165
Cecidomyiidae|[5912]|CNWBG1173-13|Cecidomyiidae|659|BOLD:AAH3724
Cecidomyiidae|[5913]|CNPKI546-14|Cecidomyiidae|661|BOLD:ACN0756
Cecidomyiidae|[5914]|SSPAB3750-13|Cecidomyiidae|658|BOLD:ACN0194
Cecidomyiidae|[5915]|CNKOO206-14|Cecidomyiidae|588|BOLD:ACN1748
Cecidomyiidae|[5916]|CNKJM1322-14|Cecidomyiidae|610|BOLD:ACN0758
Cecidomyiidae|[5917]|CNWLN932-13|Cecidomyiidae|661|BOLD:ACG6101
Cecidomyiidae|[5918]|CNPAD888-13|Cecidomyiidae|658|BOLD:ACE1190
Cecidomyiidae|[5919]|CNEID2880-12|Cecidomyiidae|658|BOLD:ACB0251
Cecidomyiidae|[5920]|CNFNF1162-14|Cecidomyiidae|664|BOLD:ACL3333
Cecidomyiidae|[5921]|CNBRD972-14|Cecidomyiidae|658|BOLD:ACL4537
Cecidomyiidae|[5922]|CNKJM1097-14|Cecidomyiidae|564|BOLD:ACN4913
Cecidomyiidae|[5923]|CNSLD379-12|Cecidomyiidae|658|BOLD:ABV1387
Cecidomyiidae|[5924]|CNNHB2247-14|Cecidomyiidae|633|BOLD:ACR3970
Cecidomyiidae|[5925]|CNKOE1683-14|Cecidomyiidae|661|BOLD:ACL9620
Cecidomyiidae|[5926]|CNGIK015-13|Cecidomyiidae|661|BOLD:ACH4285
Cecidomyiidae|[5927]|CNWLF1301-12|Cecidomyiidae|658|BOLD:ACD1357
Cecidomyiidae|[5928]|CNGUE134-15|Cecidomyiidae|633|BOLD:ACS6691
Cecidomyiidae|[5929]|SSWLF2053-13|Cecidomyiidae|658|BOLD:ACL0379
Cecidomyiidae|[5930]|SSBAF1495-13|Cecidomyiidae|597|BOLD:ACI4652
Cecidomyiidae|[5931]|SSBAE258-13|Cecidomyiidae|658|BOLD:ACG9075
Cecidomyiidae|[5932]|SSBAE3377-13|Cecidomyiidae|661|BOLD:ACG8830
Cecidomyiidae|[5933]|SSEIA386-13|Cecidomyiidae|670|BOLD:ACC8087
Cecidomyiidae|[5934]|CNBFL1228-15|Cecidomyiidae|591|BOLD:ACT3175
Cecidomyiidae|[5935]|CNSLE184-12|Cecidomyiidae|673|BOLD:ACL4464
Cecidomyiidae|[5936]|CNSLF395-12|Cecidomyiidae|664|BOLD:ABV0500
Cecidomyiidae|[5937]|CNBPQ055-13|Cecidomyiidae|658|BOLD:ACG9042
Cecidomyiidae|[5938]|CNSLJ074-12|Cecidomyiidae|670|BOLD:ACA6367
Cecidomyiidae|[5939]|CNGAG396-15|Cecidomyiidae|549|BOLD:ACL2676
Cecidomyiidae|[5940]|CNRME127-12|Cecidomyiidae|661|BOLD:ACC7079
Cecidomyiidae|[5941]|SSWLD5585-13|Cecidomyiidae|658|BOLD:ACJ1267
Cecidomyiidae|[5942]|CNBFM699-15|Cecidomyiidae|582|BOLD:ACT9558
Cecidomyiidae|[5943]|PHJUN838-11|Cecidomyiidae|658|BOLD:ABV9358
Cecidomyiidae|[5944]|JSAUG1161-11|Cecidomyiidae|658|BOLD:ABV1389
Cecidomyiidae|[5945]|CNRME4406-12|Cecidomyiidae|673|BOLD:ACC8756
Cecidomyiidae|[5946]|CNROM1381-13|Cecidomyiidae|658|BOLD:ABV1279
Cecidomyiidae|[5947]|BBDCM296-10|Cecidomyiidae|658|BOLD:AAP9027
Cecidomyiidae|[5948]|CNNHA326-14|Cecidomyiidae|591|BOLD:ACR4265
Cecidomyiidae|[5949]|HESEP613-12|Cecidomyiidae|681|BOLD:AAN5260
Cecidomyiidae|[5950]|CNPPJ769-12|Cecidomyiidae|670|BOLD:ACA7583
Cecidomyiidae|[5951]|SSEIB5376-13|Cecidomyiidae|658|BOLD:ACG6333
Cecidomyiidae|[5952]|SSBAD1029-12|Cecidomyiidae|670|BOLD:ACC7515
Cecidomyiidae|[5953]|SSJAD1915-13|Cecidomyiidae|661|BOLD:ACD0168
Cecidomyiidae|[5954]|CNPAO388-13|Cecidomyiidae|670|BOLD:ACD1658
Cecidomyiidae|[5955]|CNBAI236-13|Cecidomyiidae|670|BOLD:ACC8045
Cecidomyiidae|[5956]|CNRMF1561-12|Cecidomyiidae|670|BOLD:ACC7844
Cecidomyiidae|[5957]|SSPAB2725-13|Cecidomyiidae|654|BOLD:ACI5230
Cecidomyiidae|[5958]|HESEP707-12|Cecidomyiidae|664|BOLD:ABY1096
Cecidomyiidae|[5959]|CNKOS334-14|Cecidomyiidae|581|BOLD:ACM8068
Cecidomyiidae|[5960]|PAAUG1986-12|Cecidomyiidae|670|BOLD:ACB4104
Cecidomyiidae|[5961]|PHJUN042-11|Cecidomyiidae|658|BOLD:ABV0474
Cecidomyiidae|[5962]|PHNXN1343-14|Cecidomyiidae|604|BOLD:AAZ0273
Cecidomyiidae|[5963]|CNKOS945-14|Cecidomyiidae|622|BOLD:ACM9802
Cecidomyiidae|[5964]|CNKOR1210-14|Cecidomyiidae|599|BOLD:ACM7585
Cecidomyiidae|[5965]|CNROH217-13|Cecidomyiidae|661|BOLD:ACK2599
Cecidomyiidae|[5966]|CNFNQ927-14|Cecidomyiidae|658|BOLD:ACM2189
Cecidomyiidae|[5967]|SSROA084-14|Cecidomyiidae|585|BOLD:ACM7196
Cecidomyiidae|[5968]|PHJUN578-11|Cecidomyiidae|658|BOLD:ABX4079
Cecidomyiidae|[5969]|CNGUF458-15|Cecidomyiidae|567|BOLD:ACS5673
Cecidomyiidae|[5970]|DARC539-11|Cecidomyiidae|658|BOLD:AAU6765
Cecidomyiidae|[5971]|CNWLD688-12|Cecidomyiidae|673|BOLD:ACC9284
Cecidomyiidae|[5972]|JSDIS012-11|Cecidomyiidae|658|BOLD:AAY6450
Cecidomyiidae|[5973]|JSSEP384-11|Cecidomyiidae|605|BOLD:ABV1264
Cecidomyiidae|[5974]|TTMDI335-10|Cecidomyiidae|658|BOLD:AAN5211
Cecidomyiidae|[5975]|CNPPG122-12|Cecidomyiidae|664|BOLD:ABV0495
Cecidomyiidae|[5976]|PAAUG1688-12|Cecidomyiidae|670|BOLD:AAY6456
Cecidomyiidae|[5977]|CNROE894-13|Cecidomyiidae|603|BOLD:ACK1889
Cecidomyiidae|[5978]|CNMIH1343-14|Cecidomyiidae|594|BOLD:ACN4172
Cecidomyiidae|[5979]|CNGIL111-13|Cecidomyiidae|658|BOLD:ACH4373
Cecidomyiidae|[5980]|SSBAE510-13|Cecidomyiidae|664|BOLD:AAH3626
Cecidomyiidae|[5981]|CNFDD395-14|Cecidomyiidae|664|BOLD:ACO2730
Cecidomyiidae|[5982]|HESEP431-12|Cecidomyiidae|703|BOLD:ABZ3625
Cecidomyiidae|[5983]|PHJUL2174-11|Cecidomyiidae|658|BOLD:AAY6428
Cecidomyiidae|[5984]|CNEIE735-12|Cecidomyiidae|640|BOLD:ACO2728
Cecidomyiidae|[5985]|HEJUL924-12|Cecidomyiidae|703|BOLD:AAH3755
Cecidomyiidae|[5986]|SSWLA3003-13|Cecidomyiidae|630|BOLD:ACO2751
Cecidomyiidae|[5987]|HEAUG991-12|Cecidomyiidae|664|BOLD:ABW8024
Cecidomyiidae|[5988]|SSJAD1022-13|Cecidomyiidae|661|BOLD:ACO2727
Cecidomyiidae|[5989]|CNPKJ704-14|Cecidomyiidae|636|BOLD:ACM5655
Cecidomyiidae|[5990]|CNBPO353-13|Cecidomyiidae|658|BOLD:AAN5248
Cecidomyiidae|[5991]|CNNHE540-14|Cecidomyiidae|618|BOLD:ACR7357
Cecidomyiidae|[5992]|CNBPH257-12|Cecidomyiidae|658|BOLD:AAY6394
Cecidomyiidae|[5993]|SSPAC7571-13|Cecidomyiidae|664|BOLD:ACI9166
Cecidomyiidae|[5994]|CNBPP131-13|Cecidomyiidae|661|BOLD:ACC8702
Cecidomyiidae|[5995]|CNGMG303-14|Cecidomyiidae|660|BOLD:ACL4712
Cecidomyiidae|[5996]|CNWAF876-15|Cecidomyiidae|588|BOLD:ACS0465
Cecidomyiidae|[5997]|SSKJC3851-15|Cecidomyiidae|573|BOLD:ACT7868
Cecidomyiidae|[5998]|NGNAB824-13|Cecidomyiidae|664|BOLD:ACI3581
Cecidomyiidae|[5999]|SSWLF800-13|Cecidomyiidae|680|BOLD:ACK6460

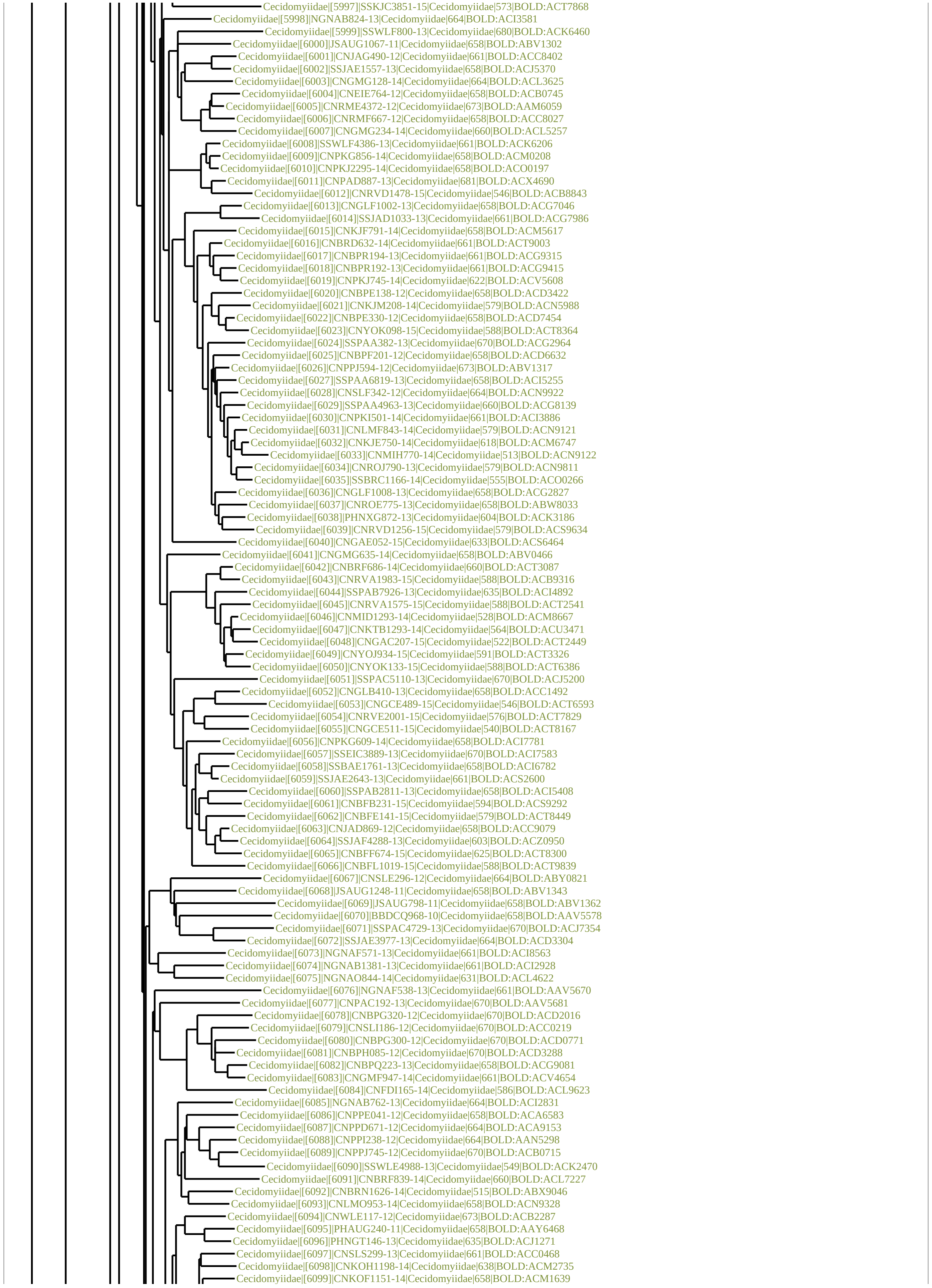
Cecidomyiidae|[5997]|SSKJC3851-15|Cecidomyiidae|573|BOLD:ACT7868
Cecidomyiidae|[5998]|NGNAB824-13|Cecidomyiidae|664|BOLD:ACI3581
Cecidomyiidae|[5999]|SSWLF800-13|Cecidomyiidae|680|BOLD:ACK6460
Cecidomyiidae|[6000]|JSAUG1067-11|Cecidomyiidae|658|BOLD:ABV1302
Cecidomyiidae|[6001]|CNJAG490-12|Cecidomyiidae|661|BOLD:ACC8402
Cecidomyiidae|[6002]|SSJAE1557-13|Cecidomyiidae|658|BOLD:ACJ5370
Cecidomyiidae|[6003]|CNGMG128-14|Cecidomyiidae|664|BOLD:ACL3625
Cecidomyiidae|[6004]|CNEIE764-12|Cecidomyiidae|658|BOLD:ACB0745
Cecidomyiidae|[6005]|CNRME4372-12|Cecidomyiidae|673|BOLD:AAM6059
Cecidomyiidae|[6006]|CNRMF667-12|Cecidomyiidae|658|BOLD:ACC8027
Cecidomyiidae|[6007]|CNGMG234-14|Cecidomyiidae|660|BOLD:ACL5257
Cecidomyiidae|[6008]|SSWLF4386-13|Cecidomyiidae|661|BOLD:ACK6206
Cecidomyiidae|[6009]|CNPKG856-14|Cecidomyiidae|658|BOLD:ACM0208
Cecidomyiidae|[6010]|CNPKJ2295-14|Cecidomyiidae|658|BOLD:ACO0197
Cecidomyiidae|[6011]|CNPAD887-13|Cecidomyiidae|681|BOLD:ACX4690
Cecidomyiidae|[6012]|CNRVD1478-15|Cecidomyiidae|546|BOLD:ACB8843
Cecidomyiidae|[6013]|CNGLF1002-13|Cecidomyiidae|658|BOLD:ACG7046
Cecidomyiidae|[6014]|SSJAD1033-13|Cecidomyiidae|661|BOLD:ACG7986
Cecidomyiidae|[6015]|CNKJF791-14|Cecidomyiidae|658|BOLD:ACM5617
Cecidomyiidae|[6016]|CNBRD632-14|Cecidomyiidae|661|BOLD:ACT9003
Cecidomyiidae|[6017]|CNBPR194-13|Cecidomyiidae|661|BOLD:ACG9315
Cecidomyiidae|[6018]|CNBPR192-13|Cecidomyiidae|661|BOLD:ACG9415
Cecidomyiidae|[6019]|CNPKJ745-14|Cecidomyiidae|622|BOLD:ACV5608
Cecidomyiidae|[6020]|CNBPE138-12|Cecidomyiidae|658|BOLD:ACD3422
Cecidomyiidae|[6021]|CNKJM208-14|Cecidomyiidae|579|BOLD:ACN5988
Cecidomyiidae|[6022]|CNBPE330-12|Cecidomyiidae|658|BOLD:ACD7454
Cecidomyiidae|[6023]|CNYOK098-15|Cecidomyiidae|588|BOLD:ACT8364
Cecidomyiidae|[6024]|SSPAA382-13|Cecidomyiidae|670|BOLD:ACG2964
Cecidomyiidae|[6025]|CNBPF201-12|Cecidomyiidae|658|BOLD:ACD6632
Cecidomyiidae|[6026]|CNPPJ594-12|Cecidomyiidae|673|BOLD:ABV1317
Cecidomyiidae|[6027]|SSPAA6819-13|Cecidomyiidae|658|BOLD:ACI5255
Cecidomyiidae|[6028]|CNSLF342-12|Cecidomyiidae|664|BOLD:ACN9922
Cecidomyiidae|[6029]|SSPAA4963-13|Cecidomyiidae|660|BOLD:ACG8139
Cecidomyiidae|[6030]|CNPKI501-14|Cecidomyiidae|661|BOLD:ACI3886
Cecidomyiidae|[6031]|CNLMF843-14|Cecidomyiidae|579|BOLD:ACN9121
Cecidomyiidae|[6032]|CNKJE750-14|Cecidomyiidae|618|BOLD:ACM6747
Cecidomyiidae|[6033]|CNMIH770-14|Cecidomyiidae|513|BOLD:ACN9122
Cecidomyiidae|[6034]|CNROJ790-13|Cecidomyiidae|579|BOLD:ACN9811
Cecidomyiidae|[6035]|SSBRC1166-14|Cecidomyiidae|555|BOLD:ACO0266
Cecidomyiidae|[6036]|CNGLF1008-13|Cecidomyiidae|658|BOLD:ACG2827
Cecidomyiidae|[6037]|CNROE775-13|Cecidomyiidae|658|BOLD:ABW8033
Cecidomyiidae|[6038]|PHNXG872-13|Cecidomyiidae|604|BOLD:ACK3186
Cecidomyiidae|[6039]|CNRVD1256-15|Cecidomyiidae|579|BOLD:ACS9634
Cecidomyiidae|[6040]|CNGAE052-15|Cecidomyiidae|633|BOLD:ACS6464
Cecidomyiidae|[6041]|CNGMG635-14|Cecidomyiidae|658|BOLD:ABV0466
Cecidomyiidae|[6042]|CNBRF686-14|Cecidomyiidae|660|BOLD:ACT3087
Cecidomyiidae|[6043]|CNRVA1983-15|Cecidomyiidae|588|BOLD:ACB9316
Cecidomyiidae|[6044]|SSPAB7926-13|Cecidomyiidae|635|BOLD:ACI4892
Cecidomyiidae|[6045]|CNRVA1575-15|Cecidomyiidae|588|BOLD:ACT2541
Cecidomyiidae|[6046]|CNMID1293-14|Cecidomyiidae|528|BOLD:ACM8667
Cecidomyiidae|[6047]|CNKTB1293-14|Cecidomyiidae|564|BOLD:ACU3471
Cecidomyiidae|[6048]|CNGAC207-15|Cecidomyiidae|522|BOLD:ACT2449
Cecidomyiidae|[6049]|CNYOJ934-15|Cecidomyiidae|591|BOLD:ACT3326
Cecidomyiidae|[6050]|CNYOK133-15|Cecidomyiidae|588|BOLD:ACT6386
Cecidomyiidae|[6051]|SSPAC5110-13|Cecidomyiidae|670|BOLD:ACJ5200
Cecidomyiidae|[6052]|CNGLB410-13|Cecidomyiidae|658|BOLD:ACC1492
Cecidomyiidae|[6053]|CNGCE489-15|Cecidomyiidae|546|BOLD:ACT6593
Cecidomyiidae|[6054]|CNRVE2001-15|Cecidomyiidae|576|BOLD:ACT7829
Cecidomyiidae|[6055]|CNGCE511-15|Cecidomyiidae|540|BOLD:ACT8167
Cecidomyiidae|[6056]|CNPKG609-14|Cecidomyiidae|658|BOLD:ACI7781
Cecidomyiidae|[6057]|SSEIC3889-13|Cecidomyiidae|670|BOLD:ACI7583
Cecidomyiidae|[6058]|SSBAE1761-13|Cecidomyiidae|658|BOLD:ACI6782
Cecidomyiidae|[6059]|SSJAE2643-13|Cecidomyiidae|661|BOLD:ACS2600
Cecidomyiidae|[6060]|SSPAB2811-13|Cecidomyiidae|658|BOLD:ACI5408
Cecidomyiidae|[6061]|CNBFB231-15|Cecidomyiidae|594|BOLD:ACS9292
Cecidomyiidae|[6062]|CNBFE141-15|Cecidomyiidae|579|BOLD:ACT8449
Cecidomyiidae|[6063]|CNJAD869-12|Cecidomyiidae|658|BOLD:ACC9079
Cecidomyiidae|[6064]|SSJAF4288-13|Cecidomyiidae|603|BOLD:ACZ0950
Cecidomyiidae|[6065]|CNBFF674-15|Cecidomyiidae|625|BOLD:ACT8300
Cecidomyiidae|[6066]|CNBFL1019-15|Cecidomyiidae|588|BOLD:ACT9839
Cecidomyiidae|[6067]|CNSLE296-12|Cecidomyiidae|664|BOLD:ABY0821
Cecidomyiidae|[6068]|JSAUG1248-11|Cecidomyiidae|658|BOLD:ABV1343
Cecidomyiidae|[6069]|JSAUG798-11|Cecidomyiidae|658|BOLD:ABV1362
Cecidomyiidae|[6070]|BBDCQ968-10|Cecidomyiidae|658|BOLD:AAV5578
Cecidomyiidae|[6071]|SSPAC4729-13|Cecidomyiidae|670|BOLD:ACJ7354
Cecidomyiidae|[6072]|SSJAE3977-13|Cecidomyiidae|664|BOLD:ACD3304
Cecidomyiidae|[6073]|NGNAF571-13|Cecidomyiidae|661|BOLD:ACI8563
Cecidomyiidae|[6074]|NGNAB1381-13|Cecidomyiidae|661|BOLD:ACI2928
Cecidomyiidae|[6075]|NGNAO844-14|Cecidomyiidae|631|BOLD:ACL4622
Cecidomyiidae|[6076]|NGNAF538-13|Cecidomyiidae|661|BOLD:AAV5670
Cecidomyiidae|[6077]|CNPAC192-13|Cecidomyiidae|670|BOLD:AAV5681
Cecidomyiidae|[6078]|CNBPG320-12|Cecidomyiidae|670|BOLD:ACD2016
Cecidomyiidae|[6079]|CNSLI186-12|Cecidomyiidae|670|BOLD:ACC0219
Cecidomyiidae|[6080]|CNBPG300-12|Cecidomyiidae|670|BOLD:ACD0771
Cecidomyiidae|[6081]|CNBPH085-12|Cecidomyiidae|670|BOLD:ACD3288
Cecidomyiidae|[6082]|CNBPQ223-13|Cecidomyiidae|658|BOLD:ACG9081
Cecidomyiidae|[6083]|CNGMF947-14|Cecidomyiidae|661|BOLD:ACV4654
Cecidomyiidae|[6084]|CNFDI165-14|Cecidomyiidae|586|BOLD:ACL9623
Cecidomyiidae|[6085]|NGNAB762-13|Cecidomyiidae|664|BOLD:ACI2831
Cecidomyiidae|[6086]|CNPPE041-12|Cecidomyiidae|658|BOLD:ACA6583
Cecidomyiidae|[6087]|CNPPD671-12|Cecidomyiidae|664|BOLD:ACA9153
Cecidomyiidae|[6088]|CNPPI238-12|Cecidomyiidae|664|BOLD:AAN5298
Cecidomyiidae|[6089]|CNPPJ745-12|Cecidomyiidae|670|BOLD:ACB0715
Cecidomyiidae|[6090]|SSWLE4988-13|Cecidomyiidae|549|BOLD:ACK2470
Cecidomyiidae|[6091]|CNBRF839-14|Cecidomyiidae|660|BOLD:ACL7227
Cecidomyiidae|[6092]|CNBRN1626-14|Cecidomyiidae|515|BOLD:ABX9046
Cecidomyiidae|[6093]|CNLMO953-14|Cecidomyiidae|658|BOLD:ACN9328
Cecidomyiidae|[6094]|CNWLE117-12|Cecidomyiidae|673|BOLD:ACB2287
Cecidomyiidae|[6095]|PHAUG240-11|Cecidomyiidae|658|BOLD:AAY6468
Cecidomyiidae|[6096]|PHNGT146-13|Cecidomyiidae|635|BOLD:ACJ1271
Cecidomyiidae|[6097]|CNSLS299-13|Cecidomyiidae|661|BOLD:ACC0468
Cecidomyiidae|[6098]|CNKOH1198-14|Cecidomyiidae|638|BOLD:ACM2735
Cecidomyiidae|[6099]|CNKOF1151-14|Cecidomyiidae|658|BOLD:ACM1639

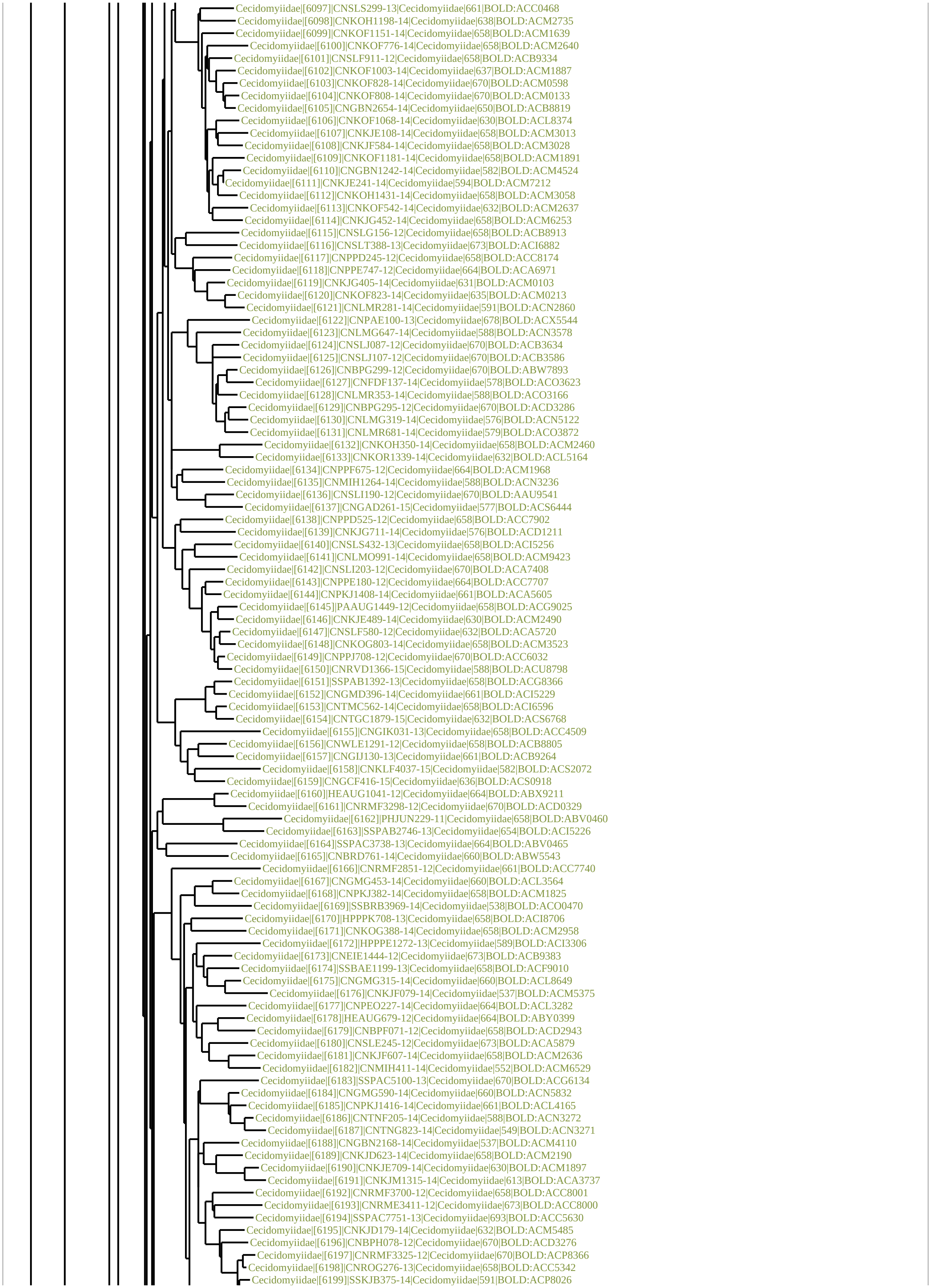
Cecidomyiidae|[6097]|CNSLS299-13|Cecidomyiidae|661|BOLD:ACC0468
Cecidomyiidae|[6098]|CNKOH1198-14|Cecidomyiidae|638|BOLD:ACM2735
Cecidomyiidae|[6099]|CNKOF1151-14|Cecidomyiidae|658|BOLD:ACM1639
Cecidomyiidae|[6100]|CNKOF776-14|Cecidomyiidae|658|BOLD:ACM2640
Cecidomyiidae|[6101]|CNSLF911-12|Cecidomyiidae|658|BOLD:ACB9334
Cecidomyiidae|[6102]|CNKOF1003-14|Cecidomyiidae|637|BOLD:ACM1887
Cecidomyiidae|[6103]|CNKOF828-14|Cecidomyiidae|670|BOLD:ACM0598
Cecidomyiidae|[6104]|CNKOF808-14|Cecidomyiidae|670|BOLD:ACM0133
Cecidomyiidae|[6105]|CNGBN2654-14|Cecidomyiidae|650|BOLD:ACB8819
Cecidomyiidae|[6106]|CNKOF1068-14|Cecidomyiidae|630|BOLD:ACL8374
Cecidomyiidae|[6107]|CNKJE108-14|Cecidomyiidae|658|BOLD:ACM3013
Cecidomyiidae|[6108]|CNKJF584-14|Cecidomyiidae|658|BOLD:ACM3028
Cecidomyiidae|[6109]|CNKOF1181-14|Cecidomyiidae|658|BOLD:ACM1891
Cecidomyiidae|[6110]|CNGBN1242-14|Cecidomyiidae|582|BOLD:ACM4524
Cecidomyiidae|[6111]|CNKJE241-14|Cecidomyiidae|594|BOLD:ACM7212
Cecidomyiidae|[6112]|CNKOH1431-14|Cecidomyiidae|658|BOLD:ACM3058
Cecidomyiidae|[6113]|CNKOF542-14|Cecidomyiidae|632|BOLD:ACM2637
Cecidomyiidae|[6114]|CNKJG452-14|Cecidomyiidae|658|BOLD:ACM6253
Cecidomyiidae|[6115]|CNSLG156-12|Cecidomyiidae|658|BOLD:ACB8913
Cecidomyiidae|[6116]|CNSLT388-13|Cecidomyiidae|673|BOLD:ACI6882
Cecidomyiidae|[6117]|CNPPD245-12|Cecidomyiidae|658|BOLD:ACC8174
Cecidomyiidae|[6118]|CNPPE747-12|Cecidomyiidae|664|BOLD:ACA6971
Cecidomyiidae|[6119]|CNKJG405-14|Cecidomyiidae|631|BOLD:ACM0103
Cecidomyiidae|[6120]|CNKOF823-14|Cecidomyiidae|635|BOLD:ACM0213
Cecidomyiidae|[6121]|CNLMR281-14|Cecidomyiidae|591|BOLD:ACN2860
Cecidomyiidae|[6122]|CNPAE100-13|Cecidomyiidae|678|BOLD:ACX5544
Cecidomyiidae|[6123]|CNLMG647-14|Cecidomyiidae|588|BOLD:ACN3578
Cecidomyiidae|[6124]|CNSLJ087-12|Cecidomyiidae|670|BOLD:ACB3634
Cecidomyiidae|[6125]|CNSLJ107-12|Cecidomyiidae|670|BOLD:ACB3586
Cecidomyiidae|[6126]|CNBPG299-12|Cecidomyiidae|670|BOLD:ABW7893
Cecidomyiidae|[6127]|CNFDF137-14|Cecidomyiidae|578|BOLD:ACO3623
Cecidomyiidae|[6128]|CNLMR353-14|Cecidomyiidae|588|BOLD:ACO3166
Cecidomyiidae|[6129]|CNBPG295-12|Cecidomyiidae|670|BOLD:ACD3286
Cecidomyiidae|[6130]|CNLMG319-14|Cecidomyiidae|576|BOLD:ACN5122
Cecidomyiidae|[6131]|CNLMR681-14|Cecidomyiidae|579|BOLD:ACO3872
Cecidomyiidae|[6132]|CNKOH350-14|Cecidomyiidae|658|BOLD:ACM2460
Cecidomyiidae|[6133]|CNKOR1339-14|Cecidomyiidae|632|BOLD:ACL5164
Cecidomyiidae|[6134]|CNPPF675-12|Cecidomyiidae|664|BOLD:ACM1968
Cecidomyiidae|[6135]|CNMIH1264-14|Cecidomyiidae|588|BOLD:ACN3236
Cecidomyiidae|[6136]|CNSLI190-12|Cecidomyiidae|670|BOLD:AAU9541
Cecidomyiidae|[6137]|CNGAD261-15|Cecidomyiidae|577|BOLD:ACS6444
Cecidomyiidae|[6138]|CNPPD525-12|Cecidomyiidae|658|BOLD:ACC7902
Cecidomyiidae|[6139]|CNKJG711-14|Cecidomyiidae|576|BOLD:ACD1211
Cecidomyiidae|[6140]|CNSLS432-13|Cecidomyiidae|658|BOLD:ACI5256
Cecidomyiidae|[6141]|CNLMO991-14|Cecidomyiidae|658|BOLD:ACM9423
Cecidomyiidae|[6142]|CNSLI203-12|Cecidomyiidae|670|BOLD:ACA7408
Cecidomyiidae|[6143]|CNPPE180-12|Cecidomyiidae|664|BOLD:ACC7707
Cecidomyiidae|[6144]|CNPKJ1408-14|Cecidomyiidae|661|BOLD:ACA5605
Cecidomyiidae|[6145]|PAAUG1449-12|Cecidomyiidae|658|BOLD:ACG9025
Cecidomyiidae|[6146]|CNKJE489-14|Cecidomyiidae|630|BOLD:ACM2490
Cecidomyiidae|[6147]|CNSLF580-12|Cecidomyiidae|632|BOLD:ACA5720
Cecidomyiidae|[6148]|CNKOG803-14|Cecidomyiidae|658|BOLD:ACM3523
Cecidomyiidae|[6149]|CNPPJ708-12|Cecidomyiidae|670|BOLD:ACC6032
Cecidomyiidae|[6150]|CNRVD1366-15|Cecidomyiidae|588|BOLD:ACU8798
Cecidomyiidae|[6151]|SSPAB1392-13|Cecidomyiidae|658|BOLD:ACG8366
Cecidomyiidae|[6152]|CNGMD396-14|Cecidomyiidae|661|BOLD:ACI5229
Cecidomyiidae|[6153]|CNTMC562-14|Cecidomyiidae|658|BOLD:ACI6596
Cecidomyiidae|[6154]|CNTGC1879-15|Cecidomyiidae|632|BOLD:ACS6768
Cecidomyiidae|[6155]|CNGIK031-13|Cecidomyiidae|658|BOLD:ACC4509
Cecidomyiidae|[6156]|CNWLE1291-12|Cecidomyiidae|658|BOLD:ACB8805
Cecidomyiidae|[6157]|CNGIJ130-13|Cecidomyiidae|661|BOLD:ACB9264
Cecidomyiidae|[6158]|CNKLF4037-15|Cecidomyiidae|582|BOLD:ACS2072
Cecidomyiidae|[6159]|CNGCF416-15|Cecidomyiidae|636|BOLD:ACS0918
Cecidomyiidae|[6160]|HEAUG1041-12|Cecidomyiidae|664|BOLD:ABX9211
Cecidomyiidae|[6161]|CNRMF3298-12|Cecidomyiidae|670|BOLD:ACD0329
Cecidomyiidae|[6162]|PHJUN229-11|Cecidomyiidae|658|BOLD:ABV0460
Cecidomyiidae|[6163]|SSPAB2746-13|Cecidomyiidae|654|BOLD:ACI5226
Cecidomyiidae|[6164]|SSPAC3738-13|Cecidomyiidae|664|BOLD:ABV0465
Cecidomyiidae|[6165]|CNBRD761-14|Cecidomyiidae|660|BOLD:ABW5543
Cecidomyiidae|[6166]|CNRMF2851-12|Cecidomyiidae|661|BOLD:ACC7740
Cecidomyiidae|[6167]|CNGMG453-14|Cecidomyiidae|660|BOLD:ACL3564
Cecidomyiidae|[6168]|CNPKJ382-14|Cecidomyiidae|658|BOLD:ACM1825
Cecidomyiidae|[6169]|SSBRB3969-14|Cecidomyiidae|538|BOLD:ACO0470
Cecidomyiidae|[6170]|HPPPK708-13|Cecidomyiidae|658|BOLD:ACI8706
Cecidomyiidae|[6171]|CNKOG388-14|Cecidomyiidae|658|BOLD:ACM2958
Cecidomyiidae|[6172]|HPPPE1272-13|Cecidomyiidae|589|BOLD:ACI3306
Cecidomyiidae|[6173]|CNEIE1444-12|Cecidomyiidae|673|BOLD:ACB9383
Cecidomyiidae|[6174]|SSBAE1199-13|Cecidomyiidae|658|BOLD:ACF9010
Cecidomyiidae|[6175]|CNGMG315-14|Cecidomyiidae|660|BOLD:ACL8649
Cecidomyiidae|[6176]|CNKJF079-14|Cecidomyiidae|537|BOLD:ACM5375
Cecidomyiidae|[6177]|CNPEO227-14|Cecidomyiidae|664|BOLD:ACL3282
Cecidomyiidae|[6178]|HEAUG679-12|Cecidomyiidae|664|BOLD:ABY0399
Cecidomyiidae|[6179]|CNBPF071-12|Cecidomyiidae|658|BOLD:ACD2943
Cecidomyiidae|[6180]|CNSLE245-12|Cecidomyiidae|673|BOLD:ACA5879
Cecidomyiidae|[6181]|CNKJF607-14|Cecidomyiidae|658|BOLD:ACM2636
Cecidomyiidae|[6182]|CNMIH411-14|Cecidomyiidae|552|BOLD:ACM6529
Cecidomyiidae|[6183]|SSPAC5100-13|Cecidomyiidae|670|BOLD:ACG6134
Cecidomyiidae|[6184]|CNGMG590-14|Cecidomyiidae|660|BOLD:ACN5832
Cecidomyiidae|[6185]|CNPKJ1416-14|Cecidomyiidae|661|BOLD:ACL4165
Cecidomyiidae|[6186]|CNTNF205-14|Cecidomyiidae|588|BOLD:ACN3272
Cecidomyiidae|[6187]|CNTNG823-14|Cecidomyiidae|549|BOLD:ACN3271
Cecidomyiidae|[6188]|CNGBN2168-14|Cecidomyiidae|537|BOLD:ACM4110
Cecidomyiidae|[6189]|CNKJD623-14|Cecidomyiidae|658|BOLD:ACM2190
Cecidomyiidae|[6190]|CNKJE709-14|Cecidomyiidae|630|BOLD:ACM1897
Cecidomyiidae|[6191]|CNKJM1315-14|Cecidomyiidae|613|BOLD:ACA3737
Cecidomyiidae|[6192]|CNRMF3700-12|Cecidomyiidae|658|BOLD:ACC8001
Cecidomyiidae|[6193]|CNRME3411-12|Cecidomyiidae|673|BOLD:ACC8000
Cecidomyiidae|[6194]|SSPAC7751-13|Cecidomyiidae|693|BOLD:ACC5630
Cecidomyiidae|[6195]|CNKJD179-14|Cecidomyiidae|632|BOLD:ACM5485
Cecidomyiidae|[6196]|CNBPH078-12|Cecidomyiidae|670|BOLD:ACD3276
Cecidomyiidae|[6197]|CNRMF3325-12|Cecidomyiidae|670|BOLD:ACP8366
Cecidomyiidae|[6198]|CNROG276-13|Cecidomyiidae|658|BOLD:ACC5342
Cecidomyiidae|[6199]|SSKJB375-14|Cecidomyiidae|591|BOLD:ACP8026

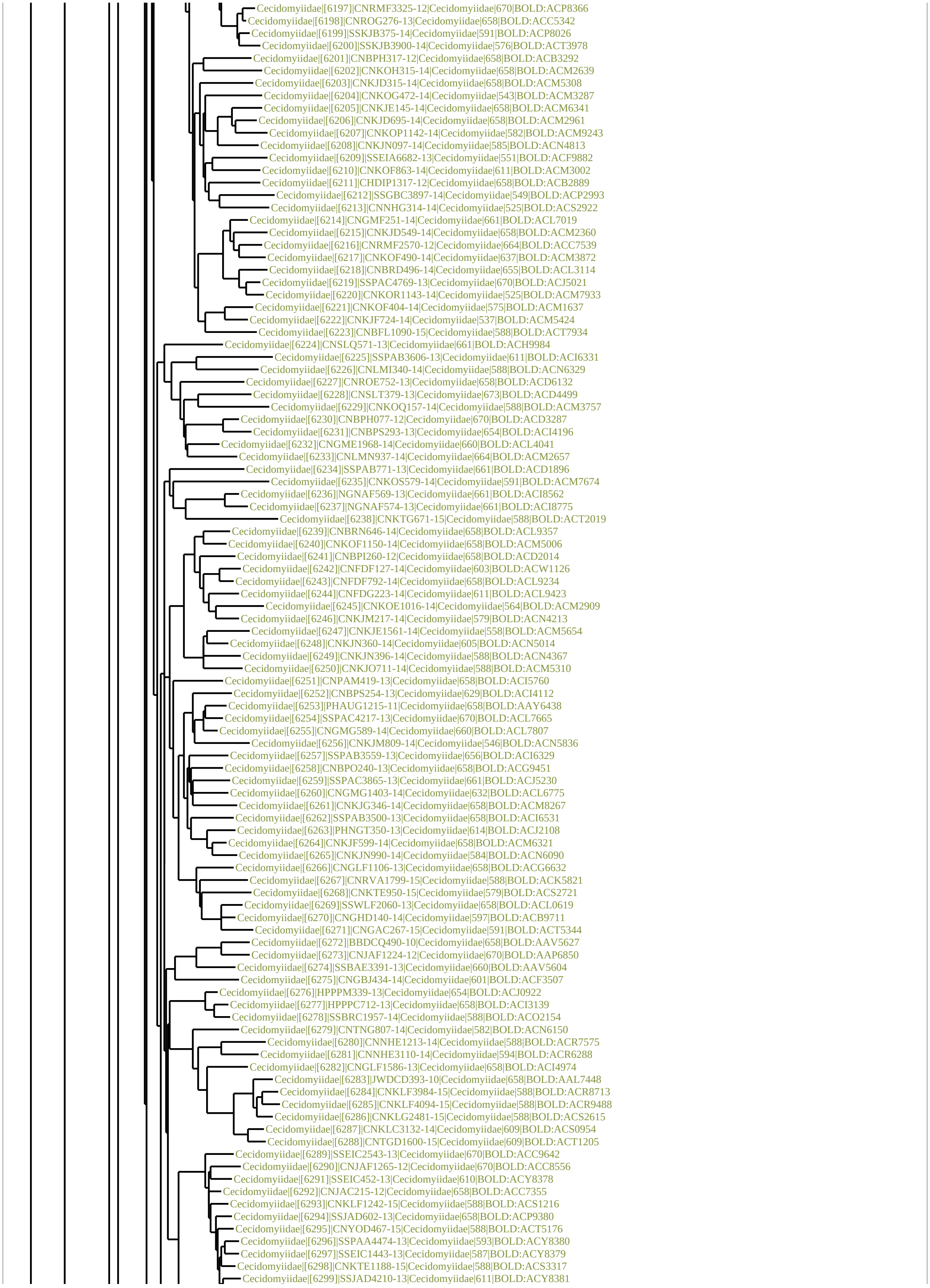

Cecidomyiidae|[6197]|CNRMF3325-12|Cecidomyiidae|670|BOLD:ACP8366
Cecidomyiidae|[6198]|CNROG276-13|Cecidomyiidae|658|BOLD:ACC5342
Cecidomyiidae|[6199]|SSKJB375-14|Cecidomyiidae|591|BOLD:ACP8026
Cecidomyiidae|[6200]|SSKJB3900-14|Cecidomyiidae|576|BOLD:ACT3978
Cecidomyiidae|[6201]|CNBPH317-12|Cecidomyiidae|658|BOLD:ACB3292
Cecidomyiidae|[6202]|CNKOH315-14|Cecidomyiidae|658|BOLD:ACM2639
Cecidomyiidae|[6203]|CNKJD315-14|Cecidomyiidae|658|BOLD:ACM5308
Cecidomyiidae|[6204]|CNKOG472-14|Cecidomyiidae|543|BOLD:ACM3287
Cecidomyiidae|[6205]|CNKJE145-14|Cecidomyiidae|658|BOLD:ACM6341
Cecidomyiidae|[6206]|CNKJD695-14|Cecidomyiidae|658|BOLD:ACM2961
Cecidomyiidae|[6207]|CNKOP1142-14|Cecidomyiidae|582|BOLD:ACM9243
Cecidomyiidae|[6208]|CNKJN097-14|Cecidomyiidae|585|BOLD:ACN4813
Cecidomyiidae|[6209]|SSEIA6682-13|Cecidomyiidae|551|BOLD:ACF9882
Cecidomyiidae|[6210]|CNKOF863-14|Cecidomyiidae|611|BOLD:ACM3002
Cecidomyiidae|[6211]|CHDIP1317-12|Cecidomyiidae|658|BOLD:ACB2889
Cecidomyiidae|[6212]|SSGBC3897-14|Cecidomyiidae|549|BOLD:ACP2993
Cecidomyiidae|[6213]|CNNHG314-14|Cecidomyiidae|525|BOLD:ACS2922
Cecidomyiidae|[6214]|CNGMF251-14|Cecidomyiidae|661|BOLD:ACL7019
Cecidomyiidae|[6215]|CNKJD549-14|Cecidomyiidae|658|BOLD:ACM2360
Cecidomyiidae|[6216]|CNRMF2570-12|Cecidomyiidae|664|BOLD:ACC7539
Cecidomyiidae|[6217]|CNKOF490-14|Cecidomyiidae|637|BOLD:ACM3872
Cecidomyiidae|[6218]|CNBRD496-14|Cecidomyiidae|655|BOLD:ACL3114
Cecidomyiidae|[6219]|SSPAC4769-13|Cecidomyiidae|670|BOLD:ACJ5021
Cecidomyiidae|[6220]|CNKOR1143-14|Cecidomyiidae|525|BOLD:ACM7933
Cecidomyiidae|[6221]|CNKOF404-14|Cecidomyiidae|575|BOLD:ACM1637
Cecidomyiidae|[6222]|CNKJF724-14|Cecidomyiidae|537|BOLD:ACM5424
Cecidomyiidae|[6223]|CNBFL1090-15|Cecidomyiidae|588|BOLD:ACT7934
Cecidomyiidae|[6224]|CNSLQ571-13|Cecidomyiidae|661|BOLD:ACH9984
Cecidomyiidae|[6225]|SSPAB3606-13|Cecidomyiidae|611|BOLD:ACI6331
Cecidomyiidae|[6226]|CNLMI340-14|Cecidomyiidae|588|BOLD:ACN6329
Cecidomyiidae|[6227]|CNROE752-13|Cecidomyiidae|658|BOLD:ACD6132
Cecidomyiidae|[6228]|CNSLT379-13|Cecidomyiidae|673|BOLD:ACD4499
Cecidomyiidae|[6229]|CNKOQ157-14|Cecidomyiidae|588|BOLD:ACM3757
Cecidomyiidae|[6230]|CNBPH077-12|Cecidomyiidae|670|BOLD:ACD3287
Cecidomyiidae|[6231]|CNBPS293-13|Cecidomyiidae|654|BOLD:ACI4196
Cecidomyiidae|[6232]|CNGME1968-14|Cecidomyiidae|660|BOLD:ACL4041
Cecidomyiidae|[6233]|CNLMN937-14|Cecidomyiidae|664|BOLD:ACM2657
Cecidomyiidae|[6234]|SSPAB771-13|Cecidomyiidae|661|BOLD:ACD1896
Cecidomyiidae|[6235]|CNKOS579-14|Cecidomyiidae|591|BOLD:ACM7674
Cecidomyiidae|[6236]|NGNAF569-13|Cecidomyiidae|661|BOLD:ACI8562
Cecidomyiidae|[6237]|NGNAF574-13|Cecidomyiidae|661|BOLD:ACI8775
Cecidomyiidae|[6238]|CNKTG671-15|Cecidomyiidae|588|BOLD:ACT2019
Cecidomyiidae|[6239]|CNBRN646-14|Cecidomyiidae|658|BOLD:ACL9357
Cecidomyiidae|[6240]|CNKOF1150-14|Cecidomyiidae|658|BOLD:ACM5006
Cecidomyiidae|[6241]|CNBPI260-12|Cecidomyiidae|658|BOLD:ACD2014
Cecidomyiidae|[6242]|CNFDF127-14|Cecidomyiidae|603|BOLD:ACW1126
Cecidomyiidae|[6243]|CNFDF792-14|Cecidomyiidae|658|BOLD:ACL9234
Cecidomyiidae|[6244]|CNFDG223-14|Cecidomyiidae|611|BOLD:ACL9423
Cecidomyiidae|[6245]|CNKOE1016-14|Cecidomyiidae|564|BOLD:ACM2909
Cecidomyiidae|[6246]|CNKJM217-14|Cecidomyiidae|579|BOLD:ACN4213
Cecidomyiidae|[6247]|CNKJE1561-14|Cecidomyiidae|558|BOLD:ACM5654
Cecidomyiidae|[6248]|CNKJN360-14|Cecidomyiidae|605|BOLD:ACN5014
Cecidomyiidae|[6249]|CNKJN396-14|Cecidomyiidae|588|BOLD:ACN4367
Cecidomyiidae|[6250]|CNKJO711-14|Cecidomyiidae|588|BOLD:ACM5310
Cecidomyiidae|[6251]|CNPAM419-13|Cecidomyiidae|658|BOLD:ACI5760
Cecidomyiidae|[6252]|CNBPS254-13|Cecidomyiidae|629|BOLD:ACI4112
Cecidomyiidae|[6253]|PHAUG1215-11|Cecidomyiidae|658|BOLD:AAY6438
Cecidomyiidae|[6254]|SSPAC4217-13|Cecidomyiidae|670|BOLD:ACL7665
Cecidomyiidae|[6255]|CNGMG589-14|Cecidomyiidae|660|BOLD:ACL7807
Cecidomyiidae|[6256]|CNKJM809-14|Cecidomyiidae|546|BOLD:ACN5836
Cecidomyiidae|[6257]|SSPAB3559-13|Cecidomyiidae|656|BOLD:ACI6329
Cecidomyiidae|[6258]|CNBPO240-13|Cecidomyiidae|658|BOLD:ACG9451
Cecidomyiidae|[6259]|SSPAC3865-13|Cecidomyiidae|661|BOLD:ACJ5230
Cecidomyiidae|[6260]|CNGMG1403-14|Cecidomyiidae|632|BOLD:ACL6775
Cecidomyiidae|[6261]|CNKJG346-14|Cecidomyiidae|658|BOLD:ACM8267
Cecidomyiidae|[6262]|SSPAB3500-13|Cecidomyiidae|658|BOLD:ACI6531
Cecidomyiidae|[6263]|PHNGT350-13|Cecidomyiidae|614|BOLD:ACJ2108
Cecidomyiidae|[6264]|CNKJF599-14|Cecidomyiidae|658|BOLD:ACM6321
Cecidomyiidae|[6265]|CNKJN990-14|Cecidomyiidae|584|BOLD:ACN6090
Cecidomyiidae|[6266]|CNGLF1106-13|Cecidomyiidae|658|BOLD:ACG6632
Cecidomyiidae|[6267]|CNRVA1799-15|Cecidomyiidae|588|BOLD:ACK5821
Cecidomyiidae|[6268]|CNKTE950-15|Cecidomyiidae|579|BOLD:ACS2721
Cecidomyiidae|[6269]|SSWLF2060-13|Cecidomyiidae|658|BOLD:ACL0619
Cecidomyiidae|[6270]|CNGHD140-14|Cecidomyiidae|597|BOLD:ACB9711
Cecidomyiidae|[6271]|CNGAC267-15|Cecidomyiidae|591|BOLD:ACT5344
Cecidomyiidae|[6272]|BBDCQ490-10|Cecidomyiidae|658|BOLD:AAV5627
Cecidomyiidae|[6273]|CNJAF1224-12|Cecidomyiidae|670|BOLD:AAP6850
Cecidomyiidae|[6274]|SSBAE3391-13|Cecidomyiidae|660|BOLD:AAV5604
Cecidomyiidae|[6275]|CNGBJ434-14|Cecidomyiidae|601|BOLD:ACF3507
Cecidomyiidae|[6276]|HPPPM339-13|Cecidomyiidae|654|BOLD:ACJ0922
Cecidomyiidae|[6277]|HPPPC712-13|Cecidomyiidae|658|BOLD:ACI3139
Cecidomyiidae|[6278]|SSBRC1957-14|Cecidomyiidae|588|BOLD:ACO2154
Cecidomyiidae|[6279]|CNTNG807-14|Cecidomyiidae|582|BOLD:ACN6150
Cecidomyiidae|[6280]|CNNHE1213-14|Cecidomyiidae|588|BOLD:ACR7575
Cecidomyiidae|[6281]|CNNHE3110-14|Cecidomyiidae|594|BOLD:ACR6288
Cecidomyiidae|[6282]|CNGLF1586-13|Cecidomyiidae|658|BOLD:ACI4974
Cecidomyiidae|[6283]|JWDCD393-10|Cecidomyiidae|658|BOLD:AAL7448
Cecidomyiidae|[6284]|CNKLF3984-15|Cecidomyiidae|588|BOLD:ACR8713
Cecidomyiidae|[6285]|CNKLF4094-15|Cecidomyiidae|588|BOLD:ACR9488
Cecidomyiidae|[6286]|CNKLG2481-15|Cecidomyiidae|588|BOLD:ACS2615
Cecidomyiidae|[6287]|CNKLC3132-14|Cecidomyiidae|609|BOLD:ACS0954
Cecidomyiidae|[6288]|CNTGD1600-15|Cecidomyiidae|609|BOLD:ACT1205
Cecidomyiidae|[6289]|SSEIC2543-13|Cecidomyiidae|670|BOLD:ACC9642
Cecidomyiidae|[6290]|CNJAF1265-12|Cecidomyiidae|670|BOLD:ACC8556
Cecidomyiidae|[6291]|SSEIC452-13|Cecidomyiidae|610|BOLD:ACY8378
Cecidomyiidae|[6292]|CNJAC215-12|Cecidomyiidae|658|BOLD:ACC7355
Cecidomyiidae|[6293]|CNKLF1242-15|Cecidomyiidae|588|BOLD:ACS1216
Cecidomyiidae|[6294]|SSJAD602-13|Cecidomyiidae|658|BOLD:ACP9380
Cecidomyiidae|[6295]|CNYOD467-15|Cecidomyiidae|588|BOLD:ACT5176
Cecidomyiidae|[6296]|SSPAA4474-13|Cecidomyiidae|593|BOLD:ACY8380
Cecidomyiidae|[6297]|SSEIC1443-13|Cecidomyiidae|587|BOLD:ACY8379
Cecidomyiidae|[6298]|CNKTE1188-15|Cecidomyiidae|588|BOLD:ACS3317
Cecidomyiidae|[6299]|SSJAD4210-13|Cecidomyiidae|611|BOLD:ACY8381

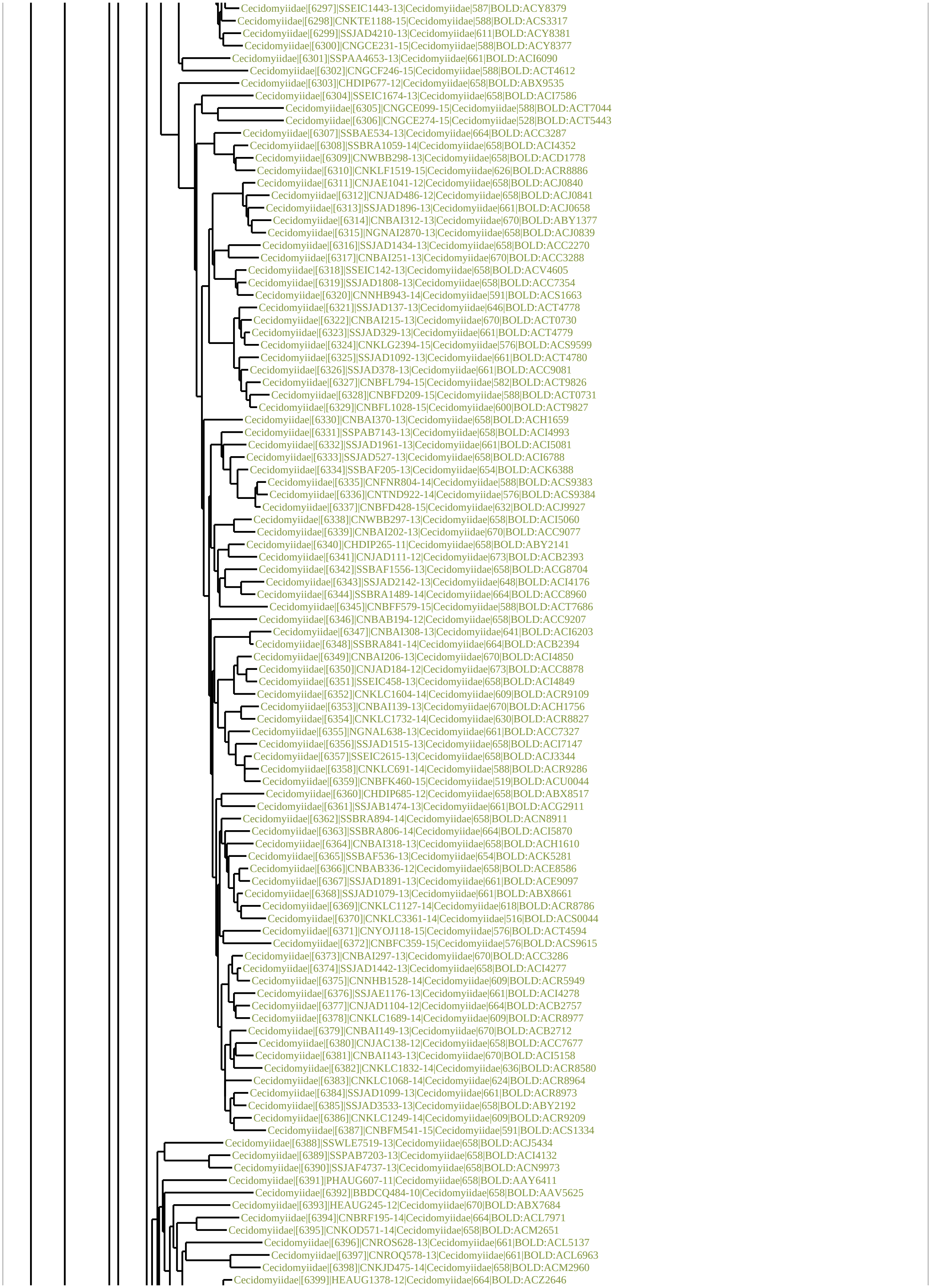
Cecidomyiidae|[6297]|SSEIC1443-13|Cecidomyiidae|587|BOLD:ACY8379
Cecidomyiidae|[6298]|CNKTE1188-15|Cecidomyiidae|588|BOLD:ACS3317
Cecidomyiidae|[6299]|SSJAD4210-13|Cecidomyiidae|611|BOLD:ACY8381
Cecidomyiidae|[6300]|CNGCE231-15|Cecidomyiidae|588|BOLD:ACY8377
Cecidomyiidae|[6301]|SSPAA4653-13|Cecidomyiidae|661|BOLD:ACI6090
Cecidomyiidae|[6302]|CNGCF246-15|Cecidomyiidae|588|BOLD:ACT4612
Cecidomyiidae|[6303]|CHDIP677-12|Cecidomyiidae|658|BOLD:ABX9535
Cecidomyiidae|[6304]|SSEIC1674-13|Cecidomyiidae|658|BOLD:ACI7586
Cecidomyiidae|[6305]|CNGCE099-15|Cecidomyiidae|588|BOLD:ACT7044
Cecidomyiidae|[6306]|CNGCE274-15|Cecidomyiidae|528|BOLD:ACT5443
Cecidomyiidae|[6307]|SSBAE534-13|Cecidomyiidae|664|BOLD:ACC3287
Cecidomyiidae|[6308]|SSBRA1059-14|Cecidomyiidae|658|BOLD:ACI4352
Cecidomyiidae|[6309]|CNWBB298-13|Cecidomyiidae|658|BOLD:ACD1778
Cecidomyiidae|[6310]|CNKLF1519-15|Cecidomyiidae|626|BOLD:ACR8886
Cecidomyiidae|[6311]|CNJAE1041-12|Cecidomyiidae|658|BOLD:ACJ0840
Cecidomyiidae|[6312]|CNJAD486-12|Cecidomyiidae|658|BOLD:ACJ0841
Cecidomyiidae|[6313]|SSJAD1896-13|Cecidomyiidae|661|BOLD:ACJ0658
Cecidomyiidae|[6314]|CNBAI312-13|Cecidomyiidae|670|BOLD:ABY1377
Cecidomyiidae|[6315]|NGNAI2870-13|Cecidomyiidae|658|BOLD:ACJ0839
Cecidomyiidae|[6316]|SSJAD1434-13|Cecidomyiidae|658|BOLD:ACC2270
Cecidomyiidae|[6317]|CNBAI251-13|Cecidomyiidae|670|BOLD:ACC3288
Cecidomyiidae|[6318]|SSEIC142-13|Cecidomyiidae|658|BOLD:ACV4605
Cecidomyiidae|[6319]|SSJAD1808-13|Cecidomyiidae|658|BOLD:ACC7354
Cecidomyiidae|[6320]|CNNHB943-14|Cecidomyiidae|591|BOLD:ACS1663
Cecidomyiidae|[6321]|SSJAD137-13|Cecidomyiidae|646|BOLD:ACT4778
Cecidomyiidae|[6322]|CNBAI215-13|Cecidomyiidae|670|BOLD:ACT0730
Cecidomyiidae|[6323]|SSJAD329-13|Cecidomyiidae|661|BOLD:ACT4779
Cecidomyiidae|[6324]|CNKLG2394-15|Cecidomyiidae|576|BOLD:ACS9599
Cecidomyiidae|[6325]|SSJAD1092-13|Cecidomyiidae|661|BOLD:ACT4780
Cecidomyiidae|[6326]|SSJAD378-13|Cecidomyiidae|661|BOLD:ACC9081
Cecidomyiidae|[6327]|CNBFL794-15|Cecidomyiidae|582|BOLD:ACT9826
Cecidomyiidae|[6328]|CNBFD209-15|Cecidomyiidae|588|BOLD:ACT0731
Cecidomyiidae|[6329]|CNBFL1028-15|Cecidomyiidae|600|BOLD:ACT9827
Cecidomyiidae|[6330]|CNBAI370-13|Cecidomyiidae|658|BOLD:ACH1659
Cecidomyiidae|[6331]|SSPAB7143-13|Cecidomyiidae|658|BOLD:ACI4993
Cecidomyiidae|[6332]|SSJAD1961-13|Cecidomyiidae|661|BOLD:ACI5081
Cecidomyiidae|[6333]|SSJAD527-13|Cecidomyiidae|658|BOLD:ACI6788
Cecidomyiidae|[6334]|SSBAF205-13|Cecidomyiidae|654|BOLD:ACK6388
Cecidomyiidae|[6335]|CNFNR804-14|Cecidomyiidae|588|BOLD:ACS9383
Cecidomyiidae|[6336]|CNTND922-14|Cecidomyiidae|576|BOLD:ACS9384
Cecidomyiidae|[6337]|CNBFD428-15|Cecidomyiidae|632|BOLD:ACJ9927
Cecidomyiidae|[6338]|CNWBB297-13|Cecidomyiidae|658|BOLD:ACI5060
Cecidomyiidae|[6339]|CNBAI202-13|Cecidomyiidae|670|BOLD:ACC9077
Cecidomyiidae|[6340]|CHDIP265-11|Cecidomyiidae|658|BOLD:ABY2141
Cecidomyiidae|[6341]|CNJAD111-12|Cecidomyiidae|673|BOLD:ACB2393
Cecidomyiidae|[6342]|SSBAF1556-13|Cecidomyiidae|658|BOLD:ACG8704
Cecidomyiidae|[6343]|SSJAD2142-13|Cecidomyiidae|648|BOLD:ACI4176
Cecidomyiidae|[6344]|SSBRA1489-14|Cecidomyiidae|664|BOLD:ACC8960
Cecidomyiidae|[6345]|CNBFF579-15|Cecidomyiidae|588|BOLD:ACT7686
Cecidomyiidae|[6346]|CNBAB194-12|Cecidomyiidae|658|BOLD:ACC9207
Cecidomyiidae|[6347]|CNBAI308-13|Cecidomyiidae|641|BOLD:ACI6203
Cecidomyiidae|[6348]|SSBRA841-14|Cecidomyiidae|664|BOLD:ACB2394
Cecidomyiidae|[6349]|CNBAI206-13|Cecidomyiidae|670|BOLD:ACI4850
Cecidomyiidae|[6350]|CNJAD184-12|Cecidomyiidae|673|BOLD:ACC8878
Cecidomyiidae|[6351]|SSEIC458-13|Cecidomyiidae|658|BOLD:ACI4849
Cecidomyiidae|[6352]|CNKLC1604-14|Cecidomyiidae|609|BOLD:ACR9109
Cecidomyiidae|[6353]|CNBAI139-13|Cecidomyiidae|670|BOLD:ACH1756
Cecidomyiidae|[6354]|CNKLC1732-14|Cecidomyiidae|630|BOLD:ACR8827
Cecidomyiidae|[6355]|NGNAL638-13|Cecidomyiidae|661|BOLD:ACC7327
Cecidomyiidae|[6356]|SSJAD1515-13|Cecidomyiidae|658|BOLD:ACI7147
Cecidomyiidae|[6357]|SSEIC2615-13|Cecidomyiidae|658|BOLD:ACJ3344
Cecidomyiidae|[6358]|CNKLC691-14|Cecidomyiidae|588|BOLD:ACR9286
Cecidomyiidae|[6359]|CNBFK460-15|Cecidomyiidae|519|BOLD:ACU0044
Cecidomyiidae|[6360]|CHDIP685-12|Cecidomyiidae|658|BOLD:ABX8517
Cecidomyiidae|[6361]|SSJAB1474-13|Cecidomyiidae|661|BOLD:ACG2911
Cecidomyiidae|[6362]|SSBRA894-14|Cecidomyiidae|658|BOLD:ACN8911
Cecidomyiidae|[6363]|SSBRA806-14|Cecidomyiidae|664|BOLD:ACI5870
Cecidomyiidae|[6364]|CNBAI318-13|Cecidomyiidae|658|BOLD:ACH1610
Cecidomyiidae|[6365]|SSBAF536-13|Cecidomyiidae|654|BOLD:ACK5281
Cecidomyiidae|[6366]|CNBAB336-12|Cecidomyiidae|658|BOLD:ACE8586
Cecidomyiidae|[6367]|SSJAD1891-13|Cecidomyiidae|661|BOLD:ACE9097
Cecidomyiidae|[6368]|SSJAD1079-13|Cecidomyiidae|661|BOLD:ABX8661
Cecidomyiidae|[6369]|CNKLC1127-14|Cecidomyiidae|618|BOLD:ACR8786
Cecidomyiidae|[6370]|CNKLC3361-14|Cecidomyiidae|516|BOLD:ACS0044
Cecidomyiidae|[6371]|CNYOJ118-15|Cecidomyiidae|576|BOLD:ACT4594
Cecidomyiidae|[6372]|CNBFC359-15|Cecidomyiidae|576|BOLD:ACS9615
Cecidomyiidae|[6373]|CNBAI297-13|Cecidomyiidae|670|BOLD:ACC3286
Cecidomyiidae|[6374]|SSJAD1442-13|Cecidomyiidae|658|BOLD:ACI4277
Cecidomyiidae|[6375]|CNNHB1528-14|Cecidomyiidae|609|BOLD:ACR5949
Cecidomyiidae|[6376]|SSJAE1176-13|Cecidomyiidae|661|BOLD:ACI4278
Cecidomyiidae|[6377]|CNJAD1104-12|Cecidomyiidae|664|BOLD:ACB2757
Cecidomyiidae|[6378]|CNKLC1689-14|Cecidomyiidae|609|BOLD:ACR8977
Cecidomyiidae|[6379]|CNBAI149-13|Cecidomyiidae|670|BOLD:ACB2712
Cecidomyiidae|[6380]|CNJAC138-12|Cecidomyiidae|658|BOLD:ACC7677
Cecidomyiidae|[6381]|CNBAI143-13|Cecidomyiidae|670|BOLD:ACI5158
Cecidomyiidae|[6382]|CNKLC1832-14|Cecidomyiidae|636|BOLD:ACR8580
Cecidomyiidae|[6383]|CNKLC1068-14|Cecidomyiidae|624|BOLD:ACR8964
Cecidomyiidae|[6384]|SSJAD1099-13|Cecidomyiidae|661|BOLD:ACR8973
Cecidomyiidae|[6385]|SSJAD3533-13|Cecidomyiidae|658|BOLD:ABY2192
Cecidomyiidae|[6386]|CNKLC1249-14|Cecidomyiidae|609|BOLD:ACR9209
Cecidomyiidae|[6387]|CNBFM541-15|Cecidomyiidae|591|BOLD:ACS1334
Cecidomyiidae|[6388]|SSWLE7519-13|Cecidomyiidae|658|BOLD:ACJ5434
Cecidomyiidae|[6389]|SSPAB7203-13|Cecidomyiidae|658|BOLD:ACI4132
Cecidomyiidae|[6390]|SSJAF4737-13|Cecidomyiidae|658|BOLD:ACN9973
Cecidomyiidae|[6391]|PHAUG607-11|Cecidomyiidae|658|BOLD:AAY6411
Cecidomyiidae|[6392]|BBDCQ484-10|Cecidomyiidae|658|BOLD:AAV5625
Cecidomyiidae|[6393]|HEAUG245-12|Cecidomyiidae|670|BOLD:ABX7684
Cecidomyiidae|[6394]|CNBRF195-14|Cecidomyiidae|664|BOLD:ACL7971
Cecidomyiidae|[6395]|CNKOD571-14|Cecidomyiidae|658|BOLD:ACM2651
Cecidomyiidae|[6396]|CNROS628-13|Cecidomyiidae|661|BOLD:ACL5137
Cecidomyiidae|[6397]|CNROQ578-13|Cecidomyiidae|661|BOLD:ACL6963
Cecidomyiidae|[6398]|CNKJD475-14|Cecidomyiidae|658|BOLD:ACM2960
Cecidomyiidae|[6399]|HEAUG1378-12|Cecidomyiidae|664|BOLD:ACZ2646

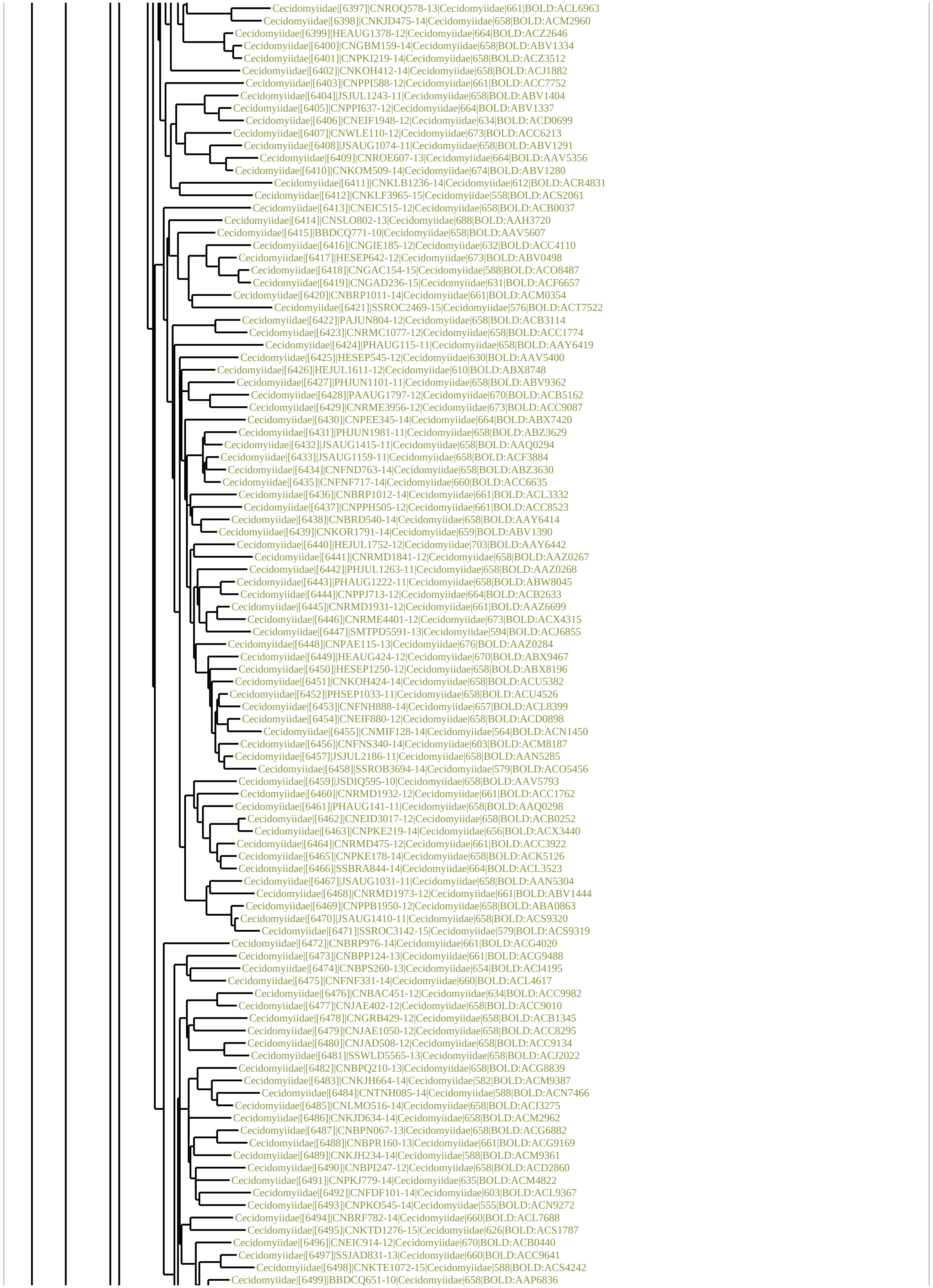
Cecidomyiidae|[6397]|CNROQ578-13|Cecidomyiidae|661|BOLD:ACL6963
Cecidomyiidae|[6398]|CNKJD475-14|Cecidomyiidae|658|BOLD:ACM2960
Cecidomyiidae|[6399]|HEAUG1378-12|Cecidomyiidae|664|BOLD:ACZ2646
Cecidomyiidae|[6400]|CNGBM159-14|Cecidomyiidae|658|BOLD:ABV1334
Cecidomyiidae|[6401]|CNPKI219-14|Cecidomyiidae|658|BOLD:ACZ3512
Cecidomyiidae|[6402]|CNKOH412-14|Cecidomyiidae|658|BOLD:ACJ1882
Cecidomyiidae|[6403]|CNPPI588-12|Cecidomyiidae|661|BOLD:ACC7752
Cecidomyiidae|[6404]|JSJUL1243-11|Cecidomyiidae|658|BOLD:ABV1404
Cecidomyiidae|[6405]|CNPPI637-12|Cecidomyiidae|664|BOLD:ABV1337
Cecidomyiidae|[6406]|CNEIF1948-12|Cecidomyiidae|634|BOLD:ACD0699
Cecidomyiidae|[6407]|CNWLE110-12|Cecidomyiidae|673|BOLD:ACC6213
Cecidomyiidae|[6408]|JSAUG1074-11|Cecidomyiidae|658|BOLD:ABV1291
Cecidomyiidae|[6409]|CNROE607-13|Cecidomyiidae|664|BOLD:AAV5356
Cecidomyiidae|[6410]|CNKOM509-14|Cecidomyiidae|674|BOLD:ABV1280
Cecidomyiidae|[6411]|CNKLB1236-14|Cecidomyiidae|612|BOLD:ACR4831
Cecidomyiidae|[6412]|CNKLF3965-15|Cecidomyiidae|558|BOLD:ACS2061
Cecidomyiidae|[6413]|CNEIC515-12|Cecidomyiidae|658|BOLD:ACB0037
Cecidomyiidae|[6414]|CNSLO802-13|Cecidomyiidae|688|BOLD:AAH3720
Cecidomyiidae|[6415]|BBDCQ771-10|Cecidomyiidae|658|BOLD:AAV5607
Cecidomyiidae|[6416]|CNGIE185-12|Cecidomyiidae|632|BOLD:ACC4110
Cecidomyiidae|[6417]|HESEP642-12|Cecidomyiidae|673|BOLD:ABV0498
Cecidomyiidae|[6418]|CNGAC154-15|Cecidomyiidae|588|BOLD:ACO8487
Cecidomyiidae|[6419]|CNGAD236-15|Cecidomyiidae|631|BOLD:ACF6657
Cecidomyiidae|[6420]|CNBRP1011-14|Cecidomyiidae|661|BOLD:ACM0354
Cecidomyiidae|[6421]|SSROC2469-15|Cecidomyiidae|576|BOLD:ACT7522
Cecidomyiidae|[6422]|PAJUN804-12|Cecidomyiidae|658|BOLD:ACB3114
Cecidomyiidae|[6423]|CNRMC1077-12|Cecidomyiidae|658|BOLD:ACC1774
Cecidomyiidae|[6424]|PHAUG115-11|Cecidomyiidae|658|BOLD:AAY6419
Cecidomyiidae|[6425]|HESEP545-12|Cecidomyiidae|630|BOLD:AAV5400
Cecidomyiidae|[6426]|HEJUL1611-12|Cecidomyiidae|610|BOLD:ABX8748
Cecidomyiidae|[6427]|PHJUN1101-11|Cecidomyiidae|658|BOLD:ABV9362
Cecidomyiidae|[6428]|PAAUG1797-12|Cecidomyiidae|670|BOLD:ACB5162
Cecidomyiidae|[6429]|CNRME3956-12|Cecidomyiidae|673|BOLD:ACC9087
Cecidomyiidae|[6430]|CNPEE345-14|Cecidomyiidae|664|BOLD:ABX7420
Cecidomyiidae|[6431]|PHJUN1981-11|Cecidomyiidae|658|BOLD:ABZ3629
Cecidomyiidae|[6432]|JSAUG1415-11|Cecidomyiidae|658|BOLD:AAQ0294
Cecidomyiidae|[6433]|JSAUG1159-11|Cecidomyiidae|658|BOLD:ACF3884
Cecidomyiidae|[6434]|CNFND763-14|Cecidomyiidae|658|BOLD:ABZ3630
Cecidomyiidae|[6435]|CNFNF717-14|Cecidomyiidae|660|BOLD:ACC6635
Cecidomyiidae|[6436]|CNBRP1012-14|Cecidomyiidae|661|BOLD:ACL3332
Cecidomyiidae|[6437]|CNPPH505-12|Cecidomyiidae|661|BOLD:ACC8523
Cecidomyiidae|[6438]|CNBRD540-14|Cecidomyiidae|658|BOLD:AAY6414
Cecidomyiidae|[6439]|CNKOR1791-14|Cecidomyiidae|659|BOLD:ABV1390
Cecidomyiidae|[6440]|HEJUL1752-12|Cecidomyiidae|703|BOLD:AAY6442
Cecidomyiidae|[6441]|CNRMD1841-12|Cecidomyiidae|658|BOLD:AAZ0267
Cecidomyiidae|[6442]|PHJUL1263-11|Cecidomyiidae|658|BOLD:AAZ0268
Cecidomyiidae|[6443]|PHAUG1222-11|Cecidomyiidae|658|BOLD:ABW8045
Cecidomyiidae|[6444]|CNPPJ713-12|Cecidomyiidae|664|BOLD:ACB2633
Cecidomyiidae|[6445]|CNRMD1931-12|Cecidomyiidae|661|BOLD:AAZ6699
Cecidomyiidae|[6446]|CNRME4401-12|Cecidomyiidae|673|BOLD:ACX4315
Cecidomyiidae|[6447]|SMTPD5591-13|Cecidomyiidae|594|BOLD:ACJ6855
Cecidomyiidae|[6448]|CNPAE115-13|Cecidomyiidae|676|BOLD:AAZ0284
Cecidomyiidae|[6449]|HEAUG424-12|Cecidomyiidae|670|BOLD:ABX9467
Cecidomyiidae|[6450]|HESEP1250-12|Cecidomyiidae|658|BOLD:ABX8196
Cecidomyiidae|[6451]|CNKOH424-14|Cecidomyiidae|658|BOLD:ACU5382
Cecidomyiidae|[6452]|PHSEP1033-11|Cecidomyiidae|658|BOLD:ACU4526
Cecidomyiidae|[6453]|CNFNH888-14|Cecidomyiidae|657|BOLD:ACL8399
Cecidomyiidae|[6454]|CNEIF880-12|Cecidomyiidae|658|BOLD:ACD0898
Cecidomyiidae|[6455]|CNMIF128-14|Cecidomyiidae|564|BOLD:ACN1450
Cecidomyiidae|[6456]|CNFNS340-14|Cecidomyiidae|603|BOLD:ACM8187
Cecidomyiidae|[6457]|JSJUL2186-11|Cecidomyiidae|658|BOLD:AAN5285
Cecidomyiidae|[6458]|SSROB3694-14|Cecidomyiidae|579|BOLD:ACO5456
Cecidomyiidae|[6459]|JSDIQ595-10|Cecidomyiidae|658|BOLD:AAV5793
Cecidomyiidae|[6460]|CNRMD1932-12|Cecidomyiidae|661|BOLD:ACC1762
Cecidomyiidae|[6461]|PHAUG141-11|Cecidomyiidae|658|BOLD:AAQ0298
Cecidomyiidae|[6462]|CNEID3017-12|Cecidomyiidae|658|BOLD:ACB0252
Cecidomyiidae|[6463]|CNPKE219-14|Cecidomyiidae|656|BOLD:ACX3440
Cecidomyiidae|[6464]|CNRMD475-12|Cecidomyiidae|661|BOLD:ACC3922
Cecidomyiidae|[6465]|CNPKE178-14|Cecidomyiidae|658|BOLD:ACK5126
Cecidomyiidae|[6466]|SSBRA844-14|Cecidomyiidae|664|BOLD:ACL3523
Cecidomyiidae|[6467]|JSAUG1031-11|Cecidomyiidae|658|BOLD:AAN5304
Cecidomyiidae|[6468]|CNRMD1973-12|Cecidomyiidae|661|BOLD:ABV1444
Cecidomyiidae|[6469]|CNPPB1950-12|Cecidomyiidae|658|BOLD:ABA0863
Cecidomyiidae|[6470]|JSAUG1410-11|Cecidomyiidae|658|BOLD:ACS9320
Cecidomyiidae|[6471]|SSROC3142-15|Cecidomyiidae|579|BOLD:ACS9319
Cecidomyiidae|[6472]|CNBRP976-14|Cecidomyiidae|661|BOLD:ACG4020
Cecidomyiidae|[6473]|CNBPP124-13|Cecidomyiidae|661|BOLD:ACG9488
Cecidomyiidae|[6474]|CNBPS260-13|Cecidomyiidae|654|BOLD:ACI4195
Cecidomyiidae|[6475]|CNFNF331-14|Cecidomyiidae|660|BOLD:ACL4617
Cecidomyiidae|[6476]|CNBAC451-12|Cecidomyiidae|634|BOLD:ACC9982
Cecidomyiidae|[6477]|CNJAE402-12|Cecidomyiidae|658|BOLD:ACC9010
Cecidomyiidae|[6478]|CNGRB429-12|Cecidomyiidae|658|BOLD:ACB1345
Cecidomyiidae|[6479]|CNJAE1050-12|Cecidomyiidae|658|BOLD:ACC8295
Cecidomyiidae|[6480]|CNJAD508-12|Cecidomyiidae|658|BOLD:ACC9134
Cecidomyiidae|[6481]|SSWLD5565-13|Cecidomyiidae|658|BOLD:ACJ2022
Cecidomyiidae|[6482]|CNBPQ210-13|Cecidomyiidae|658|BOLD:ACG8839
Cecidomyiidae|[6483]|CNKJH664-14|Cecidomyiidae|582|BOLD:ACM9387
Cecidomyiidae|[6484]|CNTNH085-14|Cecidomyiidae|588|BOLD:ACN7466
Cecidomyiidae|[6485]|CNLMO516-14|Cecidomyiidae|658|BOLD:ACI3275
Cecidomyiidae|[6486]|CNKJD634-14|Cecidomyiidae|658|BOLD:ACM2962
Cecidomyiidae|[6487]|CNBPN067-13|Cecidomyiidae|658|BOLD:ACG6882
Cecidomyiidae|[6488]|CNBPR160-13|Cecidomyiidae|661|BOLD:ACG9169
Cecidomyiidae|[6489]|CNKJH234-14|Cecidomyiidae|588|BOLD:ACM9361
Cecidomyiidae|[6490]|CNBPI247-12|Cecidomyiidae|658|BOLD:ACD2860
Cecidomyiidae|[6491]|CNPKJ779-14|Cecidomyiidae|635|BOLD:ACM4822
Cecidomyiidae|[6492]|CNFDF101-14|Cecidomyiidae|603|BOLD:ACL9367
Cecidomyiidae|[6493]|CNPKO545-14|Cecidomyiidae|555|BOLD:ACN9272
Cecidomyiidae|[6494]|CNBRF782-14|Cecidomyiidae|660|BOLD:ACL7688
Cecidomyiidae|[6495]|CNKTD1276-15|Cecidomyiidae|626|BOLD:ACS1787
Cecidomyiidae|[6496]|CNEIC914-12|Cecidomyiidae|670|BOLD:ACB0440
Cecidomyiidae|[6497]|SSJAD831-13|Cecidomyiidae|660|BOLD:ACC9641
Cecidomyiidae|[6498]|CNKTE1072-15|Cecidomyiidae|588|BOLD:ACS4242
Cecidomyiidae|[6499]|BBDCQ651-10|Cecidomyiidae|658|BOLD:AAP6836

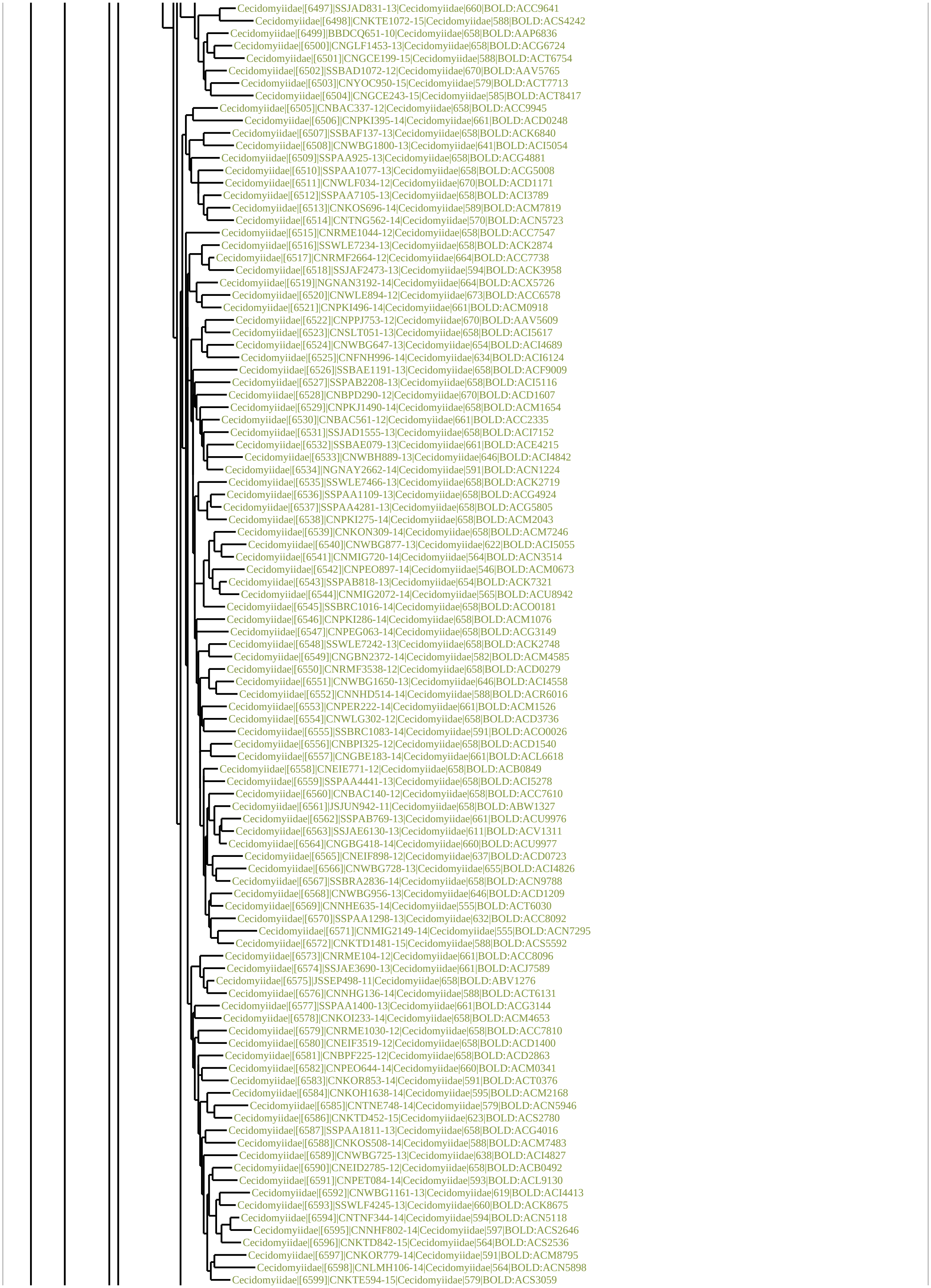
Cecidomyiidae|[6497]|SSJAD831-13|Cecidomyiidae|660|BOLD:ACC9641
Cecidomyiidae|[6498]|CNKTE1072-15|Cecidomyiidae|588|BOLD:ACS4242
Cecidomyiidae|[6499]|BBDCQ651-10|Cecidomyiidae|658|BOLD:AAP6836
Cecidomyiidae|[6500]|CNGLF1453-13|Cecidomyiidae|658|BOLD:ACG6724
Cecidomyiidae|[6501]|CNGCE199-15|Cecidomyiidae|588|BOLD:ACT6754
Cecidomyiidae|[6502]|SSBAD1072-12|Cecidomyiidae|670|BOLD:AAV5765
Cecidomyiidae|[6503]|CNYOC950-15|Cecidomyiidae|579|BOLD:ACT7713
Cecidomyiidae|[6504]|CNGCE243-15|Cecidomyiidae|585|BOLD:ACT8417
Cecidomyiidae|[6505]|CNBAC337-12|Cecidomyiidae|658|BOLD:ACC9945
Cecidomyiidae|[6506]|CNPKI395-14|Cecidomyiidae|661|BOLD:ACD0248
Cecidomyiidae|[6507]|SSBAF137-13|Cecidomyiidae|658|BOLD:ACK6840
Cecidomyiidae|[6508]|CNWBG1800-13|Cecidomyiidae|641|BOLD:ACI5054
Cecidomyiidae|[6509]|SSPAA925-13|Cecidomyiidae|658|BOLD:ACG4881
Cecidomyiidae|[6510]|SSPAA1077-13|Cecidomyiidae|658|BOLD:ACG5008
Cecidomyiidae|[6511]|CNWLF034-12|Cecidomyiidae|670|BOLD:ACD1171
Cecidomyiidae|[6512]|SSPAA7105-13|Cecidomyiidae|658|BOLD:ACI3789
Cecidomyiidae|[6513]|CNKOS696-14|Cecidomyiidae|589|BOLD:ACM7819
Cecidomyiidae|[6514]|CNTNG562-14|Cecidomyiidae|570|BOLD:ACN5723
Cecidomyiidae|[6515]|CNRME1044-12|Cecidomyiidae|658|BOLD:ACC7547
Cecidomyiidae|[6516]|SSWLE7234-13|Cecidomyiidae|658|BOLD:ACK2874
Cecidomyiidae|[6517]|CNRMF2664-12|Cecidomyiidae|664|BOLD:ACC7738
Cecidomyiidae|[6518]|SSJAF2473-13|Cecidomyiidae|594|BOLD:ACK3958
Cecidomyiidae|[6519]|NGNAN3192-14|Cecidomyiidae|664|BOLD:ACX5726
Cecidomyiidae|[6520]|CNWLE894-12|Cecidomyiidae|673|BOLD:ACC6578
Cecidomyiidae|[6521]|CNPKI496-14|Cecidomyiidae|661|BOLD:ACM0918
Cecidomyiidae|[6522]|CNPPJ753-12|Cecidomyiidae|670|BOLD:AAV5609
Cecidomyiidae|[6523]|CNSLT051-13|Cecidomyiidae|658|BOLD:ACI5617
Cecidomyiidae|[6524]|CNWBG647-13|Cecidomyiidae|654|BOLD:ACI4689
Cecidomyiidae|[6525]|CNFNH996-14|Cecidomyiidae|634|BOLD:ACI6124
Cecidomyiidae|[6526]|SSBAE1191-13|Cecidomyiidae|658|BOLD:ACF9009
Cecidomyiidae|[6527]|SSPAB2208-13|Cecidomyiidae|658|BOLD:ACI5116
Cecidomyiidae|[6528]|CNBPD290-12|Cecidomyiidae|670|BOLD:ACD1607
Cecidomyiidae|[6529]|CNPKJ1490-14|Cecidomyiidae|658|BOLD:ACM1654
Cecidomyiidae|[6530]|CNBAC561-12|Cecidomyiidae|661|BOLD:ACC2335
Cecidomyiidae|[6531]|SSJAD1555-13|Cecidomyiidae|658|BOLD:ACI7152
Cecidomyiidae|[6532]|SSBAE079-13|Cecidomyiidae|661|BOLD:ACE4215
Cecidomyiidae|[6533]|CNWBH889-13|Cecidomyiidae|646|BOLD:ACI4842
Cecidomyiidae|[6534]|NGNAY2662-14|Cecidomyiidae|591|BOLD:ACN1224
Cecidomyiidae|[6535]|SSWLE7466-13|Cecidomyiidae|658|BOLD:ACK2719
Cecidomyiidae|[6536]|SSPAA1109-13|Cecidomyiidae|658|BOLD:ACG4924
Cecidomyiidae|[6537]|SSPAA4281-13|Cecidomyiidae|658|BOLD:ACG5805
Cecidomyiidae|[6538]|CNPKI275-14|Cecidomyiidae|658|BOLD:ACM2043
Cecidomyiidae|[6539]|CNKON309-14|Cecidomyiidae|658|BOLD:ACM7246
Cecidomyiidae|[6540]|CNWBG877-13|Cecidomyiidae|622|BOLD:ACI5055
Cecidomyiidae|[6541]|CNMIG720-14|Cecidomyiidae|564|BOLD:ACN3514
Cecidomyiidae|[6542]|CNPEO897-14|Cecidomyiidae|546|BOLD:ACM0673
Cecidomyiidae|[6543]|SSPAB818-13|Cecidomyiidae|654|BOLD:ACK7321
Cecidomyiidae|[6544]|CNMIG2072-14|Cecidomyiidae|565|BOLD:ACU8942
Cecidomyiidae|[6545]|SSBRC1016-14|Cecidomyiidae|658|BOLD:ACO0181
Cecidomyiidae|[6546]|CNPKI286-14|Cecidomyiidae|658|BOLD:ACM1076
Cecidomyiidae|[6547]|CNPEG063-14|Cecidomyiidae|658|BOLD:ACG3149
Cecidomyiidae|[6548]|SSWLE7242-13|Cecidomyiidae|658|BOLD:ACK2748
Cecidomyiidae|[6549]|CNGBN2372-14|Cecidomyiidae|582|BOLD:ACM4585
Cecidomyiidae|[6550]|CNRMF3538-12|Cecidomyiidae|658|BOLD:ACD0279
Cecidomyiidae|[6551]|CNWBG1650-13|Cecidomyiidae|646|BOLD:ACI4558
Cecidomyiidae|[6552]|CNNHD514-14|Cecidomyiidae|588|BOLD:ACR6016
Cecidomyiidae|[6553]|CNPER222-14|Cecidomyiidae|661|BOLD:ACM1526
Cecidomyiidae|[6554]|CNWLG302-12|Cecidomyiidae|658|BOLD:ACD3736
Cecidomyiidae|[6555]|SSBRC1083-14|Cecidomyiidae|591|BOLD:ACO0026
Cecidomyiidae|[6556]|CNBPI325-12|Cecidomyiidae|658|BOLD:ACD1540
Cecidomyiidae|[6557]|CNGBE183-14|Cecidomyiidae|661|BOLD:ACL6618
Cecidomyiidae|[6558]|CNEIE771-12|Cecidomyiidae|658|BOLD:ACB0849
Cecidomyiidae|[6559]|SSPAA4441-13|Cecidomyiidae|658|BOLD:ACI5278
Cecidomyiidae|[6560]|CNBAC140-12|Cecidomyiidae|658|BOLD:ACC7610
Cecidomyiidae|[6561]|JSJUN942-11|Cecidomyiidae|658|BOLD:ABW1327
Cecidomyiidae|[6562]|SSPAB769-13|Cecidomyiidae|661|BOLD:ACU9976
Cecidomyiidae|[6563]|SSJAE6130-13|Cecidomyiidae|611|BOLD:ACV1311
Cecidomyiidae|[6564]|CNGBG418-14|Cecidomyiidae|660|BOLD:ACU9977
Cecidomyiidae|[6565]|CNEIF898-12|Cecidomyiidae|637|BOLD:ACD0723
Cecidomyiidae|[6566]|CNWBG728-13|Cecidomyiidae|655|BOLD:ACI4826
Cecidomyiidae|[6567]|SSBRA2836-14|Cecidomyiidae|658|BOLD:ACN9788
Cecidomyiidae|[6568]|CNWBG956-13|Cecidomyiidae|646|BOLD:ACD1209
Cecidomyiidae|[6569]|CNNHE635-14|Cecidomyiidae|555|BOLD:ACT6030
Cecidomyiidae|[6570]|SSPAA1298-13|Cecidomyiidae|632|BOLD:ACC8092
Cecidomyiidae|[6571]|CNMIG2149-14|Cecidomyiidae|555|BOLD:ACN7295
Cecidomyiidae|[6572]|CNKTD1481-15|Cecidomyiidae|588|BOLD:ACS5592
Cecidomyiidae|[6573]|CNRME104-12|Cecidomyiidae|661|BOLD:ACC8096
Cecidomyiidae|[6574]|SSJAE3690-13|Cecidomyiidae|661|BOLD:ACJ7589
Cecidomyiidae|[6575]|JSSEP498-11|Cecidomyiidae|658|BOLD:ABV1276
Cecidomyiidae|[6576]|CNNHG136-14|Cecidomyiidae|588|BOLD:ACT6131
Cecidomyiidae|[6577]|SSPAA1400-13|Cecidomyiidae|661|BOLD:ACG3144
Cecidomyiidae|[6578]|CNKOI233-14|Cecidomyiidae|658|BOLD:ACM4653
Cecidomyiidae|[6579]|CNRME1030-12|Cecidomyiidae|658|BOLD:ACC7810
Cecidomyiidae|[6580]|CNEIF3519-12|Cecidomyiidae|658|BOLD:ACD1400
Cecidomyiidae|[6581]|CNBPF225-12|Cecidomyiidae|658|BOLD:ACD2863
Cecidomyiidae|[6582]|CNPEO644-14|Cecidomyiidae|660|BOLD:ACM0341
Cecidomyiidae|[6583]|CNKOR853-14|Cecidomyiidae|591|BOLD:ACT0376
Cecidomyiidae|[6584]|CNKOH1638-14|Cecidomyiidae|595|BOLD:ACM2168
Cecidomyiidae|[6585]|CNTNE748-14|Cecidomyiidae|579|BOLD:ACN5946
Cecidomyiidae|[6586]|CNKTD452-15|Cecidomyiidae|623|BOLD:ACS2780
Cecidomyiidae|[6587]|SSPAA1811-13|Cecidomyiidae|658|BOLD:ACG4016
Cecidomyiidae|[6588]|CNKOS508-14|Cecidomyiidae|588|BOLD:ACM7483
Cecidomyiidae|[6589]|CNWBG725-13|Cecidomyiidae|638|BOLD:ACI4827
Cecidomyiidae|[6590]|CNEID2785-12|Cecidomyiidae|658|BOLD:ACB0492
Cecidomyiidae|[6591]|CNPET084-14|Cecidomyiidae|593|BOLD:ACL9130
Cecidomyiidae|[6592]|CNWBG1161-13|Cecidomyiidae|619|BOLD:ACI4413
Cecidomyiidae|[6593]|SSWLF4245-13|Cecidomyiidae|660|BOLD:ACK8675
Cecidomyiidae|[6594]|CNTNF344-14|Cecidomyiidae|594|BOLD:ACN5118
Cecidomyiidae|[6595]|CNNHF802-14|Cecidomyiidae|597|BOLD:ACS2646
Cecidomyiidae|[6596]|CNKTD842-15|Cecidomyiidae|564|BOLD:ACS2536
Cecidomyiidae|[6597]|CNKOR779-14|Cecidomyiidae|591|BOLD:ACM8795
Cecidomyiidae|[6598]|CNLMH106-14|Cecidomyiidae|564|BOLD:ACN5898
Cecidomyiidae|[6599]|CNKTE594-15|Cecidomyiidae|579|BOLD:ACS3059

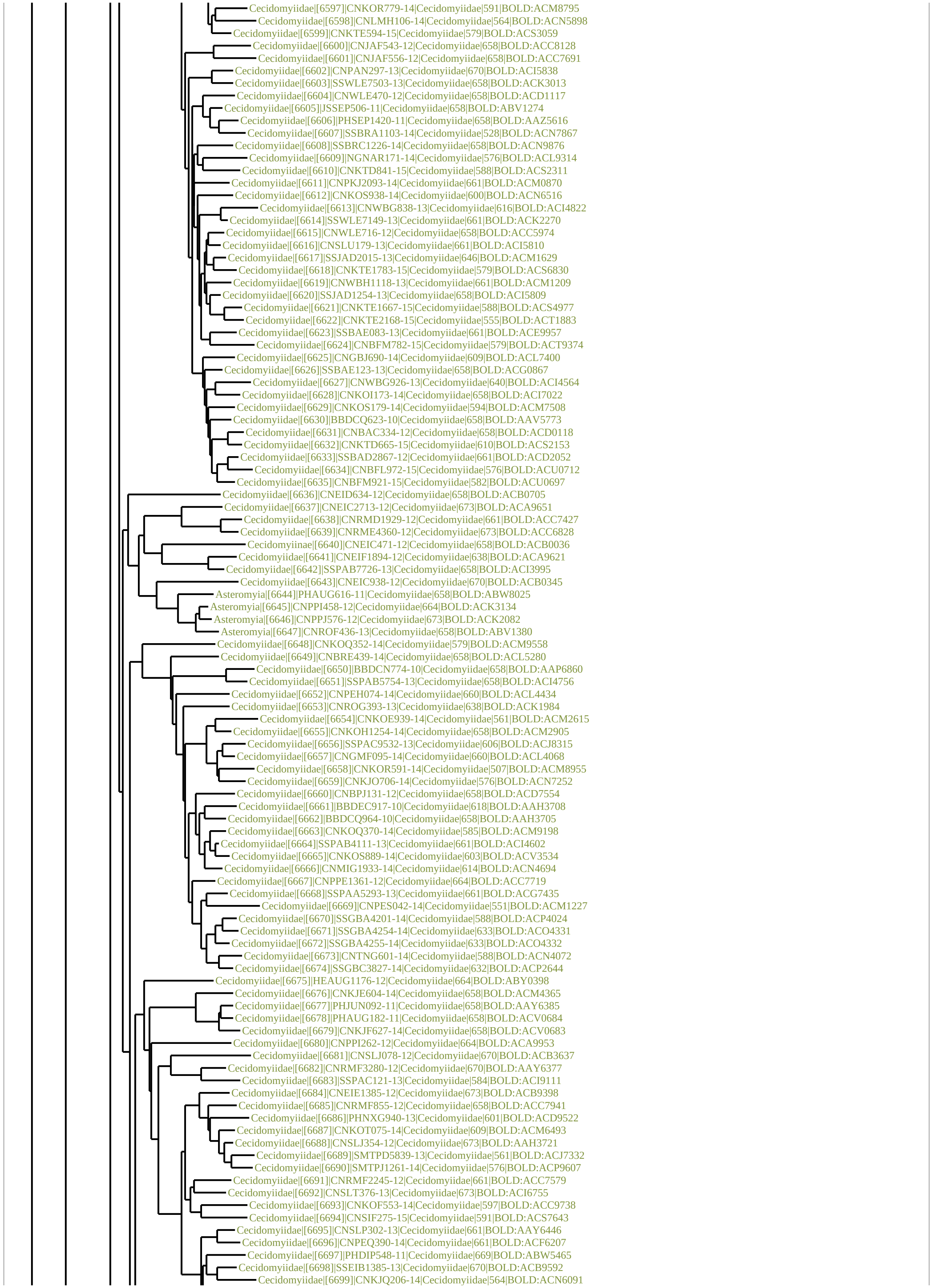

Cecidomyiidae|[6597]|CNKOR779-14|Cecidomyiidae|591|BOLD:ACM8795
Cecidomyiidae|[6598]|CNLMH106-14|Cecidomyiidae|564|BOLD:ACN5898
Cecidomyiidae|[6599]|CNKTE594-15|Cecidomyiidae|579|BOLD:ACS3059
Cecidomyiidae|[6600]|CNJAF543-12|Cecidomyiidae|658|BOLD:ACC8128
Cecidomyiidae|[6601]|CNJAF556-12|Cecidomyiidae|658|BOLD:ACC7691
Cecidomyiidae|[6602]|CNPAN297-13|Cecidomyiidae|670|BOLD:ACI5838
Cecidomyiidae|[6603]|SSWLE7503-13|Cecidomyiidae|658|BOLD:ACK3013
Cecidomyiidae|[6604]|CNWLE470-12|Cecidomyiidae|658|BOLD:ACD1117
Cecidomyiidae|[6605]|JSSEP506-11|Cecidomyiidae|658|BOLD:ABV1274
Cecidomyiidae|[6606]|PHSEP1420-11|Cecidomyiidae|658|BOLD:AAZ5616
Cecidomyiidae|[6607]|SSBRA1103-14|Cecidomyiidae|528|BOLD:ACN7867
Cecidomyiidae|[6608]|SSBRC1226-14|Cecidomyiidae|658|BOLD:ACN9876
Cecidomyiidae|[6609]|NGNAR171-14|Cecidomyiidae|576|BOLD:ACL9314
Cecidomyiidae|[6610]|CNKTD841-15|Cecidomyiidae|588|BOLD:ACS2311
Cecidomyiidae|[6611]|CNPKJ2093-14|Cecidomyiidae|661|BOLD:ACM0870
Cecidomyiidae|[6612]|CNKOS938-14|Cecidomyiidae|600|BOLD:ACN6516
Cecidomyiidae|[6613]|CNWBG838-13|Cecidomyiidae|616|BOLD:ACI4822
Cecidomyiidae|[6614]|SSWLE7149-13|Cecidomyiidae|661|BOLD:ACK2270
Cecidomyiidae|[6615]|CNWLE716-12|Cecidomyiidae|658|BOLD:ACC5974
Cecidomyiidae|[6616]|CNSLU179-13|Cecidomyiidae|661|BOLD:ACI5810
Cecidomyiidae|[6617]|SSJAD2015-13|Cecidomyiidae|646|BOLD:ACM1629
Cecidomyiidae|[6618]|CNKTE1783-15|Cecidomyiidae|579|BOLD:ACS6830
Cecidomyiidae|[6619]|CNWBH1118-13|Cecidomyiidae|661|BOLD:ACM1209
Cecidomyiidae|[6620]|SSJAD1254-13|Cecidomyiidae|658|BOLD:ACI5809
Cecidomyiidae|[6621]|CNKTE1667-15|Cecidomyiidae|588|BOLD:ACS4977
Cecidomyiidae|[6622]|CNKTE2168-15|Cecidomyiidae|555|BOLD:ACT1883
Cecidomyiidae|[6623]|SSBAE083-13|Cecidomyiidae|661|BOLD:ACE9957
Cecidomyiidae|[6624]|CNBFM782-15|Cecidomyiidae|579|BOLD:ACT9374
Cecidomyiidae|[6625]|CNGBJ690-14|Cecidomyiidae|609|BOLD:ACL7400
Cecidomyiidae|[6626]|SSBAE123-13|Cecidomyiidae|658|BOLD:ACG0867
Cecidomyiidae|[6627]|CNWBG926-13|Cecidomyiidae|640|BOLD:ACI4564
Cecidomyiidae|[6628]|CNKOI173-14|Cecidomyiidae|658|BOLD:ACI7022
Cecidomyiidae|[6629]|CNKOS179-14|Cecidomyiidae|594|BOLD:ACM7508
Cecidomyiidae|[6630]|BBDCQ623-10|Cecidomyiidae|658|BOLD:AAV5773
Cecidomyiidae|[6631]|CNBAC334-12|Cecidomyiidae|658|BOLD:ACD0118
Cecidomyiidae|[6632]|CNKTD665-15|Cecidomyiidae|610|BOLD:ACS2153
Cecidomyiidae|[6633]|SSBAD2867-12|Cecidomyiidae|661|BOLD:ACD2052
Cecidomyiidae|[6634]|CNBFL972-15|Cecidomyiidae|576|BOLD:ACU0712
Cecidomyiidae|[6635]|CNBFM921-15|Cecidomyiidae|582|BOLD:ACU0697
Cecidomyiidae|[6636]|CNEID634-12|Cecidomyiidae|658|BOLD:ACB0705
Cecidomyiidae|[6637]|CNEIC2713-12|Cecidomyiidae|673|BOLD:ACA9651
Cecidomyiidae|[6638]|CNRMD1929-12|Cecidomyiidae|661|BOLD:ACC7427
Cecidomyiidae|[6639]|CNRME4360-12|Cecidomyiidae|673|BOLD:ACC6828
Cecidomyiinae|[6640]|CNEIC471-12|Cecidomyiidae|658|BOLD:ACB0036
Cecidomyiidae|[6641]|CNEIF1894-12|Cecidomyiidae|638|BOLD:ACA9621
Cecidomyiidae|[6642]|SSPAB7726-13|Cecidomyiidae|658|BOLD:ACI3995
Cecidomyiidae|[6643]|CNEIC938-12|Cecidomyiidae|670|BOLD:ACB0345
Asteromyia|[6644]|PHAUG616-11|Cecidomyiidae|658|BOLD:ABW8025
Asteromyia|[6645]|CNPPI458-12|Cecidomyiidae|664|BOLD:ACK3134
Asteromyia|[6646]|CNPPJ576-12|Cecidomyiidae|673|BOLD:ACK2082
Asteromyia|[6647]|CNROF436-13|Cecidomyiidae|658|BOLD:ABV1380
Cecidomyiidae|[6648]|CNKOQ352-14|Cecidomyiidae|579|BOLD:ACM9558
Cecidomyiidae|[6649]|CNBRE439-14|Cecidomyiidae|658|BOLD:ACL5280
Cecidomyiidae|[6650]|BBDCN774-10|Cecidomyiidae|658|BOLD:AAP6860
Cecidomyiidae|[6651]|SSPAB5754-13|Cecidomyiidae|658|BOLD:ACI4756
Cecidomyiidae|[6652]|CNPEH074-14|Cecidomyiidae|660|BOLD:ACL4434
Cecidomyiidae|[6653]|CNROG393-13|Cecidomyiidae|638|BOLD:ACK1984
Cecidomyiidae|[6654]|CNKOE939-14|Cecidomyiidae|561|BOLD:ACM2615
Cecidomyiidae|[6655]|CNKOH1254-14|Cecidomyiidae|658|BOLD:ACM2905
Cecidomyiidae|[6656]|SSPAC9532-13|Cecidomyiidae|606|BOLD:ACJ8315
Cecidomyiidae|[6657]|CNGMF095-14|Cecidomyiidae|660|BOLD:ACL4068
Cecidomyiidae|[6658]|CNKOR591-14|Cecidomyiidae|507|BOLD:ACM8955
Cecidomyiidae|[6659]|CNKJO706-14|Cecidomyiidae|576|BOLD:ACN7252
Cecidomyiidae|[6660]|CNBPJ131-12|Cecidomyiidae|658|BOLD:ACD7554
Cecidomyiidae|[6661]|BBDEC917-10|Cecidomyiidae|618|BOLD:AAH3708
Cecidomyiidae|[6662]|BBDCQ964-10|Cecidomyiidae|658|BOLD:AAH3705
Cecidomyiidae|[6663]|CNKOQ370-14|Cecidomyiidae|585|BOLD:ACM9198
Cecidomyiidae|[6664]|SSPAB4111-13|Cecidomyiidae|661|BOLD:ACI4602
Cecidomyiidae|[6665]|CNKOS889-14|Cecidomyiidae|603|BOLD:ACV3534
Cecidomyiidae|[6666]|CNMIG1933-14|Cecidomyiidae|614|BOLD:ACN4694
Cecidomyiidae|[6667]|CNPPE1361-12|Cecidomyiidae|664|BOLD:ACC7719
Cecidomyiidae|[6668]|SSPAA5293-13|Cecidomyiidae|661|BOLD:ACG7435
Cecidomyiidae|[6669]|CNPES042-14|Cecidomyiidae|551|BOLD:ACM1227
Cecidomyiidae|[6670]|SSGBA4201-14|Cecidomyiidae|588|BOLD:ACP4024
Cecidomyiidae|[6671]|SSGBA4254-14|Cecidomyiidae|633|BOLD:ACO4331
Cecidomyiidae|[6672]|SSGBA4255-14|Cecidomyiidae|633|BOLD:ACO4332
Cecidomyiidae|[6673]|CNTNG601-14|Cecidomyiidae|588|BOLD:ACN4072
Cecidomyiidae|[6674]|SSGBC3827-14|Cecidomyiidae|632|BOLD:ACP2644
Cecidomyiidae|[6675]|HEAUG1176-12|Cecidomyiidae|664|BOLD:ABY0398
Cecidomyiidae|[6676]|CNKJE604-14|Cecidomyiidae|658|BOLD:ACM4365
Cecidomyiidae|[6677]|PHJUN092-11|Cecidomyiidae|658|BOLD:AAY6385
Cecidomyiidae|[6678]|PHAUG182-11|Cecidomyiidae|658|BOLD:ACV0684
Cecidomyiidae|[6679]|CNKJF627-14|Cecidomyiidae|658|BOLD:ACV0683
Cecidomyiidae|[6680]|CNPPI262-12|Cecidomyiidae|664|BOLD:ACA9953
Cecidomyiidae|[6681]|CNSLJ078-12|Cecidomyiidae|670|BOLD:ACB3637
Cecidomyiidae|[6682]|CNRMF3280-12|Cecidomyiidae|670|BOLD:AAY6377
Cecidomyiidae|[6683]|SSPAC121-13|Cecidomyiidae|584|BOLD:ACI9111
Cecidomyiidae|[6684]|CNEIE1385-12|Cecidomyiidae|673|BOLD:ACB9398
Cecidomyiidae|[6685]|CNRMF855-12|Cecidomyiidae|658|BOLD:ACC7941
Cecidomyiidae|[6686]|PHNXG940-13|Cecidomyiidae|601|BOLD:ACD9522
Cecidomyiidae|[6687]|CNKOT075-14|Cecidomyiidae|609|BOLD:ACM6493
Cecidomyiidae|[6688]|CNSLJ354-12|Cecidomyiidae|673|BOLD:AAH3721
Cecidomyiidae|[6689]|SMTPD5839-13|Cecidomyiidae|561|BOLD:ACJ7332
Cecidomyiidae|[6690]|SMTPJ1261-14|Cecidomyiidae|576|BOLD:ACP9607
Cecidomyiidae|[6691]|CNRMF2245-12|Cecidomyiidae|661|BOLD:ACC7579
Cecidomyiidae|[6692]|CNSLT376-13|Cecidomyiidae|673|BOLD:ACI6755
Cecidomyiidae|[6693]|CNKOF553-14|Cecidomyiidae|597|BOLD:ACC9738
Cecidomyiidae|[6694]|CNSIF275-15|Cecidomyiidae|591|BOLD:ACS7643
Cecidomyiidae|[6695]|CNSLP302-13|Cecidomyiidae|661|BOLD:AAY6446
Cecidomyiidae|[6696]|CNPEQ390-14|Cecidomyiidae|661|BOLD:ACF6207
Cecidomyiidae|[6697]|PHDIP548-11|Cecidomyiidae|669|BOLD:ABW5465
Cecidomyiidae|[6698]|SSEIB1385-13|Cecidomyiidae|670|BOLD:ACB9592
Cecidomyiidae|[6699]|CNKJQ206-14|Cecidomyiidae|564|BOLD:ACN6091

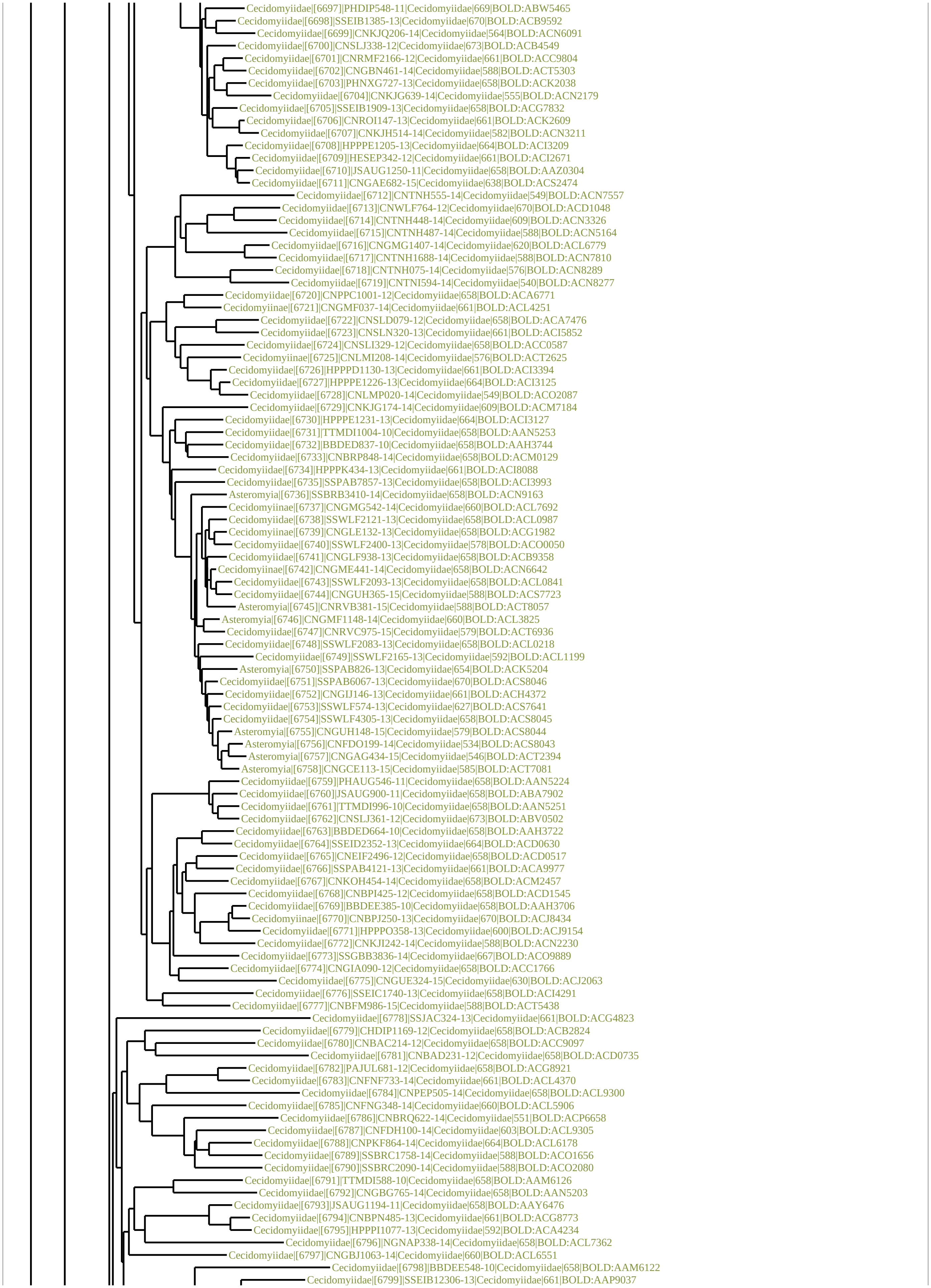
Cecidomyiidae|[6697]|PHDIP548-11|Cecidomyiidae|669|BOLD:ABW5465
Cecidomyiidae|[6698]|SSEIB1385-13|Cecidomyiidae|670|BOLD:ACB9592
Cecidomyiidae|[6699]|CNKJQ206-14|Cecidomyiidae|564|BOLD:ACN6091
Cecidomyiidae|[6700]|CNSLJ338-12|Cecidomyiidae|673|BOLD:ACB4549
Cecidomyiidae|[6701]|CNRMF2166-12|Cecidomyiidae|661|BOLD:ACC9804
Cecidomyiidae|[6702]|CNGBN461-14|Cecidomyiidae|588|BOLD:ACT5303
Cecidomyiidae|[6703]|PHNXG727-13|Cecidomyiidae|658|BOLD:ACK2038
Cecidomyiidae|[6704]|CNKJG639-14|Cecidomyiidae|555|BOLD:ACN2179
Cecidomyiidae|[6705]|SSEIB1909-13|Cecidomyiidae|658|BOLD:ACG7832
Cecidomyiidae|[6706]|CNROI147-13|Cecidomyiidae|661|BOLD:ACK2609
Cecidomyiidae|[6707]|CNKJH514-14|Cecidomyiidae|582|BOLD:ACN3211
Cecidomyiidae|[6708]|HPPPE1205-13|Cecidomyiidae|664|BOLD:ACI3209
Cecidomyiidae|[6709]|HESEP342-12|Cecidomyiidae|661|BOLD:ACI2671
Cecidomyiidae|[6710]|JSAUG1250-11|Cecidomyiidae|658|BOLD:AAZ0304
Cecidomyiidae|[6711]|CNGAE682-15|Cecidomyiidae|638|BOLD:ACS2474
Cecidomyiidae|[6712]|CNTNH555-14|Cecidomyiidae|549|BOLD:ACN7557
Cecidomyiidae|[6713]|CNWLF764-12|Cecidomyiidae|670|BOLD:ACD1048
Cecidomyiidae|[6714]|CNTNH448-14|Cecidomyiidae|609|BOLD:ACN3326
Cecidomyiidae|[6715]|CNTNH487-14|Cecidomyiidae|588|BOLD:ACN5164
Cecidomyiidae|[6716]|CNGMG1407-14|Cecidomyiidae|620|BOLD:ACL6779
Cecidomyiidae|[6717]|CNTNH1688-14|Cecidomyiidae|588|BOLD:ACN7810
Cecidomyiidae|[6718]|CNTNH075-14|Cecidomyiidae|576|BOLD:ACN8289
Cecidomyiidae|[6719]|CNTNI594-14|Cecidomyiidae|540|BOLD:ACN8277
Cecidomyiidae|[6720]|CNPPC1001-12|Cecidomyiidae|658|BOLD:ACA6771
Cecidomyiinae|[6721]|CNGMF037-14|Cecidomyiidae|661|BOLD:ACL4251
Cecidomyiidae|[6722]|CNSLD079-12|Cecidomyiidae|658|BOLD:ACA7476
Cecidomyiidae|[6723]|CNSLN320-13|Cecidomyiidae|661|BOLD:ACI5852
Cecidomyiidae|[6724]|CNSLI329-12|Cecidomyiidae|658|BOLD:ACC0587
Cecidomyiinae|[6725]|CNLMI208-14|Cecidomyiidae|576|BOLD:ACT2625
Cecidomyiidae|[6726]|HPPPD1130-13|Cecidomyiidae|661|BOLD:ACI3394
Cecidomyiidae|[6727]|HPPPE1226-13|Cecidomyiidae|664|BOLD:ACI3125
Cecidomyiidae|[6728]|CNLMP020-14|Cecidomyiidae|549|BOLD:ACO2087
Cecidomyiidae|[6729]|CNKJG174-14|Cecidomyiidae|609|BOLD:ACM7184
Cecidomyiidae|[6730]|HPPPE1231-13|Cecidomyiidae|664|BOLD:ACI3127
Cecidomyiidae|[6731]|TTMDI1004-10|Cecidomyiidae|658|BOLD:AAN5253
Cecidomyiidae|[6732]|BBDED837-10|Cecidomyiidae|658|BOLD:AAH3744
Cecidomyiidae|[6733]|CNBRP848-14|Cecidomyiidae|658|BOLD:ACM0129
Cecidomyiidae|[6734]|HPPPK434-13|Cecidomyiidae|661|BOLD:ACI8088
Cecidomyiidae|[6735]|SSPAB7857-13|Cecidomyiidae|658|BOLD:ACI3993
Asteromyia|[6736]|SSBRB3410-14|Cecidomyiidae|658|BOLD:ACN9163
Cecidomyiinae|[6737]|CNGMG542-14|Cecidomyiidae|660|BOLD:ACL7692
Cecidomyiidae|[6738]|SSWLF2121-13|Cecidomyiidae|658|BOLD:ACL0987
Cecidomyiinae|[6739]|CNGLE132-13|Cecidomyiidae|658|BOLD:ACG1982
Cecidomyiidae|[6740]|SSWLF2400-13|Cecidomyiidae|578|BOLD:ACO0050
Cecidomyiidae|[6741]|CNGLF938-13|Cecidomyiidae|658|BOLD:ACB9358
Cecidomyiinae|[6742]|CNGME441-14|Cecidomyiidae|658|BOLD:ACN6642
Cecidomyiidae|[6743]|SSWLF2093-13|Cecidomyiidae|658|BOLD:ACL0841
Cecidomyiidae|[6744]|CNGUH365-15|Cecidomyiidae|588|BOLD:ACS7723
Asteromyia|[6745]|CNRVB381-15|Cecidomyiidae|588|BOLD:ACT8057
Asteromyia|[6746]|CNGMF1148-14|Cecidomyiidae|660|BOLD:ACL3825
Cecidomyiidae|[6747]|CNRVC975-15|Cecidomyiidae|579|BOLD:ACT6936
Cecidomyiidae|[6748]|SSWLF2083-13|Cecidomyiidae|658|BOLD:ACL0218
Cecidomyiidae|[6749]|SSWLF2165-13|Cecidomyiidae|592|BOLD:ACL1199
Asteromyia|[6750]|SSPAB826-13|Cecidomyiidae|654|BOLD:ACK5204
Cecidomyiidae|[6751]|SSPAB6067-13|Cecidomyiidae|670|BOLD:ACS8046
Cecidomyiidae|[6752]|CNGIJ146-13|Cecidomyiidae|661|BOLD:ACH4372
Cecidomyiidae|[6753]|SSWLF574-13|Cecidomyiidae|627|BOLD:ACS7641
Cecidomyiidae|[6754]|SSWLF4305-13|Cecidomyiidae|658|BOLD:ACS8045
Asteromyia|[6755]|CNGUH148-15|Cecidomyiidae|579|BOLD:ACS8044
Asteromyia|[6756]|CNFDO199-14|Cecidomyiidae|534|BOLD:ACS8043
Asteromyia|[6757]|CNGAG434-15|Cecidomyiidae|546|BOLD:ACT2394
Asteromyia|[6758]|CNGCE113-15|Cecidomyiidae|585|BOLD:ACT7081
Cecidomyiidae|[6759]|PHAUG546-11|Cecidomyiidae|658|BOLD:AAN5224
Cecidomyiidae|[6760]|JSAUG900-11|Cecidomyiidae|658|BOLD:ABA7902
Cecidomyiidae|[6761]|TTMDI996-10|Cecidomyiidae|658|BOLD:AAN5251
Cecidomyiidae|[6762]|CNSLJ361-12|Cecidomyiidae|673|BOLD:ABV0502
Cecidomyiidae|[6763]|BBDED664-10|Cecidomyiidae|658|BOLD:AAH3722
Cecidomyiidae|[6764]|SSEID2352-13|Cecidomyiidae|664|BOLD:ACD0630
Cecidomyiidae|[6765]|CNEIF2496-12|Cecidomyiidae|658|BOLD:ACD0517
Cecidomyiidae|[6766]|SSPAB4121-13|Cecidomyiidae|661|BOLD:ACA9977
Cecidomyiidae|[6767]|CNKOH454-14|Cecidomyiidae|658|BOLD:ACM2457
Cecidomyiidae|[6768]|CNBPI425-12|Cecidomyiidae|658|BOLD:ACD1545
Cecidomyiidae|[6769]|BBDEE385-10|Cecidomyiidae|658|BOLD:AAH3706
Cecidomyiinae|[6770]|CNBPJ250-13|Cecidomyiidae|670|BOLD:ACJ8434
Cecidomyiidae|[6771]|HPPPO358-13|Cecidomyiidae|600|BOLD:ACJ9154
Cecidomyiidae|[6772]|CNKJI242-14|Cecidomyiidae|588|BOLD:ACN2230
Cecidomyiidae|[6773]|SSGBB3836-14|Cecidomyiidae|667|BOLD:ACO9889
Cecidomyiidae|[6774]|CNGIA090-12|Cecidomyiidae|658|BOLD:ACC1766
Cecidomyiidae|[6775]|CNGUE324-15|Cecidomyiidae|630|BOLD:ACJ2063
Cecidomyiidae|[6776]|SSEIC1740-13|Cecidomyiidae|658|BOLD:ACI4291
Cecidomyiidae|[6777]|CNBFM986-15|Cecidomyiidae|588|BOLD:ACT5438
Cecidomyiidae|[6778]|SSJAC324-13|Cecidomyiidae|661|BOLD:ACG4823
Cecidomyiidae|[6779]|CHDIP1169-12|Cecidomyiidae|658|BOLD:ACB2824
Cecidomyiidae|[6780]|CNBAC214-12|Cecidomyiidae|658|BOLD:ACC9097
Cecidomyiidae|[6781]|CNBAD231-12|Cecidomyiidae|658|BOLD:ACD0735
Cecidomyiidae|[6782]|PAJUL681-12|Cecidomyiidae|658|BOLD:ACG8921
Cecidomyiidae|[6783]|CNFNF733-14|Cecidomyiidae|661|BOLD:ACL4370
Cecidomyiidae|[6784]|CNPEP505-14|Cecidomyiidae|658|BOLD:ACL9300
Cecidomyiidae|[6785]|CNFNG348-14|Cecidomyiidae|660|BOLD:ACL5906
Cecidomyiidae|[6786]|CNBRQ622-14|Cecidomyiidae|551|BOLD:ACP6658
Cecidomyiidae|[6787]|CNFDH100-14|Cecidomyiidae|603|BOLD:ACL9305
Cecidomyiidae|[6788]|CNPKF864-14|Cecidomyiidae|664|BOLD:ACL6178
Cecidomyiidae|[6789]|SSBRC1758-14|Cecidomyiidae|588|BOLD:ACO1656
Cecidomyiidae|[6790]|SSBRC2090-14|Cecidomyiidae|588|BOLD:ACO2080
Cecidomyiidae|[6791]|TTMDI588-10|Cecidomyiidae|658|BOLD:AAM6126
Cecidomyiidae|[6792]|CNGBG765-14|Cecidomyiidae|658|BOLD:AAN5203
Cecidomyiidae|[6793]|JSAUG1194-11|Cecidomyiidae|658|BOLD:AAY6476
Cecidomyiidae|[6794]|CNBPN485-13|Cecidomyiidae|661|BOLD:ACG8773
Cecidomyiidae|[6795]|HPPPI1077-13|Cecidomyiidae|592|BOLD:ACA4234
Cecidomyiidae|[6796]|NGNAP338-14|Cecidomyiidae|658|BOLD:ACL7362
Cecidomyiidae|[6797]|CNGBJ1063-14|Cecidomyiidae|660|BOLD:ACL6551
Cecidomyiidae|[6798]|BBDEE548-10|Cecidomyiidae|658|BOLD:AAM6122
Cecidomyiidae|[6799]|SSEIB12306-13|Cecidomyiidae|661|BOLD:AAP9037

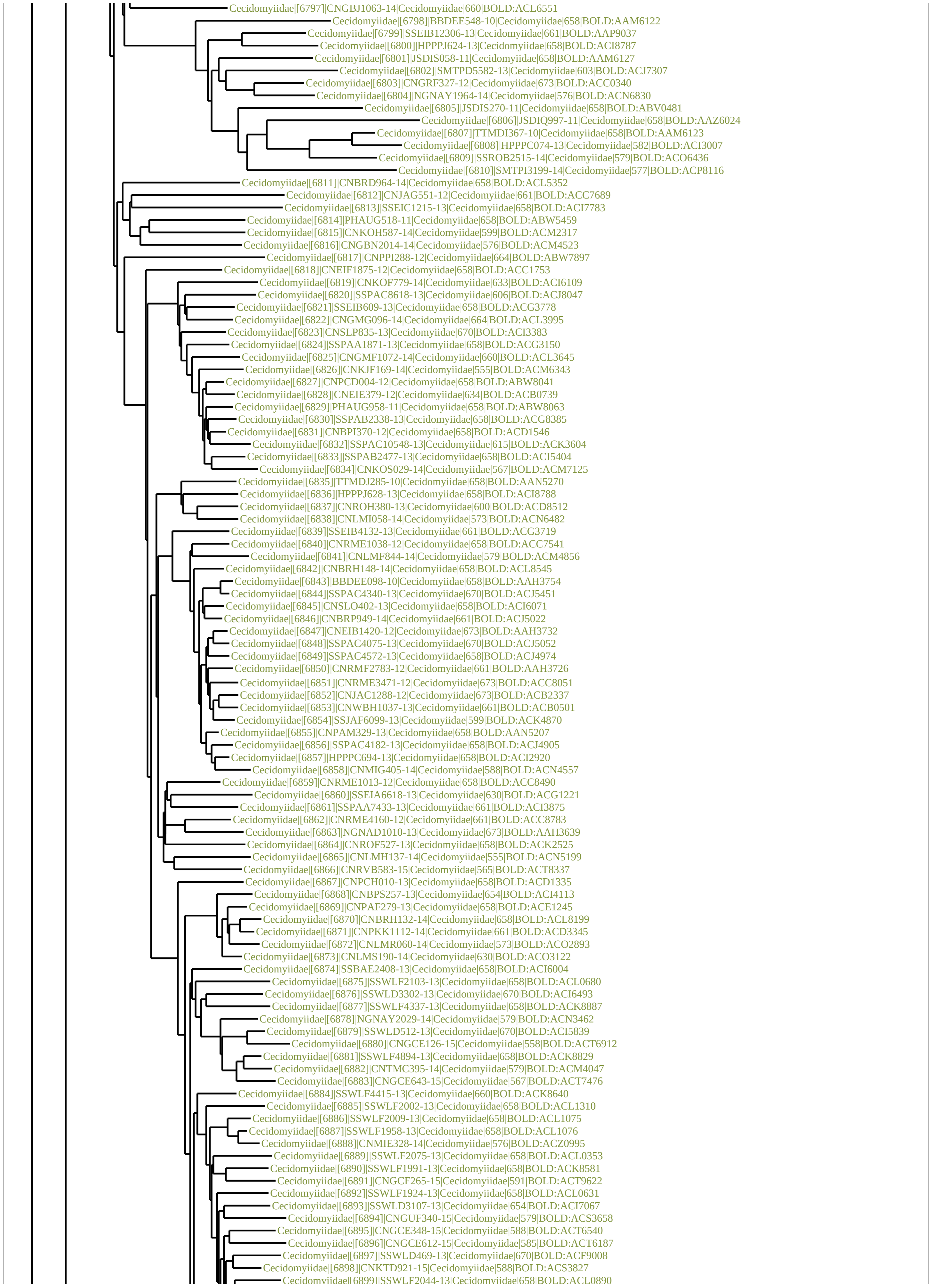

Cecidomyiidae|[6797]|CNGBJ1063-14|Cecidomyiidae|660|BOLD:ACL6551
Cecidomyiidae|[6798]|BBDEE548-10|Cecidomyiidae|658|BOLD:AAM6122
Cecidomyiidae|[6799]|SSEIB12306-13|Cecidomyiidae|661|BOLD:AAP9037
Cecidomyiidae|[6800]|HPPPJ624-13|Cecidomyiidae|658|BOLD:ACI8787
Cecidomyiidae|[6801]|JSDIS058-11|Cecidomyiidae|658|BOLD:AAM6127
Cecidomyiidae|[6802]|SMTPD5582-13|Cecidomyiidae|603|BOLD:ACJ7307
Cecidomyiidae|[6803]|CNGRF327-12|Cecidomyiidae|673|BOLD:ACC0340
Cecidomyiidae|[6804]|NGNAY1964-14|Cecidomyiidae|576|BOLD:ACN6830
Cecidomyiidae|[6805]|JSDIS270-11|Cecidomyiidae|658|BOLD:ABV0481
Cecidomyiidae|[6806]|JSDIQ997-11|Cecidomyiidae|658|BOLD:AAZ6024
Cecidomyiidae|[6807]|TTMDI367-10|Cecidomyiidae|658|BOLD:AAM6123
Cecidomyiidae|[6808]|HPPPC074-13|Cecidomyiidae|582|BOLD:ACI3007
Cecidomyiidae|[6809]|SSROB2515-14|Cecidomyiidae|579|BOLD:ACO6436
Cecidomyiidae|[6810]|SMTPI3199-14|Cecidomyiidae|577|BOLD:ACP8116
Cecidomyiidae|[6811]|CNBRD964-14|Cecidomyiidae|658|BOLD:ACL5352
Cecidomyiidae|[6812]|CNJAG551-12|Cecidomyiidae|661|BOLD:ACC7689
Cecidomyiidae|[6813]|SSEIC1215-13|Cecidomyiidae|658|BOLD:ACI7783
Cecidomyiidae|[6814]|PHAUG518-11|Cecidomyiidae|658|BOLD:ABW5459
Cecidomyiidae|[6815]|CNKOH587-14|Cecidomyiidae|599|BOLD:ACM2317
Cecidomyiidae|[6816]|CNGBN2014-14|Cecidomyiidae|576|BOLD:ACM4523
Cecidomyiidae|[6817]|CNPPI288-12|Cecidomyiidae|664|BOLD:ABW7897
Cecidomyiidae|[6818]|CNEIF1875-12|Cecidomyiidae|658|BOLD:ACC1753
Cecidomyiidae|[6819]|CNKOF779-14|Cecidomyiidae|633|BOLD:ACI6109
Cecidomyiidae|[6820]|SSPAC8618-13|Cecidomyiidae|606|BOLD:ACJ8047
Cecidomyiidae|[6821]|SSEIB609-13|Cecidomyiidae|658|BOLD:ACG3778
Cecidomyiidae|[6822]|CNGMG096-14|Cecidomyiidae|664|BOLD:ACL3995
Cecidomyiidae|[6823]|CNSLP835-13|Cecidomyiidae|670|BOLD:ACI3383
Cecidomyiidae|[6824]|SSPAA1871-13|Cecidomyiidae|658|BOLD:ACG3150
Cecidomyiidae|[6825]|CNGMF1072-14|Cecidomyiidae|660|BOLD:ACL3645
Cecidomyiidae|[6826]|CNKJF169-14|Cecidomyiidae|555|BOLD:ACM6343
Cecidomyiidae|[6827]|CNPCD004-12|Cecidomyiidae|658|BOLD:ABW8041
Cecidomyiidae|[6828]|CNEIE379-12|Cecidomyiidae|634|BOLD:ACB0739
Cecidomyiidae|[6829]|PHAUG958-11|Cecidomyiidae|658|BOLD:ABW8063
Cecidomyiidae|[6830]|SSPAB2338-13|Cecidomyiidae|658|BOLD:ACG8385
Cecidomyiidae|[6831]|CNBPI370-12|Cecidomyiidae|658|BOLD:ACD1546
Cecidomyiidae|[6832]|SSPAC10548-13|Cecidomyiidae|615|BOLD:ACK3604
Cecidomyiidae|[6833]|SSPAB2477-13|Cecidomyiidae|658|BOLD:ACI5404
Cecidomyiidae|[6834]|CNKOS029-14|Cecidomyiidae|567|BOLD:ACM7125
Cecidomyiidae|[6835]|TTMDJ285-10|Cecidomyiidae|658|BOLD:AAN5270
Cecidomyiidae|[6836]|HPPPJ628-13|Cecidomyiidae|658|BOLD:ACI8788
Cecidomyiidae|[6837]|CNROH380-13|Cecidomyiidae|600|BOLD:ACD8512
Cecidomyiidae|[6838]|CNLMI058-14|Cecidomyiidae|573|BOLD:ACN6482
Cecidomyiidae|[6839]|SSEIB4132-13|Cecidomyiidae|661|BOLD:ACG3719
Cecidomyiidae|[6840]|CNRME1038-12|Cecidomyiidae|658|BOLD:ACC7541
Cecidomyiidae|[6841]|CNLMF844-14|Cecidomyiidae|579|BOLD:ACM4856
Cecidomyiidae|[6842]|CNBRH148-14|Cecidomyiidae|658|BOLD:ACL8545
Cecidomyiidae|[6843]|BBDEE098-10|Cecidomyiidae|658|BOLD:AAH3754
Cecidomyiidae|[6844]|SSPAC4340-13|Cecidomyiidae|670|BOLD:ACJ5451
Cecidomyiidae|[6845]|CNSLO402-13|Cecidomyiidae|658|BOLD:ACI6071
Cecidomyiidae|[6846]|CNBRP949-14|Cecidomyiidae|661|BOLD:ACJ5022
Cecidomyiidae|[6847]|CNEIB1420-12|Cecidomyiidae|673|BOLD:AAH3732
Cecidomyiidae|[6848]|SSPAC4075-13|Cecidomyiidae|670|BOLD:ACJ5052
Cecidomyiidae|[6849]|SSPAC4572-13|Cecidomyiidae|658|BOLD:ACJ4974
Cecidomyiidae|[6850]|CNRMF2783-12|Cecidomyiidae|661|BOLD:AAH3726
Cecidomyiidae|[6851]|CNRME3471-12|Cecidomyiidae|673|BOLD:ACC8051
Cecidomyiidae|[6852]|CNJAC1288-12|Cecidomyiidae|673|BOLD:ACB2337
Cecidomyiidae|[6853]|CNWBH1037-13|Cecidomyiidae|661|BOLD:ACB0501
Cecidomyiidae|[6854]|SSJAF6099-13|Cecidomyiidae|599|BOLD:ACK4870
Cecidomyiidae|[6855]|CNPAM329-13|Cecidomyiidae|658|BOLD:AAN5207
Cecidomyiidae|[6856]|SSPAC4182-13|Cecidomyiidae|658|BOLD:ACJ4905
Cecidomyiidae|[6857]|HPPPC694-13|Cecidomyiidae|658|BOLD:ACI2920
Cecidomyiidae|[6858]|CNMIG405-14|Cecidomyiidae|588|BOLD:ACN4557
Cecidomyiidae|[6859]|CNRME1013-12|Cecidomyiidae|658|BOLD:ACC8490
Cecidomyiidae|[6860]|SSEIA6618-13|Cecidomyiidae|630|BOLD:ACG1221
Cecidomyiidae|[6861]|SSPAA7433-13|Cecidomyiidae|661|BOLD:ACI3875
Cecidomyiidae|[6862]|CNRME4160-12|Cecidomyiidae|661|BOLD:ACC8783
Cecidomyiidae|[6863]|NGNAD1010-13|Cecidomyiidae|673|BOLD:AAH3639
Cecidomyiidae|[6864]|CNROF527-13|Cecidomyiidae|658|BOLD:ACK2525
Cecidomyiidae|[6865]|CNLMH137-14|Cecidomyiidae|555|BOLD:ACN5199
Cecidomyiidae|[6866]|CNRVB583-15|Cecidomyiidae|565|BOLD:ACT8337
Cecidomyiidae|[6867]|CNPCH010-13|Cecidomyiidae|658|BOLD:ACD1335
Cecidomyiidae|[6868]|CNBPS257-13|Cecidomyiidae|654|BOLD:ACI4113
Cecidomyiidae|[6869]|CNPAF279-13|Cecidomyiidae|658|BOLD:ACE1245
Cecidomyiidae|[6870]|CNBRH132-14|Cecidomyiidae|658|BOLD:ACL8199
Cecidomyiidae|[6871]|CNPKK1112-14|Cecidomyiidae|661|BOLD:ACD3345
Cecidomyiidae|[6872]|CNLMR060-14|Cecidomyiidae|573|BOLD:ACO2893
Cecidomyiidae|[6873]|CNLMS190-14|Cecidomyiidae|630|BOLD:ACO3122
Cecidomyiidae|[6874]|SSBAE2408-13|Cecidomyiidae|658|BOLD:ACI6004
Cecidomyiidae|[6875]|SSWLF2103-13|Cecidomyiidae|658|BOLD:ACL0680
Cecidomyiidae|[6876]|SSWLD3302-13|Cecidomyiidae|670|BOLD:ACI6493
Cecidomyiidae|[6877]|SSWLF4337-13|Cecidomyiidae|658|BOLD:ACK8887
Cecidomyiidae|[6878]|NGNAY2029-14|Cecidomyiidae|579|BOLD:ACN3462
Cecidomyiidae|[6879]|SSWLD512-13|Cecidomyiidae|670|BOLD:ACI5839
Cecidomyiidae|[6880]|CNGCE126-15|Cecidomyiidae|558|BOLD:ACT6912
Cecidomyiidae|[6881]|SSWLF4894-13|Cecidomyiidae|658|BOLD:ACK8829
Cecidomyiidae|[6882]|CNTMC395-14|Cecidomyiidae|579|BOLD:ACM4047
Cecidomyiidae|[6883]|CNGCE643-15|Cecidomyiidae|567|BOLD:ACT7476
Cecidomyiidae|[6884]|SSWLF4415-13|Cecidomyiidae|660|BOLD:ACK8640
Cecidomyiidae|[6885]|SSWLF2002-13|Cecidomyiidae|658|BOLD:ACL1310
Cecidomyiidae|[6886]|SSWLF2009-13|Cecidomyiidae|658|BOLD:ACL1075
Cecidomyiidae|[6887]|SSWLF1958-13|Cecidomyiidae|658|BOLD:ACL1076
Cecidomyiidae|[6888]|CNMIE328-14|Cecidomyiidae|576|BOLD:ACZ0995
Cecidomyiidae|[6889]|SSWLF2075-13|Cecidomyiidae|658|BOLD:ACL0353
Cecidomyiidae|[6890]|SSWLF1991-13|Cecidomyiidae|658|BOLD:ACK8581
Cecidomyiidae|[6891]|CNGCF265-15|Cecidomyiidae|591|BOLD:ACT9622
Cecidomyiidae|[6892]|SSWLF1924-13|Cecidomyiidae|658|BOLD:ACL0631
Cecidomyiidae|[6893]|SSWLD3107-13|Cecidomyiidae|654|BOLD:ACI7067
Cecidomyiidae|[6894]|CNGUF340-15|Cecidomyiidae|579|BOLD:ACS3658
Cecidomyiidae|[6895]|CNGCE348-15|Cecidomyiidae|588|BOLD:ACT6540
Cecidomyiidae|[6896]|CNGCE612-15|Cecidomyiidae|585|BOLD:ACT6187
Cecidomyiidae|[6897]|SSWLD469-13|Cecidomyiidae|670|BOLD:ACF9008
Cecidomyiidae|[6898]|CNKTD921-15|Cecidomyiidae|588|BOLD:ACS3827
Cecidomyiidae|[6899]|SSWLF2044-13|Cecidomyiidae|658|BOLD:ACL0890

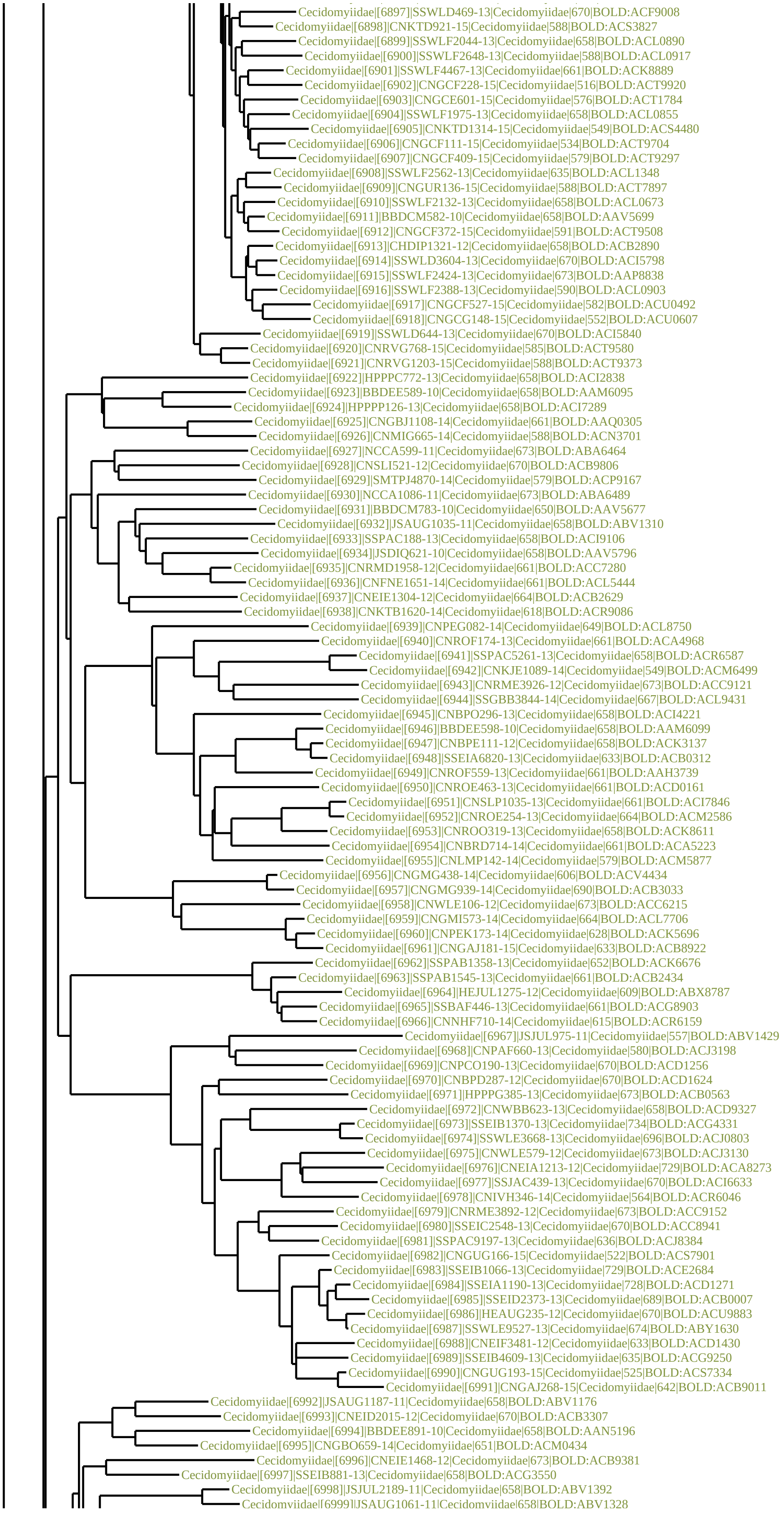

Cecidomyiidae|[6897]|SSWLD469-13|Cecidomyiidae|670|BOLD:ACF9008
Cecidomyiidae|[6898]|CNKTD921-15|Cecidomyiidae|588|BOLD:ACS3827
Cecidomyiidae|[6899]|SSWLF2044-13|Cecidomyiidae|658|BOLD:ACL0890
Cecidomyiidae|[6900]|SSWLF2648-13|Cecidomyiidae|588|BOLD:ACL0917
Cecidomyiidae|[6901]|SSWLF4467-13|Cecidomyiidae|661|BOLD:ACK8889
Cecidomyiidae|[6902]|CNGCF228-15|Cecidomyiidae|516|BOLD:ACT9920
Cecidomyiidae|[6903]|CNGCE601-15|Cecidomyiidae|576|BOLD:ACT1784
Cecidomyiidae|[6904]|SSWLF1975-13|Cecidomyiidae|658|BOLD:ACL0855
Cecidomyiidae|[6905]|CNKTD1314-15|Cecidomyiidae|549|BOLD:ACS4480
Cecidomyiidae|[6906]|CNGCF111-15|Cecidomyiidae|534|BOLD:ACT9704
Cecidomyiidae|[6907]|CNGCF409-15|Cecidomyiidae|579|BOLD:ACT9297
Cecidomyiidae|[6908]|SSWLF2562-13|Cecidomyiidae|635|BOLD:ACL1348
Cecidomyiidae|[6909]|CNGUR136-15|Cecidomyiidae|588|BOLD:ACT7897
Cecidomyiidae|[6910]|SSWLF2132-13|Cecidomyiidae|658|BOLD:ACL0673
Cecidomyiidae|[6911]|BBDCM582-10|Cecidomyiidae|658|BOLD:AAV5699
Cecidomyiidae|[6912]|CNGCF372-15|Cecidomyiidae|591|BOLD:ACT9508
Cecidomyiidae|[6913]|CHDIP1321-12|Cecidomyiidae|658|BOLD:ACB2890
Cecidomyiidae|[6914]|SSWLD3604-13|Cecidomyiidae|670|BOLD:ACI5798
Cecidomyiidae|[6915]|SSWLF2424-13|Cecidomyiidae|673|BOLD:AAP8838
Cecidomyiidae|[6916]|SSWLF2388-13|Cecidomyiidae|590|BOLD:ACL0903
Cecidomyiidae|[6917]|CNGCF527-15|Cecidomyiidae|582|BOLD:ACU0492
Cecidomyiidae|[6918]|CNGCG148-15|Cecidomyiidae|552|BOLD:ACU0607
Cecidomyiidae|[6919]|SSWLD644-13|Cecidomyiidae|670|BOLD:ACI5840
Cecidomyiidae|[6920]|CNRVG768-15|Cecidomyiidae|585|BOLD:ACT9580
Cecidomyiidae|[6921]|CNRVG1203-15|Cecidomyiidae|588|BOLD:ACT9373
Cecidomyiidae|[6922]|HPPPC772-13|Cecidomyiidae|658|BOLD:ACI2838
Cecidomyiidae|[6923]|BBDEE589-10|Cecidomyiidae|658|BOLD:AAM6095
Cecidomyiidae|[6924]|HPPPP126-13|Cecidomyiidae|658|BOLD:ACI7289
Cecidomyiidae|[6925]|CNGBJ1108-14|Cecidomyiidae|661|BOLD:AAQ0305
Cecidomyiidae|[6926]|CNMIG665-14|Cecidomyiidae|588|BOLD:ACN3701
Cecidomyiidae|[6927]|NCCA599-11|Cecidomyiidae|673|BOLD:ABA6464
Cecidomyiidae|[6928]|CNSLI521-12|Cecidomyiidae|670|BOLD:ACB9806
Cecidomyiidae|[6929]|SMTPJ4870-14|Cecidomyiidae|579|BOLD:ACP9167
Cecidomyiidae|[6930]|NCCA1086-11|Cecidomyiidae|673|BOLD:ABA6489
Cecidomyiidae|[6931]|BBDCM783-10|Cecidomyiidae|650|BOLD:AAV5677
Cecidomyiidae|[6932]|JSAUG1035-11|Cecidomyiidae|658|BOLD:ABV1310
Cecidomyiidae|[6933]|SSPAC188-13|Cecidomyiidae|658|BOLD:ACI9106
Cecidomyiidae|[6934]|JSDIQ621-10|Cecidomyiidae|658|BOLD:AAV5796
Cecidomyiidae|[6935]|CNRMD1958-12|Cecidomyiidae|661|BOLD:ACC7280
Cecidomyiidae|[6936]|CNFNE1651-14|Cecidomyiidae|661|BOLD:ACL5444
Cecidomyiidae|[6937]|CNEIE1304-12|Cecidomyiidae|664|BOLD:ACB2629
Cecidomyiidae|[6938]|CNKTB1620-14|Cecidomyiidae|618|BOLD:ACR9086
Cecidomyiidae|[6939]|CNPEG082-14|Cecidomyiidae|649|BOLD:ACL8750
Cecidomyiidae|[6940]|CNROF174-13|Cecidomyiidae|661|BOLD:ACA4968
Cecidomyiidae|[6941]|SSPAC5261-13|Cecidomyiidae|658|BOLD:ACR6587
Cecidomyiidae|[6942]|CNKJE1089-14|Cecidomyiidae|549|BOLD:ACM6499
Cecidomyiidae|[6943]|CNRME3926-12|Cecidomyiidae|673|BOLD:ACC9121
Cecidomyiidae|[6944]|SSGBB3844-14|Cecidomyiidae|667|BOLD:ACL9431
Cecidomyiidae|[6945]|CNBPO296-13|Cecidomyiidae|658|BOLD:ACI4221
Cecidomyiidae|[6946]|BBDEE598-10|Cecidomyiidae|658|BOLD:AAM6099
Cecidomyiidae|[6947]|CNBPE111-12|Cecidomyiidae|658|BOLD:ACK3137
Cecidomyiidae|[6948]|SSEIA6820-13|Cecidomyiidae|633|BOLD:ACB0312
Cecidomyiidae|[6949]|CNROF559-13|Cecidomyiidae|661|BOLD:AAH3739
Cecidomyiidae|[6950]|CNROE463-13|Cecidomyiidae|661|BOLD:ACD0161
Cecidomyiidae|[6951]|CNSLP1035-13|Cecidomyiidae|661|BOLD:ACI7846
Cecidomyiidae|[6952]|CNROE254-13|Cecidomyiidae|664|BOLD:ACM2586
Cecidomyiidae|[6953]|CNROO319-13|Cecidomyiidae|658|BOLD:ACK8611
Cecidomyiidae|[6954]|CNBRD714-14|Cecidomyiidae|661|BOLD:ACA5223
Cecidomyiidae|[6955]|CNLMP142-14|Cecidomyiidae|579|BOLD:ACM5877
Cecidomyiidae|[6956]|CNGMG438-14|Cecidomyiidae|606|BOLD:ACV4434
Cecidomyiidae|[6957]|CNGMG939-14|Cecidomyiidae|690|BOLD:ACB3033
Cecidomyiidae|[6958]|CNWLE106-12|Cecidomyiidae|673|BOLD:ACC6215
Cecidomyiidae|[6959]|CNGMI573-14|Cecidomyiidae|664|BOLD:ACL7706
Cecidomyiidae|[6960]|CNPEK173-14|Cecidomyiidae|628|BOLD:ACK5696
Cecidomyiidae|[6961]|CNGAJ181-15|Cecidomyiidae|633|BOLD:ACB8922
Cecidomyiidae|[6962]|SSPAB1358-13|Cecidomyiidae|652|BOLD:ACK6676
Cecidomyiidae|[6963]|SSPAB1545-13|Cecidomyiidae|661|BOLD:ACB2434
Cecidomyiidae|[6964]|HEJUL1275-12|Cecidomyiidae|609|BOLD:ABX8787
Cecidomyiidae|[6965]|SSBAF446-13|Cecidomyiidae|661|BOLD:ACG8903
Cecidomyiidae|[6966]|CNNHF710-14|Cecidomyiidae|615|BOLD:ACR6159
Cecidomyiidae|[6967]|JSJUL975-11|Cecidomyiidae|557|BOLD:ABV1429
Cecidomyiidae|[6968]|CNPAF660-13|Cecidomyiidae|580|BOLD:ACJ3198
Cecidomyiidae|[6969]|CNPCO190-13|Cecidomyiidae|670|BOLD:ACD1256
Cecidomyiidae|[6970]|CNBPD287-12|Cecidomyiidae|670|BOLD:ACD1624
Cecidomyiidae|[6971]|HPPPG385-13|Cecidomyiidae|673|BOLD:ACB0563
Cecidomyiidae|[6972]|CNWBB623-13|Cecidomyiidae|658|BOLD:ACD9327
Cecidomyiidae|[6973]|SSEIB1370-13|Cecidomyiidae|734|BOLD:ACG4331
Cecidomyiidae|[6974]|SSWLE3668-13|Cecidomyiidae|696|BOLD:ACJ0803
Cecidomyiidae|[6975]|CNWLE579-12|Cecidomyiidae|673|BOLD:ACJ3130
Cecidomyiidae|[6976]|CNEIA1213-12|Cecidomyiidae|729|BOLD:ACA8273
Cecidomyiidae|[6977]|SSJAC439-13|Cecidomyiidae|670|BOLD:ACI6633
Cecidomyiidae|[6978]|CNIVH346-14|Cecidomyiidae|564|BOLD:ACR6046
Cecidomyiidae|[6979]|CNRME3892-12|Cecidomyiidae|673|BOLD:ACC9152
Cecidomyiidae|[6980]|SSEIC2548-13|Cecidomyiidae|670|BOLD:ACC8941
Cecidomyiidae|[6981]|SSPAC9197-13|Cecidomyiidae|636|BOLD:ACJ8384
Cecidomyiidae|[6982]|CNGUG166-15|Cecidomyiidae|522|BOLD:ACS7901
Cecidomyiidae|[6983]|SSEIB1066-13|Cecidomyiidae|729|BOLD:ACE2684
Cecidomyiidae|[6984]|SSEIA1190-13|Cecidomyiidae|728|BOLD:ACD1271
Cecidomyiidae|[6985]|SSEID2373-13|Cecidomyiidae|689|BOLD:ACB0007
Cecidomyiidae|[6986]|HEAUG235-12|Cecidomyiidae|670|BOLD:ACU9883
Cecidomyiidae|[6987]|SSWLE9527-13|Cecidomyiidae|674|BOLD:ABY1630
Cecidomyiidae|[6988]|CNEIF3481-12|Cecidomyiidae|633|BOLD:ACD1430
Cecidomyiidae|[6989]|SSEIB4609-13|Cecidomyiidae|635|BOLD:ACG9250
Cecidomyiidae|[6990]|CNGUG193-15|Cecidomyiidae|525|BOLD:ACS7334
Cecidomyiidae|[6991]|CNGAJ268-15|Cecidomyiidae|642|BOLD:ACB9011
Cecidomyiidae|[6992]|JSAUG1187-11|Cecidomyiidae|658|BOLD:ABV1176
Cecidomyiidae|[6993]|CNEID2015-12|Cecidomyiidae|670|BOLD:ACB3307
Cecidomyiidae|[6994]|BBDEE891-10|Cecidomyiidae|658|BOLD:AAN5196
Cecidomyiidae|[6995]|CNGBO659-14|Cecidomyiidae|651|BOLD:ACM0434
Cecidomyiidae|[6996]|CNEIE1468-12|Cecidomyiidae|673|BOLD:ACB9381
Cecidomyiidae|[6997]|SSEIB881-13|Cecidomyiidae|658|BOLD:ACG3550
Cecidomyiidae|[6998]|JSJUL2189-11|Cecidomyiidae|658|BOLD:ABV1392
Cecidomyiidae|[6999]|JSAUG1061-11|Cecidomyiidae|658|BOLD:ABV1328

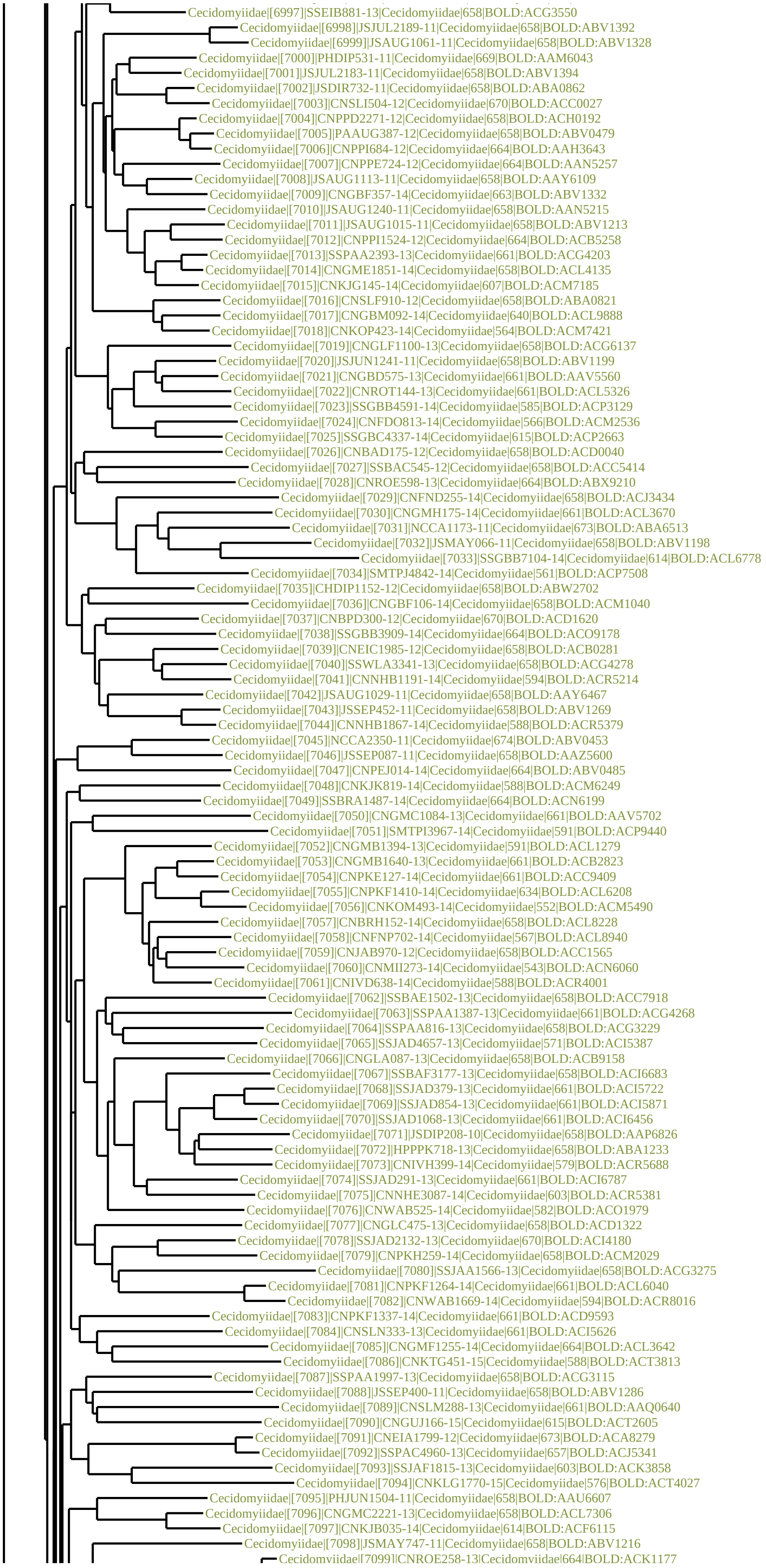
Cecidomyiidae|[6997]|SSEIB881-13|Cecidomyiidae|658|BOLD:ACG3550
Cecidomyiidae|[6998]|JSJUL2189-11|Cecidomyiidae|658|BOLD:ABV1392
Cecidomyiidae|[6999]|JSAUG1061-11|Cecidomyiidae|658|BOLD:ABV1328
Cecidomyiidae|[7000]|PHDIP531-11|Cecidomyiidae|669|BOLD:AAM6043
Cecidomyiidae|[7001]|JSJUL2183-11|Cecidomyiidae|658|BOLD:ABV1394
Cecidomyiidae|[7002]|JSDIR732-11|Cecidomyiidae|658|BOLD:ABA0862
Cecidomyiidae|[7003]|CNSLI504-12|Cecidomyiidae|670|BOLD:ACC0027
Cecidomyiidae|[7004]|CNPPD2271-12|Cecidomyiidae|658|BOLD:ACH0192
Cecidomyiidae|[7005]|PAAUG387-12|Cecidomyiidae|658|BOLD:ABV0479
Cecidomyiidae|[7006]|CNPPI684-12|Cecidomyiidae|664|BOLD:AAH3643
Cecidomyiidae|[7007]|CNPPE724-12|Cecidomyiidae|664|BOLD:AAN5257
Cecidomyiidae|[7008]|JSAUG1113-11|Cecidomyiidae|658|BOLD:AAY6109
Cecidomyiidae|[7009]|CNGBF357-14|Cecidomyiidae|663|BOLD:ABV1332
Cecidomyiidae|[7010]|JSAUG1240-11|Cecidomyiidae|658|BOLD:AAN5215
Cecidomyiidae|[7011]|JSAUG1015-11|Cecidomyiidae|658|BOLD:ABV1213
Cecidomyiidae|[7012]|CNPPI1524-12|Cecidomyiidae|664|BOLD:ACB5258
Cecidomyiidae|[7013]|SSPAA2393-13|Cecidomyiidae|661|BOLD:ACG4203
Cecidomyiidae|[7014]|CNGME1851-14|Cecidomyiidae|658|BOLD:ACL4135
Cecidomyiidae|[7015]|CNKJG145-14|Cecidomyiidae|607|BOLD:ACM7185
Cecidomyiidae|[7016]|CNSLF910-12|Cecidomyiidae|658|BOLD:ABA0821
Cecidomyiidae|[7017]|CNGBM092-14|Cecidomyiidae|640|BOLD:ACL9888
Cecidomyiidae|[7018]|CNKOP423-14|Cecidomyiidae|564|BOLD:ACM7421
Cecidomyiidae|[7019]|CNGLF1100-13|Cecidomyiidae|658|BOLD:ACG6137
Cecidomyiidae|[7020]|JSJUN1241-11|Cecidomyiidae|658|BOLD:ABV1199
Cecidomyiidae|[7021]|CNGBD575-13|Cecidomyiidae|661|BOLD:AAV5560
Cecidomyiidae|[7022]|CNROT144-13|Cecidomyiidae|661|BOLD:ACL5326
Cecidomyiidae|[7023]|SSGBB4591-14|Cecidomyiidae|585|BOLD:ACP3129
Cecidomyiidae|[7024]|CNFDO813-14|Cecidomyiidae|566|BOLD:ACM2536
Cecidomyiidae|[7025]|SSGBC4337-14|Cecidomyiidae|615|BOLD:ACP2663
Cecidomyiidae|[7026]|CNBAD175-12|Cecidomyiidae|658|BOLD:ACD0040
Cecidomyiidae|[7027]|SSBAC545-12|Cecidomyiidae|658|BOLD:ACC5414
Cecidomyiidae|[7028]|CNROE598-13|Cecidomyiidae|664|BOLD:ABX9210
Cecidomyiidae|[7029]|CNFND255-14|Cecidomyiidae|658|BOLD:ACJ3434
Cecidomyiidae|[7030]|CNGMH175-14|Cecidomyiidae|661|BOLD:ACL3670
Cecidomyiidae|[7031]|NCCA1173-11|Cecidomyiidae|673|BOLD:ABA6513
Cecidomyiidae|[7032]|JSMAY066-11|Cecidomyiidae|658|BOLD:ABV1198
Cecidomyiidae|[7033]|SSGBB7104-14|Cecidomyiidae|614|BOLD:ACL6778
Cecidomyiidae|[7034]|SMTPJ4842-14|Cecidomyiidae|561|BOLD:ACP7508
Cecidomyiidae|[7035]|CHDIP1152-12|Cecidomyiidae|658|BOLD:ABW2702
Cecidomyiidae|[7036]|CNGBF106-14|Cecidomyiidae|658|BOLD:ACM1040
Cecidomyiidae|[7037]|CNBPD300-12|Cecidomyiidae|670|BOLD:ACD1620
Cecidomyiidae|[7038]|SSGBB3909-14|Cecidomyiidae|664|BOLD:ACO9178
Cecidomyiidae|[7039]|CNEIC1985-12|Cecidomyiidae|658|BOLD:ACB0281
Cecidomyiidae|[7040]|SSWLA3341-13|Cecidomyiidae|658|BOLD:ACG4278
Cecidomyiidae|[7041]|CNNHB1191-14|Cecidomyiidae|594|BOLD:ACR5214
Cecidomyiidae|[7042]|JSAUG1029-11|Cecidomyiidae|658|BOLD:AAY6467
Cecidomyiidae|[7043]|JSSEP452-11|Cecidomyiidae|658|BOLD:ABV1269
Cecidomyiidae|[7044]|CNNHB1867-14|Cecidomyiidae|588|BOLD:ACR5379
Cecidomyiidae|[7045]|NCCA2350-11|Cecidomyiidae|674|BOLD:ABV0453
Cecidomyiidae|[7046]|JSSEP087-11|Cecidomyiidae|658|BOLD:AAZ5600
Cecidomyiidae|[7047]|CNPEJ014-14|Cecidomyiidae|664|BOLD:ABV0485
Cecidomyiidae|[7048]|CNKJK819-14|Cecidomyiidae|588|BOLD:ACM6249
Cecidomyiidae|[7049]|SSBRA1487-14|Cecidomyiidae|664|BOLD:ACN6199
Cecidomyiidae|[7050]|CNGMC1084-13|Cecidomyiidae|661|BOLD:AAV5702
Cecidomyiidae|[7051]|SMTPI3967-14|Cecidomyiidae|591|BOLD:ACP9440
Cecidomyiidae|[7052]|CNGMB1394-13|Cecidomyiidae|591|BOLD:ACL1279
Cecidomyiidae|[7053]|CNGMB1640-13|Cecidomyiidae|661|BOLD:ACB2823
Cecidomyiidae|[7054]|CNPKE127-14|Cecidomyiidae|661|BOLD:ACC9409
Cecidomyiidae|[7055]|CNPKF1410-14|Cecidomyiidae|634|BOLD:ACL6208
Cecidomyiidae|[7056]|CNKOM493-14|Cecidomyiidae|552|BOLD:ACM5490
Cecidomyiidae|[7057]|CNBRH152-14|Cecidomyiidae|658|BOLD:ACL8228
Cecidomyiidae|[7058]|CNFNP702-14|Cecidomyiidae|567|BOLD:ACL8940
Cecidomyiidae|[7059]|CNJAB970-12|Cecidomyiidae|658|BOLD:ACC1565
Cecidomyiidae|[7060]|CNMII273-14|Cecidomyiidae|543|BOLD:ACN6060
Cecidomyiidae|[7061]|CNIVD638-14|Cecidomyiidae|588|BOLD:ACR4001
Cecidomyiidae|[7062]|SSBAE1502-13|Cecidomyiidae|658|BOLD:ACC7918
Cecidomyiidae|[7063]|SSPAA1387-13|Cecidomyiidae|661|BOLD:ACG4268
Cecidomyiidae|[7064]|SSPAA816-13|Cecidomyiidae|658|BOLD:ACG3229
Cecidomyiidae|[7065]|SSJAD4657-13|Cecidomyiidae|571|BOLD:ACI5387
Cecidomyiidae|[7066]|CNGLA087-13|Cecidomyiidae|658|BOLD:ACB9158
Cecidomyiidae|[7067]|SSBAF3177-13|Cecidomyiidae|658|BOLD:ACI6683
Cecidomyiidae|[7068]|SSJAD379-13|Cecidomyiidae|661|BOLD:ACI5722
Cecidomyiidae|[7069]|SSJAD854-13|Cecidomyiidae|661|BOLD:ACI5871
Cecidomyiidae|[7070]|SSJAD1068-13|Cecidomyiidae|661|BOLD:ACI6456
Cecidomyiidae|[7071]|JSDIP208-10|Cecidomyiidae|658|BOLD:AAP6826
Cecidomyiidae|[7072]|HPPPK718-13|Cecidomyiidae|658|BOLD:ABA1233
Cecidomyiidae|[7073]|CNIVH399-14|Cecidomyiidae|579|BOLD:ACR5688
Cecidomyiidae|[7074]|SSJAD291-13|Cecidomyiidae|661|BOLD:ACI6787
Cecidomyiidae|[7075]|CNNHE3087-14|Cecidomyiidae|603|BOLD:ACR5381
Cecidomyiidae|[7076]|CNWAB525-14|Cecidomyiidae|582|BOLD:ACO1979
Cecidomyiidae|[7077]|CNGLC475-13|Cecidomyiidae|658|BOLD:ACD1322
Cecidomyiidae|[7078]|SSJAD2132-13|Cecidomyiidae|670|BOLD:ACI4180
Cecidomyiidae|[7079]|CNPKH259-14|Cecidomyiidae|658|BOLD:ACM2029
Cecidomyiidae|[7080]|SSJAA1566-13|Cecidomyiidae|658|BOLD:ACG3275
Cecidomyiidae|[7081]|CNPKF1264-14|Cecidomyiidae|661|BOLD:ACL6040
Cecidomyiidae|[7082]|CNWAB1669-14|Cecidomyiidae|594|BOLD:ACR8016
Cecidomyiidae|[7083]|CNPKF1337-14|Cecidomyiidae|661|BOLD:ACD9593
Cecidomyiidae|[7084]|CNSLN333-13|Cecidomyiidae|661|BOLD:ACI5626
Cecidomyiidae|[7085]|CNGMF1255-14|Cecidomyiidae|664|BOLD:ACL3642
Cecidomyiidae|[7086]|CNKTG451-15|Cecidomyiidae|588|BOLD:ACT3813
Cecidomyiidae|[7087]|SSPAA1997-13|Cecidomyiidae|658|BOLD:ACG3115
Cecidomyiidae|[7088]|JSSEP400-11|Cecidomyiidae|658|BOLD:ABV1286
Cecidomyiidae|[7089]|CNSLM288-13|Cecidomyiidae|661|BOLD:AAQ0640
Cecidomyiidae|[7090]|CNGUJ166-15|Cecidomyiidae|615|BOLD:ACT2605
Cecidomyiidae|[7091]|CNEIA1799-12|Cecidomyiidae|673|BOLD:ACA8279
Cecidomyiidae|[7092]|SSPAC4960-13|Cecidomyiidae|657|BOLD:ACJ5341
Cecidomyiidae|[7093]|SSJAF1815-13|Cecidomyiidae|603|BOLD:ACK3858
Cecidomyiidae|[7094]|CNKLG1770-15|Cecidomyiidae|576|BOLD:ACT4027
Cecidomyiidae|[7095]|PHJUN1504-11|Cecidomyiidae|658|BOLD:AAU6607
Cecidomyiidae|[7096]|CNGMC2221-13|Cecidomyiidae|658|BOLD:ACL7306
Cecidomyiidae|[7097]|CNKJB035-14|Cecidomyiidae|614|BOLD:ACF6115
Cecidomyiidae|[7098]|JSMAY747-11|Cecidomyiidae|658|BOLD:ABV1216
Cecidomyiidae|[7099]|CNROE258-13|Cecidomyiidae|664|BOLD:ACK1177

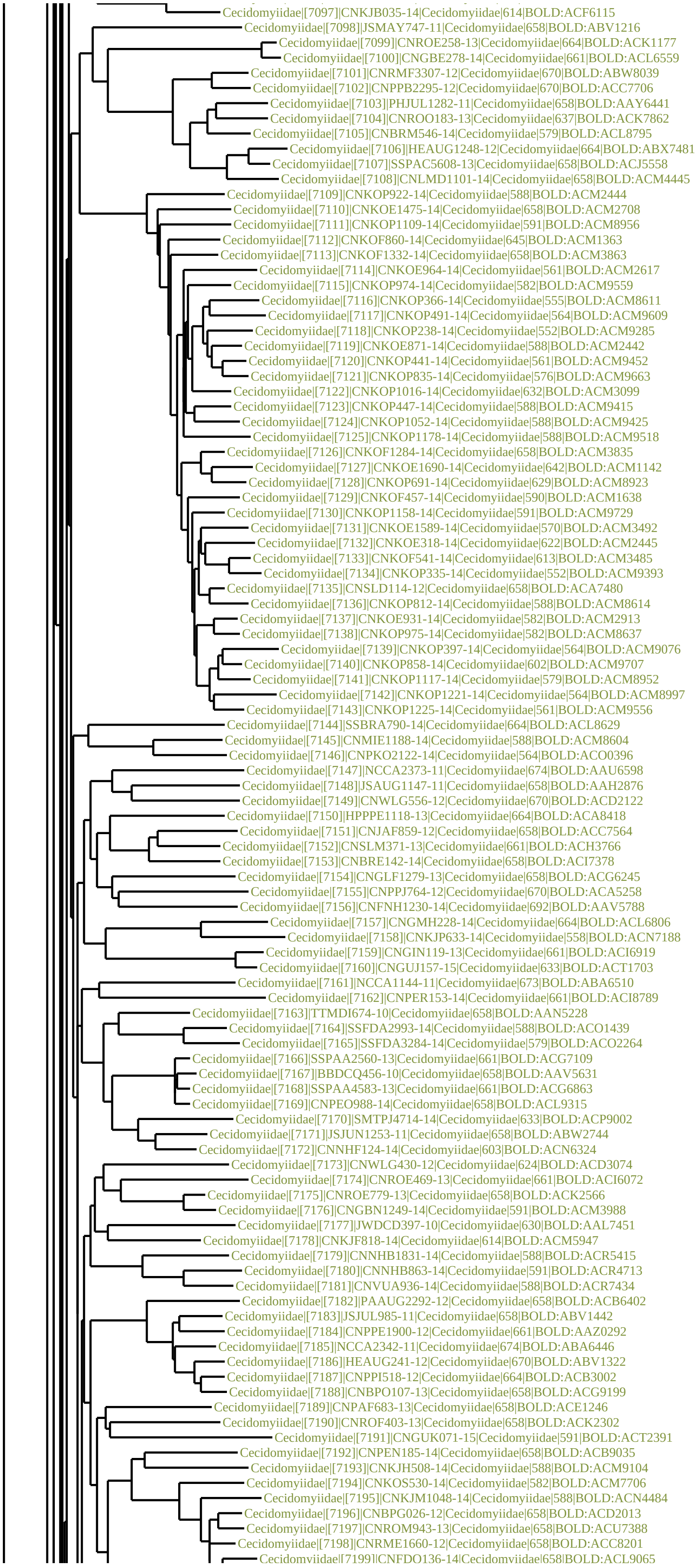

Cecidomyiidae|[7097]|CNKJB035-14|Cecidomyiidae|614|BOLD:ACF6115
Cecidomyiidae|[7098]|JSMAY747-11|Cecidomyiidae|658|BOLD:ABV1216
Cecidomyiidae|[7099]|CNROE258-13|Cecidomyiidae|664|BOLD:ACK1177
Cecidomyiidae|[7100]|CNGBE278-14|Cecidomyiidae|661|BOLD:ACL6559
Cecidomyiidae|[7101]|CNRMF3307-12|Cecidomyiidae|670|BOLD:ABW8039
Cecidomyiidae|[7102]|CNPPB2295-12|Cecidomyiidae|670|BOLD:ACC7706
Cecidomyiidae|[7103]|PHJUL1282-11|Cecidomyiidae|658|BOLD:AAY6441
Cecidomyiidae|[7104]|CNROO183-13|Cecidomyiidae|637|BOLD:ACK7862
Cecidomyiidae|[7105]|CNBRM546-14|Cecidomyiidae|579|BOLD:ACL8795
Cecidomyiidae|[7106]|HEAUG1248-12|Cecidomyiidae|664|BOLD:ABX7481
Cecidomyiidae|[7107]|SSPAC5608-13|Cecidomyiidae|658|BOLD:ACJ5558
Cecidomyiidae|[7108]|CNLMD1101-14|Cecidomyiidae|658|BOLD:ACM4445
Cecidomyiidae|[7109]|CNKOP922-14|Cecidomyiidae|588|BOLD:ACM2444
Cecidomyiidae|[7110]|CNKOE1475-14|Cecidomyiidae|658|BOLD:ACM2708
Cecidomyiidae|[7111]|CNKOP1109-14|Cecidomyiidae|591|BOLD:ACM8956
Cecidomyiidae|[7112]|CNKOF860-14|Cecidomyiidae|645|BOLD:ACM1363
Cecidomyiidae|[7113]|CNKOF1332-14|Cecidomyiidae|658|BOLD:ACM3863
Cecidomyiidae|[7114]|CNKOE964-14|Cecidomyiidae|561|BOLD:ACM2617
Cecidomyiidae|[7115]|CNKOP974-14|Cecidomyiidae|582|BOLD:ACM9559
Cecidomyiidae|[7116]|CNKOP366-14|Cecidomyiidae|555|BOLD:ACM8611
Cecidomyiidae|[7117]|CNKOP491-14|Cecidomyiidae|564|BOLD:ACM9609
Cecidomyiidae|[7118]|CNKOP238-14|Cecidomyiidae|552|BOLD:ACM9285
Cecidomyiidae|[7119]|CNKOE871-14|Cecidomyiidae|588|BOLD:ACM2442
Cecidomyiidae|[7120]|CNKOP441-14|Cecidomyiidae|561|BOLD:ACM9452
Cecidomyiidae|[7121]|CNKOP835-14|Cecidomyiidae|576|BOLD:ACM9663
Cecidomyiidae|[7122]|CNKOP1016-14|Cecidomyiidae|632|BOLD:ACM3099
Cecidomyiidae|[7123]|CNKOP447-14|Cecidomyiidae|588|BOLD:ACM9415
Cecidomyiidae|[7124]|CNKOP1052-14|Cecidomyiidae|588|BOLD:ACM9425
Cecidomyiidae|[7125]|CNKOP1178-14|Cecidomyiidae|588|BOLD:ACM9518
Cecidomyiidae|[7126]|CNKOF1284-14|Cecidomyiidae|658|BOLD:ACM3835
Cecidomyiidae|[7127]|CNKOE1690-14|Cecidomyiidae|642|BOLD:ACM1142
Cecidomyiidae|[7128]|CNKOP691-14|Cecidomyiidae|629|BOLD:ACM8923
Cecidomyiidae|[7129]|CNKOF457-14|Cecidomyiidae|590|BOLD:ACM1638
Cecidomyiidae|[7130]|CNKOP1158-14|Cecidomyiidae|591|BOLD:ACM9729
Cecidomyiidae|[7131]|CNKOE1589-14|Cecidomyiidae|570|BOLD:ACM3492
Cecidomyiidae|[7132]|CNKOE318-14|Cecidomyiidae|622|BOLD:ACM2445
Cecidomyiidae|[7133]|CNKOF541-14|Cecidomyiidae|613|BOLD:ACM3485
Cecidomyiidae|[7134]|CNKOP335-14|Cecidomyiidae|552|BOLD:ACM9393
Cecidomyiidae|[7135]|CNSLD114-12|Cecidomyiidae|658|BOLD:ACA7480
Cecidomyiidae|[7136]|CNKOP812-14|Cecidomyiidae|588|BOLD:ACM8614
Cecidomyiidae|[7137]|CNKOE931-14|Cecidomyiidae|582|BOLD:ACM2913
Cecidomyiidae|[7138]|CNKOP975-14|Cecidomyiidae|582|BOLD:ACM8637
Cecidomyiidae|[7139]|CNKOP397-14|Cecidomyiidae|564|BOLD:ACM9076
Cecidomyiidae|[7140]|CNKOP858-14|Cecidomyiidae|602|BOLD:ACM9707
Cecidomyiidae|[7141]|CNKOP1117-14|Cecidomyiidae|579|BOLD:ACM8952
Cecidomyiidae|[7142]|CNKOP1221-14|Cecidomyiidae|564|BOLD:ACM8997
Cecidomyiidae|[7143]|CNKOP1225-14|Cecidomyiidae|561|BOLD:ACM9556
Cecidomyiidae|[7144]|SSBRA790-14|Cecidomyiidae|664|BOLD:ACL8629
Cecidomyiidae|[7145]|CNMIE1188-14|Cecidomyiidae|588|BOLD:ACM8604
Cecidomyiidae|[7146]|CNPKO2122-14|Cecidomyiidae|564|BOLD:ACO0396
Cecidomyiidae|[7147]|NCCA2373-11|Cecidomyiidae|674|BOLD:AAU6598
Cecidomyiidae|[7148]|JSAUG1147-11|Cecidomyiidae|658|BOLD:AAH2876
Cecidomyiidae|[7149]|CNWLG556-12|Cecidomyiidae|670|BOLD:ACD2122
Cecidomyiidae|[7150]|HPPPE1118-13|Cecidomyiidae|664|BOLD:ACA8418
Cecidomyiidae|[7151]|CNJAF859-12|Cecidomyiidae|658|BOLD:ACC7564
Cecidomyiidae|[7152]|CNSLM371-13|Cecidomyiidae|661|BOLD:ACH3766
Cecidomyiidae|[7153]|CNBRE142-14|Cecidomyiidae|658|BOLD:ACI7378
Cecidomyiidae|[7154]|CNGLF1279-13|Cecidomyiidae|658|BOLD:ACG6245
Cecidomyiidae|[7155]|CNPPJ764-12|Cecidomyiidae|670|BOLD:ACA5258
Cecidomyiidae|[7156]|CNFNH1230-14|Cecidomyiidae|692|BOLD:AAV5788
Cecidomyiidae|[7157]|CNGMH228-14|Cecidomyiidae|664|BOLD:ACL6806
Cecidomyiidae|[7158]|CNKJP633-14|Cecidomyiidae|558|BOLD:ACN7188
Cecidomyiidae|[7159]|CNGIN119-13|Cecidomyiidae|661|BOLD:ACI6919
Cecidomyiidae|[7160]|CNGUJ157-15|Cecidomyiidae|633|BOLD:ACT1703
Cecidomyiidae|[7161]|NCCA1144-11|Cecidomyiidae|673|BOLD:ABA6510
Cecidomyiidae|[7162]|CNPER153-14|Cecidomyiidae|661|BOLD:ACI8789
Cecidomyiidae|[7163]|TTMDI674-10|Cecidomyiidae|658|BOLD:AAN5228
Cecidomyiidae|[7164]|SSFDA2993-14|Cecidomyiidae|588|BOLD:ACO1439
Cecidomyiidae|[7165]|SSFDA3284-14|Cecidomyiidae|579|BOLD:ACO2264
Cecidomyiidae|[7166]|SSPAA2560-13|Cecidomyiidae|661|BOLD:ACG7109
Cecidomyiidae|[7167]|BBDCQ456-10|Cecidomyiidae|658|BOLD:AAV5631
Cecidomyiidae|[7168]|SSPAA4583-13|Cecidomyiidae|661|BOLD:ACG6863
Cecidomyiidae|[7169]|CNPEO988-14|Cecidomyiidae|658|BOLD:ACL9315
Cecidomyiidae|[7170]|SMTPJ4714-14|Cecidomyiidae|633|BOLD:ACP9002
Cecidomyiidae|[7171]|JSJUN1253-11|Cecidomyiidae|658|BOLD:ABW2744
Cecidomyiidae|[7172]|CNNHF124-14|Cecidomyiidae|603|BOLD:ACN6324
Cecidomyiidae|[7173]|CNWLG430-12|Cecidomyiidae|624|BOLD:ACD3074
Cecidomyiidae|[7174]|CNROE469-13|Cecidomyiidae|661|BOLD:ACI6072
Cecidomyiidae|[7175]|CNROE779-13|Cecidomyiidae|658|BOLD:ACK2566
Cecidomyiidae|[7176]|CNGBN1249-14|Cecidomyiidae|591|BOLD:ACM3988
Cecidomyiidae|[7177]|JWDCD397-10|Cecidomyiidae|630|BOLD:AAL7451
Cecidomyiidae|[7178]|CNKJF818-14|Cecidomyiidae|614|BOLD:ACM5947
Cecidomyiidae|[7179]|CNNHB1831-14|Cecidomyiidae|588|BOLD:ACR5415
Cecidomyiidae|[7180]|CNNHB863-14|Cecidomyiidae|591|BOLD:ACR4713
Cecidomyiidae|[7181]|CNVUA936-14|Cecidomyiidae|588|BOLD:ACR7434
Cecidomyiidae|[7182]|PAAUG2292-12|Cecidomyiidae|658|BOLD:ACB6402
Cecidomyiidae|[7183]|JSJUL985-11|Cecidomyiidae|658|BOLD:ABV1442
Cecidomyiidae|[7184]|CNPPE1900-12|Cecidomyiidae|661|BOLD:AAZ0292
Cecidomyiidae|[7185]|NCCA2342-11|Cecidomyiidae|674|BOLD:ABA6446
Cecidomyiidae|[7186]|HEAUG241-12|Cecidomyiidae|670|BOLD:ABV1322
Cecidomyiidae|[7187]|CNPPI518-12|Cecidomyiidae|664|BOLD:ACB3002
Cecidomyiidae|[7188]|CNBPO107-13|Cecidomyiidae|658|BOLD:ACG9199
Cecidomyiidae|[7189]|CNPAF683-13|Cecidomyiidae|658|BOLD:ACE1246
Cecidomyiidae|[7190]|CNROF403-13|Cecidomyiidae|658|BOLD:ACK2302
Cecidomyiidae|[7191]|CNGUK071-15|Cecidomyiidae|591|BOLD:ACT2391
Cecidomyiidae|[7192]|CNPEN185-14|Cecidomyiidae|658|BOLD:ACB9035
Cecidomyiidae|[7193]|CNKJH508-14|Cecidomyiidae|588|BOLD:ACM9104
Cecidomyiidae|[7194]|CNKOS530-14|Cecidomyiidae|582|BOLD:ACM7706
Cecidomyiidae|[7195]|CNKJM1048-14|Cecidomyiidae|588|BOLD:ACN4484
Cecidomyiidae|[7196]|CNBPG026-12|Cecidomyiidae|658|BOLD:ACD2013
Cecidomyiidae|[7197]|CNROM943-13|Cecidomyiidae|658|BOLD:ACU7388
Cecidomyiidae|[7198]|CNRME1660-12|Cecidomyiidae|658|BOLD:ACC8201
Cecidomyiidae|[7199]|CNFDO136-14|Cecidomyiidae|658|BOLD:ACL9065

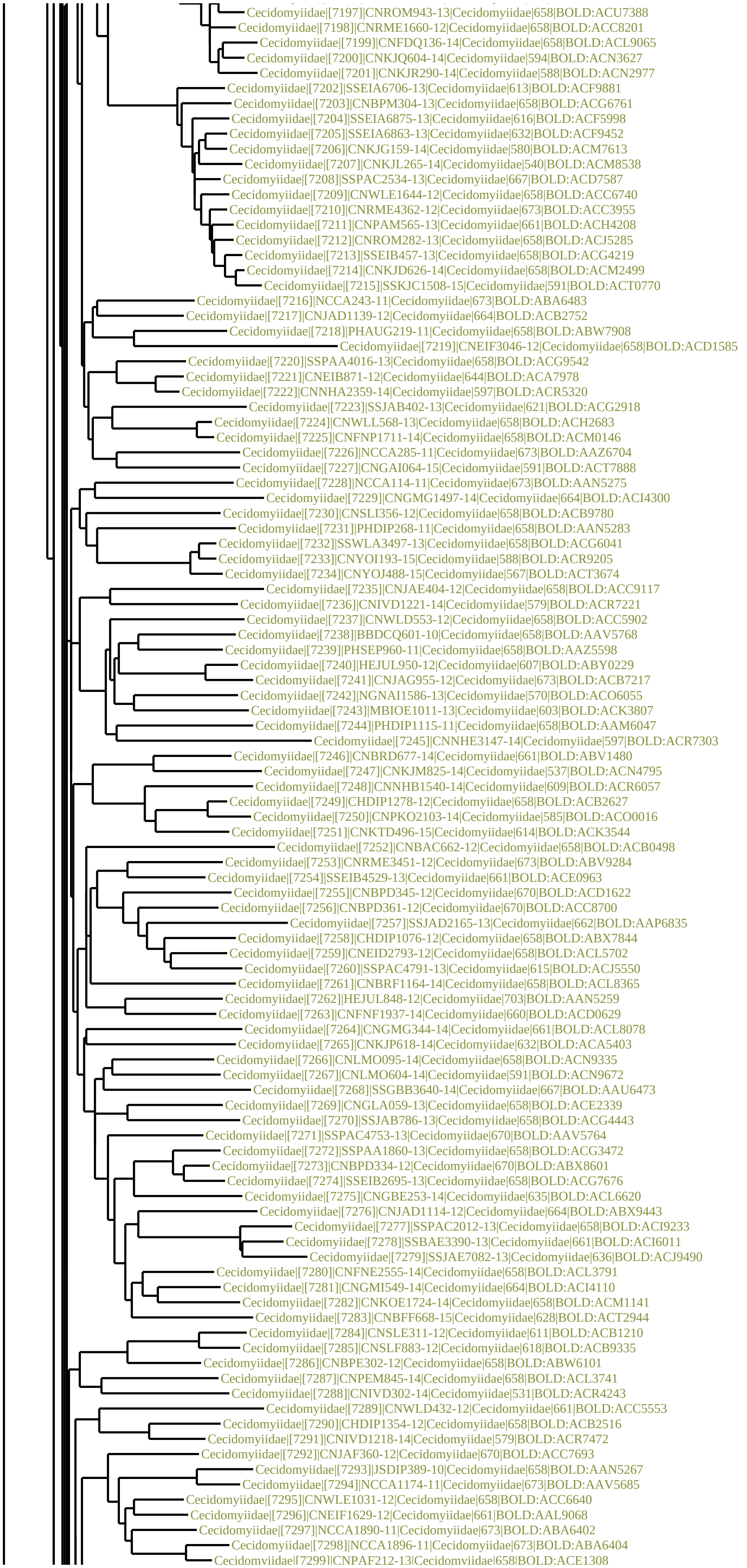
Cecidomyiidae|[7197]|CNROM943-13|Cecidomyiidae|658|BOLD:ACU7388
Cecidomyiidae|[7198]|CNRME1660-12|Cecidomyiidae|658|BOLD:ACC8201
Cecidomyiidae|[7199]|CNFDQ136-14|Cecidomyiidae|658|BOLD:ACL9065
Cecidomyiidae|[7200]|CNKJQ604-14|Cecidomyiidae|594|BOLD:ACN3627
Cecidomyiidae|[7201]|CNKJR290-14|Cecidomyiidae|588|BOLD:ACN2977
Cecidomyiidae|[7202]|SSEIA6706-13|Cecidomyiidae|613|BOLD:ACF9881
Cecidomyiidae|[7203]|CNBPM304-13|Cecidomyiidae|658|BOLD:ACG6761
Cecidomyiidae|[7204]|SSEIA6875-13|Cecidomyiidae|616|BOLD:ACF5998
Cecidomyiidae|[7205]|SSEIA6863-13|Cecidomyiidae|632|BOLD:ACF9452
Cecidomyiidae|[7206]|CNKJG159-14|Cecidomyiidae|580|BOLD:ACM7613
Cecidomyiidae|[7207]|CNKJL265-14|Cecidomyiidae|540|BOLD:ACM8538
Cecidomyiidae|[7208]|SSPAC2534-13|Cecidomyiidae|667|BOLD:ACD7587
Cecidomyiidae|[7209]|CNWLE1644-12|Cecidomyiidae|658|BOLD:ACC6740
Cecidomyiidae|[7210]|CNRME4362-12|Cecidomyiidae|673|BOLD:ACC3955
Cecidomyiidae|[7211]|CNPAM565-13|Cecidomyiidae|661|BOLD:ACH4208
Cecidomyiidae|[7212]|CNROM282-13|Cecidomyiidae|658|BOLD:ACJ5285
Cecidomyiidae|[7213]|SSEIB457-13|Cecidomyiidae|658|BOLD:ACG4219
Cecidomyiidae|[7214]|CNKJD626-14|Cecidomyiidae|658|BOLD:ACM2499
Cecidomyiidae|[7215]|SSKJC1508-15|Cecidomyiidae|591|BOLD:ACT0770
Cecidomyiidae|[7216]|NCCA243-11|Cecidomyiidae|673|BOLD:ABA6483
Cecidomyiidae|[7217]|CNJAD1139-12|Cecidomyiidae|664|BOLD:ACB2752
Cecidomyiidae|[7218]|PHAUG219-11|Cecidomyiidae|658|BOLD:ABW7908
Cecidomyiidae|[7219]|CNEIF3046-12|Cecidomyiidae|658|BOLD:ACD1585
Cecidomyiidae|[7220]|SSPAA4016-13|Cecidomyiidae|658|BOLD:ACG9542
Cecidomyiidae|[7221]|CNEIB871-12|Cecidomyiidae|644|BOLD:ACA7978
Cecidomyiidae|[7222]|CNNHA2359-14|Cecidomyiidae|597|BOLD:ACR5320
Cecidomyiidae|[7223]|SSJAB402-13|Cecidomyiidae|621|BOLD:ACG2918
Cecidomyiidae|[7224]|CNWLL568-13|Cecidomyiidae|658|BOLD:ACH2683
Cecidomyiidae|[7225]|CNFNP1711-14|Cecidomyiidae|658|BOLD:ACM0146
Cecidomyiidae|[7226]|NCCA285-11|Cecidomyiidae|673|BOLD:AAZ6704
Cecidomyiidae|[7227]|CNGAI064-15|Cecidomyiidae|591|BOLD:ACT7888
Cecidomyiidae|[7228]|NCCA114-11|Cecidomyiidae|673|BOLD:AAN5275
Cecidomyiidae|[7229]|CNGMG1497-14|Cecidomyiidae|664|BOLD:ACI4300
Cecidomyiidae|[7230]|CNSLI356-12|Cecidomyiidae|658|BOLD:ACB9780
Cecidomyiidae|[7231]|PHDIP268-11|Cecidomyiidae|658|BOLD:AAN5283
Cecidomyiidae|[7232]|SSWLA3497-13|Cecidomyiidae|658|BOLD:ACG6041
Cecidomyiidae|[7233]|CNYOI193-15|Cecidomyiidae|588|BOLD:ACR9205
Cecidomyiidae|[7234]|CNYOJ488-15|Cecidomyiidae|567|BOLD:ACT3674
Cecidomyiidae|[7235]|CNJAE404-12|Cecidomyiidae|658|BOLD:ACC9117
Cecidomyiidae|[7236]|CNIVD1221-14|Cecidomyiidae|579|BOLD:ACR7221
Cecidomyiidae|[7237]|CNWLD553-12|Cecidomyiidae|658|BOLD:ACC5902
Cecidomyiidae|[7238]|BBDCQ601-10|Cecidomyiidae|658|BOLD:AAV5768
Cecidomyiidae|[7239]|PHSEP960-11|Cecidomyiidae|658|BOLD:AAZ5598
Cecidomyiidae|[7240]|HEJUL950-12|Cecidomyiidae|607|BOLD:ABY0229
Cecidomyiidae|[7241]|CNJAG955-12|Cecidomyiidae|673|BOLD:ACB7217
Cecidomyiidae|[7242]|NGNAI1586-13|Cecidomyiidae|570|BOLD:ACO6055
Cecidomyiidae|[7243]|MBIOE1011-13|Cecidomyiidae|603|BOLD:ACK3807
Cecidomyiidae|[7244]|PHDIP1115-11|Cecidomyiidae|658|BOLD:AAM6047
Cecidomyiidae|[7245]|CNNHE3147-14|Cecidomyiidae|597|BOLD:ACR7303
Cecidomyiidae|[7246]|CNBRD677-14|Cecidomyiidae|661|BOLD:ABV1480
Cecidomyiidae|[7247]|CNKJM825-14|Cecidomyiidae|537|BOLD:ACN4795
Cecidomyiidae|[7248]|CNNHB1540-14|Cecidomyiidae|609|BOLD:ACR6057
Cecidomyiidae|[7249]|CHDIP1278-12|Cecidomyiidae|658|BOLD:ACB2627
Cecidomyiidae|[7250]|CNPKO2103-14|Cecidomyiidae|585|BOLD:ACO0016
Cecidomyiidae|[7251]|CNKTD496-15|Cecidomyiidae|614|BOLD:ACK3544
Cecidomyiidae|[7252]|CNBAC662-12|Cecidomyiidae|658|BOLD:ACB0498
Cecidomyiidae|[7253]|CNRME3451-12|Cecidomyiidae|673|BOLD:ABV9284
Cecidomyiidae|[7254]|SSEIB4529-13|Cecidomyiidae|661|BOLD:ACE0963
Cecidomyiidae|[7255]|CNBPD345-12|Cecidomyiidae|670|BOLD:ACD1622
Cecidomyiidae|[7256]|CNBPD361-12|Cecidomyiidae|670|BOLD:ACC8700
Cecidomyiidae|[7257]|SSJAD2165-13|Cecidomyiidae|662|BOLD:AAP6835
Cecidomyiidae|[7258]|CHDIP1076-12|Cecidomyiidae|658|BOLD:ABX7844
Cecidomyiidae|[7259]|CNEID2793-12|Cecidomyiidae|658|BOLD:ACL5702
Cecidomyiidae|[7260]|SSPAC4791-13|Cecidomyiidae|615|BOLD:ACJ5550
Cecidomyiidae|[7261]|CNBRF1164-14|Cecidomyiidae|658|BOLD:ACL8365
Cecidomyiidae|[7262]|HEJUL848-12|Cecidomyiidae|703|BOLD:AAN5259
Cecidomyiidae|[7263]|CNFNF1937-14|Cecidomyiidae|660|BOLD:ACD0629
Cecidomyiidae|[7264]|CNGMG344-14|Cecidomyiidae|661|BOLD:ACL8078
Cecidomyiidae|[7265]|CNKJP618-14|Cecidomyiidae|632|BOLD:ACA5403
Cecidomyiidae|[7266]|CNLMO095-14|Cecidomyiidae|658|BOLD:ACN9335
Cecidomyiidae|[7267]|CNLMO604-14|Cecidomyiidae|591|BOLD:ACN9672
Cecidomyiidae|[7268]|SSGBB3640-14|Cecidomyiidae|667|BOLD:AAU6473
Cecidomyiidae|[7269]|CNGLA059-13|Cecidomyiidae|658|BOLD:ACE2339
Cecidomyiidae|[7270]|SSJAB786-13|Cecidomyiidae|658|BOLD:ACG4443
Cecidomyiidae|[7271]|SSPAC4753-13|Cecidomyiidae|670|BOLD:AAV5764
Cecidomyiidae|[7272]|SSPAA1860-13|Cecidomyiidae|658|BOLD:ACG3472
Cecidomyiidae|[7273]|CNBPD334-12|Cecidomyiidae|670|BOLD:ABX8601
Cecidomyiidae|[7274]|SSEIB2695-13|Cecidomyiidae|658|BOLD:ACG7676
Cecidomyiidae|[7275]|CNGBE253-14|Cecidomyiidae|635|BOLD:ACL6620
Cecidomyiidae|[7276]|CNJAD1114-12|Cecidomyiidae|664|BOLD:ABX9443
Cecidomyiidae|[7277]|SSPAC2012-13|Cecidomyiidae|658|BOLD:ACI9233
Cecidomyiidae|[7278]|SSBAE3390-13|Cecidomyiidae|661|BOLD:ACI6011
Cecidomyiidae|[7279]|SSJAE7082-13|Cecidomyiidae|636|BOLD:ACJ9490
Cecidomyiidae|[7280]|CNFNE2555-14|Cecidomyiidae|658|BOLD:ACL3791
Cecidomyiidae|[7281]|CNGMI549-14|Cecidomyiidae|664|BOLD:ACI4110
Cecidomyiidae|[7282]|CNKOE1724-14|Cecidomyiidae|658|BOLD:ACM1141
Cecidomyiidae|[7283]|CNBFF668-15|Cecidomyiidae|628|BOLD:ACT2944
Cecidomyiidae|[7284]|CNSLE311-12|Cecidomyiidae|611|BOLD:ACB1210
Cecidomyiidae|[7285]|CNSLF883-12|Cecidomyiidae|618|BOLD:ACB9335
Cecidomyiidae|[7286]|CNBPE302-12|Cecidomyiidae|658|BOLD:ABW6101
Cecidomyiidae|[7287]|CNPEM845-14|Cecidomyiidae|658|BOLD:ACL3741
Cecidomyiidae|[7288]|CNIVD302-14|Cecidomyiidae|531|BOLD:ACR4243
Cecidomyiidae|[7289]|CNWLD432-12|Cecidomyiidae|661|BOLD:ACC5553
Cecidomyiidae|[7290]|CHDIP1354-12|Cecidomyiidae|658|BOLD:ACB2516
Cecidomyiidae|[7291]|CNIVD1218-14|Cecidomyiidae|579|BOLD:ACR7472
Cecidomyiidae|[7292]|CNJAF360-12|Cecidomyiidae|670|BOLD:ACC7693
Cecidomyiidae|[7293]|JSDIP389-10|Cecidomyiidae|658|BOLD:AAN5267
Cecidomyiidae|[7294]|NCCA1174-11|Cecidomyiidae|673|BOLD:AAV5685
Cecidomyiidae|[7295]|CNWLE1031-12|Cecidomyiidae|658|BOLD:ACC6640
Cecidomyiidae|[7296]|CNEIF1629-12|Cecidomyiidae|661|BOLD:AAL9068
Cecidomyiidae|[7297]|NCCA1890-11|Cecidomyiidae|673|BOLD:ABA6402
Cecidomyiidae|[7298]|NCCA1896-11|Cecidomyiidae|673|BOLD:ABA6404
Cecidomyiidae|[7299]|CNPAF212-13|Cecidomyiidae|658|BOLD:ACE1308

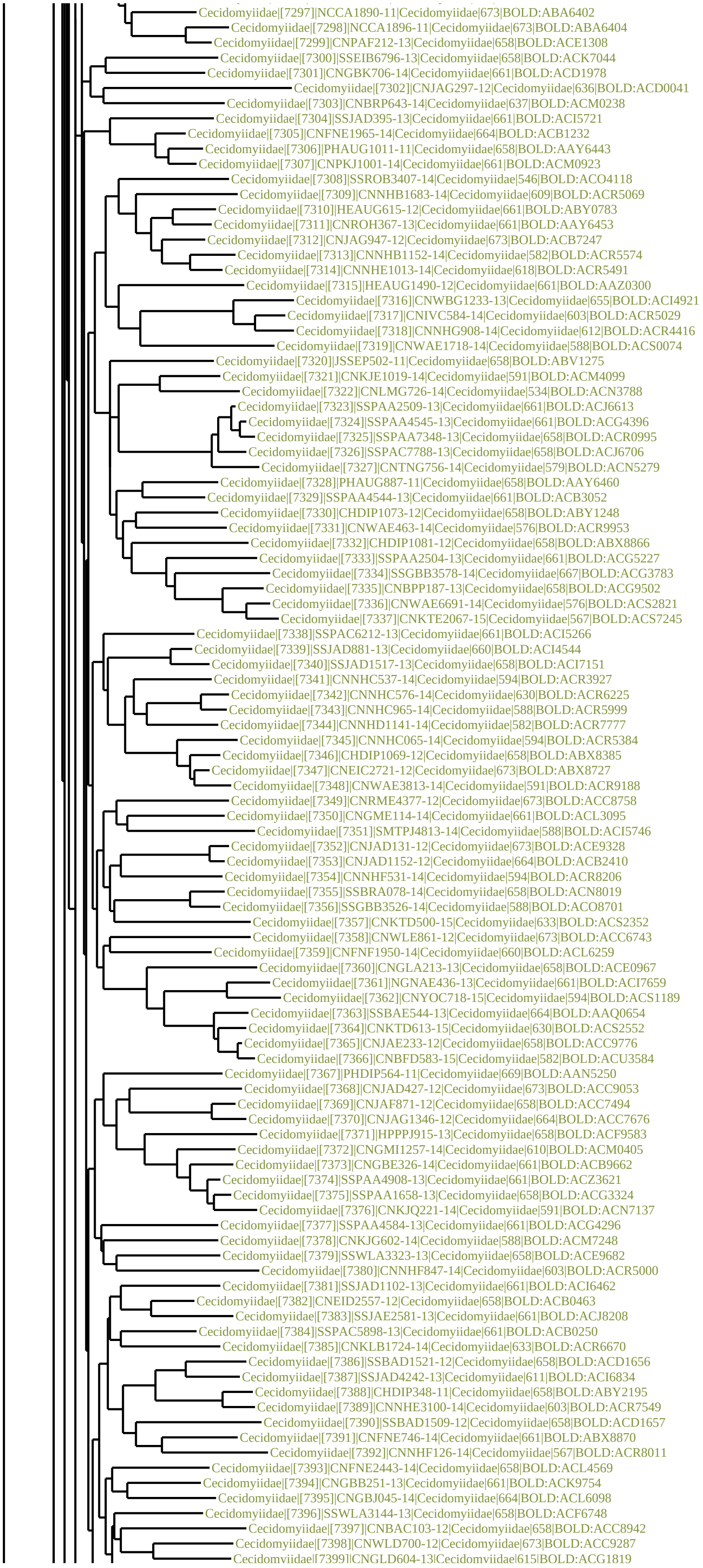
Cecidomyiidae|[7297]|NCCA1890-11|Cecidomyiidae|673|BOLD:ABA6402
Cecidomyiidae|[7298]|NCCA1896-11|Cecidomyiidae|673|BOLD:ABA6404
Cecidomyiidae|[7299]|CNPAF212-13|Cecidomyiidae|658|BOLD:ACE1308
Cecidomyiidae|[7300]|SSEIB6796-13|Cecidomyiidae|658|BOLD:ACK7044
Cecidomyiidae|[7301]|CNGBK706-14|Cecidomyiidae|661|BOLD:ACD1978
Cecidomyiidae|[7302]|CNJAG297-12|Cecidomyiidae|636|BOLD:ACD0041
Cecidomyiidae|[7303]|CNBRP643-14|Cecidomyiidae|637|BOLD:ACM0238
Cecidomyiidae|[7304]|SSJAD395-13|Cecidomyiidae|661|BOLD:ACI5721
Cecidomyiidae|[7305]|CNFNE1965-14|Cecidomyiidae|664|BOLD:ACB1232
Cecidomyiidae|[7306]|PHAUG1011-11|Cecidomyiidae|658|BOLD:AAY6443
Cecidomyiidae|[7307]|CNPKJ1001-14|Cecidomyiidae|661|BOLD:ACM0923
Cecidomyiidae|[7308]|SSROB3407-14|Cecidomyiidae|546|BOLD:ACO4118
Cecidomyiidae|[7309]|CNNHB1683-14|Cecidomyiidae|609|BOLD:ACR5069
Cecidomyiidae|[7310]|HEAUG615-12|Cecidomyiidae|661|BOLD:ABY0783
Cecidomyiidae|[7311]|CNROH367-13|Cecidomyiidae|661|BOLD:AAY6453
Cecidomyiidae|[7312]|CNJAG947-12|Cecidomyiidae|673|BOLD:ACB7247
Cecidomyiidae|[7313]|CNNHB1152-14|Cecidomyiidae|582|BOLD:ACR5574
Cecidomyiidae|[7314]|CNNHE1013-14|Cecidomyiidae|618|BOLD:ACR5491
Cecidomyiidae|[7315]|HEAUG1490-12|Cecidomyiidae|661|BOLD:AAZ0300
Cecidomyiidae|[7316]|CNWBG1233-13|Cecidomyiidae|655|BOLD:ACI4921
Cecidomyiidae|[7317]|CNIVC584-14|Cecidomyiidae|603|BOLD:ACR5029
Cecidomyiidae|[7318]|CNNHG908-14|Cecidomyiidae|612|BOLD:ACR4416
Cecidomyiidae|[7319]|CNWAE1718-14|Cecidomyiidae|588|BOLD:ACS0074
Cecidomyiidae|[7320]|JSSEP502-11|Cecidomyiidae|658|BOLD:ABV1275
Cecidomyiidae|[7321]|CNKJE1019-14|Cecidomyiidae|591|BOLD:ACM4099
Cecidomyiidae|[7322]|CNLMG726-14|Cecidomyiidae|534|BOLD:ACN3788
Cecidomyiidae|[7323]|SSPAA2509-13|Cecidomyiidae|661|BOLD:ACJ6613
Cecidomyiidae|[7324]|SSPAA4545-13|Cecidomyiidae|661|BOLD:ACG4396
Cecidomyiidae|[7325]|SSPAA7348-13|Cecidomyiidae|658|BOLD:ACR0995
Cecidomyiidae|[7326]|SSPAC7788-13|Cecidomyiidae|658|BOLD:ACJ6706
Cecidomyiidae|[7327]|CNTNG756-14|Cecidomyiidae|579|BOLD:ACN5279
Cecidomyiidae|[7328]|PHAUG887-11|Cecidomyiidae|658|BOLD:AAY6460
Cecidomyiidae|[7329]|SSPAA4544-13|Cecidomyiidae|661|BOLD:ACB3052
Cecidomyiidae|[7330]|CHDIP1073-12|Cecidomyiidae|658|BOLD:ABY1248
Cecidomyiidae|[7331]|CNWAE463-14|Cecidomyiidae|576|BOLD:ACR9953
Cecidomyiidae|[7332]|CHDIP1081-12|Cecidomyiidae|658|BOLD:ABX8866
Cecidomyiidae|[7333]|SSPAA2504-13|Cecidomyiidae|661|BOLD:ACG5227
Cecidomyiidae|[7334]|SSGBB3578-14|Cecidomyiidae|667|BOLD:ACG3783
Cecidomyiidae|[7335]|CNBPP187-13|Cecidomyiidae|658|BOLD:ACG9502
Cecidomyiidae|[7336]|CNWAE6691-14|Cecidomyiidae|576|BOLD:ACS2821
Cecidomyiidae|[7337]|CNKTE2067-15|Cecidomyiidae|567|BOLD:ACS7245
Cecidomyiidae|[7338]|SSPAC6212-13|Cecidomyiidae|661|BOLD:ACI5266
Cecidomyiidae|[7339]|SSJAD881-13|Cecidomyiidae|660|BOLD:ACI4544
Cecidomyiidae|[7340]|SSJAD1517-13|Cecidomyiidae|658|BOLD:ACI7151
Cecidomyiidae|[7341]|CNNHC537-14|Cecidomyiidae|594|BOLD:ACR3927
Cecidomyiidae|[7342]|CNNHC576-14|Cecidomyiidae|630|BOLD:ACR6225
Cecidomyiidae|[7343]|CNNHC965-14|Cecidomyiidae|588|BOLD:ACR5999
Cecidomyiidae|[7344]|CNNHD1141-14|Cecidomyiidae|582|BOLD:ACR7777
Cecidomyiidae|[7345]|CNNHC065-14|Cecidomyiidae|594|BOLD:ACR5384
Cecidomyiidae|[7346]|CHDIP1069-12|Cecidomyiidae|658|BOLD:ABX8385
Cecidomyiidae|[7347]|CNEIC2721-12|Cecidomyiidae|673|BOLD:ABX8727
Cecidomyiidae|[7348]|CNWAE3813-14|Cecidomyiidae|591|BOLD:ACR9188
Cecidomyiidae|[7349]|CNRME4377-12|Cecidomyiidae|673|BOLD:ACC8758
Cecidomyiidae|[7350]|CNGME114-14|Cecidomyiidae|661|BOLD:ACL3095
Cecidomyiidae|[7351]|SMTPJ4813-14|Cecidomyiidae|588|BOLD:ACI5746
Cecidomyiidae|[7352]|CNJAD131-12|Cecidomyiidae|673|BOLD:ACE9328
Cecidomyiidae|[7353]|CNJAD1152-12|Cecidomyiidae|664|BOLD:ACB2410
Cecidomyiidae|[7354]|CNNHF531-14|Cecidomyiidae|594|BOLD:ACR8206
Cecidomyiidae|[7355]|SSBRA078-14|Cecidomyiidae|658|BOLD:ACN8019
Cecidomyiidae|[7356]|SSGBB3526-14|Cecidomyiidae|588|BOLD:ACO8701
Cecidomyiidae|[7357]|CNKTD500-15|Cecidomyiidae|633|BOLD:ACS2352
Cecidomyiidae|[7358]|CNWLE861-12|Cecidomyiidae|673|BOLD:ACC6743
Cecidomyiidae|[7359]|CNFNF1950-14|Cecidomyiidae|660|BOLD:ACL6259
Cecidomyiidae|[7360]|CNGLA213-13|Cecidomyiidae|658|BOLD:ACE0967
Cecidomyiidae|[7361]|NGNAE436-13|Cecidomyiidae|661|BOLD:ACI7659
Cecidomyiidae|[7362]|CNYOC718-15|Cecidomyiidae|594|BOLD:ACS1189
Cecidomyiidae|[7363]|SSBAE544-13|Cecidomyiidae|664|BOLD:AAQ0654
Cecidomyiidae|[7364]|CNKTD613-15|Cecidomyiidae|630|BOLD:ACS2552
Cecidomyiidae|[7365]|CNJAE233-12|Cecidomyiidae|658|BOLD:ACC9776
Cecidomyiidae|[7366]|CNBFD583-15|Cecidomyiidae|582|BOLD:ACU3584
Cecidomyiidae|[7367]|PHDIP564-11|Cecidomyiidae|669|BOLD:AAN5250
Cecidomyiidae|[7368]|CNJAD427-12|Cecidomyiidae|673|BOLD:ACC9053
Cecidomyiidae|[7369]|CNJAF871-12|Cecidomyiidae|658|BOLD:ACC7494
Cecidomyiidae|[7370]|CNJAG1346-12|Cecidomyiidae|664|BOLD:ACC7676
Cecidomyiidae|[7371]|HPPPJ915-13|Cecidomyiidae|658|BOLD:ACF9583
Cecidomyiidae|[7372]|CNGMI1257-14|Cecidomyiidae|610|BOLD:ACM0405
Cecidomyiidae|[7373]|CNGBE326-14|Cecidomyiidae|661|BOLD:ACB9662
Cecidomyiidae|[7374]|SSPAA4908-13|Cecidomyiidae|661|BOLD:ACZ3621
Cecidomyiidae|[7375]|SSPAA1658-13|Cecidomyiidae|658|BOLD:ACG3324
Cecidomyiidae|[7376]|CNKJQ221-14|Cecidomyiidae|591|BOLD:ACN7137
Cecidomyiidae|[7377]|SSPAA4584-13|Cecidomyiidae|661|BOLD:ACG4296
Cecidomyiidae|[7378]|CNKJG602-14|Cecidomyiidae|588|BOLD:ACM7248
Cecidomyiidae|[7379]|SSWLA3323-13|Cecidomyiidae|658|BOLD:ACE9682
Cecidomyiidae|[7380]|CNNHF847-14|Cecidomyiidae|603|BOLD:ACR5000
Cecidomyiidae|[7381]|SSJAD1102-13|Cecidomyiidae|661|BOLD:ACI6462
Cecidomyiidae|[7382]|CNEID2557-12|Cecidomyiidae|658|BOLD:ACB0463
Cecidomyiidae|[7383]|SSJAE2581-13|Cecidomyiidae|661|BOLD:ACJ8208
Cecidomyiidae|[7384]|SSPAC5898-13|Cecidomyiidae|661|BOLD:ACB0250
Cecidomyiidae|[7385]|CNKLB1724-14|Cecidomyiidae|633|BOLD:ACR6670
Cecidomyiidae|[7386]|SSBAD1521-12|Cecidomyiidae|658|BOLD:ACD1656
Cecidomyiidae|[7387]|SSJAD4242-13|Cecidomyiidae|611|BOLD:ACI6834
Cecidomyiidae|[7388]|CHDIP348-11|Cecidomyiidae|658|BOLD:ABY2195
Cecidomyiidae|[7389]|CNNHE3100-14|Cecidomyiidae|603|BOLD:ACR7549
Cecidomyiidae|[7390]|SSBAD1509-12|Cecidomyiidae|658|BOLD:ACD1657
Cecidomyiidae|[7391]|CNFNE746-14|Cecidomyiidae|661|BOLD:ABX8870
Cecidomyiidae|[7392]|CNNHF126-14|Cecidomyiidae|567|BOLD:ACR8011
Cecidomyiidae|[7393]|CNFNE2443-14|Cecidomyiidae|658|BOLD:ACL4569
Cecidomyiidae|[7394]|CNGBB251-13|Cecidomyiidae|661|BOLD:ACK9754
Cecidomyiidae|[7395]|CNGBJ045-14|Cecidomyiidae|664|BOLD:ACL6098
Cecidomyiidae|[7396]|SSWLA3144-13|Cecidomyiidae|658|BOLD:ACF6748
Cecidomyiidae|[7397]|CNBAC103-12|Cecidomyiidae|658|BOLD:ACC8942
Cecidomyiidae|[7398]|CNWLD700-12|Cecidomyiidae|673|BOLD:ACC9287
Cecidomyiidae|[7399]|CNGLD604-13|Cecidomyiidae|615|BOLD:ACG1819

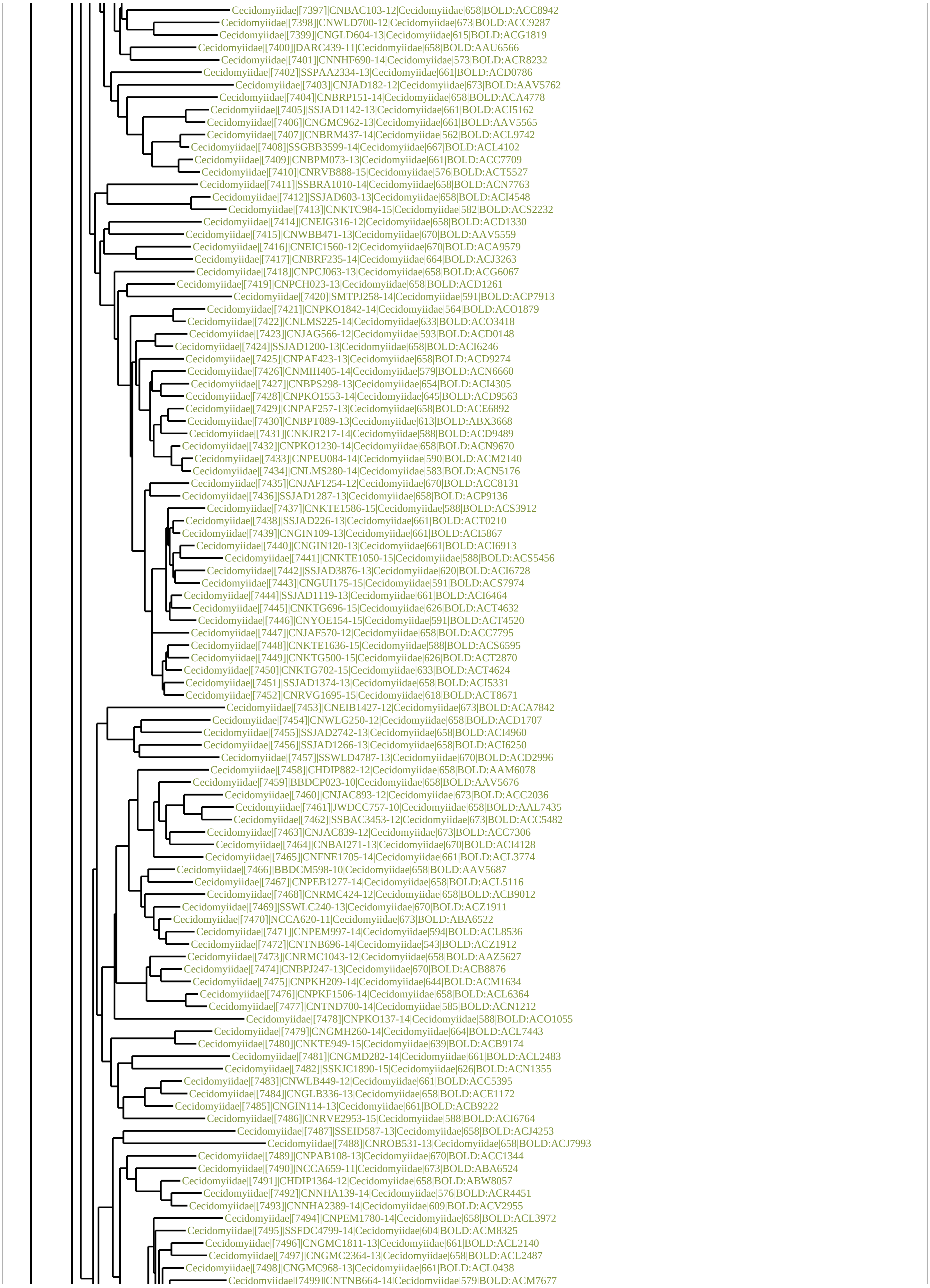
Cecidomyiidae|[7397]|CNBAC103-12|Cecidomyiidae|658|BOLD:ACC8942
Cecidomyiidae|[7398]|CNWLD700-12|Cecidomyiidae|673|BOLD:ACC9287
Cecidomyiidae|[7399]|CNGLD604-13|Cecidomyiidae|615|BOLD:ACG1819
Cecidomyiidae|[7400]|DARC439-11|Cecidomyiidae|658|BOLD:AAU6566
Cecidomyiidae|[7401]|CNNHF690-14|Cecidomyiidae|573|BOLD:ACR8232
Cecidomyiidae|[7402]|SSPAA2334-13|Cecidomyiidae|661|BOLD:ACD0786
Cecidomyiidae|[7403]|CNJAD182-12|Cecidomyiidae|673|BOLD:AAV5762
Cecidomyiidae|[7404]|CNBRP151-14|Cecidomyiidae|658|BOLD:ACA4778
Cecidomyiidae|[7405]|SSJAD1142-13|Cecidomyiidae|661|BOLD:ACI5162
Cecidomyiidae|[7406]|CNGMC962-13|Cecidomyiidae|661|BOLD:AAV5565
Cecidomyiidae|[7407]|CNBRM437-14|Cecidomyiidae|562|BOLD:ACL9742
Cecidomyiidae|[7408]|SSGBB3599-14|Cecidomyiidae|667|BOLD:ACL4102
Cecidomyiidae|[7409]|CNBPM073-13|Cecidomyiidae|661|BOLD:ACC7709
Cecidomyiidae|[7410]|CNRVB888-15|Cecidomyiidae|576|BOLD:ACT5527
Cecidomyiidae|[7411]|SSBRA1010-14|Cecidomyiidae|658|BOLD:ACN7763
Cecidomyiidae|[7412]|SSJAD603-13|Cecidomyiidae|658|BOLD:ACI4548
Cecidomyiidae|[7413]|CNKTC984-15|Cecidomyiidae|582|BOLD:ACS2232
Cecidomyiidae|[7414]|CNEIG316-12|Cecidomyiidae|658|BOLD:ACD1330
Cecidomyiidae|[7415]|CNWBB471-13|Cecidomyiidae|670|BOLD:AAV5559
Cecidomyiidae|[7416]|CNEIC1560-12|Cecidomyiidae|670|BOLD:ACA9579
Cecidomyiidae|[7417]|CNBRF235-14|Cecidomyiidae|664|BOLD:ACJ3263
Cecidomyiidae|[7418]|CNPCJ063-13|Cecidomyiidae|658|BOLD:ACG6067
Cecidomyiidae|[7419]|CNPCH023-13|Cecidomyiidae|658|BOLD:ACD1261
Cecidomyiidae|[7420]|SMTPJ258-14|Cecidomyiidae|591|BOLD:ACP7913
Cecidomyiidae|[7421]|CNPKO1842-14|Cecidomyiidae|564|BOLD:ACO1879
Cecidomyiidae|[7422]|CNLMS225-14|Cecidomyiidae|633|BOLD:ACO3418
Cecidomyiidae|[7423]|CNJAG566-12|Cecidomyiidae|593|BOLD:ACD0148
Cecidomyiidae|[7424]|SSJAD1200-13|Cecidomyiidae|658|BOLD:ACI6246
Cecidomyiidae|[7425]|CNPAF423-13|Cecidomyiidae|658|BOLD:ACD9274
Cecidomyiidae|[7426]|CNMIH405-14|Cecidomyiidae|579|BOLD:ACN6660
Cecidomyiidae|[7427]|CNBPS298-13|Cecidomyiidae|654|BOLD:ACI4305
Cecidomyiidae|[7428]|CNPKO1553-14|Cecidomyiidae|645|BOLD:ACD9563
Cecidomyiidae|[7429]|CNPAF257-13|Cecidomyiidae|658|BOLD:ACE6892
Cecidomyiidae|[7430]|CNBPT089-13|Cecidomyiidae|613|BOLD:ABX3668
Cecidomyiidae|[7431]|CNKJR217-14|Cecidomyiidae|588|BOLD:ACD9489
Cecidomyiidae|[7432]|CNPKO1230-14|Cecidomyiidae|658|BOLD:ACN9670
Cecidomyiidae|[7433]|CNPEU084-14|Cecidomyiidae|590|BOLD:ACM2140
Cecidomyiidae|[7434]|CNLMS280-14|Cecidomyiidae|583|BOLD:ACN5176
Cecidomyiidae|[7435]|CNJAF1254-12|Cecidomyiidae|670|BOLD:ACC8131
Cecidomyiidae|[7436]|SSJAD1287-13|Cecidomyiidae|658|BOLD:ACP9136
Cecidomyiidae|[7437]|CNKTE1586-15|Cecidomyiidae|588|BOLD:ACS3912
Cecidomyiidae|[7438]|SSJAD226-13|Cecidomyiidae|661|BOLD:ACT0210
Cecidomyiidae|[7439]|CNGIN109-13|Cecidomyiidae|661|BOLD:ACI5867
Cecidomyiidae|[7440]|CNGIN120-13|Cecidomyiidae|661|BOLD:ACI6913
Cecidomyiidae|[7441]|CNKTE1050-15|Cecidomyiidae|588|BOLD:ACS5456
Cecidomyiidae|[7442]|SSJAD3876-13|Cecidomyiidae|620|BOLD:ACI6728
Cecidomyiidae|[7443]|CNGUI175-15|Cecidomyiidae|591|BOLD:ACS7974
Cecidomyiidae|[7444]|SSJAD1119-13|Cecidomyiidae|661|BOLD:ACI6464
Cecidomyiidae|[7445]|CNKTG696-15|Cecidomyiidae|626|BOLD:ACT4632
Cecidomyiidae|[7446]|CNYOE154-15|Cecidomyiidae|591|BOLD:ACT4520
Cecidomyiidae|[7447]|CNJAF570-12|Cecidomyiidae|658|BOLD:ACC7795
Cecidomyiidae|[7448]|CNKTE1636-15|Cecidomyiidae|588|BOLD:ACS6595
Cecidomyiidae|[7449]|CNKTG500-15|Cecidomyiidae|626|BOLD:ACT2870
Cecidomyiidae|[7450]|CNKTG702-15|Cecidomyiidae|633|BOLD:ACT4624
Cecidomyiidae|[7451]|SSJAD1374-13|Cecidomyiidae|658|BOLD:ACI5331
Cecidomyiidae|[7452]|CNRVG1695-15|Cecidomyiidae|618|BOLD:ACT8671
Cecidomyiidae|[7453]|CNEIB1427-12|Cecidomyiidae|673|BOLD:ACA7842
Cecidomyiidae|[7454]|CNWLG250-12|Cecidomyiidae|658|BOLD:ACD1707
Cecidomyiidae|[7455]|SSJAD2742-13|Cecidomyiidae|658|BOLD:ACI4960
Cecidomyiidae|[7456]|SSJAD1266-13|Cecidomyiidae|658|BOLD:ACI6250
Cecidomyiidae|[7457]|SSWLD4787-13|Cecidomyiidae|670|BOLD:ACD2996
Cecidomyiidae|[7458]|CHDIP882-12|Cecidomyiidae|658|BOLD:AAM6078
Cecidomyiidae|[7459]|BBDCP023-10|Cecidomyiidae|658|BOLD:AAV5676
Cecidomyiidae|[7460]|CNJAC893-12|Cecidomyiidae|673|BOLD:ACC2036
Cecidomyiidae|[7461]|JWDCC757-10|Cecidomyiidae|658|BOLD:AAL7435
Cecidomyiidae|[7462]|SSBAC3453-12|Cecidomyiidae|673|BOLD:ACC5482
Cecidomyiidae|[7463]|CNJAC839-12|Cecidomyiidae|673|BOLD:ACC7306
Cecidomyiidae|[7464]|CNBAI271-13|Cecidomyiidae|670|BOLD:ACI4128
Cecidomyiidae|[7465]|CNFNE1705-14|Cecidomyiidae|661|BOLD:ACL3774
Cecidomyiidae|[7466]|BBDCM598-10|Cecidomyiidae|658|BOLD:AAV5687
Cecidomyiidae|[7467]|CNPEB1277-14|Cecidomyiidae|658|BOLD:ACL5116
Cecidomyiidae|[7468]|CNRMC424-12|Cecidomyiidae|658|BOLD:ACB9012
Cecidomyiidae|[7469]|SSWLC240-13|Cecidomyiidae|670|BOLD:ACZ1911
Cecidomyiidae|[7470]|NCCA620-11|Cecidomyiidae|673|BOLD:ABA6522
Cecidomyiidae|[7471]|CNPEM997-14|Cecidomyiidae|594|BOLD:ACL8536
Cecidomyiidae|[7472]|CNTNB696-14|Cecidomyiidae|543|BOLD:ACZ1912
Cecidomyiidae|[7473]|CNRMC1043-12|Cecidomyiidae|658|BOLD:AAZ5627
Cecidomyiidae|[7474]|CNBPJ247-13|Cecidomyiidae|670|BOLD:ACB8876
Cecidomyiidae|[7475]|CNPKH209-14|Cecidomyiidae|644|BOLD:ACM1634
Cecidomyiidae|[7476]|CNPKF1506-14|Cecidomyiidae|658|BOLD:ACL6364
Cecidomyiidae|[7477]|CNTND700-14|Cecidomyiidae|585|BOLD:ACN1212
Cecidomyiidae|[7478]|CNPKO137-14|Cecidomyiidae|588|BOLD:ACO1055
Cecidomyiidae|[7479]|CNGMH260-14|Cecidomyiidae|664|BOLD:ACL7443
Cecidomyiidae|[7480]|CNKTE949-15|Cecidomyiidae|639|BOLD:ACB9174
Cecidomyiidae|[7481]|CNGMD282-14|Cecidomyiidae|661|BOLD:ACL2483
Cecidomyiidae|[7482]|SSKJC1890-15|Cecidomyiidae|626|BOLD:ACN1355
Cecidomyiidae|[7483]|CNWLB449-12|Cecidomyiidae|661|BOLD:ACC5395
Cecidomyiidae|[7484]|CNGLB336-13|Cecidomyiidae|658|BOLD:ACE1172
Cecidomyiidae|[7485]|CNGIN114-13|Cecidomyiidae|661|BOLD:ACB9222
Cecidomyiidae|[7486]|CNRVE2953-15|Cecidomyiidae|588|BOLD:ACI6764
Cecidomyiidae|[7487]|SSEID587-13|Cecidomyiidae|658|BOLD:ACJ4253
Cecidomyiidae|[7488]|CNROB531-13|Cecidomyiidae|658|BOLD:ACJ7993
Cecidomyiidae|[7489]|CNPAB108-13|Cecidomyiidae|670|BOLD:ACC1344
Cecidomyiidae|[7490]|NCCA659-11|Cecidomyiidae|673|BOLD:ABA6524
Cecidomyiidae|[7491]|CHDIP1364-12|Cecidomyiidae|658|BOLD:ABW8057
Cecidomyiidae|[7492]|CNNHA139-14|Cecidomyiidae|576|BOLD:ACR4451
Cecidomyiidae|[7493]|CNNHA2389-14|Cecidomyiidae|609|BOLD:ACV2955
Cecidomyiidae|[7494]|CNPEM1780-14|Cecidomyiidae|658|BOLD:ACL3972
Cecidomyiidae|[7495]|SSFDC4799-14|Cecidomyiidae|604|BOLD:ACM8325
Cecidomyiidae|[7496]|CNGMC1811-13|Cecidomyiidae|661|BOLD:ACL2140
Cecidomyiidae|[7497]|CNGMC2364-13|Cecidomyiidae|658|BOLD:ACL2487
Cecidomyiidae|[7498]|CNGMC968-13|Cecidomyiidae|661|BOLD:ACL0438
Cecidomyiidae|[7499]|CNTNB664-14|Cecidomyiidae|579|BOLD:ACM7677

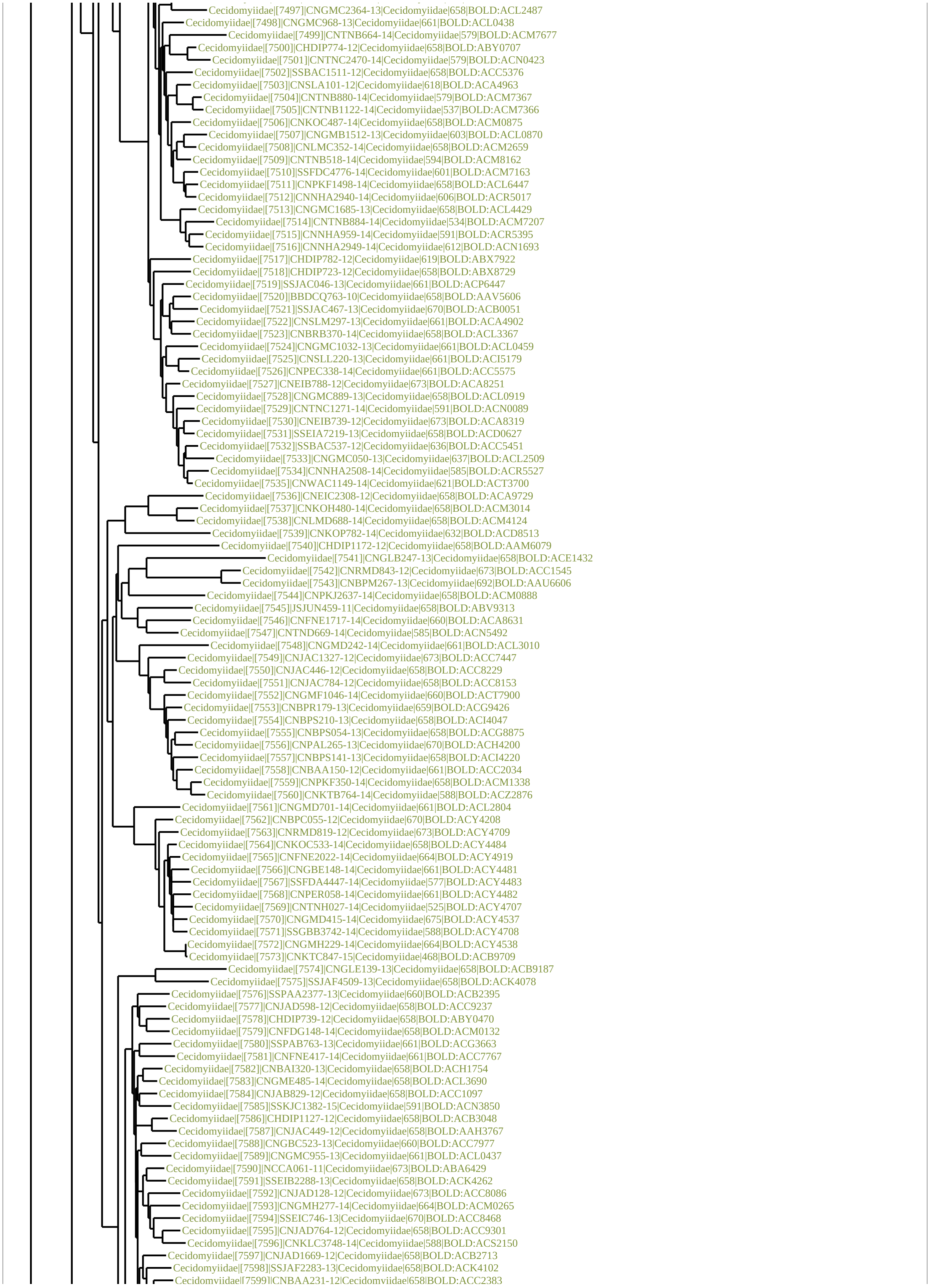

Cecidomyiidae|[7497]|CNGMC2364-13|Cecidomyiidae|658|BOLD:ACL2487
Cecidomyiidae|[7498]|CNGMC968-13|Cecidomyiidae|661|BOLD:ACL0438
Cecidomyiidae|[7499]|CNTNB664-14|Cecidomyiidae|579|BOLD:ACM7677
Cecidomyiidae|[7500]|CHDIP774-12|Cecidomyiidae|658|BOLD:ABY0707
Cecidomyiidae|[7501]|CNTNC2470-14|Cecidomyiidae|579|BOLD:ACN0423
Cecidomyiidae|[7502]|SSBAC1511-12|Cecidomyiidae|658|BOLD:ACC5376
Cecidomyiidae|[7503]|CNSLA101-12|Cecidomyiidae|618|BOLD:ACA4963
Cecidomyiidae|[7504]|CNTNB880-14|Cecidomyiidae|579|BOLD:ACM7367
Cecidomyiidae|[7505]|CNTNB1122-14|Cecidomyiidae|537|BOLD:ACM7366
Cecidomyiidae|[7506]|CNKOC487-14|Cecidomyiidae|658|BOLD:ACM0875
Cecidomyiidae|[7507]|CNGMB1512-13|Cecidomyiidae|603|BOLD:ACL0870
Cecidomyiidae|[7508]|CNLMC352-14|Cecidomyiidae|658|BOLD:ACM2659
Cecidomyiidae|[7509]|CNTNB518-14|Cecidomyiidae|594|BOLD:ACM8162
Cecidomyiidae|[7510]|SSFDC4776-14|Cecidomyiidae|601|BOLD:ACM7163
Cecidomyiidae|[7511]|CNPKF1498-14|Cecidomyiidae|658|BOLD:ACL6447
Cecidomyiidae|[7512]|CNNHA2940-14|Cecidomyiidae|606|BOLD:ACR5017
Cecidomyiidae|[7513]|CNGMC1685-13|Cecidomyiidae|658|BOLD:ACL4429
Cecidomyiidae|[7514]|CNTNB884-14|Cecidomyiidae|534|BOLD:ACM7207
Cecidomyiidae|[7515]|CNNHA959-14|Cecidomyiidae|591|BOLD:ACR5395
Cecidomyiidae|[7516]|CNNHA2949-14|Cecidomyiidae|612|BOLD:ACN1693
Cecidomyiidae|[7517]|CHDIP782-12|Cecidomyiidae|619|BOLD:ABX7922
Cecidomyiidae|[7518]|CHDIP723-12|Cecidomyiidae|658|BOLD:ABX8729
Cecidomyiidae|[7519]|SSJAC046-13|Cecidomyiidae|661|BOLD:ACP6447
Cecidomyiidae|[7520]|BBDCQ763-10|Cecidomyiidae|658|BOLD:AAV5606
Cecidomyiidae|[7521]|SSJAC467-13|Cecidomyiidae|670|BOLD:ACB0051
Cecidomyiidae|[7522]|CNSLM297-13|Cecidomyiidae|661|BOLD:ACA4902
Cecidomyiidae|[7523]|CNBRB370-14|Cecidomyiidae|658|BOLD:ACL3367
Cecidomyiidae|[7524]|CNGMC1032-13|Cecidomyiidae|661|BOLD:ACL0459
Cecidomyiidae|[7525]|CNSLL220-13|Cecidomyiidae|661|BOLD:ACI5179
Cecidomyiidae|[7526]|CNPEC338-14|Cecidomyiidae|661|BOLD:ACC5575
Cecidomyiidae|[7527]|CNEIB788-12|Cecidomyiidae|673|BOLD:ACA8251
Cecidomyiidae|[7528]|CNGMC889-13|Cecidomyiidae|658|BOLD:ACL0919
Cecidomyiidae|[7529]|CNTNC1271-14|Cecidomyiidae|591|BOLD:ACN0089
Cecidomyiidae|[7530]|CNEIB739-12|Cecidomyiidae|673|BOLD:ACA8319
Cecidomyiidae|[7531]|SSEIA7219-13|Cecidomyiidae|658|BOLD:ACD0627
Cecidomyiidae|[7532]|SSBAC537-12|Cecidomyiidae|636|BOLD:ACC5451
Cecidomyiidae|[7533]|CNGMC050-13|Cecidomyiidae|637|BOLD:ACL2509
Cecidomyiidae|[7534]|CNNHA2508-14|Cecidomyiidae|585|BOLD:ACR5527
Cecidomyiidae|[7535]|CNWAC1149-14|Cecidomyiidae|621|BOLD:ACT3700
Cecidomyiidae|[7536]|CNEIC2308-12|Cecidomyiidae|658|BOLD:ACA9729
Cecidomyiidae|[7537]|CNKOH480-14|Cecidomyiidae|658|BOLD:ACM3014
Cecidomyiidae|[7538]|CNLMD688-14|Cecidomyiidae|658|BOLD:ACM4124
Cecidomyiidae|[7539]|CNKOP782-14|Cecidomyiidae|632|BOLD:ACD8513
Cecidomyiidae|[7540]|CHDIP1172-12|Cecidomyiidae|658|BOLD:AAM6079
Cecidomyiidae|[7541]|CNGLB247-13|Cecidomyiidae|658|BOLD:ACE1432
Cecidomyiidae|[7542]|CNRMD843-12|Cecidomyiidae|673|BOLD:ACC1545
Cecidomyiidae|[7543]|CNBPM267-13|Cecidomyiidae|692|BOLD:AAU6606
Cecidomyiidae|[7544]|CNPKJ2637-14|Cecidomyiidae|658|BOLD:ACM0888
Cecidomyiidae|[7545]|JSJUN459-11|Cecidomyiidae|658|BOLD:ABV9313
Cecidomyiidae|[7546]|CNFNE1717-14|Cecidomyiidae|660|BOLD:ACA8631
Cecidomyiidae|[7547]|CNTND669-14|Cecidomyiidae|585|BOLD:ACN5492
Cecidomyiidae|[7548]|CNGMD242-14|Cecidomyiidae|661|BOLD:ACL3010
Cecidomyiidae|[7549]|CNJAC1327-12|Cecidomyiidae|673|BOLD:ACC7447
Cecidomyiidae|[7550]|CNJAC446-12|Cecidomyiidae|658|BOLD:ACC8229
Cecidomyiidae|[7551]|CNJAC784-12|Cecidomyiidae|658|BOLD:ACC8153
Cecidomyiidae|[7552]|CNGMF1046-14|Cecidomyiidae|660|BOLD:ACT7900
Cecidomyiidae|[7553]|CNBPR179-13|Cecidomyiidae|659|BOLD:ACG9426
Cecidomyiidae|[7554]|CNBPS210-13|Cecidomyiidae|658|BOLD:ACI4047
Cecidomyiidae|[7555]|CNBPS054-13|Cecidomyiidae|658|BOLD:ACG8875
Cecidomyiidae|[7556]|CNPAL265-13|Cecidomyiidae|670|BOLD:ACH4200
Cecidomyiidae|[7557]|CNBPS141-13|Cecidomyiidae|658|BOLD:ACI4220
Cecidomyiidae|[7558]|CNBAA150-12|Cecidomyiidae|661|BOLD:ACC2034
Cecidomyiidae|[7559]|CNPKF350-14|Cecidomyiidae|658|BOLD:ACM1338
Cecidomyiidae|[7560]|CNKTB764-14|Cecidomyiidae|588|BOLD:ACZ2876
Cecidomyiidae|[7561]|CNGMD701-14|Cecidomyiidae|661|BOLD:ACL2804
Cecidomyiidae|[7562]|CNBPC055-12|Cecidomyiidae|670|BOLD:ACY4208
Cecidomyiidae|[7563]|CNRMD819-12|Cecidomyiidae|673|BOLD:ACY4709
Cecidomyiidae|[7564]|CNKOC533-14|Cecidomyiidae|658|BOLD:ACY4484
Cecidomyiidae|[7565]|CNFNE2022-14|Cecidomyiidae|664|BOLD:ACY4919
Cecidomyiidae|[7566]|CNGBE148-14|Cecidomyiidae|661|BOLD:ACY4481
Cecidomyiidae|[7567]|SSFDA4447-14|Cecidomyiidae|577|BOLD:ACY4483
Cecidomyiidae|[7568]|CNPER058-14|Cecidomyiidae|661|BOLD:ACY4482
Cecidomyiidae|[7569]|CNTNH027-14|Cecidomyiidae|525|BOLD:ACY4707
Cecidomyiidae|[7570]|CNGMD415-14|Cecidomyiidae|675|BOLD:ACY4537
Cecidomyiidae|[7571]|SSGBB3742-14|Cecidomyiidae|588|BOLD:ACY4708
Cecidomyiidae|[7572]|CNGMH229-14|Cecidomyiidae|664|BOLD:ACY4538
Cecidomyiidae|[7573]|CNKTC847-15|Cecidomyiidae|468|BOLD:ACB9709
Cecidomyiidae|[7574]|CNGLE139-13|Cecidomyiidae|658|BOLD:ACB9187
Cecidomyiidae|[7575]|SSJAF4509-13|Cecidomyiidae|658|BOLD:ACK4078
Cecidomyiidae|[7576]|SSPAA2377-13|Cecidomyiidae|660|BOLD:ACB2395
Cecidomyiidae|[7577]|CNJAD598-12|Cecidomyiidae|658|BOLD:ACC9237
Cecidomyiidae|[7578]|CHDIP739-12|Cecidomyiidae|658|BOLD:ABY0470
Cecidomyiidae|[7579]|CNFDG148-14|Cecidomyiidae|658|BOLD:ACM0132
Cecidomyiidae|[7580]|SSPAB763-13|Cecidomyiidae|661|BOLD:ACG3663
Cecidomyiidae|[7581]|CNFNE417-14|Cecidomyiidae|661|BOLD:ACC7767
Cecidomyiidae|[7582]|CNBAI320-13|Cecidomyiidae|658|BOLD:ACH1754
Cecidomyiidae|[7583]|CNGME485-14|Cecidomyiidae|658|BOLD:ACL3690
Cecidomyiidae|[7584]|CNJAB829-12|Cecidomyiidae|658|BOLD:ACC1097
Cecidomyiidae|[7585]|SSKJC1382-15|Cecidomyiidae|591|BOLD:ACN3850
Cecidomyiidae|[7586]|CHDIP1127-12|Cecidomyiidae|658|BOLD:ACB3048
Cecidomyiidae|[7587]|CNJAC449-12|Cecidomyiidae|658|BOLD:AAH3767
Cecidomyiidae|[7588]|CNGBC523-13|Cecidomyiidae|660|BOLD:ACC7977
Cecidomyiidae|[7589]|CNGMC955-13|Cecidomyiidae|661|BOLD:ACL0437
Cecidomyiidae|[7590]|NCCA061-11|Cecidomyiidae|673|BOLD:ABA6429
Cecidomyiidae|[7591]|SSEIB2288-13|Cecidomyiidae|658|BOLD:ACK4262
Cecidomyiidae|[7592]|CNJAD128-12|Cecidomyiidae|673|BOLD:ACC8086
Cecidomyiidae|[7593]|CNGMH277-14|Cecidomyiidae|664|BOLD:ACM0265
Cecidomyiidae|[7594]|SSEIC746-13|Cecidomyiidae|670|BOLD:ACC8468
Cecidomyiidae|[7595]|CNJAD764-12|Cecidomyiidae|658|BOLD:ACC9301
Cecidomyiidae|[7596]|CNKLC3748-14|Cecidomyiidae|588|BOLD:ACS2150
Cecidomyiidae|[7597]|CNJAD1669-12|Cecidomyiidae|658|BOLD:ACB2713
Cecidomyiidae|[7598]|SSJAF2283-13|Cecidomyiidae|658|BOLD:ACK4102
Cecidomyiidae|[7599]|CNBAA231-12|Cecidomyiidae|658|BOLD:ACC2383

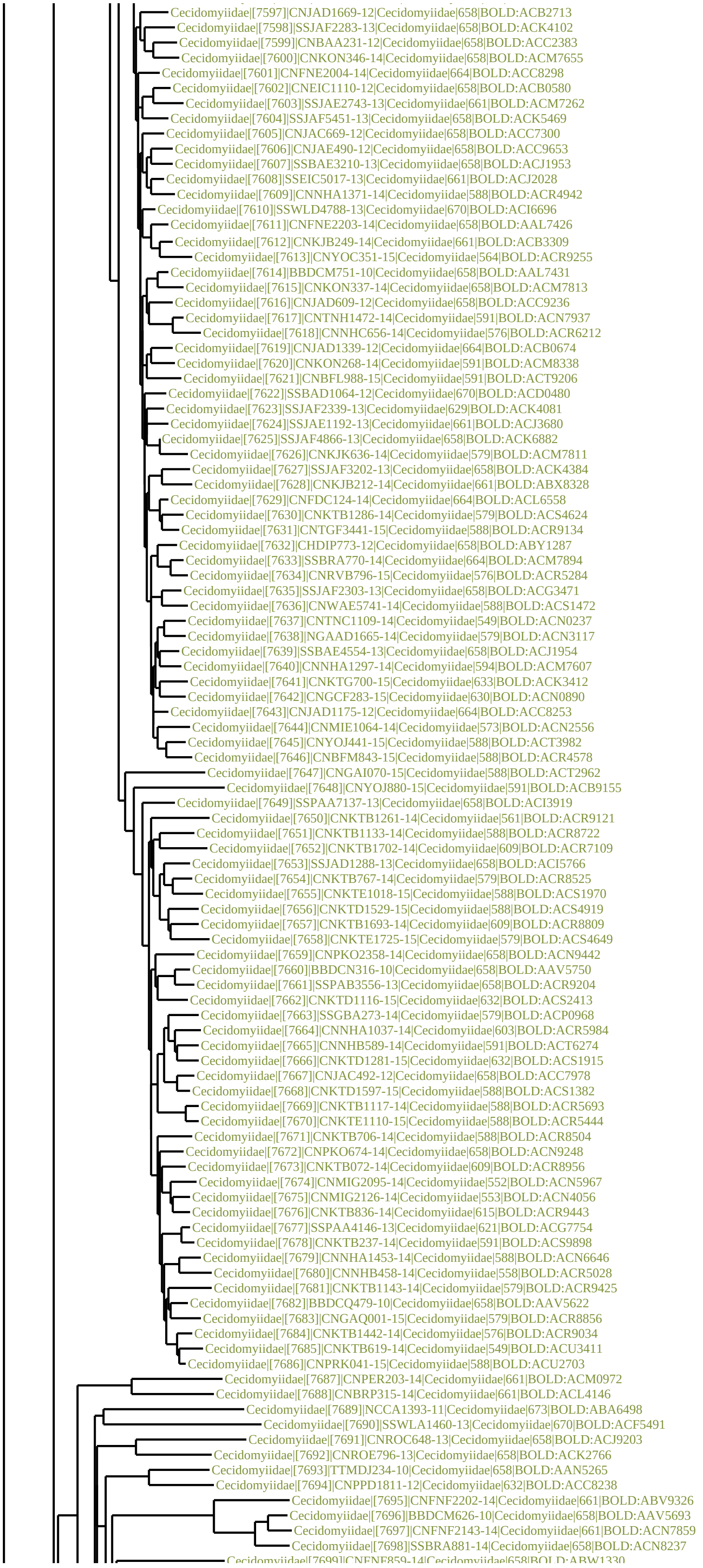
Cecidomyiidae|[7597]|CNJAD1669-12|Cecidomyiidae|658|BOLD:ACB2713
Cecidomyiidae|[7598]|SSJAF2283-13|Cecidomyiidae|658|BOLD:ACK4102
Cecidomyiidae|[7599]|CNBAA231-12|Cecidomyiidae|658|BOLD:ACC2383
Cecidomyiidae|[7600]|CNKON346-14|Cecidomyiidae|658|BOLD:ACM7655
Cecidomyiidae|[7601]|CNFNE2004-14|Cecidomyiidae|664|BOLD:ACC8298
Cecidomyiidae|[7602]|CNEIC1110-12|Cecidomyiidae|658|BOLD:ACB0580
Cecidomyiidae|[7603]|SSJAE2743-13|Cecidomyiidae|661|BOLD:ACM7262
Cecidomyiidae|[7604]|SSJAF5451-13|Cecidomyiidae|658|BOLD:ACK5469
Cecidomyiidae|[7605]|CNJAC669-12|Cecidomyiidae|658|BOLD:ACC7300
Cecidomyiidae|[7606]|CNJAE490-12|Cecidomyiidae|658|BOLD:ACC9653
Cecidomyiidae|[7607]|SSBAE3210-13|Cecidomyiidae|658|BOLD:ACJ1953
Cecidomyiidae|[7608]|SSEIC5017-13|Cecidomyiidae|661|BOLD:ACJ2028
Cecidomyiidae|[7609]|CNNHA1371-14|Cecidomyiidae|588|BOLD:ACR4942
Cecidomyiidae|[7610]|SSWLD4788-13|Cecidomyiidae|670|BOLD:ACI6696
Cecidomyiidae|[7611]|CNFNE2203-14|Cecidomyiidae|658|BOLD:AAL7426
Cecidomyiidae|[7612]|CNKJB249-14|Cecidomyiidae|661|BOLD:ACB3309
Cecidomyiidae|[7613]|CNYOC351-15|Cecidomyiidae|564|BOLD:ACR9255
Cecidomyiidae|[7614]|BBDCM751-10|Cecidomyiidae|658|BOLD:AAL7431
Cecidomyiidae|[7615]|CNKON337-14|Cecidomyiidae|658|BOLD:ACM7813
Cecidomyiidae|[7616]|CNJAD609-12|Cecidomyiidae|658|BOLD:ACC9236
Cecidomyiidae|[7617]|CNTNH1472-14|Cecidomyiidae|591|BOLD:ACN7937
Cecidomyiidae|[7618]|CNNHC656-14|Cecidomyiidae|576|BOLD:ACR6212
Cecidomyiidae|[7619]|CNJAD1339-12|Cecidomyiidae|664|BOLD:ACB0674
Cecidomyiidae|[7620]|CNKON268-14|Cecidomyiidae|591|BOLD:ACM8338
Cecidomyiidae|[7621]|CNBFL988-15|Cecidomyiidae|591|BOLD:ACT9206
Cecidomyiidae|[7622]|SSBAD1064-12|Cecidomyiidae|670|BOLD:ACD0480
Cecidomyiidae|[7623]|SSJAF2339-13|Cecidomyiidae|629|BOLD:ACK4081
Cecidomyiidae|[7624]|SSJAE1192-13|Cecidomyiidae|661|BOLD:ACJ3680
Cecidomyiidae|[7625]|SSJAF4866-13|Cecidomyiidae|658|BOLD:ACK6882
Cecidomyiidae|[7626]|CNKJK636-14|Cecidomyiidae|579|BOLD:ACM7811
Cecidomyiidae|[7627]|SSJAF3202-13|Cecidomyiidae|658|BOLD:ACK4384
Cecidomyiidae|[7628]|CNKJB212-14|Cecidomyiidae|661|BOLD:ABX8328
Cecidomyiidae|[7629]|CNFDC124-14|Cecidomyiidae|664|BOLD:ACL6558
Cecidomyiidae|[7630]|CNKTB1286-14|Cecidomyiidae|579|BOLD:ACS4624
Cecidomyiidae|[7631]|CNTGF3441-15|Cecidomyiidae|588|BOLD:ACR9134
Cecidomyiidae|[7632]|CHDIP773-12|Cecidomyiidae|658|BOLD:ABY1287
Cecidomyiidae|[7633]|SSBRA770-14|Cecidomyiidae|664|BOLD:ACM7894
Cecidomyiidae|[7634]|CNRVB796-15|Cecidomyiidae|576|BOLD:ACR5284
Cecidomyiidae|[7635]|SSJAF2303-13|Cecidomyiidae|658|BOLD:ACG3471
Cecidomyiidae|[7636]|CNWAE5741-14|Cecidomyiidae|588|BOLD:ACS1472
Cecidomyiidae|[7637]|CNTNC1109-14|Cecidomyiidae|549|BOLD:ACN0237
Cecidomyiidae|[7638]|NGAAD1665-14|Cecidomyiidae|579|BOLD:ACN3117
Cecidomyiidae|[7639]|SSBAE4554-13|Cecidomyiidae|658|BOLD:ACJ1954
Cecidomyiidae|[7640]|CNNHA1297-14|Cecidomyiidae|594|BOLD:ACM7607
Cecidomyiidae|[7641]|CNKTG700-15|Cecidomyiidae|633|BOLD:ACK3412
Cecidomyiidae|[7642]|CNGCF283-15|Cecidomyiidae|630|BOLD:ACN0890
Cecidomyiidae|[7643]|CNJAD1175-12|Cecidomyiidae|664|BOLD:ACC8253
Cecidomyiidae|[7644]|CNMIE1064-14|Cecidomyiidae|573|BOLD:ACN2556
Cecidomyiidae|[7645]|CNYOJ441-15|Cecidomyiidae|588|BOLD:ACT3982
Cecidomyiidae|[7646]|CNBFM843-15|Cecidomyiidae|588|BOLD:ACR4578
Cecidomyiidae|[7647]|CNGAI070-15|Cecidomyiidae|588|BOLD:ACT2962
Cecidomyiidae|[7648]|CNYOJ880-15|Cecidomyiidae|591|BOLD:ACB9155
Cecidomyiidae|[7649]|SSPAA7137-13|Cecidomyiidae|658|BOLD:ACI3919
Cecidomyiidae|[7650]|CNKTB1261-14|Cecidomyiidae|561|BOLD:ACR9121
Cecidomyiidae|[7651]|CNKTB1133-14|Cecidomyiidae|588|BOLD:ACR8722
Cecidomyiidae|[7652]|CNKTB1702-14|Cecidomyiidae|609|BOLD:ACR7109
Cecidomyiidae|[7653]|SSJAD1288-13|Cecidomyiidae|658|BOLD:ACI5766
Cecidomyiidae|[7654]|CNKTB767-14|Cecidomyiidae|579|BOLD:ACR8525
Cecidomyiidae|[7655]|CNKTE1018-15|Cecidomyiidae|588|BOLD:ACS1970
Cecidomyiidae|[7656]|CNKTD1529-15|Cecidomyiidae|588|BOLD:ACS4919
Cecidomyiidae|[7657]|CNKTB1693-14|Cecidomyiidae|609|BOLD:ACR8809
Cecidomyiidae|[7658]|CNKTE1725-15|Cecidomyiidae|579|BOLD:ACS4649
Cecidomyiidae|[7659]|CNPKO2358-14|Cecidomyiidae|658|BOLD:ACN9442
Cecidomyiidae|[7660]|BBDCN316-10|Cecidomyiidae|658|BOLD:AAV5750
Cecidomyiidae|[7661]|SSPAB3556-13|Cecidomyiidae|658|BOLD:ACR9204
Cecidomyiidae|[7662]|CNKTD1116-15|Cecidomyiidae|632|BOLD:ACS2413
Cecidomyiidae|[7663]|SSGBA273-14|Cecidomyiidae|579|BOLD:ACP0968
Cecidomyiidae|[7664]|CNNHA1037-14|Cecidomyiidae|603|BOLD:ACR5984
Cecidomyiidae|[7665]|CNNHB589-14|Cecidomyiidae|591|BOLD:ACT6274
Cecidomyiidae|[7666]|CNKTD1281-15|Cecidomyiidae|632|BOLD:ACS1915
Cecidomyiidae|[7667]|CNJAC492-12|Cecidomyiidae|658|BOLD:ACC7978
Cecidomyiidae|[7668]|CNKTD1597-15|Cecidomyiidae|588|BOLD:ACS1382
Cecidomyiidae|[7669]|CNKTB1117-14|Cecidomyiidae|588|BOLD:ACR5693
Cecidomyiidae|[7670]|CNKTE1110-15|Cecidomyiidae|588|BOLD:ACR5444
Cecidomyiidae|[7671]|CNKTB706-14|Cecidomyiidae|588|BOLD:ACR8504
Cecidomyiidae|[7672]|CNPKO674-14|Cecidomyiidae|658|BOLD:ACN9248
Cecidomyiidae|[7673]|CNKTB072-14|Cecidomyiidae|609|BOLD:ACR8956
Cecidomyiidae|[7674]|CNMIG2095-14|Cecidomyiidae|552|BOLD:ACN5967
Cecidomyiidae|[7675]|CNMIG2126-14|Cecidomyiidae|553|BOLD:ACN4056
Cecidomyiidae|[7676]|CNKTB836-14|Cecidomyiidae|615|BOLD:ACR9443
Cecidomyiidae|[7677]|SSPAA4146-13|Cecidomyiidae|621|BOLD:ACG7754
Cecidomyiidae|[7678]|CNKTB237-14|Cecidomyiidae|591|BOLD:ACS9898
Cecidomyiidae|[7679]|CNNHA1453-14|Cecidomyiidae|588|BOLD:ACN6646
Cecidomyiidae|[7680]|CNNHB458-14|Cecidomyiidae|558|BOLD:ACR5028
Cecidomyiidae|[7681]|CNKTB1143-14|Cecidomyiidae|579|BOLD:ACR9425
Cecidomyiidae|[7682]|BBDCQ479-10|Cecidomyiidae|658|BOLD:AAV5622
Cecidomyiidae|[7683]|CNGAQ001-15|Cecidomyiidae|579|BOLD:ACR8856
Cecidomyiidae|[7684]|CNKTB1442-14|Cecidomyiidae|576|BOLD:ACR9034
Cecidomyiidae|[7685]|CNKTB619-14|Cecidomyiidae|549|BOLD:ACU3411
Cecidomyiidae|[7686]|CNPRK041-15|Cecidomyiidae|588|BOLD:ACU2703
Cecidomyiidae|[7687]|CNPER203-14|Cecidomyiidae|661|BOLD:ACM0972
Cecidomyiidae|[7688]|CNBRP315-14|Cecidomyiidae|661|BOLD:ACL4146
Cecidomyiidae|[7689]|NCCA1393-11|Cecidomyiidae|673|BOLD:ABA6498
Cecidomyiidae|[7690]|SSWLA1460-13|Cecidomyiidae|670|BOLD:ACF5491
Cecidomyiidae|[7691]|CNROC648-13|Cecidomyiidae|658|BOLD:ACJ9203
Cecidomyiidae|[7692]|CNROE796-13|Cecidomyiidae|658|BOLD:ACK2766
Cecidomyiidae|[7693]|TTMDJ234-10|Cecidomyiidae|658|BOLD:AAN5265
Cecidomyiidae|[7694]|CNPPD1811-12|Cecidomyiidae|632|BOLD:ACC8238
Cecidomyiidae|[7695]|CNFNF2202-14|Cecidomyiidae|661|BOLD:ABV9326
Cecidomyiidae|[7696]|BBDCM626-10|Cecidomyiidae|658|BOLD:AAV5693
Cecidomyiidae|[7697]|CNFNF2143-14|Cecidomyiidae|661|BOLD:ACN7859
Cecidomyiidae|[7698]|SSBRA881-14|Cecidomyiidae|658|BOLD:ACN8237
Cecidomyiidae|[7699]|CNFNF859-14|Cecidomyiidae|658|BOLD:ABW1330

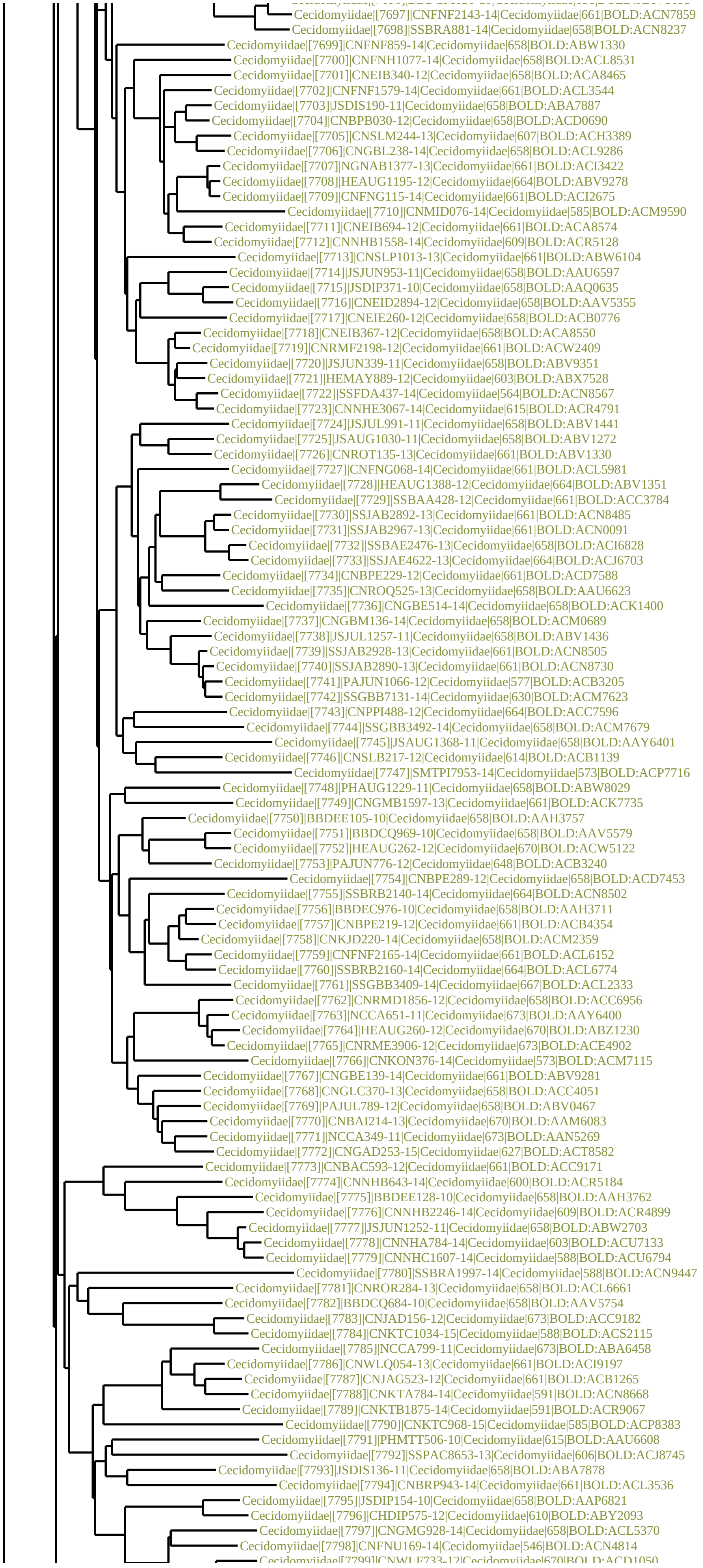

Cecidomyiidae|[7697]|CNFNF2143-14|Cecidomyiidae|661|BOLD:ACN7859
Cecidomyiidae|[7698]|SSBRA881-14|Cecidomyiidae|658|BOLD:ACN8237
Cecidomyiidae|[7699]|CNFNF859-14|Cecidomyiidae|658|BOLD:ABW1330
Cecidomyiidae|[7700]|CNFNH1077-14|Cecidomyiidae|658|BOLD:ACL8531
Cecidomyiidae|[7701]|CNEIB340-12|Cecidomyiidae|658|BOLD:ACA8465
Cecidomyiidae|[7702]|CNFNF1579-14|Cecidomyiidae|661|BOLD:ACL3544
Cecidomyiidae|[7703]|JSDIS190-11|Cecidomyiidae|658|BOLD:ABA7887
Cecidomyiidae|[7704]|CNBPB030-12|Cecidomyiidae|658|BOLD:ACD0690
Cecidomyiidae|[7705]|CNSLM244-13|Cecidomyiidae|607|BOLD:ACH3389
Cecidomyiidae|[7706]|CNGBL238-14|Cecidomyiidae|658|BOLD:ACL9286
Cecidomyiidae|[7707]|NGNAB1377-13|Cecidomyiidae|661|BOLD:ACI3422
Cecidomyiidae|[7708]|HEAUG1195-12|Cecidomyiidae|664|BOLD:ABV9278
Cecidomyiidae|[7709]|CNFNG115-14|Cecidomyiidae|661|BOLD:ACI2675
Cecidomyiidae|[7710]|CNMID076-14|Cecidomyiidae|585|BOLD:ACM9590
Cecidomyiidae|[7711]|CNEIB694-12|Cecidomyiidae|661|BOLD:ACA8574
Cecidomyiidae|[7712]|CNNHB1558-14|Cecidomyiidae|609|BOLD:ACR5128
Cecidomyiidae|[7713]|CNSLP1013-13|Cecidomyiidae|661|BOLD:ABW6104
Cecidomyiidae|[7714]|JSJUN953-11|Cecidomyiidae|658|BOLD:AAU6597
Cecidomyiidae|[7715]|JSDIP371-10|Cecidomyiidae|658|BOLD:AAQ0635
Cecidomyiidae|[7716]|CNEID2894-12|Cecidomyiidae|658|BOLD:AAV5355
Cecidomyiidae|[7717]|CNEIE260-12|Cecidomyiidae|658|BOLD:ACB0776
Cecidomyiidae|[7718]|CNEIB367-12|Cecidomyiidae|658|BOLD:ACA8550
Cecidomyiidae|[7719]|CNRMF2198-12|Cecidomyiidae|661|BOLD:ACW2409
Cecidomyiidae|[7720]|JSJUN339-11|Cecidomyiidae|658|BOLD:ABV9351
Cecidomyiidae|[7721]|HEMAY889-12|Cecidomyiidae|603|BOLD:ABX7528
Cecidomyiidae|[7722]|SSFDA437-14|Cecidomyiidae|564|BOLD:ACN8567
Cecidomyiidae|[7723]|CNNHE3067-14|Cecidomyiidae|615|BOLD:ACR4791
Cecidomyiidae|[7724]|JSJUL991-11|Cecidomyiidae|658|BOLD:ABV1441
Cecidomyiidae|[7725]|JSAUG1030-11|Cecidomyiidae|658|BOLD:ABV1272
Cecidomyiidae|[7726]|CNROT135-13|Cecidomyiidae|661|BOLD:ABV1330
Cecidomyiidae|[7727]|CNFNG068-14|Cecidomyiidae|661|BOLD:ACL5981
Cecidomyiidae|[7728]|HEAUG1388-12|Cecidomyiidae|664|BOLD:ABV1351
Cecidomyiidae|[7729]|SSBAA428-12|Cecidomyiidae|661|BOLD:ACC3784
Cecidomyiidae|[7730]|SSJAB2892-13|Cecidomyiidae|661|BOLD:ACN8485
Cecidomyiidae|[7731]|SSJAB2967-13|Cecidomyiidae|661|BOLD:ACN0091
Cecidomyiidae|[7732]|SSBAE2476-13|Cecidomyiidae|658|BOLD:ACI6828
Cecidomyiidae|[7733]|SSJAE4622-13|Cecidomyiidae|664|BOLD:ACJ6703
Cecidomyiidae|[7734]|CNBPE229-12|Cecidomyiidae|661|BOLD:ACD7588
Cecidomyiidae|[7735]|CNROQ525-13|Cecidomyiidae|658|BOLD:AAU6623
Cecidomyiidae|[7736]|CNGBE514-14|Cecidomyiidae|658|BOLD:ACK1400
Cecidomyiidae|[7737]|CNGBM136-14|Cecidomyiidae|658|BOLD:ACM0689
Cecidomyiidae|[7738]|JSJUL1257-11|Cecidomyiidae|658|BOLD:ABV1436
Cecidomyiidae|[7739]|SSJAB2928-13|Cecidomyiidae|661|BOLD:ACN8505
Cecidomyiidae|[7740]|SSJAB2890-13|Cecidomyiidae|661|BOLD:ACN8730
Cecidomyiidae|[7741]|PAJUN1066-12|Cecidomyiidae|577|BOLD:ACB3205
Cecidomyiidae|[7742]|SSGBB7131-14|Cecidomyiidae|630|BOLD:ACM7623
Cecidomyiidae|[7743]|CNPPI488-12|Cecidomyiidae|664|BOLD:ACC7596
Cecidomyiidae|[7744]|SSGBB3492-14|Cecidomyiidae|658|BOLD:ACM7679
Cecidomyiidae|[7745]|JSAUG1368-11|Cecidomyiidae|658|BOLD:AAY6401
Cecidomyiidae|[7746]|CNSLB217-12|Cecidomyiidae|614|BOLD:ACB1139
Cecidomyiidae|[7747]|SMTPI7953-14|Cecidomyiidae|573|BOLD:ACP7716
Cecidomyiidae|[7748]|PHAUG1229-11|Cecidomyiidae|658|BOLD:ABW8029
Cecidomyiidae|[7749]|CNGMB1597-13|Cecidomyiidae|661|BOLD:ACK7735
Cecidomyiidae|[7750]|BBDEE105-10|Cecidomyiidae|658|BOLD:AAH3757
Cecidomyiidae|[7751]|BBDCQ969-10|Cecidomyiidae|658|BOLD:AAV5579
Cecidomyiidae|[7752]|HEAUG262-12|Cecidomyiidae|670|BOLD:ACW5122
Cecidomyiidae|[7753]|PAJUN776-12|Cecidomyiidae|648|BOLD:ACB3240
Cecidomyiidae|[7754]|CNBPE289-12|Cecidomyiidae|658|BOLD:ACD7453
Cecidomyiidae|[7755]|SSBRB2140-14|Cecidomyiidae|664|BOLD:ACN8502
Cecidomyiidae|[7756]|BBDEC976-10|Cecidomyiidae|658|BOLD:AAH3711
Cecidomyiidae|[7757]|CNBPE219-12|Cecidomyiidae|661|BOLD:ACB4354
Cecidomyiidae|[7758]|CNKJD220-14|Cecidomyiidae|658|BOLD:ACM2359
Cecidomyiidae|[7759]|CNFNF2165-14|Cecidomyiidae|661|BOLD:ACL6152
Cecidomyiidae|[7760]|SSBRB2160-14|Cecidomyiidae|664|BOLD:ACL6774
Cecidomyiidae|[7761]|SSGBB3409-14|Cecidomyiidae|667|BOLD:ACL2333
Cecidomyiidae|[7762]|CNRMD1856-12|Cecidomyiidae|658|BOLD:ACC6956
Cecidomyiidae|[7763]|NCCA651-11|Cecidomyiidae|673|BOLD:AAY6400
Cecidomyiidae|[7764]|HEAUG260-12|Cecidomyiidae|670|BOLD:ABZ1230
Cecidomyiidae|[7765]|CNRME3906-12|Cecidomyiidae|673|BOLD:ACE4902
Cecidomyiidae|[7766]|CNKON376-14|Cecidomyiidae|573|BOLD:ACM7115
Cecidomyiidae|[7767]|CNGBE139-14|Cecidomyiidae|661|BOLD:ABV9281
Cecidomyiidae|[7768]|CNGLC370-13|Cecidomyiidae|658|BOLD:ACC4051
Cecidomyiidae|[7769]|PAJUL789-12|Cecidomyiidae|658|BOLD:ABV0467
Cecidomyiidae|[7770]|CNBAI214-13|Cecidomyiidae|670|BOLD:AAM6083
Cecidomyiidae|[7771]|NCCA349-11|Cecidomyiidae|673|BOLD:AAN5269
Cecidomyiidae|[7772]|CNGAD253-15|Cecidomyiidae|627|BOLD:ACT8582
Cecidomyiidae|[7773]|CNBAC593-12|Cecidomyiidae|661|BOLD:ACC9171
Cecidomyiidae|[7774]|CNNHB643-14|Cecidomyiidae|600|BOLD:ACR5184
Cecidomyiidae|[7775]|BBDEE128-10|Cecidomyiidae|658|BOLD:AAH3762
Cecidomyiidae|[7776]|CNNHB2246-14|Cecidomyiidae|609|BOLD:ACR4899
Cecidomyiidae|[7777]|JSJUN1252-11|Cecidomyiidae|658|BOLD:ABW2703
Cecidomyiidae|[7778]|CNNHA784-14|Cecidomyiidae|603|BOLD:ACU7133
Cecidomyiidae|[7779]|CNNHC1607-14|Cecidomyiidae|588|BOLD:ACU6794
Cecidomyiidae|[7780]|SSBRA1997-14|Cecidomyiidae|588|BOLD:ACN9447
Cecidomyiidae|[7781]|CNROR284-13|Cecidomyiidae|658|BOLD:ACL6661
Cecidomyiidae|[7782]|BBDCQ684-10|Cecidomyiidae|658|BOLD:AAV5754
Cecidomyiidae|[7783]|CNJAD156-12|Cecidomyiidae|673|BOLD:ACC9182
Cecidomyiidae|[7784]|CNKTC1034-15|Cecidomyiidae|588|BOLD:ACS2115
Cecidomyiidae|[7785]|NCCA799-11|Cecidomyiidae|673|BOLD:ABA6458
Cecidomyiidae|[7786]|CNWLQ054-13|Cecidomyiidae|661|BOLD:ACI9197
Cecidomyiidae|[7787]|CNJAG523-12|Cecidomyiidae|661|BOLD:ACB1265
Cecidomyiidae|[7788]|CNKTA784-14|Cecidomyiidae|591|BOLD:ACN8668
Cecidomyiidae|[7789]|CNKTB1875-14|Cecidomyiidae|591|BOLD:ACR9067
Cecidomyiidae|[7790]|CNKTC968-15|Cecidomyiidae|585|BOLD:ACP8383
Cecidomyiidae|[7791]|PHMTT506-10|Cecidomyiidae|615|BOLD:AAU6608
Cecidomyiidae|[7792]|SSPAC8653-13|Cecidomyiidae|606|BOLD:ACJ8745
Cecidomyiidae|[7793]|JSDIS136-11|Cecidomyiidae|658|BOLD:ABA7878
Cecidomyiidae|[7794]|CNBRP943-14|Cecidomyiidae|661|BOLD:ACL3536
Cecidomyiidae|[7795]|JSDIP154-10|Cecidomyiidae|658|BOLD:AAP6821
Cecidomyiidae|[7796]|CHDIP575-12|Cecidomyiidae|610|BOLD:ABY2093
Cecidomyiidae|[7797]|CNGMG928-14|Cecidomyiidae|658|BOLD:ACL5370
Cecidomyiidae|[7798]|CNFNU169-14|Cecidomyiidae|546|BOLD:ACN4814
Cecidomyiidae|[7799]|CNWLF733-12|Cecidomyiidae|670|BOLD:ACD1050

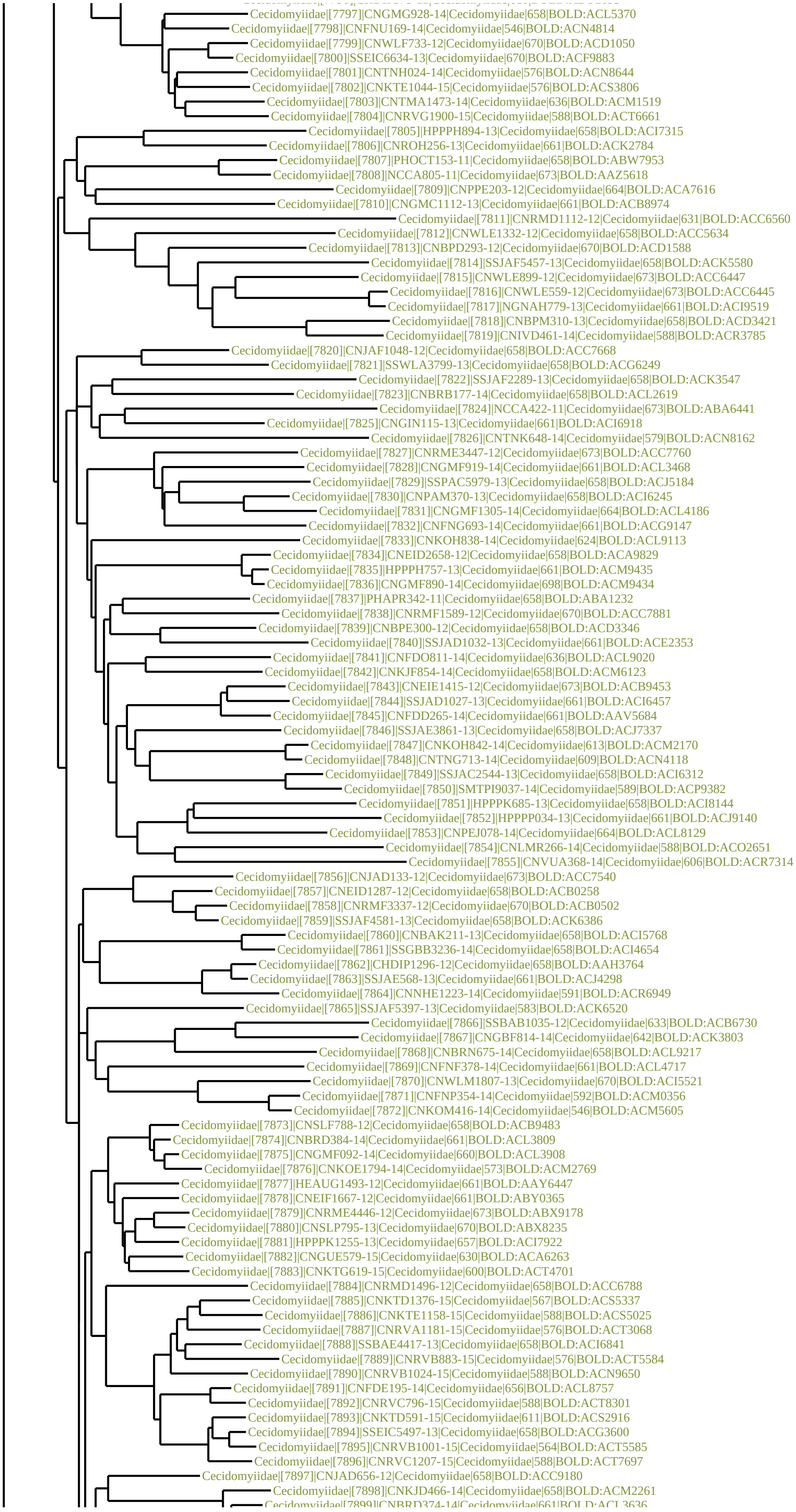
Cecidomyiidae|[7797]|CNGMG928-14|Cecidomyiidae|658|BOLD:ACL5370
Cecidomyiidae|[7798]|CNFNU169-14|Cecidomyiidae|546|BOLD:ACN4814
Cecidomyiidae|[7799]|CNWLF733-12|Cecidomyiidae|670|BOLD:ACD1050
Cecidomyiidae|[7800]|SSEIC6634-13|Cecidomyiidae|670|BOLD:ACF9883
Cecidomyiidae|[7801]|CNTNH024-14|Cecidomyiidae|576|BOLD:ACN8644
Cecidomyiidae|[7802]|CNKTE1044-15|Cecidomyiidae|576|BOLD:ACS3806
Cecidomyiidae|[7803]|CNTMA1473-14|Cecidomyiidae|636|BOLD:ACM1519
Cecidomyiidae|[7804]|CNRVG1900-15|Cecidomyiidae|588|BOLD:ACT6661
Cecidomyiidae|[7805]|HPPPH894-13|Cecidomyiidae|658|BOLD:ACI7315
Cecidomyiidae|[7806]|CNROH256-13|Cecidomyiidae|661|BOLD:ACK2784
Cecidomyiidae|[7807]|PHOCT153-11|Cecidomyiidae|658|BOLD:ABW7953
Cecidomyiidae|[7808]|NCCA805-11|Cecidomyiidae|673|BOLD:AAZ5618
Cecidomyiidae|[7809]|CNPPE203-12|Cecidomyiidae|664|BOLD:ACA7616
Cecidomyiidae|[7810]|CNGMC1112-13|Cecidomyiidae|661|BOLD:ACB8974
Cecidomyiidae|[7811]|CNRMD1112-12|Cecidomyiidae|631|BOLD:ACC6560
Cecidomyiidae|[7812]|CNWLE1332-12|Cecidomyiidae|658|BOLD:ACC5634
Cecidomyiidae|[7813]|CNBPD293-12|Cecidomyiidae|670|BOLD:ACD1588
Cecidomyiidae|[7814]|SSJAF5457-13|Cecidomyiidae|658|BOLD:ACK5580
Cecidomyiidae|[7815]|CNWLE899-12|Cecidomyiidae|673|BOLD:ACC6447
Cecidomyiidae|[7816]|CNWLE559-12|Cecidomyiidae|673|BOLD:ACC6445
Cecidomyiidae|[7817]|NGNAH779-13|Cecidomyiidae|661|BOLD:ACI9519
Cecidomyiidae|[7818]|CNBPM310-13|Cecidomyiidae|658|BOLD:ACD3421
Cecidomyiidae|[7819]|CNIVD461-14|Cecidomyiidae|588|BOLD:ACR3785
Cecidomyiidae|[7820]|CNJAF1048-12|Cecidomyiidae|658|BOLD:ACC7668
Cecidomyiidae|[7821]|SSWLA3799-13|Cecidomyiidae|658|BOLD:ACG6249
Cecidomyiidae|[7822]|SSJAF2289-13|Cecidomyiidae|658|BOLD:ACK3547
Cecidomyiidae|[7823]|CNBRB177-14|Cecidomyiidae|658|BOLD:ACL2619
Cecidomyiidae|[7824]|NCCA422-11|Cecidomyiidae|673|BOLD:ABA6441
Cecidomyiidae|[7825]|CNGIN115-13|Cecidomyiidae|661|BOLD:ACI6918
Cecidomyiidae|[7826]|CNTNK648-14|Cecidomyiidae|579|BOLD:ACN8162
Cecidomyiidae|[7827]|CNRME3447-12|Cecidomyiidae|673|BOLD:ACC7760
Cecidomyiidae|[7828]|CNGMF919-14|Cecidomyiidae|661|BOLD:ACL3468
Cecidomyiidae|[7829]|SSPAC5979-13|Cecidomyiidae|658|BOLD:ACJ5184
Cecidomyiidae|[7830]|CNPAM370-13|Cecidomyiidae|658|BOLD:ACI6245
Cecidomyiidae|[7831]|CNGMF1305-14|Cecidomyiidae|664|BOLD:ACL4186
Cecidomyiidae|[7832]|CNFNG693-14|Cecidomyiidae|661|BOLD:ACG9147
Cecidomyiidae|[7833]|CNKOH838-14|Cecidomyiidae|624|BOLD:ACL9113
Cecidomyiidae|[7834]|CNEID2658-12|Cecidomyiidae|658|BOLD:ACA9829
Cecidomyiidae|[7835]|HPPPH757-13|Cecidomyiidae|661|BOLD:ACM9435
Cecidomyiidae|[7836]|CNGMF890-14|Cecidomyiidae|698|BOLD:ACM9434
Cecidomyiidae|[7837]|PHAPR342-11|Cecidomyiidae|658|BOLD:ABA1232
Cecidomyiidae|[7838]|CNRMF1589-12|Cecidomyiidae|670|BOLD:ACC7881
Cecidomyiidae|[7839]|CNBPE300-12|Cecidomyiidae|658|BOLD:ACD3346
Cecidomyiidae|[7840]|SSJAD1032-13|Cecidomyiidae|661|BOLD:ACE2353
Cecidomyiidae|[7841]|CNFDO811-14|Cecidomyiidae|636|BOLD:ACL9020
Cecidomyiidae|[7842]|CNKJF854-14|Cecidomyiidae|658|BOLD:ACM6123
Cecidomyiidae|[7843]|CNEIE1415-12|Cecidomyiidae|673|BOLD:ACB9453
Cecidomyiidae|[7844]|SSJAD1027-13|Cecidomyiidae|661|BOLD:ACI6457
Cecidomyiidae|[7845]|CNFDD265-14|Cecidomyiidae|661|BOLD:AAV5684
Cecidomyiidae|[7846]|SSJAE3861-13|Cecidomyiidae|658|BOLD:ACJ7337
Cecidomyiidae|[7847]|CNKOH842-14|Cecidomyiidae|613|BOLD:ACM2170
Cecidomyiidae|[7848]|CNTNG713-14|Cecidomyiidae|609|BOLD:ACN4118
Cecidomyiidae|[7849]|SSJAC2544-13|Cecidomyiidae|658|BOLD:ACI6312
Cecidomyiidae|[7850]|SMTPI9037-14|Cecidomyiidae|589|BOLD:ACP9382
Cecidomyiidae|[7851]|HPPPK685-13|Cecidomyiidae|658|BOLD:ACI8144
Cecidomyiidae|[7852]|HPPPP034-13|Cecidomyiidae|661|BOLD:ACJ9140
Cecidomyiidae|[7853]|CNPEJ078-14|Cecidomyiidae|664|BOLD:ACL8129
Cecidomyiidae|[7854]|CNLMR266-14|Cecidomyiidae|588|BOLD:ACO2651
Cecidomyiidae|[7855]|CNVUA368-14|Cecidomyiidae|606|BOLD:ACR7314
Cecidomyiidae|[7856]|CNJAD133-12|Cecidomyiidae|673|BOLD:ACC7540
Cecidomyiidae|[7857]|CNEID1287-12|Cecidomyiidae|658|BOLD:ACB0258
Cecidomyiidae|[7858]|CNRMF3337-12|Cecidomyiidae|670|BOLD:ACB0502
Cecidomyiidae|[7859]|SSJAF4581-13|Cecidomyiidae|658|BOLD:ACK6386
Cecidomyiidae|[7860]|CNBAK211-13|Cecidomyiidae|658|BOLD:ACI5768
Cecidomyiidae|[7861]|SSGBB3236-14|Cecidomyiidae|658|BOLD:ACI4654
Cecidomyiidae|[7862]|CHDIP1296-12|Cecidomyiidae|658|BOLD:AAH3764
Cecidomyiidae|[7863]|SSJAE568-13|Cecidomyiidae|661|BOLD:ACJ4298
Cecidomyiidae|[7864]|CNNHE1223-14|Cecidomyiidae|591|BOLD:ACR6949
Cecidomyiidae|[7865]|SSJAF5397-13|Cecidomyiidae|583|BOLD:ACK6520
Cecidomyiidae|[7866]|SSBAB1035-12|Cecidomyiidae|633|BOLD:ACB6730
Cecidomyiidae|[7867]|CNGBF814-14|Cecidomyiidae|642|BOLD:ACK3803
Cecidomyiidae|[7868]|CNBRN675-14|Cecidomyiidae|658|BOLD:ACL9217
Cecidomyiidae|[7869]|CNFNF378-14|Cecidomyiidae|661|BOLD:ACL4717
Cecidomyiidae|[7870]|CNWLM1807-13|Cecidomyiidae|670|BOLD:ACI5521
Cecidomyiidae|[7871]|CNFNP354-14|Cecidomyiidae|592|BOLD:ACM0356
Cecidomyiidae|[7872]|CNKOM416-14|Cecidomyiidae|546|BOLD:ACM5605
Cecidomyiidae|[7873]|CNSLF788-12|Cecidomyiidae|658|BOLD:ACB9483
Cecidomyiidae|[7874]|CNBRD384-14|Cecidomyiidae|661|BOLD:ACL3809
Cecidomyiidae|[7875]|CNGMF092-14|Cecidomyiidae|660|BOLD:ACL3908
Cecidomyiidae|[7876]|CNKOE1794-14|Cecidomyiidae|573|BOLD:ACM2769
Cecidomyiidae|[7877]|HEAUG1493-12|Cecidomyiidae|661|BOLD:AAY6447
Cecidomyiidae|[7878]|CNEIF1667-12|Cecidomyiidae|661|BOLD:ABY0365
Cecidomyiidae|[7879]|CNRME4446-12|Cecidomyiidae|673|BOLD:ABX9178
Cecidomyiidae|[7880]|CNSLP795-13|Cecidomyiidae|670|BOLD:ABX8235
Cecidomyiidae|[7881]|HPPPK1255-13|Cecidomyiidae|657|BOLD:ACI7922
Cecidomyiidae|[7882]|CNGUE579-15|Cecidomyiidae|630|BOLD:ACA6263
Cecidomyiidae|[7883]|CNKTG619-15|Cecidomyiidae|600|BOLD:ACT4701
Cecidomyiidae|[7884]|CNRMD1496-12|Cecidomyiidae|658|BOLD:ACC6788
Cecidomyiidae|[7885]|CNKTD1376-15|Cecidomyiidae|567|BOLD:ACS5337
Cecidomyiidae|[7886]|CNKTE1158-15|Cecidomyiidae|588|BOLD:ACS5025
Cecidomyiidae|[7887]|CNRVA1181-15|Cecidomyiidae|576|BOLD:ACT3068
Cecidomyiidae|[7888]|SSBAE4417-13|Cecidomyiidae|658|BOLD:ACI6841
Cecidomyiidae|[7889]|CNRVB883-15|Cecidomyiidae|576|BOLD:ACT5584
Cecidomyiidae|[7890]|CNRVB1024-15|Cecidomyiidae|588|BOLD:ACN9650
Cecidomyiidae|[7891]|CNFDE195-14|Cecidomyiidae|656|BOLD:ACL8757
Cecidomyiidae|[7892]|CNRVC796-15|Cecidomyiidae|588|BOLD:ACT8301
Cecidomyiidae|[7893]|CNKTD591-15|Cecidomyiidae|611|BOLD:ACS2916
Cecidomyiidae|[7894]|SSEIC5497-13|Cecidomyiidae|658|BOLD:ACG3600
Cecidomyiidae|[7895]|CNRVB1001-15|Cecidomyiidae|564|BOLD:ACT5585
Cecidomyiidae|[7896]|CNRVC1207-15|Cecidomyiidae|588|BOLD:ACT7697
Cecidomyiidae|[7897]|CNJAD656-12|Cecidomyiidae|658|BOLD:ACC9180
Cecidomyiidae|[7898]|CNKJD466-14|Cecidomyiidae|658|BOLD:ACM2261
Cecidomyiidae|[7899]|CNBRD374-14|Cecidomyiidae|661|BOLD:ACL3636

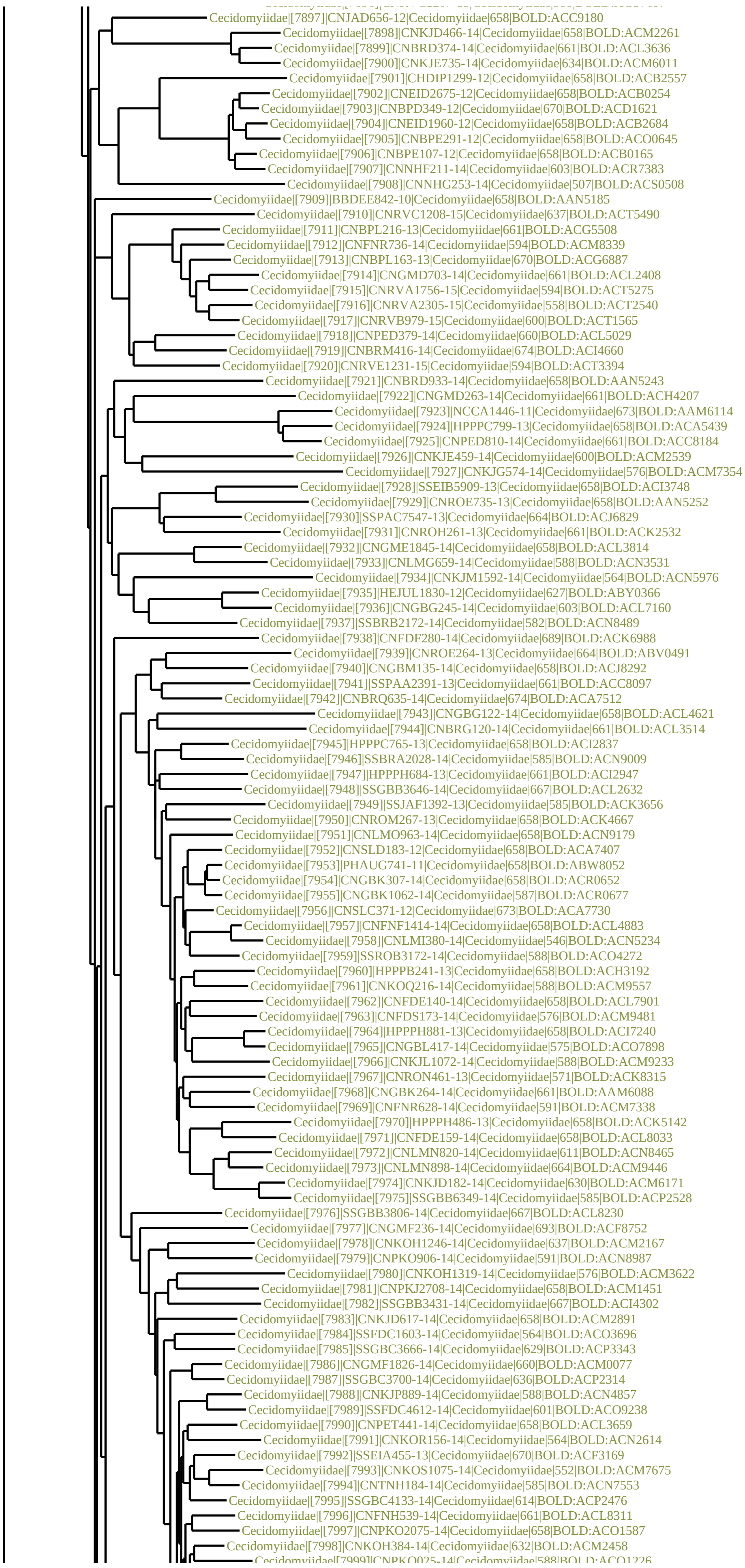
Cecidomyiidae|[7897]|CNJAD656-12|Cecidomyiidae|658|BOLD:ACC9180
Cecidomyiidae|[7898]|CNKJD466-14|Cecidomyiidae|658|BOLD:ACM2261
Cecidomyiidae|[7899]|CNBRD374-14|Cecidomyiidae|661|BOLD:ACL3636
Cecidomyiidae|[7900]|CNKJE735-14|Cecidomyiidae|634|BOLD:ACM6011
Cecidomyiidae|[7901]|CHDIP1299-12|Cecidomyiidae|658|BOLD:ACB2557
Cecidomyiidae|[7902]|CNEID2675-12|Cecidomyiidae|658|BOLD:ACB0254
Cecidomyiidae|[7903]|CNBPD349-12|Cecidomyiidae|670|BOLD:ACD1621
Cecidomyiidae|[7904]|CNEID1960-12|Cecidomyiidae|658|BOLD:ACB2684
Cecidomyiidae|[7905]|CNBPE291-12|Cecidomyiidae|658|BOLD:ACO0645
Cecidomyiidae|[7906]|CNBPE107-12|Cecidomyiidae|658|BOLD:ACB0165
Cecidomyiidae|[7907]|CNNHF211-14|Cecidomyiidae|603|BOLD:ACR7383
Cecidomyiidae|[7908]|CNNHG253-14|Cecidomyiidae|507|BOLD:ACS0508
Cecidomyiidae|[7909]|BBDEE842-10|Cecidomyiidae|658|BOLD:AAN5185
Cecidomyiidae|[7910]|CNRVC1208-15|Cecidomyiidae|637|BOLD:ACT5490
Cecidomyiidae|[7911]|CNBPL216-13|Cecidomyiidae|661|BOLD:ACG5508
Cecidomyiidae|[7912]|CNFNR736-14|Cecidomyiidae|594|BOLD:ACM8339
Cecidomyiidae|[7913]|CNBPL163-13|Cecidomyiidae|670|BOLD:ACG6887
Cecidomyiidae|[7914]|CNGMD703-14|Cecidomyiidae|661|BOLD:ACL2408
Cecidomyiidae|[7915]|CNRVA1756-15|Cecidomyiidae|594|BOLD:ACT5275
Cecidomyiidae|[7916]|CNRVA2305-15|Cecidomyiidae|558|BOLD:ACT2540
Cecidomyiidae|[7917]|CNRVB979-15|Cecidomyiidae|600|BOLD:ACT1565
Cecidomyiidae|[7918]|CNPED379-14|Cecidomyiidae|660|BOLD:ACL5029
Cecidomyiidae|[7919]|CNBRM416-14|Cecidomyiidae|674|BOLD:ACI4660
Cecidomyiidae|[7920]|CNRVE1231-15|Cecidomyiidae|594|BOLD:ACT3394
Cecidomyiidae|[7921]|CNBRD933-14|Cecidomyiidae|658|BOLD:AAN5243
Cecidomyiidae|[7922]|CNGMD263-14|Cecidomyiidae|661|BOLD:ACH4207
Cecidomyiidae|[7923]|NCCA1446-11|Cecidomyiidae|673|BOLD:AAM6114
Cecidomyiidae|[7924]|HPPPC799-13|Cecidomyiidae|658|BOLD:ACA5439
Cecidomyiidae|[7925]|CNPED810-14|Cecidomyiidae|661|BOLD:ACC8184
Cecidomyiidae|[7926]|CNKJE459-14|Cecidomyiidae|600|BOLD:ACM2539
Cecidomyiidae|[7927]|CNKJG574-14|Cecidomyiidae|576|BOLD:ACM7354
Cecidomyiidae|[7928]|SSEIB5909-13|Cecidomyiidae|658|BOLD:ACI3748
Cecidomyiidae|[7929]|CNROE735-13|Cecidomyiidae|658|BOLD:AAN5252
Cecidomyiidae|[7930]|SSPAC7547-13|Cecidomyiidae|664|BOLD:ACJ6829
Cecidomyiidae|[7931]|CNROH261-13|Cecidomyiidae|661|BOLD:ACK2532
Cecidomyiidae|[7932]|CNGME1845-14|Cecidomyiidae|658|BOLD:ACL3814
Cecidomyiidae|[7933]|CNLMG659-14|Cecidomyiidae|588|BOLD:ACN3531
Cecidomyiidae|[7934]|CNKJM1592-14|Cecidomyiidae|564|BOLD:ACN5976
Cecidomyiidae|[7935]|HEJUL1830-12|Cecidomyiidae|627|BOLD:ABY0366
Cecidomyiidae|[7936]|CNGBG245-14|Cecidomyiidae|603|BOLD:ACL7160
Cecidomyiidae|[7937]|SSBRB2172-14|Cecidomyiidae|582|BOLD:ACN8489
Cecidomyiidae|[7938]|CNFDF280-14|Cecidomyiidae|689|BOLD:ACK6988
Cecidomyiidae|[7939]|CNROE264-13|Cecidomyiidae|664|BOLD:ABV0491
Cecidomyiidae|[7940]|CNGBM135-14|Cecidomyiidae|658|BOLD:ACJ8292
Cecidomyiidae|[7941]|SSPAA2391-13|Cecidomyiidae|661|BOLD:ACC8097
Cecidomyiidae|[7942]|CNBRQ635-14|Cecidomyiidae|674|BOLD:ACA7512
Cecidomyiidae|[7943]|CNGBG122-14|Cecidomyiidae|658|BOLD:ACL4621
Cecidomyiidae|[7944]|CNBRG120-14|Cecidomyiidae|661|BOLD:ACL3514
Cecidomyiidae|[7945]|HPPPC765-13|Cecidomyiidae|658|BOLD:ACI2837
Cecidomyiidae|[7946]|SSBRA2028-14|Cecidomyiidae|585|BOLD:ACN9009
Cecidomyiidae|[7947]|HPPPH684-13|Cecidomyiidae|661|BOLD:ACI2947
Cecidomyiidae|[7948]|SSGBB3646-14|Cecidomyiidae|667|BOLD:ACL2632
Cecidomyiidae|[7949]|SSJAF1392-13|Cecidomyiidae|585|BOLD:ACK3656
Cecidomyiidae|[7950]|CNROM267-13|Cecidomyiidae|658|BOLD:ACK4667
Cecidomyiidae|[7951]|CNLMO963-14|Cecidomyiidae|658|BOLD:ACN9179
Cecidomyiidae|[7952]|CNSLD183-12|Cecidomyiidae|658|BOLD:ACA7407
Cecidomyiidae|[7953]|PHAUG741-11|Cecidomyiidae|658|BOLD:ABW8052
Cecidomyiidae|[7954]|CNGBK307-14|Cecidomyiidae|658|BOLD:ACR0652
Cecidomyiidae|[7955]|CNGBK1062-14|Cecidomyiidae|587|BOLD:ACR0677
Cecidomyiidae|[7956]|CNSLC371-12|Cecidomyiidae|673|BOLD:ACA7730
Cecidomyiidae|[7957]|CNFNF1414-14|Cecidomyiidae|658|BOLD:ACL4883
Cecidomyiidae|[7958]|CNLMI380-14|Cecidomyiidae|546|BOLD:ACN5234
Cecidomyiidae|[7959]|SSROB3172-14|Cecidomyiidae|588|BOLD:ACO4272
Cecidomyiidae|[7960]|HPPPB241-13|Cecidomyiidae|658|BOLD:ACH3192
Cecidomyiidae|[7961]|CNKOQ216-14|Cecidomyiidae|588|BOLD:ACM9557
Cecidomyiidae|[7962]|CNFDE140-14|Cecidomyiidae|658|BOLD:ACL7901
Cecidomyiidae|[7963]|CNFDS173-14|Cecidomyiidae|576|BOLD:ACM9481
Cecidomyiidae|[7964]|HPPPH881-13|Cecidomyiidae|658|BOLD:ACI7240
Cecidomyiidae|[7965]|CNGBL417-14|Cecidomyiidae|575|BOLD:ACO7898
Cecidomyiidae|[7966]|CNKJL1072-14|Cecidomyiidae|588|BOLD:ACM9233
Cecidomyiidae|[7967]|CNRON461-13|Cecidomyiidae|571|BOLD:ACK8315
Cecidomyiidae|[7968]|CNGBK264-14|Cecidomyiidae|661|BOLD:AAM6088
Cecidomyiidae|[7969]|CNFNR628-14|Cecidomyiidae|591|BOLD:ACM7338
Cecidomyiidae|[7970]|HPPPH486-13|Cecidomyiidae|658|BOLD:ACK5142
Cecidomyiidae|[7971]|CNFDE159-14|Cecidomyiidae|658|BOLD:ACL8033
Cecidomyiidae|[7972]|CNLMN820-14|Cecidomyiidae|611|BOLD:ACN8465
Cecidomyiidae|[7973]|CNLMN898-14|Cecidomyiidae|664|BOLD:ACM9446
Cecidomyiidae|[7974]|CNKJD182-14|Cecidomyiidae|630|BOLD:ACM6171
Cecidomyiidae|[7975]|SSGBB6349-14|Cecidomyiidae|585|BOLD:ACP2528
Cecidomyiidae|[7976]|SSGBB3806-14|Cecidomyiidae|667|BOLD:ACL8230
Cecidomyiidae|[7977]|CNGMF236-14|Cecidomyiidae|693|BOLD:ACF8752
Cecidomyiidae|[7978]|CNKOH1246-14|Cecidomyiidae|637|BOLD:ACM2167
Cecidomyiidae|[7979]|CNPKO906-14|Cecidomyiidae|591|BOLD:ACN8987
Cecidomyiidae|[7980]|CNKOH1319-14|Cecidomyiidae|576|BOLD:ACM3622
Cecidomyiidae|[7981]|CNPKJ2708-14|Cecidomyiidae|658|BOLD:ACM1451
Cecidomyiidae|[7982]|SSGBB3431-14|Cecidomyiidae|667|BOLD:ACI4302
Cecidomyiidae|[7983]|CNKJD617-14|Cecidomyiidae|658|BOLD:ACM2891
Cecidomyiidae|[7984]|SSFDC1603-14|Cecidomyiidae|564|BOLD:ACO3696
Cecidomyiidae|[7985]|SSGBC3666-14|Cecidomyiidae|629|BOLD:ACP3343
Cecidomyiidae|[7986]|CNGMF1826-14|Cecidomyiidae|660|BOLD:ACM0077
Cecidomyiidae|[7987]|SSGBC3700-14|Cecidomyiidae|636|BOLD:ACP2314
Cecidomyiidae|[7988]|CNKJP889-14|Cecidomyiidae|588|BOLD:ACN4857
Cecidomyiidae|[7989]|SSFDC4612-14|Cecidomyiidae|601|BOLD:ACO9238
Cecidomyiidae|[7990]|CNPET441-14|Cecidomyiidae|658|BOLD:ACL3659
Cecidomyiidae|[7991]|CNKOR156-14|Cecidomyiidae|564|BOLD:ACN2614
Cecidomyiidae|[7992]|SSEIA455-13|Cecidomyiidae|670|BOLD:ACF3169
Cecidomyiidae|[7993]|CNKOS1075-14|Cecidomyiidae|552|BOLD:ACM7675
Cecidomyiidae|[7994]|CNTNH184-14|Cecidomyiidae|585|BOLD:ACN7553
Cecidomyiidae|[7995]|SSGBC4133-14|Cecidomyiidae|614|BOLD:ACP2476
Cecidomyiidae|[7996]|CNFNH539-14|Cecidomyiidae|661|BOLD:ACL8311
Cecidomyiidae|[7997]|CNPKO2075-14|Cecidomyiidae|658|BOLD:ACO1587
Cecidomyiidae|[7998]|CNKOH384-14|Cecidomyiidae|632|BOLD:ACM2458

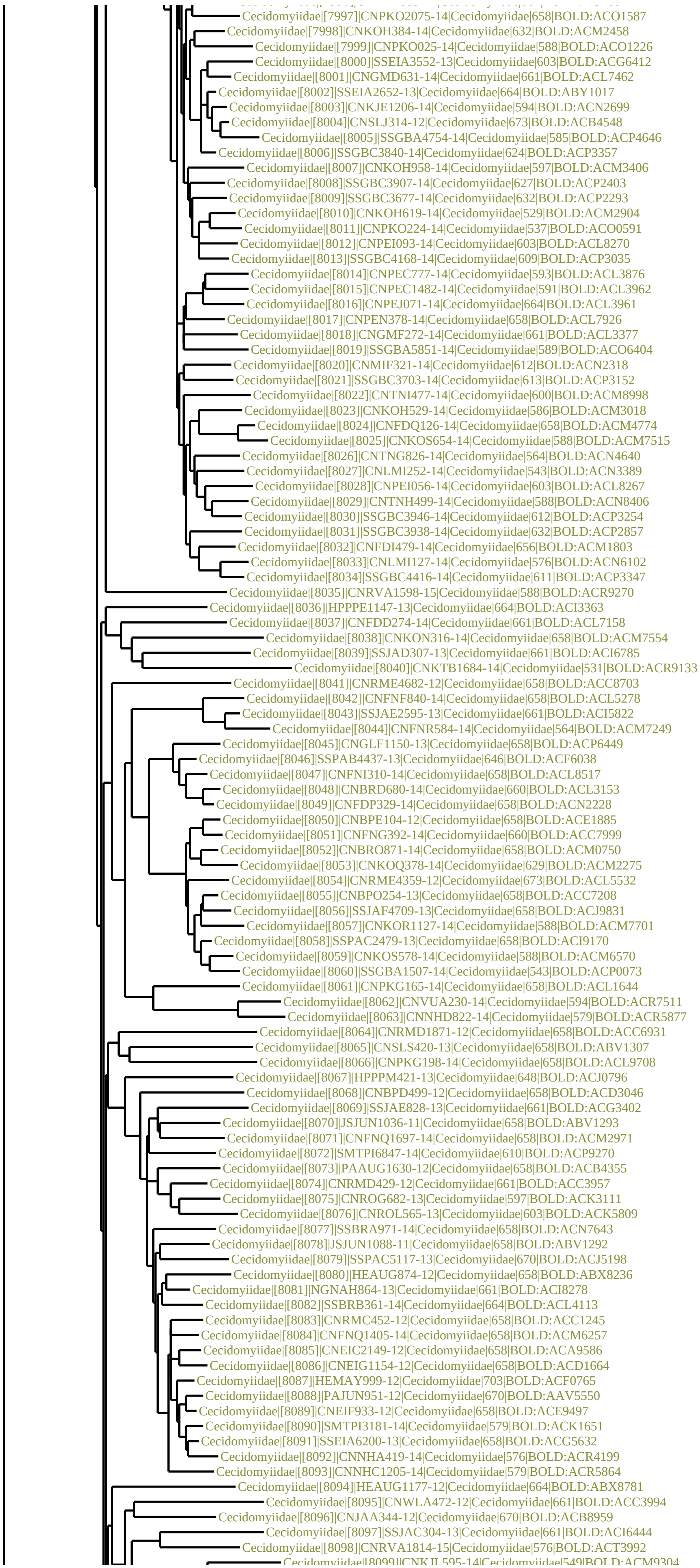
Cecidomyiidae|[7997]|CNPKO2075-14|Cecidomyiidae|658|BOLD:ACO1587
Cecidomyiidae|[7998]|CNKOH384-14|Cecidomyiidae|632|BOLD:ACM2458
Cecidomyiidae|[7999]|CNPKO025-14|Cecidomyiidae|588|BOLD:ACO1226
Cecidomyiidae|[8000]|SSEIA3552-13|Cecidomyiidae|603|BOLD:ACG6412
Cecidomyiidae|[8001]|CNGMD631-14|Cecidomyiidae|661|BOLD:ACL7462
Cecidomyiidae|[8002]|SSEIA2652-13|Cecidomyiidae|664|BOLD:ABY1017
Cecidomyiidae|[8003]|CNKJE1206-14|Cecidomyiidae|594|BOLD:ACN2699
Cecidomyiidae|[8004]|CNSLJ314-12|Cecidomyiidae|673|BOLD:ACB4548
Cecidomyiidae|[8005]|SSGBA4754-14|Cecidomyiidae|585|BOLD:ACP4646
Cecidomyiidae|[8006]|SSGBC3840-14|Cecidomyiidae|624|BOLD:ACP3357
Cecidomyiidae|[8007]|CNKOH958-14|Cecidomyiidae|597|BOLD:ACM3406
Cecidomyiidae|[8008]|SSGBC3907-14|Cecidomyiidae|627|BOLD:ACP2403
Cecidomyiidae|[8009]|SSGBC3677-14|Cecidomyiidae|632|BOLD:ACP2293
Cecidomyiidae|[8010]|CNKOH619-14|Cecidomyiidae|529|BOLD:ACM2904
Cecidomyiidae|[8011]|CNPKO224-14|Cecidomyiidae|537|BOLD:ACO0591
Cecidomyiidae|[8012]|CNPEI093-14|Cecidomyiidae|603|BOLD:ACL8270
Cecidomyiidae|[8013]|SSGBC4168-14|Cecidomyiidae|609|BOLD:ACP3035
Cecidomyiidae|[8014]|CNPEC777-14|Cecidomyiidae|593|BOLD:ACL3876
Cecidomyiidae|[8015]|CNPEC1482-14|Cecidomyiidae|591|BOLD:ACL3962
Cecidomyiidae|[8016]|CNPEJ071-14|Cecidomyiidae|664|BOLD:ACL3961
Cecidomyiidae|[8017]|CNPEN378-14|Cecidomyiidae|658|BOLD:ACL7926
Cecidomyiidae|[8018]|CNGMF272-14|Cecidomyiidae|661|BOLD:ACL3377
Cecidomyiidae|[8019]|SSGBA5851-14|Cecidomyiidae|589|BOLD:ACO6404
Cecidomyiidae|[8020]|CNMIF321-14|Cecidomyiidae|612|BOLD:ACN2318
Cecidomyiidae|[8021]|SSGBC3703-14|Cecidomyiidae|613|BOLD:ACP3152
Cecidomyiidae|[8022]|CNTNI477-14|Cecidomyiidae|600|BOLD:ACM8998
Cecidomyiidae|[8023]|CNKOH529-14|Cecidomyiidae|586|BOLD:ACM3018
Cecidomyiidae|[8024]|CNFDQ126-14|Cecidomyiidae|658|BOLD:ACM4774
Cecidomyiidae|[8025]|CNKOS654-14|Cecidomyiidae|588|BOLD:ACM7515
Cecidomyiidae|[8026]|CNTNG826-14|Cecidomyiidae|564|BOLD:ACN4640
Cecidomyiidae|[8027]|CNLMI252-14|Cecidomyiidae|543|BOLD:ACN3389
Cecidomyiidae|[8028]|CNPEI056-14|Cecidomyiidae|603|BOLD:ACL8267
Cecidomyiidae|[8029]|CNTNH499-14|Cecidomyiidae|588|BOLD:ACN8406
Cecidomyiidae|[8030]|SSGBC3946-14|Cecidomyiidae|612|BOLD:ACP3254
Cecidomyiidae|[8031]|SSGBC3938-14|Cecidomyiidae|632|BOLD:ACP2857
Cecidomyiidae|[8032]|CNFDI479-14|Cecidomyiidae|656|BOLD:ACM1803
Cecidomyiidae|[8033]|CNLMI127-14|Cecidomyiidae|576|BOLD:ACN6102
Cecidomyiidae|[8034]|SSGBC4416-14|Cecidomyiidae|611|BOLD:ACP3347
Cecidomyiidae|[8035]|CNRVA1598-15|Cecidomyiidae|588|BOLD:ACR9270
Cecidomyiidae|[8036]|HPPPE1147-13|Cecidomyiidae|664|BOLD:ACI3363
Cecidomyiidae|[8037]|CNFDD274-14|Cecidomyiidae|661|BOLD:ACL7158
Cecidomyiidae|[8038]|CNKON316-14|Cecidomyiidae|658|BOLD:ACM7554
Cecidomyiidae|[8039]|SSJAD307-13|Cecidomyiidae|661|BOLD:ACI6785
Cecidomyiidae|[8040]|CNKTB1684-14|Cecidomyiidae|531|BOLD:ACR9133
Cecidomyiidae|[8041]|CNRME4682-12|Cecidomyiidae|658|BOLD:ACC8703
Cecidomyiidae|[8042]|CNFNF840-14|Cecidomyiidae|658|BOLD:ACL5278
Cecidomyiidae|[8043]|SSJAE2595-13|Cecidomyiidae|661|BOLD:ACI5822
Cecidomyiidae|[8044]|CNFNR584-14|Cecidomyiidae|564|BOLD:ACM7249
Cecidomyiidae|[8045]|CNGLF1150-13|Cecidomyiidae|658|BOLD:ACP6449
Cecidomyiidae|[8046]|SSPAB4437-13|Cecidomyiidae|646|BOLD:ACF6038
Cecidomyiidae|[8047]|CNFNI310-14|Cecidomyiidae|658|BOLD:ACL8517
Cecidomyiidae|[8048]|CNBRD680-14|Cecidomyiidae|660|BOLD:ACL3153
Cecidomyiidae|[8049]|CNFDP329-14|Cecidomyiidae|658|BOLD:ACN2228
Cecidomyiidae|[8050]|CNBPE104-12|Cecidomyiidae|658|BOLD:ACE1885
Cecidomyiidae|[8051]|CNFNG392-14|Cecidomyiidae|660|BOLD:ACC7999
Cecidomyiidae|[8052]|CNBRO871-14|Cecidomyiidae|658|BOLD:ACM0750
Cecidomyiidae|[8053]|CNKOQ378-14|Cecidomyiidae|629|BOLD:ACM2275
Cecidomyiidae|[8054]|CNRME4359-12|Cecidomyiidae|673|BOLD:ACL5532
Cecidomyiidae|[8055]|CNBPO254-13|Cecidomyiidae|658|BOLD:ACC7208
Cecidomyiidae|[8056]|SSJAF4709-13|Cecidomyiidae|658|BOLD:ACJ9831
Cecidomyiidae|[8057]|CNKOR1127-14|Cecidomyiidae|588|BOLD:ACM7701
Cecidomyiidae|[8058]|SSPAC2479-13|Cecidomyiidae|658|BOLD:ACI9170
Cecidomyiidae|[8059]|CNKOS578-14|Cecidomyiidae|588|BOLD:ACM6570
Cecidomyiidae|[8060]|SSGBA1507-14|Cecidomyiidae|543|BOLD:ACP0073
Cecidomyiidae|[8061]|CNPKG165-14|Cecidomyiidae|658|BOLD:ACL1644
Cecidomyiidae|[8062]|CNVUA230-14|Cecidomyiidae|594|BOLD:ACR7511
Cecidomyiidae|[8063]|CNNHD822-14|Cecidomyiidae|579|BOLD:ACR5877
Cecidomyiidae|[8064]|CNRMD1871-12|Cecidomyiidae|658|BOLD:ACC6931
Cecidomyiidae|[8065]|CNSLS420-13|Cecidomyiidae|658|BOLD:ABV1307
Cecidomyiidae|[8066]|CNPKG198-14|Cecidomyiidae|658|BOLD:ACL9708
Cecidomyiidae|[8067]|HPPPM421-13|Cecidomyiidae|648|BOLD:ACJ0796
Cecidomyiidae|[8068]|CNBPD499-12|Cecidomyiidae|658|BOLD:ACD3046
Cecidomyiidae|[8069]|SSJAE828-13|Cecidomyiidae|661|BOLD:ACG3402
Cecidomyiidae|[8070]|JSJUN1036-11|Cecidomyiidae|658|BOLD:ABV1293
Cecidomyiidae|[8071]|CNFNQ1697-14|Cecidomyiidae|658|BOLD:ACM2971
Cecidomyiidae|[8072]|SMTPI6847-14|Cecidomyiidae|610|BOLD:ACP9270
Cecidomyiidae|[8073]|PAAUG1630-12|Cecidomyiidae|658|BOLD:ACB4355
Cecidomyiidae|[8074]|CNRMD429-12|Cecidomyiidae|661|BOLD:ACC3957
Cecidomyiidae|[8075]|CNROG682-13|Cecidomyiidae|597|BOLD:ACK3111
Cecidomyiidae|[8076]|CNROL565-13|Cecidomyiidae|603|BOLD:ACK5809
Cecidomyiidae|[8077]|SSBRA971-14|Cecidomyiidae|658|BOLD:ACN7643
Cecidomyiidae|[8078]|JSJUN1088-11|Cecidomyiidae|658|BOLD:ABV1292
Cecidomyiidae|[8079]|SSPAC5117-13|Cecidomyiidae|670|BOLD:ACJ5198
Cecidomyiidae|[8080]|HEAUG874-12|Cecidomyiidae|658|BOLD:ABX8236
Cecidomyiidae|[8081]|NGNAH864-13|Cecidomyiidae|661|BOLD:ACI8278
Cecidomyiidae|[8082]|SSBRB361-14|Cecidomyiidae|664|BOLD:ACL4113
Cecidomyiidae|[8083]|CNRMC452-12|Cecidomyiidae|658|BOLD:ACC1245
Cecidomyiidae|[8084]|CNFNQ1405-14|Cecidomyiidae|658|BOLD:ACM6257
Cecidomyiidae|[8085]|CNEIC2149-12|Cecidomyiidae|658|BOLD:ACA9586
Cecidomyiidae|[8086]|CNEIG1154-12|Cecidomyiidae|658|BOLD:ACD1664
Cecidomyiidae|[8087]|HEMAY999-12|Cecidomyiidae|703|BOLD:ACF0765
Cecidomyiidae|[8088]|PAJUN951-12|Cecidomyiidae|670|BOLD:AAV5550
Cecidomyiidae|[8089]|CNEIF933-12|Cecidomyiidae|658|BOLD:ACE9497
Cecidomyiidae|[8090]|SMTPI3181-14|Cecidomyiidae|579|BOLD:ACK1651
Cecidomyiidae|[8091]|SSEIA6200-13|Cecidomyiidae|658|BOLD:ACG5632
Cecidomyiidae|[8092]|CNNHA419-14|Cecidomyiidae|576|BOLD:ACR4199
Cecidomyiidae|[8093]|CNNHC1205-14|Cecidomyiidae|579|BOLD:ACR5864
Cecidomyiidae|[8094]|HEAUG1177-12|Cecidomyiidae|664|BOLD:ABX8781
Cecidomyiidae|[8095]|CNWLA472-12|Cecidomyiidae|661|BOLD:ACC3994
Cecidomyiidae|[8096]|CNJAA344-12|Cecidomyiidae|670|BOLD:ACB8959
Cecidomyiidae|[8097]|SSJAC304-13|Cecidomyiidae|661|BOLD:ACI6444
Cecidomyiidae|[8098]|CNRVA1814-15|Cecidomyiidae|576|BOLD:ACT3992
Cecidomyiidae|[8099]|CNKJL595-14|Cecidomyiidae|549|BOLD:ACM9304

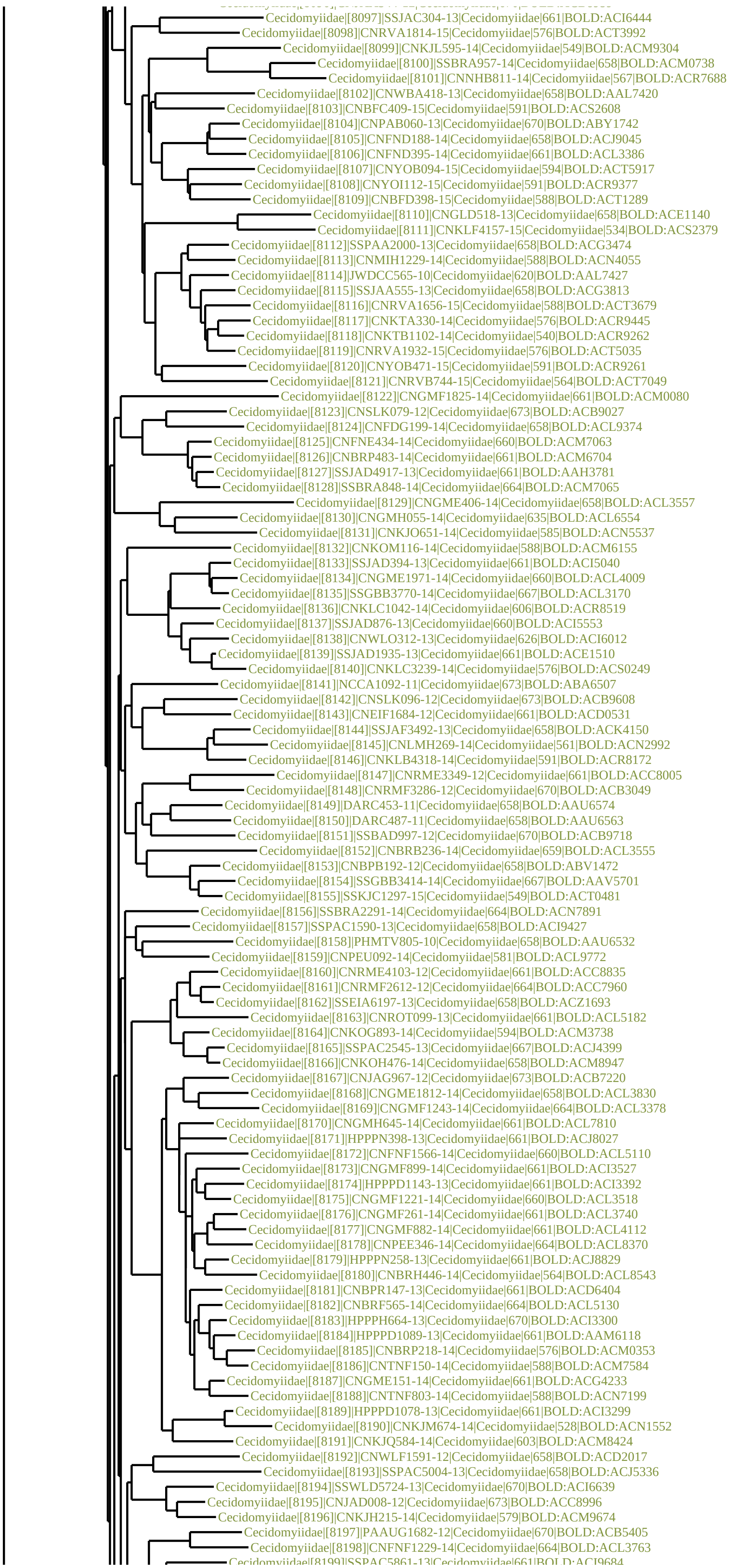
Cecidomyiidae|[8097]|SSJAC304-13|Cecidomyiidae|661|BOLD:ACI6444
Cecidomyiidae|[8098]|CNRVA1814-15|Cecidomyiidae|576|BOLD:ACT3992
Cecidomyiidae|[8099]|CNKJL595-14|Cecidomyiidae|549|BOLD:ACM9304
Cecidomyiidae|[8100]|SSBRA957-14|Cecidomyiidae|658|BOLD:ACM0738
Cecidomyiidae|[8101]|CNNHB811-14|Cecidomyiidae|567|BOLD:ACR7688
Cecidomyiidae|[8102]|CNWBA418-13|Cecidomyiidae|658|BOLD:AAL7420
Cecidomyiidae|[8103]|CNBFC409-15|Cecidomyiidae|591|BOLD:ACS2608
Cecidomyiidae|[8104]|CNPAB060-13|Cecidomyiidae|670|BOLD:ABY1742
Cecidomyiidae|[8105]|CNFND188-14|Cecidomyiidae|658|BOLD:ACJ9045
Cecidomyiidae|[8106]|CNFND395-14|Cecidomyiidae|661|BOLD:ACL3386
Cecidomyiidae|[8107]|CNYOB094-15|Cecidomyiidae|594|BOLD:ACT5917
Cecidomyiidae|[8108]|CNYOI112-15|Cecidomyiidae|591|BOLD:ACR9377
Cecidomyiidae|[8109]|CNBFD398-15|Cecidomyiidae|588|BOLD:ACT1289
Cecidomyiidae|[8110]|CNGLD518-13|Cecidomyiidae|658|BOLD:ACE1140
Cecidomyiidae|[8111]|CNKLF4157-15|Cecidomyiidae|534|BOLD:ACS2379
Cecidomyiidae|[8112]|SSPAA2000-13|Cecidomyiidae|658|BOLD:ACG3474
Cecidomyiidae|[8113]|CNMIH1229-14|Cecidomyiidae|588|BOLD:ACN4055
Cecidomyiidae|[8114]|JWDCC565-10|Cecidomyiidae|620|BOLD:AAL7427
Cecidomyiidae|[8115]|SSJAA555-13|Cecidomyiidae|658|BOLD:ACG3813
Cecidomyiidae|[8116]|CNRVA1656-15|Cecidomyiidae|588|BOLD:ACT3679
Cecidomyiidae|[8117]|CNKTA330-14|Cecidomyiidae|576|BOLD:ACR9445
Cecidomyiidae|[8118]|CNKTB1102-14|Cecidomyiidae|540|BOLD:ACR9262
Cecidomyiidae|[8119]|CNRVA1932-15|Cecidomyiidae|576|BOLD:ACT5035
Cecidomyiidae|[8120]|CNYOB471-15|Cecidomyiidae|591|BOLD:ACR9261
Cecidomyiidae|[8121]|CNRVB744-15|Cecidomyiidae|564|BOLD:ACT7049
Cecidomyiidae|[8122]|CNGMF1825-14|Cecidomyiidae|661|BOLD:ACM0080
Cecidomyiidae|[8123]|CNSLK079-12|Cecidomyiidae|673|BOLD:ACB9027
Cecidomyiidae|[8124]|CNFDG199-14|Cecidomyiidae|658|BOLD:ACL9374
Cecidomyiidae|[8125]|CNFNE434-14|Cecidomyiidae|660|BOLD:ACM7063
Cecidomyiidae|[8126]|CNBRP483-14|Cecidomyiidae|661|BOLD:ACM6704
Cecidomyiidae|[8127]|SSJAD4917-13|Cecidomyiidae|661|BOLD:AAH3781
Cecidomyiidae|[8128]|SSBRA848-14|Cecidomyiidae|664|BOLD:ACM7065
Cecidomyiidae|[8129]|CNGME406-14|Cecidomyiidae|658|BOLD:ACL3557
Cecidomyiidae|[8130]|CNGMH055-14|Cecidomyiidae|635|BOLD:ACL6554
Cecidomyiidae|[8131]|CNKJO651-14|Cecidomyiidae|585|BOLD:ACN5537
Cecidomyiidae|[8132]|CNKOM116-14|Cecidomyiidae|588|BOLD:ACM6155
Cecidomyiidae|[8133]|SSJAD394-13|Cecidomyiidae|661|BOLD:ACI5040
Cecidomyiidae|[8134]|CNGME1971-14|Cecidomyiidae|660|BOLD:ACL4009
Cecidomyiidae|[8135]|SSGBB3770-14|Cecidomyiidae|667|BOLD:ACL3170
Cecidomyiidae|[8136]|CNKLC1042-14|Cecidomyiidae|606|BOLD:ACR8519
Cecidomyiidae|[8137]|SSJAD876-13|Cecidomyiidae|660|BOLD:ACI5553
Cecidomyiidae|[8138]|CNWLO312-13|Cecidomyiidae|626|BOLD:ACI6012
Cecidomyiidae|[8139]|SSJAD1935-13|Cecidomyiidae|661|BOLD:ACE1510
Cecidomyiidae|[8140]|CNKLC3239-14|Cecidomyiidae|576|BOLD:ACS0249
Cecidomyiidae|[8141]|NCCA1092-11|Cecidomyiidae|673|BOLD:ABA6507
Cecidomyiidae|[8142]|CNSLK096-12|Cecidomyiidae|673|BOLD:ACB9608
Cecidomyiidae|[8143]|CNEIF1684-12|Cecidomyiidae|661|BOLD:ACD0531
Cecidomyiidae|[8144]|SSJAF3492-13|Cecidomyiidae|658|BOLD:ACK4150
Cecidomyiidae|[8145]|CNLMH269-14|Cecidomyiidae|561|BOLD:ACN2992
Cecidomyiidae|[8146]|CNKLB4318-14|Cecidomyiidae|591|BOLD:ACR8172
Cecidomyiidae|[8147]|CNRME3349-12|Cecidomyiidae|661|BOLD:ACC8005
Cecidomyiidae|[8148]|CNRMF3286-12|Cecidomyiidae|670|BOLD:ACB3049
Cecidomyiidae|[8149]|DARC453-11|Cecidomyiidae|658|BOLD:AAU6574
Cecidomyiidae|[8150]|DARC487-11|Cecidomyiidae|658|BOLD:AAU6563
Cecidomyiidae|[8151]|SSBAD997-12|Cecidomyiidae|670|BOLD:ACB9718
Cecidomyiidae|[8152]|CNBRB236-14|Cecidomyiidae|659|BOLD:ACL3555
Cecidomyiidae|[8153]|CNBPB192-12|Cecidomyiidae|658|BOLD:ABV1472
Cecidomyiidae|[8154]|SSGBB3414-14|Cecidomyiidae|667|BOLD:AAV5701
Cecidomyiidae|[8155]|SSKJC1297-15|Cecidomyiidae|549|BOLD:ACT0481
Cecidomyiidae|[8156]|SSBRA2291-14|Cecidomyiidae|664|BOLD:ACN7891
Cecidomyiidae|[8157]|SSPAC1590-13|Cecidomyiidae|658|BOLD:ACI9427
Cecidomyiidae|[8158]|PHMTV805-10|Cecidomyiidae|658|BOLD:AAU6532
Cecidomyiidae|[8159]|CNPEU092-14|Cecidomyiidae|581|BOLD:ACL9772
Cecidomyiidae|[8160]|CNRME4103-12|Cecidomyiidae|661|BOLD:ACC8835
Cecidomyiidae|[8161]|CNRMF2612-12|Cecidomyiidae|664|BOLD:ACC7960
Cecidomyiidae|[8162]|SSEIA6197-13|Cecidomyiidae|658|BOLD:ACZ1693
Cecidomyiidae|[8163]|CNROT099-13|Cecidomyiidae|661|BOLD:ACL5182
Cecidomyiidae|[8164]|CNKOG893-14|Cecidomyiidae|594|BOLD:ACM3738
Cecidomyiidae|[8165]|SSPAC2545-13|Cecidomyiidae|667|BOLD:ACJ4399
Cecidomyiidae|[8166]|CNKOH476-14|Cecidomyiidae|658|BOLD:ACM8947
Cecidomyiidae|[8167]|CNJAG967-12|Cecidomyiidae|673|BOLD:ACB7220
Cecidomyiidae|[8168]|CNGME1812-14|Cecidomyiidae|658|BOLD:ACL3830
Cecidomyiidae|[8169]|CNGMF1243-14|Cecidomyiidae|664|BOLD:ACL3378
Cecidomyiidae|[8170]|CNGMH645-14|Cecidomyiidae|661|BOLD:ACL7810
Cecidomyiidae|[8171]|HPPPN398-13|Cecidomyiidae|661|BOLD:ACJ8027
Cecidomyiidae|[8172]|CNFNF1566-14|Cecidomyiidae|660|BOLD:ACL5110
Cecidomyiidae|[8173]|CNGMF899-14|Cecidomyiidae|661|BOLD:ACI3527
Cecidomyiidae|[8174]|HPPPD1143-13|Cecidomyiidae|661|BOLD:ACI3392
Cecidomyiidae|[8175]|CNGMF1221-14|Cecidomyiidae|660|BOLD:ACL3518
Cecidomyiidae|[8176]|CNGMF261-14|Cecidomyiidae|661|BOLD:ACL3740
Cecidomyiidae|[8177]|CNGMF882-14|Cecidomyiidae|661|BOLD:ACL4112
Cecidomyiidae|[8178]|CNPEE346-14|Cecidomyiidae|664|BOLD:ACL8370
Cecidomyiidae|[8179]|HPPPN258-13|Cecidomyiidae|661|BOLD:ACJ8829
Cecidomyiidae|[8180]|CNBRH446-14|Cecidomyiidae|564|BOLD:ACL8543
Cecidomyiidae|[8181]|CNBPR147-13|Cecidomyiidae|661|BOLD:ACD6404
Cecidomyiidae|[8182]|CNBRF565-14|Cecidomyiidae|664|BOLD:ACL5130
Cecidomyiidae|[8183]|HPPPH664-13|Cecidomyiidae|670|BOLD:ACI3300
Cecidomyiidae|[8184]|HPPPD1089-13|Cecidomyiidae|661|BOLD:AAM6118
Cecidomyiidae|[8185]|CNBRP218-14|Cecidomyiidae|576|BOLD:ACM0353
Cecidomyiidae|[8186]|CNTNF150-14|Cecidomyiidae|588|BOLD:ACM7584
Cecidomyiidae|[8187]|CNGME151-14|Cecidomyiidae|661|BOLD:ACG4233
Cecidomyiidae|[8188]|CNTNF803-14|Cecidomyiidae|588|BOLD:ACN7199
Cecidomyiidae|[8189]|HPPPD1078-13|Cecidomyiidae|661|BOLD:ACI3299
Cecidomyiidae|[8190]|CNKJM674-14|Cecidomyiidae|528|BOLD:ACN1552
Cecidomyiidae|[8191]|CNKJQ584-14|Cecidomyiidae|603|BOLD:ACM8424
Cecidomyiidae|[8192]|CNWLF1591-12|Cecidomyiidae|658|BOLD:ACD2017
Cecidomyiidae|[8193]|SSPAC5004-13|Cecidomyiidae|658|BOLD:ACJ5336
Cecidomyiidae|[8194]|SSWLD5724-13|Cecidomyiidae|670|BOLD:ACI6639
Cecidomyiidae|[8195]|CNJAD008-12|Cecidomyiidae|673|BOLD:ACC8996
Cecidomyiidae|[8196]|CNKJH215-14|Cecidomyiidae|579|BOLD:ACM9674
Cecidomyiidae|[8197]|PAAUG1682-12|Cecidomyiidae|670|BOLD:ACB5405
Cecidomyiidae|[8198]|CNFNF1229-14|Cecidomyiidae|664|BOLD:ACL3763
Cecidomyiidae|[8199]|SSPAC5861-13|Cecidomyiidae|661|BOLD:ACI9684

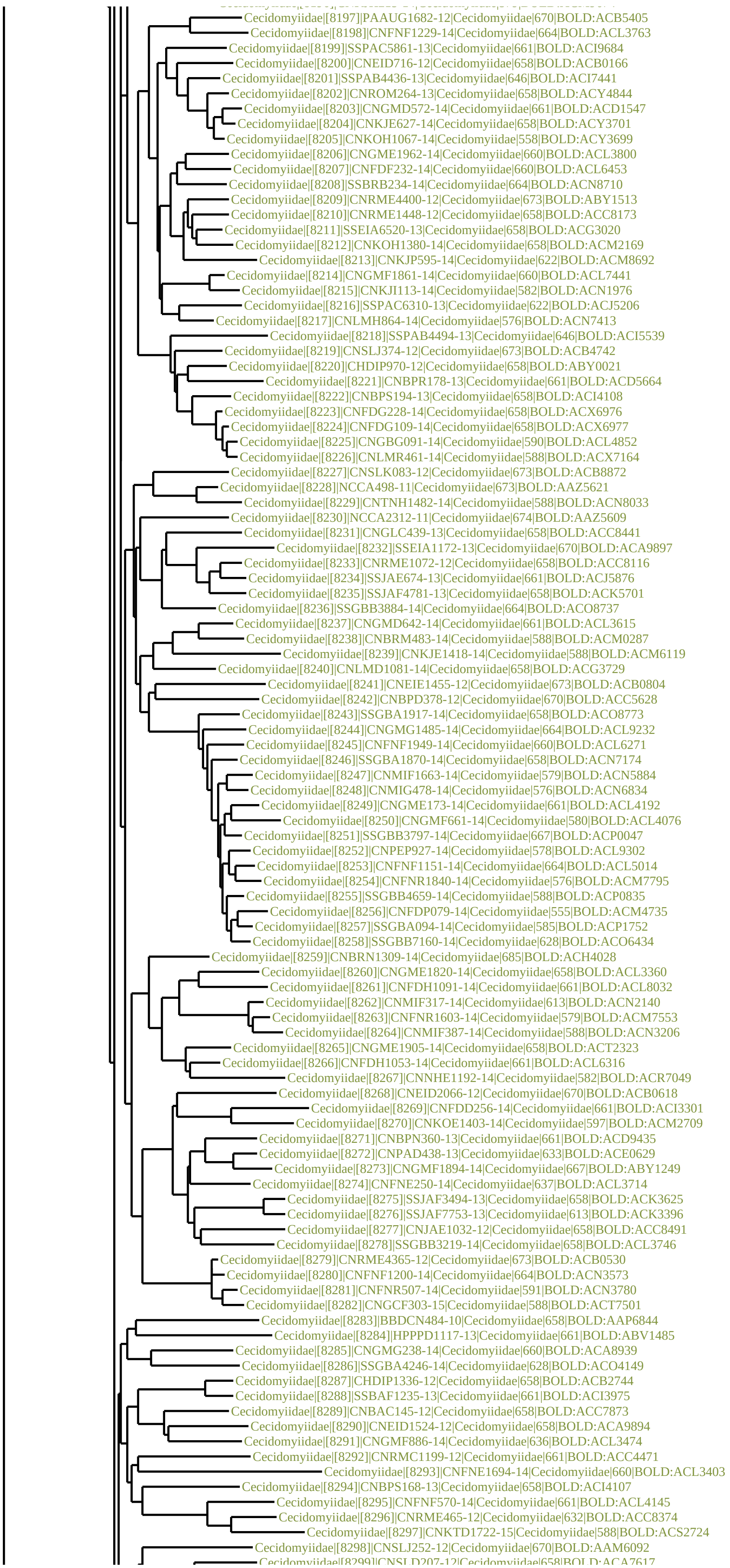
Cecidomyiidae|[8197]|PAAUG1682-12|Cecidomyiidae|670|BOLD:ACB5405
Cecidomyiidae|[8198]|CNFNF1229-14|Cecidomyiidae|664|BOLD:ACL3763
Cecidomyiidae|[8199]|SSPAC5861-13|Cecidomyiidae|661|BOLD:ACI9684
Cecidomyiidae|[8200]|CNEID716-12|Cecidomyiidae|658|BOLD:ACB0166
Cecidomyiidae|[8201]|SSPAB4436-13|Cecidomyiidae|646|BOLD:ACI7441
Cecidomyiidae|[8202]|CNROM264-13|Cecidomyiidae|658|BOLD:ACY4844
Cecidomyiidae|[8203]|CNGMD572-14|Cecidomyiidae|661|BOLD:ACD1547
Cecidomyiidae|[8204]|CNKJE627-14|Cecidomyiidae|658|BOLD:ACY3701
Cecidomyiidae|[8205]|CNKOH1067-14|Cecidomyiidae|558|BOLD:ACY3699
Cecidomyiidae|[8206]|CNGME1962-14|Cecidomyiidae|660|BOLD:ACL3800
Cecidomyiidae|[8207]|CNFDF232-14|Cecidomyiidae|660|BOLD:ACL6453
Cecidomyiidae|[8208]|SSBRB234-14|Cecidomyiidae|664|BOLD:ACN8710
Cecidomyiidae|[8209]|CNRME4400-12|Cecidomyiidae|673|BOLD:ABY1513
Cecidomyiidae|[8210]|CNRME1448-12|Cecidomyiidae|658|BOLD:ACC8173
Cecidomyiidae|[8211]|SSEIA6520-13|Cecidomyiidae|658|BOLD:ACG3020
Cecidomyiidae|[8212]|CNKOH1380-14|Cecidomyiidae|658|BOLD:ACM2169
Cecidomyiidae|[8213]|CNKJP595-14|Cecidomyiidae|622|BOLD:ACM8692
Cecidomyiidae|[8214]|CNGMF1861-14|Cecidomyiidae|660|BOLD:ACL7441
Cecidomyiidae|[8215]|CNKJI113-14|Cecidomyiidae|582|BOLD:ACN1976
Cecidomyiidae|[8216]|SSPAC6310-13|Cecidomyiidae|622|BOLD:ACJ5206
Cecidomyiidae|[8217]|CNLMH864-14|Cecidomyiidae|576|BOLD:ACN7413
Cecidomyiidae|[8218]|SSPAB4494-13|Cecidomyiidae|646|BOLD:ACI5539
Cecidomyiidae|[8219]|CNSLJ374-12|Cecidomyiidae|673|BOLD:ACB4742
Cecidomyiidae|[8220]|CHDIP970-12|Cecidomyiidae|658|BOLD:ABY0021
Cecidomyiidae|[8221]|CNBPR178-13|Cecidomyiidae|661|BOLD:ACD5664
Cecidomyiidae|[8222]|CNBPS194-13|Cecidomyiidae|658|BOLD:ACI4108
Cecidomyiidae|[8223]|CNFDG228-14|Cecidomyiidae|658|BOLD:ACX6976
Cecidomyiidae|[8224]|CNFDG109-14|Cecidomyiidae|658|BOLD:ACX6977
Cecidomyiidae|[8225]|CNGBG091-14|Cecidomyiidae|590|BOLD:ACL4852
Cecidomyiidae|[8226]|CNLMR461-14|Cecidomyiidae|588|BOLD:ACX7164
Cecidomyiidae|[8227]|CNSLK083-12|Cecidomyiidae|673|BOLD:ACB8872
Cecidomyiidae|[8228]|NCCA498-11|Cecidomyiidae|673|BOLD:AAZ5621
Cecidomyiidae|[8229]|CNTNH1482-14|Cecidomyiidae|588|BOLD:ACN8033
Cecidomyiidae|[8230]|NCCA2312-11|Cecidomyiidae|674|BOLD:AAZ5609
Cecidomyiidae|[8231]|CNGLC439-13|Cecidomyiidae|658|BOLD:ACC8441
Cecidomyiidae|[8232]|SSEIA1172-13|Cecidomyiidae|670|BOLD:ACA9897
Cecidomyiidae|[8233]|CNRME1072-12|Cecidomyiidae|658|BOLD:ACC8116
Cecidomyiidae|[8234]|SSJAE674-13|Cecidomyiidae|661|BOLD:ACJ5876
Cecidomyiidae|[8235]|SSJAF4781-13|Cecidomyiidae|658|BOLD:ACK5701
Cecidomyiidae|[8236]|SSGBB3884-14|Cecidomyiidae|664|BOLD:ACO8737
Cecidomyiidae|[8237]|CNGMD642-14|Cecidomyiidae|661|BOLD:ACL3615
Cecidomyiidae|[8238]|CNBRM483-14|Cecidomyiidae|588|BOLD:ACM0287
Cecidomyiidae|[8239]|CNKJE1418-14|Cecidomyiidae|588|BOLD:ACM6119
Cecidomyiidae|[8240]|CNLMD1081-14|Cecidomyiidae|658|BOLD:ACG3729
Cecidomyiidae|[8241]|CNEIE1455-12|Cecidomyiidae|673|BOLD:ACB0804
Cecidomyiidae|[8242]|CNBPD378-12|Cecidomyiidae|670|BOLD:ACC5628
Cecidomyiidae|[8243]|SSGBA1917-14|Cecidomyiidae|658|BOLD:ACO8773
Cecidomyiidae|[8244]|CNGMG1485-14|Cecidomyiidae|664|BOLD:ACL9232
Cecidomyiidae|[8245]|CNFNF1949-14|Cecidomyiidae|660|BOLD:ACL6271
Cecidomyiidae|[8246]|SSGBA1870-14|Cecidomyiidae|658|BOLD:ACN7174
Cecidomyiidae|[8247]|CNMIF1663-14|Cecidomyiidae|579|BOLD:ACN5884
Cecidomyiidae|[8248]|CNMIG478-14|Cecidomyiidae|576|BOLD:ACN6834
Cecidomyiidae|[8249]|CNGME173-14|Cecidomyiidae|661|BOLD:ACL4192
Cecidomyiidae|[8250]|CNGMF661-14|Cecidomyiidae|580|BOLD:ACL4076
Cecidomyiidae|[8251]|SSGBB3797-14|Cecidomyiidae|667|BOLD:ACP0047
Cecidomyiidae|[8252]|CNPEP927-14|Cecidomyiidae|578|BOLD:ACL9302
Cecidomyiidae|[8253]|CNFNF1151-14|Cecidomyiidae|664|BOLD:ACL5014
Cecidomyiidae|[8254]|CNFNR1840-14|Cecidomyiidae|576|BOLD:ACM7795
Cecidomyiidae|[8255]|SSGBB4659-14|Cecidomyiidae|588|BOLD:ACP0835
Cecidomyiidae|[8256]|CNFDP079-14|Cecidomyiidae|555|BOLD:ACM4735
Cecidomyiidae|[8257]|SSGBA094-14|Cecidomyiidae|585|BOLD:ACP1752
Cecidomyiidae|[8258]|SSGBB7160-14|Cecidomyiidae|628|BOLD:ACO6434
Cecidomyiidae|[8259]|CNBRN1309-14|Cecidomyiidae|685|BOLD:ACH4028
Cecidomyiidae|[8260]|CNGME1820-14|Cecidomyiidae|658|BOLD:ACL3360
Cecidomyiidae|[8261]|CNFDH1091-14|Cecidomyiidae|661|BOLD:ACL8032
Cecidomyiidae|[8262]|CNMIF317-14|Cecidomyiidae|613|BOLD:ACN2140
Cecidomyiidae|[8263]|CNFNR1603-14|Cecidomyiidae|579|BOLD:ACM7553
Cecidomyiidae|[8264]|CNMIF387-14|Cecidomyiidae|588|BOLD:ACN3206
Cecidomyiidae|[8265]|CNGME1905-14|Cecidomyiidae|658|BOLD:ACT2323
Cecidomyiidae|[8266]|CNFDH1053-14|Cecidomyiidae|661|BOLD:ACL6316
Cecidomyiidae|[8267]|CNNHE1192-14|Cecidomyiidae|582|BOLD:ACR7049
Cecidomyiidae|[8268]|CNEID2066-12|Cecidomyiidae|670|BOLD:ACB0618
Cecidomyiidae|[8269]|CNFDD256-14|Cecidomyiidae|661|BOLD:ACI3301
Cecidomyiidae|[8270]|CNKOE1403-14|Cecidomyiidae|597|BOLD:ACM2709
Cecidomyiidae|[8271]|CNBPN360-13|Cecidomyiidae|661|BOLD:ACD9435
Cecidomyiidae|[8272]|CNPAD438-13|Cecidomyiidae|633|BOLD:ACE0629
Cecidomyiidae|[8273]|CNGMF1894-14|Cecidomyiidae|667|BOLD:ABY1249
Cecidomyiidae|[8274]|CNFNE250-14|Cecidomyiidae|637|BOLD:ACL3714
Cecidomyiidae|[8275]|SSJAF3494-13|Cecidomyiidae|658|BOLD:ACK3625
Cecidomyiidae|[8276]|SSJAF7753-13|Cecidomyiidae|613|BOLD:ACK3396
Cecidomyiidae|[8277]|CNJAE1032-12|Cecidomyiidae|658|BOLD:ACC8491
Cecidomyiidae|[8278]|SSGBB3219-14|Cecidomyiidae|658|BOLD:ACL3746
Cecidomyiidae|[8279]|CNRME4365-12|Cecidomyiidae|673|BOLD:ACB0530
Cecidomyiidae|[8280]|CNFNF1200-14|Cecidomyiidae|664|BOLD:ACN3573
Cecidomyiidae|[8281]|CNFNR507-14|Cecidomyiidae|591|BOLD:ACN3780
Cecidomyiidae|[8282]|CNGCF303-15|Cecidomyiidae|588|BOLD:ACT7501
Cecidomyiidae|[8283]|BBDCN484-10|Cecidomyiidae|658|BOLD:AAP6844
Cecidomyiidae|[8284]|HPPPD1117-13|Cecidomyiidae|661|BOLD:ABV1485
Cecidomyiidae|[8285]|CNGMG238-14|Cecidomyiidae|660|BOLD:ACA8939
Cecidomyiidae|[8286]|SSGBA4246-14|Cecidomyiidae|628|BOLD:ACO4149
Cecidomyiidae|[8287]|CHDIP1336-12|Cecidomyiidae|658|BOLD:ACB2744
Cecidomyiidae|[8288]|SSBAF1235-13|Cecidomyiidae|661|BOLD:ACI3975
Cecidomyiidae|[8289]|CNBAC145-12|Cecidomyiidae|658|BOLD:ACC7873
Cecidomyiidae|[8290]|CNEID1524-12|Cecidomyiidae|658|BOLD:ACA9894
Cecidomyiidae|[8291]|CNGMF886-14|Cecidomyiidae|636|BOLD:ACL3474
Cecidomyiidae|[8292]|CNRMC1199-12|Cecidomyiidae|661|BOLD:ACC4471
Cecidomyiidae|[8293]|CNFNE1694-14|Cecidomyiidae|660|BOLD:ACL3403
Cecidomyiidae|[8294]|CNBPS168-13|Cecidomyiidae|658|BOLD:ACI4107
Cecidomyiidae|[8295]|CNFNF570-14|Cecidomyiidae|661|BOLD:ACL4145
Cecidomyiidae|[8296]|CNRME465-12|Cecidomyiidae|632|BOLD:ACC8374
Cecidomyiidae|[8297]|CNKTD1722-15|Cecidomyiidae|588|BOLD:ACS2724
Cecidomyiidae|[8298]|CNSLJ252-12|Cecidomyiidae|670|BOLD:AAM6092
Cecidomyiidae|[8299]|CNSLD207-12|Cecidomyiidae|658|BOLD:ACA7617

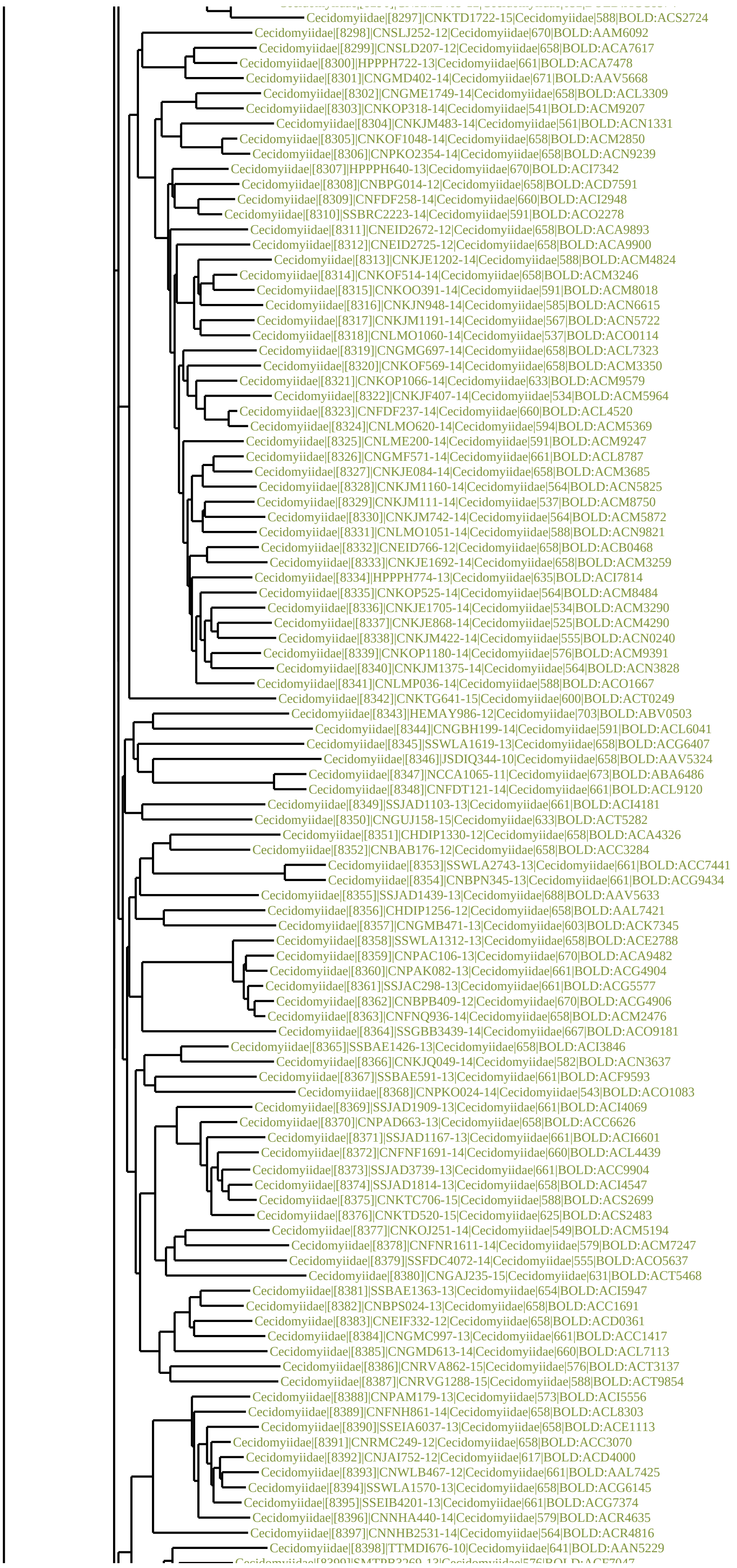

Cecidomyiidae|[8297]|CNKTD1722-15|Cecidomyiidae|588|BOLD:ACS2724
Cecidomyiidae|[8298]|CNSLJ252-12|Cecidomyiidae|670|BOLD:AAM6092
Cecidomyiidae|[8299]|CNSLD207-12|Cecidomyiidae|658|BOLD:ACA7617
Cecidomyiidae|[8300]|HPPPH722-13|Cecidomyiidae|661|BOLD:ACA7478
Cecidomyiidae|[8301]|CNGMD402-14|Cecidomyiidae|671|BOLD:AAV5668
Cecidomyiidae|[8302]|CNGME1749-14|Cecidomyiidae|658|BOLD:ACL3309
Cecidomyiidae|[8303]|CNKOP318-14|Cecidomyiidae|541|BOLD:ACM9207
Cecidomyiidae|[8304]|CNKJM483-14|Cecidomyiidae|561|BOLD:ACN1331
Cecidomyiidae|[8305]|CNKOF1048-14|Cecidomyiidae|658|BOLD:ACM2850
Cecidomyiidae|[8306]|CNPKO2354-14|Cecidomyiidae|658|BOLD:ACN9239
Cecidomyiidae|[8307]|HPPPH640-13|Cecidomyiidae|670|BOLD:ACI7342
Cecidomyiidae|[8308]|CNBPG014-12|Cecidomyiidae|658|BOLD:ACD7591
Cecidomyiidae|[8309]|CNFDF258-14|Cecidomyiidae|660|BOLD:ACI2948
Cecidomyiidae|[8310]|SSBRC2223-14|Cecidomyiidae|591|BOLD:ACO2278
Cecidomyiidae|[8311]|CNEID2672-12|Cecidomyiidae|658|BOLD:ACA9893
Cecidomyiidae|[8312]|CNEID2725-12|Cecidomyiidae|658|BOLD:ACA9900
Cecidomyiidae|[8313]|CNKJE1202-14|Cecidomyiidae|588|BOLD:ACM4824
Cecidomyiidae|[8314]|CNKOF514-14|Cecidomyiidae|658|BOLD:ACM3246
Cecidomyiidae|[8315]|CNKOO391-14|Cecidomyiidae|591|BOLD:ACM8018
Cecidomyiidae|[8316]|CNKJN948-14|Cecidomyiidae|585|BOLD:ACN6615
Cecidomyiidae|[8317]|CNKJM1191-14|Cecidomyiidae|567|BOLD:ACN5722
Cecidomyiidae|[8318]|CNLMO1060-14|Cecidomyiidae|537|BOLD:ACO0114
Cecidomyiidae|[8319]|CNGMG697-14|Cecidomyiidae|658|BOLD:ACL7323
Cecidomyiidae|[8320]|CNKOF569-14|Cecidomyiidae|658|BOLD:ACM3350
Cecidomyiidae|[8321]|CNKOP1066-14|Cecidomyiidae|633|BOLD:ACM9579
Cecidomyiidae|[8322]|CNKJF407-14|Cecidomyiidae|534|BOLD:ACM5964
Cecidomyiidae|[8323]|CNFDF237-14|Cecidomyiidae|660|BOLD:ACL4520
Cecidomyiidae|[8324]|CNLMO620-14|Cecidomyiidae|594|BOLD:ACM5369
Cecidomyiidae|[8325]|CNLME200-14|Cecidomyiidae|591|BOLD:ACM9247
Cecidomyiidae|[8326]|CNGMF571-14|Cecidomyiidae|661|BOLD:ACL8787
Cecidomyiidae|[8327]|CNKJE084-14|Cecidomyiidae|658|BOLD:ACM3685
Cecidomyiidae|[8328]|CNKJM1160-14|Cecidomyiidae|564|BOLD:ACN5825
Cecidomyiidae|[8329]|CNKJM111-14|Cecidomyiidae|537|BOLD:ACM8750
Cecidomyiidae|[8330]|CNKJM742-14|Cecidomyiidae|564|BOLD:ACM5872
Cecidomyiidae|[8331]|CNLMO1051-14|Cecidomyiidae|588|BOLD:ACN9821
Cecidomyiidae|[8332]|CNEID766-12|Cecidomyiidae|658|BOLD:ACB0468
Cecidomyiidae|[8333]|CNKJE1692-14|Cecidomyiidae|658|BOLD:ACM3259
Cecidomyiidae|[8334]|HPPPH774-13|Cecidomyiidae|635|BOLD:ACI7814
Cecidomyiidae|[8335]|CNKOP525-14|Cecidomyiidae|564|BOLD:ACM8484
Cecidomyiidae|[8336]|CNKJE1705-14|Cecidomyiidae|534|BOLD:ACM3290
Cecidomyiidae|[8337]|CNKJE868-14|Cecidomyiidae|525|BOLD:ACM4290
Cecidomyiidae|[8338]|CNKJM422-14|Cecidomyiidae|555|BOLD:ACN0240
Cecidomyiidae|[8339]|CNKOP1180-14|Cecidomyiidae|576|BOLD:ACM9391
Cecidomyiidae|[8340]|CNKJM1375-14|Cecidomyiidae|564|BOLD:ACN3828
Cecidomyiidae|[8341]|CNLMP036-14|Cecidomyiidae|588|BOLD:ACO1667
Cecidomyiidae|[8342]|CNKTG641-15|Cecidomyiidae|600|BOLD:ACT0249
Cecidomyiidae|[8343]|HEMAY986-12|Cecidomyiidae|703|BOLD:ABV0503
Cecidomyiidae|[8344]|CNGBH199-14|Cecidomyiidae|591|BOLD:ACL6041
Cecidomyiidae|[8345]|SSWLA1619-13|Cecidomyiidae|658|BOLD:ACG6407
Cecidomyiidae|[8346]|JSDIQ344-10|Cecidomyiidae|658|BOLD:AAV5324
Cecidomyiidae|[8347]|NCCA1065-11|Cecidomyiidae|673|BOLD:ABA6486
Cecidomyiidae|[8348]|CNFDT121-14|Cecidomyiidae|661|BOLD:ACL9120
Cecidomyiidae|[8349]|SSJAD1103-13|Cecidomyiidae|661|BOLD:ACI4181
Cecidomyiidae|[8350]|CNGUJ158-15|Cecidomyiidae|633|BOLD:ACT5282
Cecidomyiidae|[8351]|CHDIP1330-12|Cecidomyiidae|658|BOLD:ACA4326
Cecidomyiidae|[8352]|CNBAB176-12|Cecidomyiidae|658|BOLD:ACC3284
Cecidomyiidae|[8353]|SSWLA2743-13|Cecidomyiidae|661|BOLD:ACC7441
Cecidomyiidae|[8354]|CNBPN345-13|Cecidomyiidae|661|BOLD:ACG9434
Cecidomyiidae|[8355]|SSJAD1439-13|Cecidomyiidae|688|BOLD:AAV5633
Cecidomyiidae|[8356]|CHDIP1256-12|Cecidomyiidae|658|BOLD:AAL7421
Cecidomyiidae|[8357]|CNGMB471-13|Cecidomyiidae|603|BOLD:ACK7345
Cecidomyiidae|[8358]|SSWLA1312-13|Cecidomyiidae|658|BOLD:ACE2788
Cecidomyiidae|[8359]|CNPAC106-13|Cecidomyiidae|670|BOLD:ACA9482
Cecidomyiidae|[8360]|CNPAK082-13|Cecidomyiidae|661|BOLD:ACG4904
Cecidomyiidae|[8361]|SSJAC298-13|Cecidomyiidae|661|BOLD:ACG5577
Cecidomyiidae|[8362]|CNBPB409-12|Cecidomyiidae|670|BOLD:ACG4906
Cecidomyiidae|[8363]|CNFNQ936-14|Cecidomyiidae|658|BOLD:ACM2476
Cecidomyiidae|[8364]|SSGBB3439-14|Cecidomyiidae|667|BOLD:ACO9181
Cecidomyiidae|[8365]|SSBAE1426-13|Cecidomyiidae|658|BOLD:ACI3846
Cecidomyiidae|[8366]|CNKJQ049-14|Cecidomyiidae|582|BOLD:ACN3637
Cecidomyiidae|[8367]|SSBAE591-13|Cecidomyiidae|661|BOLD:ACF9593
Cecidomyiidae|[8368]|CNPKO024-14|Cecidomyiidae|543|BOLD:ACO1083
Cecidomyiidae|[8369]|SSJAD1909-13|Cecidomyiidae|661|BOLD:ACI4069
Cecidomyiidae|[8370]|CNPAD663-13|Cecidomyiidae|658|BOLD:ACC6626
Cecidomyiidae|[8371]|SSJAD1167-13|Cecidomyiidae|661|BOLD:ACI6601
Cecidomyiidae|[8372]|CNFNF1691-14|Cecidomyiidae|660|BOLD:ACL4439
Cecidomyiidae|[8373]|SSJAD3739-13|Cecidomyiidae|661|BOLD:ACC9904
Cecidomyiidae|[8374]|SSJAD1814-13|Cecidomyiidae|658|BOLD:ACI4547
Cecidomyiidae|[8375]|CNKTC706-15|Cecidomyiidae|588|BOLD:ACS2699
Cecidomyiidae|[8376]|CNKTD520-15|Cecidomyiidae|625|BOLD:ACS2483
Cecidomyiidae|[8377]|CNKOJ251-14|Cecidomyiidae|549|BOLD:ACM5194
Cecidomyiidae|[8378]|CNFNR1611-14|Cecidomyiidae|579|BOLD:ACM7247
Cecidomyiidae|[8379]|SSFDC4072-14|Cecidomyiidae|555|BOLD:ACO5637
Cecidomyiidae|[8380]|CNGAJ235-15|Cecidomyiidae|631|BOLD:ACT5468
Cecidomyiidae|[8381]|SSBAE1363-13|Cecidomyiidae|654|BOLD:ACI5947
Cecidomyiidae|[8382]|CNBPS024-13|Cecidomyiidae|658|BOLD:ACC1691
Cecidomyiidae|[8383]|CNEIF332-12|Cecidomyiidae|658|BOLD:ACD0361
Cecidomyiidae|[8384]|CNGMC997-13|Cecidomyiidae|661|BOLD:ACC1417
Cecidomyiidae|[8385]|CNGMD613-14|Cecidomyiidae|660|BOLD:ACL7113
Cecidomyiidae|[8386]|CNRVA862-15|Cecidomyiidae|576|BOLD:ACT3137
Cecidomyiidae|[8387]|CNRVG1288-15|Cecidomyiidae|588|BOLD:ACT9854
Cecidomyiidae|[8388]|CNPAM179-13|Cecidomyiidae|573|BOLD:ACI5556
Cecidomyiidae|[8389]|CNFNH861-14|Cecidomyiidae|658|BOLD:ACL8303
Cecidomyiidae|[8390]|SSEIA6037-13|Cecidomyiidae|658|BOLD:ACE1113
Cecidomyiidae|[8391]|CNRMC249-12|Cecidomyiidae|658|BOLD:ACC3070
Cecidomyiidae|[8392]|CNJAI752-12|Cecidomyiidae|617|BOLD:ACD4000
Cecidomyiidae|[8393]|CNWLB467-12|Cecidomyiidae|661|BOLD:AAL7425
Cecidomyiidae|[8394]|SSWLA1570-13|Cecidomyiidae|658|BOLD:ACG6145
Cecidomyiidae|[8395]|SSEIB4201-13|Cecidomyiidae|661|BOLD:ACG7374
Cecidomyiidae|[8396]|CNNHA440-14|Cecidomyiidae|579|BOLD:ACR4635
Cecidomyiidae|[8397]|CNNHB2531-14|Cecidomyiidae|564|BOLD:ACR4816
Cecidomyiidae|[8398]|TTMDI676-10|Cecidomyiidae|641|BOLD:AAN5229

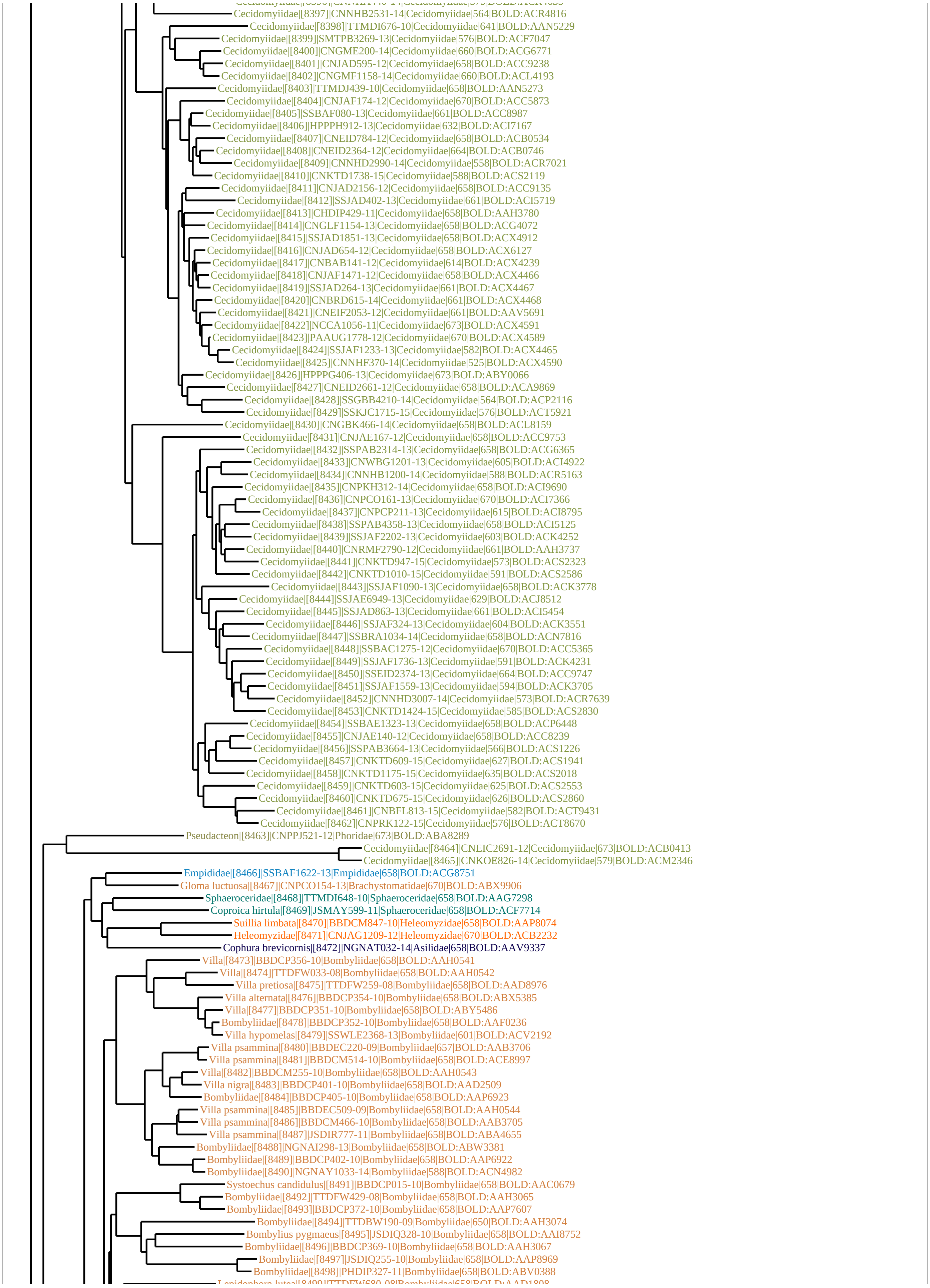

Cecidomyiidae|[8397]|CNNHB2531-14|Cecidomyiidae|564|BOLD:ACR4816
Cecidomyiidae|[8398]|TTMDI676-10|Cecidomyiidae|641|BOLD:AAN5229
Cecidomyiidae|[8399]|SMTPB3269-13|Cecidomyiidae|576|BOLD:ACF7047
Cecidomyiidae|[8400]|CNGME200-14|Cecidomyiidae|660|BOLD:ACG6771
Cecidomyiidae|[8401]|CNJAD595-12|Cecidomyiidae|658|BOLD:ACC9238
Cecidomyiidae|[8402]|CNGMF1158-14|Cecidomyiidae|660|BOLD:ACL4193
Cecidomyiidae|[8403]|TTMDJ439-10|Cecidomyiidae|658|BOLD:AAN5273
Cecidomyiidae|[8404]|CNJAF174-12|Cecidomyiidae|670|BOLD:ACC5873
Cecidomyiidae|[8405]|SSBAF080-13|Cecidomyiidae|661|BOLD:ACC8987
Cecidomyiidae|[8406]|HPPPH912-13|Cecidomyiidae|632|BOLD:ACI7167
Cecidomyiidae|[8407]|CNEID784-12|Cecidomyiidae|658|BOLD:ACB0534
Cecidomyiidae|[8408]|CNEID2364-12|Cecidomyiidae|664|BOLD:ACB0746
Cecidomyiidae|[8409]|CNNHD2990-14|Cecidomyiidae|558|BOLD:ACR7021
Cecidomyiidae|[8410]|CNKTD1738-15|Cecidomyiidae|588|BOLD:ACS2119
Cecidomyiidae|[8411]|CNJAD2156-12|Cecidomyiidae|658|BOLD:ACC9135
Cecidomyiidae|[8412]|SSJAD402-13|Cecidomyiidae|661|BOLD:ACI5719
Cecidomyiidae|[8413]|CHDIP429-11|Cecidomyiidae|658|BOLD:AAH3780
Cecidomyiidae|[8414]|CNGLF1154-13|Cecidomyiidae|658|BOLD:ACG4072
Cecidomyiidae|[8415]|SSJAD1851-13|Cecidomyiidae|658|BOLD:ACX4912
Cecidomyiidae|[8416]|CNJAD654-12|Cecidomyiidae|658|BOLD:ACX6127
Cecidomyiidae|[8417]|CNBAB141-12|Cecidomyiidae|614|BOLD:ACX4239
Cecidomyiidae|[8418]|CNJAF1471-12|Cecidomyiidae|658|BOLD:ACX4466
Cecidomyiidae|[8419]|SSJAD264-13|Cecidomyiidae|661|BOLD:ACX4467
Cecidomyiidae|[8420]|CNBRD615-14|Cecidomyiidae|661|BOLD:ACX4468
Cecidomyiidae|[8421]|CNEIF2053-12|Cecidomyiidae|661|BOLD:AAV5691
Cecidomyiidae|[8422]|NCCA1056-11|Cecidomyiidae|673|BOLD:ACX4591
Cecidomyiidae|[8423]|PAAUG1778-12|Cecidomyiidae|670|BOLD:ACX4589
Cecidomyiidae|[8424]|SSJAF1233-13|Cecidomyiidae|582|BOLD:ACX4465
Cecidomyiidae|[8425]|CNNHF370-14|Cecidomyiidae|525|BOLD:ACX4590
Cecidomyiidae|[8426]|HPPPG406-13|Cecidomyiidae|673|BOLD:ABY0066
Cecidomyiidae|[8427]|CNEID2661-12|Cecidomyiidae|658|BOLD:ACA9869
Cecidomyiidae|[8428]|SSGBB4210-14|Cecidomyiidae|564|BOLD:ACP2116
Cecidomyiidae|[8429]|SSKJC1715-15|Cecidomyiidae|576|BOLD:ACT5921
Cecidomyiidae|[8430]|CNGBK466-14|Cecidomyiidae|658|BOLD:ACL8159
Cecidomyiidae|[8431]|CNJAE167-12|Cecidomyiidae|658|BOLD:ACC9753
Cecidomyiidae|[8432]|SSPAB2314-13|Cecidomyiidae|658|BOLD:ACG6365
Cecidomyiidae|[8433]|CNWBG1201-13|Cecidomyiidae|605|BOLD:ACI4922
Cecidomyiidae|[8434]|CNNHB1200-14|Cecidomyiidae|588|BOLD:ACR5163
Cecidomyiidae|[8435]|CNPKH312-14|Cecidomyiidae|658|BOLD:ACI9690
Cecidomyiidae|[8436]|CNPCO161-13|Cecidomyiidae|670|BOLD:ACI7366
Cecidomyiidae|[8437]|CNPCP211-13|Cecidomyiidae|615|BOLD:ACI8795
Cecidomyiidae|[8438]|SSPAB4358-13|Cecidomyiidae|658|BOLD:ACI5125
Cecidomyiidae|[8439]|SSJAF2202-13|Cecidomyiidae|603|BOLD:ACK4252
Cecidomyiidae|[8440]|CNRMF2790-12|Cecidomyiidae|661|BOLD:AAH3737
Cecidomyiidae|[8441]|CNKTD947-15|Cecidomyiidae|573|BOLD:ACS2323
Cecidomyiidae|[8442]|CNKTD1010-15|Cecidomyiidae|591|BOLD:ACS2586
Cecidomyiidae|[8443]|SSJAF1090-13|Cecidomyiidae|658|BOLD:ACK3778
Cecidomyiidae|[8444]|SSJAE6949-13|Cecidomyiidae|629|BOLD:ACJ8512
Cecidomyiidae|[8445]|SSJAD863-13|Cecidomyiidae|661|BOLD:ACI5454
Cecidomyiidae|[8446]|SSJAF324-13|Cecidomyiidae|604|BOLD:ACK3551
Cecidomyiidae|[8447]|SSBRA1034-14|Cecidomyiidae|658|BOLD:ACN7816
Cecidomyiidae|[8448]|SSBAC1275-12|Cecidomyiidae|670|BOLD:ACC5365
Cecidomyiidae|[8449]|SSJAF1736-13|Cecidomyiidae|591|BOLD:ACK4231
Cecidomyiidae|[8450]|SSEID2374-13|Cecidomyiidae|664|BOLD:ACC9747
Cecidomyiidae|[8451]|SSJAF1559-13|Cecidomyiidae|594|BOLD:ACK3705
Cecidomyiidae|[8452]|CNNHD3007-14|Cecidomyiidae|573|BOLD:ACR7639
Cecidomyiidae|[8453]|CNKTD1424-15|Cecidomyiidae|585|BOLD:ACS2830
Cecidomyiidae|[8454]|SSBAE1323-13|Cecidomyiidae|658|BOLD:ACP6448
Cecidomyiidae|[8455]|CNJAE140-12|Cecidomyiidae|658|BOLD:ACC8239
Cecidomyiidae|[8456]|SSPAB3664-13|Cecidomyiidae|566|BOLD:ACS1226
Cecidomyiidae|[8457]|CNKTD609-15|Cecidomyiidae|627|BOLD:ACS1941
Cecidomyiidae|[8458]|CNKTD1175-15|Cecidomyiidae|635|BOLD:ACS2018
Cecidomyiidae|[8459]|CNKTD603-15|Cecidomyiidae|625|BOLD:ACS2553
Cecidomyiidae|[8460]|CNKTD675-15|Cecidomyiidae|626|BOLD:ACS2860
Cecidomyiidae|[8461]|CNBFL813-15|Cecidomyiidae|582|BOLD:ACT9431
Cecidomyiidae|[8462]|CNPRK122-15|Cecidomyiidae|576|BOLD:ACT8670
Pseudacteon|[8463]|CNPPJ521-12|Phoridae|673|BOLD:ABA8289
Cecidomyiidae|[8464]|CNEIC2691-12|Cecidomyiidae|673|BOLD:ACB0413
Cecidomyiidae|[8465]|CNKOE826-14|Cecidomyiidae|579|BOLD:ACM2346
Empididae|[8466]|SSBAF1622-13|Empididae|658|BOLD:ACG8751
Gloma luctuosa|[8467]|CNPCO154-13|Brachystomatidae|670|BOLD:ABX9906
Sphaeroceridae|[8468]|TTMDI648-10|Sphaeroceridae|658|BOLD:AAG7298
Coproica hirtula|[8469]|JSMAY599-11|Sphaeroceridae|658|BOLD:ACF7714
Suillia limbata|[8470]|BBDCM847-10|Heleomyzidae|658|BOLD:AAP8074
Heleomyzidae|[8471]|CNJAG1209-12|Heleomyzidae|670|BOLD:ACB2232
Cophura brevicornis|[8472]|NGNAT032-14|Asilidae|658|BOLD:AAV9337
Villa|[8473]|BBDCP356-10|Bombyliidae|658|BOLD:AAH0541
Villa|[8474]|TTDFW033-08|Bombyliidae|658|BOLD:AAH0542
Villa pretiosa|[8475]|TTDFW259-08|Bombyliidae|658|BOLD:AAD8976
Villa alternata|[8476]|BBDCP354-10|Bombyliidae|658|BOLD:ABX5385
Villa|[8477]|BBDCP351-10|Bombyliidae|658|BOLD:ABY5486
Bombyliidae|[8478]|BBDCP352-10|Bombyliidae|658|BOLD:AAF0236
Villa hypomelas|[8479]|SSWLE2368-13|Bombyliidae|601|BOLD:ACV2192
Villa psammina|[8480]|BBDEC220-09|Bombyliidae|657|BOLD:AAB3706
Villa psammina|[8481]|BBDCM514-10|Bombyliidae|658|BOLD:ACE8997
Villa|[8482]|BBDCM255-10|Bombyliidae|658|BOLD:AAH0543
Villa nigra|[8483]|BBDCP401-10|Bombyliidae|658|BOLD:AAD2509
Bombyliidae|[8484]|BBDCP405-10|Bombyliidae|658|BOLD:AAP6923
Villa psammina|[8485]|BBDEC509-09|Bombyliidae|658|BOLD:AAH0544
Villa psammina|[8486]|BBDCM466-10|Bombyliidae|658|BOLD:AAB3705
Villa psammina|[8487]|JSDIR777-11|Bombyliidae|658|BOLD:ABA4655
Bombyliidae|[8488]|NGNAI298-13|Bombyliidae|658|BOLD:ABW3381
Bombyliidae|[8489]|BBDCP402-10|Bombyliidae|658|BOLD:AAP6922
Bombyliidae|[8490]|NGNAY1033-14|Bombyliidae|588|BOLD:ACN4982
Systoechus candidulus|[8491]|BBDCP015-10|Bombyliidae|658|BOLD:AAC0679
Bombyliidae|[8492]|TTDFW429-08|Bombyliidae|658|BOLD:AAH3065
Bombyliidae|[8493]|BBDCP372-10|Bombyliidae|658|BOLD:AAP7607
Bombyliidae|[8494]|TTDBW190-09|Bombyliidae|650|BOLD:AAH3074
Bombylius pygmaeus|[8495]|JSDIQ328-10|Bombyliidae|658|BOLD:AAI8752
Bombyliidae|[8496]|BBDCP369-10|Bombyliidae|658|BOLD:AAH3067
Bombyliidae|[8497]|JSDIQ255-10|Bombyliidae|658|BOLD:AAP8969
Bombyliidae|[8498]|PHDIP327-11|Bombyliidae|658|BOLD:ABV0388

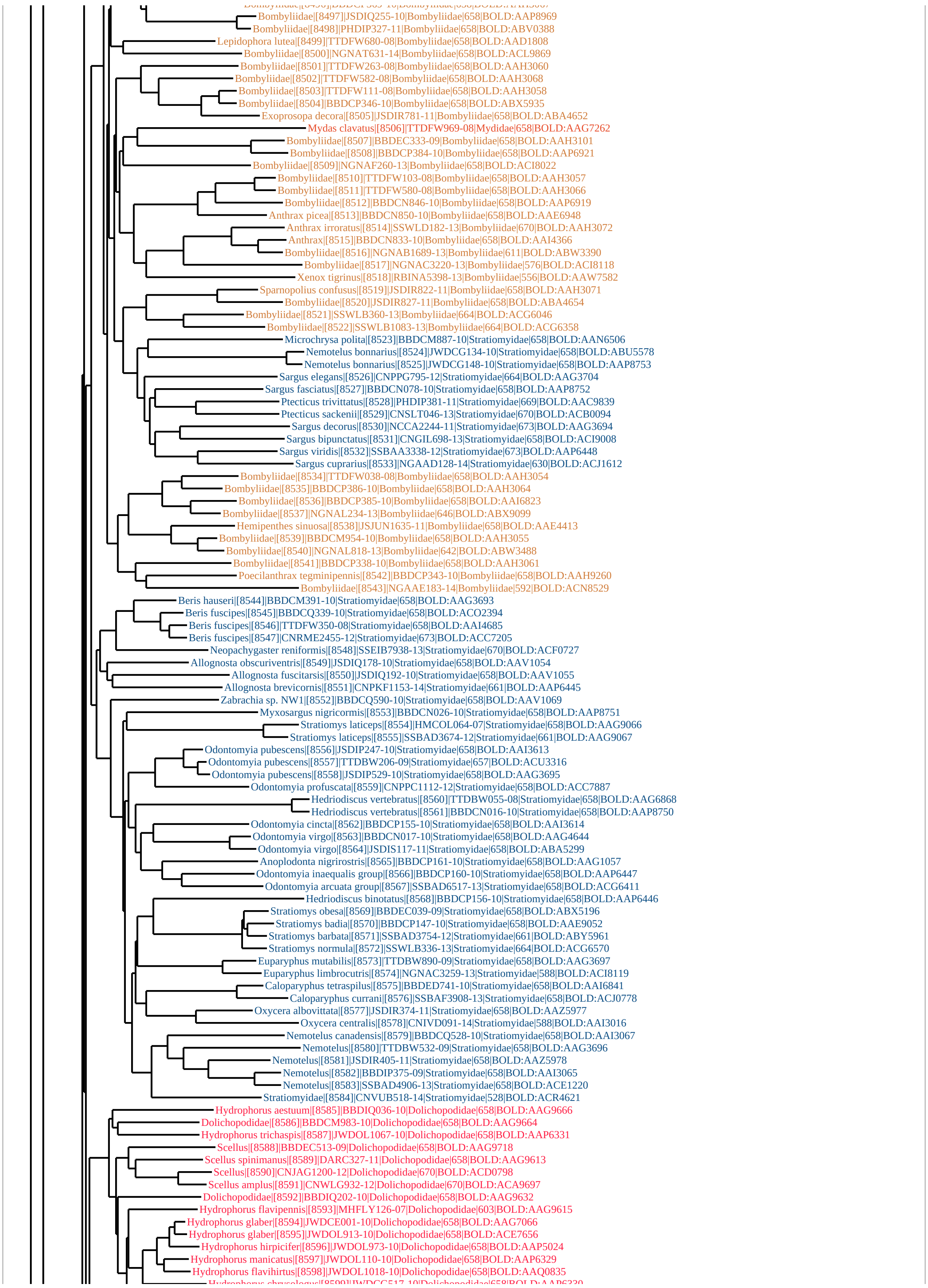

Bombyliidae|[8497]|JSDIQ255-10|Bombyliidae|658|BOLD:AAP8969
Bombyliidae|[8498]|PHDIP327-11|Bombyliidae|658|BOLD:ABV0388
Lepidophora lutea|[8499]|TTDFW680-08|Bombyliidae|658|BOLD:AAD1808
Bombyliidae|[8500]|NGNAT631-14|Bombyliidae|658|BOLD:ACL9869
Bombyliidae|[8501]|TTDFW263-08|Bombyliidae|658|BOLD:AAH3060
Bombyliidae|[8502]|TTDFW582-08|Bombyliidae|658|BOLD:AAH3068
Bombyliidae|[8503]|TTDFW111-08|Bombyliidae|658|BOLD:AAH3058
Bombyliidae|[8504]|BBDCP346-10|Bombyliidae|658|BOLD:ABX5935
Exoprosopa decora|[8505]|JSDIR781-11|Bombyliidae|658|BOLD:ABA4652
Mydas clavatus|[8506]|TTDFW969-08|Mydidae|658|BOLD:AAG7262
Bombyliidae|[8507]|BBDEC333-09|Bombyliidae|658|BOLD:AAH3101
Bombyliidae|[8508]|BBDCP384-10|Bombyliidae|658|BOLD:AAP6921
Bombyliidae|[8509]|NGNAF260-13|Bombyliidae|658|BOLD:ACI8022
Bombyliidae|[8510]|TTDFW103-08|Bombyliidae|658|BOLD:AAH3057
Bombyliidae|[8511]|TTDFW580-08|Bombyliidae|658|BOLD:AAH3066
Bombyliidae|[8512]|BBDCN846-10|Bombyliidae|658|BOLD:AAP6919
Anthrax picea|[8513]|BBDCN850-10|Bombyliidae|658|BOLD:AAE6948
Anthrax irroratus|[8514]|SSWLD182-13|Bombyliidae|670|BOLD:AAH3072
Anthrax|[8515]|BBDCN833-10|Bombyliidae|658|BOLD:AAI4366
Bombyliidae|[8516]|NGNAB1689-13|Bombyliidae|611|BOLD:ABW3390
Bombyliidae|[8517]|NGNAC3220-13|Bombyliidae|576|BOLD:ACI8118
Xenox tigrinus|[8518]|RBINA5398-13|Bombyliidae|556|BOLD:AAW7582
Sparnopolius confusus|[8519]|JSDIR822-11|Bombyliidae|658|BOLD:AAH3071
Bombyliidae|[8520]|JSDIR827-11|Bombyliidae|658|BOLD:ABA4654
Bombyliidae|[8521]|SSWLB360-13|Bombyliidae|664|BOLD:ACG6046
Bombyliidae|[8522]|SSWLB1083-13|Bombyliidae|664|BOLD:ACG6358
Microchrysa polita|[8523]|BBDCM887-10|Stratiomyidae|658|BOLD:AAN6506
Nemotelus bonnarius|[8524]|JWDCG134-10|Stratiomyidae|658|BOLD:ABU5578
Nemotelus bonnarius|[8525]|JWDCG148-10|Stratiomyidae|658|BOLD:AAP8753
Sargus elegans|[8526]|CNPPG795-12|Stratiomyidae|664|BOLD:AAG3704
Sargus fasciatus|[8527]|BBDCN078-10|Stratiomyidae|658|BOLD:AAP8752
Ptecticus trivittatus|[8528]|PHDIP381-11|Stratiomyidae|669|BOLD:AAC9839
Ptecticus sackenii|[8529]|CNSLT046-13|Stratiomyidae|670|BOLD:ACB0094
Sargus decorus|[8530]|NCCA2244-11|Stratiomyidae|673|BOLD:AAG3694
Sargus bipunctatus|[8531]|CNGIL698-13|Stratiomyidae|658|BOLD:ACI9008
Sargus viridis|[8532]|SSBAA3338-12|Stratiomyidae|673|BOLD:AAP6448
Sargus cuprarius|[8533]|NGAAD128-14|Stratiomyidae|630|BOLD:ACJ1612
Bombyliidae|[8534]|TTDFW038-08|Bombyliidae|658|BOLD:AAH3054
Bombyliidae|[8535]|BBDCP386-10|Bombyliidae|658|BOLD:AAH3064
Bombyliidae|[8536]|BBDCP385-10|Bombyliidae|658|BOLD:AAI6823
Bombyliidae|[8537]|NGNAL234-13|Bombyliidae|646|BOLD:ABX9099
Hemipenthes sinuosa|[8538]|JSJUN1635-11|Bombyliidae|658|BOLD:AAE4413
Bombyliidae|[8539]|BBDCM954-10|Bombyliidae|658|BOLD:AAH3055
Bombyliidae|[8540]|NGNAL818-13|Bombyliidae|642|BOLD:ABW3488
Bombyliidae|[8541]|BBDCP338-10|Bombyliidae|658|BOLD:AAH3061
Poecilanthrax tegminipennis|[8542]|BBDCP343-10|Bombyliidae|658|BOLD:AAH9260
Bombyliidae|[8543]|NGAAE183-14|Bombyliidae|592|BOLD:ACN8529
Beris hauseri|[8544]|BBDCM391-10|Stratiomyidae|658|BOLD:AAG3693
Beris fuscipes|[8545]|BBDCQ339-10|Stratiomyidae|658|BOLD:ACO2394
Beris fuscipes|[8546]|TTDFW350-08|Stratiomyidae|658|BOLD:AAI4685
Beris fuscipes|[8547]|CNRME2455-12|Stratiomyidae|673|BOLD:ACC7205
Neopachygaster reniformis|[8548]|SSEIB7938-13|Stratiomyidae|670|BOLD:ACF0727
Allognosta obscuriventris|[8549]|JSDIQ178-10|Stratiomyidae|658|BOLD:AAV1054
Allognosta fuscitarsis|[8550]|JSDIQ192-10|Stratiomyidae|658|BOLD:AAV1055
Allognosta brevicornis|[8551]|CNPKF1153-14|Stratiomyidae|661|BOLD:AAP6445
Zabrachia sp. NW1|[8552]|BBDCQ590-10|Stratiomyidae|658|BOLD:AAV1069
Myxosargus nigricormis|[8553]|BBDCN026-10|Stratiomyidae|658|BOLD:AAP8751
Stratiomys laticeps|[8554]|HMCOL064-07|Stratiomyidae|658|BOLD:AAG9066
Stratiomys laticeps|[8555]|SSBAD3674-12|Stratiomyidae|661|BOLD:AAG9067
Odontomyia pubescens|[8556]|JSDIP247-10|Stratiomyidae|658|BOLD:AAI3613
Odontomyia pubescens|[8557]|TTDBW206-09|Stratiomyidae|657|BOLD:ACU3316
Odontomyia pubescens|[8558]|JSDIP529-10|Stratiomyidae|658|BOLD:AAG3695
Odontomyia profuscata|[8559]|CNPPC1112-12|Stratiomyidae|658|BOLD:ACC7887
Hedriodiscus vertebratus|[8560]|TTDBW055-08|Stratiomyidae|658|BOLD:AAG6868
Hedriodiscus vertebratus|[8561]|BBDCN016-10|Stratiomyidae|658|BOLD:AAP8750
Odontomyia cincta|[8562]|BBDCP155-10|Stratiomyidae|658|BOLD:AAI3614
Odontomyia virgo|[8563]|BBDCN017-10|Stratiomyidae|658|BOLD:AAG4644
Odontomyia virgo|[8564]|JSDIS117-11|Stratiomyidae|658|BOLD:ABA5299
Anoplodonta nigrirostris|[8565]|BBDCP161-10|Stratiomyidae|658|BOLD:AAG1057
Odontomyia inaequalis group|[8566]|BBDCP160-10|Stratiomyidae|658|BOLD:AAP6447
Odontomyia arcuata group|[8567]|SSBAD6517-13|Stratiomyidae|658|BOLD:ACG6411
Hedriodiscus binotatus|[8568]|BBDCP156-10|Stratiomyidae|658|BOLD:AAP6446
Stratiomys obesa|[8569]|BBDEC039-09|Stratiomyidae|658|BOLD:ABX5196
Stratiomys badia|[8570]|BBDCP147-10|Stratiomyidae|658|BOLD:AAE9052
Stratiomys barbata|[8571]|SSBAD3754-12|Stratiomyidae|661|BOLD:ABY5961
Stratiomys normula|[8572]|SSWLB336-13|Stratiomyidae|664|BOLD:ACG6570
Euparyphus mutabilis|[8573]|TTDBW890-09|Stratiomyidae|658|BOLD:AAG3697
Euparyphus limbrocutris|[8574]|NGNAC3259-13|Stratiomyidae|588|BOLD:ACI8119
Caloparyphus tetraspilus|[8575]|BBDED741-10|Stratiomyidae|658|BOLD:AAI6841
Caloparyphus currani|[8576]|SSBAF3908-13|Stratiomyidae|658|BOLD:ACJ0778
Oxycera albovittata|[8577]|JSDIR374-11|Stratiomyidae|658|BOLD:AAZ5977
Oxycera centralis|[8578]|CNIVD091-14|Stratiomyidae|588|BOLD:AAI3016
Nemotelus canadensis|[8579]|BBDCQ528-10|Stratiomyidae|658|BOLD:AAI3067
Nemotelus|[8580]|TTDBW532-09|Stratiomyidae|658|BOLD:AAG3696
Nemotelus|[8581]|JSDIR405-11|Stratiomyidae|658|BOLD:AAZ5978
Nemotelus|[8582]|BBDIP375-09|Stratiomyidae|658|BOLD:AAI3065
Nemotelus|[8583]|SSBAD4906-13|Stratiomyidae|658|BOLD:ACE1220
Stratiomyidae|[8584]|CNVUB518-14|Stratiomyidae|528|BOLD:ACR4621
Hydrophorus aestuum|[8585]|BBDIQ036-10|Dolichopodidae|658|BOLD:AAG9666
Dolichopodidae|[8586]|BBDCM983-10|Dolichopodidae|658|BOLD:AAG9664
Hydrophorus trichaspis|[8587]|JWDOL1067-10|Dolichopodidae|658|BOLD:AAP6331
Scellus|[8588]|BBDEC513-09|Dolichopodidae|658|BOLD:AAG9718
Scellus spinimanus|[8589]|DARC327-11|Dolichopodidae|658|BOLD:AAG9613
Scellus|[8590]|CNJAG1200-12|Dolichopodidae|670|BOLD:ACD0798
Scellus amplus|[8591]|CNWLG932-12|Dolichopodidae|670|BOLD:ACA9697
Dolichopodidae|[8592]|BBDIQ202-10|Dolichopodidae|658|BOLD:AAG9632
Hydrophorus flavipennis|[8593]|MHFLY126-07|Dolichopodidae|603|BOLD:AAG9615
Hydrophorus glaber|[8594]|JWDCE001-10|Dolichopodidae|658|BOLD:AAG7066
Hydrophorus glaber|[8595]|JWDOL913-10|Dolichopodidae|658|BOLD:ACE7656
Hydrophorus hirpicifer|[8596]|JWDOL973-10|Dolichopodidae|658|BOLD:AAP5024
Hydrophorus manicatus|[8597]|JWDOL110-10|Dolichopodidae|658|BOLD:AAP6329
Hydrophorus flavihirtus|[8598]|JWDOL1018-10|Dolichopodidae|658|BOLD:AAQ0835

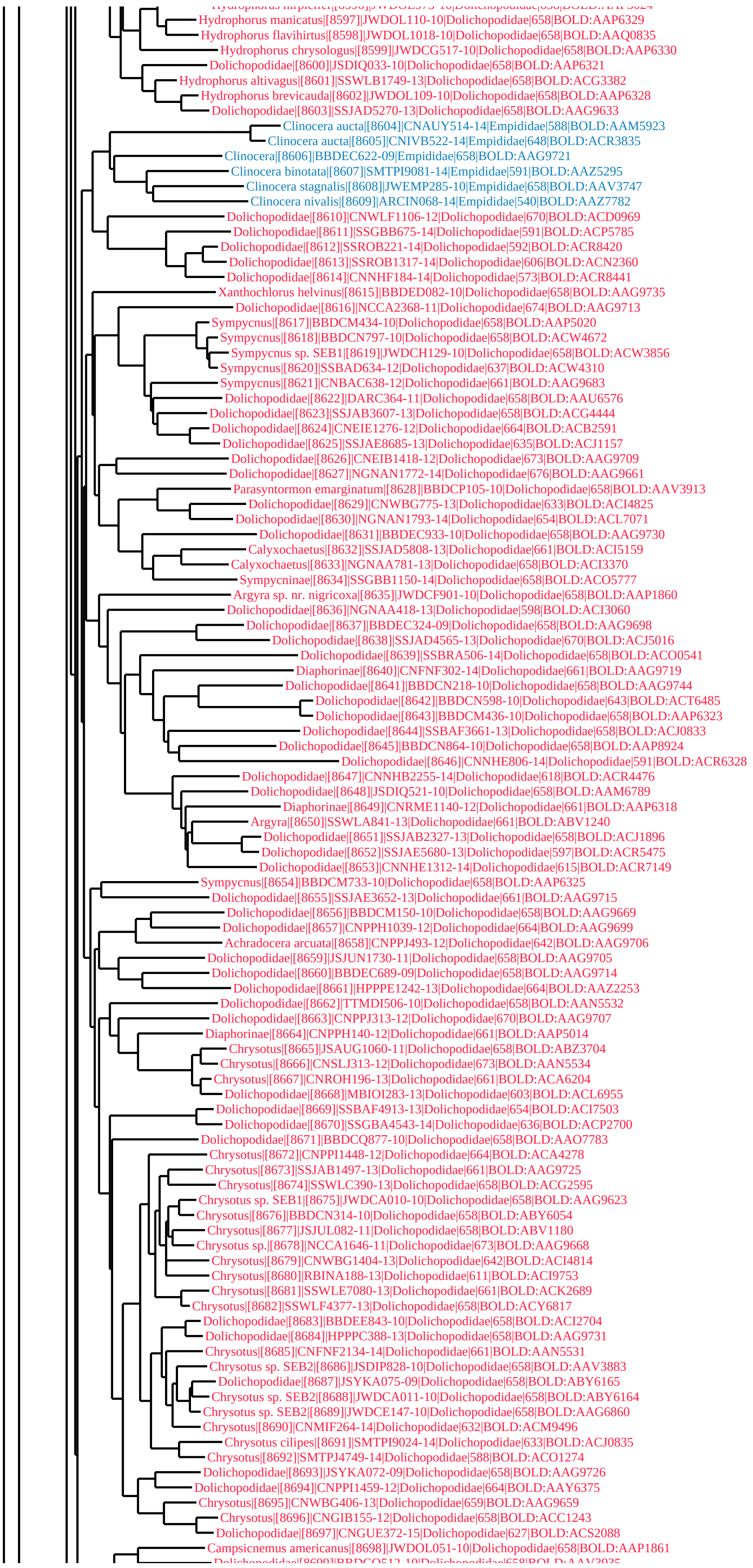
Hydrophorus manicatus|[8597]|JWDOL110-10|Dolichopodidae|658|BOLD:AAP6329
Hydrophorus flavihirtus|[8598]|JWDOL1018-10|Dolichopodidae|658|BOLD:AAQ0835
Hydrophorus chrysologus|[8599]|JWDCG517-10|Dolichopodidae|658|BOLD:AAP6330
Dolichopodidae|[8600]|JSDIQ033-10|Dolichopodidae|658|BOLD:AAP6321
Hydrophorus altivagus|[8601]|SSWLB1749-13|Dolichopodidae|658|BOLD:ACG3382
Hydrophorus brevicauda|[8602]|JWDOL109-10|Dolichopodidae|658|BOLD:AAP6328
Dolichopodidae|[8603]|SSJAD5270-13|Dolichopodidae|658|BOLD:AAG9633
Clinocera aucta|[8604]|CNAUY514-14|Empididae|588|BOLD:AAM5923
Clinocera aucta|[8605]|CNIVB522-14|Empididae|648|BOLD:ACR3835
Clinocera|[8606]|BBDEC622-09|Empididae|658|BOLD:AAG9721
Clinocera binotata|[8607]|SMTPI9081-14|Empididae|591|BOLD:AAZ5295
Clinocera stagnalis|[8608]|JWEMP285-10|Empididae|658|BOLD:AAV3747
Clinocera nivalis|[8609]|ARCIN068-14|Empididae|540|BOLD:AAZ7782
Dolichopodidae|[8610]|CNWLF1106-12|Dolichopodidae|670|BOLD:ACD0969
Dolichopodidae|[8611]|SSGBB675-14|Dolichopodidae|591|BOLD:ACP5785
Dolichopodidae|[8612]|SSROB221-14|Dolichopodidae|592|BOLD:ACR8420
Dolichopodidae|[8613]|SSROB1317-14|Dolichopodidae|606|BOLD:ACN2360
Dolichopodidae|[8614]|CNNHF184-14|Dolichopodidae|573|BOLD:ACR8441
Xanthochlorus helvinus|[8615]|BBDED082-10|Dolichopodidae|658|BOLD:AAG9735
Dolichopodidae|[8616]|NCCA2368-11|Dolichopodidae|674|BOLD:AAG9713
Sympycnus|[8617]|BBDCM434-10|Dolichopodidae|658|BOLD:AAP5020
Sympycnus|[8618]|BBDCN797-10|Dolichopodidae|658|BOLD:ACW4672
Sympycnus sp. SEB1|[8619]|JWDCH129-10|Dolichopodidae|658|BOLD:ACW3856
Sympycnus|[8620]|SSBAD634-12|Dolichopodidae|637|BOLD:ACW4310
Sympycnus|[8621]|CNBAC638-12|Dolichopodidae|661|BOLD:AAG9683
Dolichopodidae|[8622]|DARC364-11|Dolichopodidae|658|BOLD:AAU6576
Dolichopodidae|[8623]|SSJAB3607-13|Dolichopodidae|658|BOLD:ACG4444
Dolichopodidae|[8624]|CNEIE1276-12|Dolichopodidae|664|BOLD:ACB2591
Dolichopodidae|[8625]|SSJAE8685-13|Dolichopodidae|635|BOLD:ACJ1157
Dolichopodidae|[8626]|CNEIB1418-12|Dolichopodidae|673|BOLD:AAG9709
Dolichopodidae|[8627]|NGNAN1772-14|Dolichopodidae|676|BOLD:AAG9661
Parasyntormon emarginatum|[8628]|BBDCP105-10|Dolichopodidae|658|BOLD:AAV3913
Dolichopodidae|[8629]|CNWBG775-13|Dolichopodidae|633|BOLD:ACI4825
Dolichopodidae|[8630]|NGNAN1793-14|Dolichopodidae|654|BOLD:ACL7071
Dolichopodidae|[8631]|BBDEC933-10|Dolichopodidae|658|BOLD:AAG9730
Calyxochaetus|[8632]|SSJAD5808-13|Dolichopodidae|661|BOLD:ACI5159
Calyxochaetus|[8633]|NGNAA781-13|Dolichopodidae|658|BOLD:ACI3370
Sympycninae|[8634]|SSGBB1150-14|Dolichopodidae|658|BOLD:ACO5777
Argyra sp. nr. nigricoxa|[8635]|JWDCF901-10|Dolichopodidae|658|BOLD:AAP1860
Dolichopodidae|[8636]|NGNAA418-13|Dolichopodidae|598|BOLD:ACI3060
Dolichopodidae|[8637]|BBDEC324-09|Dolichopodidae|658|BOLD:AAG9698
Dolichopodidae|[8638]|SSJAD4565-13|Dolichopodidae|670|BOLD:ACJ5016
Dolichopodidae|[8639]|SSBRA506-14|Dolichopodidae|658|BOLD:ACO0541
Diaphorinae|[8640]|CNFNF302-14|Dolichopodidae|661|BOLD:AAG9719
Dolichopodidae|[8641]|BBDCN218-10|Dolichopodidae|658|BOLD:AAG9744
Dolichopodidae|[8642]|BBDCN598-10|Dolichopodidae|643|BOLD:ACT6485
Dolichopodidae|[8643]|BBDCM436-10|Dolichopodidae|658|BOLD:AAP6323
Dolichopodidae|[8644]|SSBAF3661-13|Dolichopodidae|658|BOLD:ACJ0833
Dolichopodidae|[8645]|BBDCN864-10|Dolichopodidae|658|BOLD:AAP8924
Dolichopodidae|[8646]|CNNHE806-14|Dolichopodidae|591|BOLD:ACR6328
Dolichopodidae|[8647]|CNNHB2255-14|Dolichopodidae|618|BOLD:ACR4476
Dolichopodidae|[8648]|JSDIQ521-10|Dolichopodidae|658|BOLD:AAM6789
Diaphorinae|[8649]|CNRME1140-12|Dolichopodidae|661|BOLD:AAP6318
Argyra|[8650]|SSWLA841-13|Dolichopodidae|661|BOLD:ABV1240
Dolichopodidae|[8651]|SSJAB2327-13|Dolichopodidae|658|BOLD:ACJ1896
Dolichopodidae|[8652]|SSJAE5680-13|Dolichopodidae|597|BOLD:ACR5475
Dolichopodidae|[8653]|CNNHE1312-14|Dolichopodidae|615|BOLD:ACR7149
Sympycnus|[8654]|BBDCM733-10|Dolichopodidae|658|BOLD:AAP6325
Dolichopodidae|[8655]|SSJAE3652-13|Dolichopodidae|661|BOLD:AAG9715
Dolichopodidae|[8656]|BBDCM150-10|Dolichopodidae|658|BOLD:AAG9669
Dolichopodidae|[8657]|CNPPH1039-12|Dolichopodidae|664|BOLD:AAG9699
Achradocera arcuata|[8658]|CNPPJ493-12|Dolichopodidae|642|BOLD:AAG9706
Dolichopodidae|[8659]|JSJUN1730-11|Dolichopodidae|658|BOLD:AAG9705
Dolichopodidae|[8660]|BBDEC689-09|Dolichopodidae|658|BOLD:AAG9714
Dolichopodidae|[8661]|HPPPE1242-13|Dolichopodidae|664|BOLD:AAZ2253
Dolichopodidae|[8662]|TTMDI506-10|Dolichopodidae|658|BOLD:AAN5532
Dolichopodidae|[8663]|CNPPJ313-12|Dolichopodidae|670|BOLD:AAG9707
Diaphorinae|[8664]|CNPPH140-12|Dolichopodidae|661|BOLD:AAP5014
Chrysotus|[8665]|JSAUG1060-11|Dolichopodidae|658|BOLD:ABZ3704
Chrysotus|[8666]|CNSLJ313-12|Dolichopodidae|673|BOLD:AAN5534
Chrysotus|[8667]|CNROH196-13|Dolichopodidae|661|BOLD:ACA6204
Dolichopodidae|[8668]|MBIOI283-13|Dolichopodidae|603|BOLD:ACL6955
Dolichopodidae|[8669]|SSBAF4913-13|Dolichopodidae|654|BOLD:ACI7503
Dolichopodidae|[8670]|SSGBA4543-14|Dolichopodidae|636|BOLD:ACP2700
Dolichopodidae|[8671]|BBDCQ877-10|Dolichopodidae|658|BOLD:AAO7783
Chrysotus|[8672]|CNPPI1448-12|Dolichopodidae|664|BOLD:ACA4278
Chrysotus|[8673]|SSJAB1497-13|Dolichopodidae|661|BOLD:AAG9725
Chrysotus|[8674]|SSWLC390-13|Dolichopodidae|658|BOLD:ACG2595
Chrysotus sp. SEB1|[8675]|JWDCA010-10|Dolichopodidae|658|BOLD:AAG9623
Chrysotus|[8676]|BBDCN314-10|Dolichopodidae|658|BOLD:ABY6054
Chrysotus|[8677]|JSJUL082-11|Dolichopodidae|658|BOLD:ABV1180
Chrysotus sp.|[8678]|NCCA1646-11|Dolichopodidae|673|BOLD:AAG9668
Chrysotus|[8679]|CNWBG1404-13|Dolichopodidae|642|BOLD:ACI4814
Chrysotus|[8680]|RBINA188-13|Dolichopodidae|611|BOLD:ACI9753
Chrysotus|[8681]|SSWLE7080-13|Dolichopodidae|661|BOLD:ACK2689
Chrysotus|[8682]|SSWLF4377-13|Dolichopodidae|658|BOLD:ACY6817
Dolichopodidae|[8683]|BBDEE843-10|Dolichopodidae|658|BOLD:ACI2704
Dolichopodidae|[8684]|HPPPC388-13|Dolichopodidae|658|BOLD:AAG9731
Chrysotus|[8685]|CNFNF2134-14|Dolichopodidae|661|BOLD:AAN5531
Chrysotus sp. SEB2|[8686]|JSDIP828-10|Dolichopodidae|658|BOLD:AAV3883
Dolichopodidae|[8687]|JSYKA075-09|Dolichopodidae|658|BOLD:ABY6165
Chrysotus sp. SEB2|[8688]|JWDCA011-10|Dolichopodidae|658|BOLD:ABY6164
Chrysotus sp. SEB2|[8689]|JWDCE147-10|Dolichopodidae|658|BOLD:AAG6860
Chrysotus|[8690]|CNMIF264-14|Dolichopodidae|632|BOLD:ACM9496
Chrysotus cilipes|[8691]|SMTPI9024-14|Dolichopodidae|633|BOLD:ACJ0835
Chrysotus|[8692]|SMTPJ4749-14|Dolichopodidae|588|BOLD:ACO1274
Dolichopodidae|[8693]|JSYKA072-09|Dolichopodidae|658|BOLD:AAG9726
Dolichopodidae|[8694]|CNPPI1459-12|Dolichopodidae|664|BOLD:AAY6375
Chrysotus|[8695]|CNWBG406-13|Dolichopodidae|659|BOLD:AAG9659
Chrysotus|[8696]|CNGIB155-12|Dolichopodidae|658|BOLD:ACC1243
Dolichopodidae|[8697]|CNGUE372-15|Dolichopodidae|627|BOLD:ACS2088
Campsicnemus americanus|[8698]|JWDOL051-10|Dolichopodidae|658|BOLD:AAP1861

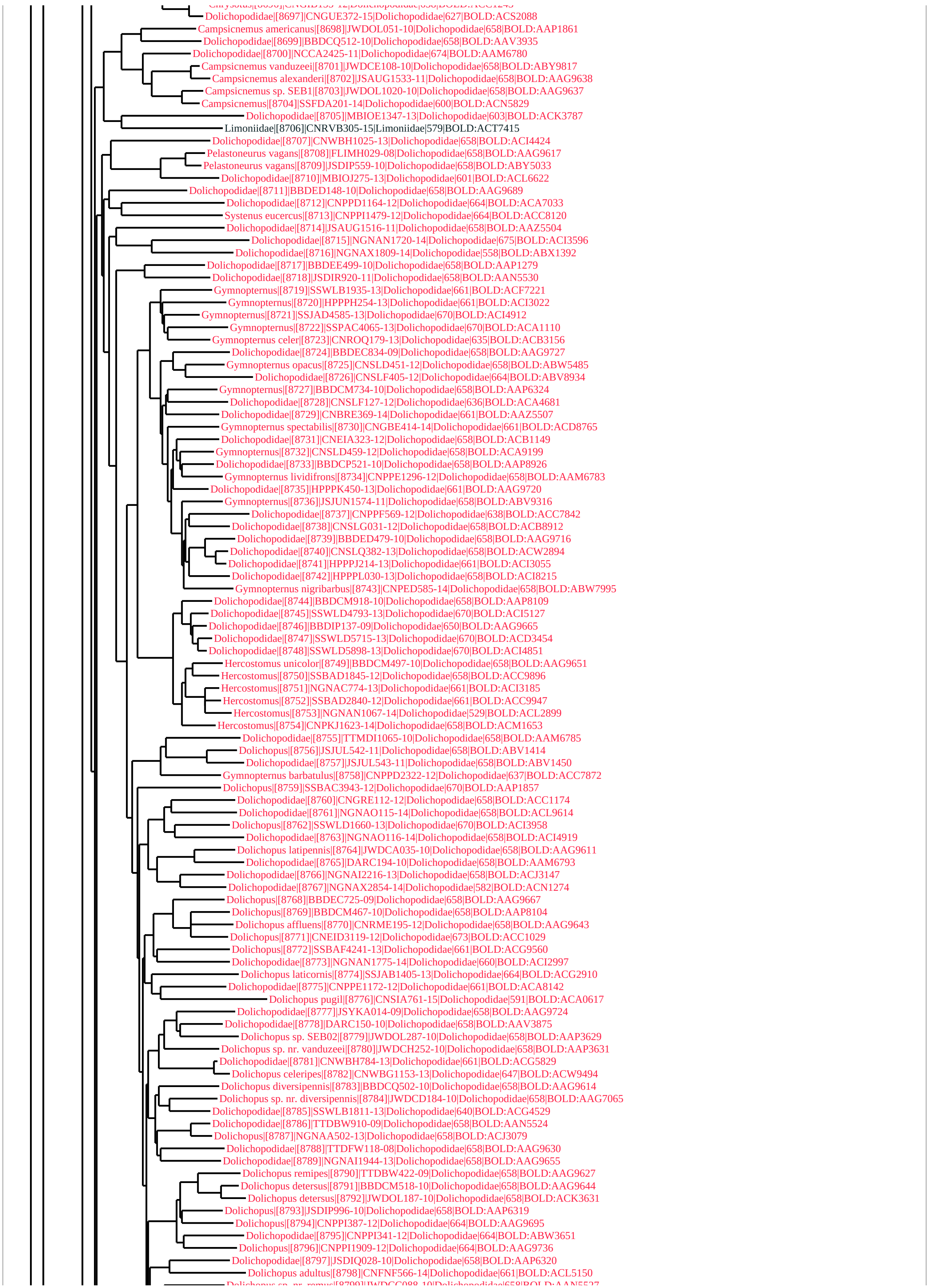

Dolichopodidae|[8697]|CNGUE372-15|Dolichopodidae|627|BOLD:ACS2088
Campsicnemus americanus|[8698]|JWDOL051-10|Dolichopodidae|658|BOLD:AAP1861
Dolichopodidae|[8699]|BBDCQ512-10|Dolichopodidae|658|BOLD:AAV3935
Dolichopodidae|[8700]|NCCA2425-11|Dolichopodidae|674|BOLD:AAM6780
Campsicnemus vanduzeei|[8701]|JWDCE108-10|Dolichopodidae|658|BOLD:ABY9817
Campsicnemus alexanderi|[8702]|JSAUG1533-11|Dolichopodidae|658|BOLD:AAG9638
Campsicnemus sp. SEB1|[8703]|JWDOL1020-10|Dolichopodidae|658|BOLD:AAG9637
Campsicnemus|[8704]|SSFDA201-14|Dolichopodidae|600|BOLD:ACN5829
Dolichopodidae|[8705]|MBIOE1347-13|Dolichopodidae|603|BOLD:ACK3787
Limoniidae|[8706]|CNRVB305-15|Limoniidae|579|BOLD:ACT7415
Dolichopodidae|[8707]|CNWBH1025-13|Dolichopodidae|658|BOLD:ACI4424
Pelastoneurus vagans|[8708]|FLIMH029-08|Dolichopodidae|658|BOLD:AAG9617
Pelastoneurus vagans|[8709]|JSDIP559-10|Dolichopodidae|658|BOLD:ABY5033
Dolichopodidae|[8710]|MBIOJ275-13|Dolichopodidae|601|BOLD:ACL6622
Dolichopodidae|[8711]|BBDED148-10|Dolichopodidae|658|BOLD:AAG9689
Dolichopodidae|[8712]|CNPPD1164-12|Dolichopodidae|664|BOLD:ACA7033
Systenus eucercus|[8713]|CNPPI1479-12|Dolichopodidae|664|BOLD:ACC8120
Dolichopodidae|[8714]|JSAUG1516-11|Dolichopodidae|658|BOLD:AAZ5504
Dolichopodidae|[8715]|NGNAN1720-14|Dolichopodidae|675|BOLD:ACI3596
Dolichopodidae|[8716]|NGNAX1809-14|Dolichopodidae|558|BOLD:ABX1392
Dolichopodidae|[8717]|BBDEE499-10|Dolichopodidae|658|BOLD:AAP1279
Dolichopodidae|[8718]|JSDIR920-11|Dolichopodidae|658|BOLD:AAN5530
Gymnopternus|[8719]|SSWLB1935-13|Dolichopodidae|661|BOLD:ACF7221
Gymnopternus|[8720]|HPPPH254-13|Dolichopodidae|661|BOLD:ACI3022
Gymnopternus|[8721]|SSJAD4585-13|Dolichopodidae|670|BOLD:ACI4912
Gymnopternus|[8722]|SSPAC4065-13|Dolichopodidae|670|BOLD:ACA1110
Gymnopternus celer|[8723]|CNROQ179-13|Dolichopodidae|635|BOLD:ACB3156
Dolichopodidae|[8724]|BBDEC834-09|Dolichopodidae|658|BOLD:AAG9727
Gymnopternus opacus|[8725]|CNSLD451-12|Dolichopodidae|658|BOLD:ABW5485
Dolichopodidae|[8726]|CNSLF405-12|Dolichopodidae|664|BOLD:ABV8934
Gymnopternus|[8727]|BBDCM734-10|Dolichopodidae|658|BOLD:AAP6324
Dolichopodidae|[8728]|CNSLF127-12|Dolichopodidae|636|BOLD:ACA4681
Dolichopodidae|[8729]|CNBRE369-14|Dolichopodidae|661|BOLD:AAZ5507
Gymnopternus spectabilis|[8730]|CNGBE414-14|Dolichopodidae|661|BOLD:ACD8765
Dolichopodidae|[8731]|CNEIA323-12|Dolichopodidae|658|BOLD:ACB1149
Gymnopternus|[8732]|CNSLD459-12|Dolichopodidae|658|BOLD:ACA9199
Dolichopodidae|[8733]|BBDCP521-10|Dolichopodidae|658|BOLD:AAP8926
Gymnopternus lividifrons|[8734]|CNPPE1296-12|Dolichopodidae|658|BOLD:AAM6783
Dolichopodidae|[8735]|HPPPK450-13|Dolichopodidae|661|BOLD:AAG9720
Gymnopternus|[8736]|JSJUN1574-11|Dolichopodidae|658|BOLD:ABV9316
Dolichopodidae|[8737]|CNPPF569-12|Dolichopodidae|638|BOLD:ACC7842
Dolichopodidae|[8738]|CNSLG031-12|Dolichopodidae|658|BOLD:ACB8912
Dolichopodidae|[8739]|BBDED479-10|Dolichopodidae|658|BOLD:AAG9716
Dolichopodidae|[8740]|CNSLQ382-13|Dolichopodidae|658|BOLD:ACW2894
Dolichopodidae|[8741]|HPPPJ214-13|Dolichopodidae|661|BOLD:ACI3055
Dolichopodidae|[8742]|HPPPL030-13|Dolichopodidae|658|BOLD:ACI8215
Gymnopternus nigribarbus|[8743]|CNPED585-14|Dolichopodidae|658|BOLD:ABW7995
Dolichopodidae|[8744]|BBDCM918-10|Dolichopodidae|658|BOLD:AAP8109
Dolichopodidae|[8745]|SSWLD4793-13|Dolichopodidae|670|BOLD:ACI5127
Dolichopodidae|[8746]|BBDIP137-09|Dolichopodidae|650|BOLD:AAG9665
Dolichopodidae|[8747]|SSWLD5715-13|Dolichopodidae|670|BOLD:ACD3454
Dolichopodidae|[8748]|SSWLD5898-13|Dolichopodidae|670|BOLD:ACI4851
Hercostomus unicolor|[8749]|BBDCM497-10|Dolichopodidae|658|BOLD:AAG9651
Hercostomus|[8750]|SSBAD1845-12|Dolichopodidae|658|BOLD:ACC9896
Hercostomus|[8751]|NGNAC774-13|Dolichopodidae|661|BOLD:ACI3185
Hercostomus|[8752]|SSBAD2840-12|Dolichopodidae|661|BOLD:ACC9947
Hercostomus|[8753]|NGNAN1067-14|Dolichopodidae|529|BOLD:ACL2899
Hercostomus|[8754]|CNPKJ1623-14|Dolichopodidae|658|BOLD:ACM1653
Dolichopodidae|[8755]|TTMDI1065-10|Dolichopodidae|658|BOLD:AAM6785
Dolichopus|[8756]|JSJUL542-11|Dolichopodidae|658|BOLD:ABV1414
Dolichopodidae|[8757]|JSJUL543-11|Dolichopodidae|658|BOLD:ABV1450
Gymnopternus barbatulus|[8758]|CNPPD2322-12|Dolichopodidae|637|BOLD:ACC7872
Dolichopus|[8759]|SSBAC3943-12|Dolichopodidae|670|BOLD:AAP1857
Dolichopodidae|[8760]|CNGRE112-12|Dolichopodidae|658|BOLD:ACC1174
Dolichopodidae|[8761]|NGNAO115-14|Dolichopodidae|658|BOLD:ACL9614
Dolichopus|[8762]|SSWLD1660-13|Dolichopodidae|670|BOLD:ACI3958
Dolichopodidae|[8763]|NGNAO116-14|Dolichopodidae|658|BOLD:ACI4919
Dolichopus latipennis|[8764]|JWDCA035-10|Dolichopodidae|658|BOLD:AAG9611
Dolichopodidae|[8765]|DARC194-10|Dolichopodidae|658|BOLD:AAM6793
Dolichopodidae|[8766]|NGNAI2216-13|Dolichopodidae|658|BOLD:ACJ3147
Dolichopodidae|[8767]|NGNAX2854-14|Dolichopodidae|582|BOLD:ACN1274
Dolichopus|[8768]|BBDEC725-09|Dolichopodidae|658|BOLD:AAG9667
Dolichopus|[8769]|BBDCM467-10|Dolichopodidae|658|BOLD:AAP8104
Dolichopus affluens|[8770]|CNRME195-12|Dolichopodidae|658|BOLD:AAG9643
Dolichopus|[8771]|CNEID3119-12|Dolichopodidae|673|BOLD:ACC1029
Dolichopus|[8772]|SSBAF4241-13|Dolichopodidae|661|BOLD:ACG9560
Dolichopodidae|[8773]|NGNAN1775-14|Dolichopodidae|660|BOLD:ACI2997
Dolichopus laticornis|[8774]|SSJAB1405-13|Dolichopodidae|664|BOLD:ACG2910
Dolichopodidae|[8775]|CNPPE1172-12|Dolichopodidae|661|BOLD:ACA8142
Dolichopus pugil|[8776]|CNSIA761-15|Dolichopodidae|591|BOLD:ACA0617
Dolichopodidae|[8777]|JSYKA014-09|Dolichopodidae|658|BOLD:AAG9724
Dolichopodidae|[8778]|DARC150-10|Dolichopodidae|658|BOLD:AAV3875
Dolichopus sp. SEB02|[8779]|JWDOL287-10|Dolichopodidae|658|BOLD:AAP3629
Dolichopus sp. nr. vanduzeei|[8780]|JWDCH252-10|Dolichopodidae|658|BOLD:AAP3631
Dolichopodidae|[8781]|CNWBH784-13|Dolichopodidae|661|BOLD:ACG5829
Dolichopus celeripes|[8782]|CNWBG1153-13|Dolichopodidae|647|BOLD:ACW9494
Dolichopus diversipennis|[8783]|BBDCQ502-10|Dolichopodidae|658|BOLD:AAG9614
Dolichopus sp. nr. diversipennis|[8784]|JWDCD184-10|Dolichopodidae|658|BOLD:AAG7065
Dolichopodidae|[8785]|SSWLB1811-13|Dolichopodidae|640|BOLD:ACG4529
Dolichopodidae|[8786]|TTDBW910-09|Dolichopodidae|658|BOLD:AAN5524
Dolichopus|[8787]|NGNAA502-13|Dolichopodidae|658|BOLD:ACJ3079
Dolichopodidae|[8788]|TTDFW118-08|Dolichopodidae|658|BOLD:AAG9630
Dolichopodidae|[8789]|NGNAI1944-13|Dolichopodidae|658|BOLD:AAG9655
Dolichopus remipes|[8790]|TTDBW422-09|Dolichopodidae|658|BOLD:AAG9627
Dolichopus detersus|[8791]|BBDCM518-10|Dolichopodidae|658|BOLD:AAG9644
Dolichopus detersus|[8792]|JWDOL187-10|Dolichopodidae|658|BOLD:ACK3631
Dolichopus|[8793]|JSDIP996-10|Dolichopodidae|658|BOLD:AAP6319
Dolichopus|[8794]|CNPPI387-12|Dolichopodidae|664|BOLD:AAG9695
Dolichopodidae|[8795]|CNPPI341-12|Dolichopodidae|664|BOLD:ABW3651
Dolichopus|[8796]|CNPPI1909-12|Dolichopodidae|664|BOLD:AAG9736
Dolichopodidae|[8797]|JSDIQ028-10|Dolichopodidae|658|BOLD:AAP6320
Dolichopus adultus|[8798]|CNFNF566-14|Dolichopodidae|661|BOLD:ACL5150

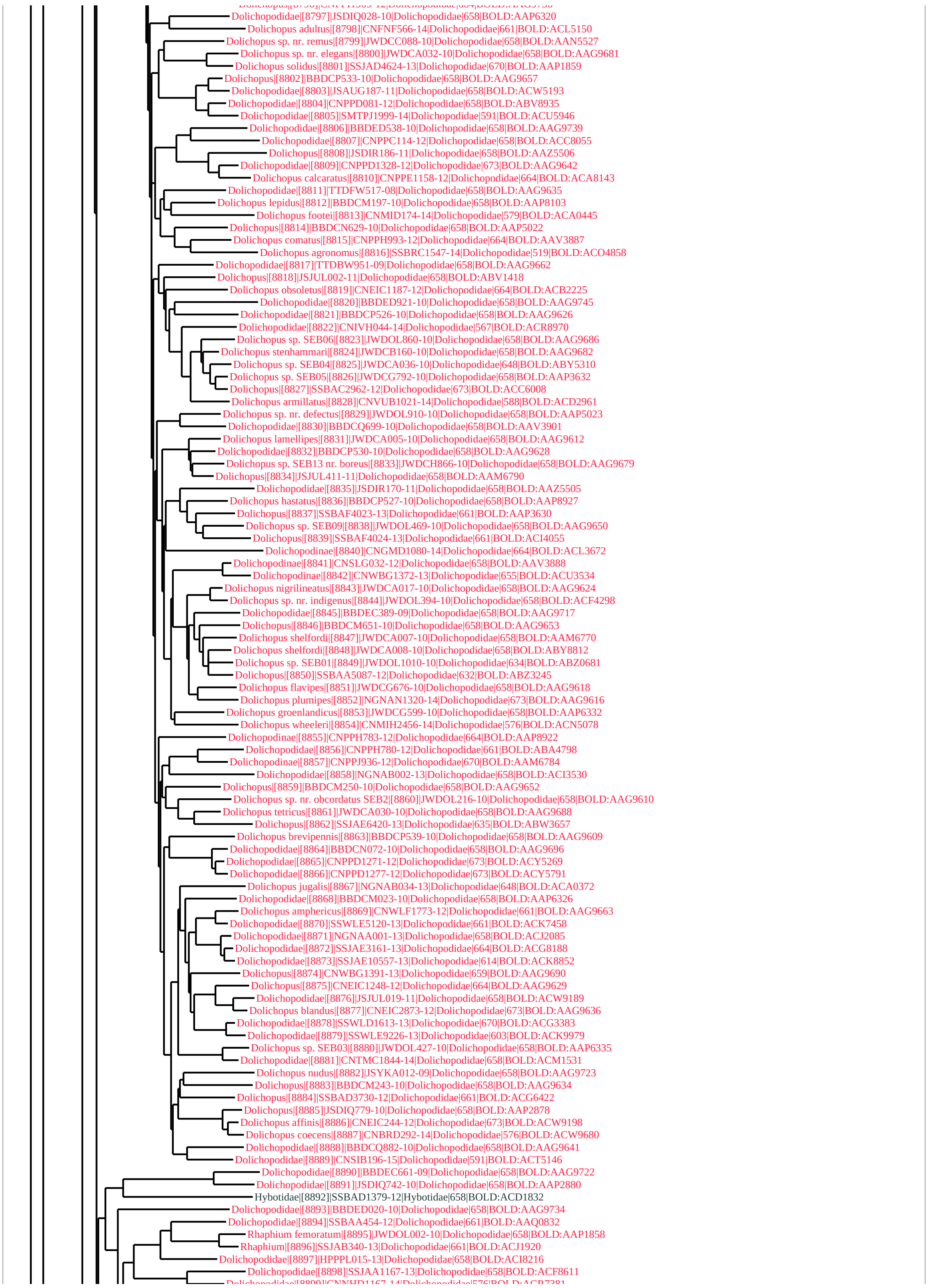
Dolichopodidae|[8797]|JSDIQ028-10|Dolichopodidae|658|BOLD:AAP6320
Dolichopus adultus|[8798]|CNFNF566-14|Dolichopodidae|661|BOLD:ACL5150
Dolichopus sp. nr. remus|[8799]|JWDCC088-10|Dolichopodidae|658|BOLD:AAN5527
Dolichopus sp. nr. elegans|[8800]|JWDCA032-10|Dolichopodidae|658|BOLD:AAG9681
Dolichopus solidus|[8801]|SSJAD4624-13|Dolichopodidae|670|BOLD:AAP1859
Dolichopus|[8802]|BBDCP533-10|Dolichopodidae|658|BOLD:AAG9657
Dolichopodidae|[8803]|JSAUG187-11|Dolichopodidae|658|BOLD:ACW5193
Dolichopodidae|[8804]|CNPPD081-12|Dolichopodidae|658|BOLD:ABV8935
Dolichopodidae|[8805]|SMTPJ1999-14|Dolichopodidae|591|BOLD:ACU5946
Dolichopodidae|[8806]|BBDED538-10|Dolichopodidae|658|BOLD:AAG9739
Dolichopodidae|[8807]|CNPPC114-12|Dolichopodidae|658|BOLD:ACC8055
Dolichopus|[8808]|JSDIR186-11|Dolichopodidae|658|BOLD:AAZ5506
Dolichopodidae|[8809]|CNPPD1328-12|Dolichopodidae|673|BOLD:AAG9642
Dolichopus calcaratus|[8810]|CNPPE1158-12|Dolichopodidae|664|BOLD:ACA8143
Dolichopodidae|[8811]|TTDFW517-08|Dolichopodidae|658|BOLD:AAG9635
Dolichopus lepidus|[8812]|BBDCM197-10|Dolichopodidae|658|BOLD:AAP8103
Dolichopus footei|[8813]|CNMID174-14|Dolichopodidae|579|BOLD:ACA0445
Dolichopus|[8814]|BBDCN629-10|Dolichopodidae|658|BOLD:AAP5022
Dolichopus comatus|[8815]|CNPPH993-12|Dolichopodidae|664|BOLD:AAV3887
Dolichopus agronomus|[8816]|SSBRC1547-14|Dolichopodidae|519|BOLD:ACO4858
Dolichopodidae|[8817]|TTDBW951-09|Dolichopodidae|658|BOLD:AAG9662
Dolichopus|[8818]|JSJUL002-11|Dolichopodidae|658|BOLD:ABV1418
Dolichopus obsoletus|[8819]|CNEIC1187-12|Dolichopodidae|664|BOLD:ACB2225
Dolichopodidae|[8820]|BBDED921-10|Dolichopodidae|658|BOLD:AAG9745
Dolichopodidae|[8821]|BBDCP526-10|Dolichopodidae|658|BOLD:AAG9626
Dolichopodidae|[8822]|CNIVH044-14|Dolichopodidae|567|BOLD:ACR8970
Dolichopus sp. SEB06|[8823]|JWDOL860-10|Dolichopodidae|658|BOLD:AAG9686
Dolichopus stenhammari|[8824]|JWDCB160-10|Dolichopodidae|658|BOLD:AAG9682
Dolichopus sp. SEB04|[8825]|JWDCA036-10|Dolichopodidae|648|BOLD:ABY5310
Dolichopus sp. SEB05|[8826]|JWDCG792-10|Dolichopodidae|658|BOLD:AAP3632
Dolichopus|[8827]|SSBAC2962-12|Dolichopodidae|673|BOLD:ACC6008
Dolichopus armillatus|[8828]|CNVUB1021-14|Dolichopodidae|588|BOLD:ACD2961
Dolichopus sp. nr. defectus|[8829]|JWDOL910-10|Dolichopodidae|658|BOLD:AAP5023
Dolichopodidae|[8830]|BBDCQ699-10|Dolichopodidae|658|BOLD:AAV3901
Dolichopus lamellipes|[8831]|JWDCA005-10|Dolichopodidae|658|BOLD:AAG9612
Dolichopodidae|[8832]|BBDCP530-10|Dolichopodidae|658|BOLD:AAG9628
Dolichopus sp. SEB13 nr. boreus|[8833]|JWDCH866-10|Dolichopodidae|658|BOLD:AAG9679
Dolichopus|[8834]|JSJUL411-11|Dolichopodidae|658|BOLD:AAM6790
Dolichopodidae|[8835]|JSDIR170-11|Dolichopodidae|658|BOLD:AAZ5505
Dolichopus hastatus|[8836]|BBDCP527-10|Dolichopodidae|658|BOLD:AAP8927
Dolichopus|[8837]|SSBAF4023-13|Dolichopodidae|661|BOLD:AAP3630
Dolichopus sp. SEB09|[8838]|JWDOL469-10|Dolichopodidae|658|BOLD:AAG9650
Dolichopus|[8839]|SSBAF4024-13|Dolichopodidae|661|BOLD:ACI4055
Dolichopodinae|[8840]|CNGMD1080-14|Dolichopodidae|664|BOLD:ACL3672
Dolichopodinae|[8841]|CNSLG032-12|Dolichopodidae|658|BOLD:AAV3888
Dolichopodinae|[8842]|CNWBG1372-13|Dolichopodidae|655|BOLD:ACU3534
Dolichopus nigrilineatus|[8843]|JWDCA017-10|Dolichopodidae|658|BOLD:AAG9624
Dolichopus sp. nr. indigenus|[8844]|JWDOL394-10|Dolichopodidae|658|BOLD:ACF4298
Dolichopodidae|[8845]|BBDEC389-09|Dolichopodidae|658|BOLD:AAG9717
Dolichopus|[8846]|BBDCM651-10|Dolichopodidae|658|BOLD:AAG9653
Dolichopus shelfordi|[8847]|JWDCA007-10|Dolichopodidae|658|BOLD:AAM6770
Dolichopus shelfordi|[8848]|JWDCA008-10|Dolichopodidae|658|BOLD:ABY8812
Dolichopus sp. SEB01|[8849]|JWDOL1010-10|Dolichopodidae|634|BOLD:ABZ0681
Dolichopus|[8850]|SSBAA5087-12|Dolichopodidae|632|BOLD:ABZ3245
Dolichopus flavipes|[8851]|JWDCG676-10|Dolichopodidae|658|BOLD:AAG9618
Dolichopus plumipes|[8852]|NGNAN1320-14|Dolichopodidae|673|BOLD:AAG9616
Dolichopus groenlandicus|[8853]|JWDCG599-10|Dolichopodidae|658|BOLD:AAP6332
Dolichopus wheeleri|[8854]|CNMIH2456-14|Dolichopodidae|576|BOLD:ACN5078
Dolichopodinae|[8855]|CNPPH783-12|Dolichopodidae|664|BOLD:AAP8922
Dolichopodidae|[8856]|CNPPH780-12|Dolichopodidae|661|BOLD:ABA4798
Dolichopodinae|[8857]|CNPPJ936-12|Dolichopodidae|670|BOLD:AAM6784
Dolichopodidae|[8858]|NGNAB002-13|Dolichopodidae|658|BOLD:ACI3530
Dolichopus|[8859]|BBDCM250-10|Dolichopodidae|658|BOLD:AAG9652
Dolichopus sp. nr. obcordatus SEB2|[8860]|JWDOL216-10|Dolichopodidae|658|BOLD:AAG9610
Dolichopus tetricus|[8861]|JWDCA030-10|Dolichopodidae|658|BOLD:AAG9688
Dolichopus|[8862]|SSJAE6420-13|Dolichopodidae|635|BOLD:ABW3657
Dolichopus brevipennis|[8863]|BBDCP539-10|Dolichopodidae|658|BOLD:AAG9609
Dolichopodidae|[8864]|BBDCN072-10|Dolichopodidae|658|BOLD:AAG9696
Dolichopodidae|[8865]|CNPPD1271-12|Dolichopodidae|673|BOLD:ACY5269
Dolichopodidae|[8866]|CNPPD1277-12|Dolichopodidae|673|BOLD:ACY5791
Dolichopus jugalis|[8867]|NGNAB034-13|Dolichopodidae|648|BOLD:ACA0372
Dolichopodidae|[8868]|BBDCM023-10|Dolichopodidae|658|BOLD:AAP6326
Dolichopus amphericus|[8869]|CNWLF1773-12|Dolichopodidae|661|BOLD:AAG9663
Dolichopodidae|[8870]|SSWLE5120-13|Dolichopodidae|661|BOLD:ACK7458
Dolichopodidae|[8871]|NGNAA001-13|Dolichopodidae|658|BOLD:ACJ2085
Dolichopodidae|[8872]|SSJAE3161-13|Dolichopodidae|664|BOLD:ACG8188
Dolichopodidae|[8873]|SSJAE10557-13|Dolichopodidae|614|BOLD:ACK8852
Dolichopus|[8874]|CNWBG1391-13|Dolichopodidae|659|BOLD:AAG9690
Dolichopus|[8875]|CNEIC1248-12|Dolichopodidae|664|BOLD:AAG9629
Dolichopodidae|[8876]|JSJUL019-11|Dolichopodidae|658|BOLD:ACW9189
Dolichopus blandus|[8877]|CNEIC2873-12|Dolichopodidae|673|BOLD:AAG9636
Dolichopodidae|[8878]|SSWLD1613-13|Dolichopodidae|670|BOLD:ACG3383
Dolichopodidae|[8879]|SSWLE9226-13|Dolichopodidae|603|BOLD:ACK9979
Dolichopus sp. SEB03|[8880]|JWDOL427-10|Dolichopodidae|658|BOLD:AAP6335
Dolichopodidae|[8881]|CNTMC1844-14|Dolichopodidae|658|BOLD:ACM1531
Dolichopus nudus|[8882]|JSYKA012-09|Dolichopodidae|658|BOLD:AAG9723
Dolichopus|[8883]|BBDCM243-10|Dolichopodidae|658|BOLD:AAG9634
Dolichopus|[8884]|SSBAD3730-12|Dolichopodidae|661|BOLD:ACG6422
Dolichopus|[8885]|JSDIQ779-10|Dolichopodidae|658|BOLD:AAP2878
Dolichopus affinis|[8886]|CNEIC244-12|Dolichopodidae|673|BOLD:ACW9198
Dolichopus coecens|[8887]|CNBRD292-14|Dolichopodidae|576|BOLD:ACW9680
Dolichopodidae|[8888]|BBDCQ882-10|Dolichopodidae|658|BOLD:AAG9641
Dolichopodidae|[8889]|CNSIB196-15|Dolichopodidae|591|BOLD:ACT5146
Dolichopodidae|[8890]|BBDEC661-09|Dolichopodidae|658|BOLD:AAG9722
Dolichopodidae|[8891]|JSDIQ742-10|Dolichopodidae|658|BOLD:AAP2880
Hybotidae|[8892]|SSBAD1379-12|Hybotidae|658|BOLD:ACD1832
Dolichopodidae|[8893]|BBDED020-10|Dolichopodidae|658|BOLD:AAG9734
Dolichopodidae|[8894]|SSBAA454-12|Dolichopodidae|661|BOLD:AAQ0832
Rhaphium femoratum|[8895]|JWDOL002-10|Dolichopodidae|658|BOLD:AAP1858
Rhaphium|[8896]|SSJAB340-13|Dolichopodidae|661|BOLD:ACJ1920
Dolichopodidae|[8897]|HPPPL015-13|Dolichopodidae|658|BOLD:ACI8216
Dolichopodidae|[8898]|SSJAA1167-13|Dolichopodidae|658|BOLD:ACF8611

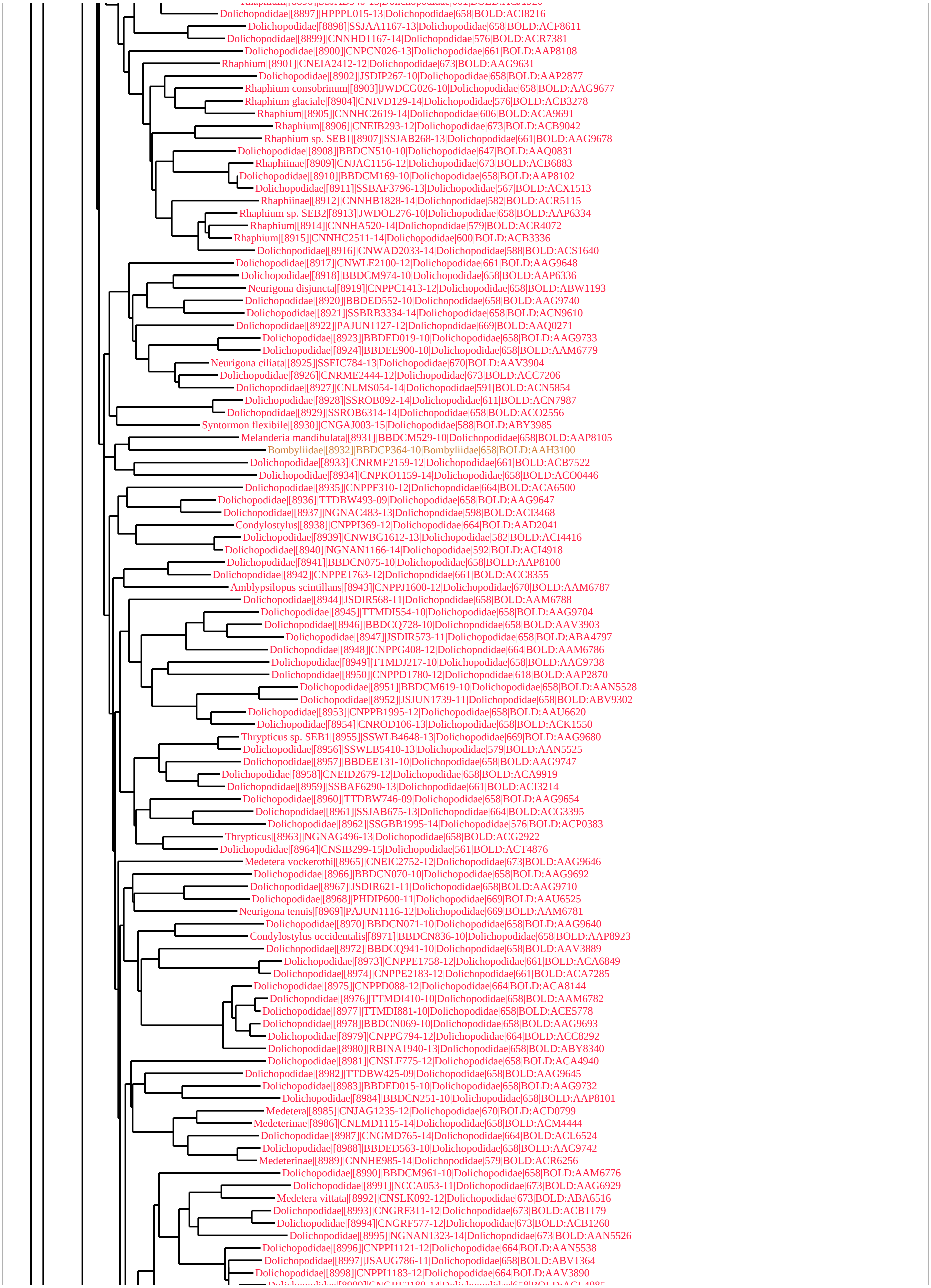
Dolichopodidae|[8897]|HPPPL015-13|Dolichopodidae|658|BOLD:ACI8216
Dolichopodidae|[8898]|SSJAA1167-13|Dolichopodidae|658|BOLD:ACF8611
Dolichopodidae|[8899]|CNNHD1167-14|Dolichopodidae|576|BOLD:ACR7381
Dolichopodidae|[8900]|CNPCN026-13|Dolichopodidae|661|BOLD:AAP8108
Rhaphium|[8901]|CNEIA2412-12|Dolichopodidae|673|BOLD:AAG9631
Dolichopodidae|[8902]|JSDIP267-10|Dolichopodidae|658|BOLD:AAP2877
Rhaphium consobrinum|[8903]|JWDCG026-10|Dolichopodidae|658|BOLD:AAG9677
Rhaphium glaciale|[8904]|CNIVD129-14|Dolichopodidae|576|BOLD:ACB3278
Rhaphium|[8905]|CNNHC2619-14|Dolichopodidae|606|BOLD:ACA9691
Rhaphium|[8906]|CNEIB293-12|Dolichopodidae|673|BOLD:ACB9042
Rhaphium sp. SEB1|[8907]|SSJAB268-13|Dolichopodidae|661|BOLD:AAG9678
Dolichopodidae|[8908]|BBDCN510-10|Dolichopodidae|647|BOLD:AAQ0831
Rhaphiinae|[8909]|CNJAC1156-12|Dolichopodidae|673|BOLD:ACB6883
Dolichopodidae|[8910]|BBDCM169-10|Dolichopodidae|658|BOLD:AAP8102
Dolichopodidae|[8911]|SSBAF3796-13|Dolichopodidae|567|BOLD:ACX1513
Rhaphiinae|[8912]|CNNHB1828-14|Dolichopodidae|582|BOLD:ACR5115
Rhaphium sp. SEB2|[8913]|JWDOL276-10|Dolichopodidae|658|BOLD:AAP6334
Rhaphium|[8914]|CNNHA520-14|Dolichopodidae|579|BOLD:ACR4072
Rhaphium|[8915]|CNNHC2511-14|Dolichopodidae|600|BOLD:ACB3336
Dolichopodidae|[8916]|CNWAD2033-14|Dolichopodidae|588|BOLD:ACS1640
Dolichopodidae|[8917]|CNWLE2100-12|Dolichopodidae|661|BOLD:AAG9648
Dolichopodidae|[8918]|BBDCM974-10|Dolichopodidae|658|BOLD:AAP6336
Neurigona disjuncta|[8919]|CNPPC1413-12|Dolichopodidae|658|BOLD:ABW1193
Dolichopodidae|[8920]|BBDED552-10|Dolichopodidae|658|BOLD:AAG9740
Dolichopodidae|[8921]|SSBRB3334-14|Dolichopodidae|658|BOLD:ACN9610
Dolichopodidae|[8922]|PAJUN1127-12|Dolichopodidae|669|BOLD:AAQ0271
Dolichopodidae|[8923]|BBDED019-10|Dolichopodidae|658|BOLD:AAG9733
Dolichopodidae|[8924]|BBDEE900-10|Dolichopodidae|658|BOLD:AAM6779
Neurigona ciliata|[8925]|SSEIC784-13|Dolichopodidae|670|BOLD:AAV3904
Dolichopodidae|[8926]|CNRME2444-12|Dolichopodidae|673|BOLD:ACC7206
Dolichopodidae|[8927]|CNLMS054-14|Dolichopodidae|591|BOLD:ACN5854
Dolichopodidae|[8928]|SSROB092-14|Dolichopodidae|611|BOLD:ACN7987
Dolichopodidae|[8929]|SSROB6314-14|Dolichopodidae|658|BOLD:ACO2556
Syntormon flexibile|[8930]|CNGAJ003-15|Dolichopodidae|588|BOLD:ABY3985
Melanderia mandibulata|[8931]|BBDCM529-10|Dolichopodidae|658|BOLD:AAP8105
Bombyliidae|[8932]|BBDCP364-10|Bombyliidae|658|BOLD:AAH3100
Dolichopodidae|[8933]|CNRMF2159-12|Dolichopodidae|661|BOLD:ACB7522
Dolichopodidae|[8934]|CNPKO1159-14|Dolichopodidae|658|BOLD:ACO0446
Dolichopodidae|[8935]|CNPPF310-12|Dolichopodidae|664|BOLD:ACA6500
Dolichopodidae|[8936]|TTDBW493-09|Dolichopodidae|658|BOLD:AAG9647
Dolichopodidae|[8937]|NGNAC483-13|Dolichopodidae|598|BOLD:ACI3468
Condylostylus|[8938]|CNPPI369-12|Dolichopodidae|664|BOLD:AAD2041
Dolichopodidae|[8939]|CNWBG1612-13|Dolichopodidae|582|BOLD:ACI4416
Dolichopodidae|[8940]|NGNAN1166-14|Dolichopodidae|592|BOLD:ACI4918
Dolichopodidae|[8941]|BBDCN075-10|Dolichopodidae|658|BOLD:AAP8100
Dolichopodidae|[8942]|CNPPE1763-12|Dolichopodidae|661|BOLD:ACC8355
Amblypsilopus scintillans|[8943]|CNPPJ1600-12|Dolichopodidae|670|BOLD:AAM6787
Dolichopodidae|[8944]|JSDIR568-11|Dolichopodidae|658|BOLD:AAM6788
Dolichopodidae|[8945]|TTMDI554-10|Dolichopodidae|658|BOLD:AAG9704
Dolichopodidae|[8946]|BBDCQ728-10|Dolichopodidae|658|BOLD:AAV3903
Dolichopodidae|[8947]|JSDIR573-11|Dolichopodidae|658|BOLD:ABA4797
Dolichopodidae|[8948]|CNPPG408-12|Dolichopodidae|664|BOLD:AAM6786
Dolichopodidae|[8949]|TTMDJ217-10|Dolichopodidae|658|BOLD:AAG9738
Dolichopodidae|[8950]|CNPPD1780-12|Dolichopodidae|618|BOLD:AAP2870
Dolichopodidae|[8951]|BBDCM619-10|Dolichopodidae|658|BOLD:AAN5528
Dolichopodidae|[8952]|JSJUN1739-11|Dolichopodidae|658|BOLD:ABV9302
Dolichopodidae|[8953]|CNPPB1995-12|Dolichopodidae|658|BOLD:AAU6620
Dolichopodidae|[8954]|CNROD106-13|Dolichopodidae|658|BOLD:ACK1550
Thrypticus sp. SEB1|[8955]|SSWLB4648-13|Dolichopodidae|669|BOLD:AAG9680
Dolichopodidae|[8956]|SSWLB5410-13|Dolichopodidae|579|BOLD:AAN5525
Dolichopodidae|[8957]|BBDEE131-10|Dolichopodidae|658|BOLD:AAG9747
Dolichopodidae|[8958]|CNEID2679-12|Dolichopodidae|658|BOLD:ACA9919
Dolichopodidae|[8959]|SSBAF6290-13|Dolichopodidae|661|BOLD:ACI3214
Dolichopodidae|[8960]|TTDBW746-09|Dolichopodidae|658|BOLD:AAG9654
Dolichopodidae|[8961]|SSJAB675-13|Dolichopodidae|664|BOLD:ACG3395
Dolichopodidae|[8962]|SSGBB1995-14|Dolichopodidae|576|BOLD:ACP0383
Thrypticus|[8963]|NGNAG496-13|Dolichopodidae|658|BOLD:ACG2922
Dolichopodidae|[8964]|CNSIB299-15|Dolichopodidae|561|BOLD:ACT4876
Medetera vockerothi|[8965]|CNEIC2752-12|Dolichopodidae|673|BOLD:AAG9646
Dolichopodidae|[8966]|BBDCN070-10|Dolichopodidae|658|BOLD:AAG9692
Dolichopodidae|[8967]|JSDIR621-11|Dolichopodidae|658|BOLD:AAG9710
Dolichopodidae|[8968]|PHDIP600-11|Dolichopodidae|669|BOLD:AAU6525
Neurigona tenuis|[8969]|PAJUN1116-12|Dolichopodidae|669|BOLD:AAM6781
Dolichopodidae|[8970]|BBDCN071-10|Dolichopodidae|658|BOLD:AAG9640
Condylostylus occidentalis|[8971]|BBDCN836-10|Dolichopodidae|658|BOLD:AAP8923
Dolichopodidae|[8972]|BBDCQ941-10|Dolichopodidae|658|BOLD:AAV3889
Dolichopodidae|[8973]|CNPPE1758-12|Dolichopodidae|661|BOLD:ACA6849
Dolichopodidae|[8974]|CNPPE2183-12|Dolichopodidae|661|BOLD:ACA7285
Dolichopodidae|[8975]|CNPPD088-12|Dolichopodidae|664|BOLD:ACA8144
Dolichopodidae|[8976]|TTMDI410-10|Dolichopodidae|658|BOLD:AAM6782
Dolichopodidae|[8977]|TTMDI881-10|Dolichopodidae|658|BOLD:ACE5778
Dolichopodidae|[8978]|BBDCN069-10|Dolichopodidae|658|BOLD:AAG9693
Dolichopodidae|[8979]|CNPPG794-12|Dolichopodidae|664|BOLD:ACC8292
Dolichopodidae|[8980]|RBINA1940-13|Dolichopodidae|658|BOLD:ABY8340
Dolichopodidae|[8981]|CNSLF775-12|Dolichopodidae|658|BOLD:ACA4940
Dolichopodidae|[8982]|TTDBW425-09|Dolichopodidae|658|BOLD:AAG9645
Dolichopodidae|[8983]|BBDED015-10|Dolichopodidae|658|BOLD:AAG9732
Dolichopodidae|[8984]|BBDCN251-10|Dolichopodidae|658|BOLD:AAP8101
Medetera|[8985]|CNJAG1235-12|Dolichopodidae|670|BOLD:ACD0799
Medeterinae|[8986]|CNLMD1115-14|Dolichopodidae|658|BOLD:ACM4444
Dolichopodidae|[8987]|CNGMD765-14|Dolichopodidae|664|BOLD:ACL6524
Dolichopodidae|[8988]|BBDED563-10|Dolichopodidae|658|BOLD:AAG9742
Medeterinae|[8989]|CNNHE985-14|Dolichopodidae|579|BOLD:ACR6256
Dolichopodidae|[8990]|BBDCM961-10|Dolichopodidae|658|BOLD:AAM6776
Dolichopodidae|[8991]|NCCA053-11|Dolichopodidae|673|BOLD:AAG6929
Medetera vittata|[8992]|CNSLK092-12|Dolichopodidae|673|BOLD:ABA6516
Dolichopodidae|[8993]|CNGRF311-12|Dolichopodidae|673|BOLD:ACB1179
Dolichopodidae|[8994]|CNGRF577-12|Dolichopodidae|673|BOLD:ACB1260
Dolichopodidae|[8995]|NGNAN1323-14|Dolichopodidae|673|BOLD:AAN5526
Dolichopodidae|[8996]|CNPPI1121-12|Dolichopodidae|664|BOLD:AAN5538
Dolichopodidae|[8997]|JSAUG786-11|Dolichopodidae|658|BOLD:ABV1364
Dolichopodidae|[8998]|CNPPI1183-12|Dolichopodidae|664|BOLD:AAV3890

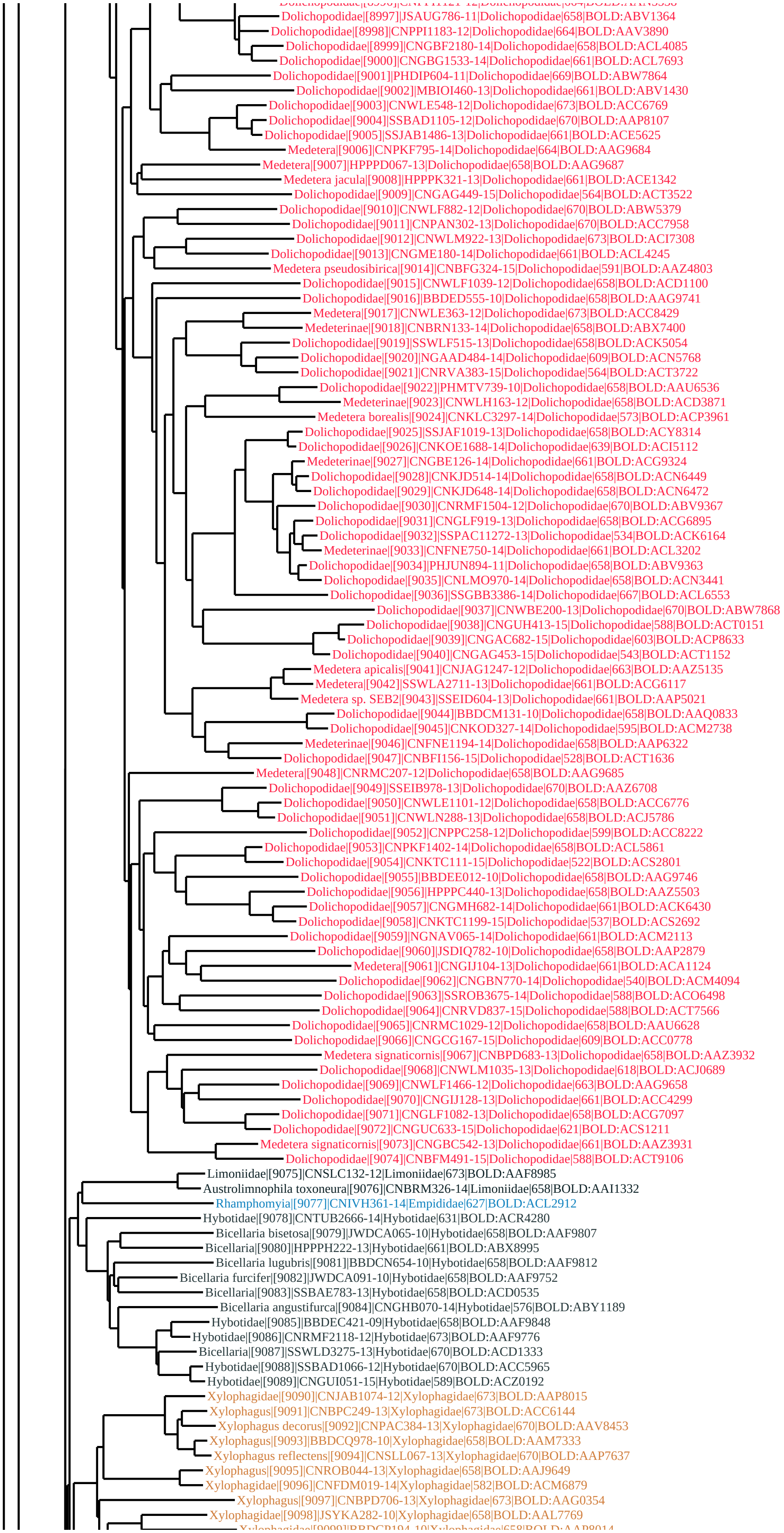
Dolichopodidae|[8997]|JSAUG786-11|Dolichopodidae|658|BOLD:ABV1364
Dolichopodidae|[8998]|CNPPI1183-12|Dolichopodidae|664|BOLD:AAV3890
Dolichopodidae|[8999]|CNGBF2180-14|Dolichopodidae|658|BOLD:ACL4085
Dolichopodidae|[9000]|CNGBG1533-14|Dolichopodidae|661|BOLD:ACL7693
Dolichopodidae|[9001]|PHDIP604-11|Dolichopodidae|669|BOLD:ABW7864
Dolichopodidae|[9002]|MBIOI460-13|Dolichopodidae|661|BOLD:ABV1430
Dolichopodidae|[9003]|CNWLE548-12|Dolichopodidae|673|BOLD:ACC6769
Dolichopodidae|[9004]|SSBAD1105-12|Dolichopodidae|670|BOLD:AAP8107
Dolichopodidae|[9005]|SSJAB1486-13|Dolichopodidae|661|BOLD:ACE5625
Medetera|[9006]|CNPKF795-14|Dolichopodidae|664|BOLD:AAG9684
Medetera|[9007]|HPPPD067-13|Dolichopodidae|658|BOLD:AAG9687
Medetera jacula|[9008]|HPPPK321-13|Dolichopodidae|661|BOLD:ACE1342
Dolichopodidae|[9009]|CNGAG449-15|Dolichopodidae|564|BOLD:ACT3522
Dolichopodidae|[9010]|CNWLF882-12|Dolichopodidae|670|BOLD:ABW5379
Dolichopodidae|[9011]|CNPAN302-13|Dolichopodidae|670|BOLD:ACC7958
Dolichopodidae|[9012]|CNWLM922-13|Dolichopodidae|673|BOLD:ACI7308
Dolichopodidae|[9013]|CNGME180-14|Dolichopodidae|661|BOLD:ACL4245
Medetera pseudosibirica|[9014]|CNBFG324-15|Dolichopodidae|591|BOLD:AAZ4803
Dolichopodidae|[9015]|CNWLF1039-12|Dolichopodidae|658|BOLD:ACD1100
Dolichopodidae|[9016]|BBDED555-10|Dolichopodidae|658|BOLD:AAG9741
Medetera|[9017]|CNWLE363-12|Dolichopodidae|673|BOLD:ACC8429
Medeterinae|[9018]|CNBRN133-14|Dolichopodidae|658|BOLD:ABX7400
Dolichopodidae|[9019]|SSWLF515-13|Dolichopodidae|658|BOLD:ACK5054
Dolichopodidae|[9020]|NGAAD484-14|Dolichopodidae|609|BOLD:ACN5768
Dolichopodidae|[9021]|CNRVA383-15|Dolichopodidae|564|BOLD:ACT3722
Dolichopodidae|[9022]|PHMTV739-10|Dolichopodidae|658|BOLD:AAU6536
Medeterinae|[9023]|CNWLH163-12|Dolichopodidae|658|BOLD:ACD3871
Medetera borealis|[9024]|CNKLC3297-14|Dolichopodidae|573|BOLD:ACP3961
Dolichopodidae|[9025]|SSJAF1019-13|Dolichopodidae|658|BOLD:ACY8314
Dolichopodidae|[9026]|CNKOE1688-14|Dolichopodidae|639|BOLD:ACI5112
Medeterinae|[9027]|CNGBE126-14|Dolichopodidae|661|BOLD:ACG9324
Dolichopodidae|[9028]|CNKJD514-14|Dolichopodidae|658|BOLD:ACN6449
Dolichopodidae|[9029]|CNKJD648-14|Dolichopodidae|658|BOLD:ACN6472
Dolichopodidae|[9030]|CNRMF1504-12|Dolichopodidae|670|BOLD:ABV9367
Dolichopodidae|[9031]|CNGLF919-13|Dolichopodidae|658|BOLD:ACG6895
Dolichopodidae|[9032]|SSPAC11272-13|Dolichopodidae|534|BOLD:ACK6164
Medeterinae|[9033]|CNFNE750-14|Dolichopodidae|661|BOLD:ACL3202
Dolichopodidae|[9034]|PHJUN894-11|Dolichopodidae|658|BOLD:ABV9363
Dolichopodidae|[9035]|CNLMO970-14|Dolichopodidae|658|BOLD:ACN3441
Dolichopodidae|[9036]|SSGBB3386-14|Dolichopodidae|667|BOLD:ACL6553
Dolichopodidae|[9037]|CNWBE200-13|Dolichopodidae|670|BOLD:ABW7868
Dolichopodidae|[9038]|CNGUH413-15|Dolichopodidae|588|BOLD:ACT0151
Dolichopodidae|[9039]|CNGAC682-15|Dolichopodidae|603|BOLD:ACP8633
Dolichopodidae|[9040]|CNGAG453-15|Dolichopodidae|543|BOLD:ACT1152
Medetera apicalis|[9041]|CNJAG1247-12|Dolichopodidae|663|BOLD:AAZ5135
Medetera|[9042]|SSWLA2711-13|Dolichopodidae|661|BOLD:ACG6117
Medetera sp. SEB2|[9043]|SSEID604-13|Dolichopodidae|661|BOLD:AAP5021
Dolichopodidae|[9044]|BBDCM131-10|Dolichopodidae|658|BOLD:AAQ0833
Dolichopodidae|[9045]|CNKOD327-14|Dolichopodidae|595|BOLD:ACM2738
Medeterinae|[9046]|CNFNE1194-14|Dolichopodidae|658|BOLD:AAP6322
Dolichopodidae|[9047]|CNBFI156-15|Dolichopodidae|528|BOLD:ACT1636
Medetera|[9048]|CNRMC207-12|Dolichopodidae|658|BOLD:AAG9685
Dolichopodidae|[9049]|SSEIB978-13|Dolichopodidae|670|BOLD:AAZ6708
Dolichopodidae|[9050]|CNWLE1101-12|Dolichopodidae|658|BOLD:ACC6776
Dolichopodidae|[9051]|CNWLN288-13|Dolichopodidae|658|BOLD:ACJ5786
Dolichopodidae|[9052]|CNPPC258-12|Dolichopodidae|599|BOLD:ACC8222
Dolichopodidae|[9053]|CNPKF1402-14|Dolichopodidae|658|BOLD:ACL5861
Dolichopodidae|[9054]|CNKTC111-15|Dolichopodidae|522|BOLD:ACS2801
Dolichopodidae|[9055]|BBDEE012-10|Dolichopodidae|658|BOLD:AAG9746
Dolichopodidae|[9056]|HPPPC440-13|Dolichopodidae|658|BOLD:AAZ5503
Dolichopodidae|[9057]|CNGMH682-14|Dolichopodidae|661|BOLD:ACK6430
Dolichopodidae|[9058]|CNKTC1199-15|Dolichopodidae|537|BOLD:ACS2692
Dolichopodidae|[9059]|NGNAV065-14|Dolichopodidae|661|BOLD:ACM2113
Dolichopodidae|[9060]|JSDIQ782-10|Dolichopodidae|658|BOLD:AAP2879
Medetera|[9061]|CNGIJ104-13|Dolichopodidae|661|BOLD:ACA1124
Dolichopodidae|[9062]|CNGBN770-14|Dolichopodidae|540|BOLD:ACM4094
Dolichopodidae|[9063]|SSROB3675-14|Dolichopodidae|588|BOLD:ACO6498
Dolichopodidae|[9064]|CNRVD837-15|Dolichopodidae|588|BOLD:ACT7566
Dolichopodidae|[9065]|CNRMC1029-12|Dolichopodidae|658|BOLD:AAU6628
Dolichopodidae|[9066]|CNGCG167-15|Dolichopodidae|609|BOLD:ACC0778
Medetera signaticornis|[9067]|CNBPD683-13|Dolichopodidae|658|BOLD:AAZ3932
Dolichopodidae|[9068]|CNWLM1035-13|Dolichopodidae|618|BOLD:ACJ0689
Dolichopodidae|[9069]|CNWLF1466-12|Dolichopodidae|663|BOLD:AAG9658
Dolichopodidae|[9070]|CNGIJ128-13|Dolichopodidae|661|BOLD:ACC4299
Dolichopodidae|[9071]|CNGLF1082-13|Dolichopodidae|658|BOLD:ACG7097
Dolichopodidae|[9072]|CNGUC633-15|Dolichopodidae|621|BOLD:ACS1211
Medetera signaticornis|[9073]|CNGBC542-13|Dolichopodidae|661|BOLD:AAZ3931
Dolichopodidae|[9074]|CNBFM491-15|Dolichopodidae|588|BOLD:ACT9106
Limoniidae|[9075]|CNSLC132-12|Limoniidae|673|BOLD:AAF8985
Austrolimnophila toxoneura|[9076]|CNBRM326-14|Limoniidae|658|BOLD:AAI1332
Rhamphomyia|[9077]|CNIVH361-14|Empididae|627|BOLD:ACL2912
Hybotidae|[9078]|CNTUB2666-14|Hybotidae|631|BOLD:ACR4280
Bicellaria bisetosa|[9079]|JWDCA065-10|Hybotidae|658|BOLD:AAF9807
Bicellaria|[9080]|HPPPH222-13|Hybotidae|661|BOLD:ABX8995
Bicellaria lugubris|[9081]|BBDCN654-10|Hybotidae|658|BOLD:AAF9812
Bicellaria furcifer|[9082]|JWDCA091-10|Hybotidae|658|BOLD:AAF9752
Bicellaria|[9083]|SSBAE783-13|Hybotidae|658|BOLD:ACD0535
Bicellaria angustifurca|[9084]|CNGHB070-14|Hybotidae|576|BOLD:ABY1189
Hybotidae|[9085]|BBDEC421-09|Hybotidae|658|BOLD:AAF9848
Hybotidae|[9086]|CNRMF2118-12|Hybotidae|673|BOLD:AAF9776
Bicellaria|[9087]|SSWLD3275-13|Hybotidae|670|BOLD:ACD1333
Hybotidae|[9088]|SSBAD1066-12|Hybotidae|670|BOLD:ACC5965
Hybotidae|[9089]|CNGUI051-15|Hybotidae|589|BOLD:ACZ0192
Xylophagidae|[9090]|CNJAB1074-12|Xylophagidae|673|BOLD:AAP8015
Xylophagus|[9091]|CNBPC249-13|Xylophagidae|673|BOLD:ACC6144
Xylophagus decorus|[9092]|CNPAC384-13|Xylophagidae|670|BOLD:AAV8453
Xylophagus|[9093]|BBDCQ978-10|Xylophagidae|658|BOLD:AAM7333
Xylophagus reflectens|[9094]|CNSLL067-13|Xylophagidae|670|BOLD:AAP7637
Xylophagus|[9095]|CNROB044-13|Xylophagidae|658|BOLD:AAJ9649
Xylophagidae|[9096]|CNFDM019-14|Xylophagidae|582|BOLD:ACM6879
Xylophagus|[9097]|CNBPD706-13|Xylophagidae|673|BOLD:AAG0354
Xylophagus|[9098]|JSYKA282-10|Xylophagidae|658|BOLD:AAL7769

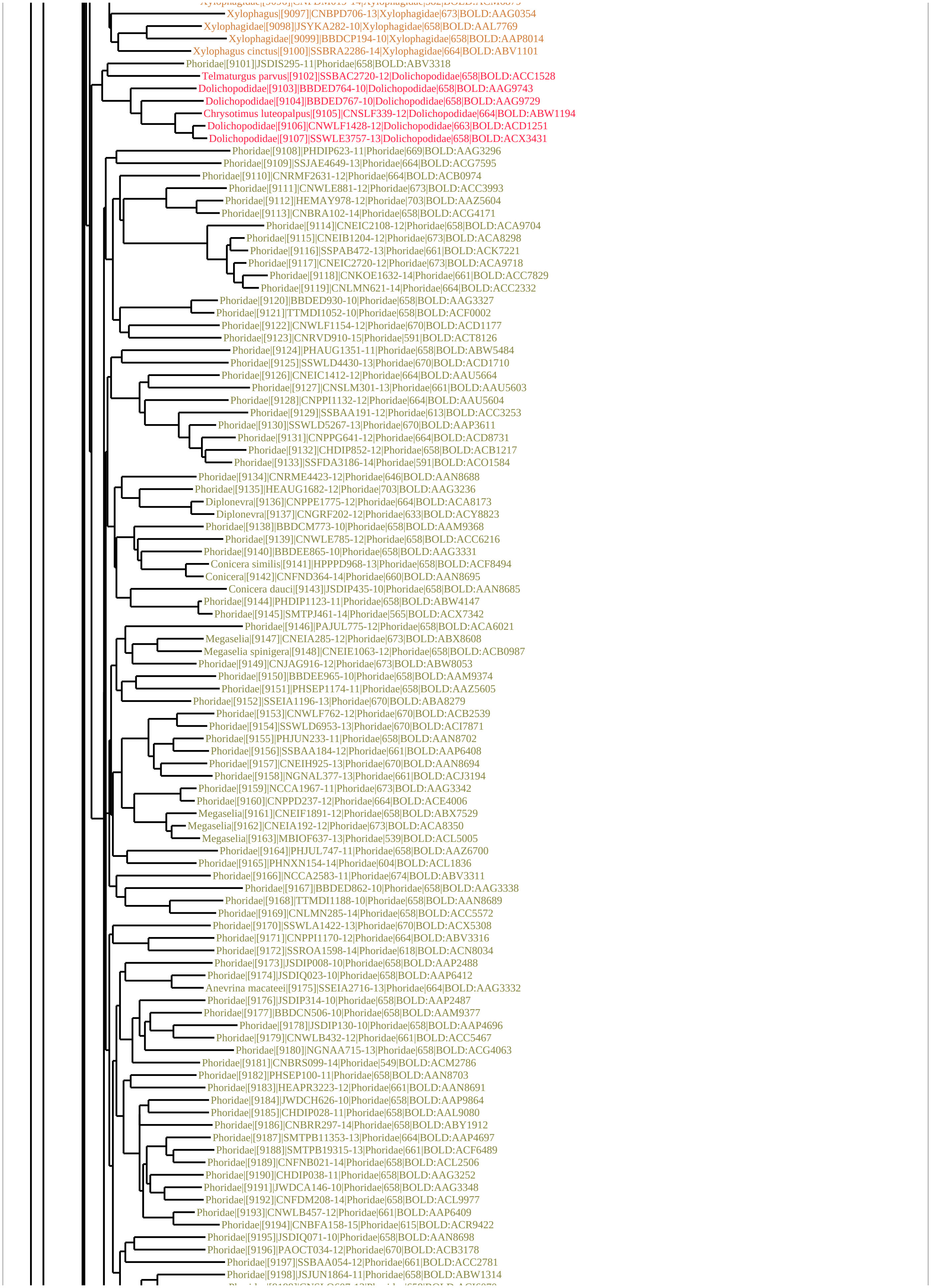
Xylophagus|[9097]|CNBPD706-13|Xylophagidae|673|BOLD:AAG0354
Xylophagidae|[9098]|JSYKA282-10|Xylophagidae|658|BOLD:AAL7769
Xylophagidae|[9099]|BBDCP194-10|Xylophagidae|658|BOLD:AAP8014
Xylophagus cinctus|[9100]|SSBRA2286-14|Xylophagidae|664|BOLD:ABV1101
Phoridae|[9101]|JSDIS295-11|Phoridae|658|BOLD:ABV3318
Telmaturgus parvus|[9102]|SSBAC2720-12|Dolichopodidae|658|BOLD:ACC1528
Dolichopodidae|[9103]|BBDED764-10|Dolichopodidae|658|BOLD:AAG9743
Dolichopodidae|[9104]|BBDED767-10|Dolichopodidae|658|BOLD:AAG9729
Chrysotimus luteopalpus|[9105]|CNSLF339-12|Dolichopodidae|664|BOLD:ABW1194
Dolichopodidae|[9106]|CNWLF1428-12|Dolichopodidae|663|BOLD:ACD1251
Dolichopodidae|[9107]|SSWLE3757-13|Dolichopodidae|658|BOLD:ACX3431
Phoridae|[9108]|PHDIP623-11|Phoridae|669|BOLD:AAG3296
Phoridae|[9109]|SSJAE4649-13|Phoridae|664|BOLD:ACG7595
Phoridae|[9110]|CNRMF2631-12|Phoridae|664|BOLD:ACB0974
Phoridae|[9111]|CNWLE881-12|Phoridae|673|BOLD:ACC3993
Phoridae|[9112]|HEMAY978-12|Phoridae|703|BOLD:AAZ5604
Phoridae|[9113]|CNBRA102-14|Phoridae|658|BOLD:ACG4171
Phoridae|[9114]|CNEIC2108-12|Phoridae|658|BOLD:ACA9704
Phoridae|[9115]|CNEIB1204-12|Phoridae|673|BOLD:ACA8298
Phoridae|[9116]|SSPAB472-13|Phoridae|661|BOLD:ACK7221
Phoridae|[9117]|CNEIC2720-12|Phoridae|673|BOLD:ACA9718
Phoridae|[9118]|CNKOE1632-14|Phoridae|661|BOLD:ACC7829
Phoridae|[9119]|CNLMN621-14|Phoridae|664|BOLD:ACC2332
Phoridae|[9120]|BBDED930-10|Phoridae|658|BOLD:AAG3327
Phoridae|[9121]|TTMDI1052-10|Phoridae|658|BOLD:ACF0002
Phoridae|[9122]|CNWLF1154-12|Phoridae|670|BOLD:ACD1177
Phoridae|[9123]|CNRVD910-15|Phoridae|591|BOLD:ACT8126
Phoridae|[9124]|PHAUG1351-11|Phoridae|658|BOLD:ABW5484
Phoridae|[9125]|SSWLD4430-13|Phoridae|670|BOLD:ACD1710
Phoridae|[9126]|CNEIC1412-12|Phoridae|664|BOLD:AAU5664
Phoridae|[9127]|CNSLM301-13|Phoridae|661|BOLD:AAU5603
Phoridae|[9128]|CNPPI1132-12|Phoridae|664|BOLD:AAU5604
Phoridae|[9129]|SSBAA191-12|Phoridae|613|BOLD:ACC3253
Phoridae|[9130]|SSWLD5267-13|Phoridae|670|BOLD:AAP3611
Phoridae|[9131]|CNPPG641-12|Phoridae|664|BOLD:ACD8731
Phoridae|[9132]|CHDIP852-12|Phoridae|658|BOLD:ACB1217
Phoridae|[9133]|SSFDA3186-14|Phoridae|591|BOLD:ACO1584
Phoridae|[9134]|CNRME4423-12|Phoridae|646|BOLD:AAN8688
Phoridae|[9135]|HEAUG1682-12|Phoridae|703|BOLD:AAG3236
Diplonevra|[9136]|CNPPE1775-12|Phoridae|664|BOLD:ACA8173
Diplonevra|[9137]|CNGRF202-12|Phoridae|633|BOLD:ACY8823
Phoridae|[9138]|BBDCM773-10|Phoridae|658|BOLD:AAM9368
Phoridae|[9139]|CNWLE785-12|Phoridae|658|BOLD:ACC6216
Phoridae|[9140]|BBDEE865-10|Phoridae|658|BOLD:AAG3331
Conicera similis|[9141]|HPPPD968-13|Phoridae|658|BOLD:ACF8494
Conicera|[9142]|CNFND364-14|Phoridae|660|BOLD:AAN8695
Conicera dauci|[9143]|JSDIP435-10|Phoridae|658|BOLD:AAN8685
Phoridae|[9144]|PHDIP1123-11|Phoridae|658|BOLD:ABW4147
Phoridae|[9145]|SMTPJ461-14|Phoridae|565|BOLD:ACX7342
Phoridae|[9146]|PAJUL775-12|Phoridae|658|BOLD:ACA6021
Megaselia|[9147]|CNEIA285-12|Phoridae|673|BOLD:ABX8608
Megaselia spinigera|[9148]|CNEIE1063-12|Phoridae|658|BOLD:ACB0987
Phoridae|[9149]|CNJAG916-12|Phoridae|673|BOLD:ABW8053
Phoridae|[9150]|BBDEE965-10|Phoridae|658|BOLD:AAM9374
Phoridae|[9151]|PHSEP1174-11|Phoridae|658|BOLD:AAZ5605
Phoridae|[9152]|SSEIA1196-13|Phoridae|670|BOLD:ABA8279
Phoridae|[9153]|CNWLF762-12|Phoridae|670|BOLD:ACB2539
Phoridae|[9154]|SSWLD6953-13|Phoridae|670|BOLD:ACI7871
Phoridae|[9155]|PHJUN233-11|Phoridae|658|BOLD:AAN8702
Phoridae|[9156]|SSBAA184-12|Phoridae|661|BOLD:AAP6408
Phoridae|[9157]|CNEIH925-13|Phoridae|670|BOLD:AAN8694
Phoridae|[9158]|NGNAL377-13|Phoridae|661|BOLD:ACJ3194
Phoridae|[9159]|NCCA1967-11|Phoridae|673|BOLD:AAG3342
Phoridae|[9160]|CNPPD237-12|Phoridae|664|BOLD:ACE4006
Megaselia|[9161]|CNEIF1891-12|Phoridae|658|BOLD:ABX7529
Megaselia|[9162]|CNEIA192-12|Phoridae|673|BOLD:ACA8350
Megaselia|[9163]|MBIOF637-13|Phoridae|539|BOLD:ACL5005
Phoridae|[9164]|PHJUL747-11|Phoridae|658|BOLD:AAZ6700
Phoridae|[9165]|PHNXN154-14|Phoridae|604|BOLD:ACL1836
Phoridae|[9166]|NCCA2583-11|Phoridae|674|BOLD:ABV3311
Phoridae|[9167]|BBDED862-10|Phoridae|658|BOLD:AAG3338
Phoridae|[9168]|TTMDI1188-10|Phoridae|658|BOLD:AAN8689
Phoridae|[9169]|CNLMN285-14|Phoridae|658|BOLD:ACC5572
Phoridae|[9170]|SSWLA1422-13|Phoridae|670|BOLD:ACX5308
Phoridae|[9171]|CNPPI1170-12|Phoridae|664|BOLD:ABV3316
Phoridae|[9172]|SSROA1598-14|Phoridae|618|BOLD:ACN8034
Phoridae|[9173]|JSDIP008-10|Phoridae|658|BOLD:AAP2488
Phoridae|[9174]|JSDIQ023-10|Phoridae|658|BOLD:AAP6412
Anevrina macateei|[9175]|SSEIA2716-13|Phoridae|664|BOLD:AAG3332
Phoridae|[9176]|JSDIP314-10|Phoridae|658|BOLD:AAP2487
Phoridae|[9177]|BBDCN506-10|Phoridae|658|BOLD:AAM9377
Phoridae|[9178]|JSDIP130-10|Phoridae|658|BOLD:AAP4696
Phoridae|[9179]|CNWLB432-12|Phoridae|661|BOLD:ACC5467
Phoridae|[9180]|NGNAA715-13|Phoridae|658|BOLD:ACG4063
Phoridae|[9181]|CNBRS099-14|Phoridae|549|BOLD:ACM2786
Phoridae|[9182]|PHSEP100-11|Phoridae|658|BOLD:AAN8703
Phoridae|[9183]|HEAPR3223-12|Phoridae|661|BOLD:AAN8691
Phoridae|[9184]|JWDCH626-10|Phoridae|658|BOLD:AAP9864
Phoridae|[9185]|CHDIP028-11|Phoridae|658|BOLD:AAL9080
Phoridae|[9186]|CNBRR297-14|Phoridae|658|BOLD:ABY1912
Phoridae|[9187]|SMTPB11353-13|Phoridae|664|BOLD:AAP4697
Phoridae|[9188]|SMTPB19315-13|Phoridae|661|BOLD:ACF6489
Phoridae|[9189]|CNFNB021-14|Phoridae|658|BOLD:ACL2506
Phoridae|[9190]|CHDIP038-11|Phoridae|658|BOLD:AAG3252
Phoridae|[9191]|JWDCA146-10|Phoridae|658|BOLD:AAG3348
Phoridae|[9192]|CNFDM208-14|Phoridae|658|BOLD:ACL9977
Phoridae|[9193]|CNWLB457-12|Phoridae|661|BOLD:AAP6409
Phoridae|[9194]|CNBFA158-15|Phoridae|615|BOLD:ACR9422
Phoridae|[9195]|JSDIQ071-10|Phoridae|658|BOLD:AAN8698
Phoridae|[9196]|PAOCT034-12|Phoridae|670|BOLD:ACB3178
Phoridae|[9197]|SSBAA054-12|Phoridae|661|BOLD:ACC2781
Phoridae|[9198]|JSJUN1864-11|Phoridae|658|BOLD:ABW1314

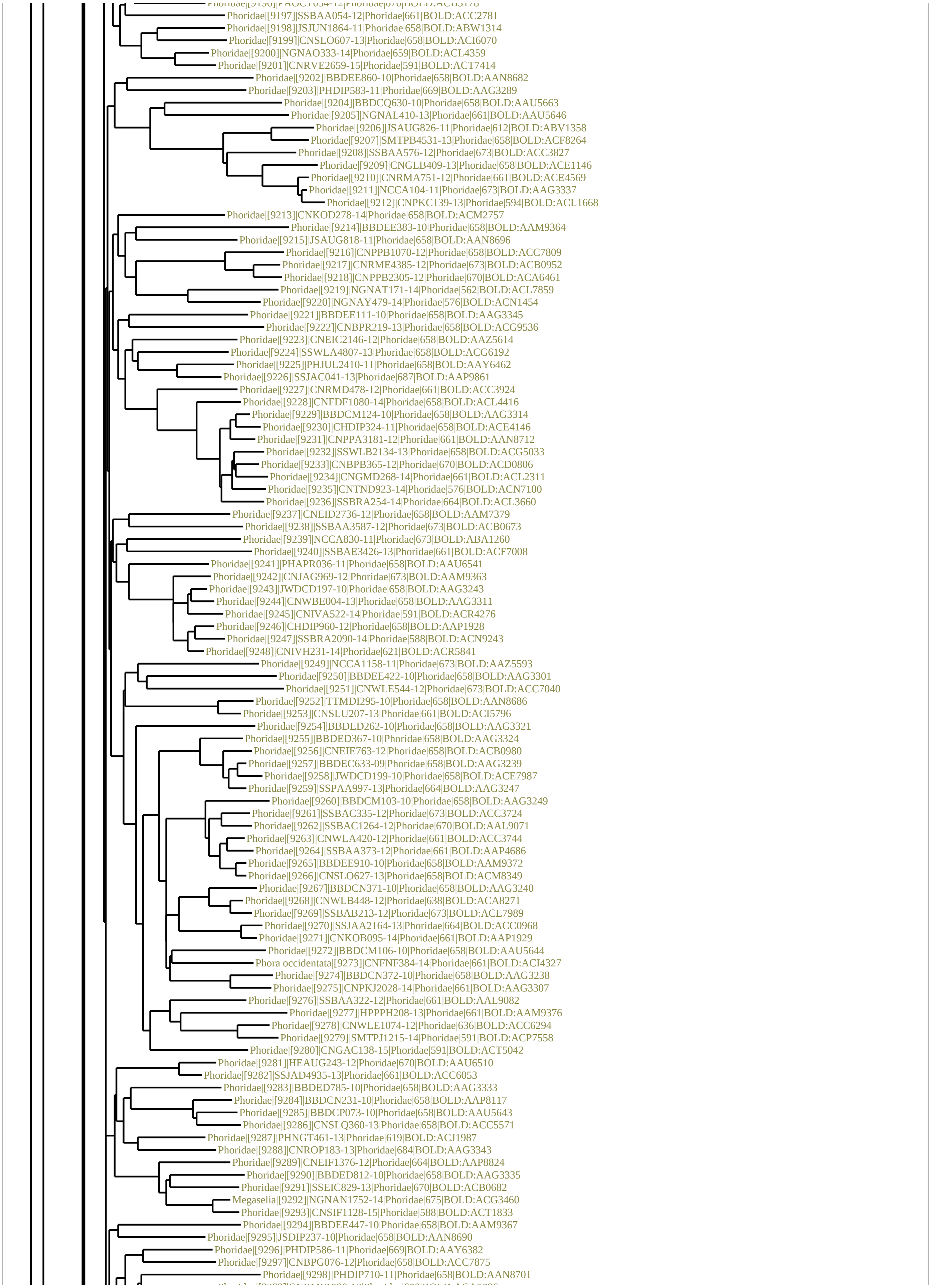

Phoridae|[9197]|SSBAA054-12|Phoridae|661|BOLD:ACC2781
Phoridae|[9198]|JSJUN1864-11|Phoridae|658|BOLD:ABW1314
Phoridae|[9199]|CNSLO607-13|Phoridae|658|BOLD:ACI6070
Phoridae|[9200]|NGNAO333-14|Phoridae|659|BOLD:ACL4359
Phoridae|[9201]|CNRVE2659-15|Phoridae|591|BOLD:ACT7414
Phoridae|[9202]|BBDEE860-10|Phoridae|658|BOLD:AAN8682
Phoridae|[9203]|PHDIP583-11|Phoridae|669|BOLD:AAG3289
Phoridae|[9204]|BBDCQ630-10|Phoridae|658|BOLD:AAU5663
Phoridae|[9205]|NGNAL410-13|Phoridae|661|BOLD:AAU5646
Phoridae|[9206]|JSAUG826-11|Phoridae|612|BOLD:ABV1358
Phoridae|[9207]|SMTPB4531-13|Phoridae|658|BOLD:ACF8264
Phoridae|[9208]|SSBAA576-12|Phoridae|673|BOLD:ACC3827
Phoridae|[9209]|CNGLB409-13|Phoridae|658|BOLD:ACE1146
Phoridae|[9210]|CNRMA751-12|Phoridae|661|BOLD:ACE4569
Phoridae|[9211]|NCCA104-11|Phoridae|673|BOLD:AAG3337
Phoridae|[9212]|CNPKC139-13|Phoridae|594|BOLD:ACL1668
Phoridae|[9213]|CNKOD278-14|Phoridae|658|BOLD:ACM2757
Phoridae|[9214]|BBDEE383-10|Phoridae|658|BOLD:AAM9364
Phoridae|[9215]|JSAUG818-11|Phoridae|658|BOLD:AAN8696
Phoridae|[9216]|CNPPB1070-12|Phoridae|658|BOLD:ACC7809
Phoridae|[9217]|CNRME4385-12|Phoridae|673|BOLD:ACB0952
Phoridae|[9218]|CNPPB2305-12|Phoridae|670|BOLD:ACA6461
Phoridae|[9219]|NGNAT171-14|Phoridae|562|BOLD:ACL7859
Phoridae|[9220]|NGNAY479-14|Phoridae|576|BOLD:ACN1454
Phoridae|[9221]|BBDEE111-10|Phoridae|658|BOLD:AAG3345
Phoridae|[9222]|CNBPR219-13|Phoridae|658|BOLD:ACG9536
Phoridae|[9223]|CNEIC2146-12|Phoridae|658|BOLD:AAZ5614
Phoridae|[9224]|SSWLA4807-13|Phoridae|658|BOLD:ACG6192
Phoridae|[9225]|PHJUL2410-11|Phoridae|658|BOLD:AAY6462
Phoridae|[9226]|SSJAC041-13|Phoridae|687|BOLD:AAP9861
Phoridae|[9227]|CNRMD478-12|Phoridae|661|BOLD:ACC3924
Phoridae|[9228]|CNFDF1080-14|Phoridae|658|BOLD:ACL4416
Phoridae|[9229]|BBDCM124-10|Phoridae|658|BOLD:AAG3314
Phoridae|[9230]|CHDIP324-11|Phoridae|658|BOLD:ACE4146
Phoridae|[9231]|CNPPA3181-12|Phoridae|661|BOLD:AAN8712
Phoridae|[9232]|SSWLB2134-13|Phoridae|658|BOLD:ACG5033
Phoridae|[9233]|CNBPB365-12|Phoridae|670|BOLD:ACD0806
Phoridae|[9234]|CNGMD268-14|Phoridae|661|BOLD:ACL2311
Phoridae|[9235]|CNTND923-14|Phoridae|576|BOLD:ACN7100
Phoridae|[9236]|SSBRA254-14|Phoridae|664|BOLD:ACL3660
Phoridae|[9237]|CNEID2736-12|Phoridae|658|BOLD:AAM7379
Phoridae|[9238]|SSBAA3587-12|Phoridae|673|BOLD:ACB0673
Phoridae|[9239]|NCCA830-11|Phoridae|673|BOLD:ABA1260
Phoridae|[9240]|SSBAE3426-13|Phoridae|661|BOLD:ACF7008
Phoridae|[9241]|PHAPR036-11|Phoridae|658|BOLD:AAU6541
Phoridae|[9242]|CNJAG969-12|Phoridae|673|BOLD:AAM9363
Phoridae|[9243]|JWDCD197-10|Phoridae|658|BOLD:AAG3243
Phoridae|[9244]|CNWBE004-13|Phoridae|658|BOLD:AAG3311
Phoridae|[9245]|CNIVA522-14|Phoridae|591|BOLD:ACR4276
Phoridae|[9246]|CHDIP960-12|Phoridae|658|BOLD:AAP1928
Phoridae|[9247]|SSBRA2090-14|Phoridae|588|BOLD:ACN9243
Phoridae|[9248]|CNIVH231-14|Phoridae|621|BOLD:ACR5841
Phoridae|[9249]|NCCA1158-11|Phoridae|673|BOLD:AAZ5593
Phoridae|[9250]|BBDEE422-10|Phoridae|658|BOLD:AAG3301
Phoridae|[9251]|CNWLE544-12|Phoridae|673|BOLD:ACC7040
Phoridae|[9252]|TTMDI295-10|Phoridae|658|BOLD:AAN8686
Phoridae|[9253]|CNSLU207-13|Phoridae|661|BOLD:ACI5796
Phoridae|[9254]|BBDED262-10|Phoridae|658|BOLD:AAG3321
Phoridae|[9255]|BBDED367-10|Phoridae|658|BOLD:AAG3324
Phoridae|[9256]|CNEIE763-12|Phoridae|658|BOLD:ACB0980
Phoridae|[9257]|BBDEC633-09|Phoridae|658|BOLD:AAG3239
Phoridae|[9258]|JWDCD199-10|Phoridae|658|BOLD:ACE7987
Phoridae|[9259]|SSPAA997-13|Phoridae|664|BOLD:AAG3247
Phoridae|[9260]|BBDCM103-10|Phoridae|658|BOLD:AAG3249
Phoridae|[9261]|SSBAC335-12|Phoridae|673|BOLD:ACC3724
Phoridae|[9262]|SSBAC1264-12|Phoridae|670|BOLD:AAL9071
Phoridae|[9263]|CNWLA420-12|Phoridae|661|BOLD:ACC3744
Phoridae|[9264]|SSBAA373-12|Phoridae|661|BOLD:AAP4686
Phoridae|[9265]|BBDEE910-10|Phoridae|658|BOLD:AAM9372
Phoridae|[9266]|CNSLO627-13|Phoridae|658|BOLD:ACM8349
Phoridae|[9267]|BBDCN371-10|Phoridae|658|BOLD:AAG3240
Phoridae|[9268]|CNWLB448-12|Phoridae|638|BOLD:ACA8271
Phoridae|[9269]|SSBAB213-12|Phoridae|673|BOLD:ACE7989
Phoridae|[9270]|SSJAA2164-13|Phoridae|664|BOLD:ACC0968
Phoridae|[9271]|CNKOB095-14|Phoridae|661|BOLD:AAP1929
Phoridae|[9272]|BBDCM106-10|Phoridae|658|BOLD:AAU5644
Phora occidentata|[9273]|CNFNF384-14|Phoridae|661|BOLD:ACI4327
Phoridae|[9274]|BBDCN372-10|Phoridae|658|BOLD:AAG3238
Phoridae|[9275]|CNPKJ2028-14|Phoridae|661|BOLD:AAG3307
Phoridae|[9276]|SSBAA322-12|Phoridae|661|BOLD:AAL9082
Phoridae|[9277]|HPPPH208-13|Phoridae|661|BOLD:AAM9376
Phoridae|[9278]|CNWLE1074-12|Phoridae|636|BOLD:ACC6294
Phoridae|[9279]|SMTPJ1215-14|Phoridae|591|BOLD:ACP7558
Phoridae|[9280]|CNGAC138-15|Phoridae|591|BOLD:ACT5042
Phoridae|[9281]|HEAUG243-12|Phoridae|670|BOLD:AAU6510
Phoridae|[9282]|SSJAD4935-13|Phoridae|661|BOLD:ACC6053
Phoridae|[9283]|BBDED785-10|Phoridae|658|BOLD:AAG3333
Phoridae|[9284]|BBDCN231-10|Phoridae|658|BOLD:AAP8117
Phoridae|[9285]|BBDCP073-10|Phoridae|658|BOLD:AAU5643
Phoridae|[9286]|CNSLQ360-13|Phoridae|658|BOLD:ACC5571
Phoridae|[9287]|PHNGT461-13|Phoridae|619|BOLD:ACJ1987
Phoridae|[9288]|CNROP183-13|Phoridae|684|BOLD:AAG3343
Phoridae|[9289]|CNEIF1376-12|Phoridae|664|BOLD:AAP8824
Phoridae|[9290]|BBDED812-10|Phoridae|658|BOLD:AAG3335
Phoridae|[9291]|SSEIC829-13|Phoridae|670|BOLD:ACB0682
Megaselia|[9292]|NGNAN1752-14|Phoridae|675|BOLD:ACG3460
Phoridae|[9293]|CNSIF1128-15|Phoridae|588|BOLD:ACT1833
Phoridae|[9294]|BBDEE447-10|Phoridae|658|BOLD:AAM9367
Phoridae|[9295]|JSDIP237-10|Phoridae|658|BOLD:AAN8690
Phoridae|[9296]|PHDIP586-11|Phoridae|669|BOLD:AAY6382
Phoridae|[9297]|CNBPG076-12|Phoridae|658|BOLD:ACC7875
Phoridae|[9298]|PHDIP710-11|Phoridae|658|BOLD:AAN8701

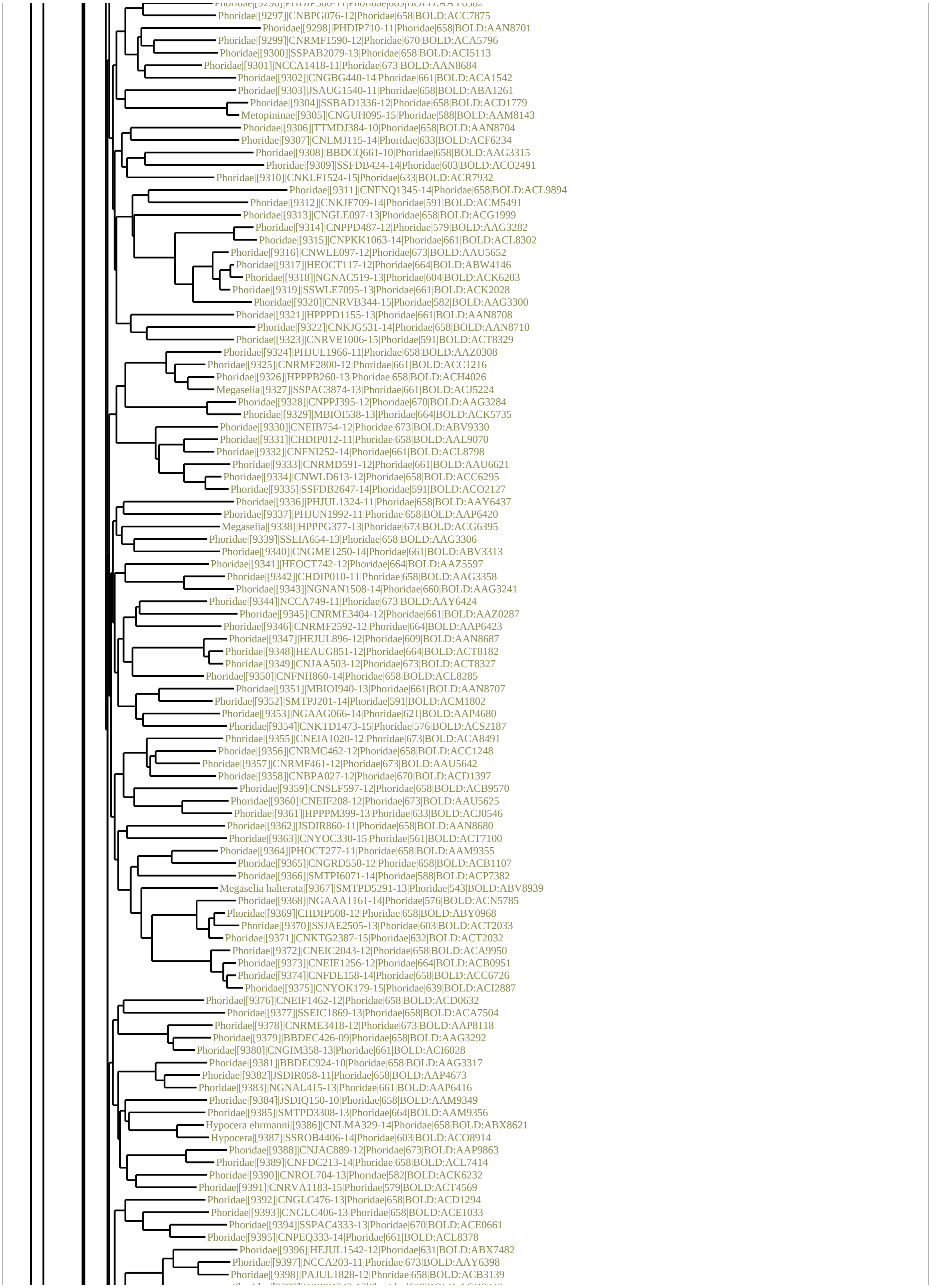
Phoridae|[9297]|CNBPG076-12|Phoridae|658|BOLD:ACC7875
Phoridae|[9298]|PHDIP710-11|Phoridae|658|BOLD:AAN8701
Phoridae|[9299]|CNRMF1590-12|Phoridae|670|BOLD:ACA5796
Phoridae|[9300]|SSPAB2079-13|Phoridae|658|BOLD:ACI5113
Phoridae|[9301]|NCCA1418-11|Phoridae|673|BOLD:AAN8684
Phoridae|[9302]|CNGBG440-14|Phoridae|661|BOLD:ACA1542
Phoridae|[9303]|JSAUG1540-11|Phoridae|658|BOLD:ABA1261
Phoridae|[9304]|SSBAD1336-12|Phoridae|658|BOLD:ACD1779
Metopininae|[9305]|CNGUH095-15|Phoridae|588|BOLD:AAM8143
Phoridae|[9306]|TTMDJ384-10|Phoridae|658|BOLD:AAN8704
Phoridae|[9307]|CNLMJ115-14|Phoridae|633|BOLD:ACF6234
Phoridae|[9308]|BBDCQ661-10|Phoridae|658|BOLD:AAG3315
Phoridae|[9309]|SSFDB424-14|Phoridae|603|BOLD:ACO2491
Phoridae|[9310]|CNKLF1524-15|Phoridae|633|BOLD:ACR7932
Phoridae|[9311]|CNFNQ1345-14|Phoridae|658|BOLD:ACL9894
Phoridae|[9312]|CNKJF709-14|Phoridae|591|BOLD:ACM5491
Phoridae|[9313]|CNGLE097-13|Phoridae|658|BOLD:ACG1999
Phoridae|[9314]|CNPPD487-12|Phoridae|579|BOLD:AAG3282
Phoridae|[9315]|CNPKK1063-14|Phoridae|661|BOLD:ACL8302
Phoridae|[9316]|CNWLE097-12|Phoridae|673|BOLD:AAU5652
Phoridae|[9317]|HEOCT117-12|Phoridae|664|BOLD:ABW4146
Phoridae|[9318]|NGNAC519-13|Phoridae|604|BOLD:ACK6203
Phoridae|[9319]|SSWLE7095-13|Phoridae|661|BOLD:ACK2028
Phoridae|[9320]|CNRVB344-15|Phoridae|582|BOLD:AAG3300
Phoridae|[9321]|HPPPD1155-13|Phoridae|661|BOLD:AAN8708
Phoridae|[9322]|CNKJG531-14|Phoridae|658|BOLD:AAN8710
Phoridae|[9323]|CNRVE1006-15|Phoridae|591|BOLD:ACT8329
Phoridae|[9324]|PHJUL1966-11|Phoridae|658|BOLD:AAZ0308
Phoridae|[9325]|CNRMF2800-12|Phoridae|661|BOLD:ACC1216
Phoridae|[9326]|HPPPB260-13|Phoridae|658|BOLD:ACH4026
Megaselia|[9327]|SSPAC3874-13|Phoridae|661|BOLD:ACJ5224
Phoridae|[9328]|CNPPJ395-12|Phoridae|670|BOLD:AAG3284
Phoridae|[9329]|MBIOI538-13|Phoridae|664|BOLD:ACK5735
Phoridae|[9330]|CNEIB754-12|Phoridae|673|BOLD:ABV9330
Phoridae|[9331]|CHDIP012-11|Phoridae|658|BOLD:AAL9070
Phoridae|[9332]|CNFNI252-14|Phoridae|661|BOLD:ACL8798
Phoridae|[9333]|CNRMD591-12|Phoridae|661|BOLD:AAU6621
Phoridae|[9334]|CNWLD613-12|Phoridae|658|BOLD:ACC6295
Phoridae|[9335]|SSFDB2647-14|Phoridae|591|BOLD:ACO2127
Phoridae|[9336]|PHJUL1324-11|Phoridae|658|BOLD:AAY6437
Phoridae|[9337]|PHJUN1992-11|Phoridae|658|BOLD:AAP6420
Megaselia|[9338]|HPPPG377-13|Phoridae|673|BOLD:ACG6395
Phoridae|[9339]|SSEIA654-13|Phoridae|658|BOLD:AAG3306
Phoridae|[9340]|CNGME1250-14|Phoridae|661|BOLD:ABV3313
Phoridae|[9341]|HEOCT742-12|Phoridae|664|BOLD:AAZ5597
Phoridae|[9342]|CHDIP010-11|Phoridae|658|BOLD:AAG3358
Phoridae|[9343]|NGNAN1508-14|Phoridae|660|BOLD:AAG3241
Phoridae|[9344]|NCCA749-11|Phoridae|673|BOLD:AAY6424
Phoridae|[9345]|CNRME3404-12|Phoridae|661|BOLD:AAZ0287
Phoridae|[9346]|CNRMF2592-12|Phoridae|664|BOLD:AAP6423
Phoridae|[9347]|HEJUL896-12|Phoridae|609|BOLD:AAN8687
Phoridae|[9348]|HEAUG851-12|Phoridae|664|BOLD:ACT8182
Phoridae|[9349]|CNJAA503-12|Phoridae|673|BOLD:ACT8327
Phoridae|[9350]|CNFNH860-14|Phoridae|658|BOLD:ACL8285
Phoridae|[9351]|MBIOI940-13|Phoridae|661|BOLD:AAN8707
Phoridae|[9352]|SMTPJ201-14|Phoridae|591|BOLD:ACM1802
Phoridae|[9353]|NGAAG066-14|Phoridae|621|BOLD:AAP4680
Phoridae|[9354]|CNKTD1473-15|Phoridae|576|BOLD:ACS2187
Phoridae|[9355]|CNEIA1020-12|Phoridae|673|BOLD:ACA8491
Phoridae|[9356]|CNRMC462-12|Phoridae|658|BOLD:ACC1248
Phoridae|[9357]|CNRMF461-12|Phoridae|673|BOLD:AAU5642
Phoridae|[9358]|CNBPA027-12|Phoridae|670|BOLD:ACD1397
Phoridae|[9359]|CNSLF597-12|Phoridae|658|BOLD:ACB9570
Phoridae|[9360]|CNEIF208-12|Phoridae|673|BOLD:AAU5625
Phoridae|[9361]|HPPPM399-13|Phoridae|633|BOLD:ACJ0546
Phoridae|[9362]|JSDIR860-11|Phoridae|658|BOLD:AAN8680
Phoridae|[9363]|CNYOC330-15|Phoridae|561|BOLD:ACT7100
Phoridae|[9364]|PHOCT277-11|Phoridae|658|BOLD:AAM9355
Phoridae|[9365]|CNGRD550-12|Phoridae|658|BOLD:ACB1107
Phoridae|[9366]|SMTPI6071-14|Phoridae|588|BOLD:ACP7382
Megaselia halterata|[9367]|SMTPD5291-13|Phoridae|543|BOLD:ABV8939
Phoridae|[9368]|NGAAA1161-14|Phoridae|576|BOLD:ACN5785
Phoridae|[9369]|CHDIP508-12|Phoridae|658|BOLD:ABY0968
Phoridae|[9370]|SSJAE2505-13|Phoridae|603|BOLD:ACT2033
Phoridae|[9371]|CNKTG2387-15|Phoridae|632|BOLD:ACT2032
Phoridae|[9372]|CNEIC2043-12|Phoridae|658|BOLD:ACA9950
Phoridae|[9373]|CNEIE1256-12|Phoridae|664|BOLD:ACB0951
Phoridae|[9374]|CNFDE158-14|Phoridae|658|BOLD:ACC6726
Phoridae|[9375]|CNYOK179-15|Phoridae|639|BOLD:ACI2887
Phoridae|[9376]|CNEIF1462-12|Phoridae|658|BOLD:ACD0632
Phoridae|[9377]|SSEIC1869-13|Phoridae|658|BOLD:ACA7504
Phoridae|[9378]|CNRME3418-12|Phoridae|673|BOLD:AAP8118
Phoridae|[9379]|BBDEC426-09|Phoridae|658|BOLD:AAG3292
Phoridae|[9380]|CNGIM358-13|Phoridae|661|BOLD:ACI6028
Phoridae|[9381]|BBDEC924-10|Phoridae|658|BOLD:AAG3317
Phoridae|[9382]|JSDIR058-11|Phoridae|658|BOLD:AAP4673
Phoridae|[9383]|NGNAL415-13|Phoridae|661|BOLD:AAP6416
Phoridae|[9384]|JSDIQ150-10|Phoridae|658|BOLD:AAM9349
Phoridae|[9385]|SMTPD3308-13|Phoridae|664|BOLD:AAM9356
Hypocera ehrmanni|[9386]|CNLMA329-14|Phoridae|658|BOLD:ABX8621
Hypocera|[9387]|SSROB4406-14|Phoridae|603|BOLD:ACO8914
Phoridae|[9388]|CNJAC889-12|Phoridae|673|BOLD:AAP9863
Phoridae|[9389]|CNFDC213-14|Phoridae|658|BOLD:ACL7414
Phoridae|[9390]|CNROL704-13|Phoridae|582|BOLD:ACK6232
Phoridae|[9391]|CNRVA1183-15|Phoridae|579|BOLD:ACT4569
Phoridae|[9392]|CNGLC476-13|Phoridae|658|BOLD:ACD1294
Phoridae|[9393]|CNGLC406-13|Phoridae|658|BOLD:ACE1033
Phoridae|[9394]|SSPAC4333-13|Phoridae|670|BOLD:ACE0661
Phoridae|[9395]|CNPEQ333-14|Phoridae|661|BOLD:ACL8378
Phoridae|[9396]|HEJUL1542-12|Phoridae|631|BOLD:ABX7482
Phoridae|[9397]|NCCA203-11|Phoridae|673|BOLD:AAY6398
Phoridae|[9398]|PAJUL1828-12|Phoridae|658|BOLD:ACB3139

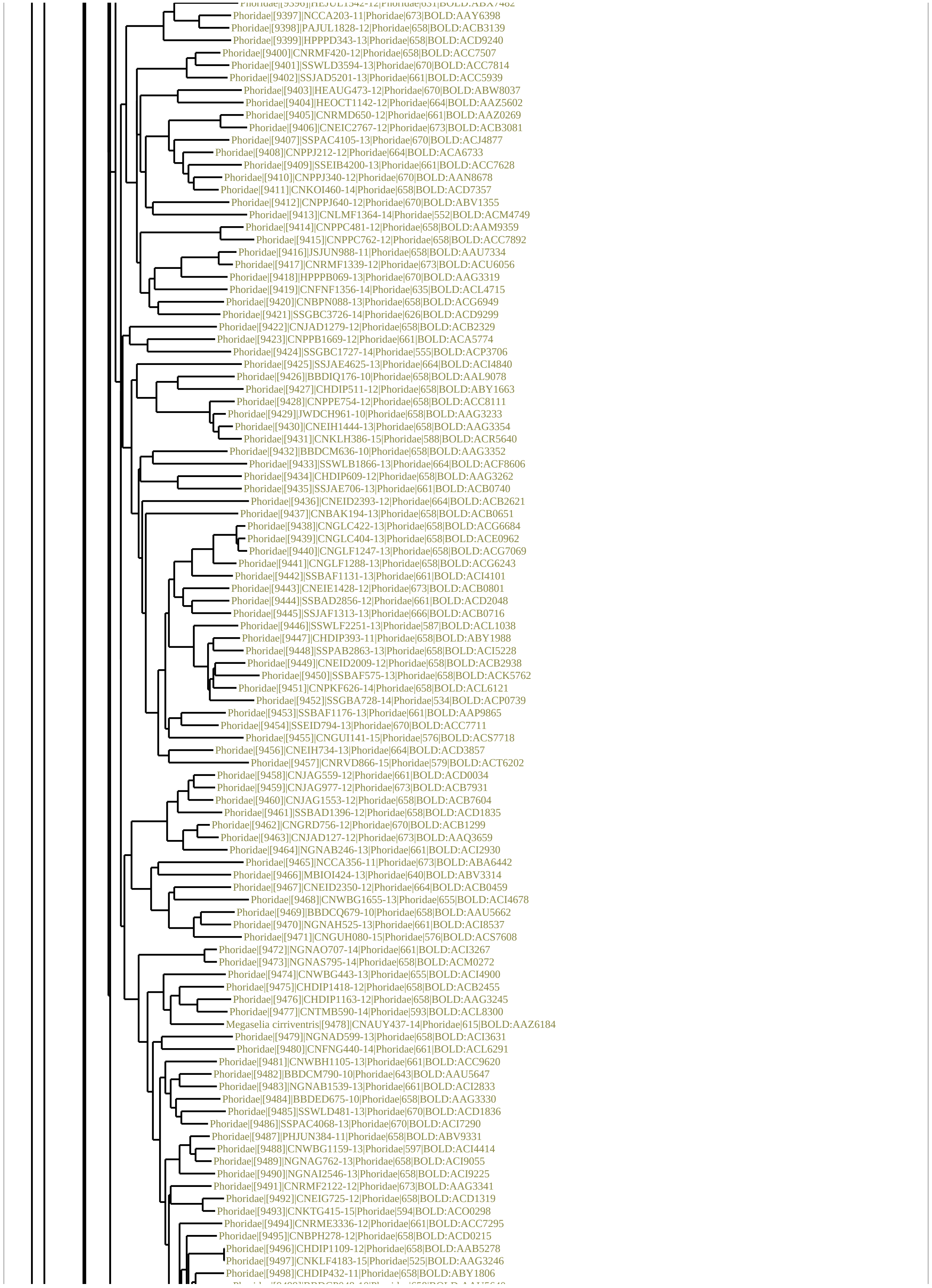
Phoridae|[9397]|NCCA203-11|Phoridae|673|BOLD:AAY6398
Phoridae|[9398]|PAJUL1828-12|Phoridae|658|BOLD:ACB3139
Phoridae|[9399]|HPPPD343-13|Phoridae|658|BOLD:ACD9240
Phoridae|[9400]|CNRMF420-12|Phoridae|658|BOLD:ACC7507
Phoridae|[9401]|SSWLD3594-13|Phoridae|670|BOLD:ACC7814
Phoridae|[9402]|SSJAD5201-13|Phoridae|661|BOLD:ACC5939
Phoridae|[9403]|HEAUG473-12|Phoridae|670|BOLD:ABW8037
Phoridae|[9404]|HEOCT1142-12|Phoridae|664|BOLD:AAZ5602
Phoridae|[9405]|CNRMD650-12|Phoridae|661|BOLD:AAZ0269
Phoridae|[9406]|CNEIC2767-12|Phoridae|673|BOLD:ACB3081
Phoridae|[9407]|SSPAC4105-13|Phoridae|670|BOLD:ACJ4877
Phoridae|[9408]|CNPPJ212-12|Phoridae|664|BOLD:ACA6733
Phoridae|[9409]|SSEIB4200-13|Phoridae|661|BOLD:ACC7628
Phoridae|[9410]|CNPPJ340-12|Phoridae|670|BOLD:AAN8678
Phoridae|[9411]|CNKOI460-14|Phoridae|658|BOLD:ACD7357
Phoridae|[9412]|CNPPJ640-12|Phoridae|670|BOLD:ABV1355
Phoridae|[9413]|CNLMF1364-14|Phoridae|552|BOLD:ACM4749
Phoridae|[9414]|CNPPC481-12|Phoridae|658|BOLD:AAM9359
Phoridae|[9415]|CNPPC762-12|Phoridae|658|BOLD:ACC7892
Phoridae|[9416]|JSJUN988-11|Phoridae|658|BOLD:AAU7334
Phoridae|[9417]|CNRMF1339-12|Phoridae|673|BOLD:ACU6056
Phoridae|[9418]|HPPPB069-13|Phoridae|670|BOLD:AAG3319
Phoridae|[9419]|CNFNF1356-14|Phoridae|635|BOLD:ACL4715
Phoridae|[9420]|CNBPN088-13|Phoridae|658|BOLD:ACG6949
Phoridae|[9421]|SSGBC3726-14|Phoridae|626|BOLD:ACD9299
Phoridae|[9422]|CNJAD1279-12|Phoridae|658|BOLD:ACB2329
Phoridae|[9423]|CNPPB1669-12|Phoridae|661|BOLD:ACA5774
Phoridae|[9424]|SSGBC1727-14|Phoridae|555|BOLD:ACP3706
Phoridae|[9425]|SSJAE4625-13|Phoridae|664|BOLD:ACI4840
Phoridae|[9426]|BBDIQ176-10|Phoridae|658|BOLD:AAL9078
Phoridae|[9427]|CHDIP511-12|Phoridae|658|BOLD:ABY1663
Phoridae|[9428]|CNPPE754-12|Phoridae|658|BOLD:ACC8111
Phoridae|[9429]|JWDCH961-10|Phoridae|658|BOLD:AAG3233
Phoridae|[9430]|CNEIH1444-13|Phoridae|658|BOLD:AAG3354
Phoridae|[9431]|CNKLH386-15|Phoridae|588|BOLD:ACR5640
Phoridae|[9432]|BBDCM636-10|Phoridae|658|BOLD:AAG3352
Phoridae|[9433]|SSWLB1866-13|Phoridae|664|BOLD:ACF8606
Phoridae|[9434]|CHDIP609-12|Phoridae|658|BOLD:AAG3262
Phoridae|[9435]|SSJAE706-13|Phoridae|661|BOLD:ACB0740
Phoridae|[9436]|CNEID2393-12|Phoridae|664|BOLD:ACB2621
Phoridae|[9437]|CNBAK194-13|Phoridae|658|BOLD:ACB0651
Phoridae|[9438]|CNGLC422-13|Phoridae|658|BOLD:ACG6684
Phoridae|[9439]|CNGLC404-13|Phoridae|658|BOLD:ACE0962
Phoridae|[9440]|CNGLF1247-13|Phoridae|658|BOLD:ACG7069
Phoridae|[9441]|CNGLF1288-13|Phoridae|658|BOLD:ACG6243
Phoridae|[9442]|SSBAF1131-13|Phoridae|661|BOLD:ACI4101
Phoridae|[9443]|CNEIE1428-12|Phoridae|673|BOLD:ACB0801
Phoridae|[9444]|SSBAD2856-12|Phoridae|661|BOLD:ACD2048
Phoridae|[9445]|SSJAF1313-13|Phoridae|666|BOLD:ACB0716
Phoridae|[9446]|SSWLF2251-13|Phoridae|587|BOLD:ACL1038
Phoridae|[9447]|CHDIP393-11|Phoridae|658|BOLD:ABY1988
Phoridae|[9448]|SSPAB2863-13|Phoridae|658|BOLD:ACI5228
Phoridae|[9449]|CNEID2009-12|Phoridae|658|BOLD:ACB2938
Phoridae|[9450]|SSBAF575-13|Phoridae|658|BOLD:ACK5762
Phoridae|[9451]|CNPKF626-14|Phoridae|658|BOLD:ACL6121
Phoridae|[9452]|SSGBA728-14|Phoridae|534|BOLD:ACP0739
Phoridae|[9453]|SSBAF1176-13|Phoridae|661|BOLD:AAP9865
Phoridae|[9454]|SSEID794-13|Phoridae|670|BOLD:ACC7711
Phoridae|[9455]|CNGUI141-15|Phoridae|576|BOLD:ACS7718
Phoridae|[9456]|CNEIH734-13|Phoridae|664|BOLD:ACD3857
Phoridae|[9457]|CNRVD866-15|Phoridae|579|BOLD:ACT6202
Phoridae|[9458]|CNJAG559-12|Phoridae|661|BOLD:ACD0034
Phoridae|[9459]|CNJAG977-12|Phoridae|673|BOLD:ACB7931
Phoridae|[9460]|CNJAG1553-12|Phoridae|658|BOLD:ACB7604
Phoridae|[9461]|SSBAD1396-12|Phoridae|658|BOLD:ACD1835
Phoridae|[9462]|CNGRD756-12|Phoridae|670|BOLD:ACB1299
Phoridae|[9463]|CNJAD127-12|Phoridae|673|BOLD:AAQ3659
Phoridae|[9464]|NGNAB246-13|Phoridae|661|BOLD:ACI2930
Phoridae|[9465]|NCCA356-11|Phoridae|673|BOLD:ABA6442
Phoridae|[9466]|MBIOI424-13|Phoridae|640|BOLD:ABV3314
Phoridae|[9467]|CNEID2350-12|Phoridae|664|BOLD:ACB0459
Phoridae|[9468]|CNWBG1655-13|Phoridae|655|BOLD:ACI4678
Phoridae|[9469]|BBDCQ679-10|Phoridae|658|BOLD:AAU5662
Phoridae|[9470]|NGNAH525-13|Phoridae|661|BOLD:ACI8537
Phoridae|[9471]|CNGUH080-15|Phoridae|576|BOLD:ACS7608
Phoridae|[9472]|NGNAO707-14|Phoridae|661|BOLD:ACI3267
Phoridae|[9473]|NGNAS795-14|Phoridae|658|BOLD:ACM0272
Phoridae|[9474]|CNWBG443-13|Phoridae|655|BOLD:ACI4900
Phoridae|[9475]|CHDIP1418-12|Phoridae|658|BOLD:ACB2455
Phoridae|[9476]|CHDIP1163-12|Phoridae|658|BOLD:AAG3245
Phoridae|[9477]|CNTMB590-14|Phoridae|593|BOLD:ACL8300
Megaselia cirriventris|[9478]|CNAUY437-14|Phoridae|615|BOLD:AAZ6184
Phoridae|[9479]|NGNAD599-13|Phoridae|658|BOLD:ACI3631
Phoridae|[9480]|CNFNG440-14|Phoridae|661|BOLD:ACL6291
Phoridae|[9481]|CNWBH1105-13|Phoridae|661|BOLD:ACC9620
Phoridae|[9482]|BBDCM790-10|Phoridae|643|BOLD:AAU5647
Phoridae|[9483]|NGNAB1539-13|Phoridae|661|BOLD:ACI2833
Phoridae|[9484]|BBDED675-10|Phoridae|658|BOLD:AAG3330
Phoridae|[9485]|SSWLD481-13|Phoridae|670|BOLD:ACD1836
Phoridae|[9486]|SSPAC4068-13|Phoridae|670|BOLD:ACI7290
Phoridae|[9487]|PHJUN384-11|Phoridae|658|BOLD:ABV9331
Phoridae|[9488]|CNWBG1159-13|Phoridae|597|BOLD:ACI4414
Phoridae|[9489]|NGNAG762-13|Phoridae|658|BOLD:ACI9055
Phoridae|[9490]|NGNAI2546-13|Phoridae|658|BOLD:ACI9225
Phoridae|[9491]|CNRMF2122-12|Phoridae|673|BOLD:AAG3341
Phoridae|[9492]|CNEIG725-12|Phoridae|658|BOLD:ACD1319
Phoridae|[9493]|CNKTG415-15|Phoridae|594|BOLD:ACO0298
Phoridae|[9494]|CNRME3336-12|Phoridae|661|BOLD:ACC7295
Phoridae|[9495]|CNBPH278-12|Phoridae|658|BOLD:ACD0215
Phoridae|[9496]|CHDIP1109-12|Phoridae|658|BOLD:AAB5278
Phoridae|[9497]|CNKLF4183-15|Phoridae|525|BOLD:AAG3246
Phoridae|[9498]|CHDIP432-11|Phoridae|658|BOLD:ABY1806

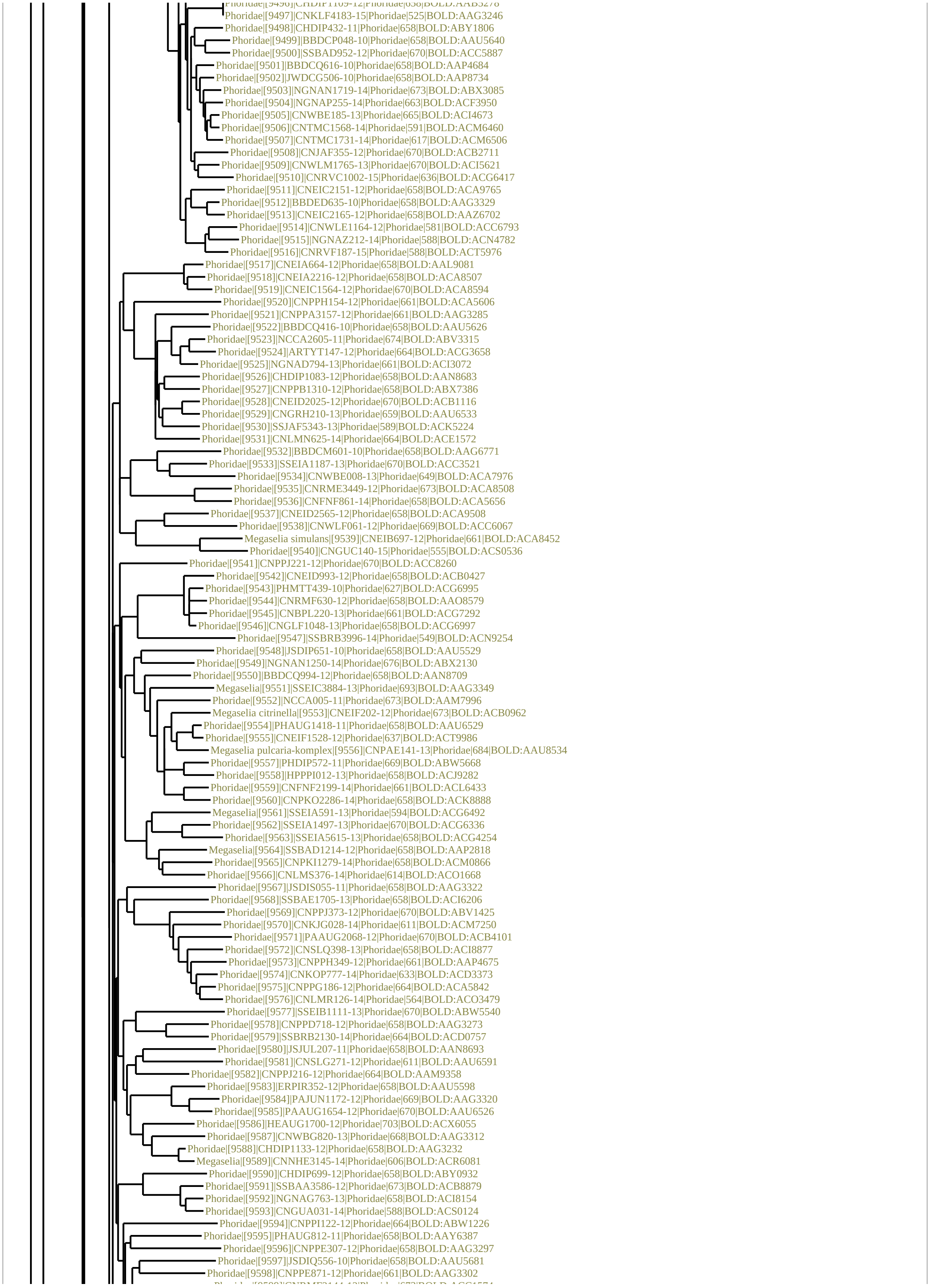

Phoridae|[9496]|CHDIP1109-12|Phoridae|658|BOLD:AAB5278
Phoridae|[9497]|CNKLF4183-15|Phoridae|525|BOLD:AAG3246
Phoridae|[9498]|CHDIP432-11|Phoridae|658|BOLD:ABY1806
Phoridae|[9499]|BBDCP048-10|Phoridae|658|BOLD:AAU5640
Phoridae|[9500]|SSBAD952-12|Phoridae|670|BOLD:ACC5887
Phoridae|[9501]|BBDCQ616-10|Phoridae|658|BOLD:AAP4684
Phoridae|[9502]|JWDCG506-10|Phoridae|658|BOLD:AAP8734
Phoridae|[9503]|NGNAN1719-14|Phoridae|673|BOLD:ABX3085
Phoridae|[9504]|NGNAP255-14|Phoridae|663|BOLD:ACF3950
Phoridae|[9505]|CNWBE185-13|Phoridae|665|BOLD:ACI4673
Phoridae|[9506]|CNTMC1568-14|Phoridae|591|BOLD:ACM6460
Phoridae|[9507]|CNTMC1731-14|Phoridae|617|BOLD:ACM6506
Phoridae|[9508]|CNJAF355-12|Phoridae|670|BOLD:ACB2711
Phoridae|[9509]|CNWLM1765-13|Phoridae|670|BOLD:ACI5621
Phoridae|[9510]|CNRVC1002-15|Phoridae|636|BOLD:ACG6417
Phoridae|[9511]|CNEIC2151-12|Phoridae|658|BOLD:ACA9765
Phoridae|[9512]|BBDED635-10|Phoridae|658|BOLD:AAG3329
Phoridae|[9513]|CNEIC2165-12|Phoridae|658|BOLD:AAZ6702
Phoridae|[9514]|CNWLE1164-12|Phoridae|581|BOLD:ACC6793
Phoridae|[9515]|NGNAZ212-14|Phoridae|588|BOLD:ACN4782
Phoridae|[9516]|CNRVF187-15|Phoridae|588|BOLD:ACT5976
Phoridae|[9517]|CNEIA664-12|Phoridae|658|BOLD:AAL9081
Phoridae|[9518]|CNEIA2216-12|Phoridae|658|BOLD:ACA8507
Phoridae|[9519]|CNEIC1564-12|Phoridae|670|BOLD:ACA8594
Phoridae|[9520]|CNPPH154-12|Phoridae|661|BOLD:ACA5606
Phoridae|[9521]|CNPPA3157-12|Phoridae|661|BOLD:AAG3285
Phoridae|[9522]|BBDCQ416-10|Phoridae|658|BOLD:AAU5626
Phoridae|[9523]|NCCA2605-11|Phoridae|674|BOLD:ABV3315
Phoridae|[9524]|ARTYT147-12|Phoridae|664|BOLD:ACG3658
Phoridae|[9525]|NGNAD794-13|Phoridae|661|BOLD:ACI3072
Phoridae|[9526]|CHDIP1083-12|Phoridae|658|BOLD:AAN8683
Phoridae|[9527]|CNPPB1310-12|Phoridae|658|BOLD:ABX7386
Phoridae|[9528]|CNEID2025-12|Phoridae|670|BOLD:ACB1116
Phoridae|[9529]|CNGRH210-13|Phoridae|659|BOLD:AAU6533
Phoridae|[9530]|SSJAF5343-13|Phoridae|589|BOLD:ACK5224
Phoridae|[9531]|CNLMN625-14|Phoridae|664|BOLD:ACE1572
Phoridae|[9532]|BBDCM601-10|Phoridae|658|BOLD:AAG6771
Phoridae|[9533]|SSEIA1187-13|Phoridae|670|BOLD:ACC3521
Phoridae|[9534]|CNWBE008-13|Phoridae|649|BOLD:ACA7976
Phoridae|[9535]|CNRME3449-12|Phoridae|673|BOLD:ACA8508
Phoridae|[9536]|CNFNF861-14|Phoridae|658|BOLD:ACA5656
Phoridae|[9537]|CNEID2565-12|Phoridae|658|BOLD:ACA9508
Phoridae|[9538]|CNWLF061-12|Phoridae|669|BOLD:ACC6067
Megaselia simulans|[9539]|CNEIB697-12|Phoridae|661|BOLD:ACA8452
Phoridae|[9540]|CNGUC140-15|Phoridae|555|BOLD:ACS0536
Phoridae|[9541]|CNPPJ221-12|Phoridae|670|BOLD:ACC8260
Phoridae|[9542]|CNEID993-12|Phoridae|658|BOLD:ACB0427
Phoridae|[9543]|PHMTT439-10|Phoridae|627|BOLD:ACG6995
Phoridae|[9544]|CNRMF630-12|Phoridae|658|BOLD:AAO8579
Phoridae|[9545]|CNBPL220-13|Phoridae|661|BOLD:ACG7292
Phoridae|[9546]|CNGLF1048-13|Phoridae|658|BOLD:ACG6997
Phoridae|[9547]|SSBRB3996-14|Phoridae|549|BOLD:ACN9254
Phoridae|[9548]|JSDIP651-10|Phoridae|658|BOLD:AAU5529
Phoridae|[9549]|NGNAN1250-14|Phoridae|676|BOLD:ABX2130
Phoridae|[9550]|BBDCQ994-12|Phoridae|658|BOLD:AAN8709
Megaselia|[9551]|SSEIC3884-13|Phoridae|693|BOLD:AAG3349
Phoridae|[9552]|NCCA005-11|Phoridae|673|BOLD:AAM7996
Megaselia citrinella|[9553]|CNEIF202-12|Phoridae|673|BOLD:ACB0962
Phoridae|[9554]|PHAUG1418-11|Phoridae|658|BOLD:AAU6529
Phoridae|[9555]|CNEIF1528-12|Phoridae|637|BOLD:ACT9986
Megaselia pulcaria-komplex|[9556]|CNPAE141-13|Phoridae|684|BOLD:AAU8534
Phoridae|[9557]|PHDIP572-11|Phoridae|669|BOLD:ABW5668
Phoridae|[9558]|HPPPI012-13|Phoridae|658|BOLD:ACJ9282
Phoridae|[9559]|CNFNF2199-14|Phoridae|661|BOLD:ACL6433
Phoridae|[9560]|CNPKO2286-14|Phoridae|658|BOLD:ACK8888
Megaselia|[9561]|SSEIA591-13|Phoridae|594|BOLD:ACG6492
Phoridae|[9562]|SSEIA1497-13|Phoridae|670|BOLD:ACG6336
Phoridae|[9563]|SSEIA5615-13|Phoridae|658|BOLD:ACG4254
Megaselia|[9564]|SSBAD1214-12|Phoridae|658|BOLD:AAP2818
Phoridae|[9565]|CNPKI1279-14|Phoridae|658|BOLD:ACM0866
Phoridae|[9566]|CNLMS376-14|Phoridae|614|BOLD:ACO1668
Phoridae|[9567]|JSDIS055-11|Phoridae|658|BOLD:AAG3322
Phoridae|[9568]|SSBAE1705-13|Phoridae|658|BOLD:ACI6206
Phoridae|[9569]|CNPPJ373-12|Phoridae|670|BOLD:ABV1425
Phoridae|[9570]|CNKJG028-14|Phoridae|611|BOLD:ACM7250
Phoridae|[9571]|PAAUG2068-12|Phoridae|670|BOLD:ACB4101
Phoridae|[9572]|CNSLQ398-13|Phoridae|658|BOLD:ACI8877
Phoridae|[9573]|CNPPH349-12|Phoridae|661|BOLD:AAP4675
Phoridae|[9574]|CNKOP777-14|Phoridae|633|BOLD:ACD3373
Phoridae|[9575]|CNPPG186-12|Phoridae|664|BOLD:ACA5842
Phoridae|[9576]|CNLMR126-14|Phoridae|564|BOLD:ACO3479
Phoridae|[9577]|SSEIB1111-13|Phoridae|670|BOLD:ABW5540
Phoridae|[9578]|CNPPD718-12|Phoridae|658|BOLD:AAG3273
Phoridae|[9579]|SSBRB2130-14|Phoridae|664|BOLD:ACD0757
Phoridae|[9580]|JSJUL207-11|Phoridae|658|BOLD:AAN8693
Phoridae|[9581]|CNSLG271-12|Phoridae|611|BOLD:AAU6591
Phoridae|[9582]|CNPPJ216-12|Phoridae|664|BOLD:AAM9358
Phoridae|[9583]|ERPIR352-12|Phoridae|658|BOLD:AAU5598
Phoridae|[9584]|PAJUN1172-12|Phoridae|669|BOLD:AAG3320
Phoridae|[9585]|PAAUG1654-12|Phoridae|670|BOLD:AAU6526
Phoridae|[9586]|HEAUG1700-12|Phoridae|703|BOLD:ACX6055
Phoridae|[9587]|CNWBG820-13|Phoridae|668|BOLD:AAG3312
Phoridae|[9588]|CHDIP1133-12|Phoridae|658|BOLD:AAG3232
Megaselia|[9589]|CNNHE3145-14|Phoridae|606|BOLD:ACR6081
Phoridae|[9590]|CHDIP699-12|Phoridae|658|BOLD:ABY0932
Phoridae|[9591]|SSBAA3586-12|Phoridae|673|BOLD:ACB8879
Phoridae|[9592]|NGNAG763-13|Phoridae|658|BOLD:ACI8154
Phoridae|[9593]|CNGUA031-14|Phoridae|588|BOLD:ACS0124
Phoridae|[9594]|CNPPI122-12|Phoridae|664|BOLD:ABW1226
Phoridae|[9595]|PHAUG812-11|Phoridae|658|BOLD:AAY6387
Phoridae|[9596]|CNPPE307-12|Phoridae|658|BOLD:AAG3297
Phoridae|[9597]|JSDIQ556-10|Phoridae|658|BOLD:AAU5681
Phoridae|[9598]|CNPPE871-12|Phoridae|661|BOLD:AAG3302

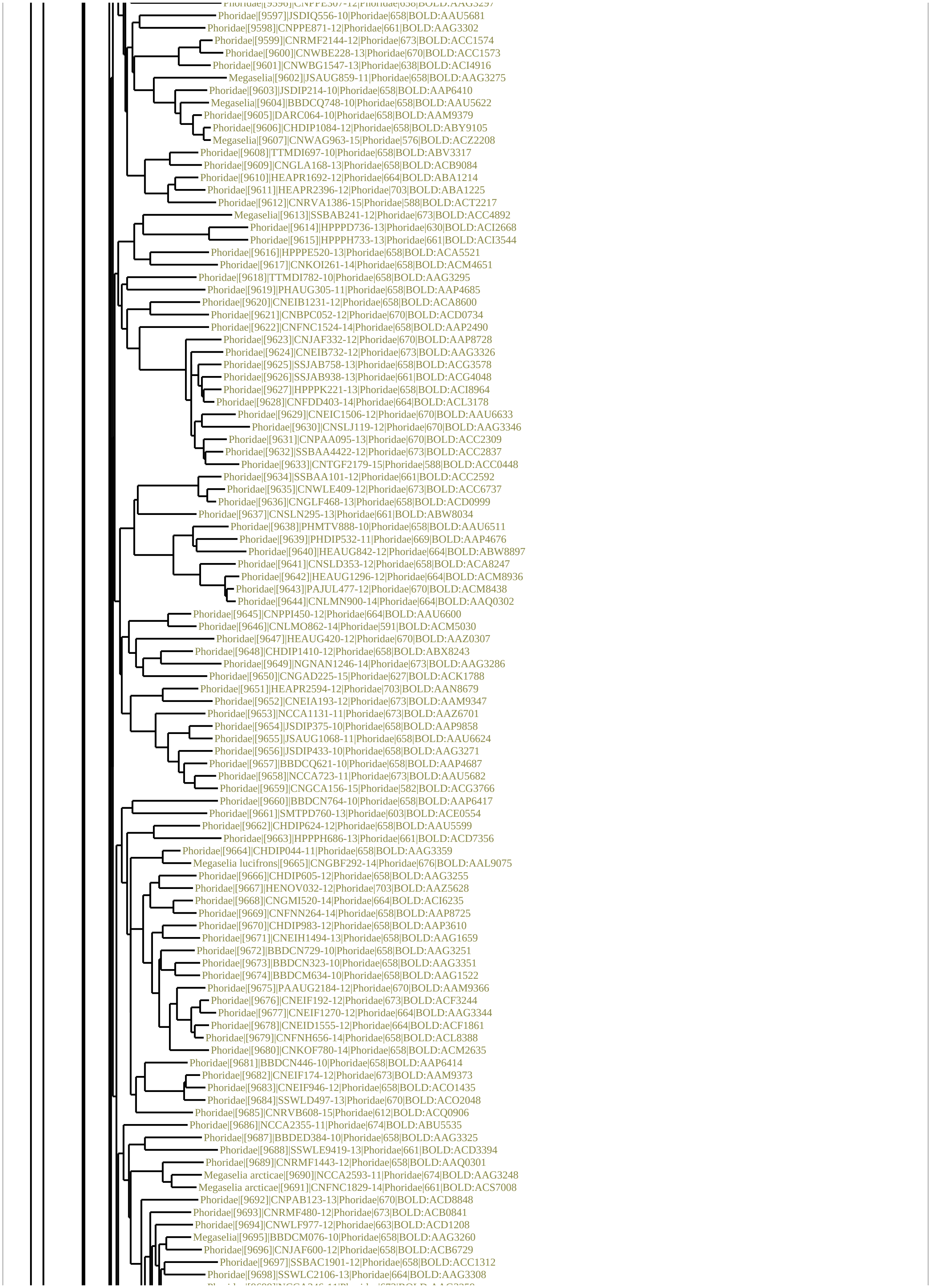
Phoridae|[9596]|CNPPE307-12|Phoridae|658|BOLD:AAG3297
Phoridae|[9597]|JSDIQ556-10|Phoridae|658|BOLD:AAU5681
Phoridae|[9598]|CNPPE871-12|Phoridae|661|BOLD:AAG3302
Phoridae|[9599]|CNRMF2144-12|Phoridae|673|BOLD:ACC1574
Phoridae|[9600]|CNWBE228-13|Phoridae|670|BOLD:ACC1573
Phoridae|[9601]|CNWBG1547-13|Phoridae|638|BOLD:ACI4916
Megaselia|[9602]|JSAUG859-11|Phoridae|658|BOLD:AAG3275
Phoridae|[9603]|JSDIP214-10|Phoridae|658|BOLD:AAP6410
Megaselia|[9604]|BBDCQ748-10|Phoridae|658|BOLD:AAU5622
Phoridae|[9605]|DARC064-10|Phoridae|658|BOLD:AAM9379
Phoridae|[9606]|CHDIP1084-12|Phoridae|658|BOLD:ABY9105
Megaselia|[9607]|CNWAG963-15|Phoridae|576|BOLD:ACZ2208
Phoridae|[9608]|TTMDI697-10|Phoridae|658|BOLD:ABV3317
Phoridae|[9609]|CNGLA168-13|Phoridae|658|BOLD:ACB9084
Phoridae|[9610]|HEAPR1692-12|Phoridae|664|BOLD:ABA1214
Phoridae|[9611]|HEAPR2396-12|Phoridae|703|BOLD:ABA1225
Phoridae|[9612]|CNRVA1386-15|Phoridae|588|BOLD:ACT2217
Megaselia|[9613]|SSBAB241-12|Phoridae|673|BOLD:ACC4892
Phoridae|[9614]|HPPPD736-13|Phoridae|630|BOLD:ACI2668
Phoridae|[9615]|HPPPH733-13|Phoridae|661|BOLD:ACI3544
Phoridae|[9616]|HPPPE520-13|Phoridae|658|BOLD:ACA5521
Phoridae|[9617]|CNKOI261-14|Phoridae|658|BOLD:ACM4651
Phoridae|[9618]|TTMDI782-10|Phoridae|658|BOLD:AAG3295
Phoridae|[9619]|PHAUG305-11|Phoridae|658|BOLD:AAP4685
Phoridae|[9620]|CNEIB1231-12|Phoridae|658|BOLD:ACA8600
Phoridae|[9621]|CNBPC052-12|Phoridae|670|BOLD:ACD0734
Phoridae|[9622]|CNFNC1524-14|Phoridae|658|BOLD:AAP2490
Phoridae|[9623]|CNJAF332-12|Phoridae|670|BOLD:AAP8728
Phoridae|[9624]|CNEIB732-12|Phoridae|673|BOLD:AAG3326
Phoridae|[9625]|SSJAB758-13|Phoridae|658|BOLD:ACG3578
Phoridae|[9626]|SSJAB938-13|Phoridae|661|BOLD:ACG4048
Phoridae|[9627]|HPPPK221-13|Phoridae|658|BOLD:ACI8964
Phoridae|[9628]|CNFDD403-14|Phoridae|664|BOLD:ACL3178
Phoridae|[9629]|CNEIC1506-12|Phoridae|670|BOLD:AAU6633
Phoridae|[9630]|CNSLJ119-12|Phoridae|670|BOLD:AAG3346
Phoridae|[9631]|CNPAA095-13|Phoridae|670|BOLD:ACC2309
Phoridae|[9632]|SSBAA4422-12|Phoridae|673|BOLD:ACC2837
Phoridae|[9633]|CNTGF2179-15|Phoridae|588|BOLD:ACC0448
Phoridae|[9634]|SSBAA101-12|Phoridae|661|BOLD:ACC2592
Phoridae|[9635]|CNWLE409-12|Phoridae|673|BOLD:ACC6737
Phoridae|[9636]|CNGLF468-13|Phoridae|658|BOLD:ACD0999
Phoridae|[9637]|CNSLN295-13|Phoridae|661|BOLD:ABW8034
Phoridae|[9638]|PHMTV888-10|Phoridae|658|BOLD:AAU6511
Phoridae|[9639]|PHDIP532-11|Phoridae|669|BOLD:AAP4676
Phoridae|[9640]|HEAUG842-12|Phoridae|664|BOLD:ABW8897
Phoridae|[9641]|CNSLD353-12|Phoridae|658|BOLD:ACA8247
Phoridae|[9642]|HEAUG1296-12|Phoridae|664|BOLD:ACM8936
Phoridae|[9643]|PAJUL477-12|Phoridae|670|BOLD:ACM8438
Phoridae|[9644]|CNLMN900-14|Phoridae|664|BOLD:AAQ0302
Phoridae|[9645]|CNPPI450-12|Phoridae|664|BOLD:AAU6600
Phoridae|[9646]|CNLMO862-14|Phoridae|591|BOLD:ACM5030
Phoridae|[9647]|HEAUG420-12|Phoridae|670|BOLD:AAZ0307
Phoridae|[9648]|CHDIP1410-12|Phoridae|658|BOLD:ABX8243
Phoridae|[9649]|NGNAN1246-14|Phoridae|673|BOLD:AAG3286
Phoridae|[9650]|CNGAD225-15|Phoridae|627|BOLD:ACK1788
Phoridae|[9651]|HEAPR2594-12|Phoridae|703|BOLD:AAN8679
Phoridae|[9652]|CNEIA193-12|Phoridae|673|BOLD:AAM9347
Phoridae|[9653]|NCCA1131-11|Phoridae|673|BOLD:AAZ6701
Phoridae|[9654]|JSDIP375-10|Phoridae|658|BOLD:AAP9858
Phoridae|[9655]|JSAUG1068-11|Phoridae|658|BOLD:AAU6624
Phoridae|[9656]|JSDIP433-10|Phoridae|658|BOLD:AAG3271
Phoridae|[9657]|BBDCQ621-10|Phoridae|658|BOLD:AAP4687
Phoridae|[9658]|NCCA723-11|Phoridae|673|BOLD:AAU5682
Phoridae|[9659]|CNGCA156-15|Phoridae|582|BOLD:ACG3766
Phoridae|[9660]|BBDCN764-10|Phoridae|658|BOLD:AAP6417
Phoridae|[9661]|SMTPD760-13|Phoridae|603|BOLD:ACE0554
Phoridae|[9662]|CHDIP624-12|Phoridae|658|BOLD:AAU5599
Phoridae|[9663]|HPPPH686-13|Phoridae|661|BOLD:ACD7356
Phoridae|[9664]|CHDIP044-11|Phoridae|658|BOLD:AAG3359
Megaselia lucifrons|[9665]|CNGBF292-14|Phoridae|676|BOLD:AAL9075
Phoridae|[9666]|CHDIP605-12|Phoridae|658|BOLD:AAG3255
Phoridae|[9667]|HENOV032-12|Phoridae|703|BOLD:AAZ5628
Phoridae|[9668]|CNGMI520-14|Phoridae|664|BOLD:ACI6235
Phoridae|[9669]|CNFNN264-14|Phoridae|658|BOLD:AAP8725
Phoridae|[9670]|CHDIP983-12|Phoridae|658|BOLD:AAP3610
Phoridae|[9671]|CNEIH1494-13|Phoridae|658|BOLD:AAG1659
Phoridae|[9672]|BBDCN729-10|Phoridae|658|BOLD:AAG3251
Phoridae|[9673]|BBDCN323-10|Phoridae|658|BOLD:AAG3351
Phoridae|[9674]|BBDCM634-10|Phoridae|658|BOLD:AAG1522
Phoridae|[9675]|PAAUG2184-12|Phoridae|670|BOLD:AAM9366
Phoridae|[9676]|CNEIF192-12|Phoridae|673|BOLD:ACF3244
Phoridae|[9677]|CNEIF1270-12|Phoridae|664|BOLD:AAG3344
Phoridae|[9678]|CNEID1555-12|Phoridae|664|BOLD:ACF1861
Phoridae|[9679]|CNFNH656-14|Phoridae|658|BOLD:ACL8388
Phoridae|[9680]|CNKOF780-14|Phoridae|658|BOLD:ACM2635
Phoridae|[9681]|BBDCN446-10|Phoridae|658|BOLD:AAP6414
Phoridae|[9682]|CNEIF174-12|Phoridae|673|BOLD:AAM9373
Phoridae|[9683]|CNEIF946-12|Phoridae|658|BOLD:ACO1435
Phoridae|[9684]|SSWLD497-13|Phoridae|670|BOLD:ACO2048
Phoridae|[9685]|CNRVB608-15|Phoridae|612|BOLD:ACQ0906
Phoridae|[9686]|NCCA2355-11|Phoridae|674|BOLD:ABU5535
Phoridae|[9687]|BBDED384-10|Phoridae|658|BOLD:AAG3325
Phoridae|[9688]|SSWLE9419-13|Phoridae|661|BOLD:ACD3394
Phoridae|[9689]|CNRMF1443-12|Phoridae|658|BOLD:AAQ0301
Megaselia arcticae|[9690]|NCCA2593-11|Phoridae|674|BOLD:AAG3248
Megaselia arcticae|[9691]|CNFNC1829-14|Phoridae|661|BOLD:ACS7008
Phoridae|[9692]|CNPAB123-13|Phoridae|670|BOLD:ACD8848
Phoridae|[9693]|CNRMF480-12|Phoridae|673|BOLD:ACB0841
Phoridae|[9694]|CNWLF977-12|Phoridae|663|BOLD:ACD1208
Megaselia|[9695]|BBDCM076-10|Phoridae|658|BOLD:AAG3260
Phoridae|[9696]|CNJAF600-12|Phoridae|658|BOLD:ACB6729
Phoridae|[9697]|SSBAC1901-12|Phoridae|658|BOLD:ACC1312
Phoridae|[9698]|SSWLC2106-13|Phoridae|664|BOLD:AAG3308

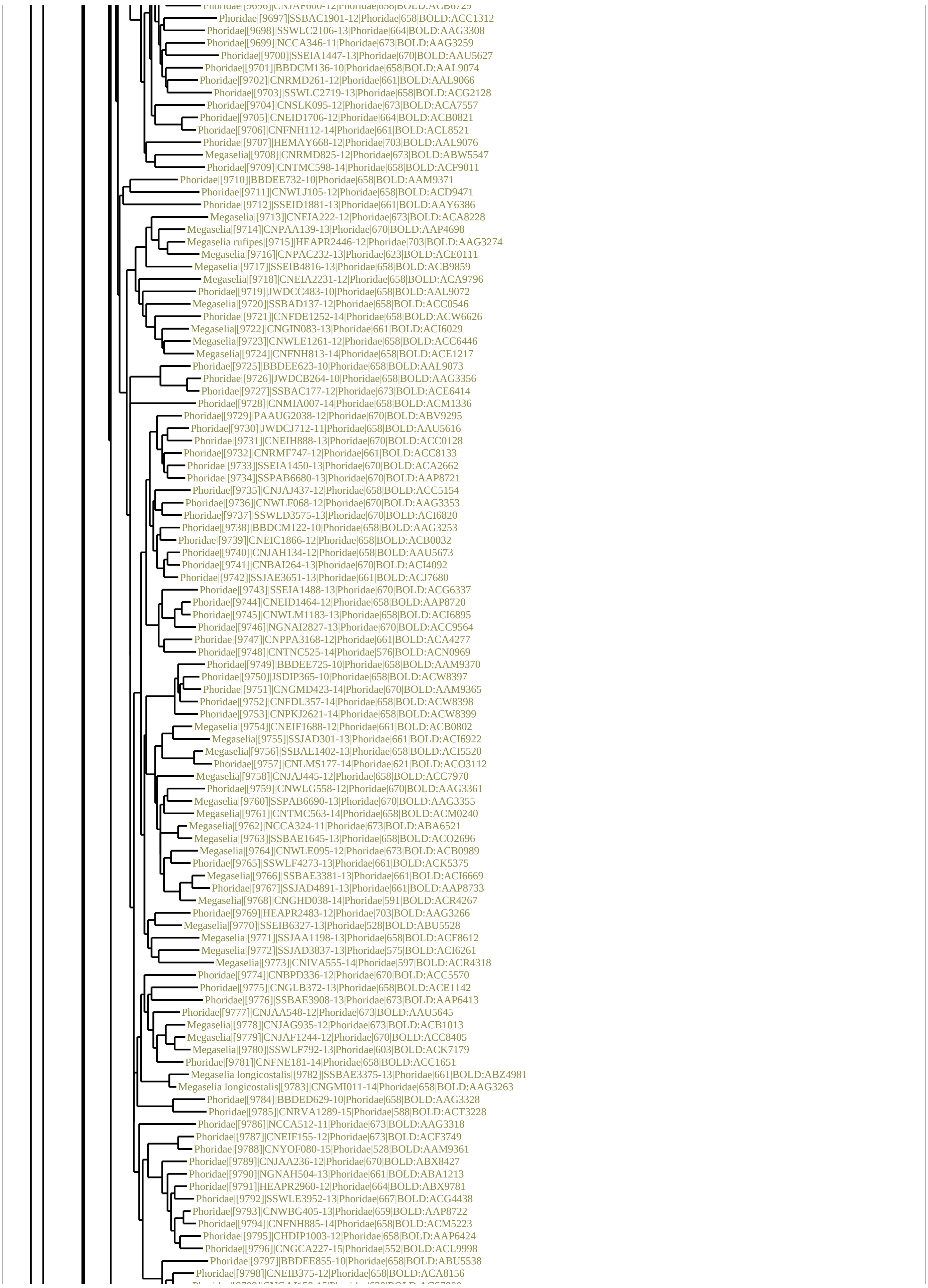

Phoridae|[9697]|SSBAC1901-12|Phoridae|658|BOLD:ACC1312
Phoridae|[9698]|SSWLC2106-13|Phoridae|664|BOLD:AAG3308
Phoridae|[9699]|NCCA346-11|Phoridae|673|BOLD:AAG3259
Phoridae|[9700]|SSEIA1447-13|Phoridae|670|BOLD:AAU5627
Phoridae|[9701]|BBDCM136-10|Phoridae|658|BOLD:AAL9074
Phoridae|[9702]|CNRMD261-12|Phoridae|661|BOLD:AAL9066
Phoridae|[9703]|SSWLC2719-13|Phoridae|658|BOLD:ACG2128
Phoridae|[9704]|CNSLK095-12|Phoridae|673|BOLD:ACA7557
Phoridae|[9705]|CNEID1706-12|Phoridae|664|BOLD:ACB0821
Phoridae|[9706]|CNFNH112-14|Phoridae|661|BOLD:ACL8521
Phoridae|[9707]|HEMAY668-12|Phoridae|703|BOLD:AAL9076
Megaselia|[9708]|CNRMD825-12|Phoridae|673|BOLD:ABW5547
Phoridae|[9709]|CNTMC598-14|Phoridae|658|BOLD:ACF9011
Phoridae|[9710]|BBDEE732-10|Phoridae|658|BOLD:AAM9371
Phoridae|[9711]|CNWLJ105-12|Phoridae|658|BOLD:ACD9471
Phoridae|[9712]|SSEID1881-13|Phoridae|661|BOLD:AAY6386
Megaselia|[9713]|CNEIA222-12|Phoridae|673|BOLD:ACA8228
Megaselia|[9714]|CNPAA139-13|Phoridae|670|BOLD:AAP4698
Megaselia rufipes|[9715]|HEAPR2446-12|Phoridae|703|BOLD:AAG3274
Megaselia|[9716]|CNPAC232-13|Phoridae|623|BOLD:ACE0111
Megaselia|[9717]|SSEIB4816-13|Phoridae|658|BOLD:ACB9859
Megaselia|[9718]|CNEIA2231-12|Phoridae|658|BOLD:ACA9796
Phoridae|[9719]|JWDCC483-10|Phoridae|658|BOLD:AAL9072
Megaselia|[9720]|SSBAD137-12|Phoridae|658|BOLD:ACC0546
Phoridae|[9721]|CNFDE1252-14|Phoridae|658|BOLD:ACW6626
Megaselia|[9722]|CNGIN083-13|Phoridae|661|BOLD:ACI6029
Megaselia|[9723]|CNWLE1261-12|Phoridae|658|BOLD:ACC6446
Megaselia|[9724]|CNFNH813-14|Phoridae|658|BOLD:ACE1217
Phoridae|[9725]|BBDEE623-10|Phoridae|658|BOLD:AAL9073
Phoridae|[9726]|JWDCB264-10|Phoridae|658|BOLD:AAG3356
Phoridae|[9727]|SSBAC177-12|Phoridae|673|BOLD:ACE6414
Phoridae|[9728]|CNMIA007-14|Phoridae|658|BOLD:ACM1336
Phoridae|[9729]|PAAUG2038-12|Phoridae|670|BOLD:ABV9295
Phoridae|[9730]|JWDCJ712-11|Phoridae|658|BOLD:AAU5616
Phoridae|[9731]|CNEIH888-13|Phoridae|670|BOLD:ACC0128
Phoridae|[9732]|CNRMF747-12|Phoridae|661|BOLD:ACC8133
Phoridae|[9733]|SSEIA1450-13|Phoridae|670|BOLD:ACA2662
Phoridae|[9734]|SSPAB6680-13|Phoridae|670|BOLD:AAP8721
Phoridae|[9735]|CNJAJ437-12|Phoridae|658|BOLD:ACC5154
Phoridae|[9736]|CNWLF068-12|Phoridae|670|BOLD:AAG3353
Phoridae|[9737]|SSWLD3575-13|Phoridae|670|BOLD:ACI6820
Phoridae|[9738]|BBDCM122-10|Phoridae|658|BOLD:AAG3253
Phoridae|[9739]|CNEIC1866-12|Phoridae|658|BOLD:ACB0032
Phoridae|[9740]|CNJAH134-12|Phoridae|658|BOLD:AAU5673
Phoridae|[9741]|CNBAI264-13|Phoridae|670|BOLD:ACI4092
Phoridae|[9742]|SSJAE3651-13|Phoridae|661|BOLD:ACJ7680
Phoridae|[9743]|SSEIA1488-13|Phoridae|670|BOLD:ACG6337
Phoridae|[9744]|CNEID1464-12|Phoridae|658|BOLD:AAP8720
Phoridae|[9745]|CNWLM1183-13|Phoridae|658|BOLD:ACI6895
Phoridae|[9746]|NGNAI2827-13|Phoridae|670|BOLD:ACC9564
Phoridae|[9747]|CNPPA3168-12|Phoridae|661|BOLD:ACA4277
Phoridae|[9748]|CNTNC525-14|Phoridae|576|BOLD:ACN0969
Phoridae|[9749]|BBDEE725-10|Phoridae|658|BOLD:AAM9370
Phoridae|[9750]|JSDIP365-10|Phoridae|658|BOLD:ACW8397
Phoridae|[9751]|CNGMD423-14|Phoridae|670|BOLD:AAM9365
Phoridae|[9752]|CNFDL357-14|Phoridae|658|BOLD:ACW8398
Phoridae|[9753]|CNPKJ2621-14|Phoridae|658|BOLD:ACW8399
Megaselia|[9754]|CNEIF1688-12|Phoridae|661|BOLD:ACB0802
Megaselia|[9755]|SSJAD301-13|Phoridae|661|BOLD:ACI6922
Megaselia|[9756]|SSBAE1402-13|Phoridae|658|BOLD:ACI5520
Phoridae|[9757]|CNLMS177-14|Phoridae|621|BOLD:ACO3112
Megaselia|[9758]|CNJAJ445-12|Phoridae|658|BOLD:ACC7970
Phoridae|[9759]|CNWLG558-12|Phoridae|670|BOLD:AAG3361
Megaselia|[9760]|SSPAB6690-13|Phoridae|670|BOLD:AAG3355
Megaselia|[9761]|CNTMC563-14|Phoridae|658|BOLD:ACM0240
Megaselia|[9762]|NCCA324-11|Phoridae|673|BOLD:ABA6521
Megaselia|[9763]|SSBAE1645-13|Phoridae|658|BOLD:ACO2696
Megaselia|[9764]|CNWLE095-12|Phoridae|673|BOLD:ACB0989
Phoridae|[9765]|SSWLF4273-13|Phoridae|661|BOLD:ACK5375
Megaselia|[9766]|SSBAE3381-13|Phoridae|661|BOLD:ACI6669
Phoridae|[9767]|SSJAD4891-13|Phoridae|661|BOLD:AAP8733
Megaselia|[9768]|CNGHD038-14|Phoridae|591|BOLD:ACR4267
Phoridae|[9769]|HEAPR2483-12|Phoridae|703|BOLD:AAG3266
Megaselia|[9770]|SSEIB6327-13|Phoridae|528|BOLD:ABU5528
Megaselia|[9771]|SSJAA1198-13|Phoridae|658|BOLD:ACF8612
Megaselia|[9772]|SSJAD3837-13|Phoridae|575|BOLD:ACI6261
Megaselia|[9773]|CNIVA555-14|Phoridae|597|BOLD:ACR4318
Phoridae|[9774]|CNBPD336-12|Phoridae|670|BOLD:ACC5570
Phoridae|[9775]|CNGLB372-13|Phoridae|658|BOLD:ACE1142
Phoridae|[9776]|SSBAE3908-13|Phoridae|673|BOLD:AAP6413
Phoridae|[9777]|CNJAA548-12|Phoridae|673|BOLD:AAU5645
Megaselia|[9778]|CNJAG935-12|Phoridae|673|BOLD:ACB1013
Megaselia|[9779]|CNJAF1244-12|Phoridae|670|BOLD:ACC8405
Megaselia|[9780]|SSWLF792-13|Phoridae|603|BOLD:ACK7179
Phoridae|[9781]|CNFNE181-14|Phoridae|658|BOLD:ACC1651
Megaselia longicostalis|[9782]|SSBAE3375-13|Phoridae|661|BOLD:ABZ4981
Megaselia longicostalis|[9783]|CNGMI011-14|Phoridae|658|BOLD:AAG3263
Phoridae|[9784]|BBDED629-10|Phoridae|658|BOLD:AAG3328
Phoridae|[9785]|CNRVA1289-15|Phoridae|588|BOLD:ACT3228
Phoridae|[9786]|NCCA512-11|Phoridae|673|BOLD:AAG3318
Phoridae|[9787]|CNEIF155-12|Phoridae|673|BOLD:ACF3749
Phoridae|[9788]|CNYOF080-15|Phoridae|528|BOLD:AAM9361
Phoridae|[9789]|CNJAA236-12|Phoridae|670|BOLD:ABX8427
Phoridae|[9790]|NGNAH504-13|Phoridae|661|BOLD:ABA1213
Phoridae|[9791]|HEAPR2960-12|Phoridae|664|BOLD:ABX9781
Phoridae|[9792]|SSWLE3952-13|Phoridae|667|BOLD:ACG4438
Phoridae|[9793]|CNWBG405-13|Phoridae|659|BOLD:AAP8722
Phoridae|[9794]|CNFNH885-14|Phoridae|658|BOLD:ACM5223
Phoridae|[9795]|CHDIP1003-12|Phoridae|658|BOLD:AAP6424
Phoridae|[9796]|CNGCA227-15|Phoridae|552|BOLD:ACL9998
Phoridae|[9797]|BBDEE855-10|Phoridae|658|BOLD:ABU5538
Phoridae|[9798]|CNEIB375-12|Phoridae|658|BOLD:ACA8156

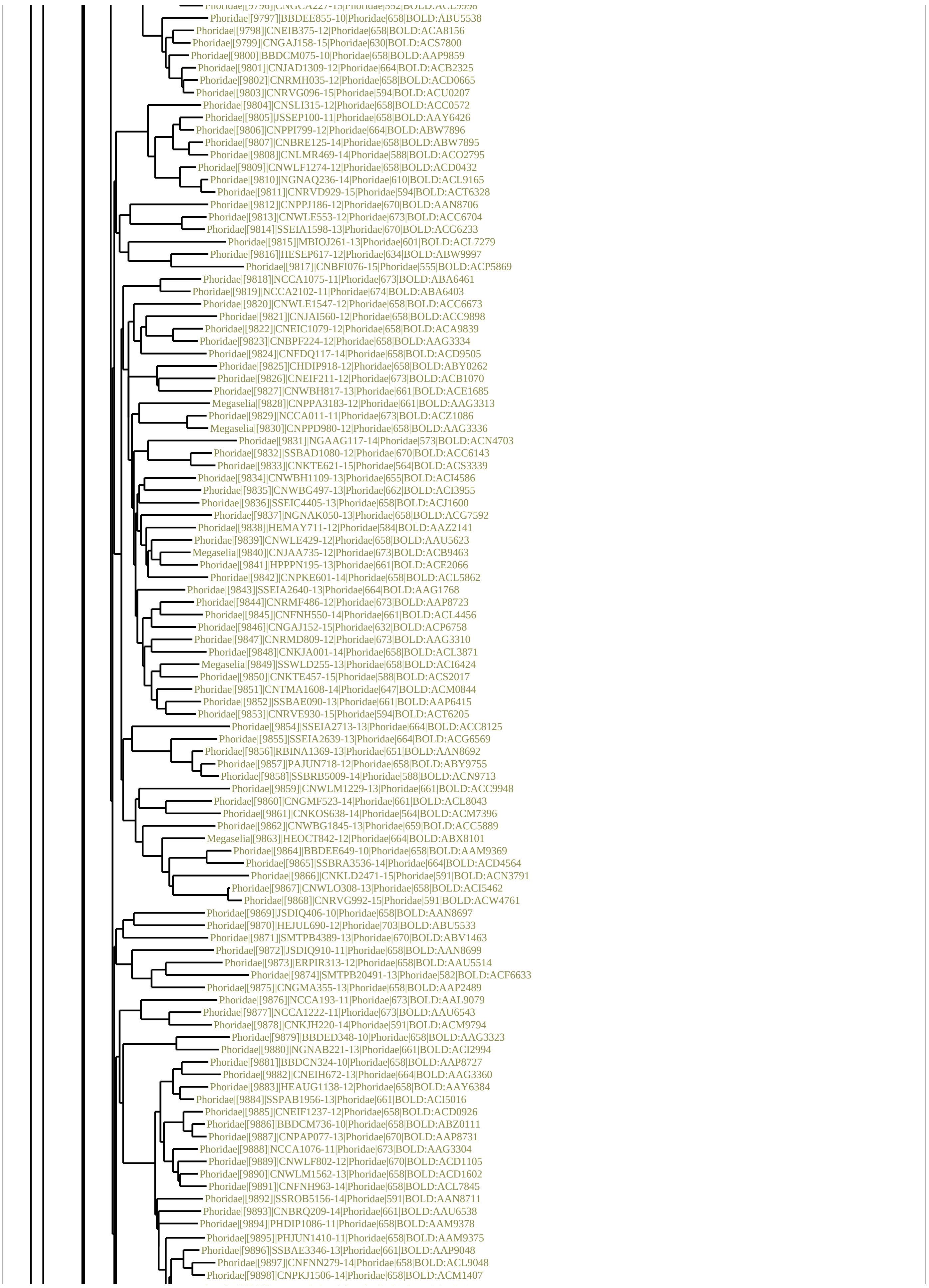

Phoridae|[9796]|CNGCA227-13|Phoridae|552|BOLD:ACL9998
Phoridae|[9797]|BBDEE855-10|Phoridae|658|BOLD:ABU5538
Phoridae|[9798]|CNEIB375-12|Phoridae|658|BOLD:ACA8156
Phoridae|[9799]|CNGAJ158-15|Phoridae|630|BOLD:ACS7800
Phoridae|[9800]|BBDCM075-10|Phoridae|658|BOLD:AAP9859
Phoridae|[9801]|CNJAD1309-12|Phoridae|664|BOLD:ACB2325
Phoridae|[9802]|CNRMH035-12|Phoridae|658|BOLD:ACD0665
Phoridae|[9803]|CNRVG096-15|Phoridae|594|BOLD:ACU0207
Phoridae|[9804]|CNSLI315-12|Phoridae|658|BOLD:ACC0572
Phoridae|[9805]|JSSEP100-11|Phoridae|658|BOLD:AAY6426
Phoridae|[9806]|CNPPI799-12|Phoridae|664|BOLD:ABW7896
Phoridae|[9807]|CNBRE125-14|Phoridae|658|BOLD:ABW7895
Phoridae|[9808]|CNLMR469-14|Phoridae|588|BOLD:ACO2795
Phoridae|[9809]|CNWLF1274-12|Phoridae|658|BOLD:ACD0432
Phoridae|[9810]|NGNAQ236-14|Phoridae|610|BOLD:ACL9165
Phoridae|[9811]|CNRVD929-15|Phoridae|594|BOLD:ACT6328
Phoridae|[9812]|CNPPJ186-12|Phoridae|670|BOLD:AAN8706
Phoridae|[9813]|CNWLE553-12|Phoridae|673|BOLD:ACC6704
Phoridae|[9814]|SSEIA1598-13|Phoridae|670|BOLD:ACG6233
Phoridae|[9815]|MBIOJ261-13|Phoridae|601|BOLD:ACL7279
Phoridae|[9816]|HESEP617-12|Phoridae|634|BOLD:ABW9997
Phoridae|[9817]|CNBFI076-15|Phoridae|555|BOLD:ACP5869
Phoridae|[9818]|NCCA1075-11|Phoridae|673|BOLD:ABA6461
Phoridae|[9819]|NCCA2102-11|Phoridae|674|BOLD:ABA6403
Phoridae|[9820]|CNWLE1547-12|Phoridae|658|BOLD:ACC6673
Phoridae|[9821]|CNJAI560-12|Phoridae|658|BOLD:ACC9898
Phoridae|[9822]|CNEIC1079-12|Phoridae|658|BOLD:ACA9839
Phoridae|[9823]|CNBPF224-12|Phoridae|658|BOLD:AAG3334
Phoridae|[9824]|CNFDQ117-14|Phoridae|658|BOLD:ACD9505
Phoridae|[9825]|CHDIP918-12|Phoridae|658|BOLD:ABY0262
Phoridae|[9826]|CNEIF211-12|Phoridae|673|BOLD:ACB1070
Phoridae|[9827]|CNWBH817-13|Phoridae|661|BOLD:ACE1685
Megaselia|[9828]|CNPPA3183-12|Phoridae|661|BOLD:AAG3313
Phoridae|[9829]|NCCA011-11|Phoridae|673|BOLD:ACZ1086
Megaselia|[9830]|CNPPD980-12|Phoridae|658|BOLD:AAG3336
Phoridae|[9831]|NGAAG117-14|Phoridae|573|BOLD:ACN4703
Phoridae|[9832]|SSBAD1080-12|Phoridae|670|BOLD:ACC6143
Phoridae|[9833]|CNKTE621-15|Phoridae|564|BOLD:ACS3339
Phoridae|[9834]|CNWBH1109-13|Phoridae|655|BOLD:ACI4586
Phoridae|[9835]|CNWBG497-13|Phoridae|662|BOLD:ACI3955
Phoridae|[9836]|SSEIC4405-13|Phoridae|658|BOLD:ACJ1600
Phoridae|[9837]|NGNAK050-13|Phoridae|658|BOLD:ACG7592
Phoridae|[9838]|HEMAY711-12|Phoridae|584|BOLD:AAZ2141
Phoridae|[9839]|CNWLE429-12|Phoridae|658|BOLD:AAU5623
Megaselia|[9840]|CNJAA735-12|Phoridae|673|BOLD:ACB9463
Phoridae|[9841]|HPPPN195-13|Phoridae|661|BOLD:ACE2066
Phoridae|[9842]|CNPKE601-14|Phoridae|658|BOLD:ACL5862
Phoridae|[9843]|SSEIA2640-13|Phoridae|664|BOLD:AAG1768
Phoridae|[9844]|CNRMF486-12|Phoridae|673|BOLD:AAP8723
Phoridae|[9845]|CNFNH550-14|Phoridae|661|BOLD:ACL4456
Phoridae|[9846]|CNGAJ152-15|Phoridae|632|BOLD:ACP6758
Phoridae|[9847]|CNRMD809-12|Phoridae|673|BOLD:AAG3310
Phoridae|[9848]|CNKJA001-14|Phoridae|658|BOLD:ACL3871
Megaselia|[9849]|SSWLD255-13|Phoridae|658|BOLD:ACI6424
Phoridae|[9850]|CNKTE457-15|Phoridae|588|BOLD:ACS2017
Phoridae|[9851]|CNTMA1608-14|Phoridae|647|BOLD:ACM0844
Phoridae|[9852]|SSBAE090-13|Phoridae|661|BOLD:AAP6415
Phoridae|[9853]|CNRVE930-15|Phoridae|594|BOLD:ACT6205
Phoridae|[9854]|SSEIA2713-13|Phoridae|664|BOLD:ACC8125
Phoridae|[9855]|SSEIA2639-13|Phoridae|664|BOLD:ACG6569
Phoridae|[9856]|RBINA1369-13|Phoridae|651|BOLD:AAN8692
Phoridae|[9857]|PAJUN718-12|Phoridae|658|BOLD:ABY9755
Phoridae|[9858]|SSBRB5009-14|Phoridae|588|BOLD:ACN9713
Phoridae|[9859]|CNWLM1229-13|Phoridae|661|BOLD:ACC9948
Phoridae|[9860]|CNGMF523-14|Phoridae|661|BOLD:ACL8043
Phoridae|[9861]|CNKOS638-14|Phoridae|564|BOLD:ACM7396
Phoridae|[9862]|CNWBG1845-13|Phoridae|659|BOLD:ACC5889
Megaselia|[9863]|HEOCT842-12|Phoridae|664|BOLD:ABX8101
Phoridae|[9864]|BBDEE649-10|Phoridae|658|BOLD:AAM9369
Phoridae|[9865]|SSBRA3536-14|Phoridae|664|BOLD:ACD4564
Phoridae|[9866]|CNKLD2471-15|Phoridae|591|BOLD:ACN3791
Phoridae|[9867]|CNWLO308-13|Phoridae|658|BOLD:ACI5462
Phoridae|[9868]|CNRVG992-15|Phoridae|591|BOLD:ACW4761
Phoridae|[9869]|JSDIQ406-10|Phoridae|658|BOLD:AAN8697
Phoridae|[9870]|HEJUL690-12|Phoridae|703|BOLD:ABU5533
Phoridae|[9871]|SMTPB4389-13|Phoridae|670|BOLD:ABV1463
Phoridae|[9872]|JSDIQ910-11|Phoridae|658|BOLD:AAN8699
Phoridae|[9873]|ERPIR313-12|Phoridae|658|BOLD:AAU5514
Phoridae|[9874]|SMTPB20491-13|Phoridae|582|BOLD:ACF6633
Phoridae|[9875]|CNGMA355-13|Phoridae|658|BOLD:AAP2489
Phoridae|[9876]|NCCA193-11|Phoridae|673|BOLD:AAL9079
Phoridae|[9877]|NCCA1222-11|Phoridae|673|BOLD:AAU6543
Phoridae|[9878]|CNKJH220-14|Phoridae|591|BOLD:ACM9794
Phoridae|[9879]|BBDED348-10|Phoridae|658|BOLD:AAG3323
Phoridae|[9880]|NGNAB221-13|Phoridae|661|BOLD:ACI2994
Phoridae|[9881]|BBDCN324-10|Phoridae|658|BOLD:AAP8727
Phoridae|[9882]|CNEIH672-13|Phoridae|664|BOLD:AAG3360
Phoridae|[9883]|HEAUG1138-12|Phoridae|658|BOLD:AAY6384
Phoridae|[9884]|SSPAB1956-13|Phoridae|661|BOLD:ACI5016
Phoridae|[9885]|CNEIF1237-12|Phoridae|658|BOLD:ACD0926
Phoridae|[9886]|BBDCM736-10|Phoridae|658|BOLD:ABZ0111
Phoridae|[9887]|CNPAP077-13|Phoridae|670|BOLD:AAP8731
Phoridae|[9888]|NCCA1076-11|Phoridae|673|BOLD:AAG3304
Phoridae|[9889]|CNWLF802-12|Phoridae|670|BOLD:ACD1105
Phoridae|[9890]|CNWLM1562-13|Phoridae|658|BOLD:ACD1602
Phoridae|[9891]|CNFNH963-14|Phoridae|658|BOLD:ACL7845
Phoridae|[9892]|SSROB5156-14|Phoridae|591|BOLD:AAN8711
Phoridae|[9893]|CNBRQ209-14|Phoridae|661|BOLD:AAU6538
Phoridae|[9894]|PHDIP1086-11|Phoridae|658|BOLD:AAM9378
Phoridae|[9895]|PHJUN1410-11|Phoridae|658|BOLD:AAM9375
Phoridae|[9896]|SSBAE3346-13|Phoridae|661|BOLD:AAP9048
Phoridae|[9897]|CNFNN279-14|Phoridae|658|BOLD:ACL9048
Phoridae|[9898]|CNPKJ1506-14|Phoridae|658|BOLD:ACM1407

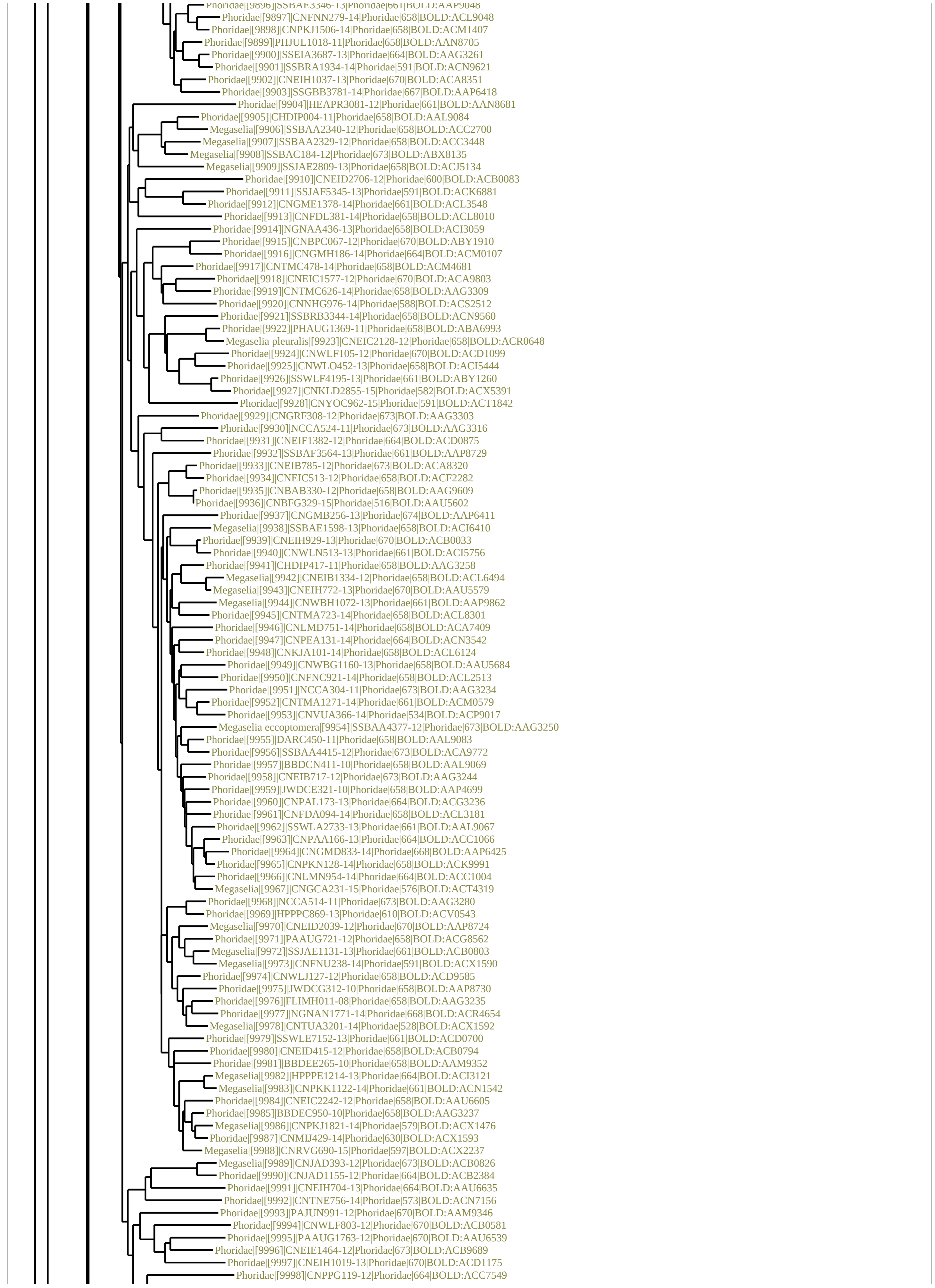
Phoridae|[9896]|SSBAE3346-13|Phoridae|661|BOLD:AAP9048
Phoridae|[9897]|CNFNN279-14|Phoridae|658|BOLD:ACL9048
Phoridae|[9898]|CNPKJ1506-14|Phoridae|658|BOLD:ACM1407
Phoridae|[9899]|PHJUL1018-11|Phoridae|658|BOLD:AAN8705
Phoridae|[9900]|SSEIA3687-13|Phoridae|664|BOLD:AAG3261
Phoridae|[9901]|SSBRA1934-14|Phoridae|591|BOLD:ACN9621
Phoridae|[9902]|CNEIH1037-13|Phoridae|670|BOLD:ACA8351
Phoridae|[9903]|SSGBB3781-14|Phoridae|667|BOLD:AAP6418
Phoridae|[9904]|HEAPR3081-12|Phoridae|661|BOLD:AAN8681
Phoridae|[9905]|CHDIP004-11|Phoridae|658|BOLD:AAL9084
Megaselia|[9906]|SSBAA2340-12|Phoridae|658|BOLD:ACC2700
Megaselia|[9907]|SSBAA2329-12|Phoridae|658|BOLD:ACC3448
Megaselia|[9908]|SSBAC184-12|Phoridae|673|BOLD:ABX8135
Megaselia|[9909]|SSJAE2809-13|Phoridae|658|BOLD:ACJ5134
Phoridae|[9910]|CNEID2706-12|Phoridae|600|BOLD:ACB0083
Phoridae|[9911]|SSJAF5345-13|Phoridae|591|BOLD:ACK6881
Phoridae|[9912]|CNGME1378-14|Phoridae|661|BOLD:ACL3548
Phoridae|[9913]|CNFDL381-14|Phoridae|658|BOLD:ACL8010
Phoridae|[9914]|NGNAA436-13|Phoridae|658|BOLD:ACI3059
Phoridae|[9915]|CNBPC067-12|Phoridae|670|BOLD:ABY1910
Phoridae|[9916]|CNGMH186-14|Phoridae|664|BOLD:ACM0107
Phoridae|[9917]|CNTMC478-14|Phoridae|658|BOLD:ACM4681
Phoridae|[9918]|CNEIC1577-12|Phoridae|670|BOLD:ACA9803
Phoridae|[9919]|CNTMC626-14|Phoridae|658|BOLD:AAG3309
Phoridae|[9920]|CNNHG976-14|Phoridae|588|BOLD:ACS2512
Phoridae|[9921]|SSBRB3344-14|Phoridae|658|BOLD:ACN9560
Phoridae|[9922]|PHAUG1369-11|Phoridae|658|BOLD:ABA6993
Megaselia pleuralis|[9923]|CNEIC2128-12|Phoridae|658|BOLD:ACR0648
Phoridae|[9924]|CNWLF105-12|Phoridae|670|BOLD:ACD1099
Phoridae|[9925]|CNWLO452-13|Phoridae|658|BOLD:ACI5444
Phoridae|[9926]|SSWLF4195-13|Phoridae|661|BOLD:ABY1260
Phoridae|[9927]|CNKLD2855-15|Phoridae|582|BOLD:ACX5391
Phoridae|[9928]|CNYOC962-15|Phoridae|591|BOLD:ACT1842
Phoridae|[9929]|CNGRF308-12|Phoridae|673|BOLD:AAG3303
Phoridae|[9930]|NCCA524-11|Phoridae|673|BOLD:AAG3316
Phoridae|[9931]|CNEIF1382-12|Phoridae|664|BOLD:ACD0875
Phoridae|[9932]|SSBAF3564-13|Phoridae|661|BOLD:AAP8729
Phoridae|[9933]|CNEIB785-12|Phoridae|673|BOLD:ACA8320
Phoridae|[9934]|CNEIC513-12|Phoridae|658|BOLD:ACF2282
Phoridae|[9935]|CNBAB330-12|Phoridae|658|BOLD:AAG9609
Phoridae|[9936]|CNBFG329-15|Phoridae|516|BOLD:AAU5602
Phoridae|[9937]|CNGMB256-13|Phoridae|674|BOLD:AAP6411
Megaselia|[9938]|SSBAE1598-13|Phoridae|658|BOLD:ACI6410
Phoridae|[9939]|CNEIH929-13|Phoridae|670|BOLD:ACB0033
Phoridae|[9940]|CNWLN513-13|Phoridae|661|BOLD:ACI5756
Phoridae|[9941]|CHDIP417-11|Phoridae|658|BOLD:AAG3258
Megaselia|[9942]|CNEIB1334-12|Phoridae|658|BOLD:ACL6494
Megaselia|[9943]|CNEIH772-13|Phoridae|670|BOLD:AAU5579
Megaselia|[9944]|CNWBH1072-13|Phoridae|661|BOLD:AAP9862
Phoridae|[9945]|CNTMA723-14|Phoridae|658|BOLD:ACL8301
Phoridae|[9946]|CNLMD751-14|Phoridae|658|BOLD:ACA7409
Phoridae|[9947]|CNPEA131-14|Phoridae|664|BOLD:ACN3542
Phoridae|[9948]|CNKJA101-14|Phoridae|658|BOLD:ACL6124
Phoridae|[9949]|CNWBG1160-13|Phoridae|658|BOLD:AAU5684
Phoridae|[9950]|CNFNC921-14|Phoridae|658|BOLD:ACL2513
Phoridae|[9951]|NCCA304-11|Phoridae|673|BOLD:AAG3234
Phoridae|[9952]|CNTMA1271-14|Phoridae|661|BOLD:ACM0579
Phoridae|[9953]|CNVUA366-14|Phoridae|534|BOLD:ACP9017
Megaselia eccoptomera|[9954]|SSBAA4377-12|Phoridae|673|BOLD:AAG3250
Phoridae|[9955]|DARC450-11|Phoridae|658|BOLD:AAL9083
Phoridae|[9956]|SSBAA4415-12|Phoridae|673|BOLD:ACA9772
Phoridae|[9957]|BBDCN411-10|Phoridae|658|BOLD:AAL9069
Phoridae|[9958]|CNEIB717-12|Phoridae|673|BOLD:AAG3244
Phoridae|[9959]|JWDCE321-10|Phoridae|658|BOLD:AAP4699
Phoridae|[9960]|CNPAL173-13|Phoridae|664|BOLD:ACG3236
Phoridae|[9961]|CNFDA094-14|Phoridae|658|BOLD:ACL3181
Phoridae|[9962]|SSWLA2733-13|Phoridae|661|BOLD:AAL9067
Phoridae|[9963]|CNPAA166-13|Phoridae|664|BOLD:ACC1066
Phoridae|[9964]|CNGMD833-14|Phoridae|668|BOLD:AAP6425
Phoridae|[9965]|CNPKN128-14|Phoridae|658|BOLD:ACK9991
Phoridae|[9966]|CNLMN954-14|Phoridae|664|BOLD:ACC1004
Megaselia|[9967]|CNGCA231-15|Phoridae|576|BOLD:ACT4319
Phoridae|[9968]|NCCA514-11|Phoridae|673|BOLD:AAG3280
Phoridae|[9969]|HPPPC869-13|Phoridae|610|BOLD:ACV0543
Megaselia|[9970]|CNEID2039-12|Phoridae|670|BOLD:AAP8724
Phoridae|[9971]|PAAUG721-12|Phoridae|658|BOLD:ACG8562
Megaselia|[9972]|SSJAE1131-13|Phoridae|661|BOLD:ACB0803
Megaselia|[9973]|CNFNU238-14|Phoridae|591|BOLD:ACX1590
Phoridae|[9974]|CNWLJ127-12|Phoridae|658|BOLD:ACD9585
Phoridae|[9975]|JWDCG312-10|Phoridae|658|BOLD:AAP8730
Phoridae|[9976]|FLIMH011-08|Phoridae|658|BOLD:AAG3235
Phoridae|[9977]|NGNAN1771-14|Phoridae|668|BOLD:ACR4654
Megaselia|[9978]|CNTUA3201-14|Phoridae|528|BOLD:ACX1592
Phoridae|[9979]|SSWLE7152-13|Phoridae|661|BOLD:ACD0700
Phoridae|[9980]|CNEID415-12|Phoridae|658|BOLD:ACB0794
Phoridae|[9981]|BBDEE265-10|Phoridae|658|BOLD:AAM9352
Megaselia|[9982]|HPPPE1214-13|Phoridae|664|BOLD:ACI3121
Megaselia|[9983]|CNPKK1122-14|Phoridae|661|BOLD:ACN1542
Phoridae|[9984]|CNEIC2242-12|Phoridae|658|BOLD:AAU6605
Phoridae|[9985]|BBDEC950-10|Phoridae|658|BOLD:AAG3237
Megaselia|[9986]|CNPKJ1821-14|Phoridae|579|BOLD:ACX1476
Phoridae|[9987]|CNMIJ429-14|Phoridae|630|BOLD:ACX1593
Megaselia|[9988]|CNRVG690-15|Phoridae|597|BOLD:ACX2237
Megaselia|[9989]|CNJAD393-12|Phoridae|673|BOLD:ACB0826
Phoridae|[9990]|CNJAD1155-12|Phoridae|664|BOLD:ACB2384
Phoridae|[9991]|CNEIH704-13|Phoridae|664|BOLD:AAU6635
Phoridae|[9992]|CNTNE756-14|Phoridae|573|BOLD:ACN7156
Phoridae|[9993]|PAJUN991-12|Phoridae|670|BOLD:AAM9346
Phoridae|[9994]|CNWLF803-12|Phoridae|670|BOLD:ACB0581
Phoridae|[9995]|PAAUG1763-12|Phoridae|670|BOLD:AAU6539
Phoridae|[9996]|CNEIE1464-12|Phoridae|673|BOLD:ACB9689
Phoridae|[9997]|CNEIH1019-13|Phoridae|670|BOLD:ACD1175
Phoridae|[9998]|CNPPG119-12|Phoridae|664|BOLD:ACC7549

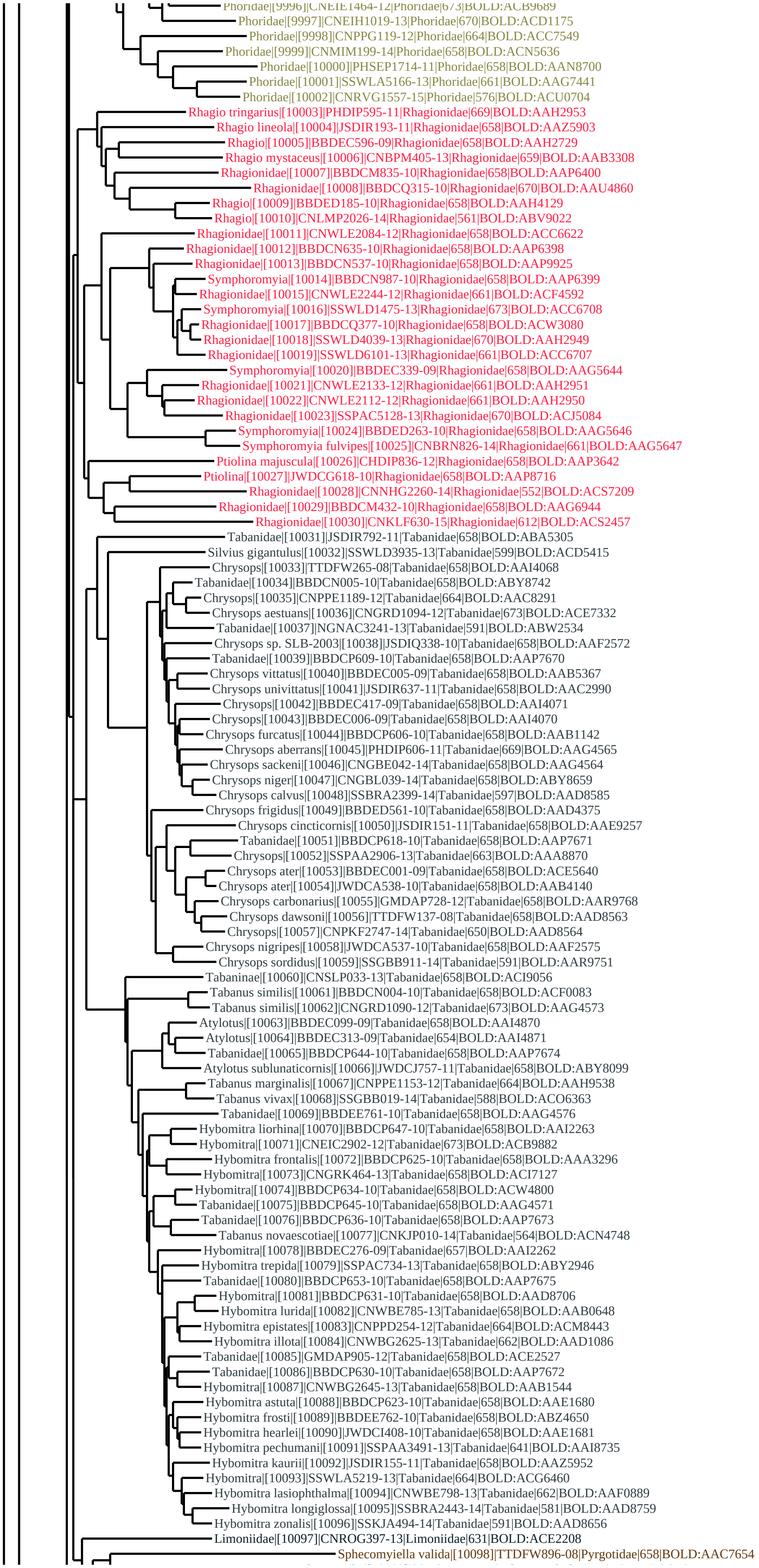
Phoridae|[9996]|CNEIE1464-12|Phoridae|673|BOLD:ACB9689
Phoridae|[9997]|CNEIH1019-13|Phoridae|670|BOLD:ACD1175
Phoridae|[9998]|CNPPG119-12|Phoridae|664|BOLD:ACC7549
Phoridae|[9999]|CNMIM199-14|Phoridae|658|BOLD:ACN5636
Phoridae|[10000]|PHSEP1714-11|Phoridae|658|BOLD:AAN8700
Phoridae|[10001]|SSWLA5166-13|Phoridae|661|BOLD:AAG7441
Phoridae|[10002]|CNRVG1557-15|Phoridae|576|BOLD:ACU0704
Rhagio tringarius|[10003]|PHDIP595-11|Rhagionidae|669|BOLD:AAH2953
Rhagio lineola|[10004]|JSDIR193-11|Rhagionidae|658|BOLD:AAZ5903
Rhagio|[10005]|BBDEC596-09|Rhagionidae|658|BOLD:AAH2729
Rhagio mystaceus|[10006]|CNBPM405-13|Rhagionidae|659|BOLD:AAB3308
Rhagionidae|[10007]|BBDCM835-10|Rhagionidae|658|BOLD:AAP6400
Rhagionidae|[10008]|BBDCQ315-10|Rhagionidae|670|BOLD:AAU4860
Rhagio|[10009]|BBDED185-10|Rhagionidae|658|BOLD:AAH4129
Rhagio|[10010]|CNLMP2026-14|Rhagionidae|561|BOLD:ABV9022
Rhagionidae|[10011]|CNWLE2084-12|Rhagionidae|658|BOLD:ACC6622
Rhagionidae|[10012]|BBDCN635-10|Rhagionidae|658|BOLD:AAP6398
Rhagionidae|[10013]|BBDCN537-10|Rhagionidae|658|BOLD:AAP9925
Symphoromyia|[10014]|BBDCN987-10|Rhagionidae|658|BOLD:AAP6399
Rhagionidae|[10015]|CNWLE2244-12|Rhagionidae|661|BOLD:ACF4592
Symphoromyia|[10016]|SSWLD1475-13|Rhagionidae|673|BOLD:ACC6708
Rhagionidae|[10017]|BBDCQ377-10|Rhagionidae|658|BOLD:ACW3080
Rhagionidae|[10018]|SSWLD4039-13|Rhagionidae|670|BOLD:AAH2949
Rhagionidae|[10019]|SSWLD6101-13|Rhagionidae|661|BOLD:ACC6707
Symphoromyia|[10020]|BBDEC339-09|Rhagionidae|658|BOLD:AAG5644
Rhagionidae|[10021]|CNWLE2133-12|Rhagionidae|661|BOLD:AAH2951
Rhagionidae|[10022]|CNWLE2112-12|Rhagionidae|661|BOLD:AAH2950
Rhagionidae|[10023]|SSPAC5128-13|Rhagionidae|670|BOLD:ACJ5084
Symphoromyia|[10024]|BBDED263-10|Rhagionidae|658|BOLD:AAG5646
Symphoromyia fulvipes|[10025]|CNBRN826-14|Rhagionidae|661|BOLD:AAG5647
Ptiolina majuscula|[10026]|CHDIP836-12|Rhagionidae|658|BOLD:AAP3642
Ptiolina|[10027]|JWDCG618-10|Rhagionidae|658|BOLD:AAP8716
Rhagionidae|[10028]|CNNHG2260-14|Rhagionidae|552|BOLD:ACS7209
Rhagionidae|[10029]|BBDCM432-10|Rhagionidae|658|BOLD:AAG6944
Rhagionidae|[10030]|CNKLF630-15|Rhagionidae|612|BOLD:ACS2457
Tabanidae|[10031]|JSDIR792-11|Tabanidae|658|BOLD:ABA5305
Silvius gigantulus|[10032]|SSWLD3935-13|Tabanidae|599|BOLD:ACD5415
Chrysops|[10033]|TTDFW265-08|Tabanidae|658|BOLD:AAI4068
Tabanidae|[10034]|BBDCN005-10|Tabanidae|658|BOLD:ABY8742
Chrysops|[10035]|CNPPE1189-12|Tabanidae|664|BOLD:AAC8291
Chrysops aestuans|[10036]|CNGRD1094-12|Tabanidae|673|BOLD:ACE7332
Tabanidae|[10037]|NGNAC3241-13|Tabanidae|591|BOLD:ABW2534
Chrysops sp. SLB-2003|[10038]|JSDIQ338-10|Tabanidae|658|BOLD:AAF2572
Tabanidae|[10039]|BBDCP609-10|Tabanidae|658|BOLD:AAP7670
Chrysops vittatus|[10040]|BBDEC005-09|Tabanidae|658|BOLD:AAB5367
Chrysops univittatus|[10041]|JSDIR637-11|Tabanidae|658|BOLD:AAC2990
Chrysops|[10042]|BBDEC417-09|Tabanidae|658|BOLD:AAI4071
Chrysops|[10043]|BBDEC006-09|Tabanidae|658|BOLD:AAI4070
Chrysops furcatus|[10044]|BBDCP606-10|Tabanidae|658|BOLD:AAB1142
Chrysops aberrans|[10045]|PHDIP606-11|Tabanidae|669|BOLD:AAG4565
Chrysops sackeni|[10046]|CNGBE042-14|Tabanidae|658|BOLD:AAG4564
Chrysops niger|[10047]|CNGBL039-14|Tabanidae|658|BOLD:ABY8659
Chrysops calvus|[10048]|SSBRA2399-14|Tabanidae|597|BOLD:AAD8585
Chrysops frigidus|[10049]|BBDED561-10|Tabanidae|658|BOLD:AAD4375
Chrysops cincticornis|[10050]|JSDIR151-11|Tabanidae|658|BOLD:AAE9257
Tabanidae|[10051]|BBDCP618-10|Tabanidae|658|BOLD:AAP7671
Chrysops|[10052]|SSPAA2906-13|Tabanidae|663|BOLD:AAA8870
Chrysops ater|[10053]|BBDEC001-09|Tabanidae|658|BOLD:ACE5640
Chrysops ater|[10054]|JWDCA538-10|Tabanidae|658|BOLD:AAB4140
Chrysops carbonarius|[10055]|GMDAP728-12|Tabanidae|658|BOLD:AAR9768
Chrysops dawsoni|[10056]|TTDFW137-08|Tabanidae|658|BOLD:AAD8563
Chrysops|[10057]|CNPKF2747-14|Tabanidae|650|BOLD:AAD8564
Chrysops nigripes|[10058]|JWDCA537-10|Tabanidae|658|BOLD:AAF2575
Chrysops sordidus|[10059]|SSGBB911-14|Tabanidae|591|BOLD:AAR9751
Tabaninae|[10060]|CNSLP033-13|Tabanidae|658|BOLD:ACI9056
Tabanus similis|[10061]|BBDCN004-10|Tabanidae|658|BOLD:ACF0083
Tabanus similis|[10062]|CNGRD1090-12|Tabanidae|673|BOLD:AAG4573
Atylotus|[10063]|BBDEC099-09|Tabanidae|658|BOLD:AAI4870
Atylotus|[10064]|BBDEC313-09|Tabanidae|654|BOLD:AAI4871
Tabanidae|[10065]|BBDCP644-10|Tabanidae|658|BOLD:AAP7674
Atylotus sublunaticornis|[10066]|JWDCJ757-11|Tabanidae|658|BOLD:ABY8099
Tabanus marginalis|[10067]|CNPPE1153-12|Tabanidae|664|BOLD:AAH9538
Tabanus vivax|[10068]|SSGBB019-14|Tabanidae|588|BOLD:ACO6363
Tabanidae|[10069]|BBDEE761-10|Tabanidae|658|BOLD:AAG4576
Hybomitra liorhina|[10070]|BBDCP647-10|Tabanidae|658|BOLD:AAI2263
Hybomitra|[10071]|CNEIC2902-12|Tabanidae|673|BOLD:ACB9882
Hybomitra frontalis|[10072]|BBDCP625-10|Tabanidae|658|BOLD:AAA3296
Hybomitra|[10073]|CNGRK464-13|Tabanidae|658|BOLD:ACI7127
Hybomitra|[10074]|BBDCP634-10|Tabanidae|658|BOLD:ACW4800
Tabanidae|[10075]|BBDCP645-10|Tabanidae|658|BOLD:AAG4571
Tabanidae|[10076]|BBDCP636-10|Tabanidae|658|BOLD:AAP7673
Tabanus novaescotiae|[10077]|CNKJP010-14|Tabanidae|564|BOLD:ACN4748
Hybomitra|[10078]|BBDEC276-09|Tabanidae|657|BOLD:AAI2262
Hybomitra trepida|[10079]|SSPAC734-13|Tabanidae|658|BOLD:ABY2946
Tabanidae|[10080]|BBDCP653-10|Tabanidae|658|BOLD:AAP7675
Hybomitra|[10081]|BBDCP631-10|Tabanidae|658|BOLD:AAD8706
Hybomitra lurida|[10082]|CNWBE785-13|Tabanidae|658|BOLD:AAB0648
Hybomitra epistates|[10083]|CNPPD254-12|Tabanidae|664|BOLD:ACM8443
Hybomitra illota|[10084]|CNWBG2625-13|Tabanidae|662|BOLD:AAD1086
Tabanidae|[10085]|GMDAP905-12|Tabanidae|658|BOLD:ACE2527
Tabanidae|[10086]|BBDCP630-10|Tabanidae|658|BOLD:AAP7672
Hybomitra|[10087]|CNWBG2645-13|Tabanidae|658|BOLD:AAB1544
Hybomitra astuta|[10088]|BBDCP623-10|Tabanidae|658|BOLD:AAE1680
Hybomitra frosti|[10089]|BBDEE762-10|Tabanidae|658|BOLD:ABZ4650
Hybomitra hearlei|[10090]|JWDCI408-10|Tabanidae|658|BOLD:AAE1681
Hybomitra pechumani|[10091]|SSPAA3491-13|Tabanidae|641|BOLD:AAI8735
Hybomitra kaurii|[10092]|JSDIR155-11|Tabanidae|658|BOLD:AAZ5952
Hybomitra|[10093]|SSWLA5219-13|Tabanidae|664|BOLD:ACG6460
Hybomitra lasiophthalma|[10094]|CNWBE798-13|Tabanidae|662|BOLD:AAF0889
Hybomitra longiglossa|[10095]|SSBRA2443-14|Tabanidae|581|BOLD:AAD8759
Hybomitra zonalis|[10096]|SSKJA494-14|Tabanidae|591|BOLD:AAD8656
Limoniidae|[10097]|CNROG397-13|Limoniidae|631|BOLD:ACE2208
Sphecomyiella valida|[10098]|TTDFW896-08|Pyrgotidae|658|BOLD:AAC7654

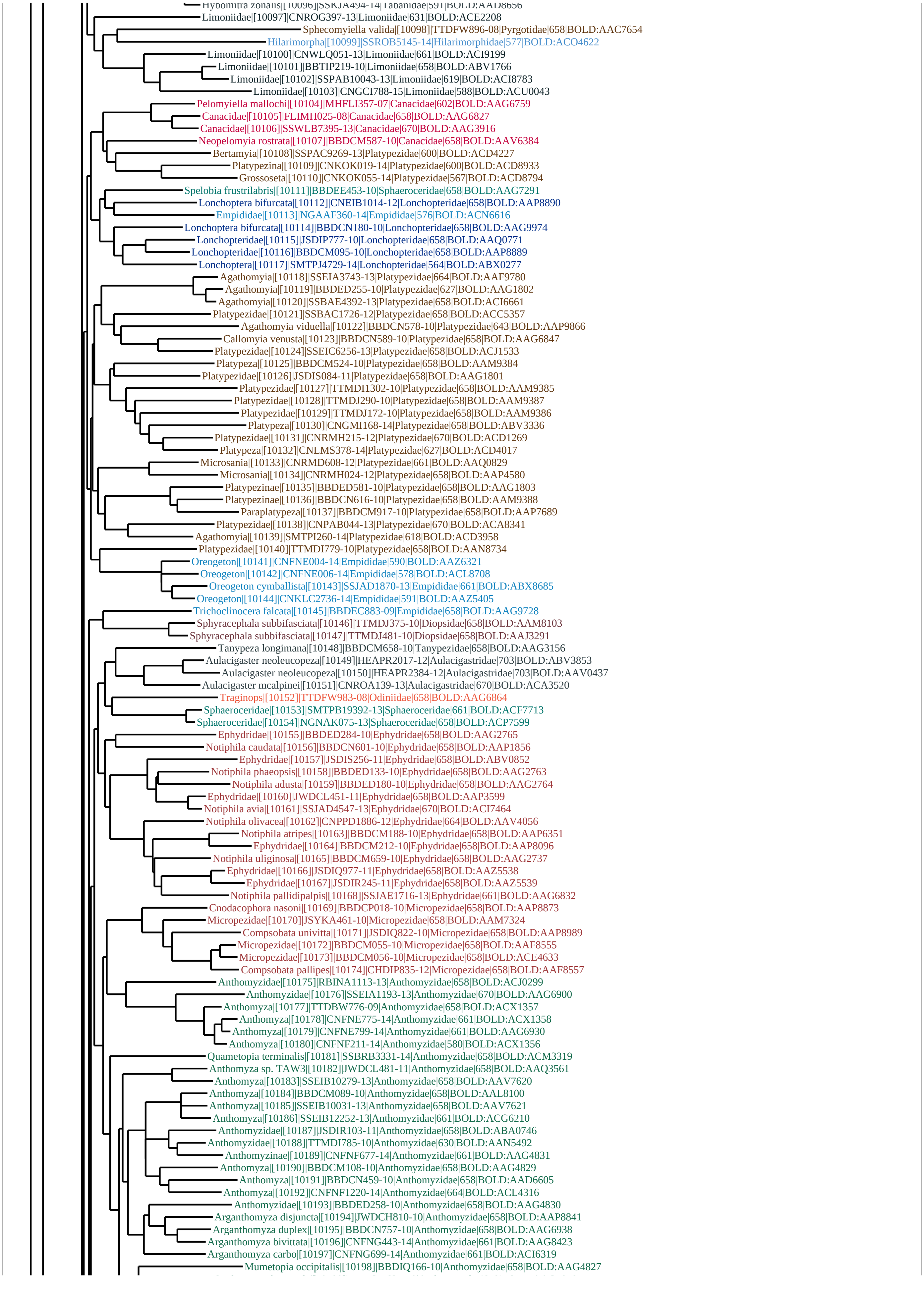
Hybomitra zonalis|[10096]|SSKJA494-14|Tabanidae|591|BOLD:AAD8656
Limoniidae|[10097]|CNROG397-13|Limoniidae|631|BOLD:ACE2208
Sphecomyiella valida|[10098]|TTDFW896-08|Pyrgotidae|658|BOLD:AAC7654
Hilarimorpha|[10099]|SSROB5145-14|Hilarimorphidae|577|BOLD:ACO4622
Limoniidae|[10100]|CNWLQ051-13|Limoniidae|661|BOLD:ACI9199
Limoniidae|[10101]|BBTIP219-10|Limoniidae|658|BOLD:ABV1766
Limoniidae|[10102]|SSPAB10043-13|Limoniidae|619|BOLD:ACI8783
Limoniidae|[10103]|CNGCI788-15|Limoniidae|588|BOLD:ACU0043
Pelomyiella mallochi|[10104]|MHFLI357-07|Canacidae|602|BOLD:AAG6759
Canacidae|[10105]|FLIMH025-08|Canacidae|658|BOLD:AAG6827
Canacidae|[10106]|SSWLB7395-13|Canacidae|670|BOLD:AAG3916
Neopelomyia rostrata|[10107]|BBDCM587-10|Canacidae|658|BOLD:AAV6384
Bertamyia|[10108]|SSPAC9269-13|Platypezidae|600|BOLD:ACD4227
Platypezina|[10109]|CNKOK019-14|Platypezidae|600|BOLD:ACD8933
Grossoseta|[10110]|CNKOK055-14|Platypezidae|567|BOLD:ACD8794
Spelobia frustrilabris|[10111]|BBDEE453-10|Sphaeroceridae|658|BOLD:AAG7291
Lonchoptera bifurcata|[10112]|CNEIB1014-12|Lonchopteridae|658|BOLD:AAP8890
Empididae|[10113]|NGAAF360-14|Empididae|576|BOLD:ACN6616
Lonchoptera bifurcata|[10114]|BBDCN180-10|Lonchopteridae|658|BOLD:AAG9974
Lonchopteridae|[10115]|JSDIP777-10|Lonchopteridae|658|BOLD:AAQ0771
Lonchopteridae|[10116]|BBDCM095-10|Lonchopteridae|658|BOLD:AAP8889
Lonchoptera|[10117]|SMTPJ4729-14|Lonchopteridae|564|BOLD:ABX0277
Agathomyia|[10118]|SSEIA3743-13|Platypezidae|664|BOLD:AAF9780
Agathomyia|[10119]|BBDED255-10|Platypezidae|627|BOLD:AAG1802
Agathomyia|[10120]|SSBAE4392-13|Platypezidae|658|BOLD:ACI6661
Platypezidae|[10121]|SSBAC1726-12|Platypezidae|658|BOLD:ACC5357
Agathomyia viduella|[10122]|BBDCN578-10|Platypezidae|643|BOLD:AAP9866
Callomyia venusta|[10123]|BBDCN589-10|Platypezidae|658|BOLD:AAG6847
Platypezidae|[10124]|SSEIC6256-13|Platypezidae|658|BOLD:ACJ1533
Platypeza|[10125]|BBDCM524-10|Platypezidae|658|BOLD:AAM9384
Platypezidae|[10126]|JSDIS084-11|Platypezidae|658|BOLD:AAG1801
Platypezidae|[10127]|TTMDI1302-10|Platypezidae|658|BOLD:AAM9385
Platypezidae|[10128]|TTMDJ290-10|Platypezidae|658|BOLD:AAM9387
Platypezidae|[10129]|TTMDJ172-10|Platypezidae|658|BOLD:AAM9386
Platypeza|[10130]|CNGMI168-14|Platypezidae|658|BOLD:ABV3336
Platypezidae|[10131]|CNRMH215-12|Platypezidae|670|BOLD:ACD1269
Platypeza|[10132]|CNLMS378-14|Platypezidae|627|BOLD:ACD4017
Microsania|[10133]|CNRMD608-12|Platypezidae|661|BOLD:AAQ0829
Microsania|[10134]|CNRMH024-12|Platypezidae|658|BOLD:AAP4580
Platypezinae|[10135]|BBDED581-10|Platypezidae|658|BOLD:AAG1803
Platypezinae|[10136]|BBDCN616-10|Platypezidae|658|BOLD:AAM9388
Paraplatypeza|[10137]|BBDCM917-10|Platypezidae|658|BOLD:AAP7689
Platypezidae|[10138]|CNPAB044-13|Platypezidae|670|BOLD:ACA8341
Agathomyia|[10139]|SMTPI260-14|Platypezidae|618|BOLD:ACD3958
Platypezidae|[10140]|TTMDI779-10|Platypezidae|658|BOLD:AAN8734
Oreogeton|[10141]|CNFNE004-14|Empididae|590|BOLD:AAZ6321
Oreogeton|[10142]|CNFNE006-14|Empididae|578|BOLD:ACL8708
Oreogeton cymballista|[10143]|SSJAD1870-13|Empididae|661|BOLD:ABX8685
Oreogeton|[10144]|CNKLC2736-14|Empididae|591|BOLD:AAZ5405
Trichoclinocera falcata|[10145]|BBDEC883-09|Empididae|658|BOLD:AAG9728
Sphyracephala subbifasciata|[10146]|TTMDJ375-10|Diopsidae|658|BOLD:AAM8103
Sphyracephala subbifasciata|[10147]|TTMDJ481-10|Diopsidae|658|BOLD:AAJ3291
Tanypeza longimana|[10148]|BBDCM658-10|Tanypezidae|658|BOLD:AAG3156
Aulacigaster neoleucopeza|[10149]|HEAPR2017-12|Aulacigastridae|703|BOLD:ABV3853
Aulacigaster neoleucopeza|[10150]|HEAPR2384-12|Aulacigastridae|703|BOLD:AAV0437
Aulacigaster mcalpinei|[10151]|CNROA139-13|Aulacigastridae|670|BOLD:ACA3520
Traginops|[10152]|TTDFW983-08|Odiniidae|658|BOLD:AAG6864
Sphaeroceridae|[10153]|SMTPB19392-13|Sphaeroceridae|661|BOLD:ACF7713
Sphaeroceridae|[10154]|NGNAK075-13|Sphaeroceridae|658|BOLD:ACP7599
Ephydridae|[10155]|BBDED284-10|Ephydridae|658|BOLD:AAG2765
Notiphila caudata|[10156]|BBDCN601-10|Ephydridae|658|BOLD:AAP1856
Ephydridae|[10157]|JSDIS256-11|Ephydridae|658|BOLD:ABV0852
Notiphila phaeopsis|[10158]|BBDED133-10|Ephydridae|658|BOLD:AAG2763
Notiphila adusta|[10159]|BBDED180-10|Ephydridae|658|BOLD:AAG2764
Ephydridae|[10160]|JWDCL451-11|Ephydridae|658|BOLD:AAP3599
Notiphila avia|[10161]|SSJAD4547-13|Ephydridae|670|BOLD:ACI7464
Notiphila olivacea|[10162]|CNPPD1886-12|Ephydridae|664|BOLD:AAV4056
Notiphila atripes|[10163]|BBDCM188-10|Ephydridae|658|BOLD:AAP6351
Ephydridae|[10164]|BBDCM212-10|Ephydridae|658|BOLD:AAP8096
Notiphila uliginosa|[10165]|BBDCM659-10|Ephydridae|658|BOLD:AAG2737
Ephydridae|[10166]|JSDIQ977-11|Ephydridae|658|BOLD:AAZ5538
Ephydridae|[10167]|JSDIR245-11|Ephydridae|658|BOLD:AAZ5539
Notiphila pallidipalpis|[10168]|SSJAE1716-13|Ephydridae|661|BOLD:AAG6832
Cnodacophora nasoni|[10169]|BBDCP018-10|Micropezidae|658|BOLD:AAP8873
Micropezidae|[10170]|JSYKA461-10|Micropezidae|658|BOLD:AAM7324
Compsobata univitta|[10171]|JSDIQ822-10|Micropezidae|658|BOLD:AAP8989
Micropezidae|[10172]|BBDCM055-10|Micropezidae|658|BOLD:AAF8555
Micropezidae|[10173]|BBDCM056-10|Micropezidae|658|BOLD:ACE4633
Compsobata pallipes|[10174]|CHDIP835-12|Micropezidae|658|BOLD:AAF8557
Anthomyzidae|[10175]|RBINA1113-13|Anthomyzidae|658|BOLD:ACJ0299
Anthomyzidae|[10176]|SSEIA1193-13|Anthomyzidae|670|BOLD:AAG6900
Anthomyza|[10177]|TTDBW776-09|Anthomyzidae|658|BOLD:ACX1357
Anthomyza|[10178]|CNFNE775-14|Anthomyzidae|661|BOLD:ACX1358
Anthomyza|[10179]|CNFNE799-14|Anthomyzidae|661|BOLD:AAG6930
Anthomyza|[10180]|CNFNF211-14|Anthomyzidae|580|BOLD:ACX1356
Quametopia terminalis|[10181]|SSBRB3331-14|Anthomyzidae|658|BOLD:ACM3319
Anthomyza sp. TAW3|[10182]|JWDCL481-11|Anthomyzidae|658|BOLD:AAQ3561
Anthomyza|[10183]|SSEIB10279-13|Anthomyzidae|658|BOLD:AAV7620
Anthomyza|[10184]|BBDCM089-10|Anthomyzidae|658|BOLD:AAL8100
Anthomyza|[10185]|SSEIB10031-13|Anthomyzidae|658|BOLD:AAV7621
Anthomyza|[10186]|SSEIB12252-13|Anthomyzidae|661|BOLD:ACG6210
Anthomyzidae|[10187]|JSDIR103-11|Anthomyzidae|658|BOLD:ABA0746
Anthomyzidae|[10188]|TTMDI785-10|Anthomyzidae|630|BOLD:AAN5492
Anthomyzinae|[10189]|CNFNF677-14|Anthomyzidae|661|BOLD:AAG4831
Anthomyza|[10190]|BBDCM108-10|Anthomyzidae|658|BOLD:AAG4829
Anthomyza|[10191]|BBDCN459-10|Anthomyzidae|658|BOLD:AAD6605
Anthomyza|[10192]|CNFNF1220-14|Anthomyzidae|664|BOLD:ACL4316
Anthomyzidae|[10193]|BBDED258-10|Anthomyzidae|658|BOLD:AAG4830
Arganthomyza disjuncta|[10194]|JWDCH810-10|Anthomyzidae|658|BOLD:AAP8841
Arganthomyza duplex|[10195]|BBDCN757-10|Anthomyzidae|658|BOLD:AAG6938
Arganthomyza bivittata|[10196]|CNFNG443-14|Anthomyzidae|661|BOLD:AAG8423
Arganthomyza carbo|[10197]|CNFNG699-14|Anthomyzidae|661|BOLD:ACI6319
Mumetopia occipitalis|[10198]|BBDIQ166-10|Anthomyzidae|658|BOLD:AAG4827

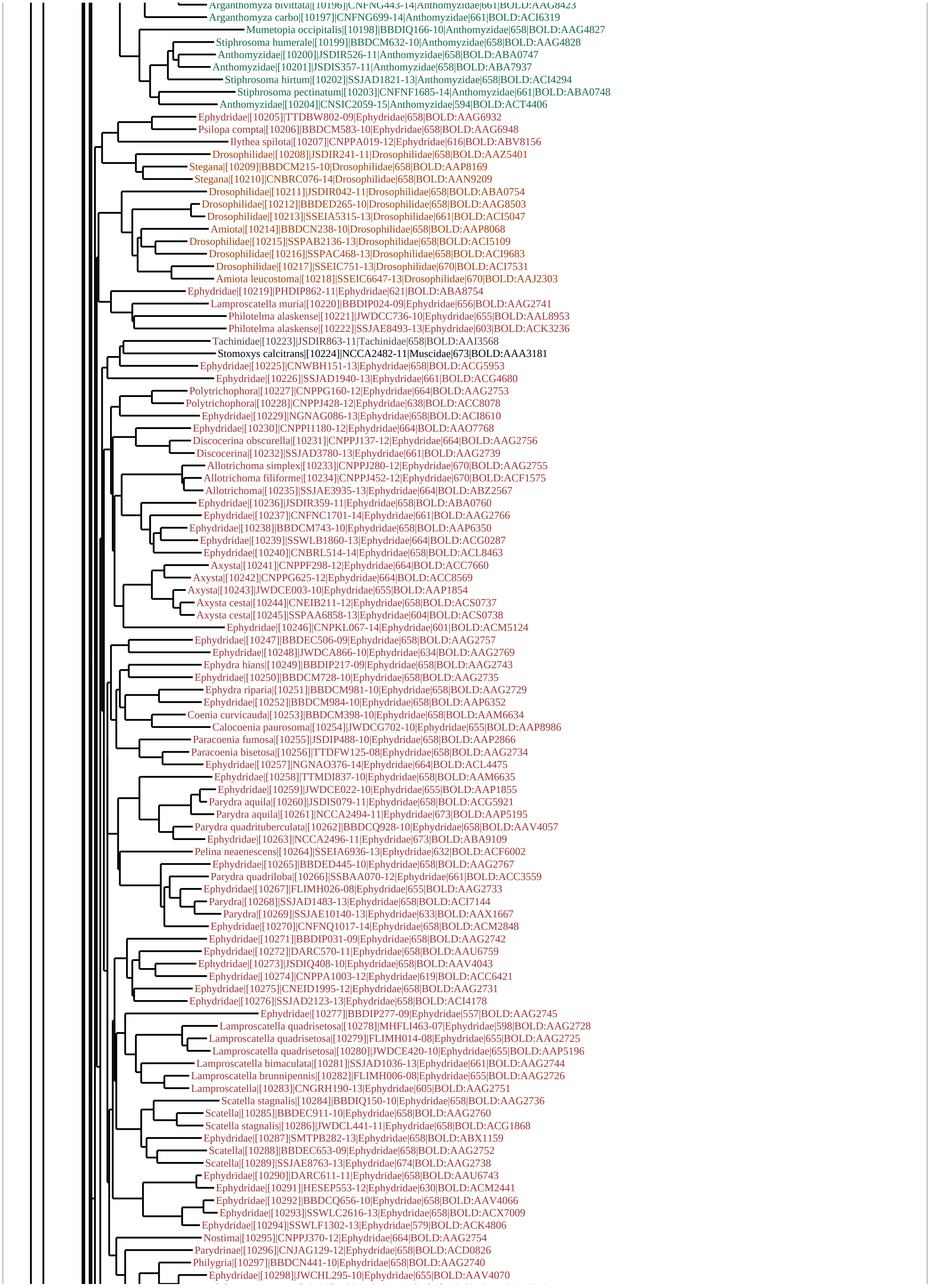

Arganthomyza bivittata|[10196]|CNFNG443-14|Anthomyzidae|661|BOLD:AAG8423
Arganthomyza carbo|[10197]|CNFNG699-14|Anthomyzidae|661|BOLD:ACI6319
Mumetopia occipitalis|[10198]|BBDIQ166-10|Anthomyzidae|658|BOLD:AAG4827
Stiphrosoma humerale|[10199]|BBDCM632-10|Anthomyzidae|658|BOLD:AAG4828
Anthomyzidae|[10200]|JSDIR526-11|Anthomyzidae|658|BOLD:ABA0747
Anthomyzidae|[10201]|JSDIS357-11|Anthomyzidae|658|BOLD:ABA7937
Stiphrosoma hirtum|[10202]|SSJAD1821-13|Anthomyzidae|658|BOLD:ACI4294
Stiphrosoma pectinatum|[10203]|CNFNF1685-14|Anthomyzidae|661|BOLD:ABA0748
Anthomyzidae|[10204]|CNSIC2059-15|Anthomyzidae|594|BOLD:ACT4406
Ephydridae|[10205]|TTDBW802-09|Ephydridae|658|BOLD:AAG6932
Psilopa compta|[10206]|BBDCM583-10|Ephydridae|658|BOLD:AAG6948
Ilythea spilota|[10207]|CNPPA019-12|Ephydridae|616|BOLD:ABV8156
Drosophilidae|[10208]|JSDIR241-11|Drosophilidae|658|BOLD:AAZ5401
Stegana|[10209]|BBDCM215-10|Drosophilidae|658|BOLD:AAP8169
Stegana|[10210]|CNBRC076-14|Drosophilidae|658|BOLD:AAN9209
Drosophilidae|[10211]|JSDIR042-11|Drosophilidae|658|BOLD:ABA0754
Drosophilidae|[10212]|BBDED265-10|Drosophilidae|658|BOLD:AAG8503
Drosophilidae|[10213]|SSEIA5315-13|Drosophilidae|661|BOLD:ACI5047
Amiota|[10214]|BBDCN238-10|Drosophilidae|658|BOLD:AAP8068
Drosophilidae|[10215]|SSPAB2136-13|Drosophilidae|658|BOLD:ACI5109
Drosophilidae|[10216]|SSPAC468-13|Drosophilidae|658|BOLD:ACI9683
Drosophilidae|[10217]|SSEIC751-13|Drosophilidae|670|BOLD:ACI7531
Amiota leucostoma|[10218]|SSEIC6647-13|Drosophilidae|670|BOLD:AAJ2303
Ephydridae|[10219]|PHDIP862-11|Ephydridae|621|BOLD:ABA8754
Lamproscatella muria|[10220]|BBDIP024-09|Ephydridae|656|BOLD:AAG2741
Philotelma alaskense|[10221]|JWDCC736-10|Ephydridae|655|BOLD:AAL8953
Philotelma alaskense|[10222]|SSJAE8493-13|Ephydridae|603|BOLD:ACK3236
Tachinidae|[10223]|JSDIR863-11|Tachinidae|658|BOLD:AAI3568
Stomoxys calcitrans|[10224]|NCCA2482-11|Muscidae|673|BOLD:AAA3181
Ephydridae|[10225]|CNWBH151-13|Ephydridae|658|BOLD:ACG5953
Ephydridae|[10226]|SSJAD1940-13|Ephydridae|661|BOLD:ACG4680
Polytrichophora|[10227]|CNPPG160-12|Ephydridae|664|BOLD:AAG2753
Polytrichophora|[10228]|CNPPJ428-12|Ephydridae|638|BOLD:ACC8078
Ephydridae|[10229]|NGNAG086-13|Ephydridae|658|BOLD:ACI8610
Ephydridae|[10230]|CNPPI1180-12|Ephydridae|664|BOLD:AAO7768
Discocerina obscurella|[10231]|CNPPJ137-12|Ephydridae|664|BOLD:AAG2756
Discocerina|[10232]|SSJAD3780-13|Ephydridae|661|BOLD:AAG2739
Allotrichoma simplex|[10233]|CNPPJ280-12|Ephydridae|670|BOLD:AAG2755
Allotrichoma filiforme|[10234]|CNPPJ452-12|Ephydridae|670|BOLD:ACF1575
Allotrichoma|[10235]|SSJAE3935-13|Ephydridae|664|BOLD:ABZ2567
Ephydridae|[10236]|JSDIR359-11|Ephydridae|658|BOLD:ABA0760
Ephydridae|[10237]|CNFNC1701-14|Ephydridae|661|BOLD:AAG2766
Ephydridae|[10238]|BBDCM743-10|Ephydridae|658|BOLD:AAP6350
Ephydridae|[10239]|SSWLB1860-13|Ephydridae|664|BOLD:ACG0287
Ephydridae|[10240]|CNBRL514-14|Ephydridae|658|BOLD:ACL8463
Axysta|[10241]|CNPPF298-12|Ephydridae|664|BOLD:ACC7660
Axysta|[10242]|CNPPG625-12|Ephydridae|664|BOLD:ACC8569
Axysta|[10243]|JWDCE003-10|Ephydridae|655|BOLD:AAP1854
Axysta cesta|[10244]|CNEIB211-12|Ephydridae|658|BOLD:ACS0737
Axysta cesta|[10245]|SSPAA6858-13|Ephydridae|604|BOLD:ACS0738
Ephydridae|[10246]|CNPKL067-14|Ephydridae|601|BOLD:ACM5124
Ephydridae|[10247]|BBDEC506-09|Ephydridae|658|BOLD:AAG2757
Ephydridae|[10248]|JWDCA866-10|Ephydridae|634|BOLD:AAG2769
Ephydra hians|[10249]|BBDIP217-09|Ephydridae|658|BOLD:AAG2743
Ephydridae|[10250]|BBDCM728-10|Ephydridae|658|BOLD:AAG2735
Ephydra riparia|[10251]|BBDCM981-10|Ephydridae|658|BOLD:AAG2729
Ephydridae|[10252]|BBDCM984-10|Ephydridae|658|BOLD:AAP6352
Coenia curvicauda|[10253]|BBDCM398-10|Ephydridae|658|BOLD:AAM6634
Calocoenia paurosoma|[10254]|JWDCG702-10|Ephydridae|655|BOLD:AAP8986
Paracoenia fumosa|[10255]|JSDIP488-10|Ephydridae|658|BOLD:AAP2866
Paracoenia bisetosa|[10256]|TTDFW125-08|Ephydridae|658|BOLD:AAG2734
Ephydridae|[10257]|NGNAO376-14|Ephydridae|664|BOLD:ACL4475
Ephydridae|[10258]|TTMDI837-10|Ephydridae|658|BOLD:AAM6635
Ephydridae|[10259]|JWDCE022-10|Ephydridae|655|BOLD:AAP1855
Parydra aquila|[10260]|JSDIS079-11|Ephydridae|658|BOLD:ACG5921
Parydra aquila|[10261]|NCCA2494-11|Ephydridae|673|BOLD:AAP5195
Parydra quadrituberculata|[10262]|BBDCQ928-10|Ephydridae|658|BOLD:AAV4057
Ephydridae|[10263]|NCCA2496-11|Ephydridae|673|BOLD:ABA9109
Pelina neaenescens|[10264]|SSEIA6936-13|Ephydridae|632|BOLD:ACF6002
Ephydridae|[10265]|BBDED445-10|Ephydridae|658|BOLD:AAG2767
Parydra quadriloba|[10266]|SSBAA070-12|Ephydridae|661|BOLD:ACC3559
Ephydridae|[10267]|FLIMH026-08|Ephydridae|655|BOLD:AAG2733
Parydra|[10268]|SSJAD1483-13|Ephydridae|658|BOLD:ACI7144
Parydra|[10269]|SSJAE10140-13|Ephydridae|633|BOLD:AAX1667
Ephydridae|[10270]|CNFNQ1017-14|Ephydridae|658|BOLD:ACM2848
Ephydridae|[10271]|BBDIP031-09|Ephydridae|658|BOLD:AAG2742
Ephydridae|[10272]|DARC570-11|Ephydridae|658|BOLD:AAU6759
Ephydridae|[10273]|JSDIQ408-10|Ephydridae|658|BOLD:AAV4043
Ephydridae|[10274]|CNPPA1003-12|Ephydridae|619|BOLD:ACC6421
Ephydridae|[10275]|CNEID1995-12|Ephydridae|658|BOLD:AAG2731
Ephydridae|[10276]|SSJAD2123-13|Ephydridae|658|BOLD:ACI4178
Ephydridae|[10277]|BBDIP277-09|Ephydridae|557|BOLD:AAG2745
Lamproscatella quadrisetosa|[10278]|MHFLI463-07|Ephydridae|598|BOLD:AAG2728
Lamproscatella quadrisetosa|[10279]|FLIMH014-08|Ephydridae|655|BOLD:AAG2725
Lamproscatella quadrisetosa|[10280]|JWDCE420-10|Ephydridae|655|BOLD:AAP5196
Lamproscatella bimaculata|[10281]|SSJAD1036-13|Ephydridae|661|BOLD:AAG2744
Lamproscatella brunnipennis|[10282]|FLIMH006-08|Ephydridae|655|BOLD:AAG2726
Lamproscatella|[10283]|CNGRH190-13|Ephydridae|605|BOLD:AAG2751
Scatella stagnalis|[10284]|BBDIQ150-10|Ephydridae|658|BOLD:AAG2736
Scatella|[10285]|BBDEC911-10|Ephydridae|658|BOLD:AAG2760
Scatella stagnalis|[10286]|JWDCL441-11|Ephydridae|658|BOLD:ACG1868
Ephydridae|[10287]|SMTPB282-13|Ephydridae|658|BOLD:ABX1159
Scatella|[10288]|BBDEC653-09|Ephydridae|658|BOLD:AAG2752
Scatella|[10289]|SSJAE8763-13|Ephydridae|674|BOLD:AAG2738
Ephydridae|[10290]|DARC611-11|Ephydridae|658|BOLD:AAU6743
Ephydridae|[10291]|HESEP553-12|Ephydridae|630|BOLD:ACM2441
Ephydridae|[10292]|BBDCQ656-10|Ephydridae|658|BOLD:AAV4066
Ephydridae|[10293]|SSWLC2616-13|Ephydridae|658|BOLD:ACX7009
Ephydridae|[10294]|SSWLF1302-13|Ephydridae|579|BOLD:ACK4806
Nostima|[10295]|CNPPJ370-12|Ephydridae|664|BOLD:AAG2754
Parydrinae|[10296]|CNJAG129-12|Ephydridae|658|BOLD:ACD0826
Philygria|[10297]|BBDCN441-10|Ephydridae|658|BOLD:AAG2740
Ephydridae|[10298]|JWCHL295-10|Ephydridae|655|BOLD:AAV4070

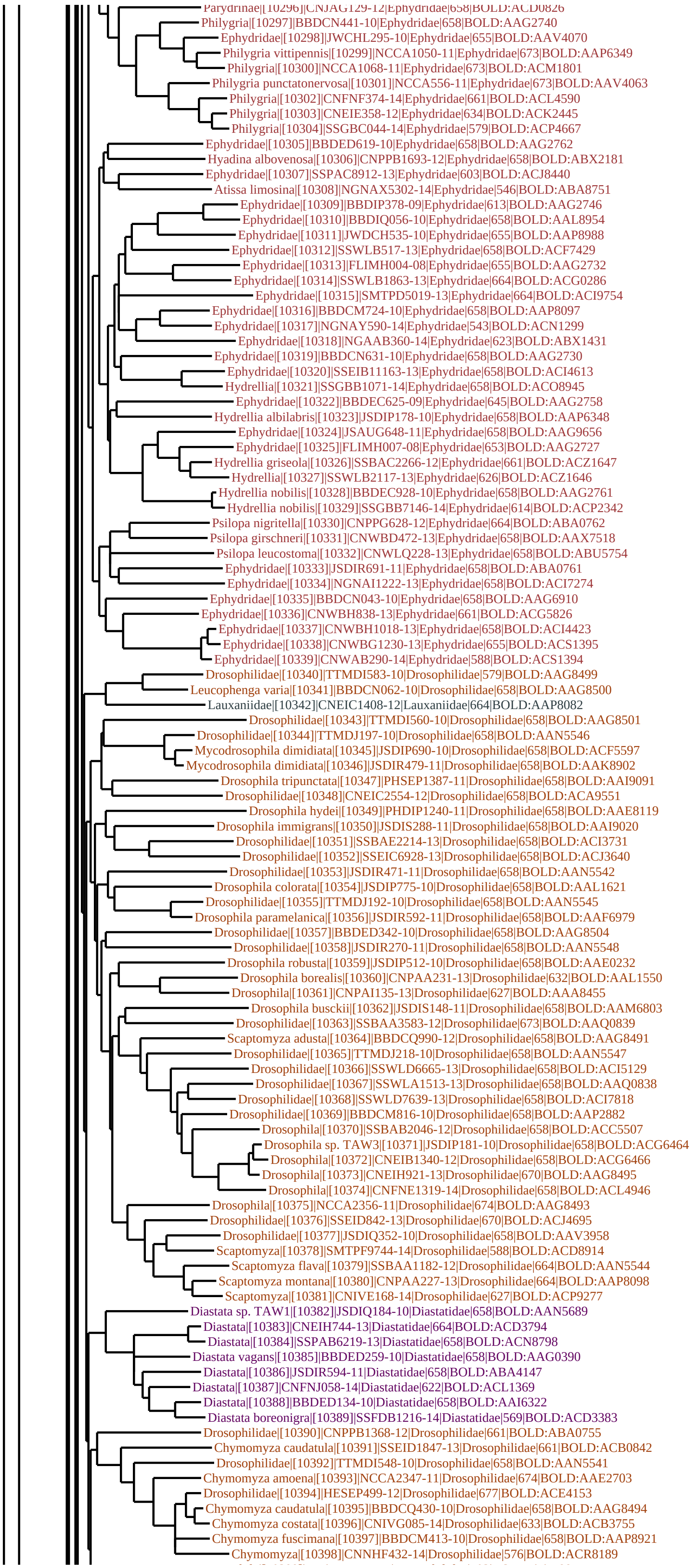

Parydrinae|[10296]|CNJAG129-12|Ephydridae|658|BOLD:ACD0826
Philygria|[10297]|BBDCN441-10|Ephydridae|658|BOLD:AAG2740
Ephydridae|[10298]|JWCHL295-10|Ephydridae|655|BOLD:AAV4070
Philygria vittipennis|[10299]|NCCA1050-11|Ephydridae|673|BOLD:AAP6349
Philygria|[10300]|NCCA1068-11|Ephydridae|673|BOLD:ACM1801
Philygria punctatonervosa|[10301]|NCCA556-11|Ephydridae|673|BOLD:AAV4063
Philygria|[10302]|CNFNF374-14|Ephydridae|661|BOLD:ACL4590
Philygria|[10303]|CNEIE358-12|Ephydridae|634|BOLD:ACK2445
Philygria|[10304]|SSGBC044-14|Ephydridae|579|BOLD:ACP4667
Ephydridae|[10305]|BBDED619-10|Ephydridae|658|BOLD:AAG2762
Hyadina albovenosa|[10306]|CNPPB1693-12|Ephydridae|658|BOLD:ABX2181
Ephydridae|[10307]|SSPAC8912-13|Ephydridae|603|BOLD:ACJ8440
Atissa limosina|[10308]|NGNAX5302-14|Ephydridae|546|BOLD:ABA8751
Ephydridae|[10309]|BBDIP378-09|Ephydridae|613|BOLD:AAG2746
Ephydridae|[10310]|BBDIQ056-10|Ephydridae|658|BOLD:AAL8954
Ephydridae|[10311]|JWDCH535-10|Ephydridae|655|BOLD:AAP8988
Ephydridae|[10312]|SSWLB517-13|Ephydridae|658|BOLD:ACF7429
Ephydridae|[10313]|FLIMH004-08|Ephydridae|655|BOLD:AAG2732
Ephydridae|[10314]|SSWLB1863-13|Ephydridae|664|BOLD:ACG0286
Ephydridae|[10315]|SMTPD5019-13|Ephydridae|664|BOLD:ACI9754
Ephydridae|[10316]|BBDCM724-10|Ephydridae|658|BOLD:AAP8097
Ephydridae|[10317]|NGNAY590-14|Ephydridae|543|BOLD:ACN1299
Ephydridae|[10318]|NGAAB360-14|Ephydridae|623|BOLD:ABX1431
Ephydridae|[10319]|BBDCN631-10|Ephydridae|658|BOLD:AAG2730
Ephydridae|[10320]|SSEIB11163-13|Ephydridae|658|BOLD:ACI4613
Hydrellia|[10321]|SSGBB1071-14|Ephydridae|658|BOLD:ACO8945
Ephydridae|[10322]|BBDEC625-09|Ephydridae|645|BOLD:AAG2758
Hydrellia albilabris|[10323]|JSDIP178-10|Ephydridae|658|BOLD:AAP6348
Ephydridae|[10324]|JSAUG648-11|Ephydridae|658|BOLD:AAG9656
Ephydridae|[10325]|FLIMH007-08|Ephydridae|653|BOLD:AAG2727
Hydrellia griseola|[10326]|SSBAC2266-12|Ephydridae|661|BOLD:ACZ1647
Hydrellia|[10327]|SSWLB2117-13|Ephydridae|626|BOLD:ACZ1646
Hydrellia nobilis|[10328]|BBDEC928-10|Ephydridae|658|BOLD:AAG2761
Hydrellia nobilis|[10329]|SSGBB7146-14|Ephydridae|614|BOLD:ACP2342
Psilopa nigritella|[10330]|CNPPG628-12|Ephydridae|664|BOLD:ABA0762
Psilopa girschneri|[10331]|CNWBD472-13|Ephydridae|658|BOLD:AAX7518
Psilopa leucostoma|[10332]|CNWLQ228-13|Ephydridae|658|BOLD:ABU5754
Ephydridae|[10333]|JSDIR691-11|Ephydridae|658|BOLD:ABA0761
Ephydridae|[10334]|NGNAI1222-13|Ephydridae|658|BOLD:ACI7274
Ephydridae|[10335]|BBDCN043-10|Ephydridae|658|BOLD:AAG6910
Ephydridae|[10336]|CNWBH838-13|Ephydridae|661|BOLD:ACG5826
Ephydridae|[10337]|CNWBH1018-13|Ephydridae|658|BOLD:ACI4423
Ephydridae|[10338]|CNWBG1230-13|Ephydridae|655|BOLD:ACS1395
Ephydridae|[10339]|CNWAB290-14|Ephydridae|588|BOLD:ACS1394
Drosophilidae|[10340]|TTMDI583-10|Drosophilidae|579|BOLD:AAG8499
Leucophenga varia|[10341]|BBDCN062-10|Drosophilidae|658|BOLD:AAG8500
Lauxaniidae|[10342]|CNEIC1408-12|Lauxaniidae|664|BOLD:AAP8082
Drosophilidae|[10343]|TTMDI560-10|Drosophilidae|658|BOLD:AAG8501
Drosophilidae|[10344]|TTMDJ197-10|Drosophilidae|658|BOLD:AAN5546
Mycodrosophila dimidiata|[10345]|JSDIP690-10|Drosophilidae|658|BOLD:ACF5597
Mycodrosophila dimidiata|[10346]|JSDIR479-11|Drosophilidae|658|BOLD:AAK8902
Drosophila tripunctata|[10347]|PHSEP1387-11|Drosophilidae|658|BOLD:AAI9091
Drosophilidae|[10348]|CNEIC2554-12|Drosophilidae|658|BOLD:ACA9551
Drosophila hydei|[10349]|PHDIP1240-11|Drosophilidae|658|BOLD:AAE8119
Drosophila immigrans|[10350]|JSDIS288-11|Drosophilidae|658|BOLD:AAI9020
Drosophilidae|[10351]|SSBAE2214-13|Drosophilidae|658|BOLD:ACI3731
Drosophilidae|[10352]|SSEIC6928-13|Drosophilidae|658|BOLD:ACJ3640
Drosophilidae|[10353]|JSDIR471-11|Drosophilidae|658|BOLD:AAN5542
Drosophila colorata|[10354]|JSDIP775-10|Drosophilidae|658|BOLD:AAL1621
Drosophilidae|[10355]|TTMDJ192-10|Drosophilidae|658|BOLD:AAN5545
Drosophila paramelanica|[10356]|JSDIR592-11|Drosophilidae|658|BOLD:AAF6979
Drosophilidae|[10357]|BBDED342-10|Drosophilidae|658|BOLD:AAG8504
Drosophilidae|[10358]|JSDIR270-11|Drosophilidae|658|BOLD:AAN5548
Drosophila robusta|[10359]|JSDIP512-10|Drosophilidae|658|BOLD:AAE0232
Drosophila borealis|[10360]|CNPAA231-13|Drosophilidae|632|BOLD:AAL1550
Drosophila|[10361]|CNPAI135-13|Drosophilidae|627|BOLD:AAA8455
Drosophila busckii|[10362]|JSDIS148-11|Drosophilidae|658|BOLD:AAM6803
Drosophilidae|[10363]|SSBAA3583-12|Drosophilidae|673|BOLD:AAQ0839
Scaptomyza adusta|[10364]|BBDCQ990-12|Drosophilidae|658|BOLD:AAG8491
Drosophilidae|[10365]|TTMDJ218-10|Drosophilidae|658|BOLD:AAN5547
Drosophilidae|[10366]|SSWLD6665-13|Drosophilidae|658|BOLD:ACI5129
Drosophilidae|[10367]|SSWLA1513-13|Drosophilidae|658|BOLD:AAQ0838
Drosophilidae|[10368]|SSWLD7639-13|Drosophilidae|658|BOLD:ACI7818
Drosophilidae|[10369]|BBDCM816-10|Drosophilidae|658|BOLD:AAP2882
Drosophila|[10370]|SSBAB2046-12|Drosophilidae|658|BOLD:ACC5507
Drosophila sp. TAW3|[10371]|JSDIP181-10|Drosophilidae|658|BOLD:ACG6464
Drosophila|[10372]|CNEIB1340-12|Drosophilidae|658|BOLD:ACG6466
Drosophila|[10373]|CNEIH921-13|Drosophilidae|670|BOLD:AAG8495
Drosophila|[10374]|CNFNE1319-14|Drosophilidae|658|BOLD:ACL4946
Drosophila|[10375]|NCCA2356-11|Drosophilidae|674|BOLD:AAG8493
Drosophilidae|[10376]|SSEID842-13|Drosophilidae|670|BOLD:ACJ4695
Drosophilidae|[10377]|JSDIQ352-10|Drosophilidae|658|BOLD:AAV3958
Scaptomyza|[10378]|SMTPF9744-14|Drosophilidae|588|BOLD:ACD8914
Scaptomyza flava|[10379]|SSBAA1182-12|Drosophilidae|664|BOLD:AAN5544
Scaptomyza montana|[10380]|CNPAA227-13|Drosophilidae|664|BOLD:AAP8098
Scaptomyza|[10381]|CNIVE168-14|Drosophilidae|627|BOLD:ACP9277
Diastata sp. TAW1|[10382]|JSDIQ184-10|Diastatidae|658|BOLD:AAN5689
Diastata|[10383]|CNEIH744-13|Diastatidae|664|BOLD:ACD3794
Diastata|[10384]|SSPAB6219-13|Diastatidae|658|BOLD:ACN8798
Diastata vagans|[10385]|BBDED259-10|Diastatidae|658|BOLD:AAG0390
Diastata|[10386]|JSDIR594-11|Diastatidae|658|BOLD:ABA4147
Diastata|[10387]|CNFNJ058-14|Diastatidae|622|BOLD:ACL1369
Diastata|[10388]|BBDED134-10|Diastatidae|658|BOLD:AAI6322
Diastata boreonigra|[10389]|SSFDB1216-14|Diastatidae|569|BOLD:ACD3383
Drosophilidae|[10390]|CNPPB1368-12|Drosophilidae|661|BOLD:ABA0755
Chymomyza caudatula|[10391]|SSEID1847-13|Drosophilidae|661|BOLD:ACB0842
Drosophilidae|[10392]|TTMDI548-10|Drosophilidae|658|BOLD:AAN5541
Chymomyza amoena|[10393]|NCCA2347-11|Drosophilidae|674|BOLD:AAE2703
Drosophilidae|[10394]|HESEP499-12|Drosophilidae|677|BOLD:ACE4153
Chymomyza caudatula|[10395]|BBDCQ430-10|Drosophilidae|658|BOLD:AAG8494
Chymomyza costata|[10396]|CNIVG085-14|Drosophilidae|633|BOLD:ACB3755
Chymomyza fuscimana|[10397]|BBDCM413-10|Drosophilidae|658|BOLD:AAP8921
Chymomyza|[10398]|CNNHF432-14|Drosophilidae|576|BOLD:ACR8189

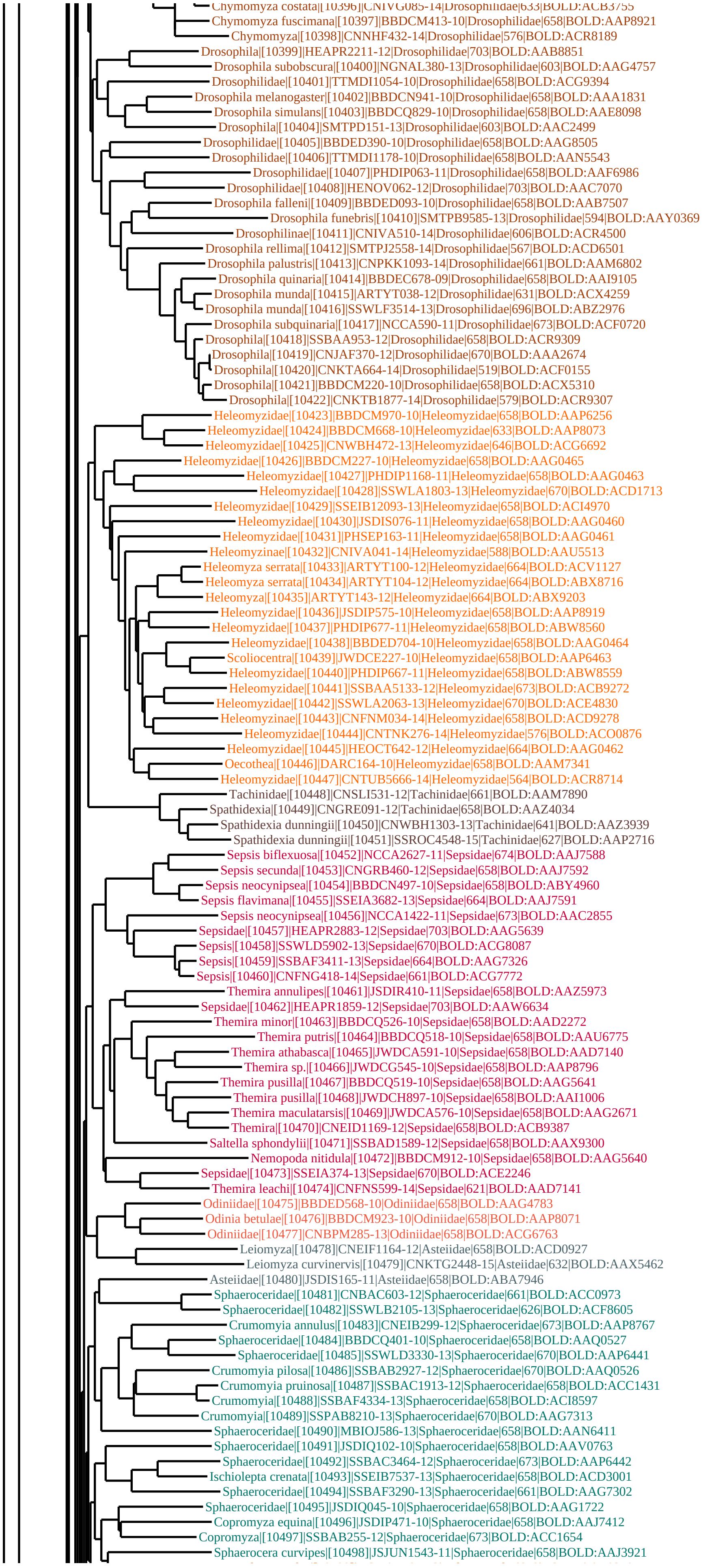
Chymomyza costata|[10396]|CNIVG085-14|Drosophilidae|633|BOLD:ACB3755
Chymomyza fuscimana|[10397]|BBDCM413-10|Drosophilidae|658|BOLD:AAP8921
Chymomyza|[10398]|CNNHF432-14|Drosophilidae|576|BOLD:ACR8189
Drosophila|[10399]|HEAPR2211-12|Drosophilidae|703|BOLD:AAB8851
Drosophila subobscura|[10400]|NGNAL380-13|Drosophilidae|603|BOLD:AAG4757
Drosophilidae|[10401]|TTMDI1054-10|Drosophilidae|658|BOLD:ACG9394
Drosophila melanogaster|[10402]|BBDCN941-10|Drosophilidae|658|BOLD:AAA1831
Drosophila simulans|[10403]|BBDCQ829-10|Drosophilidae|658|BOLD:AAE8098
Drosophila|[10404]|SMTPD151-13|Drosophilidae|603|BOLD:AAC2499
Drosophilidae|[10405]|BBDED390-10|Drosophilidae|658|BOLD:AAG8505
Drosophilidae|[10406]|TTMDI1178-10|Drosophilidae|658|BOLD:AAN5543
Drosophilidae|[10407]|PHDIP063-11|Drosophilidae|658|BOLD:AAF6986
Drosophilidae|[10408]|HENOV062-12|Drosophilidae|703|BOLD:AAC7070
Drosophila falleni|[10409]|BBDED093-10|Drosophilidae|658|BOLD:AAB7507
Drosophila funebris|[10410]|SMTPB9585-13|Drosophilidae|594|BOLD:AAY0369
Drosophilinae|[10411]|CNIVA510-14|Drosophilidae|606|BOLD:ACR4500
Drosophila rellima|[10412]|SMTPJ2558-14|Drosophilidae|567|BOLD:ACD6501
Drosophila palustris|[10413]|CNPKK1093-14|Drosophilidae|661|BOLD:AAM6802
Drosophila quinaria|[10414]|BBDEC678-09|Drosophilidae|658|BOLD:AAI9105
Drosophila munda|[10415]|ARTYT038-12|Drosophilidae|631|BOLD:ACX4259
Drosophila munda|[10416]|SSWLF3514-13|Drosophilidae|696|BOLD:ABZ2976
Drosophila subquinaria|[10417]|NCCA590-11|Drosophilidae|673|BOLD:ACF0720
Drosophila|[10418]|SSBAA953-12|Drosophilidae|658|BOLD:ACR9309
Drosophila|[10419]|CNJAF370-12|Drosophilidae|670|BOLD:AAA2674
Drosophila|[10420]|CNKTA664-14|Drosophilidae|519|BOLD:ACF0155
Drosophila|[10421]|BBDCM220-10|Drosophilidae|658|BOLD:ACX5310
Drosophila|[10422]|CNKTB1877-14|Drosophilidae|579|BOLD:ACR9307
Heleomyzidae|[10423]|BBDCM970-10|Heleomyzidae|658|BOLD:AAP6256
Heleomyzidae|[10424]|BBDCM668-10|Heleomyzidae|633|BOLD:AAP8073
Heleomyzidae|[10425]|CNWBH472-13|Heleomyzidae|646|BOLD:ACG6692
Heleomyzidae|[10426]|BBDCM227-10|Heleomyzidae|658|BOLD:AAG0465
Heleomyzidae|[10427]|PHDIP1168-11|Heleomyzidae|658|BOLD:AAG0463
Heleomyzidae|[10428]|SSWLA1803-13|Heleomyzidae|670|BOLD:ACD1713
Heleomyzidae|[10429]|SSEIB12093-13|Heleomyzidae|658|BOLD:ACI4970
Heleomyzidae|[10430]|JSDIS076-11|Heleomyzidae|658|BOLD:AAG0460
Heleomyzidae|[10431]|PHSEP163-11|Heleomyzidae|658|BOLD:AAG0461
Heleomyzinae|[10432]|CNIVA041-14|Heleomyzidae|588|BOLD:AAU5513
Heleomyza serrata|[10433]|ARTYT100-12|Heleomyzidae|664|BOLD:ACV1127
Heleomyza serrata|[10434]|ARTYT104-12|Heleomyzidae|664|BOLD:ABX8716
Heleomyza|[10435]|ARTYT143-12|Heleomyzidae|664|BOLD:ABX9203
Heleomyzidae|[10436]|JSDIP575-10|Heleomyzidae|658|BOLD:AAP8919
Heleomyzidae|[10437]|PHDIP677-11|Heleomyzidae|658|BOLD:ABW8560
Heleomyzidae|[10438]|BBDED704-10|Heleomyzidae|658|BOLD:AAG0464
Scoliocentra|[10439]|JWDCE227-10|Heleomyzidae|658|BOLD:AAP6463
Heleomyzidae|[10440]|PHDIP667-11|Heleomyzidae|658|BOLD:ABW8559
Heleomyzidae|[10441]|SSBAA5133-12|Heleomyzidae|673|BOLD:ACB9272
Heleomyzidae|[10442]|SSWLA2063-13|Heleomyzidae|670|BOLD:ACE4830
Heleomyzinae|[10443]|CNFNM034-14|Heleomyzidae|658|BOLD:ACD9278
Heleomyzidae|[10444]|CNTNK276-14|Heleomyzidae|576|BOLD:ACO0876
Heleomyzidae|[10445]|HEOCT642-12|Heleomyzidae|664|BOLD:AAG0462
Oecothea|[10446]|DARC164-10|Heleomyzidae|658|BOLD:AAM7341
Heleomyzidae|[10447]|CNTUB5666-14|Heleomyzidae|564|BOLD:ACR8714
Tachinidae|[10448]|CNSLI531-12|Tachinidae|661|BOLD:AAM7890
Spathidexia|[10449]|CNGRE091-12|Tachinidae|658|BOLD:AAZ4034
Spathidexia dunningii|[10450]|CNWBH1303-13|Tachinidae|641|BOLD:AAZ3939
Spathidexia dunningii|[10451]|SSROC4548-15|Tachinidae|627|BOLD:AAP2716
Sepsis biflexuosa|[10452]|NCCA2627-11|Sepsidae|674|BOLD:AAJ7588
Sepsis secunda|[10453]|CNGRB460-12|Sepsidae|658|BOLD:AAJ7592
Sepsis neocynipsea|[10454]|BBDCN497-10|Sepsidae|658|BOLD:ABY4960
Sepsis flavimana|[10455]|SSEIA3682-13|Sepsidae|664|BOLD:AAJ7591
Sepsis neocynipsea|[10456]|NCCA1422-11|Sepsidae|673|BOLD:AAC2855
Sepsidae|[10457]|HEAPR2883-12|Sepsidae|703|BOLD:AAG5639
Sepsis|[10458]|SSWLD5902-13|Sepsidae|670|BOLD:ACG8087
Sepsis|[10459]|SSBAF3411-13|Sepsidae|664|BOLD:AAG7326
Sepsis|[10460]|CNFNG418-14|Sepsidae|661|BOLD:ACG7772
Themira annulipes|[10461]|JSDIR410-11|Sepsidae|658|BOLD:AAZ5973
Sepsidae|[10462]|HEAPR1859-12|Sepsidae|703|BOLD:AAW6634
Themira minor|[10463]|BBDCQ526-10|Sepsidae|658|BOLD:AAD2272
Themira putris|[10464]|BBDCQ518-10|Sepsidae|658|BOLD:AAU6775
Themira athabasca|[10465]|JWDCA591-10|Sepsidae|658|BOLD:AAD7140
Themira sp.|[10466]|JWDCG545-10|Sepsidae|658|BOLD:AAP8796
Themira pusilla|[10467]|BBDCQ519-10|Sepsidae|658|BOLD:AAG5641
Themira pusilla|[10468]|JWDCH897-10|Sepsidae|658|BOLD:AAI1006
Themira maculatarsis|[10469]|JWDCA576-10|Sepsidae|658|BOLD:AAG2671
Themira|[10470]|CNEID1169-12|Sepsidae|658|BOLD:ACB9387
Saltella sphondylii|[10471]|SSBAD1589-12|Sepsidae|658|BOLD:AAX9300
Nemopoda nitidula|[10472]|BBDCM912-10|Sepsidae|658|BOLD:AAG5640
Sepsidae|[10473]|SSEIA374-13|Sepsidae|670|BOLD:ACE2246
Themira leachi|[10474]|CNFNS599-14|Sepsidae|621|BOLD:AAD7141
Odiniidae|[10475]|BBDED568-10|Odiniidae|658|BOLD:AAG4783
Odinia betulae|[10476]|BBDCM923-10|Odiniidae|658|BOLD:AAP8071
Odiniidae|[10477]|CNBPM285-13|Odiniidae|658|BOLD:ACG6763
Leiomyza|[10478]|CNEIF1164-12|Asteiidae|658|BOLD:ACD0927
Leiomyza curvinervis|[10479]|CNKTG2448-15|Asteiidae|632|BOLD:AAX5462
Asteiidae|[10480]|JSDIS165-11|Asteiidae|658|BOLD:ABA7946
Sphaeroceridae|[10481]|CNBAC603-12|Sphaeroceridae|661|BOLD:ACC0973
Sphaeroceridae|[10482]|SSWLB2105-13|Sphaeroceridae|626|BOLD:ACF8605
Crumomyia annulus|[10483]|CNEIB299-12|Sphaeroceridae|673|BOLD:AAP8767
Sphaeroceridae|[10484]|BBDCQ401-10|Sphaeroceridae|658|BOLD:AAQ0527
Sphaeroceridae|[10485]|SSWLD3330-13|Sphaeroceridae|670|BOLD:AAP6441
Crumomyia pilosa|[10486]|SSBAB2927-12|Sphaeroceridae|670|BOLD:AAQ0526
Crumomyia pruinosa|[10487]|SSBAC1913-12|Sphaeroceridae|658|BOLD:ACC1431
Crumomyia|[10488]|SSBAF4334-13|Sphaeroceridae|658|BOLD:ACI8597
Crumomyia|[10489]|SSPAB8210-13|Sphaeroceridae|670|BOLD:AAG7313
Sphaeroceridae|[10490]|MBIOJ586-13|Sphaeroceridae|658|BOLD:AAN6411
Sphaeroceridae|[10491]|JSDIQ102-10|Sphaeroceridae|658|BOLD:AAV0763
Sphaeroceridae|[10492]|SSBAC3464-12|Sphaeroceridae|673|BOLD:AAP6442
Ischiolepta crenata|[10493]|SSEIB7537-13|Sphaeroceridae|658|BOLD:ACD3001
Sphaeroceridae|[10494]|SSBAF3290-13|Sphaeroceridae|661|BOLD:AAG7302
Sphaeroceridae|[10495]|JSDIQ045-10|Sphaeroceridae|658|BOLD:AAG1722
Copromyza equina|[10496]|JSDIP471-10|Sphaeroceridae|658|BOLD:AAJ7412
Copromyza|[10497]|SSBAB255-12|Sphaeroceridae|673|BOLD:ACC1654
Sphaerocera curvipes|[10498]|JSJUN1543-11|Sphaeroceridae|658|BOLD:AAJ3921

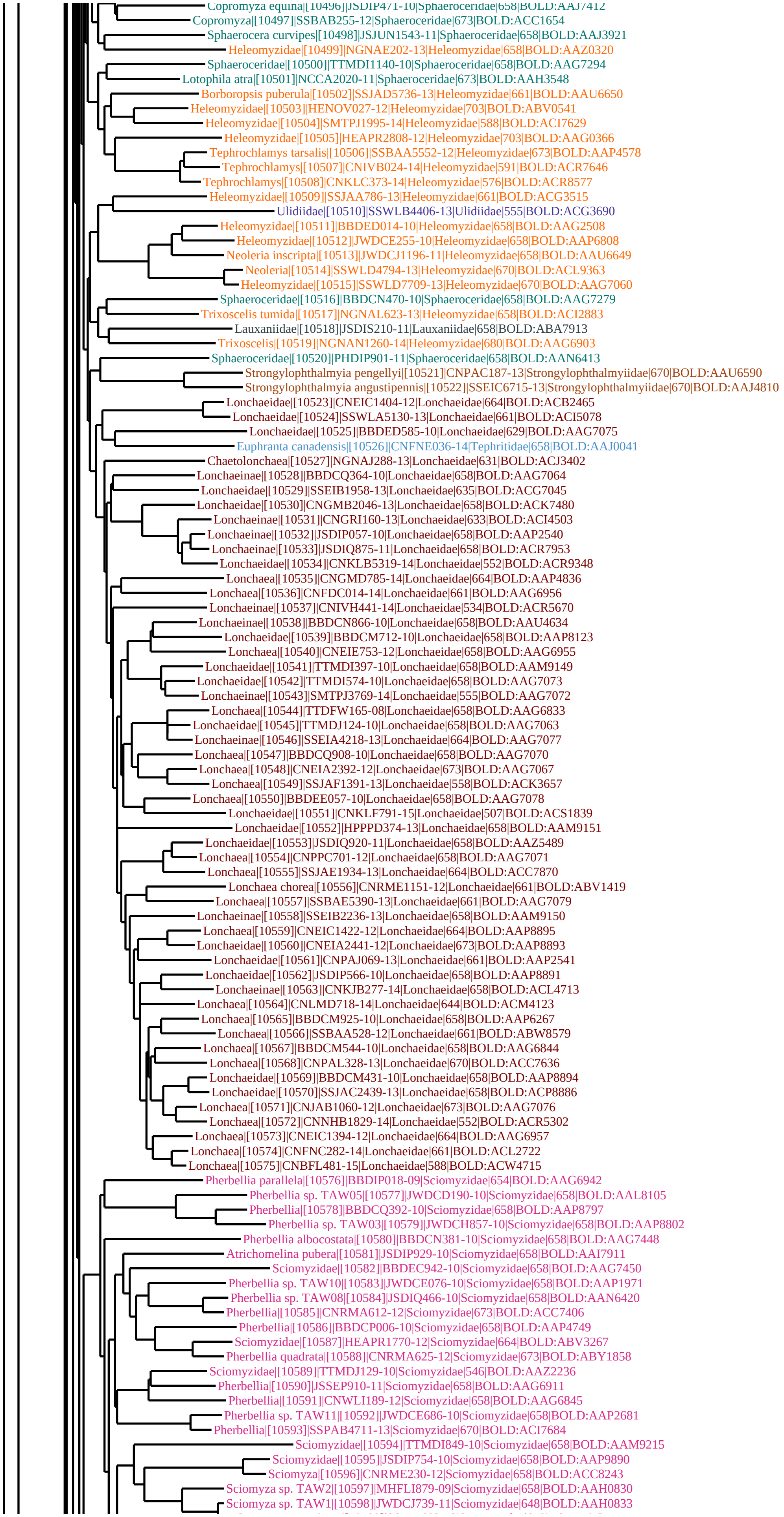
Copromyza equina|[10496]|JSDIP471-10|Sphaeroceridae|658|BOLD:AAJ7412
Copromyza|[10497]|SSBAB255-12|Sphaeroceridae|673|BOLD:ACC1654
Sphaerocera curvipes|[10498]|JSJUN1543-11|Sphaeroceridae|658|BOLD:AAJ3921
Heleomyzidae|[10499]|NGNAE202-13|Heleomyzidae|658|BOLD:AAZ0320
Sphaeroceridae|[10500]|TTMDI1140-10|Sphaeroceridae|658|BOLD:AAG7294
Lotophila atra|[10501]|NCCA2020-11|Sphaeroceridae|673|BOLD:AAH3548
Borboropsis puberula|[10502]|SSJAD5736-13|Heleomyzidae|661|BOLD:AAU6650
Heleomyzidae|[10503]|HENOV027-12|Heleomyzidae|703|BOLD:ABV0541
Heleomyzidae|[10504]|SMTPJ1995-14|Heleomyzidae|588|BOLD:ACI7629
Heleomyzidae|[10505]|HEAPR2808-12|Heleomyzidae|703|BOLD:AAG0366
Tephrochlamys tarsalis|[10506]|SSBAA5552-12|Heleomyzidae|673|BOLD:AAP4578
Tephrochlamys|[10507]|CNIVB024-14|Heleomyzidae|591|BOLD:ACR7646
Tephrochlamys|[10508]|CNKLC373-14|Heleomyzidae|576|BOLD:ACR8577
Heleomyzidae|[10509]|SSJAA786-13|Heleomyzidae|661|BOLD:ACG3515
Ulidiidae|[10510]|SSWLB4406-13|Ulidiidae|555|BOLD:ACG3690
Heleomyzidae|[10511]|BBDED014-10|Heleomyzidae|658|BOLD:AAG2508
Heleomyzidae|[10512]|JWDCE255-10|Heleomyzidae|658|BOLD:AAP6808
Neoleria inscripta|[10513]|JWDCJ1196-11|Heleomyzidae|658|BOLD:AAU6649
Neoleria|[10514]|SSWLD4794-13|Heleomyzidae|670|BOLD:ACL9363
Heleomyzidae|[10515]|SSWLD7709-13|Heleomyzidae|670|BOLD:AAG7060
Sphaeroceridae|[10516]|BBDCN470-10|Sphaeroceridae|658|BOLD:AAG7279
Trixoscelis tumida|[10517]|NGNAL623-13|Heleomyzidae|658|BOLD:ACI2883
Lauxaniidae|[10518]|JSDIS210-11|Lauxaniidae|658|BOLD:ABA7913
Trixoscelis|[10519]|NGNAN1260-14|Heleomyzidae|680|BOLD:AAG6903
Sphaeroceridae|[10520]|PHDIP901-11|Sphaeroceridae|658|BOLD:AAN6413
Strongylophthalmyia pengellyi|[10521]|CNPAC187-13|Strongylophthalmyiidae|670|BOLD:AAU6590
Strongylophthalmyia angustipennis|[10522]|SSEIC6715-13|Strongylophthalmyiidae|670|BOLD:AAJ4810
Lonchaeidae|[10523]|CNEIC1404-12|Lonchaeidae|664|BOLD:ACB2465
Lonchaeidae|[10524]|SSWLA5130-13|Lonchaeidae|661|BOLD:ACI5078
Lonchaeidae|[10525]|BBDED585-10|Lonchaeidae|629|BOLD:AAG7075
Euphranta canadensis|[10526]|CNFNE036-14|Tephritidae|658|BOLD:AAJ0041
Chaetolonchaea|[10527]|NGNAJ288-13|Lonchaeidae|631|BOLD:ACJ3402
Lonchaeinae|[10528]|BBDCQ364-10|Lonchaeidae|658|BOLD:AAG7064
Lonchaeidae|[10529]|SSEIB1958-13|Lonchaeidae|635|BOLD:ACG7045
Lonchaeidae|[10530]|CNGMB2046-13|Lonchaeidae|658|BOLD:ACK7480
Lonchaeinae|[10531]|CNGRI160-13|Lonchaeidae|633|BOLD:ACI4503
Lonchaeinae|[10532]|JSDIP057-10|Lonchaeidae|658|BOLD:AAP2540
Lonchaeinae|[10533]|JSDIQ875-11|Lonchaeidae|658|BOLD:ACR7953
Lonchaeidae|[10534]|CNKLB5319-14|Lonchaeidae|552|BOLD:ACR9348
Lonchaea|[10535]|CNGMD785-14|Lonchaeidae|664|BOLD:AAP4836
Lonchaea|[10536]|CNFDC014-14|Lonchaeidae|661|BOLD:AAG6956
Lonchaeinae|[10537]|CNIVH441-14|Lonchaeidae|534|BOLD:ACR5670
Lonchaeinae|[10538]|BBDCN866-10|Lonchaeidae|658|BOLD:AAU4634
Lonchaeidae|[10539]|BBDCM712-10|Lonchaeidae|658|BOLD:AAP8123
Lonchaea|[10540]|CNEIE753-12|Lonchaeidae|658|BOLD:AAG6955
Lonchaeidae|[10541]|TTMDI397-10|Lonchaeidae|658|BOLD:AAM9149
Lonchaeidae|[10542]|TTMDI574-10|Lonchaeidae|658|BOLD:AAG7073
Lonchaeinae|[10543]|SMTPJ3769-14|Lonchaeidae|555|BOLD:AAG7072
Lonchaea|[10544]|TTDFW165-08|Lonchaeidae|658|BOLD:AAG6833
Lonchaeidae|[10545]|TTMDJ124-10|Lonchaeidae|658|BOLD:AAG7063
Lonchaeinae|[10546]|SSEIA4218-13|Lonchaeidae|664|BOLD:AAG7077
Lonchaea|[10547]|BBDCQ908-10|Lonchaeidae|658|BOLD:AAG7070
Lonchaea|[10548]|CNEIA2392-12|Lonchaeidae|673|BOLD:AAG7067
Lonchaea|[10549]|SSJAF1391-13|Lonchaeidae|558|BOLD:ACK3657
Lonchaea|[10550]|BBDEE057-10|Lonchaeidae|658|BOLD:AAG7078
Lonchaeidae|[10551]|CNKLF791-15|Lonchaeidae|507|BOLD:ACS1839
Lonchaeidae|[10552]|HPPPD374-13|Lonchaeidae|658|BOLD:AAM9151
Lonchaeidae|[10553]|JSDIQ920-11|Lonchaeidae|658|BOLD:AAZ5489
Lonchaea|[10554]|CNPPC701-12|Lonchaeidae|658|BOLD:AAG7071
Lonchaea|[10555]|SSJAE1934-13|Lonchaeidae|664|BOLD:ACC7870
Lonchaea chorea|[10556]|CNRME1151-12|Lonchaeidae|661|BOLD:ABV1419
Lonchaea|[10557]|SSBAE5390-13|Lonchaeidae|661|BOLD:AAG7079
Lonchaeinae|[10558]|SSEIB2236-13|Lonchaeidae|658|BOLD:AAM9150
Lonchaea|[10559]|CNEIC1422-12|Lonchaeidae|664|BOLD:AAP8895
Lonchaeidae|[10560]|CNEIA2441-12|Lonchaeidae|673|BOLD:AAP8893
Lonchaeidae|[10561]|CNPAJ069-13|Lonchaeidae|661|BOLD:AAP2541
Lonchaeidae|[10562]|JSDIP566-10|Lonchaeidae|658|BOLD:AAP8891
Lonchaeinae|[10563]|CNKJB277-14|Lonchaeidae|658|BOLD:ACL4713
Lonchaea|[10564]|CNLMD718-14|Lonchaeidae|644|BOLD:ACM4123
Lonchaea|[10565]|BBDCM925-10|Lonchaeidae|658|BOLD:AAP6267
Lonchaea|[10566]|SSBAA528-12|Lonchaeidae|661|BOLD:ABW8579
Lonchaea|[10567]|BBDCM544-10|Lonchaeidae|658|BOLD:AAG6844
Lonchaea|[10568]|CNPAL328-13|Lonchaeidae|670|BOLD:ACC7636
Lonchaeidae|[10569]|BBDCM431-10|Lonchaeidae|658|BOLD:AAP8894
Lonchaeidae|[10570]|SSJAC2439-13|Lonchaeidae|658|BOLD:ACP8886
Lonchaea|[10571]|CNJAB1060-12|Lonchaeidae|673|BOLD:AAG7076
Lonchaea|[10572]|CNNHB1829-14|Lonchaeidae|552|BOLD:ACR5302
Lonchaea|[10573]|CNEIC1394-12|Lonchaeidae|664|BOLD:AAG6957
Lonchaea|[10574]|CNFNC282-14|Lonchaeidae|661|BOLD:ACL2722
Lonchaea|[10575]|CNBFL481-15|Lonchaeidae|588|BOLD:ACW4715
Pherbellia parallela|[10576]|BBDIP018-09|Sciomyzidae|654|BOLD:AAG6942
Pherbellia sp. TAW05|[10577]|JWDCD190-10|Sciomyzidae|658|BOLD:AAL8105
Pherbellia|[10578]|BBDCQ392-10|Sciomyzidae|658|BOLD:AAP8797
Pherbellia sp. TAW03|[10579]|JWDCH857-10|Sciomyzidae|658|BOLD:AAP8802
Pherbellia albocostata|[10580]|BBDCN381-10|Sciomyzidae|658|BOLD:AAG7448
Atrichomelina pubera|[10581]|JSDIP929-10|Sciomyzidae|658|BOLD:AAI7911
Sciomyzidae|[10582]|BBDEC942-10|Sciomyzidae|658|BOLD:AAG7450
Pherbellia sp. TAW10|[10583]|JWDCE076-10|Sciomyzidae|658|BOLD:AAP1971
Pherbellia sp. TAW08|[10584]|JSDIQ466-10|Sciomyzidae|658|BOLD:AAN6420
Pherbellia|[10585]|CNRMA612-12|Sciomyzidae|673|BOLD:ACC7406
Pherbellia|[10586]|BBDCP006-10|Sciomyzidae|658|BOLD:AAP4749
Sciomyzidae|[10587]|HEAPR1770-12|Sciomyzidae|664|BOLD:ABV3267
Pherbellia quadrata|[10588]|CNRMA625-12|Sciomyzidae|673|BOLD:ABY1858
Sciomyzidae|[10589]|TTMDJ129-10|Sciomyzidae|546|BOLD:AAZ2236
Pherbellia|[10590]|JSSEP910-11|Sciomyzidae|658|BOLD:AAG6911
Pherbellia|[10591]|CNWLI189-12|Sciomyzidae|658|BOLD:AAG6845
Pherbellia sp. TAW11|[10592]|JWDCE686-10|Sciomyzidae|658|BOLD:AAP2681
Pherbellia|[10593]|SSPAB4711-13|Sciomyzidae|670|BOLD:ACI7684
Sciomyzidae|[10594]|TTMDI849-10|Sciomyzidae|658|BOLD:AAM9215
Sciomyzidae|[10595]|JSDIP754-10|Sciomyzidae|658|BOLD:AAP9890
Sciomyza|[10596]|CNRME230-12|Sciomyzidae|658|BOLD:ACC8243
Sciomyza sp. TAW2|[10597]|MHFLI879-09|Sciomyzidae|658|BOLD:AAH0830
Sciomyza sp. TAW1|[10598]|JWDCJ739-11|Sciomyzidae|648|BOLD:AAH0833

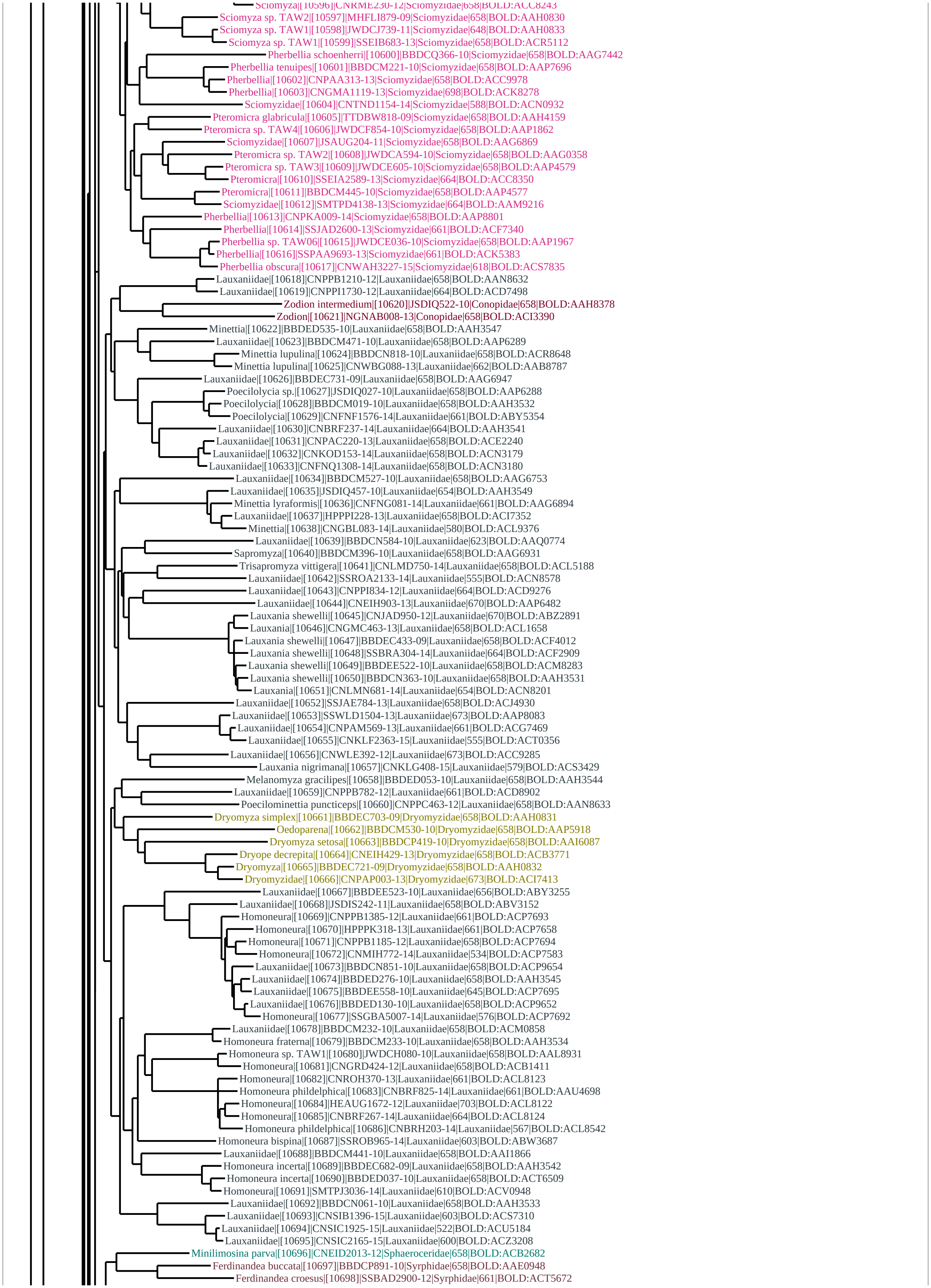
Sciomyza|[10596]|CNRME230-12|Sciomyzidae|658|BOLD:ACC8243
Sciomyza sp. TAW2|[10597]|MHFLI879-09|Sciomyzidae|658|BOLD:AAH0830
Sciomyza sp. TAW1|[10598]|JWDCJ739-11|Sciomyzidae|648|BOLD:AAH0833
Sciomyza sp. TAW1|[10599]|SSEIB683-13|Sciomyzidae|658|BOLD:ACR5112
Pherbellia schoenherri|[10600]|BBDCQ366-10|Sciomyzidae|658|BOLD:AAG7442
Pherbellia tenuipes|[10601]|BBDCM221-10|Sciomyzidae|658|BOLD:AAP7696
Pherbellia|[10602]|CNPAA313-13|Sciomyzidae|658|BOLD:ACC9978
Pherbellia|[10603]|CNGMA1119-13|Sciomyzidae|698|BOLD:ACK8278
Sciomyzidae|[10604]|CNTND1154-14|Sciomyzidae|588|BOLD:ACN0932
Pteromicra glabricula|[10605]|TTDBW818-09|Sciomyzidae|658|BOLD:AAH4159
Pteromicra sp. TAW4|[10606]|JWDCF854-10|Sciomyzidae|658|BOLD:AAP1862
Sciomyzidae|[10607]|JSAUG204-11|Sciomyzidae|658|BOLD:AAG6869
Pteromicra sp. TAW2|[10608]|JWDCA594-10|Sciomyzidae|658|BOLD:AAG0358
Pteromicra sp. TAW3|[10609]|JWDCE605-10|Sciomyzidae|658|BOLD:AAP4579
Pteromicra|[10610]|SSEIA2589-13|Sciomyzidae|664|BOLD:ACC8350
Pteromicra|[10611]|BBDCM445-10|Sciomyzidae|658|BOLD:AAP4577
Sciomyzidae|[10612]|SMTPD4138-13|Sciomyzidae|664|BOLD:AAM9216
Pherbellia|[10613]|CNPKA009-14|Sciomyzidae|658|BOLD:AAP8801
Pherbellia|[10614]|SSJAD2600-13|Sciomyzidae|661|BOLD:ACF7340
Pherbellia sp. TAW06|[10615]|JWDCE036-10|Sciomyzidae|658|BOLD:AAP1967
Pherbellia|[10616]|SSPAA9693-13|Sciomyzidae|661|BOLD:ACK5383
Pherbellia obscura|[10617]|CNWAH3227-15|Sciomyzidae|618|BOLD:ACS7835
Lauxaniidae|[10618]|CNPPB1210-12|Lauxaniidae|658|BOLD:AAN8632
Lauxaniidae|[10619]|CNPPI1730-12|Lauxaniidae|664|BOLD:ACD7498
Zodion intermedium|[10620]|JSDIQ522-10|Conopidae|658|BOLD:AAH8378
Zodion|[10621]|NGNAB008-13|Conopidae|658|BOLD:ACI3390
Minettia|[10622]|BBDED535-10|Lauxaniidae|658|BOLD:AAH3547
Lauxaniidae|[10623]|BBDCM471-10|Lauxaniidae|658|BOLD:AAP6289
Minettia lupulina|[10624]|BBDCN818-10|Lauxaniidae|658|BOLD:ACR8648
Minettia lupulina|[10625]|CNWBG088-13|Lauxaniidae|662|BOLD:AAB8787
Lauxaniidae|[10626]|BBDEC731-09|Lauxaniidae|658|BOLD:AAG6947
Poecilolycia sp.|[10627]|JSDIQ027-10|Lauxaniidae|658|BOLD:AAP6288
Poecilolycia|[10628]|BBDCM019-10|Lauxaniidae|658|BOLD:AAH3532
Poecilolycia|[10629]|CNFNF1576-14|Lauxaniidae|661|BOLD:ABY5354
Lauxaniidae|[10630]|CNBRF237-14|Lauxaniidae|664|BOLD:AAH3541
Lauxaniidae|[10631]|CNPAC220-13|Lauxaniidae|658|BOLD:ACE2240
Lauxaniidae|[10632]|CNKOD153-14|Lauxaniidae|658|BOLD:ACN3179
Lauxaniidae|[10633]|CNFNQ1308-14|Lauxaniidae|658|BOLD:ACN3180
Lauxaniidae|[10634]|BBDCM527-10|Lauxaniidae|658|BOLD:AAG6753
Lauxaniidae|[10635]|JSDIQ457-10|Lauxaniidae|654|BOLD:AAH3549
Minettia lyraformis|[10636]|CNFNG081-14|Lauxaniidae|661|BOLD:AAG6894
Lauxaniidae|[10637]|HPPPI228-13|Lauxaniidae|658|BOLD:ACI7352
Minettia|[10638]|CNGBL083-14|Lauxaniidae|580|BOLD:ACL9376
Lauxaniidae|[10639]|BBDCN584-10|Lauxaniidae|623|BOLD:AAQ0774
Sapromyza|[10640]|BBDCM396-10|Lauxaniidae|658|BOLD:AAG6931
Trisapromyza vittigera|[10641]|CNLMD750-14|Lauxaniidae|658|BOLD:ACL5188
Lauxaniidae|[10642]|SSROA2133-14|Lauxaniidae|555|BOLD:ACN8578
Lauxaniidae|[10643]|CNPPI834-12|Lauxaniidae|664|BOLD:ACD9276
Lauxaniidae|[10644]|CNEIH903-13|Lauxaniidae|670|BOLD:AAP6482
Lauxania shewelli|[10645]|CNJAD950-12|Lauxaniidae|670|BOLD:ABZ2891
Lauxania|[10646]|CNGMC463-13|Lauxaniidae|658|BOLD:ACL1658
Lauxania shewelli|[10647]|BBDEC433-09|Lauxaniidae|658|BOLD:ACF4012
Lauxania shewelli|[10648]|SSBRA304-14|Lauxaniidae|664|BOLD:ACF2909
Lauxania shewelli|[10649]|BBDEE522-10|Lauxaniidae|658|BOLD:ACM8283
Lauxania shewelli|[10650]|BBDCN363-10|Lauxaniidae|658|BOLD:AAH3531
Lauxania|[10651]|CNLMN681-14|Lauxaniidae|654|BOLD:ACN8201
Lauxaniidae|[10652]|SSJAE784-13|Lauxaniidae|658|BOLD:ACJ4930
Lauxaniidae|[10653]|SSWLD1504-13|Lauxaniidae|673|BOLD:AAP8083
Lauxaniidae|[10654]|CNPAM569-13|Lauxaniidae|661|BOLD:ACG7469
Lauxaniidae|[10655]|CNKLF2363-15|Lauxaniidae|555|BOLD:ACT0356
Lauxaniidae|[10656]|CNWLE392-12|Lauxaniidae|673|BOLD:ACC9285
Lauxania nigrimana|[10657]|CNKLG408-15|Lauxaniidae|579|BOLD:ACS3429
Melanomyza gracilipes|[10658]|BBDED053-10|Lauxaniidae|658|BOLD:AAH3544
Lauxaniidae|[10659]|CNPPB782-12|Lauxaniidae|661|BOLD:ACD8902
Poecilominettia puncticeps|[10660]|CNPPC463-12|Lauxaniidae|658|BOLD:AAN8633
Dryomyza simplex|[10661]|BBDEC703-09|Dryomyzidae|658|BOLD:AAH0831
Oedoparena|[10662]|BBDCM530-10|Dryomyzidae|658|BOLD:AAP5918
Dryomyza setosa|[10663]|BBDCP419-10|Dryomyzidae|658|BOLD:AAI6087
Dryope decrepita|[10664]|CNEIH429-13|Dryomyzidae|658|BOLD:ACB3771
Dryomyza|[10665]|BBDEC721-09|Dryomyzidae|658|BOLD:AAH0832
Dryomyzidae|[10666]|CNPAP003-13|Dryomyzidae|673|BOLD:ACI7413
Lauxaniidae|[10667]|BBDEE523-10|Lauxaniidae|656|BOLD:ABY3255
Lauxaniidae|[10668]|JSDIS242-11|Lauxaniidae|658|BOLD:ABV3152
Homoneura|[10669]|CNPPB1385-12|Lauxaniidae|661|BOLD:ACP7693
Homoneura|[10670]|HPPPK318-13|Lauxaniidae|661|BOLD:ACP7658
Homoneura|[10671]|CNPPB1185-12|Lauxaniidae|658|BOLD:ACP7694
Homoneura|[10672]|CNMIH772-14|Lauxaniidae|534|BOLD:ACP7583
Lauxaniidae|[10673]|BBDCN851-10|Lauxaniidae|658|BOLD:ACP9654
Lauxaniidae|[10674]|BBDED276-10|Lauxaniidae|658|BOLD:AAH3545
Lauxaniidae|[10675]|BBDEE558-10|Lauxaniidae|645|BOLD:ACP7695
Lauxaniidae|[10676]|BBDED130-10|Lauxaniidae|658|BOLD:ACP9652
Homoneura|[10677]|SSGBA5007-14|Lauxaniidae|576|BOLD:ACP7692
Lauxaniidae|[10678]|BBDCM232-10|Lauxaniidae|658|BOLD:ACM0858
Homoneura fraterna|[10679]|BBDCM233-10|Lauxaniidae|658|BOLD:AAH3534
Homoneura sp. TAW1|[10680]|JWDCH080-10|Lauxaniidae|658|BOLD:AAL8931
Homoneura|[10681]|CNGRD424-12|Lauxaniidae|658|BOLD:ACB1411
Homoneura|[10682]|CNROH370-13|Lauxaniidae|661|BOLD:ACL8123
Homoneura phildelphica|[10683]|CNBRF825-14|Lauxaniidae|661|BOLD:AAU4698
Homoneura|[10684]|HEAUG1672-12|Lauxaniidae|703|BOLD:ACL8122
Homoneura|[10685]|CNBRF267-14|Lauxaniidae|664|BOLD:ACL8124
Homoneura phildelphica|[10686]|CNBRH203-14|Lauxaniidae|567|BOLD:ACL8542
Homoneura bispina|[10687]|SSROB965-14|Lauxaniidae|603|BOLD:ABW3687
Lauxaniidae|[10688]|BBDCM441-10|Lauxaniidae|658|BOLD:AAI1866
Homoneura incerta|[10689]|BBDEC682-09|Lauxaniidae|658|BOLD:AAH3542
Homoneura incerta|[10690]|BBDED037-10|Lauxaniidae|658|BOLD:ACT6509
Homoneura|[10691]|SMTPJ3036-14|Lauxaniidae|610|BOLD:ACV0948
Lauxaniidae|[10692]|BBDCN061-10|Lauxaniidae|658|BOLD:AAH3533
Lauxaniidae|[10693]|CNSIB1396-15|Lauxaniidae|603|BOLD:ACS7310
Lauxaniidae|[10694]|CNSIC1925-15|Lauxaniidae|522|BOLD:ACU5184
Lauxaniidae|[10695]|CNSIC2165-15|Lauxaniidae|600|BOLD:ACZ3208
Minilimosina parva|[10696]|CNEID2013-12|Sphaeroceridae|658|BOLD:ACB2682
Ferdinandea buccata|[10697]|BBDCP891-10|Syrphidae|658|BOLD:AAE0948
Ferdinandea croesus|[10698]|SSBAD2900-12|Syrphidae|661|BOLD:ACT5672

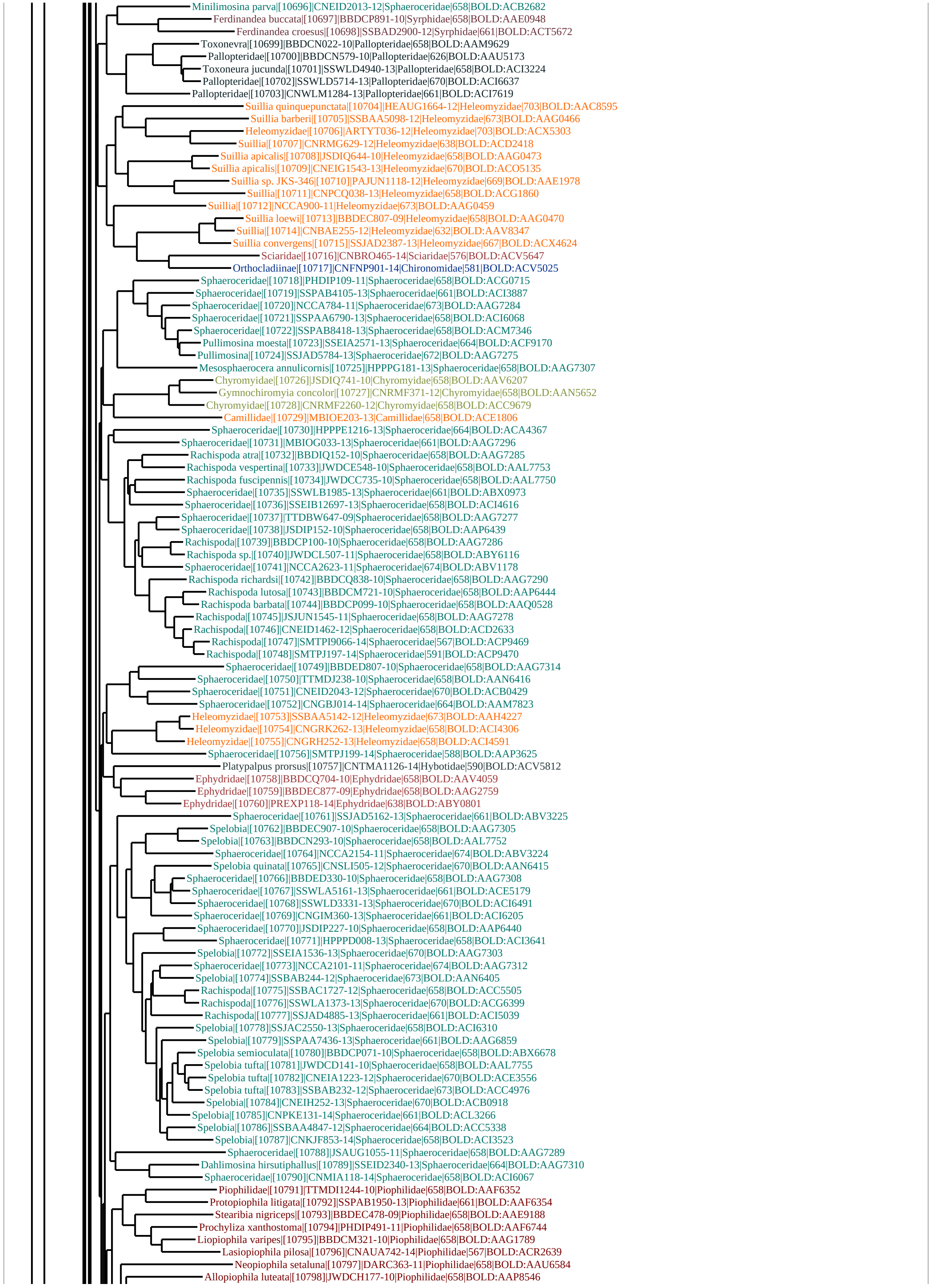

Minilimosina parva|[10696]|CNEID2013-12|Sphaeroceridae|658|BOLD:ACB2682
Ferdinandea buccata|[10697]|BBDCP891-10|Syrphidae|658|BOLD:AAE0948
Ferdinandea croesus|[10698]|SSBAD2900-12|Syrphidae|661|BOLD:ACT5672
Toxonevra|[10699]|BBDCN022-10|Pallopteridae|658|BOLD:AAM9629
Pallopteridae|[10700]|BBDCN579-10|Pallopteridae|626|BOLD:AAU5173
Toxoneura jucunda|[10701]|SSWLD4940-13|Pallopteridae|658|BOLD:ACI3224
Pallopteridae|[10702]|SSWLD5714-13|Pallopteridae|670|BOLD:ACI6637
Pallopteridae|[10703]|CNWLM1284-13|Pallopteridae|661|BOLD:ACI7619
Suillia quinquepunctata|[10704]|HEAUG1664-12|Heleomyzidae|703|BOLD:AAC8595
Suillia barberi|[10705]|SSBAA5098-12|Heleomyzidae|673|BOLD:AAG0466
Heleomyzidae|[10706]|ARTYT036-12|Heleomyzidae|703|BOLD:ACX5303
Suillia|[10707]|CNRMG629-12|Heleomyzidae|638|BOLD:ACD2418
Suillia apicalis|[10708]|JSDIQ644-10|Heleomyzidae|658|BOLD:AAG0473
Suillia apicalis|[10709]|CNEIG1543-13|Heleomyzidae|670|BOLD:ACO5135
Suillia sp. JKS-346|[10710]|PAJUN1118-12|Heleomyzidae|669|BOLD:AAE1978
Suillia|[10711]|CNPCQ038-13|Heleomyzidae|658|BOLD:ACG1860
Suillia|[10712]|NCCA900-11|Heleomyzidae|673|BOLD:AAG0459
Suillia loewi|[10713]|BBDEC807-09|Heleomyzidae|658|BOLD:AAG0470
Suillia|[10714]|CNBAE255-12|Heleomyzidae|632|BOLD:AAV8347
Suillia convergens|[10715]|SSJAD2387-13|Heleomyzidae|667|BOLD:ACX4624
Sciaridae|[10716]|CNBRO465-14|Sciaridae|576|BOLD:ACV5647
Orthocladiinae|[10717]|CNFNP901-14|Chironomidae|581|BOLD:ACV5025
Sphaeroceridae|[10718]|PHDIP109-11|Sphaeroceridae|658|BOLD:ACG0715
Sphaeroceridae|[10719]|SSPAB4105-13|Sphaeroceridae|661|BOLD:ACI3887
Sphaeroceridae|[10720]|NCCA784-11|Sphaeroceridae|673|BOLD:AAG7284
Sphaeroceridae|[10721]|SSPAA6790-13|Sphaeroceridae|658|BOLD:ACI6068
Sphaeroceridae|[10722]|SSPAB8418-13|Sphaeroceridae|658|BOLD:ACM7346
Pullimosina moesta|[10723]|SSEIA2571-13|Sphaeroceridae|664|BOLD:ACF9170
Pullimosina|[10724]|SSJAD5784-13|Sphaeroceridae|672|BOLD:AAG7275
Mesosphaerocera annulicornis|[10725]|HPPPG181-13|Sphaeroceridae|658|BOLD:AAG7307
Chyromyidae|[10726]|JSDIQ741-10|Chyromyidae|658|BOLD:AAV6207
Gymnochiromyia concolor|[10727]|CNRMF371-12|Chyromyidae|658|BOLD:AAN5652
Chyromyidae|[10728]|CNRMF2260-12|Chyromyidae|658|BOLD:ACC9679
Camillidae|[10729]|MBIOE203-13|Camillidae|658|BOLD:ACE1806
Sphaeroceridae|[10730]|HPPPE1216-13|Sphaeroceridae|664|BOLD:ACA4367
Sphaeroceridae|[10731]|MBIOG033-13|Sphaeroceridae|661|BOLD:AAG7296
Rachispoda atra|[10732]|BBDIQ152-10|Sphaeroceridae|658|BOLD:AAG7285
Rachispoda vespertina|[10733]|JWDCE548-10|Sphaeroceridae|658|BOLD:AAL7753
Rachispoda fuscipennis|[10734]|JWDCC735-10|Sphaeroceridae|658|BOLD:AAL7750
Sphaeroceridae|[10735]|SSWLB1985-13|Sphaeroceridae|661|BOLD:ABX0973
Sphaeroceridae|[10736]|SSEIB12697-13|Sphaeroceridae|658|BOLD:ACI4616
Sphaeroceridae|[10737]|TTDBW647-09|Sphaeroceridae|658|BOLD:AAG7277
Sphaeroceridae|[10738]|JSDIP152-10|Sphaeroceridae|658|BOLD:AAP6439
Rachispoda|[10739]|BBDCP100-10|Sphaeroceridae|658|BOLD:AAG7286
Rachispoda sp.|[10740]|JWDCL507-11|Sphaeroceridae|658|BOLD:ABY6116
Sphaeroceridae|[10741]|NCCA2623-11|Sphaeroceridae|674|BOLD:ABV1178
Rachispoda richardsi|[10742]|BBDCQ838-10|Sphaeroceridae|658|BOLD:AAG7290
Rachispoda lutosa|[10743]|BBDCM721-10|Sphaeroceridae|658|BOLD:AAP6444
Rachispoda barbata|[10744]|BBDCP099-10|Sphaeroceridae|658|BOLD:AAQ0528
Rachispoda|[10745]|JSJUN1545-11|Sphaeroceridae|658|BOLD:AAG7278
Rachispoda|[10746]|CNEID1462-12|Sphaeroceridae|658|BOLD:ACD2633
Rachispoda|[10747]|SMTPI9066-14|Sphaeroceridae|567|BOLD:ACP9469
Rachispoda|[10748]|SMTPJ197-14|Sphaeroceridae|591|BOLD:ACP9470
Sphaeroceridae|[10749]|BBDED807-10|Sphaeroceridae|658|BOLD:AAG7314
Sphaeroceridae|[10750]|TTMDJ238-10|Sphaeroceridae|658|BOLD:AAN6416
Sphaeroceridae|[10751]|CNEID2043-12|Sphaeroceridae|670|BOLD:ACB0429
Sphaeroceridae|[10752]|CNGBJ014-14|Sphaeroceridae|664|BOLD:AAM7823
Heleomyzidae|[10753]|SSBAA5142-12|Heleomyzidae|673|BOLD:AAH4227
Heleomyzidae|[10754]|CNGRK262-13|Heleomyzidae|658|BOLD:ACI4306
Heleomyzidae|[10755]|CNGRH252-13|Heleomyzidae|658|BOLD:ACI4591
Sphaeroceridae|[10756]|SMTPJ199-14|Sphaeroceridae|588|BOLD:AAP3625
Platypalpus prorsus|[10757]|CNTMA1126-14|Hybotidae|590|BOLD:ACV5812
Ephydridae|[10758]|BBDCQ704-10|Ephydridae|658|BOLD:AAV4059
Ephydridae|[10759]|BBDEC877-09|Ephydridae|658|BOLD:AAG2759
Ephydridae|[10760]|PREXP118-14|Ephydridae|638|BOLD:ABY0801
Sphaeroceridae|[10761]|SSJAD5162-13|Sphaeroceridae|661|BOLD:ABV3225
Spelobia|[10762]|BBDEC907-10|Sphaeroceridae|658|BOLD:AAG7305
Spelobia|[10763]|BBDCN293-10|Sphaeroceridae|658|BOLD:AAL7752
Sphaeroceridae|[10764]|NCCA2154-11|Sphaeroceridae|674|BOLD:ABV3224
Spelobia quinata|[10765]|CNSLI505-12|Sphaeroceridae|670|BOLD:AAN6415
Sphaeroceridae|[10766]|BBDED330-10|Sphaeroceridae|658|BOLD:AAG7308
Sphaeroceridae|[10767]|SSWLA5161-13|Sphaeroceridae|661|BOLD:ACE5179
Sphaeroceridae|[10768]|SSWLD3331-13|Sphaeroceridae|670|BOLD:ACI6491
Sphaeroceridae|[10769]|CNGIM360-13|Sphaeroceridae|661|BOLD:ACI6205
Sphaeroceridae|[10770]|JSDIP227-10|Sphaeroceridae|658|BOLD:AAP6440
Sphaeroceridae|[10771]|HPPPD008-13|Sphaeroceridae|658|BOLD:ACI3641
Spelobia|[10772]|SSEIA1536-13|Sphaeroceridae|670|BOLD:AAG7303
Sphaeroceridae|[10773]|NCCA2101-11|Sphaeroceridae|674|BOLD:AAG7312
Spelobia|[10774]|SSBAB244-12|Sphaeroceridae|673|BOLD:AAN6405
Rachispoda|[10775]|SSBAC1727-12|Sphaeroceridae|658|BOLD:ACC5505
Rachispoda|[10776]|SSWLA1373-13|Sphaeroceridae|670|BOLD:ACG6399
Rachispoda|[10777]|SSJAD4885-13|Sphaeroceridae|661|BOLD:ACI5039
Spelobia|[10778]|SSJAC2550-13|Sphaeroceridae|658|BOLD:ACI6310
Spelobia|[10779]|SSPAA7436-13|Sphaeroceridae|661|BOLD:AAG6859
Spelobia semioculata|[10780]|BBDCP071-10|Sphaeroceridae|658|BOLD:ABX6678
Spelobia tufta|[10781]|JWDCD141-10|Sphaeroceridae|658|BOLD:AAL7755
Spelobia tufta|[10782]|CNEIA1223-12|Sphaeroceridae|670|BOLD:ACE3556
Spelobia tufta|[10783]|SSBAB232-12|Sphaeroceridae|673|BOLD:ACC4976
Spelobia|[10784]|CNEIH252-13|Sphaeroceridae|670|BOLD:ACB0918
Spelobia|[10785]|CNPKE131-14|Sphaeroceridae|661|BOLD:ACL3266
Spelobia|[10786]|SSBAA4847-12|Sphaeroceridae|664|BOLD:ACC5338
Spelobia|[10787]|CNKJF853-14|Sphaeroceridae|658|BOLD:ACI3523
Sphaeroceridae|[10788]|JSAUG1055-11|Sphaeroceridae|658|BOLD:AAG7289
Dahlimosina hirsutiphallus|[10789]|SSEID2340-13|Sphaeroceridae|664|BOLD:AAG7310
Sphaeroceridae|[10790]|CNMIA118-14|Sphaeroceridae|658|BOLD:ACI6067
Piophilidae|[10791]|TTMDI1244-10|Piophilidae|658|BOLD:AAF6352
Protopiophila litigata|[10792]|SSPAB1950-13|Piophilidae|661|BOLD:AAF6354
Stearibia nigriceps|[10793]|BBDEC478-09|Piophilidae|658|BOLD:AAE9188
Prochyliza xanthostoma|[10794]|PHDIP491-11|Piophilidae|658|BOLD:AAF6744
Liopiophila varipes|[10795]|BBDCM321-10|Piophilidae|658|BOLD:AAG1789
Lasiopiophila pilosa|[10796]|CNAUA742-14|Piophilidae|567|BOLD:ACR2639
Neopiophila setaluna|[10797]|DARC363-11|Piophilidae|658|BOLD:AAU6584
Allopiophila luteata|[10798]|JWDCH177-10|Piophilidae|658|BOLD:AAP8546

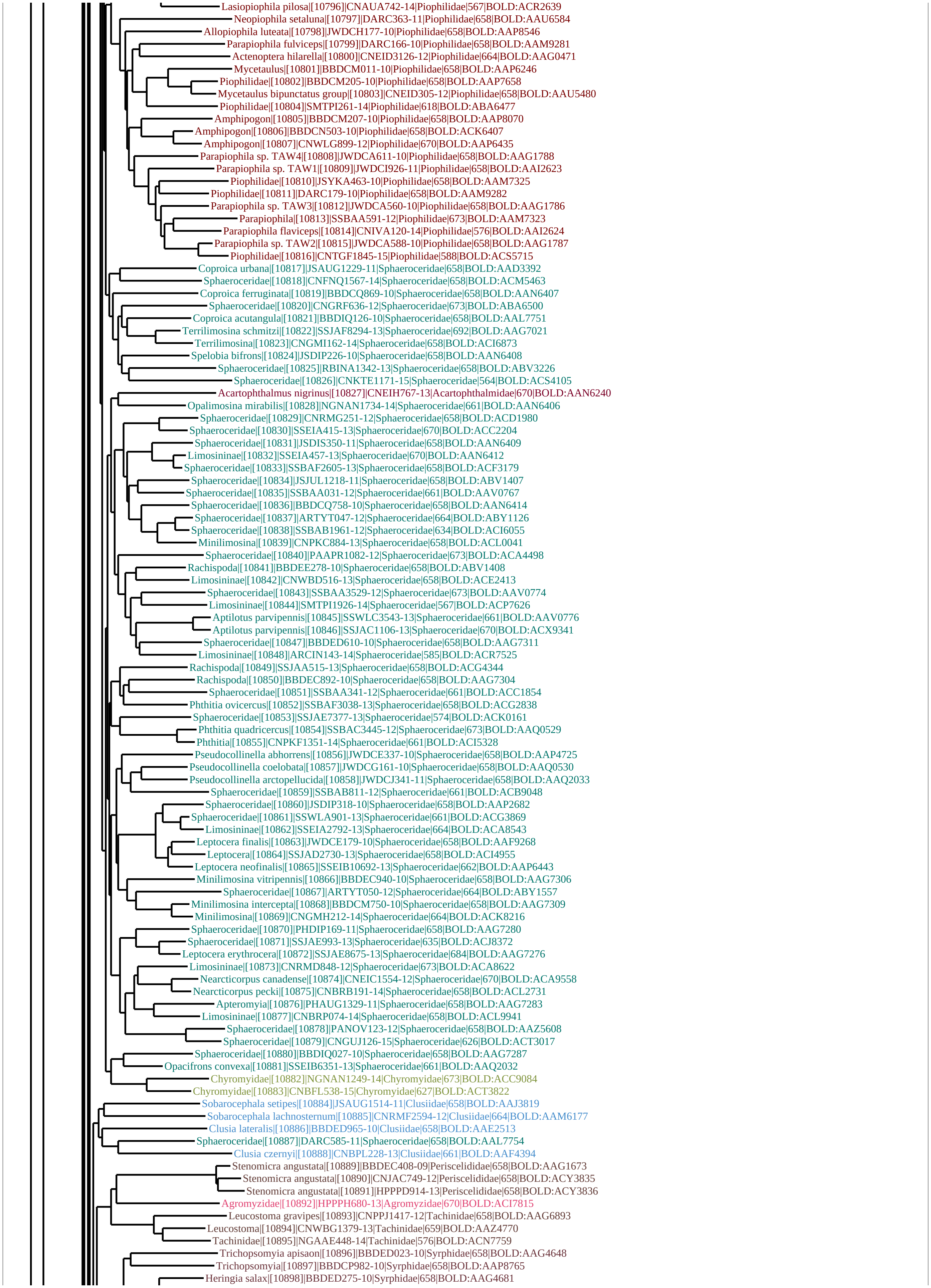

Lasiopiophila pilosa|[10796]|CNAUA742-14|Piophilidae|567|BOLD:ACR2639
Neopiophila setaluna|[10797]|DARC363-11|Piophilidae|658|BOLD:AAU6584
Allopiophila luteata|[10798]|JWDCH177-10|Piophilidae|658|BOLD:AAP8546
Parapiophila fulviceps|[10799]|DARC166-10|Piophilidae|658|BOLD:AAM9281
Actenoptera hilarella|[10800]|CNEID3126-12|Piophilidae|664|BOLD:AAG0471
Mycetaulus|[10801]|BBDCM011-10|Piophilidae|658|BOLD:AAP6246
Piophilidae|[10802]|BBDCM205-10|Piophilidae|658|BOLD:AAP7658
Mycetaulus bipunctatus group|[10803]|CNEID305-12|Piophilidae|658|BOLD:AAU5480
Piophilidae|[10804]|SMTPI261-14|Piophilidae|618|BOLD:ABA6477
Amphipogon|[10805]|BBDCM207-10|Piophilidae|658|BOLD:AAP8070
Amphipogon|[10806]|BBDCN503-10|Piophilidae|658|BOLD:ACK6407
Amphipogon|[10807]|CNWLG899-12|Piophilidae|670|BOLD:AAP6435
Parapiophila sp. TAW4|[10808]|JWDCA611-10|Piophilidae|658|BOLD:AAG1788
Parapiophila sp. TAW1|[10809]|JWDCI926-11|Piophilidae|658|BOLD:AAI2623
Piophilidae|[10810]|JSYKA463-10|Piophilidae|658|BOLD:AAM7325
Piophilidae|[10811]|DARC179-10|Piophilidae|658|BOLD:AAM9282
Parapiophila sp. TAW3|[10812]|JWDCA560-10|Piophilidae|658|BOLD:AAG1786
Parapiophila|[10813]|SSBAA591-12|Piophilidae|673|BOLD:AAM7323
Parapiophila flaviceps|[10814]|CNIVA120-14|Piophilidae|576|BOLD:AAI2624
Parapiophila sp. TAW2|[10815]|JWDCA588-10|Piophilidae|658|BOLD:AAG1787
Piophilidae|[10816]|CNTGF1845-15|Piophilidae|588|BOLD:ACS5715
Coproica urbana|[10817]|JSAUG1229-11|Sphaeroceridae|658|BOLD:AAD3392
Sphaeroceridae|[10818]|CNFNQ1567-14|Sphaeroceridae|658|BOLD:ACM5463
Coproica ferruginata|[10819]|BBDCQ869-10|Sphaeroceridae|658|BOLD:AAN6407
Sphaeroceridae|[10820]|CNGRF636-12|Sphaeroceridae|673|BOLD:ABA6500
Coproica acutangula|[10821]|BBDIQ126-10|Sphaeroceridae|658|BOLD:AAL7751
Terrilimosina schmitzi|[10822]|SSJAF8294-13|Sphaeroceridae|692|BOLD:AAG7021
Terrilimosina|[10823]|CNGMI162-14|Sphaeroceridae|658|BOLD:ACI6873
Spelobia bifrons|[10824]|JSDIP226-10|Sphaeroceridae|658|BOLD:AAN6408
Sphaeroceridae|[10825]|RBINA1342-13|Sphaeroceridae|658|BOLD:ABV3226
Sphaeroceridae|[10826]|CNKTE1171-15|Sphaeroceridae|564|BOLD:ACS4105
Acartophthalmus nigrinus|[10827]|CNEIH767-13|Acartophthalmidae|670|BOLD:AAN6240
Opalimosina mirabilis|[10828]|NGNAN1734-14|Sphaeroceridae|661|BOLD:AAN6406
Sphaeroceridae|[10829]|CNRMG251-12|Sphaeroceridae|658|BOLD:ACD1980
Sphaeroceridae|[10830]|SSEIA415-13|Sphaeroceridae|670|BOLD:ACC2204
Sphaeroceridae|[10831]|JSDIS350-11|Sphaeroceridae|658|BOLD:AAN6409
Limosininae|[10832]|SSEIA457-13|Sphaeroceridae|670|BOLD:AAN6412
Sphaeroceridae|[10833]|SSBAF2605-13|Sphaeroceridae|658|BOLD:ACF3179
Sphaeroceridae|[10834]|JSJUL1218-11|Sphaeroceridae|658|BOLD:ABV1407
Sphaeroceridae|[10835]|SSBAA031-12|Sphaeroceridae|661|BOLD:AAV0767
Sphaeroceridae|[10836]|BBDCQ758-10|Sphaeroceridae|658|BOLD:AAN6414
Sphaeroceridae|[10837]|ARTYT047-12|Sphaeroceridae|664|BOLD:ABY1126
Sphaeroceridae|[10838]|SSBAB1961-12|Sphaeroceridae|634|BOLD:ACI6055
Minilimosina|[10839]|CNPKC884-13|Sphaeroceridae|658|BOLD:ACL0041
Sphaeroceridae|[10840]|PAAPR1082-12|Sphaeroceridae|673|BOLD:ACA4498
Rachispoda|[10841]|BBDEE278-10|Sphaeroceridae|658|BOLD:ABV1408
Limosininae|[10842]|CNWBD516-13|Sphaeroceridae|658|BOLD:ACE2413
Sphaeroceridae|[10843]|SSBAA3529-12|Sphaeroceridae|673|BOLD:AAV0774
Limosininae|[10844]|SMTPI1926-14|Sphaeroceridae|567|BOLD:ACP7626
Aptilotus parvipennis|[10845]|SSWLC3543-13|Sphaeroceridae|661|BOLD:AAV0776
Aptilotus parvipennis|[10846]|SSJAC1106-13|Sphaeroceridae|670|BOLD:ACX9341
Sphaeroceridae|[10847]|BBDED610-10|Sphaeroceridae|658|BOLD:AAG7311
Limosininae|[10848]|ARCIN143-14|Sphaeroceridae|585|BOLD:ACR7525
Rachispoda|[10849]|SSJAA515-13|Sphaeroceridae|658|BOLD:ACG4344
Rachispoda|[10850]|BBDEC892-10|Sphaeroceridae|658|BOLD:AAG7304
Sphaeroceridae|[10851]|SSBAA341-12|Sphaeroceridae|661|BOLD:ACC1854
Phthitia ovicercus|[10852]|SSBAF3038-13|Sphaeroceridae|658|BOLD:ACG2838
Sphaeroceridae|[10853]|SSJAE7377-13|Sphaeroceridae|574|BOLD:ACK0161
Phthitia quadricercus|[10854]|SSBAC3445-12|Sphaeroceridae|673|BOLD:AAQ0529
Phthitia|[10855]|CNPKF1351-14|Sphaeroceridae|661|BOLD:ACI5328
Pseudocollinella abhorrens|[10856]|JWDCE337-10|Sphaeroceridae|658|BOLD:AAP4725
Pseudocollinella coelobata|[10857]|JWDCG161-10|Sphaeroceridae|658|BOLD:AAQ0530
Pseudocollinella arctopellucida|[10858]|JWDCJ341-11|Sphaeroceridae|658|BOLD:AAQ2033
Sphaeroceridae|[10859]|SSBAB811-12|Sphaeroceridae|661|BOLD:ACB9048
Sphaeroceridae|[10860]|JSDIP318-10|Sphaeroceridae|658|BOLD:AAP2682
Sphaeroceridae|[10861]|SSWLA901-13|Sphaeroceridae|661|BOLD:ACG3869
Limosininae|[10862]|SSEIA2792-13|Sphaeroceridae|664|BOLD:ACA8543
Leptocera finalis|[10863]|JWDCE179-10|Sphaeroceridae|658|BOLD:AAF9268
Leptocera|[10864]|SSJAD2730-13|Sphaeroceridae|658|BOLD:ACI4955
Leptocera neofinalis|[10865]|SSEIB10692-13|Sphaeroceridae|662|BOLD:AAP6443
Minilimosina vitripennis|[10866]|BBDEC940-10|Sphaeroceridae|658|BOLD:AAG7306
Sphaeroceridae|[10867]|ARTYT050-12|Sphaeroceridae|664|BOLD:ABY1557
Minilimosina intercepta|[10868]|BBDCM750-10|Sphaeroceridae|658|BOLD:AAG7309
Minilimosina|[10869]|CNGMH212-14|Sphaeroceridae|664|BOLD:ACK8216
Sphaeroceridae|[10870]|PHDIP169-11|Sphaeroceridae|658|BOLD:AAG7280
Sphaeroceridae|[10871]|SSJAE993-13|Sphaeroceridae|635|BOLD:ACJ8372
Leptocera erythrocera|[10872]|SSJAE8675-13|Sphaeroceridae|684|BOLD:AAG7276
Limosininae|[10873]|CNRMD848-12|Sphaeroceridae|673|BOLD:ACA8622
Nearcticorpus canadense|[10874]|CNEIC1554-12|Sphaeroceridae|670|BOLD:ACA9558
Nearcticorpus pecki|[10875]|CNBRB191-14|Sphaeroceridae|658|BOLD:ACL2731
Apteromyia|[10876]|PHAUG1329-11|Sphaeroceridae|658|BOLD:AAG7283
Limosininae|[10877]|CNBRP074-14|Sphaeroceridae|658|BOLD:ACL9941
Sphaeroceridae|[10878]|PANOV123-12|Sphaeroceridae|658|BOLD:AAZ5608
Sphaeroceridae|[10879]|CNGUJ126-15|Sphaeroceridae|626|BOLD:ACT3017
Sphaeroceridae|[10880]|BBDIQ027-10|Sphaeroceridae|658|BOLD:AAG7287
Opacifrons convexa|[10881]|SSEIB6351-13|Sphaeroceridae|661|BOLD:AAQ2032
Chyromyidae|[10882]|NGNAN1249-14|Chyromyidae|673|BOLD:ACC9084
Chyromyidae|[10883]|CNBFL538-15|Chyromyidae|627|BOLD:ACT3822
Sobarocephala setipes|[10884]|JSAUG1514-11|Clusiidae|658|BOLD:AAJ3819
Sobarocephala lachnosternum|[10885]|CNRMF2594-12|Clusiidae|664|BOLD:AAM6177
Clusia lateralis|[10886]|BBDED965-10|Clusiidae|658|BOLD:AAE2513
Sphaeroceridae|[10887]|DARC585-11|Sphaeroceridae|658|BOLD:AAL7754
Clusia czernyi|[10888]|CNBPL228-13|Clusiidae|661|BOLD:AAF4394
Stenomicra angustata|[10889]|BBDEC408-09|Periscelididae|658|BOLD:AAG1673
Stenomicra angustata|[10890]|CNJAC749-12|Periscelididae|658|BOLD:ACY3835
Stenomicra angustata|[10891]|HPPPD914-13|Periscelididae|658|BOLD:ACY3836
Agromyzidae|[10892]|HPPPH680-13|Agromyzidae|670|BOLD:ACI7815
Leucostoma gravipes|[10893]|CNPPJ1417-12|Tachinidae|658|BOLD:AAG6893
Leucostoma|[10894]|CNWBG1379-13|Tachinidae|659|BOLD:AAZ4770
Tachinidae|[10895]|NGAAE448-14|Tachinidae|576|BOLD:ACN7759
Trichopsomyia apisaon|[10896]|BBDED023-10|Syrphidae|658|BOLD:AAG4648
Trichopsomyia|[10897]|BBDCP982-10|Syrphidae|658|BOLD:AAP8765
Heringia salax|[10898]|BBDED275-10|Syrphidae|658|BOLD:AAG4681

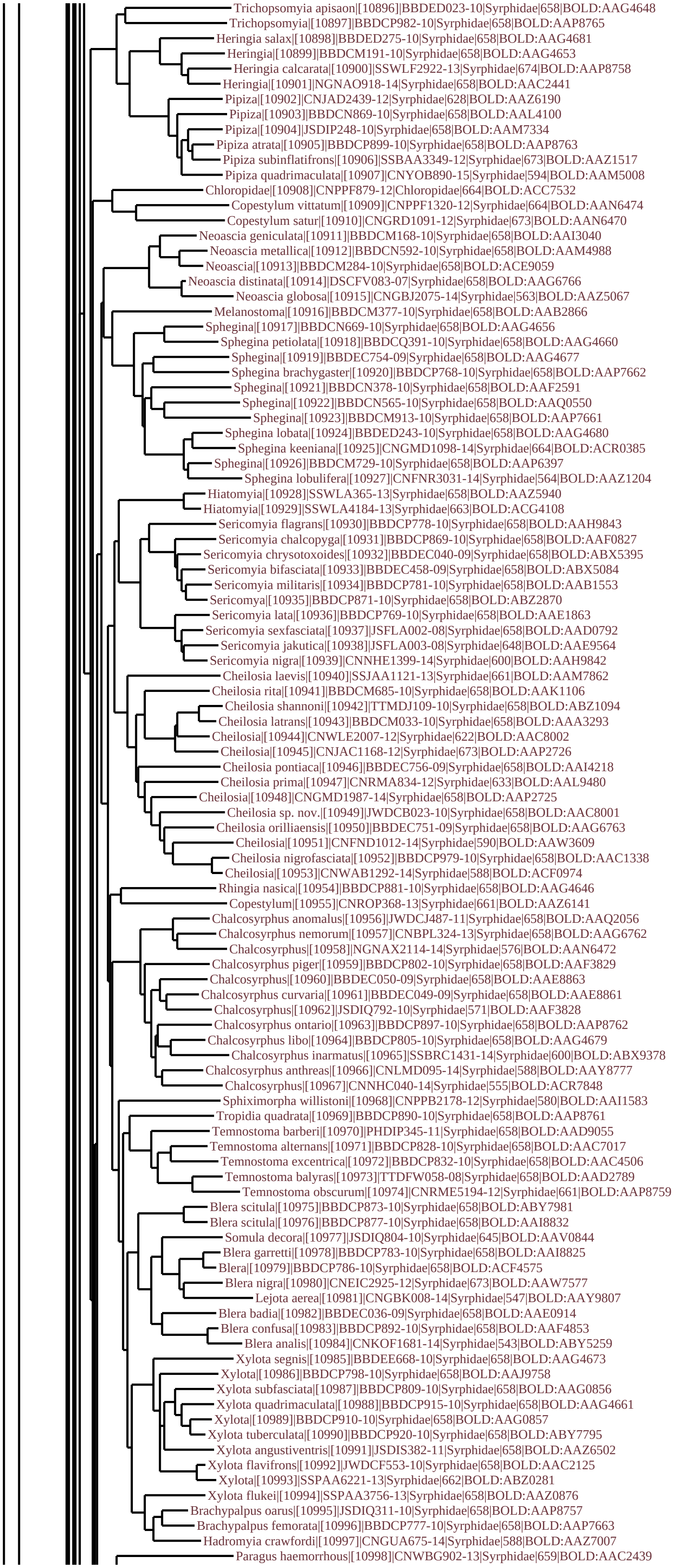
Trichopsomyia apisaon|[10896]|BBDED023-10|Syrphidae|658|BOLD:AAG4648
Trichopsomyia|[10897]|BBDCP982-10|Syrphidae|658|BOLD:AAP8765
Heringia salax|[10898]|BBDED275-10|Syrphidae|658|BOLD:AAG4681
Heringia|[10899]|BBDCM191-10|Syrphidae|658|BOLD:AAG4653
Heringia calcarata|[10900]|SSWLF2922-13|Syrphidae|674|BOLD:AAP8758
Heringia|[10901]|NGNAO918-14|Syrphidae|658|BOLD:AAC2441
Pipiza|[10902]|CNJAD2439-12|Syrphidae|628|BOLD:AAZ6190
Pipiza|[10903]|BBDCN869-10|Syrphidae|658|BOLD:AAL4100
Pipiza|[10904]|JSDIP248-10|Syrphidae|658|BOLD:AAM7334
Pipiza atrata|[10905]|BBDCP899-10|Syrphidae|658|BOLD:AAP8763
Pipiza subinflatifrons|[10906]|SSBAA3349-12|Syrphidae|673|BOLD:AAZ1517
Pipiza quadrimaculata|[10907]|CNYOB890-15|Syrphidae|594|BOLD:AAM5008
Chloropidae|[10908]|CNPPF879-12|Chloropidae|664|BOLD:ACC7532
Copestylum vittatum|[10909]|CNPPF1320-12|Syrphidae|664|BOLD:AAN6474
Copestylum satur|[10910]|CNGRD1091-12|Syrphidae|673|BOLD:AAN6470
Neoascia geniculata|[10911]|BBDCM168-10|Syrphidae|658|BOLD:AAI3040
Neoascia metallica|[10912]|BBDCN592-10|Syrphidae|658|BOLD:AAM4988
Neoascia|[10913]|BBDCM284-10|Syrphidae|658|BOLD:ACE9059
Neoascia distinata|[10914]|DSCFV083-07|Syrphidae|658|BOLD:AAG6766
Neoascia globosa|[10915]|CNGBJ2075-14|Syrphidae|563|BOLD:AAZ5067
Melanostoma|[10916]|BBDCM377-10|Syrphidae|658|BOLD:AAB2866
Sphegina|[10917]|BBDCN669-10|Syrphidae|658|BOLD:AAG4656
Sphegina petiolata|[10918]|BBDCQ391-10|Syrphidae|658|BOLD:AAG4660
Sphegina|[10919]|BBDEC754-09|Syrphidae|658|BOLD:AAG4677
Sphegina brachygaster|[10920]|BBDCP768-10|Syrphidae|658|BOLD:AAP7662
Sphegina|[10921]|BBDCN378-10|Syrphidae|658|BOLD:AAF2591
Sphegina|[10922]|BBDCN565-10|Syrphidae|658|BOLD:AAQ0550
Sphegina|[10923]|BBDCM913-10|Syrphidae|658|BOLD:AAP7661
Sphegina lobata|[10924]|BBDED243-10|Syrphidae|658|BOLD:AAG4680
Sphegina keeniana|[10925]|CNGMD1098-14|Syrphidae|664|BOLD:ACR0385
Sphegina|[10926]|BBDCM729-10|Syrphidae|658|BOLD:AAP6397
Sphegina lobulifera|[10927]|CNFNR3031-14|Syrphidae|564|BOLD:AAZ1204
Hiatomyia|[10928]|SSWLA365-13|Syrphidae|658|BOLD:AAZ5940
Hiatomyia|[10929]|SSWLA4184-13|Syrphidae|663|BOLD:ACG4108
Sericomyia flagrans|[10930]|BBDCP778-10|Syrphidae|658|BOLD:AAH9843
Sericomyia chalcopyga|[10931]|BBDCP869-10|Syrphidae|658|BOLD:AAF0827
Sericomyia chrysotoxoides|[10932]|BBDEC040-09|Syrphidae|658|BOLD:ABX5395
Sericomyia bifasciata|[10933]|BBDEC458-09|Syrphidae|658|BOLD:ABX5084
Sericomyia militaris|[10934]|BBDCP781-10|Syrphidae|658|BOLD:AAB1553
Sericomya|[10935]|BBDCP871-10|Syrphidae|658|BOLD:ABZ2870
Sericomyia lata|[10936]|BBDCP769-10|Syrphidae|658|BOLD:AAE1863
Sericomyia sexfasciata|[10937]|JSFLA002-08|Syrphidae|658|BOLD:AAD0792
Sericomyia jakutica|[10938]|JSFLA003-08|Syrphidae|648|BOLD:AAE9564
Sericomyia nigra|[10939]|CNNHE1399-14|Syrphidae|600|BOLD:AAH9842
Cheilosia laevis|[10940]|SSJAA1121-13|Syrphidae|661|BOLD:AAM7862
Cheilosia rita|[10941]|BBDCM685-10|Syrphidae|658|BOLD:AAK1106
Cheilosia shannoni|[10942]|TTMDJ109-10|Syrphidae|658|BOLD:ABZ1094
Cheilosia latrans|[10943]|BBDCM033-10|Syrphidae|658|BOLD:AAA3293
Cheilosia|[10944]|CNWLE2007-12|Syrphidae|622|BOLD:AAC8002
Cheilosia|[10945]|CNJAC1168-12|Syrphidae|673|BOLD:AAP2726
Cheilosia pontiaca|[10946]|BBDEC756-09|Syrphidae|658|BOLD:AAI4218
Cheilosia prima|[10947]|CNRMA834-12|Syrphidae|633|BOLD:AAL9480
Cheilosia|[10948]|CNGMD1987-14|Syrphidae|658|BOLD:AAP2725
Cheilosia sp. nov.|[10949]|JWDCB023-10|Syrphidae|658|BOLD:AAC8001
Cheilosia orilliaensis|[10950]|BBDEC751-09|Syrphidae|658|BOLD:AAG6763
Cheilosia|[10951]|CNFND1012-14|Syrphidae|590|BOLD:AAW3609
Cheilosia nigrofasciata|[10952]|BBDCP979-10|Syrphidae|658|BOLD:AAC1338
Cheilosia|[10953]|CNWAB1292-14|Syrphidae|588|BOLD:ACF0974
Rhingia nasica|[10954]|BBDCP881-10|Syrphidae|658|BOLD:AAG4646
Copestylum|[10955]|CNROP368-13|Syrphidae|661|BOLD:AAZ6141
Chalcosyrphus anomalus|[10956]|JWDCJ487-11|Syrphidae|658|BOLD:AAQ2056
Chalcosyrphus nemorum|[10957]|CNBPL324-13|Syrphidae|658|BOLD:AAG6762
Chalcosyrphus|[10958]|NGNAX2114-14|Syrphidae|576|BOLD:AAN6472
Chalcosyrphus piger|[10959]|BBDCP802-10|Syrphidae|658|BOLD:AAF3829
Chalcosyrphus|[10960]|BBDEC050-09|Syrphidae|658|BOLD:AAE8863
Chalcosyrphus curvaria|[10961]|BBDEC049-09|Syrphidae|658|BOLD:AAE8861
Chalcosyrphus|[10962]|JSDIQ792-10|Syrphidae|571|BOLD:AAF3828
Chalcosyrphus ontario|[10963]|BBDCP897-10|Syrphidae|658|BOLD:AAP8762
Chalcosyrphus libo|[10964]|BBDCP805-10|Syrphidae|658|BOLD:AAG4679
Chalcosyrphus inarmatus|[10965]|SSBRC1431-14|Syrphidae|600|BOLD:ABX9378
Chalcosyrphus anthreas|[10966]|CNLMD095-14|Syrphidae|588|BOLD:AAY8777
Chalcosyrphus|[10967]|CNNHC040-14|Syrphidae|555|BOLD:ACR7848
Sphiximorpha willistoni|[10968]|CNPPB2178-12|Syrphidae|580|BOLD:AAI1583
Tropidia quadrata|[10969]|BBDCP890-10|Syrphidae|658|BOLD:AAP8761
Temnostoma barberi|[10970]|PHDIP345-11|Syrphidae|658|BOLD:AAD9055
Temnostoma alternans|[10971]|BBDCP828-10|Syrphidae|658|BOLD:AAC7017
Temnostoma excentrica|[10972]|BBDCP832-10|Syrphidae|658|BOLD:AAC4506
Temnostoma balyras|[10973]|TTDFW058-08|Syrphidae|658|BOLD:AAD2789
Temnostoma obscurum|[10974]|CNRME5194-12|Syrphidae|661|BOLD:AAP8759
Blera scitula|[10975]|BBDCP873-10|Syrphidae|658|BOLD:ABY7981
Blera scitula|[10976]|BBDCP877-10|Syrphidae|658|BOLD:AAI8832
Somula decora|[10977]|JSDIQ804-10|Syrphidae|645|BOLD:AAV0844
Blera garretti|[10978]|BBDCP783-10|Syrphidae|658|BOLD:AAI8825
Blera|[10979]|BBDCP786-10|Syrphidae|658|BOLD:ACF4575
Blera nigra|[10980]|CNEIC2925-12|Syrphidae|673|BOLD:AAW7577
Lejota aerea|[10981]|CNGBK008-14|Syrphidae|547|BOLD:AAY9807
Blera badia|[10982]|BBDEC036-09|Syrphidae|658|BOLD:AAE0914
Blera confusa|[10983]|BBDCP892-10|Syrphidae|658|BOLD:AAF4853
Blera analis|[10984]|CNKOF1681-14|Syrphidae|543|BOLD:ABY5259
Xylota segnis|[10985]|BBDEE668-10|Syrphidae|658|BOLD:AAG4673
Xylota|[10986]|BBDCP798-10|Syrphidae|658|BOLD:AAJ9758
Xylota subfasciata|[10987]|BBDCP809-10|Syrphidae|658|BOLD:AAG0856
Xylota quadrimaculata|[10988]|BBDCP915-10|Syrphidae|658|BOLD:AAG4661
Xylota|[10989]|BBDCP910-10|Syrphidae|658|BOLD:AAG0857
Xylota tuberculata|[10990]|BBDCP920-10|Syrphidae|658|BOLD:ABY7795
Xylota angustiventris|[10991]|JSDIS382-11|Syrphidae|658|BOLD:AAZ6502
Xylota flavifrons|[10992]|JWDCF553-10|Syrphidae|658|BOLD:AAC2125
Xylota|[10993]|SSPAA6221-13|Syrphidae|662|BOLD:ABZ0281
Xylota flukei|[10994]|SSPAA3756-13|Syrphidae|658|BOLD:AAZ0876
Brachypalpus oarus|[10995]|JSDIQ311-10|Syrphidae|658|BOLD:AAP8757
Brachypalpus femorata|[10996]|BBDCP777-10|Syrphidae|658|BOLD:AAP7663
Hadromyia crawfordi|[10997]|CNGUA675-14|Syrphidae|588|BOLD:AAZ7007
Paragus haemorrhous|[10998]|CNWBG902-13|Syrphidae|659|BOLD:AAC2439

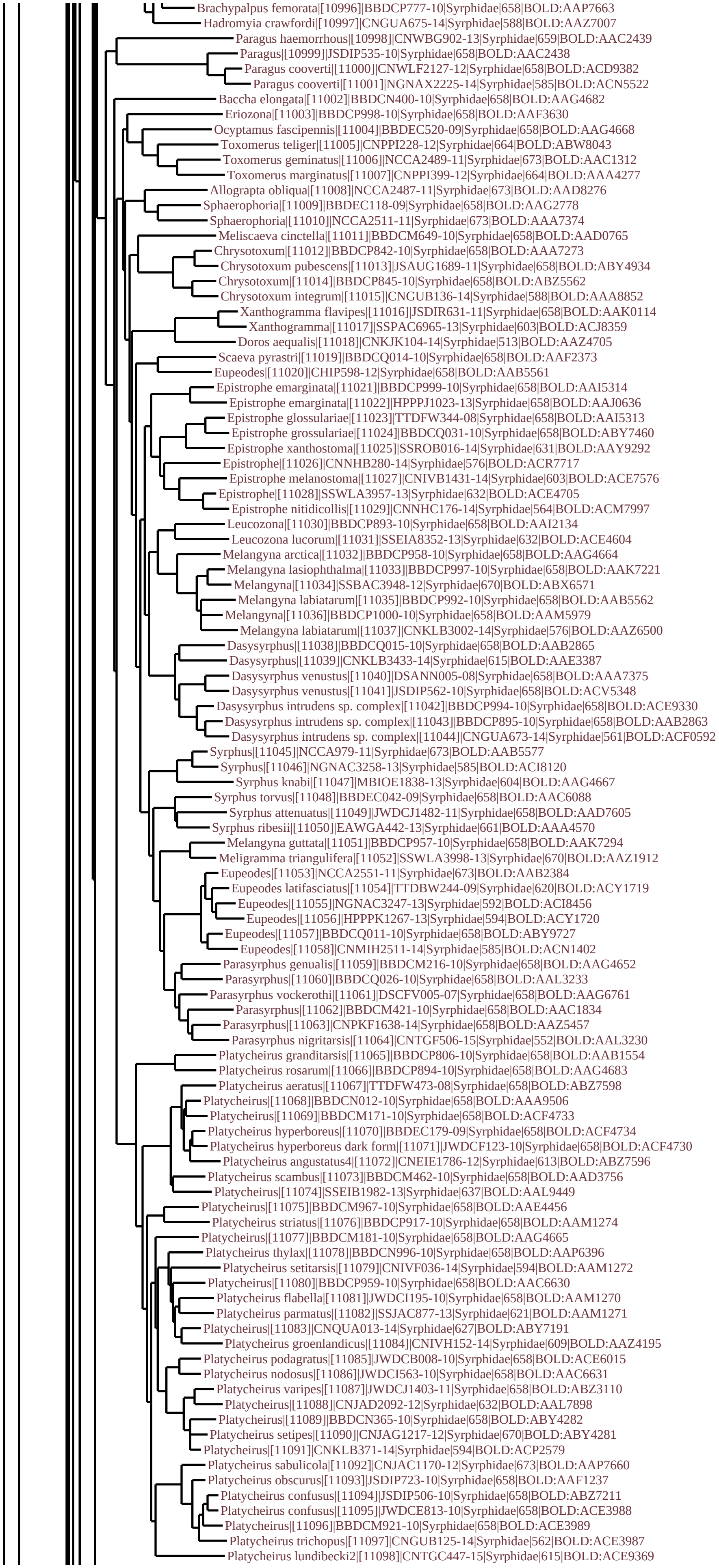

Brachypalpus femorata|[10996]|BBDCP777-10|Syrphidae|658|BOLD:AAP7663
Hadromyia crawfordi|[10997]|CNGUA675-14|Syrphidae|588|BOLD:AAZ7007
Paragus haemorrhous|[10998]|CNWBG902-13|Syrphidae|659|BOLD:AAC2439
Paragus|[10999]|JSDIP535-10|Syrphidae|658|BOLD:AAC2438
Paragus cooverti|[11000]|CNWLF2127-12|Syrphidae|658|BOLD:ACD9382
Paragus cooverti|[11001]|NGNAX2225-14|Syrphidae|585|BOLD:ACN5522
Baccha elongata|[11002]|BBDCN400-10|Syrphidae|658|BOLD:AAG4682
Eriozona|[11003]|BBDCP998-10|Syrphidae|658|BOLD:AAF3630
Ocyptamus fascipennis|[11004]|BBDEC520-09|Syrphidae|658|BOLD:AAG4668
Toxomerus teliger|[11005]|CNPPI228-12|Syrphidae|664|BOLD:ABW8043
Toxomerus geminatus|[11006]|NCCA2489-11|Syrphidae|673|BOLD:AAC1312
Toxomerus marginatus|[11007]|CNPPI399-12|Syrphidae|664|BOLD:AAA4277
Allograpta obliqua|[11008]|NCCA2487-11|Syrphidae|673|BOLD:AAD8276
Sphaerophoria|[11009]|BBDEC118-09|Syrphidae|658|BOLD:AAG2778
Sphaerophoria|[11010]|NCCA2511-11|Syrphidae|673|BOLD:AAA7374
Meliscaeva cinctella|[11011]|BBDCM649-10|Syrphidae|658|BOLD:AAD0765
Chrysotoxum|[11012]|BBDCP842-10|Syrphidae|658|BOLD:AAA7273
Chrysotoxum pubescens|[11013]|JSAUG1689-11|Syrphidae|658|BOLD:ABY4934
Chrysotoxum|[11014]|BBDCP845-10|Syrphidae|658|BOLD:ABZ5562
Chrysotoxum integrum|[11015]|CNGUB136-14|Syrphidae|588|BOLD:AAA8852
Xanthogramma flavipes|[11016]|JSDIR631-11|Syrphidae|658|BOLD:AAK0114
Xanthogramma|[11017]|SSPAC6965-13|Syrphidae|603|BOLD:ACJ8359
Doros aequalis|[11018]|CNKJK104-14|Syrphidae|513|BOLD:AAZ4705
Scaeva pyrastri|[11019]|BBDCQ014-10|Syrphidae|658|BOLD:AAF2373
Eupeodes|[11020]|CHIP598-12|Syrphidae|658|BOLD:AAB5561
Epistrophe emarginata|[11021]|BBDCP999-10|Syrphidae|658|BOLD:AAI5314
Epistrophe emarginata|[11022]|HPPPJ1023-13|Syrphidae|658|BOLD:AAJ0636
Epistrophe glossulariae|[11023]|TTDFW344-08|Syrphidae|658|BOLD:AAI5313
Epistrophe grossulariae|[11024]|BBDCQ031-10|Syrphidae|658|BOLD:ABY7460
Epistrophe xanthostoma|[11025]|SSROB016-14|Syrphidae|631|BOLD:AAY9292
Epistrophe|[11026]|CNNHB280-14|Syrphidae|576|BOLD:ACR7717
Epistrophe melanostoma|[11027]|CNIVB1431-14|Syrphidae|603|BOLD:ACE7576
Epistrophe|[11028]|SSWLA3957-13|Syrphidae|632|BOLD:ACE4705
Epistrophe nitidicollis|[11029]|CNNHC176-14|Syrphidae|564|BOLD:ACM7997
Leucozona|[11030]|BBDCP893-10|Syrphidae|658|BOLD:AAI2134
Leucozona lucorum|[11031]|SSEIA8352-13|Syrphidae|632|BOLD:ACE4604
Melangyna arctica|[11032]|BBDCP958-10|Syrphidae|658|BOLD:AAG4664
Melangyna lasiophthalma|[11033]|BBDCP997-10|Syrphidae|658|BOLD:AAK7221
Melangyna|[11034]|SSBAC3948-12|Syrphidae|670|BOLD:ABX6571
Melangyna labiatarum|[11035]|BBDCP992-10|Syrphidae|658|BOLD:AAB5562
Melangyna|[11036]|BBDCP1000-10|Syrphidae|658|BOLD:AAM5979
Melangyna labiatarum|[11037]|CNKLB3002-14|Syrphidae|576|BOLD:AAZ6500
Dasysyrphus|[11038]|BBDCQ015-10|Syrphidae|658|BOLD:AAB2865
Dasysyrphus|[11039]|CNKLB3433-14|Syrphidae|615|BOLD:AAE3387
Dasysyrphus venustus|[11040]|DSANN005-08|Syrphidae|658|BOLD:AAA7375
Dasysyrphus venustus|[11041]|JSDIP562-10|Syrphidae|658|BOLD:ACV5348
Dasysyrphus intrudens sp. complex|[11042]|BBDCP994-10|Syrphidae|658|BOLD:ACE9330
Dasysyrphus intrudens sp. complex|[11043]|BBDCP895-10|Syrphidae|658|BOLD:AAB2863
Dasysyrphus intrudens sp. complex|[11044]|CNGUA673-14|Syrphidae|561|BOLD:ACF0592
Syrphus|[11045]|NCCA979-11|Syrphidae|673|BOLD:AAB5577
Syrphus|[11046]|NGNAC3258-13|Syrphidae|585|BOLD:ACI8120
Syrphus knabi|[11047]|MBIOE1838-13|Syrphidae|604|BOLD:AAG4667
Syrphus torvus|[11048]|BBDEC042-09|Syrphidae|658|BOLD:AAC6088
Syrphus attenuatus|[11049]|JWDCJ1482-11|Syrphidae|658|BOLD:AAD7605
Syrphus ribesii|[11050]|EAWGA442-13|Syrphidae|661|BOLD:AAA4570
Melangyna guttata|[11051]|BBDCP957-10|Syrphidae|658|BOLD:AAK7294
Meligramma triangulifera|[11052]|SSWLA3998-13|Syrphidae|670|BOLD:AAZ1912
Eupeodes|[11053]|NCCA2551-11|Syrphidae|673|BOLD:AAB2384
Eupeodes latifasciatus|[11054]|TTDBW244-09|Syrphidae|620|BOLD:ACY1719
Eupeodes|[11055]|NGNAC3247-13|Syrphidae|592|BOLD:ACI8456
Eupeodes|[11056]|HPPPK1267-13|Syrphidae|594|BOLD:ACY1720
Eupeodes|[11057]|BBDCQ011-10|Syrphidae|658|BOLD:ABY9727
Eupeodes|[11058]|CNMIH2511-14|Syrphidae|585|BOLD:ACN1402
Parasyrphus genualis|[11059]|BBDCM216-10|Syrphidae|658|BOLD:AAG4652
Parasyrphus|[11060]|BBDCQ026-10|Syrphidae|658|BOLD:AAL3233
Parasyrphus vockerothi|[11061]|DSCFV005-07|Syrphidae|658|BOLD:AAG6761
Parasyrphus|[11062]|BBDCM421-10|Syrphidae|658|BOLD:AAC1834
Parasyrphus|[11063]|CNPKF1638-14|Syrphidae|658|BOLD:AAZ5457
Parasyrphus nigritarsis|[11064]|CNTGF506-15|Syrphidae|552|BOLD:AAL3230
Platycheirus granditarsis|[11065]|BBDCP806-10|Syrphidae|658|BOLD:AAB1554
Platycheirus rosarum|[11066]|BBDCP894-10|Syrphidae|658|BOLD:AAG4683
Platycheirus aeratus|[11067]|TTDFW473-08|Syrphidae|658|BOLD:ABZ7598
Platycheirus|[11068]|BBDCN012-10|Syrphidae|658|BOLD:AAA9506
Platycheirus|[11069]|BBDCM171-10|Syrphidae|658|BOLD:ACF4733
Platycheirus hyperboreus|[11070]|BBDEC179-09|Syrphidae|658|BOLD:ACF4734
Platycheirus hyperboreus dark form|[11071]|JWDCF123-10|Syrphidae|658|BOLD:ACF4730
Platycheirus angustatus4|[11072]|CNEIE1786-12|Syrphidae|613|BOLD:ABZ7596
Platycheirus scambus|[11073]|BBDCM462-10|Syrphidae|658|BOLD:AAD3756
Platycheirus|[11074]|SSEIB1982-13|Syrphidae|637|BOLD:AAL9449
Platycheirus|[11075]|BBDCM967-10|Syrphidae|658|BOLD:AAE4456
Platycheirus striatus|[11076]|BBDCP917-10|Syrphidae|658|BOLD:AAM1274
Platycheirus|[11077]|BBDCM181-10|Syrphidae|658|BOLD:AAG4665
Platycheirus thylax|[11078]|BBDCN996-10|Syrphidae|658|BOLD:AAP6396
Platycheirus setitarsis|[11079]|CNIVF036-14|Syrphidae|594|BOLD:AAM1272
Platycheirus|[11080]|BBDCP959-10|Syrphidae|658|BOLD:AAC6630
Platycheirus flabella|[11081]|JWDCI195-10|Syrphidae|658|BOLD:AAM1270
Platycheirus parmatus|[11082]|SSJAC877-13|Syrphidae|621|BOLD:AAM1271
Platycheirus|[11083]|CNQUA013-14|Syrphidae|627|BOLD:ABY7191
Platycheirus groenlandicus|[11084]|CNIVH152-14|Syrphidae|609|BOLD:AAZ4195
Platycheirus podagratus|[11085]|JWDCB008-10|Syrphidae|658|BOLD:ACE6015
Platycheirus nodosus|[11086]|JWDCI563-10|Syrphidae|658|BOLD:AAC6631
Platycheirus varipes|[11087]|JWDCJ1403-11|Syrphidae|658|BOLD:ABZ3110
Platycheirus|[11088]|CNJAD2092-12|Syrphidae|632|BOLD:AAL7898
Platycheirus|[11089]|BBDCN365-10|Syrphidae|658|BOLD:ABY4282
Platycheirus setipes|[11090]|CNJAG1217-12|Syrphidae|670|BOLD:ABY4281
Platycheirus|[11091]|CNKLB371-14|Syrphidae|594|BOLD:ACP2579
Platycheirus sabulicola|[11092]|CNJAC1170-12|Syrphidae|673|BOLD:AAP7660
Platycheirus obscurus|[11093]|JSDIP723-10|Syrphidae|658|BOLD:AAF1237
Platycheirus confusus|[11094]|JSDIP506-10|Syrphidae|658|BOLD:ABZ7211
Platycheirus confusus|[11095]|JWDCE813-10|Syrphidae|658|BOLD:ACE3988
Platycheirus|[11096]|BBDCM921-10|Syrphidae|658|BOLD:ACE3989
Platycheirus trichopus|[11097]|CNGUB125-14|Syrphidae|562|BOLD:ACE3987
Platycheirus lundibecki2|[11098]|CNTGC447-15|Syrphidae|615|BOLD:ACE9369

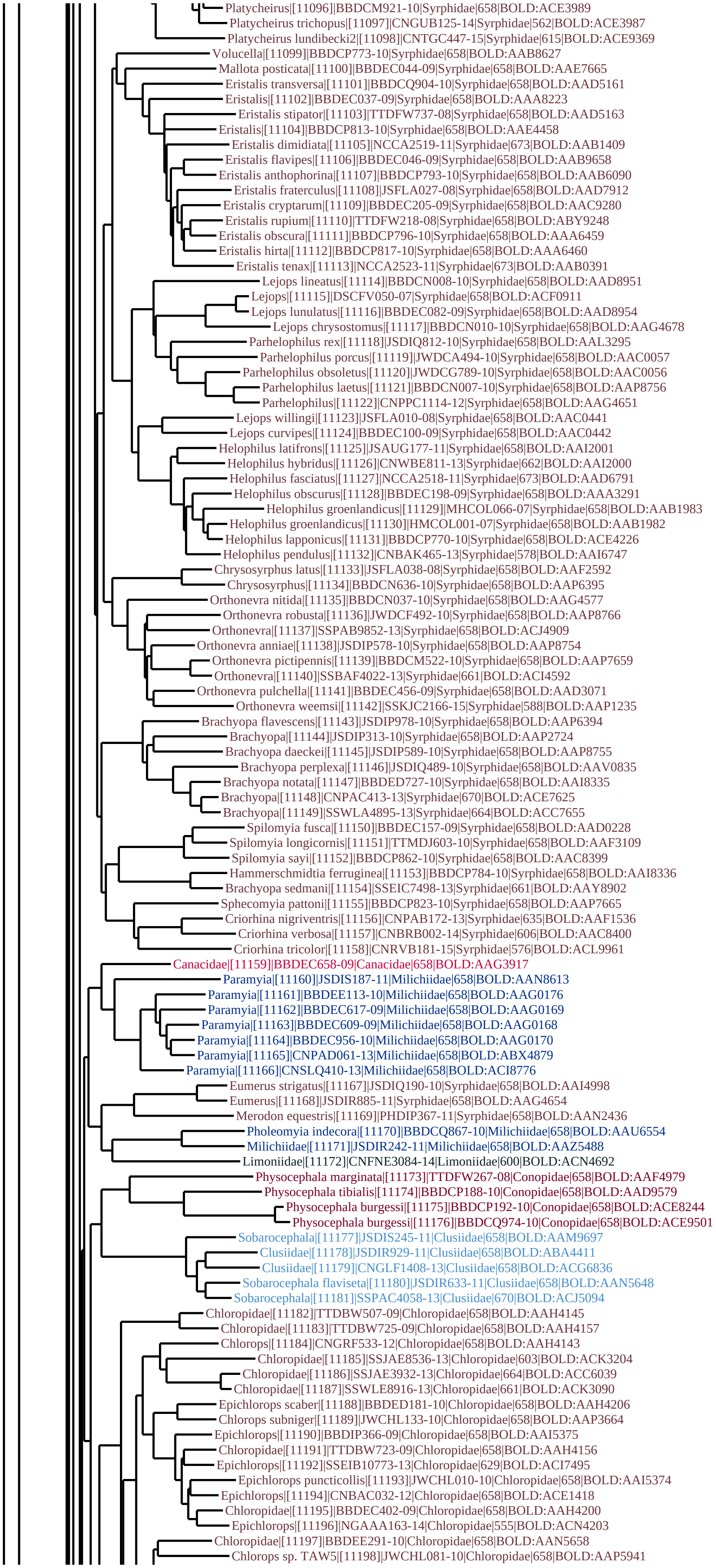

Platycheirus|[11096]|BBDCM921-10|Syrphidae|658|BOLD:ACE3989
Platycheirus trichopus|[11097]|CNGUB125-14|Syrphidae|562|BOLD:ACE3987
Platycheirus lundibecki2|[11098]|CNTGC447-15|Syrphidae|615|BOLD:ACE9369
Volucella|[11099]|BBDCP773-10|Syrphidae|658|BOLD:AAB8627
Mallota posticata|[11100]|BBDEC044-09|Syrphidae|658|BOLD:AAE7665
Eristalis transversa|[11101]|BBDCQ904-10|Syrphidae|658|BOLD:AAD5161
Eristalis|[11102]|BBDEC037-09|Syrphidae|658|BOLD:AAA8223
Eristalis stipator|[11103]|TTDFW737-08|Syrphidae|658|BOLD:AAD5163
Eristalis|[11104]|BBDCP813-10|Syrphidae|658|BOLD:AAE4458
Eristalis dimidiata|[11105]|NCCA2519-11|Syrphidae|673|BOLD:AAB1409
Eristalis flavipes|[11106]|BBDEC046-09|Syrphidae|658|BOLD:AAB9658
Eristalis anthophorina|[11107]|BBDCP793-10|Syrphidae|658|BOLD:AAB6090
Eristalis fraterculus|[11108]|JSFLA027-08|Syrphidae|658|BOLD:AAD7912
Eristalis cryptarum|[11109]|BBDEC205-09|Syrphidae|658|BOLD:AAC9280
Eristalis rupium|[11110]|TTDFW218-08|Syrphidae|658|BOLD:ABY9248
Eristalis obscura|[11111]|BBDCP796-10|Syrphidae|658|BOLD:AAA6459
Eristalis hirta|[11112]|BBDCP817-10|Syrphidae|658|BOLD:AAA6460
Eristalis tenax|[11113]|NCCA2523-11|Syrphidae|673|BOLD:AAB0391
Lejops lineatus|[11114]|BBDCN008-10|Syrphidae|658|BOLD:AAD8951
Lejops|[11115]|DSCFV050-07|Syrphidae|658|BOLD:ACF0911
Lejops lunulatus|[11116]|BBDEC082-09|Syrphidae|658|BOLD:AAD8954
Lejops chrysostomus|[11117]|BBDCN010-10|Syrphidae|658|BOLD:AAG4678
Parhelophilus rex|[11118]|JSDIQ812-10|Syrphidae|658|BOLD:AAL3295
Parhelophilus porcus|[11119]|JWDCA494-10|Syrphidae|658|BOLD:AAC0057
Parhelophilus obsoletus|[11120]|JWDCG789-10|Syrphidae|658|BOLD:AAC0056
Parhelophilus laetus|[11121]|BBDCN007-10|Syrphidae|658|BOLD:AAP8756
Parhelophilus|[11122]|CNPPC1114-12|Syrphidae|658|BOLD:AAG4651
Lejops willingi|[11123]|JSFLA010-08|Syrphidae|658|BOLD:AAC0441
Lejops curvipes|[11124]|BBDEC100-09|Syrphidae|658|BOLD:AAC0442
Helophilus latifrons|[11125]|JSAUG177-11|Syrphidae|658|BOLD:AAI2001
Helophilus hybridus|[11126]|CNWBE811-13|Syrphidae|662|BOLD:AAI2000
Helophilus fasciatus|[11127]|NCCA2518-11|Syrphidae|673|BOLD:AAD6791
Helophilus obscurus|[11128]|BBDEC198-09|Syrphidae|658|BOLD:AAA3291
Helophilus groenlandicus|[11129]|MHCOL066-07|Syrphidae|658|BOLD:AAB1983
Helophilus groenlandicus|[11130]|HMCOL001-07|Syrphidae|658|BOLD:AAB1982
Helophilus lapponicus|[11131]|BBDCP770-10|Syrphidae|658|BOLD:ACE4226
Helophilus pendulus|[11132]|CNBAK465-13|Syrphidae|578|BOLD:AAI6747
Chrysosyrphus latus|[11133]|JSFLA038-08|Syrphidae|658|BOLD:AAF2592
Chrysosyrphus|[11134]|BBDCN636-10|Syrphidae|658|BOLD:AAP6395
Orthonevra nitida|[11135]|BBDCN037-10|Syrphidae|658|BOLD:AAG4577
Orthonevra robusta|[11136]|JWDCF492-10|Syrphidae|658|BOLD:AAP8766
Orthonevra|[11137]|SSPAB9852-13|Syrphidae|658|BOLD:ACJ4909
Orthonevra anniae|[11138]|JSDIP578-10|Syrphidae|658|BOLD:AAP8754
Orthonevra pictipennis|[11139]|BBDCM522-10|Syrphidae|658|BOLD:AAP7659
Orthonevra|[11140]|SSBAF4022-13|Syrphidae|661|BOLD:ACI4592
Orthonevra pulchella|[11141]|BBDEC456-09|Syrphidae|658|BOLD:AAD3071
Orthonevra weemsi|[11142]|SSKJC2166-15|Syrphidae|588|BOLD:AAP1235
Brachyopa flavescens|[11143]|JSDIP978-10|Syrphidae|658|BOLD:AAP6394
Brachyopa|[11144]|JSDIP313-10|Syrphidae|658|BOLD:AAP2724
Brachyopa daeckei|[11145]|JSDIP589-10|Syrphidae|658|BOLD:AAP8755
Brachyopa perplexa|[11146]|JSDIQ489-10|Syrphidae|658|BOLD:AAV0835
Brachyopa notata|[11147]|BBDED727-10|Syrphidae|658|BOLD:AAI8335
Brachyopa|[11148]|CNPAC413-13|Syrphidae|670|BOLD:ACE7625
Brachyopa|[11149]|SSWLA4895-13|Syrphidae|664|BOLD:ACC7655
Spilomyia fusca|[11150]|BBDEC157-09|Syrphidae|658|BOLD:AAD0228
Spilomyia longicornis|[11151]|TTMDJ603-10|Syrphidae|658|BOLD:AAF3109
Spilomyia sayi|[11152]|BBDCP862-10|Syrphidae|658|BOLD:AAC8399
Hammerschmidtia ferruginea|[11153]|BBDCP784-10|Syrphidae|658|BOLD:AAI8336
Brachyopa sedmani|[11154]|SSEIC7498-13|Syrphidae|661|BOLD:AAY8902
Sphecomyia pattoni|[11155]|BBDCP823-10|Syrphidae|658|BOLD:AAP7665
Criorhina nigriventris|[11156]|CNPAB172-13|Syrphidae|635|BOLD:AAF1536
Criorhina verbosa|[11157]|CNBRB002-14|Syrphidae|606|BOLD:AAC8400
Criorhina tricolor|[11158]|CNRVB181-15|Syrphidae|576|BOLD:ACL9961
Canacidae|[11159]|BBDEC658-09|Canacidae|658|BOLD:AAG3917
Paramyia|[11160]|JSDIS187-11|Milichiidae|658|BOLD:AAN8613
Paramyia|[11161]|BBDEE113-10|Milichiidae|658|BOLD:AAG0176
Paramyia|[11162]|BBDEC617-09|Milichiidae|658|BOLD:AAG0169
Paramyia|[11163]|BBDEC609-09|Milichiidae|658|BOLD:AAG0168
Paramyia|[11164]|BBDEC956-10|Milichiidae|658|BOLD:AAG0170
Paramyia|[11165]|CNPAD061-13|Milichiidae|658|BOLD:ABX4879
Paramyia|[11166]|CNSLQ410-13|Milichiidae|658|BOLD:ACI8776
Eumerus strigatus|[11167]|JSDIQ190-10|Syrphidae|658|BOLD:AAI4998
Eumerus|[11168]|JSDIR885-11|Syrphidae|658|BOLD:AAG4654
Merodon equestris|[11169]|PHDIP367-11|Syrphidae|658|BOLD:AAN2436
Pholeomyia indecora|[11170]|BBDCQ867-10|Milichiidae|658|BOLD:AAU6554
Milichiidae|[11171]|JSDIR242-11|Milichiidae|658|BOLD:AAZ5488
Limoniidae|[11172]|CNFNE3084-14|Limoniidae|600|BOLD:ACN4692
Physocephala marginata|[11173]|TTDFW267-08|Conopidae|658|BOLD:AAF4979
Physocephala tibialis|[11174]|BBDCP188-10|Conopidae|658|BOLD:AAD9579
Physocephala burgessi|[11175]|BBDCP192-10|Conopidae|658|BOLD:ACE8244
Physocephala burgessi|[11176]|BBDCQ974-10|Conopidae|658|BOLD:ACE9501
Sobarocephala|[11177]|JSDIS245-11|Clusiidae|658|BOLD:AAM9697
Clusiidae|[11178]|JSDIR929-11|Clusiidae|658|BOLD:ABA4411
Clusiidae|[11179]|CNGLF1408-13|Clusiidae|658|BOLD:ACG6836
Sobarocephala flaviseta|[11180]|JSDIR633-11|Clusiidae|658|BOLD:AAN5648
Sobarocephala|[11181]|SSPAC4058-13|Clusiidae|670|BOLD:ACJ5094
Chloropidae|[11182]|TTDBW507-09|Chloropidae|658|BOLD:AAH4145
Chloropidae|[11183]|TTDBW725-09|Chloropidae|658|BOLD:AAH4157
Chlorops|[11184]|CNGRF533-12|Chloropidae|658|BOLD:AAH4143
Chloropidae|[11185]|SSJAE8536-13|Chloropidae|603|BOLD:ACK3204
Chloropidae|[11186]|SSJAE3932-13|Chloropidae|664|BOLD:ACC6039
Chloropidae|[11187]|SSWLE8916-13|Chloropidae|661|BOLD:ACK3090
Epichlorops scaber|[11188]|BBDED181-10|Chloropidae|658|BOLD:AAH4206
Chlorops subniger|[11189]|JWCHL133-10|Chloropidae|658|BOLD:AAP3664
Epichlorops|[11190]|BBDIP366-09|Chloropidae|658|BOLD:AAI5375
Chloropidae|[11191]|TTDBW723-09|Chloropidae|658|BOLD:AAH4156
Epichlorops|[11192]|SSEIB10773-13|Chloropidae|629|BOLD:ACI7495
Epichlorops puncticollis|[11193]|JWCHL010-10|Chloropidae|658|BOLD:AAI5374
Epichlorops|[11194]|CNBAC032-12|Chloropidae|658|BOLD:ACE1418
Chloropidae|[11195]|BBDEC402-09|Chloropidae|658|BOLD:AAH4200
Epichlorops|[11196]|NGAAA163-14|Chloropidae|555|BOLD:ACN4203
Chloropidae|[11197]|BBDEE291-10|Chloropidae|658|BOLD:AAN5658
Chlorops sp. TAW5|[11198]|JWCHL081-10|Chloropidae|658|BOLD:AAP5941

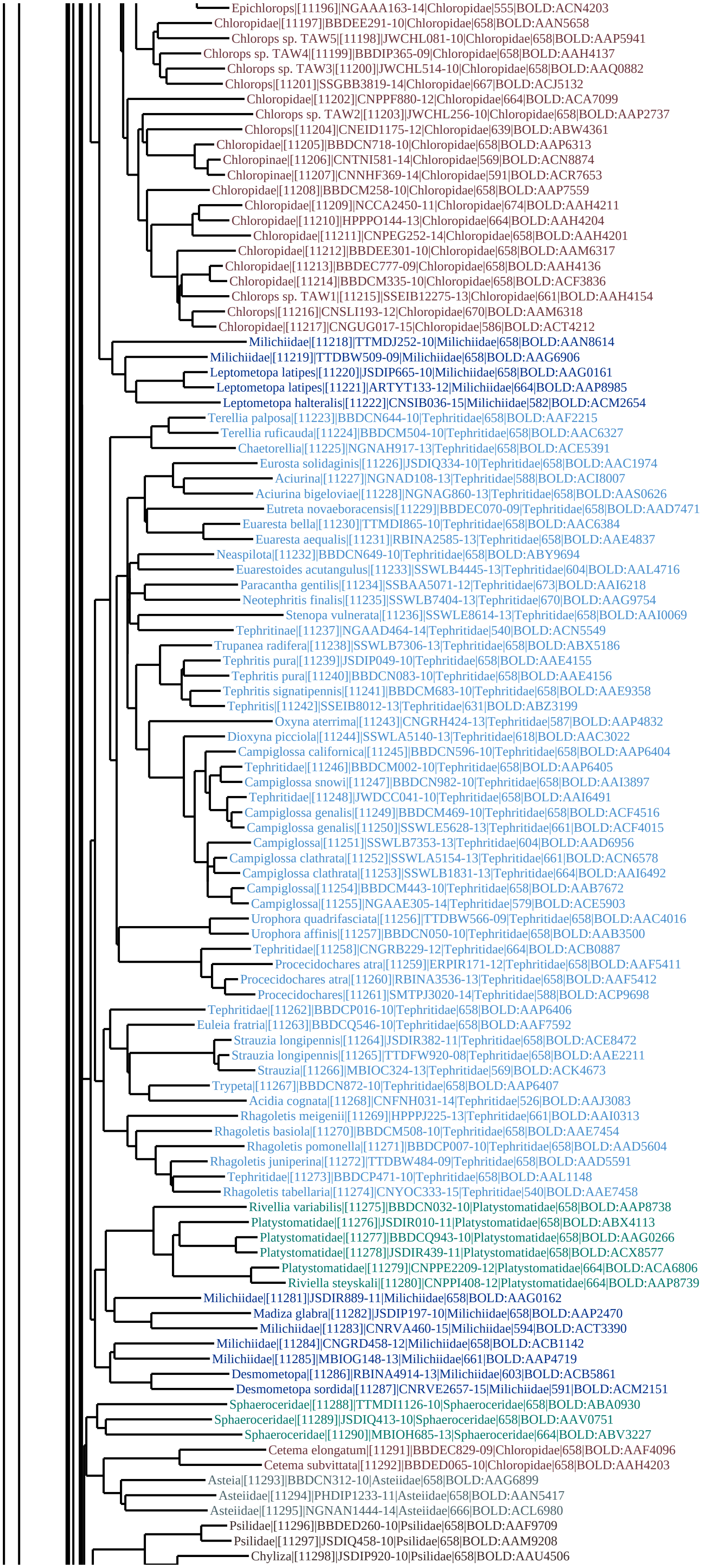

Epichlorops|[11196]|NGAAA163-14|Chloropidae|555|BOLD:ACN4203
Chloropidae|[11197]|BBDEE291-10|Chloropidae|658|BOLD:AAN5658
Chlorops sp. TAW5|[11198]|JWCHL081-10|Chloropidae|658|BOLD:AAP5941
Chlorops sp. TAW4|[11199]|BBDIP365-09|Chloropidae|658|BOLD:AAH4137
Chlorops sp. TAW3|[11200]|JWCHL514-10|Chloropidae|658|BOLD:AAQ0882
Chlorops|[11201]|SSGBB3819-14|Chloropidae|667|BOLD:ACJ5132
Chloropidae|[11202]|CNPPF880-12|Chloropidae|664|BOLD:ACA7099
Chlorops sp. TAW2|[11203]|JWCHL256-10|Chloropidae|658|BOLD:AAP2737
Chlorops|[11204]|CNEID1175-12|Chloropidae|639|BOLD:ABW4361
Chloropidae|[11205]|BBDCN718-10|Chloropidae|658|BOLD:AAP6313
Chloropinae|[11206]|CNTNI581-14|Chloropidae|569|BOLD:ACN8874
Chloropinae|[11207]|CNNHF369-14|Chloropidae|591|BOLD:ACR7653
Chloropidae|[11208]|BBDCM258-10|Chloropidae|658|BOLD:AAP7559
Chloropidae|[11209]|NCCA2450-11|Chloropidae|674|BOLD:AAH4211
Chloropidae|[11210]|HPPPO144-13|Chloropidae|664|BOLD:AAH4204
Chloropidae|[11211]|CNPEG252-14|Chloropidae|658|BOLD:AAH4201
Chloropidae|[11212]|BBDEE301-10|Chloropidae|658|BOLD:AAM6317
Chloropidae|[11213]|BBDEC777-09|Chloropidae|658|BOLD:AAH4136
Chloropidae|[11214]|BBDCM335-10|Chloropidae|658|BOLD:ACF3836
Chlorops sp. TAW1|[11215]|SSEIB12275-13|Chloropidae|661|BOLD:AAH4154
Chlorops|[11216]|CNSLI193-12|Chloropidae|670|BOLD:AAM6318
Chloropidae|[11217]|CNGUG017-15|Chloropidae|586|BOLD:ACT4212
Milichiidae|[11218]|TTMDJ252-10|Milichiidae|658|BOLD:AAN8614
Milichiidae|[11219]|TTDBW509-09|Milichiidae|658|BOLD:AAG6906
Leptometopa latipes|[11220]|JSDIP665-10|Milichiidae|658|BOLD:AAG0161
Leptometopa latipes|[11221]|ARTYT133-12|Milichiidae|664|BOLD:AAP8985
Leptometopa halteralis|[11222]|CNSIB036-15|Milichiidae|582|BOLD:ACM2654
Terellia palposa|[11223]|BBDCN644-10|Tephritidae|658|BOLD:AAF2215
Terellia ruficauda|[11224]|BBDCM504-10|Tephritidae|658|BOLD:AAC6327
Chaetorellia|[11225]|NGNAH917-13|Tephritidae|658|BOLD:ACE5391
Eurosta solidaginis|[11226]|JSDIQ334-10|Tephritidae|658|BOLD:AAC1974
Aciurina|[11227]|NGNAD108-13|Tephritidae|588|BOLD:ACI8007
Aciurina bigeloviae|[11228]|NGNAG860-13|Tephritidae|658|BOLD:AAS0626
Eutreta novaeboracensis|[11229]|BBDEC070-09|Tephritidae|658|BOLD:AAD7471
Euaresta bella|[11230]|TTMDI865-10|Tephritidae|658|BOLD:AAC6384
Euaresta aequalis|[11231]|RBINA2585-13|Tephritidae|658|BOLD:AAE4837
Neaspilota|[11232]|BBDCN649-10|Tephritidae|658|BOLD:ABY9694
Euarestoides acutangulus|[11233]|SSWLB4445-13|Tephritidae|604|BOLD:AAL4716
Paracantha gentilis|[11234]|SSBAA5071-12|Tephritidae|673|BOLD:AAI6218
Neotephritis finalis|[11235]|SSWLB7404-13|Tephritidae|670|BOLD:AAG9754
Stenopa vulnerata|[11236]|SSWLE8614-13|Tephritidae|658|BOLD:AAI0069
Tephritinae|[11237]|NGAAD464-14|Tephritidae|540|BOLD:ACN5549
Trupanea radifera|[11238]|SSWLB7306-13|Tephritidae|658|BOLD:ABX5186
Tephritis pura|[11239]|JSDIP049-10|Tephritidae|658|BOLD:AAE4155
Tephritis pura|[11240]|BBDCN083-10|Tephritidae|658|BOLD:AAE4156
Tephritis signatipennis|[11241]|BBDCM683-10|Tephritidae|658|BOLD:AAE9358
Tephritis|[11242]|SSEIB8012-13|Tephritidae|631|BOLD:ABZ3199
Oxyna aterrima|[11243]|CNGRH424-13|Tephritidae|587|BOLD:AAP4832
Dioxyna picciola|[11244]|SSWLA5140-13|Tephritidae|618|BOLD:AAC3022
Campiglossa californica|[11245]|BBDCN596-10|Tephritidae|658|BOLD:AAP6404
Tephritidae|[11246]|BBDCM002-10|Tephritidae|658|BOLD:AAP6405
Campiglossa snowi|[11247]|BBDCN982-10|Tephritidae|658|BOLD:AAI3897
Tephritidae|[11248]|JWDCC041-10|Tephritidae|658|BOLD:AAI6491
Campiglossa genalis|[11249]|BBDCM469-10|Tephritidae|658|BOLD:ACF4516
Campiglossa genalis|[11250]|SSWLE5628-13|Tephritidae|661|BOLD:ACF4015
Campiglossa|[11251]|SSWLB7353-13|Tephritidae|604|BOLD:AAD6956
Campiglossa clathrata|[11252]|SSWLA5154-13|Tephritidae|661|BOLD:ACN6578
Campiglossa clathrata|[11253]|SSWLB1831-13|Tephritidae|664|BOLD:AAI6492
Campiglossa|[11254]|BBDCM443-10|Tephritidae|658|BOLD:AAB7672
Campiglossa|[11255]|NGAAE305-14|Tephritidae|579|BOLD:ACE5903
Urophora quadrifasciata|[11256]|TTDBW566-09|Tephritidae|658|BOLD:AAC4016
Urophora affinis|[11257]|BBDCN050-10|Tephritidae|658|BOLD:AAB3500
Tephritidae|[11258]|CNGRB229-12|Tephritidae|664|BOLD:ACB0887
Procecidochares atra|[11259]|ERPIR171-12|Tephritidae|658|BOLD:AAF5411
Procecidochares atra|[11260]|RBINA3536-13|Tephritidae|658|BOLD:AAF5412
Procecidochares|[11261]|SMTPJ3020-14|Tephritidae|588|BOLD:ACP9698
Tephritidae|[11262]|BBDCP016-10|Tephritidae|658|BOLD:AAP6406
Euleia fratria|[11263]|BBDCQ546-10|Tephritidae|658|BOLD:AAF7592
Strauzia longipennis|[11264]|JSDIR382-11|Tephritidae|658|BOLD:ACE8472
Strauzia longipennis|[11265]|TTDFW920-08|Tephritidae|658|BOLD:AAE2211
Strauzia|[11266]|MBIOC324-13|Tephritidae|569|BOLD:ACK4673
Trypeta|[11267]|BBDCN872-10|Tephritidae|658|BOLD:AAP6407
Acidia cognata|[11268]|CNFNH031-14|Tephritidae|526|BOLD:AAJ3083
Rhagoletis meigenii|[11269]|HPPPJ225-13|Tephritidae|661|BOLD:AAI0313
Rhagoletis basiola|[11270]|BBDCM508-10|Tephritidae|658|BOLD:AAE7454
Rhagoletis pomonella|[11271]|BBDCP007-10|Tephritidae|658|BOLD:AAD5604
Rhagoletis juniperina|[11272]|TTDBW484-09|Tephritidae|658|BOLD:AAD5591
Tephritidae|[11273]|BBDCP471-10|Tephritidae|658|BOLD:AAL1148
Rhagoletis tabellaria|[11274]|CNYOC333-15|Tephritidae|540|BOLD:AAE7458
Rivellia variabilis|[11275]|BBDCN032-10|Platystomatidae|658|BOLD:AAP8738
Platystomatidae|[11276]|JSDIR010-11|Platystomatidae|658|BOLD:ABX4113
Platystomatidae|[11277]|BBDCQ943-10|Platystomatidae|658|BOLD:AAG0266
Platystomatidae|[11278]|JSDIR439-11|Platystomatidae|658|BOLD:ACX8577
Platystomatidae|[11279]|CNPPE2209-12|Platystomatidae|664|BOLD:ACA6806
Riviella steyskali|[11280]|CNPPI408-12|Platystomatidae|664|BOLD:AAP8739
Milichiidae|[11281]|JSDIR889-11|Milichiidae|658|BOLD:AAG0162
Madiza glabra|[11282]|JSDIP197-10|Milichiidae|658|BOLD:AAP2470
Milichiidae|[11283]|CNRVA460-15|Milichiidae|594|BOLD:ACT3390
Milichiidae|[11284]|CNGRD458-12|Milichiidae|658|BOLD:ACB1142
Milichiidae|[11285]|MBIOG148-13|Milichiidae|661|BOLD:AAP4719
Desmometopa|[11286]|RBINA4914-13|Milichiidae|603|BOLD:ACB5861
Desmometopa sordida|[11287]|CNRVE2657-15|Milichiidae|591|BOLD:ACM2151
Sphaeroceridae|[11288]|TTMDI1126-10|Sphaeroceridae|658|BOLD:ABA0930
Sphaeroceridae|[11289]|JSDIQ413-10|Sphaeroceridae|658|BOLD:AAV0751
Sphaeroceridae|[11290]|MBIOH685-13|Sphaeroceridae|664|BOLD:ABV3227
Cetema elongatum|[11291]|BBDEC829-09|Chloropidae|658|BOLD:AAF4096
Cetema subvittata|[11292]|BBDED065-10|Chloropidae|658|BOLD:AAH4203
Asteia|[11293]|BBDCN312-10|Asteiidae|658|BOLD:AAG6899
Asteiidae|[11294]|PHDIP1233-11|Asteiidae|658|BOLD:AAN5417
Asteiidae|[11295]|NGNAN1444-14|Asteiidae|666|BOLD:ACL6980
Psilidae|[11296]|BBDED260-10|Psilidae|658|BOLD:AAF9709
Psilidae|[11297]|JSDIQ458-10|Psilidae|658|BOLD:AAM9208
Chyliza|[11298]|JSDIP920-10|Psilidae|658|BOLD:AAU4506

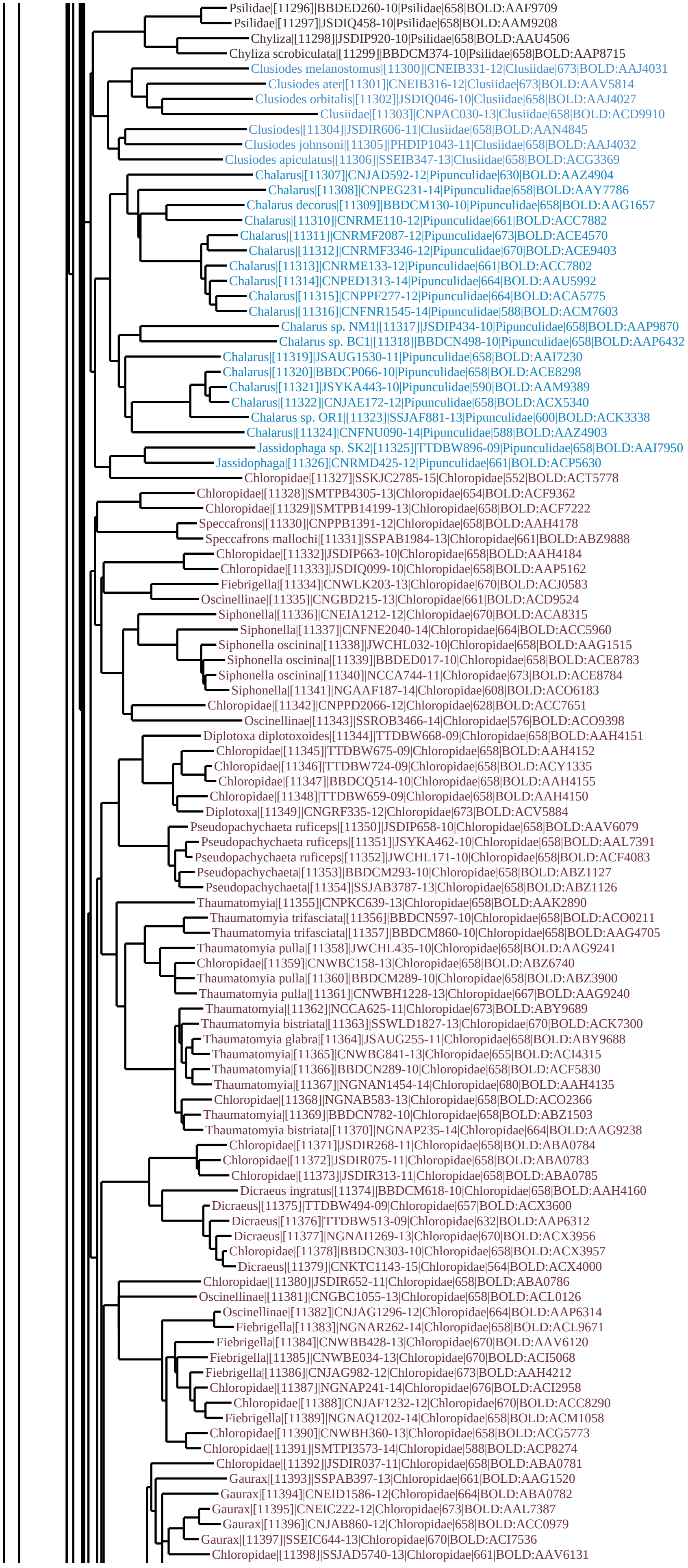

Psilidae|[11296]|BBDED260-10|Psilidae|658|BOLD:AAF9709
Psilidae|[11297]|JSDIQ458-10|Psilidae|658|BOLD:AAM9208
Chyliza|[11298]|JSDIP920-10|Psilidae|658|BOLD:AAU4506
Chyliza scrobiculata|[11299]|BBDCM374-10|Psilidae|658|BOLD:AAP8715
Clusiodes melanostomus|[11300]|CNEIB331-12|Clusiidae|673|BOLD:AAJ4031
Clusiodes ater|[11301]|CNEIB316-12|Clusiidae|673|BOLD:AAV5814
Clusiodes orbitalis|[11302]|JSDIQ046-10|Clusiidae|658|BOLD:AAJ4027
Clusiidae|[11303]|CNPAC030-13|Clusiidae|658|BOLD:ACD9910
Clusiodes|[11304]|JSDIR606-11|Clusiidae|658|BOLD:AAN4845
Clusiodes johnsoni|[11305]|PHDIP1043-11|Clusiidae|658|BOLD:AAJ4032
Clusiodes apiculatus|[11306]|SSEIB347-13|Clusiidae|658|BOLD:ACG3369
Chalarus|[11307]|CNJAD592-12|Pipunculidae|630|BOLD:AAZ4904
Chalarus|[11308]|CNPEG231-14|Pipunculidae|658|BOLD:AAY7786
Chalarus decorus|[11309]|BBDCM130-10|Pipunculidae|658|BOLD:AAG1657
Chalarus|[11310]|CNRME110-12|Pipunculidae|661|BOLD:ACC7882
Chalarus|[11311]|CNRMF2087-12|Pipunculidae|673|BOLD:ACE4570
Chalarus|[11312]|CNRMF3346-12|Pipunculidae|670|BOLD:ACE9403
Chalarus|[11313]|CNRME133-12|Pipunculidae|661|BOLD:ACC7802
Chalarus|[11314]|CNPED1313-14|Pipunculidae|664|BOLD:AAU5992
Chalarus|[11315]|CNPPF277-12|Pipunculidae|664|BOLD:ACA5775
Chalarus|[11316]|CNFNR1545-14|Pipunculidae|588|BOLD:ACM7603
Chalarus sp. NM1|[11317]|JSDIP434-10|Pipunculidae|658|BOLD:AAP9870
Chalarus sp. BC1|[11318]|BBDCN498-10|Pipunculidae|658|BOLD:AAP6432
Chalarus|[11319]|JSAUG1530-11|Pipunculidae|658|BOLD:AAI7230
Chalarus|[11320]|BBDCP066-10|Pipunculidae|658|BOLD:ACE8298
Chalarus|[11321]|JSYKA443-10|Pipunculidae|590|BOLD:AAM9389
Chalarus|[11322]|CNJAE172-12|Pipunculidae|658|BOLD:ACX5340
Chalarus sp. OR1|[11323]|SSJAF881-13|Pipunculidae|600|BOLD:ACK3338
Chalarus|[11324]|CNFNU090-14|Pipunculidae|588|BOLD:AAZ4903
Jassidophaga sp. SK2|[11325]|TTDBW896-09|Pipunculidae|658|BOLD:AAI7950
Jassidophaga|[11326]|CNRMD425-12|Pipunculidae|661|BOLD:ACP5630
Chloropidae|[11327]|SSKJC2785-15|Chloropidae|552|BOLD:ACT5778
Chloropidae|[11328]|SMTPB4305-13|Chloropidae|654|BOLD:ACF9362
Chloropidae|[11329]|SMTPB14199-13|Chloropidae|658|BOLD:ACF7222
Speccafrons|[11330]|CNPPB1391-12|Chloropidae|658|BOLD:AAH4178
Speccafrons mallochi|[11331]|SSPAB1984-13|Chloropidae|661|BOLD:ABZ9888
Chloropidae|[11332]|JSDIP663-10|Chloropidae|658|BOLD:AAH4184
Chloropidae|[11333]|JSDIQ099-10|Chloropidae|658|BOLD:AAP5162
Fiebrigella|[11334]|CNWLK203-13|Chloropidae|670|BOLD:ACJ0583
Oscinellinae|[11335]|CNGBD215-13|Chloropidae|661|BOLD:ACD9524
Siphonella|[11336]|CNEIA1212-12|Chloropidae|670|BOLD:ACA8315
Siphonella|[11337]|CNFNE2040-14|Chloropidae|664|BOLD:ACC5960
Siphonella oscinina|[11338]|JWCHL032-10|Chloropidae|658|BOLD:AAG1515
Siphonella oscinina|[11339]|BBDED017-10|Chloropidae|658|BOLD:ACE8783
Siphonella oscinina|[11340]|NCCA744-11|Chloropidae|673|BOLD:ACE8784
Siphonella|[11341]|NGAAF187-14|Chloropidae|608|BOLD:ACO6183
Chloropidae|[11342]|CNPPD2066-12|Chloropidae|628|BOLD:ACC7651
Oscinellinae|[11343]|SSROB3466-14|Chloropidae|576|BOLD:ACO9398
Diplotoxa diplotoxoides|[11344]|TTDBW668-09|Chloropidae|658|BOLD:AAH4151
Chloropidae|[11345]|TTDBW675-09|Chloropidae|658|BOLD:AAH4152
Chloropidae|[11346]|TTDBW724-09|Chloropidae|658|BOLD:ACY1335
Chloropidae|[11347]|BBDCQ514-10|Chloropidae|658|BOLD:AAH4155
Chloropidae|[11348]|TTDBW659-09|Chloropidae|658|BOLD:AAH4150
Diplotoxa|[11349]|CNGRF335-12|Chloropidae|673|BOLD:ACV5884
Pseudopachychaeta ruficeps|[11350]|JSDIP658-10|Chloropidae|658|BOLD:AAV6079
Pseudopachychaeta ruficeps|[11351]|JSYKA462-10|Chloropidae|658|BOLD:AAL7391
Pseudopachychaeta ruficeps|[11352]|JWCHL171-10|Chloropidae|658|BOLD:ACF4083
Pseudopachychaeta|[11353]|BBDCM293-10|Chloropidae|658|BOLD:ABZ1127
Pseudopachychaeta|[11354]|SSJAB3787-13|Chloropidae|658|BOLD:ABZ1126
Thaumatomyia|[11355]|CNPKC639-13|Chloropidae|658|BOLD:AAK2890
Thaumatomyia trifasciata|[11356]|BBDCN597-10|Chloropidae|658|BOLD:ACO0211
Thaumatomyia trifasciata|[11357]|BBDCM860-10|Chloropidae|658|BOLD:AAG4705
Thaumatomyia pulla|[11358]|JWCHL435-10|Chloropidae|658|BOLD:AAG9241
Chloropidae|[11359]|CNWBC158-13|Chloropidae|658|BOLD:ABZ6740
Thaumatomyia pulla|[11360]|BBDCM289-10|Chloropidae|658|BOLD:ABZ3900
Thaumatomyia pulla|[11361]|CNWBH1228-13|Chloropidae|667|BOLD:AAG9240
Thaumatomyia|[11362]|NCCA625-11|Chloropidae|673|BOLD:ABY9689
Thaumatomyia bistriata|[11363]|SSWLD1827-13|Chloropidae|670|BOLD:ACK7300
Thaumatomyia glabra|[11364]|JSAUG255-11|Chloropidae|658|BOLD:ABY9688
Thaumatomyia|[11365]|CNWBG841-13|Chloropidae|655|BOLD:ACI4315
Thaumatomyia|[11366]|BBDCN289-10|Chloropidae|658|BOLD:ACF5830
Thaumatomyia|[11367]|NGNAN1454-14|Chloropidae|680|BOLD:AAH4135
Chloropidae|[11368]|NGNAB583-13|Chloropidae|658|BOLD:ACO2366
Thaumatomyia|[11369]|BBDCN782-10|Chloropidae|658|BOLD:ABZ1503
Thaumatomyia bistriata|[11370]|NGNAP235-14|Chloropidae|664|BOLD:AAG9238
Chloropidae|[11371]|JSDIR268-11|Chloropidae|658|BOLD:ABA0784
Chloropidae|[11372]|JSDIR075-11|Chloropidae|658|BOLD:ABA0783
Chloropidae|[11373]|JSDIR313-11|Chloropidae|658|BOLD:ABA0785
Dicraeus ingratus|[11374]|BBDCM618-10|Chloropidae|658|BOLD:AAH4160
Dicraeus|[11375]|TTDBW494-09|Chloropidae|657|BOLD:ACX3600
Dicraeus|[11376]|TTDBW513-09|Chloropidae|632|BOLD:AAP6312
Dicraeus|[11377]|NGNAI1269-13|Chloropidae|670|BOLD:ACX3956
Chloropidae|[11378]|BBDCN303-10|Chloropidae|658|BOLD:ACX3957
Dicraeus|[11379]|CNKTC1143-15|Chloropidae|564|BOLD:ACX4000
Chloropidae|[11380]|JSDIR652-11|Chloropidae|658|BOLD:ABA0786
Oscinellinae|[11381]|CNGBC1055-13|Chloropidae|658|BOLD:ACL0126
Oscinellinae|[11382]|CNJAG1296-12|Chloropidae|664|BOLD:AAP6314
Fiebrigella|[11383]|NGNAR262-14|Chloropidae|658|BOLD:ACL9671
Fiebrigella|[11384]|CNWBB428-13|Chloropidae|670|BOLD:AAV6120
Fiebrigella|[11385]|CNWBE034-13|Chloropidae|670|BOLD:ACI5068
Fiebrigella|[11386]|CNJAG982-12|Chloropidae|673|BOLD:AAH4212
Chloropidae|[11387]|NGNAP241-14|Chloropidae|676|BOLD:ACI2958
Chloropidae|[11388]|CNJAF1232-12|Chloropidae|670|BOLD:ACC8290
Fiebrigella|[11389]|NGNAQ1202-14|Chloropidae|658|BOLD:ACM1058
Chloropidae|[11390]|CNWBH360-13|Chloropidae|658|BOLD:ACG5773
Chloropidae|[11391]|SMTPI3573-14|Chloropidae|588|BOLD:ACP8274
Chloropidae|[11392]|JSDIR037-11|Chloropidae|658|BOLD:ABA0781
Gaurax|[11393]|SSPAB397-13|Chloropidae|661|BOLD:AAG1520
Gaurax|[11394]|CNEID1586-12|Chloropidae|664|BOLD:ABA0782
Gaurax|[11395]|CNEIC222-12|Chloropidae|673|BOLD:AAL7387
Gaurax|[11396]|CNJAB860-12|Chloropidae|658|BOLD:ACC0979
Gaurax|[11397]|SSEIC644-13|Chloropidae|670|BOLD:ACI7536
Chloropidae|[11398]|SSJAD5740-13|Chloropidae|661|BOLD:AAV6131

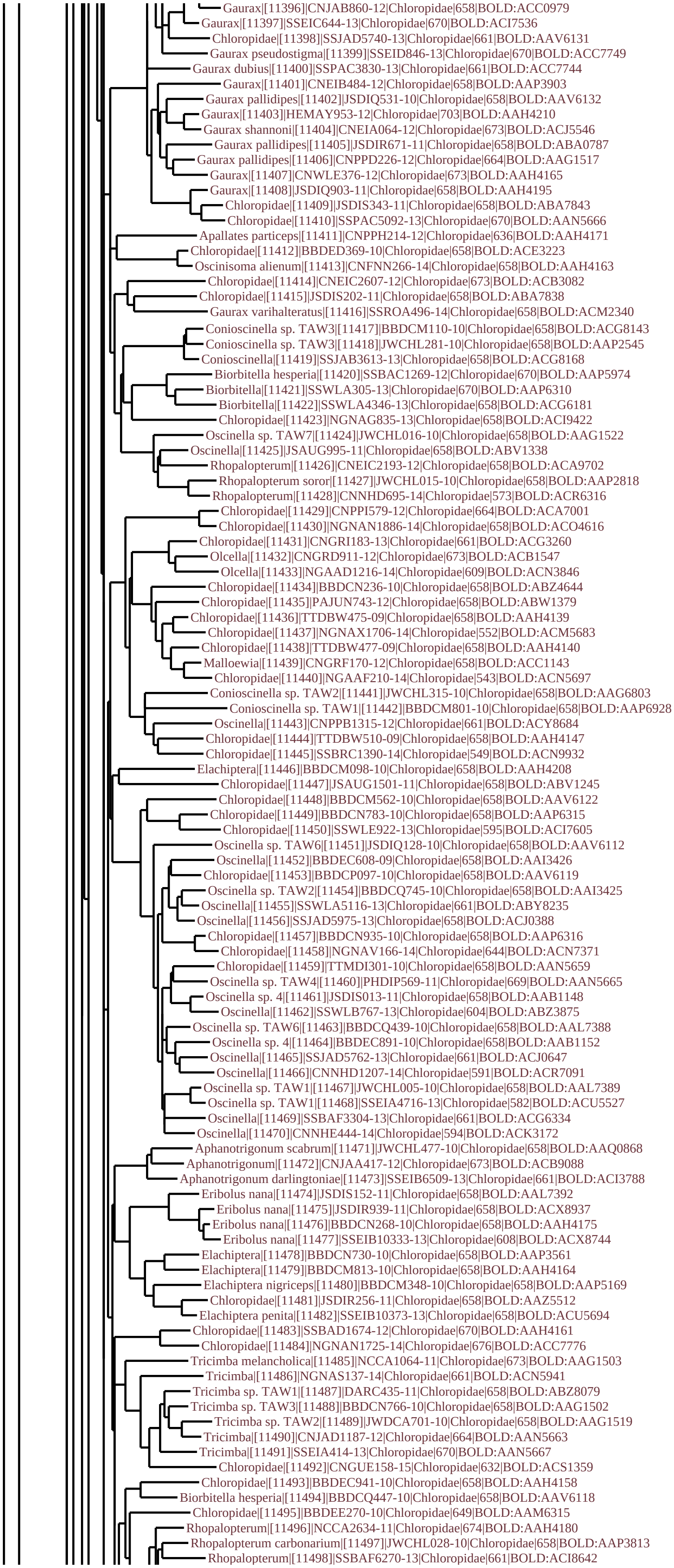
Gaurax|[11396]|CNJAB860-12|Chloropidae|658|BOLD:ACC0979
Gaurax|[11397]|SSEIC644-13|Chloropidae|670|BOLD:ACI7536
Chloropidae|[11398]|SSJAD5740-13|Chloropidae|661|BOLD:AAV6131
Gaurax pseudostigma|[11399]|SSEID846-13|Chloropidae|670|BOLD:ACC7749
Gaurax dubius|[11400]|SSPAC3830-13|Chloropidae|661|BOLD:ACC7744
Gaurax|[11401]|CNEIB484-12|Chloropidae|658|BOLD:AAP3903
Gaurax pallidipes|[11402]|JSDIQ531-10|Chloropidae|658|BOLD:AAV6132
Gaurax|[11403]|HEMAY953-12|Chloropidae|703|BOLD:AAH4210
Gaurax shannoni|[11404]|CNEIA064-12|Chloropidae|673|BOLD:ACJ5546
Gaurax pallidipes|[11405]|JSDIR671-11|Chloropidae|658|BOLD:ABA0787
Gaurax pallidipes|[11406]|CNPPD226-12|Chloropidae|664|BOLD:AAG1517
Gaurax|[11407]|CNWLE376-12|Chloropidae|673|BOLD:AAH4165
Gaurax|[11408]|JSDIQ903-11|Chloropidae|658|BOLD:AAH4195
Chloropidae|[11409]|JSDIS343-11|Chloropidae|658|BOLD:ABA7843
Chloropidae|[11410]|SSPAC5092-13|Chloropidae|670|BOLD:AAN5666
Apallates particeps|[11411]|CNPPH214-12|Chloropidae|636|BOLD:AAH4171
Chloropidae|[11412]|BBDED369-10|Chloropidae|658|BOLD:ACE3223
Oscinisoma alienum|[11413]|CNFNN266-14|Chloropidae|658|BOLD:AAH4163
Chloropidae|[11414]|CNEIC2607-12|Chloropidae|673|BOLD:ACB3082
Chloropidae|[11415]|JSDIS202-11|Chloropidae|658|BOLD:ABA7838
Gaurax varihalteratus|[11416]|SSROA496-14|Chloropidae|658|BOLD:ACM2340
Conioscinella sp. TAW3|[11417]|BBDCM110-10|Chloropidae|658|BOLD:ACG8143
Conioscinella sp. TAW3|[11418]|JWCHL281-10|Chloropidae|658|BOLD:AAP2545
Conioscinella|[11419]|SSJAB3613-13|Chloropidae|658|BOLD:ACG8168
Biorbitella hesperia|[11420]|SSBAC1269-12|Chloropidae|670|BOLD:AAP5974
Biorbitella|[11421]|SSWLA305-13|Chloropidae|670|BOLD:AAP6310
Biorbitella|[11422]|SSWLA4346-13|Chloropidae|658|BOLD:ACG6181
Chloropidae|[11423]|NGNAG835-13|Chloropidae|658|BOLD:ACI9422
Oscinella sp. TAW7|[11424]|JWCHL016-10|Chloropidae|658|BOLD:AAG1522
Oscinella|[11425]|JSAUG995-11|Chloropidae|658|BOLD:ABV1338
Rhopalopterum|[11426]|CNEIC2193-12|Chloropidae|658|BOLD:ACA9702
Rhopalopterum soror|[11427]|JWCHL015-10|Chloropidae|658|BOLD:AAP2818
Rhopalopterum|[11428]|CNNHD695-14|Chloropidae|573|BOLD:ACR6316
Chloropidae|[11429]|CNPPI579-12|Chloropidae|664|BOLD:ACA7001
Chloropidae|[11430]|NGNAN1886-14|Chloropidae|658|BOLD:ACO4616
Chloropidae|[11431]|CNGRI183-13|Chloropidae|661|BOLD:ACG3260
Olcella|[11432]|CNGRD911-12|Chloropidae|673|BOLD:ACB1547
Olcella|[11433]|NGAAD1216-14|Chloropidae|609|BOLD:ACN3846
Chloropidae|[11434]|BBDCN236-10|Chloropidae|658|BOLD:ABZ4644
Chloropidae|[11435]|PAJUN743-12|Chloropidae|658|BOLD:ABW1379
Chloropidae|[11436]|TTDBW475-09|Chloropidae|658|BOLD:AAH4139
Chloropidae|[11437]|NGNAX1706-14|Chloropidae|552|BOLD:ACM5683
Chloropidae|[11438]|TTDBW477-09|Chloropidae|658|BOLD:AAH4140
Malloewia|[11439]|CNGRF170-12|Chloropidae|658|BOLD:ACC1143
Chloropidae|[11440]|NGAAF210-14|Chloropidae|543|BOLD:ACN5697
Conioscinella sp. TAW2|[11441]|JWCHL315-10|Chloropidae|658|BOLD:AAG6803
Conioscinella sp. TAW1|[11442]|BBDCM801-10|Chloropidae|658|BOLD:AAP6928
Oscinella|[11443]|CNPPB1315-12|Chloropidae|661|BOLD:ACY8684
Chloropidae|[11444]|TTDBW510-09|Chloropidae|658|BOLD:AAH4147
Chloropidae|[11445]|SSBRC1390-14|Chloropidae|549|BOLD:ACN9932
Elachiptera|[11446]|BBDCM098-10|Chloropidae|658|BOLD:AAH4208
Chloropidae|[11447]|JSAUG1501-11|Chloropidae|658|BOLD:ABV1245
Chloropidae|[11448]|BBDCM562-10|Chloropidae|658|BOLD:AAV6122
Chloropidae|[11449]|BBDCN783-10|Chloropidae|658|BOLD:AAP6315
Chloropidae|[11450]|SSWLE922-13|Chloropidae|595|BOLD:ACI7605
Oscinella sp. TAW6|[11451]|JSDIQ128-10|Chloropidae|658|BOLD:AAV6112
Oscinella|[11452]|BBDEC608-09|Chloropidae|658|BOLD:AAI3426
Chloropidae|[11453]|BBDCP097-10|Chloropidae|658|BOLD:AAV6119
Oscinella sp. TAW2|[11454]|BBDCQ745-10|Chloropidae|658|BOLD:AAI3425
Oscinella|[11455]|SSWLA5116-13|Chloropidae|661|BOLD:ABY8235
Oscinella|[11456]|SSJAD5975-13|Chloropidae|658|BOLD:ACJ0388
Chloropidae|[11457]|BBDCN935-10|Chloropidae|658|BOLD:AAP6316
Chloropidae|[11458]|NGNAV166-14|Chloropidae|644|BOLD:ACN7371
Chloropidae|[11459]|TTMDI301-10|Chloropidae|658|BOLD:AAN5659
Oscinella sp. TAW4|[11460]|PHDIP569-11|Chloropidae|669|BOLD:AAN5665
Oscinella sp. 4|[11461]|JSDIS013-11|Chloropidae|658|BOLD:AAB1148
Oscinella|[11462]|SSWLB767-13|Chloropidae|604|BOLD:ABZ3875
Oscinella sp. TAW6|[11463]|BBDCQ439-10|Chloropidae|658|BOLD:AAL7388
Oscinella sp. 4|[11464]|BBDEC891-10|Chloropidae|658|BOLD:AAB1152
Oscinella|[11465]|SSJAD5762-13|Chloropidae|661|BOLD:ACJ0647
Oscinella|[11466]|CNNHD1207-14|Chloropidae|591|BOLD:ACR7091
Oscinella sp. TAW1|[11467]|JWCHL005-10|Chloropidae|658|BOLD:AAL7389
Oscinella sp. TAW1|[11468]|SSEIA4716-13|Chloropidae|582|BOLD:ACU5527
Oscinella|[11469]|SSBAF3304-13|Chloropidae|661|BOLD:ACG6334
Oscinella|[11470]|CNNHE444-14|Chloropidae|594|BOLD:ACK3172
Aphanotrigonum scabrum|[11471]|JWCHL477-10|Chloropidae|658|BOLD:AAQ0868
Aphanotrigonum|[11472]|CNJAA417-12|Chloropidae|673|BOLD:ACB9088
Aphanotrigonum darlingtoniae|[11473]|SSEIB6509-13|Chloropidae|661|BOLD:ACI3788
Eribolus nana|[11474]|JSDIS152-11|Chloropidae|658|BOLD:AAL7392
Eribolus nana|[11475]|JSDIR939-11|Chloropidae|658|BOLD:ACX8937
Eribolus nana|[11476]|BBDCN268-10|Chloropidae|658|BOLD:AAH4175
Eribolus nana|[11477]|SSEIB10333-13|Chloropidae|608|BOLD:ACX8744
Elachiptera|[11478]|BBDCN730-10|Chloropidae|658|BOLD:AAP3561
Elachiptera|[11479]|BBDCM813-10|Chloropidae|658|BOLD:AAH4164
Elachiptera nigriceps|[11480]|BBDCM348-10|Chloropidae|658|BOLD:AAP5169
Chloropidae|[11481]|JSDIR256-11|Chloropidae|658|BOLD:AAZ5512
Elachiptera penita|[11482]|SSEIB10373-13|Chloropidae|658|BOLD:ACU5694
Chloropidae|[11483]|SSBAD1674-12|Chloropidae|670|BOLD:AAH4161
Chloropidae|[11484]|NGNAN1725-14|Chloropidae|676|BOLD:ACC7776
Tricimba melancholica|[11485]|NCCA1064-11|Chloropidae|673|BOLD:AAG1503
Tricimba|[11486]|NGNAS137-14|Chloropidae|661|BOLD:ACN5941
Tricimba sp. TAW1|[11487]|DARC435-11|Chloropidae|658|BOLD:ABZ8079
Tricimba sp. TAW3|[11488]|BBDCN766-10|Chloropidae|658|BOLD:AAG1502
Tricimba sp. TAW2|[11489]|JWDCA701-10|Chloropidae|658|BOLD:AAG1519
Tricimba|[11490]|CNJAD1187-12|Chloropidae|664|BOLD:AAN5663
Tricimba|[11491]|SSEIA414-13|Chloropidae|670|BOLD:AAN5667
Chloropidae|[11492]|CNGUE158-15|Chloropidae|632|BOLD:ACS1359
Chloropidae|[11493]|BBDEC941-10|Chloropidae|658|BOLD:AAH4158
Biorbitella hesperia|[11494]|BBDCQ447-10|Chloropidae|658|BOLD:AAV6118
Chloropidae|[11495]|BBDEE270-10|Chloropidae|649|BOLD:AAM6315
Rhopalopterum|[11496]|NCCA2634-11|Chloropidae|674|BOLD:AAH4180
Rhopalopterum carbonarium|[11497]|JWCHL028-10|Chloropidae|658|BOLD:AAP3813
Rhopalopterum|[11498]|SSBAF6270-13|Chloropidae|661|BOLD:ACI8642

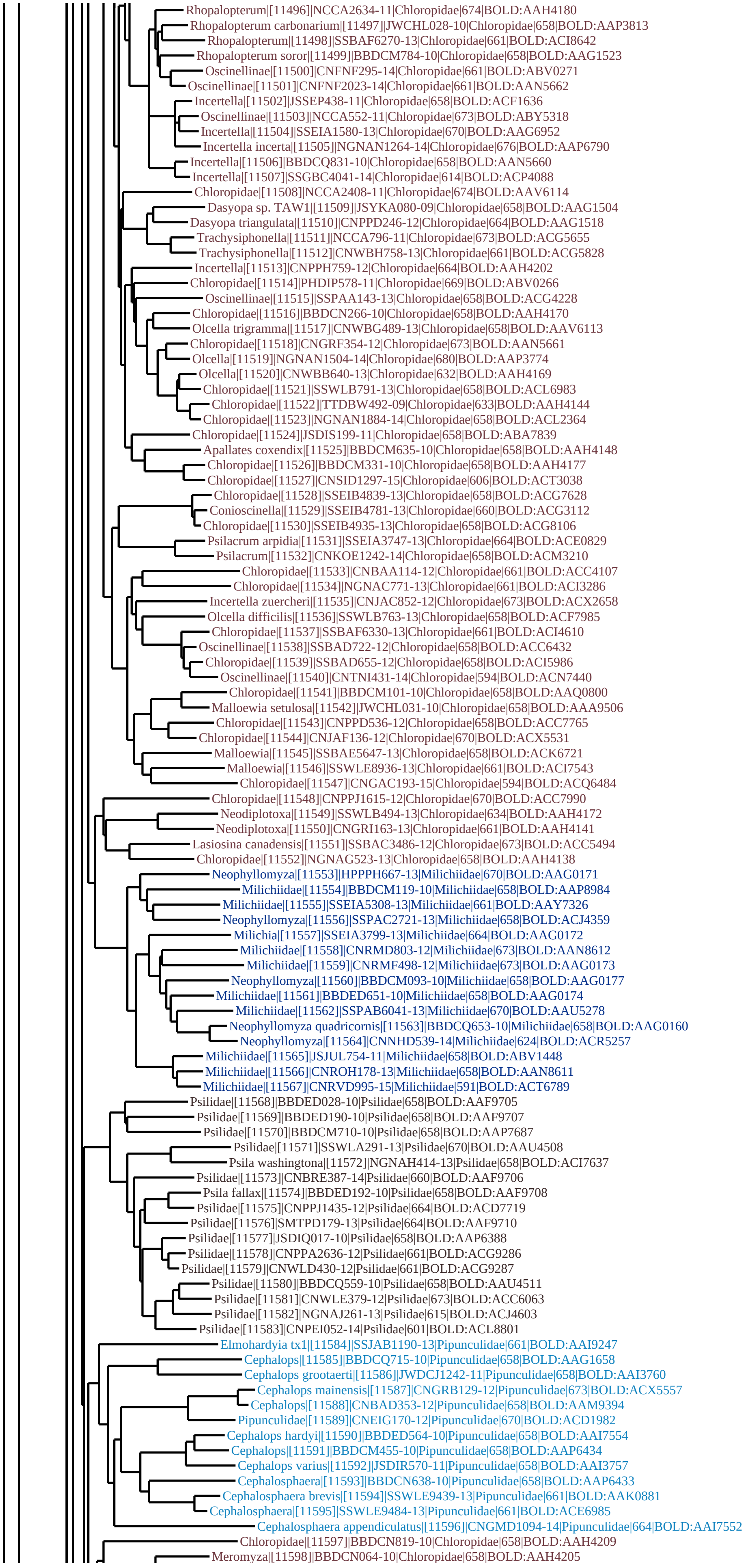
Rhopalopterum|[11496]|NCCA2634-11|Chloropidae|674|BOLD:AAH4180
Rhopalopterum carbonarium|[11497]|JWCHL028-10|Chloropidae|658|BOLD:AAP3813
Rhopalopterum|[11498]|SSBAF6270-13|Chloropidae|661|BOLD:ACI8642
Rhopalopterum soror|[11499]|BBDCM784-10|Chloropidae|658|BOLD:AAG1523
Oscinellinae|[11500]|CNFNF295-14|Chloropidae|661|BOLD:ABV0271
Oscinellinae|[11501]|CNFNF2023-14|Chloropidae|661|BOLD:AAN5662
Incertella|[11502]|JSSEP438-11|Chloropidae|658|BOLD:ACF1636
Oscinellinae|[11503]|NCCA552-11|Chloropidae|673|BOLD:ABY5318
Incertella|[11504]|SSEIA1580-13|Chloropidae|670|BOLD:AAG6952
Incertella incerta|[11505]|NGNAN1264-14|Chloropidae|676|BOLD:AAP6790
Incertella|[11506]|BBDCQ831-10|Chloropidae|658|BOLD:AAN5660
Incertella|[11507]|SSGBC4041-14|Chloropidae|614|BOLD:ACP4088
Chloropidae|[11508]|NCCA2408-11|Chloropidae|674|BOLD:AAV6114
Dasyopa sp. TAW1|[11509]|JSYKA080-09|Chloropidae|658|BOLD:AAG1504
Dasyopa triangulata|[11510]|CNPPD246-12|Chloropidae|664|BOLD:AAG1518
Trachysiphonella|[11511]|NCCA796-11|Chloropidae|673|BOLD:ACG5655
Trachysiphonella|[11512]|CNWBH758-13|Chloropidae|661|BOLD:ACG5828
Incertella|[11513]|CNPPH759-12|Chloropidae|664|BOLD:AAH4202
Chloropidae|[11514]|PHDIP578-11|Chloropidae|669|BOLD:ABV0266
Oscinellinae|[11515]|SSPAA143-13|Chloropidae|658|BOLD:ACG4228
Chloropidae|[11516]|BBDCN266-10|Chloropidae|658|BOLD:AAH4170
Olcella trigramma|[11517]|CNWBG489-13|Chloropidae|658|BOLD:AAV6113
Chloropidae|[11518]|CNGRF354-12|Chloropidae|673|BOLD:AAN5661
Olcella|[11519]|NGNAN1504-14|Chloropidae|680|BOLD:AAP3774
Olcella|[11520]|CNWBB640-13|Chloropidae|632|BOLD:AAH4169
Chloropidae|[11521]|SSWLB791-13|Chloropidae|658|BOLD:ACL6983
Chloropidae|[11522]|TTDBW492-09|Chloropidae|633|BOLD:AAH4144
Chloropidae|[11523]|NGNAN1884-14|Chloropidae|658|BOLD:ACL2364
Chloropidae|[11524]|JSDIS199-11|Chloropidae|658|BOLD:ABA7839
Apallates coxendix|[11525]|BBDCM635-10|Chloropidae|658|BOLD:AAH4148
Chloropidae|[11526]|BBDCM331-10|Chloropidae|658|BOLD:AAH4177
Chloropidae|[11527]|CNSID1297-15|Chloropidae|606|BOLD:ACT3038
Chloropidae|[11528]|SSEIB4839-13|Chloropidae|658|BOLD:ACG7628
Conioscinella|[11529]|SSEIB4781-13|Chloropidae|660|BOLD:ACG3112
Chloropidae|[11530]|SSEIB4935-13|Chloropidae|658|BOLD:ACG8106
Psilacrum arpidia|[11531]|SSEIA3747-13|Chloropidae|664|BOLD:ACE0829
Psilacrum|[11532]|CNKOE1242-14|Chloropidae|658|BOLD:ACM3210
Chloropidae|[11533]|CNBAA114-12|Chloropidae|661|BOLD:ACC4107
Chloropidae|[11534]|NGNAC771-13|Chloropidae|661|BOLD:ACI3286
Incertella zuercheri|[11535]|CNJAC852-12|Chloropidae|673|BOLD:ACX2658
Olcella difficilis|[11536]|SSWLB763-13|Chloropidae|658|BOLD:ACF7985
Chloropidae|[11537]|SSBAF6330-13|Chloropidae|661|BOLD:ACI4610
Oscinellinae|[11538]|SSBAD722-12|Chloropidae|658|BOLD:ACC6432
Chloropidae|[11539]|SSBAD655-12|Chloropidae|658|BOLD:ACI5986
Oscinellinae|[11540]|CNTNI431-14|Chloropidae|594|BOLD:ACN7440
Chloropidae|[11541]|BBDCM101-10|Chloropidae|658|BOLD:AAQ0800
Malloewia setulosa|[11542]|JWCHL031-10|Chloropidae|658|BOLD:AAA9506
Chloropidae|[11543]|CNPPD536-12|Chloropidae|658|BOLD:ACC7765
Chloropidae|[11544]|CNJAF136-12|Chloropidae|670|BOLD:ACX5531
Malloewia|[11545]|SSBAE5647-13|Chloropidae|658|BOLD:ACK6721
Malloewia|[11546]|SSWLE8936-13|Chloropidae|661|BOLD:ACI7543
Chloropidae|[11547]|CNGAC193-15|Chloropidae|594|BOLD:ACQ6484
Chloropidae|[11548]|CNPPJ1615-12|Chloropidae|670|BOLD:ACC7990
Neodiplotoxa|[11549]|SSWLB494-13|Chloropidae|634|BOLD:AAH4172
Neodiplotoxa|[11550]|CNGRI163-13|Chloropidae|661|BOLD:AAH4141
Lasiosina canadensis|[11551]|SSBAC3486-12|Chloropidae|673|BOLD:ACC5494
Chloropidae|[11552]|NGNAG523-13|Chloropidae|658|BOLD:AAH4138
Neophyllomyza|[11553]|HPPPH667-13|Milichiidae|670|BOLD:AAG0171
Milichiidae|[11554]|BBDCM119-10|Milichiidae|658|BOLD:AAP8984
Milichiidae|[11555]|SSEIA5308-13|Milichiidae|661|BOLD:AAY7326
Neophyllomyza|[11556]|SSPAC2721-13|Milichiidae|658|BOLD:ACJ4359
Milichia|[11557]|SSEIA3799-13|Milichiidae|664|BOLD:AAG0172
Milichiidae|[11558]|CNRMD803-12|Milichiidae|673|BOLD:AAN8612
Milichiidae|[11559]|CNRMF498-12|Milichiidae|673|BOLD:AAG0173
Neophyllomyza|[11560]|BBDCM093-10|Milichiidae|658|BOLD:AAG0177
Milichiidae|[11561]|BBDED651-10|Milichiidae|658|BOLD:AAG0174
Milichiidae|[11562]|SSPAB6041-13|Milichiidae|670|BOLD:AAU5278
Neophyllomyza quadricornis|[11563]|BBDCQ653-10|Milichiidae|658|BOLD:AAG0160
Neophyllomyza|[11564]|CNNHD539-14|Milichiidae|624|BOLD:ACR5257
Milichiidae|[11565]|JSJUL754-11|Milichiidae|658|BOLD:ABV1448
Milichiidae|[11566]|CNROH178-13|Milichiidae|658|BOLD:AAN8611
Milichiidae|[11567]|CNRVD995-15|Milichiidae|591|BOLD:ACT6789
Psilidae|[11568]|BBDED028-10|Psilidae|658|BOLD:AAF9705
Psilidae|[11569]|BBDED190-10|Psilidae|658|BOLD:AAF9707
Psilidae|[11570]|BBDCM710-10|Psilidae|658|BOLD:AAP7687
Psilidae|[11571]|SSWLA291-13|Psilidae|670|BOLD:AAU4508
Psila washingtona|[11572]|NGNAH414-13|Psilidae|658|BOLD:ACI7637
Psilidae|[11573]|CNBRE387-14|Psilidae|660|BOLD:AAF9706
Psila fallax|[11574]|BBDED192-10|Psilidae|658|BOLD:AAF9708
Psilidae|[11575]|CNPPJ1435-12|Psilidae|664|BOLD:ACD7719
Psilidae|[11576]|SMTPD179-13|Psilidae|664|BOLD:AAF9710
Psilidae|[11577]|JSDIQ017-10|Psilidae|658|BOLD:AAP6388
Psilidae|[11578]|CNPPA2636-12|Psilidae|661|BOLD:ACG9286
Psilidae|[11579]|CNWLD430-12|Psilidae|661|BOLD:ACG9287
Psilidae|[11580]|BBDCQ559-10|Psilidae|658|BOLD:AAU4511
Psilidae|[11581]|CNWLE379-12|Psilidae|673|BOLD:ACC6063
Psilidae|[11582]|NGNAJ261-13|Psilidae|615|BOLD:ACJ4603
Psilidae|[11583]|CNPEI052-14|Psilidae|601|BOLD:ACL8801
Elmohardyia tx1|[11584]|SSJAB1190-13|Pipunculidae|661|BOLD:AAI9247
Cephalops|[11585]|BBDCQ715-10|Pipunculidae|658|BOLD:AAG1658
Cephalops grootaerti|[11586]|JWDCJ1242-11|Pipunculidae|658|BOLD:AAI3760
Cephalops mainensis|[11587]|CNGRB129-12|Pipunculidae|673|BOLD:ACX5557
Cephalops|[11588]|CNBAD353-12|Pipunculidae|658|BOLD:AAM9394
Pipunculidae|[11589]|CNEIG170-12|Pipunculidae|670|BOLD:ACD1982
Cephalops hardyi|[11590]|BBDED564-10|Pipunculidae|658|BOLD:AAI7554
Cephalops|[11591]|BBDCM455-10|Pipunculidae|658|BOLD:AAP6434
Cephalops varius|[11592]|JSDIR570-11|Pipunculidae|658|BOLD:AAI3757
Cephalosphaera|[11593]|BBDCN638-10|Pipunculidae|658|BOLD:AAP6433
Cephalosphaera brevis|[11594]|SSWLE9439-13|Pipunculidae|661|BOLD:AAK0881
Cephalosphaera|[11595]|SSWLE9484-13|Pipunculidae|661|BOLD:ACE6985
Cephalosphaera appendiculatus|[11596]|CNGMD1094-14|Pipunculidae|664|BOLD:AAI7552
Chloropidae|[11597]|BBDCN819-10|Chloropidae|658|BOLD:AAH4209
Meromyza|[11598]|BBDCN064-10|Chloropidae|658|BOLD:AAH4205

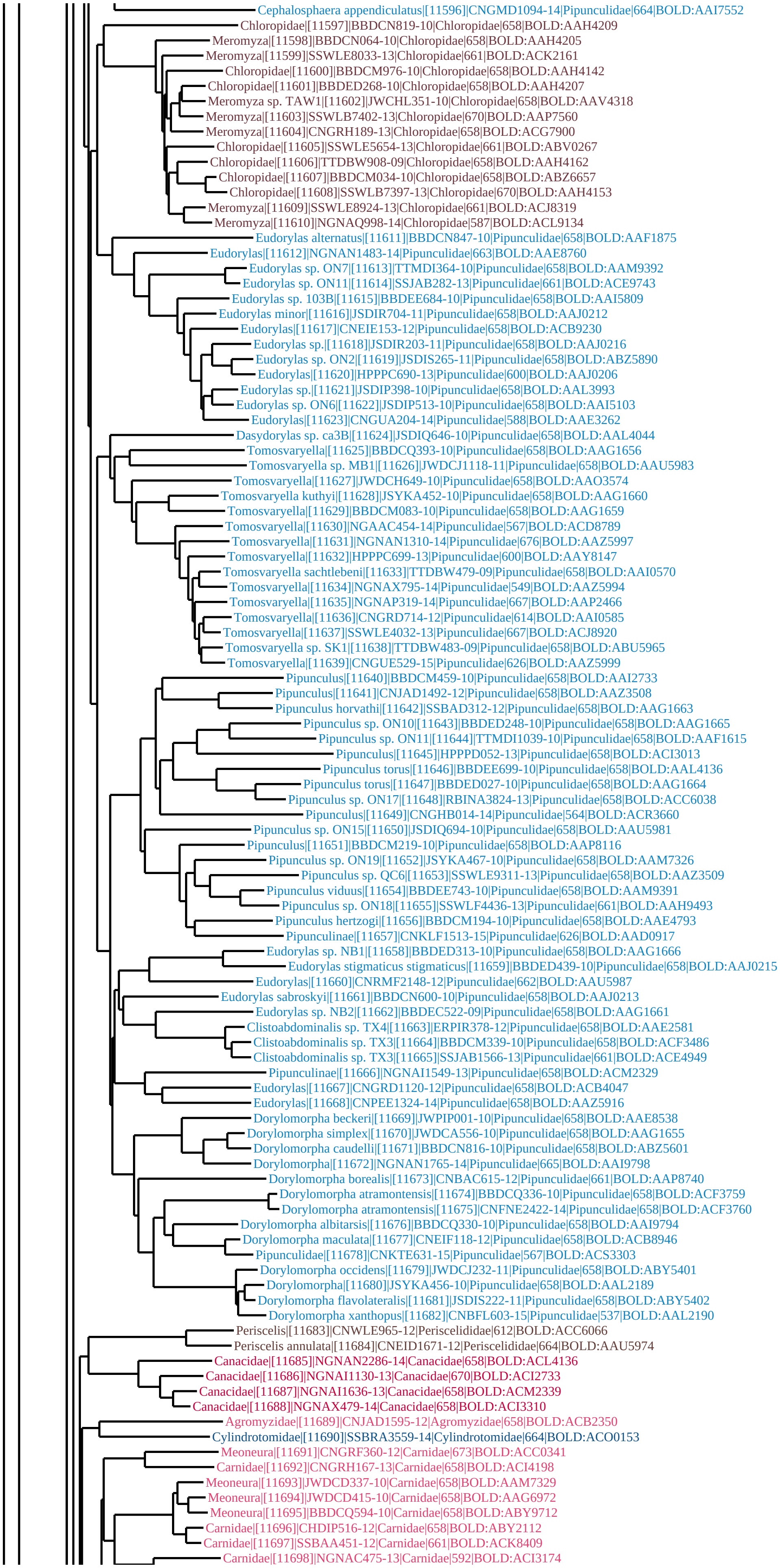

Cephalosphaera appendiculatus|[11596]|CNGMD1094-14|Pipunculidae|664|BOLD:AAI7552
Chloropidae|[11597]|BBDCN819-10|Chloropidae|658|BOLD:AAH4209
Meromyza|[11598]|BBDCN064-10|Chloropidae|658|BOLD:AAH4205
Meromyza|[11599]|SSWLE8033-13|Chloropidae|661|BOLD:ACK2161
Chloropidae|[11600]|BBDCM976-10|Chloropidae|658|BOLD:AAH4142
Chloropidae|[11601]|BBDED268-10|Chloropidae|658|BOLD:AAH4207
Meromyza sp. TAW1|[11602]|JWCHL351-10|Chloropidae|658|BOLD:AAV4318
Meromyza|[11603]|SSWLB7402-13|Chloropidae|670|BOLD:AAP7560
Meromyza|[11604]|CNGRH189-13|Chloropidae|658|BOLD:ACG7900
Chloropidae|[11605]|SSWLE5654-13|Chloropidae|661|BOLD:ABV0267
Chloropidae|[11606]|TTDBW908-09|Chloropidae|658|BOLD:AAH4162
Chloropidae|[11607]|BBDCM034-10|Chloropidae|658|BOLD:ABZ6657
Chloropidae|[11608]|SSWLB7397-13|Chloropidae|670|BOLD:AAH4153
Meromyza|[11609]|SSWLE8924-13|Chloropidae|661|BOLD:ACJ8319
Meromyza|[11610]|NGNAQ998-14|Chloropidae|587|BOLD:ACL9134
Eudorylas alternatus|[11611]|BBDCN847-10|Pipunculidae|658|BOLD:AAF1875
Eudorylas|[11612]|NGNAN1483-14|Pipunculidae|663|BOLD:AAE8760
Eudorylas sp. ON7|[11613]|TTMDI364-10|Pipunculidae|658|BOLD:AAM9392
Eudorylas sp. ON11|[11614]|SSJAB282-13|Pipunculidae|661|BOLD:ACE9743
Eudorylas sp. 103B|[11615]|BBDEE684-10|Pipunculidae|658|BOLD:AAI5809
Eudorylas minor|[11616]|JSDIR704-11|Pipunculidae|658|BOLD:AAJ0212
Eudorylas|[11617]|CNEIE153-12|Pipunculidae|658|BOLD:ACB9230
Eudorylas sp.|[11618]|JSDIR203-11|Pipunculidae|658|BOLD:AAJ0216
Eudorylas sp. ON2|[11619]|JSDIS265-11|Pipunculidae|658|BOLD:ABZ5890
Eudorylas|[11620]|HPPPC690-13|Pipunculidae|600|BOLD:AAJ0206
Eudorylas sp.|[11621]|JSDIP398-10|Pipunculidae|658|BOLD:AAL3993
Eudorylas sp. ON6|[11622]|JSDIP513-10|Pipunculidae|658|BOLD:AAI5103
Eudorylas|[11623]|CNGUA204-14|Pipunculidae|588|BOLD:AAE3262
Dasydorylas sp. ca3B|[11624]|JSDIQ646-10|Pipunculidae|658|BOLD:AAL4044
Tomosvaryella|[11625]|BBDCQ393-10|Pipunculidae|658|BOLD:AAG1656
Tomosvaryella sp. MB1|[11626]|JWDCJ1118-11|Pipunculidae|658|BOLD:AAU5983
Tomosvaryella|[11627]|JWDCH649-10|Pipunculidae|658|BOLD:AAO3574
Tomosvaryella kuthyi|[11628]|JSYKA452-10|Pipunculidae|658|BOLD:AAG1660
Tomosvaryella|[11629]|BBDCM083-10|Pipunculidae|658|BOLD:AAG1659
Tomosvaryella|[11630]|NGAAC454-14|Pipunculidae|567|BOLD:ACD8789
Tomosvaryella|[11631]|NGNAN1310-14|Pipunculidae|676|BOLD:AAZ5997
Tomosvaryella|[11632]|HPPPC699-13|Pipunculidae|600|BOLD:AAY8147
Tomosvaryella sachtlebeni|[11633]|TTDBW479-09|Pipunculidae|658|BOLD:AAI0570
Tomosvaryella|[11634]|NGNAX795-14|Pipunculidae|549|BOLD:AAZ5994
Tomosvaryella|[11635]|NGNAP319-14|Pipunculidae|667|BOLD:AAP2466
Tomosvaryella|[11636]|CNGRD714-12|Pipunculidae|614|BOLD:AAI0585
Tomosvaryella|[11637]|SSWLE4032-13|Pipunculidae|667|BOLD:ACJ8920
Tomosvaryella sp. SK1|[11638]|TTDBW483-09|Pipunculidae|658|BOLD:ABU5965
Tomosvaryella|[11639]|CNGUE529-15|Pipunculidae|626|BOLD:AAZ5999
Pipunculus|[11640]|BBDCM459-10|Pipunculidae|658|BOLD:AAI2733
Pipunculus|[11641]|CNJAD1492-12|Pipunculidae|658|BOLD:AAZ3508
Pipunculus horvathi|[11642]|SSBAD312-12|Pipunculidae|658|BOLD:AAG1663
Pipunculus sp. ON10|[11643]|BBDED248-10|Pipunculidae|658|BOLD:AAG1665
Pipunculus sp. ON11|[11644]|TTMDI1039-10|Pipunculidae|658|BOLD:AAF1615
Pipunculus|[11645]|HPPPD052-13|Pipunculidae|658|BOLD:ACI3013
Pipunculus torus|[11646]|BBDEE699-10|Pipunculidae|658|BOLD:AAL4136
Pipunculus torus|[11647]|BBDED027-10|Pipunculidae|658|BOLD:AAG1664
Pipunculus sp. ON17|[11648]|RBINA3824-13|Pipunculidae|658|BOLD:ACC6038
Pipunculus|[11649]|CNGHB014-14|Pipunculidae|564|BOLD:ACR3660
Pipunculus sp. ON15|[11650]|JSDIQ694-10|Pipunculidae|658|BOLD:AAU5981
Pipunculus|[11651]|BBDCM219-10|Pipunculidae|658|BOLD:AAP8116
Pipunculus sp. ON19|[11652]|JSYKA467-10|Pipunculidae|658|BOLD:AAM7326
Pipunculus sp. QC6|[11653]|SSWLE9311-13|Pipunculidae|658|BOLD:AAZ3509
Pipunculus viduus|[11654]|BBDEE743-10|Pipunculidae|658|BOLD:AAM9391
Pipunculus sp. ON18|[11655]|SSWLF4436-13|Pipunculidae|661|BOLD:AAH9493
Pipunculus hertzogi|[11656]|BBDCM194-10|Pipunculidae|658|BOLD:AAE4793
Pipunculinae|[11657]|CNKLF1513-15|Pipunculidae|626|BOLD:AAD0917
Eudorylas sp. NB1|[11658]|BBDED313-10|Pipunculidae|658|BOLD:AAG1666
Eudorylas stigmaticus stigmaticus|[11659]|BBDED439-10|Pipunculidae|658|BOLD:AAJ0215
Eudorylas|[11660]|CNRMF2148-12|Pipunculidae|662|BOLD:AAU5987
Eudorylas sabroskyi|[11661]|BBDCN600-10|Pipunculidae|658|BOLD:AAJ0213
Eudorylas sp. NB2|[11662]|BBDEC522-09|Pipunculidae|658|BOLD:AAG1661
Clistoabdominalis sp. TX4|[11663]|ERPIR378-12|Pipunculidae|658|BOLD:AAE2581
Clistoabdominalis sp. TX3|[11664]|BBDCM339-10|Pipunculidae|658|BOLD:ACF3486
Clistoabdominalis sp. TX3|[11665]|SSJAB1566-13|Pipunculidae|661|BOLD:ACE4949
Pipunculinae|[11666]|NGNAI1549-13|Pipunculidae|658|BOLD:ACM2329
Eudorylas|[11667]|CNGRD1120-12|Pipunculidae|658|BOLD:ACB4047
Eudorylas|[11668]|CNPEE1324-14|Pipunculidae|658|BOLD:AAZ5916
Dorylomorpha beckeri|[11669]|JWPIP001-10|Pipunculidae|658|BOLD:AAE8538
Dorylomorpha simplex|[11670]|JWDCA556-10|Pipunculidae|658|BOLD:AAG1655
Dorylomorpha caudelli|[11671]|BBDCN816-10|Pipunculidae|658|BOLD:ABZ5601
Dorylomorpha|[11672]|NGNAN1765-14|Pipunculidae|665|BOLD:AAI9798
Dorylomorpha borealis|[11673]|CNBAC615-12|Pipunculidae|661|BOLD:AAP8740
Dorylomorpha atramontensis|[11674]|BBDCQ336-10|Pipunculidae|658|BOLD:ACF3759
Dorylomorpha atramontensis|[11675]|CNFNE2422-14|Pipunculidae|658|BOLD:ACF3760
Dorylomorpha albitarsis|[11676]|BBDCQ330-10|Pipunculidae|658|BOLD:AAI9794
Dorylomorpha maculata|[11677]|CNEIF118-12|Pipunculidae|658|BOLD:ACB8946
Pipunculidae|[11678]|CNKTE631-15|Pipunculidae|567|BOLD:ACS3303
Dorylomorpha occidens|[11679]|JWDCJ232-11|Pipunculidae|658|BOLD:ABY5401
Dorylomorpha|[11680]|JSYKA456-10|Pipunculidae|658|BOLD:AAL2189
Dorylomorpha flavolateralis|[11681]|JSDIS222-11|Pipunculidae|658|BOLD:ABY5402
Dorylomorpha xanthopus|[11682]|CNBFL603-15|Pipunculidae|537|BOLD:AAL2190
Periscelis|[11683]|CNWLE965-12|Periscelididae|612|BOLD:ACC6066
Periscelis annulata|[11684]|CNEID1671-12|Periscelididae|664|BOLD:AAU5974
Canacidae|[11685]|NGNAN2286-14|Canacidae|658|BOLD:ACL4136
Canacidae|[11686]|NGNAI1130-13|Canacidae|670|BOLD:ACI2733
Canacidae|[11687]|NGNAI1636-13|Canacidae|658|BOLD:ACM2339
Canacidae|[11688]|NGNAX479-14|Canacidae|658|BOLD:ACI3310
Agromyzidae|[11689]|CNJAD1595-12|Agromyzidae|658|BOLD:ACB2350
Cylindrotomidae|[11690]|SSBRA3559-14|Cylindrotomidae|664|BOLD:ACO0153
Meoneura|[11691]|CNGRF360-12|Carnidae|673|BOLD:ACC0341
Carnidae|[11692]|CNGRH167-13|Carnidae|658|BOLD:ACI4198
Meoneura|[11693]|JWDCD337-10|Carnidae|658|BOLD:AAM7329
Meoneura|[11694]|JWDCD415-10|Carnidae|658|BOLD:AAG6972
Meoneura|[11695]|BBDCQ594-10|Carnidae|658|BOLD:ABY9712
Carnidae|[11696]|CHDIP516-12|Carnidae|658|BOLD:ABY2112
Carnidae|[11697]|SSBAA451-12|Carnidae|661|BOLD:ACK8409
Carnidae|[11698]|NGNAC475-13|Carnidae|592|BOLD:ACI3174

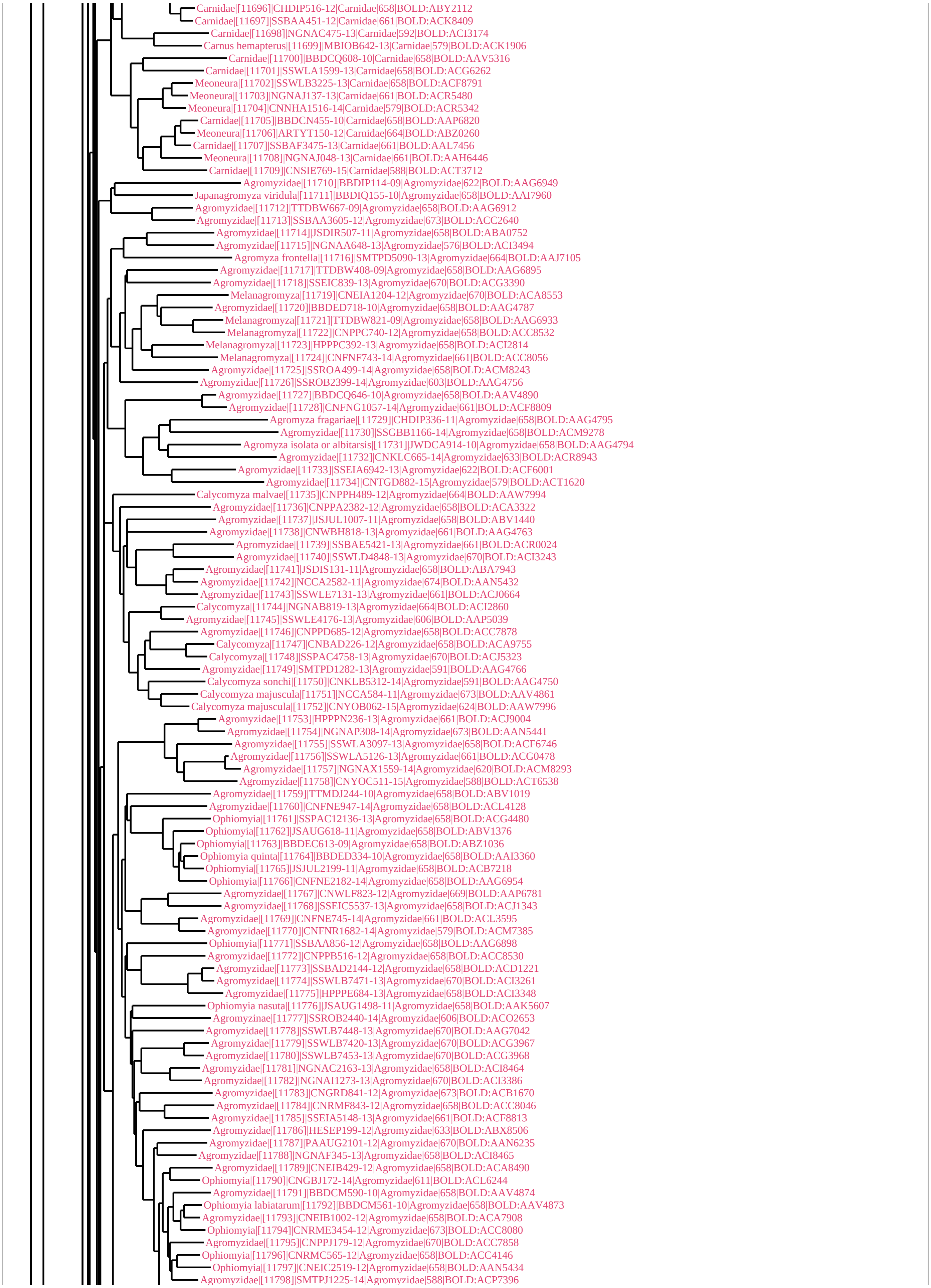
Carnidae|[11696]|CHDIP516-12|Carnidae|658|BOLD:ABY2112
Carnidae|[11697]|SSBAA451-12|Carnidae|661|BOLD:ACK8409
Carnidae|[11698]|NGNAC475-13|Carnidae|592|BOLD:ACI3174
Carnus hemapterus|[11699]|MBIOB642-13|Carnidae|579|BOLD:ACK1906
Carnidae|[11700]|BBDCQ608-10|Carnidae|658|BOLD:AAV5316
Carnidae|[11701]|SSWLA1599-13|Carnidae|658|BOLD:ACG6262
Meoneura|[11702]|SSWLB3225-13|Carnidae|658|BOLD:ACF8791
Meoneura|[11703]|NGNAJ137-13|Carnidae|661|BOLD:ACR5480
Meoneura|[11704]|CNNHA1516-14|Carnidae|579|BOLD:ACR5342
Carnidae|[11705]|BBDCN455-10|Carnidae|658|BOLD:AAP6820
Meoneura|[11706]|ARTYT150-12|Carnidae|664|BOLD:ABZ0260
Carnidae|[11707]|SSBAF3475-13|Carnidae|661|BOLD:AAL7456
Meoneura|[11708]|NGNAJ048-13|Carnidae|661|BOLD:AAH6446
Carnidae|[11709]|CNSIE769-15|Carnidae|588|BOLD:ACT3712
Agromyzidae|[11710]|BBDIP114-09|Agromyzidae|622|BOLD:AAG6949
Japanagromyza viridula|[11711]|BBDIQ155-10|Agromyzidae|658|BOLD:AAI7960
Agromyzidae|[11712]|TTDBW667-09|Agromyzidae|658|BOLD:AAG6912
Agromyzidae|[11713]|SSBAA3605-12|Agromyzidae|673|BOLD:ACC2640
Agromyzidae|[11714]|JSDIR507-11|Agromyzidae|658|BOLD:ABA0752
Agromyzidae|[11715]|NGNAA648-13|Agromyzidae|576|BOLD:ACI3494
Agromyza frontella|[11716]|SMTPD5090-13|Agromyzidae|664|BOLD:AAJ7105
Agromyzidae|[11717]|TTDBW408-09|Agromyzidae|658|BOLD:AAG6895
Agromyzidae|[11718]|SSEIC839-13|Agromyzidae|670|BOLD:ACG3390
Melanagromyza|[11719]|CNEIA1204-12|Agromyzidae|670|BOLD:ACA8553
Agromyzidae|[11720]|BBDED718-10|Agromyzidae|658|BOLD:AAG4787
Melanagromyza|[11721]|TTDBW821-09|Agromyzidae|658|BOLD:AAG6933
Melanagromyza|[11722]|CNPPC740-12|Agromyzidae|658|BOLD:ACC8532
Melanagromyza|[11723]|HPPPC392-13|Agromyzidae|658|BOLD:ACI2814
Melanagromyza|[11724]|CNFNF743-14|Agromyzidae|661|BOLD:ACC8056
Agromyzidae|[11725]|SSROA499-14|Agromyzidae|658|BOLD:ACM8243
Agromyzidae|[11726]|SSROB2399-14|Agromyzidae|603|BOLD:AAG4756
Agromyzidae|[11727]|BBDCQ646-10|Agromyzidae|658|BOLD:AAV4890
Agromyzidae|[11728]|CNFNG1057-14|Agromyzidae|661|BOLD:ACF8809
Agromyza fragariae|[11729]|CHDIP336-11|Agromyzidae|658|BOLD:AAG4795
Agromyzidae|[11730]|SSGBB1166-14|Agromyzidae|658|BOLD:ACM9278
Agromyza isolata or albitarsis|[11731]|JWDCA914-10|Agromyzidae|658|BOLD:AAG4794
Agromyzidae|[11732]|CNKLC665-14|Agromyzidae|633|BOLD:ACR8943
Agromyzidae|[11733]|SSEIA6942-13|Agromyzidae|622|BOLD:ACF6001
Agromyzidae|[11734]|CNTGD882-15|Agromyzidae|579|BOLD:ACT1620
Calycomyza malvae|[11735]|CNPPH489-12|Agromyzidae|664|BOLD:AAW7994
Agromyzidae|[11736]|CNPPA2382-12|Agromyzidae|658|BOLD:ACA3322
Agromyzidae|[11737]|JSJUL1007-11|Agromyzidae|658|BOLD:ABV1440
Agromyzidae|[11738]|CNWBH818-13|Agromyzidae|661|BOLD:AAG4763
Agromyzidae|[11739]|SSBAE5421-13|Agromyzidae|661|BOLD:ACR0024
Agromyzidae|[11740]|SSWLD4848-13|Agromyzidae|670|BOLD:ACI3243
Agromyzidae|[11741]|JSDIS131-11|Agromyzidae|658|BOLD:ABA7943
Agromyzidae|[11742]|NCCA2582-11|Agromyzidae|674|BOLD:AAN5432
Agromyzidae|[11743]|SSWLE7131-13|Agromyzidae|661|BOLD:ACJ0664
Calycomyza|[11744]|NGNAB819-13|Agromyzidae|664|BOLD:ACI2860
Agromyzidae|[11745]|SSWLE4176-13|Agromyzidae|606|BOLD:AAP5039
Agromyzidae|[11746]|CNPPD685-12|Agromyzidae|658|BOLD:ACC7878
Calycomyza|[11747]|CNBAD226-12|Agromyzidae|658|BOLD:ACA9755
Calycomyza|[11748]|SSPAC4758-13|Agromyzidae|670|BOLD:ACJ5323
Agromyzidae|[11749]|SMTPD1282-13|Agromyzidae|591|BOLD:AAG4766
Calycomyza sonchi|[11750]|CNKLB5312-14|Agromyzidae|591|BOLD:AAG4750
Calycomyza majuscula|[11751]|NCCA584-11|Agromyzidae|673|BOLD:AAV4861
Calycomyza majuscula|[11752]|CNYOB062-15|Agromyzidae|624|BOLD:AAW7996
Agromyzidae|[11753]|HPPPN236-13|Agromyzidae|661|BOLD:ACJ9004
Agromyzidae|[11754]|NGNAP308-14|Agromyzidae|673|BOLD:AAN5441
Agromyzidae|[11755]|SSWLA3097-13|Agromyzidae|658|BOLD:ACF6746
Agromyzidae|[11756]|SSWLA5126-13|Agromyzidae|661|BOLD:ACG0478
Agromyzidae|[11757]|NGNAX1559-14|Agromyzidae|620|BOLD:ACM8293
Agromyzidae|[11758]|CNYOC511-15|Agromyzidae|588|BOLD:ACT6538
Agromyzidae|[11759]|TTMDJ244-10|Agromyzidae|658|BOLD:ABV1019
Agromyzidae|[11760]|CNFNE947-14|Agromyzidae|658|BOLD:ACL4128
Ophiomyia|[11761]|SSPAC12136-13|Agromyzidae|658|BOLD:ACG4480
Ophiomyia|[11762]|JSAUG618-11|Agromyzidae|658|BOLD:ABV1376
Ophiomyia|[11763]|BBDEC613-09|Agromyzidae|658|BOLD:ABZ1036
Ophiomyia quinta|[11764]|BBDED334-10|Agromyzidae|658|BOLD:AAI3360
Ophiomyia|[11765]|JSJUL2199-11|Agromyzidae|658|BOLD:ACB7218
Ophiomyia|[11766]|CNFNE2182-14|Agromyzidae|658|BOLD:AAG6954
Agromyzidae|[11767]|CNWLF823-12|Agromyzidae|669|BOLD:AAP6781
Agromyzidae|[11768]|SSEIC5537-13|Agromyzidae|658|BOLD:ACJ1343
Agromyzidae|[11769]|CNFNE745-14|Agromyzidae|661|BOLD:ACL3595
Agromyzidae|[11770]|CNFNR1682-14|Agromyzidae|579|BOLD:ACM7385
Ophiomyia|[11771]|SSBAA856-12|Agromyzidae|658|BOLD:AAG6898
Agromyzidae|[11772]|CNPPB516-12|Agromyzidae|658|BOLD:ACC8530
Agromyzidae|[11773]|SSBAD2144-12|Agromyzidae|658|BOLD:ACD1221
Agromyzidae|[11774]|SSWLB7471-13|Agromyzidae|670|BOLD:ACI3261
Agromyzidae|[11775]|HPPPE684-13|Agromyzidae|658|BOLD:ACI3348
Ophiomyia nasuta|[11776]|JSAUG1498-11|Agromyzidae|658|BOLD:AAK5607
Agromyzinae|[11777]|SSROB2440-14|Agromyzidae|606|BOLD:ACO2653
Agromyzidae|[11778]|SSWLB7448-13|Agromyzidae|670|BOLD:AAG7042
Agromyzidae|[11779]|SSWLB7420-13|Agromyzidae|670|BOLD:ACG3967
Agromyzidae|[11780]|SSWLB7453-13|Agromyzidae|670|BOLD:ACG3968
Agromyzidae|[11781]|NGNAC2163-13|Agromyzidae|658|BOLD:ACI8464
Agromyzidae|[11782]|NGNAI1273-13|Agromyzidae|670|BOLD:ACI3386
Agromyzidae|[11783]|CNGRD841-12|Agromyzidae|673|BOLD:ACB1670
Agromyzidae|[11784]|CNRMF843-12|Agromyzidae|658|BOLD:ACC8046
Agromyzidae|[11785]|SSEIA5148-13|Agromyzidae|661|BOLD:ACF8813
Agromyzidae|[11786]|HESEP199-12|Agromyzidae|633|BOLD:ABX8506
Agromyzidae|[11787]|PAAUG2101-12|Agromyzidae|670|BOLD:AAN6235
Agromyzidae|[11788]|NGNAF345-13|Agromyzidae|658|BOLD:ACI8465
Agromyzidae|[11789]|CNEIB429-12|Agromyzidae|658|BOLD:ACA8490
Ophiomyia|[11790]|CNGBJ172-14|Agromyzidae|611|BOLD:ACL6244
Agromyzidae|[11791]|BBDCM590-10|Agromyzidae|658|BOLD:AAV4874
Ophiomyia labiatarum|[11792]|BBDCM561-10|Agromyzidae|658|BOLD:AAV4873
Agromyzidae|[11793]|CNEIB1002-12|Agromyzidae|658|BOLD:ACA7908
Ophiomyia|[11794]|CNRME3454-12|Agromyzidae|673|BOLD:ACC8080
Agromyzidae|[11795]|CNPPJ179-12|Agromyzidae|670|BOLD:ACC7858
Ophiomyia|[11796]|CNRMC565-12|Agromyzidae|658|BOLD:ACC4146
Ophiomyia|[11797]|CNEIC2519-12|Agromyzidae|658|BOLD:AAN5434
Agromyzidae|[11798]|SMTPJ1225-14|Agromyzidae|588|BOLD:ACP7396

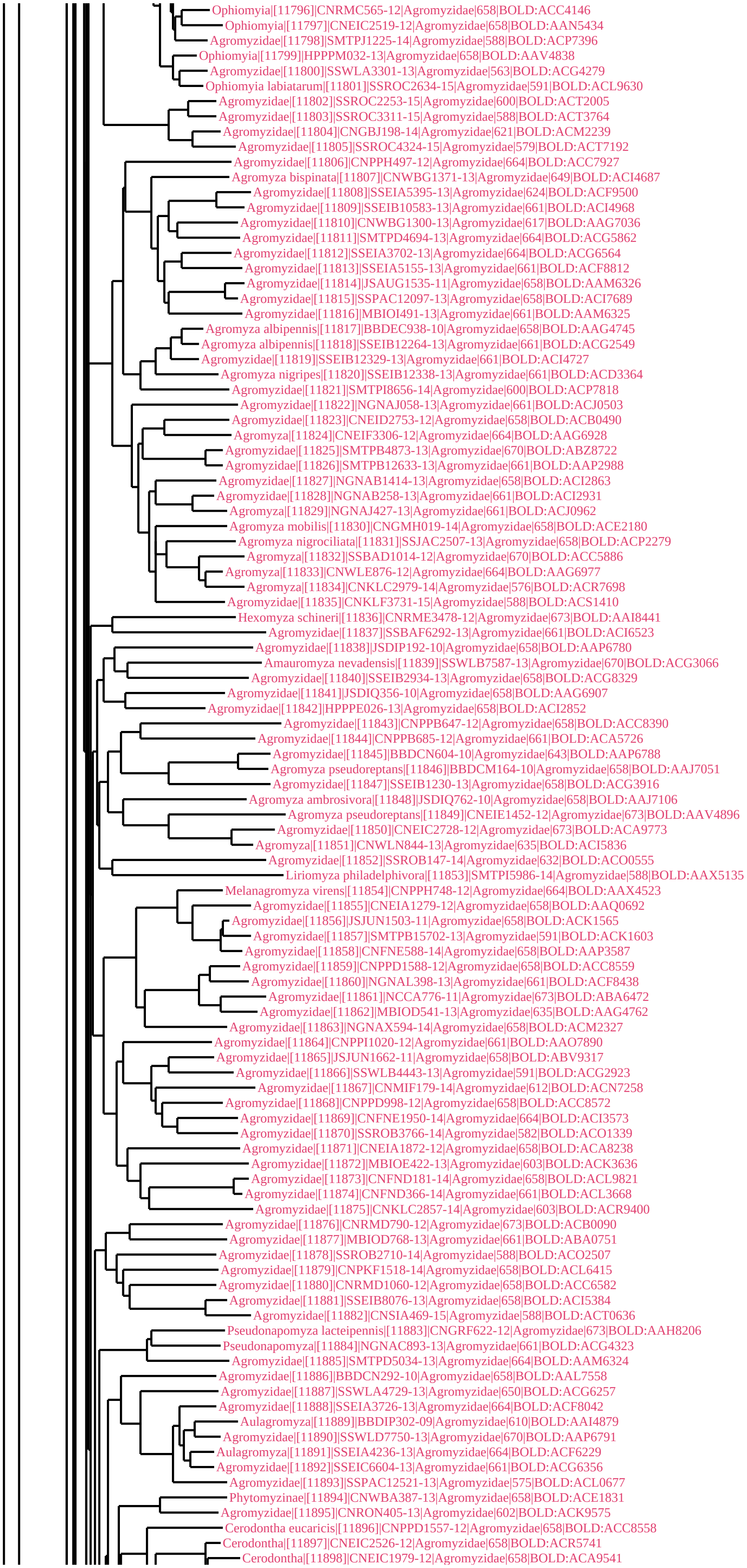

Ophiomyia|[11796]|CNRMC565-12|Agromyzidae|658|BOLD:ACC4146
Ophiomyia|[11797]|CNEIC2519-12|Agromyzidae|658|BOLD:AAN5434
Agromyzidae|[11798]|SMTPJ1225-14|Agromyzidae|588|BOLD:ACP7396
Ophiomyia|[11799]|HPPPM032-13|Agromyzidae|658|BOLD:AAV4838
Agromyzidae|[11800]|SSWLA3301-13|Agromyzidae|563|BOLD:ACG4279
Ophiomyia labiatarum|[11801]|SSROC2634-15|Agromyzidae|591|BOLD:ACL9630
Agromyzidae|[11802]|SSROC2253-15|Agromyzidae|600|BOLD:ACT2005
Agromyzidae|[11803]|SSROC3311-15|Agromyzidae|588|BOLD:ACT3764
Agromyzidae|[11804]|CNGBJ198-14|Agromyzidae|621|BOLD:ACM2239
Agromyzidae|[11805]|SSROC4324-15|Agromyzidae|579|BOLD:ACT7192
Agromyzidae|[11806]|CNPPH497-12|Agromyzidae|664|BOLD:ACC7927
Agromyza bispinata|[11807]|CNWBG1371-13|Agromyzidae|649|BOLD:ACI4687
Agromyzidae|[11808]|SSEIA5395-13|Agromyzidae|624|BOLD:ACF9500
Agromyzidae|[11809]|SSEIB10583-13|Agromyzidae|661|BOLD:ACI4968
Agromyzidae|[11810]|CNWBG1300-13|Agromyzidae|617|BOLD:AAG7036
Agromyzidae|[11811]|SMTPD4694-13|Agromyzidae|664|BOLD:ACG5862
Agromyzidae|[11812]|SSEIA3702-13|Agromyzidae|664|BOLD:ACG6564
Agromyzidae|[11813]|SSEIA5155-13|Agromyzidae|661|BOLD:ACF8812
Agromyzidae|[11814]|JSAUG1535-11|Agromyzidae|658|BOLD:AAM6326
Agromyzidae|[11815]|SSPAC12097-13|Agromyzidae|658|BOLD:ACI7689
Agromyzidae|[11816]|MBIOI491-13|Agromyzidae|661|BOLD:AAM6325
Agromyza albipennis|[11817]|BBDEC938-10|Agromyzidae|658|BOLD:AAG4745
Agromyza albipennis|[11818]|SSEIB12264-13|Agromyzidae|661|BOLD:ACG2549
Agromyzidae|[11819]|SSEIB12329-13|Agromyzidae|661|BOLD:ACI4727
Agromyza nigripes|[11820]|SSEIB12338-13|Agromyzidae|661|BOLD:ACD3364
Agromyzidae|[11821]|SMTPI8656-14|Agromyzidae|600|BOLD:ACP7818
Agromyzidae|[11822]|NGNAJ058-13|Agromyzidae|661|BOLD:ACJ0503
Agromyzidae|[11823]|CNEID2753-12|Agromyzidae|658|BOLD:ACB0490
Agromyza|[11824]|CNEIF3306-12|Agromyzidae|664|BOLD:AAG6928
Agromyzidae|[11825]|SMTPB4873-13|Agromyzidae|670|BOLD:ABZ8722
Agromyzidae|[11826]|SMTPB12633-13|Agromyzidae|661|BOLD:AAP2988
Agromyzidae|[11827]|NGNAB1414-13|Agromyzidae|658|BOLD:ACI2863
Agromyzidae|[11828]|NGNAB258-13|Agromyzidae|661|BOLD:ACI2931
Agromyza|[11829]|NGNAJ427-13|Agromyzidae|661|BOLD:ACJ0962
Agromyza mobilis|[11830]|CNGMH019-14|Agromyzidae|658|BOLD:ACE2180
Agromyza nigrociliata|[11831]|SSJAC2507-13|Agromyzidae|658|BOLD:ACP2279
Agromyza|[11832]|SSBAD1014-12|Agromyzidae|670|BOLD:ACC5886
Agromyza|[11833]|CNWLE876-12|Agromyzidae|664|BOLD:AAG6977
Agromyza|[11834]|CNKLC2979-14|Agromyzidae|576|BOLD:ACR7698
Agromyzidae|[11835]|CNKLF3731-15|Agromyzidae|588|BOLD:ACS1410
Hexomyza schineri|[11836]|CNRME3478-12|Agromyzidae|673|BOLD:AAI8441
Agromyzidae|[11837]|SSBAF6292-13|Agromyzidae|661|BOLD:ACI6523
Agromyzidae|[11838]|JSDIP192-10|Agromyzidae|658|BOLD:AAP6780
Amauromyza nevadensis|[11839]|SSWLB7587-13|Agromyzidae|670|BOLD:ACG3066
Agromyzidae|[11840]|SSEIB2934-13|Agromyzidae|658|BOLD:ACG8329
Agromyzidae|[11841]|JSDIQ356-10|Agromyzidae|658|BOLD:AAG6907
Agromyzidae|[11842]|HPPPE026-13|Agromyzidae|658|BOLD:ACI2852
Agromyzidae|[11843]|CNPPB647-12|Agromyzidae|658|BOLD:ACC8390
Agromyzidae|[11844]|CNPPB685-12|Agromyzidae|661|BOLD:ACA5726
Agromyzidae|[11845]|BBDCN604-10|Agromyzidae|643|BOLD:AAP6788
Agromyza pseudoreptans|[11846]|BBDCM164-10|Agromyzidae|658|BOLD:AAJ7051
Agromyzidae|[11847]|SSEIB1230-13|Agromyzidae|658|BOLD:ACG3916
Agromyza ambrosivora|[11848]|JSDIQ762-10|Agromyzidae|658|BOLD:AAJ7106
Agromyza pseudoreptans|[11849]|CNEIE1452-12|Agromyzidae|673|BOLD:AAV4896
Agromyzidae|[11850]|CNEIC2728-12|Agromyzidae|673|BOLD:ACA9773
Agromyza|[11851]|CNWLN844-13|Agromyzidae|635|BOLD:ACI5836
Agromyzidae|[11852]|SSROB147-14|Agromyzidae|632|BOLD:ACO0555
Liriomyza philadelphivora|[11853]|SMTPI5986-14|Agromyzidae|588|BOLD:AAX5135
Melanagromyza virens|[11854]|CNPPH748-12|Agromyzidae|664|BOLD:AAX4523
Agromyzidae|[11855]|CNEIA1279-12|Agromyzidae|658|BOLD:AAQ0692
Agromyzidae|[11856]|JSJUN1503-11|Agromyzidae|658|BOLD:ACK1565
Agromyzidae|[11857]|SMTPB15702-13|Agromyzidae|591|BOLD:ACK1603
Agromyzidae|[11858]|CNFNE588-14|Agromyzidae|658|BOLD:AAP3587
Agromyzidae|[11859]|CNPPD1588-12|Agromyzidae|658|BOLD:ACC8559
Agromyzidae|[11860]|NGNAL398-13|Agromyzidae|661|BOLD:ACF8438
Agromyzidae|[11861]|NCCA776-11|Agromyzidae|673|BOLD:ABA6472
Agromyzidae|[11862]|MBIOD541-13|Agromyzidae|635|BOLD:AAG4762
Agromyzidae|[11863]|NGNAX594-14|Agromyzidae|658|BOLD:ACM2327
Agromyzidae|[11864]|CNPPI1020-12|Agromyzidae|661|BOLD:AAO7890
Agromyzidae|[11865]|JSJUN1662-11|Agromyzidae|658|BOLD:ABV9317
Agromyzidae|[11866]|SSWLB4443-13|Agromyzidae|591|BOLD:ACG2923
Agromyzidae|[11867]|CNMIF179-14|Agromyzidae|612|BOLD:ACN7258
Agromyzidae|[11868]|CNPPD998-12|Agromyzidae|658|BOLD:ACC8572
Agromyzidae|[11869]|CNFNE1950-14|Agromyzidae|664|BOLD:ACI3573
Agromyzidae|[11870]|SSROB3766-14|Agromyzidae|582|BOLD:ACO1339
Agromyzidae|[11871]|CNEIA1872-12|Agromyzidae|658|BOLD:ACA8238
Agromyzidae|[11872]|MBIOE422-13|Agromyzidae|603|BOLD:ACK3636
Agromyzidae|[11873]|CNFND181-14|Agromyzidae|658|BOLD:ACL9821
Agromyzidae|[11874]|CNFND366-14|Agromyzidae|661|BOLD:ACL3668
Agromyzidae|[11875]|CNKLC2857-14|Agromyzidae|603|BOLD:ACR9400
Agromyzidae|[11876]|CNRMD790-12|Agromyzidae|673|BOLD:ACB0090
Agromyzidae|[11877]|MBIOD768-13|Agromyzidae|661|BOLD:ABA0751
Agromyzidae|[11878]|SSROB2710-14|Agromyzidae|588|BOLD:ACO2507
Agromyzidae|[11879]|CNPKF1518-14|Agromyzidae|658|BOLD:ACL6415
Agromyzidae|[11880]|CNRMD1060-12|Agromyzidae|658|BOLD:ACC6582
Agromyzidae|[11881]|SSEIB8076-13|Agromyzidae|658|BOLD:ACI5384
Agromyzidae|[11882]|CNSIA469-15|Agromyzidae|588|BOLD:ACT0636
Pseudonapomyza lacteipennis|[11883]|CNGRF622-12|Agromyzidae|673|BOLD:AAH8206
Pseudonapomyza|[11884]|NGNAC893-13|Agromyzidae|661|BOLD:ACG4323
Agromyzidae|[11885]|SMTPD5034-13|Agromyzidae|664|BOLD:AAM6324
Agromyzidae|[11886]|BBDCN292-10|Agromyzidae|658|BOLD:AAL7558
Agromyzidae|[11887]|SSWLA4729-13|Agromyzidae|650|BOLD:ACG6257
Agromyzidae|[11888]|SSEIA3726-13|Agromyzidae|664|BOLD:ACF8042
Aulagromyza|[11889]|BBDIP302-09|Agromyzidae|610|BOLD:AAI4879
Agromyzidae|[11890]|SSWLD7750-13|Agromyzidae|670|BOLD:AAP6791
Aulagromyza|[11891]|SSEIA4236-13|Agromyzidae|664|BOLD:ACF6229
Agromyzidae|[11892]|SSEIC6604-13|Agromyzidae|661|BOLD:ACG6356
Agromyzidae|[11893]|SSPAC12521-13|Agromyzidae|575|BOLD:ACL0677
Phytomyzinae|[11894]|CNWBA387-13|Agromyzidae|658|BOLD:ACE1831
Agromyzidae|[11895]|CNRON405-13|Agromyzidae|602|BOLD:ACK9575
Cerodontha eucaricis|[11896]|CNPPD1557-12|Agromyzidae|658|BOLD:ACC8558
Cerodontha|[11897]|CNEIC2526-12|Agromyzidae|658|BOLD:ACR5741
Cerodontha|[11898]|CNEIC1979-12|Agromyzidae|658|BOLD:ACA9541

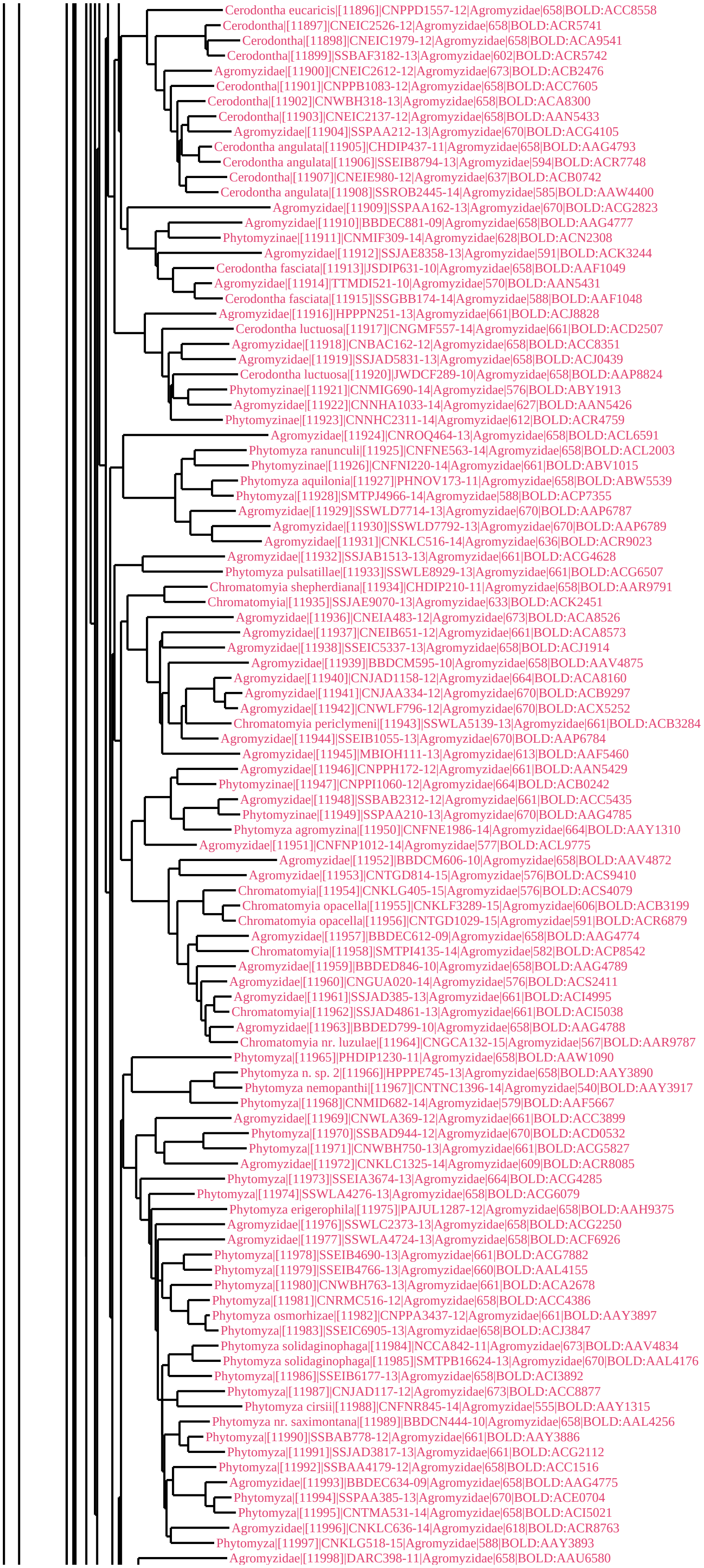
Cerodontha eucaricis|[11896]|CNPPD1557-12|Agromyzidae|658|BOLD:ACC8558
Cerodontha|[11897]|CNEIC2526-12|Agromyzidae|658|BOLD:ACR5741
Cerodontha|[11898]|CNEIC1979-12|Agromyzidae|658|BOLD:ACA9541
Cerodontha|[11899]|SSBAF3182-13|Agromyzidae|602|BOLD:ACR5742
Agromyzidae|[11900]|CNEIC2612-12|Agromyzidae|673|BOLD:ACB2476
Cerodontha|[11901]|CNPPB1083-12|Agromyzidae|658|BOLD:ACC7605
Cerodontha|[11902]|CNWBH318-13|Agromyzidae|658|BOLD:ACA8300
Cerodontha|[11903]|CNEIC2137-12|Agromyzidae|658|BOLD:AAN5433
Agromyzidae|[11904]|SSPAA212-13|Agromyzidae|670|BOLD:ACG4105
Cerodontha angulata|[11905]|CHDIP437-11|Agromyzidae|658|BOLD:AAG4793
Cerodontha angulata|[11906]|SSEIB8794-13|Agromyzidae|594|BOLD:ACR7748
Cerodontha|[11907]|CNEIE980-12|Agromyzidae|637|BOLD:ACB0742
Cerodontha angulata|[11908]|SSROB2445-14|Agromyzidae|585|BOLD:AAW4400
Agromyzidae|[11909]|SSPAA162-13|Agromyzidae|670|BOLD:ACG2823
Agromyzidae|[11910]|BBDEC881-09|Agromyzidae|658|BOLD:AAG4777
Phytomyzinae|[11911]|CNMIF309-14|Agromyzidae|628|BOLD:ACN2308
Agromyzidae|[11912]|SSJAE8358-13|Agromyzidae|591|BOLD:ACK3244
Cerodontha fasciata|[11913]|JSDIP631-10|Agromyzidae|658|BOLD:AAF1049
Agromyzidae|[11914]|TTMDI521-10|Agromyzidae|570|BOLD:AAN5431
Cerodontha fasciata|[11915]|SSGBB174-14|Agromyzidae|588|BOLD:AAF1048
Agromyzidae|[11916]|HPPPN251-13|Agromyzidae|661|BOLD:ACJ8828
Cerodontha luctuosa|[11917]|CNGMF557-14|Agromyzidae|661|BOLD:ACD2507
Agromyzidae|[11918]|CNBAC162-12|Agromyzidae|658|BOLD:ACC8351
Agromyzidae|[11919]|SSJAD5831-13|Agromyzidae|658|BOLD:ACJ0439
Cerodontha luctuosa|[11920]|JWDCF289-10|Agromyzidae|658|BOLD:AAP8824
Phytomyzinae|[11921]|CNMIG690-14|Agromyzidae|576|BOLD:ABY1913
Agromyzidae|[11922]|CNNHA1033-14|Agromyzidae|627|BOLD:AAN5426
Phytomyzinae|[11923]|CNNHC2311-14|Agromyzidae|612|BOLD:ACR4759
Agromyzidae|[11924]|CNROQ464-13|Agromyzidae|658|BOLD:ACL6591
Phytomyza ranunculi|[11925]|CNFNE563-14|Agromyzidae|658|BOLD:ACL2003
Phytomyzinae|[11926]|CNFNI220-14|Agromyzidae|661|BOLD:ABV1015
Phytomyza aquilonia|[11927]|PHNOV173-11|Agromyzidae|658|BOLD:ABW5539
Phytomyza|[11928]|SMTPJ4966-14|Agromyzidae|588|BOLD:ACP7355
Agromyzidae|[11929]|SSWLD7714-13|Agromyzidae|670|BOLD:AAP6787
Agromyzidae|[11930]|SSWLD7792-13|Agromyzidae|670|BOLD:AAP6789
Agromyzidae|[11931]|CNKLC516-14|Agromyzidae|636|BOLD:ACR9023
Agromyzidae|[11932]|SSJAB1513-13|Agromyzidae|661|BOLD:ACG4628
Phytomyza pulsatillae|[11933]|SSWLE8929-13|Agromyzidae|661|BOLD:ACG6507
Chromatomyia shepherdiana|[11934]|CHDIP210-11|Agromyzidae|658|BOLD:AAR9791
Chromatomyia|[11935]|SSJAE9070-13|Agromyzidae|633|BOLD:ACK2451
Agromyzidae|[11936]|CNEIA483-12|Agromyzidae|673|BOLD:ACA8526
Agromyzidae|[11937]|CNEIB651-12|Agromyzidae|661|BOLD:ACA8573
Agromyzidae|[11938]|SSEIC5337-13|Agromyzidae|658|BOLD:ACJ1914
Agromyzidae|[11939]|BBDCM595-10|Agromyzidae|658|BOLD:AAV4875
Agromyzidae|[11940]|CNJAD1158-12|Agromyzidae|664|BOLD:ACA8160
Agromyzidae|[11941]|CNJAA334-12|Agromyzidae|670|BOLD:ACB9297
Agromyzidae|[11942]|CNWLF796-12|Agromyzidae|670|BOLD:ACX5252
Chromatomyia periclymeni|[11943]|SSWLA5139-13|Agromyzidae|661|BOLD:ACB3284
Agromyzidae|[11944]|SSEIB1055-13|Agromyzidae|670|BOLD:AAP6784
Agromyzidae|[11945]|MBIOH111-13|Agromyzidae|613|BOLD:AAF5460
Agromyzidae|[11946]|CNPPH172-12|Agromyzidae|661|BOLD:AAN5429
Phytomyzinae|[11947]|CNPPI1060-12|Agromyzidae|664|BOLD:ACB0242
Agromyzidae|[11948]|SSBAB2312-12|Agromyzidae|661|BOLD:ACC5435
Phytomyzinae|[11949]|SSPAA210-13|Agromyzidae|670|BOLD:AAG4785
Phytomyza agromyzina|[11950]|CNFNE1986-14|Agromyzidae|664|BOLD:AAY1310
Agromyzidae|[11951]|CNFNP1012-14|Agromyzidae|577|BOLD:ACL9775
Agromyzidae|[11952]|BBDCM606-10|Agromyzidae|658|BOLD:AAV4872
Agromyzidae|[11953]|CNTGD814-15|Agromyzidae|576|BOLD:ACS9410
Chromatomyia|[11954]|CNKLG405-15|Agromyzidae|576|BOLD:ACS4079
Chromatomyia opacella|[11955]|CNKLF3289-15|Agromyzidae|606|BOLD:ACB3199
Chromatomyia opacella|[11956]|CNTGD1029-15|Agromyzidae|591|BOLD:ACR6879
Agromyzidae|[11957]|BBDEC612-09|Agromyzidae|658|BOLD:AAG4774
Chromatomyia|[11958]|SMTPI4135-14|Agromyzidae|582|BOLD:ACP8542
Agromyzidae|[11959]|BBDED846-10|Agromyzidae|658|BOLD:AAG4789
Agromyzidae|[11960]|CNGUA020-14|Agromyzidae|576|BOLD:ACS2411
Agromyzidae|[11961]|SSJAD385-13|Agromyzidae|661|BOLD:ACI4995
Chromatomyia|[11962]|SSJAD4861-13|Agromyzidae|661|BOLD:ACI5038
Agromyzidae|[11963]|BBDED799-10|Agromyzidae|658|BOLD:AAG4788
Chromatomyia nr. luzulae|[11964]|CNGCA132-15|Agromyzidae|567|BOLD:AAR9787
Phytomyza|[11965]|PHDIP1230-11|Agromyzidae|658|BOLD:AAW1090
Phytomyza n. sp. 2|[11966]|HPPPE745-13|Agromyzidae|658|BOLD:AAY3890
Phytomyza nemopanthi|[11967]|CNTNC1396-14|Agromyzidae|540|BOLD:AAY3917
Phytomyza|[11968]|CNMID682-14|Agromyzidae|579|BOLD:AAF5667
Agromyzidae|[11969]|CNWLA369-12|Agromyzidae|661|BOLD:ACC3899
Phytomyza|[11970]|SSBAD944-12|Agromyzidae|670|BOLD:ACD0532
Phytomyza|[11971]|CNWBH750-13|Agromyzidae|661|BOLD:ACG5827
Agromyzidae|[11972]|CNKLC1325-14|Agromyzidae|609|BOLD:ACR8085
Phytomyza|[11973]|SSEIA3674-13|Agromyzidae|664|BOLD:ACG4285
Phytomyza|[11974]|SSWLA4276-13|Agromyzidae|658|BOLD:ACG6079
Phytomyza erigerophila|[11975]|PAJUL1287-12|Agromyzidae|658|BOLD:AAH9375
Agromyzidae|[11976]|SSWLC2373-13|Agromyzidae|658|BOLD:ACG2250
Agromyzidae|[11977]|SSWLA4724-13|Agromyzidae|658|BOLD:ACF6926
Phytomyza|[11978]|SSEIB4690-13|Agromyzidae|661|BOLD:ACG7882
Phytomyza|[11979]|SSEIB4766-13|Agromyzidae|660|BOLD:AAL4155
Phytomyza|[11980]|CNWBH763-13|Agromyzidae|661|BOLD:ACA2678
Phytomyza|[11981]|CNRMC516-12|Agromyzidae|658|BOLD:ACC4386
Phytomyza osmorhizae|[11982]|CNPPA3437-12|Agromyzidae|661|BOLD:AAY3897
Phytomyza|[11983]|SSEIC6905-13|Agromyzidae|658|BOLD:ACJ3847
Phytomyza solidaginophaga|[11984]|NCCA842-11|Agromyzidae|673|BOLD:AAV4834
Phytomyza solidaginophaga|[11985]|SMTPB16624-13|Agromyzidae|670|BOLD:AAL4176
Phytomyza|[11986]|SSEIB6177-13|Agromyzidae|658|BOLD:ACI3892
Phytomyza|[11987]|CNJAD117-12|Agromyzidae|673|BOLD:ACC8877
Phytomyza cirsii|[11988]|CNFNR845-14|Agromyzidae|555|BOLD:AAY1315
Phytomyza nr. saximontana|[11989]|BBDCN444-10|Agromyzidae|658|BOLD:AAL4256
Phytomyza|[11990]|SSBAB778-12|Agromyzidae|661|BOLD:AAY3886
Phytomyza|[11991]|SSJAD3817-13|Agromyzidae|661|BOLD:ACG2112
Phytomyza|[11992]|SSBAA4179-12|Agromyzidae|658|BOLD:ACC1516
Agromyzidae|[11993]|BBDEC634-09|Agromyzidae|658|BOLD:AAG4775
Phytomyza|[11994]|SSPAA385-13|Agromyzidae|670|BOLD:ACE0704
Phytomyza|[11995]|CNTMA531-14|Agromyzidae|658|BOLD:ACI5021
Agromyzidae|[11996]|CNKLC636-14|Agromyzidae|618|BOLD:ACR8763
Phytomyza|[11997]|CNKLG518-15|Agromyzidae|588|BOLD:AAY3893
Agromyzidae|[11998]|DARC398-11|Agromyzidae|658|BOLD:AAU6580

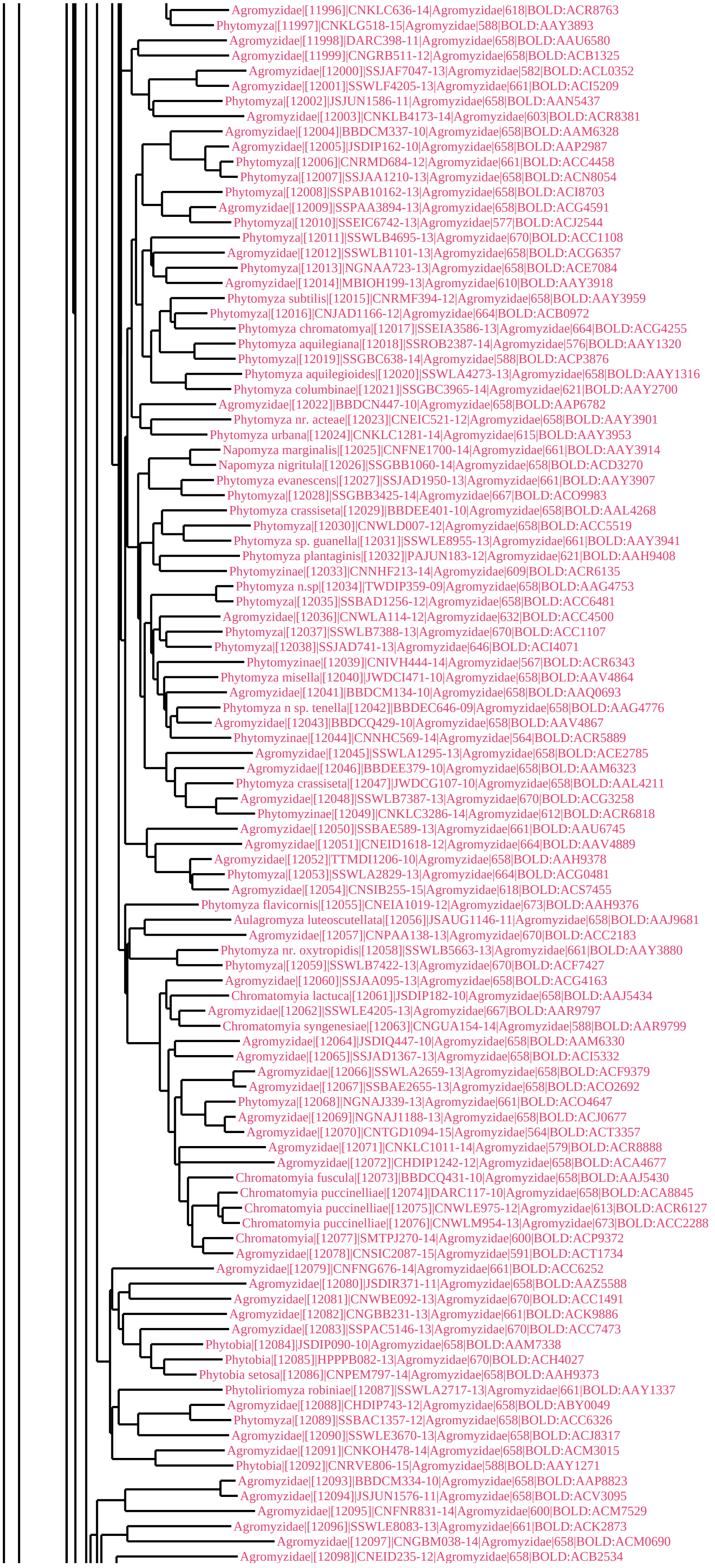

Agromyzidae|[11996]|CNKLC636-14|Agromyzidae|618|BOLD:ACR8763
Phytomyza|[11997]|CNKLG518-15|Agromyzidae|588|BOLD:AAY3893
Agromyzidae|[11998]|DARC398-11|Agromyzidae|658|BOLD:AAU6580
Agromyzidae|[11999]|CNGRB511-12|Agromyzidae|658|BOLD:ACB1325
Agromyzidae|[12000]|SSJAF7047-13|Agromyzidae|582|BOLD:ACL0352
Agromyzidae|[12001]|SSWLF4205-13|Agromyzidae|661|BOLD:ACI5209
Phytomyza|[12002]|JSJUN1586-11|Agromyzidae|658|BOLD:AAN5437
Agromyzidae|[12003]|CNKLB4173-14|Agromyzidae|603|BOLD:ACR8381
Agromyzidae|[12004]|BBDCM337-10|Agromyzidae|658|BOLD:AAM6328
Agromyzidae|[12005]|JSDIP162-10|Agromyzidae|658|BOLD:AAP2987
Phytomyza|[12006]|CNRMD684-12|Agromyzidae|661|BOLD:ACC4458
Phytomyza|[12007]|SSJAA1210-13|Agromyzidae|658|BOLD:ACN8054
Phytomyza|[12008]|SSPAB10162-13|Agromyzidae|658|BOLD:ACI8703
Agromyzidae|[12009]|SSPAA3894-13|Agromyzidae|658|BOLD:ACG4591
Phytomyza|[12010]|SSEIC6742-13|Agromyzidae|577|BOLD:ACJ2544
Phytomyza|[12011]|SSWLB4695-13|Agromyzidae|670|BOLD:ACC1108
Agromyzidae|[12012]|SSWLB1101-13|Agromyzidae|658|BOLD:ACG6357
Phytomyza|[12013]|NGNAA723-13|Agromyzidae|658|BOLD:ACE7084
Agromyzidae|[12014]|MBIOH199-13|Agromyzidae|610|BOLD:AAY3918
Phytomyza subtilis|[12015]|CNRMF394-12|Agromyzidae|658|BOLD:AAY3959
Phytomyza|[12016]|CNJAD1166-12|Agromyzidae|664|BOLD:ACB0972
Phytomyza chromatomyia|[12017]|SSEIA3586-13|Agromyzidae|664|BOLD:ACG4255
Phytomyza aquilegiana|[12018]|SSROB2387-14|Agromyzidae|576|BOLD:AAY1320
Phytomyza|[12019]|SSGBC638-14|Agromyzidae|588|BOLD:ACP3876
Phytomyza aquilegioides|[12020]|SSWLA4273-13|Agromyzidae|658|BOLD:AAY1316
Phytomyza columbinae|[12021]|SSGBC3965-14|Agromyzidae|621|BOLD:AAY2700
Agromyzidae|[12022]|BBDCN447-10|Agromyzidae|658|BOLD:AAP6782
Phytomyza nr. acteae|[12023]|CNEIC521-12|Agromyzidae|658|BOLD:AAY3901
Phytomyza urbana|[12024]|CNKLC1281-14|Agromyzidae|615|BOLD:AAY3953
Napomyza marginalis|[12025]|CNFNE1700-14|Agromyzidae|661|BOLD:AAY3914
Napomyza nigritula|[12026]|SSGBB1060-14|Agromyzidae|658|BOLD:ACD3270
Phytomyza evanescens|[12027]|SSJAD1950-13|Agromyzidae|661|BOLD:AAY3907
Phytomyza|[12028]|SSGBB3425-14|Agromyzidae|667|BOLD:ACO9983
Phytomyza crassiseta|[12029]|BBDEE401-10|Agromyzidae|658|BOLD:AAL4268
Phytomyza|[12030]|CNWLD007-12|Agromyzidae|658|BOLD:ACC5519
Phytomyza sp. guanella|[12031]|SSWLE8955-13|Agromyzidae|661|BOLD:AAY3941
Phytomyza plantaginis|[12032]|PAJUN183-12|Agromyzidae|621|BOLD:AAH9408
Phytomyzinae|[12033]|CNNHF213-14|Agromyzidae|609|BOLD:ACR6135
Phytomyza n.sp|[12034]|TWDIP359-09|Agromyzidae|658|BOLD:AAG4753
Phytomyza|[12035]|SSBAD1256-12|Agromyzidae|658|BOLD:ACC6481
Agromyzidae|[12036]|CNWLA114-12|Agromyzidae|632|BOLD:ACC4500
Phytomyza|[12037]|SSWLB7388-13|Agromyzidae|670|BOLD:ACC1107
Phytomyza|[12038]|SSJAD741-13|Agromyzidae|646|BOLD:ACI4071
Phytomyzinae|[12039]|CNIVH444-14|Agromyzidae|567|BOLD:ACR6343
Phytomyza misella|[12040]|JWDCI471-10|Agromyzidae|658|BOLD:AAV4864
Agromyzidae|[12041]|BBDCM134-10|Agromyzidae|658|BOLD:AAQ0693
Phytomyza n sp. tenella|[12042]|BBDEC646-09|Agromyzidae|658|BOLD:AAG4776
Agromyzidae|[12043]|BBDCQ429-10|Agromyzidae|658|BOLD:AAV4867
Phytomyzinae|[12044]|CNNHC569-14|Agromyzidae|564|BOLD:ACR5889
Agromyzidae|[12045]|SSWLA1295-13|Agromyzidae|658|BOLD:ACE2785
Agromyzidae|[12046]|BBDEE379-10|Agromyzidae|658|BOLD:AAM6323
Phytomyza crassiseta|[12047]|JWDCG107-10|Agromyzidae|658|BOLD:AAL4211
Agromyzidae|[12048]|SSWLB7387-13|Agromyzidae|670|BOLD:ACG3258
Phytomyzinae|[12049]|CNKLC3286-14|Agromyzidae|612|BOLD:ACR6818
Agromyzidae|[12050]|SSBAE589-13|Agromyzidae|661|BOLD:AAU6745
Agromyzidae|[12051]|CNEID1618-12|Agromyzidae|664|BOLD:AAV4889
Agromyzidae|[12052]|TTMDI1206-10|Agromyzidae|658|BOLD:AAH9378
Phytomyza|[12053]|SSWLA2829-13|Agromyzidae|664|BOLD:ACG0481
Agromyzidae|[12054]|CNSIB255-15|Agromyzidae|618|BOLD:ACS7455
Phytomyza flavicornis|[12055]|CNEIA1019-12|Agromyzidae|673|BOLD:AAH9376
Aulagromyza luteoscutellata|[12056]|JSAUG1146-11|Agromyzidae|658|BOLD:AAJ9681
Agromyzidae|[12057]|CNPAA138-13|Agromyzidae|670|BOLD:ACC2183
Phytomyza nr. oxytropidis|[12058]|SSWLB5663-13|Agromyzidae|661|BOLD:AAY3880
Phytomyza|[12059]|SSWLB7422-13|Agromyzidae|670|BOLD:ACF7427
Agromyzidae|[12060]|SSJAA095-13|Agromyzidae|658|BOLD:ACG4163
Chromatomyia lactuca|[12061]|JSDIP182-10|Agromyzidae|658|BOLD:AAJ5434
Agromyzidae|[12062]|SSWLE4205-13|Agromyzidae|667|BOLD:AAR9797
Chromatomyia syngenesiae|[12063]|CNGUA154-14|Agromyzidae|588|BOLD:AAR9799
Agromyzidae|[12064]|JSDIQ447-10|Agromyzidae|658|BOLD:AAM6330
Agromyzidae|[12065]|SSJAD1367-13|Agromyzidae|658|BOLD:ACI5332
Agromyzidae|[12066]|SSWLA2659-13|Agromyzidae|658|BOLD:ACF9379
Agromyzidae|[12067]|SSBAE2655-13|Agromyzidae|658|BOLD:ACO2692
Phytomyza|[12068]|NGNAJ339-13|Agromyzidae|661|BOLD:ACO4647
Agromyzidae|[12069]|NGNAJ1188-13|Agromyzidae|658|BOLD:ACJ0677
Agromyzidae|[12070]|CNTGD1094-15|Agromyzidae|564|BOLD:ACT3357
Agromyzidae|[12071]|CNKLC1011-14|Agromyzidae|579|BOLD:ACR8888
Agromyzidae|[12072]|CHDIP1242-12|Agromyzidae|658|BOLD:ACA4677
Chromatomyia fuscula|[12073]|BBDCQ431-10|Agromyzidae|658|BOLD:AAJ5430
Chromatomyia puccinelliae|[12074]|DARC117-10|Agromyzidae|658|BOLD:ACA8845
Chromatomyia puccinelliae|[12075]|CNWLE975-12|Agromyzidae|613|BOLD:ACR6127
Chromatomyia puccinelliae|[12076]|CNWLM954-13|Agromyzidae|673|BOLD:ACC2288
Chromatomyia|[12077]|SMTPJ270-14|Agromyzidae|600|BOLD:ACP9372
Agromyzidae|[12078]|CNSIC2087-15|Agromyzidae|591|BOLD:ACT1734
Agromyzidae|[12079]|CNFNG676-14|Agromyzidae|661|BOLD:ACC6252
Agromyzidae|[12080]|JSDIR371-11|Agromyzidae|658|BOLD:AAZ5588
Agromyzidae|[12081]|CNWBE092-13|Agromyzidae|670|BOLD:ACC1491
Agromyzidae|[12082]|CNGBB231-13|Agromyzidae|661|BOLD:ACK9886
Agromyzidae|[12083]|SSPAC5146-13|Agromyzidae|670|BOLD:ACC7473
Phytobia|[12084]|JSDIP090-10|Agromyzidae|658|BOLD:AAM7338
Phytobia|[12085]|HPPPB082-13|Agromyzidae|670|BOLD:ACH4027
Phytobia setosa|[12086]|CNPEM797-14|Agromyzidae|658|BOLD:AAH9373
Phytoliriomyza robiniae|[12087]|SSWLA2717-13|Agromyzidae|661|BOLD:AAY1337
Agromyzidae|[12088]|CHDIP743-12|Agromyzidae|658|BOLD:ABY0049
Phytomyza|[12089]|SSBAC1357-12|Agromyzidae|658|BOLD:ACC6326
Agromyzidae|[12090]|SSWLE3670-13|Agromyzidae|658|BOLD:ACJ8317
Agromyzidae|[12091]|CNKOH478-14|Agromyzidae|658|BOLD:ACM3015
Phytobia|[12092]|CNRVE806-15|Agromyzidae|588|BOLD:AAY1271
Agromyzidae|[12093]|BBDCM334-10|Agromyzidae|658|BOLD:AAP8823
Agromyzidae|[12094]|JSJUN1576-11|Agromyzidae|658|BOLD:ACV3095
Agromyzidae|[12095]|CNFNR831-14|Agromyzidae|600|BOLD:ACM7529
Agromyzidae|[12096]|SSWLE8083-13|Agromyzidae|661|BOLD:ACK2873
Agromyzidae|[12097]|CNGBM038-14|Agromyzidae|658|BOLD:ACM0690
Agromyzidae|[12098]|CNEID235-12|Agromyzidae|658|BOLD:ACB2534

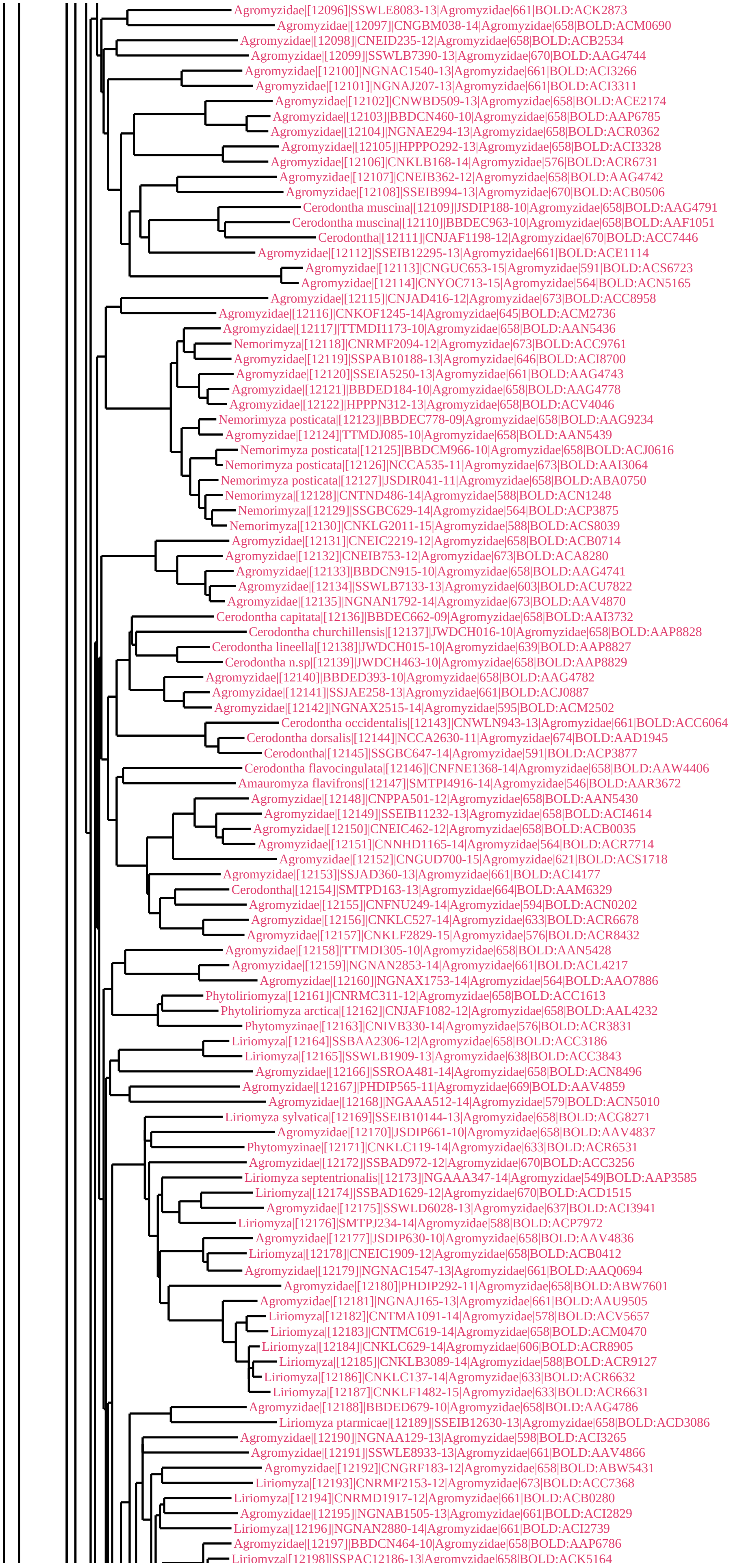

Agromyzidae|[12096]|SSWLE8083-13|Agromyzidae|661|BOLD:ACK2873
Agromyzidae|[12097]|CNGBM038-14|Agromyzidae|658|BOLD:ACM0690
Agromyzidae|[12098]|CNEID235-12|Agromyzidae|658|BOLD:ACB2534
Agromyzidae|[12099]|SSWLB7390-13|Agromyzidae|670|BOLD:AAG4744
Agromyzidae|[12100]|NGNAC1540-13|Agromyzidae|661|BOLD:ACI3266
Agromyzidae|[12101]|NGNAJ207-13|Agromyzidae|661|BOLD:ACI3311
Agromyzidae|[12102]|CNWBD509-13|Agromyzidae|658|BOLD:ACE2174
Agromyzidae|[12103]|BBDCN460-10|Agromyzidae|658|BOLD:AAP6785
Agromyzidae|[12104]|NGNAE294-13|Agromyzidae|658|BOLD:ACR0362
Agromyzidae|[12105]|HPPPO292-13|Agromyzidae|658|BOLD:ACI3328
Agromyzidae|[12106]|CNKLB168-14|Agromyzidae|576|BOLD:ACR6731
Agromyzidae|[12107]|CNEIB362-12|Agromyzidae|658|BOLD:AAG4742
Agromyzidae|[12108]|SSEIB994-13|Agromyzidae|670|BOLD:ACB0506
Cerodontha muscina|[12109]|JSDIP188-10|Agromyzidae|658|BOLD:AAG4791
Cerodontha muscina|[12110]|BBDEC963-10|Agromyzidae|658|BOLD:AAF1051
Cerodontha|[12111]|CNJAF1198-12|Agromyzidae|670|BOLD:ACC7446
Agromyzidae|[12112]|SSEIB12295-13|Agromyzidae|661|BOLD:ACE1114
Agromyzidae|[12113]|CNGUC653-15|Agromyzidae|591|BOLD:ACS6723
Agromyzidae|[12114]|CNYOC713-15|Agromyzidae|564|BOLD:ACN5165
Agromyzidae|[12115]|CNJAD416-12|Agromyzidae|673|BOLD:ACC8958
Agromyzidae|[12116]|CNKOF1245-14|Agromyzidae|645|BOLD:ACM2736
Agromyzidae|[12117]|TTMDI1173-10|Agromyzidae|658|BOLD:AAN5436
Nemorimyza|[12118]|CNRMF2094-12|Agromyzidae|673|BOLD:ACC9761
Agromyzidae|[12119]|SSPAB10188-13|Agromyzidae|646|BOLD:ACI8700
Agromyzidae|[12120]|SSEIA5250-13|Agromyzidae|661|BOLD:AAG4743
Agromyzidae|[12121]|BBDED184-10|Agromyzidae|658|BOLD:AAG4778
Agromyzidae|[12122]|HPPPN312-13|Agromyzidae|658|BOLD:ACV4046
Nemorimyza posticata|[12123]|BBDEC778-09|Agromyzidae|658|BOLD:AAG9234
Agromyzidae|[12124]|TTMDJ085-10|Agromyzidae|658|BOLD:AAN5439
Nemorimyza posticata|[12125]|BBDCM966-10|Agromyzidae|658|BOLD:ACJ0616
Nemorimyza posticata|[12126]|NCCA535-11|Agromyzidae|673|BOLD:AAI3064
Nemorimyza posticata|[12127]|JSDIR041-11|Agromyzidae|658|BOLD:ABA0750
Nemorimyza|[12128]|CNTND486-14|Agromyzidae|588|BOLD:ACN1248
Nemorimyza|[12129]|SSGBC629-14|Agromyzidae|564|BOLD:ACP3875
Nemorimyza|[12130]|CNKLG2011-15|Agromyzidae|588|BOLD:ACS8039
Agromyzidae|[12131]|CNEIC2219-12|Agromyzidae|658|BOLD:ACB0714
Agromyzidae|[12132]|CNEIB753-12|Agromyzidae|673|BOLD:ACA8280
Agromyzidae|[12133]|BBDCN915-10|Agromyzidae|658|BOLD:AAG4741
Agromyzidae|[12134]|SSWLB7133-13|Agromyzidae|603|BOLD:ACU7822
Agromyzidae|[12135]|NGNAN1792-14|Agromyzidae|673|BOLD:AAV4870
Cerodontha capitata|[12136]|BBDEC662-09|Agromyzidae|658|BOLD:AAI3732
Cerodontha churchillensis|[12137]|JWDCH016-10|Agromyzidae|658|BOLD:AAP8828
Cerodontha lineella|[12138]|JWDCH015-10|Agromyzidae|639|BOLD:AAP8827
Cerodontha n.sp|[12139]|JWDCH463-10|Agromyzidae|658|BOLD:AAP8829
Agromyzidae|[12140]|BBDED393-10|Agromyzidae|658|BOLD:AAG4782
Agromyzidae|[12141]|SSJAE258-13|Agromyzidae|661|BOLD:ACJ0887
Agromyzidae|[12142]|NGNAX2515-14|Agromyzidae|595|BOLD:ACM2502
Cerodontha occidentalis|[12143]|CNWLN943-13|Agromyzidae|661|BOLD:ACC6064
Cerodontha dorsalis|[12144]|NCCA2630-11|Agromyzidae|674|BOLD:AAD1945
Cerodontha|[12145]|SSGBC647-14|Agromyzidae|591|BOLD:ACP3877
Cerodontha flavocingulata|[12146]|CNFNE1368-14|Agromyzidae|658|BOLD:AAW4406
Amauromyza flavifrons|[12147]|SMTPI4916-14|Agromyzidae|546|BOLD:AAR3672
Agromyzidae|[12148]|CNPPA501-12|Agromyzidae|658|BOLD:AAN5430
Agromyzidae|[12149]|SSEIB11232-13|Agromyzidae|658|BOLD:ACI4614
Agromyzidae|[12150]|CNEIC462-12|Agromyzidae|658|BOLD:ACB0035
Agromyzidae|[12151]|CNNHD1165-14|Agromyzidae|564|BOLD:ACR7714
Agromyzidae|[12152]|CNGUD700-15|Agromyzidae|621|BOLD:ACS1718
Agromyzidae|[12153]|SSJAD360-13|Agromyzidae|661|BOLD:ACI4177
Cerodontha|[12154]|SMTPD163-13|Agromyzidae|664|BOLD:AAM6329
Agromyzidae|[12155]|CNFNU249-14|Agromyzidae|594|BOLD:ACN0202
Agromyzidae|[12156]|CNKLC527-14|Agromyzidae|633|BOLD:ACR6678
Agromyzidae|[12157]|CNKLF2829-15|Agromyzidae|576|BOLD:ACR8432
Agromyzidae|[12158]|TTMDI305-10|Agromyzidae|658|BOLD:AAN5428
Agromyzidae|[12159]|NGNAN2853-14|Agromyzidae|661|BOLD:ACL4217
Agromyzidae|[12160]|NGNAX1753-14|Agromyzidae|564|BOLD:AAO7886
Phytoliriomyza|[12161]|CNRMC311-12|Agromyzidae|658|BOLD:ACC1613
Phytoliriomyza arctica|[12162]|CNJAF1082-12|Agromyzidae|658|BOLD:AAL4232
Phytomyzinae|[12163]|CNIVB330-14|Agromyzidae|576|BOLD:ACR3831
Liriomyza|[12164]|SSBAA2306-12|Agromyzidae|658|BOLD:ACC3186
Liriomyza|[12165]|SSWLB1909-13|Agromyzidae|638|BOLD:ACC3843
Agromyzidae|[12166]|SSROA481-14|Agromyzidae|658|BOLD:ACN8496
Agromyzidae|[12167]|PHDIP565-11|Agromyzidae|669|BOLD:AAV4859
Agromyzidae|[12168]|NGAAA512-14|Agromyzidae|579|BOLD:ACN5010
Liriomyza sylvatica|[12169]|SSEIB10144-13|Agromyzidae|658|BOLD:ACG8271
Agromyzidae|[12170]|JSDIP661-10|Agromyzidae|658|BOLD:AAV4837
Phytomyzinae|[12171]|CNKLC119-14|Agromyzidae|633|BOLD:ACR6531
Agromyzidae|[12172]|SSBAD972-12|Agromyzidae|670|BOLD:ACC3256
Liriomyza septentrionalis|[12173]|NGAAA347-14|Agromyzidae|549|BOLD:AAP3585
Liriomyza|[12174]|SSBAD1629-12|Agromyzidae|670|BOLD:ACD1515
Agromyzidae|[12175]|SSWLD6028-13|Agromyzidae|637|BOLD:ACI3941
Liriomyza|[12176]|SMTPJ234-14|Agromyzidae|588|BOLD:ACP7972
Agromyzidae|[12177]|JSDIP630-10|Agromyzidae|658|BOLD:AAV4836
Liriomyza|[12178]|CNEIC1909-12|Agromyzidae|658|BOLD:ACB0412
Agromyzidae|[12179]|NGNAC1547-13|Agromyzidae|661|BOLD:AAQ0694
Agromyzidae|[12180]|PHDIP292-11|Agromyzidae|658|BOLD:ABW7601
Agromyzidae|[12181]|NGNAJ165-13|Agromyzidae|661|BOLD:AAU9505
Liriomyza|[12182]|CNTMA1091-14|Agromyzidae|578|BOLD:ACV5657
Liriomyza|[12183]|CNTMC619-14|Agromyzidae|658|BOLD:ACM0470
Liriomyza|[12184]|CNKLC629-14|Agromyzidae|606|BOLD:ACR8905
Liriomyza|[12185]|CNKLB3089-14|Agromyzidae|588|BOLD:ACR9127
Liriomyza|[12186]|CNKLC137-14|Agromyzidae|633|BOLD:ACR6632
Liriomyza|[12187]|CNKLF1482-15|Agromyzidae|633|BOLD:ACR6631
Agromyzidae|[12188]|BBDED679-10|Agromyzidae|658|BOLD:AAG4786
Liriomyza ptarmicae|[12189]|SSEIB12630-13|Agromyzidae|658|BOLD:ACD3086
Agromyzidae|[12190]|NGNAA129-13|Agromyzidae|598|BOLD:ACI3265
Agromyzidae|[12191]|SSWLE8933-13|Agromyzidae|661|BOLD:AAV4866
Agromyzidae|[12192]|CNGRF183-12|Agromyzidae|658|BOLD:ABW5431
Liriomyza|[12193]|CNRMF2153-12|Agromyzidae|673|BOLD:ACC7368
Liriomyza|[12194]|CNRMD1917-12|Agromyzidae|661|BOLD:ACB0280
Agromyzidae|[12195]|NGNAB1505-13|Agromyzidae|661|BOLD:ACI2829
Liriomyza|[12196]|NGNAN2880-14|Agromyzidae|661|BOLD:ACI2739
Agromyzidae|[12197]|BBDCN464-10|Agromyzidae|658|BOLD:AAP6786
Liriomyza|[12198]|SSPAC12186-13|Agromyzidae|658|BOLD:ACK5164

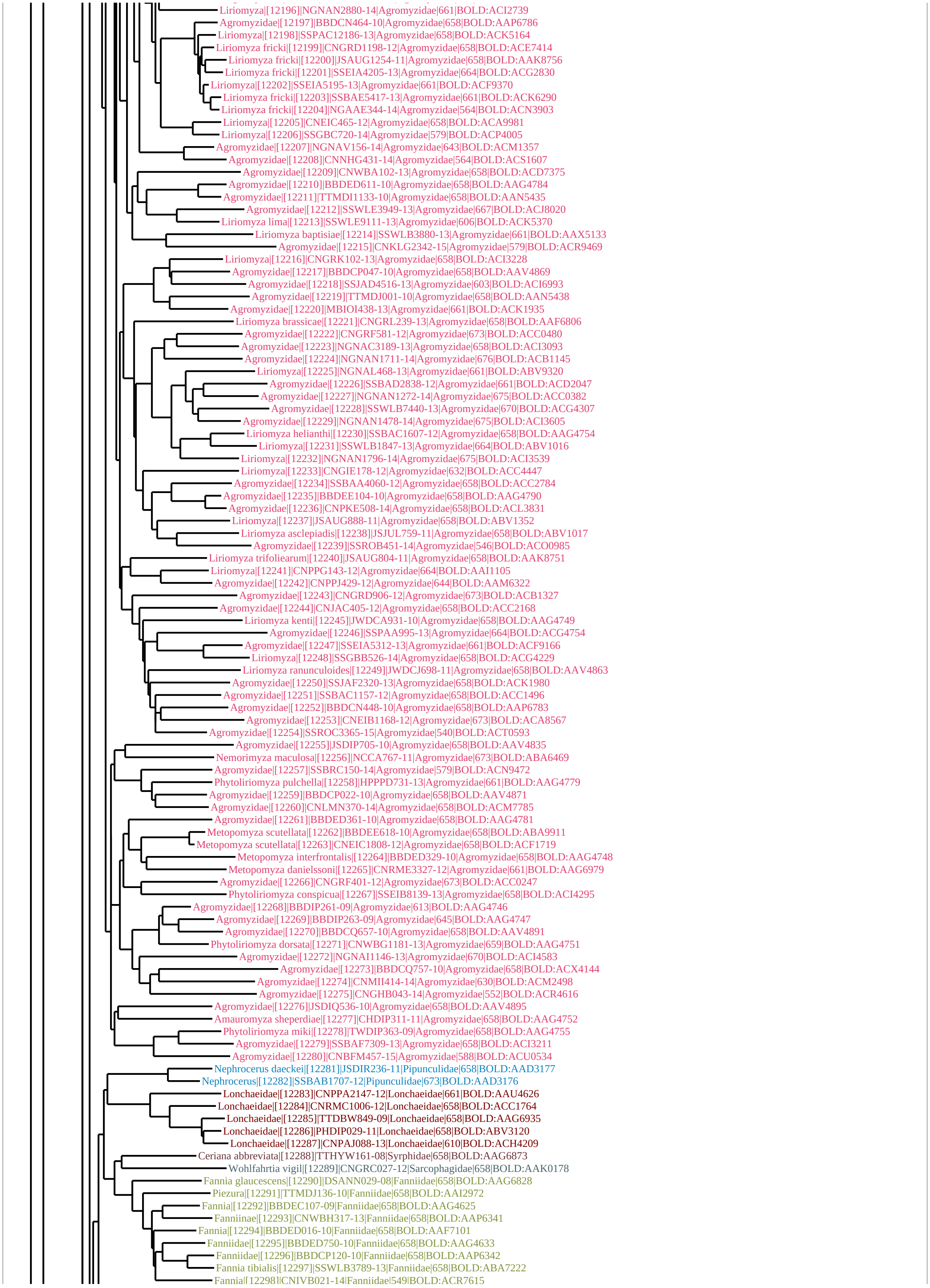
Liriomyza|[12196]|NGNAN2880-14|Agromyzidae|661|BOLD:ACI2739
Agromyzidae|[12197]|BBDCN464-10|Agromyzidae|658|BOLD:AAP6786
Liriomyza|[12198]|SSPAC12186-13|Agromyzidae|658|BOLD:ACK5164
Liriomyza fricki|[12199]|CNGRD1198-12|Agromyzidae|658|BOLD:ACE7414
Liriomyza fricki|[12200]|JSAUG1254-11|Agromyzidae|658|BOLD:AAK8756
Liriomyza fricki|[12201]|SSEIA4205-13|Agromyzidae|664|BOLD:ACG2830
Liriomyza|[12202]|SSEIA5195-13|Agromyzidae|661|BOLD:ACF9370
Liriomyza fricki|[12203]|SSBAE5417-13|Agromyzidae|661|BOLD:ACK6290
Liriomyza fricki|[12204]|NGAAE344-14|Agromyzidae|564|BOLD:ACN3903
Liriomyza|[12205]|CNEIC465-12|Agromyzidae|658|BOLD:ACA9981
Liriomyza|[12206]|SSGBC720-14|Agromyzidae|579|BOLD:ACP4005
Agromyzidae|[12207]|NGNAV156-14|Agromyzidae|643|BOLD:ACM1357
Agromyzidae|[12208]|CNNHG431-14|Agromyzidae|564|BOLD:ACS1607
Agromyzidae|[12209]|CNWBA102-13|Agromyzidae|658|BOLD:ACD7375
Agromyzidae|[12210]|BBDED611-10|Agromyzidae|658|BOLD:AAG4784
Agromyzidae|[12211]|TTMDI1133-10|Agromyzidae|658|BOLD:AAN5435
Agromyzidae|[12212]|SSWLE3949-13|Agromyzidae|667|BOLD:ACJ8020
Liriomyza lima|[12213]|SSWLE9111-13|Agromyzidae|606|BOLD:ACK5370
Liriomyza baptisiae|[12214]|SSWLB3880-13|Agromyzidae|661|BOLD:AAX5133
Agromyzidae|[12215]|CNKLG2342-15|Agromyzidae|579|BOLD:ACR9469
Liriomyza|[12216]|CNGRK102-13|Agromyzidae|658|BOLD:ACI3228
Agromyzidae|[12217]|BBDCP047-10|Agromyzidae|658|BOLD:AAV4869
Agromyzidae|[12218]|SSJAD4516-13|Agromyzidae|603|BOLD:ACI6993
Agromyzidae|[12219]|TTMDJ001-10|Agromyzidae|658|BOLD:AAN5438
Agromyzidae|[12220]|MBIOI438-13|Agromyzidae|661|BOLD:ACK1935
Liriomyza brassicae|[12221]|CNGRL239-13|Agromyzidae|658|BOLD:AAF6806
Agromyzidae|[12222]|CNGRF581-12|Agromyzidae|673|BOLD:ACC0480
Agromyzidae|[12223]|NGNAC3189-13|Agromyzidae|658|BOLD:ACI3093
Agromyzidae|[12224]|NGNAN1711-14|Agromyzidae|676|BOLD:ACB1145
Liriomyza|[12225]|NGNAL468-13|Agromyzidae|661|BOLD:ABV9320
Agromyzidae|[12226]|SSBAD2838-12|Agromyzidae|661|BOLD:ACD2047
Agromyzidae|[12227]|NGNAN1272-14|Agromyzidae|675|BOLD:ACC0382
Agromyzidae|[12228]|SSWLB7440-13|Agromyzidae|670|BOLD:ACG4307
Agromyzidae|[12229]|NGNAN1478-14|Agromyzidae|675|BOLD:ACI3605
Liriomyza helianthi|[12230]|SSBAC1607-12|Agromyzidae|658|BOLD:AAG4754
Liriomyza|[12231]|SSWLB1847-13|Agromyzidae|664|BOLD:ABV1016
Liriomyza|[12232]|NGNAN1796-14|Agromyzidae|675|BOLD:ACI3539
Liriomyza|[12233]|CNGIE178-12|Agromyzidae|632|BOLD:ACC4447
Agromyzidae|[12234]|SSBAA4060-12|Agromyzidae|658|BOLD:ACC2784
Agromyzidae|[12235]|BBDEE104-10|Agromyzidae|658|BOLD:AAG4790
Agromyzidae|[12236]|CNPKE508-14|Agromyzidae|658|BOLD:ACL3831
Liriomyza|[12237]|JSAUG888-11|Agromyzidae|658|BOLD:ABV1352
Liriomyza asclepiadis|[12238]|JSJUL759-11|Agromyzidae|658|BOLD:ABV1017
Agromyzidae|[12239]|SSROB451-14|Agromyzidae|546|BOLD:ACO0985
Liriomyza trifoliearum|[12240]|JSAUG804-11|Agromyzidae|658|BOLD:AAK8751
Liriomyza|[12241]|CNPPG143-12|Agromyzidae|664|BOLD:AAI1105
Agromyzidae|[12242]|CNPPJ429-12|Agromyzidae|644|BOLD:AAM6322
Agromyzidae|[12243]|CNGRD906-12|Agromyzidae|673|BOLD:ACB1327
Agromyzidae|[12244]|CNJAC405-12|Agromyzidae|658|BOLD:ACC2168
Liriomyza kenti|[12245]|JWDCA931-10|Agromyzidae|658|BOLD:AAG4749
Agromyzidae|[12246]|SSPAA995-13|Agromyzidae|664|BOLD:ACG4754
Agromyzidae|[12247]|SSEIA5312-13|Agromyzidae|661|BOLD:ACF9166
Liriomyza|[12248]|SSGBB526-14|Agromyzidae|658|BOLD:ACG4229
Liriomyza ranunculoides|[12249]|JWDCJ698-11|Agromyzidae|658|BOLD:AAV4863
Agromyzidae|[12250]|SSJAF2320-13|Agromyzidae|658|BOLD:ACK1980
Agromyzidae|[12251]|SSBAC1157-12|Agromyzidae|658|BOLD:ACC1496
Agromyzidae|[12252]|BBDCN448-10|Agromyzidae|658|BOLD:AAP6783
Agromyzidae|[12253]|CNEIB1168-12|Agromyzidae|673|BOLD:ACA8567
Agromyzidae|[12254]|SSROC3365-15|Agromyzidae|540|BOLD:ACT0593
Agromyzidae|[12255]|JSDIP705-10|Agromyzidae|658|BOLD:AAV4835
Nemorimyza maculosa|[12256]|NCCA767-11|Agromyzidae|673|BOLD:ABA6469
Agromyzidae|[12257]|SSBRC150-14|Agromyzidae|579|BOLD:ACN9472
Phytoliriomyza pulchella|[12258]|HPPPD731-13|Agromyzidae|661|BOLD:AAG4779
Agromyzidae|[12259]|BBDCP022-10|Agromyzidae|658|BOLD:AAV4871
Agromyzidae|[12260]|CNLMN370-14|Agromyzidae|658|BOLD:ACM7785
Agromyzidae|[12261]|BBDED361-10|Agromyzidae|658|BOLD:AAG4781
Metopomyza scutellata|[12262]|BBDEE618-10|Agromyzidae|658|BOLD:ABA9911
Metopomyza scutellata|[12263]|CNEIC1808-12|Agromyzidae|658|BOLD:ACF1719
Metopomyza interfrontalis|[12264]|BBDED329-10|Agromyzidae|658|BOLD:AAG4748
Metopomyza danielssoni|[12265]|CNRME3327-12|Agromyzidae|661|BOLD:AAG6979
Agromyzidae|[12266]|CNGRF401-12|Agromyzidae|673|BOLD:ACC0247
Phytoliriomyza conspicua|[12267]|SSEIB8139-13|Agromyzidae|658|BOLD:ACI4295
Agromyzidae|[12268]|BBDIP261-09|Agromyzidae|613|BOLD:AAG4746
Agromyzidae|[12269]|BBDIP263-09|Agromyzidae|645|BOLD:AAG4747
Agromyzidae|[12270]|BBDCQ657-10|Agromyzidae|658|BOLD:AAV4891
Phytoliriomyza dorsata|[12271]|CNWBG1181-13|Agromyzidae|659|BOLD:AAG4751
Agromyzidae|[12272]|NGNAI1146-13|Agromyzidae|670|BOLD:ACI4583
Agromyzidae|[12273]|BBDCQ757-10|Agromyzidae|658|BOLD:ACX4144
Agromyzidae|[12274]|CNMII414-14|Agromyzidae|630|BOLD:ACM2498
Agromyzidae|[12275]|CNGHB043-14|Agromyzidae|552|BOLD:ACR4616
Agromyzidae|[12276]|JSDIQ536-10|Agromyzidae|658|BOLD:AAV4895
Amauromyza sheperdiae|[12277]|CHDIP311-11|Agromyzidae|658|BOLD:AAG4752
Phytoliriomyza miki|[12278]|TWDIP363-09|Agromyzidae|658|BOLD:AAG4755
Agromyzidae|[12279]|SSBAF7309-13|Agromyzidae|658|BOLD:ACI3211
Agromyzidae|[12280]|CNBFM457-15|Agromyzidae|588|BOLD:ACU0534
Nephrocerus daeckei|[12281]|JSDIR236-11|Pipunculidae|658|BOLD:AAD3177
Nephrocerus|[12282]|SSBAB1707-12|Pipunculidae|673|BOLD:AAD3176
Lonchaeidae|[12283]|CNPPA2147-12|Lonchaeidae|661|BOLD:AAU4626
Lonchaeidae|[12284]|CNRMC1006-12|Lonchaeidae|658|BOLD:ACC1764
Lonchaeidae|[12285]|TTDBW849-09|Lonchaeidae|658|BOLD:AAG6935
Lonchaeidae|[12286]|PHDIP029-11|Lonchaeidae|658|BOLD:ABV3120
Lonchaeidae|[12287]|CNPAJ088-13|Lonchaeidae|610|BOLD:ACH4209
Ceriana abbreviata|[12288]|TTHYW161-08|Syrphidae|658|BOLD:AAG6873
Wohlfahrtia vigil|[12289]|CNGRC027-12|Sarcophagidae|658|BOLD:AAK0178
Fannia glaucescens|[12290]|DSANN029-08|Fanniidae|658|BOLD:AAG6828
Piezura|[12291]|TTMDJ136-10|Fanniidae|658|BOLD:AAI2972
Fannia|[12292]|BBDEC107-09|Fanniidae|658|BOLD:AAG4625
Fanniinae|[12293]|CNWBH317-13|Fanniidae|658|BOLD:AAP6341
Fannia|[12294]|BBDED016-10|Fanniidae|658|BOLD:AAF7101
Fanniidae|[12295]|BBDED750-10|Fanniidae|658|BOLD:AAG4633
Fanniidae|[12296]|BBDCP120-10|Fanniidae|658|BOLD:AAP6342
Fannia tibialis|[12297]|SSWLB3789-13|Fanniidae|658|BOLD:ABA7222
Fannia|[12298]|CNIVB021-14|Fanniidae|549|BOLD:ACR7615

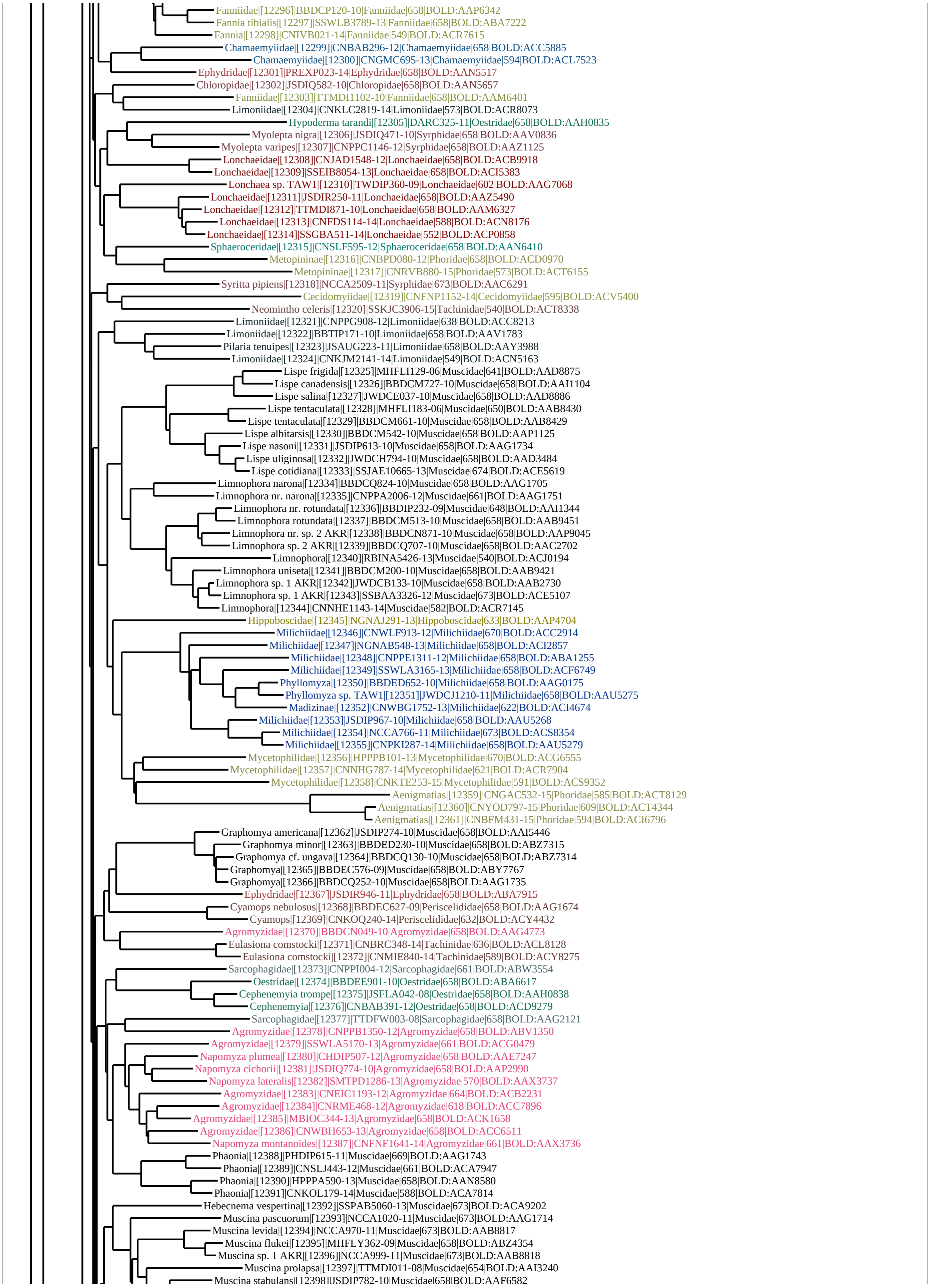

Fanniidae|[12296]|BBDCP120-10|Fanniidae|658|BOLD:AAP6342
Fannia tibialis|[12297]|SSWLB3789-13|Fanniidae|658|BOLD:ABA7222
Fannia|[12298]|CNIVB021-14|Fanniidae|549|BOLD:ACR7615
Chamaemyiidae|[12299]|CNBAB296-12|Chamaemyiidae|658|BOLD:ACC5885
Chamaemyiidae|[12300]|CNGMC695-13|Chamaemyiidae|594|BOLD:ACL7523
Ephydridae|[12301]|PREXP023-14|Ephydridae|658|BOLD:AAN5517
Chloropidae|[12302]|JSDIQ582-10|Chloropidae|658|BOLD:AAN5657
Fanniidae|[12303]|TTMDI1102-10|Fanniidae|658|BOLD:AAM6401
Limoniidae|[12304]|CNKLC2819-14|Limoniidae|573|BOLD:ACR8073
Hypoderma tarandi|[12305]|DARC325-11|Oestridae|658|BOLD:AAH0835
Myolepta nigra|[12306]|JSDIQ471-10|Syrphidae|658|BOLD:AAV0836
Myolepta varipes|[12307]|CNPPC1146-12|Syrphidae|658|BOLD:AAZ1125
Lonchaeidae|[12308]|CNJAD1548-12|Lonchaeidae|658|BOLD:ACB9918
Lonchaeidae|[12309]|SSEIB8054-13|Lonchaeidae|658|BOLD:ACI5383
Lonchaea sp. TAW1|[12310]|TWDIP360-09|Lonchaeidae|602|BOLD:AAG7068
Lonchaeidae|[12311]|JSDIR250-11|Lonchaeidae|658|BOLD:AAZ5490
Lonchaeidae|[12312]|TTMDI871-10|Lonchaeidae|658|BOLD:AAM6327
Lonchaeidae|[12313]|CNFDS114-14|Lonchaeidae|588|BOLD:ACN8176
Lonchaeidae|[12314]|SSGBA511-14|Lonchaeidae|552|BOLD:ACP0858
Sphaeroceridae|[12315]|CNSLF595-12|Sphaeroceridae|658|BOLD:AAN6410
Metopininae|[12316]|CNBPD080-12|Phoridae|658|BOLD:ACD0970
Metopininae|[12317]|CNRVB880-15|Phoridae|573|BOLD:ACT6155
Syritta pipiens|[12318]|NCCA2509-11|Syrphidae|673|BOLD:AAC6291
Cecidomyiidae|[12319]|CNFNP1152-14|Cecidomyiidae|595|BOLD:ACV5400
Neomintho celeris|[12320]|SSKJC3906-15|Tachinidae|540|BOLD:ACT8338
Limoniidae|[12321]|CNPPG908-12|Limoniidae|638|BOLD:ACC8213
Limoniidae|[12322]|BBTIP171-10|Limoniidae|658|BOLD:AAV1783
Pilaria tenuipes|[12323]|JSAUG223-11|Limoniidae|658|BOLD:AAY3988
Limoniidae|[12324]|CNKJM2141-14|Limoniidae|549|BOLD:ACN5163
Lispe frigida|[12325]|MHFLI129-06|Muscidae|641|BOLD:AAD8875
Lispe canadensis|[12326]|BBDCM727-10|Muscidae|658|BOLD:AAI1104
Lispe salina|[12327]|JWDCE037-10|Muscidae|658|BOLD:AAD8886
Lispe tentaculata|[12328]|MHFLI183-06|Muscidae|650|BOLD:AAB8430
Lispe tentaculata|[12329]|BBDCM661-10|Muscidae|658|BOLD:AAB8429
Lispe albitarsis|[12330]|BBDCM542-10|Muscidae|658|BOLD:AAP1125
Lispe nasoni|[12331]|JSDIP613-10|Muscidae|658|BOLD:AAG1734
Lispe uliginosa|[12332]|JWDCH794-10|Muscidae|658|BOLD:AAD3484
Lispe cotidiana|[12333]|SSJAE10665-13|Muscidae|674|BOLD:ACE5619
Limnophora narona|[12334]|BBDCQ824-10|Muscidae|658|BOLD:AAG1705
Limnophora nr. narona|[12335]|CNPPA2006-12|Muscidae|661|BOLD:AAG1751
Limnophora nr. rotundata|[12336]|BBDIP232-09|Muscidae|648|BOLD:AAI1344
Limnophora rotundata|[12337]|BBDCM513-10|Muscidae|658|BOLD:AAB9451
Limnophora nr. sp. 2 AKR|[12338]|BBDCN871-10|Muscidae|658|BOLD:AAP9045
Limnophora sp. 2 AKR|[12339]|BBDCQ707-10|Muscidae|658|BOLD:AAC2702
Limnophora|[12340]|RBINA5426-13|Muscidae|540|BOLD:ACJ0194
Limnophora uniseta|[12341]|BBDCM200-10|Muscidae|658|BOLD:AAB9421
Limnophora sp. 1 AKR|[12342]|JWDCB133-10|Muscidae|658|BOLD:AAB2730
Limnophora sp. 1 AKR|[12343]|SSBAA3326-12|Muscidae|673|BOLD:ACE5107
Limnophora|[12344]|CNNHE1143-14|Muscidae|582|BOLD:ACR7145
Hippoboscidae|[12345]|NGNAJ291-13|Hippoboscidae|633|BOLD:AAP4704
Milichiidae|[12346]|CNWLF913-12|Milichiidae|670|BOLD:ACC2914
Milichiidae|[12347]|NGNAB548-13|Milichiidae|658|BOLD:ACI2857
Milichiidae|[12348]|CNPPE1311-12|Milichiidae|658|BOLD:ABA1255
Milichiidae|[12349]|SSWLA3165-13|Milichiidae|658|BOLD:ACF6749
Phyllomyza|[12350]|BBDED652-10|Milichiidae|658|BOLD:AAG0175
Phyllomyza sp. TAW1|[12351]|JWDCJ1210-11|Milichiidae|658|BOLD:AAU5275
Madizinae|[12352]|CNWBG1752-13|Milichiidae|622|BOLD:ACI4674
Milichiidae|[12353]|JSDIP967-10|Milichiidae|658|BOLD:AAU5268
Milichiidae|[12354]|NCCA766-11|Milichiidae|673|BOLD:ACS8354
Milichiidae|[12355]|CNPKI287-14|Milichiidae|658|BOLD:AAU5279
Mycetophilidae|[12356]|HPPPB101-13|Mycetophilidae|670|BOLD:ACG6555
Mycetophilidae|[12357]|CNNHG787-14|Mycetophilidae|621|BOLD:ACR7904
Mycetophilidae|[12358]|CNKTE253-15|Mycetophilidae|591|BOLD:ACS9352
Aenigmatias|[12359]|CNGAC532-15|Phoridae|585|BOLD:ACT8129
Aenigmatias|[12360]|CNYOD797-15|Phoridae|609|BOLD:ACT4344
Aenigmatias|[12361]|CNBFM431-15|Phoridae|594|BOLD:ACI6796
Graphomya americana|[12362]|JSDIP274-10|Muscidae|658|BOLD:AAI5446
Graphomya minor|[12363]|BBDED230-10|Muscidae|658|BOLD:ABZ7315
Graphomya cf. ungava|[12364]|BBDCQ130-10|Muscidae|658|BOLD:ABZ7314
Graphomya|[12365]|BBDEC576-09|Muscidae|658|BOLD:ABY7767
Graphomya|[12366]|BBDCQ252-10|Muscidae|658|BOLD:AAG1735
Ephydridae|[12367]|JSDIR946-11|Ephydridae|658|BOLD:ABA7915
Cyamops nebulosus|[12368]|BBDEC627-09|Periscelididae|658|BOLD:AAG1674
Cyamops|[12369]|CNKOQ240-14|Periscelididae|632|BOLD:ACY4432
Agromyzidae|[12370]|BBDCN049-10|Agromyzidae|658|BOLD:AAG4773
Eulasiona comstocki|[12371]|CNBRC348-14|Tachinidae|636|BOLD:ACL8128
Eulasiona comstocki|[12372]|CNMIE840-14|Tachinidae|589|BOLD:ACY8275
Sarcophagidae|[12373]|CNPPI004-12|Sarcophagidae|661|BOLD:ABW3554
Oestridae|[12374]|BBDEE901-10|Oestridae|658|BOLD:ABA6617
Cephenemyia trompe|[12375]|JSFLA042-08|Oestridae|658|BOLD:AAH0838
Cephenemyia|[12376]|CNBAB391-12|Oestridae|658|BOLD:ACD9279
Sarcophagidae|[12377]|TTDFW003-08|Sarcophagidae|658|BOLD:AAG2121
Agromyzidae|[12378]|CNPPB1350-12|Agromyzidae|658|BOLD:ABV1350
Agromyzidae|[12379]|SSWLA5170-13|Agromyzidae|661|BOLD:ACG0479
Napomyza plumea|[12380]|CHDIP507-12|Agromyzidae|658|BOLD:AAE7247
Napomyza cichorii|[12381]|JSDIQ774-10|Agromyzidae|658|BOLD:AAP2990
Napomyza lateralis|[12382]|SMTPD1286-13|Agromyzidae|570|BOLD:AAX3737
Agromyzidae|[12383]|CNEIC1193-12|Agromyzidae|664|BOLD:ACB2231
Agromyzidae|[12384]|CNRME468-12|Agromyzidae|618|BOLD:ACC7896
Agromyzidae|[12385]|MBIOC344-13|Agromyzidae|658|BOLD:ACK1658
Agromyzidae|[12386]|CNWBH653-13|Agromyzidae|658|BOLD:ACC6511
Napomyza montanoides|[12387]|CNFNF1641-14|Agromyzidae|661|BOLD:AAX3736
Phaonia|[12388]|PHDIP615-11|Muscidae|669|BOLD:AAG1743
Phaonia|[12389]|CNSLJ443-12|Muscidae|661|BOLD:ACA7947
Phaonia|[12390]|HPPPA590-13|Muscidae|658|BOLD:AAN8580
Phaonia|[12391]|CNKOL179-14|Muscidae|588|BOLD:ACA7814
Hebecnema vespertina|[12392]|SSPAB5060-13|Muscidae|673|BOLD:ACA9202
Muscina pascuorum|[12393]|NCCA1020-11|Muscidae|673|BOLD:AAG1714
Muscina levida|[12394]|NCCA970-11|Muscidae|673|BOLD:AAB8817
Muscina flukei|[12395]|MHFLY362-09|Muscidae|658|BOLD:ABZ4354
Muscina sp. 1 AKR|[12396]|NCCA999-11|Muscidae|673|BOLD:AAB8818
Muscina prolapsa|[12397]|TTMDI011-08|Muscidae|654|BOLD:AAI3240
Muscina stabulans|[12398]|JSDIP782-10|Muscidae|658|BOLD:AAF6582

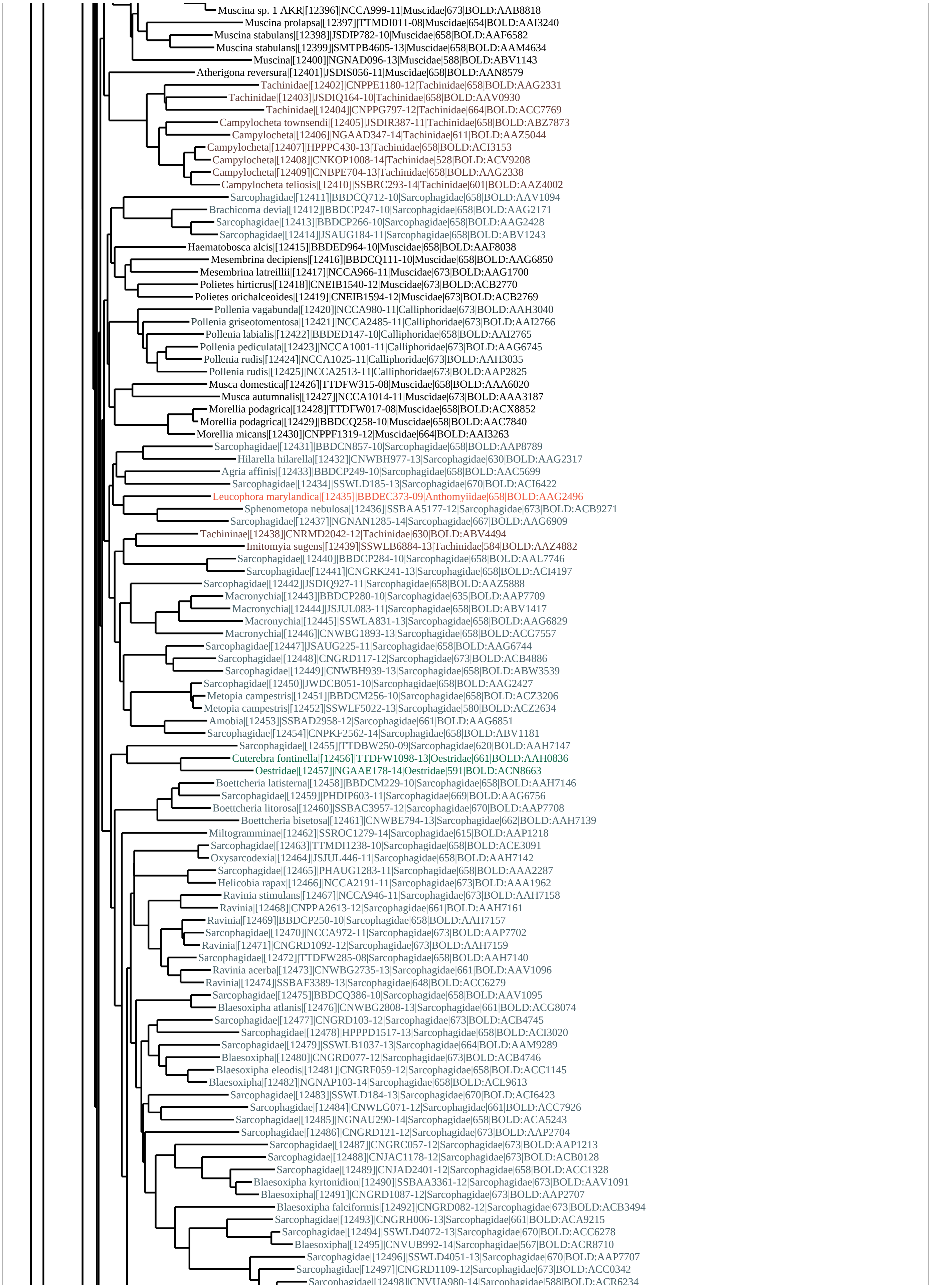
Muscina sp. 1 AKR|[12396]|NCCA999-11|Muscidae|673|BOLD:AAB8818
Muscina prolapsa|[12397]|TTMDI011-08|Muscidae|654|BOLD:AAI3240
Muscina stabulans|[12398]|JSDIP782-10|Muscidae|658|BOLD:AAF6582
Muscina stabulans|[12399]|SMTPB4605-13|Muscidae|658|BOLD:AAM4634
Muscina|[12400]|NGNAD096-13|Muscidae|588|BOLD:ABV1143
Atherigona reversura|[12401]|JSDIS056-11|Muscidae|658|BOLD:AAN8579
Tachinidae|[12402]|CNPPE1180-12|Tachinidae|658|BOLD:AAG2331
Tachinidae|[12403]|JSDIQ164-10|Tachinidae|658|BOLD:AAV0930
Tachinidae|[12404]|CNPPG797-12|Tachinidae|664|BOLD:ACC7769
Campylocheta townsendi|[12405]|JSDIR387-11|Tachinidae|658|BOLD:ABZ7873
Campylocheta|[12406]|NGAAD347-14|Tachinidae|611|BOLD:AAZ5044
Campylocheta|[12407]|HPPPC430-13|Tachinidae|658|BOLD:ACI3153
Campylocheta|[12408]|CNKOP1008-14|Tachinidae|528|BOLD:ACV9208
Campylocheta|[12409]|CNBPE704-13|Tachinidae|658|BOLD:AAG2338
Campylocheta teliosis|[12410]|SSBRC293-14|Tachinidae|601|BOLD:AAZ4002
Sarcophagidae|[12411]|BBDCQ712-10|Sarcophagidae|658|BOLD:AAV1094
Brachicoma devia|[12412]|BBDCP247-10|Sarcophagidae|658|BOLD:AAG2171
Sarcophagidae|[12413]|BBDCP266-10|Sarcophagidae|658|BOLD:AAG2428
Sarcophagidae|[12414]|JSAUG184-11|Sarcophagidae|658|BOLD:ABV1243
Haematobosca alcis|[12415]|BBDED964-10|Muscidae|658|BOLD:AAF8038
Mesembrina decipiens|[12416]|BBDCQ111-10|Muscidae|658|BOLD:AAG6850
Mesembrina latreillii|[12417]|NCCA966-11|Muscidae|673|BOLD:AAG1700
Polietes hirticrus|[12418]|CNEIB1540-12|Muscidae|673|BOLD:ACB2770
Polietes orichalceoides|[12419]|CNEIB1594-12|Muscidae|673|BOLD:ACB2769
Pollenia vagabunda|[12420]|NCCA980-11|Calliphoridae|673|BOLD:AAH3040
Pollenia griseotomentosa|[12421]|NCCA2485-11|Calliphoridae|673|BOLD:AAI2766
Pollenia labialis|[12422]|BBDED147-10|Calliphoridae|658|BOLD:AAI2765
Pollenia pediculata|[12423]|NCCA1001-11|Calliphoridae|673|BOLD:AAG6745
Pollenia rudis|[12424]|NCCA1025-11|Calliphoridae|673|BOLD:AAH3035
Pollenia rudis|[12425]|NCCA2513-11|Calliphoridae|673|BOLD:AAP2825
Musca domestica|[12426]|TTDFW315-08|Muscidae|658|BOLD:AAA6020
Musca autumnalis|[12427]|NCCA1014-11|Muscidae|673|BOLD:AAA3187
Morellia podagrica|[12428]|TTDFW017-08|Muscidae|658|BOLD:ACX8852
Morellia podagrica|[12429]|BBDCQ258-10|Muscidae|658|BOLD:AAC7840
Morellia micans|[12430]|CNPPF1319-12|Muscidae|664|BOLD:AAI3263
Sarcophagidae|[12431]|BBDCN857-10|Sarcophagidae|658|BOLD:AAP8789
Hilarella hilarella|[12432]|CNWBH977-13|Sarcophagidae|630|BOLD:AAG2317
Agria affinis|[12433]|BBDCP249-10|Sarcophagidae|658|BOLD:AAC5699
Sarcophagidae|[12434]|SSWLD185-13|Sarcophagidae|670|BOLD:ACI6422
Leucophora marylandica|[12435]|BBDEC373-09|Anthomyiidae|658|BOLD:AAG2496
Sphenometopa nebulosa|[12436]|SSBAA5177-12|Sarcophagidae|673|BOLD:ACB9271
Sarcophagidae|[12437]|NGNAN1285-14|Sarcophagidae|667|BOLD:AAG6909
Tachininae|[12438]|CNRMD2042-12|Tachinidae|630|BOLD:ABV4494
Imitomyia sugens|[12439]|SSWLB6884-13|Tachinidae|584|BOLD:AAZ4882
Sarcophagidae|[12440]|BBDCP284-10|Sarcophagidae|658|BOLD:AAL7746
Sarcophagidae|[12441]|CNGRK241-13|Sarcophagidae|658|BOLD:ACI4197
Sarcophagidae|[12442]|JSDIQ927-11|Sarcophagidae|658|BOLD:AAZ5888
Macronychia|[12443]|BBDCP280-10|Sarcophagidae|635|BOLD:AAP7709
Macronychia|[12444]|JSJUL083-11|Sarcophagidae|658|BOLD:ABV1417
Macronychia|[12445]|SSWLA831-13|Sarcophagidae|658|BOLD:AAG6829
Macronychia|[12446]|CNWBG1893-13|Sarcophagidae|658|BOLD:ACG7557
Sarcophagidae|[12447]|JSAUG225-11|Sarcophagidae|658|BOLD:AAG6744
Sarcophagidae|[12448]|CNGRD117-12|Sarcophagidae|673|BOLD:ACB4886
Sarcophagidae|[12449]|CNWBH939-13|Sarcophagidae|658|BOLD:ABW3539
Sarcophagidae|[12450]|JWDCB051-10|Sarcophagidae|658|BOLD:AAG2427
Metopia campestris|[12451]|BBDCM256-10|Sarcophagidae|658|BOLD:ACZ3206
Metopia campestris|[12452]|SSWLF5022-13|Sarcophagidae|580|BOLD:ACZ2634
Amobia|[12453]|SSBAD2958-12|Sarcophagidae|661|BOLD:AAG6851
Sarcophagidae|[12454]|CNPKF2562-14|Sarcophagidae|658|BOLD:ABV1181
Sarcophagidae|[12455]|TTDBW250-09|Sarcophagidae|620|BOLD:AAH7147
Cuterebra fontinella|[12456]|TTDFW1098-13|Oestridae|661|BOLD:AAH0836
Oestridae|[12457]|NGAAE178-14|Oestridae|591|BOLD:ACN8663
Boettcheria latisterna|[12458]|BBDCM229-10|Sarcophagidae|658|BOLD:AAH7146
Sarcophagidae|[12459]|PHDIP603-11|Sarcophagidae|669|BOLD:AAG6756
Boettcheria litorosa|[12460]|SSBAC3957-12|Sarcophagidae|670|BOLD:AAP7708
Boettcheria bisetosa|[12461]|CNWBE794-13|Sarcophagidae|662|BOLD:AAH7139
Miltogramminae|[12462]|SSROC1279-14|Sarcophagidae|615|BOLD:AAP1218
Sarcophagidae|[12463]|TTMDI1238-10|Sarcophagidae|658|BOLD:ACE3091
Oxysarcodexia|[12464]|JSJUL446-11|Sarcophagidae|658|BOLD:AAH7142
Sarcophagidae|[12465]|PHAUG1283-11|Sarcophagidae|658|BOLD:AAA2287
Helicobia rapax|[12466]|NCCA2191-11|Sarcophagidae|673|BOLD:AAA1962
Ravinia stimulans|[12467]|NCCA946-11|Sarcophagidae|673|BOLD:AAH7158
Ravinia|[12468]|CNPPA2613-12|Sarcophagidae|661|BOLD:AAH7161
Ravinia|[12469]|BBDCP250-10|Sarcophagidae|658|BOLD:AAH7157
Sarcophagidae|[12470]|NCCA972-11|Sarcophagidae|673|BOLD:AAP7702
Ravinia|[12471]|CNGRD1092-12|Sarcophagidae|673|BOLD:AAH7159
Sarcophagidae|[12472]|TTDFW285-08|Sarcophagidae|658|BOLD:AAH7140
Ravinia acerba|[12473]|CNWBG2735-13|Sarcophagidae|661|BOLD:AAV1096
Ravinia|[12474]|SSBAF3389-13|Sarcophagidae|648|BOLD:ACC6279
Sarcophagidae|[12475]|BBDCQ386-10|Sarcophagidae|658|BOLD:AAV1095
Blaesoxipha atlanis|[12476]|CNWBG2808-13|Sarcophagidae|661|BOLD:ACG8074
Sarcophagidae|[12477]|CNGRD103-12|Sarcophagidae|673|BOLD:ACB4745
Sarcophagidae|[12478]|HPPPD1517-13|Sarcophagidae|658|BOLD:ACI3020
Sarcophagidae|[12479]|SSWLB1037-13|Sarcophagidae|664|BOLD:AAM9289
Blaesoxipha|[12480]|CNGRD077-12|Sarcophagidae|673|BOLD:ACB4746
Blaesoxipha eleodis|[12481]|CNGRF059-12|Sarcophagidae|658|BOLD:ACC1145
Blaesoxipha|[12482]|NGNAP103-14|Sarcophagidae|658|BOLD:ACL9613
Sarcophagidae|[12483]|SSWLD184-13|Sarcophagidae|670|BOLD:ACI6423
Sarcophagidae|[12484]|CNWLG071-12|Sarcophagidae|661|BOLD:ACC7926
Sarcophagidae|[12485]|NGNAU290-14|Sarcophagidae|658|BOLD:ACA5243
Sarcophagidae|[12486]|CNGRD121-12|Sarcophagidae|673|BOLD:AAP2704
Sarcophagidae|[12487]|CNGRC057-12|Sarcophagidae|673|BOLD:AAP1213
Sarcophagidae|[12488]|CNJAC1178-12|Sarcophagidae|673|BOLD:ACB0128
Sarcophagidae|[12489]|CNJAD2401-12|Sarcophagidae|658|BOLD:ACC1328
Blaesoxipha kyrtonidion|[12490]|SSBAA3361-12|Sarcophagidae|673|BOLD:AAV1091
Blaesoxipha|[12491]|CNGRD1087-12|Sarcophagidae|673|BOLD:AAP2707
Blaesoxipha falciformis|[12492]|CNGRD082-12|Sarcophagidae|673|BOLD:ACB3494
Sarcophagidae|[12493]|CNGRH006-13|Sarcophagidae|661|BOLD:ACA9215
Sarcophagidae|[12494]|SSWLD4072-13|Sarcophagidae|670|BOLD:ACC6278
Blaesoxipha|[12495]|CNVUB992-14|Sarcophagidae|567|BOLD:ACR8710
Sarcophagidae|[12496]|SSWLD4051-13|Sarcophagidae|670|BOLD:AAP7707
Sarcophagidae|[12497]|CNGRD1109-12|Sarcophagidae|673|BOLD:ACC0342
Sarcophagidae|[12498]|CNVUA980-14|Sarcophagidae|588|BOLD:ACR6234

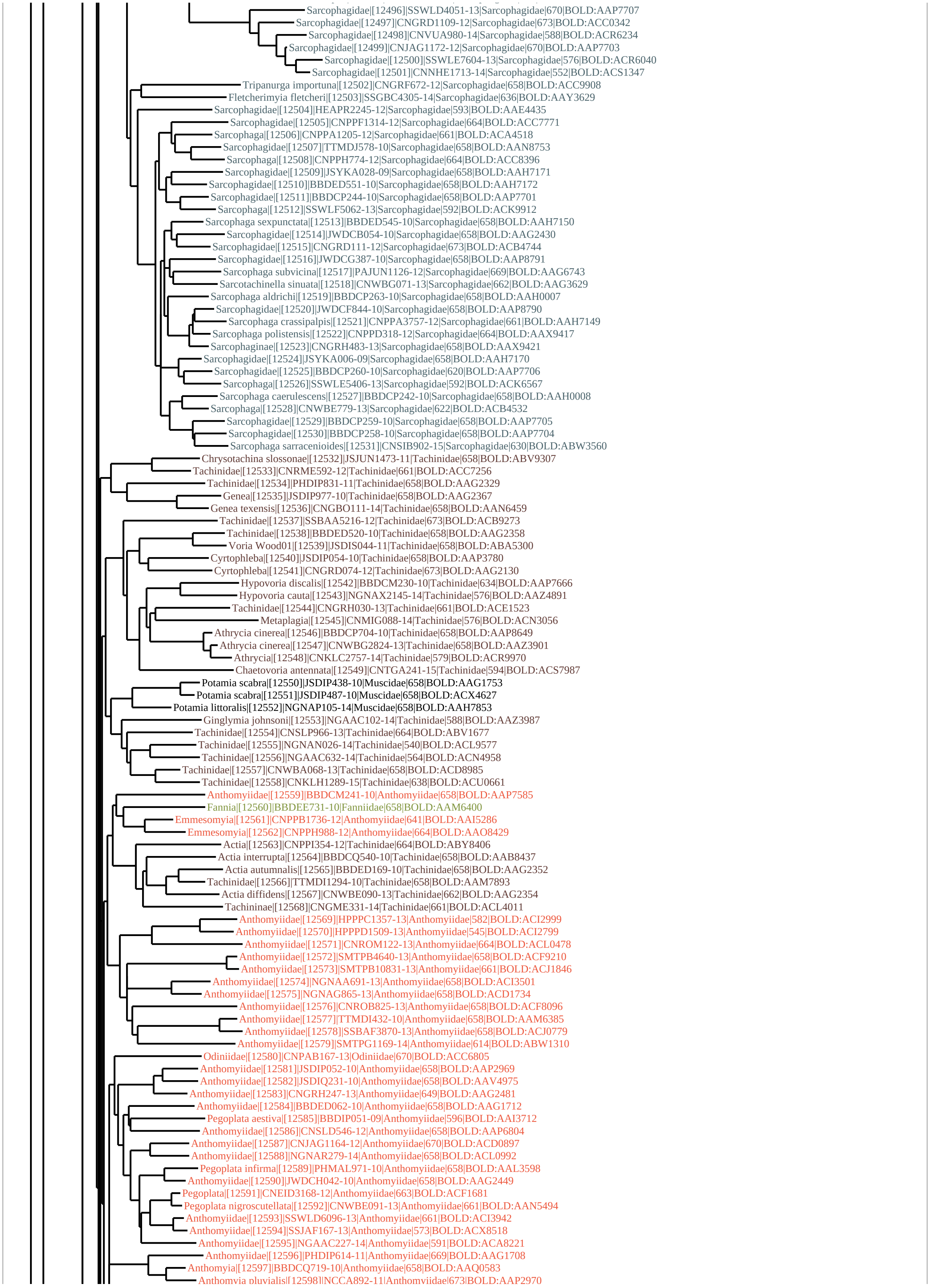
Sarcophagidae|[12496]|SSWLD4051-13|Sarcophagidae|670|BOLD:AAP7707
Sarcophagidae|[12497]|CNGRD1109-12|Sarcophagidae|673|BOLD:ACC0342
Sarcophagidae|[12498]|CNVUA980-14|Sarcophagidae|588|BOLD:ACR6234
Sarcophagidae|[12499]|CNJAG1172-12|Sarcophagidae|670|BOLD:AAP7703
Sarcophagidae|[12500]|SSWLE7604-13|Sarcophagidae|576|BOLD:ACR6040
Sarcophagidae|[12501]|CNNHE1713-14|Sarcophagidae|552|BOLD:ACS1347
Tripanurga importuna|[12502]|CNGRF672-12|Sarcophagidae|658|BOLD:ACC9908
Fletcherimyia fletcheri|[12503]|SSGBC4305-14|Sarcophagidae|636|BOLD:AAY3629
Sarcophagidae|[12504]|HEAPR2245-12|Sarcophagidae|593|BOLD:AAE4435
Sarcophagidae|[12505]|CNPPF1314-12|Sarcophagidae|664|BOLD:ACC7771
Sarcophaga|[12506]|CNPPA1205-12|Sarcophagidae|661|BOLD:ACA4518
Sarcophagidae|[12507]|TTMDJ578-10|Sarcophagidae|658|BOLD:AAN8753
Sarcophaga|[12508]|CNPPH774-12|Sarcophagidae|664|BOLD:ACC8396
Sarcophagidae|[12509]|JSYKA028-09|Sarcophagidae|658|BOLD:AAH7171
Sarcophagidae|[12510]|BBDED551-10|Sarcophagidae|658|BOLD:AAH7172
Sarcophagidae|[12511]|BBDCP244-10|Sarcophagidae|658|BOLD:AAP7701
Sarcophaga|[12512]|SSWLF5062-13|Sarcophagidae|592|BOLD:ACK9912
Sarcophaga sexpunctata|[12513]|BBDED545-10|Sarcophagidae|658|BOLD:AAH7150
Sarcophagidae|[12514]|JWDCB054-10|Sarcophagidae|658|BOLD:AAG2430
Sarcophagidae|[12515]|CNGRD111-12|Sarcophagidae|673|BOLD:ACB4744
Sarcophagidae|[12516]|JWDCG387-10|Sarcophagidae|658|BOLD:AAP8791
Sarcophaga subvicina|[12517]|PAJUN1126-12|Sarcophagidae|669|BOLD:AAG6743
Sarcotachinella sinuata|[12518]|CNWBG071-13|Sarcophagidae|662|BOLD:AAG3629
Sarcophaga aldrichi|[12519]|BBDCP263-10|Sarcophagidae|658|BOLD:AAH0007
Sarcophagidae|[12520]|JWDCF844-10|Sarcophagidae|658|BOLD:AAP8790
Sarcophaga crassipalpis|[12521]|CNPPA3757-12|Sarcophagidae|661|BOLD:AAH7149
Sarcophaga polistensis|[12522]|CNPPD318-12|Sarcophagidae|664|BOLD:AAX9417
Sarcophaginae|[12523]|CNGRH483-13|Sarcophagidae|658|BOLD:AAX9421
Sarcophagidae|[12524]|JSYKA006-09|Sarcophagidae|658|BOLD:AAH7170
Sarcophagidae|[12525]|BBDCP260-10|Sarcophagidae|620|BOLD:AAP7706
Sarcophaga|[12526]|SSWLE5406-13|Sarcophagidae|592|BOLD:ACK6567
Sarcophaga caerulescens|[12527]|BBDCP242-10|Sarcophagidae|658|BOLD:AAH0008
Sarcophaga|[12528]|CNWBE779-13|Sarcophagidae|622|BOLD:ACB4532
Sarcophagidae|[12529]|BBDCP259-10|Sarcophagidae|658|BOLD:AAP7705
Sarcophagidae|[12530]|BBDCP258-10|Sarcophagidae|658|BOLD:AAP7704
Sarcophaga sarracenioides|[12531]|CNSIB902-15|Sarcophagidae|630|BOLD:ABW3560
Chrysotachina slossonae|[12532]|JSJUN1473-11|Tachinidae|658|BOLD:ABV9307
Tachinidae|[12533]|CNRME592-12|Tachinidae|661|BOLD:ACC7256
Tachinidae|[12534]|PHDIP831-11|Tachinidae|658|BOLD:AAG2329
Genea|[12535]|JSDIP977-10|Tachinidae|658|BOLD:AAG2367
Genea texensis|[12536]|CNGBO111-14|Tachinidae|658|BOLD:AAN6459
Tachinidae|[12537]|SSBAA5216-12|Tachinidae|673|BOLD:ACB9273
Tachinidae|[12538]|BBDED520-10|Tachinidae|658|BOLD:AAG2358
Voria Wood01|[12539]|JSDIS044-11|Tachinidae|658|BOLD:ABA5300
Cyrtophleba|[12540]|JSDIP054-10|Tachinidae|658|BOLD:AAP3780
Cyrtophleba|[12541]|CNGRD074-12|Tachinidae|673|BOLD:AAG2130
Hypovoria discalis|[12542]|BBDCM230-10|Tachinidae|634|BOLD:AAP7666
Hypovoria cauta|[12543]|NGNAX2145-14|Tachinidae|576|BOLD:AAZ4891
Tachinidae|[12544]|CNGRH030-13|Tachinidae|661|BOLD:ACE1523
Metaplagia|[12545]|CNMIG088-14|Tachinidae|576|BOLD:ACN3056
Athrycia cinerea|[12546]|BBDCP704-10|Tachinidae|658|BOLD:AAP8649
Athrycia cinerea|[12547]|CNWBG2824-13|Tachinidae|658|BOLD:AAZ3901
Athrycia|[12548]|CNKLC2757-14|Tachinidae|579|BOLD:ACR9970
Chaetovoria antennata|[12549]|CNTGA241-15|Tachinidae|594|BOLD:ACS7987
Potamia scabra|[12550]|JSDIP438-10|Muscidae|658|BOLD:AAG1753
Potamia scabra|[12551]|JSDIP487-10|Muscidae|658|BOLD:ACX4627
Potamia littoralis|[12552]|NGNAP105-14|Muscidae|658|BOLD:AAH7853
Ginglymia johnsoni|[12553]|NGAAC102-14|Tachinidae|588|BOLD:AAZ3987
Tachinidae|[12554]|CNSLP966-13|Tachinidae|664|BOLD:ABV1677
Tachinidae|[12555]|NGNAN026-14|Tachinidae|540|BOLD:ACL9577
Tachinidae|[12556]|NGAAC632-14|Tachinidae|564|BOLD:ACN4958
Tachinidae|[12557]|CNWBA068-13|Tachinidae|658|BOLD:ACD8985
Tachinidae|[12558]|CNKLH1289-15|Tachinidae|638|BOLD:ACU0661
Anthomyiidae|[12559]|BBDCM241-10|Anthomyiidae|658|BOLD:AAP7585
Fannia|[12560]|BBDEE731-10|Fanniidae|658|BOLD:AAM6400
Emmesomyia|[12561]|CNPPB1736-12|Anthomyiidae|641|BOLD:AAI5286
Emmesomyia|[12562]|CNPPH988-12|Anthomyiidae|664|BOLD:AAO8429
Actia|[12563]|CNPPI354-12|Tachinidae|664|BOLD:ABY8406
Actia interrupta|[12564]|BBDCQ540-10|Tachinidae|658|BOLD:AAB8437
Actia autumnalis|[12565]|BBDED169-10|Tachinidae|658|BOLD:AAG2352
Tachinidae|[12566]|TTMDI1294-10|Tachinidae|658|BOLD:AAM7893
Actia diffidens|[12567]|CNWBE090-13|Tachinidae|662|BOLD:AAG2354
Tachininae|[12568]|CNGME331-14|Tachinidae|661|BOLD:ACL4011
Anthomyiidae|[12569]|HPPPC1357-13|Anthomyiidae|582|BOLD:ACI2999
Anthomyiidae|[12570]|HPPPD1509-13|Anthomyiidae|545|BOLD:ACI2799
Anthomyiidae|[12571]|CNROM122-13|Anthomyiidae|664|BOLD:ACL0478
Anthomyiidae|[12572]|SMTPB4640-13|Anthomyiidae|658|BOLD:ACF9210
Anthomyiidae|[12573]|SMTPB10831-13|Anthomyiidae|661|BOLD:ACJ1846
Anthomyiidae|[12574]|NGNAA691-13|Anthomyiidae|658|BOLD:ACI3501
Anthomyiidae|[12575]|NGNAG865-13|Anthomyiidae|658|BOLD:ACD1734
Anthomyiidae|[12576]|CNROB825-13|Anthomyiidae|658|BOLD:ACF8096
Anthomyiidae|[12577]|TTMDI432-10|Anthomyiidae|658|BOLD:AAM6385
Anthomyiidae|[12578]|SSBAF3870-13|Anthomyiidae|658|BOLD:ACJ0779
Anthomyiidae|[12579]|SMTPG1169-14|Anthomyiidae|614|BOLD:ABW1310
Odiniidae|[12580]|CNPAB167-13|Odiniidae|670|BOLD:ACC6805
Anthomyiidae|[12581]|JSDIP052-10|Anthomyiidae|658|BOLD:AAP2969
Anthomyiidae|[12582]|JSDIQ231-10|Anthomyiidae|658|BOLD:AAV4975
Anthomyiidae|[12583]|CNGRH247-13|Anthomyiidae|649|BOLD:AAG2481
Anthomyiidae|[12584]|BBDED062-10|Anthomyiidae|658|BOLD:AAG1712
Pegoplata aestiva|[12585]|BBDIP051-09|Anthomyiidae|596|BOLD:AAI3712
Anthomyiidae|[12586]|CNSLD546-12|Anthomyiidae|658|BOLD:AAP6804
Anthomyiidae|[12587]|CNJAG1164-12|Anthomyiidae|670|BOLD:ACD0897
Anthomyiidae|[12588]|NGNAR279-14|Anthomyiidae|658|BOLD:ACL0992
Pegoplata infirma|[12589]|PHMAL971-10|Anthomyiidae|658|BOLD:AAL3598
Anthomyiidae|[12590]|JWDCH042-10|Anthomyiidae|658|BOLD:AAG2449
Pegoplata|[12591]|CNEID3168-12|Anthomyiidae|663|BOLD:ACF1681
Pegoplata nigroscutellata|[12592]|CNWBE091-13|Anthomyiidae|661|BOLD:AAN5494
Anthomyiidae|[12593]|SSWLD6096-13|Anthomyiidae|661|BOLD:ACI3942
Anthomyiidae|[12594]|SSJAF167-13|Anthomyiidae|573|BOLD:ACX8518
Anthomyiidae|[12595]|NGAAC227-14|Anthomyiidae|591|BOLD:ACA8221
Anthomyiidae|[12596]|PHDIP614-11|Anthomyiidae|669|BOLD:AAG1708
Anthomyia|[12597]|BBDCQ719-10|Anthomyiidae|658|BOLD:AAQ0583
Anthomyia pluvialis|[12598]|NCCA892-11|Anthomyiidae|673|BOLD:AAP2970

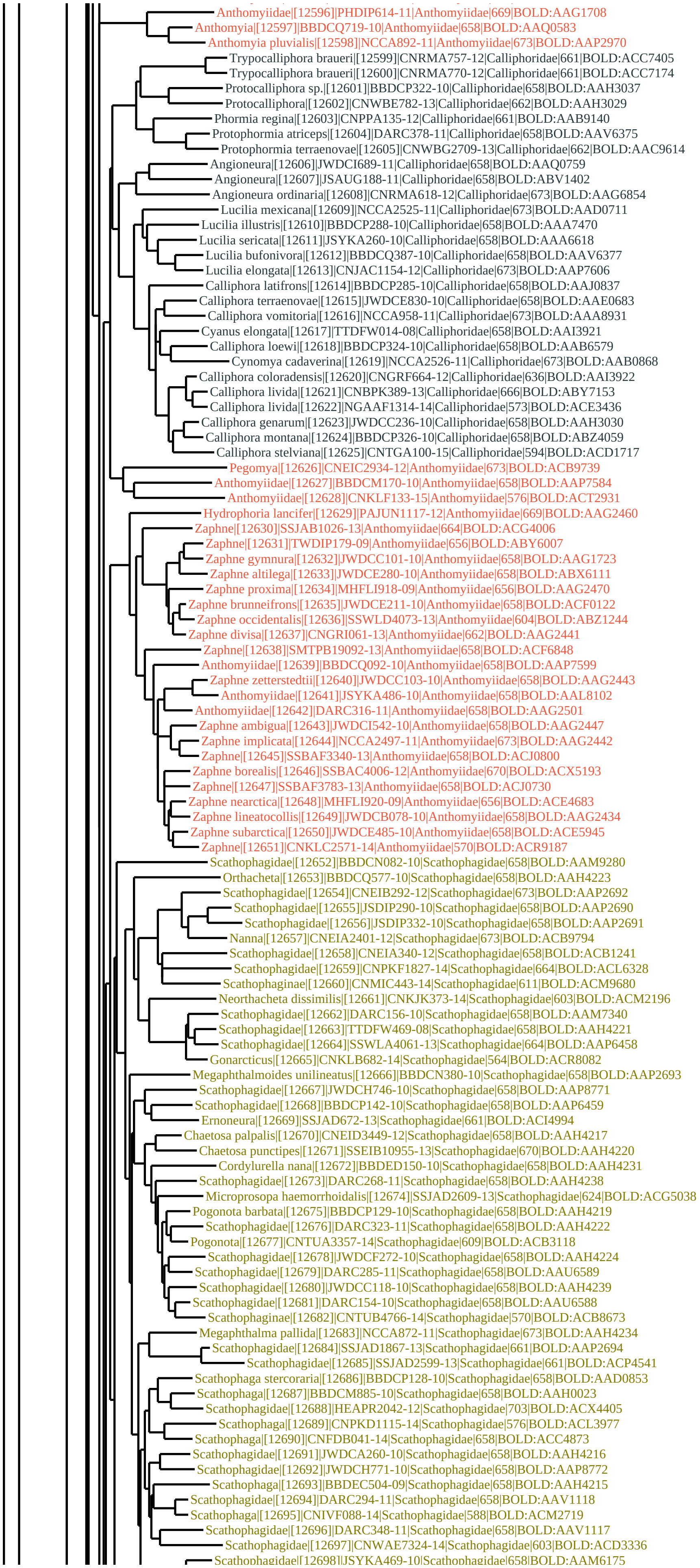

Anthomyiidae|[12596]|PHDIP614-11|Anthomyiidae|669|BOLD:AAG1708
Anthomyia|[12597]|BBDCQ719-10|Anthomyiidae|658|BOLD:AAQ0583
Anthomyia pluvialis|[12598]|NCCA892-11|Anthomyiidae|673|BOLD:AAP2970
Trypocalliphora braueri|[12599]|CNRMA757-12|Calliphoridae|661|BOLD:ACC7405
Trypocalliphora braueri|[12600]|CNRMA770-12|Calliphoridae|661|BOLD:ACC7174
Protocalliphora sp.|[12601]|BBDCP322-10|Calliphoridae|658|BOLD:AAH3037
Protocalliphora|[12602]|CNWBE782-13|Calliphoridae|662|BOLD:AAH3029
Phormia regina|[12603]|CNPPA135-12|Calliphoridae|661|BOLD:AAB9140
Protophormia atriceps|[12604]|DARC378-11|Calliphoridae|658|BOLD:AAV6375
Protophormia terraenovae|[12605]|CNWBG2709-13|Calliphoridae|662|BOLD:AAC9614
Angioneura|[12606]|JWDCI689-11|Calliphoridae|658|BOLD:AAQ0759
Angioneura|[12607]|JSAUG188-11|Calliphoridae|658|BOLD:ABV1402
Angioneura ordinaria|[12608]|CNRMA618-12|Calliphoridae|673|BOLD:AAG6854
Lucilia mexicana|[12609]|NCCA2525-11|Calliphoridae|673|BOLD:AAD0711
Lucilia illustris|[12610]|BBDCP288-10|Calliphoridae|658|BOLD:AAA7470
Lucilia sericata|[12611]|JSYKA260-10|Calliphoridae|658|BOLD:AAA6618
Lucilia bufonivora|[12612]|BBDCQ387-10|Calliphoridae|658|BOLD:AAV6377
Lucilia elongata|[12613]|CNJAC1154-12|Calliphoridae|673|BOLD:AAP7606
Calliphora latifrons|[12614]|BBDCP285-10|Calliphoridae|658|BOLD:AAJ0837
Calliphora terraenovae|[12615]|JWDCE830-10|Calliphoridae|658|BOLD:AAE0683
Calliphora vomitoria|[12616]|NCCA958-11|Calliphoridae|673|BOLD:AAA8931
Cyanus elongata|[12617]|TTDFW014-08|Calliphoridae|658|BOLD:AAI3921
Calliphora loewi|[12618]|BBDCP324-10|Calliphoridae|658|BOLD:AAB6579
Cynomya cadaverina|[12619]|NCCA2526-11|Calliphoridae|673|BOLD:AAB0868
Calliphora coloradensis|[12620]|CNGRF664-12|Calliphoridae|636|BOLD:AAI3922
Calliphora livida|[12621]|CNBPK389-13|Calliphoridae|666|BOLD:ABY7153
Calliphora livida|[12622]|NGAAF1314-14|Calliphoridae|573|BOLD:ACE3436
Calliphora genarum|[12623]|JWDCC236-10|Calliphoridae|658|BOLD:AAH3030
Calliphora montana|[12624]|BBDCP326-10|Calliphoridae|658|BOLD:ABZ4059
Calliphora stelviana|[12625]|CNTGA100-15|Calliphoridae|594|BOLD:ACD1717
Pegomya|[12626]|CNEIC2934-12|Anthomyiidae|673|BOLD:ACB9739
Anthomyiidae|[12627]|BBDCM170-10|Anthomyiidae|658|BOLD:AAP7584
Anthomyiidae|[12628]|CNKLF133-15|Anthomyiidae|576|BOLD:ACT2931
Hydrophoria lancifer|[12629]|PAJUN1117-12|Anthomyiidae|669|BOLD:AAG2460
Zaphne|[12630]|SSJAB1026-13|Anthomyiidae|664|BOLD:ACG4006
Zaphne|[12631]|TWDIP179-09|Anthomyiidae|656|BOLD:ABY6007
Zaphne gymnura|[12632]|JWDCC101-10|Anthomyiidae|658|BOLD:AAG1723
Zaphne altilega|[12633]|JWDCE280-10|Anthomyiidae|658|BOLD:ABX6111
Zaphne proxima|[12634]|MHFLI918-09|Anthomyiidae|656|BOLD:AAG2470
Zaphne brunneifrons|[12635]|JWDCE211-10|Anthomyiidae|658|BOLD:ACF0122
Zaphne occidentalis|[12636]|SSWLD4073-13|Anthomyiidae|604|BOLD:ABZ1244
Zaphne divisa|[12637]|CNGRI061-13|Anthomyiidae|662|BOLD:AAG2441
Zaphne|[12638]|SMTPB19092-13|Anthomyiidae|658|BOLD:ACF6848
Anthomyiidae|[12639]|BBDCQ092-10|Anthomyiidae|658|BOLD:AAP7599
Zaphne zetterstedtii|[12640]|JWDCC103-10|Anthomyiidae|658|BOLD:AAG2443
Anthomyiidae|[12641]|JSYKA486-10|Anthomyiidae|658|BOLD:AAL8102
Anthomyiidae|[12642]|DARC316-11|Anthomyiidae|658|BOLD:AAG2501
Zaphne ambigua|[12643]|JWDCI542-10|Anthomyiidae|658|BOLD:AAG2447
Zaphne implicata|[12644]|NCCA2497-11|Anthomyiidae|673|BOLD:AAG2442
Zaphne|[12645]|SSBAF3340-13|Anthomyiidae|658|BOLD:ACJ0800
Zaphne borealis|[12646]|SSBAC4006-12|Anthomyiidae|670|BOLD:ACX5193
Zaphne|[12647]|SSBAF3783-13|Anthomyiidae|658|BOLD:ACJ0730
Zaphne nearctica|[12648]|MHFLI920-09|Anthomyiidae|656|BOLD:ACE4683
Zaphne lineatocollis|[12649]|JWDCB078-10|Anthomyiidae|658|BOLD:AAG2434
Zaphne subarctica|[12650]|JWDCE485-10|Anthomyiidae|658|BOLD:ACE5945
Zaphne|[12651]|CNKLC2571-14|Anthomyiidae|570|BOLD:ACR9187
Scathophagidae|[12652]|BBDCN082-10|Scathophagidae|658|BOLD:AAM9280
Orthacheta|[12653]|BBDCQ577-10|Scathophagidae|658|BOLD:AAH4223
Scathophagidae|[12654]|CNEIB292-12|Scathophagidae|673|BOLD:AAP2692
Scathophagidae|[12655]|JSDIP290-10|Scathophagidae|658|BOLD:AAP2690
Scathophagidae|[12656]|JSDIP332-10|Scathophagidae|658|BOLD:AAP2691
Nanna|[12657]|CNEIA2401-12|Scathophagidae|673|BOLD:ACB9794
Scathophagidae|[12658]|CNEIA340-12|Scathophagidae|658|BOLD:ACB1241
Scathophagidae|[12659]|CNPKF1827-14|Scathophagidae|664|BOLD:ACL6328
Scathophaginae|[12660]|CNMIC443-14|Scathophagidae|611|BOLD:ACM9680
Neorthacheta dissimilis|[12661]|CNKJK373-14|Scathophagidae|603|BOLD:ACM2196
Scathophagidae|[12662]|DARC156-10|Scathophagidae|658|BOLD:AAM7340
Scathophagidae|[12663]|TTDFW469-08|Scathophagidae|658|BOLD:AAH4221
Scathophagidae|[12664]|SSWLA4061-13|Scathophagidae|664|BOLD:AAP6458
Gonarcticus|[12665]|CNKLB682-14|Scathophagidae|564|BOLD:ACR8082
Megaphthalmoides unilineatus|[12666]|BBDCN380-10|Scathophagidae|658|BOLD:AAP2693
Scathophagidae|[12667]|JWDCH746-10|Scathophagidae|658|BOLD:AAP8771
Scathophagidae|[12668]|BBDCP142-10|Scathophagidae|658|BOLD:AAP6459
Ernoneura|[12669]|SSJAD672-13|Scathophagidae|661|BOLD:ACI4994
Chaetosa palpalis|[12670]|CNEID3449-12|Scathophagidae|658|BOLD:AAH4217
Chaetosa punctipes|[12671]|SSEIB10955-13|Scathophagidae|670|BOLD:AAH4220
Cordylurella nana|[12672]|BBDED150-10|Scathophagidae|658|BOLD:AAH4231
Scathophagidae|[12673]|DARC268-11|Scathophagidae|658|BOLD:AAH4238
Microprosopa haemorrhoidalis|[12674]|SSJAD2609-13|Scathophagidae|624|BOLD:ACG5038
Pogonota barbata|[12675]|BBDCP129-10|Scathophagidae|658|BOLD:AAH4219
Scathophagidae|[12676]|DARC323-11|Scathophagidae|658|BOLD:AAH4222
Pogonota|[12677]|CNTUA3357-14|Scathophagidae|609|BOLD:ACB3118
Scathophagidae|[12678]|JWDCF272-10|Scathophagidae|658|BOLD:AAH4224
Scathophagidae|[12679]|DARC285-11|Scathophagidae|658|BOLD:AAU6589
Scathophagidae|[12680]|JWDCC118-10|Scathophagidae|658|BOLD:AAH4239
Scathophagidae|[12681]|DARC154-10|Scathophagidae|658|BOLD:AAU6588
Scathophaginae|[12682]|CNTUB4766-14|Scathophagidae|570|BOLD:ACB8673
Megaphthalma pallida|[12683]|NCCA872-11|Scathophagidae|673|BOLD:AAH4234
Scathophagidae|[12684]|SSJAD1867-13|Scathophagidae|661|BOLD:AAP2694
Scathophagidae|[12685]|SSJAD2599-13|Scathophagidae|661|BOLD:ACP4541
Scathophaga stercoraria|[12686]|BBDCP128-10|Scathophagidae|658|BOLD:AAD0853
Scathophaga|[12687]|BBDCM885-10|Scathophagidae|658|BOLD:AAH0023
Scathophagidae|[12688]|HEAPR2042-12|Scathophagidae|703|BOLD:ACX4405
Scathophaga|[12689]|CNPKD1115-14|Scathophagidae|576|BOLD:ACL3977
Scathophaga|[12690]|CNFDB041-14|Scathophagidae|658|BOLD:ACC4873
Scathophagidae|[12691]|JWDCA260-10|Scathophagidae|658|BOLD:AAH4216
Scathophagidae|[12692]|JWDCH771-10|Scathophagidae|658|BOLD:AAP8772
Scathophaga|[12693]|BBDEC504-09|Scathophagidae|658|BOLD:AAH4215
Scathophagidae|[12694]|DARC294-11|Scathophagidae|658|BOLD:AAV1118
Scathophaga|[12695]|CNIVF088-14|Scathophagidae|588|BOLD:ACM2719
Scathophagidae|[12696]|DARC348-11|Scathophagidae|658|BOLD:AAV1117
Scathophagidae|[12697]|CNWAE7324-14|Scathophagidae|603|BOLD:ACD3336
Scathophagidae|[12698]|JSYKA469-10|Scathophagidae|658|BOLD:AAM6175

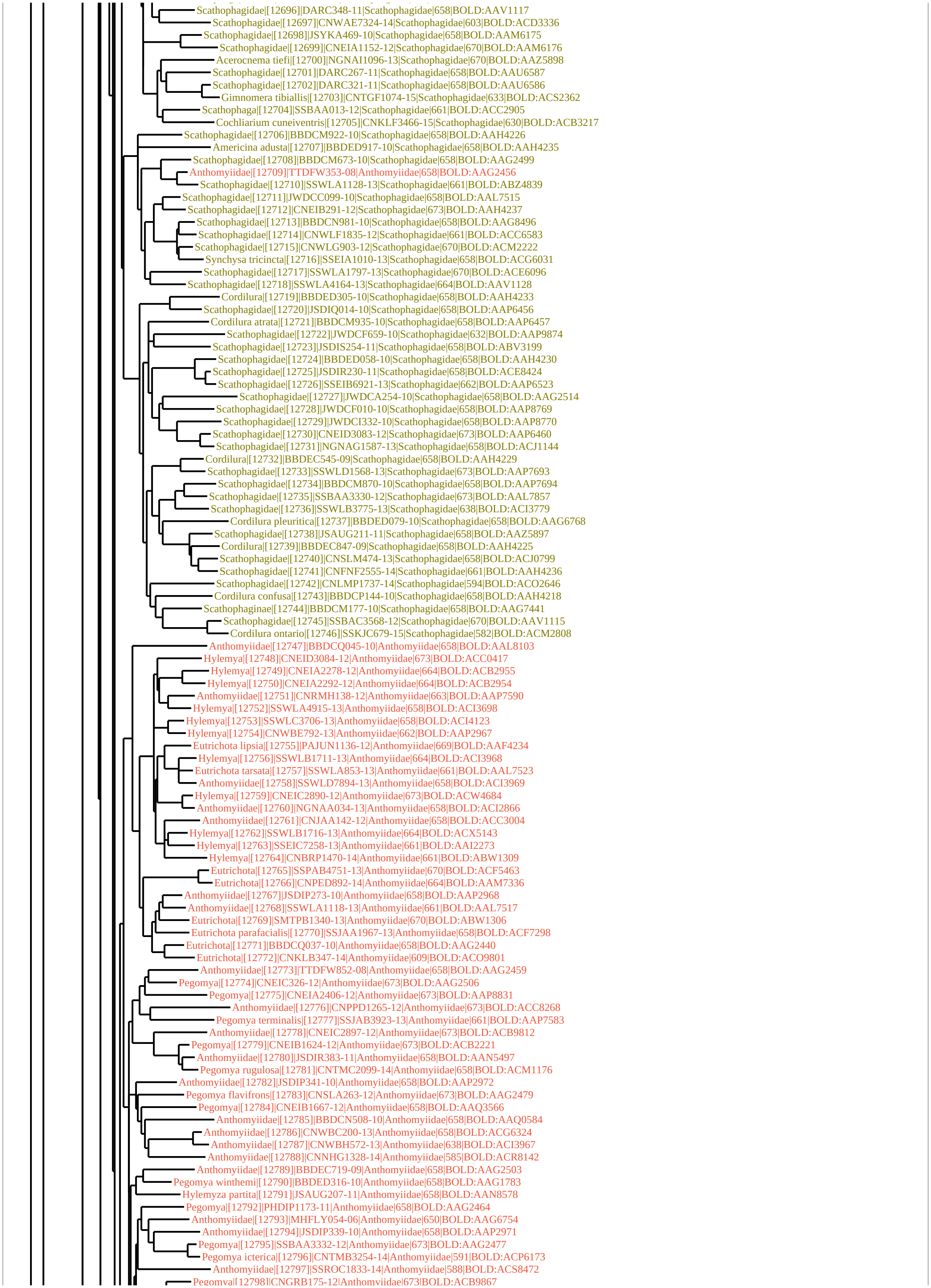
Scathophagidae|[12696]|DARC348-11|Scathophagidae|658|BOLD:AAV1117
Scathophagidae|[12697]|CNWAE7324-14|Scathophagidae|603|BOLD:ACD3336
Scathophagidae|[12698]|JSYKA469-10|Scathophagidae|658|BOLD:AAM6175
Scathophagidae|[12699]|CNEIA1152-12|Scathophagidae|670|BOLD:AAM6176
Acerocnema tiefi|[12700]|NGNAI1096-13|Scathophagidae|670|BOLD:AAZ5898
Scathophagidae|[12701]|DARC267-11|Scathophagidae|658|BOLD:AAU6587
Scathophagidae|[12702]|DARC321-11|Scathophagidae|658|BOLD:AAU6586
Gimnomera tibiallis|[12703]|CNTGF1074-15|Scathophagidae|633|BOLD:ACS2362
Scathophaga|[12704]|SSBAA013-12|Scathophagidae|661|BOLD:ACC2905
Cochliarium cuneiventris|[12705]|CNKLF3466-15|Scathophagidae|630|BOLD:ACB3217
Scathophagidae|[12706]|BBDCM922-10|Scathophagidae|658|BOLD:AAH4226
Americina adusta|[12707]|BBDED917-10|Scathophagidae|658|BOLD:AAH4235
Scathophagidae|[12708]|BBDCM673-10|Scathophagidae|658|BOLD:AAG2499
Anthomyiidae|[12709]|TTDFW353-08|Anthomyiidae|658|BOLD:AAG2456
Scathophagidae|[12710]|SSWLA1128-13|Scathophagidae|661|BOLD:ABZ4839
Scathophagidae|[12711]|JWDCC099-10|Scathophagidae|658|BOLD:AAL7515
Scathophagidae|[12712]|CNEIB291-12|Scathophagidae|673|BOLD:AAH4237
Scathophagidae|[12713]|BBDCN981-10|Scathophagidae|658|BOLD:AAG8496
Scathophagidae|[12714]|CNWLF1835-12|Scathophagidae|661|BOLD:ACC6583
Scathophagidae|[12715]|CNWLG903-12|Scathophagidae|670|BOLD:ACM2222
Synchysa tricincta|[12716]|SSEIA1010-13|Scathophagidae|658|BOLD:ACG6031
Scathophagidae|[12717]|SSWLA1797-13|Scathophagidae|670|BOLD:ACE6096
Scathophagidae|[12718]|SSWLA4164-13|Scathophagidae|664|BOLD:AAV1128
Cordilura|[12719]|BBDED305-10|Scathophagidae|658|BOLD:AAH4233
Scathophagidae|[12720]|JSDIQ014-10|Scathophagidae|658|BOLD:AAP6456
Cordilura atrata|[12721]|BBDCM935-10|Scathophagidae|658|BOLD:AAP6457
Scathophagidae|[12722]|JWDCF659-10|Scathophagidae|632|BOLD:AAP9874
Scathophagidae|[12723]|JSDIS254-11|Scathophagidae|658|BOLD:ABV3199
Scathophagidae|[12724]|BBDED058-10|Scathophagidae|658|BOLD:AAH4230
Scathophagidae|[12725]|JSDIR230-11|Scathophagidae|658|BOLD:ACE8424
Scathophagidae|[12726]|SSEIB6921-13|Scathophagidae|662|BOLD:AAP6523
Scathophagidae|[12727]|JWDCA254-10|Scathophagidae|658|BOLD:AAG2514
Scathophagidae|[12728]|JWDCF010-10|Scathophagidae|658|BOLD:AAP8769
Scathophagidae|[12729]|JWDCI332-10|Scathophagidae|658|BOLD:AAP8770
Scathophagidae|[12730]|CNEID3083-12|Scathophagidae|673|BOLD:AAP6460
Scathophagidae|[12731]|NGNAG1587-13|Scathophagidae|658|BOLD:ACJ1144
Cordilura|[12732]|BBDEC545-09|Scathophagidae|658|BOLD:AAH4229
Scathophagidae|[12733]|SSWLD1568-13|Scathophagidae|673|BOLD:AAP7693
Scathophagidae|[12734]|BBDCM870-10|Scathophagidae|658|BOLD:AAP7694
Scathophagidae|[12735]|SSBAA3330-12|Scathophagidae|673|BOLD:AAL7857
Scathophagidae|[12736]|SSWLB3775-13|Scathophagidae|638|BOLD:ACI3779
Cordilura pleuritica|[12737]|BBDED079-10|Scathophagidae|658|BOLD:AAG6768
Scathophagidae|[12738]|JSAUG211-11|Scathophagidae|658|BOLD:AAZ5897
Cordilura|[12739]|BBDEC847-09|Scathophagidae|658|BOLD:AAH4225
Scathophagidae|[12740]|CNSLM474-13|Scathophagidae|658|BOLD:ACJ0799
Scathophagidae|[12741]|CNFNF2555-14|Scathophagidae|661|BOLD:AAH4236
Scathophagidae|[12742]|CNLMP1737-14|Scathophagidae|594|BOLD:ACO2646
Cordilura confusa|[12743]|BBDCP144-10|Scathophagidae|658|BOLD:AAH4218
Scathophaginae|[12744]|BBDCM177-10|Scathophagidae|658|BOLD:AAG7441
Scathophagidae|[12745]|SSBAC3568-12|Scathophagidae|670|BOLD:AAV1115
Cordilura ontario|[12746]|SSKJC679-15|Scathophagidae|582|BOLD:ACM2808
Anthomyiidae|[12747]|BBDCQ045-10|Anthomyiidae|658|BOLD:AAL8103
Hylemya|[12748]|CNEID3084-12|Anthomyiidae|673|BOLD:ACC0417
Hylemya|[12749]|CNEIA2278-12|Anthomyiidae|664|BOLD:ACB2955
Hylemya|[12750]|CNEIA2292-12|Anthomyiidae|664|BOLD:ACB2954
Anthomyiidae|[12751]|CNRMH138-12|Anthomyiidae|663|BOLD:AAP7590
Hylemya|[12752]|SSWLA4915-13|Anthomyiidae|658|BOLD:ACI3698
Hylemya|[12753]|SSWLC3706-13|Anthomyiidae|658|BOLD:ACI4123
Hylemya|[12754]|CNWBE792-13|Anthomyiidae|662|BOLD:AAP2967
Eutrichota lipsia|[12755]|PAJUN1136-12|Anthomyiidae|669|BOLD:AAF4234
Hylemya|[12756]|SSWLB1711-13|Anthomyiidae|664|BOLD:ACI3968
Eutrichota tarsata|[12757]|SSWLA853-13|Anthomyiidae|661|BOLD:AAL7523
Anthomyiidae|[12758]|SSWLD7894-13|Anthomyiidae|658|BOLD:ACI3969
Hylemya|[12759]|CNEIC2890-12|Anthomyiidae|673|BOLD:ACW4684
Anthomyiidae|[12760]|NGNAA034-13|Anthomyiidae|658|BOLD:ACI2866
Anthomyiidae|[12761]|CNJAA142-12|Anthomyiidae|658|BOLD:ACC3004
Hylemya|[12762]|SSWLB1716-13|Anthomyiidae|664|BOLD:ACX5143
Hylemya|[12763]|SSEIC7258-13|Anthomyiidae|661|BOLD:AAI2273
Hylemya|[12764]|CNBRP1470-14|Anthomyiidae|661|BOLD:ABW1309
Eutrichota|[12765]|SSPAB4751-13|Anthomyiidae|670|BOLD:ACF5463
Eutrichota|[12766]|CNPED892-14|Anthomyiidae|664|BOLD:AAM7336
Anthomyiidae|[12767]|JSDIP273-10|Anthomyiidae|658|BOLD:AAP2968
Anthomyiidae|[12768]|SSWLA1118-13|Anthomyiidae|661|BOLD:AAL7517
Eutrichota|[12769]|SMTPB1340-13|Anthomyiidae|670|BOLD:ABW1306
Eutrichota parafacialis|[12770]|SSJAA1967-13|Anthomyiidae|658|BOLD:ACF7298
Eutrichota|[12771]|BBDCQ037-10|Anthomyiidae|658|BOLD:AAG2440
Eutrichota|[12772]|CNKLB347-14|Anthomyiidae|609|BOLD:ACO9801
Anthomyiidae|[12773]|TTDFW852-08|Anthomyiidae|658|BOLD:AAG2459
Pegomya|[12774]|CNEIC326-12|Anthomyiidae|673|BOLD:AAG2506
Pegomya|[12775]|CNEIA2406-12|Anthomyiidae|673|BOLD:AAP8831
Anthomyiidae|[12776]|CNPPD1265-12|Anthomyiidae|673|BOLD:ACC8268
Pegomya terminalis|[12777]|SSJAB3923-13|Anthomyiidae|661|BOLD:AAP7583
Anthomyiidae|[12778]|CNEIC2897-12|Anthomyiidae|673|BOLD:ACB9812
Pegomya|[12779]|CNEIB1624-12|Anthomyiidae|673|BOLD:ACB2221
Anthomyiidae|[12780]|JSDIR383-11|Anthomyiidae|658|BOLD:AAN5497
Pegomya rugulosa|[12781]|CNTMC2099-14|Anthomyiidae|658|BOLD:ACM1176
Anthomyiidae|[12782]|JSDIP341-10|Anthomyiidae|658|BOLD:AAP2972
Pegomya flavifrons|[12783]|CNSLA263-12|Anthomyiidae|673|BOLD:AAG2479
Pegomya|[12784]|CNEIB1667-12|Anthomyiidae|658|BOLD:AAQ3566
Anthomyiidae|[12785]|BBDCN508-10|Anthomyiidae|658|BOLD:AAQ0584
Anthomyiidae|[12786]|CNWBC200-13|Anthomyiidae|658|BOLD:ACG6324
Anthomyiidae|[12787]|CNWBH572-13|Anthomyiidae|638|BOLD:ACI3967
Anthomyiidae|[12788]|CNNHG1328-14|Anthomyiidae|585|BOLD:ACR8142
Anthomyiidae|[12789]|BBDEC719-09|Anthomyiidae|658|BOLD:AAG2503
Pegomya winthemi|[12790]|BBDED316-10|Anthomyiidae|658|BOLD:AAG1783
Hylemyza partita|[12791]|JSAUG207-11|Anthomyiidae|658|BOLD:AAN8578
Pegomya|[12792]|PHDIP1173-11|Anthomyiidae|658|BOLD:AAG2464
Anthomyiidae|[12793]|MHFLY054-06|Anthomyiidae|650|BOLD:AAG6754
Anthomyiidae|[12794]|JSDIP339-10|Anthomyiidae|658|BOLD:AAP2971
Pegomya|[12795]|SSBAA3332-12|Anthomyiidae|673|BOLD:AAG2477
Pegomya icterica|[12796]|CNTMB3254-14|Anthomyiidae|591|BOLD:ACP6173
Anthomyiidae|[12797]|SSROC1833-14|Anthomyiidae|588|BOLD:ACS8472
Pegomya|[12798]|CNGRB175-12|Anthomyiidae|673|BOLD:ACB9867

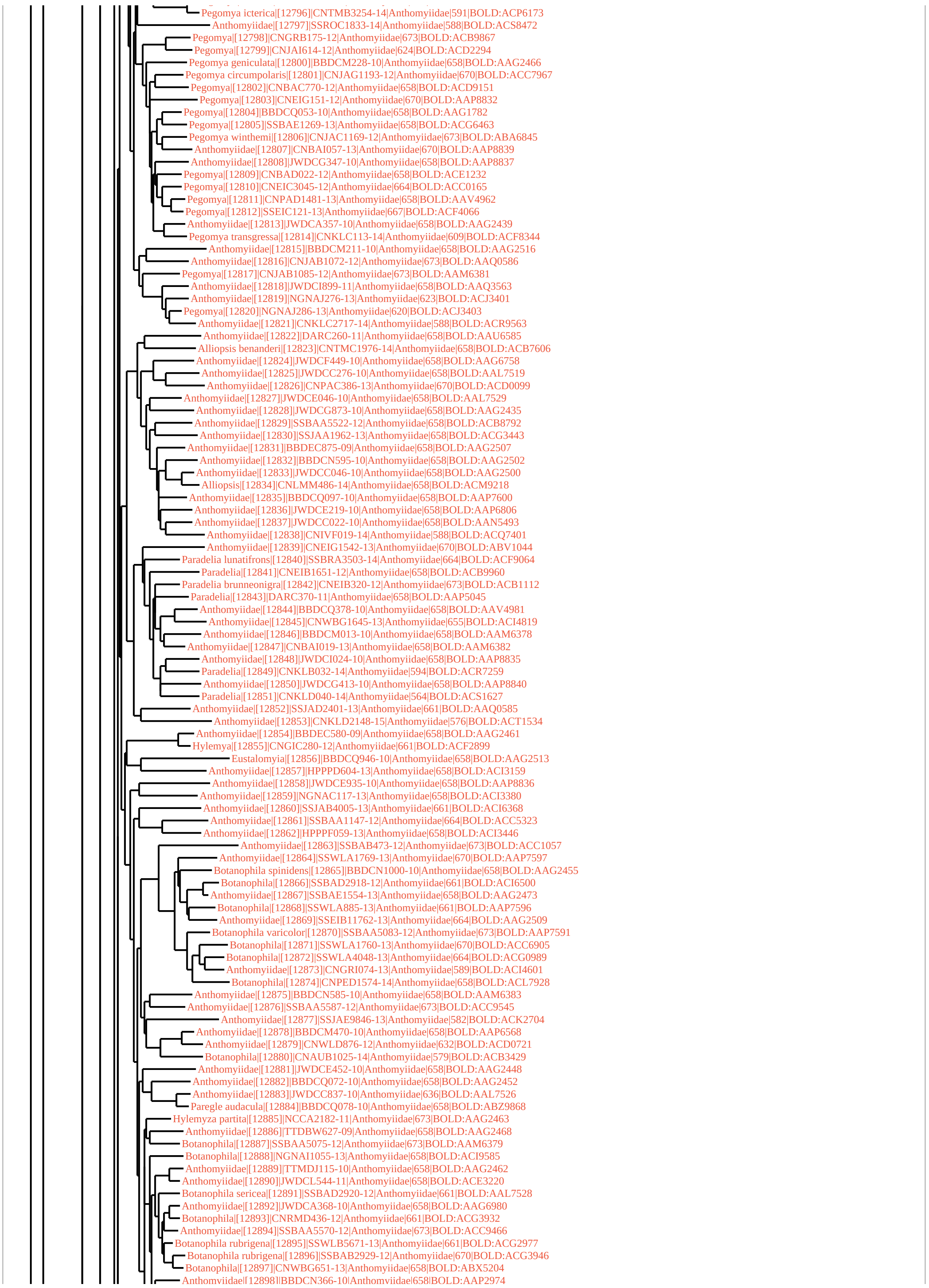
Pegomya icterica|[12796]|CNTMB3254-14|Anthomyiidae|591|BOLD:ACP6173
Anthomyiidae|[12797]|SSROC1833-14|Anthomyiidae|588|BOLD:ACS8472
Pegomya|[12798]|CNGRB175-12|Anthomyiidae|673|BOLD:ACB9867
Pegomya|[12799]|CNJAI614-12|Anthomyiidae|624|BOLD:ACD2294
Pegomya geniculata|[12800]|BBDCM228-10|Anthomyiidae|658|BOLD:AAG2466
Pegomya circumpolaris|[12801]|CNJAG1193-12|Anthomyiidae|670|BOLD:ACC7967
Pegomya|[12802]|CNBAC770-12|Anthomyiidae|658|BOLD:ACD9151
Pegomya|[12803]|CNEIG151-12|Anthomyiidae|670|BOLD:AAP8832
Pegomya|[12804]|BBDCQ053-10|Anthomyiidae|658|BOLD:AAG1782
Pegomya|[12805]|SSBAE1269-13|Anthomyiidae|658|BOLD:ACG6463
Pegomya winthemi|[12806]|CNJAC1169-12|Anthomyiidae|673|BOLD:ABA6845
Anthomyiidae|[12807]|CNBAI057-13|Anthomyiidae|670|BOLD:AAP8839
Anthomyiidae|[12808]|JWDCG347-10|Anthomyiidae|658|BOLD:AAP8837
Pegomya|[12809]|CNBAD022-12|Anthomyiidae|658|BOLD:ACE1232
Pegomya|[12810]|CNEIC3045-12|Anthomyiidae|664|BOLD:ACC0165
Pegomya|[12811]|CNPAD1481-13|Anthomyiidae|658|BOLD:AAV4962
Pegomya|[12812]|SSEIC121-13|Anthomyiidae|667|BOLD:ACF4066
Anthomyiidae|[12813]|JWDCA357-10|Anthomyiidae|658|BOLD:AAG2439
Pegomya transgressa|[12814]|CNKLC113-14|Anthomyiidae|609|BOLD:ACF8344
Anthomyiidae|[12815]|BBDCM211-10|Anthomyiidae|658|BOLD:AAG2516
Anthomyiidae|[12816]|CNJAB1072-12|Anthomyiidae|673|BOLD:AAQ0586
Pegomya|[12817]|CNJAB1085-12|Anthomyiidae|673|BOLD:AAM6381
Anthomyiidae|[12818]|JWDCI899-11|Anthomyiidae|658|BOLD:AAQ3563
Anthomyiidae|[12819]|NGNAJ276-13|Anthomyiidae|623|BOLD:ACJ3401
Pegomya|[12820]|NGNAJ286-13|Anthomyiidae|620|BOLD:ACJ3403
Anthomyiidae|[12821]|CNKLC2717-14|Anthomyiidae|588|BOLD:ACR9563
Anthomyiidae|[12822]|DARC260-11|Anthomyiidae|658|BOLD:AAU6585
Alliopsis benanderi|[12823]|CNTMC1976-14|Anthomyiidae|658|BOLD:ACB7606
Anthomyiidae|[12824]|JWDCF449-10|Anthomyiidae|658|BOLD:AAG6758
Anthomyiidae|[12825]|JWDCC276-10|Anthomyiidae|658|BOLD:AAL7519
Anthomyiidae|[12826]|CNPAC386-13|Anthomyiidae|670|BOLD:ACD0099
Anthomyiidae|[12827]|JWDCE046-10|Anthomyiidae|658|BOLD:AAL7529
Anthomyiidae|[12828]|JWDCG873-10|Anthomyiidae|658|BOLD:AAG2435
Anthomyiidae|[12829]|SSBAA5522-12|Anthomyiidae|658|BOLD:ACB8792
Anthomyiidae|[12830]|SSJAA1962-13|Anthomyiidae|658|BOLD:ACG3443
Anthomyiidae|[12831]|BBDEC875-09|Anthomyiidae|658|BOLD:AAG2507
Anthomyiidae|[12832]|BBDCN595-10|Anthomyiidae|658|BOLD:AAG2502
Anthomyiidae|[12833]|JWDCC046-10|Anthomyiidae|658|BOLD:AAG2500
Alliopsis|[12834]|CNLMM486-14|Anthomyiidae|658|BOLD:ACM9218
Anthomyiidae|[12835]|BBDCQ097-10|Anthomyiidae|658|BOLD:AAP7600
Anthomyiidae|[12836]|JWDCE219-10|Anthomyiidae|658|BOLD:AAP6806
Anthomyiidae|[12837]|JWDCC022-10|Anthomyiidae|658|BOLD:AAN5493
Anthomyiidae|[12838]|CNIVF019-14|Anthomyiidae|588|BOLD:ACQ7401
Anthomyiidae|[12839]|CNEIG1542-13|Anthomyiidae|670|BOLD:ABV1044
Paradelia lunatifrons|[12840]|SSBRA3503-14|Anthomyiidae|664|BOLD:ACF9064
Paradelia|[12841]|CNEIB1651-12|Anthomyiidae|658|BOLD:ACB9960
Paradelia brunneonigra|[12842]|CNEIB320-12|Anthomyiidae|673|BOLD:ACB1112
Paradelia|[12843]|DARC370-11|Anthomyiidae|658|BOLD:AAP5045
Anthomyiidae|[12844]|BBDCQ378-10|Anthomyiidae|658|BOLD:AAV4981
Anthomyiidae|[12845]|CNWBG1645-13|Anthomyiidae|655|BOLD:ACI4819
Anthomyiidae|[12846]|BBDCM013-10|Anthomyiidae|658|BOLD:AAM6378
Anthomyiidae|[12847]|CNBAI019-13|Anthomyiidae|658|BOLD:AAM6382
Anthomyiidae|[12848]|JWDCI024-10|Anthomyiidae|658|BOLD:AAP8835
Paradelia|[12849]|CNKLB032-14|Anthomyiidae|594|BOLD:ACR7259
Anthomyiidae|[12850]|JWDCG413-10|Anthomyiidae|658|BOLD:AAP8840
Paradelia|[12851]|CNKLD040-14|Anthomyiidae|564|BOLD:ACS1627
Anthomyiidae|[12852]|SSJAD2401-13|Anthomyiidae|661|BOLD:AAQ0585
Anthomyiidae|[12853]|CNKLD2148-15|Anthomyiidae|576|BOLD:ACT1534
Anthomyiidae|[12854]|BBDEC580-09|Anthomyiidae|658|BOLD:AAG2461
Hylemya|[12855]|CNGIC280-12|Anthomyiidae|661|BOLD:ACF2899
Eustalomyia|[12856]|BBDCQ946-10|Anthomyiidae|658|BOLD:AAG2513
Anthomyiidae|[12857]|HPPPD604-13|Anthomyiidae|658|BOLD:ACI3159
Anthomyiidae|[12858]|JWDCE935-10|Anthomyiidae|658|BOLD:AAP8836
Anthomyiidae|[12859]|NGNAC117-13|Anthomyiidae|658|BOLD:ACI3380
Anthomyiidae|[12860]|SSJAB4005-13|Anthomyiidae|661|BOLD:ACI6368
Anthomyiidae|[12861]|SSBAA1147-12|Anthomyiidae|664|BOLD:ACC5323
Anthomyiidae|[12862]|HPPPF059-13|Anthomyiidae|658|BOLD:ACI3446
Anthomyiidae|[12863]|SSBAB473-12|Anthomyiidae|673|BOLD:ACC1057
Anthomyiidae|[12864]|SSWLA1769-13|Anthomyiidae|670|BOLD:AAP7597
Botanophila spinidens|[12865]|BBDCN1000-10|Anthomyiidae|658|BOLD:AAG2455
Botanophila|[12866]|SSBAD2918-12|Anthomyiidae|661|BOLD:ACI6500
Anthomyiidae|[12867]|SSBAE1554-13|Anthomyiidae|658|BOLD:AAG2473
Botanophila|[12868]|SSWLA885-13|Anthomyiidae|661|BOLD:AAP7596
Anthomyiidae|[12869]|SSEIB11762-13|Anthomyiidae|664|BOLD:AAG2509
Botanophila varicolor|[12870]|SSBAA5083-12|Anthomyiidae|673|BOLD:AAP7591
Botanophila|[12871]|SSWLA1760-13|Anthomyiidae|670|BOLD:ACC6905
Botanophila|[12872]|SSWLA4048-13|Anthomyiidae|664|BOLD:ACG0989
Anthomyiidae|[12873]|CNGRI074-13|Anthomyiidae|589|BOLD:ACI4601
Botanophila|[12874]|CNPED1574-14|Anthomyiidae|658|BOLD:ACL7928
Anthomyiidae|[12875]|BBDCN585-10|Anthomyiidae|658|BOLD:AAM6383
Anthomyiidae|[12876]|SSBAA5587-12|Anthomyiidae|673|BOLD:ACC9545
Anthomyiidae|[12877]|SSJAE9846-13|Anthomyiidae|582|BOLD:ACK2704
Anthomyiidae|[12878]|BBDCM470-10|Anthomyiidae|658|BOLD:AAP6568
Anthomyiidae|[12879]|CNWLD876-12|Anthomyiidae|632|BOLD:ACD0721
Botanophila|[12880]|CNAUB1025-14|Anthomyiidae|579|BOLD:ACB3429
Anthomyiidae|[12881]|JWDCE452-10|Anthomyiidae|658|BOLD:AAG2448
Anthomyiidae|[12882]|BBDCQ072-10|Anthomyiidae|658|BOLD:AAG2452
Anthomyiidae|[12883]|JWDCC837-10|Anthomyiidae|636|BOLD:AAL7526
Paregle audacula|[12884]|BBDCQ078-10|Anthomyiidae|658|BOLD:ABZ9868
Hylemyza partita|[12885]|NCCA2182-11|Anthomyiidae|673|BOLD:AAG2463
Anthomyiidae|[12886]|TTDBW627-09|Anthomyiidae|658|BOLD:AAG2468
Botanophila|[12887]|SSBAA5075-12|Anthomyiidae|673|BOLD:AAM6379
Botanophila|[12888]|NGNAI1055-13|Anthomyiidae|658|BOLD:ACI9585
Anthomyiidae|[12889]|TTMDJ115-10|Anthomyiidae|658|BOLD:AAG2462
Anthomyiidae|[12890]|JWDCL544-11|Anthomyiidae|658|BOLD:ACE3220
Botanophila sericea|[12891]|SSBAD2920-12|Anthomyiidae|661|BOLD:AAL7528
Anthomyiidae|[12892]|JWDCA368-10|Anthomyiidae|658|BOLD:AAG6980
Botanophila|[12893]|CNRMD436-12|Anthomyiidae|661|BOLD:ACG3932
Anthomyiidae|[12894]|SSBAA5570-12|Anthomyiidae|673|BOLD:ACC9466
Botanophila rubrigena|[12895]|SSWLB5671-13|Anthomyiidae|661|BOLD:ACG2977
Botanophila rubrigena|[12896]|SSBAB2929-12|Anthomyiidae|670|BOLD:ACG3946
Botanophila|[12897]|CNWBG651-13|Anthomyiidae|658|BOLD:ABX5204
Anthomyiidae|[12898]|BBDCN366-10|Anthomyiidae|658|BOLD:AAP2974

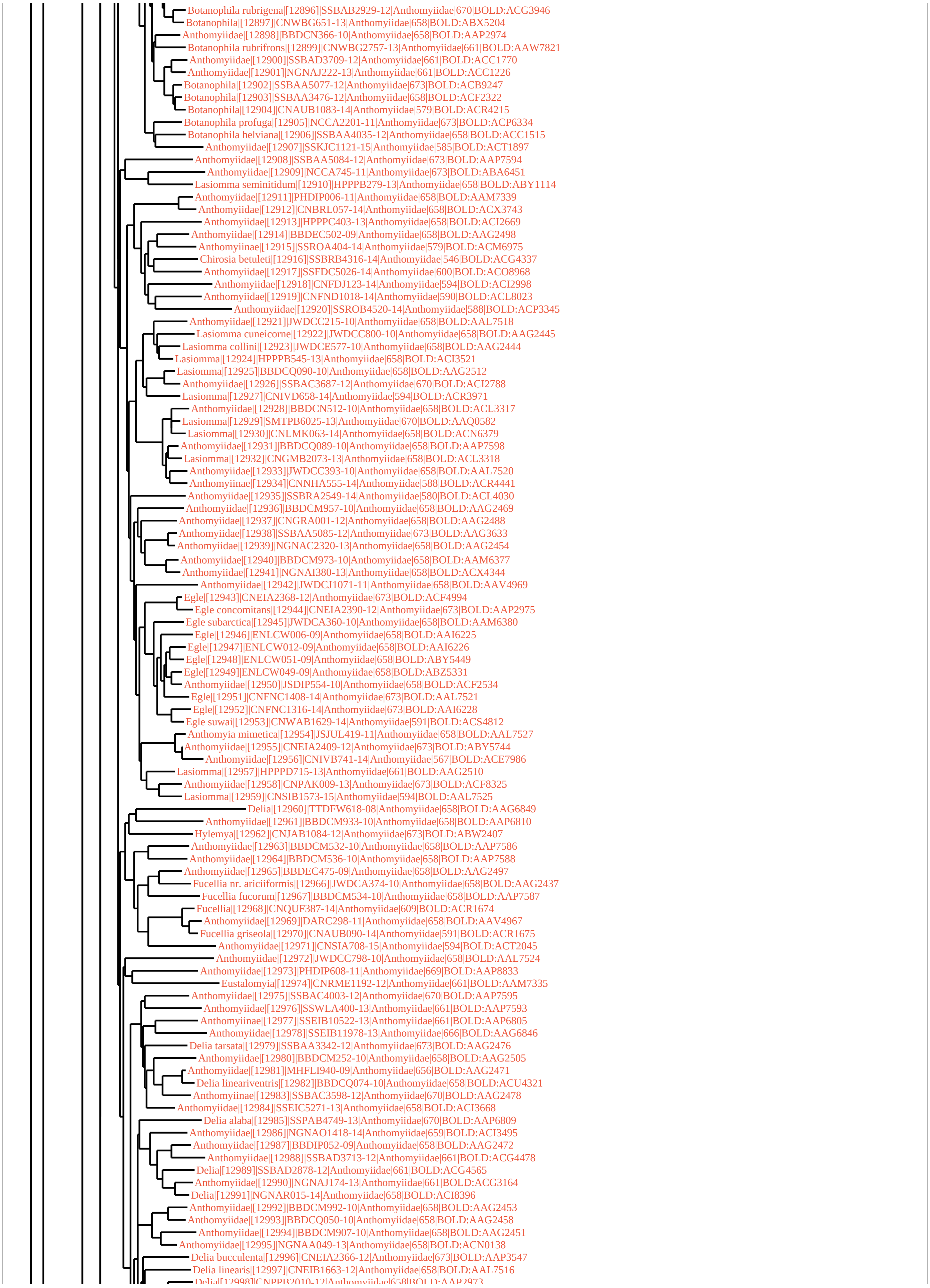
Botanophila rubrigena|[12896]|SSBAB2929-12|Anthomyiidae|670|BOLD:ACG3946
Botanophila|[12897]|CNWBG651-13|Anthomyiidae|658|BOLD:ABX5204
Anthomyiidae|[12898]|BBDCN366-10|Anthomyiidae|658|BOLD:AAP2974
Botanophila rubrifrons|[12899]|CNWBG2757-13|Anthomyiidae|661|BOLD:AAW7821
Anthomyiidae|[12900]|SSBAD3709-12|Anthomyiidae|661|BOLD:ACC1770
Anthomyiidae|[12901]|NGNAJ222-13|Anthomyiidae|661|BOLD:ACC1226
Botanophila|[12902]|SSBAA5077-12|Anthomyiidae|673|BOLD:ACB9247
Botanophila|[12903]|SSBAA3476-12|Anthomyiidae|658|BOLD:ACF2322
Botanophila|[12904]|CNAUB1083-14|Anthomyiidae|579|BOLD:ACR4215
Botanophila profuga|[12905]|NCCA2201-11|Anthomyiidae|673|BOLD:ACP6334
Botanophila helviana|[12906]|SSBAA4035-12|Anthomyiidae|658|BOLD:ACC1515
Anthomyiidae|[12907]|SSKJC1121-15|Anthomyiidae|585|BOLD:ACT1897
Anthomyiidae|[12908]|SSBAA5084-12|Anthomyiidae|673|BOLD:AAP7594
Anthomyiidae|[12909]|NCCA745-11|Anthomyiidae|673|BOLD:ABA6451
Lasiomma seminitidum|[12910]|HPPPB279-13|Anthomyiidae|658|BOLD:ABY1114
Anthomyiidae|[12911]|PHDIP006-11|Anthomyiidae|658|BOLD:AAM7339
Anthomyiidae|[12912]|CNBRL057-14|Anthomyiidae|658|BOLD:ACX3743
Anthomyiidae|[12913]|HPPPC403-13|Anthomyiidae|658|BOLD:ACI2669
Anthomyiidae|[12914]|BBDEC502-09|Anthomyiidae|658|BOLD:AAG2498
Anthomyiinae|[12915]|SSROA404-14|Anthomyiidae|579|BOLD:ACM6975
Chirosia betuleti|[12916]|SSBRB4316-14|Anthomyiidae|546|BOLD:ACG4337
Anthomyiidae|[12917]|SSFDC5026-14|Anthomyiidae|600|BOLD:ACO8968
Anthomyiidae|[12918]|CNFDJ123-14|Anthomyiidae|594|BOLD:ACI2998
Anthomyiidae|[12919]|CNFND1018-14|Anthomyiidae|590|BOLD:ACL8023
Anthomyiidae|[12920]|SSROB4520-14|Anthomyiidae|588|BOLD:ACP3345
Anthomyiidae|[12921]|JWDCC215-10|Anthomyiidae|658|BOLD:AAL7518
Lasiomma cuneicorne|[12922]|JWDCC800-10|Anthomyiidae|658|BOLD:AAG2445
Lasiomma collini|[12923]|JWDCE577-10|Anthomyiidae|658|BOLD:AAG2444
Lasiomma|[12924]|HPPPB545-13|Anthomyiidae|658|BOLD:ACI3521
Lasiomma|[12925]|BBDCQ090-10|Anthomyiidae|658|BOLD:AAG2512
Anthomyiidae|[12926]|SSBAC3687-12|Anthomyiidae|670|BOLD:ACI2788
Lasiomma|[12927]|CNIVD658-14|Anthomyiidae|594|BOLD:ACR3971
Anthomyiidae|[12928]|BBDCN512-10|Anthomyiidae|658|BOLD:ACL3317
Lasiomma|[12929]|SMTPB6025-13|Anthomyiidae|670|BOLD:AAQ0582
Lasiomma|[12930]|CNLMK063-14|Anthomyiidae|658|BOLD:ACN6379
Anthomyiidae|[12931]|BBDCQ089-10|Anthomyiidae|658|BOLD:AAP7598
Lasiomma|[12932]|CNGMB2073-13|Anthomyiidae|658|BOLD:ACL3318
Anthomyiidae|[12933]|JWDCC393-10|Anthomyiidae|658|BOLD:AAL7520
Anthomyiinae|[12934]|CNNHA555-14|Anthomyiidae|588|BOLD:ACR4441
Anthomyiidae|[12935]|SSBRA2549-14|Anthomyiidae|580|BOLD:ACL4030
Anthomyiidae|[12936]|BBDCM957-10|Anthomyiidae|658|BOLD:AAG2469
Anthomyiidae|[12937]|CNGRA001-12|Anthomyiidae|658|BOLD:AAG2488
Anthomyiidae|[12938]|SSBAA5085-12|Anthomyiidae|673|BOLD:AAG3633
Anthomyiidae|[12939]|NGNAC2320-13|Anthomyiidae|658|BOLD:AAG2454
Anthomyiidae|[12940]|BBDCM973-10|Anthomyiidae|658|BOLD:AAM6377
Anthomyiidae|[12941]|NGNAI380-13|Anthomyiidae|658|BOLD:ACX4344
Anthomyiidae|[12942]|JWDCJ1071-11|Anthomyiidae|658|BOLD:AAV4969
Egle|[12943]|CNEIA2368-12|Anthomyiidae|673|BOLD:ACF4994
Egle concomitans|[12944]|CNEIA2390-12|Anthomyiidae|673|BOLD:AAP2975
Egle subarctica|[12945]|JWDCA360-10|Anthomyiidae|658|BOLD:AAM6380
Egle|[12946]|ENLCW006-09|Anthomyiidae|658|BOLD:AAI6225
Egle|[12947]|ENLCW012-09|Anthomyiidae|658|BOLD:AAI6226
Egle|[12948]|ENLCW051-09|Anthomyiidae|658|BOLD:ABY5449
Egle|[12949]|ENLCW049-09|Anthomyiidae|658|BOLD:ABZ5331
Anthomyiidae|[12950]|JSDIP554-10|Anthomyiidae|658|BOLD:ACF2534
Egle|[12951]|CNFNC1408-14|Anthomyiidae|673|BOLD:AAL7521
Egle|[12952]|CNFNC1316-14|Anthomyiidae|673|BOLD:AAI6228
Egle suwai|[12953]|CNWAB1629-14|Anthomyiidae|591|BOLD:ACS4812
Anthomyia mimetica|[12954]|JSJUL419-11|Anthomyiidae|658|BOLD:AAL7527
Anthomyiidae|[12955]|CNEIA2409-12|Anthomyiidae|673|BOLD:ABY5744
Anthomyiidae|[12956]|CNIVB741-14|Anthomyiidae|567|BOLD:ACE7986
Lasiomma|[12957]|HPPPD715-13|Anthomyiidae|661|BOLD:AAG2510
Anthomyiidae|[12958]|CNPAK009-13|Anthomyiidae|673|BOLD:ACF8325
Lasiomma|[12959]|CNSIB1573-15|Anthomyiidae|594|BOLD:AAL7525
Delia|[12960]|TTDFW618-08|Anthomyiidae|658|BOLD:AAG6849
Anthomyiidae|[12961]|BBDCM933-10|Anthomyiidae|658|BOLD:AAP6810
Hylemya|[12962]|CNJAB1084-12|Anthomyiidae|673|BOLD:ABW2407
Anthomyiidae|[12963]|BBDCM532-10|Anthomyiidae|658|BOLD:AAP7586
Anthomyiidae|[12964]|BBDCM536-10|Anthomyiidae|658|BOLD:AAP7588
Anthomyiidae|[12965]|BBDEC475-09|Anthomyiidae|658|BOLD:AAG2497
Fucellia nr. ariciiformis|[12966]|JWDCA374-10|Anthomyiidae|658|BOLD:AAG2437
Fucellia fucorum|[12967]|BBDCM534-10|Anthomyiidae|658|BOLD:AAP7587
Fucellia|[12968]|CNQUF387-14|Anthomyiidae|609|BOLD:ACR1674
Anthomyiidae|[12969]|DARC298-11|Anthomyiidae|658|BOLD:AAV4967
Fucellia griseola|[12970]|CNAUB090-14|Anthomyiidae|591|BOLD:ACR1675
Anthomyiidae|[12971]|CNSIA708-15|Anthomyiidae|594|BOLD:ACT2045
Anthomyiidae|[12972]|JWDCC798-10|Anthomyiidae|658|BOLD:AAL7524
Anthomyiidae|[12973]|PHDIP608-11|Anthomyiidae|669|BOLD:AAP8833
Eustalomyia|[12974]|CNRME1192-12|Anthomyiidae|661|BOLD:AAM7335
Anthomyiidae|[12975]|SSBAC4003-12|Anthomyiidae|670|BOLD:AAP7595
Anthomyiidae|[12976]|SSWLA400-13|Anthomyiidae|661|BOLD:AAP7593
Anthomyiinae|[12977]|SSEIB10522-13|Anthomyiidae|661|BOLD:AAP6805
Anthomyiidae|[12978]|SSEIB11978-13|Anthomyiidae|666|BOLD:AAG6846
Delia tarsata|[12979]|SSBAA3342-12|Anthomyiidae|673|BOLD:AAG2476
Anthomyiidae|[12980]|BBDCM252-10|Anthomyiidae|658|BOLD:AAG2505
Anthomyiidae|[12981]|MHFLI940-09|Anthomyiidae|656|BOLD:AAG2471
Delia lineariventris|[12982]|BBDCQ074-10|Anthomyiidae|658|BOLD:ACU4321
Anthomyiinae|[12983]|SSBAC3598-12|Anthomyiidae|670|BOLD:AAG2478
Anthomyiidae|[12984]|SSEIC5271-13|Anthomyiidae|658|BOLD:ACI3668
Delia alaba|[12985]|SSPAB4749-13|Anthomyiidae|670|BOLD:AAP6809
Anthomyiidae|[12986]|NGNAO1418-14|Anthomyiidae|659|BOLD:ACI3495
Anthomyiidae|[12987]|BBDIP052-09|Anthomyiidae|658|BOLD:AAG2472
Anthomyiidae|[12988]|SSBAD3713-12|Anthomyiidae|661|BOLD:ACG4478
Delia|[12989]|SSBAD2878-12|Anthomyiidae|661|BOLD:ACG4565
Anthomyiidae|[12990]|NGNAJ174-13|Anthomyiidae|661|BOLD:ACG3164
Delia|[12991]|NGNAR015-14|Anthomyiidae|658|BOLD:ACI8396
Anthomyiidae|[12992]|BBDCM992-10|Anthomyiidae|658|BOLD:AAG2453
Anthomyiidae|[12993]|BBDCQ050-10|Anthomyiidae|658|BOLD:AAG2458
Anthomyiidae|[12994]|BBDCM907-10|Anthomyiidae|658|BOLD:AAG2451
Anthomyiidae|[12995]|NGNAA049-13|Anthomyiidae|658|BOLD:ACN0138
Delia bucculenta|[12996]|CNEIA2366-12|Anthomyiidae|673|BOLD:AAP3547
Delia linearis|[12997]|CNEIB1663-12|Anthomyiidae|658|BOLD:AAL7516
Delia|[12998]|CNPPB2010-12|Anthomyiidae|658|BOLD:AAP2973

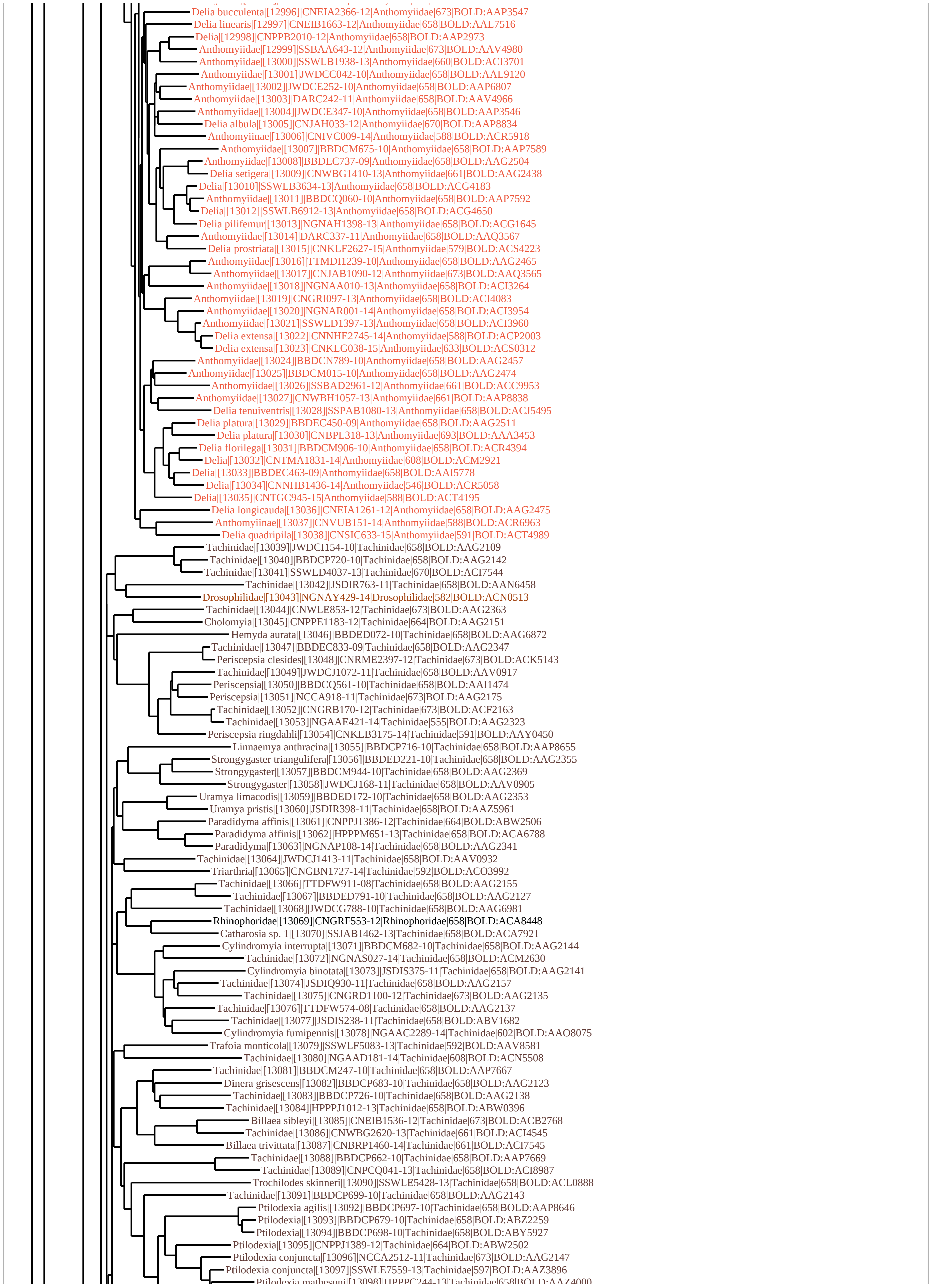

Delia bucculenta|[12996]|CNEIA2366-12|Anthomyiidae|673|BOLD:AAP3547
Delia linearis|[12997]|CNEIB1663-12|Anthomyiidae|658|BOLD:AAL7516
Delia|[12998]|CNPPB2010-12|Anthomyiidae|658|BOLD:AAP2973
Anthomyiidae|[12999]|SSBAA643-12|Anthomyiidae|673|BOLD:AAV4980
Anthomyiidae|[13000]|SSWLB1938-13|Anthomyiidae|660|BOLD:ACI3701
Anthomyiidae|[13001]|JWDCC042-10|Anthomyiidae|658|BOLD:AAL9120
Anthomyiidae|[13002]|JWDCE252-10|Anthomyiidae|658|BOLD:AAP6807
Anthomyiidae|[13003]|DARC242-11|Anthomyiidae|658|BOLD:AAV4966
Anthomyiidae|[13004]|JWDCE347-10|Anthomyiidae|658|BOLD:AAP3546
Delia albula|[13005]|CNJAH033-12|Anthomyiidae|670|BOLD:AAP8834
Anthomyiinae|[13006]|CNIVC009-14|Anthomyiidae|588|BOLD:ACR5918
Anthomyiidae|[13007]|BBDCM675-10|Anthomyiidae|658|BOLD:AAP7589
Anthomyiidae|[13008]|BBDEC737-09|Anthomyiidae|658|BOLD:AAG2504
Delia setigera|[13009]|CNWBG1410-13|Anthomyiidae|661|BOLD:AAG2438
Delia|[13010]|SSWLB3634-13|Anthomyiidae|658|BOLD:ACG4183
Anthomyiidae|[13011]|BBDCQ060-10|Anthomyiidae|658|BOLD:AAP7592
Delia|[13012]|SSWLB6912-13|Anthomyiidae|658|BOLD:ACG4650
Delia pilifemur|[13013]|NGNAH1398-13|Anthomyiidae|658|BOLD:ACG1645
Anthomyiidae|[13014]|DARC337-11|Anthomyiidae|658|BOLD:AAQ3567
Delia prostriata|[13015]|CNKLF2627-15|Anthomyiidae|579|BOLD:ACS4223
Anthomyiidae|[13016]|TTMDI1239-10|Anthomyiidae|658|BOLD:AAG2465
Anthomyiidae|[13017]|CNJAB1090-12|Anthomyiidae|673|BOLD:AAQ3565
Anthomyiidae|[13018]|NGNAA010-13|Anthomyiidae|658|BOLD:ACI3264
Anthomyiidae|[13019]|CNGRI097-13|Anthomyiidae|658|BOLD:ACI4083
Anthomyiidae|[13020]|NGNAR001-14|Anthomyiidae|658|BOLD:ACI3954
Anthomyiidae|[13021]|SSWLD1397-13|Anthomyiidae|658|BOLD:ACI3960
Delia extensa|[13022]|CNNHE2745-14|Anthomyiidae|588|BOLD:ACP2003
Delia extensa|[13023]|CNKLG038-15|Anthomyiidae|633|BOLD:ACS0312
Anthomyiidae|[13024]|BBDCN789-10|Anthomyiidae|658|BOLD:AAG2457
Anthomyiidae|[13025]|BBDCM015-10|Anthomyiidae|658|BOLD:AAG2474
Anthomyiidae|[13026]|SSBAD2961-12|Anthomyiidae|661|BOLD:ACC9953
Anthomyiidae|[13027]|CNWBH1057-13|Anthomyiidae|661|BOLD:AAP8838
Delia tenuiventris|[13028]|SSPAB1080-13|Anthomyiidae|658|BOLD:ACJ5495
Delia platura|[13029]|BBDEC450-09|Anthomyiidae|658|BOLD:AAG2511
Delia platura|[13030]|CNBPL318-13|Anthomyiidae|693|BOLD:AAA3453
Delia florilega|[13031]|BBDCM906-10|Anthomyiidae|658|BOLD:ACR4394
Delia|[13032]|CNTMA1831-14|Anthomyiidae|608|BOLD:ACM2921
Delia|[13033]|BBDEC463-09|Anthomyiidae|658|BOLD:AAI5778
Delia|[13034]|CNNHB1436-14|Anthomyiidae|546|BOLD:ACR5058
Delia|[13035]|CNTGC945-15|Anthomyiidae|588|BOLD:ACT4195
Delia longicauda|[13036]|CNEIA1261-12|Anthomyiidae|658|BOLD:AAG2475
Anthomyiinae|[13037]|CNVUB151-14|Anthomyiidae|588|BOLD:ACR6963
Delia quadripila|[13038]|CNSIC633-15|Anthomyiidae|591|BOLD:ACT4989
Tachinidae|[13039]|JWDCI154-10|Tachinidae|658|BOLD:AAG2109
Tachinidae|[13040]|BBDCP720-10|Tachinidae|658|BOLD:AAG2142
Tachinidae|[13041]|SSWLD4037-13|Tachinidae|670|BOLD:ACI7544
Tachinidae|[13042]|JSDIR763-11|Tachinidae|658|BOLD:AAN6458
Drosophilidae|[13043]|NGNAY429-14|Drosophilidae|582|BOLD:ACN0513
Tachinidae|[13044]|CNWLE853-12|Tachinidae|673|BOLD:AAG2363
Cholomyia|[13045]|CNPPE1183-12|Tachinidae|664|BOLD:AAG2151
Hemyda aurata|[13046]|BBDED072-10|Tachinidae|658|BOLD:AAG6872
Tachinidae|[13047]|BBDEC833-09|Tachinidae|658|BOLD:AAG2347
Periscepsia clesides|[13048]|CNRME2397-12|Tachinidae|673|BOLD:ACK5143
Tachinidae|[13049]|JWDCJ1072-11|Tachinidae|658|BOLD:AAV0917
Periscepsia|[13050]|BBDCQ561-10|Tachinidae|658|BOLD:AAI1474
Periscepsia|[13051]|NCCA918-11|Tachinidae|673|BOLD:AAG2175
Tachinidae|[13052]|CNGRB170-12|Tachinidae|673|BOLD:ACF2163
Tachinidae|[13053]|NGAAE421-14|Tachinidae|555|BOLD:AAG2323
Periscepsia ringdahli|[13054]|CNKLB3175-14|Tachinidae|591|BOLD:AAY0450
Linnaemya anthracina|[13055]|BBDCP716-10|Tachinidae|658|BOLD:AAP8655
Strongygaster triangulifera|[13056]|BBDED221-10|Tachinidae|658|BOLD:AAG2355
Strongygaster|[13057]|BBDCM944-10|Tachinidae|658|BOLD:AAG2369
Strongygaster|[13058]|JWDCJ168-11|Tachinidae|658|BOLD:AAV0905
Uramya limacodis|[13059]|BBDED172-10|Tachinidae|658|BOLD:AAG2353
Uramya pristis|[13060]|JSDIR398-11|Tachinidae|658|BOLD:AAZ5961
Paradidyma affinis|[13061]|CNPPJ1386-12|Tachinidae|664|BOLD:ABW2506
Paradidyma affinis|[13062]|HPPPM651-13|Tachinidae|658|BOLD:ACA6788
Paradidyma|[13063]|NGNAP108-14|Tachinidae|658|BOLD:AAG2341
Tachinidae|[13064]|JWDCJ1413-11|Tachinidae|658|BOLD:AAV0932
Triarthria|[13065]|CNGBN1727-14|Tachinidae|592|BOLD:ACO3992
Tachinidae|[13066]|TTDFW911-08|Tachinidae|658|BOLD:AAG2155
Tachinidae|[13067]|BBDED791-10|Tachinidae|658|BOLD:AAG2127
Tachinidae|[13068]|JWDCG788-10|Tachinidae|658|BOLD:AAG6981
Rhinophoridae|[13069]|CNGRF553-12|Rhinophoridae|658|BOLD:ACA8448
Catharosia sp. 1|[13070]|SSJAB1462-13|Tachinidae|658|BOLD:ACA7921
Cylindromyia interrupta|[13071]|BBDCM682-10|Tachinidae|658|BOLD:AAG2144
Tachinidae|[13072]|NGNAS027-14|Tachinidae|658|BOLD:ACM2630
Cylindromyia binotata|[13073]|JSDIS375-11|Tachinidae|658|BOLD:AAG2141
Tachinidae|[13074]|JSDIQ930-11|Tachinidae|658|BOLD:AAG2157
Tachinidae|[13075]|CNGRD1100-12|Tachinidae|673|BOLD:AAG2135
Tachinidae|[13076]|TTDFW574-08|Tachinidae|658|BOLD:AAG2137
Tachinidae|[13077]|JSDIS238-11|Tachinidae|658|BOLD:ABV1682
Cylindromyia fumipennis|[13078]|NGAAC2289-14|Tachinidae|602|BOLD:AAO8075
Trafoia monticola|[13079]|SSWLF5083-13|Tachinidae|592|BOLD:AAV8581
Tachinidae|[13080]|NGAAD181-14|Tachinidae|608|BOLD:ACN5508
Tachinidae|[13081]|BBDCM247-10|Tachinidae|658|BOLD:AAP7667
Dinera grisescens|[13082]|BBDCP683-10|Tachinidae|658|BOLD:AAG2123
Tachinidae|[13083]|BBDCP726-10|Tachinidae|658|BOLD:AAG2138
Tachinidae|[13084]|HPPPJ1012-13|Tachinidae|658|BOLD:ABW0396
Billaea sibleyi|[13085]|CNEIB1536-12|Tachinidae|673|BOLD:ACB2768
Tachinidae|[13086]|CNWBG2620-13|Tachinidae|661|BOLD:ACI4545
Billaea trivittata|[13087]|CNBRP1460-14|Tachinidae|661|BOLD:ACI7545
Tachinidae|[13088]|BBDCP662-10|Tachinidae|658|BOLD:AAP7669
Tachinidae|[13089]|CNPCQ041-13|Tachinidae|658|BOLD:ACI8987
Trochilodes skinneri|[13090]|SSWLE5428-13|Tachinidae|658|BOLD:ACL0888
Tachinidae|[13091]|BBDCP699-10|Tachinidae|658|BOLD:AAG2143
Ptilodexia agilis|[13092]|BBDCP697-10|Tachinidae|658|BOLD:AAP8646
Ptilodexia|[13093]|BBDCP679-10|Tachinidae|658|BOLD:ABZ2259
Ptilodexia|[13094]|BBDCP698-10|Tachinidae|658|BOLD:ABY5927
Ptilodexia|[13095]|CNPPJ1389-12|Tachinidae|664|BOLD:ABW2502
Ptilodexia conjuncta|[13096]|NCCA2512-11|Tachinidae|673|BOLD:AAG2147
Ptilodexia conjuncta|[13097]|SSWLE7559-13|Tachinidae|597|BOLD:AAZ3896
Ptilodexia mathesoni|[13098]|HPPPC244-13|Tachinidae|658|BOLD:AAZ4000

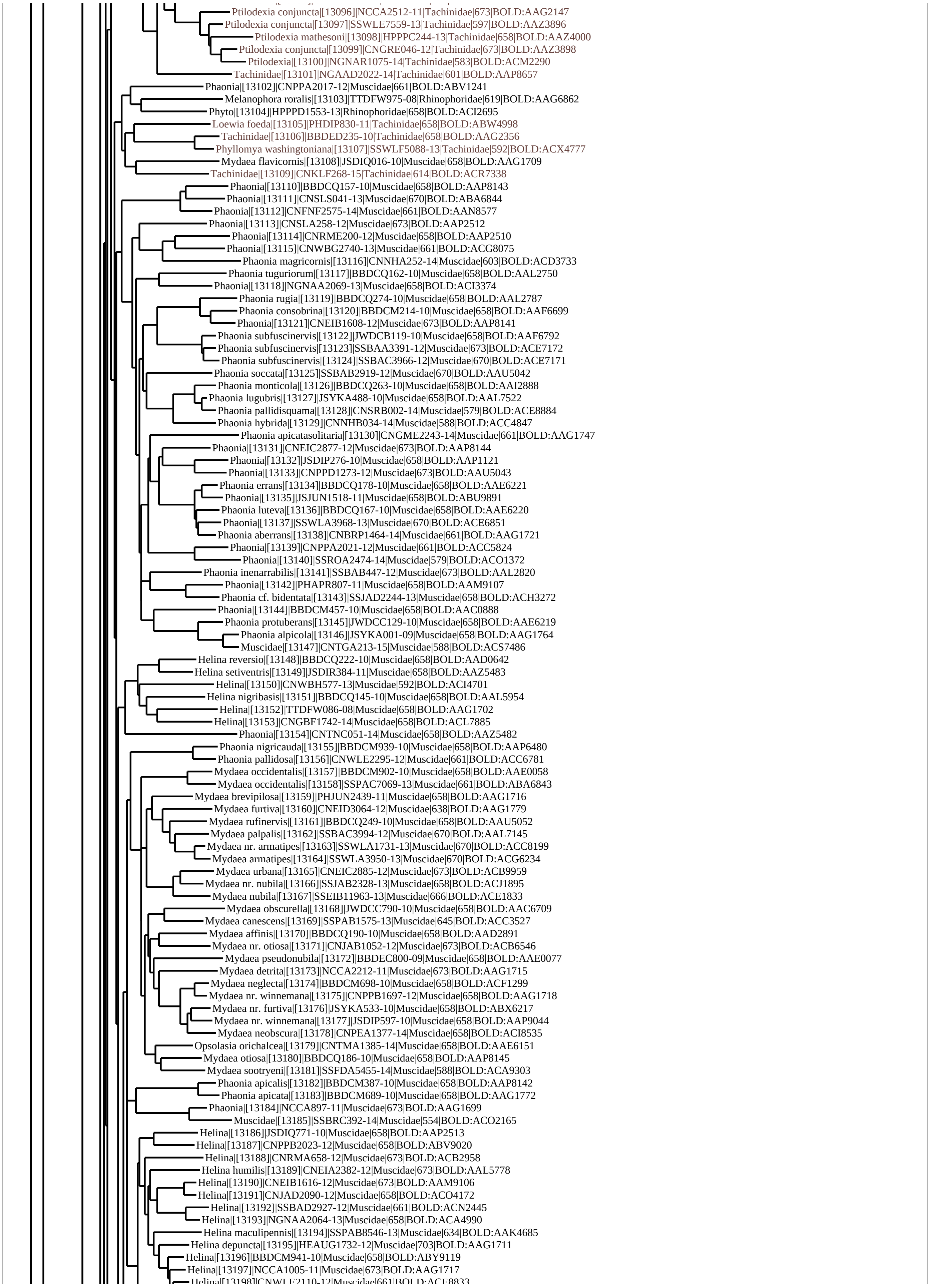
Ptilodexia conjuncta|[13096]|NCCA2512-11|Tachinidae|673|BOLD:AAG2147
Ptilodexia conjuncta|[13097]|SSWLE7559-13|Tachinidae|597|BOLD:AAZ3896
Ptilodexia mathesoni|[13098]|HPPPC244-13|Tachinidae|658|BOLD:AAZ4000
Ptilodexia conjuncta|[13099]|CNGRE046-12|Tachinidae|673|BOLD:AAZ3898
Ptilodexia|[13100]|NGNAR1075-14|Tachinidae|583|BOLD:ACM2290
Tachinidae|[13101]|NGAAD2022-14|Tachinidae|601|BOLD:AAP8657
Phaonia|[13102]|CNPPA2017-12|Muscidae|661|BOLD:ABV1241
Melanophora roralis|[13103]|TTDFW975-08|Rhinophoridae|619|BOLD:AAG6862
Phyto|[13104]|HPPPD1553-13|Rhinophoridae|658|BOLD:ACI2695
Loewia foeda|[13105]|PHDIP830-11|Tachinidae|658|BOLD:ABW4998
Tachinidae|[13106]|BBDED235-10|Tachinidae|658|BOLD:AAG2356
Phyllomya washingtoniana|[13107]|SSWLF5088-13|Tachinidae|592|BOLD:ACX4777
Mydaea flavicornis|[13108]|JSDIQ016-10|Muscidae|658|BOLD:AAG1709
Tachinidae|[13109]|CNKLF268-15|Tachinidae|614|BOLD:ACR7338
Phaonia|[13110]|BBDCQ157-10|Muscidae|658|BOLD:AAP8143
Phaonia|[13111]|CNSLS041-13|Muscidae|670|BOLD:ABA6844
Phaonia|[13112]|CNFNF2575-14|Muscidae|661|BOLD:AAN8577
Phaonia|[13113]|CNSLA258-12|Muscidae|673|BOLD:AAP2512
Phaonia|[13114]|CNRME200-12|Muscidae|658|BOLD:AAP2510
Phaonia|[13115]|CNWBG2740-13|Muscidae|661|BOLD:ACG8075
Phaonia magricornis|[13116]|CNNHA252-14|Muscidae|603|BOLD:ACD3733
Phaonia tuguriorum|[13117]|BBDCQ162-10|Muscidae|658|BOLD:AAL2750
Phaonia|[13118]|NGNAA2069-13|Muscidae|658|BOLD:ACI3374
Phaonia rugia|[13119]|BBDCQ274-10|Muscidae|658|BOLD:AAL2787
Phaonia consobrina|[13120]|BBDCM214-10|Muscidae|658|BOLD:AAF6699
Phaonia|[13121]|CNEIB1608-12|Muscidae|673|BOLD:AAP8141
Phaonia subfuscinervis|[13122]|JWDCB119-10|Muscidae|658|BOLD:AAF6792
Phaonia subfuscinervis|[13123]|SSBAA3391-12|Muscidae|673|BOLD:ACE7172
Phaonia subfuscinervis|[13124]|SSBAC3966-12|Muscidae|670|BOLD:ACE7171
Phaonia soccata|[13125]|SSBAB2919-12|Muscidae|670|BOLD:AAU5042
Phaonia monticola|[13126]|BBDCQ263-10|Muscidae|658|BOLD:AAI2888
Phaonia lugubris|[13127]|JSYKA488-10|Muscidae|658|BOLD:AAL7522
Phaonia pallidisquama|[13128]|CNSRB002-14|Muscidae|579|BOLD:ACE8884
Phaonia hybrida|[13129]|CNNHB034-14|Muscidae|588|BOLD:ACC4847
Phaonia apicatasolitaria|[13130]|CNGME2243-14|Muscidae|661|BOLD:AAG1747
Phaonia|[13131]|CNEIC2877-12|Muscidae|673|BOLD:AAP8144
Phaonia|[13132]|JSDIP276-10|Muscidae|658|BOLD:AAP1121
Phaonia|[13133]|CNPPD1273-12|Muscidae|673|BOLD:AAU5043
Phaonia errans|[13134]|BBDCQ178-10|Muscidae|658|BOLD:AAE6221
Phaonia|[13135]|JSJUN1518-11|Muscidae|658|BOLD:ABU9891
Phaonia luteva|[13136]|BBDCQ167-10|Muscidae|658|BOLD:AAE6220
Phaonia|[13137]|SSWLA3968-13|Muscidae|670|BOLD:ACE6851
Phaonia aberrans|[13138]|CNBRP1464-14|Muscidae|661|BOLD:AAG1721
Phaonia|[13139]|CNPPA2021-12|Muscidae|661|BOLD:ACC5824
Phaonia|[13140]|SSROA2474-14|Muscidae|579|BOLD:ACO1372
Phaonia inenarrabilis|[13141]|SSBAB447-12|Muscidae|673|BOLD:AAL2820
Phaonia|[13142]|PHAPR807-11|Muscidae|658|BOLD:AAM9107
Phaonia cf. bidentata|[13143]|SSJAD2244-13|Muscidae|658|BOLD:ACH3272
Phaonia|[13144]|BBDCM457-10|Muscidae|658|BOLD:AAC0888
Phaonia protuberans|[13145]|JWDCC129-10|Muscidae|658|BOLD:AAE6219
Phaonia alpicola|[13146]|JSYKA001-09|Muscidae|658|BOLD:AAG1764
Muscidae|[13147]|CNTGA213-15|Muscidae|588|BOLD:ACS7486
Helina reversio|[13148]|BBDCQ222-10|Muscidae|658|BOLD:AAD0642
Helina setiventris|[13149]|JSDIR384-11|Muscidae|658|BOLD:AAZ5483
Helina|[13150]|CNWBH577-13|Muscidae|592|BOLD:ACI4701
Helina nigribasis|[13151]|BBDCQ145-10|Muscidae|658|BOLD:AAL5954
Helina|[13152]|TTDFW086-08|Muscidae|658|BOLD:AAG1702
Helina|[13153]|CNGBF1742-14|Muscidae|658|BOLD:ACL7885
Phaonia|[13154]|CNTNC051-14|Muscidae|658|BOLD:AAZ5482
Phaonia nigricauda|[13155]|BBDCM939-10|Muscidae|658|BOLD:AAP6480
Phaonia pallidosa|[13156]|CNWLE2295-12|Muscidae|661|BOLD:ACC6781
Mydaea occidentalis|[13157]|BBDCM902-10|Muscidae|658|BOLD:AAE0058
Mydaea occidentalis|[13158]|SSPAC7069-13|Muscidae|661|BOLD:ABA6843
Mydaea brevipilosa|[13159]|PHJUN2439-11|Muscidae|658|BOLD:AAG1716
Mydaea furtiva|[13160]|CNEID3064-12|Muscidae|638|BOLD:AAG1779
Mydaea rufinervis|[13161]|BBDCQ249-10|Muscidae|658|BOLD:AAU5052
Mydaea palpalis|[13162]|SSBAC3994-12|Muscidae|670|BOLD:AAL7145
Mydaea nr. armatipes|[13163]|SSWLA1731-13|Muscidae|670|BOLD:ACC8199
Mydaea armatipes|[13164]|SSWLA3950-13|Muscidae|670|BOLD:ACG6234
Mydaea urbana|[13165]|CNEIC2885-12|Muscidae|673|BOLD:ACB9959
Mydaea nr. nubila|[13166]|SSJAB2328-13|Muscidae|658|BOLD:ACJ1895
Mydaea nubila|[13167]|SSEIB11963-13|Muscidae|666|BOLD:ACE1833
Mydaea obscurella|[13168]|JWDCC790-10|Muscidae|658|BOLD:AAC6709
Mydaea canescens|[13169]|SSPAB1575-13|Muscidae|645|BOLD:ACC3527
Mydaea affinis|[13170]|BBDCQ190-10|Muscidae|658|BOLD:AAD2891
Mydaea nr. otiosa|[13171]|CNJAB1052-12|Muscidae|673|BOLD:ACB6546
Mydaea pseudonubila|[13172]|BBDEC800-09|Muscidae|658|BOLD:AAE0077
Mydaea detrita|[13173]|NCCA2212-11|Muscidae|673|BOLD:AAG1715
Mydaea neglecta|[13174]|BBDCM698-10|Muscidae|658|BOLD:ACF1299
Mydaea nr. winnemana|[13175]|CNPPB1697-12|Muscidae|658|BOLD:AAG1718
Mydaea nr. furtiva|[13176]|JSYKA533-10|Muscidae|658|BOLD:ABX6217
Mydaea nr. winnemana|[13177]|JSDIP597-10|Muscidae|658|BOLD:AAP9044
Mydaea neobscura|[13178]|CNPEA1377-14|Muscidae|658|BOLD:ACI8535
Opsolasia orichalcea|[13179]|CNTMA1385-14|Muscidae|658|BOLD:AAE6151
Mydaea otiosa|[13180]|BBDCQ186-10|Muscidae|658|BOLD:AAP8145
Mydaea sootryeni|[13181]|SSFDA5455-14|Muscidae|588|BOLD:ACA9303
Phaonia apicalis|[13182]|BBDCM387-10|Muscidae|658|BOLD:AAP8142
Phaonia apicata|[13183]|BBDCM689-10|Muscidae|658|BOLD:AAG1772
Phaonia|[13184]|NCCA897-11|Muscidae|673|BOLD:AAG1699
Muscidae|[13185]|SSBRC392-14|Muscidae|554|BOLD:ACO2165
Helina|[13186]|JSDIQ771-10|Muscidae|658|BOLD:AAP2513
Helina|[13187]|CNPPB2023-12|Muscidae|658|BOLD:ABV9020
Helina|[13188]|CNRMA658-12|Muscidae|673|BOLD:ACB2958
Helina humilis|[13189]|CNEIA2382-12|Muscidae|673|BOLD:AAL5778
Helina|[13190]|CNEIB1616-12|Muscidae|673|BOLD:AAM9106
Helina|[13191]|CNJAD2090-12|Muscidae|658|BOLD:ACO4172
Helina|[13192]|SSBAD2927-12|Muscidae|661|BOLD:ACN2445
Helina|[13193]|NGNAA2064-13|Muscidae|658|BOLD:ACA4990
Helina maculipennis|[13194]|SSPAB8546-13|Muscidae|634|BOLD:AAK4685
Helina depuncta|[13195]|HEAUG1732-12|Muscidae|703|BOLD:AAG1711
Helina|[13196]|BBDCM941-10|Muscidae|658|BOLD:ABY9119
Helina|[13197]|NCCA1005-11|Muscidae|673|BOLD:AAG1717
Helina|[13198]|CNWLF2110-12|Muscidae|661|BOLD:ACF8833

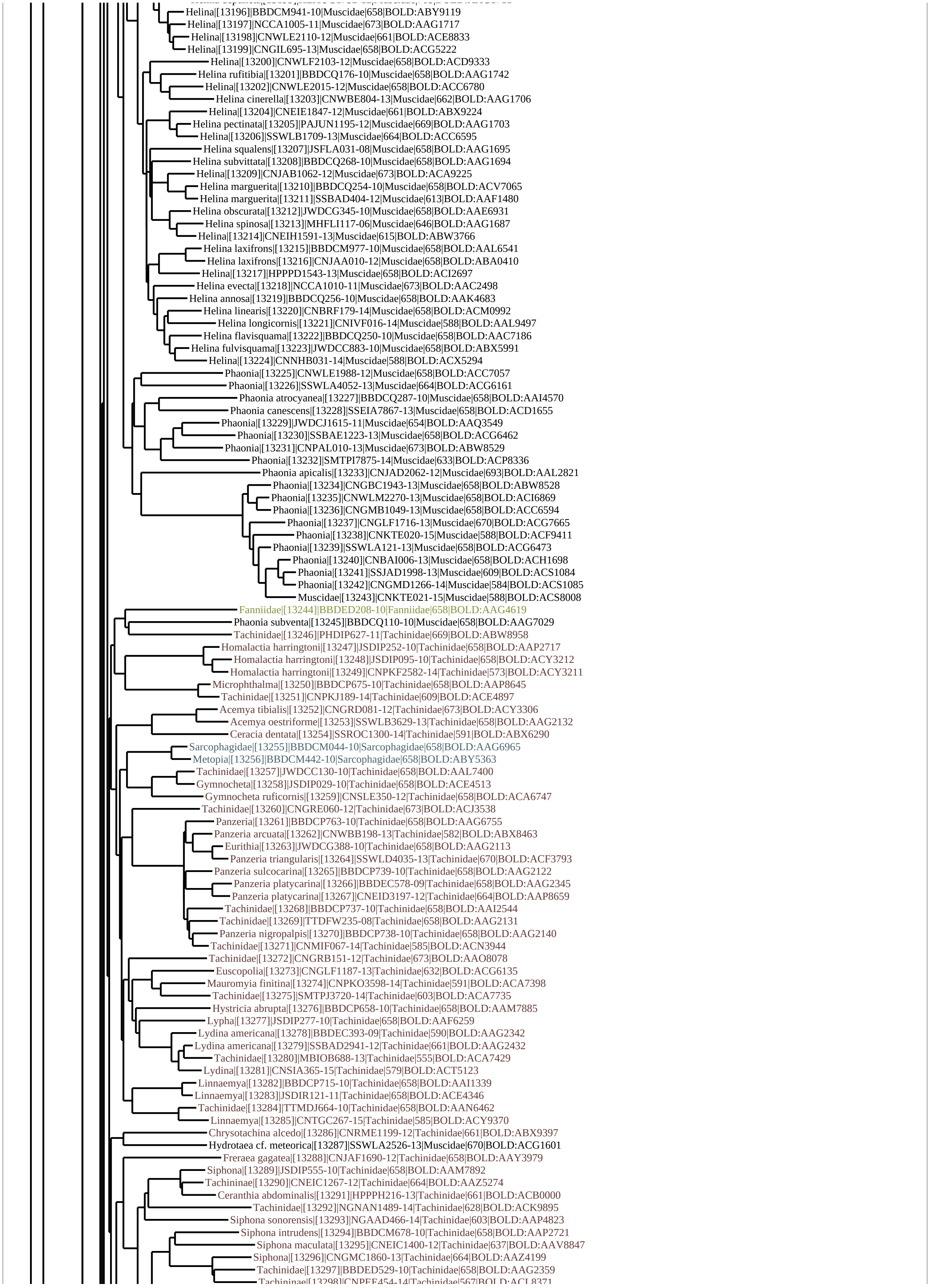
Helina|[13196]|BBDCM941-10|Muscidae|658|BOLD:ABY9119
Helina|[13197]|NCCA1005-11|Muscidae|673|BOLD:AAG1717
Helina|[13198]|CNWLE2110-12|Muscidae|661|BOLD:ACE8833
Helina|[13199]|CNGIL695-13|Muscidae|658|BOLD:ACG5222
Helina|[13200]|CNWLF2103-12|Muscidae|658|BOLD:ACD9333
Helina rufitibia|[13201]|BBDCQ176-10|Muscidae|658|BOLD:AAG1742
Helina|[13202]|CNWLE2015-12|Muscidae|658|BOLD:ACC6780
Helina cinerella|[13203]|CNWBE804-13|Muscidae|662|BOLD:AAG1706
Helina|[13204]|CNEIE1847-12|Muscidae|661|BOLD:ABX9224
Helina pectinata|[13205]|PAJUN1195-12|Muscidae|669|BOLD:AAG1703
Helina|[13206]|SSWLB1709-13|Muscidae|664|BOLD:ACC6595
Helina squalens|[13207]|JSFLA031-08|Muscidae|658|BOLD:AAG1695
Helina subvittata|[13208]|BBDCQ268-10|Muscidae|658|BOLD:AAG1694
Helina|[13209]|CNJAB1062-12|Muscidae|673|BOLD:ACA9225
Helina marguerita|[13210]|BBDCQ254-10|Muscidae|658|BOLD:ACV7065
Helina marguerita|[13211]|SSBAD404-12|Muscidae|613|BOLD:AAF1480
Helina obscurata|[13212]|JWDCG345-10|Muscidae|658|BOLD:AAE6931
Helina spinosa|[13213]|MHFLI117-06|Muscidae|646|BOLD:AAG1687
Helina|[13214]|CNEIH1591-13|Muscidae|615|BOLD:ABW3766
Helina laxifrons|[13215]|BBDCM977-10|Muscidae|658|BOLD:AAL6541
Helina laxifrons|[13216]|CNJAA010-12|Muscidae|658|BOLD:ABA0410
Helina|[13217]|HPPPD1543-13|Muscidae|658|BOLD:ACI2697
Helina evecta|[13218]|NCCA1010-11|Muscidae|673|BOLD:AAC2498
Helina annosa|[13219]|BBDCQ256-10|Muscidae|658|BOLD:AAK4683
Helina linearis|[13220]|CNBRF179-14|Muscidae|658|BOLD:ACM0992
Helina longicornis|[13221]|CNIVF016-14|Muscidae|588|BOLD:AAL9497
Helina flavisquama|[13222]|BBDCQ250-10|Muscidae|658|BOLD:AAC7186
Helina fulvisquama|[13223]|JWDCC883-10|Muscidae|658|BOLD:ABX5991
Helina|[13224]|CNNHB031-14|Muscidae|588|BOLD:ACX5294
Phaonia|[13225]|CNWLE1988-12|Muscidae|658|BOLD:ACC7057
Phaonia|[13226]|SSWLA4052-13|Muscidae|664|BOLD:ACG6161
Phaonia atrocyanea|[13227]|BBDCQ287-10|Muscidae|658|BOLD:AAI4570
Phaonia canescens|[13228]|SSEIA7867-13|Muscidae|658|BOLD:ACD1655
Phaonia|[13229]|JWDCJ1615-11|Muscidae|654|BOLD:AAQ3549
Phaonia|[13230]|SSBAE1223-13|Muscidae|658|BOLD:ACG6462
Phaonia|[13231]|CNPAL010-13|Muscidae|673|BOLD:ABW8529
Phaonia|[13232]|SMTPI7875-14|Muscidae|633|BOLD:ACP8336
Phaonia apicalis|[13233]|CNJAD2062-12|Muscidae|693|BOLD:AAL2821
Phaonia|[13234]|CNGBC1943-13|Muscidae|658|BOLD:ABW8528
Phaonia|[13235]|CNWLM2270-13|Muscidae|658|BOLD:ACI6869
Phaonia|[13236]|CNGMB1049-13|Muscidae|658|BOLD:ACC6594
Phaonia|[13237]|CNGLF1716-13|Muscidae|670|BOLD:ACG7665
Phaonia|[13238]|CNKTE020-15|Muscidae|588|BOLD:ACF9411
Phaonia|[13239]|SSWLA121-13|Muscidae|658|BOLD:ACG6473
Phaonia|[13240]|CNBAI006-13|Muscidae|658|BOLD:ACH1698
Phaonia|[13241]|SSJAD1998-13|Muscidae|609|BOLD:ACS1084
Phaonia|[13242]|CNGMD1266-14|Muscidae|584|BOLD:ACS1085
Muscidae|[13243]|CNKTE021-15|Muscidae|588|BOLD:ACS8008
Fanniidae|[13244]|BBDED208-10|Fanniidae|658|BOLD:AAG4619
Phaonia subventa|[13245]|BBDCQ110-10|Muscidae|658|BOLD:AAG7029
Tachinidae|[13246]|PHDIP627-11|Tachinidae|669|BOLD:ABW8958
Homalactia harringtoni|[13247]|JSDIP252-10|Tachinidae|658|BOLD:AAP2717
Homalactia harringtoni|[13248]|JSDIP095-10|Tachinidae|658|BOLD:ACY3212
Homalactia harringtoni|[13249]|CNPKF2582-14|Tachinidae|573|BOLD:ACY3211
Microphthalma|[13250]|BBDCP675-10|Tachinidae|658|BOLD:AAP8645
Tachinidae|[13251]|CNPKJ189-14|Tachinidae|609|BOLD:ACE4897
Acemya tibialis|[13252]|CNGRD081-12|Tachinidae|673|BOLD:ACY3306
Acemya oestriforme|[13253]|SSWLB3629-13|Tachinidae|658|BOLD:AAG2132
Ceracia dentata|[13254]|SSROC1300-14|Tachinidae|591|BOLD:ABX6290
Sarcophagidae|[13255]|BBDCM044-10|Sarcophagidae|658|BOLD:AAG6965
Metopia|[13256]|BBDCM442-10|Sarcophagidae|658|BOLD:ABY5363
Tachinidae|[13257]|JWDCC130-10|Tachinidae|658|BOLD:AAL7400
Gymnocheta|[13258]|JSDIP029-10|Tachinidae|658|BOLD:ACE4513
Gymnocheta ruficornis|[13259]|CNSLE350-12|Tachinidae|658|BOLD:ACA6747
Tachinidae|[13260]|CNGRE060-12|Tachinidae|673|BOLD:ACJ3538
Panzeria|[13261]|BBDCP763-10|Tachinidae|658|BOLD:AAG6755
Panzeria arcuata|[13262]|CNWBB198-13|Tachinidae|582|BOLD:ABX8463
Eurithia|[13263]|JWDCG388-10|Tachinidae|658|BOLD:AAG2113
Panzeria triangularis|[13264]|SSWLD4035-13|Tachinidae|670|BOLD:ACF3793
Panzeria sulcocarina|[13265]|BBDCP739-10|Tachinidae|658|BOLD:AAG2122
Panzeria platycarina|[13266]|BBDEC578-09|Tachinidae|658|BOLD:AAG2345
Panzeria platycarina|[13267]|CNEID3197-12|Tachinidae|664|BOLD:AAP8659
Tachinidae|[13268]|BBDCP737-10|Tachinidae|658|BOLD:AAI2544
Tachinidae|[13269]|TTDFW235-08|Tachinidae|658|BOLD:AAG2131
Panzeria nigropalpis|[13270]|BBDCP738-10|Tachinidae|658|BOLD:AAG2140
Tachinidae|[13271]|CNMIF067-14|Tachinidae|585|BOLD:ACN3944
Tachinidae|[13272]|CNGRB151-12|Tachinidae|673|BOLD:AAO8078
Euscopolia|[13273]|CNGLF1187-13|Tachinidae|632|BOLD:ACG6135
Mauromyia finitina|[13274]|CNPKO3598-14|Tachinidae|591|BOLD:ACA7398
Tachinidae|[13275]|SMTPJ3720-14|Tachinidae|603|BOLD:ACA7735
Hystricia abrupta|[13276]|BBDCP658-10|Tachinidae|658|BOLD:AAM7885
Lypha|[13277]|JSDIP277-10|Tachinidae|658|BOLD:AAF6259
Lydina americana|[13278]|BBDEC393-09|Tachinidae|590|BOLD:AAG2342
Lydina americana|[13279]|SSBAD2941-12|Tachinidae|661|BOLD:AAG2432
Tachinidae|[13280]|MBIOB688-13|Tachinidae|555|BOLD:ACA7429
Lydina|[13281]|CNSIA365-15|Tachinidae|579|BOLD:ACT5123
Linnaemya|[13282]|BBDCP715-10|Tachinidae|658|BOLD:AAI1339
Linnaemya|[13283]|JSDIR121-11|Tachinidae|658|BOLD:ACE4346
Tachinidae|[13284]|TTMDJ664-10|Tachinidae|658|BOLD:AAN6462
Linnaemya|[13285]|CNTGC267-15|Tachinidae|585|BOLD:ACY9370
Chrysotachina alcedo|[13286]|CNRME1199-12|Tachinidae|661|BOLD:ABX9397
Hydrotaea cf. meteorica|[13287]|SSWLA2526-13|Muscidae|670|BOLD:ACG1601
Freraea gagatea|[13288]|CNJAF1690-12|Tachinidae|658|BOLD:AAY3979
Siphona|[13289]|JSDIP555-10|Tachinidae|658|BOLD:AAM7892
Tachininae|[13290]|CNEIC1267-12|Tachinidae|664|BOLD:AAZ5274
Ceranthia abdominalis|[13291]|HPPPH216-13|Tachinidae|661|BOLD:ACB0000
Tachinidae|[13292]|NGNAN1489-14|Tachinidae|628|BOLD:ACK9895
Siphona sonorensis|[13293]|NGAAD466-14|Tachinidae|603|BOLD:AAP4823
Siphona intrudens|[13294]|BBDCM678-10|Tachinidae|658|BOLD:AAP2721
Siphona maculata|[13295]|CNEIC1400-12|Tachinidae|637|BOLD:AAV8847
Siphona|[13296]|CNGMC1860-13|Tachinidae|664|BOLD:AAZ4199
Tachinidae|[13297]|BBDED529-10|Tachinidae|658|BOLD:AAG2359
Tachininae|[13298]|CNPEF454-14|Tachinidae|567|BOLD:ACL8371

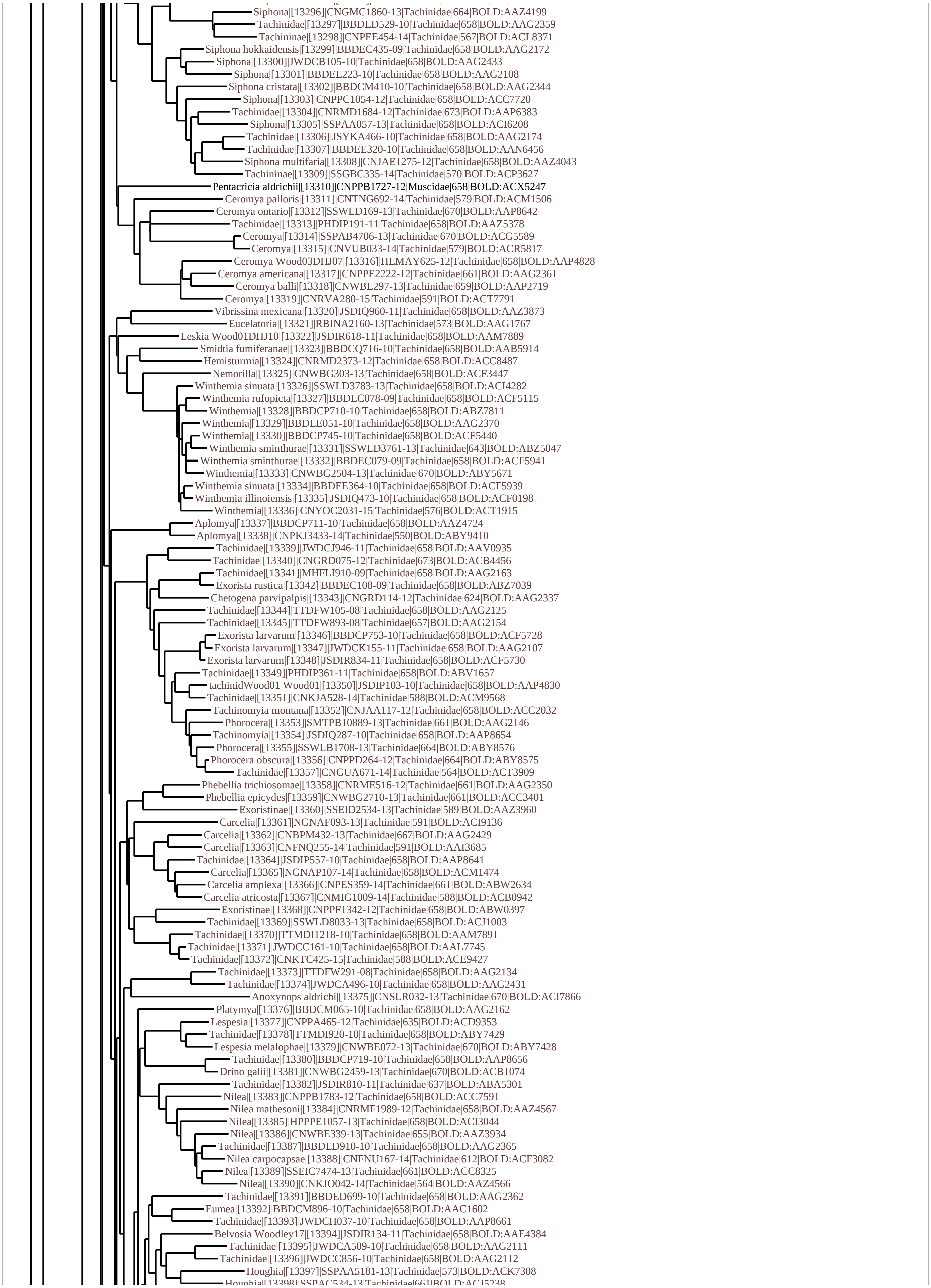

Siphona|[13296]|CNGMC1860-13|Tachinidae|664|BOLD:AAZ4199
Tachinidae|[13297]|BBDED529-10|Tachinidae|658|BOLD:AAG2359
Tachininae|[13298]|CNPEE454-14|Tachinidae|567|BOLD:ACL8371
Siphona hokkaidensis|[13299]|BBDEC435-09|Tachinidae|658|BOLD:AAG2172
Siphona|[13300]|JWDCB105-10|Tachinidae|658|BOLD:AAG2433
Siphona|[13301]|BBDEE223-10|Tachinidae|658|BOLD:AAG2108
Siphona cristata|[13302]|BBDCM410-10|Tachinidae|658|BOLD:AAG2344
Siphona|[13303]|CNPPC1054-12|Tachinidae|658|BOLD:ACC7720
Tachinidae|[13304]|CNRMD1684-12|Tachinidae|673|BOLD:AAP6383
Siphona|[13305]|SSPAA057-13|Tachinidae|658|BOLD:ACI6208
Tachinidae|[13306]|JSYKA466-10|Tachinidae|658|BOLD:AAG2174
Tachinidae|[13307]|BBDEE320-10|Tachinidae|658|BOLD:AAN6456
Siphona multifaria|[13308]|CNJAE1275-12|Tachinidae|658|BOLD:AAZ4043
Tachininae|[13309]|SSGBC335-14|Tachinidae|570|BOLD:ACP3627
Pentacricia aldrichii|[13310]|CNPPB1727-12|Muscidae|658|BOLD:ACX5247
Ceromya palloris|[13311]|CNTNG692-14|Tachinidae|579|BOLD:ACM1506
Ceromya ontario|[13312]|SSWLD169-13|Tachinidae|670|BOLD:AAP8642
Tachinidae|[13313]|PHDIP191-11|Tachinidae|658|BOLD:AAZ5378
Ceromya|[13314]|SSPAB4706-13|Tachinidae|670|BOLD:ACG5589
Ceromya|[13315]|CNVUB033-14|Tachinidae|579|BOLD:ACR5817
Ceromya Wood03DHJ07|[13316]|HEMAY625-12|Tachinidae|658|BOLD:AAP4828
Ceromya americana|[13317]|CNPPE2222-12|Tachinidae|661|BOLD:AAG2361
Ceromya balli|[13318]|CNWBE297-13|Tachinidae|659|BOLD:AAP2719
Ceromya|[13319]|CNRVA280-15|Tachinidae|591|BOLD:ACT7791
Vibrissina mexicana|[13320]|JSDIQ960-11|Tachinidae|658|BOLD:AAZ3873
Eucelatoria|[13321]|RBINA2160-13|Tachinidae|573|BOLD:AAG1767
Leskia Wood01DHJ10|[13322]|JSDIR618-11|Tachinidae|658|BOLD:AAM7889
Smidtia fumiferanae|[13323]|BBDCQ716-10|Tachinidae|658|BOLD:AAB5914
Hemisturmia|[13324]|CNRMD2373-12|Tachinidae|658|BOLD:ACC8487
Nemorilla|[13325]|CNWBG303-13|Tachinidae|658|BOLD:ACF3447
Winthemia sinuata|[13326]|SSWLD3783-13|Tachinidae|658|BOLD:ACI4282
Winthemia rufopicta|[13327]|BBDEC078-09|Tachinidae|658|BOLD:ACF5115
Winthemia|[13328]|BBDCP710-10|Tachinidae|658|BOLD:ABZ7811
Winthemia|[13329]|BBDEE051-10|Tachinidae|658|BOLD:AAG2370
Winthemia|[13330]|BBDCP745-10|Tachinidae|658|BOLD:ACF5440
Winthemia sminthurae|[13331]|SSWLD3761-13|Tachinidae|643|BOLD:ABZ5047
Winthemia sminthurae|[13332]|BBDEC079-09|Tachinidae|658|BOLD:ACF5941
Winthemia|[13333]|CNWBG2504-13|Tachinidae|670|BOLD:ABY5671
Winthemia sinuata|[13334]|BBDEE364-10|Tachinidae|658|BOLD:ACF5939
Winthemia illinoiensis|[13335]|JSDIQ473-10|Tachinidae|658|BOLD:ACF0198
Winthemia|[13336]|CNYOC2031-15|Tachinidae|576|BOLD:ACT1915
Aplomya|[13337]|BBDCP711-10|Tachinidae|658|BOLD:AAZ4724
Aplomya|[13338]|CNPKJ3433-14|Tachinidae|550|BOLD:ABY9410
Tachinidae|[13339]|JWDCJ946-11|Tachinidae|658|BOLD:AAV0935
Tachinidae|[13340]|CNGRD075-12|Tachinidae|673|BOLD:ACB4456
Tachinidae|[13341]|MHFLI910-09|Tachinidae|658|BOLD:AAG2163
Exorista rustica|[13342]|BBDEC108-09|Tachinidae|658|BOLD:ABZ7039
Chetogena parvipalpis|[13343]|CNGRD114-12|Tachinidae|624|BOLD:AAG2337
Tachinidae|[13344]|TTDFW105-08|Tachinidae|658|BOLD:AAG2125
Tachinidae|[13345]|TTDFW893-08|Tachinidae|657|BOLD:AAG2154
Exorista larvarum|[13346]|BBDCP753-10|Tachinidae|658|BOLD:ACF5728
Exorista larvarum|[13347]|JWDCK155-11|Tachinidae|658|BOLD:AAG2107
Exorista larvarum|[13348]|JSDIR834-11|Tachinidae|658|BOLD:ACF5730
Tachinidae|[13349]|PHDIP361-11|Tachinidae|658|BOLD:ABV1657
tachinidWood01 Wood01|[13350]|JSDIP103-10|Tachinidae|658|BOLD:AAP4830
Tachinidae|[13351]|CNKJA528-14|Tachinidae|588|BOLD:ACM9568
Tachinomyia montana|[13352]|CNJAA117-12|Tachinidae|658|BOLD:ACC2032
Phorocera|[13353]|SMTPB10889-13|Tachinidae|661|BOLD:AAG2146
Tachinomyia|[13354]|JSDIQ287-10|Tachinidae|658|BOLD:AAP8654
Phorocera|[13355]|SSWLB1708-13|Tachinidae|664|BOLD:ABY8576
Phorocera obscura|[13356]|CNPPD264-12|Tachinidae|664|BOLD:ABY8575
Tachinidae|[13357]|CNGUA671-14|Tachinidae|564|BOLD:ACT3909
Phebellia trichiosomae|[13358]|CNRME516-12|Tachinidae|661|BOLD:AAG2350
Phebellia epicydes|[13359]|CNWBG2710-13|Tachinidae|661|BOLD:ACC3401
Exoristinae|[13360]|SSEID2534-13|Tachinidae|589|BOLD:AAZ3960
Carcelia|[13361]|NGNAF093-13|Tachinidae|591|BOLD:ACI9136
Carcelia|[13362]|CNBPM432-13|Tachinidae|667|BOLD:AAG2429
Carcelia|[13363]|CNFNQ255-14|Tachinidae|591|BOLD:AAI3685
Tachinidae|[13364]|JSDIP557-10|Tachinidae|658|BOLD:AAP8641
Carcelia|[13365]|NGNAP107-14|Tachinidae|658|BOLD:ACM1474
Carcelia amplexa|[13366]|CNPES359-14|Tachinidae|661|BOLD:ABW2634
Carcelia atricosta|[13367]|CNMIG1009-14|Tachinidae|588|BOLD:ACB0942
Exoristinae|[13368]|CNPPF1342-12|Tachinidae|658|BOLD:ABW0397
Tachinidae|[13369]|SSWLD8033-13|Tachinidae|658|BOLD:ACJ1003
Tachinidae|[13370]|TTMDI1218-10|Tachinidae|658|BOLD:AAM7891
Tachinidae|[13371]|JWDCC161-10|Tachinidae|658|BOLD:AAL7745
Tachinidae|[13372]|CNKTC425-15|Tachinidae|588|BOLD:ACE9427
Tachinidae|[13373]|TTDFW291-08|Tachinidae|658|BOLD:AAG2134
Tachinidae|[13374]|JWDCA496-10|Tachinidae|658|BOLD:AAG2431
Anoxynops aldrichi|[13375]|CNSLR032-13|Tachinidae|670|BOLD:ACI7866
Platymya|[13376]|BBDCM065-10|Tachinidae|658|BOLD:AAG2162
Lespesia|[13377]|CNPPA465-12|Tachinidae|635|BOLD:ACD9353
Tachinidae|[13378]|TTMDI920-10|Tachinidae|658|BOLD:ABY7429
Lespesia melalophae|[13379]|CNWBE072-13|Tachinidae|670|BOLD:ABY7428
Tachinidae|[13380]|BBDCP719-10|Tachinidae|658|BOLD:AAP8656
Drino galii|[13381]|CNWBG2459-13|Tachinidae|670|BOLD:ACB1074
Tachinidae|[13382]|JSDIR810-11|Tachinidae|637|BOLD:ABA5301
Nilea|[13383]|CNPPB1783-12|Tachinidae|658|BOLD:ACC7591
Nilea mathesoni|[13384]|CNRMF1989-12|Tachinidae|658|BOLD:AAZ4567
Nilea|[13385]|HPPPE1057-13|Tachinidae|658|BOLD:ACI3044
Nilea|[13386]|CNWBE339-13|Tachinidae|655|BOLD:AAZ3934
Tachinidae|[13387]|BBDED910-10|Tachinidae|658|BOLD:AAG2365
Nilea carpocapsae|[13388]|CNFNU167-14|Tachinidae|612|BOLD:ACF3082
Nilea|[13389]|SSEIC7474-13|Tachinidae|661|BOLD:ACC8325
Nilea|[13390]|CNKJO042-14|Tachinidae|564|BOLD:AAZ4566
Tachinidae|[13391]|BBDED699-10|Tachinidae|658|BOLD:AAG2362
Eumea|[13392]|BBDCM896-10|Tachinidae|658|BOLD:AAC1602
Tachinidae|[13393]|JWDCH037-10|Tachinidae|658|BOLD:AAP8661
Belvosia Woodley17|[13394]|JSDIR134-11|Tachinidae|658|BOLD:AAE4384
Tachinidae|[13395]|JWDCA509-10|Tachinidae|658|BOLD:AAG2111
Tachinidae|[13396]|JWDCC856-10|Tachinidae|658|BOLD:AAG2112
Houghia|[13397]|SSPAA5181-13|Tachinidae|573|BOLD:ACK7308
Houghia|[13398]|SSPAC534-13|Tachinidae|661|BOLD:ACJ5238

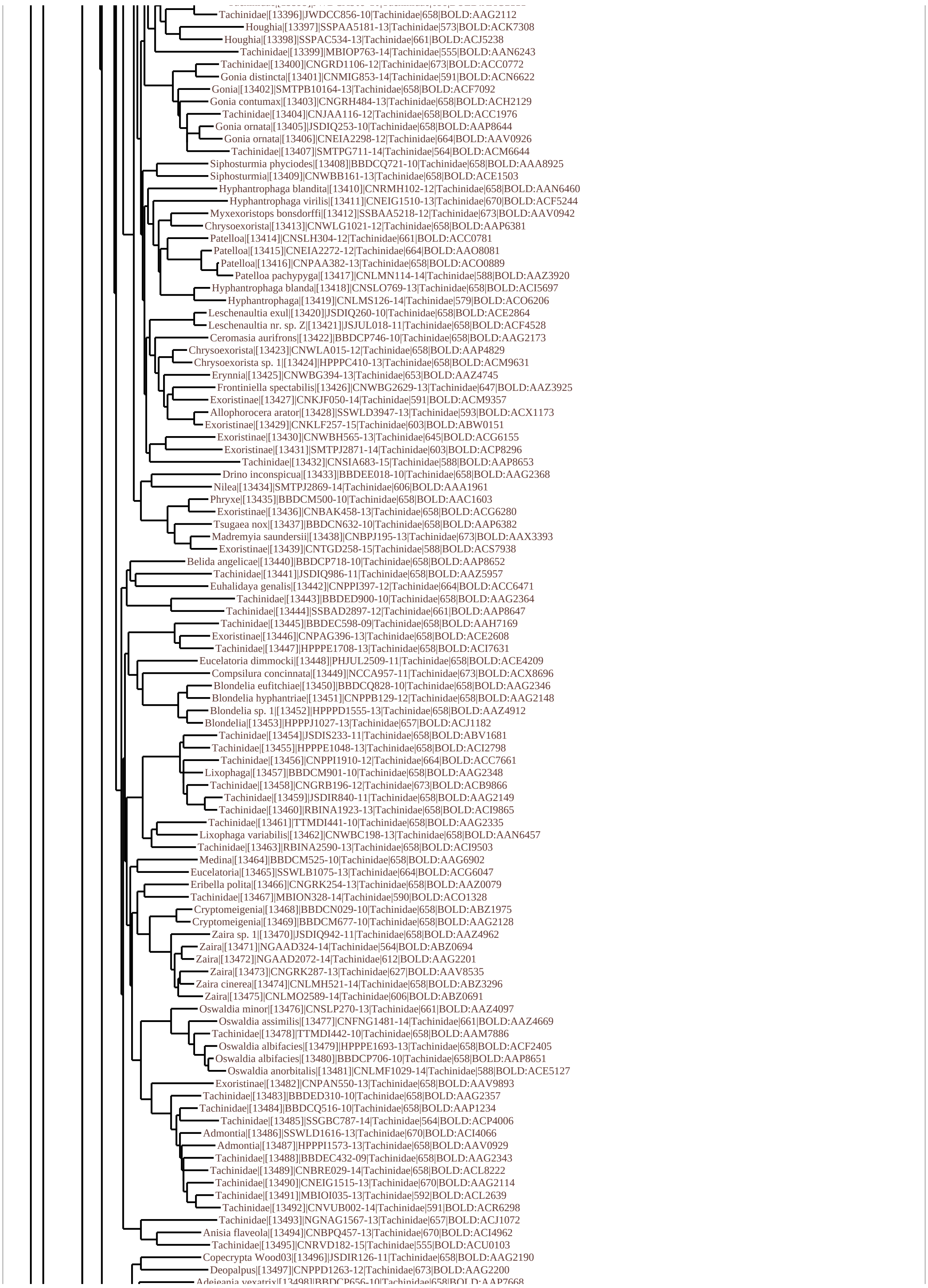

Tachinidae|[13396]|JWDCC856-10|Tachinidae|658|BOLD:AAG2112
Houghia|[13397]|SSPAA5181-13|Tachinidae|573|BOLD:ACK7308
Houghia|[13398]|SSPAC534-13|Tachinidae|661|BOLD:ACJ5238
Tachinidae|[13399]|MBIOP763-14|Tachinidae|555|BOLD:AAN6243
Tachinidae|[13400]|CNGRD1106-12|Tachinidae|673|BOLD:ACC0772
Gonia distincta|[13401]|CNMIG853-14|Tachinidae|591|BOLD:ACN6622
Gonia|[13402]|SMTPB10164-13|Tachinidae|658|BOLD:ACF7092
Gonia contumax|[13403]|CNGRH484-13|Tachinidae|658|BOLD:ACH2129
Tachinidae|[13404]|CNJAA116-12|Tachinidae|658|BOLD:ACC1976
Gonia ornata|[13405]|JSDIQ253-10|Tachinidae|658|BOLD:AAP8644
Gonia ornata|[13406]|CNEIA2298-12|Tachinidae|664|BOLD:AAV0926
Tachinidae|[13407]|SMTPG711-14|Tachinidae|564|BOLD:ACM6644
Siphosturmia phyciodes|[13408]|BBDCQ721-10|Tachinidae|658|BOLD:AAA8925
Siphosturmia|[13409]|CNWBB161-13|Tachinidae|658|BOLD:ACE1503
Hyphantrophaga blandita|[13410]|CNRMH102-12|Tachinidae|658|BOLD:AAN6460
Hyphantrophaga virilis|[13411]|CNEIG1510-13|Tachinidae|670|BOLD:ACF5244
Myxexoristops bonsdorffi|[13412]|SSBAA5218-12|Tachinidae|673|BOLD:AAV0942
Chrysoexorista|[13413]|CNWLG1021-12|Tachinidae|658|BOLD:AAP6381
Patelloa|[13414]|CNSLH304-12|Tachinidae|661|BOLD:ACC0781
Patelloa|[13415]|CNEIA2272-12|Tachinidae|664|BOLD:AAO8081
Patelloa|[13416]|CNPAA382-13|Tachinidae|658|BOLD:ACO0889
Patelloa pachypyga|[13417]|CNLMN114-14|Tachinidae|588|BOLD:AAZ3920
Hyphantrophaga blanda|[13418]|CNSLO769-13|Tachinidae|658|BOLD:ACI5697
Hyphantrophaga|[13419]|CNLMS126-14|Tachinidae|579|BOLD:ACO6206
Leschenaultia exul|[13420]|JSDIQ260-10|Tachinidae|658|BOLD:ACE2864
Leschenaultia nr. sp. Z|[13421]|JSJUL018-11|Tachinidae|658|BOLD:ACF4528
Ceromasia aurifrons|[13422]|BBDCP746-10|Tachinidae|658|BOLD:AAG2173
Chrysoexorista|[13423]|CNWLA015-12|Tachinidae|658|BOLD:AAP4829
Chrysoexorista sp. 1|[13424]|HPPPC410-13|Tachinidae|658|BOLD:ACM9631
Erynnia|[13425]|CNWBG394-13|Tachinidae|653|BOLD:AAZ4745
Frontiniella spectabilis|[13426]|CNWBG2629-13|Tachinidae|647|BOLD:AAZ3925
Exoristinae|[13427]|CNKJF050-14|Tachinidae|591|BOLD:ACM9357
Allophorocera arator|[13428]|SSWLD3947-13|Tachinidae|593|BOLD:ACX1173
Exoristinae|[13429]|CNKLF257-15|Tachinidae|603|BOLD:ABW0151
Exoristinae|[13430]|CNWBH565-13|Tachinidae|645|BOLD:ACG6155
Exoristinae|[13431]|SMTPJ2871-14|Tachinidae|603|BOLD:ACP8296
Tachinidae|[13432]|CNSIA683-15|Tachinidae|588|BOLD:AAP8653
Drino inconspicua|[13433]|BBDEE018-10|Tachinidae|658|BOLD:AAG2368
Nilea|[13434]|SMTPJ2869-14|Tachinidae|606|BOLD:AAA1961
Phryxe|[13435]|BBDCM500-10|Tachinidae|658|BOLD:AAC1603
Exoristinae|[13436]|CNBAK458-13|Tachinidae|658|BOLD:ACG6280
Tsugaea nox|[13437]|BBDCN632-10|Tachinidae|658|BOLD:AAP6382
Madremyia saundersii|[13438]|CNBPJ195-13|Tachinidae|673|BOLD:AAX3393
Exoristinae|[13439]|CNTGD258-15|Tachinidae|588|BOLD:ACS7938
Belida angelicae|[13440]|BBDCP718-10|Tachinidae|658|BOLD:AAP8652
Tachinidae|[13441]|JSDIQ986-11|Tachinidae|658|BOLD:AAZ5957
Euhalidaya genalis|[13442]|CNPPI397-12|Tachinidae|664|BOLD:ACC6471
Tachinidae|[13443]|BBDED900-10|Tachinidae|658|BOLD:AAG2364
Tachinidae|[13444]|SSBAD2897-12|Tachinidae|661|BOLD:AAP8647
Tachinidae|[13445]|BBDEC598-09|Tachinidae|658|BOLD:AAH7169
Exoristinae|[13446]|CNPAG396-13|Tachinidae|658|BOLD:ACE2608
Tachinidae|[13447]|HPPPE1708-13|Tachinidae|658|BOLD:ACI7631
Eucelatoria dimmocki|[13448]|PHJUL2509-11|Tachinidae|658|BOLD:ACE4209
Compsilura concinnata|[13449]|NCCA957-11|Tachinidae|673|BOLD:ACX8696
Blondelia eufitchiae|[13450]|BBDCQ828-10|Tachinidae|658|BOLD:AAG2346
Blondelia hyphantriae|[13451]|CNPPB129-12|Tachinidae|658|BOLD:AAG2148
Blondelia sp. 1|[13452]|HPPPD1555-13|Tachinidae|658|BOLD:AAZ4912
Blondelia|[13453]|HPPPJ1027-13|Tachinidae|657|BOLD:ACJ1182
Tachinidae|[13454]|JSDIS233-11|Tachinidae|658|BOLD:ABV1681
Tachinidae|[13455]|HPPPE1048-13|Tachinidae|658|BOLD:ACI2798
Tachinidae|[13456]|CNPPI1910-12|Tachinidae|664|BOLD:ACC7661
Lixophaga|[13457]|BBDCM901-10|Tachinidae|658|BOLD:AAG2348
Tachinidae|[13458]|CNGRB196-12|Tachinidae|673|BOLD:ACB9866
Tachinidae|[13459]|JSDIR840-11|Tachinidae|658|BOLD:AAG2149
Tachinidae|[13460]|RBINA1923-13|Tachinidae|658|BOLD:ACI9865
Tachinidae|[13461]|TTMDI441-10|Tachinidae|658|BOLD:AAG2335
Lixophaga variabilis|[13462]|CNWBC198-13|Tachinidae|658|BOLD:AAN6457
Tachinidae|[13463]|RBINA2590-13|Tachinidae|658|BOLD:ACI9503
Medina|[13464]|BBDCM525-10|Tachinidae|658|BOLD:AAG6902
Eucelatoria|[13465]|SSWLB1075-13|Tachinidae|664|BOLD:ACG6047
Eribella polita|[13466]|CNGRK254-13|Tachinidae|658|BOLD:AAZ0079
Tachinidae|[13467]|MBION328-14|Tachinidae|590|BOLD:ACO1328
Cryptomeigenia|[13468]|BBDCN029-10|Tachinidae|658|BOLD:ABZ1975
Cryptomeigenia|[13469]|BBDCM677-10|Tachinidae|658|BOLD:AAG2128
Zaira sp. 1|[13470]|JSDIQ942-11|Tachinidae|658|BOLD:AAZ4962
Zaira|[13471]|NGAAD324-14|Tachinidae|564|BOLD:ABZ0694
Zaira|[13472]|NGAAD2072-14|Tachinidae|612|BOLD:AAG2201
Zaira|[13473]|CNGRK287-13|Tachinidae|627|BOLD:AAV8535
Zaira cinerea|[13474]|CNLMH521-14|Tachinidae|658|BOLD:ABZ3296
Zaira|[13475]|CNLMO2589-14|Tachinidae|606|BOLD:ABZ0691
Oswaldia minor|[13476]|CNSLP270-13|Tachinidae|661|BOLD:AAZ4097
Oswaldia assimilis|[13477]|CNFNG1481-14|Tachinidae|661|BOLD:AAZ4669
Tachinidae|[13478]|TTMDI442-10|Tachinidae|658|BOLD:AAM7886
Oswaldia albifacies|[13479]|HPPPE1693-13|Tachinidae|658|BOLD:ACF2405
Oswaldia albifacies|[13480]|BBDCP706-10|Tachinidae|658|BOLD:AAP8651
Oswaldia anorbitalis|[13481]|CNLMF1029-14|Tachinidae|588|BOLD:ACE5127
Exoristinae|[13482]|CNPAN550-13|Tachinidae|658|BOLD:AAV9893
Tachinidae|[13483]|BBDED310-10|Tachinidae|658|BOLD:AAG2357
Tachinidae|[13484]|BBDCQ516-10|Tachinidae|658|BOLD:AAP1234
Tachinidae|[13485]|SSGBC787-14|Tachinidae|564|BOLD:ACP4006
Admontia|[13486]|SSWLD1616-13|Tachinidae|670|BOLD:ACI4066
Admontia|[13487]|HPPPI1573-13|Tachinidae|658|BOLD:AAV0929
Tachinidae|[13488]|BBDEC432-09|Tachinidae|658|BOLD:AAG2343
Tachinidae|[13489]|CNBRE029-14|Tachinidae|658|BOLD:ACL8222
Tachinidae|[13490]|CNEIG1515-13|Tachinidae|670|BOLD:AAG2114
Tachinidae|[13491]|MBIOI035-13|Tachinidae|592|BOLD:ACL2639
Tachinidae|[13492]|CNVUB002-14|Tachinidae|591|BOLD:ACR6298
Tachinidae|[13493]|NGNAG1567-13|Tachinidae|657|BOLD:ACJ1072
Anisia flaveola|[13494]|CNBPQ457-13|Tachinidae|670|BOLD:ACI4962
Tachinidae|[13495]|CNRVD182-15|Tachinidae|555|BOLD:ACU0103
Copecrypta Wood03|[13496]|JSDIR126-11|Tachinidae|658|BOLD:AAG2190
Deopalpus|[13497]|CNPPD1263-12|Tachinidae|673|BOLD:AAG2200
Adejeania vexatrix|[13498]|BBDCP656-10|Tachinidae|658|BOLD:AAP7668

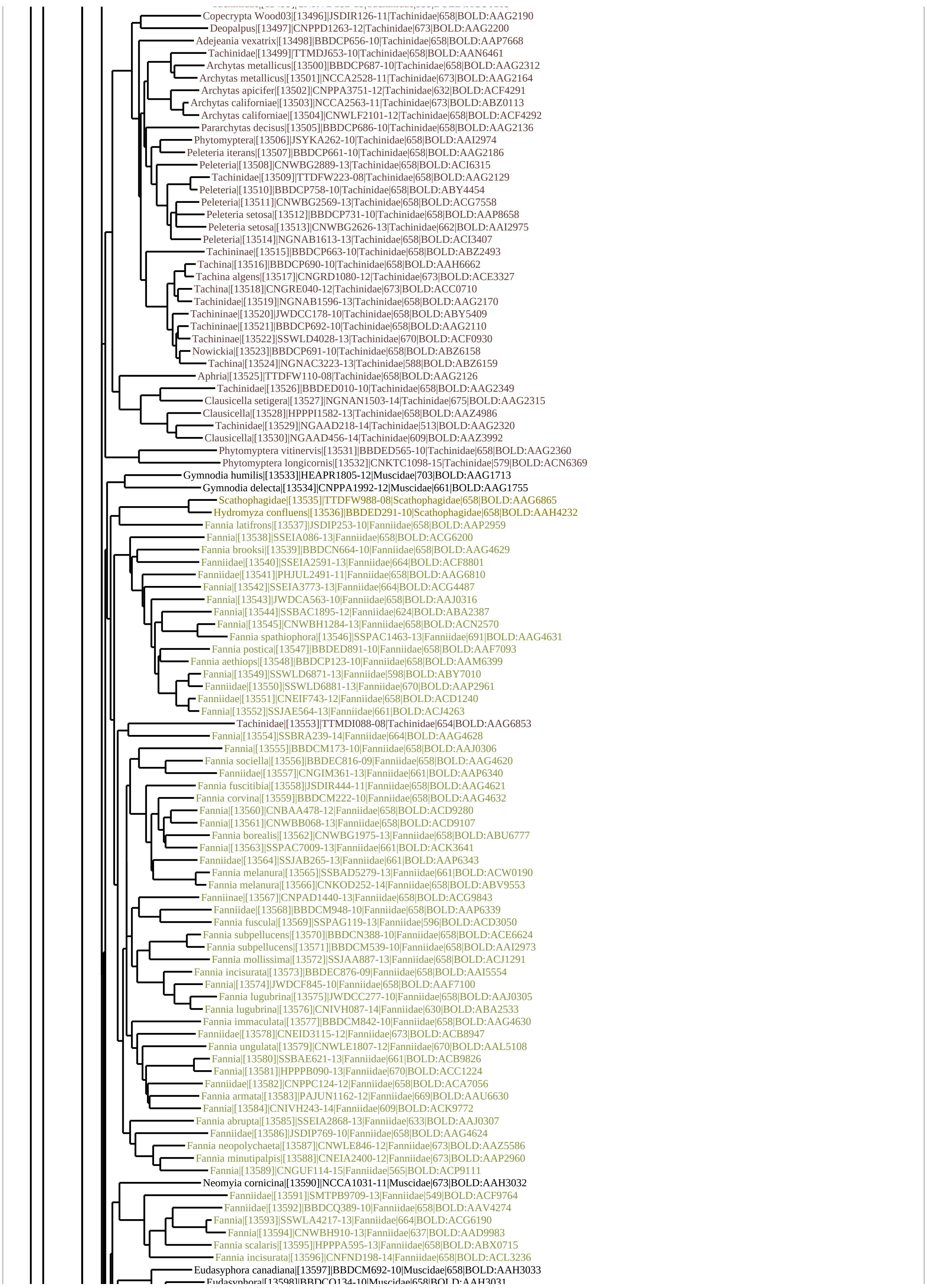

Copecrypta Wood03|[13496]|JSDIR126-11|Tachinidae|658|BOLD:AAG2190
Deopalpus|[13497]|CNPPD1263-12|Tachinidae|673|BOLD:AAG2200
Adejeania vexatrix|[13498]|BBDCP656-10|Tachinidae|658|BOLD:AAP7668
Tachinidae|[13499]|TTMDJ653-10|Tachinidae|658|BOLD:AAN6461
Archytas metallicus|[13500]|BBDCP687-10|Tachinidae|658|BOLD:AAG2312
Archytas metallicus|[13501]|NCCA2528-11|Tachinidae|673|BOLD:AAG2164
Archytas apicifer|[13502]|CNPPA3751-12|Tachinidae|632|BOLD:ACF4291
Archytas californiae|[13503]|NCCA2563-11|Tachinidae|673|BOLD:ABZ0113
Archytas californiae|[13504]|CNWLF2101-12|Tachinidae|658|BOLD:ACF4292
Pararchytas decisus|[13505]|BBDCP686-10|Tachinidae|658|BOLD:AAG2136
Phytomyptera|[13506]|JSYKA262-10|Tachinidae|658|BOLD:AAI2974
Peleteria iterans|[13507]|BBDCP661-10|Tachinidae|658|BOLD:AAG2186
Peleteria|[13508]|CNWBG2889-13|Tachinidae|658|BOLD:ACI6315
Tachinidae|[13509]|TTDFW223-08|Tachinidae|658|BOLD:AAG2129
Peleteria|[13510]|BBDCP758-10|Tachinidae|658|BOLD:ABY4454
Peleteria|[13511]|CNWBG2569-13|Tachinidae|658|BOLD:ACG7558
Peleteria setosa|[13512]|BBDCP731-10|Tachinidae|658|BOLD:AAP8658
Peleteria setosa|[13513]|CNWBG2626-13|Tachinidae|662|BOLD:AAI2975
Peleteria|[13514]|NGNAB1613-13|Tachinidae|658|BOLD:ACI3407
Tachininae|[13515]|BBDCP663-10|Tachinidae|658|BOLD:ABZ2493
Tachina|[13516]|BBDCP690-10|Tachinidae|658|BOLD:AAH6662
Tachina algens|[13517]|CNGRD1080-12|Tachinidae|673|BOLD:ACE3327
Tachina|[13518]|CNGRE040-12|Tachinidae|673|BOLD:ACC0710
Tachinidae|[13519]|NGNAB1596-13|Tachinidae|658|BOLD:AAG2170
Tachininae|[13520]|JWDCC178-10|Tachinidae|658|BOLD:ABY5409
Tachininae|[13521]|BBDCP692-10|Tachinidae|658|BOLD:AAG2110
Tachininae|[13522]|SSWLD4028-13|Tachinidae|670|BOLD:ACF0930
Nowickia|[13523]|BBDCP691-10|Tachinidae|658|BOLD:ABZ6158
Tachina|[13524]|NGNAC3223-13|Tachinidae|588|BOLD:ABZ6159
Aphria|[13525]|TTDFW110-08|Tachinidae|658|BOLD:AAG2126
Tachinidae|[13526]|BBDED010-10|Tachinidae|658|BOLD:AAG2349
Clausicella setigera|[13527]|NGNAN1503-14|Tachinidae|675|BOLD:AAG2315
Clausicella|[13528]|HPPPI1582-13|Tachinidae|658|BOLD:AAZ4986
Tachinidae|[13529]|NGAAD218-14|Tachinidae|513|BOLD:AAG2320
Clausicella|[13530]|NGAAD456-14|Tachinidae|609|BOLD:AAZ3992
Phytomyptera vitinervis|[13531]|BBDED565-10|Tachinidae|658|BOLD:AAG2360
Phytomyptera longicornis|[13532]|CNKTC1098-15|Tachinidae|579|BOLD:ACN6369
Gymnodia humilis|[13533]|HEAPR1805-12|Muscidae|703|BOLD:AAG1713
Gymnodia delecta|[13534]|CNPPA1992-12|Muscidae|661|BOLD:AAG1755
Scathophagidae|[13535]|TTDFW988-08|Scathophagidae|658|BOLD:AAG6865
Hydromyza confluens|[13536]|BBDED291-10|Scathophagidae|658|BOLD:AAH4232
Fannia latifrons|[13537]|JSDIP253-10|Fanniidae|658|BOLD:AAP2959
Fannia|[13538]|SSEIA086-13|Fanniidae|658|BOLD:ACG6200
Fannia brooksi|[13539]|BBDCN664-10|Fanniidae|658|BOLD:AAG4629
Fanniidae|[13540]|SSEIA2591-13|Fanniidae|664|BOLD:ACF8801
Fanniidae|[13541]|PHJUL2491-11|Fanniidae|658|BOLD:AAG6810
Fannia|[13542]|SSEIA3773-13|Fanniidae|664|BOLD:ACG4487
Fannia|[13543]|JWDCA563-10|Fanniidae|658|BOLD:AAJ0316
Fannia|[13544]|SSBAC1895-12|Fanniidae|624|BOLD:ABA2387
Fannia|[13545]|CNWBH1284-13|Fanniidae|658|BOLD:ACN2570
Fannia spathiophora|[13546]|SSPAC1463-13|Fanniidae|691|BOLD:AAG4631
Fannia postica|[13547]|BBDED891-10|Fanniidae|658|BOLD:AAF7093
Fannia aethiops|[13548]|BBDCP123-10|Fanniidae|658|BOLD:AAM6399
Fannia|[13549]|SSWLD6871-13|Fanniidae|598|BOLD:ABY7010
Fanniidae|[13550]|SSWLD6881-13|Fanniidae|670|BOLD:AAP2961
Fanniidae|[13551]|CNEIF743-12|Fanniidae|658|BOLD:ACD1240
Fannia|[13552]|SSJAE564-13|Fanniidae|661|BOLD:ACJ4263
Tachinidae|[13553]|TTMDI088-08|Tachinidae|654|BOLD:AAG6853
Fannia|[13554]|SSBRA239-14|Fanniidae|664|BOLD:AAG4628
Fannia|[13555]|BBDCM173-10|Fanniidae|658|BOLD:AAJ0306
Fannia sociella|[13556]|BBDEC816-09|Fanniidae|658|BOLD:AAG4620
Fanniidae|[13557]|CNGIM361-13|Fanniidae|661|BOLD:AAP6340
Fannia fuscitibia|[13558]|JSDIR444-11|Fanniidae|658|BOLD:AAG4621
Fannia corvina|[13559]|BBDCM222-10|Fanniidae|658|BOLD:AAG4632
Fannia|[13560]|CNBAA478-12|Fanniidae|658|BOLD:ACD9280
Fannia|[13561]|CNWBB068-13|Fanniidae|658|BOLD:ACD9107
Fannia borealis|[13562]|CNWBG1975-13|Fanniidae|658|BOLD:ABU6777
Fannia|[13563]|SSPAC7009-13|Fanniidae|661|BOLD:ACK3641
Fanniidae|[13564]|SSJAB265-13|Fanniidae|661|BOLD:AAP6343
Fannia melanura|[13565]|SSBAD5279-13|Fanniidae|661|BOLD:ACW0190
Fannia melanura|[13566]|CNKOD252-14|Fanniidae|658|BOLD:ABV9553
Fanniinae|[13567]|CNPAD1440-13|Fanniidae|658|BOLD:ACG9843
Fanniidae|[13568]|BBDCM948-10|Fanniidae|658|BOLD:AAP6339
Fannia fuscula|[13569]|SSPAG119-13|Fanniidae|596|BOLD:ACD3050
Fannia subpellucens|[13570]|BBDCN388-10|Fanniidae|658|BOLD:ACE6624
Fannia subpellucens|[13571]|BBDCM539-10|Fanniidae|658|BOLD:AAI2973
Fannia mollissima|[13572]|SSJAA887-13|Fanniidae|658|BOLD:ACJ1291
Fannia incisurata|[13573]|BBDEC876-09|Fanniidae|658|BOLD:AAI5554
Fannia|[13574]|JWDCF845-10|Fanniidae|658|BOLD:AAF7100
Fannia lugubrina|[13575]|JWDCC277-10|Fanniidae|658|BOLD:AAJ0305
Fannia lugubrina|[13576]|CNIVH087-14|Fanniidae|630|BOLD:ABA2533
Fannia immaculata|[13577]|BBDCM842-10|Fanniidae|658|BOLD:AAG4630
Fanniidae|[13578]|CNEID3115-12|Fanniidae|673|BOLD:ACB8947
Fannia ungulata|[13579]|CNWLE1807-12|Fanniidae|670|BOLD:AAL5108
Fannia|[13580]|SSBAE621-13|Fanniidae|661|BOLD:ACB9826
Fannia|[13581]|HPPPB090-13|Fanniidae|670|BOLD:ACC1224
Fanniidae|[13582]|CNPPC124-12|Fanniidae|658|BOLD:ACA7056
Fannia armata|[13583]|PAJUN1162-12|Fanniidae|669|BOLD:AAU6630
Fannia|[13584]|CNIVH243-14|Fanniidae|609|BOLD:ACK9772
Fannia abrupta|[13585]|SSEIA2868-13|Fanniidae|633|BOLD:AAJ0307
Fanniidae|[13586]|JSDIP769-10|Fanniidae|658|BOLD:AAG4624
Fannia neopolychaeta|[13587]|CNWLE846-12|Fanniidae|673|BOLD:AAZ5586
Fannia minutipalpis|[13588]|CNEIA2400-12|Fanniidae|673|BOLD:AAP2960
Fannia|[13589]|CNGUF114-15|Fanniidae|565|BOLD:ACP9111
Neomyia cornicina|[13590]|NCCA1031-11|Muscidae|673|BOLD:AAH3032
Fanniidae|[13591]|SMTPB9709-13|Fanniidae|549|BOLD:ACF9764
Fanniidae|[13592]|BBDCQ389-10|Fanniidae|658|BOLD:AAV4274
Fannia|[13593]|SSWLA4217-13|Fanniidae|664|BOLD:ACG6190
Fannia|[13594]|CNWBH910-13|Fanniidae|637|BOLD:AAD9983
Fannia scalaris|[13595]|HPPPA595-13|Fanniidae|658|BOLD:ABX0715
Fannia incisurata|[13596]|CNFND198-14|Fanniidae|658|BOLD:ACL3236
Eudasyphora canadiana|[13597]|BBDCM692-10|Muscidae|658|BOLD:AAH3033
Eudasyphora|[13598]|BBDCQ134-10|Muscidae|658|BOLD:AAH3031

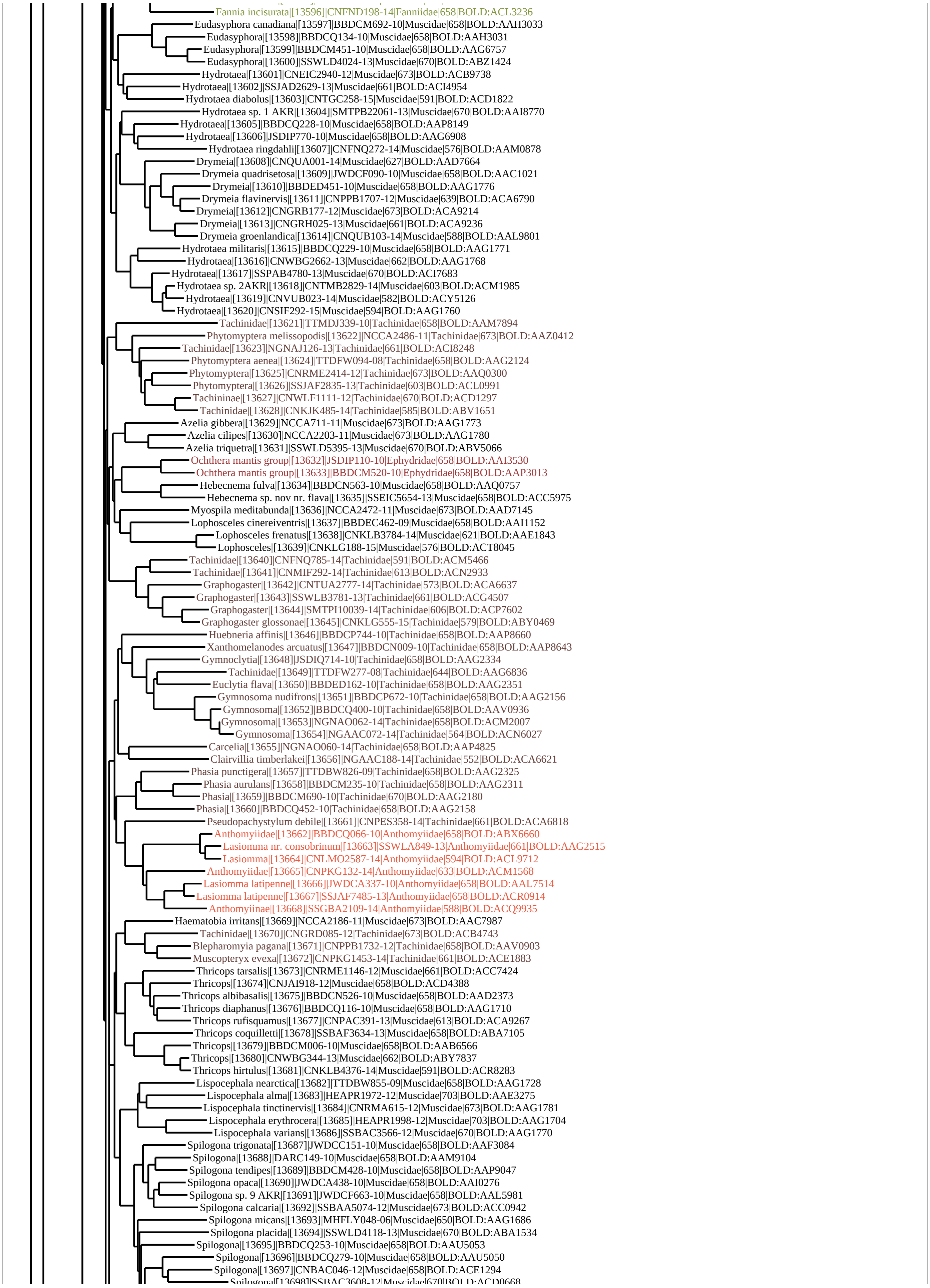
Fannia incisurata|[13596]|CNFND198-14|Fanniidae|658|BOLD:ACL3236
Eudasyphora canadiana|[13597]|BBDCM692-10|Muscidae|658|BOLD:AAH3033
Eudasyphora|[13598]|BBDCQ134-10|Muscidae|658|BOLD:AAH3031
Eudasyphora|[13599]|BBDCM451-10|Muscidae|658|BOLD:AAG6757
Eudasyphora|[13600]|SSWLD4024-13|Muscidae|670|BOLD:ABZ1424
Hydrotaea|[13601]|CNEIC2940-12|Muscidae|673|BOLD:ACB9738
Hydrotaea|[13602]|SSJAD2629-13|Muscidae|661|BOLD:ACI4954
Hydrotaea diabolus|[13603]|CNTGC258-15|Muscidae|591|BOLD:ACD1822
Hydrotaea sp. 1 AKR|[13604]|SMTPB22061-13|Muscidae|670|BOLD:AAI8770
Hydrotaea|[13605]|BBDCQ228-10|Muscidae|658|BOLD:AAP8149
Hydrotaea|[13606]|JSDIP770-10|Muscidae|658|BOLD:AAG6908
Hydrotaea ringdahli|[13607]|CNFNQ272-14|Muscidae|576|BOLD:AAM0878
Drymeia|[13608]|CNQUA001-14|Muscidae|627|BOLD:AAD7664
Drymeia quadrisetosa|[13609]|JWDCF090-10|Muscidae|658|BOLD:AAC1021
Drymeia|[13610]|BBDED451-10|Muscidae|658|BOLD:AAG1776
Drymeia flavinervis|[13611]|CNPPB1707-12|Muscidae|639|BOLD:ACA6790
Drymeia|[13612]|CNGRB177-12|Muscidae|673|BOLD:ACA9214
Drymeia|[13613]|CNGRH025-13|Muscidae|661|BOLD:ACA9236
Drymeia groenlandica|[13614]|CNQUB103-14|Muscidae|588|BOLD:AAL9801
Hydrotaea militaris|[13615]|BBDCQ229-10|Muscidae|658|BOLD:AAG1771
Hydrotaea|[13616]|CNWBG2662-13|Muscidae|662|BOLD:AAG1768
Hydrotaea|[13617]|SSPAB4780-13|Muscidae|670|BOLD:ACI7683
Hydrotaea sp. 2AKR|[13618]|CNTMB2829-14|Muscidae|603|BOLD:ACM1985
Hydrotaea|[13619]|CNVUB023-14|Muscidae|582|BOLD:ACY5126
Hydrotaea|[13620]|CNSIF292-15|Muscidae|594|BOLD:AAG1760
Tachinidae|[13621]|TTMDJ339-10|Tachinidae|658|BOLD:AAM7894
Phytomyptera melissopodis|[13622]|NCCA2486-11|Tachinidae|673|BOLD:AAZ0412
Tachinidae|[13623]|NGNAJ126-13|Tachinidae|661|BOLD:ACI8248
Phytomyptera aenea|[13624]|TTDFW094-08|Tachinidae|658|BOLD:AAG2124
Phytomyptera|[13625]|CNRME2414-12|Tachinidae|673|BOLD:AAQ0300
Phytomyptera|[13626]|SSJAF2835-13|Tachinidae|603|BOLD:ACL0991
Tachininae|[13627]|CNWLF1111-12|Tachinidae|670|BOLD:ACD1297
Tachinidae|[13628]|CNKJK485-14|Tachinidae|585|BOLD:ABV1651
Azelia gibbera|[13629]|NCCA711-11|Muscidae|673|BOLD:AAG1773
Azelia cilipes|[13630]|NCCA2203-11|Muscidae|673|BOLD:AAG1780
Azelia triquetra|[13631]|SSWLD5395-13|Muscidae|670|BOLD:ABV5066
Ochthera mantis group|[13632]|JSDIP110-10|Ephydridae|658|BOLD:AAI3530
Ochthera mantis group|[13633]|BBDCM520-10|Ephydridae|658|BOLD:AAP3013
Hebecnema fulva|[13634]|BBDCN563-10|Muscidae|658|BOLD:AAQ0757
Hebecnema sp. nov nr. flava|[13635]|SSEIC5654-13|Muscidae|658|BOLD:ACC5975
Myospila meditabunda|[13636]|NCCA2472-11|Muscidae|673|BOLD:AAD7145
Lophosceles cinereiventris|[13637]|BBDEC462-09|Muscidae|658|BOLD:AAI1152
Lophosceles frenatus|[13638]|CNKLB3784-14|Muscidae|621|BOLD:AAE1843
Lophosceles|[13639]|CNKLG188-15|Muscidae|576|BOLD:ACT8045
Tachinidae|[13640]|CNFNQ785-14|Tachinidae|591|BOLD:ACM5466
Tachinidae|[13641]|CNMIF292-14|Tachinidae|613|BOLD:ACN2933
Graphogaster|[13642]|CNTUA2777-14|Tachinidae|573|BOLD:ACA6637
Graphogaster|[13643]|SSWLB3781-13|Tachinidae|661|BOLD:ACG4507
Graphogaster|[13644]|SMTPI10039-14|Tachinidae|606|BOLD:ACP7602
Graphogaster glossonae|[13645]|CNKLG555-15|Tachinidae|579|BOLD:ABY0469
Huebneria affinis|[13646]|BBDCP744-10|Tachinidae|658|BOLD:AAP8660
Xanthomelanodes arcuatus|[13647]|BBDCN009-10|Tachinidae|658|BOLD:AAP8643
Gymnoclytia|[13648]|JSDIQ714-10|Tachinidae|658|BOLD:AAG2334
Tachinidae|[13649]|TTDFW277-08|Tachinidae|644|BOLD:AAG6836
Euclytia flava|[13650]|BBDED162-10|Tachinidae|658|BOLD:AAG2351
Gymnosoma nudifrons|[13651]|BBDCP672-10|Tachinidae|658|BOLD:AAG2156
Gymnosoma|[13652]|BBDCQ400-10|Tachinidae|658|BOLD:AAV0936
Gymnosoma|[13653]|NGNAO062-14|Tachinidae|658|BOLD:ACM2007
Gymnosoma|[13654]|NGAAC072-14|Tachinidae|564|BOLD:ACN6027
Carcelia|[13655]|NGNAO060-14|Tachinidae|658|BOLD:AAP4825
Clairvillia timberlakei|[13656]|NGAAC188-14|Tachinidae|552|BOLD:ACA6621
Phasia punctigera|[13657]|TTDBW826-09|Tachinidae|658|BOLD:AAG2325
Phasia aurulans|[13658]|BBDCM235-10|Tachinidae|658|BOLD:AAG2311
Phasia|[13659]|BBDCM690-10|Tachinidae|670|BOLD:AAG2180
Phasia|[13660]|BBDCQ452-10|Tachinidae|658|BOLD:AAG2158
Pseudopachystylum debile|[13661]|CNPES358-14|Tachinidae|661|BOLD:ACA6818
Anthomyiidae|[13662]|BBDCQ066-10|Anthomyiidae|658|BOLD:ABX6660
Lasiomma nr. consobrinum|[13663]|SSWLA849-13|Anthomyiidae|661|BOLD:AAG2515
Lasiomma|[13664]|CNLMO2587-14|Anthomyiidae|594|BOLD:ACL9712
Anthomyiidae|[13665]|CNPKG132-14|Anthomyiidae|633|BOLD:ACM1568
Lasiomma latipenne|[13666]|JWDCA337-10|Anthomyiidae|658|BOLD:AAL7514
Lasiomma latipenne|[13667]|SSJAF7485-13|Anthomyiidae|658|BOLD:ACR0914
Anthomyiinae|[13668]|SSGBA2109-14|Anthomyiidae|588|BOLD:ACQ9935
Haematobia irritans|[13669]|NCCA2186-11|Muscidae|673|BOLD:AAC7987
Tachinidae|[13670]|CNGRD085-12|Tachinidae|673|BOLD:ACB4743
Blepharomyia pagana|[13671]|CNPPB1732-12|Tachinidae|658|BOLD:AAV0903
Muscopteryx evexa|[13672]|CNPKG1453-14|Tachinidae|661|BOLD:ACE1883
Thricops tarsalis|[13673]|CNRME1146-12|Muscidae|661|BOLD:ACC7424
Thricops|[13674]|CNJAI918-12|Muscidae|658|BOLD:ACD4388
Thricops albibasalis|[13675]|BBDCN526-10|Muscidae|658|BOLD:AAD2373
Thricops diaphanus|[13676]|BBDCQ116-10|Muscidae|658|BOLD:AAG1710
Thricops rufisquamus|[13677]|CNPAC391-13|Muscidae|613|BOLD:ACA9267
Thricops coquilletti|[13678]|SSBAF3634-13|Muscidae|658|BOLD:ABA7105
Thricops|[13679]|BBDCM006-10|Muscidae|658|BOLD:AAB6566
Thricops|[13680]|CNWBG344-13|Muscidae|662|BOLD:ABY7837
Thricops hirtulus|[13681]|CNKLB4376-14|Muscidae|591|BOLD:ACR8283
Lispocephala nearctica|[13682]|TTDBW855-09|Muscidae|658|BOLD:AAG1728
Lispocephala alma|[13683]|HEAPR1972-12|Muscidae|703|BOLD:AAE3275
Lispocephala tinctinervis|[13684]|CNRMA615-12|Muscidae|673|BOLD:AAG1781
Lispocephala erythrocera|[13685]|HEAPR1998-12|Muscidae|703|BOLD:AAG1704
Lispocephala varians|[13686]|SSBAC3566-12|Muscidae|670|BOLD:AAG1770
Spilogona trigonata|[13687]|JWDCC151-10|Muscidae|658|BOLD:AAF3084
Spilogona|[13688]|DARC149-10|Muscidae|658|BOLD:AAM9104
Spilogona tendipes|[13689]|BBDCM428-10|Muscidae|658|BOLD:AAP9047
Spilogona opaca|[13690]|JWDCA438-10|Muscidae|658|BOLD:AAI0276
Spilogona sp. 9 AKR|[13691]|JWDCF663-10|Muscidae|658|BOLD:AAL5981
Spilogona calcaria|[13692]|SSBAA5074-12|Muscidae|673|BOLD:ACC0942
Spilogona micans|[13693]|MHFLY048-06|Muscidae|650|BOLD:AAG1686
Spilogona placida|[13694]|SSWLD4118-13|Muscidae|670|BOLD:ABA1534
Spilogona|[13695]|BBDCQ253-10|Muscidae|658|BOLD:AAU5053
Spilogona|[13696]|BBDCQ279-10|Muscidae|658|BOLD:AAU5050
Spilogona|[13697]|CNBAC046-12|Muscidae|658|BOLD:ACE1294
Spilogona|[13698]|SSBAC3608-12|Muscidae|670|BOLD:ACD0668

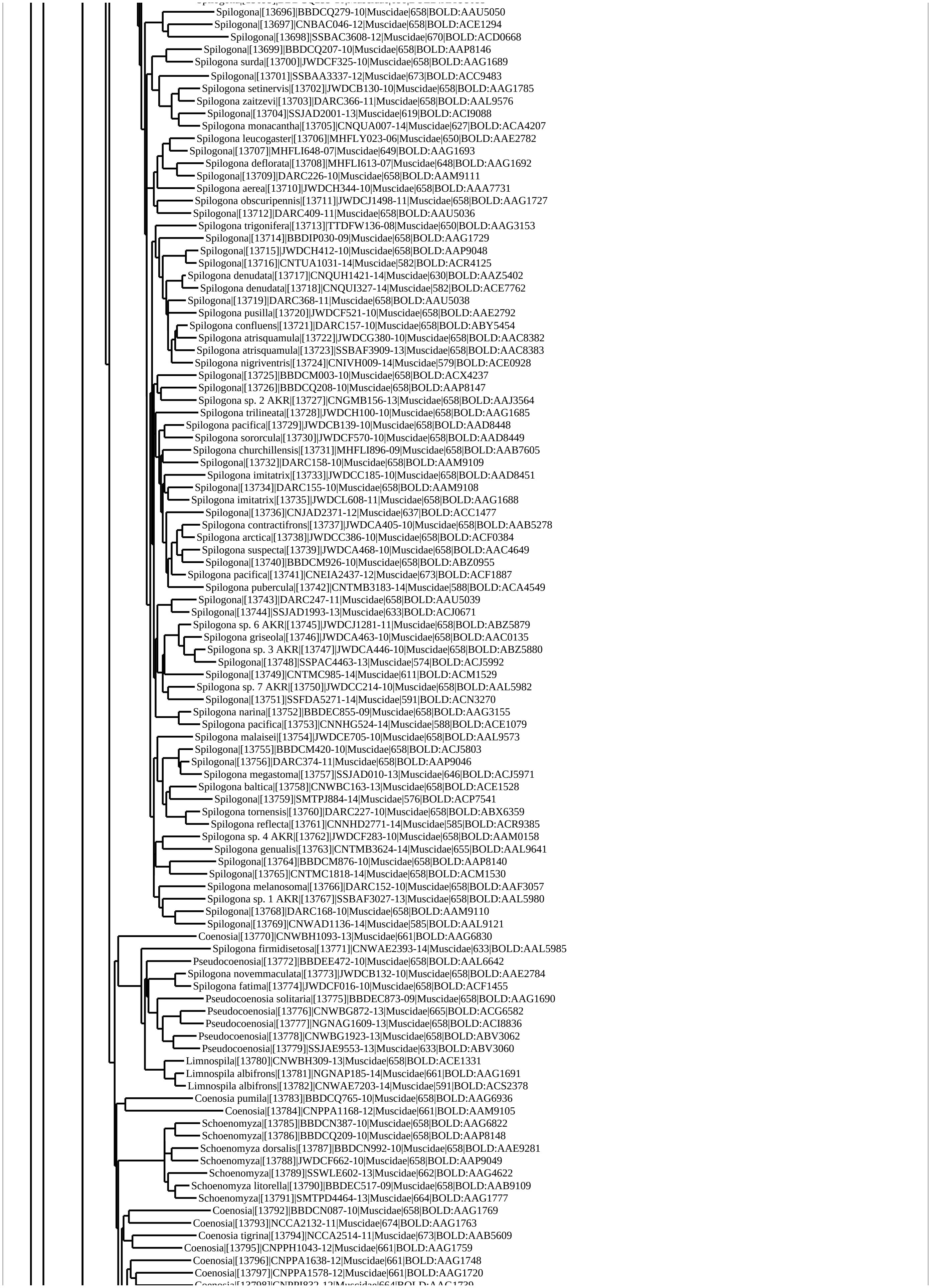
Spilogona|[13696]|BBDCQ279-10|Muscidae|658|BOLD:AAU5050
Spilogona|[13697]|CNBAC046-12|Muscidae|658|BOLD:ACE1294
Spilogona|[13698]|SSBAC3608-12|Muscidae|670|BOLD:ACD0668
Spilogona|[13699]|BBDCQ207-10|Muscidae|658|BOLD:AAP8146
Spilogona surda|[13700]|JWDCF325-10|Muscidae|658|BOLD:AAG1689
Spilogona|[13701]|SSBAA3337-12|Muscidae|673|BOLD:ACC9483
Spilogona setinervis|[13702]|JWDCB130-10|Muscidae|658|BOLD:AAG1785
Spilogona zaitzevi|[13703]|DARC366-11|Muscidae|658|BOLD:AAL9576
Spilogona|[13704]|SSJAD2001-13|Muscidae|619|BOLD:ACI9088
Spilogona monacantha|[13705]|CNQUA007-14|Muscidae|627|BOLD:ACA4207
Spilogona leucogaster|[13706]|MHFLY023-06|Muscidae|650|BOLD:AAE2782
Spilogona|[13707]|MHFLI648-07|Muscidae|649|BOLD:AAG1693
Spilogona deflorata|[13708]|MHFLI613-07|Muscidae|648|BOLD:AAG1692
Spilogona|[13709]|DARC226-10|Muscidae|658|BOLD:AAM9111
Spilogona aerea|[13710]|JWDCH344-10|Muscidae|658|BOLD:AAA7731
Spilogona obscuripennis|[13711]|JWDCJ1498-11|Muscidae|658|BOLD:AAG1727
Spilogona|[13712]|DARC409-11|Muscidae|658|BOLD:AAU5036
Spilogona trigonifera|[13713]|TTDFW136-08|Muscidae|650|BOLD:AAG3153
Spilogona|[13714]|BBDIP030-09|Muscidae|658|BOLD:AAG1729
Spilogona|[13715]|JWDCH412-10|Muscidae|658|BOLD:AAP9048
Spilogona|[13716]|CNTUA1031-14|Muscidae|582|BOLD:ACR4125
Spilogona denudata|[13717]|CNQUH1421-14|Muscidae|630|BOLD:AAZ5402
Spilogona denudata|[13718]|CNQUI327-14|Muscidae|582|BOLD:ACE7762
Spilogona|[13719]|DARC368-11|Muscidae|658|BOLD:AAU5038
Spilogona pusilla|[13720]|JWDCF521-10|Muscidae|658|BOLD:AAE2792
Spilogona confluens|[13721]|DARC157-10|Muscidae|658|BOLD:ABY5454
Spilogona atrisquamula|[13722]|JWDCG380-10|Muscidae|658|BOLD:AAC8382
Spilogona atrisquamula|[13723]|SSBAF3909-13|Muscidae|658|BOLD:AAC8383
Spilogona nigriventris|[13724]|CNIVH009-14|Muscidae|579|BOLD:ACE0928
Spilogona|[13725]|BBDCM003-10|Muscidae|658|BOLD:ACX4237
Spilogona|[13726]|BBDCQ208-10|Muscidae|658|BOLD:AAP8147
Spilogona sp. 2 AKR|[13727]|CNGMB156-13|Muscidae|658|BOLD:AAJ3564
Spilogona trilineata|[13728]|JWDCH100-10|Muscidae|658|BOLD:AAG1685
Spilogona pacifica|[13729]|JWDCB139-10|Muscidae|658|BOLD:AAD8448
Spilogona sororcula|[13730]|JWDCF570-10|Muscidae|658|BOLD:AAD8449
Spilogona churchillensis|[13731]|MHFLI896-09|Muscidae|658|BOLD:AAB7605
Spilogona|[13732]|DARC158-10|Muscidae|658|BOLD:AAM9109
Spilogona imitatrix|[13733]|JWDCC185-10|Muscidae|658|BOLD:AAD8451
Spilogona|[13734]|DARC155-10|Muscidae|658|BOLD:AAM9108
Spilogona imitatrix|[13735]|JWDCL608-11|Muscidae|658|BOLD:AAG1688
Spilogona|[13736]|CNJAD2371-12|Muscidae|637|BOLD:ACC1477
Spilogona contractifrons|[13737]|JWDCA405-10|Muscidae|658|BOLD:AAB5278
Spilogona arctica|[13738]|JWDCC386-10|Muscidae|658|BOLD:ACF0384
Spilogona suspecta|[13739]|JWDCA468-10|Muscidae|658|BOLD:AAC4649
Spilogona|[13740]|BBDCM926-10|Muscidae|658|BOLD:ABZ0955
Spilogona pacifica|[13741]|CNEIA2437-12|Muscidae|673|BOLD:ACF1887
Spilogona pubercula|[13742]|CNTMB3183-14|Muscidae|588|BOLD:ACA4549
Spilogona|[13743]|DARC247-11|Muscidae|658|BOLD:AAU5039
Spilogona|[13744]|SSJAD1993-13|Muscidae|633|BOLD:ACJ0671
Spilogona sp. 6 AKR|[13745]|JWDCJ1281-11|Muscidae|658|BOLD:ABZ5879
Spilogona griseola|[13746]|JWDCA463-10|Muscidae|658|BOLD:AAC0135
Spilogona sp. 3 AKR|[13747]|JWDCA446-10|Muscidae|658|BOLD:ABZ5880
Spilogona|[13748]|SSPAC4463-13|Muscidae|574|BOLD:ACJ5992
Spilogona|[13749]|CNTMC985-14|Muscidae|611|BOLD:ACM1529
Spilogona sp. 7 AKR|[13750]|JWDCC214-10|Muscidae|658|BOLD:AAL5982
Spilogona|[13751]|SSFDA5271-14|Muscidae|591|BOLD:ACN3270
Spilogona narina|[13752]|BBDEC855-09|Muscidae|658|BOLD:AAG3155
Spilogona pacifica|[13753]|CNNHG524-14|Muscidae|588|BOLD:ACE1079
Spilogona malaisei|[13754]|JWDCE705-10|Muscidae|658|BOLD:AAL9573
Spilogona|[13755]|BBDCM420-10|Muscidae|658|BOLD:ACJ5803
Spilogona|[13756]|DARC374-11|Muscidae|658|BOLD:AAP9046
Spilogona megastoma|[13757]|SSJAD010-13|Muscidae|646|BOLD:ACJ5971
Spilogona baltica|[13758]|CNWBC163-13|Muscidae|658|BOLD:ACE1528
Spilogona|[13759]|SMTPJ884-14|Muscidae|576|BOLD:ACP7541
Spilogona tornensis|[13760]|DARC227-10|Muscidae|658|BOLD:ABX6359
Spilogona reflecta|[13761]|CNNHD2771-14|Muscidae|585|BOLD:ACR9385
Spilogona sp. 4 AKR|[13762]|JWDCF283-10|Muscidae|658|BOLD:AAM0158
Spilogona genualis|[13763]|CNTMB3624-14|Muscidae|655|BOLD:AAL9641
Spilogona|[13764]|BBDCM876-10|Muscidae|658|BOLD:AAP8140
Spilogona|[13765]|CNTMC1818-14|Muscidae|658|BOLD:ACM1530
Spilogona melanosoma|[13766]|DARC152-10|Muscidae|658|BOLD:AAF3057
Spilogona sp. 1 AKR|[13767]|SSBAF3027-13|Muscidae|658|BOLD:AAL5980
Spilogona|[13768]|DARC168-10|Muscidae|658|BOLD:AAM9110
Spilogona|[13769]|CNWAD1136-14|Muscidae|585|BOLD:AAL9121
Coenosia|[13770]|CNWBH1093-13|Muscidae|661|BOLD:AAG6830
Spilogona firmidisetosa|[13771]|CNWAE2393-14|Muscidae|633|BOLD:AAL5985
Pseudocoenosia|[13772]|BBDEE472-10|Muscidae|658|BOLD:AAL6642
Spilogona novemmaculata|[13773]|JWDCB132-10|Muscidae|658|BOLD:AAE2784
Spilogona fatima|[13774]|JWDCF016-10|Muscidae|658|BOLD:ACF1455
Pseudocoenosia solitaria|[13775]|BBDEC873-09|Muscidae|658|BOLD:AAG1690
Pseudocoenosia|[13776]|CNWBG872-13|Muscidae|665|BOLD:ACG6582
Pseudocoenosia|[13777]|NGNAG1609-13|Muscidae|658|BOLD:ACI8836
Pseudocoenosia|[13778]|CNWBG1923-13|Muscidae|658|BOLD:ABV3062
Pseudocoenosia|[13779]|SSJAE9553-13|Muscidae|633|BOLD:ABV3060
Limnospila|[13780]|CNWBH309-13|Muscidae|658|BOLD:ACE1331
Limnospila albifrons|[13781]|NGNAP185-14|Muscidae|661|BOLD:AAG1691
Limnospila albifrons|[13782]|CNWAE7203-14|Muscidae|591|BOLD:ACS2378
Coenosia pumila|[13783]|BBDCQ765-10|Muscidae|658|BOLD:AAG6936
Coenosia|[13784]|CNPPA1168-12|Muscidae|661|BOLD:AAM9105
Schoenomyza|[13785]|BBDCN387-10|Muscidae|658|BOLD:AAG6822
Schoenomyza|[13786]|BBDCQ209-10|Muscidae|658|BOLD:AAP8148
Schoenomyza dorsalis|[13787]|BBDCN992-10|Muscidae|658|BOLD:AAE9281
Schoenomyza|[13788]|JWDCF662-10|Muscidae|658|BOLD:AAP9049
Schoenomyza|[13789]|SSWLE602-13|Muscidae|662|BOLD:AAG4622
Schoenomyza litorella|[13790]|BBDEC517-09|Muscidae|658|BOLD:AAB9109
Schoenomyza|[13791]|SMTPD4464-13|Muscidae|664|BOLD:AAG1777
Coenosia|[13792]|BBDCN087-10|Muscidae|658|BOLD:AAG1769
Coenosia|[13793]|NCCA2132-11|Muscidae|674|BOLD:AAG1763
Coenosia tigrina|[13794]|NCCA2514-11|Muscidae|673|BOLD:AAB5609
Coenosia|[13795]|CNPPH1043-12|Muscidae|661|BOLD:AAG1759
Coenosia|[13796]|CNPPA1638-12|Muscidae|661|BOLD:AAG1748
Coenosia|[13797]|CNPPA1578-12|Muscidae|661|BOLD:AAG1720

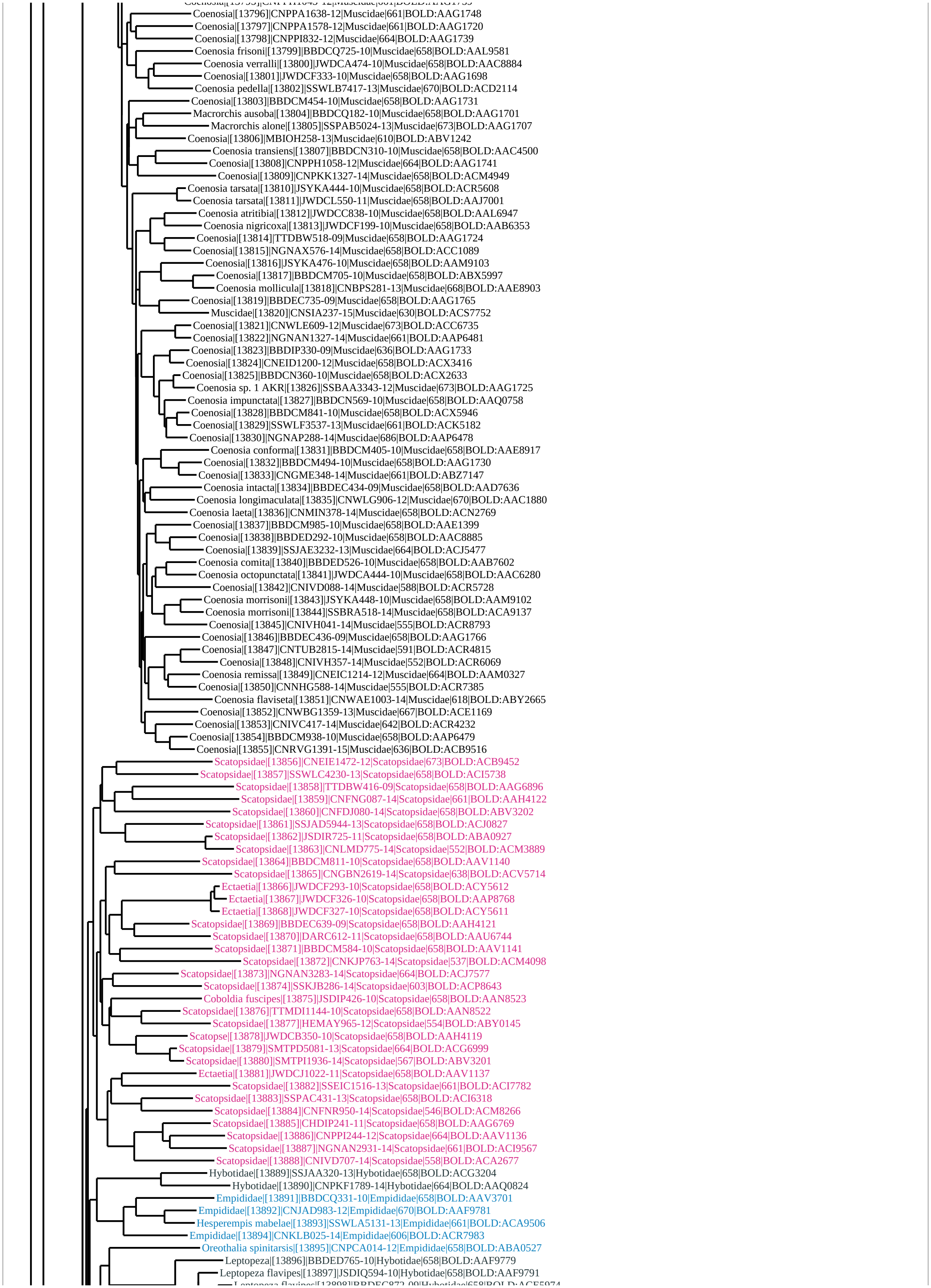
Coenosia|[13796]|CNPPA1638-12|Muscidae|661|BOLD:AAG1748
Coenosia|[13797]|CNPPA1578-12|Muscidae|661|BOLD:AAG1720
Coenosia|[13798]|CNPPI832-12|Muscidae|664|BOLD:AAG1739
Coenosia frisoni|[13799]|BBDCQ725-10|Muscidae|658|BOLD:AAL9581
Coenosia verralli|[13800]|JWDCA474-10|Muscidae|658|BOLD:AAC8884
Coenosia|[13801]|JWDCF333-10|Muscidae|658|BOLD:AAG1698
Coenosia pedella|[13802]|SSWLB7417-13|Muscidae|670|BOLD:ACD2114
Coenosia|[13803]|BBDCM454-10|Muscidae|658|BOLD:AAG1731
Macrorchis ausoba|[13804]|BBDCQ182-10|Muscidae|658|BOLD:AAG1701
Macrorchis alone|[13805]|SSPAB5024-13|Muscidae|673|BOLD:AAG1707
Coenosia|[13806]|MBIOH258-13|Muscidae|610|BOLD:ABV1242
Coenosia transiens|[13807]|BBDCN310-10|Muscidae|658|BOLD:AAC4500
Coenosia|[13808]|CNPPH1058-12|Muscidae|664|BOLD:AAG1741
Coenosia|[13809]|CNPKK1327-14|Muscidae|658|BOLD:ACM4949
Coenosia tarsata|[13810]|JSYKA444-10|Muscidae|658|BOLD:ACR5608
Coenosia tarsata|[13811]|JWDCL550-11|Muscidae|658|BOLD:AAJ7001
Coenosia atritibia|[13812]|JWDCC838-10|Muscidae|658|BOLD:AAL6947
Coenosia nigricoxa|[13813]|JWDCF199-10|Muscidae|658|BOLD:AAB6353
Coenosia|[13814]|TTDBW518-09|Muscidae|658|BOLD:AAG1724
Coenosia|[13815]|NGNAX576-14|Muscidae|658|BOLD:ACC1089
Coenosia|[13816]|JSYKA476-10|Muscidae|658|BOLD:AAM9103
Coenosia|[13817]|BBDCM705-10|Muscidae|658|BOLD:ABX5997
Coenosia mollicula|[13818]|CNBPS281-13|Muscidae|668|BOLD:AAE8903
Coenosia|[13819]|BBDEC735-09|Muscidae|658|BOLD:AAG1765
Muscidae|[13820]|CNSIA237-15|Muscidae|630|BOLD:ACS7752
Coenosia|[13821]|CNWLE609-12|Muscidae|673|BOLD:ACC6735
Coenosia|[13822]|NGNAN1327-14|Muscidae|661|BOLD:AAP6481
Coenosia|[13823]|BBDIP330-09|Muscidae|636|BOLD:AAG1733
Coenosia|[13824]|CNEID1200-12|Muscidae|658|BOLD:ACX3416
Coenosia|[13825]|BBDCN360-10|Muscidae|658|BOLD:ACX2633
Coenosia sp. 1 AKR|[13826]|SSBAA3343-12|Muscidae|673|BOLD:AAG1725
Coenosia impunctata|[13827]|BBDCN569-10|Muscidae|658|BOLD:AAQ0758
Coenosia|[13828]|BBDCM841-10|Muscidae|658|BOLD:ACX5946
Coenosia|[13829]|SSWLF3537-13|Muscidae|661|BOLD:ACK5182
Coenosia|[13830]|NGNAP288-14|Muscidae|686|BOLD:AAP6478
Coenosia conforma|[13831]|BBDCM405-10|Muscidae|658|BOLD:AAE8917
Coenosia|[13832]|BBDCM494-10|Muscidae|658|BOLD:AAG1730
Coenosia|[13833]|CNGME348-14|Muscidae|661|BOLD:ABZ7147
Coenosia intacta|[13834]|BBDEC434-09|Muscidae|658|BOLD:AAD7636
Coenosia longimaculata|[13835]|CNWLG906-12|Muscidae|670|BOLD:AAC1880
Coenosia laeta|[13836]|CNMIN378-14|Muscidae|658|BOLD:ACN2769
Coenosia|[13837]|BBDCM985-10|Muscidae|658|BOLD:AAE1399
Coenosia|[13838]|BBDED292-10|Muscidae|658|BOLD:AAC8885
Coenosia|[13839]|SSJAE3232-13|Muscidae|664|BOLD:ACJ5477
Coenosia comita|[13840]|BBDED526-10|Muscidae|658|BOLD:AAB7602
Coenosia octopunctata|[13841]|JWDCA444-10|Muscidae|658|BOLD:AAC6280
Coenosia|[13842]|CNIVD088-14|Muscidae|588|BOLD:ACR5728
Coenosia morrisoni|[13843]|JSYKA448-10|Muscidae|658|BOLD:AAM9102
Coenosia morrisoni|[13844]|SSBRA518-14|Muscidae|658|BOLD:ACA9137
Coenosia|[13845]|CNIVH041-14|Muscidae|555|BOLD:ACR8793
Coenosia|[13846]|BBDEC436-09|Muscidae|658|BOLD:AAG1766
Coenosia|[13847]|CNTUB2815-14|Muscidae|591|BOLD:ACR4815
Coenosia|[13848]|CNIVH357-14|Muscidae|552|BOLD:ACR6069
Coenosia remissa|[13849]|CNEIC1214-12|Muscidae|664|BOLD:AAM0327
Coenosia|[13850]|CNNHG588-14|Muscidae|555|BOLD:ACR7385
Coenosia flaviseta|[13851]|CNWAE1003-14|Muscidae|618|BOLD:ABY2665
Coenosia|[13852]|CNWBG1359-13|Muscidae|667|BOLD:ACE1169
Coenosia|[13853]|CNIVC417-14|Muscidae|642|BOLD:ACR4232
Coenosia|[13854]|BBDCM938-10|Muscidae|658|BOLD:AAP6479
Coenosia|[13855]|CNRVG1391-15|Muscidae|636|BOLD:ACB9516
Scatopsidae|[13856]|CNEIE1472-12|Scatopsidae|673|BOLD:ACB9452
Scatopsidae|[13857]|SSWLC4230-13|Scatopsidae|658|BOLD:ACI5738
Scatopsidae|[13858]|TTDBW416-09|Scatopsidae|658|BOLD:AAG6896
Scatopsidae|[13859]|CNFNG087-14|Scatopsidae|661|BOLD:AAH4122
Scatopsidae|[13860]|CNFDJ080-14|Scatopsidae|658|BOLD:ABV3202
Scatopsidae|[13861]|SSJAD5944-13|Scatopsidae|658|BOLD:ACJ0827
Scatopsidae|[13862]|JSDIR725-11|Scatopsidae|658|BOLD:ABA0927
Scatopsidae|[13863]|CNLMD775-14|Scatopsidae|552|BOLD:ACM3889
Scatopsidae|[13864]|BBDCM811-10|Scatopsidae|658|BOLD:AAV1140
Scatopsidae|[13865]|CNGBN2619-14|Scatopsidae|638|BOLD:ACV5714
Ectaetia|[13866]|JWDCF293-10|Scatopsidae|658|BOLD:ACY5612
Ectaetia|[13867]|JWDCF326-10|Scatopsidae|658|BOLD:AAP8768
Ectaetia|[13868]|JWDCF327-10|Scatopsidae|658|BOLD:ACY5611
Scatopsidae|[13869]|BBDEC639-09|Scatopsidae|658|BOLD:AAH4121
Scatopsidae|[13870]|DARC612-11|Scatopsidae|658|BOLD:AAU6744
Scatopsidae|[13871]|BBDCM584-10|Scatopsidae|658|BOLD:AAV1141
Scatopsidae|[13872]|CNKJP763-14|Scatopsidae|537|BOLD:ACM4098
Scatopsidae|[13873]|NGNAN3283-14|Scatopsidae|664|BOLD:ACJ7577
Scatopsidae|[13874]|SSKJB286-14|Scatopsidae|603|BOLD:ACP8643
Coboldia fuscipes|[13875]|JSDIP426-10|Scatopsidae|658|BOLD:AAN8523
Scatopsidae|[13876]|TTMDI1144-10|Scatopsidae|658|BOLD:AAN8522
Scatopsidae|[13877]|HEMAY965-12|Scatopsidae|554|BOLD:ABY0145
Scatopse|[13878]|JWDCB350-10|Scatopsidae|658|BOLD:AAH4119
Scatopsidae|[13879]|SMTPD5081-13|Scatopsidae|664|BOLD:ACG6999
Scatopsidae|[13880]|SMTPI1936-14|Scatopsidae|567|BOLD:ABV3201
Ectaetia|[13881]|JWDCJ1022-11|Scatopsidae|658|BOLD:AAV1137
Scatopsidae|[13882]|SSEIC1516-13|Scatopsidae|661|BOLD:ACI7782
Scatopsidae|[13883]|SSPAC431-13|Scatopsidae|658|BOLD:ACI6318
Scatopsidae|[13884]|CNFNR950-14|Scatopsidae|546|BOLD:ACM8266
Scatopsidae|[13885]|CHDIP241-11|Scatopsidae|658|BOLD:AAG6769
Scatopsidae|[13886]|CNPPI244-12|Scatopsidae|664|BOLD:AAV1136
Scatopsidae|[13887]|NGNAN2931-14|Scatopsidae|661|BOLD:ACI9567
Scatopsidae|[13888]|CNIVD707-14|Scatopsidae|558|BOLD:ACA2677
Hybotidae|[13889]|SSJAA320-13|Hybotidae|658|BOLD:ACG3204
Hybotidae|[13890]|CNPKF1789-14|Hybotidae|664|BOLD:AAQ0824
Empididae|[13891]|BBDCQ331-10|Empididae|658|BOLD:AAV3701
Empididae|[13892]|CNJAD983-12|Empididae|670|BOLD:AAF9781
Hesperempis mabelae|[13893]|SSWLA5131-13|Empididae|661|BOLD:ACA9506
Empididae|[13894]|CNKLB025-14|Empididae|606|BOLD:ACR7983
Oreothalia spinitarsis|[13895]|CNPCA014-12|Empididae|658|BOLD:ABA0527
Leptopeza|[13896]|BBDED765-10|Hybotidae|658|BOLD:AAF9779
Leptopeza flavipes|[13897]|JSDIQ594-10|Hybotidae|658|BOLD:AAF9791

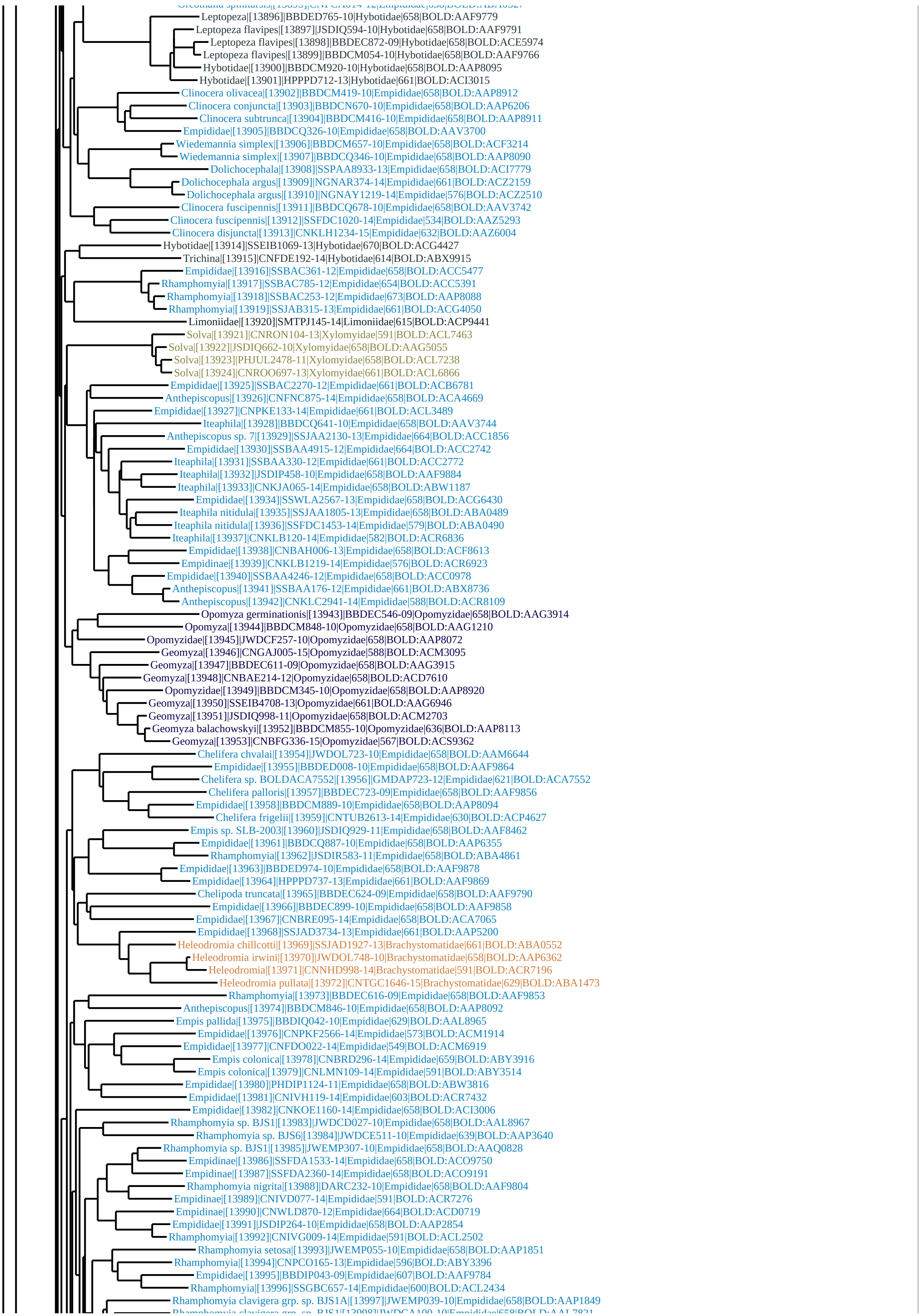

Leptopeza|[13896]|BBDED765-10|Hybotidae|658|BOLD:AAF9779
Leptopeza flavipes|[13897]|JSDIQ594-10|Hybotidae|658|BOLD:AAF9791
Leptopeza flavipes|[13898]|BBDEC872-09|Hybotidae|658|BOLD:ACE5974
Leptopeza flavipes|[13899]|BBDCM054-10|Hybotidae|658|BOLD:AAF9766
Hybotidae|[13900]|BBDCM920-10|Hybotidae|658|BOLD:AAP8095
Hybotidae|[13901]|HPPPD712-13|Hybotidae|661|BOLD:ACI3015
Clinocera olivacea|[13902]|BBDCM419-10|Empididae|658|BOLD:AAP8912
Clinocera conjuncta|[13903]|BBDCN670-10|Empididae|658|BOLD:AAP6206
Clinocera subtrunca|[13904]|BBDCM416-10|Empididae|658|BOLD:AAP8911
Empididae|[13905]|BBDCQ326-10|Empididae|658|BOLD:AAV3700
Wiedemannia simplex|[13906]|BBDCM657-10|Empididae|658|BOLD:ACF3214
Wiedemannia simplex|[13907]|BBDCQ346-10|Empididae|658|BOLD:AAP8090
Dolichocephala|[13908]|SSPAA8933-13|Empididae|658|BOLD:ACI7779
Dolichocephala argus|[13909]|NGNAR374-14|Empididae|661|BOLD:ACZ2159
Dolichocephala argus|[13910]|NGNAY1219-14|Empididae|576|BOLD:ACZ2510
Clinocera fuscipennis|[13911]|BBDCQ678-10|Empididae|658|BOLD:AAV3742
Clinocera fuscipennis|[13912]|SSFDC1020-14|Empididae|534|BOLD:AAZ5293
Clinocera disjuncta|[13913]|CNKLH1234-15|Empididae|632|BOLD:AAZ6004
Hybotidae|[13914]|SSEIB1069-13|Hybotidae|670|BOLD:ACG4427
Trichina|[13915]|CNFDE192-14|Hybotidae|614|BOLD:ABX9915
Empididae|[13916]|SSBAC361-12|Empididae|658|BOLD:ACC5477
Rhamphomyia|[13917]|SSBAC785-12|Empididae|654|BOLD:ACC5391
Rhamphomyia|[13918]|SSBAC253-12|Empididae|673|BOLD:AAP8088
Rhamphomyia|[13919]|SSJAB315-13|Empididae|661|BOLD:ACG4050
Limoniidae|[13920]|SMTPJ145-14|Limoniidae|615|BOLD:ACP9441
Solva|[13921]|CNRON104-13|Xylomyidae|591|BOLD:ACL7463
Solva|[13922]|JSDIQ662-10|Xylomyidae|658|BOLD:AAG5055
Solva|[13923]|PHJUL2478-11|Xylomyidae|658|BOLD:ACL7238
Solva|[13924]|CNROO697-13|Xylomyidae|661|BOLD:ACL6866
Empididae|[13925]|SSBAC2270-12|Empididae|661|BOLD:ACB6781
Anthepiscopus|[13926]|CNFNC875-14|Empididae|658|BOLD:ACA4669
Empididae|[13927]|CNPKE133-14|Empididae|661|BOLD:ACL3489
Iteaphila|[13928]|BBDCQ641-10|Empididae|658|BOLD:AAV3744
Anthepiscopus sp. 7|[13929]|SSJAA2130-13|Empididae|664|BOLD:ACC1856
Empididae|[13930]|SSBAA4915-12|Empididae|664|BOLD:ACC2742
Iteaphila|[13931]|SSBAA330-12|Empididae|661|BOLD:ACC2772
Iteaphila|[13932]|JSDIP458-10|Empididae|658|BOLD:AAF9884
Iteaphila|[13933]|CNKJA065-14|Empididae|658|BOLD:ABW1187
Empididae|[13934]|SSWLA2567-13|Empididae|658|BOLD:ACG6430
Iteaphila nitidula|[13935]|SSJAA1805-13|Empididae|658|BOLD:ABA0489
Iteaphila nitidula|[13936]|SSFDC1453-14|Empididae|579|BOLD:ABA0490
Iteaphila|[13937]|CNKLB120-14|Empididae|582|BOLD:ACR6836
Empididae|[13938]|CNBAH006-13|Empididae|658|BOLD:ACF8613
Empidinae|[13939]|CNKLB1219-14|Empididae|576|BOLD:ACR6923
Empididae|[13940]|SSBAA4246-12|Empididae|658|BOLD:ACC0978
Anthepiscopus|[13941]|SSBAA176-12|Empididae|661|BOLD:ABX8736
Anthepiscopus|[13942]|CNKLC2941-14|Empididae|588|BOLD:ACR8109
Opomyza germinationis|[13943]|BBDEC546-09|Opomyzidae|658|BOLD:AAG3914
Opomyza|[13944]|BBDCM848-10|Opomyzidae|658|BOLD:AAG1210
Opomyzidae|[13945]|JWDCF257-10|Opomyzidae|658|BOLD:AAP8072
Geomyza|[13946]|CNGAJ005-15|Opomyzidae|588|BOLD:ACM3095
Geomyza|[13947]|BBDEC611-09|Opomyzidae|658|BOLD:AAG3915
Geomyza|[13948]|CNBAE214-12|Opomyzidae|658|BOLD:ACD7610
Opomyzidae|[13949]|BBDCM345-10|Opomyzidae|658|BOLD:AAP8920
Geomyza|[13950]|SSEIB4708-13|Opomyzidae|661|BOLD:AAG6946
Geomyza|[13951]|JSDIQ998-11|Opomyzidae|658|BOLD:ACM2703
Geomyza balachowskyi|[13952]|BBDCM855-10|Opomyzidae|636|BOLD:AAP8113
Geomyza|[13953]|CNBFG336-15|Opomyzidae|567|BOLD:ACS9362
Chelifera chvalai|[13954]|JWDOL723-10|Empididae|658|BOLD:AAM6644
Empididae|[13955]|BBDED008-10|Empididae|658|BOLD:AAF9864
Chelifera sp. BOLDACA7552|[13956]|GMDAP723-12|Empididae|621|BOLD:ACA7552
Chelifera palloris|[13957]|BBDEC723-09|Empididae|658|BOLD:AAF9856
Empididae|[13958]|BBDCM889-10|Empididae|658|BOLD:AAP8094
Chelifera frigelii|[13959]|CNTUB2613-14|Empididae|630|BOLD:ACP4627
Empis sp. SLB-2003|[13960]|JSDIQ929-11|Empididae|658|BOLD:AAF8462
Empididae|[13961]|BBDCQ887-10|Empididae|658|BOLD:AAP6355
Rhamphomyia|[13962]|JSDIR583-11|Empididae|658|BOLD:ABA4861
Empididae|[13963]|BBDED974-10|Empididae|658|BOLD:AAF9878
Empididae|[13964]|HPPPD737-13|Empididae|661|BOLD:AAF9869
Chelipoda truncata|[13965]|BBDEC624-09|Empididae|658|BOLD:AAF9790
Empididae|[13966]|BBDEC899-10|Empididae|658|BOLD:AAF9858
Empididae|[13967]|CNBRE095-14|Empididae|658|BOLD:ACA7065
Empididae|[13968]|SSJAD3734-13|Empididae|661|BOLD:AAP5200
Heleodromia chillcotti|[13969]|SSJAD1927-13|Brachystomatidae|661|BOLD:ABA0552
Heleodromia irwini|[13970]|JWDOL748-10|Brachystomatidae|658|BOLD:AAP6362
Heleodromia|[13971]|CNNHD998-14|Brachystomatidae|591|BOLD:ACR7196
Heleodromia pullata|[13972]|CNTGC1646-15|Brachystomatidae|629|BOLD:ABA1473
Rhamphomyia|[13973]|BBDEC616-09|Empididae|658|BOLD:AAF9853
Anthepiscopus|[13974]|BBDCM846-10|Empididae|658|BOLD:AAP8092
Empis pallida|[13975]|BBDIQ042-10|Empididae|629|BOLD:AAL8965
Empididae|[13976]|CNPKF2566-14|Empididae|573|BOLD:ACM1914
Empididae|[13977]|CNFDO022-14|Empididae|549|BOLD:ACM6919
Empis colonica|[13978]|CNBRD296-14|Empididae|659|BOLD:ABY3916
Empis colonica|[13979]|CNLMN109-14|Empididae|591|BOLD:ABY3514
Empididae|[13980]|PHDIP1124-11|Empididae|658|BOLD:ABW3816
Empididae|[13981]|CNIVH119-14|Empididae|603|BOLD:ACR7432
Empididae|[13982]|CNKOE1160-14|Empididae|658|BOLD:ACI3006
Rhamphomyia sp. BJS1|[13983]|JWDCD027-10|Empididae|658|BOLD:AAL8967
Rhamphomyia sp. BJS6|[13984]|JWDCE511-10|Empididae|639|BOLD:AAP3640
Rhamphomyia sp. BJS1|[13985]|JWEMP307-10|Empididae|658|BOLD:AAQ0828
Empidinae|[13986]|SSFDA1533-14|Empididae|658|BOLD:ACO9750
Empidinae|[13987]|SSFDA2360-14|Empididae|658|BOLD:ACO9191
Rhamphomyia nigrita|[13988]|DARC232-10|Empididae|658|BOLD:AAF9804
Empidinae|[13989]|CNIVD077-14|Empididae|591|BOLD:ACR7276
Empidinae|[13990]|CNWLD870-12|Empididae|664|BOLD:ACD0719
Empididae|[13991]|JSDIP264-10|Empididae|658|BOLD:AAP2854
Rhamphomyia|[13992]|CNIVG009-14|Empididae|591|BOLD:ACL2502
Rhamphomyia setosa|[13993]|JWEMP055-10|Empididae|658|BOLD:AAP1851
Rhamphomyia|[13994]|CNPCO165-13|Empididae|596|BOLD:ABY3396
Empididae|[13995]|BBDIP043-09|Empididae|607|BOLD:AAF9784
Rhamphomyia|[13996]|SSGBC657-14|Empididae|600|BOLD:ACL2434
Rhamphomyia clavigera grp. sp. BJS1A|[13997]|JWEMP039-10|Empididae|658|BOLD:AAP1849

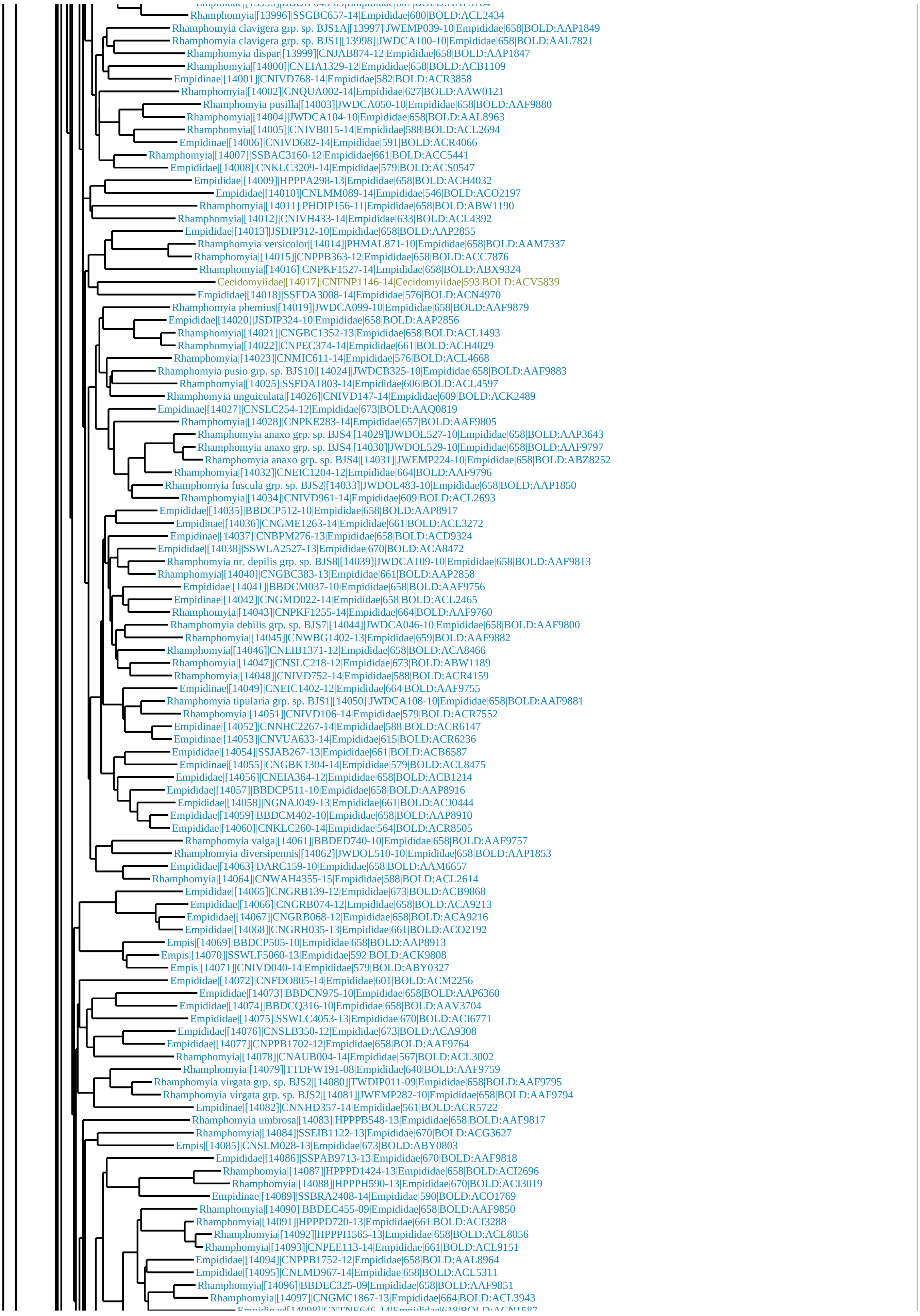
Rhamphomyia|[13996]|SSGBC657-14|Empididae|600|BOLD:ACL2434
Rhamphomyia clavigera grp. sp. BJS1A|[13997]|JWEMP039-10|Empididae|658|BOLD:AAP1849
Rhamphomyia clavigera grp. sp. BJS1|[13998]|JWDCA100-10|Empididae|658|BOLD:AAL7821
Rhamphomyia dispar|[13999]|CNJAB874-12|Empididae|658|BOLD:AAP1847
Rhamphomyia|[14000]|CNEIA1329-12|Empididae|658|BOLD:ACB1109
Empidinae|[14001]|CNIVD768-14|Empididae|582|BOLD:ACR3858
Rhamphomyia|[14002]|CNQUA002-14|Empididae|627|BOLD:AAW0121
Rhamphomyia pusilla|[14003]|JWDCA050-10|Empididae|658|BOLD:AAF9880
Rhamphomyia|[14004]|JWDCA104-10|Empididae|658|BOLD:AAL8963
Rhamphomyia|[14005]|CNIVB015-14|Empididae|588|BOLD:ACL2694
Empidinae|[14006]|CNIVD682-14|Empididae|591|BOLD:ACR4066
Rhamphomyia|[14007]|SSBAC3160-12|Empididae|661|BOLD:ACC5441
Empididae|[14008]|CNKLC3209-14|Empididae|579|BOLD:ACS0547
Empididae|[14009]|HPPPA298-13|Empididae|658|BOLD:ACH4032
Empididae|[14010]|CNLMM089-14|Empididae|546|BOLD:ACO2197
Rhamphomyia|[14011]|PHDIP156-11|Empididae|658|BOLD:ABW1190
Rhamphomyia|[14012]|CNIVH433-14|Empididae|633|BOLD:ACL4392
Empididae|[14013]|JSDIP312-10|Empididae|658|BOLD:AAP2855
Rhamphomyia versicolor|[14014]|PHMAL871-10|Empididae|658|BOLD:AAM7337
Rhamphomyia|[14015]|CNPPB363-12|Empididae|658|BOLD:ACC7876
Rhamphomyia|[14016]|CNPKF1527-14|Empididae|658|BOLD:ABX9324
Cecidomyiidae|[14017]|CNFNP1146-14|Cecidomyiidae|593|BOLD:ACV5839
Empididae|[14018]|SSFDA3008-14|Empididae|576|BOLD:ACN4970
Rhamphomyia phemius|[14019]|JWDCA099-10|Empididae|658|BOLD:AAF9879
Empididae|[14020]|JSDIP324-10|Empididae|658|BOLD:AAP2856
Rhamphomyia|[14021]|CNGBC1352-13|Empididae|658|BOLD:ACL1493
Rhamphomyia|[14022]|CNPEC374-14|Empididae|661|BOLD:ACH4029
Rhamphomyia|[14023]|CNMIC611-14|Empididae|576|BOLD:ACL4668
Rhamphomyia pusio grp. sp. BJS10|[14024]|JWDCB325-10|Empididae|658|BOLD:AAF9883
Rhamphomyia|[14025]|SSFDA1803-14|Empididae|606|BOLD:ACL4597
Rhamphomyia unguiculata|[14026]|CNIVD147-14|Empididae|609|BOLD:ACK2489
Empidinae|[14027]|CNSLC254-12|Empididae|673|BOLD:AAQ0819
Rhamphomyia|[14028]|CNPKE283-14|Empididae|657|BOLD:AAF9805
Rhamphomyia anaxo grp. sp. BJS4|[14029]|JWDOL527-10|Empididae|658|BOLD:AAP3643
Rhamphomyia anaxo grp. sp. BJS4|[14030]|JWDOL529-10|Empididae|658|BOLD:AAF9797
Rhamphomyia anaxo grp. sp. BJS4|[14031]|JWEMP224-10|Empididae|658|BOLD:ABZ8252
Rhamphomyia|[14032]|CNEIC1204-12|Empididae|664|BOLD:AAF9796
Rhamphomyia fuscula grp. sp. BJS2|[14033]|JWDOL483-10|Empididae|658|BOLD:AAP1850
Rhamphomyia|[14034]|CNIVD961-14|Empididae|609|BOLD:ACL2693
Empididae|[14035]|BBDCP512-10|Empididae|658|BOLD:AAP8917
Empidinae|[14036]|CNGME1263-14|Empididae|661|BOLD:ACL3272
Empididae|[14037]|CNBPM276-13|Empididae|658|BOLD:ACD9324
Empididae|[14038]|SSWLA2527-13|Empididae|670|BOLD:ACA8472
Rhamphomyia nr. depilis grp. sp. BJS8|[14039]|JWDCA109-10|Empididae|658|BOLD:AAF9813
Rhamphomyia|[14040]|CNGBC383-13|Empididae|661|BOLD:AAP2858
Empididae|[14041]|BBDCM037-10|Empididae|658|BOLD:AAF9756
Empidinae|[14042]|CNGMD022-14|Empididae|658|BOLD:ACL2465
Rhamphomyia|[14043]|CNPKF1255-14|Empididae|664|BOLD:AAF9760
Rhamphomyia debilis grp. sp. BJS7|[14044]|JWDCA046-10|Empididae|658|BOLD:AAF9800
Rhamphomyia|[14045]|CNWBG1402-13|Empididae|659|BOLD:AAF9882
Rhamphomyia|[14046]|CNEIB1371-12|Empididae|658|BOLD:ACA8466
Rhamphomyia|[14047]|CNSLC218-12|Empididae|673|BOLD:ABW1189
Rhamphomyia|[14048]|CNIVD752-14|Empididae|588|BOLD:ACR4159
Empidinae|[14049]|CNEIC1402-12|Empididae|664|BOLD:AAF9755
Rhamphomyia tipularia grp. sp. BJS1|[14050]|JWDCA108-10|Empididae|658|BOLD:AAF9881
Rhamphomyia|[14051]|CNIVD106-14|Empididae|579|BOLD:ACR7552
Empidinae|[14052]|CNNHC2267-14|Empididae|588|BOLD:ACR6147
Empidinae|[14053]|CNVUA633-14|Empididae|615|BOLD:ACR6236
Empididae|[14054]|SSJAB267-13|Empididae|661|BOLD:ACB6587
Empidinae|[14055]|CNGBK1304-14|Empididae|579|BOLD:ACL8475
Empididae|[14056]|CNEIA364-12|Empididae|658|BOLD:ACB1214
Empididae|[14057]|BBDCP511-10|Empididae|658|BOLD:AAP8916
Empididae|[14058]|NGNAJ049-13|Empididae|661|BOLD:ACJ0444
Empididae|[14059]|BBDCM402-10|Empididae|658|BOLD:AAP8910
Empididae|[14060]|CNKLC260-14|Empididae|564|BOLD:ACR8505
Rhamphomyia valga|[14061]|BBDED740-10|Empididae|658|BOLD:AAF9757
Rhamphomyia diversipennis|[14062]|JWDOL510-10|Empididae|658|BOLD:AAP1853
Empididae|[14063]|DARC159-10|Empididae|658|BOLD:AAM6657
Rhamphomyia|[14064]|CNWAH4355-15|Empididae|588|BOLD:ACL2614
Empididae|[14065]|CNGRB139-12|Empididae|673|BOLD:ACB9868
Empididae|[14066]|CNGRB074-12|Empididae|658|BOLD:ACA9213
Empididae|[14067]|CNGRB068-12|Empididae|658|BOLD:ACA9216
Empididae|[14068]|CNGRH035-13|Empididae|661|BOLD:ACO2192
Empis|[14069]|BBDCP505-10|Empididae|658|BOLD:AAP8913
Empis|[14070]|SSWLF5060-13|Empididae|592|BOLD:ACK9808
Empis|[14071]|CNIVD040-14|Empididae|579|BOLD:ABY0327
Empididae|[14072]|CNFDO805-14|Empididae|601|BOLD:ACM2256
Empididae|[14073]|BBDCN975-10|Empididae|658|BOLD:AAP6360
Empididae|[14074]|BBDCQ316-10|Empididae|658|BOLD:AAV3704
Empididae|[14075]|SSWLC4053-13|Empididae|670|BOLD:ACI6771
Empididae|[14076]|CNSLB350-12|Empididae|673|BOLD:ACA9308
Empididae|[14077]|CNPPB1702-12|Empididae|658|BOLD:AAF9764
Rhamphomyia|[14078]|CNAUB004-14|Empididae|567|BOLD:ACL3002
Rhamphomyia|[14079]|TTDFW191-08|Empididae|640|BOLD:AAF9759
Rhamphomyia virgata grp. sp. BJS2|[14080]|TWDIP011-09|Empididae|658|BOLD:AAF9795
Rhamphomyia virgata grp. sp. BJS2|[14081]|JWEMP282-10|Empididae|658|BOLD:AAF9794
Empidinae|[14082]|CNNHD357-14|Empididae|561|BOLD:ACR5722
Rhamphomyia umbrosa|[14083]|HPPPB548-13|Empididae|658|BOLD:AAF9817
Rhamphomyia|[14084]|SSEIB1122-13|Empididae|670|BOLD:ACG3627
Empis|[14085]|CNSLM028-13|Empididae|673|BOLD:ABY0803
Empididae|[14086]|SSPAB9713-13|Empididae|670|BOLD:AAF9818
Rhamphomyia|[14087]|HPPPD1424-13|Empididae|658|BOLD:ACI2696
Rhamphomyia|[14088]|HPPPH590-13|Empididae|670|BOLD:ACI3019
Empidinae|[14089]|SSBRA2408-14|Empididae|590|BOLD:ACO1769
Rhamphomyia|[14090]|BBDEC455-09|Empididae|658|BOLD:AAF9850
Rhamphomyia|[14091]|HPPPD720-13|Empididae|661|BOLD:ACI3288
Rhamphomyia|[14092]|HPPPI1565-13|Empididae|658|BOLD:ACL8056
Rhamphomyia|[14093]|CNPEE113-14|Empididae|661|BOLD:ACL9151
Empididae|[14094]|CNPPB1752-12|Empididae|658|BOLD:AAL8964
Empididae|[14095]|CNLMD967-14|Empididae|658|BOLD:ACL5311
Rhamphomyia|[14096]|BBDEC325-09|Empididae|658|BOLD:AAF9851
Rhamphomyia|[14097]|CNGMC1867-13|Empididae|664|BOLD:ACL3943
Empidinae|[14098]|CNTNE646-14|Empididae|618|BOLD:ACN1587

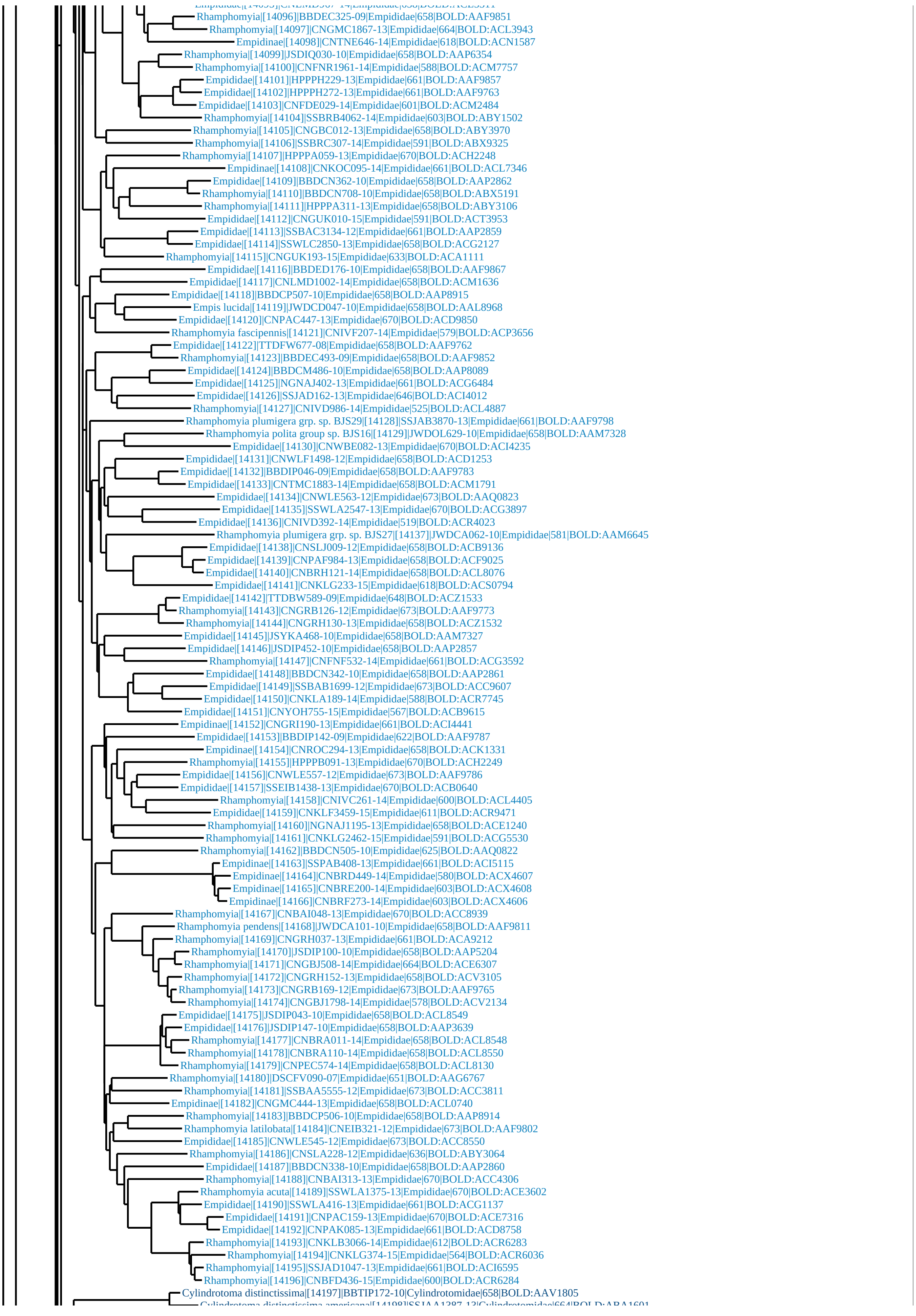

Rhamphomyia|[14096]|BBDEC325-09|Empididae|658|BOLD:AAF9851
Rhamphomyia|[14097]|CNGMC1867-13|Empididae|664|BOLD:ACL3943
Empidinae|[14098]|CNTNE646-14|Empididae|618|BOLD:ACN1587
Rhamphomyia|[14099]|JSDIQ030-10|Empididae|658|BOLD:AAP6354
Rhamphomyia|[14100]|CNFNR1961-14|Empididae|588|BOLD:ACM7757
Empididae|[14101]|HPPPH229-13|Empididae|661|BOLD:AAF9857
Empididae|[14102]|HPPPH272-13|Empididae|661|BOLD:AAF9763
Empididae|[14103]|CNFDE029-14|Empididae|601|BOLD:ACM2484
Rhamphomyia|[14104]|SSBRB4062-14|Empididae|603|BOLD:ABY1502
Rhamphomyia|[14105]|CNGBC012-13|Empididae|658|BOLD:ABY3970
Rhamphomyia|[14106]|SSBRC307-14|Empididae|591|BOLD:ABX9325
Rhamphomyia|[14107]|HPPPA059-13|Empididae|670|BOLD:ACH2248
Empidinae|[14108]|CNKOC095-14|Empididae|661|BOLD:ACL7346
Empididae|[14109]|BBDCN362-10|Empididae|658|BOLD:AAP2862
Rhamphomyia|[14110]|BBDCN708-10|Empididae|658|BOLD:ABX5191
Rhamphomyia|[14111]|HPPPA311-13|Empididae|658|BOLD:ABY3106
Empididae|[14112]|CNGUK010-15|Empididae|591|BOLD:ACT3953
Empididae|[14113]|SSBAC3134-12|Empididae|661|BOLD:AAP2859
Empididae|[14114]|SSWLC2850-13|Empididae|658|BOLD:ACG2127
Rhamphomyia|[14115]|CNGUK193-15|Empididae|633|BOLD:ACA1111
Empididae|[14116]|BBDED176-10|Empididae|658|BOLD:AAF9867
Empididae|[14117]|CNLMD1002-14|Empididae|658|BOLD:ACM1636
Empididae|[14118]|BBDCP507-10|Empididae|658|BOLD:AAP8915
Empis lucida|[14119]|JWDCD047-10|Empididae|658|BOLD:AAL8968
Empididae|[14120]|CNPAC447-13|Empididae|670|BOLD:ACD9850
Rhamphomyia fascipennis|[14121]|CNIVF207-14|Empididae|579|BOLD:ACP3656
Empididae|[14122]|TTDFW677-08|Empididae|658|BOLD:AAF9762
Rhamphomyia|[14123]|BBDEC493-09|Empididae|658|BOLD:AAF9852
Empididae|[14124]|BBDCM486-10|Empididae|658|BOLD:AAP8089
Empididae|[14125]|NGNAJ402-13|Empididae|661|BOLD:ACG6484
Empididae|[14126]|SSJAD162-13|Empididae|646|BOLD:ACI4012
Rhamphomyia|[14127]|CNIVD986-14|Empididae|525|BOLD:ACL4887
Rhamphomyia plumigera grp. sp. BJS29|[14128]|SSJAB3870-13|Empididae|661|BOLD:AAF9798
Rhamphomyia polita group sp. BJS16|[14129]|JWDOL629-10|Empididae|658|BOLD:AAM7328
Empididae|[14130]|CNWBE082-13|Empididae|670|BOLD:ACI4235
Empididae|[14131]|CNWLF1498-12|Empididae|658|BOLD:ACD1253
Empididae|[14132]|BBDIP046-09|Empididae|658|BOLD:AAF9783
Empididae|[14133]|CNTMC1883-14|Empididae|658|BOLD:ACM1791
Empididae|[14134]|CNWLE563-12|Empididae|673|BOLD:AAQ0823
Empididae|[14135]|SSWLA2547-13|Empididae|670|BOLD:ACG3897
Empididae|[14136]|CNIVD392-14|Empididae|519|BOLD:ACR4023
Rhamphomyia plumigera grp. sp. BJS27|[14137]|JWDCA062-10|Empididae|581|BOLD:AAM6645
Empididae|[14138]|CNSLJ009-12|Empididae|658|BOLD:ACB9136
Empididae|[14139]|CNPAF984-13|Empididae|658|BOLD:ACF9025
Empididae|[14140]|CNBRH121-14|Empididae|658|BOLD:ACL8076
Empididae|[14141]|CNKLG233-15|Empididae|618|BOLD:ACS0794
Empididae|[14142]|TTDBW589-09|Empididae|648|BOLD:ACZ1533
Rhamphomyia|[14143]|CNGRB126-12|Empididae|673|BOLD:AAF9773
Rhamphomyia|[14144]|CNGRH130-13|Empididae|658|BOLD:ACZ1532
Empididae|[14145]|JSYKA468-10|Empididae|658|BOLD:AAM7327
Empididae|[14146]|JSDIP452-10|Empididae|658|BOLD:AAP2857
Rhamphomyia|[14147]|CNFNF532-14|Empididae|661|BOLD:ACG3592
Empididae|[14148]|BBDCN342-10|Empididae|658|BOLD:AAP2861
Empididae|[14149]|SSBAB1699-12|Empididae|673|BOLD:ACC9607
Empididae|[14150]|CNKLA189-14|Empididae|588|BOLD:ACR7745
Empididae|[14151]|CNYOH755-15|Empididae|567|BOLD:ACB9615
Empidinae|[14152]|CNGRI190-13|Empididae|661|BOLD:ACI4441
Empididae|[14153]|BBDIP142-09|Empididae|622|BOLD:AAF9787
Empidinae|[14154]|CNROC294-13|Empididae|658|BOLD:ACK1331
Rhamphomyia|[14155]|HPPPB091-13|Empididae|670|BOLD:ACH2249
Empididae|[14156]|CNWLE557-12|Empididae|673|BOLD:AAF9786
Empididae|[14157]|SSEIB1438-13|Empididae|670|BOLD:ACB0640
Rhamphomyia|[14158]|CNIVC261-14|Empididae|600|BOLD:ACL4405
Empididae|[14159]|CNKLF3459-15|Empididae|611|BOLD:ACR9471
Rhamphomyia|[14160]|NGNAJ1195-13|Empididae|658|BOLD:ACE1240
Rhamphomyia|[14161]|CNKLG2462-15|Empididae|591|BOLD:ACG5530
Rhamphomyia|[14162]|BBDCN505-10|Empididae|625|BOLD:AAQ0822
Empidinae|[14163]|SSPAB408-13|Empididae|661|BOLD:ACI5115
Empidinae|[14164]|CNBRD449-14|Empididae|580|BOLD:ACX4607
Empidinae|[14165]|CNBRE200-14|Empididae|603|BOLD:ACX4608
Empidinae|[14166]|CNBRF273-14|Empididae|603|BOLD:ACX4606
Rhamphomyia|[14167]|CNBAI048-13|Empididae|670|BOLD:ACC8939
Rhamphomyia pendens|[14168]|JWDCA101-10|Empididae|658|BOLD:AAF9811
Rhamphomyia|[14169]|CNGRH037-13|Empididae|661|BOLD:ACA9212
Rhamphomyia|[14170]|JSDIP100-10|Empididae|658|BOLD:AAP5204
Rhamphomyia|[14171]|CNGBJ508-14|Empididae|664|BOLD:ACE6307
Rhamphomyia|[14172]|CNGRH152-13|Empididae|658|BOLD:ACV3105
Rhamphomyia|[14173]|CNGRB169-12|Empididae|673|BOLD:AAF9765
Rhamphomyia|[14174]|CNGBJ1798-14|Empididae|578|BOLD:ACV2134
Empididae|[14175]|JSDIP043-10|Empididae|658|BOLD:ACL8549
Empididae|[14176]|JSDIP147-10|Empididae|658|BOLD:AAP3639
Rhamphomyia|[14177]|CNBRA011-14|Empididae|658|BOLD:ACL8548
Rhamphomyia|[14178]|CNBRA110-14|Empididae|658|BOLD:ACL8550
Rhamphomyia|[14179]|CNPEC574-14|Empididae|658|BOLD:ACL8130
Rhamphomyia|[14180]|DSCFV090-07|Empididae|651|BOLD:AAG6767
Rhamphomyia|[14181]|SSBAA5555-12|Empididae|673|BOLD:ACC3811
Empidinae|[14182]|CNGMC444-13|Empididae|658|BOLD:ACL0740
Rhamphomyia|[14183]|BBDCP506-10|Empididae|658|BOLD:AAP8914
Rhamphomyia latilobata|[14184]|CNEIB321-12|Empididae|673|BOLD:AAF9802
Empididae|[14185]|CNWLE545-12|Empididae|673|BOLD:ACC8550
Rhamphomyia|[14186]|CNSLA228-12|Empididae|636|BOLD:ABY3064
Empididae|[14187]|BBDCN338-10|Empididae|658|BOLD:AAP2860
Rhamphomyia|[14188]|CNBAI313-13|Empididae|670|BOLD:ACC4306
Rhamphomyia acuta|[14189]|SSWLA1375-13|Empididae|670|BOLD:ACE3602
Empididae|[14190]|SSWLA416-13|Empididae|661|BOLD:ACG1137
Empididae|[14191]|CNPAC159-13|Empididae|670|BOLD:ACE7316
Empididae|[14192]|CNPAK085-13|Empididae|661|BOLD:ACD8758
Rhamphomyia|[14193]|CNKLB3066-14|Empididae|612|BOLD:ACR6283
Rhamphomyia|[14194]|CNKLG374-15|Empididae|564|BOLD:ACR6036
Rhamphomyia|[14195]|SSJAD1047-13|Empididae|661|BOLD:ACI6595
Rhamphomyia|[14196]|CNBFD436-15|Empididae|600|BOLD:ACR6284
Cylindrotoma distinctissima|[14197]|BBTIP172-10|Cylindrotomidae|658|BOLD:AAV1805

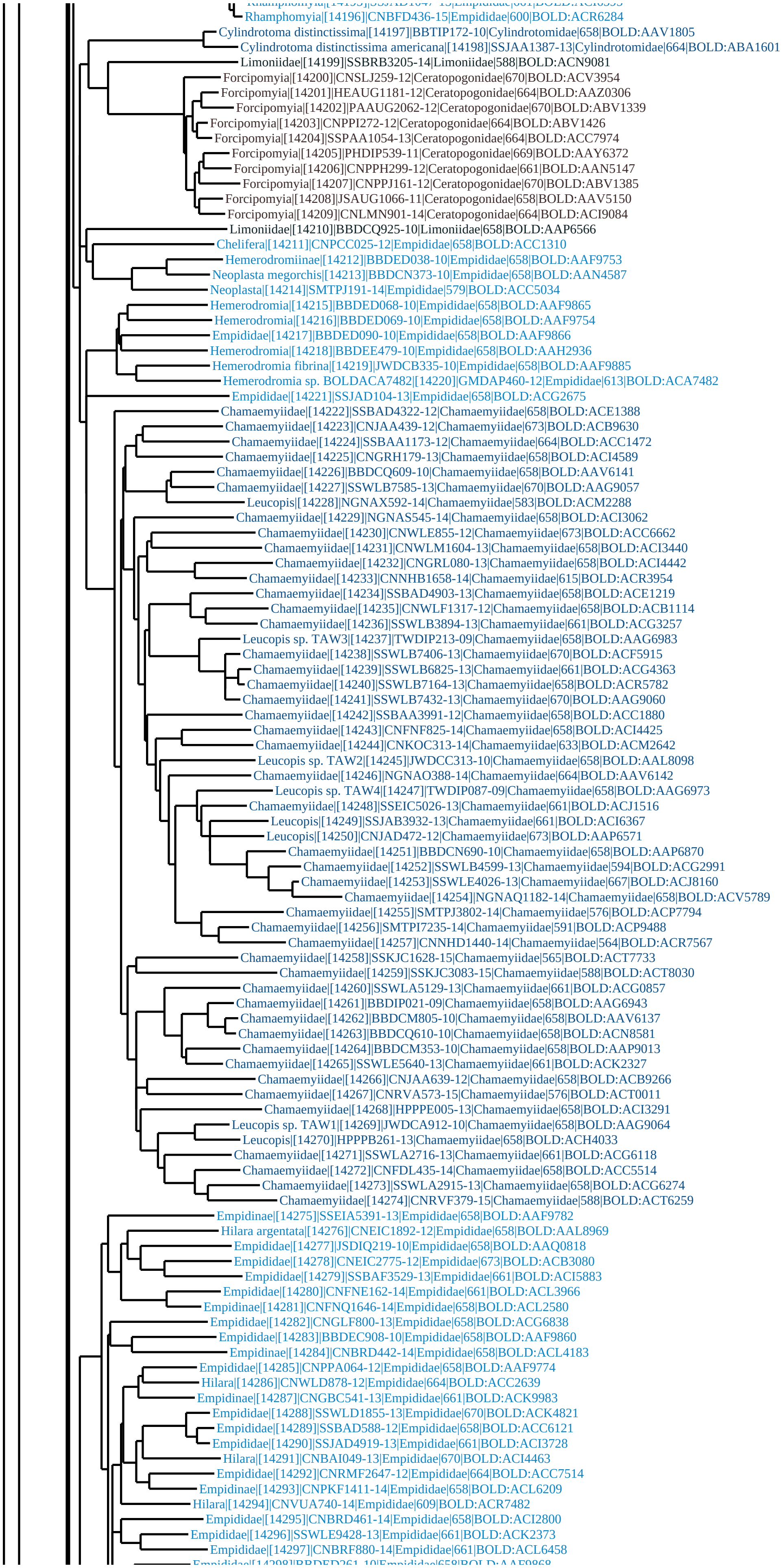

Rhamphomyia|[14196]|CNBFD436-15|Empididae|600|BOLD:ACR6284
Cylindrotoma distinctissima|[14197]|BBTIP172-10|Cylindrotomidae|658|BOLD:AAV1805
Cylindrotoma distinctissima americana|[14198]|SSJAA1387-13|Cylindrotomidae|664|BOLD:ABA1601
Limoniidae|[14199]|SSBRB3205-14|Limoniidae|588|BOLD:ACN9081
Forcipomyia|[14200]|CNSLJ259-12|Ceratopogonidae|670|BOLD:ACV3954
Forcipomyia|[14201]|HEAUG1181-12|Ceratopogonidae|664|BOLD:AAZ0306
Forcipomyia|[14202]|PAAUG2062-12|Ceratopogonidae|670|BOLD:ABV1339
Forcipomyia|[14203]|CNPPI272-12|Ceratopogonidae|664|BOLD:ABV1426
Forcipomyia|[14204]|SSPAA1054-13|Ceratopogonidae|664|BOLD:ACC7974
Forcipomyia|[14205]|PHDIP539-11|Ceratopogonidae|669|BOLD:AAY6372
Forcipomyia|[14206]|CNPPH299-12|Ceratopogonidae|661|BOLD:AAN5147
Forcipomyia|[14207]|CNPPJ161-12|Ceratopogonidae|670|BOLD:ABV1385
Forcipomyia|[14208]|JSAUG1066-11|Ceratopogonidae|658|BOLD:AAV5150
Forcipomyia|[14209]|CNLMN901-14|Ceratopogonidae|664|BOLD:ACI9084
Limoniidae|[14210]|BBDCQ925-10|Limoniidae|658|BOLD:AAP6566
Chelifera|[14211]|CNPCC025-12|Empididae|658|BOLD:ACC1310
Hemerodromiinae|[14212]|BBDED038-10|Empididae|658|BOLD:AAF9753
Neoplasta megorchis|[14213]|BBDCN373-10|Empididae|658|BOLD:AAN4587
Neoplasta|[14214]|SMTPJ191-14|Empididae|579|BOLD:ACC5034
Hemerodromia|[14215]|BBDED068-10|Empididae|658|BOLD:AAF9865
Hemerodromia|[14216]|BBDED069-10|Empididae|658|BOLD:AAF9754
Empididae|[14217]|BBDED090-10|Empididae|658|BOLD:AAF9866
Hemerodromia|[14218]|BBDEE479-10|Empididae|658|BOLD:AAH2936
Hemerodromia fibrina|[14219]|JWDCB335-10|Empididae|658|BOLD:AAF9885
Hemerodromia sp. BOLDACA7482|[14220]|GMDAP460-12|Empididae|613|BOLD:ACA7482
Empididae|[14221]|SSJAD104-13|Empididae|658|BOLD:ACG2675
Chamaemyiidae|[14222]|SSBAD4322-12|Chamaemyiidae|658|BOLD:ACE1388
Chamaemyiidae|[14223]|CNJAA439-12|Chamaemyiidae|673|BOLD:ACB9630
Chamaemyiidae|[14224]|SSBAA1173-12|Chamaemyiidae|664|BOLD:ACC1472
Chamaemyiidae|[14225]|CNGRH179-13|Chamaemyiidae|658|BOLD:ACI4589
Chamaemyiidae|[14226]|BBDCQ609-10|Chamaemyiidae|658|BOLD:AAV6141
Chamaemyiidae|[14227]|SSWLB7585-13|Chamaemyiidae|670|BOLD:AAG9057
Leucopis|[14228]|NGNAX592-14|Chamaemyiidae|583|BOLD:ACM2288
Chamaemyiidae|[14229]|NGNAS545-14|Chamaemyiidae|658|BOLD:ACI3062
Chamaemyiidae|[14230]|CNWLE855-12|Chamaemyiidae|673|BOLD:ACC6662
Chamaemyiidae|[14231]|CNWLM1604-13|Chamaemyiidae|658|BOLD:ACI3440
Chamaemyiidae|[14232]|CNGRL080-13|Chamaemyiidae|658|BOLD:ACI4442
Chamaemyiidae|[14233]|CNNHB1658-14|Chamaemyiidae|615|BOLD:ACR3954
Chamaemyiidae|[14234]|SSBAD4903-13|Chamaemyiidae|658|BOLD:ACE1219
Chamaemyiidae|[14235]|CNWLF1317-12|Chamaemyiidae|658|BOLD:ACB1114
Chamaemyiidae|[14236]|SSWLB3894-13|Chamaemyiidae|661|BOLD:ACG3257
Leucopis sp. TAW3|[14237]|TWDIP213-09|Chamaemyiidae|658|BOLD:AAG6983
Chamaemyiidae|[14238]|SSWLB7406-13|Chamaemyiidae|670|BOLD:ACF5915
Chamaemyiidae|[14239]|SSWLB6825-13|Chamaemyiidae|661|BOLD:ACG4363
Chamaemyiidae|[14240]|SSWLB7164-13|Chamaemyiidae|658|BOLD:ACR5782
Chamaemyiidae|[14241]|SSWLB7432-13|Chamaemyiidae|670|BOLD:AAG9060
Chamaemyiidae|[14242]|SSBAA3991-12|Chamaemyiidae|658|BOLD:ACC1880
Chamaemyiidae|[14243]|CNFNF825-14|Chamaemyiidae|658|BOLD:ACI4425
Chamaemyiidae|[14244]|CNKOC313-14|Chamaemyiidae|633|BOLD:ACM2642
Leucopis sp. TAW2|[14245]|JWDCC313-10|Chamaemyiidae|658|BOLD:AAL8098
Chamaemyiidae|[14246]|NGNAO388-14|Chamaemyiidae|664|BOLD:AAV6142
Leucopis sp. TAW4|[14247]|TWDIP087-09|Chamaemyiidae|658|BOLD:AAG6973
Chamaemyiidae|[14248]|SSEIC5026-13|Chamaemyiidae|661|BOLD:ACJ1516
Leucopis|[14249]|SSJAB3932-13|Chamaemyiidae|661|BOLD:ACI6367
Leucopis|[14250]|CNJAD472-12|Chamaemyiidae|673|BOLD:AAP6571
Chamaemyiidae|[14251]|BBDCN690-10|Chamaemyiidae|658|BOLD:AAP6870
Chamaemyiidae|[14252]|SSWLB4599-13|Chamaemyiidae|594|BOLD:ACG2991
Chamaemyiidae|[14253]|SSWLE4026-13|Chamaemyiidae|667|BOLD:ACJ8160
Chamaemyiidae|[14254]|NGNAQ1182-14|Chamaemyiidae|658|BOLD:ACV5789
Chamaemyiidae|[14255]|SMTPJ3802-14|Chamaemyiidae|576|BOLD:ACP7794
Chamaemyiidae|[14256]|SMTPI7235-14|Chamaemyiidae|591|BOLD:ACP9488
Chamaemyiidae|[14257]|CNNHD1440-14|Chamaemyiidae|564|BOLD:ACR7567
Chamaemyiidae|[14258]|SSKJC1628-15|Chamaemyiidae|565|BOLD:ACT7733
Chamaemyiidae|[14259]|SSKJC3083-15|Chamaemyiidae|588|BOLD:ACT8030
Chamaemyiidae|[14260]|SSWLA5129-13|Chamaemyiidae|661|BOLD:ACG0857
Chamaemyiidae|[14261]|BBDIP021-09|Chamaemyiidae|658|BOLD:AAG6943
Chamaemyiidae|[14262]|BBDCM805-10|Chamaemyiidae|658|BOLD:AAV6137
Chamaemyiidae|[14263]|BBDCQ610-10|Chamaemyiidae|658|BOLD:ACN8581
Chamaemyiidae|[14264]|BBDCM353-10|Chamaemyiidae|658|BOLD:AAP9013
Chamaemyiidae|[14265]|SSWLE5640-13|Chamaemyiidae|661|BOLD:ACK2327
Chamaemyiidae|[14266]|CNJAA639-12|Chamaemyiidae|658|BOLD:ACB9266
Chamaemyiidae|[14267]|CNRVA573-15|Chamaemyiidae|576|BOLD:ACT0011
Chamaemyiidae|[14268]|HPPPE005-13|Chamaemyiidae|658|BOLD:ACI3291
Leucopis sp. TAW1|[14269]|JWDCA912-10|Chamaemyiidae|658|BOLD:AAG9064
Leucopis|[14270]|HPPPB261-13|Chamaemyiidae|658|BOLD:ACH4033
Chamaemyiidae|[14271]|SSWLA2716-13|Chamaemyiidae|661|BOLD:ACG6118
Chamaemyiidae|[14272]|CNFDL435-14|Chamaemyiidae|658|BOLD:ACC5514
Chamaemyiidae|[14273]|SSWLA2915-13|Chamaemyiidae|658|BOLD:ACG6274
Chamaemyiidae|[14274]|CNRVF379-15|Chamaemyiidae|588|BOLD:ACT6259
Empidinae|[14275]|SSEIA5391-13|Empididae|658|BOLD:AAF9782
Hilara argentata|[14276]|CNEIC1892-12|Empididae|658|BOLD:AAL8969
Empididae|[14277]|JSDIQ219-10|Empididae|658|BOLD:AAQ0818
Empididae|[14278]|CNEIC2775-12|Empididae|673|BOLD:ACB3080
Empididae|[14279]|SSBAF3529-13|Empididae|661|BOLD:ACI5883
Empididae|[14280]|CNFNE162-14|Empididae|661|BOLD:ACL3966
Empidinae|[14281]|CNFNQ1646-14|Empididae|658|BOLD:ACL2580
Empididae|[14282]|CNGLF800-13|Empididae|658|BOLD:ACG6838
Empididae|[14283]|BBDEC908-10|Empididae|658|BOLD:AAF9860
Empidinae|[14284]|CNBRD442-14|Empididae|658|BOLD:ACL4183
Empididae|[14285]|CNPPA064-12|Empididae|658|BOLD:AAF9774
Hilara|[14286]|CNWLD878-12|Empididae|664|BOLD:ACC2639
Empidinae|[14287]|CNGBC541-13|Empididae|661|BOLD:ACK9983
Empididae|[14288]|SSWLD1855-13|Empididae|670|BOLD:ACK4821
Empididae|[14289]|SSBAD588-12|Empididae|658|BOLD:ACC6121
Empididae|[14290]|SSJAD4919-13|Empididae|661|BOLD:ACI3728
Hilara|[14291]|CNBAI049-13|Empididae|670|BOLD:ACI4463
Empididae|[14292]|CNRMF2647-12|Empididae|664|BOLD:ACC7514
Empidinae|[14293]|CNPKF1411-14|Empididae|658|BOLD:ACL6209
Hilara|[14294]|CNVUA740-14|Empididae|609|BOLD:ACR7482
Empididae|[14295]|CNBRD461-14|Empididae|658|BOLD:ACI2800
Empididae|[14296]|SSWLE9428-13|Empididae|661|BOLD:ACK2373
Empididae|[14297]|CNBRF880-14|Empididae|661|BOLD:ACL6458

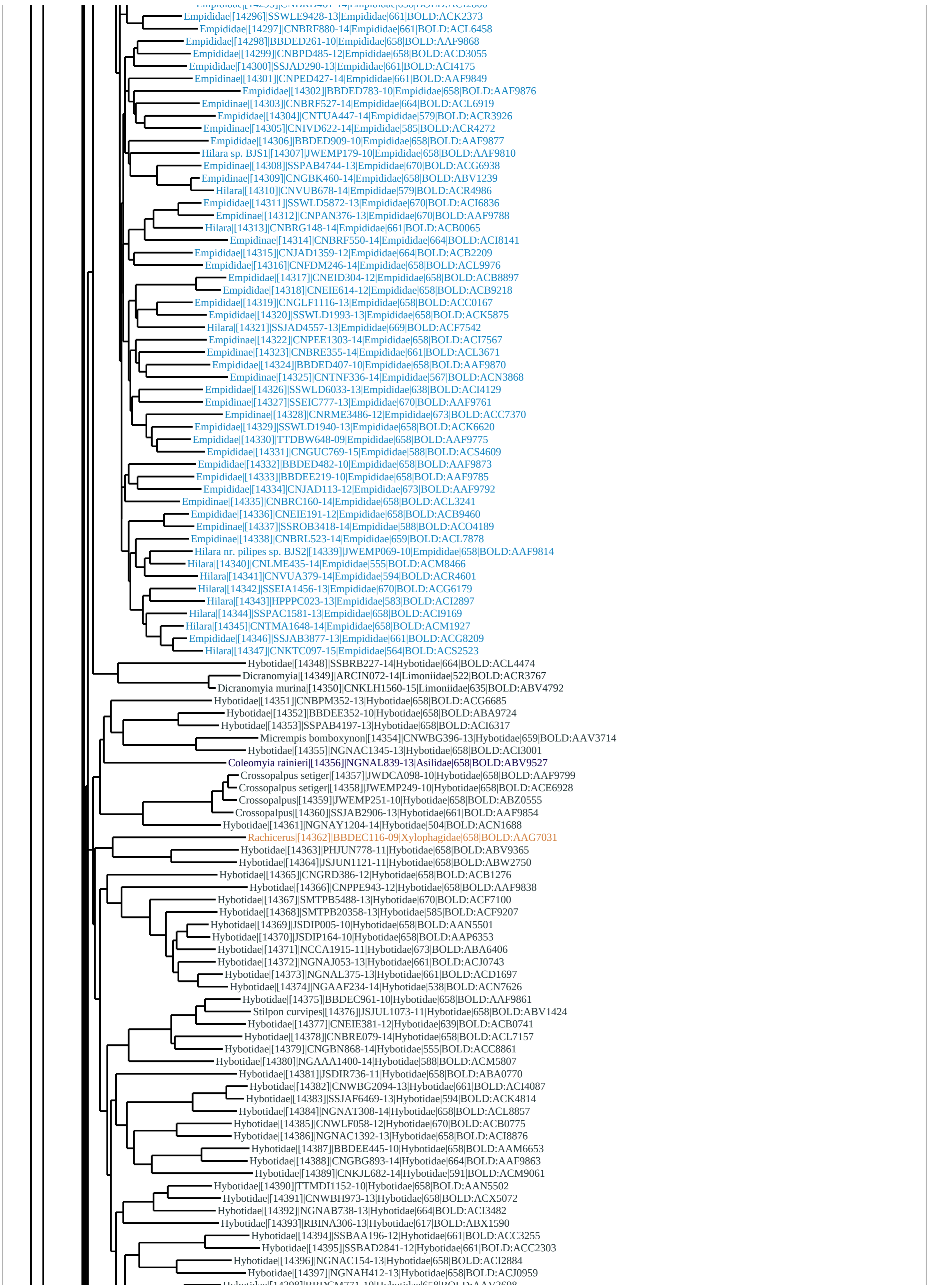
Empididae|[14296]|SSWLE9428-13|Empididae|661|BOLD:ACK2373
Empididae|[14297]|CNBRF880-14|Empididae|661|BOLD:ACL6458
Empididae|[14298]|BBDED261-10|Empididae|658|BOLD:AAF9868
Empididae|[14299]|CNBPD485-12|Empididae|658|BOLD:ACD3055
Empididae|[14300]|SSJAD290-13|Empididae|661|BOLD:ACI4175
Empidinae|[14301]|CNPED427-14|Empididae|661|BOLD:AAF9849
Empididae|[14302]|BBDED783-10|Empididae|658|BOLD:AAF9876
Empidinae|[14303]|CNBRF527-14|Empididae|664|BOLD:ACL6919
Empididae|[14304]|CNTUA447-14|Empididae|579|BOLD:ACR3926
Empidinae|[14305]|CNIVD622-14|Empididae|585|BOLD:ACR4272
Empididae|[14306]|BBDED909-10|Empididae|658|BOLD:AAF9877
Hilara sp. BJS1|[14307]|JWEMP179-10|Empididae|658|BOLD:AAF9810
Empidinae|[14308]|SSPAB4744-13|Empididae|670|BOLD:ACG6938
Empidinae|[14309]|CNGBK460-14|Empididae|658|BOLD:ABV1239
Hilara|[14310]|CNVUB678-14|Empididae|579|BOLD:ACR4986
Empidinae|[14311]|SSWLD5872-13|Empididae|670|BOLD:ACI6836
Empidinae|[14312]|CNPAN376-13|Empididae|670|BOLD:AAF9788
Hilara|[14313]|CNBRG148-14|Empididae|661|BOLD:ACB0065
Empidinae|[14314]|CNBRF550-14|Empididae|664|BOLD:ACI8141
Empididae|[14315]|CNJAD1359-12|Empididae|664|BOLD:ACB2209
Empidinae|[14316]|CNFDM246-14|Empididae|658|BOLD:ACL9976
Empididae|[14317]|CNEID304-12|Empididae|658|BOLD:ACB8897
Empididae|[14318]|CNEIE614-12|Empididae|658|BOLD:ACB9218
Empididae|[14319]|CNGLF1116-13|Empididae|658|BOLD:ACC0167
Empidinae|[14320]|SSWLD1993-13|Empididae|658|BOLD:ACK5875
Hilara|[14321]|SSJAD4557-13|Empididae|669|BOLD:ACF7542
Empidinae|[14322]|CNPEE1303-14|Empididae|658|BOLD:ACI7567
Empidinae|[14323]|CNBRE355-14|Empididae|661|BOLD:ACL3671
Empididae|[14324]|BBDED407-10|Empididae|658|BOLD:AAF9870
Empidinae|[14325]|CNTNF336-14|Empididae|567|BOLD:ACN3868
Empidinae|[14326]|SSWLD6033-13|Empididae|638|BOLD:ACI4129
Empidinae|[14327]|SSEIC777-13|Empididae|670|BOLD:AAF9761
Empidinae|[14328]|CNRME3486-12|Empididae|673|BOLD:ACC7370
Empididae|[14329]|SSWLD1940-13|Empididae|658|BOLD:ACK6620
Empididae|[14330]|TTDBW648-09|Empididae|658|BOLD:AAF9775
Empidinae|[14331]|CNGUC769-15|Empididae|588|BOLD:ACS4609
Empididae|[14332]|BBDED482-10|Empididae|658|BOLD:AAF9873
Empididae|[14333]|BBDEE219-10|Empididae|658|BOLD:AAF9785
Empidinae|[14334]|CNJAD113-12|Empididae|673|BOLD:AAF9792
Empidinae|[14335]|CNBRC160-14|Empididae|658|BOLD:ACL3241
Empididae|[14336]|CNEIE191-12|Empididae|658|BOLD:ACB9460
Empidinae|[14337]|SSROB3418-14|Empididae|588|BOLD:ACO4189
Empidinae|[14338]|CNBRL523-14|Empididae|659|BOLD:ACL7878
Hilara nr. pilipes sp. BJS2|[14339]|JWEMP069-10|Empididae|658|BOLD:AAF9814
Hilara|[14340]|CNLME435-14|Empididae|555|BOLD:ACM8466
Hilara|[14341]|CNVUA379-14|Empididae|594|BOLD:ACR4601
Hilara|[14342]|SSEIA1456-13|Empididae|670|BOLD:ACG6179
Hilara|[14343]|HPPPC023-13|Empididae|583|BOLD:ACI2897
Hilara|[14344]|SSPAC1581-13|Empididae|658|BOLD:ACI9169
Hilara|[14345]|CNTMA1648-14|Empididae|658|BOLD:ACM1927
Empididae|[14346]|SSJAB3877-13|Empididae|661|BOLD:ACG8209
Hilara|[14347]|CNKTC097-15|Empididae|564|BOLD:ACS2523
Hybotidae|[14348]|SSBRB227-14|Hybotidae|664|BOLD:ACL4474
Dicranomyia|[14349]|ARCIN072-14|Limoniidae|522|BOLD:ACR3767
Dicranomyia murina|[14350]|CNKLH1560-15|Limoniidae|635|BOLD:ABV4792
Hybotidae|[14351]|CNBPM352-13|Hybotidae|658|BOLD:ACG6685
Hybotidae|[14352]|BBDEE352-10|Hybotidae|658|BOLD:ABA9724
Hybotidae|[14353]|SSPAB4197-13|Hybotidae|658|BOLD:ACI6317
Micrempis bomboxynon|[14354]|CNWBG396-13|Hybotidae|659|BOLD:AAV3714
Hybotidae|[14355]|NGNAC1345-13|Hybotidae|658|BOLD:ACI3001
Coleomyia rainieri|[14356]|NGNAL839-13|Asilidae|658|BOLD:ABV9527
Crossopalpus setiger|[14357]|JWDCA098-10|Hybotidae|658|BOLD:AAF9799
Crossopalpus setiger|[14358]|JWEMP249-10|Hybotidae|658|BOLD:ACE6928
Crossopalpus|[14359]|JWEMP251-10|Hybotidae|658|BOLD:ABZ0555
Crossopalpus|[14360]|SSJAB2906-13|Hybotidae|661|BOLD:AAF9854
Hybotidae|[14361]|NGNAY1204-14|Hybotidae|504|BOLD:ACN1688
Rachicerus|[14362]|BBDEC116-09|Xylophagidae|658|BOLD:AAG7031
Hybotidae|[14363]|PHJUN778-11|Hybotidae|658|BOLD:ABV9365
Hybotidae|[14364]|JSJUN1121-11|Hybotidae|658|BOLD:ABW2750
Hybotidae|[14365]|CNGRD386-12|Hybotidae|658|BOLD:ACB1276
Hybotidae|[14366]|CNPPE943-12|Hybotidae|658|BOLD:AAF9838
Hybotidae|[14367]|SMTPB5488-13|Hybotidae|670|BOLD:ACF7100
Hybotidae|[14368]|SMTPB20358-13|Hybotidae|585|BOLD:ACF9207
Hybotidae|[14369]|JSDIP005-10|Hybotidae|658|BOLD:AAN5501
Hybotidae|[14370]|JSDIP164-10|Hybotidae|658|BOLD:AAP6353
Hybotidae|[14371]|NCCA1915-11|Hybotidae|673|BOLD:ABA6406
Hybotidae|[14372]|NGNAJ053-13|Hybotidae|661|BOLD:ACJ0743
Hybotidae|[14373]|NGNAL375-13|Hybotidae|661|BOLD:ACD1697
Hybotidae|[14374]|NGAAF234-14|Hybotidae|538|BOLD:ACN7626
Hybotidae|[14375]|BBDEC961-10|Hybotidae|658|BOLD:AAF9861
Stilpon curvipes|[14376]|JSJUL1073-11|Hybotidae|658|BOLD:ABV1424
Hybotidae|[14377]|CNEIE381-12|Hybotidae|639|BOLD:ACB0741
Hybotidae|[14378]|CNBRE079-14|Hybotidae|658|BOLD:ACL7157
Hybotidae|[14379]|CNGBN868-14|Hybotidae|555|BOLD:ACC8861
Hybotidae|[14380]|NGAAA1400-14|Hybotidae|588|BOLD:ACM5807
Hybotidae|[14381]|JSDIR736-11|Hybotidae|658|BOLD:ABA0770
Hybotidae|[14382]|CNWBG2094-13|Hybotidae|661|BOLD:ACI4087
Hybotidae|[14383]|SSJAF6469-13|Hybotidae|594|BOLD:ACK4814
Hybotidae|[14384]|NGNAT308-14|Hybotidae|658|BOLD:ACL8857
Hybotidae|[14385]|CNWLF058-12|Hybotidae|670|BOLD:ACB0775
Hybotidae|[14386]|NGNAC1392-13|Hybotidae|658|BOLD:ACI8876
Hybotidae|[14387]|BBDEE445-10|Hybotidae|658|BOLD:AAM6653
Hybotidae|[14388]|CNGBG893-14|Hybotidae|664|BOLD:AAF9863
Hybotidae|[14389]|CNKJL682-14|Hybotidae|591|BOLD:ACM9061
Hybotidae|[14390]|TTMDI1152-10|Hybotidae|658|BOLD:AAN5502
Hybotidae|[14391]|CNWBH973-13|Hybotidae|658|BOLD:ACX5072
Hybotidae|[14392]|NGNAB738-13|Hybotidae|664|BOLD:ACI3482
Hybotidae|[14393]|RBINA306-13|Hybotidae|617|BOLD:ABX1590
Hybotidae|[14394]|SSBAA196-12|Hybotidae|661|BOLD:ACC3255
Hybotidae|[14395]|SSBAD2841-12|Hybotidae|661|BOLD:ACC2303
Hybotidae|[14396]|NGNAC154-13|Hybotidae|658|BOLD:ACI2884
Hybotidae|[14397]|NGNAH412-13|Hybotidae|658|BOLD:ACJ0959

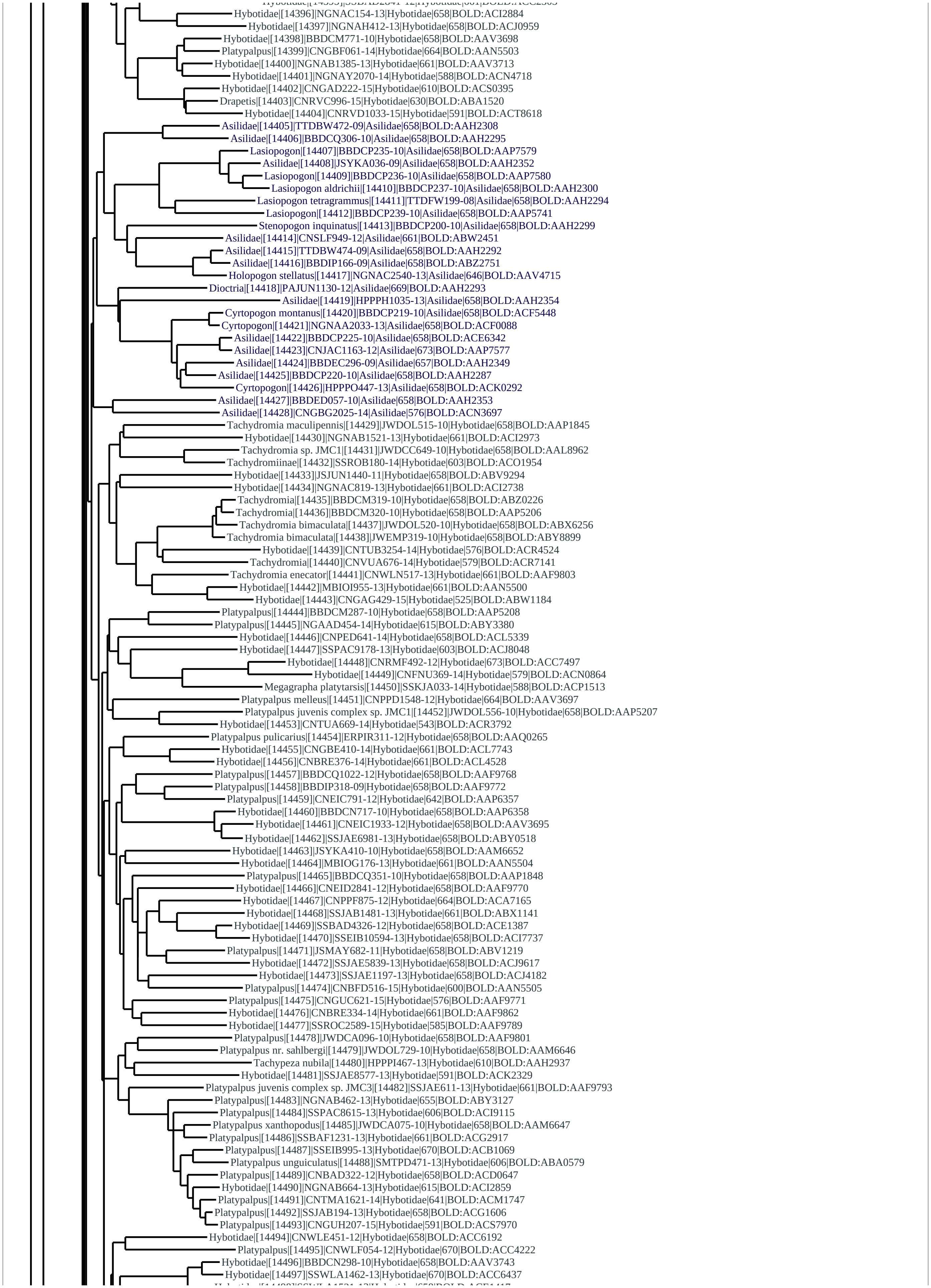

Hybotidae|[14396]|NGNAC154-13|Hybotidae|658|BOLD:ACI2884
Hybotidae|[14397]|NGNAH412-13|Hybotidae|658|BOLD:ACJ0959
Hybotidae|[14398]|BBDCM771-10|Hybotidae|658|BOLD:AAV3698
Platypalpus|[14399]|CNGBF061-14|Hybotidae|664|BOLD:AAN5503
Hybotidae|[14400]|NGNAB1385-13|Hybotidae|661|BOLD:AAV3713
Hybotidae|[14401]|NGNAY2070-14|Hybotidae|588|BOLD:ACN4718
Hybotidae|[14402]|CNGAD222-15|Hybotidae|610|BOLD:ACS0395
Drapetis|[14403]|CNRVC996-15|Hybotidae|630|BOLD:ABA1520
Hybotidae|[14404]|CNRVD1033-15|Hybotidae|591|BOLD:ACT8618
Asilidae|[14405]|TTDBW472-09|Asilidae|658|BOLD:AAH2308
Asilidae|[14406]|BBDCQ306-10|Asilidae|658|BOLD:AAH2295
Lasiopogon|[14407]|BBDCP235-10|Asilidae|658|BOLD:AAP7579
Asilidae|[14408]|JSYKA036-09|Asilidae|658|BOLD:AAH2352
Lasiopogon|[14409]|BBDCP236-10|Asilidae|658|BOLD:AAP7580
Lasiopogon aldrichii|[14410]|BBDCP237-10|Asilidae|658|BOLD:AAH2300
Lasiopogon tetragrammus|[14411]|TTDFW199-08|Asilidae|658|BOLD:AAH2294
Lasiopogon|[14412]|BBDCP239-10|Asilidae|658|BOLD:AAP5741
Stenopogon inquinatus|[14413]|BBDCP200-10|Asilidae|658|BOLD:AAH2299
Asilidae|[14414]|CNSLF949-12|Asilidae|661|BOLD:ABW2451
Asilidae|[14415]|TTDBW474-09|Asilidae|658|BOLD:AAH2292
Asilidae|[14416]|BBDIP166-09|Asilidae|658|BOLD:ABZ2751
Holopogon stellatus|[14417]|NGNAC2540-13|Asilidae|646|BOLD:AAV4715
Dioctria|[14418]|PAJUN1130-12|Asilidae|669|BOLD:AAH2293
Asilidae|[14419]|HPPPH1035-13|Asilidae|658|BOLD:AAH2354
Cyrtopogon montanus|[14420]|BBDCP219-10|Asilidae|658|BOLD:ACF5448
Cyrtopogon|[14421]|NGNAA2033-13|Asilidae|658|BOLD:ACF0088
Asilidae|[14422]|BBDCP225-10|Asilidae|658|BOLD:ACE6342
Asilidae|[14423]|CNJAC1163-12|Asilidae|673|BOLD:AAP7577
Asilidae|[14424]|BBDEC296-09|Asilidae|657|BOLD:AAH2349
Asilidae|[14425]|BBDCP220-10|Asilidae|658|BOLD:AAH2287
Cyrtopogon|[14426]|HPPPO447-13|Asilidae|658|BOLD:ACK0292
Asilidae|[14427]|BBDED057-10|Asilidae|658|BOLD:AAH2353
Asilidae|[14428]|CNGBG2025-14|Asilidae|576|BOLD:ACN3697
Tachydromia maculipennis|[14429]|JWDOL515-10|Hybotidae|658|BOLD:AAP1845
Hybotidae|[14430]|NGNAB1521-13|Hybotidae|661|BOLD:ACI2973
Tachydromia sp. JMC1|[14431]|JWDCC649-10|Hybotidae|658|BOLD:AAL8962
Tachydromiinae|[14432]|SSROB180-14|Hybotidae|603|BOLD:ACO1954
Hybotidae|[14433]|JSJUN1440-11|Hybotidae|658|BOLD:ABV9294
Hybotidae|[14434]|NGNAC819-13|Hybotidae|661|BOLD:ACI2738
Tachydromia|[14435]|BBDCM319-10|Hybotidae|658|BOLD:ABZ0226
Tachydromia|[14436]|BBDCM320-10|Hybotidae|658|BOLD:AAP5206
Tachydromia bimaculata|[14437]|JWDOL520-10|Hybotidae|658|BOLD:ABX6256
Tachydromia bimaculata|[14438]|JWEMP319-10|Hybotidae|658|BOLD:ABY8899
Hybotidae|[14439]|CNTUB3254-14|Hybotidae|576|BOLD:ACR4524
Tachydromia|[14440]|CNVUA676-14|Hybotidae|579|BOLD:ACR7141
Tachydromia enecator|[14441]|CNWLN517-13|Hybotidae|661|BOLD:AAF9803
Hybotidae|[14442]|MBIOI955-13|Hybotidae|661|BOLD:AAN5500
Hybotidae|[14443]|CNGAG429-15|Hybotidae|525|BOLD:ABW1184
Platypalpus|[14444]|BBDCM287-10|Hybotidae|658|BOLD:AAP5208
Platypalpus|[14445]|NGAAD454-14|Hybotidae|615|BOLD:ABY3380
Hybotidae|[14446]|CNPED641-14|Hybotidae|658|BOLD:ACL5339
Hybotidae|[14447]|SSPAC9178-13|Hybotidae|603|BOLD:ACJ8048
Hybotidae|[14448]|CNRMF492-12|Hybotidae|673|BOLD:ACC7497
Hybotidae|[14449]|CNFNU369-14|Hybotidae|579|BOLD:ACN0864
Megagrapha platytarsis|[14450]|SSKJA033-14|Hybotidae|588|BOLD:ACP1513
Platypalpus melleus|[14451]|CNPPD1548-12|Hybotidae|664|BOLD:AAV3697
Platypalpus juvenis complex sp. JMC1|[14452]|JWDOL556-10|Hybotidae|658|BOLD:AAP5207
Hybotidae|[14453]|CNTUA669-14|Hybotidae|543|BOLD:ACR3792
Platypalpus pulicarius|[14454]|ERPIR311-12|Hybotidae|658|BOLD:AAQ0265
Hybotidae|[14455]|CNGBE410-14|Hybotidae|661|BOLD:ACL7743
Hybotidae|[14456]|CNBRE376-14|Hybotidae|661|BOLD:ACL4528
Platypalpus|[14457]|BBDCQ1022-12|Hybotidae|658|BOLD:AAF9768
Platypalpus|[14458]|BBDIP318-09|Hybotidae|658|BOLD:AAF9772
Platypalpus|[14459]|CNEIC791-12|Hybotidae|642|BOLD:AAP6357
Hybotidae|[14460]|BBDCN717-10|Hybotidae|658|BOLD:AAP6358
Hybotidae|[14461]|CNEIC1933-12|Hybotidae|658|BOLD:AAV3695
Hybotidae|[14462]|SSJAE6981-13|Hybotidae|658|BOLD:ABY0518
Hybotidae|[14463]|JSYKA410-10|Hybotidae|658|BOLD:AAM6652
Hybotidae|[14464]|MBIOG176-13|Hybotidae|661|BOLD:AAN5504
Platypalpus|[14465]|BBDCQ351-10|Hybotidae|658|BOLD:AAP1848
Hybotidae|[14466]|CNEID2841-12|Hybotidae|658|BOLD:AAF9770
Hybotidae|[14467]|CNPPF875-12|Hybotidae|664|BOLD:ACA7165
Hybotidae|[14468]|SSJAB1481-13|Hybotidae|661|BOLD:ABX1141
Hybotidae|[14469]|SSBAD4326-12|Hybotidae|658|BOLD:ACE1387
Hybotidae|[14470]|SSEIB10594-13|Hybotidae|658|BOLD:ACI7737
Platypalpus|[14471]|JSMAY682-11|Hybotidae|658|BOLD:ABV1219
Hybotidae|[14472]|SSJAE5839-13|Hybotidae|658|BOLD:ACJ9617
Hybotidae|[14473]|SSJAE1197-13|Hybotidae|658|BOLD:ACJ4182
Platypalpus|[14474]|CNBFD516-15|Hybotidae|600|BOLD:AAN5505
Platypalpus|[14475]|CNGUC621-15|Hybotidae|576|BOLD:AAF9771
Hybotidae|[14476]|CNBRE334-14|Hybotidae|661|BOLD:AAF9862
Hybotidae|[14477]|SSROC2589-15|Hybotidae|585|BOLD:AAF9789
Platypalpus|[14478]|JWDCA096-10|Hybotidae|658|BOLD:AAF9801
Platypalpus nr. sahlbergi|[14479]|JWDOL729-10|Hybotidae|658|BOLD:AAM6646
Tachypeza nubila|[14480]|HPPPI467-13|Hybotidae|610|BOLD:AAH2937
Hybotidae|[14481]|SSJAE8577-13|Hybotidae|591|BOLD:ACK2329
Platypalpus juvenis complex sp. JMC3|[14482]|SSJAE611-13|Hybotidae|661|BOLD:AAF9793
Platypalpus|[14483]|NGNAB462-13|Hybotidae|655|BOLD:ABY3127
Platypalpus|[14484]|SSPAC8615-13|Hybotidae|606|BOLD:ACI9115
Platypalpus xanthopodus|[14485]|JWDCA075-10|Hybotidae|658|BOLD:AAM6647
Platypalpus|[14486]|SSBAF1231-13|Hybotidae|661|BOLD:ACG2917
Platypalpus|[14487]|SSEIB995-13|Hybotidae|670|BOLD:ACB1069
Platypalpus unguiculatus|[14488]|SMTPD471-13|Hybotidae|606|BOLD:ABA0579
Platypalpus|[14489]|CNBAD322-12|Hybotidae|658|BOLD:ACD0647
Hybotidae|[14490]|NGNAB664-13|Hybotidae|615|BOLD:ACI2859
Platypalpus|[14491]|CNTMA1621-14|Hybotidae|641|BOLD:ACM1747
Platypalpus|[14492]|SSJAB194-13|Hybotidae|658|BOLD:ACG1606
Platypalpus|[14493]|CNGUH207-15|Hybotidae|591|BOLD:ACS7970
Hybotidae|[14494]|CNWLE451-12|Hybotidae|658|BOLD:ACC6192
Platypalpus|[14495]|CNWLF054-12|Hybotidae|670|BOLD:ACC4222
Hybotidae|[14496]|BBDCN298-10|Hybotidae|658|BOLD:AAV3743
Hybotidae|[14497]|SSWLA1462-13|Hybotidae|670|BOLD:ACC6437

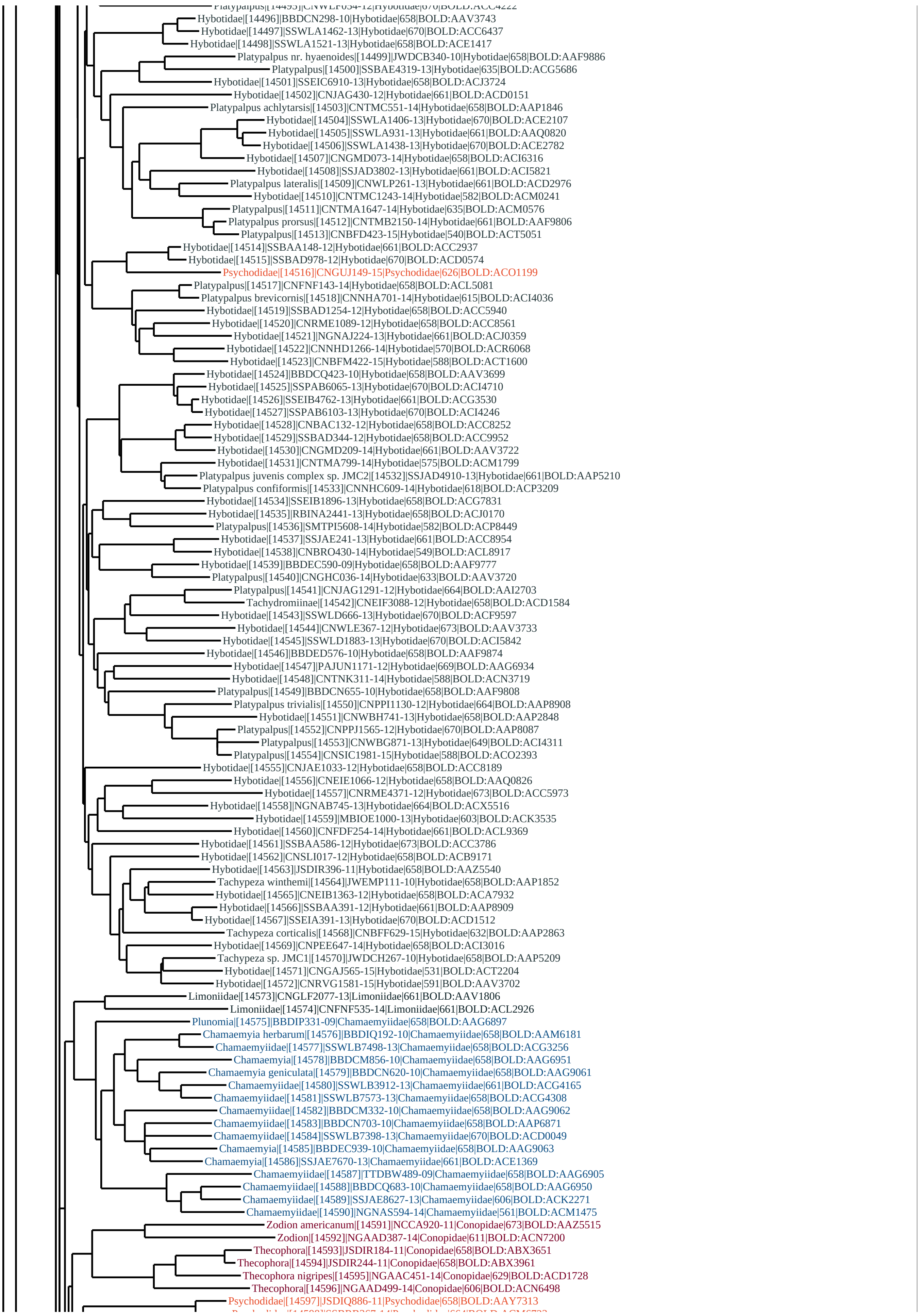

Hybotidae|[14496]|BBDCN298-10|Hybotidae|658|BOLD:AAV3743
Hybotidae|[14497]|SSWLA1462-13|Hybotidae|670|BOLD:ACC6437
Hybotidae|[14498]|SSWLA1521-13|Hybotidae|658|BOLD:ACE1417
Platypalpus nr. hyaenoides|[14499]|JWDCB340-10|Hybotidae|658|BOLD:AAF9886
Platypalpus|[14500]|SSBAE4319-13|Hybotidae|635|BOLD:ACG5686
Hybotidae|[14501]|SSEIC6910-13|Hybotidae|658|BOLD:ACJ3724
Hybotidae|[14502]|CNJAG430-12|Hybotidae|661|BOLD:ACD0151
Platypalpus achlytarsis|[14503]|CNTMC551-14|Hybotidae|658|BOLD:AAP1846
Hybotidae|[14504]|SSWLA1406-13|Hybotidae|670|BOLD:ACE2107
Hybotidae|[14505]|SSWLA931-13|Hybotidae|661|BOLD:AAQ0820
Hybotidae|[14506]|SSWLA1438-13|Hybotidae|670|BOLD:ACE2782
Hybotidae|[14507]|CNGMD073-14|Hybotidae|658|BOLD:ACI6316
Hybotidae|[14508]|SSJAD3802-13|Hybotidae|661|BOLD:ACI5821
Platypalpus lateralis|[14509]|CNWLP261-13|Hybotidae|661|BOLD:ACD2976
Hybotidae|[14510]|CNTMC1243-14|Hybotidae|582|BOLD:ACM0241
Platypalpus|[14511]|CNTMA1647-14|Hybotidae|635|BOLD:ACM0576
Platypalpus prorsus|[14512]|CNTMB2150-14|Hybotidae|661|BOLD:AAF9806
Platypalpus|[14513]|CNBFD423-15|Hybotidae|540|BOLD:ACT5051
Hybotidae|[14514]|SSBAA148-12|Hybotidae|661|BOLD:ACC2937
Hybotidae|[14515]|SSBAD978-12|Hybotidae|670|BOLD:ACD0574
Psychodidae|[14516]|CNGUJ149-15|Psychodidae|626|BOLD:ACO1199
Platypalpus|[14517]|CNFNF143-14|Hybotidae|658|BOLD:ACL5081
Platypalpus brevicornis|[14518]|CNNHA701-14|Hybotidae|615|BOLD:ACI4036
Hybotidae|[14519]|SSBAD1254-12|Hybotidae|658|BOLD:ACC5940
Hybotidae|[14520]|CNRME1089-12|Hybotidae|658|BOLD:ACC8561
Hybotidae|[14521]|NGNAJ224-13|Hybotidae|661|BOLD:ACJ0359
Hybotidae|[14522]|CNNHD1266-14|Hybotidae|570|BOLD:ACR6068
Hybotidae|[14523]|CNBFM422-15|Hybotidae|588|BOLD:ACT1600
Hybotidae|[14524]|BBDCQ423-10|Hybotidae|658|BOLD:AAV3699
Hybotidae|[14525]|SSPAB6065-13|Hybotidae|670|BOLD:ACI4710
Hybotidae|[14526]|SSEIB4762-13|Hybotidae|661|BOLD:ACG3530
Hybotidae|[14527]|SSPAB6103-13|Hybotidae|670|BOLD:ACI4246
Hybotidae|[14528]|CNBAC132-12|Hybotidae|658|BOLD:ACC8252
Hybotidae|[14529]|SSBAD344-12|Hybotidae|658|BOLD:ACC9952
Hybotidae|[14530]|CNGMD209-14|Hybotidae|661|BOLD:AAV3722
Hybotidae|[14531]|CNTMA799-14|Hybotidae|575|BOLD:ACM1799
Platypalpus juvenis complex sp. JMC2|[14532]|SSJAD4910-13|Hybotidae|661|BOLD:AAP5210
Platypalpus confiformis|[14533]|CNNHC609-14|Hybotidae|618|BOLD:ACP3209
Hybotidae|[14534]|SSEIB1896-13|Hybotidae|658|BOLD:ACG7831
Hybotidae|[14535]|RBINA2441-13|Hybotidae|658|BOLD:ACJ0170
Platypalpus|[14536]|SMTPI5608-14|Hybotidae|582|BOLD:ACP8449
Hybotidae|[14537]|SSJAE241-13|Hybotidae|661|BOLD:ACC8954
Hybotidae|[14538]|CNBRO430-14|Hybotidae|549|BOLD:ACL8917
Hybotidae|[14539]|BBDEC590-09|Hybotidae|658|BOLD:AAF9777
Platypalpus|[14540]|CNGHC036-14|Hybotidae|633|BOLD:AAV3720
Platypalpus|[14541]|CNJAG1291-12|Hybotidae|664|BOLD:AAI2703
Tachydromiinae|[14542]|CNEIF3088-12|Hybotidae|658|BOLD:ACD1584
Hybotidae|[14543]|SSWLD666-13|Hybotidae|670|BOLD:ACF9597
Hybotidae|[14544]|CNWLE367-12|Hybotidae|673|BOLD:AAV3733
Hybotidae|[14545]|SSWLD1883-13|Hybotidae|670|BOLD:ACI5842
Hybotidae|[14546]|BBDED576-10|Hybotidae|658|BOLD:AAF9874
Hybotidae|[14547]|PAJUN1171-12|Hybotidae|669|BOLD:AAG6934
Hybotidae|[14548]|CNTNK311-14|Hybotidae|588|BOLD:ACN3719
Platypalpus|[14549]|BBDCN655-10|Hybotidae|658|BOLD:AAF9808
Platypalpus trivialis|[14550]|CNPPI1130-12|Hybotidae|664|BOLD:AAP8908
Hybotidae|[14551]|CNWBH741-13|Hybotidae|658|BOLD:AAP2848
Platypalpus|[14552]|CNPPJ1565-12|Hybotidae|670|BOLD:AAP8087
Platypalpus|[14553]|CNWBG871-13|Hybotidae|649|BOLD:ACI4311
Platypalpus|[14554]|CNSIC1981-15|Hybotidae|588|BOLD:ACO2393
Hybotidae|[14555]|CNJAE1033-12|Hybotidae|658|BOLD:ACC8189
Hybotidae|[14556]|CNEIE1066-12|Hybotidae|658|BOLD:AAQ0826
Hybotidae|[14557]|CNRME4371-12|Hybotidae|673|BOLD:ACC5973
Hybotidae|[14558]|NGNAB745-13|Hybotidae|664|BOLD:ACX5516
Hybotidae|[14559]|MBIOE1000-13|Hybotidae|603|BOLD:ACK3535
Hybotidae|[14560]|CNFDF254-14|Hybotidae|661|BOLD:ACL9369
Hybotidae|[14561]|SSBAA586-12|Hybotidae|673|BOLD:ACC3786
Hybotidae|[14562]|CNSLI017-12|Hybotidae|658|BOLD:ACB9171
Hybotidae|[14563]|JSDIR396-11|Hybotidae|658|BOLD:AAZ5540
Tachypeza winthemi|[14564]|JWEMP111-10|Hybotidae|658|BOLD:AAP1852
Hybotidae|[14565]|CNEIB1363-12|Hybotidae|658|BOLD:ACA7932
Hybotidae|[14566]|SSBAA391-12|Hybotidae|661|BOLD:AAP8909
Hybotidae|[14567]|SSEIA391-13|Hybotidae|670|BOLD:ACD1512
Tachypeza corticalis|[14568]|CNBFF629-15|Hybotidae|632|BOLD:AAP2863
Hybotidae|[14569]|CNPEE647-14|Hybotidae|658|BOLD:ACI3016
Tachypeza sp. JMC1|[14570]|JWDCH267-10|Hybotidae|658|BOLD:AAP5209
Hybotidae|[14571]|CNGAJ565-15|Hybotidae|531|BOLD:ACT2204
Hybotidae|[14572]|CNRVG1581-15|Hybotidae|591|BOLD:AAV3702
Limoniidae|[14573]|CNGLF2077-13|Limoniidae|661|BOLD:AAV1806
Limoniidae|[14574]|CNFNF535-14|Limoniidae|661|BOLD:ACL2926
Plunomia|[14575]|BBDIP331-09|Chamaemyiidae|658|BOLD:AAG6897
Chamaemyia herbarum|[14576]|BBDIQ192-10|Chamaemyiidae|658|BOLD:AAM6181
Chamaemyiidae|[14577]|SSWLB7498-13|Chamaemyiidae|658|BOLD:ACG3256
Chamaemyia|[14578]|BBDCM856-10|Chamaemyiidae|658|BOLD:AAG6951
Chamaemyia geniculata|[14579]|BBDCN620-10|Chamaemyiidae|658|BOLD:AAG9061
Chamaemyiidae|[14580]|SSWLB3912-13|Chamaemyiidae|661|BOLD:ACG4165
Chamaemyiidae|[14581]|SSWLB7573-13|Chamaemyiidae|658|BOLD:ACG4308
Chamaemyiidae|[14582]|BBDCM332-10|Chamaemyiidae|658|BOLD:AAG9062
Chamaemyiidae|[14583]|BBDCN703-10|Chamaemyiidae|658|BOLD:AAP6871
Chamaemyiidae|[14584]|SSWLB7398-13|Chamaemyiidae|670|BOLD:ACD0049
Chamaemyia|[14585]|BBDEC939-10|Chamaemyiidae|658|BOLD:AAG9063
Chamaemyia|[14586]|SSJAE7670-13|Chamaemyiidae|661|BOLD:ACE1369
Chamaemyiidae|[14587]|TTDBW489-09|Chamaemyiidae|658|BOLD:AAG6905
Chamaemyiidae|[14588]|BBDCQ683-10|Chamaemyiidae|658|BOLD:AAG6950
Chamaemyiidae|[14589]|SSJAE8627-13|Chamaemyiidae|606|BOLD:ACK2271
Chamaemyiidae|[14590]|NGNAS594-14|Chamaemyiidae|561|BOLD:ACM1475
Zodion americanum|[14591]|NCCA920-11|Conopidae|673|BOLD:AAZ5515
Zodion|[14592]|NGAAD387-14|Conopidae|611|BOLD:ACN7200
Thecophora|[14593]|JSDIR184-11|Conopidae|658|BOLD:ABX3651
Thecophora|[14594]|JSDIR244-11|Conopidae|658|BOLD:ABX3961
Thecophora nigripes|[14595]|NGAAC451-14|Conopidae|629|BOLD:ACD1728
Thecophora|[14596]|NGAAD499-14|Conopidae|606|BOLD:ACN6498
Psychodidae|[14597]|JSDIQ886-11|Psychodidae|658|BOLD:AAY7313

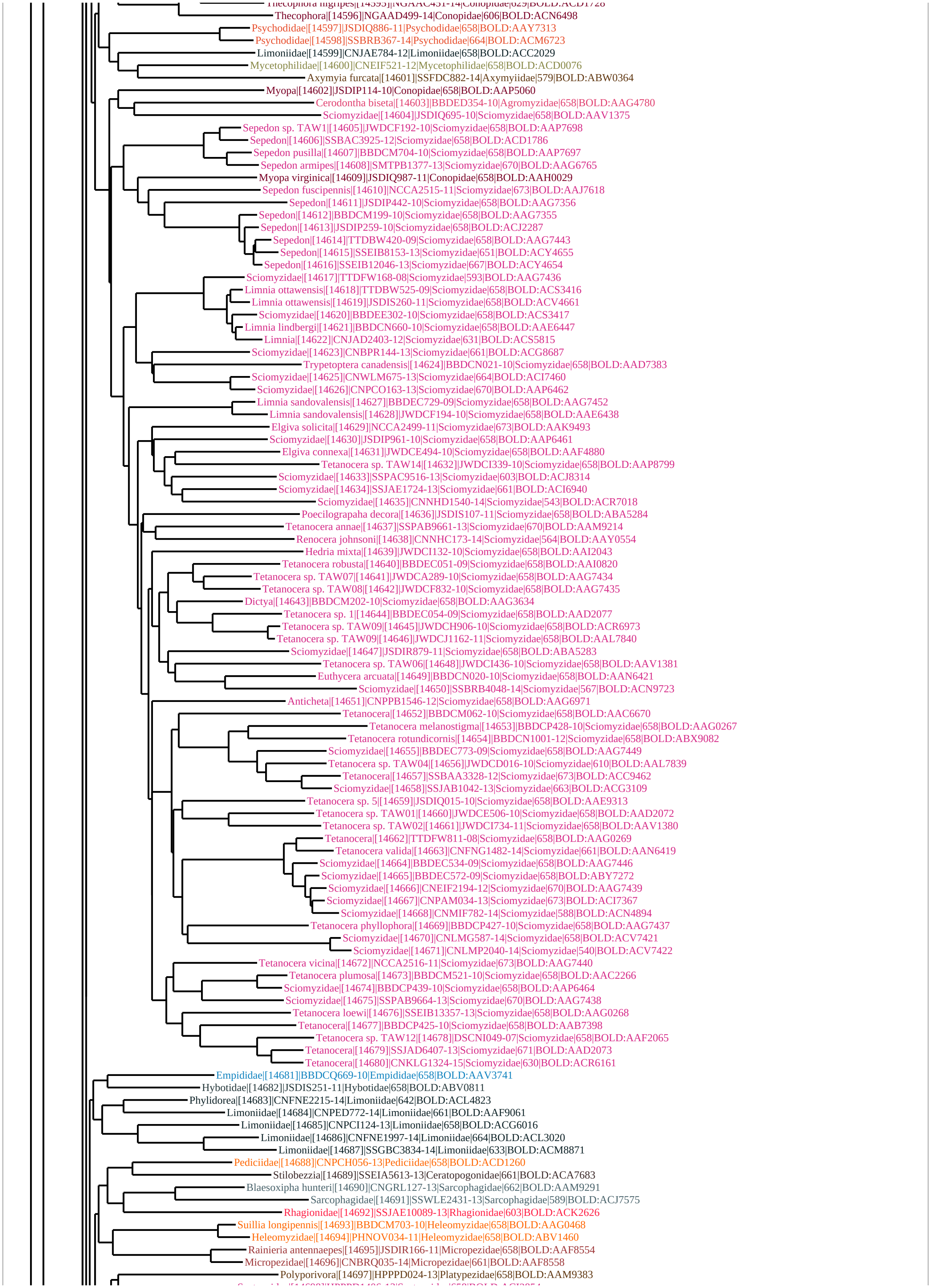
Thecophora|[14596]|NGAAD499-14|Conopidae|606|BOLD:ACN6498
Psychodidae|[14597]|JSDIQ886-11|Psychodidae|658|BOLD:AAY7313
Psychodidae|[14598]|SSBRB367-14|Psychodidae|664|BOLD:ACM6723
Limoniidae|[14599]|CNJAE784-12|Limoniidae|658|BOLD:ACC2029
Mycetophilidae|[14600]|CNEIF521-12|Mycetophilidae|658|BOLD:ACD0076
Axymyia furcata|[14601]|SSFDC882-14|Axymyiidae|579|BOLD:ABW0364
Myopa|[14602]|JSDIP114-10|Conopidae|658|BOLD:AAP5060
Cerodontha biseta|[14603]|BBDED354-10|Agromyzidae|658|BOLD:AAG4780
Sciomyzidae|[14604]|JSDIQ695-10|Sciomyzidae|658|BOLD:AAV1375
Sepedon sp. TAW1|[14605]|JWDCF192-10|Sciomyzidae|658|BOLD:AAP7698
Sepedon|[14606]|SSBAC3925-12|Sciomyzidae|658|BOLD:ACD1786
Sepedon pusilla|[14607]|BBDCM704-10|Sciomyzidae|658|BOLD:AAP7697
Sepedon armipes|[14608]|SMTPB1377-13|Sciomyzidae|670|BOLD:AAG6765
Myopa virginica|[14609]|JSDIQ987-11|Conopidae|658|BOLD:AAH0029
Sepedon fuscipennis|[14610]|NCCA2515-11|Sciomyzidae|673|BOLD:AAJ7618
Sepedon|[14611]|JSDIP442-10|Sciomyzidae|658|BOLD:AAG7356
Sepedon|[14612]|BBDCM199-10|Sciomyzidae|658|BOLD:AAG7355
Sepedon|[14613]|JSDIP259-10|Sciomyzidae|658|BOLD:ACJ2287
Sepedon|[14614]|TTDBW420-09|Sciomyzidae|658|BOLD:AAG7443
Sepedon|[14615]|SSEIB8153-13|Sciomyzidae|651|BOLD:ACY4655
Sepedon|[14616]|SSEIB12046-13|Sciomyzidae|667|BOLD:ACY4654
Sciomyzidae|[14617]|TTDFW168-08|Sciomyzidae|593|BOLD:AAG7436
Limnia ottawensis|[14618]|TTDBW525-09|Sciomyzidae|658|BOLD:ACS3416
Limnia ottawensis|[14619]|JSDIS260-11|Sciomyzidae|658|BOLD:ACV4661
Sciomyzidae|[14620]|BBDEE302-10|Sciomyzidae|658|BOLD:ACS3417
Limnia lindbergi|[14621]|BBDCN660-10|Sciomyzidae|658|BOLD:AAE6447
Limnia|[14622]|CNJAD2403-12|Sciomyzidae|631|BOLD:ACS5815
Sciomyzidae|[14623]|CNBPR144-13|Sciomyzidae|661|BOLD:ACG8687
Trypetoptera canadensis|[14624]|BBDCN021-10|Sciomyzidae|658|BOLD:AAD7383
Sciomyzidae|[14625]|CNWLM675-13|Sciomyzidae|664|BOLD:ACI7460
Sciomyzidae|[14626]|CNPCO163-13|Sciomyzidae|670|BOLD:AAP6462
Limnia sandovalensis|[14627]|BBDEC729-09|Sciomyzidae|658|BOLD:AAG7452
Limnia sandovalensis|[14628]|JWDCF194-10|Sciomyzidae|658|BOLD:AAE6438
Elgiva solicita|[14629]|NCCA2499-11|Sciomyzidae|673|BOLD:AAK9493
Sciomyzidae|[14630]|JSDIP961-10|Sciomyzidae|658|BOLD:AAP6461
Elgiva connexa|[14631]|JWDCE494-10|Sciomyzidae|658|BOLD:AAF4880
Tetanocera sp. TAW14|[14632]|JWDCI339-10|Sciomyzidae|658|BOLD:AAP8799
Sciomyzidae|[14633]|SSPAC9516-13|Sciomyzidae|603|BOLD:ACJ8314
Sciomyzidae|[14634]|SSJAE1724-13|Sciomyzidae|661|BOLD:ACI6940
Sciomyzidae|[14635]|CNNHD1540-14|Sciomyzidae|543|BOLD:ACR7018
Poecilographa decora|[14636]|JSDIS107-11|Sciomyzidae|658|BOLD:ABA5284
Tetanocera annae|[14637]|SSPAB9661-13|Sciomyzidae|670|BOLD:AAM9214
Renocera johnsoni|[14638]|CNNHC173-14|Sciomyzidae|564|BOLD:AAY0554
Hedria mixta|[14639]|JWDCI132-10|Sciomyzidae|658|BOLD:AAI2043
Tetanocera robusta|[14640]|BBDEC051-09|Sciomyzidae|658|BOLD:AAI0820
Tetanocera sp. TAW07|[14641]|JWDCA289-10|Sciomyzidae|658|BOLD:AAG7434
Tetanocera sp. TAW08|[14642]|JWDCF832-10|Sciomyzidae|658|BOLD:AAG7435
Dictya|[14643]|BBDCM202-10|Sciomyzidae|658|BOLD:AAG3634
Tetanocera sp. 1|[14644]|BBDEC054-09|Sciomyzidae|658|BOLD:AAD2077
Tetanocera sp. TAW09|[14645]|JWDCH906-10|Sciomyzidae|658|BOLD:ACR6973
Tetanocera sp. TAW09|[14646]|JWDCJ1162-11|Sciomyzidae|658|BOLD:AAL7840
Sciomyzidae|[14647]|JSDIR879-11|Sciomyzidae|658|BOLD:ABA5283
Tetanocera sp. TAW06|[14648]|JWDCI436-10|Sciomyzidae|658|BOLD:AAV1381
Euthycera arcuata|[14649]|BBDCN020-10|Sciomyzidae|658|BOLD:AAN6421
Sciomyzidae|[14650]|SSBRB4048-14|Sciomyzidae|567|BOLD:ACN9723
Anticheta|[14651]|CNPPB1546-12|Sciomyzidae|658|BOLD:AAG6971
Tetanocera|[14652]|BBDCM062-10|Sciomyzidae|658|BOLD:AAC6670
Tetanocera melanostigma|[14653]|BBDCP428-10|Sciomyzidae|658|BOLD:AAG0267
Tetanocera rotundicornis|[14654]|BBDCN1001-12|Sciomyzidae|658|BOLD:ABX9082
Sciomyzidae|[14655]|BBDEC773-09|Sciomyzidae|658|BOLD:AAG7449
Tetanocera sp. TAW04|[14656]|JWDCD016-10|Sciomyzidae|610|BOLD:AAL7839
Tetanocera|[14657]|SSBAA3328-12|Sciomyzidae|673|BOLD:ACC9462
Sciomyzidae|[14658]|SSJAB1042-13|Sciomyzidae|663|BOLD:ACG3109
Tetanocera sp. 5|[14659]|JSDIQ015-10|Sciomyzidae|658|BOLD:AAE9313
Tetanocera sp. TAW01|[14660]|JWDCE506-10|Sciomyzidae|658|BOLD:AAD2072
Tetanocera sp. TAW02|[14661]|JWDCI734-11|Sciomyzidae|658|BOLD:AAV1380
Tetanocera|[14662]|TTDFW811-08|Sciomyzidae|658|BOLD:AAG0269
Tetanocera valida|[14663]|CNFNG1482-14|Sciomyzidae|661|BOLD:AAN6419
Sciomyzidae|[14664]|BBDEC534-09|Sciomyzidae|658|BOLD:AAG7446
Sciomyzidae|[14665]|BBDEC572-09|Sciomyzidae|658|BOLD:ABY7272
Sciomyzidae|[14666]|CNEIF2194-12|Sciomyzidae|670|BOLD:AAG7439
Sciomyzidae|[14667]|CNPAM034-13|Sciomyzidae|673|BOLD:ACI7367
Sciomyzidae|[14668]|CNMIF782-14|Sciomyzidae|588|BOLD:ACN4894
Tetanocera phyllophora|[14669]|BBDCP427-10|Sciomyzidae|658|BOLD:AAG7437
Sciomyzidae|[14670]|CNLMG587-14|Sciomyzidae|658|BOLD:ACV7421
Sciomyzidae|[14671]|CNLMP2040-14|Sciomyzidae|540|BOLD:ACV7422
Tetanocera vicina|[14672]|NCCA2516-11|Sciomyzidae|673|BOLD:AAG7440
Tetanocera plumosa|[14673]|BBDCM521-10|Sciomyzidae|658|BOLD:AAC2266
Sciomyzidae|[14674]|BBDCP439-10|Sciomyzidae|658|BOLD:AAP6464
Sciomyzidae|[14675]|SSPAB9664-13|Sciomyzidae|670|BOLD:AAG7438
Tetanocera loewi|[14676]|SSEIB13357-13|Sciomyzidae|658|BOLD:AAG0268
Tetanocera|[14677]|BBDCP425-10|Sciomyzidae|658|BOLD:AAB7398
Tetanocera sp. TAW12|[14678]|DSCNI049-07|Sciomyzidae|658|BOLD:AAF2065
Tetanocera|[14679]|SSJAD6407-13|Sciomyzidae|671|BOLD:AAD2073
Tetanocera|[14680]|CNKLG1324-15|Sciomyzidae|630|BOLD:ACR6161
Empididae|[14681]|BBDCQ669-10|Empididae|658|BOLD:AAV3741
Hybotidae|[14682]|JSDIS251-11|Hybotidae|658|BOLD:ABV0811
Phylidorea|[14683]|CNFNE2215-14|Limoniidae|642|BOLD:ACL4823
Limoniidae|[14684]|CNPED772-14|Limoniidae|661|BOLD:AAF9061
Limoniidae|[14685]|CNPCI124-13|Limoniidae|658|BOLD:ACG6016
Limoniidae|[14686]|CNFNE1997-14|Limoniidae|664|BOLD:ACL3020
Limoniidae|[14687]|SSGBC3834-14|Limoniidae|633|BOLD:ACM8871
Pediciidae|[14688]|CNPCH056-13|Pediciidae|658|BOLD:ACD1260
Stilobezzia|[14689]|SSEIA5613-13|Ceratopogonidae|661|BOLD:ACA7683
Blaesoxipha hunteri|[14690]|CNGRL127-13|Sarcophagidae|662|BOLD:AAM9291
Sarcophagidae|[14691]|SSWLE2431-13|Sarcophagidae|589|BOLD:ACJ7575
Rhagionidae|[14692]|SSJAE10089-13|Rhagionidae|603|BOLD:ACK2626
Suillia longipennis|[14693]|BBDCM703-10|Heleomyzidae|658|BOLD:AAG0468
Heleomyzidae|[14694]|PHNOV034-11|Heleomyzidae|658|BOLD:ABV1460
Rainieria antennaepes|[14695]|JSDIR166-11|Micropezidae|658|BOLD:AAF8554
Micropezidae|[14696]|CNBRQ035-14|Micropezidae|661|BOLD:AAF8558
Polyporivora|[14697]|HPPPD024-13|Platypezidae|658|BOLD:AAM9383

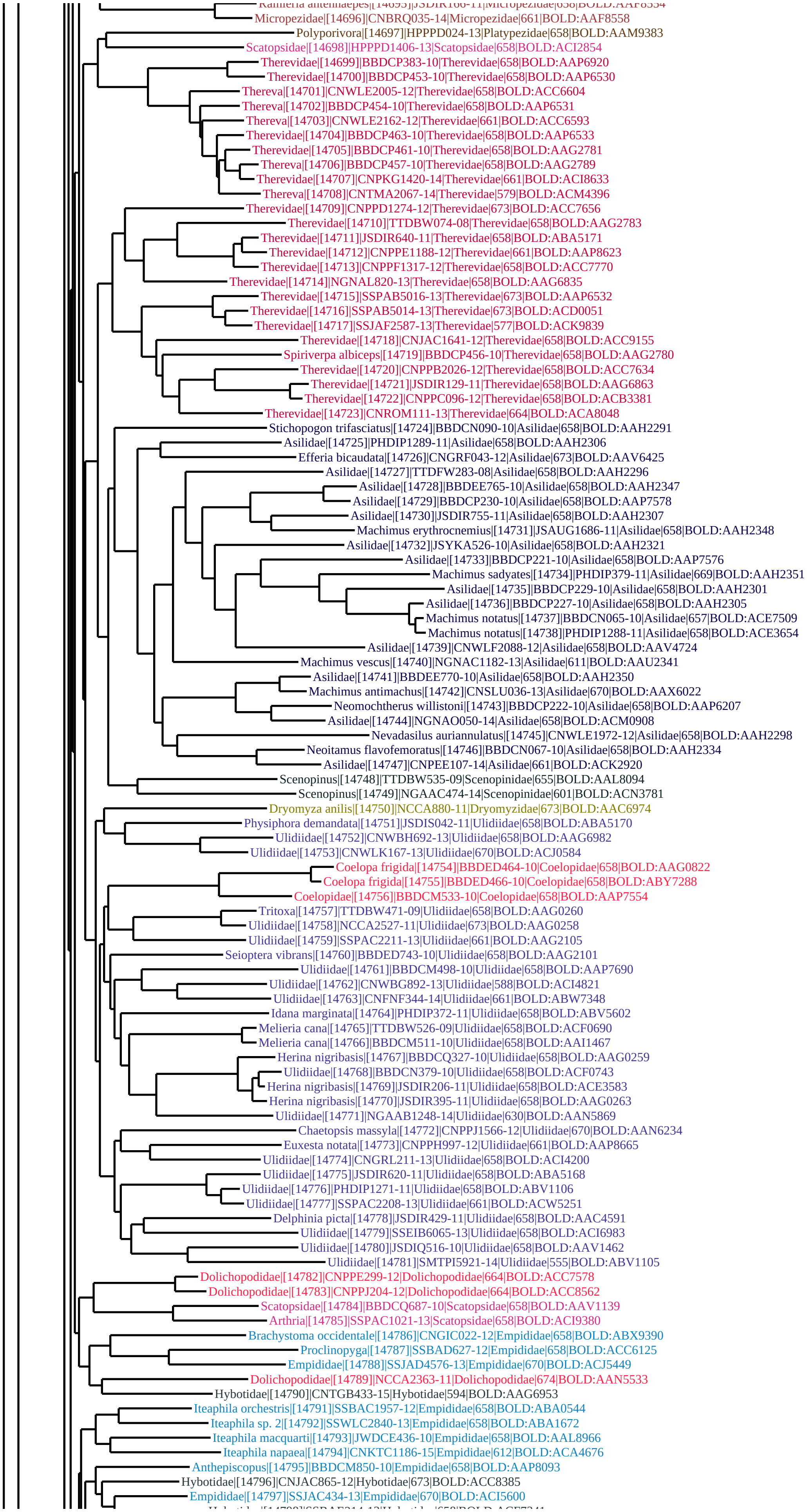
Micropezidae|[14696]|CNBRQ035-14|Micropezidae|661|BOLD:AAF8558
Polyporivora|[14697]|HPPPD024-13|Platypezidae|658|BOLD:AAM9383
Scatopsidae|[14698]|HPPPD1406-13|Scatopsidae|658|BOLD:ACI2854
Therevidae|[14699]|BBDCP383-10|Therevidae|658|BOLD:AAP6920
Therevidae|[14700]|BBDCP453-10|Therevidae|658|BOLD:AAP6530
Thereva|[14701]|CNWLE2005-12|Therevidae|658|BOLD:ACC6604
Thereva|[14702]|BBDCP454-10|Therevidae|658|BOLD:AAP6531
Thereva|[14703]|CNWLE2162-12|Therevidae|661|BOLD:ACC6593
Therevidae|[14704]|BBDCP463-10|Therevidae|658|BOLD:AAP6533
Therevidae|[14705]|BBDCP461-10|Therevidae|658|BOLD:AAG2781
Thereva|[14706]|BBDCP457-10|Therevidae|658|BOLD:AAG2789
Therevidae|[14707]|CNPKG1420-14|Therevidae|661|BOLD:ACI8633
Thereva|[14708]|CNTMA2067-14|Therevidae|579|BOLD:ACM4396
Therevidae|[14709]|CNPPD1274-12|Therevidae|673|BOLD:ACC7656
Therevidae|[14710]|TTDBW074-08|Therevidae|658|BOLD:AAG2783
Therevidae|[14711]|JSDIR640-11|Therevidae|658|BOLD:ABA5171
Therevidae|[14712]|CNPPE1188-12|Therevidae|661|BOLD:AAP8623
Therevidae|[14713]|CNPPF1317-12|Therevidae|658|BOLD:ACC7770
Therevidae|[14714]|NGNAL820-13|Therevidae|658|BOLD:AAG6835
Therevidae|[14715]|SSPAB5016-13|Therevidae|673|BOLD:AAP6532
Therevidae|[14716]|SSPAB5014-13|Therevidae|673|BOLD:ACD0051
Therevidae|[14717]|SSJAF2587-13|Therevidae|577|BOLD:ACK9839
Therevidae|[14718]|CNJAC1641-12|Therevidae|658|BOLD:ACC9155
Spiriverpa albiceps|[14719]|BBDCP456-10|Therevidae|658|BOLD:AAG2780
Therevidae|[14720]|CNPPB2026-12|Therevidae|658|BOLD:ACC7634
Therevidae|[14721]|JSDIR129-11|Therevidae|658|BOLD:AAG6863
Therevidae|[14722]|CNPPC096-12|Therevidae|658|BOLD:ACB3381
Therevidae|[14723]|CNROM111-13|Therevidae|664|BOLD:ACA8048
Stichopogon trifasciatus|[14724]|BBDCN090-10|Asilidae|658|BOLD:AAH2291
Asilidae|[14725]|PHDIP1289-11|Asilidae|658|BOLD:AAH2306
Efferia bicaudata|[14726]|CNGRF043-12|Asilidae|673|BOLD:AAV6425
Asilidae|[14727]|TTDFW283-08|Asilidae|658|BOLD:AAH2296
Asilidae|[14728]|BBDEE765-10|Asilidae|658|BOLD:AAH2347
Asilidae|[14729]|BBDCP230-10|Asilidae|658|BOLD:AAP7578
Asilidae|[14730]|JSDIR755-11|Asilidae|658|BOLD:AAH2307
Machimus erythrocnemius|[14731]|JSAUG1686-11|Asilidae|658|BOLD:AAH2348
Asilidae|[14732]|JSYKA526-10|Asilidae|658|BOLD:AAH2321
Asilidae|[14733]|BBDCP221-10|Asilidae|658|BOLD:AAP7576
Machimus sadyates|[14734]|PHDIP379-11|Asilidae|669|BOLD:AAH2351
Asilidae|[14735]|BBDCP229-10|Asilidae|658|BOLD:AAH2301
Asilidae|[14736]|BBDCP227-10|Asilidae|658|BOLD:AAH2305
Machimus notatus|[14737]|BBDCN065-10|Asilidae|657|BOLD:ACE7509
Machimus notatus|[14738]|PHDIP1288-11|Asilidae|658|BOLD:ACE3654
Asilidae|[14739]|CNWLF2088-12|Asilidae|658|BOLD:AAV4724
Machimus vescus|[14740]|NGNAC1182-13|Asilidae|611|BOLD:AAU2341
Asilidae|[14741]|BBDEE770-10|Asilidae|658|BOLD:AAH2350
Machimus antimachus|[14742]|CNSLU036-13|Asilidae|670|BOLD:AAX6022
Neomochtherus willistoni|[14743]|BBDCP222-10|Asilidae|658|BOLD:AAP6207
Asilidae|[14744]|NGNAO050-14|Asilidae|658|BOLD:ACM0908
Nevadasilus auriannulatus|[14745]|CNWLE1972-12|Asilidae|658|BOLD:AAH2298
Neoitamus flavofemoratus|[14746]|BBDCN067-10|Asilidae|658|BOLD:AAH2334
Asilidae|[14747]|CNPEE107-14|Asilidae|661|BOLD:ACK2920
Scenopinus|[14748]|TTDBW535-09|Scenopinidae|655|BOLD:AAL8094
Scenopinus|[14749]|NGAAC474-14|Scenopinidae|601|BOLD:ACN3781
Dryomyza anilis|[14750]|NCCA880-11|Dryomyzidae|673|BOLD:AAC6974
Physiphora demandata|[14751]|JSDIS042-11|Ulidiidae|658|BOLD:ABA5170
Ulidiidae|[14752]|CNWBH692-13|Ulidiidae|658|BOLD:AAG6982
Ulidiidae|[14753]|CNWLK167-13|Ulidiidae|670|BOLD:ACJ0584
Coelopa frigida|[14754]|BBDED464-10|Coelopidae|658|BOLD:AAG0822
Coelopa frigida|[14755]|BBDED466-10|Coelopidae|658|BOLD:ABY7288
Coelopidae|[14756]|BBDCM533-10|Coelopidae|658|BOLD:AAP7554
Tritoxa|[14757]|TTDBW471-09|Ulidiidae|658|BOLD:AAG0260
Ulidiidae|[14758]|NCCA2527-11|Ulidiidae|673|BOLD:AAG0258
Ulidiidae|[14759]|SSPAC2211-13|Ulidiidae|661|BOLD:AAG2105
Seioptera vibrans|[14760]|BBDED743-10|Ulidiidae|658|BOLD:AAG2101
Ulidiidae|[14761]|BBDCM498-10|Ulidiidae|658|BOLD:AAP7690
Ulidiidae|[14762]|CNWBG892-13|Ulidiidae|588|BOLD:ACI4821
Ulidiidae|[14763]|CNFNF344-14|Ulidiidae|661|BOLD:ABW7348
Idana marginata|[14764]|PHDIP372-11|Ulidiidae|658|BOLD:ABV5602
Melieria cana|[14765]|TTDBW526-09|Ulidiidae|658|BOLD:ACF0690
Melieria cana|[14766]|BBDCM511-10|Ulidiidae|658|BOLD:AAI1467
Herina nigribasis|[14767]|BBDCQ327-10|Ulidiidae|658|BOLD:AAG0259
Ulidiidae|[14768]|BBDCN379-10|Ulidiidae|658|BOLD:ACF0743
Herina nigribasis|[14769]|JSDIR206-11|Ulidiidae|658|BOLD:ACE3583
Herina nigribasis|[14770]|JSDIR395-11|Ulidiidae|658|BOLD:AAG0263
Ulidiidae|[14771]|NGAAB1248-14|Ulidiidae|630|BOLD:AAN5869
Chaetopsis massyla|[14772]|CNPPJ1566-12|Ulidiidae|670|BOLD:AAN6234
Euxesta notata|[14773]|CNPPH997-12|Ulidiidae|661|BOLD:AAP8665
Ulidiidae|[14774]|CNGRL211-13|Ulidiidae|658|BOLD:ACI4200
Ulidiidae|[14775]|JSDIR620-11|Ulidiidae|658|BOLD:ABA5168
Ulidiidae|[14776]|PHDIP1271-11|Ulidiidae|658|BOLD:ABV1106
Ulidiidae|[14777]|SSPAC2208-13|Ulidiidae|661|BOLD:ACW5251
Delphinia picta|[14778]|JSDIR429-11|Ulidiidae|658|BOLD:AAC4591
Ulidiidae|[14779]|SSEIB6065-13|Ulidiidae|658|BOLD:ACI6983
Ulidiidae|[14780]|JSDIQ516-10|Ulidiidae|658|BOLD:AAV1462
Ulidiidae|[14781]|SMTPI5921-14|Ulidiidae|555|BOLD:ABV1105
Dolichopodidae|[14782]|CNPPE299-12|Dolichopodidae|664|BOLD:ACC7578
Dolichopodidae|[14783]|CNPPJ204-12|Dolichopodidae|664|BOLD:ACC8562
Scatopsidae|[14784]|BBDCQ687-10|Scatopsidae|658|BOLD:AAV1139
Arthria|[14785]|SSPAC1021-13|Scatopsidae|658|BOLD:ACI9380
Brachystoma occidentale|[14786]|CNGIC022-12|Empididae|658|BOLD:ABX9390
Proclinopyga|[14787]|SSBAD627-12|Empididae|658|BOLD:ACC6125
Empididae|[14788]|SSJAD4576-13|Empididae|670|BOLD:ACJ5449
Dolichopodidae|[14789]|NCCA2363-11|Dolichopodidae|674|BOLD:AAN5533
Hybotidae|[14790]|CNTGB433-15|Hybotidae|594|BOLD:AAG6953
Iteaphila orchestris|[14791]|SSBAC1957-12|Empididae|658|BOLD:ABA0544
Iteaphila sp. 2|[14792]|SSWLC2840-13|Empididae|658|BOLD:ABA1672
Iteaphila macquarti|[14793]|JWDCE436-10|Empididae|658|BOLD:AAL8966
Iteaphila napaea|[14794]|CNKTC1186-15|Empididae|612|BOLD:ACA4676
Anthepiscopus|[14795]|BBDCM850-10|Empididae|658|BOLD:AAP8093
Hybotidae|[14796]|CNJAC865-12|Hybotidae|673|BOLD:ACC8385
Empididae|[14797]|SSJAC434-13|Empididae|670|BOLD:ACI5600

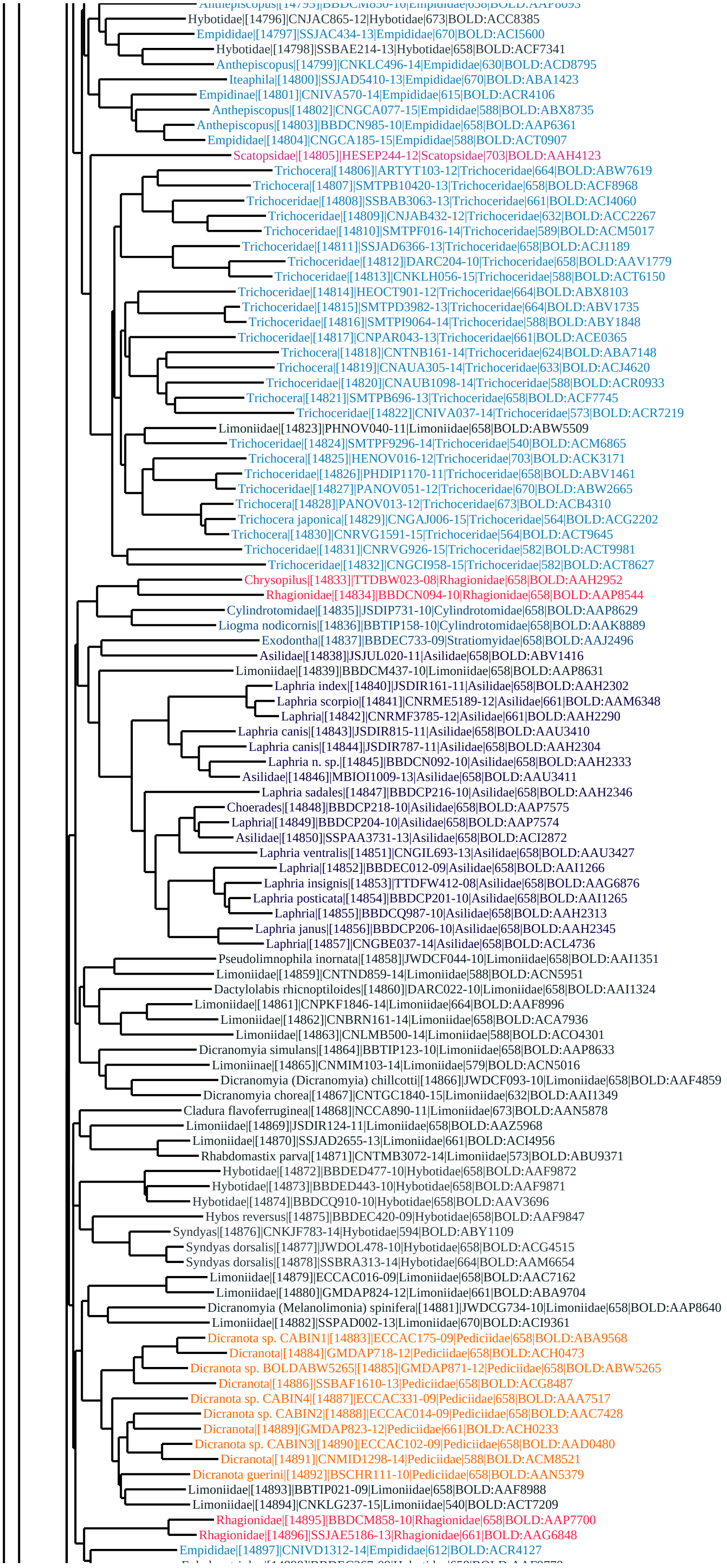

Hybotidae|[14796]|CNJAC865-12|Hybotidae|673|BOLD:ACC8385
Empididae|[14797]|SSJAC434-13|Empididae|670|BOLD:ACI5600
Hybotidae|[14798]|SSBAE214-13|Hybotidae|658|BOLD:ACF7341
Anthepiscopus|[14799]|CNKLC496-14|Empididae|630|BOLD:ACD8795
Iteaphila|[14800]|SSJAD5410-13|Empididae|670|BOLD:ABA1423
Empidinae|[14801]|CNIVA570-14|Empididae|615|BOLD:ACR4106
Anthepiscopus|[14802]|CNGCA077-15|Empididae|588|BOLD:ABX8735
Anthepiscopus|[14803]|BBDCN985-10|Empididae|658|BOLD:AAP6361
Empididae|[14804]|CNGCA185-15|Empididae|588|BOLD:ACT0907
Scatopsidae|[14805]|HESEP244-12|Scatopsidae|703|BOLD:AAH4123
Trichocera|[14806]|ARTYT103-12|Trichoceridae|664|BOLD:ABW7619
Trichocera|[14807]|SMTPB10420-13|Trichoceridae|658|BOLD:ACF8968
Trichoceridae|[14808]|SSBAB3063-13|Trichoceridae|661|BOLD:ACI4060
Trichoceridae|[14809]|CNJAB432-12|Trichoceridae|632|BOLD:ACC2267
Trichoceridae|[14810]|SMTPF016-14|Trichoceridae|589|BOLD:ACM5017
Trichoceridae|[14811]|SSJAD6366-13|Trichoceridae|658|BOLD:ACJ1189
Trichoceridae|[14812]|DARC204-10|Trichoceridae|658|BOLD:AAV1779
Trichoceridae|[14813]|CNKLH056-15|Trichoceridae|588|BOLD:ACT6150
Trichoceridae|[14814]|HEOCT901-12|Trichoceridae|664|BOLD:ABX8103
Trichoceridae|[14815]|SMTPD3982-13|Trichoceridae|664|BOLD:ABV1735
Trichoceridae|[14816]|SMTPI9064-14|Trichoceridae|588|BOLD:ABY1848
Trichoceridae|[14817]|CNPAR043-13|Trichoceridae|661|BOLD:ACE0365
Trichocera|[14818]|CNTNB161-14|Trichoceridae|624|BOLD:ABA7148
Trichocera|[14819]|CNAUA305-14|Trichoceridae|633|BOLD:ACJ4620
Trichoceridae|[14820]|CNAUB1098-14|Trichoceridae|588|BOLD:ACR0933
Trichocera|[14821]|SMTPB696-13|Trichoceridae|658|BOLD:ACF7745
Trichoceridae|[14822]|CNIVA037-14|Trichoceridae|573|BOLD:ACR7219
Limoniidae|[14823]|PHNOV040-11|Limoniidae|658|BOLD:ABW5509
Trichoceridae|[14824]|SMTPF9296-14|Trichoceridae|540|BOLD:ACM6865
Trichocera|[14825]|HENOV016-12|Trichoceridae|703|BOLD:ACK3171
Trichoceridae|[14826]|PHDIP1170-11|Trichoceridae|658|BOLD:ABV1461
Trichoceridae|[14827]|PANOV051-12|Trichoceridae|670|BOLD:ABW2665
Trichocera|[14828]|PANOV013-12|Trichoceridae|673|BOLD:ACB4310
Trichocera japonica|[14829]|CNGAJ006-15|Trichoceridae|564|BOLD:ACG2202
Trichocera|[14830]|CNRVG1591-15|Trichoceridae|564|BOLD:ACT9645
Trichoceridae|[14831]|CNRVG926-15|Trichoceridae|582|BOLD:ACT9981
Trichoceridae|[14832]|CNGCI958-15|Trichoceridae|582|BOLD:ACT8627
Chrysopilus|[14833]|TTDBW023-08|Rhagionidae|658|BOLD:AAH2952
Rhagionidae|[14834]|BBDCN094-10|Rhagionidae|658|BOLD:AAP8544
Cylindrotomidae|[14835]|JSDIP731-10|Cylindrotomidae|658|BOLD:AAP8629
Liogma nodicornis|[14836]|BBTIP158-10|Cylindrotomidae|658|BOLD:AAK8889
Exodontha|[14837]|BBDEC733-09|Stratiomyidae|658|BOLD:AAJ2496
Asilidae|[14838]|JSJUL020-11|Asilidae|658|BOLD:ABV1416
Limoniidae|[14839]|BBDCM437-10|Limoniidae|658|BOLD:AAP8631
Laphria index|[14840]|JSDIR161-11|Asilidae|658|BOLD:AAH2302
Laphria scorpio|[14841]|CNRME5189-12|Asilidae|661|BOLD:AAM6348
Laphria|[14842]|CNRMF3785-12|Asilidae|661|BOLD:AAH2290
Laphria canis|[14843]|JSDIR815-11|Asilidae|658|BOLD:AAU3410
Laphria canis|[14844]|JSDIR787-11|Asilidae|658|BOLD:AAH2304
Laphria n. sp.|[14845]|BBDCN092-10|Asilidae|658|BOLD:AAH2333
Asilidae|[14846]|MBIOI1009-13|Asilidae|658|BOLD:AAU3411
Laphria sadales|[14847]|BBDCP216-10|Asilidae|658|BOLD:AAH2346
Choerades|[14848]|BBDCP218-10|Asilidae|658|BOLD:AAP7575
Laphria|[14849]|BBDCP204-10|Asilidae|658|BOLD:AAP7574
Asilidae|[14850]|SSPAA3731-13|Asilidae|658|BOLD:ACI2872
Laphria ventralis|[14851]|CNGIL693-13|Asilidae|658|BOLD:AAU3427
Laphria|[14852]|BBDEC012-09|Asilidae|658|BOLD:AAI1266
Laphria insignis|[14853]|TTDFW412-08|Asilidae|658|BOLD:AAG6876
Laphria posticata|[14854]|BBDCP201-10|Asilidae|658|BOLD:AAI1265
Laphria|[14855]|BBDCQ987-10|Asilidae|658|BOLD:AAH2313
Laphria janus|[14856]|BBDCP206-10|Asilidae|658|BOLD:AAH2345
Laphria|[14857]|CNGBE037-14|Asilidae|658|BOLD:ACL4736
Pseudolimnophila inornata|[14858]|JWDCF044-10|Limoniidae|658|BOLD:AAI1351
Limoniidae|[14859]|CNTND859-14|Limoniidae|588|BOLD:ACN5951
Dactylolabis rhicnoptiloides|[14860]|DARC022-10|Limoniidae|658|BOLD:AAI1324
Limoniidae|[14861]|CNPKF1846-14|Limoniidae|664|BOLD:AAF8996
Limoniidae|[14862]|CNBRN161-14|Limoniidae|658|BOLD:ACA7936
Limoniidae|[14863]|CNLMB500-14|Limoniidae|588|BOLD:ACO4301
Dicranomyia simulans|[14864]|BBTIP123-10|Limoniidae|658|BOLD:AAP8633
Limoniinae|[14865]|CNMIM103-14|Limoniidae|579|BOLD:ACN5016
Dicranomyia (Dicranomyia) chillcotti|[14866]|JWDCF093-10|Limoniidae|658|BOLD:AAF4859
Dicranomyia chorea|[14867]|CNTGC1840-15|Limoniidae|632|BOLD:AAI1349
Cladura flavoferruginea|[14868]|NCCA890-11|Limoniidae|673|BOLD:AAN5878
Limoniidae|[14869]|JSDIR124-11|Limoniidae|658|BOLD:AAZ5968
Limoniidae|[14870]|SSJAD2655-13|Limoniidae|661|BOLD:ACI4956
Rhabdomastix parva|[14871]|CNTMB3072-14|Limoniidae|573|BOLD:ABU9371
Hybotidae|[14872]|BBDED477-10|Hybotidae|658|BOLD:AAF9872
Hybotidae|[14873]|BBDED443-10|Hybotidae|658|BOLD:AAF9871
Hybotidae|[14874]|BBDCQ910-10|Hybotidae|658|BOLD:AAV3696
Hybos reversus|[14875]|BBDEC420-09|Hybotidae|658|BOLD:AAF9847
Syndyas|[14876]|CNKJF783-14|Hybotidae|594|BOLD:ABY1109
Syndyas dorsalis|[14877]|JWDOL478-10|Hybotidae|658|BOLD:ACG4515
Syndyas dorsalis|[14878]|SSBRA313-14|Hybotidae|664|BOLD:AAM6654
Limoniidae|[14879]|ECCAC016-09|Limoniidae|658|BOLD:AAC7162
Limoniidae|[14880]|GMDAP824-12|Limoniidae|661|BOLD:ABA9704
Dicranomyia (Melanolimonia) spinifera|[14881]|JWDCG734-10|Limoniidae|658|BOLD:AAP8640
Limoniidae|[14882]|SSPAD002-13|Limoniidae|670|BOLD:ACI9361
Dicranota sp. CABIN1|[14883]|ECCAC175-09|Pediciidae|658|BOLD:ABA9568
Dicranota|[14884]|GMDAP718-12|Pediciidae|658|BOLD:ACH0473
Dicranota sp. BOLDABW5265|[14885]|GMDAP871-12|Pediciidae|658|BOLD:ABW5265
Dicranota|[14886]|SSBAF1610-13|Pediciidae|658|BOLD:ACG8487
Dicranota sp. CABIN4|[14887]|ECCAC331-09|Pediciidae|658|BOLD:AAA7517
Dicranota sp. CABIN2|[14888]|ECCAC014-09|Pediciidae|658|BOLD:AAC7428
Dicranota|[14889]|GMDAP823-12|Pediciidae|661|BOLD:ACH0233
Dicranota sp. CABIN3|[14890]|ECCAC102-09|Pediciidae|658|BOLD:AAD0480
Dicranota|[14891]|CNMID1298-14|Pediciidae|588|BOLD:ACM8521
Dicranota guerini|[14892]|BSCHR111-10|Pediciidae|658|BOLD:AAN5379
Limoniidae|[14893]|BBTIP021-09|Limoniidae|658|BOLD:AAF8988
Limoniidae|[14894]|CNKLG237-15|Limoniidae|540|BOLD:ACT7209
Rhagionidae|[14895]|BBDCM858-10|Rhagionidae|658|BOLD:AAP7700
Rhagionidae|[14896]|SSJAE5186-13|Rhagionidae|661|BOLD:AAG6848
Empididae|[14897]|CNIVD1312-14|Empididae|612|BOLD:ACR4127

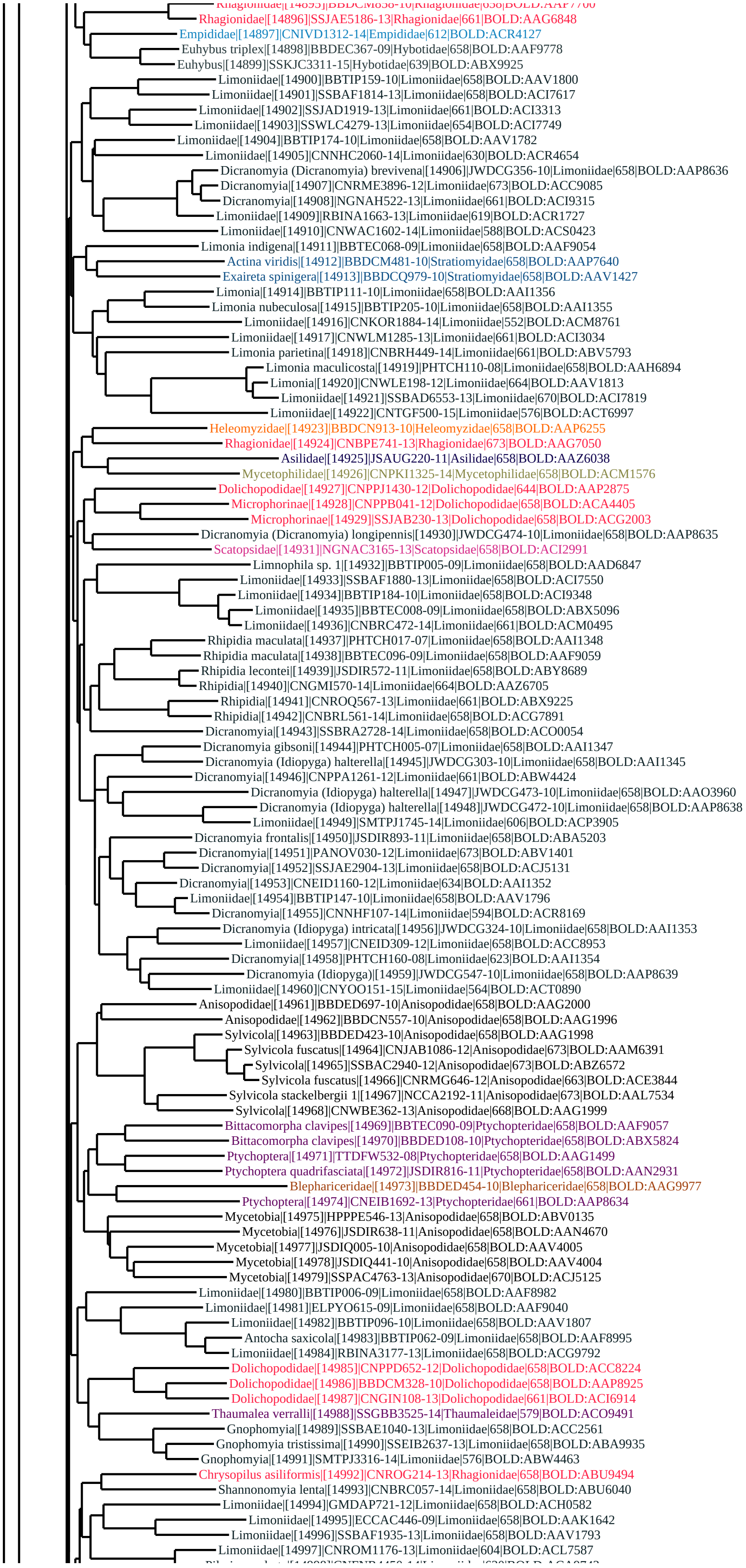
Rhagionidae|[14896]|SSJAE5186-13|Rhagionidae|661|BOLD:AAG6848
Empididae|[14897]|CNIVD1312-14|Empididae|612|BOLD:ACR4127
Euhybus triplex|[14898]|BBDEC367-09|Hybotidae|658|BOLD:AAF9778
Euhybus|[14899]|SSKJC3311-15|Hybotidae|639|BOLD:ABX9925
Limoniidae|[14900]|BBTIP159-10|Limoniidae|658|BOLD:AAV1800
Limoniidae|[14901]|SSBAF1814-13|Limoniidae|658|BOLD:ACI7617
Limoniidae|[14902]|SSJAD1919-13|Limoniidae|661|BOLD:ACI3313
Limoniidae|[14903]|SSWLC4279-13|Limoniidae|654|BOLD:ACI7749
Limoniidae|[14904]|BBTIP174-10|Limoniidae|658|BOLD:AAV1782
Limoniidae|[14905]|CNNHC2060-14|Limoniidae|630|BOLD:ACR4654
Dicranomyia (Dicranomyia) brevivena|[14906]|JWDCG356-10|Limoniidae|658|BOLD:AAP8636
Dicranomyia|[14907]|CNRME3896-12|Limoniidae|673|BOLD:ACC9085
Dicranomyia|[14908]|NGNAH522-13|Limoniidae|661|BOLD:ACI9315
Limoniidae|[14909]|RBINA1663-13|Limoniidae|619|BOLD:ACR1727
Limoniidae|[14910]|CNWAC1602-14|Limoniidae|588|BOLD:ACS0423
Limonia indigena|[14911]|BBTEC068-09|Limoniidae|658|BOLD:AAF9054
Actina viridis|[14912]|BBDCM481-10|Stratiomyidae|658|BOLD:AAP7640
Exaireta spinigera|[14913]|BBDCQ979-10|Stratiomyidae|658|BOLD:AAV1427
Limonia|[14914]|BBTIP111-10|Limoniidae|658|BOLD:AAI1356
Limonia nubeculosa|[14915]|BBTIP205-10|Limoniidae|658|BOLD:AAI1355
Limoniidae|[14916]|CNKOR1884-14|Limoniidae|552|BOLD:ACM8761
Limoniidae|[14917]|CNWLM1285-13|Limoniidae|661|BOLD:ACI3034
Limonia parietina|[14918]|CNBRH449-14|Limoniidae|661|BOLD:ABV5793
Limonia maculicosta|[14919]|PHTCH110-08|Limoniidae|658|BOLD:AAH6894
Limonia|[14920]|CNWLE198-12|Limoniidae|664|BOLD:AAV1813
Limoniidae|[14921]|SSBAD6553-13|Limoniidae|670|BOLD:ACI7819
Limoniidae|[14922]|CNTGF500-15|Limoniidae|576|BOLD:ACT6997
Heleomyzidae|[14923]|BBDCN913-10|Heleomyzidae|658|BOLD:AAP6255
Rhagionidae|[14924]|CNBPE741-13|Rhagionidae|673|BOLD:AAG7050
Asilidae|[14925]|JSAUG220-11|Asilidae|658|BOLD:AAZ6038
Mycetophilidae|[14926]|CNPKI1325-14|Mycetophilidae|658|BOLD:ACM1576
Dolichopodidae|[14927]|CNPPJ1430-12|Dolichopodidae|644|BOLD:AAP2875
Microphorinae|[14928]|CNPPB041-12|Dolichopodidae|658|BOLD:ACA4405
Microphorinae|[14929]|SSJAB230-13|Dolichopodidae|658|BOLD:ACG2003
Dicranomyia (Dicranomyia) longipennis|[14930]|JWDCG474-10|Limoniidae|658|BOLD:AAP8635
Scatopsidae|[14931]|NGNAC3165-13|Scatopsidae|658|BOLD:ACI2991
Limnophila sp. 1|[14932]|BBTIP005-09|Limoniidae|658|BOLD:AAD6847
Limoniidae|[14933]|SSBAF1880-13|Limoniidae|658|BOLD:ACI7550
Limoniidae|[14934]|BBTIP184-10|Limoniidae|658|BOLD:ACI9348
Limoniidae|[14935]|BBTEC008-09|Limoniidae|658|BOLD:ABX5096
Limoniidae|[14936]|CNBRC472-14|Limoniidae|661|BOLD:ACM0495
Rhipidia maculata|[14937]|PHTCH017-07|Limoniidae|658|BOLD:AAI1348
Rhipidia maculata|[14938]|BBTEC096-09|Limoniidae|658|BOLD:AAF9059
Rhipidia lecontei|[14939]|JSDIR572-11|Limoniidae|658|BOLD:ABY8689
Rhipidia|[14940]|CNGMI570-14|Limoniidae|664|BOLD:AAZ6705
Rhipidia|[14941]|CNROQ567-13|Limoniidae|661|BOLD:ABX9225
Rhipidia|[14942]|CNBRL561-14|Limoniidae|658|BOLD:ACG7891
Dicranomyia|[14943]|SSBRA2728-14|Limoniidae|658|BOLD:ACO0054
Dicranomyia gibsoni|[14944]|PHTCH005-07|Limoniidae|658|BOLD:AAI1347
Dicranomyia (Idiopyga) halterella|[14945]|JWDCG303-10|Limoniidae|658|BOLD:AAI1345
Dicranomyia|[14946]|CNPPA1261-12|Limoniidae|661|BOLD:ABW4424
Dicranomyia (Idiopyga) halterella|[14947]|JWDCG473-10|Limoniidae|658|BOLD:AAO3960
Dicranomyia (Idiopyga) halterella|[14948]|JWDCG472-10|Limoniidae|658|BOLD:AAP8638
Limoniidae|[14949]|SMTPJ1745-14|Limoniidae|606|BOLD:ACP3905
Dicranomyia frontalis|[14950]|JSDIR893-11|Limoniidae|658|BOLD:ABA5203
Dicranomyia|[14951]|PANOV030-12|Limoniidae|673|BOLD:ABV1401
Dicranomyia|[14952]|SSJAE2904-13|Limoniidae|658|BOLD:ACJ5131
Dicranomyia|[14953]|CNEID1160-12|Limoniidae|634|BOLD:AAI1352
Limoniidae|[14954]|BBTIP147-10|Limoniidae|658|BOLD:AAV1796
Dicranomyia|[14955]|CNNHF107-14|Limoniidae|594|BOLD:ACR8169
Dicranomyia (Idiopyga) intricata|[14956]|JWDCG324-10|Limoniidae|658|BOLD:AAI1353
Limoniidae|[14957]|CNEID309-12|Limoniidae|658|BOLD:ACC8953
Dicranomyia|[14958]|PHTCH160-08|Limoniidae|623|BOLD:AAI1354
Dicranomyia (Idiopyga)|[14959]|JWDCG547-10|Limoniidae|658|BOLD:AAP8639
Limoniidae|[14960]|CNYOO151-15|Limoniidae|564|BOLD:ACT0890
Anisopodidae|[14961]|BBDED697-10|Anisopodidae|658|BOLD:AAG2000
Anisopodidae|[14962]|BBDCN557-10|Anisopodidae|658|BOLD:AAG1996
Sylvicola|[14963]|BBDED423-10|Anisopodidae|658|BOLD:AAG1998
Sylvicola fuscatus|[14964]|CNJAB1086-12|Anisopodidae|673|BOLD:AAM6391
Sylvicola|[14965]|SSBAC2940-12|Anisopodidae|673|BOLD:ABZ6572
Sylvicola fuscatus|[14966]|CNRMG646-12|Anisopodidae|663|BOLD:ACE3844
Sylvicola stackelbergii 1|[14967]|NCCA2192-11|Anisopodidae|673|BOLD:AAL7534
Sylvicola|[14968]|CNWBE362-13|Anisopodidae|668|BOLD:AAG1999
Bittacomorpha clavipes|[14969]|BBTEC090-09|Ptychopteridae|658|BOLD:AAF9057
Bittacomorpha clavipes|[14970]|BBDED108-10|Ptychopteridae|658|BOLD:ABX5824
Ptychoptera|[14971]|TTDFW532-08|Ptychopteridae|658|BOLD:AAG1499
Ptychoptera quadrifasciata|[14972]|JSDIR816-11|Ptychopteridae|658|BOLD:AAN2931
Blephariceridae|[14973]|BBDED454-10|Blephariceridae|658|BOLD:AAG9977
Ptychoptera|[14974]|CNEIB1692-13|Ptychopteridae|661|BOLD:AAP8634
Mycetobia|[14975]|HPPPE546-13|Anisopodidae|658|BOLD:ABV0135
Mycetobia|[14976]|JSDIR638-11|Anisopodidae|658|BOLD:AAN4670
Mycetobia|[14977]|JSDIQ005-10|Anisopodidae|658|BOLD:AAV4005
Mycetobia|[14978]|JSDIQ441-10|Anisopodidae|658|BOLD:AAV4004
Mycetobia|[14979]|SSPAC4763-13|Anisopodidae|670|BOLD:ACJ5125
Limoniidae|[14980]|BBTIP006-09|Limoniidae|658|BOLD:AAF8982
Limoniidae|[14981]|ELPYO615-09|Limoniidae|658|BOLD:AAF9040
Limoniidae|[14982]|BBTIP096-10|Limoniidae|658|BOLD:AAV1807
Antocha saxicola|[14983]|BBTIP062-09|Limoniidae|658|BOLD:AAF8995
Limoniidae|[14984]|RBINA3177-13|Limoniidae|658|BOLD:ACG9792
Dolichopodidae|[14985]|CNPPD652-12|Dolichopodidae|658|BOLD:ACC8224
Dolichopodidae|[14986]|BBDCM328-10|Dolichopodidae|658|BOLD:AAP8925
Dolichopodidae|[14987]|CNGIN108-13|Dolichopodidae|661|BOLD:ACI6914
Thaumalea verralli|[14988]|SSGBB3525-14|Thaumaleidae|579|BOLD:ACO9491
Gnophomyia|[14989]|SSBAE1040-13|Limoniidae|658|BOLD:ACC2561
Gnophomyia tristissima|[14990]|SSEIB2637-13|Limoniidae|658|BOLD:ABA9935
Gnophomyia|[14991]|SMTPJ3316-14|Limoniidae|576|BOLD:ABW4463
Chrysopilus asiliformis|[14992]|CNROG214-13|Rhagionidae|658|BOLD:ABU9494
Shannonomyia lenta|[14993]|CNBRC057-14|Limoniidae|658|BOLD:ABU6040
Limoniidae|[14994]|GMDAP721-12|Limoniidae|658|BOLD:ACH0582
Limoniidae|[14995]|ECCAC446-09|Limoniidae|658|BOLD:AAK1642
Limoniidae|[14996]|SSBAF1935-13|Limoniidae|658|BOLD:AAV1793
Limoniidae|[14997]|CNROM1176-13|Limoniidae|604|BOLD:ACL7587

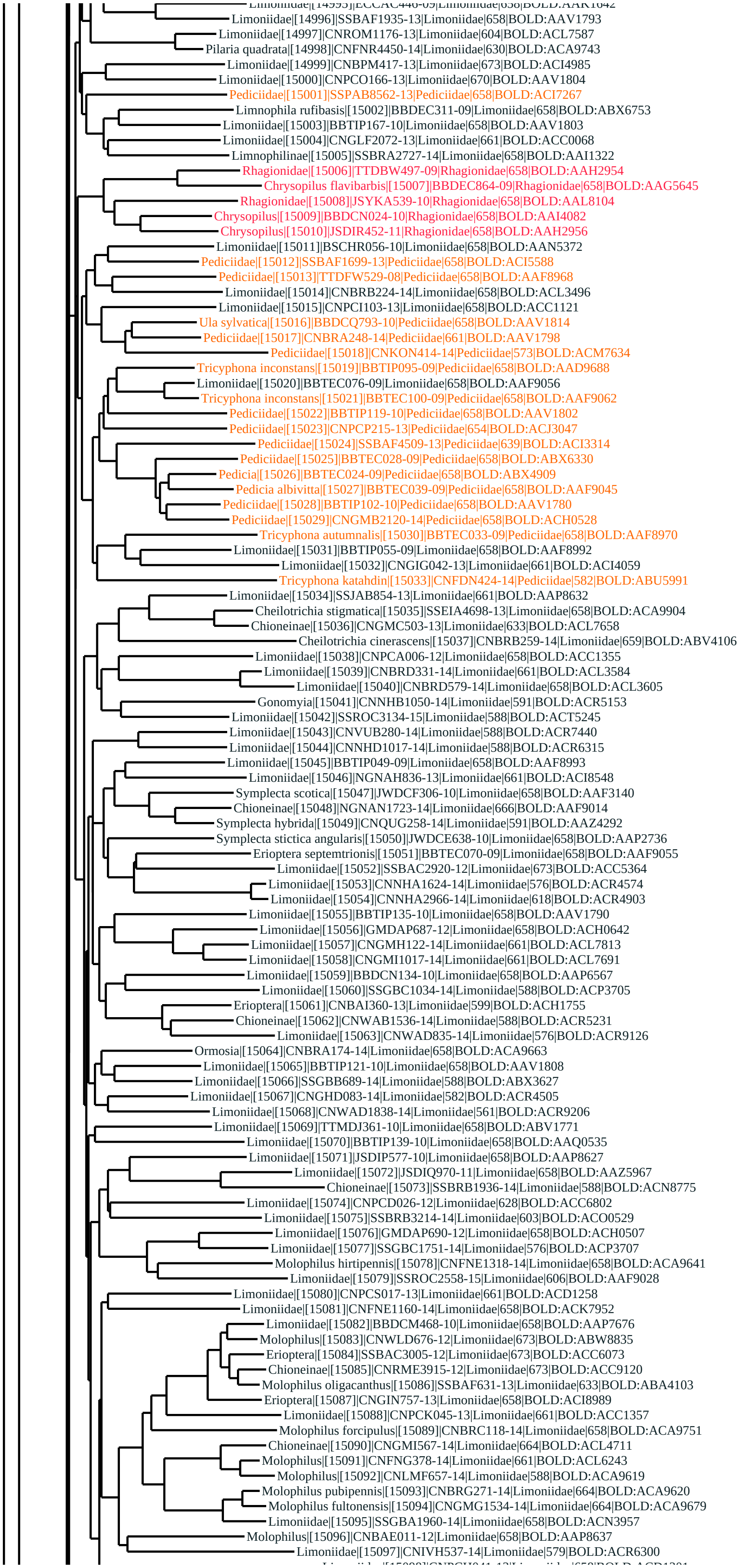
Limoniidae|[14996]|SSBAF1935-13|Limoniidae|658|BOLD:AAV1793
Limoniidae|[14997]|CNROM1176-13|Limoniidae|604|BOLD:ACL7587
Pilaria quadrata|[14998]|CNFNR4450-14|Limoniidae|630|BOLD:ACA9743
Limoniidae|[14999]|CNBPM417-13|Limoniidae|673|BOLD:ACI4985
Limoniidae|[15000]|CNPCO166-13|Limoniidae|670|BOLD:AAV1804
Pediciidae|[15001]|SSPAB8562-13|Pediciidae|658|BOLD:ACI7267
Limnophila rufibasis|[15002]|BBDEC311-09|Limoniidae|658|BOLD:ABX6753
Limoniidae|[15003]|BBTIP167-10|Limoniidae|658|BOLD:AAV1803
Limoniidae|[15004]|CNGLF2072-13|Limoniidae|661|BOLD:ACC0068
Limnophilinae|[15005]|SSBRA2727-14|Limoniidae|658|BOLD:AAI1322
Rhagionidae|[15006]|TTDBW497-09|Rhagionidae|658|BOLD:AAH2954
Chrysopilus flavibarbis|[15007]|BBDEC864-09|Rhagionidae|658|BOLD:AAG5645
Rhagionidae|[15008]|JSYKA539-10|Rhagionidae|658|BOLD:AAL8104
Chrysopilus|[15009]|BBDCN024-10|Rhagionidae|658|BOLD:AAI4082
Chrysopilus|[15010]|JSDIR452-11|Rhagionidae|658|BOLD:AAH2956
Limoniidae|[15011]|BSCHR056-10|Limoniidae|658|BOLD:AAN5372
Pediciidae|[15012]|SSBAF1699-13|Pediciidae|658|BOLD:ACI5588
Pediciidae|[15013]|TTDFW529-08|Pediciidae|658|BOLD:AAF8968
Limoniidae|[15014]|CNBRB224-14|Limoniidae|658|BOLD:ACL3496
Limoniidae|[15015]|CNPCI103-13|Limoniidae|658|BOLD:ACC1121
Ula sylvatica|[15016]|BBDCQ793-10|Pediciidae|658|BOLD:AAV1814
Pediciidae|[15017]|CNBRA248-14|Pediciidae|661|BOLD:AAV1798
Pediciidae|[15018]|CNKON414-14|Pediciidae|573|BOLD:ACM7634
Tricyphona inconstans|[15019]|BBTIP095-09|Pediciidae|658|BOLD:AAD9688
Limoniidae|[15020]|BBTEC076-09|Limoniidae|658|BOLD:AAF9056
Tricyphona inconstans|[15021]|BBTEC100-09|Pediciidae|658|BOLD:AAF9062
Pediciidae|[15022]|BBTIP119-10|Pediciidae|658|BOLD:AAV1802
Pediciidae|[15023]|CNPCP215-13|Pediciidae|654|BOLD:ACJ3047
Pediciidae|[15024]|SSBAF4509-13|Pediciidae|639|BOLD:ACI3314
Pediciidae|[15025]|BBTEC028-09|Pediciidae|658|BOLD:ABX6330
Pedicia|[15026]|BBTEC024-09|Pediciidae|658|BOLD:ABX4909
Pedicia albivitta|[15027]|BBTEC039-09|Pediciidae|658|BOLD:AAF9045
Pediciidae|[15028]|BBTIP102-10|Pediciidae|658|BOLD:AAV1780
Pediciidae|[15029]|CNGMB2120-14|Pediciidae|658|BOLD:ACH0528
Tricyphona autumnalis|[15030]|BBTEC033-09|Pediciidae|658|BOLD:AAF8970
Limoniidae|[15031]|BBTIP055-09|Limoniidae|658|BOLD:AAF8992
Limoniidae|[15032]|CNGIG042-13|Limoniidae|661|BOLD:ACI4059
Tricyphona katahdin|[15033]|CNFDN424-14|Pediciidae|582|BOLD:ABU5991
Limoniidae|[15034]|SSJAB854-13|Limoniidae|661|BOLD:AAP8632
Cheilotrichia stigmatica|[15035]|SSEIA4698-13|Limoniidae|658|BOLD:ACA9904
Chioneinae|[15036]|CNGMC503-13|Limoniidae|633|BOLD:ACL7658
Cheilotrichia cinerascens|[15037]|CNBRB259-14|Limoniidae|659|BOLD:ABV4106
Limoniidae|[15038]|CNPCA006-12|Limoniidae|658|BOLD:ACC1355
Limoniidae|[15039]|CNBRD331-14|Limoniidae|661|BOLD:ACL3584
Limoniidae|[15040]|CNBRD579-14|Limoniidae|658|BOLD:ACL3605
Gonomyia|[15041]|CNNHB1050-14|Limoniidae|591|BOLD:ACR5153
Limoniidae|[15042]|SSROC3134-15|Limoniidae|588|BOLD:ACT5245
Limoniidae|[15043]|CNVUB280-14|Limoniidae|588|BOLD:ACR7440
Limoniidae|[15044]|CNNHD1017-14|Limoniidae|588|BOLD:ACR6315
Limoniidae|[15045]|BBTIP049-09|Limoniidae|658|BOLD:AAF8993
Limoniidae|[15046]|NGNAH836-13|Limoniidae|661|BOLD:ACI8548
Symplecta scotica|[15047]|JWDCF306-10|Limoniidae|658|BOLD:AAF3140
Chioneinae|[15048]|NGNAN1723-14|Limoniidae|666|BOLD:AAF9014
Symplecta hybrida|[15049]|CNQUG258-14|Limoniidae|591|BOLD:AAZ4292
Symplecta stictica angularis|[15050]|JWDCE638-10|Limoniidae|658|BOLD:AAP2736
Erioptera septemtrionis|[15051]|BBTEC070-09|Limoniidae|658|BOLD:AAF9055
Limoniidae|[15052]|SSBAC2920-12|Limoniidae|673|BOLD:ACC5364
Limoniidae|[15053]|CNNHA1624-14|Limoniidae|576|BOLD:ACR4574
Limoniidae|[15054]|CNNHA2966-14|Limoniidae|618|BOLD:ACR4903
Limoniidae|[15055]|BBTIP135-10|Limoniidae|658|BOLD:AAV1790
Limoniidae|[15056]|GMDAP687-12|Limoniidae|658|BOLD:ACH0642
Limoniidae|[15057]|CNGMH122-14|Limoniidae|661|BOLD:ACL7813
Limoniidae|[15058]|CNGMI1017-14|Limoniidae|661|BOLD:ACL7691
Limoniidae|[15059]|BBDCN134-10|Limoniidae|658|BOLD:AAP6567
Limoniidae|[15060]|SSGBC1034-14|Limoniidae|588|BOLD:ACP3705
Erioptera|[15061]|CNBAI360-13|Limoniidae|599|BOLD:ACH1755
Chioneinae|[15062]|CNWAB1536-14|Limoniidae|588|BOLD:ACR5231
Limoniidae|[15063]|CNWAD835-14|Limoniidae|576|BOLD:ACR9126
Ormosia|[15064]|CNBRA174-14|Limoniidae|658|BOLD:ACA9663
Limoniidae|[15065]|BBTIP121-10|Limoniidae|658|BOLD:AAV1808
Limoniidae|[15066]|SSGBB689-14|Limoniidae|588|BOLD:ABX3627
Limoniidae|[15067]|CNGHD083-14|Limoniidae|582|BOLD:ACR4505
Limoniidae|[15068]|CNWAD1838-14|Limoniidae|561|BOLD:ACR9206
Limoniidae|[15069]|TTMDJ361-10|Limoniidae|658|BOLD:ABV1771
Limoniidae|[15070]|BBTIP139-10|Limoniidae|658|BOLD:AAQ0535
Limoniidae|[15071]|JSDIP577-10|Limoniidae|658|BOLD:AAP8627
Limoniidae|[15072]|JSDIQ970-11|Limoniidae|658|BOLD:AAZ5967
Chioneinae|[15073]|SSBRB1936-14|Limoniidae|588|BOLD:ACN8775
Limoniidae|[15074]|CNPCD026-12|Limoniidae|628|BOLD:ACC6802
Limoniidae|[15075]|SSBRB3214-14|Limoniidae|603|BOLD:ACO0529
Limoniidae|[15076]|GMDAP690-12|Limoniidae|658|BOLD:ACH0507
Limoniidae|[15077]|SSGBC1751-14|Limoniidae|576|BOLD:ACP3707
Molophilus hirtipennis|[15078]|CNFNE1318-14|Limoniidae|658|BOLD:ACA9641
Limoniidae|[15079]|SSROC2558-15|Limoniidae|606|BOLD:AAF9028
Limoniidae|[15080]|CNPCS017-13|Limoniidae|661|BOLD:ACD1258
Limoniidae|[15081]|CNFNE1160-14|Limoniidae|658|BOLD:ACK7952
Limoniidae|[15082]|BBDCM468-10|Limoniidae|658|BOLD:AAP7676
Molophilus|[15083]|CNWLD676-12|Limoniidae|673|BOLD:ABW8835
Erioptera|[15084]|SSBAC3005-12|Limoniidae|673|BOLD:ACC6073
Chioneinae|[15085]|CNRME3915-12|Limoniidae|673|BOLD:ACC9120
Molophilus oligacanthus|[15086]|SSBAF631-13|Limoniidae|633|BOLD:ABA4103
Erioptera|[15087]|CNGIN757-13|Limoniidae|658|BOLD:ACI8989
Limoniidae|[15088]|CNPCK045-13|Limoniidae|661|BOLD:ACC1357
Molophilus forcipulus|[15089]|CNBRC118-14|Limoniidae|658|BOLD:ACA9751
Chioneinae|[15090]|CNGMI567-14|Limoniidae|664|BOLD:ACL4711
Molophilus|[15091]|CNFNG378-14|Limoniidae|661|BOLD:ACL6243
Molophilus|[15092]|CNLMF657-14|Limoniidae|588|BOLD:ACA9619
Molophilus pubipennis|[15093]|CNBRG271-14|Limoniidae|664|BOLD:ACA9620
Molophilus fultonensis|[15094]|CNGMG1534-14|Limoniidae|664|BOLD:ACA9679
Limoniidae|[15095]|SSGBA1960-14|Limoniidae|658|BOLD:ACN3957
Molophilus|[15096]|CNBAE011-12|Limoniidae|658|BOLD:AAP8637
Limoniidae|[15097]|CNIVH537-14|Limoniidae|579|BOLD:ACR6300

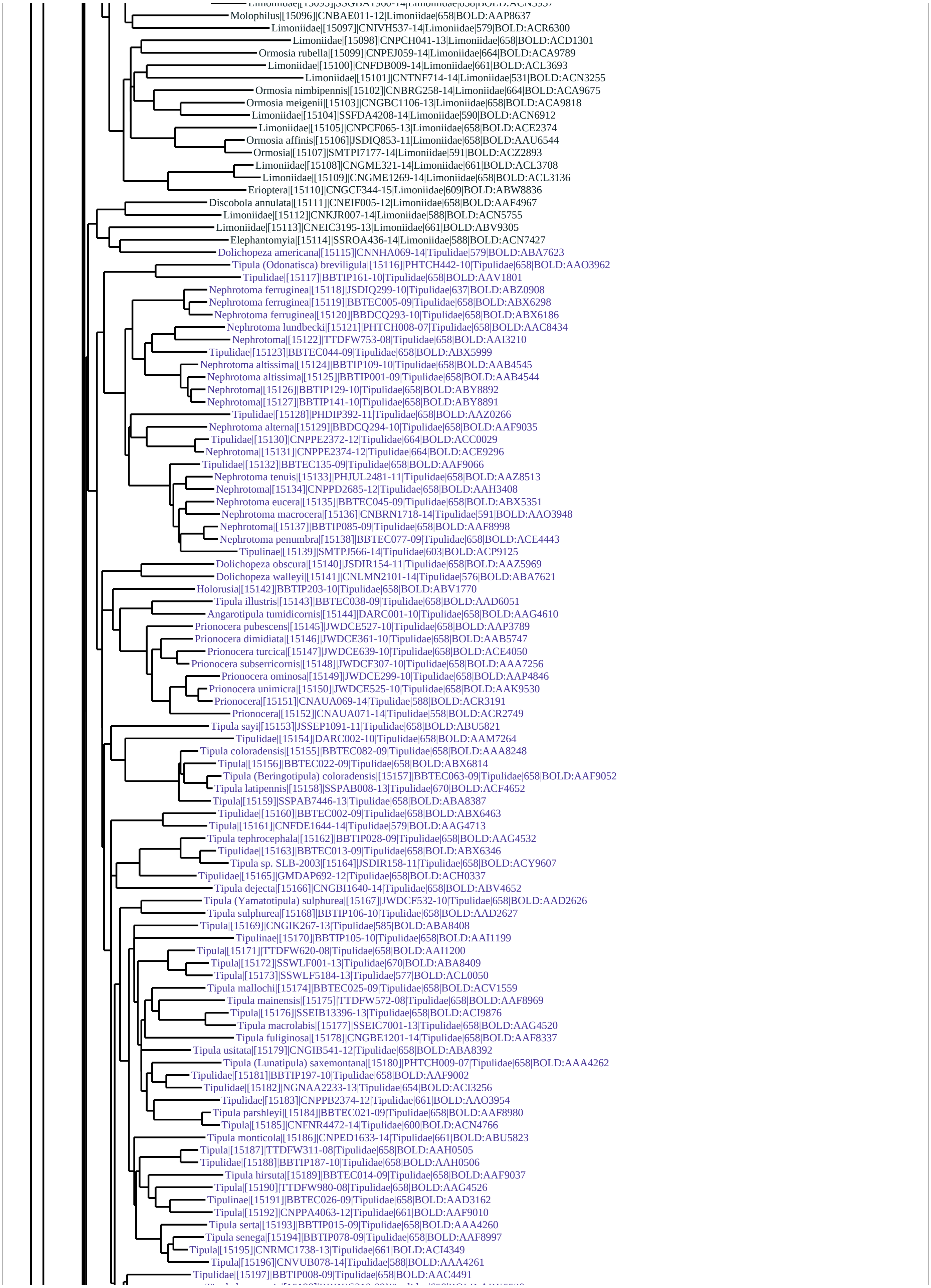
Molophilus|[15096]|CNBAE011-12|Limoniidae|658|BOLD:AAP8637
Limoniidae|[15097]|CNIVH537-14|Limoniidae|579|BOLD:ACR6300
Limoniidae|[15098]|CNPCH041-13|Limoniidae|658|BOLD:ACD1301
Ormosia rubella|[15099]|CNPEJ059-14|Limoniidae|664|BOLD:ACA9789
Limoniidae|[15100]|CNFDB009-14|Limoniidae|661|BOLD:ACL3693
Limoniidae|[15101]|CNTNF714-14|Limoniidae|531|BOLD:ACN3255
Ormosia nimbipennis|[15102]|CNBRG258-14|Limoniidae|664|BOLD:ACA9675
Ormosia meigenii|[15103]|CNGBC1106-13|Limoniidae|658|BOLD:ACA9818
Limoniidae|[15104]|SSFDA4208-14|Limoniidae|590|BOLD:ACN6912
Limoniidae|[15105]|CNPCF065-13|Limoniidae|658|BOLD:ACE2374
Ormosia affinis|[15106]|JSDIQ853-11|Limoniidae|658|BOLD:AAU6544
Ormosia|[15107]|SMTPI7177-14|Limoniidae|591|BOLD:ACZ2893
Limoniidae|[15108]|CNGME321-14|Limoniidae|661|BOLD:ACL3708
Limoniidae|[15109]|CNGME1269-14|Limoniidae|658|BOLD:ACL3136
Erioptera|[15110]|CNGCF344-15|Limoniidae|609|BOLD:ABW8836
Discobola annulata|[15111]|CNEIF005-12|Limoniidae|658|BOLD:AAF4967
Limoniidae|[15112]|CNKJR007-14|Limoniidae|588|BOLD:ACN5755
Limoniidae|[15113]|CNEIC3195-13|Limoniidae|661|BOLD:ABV9305
Elephantomyia|[15114]|SSROA436-14|Limoniidae|588|BOLD:ACN7427
Dolichopeza americana|[15115]|CNNHA069-14|Tipulidae|579|BOLD:ABA7623
Tipula (Odonatisca) breviligula|[15116]|PHTCH442-10|Tipulidae|658|BOLD:AAO3962
Tipulidae|[15117]|BBTIP161-10|Tipulidae|658|BOLD:AAV1801
Nephrotoma ferruginea|[15118]|JSDIQ299-10|Tipulidae|637|BOLD:ABZ0908
Nephrotoma ferruginea|[15119]|BBTEC005-09|Tipulidae|658|BOLD:ABX6298
Nephrotoma ferruginea|[15120]|BBDCQ293-10|Tipulidae|658|BOLD:ABX6186
Nephrotoma lundbecki|[15121]|PHTCH008-07|Tipulidae|658|BOLD:AAC8434
Nephrotoma|[15122]|TTDFW753-08|Tipulidae|658|BOLD:AAI3210
Tipulidae|[15123]|BBTEC044-09|Tipulidae|658|BOLD:ABX5999
Nephrotoma altissima|[15124]|BBTIP109-10|Tipulidae|658|BOLD:AAB4545
Nephrotoma altissima|[15125]|BBTIP001-09|Tipulidae|658|BOLD:AAB4544
Nephrotoma|[15126]|BBTIP129-10|Tipulidae|658|BOLD:ABY8892
Nephrotoma|[15127]|BBTIP141-10|Tipulidae|658|BOLD:ABY8891
Tipulidae|[15128]|PHDIP392-11|Tipulidae|658|BOLD:AAZ0266
Nephrotoma alterna|[15129]|BBDCQ294-10|Tipulidae|658|BOLD:AAF9035
Tipulidae|[15130]|CNPPE2372-12|Tipulidae|664|BOLD:ACC0029
Nephrotoma|[15131]|CNPPE2374-12|Tipulidae|664|BOLD:ACE9296
Tipulidae|[15132]|BBTEC135-09|Tipulidae|658|BOLD:AAF9066
Nephrotoma tenuis|[15133]|PHJUL2481-11|Tipulidae|658|BOLD:AAZ8513
Nephrotoma|[15134]|CNPPD2685-12|Tipulidae|658|BOLD:AAH3408
Nephrotoma eucera|[15135]|BBTEC045-09|Tipulidae|658|BOLD:ABX5351
Nephrotoma macrocera|[15136]|CNBRN1718-14|Tipulidae|591|BOLD:AAO3948
Nephrotoma|[15137]|BBTIP085-09|Tipulidae|658|BOLD:AAF8998
Nephrotoma penumbra|[15138]|BBTEC077-09|Tipulidae|658|BOLD:ACE4443
Tipulinae|[15139]|SMTPJ566-14|Tipulidae|603|BOLD:ACP9125
Dolichopeza obscura|[15140]|JSDIR154-11|Tipulidae|658|BOLD:AAZ5969
Dolichopeza walleyi|[15141]|CNLMN2101-14|Tipulidae|576|BOLD:ABA7621
Holorusia|[15142]|BBTIP203-10|Tipulidae|658|BOLD:ABV1770
Tipula illustris|[15143]|BBTEC038-09|Tipulidae|658|BOLD:AAD6051
Angarotipula tumidicornis|[15144]|DARC001-10|Tipulidae|658|BOLD:AAG4610
Prionocera pubescens|[15145]|JWDCE527-10|Tipulidae|658|BOLD:AAP3789
Prionocera dimidiata|[15146]|JWDCE361-10|Tipulidae|658|BOLD:AAB5747
Prionocera turcica|[15147]|JWDCE639-10|Tipulidae|658|BOLD:ACE4050
Prionocera subserricornis|[15148]|JWDCF307-10|Tipulidae|658|BOLD:AAA7256
Prionocera ominosa|[15149]|JWDCE299-10|Tipulidae|658|BOLD:AAP4846
Prionocera unimicra|[15150]|JWDCE525-10|Tipulidae|658|BOLD:AAK9530
Prionocera|[15151]|CNAUA069-14|Tipulidae|588|BOLD:ACR3191
Prionocera|[15152]|CNAUA071-14|Tipulidae|558|BOLD:ACR2749
Tipula sayi|[15153]|JSSEP1091-11|Tipulidae|658|BOLD:ABU5821
Tipulidae|[15154]|DARC002-10|Tipulidae|658|BOLD:AAM7264
Tipula coloradensis|[15155]|BBTEC082-09|Tipulidae|658|BOLD:AAA8248
Tipula|[15156]|BBTEC022-09|Tipulidae|658|BOLD:ABX6814
Tipula (Beringotipula) coloradensis|[15157]|BBTEC063-09|Tipulidae|658|BOLD:AAF9052
Tipula latipennis|[15158]|SSPAB008-13|Tipulidae|670|BOLD:ACF4652
Tipula|[15159]|SSPAB7446-13|Tipulidae|658|BOLD:ABA8387
Tipulidae|[15160]|BBTEC002-09|Tipulidae|658|BOLD:ABX6463
Tipula|[15161]|CNFDE1644-14|Tipulidae|579|BOLD:AAG4713
Tipula tephrocephala|[15162]|BBTIP028-09|Tipulidae|658|BOLD:AAG4532
Tipulidae|[15163]|BBTEC013-09|Tipulidae|658|BOLD:ABX6346
Tipula sp. SLB-2003|[15164]|JSDIR158-11|Tipulidae|658|BOLD:ACY9607
Tipulidae|[15165]|GMDAP692-12|Tipulidae|658|BOLD:ACH0337
Tipula dejecta|[15166]|CNGBI1640-14|Tipulidae|658|BOLD:ABV4652
Tipula (Yamatotipula) sulphurea|[15167]|JWDCF532-10|Tipulidae|658|BOLD:AAD2626
Tipula sulphurea|[15168]|BBTIP106-10|Tipulidae|658|BOLD:AAD2627
Tipula|[15169]|CNGIK267-13|Tipulidae|585|BOLD:ABA8408
Tipulinae|[15170]|BBTIP105-10|Tipulidae|658|BOLD:AAI1199
Tipula|[15171]|TTDFW620-08|Tipulidae|658|BOLD:AAI1200
Tipula|[15172]|SSWLF001-13|Tipulidae|670|BOLD:ABA8409
Tipula|[15173]|SSWLF5184-13|Tipulidae|577|BOLD:ACL0050
Tipula mallochi|[15174]|BBTEC025-09|Tipulidae|658|BOLD:ACV1559
Tipula mainensis|[15175]|TTDFW572-08|Tipulidae|658|BOLD:AAF8969
Tipula|[15176]|SSEIB13396-13|Tipulidae|658|BOLD:ACI9876
Tipula macrolabis|[15177]|SSEIC7001-13|Tipulidae|658|BOLD:AAG4520
Tipula fuliginosa|[15178]|CNGBE1201-14|Tipulidae|658|BOLD:AAF8337
Tipula usitata|[15179]|CNGIB541-12|Tipulidae|658|BOLD:ABA8392
Tipula (Lunatipula) saxemontana|[15180]|PHTCH009-07|Tipulidae|658|BOLD:AAA4262
Tipulidae|[15181]|BBTIP197-10|Tipulidae|658|BOLD:AAF9002
Tipulidae|[15182]|NGNAA2233-13|Tipulidae|654|BOLD:ACI3256
Tipulidae|[15183]|CNPPB2374-12|Tipulidae|661|BOLD:AAO3954
Tipula parshleyi|[15184]|BBTEC021-09|Tipulidae|658|BOLD:AAF8980
Tipula|[15185]|CNFNR4472-14|Tipulidae|600|BOLD:ACN4766
Tipula monticola|[15186]|CNPED1633-14|Tipulidae|661|BOLD:ABU5823
Tipula|[15187]|TTDFW311-08|Tipulidae|658|BOLD:AAH0505
Tipulidae|[15188]|BBTIP187-10|Tipulidae|658|BOLD:AAH0506
Tipula hirsuta|[15189]|BBTEC014-09|Tipulidae|658|BOLD:AAF9037
Tipula|[15190]|TTDFW980-08|Tipulidae|658|BOLD:AAG4526
Tipulinae|[15191]|BBTEC026-09|Tipulidae|658|BOLD:AAD3162
Tipula|[15192]|CNPPA4063-12|Tipulidae|661|BOLD:AAF9010
Tipula serta|[15193]|BBTIP015-09|Tipulidae|658|BOLD:AAA4260
Tipula senega|[15194]|BBTIP078-09|Tipulidae|658|BOLD:AAF8997
Tipula|[15195]|CNRMC1738-13|Tipulidae|661|BOLD:ACI4349
Tipula|[15196]|CNVUB078-14|Tipulidae|588|BOLD:AAA4261
Tipulidae|[15197]|BBTIP008-09|Tipulidae|658|BOLD:AAC4491

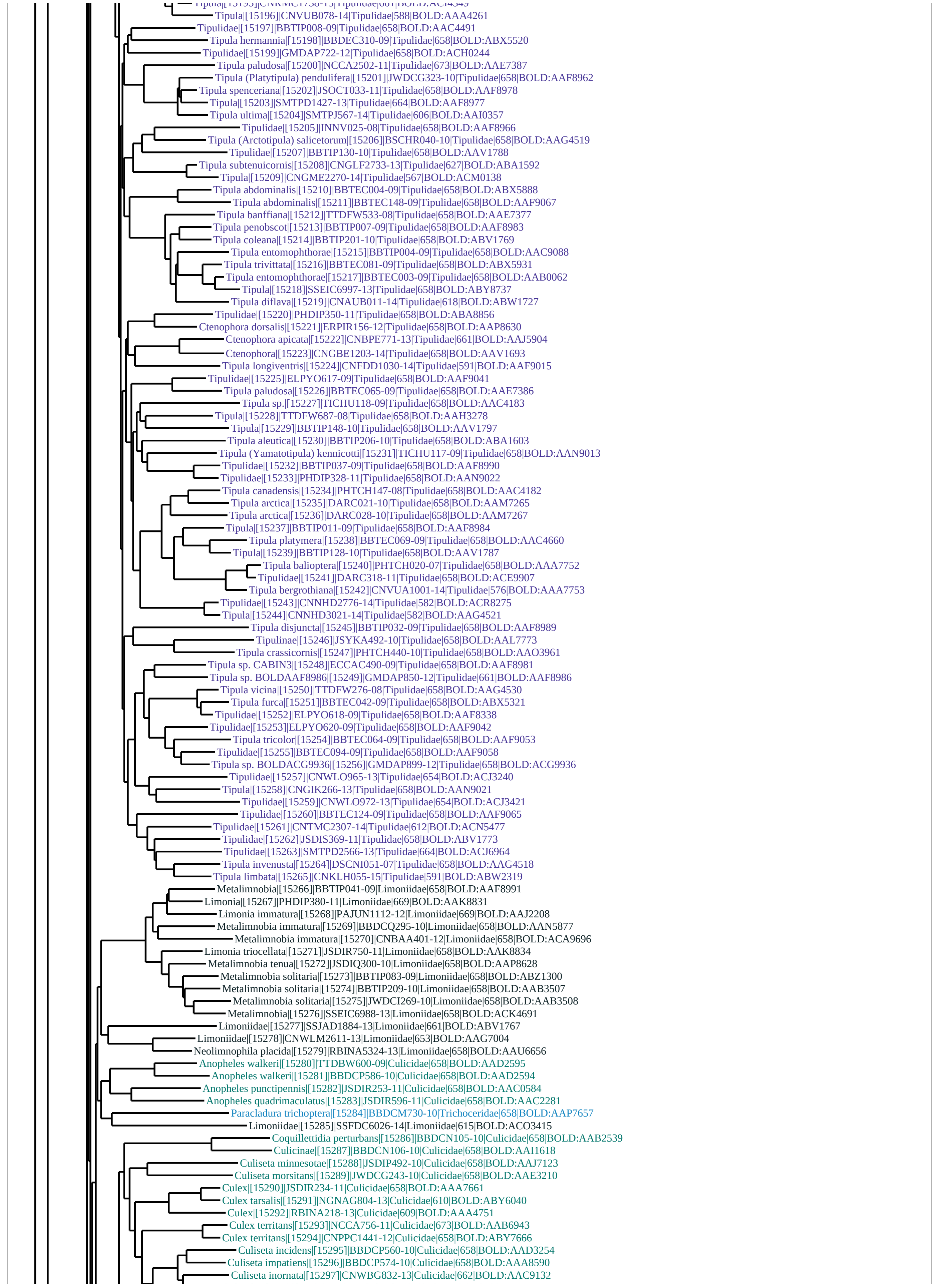
Tipula|[15195]|CNRMC1738-13|Tipulidae|661|BOLD:ACI4349
Tipula|[15196]|CNVUB078-14|Tipulidae|588|BOLD:AAA4261
Tipulidae|[15197]|BBTIP008-09|Tipulidae|658|BOLD:AAC4491
Tipula hermannia|[15198]|BBDEC310-09|Tipulidae|658|BOLD:ABX5520
Tipulidae|[15199]|GMDAP722-12|Tipulidae|658|BOLD:ACH0244
Tipula paludosa|[15200]|NCCA2502-11|Tipulidae|673|BOLD:AAE7387
Tipula (Platytipula) pendulifera|[15201]|JWDCG323-10|Tipulidae|658|BOLD:AAF8962
Tipula spenceriana|[15202]|JSOCT033-11|Tipulidae|658|BOLD:AAF8978
Tipula|[15203]|SMTPD1427-13|Tipulidae|664|BOLD:AAF8977
Tipula ultima|[15204]|SMTPJ567-14|Tipulidae|606|BOLD:AAI0357
Tipulidae|[15205]|INNV025-08|Tipulidae|658|BOLD:AAF8966
Tipula (Arctotipula) salicetorum|[15206]|BSCHR040-10|Tipulidae|658|BOLD:AAG4519
Tipulidae|[15207]|BBTIP130-10|Tipulidae|658|BOLD:AAV1788
Tipula subtenuicornis|[15208]|CNGLF2733-13|Tipulidae|627|BOLD:ABA1592
Tipula|[15209]|CNGME2270-14|Tipulidae|567|BOLD:ACM0138
Tipula abdominalis|[15210]|BBTEC004-09|Tipulidae|658|BOLD:ABX5888
Tipula abdominalis|[15211]|BBTEC148-09|Tipulidae|658|BOLD:AAF9067
Tipula banffiana|[15212]|TTDFW533-08|Tipulidae|658|BOLD:AAE7377
Tipula penobscot|[15213]|BBTIP007-09|Tipulidae|658|BOLD:AAF8983
Tipula coleana|[15214]|BBTIP201-10|Tipulidae|658|BOLD:ABV1769
Tipula entomophthorae|[15215]|BBTIP004-09|Tipulidae|658|BOLD:AAC9088
Tipula trivittata|[15216]|BBTEC081-09|Tipulidae|658|BOLD:ABX5931
Tipula entomophthorae|[15217]|BBTEC003-09|Tipulidae|658|BOLD:AAB0062
Tipula|[15218]|SSEIC6997-13|Tipulidae|658|BOLD:ABY8737
Tipula diflava|[15219]|CNAUB011-14|Tipulidae|618|BOLD:ABW1727
Tipulidae|[15220]|PHDIP350-11|Tipulidae|658|BOLD:ABA8856
Ctenophora dorsalis|[15221]|ERPIR156-12|Tipulidae|658|BOLD:AAP8630
Ctenophora apicata|[15222]|CNBPE771-13|Tipulidae|661|BOLD:AAJ5904
Ctenophora|[15223]|CNGBE1203-14|Tipulidae|658|BOLD:AAV1693
Tipula longiventris|[15224]|CNFDD1030-14|Tipulidae|591|BOLD:AAF9015
Tipulidae|[15225]|ELPYO617-09|Tipulidae|658|BOLD:AAF9041
Tipula paludosa|[15226]|BBTEC065-09|Tipulidae|658|BOLD:AAE7386
Tipula sp.|[15227]|TICHU118-09|Tipulidae|658|BOLD:AAC4183
Tipula|[15228]|TTDFW687-08|Tipulidae|658|BOLD:AAH3278
Tipula|[15229]|BBTIP148-10|Tipulidae|658|BOLD:AAV1797
Tipula aleutica|[15230]|BBTIP206-10|Tipulidae|658|BOLD:ABA1603
Tipula (Yamatotipula) kennicotti|[15231]|TICHU117-09|Tipulidae|658|BOLD:AAN9013
Tipulidae|[15232]|BBTIP037-09|Tipulidae|658|BOLD:AAF8990
Tipulidae|[15233]|PHDIP328-11|Tipulidae|658|BOLD:AAN9022
Tipula canadensis|[15234]|PHTCH147-08|Tipulidae|658|BOLD:AAC4182
Tipula arctica|[15235]|DARC021-10|Tipulidae|658|BOLD:AAM7265
Tipula arctica|[15236]|DARC028-10|Tipulidae|658|BOLD:AAM7267
Tipula|[15237]|BBTIP011-09|Tipulidae|658|BOLD:AAF8984
Tipula platymera|[15238]|BBTEC069-09|Tipulidae|658|BOLD:AAC4660
Tipula|[15239]|BBTIP128-10|Tipulidae|658|BOLD:AAV1787
Tipula balioptera|[15240]|PHTCH020-07|Tipulidae|658|BOLD:AAA7752
Tipulidae|[15241]|DARC318-11|Tipulidae|658|BOLD:ACE9907
Tipula bergrothiana|[15242]|CNVUA1001-14|Tipulidae|576|BOLD:AAA7753
Tipulidae|[15243]|CNNHD2776-14|Tipulidae|582|BOLD:ACR8275
Tipula|[15244]|CNNHD3021-14|Tipulidae|582|BOLD:AAG4521
Tipula disjuncta|[15245]|BBTIP032-09|Tipulidae|658|BOLD:AAF8989
Tipulinae|[15246]|JSYKA492-10|Tipulidae|658|BOLD:AAL7773
Tipula crassicornis|[15247]|PHTCH440-10|Tipulidae|658|BOLD:AAO3961
Tipula sp. CABIN3|[15248]|ECCAC490-09|Tipulidae|658|BOLD:AAF8981
Tipula sp. BOLDAAF8986|[15249]|GMDAP850-12|Tipulidae|661|BOLD:AAF8986
Tipula vicina|[15250]|TTDFW276-08|Tipulidae|658|BOLD:AAG4530
Tipula furca|[15251]|BBTEC042-09|Tipulidae|658|BOLD:ABX5321
Tipulidae|[15252]|ELPYO618-09|Tipulidae|658|BOLD:AAF8338
Tipulidae|[15253]|ELPYO620-09|Tipulidae|658|BOLD:AAF9042
Tipula tricolor|[15254]|BBTEC064-09|Tipulidae|658|BOLD:AAF9053
Tipulidae|[15255]|BBTEC094-09|Tipulidae|658|BOLD:AAF9058
Tipula sp. BOLDACG9936|[15256]|GMDAP899-12|Tipulidae|658|BOLD:ACG9936
Tipulidae|[15257]|CNWLO965-13|Tipulidae|654|BOLD:ACJ3240
Tipula|[15258]|CNGIK266-13|Tipulidae|658|BOLD:AAN9021
Tipulidae|[15259]|CNWLO972-13|Tipulidae|654|BOLD:ACJ3421
Tipulidae|[15260]|BBTEC124-09|Tipulidae|658|BOLD:AAF9065
Tipulidae|[15261]|CNTMC2307-14|Tipulidae|612|BOLD:ACN5477
Tipulidae|[15262]|JSDIS369-11|Tipulidae|658|BOLD:ABV1773
Tipulidae|[15263]|SMTPD2566-13|Tipulidae|664|BOLD:ACJ6964
Tipula invenusta|[15264]|DSCNI051-07|Tipulidae|658|BOLD:AAG4518
Tipula limbata|[15265]|CNKLH055-15|Tipulidae|591|BOLD:ABW2319
Metalimnobia|[15266]|BBTIP041-09|Limoniidae|658|BOLD:AAF8991
Limonia|[15267]|PHDIP380-11|Limoniidae|669|BOLD:AAK8831
Limonia immatura|[15268]|PAJUN1112-12|Limoniidae|669|BOLD:AAJ2208
Metalimnobia immatura|[15269]|BBDCQ295-10|Limoniidae|658|BOLD:AAN5877
Metalimnobia immatura|[15270]|CNBAA401-12|Limoniidae|658|BOLD:ACA9696
Limonia triocellata|[15271]|JSDIR750-11|Limoniidae|658|BOLD:AAK8834
Metalimnobia tenua|[15272]|JSDIQ300-10|Limoniidae|658|BOLD:AAP8628
Metalimnobia solitaria|[15273]|BBTIP083-09|Limoniidae|658|BOLD:ABZ1300
Metalimnobia solitaria|[15274]|BBTIP209-10|Limoniidae|658|BOLD:AAB3507
Metalimnobia solitaria|[15275]|JWDCI269-10|Limoniidae|658|BOLD:AAB3508
Metalimnobia|[15276]|SSEIC6988-13|Limoniidae|658|BOLD:ACK4691
Limoniidae|[15277]|SSJAD1884-13|Limoniidae|661|BOLD:ABV1767
Limoniidae|[15278]|CNWLM2611-13|Limoniidae|653|BOLD:AAG7004
Neolimnophila placida|[15279]|RBINA5324-13|Limoniidae|658|BOLD:AAU6656
Anopheles walkeri|[15280]|TTDBW600-09|Culicidae|658|BOLD:AAD2595
Anopheles walkeri|[15281]|BBDCP586-10|Culicidae|658|BOLD:AAD2594
Anopheles punctipennis|[15282]|JSDIR253-11|Culicidae|658|BOLD:AAC0584
Anopheles quadrimaculatus|[15283]|JSDIR596-11|Culicidae|658|BOLD:AAC2281
Paracladura trichoptera|[15284]|BBDCM730-10|Trichoceridae|658|BOLD:AAP7657
Limoniidae|[15285]|SSFDC6026-14|Limoniidae|615|BOLD:ACO3415
Coquillettidia perturbans|[15286]|BBDCN105-10|Culicidae|658|BOLD:AAB2539
Culicinae|[15287]|BBDCN106-10|Culicidae|658|BOLD:AAI1618
Culiseta minnesotae|[15288]|JSDIP492-10|Culicidae|658|BOLD:AAJ7123
Culiseta morsitans|[15289]|JWDCG243-10|Culicidae|658|BOLD:AAE3210
Culex|[15290]|JSDIR234-11|Culicidae|658|BOLD:AAA7661
Culex tarsalis|[15291]|NGNAG804-13|Culicidae|610|BOLD:ABY6040
Culex|[15292]|RBINA218-13|Culicidae|609|BOLD:AAA4751
Culex territans|[15293]|NCCA756-11|Culicidae|673|BOLD:AAB6943
Culex territans|[15294]|CNPPC1441-12|Culicidae|658|BOLD:ABY7666
Culiseta incidens|[15295]|BBDCP560-10|Culicidae|658|BOLD:AAD3254
Culiseta impatiens|[15296]|BBDCP574-10|Culicidae|658|BOLD:AAA8590
Culiseta inornata|[15297]|CNWBG832-13|Culicidae|662|BOLD:AAC9132

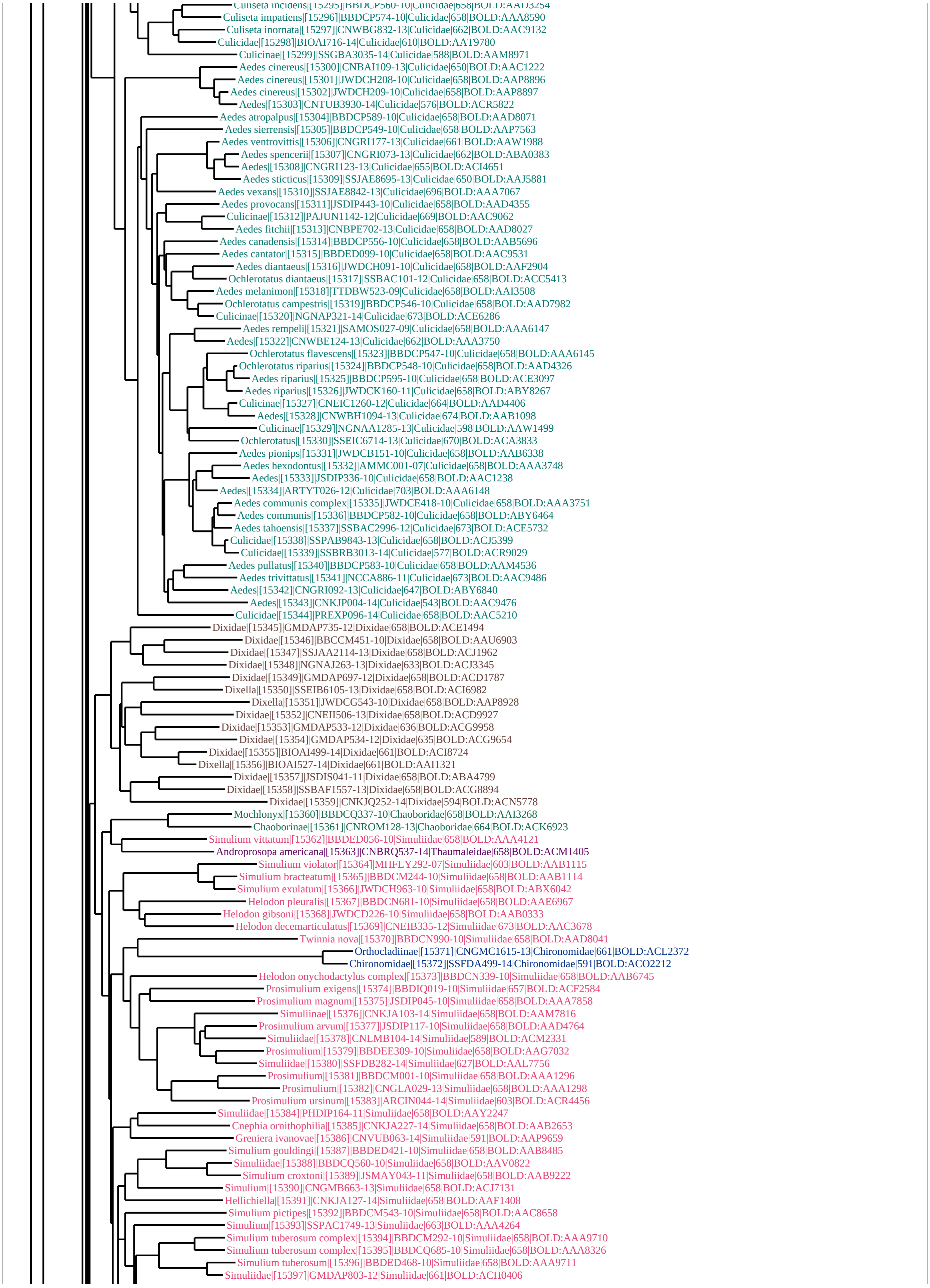
Culiseta incidens|[15295]|BBDCP560-10|Culicidae|658|BOLD:AAD3254
Culiseta impatiens|[15296]|BBDCP574-10|Culicidae|658|BOLD:AAA8590
Culiseta inornata|[15297]|CNWBG832-13|Culicidae|662|BOLD:AAC9132
Culicidae|[15298]|BIOAI716-14|Culicidae|610|BOLD:AAT9780
Culicinae|[15299]|SSGBA3035-14|Culicidae|588|BOLD:AAM8971
Aedes cinereus|[15300]|CNBAI109-13|Culicidae|650|BOLD:AAC1222
Aedes cinereus|[15301]|JWDCH208-10|Culicidae|658|BOLD:AAP8896
Aedes cinereus|[15302]|JWDCH209-10|Culicidae|658|BOLD:AAP8897
Aedes|[15303]|CNTUB3930-14|Culicidae|576|BOLD:ACR5822
Aedes atropalpus|[15304]|BBDCP589-10|Culicidae|658|BOLD:AAD8071
Aedes sierrensis|[15305]|BBDCP549-10|Culicidae|658|BOLD:AAP7563
Aedes ventrovittis|[15306]|CNGRI177-13|Culicidae|661|BOLD:AAW1988
Aedes spencerii|[15307]|CNGRI073-13|Culicidae|662|BOLD:ABA0383
Aedes|[15308]|CNGRI123-13|Culicidae|655|BOLD:ACI4651
Aedes sticticus|[15309]|SSJAE8695-13|Culicidae|650|BOLD:AAJ5881
Aedes vexans|[15310]|SSJAE8842-13|Culicidae|696|BOLD:AAA7067
Aedes provocans|[15311]|JSDIP443-10|Culicidae|658|BOLD:AAD4355
Culicinae|[15312]|PAJUN1142-12|Culicidae|669|BOLD:AAC9062
Aedes fitchii|[15313]|CNBPE702-13|Culicidae|658|BOLD:AAD8027
Aedes canadensis|[15314]|BBDCP556-10|Culicidae|658|BOLD:AAB5696
Aedes cantator|[15315]|BBDED099-10|Culicidae|658|BOLD:AAC9531
Aedes diantaeus|[15316]|JWDCH091-10|Culicidae|658|BOLD:AAF2904
Ochlerotatus diantaeus|[15317]|SSBAC101-12|Culicidae|658|BOLD:ACC5413
Aedes melanimon|[15318]|TTDBW523-09|Culicidae|658|BOLD:AAI3508
Ochlerotatus campestris|[15319]|BBDCP546-10|Culicidae|658|BOLD:AAD7982
Culicinae|[15320]|NGNAP321-14|Culicidae|673|BOLD:ACE6286
Aedes rempeli|[15321]|SAMOS027-09|Culicidae|658|BOLD:AAA6147
Aedes|[15322]|CNWBE124-13|Culicidae|662|BOLD:AAA3750
Ochlerotatus flavescens|[15323]|BBDCP547-10|Culicidae|658|BOLD:AAA6145
Ochlerotatus riparius|[15324]|BBDCP548-10|Culicidae|658|BOLD:AAD4326
Aedes riparius|[15325]|BBDCP595-10|Culicidae|658|BOLD:ACE3097
Aedes riparius|[15326]|JWDCK160-11|Culicidae|658|BOLD:ABY8267
Culicinae|[15327]|CNEIC1260-12|Culicidae|664|BOLD:AAD4406
Aedes|[15328]|CNWBH1094-13|Culicidae|674|BOLD:AAB1098
Culicinae|[15329]|NGNAA1285-13|Culicidae|598|BOLD:AAW1499
Ochlerotatus|[15330]|SSEIC6714-13|Culicidae|670|BOLD:ACA3833
Aedes pionips|[15331]|JWDCB151-10|Culicidae|658|BOLD:AAB6338
Aedes hexodontus|[15332]|AMMC001-07|Culicidae|658|BOLD:AAA3748
Aedes|[15333]|JSDIP336-10|Culicidae|658|BOLD:AAC1238
Aedes|[15334]|ARTYT026-12|Culicidae|703|BOLD:AAA6148
Aedes communis complex|[15335]|JWDCE418-10|Culicidae|658|BOLD:AAA3751
Aedes communis|[15336]|BBDCP582-10|Culicidae|658|BOLD:ABY6464
Aedes tahoensis|[15337]|SSBAC2996-12|Culicidae|673|BOLD:ACE5732
Culicidae|[15338]|SSPAB9843-13|Culicidae|658|BOLD:ACJ5399
Culicidae|[15339]|SSBRB3013-14|Culicidae|577|BOLD:ACR9029
Aedes pullatus|[15340]|BBDCP583-10|Culicidae|658|BOLD:AAM4536
Aedes trivittatus|[15341]|NCCA886-11|Culicidae|673|BOLD:AAC9486
Aedes|[15342]|CNGRI092-13|Culicidae|647|BOLD:ABY6840
Aedes|[15343]|CNKJP004-14|Culicidae|543|BOLD:AAC9476
Culicidae|[15344]|PREXP096-14|Culicidae|658|BOLD:AAC5210
Dixidae|[15345]|GMDAP735-12|Dixidae|658|BOLD:ACE1494
Dixidae|[15346]|BBCCM451-10|Dixidae|658|BOLD:AAU6903
Dixidae|[15347]|SSJAA2114-13|Dixidae|658|BOLD:ACJ1962
Dixidae|[15348]|NGNAJ263-13|Dixidae|633|BOLD:ACJ3345
Dixidae|[15349]|GMDAP697-12|Dixidae|658|BOLD:ACD1787
Dixella|[15350]|SSEIB6105-13|Dixidae|658|BOLD:ACI6982
Dixella|[15351]|JWDCG543-10|Dixidae|658|BOLD:AAP8928
Dixidae|[15352]|CNEII506-13|Dixidae|658|BOLD:ACD9927
Dixidae|[15353]|GMDAP533-12|Dixidae|636|BOLD:ACG9958
Dixidae|[15354]|GMDAP534-12|Dixidae|635|BOLD:ACG9654
Dixidae|[15355]|BIOAI499-14|Dixidae|661|BOLD:ACI8724
Dixella|[15356]|BIOAI527-14|Dixidae|661|BOLD:AAI1321
Dixidae|[15357]|JSDIS041-11|Dixidae|658|BOLD:ABA4799
Dixidae|[15358]|SSBAF1557-13|Dixidae|658|BOLD:ACG8894
Dixidae|[15359]|CNKJQ252-14|Dixidae|594|BOLD:ACN5778
Mochlonyx|[15360]|BBDCQ337-10|Chaoboridae|658|BOLD:AAI3268
Chaoborinae|[15361]|CNROM128-13|Chaoboridae|664|BOLD:ACK6923
Simulium vittatum|[15362]|BBDED056-10|Simuliidae|658|BOLD:AAA4121
Androprosopa americana|[15363]|CNBRQ537-14|Thaumaleidae|658|BOLD:ACM1405
Simulium violator|[15364]|MHFLY292-07|Simuliidae|603|BOLD:AAB1115
Simulium bracteatum|[15365]|BBDCM244-10|Simuliidae|658|BOLD:AAB1114
Simulium exulatum|[15366]|JWDCH963-10|Simuliidae|658|BOLD:ABX6042
Helodon pleuralis|[15367]|BBDCN681-10|Simuliidae|658|BOLD:AAE6967
Helodon gibsoni|[15368]|JWDCD226-10|Simuliidae|658|BOLD:AAB0333
Helodon decemarticulatus|[15369]|CNEIB335-12|Simuliidae|673|BOLD:AAC3678
Twinnia nova|[15370]|BBDCN990-10|Simuliidae|658|BOLD:AAD8041
Orthocladiinae|[15371]|CNGMC1615-13|Chironomidae|661|BOLD:ACL2372
Chironomidae|[15372]|SSFDA499-14|Chironomidae|591|BOLD:ACO2212
Helodon onychodactylus complex|[15373]|BBDCN339-10|Simuliidae|658|BOLD:AAB6745
Prosimulium exigens|[15374]|BBDIQ019-10|Simuliidae|657|BOLD:ACF2584
Prosimulium magnum|[15375]|JSDIP045-10|Simuliidae|658|BOLD:AAA7858
Simuliinae|[15376]|CNKJA103-14|Simuliidae|658|BOLD:AAM7816
Prosimulium arvum|[15377]|JSDIP117-10|Simuliidae|658|BOLD:AAD4764
Simuliidae|[15378]|CNLMB104-14|Simuliidae|589|BOLD:ACM2331
Prosimulium|[15379]|BBDEE309-10|Simuliidae|658|BOLD:AAG7032
Simuliidae|[15380]|SSFDB282-14|Simuliidae|627|BOLD:AAL7756
Prosimulium|[15381]|BBDCM001-10|Simuliidae|658|BOLD:AAA1296
Prosimulium|[15382]|CNGLA029-13|Simuliidae|658|BOLD:AAA1298
Prosimulium ursinum|[15383]|ARCIN044-14|Simuliidae|603|BOLD:ACR4456
Simuliidae|[15384]|PHDIP164-11|Simuliidae|658|BOLD:AAY2247
Cnephia ornithophilia|[15385]|CNKJA227-14|Simuliidae|658|BOLD:AAB2653
Greniera ivanovae|[15386]|CNVUB063-14|Simuliidae|591|BOLD:AAP9659
Simulium gouldingi|[15387]|BBDED421-10|Simuliidae|658|BOLD:AAB8485
Simuliidae|[15388]|BBDCQ560-10|Simuliidae|658|BOLD:AAV0822
Simulium croxtoni|[15389]|JSMAY043-11|Simuliidae|658|BOLD:AAB9222
Simulium|[15390]|CNGMB663-13|Simuliidae|658|BOLD:ACJ7131
Hellichiella|[15391]|CNKJA127-14|Simuliidae|658|BOLD:AAF1408
Simulium pictipes|[15392]|BBDCM543-10|Simuliidae|658|BOLD:AAC8658
Simulium|[15393]|SSPAC1749-13|Simuliidae|663|BOLD:AAA4264
Simulium tuberosum complex|[15394]|BBDCM292-10|Simuliidae|658|BOLD:AAA9710
Simulium tuberosum complex|[15395]|BBDCQ685-10|Simuliidae|658|BOLD:AAA8326
Simulium tuberosum|[15396]|BBDED468-10|Simuliidae|658|BOLD:AAA9711
Simuliidae|[15397]|GMDAP803-12|Simuliidae|661|BOLD:ACH0406

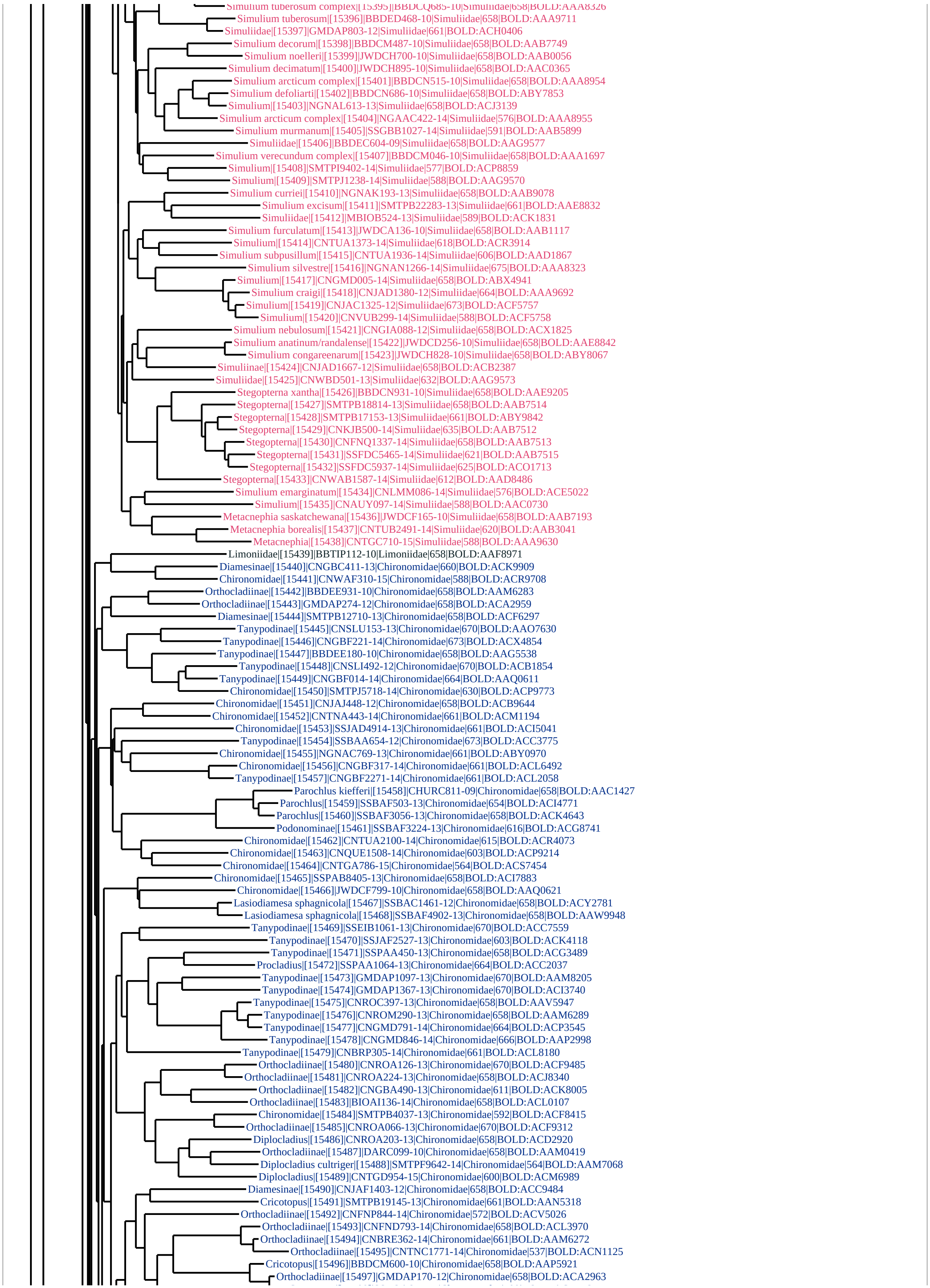

Simulium tuberosum complex|[15395]|BBDCQ685-10|Simuliidae|658|BOLD:AAA8326
Simulium tuberosum|[15396]|BBDED468-10|Simuliidae|658|BOLD:AAA9711
Simuliidae|[15397]|GMDAP803-12|Simuliidae|661|BOLD:ACH0406
Simulium decorum|[15398]|BBDCM487-10|Simuliidae|658|BOLD:AAB7749
Simulium noelleri|[15399]|JWDCH700-10|Simuliidae|658|BOLD:AAB0056
Simulium decimatum|[15400]|JWDCH895-10|Simuliidae|658|BOLD:AAC0365
Simulium arcticum complex|[15401]|BBDCN515-10|Simuliidae|658|BOLD:AAA8954
Simulium defoliarti|[15402]|BBDCN686-10|Simuliidae|658|BOLD:ABY7853
Simulium|[15403]|NGNAL613-13|Simuliidae|658|BOLD:ACJ3139
Simulium arcticum complex|[15404]|NGAAC422-14|Simuliidae|576|BOLD:AAA8955
Simulium murmanum|[15405]|SSGBB1027-14|Simuliidae|591|BOLD:AAB5899
Simuliidae|[15406]|BBDEC604-09|Simuliidae|658|BOLD:AAG9577
Simulium verecundum complex|[15407]|BBDCM046-10|Simuliidae|658|BOLD:AAA1697
Simulium|[15408]|SMTPI9402-14|Simuliidae|577|BOLD:ACP8859
Simulium|[15409]|SMTPJ1238-14|Simuliidae|588|BOLD:AAG9570
Simulium curriei|[15410]|NGNAK193-13|Simuliidae|658|BOLD:AAB9078
Simulium excisum|[15411]|SMTPB22283-13|Simuliidae|661|BOLD:AAE8832
Simuliidae|[15412]|MBIOB524-13|Simuliidae|589|BOLD:ACK1831
Simulium furculatum|[15413]|JWDCA136-10|Simuliidae|658|BOLD:AAB1117
Simulium|[15414]|CNTUA1373-14|Simuliidae|618|BOLD:ACR3914
Simulium subpusillum|[15415]|CNTUA1936-14|Simuliidae|606|BOLD:AAD1867
Simulium silvestre|[15416]|NGNAN1266-14|Simuliidae|675|BOLD:AAA8323
Simulium|[15417]|CNGMD005-14|Simuliidae|658|BOLD:ABX4941
Simulium craigi|[15418]|CNJAD1380-12|Simuliidae|664|BOLD:AAA9692
Simulium|[15419]|CNJAC1325-12|Simuliidae|673|BOLD:ACF5757
Simulium|[15420]|CNVUB299-14|Simuliidae|588|BOLD:ACF5758
Simulium nebulosum|[15421]|CNGIA088-12|Simuliidae|658|BOLD:ACX1825
Simulium anatinum/randalense|[15422]|JWDCD256-10|Simuliidae|658|BOLD:AAE8842
Simulium congareenarum|[15423]|JWDCH828-10|Simuliidae|658|BOLD:ABY8067
Simuliinae|[15424]|CNJAD1667-12|Simuliidae|658|BOLD:ACB2387
Simuliidae|[15425]|CNWBD501-13|Simuliidae|632|BOLD:AAG9573
Stegopterna xantha|[15426]|BBDCN931-10|Simuliidae|658|BOLD:AAE9205
Stegopterna|[15427]|SMTPB18814-13|Simuliidae|658|BOLD:AAB7514
Stegopterna|[15428]|SMTPB17153-13|Simuliidae|661|BOLD:ABY9842
Stegopterna|[15429]|CNKJB500-14|Simuliidae|635|BOLD:AAB7512
Stegopterna|[15430]|CNFNQ1337-14|Simuliidae|658|BOLD:AAB7513
Stegopterna|[15431]|SSFDC5465-14|Simuliidae|621|BOLD:AAB7515
Stegopterna|[15432]|SSFDC5937-14|Simuliidae|625|BOLD:ACO1713
Stegopterna|[15433]|CNWAB1587-14|Simuliidae|612|BOLD:AAD8486
Simulium emarginatum|[15434]|CNLMM086-14|Simuliidae|576|BOLD:ACE5022
Simulium|[15435]|CNAUY097-14|Simuliidae|588|BOLD:AAC0730
Metacnephia saskatchewana|[15436]|JWDCF165-10|Simuliidae|658|BOLD:AAB7193
Metacnephia borealis|[15437]|CNTUB2491-14|Simuliidae|620|BOLD:AAB3041
Metacnephia|[15438]|CNTGC710-15|Simuliidae|588|BOLD:AAA9630
Limoniidae|[15439]|BBTIP112-10|Limoniidae|658|BOLD:AAF8971
Diamesinae|[15440]|CNGBC411-13|Chironomidae|660|BOLD:ACK9909
Chironomidae|[15441]|CNWAF310-15|Chironomidae|588|BOLD:ACR9708
Orthocladiinae|[15442]|BBDEE931-10|Chironomidae|658|BOLD:AAM6283
Orthocladiinae|[15443]|GMDAP274-12|Chironomidae|658|BOLD:ACA2959
Diamesinae|[15444]|SMTPB12710-13|Chironomidae|658|BOLD:ACF6297
Tanypodinae|[15445]|CNSLU153-13|Chironomidae|670|BOLD:AAO7630
Tanypodinae|[15446]|CNGBF221-14|Chironomidae|673|BOLD:ACX4854
Tanypodinae|[15447]|BBDEE180-10|Chironomidae|658|BOLD:AAG5538
Tanypodinae|[15448]|CNSLI492-12|Chironomidae|670|BOLD:ACB1854
Tanypodinae|[15449]|CNGBF014-14|Chironomidae|664|BOLD:AAQ0611
Chironomidae|[15450]|SMTPJ5718-14|Chironomidae|630|BOLD:ACP9773
Chironomidae|[15451]|CNJAJ448-12|Chironomidae|658|BOLD:ACB9644
Chironomidae|[15452]|CNTNA443-14|Chironomidae|661|BOLD:ACM1194
Chironomidae|[15453]|SSJAD4914-13|Chironomidae|661|BOLD:ACI5041
Tanypodinae|[15454]|SSBAA654-12|Chironomidae|673|BOLD:ACC3775
Chironomidae|[15455]|NGNAC769-13|Chironomidae|661|BOLD:ABY0970
Chironomidae|[15456]|CNGBF317-14|Chironomidae|661|BOLD:ACL6492
Tanypodinae|[15457]|CNGBF2271-14|Chironomidae|661|BOLD:ACL2058
Parochlus kiefferi|[15458]|CHURC811-09|Chironomidae|658|BOLD:AAC1427
Parochlus|[15459]|SSBAF503-13|Chironomidae|654|BOLD:ACI4771
Parochlus|[15460]|SSBAF3056-13|Chironomidae|658|BOLD:ACK4643
Podonominae|[15461]|SSBAF3224-13|Chironomidae|616|BOLD:ACG8741
Chironomidae|[15462]|CNTUA2100-14|Chironomidae|615|BOLD:ACR4073
Chironomidae|[15463]|CNQUE1508-14|Chironomidae|603|BOLD:ACP9214
Chironomidae|[15464]|CNTGA786-15|Chironomidae|564|BOLD:ACS7454
Chironomidae|[15465]|SSPAB8405-13|Chironomidae|658|BOLD:ACI7883
Chironomidae|[15466]|JWDCF799-10|Chironomidae|658|BOLD:AAQ0621
Lasiodiamesa sphagnicola|[15467]|SSBAC1461-12|Chironomidae|658|BOLD:ACY2781
Lasiodiamesa sphagnicola|[15468]|SSBAF4902-13|Chironomidae|658|BOLD:AAW9948
Tanypodinae|[15469]|SSEIB1061-13|Chironomidae|670|BOLD:ACC7559
Tanypodinae|[15470]|SSJAF2527-13|Chironomidae|603|BOLD:ACK4118
Tanypodinae|[15471]|SSPAA450-13|Chironomidae|658|BOLD:ACG3489
Procladius|[15472]|SSPAA1064-13|Chironomidae|664|BOLD:ACC2037
Tanypodinae|[15473]|GMDAP1097-13|Chironomidae|670|BOLD:AAM8205
Tanypodinae|[15474]|GMDAP1367-13|Chironomidae|670|BOLD:ACI3740
Tanypodinae|[15475]|CNROC397-13|Chironomidae|658|BOLD:AAV5947
Tanypodinae|[15476]|CNROM290-13|Chironomidae|658|BOLD:AAM6289
Tanypodinae|[15477]|CNGMD791-14|Chironomidae|664|BOLD:ACP3545
Tanypodinae|[15478]|CNGMD846-14|Chironomidae|666|BOLD:AAP2998
Tanypodinae|[15479]|CNBRP305-14|Chironomidae|661|BOLD:ACL8180
Orthocladiinae|[15480]|CNROA126-13|Chironomidae|670|BOLD:ACF9485
Orthocladiinae|[15481]|CNROA224-13|Chironomidae|658|BOLD:ACJ8340
Orthocladiinae|[15482]|CNGBA490-13|Chironomidae|611|BOLD:ACK8005
Orthocladiinae|[15483]|BIOAI136-14|Chironomidae|658|BOLD:ACL0107
Chironomidae|[15484]|SMTPB4037-13|Chironomidae|592|BOLD:ACF8415
Orthocladiinae|[15485]|CNROA066-13|Chironomidae|670|BOLD:ACF9312
Diplocladius|[15486]|CNROA203-13|Chironomidae|658|BOLD:ACD2920
Orthocladiinae|[15487]|DARC099-10|Chironomidae|658|BOLD:AAM0419
Diplocladius cultriger|[15488]|SMTPF9642-14|Chironomidae|564|BOLD:AAM7068
Diplocladius|[15489]|CNTGD954-15|Chironomidae|600|BOLD:ACM6989
Diamesinae|[15490]|CNJAF1403-12|Chironomidae|658|BOLD:ACC9484
Cricotopus|[15491]|SMTPB19145-13|Chironomidae|661|BOLD:AAN5318
Orthocladiinae|[15492]|CNFNP844-14|Chironomidae|572|BOLD:ACV5026
Orthocladiinae|[15493]|CNFND793-14|Chironomidae|658|BOLD:ACL3970
Orthocladiinae|[15494]|CNBRE362-14|Chironomidae|661|BOLD:AAM6272
Orthocladiinae|[15495]|CNTNC1771-14|Chironomidae|537|BOLD:ACN1125
Cricotopus|[15496]|BBDCM600-10|Chironomidae|658|BOLD:AAP5921
Orthocladiinae|[15497]|GMDAP170-12|Chironomidae|658|BOLD:ACA2963

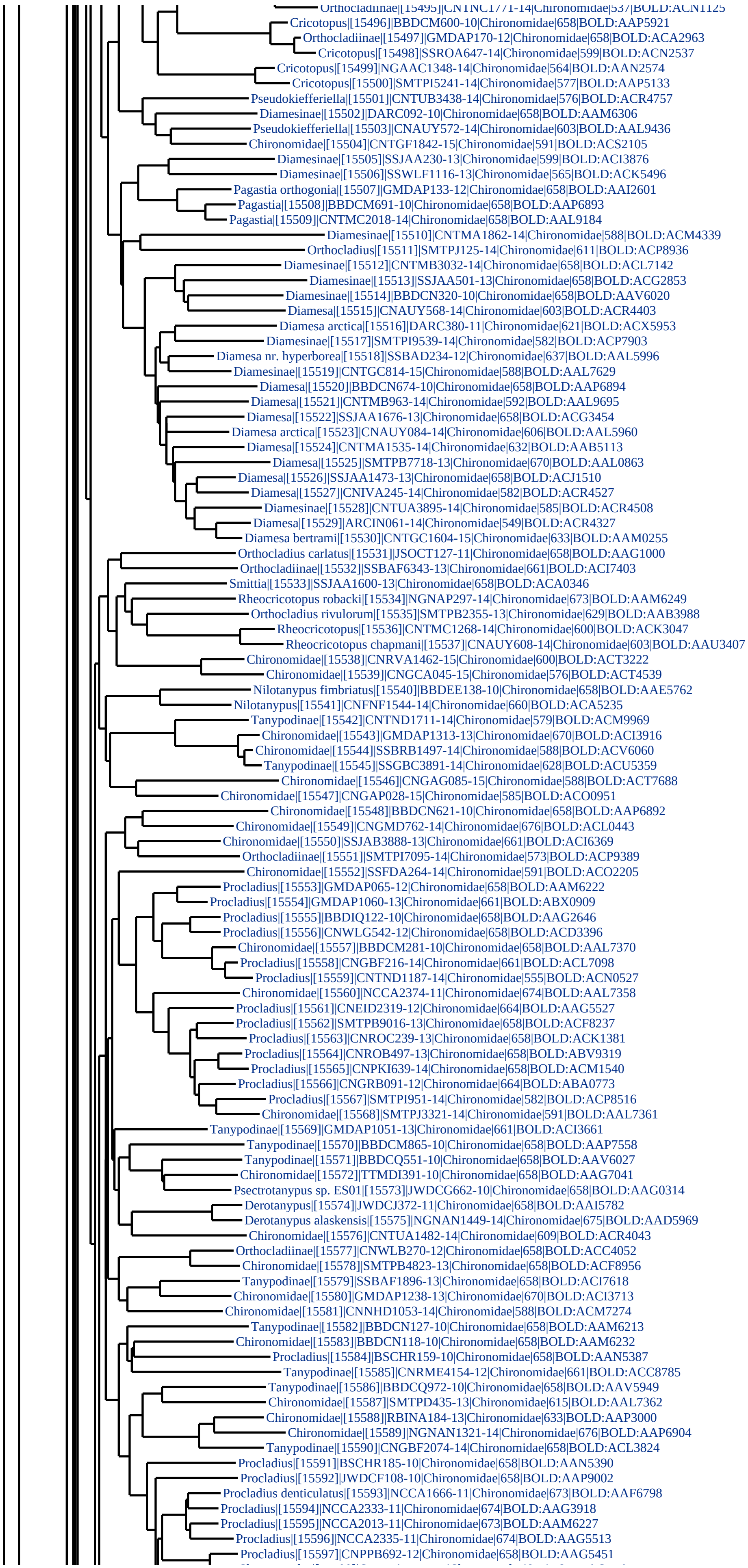

Orthocladiinae|[15495]|CNTNC1771-14|Chironomidae|537|BOLD:ACN1125
Cricotopus|[15496]|BBDCM600-10|Chironomidae|658|BOLD:AAP5921
Orthocladiinae|[15497]|GMDAP170-12|Chironomidae|658|BOLD:ACA2963
Cricotopus|[15498]|SSROA647-14|Chironomidae|599|BOLD:ACN2537
Cricotopus|[15499]|NGAAC1348-14|Chironomidae|564|BOLD:AAN2574
Cricotopus|[15500]|SMTPI5241-14|Chironomidae|577|BOLD:AAP5133
Pseudokiefferiella|[15501]|CNTUB3438-14|Chironomidae|576|BOLD:ACR4757
Diamesinae|[15502]|DARC092-10|Chironomidae|658|BOLD:AAM6306
Pseudokiefferiella|[15503]|CNAUY572-14|Chironomidae|603|BOLD:AAL9436
Chironomidae|[15504]|CNTGF1842-15|Chironomidae|591|BOLD:ACS2105
Diamesinae|[15505]|SSJAA230-13|Chironomidae|599|BOLD:ACI3876
Diamesinae|[15506]|SSWLF1116-13|Chironomidae|565|BOLD:ACK5496
Pagastia orthogonia|[15507]|GMDAP133-12|Chironomidae|658|BOLD:AAI2601
Pagastia|[15508]|BBDCM691-10|Chironomidae|658|BOLD:AAP6893
Pagastia|[15509]|CNTMC2018-14|Chironomidae|658|BOLD:AAL9184
Diamesinae|[15510]|CNTMA1862-14|Chironomidae|588|BOLD:ACM4339
Orthocladius|[15511]|SMTPJ125-14|Chironomidae|611|BOLD:ACP8936
Diamesinae|[15512]|CNTMB3032-14|Chironomidae|658|BOLD:ACL7142
Diamesinae|[15513]|SSJAA501-13|Chironomidae|658|BOLD:ACG2853
Diamesinae|[15514]|BBDCN320-10|Chironomidae|658|BOLD:AAV6020
Diamesa|[15515]|CNAUY568-14|Chironomidae|603|BOLD:ACR4403
Diamesa arctica|[15516]|DARC380-11|Chironomidae|621|BOLD:ACX5953
Diamesinae|[15517]|SMTPI9539-14|Chironomidae|582|BOLD:ACP7903
Diamesa nr. hyperborea|[15518]|SSBAD234-12|Chironomidae|637|BOLD:AAL5996
Diamesinae|[15519]|CNTGC814-15|Chironomidae|588|BOLD:AAL7629
Diamesa|[15520]|BBDCN674-10|Chironomidae|658|BOLD:AAP6894
Diamesa|[15521]|CNTMB963-14|Chironomidae|592|BOLD:AAL9695
Diamesa|[15522]|SSJAA1676-13|Chironomidae|658|BOLD:ACG3454
Diamesa arctica|[15523]|CNAUY084-14|Chironomidae|606|BOLD:AAL5960
Diamesa|[15524]|CNTMA1535-14|Chironomidae|632|BOLD:AAB5113
Diamesa|[15525]|SMTPB7718-13|Chironomidae|670|BOLD:AAL0863
Diamesa|[15526]|SSJAA1473-13|Chironomidae|658|BOLD:ACJ1510
Diamesa|[15527]|CNIVA245-14|Chironomidae|582|BOLD:ACR4527
Diamesinae|[15528]|CNTUA3895-14|Chironomidae|585|BOLD:ACR4508
Diamesa|[15529]|ARCIN061-14|Chironomidae|549|BOLD:ACR4327
Diamesa bertrami|[15530]|CNTGC1604-15|Chironomidae|633|BOLD:AAM0255
Orthocladius carlatus|[15531]|JSOCT127-11|Chironomidae|658|BOLD:AAG1000
Orthocladiinae|[15532]|SSBAF6343-13|Chironomidae|661|BOLD:ACI7403
Smittia|[15533]|SSJAA1600-13|Chironomidae|658|BOLD:ACA0346
Rheocricotopus robacki|[15534]|NGNAP297-14|Chironomidae|673|BOLD:AAM6249
Orthocladius rivulorum|[15535]|SMTPB2355-13|Chironomidae|629|BOLD:AAB3988
Rheocricotopus|[15536]|CNTMC1268-14|Chironomidae|600|BOLD:ACK3047
Rheocricotopus chapmani|[15537]|CNAUY608-14|Chironomidae|603|BOLD:AAU3407
Chironomidae|[15538]|CNRVA1462-15|Chironomidae|600|BOLD:ACT3222
Chironomidae|[15539]|CNGCA045-15|Chironomidae|576|BOLD:ACT4539
Nilotanypus fimbriatus|[15540]|BBDEE138-10|Chironomidae|658|BOLD:AAE5762
Nilotanypus|[15541]|CNFNF1544-14|Chironomidae|660|BOLD:ACA5235
Tanypodinae|[15542]|CNTND1711-14|Chironomidae|579|BOLD:ACM9969
Chironomidae|[15543]|GMDAP1313-13|Chironomidae|670|BOLD:ACI3916
Chironomidae|[15544]|SSBRB1497-14|Chironomidae|588|BOLD:ACV6060
Tanypodinae|[15545]|SSGBC3891-14|Chironomidae|628|BOLD:ACU5359
Chironomidae|[15546]|CNGAG085-15|Chironomidae|588|BOLD:ACT7688
Chironomidae|[15547]|CNGAP028-15|Chironomidae|585|BOLD:ACO0951
Chironomidae|[15548]|BBDCN621-10|Chironomidae|658|BOLD:AAP6892
Chironomidae|[15549]|CNGMD762-14|Chironomidae|676|BOLD:ACL0443
Chironomidae|[15550]|SSJAB3888-13|Chironomidae|661|BOLD:ACI6369
Orthocladiinae|[15551]|SMTPI7095-14|Chironomidae|573|BOLD:ACP9389
Chironomidae|[15552]|SSFDA264-14|Chironomidae|591|BOLD:ACO2205
Procladius|[15553]|GMDAP065-12|Chironomidae|658|BOLD:AAM6222
Procladius|[15554]|GMDAP1060-13|Chironomidae|661|BOLD:ABX0909
Procladius|[15555]|BBDIQ122-10|Chironomidae|658|BOLD:AAG2646
Procladius|[15556]|CNWLG542-12|Chironomidae|658|BOLD:ACD3396
Chironomidae|[15557]|BBDCM281-10|Chironomidae|658|BOLD:AAL7370
Procladius|[15558]|CNGBF216-14|Chironomidae|661|BOLD:ACL7098
Procladius|[15559]|CNTND1187-14|Chironomidae|555|BOLD:ACN0527
Chironomidae|[15560]|NCCA2374-11|Chironomidae|674|BOLD:AAL7358
Procladius|[15561]|CNEID2319-12|Chironomidae|664|BOLD:AAG5527
Procladius|[15562]|SMTPB9016-13|Chironomidae|658|BOLD:ACF8237
Procladius|[15563]|CNROC239-13|Chironomidae|658|BOLD:ACK1381
Procladius|[15564]|CNROB497-13|Chironomidae|658|BOLD:ABV9319
Procladius|[15565]|CNPKI639-14|Chironomidae|658|BOLD:ACM1540
Procladius|[15566]|CNGRB091-12|Chironomidae|664|BOLD:ABA0773
Procladius|[15567]|SMTPI951-14|Chironomidae|582|BOLD:ACP8516
Chironomidae|[15568]|SMTPJ3321-14|Chironomidae|591|BOLD:AAL7361
Tanypodinae|[15569]|GMDAP1051-13|Chironomidae|661|BOLD:ACI3661
Tanypodinae|[15570]|BBDCM865-10|Chironomidae|658|BOLD:AAP7558
Tanypodinae|[15571]|BBDCQ551-10|Chironomidae|658|BOLD:AAV6027
Chironomidae|[15572]|TTMDI391-10|Chironomidae|658|BOLD:AAG7041
Psectrotanypus sp. ES01|[15573]|JWDCG662-10|Chironomidae|658|BOLD:AAG0314
Derotanypus|[15574]|JWDCJ372-11|Chironomidae|658|BOLD:AAI5782
Derotanypus alaskensis|[15575]|NGNAN1449-14|Chironomidae|675|BOLD:AAD5969
Chironomidae|[15576]|CNTUA1482-14|Chironomidae|609|BOLD:ACR4043
Orthocladiinae|[15577]|CNWLB270-12|Chironomidae|658|BOLD:ACC4052
Chironomidae|[15578]|SMTPB4823-13|Chironomidae|658|BOLD:ACF8956
Tanypodinae|[15579]|SSBAF1896-13|Chironomidae|658|BOLD:ACI7618
Chironomidae|[15580]|GMDAP1238-13|Chironomidae|670|BOLD:ACI3713
Chironomidae|[15581]|CNNHD1053-14|Chironomidae|588|BOLD:ACM7274
Tanypodinae|[15582]|BBDCN127-10|Chironomidae|658|BOLD:AAM6213
Chironomidae|[15583]|BBDCN118-10|Chironomidae|658|BOLD:AAM6232
Procladius|[15584]|BSCHR159-10|Chironomidae|658|BOLD:AAN5387
Tanypodinae|[15585]|CNRME4154-12|Chironomidae|661|BOLD:ACC8785
Tanypodinae|[15586]|BBDCQ972-10|Chironomidae|658|BOLD:AAV5949
Chironomidae|[15587]|SMTPD435-13|Chironomidae|615|BOLD:AAL7362
Chironomidae|[15588]|RBINA184-13|Chironomidae|633|BOLD:AAP3000
Chironomidae|[15589]|NGNAN1321-14|Chironomidae|676|BOLD:AAP6904
Tanypodinae|[15590]|CNGBF2074-14|Chironomidae|658|BOLD:ACL3824
Procladius|[15591]|BSCHR185-10|Chironomidae|658|BOLD:AAN5390
Procladius|[15592]|JWDCF108-10|Chironomidae|658|BOLD:AAP9002
Procladius denticulatus|[15593]|NCCA1666-11|Chironomidae|673|BOLD:AAF6798
Procladius|[15594]|NCCA2333-11|Chironomidae|674|BOLD:AAG3918
Procladius|[15595]|NCCA2013-11|Chironomidae|673|BOLD:AAM6227
Procladius|[15596]|NCCA2335-11|Chironomidae|674|BOLD:AAG5513
Procladius|[15597]|CNPPB692-12|Chironomidae|658|BOLD:AAG5451

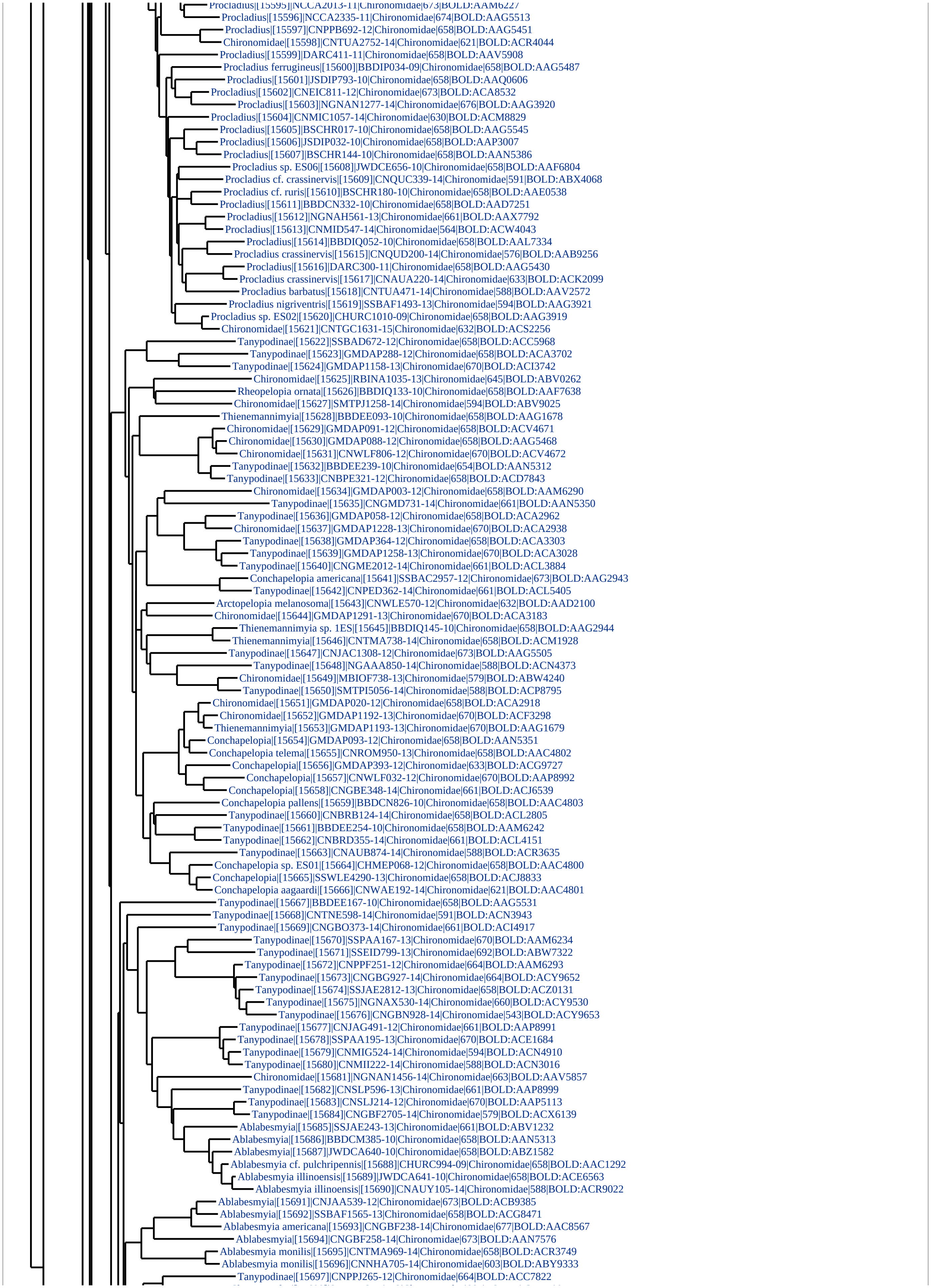
Procladius|[15595]|NCCA2013-11|Chironomidae|673|BOLD:AAM6227
Procladius|[15596]|NCCA2335-11|Chironomidae|674|BOLD:AAG5513
Procladius|[15597]|CNPPB692-12|Chironomidae|658|BOLD:AAG5451
Chironomidae|[15598]|CNTUA2752-14|Chironomidae|621|BOLD:ACR4044
Procladius|[15599]|DARC411-11|Chironomidae|658|BOLD:AAV5908
Procladius ferrugineus|[15600]|BBDIP034-09|Chironomidae|658|BOLD:AAG5487
Procladius|[15601]|JSDIP793-10|Chironomidae|658|BOLD:AAQ0606
Procladius|[15602]|CNEIC811-12|Chironomidae|673|BOLD:ACA8532
Procladius|[15603]|NGNAN1277-14|Chironomidae|676|BOLD:AAG3920
Procladius|[15604]|CNMIC1057-14|Chironomidae|630|BOLD:ACM8829
Procladius|[15605]|BSCHR017-10|Chironomidae|658|BOLD:AAG5545
Procladius|[15606]|JSDIP032-10|Chironomidae|658|BOLD:AAP3007
Procladius|[15607]|BSCHR144-10|Chironomidae|658|BOLD:AAN5386
Procladius sp. ES06|[15608]|JWDCE656-10|Chironomidae|658|BOLD:AAF6804
Procladius cf. crassinervis|[15609]|CNQUC339-14|Chironomidae|591|BOLD:ABX4068
Procladius cf. ruris|[15610]|BSCHR180-10|Chironomidae|658|BOLD:AAE0538
Procladius|[15611]|BBDCN332-10|Chironomidae|658|BOLD:AAD7251
Procladius|[15612]|NGNAH561-13|Chironomidae|661|BOLD:AAX7792
Procladius|[15613]|CNMID547-14|Chironomidae|564|BOLD:ACW4043
Procladius|[15614]|BBDIQ052-10|Chironomidae|658|BOLD:AAL7334
Procladius crassinervis|[15615]|CNQUD200-14|Chironomidae|576|BOLD:AAB9256
Procladius|[15616]|DARC300-11|Chironomidae|658|BOLD:AAG5430
Procladius crassinervis|[15617]|CNAUA220-14|Chironomidae|633|BOLD:ACK2099
Procladius barbatus|[15618]|CNTUA471-14|Chironomidae|588|BOLD:AAV2572
Procladius nigriventris|[15619]|SSBAF1493-13|Chironomidae|594|BOLD:AAG3921
Procladius sp. ES02|[15620]|CHURC1010-09|Chironomidae|658|BOLD:AAG3919
Chironomidae|[15621]|CNTGC1631-15|Chironomidae|632|BOLD:ACS2256
Tanypodinae|[15622]|SSBAD672-12|Chironomidae|658|BOLD:ACC5968
Tanypodinae|[15623]|GMDAP288-12|Chironomidae|658|BOLD:ACA3702
Tanypodinae|[15624]|GMDAP1158-13|Chironomidae|670|BOLD:ACI3742
Chironomidae|[15625]|RBINA1035-13|Chironomidae|645|BOLD:ABV0262
Rheopelopia ornata|[15626]|BBDIQ133-10|Chironomidae|658|BOLD:AAF7638
Chironomidae|[15627]|SMTPJ1258-14|Chironomidae|594|BOLD:ABV9025
Thienemannimyia|[15628]|BBDEE093-10|Chironomidae|658|BOLD:AAG1678
Chironomidae|[15629]|GMDAP091-12|Chironomidae|658|BOLD:ACV4671
Chironomidae|[15630]|GMDAP088-12|Chironomidae|658|BOLD:AAG5468
Chironomidae|[15631]|CNWLF806-12|Chironomidae|670|BOLD:ACV4672
Tanypodinae|[15632]|BBDEE239-10|Chironomidae|654|BOLD:AAN5312
Tanypodinae|[15633]|CNBPE321-12|Chironomidae|658|BOLD:ACD7843
Chironomidae|[15634]|GMDAP003-12|Chironomidae|658|BOLD:AAM6290
Tanypodinae|[15635]|CNGMD731-14|Chironomidae|661|BOLD:AAN5350
Tanypodinae|[15636]|GMDAP058-12|Chironomidae|658|BOLD:ACA2962
Chironomidae|[15637]|GMDAP1228-13|Chironomidae|670|BOLD:ACA2938
Tanypodinae|[15638]|GMDAP364-12|Chironomidae|658|BOLD:ACA3303
Tanypodinae|[15639]|GMDAP1258-13|Chironomidae|670|BOLD:ACA3028
Tanypodinae|[15640]|CNGME2012-14|Chironomidae|661|BOLD:ACL3884
Conchapelopia americana|[15641]|SSBAC2957-12|Chironomidae|673|BOLD:AAG2943
Tanypodinae|[15642]|CNPED362-14|Chironomidae|661|BOLD:ACL5405
Arctopelopia melanosoma|[15643]|CNWLE570-12|Chironomidae|632|BOLD:AAD2100
Chironomidae|[15644]|GMDAP1291-13|Chironomidae|670|BOLD:ACA3183
Thienemannimyia sp. 1ES|[15645]|BBDIQ145-10|Chironomidae|658|BOLD:AAG2944
Thienemannimyia|[15646]|CNTMA738-14|Chironomidae|658|BOLD:ACM1928
Tanypodinae|[15647]|CNJAC1308-12|Chironomidae|673|BOLD:AAG5505
Tanypodinae|[15648]|NGAAA850-14|Chironomidae|588|BOLD:ACN4373
Chironomidae|[15649]|MBIOF738-13|Chironomidae|579|BOLD:ABW4240
Tanypodinae|[15650]|SMTPI5056-14|Chironomidae|588|BOLD:ACP8795
Chironomidae|[15651]|GMDAP020-12|Chironomidae|658|BOLD:ACA2918
Chironomidae|[15652]|GMDAP1192-13|Chironomidae|670|BOLD:ACF3298
Thienemannimyia|[15653]|GMDAP1193-13|Chironomidae|670|BOLD:AAG1679
Conchapelopia|[15654]|GMDAP093-12|Chironomidae|658|BOLD:AAN5351
Conchapelopia telema|[15655]|CNROM950-13|Chironomidae|658|BOLD:AAC4802
Conchapelopia|[15656]|GMDAP393-12|Chironomidae|633|BOLD:ACG9727
Conchapelopia|[15657]|CNWLF032-12|Chironomidae|670|BOLD:AAP8992
Conchapelopia|[15658]|CNGBE348-14|Chironomidae|661|BOLD:ACJ6539
Conchapelopia pallens|[15659]|BBDCN826-10|Chironomidae|658|BOLD:AAC4803
Tanypodinae|[15660]|CNBRB124-14|Chironomidae|658|BOLD:ACL2805
Tanypodinae|[15661]|BBDEE254-10|Chironomidae|658|BOLD:AAM6242
Tanypodinae|[15662]|CNBRD355-14|Chironomidae|661|BOLD:ACL4151
Tanypodinae|[15663]|CNAUB874-14|Chironomidae|588|BOLD:ACR3635
Conchapelopia sp. ES01|[15664]|CHMEP068-12|Chironomidae|658|BOLD:AAC4800
Conchapelopia|[15665]|SSWLE4290-13|Chironomidae|658|BOLD:ACJ8833
Conchapelopia aagaardi|[15666]|CNWAE192-14|Chironomidae|621|BOLD:AAC4801
Tanypodinae|[15667]|BBDEE167-10|Chironomidae|658|BOLD:AAG5531
Tanypodinae|[15668]|CNTNE598-14|Chironomidae|591|BOLD:ACN3943
Tanypodinae|[15669]|CNGBO373-14|Chironomidae|661|BOLD:ACI4917
Tanypodinae|[15670]|SSPAA167-13|Chironomidae|670|BOLD:AAM6234
Tanypodinae|[15671]|SSEID799-13|Chironomidae|692|BOLD:ABW7322
Tanypodinae|[15672]|CNPPF251-12|Chironomidae|664|BOLD:AAM6293
Tanypodinae|[15673]|CNGBG927-14|Chironomidae|664|BOLD:ACY9652
Tanypodinae|[15674]|SSJAE2812-13|Chironomidae|658|BOLD:ACZ0131
Tanypodinae|[15675]|NGNAX530-14|Chironomidae|660|BOLD:ACY9530
Tanypodinae|[15676]|CNGBN928-14|Chironomidae|543|BOLD:ACY9653
Tanypodinae|[15677]|CNJAG491-12|Chironomidae|661|BOLD:AAP8991
Tanypodinae|[15678]|SSPAA195-13|Chironomidae|670|BOLD:ACE1684
Tanypodinae|[15679]|CNMIG524-14|Chironomidae|594|BOLD:ACN4910
Tanypodinae|[15680]|CNMII222-14|Chironomidae|588|BOLD:ACN3016
Chironomidae|[15681]|NGNAN1456-14|Chironomidae|663|BOLD:AAV5857
Tanypodinae|[15682]|CNSLP596-13|Chironomidae|661|BOLD:AAP8999
Tanypodinae|[15683]|CNSLJ214-12|Chironomidae|670|BOLD:AAP5113
Tanypodinae|[15684]|CNGBF2705-14|Chironomidae|579|BOLD:ACX6139
Ablabesmyia|[15685]|SSJAE243-13|Chironomidae|661|BOLD:ABV1232
Ablabesmyia|[15686]|BBDCM385-10|Chironomidae|658|BOLD:AAN5313
Ablabesmyia|[15687]|JWDCA640-10|Chironomidae|658|BOLD:ABZ1582
Ablabesmyia cf. pulchripennis|[15688]|CHURC994-09|Chironomidae|658|BOLD:AAC1292
Ablabesmyia illinoensis|[15689]|JWDCA641-10|Chironomidae|658|BOLD:ACE6563
Ablabesmyia illinoensis|[15690]|CNAUY105-14|Chironomidae|588|BOLD:ACR9022
Ablabesmyia|[15691]|CNJAA539-12|Chironomidae|673|BOLD:ACB9385
Ablabesmyia|[15692]|SSBAF1565-13|Chironomidae|658|BOLD:ACG8471
Ablabesmyia americana|[15693]|CNGBF238-14|Chironomidae|677|BOLD:AAC8567
Ablabesmyia|[15694]|CNGBF258-14|Chironomidae|673|BOLD:AAN7576
Ablabesmyia monilis|[15695]|CNTMA969-14|Chironomidae|658|BOLD:ACR3749
Ablabesmyia monilis|[15696]|CNNHA705-14|Chironomidae|603|BOLD:ABY9333
Tanypodinae|[15697]|CNPPJ265-12|Chironomidae|664|BOLD:ACC7822

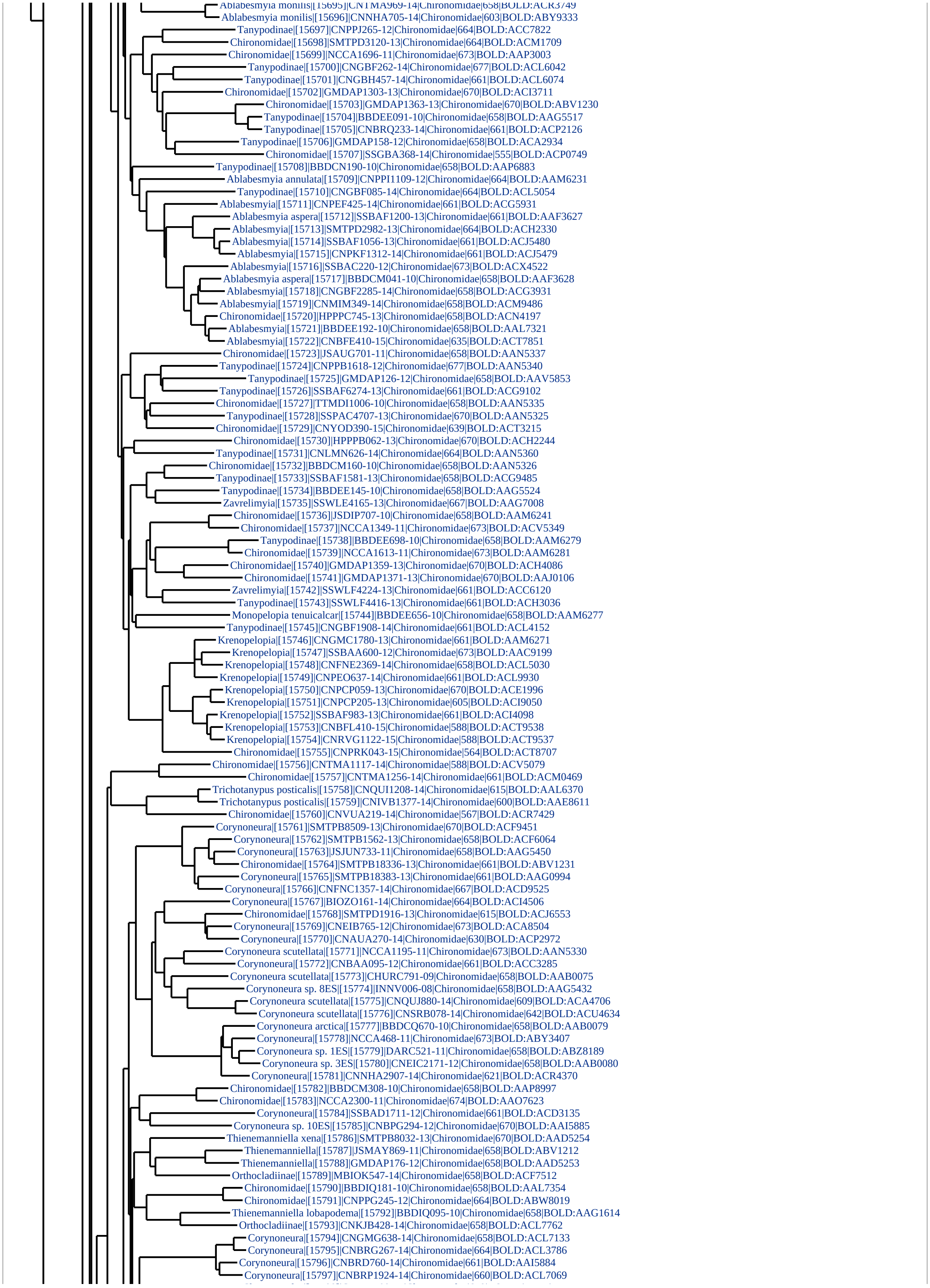
Ablabesmyia monilis|[15695]|CNTMA969-14|Chironomidae|658|BOLD:ACR3749
Ablabesmyia monilis|[15696]|CNNHA705-14|Chironomidae|603|BOLD:ABY9333
Tanypodinae|[15697]|CNPPJ265-12|Chironomidae|664|BOLD:ACC7822
Chironomidae|[15698]|SMTPD3120-13|Chironomidae|664|BOLD:ACM1709
Chironomidae|[15699]|NCCA1696-11|Chironomidae|673|BOLD:AAP3003
Tanypodinae|[15700]|CNGBF262-14|Chironomidae|677|BOLD:ACL6042
Tanypodinae|[15701]|CNGBH457-14|Chironomidae|661|BOLD:ACL6074
Chironomidae|[15702]|GMDAP1303-13|Chironomidae|670|BOLD:ACI3711
Chironomidae|[15703]|GMDAP1363-13|Chironomidae|670|BOLD:ABV1230
Tanypodinae|[15704]|BBDEE091-10|Chironomidae|658|BOLD:AAG5517
Tanypodinae|[15705]|CNBRQ233-14|Chironomidae|661|BOLD:ACP2126
Tanypodinae|[15706]|GMDAP158-12|Chironomidae|658|BOLD:ACA2934
Chironomidae|[15707]|SSGBA368-14|Chironomidae|555|BOLD:ACP0749
Tanypodinae|[15708]|BBDCN190-10|Chironomidae|658|BOLD:AAP6883
Ablabesmyia annulata|[15709]|CNPPI1109-12|Chironomidae|664|BOLD:AAM6231
Tanypodinae|[15710]|CNGBF085-14|Chironomidae|664|BOLD:ACL5054
Ablabesmyia|[15711]|CNPEF425-14|Chironomidae|661|BOLD:ACG5931
Ablabesmyia aspera|[15712]|SSBAF1200-13|Chironomidae|661|BOLD:AAF3627
Ablabesmyia|[15713]|SMTPD2982-13|Chironomidae|664|BOLD:ACH2330
Ablabesmyia|[15714]|SSBAF1056-13|Chironomidae|661|BOLD:ACJ5480
Ablabesmyia|[15715]|CNPKF1312-14|Chironomidae|661|BOLD:ACJ5479
Ablabesmyia|[15716]|SSBAC220-12|Chironomidae|673|BOLD:ACX4522
Ablabesmyia aspera|[15717]|BBDCM041-10|Chironomidae|658|BOLD:AAF3628
Ablabesmyia|[15718]|CNGBF2285-14|Chironomidae|658|BOLD:ACG3931
Ablabesmyia|[15719]|CNMIM349-14|Chironomidae|658|BOLD:ACM9486
Chironomidae|[15720]|HPPPC745-13|Chironomidae|658|BOLD:ACN4197
Ablabesmyia|[15721]|BBDEE192-10|Chironomidae|658|BOLD:AAL7321
Ablabesmyia|[15722]|CNBFE410-15|Chironomidae|635|BOLD:ACT7851
Chironomidae|[15723]|JSAUG701-11|Chironomidae|658|BOLD:AAN5337
Tanypodinae|[15724]|CNPPB1618-12|Chironomidae|677|BOLD:AAN5340
Tanypodinae|[15725]|GMDAP126-12|Chironomidae|658|BOLD:AAV5853
Tanypodinae|[15726]|SSBAF6274-13|Chironomidae|661|BOLD:ACG9102
Chironomidae|[15727]|TTMDI1006-10|Chironomidae|658|BOLD:AAN5335
Tanypodinae|[15728]|SSPAC4707-13|Chironomidae|670|BOLD:AAN5325
Chironomidae|[15729]|CNYOD390-15|Chironomidae|639|BOLD:ACT3215
Chironomidae|[15730]|HPPPB062-13|Chironomidae|670|BOLD:ACH2244
Tanypodinae|[15731]|CNLMN626-14|Chironomidae|664|BOLD:AAN5360
Chironomidae|[15732]|BBDCM160-10|Chironomidae|658|BOLD:AAN5326
Tanypodinae|[15733]|SSBAF1581-13|Chironomidae|658|BOLD:ACG9485
Tanypodinae|[15734]|BBDEE145-10|Chironomidae|658|BOLD:AAG5524
Zavrelimyia|[15735]|SSWLE4165-13|Chironomidae|667|BOLD:AAG7008
Chironomidae|[15736]|JSDIP707-10|Chironomidae|658|BOLD:AAM6241
Chironomidae|[15737]|NCCA1349-11|Chironomidae|673|BOLD:ACV5349
Tanypodinae|[15738]|BBDEE698-10|Chironomidae|658|BOLD:AAM6279
Chironomidae|[15739]|NCCA1613-11|Chironomidae|673|BOLD:AAM6281
Chironomidae|[15740]|GMDAP1359-13|Chironomidae|670|BOLD:ACH4086
Chironomidae|[15741]|GMDAP1371-13|Chironomidae|670|BOLD:AAJ0106
Zavrelimyia|[15742]|SSWLF4224-13|Chironomidae|661|BOLD:ACC6120
Tanypodinae|[15743]|SSWLF4416-13|Chironomidae|661|BOLD:ACH3036
Monopelopia tenuicalcar|[15744]|BBDEE656-10|Chironomidae|658|BOLD:AAM6277
Tanypodinae|[15745]|CNGBF1908-14|Chironomidae|661|BOLD:ACL4152
Krenopelopia|[15746]|CNGMC1780-13|Chironomidae|661|BOLD:AAM6271
Krenopelopia|[15747]|SSBAA600-12|Chironomidae|673|BOLD:AAC9199
Krenopelopia|[15748]|CNFNE2369-14|Chironomidae|658|BOLD:ACL5030
Krenopelopia|[15749]|CNPEO637-14|Chironomidae|661|BOLD:ACL9930
Krenopelopia|[15750]|CNPCP059-13|Chironomidae|670|BOLD:ACE1996
Krenopelopia|[15751]|CNPCP205-13|Chironomidae|605|BOLD:ACI9050
Krenopelopia|[15752]|SSBAF983-13|Chironomidae|661|BOLD:ACI4098
Krenopelopia|[15753]|CNBFL410-15|Chironomidae|588|BOLD:ACT9538
Krenopelopia|[15754]|CNRVG1122-15|Chironomidae|588|BOLD:ACT9537
Chironomidae|[15755]|CNPRK043-15|Chironomidae|564|BOLD:ACT8707
Chironomidae|[15756]|CNTMA1117-14|Chironomidae|588|BOLD:ACV5079
Chironomidae|[15757]|CNTMA1256-14|Chironomidae|661|BOLD:ACM0469
Trichotanypus posticalis|[15758]|CNQUI1208-14|Chironomidae|615|BOLD:AAL6370
Trichotanypus posticalis|[15759]|CNIVB1377-14|Chironomidae|600|BOLD:AAE8611
Chironomidae|[15760]|CNVUA219-14|Chironomidae|567|BOLD:ACR7429
Corynoneura|[15761]|SMTPB8509-13|Chironomidae|670|BOLD:ACF9451
Corynoneura|[15762]|SMTPB1562-13|Chironomidae|658|BOLD:ACF6064
Corynoneura|[15763]|JSJUN733-11|Chironomidae|658|BOLD:AAG5450
Chironomidae|[15764]|SMTPB18336-13|Chironomidae|661|BOLD:ABV1231
Corynoneura|[15765]|SMTPB18383-13|Chironomidae|661|BOLD:AAG0994
Corynoneura|[15766]|CNFNC1357-14|Chironomidae|667|BOLD:ACD9525
Corynoneura|[15767]|BIOZO161-14|Chironomidae|664|BOLD:ACI4506
Chironomidae|[15768]|SMTPD1916-13|Chironomidae|615|BOLD:ACJ6553
Corynoneura|[15769]|CNEIB765-12|Chironomidae|673|BOLD:ACA8504
Corynoneura|[15770]|CNAUA270-14|Chironomidae|630|BOLD:ACP2972
Corynoneura scutellata|[15771]|NCCA1195-11|Chironomidae|673|BOLD:AAN5330
Corynoneura|[15772]|CNBAA095-12|Chironomidae|661|BOLD:ACC3285
Corynoneura scutellata|[15773]|CHURC791-09|Chironomidae|658|BOLD:AAB0075
Corynoneura sp. 8ES|[15774]|INNV006-08|Chironomidae|658|BOLD:AAG5432
Corynoneura scutellata|[15775]|CNQUJ880-14|Chironomidae|609|BOLD:ACA4706
Corynoneura scutellata|[15776]|CNSRB078-14|Chironomidae|642|BOLD:ACU4634
Corynoneura arctica|[15777]|BBDCQ670-10|Chironomidae|658|BOLD:AAB0079
Corynoneura|[15778]|NCCA468-11|Chironomidae|673|BOLD:ABY3407
Corynoneura sp. 1ES|[15779]|DARC521-11|Chironomidae|658|BOLD:ABZ8189
Corynoneura sp. 3ES|[15780]|CNEIC2171-12|Chironomidae|658|BOLD:AAB0080
Corynoneura|[15781]|CNNHA2907-14|Chironomidae|621|BOLD:ACR4370
Chironomidae|[15782]|BBDCM308-10|Chironomidae|658|BOLD:AAP8997
Chironomidae|[15783]|NCCA2300-11|Chironomidae|674|BOLD:AAO7623
Corynoneura|[15784]|SSBAD1711-12|Chironomidae|661|BOLD:ACD3135
Corynoneura sp. 10ES|[15785]|CNBPG294-12|Chironomidae|670|BOLD:AAI5885
Thienemanniella xena|[15786]|SMTPB8032-13|Chironomidae|670|BOLD:AAD5254
Thienemanniella|[15787]|JSMAY869-11|Chironomidae|658|BOLD:ABV1212
Thienemanniella|[15788]|GMDAP176-12|Chironomidae|658|BOLD:AAD5253
Orthocladiinae|[15789]|MBIOK547-14|Chironomidae|658|BOLD:ACF7512
Chironomidae|[15790]|BBDIQ181-10|Chironomidae|658|BOLD:AAL7354
Chironomidae|[15791]|CNPPG245-12|Chironomidae|664|BOLD:ABW8019
Thienemanniella lobapodema|[15792]|BBDIQ095-10|Chironomidae|658|BOLD:AAG1614
Orthocladiinae|[15793]|CNKJB428-14|Chironomidae|658|BOLD:ACL7762
Corynoneura|[15794]|CNGMG638-14|Chironomidae|658|BOLD:ACL7133
Corynoneura|[15795]|CNBRG267-14|Chironomidae|664|BOLD:ACL3786
Corynoneura|[15796]|CNBRD760-14|Chironomidae|661|BOLD:AAI5884
Corynoneura|[15797]|CNBRP1924-14|Chironomidae|660|BOLD:ACL7069

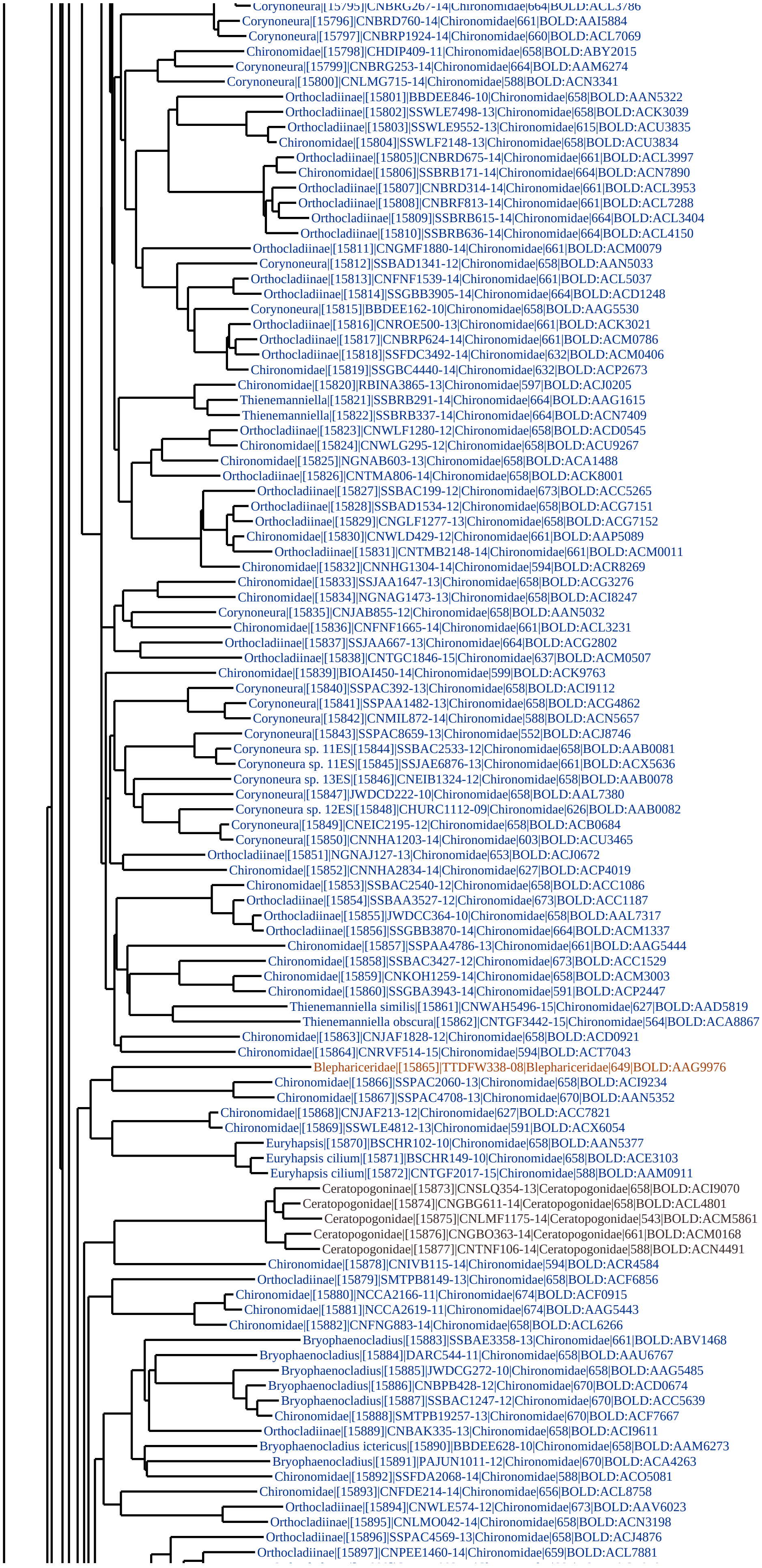

Corynoneura|[15795]|CNBRG267-14|Chironomidae|664|BOLD:ACL3786
Corynoneura|[15796]|CNBRD760-14|Chironomidae|661|BOLD:AAI5884
Corynoneura|[15797]|CNBRP1924-14|Chironomidae|660|BOLD:ACL7069
Chironomidae|[15798]|CHDIP409-11|Chironomidae|658|BOLD:ABY2015
Corynoneura|[15799]|CNBRG253-14|Chironomidae|664|BOLD:AAM6274
Corynoneura|[15800]|CNLMG715-14|Chironomidae|588|BOLD:ACN3341
Orthocladiinae|[15801]|BBDEE846-10|Chironomidae|658|BOLD:AAN5322
Orthocladiinae|[15802]|SSWLE7498-13|Chironomidae|658|BOLD:ACK3039
Orthocladiinae|[15803]|SSWLE9552-13|Chironomidae|615|BOLD:ACU3835
Chironomidae|[15804]|SSWLF2148-13|Chironomidae|658|BOLD:ACU3834
Orthocladiinae|[15805]|CNBRD675-14|Chironomidae|661|BOLD:ACL3997
Chironomidae|[15806]|SSBRB171-14|Chironomidae|664|BOLD:ACN7890
Orthocladiinae|[15807]|CNBRD314-14|Chironomidae|661|BOLD:ACL3953
Orthocladiinae|[15808]|CNBRF813-14|Chironomidae|661|BOLD:ACL7288
Orthocladiinae|[15809]|SSBRB615-14|Chironomidae|664|BOLD:ACL3404
Orthocladiinae|[15810]|SSBRB636-14|Chironomidae|664|BOLD:ACL4150
Orthocladiinae|[15811]|CNGMF1880-14|Chironomidae|661|BOLD:ACM0079
Corynoneura|[15812]|SSBAD1341-12|Chironomidae|658|BOLD:AAN5033
Orthocladiinae|[15813]|CNFNF1539-14|Chironomidae|661|BOLD:ACL5037
Orthocladiinae|[15814]|SSGBB3905-14|Chironomidae|664|BOLD:ACD1248
Corynoneura|[15815]|BBDEE162-10|Chironomidae|658|BOLD:AAG5530
Orthocladiinae|[15816]|CNROE500-13|Chironomidae|661|BOLD:ACK3021
Orthocladiinae|[15817]|CNBRP624-14|Chironomidae|661|BOLD:ACM0786
Orthocladiinae|[15818]|SSFDC3492-14|Chironomidae|632|BOLD:ACM0406
Chironomidae|[15819]|SSGBC4440-14|Chironomidae|632|BOLD:ACP2673
Chironomidae|[15820]|RBINA3865-13|Chironomidae|597|BOLD:ACJ0205
Thienemanniella|[15821]|SSBRB291-14|Chironomidae|664|BOLD:AAG1615
Thienemanniella|[15822]|SSBRB337-14|Chironomidae|664|BOLD:ACN7409
Orthocladiinae|[15823]|CNWLF1280-12|Chironomidae|658|BOLD:ACD0545
Chironomidae|[15824]|CNWLG295-12|Chironomidae|658|BOLD:ACU9267
Chironomidae|[15825]|NGNAB603-13|Chironomidae|658|BOLD:ACA1488
Orthocladiinae|[15826]|CNTMA806-14|Chironomidae|658|BOLD:ACK8001
Orthocladiinae|[15827]|SSBAC199-12|Chironomidae|673|BOLD:ACC5265
Orthocladiinae|[15828]|SSBAD1534-12|Chironomidae|658|BOLD:ACG7151
Orthocladiinae|[15829]|CNGLF1277-13|Chironomidae|658|BOLD:ACG7152
Chironomidae|[15830]|CNWLD429-12|Chironomidae|661|BOLD:AAP5089
Orthocladiinae|[15831]|CNTMB2148-14|Chironomidae|661|BOLD:ACM0011
Chironomidae|[15832]|CNNHG1304-14|Chironomidae|594|BOLD:ACR8269
Chironomidae|[15833]|SSJAA1647-13|Chironomidae|658|BOLD:ACG3276
Chironomidae|[15834]|NGNAG1473-13|Chironomidae|658|BOLD:ACI8247
Corynoneura|[15835]|CNJAB855-12|Chironomidae|658|BOLD:AAN5032
Chironomidae|[15836]|CNFNF1665-14|Chironomidae|661|BOLD:ACL3231
Orthocladiinae|[15837]|SSJAA667-13|Chironomidae|664|BOLD:ACG2802
Orthocladiinae|[15838]|CNTGC1846-15|Chironomidae|637|BOLD:ACM0507
Chironomidae|[15839]|BIOAI450-14|Chironomidae|599|BOLD:ACK9763
Corynoneura|[15840]|SSPAC392-13|Chironomidae|658|BOLD:ACI9112
Corynoneura|[15841]|SSPAA1482-13|Chironomidae|658|BOLD:ACG4862
Corynoneura|[15842]|CNMIL872-14|Chironomidae|588|BOLD:ACN5657
Corynoneura|[15843]|SSPAC8659-13|Chironomidae|552|BOLD:ACJ8746
Corynoneura sp. 11ES|[15844]|SSBAC2533-12|Chironomidae|658|BOLD:AAB0081
Corynoneura sp. 11ES|[15845]|SSJAE6876-13|Chironomidae|661|BOLD:ACX5636
Corynoneura sp. 13ES|[15846]|CNEIB1324-12|Chironomidae|658|BOLD:AAB0078
Corynoneura|[15847]|JWDCD222-10|Chironomidae|658|BOLD:AAL7380
Corynoneura sp. 12ES|[15848]|CHURC1112-09|Chironomidae|626|BOLD:AAB0082
Corynoneura|[15849]|CNEIC2195-12|Chironomidae|658|BOLD:ACB0684
Corynoneura|[15850]|CNNHA1203-14|Chironomidae|603|BOLD:ACU3465
Orthocladiinae|[15851]|NGNAJ127-13|Chironomidae|653|BOLD:ACJ0672
Chironomidae|[15852]|CNNHA2834-14|Chironomidae|627|BOLD:ACP4019
Chironomidae|[15853]|SSBAC2540-12|Chironomidae|658|BOLD:ACC1086
Orthocladiinae|[15854]|SSBAA3527-12|Chironomidae|673|BOLD:ACC1187
Orthocladiinae|[15855]|JWDCC364-10|Chironomidae|658|BOLD:AAL7317
Orthocladiinae|[15856]|SSGBB3870-14|Chironomidae|664|BOLD:ACM1337
Chironomidae|[15857]|SSPAA4786-13|Chironomidae|661|BOLD:AAG5444
Chironomidae|[15858]|SSBAC3427-12|Chironomidae|673|BOLD:ACC1529
Chironomidae|[15859]|CNKOH1259-14|Chironomidae|658|BOLD:ACM3003
Chironomidae|[15860]|SSGBA3943-14|Chironomidae|591|BOLD:ACP2447
Thienemanniella similis|[15861]|CNWAH5496-15|Chironomidae|627|BOLD:AAD5819
Thienemanniella obscura|[15862]|CNTGF3442-15|Chironomidae|564|BOLD:ACA8867
Chironomidae|[15863]|CNJAF1828-12|Chironomidae|658|BOLD:ACD0921
Chironomidae|[15864]|CNRVF514-15|Chironomidae|594|BOLD:ACT7043
Blephariceridae|[15865]|TTDFW338-08|Blephariceridae|649|BOLD:AAG9976
Chironomidae|[15866]|SSPAC2060-13|Chironomidae|658|BOLD:ACI9234
Chironomidae|[15867]|SSPAC4708-13|Chironomidae|670|BOLD:AAN5352
Chironomidae|[15868]|CNJAF213-12|Chironomidae|627|BOLD:ACC7821
Chironomidae|[15869]|SSWLE4812-13|Chironomidae|591|BOLD:ACX6054
Euryhapsis|[15870]|BSCHR102-10|Chironomidae|658|BOLD:AAN5377
Euryhapsis cilium|[15871]|BSCHR149-10|Chironomidae|658|BOLD:ACE3103
Euryhapsis cilium|[15872]|CNTGF2017-15|Chironomidae|588|BOLD:AAM0911
Ceratopogoninae|[15873]|CNSLQ354-13|Ceratopogonidae|658|BOLD:ACI9070
Ceratopogonidae|[15874]|CNGBG611-14|Ceratopogonidae|658|BOLD:ACL4801
Ceratopogonidae|[15875]|CNLMF1175-14|Ceratopogonidae|543|BOLD:ACM5861
Ceratopogonidae|[15876]|CNGBO363-14|Ceratopogonidae|661|BOLD:ACM0168
Ceratopogonidae|[15877]|CNTNF106-14|Ceratopogonidae|588|BOLD:ACN4491
Chironomidae|[15878]|CNIVB115-14|Chironomidae|594|BOLD:ACR4584
Orthocladiinae|[15879]|SMTPB8149-13|Chironomidae|658|BOLD:ACF6856
Chironomidae|[15880]|NCCA2166-11|Chironomidae|674|BOLD:ACF0915
Chironomidae|[15881]|NCCA2619-11|Chironomidae|674|BOLD:AAG5443
Chironomidae|[15882]|CNFNG883-14|Chironomidae|658|BOLD:ACL6266
Bryophaenocladius|[15883]|SSBAE3358-13|Chironomidae|661|BOLD:ABV1468
Bryophaenocladius|[15884]|DARC544-11|Chironomidae|658|BOLD:AAU6767
Bryophaenocladius|[15885]|JWDCG272-10|Chironomidae|658|BOLD:AAG5485
Bryophaenocladius|[15886]|CNBPB428-12|Chironomidae|670|BOLD:ACD0674
Bryophaenocladius|[15887]|SSBAC1247-12|Chironomidae|670|BOLD:ACC5639
Chironomidae|[15888]|SMTPB19257-13|Chironomidae|670|BOLD:ACF7667
Orthocladiinae|[15889]|CNBAK335-13|Chironomidae|658|BOLD:ACI9611
Bryophaenocladius ictericus|[15890]|BBDEE628-10|Chironomidae|658|BOLD:AAM6273
Bryophaenocladius|[15891]|PAJUN1011-12|Chironomidae|670|BOLD:ACA4263
Chironomidae|[15892]|SSFDA2068-14|Chironomidae|588|BOLD:ACO5081
Chironomidae|[15893]|CNFDE214-14|Chironomidae|656|BOLD:ACL8758
Orthocladiinae|[15894]|CNWLE574-12|Chironomidae|673|BOLD:AAV6023
Orthocladiinae|[15895]|CNLMO042-14|Chironomidae|658|BOLD:ACN3198
Orthocladiinae|[15896]|SSPAC4569-13|Chironomidae|658|BOLD:ACJ4876
Orthocladiinae|[15897]|CNPEE1460-14|Chironomidae|659|BOLD:ACL7881

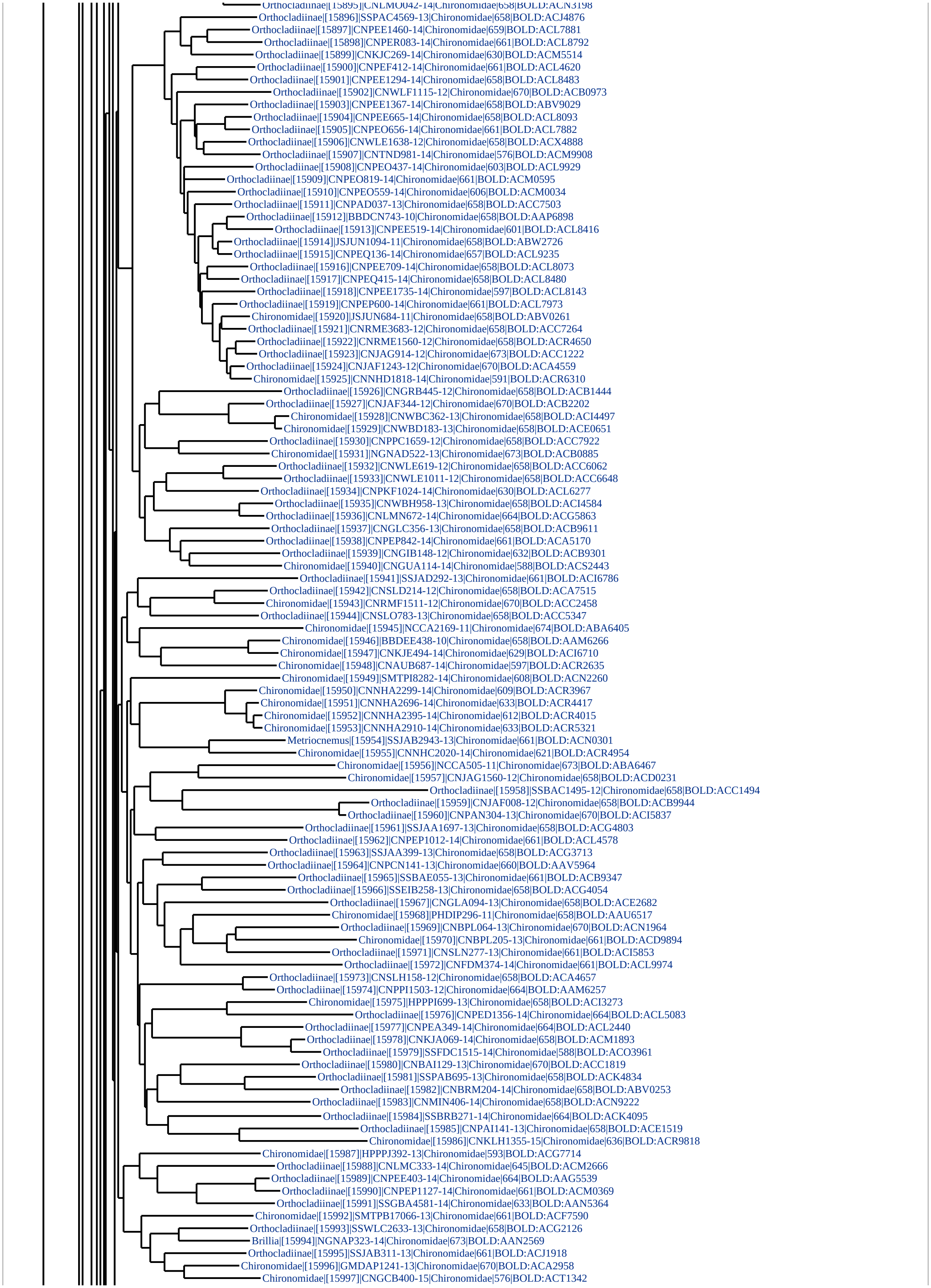
Orthocladiinae|[15895]|CNLMO042-14|Chironomidae|658|BOLD:ACN3198
Orthocladiinae|[15896]|SSPAC4569-13|Chironomidae|658|BOLD:ACJ4876
Orthocladiinae|[15897]|CNPEE1460-14|Chironomidae|659|BOLD:ACL7881
Orthocladiinae|[15898]|CNPER083-14|Chironomidae|661|BOLD:ACL8792
Orthocladiinae|[15899]|CNKJC269-14|Chironomidae|630|BOLD:ACM5514
Orthocladiinae|[15900]|CNPEF412-14|Chironomidae|661|BOLD:ACL4620
Orthocladiinae|[15901]|CNPEE1294-14|Chironomidae|658|BOLD:ACL8483
Orthocladiinae|[15902]|CNWLF1115-12|Chironomidae|670|BOLD:ACB0973
Orthocladiinae|[15903]|CNPEE1367-14|Chironomidae|658|BOLD:ABV9029
Orthocladiinae|[15904]|CNPEE665-14|Chironomidae|658|BOLD:ACL8093
Orthocladiinae|[15905]|CNPEO656-14|Chironomidae|661|BOLD:ACL7882
Orthocladiinae|[15906]|CNWLE1638-12|Chironomidae|658|BOLD:ACX4888
Orthocladiinae|[15907]|CNTND981-14|Chironomidae|576|BOLD:ACM9908
Orthocladiinae|[15908]|CNPEO437-14|Chironomidae|603|BOLD:ACL9929
Orthocladiinae|[15909]|CNPEO819-14|Chironomidae|661|BOLD:ACM0595
Orthocladiinae|[15910]|CNPEO559-14|Chironomidae|606|BOLD:ACM0034
Orthocladiinae|[15911]|CNPAD037-13|Chironomidae|658|BOLD:ACC7503
Orthocladiinae|[15912]|BBDCN743-10|Chironomidae|658|BOLD:AAP6898
Orthocladiinae|[15913]|CNPEE519-14|Chironomidae|601|BOLD:ACL8416
Orthocladiinae|[15914]|JSJUN1094-11|Chironomidae|658|BOLD:ABW2726
Orthocladiinae|[15915]|CNPEQ136-14|Chironomidae|657|BOLD:ACL9235
Orthocladiinae|[15916]|CNPEE709-14|Chironomidae|658|BOLD:ACL8073
Orthocladiinae|[15917]|CNPEQ415-14|Chironomidae|658|BOLD:ACL8480
Orthocladiinae|[15918]|CNPEE1735-14|Chironomidae|597|BOLD:ACL8143
Orthocladiinae|[15919]|CNPEP600-14|Chironomidae|661|BOLD:ACL7973
Chironomidae|[15920]|JSJUN684-11|Chironomidae|658|BOLD:ABV0261
Orthocladiinae|[15921]|CNRME3683-12|Chironomidae|658|BOLD:ACC7264
Orthocladiinae|[15922]|CNRME1560-12|Chironomidae|658|BOLD:ACR4650
Orthocladiinae|[15923]|CNJAG914-12|Chironomidae|673|BOLD:ACC1222
Orthocladiinae|[15924]|CNJAF1243-12|Chironomidae|670|BOLD:ACA4559
Chironomidae|[15925]|CNNHD1818-14|Chironomidae|591|BOLD:ACR6310
Orthocladiinae|[15926]|CNGRB445-12|Chironomidae|658|BOLD:ACB1444
Orthocladiinae|[15927]|CNJAF344-12|Chironomidae|670|BOLD:ACB2202
Chironomidae|[15928]|CNWBC362-13|Chironomidae|658|BOLD:ACI4497
Chironomidae|[15929]|CNWBD183-13|Chironomidae|658|BOLD:ACE0651
Orthocladiinae|[15930]|CNPPC1659-12|Chironomidae|658|BOLD:ACC7922
Chironomidae|[15931]|NGNAD522-13|Chironomidae|673|BOLD:ACB0885
Orthocladiinae|[15932]|CNWLE619-12|Chironomidae|658|BOLD:ACC6062
Orthocladiinae|[15933]|CNWLE1011-12|Chironomidae|658|BOLD:ACC6648
Orthocladiinae|[15934]|CNPKF1024-14|Chironomidae|630|BOLD:ACL6277
Orthocladiinae|[15935]|CNWBH958-13|Chironomidae|658|BOLD:ACI4584
Orthocladiinae|[15936]|CNLMN672-14|Chironomidae|664|BOLD:ACG5863
Orthocladiinae|[15937]|CNGLC356-13|Chironomidae|658|BOLD:ACB9611
Orthocladiinae|[15938]|CNPEP842-14|Chironomidae|661|BOLD:ACA5170
Orthocladiinae|[15939]|CNGIB148-12|Chironomidae|632|BOLD:ACB9301
Chironomidae|[15940]|CNGUA114-14|Chironomidae|588|BOLD:ACS2443
Orthocladiinae|[15941]|SSJAD292-13|Chironomidae|661|BOLD:ACI6786
Orthocladiinae|[15942]|CNSLD214-12|Chironomidae|658|BOLD:ACA7515
Chironomidae|[15943]|CNRMF1511-12|Chironomidae|670|BOLD:ACC2458
Orthocladiinae|[15944]|CNSLO783-13|Chironomidae|658|BOLD:ACC5347
Chironomidae|[15945]|NCCA2169-11|Chironomidae|674|BOLD:ABA6405
Chironomidae|[15946]|BBDEE438-10|Chironomidae|658|BOLD:AAM6266
Chironomidae|[15947]|CNKJE494-14|Chironomidae|629|BOLD:ACI6710
Chironomidae|[15948]|CNAUB687-14|Chironomidae|597|BOLD:ACR2635
Chironomidae|[15949]|SMTPI8282-14|Chironomidae|608|BOLD:ACN2260
Chironomidae|[15950]|CNNHA2299-14|Chironomidae|609|BOLD:ACR3967
Chironomidae|[15951]|CNNHA2696-14|Chironomidae|633|BOLD:ACR4417
Chironomidae|[15952]|CNNHA2395-14|Chironomidae|612|BOLD:ACR4015
Chironomidae|[15953]|CNNHA2910-14|Chironomidae|633|BOLD:ACR5321
Metriocnemus|[15954]|SSJAB2943-13|Chironomidae|661|BOLD:ACN0301
Chironomidae|[15955]|CNNHC2020-14|Chironomidae|621|BOLD:ACR4954
Chironomidae|[15956]|NCCA505-11|Chironomidae|673|BOLD:ABA6467
Chironomidae|[15957]|CNJAG1560-12|Chironomidae|658|BOLD:ACD0231
Orthocladiinae|[15958]|SSBAC1495-12|Chironomidae|658|BOLD:ACC1494
Orthocladiinae|[15959]|CNJAF008-12|Chironomidae|658|BOLD:ACB9944
Orthocladiinae|[15960]|CNPAN304-13|Chironomidae|670|BOLD:ACI5837
Orthocladiinae|[15961]|SSJAA1697-13|Chironomidae|658|BOLD:ACG4803
Orthocladiinae|[15962]|CNPEP1012-14|Chironomidae|661|BOLD:ACL4578
Orthocladiinae|[15963]|SSJAA399-13|Chironomidae|658|BOLD:ACG3713
Orthocladiinae|[15964]|CNPCN141-13|Chironomidae|660|BOLD:AAV5964
Orthocladiinae|[15965]|SSBAE055-13|Chironomidae|661|BOLD:ACB9347
Orthocladiinae|[15966]|SSEIB258-13|Chironomidae|658|BOLD:ACG4054
Orthocladiinae|[15967]|CNGLA094-13|Chironomidae|658|BOLD:ACE2682
Chironomidae|[15968]|PHDIP296-11|Chironomidae|658|BOLD:AAU6517
Orthocladiinae|[15969]|CNBPL064-13|Chironomidae|670|BOLD:ACN1964
Chironomidae|[15970]|CNBPL205-13|Chironomidae|661|BOLD:ACD9894
Orthocladiinae|[15971]|CNSLN277-13|Chironomidae|661|BOLD:ACI5853
Orthocladiinae|[15972]|CNFDM374-14|Chironomidae|661|BOLD:ACL9974
Orthocladiinae|[15973]|CNSLH158-12|Chironomidae|658|BOLD:ACA4657
Orthocladiinae|[15974]|CNPPI1503-12|Chironomidae|664|BOLD:AAM6257
Chironomidae|[15975]|HPPPI699-13|Chironomidae|658|BOLD:ACI3273
Orthocladiinae|[15976]|CNPED1356-14|Chironomidae|664|BOLD:ACL5083
Orthocladiinae|[15977]|CNPEA349-14|Chironomidae|664|BOLD:ACL2440
Orthocladiinae|[15978]|CNKJA069-14|Chironomidae|658|BOLD:ACM1893
Orthocladiinae|[15979]|SSFDC1515-14|Chironomidae|588|BOLD:ACO3961
Orthocladiinae|[15980]|CNBAI129-13|Chironomidae|670|BOLD:ACC1819
Orthocladiinae|[15981]|SSPAB695-13|Chironomidae|658|BOLD:ACK4834
Orthocladiinae|[15982]|CNBRM204-14|Chironomidae|658|BOLD:ABV0253
Orthocladiinae|[15983]|CNMIN406-14|Chironomidae|658|BOLD:ACN9222
Orthocladiinae|[15984]|SSBRB271-14|Chironomidae|664|BOLD:ACK4095
Orthocladiinae|[15985]|CNPAI141-13|Chironomidae|658|BOLD:ACE1519
Chironomidae|[15986]|CNKLH1355-15|Chironomidae|636|BOLD:ACR9818
Chironomidae|[15987]|HPPPJ392-13|Chironomidae|593|BOLD:ACG7714
Orthocladiinae|[15988]|CNLMC333-14|Chironomidae|645|BOLD:ACM2666
Orthocladiinae|[15989]|CNPEE403-14|Chironomidae|664|BOLD:AAG5539
Orthocladiinae|[15990]|CNPEP1127-14|Chironomidae|661|BOLD:ACM0369
Orthocladiinae|[15991]|SSGBA4581-14|Chironomidae|633|BOLD:AAN5364
Chironomidae|[15992]|SMTPB17066-13|Chironomidae|661|BOLD:ACF7590
Orthocladiinae|[15993]|SSWLC2633-13|Chironomidae|658|BOLD:ACG2126
Brillia|[15994]|NGNAP323-14|Chironomidae|673|BOLD:AAN2569
Orthocladiinae|[15995]|SSJAB311-13|Chironomidae|661|BOLD:ACJ1918
Chironomidae|[15996]|GMDAP1241-13|Chironomidae|670|BOLD:ACA2958
Chironomidae|[15997]|CNGCB400-15|Chironomidae|576|BOLD:ACT1342

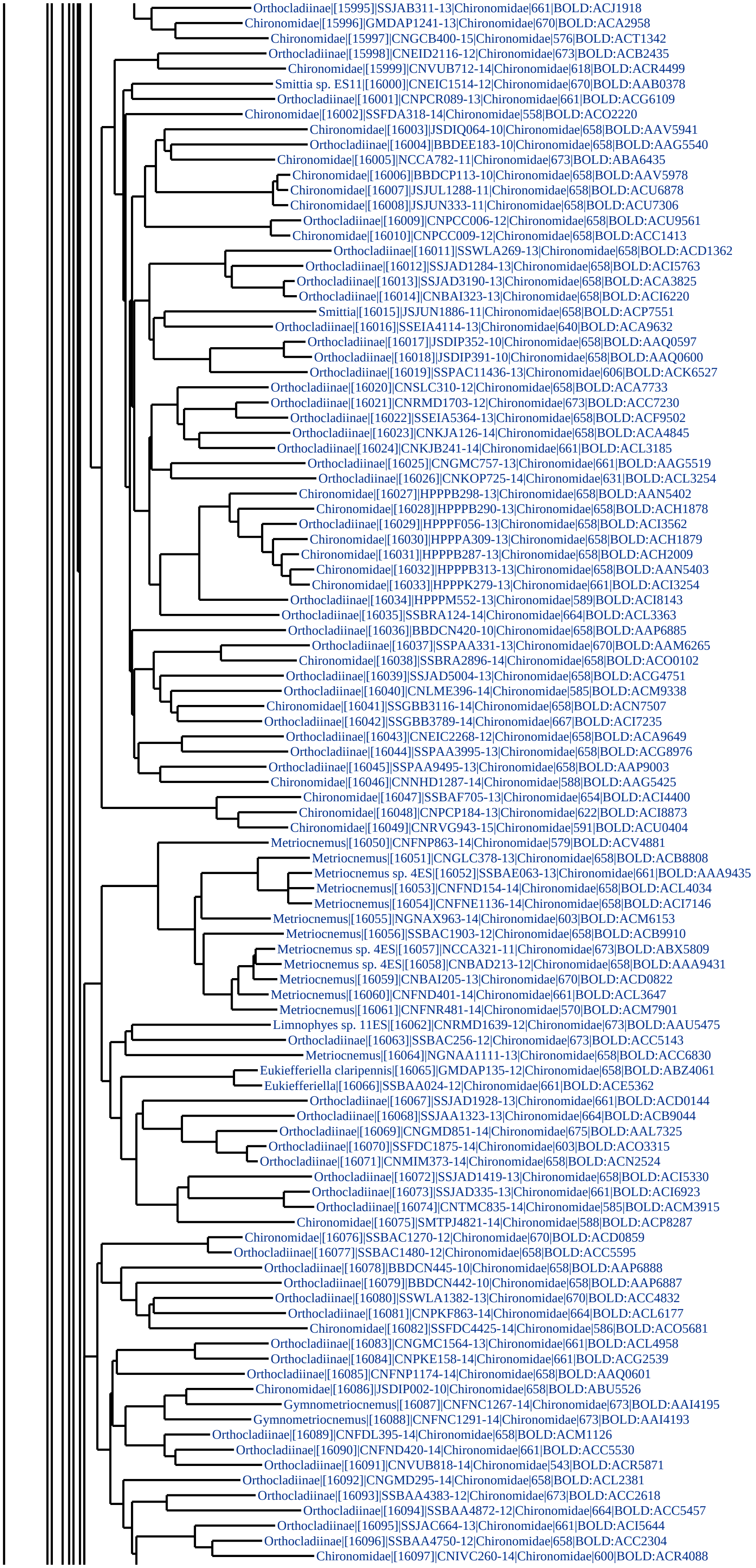

Orthocladiinae|[15995]|SSJAB311-13|Chironomidae|661|BOLD:ACJ1918
Chironomidae|[15996]|GMDAP1241-13|Chironomidae|670|BOLD:ACA2958
Chironomidae|[15997]|CNGCB400-15|Chironomidae|576|BOLD:ACT1342
Orthocladiinae|[15998]|CNEID2116-12|Chironomidae|673|BOLD:ACB2435
Chironomidae|[15999]|CNVUB712-14|Chironomidae|618|BOLD:ACR4499
Smittia sp. ES11|[16000]|CNEIC1514-12|Chironomidae|670|BOLD:AAB0378
Orthocladiinae|[16001]|CNPCR089-13|Chironomidae|661|BOLD:ACG6109
Chironomidae|[16002]|SSFDA318-14|Chironomidae|558|BOLD:ACO2220
Chironomidae|[16003]|JSDIQ064-10|Chironomidae|658|BOLD:AAV5941
Orthocladiinae|[16004]|BBDEE183-10|Chironomidae|658|BOLD:AAG5540
Chironomidae|[16005]|NCCA782-11|Chironomidae|673|BOLD:ABA6435
Chironomidae|[16006]|BBDCP113-10|Chironomidae|658|BOLD:AAV5978
Chironomidae|[16007]|JSJUL1288-11|Chironomidae|658|BOLD:ACU6878
Chironomidae|[16008]|JSJUN333-11|Chironomidae|658|BOLD:ACU7306
Orthocladiinae|[16009]|CNPCC006-12|Chironomidae|658|BOLD:ACU9561
Chironomidae|[16010]|CNPCC009-12|Chironomidae|658|BOLD:ACC1413
Orthocladiinae|[16011]|SSWLA269-13|Chironomidae|658|BOLD:ACD1362
Orthocladiinae|[16012]|SSJAD1284-13|Chironomidae|658|BOLD:ACI5763
Orthocladiinae|[16013]|SSJAD3190-13|Chironomidae|658|BOLD:ACA3825
Orthocladiinae|[16014]|CNBAI323-13|Chironomidae|658|BOLD:ACI6220
Smittia|[16015]|JSJUN1886-11|Chironomidae|658|BOLD:ACP7551
Orthocladiinae|[16016]|SSEIA4114-13|Chironomidae|640|BOLD:ACA9632
Orthocladiinae|[16017]|JSDIP352-10|Chironomidae|658|BOLD:AAQ0597
Orthocladiinae|[16018]|JSDIP391-10|Chironomidae|658|BOLD:AAQ0600
Orthocladiinae|[16019]|SSPAC11436-13|Chironomidae|606|BOLD:ACK6527
Orthocladiinae|[16020]|CNSLC310-12|Chironomidae|658|BOLD:ACA7733
Orthocladiinae|[16021]|CNRMD1703-12|Chironomidae|673|BOLD:ACC7230
Orthocladiinae|[16022]|SSEIA5364-13|Chironomidae|658|BOLD:ACF9502
Orthocladiinae|[16023]|CNKJA126-14|Chironomidae|658|BOLD:ACA4845
Orthocladiinae|[16024]|CNKJB241-14|Chironomidae|661|BOLD:ACL3185
Orthocladiinae|[16025]|CNGMC757-13|Chironomidae|661|BOLD:AAG5519
Orthocladiinae|[16026]|CNKOP725-14|Chironomidae|631|BOLD:ACL3254
Chironomidae|[16027]|HPPPB298-13|Chironomidae|658|BOLD:AAN5402
Chironomidae|[16028]|HPPPB290-13|Chironomidae|658|BOLD:ACH1878
Orthocladiinae|[16029]|HPPPF056-13|Chironomidae|658|BOLD:ACI3562
Chironomidae|[16030]|HPPPA309-13|Chironomidae|658|BOLD:ACH1879
Chironomidae|[16031]|HPPPB287-13|Chironomidae|658|BOLD:ACH2009
Chironomidae|[16032]|HPPPB313-13|Chironomidae|658|BOLD:AAN5403
Chironomidae|[16033]|HPPPK279-13|Chironomidae|661|BOLD:ACI3254
Orthocladiinae|[16034]|HPPPM552-13|Chironomidae|589|BOLD:ACI8143
Orthocladiinae|[16035]|SSBRA124-14|Chironomidae|664|BOLD:ACL3363
Orthocladiinae|[16036]|BBDCN420-10|Chironomidae|658|BOLD:AAP6885
Orthocladiinae|[16037]|SSPAA331-13|Chironomidae|670|BOLD:AAM6265
Chironomidae|[16038]|SSBRA2896-14|Chironomidae|658|BOLD:ACO0102
Orthocladiinae|[16039]|SSJAD5004-13|Chironomidae|658|BOLD:ACG4751
Orthocladiinae|[16040]|CNLME396-14|Chironomidae|585|BOLD:ACM9338
Chironomidae|[16041]|SSGBB3116-14|Chironomidae|658|BOLD:ACN7507
Orthocladiinae|[16042]|SSGBB3789-14|Chironomidae|667|BOLD:ACI7235
Orthocladiinae|[16043]|CNEIC2268-12|Chironomidae|658|BOLD:ACA9649
Orthocladiinae|[16044]|SSPAA3995-13|Chironomidae|658|BOLD:ACG8976
Orthocladiinae|[16045]|SSPAA9495-13|Chironomidae|658|BOLD:AAP9003
Chironomidae|[16046]|CNNHD1287-14|Chironomidae|588|BOLD:AAG5425
Chironomidae|[16047]|SSBAF705-13|Chironomidae|654|BOLD:ACI4400
Chironomidae|[16048]|CNPCP184-13|Chironomidae|622|BOLD:ACI8873
Chironomidae|[16049]|CNRVG943-15|Chironomidae|591|BOLD:ACU0404
Metriocnemus|[16050]|CNFNP863-14|Chironomidae|579|BOLD:ACV4881
Metriocnemus|[16051]|CNGLC378-13|Chironomidae|658|BOLD:ACB8808
Metriocnemus sp. 4ES|[16052]|SSBAE063-13|Chironomidae|661|BOLD:AAA9435
Metriocnemus|[16053]|CNFND154-14|Chironomidae|658|BOLD:ACL4034
Metriocnemus|[16054]|CNFNE1136-14|Chironomidae|658|BOLD:ACI7146
Metriocnemus|[16055]|NGNAX963-14|Chironomidae|603|BOLD:ACM6153
Metriocnemus|[16056]|SSBAC1903-12|Chironomidae|658|BOLD:ACB9910
Metriocnemus sp. 4ES|[16057]|NCCA321-11|Chironomidae|673|BOLD:ABX5809
Metriocnemus sp. 4ES|[16058]|CNBAD213-12|Chironomidae|658|BOLD:AAA9431
Metriocnemus|[16059]|CNBAI205-13|Chironomidae|670|BOLD:ACD0822
Metriocnemus|[16060]|CNFND401-14|Chironomidae|661|BOLD:ACL3647
Metriocnemus|[16061]|CNFNR481-14|Chironomidae|570|BOLD:ACM7901
Limnophyes sp. 11ES|[16062]|CNRMD1639-12|Chironomidae|673|BOLD:AAU5475
Orthocladiinae|[16063]|SSBAC256-12|Chironomidae|673|BOLD:ACC5143
Metriocnemus|[16064]|NGNAA1111-13|Chironomidae|658|BOLD:ACC6830
Eukiefferiella claripennis|[16065]|GMDAP135-12|Chironomidae|658|BOLD:ABZ4061
Eukiefferiella|[16066]|SSBAA024-12|Chironomidae|661|BOLD:ACE5362
Orthocladiinae|[16067]|SSJAD1928-13|Chironomidae|661|BOLD:ACD0144
Orthocladiinae|[16068]|SSJAA1323-13|Chironomidae|664|BOLD:ACB9044
Orthocladiinae|[16069]|CNGMD851-14|Chironomidae|675|BOLD:AAL7325
Orthocladiinae|[16070]|SSFDC1875-14|Chironomidae|603|BOLD:ACO3315
Orthocladiinae|[16071]|CNMIM373-14|Chironomidae|658|BOLD:ACN2524
Orthocladiinae|[16072]|SSJAD1419-13|Chironomidae|658|BOLD:ACI5330
Orthocladiinae|[16073]|SSJAD335-13|Chironomidae|661|BOLD:ACI6923
Orthocladiinae|[16074]|CNTMC835-14|Chironomidae|585|BOLD:ACM3915
Chironomidae|[16075]|SMTPJ4821-14|Chironomidae|588|BOLD:ACP8287
Chironomidae|[16076]|SSBAC1270-12|Chironomidae|670|BOLD:ACD0859
Orthocladiinae|[16077]|SSBAC1480-12|Chironomidae|658|BOLD:ACC5595
Orthocladiinae|[16078]|BBDCN445-10|Chironomidae|658|BOLD:AAP6888
Orthocladiinae|[16079]|BBDCN442-10|Chironomidae|658|BOLD:AAP6887
Orthocladiinae|[16080]|SSWLA1382-13|Chironomidae|670|BOLD:ACC4832
Orthocladiinae|[16081]|CNPKF863-14|Chironomidae|664|BOLD:ACL6177
Chironomidae|[16082]|SSFDC4425-14|Chironomidae|586|BOLD:ACO5681
Orthocladiinae|[16083]|CNGMC1564-13|Chironomidae|661|BOLD:ACL4958
Orthocladiinae|[16084]|CNPKE158-14|Chironomidae|661|BOLD:ACG2539
Orthocladiinae|[16085]|CNFNP1174-14|Chironomidae|658|BOLD:AAQ0601
Chironomidae|[16086]|JSDIP002-10|Chironomidae|658|BOLD:ABU5526
Gymnometriocnemus|[16087]|CNFNC1267-14|Chironomidae|673|BOLD:AAI4195
Gymnometriocnemus|[16088]|CNFNC1291-14|Chironomidae|673|BOLD:AAI4193
Orthocladiinae|[16089]|CNFDL395-14|Chironomidae|658|BOLD:ACM1126
Orthocladiinae|[16090]|CNFND420-14|Chironomidae|661|BOLD:ACC5530
Orthocladiinae|[16091]|CNVUB818-14|Chironomidae|543|BOLD:ACR5871
Orthocladiinae|[16092]|CNGMD295-14|Chironomidae|658|BOLD:ACL2381
Orthocladiinae|[16093]|SSBAA4383-12|Chironomidae|673|BOLD:ACC2618
Orthocladiinae|[16094]|SSBAA4872-12|Chironomidae|664|BOLD:ACC5457
Orthocladiinae|[16095]|SSJAC664-13|Chironomidae|661|BOLD:ACI5644
Orthocladiinae|[16096]|SSBAA4750-12|Chironomidae|658|BOLD:ACC2304
Chironomidae|[16097]|CNIVC260-14|Chironomidae|600|BOLD:ACR4088

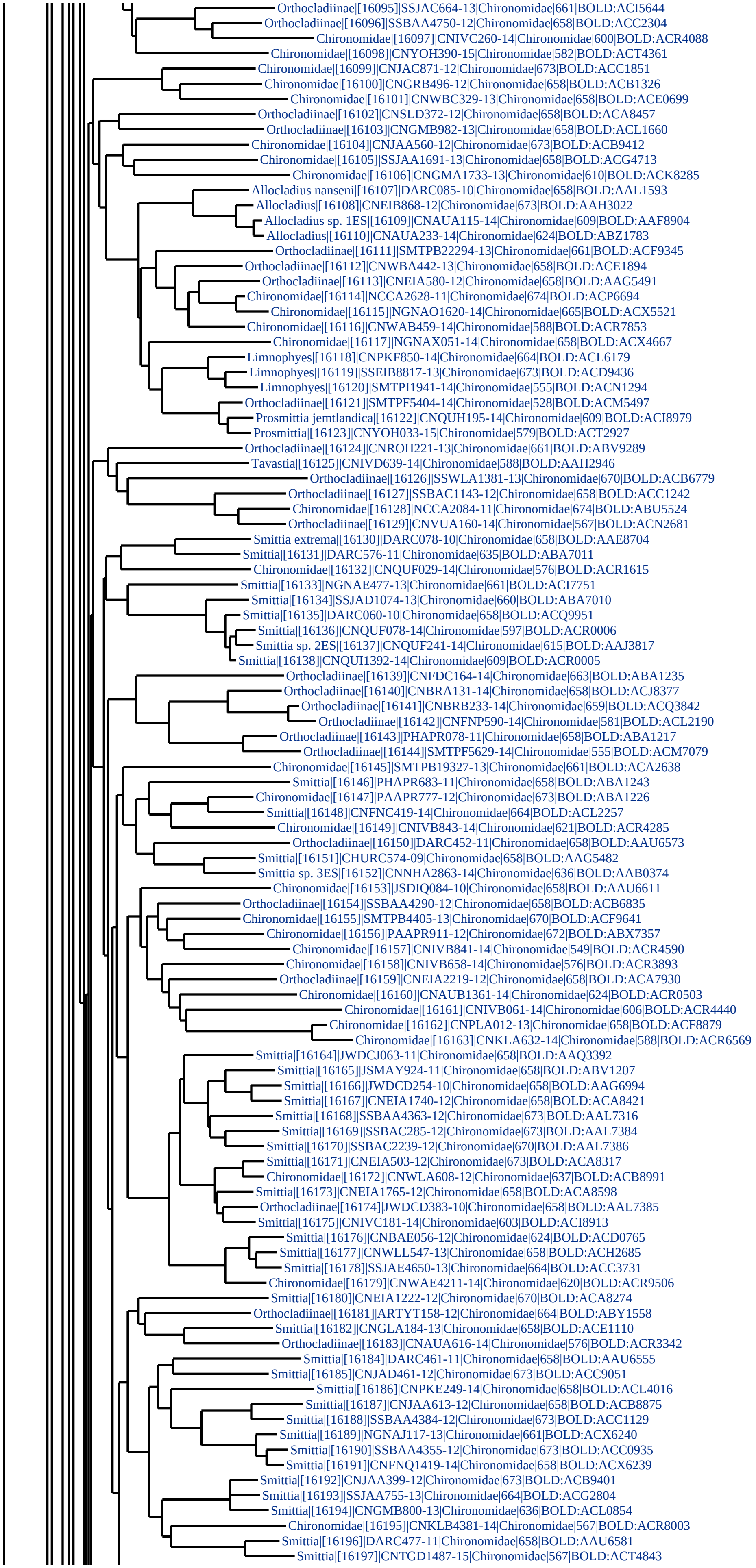

Orthocladiinae|[16095]|SSJAC664-13|Chironomidae|661|BOLD:ACI5644
Orthocladiinae|[16096]|SSBAA4750-12|Chironomidae|658|BOLD:ACC2304
Chironomidae|[16097]|CNIVC260-14|Chironomidae|600|BOLD:ACR4088
Chironomidae|[16098]|CNYOH390-15|Chironomidae|582|BOLD:ACT4361
Chironomidae|[16099]|CNJAC871-12|Chironomidae|673|BOLD:ACC1851
Chironomidae|[16100]|CNGRB496-12|Chironomidae|658|BOLD:ACB1326
Chironomidae|[16101]|CNWBC329-13|Chironomidae|658|BOLD:ACE0699
Orthocladiinae|[16102]|CNSLD372-12|Chironomidae|658|BOLD:ACA8457
Orthocladiinae|[16103]|CNGMB982-13|Chironomidae|658|BOLD:ACL1660
Chironomidae|[16104]|CNJAA560-12|Chironomidae|673|BOLD:ACB9412
Chironomidae|[16105]|SSJAA1691-13|Chironomidae|658|BOLD:ACG4713
Chironomidae|[16106]|CNGMA1733-13|Chironomidae|610|BOLD:ACK8285
Allocladius nanseni|[16107]|DARC085-10|Chironomidae|658|BOLD:AAL1593
Allocladius|[16108]|CNEIB868-12|Chironomidae|673|BOLD:AAH3022
Allocladius sp. 1ES|[16109]|CNAUA115-14|Chironomidae|609|BOLD:AAF8904
Allocladius|[16110]|CNAUA233-14|Chironomidae|624|BOLD:ABZ1783
Orthocladiinae|[16111]|SMTPB22294-13|Chironomidae|661|BOLD:ACF9345
Orthocladiinae|[16112]|CNWBA442-13|Chironomidae|658|BOLD:ACE1894
Orthocladiinae|[16113]|CNEIA580-12|Chironomidae|658|BOLD:AAG5491
Chironomidae|[16114]|NCCA2628-11|Chironomidae|674|BOLD:ACP6694
Chironomidae|[16115]|NGNAO1620-14|Chironomidae|665|BOLD:ACX5521
Chironomidae|[16116]|CNWAB459-14|Chironomidae|588|BOLD:ACR7853
Chironomidae|[16117]|NGNAX051-14|Chironomidae|658|BOLD:ACX4667
Limnophyes|[16118]|CNPKF850-14|Chironomidae|664|BOLD:ACL6179
Limnophyes|[16119]|SSEIB8817-13|Chironomidae|673|BOLD:ACD9436
Limnophyes|[16120]|SMTPI1941-14|Chironomidae|555|BOLD:ACN1294
Orthocladiinae|[16121]|SMTPF5404-14|Chironomidae|528|BOLD:ACM5497
Prosmittia jemtlandica|[16122]|CNQUH195-14|Chironomidae|609|BOLD:ACI8979
Prosmittia|[16123]|CNYOH033-15|Chironomidae|579|BOLD:ACT2927
Orthocladiinae|[16124]|CNROH221-13|Chironomidae|661|BOLD:ABV9289
Tavastia|[16125]|CNIVD639-14|Chironomidae|588|BOLD:AAH2946
Orthocladiinae|[16126]|SSWLA1381-13|Chironomidae|670|BOLD:ACB6779
Orthocladiinae|[16127]|SSBAC1143-12|Chironomidae|658|BOLD:ACC1242
Chironomidae|[16128]|NCCA2084-11|Chironomidae|674|BOLD:ABU5524
Orthocladiinae|[16129]|CNVUA160-14|Chironomidae|567|BOLD:ACN2681
Smittia extrema|[16130]|DARC078-10|Chironomidae|658|BOLD:AAE8704
Smittia|[16131]|DARC576-11|Chironomidae|635|BOLD:ABA7011
Chironomidae|[16132]|CNQUF029-14|Chironomidae|576|BOLD:ACR1615
Smittia|[16133]|NGNAE477-13|Chironomidae|661|BOLD:ACI7751
Smittia|[16134]|SSJAD1074-13|Chironomidae|660|BOLD:ABA7010
Smittia|[16135]|DARC060-10|Chironomidae|658|BOLD:ACQ9951
Smittia|[16136]|CNQUF078-14|Chironomidae|597|BOLD:ACR0006
Smittia sp. 2ES|[16137]|CNQUF241-14|Chironomidae|615|BOLD:AAJ3817
Smittia|[16138]|CNQUI1392-14|Chironomidae|609|BOLD:ACR0005
Orthocladiinae|[16139]|CNFDC164-14|Chironomidae|663|BOLD:ABA1235
Orthocladiinae|[16140]|CNBRA131-14|Chironomidae|658|BOLD:ACJ8377
Orthocladiinae|[16141]|CNBRB233-14|Chironomidae|659|BOLD:ACQ3842
Orthocladiinae|[16142]|CNFNP590-14|Chironomidae|581|BOLD:ACL2190
Orthocladiinae|[16143]|PHAPR078-11|Chironomidae|658|BOLD:ABA1217
Orthocladiinae|[16144]|SMTPF5629-14|Chironomidae|555|BOLD:ACM7079
Chironomidae|[16145]|SMTPB19327-13|Chironomidae|661|BOLD:ACA2638
Smittia|[16146]|PHAPR683-11|Chironomidae|658|BOLD:ABA1243
Chironomidae|[16147]|PAAPR777-12|Chironomidae|673|BOLD:ABA1226
Smittia|[16148]|CNFNC419-14|Chironomidae|664|BOLD:ACL2257
Chironomidae|[16149]|CNIVB843-14|Chironomidae|621|BOLD:ACR4285
Orthocladiinae|[16150]|DARC452-11|Chironomidae|658|BOLD:AAU6573
Smittia|[16151]|CHURC574-09|Chironomidae|658|BOLD:AAG5482
Smittia sp. 3ES|[16152]|CNNHA2863-14|Chironomidae|636|BOLD:AAB0374
Chironomidae|[16153]|JSDIQ084-10|Chironomidae|658|BOLD:AAU6611
Orthocladiinae|[16154]|SSBAA4290-12|Chironomidae|658|BOLD:ACB6835
Chironomidae|[16155]|SMTPB4405-13|Chironomidae|670|BOLD:ACF9641
Chironomidae|[16156]|PAAPR911-12|Chironomidae|672|BOLD:ABX7357
Chironomidae|[16157]|CNIVB841-14|Chironomidae|549|BOLD:ACR4590
Chironomidae|[16158]|CNIVB658-14|Chironomidae|576|BOLD:ACR3893
Orthocladiinae|[16159]|CNEIA2219-12|Chironomidae|658|BOLD:ACA7930
Chironomidae|[16160]|CNAUB1361-14|Chironomidae|624|BOLD:ACR0503
Chironomidae|[16161]|CNIVB061-14|Chironomidae|606|BOLD:ACR4440
Chironomidae|[16162]|CNPLA012-13|Chironomidae|658|BOLD:ACF8879
Chironomidae|[16163]|CNKLA632-14|Chironomidae|588|BOLD:ACR6569
Smittia|[16164]|JWDCJ063-11|Chironomidae|658|BOLD:AAQ3392
Smittia|[16165]|JSMAY924-11|Chironomidae|658|BOLD:ABV1207
Smittia|[16166]|JWDCD254-10|Chironomidae|658|BOLD:AAG6994
Smittia|[16167]|CNEIA1740-12|Chironomidae|658|BOLD:ACA8421
Smittia|[16168]|SSBAA4363-12|Chironomidae|673|BOLD:AAL7316
Smittia|[16169]|SSBAC285-12|Chironomidae|673|BOLD:AAL7384
Smittia|[16170]|SSBAC2239-12|Chironomidae|670|BOLD:AAL7386
Smittia|[16171]|CNEIA503-12|Chironomidae|673|BOLD:ACA8317
Chironomidae|[16172]|CNWLA608-12|Chironomidae|637|BOLD:ACB8991
Smittia|[16173]|CNEIA1765-12|Chironomidae|658|BOLD:ACA8598
Orthocladiinae|[16174]|JWDCD383-10|Chironomidae|658|BOLD:AAL7385
Smittia|[16175]|CNIVC181-14|Chironomidae|603|BOLD:ACI8913
Smittia|[16176]|CNBAE056-12|Chironomidae|624|BOLD:ACD0765
Smittia|[16177]|CNWLL547-13|Chironomidae|658|BOLD:ACH2685
Smittia|[16178]|SSJAE4650-13|Chironomidae|664|BOLD:ACC3731
Chironomidae|[16179]|CNWAE4211-14|Chironomidae|620|BOLD:ACR9506
Smittia|[16180]|CNEIA1222-12|Chironomidae|670|BOLD:ACA8274
Orthocladiinae|[16181]|ARTYT158-12|Chironomidae|664|BOLD:ABY1558
Smittia|[16182]|CNGLA184-13|Chironomidae|658|BOLD:ACE1110
Orthocladiinae|[16183]|CNAUA616-14|Chironomidae|576|BOLD:ACR3342
Smittia|[16184]|DARC461-11|Chironomidae|658|BOLD:AAU6555
Smittia|[16185]|CNJAD461-12|Chironomidae|673|BOLD:ACC9051
Smittia|[16186]|CNPKE249-14|Chironomidae|658|BOLD:ACL4016
Smittia|[16187]|CNJAA613-12|Chironomidae|658|BOLD:ACB8875
Smittia|[16188]|SSBAA4384-12|Chironomidae|673|BOLD:ACC1129
Smittia|[16189]|NGNAJ117-13|Chironomidae|661|BOLD:ACX6240
Smittia|[16190]|SSBAA4355-12|Chironomidae|673|BOLD:ACC0935
Smittia|[16191]|CNFNQ1419-14|Chironomidae|658|BOLD:ACX6239
Smittia|[16192]|CNJAA399-12|Chironomidae|673|BOLD:ACB9401
Smittia|[16193]|SSJAA755-13|Chironomidae|664|BOLD:ACG2804
Smittia|[16194]|CNGMB800-13|Chironomidae|636|BOLD:ACL0854
Chironomidae|[16195]|CNKLB4381-14|Chironomidae|567|BOLD:ACR8003
Smittia|[16196]|DARC477-11|Chironomidae|658|BOLD:AAU6581
Smittia|[16197]|CNTGD1487-15|Chironomidae|567|BOLD:ACT4843

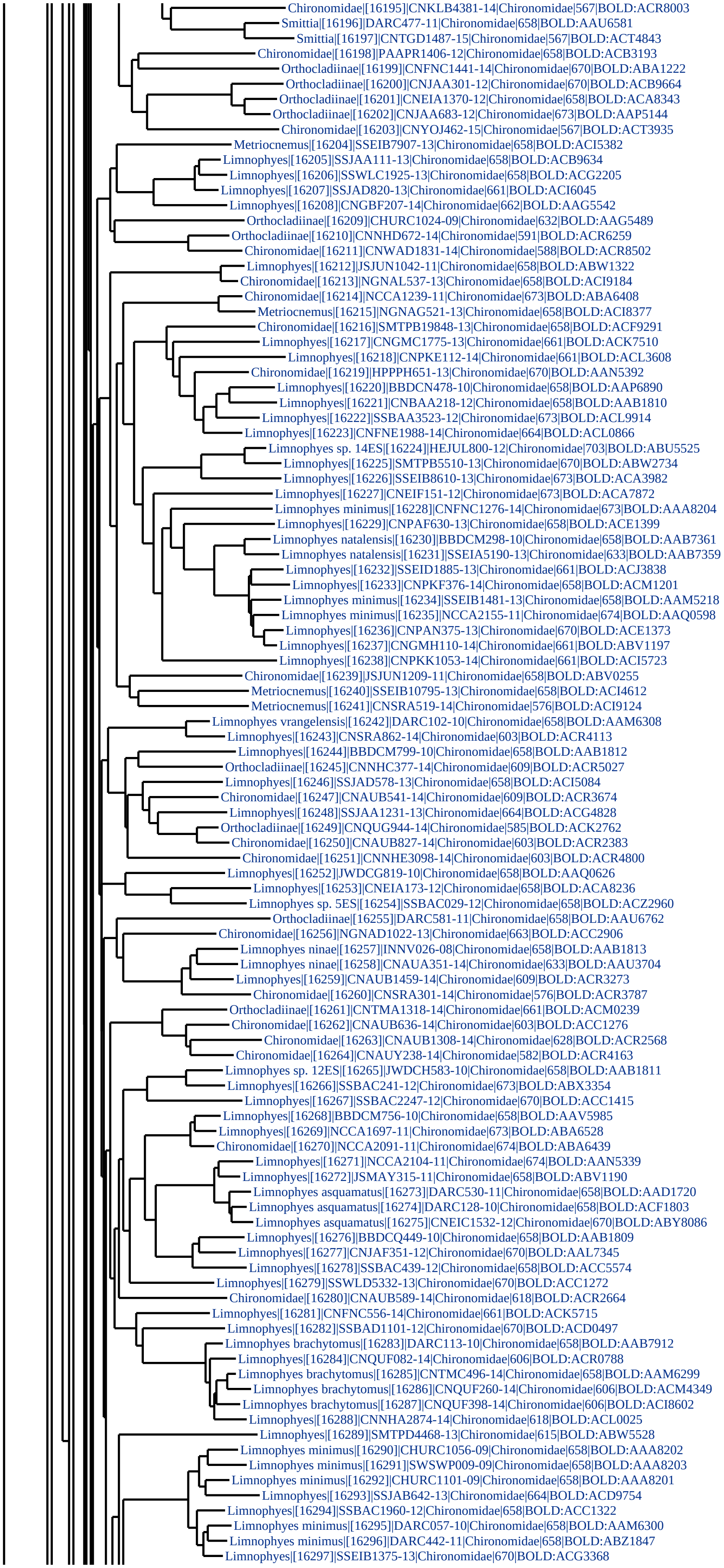

Chironomidae|[16195]|CNKLB4381-14|Chironomidae|567|BOLD:ACR8003
Smittia|[16196]|DARC477-11|Chironomidae|658|BOLD:AAU6581
Smittia|[16197]|CNTGD1487-15|Chironomidae|567|BOLD:ACT4843
Chironomidae|[16198]|PAAPR1406-12|Chironomidae|658|BOLD:ACB3193
Orthocladiinae|[16199]|CNFNC1441-14|Chironomidae|670|BOLD:ABA1222
Orthocladiinae|[16200]|CNJAA301-12|Chironomidae|670|BOLD:ACB9664
Orthocladiinae|[16201]|CNEIA1370-12|Chironomidae|658|BOLD:ACA8343
Orthocladiinae|[16202]|CNJAA683-12|Chironomidae|673|BOLD:AAP5144
Chironomidae|[16203]|CNYOJ462-15|Chironomidae|567|BOLD:ACT3935
Metriocnemus|[16204]|SSEIB7907-13|Chironomidae|658|BOLD:ACI5382
Limnophyes|[16205]|SSJAA111-13|Chironomidae|658|BOLD:ACB9634
Limnophyes|[16206]|SSWLC1925-13|Chironomidae|658|BOLD:ACG2205
Limnophyes|[16207]|SSJAD820-13|Chironomidae|661|BOLD:ACI6045
Limnophyes|[16208]|CNGBF207-14|Chironomidae|662|BOLD:AAG5542
Orthocladiinae|[16209]|CHURC1024-09|Chironomidae|632|BOLD:AAG5489
Orthocladiinae|[16210]|CNNHD672-14|Chironomidae|591|BOLD:ACR6259
Chironomidae|[16211]|CNWAD1831-14|Chironomidae|588|BOLD:ACR8502
Limnophyes|[16212]|JSJUN1042-11|Chironomidae|658|BOLD:ABW1322
Chironomidae|[16213]|NGNAL537-13|Chironomidae|658|BOLD:ACI9184
Chironomidae|[16214]|NCCA1239-11|Chironomidae|673|BOLD:ABA6408
Metriocnemus|[16215]|NGNAG521-13|Chironomidae|658|BOLD:ACI8377
Chironomidae|[16216]|SMTPB19848-13|Chironomidae|658|BOLD:ACF9291
Limnophyes|[16217]|CNGMC1775-13|Chironomidae|661|BOLD:ACK7510
Limnophyes|[16218]|CNPKE112-14|Chironomidae|661|BOLD:ACL3608
Chironomidae|[16219]|HPPPH651-13|Chironomidae|670|BOLD:AAN5392
Limnophyes|[16220]|BBDCN478-10|Chironomidae|658|BOLD:AAP6890
Limnophyes|[16221]|CNBAA218-12|Chironomidae|658|BOLD:AAB1810
Limnophyes|[16222]|SSBAA3523-12|Chironomidae|673|BOLD:ACL9914
Limnophyes|[16223]|CNFNE1988-14|Chironomidae|664|BOLD:ACL0866
Limnophyes sp. 14ES|[16224]|HEJUL800-12|Chironomidae|703|BOLD:ABU5525
Limnophyes|[16225]|SMTPB5510-13|Chironomidae|670|BOLD:ABW2734
Limnophyes|[16226]|SSEIB8610-13|Chironomidae|673|BOLD:ACA3982
Limnophyes|[16227]|CNEIF151-12|Chironomidae|673|BOLD:ACA7872
Limnophyes minimus|[16228]|CNFNC1276-14|Chironomidae|673|BOLD:AAA8204
Limnophyes|[16229]|CNPAF630-13|Chironomidae|658|BOLD:ACE1399
Limnophyes natalensis|[16230]|BBDCM298-10|Chironomidae|658|BOLD:AAB7361
Limnophyes natalensis|[16231]|SSEIA5190-13|Chironomidae|633|BOLD:AAB7359
Limnophyes|[16232]|SSEID1885-13|Chironomidae|661|BOLD:ACJ3838
Limnophyes|[16233]|CNPKF376-14|Chironomidae|658|BOLD:ACM1201
Limnophyes minimus|[16234]|SSEIB1481-13|Chironomidae|658|BOLD:AAM5218
Limnophyes minimus|[16235]|NCCA2155-11|Chironomidae|674|BOLD:AAQ0598
Limnophyes|[16236]|CNPAN375-13|Chironomidae|670|BOLD:ACE1373
Limnophyes|[16237]|CNGMH110-14|Chironomidae|661|BOLD:ABV1197
Limnophyes|[16238]|CNPKK1053-14|Chironomidae|661|BOLD:ACI5723
Chironomidae|[16239]|JSJUN1209-11|Chironomidae|658|BOLD:ABV0255
Metriocnemus|[16240]|SSEIB10795-13|Chironomidae|658|BOLD:ACI4612
Metriocnemus|[16241]|CNSRA519-14|Chironomidae|576|BOLD:ACI9124
Limnophyes vrangelensis|[16242]|DARC102-10|Chironomidae|658|BOLD:AAM6308
Limnophyes|[16243]|CNSRA862-14|Chironomidae|603|BOLD:ACR4113
Limnophyes|[16244]|BBDCM799-10|Chironomidae|658|BOLD:AAB1812
Orthocladiinae|[16245]|CNNHC377-14|Chironomidae|609|BOLD:ACR5027
Limnophyes|[16246]|SSJAD578-13|Chironomidae|658|BOLD:ACI5084
Chironomidae|[16247]|CNAUB541-14|Chironomidae|609|BOLD:ACR3674
Limnophyes|[16248]|SSJAA1231-13|Chironomidae|664|BOLD:ACG4828
Orthocladiinae|[16249]|CNQUG944-14|Chironomidae|585|BOLD:ACK2762
Chironomidae|[16250]|CNAUB827-14|Chironomidae|603|BOLD:ACR2383
Chironomidae|[16251]|CNNHE3098-14|Chironomidae|603|BOLD:ACR4800
Limnophyes|[16252]|JWDCG819-10|Chironomidae|658|BOLD:AAQ0626
Limnophyes|[16253]|CNEIA173-12|Chironomidae|658|BOLD:ACA8236
Limnophyes sp. 5ES|[16254]|SSBAC029-12|Chironomidae|658|BOLD:ACZ2960
Orthocladiinae|[16255]|DARC581-11|Chironomidae|658|BOLD:AAU6762
Chironomidae|[16256]|NGNAD1022-13|Chironomidae|663|BOLD:ACC2906
Limnophyes ninae|[16257]|INNV026-08|Chironomidae|658|BOLD:AAB1813
Limnophyes ninae|[16258]|CNAUA351-14|Chironomidae|633|BOLD:AAU3704
Limnophyes|[16259]|CNAUB1459-14|Chironomidae|609|BOLD:ACR3273
Chironomidae|[16260]|CNSRA301-14|Chironomidae|576|BOLD:ACR3787
Orthocladiinae|[16261]|CNTMA1318-14|Chironomidae|661|BOLD:ACM0239
Chironomidae|[16262]|CNAUB636-14|Chironomidae|603|BOLD:ACC1276
Chironomidae|[16263]|CNAUB1308-14|Chironomidae|628|BOLD:ACR2568
Chironomidae|[16264]|CNAUY238-14|Chironomidae|582|BOLD:ACR4163
Limnophyes sp. 12ES|[16265]|JWDCH583-10|Chironomidae|658|BOLD:AAB1811
Limnophyes|[16266]|SSBAC241-12|Chironomidae|673|BOLD:ABX3354
Limnophyes|[16267]|SSBAC2247-12|Chironomidae|670|BOLD:ACC1415
Limnophyes|[16268]|BBDCM756-10|Chironomidae|658|BOLD:AAV5985
Limnophyes|[16269]|NCCA1697-11|Chironomidae|673|BOLD:ABA6528
Chironomidae|[16270]|NCCA2091-11|Chironomidae|674|BOLD:ABA6439
Limnophyes|[16271]|NCCA2104-11|Chironomidae|674|BOLD:AAN5339
Limnophyes|[16272]|JSMAY315-11|Chironomidae|658|BOLD:ABV1190
Limnophyes asquamatus|[16273]|DARC530-11|Chironomidae|658|BOLD:AAD1720
Limnophyes asquamatus|[16274]|DARC128-10|Chironomidae|658|BOLD:ACF1803
Limnophyes asquamatus|[16275]|CNEIC1532-12|Chironomidae|670|BOLD:ABY8086
Limnophyes|[16276]|BBDCQ449-10|Chironomidae|658|BOLD:AAB1809
Limnophyes|[16277]|CNJAF351-12|Chironomidae|670|BOLD:AAL7345
Limnophyes|[16278]|SSBAC439-12|Chironomidae|658|BOLD:ACC5574
Limnophyes|[16279]|SSWLD5332-13|Chironomidae|670|BOLD:ACC1272
Chironomidae|[16280]|CNAUB589-14|Chironomidae|618|BOLD:ACR2664
Limnophyes|[16281]|CNFNC556-14|Chironomidae|661|BOLD:ACK5715
Limnophyes|[16282]|SSBAD1101-12|Chironomidae|670|BOLD:ACD0497
Limnophyes brachytomus|[16283]|DARC113-10|Chironomidae|658|BOLD:AAB7912
Limnophyes|[16284]|CNQUF082-14|Chironomidae|606|BOLD:ACR0788
Limnophyes brachytomus|[16285]|CNTMC496-14|Chironomidae|658|BOLD:AAM6299
Limnophyes brachytomus|[16286]|CNQUF260-14|Chironomidae|606|BOLD:ACM4349
Limnophyes brachytomus|[16287]|CNQUF398-14|Chironomidae|606|BOLD:ACI8602
Limnophyes|[16288]|CNNHA2874-14|Chironomidae|618|BOLD:ACL0025
Limnophyes|[16289]|SMTPD4468-13|Chironomidae|615|BOLD:ABW5528
Limnophyes minimus|[16290]|CHURC1056-09|Chironomidae|658|BOLD:AAA8202
Limnophyes minimus|[16291]|SWSWP009-09|Chironomidae|658|BOLD:AAA8203
Limnophyes minimus|[16292]|CHURC1101-09|Chironomidae|658|BOLD:AAA8201
Limnophyes|[16293]|SSJAB642-13|Chironomidae|664|BOLD:ACD9754
Limnophyes|[16294]|SSBAC1960-12|Chironomidae|658|BOLD:ACC1322
Limnophyes minimus|[16295]|DARC057-10|Chironomidae|658|BOLD:AAM6300
Limnophyes minimus|[16296]|DARC442-11|Chironomidae|658|BOLD:ABZ1847
Limnophyes|[16297]|SSEIB1375-13|Chironomidae|670|BOLD:ACG3368

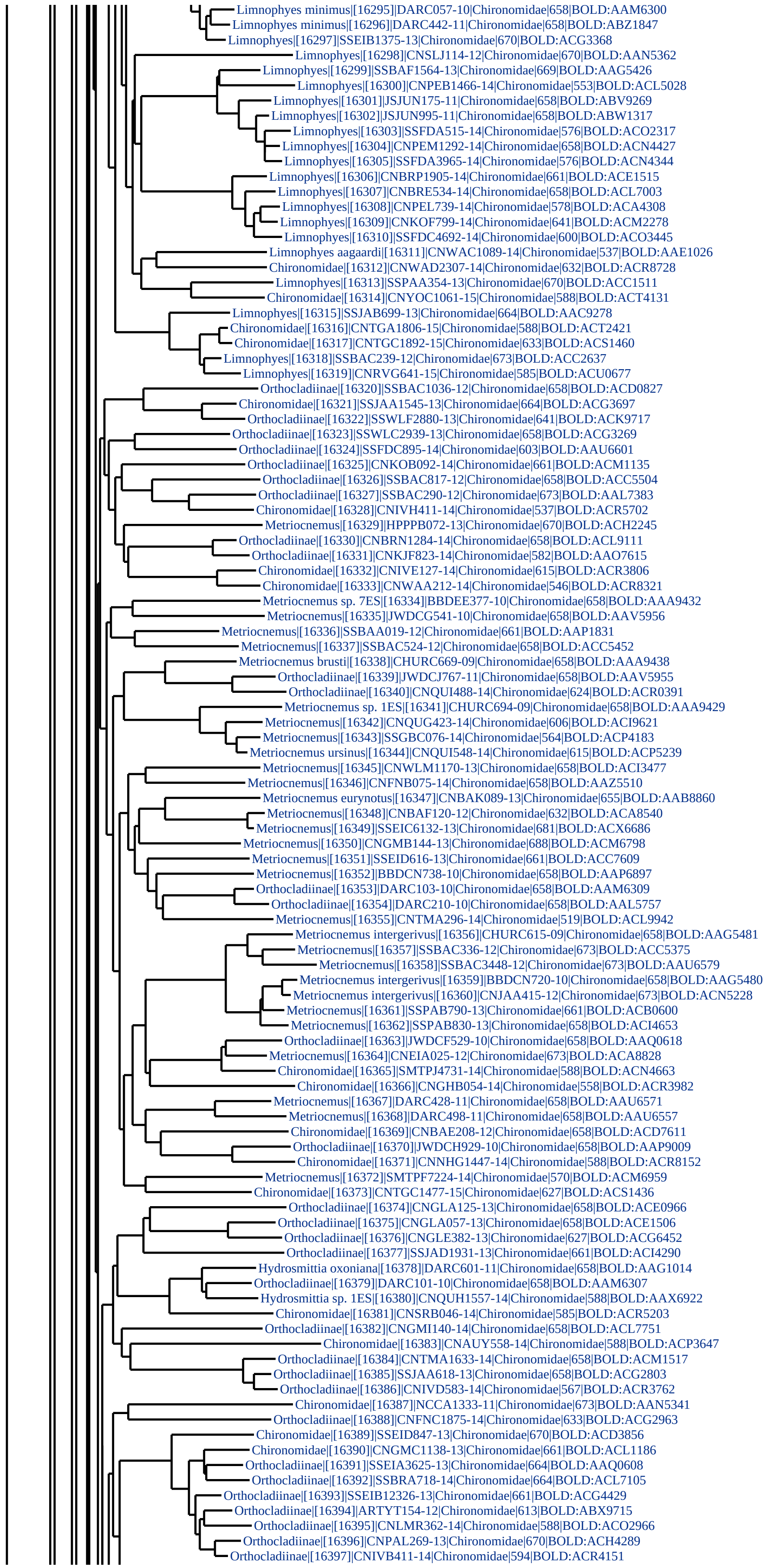
Limnophyes minimus|[16295]|DARC057-10|Chironomidae|658|BOLD:AAM6300
Limnophyes minimus|[16296]|DARC442-11|Chironomidae|658|BOLD:ABZ1847
Limnophyes|[16297]|SSEIB1375-13|Chironomidae|670|BOLD:ACG3368
Limnophyes|[16298]|CNSLJ114-12|Chironomidae|670|BOLD:AAN5362
Limnophyes|[16299]|SSBAF1564-13|Chironomidae|669|BOLD:AAG5426
Limnophyes|[16300]|CNPEB1466-14|Chironomidae|553|BOLD:ACL5028
Limnophyes|[16301]|JSJUN175-11|Chironomidae|658|BOLD:ABV9269
Limnophyes|[16302]|JSJUN995-11|Chironomidae|658|BOLD:ABW1317
Limnophyes|[16303]|SSFDA515-14|Chironomidae|576|BOLD:ACO2317
Limnophyes|[16304]|CNPEM1292-14|Chironomidae|658|BOLD:ACN4427
Limnophyes|[16305]|SSFDA3965-14|Chironomidae|576|BOLD:ACN4344
Limnophyes|[16306]|CNBRP1905-14|Chironomidae|661|BOLD:ACE1515
Limnophyes|[16307]|CNBRE534-14|Chironomidae|658|BOLD:ACL7003
Limnophyes|[16308]|CNPEL739-14|Chironomidae|578|BOLD:ACA4308
Limnophyes|[16309]|CNKOF799-14|Chironomidae|641|BOLD:ACM2278
Limnophyes|[16310]|SSFDC4692-14|Chironomidae|600|BOLD:ACO3445
Limnophyes aagaardi|[16311]|CNWAC1089-14|Chironomidae|537|BOLD:AAE1026
Chironomidae|[16312]|CNWAD2307-14|Chironomidae|632|BOLD:ACR8728
Limnophyes|[16313]|SSPAA354-13|Chironomidae|670|BOLD:ACC1511
Chironomidae|[16314]|CNYOC1061-15|Chironomidae|588|BOLD:ACT4131
Limnophyes|[16315]|SSJAB699-13|Chironomidae|664|BOLD:AAC9278
Chironomidae|[16316]|CNTGA1806-15|Chironomidae|588|BOLD:ACT2421
Chironomidae|[16317]|CNTGC1892-15|Chironomidae|633|BOLD:ACS1460
Limnophyes|[16318]|SSBAC239-12|Chironomidae|673|BOLD:ACC2637
Limnophyes|[16319]|CNRVG641-15|Chironomidae|585|BOLD:ACU0677
Orthocladiinae|[16320]|SSBAC1036-12|Chironomidae|658|BOLD:ACD0827
Chironomidae|[16321]|SSJAA1545-13|Chironomidae|664|BOLD:ACG3697
Orthocladiinae|[16322]|SSWLF2880-13|Chironomidae|641|BOLD:ACK9717
Orthocladiinae|[16323]|SSWLC2939-13|Chironomidae|658|BOLD:ACG3269
Orthocladiinae|[16324]|SSFDC895-14|Chironomidae|603|BOLD:AAU6601
Orthocladiinae|[16325]|CNKOB092-14|Chironomidae|661|BOLD:ACM1135
Orthocladiinae|[16326]|SSBAC817-12|Chironomidae|658|BOLD:ACC5504
Orthocladiinae|[16327]|SSBAC290-12|Chironomidae|673|BOLD:AAL7383
Chironomidae|[16328]|CNIVH411-14|Chironomidae|537|BOLD:ACR5702
Metriocnemus|[16329]|HPPPB072-13|Chironomidae|670|BOLD:ACH2245
Orthocladiinae|[16330]|CNBRN1284-14|Chironomidae|658|BOLD:ACL9111
Orthocladiinae|[16331]|CNKJF823-14|Chironomidae|582|BOLD:AAO7615
Chironomidae|[16332]|CNIVE127-14|Chironomidae|615|BOLD:ACR3806
Chironomidae|[16333]|CNWAA212-14|Chironomidae|546|BOLD:ACR8321
Metriocnemus sp. 7ES|[16334]|BBDEE377-10|Chironomidae|658|BOLD:AAA9432
Metriocnemus|[16335]|JWDCG541-10|Chironomidae|658|BOLD:AAV5956
Metriocnemus|[16336]|SSBAA019-12|Chironomidae|661|BOLD:AAP1831
Metriocnemus|[16337]|SSBAC524-12|Chironomidae|658|BOLD:ACC5452
Metriocnemus brusti|[16338]|CHURC669-09|Chironomidae|658|BOLD:AAA9438
Orthocladiinae|[16339]|JWDCJ767-11|Chironomidae|658|BOLD:AAV5955
Orthocladiinae|[16340]|CNQUI488-14|Chironomidae|624|BOLD:ACR0391
Metriocnemus sp. 1ES|[16341]|CHURC694-09|Chironomidae|658|BOLD:AAA9429
Metriocnemus|[16342]|CNQUG423-14|Chironomidae|606|BOLD:ACI9621
Metriocnemus|[16343]|SSGBC076-14|Chironomidae|564|BOLD:ACP4183
Metriocnemus ursinus|[16344]|CNQUI548-14|Chironomidae|615|BOLD:ACP5239
Metriocnemus|[16345]|CNWLM1170-13|Chironomidae|658|BOLD:ACI3477
Metriocnemus|[16346]|CNFNB075-14|Chironomidae|658|BOLD:AAZ5510
Metriocnemus eurynotus|[16347]|CNBAK089-13|Chironomidae|655|BOLD:AAB8860
Metriocnemus|[16348]|CNBAF120-12|Chironomidae|632|BOLD:ACA8540
Metriocnemus|[16349]|SSEIC6132-13|Chironomidae|681|BOLD:ACX6686
Metriocnemus|[16350]|CNGMB144-13|Chironomidae|688|BOLD:ACM6798
Metriocnemus|[16351]|SSEID616-13|Chironomidae|661|BOLD:ACC7609
Metriocnemus|[16352]|BBDCN738-10|Chironomidae|658|BOLD:AAP6897
Orthocladiinae|[16353]|DARC103-10|Chironomidae|658|BOLD:AAM6309
Orthocladiinae|[16354]|DARC210-10|Chironomidae|658|BOLD:AAL5757
Metriocnemus|[16355]|CNTMA296-14|Chironomidae|519|BOLD:ACL9942
Metriocnemus intergerivus|[16356]|CHURC615-09|Chironomidae|658|BOLD:AAG5481
Metriocnemus|[16357]|SSBAC336-12|Chironomidae|673|BOLD:ACC5375
Metriocnemus|[16358]|SSBAC3448-12|Chironomidae|673|BOLD:AAU6579
Metriocnemus intergerivus|[16359]|BBDCN720-10|Chironomidae|658|BOLD:AAG5480
Metriocnemus intergerivus|[16360]|CNJAA415-12|Chironomidae|673|BOLD:ACN5228
Metriocnemus|[16361]|SSPAB790-13|Chironomidae|661|BOLD:ACB0600
Metriocnemus|[16362]|SSPAB830-13|Chironomidae|658|BOLD:ACI4653
Orthocladiinae|[16363]|JWDCF529-10|Chironomidae|658|BOLD:AAQ0618
Metriocnemus|[16364]|CNEIA025-12|Chironomidae|673|BOLD:ACA8828
Chironomidae|[16365]|SMTPJ4731-14|Chironomidae|588|BOLD:ACN4663
Chironomidae|[16366]|CNGHB054-14|Chironomidae|558|BOLD:ACR3982
Metriocnemus|[16367]|DARC428-11|Chironomidae|658|BOLD:AAU6571
Metriocnemus|[16368]|DARC498-11|Chironomidae|658|BOLD:AAU6557
Chironomidae|[16369]|CNBAE208-12|Chironomidae|658|BOLD:ACD7611
Orthocladiinae|[16370]|JWDCH929-10|Chironomidae|658|BOLD:AAP9009
Chironomidae|[16371]|CNNHG1447-14|Chironomidae|588|BOLD:ACR8152
Metriocnemus|[16372]|SMTPF7224-14|Chironomidae|570|BOLD:ACM6959
Chironomidae|[16373]|CNTGC1477-15|Chironomidae|627|BOLD:ACS1436
Orthocladiinae|[16374]|CNGLA125-13|Chironomidae|658|BOLD:ACE0966
Orthocladiinae|[16375]|CNGLA057-13|Chironomidae|658|BOLD:ACE1506
Orthocladiinae|[16376]|CNGLE382-13|Chironomidae|627|BOLD:ACG6452
Orthocladiinae|[16377]|SSJAD1931-13|Chironomidae|661|BOLD:ACI4290
Hydrosmittia oxoniana|[16378]|DARC601-11|Chironomidae|658|BOLD:AAG1014
Orthocladiinae|[16379]|DARC101-10|Chironomidae|658|BOLD:AAM6307
Hydrosmittia sp. 1ES|[16380]|CNQUH1557-14|Chironomidae|588|BOLD:AAX6922
Chironomidae|[16381]|CNSRB046-14|Chironomidae|585|BOLD:ACR5203
Orthocladiinae|[16382]|CNGMI140-14|Chironomidae|658|BOLD:ACL7751
Chironomidae|[16383]|CNAUY558-14|Chironomidae|588|BOLD:ACP3647
Orthocladiinae|[16384]|CNTMA1633-14|Chironomidae|658|BOLD:ACM1517
Orthocladiinae|[16385]|SSJAA618-13|Chironomidae|658|BOLD:ACG2803
Orthocladiinae|[16386]|CNIVD583-14|Chironomidae|567|BOLD:ACR3762
Chironomidae|[16387]|NCCA1333-11|Chironomidae|673|BOLD:AAN5341
Orthocladiinae|[16388]|CNFNC1875-14|Chironomidae|633|BOLD:ACG2963
Chironomidae|[16389]|SSEID847-13|Chironomidae|670|BOLD:ACD3856
Chironomidae|[16390]|CNGMC1138-13|Chironomidae|661|BOLD:ACL1186
Orthocladiinae|[16391]|SSEIA3625-13|Chironomidae|664|BOLD:AAQ0608
Orthocladiinae|[16392]|SSBRA718-14|Chironomidae|664|BOLD:ACL7105
Orthocladiinae|[16393]|SSEIB12326-13|Chironomidae|661|BOLD:ACG4429
Orthocladiinae|[16394]|ARTYT154-12|Chironomidae|613|BOLD:ABX9715
Orthocladiinae|[16395]|CNLMR362-14|Chironomidae|588|BOLD:ACO2966
Orthocladiinae|[16396]|CNPAL269-13|Chironomidae|670|BOLD:ACH4289
Orthocladiinae|[16397]|CNIVB411-14|Chironomidae|594|BOLD:ACR4151

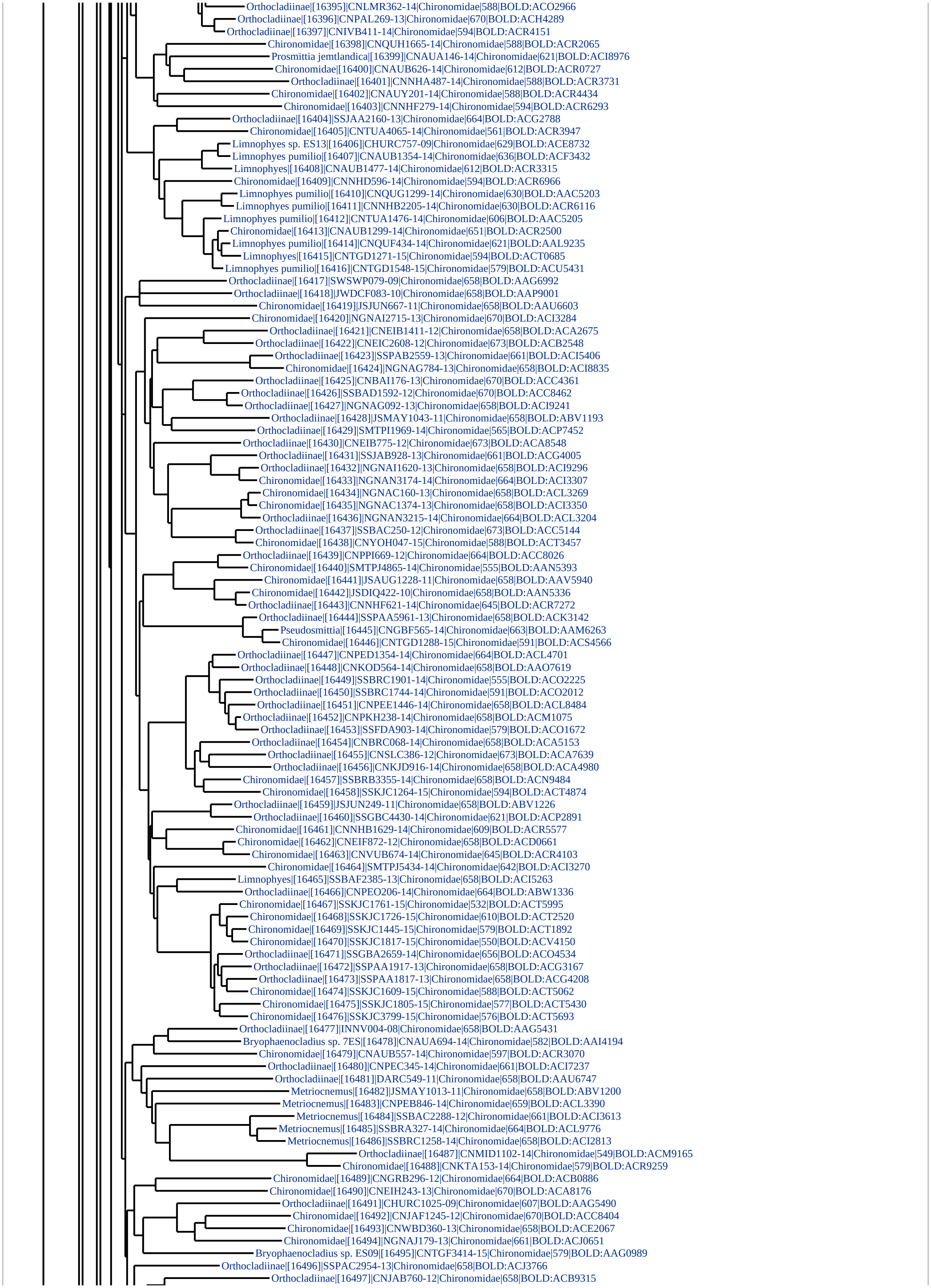

Orthocladiinae|[16395]|CNLMR362-14|Chironomidae|588|BOLD:ACO2966
Orthocladiinae|[16396]|CNPAL269-13|Chironomidae|670|BOLD:ACH4289
Orthocladiinae|[16397]|CNIVB411-14|Chironomidae|594|BOLD:ACR4151
Chironomidae|[16398]|CNQUH1665-14|Chironomidae|588|BOLD:ACR2065
Prosmittia jemtlandica|[16399]|CNAUA146-14|Chironomidae|621|BOLD:ACI8976
Chironomidae|[16400]|CNAUB626-14|Chironomidae|612|BOLD:ACR0727
Orthocladiinae|[16401]|CNNHA487-14|Chironomidae|588|BOLD:ACR3731
Chironomidae|[16402]|CNAUY201-14|Chironomidae|588|BOLD:ACR4434
Chironomidae|[16403]|CNNHF279-14|Chironomidae|594|BOLD:ACR6293
Orthocladiinae|[16404]|SSJAA2160-13|Chironomidae|664|BOLD:ACG2788
Chironomidae|[16405]|CNTUA4065-14|Chironomidae|561|BOLD:ACR3947
Limnophyes sp. ES13|[16406]|CHURC757-09|Chironomidae|629|BOLD:ACE8732
Limnophyes pumilio|[16407]|CNAUB1354-14|Chironomidae|636|BOLD:ACF3432
Limnophyes|[16408]|CNAUB1477-14|Chironomidae|612|BOLD:ACR3315
Chironomidae|[16409]|CNNHD596-14|Chironomidae|594|BOLD:ACR6966
Limnophyes pumilio|[16410]|CNQUG1299-14|Chironomidae|630|BOLD:AAC5203
Limnophyes pumilio|[16411]|CNNHB2205-14|Chironomidae|630|BOLD:ACR6116
Limnophyes pumilio|[16412]|CNTUA1476-14|Chironomidae|606|BOLD:AAC5205
Chironomidae|[16413]|CNAUB1299-14|Chironomidae|651|BOLD:ACR2500
Limnophyes pumilio|[16414]|CNQUF434-14|Chironomidae|621|BOLD:AAL9235
Limnophyes|[16415]|CNTGD1271-15|Chironomidae|594|BOLD:ACT0685
Limnophyes pumilio|[16416]|CNTGD1548-15|Chironomidae|579|BOLD:ACU5431
Orthocladiinae|[16417]|SWSWP079-09|Chironomidae|658|BOLD:AAG6992
Orthocladiinae|[16418]|JWDCF083-10|Chironomidae|658|BOLD:AAP9001
Chironomidae|[16419]|JSJUN667-11|Chironomidae|658|BOLD:AAU6603
Chironomidae|[16420]|NGNAI2715-13|Chironomidae|670|BOLD:ACI3284
Orthocladiinae|[16421]|CNEIB1411-12|Chironomidae|658|BOLD:ACA2675
Orthocladiinae|[16422]|CNEIC2608-12|Chironomidae|673|BOLD:ACB2548
Orthocladiinae|[16423]|SSPAB2559-13|Chironomidae|661|BOLD:ACI5406
Chironomidae|[16424]|NGNAG784-13|Chironomidae|658|BOLD:ACI8835
Orthocladiinae|[16425]|CNBAI176-13|Chironomidae|670|BOLD:ACC4361
Orthocladiinae|[16426]|SSBAD1592-12|Chironomidae|670|BOLD:ACC8462
Orthocladiinae|[16427]|NGNAG092-13|Chironomidae|658|BOLD:ACI9241
Orthocladiinae|[16428]|JSMAY1043-11|Chironomidae|658|BOLD:ABV1193
Orthocladiinae|[16429]|SMTPI1969-14|Chironomidae|565|BOLD:ACP7452
Orthocladiinae|[16430]|CNEIB775-12|Chironomidae|673|BOLD:ACA8548
Orthocladiinae|[16431]|SSJAB928-13|Chironomidae|661|BOLD:ACG4005
Orthocladiinae|[16432]|NGNAI1620-13|Chironomidae|658|BOLD:ACI9296
Chironomidae|[16433]|NGNAN3174-14|Chironomidae|664|BOLD:ACI3307
Chironomidae|[16434]|NGNAC160-13|Chironomidae|658|BOLD:ACL3269
Chironomidae|[16435]|NGNAC1374-13|Chironomidae|658|BOLD:ACI3350
Orthocladiinae|[16436]|NGNAN3215-14|Chironomidae|664|BOLD:ACL3204
Orthocladiinae|[16437]|SSBAC250-12|Chironomidae|673|BOLD:ACC5144
Chironomidae|[16438]|CNYOH047-15|Chironomidae|588|BOLD:ACT3457
Orthocladiinae|[16439]|CNPPI669-12|Chironomidae|664|BOLD:ACC8026
Chironomidae|[16440]|SMTPJ4865-14|Chironomidae|555|BOLD:AAN5393
Chironomidae|[16441]|JSAUG1228-11|Chironomidae|658|BOLD:AAV5940
Chironomidae|[16442]|JSDIQ422-10|Chironomidae|658|BOLD:AAN5336
Orthocladiinae|[16443]|CNNHF621-14|Chironomidae|645|BOLD:ACR7272
Orthocladiinae|[16444]|SSPAA5961-13|Chironomidae|658|BOLD:ACK3142
Pseudosmittia|[16445]|CNGBF565-14|Chironomidae|663|BOLD:AAM6263
Chironomidae|[16446]|CNTGD1288-15|Chironomidae|591|BOLD:ACS4566
Orthocladiinae|[16447]|CNPED1354-14|Chironomidae|664|BOLD:ACL4701
Orthocladiinae|[16448]|CNKOD564-14|Chironomidae|658|BOLD:AAO7619
Orthocladiinae|[16449]|SSBRC1901-14|Chironomidae|555|BOLD:ACO2225
Orthocladiinae|[16450]|SSBRC1744-14|Chironomidae|591|BOLD:ACO2012
Orthocladiinae|[16451]|CNPEE1446-14|Chironomidae|658|BOLD:ACL8484
Orthocladiinae|[16452]|CNPKH238-14|Chironomidae|658|BOLD:ACM1075
Orthocladiinae|[16453]|SSFDA903-14|Chironomidae|579|BOLD:ACO1672
Orthocladiinae|[16454]|CNBRC068-14|Chironomidae|658|BOLD:ACA5153
Orthocladiinae|[16455]|CNSLC386-12|Chironomidae|673|BOLD:ACA7639
Orthocladiinae|[16456]|CNKJD916-14|Chironomidae|658|BOLD:ACA4980
Chironomidae|[16457]|SSBRB3355-14|Chironomidae|658|BOLD:ACN9484
Chironomidae|[16458]|SSKJC1264-15|Chironomidae|594|BOLD:ACT4874
Orthocladiinae|[16459]|JSJUN249-11|Chironomidae|658|BOLD:ABV1226
Orthocladiinae|[16460]|SSGBC4430-14|Chironomidae|621|BOLD:ACP2891
Chironomidae|[16461]|CNNHB1629-14|Chironomidae|609|BOLD:ACR5577
Chironomidae|[16462]|CNEIF872-12|Chironomidae|658|BOLD:ACD0661
Chironomidae|[16463]|CNVUB674-14|Chironomidae|645|BOLD:ACR4103
Chironomidae|[16464]|SMTPJ5434-14|Chironomidae|642|BOLD:ACI3270
Limnophyes|[16465]|SSBAF2385-13|Chironomidae|658|BOLD:ACI5263
Orthocladiinae|[16466]|CNPEO206-14|Chironomidae|664|BOLD:ABW1336
Chironomidae|[16467]|SSKJC1761-15|Chironomidae|532|BOLD:ACT5995
Chironomidae|[16468]|SSKJC1726-15|Chironomidae|610|BOLD:ACT2520
Chironomidae|[16469]|SSKJC1445-15|Chironomidae|579|BOLD:ACT1892
Chironomidae|[16470]|SSKJC1817-15|Chironomidae|550|BOLD:ACV4150
Orthocladiinae|[16471]|SSGBA2659-14|Chironomidae|656|BOLD:ACO4534
Orthocladiinae|[16472]|SSPAA1917-13|Chironomidae|658|BOLD:ACG3167
Orthocladiinae|[16473]|SSPAA1817-13|Chironomidae|658|BOLD:ACG4208
Chironomidae|[16474]|SSKJC1609-15|Chironomidae|588|BOLD:ACT5062
Chironomidae|[16475]|SSKJC1805-15|Chironomidae|577|BOLD:ACT5430
Chironomidae|[16476]|SSKJC3799-15|Chironomidae|576|BOLD:ACT5693
Orthocladiinae|[16477]|INNV004-08|Chironomidae|658|BOLD:AAG5431
Bryophaenocladius sp. 7ES|[16478]|CNAUA694-14|Chironomidae|582|BOLD:AAI4194
Chironomidae|[16479]|CNAUB557-14|Chironomidae|597|BOLD:ACR3070
Orthocladiinae|[16480]|CNPEC345-14|Chironomidae|661|BOLD:ACI7237
Orthocladiinae|[16481]|DARC549-11|Chironomidae|658|BOLD:AAU6747
Metriocnemus|[16482]|JSMAY1013-11|Chironomidae|658|BOLD:ABV1200
Metriocnemus|[16483]|CNPEB846-14|Chironomidae|659|BOLD:ACL3390
Metriocnemus|[16484]|SSBAC2288-12|Chironomidae|661|BOLD:ACI3613
Metriocnemus|[16485]|SSBRA327-14|Chironomidae|664|BOLD:ACL9776
Metriocnemus|[16486]|SSBRC1258-14|Chironomidae|658|BOLD:ACI2813
Orthocladiinae|[16487]|CNMID1102-14|Chironomidae|549|BOLD:ACM9165
Chironomidae|[16488]|CNKTA153-14|Chironomidae|579|BOLD:ACR9259
Chironomidae|[16489]|CNGRB296-12|Chironomidae|664|BOLD:ACB0886
Chironomidae|[16490]|CNEIH243-13|Chironomidae|670|BOLD:ACA8176
Orthocladiinae|[16491]|CHURC1025-09|Chironomidae|607|BOLD:AAG5490
Chironomidae|[16492]|CNJAF1245-12|Chironomidae|670|BOLD:ACC8404
Chironomidae|[16493]|CNWBD360-13|Chironomidae|658|BOLD:ACE2067
Chironomidae|[16494]|NGNAJ179-13|Chironomidae|661|BOLD:ACJ0651
Bryophaenocladius sp. ES09|[16495]|CNTGF3414-15|Chironomidae|579|BOLD:AAG0989
Orthocladiinae|[16496]|SSPAC2954-13|Chironomidae|658|BOLD:ACJ3766
Orthocladiinae|[16497]|CNJAB760-12|Chironomidae|658|BOLD:ACB9315

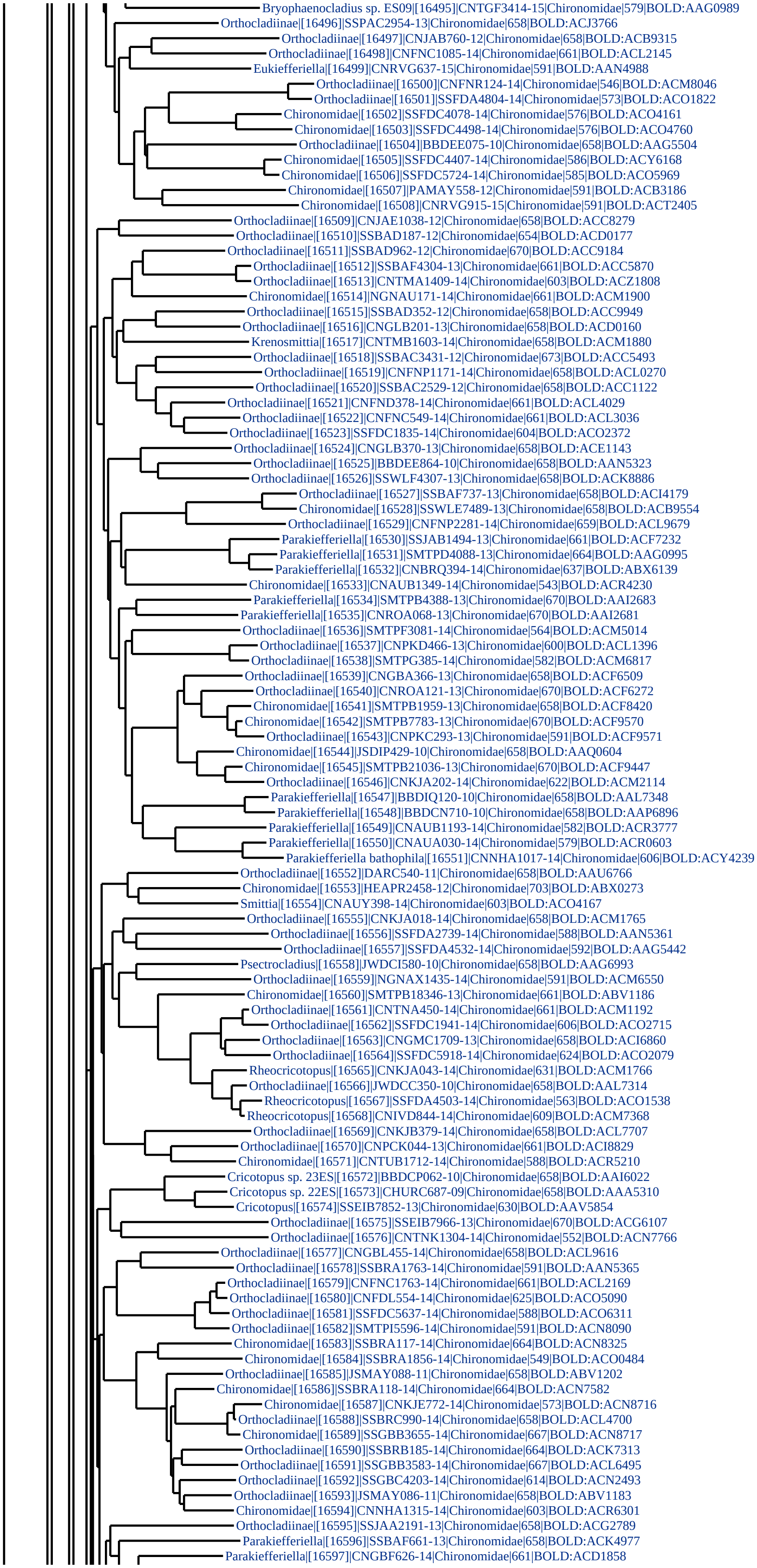

Bryophaenocladius sp. ES09|[16495]|CNTGF3414-15|Chironomidae|579|BOLD:AAG0989
Orthocladiinae|[16496]|SSPAC2954-13|Chironomidae|658|BOLD:ACJ3766
Orthocladiinae|[16497]|CNJAB760-12|Chironomidae|658|BOLD:ACB9315
Orthocladiinae|[16498]|CNFNC1085-14|Chironomidae|661|BOLD:ACL2145
Eukiefferiella|[16499]|CNRVG637-15|Chironomidae|591|BOLD:AAN4988
Orthocladiinae|[16500]|CNFNR124-14|Chironomidae|546|BOLD:ACM8046
Orthocladiinae|[16501]|SSFDA4804-14|Chironomidae|573|BOLD:ACO1822
Chironomidae|[16502]|SSFDC4078-14|Chironomidae|576|BOLD:ACO4161
Chironomidae|[16503]|SSFDC4498-14|Chironomidae|576|BOLD:ACO4760
Orthocladiinae|[16504]|BBDEE075-10|Chironomidae|658|BOLD:AAG5504
Chironomidae|[16505]|SSFDC4407-14|Chironomidae|586|BOLD:ACY6168
Chironomidae|[16506]|SSFDC5724-14|Chironomidae|585|BOLD:ACO5969
Chironomidae|[16507]|PAMAY558-12|Chironomidae|591|BOLD:ACB3186
Chironomidae|[16508]|CNRVG915-15|Chironomidae|591|BOLD:ACT2405
Orthocladiinae|[16509]|CNJAE1038-12|Chironomidae|658|BOLD:ACC8279
Orthocladiinae|[16510]|SSBAD187-12|Chironomidae|654|BOLD:ACD0177
Orthocladiinae|[16511]|SSBAD962-12|Chironomidae|670|BOLD:ACC9184
Orthocladiinae|[16512]|SSBAF4304-13|Chironomidae|661|BOLD:ACC5870
Orthocladiinae|[16513]|CNTMA1409-14|Chironomidae|603|BOLD:ACZ1808
Chironomidae|[16514]|NGNAU171-14|Chironomidae|661|BOLD:ACM1900
Orthocladiinae|[16515]|SSBAD352-12|Chironomidae|658|BOLD:ACC9949
Orthocladiinae|[16516]|CNGLB201-13|Chironomidae|658|BOLD:ACD0160
Krenosmittia|[16517]|CNTMB1603-14|Chironomidae|658|BOLD:ACM1880
Orthocladiinae|[16518]|SSBAC3431-12|Chironomidae|673|BOLD:ACC5493
Orthocladiinae|[16519]|CNFNP1171-14|Chironomidae|658|BOLD:ACL0270
Orthocladiinae|[16520]|SSBAC2529-12|Chironomidae|658|BOLD:ACC1122
Orthocladiinae|[16521]|CNFND378-14|Chironomidae|661|BOLD:ACL4029
Orthocladiinae|[16522]|CNFNC549-14|Chironomidae|661|BOLD:ACL3036
Orthocladiinae|[16523]|SSFDC1835-14|Chironomidae|604|BOLD:ACO2372
Orthocladiinae|[16524]|CNGLB370-13|Chironomidae|658|BOLD:ACE1143
Orthocladiinae|[16525]|BBDEE864-10|Chironomidae|658|BOLD:AAN5323
Orthocladiinae|[16526]|SSWLF4307-13|Chironomidae|658|BOLD:ACK8886
Orthocladiinae|[16527]|SSBAF737-13|Chironomidae|658|BOLD:ACI4179
Chironomidae|[16528]|SSWLE7489-13|Chironomidae|658|BOLD:ACB9554
Orthocladiinae|[16529]|CNFNP2281-14|Chironomidae|659|BOLD:ACL9679
Parakiefferiella|[16530]|SSJAB1494-13|Chironomidae|661|BOLD:ACF7232
Parakiefferiella|[16531]|SMTPD4088-13|Chironomidae|664|BOLD:AAG0995
Parakiefferiella|[16532]|CNBRQ394-14|Chironomidae|637|BOLD:ABX6139
Chironomidae|[16533]|CNAUB1349-14|Chironomidae|543|BOLD:ACR4230
Parakiefferiella|[16534]|SMTPB4388-13|Chironomidae|670|BOLD:AAI2683
Parakiefferiella|[16535]|CNROA068-13|Chironomidae|670|BOLD:AAI2681
Orthocladiinae|[16536]|SMTPF3081-14|Chironomidae|564|BOLD:ACM5014
Orthocladiinae|[16537]|CNPKD466-13|Chironomidae|600|BOLD:ACL1396
Orthocladiinae|[16538]|SMTPG385-14|Chironomidae|582|BOLD:ACM6817
Orthocladiinae|[16539]|CNGBA366-13|Chironomidae|658|BOLD:ACF6509
Orthocladiinae|[16540]|CNROA121-13|Chironomidae|670|BOLD:ACF6272
Chironomidae|[16541]|SMTPB1959-13|Chironomidae|658|BOLD:ACF8420
Chironomidae|[16542]|SMTPB7783-13|Chironomidae|670|BOLD:ACF9570
Orthocladiinae|[16543]|CNPKC293-13|Chironomidae|591|BOLD:ACF9571
Chironomidae|[16544]|JSDIP429-10|Chironomidae|658|BOLD:AAQ0604
Chironomidae|[16545]|SMTPB21036-13|Chironomidae|670|BOLD:ACF9447
Orthocladiinae|[16546]|CNKJA202-14|Chironomidae|622|BOLD:ACM2114
Parakiefferiella|[16547]|BBDIQ120-10|Chironomidae|658|BOLD:AAL7348
Parakiefferiella|[16548]|BBDCN710-10|Chironomidae|658|BOLD:AAP6896
Parakiefferiella|[16549]|CNAUB1193-14|Chironomidae|582|BOLD:ACR3777
Parakiefferiella|[16550]|CNAUA030-14|Chironomidae|579|BOLD:ACR0603
Parakiefferiella bathophila|[16551]|CNNHA1017-14|Chironomidae|606|BOLD:ACY4239
Orthocladiinae|[16552]|DARC540-11|Chironomidae|658|BOLD:AAU6766
Chironomidae|[16553]|HEAPR2458-12|Chironomidae|703|BOLD:ABX0273
Smittia|[16554]|CNAUY398-14|Chironomidae|603|BOLD:ACO4167
Orthocladiinae|[16555]|CNKJA018-14|Chironomidae|658|BOLD:ACM1765
Orthocladiinae|[16556]|SSFDA2739-14|Chironomidae|588|BOLD:AAN5361
Orthocladiinae|[16557]|SSFDA4532-14|Chironomidae|592|BOLD:AAG5442
Psectrocladius|[16558]|JWDCI580-10|Chironomidae|658|BOLD:AAG6993
Orthocladiinae|[16559]|NGNAX1435-14|Chironomidae|591|BOLD:ACM6550
Chironomidae|[16560]|SMTPB18346-13|Chironomidae|661|BOLD:ABV1186
Orthocladiinae|[16561]|CNTNA450-14|Chironomidae|661|BOLD:ACM1192
Orthocladiinae|[16562]|SSFDC1941-14|Chironomidae|606|BOLD:ACO2715
Orthocladiinae|[16563]|CNGMC1709-13|Chironomidae|658|BOLD:ACI6860
Orthocladiinae|[16564]|SSFDC5918-14|Chironomidae|624|BOLD:ACO2079
Rheocricotopus|[16565]|CNKJA043-14|Chironomidae|631|BOLD:ACM1766
Orthocladiinae|[16566]|JWDCC350-10|Chironomidae|658|BOLD:AAL7314
Rheocricotopus|[16567]|SSFDA4503-14|Chironomidae|563|BOLD:ACO1538
Rheocricotopus|[16568]|CNIVD844-14|Chironomidae|609|BOLD:ACM7368
Orthocladiinae|[16569]|CNKJB379-14|Chironomidae|658|BOLD:ACL7707
Orthocladiinae|[16570]|CNPCK044-13|Chironomidae|661|BOLD:ACI8829
Chironomidae|[16571]|CNTUB1712-14|Chironomidae|588|BOLD:ACR5210
Cricotopus sp. 23ES|[16572]|BBDCP062-10|Chironomidae|658|BOLD:AAI6022
Cricotopus sp. 22ES|[16573]|CHURC687-09|Chironomidae|658|BOLD:AAA5310
Cricotopus|[16574]|SSEIB7852-13|Chironomidae|630|BOLD:AAV5854
Orthocladiinae|[16575]|SSEIB7966-13|Chironomidae|670|BOLD:ACG6107
Orthocladiinae|[16576]|CNTNK1304-14|Chironomidae|552|BOLD:ACN7766
Orthocladiinae|[16577]|CNGBL455-14|Chironomidae|658|BOLD:ACL9616
Orthocladiinae|[16578]|SSBRA1763-14|Chironomidae|591|BOLD:AAN5365
Orthocladiinae|[16579]|CNFNC1763-14|Chironomidae|661|BOLD:ACL2169
Orthocladiinae|[16580]|CNFDL554-14|Chironomidae|625|BOLD:ACO5090
Orthocladiinae|[16581]|SSFDC5637-14|Chironomidae|588|BOLD:ACO6311
Orthocladiinae|[16582]|SMTPI5596-14|Chironomidae|591|BOLD:ACN8090
Chironomidae|[16583]|SSBRA117-14|Chironomidae|664|BOLD:ACN8325
Chironomidae|[16584]|SSBRA1856-14|Chironomidae|549|BOLD:ACO0484
Orthocladiinae|[16585]|JSMAY088-11|Chironomidae|658|BOLD:ABV1202
Chironomidae|[16586]|SSBRA118-14|Chironomidae|664|BOLD:ACN7582
Chironomidae|[16587]|CNKJE772-14|Chironomidae|573|BOLD:ACN8716
Orthocladiinae|[16588]|SSBRC990-14|Chironomidae|658|BOLD:ACL4700
Chironomidae|[16589]|SSGBB3655-14|Chironomidae|667|BOLD:ACN8717
Orthocladiinae|[16590]|SSBRB185-14|Chironomidae|664|BOLD:ACK7313
Orthocladiinae|[16591]|SSGBB3583-14|Chironomidae|667|BOLD:ACL6495
Orthocladiinae|[16592]|SSGBC4203-14|Chironomidae|614|BOLD:ACN2493
Orthocladiinae|[16593]|JSMAY086-11|Chironomidae|658|BOLD:ABV1183
Chironomidae|[16594]|CNNHA1315-14|Chironomidae|603|BOLD:ACR6301
Orthocladiinae|[16595]|SSJAA2191-13|Chironomidae|658|BOLD:ACG2789
Parakiefferiella|[16596]|SSBAF661-13|Chironomidae|658|BOLD:ACK4977
Parakiefferiella|[16597]|CNGBF626-14|Chironomidae|661|BOLD:ACD1858

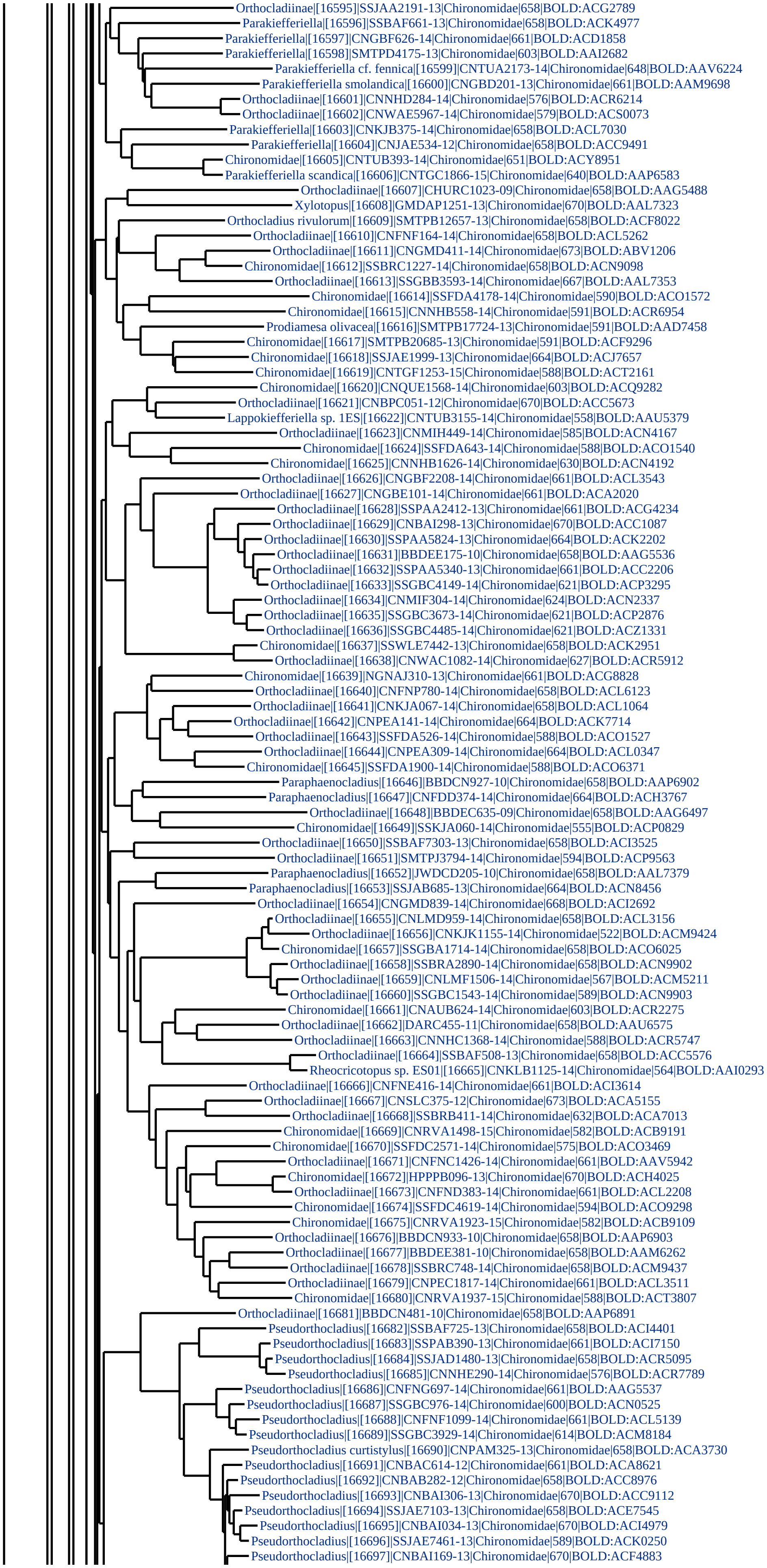

Orthocladiinae|[16595]|SSJAA2191-13|Chironomidae|658|BOLD:ACG2789
Parakiefferiella|[16596]|SSBAF661-13|Chironomidae|658|BOLD:ACK4977
Parakiefferiella|[16597]|CNGBF626-14|Chironomidae|661|BOLD:ACD1858
Parakiefferiella|[16598]|SMTPD4175-13|Chironomidae|603|BOLD:AAI2682
Parakiefferiella cf. fennica|[16599]|CNTUA2173-14|Chironomidae|648|BOLD:AAV6224
Parakiefferiella smolandica|[16600]|CNGBD201-13|Chironomidae|661|BOLD:AAM9698
Orthocladiinae|[16601]|CNNHD284-14|Chironomidae|576|BOLD:ACR6214
Orthocladiinae|[16602]|CNWAE5967-14|Chironomidae|579|BOLD:ACS0073
Parakiefferiella|[16603]|CNKJB375-14|Chironomidae|658|BOLD:ACL7030
Parakiefferiella|[16604]|CNJAE534-12|Chironomidae|658|BOLD:ACC9491
Chironomidae|[16605]|CNTUB393-14|Chironomidae|651|BOLD:ACY8951
Parakiefferiella scandica|[16606]|CNTGC1866-15|Chironomidae|640|BOLD:AAP6583
Orthocladiinae|[16607]|CHURC1023-09|Chironomidae|658|BOLD:AAG5488
Xylotopus|[16608]|GMDAP1251-13|Chironomidae|670|BOLD:AAL7323
Orthocladius rivulorum|[16609]|SMTPB12657-13|Chironomidae|658|BOLD:ACF8022
Orthocladiinae|[16610]|CNFNF164-14|Chironomidae|658|BOLD:ACL5262
Orthocladiinae|[16611]|CNGMD411-14|Chironomidae|673|BOLD:ABV1206
Chironomidae|[16612]|SSBRC1227-14|Chironomidae|658|BOLD:ACN9098
Orthocladiinae|[16613]|SSGBB3593-14|Chironomidae|667|BOLD:AAL7353
Chironomidae|[16614]|SSFDA4178-14|Chironomidae|590|BOLD:ACO1572
Chironomidae|[16615]|CNNHB558-14|Chironomidae|591|BOLD:ACR6954
Prodiamesa olivacea|[16616]|SMTPB17724-13|Chironomidae|591|BOLD:AAD7458
Chironomidae|[16617]|SMTPB20685-13|Chironomidae|591|BOLD:ACF9296
Chironomidae|[16618]|SSJAE1999-13|Chironomidae|664|BOLD:ACJ7657
Chironomidae|[16619]|CNTGF1253-15|Chironomidae|588|BOLD:ACT2161
Chironomidae|[16620]|CNQUE1568-14|Chironomidae|603|BOLD:ACQ9282
Orthocladiinae|[16621]|CNBPC051-12|Chironomidae|670|BOLD:ACC5673
Lappokiefferiella sp. 1ES|[16622]|CNTUB3155-14|Chironomidae|558|BOLD:AAU5379
Orthocladiinae|[16623]|CNMIH449-14|Chironomidae|585|BOLD:ACN4167
Chironomidae|[16624]|SSFDA643-14|Chironomidae|588|BOLD:ACO1540
Chironomidae|[16625]|CNNHB1626-14|Chironomidae|630|BOLD:ACN4192
Orthocladiinae|[16626]|CNGBF2208-14|Chironomidae|661|BOLD:ACL3543
Orthocladiinae|[16627]|CNGBE101-14|Chironomidae|661|BOLD:ACA2020
Orthocladiinae|[16628]|SSPAA2412-13|Chironomidae|661|BOLD:ACG4234
Orthocladiinae|[16629]|CNBAI298-13|Chironomidae|670|BOLD:ACC1087
Orthocladiinae|[16630]|SSPAA5824-13|Chironomidae|664|BOLD:ACK2202
Orthocladiinae|[16631]|BBDEE175-10|Chironomidae|658|BOLD:AAG5536
Orthocladiinae|[16632]|SSPAA5340-13|Chironomidae|661|BOLD:ACC2206
Orthocladiinae|[16633]|SSGBC4149-14|Chironomidae|621|BOLD:ACP3295
Orthocladiinae|[16634]|CNMIF304-14|Chironomidae|624|BOLD:ACN2337
Orthocladiinae|[16635]|SSGBC3673-14|Chironomidae|621|BOLD:ACP2876
Orthocladiinae|[16636]|SSGBC4485-14|Chironomidae|621|BOLD:ACZ1331
Chironomidae|[16637]|SSWLE7442-13|Chironomidae|658|BOLD:ACK2951
Orthocladiinae|[16638]|CNWAC1082-14|Chironomidae|627|BOLD:ACR5912
Chironomidae|[16639]|NGNAJ310-13|Chironomidae|661|BOLD:ACG8828
Orthocladiinae|[16640]|CNFNP780-14|Chironomidae|658|BOLD:ACL6123
Orthocladiinae|[16641]|CNKJA067-14|Chironomidae|658|BOLD:ACL1064
Orthocladiinae|[16642]|CNPEA141-14|Chironomidae|664|BOLD:ACK7714
Orthocladiinae|[16643]|SSFDA526-14|Chironomidae|588|BOLD:ACO1527
Orthocladiinae|[16644]|CNPEA309-14|Chironomidae|664|BOLD:ACL0347
Chironomidae|[16645]|SSFDA1900-14|Chironomidae|588|BOLD:ACO6371
Paraphaenocladius|[16646]|BBDCN927-10|Chironomidae|658|BOLD:AAP6902
Paraphaenocladius|[16647]|CNFDD374-14|Chironomidae|664|BOLD:ACH3767
Orthocladiinae|[16648]|BBDEC635-09|Chironomidae|658|BOLD:AAG6497
Chironomidae|[16649]|SSKJA060-14|Chironomidae|555|BOLD:ACP0829
Orthocladiinae|[16650]|SSBAF7303-13|Chironomidae|658|BOLD:ACI3525
Orthocladiinae|[16651]|SMTPJ3794-14|Chironomidae|594|BOLD:ACP9563
Paraphaenocladius|[16652]|JWDCD205-10|Chironomidae|658|BOLD:AAL7379
Paraphaenocladius|[16653]|SSJAB685-13|Chironomidae|664|BOLD:ACN8456
Orthocladiinae|[16654]|CNGMD839-14|Chironomidae|668|BOLD:ACI2692
Orthocladiinae|[16655]|CNLMD959-14|Chironomidae|658|BOLD:ACL3156
Orthocladiinae|[16656]|CNKJK1155-14|Chironomidae|522|BOLD:ACM9424
Chironomidae|[16657]|SSGBA1714-14|Chironomidae|658|BOLD:ACO6025
Orthocladiinae|[16658]|SSBRA2890-14|Chironomidae|658|BOLD:ACN9902
Orthocladiinae|[16659]|CNLMF1506-14|Chironomidae|567|BOLD:ACM5211
Orthocladiinae|[16660]|SSGBC1543-14|Chironomidae|589|BOLD:ACN9903
Chironomidae|[16661]|CNAUB624-14|Chironomidae|603|BOLD:ACR2275
Orthocladiinae|[16662]|DARC455-11|Chironomidae|658|BOLD:AAU6575
Orthocladiinae|[16663]|CNNHC1368-14|Chironomidae|588|BOLD:ACR5747
Orthocladiinae|[16664]|SSBAF508-13|Chironomidae|658|BOLD:ACC5576
Rheocricotopus sp. ES01|[16665]|CNKLB1125-14|Chironomidae|564|BOLD:AAI0293
Orthocladiinae|[16666]|CNFNE416-14|Chironomidae|661|BOLD:ACI3614
Orthocladiinae|[16667]|CNSLC375-12|Chironomidae|673|BOLD:ACA5155
Orthocladiinae|[16668]|SSBRB411-14|Chironomidae|632|BOLD:ACA7013
Chironomidae|[16669]|CNRVA1498-15|Chironomidae|582|BOLD:ACB9191
Chironomidae|[16670]|SSFDC2571-14|Chironomidae|575|BOLD:ACO3469
Orthocladiinae|[16671]|CNFNC1426-14|Chironomidae|661|BOLD:AAV5942
Chironomidae|[16672]|HPPPB096-13|Chironomidae|670|BOLD:ACH4025
Orthocladiinae|[16673]|CNFND383-14|Chironomidae|661|BOLD:ACL2208
Chironomidae|[16674]|SSFDC4619-14|Chironomidae|594|BOLD:ACO9298
Chironomidae|[16675]|CNRVA1923-15|Chironomidae|582|BOLD:ACB9109
Orthocladiinae|[16676]|BBDCN933-10|Chironomidae|658|BOLD:AAP6903
Orthocladiinae|[16677]|BBDEE381-10|Chironomidae|658|BOLD:AAM6262
Orthocladiinae|[16678]|SSBRC748-14|Chironomidae|658|BOLD:ACM9437
Orthocladiinae|[16679]|CNPEC1817-14|Chironomidae|661|BOLD:ACL3511
Chironomidae|[16680]|CNRVA1937-15|Chironomidae|588|BOLD:ACT3807
Orthocladiinae|[16681]|BBDCN481-10|Chironomidae|658|BOLD:AAP6891
Pseudorthocladius|[16682]|SSBAF725-13|Chironomidae|658|BOLD:ACI4401
Pseudorthocladius|[16683]|SSPAB390-13|Chironomidae|661|BOLD:ACI7150
Pseudorthocladius|[16684]|SSJAD1480-13|Chironomidae|658|BOLD:ACR5095
Pseudorthocladius|[16685]|CNNHE290-14|Chironomidae|576|BOLD:ACR7789
Pseudorthocladius|[16686]|CNFNG697-14|Chironomidae|661|BOLD:AAG5537
Pseudorthocladius|[16687]|SSGBC976-14|Chironomidae|600|BOLD:ACN0525
Pseudorthocladius|[16688]|CNFNF1099-14|Chironomidae|661|BOLD:ACL5139
Pseudorthocladius|[16689]|SSGBC3929-14|Chironomidae|614|BOLD:ACM8184
Pseudorthocladius curtistylus|[16690]|CNPAM325-13|Chironomidae|658|BOLD:ACA3730
Pseudorthocladius|[16691]|CNBAC614-12|Chironomidae|661|BOLD:ACA8621
Pseudorthocladius|[16692]|CNBAB282-12|Chironomidae|658|BOLD:ACC8976
Pseudorthocladius|[16693]|CNBAI306-13|Chironomidae|670|BOLD:ACC9112
Pseudorthocladius|[16694]|SSJAE7103-13|Chironomidae|658|BOLD:ACE7545
Pseudorthocladius|[16695]|CNBAI034-13|Chironomidae|670|BOLD:ACI4979
Pseudorthocladius|[16696]|SSJAE7461-13|Chironomidae|589|BOLD:ACK0250
Pseudorthocladius|[16697]|CNBAI169-13|Chironomidae|670|BOLD:ACF4883

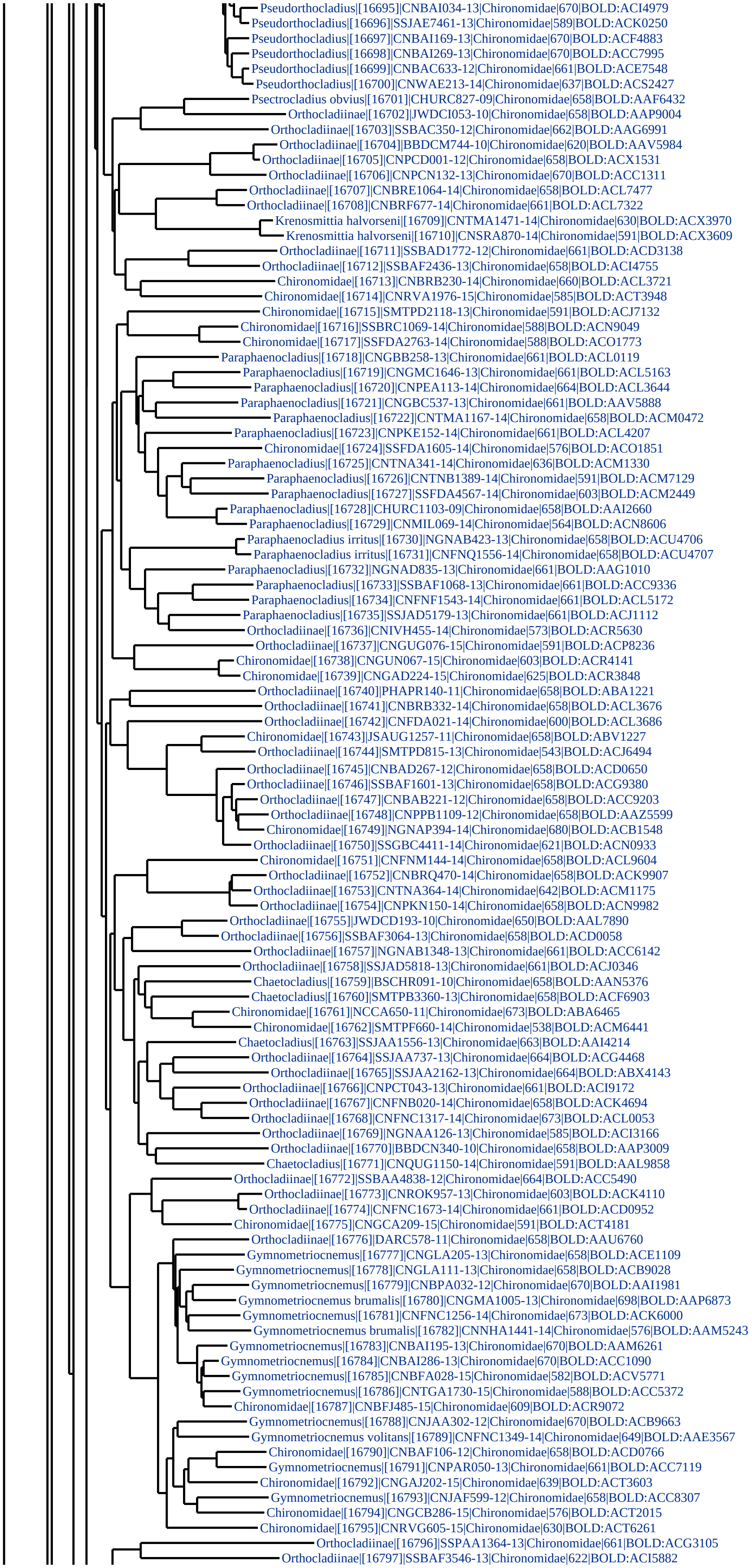
Pseudorthocladius|[16695]|CNBAI034-13|Chironomidae|670|BOLD:ACI4979
Pseudorthocladius|[16696]|SSJAE7461-13|Chironomidae|589|BOLD:ACK0250
Pseudorthocladius|[16697]|CNBAI169-13|Chironomidae|670|BOLD:ACF4883
Pseudorthocladius|[16698]|CNBAI269-13|Chironomidae|670|BOLD:ACC7995
Pseudorthocladius|[16699]|CNBAC633-12|Chironomidae|661|BOLD:ACE7548
Pseudorthocladius|[16700]|CNWAE213-14|Chironomidae|637|BOLD:ACS2427
Psectrocladius obvius|[16701]|CHURC827-09|Chironomidae|658|BOLD:AAF6432
Orthocladiinae|[16702]|JWDCI053-10|Chironomidae|658|BOLD:AAP9004
Orthocladiinae|[16703]|SSBAC350-12|Chironomidae|662|BOLD:AAG6991
Orthocladiinae|[16704]|BBDCM744-10|Chironomidae|620|BOLD:AAV5984
Orthocladiinae|[16705]|CNPCD001-12|Chironomidae|658|BOLD:ACX1531
Orthocladiinae|[16706]|CNPCN132-13|Chironomidae|670|BOLD:ACC1311
Orthocladiinae|[16707]|CNBRE1064-14|Chironomidae|658|BOLD:ACL7477
Orthocladiinae|[16708]|CNBRF677-14|Chironomidae|661|BOLD:ACL7322
Krenosmittia halvorseni|[16709]|CNTMA1471-14|Chironomidae|630|BOLD:ACX3970
Krenosmittia halvorseni|[16710]|CNSRA870-14|Chironomidae|591|BOLD:ACX3609
Orthocladiinae|[16711]|SSBAD1772-12|Chironomidae|661|BOLD:ACD3138
Orthocladiinae|[16712]|SSBAF2436-13|Chironomidae|658|BOLD:ACI4755
Chironomidae|[16713]|CNBRB230-14|Chironomidae|660|BOLD:ACL3721
Chironomidae|[16714]|CNRVA1976-15|Chironomidae|585|BOLD:ACT3948
Chironomidae|[16715]|SMTPD2118-13|Chironomidae|591|BOLD:ACJ7132
Chironomidae|[16716]|SSBRC1069-14|Chironomidae|588|BOLD:ACN9049
Chironomidae|[16717]|SSFDA2763-14|Chironomidae|588|BOLD:ACO1773
Paraphaenocladius|[16718]|CNGBB258-13|Chironomidae|661|BOLD:ACL0119
Paraphaenocladius|[16719]|CNGMC1646-13|Chironomidae|661|BOLD:ACL5163
Paraphaenocladius|[16720]|CNPEA113-14|Chironomidae|664|BOLD:ACL3644
Paraphaenocladius|[16721]|CNGBC537-13|Chironomidae|661|BOLD:AAV5888
Paraphaenocladius|[16722]|CNTMA1167-14|Chironomidae|658|BOLD:ACM0472
Paraphaenocladius|[16723]|CNPKE152-14|Chironomidae|661|BOLD:ACL4207
Chironomidae|[16724]|SSFDA1605-14|Chironomidae|576|BOLD:ACO1851
Paraphaenocladius|[16725]|CNTNA341-14|Chironomidae|636|BOLD:ACM1330
Paraphaenocladius|[16726]|CNTNB1389-14|Chironomidae|591|BOLD:ACM7129
Paraphaenocladius|[16727]|SSFDA4567-14|Chironomidae|603|BOLD:ACM2449
Paraphaenocladius|[16728]|CHURC1103-09|Chironomidae|658|BOLD:AAI2660
Paraphaenocladius|[16729]|CNMIL069-14|Chironomidae|564|BOLD:ACN8606
Paraphaenocladius irritus|[16730]|NGNAB423-13|Chironomidae|658|BOLD:ACU4706
Paraphaenocladius irritus|[16731]|CNFNQ1556-14|Chironomidae|658|BOLD:ACU4707
Paraphaenocladius|[16732]|NGNAD835-13|Chironomidae|661|BOLD:AAG1010
Paraphaenocladius|[16733]|SSBAF1068-13|Chironomidae|661|BOLD:ACC9336
Paraphaenocladius|[16734]|CNFNF1543-14|Chironomidae|661|BOLD:ACL5172
Paraphaenocladius|[16735]|SSJAD5179-13|Chironomidae|661|BOLD:ACJ1112
Orthocladiinae|[16736]|CNIVH455-14|Chironomidae|573|BOLD:ACR5630
Orthocladiinae|[16737]|CNGUG076-15|Chironomidae|591|BOLD:ACP8236
Chironomidae|[16738]|CNGUN067-15|Chironomidae|603|BOLD:ACR4141
Chironomidae|[16739]|CNGAD224-15|Chironomidae|625|BOLD:ACR3848
Orthocladiinae|[16740]|PHAPR140-11|Chironomidae|658|BOLD:ABA1221
Orthocladiinae|[16741]|CNBRB332-14|Chironomidae|658|BOLD:ACL3676
Orthocladiinae|[16742]|CNFDA021-14|Chironomidae|600|BOLD:ACL3686
Chironomidae|[16743]|JSAUG1257-11|Chironomidae|658|BOLD:ABV1227
Orthocladiinae|[16744]|SMTPD815-13|Chironomidae|543|BOLD:ACJ6494
Orthocladiinae|[16745]|CNBAD267-12|Chironomidae|658|BOLD:ACD0650
Orthocladiinae|[16746]|SSBAF1601-13|Chironomidae|658|BOLD:ACG9380
Orthocladiinae|[16747]|CNBAB221-12|Chironomidae|658|BOLD:ACC9203
Orthocladiinae|[16748]|CNPPB1109-12|Chironomidae|658|BOLD:AAZ5599
Chironomidae|[16749]|NGNAP394-14|Chironomidae|680|BOLD:ACB1548
Orthocladiinae|[16750]|SSGBC4411-14|Chironomidae|621|BOLD:ACN0933
Chironomidae|[16751]|CNFNM144-14|Chironomidae|658|BOLD:ACL9604
Orthocladiinae|[16752]|CNBRQ470-14|Chironomidae|658|BOLD:ACK9907
Orthocladiinae|[16753]|CNTNA364-14|Chironomidae|642|BOLD:ACM1175
Orthocladiinae|[16754]|CNPKN150-14|Chironomidae|658|BOLD:ACN9982
Orthocladiinae|[16755]|JWDCD193-10|Chironomidae|650|BOLD:AAL7890
Orthocladiinae|[16756]|SSBAF3064-13|Chironomidae|658|BOLD:ACD0058
Orthocladiinae|[16757]|NGNAB1348-13|Chironomidae|661|BOLD:ACC6142
Orthocladiinae|[16758]|SSJAD5818-13|Chironomidae|661|BOLD:ACJ0346
Chaetocladius|[16759]|BSCHR091-10|Chironomidae|658|BOLD:AAN5376
Chaetocladius|[16760]|SMTPB3360-13|Chironomidae|658|BOLD:ACF6903
Chironomidae|[16761]|NCCA650-11|Chironomidae|673|BOLD:ABA6465
Chironomidae|[16762]|SMTPF660-14|Chironomidae|538|BOLD:ACM6441
Chaetocladius|[16763]|SSJAA1556-13|Chironomidae|663|BOLD:AAI4214
Orthocladiinae|[16764]|SSJAA737-13|Chironomidae|664|BOLD:ACG4468
Orthocladiinae|[16765]|SSJAA2162-13|Chironomidae|664|BOLD:ABX4143
Orthocladiinae|[16766]|CNPCT043-13|Chironomidae|661|BOLD:ACI9172
Orthocladiinae|[16767]|CNFNB020-14|Chironomidae|658|BOLD:ACK4694
Orthocladiinae|[16768]|CNFNC1317-14|Chironomidae|673|BOLD:ACL0053
Orthocladiinae|[16769]|NGNAA126-13|Chironomidae|585|BOLD:ACI3166
Orthocladiinae|[16770]|BBDCN340-10|Chironomidae|658|BOLD:AAP3009
Chaetocladius|[16771]|CNQUG1150-14|Chironomidae|591|BOLD:AAL9858
Orthocladiinae|[16772]|SSBAA4838-12|Chironomidae|664|BOLD:ACC5490
Orthocladiinae|[16773]|CNROK957-13|Chironomidae|603|BOLD:ACK4110
Orthocladiinae|[16774]|CNFNC1673-14|Chironomidae|661|BOLD:ACD0952
Chironomidae|[16775]|CNGCA209-15|Chironomidae|591|BOLD:ACT4181
Orthocladiinae|[16776]|DARC578-11|Chironomidae|658|BOLD:AAU6760
Gymnometriocnemus|[16777]|CNGLA205-13|Chironomidae|658|BOLD:ACE1109
Gymnometriocnemus|[16778]|CNGLA111-13|Chironomidae|658|BOLD:ACB9028
Gymnometriocnemus|[16779]|CNBPA032-12|Chironomidae|670|BOLD:AAI1981
Gymnometriocnemus brumalis|[16780]|CNGMA1005-13|Chironomidae|698|BOLD:AAP6873
Gymnometriocnemus|[16781]|CNFNC1256-14|Chironomidae|673|BOLD:ACK6000
Gymnometriocnemus brumalis|[16782]|CNNHA1441-14|Chironomidae|576|BOLD:AAM5243
Gymnometriocnemus|[16783]|CNBAI195-13|Chironomidae|670|BOLD:AAM6261
Gymnometriocnemus|[16784]|CNBAI286-13|Chironomidae|670|BOLD:ACC1090
Gymnometriocnemus|[16785]|CNBFA028-15|Chironomidae|582|BOLD:ACV5771
Gymnometriocnemus|[16786]|CNTGA1730-15|Chironomidae|588|BOLD:ACC5372
Chironomidae|[16787]|CNBFJ485-15|Chironomidae|609|BOLD:ACR9072
Gymnometriocnemus|[16788]|CNJAA302-12|Chironomidae|670|BOLD:ACB9663
Gymnometriocnemus volitans|[16789]|CNFNC1349-14|Chironomidae|649|BOLD:AAE3567
Chironomidae|[16790]|CNBAF106-12|Chironomidae|658|BOLD:ACD0766
Gymnometriocnemus|[16791]|CNPAR050-13|Chironomidae|661|BOLD:ACC7119
Chironomidae|[16792]|CNGAJ202-15|Chironomidae|639|BOLD:ACT3603
Gymnometriocnemus|[16793]|CNJAF599-12|Chironomidae|658|BOLD:ACC8307
Chironomidae|[16794]|CNGCB286-15|Chironomidae|576|BOLD:ACT2015
Chironomidae|[16795]|CNRVG605-15|Chironomidae|630|BOLD:ACT6261
Orthocladiinae|[16796]|SSPAA1364-13|Chironomidae|661|BOLD:ACG3105
Orthocladiinae|[16797]|SSBAF3546-13|Chironomidae|622|BOLD:ACI5882

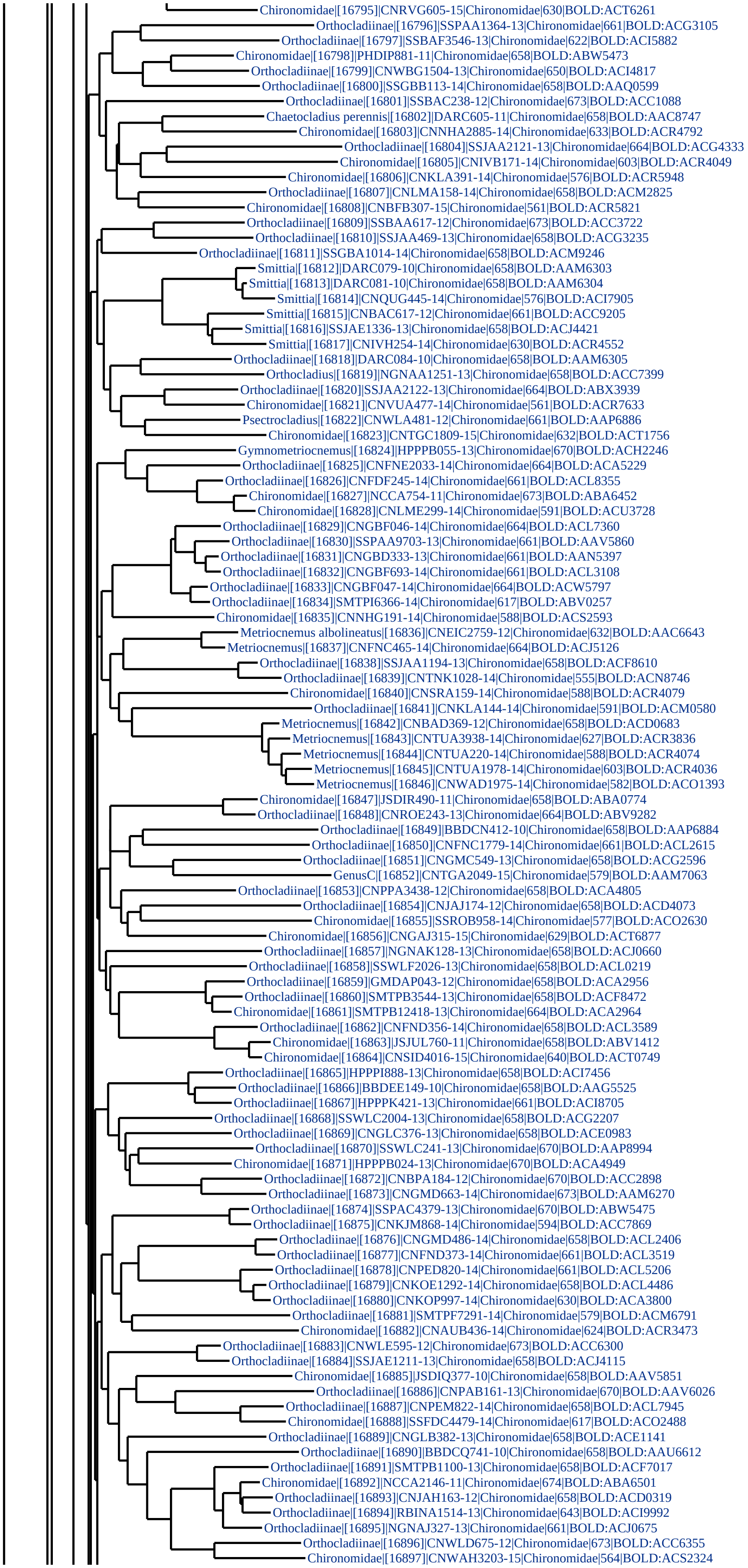
Chironomidae|[16795]|CNRVG605-15|Chironomidae|630|BOLD:ACT6261
Orthocladiinae|[16796]|SSPAA1364-13|Chironomidae|661|BOLD:ACG3105
Orthocladiinae|[16797]|SSBAF3546-13|Chironomidae|622|BOLD:ACI5882
Chironomidae|[16798]|PHDIP881-11|Chironomidae|658|BOLD:ABW5473
Orthocladiinae|[16799]|CNWBG1504-13|Chironomidae|650|BOLD:ACI4817
Orthocladiinae|[16800]|SSGBB113-14|Chironomidae|658|BOLD:AAQ0599
Orthocladiinae|[16801]|SSBAC238-12|Chironomidae|673|BOLD:ACC1088
Chaetocladius perennis|[16802]|DARC605-11|Chironomidae|658|BOLD:AAC8747
Chironomidae|[16803]|CNNHA2885-14|Chironomidae|633|BOLD:ACR4792
Orthocladiinae|[16804]|SSJAA2121-13|Chironomidae|664|BOLD:ACG4333
Chironomidae|[16805]|CNIVB171-14|Chironomidae|603|BOLD:ACR4049
Chironomidae|[16806]|CNKLA391-14|Chironomidae|576|BOLD:ACR5948
Orthocladiinae|[16807]|CNLMA158-14|Chironomidae|658|BOLD:ACM2825
Chironomidae|[16808]|CNBFB307-15|Chironomidae|561|BOLD:ACR5821
Orthocladiinae|[16809]|SSBAA617-12|Chironomidae|673|BOLD:ACC3722
Orthocladiinae|[16810]|SSJAA469-13|Chironomidae|658|BOLD:ACG3235
Orthocladiinae|[16811]|SSGBA1014-14|Chironomidae|658|BOLD:ACM9246
Smittia|[16812]|DARC079-10|Chironomidae|658|BOLD:AAM6303
Smittia|[16813]|DARC081-10|Chironomidae|658|BOLD:AAM6304
Smittia|[16814]|CNQUG445-14|Chironomidae|576|BOLD:ACI7905
Smittia|[16815]|CNBAC617-12|Chironomidae|661|BOLD:ACC9205
Smittia|[16816]|SSJAE1336-13|Chironomidae|658|BOLD:ACJ4421
Smittia|[16817]|CNIVH254-14|Chironomidae|630|BOLD:ACR4552
Orthocladiinae|[16818]|DARC084-10|Chironomidae|658|BOLD:AAM6305
Orthocladius|[16819]|NGNAA1251-13|Chironomidae|658|BOLD:ACC7399
Orthocladiinae|[16820]|SSJAA2122-13|Chironomidae|664|BOLD:ABX3939
Chironomidae|[16821]|CNVUA477-14|Chironomidae|561|BOLD:ACR7633
Psectrocladius|[16822]|CNWLA481-12|Chironomidae|661|BOLD:AAP6886
Chironomidae|[16823]|CNTGC1809-15|Chironomidae|632|BOLD:ACT1756
Gymnometriocnemus|[16824]|HPPPB055-13|Chironomidae|670|BOLD:ACH2246
Orthocladiinae|[16825]|CNFNE2033-14|Chironomidae|664|BOLD:ACA5229
Orthocladiinae|[16826]|CNFDF245-14|Chironomidae|661|BOLD:ACL8355
Chironomidae|[16827]|NCCA754-11|Chironomidae|673|BOLD:ABA6452
Chironomidae|[16828]|CNLME299-14|Chironomidae|591|BOLD:ACU3728
Orthocladiinae|[16829]|CNGBF046-14|Chironomidae|664|BOLD:ACL7360
Orthocladiinae|[16830]|SSPAA9703-13|Chironomidae|661|BOLD:AAV5860
Orthocladiinae|[16831]|CNGBD333-13|Chironomidae|661|BOLD:AAN5397
Orthocladiinae|[16832]|CNGBF693-14|Chironomidae|661|BOLD:ACL3108
Orthocladiinae|[16833]|CNGBF047-14|Chironomidae|664|BOLD:ACW5797
Orthocladiinae|[16834]|SMTPI6366-14|Chironomidae|617|BOLD:ABV0257
Chironomidae|[16835]|CNNHG191-14|Chironomidae|588|BOLD:ACS2593
Metriocnemus albolineatus|[16836]|CNEIC2759-12|Chironomidae|632|BOLD:AAC6643
Metriocnemus|[16837]|CNFNC465-14|Chironomidae|664|BOLD:ACJ5126
Orthocladiinae|[16838]|SSJAA1194-13|Chironomidae|658|BOLD:ACF8610
Orthocladiinae|[16839]|CNTNK1028-14|Chironomidae|555|BOLD:ACN8746
Chironomidae|[16840]|CNSRA159-14|Chironomidae|588|BOLD:ACR4079
Orthocladiinae|[16841]|CNKLA144-14|Chironomidae|591|BOLD:ACM0580
Metriocnemus|[16842]|CNBAD369-12|Chironomidae|658|BOLD:ACD0683
Metriocnemus|[16843]|CNTUA3938-14|Chironomidae|627|BOLD:ACR3836
Metriocnemus|[16844]|CNTUA220-14|Chironomidae|588|BOLD:ACR4074
Metriocnemus|[16845]|CNTUA1978-14|Chironomidae|603|BOLD:ACR4036
Metriocnemus|[16846]|CNWAD1975-14|Chironomidae|582|BOLD:ACO1393
Chironomidae|[16847]|JSDIR490-11|Chironomidae|658|BOLD:ABA0774
Orthocladiinae|[16848]|CNROE243-13|Chironomidae|664|BOLD:ABV9282
Orthocladiinae|[16849]|BBDCN412-10|Chironomidae|658|BOLD:AAP6884
Orthocladiinae|[16850]|CNFNC1779-14|Chironomidae|661|BOLD:ACL2615
Orthocladiinae|[16851]|CNGMC549-13|Chironomidae|658|BOLD:ACG2596
GenusC|[16852]|CNTGA2049-15|Chironomidae|579|BOLD:AAM7063
Orthocladiinae|[16853]|CNPPA3438-12|Chironomidae|658|BOLD:ACA4805
Orthocladiinae|[16854]|CNJAJ174-12|Chironomidae|658|BOLD:ACD4073
Chironomidae|[16855]|SSROB958-14|Chironomidae|577|BOLD:ACO2630
Chironomidae|[16856]|CNGAJ315-15|Chironomidae|629|BOLD:ACT6877
Orthocladiinae|[16857]|NGNAK128-13|Chironomidae|658|BOLD:ACJ0660
Orthocladiinae|[16858]|SSWLF2026-13|Chironomidae|658|BOLD:ACL0219
Orthocladiinae|[16859]|GMDAP043-12|Chironomidae|658|BOLD:ACA2956
Orthocladiinae|[16860]|SMTPB3544-13|Chironomidae|658|BOLD:ACF8472
Chironomidae|[16861]|SMTPB12418-13|Chironomidae|664|BOLD:ACA2964
Orthocladiinae|[16862]|CNFND356-14|Chironomidae|658|BOLD:ACL3589
Chironomidae|[16863]|JSJUL760-11|Chironomidae|658|BOLD:ABV1412
Chironomidae|[16864]|CNSID4016-15|Chironomidae|640|BOLD:ACT0749
Orthocladiinae|[16865]|HPPPI888-13|Chironomidae|658|BOLD:ACI7456
Orthocladiinae|[16866]|BBDEE149-10|Chironomidae|658|BOLD:AAG5525
Orthocladiinae|[16867]|HPPPK421-13|Chironomidae|661|BOLD:ACI8705
Orthocladiinae|[16868]|SSWLC2004-13|Chironomidae|658|BOLD:ACG2207
Orthocladiinae|[16869]|CNGLC376-13|Chironomidae|658|BOLD:ACE0983
Orthocladiinae|[16870]|SSWLC241-13|Chironomidae|670|BOLD:AAP8994
Chironomidae|[16871]|HPPPB024-13|Chironomidae|670|BOLD:ACA4949
Orthocladiinae|[16872]|CNBPA184-12|Chironomidae|670|BOLD:ACC2898
Orthocladiinae|[16873]|CNGMD663-14|Chironomidae|673|BOLD:AAM6270
Orthocladiinae|[16874]|SSPAC4379-13|Chironomidae|670|BOLD:ABW5475
Orthocladiinae|[16875]|CNKJM868-14|Chironomidae|594|BOLD:ACC7869
Orthocladiinae|[16876]|CNGMD486-14|Chironomidae|658|BOLD:ACL2406
Orthocladiinae|[16877]|CNFND373-14|Chironomidae|661|BOLD:ACL3519
Orthocladiinae|[16878]|CNPED820-14|Chironomidae|661|BOLD:ACL5206
Orthocladiinae|[16879]|CNKOE1292-14|Chironomidae|658|BOLD:ACL4486
Orthocladiinae|[16880]|CNKOP997-14|Chironomidae|630|BOLD:ACA3800
Orthocladiinae|[16881]|SMTPF7291-14|Chironomidae|579|BOLD:ACM6791
Chironomidae|[16882]|CNAUB436-14|Chironomidae|624|BOLD:ACR3473
Orthocladiinae|[16883]|CNWLE595-12|Chironomidae|673|BOLD:ACC6300
Orthocladiinae|[16884]|SSJAE1211-13|Chironomidae|658|BOLD:ACJ4115
Chironomidae|[16885]|JSDIQ377-10|Chironomidae|658|BOLD:AAV5851
Orthocladiinae|[16886]|CNPAB161-13|Chironomidae|670|BOLD:AAV6026
Orthocladiinae|[16887]|CNPEM822-14|Chironomidae|658|BOLD:ACL7945
Chironomidae|[16888]|SSFDC4479-14|Chironomidae|617|BOLD:ACO2488
Orthocladiinae|[16889]|CNGLB382-13|Chironomidae|658|BOLD:ACE1141
Orthocladiinae|[16890]|BBDCQ741-10|Chironomidae|658|BOLD:AAU6612
Orthocladiinae|[16891]|SMTPB1100-13|Chironomidae|658|BOLD:ACF7017
Chironomidae|[16892]|NCCA2146-11|Chironomidae|674|BOLD:ABA6501
Orthocladiinae|[16893]|CNJAH163-12|Chironomidae|658|BOLD:ACD0319
Orthocladiinae|[16894]|RBINA1514-13|Chironomidae|643|BOLD:ACI9992
Orthocladiinae|[16895]|NGNAJ327-13|Chironomidae|661|BOLD:ACJ0675
Orthocladiinae|[16896]|CNWLD675-12|Chironomidae|673|BOLD:ACC6355
Chironomidae|[16897]|CNWAH3203-15|Chironomidae|564|BOLD:ACS2324

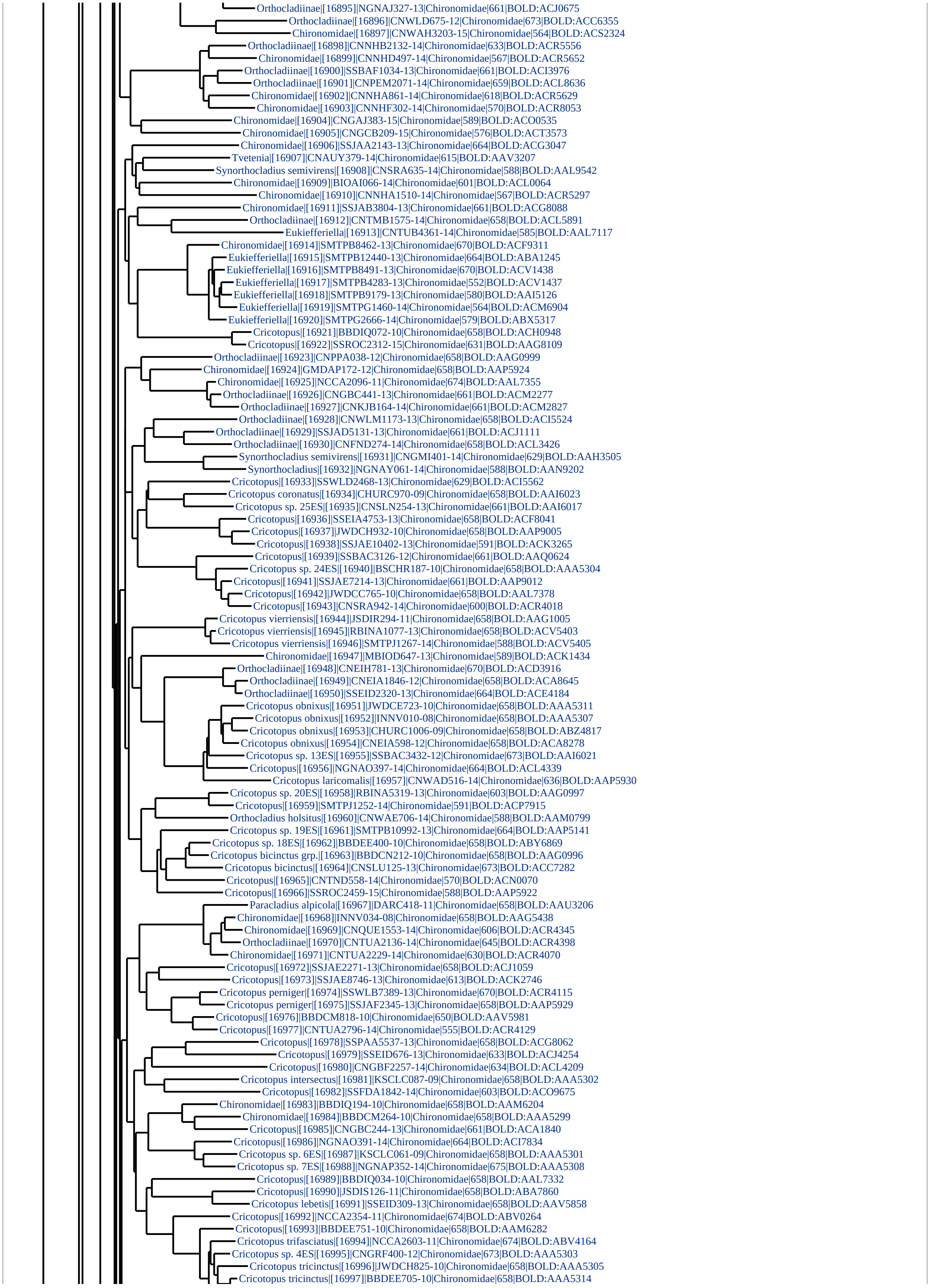

Orthocladiinae|[16895]|NGNAJ327-13|Chironomidae|661|BOLD:ACJ0675
Orthocladiinae|[16896]|CNWLD675-12|Chironomidae|673|BOLD:ACC6355
Chironomidae|[16897]|CNWAH3203-15|Chironomidae|564|BOLD:ACS2324
Orthocladiinae|[16898]|CNNHB2132-14|Chironomidae|633|BOLD:ACR5556
Chironomidae|[16899]|CNNHD497-14|Chironomidae|567|BOLD:ACR5652
Orthocladiinae|[16900]|SSBAF1034-13|Chironomidae|661|BOLD:ACI3976
Orthocladiinae|[16901]|CNPEM2071-14|Chironomidae|659|BOLD:ACL8636
Chironomidae|[16902]|CNNHA861-14|Chironomidae|618|BOLD:ACR5629
Chironomidae|[16903]|CNNHF302-14|Chironomidae|570|BOLD:ACR8053
Chironomidae|[16904]|CNGAJ383-15|Chironomidae|589|BOLD:ACO0535
Chironomidae|[16905]|CNGCB209-15|Chironomidae|576|BOLD:ACT3573
Chironomidae|[16906]|SSJAA2143-13|Chironomidae|664|BOLD:ACG3047
Tvetenia|[16907]|CNAUY379-14|Chironomidae|615|BOLD:AAV3207
Synorthocladius semivirens|[16908]|CNSRA635-14|Chironomidae|588|BOLD:AAL9542
Chironomidae|[16909]|BIOAI066-14|Chironomidae|601|BOLD:ACL0064
Chironomidae|[16910]|CNNHA1510-14|Chironomidae|567|BOLD:ACR5297
Chironomidae|[16911]|SSJAB3804-13|Chironomidae|661|BOLD:ACG8088
Orthocladiinae|[16912]|CNTMB1575-14|Chironomidae|658|BOLD:ACL5891
Eukiefferiella|[16913]|CNTUB4361-14|Chironomidae|585|BOLD:AAL7117
Chironomidae|[16914]|SMTPB8462-13|Chironomidae|670|BOLD:ACF9311
Eukiefferiella|[16915]|SMTPB12440-13|Chironomidae|664|BOLD:ABA1245
Eukiefferiella|[16916]|SMTPB8491-13|Chironomidae|670|BOLD:ACV1438
Eukiefferiella|[16917]|SMTPB4283-13|Chironomidae|552|BOLD:ACV1437
Eukiefferiella|[16918]|SMTPB9179-13|Chironomidae|580|BOLD:AAI5126
Eukiefferiella|[16919]|SMTPG1460-14|Chironomidae|564|BOLD:ACM6904
Eukiefferiella|[16920]|SMTPG2666-14|Chironomidae|579|BOLD:ABX5317
Cricotopus|[16921]|BBDIQ072-10|Chironomidae|658|BOLD:ACH0948
Cricotopus|[16922]|SSROC2312-15|Chironomidae|631|BOLD:AAG8109
Orthocladiinae|[16923]|CNPPA038-12|Chironomidae|658|BOLD:AAG0999
Chironomidae|[16924]|GMDAP172-12|Chironomidae|658|BOLD:AAP5924
Chironomidae|[16925]|NCCA2096-11|Chironomidae|674|BOLD:AAL7355
Orthocladiinae|[16926]|CNGBC441-13|Chironomidae|661|BOLD:ACM2277
Orthocladiinae|[16927]|CNKJB164-14|Chironomidae|661|BOLD:ACM2827
Orthocladiinae|[16928]|CNWLM1173-13|Chironomidae|658|BOLD:ACI5524
Orthocladiinae|[16929]|SSJAD5131-13|Chironomidae|661|BOLD:ACJ1111
Orthocladiinae|[16930]|CNFND274-14|Chironomidae|658|BOLD:ACL3426
Synorthocladius semivirens|[16931]|CNGMI401-14|Chironomidae|629|BOLD:AAH3505
Synorthocladius|[16932]|NGNAY061-14|Chironomidae|588|BOLD:AAN9202
Cricotopus|[16933]|SSWLD2468-13|Chironomidae|629|BOLD:ACI5562
Cricotopus coronatus|[16934]|CHURC970-09|Chironomidae|658|BOLD:AAI6023
Cricotopus sp. 25ES|[16935]|CNSLN254-13|Chironomidae|661|BOLD:AAI6017
Cricotopus|[16936]|SSEIA4753-13|Chironomidae|658|BOLD:ACF8041
Cricotopus|[16937]|JWDCH932-10|Chironomidae|658|BOLD:AAP9005
Cricotopus|[16938]|SSJAE10402-13|Chironomidae|591|BOLD:ACK3265
Cricotopus|[16939]|SSBAC3126-12|Chironomidae|661|BOLD:AAQ0624
Cricotopus sp. 24ES|[16940]|BSCHR187-10|Chironomidae|658|BOLD:AAA5304
Cricotopus|[16941]|SSJAE7214-13|Chironomidae|661|BOLD:AAP9012
Cricotopus|[16942]|JWDCC765-10|Chironomidae|658|BOLD:AAL7378
Cricotopus|[16943]|CNSRA942-14|Chironomidae|600|BOLD:ACR4018
Cricotopus vierriensis|[16944]|JSDIR294-11|Chironomidae|658|BOLD:AAG1005
Cricotopus vierriensis|[16945]|RBINA1077-13|Chironomidae|658|BOLD:ACV5403
Cricotopus vierriensis|[16946]|SMTPJ1267-14|Chironomidae|588|BOLD:ACV5405
Chironomidae|[16947]|MBIOD647-13|Chironomidae|589|BOLD:ACK1434
Orthocladiinae|[16948]|CNEIH781-13|Chironomidae|670|BOLD:ACD3916
Orthocladiinae|[16949]|CNEIA1846-12|Chironomidae|658|BOLD:ACA8645
Orthocladiinae|[16950]|SSEID2320-13|Chironomidae|664|BOLD:ACE4184
Cricotopus obnixus|[16951]|JWDCE723-10|Chironomidae|658|BOLD:AAA5311
Cricotopus obnixus|[16952]|INNV010-08|Chironomidae|658|BOLD:AAA5307
Cricotopus obnixus|[16953]|CHURC1006-09|Chironomidae|658|BOLD:ABZ4817
Cricotopus obnixus|[16954]|CNEIA598-12|Chironomidae|658|BOLD:ACA8278
Cricotopus sp. 13ES|[16955]|SSBAC3432-12|Chironomidae|673|BOLD:AAI6021
Cricotopus|[16956]|NGNAO397-14|Chironomidae|664|BOLD:ACL4339
Cricotopus laricomalis|[16957]|CNWAD516-14|Chironomidae|636|BOLD:AAP5930
Cricotopus sp. 20ES|[16958]|RBINA5319-13|Chironomidae|603|BOLD:AAG0997
Cricotopus|[16959]|SMTPJ1252-14|Chironomidae|591|BOLD:ACP7915
Orthocladius holsitus|[16960]|CNWAE706-14|Chironomidae|588|BOLD:AAM0799
Cricotopus sp. 19ES|[16961]|SMTPB10992-13|Chironomidae|664|BOLD:AAP5141
Cricotopus sp. 18ES|[16962]|BBDEE400-10|Chironomidae|658|BOLD:ABY6869
Cricotopus bicinctus grp.|[16963]|BBDCN212-10|Chironomidae|658|BOLD:AAG0996
Cricotopus bicinctus|[16964]|CNSLU125-13|Chironomidae|673|BOLD:ACC7282
Cricotopus|[16965]|CNTND558-14|Chironomidae|570|BOLD:ACN0070
Cricotopus|[16966]|SSROC2459-15|Chironomidae|588|BOLD:AAP5922
Paracladius alpicola|[16967]|DARC418-11|Chironomidae|658|BOLD:AAU3206
Chironomidae|[16968]|INNV034-08|Chironomidae|658|BOLD:AAG5438
Chironomidae|[16969]|CNQUE1553-14|Chironomidae|606|BOLD:ACR4345
Orthocladiinae|[16970]|CNTUA2136-14|Chironomidae|645|BOLD:ACR4398
Chironomidae|[16971]|CNTUA2229-14|Chironomidae|630|BOLD:ACR4070
Cricotopus|[16972]|SSJAE2271-13|Chironomidae|658|BOLD:ACJ1059
Cricotopus|[16973]|SSJAE8746-13|Chironomidae|613|BOLD:ACK2746
Cricotopus perniger|[16974]|SSWLB7389-13|Chironomidae|670|BOLD:ACR4115
Cricotopus perniger|[16975]|SSJAF2345-13|Chironomidae|658|BOLD:AAP5929
Cricotopus|[16976]|BBDCM818-10|Chironomidae|650|BOLD:AAV5981
Cricotopus|[16977]|CNTUA2796-14|Chironomidae|555|BOLD:ACR4129
Cricotopus|[16978]|SSPAA5537-13|Chironomidae|658|BOLD:ACG8062
Cricotopus|[16979]|SSEID676-13|Chironomidae|633|BOLD:ACJ4254
Cricotopus|[16980]|CNGBF2257-14|Chironomidae|634|BOLD:ACL4209
Cricotopus intersectus|[16981]|KSCLC087-09|Chironomidae|658|BOLD:AAA5302
Cricotopus|[16982]|SSFDA1842-14|Chironomidae|603|BOLD:ACO9675
Chironomidae|[16983]|BBDIQ194-10|Chironomidae|658|BOLD:AAM6204
Chironomidae|[16984]|BBDCM264-10|Chironomidae|658|BOLD:AAA5299
Cricotopus|[16985]|CNGBC244-13|Chironomidae|661|BOLD:ACA1840
Cricotopus|[16986]|NGNAO391-14|Chironomidae|664|BOLD:ACI7834
Cricotopus sp. 6ES|[16987]|KSCLC061-09|Chironomidae|658|BOLD:AAA5301
Cricotopus sp. 7ES|[16988]|NGNAP352-14|Chironomidae|675|BOLD:AAA5308
Cricotopus|[16989]|BBDIQ034-10|Chironomidae|658|BOLD:AAL7332
Cricotopus|[16990]|JSDIS126-11|Chironomidae|658|BOLD:ABA7860
Cricotopus lebetis|[16991]|SSEID309-13|Chironomidae|658|BOLD:AAV5858
Cricotopus|[16992]|NCCA2354-11|Chironomidae|674|BOLD:ABV0264
Cricotopus|[16993]|BBDEE751-10|Chironomidae|658|BOLD:AAM6282
Cricotopus trifasciatus|[16994]|NCCA2603-11|Chironomidae|674|BOLD:ABV4164
Cricotopus sp. 4ES|[16995]|CNGRF400-12|Chironomidae|673|BOLD:AAA5303
Cricotopus tricinctus|[16996]|JWDCH825-10|Chironomidae|658|BOLD:AAA5305
Cricotopus tricinctus|[16997]|BBDEE705-10|Chironomidae|658|BOLD:AAA5314

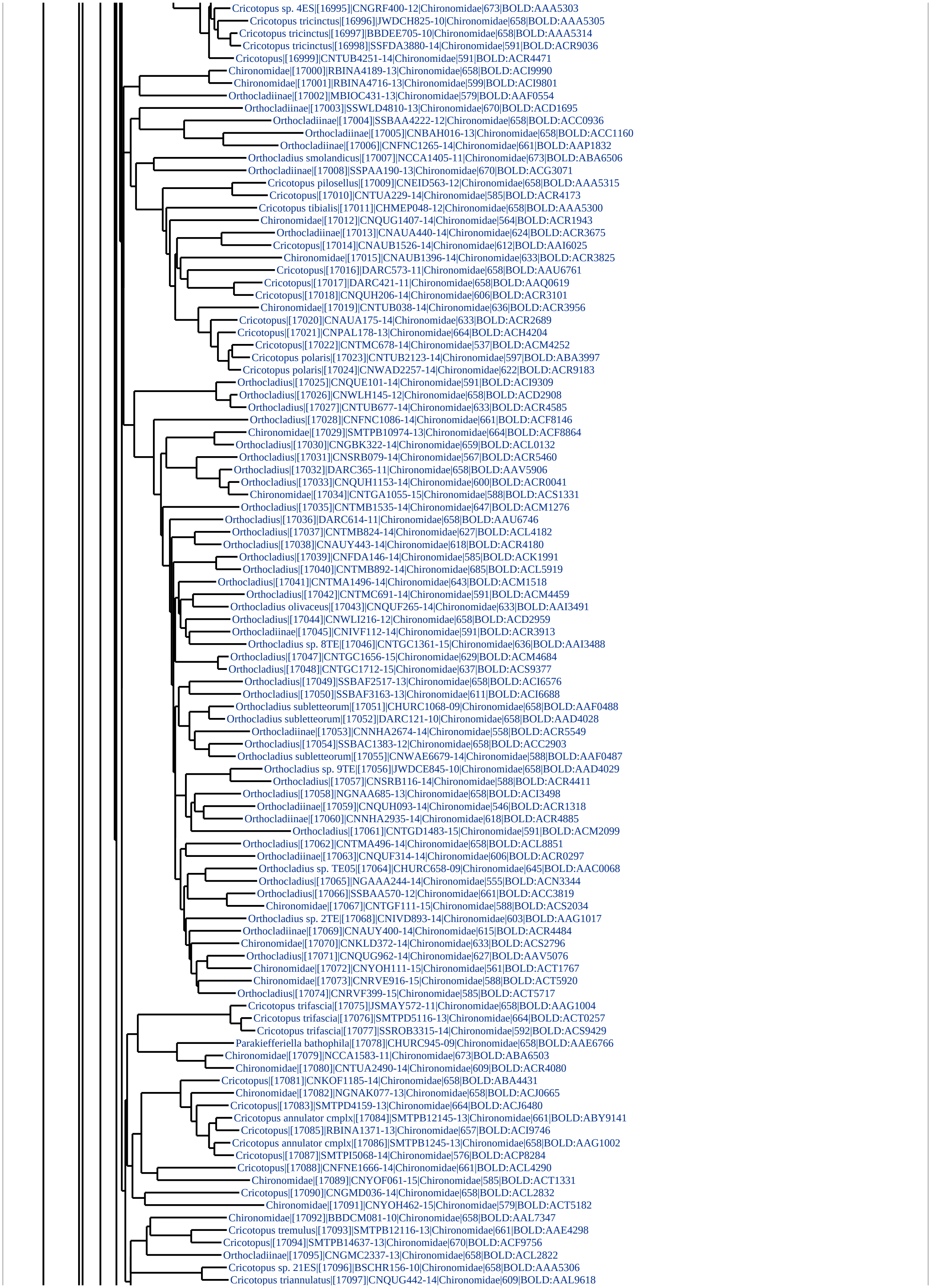
Cricotopus sp. 4ES|[16995]|CNGRF400-12|Chironomidae|673|BOLD:AAA5303
Cricotopus tricinctus|[16996]|JWDCH825-10|Chironomidae|658|BOLD:AAA5305
Cricotopus tricinctus|[16997]|BBDEE705-10|Chironomidae|658|BOLD:AAA5314
Cricotopus tricinctus|[16998]|SSFDA3880-14|Chironomidae|591|BOLD:ACR9036
Cricotopus|[16999]|CNTUB4251-14|Chironomidae|591|BOLD:ACR4471
Chironomidae|[17000]|RBINA4189-13|Chironomidae|658|BOLD:ACI9990
Chironomidae|[17001]|RBINA4716-13|Chironomidae|599|BOLD:ACI9801
Orthocladiinae|[17002]|MBIOC431-13|Chironomidae|579|BOLD:AAF0554
Orthocladiinae|[17003]|SSWLD4810-13|Chironomidae|670|BOLD:ACD1695
Orthocladiinae|[17004]|SSBAA4222-12|Chironomidae|658|BOLD:ACC0936
Orthocladiinae|[17005]|CNBAH016-13|Chironomidae|658|BOLD:ACC1160
Orthocladiinae|[17006]|CNFNC1265-14|Chironomidae|661|BOLD:AAP1832
Orthocladius smolandicus|[17007]|NCCA1405-11|Chironomidae|673|BOLD:ABA6506
Orthocladiinae|[17008]|SSPAA190-13|Chironomidae|670|BOLD:ACG3071
Cricotopus pilosellus|[17009]|CNEID563-12|Chironomidae|658|BOLD:AAA5315
Cricotopus|[17010]|CNTUA229-14|Chironomidae|585|BOLD:ACR4173
Cricotopus tibialis|[17011]|CHMEP048-12|Chironomidae|658|BOLD:AAA5300
Chironomidae|[17012]|CNQUG1407-14|Chironomidae|564|BOLD:ACR1943
Orthocladiinae|[17013]|CNAUA440-14|Chironomidae|624|BOLD:ACR3675
Cricotopus|[17014]|CNAUB1526-14|Chironomidae|612|BOLD:AAI6025
Chironomidae|[17015]|CNAUB1396-14|Chironomidae|633|BOLD:ACR3825
Cricotopus|[17016]|DARC573-11|Chironomidae|658|BOLD:AAU6761
Cricotopus|[17017]|DARC421-11|Chironomidae|658|BOLD:AAQ0619
Cricotopus|[17018]|CNQUH206-14|Chironomidae|606|BOLD:ACR3101
Chironomidae|[17019]|CNTUB038-14|Chironomidae|636|BOLD:ACR3956
Cricotopus|[17020]|CNAUA175-14|Chironomidae|633|BOLD:ACR2689
Cricotopus|[17021]|CNPAL178-13|Chironomidae|664|BOLD:ACH4204
Cricotopus|[17022]|CNTMC678-14|Chironomidae|537|BOLD:ACM4252
Cricotopus polaris|[17023]|CNTUB2123-14|Chironomidae|597|BOLD:ABA3997
Cricotopus polaris|[17024]|CNWAD2257-14|Chironomidae|622|BOLD:ACR9183
Orthocladius|[17025]|CNQUE101-14|Chironomidae|591|BOLD:ACI9309
Orthocladius|[17026]|CNWLH145-12|Chironomidae|658|BOLD:ACD2908
Orthocladius|[17027]|CNTUB677-14|Chironomidae|633|BOLD:ACR4585
Orthocladius|[17028]|CNFNC1086-14|Chironomidae|661|BOLD:ACF8146
Chironomidae|[17029]|SMTPB10974-13|Chironomidae|664|BOLD:ACF8864
Orthocladius|[17030]|CNGBK322-14|Chironomidae|659|BOLD:ACL0132
Orthocladius|[17031]|CNSRB079-14|Chironomidae|567|BOLD:ACR5460
Orthocladius|[17032]|DARC365-11|Chironomidae|658|BOLD:AAV5906
Orthocladius|[17033]|CNQUH1153-14|Chironomidae|600|BOLD:ACR0041
Chironomidae|[17034]|CNTGA1055-15|Chironomidae|588|BOLD:ACS1331
Orthocladius|[17035]|CNTMB1535-14|Chironomidae|647|BOLD:ACM1276
Orthocladius|[17036]|DARC614-11|Chironomidae|658|BOLD:AAU6746
Orthocladius|[17037]|CNTMB824-14|Chironomidae|627|BOLD:ACL4182
Orthocladius|[17038]|CNAUY443-14|Chironomidae|618|BOLD:ACR4180
Orthocladius|[17039]|CNFDA146-14|Chironomidae|585|BOLD:ACK1991
Orthocladius|[17040]|CNTMB892-14|Chironomidae|685|BOLD:ACL5919
Orthocladius|[17041]|CNTMA1496-14|Chironomidae|643|BOLD:ACM1518
Orthocladius|[17042]|CNTMC691-14|Chironomidae|591|BOLD:ACM4459
Orthocladius olivaceus|[17043]|CNQUF265-14|Chironomidae|633|BOLD:AAI3491
Orthocladius|[17044]|CNWLI216-12|Chironomidae|658|BOLD:ACD2959
Orthocladiinae|[17045]|CNIVF112-14|Chironomidae|591|BOLD:ACR3913
Orthocladius sp. 8TE|[17046]|CNTGC1361-15|Chironomidae|636|BOLD:AAI3488
Orthocladius|[17047]|CNTGC1656-15|Chironomidae|629|BOLD:ACM4684
Orthocladius|[17048]|CNTGC1712-15|Chironomidae|637|BOLD:ACS9377
Orthocladius|[17049]|SSBAF2517-13|Chironomidae|658|BOLD:ACI6576
Orthocladius|[17050]|SSBAF3163-13|Chironomidae|611|BOLD:ACI6688
Orthocladius subletteorum|[17051]|CHURC1068-09|Chironomidae|658|BOLD:AAF0488
Orthocladius subletteorum|[17052]|DARC121-10|Chironomidae|658|BOLD:AAD4028
Orthocladiinae|[17053]|CNNHA2674-14|Chironomidae|558|BOLD:ACR5549
Orthocladius|[17054]|SSBAC1383-12|Chironomidae|658|BOLD:ACC2903
Orthocladius subletteorum|[17055]|CNWAE6679-14|Chironomidae|588|BOLD:AAF0487
Orthocladius sp. 9TE|[17056]|JWDCE845-10|Chironomidae|658|BOLD:AAD4029
Orthocladius|[17057]|CNSRB116-14|Chironomidae|588|BOLD:ACR4411
Orthocladius|[17058]|NGNAA685-13|Chironomidae|658|BOLD:ACI3498
Orthocladiinae|[17059]|CNQUH093-14|Chironomidae|546|BOLD:ACR1318
Orthocladiinae|[17060]|CNNHA2935-14|Chironomidae|618|BOLD:ACR4885
Orthocladius|[17061]|CNTGD1483-15|Chironomidae|591|BOLD:ACM2099
Orthocladius|[17062]|CNTMA496-14|Chironomidae|658|BOLD:ACL8851
Orthocladiinae|[17063]|CNQUF314-14|Chironomidae|606|BOLD:ACR0297
Orthocladius sp. TE05|[17064]|CHURC658-09|Chironomidae|645|BOLD:AAC0068
Orthocladius|[17065]|NGAAA244-14|Chironomidae|555|BOLD:ACN3344
Orthocladius|[17066]|SSBAA570-12|Chironomidae|661|BOLD:ACC3819
Chironomidae|[17067]|CNTGF111-15|Chironomidae|588|BOLD:ACS2034
Orthocladius sp. 2TE|[17068]|CNIVD893-14|Chironomidae|603|BOLD:AAG1017
Orthocladiinae|[17069]|CNAUY400-14|Chironomidae|615|BOLD:ACR4484
Chironomidae|[17070]|CNKLD372-14|Chironomidae|633|BOLD:ACS2796
Orthocladius|[17071]|CNQUG962-14|Chironomidae|627|BOLD:AAV5076
Chironomidae|[17072]|CNYOH111-15|Chironomidae|561|BOLD:ACT1767
Chironomidae|[17073]|CNRVE916-15|Chironomidae|588|BOLD:ACT5920
Orthocladius|[17074]|CNRVF399-15|Chironomidae|585|BOLD:ACT5717
Cricotopus trifascia|[17075]|JSMAY572-11|Chironomidae|658|BOLD:AAG1004
Cricotopus trifascia|[17076]|SMTPD5116-13|Chironomidae|664|BOLD:ACT0257
Cricotopus trifascia|[17077]|SSROB3315-14|Chironomidae|592|BOLD:ACS9429
Parakiefferiella bathophila|[17078]|CHURC945-09|Chironomidae|658|BOLD:AAE6766
Chironomidae|[17079]|NCCA1583-11|Chironomidae|673|BOLD:ABA6503
Chironomidae|[17080]|CNTUA2490-14|Chironomidae|609|BOLD:ACR4080
Cricotopus|[17081]|CNKOF1185-14|Chironomidae|658|BOLD:ABA4431
Chironomidae|[17082]|NGNAK077-13|Chironomidae|658|BOLD:ACJ0665
Cricotopus|[17083]|SMTPD4159-13|Chironomidae|664|BOLD:ACJ6480
Cricotopus annulator cmplx|[17084]|SMTPB12145-13|Chironomidae|661|BOLD:ABY9141
Cricotopus|[17085]|RBINA1371-13|Chironomidae|657|BOLD:ACI9746
Cricotopus annulator cmplx|[17086]|SMTPB1245-13|Chironomidae|658|BOLD:AAG1002
Cricotopus|[17087]|SMTPI5068-14|Chironomidae|576|BOLD:ACP8284
Cricotopus|[17088]|CNFNE1666-14|Chironomidae|661|BOLD:ACL4290
Chironomidae|[17089]|CNYOF061-15|Chironomidae|585|BOLD:ACT1331
Cricotopus|[17090]|CNGMD036-14|Chironomidae|658|BOLD:ACL2832
Chironomidae|[17091]|CNYOH462-15|Chironomidae|579|BOLD:ACT5182
Chironomidae|[17092]|BBDCM081-10|Chironomidae|658|BOLD:AAL7347
Cricotopus tremulus|[17093]|SMTPB12116-13|Chironomidae|661|BOLD:AAE4298
Cricotopus|[17094]|SMTPB14637-13|Chironomidae|670|BOLD:ACF9756
Orthocladiinae|[17095]|CNGMC2337-13|Chironomidae|658|BOLD:ACL2822
Cricotopus sp. 21ES|[17096]|BSCHR156-10|Chironomidae|658|BOLD:AAA5306
Cricotopus triannulatus|[17097]|CNQUG442-14|Chironomidae|609|BOLD:AAL9618

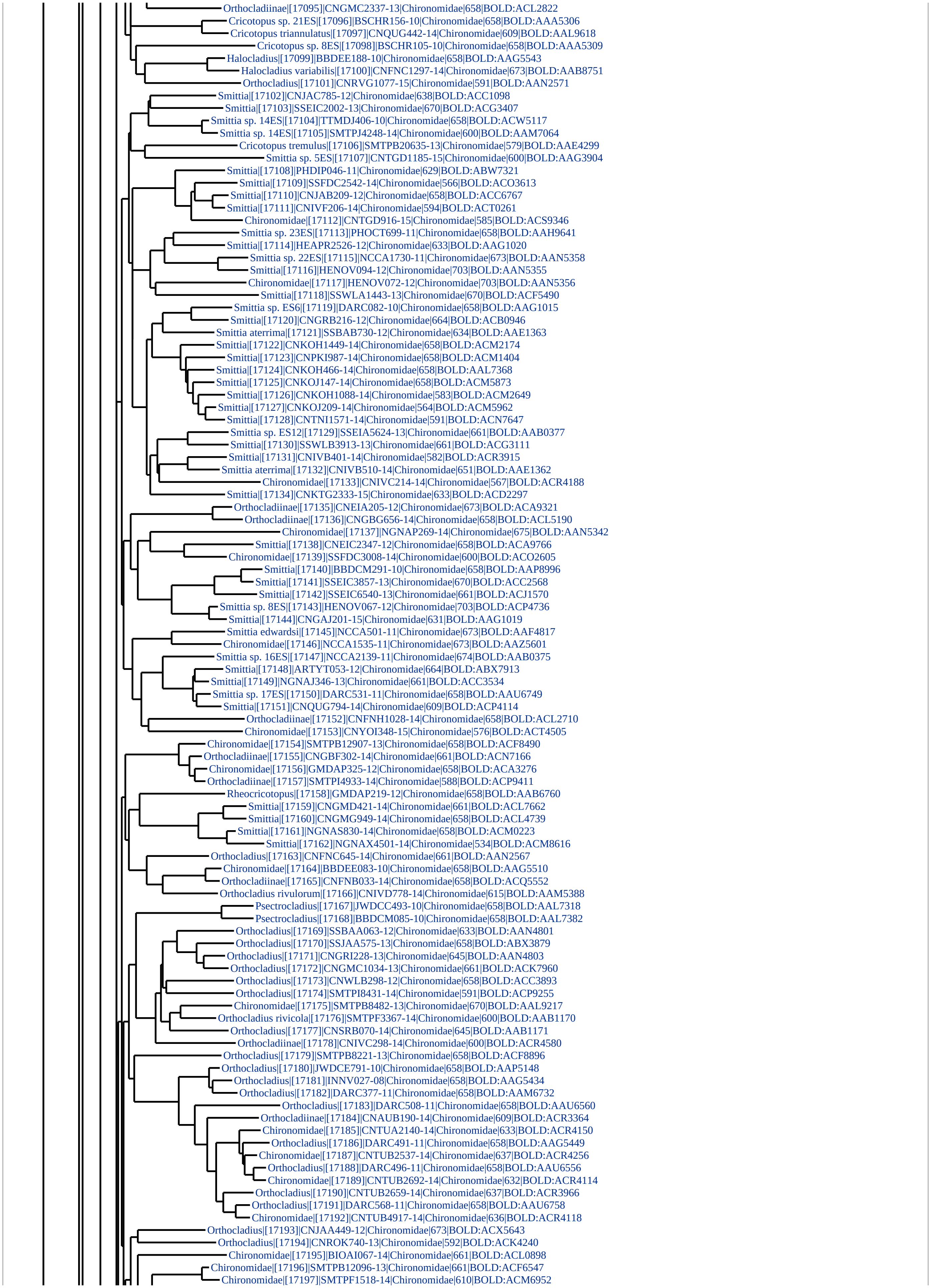
Orthocladiinae|[17095]|CNGMC2337-13|Chironomidae|658|BOLD:ACL2822
Cricotopus sp. 21ES|[17096]|BSCHR156-10|Chironomidae|658|BOLD:AAA5306
Cricotopus triannulatus|[17097]|CNQUG442-14|Chironomidae|609|BOLD:AAL9618
Cricotopus sp. 8ES|[17098]|BSCHR105-10|Chironomidae|658|BOLD:AAA5309
Halocladius|[17099]|BBDEE188-10|Chironomidae|658|BOLD:AAG5543
Halocladius variabilis|[17100]|CNFNC1297-14|Chironomidae|673|BOLD:AAB8751
Orthocladius|[17101]|CNRVG1077-15|Chironomidae|591|BOLD:AAN2571
Smittia|[17102]|CNJAC785-12|Chironomidae|638|BOLD:ACC1098
Smittia|[17103]|SSEIC2002-13|Chironomidae|670|BOLD:ACG3407
Smittia sp. 14ES|[17104]|TTMDJ406-10|Chironomidae|658|BOLD:ACW5117
Smittia sp. 14ES|[17105]|SMTPJ4248-14|Chironomidae|600|BOLD:AAM7064
Cricotopus tremulus|[17106]|SMTPB20635-13|Chironomidae|579|BOLD:AAE4299
Smittia sp. 5ES|[17107]|CNTGD1185-15|Chironomidae|600|BOLD:AAG3904
Smittia|[17108]|PHDIP046-11|Chironomidae|629|BOLD:ABW7321
Smittia|[17109]|SSFDC2542-14|Chironomidae|566|BOLD:ACO3613
Smittia|[17110]|CNJAB209-12|Chironomidae|658|BOLD:ACC6767
Smittia|[17111]|CNIVF206-14|Chironomidae|594|BOLD:ACT0261
Chironomidae|[17112]|CNTGD916-15|Chironomidae|585|BOLD:ACS9346
Smittia sp. 23ES|[17113]|PHOCT699-11|Chironomidae|658|BOLD:AAH9641
Smittia|[17114]|HEAPR2526-12|Chironomidae|633|BOLD:AAG1020
Smittia sp. 22ES|[17115]|NCCA1730-11|Chironomidae|673|BOLD:AAN5358
Smittia|[17116]|HENOV094-12|Chironomidae|703|BOLD:AAN5355
Chironomidae|[17117]|HENOV072-12|Chironomidae|703|BOLD:AAN5356
Smittia|[17118]|SSWLA1443-13|Chironomidae|670|BOLD:ACF5490
Smittia sp. ES6|[17119]|DARC082-10|Chironomidae|658|BOLD:AAG1015
Smittia|[17120]|CNGRB216-12|Chironomidae|664|BOLD:ACB0946
Smittia aterrima|[17121]|SSBAB730-12|Chironomidae|634|BOLD:AAE1363
Smittia|[17122]|CNKOH1449-14|Chironomidae|658|BOLD:ACM2174
Smittia|[17123]|CNPKI987-14|Chironomidae|658|BOLD:ACM1404
Smittia|[17124]|CNKOH466-14|Chironomidae|658|BOLD:AAL7368
Smittia|[17125]|CNKOJ147-14|Chironomidae|658|BOLD:ACM5873
Smittia|[17126]|CNKOH1088-14|Chironomidae|583|BOLD:ACM2649
Smittia|[17127]|CNKOJ209-14|Chironomidae|564|BOLD:ACM5962
Smittia|[17128]|CNTNI1571-14|Chironomidae|591|BOLD:ACN7647
Smittia sp. ES12|[17129]|SSEIA5624-13|Chironomidae|661|BOLD:AAB0377
Smittia|[17130]|SSWLB3913-13|Chironomidae|661|BOLD:ACG3111
Smittia|[17131]|CNIVB401-14|Chironomidae|582|BOLD:ACR3915
Smittia aterrima|[17132]|CNIVB510-14|Chironomidae|651|BOLD:AAE1362
Chironomidae|[17133]|CNIVC214-14|Chironomidae|567|BOLD:ACR4188
Smittia|[17134]|CNKTG2333-15|Chironomidae|633|BOLD:ACD2297
Orthocladiinae|[17135]|CNEIA205-12|Chironomidae|673|BOLD:ACA9321
Orthocladiinae|[17136]|CNGBG656-14|Chironomidae|658|BOLD:ACL5190
Chironomidae|[17137]|NGNAP269-14|Chironomidae|675|BOLD:AAN5342
Smittia|[17138]|CNEIC2347-12|Chironomidae|658|BOLD:ACA9766
Chironomidae|[17139]|SSFDC3008-14|Chironomidae|600|BOLD:ACO2605
Smittia|[17140]|BBDCM291-10|Chironomidae|658|BOLD:AAP8996
Smittia|[17141]|SSEIC3857-13|Chironomidae|670|BOLD:ACC2568
Smittia|[17142]|SSEIC6540-13|Chironomidae|661|BOLD:ACJ1570
Smittia sp. 8ES|[17143]|HENOV067-12|Chironomidae|703|BOLD:ACP4736
Smittia|[17144]|CNGAJ201-15|Chironomidae|631|BOLD:AAG1019
Smittia edwardsi|[17145]|NCCA501-11|Chironomidae|673|BOLD:AAF4817
Chironomidae|[17146]|NCCA1535-11|Chironomidae|673|BOLD:AAZ5601
Smittia sp. 16ES|[17147]|NCCA2139-11|Chironomidae|674|BOLD:AAB0375
Smittia|[17148]|ARTYT053-12|Chironomidae|664|BOLD:ABX7913
Smittia|[17149]|NGNAJ346-13|Chironomidae|661|BOLD:ACC3534
Smittia sp. 17ES|[17150]|DARC531-11|Chironomidae|658|BOLD:AAU6749
Smittia|[17151]|CNQUG794-14|Chironomidae|609|BOLD:ACP4114
Orthocladiinae|[17152]|CNFNH1028-14|Chironomidae|658|BOLD:ACL2710
Chironomidae|[17153]|CNYOI348-15|Chironomidae|576|BOLD:ACT4505
Chironomidae|[17154]|SMTPB12907-13|Chironomidae|658|BOLD:ACF8490
Orthocladiinae|[17155]|CNGBF302-14|Chironomidae|661|BOLD:ACN7166
Chironomidae|[17156]|GMDAP325-12|Chironomidae|658|BOLD:ACA3276
Orthocladiinae|[17157]|SMTPI4933-14|Chironomidae|588|BOLD:ACP9411
Rheocricotopus|[17158]|GMDAP219-12|Chironomidae|658|BOLD:AAB6760
Smittia|[17159]|CNGMD421-14|Chironomidae|661|BOLD:ACL7662
Smittia|[17160]|CNGMG949-14|Chironomidae|658|BOLD:ACL4739
Smittia|[17161]|NGNAS830-14|Chironomidae|658|BOLD:ACM0223
Smittia|[17162]|NGNAX4501-14|Chironomidae|534|BOLD:ACM8616
Orthocladius|[17163]|CNFNC645-14|Chironomidae|661|BOLD:AAN2567
Chironomidae|[17164]|BBDEE083-10|Chironomidae|658|BOLD:AAG5510
Orthocladiinae|[17165]|CNFNB033-14|Chironomidae|658|BOLD:ACQ5552
Orthocladius rivulorum|[17166]|CNIVD778-14|Chironomidae|615|BOLD:AAM5388
Psectrocladius|[17167]|JWDCC493-10|Chironomidae|658|BOLD:AAL7318
Psectrocladius|[17168]|BBDCM085-10|Chironomidae|658|BOLD:AAL7382
Orthocladius|[17169]|SSBAA063-12|Chironomidae|633|BOLD:AAN4801
Orthocladius|[17170]|SSJAA575-13|Chironomidae|658|BOLD:ABX3879
Orthocladius|[17171]|CNGRI228-13|Chironomidae|645|BOLD:AAN4803
Orthocladius|[17172]|CNGMC1034-13|Chironomidae|661|BOLD:ACK7960
Orthocladius|[17173]|CNWLB298-12|Chironomidae|658|BOLD:ACC3893
Orthocladius|[17174]|SMTPI8431-14|Chironomidae|591|BOLD:ACP9255
Chironomidae|[17175]|SMTPB8482-13|Chironomidae|670|BOLD:AAL9217
Orthocladius rivicola|[17176]|SMTPF3367-14|Chironomidae|600|BOLD:AAB1170
Orthocladius|[17177]|CNSRB070-14|Chironomidae|645|BOLD:AAB1171
Orthocladiinae|[17178]|CNIVC298-14|Chironomidae|600|BOLD:ACR4580
Orthocladius|[17179]|SMTPB8221-13|Chironomidae|658|BOLD:ACF8896
Orthocladius|[17180]|JWDCE791-10|Chironomidae|658|BOLD:AAP5148
Orthocladius|[17181]|INNV027-08|Chironomidae|658|BOLD:AAG5434
Orthocladius|[17182]|DARC377-11|Chironomidae|658|BOLD:AAM6732
Orthocladius|[17183]|DARC508-11|Chironomidae|658|BOLD:AAU6560
Orthocladiinae|[17184]|CNAUB190-14|Chironomidae|609|BOLD:ACR3364
Chironomidae|[17185]|CNTUA2140-14|Chironomidae|633|BOLD:ACR4150
Orthocladius|[17186]|DARC491-11|Chironomidae|658|BOLD:AAG5449
Chironomidae|[17187]|CNTUB2537-14|Chironomidae|637|BOLD:ACR4256
Orthocladius|[17188]|DARC496-11|Chironomidae|658|BOLD:AAU6556
Chironomidae|[17189]|CNTUB2692-14|Chironomidae|632|BOLD:ACR4114
Orthocladius|[17190]|CNTUB2659-14|Chironomidae|637|BOLD:ACR3966
Orthocladius|[17191]|DARC568-11|Chironomidae|658|BOLD:AAU6758
Chironomidae|[17192]|CNTUB4917-14|Chironomidae|636|BOLD:ACR4118
Orthocladius|[17193]|CNJAA449-12|Chironomidae|673|BOLD:ACX5643
Orthocladius|[17194]|CNROK740-13|Chironomidae|592|BOLD:ACK4240
Chironomidae|[17195]|BIOAI067-14|Chironomidae|661|BOLD:ACL0898
Chironomidae|[17196]|SMTPB12096-13|Chironomidae|661|BOLD:ACF6547
Chironomidae|[17197]|SMTPF1518-14|Chironomidae|610|BOLD:ACM6952

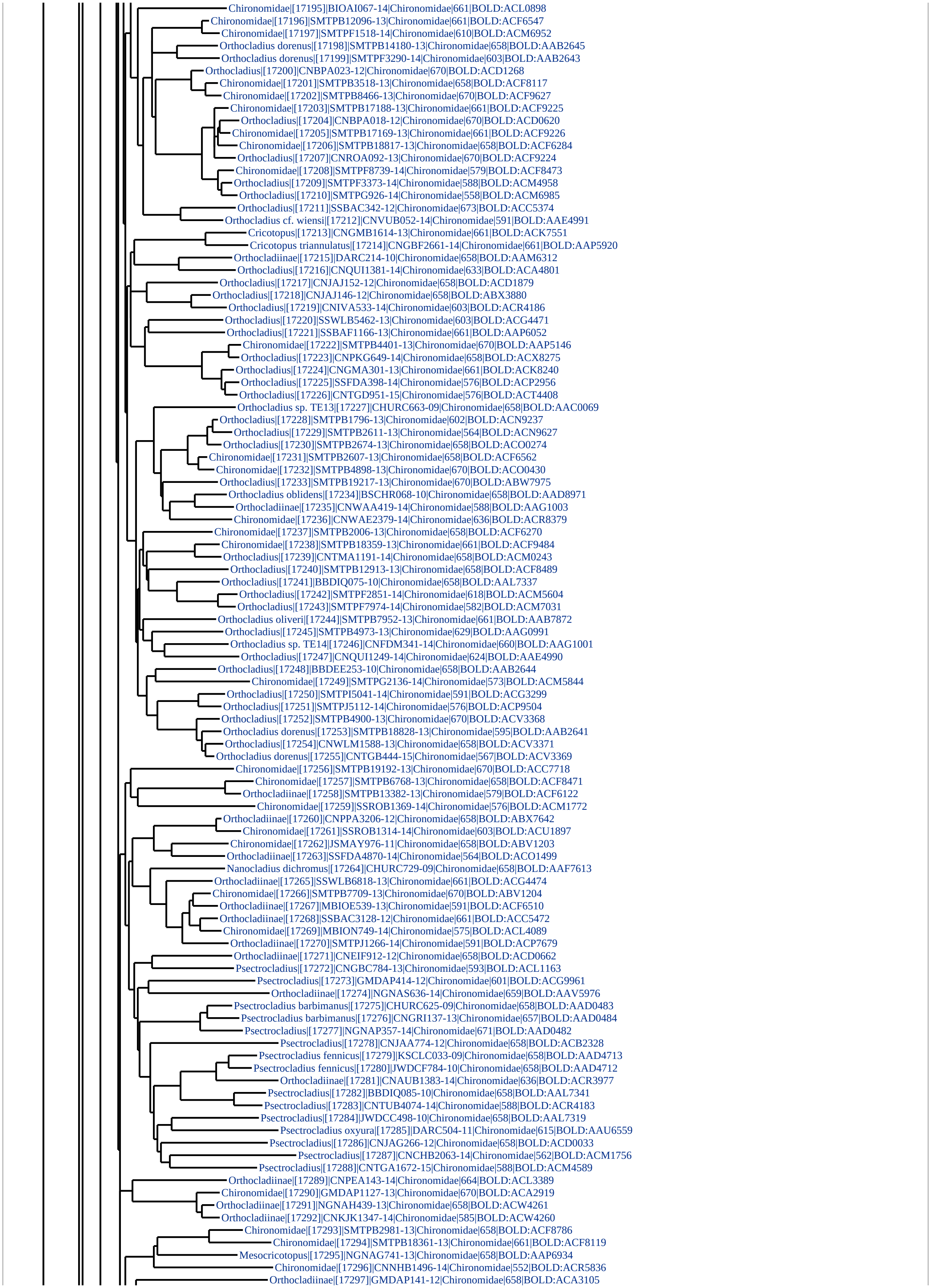
Chironomidae|[17195]|BIOAI067-14|Chironomidae|661|BOLD:ACL0898
Chironomidae|[17196]|SMTPB12096-13|Chironomidae|661|BOLD:ACF6547
Chironomidae|[17197]|SMTPF1518-14|Chironomidae|610|BOLD:ACM6952
Orthocladius dorenus|[17198]|SMTPB14180-13|Chironomidae|658|BOLD:AAB2645
Orthocladius dorenus|[17199]|SMTPF3290-14|Chironomidae|603|BOLD:AAB2643
Orthocladius|[17200]|CNBPA023-12|Chironomidae|670|BOLD:ACD1268
Chironomidae|[17201]|SMTPB3518-13|Chironomidae|658|BOLD:ACF8117
Chironomidae|[17202]|SMTPB8466-13|Chironomidae|670|BOLD:ACF9627
Chironomidae|[17203]|SMTPB17188-13|Chironomidae|661|BOLD:ACF9225
Orthocladius|[17204]|CNBPA018-12|Chironomidae|670|BOLD:ACD0620
Chironomidae|[17205]|SMTPB17169-13|Chironomidae|661|BOLD:ACF9226
Chironomidae|[17206]|SMTPB18817-13|Chironomidae|658|BOLD:ACF6284
Orthocladius|[17207]|CNROA092-13|Chironomidae|670|BOLD:ACF9224
Chironomidae|[17208]|SMTPF8739-14|Chironomidae|579|BOLD:ACF8473
Orthocladius|[17209]|SMTPF3373-14|Chironomidae|588|BOLD:ACM4958
Orthocladius|[17210]|SMTPG926-14|Chironomidae|558|BOLD:ACM6985
Orthocladius|[17211]|SSBAC342-12|Chironomidae|673|BOLD:ACC5374
Orthocladius cf. wiensi|[17212]|CNVUB052-14|Chironomidae|591|BOLD:AAE4991
Cricotopus|[17213]|CNGMB1614-13|Chironomidae|661|BOLD:ACK7551
Cricotopus triannulatus|[17214]|CNGBF2661-14|Chironomidae|661|BOLD:AAP5920
Orthocladiinae|[17215]|DARC214-10|Chironomidae|658|BOLD:AAM6312
Orthocladius|[17216]|CNQUI1381-14|Chironomidae|633|BOLD:ACA4801
Orthocladius|[17217]|CNJAJ152-12|Chironomidae|658|BOLD:ACD1879
Orthocladius|[17218]|CNJAJ146-12|Chironomidae|658|BOLD:ABX3880
Orthocladius|[17219]|CNIVA533-14|Chironomidae|603|BOLD:ACR4186
Orthocladius|[17220]|SSWLB5462-13|Chironomidae|603|BOLD:ACG4471
Orthocladius|[17221]|SSBAF1166-13|Chironomidae|661|BOLD:AAP6052
Chironomidae|[17222]|SMTPB4401-13|Chironomidae|670|BOLD:AAP5146
Orthocladius|[17223]|CNPKG649-14|Chironomidae|658|BOLD:ACX8275
Orthocladius|[17224]|CNGMA301-13|Chironomidae|661|BOLD:ACK8240
Orthocladius|[17225]|SSFDA398-14|Chironomidae|576|BOLD:ACP2956
Orthocladius|[17226]|CNTGD951-15|Chironomidae|576|BOLD:ACT4408
Orthocladius sp. TE13|[17227]|CHURC663-09|Chironomidae|658|BOLD:AAC0069
Orthocladius|[17228]|SMTPB1796-13|Chironomidae|602|BOLD:ACN9237
Orthocladius|[17229]|SMTPB2611-13|Chironomidae|564|BOLD:ACN9627
Orthocladius|[17230]|SMTPB2674-13|Chironomidae|658|BOLD:ACO0274
Chironomidae|[17231]|SMTPB2607-13|Chironomidae|658|BOLD:ACF6562
Chironomidae|[17232]|SMTPB4898-13|Chironomidae|670|BOLD:ACO0430
Orthocladius|[17233]|SMTPB19217-13|Chironomidae|670|BOLD:ABW7975
Orthocladius oblidens|[17234]|BSCHR068-10|Chironomidae|658|BOLD:AAD8971
Orthocladiinae|[17235]|CNWAA419-14|Chironomidae|588|BOLD:AAG1003
Chironomidae|[17236]|CNWAE2379-14|Chironomidae|636|BOLD:ACR8379
Chironomidae|[17237]|SMTPB2006-13|Chironomidae|658|BOLD:ACF6270
Chironomidae|[17238]|SMTPB18359-13|Chironomidae|661|BOLD:ACF9484
Orthocladius|[17239]|CNTMA1191-14|Chironomidae|658|BOLD:ACM0243
Orthocladius|[17240]|SMTPB12913-13|Chironomidae|658|BOLD:ACF8489
Orthocladius|[17241]|BBDIQ075-10|Chironomidae|658|BOLD:AAL7337
Orthocladius|[17242]|SMTPF2851-14|Chironomidae|618|BOLD:ACM5604
Orthocladius|[17243]|SMTPF7974-14|Chironomidae|582|BOLD:ACM7031
Orthocladius oliveri|[17244]|SMTPB7952-13|Chironomidae|661|BOLD:AAB7872
Orthocladius|[17245]|SMTPB4973-13|Chironomidae|629|BOLD:AAG0991
Orthocladius sp. TE14|[17246]|CNFDM341-14|Chironomidae|660|BOLD:AAG1001
Orthocladius|[17247]|CNQUI1249-14|Chironomidae|624|BOLD:AAE4990
Orthocladius|[17248]|BBDEE253-10|Chironomidae|658|BOLD:AAB2644
Chironomidae|[17249]|SMTPG2136-14|Chironomidae|573|BOLD:ACM5844
Orthocladius|[17250]|SMTPI5041-14|Chironomidae|591|BOLD:ACG3299
Orthocladius|[17251]|SMTPJ5112-14|Chironomidae|576|BOLD:ACP9504
Orthocladius|[17252]|SMTPB4900-13|Chironomidae|670|BOLD:ACV3368
Orthocladius dorenus|[17253]|SMTPB18828-13|Chironomidae|595|BOLD:AAB2641
Orthocladius|[17254]|CNWLM1588-13|Chironomidae|658|BOLD:ACV3371
Orthocladius dorenus|[17255]|CNTGB444-15|Chironomidae|567|BOLD:ACV3369
Chironomidae|[17256]|SMTPB19192-13|Chironomidae|670|BOLD:ACC7718
Chironomidae|[17257]|SMTPB6768-13|Chironomidae|658|BOLD:ACF8471
Orthocladiinae|[17258]|SMTPB13382-13|Chironomidae|579|BOLD:ACF6122
Chironomidae|[17259]|SSROB1369-14|Chironomidae|576|BOLD:ACM1772
Orthocladiinae|[17260]|CNPPA3206-12|Chironomidae|658|BOLD:ABX7642
Chironomidae|[17261]|SSROB1314-14|Chironomidae|603|BOLD:ACU1897
Chironomidae|[17262]|JSMAY976-11|Chironomidae|658|BOLD:ABV1203
Orthocladiinae|[17263]|SSFDA4870-14|Chironomidae|564|BOLD:ACO1499
Nanocladius dichromus|[17264]|CHURC729-09|Chironomidae|658|BOLD:AAF7613
Orthocladiinae|[17265]|SSWLB6818-13|Chironomidae|661|BOLD:ACG4474
Chironomidae|[17266]|SMTPB7709-13|Chironomidae|670|BOLD:ABV1204
Orthocladiinae|[17267]|MBIOE539-13|Chironomidae|591|BOLD:ACF6510
Orthocladiinae|[17268]|SSBAC3128-12|Chironomidae|661|BOLD:ACC5472
Chironomidae|[17269]|MBION749-14|Chironomidae|575|BOLD:ACL4089
Orthocladiinae|[17270]|SMTPJ1266-14|Chironomidae|591|BOLD:ACP7679
Orthocladiinae|[17271]|CNEIF912-12|Chironomidae|658|BOLD:ACD0662
Psectrocladius|[17272]|CNGBC784-13|Chironomidae|593|BOLD:ACL1163
Psectrocladius|[17273]|GMDAP414-12|Chironomidae|601|BOLD:ACG9961
Orthocladiinae|[17274]|NGNAS636-14|Chironomidae|659|BOLD:AAV5976
Psectrocladius barbimanus|[17275]|CHURC625-09|Chironomidae|658|BOLD:AAD0483
Psectrocladius barbimanus|[17276]|CNGRI137-13|Chironomidae|657|BOLD:AAD0484
Psectrocladius|[17277]|NGNAP357-14|Chironomidae|671|BOLD:AAD0482
Psectrocladius|[17278]|CNJAA774-12|Chironomidae|658|BOLD:ACB2328
Psectrocladius fennicus|[17279]|KSCLC033-09|Chironomidae|658|BOLD:AAD4713
Psectrocladius fennicus|[17280]|JWDCF784-10|Chironomidae|658|BOLD:AAD4712
Orthocladiinae|[17281]|CNAUB1383-14|Chironomidae|636|BOLD:ACR3977
Psectrocladius|[17282]|BBDIQ085-10|Chironomidae|658|BOLD:AAL7341
Psectrocladius|[17283]|CNTUB4074-14|Chironomidae|588|BOLD:ACR4183
Psectrocladius|[17284]|JWDCC498-10|Chironomidae|658|BOLD:AAL7319
Psectrocladius oxyura|[17285]|DARC504-11|Chironomidae|615|BOLD:AAU6559
Psectrocladius|[17286]|CNJAG266-12|Chironomidae|658|BOLD:ACD0033
Psectrocladius|[17287]|CNCHB2063-14|Chironomidae|562|BOLD:ACM1756
Psectrocladius|[17288]|CNTGA1672-15|Chironomidae|588|BOLD:ACM4589
Orthocladiinae|[17289]|CNPEA143-14|Chironomidae|664|BOLD:ACL3389
Chironomidae|[17290]|GMDAP1127-13|Chironomidae|670|BOLD:ACA2919
Orthocladiinae|[17291]|NGNAH439-13|Chironomidae|658|BOLD:ACW4261
Orthocladiinae|[17292]|CNKJK1347-14|Chironomidae|585|BOLD:ACW4260
Chironomidae|[17293]|SMTPB2981-13|Chironomidae|658|BOLD:ACF8786
Chironomidae|[17294]|SMTPB18361-13|Chironomidae|661|BOLD:ACF8119
Mesocricotopus|[17295]|NGNAG741-13|Chironomidae|658|BOLD:AAP6934
Chironomidae|[17296]|CNNHB1496-14|Chironomidae|552|BOLD:ACR5836
Orthocladiinae|[17297]|GMDAP141-12|Chironomidae|658|BOLD:ACA3105

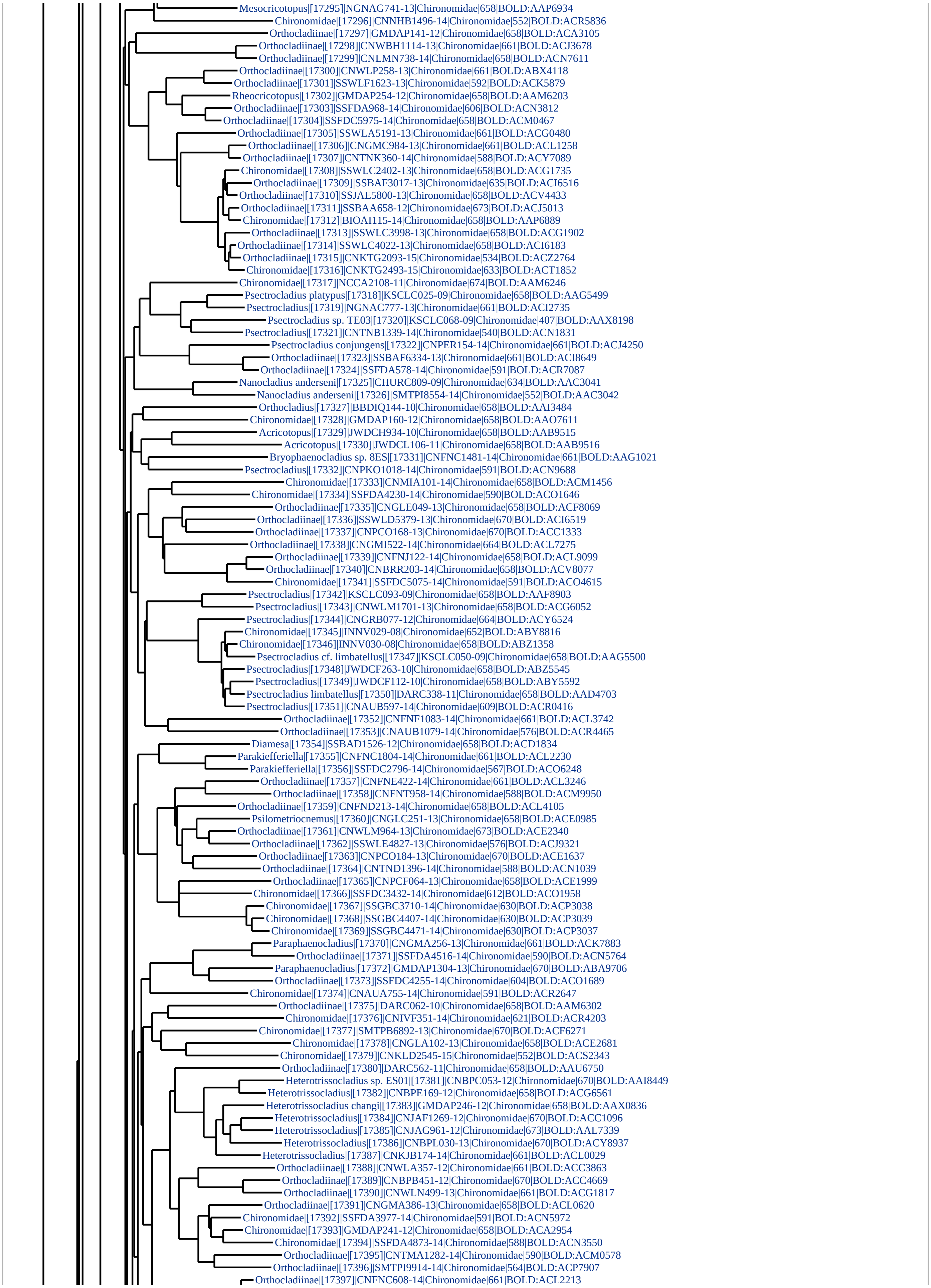
Mesocricotopus|[17295]|NGNAG741-13|Chironomidae|658|BOLD:AAP6934
Chironomidae|[17296]|CNNHB1496-14|Chironomidae|552|BOLD:ACR5836
Orthocladiinae|[17297]|GMDAP141-12|Chironomidae|658|BOLD:ACA3105
Orthocladiinae|[17298]|CNWBH1114-13|Chironomidae|661|BOLD:ACJ3678
Orthocladiinae|[17299]|CNLMN738-14|Chironomidae|658|BOLD:ACN7611
Orthocladiinae|[17300]|CNWLP258-13|Chironomidae|661|BOLD:ABX4118
Orthocladiinae|[17301]|SSWLF1623-13|Chironomidae|592|BOLD:ACK5879
Rheocricotopus|[17302]|GMDAP254-12|Chironomidae|658|BOLD:AAM6203
Orthocladiinae|[17303]|SSFDA968-14|Chironomidae|606|BOLD:ACN3812
Orthocladiinae|[17304]|SSFDC5975-14|Chironomidae|658|BOLD:ACM0467
Orthocladiinae|[17305]|SSWLA5191-13|Chironomidae|661|BOLD:ACG0480
Orthocladiinae|[17306]|CNGMC984-13|Chironomidae|661|BOLD:ACL1258
Orthocladiinae|[17307]|CNTNK360-14|Chironomidae|588|BOLD:ACY7089
Chironomidae|[17308]|SSWLC2402-13|Chironomidae|658|BOLD:ACG1735
Orthocladiinae|[17309]|SSBAF3017-13|Chironomidae|635|BOLD:ACI6516
Orthocladiinae|[17310]|SSJAE5800-13|Chironomidae|658|BOLD:ACV4433
Orthocladiinae|[17311]|SSBAA658-12|Chironomidae|673|BOLD:ACJ5013
Chironomidae|[17312]|BIOAI115-14|Chironomidae|658|BOLD:AAP6889
Orthocladiinae|[17313]|SSWLC3998-13|Chironomidae|658|BOLD:ACG1902
Orthocladiinae|[17314]|SSWLC4022-13|Chironomidae|658|BOLD:ACI6183
Orthocladiinae|[17315]|CNKTG2093-15|Chironomidae|534|BOLD:ACZ2764
Chironomidae|[17316]|CNKTG2493-15|Chironomidae|633|BOLD:ACT1852
Chironomidae|[17317]|NCCA2108-11|Chironomidae|674|BOLD:AAM6246
Psectrocladius platypus|[17318]|KSCLC025-09|Chironomidae|658|BOLD:AAG5499
Psectrocladius|[17319]|NGNAC777-13|Chironomidae|661|BOLD:ACI2735
Psectrocladius sp. TE03|[17320]|KSCLC068-09|Chironomidae|407|BOLD:AAX8198
Psectrocladius|[17321]|CNTNB1339-14|Chironomidae|540|BOLD:ACN1831
Psectrocladius conjungens|[17322]|CNPER154-14|Chironomidae|661|BOLD:ACJ4250
Orthocladiinae|[17323]|SSBAF6334-13|Chironomidae|661|BOLD:ACI8649
Orthocladiinae|[17324]|SSFDA578-14|Chironomidae|591|BOLD:ACR7087
Nanocladius anderseni|[17325]|CHURC809-09|Chironomidae|634|BOLD:AAC3041
Nanocladius anderseni|[17326]|SMTPI8554-14|Chironomidae|552|BOLD:AAC3042
Orthocladius|[17327]|BBDIQ144-10|Chironomidae|658|BOLD:AAI3484
Chironomidae|[17328]|GMDAP160-12|Chironomidae|658|BOLD:AAO7611
Acricotopus|[17329]|JWDCH934-10|Chironomidae|658|BOLD:AAB9515
Acricotopus|[17330]|JWDCL106-11|Chironomidae|658|BOLD:AAB9516
Bryophaenocladius sp. 8ES|[17331]|CNFNC1481-14|Chironomidae|661|BOLD:AAG1021
Psectrocladius|[17332]|CNPKO1018-14|Chironomidae|591|BOLD:ACN9688
Chironomidae|[17333]|CNMIA101-14|Chironomidae|658|BOLD:ACM1456
Chironomidae|[17334]|SSFDA4230-14|Chironomidae|590|BOLD:ACO1646
Orthocladiinae|[17335]|CNGLE049-13|Chironomidae|658|BOLD:ACF8069
Orthocladiinae|[17336]|SSWLD5379-13|Chironomidae|670|BOLD:ACI6519
Orthocladiinae|[17337]|CNPCO168-13|Chironomidae|670|BOLD:ACC1333
Orthocladiinae|[17338]|CNGMI522-14|Chironomidae|664|BOLD:ACL7275
Orthocladiinae|[17339]|CNFNJ122-14|Chironomidae|658|BOLD:ACL9099
Orthocladiinae|[17340]|CNBRR203-14|Chironomidae|658|BOLD:ACV8077
Chironomidae|[17341]|SSFDC5075-14|Chironomidae|591|BOLD:ACO4615
Psectrocladius|[17342]|KSCLC093-09|Chironomidae|658|BOLD:AAF8903
Psectrocladius|[17343]|CNWLM1701-13|Chironomidae|658|BOLD:ACG6052
Psectrocladius|[17344]|CNGRB077-12|Chironomidae|664|BOLD:ACY6524
Chironomidae|[17345]|INNV029-08|Chironomidae|652|BOLD:ABY8816
Chironomidae|[17346]|INNV030-08|Chironomidae|658|BOLD:ABZ1358
Psectrocladius cf. limbatellus|[17347]|KSCLC050-09|Chironomidae|658|BOLD:AAG5500
Psectrocladius|[17348]|JWDCF263-10|Chironomidae|658|BOLD:ABZ5545
Psectrocladius|[17349]|JWDCF112-10|Chironomidae|658|BOLD:ABY5592
Psectrocladius limbatellus|[17350]|DARC338-11|Chironomidae|658|BOLD:AAD4703
Psectrocladius|[17351]|CNAUB597-14|Chironomidae|609|BOLD:ACR0416
Orthocladiinae|[17352]|CNFNF1083-14|Chironomidae|661|BOLD:ACL3742
Orthocladiinae|[17353]|CNAUB1079-14|Chironomidae|576|BOLD:ACR4465
Diamesa|[17354]|SSBAD1526-12|Chironomidae|658|BOLD:ACD1834
Parakiefferiella|[17355]|CNFNC1804-14|Chironomidae|661|BOLD:ACL2230
Parakiefferiella|[17356]|SSFDC2796-14|Chironomidae|567|BOLD:ACO6248
Orthocladiinae|[17357]|CNFNE422-14|Chironomidae|661|BOLD:ACL3246
Orthocladiinae|[17358]|CNFNT958-14|Chironomidae|588|BOLD:ACM9950
Orthocladiinae|[17359]|CNFND213-14|Chironomidae|658|BOLD:ACL4105
Psilometriocnemus|[17360]|CNGLC251-13|Chironomidae|658|BOLD:ACE0985
Orthocladiinae|[17361]|CNWLM964-13|Chironomidae|673|BOLD:ACE2340
Orthocladiinae|[17362]|SSWLE4827-13|Chironomidae|576|BOLD:ACJ9321
Orthocladiinae|[17363]|CNPCO184-13|Chironomidae|670|BOLD:ACE1637
Orthocladiinae|[17364]|CNTND1396-14|Chironomidae|588|BOLD:ACN1039
Orthocladiinae|[17365]|CNPCF064-13|Chironomidae|658|BOLD:ACE1999
Chironomidae|[17366]|SSFDC3432-14|Chironomidae|612|BOLD:ACO1958
Chironomidae|[17367]|SSGBC3710-14|Chironomidae|630|BOLD:ACP3038
Chironomidae|[17368]|SSGBC4407-14|Chironomidae|630|BOLD:ACP3039
Chironomidae|[17369]|SSGBC4471-14|Chironomidae|630|BOLD:ACP3037
Paraphaenocladius|[17370]|CNGMA256-13|Chironomidae|661|BOLD:ACK7883
Orthocladiinae|[17371]|SSFDA4516-14|Chironomidae|590|BOLD:ACN5764
Paraphaenocladius|[17372]|GMDAP1304-13|Chironomidae|670|BOLD:ABA9706
Orthocladiinae|[17373]|SSFDC4255-14|Chironomidae|604|BOLD:ACO1689
Chironomidae|[17374]|CNAUA755-14|Chironomidae|591|BOLD:ACR2647
Orthocladiinae|[17375]|DARC062-10|Chironomidae|658|BOLD:AAM6302
Chironomidae|[17376]|CNIVF351-14|Chironomidae|621|BOLD:ACR4203
Chironomidae|[17377]|SMTPB6892-13|Chironomidae|670|BOLD:ACF6271
Chironomidae|[17378]|CNGLA102-13|Chironomidae|658|BOLD:ACE2681
Chironomidae|[17379]|CNKLD2545-15|Chironomidae|552|BOLD:ACS2343
Orthocladiinae|[17380]|DARC562-11|Chironomidae|658|BOLD:AAU6750
Heterotrissocladius sp. ES01|[17381]|CNBPC053-12|Chironomidae|670|BOLD:AAI8449
Heterotrissocladius|[17382]|CNBPE169-12|Chironomidae|658|BOLD:ACG6561
Heterotrissocladius changi|[17383]|GMDAP246-12|Chironomidae|658|BOLD:AAX0836
Heterotrissocladius|[17384]|CNJAF1269-12|Chironomidae|670|BOLD:ACC1096
Heterotrissocladius|[17385]|CNJAG961-12|Chironomidae|673|BOLD:AAL7339
Heterotrissocladius|[17386]|CNBPL030-13|Chironomidae|670|BOLD:ACY8937
Heterotrissocladius|[17387]|CNKJB174-14|Chironomidae|661|BOLD:ACL0029
Orthocladiinae|[17388]|CNWLA357-12|Chironomidae|661|BOLD:ACC3863
Orthocladiinae|[17389]|CNBPB451-12|Chironomidae|670|BOLD:ACC4669
Orthocladiinae|[17390]|CNWLN499-13|Chironomidae|661|BOLD:ACG1817
Orthocladiinae|[17391]|CNGMA386-13|Chironomidae|658|BOLD:ACL0620
Chironomidae|[17392]|SSFDA3977-14|Chironomidae|591|BOLD:ACN5972
Chironomidae|[17393]|GMDAP241-12|Chironomidae|658|BOLD:ACA2954
Chironomidae|[17394]|SSFDA4873-14|Chironomidae|588|BOLD:ACN3550
Orthocladiinae|[17395]|CNTMA1282-14|Chironomidae|590|BOLD:ACM0578
Orthocladiinae|[17396]|SMTPI9914-14|Chironomidae|564|BOLD:ACP7907
Orthocladiinae|[17397]|CNFNC608-14|Chironomidae|661|BOLD:ACL2213

Orthocladiinae|[17395]|CNTMA1282-14|Chironomidae|590|BOLD:ACM0578
Orthocladiinae|[17396]|SMTPI9914-14|Chironomidae|564|BOLD:ACP7907
Orthocladiinae|[17397]|CNFNC608-14|Chironomidae|661|BOLD:ACL2213
Orthocladiinae|[17398]|CNFNP454-14|Chironomidae|661|BOLD:ACO4611
Chironomidae|[17399]|BIOAI070-14|Chironomidae|603|BOLD:ACT1281
Chironomidae|[17400]|CNTGA2128-15|Chironomidae|579|BOLD:ACS2369
Orthocladiinae|[17401]|CNFNE2181-14|Chironomidae|658|BOLD:ACL4103
Orthocladiinae|[17402]|INNV035-08|Chironomidae|658|BOLD:AAG5439
Hydrobaenus conformis|[17403]|CNTUA2151-14|Chironomidae|633|BOLD:AAQ2644
Orthocladiinae|[17404]|BBDEE635-10|Chironomidae|658|BOLD:AAM6275
Chironomidae|[17405]|CNNHC2405-14|Chironomidae|615|BOLD:ACR5614
Chironomidae|[17406]|PHAPR321-11|Chironomidae|658|BOLD:ABA1230
Chironomidae|[17407]|SMTPB7538-13|Chironomidae|670|BOLD:ABA1238
Orthocladiinae|[17408]|PHAPR279-11|Chironomidae|658|BOLD:ABA1227
Orthocladiinae|[17409]|CNBPB495-12|Chironomidae|670|BOLD:ACA4749
Chironomidae|[17410]|SMTPB7543-13|Chironomidae|670|BOLD:ABA1237
Orthocladiinae|[17411]|SMTPB2024-13|Chironomidae|658|BOLD:ACF8859
Chironomidae|[17412]|SMTPB2859-13|Chironomidae|658|BOLD:ABW1509
Chironomidae|[17413]|SMTPB7517-13|Chironomidae|670|BOLD:ACF8860
Orthocladiinae|[17414]|CNGBA349-13|Chironomidae|658|BOLD:ACF8861
Orthocladiinae|[17415]|JWDCE117-10|Chironomidae|658|BOLD:AAP5143
Orthocladiinae|[17416]|CNROA019-13|Chironomidae|658|BOLD:ACK1809
Orthocladiinae|[17417]|CNROA385-13|Chironomidae|664|BOLD:ACF8943
Orthocladiinae|[17418]|DARC122-10|Chironomidae|658|BOLD:AAM6311
Oliveridia tricornis|[17419]|CNQUE816-14|Chironomidae|609|BOLD:AAX0748
Orthocladiinae|[17420]|SSJAA1539-13|Chironomidae|664|BOLD:ACG3987
Hydrobaenus|[17421]|SSJAA1617-13|Chironomidae|658|BOLD:ACG3277
Hydrobaenus fusistylus|[17422]|CNQUI626-14|Chironomidae|630|BOLD:AAM0871
Chironomidae|[17423]|SMTPB2067-13|Chironomidae|658|BOLD:ACF9313
Orthocladiinae|[17424]|SMTPB4017-13|Chironomidae|564|BOLD:ACF6680
Chironomidae|[17425]|BIOAI085-14|Chironomidae|603|BOLD:ACT8348
Chironomidae|[17426]|BIOAI112-14|Chironomidae|658|BOLD:ACK9938
Chironomidae|[17427]|CNQUH500-14|Chironomidae|588|BOLD:ACR0691
Orthocladiinae|[17428]|CNVUB071-14|Chironomidae|582|BOLD:ACR7328
Parametriocnemus boreoalpinus|[17429]|CNWLG627-12|Chironomidae|670|BOLD:AAL7338
Chironomidae|[17430]|CNYOI216-15|Chironomidae|588|BOLD:ACT2715
Orthocladiinae|[17431]|CNJAA764-12|Chironomidae|658|BOLD:ACC6768
Orthocladiinae|[17432]|CNTMB1081-14|Chironomidae|658|BOLD:ACM1608
Orthocladiinae|[17433]|SSWLC252-13|Chironomidae|670|BOLD:ACG2978
Orthocladiinae|[17434]|CNKLD3369-15|Chironomidae|594|BOLD:AAV5909
Eukiefferiella|[17435]|JSMAY1165-11|Chironomidae|658|BOLD:ABV1192
Eukiefferiella|[17436]|SMTPB6893-13|Chironomidae|670|BOLD:ABV1229
Eukiefferiella|[17437]|CNFNC510-14|Chironomidae|661|BOLD:ACL2232
Eukiefferiella|[17438]|NGAAA493-14|Chironomidae|549|BOLD:AAN4984
Orthocladiinae|[17439]|SSWLC2576-13|Chironomidae|658|BOLD:ACG1632
Orthocladiinae|[17440]|SSWLC2693-13|Chironomidae|658|BOLD:ACG2248
Orthocladiinae|[17441]|CNAUB1336-14|Chironomidae|648|BOLD:AAL7118
Tokunagaia rectangularis|[17442]|CNIVB181-14|Chironomidae|606|BOLD:ABX8388
Tokunagaia rectangularis|[17443]|CNAUY577-14|Chironomidae|594|BOLD:ACA8693
Tokunagaia|[17444]|CNIVC302-14|Chironomidae|603|BOLD:ACR3955
Chironomidae|[17445]|CNTGA1341-15|Chironomidae|588|BOLD:ACS2663
Orthocladiinae|[17446]|RBINA3478-13|Chironomidae|627|BOLD:ACJ0208
Orthocladiinae|[17447]|CNFNC1400-14|Chironomidae|662|BOLD:AAL7307
Orthocladiinae|[17448]|SSJAA248-13|Chironomidae|658|BOLD:ACG4319
Orthocladiinae|[17449]|CNTMA1329-14|Chironomidae|661|BOLD:ACM0468
Tokunagaia|[17450]|CNYOI074-15|Chironomidae|591|BOLD:ACT2263
Orthocladiinae|[17451]|SMTPB2282-13|Chironomidae|658|BOLD:ACF9382
Orthocladiinae|[17452]|SMTPB7606-13|Chironomidae|611|BOLD:ACF8763
Orthocladiinae|[17453]|CNROA198-13|Chironomidae|658|BOLD:ACJ9046
Chironomidae|[17454]|CNAUB170-14|Chironomidae|546|BOLD:ACR2166
Chaetocladius dentiforceps|[17455]|CNQUF411-14|Chironomidae|591|BOLD:AAM0566
Orthocladiinae|[17456]|CNIVC307-14|Chironomidae|576|BOLD:ACR4622
Tvetenia paucunca|[17457]|BSCHR146-10|Chironomidae|658|BOLD:AAB7489
Tvetenia paucunca|[17458]|GMDAP316-12|Chironomidae|658|BOLD:AAB7490
Orthocladiinae|[17459]|SSWLC4225-13|Chironomidae|658|BOLD:ACI5892
Orthocladiinae|[17460]|CNFNC711-14|Chironomidae|661|BOLD:ACL1522
Orthocladiinae|[17461]|CNFNC1287-14|Chironomidae|663|BOLD:ACL2656
Chironomidae|[17462]|SSFDC4246-14|Chironomidae|614|BOLD:ACO5339
Chironomidae|[17463]|CNKTE1475-15|Chironomidae|585|BOLD:ACS4494
Paraphaenocladius impensus|[17464]|NGNAP253-14|Chironomidae|676|BOLD:AAC4197
Paraphaenocladius impensus|[17465]|NGAAA1244-14|Chironomidae|567|BOLD:ACO0437
Paraphaenocladius impensus|[17466]|CHURC1096-09|Chironomidae|658|BOLD:AAC4201
Paraphaenocladius|[17467]|DARC608-11|Chironomidae|658|BOLD:AAU6742
Paraphaenocladius brevinervis|[17468]|DARC481-11|Chironomidae|658|BOLD:AAE3721
Paraphaenocladius impensus|[17469]|CNJAB093-12|Chironomidae|658|BOLD:ACX4638
Paraphaenocladius cf. nasthecus|[17470]|CNRMF2575-12|Chironomidae|664|BOLD:AAX1168
Paraphaenocladius|[17471]|JSJUN1876-11|Chironomidae|658|BOLD:ABW1311
Paraphaenocladius|[17472]|CNNHC1035-14|Chironomidae|579|BOLD:ACR5952
Paraphaenocladius impensus|[17473]|CNRMC846-12|Chironomidae|658|BOLD:AAI2661
Paraphaenocladius|[17474]|SSPAA1365-13|Chironomidae|661|BOLD:ACG3166
Orthocladiinae|[17475]|SSPAC3963-13|Chironomidae|658|BOLD:ACJ5522
Chironomidae|[17476]|CNNHA2879-14|Chironomidae|606|BOLD:ACR4311
Paraphaenocladius|[17477]|SSJAB641-13|Chironomidae|664|BOLD:ACC2059
Paraphaenocladius sp. 4ES|[17478]|CNMIA143-14|Chironomidae|658|BOLD:AAL7381
Paraphaenocladius exagitans|[17479]|SSBAF374-13|Chironomidae|658|BOLD:AAI2658
Paraphaenocladius impensus|[17480]|CNVUB321-14|Chironomidae|588|BOLD:AAQ2297
Paraphaenocladius impensus|[17481]|CNQUG1547-14|Chironomidae|621|BOLD:ACI8078
Orthocladiinae|[17482]|CNAUB1302-14|Chironomidae|648|BOLD:AAL9425
Chironomidae|[17483]|CNNHA2883-14|Chironomidae|633|BOLD:ACR3739
Chironomidae|[17484]|CNNHB2180-14|Chironomidae|633|BOLD:ACR4236
Paraphaenocladius|[17485]|DARC437-11|Chironomidae|658|BOLD:AAU6583
Paraphaenocladius|[17486]|CNIVD892-14|Chironomidae|603|BOLD:ACR5166
Paraphaenocladius|[17487]|CNVUA270-14|Chironomidae|552|BOLD:ACR7118
Chironomidae|[17488]|CNNHF258-14|Chironomidae|603|BOLD:ACR4603
Chironomidae|[17489]|CNTUA2346-14|Chironomidae|603|BOLD:ACR4174
Orthocladiinae|[17490]|CNGMB679-13|Chironomidae|658|BOLD:ACL0591
Orthocladiinae|[17491]|CNGMB625-13|Chironomidae|597|BOLD:ACK7597
Orthocladiinae|[17492]|CNGMB940-13|Chironomidae|658|BOLD:ACK8098
Orthocladiinae|[17493]|SSWLF4414-13|Chironomidae|661|BOLD:ACE1171
Orthocladiinae|[17494]|SSBRC680-14|Chironomidae|658|BOLD:ACC9735
Orthocladiinae|[17495]|BBDEE636-10|Chironomidae|658|BOLD:AAM6276
Chironomidae|[17496]|SSGBB6239-14|Chironomidae|579|BOLD:ACP2364
Orthocladiinae|[17497]|BBDEE132-10|Chironomidae|658|BOLD:AAG5518

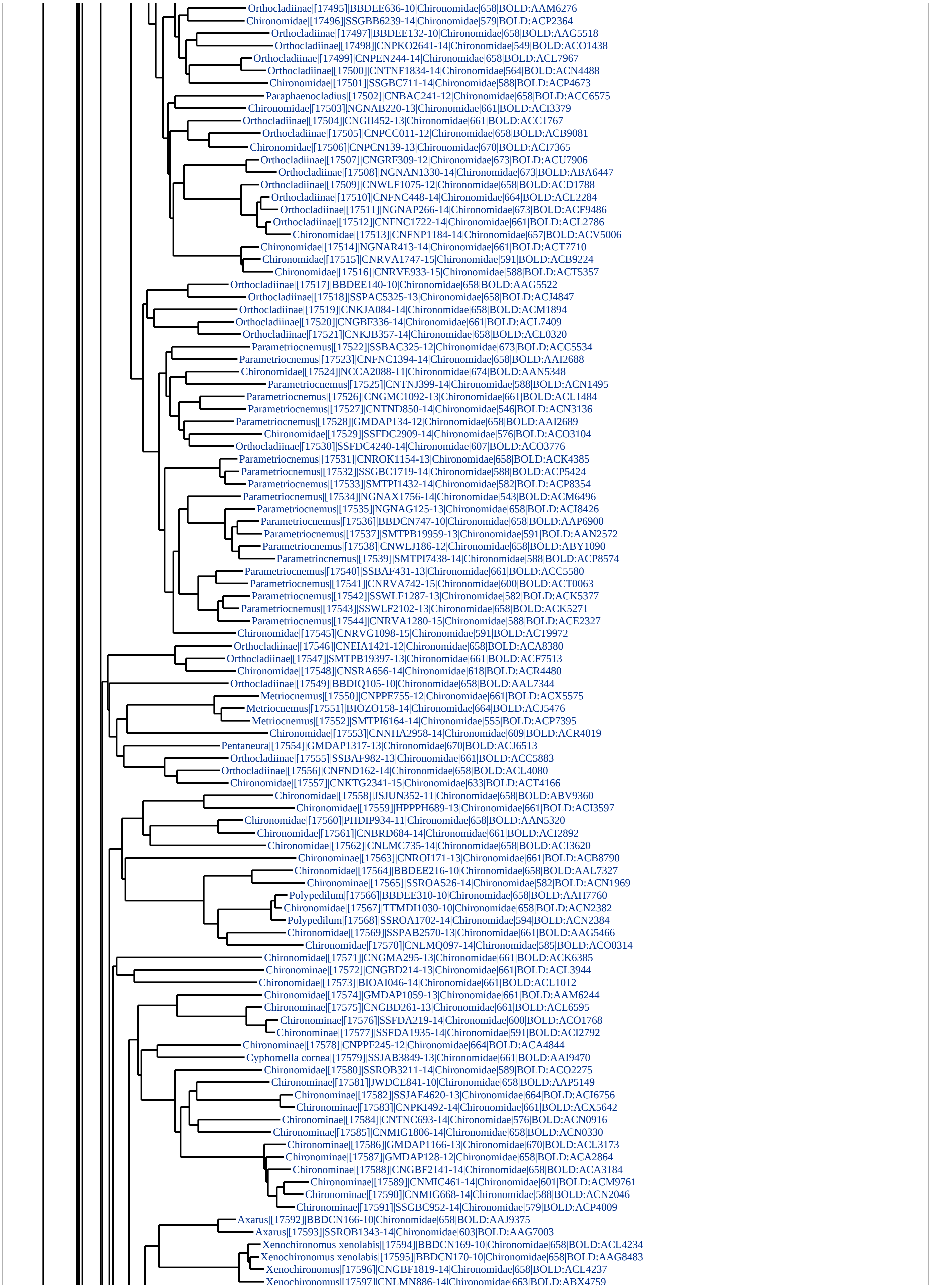

Orthocladiinae|[17495]|BBDEE636-10|Chironomidae|658|BOLD:AAM6276
Chironomidae|[17496]|SSGBB6239-14|Chironomidae|579|BOLD:ACP2364
Orthocladiinae|[17497]|BBDEE132-10|Chironomidae|658|BOLD:AAG5518
Orthocladiinae|[17498]|CNPKO2641-14|Chironomidae|549|BOLD:ACO1438
Orthocladiinae|[17499]|CNPEN244-14|Chironomidae|658|BOLD:ACL7967
Orthocladiinae|[17500]|CNTNF1834-14|Chironomidae|564|BOLD:ACN4488
Chironomidae|[17501]|SSGBC711-14|Chironomidae|588|BOLD:ACP4673
Paraphaenocladius|[17502]|CNBAC241-12|Chironomidae|658|BOLD:ACC6575
Chironomidae|[17503]|NGNAB220-13|Chironomidae|661|BOLD:ACI3379
Orthocladiinae|[17504]|CNGII452-13|Chironomidae|661|BOLD:ACC1767
Orthocladiinae|[17505]|CNPCC011-12|Chironomidae|658|BOLD:ACB9081
Chironomidae|[17506]|CNPCN139-13|Chironomidae|670|BOLD:ACI7365
Orthocladiinae|[17507]|CNGRF309-12|Chironomidae|673|BOLD:ACU7906
Orthocladiinae|[17508]|NGNAN1330-14|Chironomidae|673|BOLD:ABA6447
Orthocladiinae|[17509]|CNWLF1075-12|Chironomidae|658|BOLD:ACD1788
Orthocladiinae|[17510]|CNFNC448-14|Chironomidae|664|BOLD:ACL2284
Orthocladiinae|[17511]|NGNAP266-14|Chironomidae|673|BOLD:ACF9486
Orthocladiinae|[17512]|CNFNC1722-14|Chironomidae|661|BOLD:ACL2786
Chironomidae|[17513]|CNFNP1184-14|Chironomidae|657|BOLD:ACV5006
Chironomidae|[17514]|NGNAR413-14|Chironomidae|661|BOLD:ACT7710
Chironomidae|[17515]|CNRVA1747-15|Chironomidae|591|BOLD:ACB9224
Chironomidae|[17516]|CNRVE933-15|Chironomidae|588|BOLD:ACT5357
Orthocladiinae|[17517]|BBDEE140-10|Chironomidae|658|BOLD:AAG5522
Orthocladiinae|[17518]|SSPAC5325-13|Chironomidae|658|BOLD:ACJ4847
Orthocladiinae|[17519]|CNKJA084-14|Chironomidae|658|BOLD:ACM1894
Orthocladiinae|[17520]|CNGBF336-14|Chironomidae|661|BOLD:ACL7409
Orthocladiinae|[17521]|CNKJB357-14|Chironomidae|658|BOLD:ACL0320
Parametriocnemus|[17522]|SSBAC325-12|Chironomidae|673|BOLD:ACC5534
Parametriocnemus|[17523]|CNFNC1394-14|Chironomidae|658|BOLD:AAI2688
Chironomidae|[17524]|NCCA2088-11|Chironomidae|674|BOLD:AAN5348
Parametriocnemus|[17525]|CNTNJ399-14|Chironomidae|588|BOLD:ACN1495
Parametriocnemus|[17526]|CNGMC1092-13|Chironomidae|661|BOLD:ACL1484
Parametriocnemus|[17527]|CNTND850-14|Chironomidae|546|BOLD:ACN3136
Parametriocnemus|[17528]|GMDAP134-12|Chironomidae|658|BOLD:AAI2689
Chironomidae|[17529]|SSFDC2909-14|Chironomidae|576|BOLD:ACO3104
Orthocladiinae|[17530]|SSFDC4240-14|Chironomidae|607|BOLD:ACO3776
Parametriocnemus|[17531]|CNROK1154-13|Chironomidae|658|BOLD:ACK4385
Parametriocnemus|[17532]|SSGBC1719-14|Chironomidae|588|BOLD:ACP5424
Parametriocnemus|[17533]|SMTPI1432-14|Chironomidae|582|BOLD:ACP8354
Parametriocnemus|[17534]|NGNAX1756-14|Chironomidae|543|BOLD:ACM6496
Parametriocnemus|[17535]|NGNAG125-13|Chironomidae|658|BOLD:ACI8426
Parametriocnemus|[17536]|BBDCN747-10|Chironomidae|658|BOLD:AAP6900
Parametriocnemus|[17537]|SMTPB19959-13|Chironomidae|591|BOLD:AAN2572
Parametriocnemus|[17538]|CNWLJ186-12|Chironomidae|658|BOLD:ABY1090
Parametriocnemus|[17539]|SMTPI7438-14|Chironomidae|588|BOLD:ACP8574
Parametriocnemus|[17540]|SSBAF431-13|Chironomidae|661|BOLD:ACC5580
Parametriocnemus|[17541]|CNRVA742-15|Chironomidae|600|BOLD:ACT0063
Parametriocnemus|[17542]|SSWLF1287-13|Chironomidae|582|BOLD:ACK5377
Parametriocnemus|[17543]|SSWLF2102-13|Chironomidae|658|BOLD:ACK5271
Parametriocnemus|[17544]|CNRVA1280-15|Chironomidae|588|BOLD:ACE2327
Chironomidae|[17545]|CNRVG1098-15|Chironomidae|591|BOLD:ACT9972
Orthocladiinae|[17546]|CNEIA1421-12|Chironomidae|658|BOLD:ACA8380
Orthocladiinae|[17547]|SMTPB19397-13|Chironomidae|661|BOLD:ACF7513
Chironomidae|[17548]|CNSRA656-14|Chironomidae|618|BOLD:ACR4480
Orthocladiinae|[17549]|BBDIQ105-10|Chironomidae|658|BOLD:AAL7344
Metriocnemus|[17550]|CNPPE755-12|Chironomidae|661|BOLD:ACX5575
Metriocnemus|[17551]|BIOZO158-14|Chironomidae|664|BOLD:ACJ5476
Metriocnemus|[17552]|SMTPI6164-14|Chironomidae|555|BOLD:ACP7395
Chironomidae|[17553]|CNNHA2958-14|Chironomidae|609|BOLD:ACR4019
Pentaneura|[17554]|GMDAP1317-13|Chironomidae|670|BOLD:ACJ6513
Orthocladiinae|[17555]|SSBAF982-13|Chironomidae|661|BOLD:ACC5883
Orthocladiinae|[17556]|CNFND162-14|Chironomidae|658|BOLD:ACL4080
Chironomidae|[17557]|CNKTG2341-15|Chironomidae|633|BOLD:ACT4166
Chironomidae|[17558]|JSJUN352-11|Chironomidae|658|BOLD:ABV9360
Chironomidae|[17559]|HPPPH689-13|Chironomidae|661|BOLD:ACI3597
Chironomidae|[17560]|PHDIP934-11|Chironomidae|658|BOLD:AAN5320
Chironomidae|[17561]|CNBRD684-14|Chironomidae|661|BOLD:ACI2892
Chironomidae|[17562]|CNLMC735-14|Chironomidae|658|BOLD:ACI3620
Chironominae|[17563]|CNROI171-13|Chironomidae|661|BOLD:ACB8790
Chironomidae|[17564]|BBDEE216-10|Chironomidae|658|BOLD:AAL7327
Chironominae|[17565]|SSROA526-14|Chironomidae|582|BOLD:ACN1969
Polypedilum|[17566]|BBDEE310-10|Chironomidae|658|BOLD:AAH7760
Chironomidae|[17567]|TTMDI1030-10|Chironomidae|658|BOLD:ACN2382
Polypedilum|[17568]|SSROA1702-14|Chironomidae|594|BOLD:ACN2384
Chironomidae|[17569]|SSPAB2570-13|Chironomidae|661|BOLD:AAG5466
Chironomidae|[17570]|CNLMQ097-14|Chironomidae|585|BOLD:ACO0314
Chironomidae|[17571]|CNGMA295-13|Chironomidae|661|BOLD:ACK6385
Chironominae|[17572]|CNGBD214-13|Chironomidae|661|BOLD:ACL3944
Chironomidae|[17573]|BIOAI046-14|Chironomidae|661|BOLD:ACL1012
Chironomidae|[17574]|GMDAP1059-13|Chironomidae|661|BOLD:AAM6244
Chironominae|[17575]|CNGBD261-13|Chironomidae|661|BOLD:ACL6595
Chironominae|[17576]|SSFDA219-14|Chironomidae|600|BOLD:ACO1768
Chironominae|[17577]|SSFDA1935-14|Chironomidae|591|BOLD:ACI2792
Chironominae|[17578]|CNPPF245-12|Chironomidae|664|BOLD:ACA4844
Cyphomella cornea|[17579]|SSJAB3849-13|Chironomidae|661|BOLD:AAI9470
Chironomidae|[17580]|SSROB3211-14|Chironomidae|589|BOLD:ACO2275
Chironominae|[17581]|JWDCE841-10|Chironomidae|658|BOLD:AAP5149
Chironominae|[17582]|SSJAE4620-13|Chironomidae|664|BOLD:ACI6756
Chironominae|[17583]|CNPKI492-14|Chironomidae|661|BOLD:ACX5642
Chironominae|[17584]|CNTNC693-14|Chironomidae|576|BOLD:ACN0916
Chironominae|[17585]|CNMIG1806-14|Chironomidae|658|BOLD:ACN0330
Chironominae|[17586]|GMDAP1166-13|Chironomidae|670|BOLD:ACL3173
Chironominae|[17587]|GMDAP128-12|Chironomidae|658|BOLD:ACA2864
Chironominae|[17588]|CNGBF2141-14|Chironomidae|658|BOLD:ACA3184
Chironominae|[17589]|CNMIC461-14|Chironomidae|601|BOLD:ACM9761
Chironominae|[17590]|CNMIG668-14|Chironomidae|588|BOLD:ACN2046
Chironominae|[17591]|SSGBC952-14|Chironomidae|579|BOLD:ACP4009
Axarus|[17592]|BBDCN166-10|Chironomidae|658|BOLD:AAJ9375
Axarus|[17593]|SSROB1343-14|Chironomidae|603|BOLD:AAG7003
Xenochironomus xenolabis|[17594]|BBDCN169-10|Chironomidae|658|BOLD:ACL4234
Xenochironomus xenolabis|[17595]|BBDCN170-10|Chironomidae|658|BOLD:AAG8483
Xenochironomus|[17596]|CNGBF1819-14|Chironomidae|658|BOLD:ACL4237
Xenochironomus|[17597]|CNLMN886-14|Chironomidae|663|BOLD:ABX4759

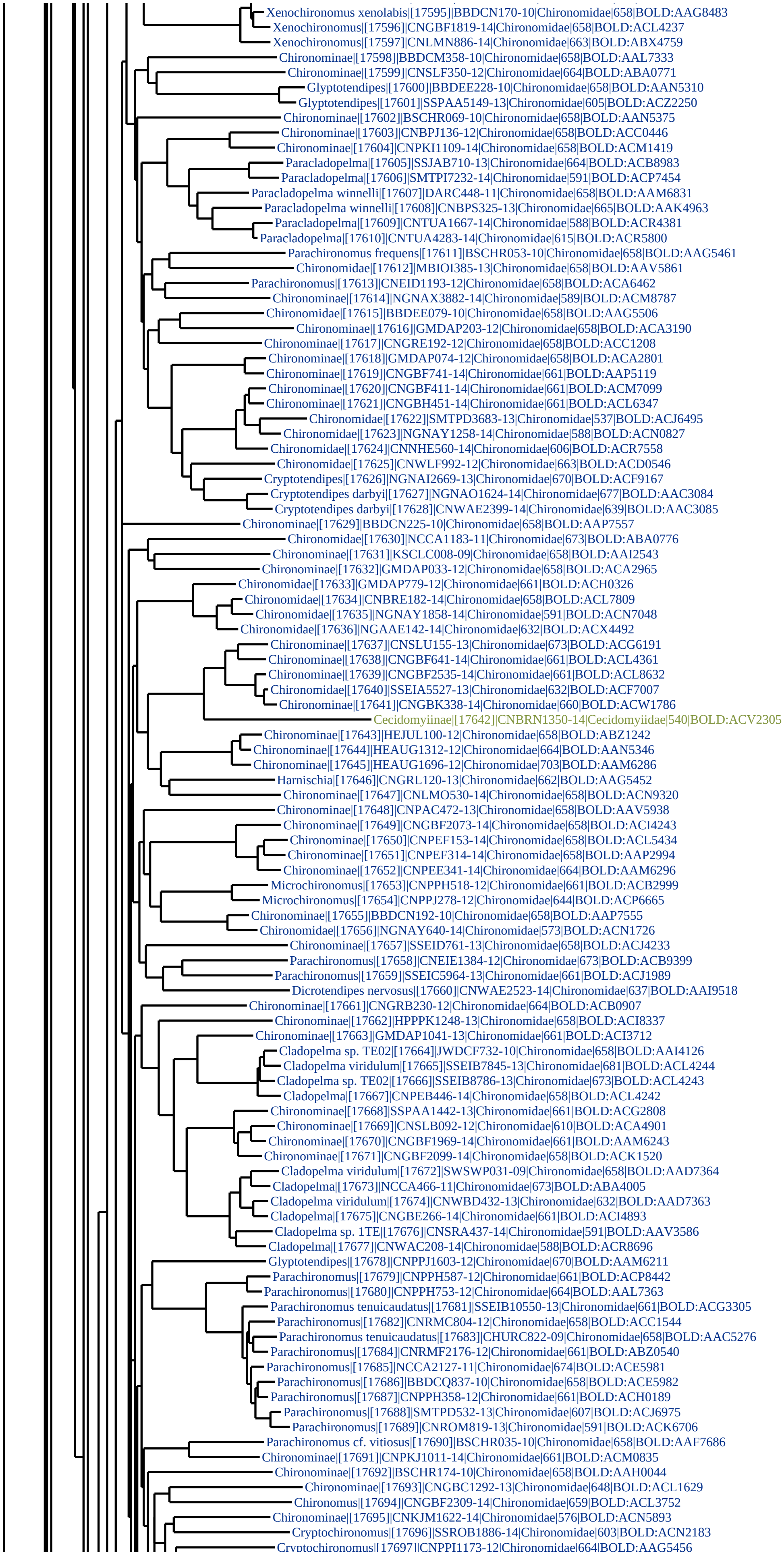
Xenochironomus xenolabis|[17595]|BBDCN170-10|Chironomidae|658|BOLD:AAG8483
Xenochironomus|[17596]|CNGBF1819-14|Chironomidae|658|BOLD:ACL4237
Xenochironomus|[17597]|CNLMN886-14|Chironomidae|663|BOLD:ABX4759
Chironominae|[17598]|BBDCM358-10|Chironomidae|658|BOLD:AAL7333
Chironominae|[17599]|CNSLF350-12|Chironomidae|664|BOLD:ABA0771
Glyptotendipes|[17600]|BBDEE228-10|Chironomidae|658|BOLD:AAN5310
Glyptotendipes|[17601]|SSPAA5149-13|Chironomidae|605|BOLD:ACZ2250
Chironominae|[17602]|BSCHR069-10|Chironomidae|658|BOLD:AAN5375
Chironominae|[17603]|CNBPJ136-12|Chironomidae|658|BOLD:ACC0446
Chironominae|[17604]|CNPKI1109-14|Chironomidae|658|BOLD:ACM1419
Paracladopelma|[17605]|SSJAB710-13|Chironomidae|664|BOLD:ACB8983
Paracladopelma|[17606]|SMTPI7232-14|Chironomidae|591|BOLD:ACP7454
Paracladopelma winnelli|[17607]|DARC448-11|Chironomidae|658|BOLD:AAM6831
Paracladopelma winnelli|[17608]|CNBPS325-13|Chironomidae|665|BOLD:AAK4963
Paracladopelma|[17609]|CNTUA1667-14|Chironomidae|588|BOLD:ACR4381
Paracladopelma|[17610]|CNTUA4283-14|Chironomidae|615|BOLD:ACR5800
Parachironomus frequens|[17611]|BSCHR053-10|Chironomidae|658|BOLD:AAG5461
Chironomidae|[17612]|MBIOI385-13|Chironomidae|658|BOLD:AAV5861
Parachironomus|[17613]|CNEID1193-12|Chironomidae|658|BOLD:ACA6462
Chironominae|[17614]|NGNAX3882-14|Chironomidae|589|BOLD:ACM8787
Chironomidae|[17615]|BBDEE079-10|Chironomidae|658|BOLD:AAG5506
Chironominae|[17616]|GMDAP203-12|Chironomidae|658|BOLD:ACA3190
Chironominae|[17617]|CNGRE192-12|Chironomidae|658|BOLD:ACC1208
Chironominae|[17618]|GMDAP074-12|Chironomidae|658|BOLD:ACA2801
Chironominae|[17619]|CNGBF741-14|Chironomidae|661|BOLD:AAP5119
Chironominae|[17620]|CNGBF411-14|Chironomidae|661|BOLD:ACM7099
Chironominae|[17621]|CNGBH451-14|Chironomidae|661|BOLD:ACL6347
Chironomidae|[17622]|SMTPD3683-13|Chironomidae|537|BOLD:ACJ6495
Chironominae|[17623]|NGNAY1258-14|Chironomidae|588|BOLD:ACN0827
Chironomidae|[17624]|CNNHE560-14|Chironomidae|606|BOLD:ACR7558
Chironomidae|[17625]|CNWLF992-12|Chironomidae|663|BOLD:ACD0546
Cryptotendipes|[17626]|NGNAI2669-13|Chironomidae|670|BOLD:ACF9167
Cryptotendipes darbyi|[17627]|NGNAO1624-14|Chironomidae|677|BOLD:AAC3084
Cryptotendipes darbyi|[17628]|CNWAE2399-14|Chironomidae|639|BOLD:AAC3085
Chironominae|[17629]|BBDCN225-10|Chironomidae|658|BOLD:AAP7557
Chironomidae|[17630]|NCCA1183-11|Chironomidae|673|BOLD:ABA0776
Chironominae|[17631]|KSCLC008-09|Chironomidae|658|BOLD:AAI2543
Chironominae|[17632]|GMDAP033-12|Chironomidae|658|BOLD:ACA2965
Chironomidae|[17633]|GMDAP779-12|Chironomidae|661|BOLD:ACH0326
Chironomidae|[17634]|CNBRE182-14|Chironomidae|658|BOLD:ACL7809
Chironomidae|[17635]|NGNAY1858-14|Chironomidae|591|BOLD:ACN7048
Chironomidae|[17636]|NGAAE142-14|Chironomidae|632|BOLD:ACX4492
Chironominae|[17637]|CNSLU155-13|Chironomidae|673|BOLD:ACG6191
Chironominae|[17638]|CNGBF641-14|Chironomidae|661|BOLD:ACL4361
Chironominae|[17639]|CNGBF2535-14|Chironomidae|661|BOLD:ACL8632
Chironomidae|[17640]|SSEIA5527-13|Chironomidae|632|BOLD:ACF7007
Chironominae|[17641]|CNGBK338-14|Chironomidae|660|BOLD:ACW1786
Cecidomyiinae|[17642]|CNBRN1350-14|Cecidomyiidae|540|BOLD:ACV2305
Chironominae|[17643]|HEJUL100-12|Chironomidae|658|BOLD:ABZ1242
Chironominae|[17644]|HEAUG1312-12|Chironomidae|664|BOLD:AAN5346
Chironominae|[17645]|HEAUG1696-12|Chironomidae|703|BOLD:AAM6286
Harnischia|[17646]|CNGRL120-13|Chironomidae|662|BOLD:AAG5452
Chironominae|[17647]|CNLMO530-14|Chironomidae|658|BOLD:ACN9320
Chironominae|[17648]|CNPAC472-13|Chironomidae|658|BOLD:AAV5938
Chironominae|[17649]|CNGBF2073-14|Chironomidae|658|BOLD:ACI4243
Chironominae|[17650]|CNPEF153-14|Chironomidae|658|BOLD:ACL5434
Chironominae|[17651]|CNPEF314-14|Chironomidae|658|BOLD:AAP2994
Chironominae|[17652]|CNPEE341-14|Chironomidae|664|BOLD:AAM6296
Microchironomus|[17653]|CNPPH518-12|Chironomidae|661|BOLD:ACB2999
Microchironomus|[17654]|CNPPJ278-12|Chironomidae|644|BOLD:ACP6665
Chironominae|[17655]|BBDCN192-10|Chironomidae|658|BOLD:AAP7555
Chironomidae|[17656]|NGNAY640-14|Chironomidae|573|BOLD:ACN1726
Chironominae|[17657]|SSEID761-13|Chironomidae|658|BOLD:ACJ4233
Parachironomus|[17658]|CNEIE1384-12|Chironomidae|673|BOLD:ACB9399
Parachironomus|[17659]|SSEIC5964-13|Chironomidae|661|BOLD:ACJ1989
Dicrotendipes nervosus|[17660]|CNWAE2523-14|Chironomidae|637|BOLD:AAI9518
Chironominae|[17661]|CNGRB230-12|Chironomidae|664|BOLD:ACB0907
Chironominae|[17662]|HPPPK1248-13|Chironomidae|658|BOLD:ACI8337
Chironominae|[17663]|GMDAP1041-13|Chironomidae|661|BOLD:ACI3712
Cladopelma sp. TE02|[17664]|JWDCF732-10|Chironomidae|658|BOLD:AAI4126
Cladopelma viridulum|[17665]|SSEIB7845-13|Chironomidae|681|BOLD:ACL4244
Cladopelma sp. TE02|[17666]|SSEIB8786-13|Chironomidae|673|BOLD:ACL4243
Cladopelma|[17667]|CNPEB446-14|Chironomidae|658|BOLD:ACL4242
Chironominae|[17668]|SSPAA1442-13|Chironomidae|661|BOLD:ACG2808
Chironominae|[17669]|CNSLB092-12|Chironomidae|610|BOLD:ACA4901
Chironominae|[17670]|CNGBF1969-14|Chironomidae|661|BOLD:AAM6243
Chironominae|[17671]|CNGBF2099-14|Chironomidae|658|BOLD:ACK1520
Cladopelma viridulum|[17672]|SWSWP031-09|Chironomidae|658|BOLD:AAD7364
Cladopelma|[17673]|NCCA466-11|Chironomidae|673|BOLD:ABA4005
Cladopelma viridulum|[17674]|CNWBD432-13|Chironomidae|632|BOLD:AAD7363
Cladopelma|[17675]|CNGBE266-14|Chironomidae|661|BOLD:ACI4893
Cladopelma sp. 1TE|[17676]|CNSRA437-14|Chironomidae|591|BOLD:AAV3586
Cladopelma|[17677]|CNWAC208-14|Chironomidae|588|BOLD:ACR8696
Glyptotendipes|[17678]|CNPPJ1603-12|Chironomidae|670|BOLD:AAM6211
Parachironomus|[17679]|CNPPH587-12|Chironomidae|661|BOLD:ACP8442
Parachironomus|[17680]|CNPPH753-12|Chironomidae|664|BOLD:AAL7363
Parachironomus tenuicaudatus|[17681]|SSEIB10550-13|Chironomidae|661|BOLD:ACG3305
Parachironomus|[17682]|CNRMC804-12|Chironomidae|658|BOLD:ACC1544
Parachironomus tenuicaudatus|[17683]|CHURC822-09|Chironomidae|658|BOLD:AAC5276
Parachironomus|[17684]|CNRMF2176-12|Chironomidae|661|BOLD:ABZ0540
Parachironomus|[17685]|NCCA2127-11|Chironomidae|674|BOLD:ACE5981
Parachironomus|[17686]|BBDCQ837-10|Chironomidae|658|BOLD:ACE5982
Parachironomus|[17687]|CNPPH358-12|Chironomidae|661|BOLD:ACH0189
Parachironomus|[17688]|SMTPD532-13|Chironomidae|607|BOLD:ACJ6975
Parachironomus|[17689]|CNROM819-13|Chironomidae|591|BOLD:ACK6706
Parachironomus cf. vitiosus|[17690]|BSCHR035-10|Chironomidae|658|BOLD:AAF7686
Chironominae|[17691]|CNPKJ1011-14|Chironomidae|661|BOLD:ACM0835
Chironominae|[17692]|BSCHR174-10|Chironomidae|658|BOLD:AAH0044
Chironominae|[17693]|CNGBC1292-13|Chironomidae|648|BOLD:ACL1629
Chironomus|[17694]|CNGBF2309-14|Chironomidae|659|BOLD:ACL3752
Chironominae|[17695]|CNKJM1622-14|Chironomidae|576|BOLD:ACN5893
Cryptochironomus|[17696]|SSROB1886-14|Chironomidae|603|BOLD:ACN2183
Cryptochironomus|[17697]|CNPPI1173-12|Chironomidae|664|BOLD:AAG5456

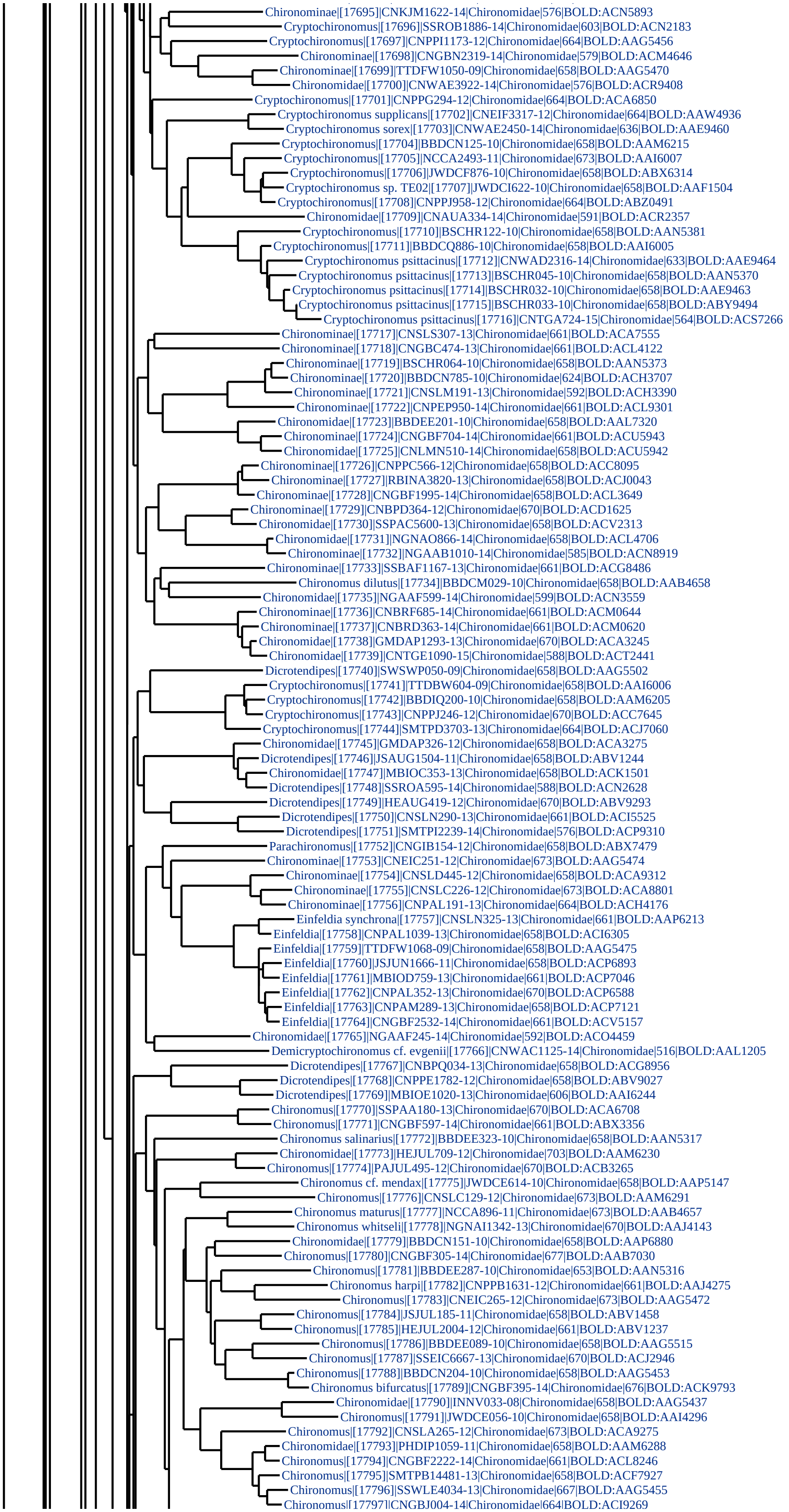
Chironominae|[17695]|CNKJM1622-14|Chironomidae|576|BOLD:ACN5893
Cryptochironomus|[17696]|SSROB1886-14|Chironomidae|603|BOLD:ACN2183
Cryptochironomus|[17697]|CNPPI1173-12|Chironomidae|664|BOLD:AAG5456
Chironominae|[17698]|CNGBN2319-14|Chironomidae|579|BOLD:ACM4646
Chironominae|[17699]|TTDFW1050-09|Chironomidae|658|BOLD:AAG5470
Chironomidae|[17700]|CNWAE3922-14|Chironomidae|576|BOLD:ACR9408
Cryptochironomus|[17701]|CNPPG294-12|Chironomidae|664|BOLD:ACA6850
Cryptochironomus supplicans|[17702]|CNEIF3317-12|Chironomidae|664|BOLD:AAW4936
Cryptochironomus sorex|[17703]|CNWAE2450-14|Chironomidae|636|BOLD:AAE9460
Cryptochironomus|[17704]|BBDCN125-10|Chironomidae|658|BOLD:AAM6215
Cryptochironomus|[17705]|NCCA2493-11|Chironomidae|673|BOLD:AAI6007
Cryptochironomus|[17706]|JWDCF876-10|Chironomidae|658|BOLD:ABX6314
Cryptochironomus sp. TE02|[17707]|JWDCI622-10|Chironomidae|658|BOLD:AAF1504
Cryptochironomus|[17708]|CNPPJ958-12|Chironomidae|664|BOLD:ABZ0491
Chironomidae|[17709]|CNAUA334-14|Chironomidae|591|BOLD:ACR2357
Cryptochironomus|[17710]|BSCHR122-10|Chironomidae|658|BOLD:AAN5381
Cryptochironomus|[17711]|BBDCQ886-10|Chironomidae|658|BOLD:AAI6005
Cryptochironomus psittacinus|[17712]|CNWAD2316-14|Chironomidae|633|BOLD:AAE9464
Cryptochironomus psittacinus|[17713]|BSCHR045-10|Chironomidae|658|BOLD:AAN5370
Cryptochironomus psittacinus|[17714]|BSCHR032-10|Chironomidae|658|BOLD:AAE9463
Cryptochironomus psittacinus|[17715]|BSCHR033-10|Chironomidae|658|BOLD:ABY9494
Cryptochironomus psittacinus|[17716]|CNTGA724-15|Chironomidae|564|BOLD:ACS7266
Chironominae|[17717]|CNSLS307-13|Chironomidae|661|BOLD:ACA7555
Chironominae|[17718]|CNGBC474-13|Chironomidae|661|BOLD:ACL4122
Chironominae|[17719]|BSCHR064-10|Chironomidae|658|BOLD:AAN5373
Chironominae|[17720]|BBDCN785-10|Chironomidae|624|BOLD:ACH3707
Chironominae|[17721]|CNSLM191-13|Chironomidae|592|BOLD:ACH3390
Chironominae|[17722]|CNPEP950-14|Chironomidae|661|BOLD:ACL9301
Chironomidae|[17723]|BBDEE201-10|Chironomidae|658|BOLD:AAL7320
Chironominae|[17724]|CNGBF704-14|Chironomidae|661|BOLD:ACU5943
Chironomidae|[17725]|CNLMN510-14|Chironomidae|658|BOLD:ACU5942
Chironominae|[17726]|CNPPC566-12|Chironomidae|658|BOLD:ACC8095
Chironominae|[17727]|RBINA3820-13|Chironomidae|658|BOLD:ACJ0043
Chironominae|[17728]|CNGBF1995-14|Chironomidae|658|BOLD:ACL3649
Chironominae|[17729]|CNBPD364-12|Chironomidae|670|BOLD:ACD1625
Chironomidae|[17730]|SSPAC5600-13|Chironomidae|658|BOLD:ACV2313
Chironomidae|[17731]|NGNAO866-14|Chironomidae|658|BOLD:ACL4706
Chironominae|[17732]|NGAAB1010-14|Chironomidae|585|BOLD:ACN8919
Chironominae|[17733]|SSBAF1167-13|Chironomidae|661|BOLD:ACG8486
Chironomus dilutus|[17734]|BBDCM029-10|Chironomidae|658|BOLD:AAB4658
Chironomidae|[17735]|NGAAF599-14|Chironomidae|599|BOLD:ACN3559
Chironominae|[17736]|CNBRF685-14|Chironomidae|661|BOLD:ACM0644
Chironominae|[17737]|CNBRD363-14|Chironomidae|661|BOLD:ACM0620
Chironomidae|[17738]|GMDAP1293-13|Chironomidae|670|BOLD:ACA3245
Chironomidae|[17739]|CNTGE1090-15|Chironomidae|588|BOLD:ACT2441
Dicrotendipes|[17740]|SWSWP050-09|Chironomidae|658|BOLD:AAG5502
Cryptochironomus|[17741]|TTDBW604-09|Chironomidae|658|BOLD:AAI6006
Cryptochironomus|[17742]|BBDIQ200-10|Chironomidae|658|BOLD:AAM6205
Cryptochironomus|[17743]|CNPPJ246-12|Chironomidae|670|BOLD:ACC7645
Cryptochironomus|[17744]|SMTPD3703-13|Chironomidae|664|BOLD:ACJ7060
Chironomidae|[17745]|GMDAP326-12|Chironomidae|658|BOLD:ACA3275
Dicrotendipes|[17746]|JSAUG1504-11|Chironomidae|658|BOLD:ABV1244
Chironomidae|[17747]|MBIOC353-13|Chironomidae|658|BOLD:ACK1501
Dicrotendipes|[17748]|SSROA595-14|Chironomidae|588|BOLD:ACN2628
Dicrotendipes|[17749]|HEAUG419-12|Chironomidae|670|BOLD:ABV9293
Dicrotendipes|[17750]|CNSLN290-13|Chironomidae|661|BOLD:ACI5525
Dicrotendipes|[17751]|SMTPI2239-14|Chironomidae|576|BOLD:ACP9310
Parachironomus|[17752]|CNGIB154-12|Chironomidae|658|BOLD:ABX7479
Chironominae|[17753]|CNEIC251-12|Chironomidae|673|BOLD:AAG5474
Chironominae|[17754]|CNSLD445-12|Chironomidae|658|BOLD:ACA9312
Chironominae|[17755]|CNSLC226-12|Chironomidae|673|BOLD:ACA8801
Chironominae|[17756]|CNPAL191-13|Chironomidae|664|BOLD:ACH4176
Einfeldia synchrona|[17757]|CNSLN325-13|Chironomidae|661|BOLD:AAP6213
Einfeldia|[17758]|CNPAL1039-13|Chironomidae|658|BOLD:ACI6305
Einfeldia|[17759]|TTDFW1068-09|Chironomidae|658|BOLD:AAG5475
Einfeldia|[17760]|JSJUN1666-11|Chironomidae|658|BOLD:ACP6893
Einfeldia|[17761]|MBIOD759-13|Chironomidae|661|BOLD:ACP7046
Einfeldia|[17762]|CNPAL352-13|Chironomidae|670|BOLD:ACP6588
Einfeldia|[17763]|CNPAM289-13|Chironomidae|658|BOLD:ACP7121
Einfeldia|[17764]|CNGBF2532-14|Chironomidae|661|BOLD:ACV5157
Chironomidae|[17765]|NGAAF245-14|Chironomidae|592|BOLD:ACO4459
Demicryptochironomus cf. evgenii|[17766]|CNWAC1125-14|Chironomidae|516|BOLD:AAL1205
Dicrotendipes|[17767]|CNBPQ034-13|Chironomidae|658|BOLD:ACG8956
Dicrotendipes|[17768]|CNPPE1782-12|Chironomidae|658|BOLD:ABV9027
Dicrotendipes|[17769]|MBIOE1020-13|Chironomidae|606|BOLD:AAI6244
Chironomus|[17770]|SSPAA180-13|Chironomidae|670|BOLD:ACA6708
Chironomus|[17771]|CNGBF597-14|Chironomidae|661|BOLD:ABX3356
Chironomus salinarius|[17772]|BBDEE323-10|Chironomidae|658|BOLD:AAN5317
Chironomidae|[17773]|HEJUL709-12|Chironomidae|703|BOLD:AAM6230
Chironomus|[17774]|PAJUL495-12|Chironomidae|670|BOLD:ACB3265
Chironomus cf. mendax|[17775]|JWDCE614-10|Chironomidae|658|BOLD:AAP5147
Chironomus|[17776]|CNSLC129-12|Chironomidae|673|BOLD:AAM6291
Chironomus maturus|[17777]|NCCA896-11|Chironomidae|673|BOLD:AAB4657
Chironomus whitseli|[17778]|NGNAI1342-13|Chironomidae|670|BOLD:AAJ4143
Chironomidae|[17779]|BBDCN151-10|Chironomidae|658|BOLD:AAP6880
Chironomus|[17780]|CNGBF305-14|Chironomidae|677|BOLD:AAB7030
Chironomus|[17781]|BBDEE287-10|Chironomidae|653|BOLD:AAN5316
Chironomus harpi|[17782]|CNPPB1631-12|Chironomidae|661|BOLD:AAJ4275
Chironomus|[17783]|CNEIC265-12|Chironomidae|673|BOLD:AAG5472
Chironomus|[17784]|JSJUL185-11|Chironomidae|658|BOLD:ABV1458
Chironomus|[17785]|HEJUL2004-12|Chironomidae|661|BOLD:ABV1237
Chironomus|[17786]|BBDEE089-10|Chironomidae|658|BOLD:AAG5515
Chironomus|[17787]|SSEIC6667-13|Chironomidae|670|BOLD:ACJ2946
Chironomus|[17788]|BBDCN204-10|Chironomidae|658|BOLD:AAG5453
Chironomus bifurcatus|[17789]|CNGBF395-14|Chironomidae|676|BOLD:ACK9793
Chironomidae|[17790]|INNV033-08|Chironomidae|658|BOLD:AAG5437
Chironomus|[17791]|JWDCE056-10|Chironomidae|658|BOLD:AAI4296
Chironomus|[17792]|CNSLA265-12|Chironomidae|673|BOLD:ACA9275
Chironomidae|[17793]|PHDIP1059-11|Chironomidae|658|BOLD:AAM6288
Chironomus|[17794]|CNGBF2222-14|Chironomidae|661|BOLD:ACL8246
Chironomus|[17795]|SMTPB14481-13|Chironomidae|658|BOLD:ACF7927
Chironomus|[17796]|SSWLE4034-13|Chironomidae|667|BOLD:AAG5455
Chironomus|[17797]|CNGBJ004-14|Chironomidae|664|BOLD:ACI9269

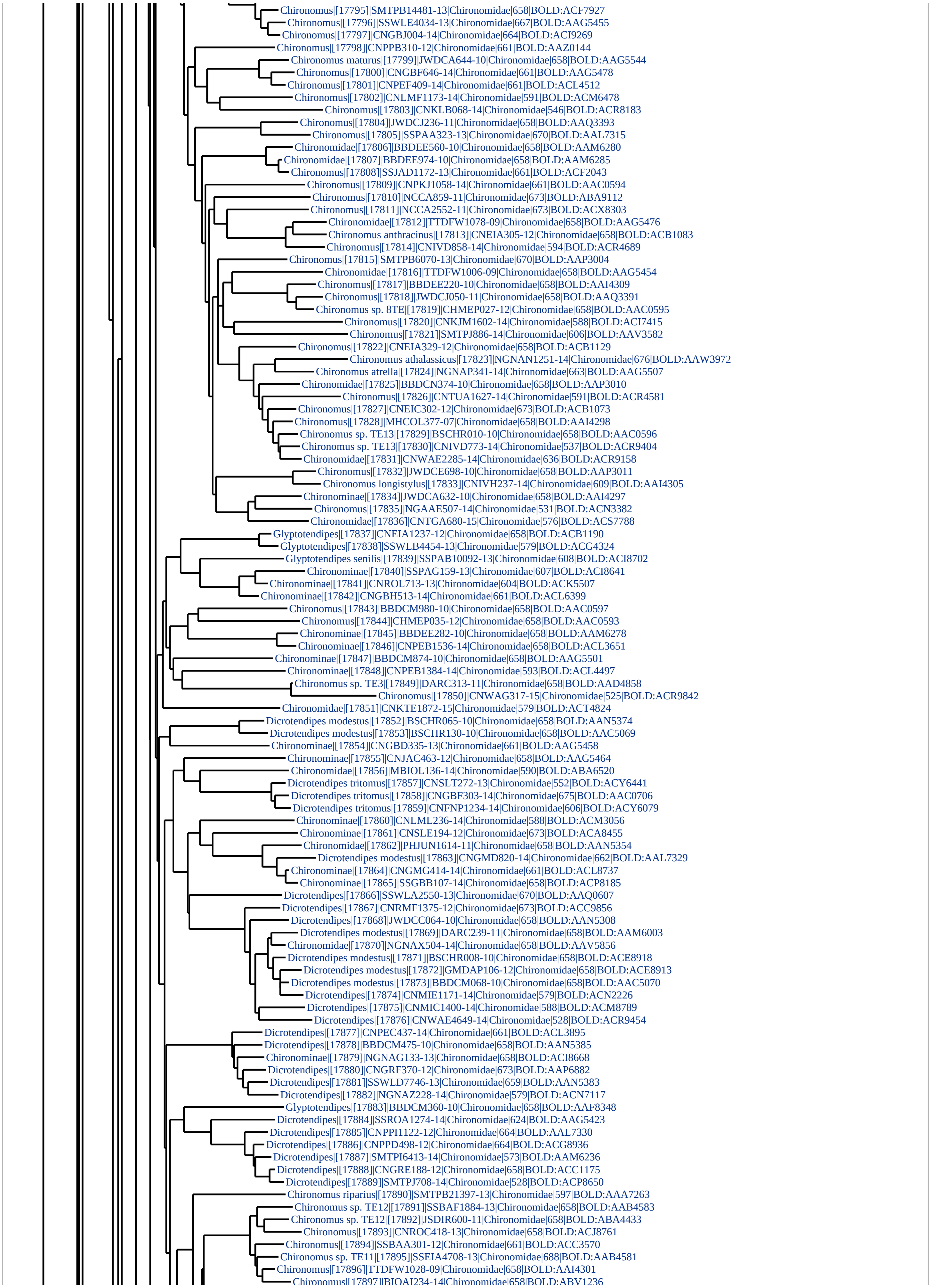
Chironomus|[17795]|SMTPB14481-13|Chironomidae|658|BOLD:ACF7927
Chironomus|[17796]|SSWLE4034-13|Chironomidae|667|BOLD:AAG5455
Chironomus|[17797]|CNGBJ004-14|Chironomidae|664|BOLD:ACI9269
Chironomus|[17798]|CNPPB310-12|Chironomidae|661|BOLD:AAZ0144
Chironomus maturus|[17799]|JWDCA644-10|Chironomidae|658|BOLD:AAG5544
Chironomus|[17800]|CNGBF646-14|Chironomidae|661|BOLD:AAG5478
Chironomus|[17801]|CNPEF409-14|Chironomidae|661|BOLD:ACL4512
Chironomus|[17802]|CNLMF1173-14|Chironomidae|591|BOLD:ACM6478
Chironomus|[17803]|CNKLB068-14|Chironomidae|546|BOLD:ACR8183
Chironomus|[17804]|JWDCJ236-11|Chironomidae|658|BOLD:AAQ3393
Chironomus|[17805]|SSPAA323-13|Chironomidae|670|BOLD:AAL7315
Chironomidae|[17806]|BBDEE560-10|Chironomidae|658|BOLD:AAM6280
Chironomidae|[17807]|BBDEE974-10|Chironomidae|658|BOLD:AAM6285
Chironomus|[17808]|SSJAD1172-13|Chironomidae|661|BOLD:ACF2043
Chironomus|[17809]|CNPKJ1058-14|Chironomidae|661|BOLD:AAC0594
Chironomus|[17810]|NCCA859-11|Chironomidae|673|BOLD:ABA9112
Chironomus|[17811]|NCCA2552-11|Chironomidae|673|BOLD:ACX8303
Chironomidae|[17812]|TTDFW1078-09|Chironomidae|658|BOLD:AAG5476
Chironomus anthracinus|[17813]|CNEIA305-12|Chironomidae|658|BOLD:ACB1083
Chironomus|[17814]|CNIVD858-14|Chironomidae|594|BOLD:ACR4689
Chironomus|[17815]|SMTPB6070-13|Chironomidae|670|BOLD:AAP3004
Chironomidae|[17816]|TTDFW1006-09|Chironomidae|658|BOLD:AAG5454
Chironomus|[17817]|BBDEE220-10|Chironomidae|658|BOLD:AAI4309
Chironomus|[17818]|JWDCJ050-11|Chironomidae|658|BOLD:AAQ3391
Chironomus sp. 8TE|[17819]|CHMEP027-12|Chironomidae|658|BOLD:AAC0595
Chironomus|[17820]|CNKJM1602-14|Chironomidae|588|BOLD:ACI7415
Chironomus|[17821]|SMTPJ886-14|Chironomidae|606|BOLD:AAV3582
Chironomus|[17822]|CNEIA329-12|Chironomidae|658|BOLD:ACB1129
Chironomus athalassicus|[17823]|NGNAN1251-14|Chironomidae|676|BOLD:AAW3972
Chironomus atrella|[17824]|NGNAP341-14|Chironomidae|663|BOLD:AAG5507
Chironomidae|[17825]|BBDCN374-10|Chironomidae|658|BOLD:AAP3010
Chironomus|[17826]|CNTUA1627-14|Chironomidae|591|BOLD:ACR4581
Chironomus|[17827]|CNEIC302-12|Chironomidae|673|BOLD:ACB1073
Chironomus|[17828]|MHCOL377-07|Chironomidae|658|BOLD:AAI4298
Chironomus sp. TE13|[17829]|BSCHR010-10|Chironomidae|658|BOLD:AAC0596
Chironomus sp. TE13|[17830]|CNIVD773-14|Chironomidae|537|BOLD:ACR9404
Chironomidae|[17831]|CNWAE2285-14|Chironomidae|636|BOLD:ACR9158
Chironomus|[17832]|JWDCE698-10|Chironomidae|658|BOLD:AAP3011
Chironomus longistylus|[17833]|CNIVH237-14|Chironomidae|609|BOLD:AAI4305
Chironominae|[17834]|JWDCA632-10|Chironomidae|658|BOLD:AAI4297
Chironomus|[17835]|NGAAE507-14|Chironomidae|531|BOLD:ACN3382
Chironomidae|[17836]|CNTGA680-15|Chironomidae|576|BOLD:ACS7788
Glyptotendipes|[17837]|CNEIA1237-12|Chironomidae|658|BOLD:ACB1190
Glyptotendipes|[17838]|SSWLB4454-13|Chironomidae|579|BOLD:ACG4324
Glyptotendipes senilis|[17839]|SSPAB10092-13|Chironomidae|608|BOLD:ACI8702
Chironominae|[17840]|SSPAG159-13|Chironomidae|607|BOLD:ACI8641
Chironominae|[17841]|CNROL713-13|Chironomidae|604|BOLD:ACK5507
Chironominae|[17842]|CNGBH513-14|Chironomidae|661|BOLD:ACL6399
Chironomus|[17843]|BBDCM980-10|Chironomidae|658|BOLD:AAC0597
Chironomus|[17844]|CHMEP035-12|Chironomidae|658|BOLD:AAC0593
Chironominae|[17845]|BBDEE282-10|Chironomidae|658|BOLD:AAM6278
Chironominae|[17846]|CNPEB1536-14|Chironomidae|658|BOLD:ACL3651
Chironominae|[17847]|BBDCM874-10|Chironomidae|658|BOLD:AAG5501
Chironominae|[17848]|CNPEB1384-14|Chironomidae|593|BOLD:ACL4497
Chironomus sp. TE3|[17849]|DARC313-11|Chironomidae|658|BOLD:AAD4858
Chironomus|[17850]|CNWAG317-15|Chironomidae|525|BOLD:ACR9842
Chironomidae|[17851]|CNKTE1872-15|Chironomidae|579|BOLD:ACT4824
Dicrotendipes modestus|[17852]|BSCHR065-10|Chironomidae|658|BOLD:AAN5374
Dicrotendipes modestus|[17853]|BSCHR130-10|Chironomidae|658|BOLD:AAC5069
Chironominae|[17854]|CNGBD335-13|Chironomidae|661|BOLD:AAG5458
Chironominae|[17855]|CNJAC463-12|Chironomidae|658|BOLD:AAG5464
Chironomidae|[17856]|MBIOL136-14|Chironomidae|590|BOLD:ABA6520
Dicrotendipes tritomus|[17857]|CNSLT272-13|Chironomidae|552|BOLD:ACY6441
Dicrotendipes tritomus|[17858]|CNGBF303-14|Chironomidae|675|BOLD:AAC0706
Dicrotendipes tritomus|[17859]|CNFNP1234-14|Chironomidae|606|BOLD:ACY6079
Chironominae|[17860]|CNLML236-14|Chironomidae|588|BOLD:ACM3056
Chironominae|[17861]|CNSLE194-12|Chironomidae|673|BOLD:ACA8455
Chironomidae|[17862]|PHJUN1614-11|Chironomidae|658|BOLD:AAN5354
Dicrotendipes modestus|[17863]|CNGMD820-14|Chironomidae|662|BOLD:AAL7329
Chironominae|[17864]|CNGMG414-14|Chironomidae|661|BOLD:ACL8737
Chironominae|[17865]|SSGBB107-14|Chironomidae|658|BOLD:ACP8185
Dicrotendipes|[17866]|SSWLA2550-13|Chironomidae|670|BOLD:AAQ0607
Dicrotendipes|[17867]|CNRMF1375-12|Chironomidae|673|BOLD:ACC9856
Dicrotendipes|[17868]|JWDCC064-10|Chironomidae|658|BOLD:AAN5308
Dicrotendipes modestus|[17869]|DARC239-11|Chironomidae|658|BOLD:AAM6003
Chironomidae|[17870]|NGNAX504-14|Chironomidae|658|BOLD:AAV5856
Dicrotendipes modestus|[17871]|BSCHR008-10|Chironomidae|658|BOLD:ACE8918
Dicrotendipes modestus|[17872]|GMDAP106-12|Chironomidae|658|BOLD:ACE8913
Dicrotendipes modestus|[17873]|BBDCM068-10|Chironomidae|658|BOLD:AAC5070
Dicrotendipes|[17874]|CNMIE1171-14|Chironomidae|579|BOLD:ACN2226
Dicrotendipes|[17875]|CNMIC1400-14|Chironomidae|588|BOLD:ACM8789
Dicrotendipes|[17876]|CNWAE4649-14|Chironomidae|528|BOLD:ACR9454
Dicrotendipes|[17877]|CNPEC437-14|Chironomidae|661|BOLD:ACL3895
Dicrotendipes|[17878]|BBDCM475-10|Chironomidae|658|BOLD:AAN5385
Chironominae|[17879]|NGNAG133-13|Chironomidae|658|BOLD:ACI8668
Dicrotendipes|[17880]|CNGRF370-12|Chironomidae|673|BOLD:AAP6882
Dicrotendipes|[17881]|SSWLD7746-13|Chironomidae|659|BOLD:AAN5383
Dicrotendipes|[17882]|NGNAZ228-14|Chironomidae|579|BOLD:ACN7117
Glyptotendipes|[17883]|BBDCM360-10|Chironomidae|658|BOLD:AAF8348
Dicrotendipes|[17884]|SSROA1274-14|Chironomidae|624|BOLD:AAG5423
Dicrotendipes|[17885]|CNPPI1122-12|Chironomidae|664|BOLD:AAL7330
Dicrotendipes|[17886]|CNPPD498-12|Chironomidae|664|BOLD:ACG8936
Dicrotendipes|[17887]|SMTPI6413-14|Chironomidae|573|BOLD:AAM6236
Dicrotendipes|[17888]|CNGRE188-12|Chironomidae|658|BOLD:ACC1175
Dicrotendipes|[17889]|SMTPJ708-14|Chironomidae|528|BOLD:ACP8650
Chironomus riparius|[17890]|SMTPB21397-13|Chironomidae|597|BOLD:AAA7263
Chironomus sp. TE12|[17891]|SSBAF1884-13|Chironomidae|658|BOLD:AAB4583
Chironomus sp. TE12|[17892]|JSDIR600-11|Chironomidae|658|BOLD:ABA4433
Chironomus|[17893]|CNROC418-13|Chironomidae|658|BOLD:ACJ8761
Chironomus|[17894]|SSBAA301-12|Chironomidae|661|BOLD:ACC3570
Chironomus sp. TE11|[17895]|SSEIA4708-13|Chironomidae|688|BOLD:AAB4581
Chironomus|[17896]|TTDFW1028-09|Chironomidae|658|BOLD:AAI4301
Chironomus|[17897]|BIOAI234-14|Chironomidae|658|BOLD:ABV1236

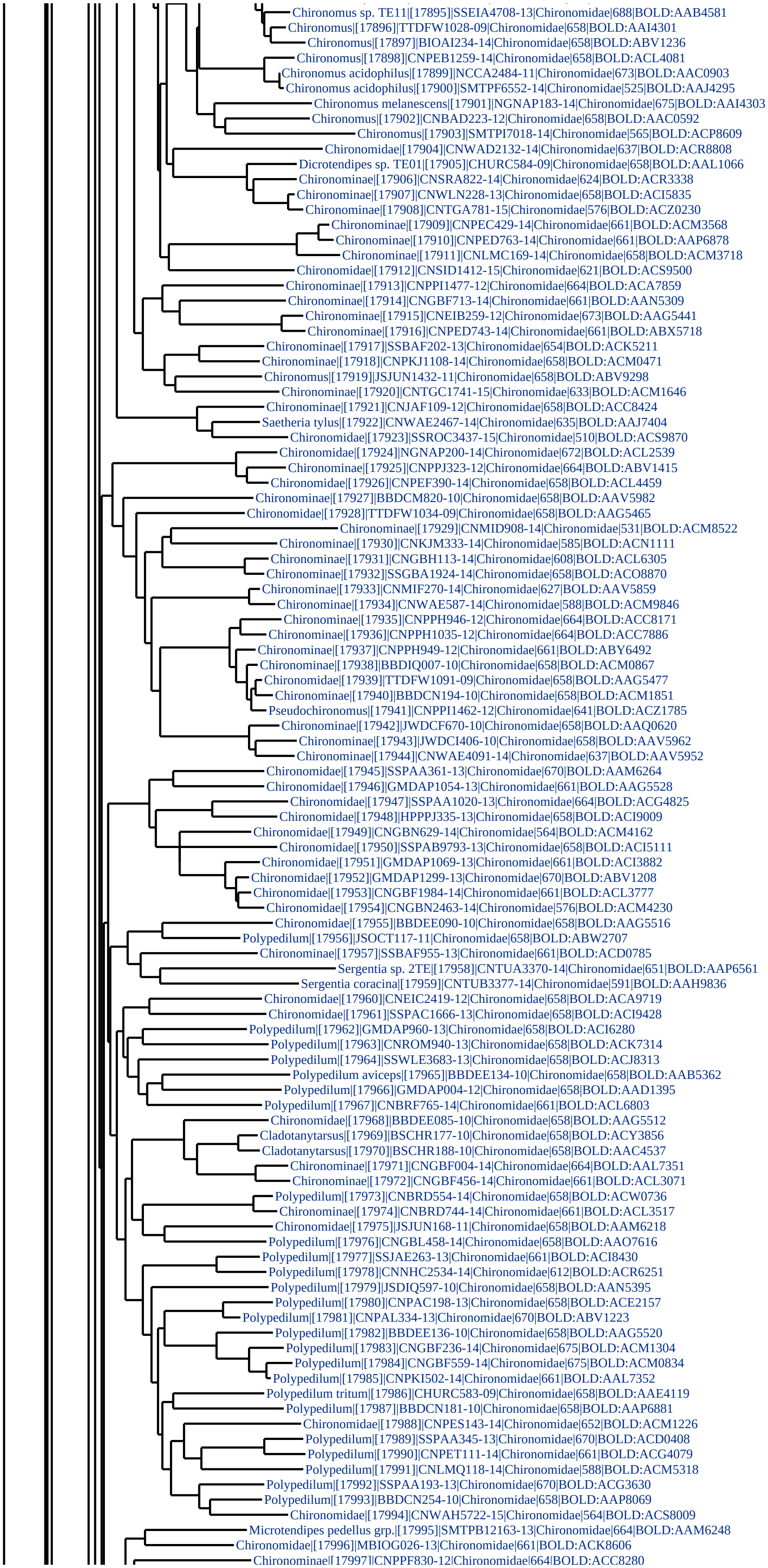

Chironomus sp. TE11|[17895]|SSEIA4708-13|Chironomidae|688|BOLD:AAB4581
Chironomus|[17896]|TTDFW1028-09|Chironomidae|658|BOLD:AAI4301
Chironomus|[17897]|BIOAI234-14|Chironomidae|658|BOLD:ABV1236
Chironomus|[17898]|CNPEB1259-14|Chironomidae|658|BOLD:ACL4081
Chironomus acidophilus|[17899]|NCCA2484-11|Chironomidae|673|BOLD:AAC0903
Chironomus acidophilus|[17900]|SMTPF6552-14|Chironomidae|525|BOLD:AAJ4295
Chironomus melanescens|[17901]|NGNAP183-14|Chironomidae|675|BOLD:AAI4303
Chironomus|[17902]|CNBAD223-12|Chironomidae|658|BOLD:AAC0592
Chironomus|[17903]|SMTPI7018-14|Chironomidae|565|BOLD:ACP8609
Chironomidae|[17904]|CNWAD2132-14|Chironomidae|637|BOLD:ACR8808
Dicrotendipes sp. TE01|[17905]|CHURC584-09|Chironomidae|658|BOLD:AAL1066
Chironominae|[17906]|CNSRA822-14|Chironomidae|624|BOLD:ACR3338
Chironominae|[17907]|CNWLN228-13|Chironomidae|658|BOLD:ACI5835
Chironominae|[17908]|CNTGA781-15|Chironomidae|576|BOLD:ACZ0230
Chironominae|[17909]|CNPEC429-14|Chironomidae|661|BOLD:ACM3568
Chironominae|[17910]|CNPED763-14|Chironomidae|661|BOLD:AAP6878
Chironominae|[17911]|CNLMC169-14|Chironomidae|658|BOLD:ACM3718
Chironomidae|[17912]|CNSID1412-15|Chironomidae|621|BOLD:ACS9500
Chironominae|[17913]|CNPPI1477-12|Chironomidae|664|BOLD:ACA7859
Chironominae|[17914]|CNGBF713-14|Chironomidae|661|BOLD:AAN5309
Chironominae|[17915]|CNEIB259-12|Chironomidae|673|BOLD:AAG5441
Chironominae|[17916]|CNPED743-14|Chironomidae|661|BOLD:ABX5718
Chironominae|[17917]|SSBAF202-13|Chironomidae|654|BOLD:ACK5211
Chironominae|[17918]|CNPKJ1108-14|Chironomidae|658|BOLD:ACM0471
Chironomus|[17919]|JSJUN1432-11|Chironomidae|658|BOLD:ABV9298
Chironominae|[17920]|CNTGC1741-15|Chironomidae|633|BOLD:ACM1646
Chironominae|[17921]|CNJAF109-12|Chironomidae|658|BOLD:ACC8424
Saetheria tylus|[17922]|CNWAE2467-14|Chironomidae|635|BOLD:AAJ7404
Chironomidae|[17923]|SSROC3437-15|Chironomidae|510|BOLD:ACS9870
Chironomidae|[17924]|NGNAP200-14|Chironomidae|672|BOLD:ACL2539
Chironominae|[17925]|CNPPJ323-12|Chironomidae|664|BOLD:ABV1415
Chironomidae|[17926]|CNPEF390-14|Chironomidae|658|BOLD:ACL4459
Chironominae|[17927]|BBDCM820-10|Chironomidae|658|BOLD:AAV5982
Chironomidae|[17928]|TTDFW1034-09|Chironomidae|658|BOLD:AAG5465
Chironominae|[17929]|CNMID908-14|Chironomidae|531|BOLD:ACM8522
Chironominae|[17930]|CNKJM333-14|Chironomidae|585|BOLD:ACN1111
Chironominae|[17931]|CNGBH113-14|Chironomidae|608|BOLD:ACL6305
Chironominae|[17932]|SSGBA1924-14|Chironomidae|658|BOLD:ACO8870
Chironominae|[17933]|CNMIF270-14|Chironomidae|627|BOLD:AAV5859
Chironominae|[17934]|CNWAE587-14|Chironomidae|588|BOLD:ACM9846
Chironominae|[17935]|CNPPH946-12|Chironomidae|664|BOLD:ACC8171
Chironominae|[17936]|CNPPH1035-12|Chironomidae|664|BOLD:ACC7886
Chironominae|[17937]|CNPPH949-12|Chironomidae|661|BOLD:ABY6492
Chironominae|[17938]|BBDIQ007-10|Chironomidae|658|BOLD:ACM0867
Chironomidae|[17939]|TTDFW1091-09|Chironomidae|658|BOLD:AAG5477
Chironominae|[17940]|BBDCN194-10|Chironomidae|658|BOLD:ACM1851
Pseudochironomus|[17941]|CNPPI1462-12|Chironomidae|641|BOLD:ACZ1785
Chironominae|[17942]|JWDCF670-10|Chironomidae|658|BOLD:AAQ0620
Chironominae|[17943]|JWDCI406-10|Chironomidae|658|BOLD:AAV5962
Chironominae|[17944]|CNWAE4091-14|Chironomidae|637|BOLD:AAV5952
Chironomidae|[17945]|SSPAA361-13|Chironomidae|670|BOLD:AAM6264
Chironomidae|[17946]|GMDAP1054-13|Chironomidae|661|BOLD:AAG5528
Chironomidae|[17947]|SSPAA1020-13|Chironomidae|664|BOLD:ACG4825
Chironomidae|[17948]|HPPPJ335-13|Chironomidae|658|BOLD:ACI9009
Chironomidae|[17949]|CNGBN629-14|Chironomidae|564|BOLD:ACM4162
Chironomidae|[17950]|SSPAB9793-13|Chironomidae|658|BOLD:ACI5111
Chironomidae|[17951]|GMDAP1069-13|Chironomidae|661|BOLD:ACI3882
Chironomidae|[17952]|GMDAP1299-13|Chironomidae|670|BOLD:ABV1208
Chironomidae|[17953]|CNGBF1984-14|Chironomidae|661|BOLD:ACL3777
Chironomidae|[17954]|CNGBN2463-14|Chironomidae|576|BOLD:ACM4230
Chironomidae|[17955]|BBDEE090-10|Chironomidae|658|BOLD:AAG5516
Polypedilum|[17956]|JSOCT117-11|Chironomidae|658|BOLD:ABW2707
Chironominae|[17957]|SSBAF955-13|Chironomidae|661|BOLD:ACD0785
Sergentia sp. 2TE|[17958]|CNTUA3370-14|Chironomidae|651|BOLD:AAP6561
Sergentia coracina|[17959]|CNTUB3377-14|Chironomidae|591|BOLD:AAH9836
Chironomidae|[17960]|CNEIC2419-12|Chironomidae|658|BOLD:ACA9719
Chironomidae|[17961]|SSPAC1666-13|Chironomidae|658|BOLD:ACI9428
Polypedilum|[17962]|GMDAP960-13|Chironomidae|658|BOLD:ACI6280
Polypedilum|[17963]|CNROM940-13|Chironomidae|658|BOLD:ACK7314
Polypedilum|[17964]|SSWLE3683-13|Chironomidae|658|BOLD:ACJ8313
Polypedilum aviceps|[17965]|BBDEE134-10|Chironomidae|658|BOLD:AAB5362
Polypedilum|[17966]|GMDAP004-12|Chironomidae|658|BOLD:AAD1395
Polypedilum|[17967]|CNBRF765-14|Chironomidae|661|BOLD:ACL6803
Chironomidae|[17968]|BBDEE085-10|Chironomidae|658|BOLD:AAG5512
Cladotanytarsus|[17969]|BSCHR177-10|Chironomidae|658|BOLD:ACY3856
Cladotanytarsus|[17970]|BSCHR188-10|Chironomidae|658|BOLD:AAC4537
Chironominae|[17971]|CNGBF004-14|Chironomidae|664|BOLD:AAL7351
Chironominae|[17972]|CNGBF456-14|Chironomidae|661|BOLD:ACL3071
Polypedilum|[17973]|CNBRD554-14|Chironomidae|658|BOLD:ACW0736
Chironominae|[17974]|CNBRD744-14|Chironomidae|661|BOLD:ACL3517
Chironomidae|[17975]|JSJUN168-11|Chironomidae|658|BOLD:AAM6218
Polypedilum|[17976]|CNGBL458-14|Chironomidae|658|BOLD:AAO7616
Polypedilum|[17977]|SSJAE263-13|Chironomidae|661|BOLD:ACI8430
Polypedilum|[17978]|CNNHC2534-14|Chironomidae|612|BOLD:ACR6251
Polypedilum|[17979]|JSDIQ597-10|Chironomidae|658|BOLD:AAN5395
Polypedilum|[17980]|CNPAC198-13|Chironomidae|658|BOLD:ACE2157
Polypedilum|[17981]|CNPAL334-13|Chironomidae|670|BOLD:ABV1223
Polypedilum|[17982]|BBDEE136-10|Chironomidae|658|BOLD:AAG5520
Polypedilum|[17983]|CNGBF236-14|Chironomidae|675|BOLD:ACM1304
Polypedilum|[17984]|CNGBF559-14|Chironomidae|675|BOLD:ACM0834
Polypedilum|[17985]|CNPKI502-14|Chironomidae|661|BOLD:AAL7352
Polypedilum tritum|[17986]|CHURC583-09|Chironomidae|658|BOLD:AAE4119
Polypedilum|[17987]|BBDCN181-10|Chironomidae|658|BOLD:AAP6881
Chironomidae|[17988]|CNPES143-14|Chironomidae|652|BOLD:ACM1226
Polypedilum|[17989]|SSPAA345-13|Chironomidae|670|BOLD:ACD0408
Polypedilum|[17990]|CNPET111-14|Chironomidae|661|BOLD:ACG4079
Polypedilum|[17991]|CNLMQ118-14|Chironomidae|588|BOLD:ACM5318
Polypedilum|[17992]|SSPAA193-13|Chironomidae|670|BOLD:ACG3630
Polypedilum|[17993]|BBDCN254-10|Chironomidae|658|BOLD:AAP8069
Chironomidae|[17994]|CNWAH5722-15|Chironomidae|564|BOLD:ACS8009
Microtendipes pedellus grp.|[17995]|SMTPB12163-13|Chironomidae|664|BOLD:AAM6248
Chironomidae|[17996]|MBIOG026-13|Chironomidae|661|BOLD:ACK8606
Chironominae|[17997]|CNPPF830-12|Chironomidae|664|BOLD:ACC8280

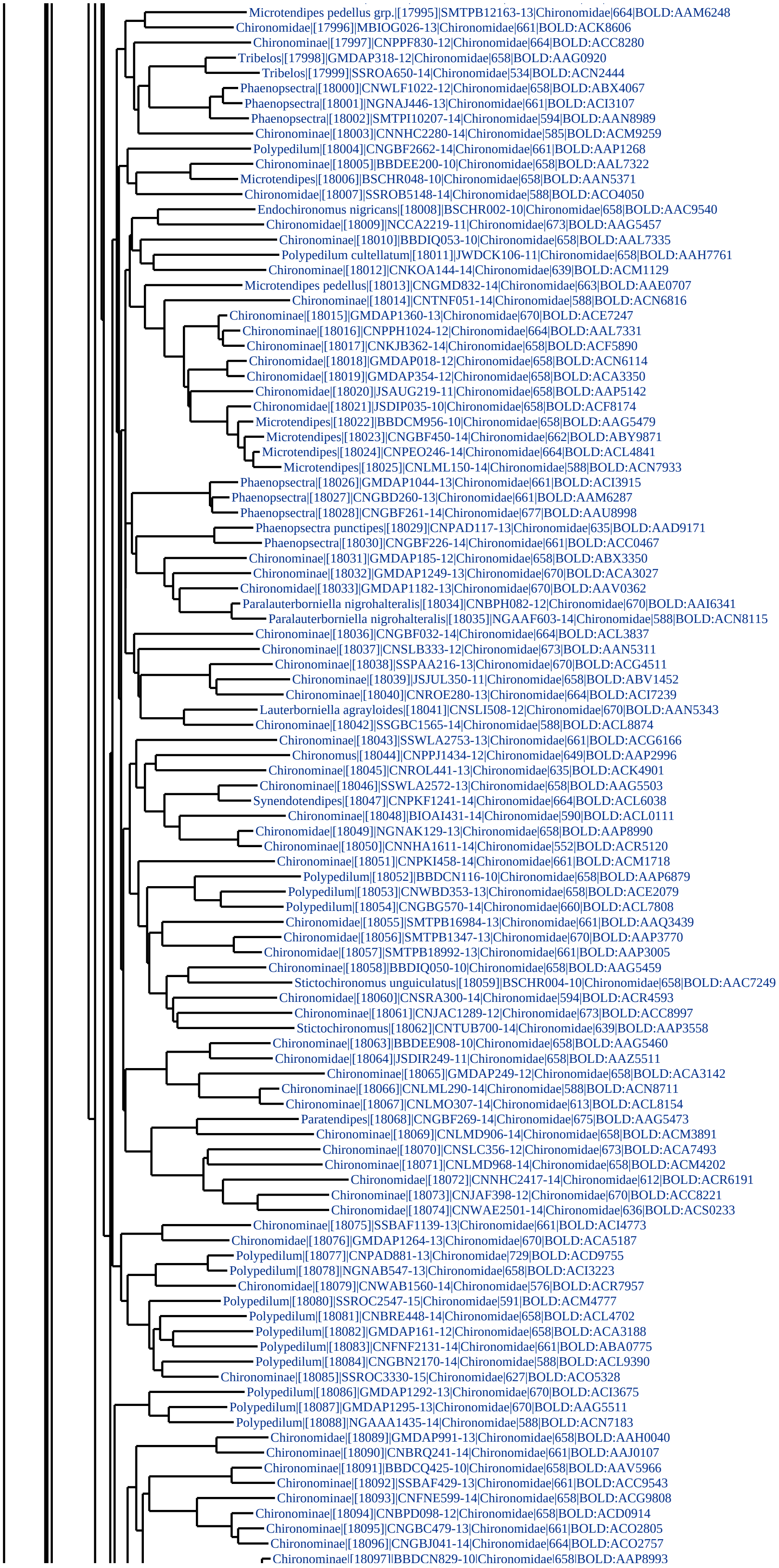
Microtendipes pedellus grp.|[17995]|SMTPB12163-13|Chironomidae|664|BOLD:AAM6248
Chironomidae|[17996]|MBIOG026-13|Chironomidae|661|BOLD:ACK8606
Chironominae|[17997]|CNPPF830-12|Chironomidae|664|BOLD:ACC8280
Tribelos|[17998]|GMDAP318-12|Chironomidae|658|BOLD:AAG0920
Tribelos|[17999]|SSROA650-14|Chironomidae|534|BOLD:ACN2444
Phaenopsectra|[18000]|CNWLF1022-12|Chironomidae|658|BOLD:ABX4067
Phaenopsectra|[18001]|NGNAJ446-13|Chironomidae|661|BOLD:ACI3107
Phaenopsectra|[18002]|SMTPI10207-14|Chironomidae|594|BOLD:AAN8989
Chironominae|[18003]|CNNHC2280-14|Chironomidae|585|BOLD:ACM9259
Polypedilum|[18004]|CNGBF2662-14|Chironomidae|661|BOLD:AAP1268
Chironominae|[18005]|BBDEE200-10|Chironomidae|658|BOLD:AAL7322
Microtendipes|[18006]|BSCHR048-10|Chironomidae|658|BOLD:AAN5371
Chironomidae|[18007]|SSROB5148-14|Chironomidae|588|BOLD:ACO4050
Endochironomus nigricans|[18008]|BSCHR002-10|Chironomidae|658|BOLD:AAC9540
Chironomidae|[18009]|NCCA2219-11|Chironomidae|673|BOLD:AAG5457
Chironominae|[18010]|BBDIQ053-10|Chironomidae|658|BOLD:AAL7335
Polypedilum cultellatum|[18011]|JWDCK106-11|Chironomidae|658|BOLD:AAH7761
Chironominae|[18012]|CNKOA144-14|Chironomidae|639|BOLD:ACM1129
Microtendipes pedellus|[18013]|CNGMD832-14|Chironomidae|663|BOLD:AAE0707
Chironominae|[18014]|CNTNF051-14|Chironomidae|588|BOLD:ACN6816
Chironominae|[18015]|GMDAP1360-13|Chironomidae|670|BOLD:ACE7247
Chironominae|[18016]|CNPPH1024-12|Chironomidae|664|BOLD:AAL7331
Chironominae|[18017]|CNKJB362-14|Chironomidae|658|BOLD:ACF5890
Chironomidae|[18018]|GMDAP018-12|Chironomidae|658|BOLD:ACN6114
Chironomidae|[18019]|GMDAP354-12|Chironomidae|658|BOLD:ACA3350
Chironomidae|[18020]|JSAUG219-11|Chironomidae|658|BOLD:AAP5142
Chironomidae|[18021]|JSDIP035-10|Chironomidae|658|BOLD:ACF8174
Microtendipes|[18022]|BBDCM956-10|Chironomidae|658|BOLD:AAG5479
Microtendipes|[18023]|CNGBF450-14|Chironomidae|662|BOLD:ABY9871
Microtendipes|[18024]|CNPEO246-14|Chironomidae|664|BOLD:ACL4841
Microtendipes|[18025]|CNLML150-14|Chironomidae|588|BOLD:ACN7933
Phaenopsectra|[18026]|GMDAP1044-13|Chironomidae|661|BOLD:ACI3915
Phaenopsectra|[18027]|CNGBD260-13|Chironomidae|661|BOLD:AAM6287
Phaenopsectra|[18028]|CNGBF261-14|Chironomidae|677|BOLD:AAU8998
Phaenopsectra punctipes|[18029]|CNPAD117-13|Chironomidae|635|BOLD:AAD9171
Phaenopsectra|[18030]|CNGBF226-14|Chironomidae|661|BOLD:ACC0467
Chironominae|[18031]|GMDAP185-12|Chironomidae|658|BOLD:ABX3350
Chironominae|[18032]|GMDAP1249-13|Chironomidae|670|BOLD:ACA3027
Chironomidae|[18033]|GMDAP1182-13|Chironomidae|670|BOLD:AAV0362
Paralauterborniella nigrohalteralis|[18034]|CNBPH082-12|Chironomidae|670|BOLD:AAI6341
Paralauterborniella nigrohalteralis|[18035]|NGAAF603-14|Chironomidae|588|BOLD:ACN8115
Chironominae|[18036]|CNGBF032-14|Chironomidae|664|BOLD:ACL3837
Chironominae|[18037]|CNSLB333-12|Chironomidae|673|BOLD:AAN5311
Chironominae|[18038]|SSPAA216-13|Chironomidae|670|BOLD:ACG4511
Chironominae|[18039]|JSJUL350-11|Chironomidae|658|BOLD:ABV1452
Chironominae|[18040]|CNROE280-13|Chironomidae|664|BOLD:ACI7239
Lauterborniella agrayloides|[18041]|CNSLI508-12|Chironomidae|670|BOLD:AAN5343
Chironominae|[18042]|SSGBC1565-14|Chironomidae|588|BOLD:ACL8874
Chironominae|[18043]|SSWLA2753-13|Chironomidae|661|BOLD:ACG6166
Chironomus|[18044]|CNPPJ1434-12|Chironomidae|649|BOLD:AAP2996
Chironominae|[18045]|CNROL441-13|Chironomidae|635|BOLD:ACK4901
Chironominae|[18046]|SSWLA2572-13|Chironomidae|658|BOLD:AAG5503
Synendotendipes|[18047]|CNPKF1241-14|Chironomidae|664|BOLD:ACL6038
Chironominae|[18048]|BIOAI431-14|Chironomidae|590|BOLD:ACL0111
Chironomidae|[18049]|NGNAK129-13|Chironomidae|658|BOLD:AAP8990
Chironominae|[18050]|CNNHA1611-14|Chironomidae|552|BOLD:ACR5120
Chironominae|[18051]|CNPKI458-14|Chironomidae|661|BOLD:ACM1718
Polypedilum|[18052]|BBDCN116-10|Chironomidae|658|BOLD:AAP6879
Polypedilum|[18053]|CNWBD353-13|Chironomidae|658|BOLD:ACE2079
Polypedilum|[18054]|CNGBG570-14|Chironomidae|660|BOLD:ACL7808
Chironomidae|[18055]|SMTPB16984-13|Chironomidae|661|BOLD:AAQ3439
Chironomidae|[18056]|SMTPB1347-13|Chironomidae|670|BOLD:AAP3770
Chironomidae|[18057]|SMTPB18992-13|Chironomidae|661|BOLD:AAP3005
Chironominae|[18058]|BBDIQ050-10|Chironomidae|658|BOLD:AAG5459
Stictochironomus unguiculatus|[18059]|BSCHR004-10|Chironomidae|658|BOLD:AAC7249
Chironomidae|[18060]|CNSRA300-14|Chironomidae|594|BOLD:ACR4593
Chironominae|[18061]|CNJAC1289-12|Chironomidae|673|BOLD:ACC8997
Stictochironomus|[18062]|CNTUB700-14|Chironomidae|639|BOLD:AAP3558
Chironominae|[18063]|BBDEE908-10|Chironomidae|658|BOLD:AAG5460
Chironomidae|[18064]|JSDIR249-11|Chironomidae|658|BOLD:AAZ5511
Chironominae|[18065]|GMDAP249-12|Chironomidae|658|BOLD:ACA3142
Chironominae|[18066]|CNLML290-14|Chironomidae|588|BOLD:ACN8711
Chironominae|[18067]|CNLMO307-14|Chironomidae|613|BOLD:ACL8154
Paratendipes|[18068]|CNGBF269-14|Chironomidae|675|BOLD:AAG5473
Chironominae|[18069]|CNLMD906-14|Chironomidae|658|BOLD:ACM3891
Chironominae|[18070]|CNSLC356-12|Chironomidae|673|BOLD:ACA7493
Chironominae|[18071]|CNLMD968-14|Chironomidae|658|BOLD:ACM4202
Chironomidae|[18072]|CNNHC2417-14|Chironomidae|612|BOLD:ACR6191
Chironominae|[18073]|CNJAF398-12|Chironomidae|670|BOLD:ACC8221
Chironomidae|[18074]|CNWAE2501-14|Chironomidae|636|BOLD:ACS0233
Chironominae|[18075]|SSBAF1139-13|Chironomidae|661|BOLD:ACI4773
Chironomidae|[18076]|GMDAP1264-13|Chironomidae|670|BOLD:ACA5187
Polypedilum|[18077]|CNPAD881-13|Chironomidae|729|BOLD:ACD9755
Polypedilum|[18078]|NGNAB547-13|Chironomidae|658|BOLD:ACI3223
Chironomidae|[18079]|CNWAB1560-14|Chironomidae|576|BOLD:ACR7957
Polypedilum|[18080]|SSROC2547-15|Chironomidae|591|BOLD:ACM4777
Polypedilum|[18081]|CNBRE448-14|Chironomidae|658|BOLD:ACL4702
Polypedilum|[18082]|GMDAP161-12|Chironomidae|658|BOLD:ACA3188
Polypedilum|[18083]|CNFNF2131-14|Chironomidae|661|BOLD:ABA0775
Polypedilum|[18084]|CNGBN2170-14|Chironomidae|588|BOLD:ACL9390
Chironominae|[18085]|SSROC3330-15|Chironomidae|627|BOLD:ACO5328
Polypedilum|[18086]|GMDAP1292-13|Chironomidae|670|BOLD:ACI3675
Polypedilum|[18087]|GMDAP1295-13|Chironomidae|670|BOLD:AAG5511
Polypedilum|[18088]|NGAAA1435-14|Chironomidae|588|BOLD:ACN7183
Chironomidae|[18089]|GMDAP991-13|Chironomidae|658|BOLD:AAH0040
Chironominae|[18090]|CNBRQ241-14|Chironomidae|661|BOLD:AAJ0107
Chironominae|[18091]|BBDCQ425-10|Chironomidae|658|BOLD:AAV5966
Chironominae|[18092]|SSBAF429-13|Chironomidae|661|BOLD:ACC9543
Chironominae|[18093]|CNFNE599-14|Chironomidae|658|BOLD:ACG9808
Chironominae|[18094]|CNBPD098-12|Chironomidae|658|BOLD:ACD0914
Chironominae|[18095]|CNGBC479-13|Chironomidae|661|BOLD:ACO2805
Chironominae|[18096]|CNGBJ041-14|Chironomidae|664|BOLD:ACO2757
Chironominae|[18097]|BBDCN829-10|Chironomidae|658|BOLD:AAP8993

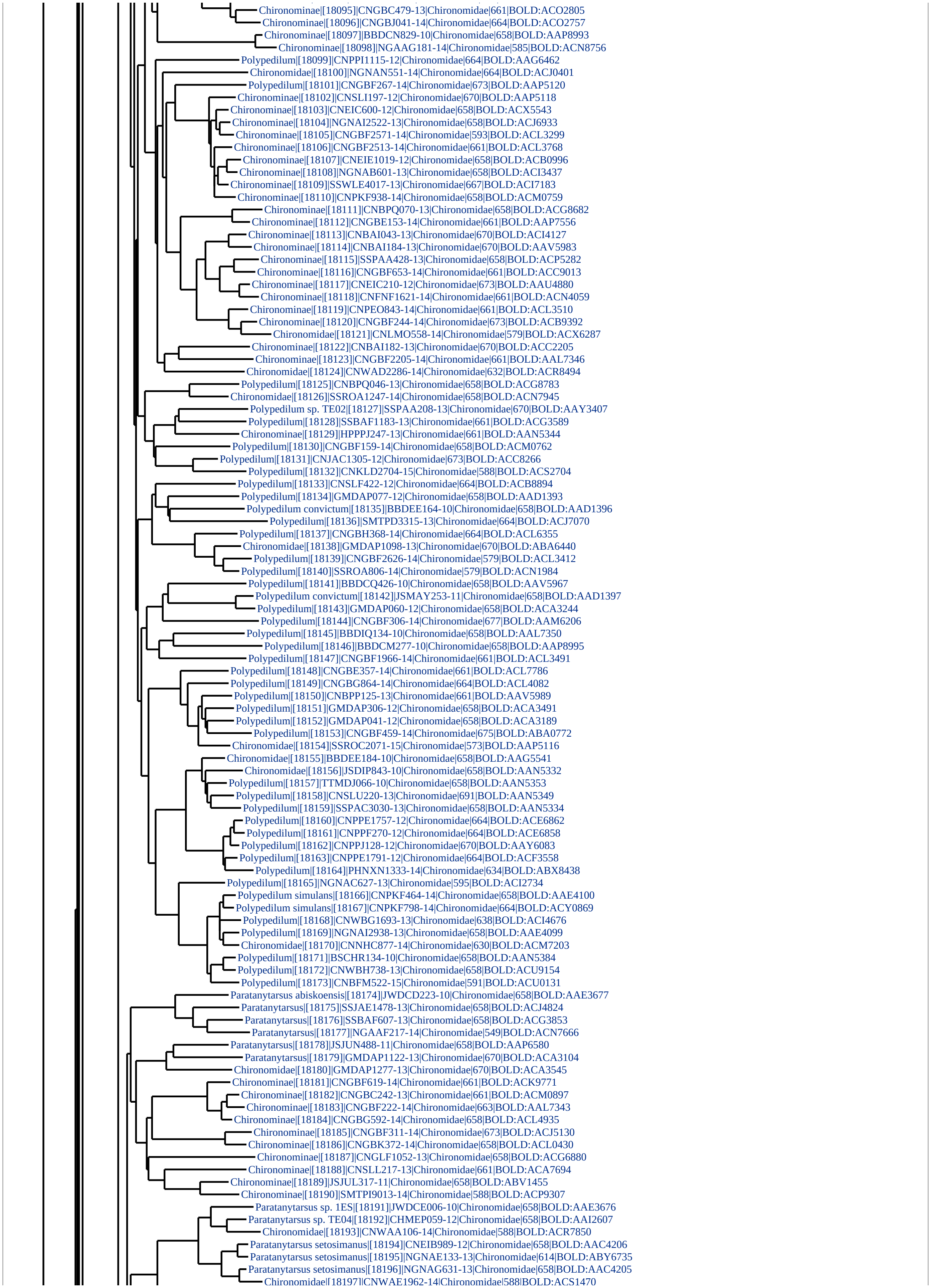
Chironominae|[18095]|CNGBC479-13|Chironomidae|661|BOLD:ACO2805
Chironominae|[18096]|CNGBJ041-14|Chironomidae|664|BOLD:ACO2757
Chironominae|[18097]|BBDCN829-10|Chironomidae|658|BOLD:AAP8993
Chironominae|[18098]|NGAAG181-14|Chironomidae|585|BOLD:ACN8756
Polypedilum|[18099]|CNPPI1115-12|Chironomidae|664|BOLD:AAG6462
Chironomidae|[18100]|NGNAN551-14|Chironomidae|664|BOLD:ACJ0401
Polypedilum|[18101]|CNGBF267-14|Chironomidae|673|BOLD:AAP5120
Chironominae|[18102]|CNSLI197-12|Chironomidae|670|BOLD:AAP5118
Chironominae|[18103]|CNEIC600-12|Chironomidae|658|BOLD:ACX5543
Chironominae|[18104]|NGNAI2522-13|Chironomidae|658|BOLD:ACJ6933
Chironominae|[18105]|CNGBF2571-14|Chironomidae|593|BOLD:ACL3299
Chironominae|[18106]|CNGBF2513-14|Chironomidae|661|BOLD:ACL3768
Chironominae|[18107]|CNEIE1019-12|Chironomidae|658|BOLD:ACB0996
Chironominae|[18108]|NGNAB601-13|Chironomidae|658|BOLD:ACI3437
Chironominae|[18109]|SSWLE4017-13|Chironomidae|667|BOLD:ACI7183
Chironominae|[18110]|CNPKF938-14|Chironomidae|658|BOLD:ACM0759
Chironominae|[18111]|CNBPQ070-13|Chironomidae|658|BOLD:ACG8682
Chironominae|[18112]|CNGBE153-14|Chironomidae|661|BOLD:AAP7556
Chironominae|[18113]|CNBAI043-13|Chironomidae|670|BOLD:ACI4127
Chironominae|[18114]|CNBAI184-13|Chironomidae|670|BOLD:AAV5983
Chironominae|[18115]|SSPAA428-13|Chironomidae|658|BOLD:ACP5282
Chironominae|[18116]|CNGBF653-14|Chironomidae|661|BOLD:ACC9013
Chironominae|[18117]|CNEIC210-12|Chironomidae|673|BOLD:AAU4880
Chironominae|[18118]|CNFNF1621-14|Chironomidae|661|BOLD:ACN4059
Chironominae|[18119]|CNPEO843-14|Chironomidae|661|BOLD:ACL3510
Chironominae|[18120]|CNGBF244-14|Chironomidae|673|BOLD:ACB9392
Chironomidae|[18121]|CNLMO558-14|Chironomidae|579|BOLD:ACX6287
Chironominae|[18122]|CNBAI182-13|Chironomidae|670|BOLD:ACC2205
Chironominae|[18123]|CNGBF2205-14|Chironomidae|661|BOLD:AAL7346
Chironomidae|[18124]|CNWAD2286-14|Chironomidae|632|BOLD:ACR8494
Polypedilum|[18125]|CNBPQ046-13|Chironomidae|658|BOLD:ACG8783
Chironomidae|[18126]|SSROA1247-14|Chironomidae|658|BOLD:ACN7945
Polypedilum sp. TE02|[18127]|SSPAA208-13|Chironomidae|670|BOLD:AAY3407
Polypedilum|[18128]|SSBAF1183-13|Chironomidae|661|BOLD:ACG3589
Chironominae|[18129]|HPPPJ247-13|Chironomidae|661|BOLD:AAN5344
Polypedilum|[18130]|CNGBF159-14|Chironomidae|658|BOLD:ACM0762
Polypedilum|[18131]|CNJAC1305-12|Chironomidae|673|BOLD:ACC8266
Polypedilum|[18132]|CNKLD2704-15|Chironomidae|588|BOLD:ACS2704
Polypedilum|[18133]|CNSLF422-12|Chironomidae|664|BOLD:ACB8894
Polypedilum|[18134]|GMDAP077-12|Chironomidae|658|BOLD:AAD1393
Polypedilum convictum|[18135]|BBDEE164-10|Chironomidae|658|BOLD:AAD1396
Polypedilum|[18136]|SMTPD3315-13|Chironomidae|664|BOLD:ACJ7070
Polypedilum|[18137]|CNGBH368-14|Chironomidae|664|BOLD:ACL6355
Chironomidae|[18138]|GMDAP1098-13|Chironomidae|670|BOLD:ABA6440
Polypedilum|[18139]|CNGBF2626-14|Chironomidae|579|BOLD:ACL3412
Polypedilum|[18140]|SSROA806-14|Chironomidae|579|BOLD:ACN1984
Polypedilum|[18141]|BBDCQ426-10|Chironomidae|658|BOLD:AAV5967
Polypedilum convictum|[18142]|JSMAY253-11|Chironomidae|658|BOLD:AAD1397
Polypedilum|[18143]|GMDAP060-12|Chironomidae|658|BOLD:ACA3244
Polypedilum|[18144]|CNGBF306-14|Chironomidae|677|BOLD:AAM6206
Polypedilum|[18145]|BBDIQ134-10|Chironomidae|658|BOLD:AAL7350
Polypedilum|[18146]|BBDCM277-10|Chironomidae|658|BOLD:AAP8995
Polypedilum|[18147]|CNGBF1966-14|Chironomidae|661|BOLD:ACL3491
Polypedilum|[18148]|CNGBE357-14|Chironomidae|661|BOLD:ACL7786
Polypedilum|[18149]|CNGBG864-14|Chironomidae|664|BOLD:ACL4082
Polypedilum|[18150]|CNBPP125-13|Chironomidae|661|BOLD:AAV5989
Polypedilum|[18151]|GMDAP306-12|Chironomidae|658|BOLD:ACA3491
Polypedilum|[18152]|GMDAP041-12|Chironomidae|658|BOLD:ACA3189
Polypedilum|[18153]|CNGBF459-14|Chironomidae|675|BOLD:ABA0772
Chironomidae|[18154]|SSROC2071-15|Chironomidae|573|BOLD:AAP5116
Chironomidae|[18155]|BBDEE184-10|Chironomidae|658|BOLD:AAG5541
Chironomidae|[18156]|JSDIP843-10|Chironomidae|658|BOLD:AAN5332
Polypedilum|[18157]|TTMDJ066-10|Chironomidae|658|BOLD:AAN5353
Polypedilum|[18158]|CNSLU220-13|Chironomidae|691|BOLD:AAN5349
Polypedilum|[18159]|SSPAC3030-13|Chironomidae|658|BOLD:AAN5334
Polypedilum|[18160]|CNPPE1757-12|Chironomidae|664|BOLD:ACE6862
Polypedilum|[18161]|CNPPF270-12|Chironomidae|664|BOLD:ACE6858
Polypedilum|[18162]|CNPPJ128-12|Chironomidae|670|BOLD:AAY6083
Polypedilum|[18163]|CNPPE1791-12|Chironomidae|664|BOLD:ACF3558
Polypedilum|[18164]|PHNXN1333-14|Chironomidae|634|BOLD:ABX8438
Polypedilum|[18165]|NGNAC627-13|Chironomidae|595|BOLD:ACI2734
Polypedilum simulans|[18166]|CNPKF464-14|Chironomidae|658|BOLD:AAE4100
Polypedilum simulans|[18167]|CNPKF798-14|Chironomidae|664|BOLD:ACY0869
Polypedilum|[18168]|CNWBG1693-13|Chironomidae|638|BOLD:ACI4676
Polypedilum|[18169]|NGNAI2938-13|Chironomidae|658|BOLD:AAE4099
Chironomidae|[18170]|CNNHC877-14|Chironomidae|630|BOLD:ACM7203
Polypedilum|[18171]|BSCHR134-10|Chironomidae|658|BOLD:AAN5384
Polypedilum|[18172]|CNWBH738-13|Chironomidae|658|BOLD:ACU9154
Polypedilum|[18173]|CNBFM522-15|Chironomidae|591|BOLD:ACU0131
Paratanytarsus abiskoensis|[18174]|JWDCD223-10|Chironomidae|658|BOLD:AAE3677
Paratanytarsus|[18175]|SSJAE1478-13|Chironomidae|658|BOLD:ACJ4824
Paratanytarsus|[18176]|SSBAF607-13|Chironomidae|658|BOLD:ACG3853
Paratanytarsus|[18177]|NGAAF217-14|Chironomidae|549|BOLD:ACN7666
Paratanytarsus|[18178]|JSJUN488-11|Chironomidae|658|BOLD:AAP6580
Paratanytarsus|[18179]|GMDAP1122-13|Chironomidae|670|BOLD:ACA3104
Chironomidae|[18180]|GMDAP1277-13|Chironomidae|670|BOLD:ACA3545
Chironominae|[18181]|CNGBF619-14|Chironomidae|661|BOLD:ACK9771
Chironominae|[18182]|CNGBC242-13|Chironomidae|661|BOLD:ACM0897
Chironominae|[18183]|CNGBF222-14|Chironomidae|663|BOLD:AAL7343
Chironominae|[18184]|CNGBG592-14|Chironomidae|658|BOLD:ACL4935
Chironominae|[18185]|CNGBF311-14|Chironomidae|673|BOLD:ACJ5130
Chironominae|[18186]|CNGBK372-14|Chironomidae|658|BOLD:ACL0430
Chironominae|[18187]|CNGLF1052-13|Chironomidae|658|BOLD:ACG6880
Chironominae|[18188]|CNSLL217-13|Chironomidae|661|BOLD:ACA7694
Chironominae|[18189]|JSJUL317-11|Chironomidae|658|BOLD:ABV1455
Chironominae|[18190]|SMTPI9013-14|Chironomidae|588|BOLD:ACP9307
Paratanytarsus sp. 1ES|[18191]|JWDCE006-10|Chironomidae|658|BOLD:AAE3676
Paratanytarsus sp. TE04|[18192]|CHMEP059-12|Chironomidae|658|BOLD:AAI2607
Chironomidae|[18193]|CNWAA106-14|Chironomidae|588|BOLD:ACR7850
Paratanytarsus setosimanus|[18194]|CNEIB989-12|Chironomidae|658|BOLD:AAC4206
Paratanytarsus setosimanus|[18195]|NGNAE133-13|Chironomidae|614|BOLD:ABY6735
Paratanytarsus setosimanus|[18196]|NGNAG631-13|Chironomidae|658|BOLD:AAC4205
Chironomidae|[18197]|CNWAE1962-14|Chironomidae|588|BOLD:ACS1470

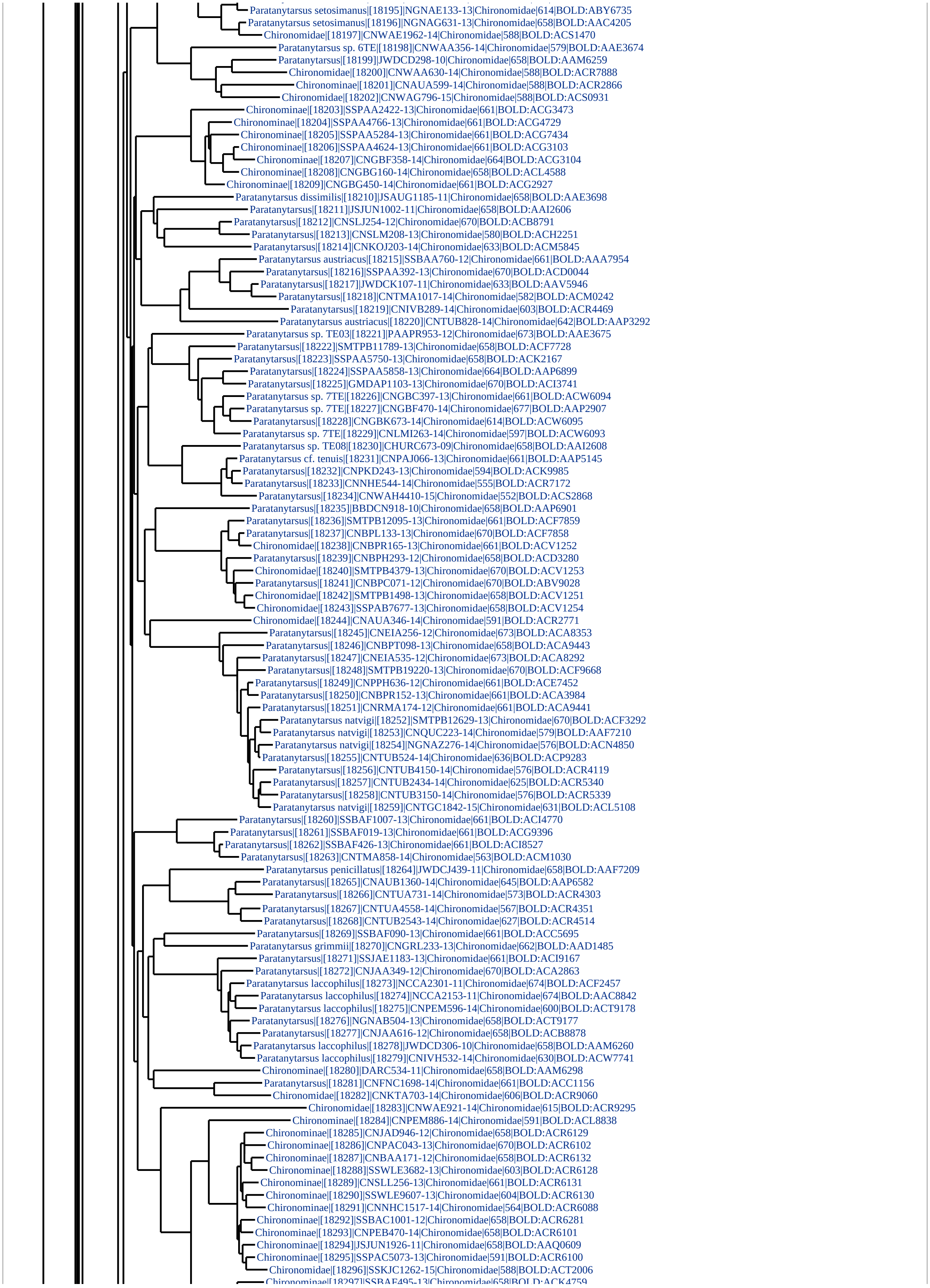

Paratanytarsus setosimanus|[18195]|NGNAE133-13|Chironomidae|614|BOLD:ABY6735
Paratanytarsus setosimanus|[18196]|NGNAG631-13|Chironomidae|658|BOLD:AAC4205
Chironomidae|[18197]|CNWAE1962-14|Chironomidae|588|BOLD:ACS1470
Paratanytarsus sp. 6TE|[18198]|CNWAA356-14|Chironomidae|579|BOLD:AAE3674
Paratanytarsus|[18199]|JWDCD298-10|Chironomidae|658|BOLD:AAM6259
Chironomidae|[18200]|CNWAA630-14|Chironomidae|588|BOLD:ACR7888
Chironominae|[18201]|CNAUA599-14|Chironomidae|588|BOLD:ACR2866
Chironomidae|[18202]|CNWAG796-15|Chironomidae|588|BOLD:ACS0931
Chironominae|[18203]|SSPAA2422-13|Chironomidae|661|BOLD:ACG3473
Chironominae|[18204]|SSPAA4766-13|Chironomidae|661|BOLD:ACG4729
Chironominae|[18205]|SSPAA5284-13|Chironomidae|661|BOLD:ACG7434
Chironominae|[18206]|SSPAA4624-13|Chironomidae|661|BOLD:ACG3103
Chironominae|[18207]|CNGBF358-14|Chironomidae|664|BOLD:ACG3104
Chironominae|[18208]|CNGBG160-14|Chironomidae|658|BOLD:ACL4588
Chironominae|[18209]|CNGBG450-14|Chironomidae|661|BOLD:ACG2927
Paratanytarsus dissimilis|[18210]|JSAUG1185-11|Chironomidae|658|BOLD:AAE3698
Paratanytarsus|[18211]|JSJUN1002-11|Chironomidae|658|BOLD:AAI2606
Paratanytarsus|[18212]|CNSLJ254-12|Chironomidae|670|BOLD:ACB8791
Paratanytarsus|[18213]|CNSLM208-13|Chironomidae|580|BOLD:ACH2251
Paratanytarsus|[18214]|CNKOJ203-14|Chironomidae|633|BOLD:ACM5845
Paratanytarsus austriacus|[18215]|SSBAA760-12|Chironomidae|661|BOLD:AAA7954
Paratanytarsus|[18216]|SSPAA392-13|Chironomidae|670|BOLD:ACD0044
Paratanytarsus|[18217]|JWDCK107-11|Chironomidae|633|BOLD:AAV5946
Paratanytarsus|[18218]|CNTMA1017-14|Chironomidae|582|BOLD:ACM0242
Paratanytarsus|[18219]|CNIVB289-14|Chironomidae|603|BOLD:ACR4469
Paratanytarsus austriacus|[18220]|CNTUB828-14|Chironomidae|642|BOLD:AAP3292
Paratanytarsus sp. TE03|[18221]|PAAPR953-12|Chironomidae|673|BOLD:AAE3675
Paratanytarsus|[18222]|SMTPB11789-13|Chironomidae|658|BOLD:ACF7728
Paratanytarsus|[18223]|SSPAA5750-13|Chironomidae|658|BOLD:ACK2167
Paratanytarsus|[18224]|SSPAA5858-13|Chironomidae|664|BOLD:AAP6899
Paratanytarsus|[18225]|GMDAP1103-13|Chironomidae|670|BOLD:ACI3741
Paratanytarsus sp. 7TE|[18226]|CNGBC397-13|Chironomidae|661|BOLD:ACW6094
Paratanytarsus sp. 7TE|[18227]|CNGBF470-14|Chironomidae|677|BOLD:AAP2907
Paratanytarsus|[18228]|CNGBK673-14|Chironomidae|614|BOLD:ACW6095
Paratanytarsus sp. 7TE|[18229]|CNLMI263-14|Chironomidae|597|BOLD:ACW6093
Paratanytarsus sp. TE08|[18230]|CHURC673-09|Chironomidae|658|BOLD:AAI2608
Paratanytarsus cf. tenuis|[18231]|CNPAJ066-13|Chironomidae|661|BOLD:AAP5145
Paratanytarsus|[18232]|CNPKD243-13|Chironomidae|594|BOLD:ACK9985
Paratanytarsus|[18233]|CNNHE544-14|Chironomidae|555|BOLD:ACR7172
Paratanytarsus|[18234]|CNWAH4410-15|Chironomidae|552|BOLD:ACS2868
Paratanytarsus|[18235]|BBDCN918-10|Chironomidae|658|BOLD:AAP6901
Paratanytarsus|[18236]|SMTPB12095-13|Chironomidae|661|BOLD:ACF7859
Paratanytarsus|[18237]|CNBPL133-13|Chironomidae|670|BOLD:ACF7858
Chironomidae|[18238]|CNBPR165-13|Chironomidae|661|BOLD:ACV1252
Paratanytarsus|[18239]|CNBPH293-12|Chironomidae|658|BOLD:ACD3280
Chironomidae|[18240]|SMTPB4379-13|Chironomidae|670|BOLD:ACV1253
Paratanytarsus|[18241]|CNBPC071-12|Chironomidae|670|BOLD:ABV9028
Chironomidae|[18242]|SMTPB1498-13|Chironomidae|658|BOLD:ACV1251
Chironomidae|[18243]|SSPAB7677-13|Chironomidae|658|BOLD:ACV1254
Chironomidae|[18244]|CNAUA346-14|Chironomidae|591|BOLD:ACR2771
Paratanytarsus|[18245]|CNEIA256-12|Chironomidae|673|BOLD:ACA8353
Paratanytarsus|[18246]|CNBPT098-13|Chironomidae|658|BOLD:ACA9443
Paratanytarsus|[18247]|CNEIA535-12|Chironomidae|673|BOLD:ACA8292
Paratanytarsus|[18248]|SMTPB19220-13|Chironomidae|670|BOLD:ACF9668
Paratanytarsus|[18249]|CNPPH636-12|Chironomidae|661|BOLD:ACE7452
Paratanytarsus|[18250]|CNBPR152-13|Chironomidae|661|BOLD:ACA3984
Paratanytarsus|[18251]|CNRMA174-12|Chironomidae|661|BOLD:ACA9441
Paratanytarsus natvigi|[18252]|SMTPB12629-13|Chironomidae|670|BOLD:ACF3292
Paratanytarsus natvigi|[18253]|CNQUC223-14|Chironomidae|579|BOLD:AAF7210
Paratanytarsus natvigi|[18254]|NGNAZ276-14|Chironomidae|576|BOLD:ACN4850
Paratanytarsus|[18255]|CNTUB524-14|Chironomidae|636|BOLD:ACP9283
Paratanytarsus|[18256]|CNTUB4150-14|Chironomidae|576|BOLD:ACR4119
Paratanytarsus|[18257]|CNTUB2434-14|Chironomidae|625|BOLD:ACR5340
Paratanytarsus|[18258]|CNTUB3150-14|Chironomidae|576|BOLD:ACR5339
Paratanytarsus natvigi|[18259]|CNTGC1842-15|Chironomidae|631|BOLD:ACL5108
Paratanytarsus|[18260]|SSBAF1007-13|Chironomidae|661|BOLD:ACI4770
Paratanytarsus|[18261]|SSBAF019-13|Chironomidae|661|BOLD:ACG9396
Paratanytarsus|[18262]|SSBAF426-13|Chironomidae|661|BOLD:ACI8527
Paratanytarsus|[18263]|CNTMA858-14|Chironomidae|563|BOLD:ACM1030
Paratanytarsus penicillatus|[18264]|JWDCJ439-11|Chironomidae|658|BOLD:AAF7209
Paratanytarsus|[18265]|CNAUB1360-14|Chironomidae|645|BOLD:AAP6582
Paratanytarsus|[18266]|CNTUA731-14|Chironomidae|573|BOLD:ACR4303
Paratanytarsus|[18267]|CNTUA4558-14|Chironomidae|567|BOLD:ACR4351
Paratanytarsus|[18268]|CNTUB2543-14|Chironomidae|627|BOLD:ACR4514
Paratanytarsus|[18269]|SSBAF090-13|Chironomidae|661|BOLD:ACC5695
Paratanytarsus grimmii|[18270]|CNGRL233-13|Chironomidae|662|BOLD:AAD1485
Paratanytarsus|[18271]|SSJAE1183-13|Chironomidae|661|BOLD:ACI9167
Paratanytarsus|[18272]|CNJAA349-12|Chironomidae|670|BOLD:ACA2863
Paratanytarsus laccophilus|[18273]|NCCA2301-11|Chironomidae|674|BOLD:ACF2457
Paratanytarsus laccophilus|[18274]|NCCA2153-11|Chironomidae|674|BOLD:AAC8842
Paratanytarsus laccophilus|[18275]|CNPEM596-14|Chironomidae|600|BOLD:ACT9178
Paratanytarsus|[18276]|NGNAB504-13|Chironomidae|658|BOLD:ACT9177
Paratanytarsus|[18277]|CNJAA616-12|Chironomidae|658|BOLD:ACB8878
Paratanytarsus laccophilus|[18278]|JWDCD306-10|Chironomidae|658|BOLD:AAM6260
Paratanytarsus laccophilus|[18279]|CNIVH532-14|Chironomidae|630|BOLD:ACW7741
Chironominae|[18280]|DARC534-11|Chironomidae|658|BOLD:AAM6298
Paratanytarsus|[18281]|CNFNC1698-14|Chironomidae|661|BOLD:ACC1156
Chironomidae|[18282]|CNKTA703-14|Chironomidae|606|BOLD:ACR9060
Chironomidae|[18283]|CNWAE921-14|Chironomidae|615|BOLD:ACR9295
Chironominae|[18284]|CNPEM886-14|Chironomidae|591|BOLD:ACL8838
Chironominae|[18285]|CNJAD946-12|Chironomidae|658|BOLD:ACR6129
Chironominae|[18286]|CNPAC043-13|Chironomidae|670|BOLD:ACR6102
Chironominae|[18287]|CNBAA171-12|Chironomidae|658|BOLD:ACR6132
Chironominae|[18288]|SSWLE3682-13|Chironomidae|603|BOLD:ACR6128
Chironominae|[18289]|CNSLL256-13|Chironomidae|661|BOLD:ACR6131
Chironominae|[18290]|SSWLE9607-13|Chironomidae|604|BOLD:ACR6130
Chironominae|[18291]|CNNHC1517-14|Chironomidae|564|BOLD:ACR6088
Chironominae|[18292]|SSBAC1001-12|Chironomidae|658|BOLD:ACR6281
Chironominae|[18293]|CNPEB470-14|Chironomidae|658|BOLD:ACR6101
Chironominae|[18294]|JSJUN1926-11|Chironomidae|658|BOLD:AAQ0609
Chironominae|[18295]|SSPAC5073-13|Chironomidae|591|BOLD:ACR6100
Chironomidae|[18296]|SSKJC1262-15|Chironomidae|588|BOLD:ACT2006
Chironominae|[18297]|SSBAF495-13|Chironomidae|658|BOLD:ACK4759

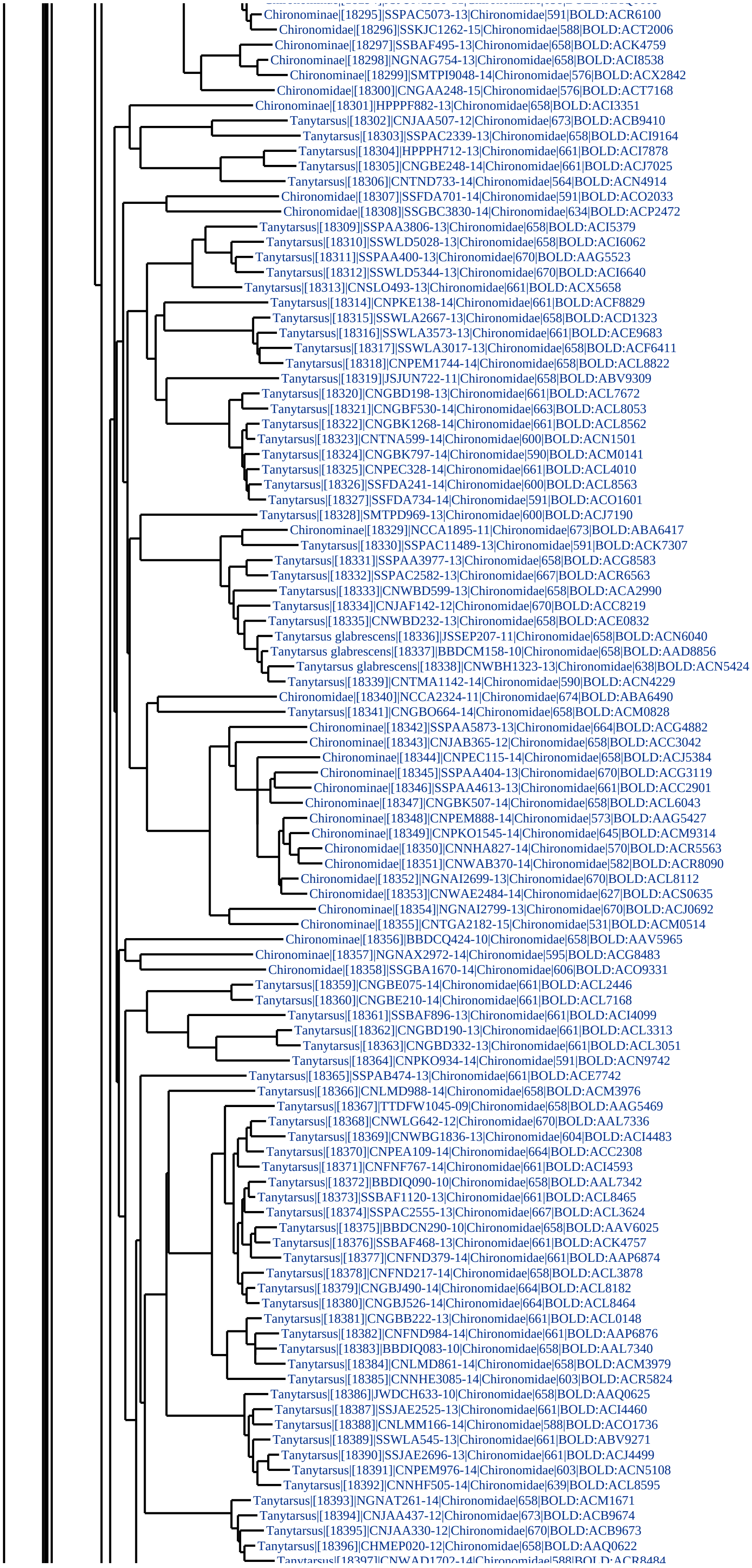
Chironominae|[18295]|SSPAC5073-13|Chironomidae|591|BOLD:ACR6100
Chironomidae|[18296]|SSKJC1262-15|Chironomidae|588|BOLD:ACT2006
Chironominae|[18297]|SSBAF495-13|Chironomidae|658|BOLD:ACK4759
Chironominae|[18298]|NGNAG754-13|Chironomidae|658|BOLD:ACI8538
Chironominae|[18299]|SMTPI9048-14|Chironomidae|576|BOLD:ACX2842
Chironomidae|[18300]|CNGAA248-15|Chironomidae|576|BOLD:ACT7168
Chironominae|[18301]|HPPPF882-13|Chironomidae|658|BOLD:ACI3351
Tanytarsus|[18302]|CNJAA507-12|Chironomidae|673|BOLD:ACB9410
Tanytarsus|[18303]|SSPAC2339-13|Chironomidae|658|BOLD:ACI9164
Tanytarsus|[18304]|HPPPH712-13|Chironomidae|661|BOLD:ACI7878
Tanytarsus|[18305]|CNGBE248-14|Chironomidae|661|BOLD:ACJ7025
Tanytarsus|[18306]|CNTND733-14|Chironomidae|564|BOLD:ACN4914
Chironomidae|[18307]|SSFDA701-14|Chironomidae|591|BOLD:ACO2033
Chironomidae|[18308]|SSGBC3830-14|Chironomidae|634|BOLD:ACP2472
Tanytarsus|[18309]|SSPAA3806-13|Chironomidae|658|BOLD:ACI5379
Tanytarsus|[18310]|SSWLD5028-13|Chironomidae|658|BOLD:ACI6062
Tanytarsus|[18311]|SSPAA400-13|Chironomidae|670|BOLD:AAG5523
Tanytarsus|[18312]|SSWLD5344-13|Chironomidae|670|BOLD:ACI6640
Tanytarsus|[18313]|CNSLO493-13|Chironomidae|661|BOLD:ACX5658
Tanytarsus|[18314]|CNPKE138-14|Chironomidae|661|BOLD:ACF8829
Tanytarsus|[18315]|SSWLA2667-13|Chironomidae|658|BOLD:ACD1323
Tanytarsus|[18316]|SSWLA3573-13|Chironomidae|661|BOLD:ACE9683
Tanytarsus|[18317]|SSWLA3017-13|Chironomidae|658|BOLD:ACF6411
Tanytarsus|[18318]|CNPEM1744-14|Chironomidae|658|BOLD:ACL8822
Tanytarsus|[18319]|JSJUN722-11|Chironomidae|658|BOLD:ABV9309
Tanytarsus|[18320]|CNGBD198-13|Chironomidae|661|BOLD:ACL7672
Tanytarsus|[18321]|CNGBF530-14|Chironomidae|663|BOLD:ACL8053
Tanytarsus|[18322]|CNGBK1268-14|Chironomidae|661|BOLD:ACL8562
Tanytarsus|[18323]|CNTNA599-14|Chironomidae|600|BOLD:ACN1501
Tanytarsus|[18324]|CNGBK797-14|Chironomidae|590|BOLD:ACM0141
Tanytarsus|[18325]|CNPEC328-14|Chironomidae|661|BOLD:ACL4010
Tanytarsus|[18326]|SSFDA241-14|Chironomidae|600|BOLD:ACL8563
Tanytarsus|[18327]|SSFDA734-14|Chironomidae|591|BOLD:ACO1601
Tanytarsus|[18328]|SMTPD969-13|Chironomidae|600|BOLD:ACJ7190
Chironominae|[18329]|NCCA1895-11|Chironomidae|673|BOLD:ABA6417
Tanytarsus|[18330]|SSPAC11489-13|Chironomidae|591|BOLD:ACK7307
Tanytarsus|[18331]|SSPAA3977-13|Chironomidae|658|BOLD:ACG8583
Tanytarsus|[18332]|SSPAC2582-13|Chironomidae|667|BOLD:ACR6563
Tanytarsus|[18333]|CNWBD599-13|Chironomidae|658|BOLD:ACA2990
Tanytarsus|[18334]|CNJAF142-12|Chironomidae|670|BOLD:ACC8219
Tanytarsus|[18335]|CNWBD232-13|Chironomidae|658|BOLD:ACE0832
Tanytarsus glabrescens|[18336]|JSSEP207-11|Chironomidae|658|BOLD:ACN6040
Tanytarsus glabrescens|[18337]|BBDCM158-10|Chironomidae|658|BOLD:AAD8856
Tanytarsus glabrescens|[18338]|CNWBH1323-13|Chironomidae|638|BOLD:ACN5424
Tanytarsus|[18339]|CNTMA1142-14|Chironomidae|590|BOLD:ACN4229
Chironomidae|[18340]|NCCA2324-11|Chironomidae|674|BOLD:ABA6490
Tanytarsus|[18341]|CNGBO664-14|Chironomidae|658|BOLD:ACM0828
Chironominae|[18342]|SSPAA5873-13|Chironomidae|664|BOLD:ACG4882
Chironominae|[18343]|CNJAB365-12|Chironomidae|658|BOLD:ACC3042
Chironominae|[18344]|CNPEC115-14|Chironomidae|658|BOLD:ACJ5384
Chironominae|[18345]|SSPAA404-13|Chironomidae|670|BOLD:ACG3119
Chironominae|[18346]|SSPAA4613-13|Chironomidae|661|BOLD:ACC2901
Chironominae|[18347]|CNGBK507-14|Chironomidae|658|BOLD:ACL6043
Chironominae|[18348]|CNPEM888-14|Chironomidae|573|BOLD:AAG5427
Chironominae|[18349]|CNPKO1545-14|Chironomidae|645|BOLD:ACM9314
Chironomidae|[18350]|CNNHA827-14|Chironomidae|570|BOLD:ACR5563
Chironomidae|[18351]|CNWAB370-14|Chironomidae|582|BOLD:ACR8090
Chironomidae|[18352]|NGNAI2699-13|Chironomidae|670|BOLD:ACL8112
Chironomidae|[18353]|CNWAE2484-14|Chironomidae|627|BOLD:ACS0635
Chironominae|[18354]|NGNAI2799-13|Chironomidae|670|BOLD:ACJ0692
Chironominae|[18355]|CNTGA2182-15|Chironomidae|531|BOLD:ACM0514
Chironominae|[18356]|BBDCQ424-10|Chironomidae|658|BOLD:AAV5965
Chironominae|[18357]|NGNAX2972-14|Chironomidae|595|BOLD:ACG8483
Chironomidae|[18358]|SSGBA1670-14|Chironomidae|606|BOLD:ACO9331
Tanytarsus|[18359]|CNGBE075-14|Chironomidae|661|BOLD:ACL2446
Tanytarsus|[18360]|CNGBE210-14|Chironomidae|661|BOLD:ACL7168
Tanytarsus|[18361]|SSBAF896-13|Chironomidae|661|BOLD:ACI4099
Tanytarsus|[18362]|CNGBD190-13|Chironomidae|661|BOLD:ACL3313
Tanytarsus|[18363]|CNGBD332-13|Chironomidae|661|BOLD:ACL3051
Tanytarsus|[18364]|CNPKO934-14|Chironomidae|591|BOLD:ACN9742
Tanytarsus|[18365]|SSPAB474-13|Chironomidae|661|BOLD:ACE7742
Tanytarsus|[18366]|CNLMD988-14|Chironomidae|658|BOLD:ACM3976
Tanytarsus|[18367]|TTDFW1045-09|Chironomidae|658|BOLD:AAG5469
Tanytarsus|[18368]|CNWLG642-12|Chironomidae|670|BOLD:AAL7336
Tanytarsus|[18369]|CNWBG1836-13|Chironomidae|604|BOLD:ACI4483
Tanytarsus|[18370]|CNPEA109-14|Chironomidae|664|BOLD:ACC2308
Tanytarsus|[18371]|CNFNF767-14|Chironomidae|661|BOLD:ACI4593
Tanytarsus|[18372]|BBDIQ090-10|Chironomidae|658|BOLD:AAL7342
Tanytarsus|[18373]|SSBAF1120-13|Chironomidae|661|BOLD:ACL8465
Tanytarsus|[18374]|SSPAC2555-13|Chironomidae|667|BOLD:ACL3624
Tanytarsus|[18375]|BBDCN290-10|Chironomidae|658|BOLD:AAV6025
Tanytarsus|[18376]|SSBAF468-13|Chironomidae|661|BOLD:ACK4757
Tanytarsus|[18377]|CNFND379-14|Chironomidae|661|BOLD:AAP6874
Tanytarsus|[18378]|CNFND217-14|Chironomidae|658|BOLD:ACL3878
Tanytarsus|[18379]|CNGBJ490-14|Chironomidae|664|BOLD:ACL8182
Tanytarsus|[18380]|CNGBJ526-14|Chironomidae|664|BOLD:ACL8464
Tanytarsus|[18381]|CNGBB222-13|Chironomidae|661|BOLD:ACL0148
Tanytarsus|[18382]|CNFND984-14|Chironomidae|661|BOLD:AAP6876
Tanytarsus|[18383]|BBDIQ083-10|Chironomidae|658|BOLD:AAL7340
Tanytarsus|[18384]|CNLMD861-14|Chironomidae|658|BOLD:ACM3979
Tanytarsus|[18385]|CNNHE3085-14|Chironomidae|603|BOLD:ACR5824
Tanytarsus|[18386]|JWDCH633-10|Chironomidae|658|BOLD:AAQ0625
Tanytarsus|[18387]|SSJAE2525-13|Chironomidae|661|BOLD:ACI4460
Tanytarsus|[18388]|CNLMM166-14|Chironomidae|588|BOLD:ACO1736
Tanytarsus|[18389]|SSWLA545-13|Chironomidae|661|BOLD:ABV9271
Tanytarsus|[18390]|SSJAE2696-13|Chironomidae|661|BOLD:ACJ4499
Tanytarsus|[18391]|CNPEM976-14|Chironomidae|603|BOLD:ACN5108
Tanytarsus|[18392]|CNNHF505-14|Chironomidae|639|BOLD:ACL8595
Tanytarsus|[18393]|NGNAT261-14|Chironomidae|658|BOLD:ACM1671
Tanytarsus|[18394]|CNJAA437-12|Chironomidae|673|BOLD:ACB9674
Tanytarsus|[18395]|CNJAA330-12|Chironomidae|670|BOLD:ACB9673
Tanytarsus|[18396]|CHMEP020-12|Chironomidae|658|BOLD:AAQ0622
Tanytarsus|[18397]|CNWAD1702-14|Chironomidae|588|BOLD:ACR8484

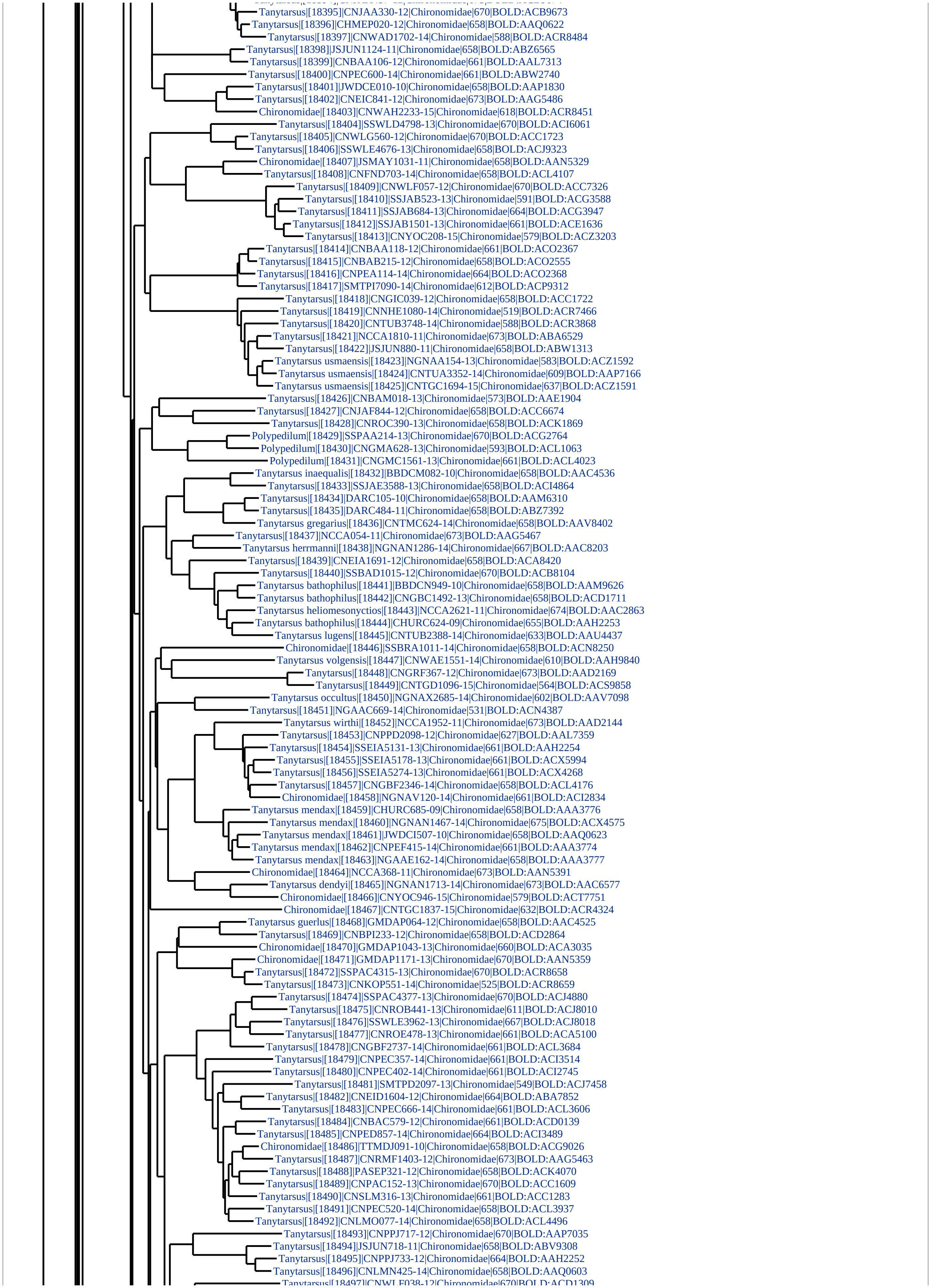
Tanytarsus|[18395]|CNJAA330-12|Chironomidae|670|BOLD:ACB9673
Tanytarsus|[18396]|CHMEP020-12|Chironomidae|658|BOLD:AAQ0622
Tanytarsus|[18397]|CNWAD1702-14|Chironomidae|588|BOLD:ACR8484
Tanytarsus|[18398]|JSJUN1124-11|Chironomidae|658|BOLD:ABZ6565
Tanytarsus|[18399]|CNBAA106-12|Chironomidae|661|BOLD:AAL7313
Tanytarsus|[18400]|CNPEC600-14|Chironomidae|661|BOLD:ABW2740
Tanytarsus|[18401]|JWDCE010-10|Chironomidae|658|BOLD:AAP1830
Tanytarsus|[18402]|CNEIC841-12|Chironomidae|673|BOLD:AAG5486
Chironomidae|[18403]|CNWAH2233-15|Chironomidae|618|BOLD:ACR8451
Tanytarsus|[18404]|SSWLD4798-13|Chironomidae|670|BOLD:ACI6061
Tanytarsus|[18405]|CNWLG560-12|Chironomidae|670|BOLD:ACC1723
Tanytarsus|[18406]|SSWLE4676-13|Chironomidae|658|BOLD:ACJ9323
Chironomidae|[18407]|JSMAY1031-11|Chironomidae|658|BOLD:AAN5329
Tanytarsus|[18408]|CNFND703-14|Chironomidae|658|BOLD:ACL4107
Tanytarsus|[18409]|CNWLF057-12|Chironomidae|670|BOLD:ACC7326
Tanytarsus|[18410]|SSJAB523-13|Chironomidae|591|BOLD:ACG3588
Tanytarsus|[18411]|SSJAB684-13|Chironomidae|664|BOLD:ACG3947
Tanytarsus|[18412]|SSJAB1501-13|Chironomidae|661|BOLD:ACE1636
Tanytarsus|[18413]|CNYOC208-15|Chironomidae|579|BOLD:ACZ3203
Tanytarsus|[18414]|CNBAA118-12|Chironomidae|661|BOLD:ACO2367
Tanytarsus|[18415]|CNBAB215-12|Chironomidae|658|BOLD:ACO2555
Tanytarsus|[18416]|CNPEA114-14|Chironomidae|664|BOLD:ACO2368
Tanytarsus|[18417]|SMTPI7090-14|Chironomidae|612|BOLD:ACP9312
Tanytarsus|[18418]|CNGIC039-12|Chironomidae|658|BOLD:ACC1722
Tanytarsus|[18419]|CNNHE1080-14|Chironomidae|519|BOLD:ACR7466
Tanytarsus|[18420]|CNTUB3748-14|Chironomidae|588|BOLD:ACR3868
Tanytarsus|[18421]|NCCA1810-11|Chironomidae|673|BOLD:ABA6529
Tanytarsus|[18422]|JSJUN880-11|Chironomidae|658|BOLD:ABW1313
Tanytarsus usmaensis|[18423]|NGNAA154-13|Chironomidae|583|BOLD:ACZ1592
Tanytarsus usmaensis|[18424]|CNTUA3352-14|Chironomidae|609|BOLD:AAP7166
Tanytarsus usmaensis|[18425]|CNTGC1694-15|Chironomidae|637|BOLD:ACZ1591
Tanytarsus|[18426]|CNBAM018-13|Chironomidae|573|BOLD:AAE1904
Tanytarsus|[18427]|CNJAF844-12|Chironomidae|658|BOLD:ACC6674
Tanytarsus|[18428]|CNROC390-13|Chironomidae|658|BOLD:ACK1869
Polypedilum|[18429]|SSPAA214-13|Chironomidae|670|BOLD:ACG2764
Polypedilum|[18430]|CNGMA628-13|Chironomidae|593|BOLD:ACL1063
Polypedilum|[18431]|CNGMC1561-13|Chironomidae|661|BOLD:ACL4023
Tanytarsus inaequalis|[18432]|BBDCM082-10|Chironomidae|658|BOLD:AAC4536
Tanytarsus|[18433]|SSJAE3588-13|Chironomidae|658|BOLD:ACI4864
Tanytarsus|[18434]|DARC105-10|Chironomidae|658|BOLD:AAM6310
Tanytarsus|[18435]|DARC484-11|Chironomidae|658|BOLD:ABZ7392
Tanytarsus gregarius|[18436]|CNTMC624-14|Chironomidae|658|BOLD:AAV8402
Tanytarsus|[18437]|NCCA054-11|Chironomidae|673|BOLD:AAG5467
Tanytarsus herrmanni|[18438]|NGNAN1286-14|Chironomidae|667|BOLD:AAC8203
Tanytarsus|[18439]|CNEIA1691-12|Chironomidae|658|BOLD:ACA8420
Tanytarsus|[18440]|SSBAD1015-12|Chironomidae|670|BOLD:ACB8104
Tanytarsus bathophilus|[18441]|BBDCN949-10|Chironomidae|658|BOLD:AAM9626
Tanytarsus bathophilus|[18442]|CNGBC1492-13|Chironomidae|658|BOLD:ACD1711
Tanytarsus heliomesonyctios|[18443]|NCCA2621-11|Chironomidae|674|BOLD:AAC2863
Tanytarsus bathophilus|[18444]|CHURC624-09|Chironomidae|655|BOLD:AAH2253
Tanytarsus lugens|[18445]|CNTUB2388-14|Chironomidae|633|BOLD:AAU4437
Chironomidae|[18446]|SSBRA1011-14|Chironomidae|658|BOLD:ACN8250
Tanytarsus volgensis|[18447]|CNWAE1551-14|Chironomidae|610|BOLD:AAH9840
Tanytarsus|[18448]|CNGRF367-12|Chironomidae|673|BOLD:AAD2169
Tanytarsus|[18449]|CNTGD1096-15|Chironomidae|564|BOLD:ACS9858
Tanytarsus occultus|[18450]|NGNAX2685-14|Chironomidae|602|BOLD:AAV7098
Tanytarsus|[18451]|NGAAC669-14|Chironomidae|531|BOLD:ACN4387
Tanytarsus wirthi|[18452]|NCCA1952-11|Chironomidae|673|BOLD:AAD2144
Tanytarsus|[18453]|CNPPD2098-12|Chironomidae|627|BOLD:AAL7359
Tanytarsus|[18454]|SSEIA5131-13|Chironomidae|661|BOLD:AAH2254
Tanytarsus|[18455]|SSEIA5178-13|Chironomidae|661|BOLD:ACX5994
Tanytarsus|[18456]|SSEIA5274-13|Chironomidae|661|BOLD:ACX4268
Tanytarsus|[18457]|CNGBF2346-14|Chironomidae|658|BOLD:ACL4176
Chironomidae|[18458]|NGNAV120-14|Chironomidae|661|BOLD:ACI2834
Tanytarsus mendax|[18459]|CHURC685-09|Chironomidae|658|BOLD:AAA3776
Tanytarsus mendax|[18460]|NGNAN1467-14|Chironomidae|675|BOLD:ACX4575
Tanytarsus mendax|[18461]|JWDCI507-10|Chironomidae|658|BOLD:AAQ0623
Tanytarsus mendax|[18462]|CNPEF415-14|Chironomidae|661|BOLD:AAA3774
Tanytarsus mendax|[18463]|NGAAE162-14|Chironomidae|658|BOLD:AAA3777
Chironomidae|[18464]|NCCA368-11|Chironomidae|673|BOLD:AAN5391
Tanytarsus dendyi|[18465]|NGNAN1713-14|Chironomidae|673|BOLD:AAC6577
Chironomidae|[18466]|CNYOC946-15|Chironomidae|579|BOLD:ACT7751
Chironomidae|[18467]|CNTGC1837-15|Chironomidae|632|BOLD:ACR4324
Tanytarsus guerlus|[18468]|GMDAP064-12|Chironomidae|658|BOLD:AAC4525
Tanytarsus|[18469]|CNBPI233-12|Chironomidae|658|BOLD:ACD2864
Chironomidae|[18470]|GMDAP1043-13|Chironomidae|660|BOLD:ACA3035
Chironomidae|[18471]|GMDAP1171-13|Chironomidae|670|BOLD:AAN5359
Tanytarsus|[18472]|SSPAC4315-13|Chironomidae|670|BOLD:ACR8658
Tanytarsus|[18473]|CNKOP551-14|Chironomidae|525|BOLD:ACR8659
Tanytarsus|[18474]|SSPAC4377-13|Chironomidae|670|BOLD:ACJ4880
Tanytarsus|[18475]|CNROB441-13|Chironomidae|611|BOLD:ACJ8010
Tanytarsus|[18476]|SSWLE3962-13|Chironomidae|667|BOLD:ACJ8018
Tanytarsus|[18477]|CNROE478-13|Chironomidae|661|BOLD:ACA5100
Tanytarsus|[18478]|CNGBF2737-14|Chironomidae|661|BOLD:ACL3684
Tanytarsus|[18479]|CNPEC357-14|Chironomidae|661|BOLD:ACI3514
Tanytarsus|[18480]|CNPEC402-14|Chironomidae|661|BOLD:ACI2745
Tanytarsus|[18481]|SMTPD2097-13|Chironomidae|549|BOLD:ACJ7458
Tanytarsus|[18482]|CNEID1604-12|Chironomidae|664|BOLD:ABA7852
Tanytarsus|[18483]|CNPEC666-14|Chironomidae|661|BOLD:ACL3606
Tanytarsus|[18484]|CNBAC579-12|Chironomidae|661|BOLD:ACD0139
Tanytarsus|[18485]|CNPED857-14|Chironomidae|664|BOLD:ACI3489
Chironomidae|[18486]|TTMDJ091-10|Chironomidae|658|BOLD:ACG9026
Tanytarsus|[18487]|CNRMF1403-12|Chironomidae|673|BOLD:AAG5463
Tanytarsus|[18488]|PASEP321-12|Chironomidae|658|BOLD:ACK4070
Tanytarsus|[18489]|CNPAC152-13|Chironomidae|670|BOLD:ACC1609
Tanytarsus|[18490]|CNSLM316-13|Chironomidae|661|BOLD:ACC1283
Tanytarsus|[18491]|CNPEC520-14|Chironomidae|658|BOLD:ACL3937
Tanytarsus|[18492]|CNLMO077-14|Chironomidae|658|BOLD:ACL4496
Tanytarsus|[18493]|CNPPJ717-12|Chironomidae|670|BOLD:AAP7035
Tanytarsus|[18494]|JSJUN718-11|Chironomidae|658|BOLD:ABV9308
Tanytarsus|[18495]|CNPPJ733-12|Chironomidae|664|BOLD:AAH2252
Tanytarsus|[18496]|CNLMN425-14|Chironomidae|658|BOLD:AAQ0603
Tanytarsus|[18497]|CNWLF038-12|Chironomidae|670|BOLD:ACD1309

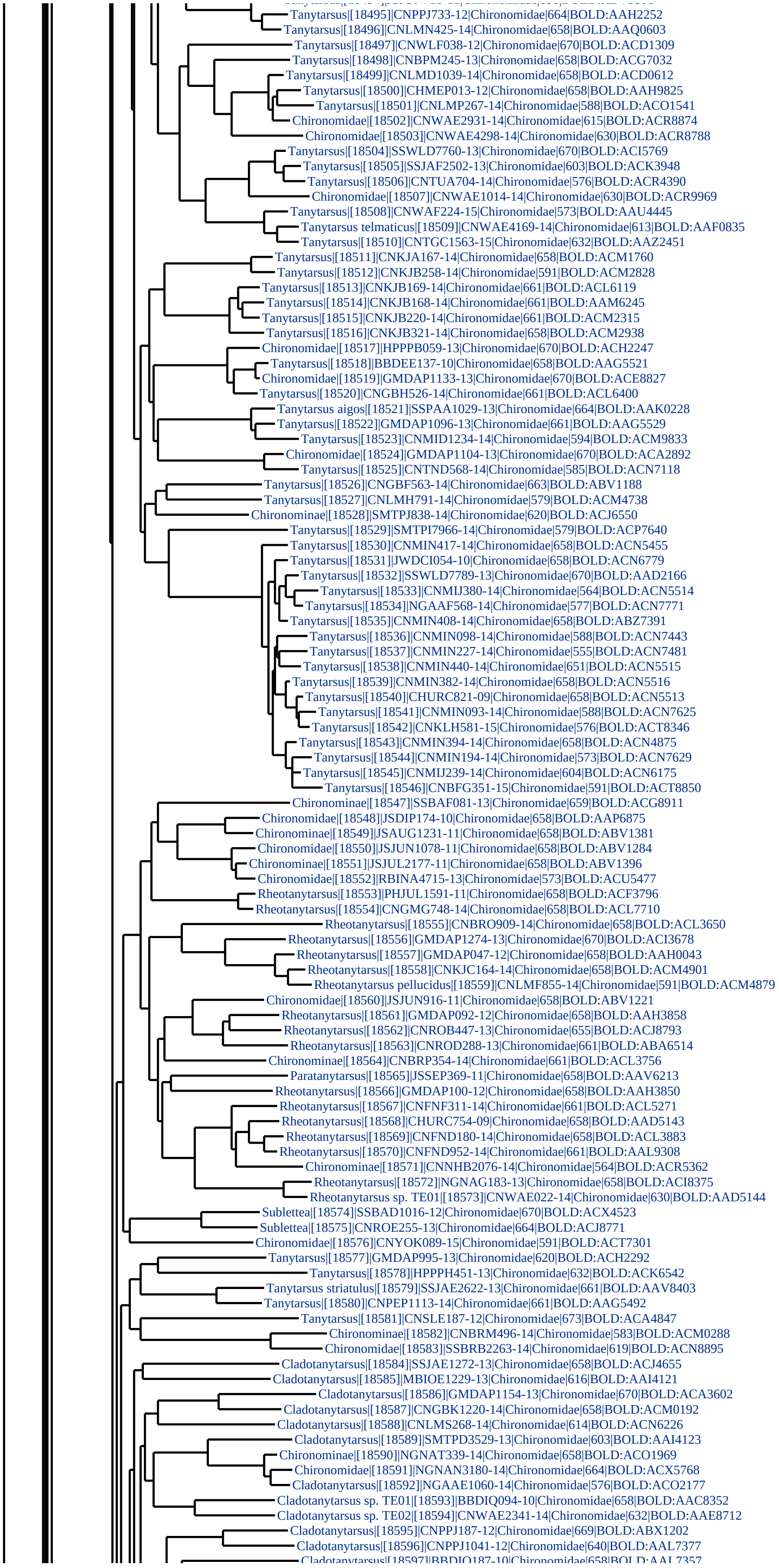
Tanytarsus|[18495]|CNPPJ733-12|Chironomidae|664|BOLD:AAH2252
Tanytarsus|[18496]|CNLMN425-14|Chironomidae|658|BOLD:AAQ0603
Tanytarsus|[18497]|CNWLF038-12|Chironomidae|670|BOLD:ACD1309
Tanytarsus|[18498]|CNBPM245-13|Chironomidae|658|BOLD:ACG7032
Tanytarsus|[18499]|CNLMD1039-14|Chironomidae|658|BOLD:ACD0612
Tanytarsus|[18500]|CHMEP013-12|Chironomidae|658|BOLD:AAH9825
Tanytarsus|[18501]|CNLMP267-14|Chironomidae|588|BOLD:ACO1541
Chironomidae|[18502]|CNWAE2931-14|Chironomidae|615|BOLD:ACR8874
Chironomidae|[18503]|CNWAE4298-14|Chironomidae|630|BOLD:ACR8788
Tanytarsus|[18504]|SSWLD7760-13|Chironomidae|670|BOLD:ACI5769
Tanytarsus|[18505]|SSJAF2502-13|Chironomidae|603|BOLD:ACK3948
Tanytarsus|[18506]|CNTUA704-14|Chironomidae|576|BOLD:ACR4390
Chironomidae|[18507]|CNWAE1014-14|Chironomidae|630|BOLD:ACR9969
Tanytarsus|[18508]|CNWAF224-15|Chironomidae|573|BOLD:AAU4445
Tanytarsus telmaticus|[18509]|CNWAE4169-14|Chironomidae|613|BOLD:AAF0835
Tanytarsus|[18510]|CNTGC1563-15|Chironomidae|632|BOLD:AAZ2451
Tanytarsus|[18511]|CNKJA167-14|Chironomidae|658|BOLD:ACM1760
Tanytarsus|[18512]|CNKJB258-14|Chironomidae|591|BOLD:ACM2828
Tanytarsus|[18513]|CNKJB169-14|Chironomidae|661|BOLD:ACL6119
Tanytarsus|[18514]|CNKJB168-14|Chironomidae|661|BOLD:AAM6245
Tanytarsus|[18515]|CNKJB220-14|Chironomidae|661|BOLD:ACM2315
Tanytarsus|[18516]|CNKJB321-14|Chironomidae|658|BOLD:ACM2938
Chironomidae|[18517]|HPPPB059-13|Chironomidae|670|BOLD:ACH2247
Tanytarsus|[18518]|BBDEE137-10|Chironomidae|658|BOLD:AAG5521
Chironomidae|[18519]|GMDAP1133-13|Chironomidae|670|BOLD:ACE8827
Tanytarsus|[18520]|CNGBH526-14|Chironomidae|661|BOLD:ACL6400
Tanytarsus aigos|[18521]|SSPAA1029-13|Chironomidae|664|BOLD:AAK0228
Tanytarsus|[18522]|GMDAP1096-13|Chironomidae|661|BOLD:AAG5529
Tanytarsus|[18523]|CNMID1234-14|Chironomidae|594|BOLD:ACM9833
Chironomidae|[18524]|GMDAP1104-13|Chironomidae|670|BOLD:ACA2892
Tanytarsus|[18525]|CNTND568-14|Chironomidae|585|BOLD:ACN7118
Tanytarsus|[18526]|CNGBF563-14|Chironomidae|663|BOLD:ABV1188
Tanytarsus|[18527]|CNLMH791-14|Chironomidae|579|BOLD:ACM4738
Chironominae|[18528]|SMTPJ838-14|Chironomidae|620|BOLD:ACJ6550
Tanytarsus|[18529]|SMTPI7966-14|Chironomidae|579|BOLD:ACP7640
Tanytarsus|[18530]|CNMIN417-14|Chironomidae|658|BOLD:ACN5455
Tanytarsus|[18531]|JWDCI054-10|Chironomidae|658|BOLD:ACN6779
Tanytarsus|[18532]|SSWLD7789-13|Chironomidae|670|BOLD:AAD2166
Tanytarsus|[18533]|CNMIJ380-14|Chironomidae|564|BOLD:ACN5514
Tanytarsus|[18534]|NGAAF568-14|Chironomidae|577|BOLD:ACN7771
Tanytarsus|[18535]|CNMIN408-14|Chironomidae|658|BOLD:ABZ7391
Tanytarsus|[18536]|CNMIN098-14|Chironomidae|588|BOLD:ACN7443
Tanytarsus|[18537]|CNMIN227-14|Chironomidae|555|BOLD:ACN7481
Tanytarsus|[18538]|CNMIN440-14|Chironomidae|651|BOLD:ACN5515
Tanytarsus|[18539]|CNMIN382-14|Chironomidae|658|BOLD:ACN5516
Tanytarsus|[18540]|CHURC821-09|Chironomidae|658|BOLD:ACN5513
Tanytarsus|[18541]|CNMIN093-14|Chironomidae|588|BOLD:ACN7625
Tanytarsus|[18542]|CNKLH581-15|Chironomidae|576|BOLD:ACT8346
Tanytarsus|[18543]|CNMIN394-14|Chironomidae|658|BOLD:ACN4875
Tanytarsus|[18544]|CNMIN194-14|Chironomidae|573|BOLD:ACN7629
Tanytarsus|[18545]|CNMIJ239-14|Chironomidae|604|BOLD:ACN6175
Tanytarsus|[18546]|CNBFG351-15|Chironomidae|591|BOLD:ACT8850
Chironominae|[18547]|SSBAF081-13|Chironomidae|659|BOLD:ACG8911
Chironomidae|[18548]|JSDIP174-10|Chironomidae|658|BOLD:AAP6875
Chironominae|[18549]|JSAUG1231-11|Chironomidae|658|BOLD:ABV1381
Chironomidae|[18550]|JSJUN1078-11|Chironomidae|658|BOLD:ABV1284
Chironominae|[18551]|JSJUL2177-11|Chironomidae|658|BOLD:ABV1396
Chironomidae|[18552]|RBINA4715-13|Chironomidae|573|BOLD:ACU5477
Rheotanytarsus|[18553]|PHJUL1591-11|Chironomidae|658|BOLD:ACF3796
Rheotanytarsus|[18554]|CNGMG748-14|Chironomidae|658|BOLD:ACL7710
Rheotanytarsus|[18555]|CNBRO909-14|Chironomidae|658|BOLD:ACL3650
Rheotanytarsus|[18556]|GMDAP1274-13|Chironomidae|670|BOLD:ACI3678
Rheotanytarsus|[18557]|GMDAP047-12|Chironomidae|658|BOLD:AAH0043
Rheotanytarsus|[18558]|CNKJC164-14|Chironomidae|658|BOLD:ACM4901
Rheotanytarsus pellucidus|[18559]|CNLMF855-14|Chironomidae|591|BOLD:ACM4879
Chironomidae|[18560]|JSJUN916-11|Chironomidae|658|BOLD:ABV1221
Rheotanytarsus|[18561]|GMDAP092-12|Chironomidae|658|BOLD:AAH3858
Rheotanytarsus|[18562]|CNROB447-13|Chironomidae|655|BOLD:ACJ8793
Rheotanytarsus|[18563]|CNROD288-13|Chironomidae|661|BOLD:ABA6514
Chironominae|[18564]|CNBRP354-14|Chironomidae|661|BOLD:ACL3756
Paratanytarsus|[18565]|JSSEP369-11|Chironomidae|658|BOLD:AAV6213
Rheotanytarsus|[18566]|GMDAP100-12|Chironomidae|658|BOLD:AAH3850
Rheotanytarsus|[18567]|CNFNF311-14|Chironomidae|661|BOLD:ACL5271
Rheotanytarsus|[18568]|CHURC754-09|Chironomidae|658|BOLD:AAD5143
Rheotanytarsus|[18569]|CNFND180-14|Chironomidae|658|BOLD:ACL3883
Rheotanytarsus|[18570]|CNFND952-14|Chironomidae|661|BOLD:AAL9308
Chironominae|[18571]|CNNHB2076-14|Chironomidae|564|BOLD:ACR5362
Rheotanytarsus|[18572]|NGNAG183-13|Chironomidae|658|BOLD:ACI8375
Rheotanytarsus sp. TE01|[18573]|CNWAE022-14|Chironomidae|630|BOLD:AAD5144
Sublettea|[18574]|SSBAD1016-12|Chironomidae|670|BOLD:ACX4523
Sublettea|[18575]|CNROE255-13|Chironomidae|664|BOLD:ACJ8771
Chironomidae|[18576]|CNYOK089-15|Chironomidae|591|BOLD:ACT7301
Tanytarsus|[18577]|GMDAP995-13|Chironomidae|620|BOLD:ACH2292
Tanytarsus|[18578]|HPPPH451-13|Chironomidae|632|BOLD:ACK6542
Tanytarsus striatulus|[18579]|SSJAE2622-13|Chironomidae|661|BOLD:AAV8403
Tanytarsus|[18580]|CNPEP1113-14|Chironomidae|661|BOLD:AAG5492
Tanytarsus|[18581]|CNSLE187-12|Chironomidae|673|BOLD:ACA4847
Chironominae|[18582]|CNBRM496-14|Chironomidae|583|BOLD:ACM0288
Chironomidae|[18583]|SSBRB2263-14|Chironomidae|619|BOLD:ACN8895
Cladotanytarsus|[18584]|SSJAE1272-13|Chironomidae|658|BOLD:ACJ4655
Cladotanytarsus|[18585]|MBIOE1229-13|Chironomidae|616|BOLD:AAI4121
Cladotanytarsus|[18586]|GMDAP1154-13|Chironomidae|670|BOLD:ACA3602
Cladotanytarsus|[18587]|CNGBK1220-14|Chironomidae|658|BOLD:ACM0192
Cladotanytarsus|[18588]|CNLMS268-14|Chironomidae|614|BOLD:ACN6226
Cladotanytarsus|[18589]|SMTPD3529-13|Chironomidae|603|BOLD:AAI4123
Chironominae|[18590]|NGNAT339-14|Chironomidae|658|BOLD:ACO1969
Chironomidae|[18591]|NGNAN3180-14|Chironomidae|664|BOLD:ACX5768
Cladotanytarsus|[18592]|NGAAE1060-14|Chironomidae|576|BOLD:ACO2177
Cladotanytarsus sp. TE01|[18593]|BBDIQ094-10|Chironomidae|658|BOLD:AAC8352
Cladotanytarsus sp. TE02|[18594]|CNWAE2341-14|Chironomidae|632|BOLD:AAE8712
Cladotanytarsus|[18595]|CNPPJ187-12|Chironomidae|669|BOLD:ABX1202
Cladotanytarsus|[18596]|CNPPJ1041-12|Chironomidae|640|BOLD:AAL7377
Cladotanytarsus|[18597]|BBDIQ187-10|Chironomidae|658|BOLD:AAL7357

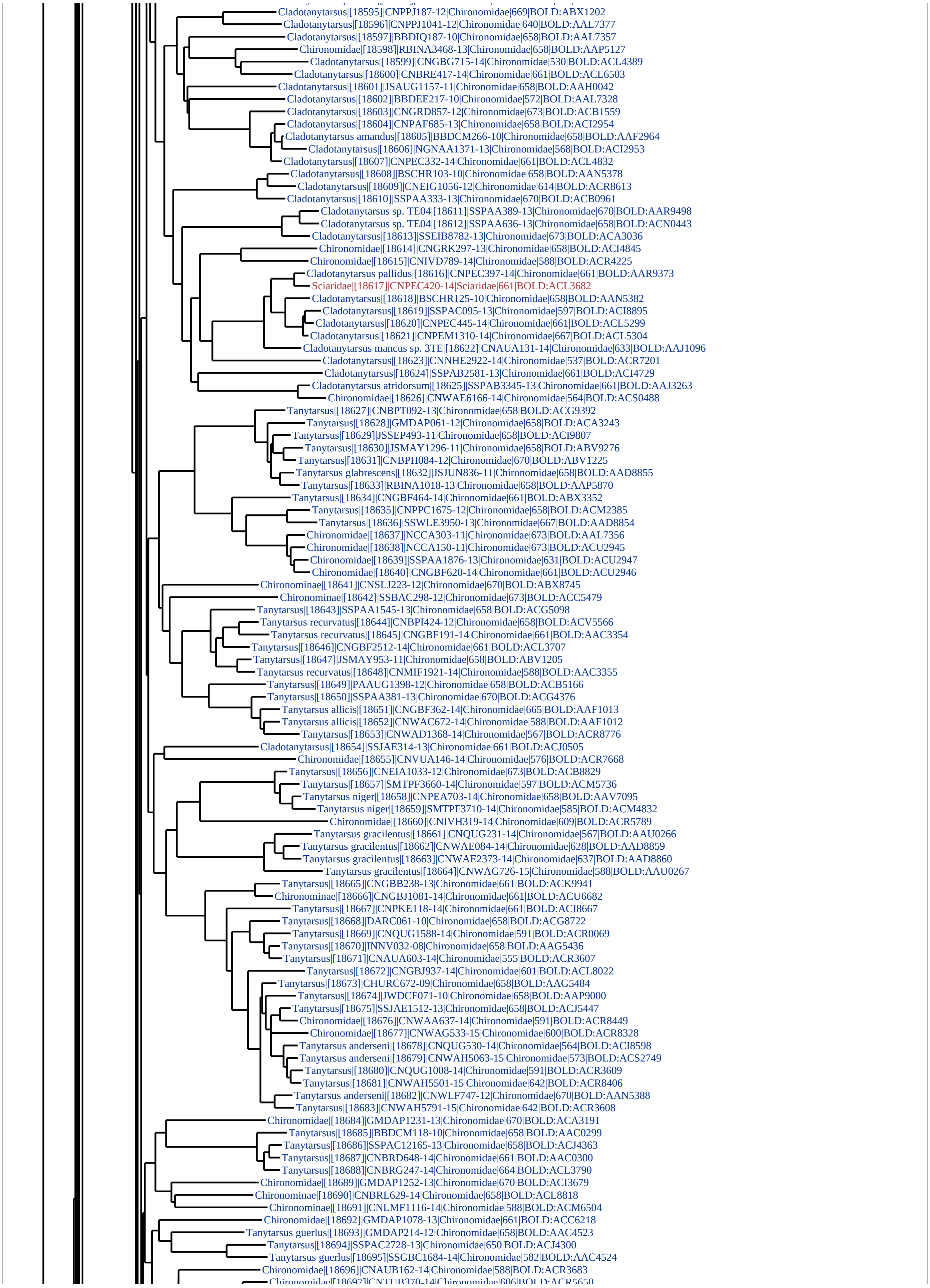
Cladotanytarsus|[18595]|CNPPJ187-12|Chironomidae|669|BOLD:ABX1202
Cladotanytarsus|[18596]|CNPPJ1041-12|Chironomidae|640|BOLD:AAL7377
Cladotanytarsus|[18597]|BBDIQ187-10|Chironomidae|658|BOLD:AAL7357
Chironomidae|[18598]|RBINA3468-13|Chironomidae|658|BOLD:AAP5127
Cladotanytarsus|[18599]|CNGBG715-14|Chironomidae|530|BOLD:ACL4389
Cladotanytarsus|[18600]|CNBRE417-14|Chironomidae|661|BOLD:ACL6503
Cladotanytarsus|[18601]|JSAUG1157-11|Chironomidae|658|BOLD:AAH0042
Cladotanytarsus|[18602]|BBDEE217-10|Chironomidae|572|BOLD:AAL7328
Cladotanytarsus|[18603]|CNGRD857-12|Chironomidae|673|BOLD:ACB1559
Cladotanytarsus|[18604]|CNPAF685-13|Chironomidae|658|BOLD:ACI2954
Cladotanytarsus amandus|[18605]|BBDCM266-10|Chironomidae|658|BOLD:AAF2964
Cladotanytarsus|[18606]|NGNAA1371-13|Chironomidae|568|BOLD:ACI2953
Cladotanytarsus|[18607]|CNPEC332-14|Chironomidae|661|BOLD:ACL4832
Cladotanytarsus|[18608]|BSCHR103-10|Chironomidae|658|BOLD:AAN5378
Cladotanytarsus|[18609]|CNEIG1056-12|Chironomidae|614|BOLD:ACR8613
Cladotanytarsus|[18610]|SSPAA333-13|Chironomidae|670|BOLD:ACB0961
Cladotanytarsus sp. TE04|[18611]|SSPAA389-13|Chironomidae|670|BOLD:AAR9498
Cladotanytarsus sp. TE04|[18612]|SSPAA636-13|Chironomidae|658|BOLD:ACN0443
Cladotanytarsus|[18613]|SSEIB8782-13|Chironomidae|673|BOLD:ACA3036
Chironomidae|[18614]|CNGRK297-13|Chironomidae|658|BOLD:ACI4845
Chironomidae|[18615]|CNIVD789-14|Chironomidae|588|BOLD:ACR4225
Cladotanytarsus pallidus|[18616]|CNPEC397-14|Chironomidae|661|BOLD:AAR9373
Sciaridae|[18617]|CNPEC420-14|Sciaridae|661|BOLD:ACL3682
Cladotanytarsus|[18618]|BSCHR125-10|Chironomidae|658|BOLD:AAN5382
Cladotanytarsus|[18619]|SSPAC095-13|Chironomidae|597|BOLD:ACI8895
Cladotanytarsus|[18620]|CNPEC445-14|Chironomidae|661|BOLD:ACL5299
Cladotanytarsus|[18621]|CNPEM1310-14|Chironomidae|667|BOLD:ACL5304
Cladotanytarsus mancus sp. 3TE|[18622]|CNAUA131-14|Chironomidae|633|BOLD:AAJ1096
Cladotanytarsus|[18623]|CNNHE2922-14|Chironomidae|537|BOLD:ACR7201
Cladotanytarsus|[18624]|SSPAB2581-13|Chironomidae|661|BOLD:ACI4729
Cladotanytarsus atridorsum|[18625]|SSPAB3345-13|Chironomidae|661|BOLD:AAJ3263
Chironomidae|[18626]|CNWAE6166-14|Chironomidae|564|BOLD:ACS0488
Tanytarsus|[18627]|CNBPT092-13|Chironomidae|658|BOLD:ACG9392
Tanytarsus|[18628]|GMDAP061-12|Chironomidae|658|BOLD:ACA3243
Tanytarsus|[18629]|JSSEP493-11|Chironomidae|658|BOLD:ACI9807
Tanytarsus|[18630]|JSMAY1296-11|Chironomidae|658|BOLD:ABV9276
Tanytarsus|[18631]|CNBPH084-12|Chironomidae|670|BOLD:ABV1225
Tanytarsus glabrescens|[18632]|JSJUN836-11|Chironomidae|658|BOLD:AAD8855
Tanytarsus|[18633]|RBINA1018-13|Chironomidae|658|BOLD:AAP5870
Tanytarsus|[18634]|CNGBF464-14|Chironomidae|661|BOLD:ABX3352
Tanytarsus|[18635]|CNPPC1675-12|Chironomidae|658|BOLD:ACM2385
Tanytarsus|[18636]|SSWLE3950-13|Chironomidae|667|BOLD:AAD8854
Chironomidae|[18637]|NCCA303-11|Chironomidae|673|BOLD:AAL7356
Chironomidae|[18638]|NCCA150-11|Chironomidae|673|BOLD:ACU2945
Chironomidae|[18639]|SSPAA1876-13|Chironomidae|631|BOLD:ACU2947
Chironomidae|[18640]|CNGBF620-14|Chironomidae|661|BOLD:ACU2946
Chironominae|[18641]|CNSLJ223-12|Chironomidae|670|BOLD:ABX8745
Chironominae|[18642]|SSBAC298-12|Chironomidae|673|BOLD:ACC5479
Tanytarsus|[18643]|SSPAA1545-13|Chironomidae|658|BOLD:ACG5098
Tanytarsus recurvatus|[18644]|CNBPI424-12|Chironomidae|658|BOLD:ACV5566
Tanytarsus recurvatus|[18645]|CNGBF191-14|Chironomidae|661|BOLD:AAC3354
Tanytarsus|[18646]|CNGBF2512-14|Chironomidae|661|BOLD:ACL3707
Tanytarsus|[18647]|JSMAY953-11|Chironomidae|658|BOLD:ABV1205
Tanytarsus recurvatus|[18648]|CNMIF1921-14|Chironomidae|588|BOLD:AAC3355
Tanytarsus|[18649]|PAAUG1398-12|Chironomidae|658|BOLD:ACB5166
Tanytarsus|[18650]|SSPAA381-13|Chironomidae|670|BOLD:ACG4376
Tanytarsus allicis|[18651]|CNGBF362-14|Chironomidae|665|BOLD:AAF1013
Tanytarsus allicis|[18652]|CNWAC672-14|Chironomidae|588|BOLD:AAF1012
Tanytarsus|[18653]|CNWAD1368-14|Chironomidae|567|BOLD:ACR8776
Cladotanytarsus|[18654]|SSJAE314-13|Chironomidae|661|BOLD:ACJ0505
Chironomidae|[18655]|CNVUA146-14|Chironomidae|576|BOLD:ACR7668
Tanytarsus|[18656]|CNEIA1033-12|Chironomidae|673|BOLD:ACB8829
Tanytarsus|[18657]|SMTPF3660-14|Chironomidae|597|BOLD:ACM5736
Tanytarsus niger|[18658]|CNPEA703-14|Chironomidae|658|BOLD:AAV7095
Tanytarsus niger|[18659]|SMTPF3710-14|Chironomidae|585|BOLD:ACM4832
Chironomidae|[18660]|CNIVH319-14|Chironomidae|609|BOLD:ACR5789
Tanytarsus gracilentus|[18661]|CNQUG231-14|Chironomidae|567|BOLD:AAU0266
Tanytarsus gracilentus|[18662]|CNWAE084-14|Chironomidae|628|BOLD:AAD8859
Tanytarsus gracilentus|[18663]|CNWAE2373-14|Chironomidae|637|BOLD:AAD8860
Tanytarsus gracilentus|[18664]|CNWAG726-15|Chironomidae|588|BOLD:AAU0267
Tanytarsus|[18665]|CNGBB238-13|Chironomidae|661|BOLD:ACK9941
Chironominae|[18666]|CNGBJ1081-14|Chironomidae|661|BOLD:ACU6682
Tanytarsus|[18667]|CNPKE118-14|Chironomidae|661|BOLD:ACI8667
Tanytarsus|[18668]|DARC061-10|Chironomidae|658|BOLD:ACG8722
Tanytarsus|[18669]|CNQUG1588-14|Chironomidae|591|BOLD:ACR0069
Tanytarsus|[18670]|INNV032-08|Chironomidae|658|BOLD:AAG5436
Tanytarsus|[18671]|CNAUA603-14|Chironomidae|555|BOLD:ACR3607
Tanytarsus|[18672]|CNGBJ937-14|Chironomidae|601|BOLD:ACL8022
Tanytarsus|[18673]|CHURC672-09|Chironomidae|658|BOLD:AAG5484
Tanytarsus|[18674]|JWDCF071-10|Chironomidae|658|BOLD:AAP9000
Tanytarsus|[18675]|SSJAE1512-13|Chironomidae|658|BOLD:ACJ5447
Chironomidae|[18676]|CNWAA637-14|Chironomidae|591|BOLD:ACR8449
Chironomidae|[18677]|CNWAG533-15|Chironomidae|600|BOLD:ACR8328
Tanytarsus anderseni|[18678]|CNQUG530-14|Chironomidae|564|BOLD:ACI8598
Tanytarsus anderseni|[18679]|CNWAH5063-15|Chironomidae|573|BOLD:ACS2749
Tanytarsus|[18680]|CNQUG1008-14|Chironomidae|591|BOLD:ACR3609
Tanytarsus|[18681]|CNWAH5501-15|Chironomidae|642|BOLD:ACR8406
Tanytarsus anderseni|[18682]|CNWLF747-12|Chironomidae|670|BOLD:AAN5388
Tanytarsus|[18683]|CNWAH5791-15|Chironomidae|642|BOLD:ACR3608
Chironomidae|[18684]|GMDAP1231-13|Chironomidae|670|BOLD:ACA3191
Tanytarsus|[18685]|BBDCM118-10|Chironomidae|658|BOLD:AAC0299
Tanytarsus|[18686]|SSPAC12165-13|Chironomidae|658|BOLD:ACJ4363
Tanytarsus|[18687]|CNBRD648-14|Chironomidae|661|BOLD:AAC0300
Tanytarsus|[18688]|CNBRG247-14|Chironomidae|664|BOLD:ACL3790
Chironomidae|[18689]|GMDAP1252-13|Chironomidae|670|BOLD:ACI3679
Chironominae|[18690]|CNBRL629-14|Chironomidae|658|BOLD:ACL8818
Chironominae|[18691]|CNLMF1116-14|Chironomidae|588|BOLD:ACM6504
Chironomidae|[18692]|GMDAP1078-13|Chironomidae|661|BOLD:ACC6218
Tanytarsus guerlus|[18693]|GMDAP214-12|Chironomidae|658|BOLD:AAC4523
Tanytarsus|[18694]|SSPAC2728-13|Chironomidae|650|BOLD:ACJ4300
Tanytarsus guerlus|[18695]|SSGBC1684-14|Chironomidae|582|BOLD:AAC4524
Chironomidae|[18696]|CNAUB162-14|Chironomidae|588|BOLD:ACR3683
Chironomidae|[18697]|CNTUB370-14|Chironomidae|606|BOLD:ACR5650

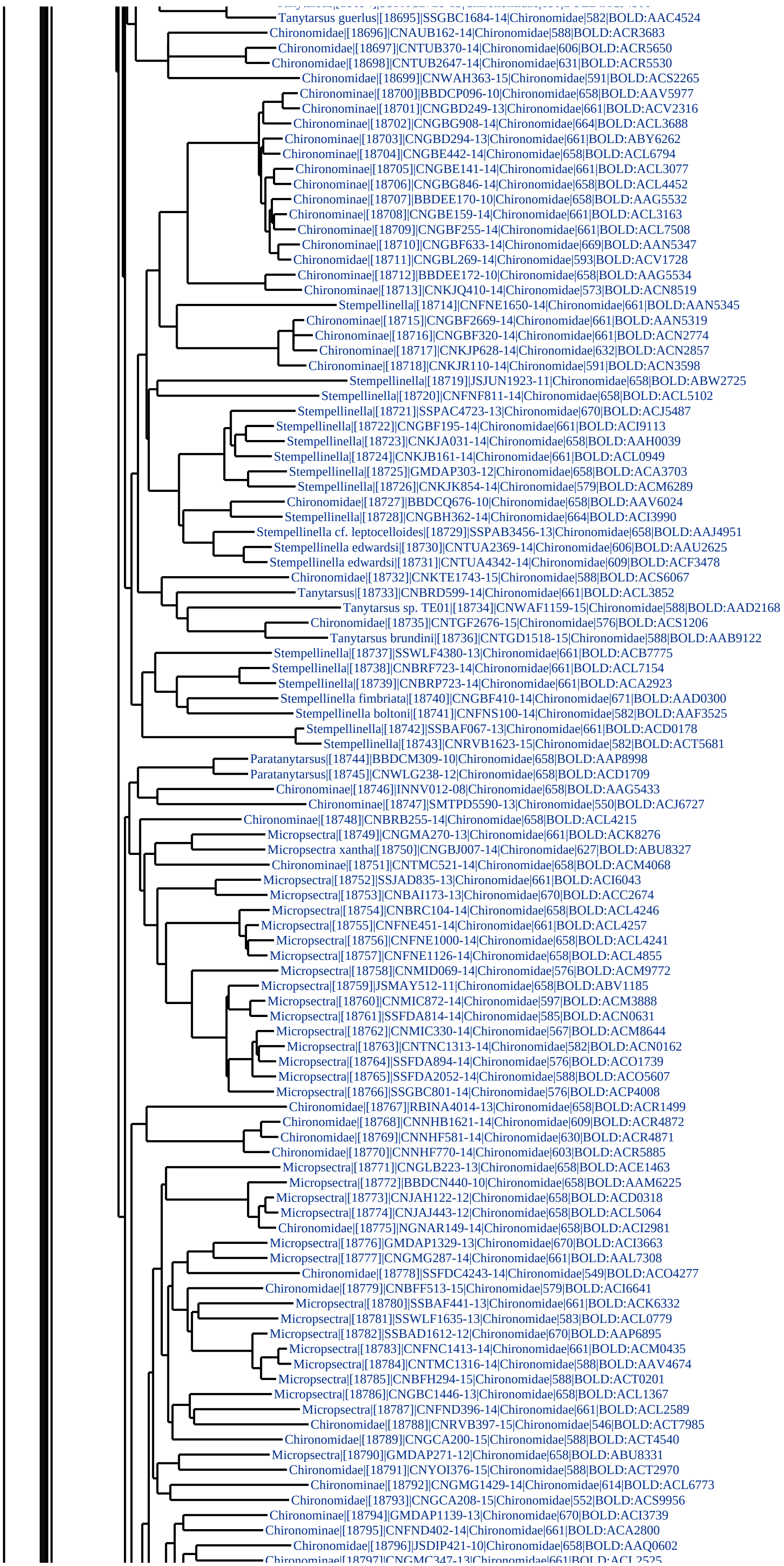

Tanytarsus guerlus|[18695]|SSGBC1684-14|Chironomidae|582|BOLD:AAC4524
Chironomidae|[18696]|CNAUB162-14|Chironomidae|588|BOLD:ACR3683
Chironomidae|[18697]|CNTUB370-14|Chironomidae|606|BOLD:ACR5650
Chironomidae|[18698]|CNTUB2647-14|Chironomidae|631|BOLD:ACR5530
Chironomidae|[18699]|CNWAH363-15|Chironomidae|591|BOLD:ACS2265
Chironominae|[18700]|BBDCP096-10|Chironomidae|658|BOLD:AAV5977
Chironominae|[18701]|CNGBD249-13|Chironomidae|661|BOLD:ACV2316
Chironominae|[18702]|CNGBG908-14|Chironomidae|664|BOLD:ACL3688
Chironominae|[18703]|CNGBD294-13|Chironomidae|661|BOLD:ABY6262
Chironominae|[18704]|CNGBE442-14|Chironomidae|658|BOLD:ACL6794
Chironominae|[18705]|CNGBE141-14|Chironomidae|661|BOLD:ACL3077
Chironominae|[18706]|CNGBG846-14|Chironomidae|658|BOLD:ACL4452
Chironominae|[18707]|BBDEE170-10|Chironomidae|658|BOLD:AAG5532
Chironominae|[18708]|CNGBE159-14|Chironomidae|661|BOLD:ACL3163
Chironominae|[18709]|CNGBF255-14|Chironomidae|661|BOLD:ACL7508
Chironominae|[18710]|CNGBF633-14|Chironomidae|669|BOLD:AAN5347
Chironomidae|[18711]|CNGBL269-14|Chironomidae|593|BOLD:ACV1728
Chironominae|[18712]|BBDEE172-10|Chironomidae|658|BOLD:AAG5534
Chironominae|[18713]|CNKJQ410-14|Chironomidae|573|BOLD:ACN8519
Stempellinella|[18714]|CNFNE1650-14|Chironomidae|661|BOLD:AAN5345
Chironominae|[18715]|CNGBF2669-14|Chironomidae|661|BOLD:AAN5319
Chironominae|[18716]|CNGBF320-14|Chironomidae|661|BOLD:ACN2774
Chironominae|[18717]|CNKJP628-14|Chironomidae|632|BOLD:ACN2857
Chironominae|[18718]|CNKJR110-14|Chironomidae|591|BOLD:ACN3598
Stempellinella|[18719]|JSJUN1923-11|Chironomidae|658|BOLD:ABW2725
Stempellinella|[18720]|CNFNF811-14|Chironomidae|658|BOLD:ACL5102
Stempellinella|[18721]|SSPAC4723-13|Chironomidae|670|BOLD:ACJ5487
Stempellinella|[18722]|CNGBF195-14|Chironomidae|661|BOLD:ACI9113
Stempellinella|[18723]|CNKJA031-14|Chironomidae|658|BOLD:AAH0039
Stempellinella|[18724]|CNKJB161-14|Chironomidae|661|BOLD:ACL0949
Stempellinella|[18725]|GMDAP303-12|Chironomidae|658|BOLD:ACA3703
Stempellinella|[18726]|CNKJK854-14|Chironomidae|579|BOLD:ACM6289
Chironomidae|[18727]|BBDCQ676-10|Chironomidae|658|BOLD:AAV6024
Stempellinella|[18728]|CNGBH362-14|Chironomidae|664|BOLD:ACI3990
Stempellinella cf. leptocelloides|[18729]|SSPAB3456-13|Chironomidae|658|BOLD:AAJ4951
Stempellinella edwardsi|[18730]|CNTUA2369-14|Chironomidae|606|BOLD:AAU2625
Stempellinella edwardsi|[18731]|CNTUA4342-14|Chironomidae|609|BOLD:ACF3478
Chironomidae|[18732]|CNKTE1743-15|Chironomidae|588|BOLD:ACS6067
Tanytarsus|[18733]|CNBRD599-14|Chironomidae|661|BOLD:ACL3852
Tanytarsus sp. TE01|[18734]|CNWAF1159-15|Chironomidae|588|BOLD:AAD2168
Chironomidae|[18735]|CNTGF2676-15|Chironomidae|576|BOLD:ACS1206
Tanytarsus brundini|[18736]|CNTGD1518-15|Chironomidae|588|BOLD:AAB9122
Stempellinella|[18737]|SSWLF4380-13|Chironomidae|661|BOLD:ACB7775
Stempellinella|[18738]|CNBRF723-14|Chironomidae|661|BOLD:ACL7154
Stempellinella|[18739]|CNBRP723-14|Chironomidae|661|BOLD:ACA2923
Stempellinella fimbriata|[18740]|CNGBF410-14|Chironomidae|671|BOLD:AAD0300
Stempellinella boltoni|[18741]|CNFNS100-14|Chironomidae|582|BOLD:AAF3525
Stempellinella|[18742]|SSBAF067-13|Chironomidae|661|BOLD:ACD0178
Stempellinella|[18743]|CNRVB1623-15|Chironomidae|582|BOLD:ACT5681
Paratanytarsus|[18744]|BBDCM309-10|Chironomidae|658|BOLD:AAP8998
Paratanytarsus|[18745]|CNWLG238-12|Chironomidae|658|BOLD:ACD1709
Chironominae|[18746]|INNV012-08|Chironomidae|658|BOLD:AAG5433
Chironominae|[18747]|SMTPD5590-13|Chironomidae|550|BOLD:ACJ6727
Chironominae|[18748]|CNBRB255-14|Chironomidae|658|BOLD:ACL4215
Micropsectra|[18749]|CNGMA270-13|Chironomidae|661|BOLD:ACK8276
Micropsectra xantha|[18750]|CNGBJ007-14|Chironomidae|627|BOLD:ABU8327
Chironominae|[18751]|CNTMC521-14|Chironomidae|658|BOLD:ACM4068
Micropsectra|[18752]|SSJAD835-13|Chironomidae|661|BOLD:ACI6043
Micropsectra|[18753]|CNBAI173-13|Chironomidae|670|BOLD:ACC2674
Micropsectra|[18754]|CNBRC104-14|Chironomidae|658|BOLD:ACL4246
Micropsectra|[18755]|CNFNE451-14|Chironomidae|661|BOLD:ACL4257
Micropsectra|[18756]|CNFNE1000-14|Chironomidae|658|BOLD:ACL4241
Micropsectra|[18757]|CNFNE1126-14|Chironomidae|658|BOLD:ACL4855
Micropsectra|[18758]|CNMID069-14|Chironomidae|576|BOLD:ACM9772
Micropsectra|[18759]|JSMAY512-11|Chironomidae|658|BOLD:ABV1185
Micropsectra|[18760]|CNMIC872-14|Chironomidae|597|BOLD:ACM3888
Micropsectra|[18761]|SSFDA814-14|Chironomidae|585|BOLD:ACN0631
Micropsectra|[18762]|CNMIC330-14|Chironomidae|567|BOLD:ACM8644
Micropsectra|[18763]|CNTNC1313-14|Chironomidae|582|BOLD:ACN0162
Micropsectra|[18764]|SSFDA894-14|Chironomidae|576|BOLD:ACO1739
Micropsectra|[18765]|SSFDA2052-14|Chironomidae|588|BOLD:ACO5607
Micropsectra|[18766]|SSGBC801-14|Chironomidae|576|BOLD:ACP4008
Chironomidae|[18767]|RBINA4014-13|Chironomidae|658|BOLD:ACR1499
Chironomidae|[18768]|CNNHB1621-14|Chironomidae|609|BOLD:ACR4872
Chironomidae|[18769]|CNNHF581-14|Chironomidae|630|BOLD:ACR4871
Chironomidae|[18770]|CNNHF770-14|Chironomidae|603|BOLD:ACR5885
Micropsectra|[18771]|CNGLB223-13|Chironomidae|658|BOLD:ACE1463
Micropsectra|[18772]|BBDCN440-10|Chironomidae|658|BOLD:AAM6225
Micropsectra|[18773]|CNJAH122-12|Chironomidae|658|BOLD:ACD0318
Micropsectra|[18774]|CNJAJ443-12|Chironomidae|658|BOLD:ACL5064
Chironomidae|[18775]|NGNAR149-14|Chironomidae|658|BOLD:ACI2981
Micropsectra|[18776]|GMDAP1329-13|Chironomidae|670|BOLD:ACI3663
Micropsectra|[18777]|CNGMG287-14|Chironomidae|661|BOLD:AAL7308
Chironomidae|[18778]|SSFDC4243-14|Chironomidae|549|BOLD:ACO4277
Chironomidae|[18779]|CNBFF513-15|Chironomidae|579|BOLD:ACI6641
Micropsectra|[18780]|SSBAF441-13|Chironomidae|661|BOLD:ACK6332
Micropsectra|[18781]|SSWLF1635-13|Chironomidae|583|BOLD:ACL0779
Micropsectra|[18782]|SSBAD1612-12|Chironomidae|670|BOLD:AAP6895
Micropsectra|[18783]|CNFNC1413-14|Chironomidae|661|BOLD:ACM0435
Micropsectra|[18784]|CNTMC1316-14|Chironomidae|588|BOLD:AAV4674
Micropsectra|[18785]|CNBFH294-15|Chironomidae|588|BOLD:ACT0201
Micropsectra|[18786]|CNGBC1446-13|Chironomidae|658|BOLD:ACL1367
Micropsectra|[18787]|CNFND396-14|Chironomidae|661|BOLD:ACL2589
Chironomidae|[18788]|CNRVB397-15|Chironomidae|546|BOLD:ACT7985
Chironomidae|[18789]|CNGCA200-15|Chironomidae|588|BOLD:ACT4540
Micropsectra|[18790]|GMDAP271-12|Chironomidae|658|BOLD:ABU8331
Chironomidae|[18791]|CNYOI376-15|Chironomidae|588|BOLD:ACT2970
Chironominae|[18792]|CNGMG1429-14|Chironomidae|614|BOLD:ACL6773
Chironomidae|[18793]|CNGCA208-15|Chironomidae|552|BOLD:ACS9956
Chironominae|[18794]|GMDAP1139-13|Chironomidae|670|BOLD:ACI3739
Chironominae|[18795]|CNFND402-14|Chironomidae|661|BOLD:ACA2800
Chironomidae|[18796]|JSDIP421-10|Chironomidae|658|BOLD:AAQ0602
Chironominae|[18797]|CNGMC347-13|Chironomidae|661|BOLD:ACL2525

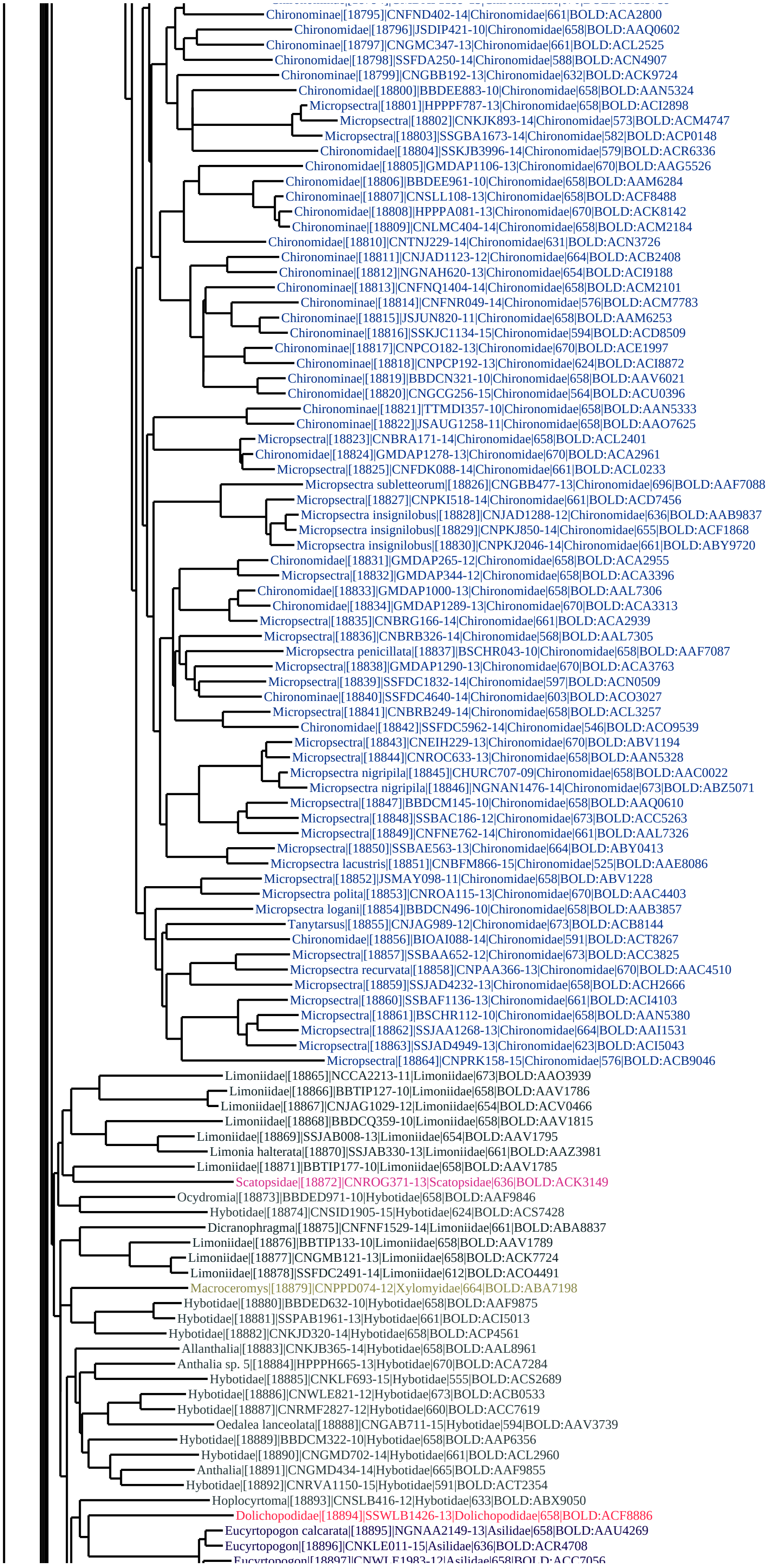

Chironominae|[18795]|CNFND402-14|Chironomidae|661|BOLD:ACA2800
Chironomidae|[18796]|JSDIP421-10|Chironomidae|658|BOLD:AAQ0602
Chironominae|[18797]|CNGMC347-13|Chironomidae|661|BOLD:ACL2525
Chironominae|[18798]|SSFDA250-14|Chironomidae|588|BOLD:ACN4907
Chironominae|[18799]|CNGBB192-13|Chironomidae|632|BOLD:ACK9724
Chironomidae|[18800]|BBDEE883-10|Chironomidae|658|BOLD:AAN5324
Micropsectra|[18801]|HPPPF787-13|Chironomidae|658|BOLD:ACI2898
Micropsectra|[18802]|CNKJK893-14|Chironomidae|573|BOLD:ACM4747
Micropsectra|[18803]|SSGBA1673-14|Chironomidae|582|BOLD:ACP0148
Chironominae|[18804]|SSKJB3996-14|Chironomidae|579|BOLD:ACR6336
Chironomidae|[18805]|GMDAP1106-13|Chironomidae|670|BOLD:AAG5526
Chironomidae|[18806]|BBDEE961-10|Chironomidae|658|BOLD:AAM6284
Chironominae|[18807]|CNSLL108-13|Chironomidae|658|BOLD:ACF8488
Chironomidae|[18808]|HPPPA081-13|Chironomidae|670|BOLD:ACK8142
Chironominae|[18809]|CNLMC404-14|Chironomidae|658|BOLD:ACM2184
Chironominae|[18810]|CNTNJ229-14|Chironomidae|631|BOLD:ACN3726
Chironominae|[18811]|CNJAD1123-12|Chironomidae|664|BOLD:ACB2408
Chironominae|[18812]|NGNAH620-13|Chironomidae|654|BOLD:ACI9188
Chironominae|[18813]|CNFNQ1404-14|Chironomidae|658|BOLD:ACM2101
Chironominae|[18814]|CNFNR049-14|Chironomidae|576|BOLD:ACM7783
Chironominae|[18815]|JSJUN820-11|Chironomidae|658|BOLD:AAM6253
Chironominae|[18816]|SSKJC1134-15|Chironomidae|594|BOLD:ACD8509
Chironominae|[18817]|CNPCO182-13|Chironomidae|670|BOLD:ACE1997
Chironominae|[18818]|CNPCP192-13|Chironomidae|624|BOLD:ACI8872
Chironominae|[18819]|BBDCN321-10|Chironomidae|658|BOLD:AAV6021
Chironomidae|[18820]|CNGCG256-15|Chironomidae|564|BOLD:ACU0396
Chironominae|[18821]|TTMDI357-10|Chironomidae|658|BOLD:AAN5333
Chironominae|[18822]|JSAUG1258-11|Chironomidae|658|BOLD:AAO7625
Micropsectra|[18823]|CNBRA171-14|Chironomidae|658|BOLD:ACL2401
Chironomidae|[18824]|GMDAP1278-13|Chironomidae|670|BOLD:ACA2961
Micropsectra|[18825]|CNFDK088-14|Chironomidae|661|BOLD:ACL0233
Micropsectra subletteorum|[18826]|CNGBB477-13|Chironomidae|696|BOLD:AAF7088
Micropsectra|[18827]|CNPKI518-14|Chironomidae|661|BOLD:ACD7456
Micropsectra insignilobus|[18828]|CNJAD1288-12|Chironomidae|636|BOLD:AAB9837
Micropsectra insignilobus|[18829]|CNPKJ850-14|Chironomidae|655|BOLD:ACF1868
Micropsectra insignilobus|[18830]|CNPKJ2046-14|Chironomidae|661|BOLD:ABY9720
Chironomidae|[18831]|GMDAP265-12|Chironomidae|658|BOLD:ACA2955
Micropsectra|[18832]|GMDAP344-12|Chironomidae|658|BOLD:ACA3396
Chironomidae|[18833]|GMDAP1000-13|Chironomidae|658|BOLD:AAL7306
Chironomidae|[18834]|GMDAP1289-13|Chironomidae|670|BOLD:ACA3313
Micropsectra|[18835]|CNBRG166-14|Chironomidae|661|BOLD:ACA2939
Micropsectra|[18836]|CNBRB326-14|Chironomidae|568|BOLD:AAL7305
Micropsectra penicillata|[18837]|BSCHR043-10|Chironomidae|658|BOLD:AAF7087
Micropsectra|[18838]|GMDAP1290-13|Chironomidae|670|BOLD:ACA3763
Micropsectra|[18839]|SSFDC1832-14|Chironomidae|597|BOLD:ACN0509
Chironominae|[18840]|SSFDC4640-14|Chironomidae|603|BOLD:ACO3027
Micropsectra|[18841]|CNBRB249-14|Chironomidae|658|BOLD:ACL3257
Chironomidae|[18842]|SSFDC5962-14|Chironomidae|546|BOLD:ACO9539
Micropsectra|[18843]|CNEIH229-13|Chironomidae|670|BOLD:ABV1194
Micropsectra|[18844]|CNROC633-13|Chironomidae|658|BOLD:AAN5328
Micropsectra nigripila|[18845]|CHURC707-09|Chironomidae|658|BOLD:AAC0022
Micropsectra nigripila|[18846]|NGNAN1476-14|Chironomidae|673|BOLD:ABZ5071
Micropsectra|[18847]|BBDCM145-10|Chironomidae|658|BOLD:AAQ0610
Micropsectra|[18848]|SSBAC186-12|Chironomidae|673|BOLD:ACC5263
Micropsectra|[18849]|CNFNE762-14|Chironomidae|661|BOLD:AAL7326
Micropsectra|[18850]|SSBAE563-13|Chironomidae|664|BOLD:ABY0413
Micropsectra lacustris|[18851]|CNBFM866-15|Chironomidae|525|BOLD:AAE8086
Micropsectra|[18852]|JSMAY098-11|Chironomidae|658|BOLD:ABV1228
Micropsectra polita|[18853]|CNROA115-13|Chironomidae|670|BOLD:AAC4403
Micropsectra logani|[18854]|BBDCN496-10|Chironomidae|658|BOLD:AAB3857
Tanytarsus|[18855]|CNJAG989-12|Chironomidae|673|BOLD:ACB8144
Chironomidae|[18856]|BIOAI088-14|Chironomidae|591|BOLD:ACT8267
Micropsectra|[18857]|SSBAA652-12|Chironomidae|673|BOLD:ACC3825
Micropsectra recurvata|[18858]|CNPAA366-13|Chironomidae|670|BOLD:AAC4510
Micropsectra|[18859]|SSJAD4232-13|Chironomidae|658|BOLD:ACH2666
Micropsectra|[18860]|SSBAF1136-13|Chironomidae|661|BOLD:ACI4103
Micropsectra|[18861]|BSCHR112-10|Chironomidae|658|BOLD:AAN5380
Micropsectra|[18862]|SSJAA1268-13|Chironomidae|664|BOLD:AAI1531
Micropsectra|[18863]|SSJAD4949-13|Chironomidae|623|BOLD:ACI5043
Micropsectra|[18864]|CNPRK158-15|Chironomidae|576|BOLD:ACB9046
Limoniidae|[18865]|NCCA2213-11|Limoniidae|673|BOLD:AAO3939
Limoniidae|[18866]|BBTIP127-10|Limoniidae|658|BOLD:AAV1786
Limoniidae|[18867]|CNJAG1029-12|Limoniidae|654|BOLD:ACV0466
Limoniidae|[18868]|BBDCQ359-10|Limoniidae|658|BOLD:AAV1815
Limoniidae|[18869]|SSJAB008-13|Limoniidae|654|BOLD:AAV1795
Limonia halterata|[18870]|SSJAB330-13|Limoniidae|661|BOLD:AAZ3981
Limoniidae|[18871]|BBTIP177-10|Limoniidae|658|BOLD:AAV1785
Scatopsidae|[18872]|CNROG371-13|Scatopsidae|636|BOLD:ACK3149
Ocydromia|[18873]|BBDED971-10|Hybotidae|658|BOLD:AAF9846
Hybotidae|[18874]|CNSID1905-15|Hybotidae|624|BOLD:ACS7428
Dicranophragma|[18875]|CNFNF1529-14|Limoniidae|661|BOLD:ABA8837
Limoniidae|[18876]|BBTIP133-10|Limoniidae|658|BOLD:AAV1789
Limoniidae|[18877]|CNGMB121-13|Limoniidae|658|BOLD:ACK7724
Limoniidae|[18878]|SSFDC2491-14|Limoniidae|612|BOLD:ACO4491
Macroceromys|[18879]|CNPPD074-12|Xylomyidae|664|BOLD:ABA7198
Hybotidae|[18880]|BBDED632-10|Hybotidae|658|BOLD:AAF9875
Hybotidae|[18881]|SSPAB1961-13|Hybotidae|661|BOLD:ACI5013
Hybotidae|[18882]|CNKJD320-14|Hybotidae|658|BOLD:ACP4561
Allanthalia|[18883]|CNKJB365-14|Hybotidae|658|BOLD:AAL8961
Anthalia sp. 5|[18884]|HPPPH665-13|Hybotidae|670|BOLD:ACA7284
Hybotidae|[18885]|CNKLF693-15|Hybotidae|555|BOLD:ACS2689
Hybotidae|[18886]|CNWLE821-12|Hybotidae|673|BOLD:ACB0533
Hybotidae|[18887]|CNRMF2827-12|Hybotidae|660|BOLD:ACC7619
Oedalea lanceolata|[18888]|CNGAB711-15|Hybotidae|594|BOLD:AAV3739
Hybotidae|[18889]|BBDCM322-10|Hybotidae|658|BOLD:AAP6356
Hybotidae|[18890]|CNGMD702-14|Hybotidae|661|BOLD:ACL2960
Anthalia|[18891]|CNGMD434-14|Hybotidae|665|BOLD:AAF9855
Hybotidae|[18892]|CNRVA1150-15|Hybotidae|591|BOLD:ACT2354
Hoplocyrtoma|[18893]|CNSLB416-12|Hybotidae|633|BOLD:ABX9050
Dolichopodidae|[18894]|SSWLB1426-13|Dolichopodidae|658|BOLD:ACF8886
Eucyrtopogon calcarata|[18895]|NGNAA2149-13|Asilidae|658|BOLD:AAU4269
Eucyrtopogon|[18896]|CNKLE011-15|Asilidae|636|BOLD:ACR4708
Eucyrtopogon|[18897]|CNWLE1983-12|Asilidae|658|BOLD:ACC7056

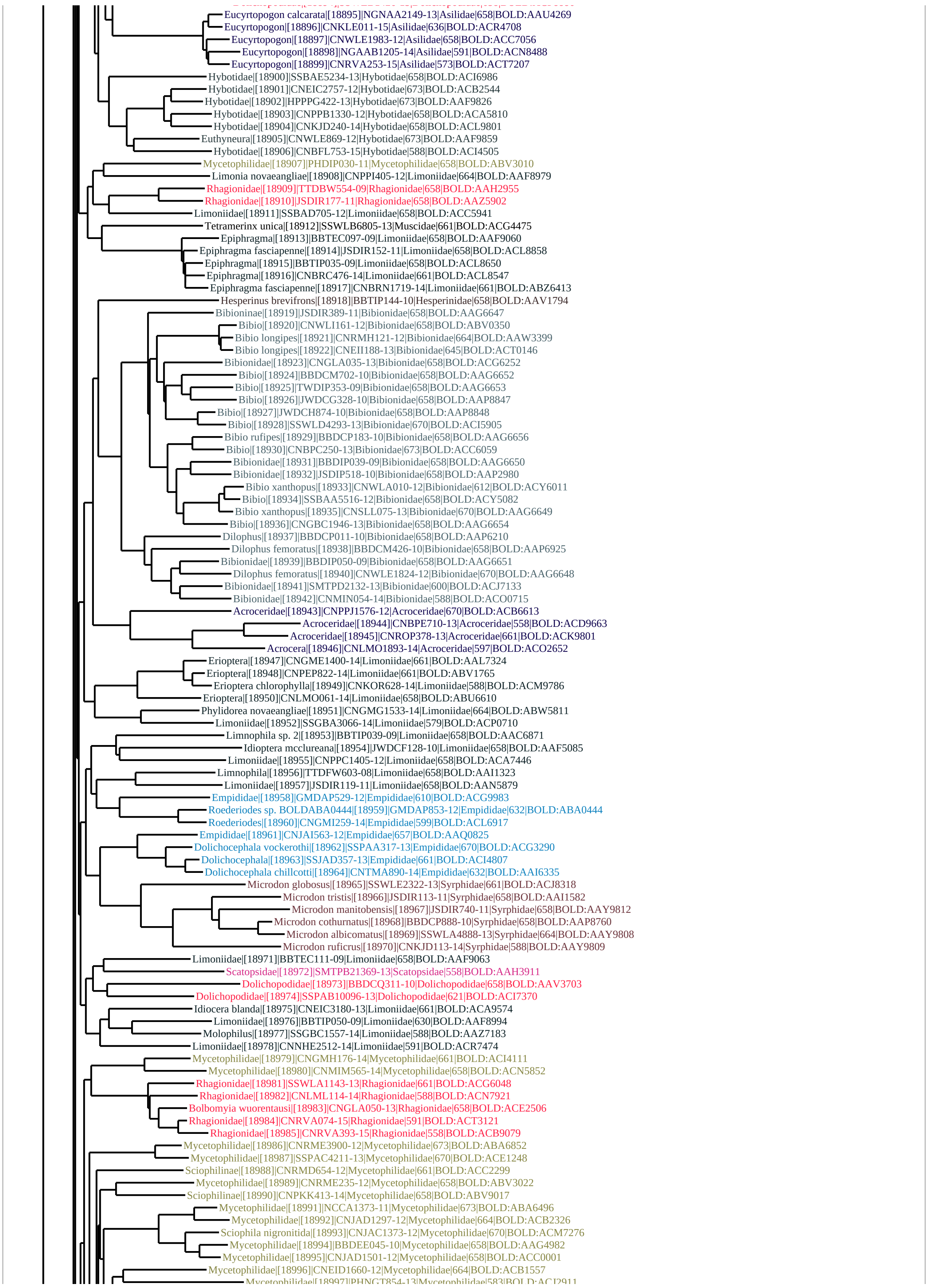

Eucyrtopogon calcarata|[18895]|NGNAA2149-13|Asilidae|658|BOLD:AAU4269
Eucyrtopogon|[18896]|CNKLE011-15|Asilidae|636|BOLD:ACR4708
Eucyrtopogon|[18897]|CNWLE1983-12|Asilidae|658|BOLD:ACC7056
Eucyrtopogon|[18898]|NGAAB1205-14|Asilidae|591|BOLD:ACN8488
Eucyrtopogon|[18899]|CNRVA253-15|Asilidae|573|BOLD:ACT7207
Hybotidae|[18900]|SSBAE5234-13|Hybotidae|658|BOLD:ACI6986
Hybotidae|[18901]|CNEIC2757-12|Hybotidae|673|BOLD:ACB2544
Hybotidae|[18902]|HPPPG422-13|Hybotidae|673|BOLD:AAF9826
Hybotidae|[18903]|CNPPB1330-12|Hybotidae|658|BOLD:ACA5810
Hybotidae|[18904]|CNKJD240-14|Hybotidae|658|BOLD:ACL9801
Euthyneura|[18905]|CNWLE869-12|Hybotidae|673|BOLD:AAF9859
Hybotidae|[18906]|CNBFL753-15|Hybotidae|588|BOLD:ACI4505
Mycetophilidae|[18907]|PHDIP030-11|Mycetophilidae|658|BOLD:ABV3010
Limonia novaeangliae|[18908]|CNPPI405-12|Limoniidae|664|BOLD:AAF8979
Rhagionidae|[18909]|TTDBW554-09|Rhagionidae|658|BOLD:AAH2955
Rhagionidae|[18910]|JSDIR177-11|Rhagionidae|658|BOLD:AAZ5902
Limoniidae|[18911]|SSBAD705-12|Limoniidae|658|BOLD:ACC5941
Tetramerinx unica|[18912]|SSWLB6805-13|Muscidae|661|BOLD:ACG4475
Epiphragma|[18913]|BBTEC097-09|Limoniidae|658|BOLD:AAF9060
Epiphragma fasciapenne|[18914]|JSDIR152-11|Limoniidae|658|BOLD:ACL8858
Epiphragma|[18915]|BBTIP035-09|Limoniidae|658|BOLD:ACL8650
Epiphragma|[18916]|CNBRC476-14|Limoniidae|661|BOLD:ACL8547
Epiphragma fasciapenne|[18917]|CNBRN1719-14|Limoniidae|661|BOLD:ABZ6413
Hesperinus brevifrons|[18918]|BBTIP144-10|Hesperinidae|658|BOLD:AAV1794
Bibioninae|[18919]|JSDIR389-11|Bibionidae|658|BOLD:AAG6647
Bibio|[18920]|CNWLI161-12|Bibionidae|658|BOLD:ABV0350
Bibio longipes|[18921]|CNRMH121-12|Bibionidae|664|BOLD:AAW3399
Bibio longipes|[18922]|CNEII188-13|Bibionidae|645|BOLD:ACT0146
Bibionidae|[18923]|CNGLA035-13|Bibionidae|658|BOLD:ACG6252
Bibio|[18924]|BBDCM702-10|Bibionidae|658|BOLD:AAG6652
Bibio|[18925]|TWDIP353-09|Bibionidae|658|BOLD:AAG6653
Bibio|[18926]|JWDCG328-10|Bibionidae|658|BOLD:AAP8847
Bibio|[18927]|JWDCH874-10|Bibionidae|658|BOLD:AAP8848
Bibio|[18928]|SSWLD4293-13|Bibionidae|670|BOLD:ACI5905
Bibio rufipes|[18929]|BBDCP183-10|Bibionidae|658|BOLD:AAG6656
Bibio|[18930]|CNBPC250-13|Bibionidae|673|BOLD:ACC6059
Bibionidae|[18931]|BBDIP039-09|Bibionidae|658|BOLD:AAG6650
Bibionidae|[18932]|JSDIP518-10|Bibionidae|658|BOLD:AAP2980
Bibio xanthopus|[18933]|CNWLA010-12|Bibionidae|612|BOLD:ACY6011
Bibio|[18934]|SSBAA5516-12|Bibionidae|658|BOLD:ACY5082
Bibio xanthopus|[18935]|CNSLL075-13|Bibionidae|670|BOLD:AAG6649
Bibio|[18936]|CNGBC1946-13|Bibionidae|658|BOLD:AAG6654
Dilophus|[18937]|BBDCP011-10|Bibionidae|658|BOLD:AAP6210
Dilophus femoratus|[18938]|BBDCM426-10|Bibionidae|658|BOLD:AAP6925
Bibionidae|[18939]|BBDIP050-09|Bibionidae|658|BOLD:AAG6651
Dilophus femoratus|[18940]|CNWLE1824-12|Bibionidae|670|BOLD:AAG6648
Bibionidae|[18941]|SMTPD2132-13|Bibionidae|600|BOLD:ACJ7133
Bibionidae|[18942]|CNMIN054-14|Bibionidae|588|BOLD:ACO0715
Acroceridae|[18943]|CNPPJ1576-12|Acroceridae|670|BOLD:ACB6613
Acroceridae|[18944]|CNBPE710-13|Acroceridae|558|BOLD:ACD9663
Acroceridae|[18945]|CNROP378-13|Acroceridae|661|BOLD:ACK9801
Acrocera|[18946]|CNLMO1893-14|Acroceridae|597|BOLD:ACO2652
Erioptera|[18947]|CNGME1400-14|Limoniidae|661|BOLD:AAL7324
Erioptera|[18948]|CNPEP822-14|Limoniidae|661|BOLD:ABV1765
Erioptera chlorophylla|[18949]|CNKOR628-14|Limoniidae|588|BOLD:ACM9786
Erioptera|[18950]|CNLMO061-14|Limoniidae|658|BOLD:ABU6610
Phylidorea novaeangliae|[18951]|CNGMG1533-14|Limoniidae|664|BOLD:ABW5811
Limoniidae|[18952]|SSGBA3066-14|Limoniidae|579|BOLD:ACP0710
Limnophila sp. 2|[18953]|BBTIP039-09|Limoniidae|658|BOLD:AAC6871
Idioptera mcclureana|[18954]|JWDCF128-10|Limoniidae|658|BOLD:AAF5085
Limoniidae|[18955]|CNPPC1405-12|Limoniidae|658|BOLD:ACA7446
Limnophila|[18956]|TTDFW603-08|Limoniidae|658|BOLD:AAI1323
Limoniidae|[18957]|JSDIR119-11|Limoniidae|658|BOLD:AAN5879
Empididae|[18958]|GMDAP529-12|Empididae|610|BOLD:ACG9983
Roederiodes sp. BOLDABA0444|[18959]|GMDAP853-12|Empididae|632|BOLD:ABA0444
Roederiodes|[18960]|CNGMI259-14|Empididae|599|BOLD:ACL6917
Empididae|[18961]|CNJAI563-12|Empididae|657|BOLD:AAQ0825
Dolichocephala vockerothi|[18962]|SSPAA317-13|Empididae|670|BOLD:ACG3290
Dolichocephala|[18963]|SSJAD357-13|Empididae|661|BOLD:ACI4807
Dolichocephala chillcotti|[18964]|CNTMA890-14|Empididae|632|BOLD:AAI6335
Microdon globosus|[18965]|SSWLE2322-13|Syrphidae|661|BOLD:ACJ8318
Microdon tristis|[18966]|JSDIR113-11|Syrphidae|658|BOLD:AAI1582
Microdon manitobensis|[18967]|JSDIR740-11|Syrphidae|658|BOLD:AAY9812
Microdon cothurnatus|[18968]|BBDCP888-10|Syrphidae|658|BOLD:AAP8760
Microdon albicomatus|[18969]|SSWLA4888-13|Syrphidae|664|BOLD:AAY9808
Microdon ruficrus|[18970]|CNKJD113-14|Syrphidae|588|BOLD:AAY9809
Limoniidae|[18971]|BBTEC111-09|Limoniidae|658|BOLD:AAF9063
Scatopsidae|[18972]|SMTPB21369-13|Scatopsidae|558|BOLD:AAH3911
Dolichopodidae|[18973]|BBDCQ311-10|Dolichopodidae|658|BOLD:AAV3703
Dolichopodidae|[18974]|SSPAB10096-13|Dolichopodidae|621|BOLD:ACI7370
Idiocera blanda|[18975]|CNEIC3180-13|Limoniidae|661|BOLD:ACA9574
Limoniidae|[18976]|BBTIP050-09|Limoniidae|630|BOLD:AAF8994
Molophilus|[18977]|SSGBC1557-14|Limoniidae|588|BOLD:AAZ7183
Limoniidae|[18978]|CNNHE2512-14|Limoniidae|591|BOLD:ACR7474
Mycetophilidae|[18979]|CNGMH176-14|Mycetophilidae|661|BOLD:ACI4111
Mycetophilidae|[18980]|CNMIM565-14|Mycetophilidae|658|BOLD:ACN5852
Rhagionidae|[18981]|SSWLA1143-13|Rhagionidae|661|BOLD:ACG6048
Rhagionidae|[18982]|CNLML114-14|Rhagionidae|588|BOLD:ACN7921
Bolbomyia wuorentausi|[18983]|CNGLA050-13|Rhagionidae|658|BOLD:ACE2506
Rhagionidae|[18984]|CNRVA074-15|Rhagionidae|591|BOLD:ACT3121
Rhagionidae|[18985]|CNRVA393-15|Rhagionidae|558|BOLD:ACB9079
Mycetophilidae|[18986]|CNRME3900-12|Mycetophilidae|673|BOLD:ABA6852
Mycetophilidae|[18987]|SSPAC4211-13|Mycetophilidae|670|BOLD:ACE1248
Sciophilinae|[18988]|CNRMD654-12|Mycetophilidae|661|BOLD:ACC2299
Mycetophilidae|[18989]|CNRME235-12|Mycetophilidae|658|BOLD:ABV3022
Sciophilinae|[18990]|CNPKK413-14|Mycetophilidae|658|BOLD:ABV9017
Mycetophilidae|[18991]|NCCA1373-11|Mycetophilidae|673|BOLD:ABA6496
Mycetophilidae|[18992]|CNJAD1297-12|Mycetophilidae|664|BOLD:ACB2326
Sciophila nigronitida|[18993]|CNJAC1373-12|Mycetophilidae|670|BOLD:ACM7276
Mycetophilidae|[18994]|BBDEE045-10|Mycetophilidae|658|BOLD:AAG4982
Mycetophilidae|[18995]|CNJAD1501-12|Mycetophilidae|658|BOLD:ACC0001
Mycetophilidae|[18996]|CNEID1660-12|Mycetophilidae|664|BOLD:ACB1557
Mycetophilidae|[18997]|PHNGT854-13|Mycetophilidae|583|BOLD:ACJ2911

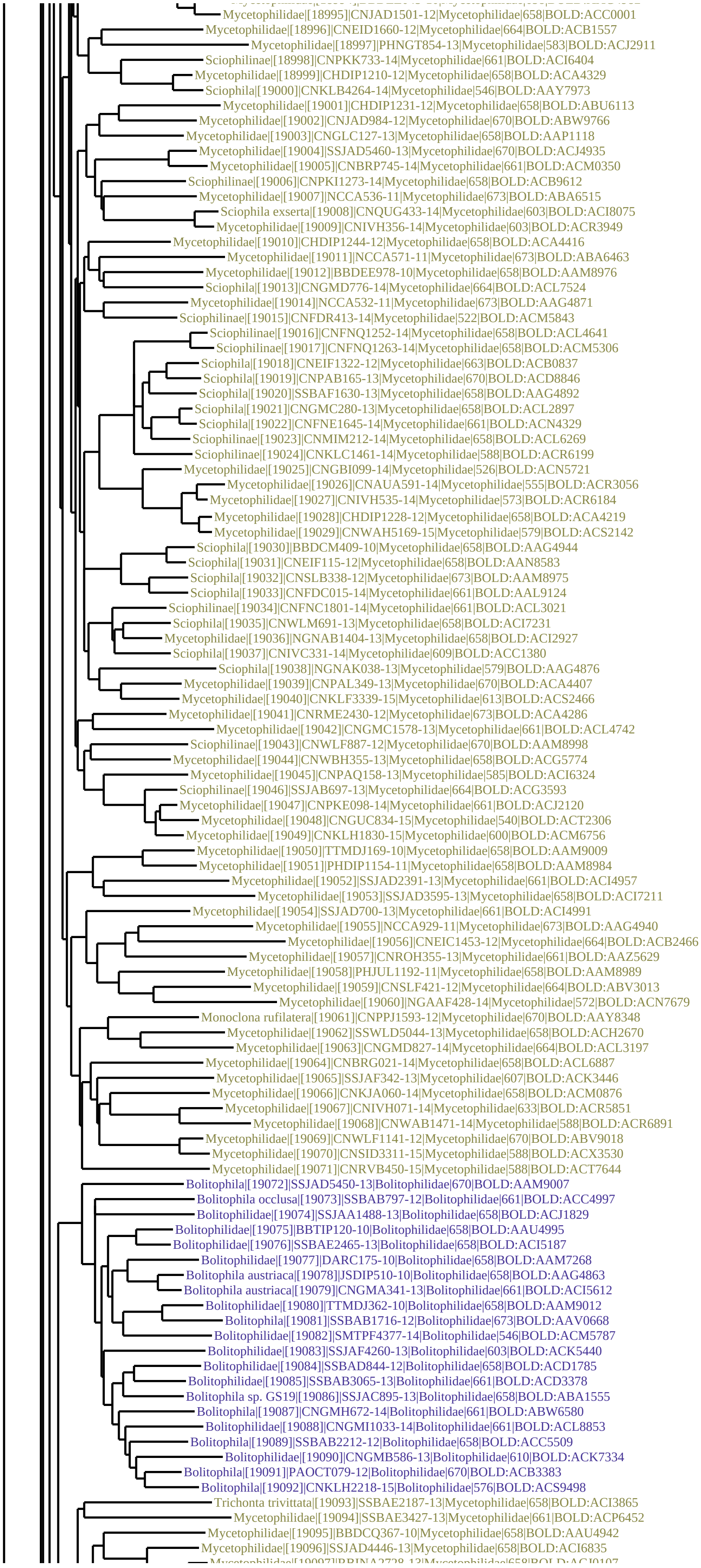
Mycetophilidae|[18995]|CNJAD1501-12|Mycetophilidae|658|BOLD:ACC0001
Mycetophilidae|[18996]|CNEID1660-12|Mycetophilidae|664|BOLD:ACB1557
Mycetophilidae|[18997]|PHNGT854-13|Mycetophilidae|583|BOLD:ACJ2911
Sciophilinae|[18998]|CNPKK733-14|Mycetophilidae|661|BOLD:ACI6404
Mycetophilidae|[18999]|CHDIP1210-12|Mycetophilidae|658|BOLD:ACA4329
Sciophila|[19000]|CNKLB4264-14|Mycetophilidae|546|BOLD:AAY7973
Mycetophilidae|[19001]|CHDIP1231-12|Mycetophilidae|658|BOLD:ABU6113
Mycetophilidae|[19002]|CNJAD984-12|Mycetophilidae|670|BOLD:ABW9766
Mycetophilidae|[19003]|CNGLC127-13|Mycetophilidae|658|BOLD:AAP1118
Mycetophilidae|[19004]|SSJAD5460-13|Mycetophilidae|670|BOLD:ACJ4935
Mycetophilidae|[19005]|CNBRP745-14|Mycetophilidae|661|BOLD:ACM0350
Sciophilinae|[19006]|CNPKI1273-14|Mycetophilidae|658|BOLD:ACB9612
Mycetophilidae|[19007]|NCCA536-11|Mycetophilidae|673|BOLD:ABA6515
Sciophila exserta|[19008]|CNQUG433-14|Mycetophilidae|603|BOLD:ACI8075
Mycetophilidae|[19009]|CNIVH356-14|Mycetophilidae|603|BOLD:ACR3949
Mycetophilidae|[19010]|CHDIP1244-12|Mycetophilidae|658|BOLD:ACA4416
Mycetophilidae|[19011]|NCCA571-11|Mycetophilidae|673|BOLD:ABA6463
Mycetophilidae|[19012]|BBDEE978-10|Mycetophilidae|658|BOLD:AAM8976
Sciophila|[19013]|CNGMD776-14|Mycetophilidae|664|BOLD:ACL7524
Mycetophilidae|[19014]|NCCA532-11|Mycetophilidae|673|BOLD:AAG4871
Sciophilinae|[19015]|CNFDR413-14|Mycetophilidae|522|BOLD:ACM5843
Sciophilinae|[19016]|CNFNQ1252-14|Mycetophilidae|658|BOLD:ACL4641
Sciophilinae|[19017]|CNFNQ1263-14|Mycetophilidae|658|BOLD:ACM5306
Sciophila|[19018]|CNEIF1322-12|Mycetophilidae|663|BOLD:ACB0837
Sciophila|[19019]|CNPAB165-13|Mycetophilidae|670|BOLD:ACD8846
Sciophila|[19020]|SSBAF1630-13|Mycetophilidae|658|BOLD:AAG4892
Sciophila|[19021]|CNGMC280-13|Mycetophilidae|658|BOLD:ACL2897
Sciophila|[19022]|CNFNE1645-14|Mycetophilidae|661|BOLD:ACN4329
Sciophilinae|[19023]|CNMIM212-14|Mycetophilidae|658|BOLD:ACL6269
Sciophilinae|[19024]|CNKLC1461-14|Mycetophilidae|588|BOLD:ACR6199
Mycetophilidae|[19025]|CNGBI099-14|Mycetophilidae|526|BOLD:ACN5721
Mycetophilidae|[19026]|CNAUA591-14|Mycetophilidae|555|BOLD:ACR3056
Mycetophilidae|[19027]|CNIVH535-14|Mycetophilidae|573|BOLD:ACR6184
Mycetophilidae|[19028]|CHDIP1228-12|Mycetophilidae|658|BOLD:ACA4219
Mycetophilidae|[19029]|CNWAH5169-15|Mycetophilidae|579|BOLD:ACS2142
Sciophila|[19030]|BBDCM409-10|Mycetophilidae|658|BOLD:AAG4944
Sciophila|[19031]|CNEIF115-12|Mycetophilidae|658|BOLD:AAN8583
Sciophila|[19032]|CNSLB338-12|Mycetophilidae|673|BOLD:AAM8975
Sciophila|[19033]|CNFDC015-14|Mycetophilidae|661|BOLD:AAL9124
Sciophilinae|[19034]|CNFNC1801-14|Mycetophilidae|661|BOLD:ACL3021
Sciophila|[19035]|CNWLM691-13|Mycetophilidae|658|BOLD:ACI7231
Mycetophilidae|[19036]|NGNAB1404-13|Mycetophilidae|658|BOLD:ACI2927
Sciophila|[19037]|CNIVC331-14|Mycetophilidae|609|BOLD:ACC1380
Sciophila|[19038]|NGNAK038-13|Mycetophilidae|579|BOLD:AAG4876
Mycetophilidae|[19039]|CNPAL349-13|Mycetophilidae|670|BOLD:ACA4407
Mycetophilidae|[19040]|CNKLF3339-15|Mycetophilidae|613|BOLD:ACS2466
Mycetophilidae|[19041]|CNRME2430-12|Mycetophilidae|673|BOLD:ACA4286
Mycetophilidae|[19042]|CNGMC1578-13|Mycetophilidae|661|BOLD:ACL4742
Sciophilinae|[19043]|CNWLF887-12|Mycetophilidae|670|BOLD:AAM8998
Mycetophilidae|[19044]|CNWBH355-13|Mycetophilidae|658|BOLD:ACG5774
Mycetophilidae|[19045]|CNPAQ158-13|Mycetophilidae|585|BOLD:ACI6324
Sciophilinae|[19046]|SSJAB697-13|Mycetophilidae|664|BOLD:ACG3593
Mycetophilidae|[19047]|CNPKE098-14|Mycetophilidae|661|BOLD:ACJ2120
Mycetophilidae|[19048]|CNGUC834-15|Mycetophilidae|540|BOLD:ACT2306
Mycetophilidae|[19049]|CNKLH1830-15|Mycetophilidae|600|BOLD:ACM6756
Mycetophilidae|[19050]|TTMDJ169-10|Mycetophilidae|658|BOLD:AAM9009
Mycetophilidae|[19051]|PHDIP1154-11|Mycetophilidae|658|BOLD:AAM8984
Mycetophilidae|[19052]|SSJAD2391-13|Mycetophilidae|661|BOLD:ACI4957
Mycetophilidae|[19053]|SSJAD3595-13|Mycetophilidae|658|BOLD:ACI7211
Mycetophilidae|[19054]|SSJAD700-13|Mycetophilidae|661|BOLD:ACI4991
Mycetophilidae|[19055]|NCCA929-11|Mycetophilidae|673|BOLD:AAG4940
Mycetophilidae|[19056]|CNEIC1453-12|Mycetophilidae|664|BOLD:ACB2466
Mycetophilidae|[19057]|CNROH355-13|Mycetophilidae|661|BOLD:AAZ5629
Mycetophilidae|[19058]|PHJUL1192-11|Mycetophilidae|658|BOLD:AAM8989
Mycetophilidae|[19059]|CNSLF421-12|Mycetophilidae|664|BOLD:ABV3013
Mycetophilidae|[19060]|NGAAF428-14|Mycetophilidae|572|BOLD:ACN7679
Monoclona rufilatera|[19061]|CNPPJ1593-12|Mycetophilidae|670|BOLD:AAY8348
Mycetophilidae|[19062]|SSWLD5044-13|Mycetophilidae|658|BOLD:ACH2670
Mycetophilidae|[19063]|CNGMD827-14|Mycetophilidae|664|BOLD:ACL3197
Mycetophilidae|[19064]|CNBRG021-14|Mycetophilidae|658|BOLD:ACL6887
Mycetophilidae|[19065]|SSJAF342-13|Mycetophilidae|607|BOLD:ACK3446
Mycetophilidae|[19066]|CNKJA060-14|Mycetophilidae|658|BOLD:ACM0876
Mycetophilidae|[19067]|CNIVH071-14|Mycetophilidae|633|BOLD:ACR5851
Mycetophilidae|[19068]|CNWAB1471-14|Mycetophilidae|588|BOLD:ACR6891
Mycetophilidae|[19069]|CNWLF1141-12|Mycetophilidae|670|BOLD:ABV9018
Mycetophilidae|[19070]|CNSID3311-15|Mycetophilidae|588|BOLD:ACX3530
Mycetophilidae|[19071]|CNRVB450-15|Mycetophilidae|588|BOLD:ACT7644
Bolitophila|[19072]|SSJAD5450-13|Bolitophilidae|670|BOLD:AAM9007
Bolitophila occlusa|[19073]|SSBAB797-12|Bolitophilidae|661|BOLD:ACC4997
Bolitophilidae|[19074]|SSJAA1488-13|Bolitophilidae|658|BOLD:ACJ1829
Bolitophilidae|[19075]|BBTIP120-10|Bolitophilidae|658|BOLD:AAU4995
Bolitophilidae|[19076]|SSBAE2465-13|Bolitophilidae|658|BOLD:ACI5187
Bolitophilidae|[19077]|DARC175-10|Bolitophilidae|658|BOLD:AAM7268
Bolitophila austriaca|[19078]|JSDIP510-10|Bolitophilidae|658|BOLD:AAG4863
Bolitophila austriaca|[19079]|CNGMA341-13|Bolitophilidae|661|BOLD:ACI5612
Bolitophilidae|[19080]|TTMDJ362-10|Bolitophilidae|658|BOLD:AAM9012
Bolitophila|[19081]|SSBAB1716-12|Bolitophilidae|673|BOLD:AAV0668
Bolitophilidae|[19082]|SMTPF4377-14|Bolitophilidae|546|BOLD:ACM5787
Bolitophilidae|[19083]|SSJAF4260-13|Bolitophilidae|603|BOLD:ACK5440
Bolitophilidae|[19084]|SSBAD844-12|Bolitophilidae|658|BOLD:ACD1785
Bolitophilidae|[19085]|SSBAB3065-13|Bolitophilidae|661|BOLD:ACD3378
Bolitophila sp. GS19|[19086]|SSJAC895-13|Bolitophilidae|658|BOLD:ABA1555
Bolitophila|[19087]|CNGMH672-14|Bolitophilidae|661|BOLD:ABW6580
Bolitophilidae|[19088]|CNGMI1033-14|Bolitophilidae|661|BOLD:ACL8853
Bolitophila|[19089]|SSBAB2212-12|Bolitophilidae|658|BOLD:ACC5509
Bolitophilidae|[19090]|CNGMB586-13|Bolitophilidae|610|BOLD:ACK7334
Bolitophila|[19091]|PAOCT079-12|Bolitophilidae|670|BOLD:ACB3383
Bolitophila|[19092]|CNKLH2218-15|Bolitophilidae|576|BOLD:ACS9498
Trichonta trivittata|[19093]|SSBAE2187-13|Mycetophilidae|658|BOLD:ACI3865
Mycetophilidae|[19094]|SSBAE3427-13|Mycetophilidae|661|BOLD:ACP6452
Mycetophilidae|[19095]|BBDCQ367-10|Mycetophilidae|658|BOLD:AAU4942
Mycetophilidae|[19096]|SSJAD4446-13|Mycetophilidae|658|BOLD:ACI6835

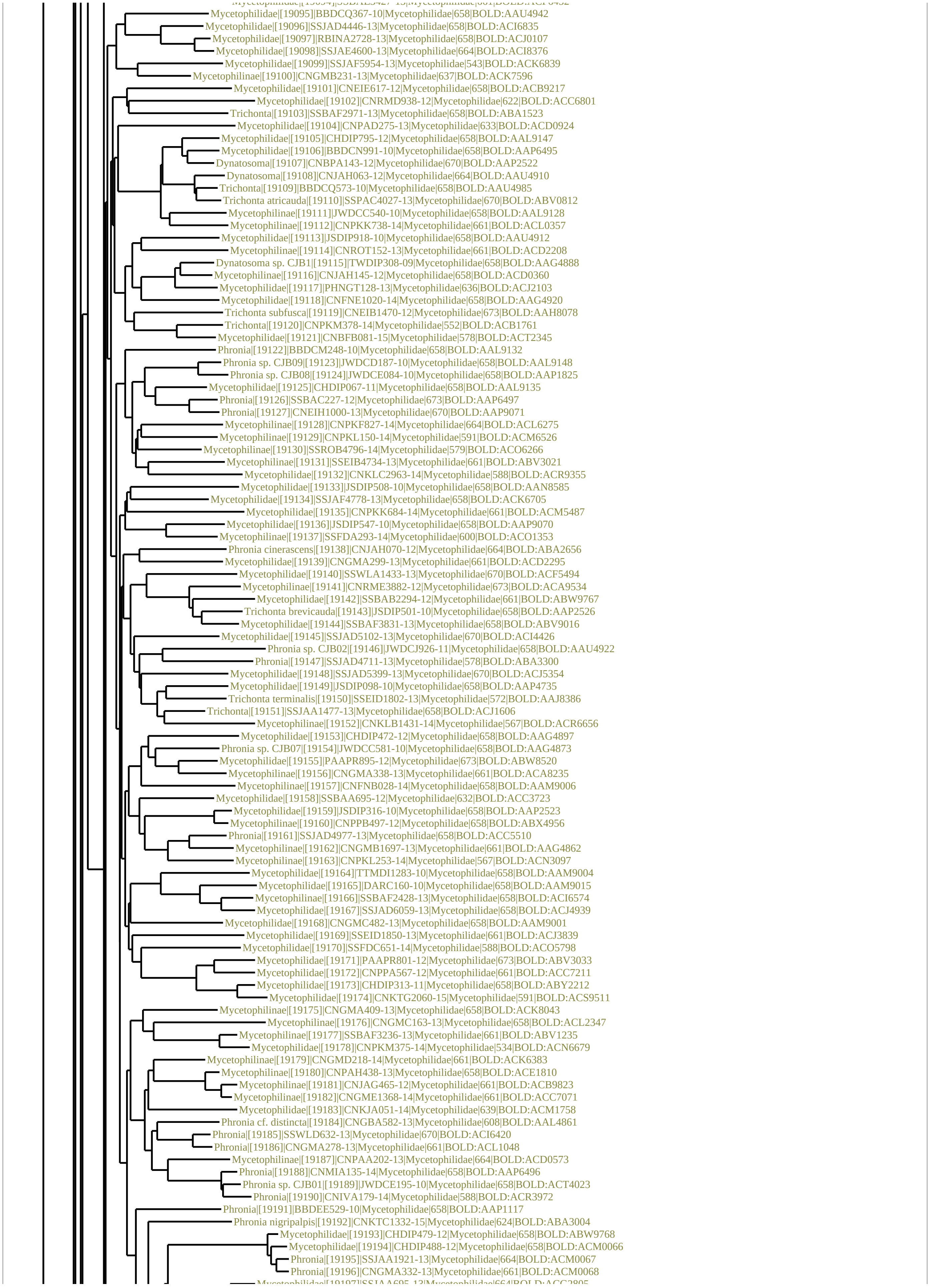
Mycetophilidae|[19095]|BBDCQ367-10|Mycetophilidae|658|BOLD:AAU4942
Mycetophilidae|[19096]|SSJAD4446-13|Mycetophilidae|658|BOLD:ACI6835
Mycetophilidae|[19097]|RBINA2728-13|Mycetophilidae|658|BOLD:ACJ0107
Mycetophilidae|[19098]|SSJAE4600-13|Mycetophilidae|664|BOLD:ACI8376
Mycetophilidae|[19099]|SSJAF5954-13|Mycetophilidae|543|BOLD:ACK6839
Mycetophilinae|[19100]|CNGMB231-13|Mycetophilidae|637|BOLD:ACK7596
Mycetophilidae|[19101]|CNEIE617-12|Mycetophilidae|658|BOLD:ACB9217
Mycetophilidae|[19102]|CNRMD938-12|Mycetophilidae|622|BOLD:ACC6801
Trichonta|[19103]|SSBAF2971-13|Mycetophilidae|658|BOLD:ABA1523
Mycetophilidae|[19104]|CNPAD275-13|Mycetophilidae|633|BOLD:ACD0924
Mycetophilidae|[19105]|CHDIP795-12|Mycetophilidae|658|BOLD:AAL9147
Mycetophilidae|[19106]|BBDCN991-10|Mycetophilidae|658|BOLD:AAP6495
Dynatosoma|[19107]|CNBPA143-12|Mycetophilidae|670|BOLD:AAP2522
Dynatosoma|[19108]|CNJAH063-12|Mycetophilidae|664|BOLD:AAU4910
Trichonta|[19109]|BBDCQ573-10|Mycetophilidae|658|BOLD:AAU4985
Trichonta atricauda|[19110]|SSPAC4027-13|Mycetophilidae|670|BOLD:ABV0812
Mycetophilinae|[19111]|JWDCC540-10|Mycetophilidae|658|BOLD:AAL9128
Mycetophilinae|[19112]|CNPKK738-14|Mycetophilidae|661|BOLD:ACL0357
Mycetophilidae|[19113]|JSDIP918-10|Mycetophilidae|658|BOLD:AAU4912
Mycetophilinae|[19114]|CNROT152-13|Mycetophilidae|661|BOLD:ACD2208
Dynatosoma sp. CJB1|[19115]|TWDIP308-09|Mycetophilidae|658|BOLD:AAG4888
Mycetophilinae|[19116]|CNJAH145-12|Mycetophilidae|658|BOLD:ACD0360
Mycetophilidae|[19117]|PHNGT128-13|Mycetophilidae|636|BOLD:ACJ2103
Mycetophilidae|[19118]|CNFNE1020-14|Mycetophilidae|658|BOLD:AAG4920
Trichonta subfusca|[19119]|CNEIB1470-12|Mycetophilidae|673|BOLD:AAH8078
Trichonta|[19120]|CNPKM378-14|Mycetophilidae|552|BOLD:ACB1761
Mycetophilidae|[19121]|CNBFB081-15|Mycetophilidae|578|BOLD:ACT2345
Phronia|[19122]|BBDCM248-10|Mycetophilidae|658|BOLD:AAL9132
Phronia sp. CJB09|[19123]|JWDCD187-10|Mycetophilidae|658|BOLD:AAL9148
Phronia sp. CJB08|[19124]|JWDCE084-10|Mycetophilidae|658|BOLD:AAP1825
Mycetophilidae|[19125]|CHDIP067-11|Mycetophilidae|658|BOLD:AAL9135
Phronia|[19126]|SSBAC227-12|Mycetophilidae|673|BOLD:AAP6497
Phronia|[19127]|CNEIH1000-13|Mycetophilidae|670|BOLD:AAP9071
Mycetophilinae|[19128]|CNPKF827-14|Mycetophilidae|664|BOLD:ACL6275
Mycetophilinae|[19129]|CNPKL150-14|Mycetophilidae|591|BOLD:ACM6526
Mycetophilinae|[19130]|SSROB4796-14|Mycetophilidae|579|BOLD:ACO6266
Mycetophilinae|[19131]|SSEIB4734-13|Mycetophilidae|661|BOLD:ABV3021
Mycetophilidae|[19132]|CNKLC2963-14|Mycetophilidae|588|BOLD:ACR9355
Mycetophilidae|[19133]|JSDIP508-10|Mycetophilidae|658|BOLD:AAN8585
Mycetophilidae|[19134]|SSJAF4778-13|Mycetophilidae|658|BOLD:ACK6705
Mycetophilidae|[19135]|CNPKK684-14|Mycetophilidae|661|BOLD:ACM5487
Mycetophilidae|[19136]|JSDIP547-10|Mycetophilidae|658|BOLD:AAP9070
Mycetophilinae|[19137]|SSFDA293-14|Mycetophilidae|600|BOLD:ACO1353
Phronia cinerascens|[19138]|CNJAH070-12|Mycetophilidae|664|BOLD:ABA2656
Mycetophilidae|[19139]|CNGMA299-13|Mycetophilidae|661|BOLD:ACD2295
Mycetophilidae|[19140]|SSWLA1433-13|Mycetophilidae|670|BOLD:ACF5494
Mycetophilinae|[19141]|CNRME3882-12|Mycetophilidae|673|BOLD:ACA9534
Mycetophilidae|[19142]|SSBAB2294-12|Mycetophilidae|661|BOLD:ABW9767
Trichonta brevicauda|[19143]|JSDIP501-10|Mycetophilidae|658|BOLD:AAP2526
Mycetophilidae|[19144]|SSBAF3831-13|Mycetophilidae|658|BOLD:ABV9016
Mycetophilidae|[19145]|SSJAD5102-13|Mycetophilidae|670|BOLD:ACI4426
Phronia sp. CJB02|[19146]|JWDCJ926-11|Mycetophilidae|658|BOLD:AAU4922
Phronia|[19147]|SSJAD4711-13|Mycetophilidae|578|BOLD:ABA3300
Mycetophilidae|[19148]|SSJAD5399-13|Mycetophilidae|670|BOLD:ACJ5354
Mycetophilidae|[19149]|JSDIP098-10|Mycetophilidae|658|BOLD:AAP4735
Trichonta terminalis|[19150]|SSEID1802-13|Mycetophilidae|572|BOLD:AAJ8386
Trichonta|[19151]|SSJAA1477-13|Mycetophilidae|658|BOLD:ACJ1606
Mycetophilinae|[19152]|CNKLB1431-14|Mycetophilidae|567|BOLD:ACR6656
Mycetophilidae|[19153]|CHDIP472-12|Mycetophilidae|658|BOLD:AAG4897
Phronia sp. CJB07|[19154]|JWDCC581-10|Mycetophilidae|658|BOLD:AAG4873
Mycetophilidae|[19155]|PAAPR895-12|Mycetophilidae|673|BOLD:ABW8520
Mycetophilinae|[19156]|CNGMA338-13|Mycetophilidae|661|BOLD:ACA8235
Mycetophilinae|[19157]|CNFNB028-14|Mycetophilidae|658|BOLD:AAM9006
Mycetophilidae|[19158]|SSBAA695-12|Mycetophilidae|632|BOLD:ACC3723
Mycetophilidae|[19159]|JSDIP316-10|Mycetophilidae|658|BOLD:AAP2523
Mycetophilinae|[19160]|CNPPB497-12|Mycetophilidae|658|BOLD:ABX4956
Phronia|[19161]|SSJAD4977-13|Mycetophilidae|658|BOLD:ACC5510
Mycetophilinae|[19162]|CNGMB1697-13|Mycetophilidae|661|BOLD:AAG4862
Mycetophilinae|[19163]|CNPKL253-14|Mycetophilidae|567|BOLD:ACN3097
Mycetophilidae|[19164]|TTMDI1283-10|Mycetophilidae|658|BOLD:AAM9004
Mycetophilidae|[19165]|DARC160-10|Mycetophilidae|658|BOLD:AAM9015
Mycetophilinae|[19166]|SSBAF2428-13|Mycetophilidae|658|BOLD:ACI6574
Mycetophilidae|[19167]|SSJAD6059-13|Mycetophilidae|658|BOLD:ACJ4939
Mycetophilidae|[19168]|CNGMC482-13|Mycetophilidae|658|BOLD:AAM9001
Mycetophilidae|[19169]|SSEID1850-13|Mycetophilidae|661|BOLD:ACJ3839
Mycetophilidae|[19170]|SSFDC651-14|Mycetophilidae|588|BOLD:ACO5798
Mycetophilidae|[19171]|PAAPR801-12|Mycetophilidae|673|BOLD:ABV3033
Mycetophilidae|[19172]|CNPPA567-12|Mycetophilidae|661|BOLD:ACC7211
Mycetophilidae|[19173]|CHDIP313-11|Mycetophilidae|658|BOLD:ABY2212
Mycetophilidae|[19174]|CNKTG2060-15|Mycetophilidae|591|BOLD:ACS9511
Mycetophilinae|[19175]|CNGMA409-13|Mycetophilidae|658|BOLD:ACK8043
Mycetophilinae|[19176]|CNGMC163-13|Mycetophilidae|658|BOLD:ACL2347
Mycetophilinae|[19177]|SSBAF3236-13|Mycetophilidae|661|BOLD:ABV1235
Mycetophilidae|[19178]|CNPKM375-14|Mycetophilidae|534|BOLD:ACN6679
Mycetophilinae|[19179]|CNGMD218-14|Mycetophilidae|661|BOLD:ACK6383
Mycetophilidae|[19180]|CNPAH438-13|Mycetophilidae|658|BOLD:ACE1810
Mycetophilinae|[19181]|CNJAG465-12|Mycetophilidae|661|BOLD:ACB9823
Mycetophilinae|[19182]|CNGME1368-14|Mycetophilidae|661|BOLD:ACC7071
Mycetophilidae|[19183]|CNKJA051-14|Mycetophilidae|639|BOLD:ACM1758
Phronia cf. distincta|[19184]|CNGBA582-13|Mycetophilidae|608|BOLD:AAL4861
Phronia|[19185]|SSWLD632-13|Mycetophilidae|670|BOLD:ACI6420
Phronia|[19186]|CNGMA278-13|Mycetophilidae|661|BOLD:ACL1048
Mycetophilinae|[19187]|CNPAA202-13|Mycetophilidae|664|BOLD:ACD0573
Phronia|[19188]|CNMIA135-14|Mycetophilidae|658|BOLD:AAP6496
Phronia sp. CJB01|[19189]|JWDCE195-10|Mycetophilidae|658|BOLD:ACT4023
Phronia|[19190]|CNIVA179-14|Mycetophilidae|588|BOLD:ACR3972
Phronia|[19191]|BBDEE529-10|Mycetophilidae|658|BOLD:AAP1117
Phronia nigripalpis|[19192]|CNKTC1332-15|Mycetophilidae|624|BOLD:ABA3004
Mycetophilidae|[19193]|CHDIP479-12|Mycetophilidae|658|BOLD:ABW9768
Mycetophilidae|[19194]|CHDIP488-12|Mycetophilidae|658|BOLD:ACM0066
Phronia|[19195]|SSJAA1921-13|Mycetophilidae|664|BOLD:ACM0067
Phronia|[19196]|CNGMA332-13|Mycetophilidae|661|BOLD:ACM0068

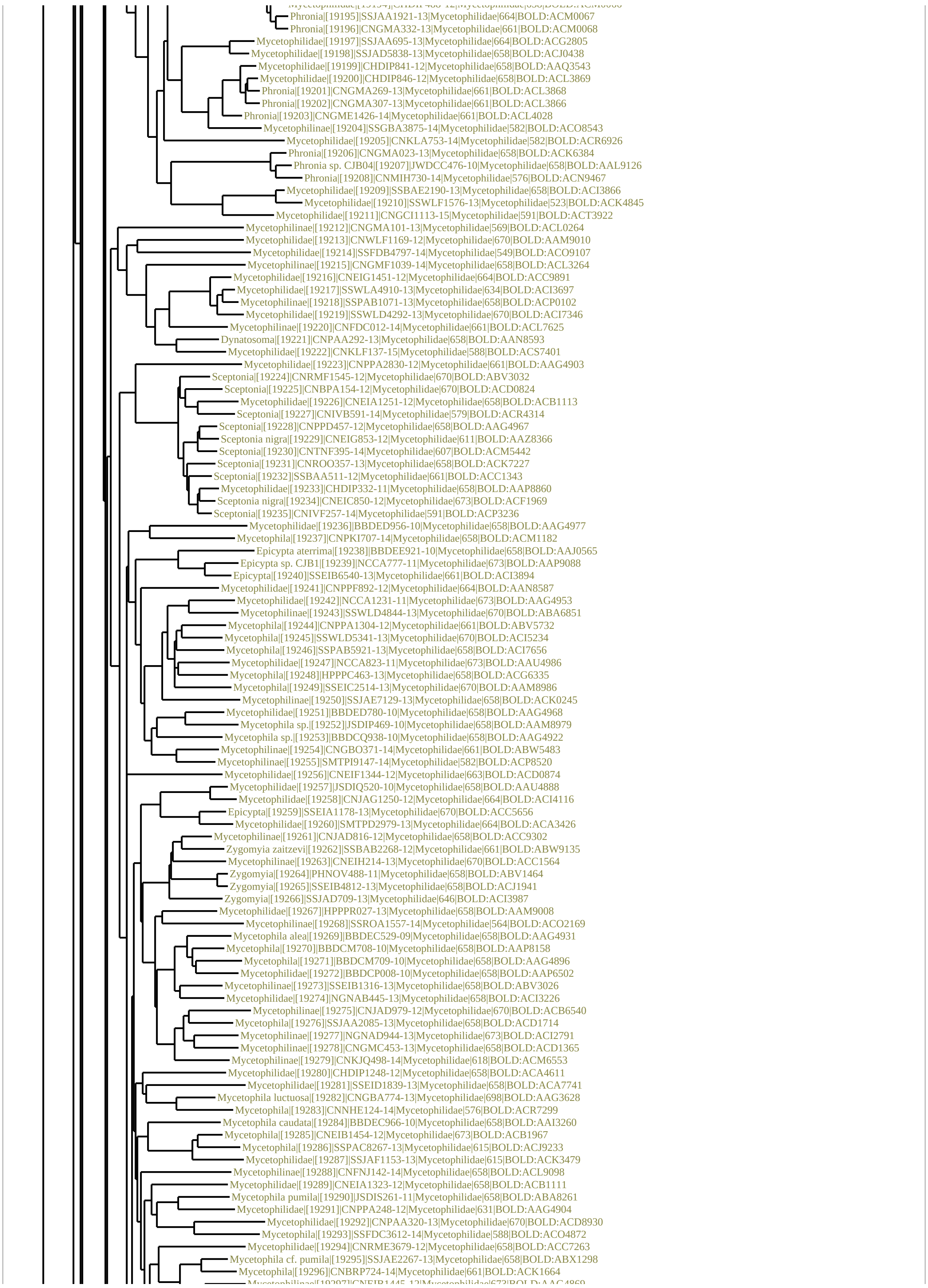

Phronia|[19195]|SSJAA1921-13|Mycetophilidae|664|BOLD:ACM0067
Phronia|[19196]|CNGMA332-13|Mycetophilidae|661|BOLD:ACM0068
Mycetophilidae|[19197]|SSJAA695-13|Mycetophilidae|664|BOLD:ACG2805
Mycetophilidae|[19198]|SSJAD5838-13|Mycetophilidae|658|BOLD:ACJ0438
Mycetophilidae|[19199]|CHDIP841-12|Mycetophilidae|658|BOLD:AAQ3543
Mycetophilidae|[19200]|CHDIP846-12|Mycetophilidae|658|BOLD:ACL3869
Phronia|[19201]|CNGMA269-13|Mycetophilidae|661|BOLD:ACL3868
Phronia|[19202]|CNGMA307-13|Mycetophilidae|661|BOLD:ACL3866
Phronia|[19203]|CNGME1426-14|Mycetophilidae|661|BOLD:ACL4028
Mycetophilinae|[19204]|SSGBA3875-14|Mycetophilidae|582|BOLD:ACO8543
Mycetophilidae|[19205]|CNKLA753-14|Mycetophilidae|582|BOLD:ACR6926
Phronia|[19206]|CNGMA023-13|Mycetophilidae|658|BOLD:ACK6384
Phronia sp. CJB04|[19207]|JWDCC476-10|Mycetophilidae|658|BOLD:AAL9126
Phronia|[19208]|CNMIH730-14|Mycetophilidae|576|BOLD:ACN9467
Mycetophilidae|[19209]|SSBAE2190-13|Mycetophilidae|658|BOLD:ACI3866
Mycetophilidae|[19210]|SSWLF1576-13|Mycetophilidae|523|BOLD:ACK4845
Mycetophilidae|[19211]|CNGCI1113-15|Mycetophilidae|591|BOLD:ACT3922
Mycetophilinae|[19212]|CNGMA101-13|Mycetophilidae|569|BOLD:ACL0264
Mycetophilidae|[19213]|CNWLF1169-12|Mycetophilidae|670|BOLD:AAM9010
Mycetophilidae|[19214]|SSFDB4797-14|Mycetophilidae|549|BOLD:ACO9107
Mycetophilinae|[19215]|CNGMF1039-14|Mycetophilidae|658|BOLD:ACL3264
Mycetophilidae|[19216]|CNEIG1451-12|Mycetophilidae|664|BOLD:ACC9891
Mycetophilidae|[19217]|SSWLA4910-13|Mycetophilidae|634|BOLD:ACI3697
Mycetophilinae|[19218]|SSPAB1071-13|Mycetophilidae|658|BOLD:ACP0102
Mycetophilidae|[19219]|SSWLD4292-13|Mycetophilidae|670|BOLD:ACI7346
Mycetophilinae|[19220]|CNFDC012-14|Mycetophilidae|661|BOLD:ACL7625
Dynatosoma|[19221]|CNPAA292-13|Mycetophilidae|658|BOLD:AAN8593
Mycetophilinae|[19222]|CNKLF137-15|Mycetophilidae|588|BOLD:ACS7401
Mycetophilidae|[19223]|CNPPA2830-12|Mycetophilidae|661|BOLD:AAG4903
Sceptonia|[19224]|CNRMF1545-12|Mycetophilidae|670|BOLD:ABV3032
Sceptonia|[19225]|CNBPA154-12|Mycetophilidae|670|BOLD:ACD0824
Mycetophilidae|[19226]|CNEIA1251-12|Mycetophilidae|658|BOLD:ACB1113
Sceptonia|[19227]|CNIVB591-14|Mycetophilidae|579|BOLD:ACR4314
Sceptonia|[19228]|CNPPD457-12|Mycetophilidae|658|BOLD:AAG4967
Sceptonia nigra|[19229]|CNEIG853-12|Mycetophilidae|611|BOLD:AAZ8366
Sceptonia|[19230]|CNTNF395-14|Mycetophilidae|607|BOLD:ACM5442
Sceptonia|[19231]|CNROO357-13|Mycetophilidae|658|BOLD:ACK7227
Sceptonia|[19232]|SSBAA511-12|Mycetophilidae|661|BOLD:ACC1343
Mycetophilidae|[19233]|CHDIP332-11|Mycetophilidae|658|BOLD:AAP8860
Sceptonia nigra|[19234]|CNEIC850-12|Mycetophilidae|673|BOLD:ACF1969
Sceptonia|[19235]|CNIVF257-14|Mycetophilidae|591|BOLD:ACP3236
Mycetophilidae|[19236]|BBDED956-10|Mycetophilidae|658|BOLD:AAG4977
Mycetophila|[19237]|CNPKI707-14|Mycetophilidae|658|BOLD:ACM1182
Epicypta aterrima|[19238]|BBDEE921-10|Mycetophilidae|658|BOLD:AAJ0565
Epicypta sp. CJB1|[19239]|NCCA777-11|Mycetophilidae|673|BOLD:AAP9088
Epicypta|[19240]|SSEIB6540-13|Mycetophilidae|661|BOLD:ACI3894
Mycetophilidae|[19241]|CNPPF892-12|Mycetophilidae|664|BOLD:AAN8587
Mycetophilidae|[19242]|NCCA1231-11|Mycetophilidae|673|BOLD:AAG4953
Mycetophilinae|[19243]|SSWLD4844-13|Mycetophilidae|670|BOLD:ABA6851
Mycetophila|[19244]|CNPPA1304-12|Mycetophilidae|661|BOLD:ABV5732
Mycetophila|[19245]|SSWLD5341-13|Mycetophilidae|670|BOLD:ACI5234
Mycetophila|[19246]|SSPAB5921-13|Mycetophilidae|658|BOLD:ACI7656
Mycetophilidae|[19247]|NCCA823-11|Mycetophilidae|673|BOLD:AAU4986
Mycetophila|[19248]|HPPPC463-13|Mycetophilidae|658|BOLD:ACG6335
Mycetophila|[19249]|SSEIC2514-13|Mycetophilidae|670|BOLD:AAM8986
Mycetophilinae|[19250]|SSJAE7129-13|Mycetophilidae|658|BOLD:ACK0245
Mycetophilidae|[19251]|BBDED780-10|Mycetophilidae|658|BOLD:AAG4968
Mycetophila sp.|[19252]|JSDIP469-10|Mycetophilidae|658|BOLD:AAM8979
Mycetophila sp.|[19253]|BBDCQ938-10|Mycetophilidae|658|BOLD:AAG4922
Mycetophilinae|[19254]|CNGBO371-14|Mycetophilidae|661|BOLD:ABW5483
Mycetophilinae|[19255]|SMTPI9147-14|Mycetophilidae|582|BOLD:ACP8520
Mycetophilidae|[19256]|CNEIF1344-12|Mycetophilidae|663|BOLD:ACD0874
Mycetophilidae|[19257]|JSDIQ520-10|Mycetophilidae|658|BOLD:AAU4888
Mycetophilidae|[19258]|CNJAG1250-12|Mycetophilidae|664|BOLD:ACI4116
Epicypta|[19259]|SSEIA1178-13|Mycetophilidae|670|BOLD:ACC5656
Mycetophilidae|[19260]|SMTPD2979-13|Mycetophilidae|664|BOLD:ACA3426
Mycetophilinae|[19261]|CNJAD816-12|Mycetophilidae|658|BOLD:ACC9302
Zygomyia zaitzevi|[19262]|SSBAB2268-12|Mycetophilidae|661|BOLD:ABW9135
Mycetophilinae|[19263]|CNEIH214-13|Mycetophilidae|670|BOLD:ACC1564
Zygomyia|[19264]|PHNOV488-11|Mycetophilidae|658|BOLD:ABV1464
Zygomyia|[19265]|SSEIB4812-13|Mycetophilidae|658|BOLD:ACJ1941
Zygomyia|[19266]|SSJAD709-13|Mycetophilidae|646|BOLD:ACI3987
Mycetophilidae|[19267]|HPPPR027-13|Mycetophilidae|658|BOLD:AAM9008
Mycetophilinae|[19268]|SSROA1557-14|Mycetophilidae|564|BOLD:ACO2169
Mycetophila alea|[19269]|BBDEC529-09|Mycetophilidae|658|BOLD:AAG4931
Mycetophila|[19270]|BBDCM708-10|Mycetophilidae|658|BOLD:AAP8158
Mycetophila|[19271]|BBDCM709-10|Mycetophilidae|658|BOLD:AAG4896
Mycetophilidae|[19272]|BBDCP008-10|Mycetophilidae|658|BOLD:AAP6502
Mycetophilinae|[19273]|SSEIB1316-13|Mycetophilidae|658|BOLD:ABV3026
Mycetophilidae|[19274]|NGNAB445-13|Mycetophilidae|658|BOLD:ACI3226
Mycetophilinae|[19275]|CNJAD979-12|Mycetophilidae|670|BOLD:ACB6540
Mycetophila|[19276]|SSJAA2085-13|Mycetophilidae|658|BOLD:ACD1714
Mycetophilinae|[19277]|NGNAD944-13|Mycetophilidae|673|BOLD:ACI2791
Mycetophilinae|[19278]|CNGMC453-13|Mycetophilidae|658|BOLD:ACD1365
Mycetophilinae|[19279]|CNKJQ498-14|Mycetophilidae|618|BOLD:ACM6553
Mycetophilidae|[19280]|CHDIP1248-12|Mycetophilidae|658|BOLD:ACA4611
Mycetophilidae|[19281]|SSEID1839-13|Mycetophilidae|658|BOLD:ACA7741
Mycetophila luctuosa|[19282]|CNGBA774-13|Mycetophilidae|698|BOLD:AAG3628
Mycetophila|[19283]|CNNHE124-14|Mycetophilidae|576|BOLD:ACR7299
Mycetophila caudata|[19284]|BBDEC966-10|Mycetophilidae|658|BOLD:AAI3260
Mycetophila|[19285]|CNEIB1454-12|Mycetophilidae|673|BOLD:ACB1967
Mycetophila|[19286]|SSPAC8267-13|Mycetophilidae|615|BOLD:ACJ9233
Mycetophilidae|[19287]|SSJAF1153-13|Mycetophilidae|615|BOLD:ACK3479
Mycetophilinae|[19288]|CNFNJ142-14|Mycetophilidae|658|BOLD:ACL9098
Mycetophilidae|[19289]|CNEIA1323-12|Mycetophilidae|658|BOLD:ACB1111
Mycetophila pumila|[19290]|JSDIS261-11|Mycetophilidae|658|BOLD:ABA8261
Mycetophilidae|[19291]|CNPPA248-12|Mycetophilidae|631|BOLD:AAG4904
Mycetophilidae|[19292]|CNPAA320-13|Mycetophilidae|670|BOLD:ACD8930
Mycetophila|[19293]|SSFDC3612-14|Mycetophilidae|588|BOLD:ACO4872
Mycetophilidae|[19294]|CNRME3679-12|Mycetophilidae|658|BOLD:ACC7263
Mycetophila cf. pumila|[19295]|SSJAE2267-13|Mycetophilidae|658|BOLD:ABX1298
Mycetophila|[19296]|CNBRP724-14|Mycetophilidae|661|BOLD:ACK1664

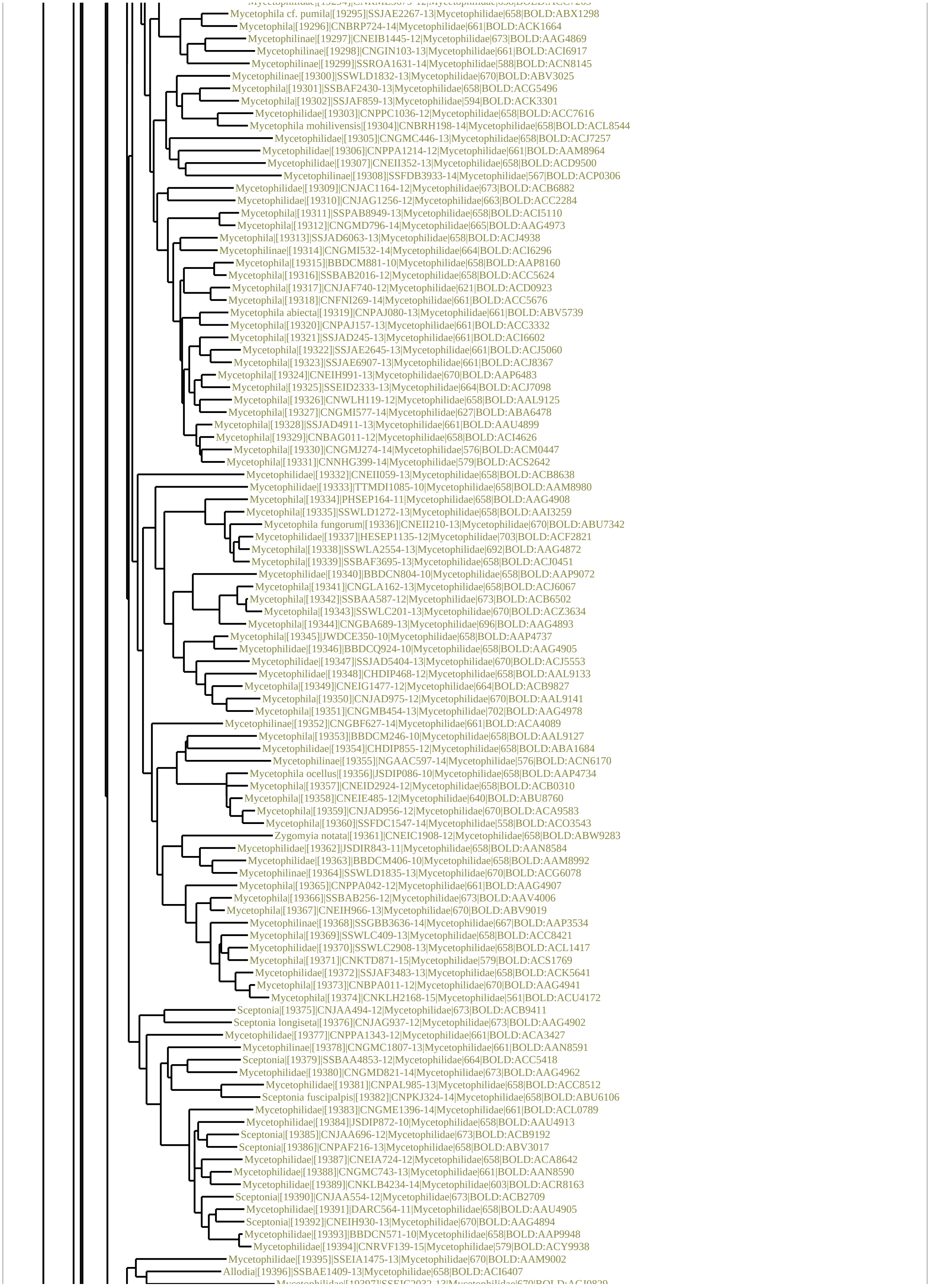
Mycetophila cf. pumila|[19295]|SSJAE2267-13|Mycetophilidae|658|BOLD:ABX1298
Mycetophila|[19296]|CNBRP724-14|Mycetophilidae|661|BOLD:ACK1664
Mycetophilinae|[19297]|CNEIB1445-12|Mycetophilidae|673|BOLD:AAG4869
Mycetophilinae|[19298]|CNGIN103-13|Mycetophilidae|661|BOLD:ACI6917
Mycetophilinae|[19299]|SSROA1631-14|Mycetophilidae|588|BOLD:ACN8145
Mycetophilinae|[19300]|SSWLD1832-13|Mycetophilidae|670|BOLD:ABV3025
Mycetophila|[19301]|SSBAF2430-13|Mycetophilidae|658|BOLD:ACG5496
Mycetophila|[19302]|SSJAF859-13|Mycetophilidae|594|BOLD:ACK3301
Mycetophilidae|[19303]|CNPPC1036-12|Mycetophilidae|658|BOLD:ACC7616
Mycetophila mohilivensis|[19304]|CNBRH198-14|Mycetophilidae|658|BOLD:ACL8544
Mycetophilidae|[19305]|CNGMC446-13|Mycetophilidae|658|BOLD:ACJ7257
Mycetophilidae|[19306]|CNPPA1214-12|Mycetophilidae|661|BOLD:AAM8964
Mycetophilidae|[19307]|CNEII352-13|Mycetophilidae|658|BOLD:ACD9500
Mycetophilinae|[19308]|SSFDB3933-14|Mycetophilidae|567|BOLD:ACP0306
Mycetophilidae|[19309]|CNJAC1164-12|Mycetophilidae|673|BOLD:ACB6882
Mycetophilidae|[19310]|CNJAG1256-12|Mycetophilidae|663|BOLD:ACC2284
Mycetophila|[19311]|SSPAB8949-13|Mycetophilidae|658|BOLD:ACI5110
Mycetophila|[19312]|CNGMD796-14|Mycetophilidae|665|BOLD:AAG4973
Mycetophila|[19313]|SSJAD6063-13|Mycetophilidae|658|BOLD:ACJ4938
Mycetophilinae|[19314]|CNGMI532-14|Mycetophilidae|664|BOLD:ACI6296
Mycetophila|[19315]|BBDCM881-10|Mycetophilidae|658|BOLD:AAP8160
Mycetophila|[19316]|SSBAB2016-12|Mycetophilidae|658|BOLD:ACC5624
Mycetophila|[19317]|CNJAF740-12|Mycetophilidae|621|BOLD:ACD0923
Mycetophila|[19318]|CNFNI269-14|Mycetophilidae|661|BOLD:ACC5676
Mycetophila abiecta|[19319]|CNPAJ080-13|Mycetophilidae|661|BOLD:ABV5739
Mycetophila|[19320]|CNPAJ157-13|Mycetophilidae|661|BOLD:ACC3332
Mycetophila|[19321]|SSJAD245-13|Mycetophilidae|661|BOLD:ACI6602
Mycetophila|[19322]|SSJAE2645-13|Mycetophilidae|661|BOLD:ACJ5060
Mycetophila|[19323]|SSJAE6907-13|Mycetophilidae|661|BOLD:ACJ8367
Mycetophila|[19324]|CNEIH991-13|Mycetophilidae|670|BOLD:AAP6483
Mycetophila|[19325]|SSEID2333-13|Mycetophilidae|664|BOLD:ACJ7098
Mycetophila|[19326]|CNWLH119-12|Mycetophilidae|658|BOLD:AAL9125
Mycetophila|[19327]|CNGMI577-14|Mycetophilidae|627|BOLD:ABA6478
Mycetophila|[19328]|SSJAD4911-13|Mycetophilidae|661|BOLD:AAU4899
Mycetophila|[19329]|CNBAG011-12|Mycetophilidae|658|BOLD:ACI4626
Mycetophila|[19330]|CNGMJ274-14|Mycetophilidae|576|BOLD:ACM0447
Mycetophila|[19331]|CNNHG399-14|Mycetophilidae|579|BOLD:ACS2642
Mycetophilidae|[19332]|CNEII059-13|Mycetophilidae|658|BOLD:ACB8638
Mycetophilidae|[19333]|TTMDI1085-10|Mycetophilidae|658|BOLD:AAM8980
Mycetophila|[19334]|PHSEP164-11|Mycetophilidae|658|BOLD:AAG4908
Mycetophila|[19335]|SSWLD1272-13|Mycetophilidae|658|BOLD:AAI3259
Mycetophila fungorum|[19336]|CNEII210-13|Mycetophilidae|670|BOLD:ABU7342
Mycetophilidae|[19337]|HESEP1135-12|Mycetophilidae|703|BOLD:ACF2821
Mycetophila|[19338]|SSWLA2554-13|Mycetophilidae|692|BOLD:AAG4872
Mycetophila|[19339]|SSBAF3695-13|Mycetophilidae|658|BOLD:ACJ0451
Mycetophilidae|[19340]|BBDCN804-10|Mycetophilidae|658|BOLD:AAP9072
Mycetophila|[19341]|CNGLA162-13|Mycetophilidae|658|BOLD:ACJ6067
Mycetophila|[19342]|SSBAA587-12|Mycetophilidae|673|BOLD:ACB6502
Mycetophila|[19343]|SSWLC201-13|Mycetophilidae|670|BOLD:ACZ3634
Mycetophila|[19344]|CNGBA689-13|Mycetophilidae|696|BOLD:AAG4893
Mycetophila|[19345]|JWDCE350-10|Mycetophilidae|658|BOLD:AAP4737
Mycetophilidae|[19346]|BBDCQ924-10|Mycetophilidae|658|BOLD:AAG4905
Mycetophilidae|[19347]|SSJAD5404-13|Mycetophilidae|670|BOLD:ACJ5553
Mycetophilidae|[19348]|CHDIP468-12|Mycetophilidae|658|BOLD:AAL9133
Mycetophila|[19349]|CNEIG1477-12|Mycetophilidae|664|BOLD:ACB9827
Mycetophila|[19350]|CNJAD975-12|Mycetophilidae|670|BOLD:AAL9141
Mycetophila|[19351]|CNGMB454-13|Mycetophilidae|702|BOLD:AAG4978
Mycetophilinae|[19352]|CNGBF627-14|Mycetophilidae|661|BOLD:ACA4089
Mycetophila|[19353]|BBDCM246-10|Mycetophilidae|658|BOLD:AAL9127
Mycetophilidae|[19354]|CHDIP855-12|Mycetophilidae|658|BOLD:ABA1684
Mycetophilinae|[19355]|NGAAC597-14|Mycetophilidae|576|BOLD:ACN6170
Mycetophila ocellus|[19356]|JSDIP086-10|Mycetophilidae|658|BOLD:AAP4734
Mycetophila|[19357]|CNEID2924-12|Mycetophilidae|658|BOLD:ACB0310
Mycetophila|[19358]|CNEIE485-12|Mycetophilidae|640|BOLD:ABU8760
Mycetophila|[19359]|CNJAD956-12|Mycetophilidae|670|BOLD:ACA9583
Mycetophila|[19360]|SSFDC1547-14|Mycetophilidae|558|BOLD:ACO3543
Zygomyia notata|[19361]|CNEIC1908-12|Mycetophilidae|658|BOLD:ABW9283
Mycetophilidae|[19362]|JSDIR843-11|Mycetophilidae|658|BOLD:AAN8584
Mycetophilidae|[19363]|BBDCM406-10|Mycetophilidae|658|BOLD:AAM8992
Mycetophilinae|[19364]|SSWLD1835-13|Mycetophilidae|670|BOLD:ACG6078
Mycetophila|[19365]|CNPPA042-12|Mycetophilidae|661|BOLD:AAG4907
Mycetophila|[19366]|SSBAB256-12|Mycetophilidae|673|BOLD:AAV4006
Mycetophila|[19367]|CNEIH966-13|Mycetophilidae|670|BOLD:ABV9019
Mycetophilinae|[19368]|SSGBB3636-14|Mycetophilidae|667|BOLD:AAP3534
Mycetophila|[19369]|SSWLC409-13|Mycetophilidae|658|BOLD:ACC8421
Mycetophilidae|[19370]|SSWLC2908-13|Mycetophilidae|658|BOLD:ACL1417
Mycetophila|[19371]|CNKTD871-15|Mycetophilidae|579|BOLD:ACS1769
Mycetophilidae|[19372]|SSJAF3483-13|Mycetophilidae|658|BOLD:ACK5641
Mycetophila|[19373]|CNBPA011-12|Mycetophilidae|670|BOLD:AAG4941
Mycetophila|[19374]|CNKLH2168-15|Mycetophilidae|561|BOLD:ACU4172
Sceptonia|[19375]|CNJAA494-12|Mycetophilidae|673|BOLD:ACB9411
Sceptonia longiseta|[19376]|CNJAG937-12|Mycetophilidae|673|BOLD:AAG4902
Mycetophilidae|[19377]|CNPPA1343-12|Mycetophilidae|661|BOLD:ACA3427
Mycetophilinae|[19378]|CNGMC1807-13|Mycetophilidae|661|BOLD:AAN8591
Sceptonia|[19379]|SSBAA4853-12|Mycetophilidae|664|BOLD:ACC5418
Mycetophilidae|[19380]|CNGMD821-14|Mycetophilidae|673|BOLD:AAG4962
Mycetophilidae|[19381]|CNPAL985-13|Mycetophilidae|658|BOLD:ACC8512
Sceptonia fuscipalpis|[19382]|CNPKJ324-14|Mycetophilidae|658|BOLD:ABU6106
Mycetophilidae|[19383]|CNGME1396-14|Mycetophilidae|661|BOLD:ACL0789
Mycetophilidae|[19384]|JSDIP872-10|Mycetophilidae|658|BOLD:AAU4913
Sceptonia|[19385]|CNJAA696-12|Mycetophilidae|673|BOLD:ACB9192
Sceptonia|[19386]|CNPAF216-13|Mycetophilidae|658|BOLD:ABV3017
Mycetophilidae|[19387]|CNEIA724-12|Mycetophilidae|658|BOLD:ACA8642
Mycetophilidae|[19388]|CNGMC743-13|Mycetophilidae|661|BOLD:AAN8590
Mycetophilidae|[19389]|CNKLB4234-14|Mycetophilidae|603|BOLD:ACR8163
Sceptonia|[19390]|CNJAA554-12|Mycetophilidae|673|BOLD:ACB2709
Mycetophilidae|[19391]|DARC564-11|Mycetophilidae|658|BOLD:AAU4905
Sceptonia|[19392]|CNEIH930-13|Mycetophilidae|670|BOLD:AAG4894
Mycetophilidae|[19393]|BBDCN571-10|Mycetophilidae|658|BOLD:AAP9948
Mycetophilidae|[19394]|CNRVF139-15|Mycetophilidae|579|BOLD:ACY9938
Mycetophilidae|[19395]|SSEIA1475-13|Mycetophilidae|670|BOLD:AAM9002
Allodia|[19396]|SSBAE1409-13|Mycetophilidae|658|BOLD:ACI6407

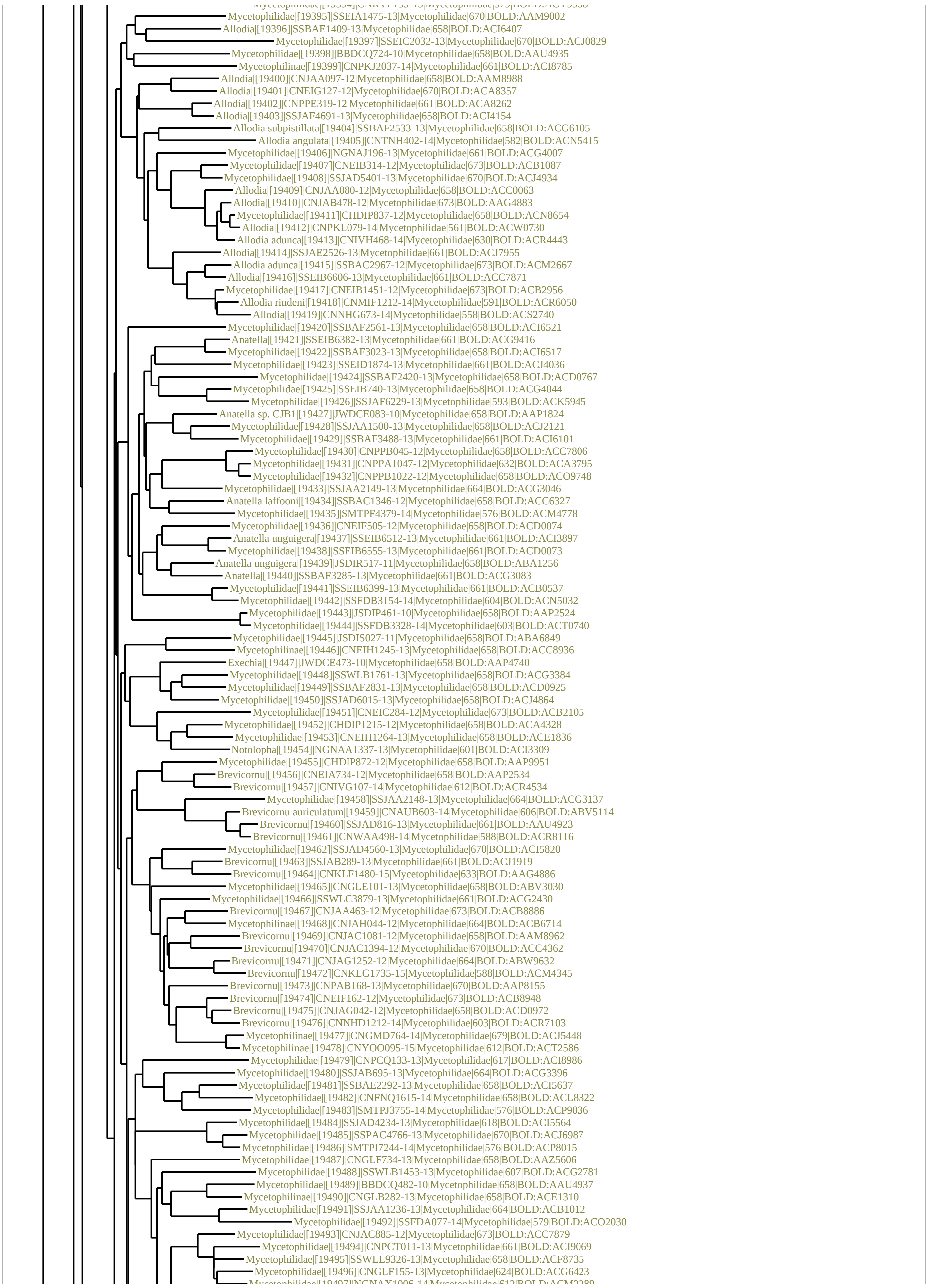

Mycetophilidae|[19395]|SSEIA1475-13|Mycetophilidae|670|BOLD:AAM9002
Allodia|[19396]|SSBAE1409-13|Mycetophilidae|658|BOLD:ACI6407
Mycetophilidae|[19397]|SSEIC2032-13|Mycetophilidae|670|BOLD:ACJ0829
Mycetophilidae|[19398]|BBDCQ724-10|Mycetophilidae|658|BOLD:AAU4935
Mycetophilinae|[19399]|CNPKJ2037-14|Mycetophilidae|661|BOLD:ACI8785
Allodia|[19400]|CNJAA097-12|Mycetophilidae|658|BOLD:AAM8988
Allodia|[19401]|CNEIG127-12|Mycetophilidae|670|BOLD:ACA8357
Allodia|[19402]|CNPPE319-12|Mycetophilidae|661|BOLD:ACA8262
Allodia|[19403]|SSJAF4691-13|Mycetophilidae|658|BOLD:ACI4154
Allodia subpistillata|[19404]|SSBAF2533-13|Mycetophilidae|658|BOLD:ACG6105
Allodia angulata|[19405]|CNTNH402-14|Mycetophilidae|582|BOLD:ACN5415
Mycetophilidae|[19406]|NGNAJ196-13|Mycetophilidae|661|BOLD:ACG4007
Mycetophilidae|[19407]|CNEIB314-12|Mycetophilidae|673|BOLD:ACB1087
Mycetophilidae|[19408]|SSJAD5401-13|Mycetophilidae|670|BOLD:ACJ4934
Allodia|[19409]|CNJAA080-12|Mycetophilidae|658|BOLD:ACC0063
Allodia|[19410]|CNJAB478-12|Mycetophilidae|673|BOLD:AAG4883
Mycetophilidae|[19411]|CHDIP837-12|Mycetophilidae|658|BOLD:ACN8654
Allodia|[19412]|CNPKL079-14|Mycetophilidae|561|BOLD:ACW0730
Allodia adunca|[19413]|CNIVH468-14|Mycetophilidae|630|BOLD:ACR4443
Allodia|[19414]|SSJAE2526-13|Mycetophilidae|661|BOLD:ACJ7955
Allodia adunca|[19415]|SSBAC2967-12|Mycetophilidae|673|BOLD:ACM2667
Allodia|[19416]|SSEIB6606-13|Mycetophilidae|661|BOLD:ACC7871
Mycetophilidae|[19417]|CNEIB1451-12|Mycetophilidae|673|BOLD:ACB2956
Allodia rindeni|[19418]|CNMIF1212-14|Mycetophilidae|591|BOLD:ACR6050
Allodia|[19419]|CNNHG673-14|Mycetophilidae|558|BOLD:ACS2740
Mycetophilidae|[19420]|SSBAF2561-13|Mycetophilidae|658|BOLD:ACI6521
Anatella|[19421]|SSEIB6382-13|Mycetophilidae|661|BOLD:ACG9416
Mycetophilidae|[19422]|SSBAF3023-13|Mycetophilidae|658|BOLD:ACI6517
Mycetophilidae|[19423]|SSEID1874-13|Mycetophilidae|661|BOLD:ACJ4036
Mycetophilidae|[19424]|SSBAF2420-13|Mycetophilidae|658|BOLD:ACD0767
Mycetophilidae|[19425]|SSEIB740-13|Mycetophilidae|658|BOLD:ACG4044
Mycetophilidae|[19426]|SSJAF6229-13|Mycetophilidae|593|BOLD:ACK5945
Anatella sp. CJB1|[19427]|JWDCE083-10|Mycetophilidae|658|BOLD:AAP1824
Mycetophilidae|[19428]|SSJAA1500-13|Mycetophilidae|658|BOLD:ACJ2121
Mycetophilidae|[19429]|SSBAF3488-13|Mycetophilidae|661|BOLD:ACI6101
Mycetophilidae|[19430]|CNPPB045-12|Mycetophilidae|658|BOLD:ACC7806
Mycetophilidae|[19431]|CNPPA1047-12|Mycetophilidae|632|BOLD:ACA3795
Mycetophilidae|[19432]|CNPPB1022-12|Mycetophilidae|658|BOLD:ACO9748
Mycetophilidae|[19433]|SSJAA2149-13|Mycetophilidae|664|BOLD:ACG3046
Anatella laffooni|[19434]|SSBAC1346-12|Mycetophilidae|658|BOLD:ACC6327
Mycetophilidae|[19435]|SMTPF4379-14|Mycetophilidae|576|BOLD:ACM4778
Mycetophilidae|[19436]|CNEIF505-12|Mycetophilidae|658|BOLD:ACD0074
Anatella unguigera|[19437]|SSEIB6512-13|Mycetophilidae|661|BOLD:ACI3897
Mycetophilidae|[19438]|SSEIB6555-13|Mycetophilidae|661|BOLD:ACD0073
Anatella unguigera|[19439]|JSDIR517-11|Mycetophilidae|658|BOLD:ABA1256
Anatella|[19440]|SSBAF3285-13|Mycetophilidae|661|BOLD:ACG3083
Mycetophilidae|[19441]|SSEIB6399-13|Mycetophilidae|661|BOLD:ACB0537
Mycetophilidae|[19442]|SSFDB3154-14|Mycetophilidae|604|BOLD:ACN5032
Mycetophilidae|[19443]|JSDIP461-10|Mycetophilidae|658|BOLD:AAP2524
Mycetophilidae|[19444]|SSFDB3328-14|Mycetophilidae|603|BOLD:ACT0740
Mycetophilidae|[19445]|JSDIS027-11|Mycetophilidae|658|BOLD:ABA6849
Mycetophilinae|[19446]|CNEIH1245-13|Mycetophilidae|658|BOLD:ACC8936
Exechia|[19447]|JWDCE473-10|Mycetophilidae|658|BOLD:AAP4740
Mycetophilidae|[19448]|SSWLB1761-13|Mycetophilidae|658|BOLD:ACG3384
Mycetophilidae|[19449]|SSBAF2831-13|Mycetophilidae|658|BOLD:ACD0925
Mycetophilidae|[19450]|SSJAD6015-13|Mycetophilidae|658|BOLD:ACJ4864
Mycetophilidae|[19451]|CNEIC284-12|Mycetophilidae|673|BOLD:ACB2105
Mycetophilidae|[19452]|CHDIP1215-12|Mycetophilidae|658|BOLD:ACA4328
Mycetophilidae|[19453]|CNEIH1264-13|Mycetophilidae|658|BOLD:ACE1836
Notolopha|[19454]|NGNAA1337-13|Mycetophilidae|601|BOLD:ACI3309
Mycetophilidae|[19455]|CHDIP872-12|Mycetophilidae|658|BOLD:AAP9951
Brevicornu|[19456]|CNEIA734-12|Mycetophilidae|658|BOLD:AAP2534
Brevicornu|[19457]|CNIVG107-14|Mycetophilidae|612|BOLD:ACR4534
Mycetophilidae|[19458]|SSJAA2148-13|Mycetophilidae|664|BOLD:ACG3137
Brevicornu auriculatum|[19459]|CNAUB603-14|Mycetophilidae|606|BOLD:ABV5114
Brevicornu|[19460]|SSJAD816-13|Mycetophilidae|661|BOLD:AAU4923
Brevicornu|[19461]|CNWAA498-14|Mycetophilidae|588|BOLD:ACR8116
Mycetophilidae|[19462]|SSJAD4560-13|Mycetophilidae|670|BOLD:ACI5820
Brevicornu|[19463]|SSJAB289-13|Mycetophilidae|661|BOLD:ACJ1919
Brevicornu|[19464]|CNKLF1480-15|Mycetophilidae|633|BOLD:AAG4886
Mycetophilidae|[19465]|CNGLE101-13|Mycetophilidae|658|BOLD:ABV3030
Mycetophilidae|[19466]|SSWLC3879-13|Mycetophilidae|661|BOLD:ACG2430
Brevicornu|[19467]|CNJAA463-12|Mycetophilidae|673|BOLD:ACB8886
Mycetophilinae|[19468]|CNJAH044-12|Mycetophilidae|664|BOLD:ACB6714
Brevicornu|[19469]|CNJAC1081-12|Mycetophilidae|658|BOLD:AAM8962
Brevicornu|[19470]|CNJAC1394-12|Mycetophilidae|670|BOLD:ACC4362
Brevicornu|[19471]|CNJAG1252-12|Mycetophilidae|664|BOLD:ABW9632
Brevicornu|[19472]|CNKLG1735-15|Mycetophilidae|588|BOLD:ACM4345
Brevicornu|[19473]|CNPAB168-13|Mycetophilidae|670|BOLD:AAP8155
Brevicornu|[19474]|CNEIF162-12|Mycetophilidae|673|BOLD:ACB8948
Brevicornu|[19475]|CNJAG042-12|Mycetophilidae|658|BOLD:ACD0972
Brevicornu|[19476]|CNNHD1212-14|Mycetophilidae|603|BOLD:ACR7103
Mycetophilinae|[19477]|CNGMD764-14|Mycetophilidae|679|BOLD:ACJ5448
Mycetophilinae|[19478]|CNYOO095-15|Mycetophilidae|612|BOLD:ACT2586
Mycetophilidae|[19479]|CNPCQ133-13|Mycetophilidae|617|BOLD:ACI8986
Mycetophilidae|[19480]|SSJAB695-13|Mycetophilidae|664|BOLD:ACG3396
Mycetophilidae|[19481]|SSBAE2292-13|Mycetophilidae|658|BOLD:ACI5637
Mycetophilidae|[19482]|CNFNQ1615-14|Mycetophilidae|658|BOLD:ACL8322
Mycetophilidae|[19483]|SMTPJ3755-14|Mycetophilidae|576|BOLD:ACP9036
Mycetophilidae|[19484]|SSJAD4234-13|Mycetophilidae|618|BOLD:ACI5564
Mycetophilidae|[19485]|SSPAC4766-13|Mycetophilidae|670|BOLD:ACJ6987
Mycetophilidae|[19486]|SMTPI7244-14|Mycetophilidae|576|BOLD:ACP8015
Mycetophilidae|[19487]|CNGLF734-13|Mycetophilidae|658|BOLD:AAZ5606
Mycetophilidae|[19488]|SSWLB1453-13|Mycetophilidae|607|BOLD:ACG2781
Mycetophilidae|[19489]|BBDCQ482-10|Mycetophilidae|658|BOLD:AAU4937
Mycetophilinae|[19490]|CNGLB282-13|Mycetophilidae|658|BOLD:ACE1310
Mycetophilidae|[19491]|SSJAA1236-13|Mycetophilidae|664|BOLD:ACB1012
Mycetophilidae|[19492]|SSFDA077-14|Mycetophilidae|579|BOLD:ACO2030
Mycetophilidae|[19493]|CNJAC885-12|Mycetophilidae|673|BOLD:ACC7879
Mycetophilidae|[19494]|CNPCT011-13|Mycetophilidae|661|BOLD:ACI9069
Mycetophilidae|[19495]|SSWLE9326-13|Mycetophilidae|658|BOLD:ACF8735
Mycetophilidae|[19496]|CNGLF155-13|Mycetophilidae|624|BOLD:ACG6423

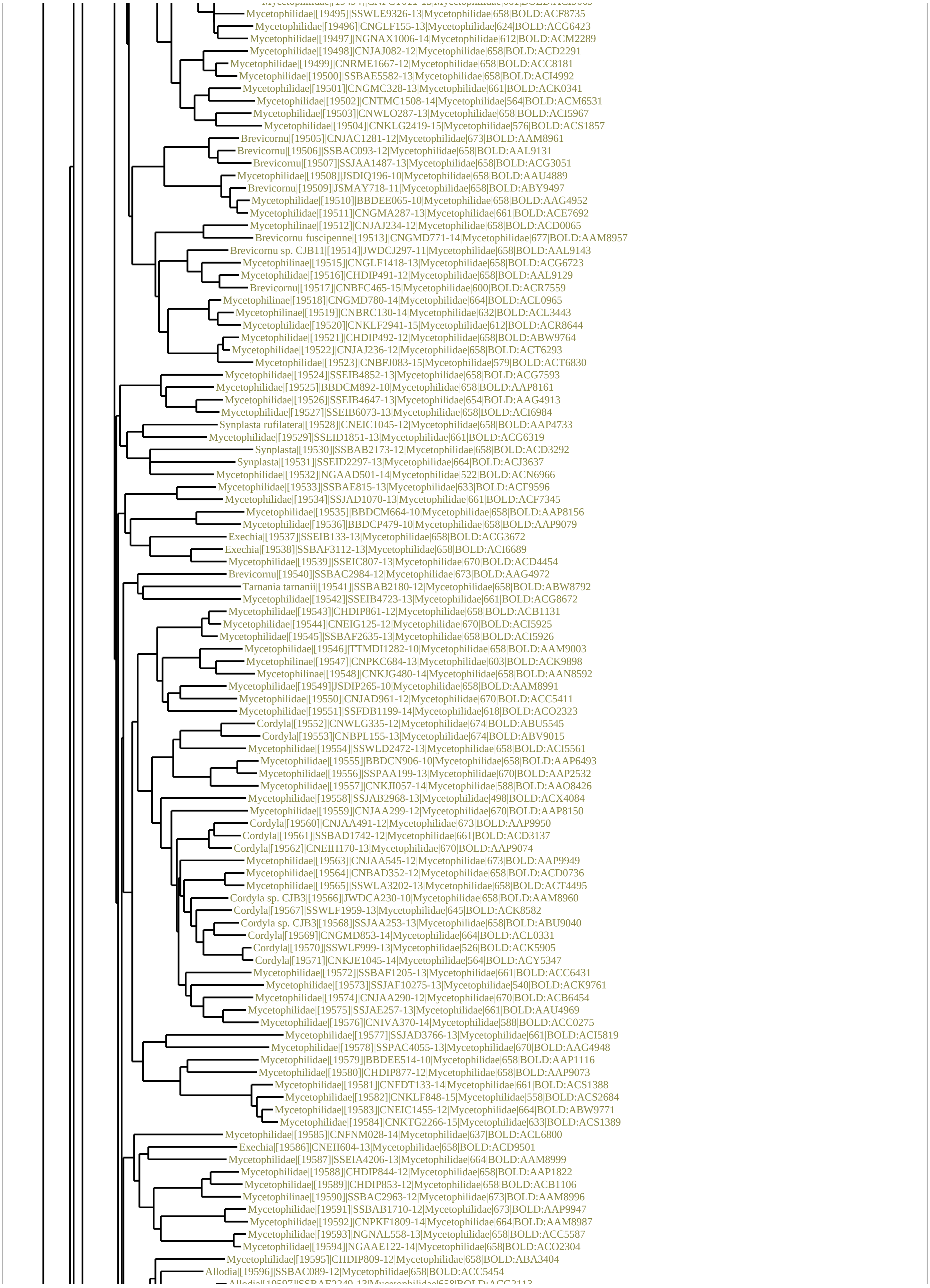
Mycetophilidae|[19495]|SSWLE9326-13|Mycetophilidae|658|BOLD:ACF8735
Mycetophilidae|[19496]|CNGLF155-13|Mycetophilidae|624|BOLD:ACG6423
Mycetophilidae|[19497]|NGNAX1006-14|Mycetophilidae|612|BOLD:ACM2289
Mycetophilidae|[19498]|CNJAJ082-12|Mycetophilidae|658|BOLD:ACD2291
Mycetophilidae|[19499]|CNRME1667-12|Mycetophilidae|658|BOLD:ACC8181
Mycetophilidae|[19500]|SSBAE5582-13|Mycetophilidae|658|BOLD:ACI4992
Mycetophilidae|[19501]|CNGMC328-13|Mycetophilidae|661|BOLD:ACK0341
Mycetophilidae|[19502]|CNTMC1508-14|Mycetophilidae|564|BOLD:ACM6531
Mycetophilidae|[19503]|CNWLO287-13|Mycetophilidae|658|BOLD:ACI5967
Mycetophilidae|[19504]|CNKLG2419-15|Mycetophilidae|576|BOLD:ACS1857
Brevicornu|[19505]|CNJAC1281-12|Mycetophilidae|673|BOLD:AAM8961
Brevicornu|[19506]|SSBAC093-12|Mycetophilidae|658|BOLD:AAL9131
Brevicornu|[19507]|SSJAA1487-13|Mycetophilidae|658|BOLD:ACG3051
Mycetophilidae|[19508]|JSDIQ196-10|Mycetophilidae|658|BOLD:AAU4889
Brevicornu|[19509]|JSMAY718-11|Mycetophilidae|658|BOLD:ABY9497
Mycetophilidae|[19510]|BBDEE065-10|Mycetophilidae|658|BOLD:AAG4952
Mycetophilidae|[19511]|CNGMA287-13|Mycetophilidae|661|BOLD:ACE7692
Mycetophilinae|[19512]|CNJAJ234-12|Mycetophilidae|658|BOLD:ACD0065
Brevicornu fuscipenne|[19513]|CNGMD771-14|Mycetophilidae|677|BOLD:AAM8957
Brevicornu sp. CJB11|[19514]|JWDCJ297-11|Mycetophilidae|658|BOLD:AAL9143
Mycetophilinae|[19515]|CNGLF1418-13|Mycetophilidae|658|BOLD:ACG6723
Mycetophilidae|[19516]|CHDIP491-12|Mycetophilidae|658|BOLD:AAL9129
Brevicornu|[19517]|CNBFC465-15|Mycetophilidae|600|BOLD:ACR7559
Mycetophilinae|[19518]|CNGMD780-14|Mycetophilidae|664|BOLD:ACL0965
Mycetophilinae|[19519]|CNBRC130-14|Mycetophilidae|632|BOLD:ACL3443
Mycetophilidae|[19520]|CNKLF2941-15|Mycetophilidae|612|BOLD:ACR8644
Mycetophilidae|[19521]|CHDIP492-12|Mycetophilidae|658|BOLD:ABW9764
Mycetophilidae|[19522]|CNJAJ236-12|Mycetophilidae|658|BOLD:ACT6293
Mycetophilidae|[19523]|CNBFJ083-15|Mycetophilidae|579|BOLD:ACT6830
Mycetophilidae|[19524]|SSEIB4852-13|Mycetophilidae|658|BOLD:ACG7593
Mycetophilidae|[19525]|BBDCM892-10|Mycetophilidae|658|BOLD:AAP8161
Mycetophilidae|[19526]|SSEIB4647-13|Mycetophilidae|654|BOLD:AAG4913
Mycetophilidae|[19527]|SSEIB6073-13|Mycetophilidae|658|BOLD:ACI6984
Synplasta rufilatera|[19528]|CNEIC1045-12|Mycetophilidae|658|BOLD:AAP4733
Mycetophilidae|[19529]|SSEID1851-13|Mycetophilidae|661|BOLD:ACG6319
Synplasta|[19530]|SSBAB2173-12|Mycetophilidae|658|BOLD:ACD3292
Synplasta|[19531]|SSEID2297-13|Mycetophilidae|664|BOLD:ACJ3637
Mycetophilidae|[19532]|NGAAD501-14|Mycetophilidae|522|BOLD:ACN6966
Mycetophilidae|[19533]|SSBAE815-13|Mycetophilidae|633|BOLD:ACF9596
Mycetophilidae|[19534]|SSJAD1070-13|Mycetophilidae|661|BOLD:ACF7345
Mycetophilidae|[19535]|BBDCM664-10|Mycetophilidae|658|BOLD:AAP8156
Mycetophilidae|[19536]|BBDCP479-10|Mycetophilidae|658|BOLD:AAP9079
Exechia|[19537]|SSEIB133-13|Mycetophilidae|658|BOLD:ACG3672
Exechia|[19538]|SSBAF3112-13|Mycetophilidae|658|BOLD:ACI6689
Mycetophilidae|[19539]|SSEIC807-13|Mycetophilidae|670|BOLD:ACD4454
Brevicornu|[19540]|SSBAC2984-12|Mycetophilidae|673|BOLD:AAG4972
Tarnania tarnanii|[19541]|SSBAB2180-12|Mycetophilidae|658|BOLD:ABW8792
Mycetophilidae|[19542]|SSEIB4723-13|Mycetophilidae|661|BOLD:ACG8672
Mycetophilidae|[19543]|CHDIP861-12|Mycetophilidae|658|BOLD:ACB1131
Mycetophilidae|[19544]|CNEIG125-12|Mycetophilidae|670|BOLD:ACI5925
Mycetophilidae|[19545]|SSBAF2635-13|Mycetophilidae|658|BOLD:ACI5926
Mycetophilidae|[19546]|TTMDI1282-10|Mycetophilidae|658|BOLD:AAM9003
Mycetophilinae|[19547]|CNPKC684-13|Mycetophilidae|603|BOLD:ACK9898
Mycetophilinae|[19548]|CNKJG480-14|Mycetophilidae|658|BOLD:AAN8592
Mycetophilidae|[19549]|JSDIP265-10|Mycetophilidae|658|BOLD:AAM8991
Mycetophilidae|[19550]|CNJAD961-12|Mycetophilidae|670|BOLD:ACC5411
Mycetophilidae|[19551]|SSFDB1199-14|Mycetophilidae|618|BOLD:ACO2323
Cordyla|[19552]|CNWLG335-12|Mycetophilidae|674|BOLD:ABU5545
Cordyla|[19553]|CNBPL155-13|Mycetophilidae|674|BOLD:ABV9015
Mycetophilidae|[19554]|SSWLD2472-13|Mycetophilidae|658|BOLD:ACI5561
Mycetophilidae|[19555]|BBDCN906-10|Mycetophilidae|658|BOLD:AAP6493
Mycetophilidae|[19556]|SSPAA199-13|Mycetophilidae|670|BOLD:AAP2532
Mycetophilidae|[19557]|CNKJI057-14|Mycetophilidae|588|BOLD:AAO8426
Mycetophilidae|[19558]|SSJAB2968-13|Mycetophilidae|498|BOLD:ACX4084
Mycetophilidae|[19559]|CNJAA299-12|Mycetophilidae|670|BOLD:AAP8150
Cordyla|[19560]|CNJAA491-12|Mycetophilidae|673|BOLD:AAP9950
Cordyla|[19561]|SSBAD1742-12|Mycetophilidae|661|BOLD:ACD3137
Cordyla|[19562]|CNEIH170-13|Mycetophilidae|670|BOLD:AAP9074
Mycetophilidae|[19563]|CNJAA545-12|Mycetophilidae|673|BOLD:AAP9949
Mycetophilidae|[19564]|CNBAD352-12|Mycetophilidae|658|BOLD:ACD0736
Mycetophilidae|[19565]|SSWLA3202-13|Mycetophilidae|658|BOLD:ACT4495
Cordyla sp. CJB3|[19566]|JWDCA230-10|Mycetophilidae|658|BOLD:AAM8960
Cordyla|[19567]|SSWLF1959-13|Mycetophilidae|645|BOLD:ACK8582
Cordyla sp. CJB3|[19568]|SSJAA253-13|Mycetophilidae|658|BOLD:ABU9040
Cordyla|[19569]|CNGMD853-14|Mycetophilidae|664|BOLD:ACL0331
Cordyla|[19570]|SSWLF999-13|Mycetophilidae|526|BOLD:ACK5905
Cordyla|[19571]|CNKJE1045-14|Mycetophilidae|564|BOLD:ACY5347
Mycetophilidae|[19572]|SSBAF1205-13|Mycetophilidae|661|BOLD:ACC6431
Mycetophilidae|[19573]|SSJAF10275-13|Mycetophilidae|540|BOLD:ACK9761
Mycetophilidae|[19574]|CNJAA290-12|Mycetophilidae|670|BOLD:ACB6454
Mycetophilidae|[19575]|SSJAE257-13|Mycetophilidae|661|BOLD:AAU4969
Mycetophilidae|[19576]|CNIVA370-14|Mycetophilidae|588|BOLD:ACC0275
Mycetophilidae|[19577]|SSJAD3766-13|Mycetophilidae|661|BOLD:ACI5819
Mycetophilidae|[19578]|SSPAC4055-13|Mycetophilidae|670|BOLD:AAG4948
Mycetophilidae|[19579]|BBDEE514-10|Mycetophilidae|658|BOLD:AAP1116
Mycetophilidae|[19580]|CHDIP877-12|Mycetophilidae|658|BOLD:AAP9073
Mycetophilidae|[19581]|CNFDT133-14|Mycetophilidae|661|BOLD:ACS1388
Mycetophilidae|[19582]|CNKLF848-15|Mycetophilidae|558|BOLD:ACS2684
Mycetophilidae|[19583]|CNEIC1455-12|Mycetophilidae|664|BOLD:ABW9771
Mycetophilidae|[19584]|CNKTG2266-15|Mycetophilidae|633|BOLD:ACS1389
Mycetophilidae|[19585]|CNFNM028-14|Mycetophilidae|637|BOLD:ACL6800
Exechia|[19586]|CNEII604-13|Mycetophilidae|658|BOLD:ACD9501
Mycetophilidae|[19587]|SSEIA4206-13|Mycetophilidae|664|BOLD:AAM8999
Mycetophilidae|[19588]|CHDIP844-12|Mycetophilidae|658|BOLD:AAP1822
Mycetophilidae|[19589]|CHDIP853-12|Mycetophilidae|658|BOLD:ACB1106
Mycetophilinae|[19590]|SSBAC2963-12|Mycetophilidae|673|BOLD:AAM8996
Mycetophilidae|[19591]|SSBAB1710-12|Mycetophilidae|673|BOLD:AAP9947
Mycetophilidae|[19592]|CNPKF1809-14|Mycetophilidae|664|BOLD:AAM8987
Mycetophilidae|[19593]|NGNAL558-13|Mycetophilidae|658|BOLD:ACC5587
Mycetophilidae|[19594]|NGAAE122-14|Mycetophilidae|658|BOLD:ACO2304
Mycetophilidae|[19595]|CHDIP809-12|Mycetophilidae|658|BOLD:ABA3404
Allodia|[19596]|SSBAC089-12|Mycetophilidae|658|BOLD:ACC5454

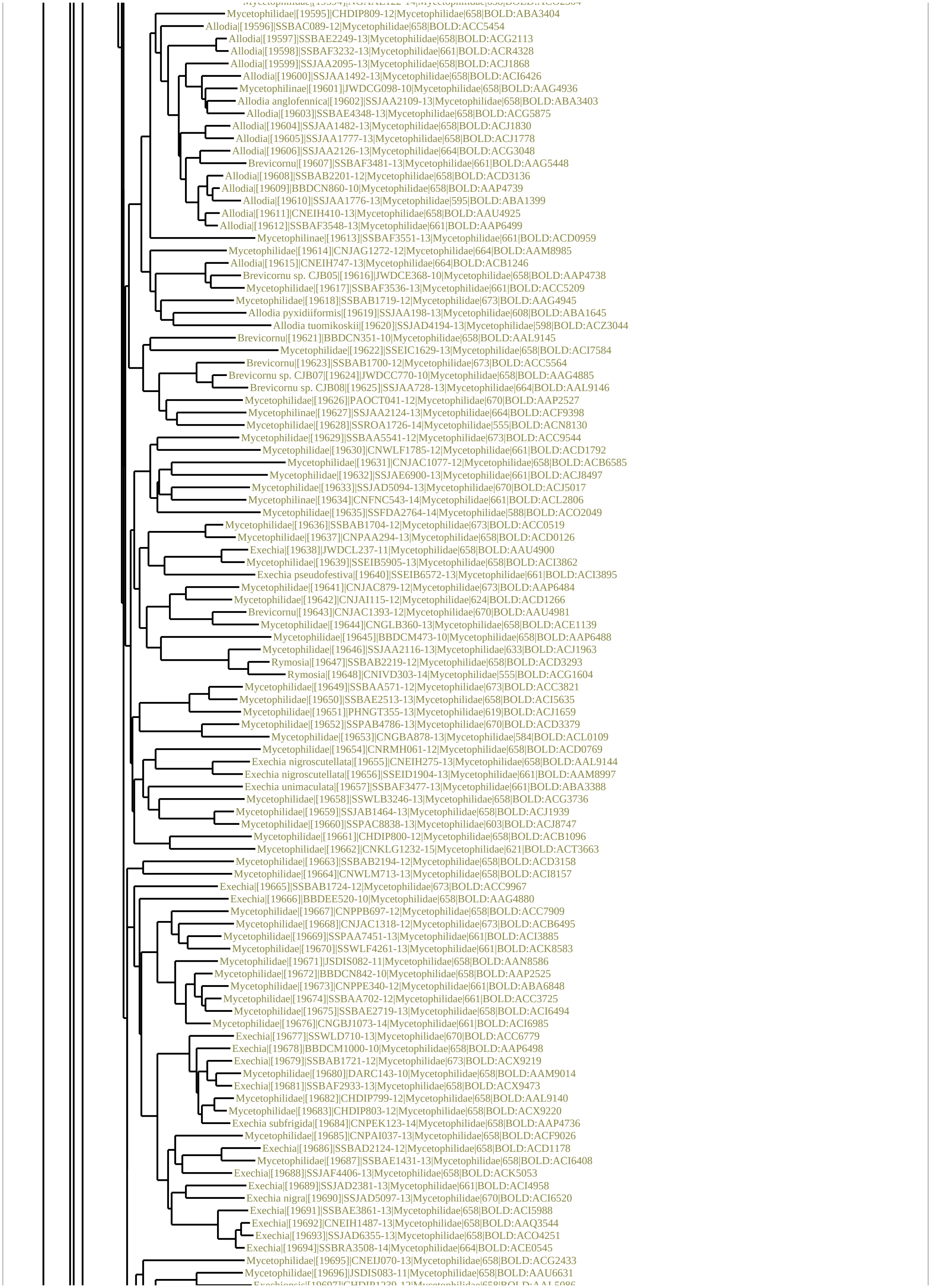

Mycetophilidae|[19595]|CHDIP809-12|Mycetophilidae|658|BOLD:ABA3404
Allodia|[19596]|SSBAC089-12|Mycetophilidae|658|BOLD:ACC5454
Allodia|[19597]|SSBAE2249-13|Mycetophilidae|658|BOLD:ACG2113
Allodia|[19598]|SSBAF3232-13|Mycetophilidae|661|BOLD:ACR4328
Allodia|[19599]|SSJAA2095-13|Mycetophilidae|658|BOLD:ACJ1868
Allodia|[19600]|SSJAA1492-13|Mycetophilidae|658|BOLD:ACI6426
Mycetophilinae|[19601]|JWDCG098-10|Mycetophilidae|658|BOLD:AAG4936
Allodia anglofennica|[19602]|SSJAA2109-13|Mycetophilidae|658|BOLD:ABA3403
Allodia|[19603]|SSBAE4348-13|Mycetophilidae|658|BOLD:ACG5875
Allodia|[19604]|SSJAA1482-13|Mycetophilidae|658|BOLD:ACJ1830
Allodia|[19605]|SSJAA1777-13|Mycetophilidae|658|BOLD:ACJ1778
Allodia|[19606]|SSJAA2126-13|Mycetophilidae|664|BOLD:ACG3048
Brevicornu|[19607]|SSBAF3481-13|Mycetophilidae|661|BOLD:AAG5448
Allodia|[19608]|SSBAB2201-12|Mycetophilidae|658|BOLD:ACD3136
Allodia|[19609]|BBDCN860-10|Mycetophilidae|658|BOLD:AAP4739
Allodia|[19610]|SSJAA1776-13|Mycetophilidae|595|BOLD:ABA1399
Allodia|[19611]|CNEIH410-13|Mycetophilidae|658|BOLD:AAU4925
Allodia|[19612]|SSBAF3548-13|Mycetophilidae|661|BOLD:AAP6499
Mycetophilinae|[19613]|SSBAF3551-13|Mycetophilidae|661|BOLD:ACD0959
Mycetophilidae|[19614]|CNJAG1272-12|Mycetophilidae|664|BOLD:AAM8985
Allodia|[19615]|CNEIH747-13|Mycetophilidae|664|BOLD:ACB1246
Brevicornu sp. CJB05|[19616]|JWDCE368-10|Mycetophilidae|658|BOLD:AAP4738
Mycetophilidae|[19617]|SSBAF3536-13|Mycetophilidae|661|BOLD:ACC5209
Mycetophilidae|[19618]|SSBAB1719-12|Mycetophilidae|673|BOLD:AAG4945
Allodia pyxidiiformis|[19619]|SSJAA198-13|Mycetophilidae|608|BOLD:ABA1645
Allodia tuomikoskii|[19620]|SSJAD4194-13|Mycetophilidae|598|BOLD:ACZ3044
Brevicornu|[19621]|BBDCN351-10|Mycetophilidae|658|BOLD:AAL9145
Mycetophilidae|[19622]|SSEIC1629-13|Mycetophilidae|658|BOLD:ACI7584
Brevicornu|[19623]|SSBAB1700-12|Mycetophilidae|673|BOLD:ACC5564
Brevicornu sp. CJB07|[19624]|JWDCC770-10|Mycetophilidae|658|BOLD:AAG4885
Brevicornu sp. CJB08|[19625]|SSJAA728-13|Mycetophilidae|664|BOLD:AAL9146
Mycetophilidae|[19626]|PAOCT041-12|Mycetophilidae|670|BOLD:AAP2527
Mycetophilinae|[19627]|SSJAA2124-13|Mycetophilidae|664|BOLD:ACF9398
Mycetophilidae|[19628]|SSROA1726-14|Mycetophilidae|555|BOLD:ACN8130
Mycetophilidae|[19629]|SSBAA5541-12|Mycetophilidae|673|BOLD:ACC9544
Mycetophilidae|[19630]|CNWLF1785-12|Mycetophilidae|661|BOLD:ACD1792
Mycetophilidae|[19631]|CNJAC1077-12|Mycetophilidae|658|BOLD:ACB6585
Mycetophilidae|[19632]|SSJAE6900-13|Mycetophilidae|661|BOLD:ACJ8497
Mycetophilidae|[19633]|SSJAD5094-13|Mycetophilidae|670|BOLD:ACJ5017
Mycetophilinae|[19634]|CNFNC543-14|Mycetophilidae|661|BOLD:ACL2806
Mycetophilidae|[19635]|SSFDA2764-14|Mycetophilidae|588|BOLD:ACO2049
Mycetophilidae|[19636]|SSBAB1704-12|Mycetophilidae|673|BOLD:ACC0519
Mycetophilidae|[19637]|CNPAA294-13|Mycetophilidae|658|BOLD:ACD0126
Exechia|[19638]|JWDCL237-11|Mycetophilidae|658|BOLD:AAU4900
Mycetophilidae|[19639]|SSEIB5905-13|Mycetophilidae|658|BOLD:ACI3862
Exechia pseudofestiva|[19640]|SSEIB6572-13|Mycetophilidae|661|BOLD:ACI3895
Mycetophilidae|[19641]|CNJAC879-12|Mycetophilidae|673|BOLD:AAP6484
Mycetophilidae|[19642]|CNJAI115-12|Mycetophilidae|624|BOLD:ACD1266
Brevicornu|[19643]|CNJAC1393-12|Mycetophilidae|670|BOLD:AAU4981
Mycetophilidae|[19644]|CNGLB360-13|Mycetophilidae|658|BOLD:ACE1139
Mycetophilidae|[19645]|BBDCM473-10|Mycetophilidae|658|BOLD:AAP6488
Mycetophilidae|[19646]|SSJAA2116-13|Mycetophilidae|633|BOLD:ACJ1963
Rymosia|[19647]|SSBAB2219-12|Mycetophilidae|658|BOLD:ACD3293
Rymosia|[19648]|CNIVD303-14|Mycetophilidae|555|BOLD:ACG1604
Mycetophilidae|[19649]|SSBAA571-12|Mycetophilidae|673|BOLD:ACC3821
Mycetophilidae|[19650]|SSBAE2513-13|Mycetophilidae|658|BOLD:ACI5635
Mycetophilidae|[19651]|PHNGT355-13|Mycetophilidae|619|BOLD:ACJ1659
Mycetophilidae|[19652]|SSPAB4786-13|Mycetophilidae|670|BOLD:ACD3379
Mycetophilidae|[19653]|CNGBA878-13|Mycetophilidae|584|BOLD:ACL0109
Mycetophilidae|[19654]|CNRMH061-12|Mycetophilidae|658|BOLD:ACD0769
Exechia nigroscutellata|[19655]|CNEIH275-13|Mycetophilidae|658|BOLD:AAL9144
Exechia nigroscutellata|[19656]|SSEID1904-13|Mycetophilidae|661|BOLD:AAM8997
Exechia unimaculata|[19657]|SSBAF3477-13|Mycetophilidae|661|BOLD:ABA3388
Mycetophilidae|[19658]|SSWLB3246-13|Mycetophilidae|658|BOLD:ACG3736
Mycetophilidae|[19659]|SSJAB1464-13|Mycetophilidae|658|BOLD:ACJ1939
Mycetophilidae|[19660]|SSPAC8838-13|Mycetophilidae|603|BOLD:ACJ8747
Mycetophilidae|[19661]|CHDIP800-12|Mycetophilidae|658|BOLD:ACB1096
Mycetophilidae|[19662]|CNKLG1232-15|Mycetophilidae|621|BOLD:ACT3663
Mycetophilidae|[19663]|SSBAB2194-12|Mycetophilidae|658|BOLD:ACD3158
Mycetophilidae|[19664]|CNWLM713-13|Mycetophilidae|658|BOLD:ACI8157
Exechia|[19665]|SSBAB1724-12|Mycetophilidae|673|BOLD:ACC9967
Exechia|[19666]|BBDEE520-10|Mycetophilidae|658|BOLD:AAG4880
Mycetophilidae|[19667]|CNPPB697-12|Mycetophilidae|658|BOLD:ACC7909
Mycetophilidae|[19668]|CNJAC1318-12|Mycetophilidae|673|BOLD:ACB6495
Mycetophilidae|[19669]|SSPAA7451-13|Mycetophilidae|661|BOLD:ACI3885
Mycetophilidae|[19670]|SSWLF4261-13|Mycetophilidae|661|BOLD:ACK8583
Mycetophilidae|[19671]|JSDIS082-11|Mycetophilidae|658|BOLD:AAN8586
Mycetophilidae|[19672]|BBDCN842-10|Mycetophilidae|658|BOLD:AAP2525
Mycetophilidae|[19673]|CNPPE340-12|Mycetophilidae|661|BOLD:ABA6848
Mycetophilidae|[19674]|SSBAA702-12|Mycetophilidae|661|BOLD:ACC3725
Mycetophilidae|[19675]|SSBAE2719-13|Mycetophilidae|658|BOLD:ACI6494
Mycetophilidae|[19676]|CNGBJ1073-14|Mycetophilidae|661|BOLD:ACI6985
Exechia|[19677]|SSWLD710-13|Mycetophilidae|670|BOLD:ACC6779
Exechia|[19678]|BBDCM1000-10|Mycetophilidae|658|BOLD:AAP6498
Exechia|[19679]|SSBAB1721-12|Mycetophilidae|673|BOLD:ACX9219
Mycetophilidae|[19680]|DARC143-10|Mycetophilidae|658|BOLD:AAM9014
Exechia|[19681]|SSBAF2933-13|Mycetophilidae|658|BOLD:ACX9473
Mycetophilidae|[19682]|CHDIP799-12|Mycetophilidae|658|BOLD:AAL9140
Mycetophilidae|[19683]|CHDIP803-12|Mycetophilidae|658|BOLD:ACX9220
Exechia subfrigida|[19684]|CNPEK123-14|Mycetophilidae|658|BOLD:AAP4736
Mycetophilidae|[19685]|CNPAI037-13|Mycetophilidae|658|BOLD:ACF9026
Exechia|[19686]|SSBAD2124-12|Mycetophilidae|658|BOLD:ACD1178
Mycetophilidae|[19687]|SSBAE1431-13|Mycetophilidae|658|BOLD:ACI6408
Exechia|[19688]|SSJAF4406-13|Mycetophilidae|658|BOLD:ACK5053
Exechia|[19689]|SSJAD2381-13|Mycetophilidae|661|BOLD:ACI4958
Exechia nigra|[19690]|SSJAD5097-13|Mycetophilidae|670|BOLD:ACI6520
Exechia|[19691]|SSBAE3861-13|Mycetophilidae|658|BOLD:ACI5988
Exechia|[19692]|CNEIH1487-13|Mycetophilidae|658|BOLD:AAQ3544
Exechia|[19693]|SSJAD6355-13|Mycetophilidae|658|BOLD:ACO4251
Exechia|[19694]|SSBRA3508-14|Mycetophilidae|664|BOLD:ACE0545
Mycetophilidae|[19695]|CNEIJ070-13|Mycetophilidae|658|BOLD:ACG2433
Mycetophilidae|[19696]|JSDIS083-11|Mycetophilidae|658|BOLD:AAU6631

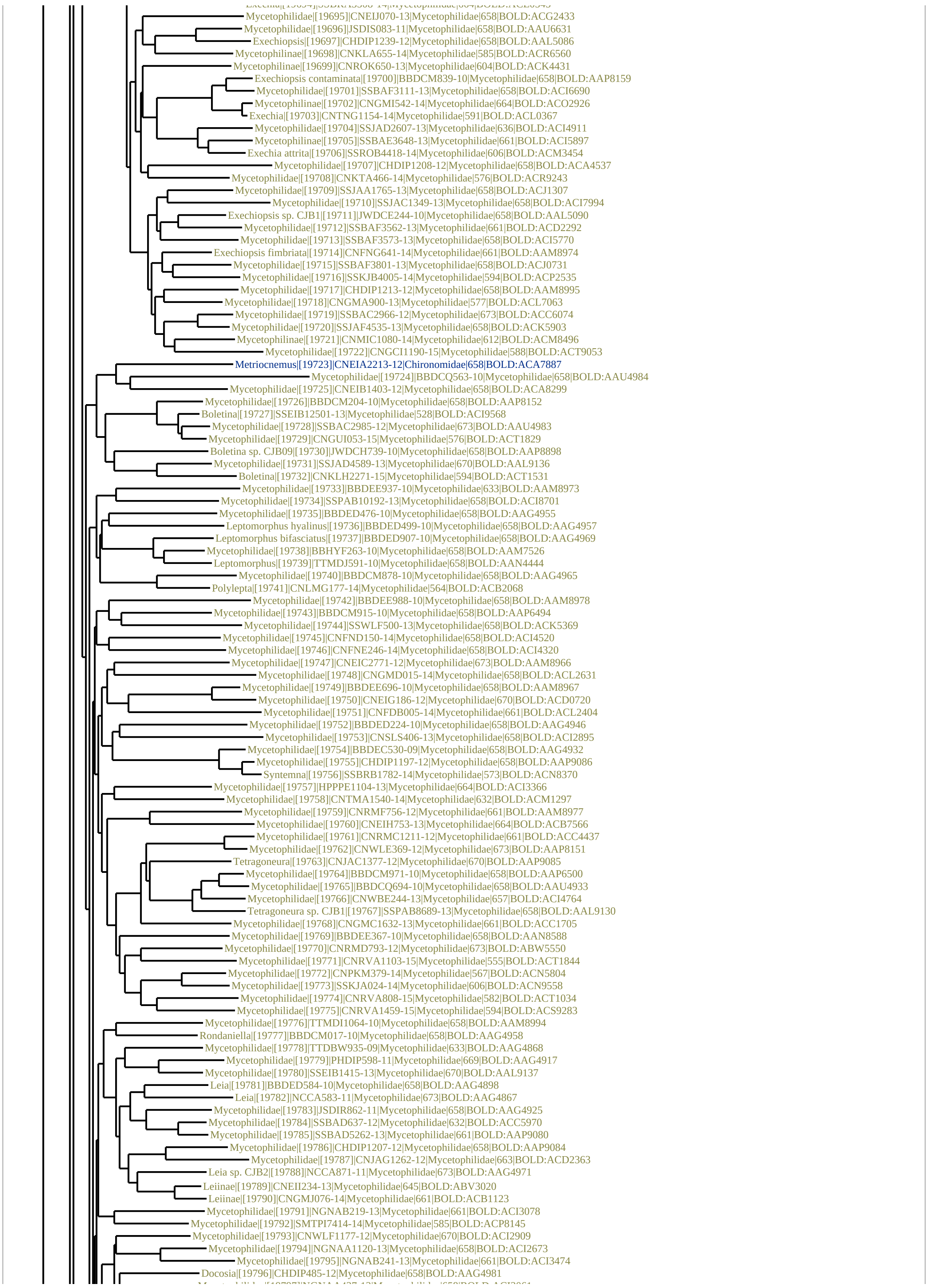

Mycetophilidae|[19695]|CNEIJ070-13|Mycetophilidae|658|BOLD:ACG2433
Mycetophilidae|[19696]|JSDIS083-11|Mycetophilidae|658|BOLD:AAU6631
Exechiopsis|[19697]|CHDIP1239-12|Mycetophilidae|658|BOLD:AAL5086
Mycetophilinae|[19698]|CNKLA655-14|Mycetophilidae|585|BOLD:ACR6560
Mycetophilinae|[19699]|CNROK650-13|Mycetophilidae|604|BOLD:ACK4431
Exechiopsis contaminata|[19700]|BBDCM839-10|Mycetophilidae|658|BOLD:AAP8159
Mycetophilidae|[19701]|SSBAF3111-13|Mycetophilidae|658|BOLD:ACI6690
Mycetophilinae|[19702]|CNGMI542-14|Mycetophilidae|664|BOLD:ACO2926
Exechia|[19703]|CNTNG1154-14|Mycetophilidae|591|BOLD:ACL0367
Mycetophilidae|[19704]|SSJAD2607-13|Mycetophilidae|636|BOLD:ACI4911
Mycetophilinae|[19705]|SSBAE3648-13|Mycetophilidae|661|BOLD:ACI5897
Exechia attrita|[19706]|SSROB4418-14|Mycetophilidae|606|BOLD:ACM3454
Mycetophilidae|[19707]|CHDIP1208-12|Mycetophilidae|658|BOLD:ACA4537
Mycetophilidae|[19708]|CNKTA466-14|Mycetophilidae|576|BOLD:ACR9243
Mycetophilidae|[19709]|SSJAA1765-13|Mycetophilidae|658|BOLD:ACJ1307
Mycetophilidae|[19710]|SSJAC1349-13|Mycetophilidae|658|BOLD:ACI7994
Exechiopsis sp. CJB1|[19711]|JWDCE244-10|Mycetophilidae|658|BOLD:AAL5090
Mycetophilidae|[19712]|SSBAF3562-13|Mycetophilidae|661|BOLD:ACD2292
Mycetophilidae|[19713]|SSBAF3573-13|Mycetophilidae|658|BOLD:ACI5770
Exechiopsis fimbriata|[19714]|CNFNG641-14|Mycetophilidae|661|BOLD:AAM8974
Mycetophilidae|[19715]|SSBAF3801-13|Mycetophilidae|658|BOLD:ACJ0731
Mycetophilidae|[19716]|SSKJB4005-14|Mycetophilidae|594|BOLD:ACP2535
Mycetophilidae|[19717]|CHDIP1213-12|Mycetophilidae|658|BOLD:AAM8995
Mycetophilidae|[19718]|CNGMA900-13|Mycetophilidae|577|BOLD:ACL7063
Mycetophilidae|[19719]|SSBAC2966-12|Mycetophilidae|673|BOLD:ACC6074
Mycetophilidae|[19720]|SSJAF4535-13|Mycetophilidae|658|BOLD:ACK5903
Mycetophilinae|[19721]|CNMIC1080-14|Mycetophilidae|612|BOLD:ACM8496
Mycetophilidae|[19722]|CNGCI1190-15|Mycetophilidae|588|BOLD:ACT9053
Metriocnemus|[19723]|CNEIA2213-12|Chironomidae|658|BOLD:ACA7887
Mycetophilidae|[19724]|BBDCQ563-10|Mycetophilidae|658|BOLD:AAU4984
Mycetophilidae|[19725]|CNEIB1403-12|Mycetophilidae|658|BOLD:ACA8299
Mycetophilidae|[19726]|BBDCM204-10|Mycetophilidae|658|BOLD:AAP8152
Boletina|[19727]|SSEIB12501-13|Mycetophilidae|528|BOLD:ACI9568
Mycetophilidae|[19728]|SSBAC2985-12|Mycetophilidae|673|BOLD:AAU4983
Mycetophilidae|[19729]|CNGUI053-15|Mycetophilidae|576|BOLD:ACT1829
Boletina sp. CJB09|[19730]|JWDCH739-10|Mycetophilidae|658|BOLD:AAP8898
Mycetophilidae|[19731]|SSJAD4589-13|Mycetophilidae|670|BOLD:AAL9136
Boletina|[19732]|CNKLH2271-15|Mycetophilidae|594|BOLD:ACT1531
Mycetophilidae|[19733]|BBDEE937-10|Mycetophilidae|633|BOLD:AAM8973
Mycetophilidae|[19734]|SSPAB10192-13|Mycetophilidae|658|BOLD:ACI8701
Mycetophilidae|[19735]|BBDED476-10|Mycetophilidae|658|BOLD:AAG4955
Leptomorphus hyalinus|[19736]|BBDED499-10|Mycetophilidae|658|BOLD:AAG4957
Leptomorphus bifasciatus|[19737]|BBDED907-10|Mycetophilidae|658|BOLD:AAG4969
Mycetophilidae|[19738]|BBHYF263-10|Mycetophilidae|658|BOLD:AAM7526
Leptomorphus|[19739]|TTMDJ591-10|Mycetophilidae|658|BOLD:AAN4444
Mycetophilidae|[19740]|BBDCM878-10|Mycetophilidae|658|BOLD:AAG4965
Polylepta|[19741]|CNLMG177-14|Mycetophilidae|564|BOLD:ACB2068
Mycetophilidae|[19742]|BBDEE988-10|Mycetophilidae|658|BOLD:AAM8978
Mycetophilidae|[19743]|BBDCM915-10|Mycetophilidae|658|BOLD:AAP6494
Mycetophilidae|[19744]|SSWLF500-13|Mycetophilidae|658|BOLD:ACK5369
Mycetophilidae|[19745]|CNFND150-14|Mycetophilidae|658|BOLD:ACI4520
Mycetophilidae|[19746]|CNFNE246-14|Mycetophilidae|658|BOLD:ACI4320
Mycetophilidae|[19747]|CNEIC2771-12|Mycetophilidae|673|BOLD:AAM8966
Mycetophilidae|[19748]|CNGMD015-14|Mycetophilidae|658|BOLD:ACL2631
Mycetophilidae|[19749]|BBDEE696-10|Mycetophilidae|658|BOLD:AAM8967
Mycetophilidae|[19750]|CNEIG186-12|Mycetophilidae|670|BOLD:ACD0720
Mycetophilidae|[19751]|CNFDB005-14|Mycetophilidae|661|BOLD:ACL2404
Mycetophilidae|[19752]|BBDED224-10|Mycetophilidae|658|BOLD:AAG4946
Mycetophilidae|[19753]|CNSLS406-13|Mycetophilidae|658|BOLD:ACI2895
Mycetophilidae|[19754]|BBDEC530-09|Mycetophilidae|658|BOLD:AAG4932
Mycetophilidae|[19755]|CHDIP1197-12|Mycetophilidae|658|BOLD:AAP9086
Syntemna|[19756]|SSBRB1782-14|Mycetophilidae|573|BOLD:ACN8370
Mycetophilidae|[19757]|HPPPE1104-13|Mycetophilidae|664|BOLD:ACI3366
Mycetophilidae|[19758]|CNTMA1540-14|Mycetophilidae|632|BOLD:ACM1297
Mycetophilidae|[19759]|CNRMF756-12|Mycetophilidae|661|BOLD:AAM8977
Mycetophilidae|[19760]|CNEIH753-13|Mycetophilidae|664|BOLD:ACB7566
Mycetophilidae|[19761]|CNRMC1211-12|Mycetophilidae|661|BOLD:ACC4437
Mycetophilidae|[19762]|CNWLE369-12|Mycetophilidae|673|BOLD:AAP8151
Tetragoneura|[19763]|CNJAC1377-12|Mycetophilidae|670|BOLD:AAP9085
Mycetophilidae|[19764]|BBDCM971-10|Mycetophilidae|658|BOLD:AAP6500
Mycetophilidae|[19765]|BBDCQ694-10|Mycetophilidae|658|BOLD:AAU4933
Mycetophilidae|[19766]|CNWBE244-13|Mycetophilidae|657|BOLD:ACI4764
Tetragoneura sp. CJB1|[19767]|SSPAB8689-13|Mycetophilidae|658|BOLD:AAL9130
Mycetophilidae|[19768]|CNGMC1632-13|Mycetophilidae|661|BOLD:ACC1705
Mycetophilidae|[19769]|BBDEE367-10|Mycetophilidae|658|BOLD:AAN8588
Mycetophilidae|[19770]|CNRMD793-12|Mycetophilidae|673|BOLD:ABW5550
Mycetophilidae|[19771]|CNRVA1103-15|Mycetophilidae|555|BOLD:ACT1844
Mycetophilidae|[19772]|CNPKM379-14|Mycetophilidae|567|BOLD:ACN5804
Mycetophilidae|[19773]|SSKJA024-14|Mycetophilidae|606|BOLD:ACN9558
Mycetophilidae|[19774]|CNRVA808-15|Mycetophilidae|582|BOLD:ACT1034
Mycetophilidae|[19775]|CNRVA1459-15|Mycetophilidae|594|BOLD:ACS9283
Mycetophilidae|[19776]|TTMDI1064-10|Mycetophilidae|658|BOLD:AAM8994
Rondaniella|[19777]|BBDCM017-10|Mycetophilidae|658|BOLD:AAG4958
Mycetophilidae|[19778]|TTDBW935-09|Mycetophilidae|633|BOLD:AAG4868
Mycetophilidae|[19779]|PHDIP598-11|Mycetophilidae|669|BOLD:AAG4917
Mycetophilidae|[19780]|SSEIB1415-13|Mycetophilidae|670|BOLD:AAL9137
Leia|[19781]|BBDED584-10|Mycetophilidae|658|BOLD:AAG4898
Leia|[19782]|NCCA583-11|Mycetophilidae|673|BOLD:AAG4867
Mycetophilidae|[19783]|JSDIR862-11|Mycetophilidae|658|BOLD:AAG4925
Mycetophilidae|[19784]|SSBAD637-12|Mycetophilidae|632|BOLD:ACC5970
Mycetophilidae|[19785]|SSBAD5262-13|Mycetophilidae|661|BOLD:AAP9080
Mycetophilidae|[19786]|CHDIP1207-12|Mycetophilidae|658|BOLD:AAP9084
Mycetophilidae|[19787]|CNJAG1262-12|Mycetophilidae|663|BOLD:ACD2363
Leia sp. CJB2|[19788]|NCCA871-11|Mycetophilidae|673|BOLD:AAG4971
Leiinae|[19789]|CNEII234-13|Mycetophilidae|645|BOLD:ABV3020
Leiinae|[19790]|CNGMJ076-14|Mycetophilidae|661|BOLD:ACB1123
Mycetophilidae|[19791]|NGNAB219-13|Mycetophilidae|661|BOLD:ACI3078
Mycetophilidae|[19792]|SMTPI7414-14|Mycetophilidae|585|BOLD:ACP8145
Mycetophilidae|[19793]|CNWLF1177-12|Mycetophilidae|670|BOLD:ACI2909
Mycetophilidae|[19794]|NGNAA1120-13|Mycetophilidae|658|BOLD:ACI2673
Mycetophilidae|[19795]|NGNAB241-13|Mycetophilidae|661|BOLD:ACI3474
Docosia|[19796]|CHDIP485-12|Mycetophilidae|658|BOLD:AAG4981

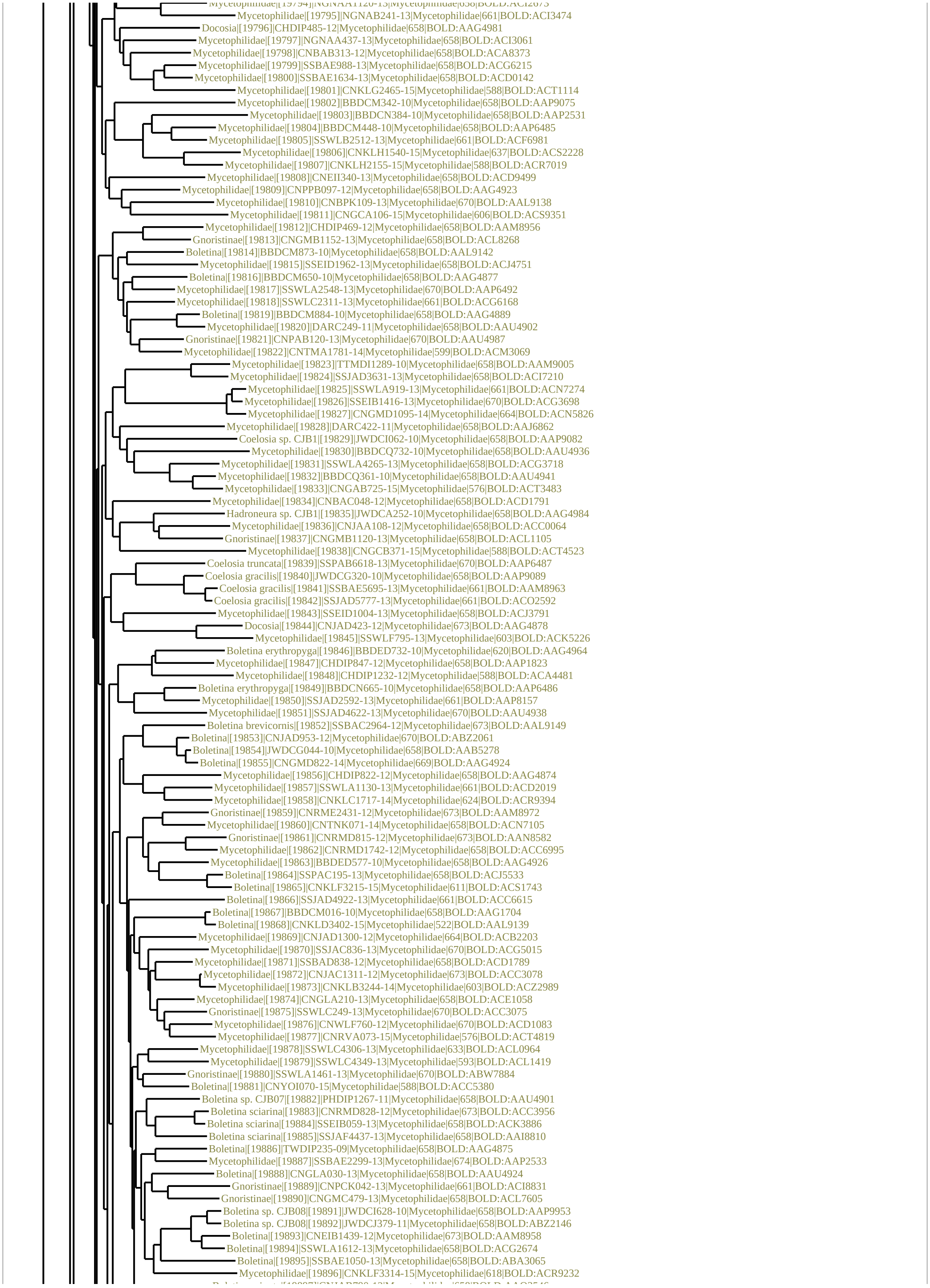
Mycetophilidae|[19795]|NGNAB241-13|Mycetophilidae|661|BOLD:ACI3474
Docosia|[19796]|CHDIP485-12|Mycetophilidae|658|BOLD:AAG4981
Mycetophilidae|[19797]|NGNAA437-13|Mycetophilidae|658|BOLD:ACI3061
Mycetophilidae|[19798]|CNBAB313-12|Mycetophilidae|658|BOLD:ACA8373
Mycetophilidae|[19799]|SSBAE988-13|Mycetophilidae|658|BOLD:ACG6215
Mycetophilidae|[19800]|SSBAE1634-13|Mycetophilidae|658|BOLD:ACD0142
Mycetophilidae|[19801]|CNKLG2465-15|Mycetophilidae|588|BOLD:ACT1114
Mycetophilidae|[19802]|BBDCM342-10|Mycetophilidae|658|BOLD:AAP9075
Mycetophilidae|[19803]|BBDCN384-10|Mycetophilidae|658|BOLD:AAP2531
Mycetophilidae|[19804]|BBDCM448-10|Mycetophilidae|658|BOLD:AAP6485
Mycetophilidae|[19805]|SSWLB2512-13|Mycetophilidae|661|BOLD:ACF6981
Mycetophilidae|[19806]|CNKLH1540-15|Mycetophilidae|637|BOLD:ACS2228
Mycetophilidae|[19807]|CNKLH2155-15|Mycetophilidae|588|BOLD:ACR7019
Mycetophilidae|[19808]|CNEII340-13|Mycetophilidae|658|BOLD:ACD9499
Mycetophilidae|[19809]|CNPPB097-12|Mycetophilidae|658|BOLD:AAG4923
Mycetophilidae|[19810]|CNBPK109-13|Mycetophilidae|670|BOLD:AAL9138
Mycetophilidae|[19811]|CNGCA106-15|Mycetophilidae|606|BOLD:ACS9351
Mycetophilidae|[19812]|CHDIP469-12|Mycetophilidae|658|BOLD:AAM8956
Gnoristinae|[19813]|CNGMB1152-13|Mycetophilidae|658|BOLD:ACL8268
Boletina|[19814]|BBDCM873-10|Mycetophilidae|658|BOLD:AAL9142
Mycetophilidae|[19815]|SSEID1962-13|Mycetophilidae|658|BOLD:ACJ4751
Boletina|[19816]|BBDCM650-10|Mycetophilidae|658|BOLD:AAG4877
Mycetophilidae|[19817]|SSWLA2548-13|Mycetophilidae|670|BOLD:AAP6492
Mycetophilidae|[19818]|SSWLC2311-13|Mycetophilidae|661|BOLD:ACG6168
Boletina|[19819]|BBDCM884-10|Mycetophilidae|658|BOLD:AAG4889
Mycetophilidae|[19820]|DARC249-11|Mycetophilidae|658|BOLD:AAU4902
Gnoristinae|[19821]|CNPAB120-13|Mycetophilidae|670|BOLD:AAU4987
Mycetophilidae|[19822]|CNTMA1781-14|Mycetophilidae|599|BOLD:ACM3069
Mycetophilidae|[19823]|TTMDI1289-10|Mycetophilidae|658|BOLD:AAM9005
Mycetophilidae|[19824]|SSJAD3631-13|Mycetophilidae|658|BOLD:ACI7210
Mycetophilidae|[19825]|SSWLA919-13|Mycetophilidae|661|BOLD:ACN7274
Mycetophilidae|[19826]|SSEIB1416-13|Mycetophilidae|670|BOLD:ACG3698
Mycetophilidae|[19827]|CNGMD1095-14|Mycetophilidae|664|BOLD:ACN5826
Mycetophilidae|[19828]|DARC422-11|Mycetophilidae|658|BOLD:AAJ6862
Coelosia sp. CJB1|[19829]|JWDCI062-10|Mycetophilidae|658|BOLD:AAP9082
Mycetophilidae|[19830]|BBDCQ732-10|Mycetophilidae|658|BOLD:AAU4936
Mycetophilidae|[19831]|SSWLA4265-13|Mycetophilidae|658|BOLD:ACG3718
Mycetophilidae|[19832]|BBDCQ361-10|Mycetophilidae|658|BOLD:AAU4941
Mycetophilidae|[19833]|CNGAB725-15|Mycetophilidae|576|BOLD:ACT3483
Mycetophilidae|[19834]|CNBAC048-12|Mycetophilidae|658|BOLD:ACD1791
Hadroneura sp. CJB1|[19835]|JWDCA252-10|Mycetophilidae|658|BOLD:AAG4984
Mycetophilidae|[19836]|CNJAA108-12|Mycetophilidae|658|BOLD:ACC0064
Gnoristinae|[19837]|CNGMB1120-13|Mycetophilidae|658|BOLD:ACL1105
Mycetophilidae|[19838]|CNGCB371-15|Mycetophilidae|588|BOLD:ACT4523
Coelosia truncata|[19839]|SSPAB6618-13|Mycetophilidae|670|BOLD:AAP6487
Coelosia gracilis|[19840]|JWDCG320-10|Mycetophilidae|658|BOLD:AAP9089
Coelosia gracilis|[19841]|SSBAE5695-13|Mycetophilidae|661|BOLD:AAM8963
Coelosia gracilis|[19842]|SSJAD5777-13|Mycetophilidae|661|BOLD:ACO2592
Mycetophilidae|[19843]|SSEID1004-13|Mycetophilidae|658|BOLD:ACJ3791
Docosia|[19844]|CNJAD423-12|Mycetophilidae|673|BOLD:AAG4878
Mycetophilidae|[19845]|SSWLF795-13|Mycetophilidae|603|BOLD:ACK5226
Boletina erythropyga|[19846]|BBDED732-10|Mycetophilidae|620|BOLD:AAG4964
Mycetophilidae|[19847]|CHDIP847-12|Mycetophilidae|658|BOLD:AAP1823
Mycetophilidae|[19848]|CHDIP1232-12|Mycetophilidae|588|BOLD:ACA4481
Boletina erythropyga|[19849]|BBDCN665-10|Mycetophilidae|658|BOLD:AAP6486
Mycetophilidae|[19850]|SSJAD2592-13|Mycetophilidae|661|BOLD:AAP8157
Mycetophilidae|[19851]|SSJAD4622-13|Mycetophilidae|670|BOLD:AAU4938
Boletina brevicornis|[19852]|SSBAC2964-12|Mycetophilidae|673|BOLD:AAL9149
Boletina|[19853]|CNJAD953-12|Mycetophilidae|670|BOLD:ABZ2061
Boletina|[19854]|JWDCG044-10|Mycetophilidae|658|BOLD:AAB5278
Boletina|[19855]|CNGMD822-14|Mycetophilidae|669|BOLD:AAG4924
Mycetophilidae|[19856]|CHDIP822-12|Mycetophilidae|658|BOLD:AAG4874
Mycetophilidae|[19857]|SSWLA1130-13|Mycetophilidae|661|BOLD:ACD2019
Mycetophilidae|[19858]|CNKLC1717-14|Mycetophilidae|624|BOLD:ACR9394
Gnoristinae|[19859]|CNRME2431-12|Mycetophilidae|673|BOLD:AAM8972
Mycetophilidae|[19860]|CNTNK071-14|Mycetophilidae|658|BOLD:ACN7105
Gnoristinae|[19861]|CNRMD815-12|Mycetophilidae|673|BOLD:AAN8582
Mycetophilidae|[19862]|CNRMD1742-12|Mycetophilidae|658|BOLD:ACC6995
Mycetophilidae|[19863]|BBDED577-10|Mycetophilidae|658|BOLD:AAG4926
Boletina|[19864]|SSPAC195-13|Mycetophilidae|658|BOLD:ACJ5533
Boletina|[19865]|CNKLF3215-15|Mycetophilidae|611|BOLD:ACS1743
Boletina|[19866]|SSJAD4922-13|Mycetophilidae|661|BOLD:ACC6615
Boletina|[19867]|BBDCM016-10|Mycetophilidae|658|BOLD:AAG1704
Boletina|[19868]|CNKLD3402-15|Mycetophilidae|522|BOLD:AAL9139
Mycetophilidae|[19869]|CNJAD1300-12|Mycetophilidae|664|BOLD:ACB2203
Mycetophilidae|[19870]|SSJAC836-13|Mycetophilidae|670|BOLD:ACG5015
Mycetophilidae|[19871]|SSBAD838-12|Mycetophilidae|658|BOLD:ACD1789
Mycetophilidae|[19872]|CNJAC1311-12|Mycetophilidae|673|BOLD:ACC3078
Mycetophilidae|[19873]|CNKLB3244-14|Mycetophilidae|603|BOLD:ACZ2989
Mycetophilidae|[19874]|CNGLA210-13|Mycetophilidae|658|BOLD:ACE1058
Gnoristinae|[19875]|SSWLC249-13|Mycetophilidae|670|BOLD:ACC3075
Mycetophilidae|[19876]|CNWLF760-12|Mycetophilidae|670|BOLD:ACD1083
Mycetophilidae|[19877]|CNRVA073-15|Mycetophilidae|576|BOLD:ACT4819
Mycetophilidae|[19878]|SSWLC4306-13|Mycetophilidae|633|BOLD:ACL0964
Mycetophilidae|[19879]|SSWLC4349-13|Mycetophilidae|593|BOLD:ACL1419
Gnoristinae|[19880]|SSWLA1461-13|Mycetophilidae|670|BOLD:ABW7884
Boletina|[19881]|CNYOI070-15|Mycetophilidae|588|BOLD:ACC5380
Boletina sp. CJB07|[19882]|PHDIP1267-11|Mycetophilidae|658|BOLD:AAU4901
Boletina sciarina|[19883]|CNRMD828-12|Mycetophilidae|673|BOLD:ACC3956
Boletina sciarina|[19884]|SSEIB059-13|Mycetophilidae|658|BOLD:ACK3886
Boletina sciarina|[19885]|SSJAF4437-13|Mycetophilidae|658|BOLD:AAI8810
Boletina|[19886]|TWDIP235-09|Mycetophilidae|658|BOLD:AAG4875
Mycetophilidae|[19887]|SSBAE2299-13|Mycetophilidae|674|BOLD:AAP2533
Boletina|[19888]|CNGLA030-13|Mycetophilidae|658|BOLD:AAU4924
Gnoristinae|[19889]|CNPCK042-13|Mycetophilidae|661|BOLD:ACI8831
Gnoristinae|[19890]|CNGMC479-13|Mycetophilidae|658|BOLD:ACL7605
Boletina sp. CJB08|[19891]|JWDCI628-10|Mycetophilidae|658|BOLD:AAP9953
Boletina sp. CJB08|[19892]|JWDCJ379-11|Mycetophilidae|658|BOLD:ABZ2146
Boletina|[19893]|CNEIB1439-12|Mycetophilidae|673|BOLD:AAM8958
Boletina|[19894]|SSWLA1612-13|Mycetophilidae|658|BOLD:ACG2674
Boletina|[19895]|SSBAE1050-13|Mycetophilidae|658|BOLD:ABA3065
Mycetophilidae|[19896]|CNKLF3314-15|Mycetophilidae|618|BOLD:ACR9232

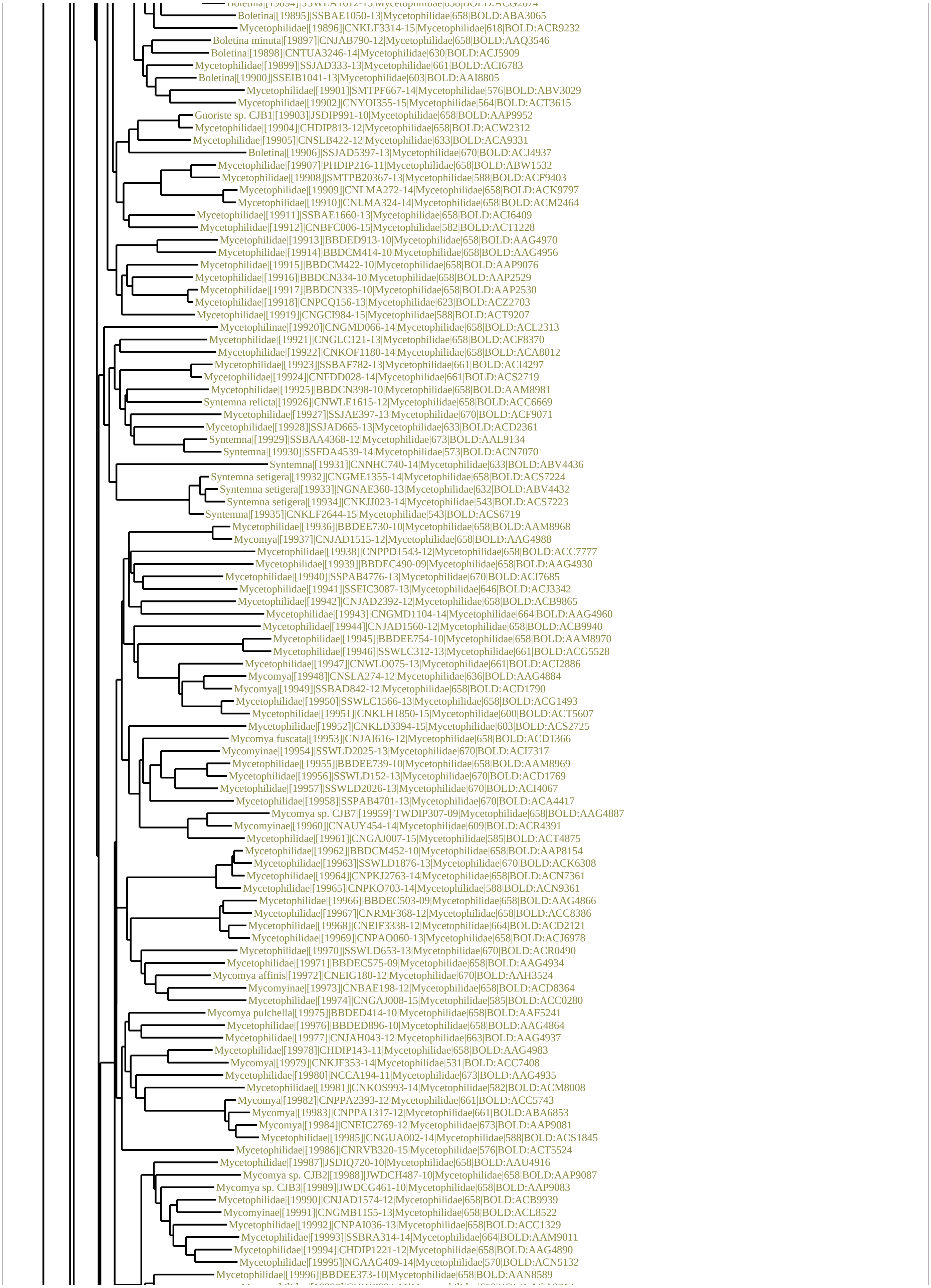

Boletina|[19895]|SSBAE1050-13|Mycetophilidae|658|BOLD:ABA3065
Mycetophilidae|[19896]|CNKLF3314-15|Mycetophilidae|618|BOLD:ACR9232
Boletina minuta|[19897]|CNJAB790-12|Mycetophilidae|658|BOLD:AAQ3546
Boletina|[19898]|CNTUA3246-14|Mycetophilidae|630|BOLD:ACJ5909
Mycetophilidae|[19899]|SSJAD333-13|Mycetophilidae|661|BOLD:ACI6783
Boletina|[19900]|SSEIB1041-13|Mycetophilidae|603|BOLD:AAI8805
Mycetophilidae|[19901]|SMTPF667-14|Mycetophilidae|576|BOLD:ABV3029
Mycetophilidae|[19902]|CNYOI355-15|Mycetophilidae|564|BOLD:ACT3615
Gnoriste sp. CJB1|[19903]|JSDIP991-10|Mycetophilidae|658|BOLD:AAP9952
Mycetophilidae|[19904]|CHDIP813-12|Mycetophilidae|658|BOLD:ACW2312
Mycetophilidae|[19905]|CNSLB422-12|Mycetophilidae|633|BOLD:ACA9331
Boletina|[19906]|SSJAD5397-13|Mycetophilidae|670|BOLD:ACJ4937
Mycetophilidae|[19907]|PHDIP216-11|Mycetophilidae|658|BOLD:ABW1532
Mycetophilidae|[19908]|SMTPB20367-13|Mycetophilidae|588|BOLD:ACF9403
Mycetophilidae|[19909]|CNLMA272-14|Mycetophilidae|658|BOLD:ACK9797
Mycetophilidae|[19910]|CNLMA324-14|Mycetophilidae|658|BOLD:ACM2464
Mycetophilidae|[19911]|SSBAE1660-13|Mycetophilidae|658|BOLD:ACI6409
Mycetophilidae|[19912]|CNBFC006-15|Mycetophilidae|582|BOLD:ACT1228
Mycetophilidae|[19913]|BBDED913-10|Mycetophilidae|658|BOLD:AAG4970
Mycetophilidae|[19914]|BBDCM414-10|Mycetophilidae|658|BOLD:AAG4956
Mycetophilidae|[19915]|BBDCM422-10|Mycetophilidae|658|BOLD:AAP9076
Mycetophilidae|[19916]|BBDCN334-10|Mycetophilidae|658|BOLD:AAP2529
Mycetophilidae|[19917]|BBDCN335-10|Mycetophilidae|658|BOLD:AAP2530
Mycetophilidae|[19918]|CNPCQ156-13|Mycetophilidae|623|BOLD:ACZ2703
Mycetophilidae|[19919]|CNGCI984-15|Mycetophilidae|588|BOLD:ACT9207
Mycetophilinae|[19920]|CNGMD066-14|Mycetophilidae|658|BOLD:ACL2313
Mycetophilidae|[19921]|CNGLC121-13|Mycetophilidae|658|BOLD:ACF8370
Mycetophilidae|[19922]|CNKOF1180-14|Mycetophilidae|658|BOLD:ACA8012
Mycetophilidae|[19923]|SSBAF782-13|Mycetophilidae|661|BOLD:ACI4297
Mycetophilidae|[19924]|CNFDD028-14|Mycetophilidae|661|BOLD:ACS2719
Mycetophilidae|[19925]|BBDCN398-10|Mycetophilidae|658|BOLD:AAM8981
Syntemna relicta|[19926]|CNWLE1615-12|Mycetophilidae|658|BOLD:ACC6669
Mycetophilidae|[19927]|SSJAE397-13|Mycetophilidae|670|BOLD:ACF9071
Mycetophilidae|[19928]|SSJAD665-13|Mycetophilidae|633|BOLD:ACD2361
Syntemna|[19929]|SSBAA4368-12|Mycetophilidae|673|BOLD:AAL9134
Syntemna|[19930]|SSFDA4539-14|Mycetophilidae|573|BOLD:ACN7070
Syntemna|[19931]|CNNHC740-14|Mycetophilidae|633|BOLD:ABV4436
Syntemna setigera|[19932]|CNGME1355-14|Mycetophilidae|658|BOLD:ACS7224
Syntemna setigera|[19933]|NGNAE360-13|Mycetophilidae|632|BOLD:ABV4432
Syntemna setigera|[19934]|CNKJJ023-14|Mycetophilidae|543|BOLD:ACS7223
Syntemna|[19935]|CNKLF2644-15|Mycetophilidae|543|BOLD:ACS6719
Mycetophilidae|[19936]|BBDEE730-10|Mycetophilidae|658|BOLD:AAM8968
Mycomya|[19937]|CNJAD1515-12|Mycetophilidae|658|BOLD:AAG4988
Mycetophilidae|[19938]|CNPPD1543-12|Mycetophilidae|658|BOLD:ACC7777
Mycetophilidae|[19939]|BBDEC490-09|Mycetophilidae|658|BOLD:AAG4930
Mycetophilidae|[19940]|SSPAB4776-13|Mycetophilidae|670|BOLD:ACI7685
Mycetophilidae|[19941]|SSEIC3087-13|Mycetophilidae|646|BOLD:ACJ3342
Mycetophilidae|[19942]|CNJAD2392-12|Mycetophilidae|658|BOLD:ACB9865
Mycetophilidae|[19943]|CNGMD1104-14|Mycetophilidae|664|BOLD:AAG4960
Mycetophilidae|[19944]|CNJAD1560-12|Mycetophilidae|658|BOLD:ACB9940
Mycetophilidae|[19945]|BBDEE754-10|Mycetophilidae|658|BOLD:AAM8970
Mycetophilidae|[19946]|SSWLC312-13|Mycetophilidae|661|BOLD:ACG5528
Mycetophilidae|[19947]|CNWLO075-13|Mycetophilidae|661|BOLD:ACI2886
Mycomya|[19948]|CNSLA274-12|Mycetophilidae|636|BOLD:AAG4884
Mycomya|[19949]|SSBAD842-12|Mycetophilidae|658|BOLD:ACD1790
Mycetophilidae|[19950]|SSWLC1566-13|Mycetophilidae|658|BOLD:ACG1493
Mycetophilidae|[19951]|CNKLH1850-15|Mycetophilidae|600|BOLD:ACT5607
Mycetophilidae|[19952]|CNKLD3394-15|Mycetophilidae|603|BOLD:ACS2725
Mycomya fuscata|[19953]|CNJAI616-12|Mycetophilidae|658|BOLD:ACD1366
Mycomyinae|[19954]|SSWLD2025-13|Mycetophilidae|670|BOLD:ACI7317
Mycetophilidae|[19955]|BBDEE739-10|Mycetophilidae|658|BOLD:AAM8969
Mycetophilidae|[19956]|SSWLD152-13|Mycetophilidae|670|BOLD:ACD1769
Mycetophilidae|[19957]|SSWLD2026-13|Mycetophilidae|670|BOLD:ACI4067
Mycetophilidae|[19958]|SSPAB4701-13|Mycetophilidae|670|BOLD:ACA4417
Mycomya sp. CJB7|[19959]|TWDIP307-09|Mycetophilidae|658|BOLD:AAG4887
Mycomyinae|[19960]|CNAUY454-14|Mycetophilidae|609|BOLD:ACR4391
Mycetophilidae|[19961]|CNGAJ007-15|Mycetophilidae|585|BOLD:ACT4875
Mycetophilidae|[19962]|BBDCM452-10|Mycetophilidae|658|BOLD:AAP8154
Mycetophilidae|[19963]|SSWLD1876-13|Mycetophilidae|670|BOLD:ACK6308
Mycetophilidae|[19964]|CNPKJ2763-14|Mycetophilidae|658|BOLD:ACN7361
Mycetophilidae|[19965]|CNPKO703-14|Mycetophilidae|588|BOLD:ACN9361
Mycetophilidae|[19966]|BBDEC503-09|Mycetophilidae|658|BOLD:AAG4866
Mycetophilidae|[19967]|CNRMF368-12|Mycetophilidae|658|BOLD:ACC8386
Mycetophilidae|[19968]|CNEIF3338-12|Mycetophilidae|664|BOLD:ACD2121
Mycetophilidae|[19969]|CNPAO060-13|Mycetophilidae|658|BOLD:ACJ6978
Mycetophilidae|[19970]|SSWLD653-13|Mycetophilidae|670|BOLD:ACR0490
Mycetophilidae|[19971]|BBDEC575-09|Mycetophilidae|658|BOLD:AAG4934
Mycomya affinis|[19972]|CNEIG180-12|Mycetophilidae|670|BOLD:AAH3524
Mycomyinae|[19973]|CNBAE198-12|Mycetophilidae|658|BOLD:ACD8364
Mycetophilidae|[19974]|CNGAJ008-15|Mycetophilidae|585|BOLD:ACC0280
Mycomya pulchella|[19975]|BBDED414-10|Mycetophilidae|658|BOLD:AAF5241
Mycetophilidae|[19976]|BBDED896-10|Mycetophilidae|658|BOLD:AAG4864
Mycetophilidae|[19977]|CNJAH043-12|Mycetophilidae|663|BOLD:AAG4937
Mycetophilidae|[19978]|CHDIP143-11|Mycetophilidae|658|BOLD:AAG4983
Mycomya|[19979]|CNKJF353-14|Mycetophilidae|531|BOLD:ACC7408
Mycetophilidae|[19980]|NCCA194-11|Mycetophilidae|673|BOLD:AAG4935
Mycetophilidae|[19981]|CNKOS993-14|Mycetophilidae|582|BOLD:ACM8008
Mycomya|[19982]|CNPPA2393-12|Mycetophilidae|661|BOLD:ACC5743
Mycomya|[19983]|CNPPA1317-12|Mycetophilidae|661|BOLD:ABA6853
Mycomya|[19984]|CNEIC2769-12|Mycetophilidae|673|BOLD:AAP9081
Mycetophilidae|[19985]|CNGUA002-14|Mycetophilidae|588|BOLD:ACS1845
Mycetophilidae|[19986]|CNRVB320-15|Mycetophilidae|576|BOLD:ACT5524
Mycetophilidae|[19987]|JSDIQ720-10|Mycetophilidae|658|BOLD:AAU4916
Mycomya sp. CJB2|[19988]|JWDCH487-10|Mycetophilidae|658|BOLD:AAP9087
Mycomya sp. CJB3|[19989]|JWDCG461-10|Mycetophilidae|658|BOLD:AAP9083
Mycetophilidae|[19990]|CNJAD1574-12|Mycetophilidae|658|BOLD:ACB9939
Mycomyinae|[19991]|CNGMB1155-13|Mycetophilidae|658|BOLD:ACL8522
Mycetophilidae|[19992]|CNPAI036-13|Mycetophilidae|658|BOLD:ACC1329
Mycetophilidae|[19993]|SSBRA314-14|Mycetophilidae|664|BOLD:AAM9011
Mycetophilidae|[19994]|CHDIP1221-12|Mycetophilidae|658|BOLD:AAG4890
Mycetophilidae|[19995]|NGAAG409-14|Mycetophilidae|570|BOLD:ACN5132
Mycetophilidae|[19996]|BBDEE373-10|Mycetophilidae|658|BOLD:AAN8589

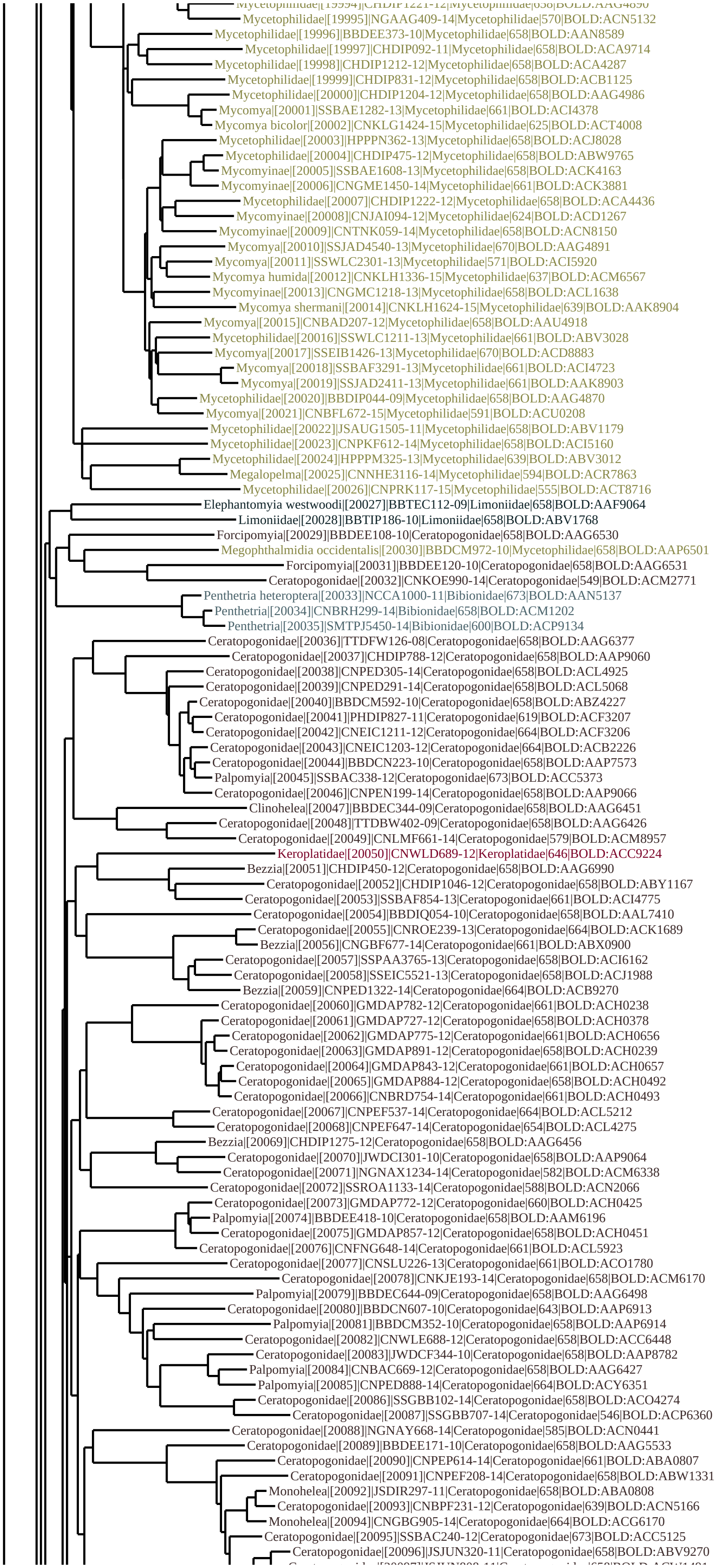

Mycetophilidae|[19994]|CHDIP1221-12|Mycetophilidae|658|BOLD:AAG4890
Mycetophilidae|[19995]|NGAAG409-14|Mycetophilidae|570|BOLD:ACN5132
Mycetophilidae|[19996]|BBDEE373-10|Mycetophilidae|658|BOLD:AAN8589
Mycetophilidae|[19997]|CHDIP092-11|Mycetophilidae|658|BOLD:ACA9714
Mycetophilidae|[19998]|CHDIP1212-12|Mycetophilidae|658|BOLD:ACA4287
Mycetophilidae|[19999]|CHDIP831-12|Mycetophilidae|658|BOLD:ACB1125
Mycetophilidae|[20000]|CHDIP1204-12|Mycetophilidae|658|BOLD:AAG4986
Mycomya|[20001]|SSBAE1282-13|Mycetophilidae|661|BOLD:ACI4378
Mycomya bicolor|[20002]|CNKLG1424-15|Mycetophilidae|625|BOLD:ACT4008
Mycetophilidae|[20003]|HPPPN362-13|Mycetophilidae|658|BOLD:ACJ8028
Mycetophilidae|[20004]|CHDIP475-12|Mycetophilidae|658|BOLD:ABW9765
Mycomyinae|[20005]|SSBAE1608-13|Mycetophilidae|658|BOLD:ACK4163
Mycomyinae|[20006]|CNGME1450-14|Mycetophilidae|661|BOLD:ACK3881
Mycetophilidae|[20007]|CHDIP1222-12|Mycetophilidae|658|BOLD:ACA4436
Mycomyinae|[20008]|CNJAI094-12|Mycetophilidae|624|BOLD:ACD1267
Mycomyinae|[20009]|CNTNK059-14|Mycetophilidae|658|BOLD:ACN8150
Mycomya|[20010]|SSJAD4540-13|Mycetophilidae|670|BOLD:AAG4891
Mycomya|[20011]|SSWLC2301-13|Mycetophilidae|571|BOLD:ACI5920
Mycomya humida|[20012]|CNKLH1336-15|Mycetophilidae|637|BOLD:ACM6567
Mycomyinae|[20013]|CNGMC1218-13|Mycetophilidae|658|BOLD:ACL1638
Mycomya shermani|[20014]|CNKLH1624-15|Mycetophilidae|639|BOLD:AAK8904
Mycomya|[20015]|CNBAD207-12|Mycetophilidae|658|BOLD:AAU4918
Mycetophilidae|[20016]|SSWLC1211-13|Mycetophilidae|661|BOLD:ABV3028
Mycomya|[20017]|SSEIB1426-13|Mycetophilidae|670|BOLD:ACD8883
Mycomya|[20018]|SSBAF3291-13|Mycetophilidae|661|BOLD:ACI4723
Mycomya|[20019]|SSJAD2411-13|Mycetophilidae|661|BOLD:AAK8903
Mycetophilidae|[20020]|BBDIP044-09|Mycetophilidae|658|BOLD:AAG4870
Mycomya|[20021]|CNBFL672-15|Mycetophilidae|591|BOLD:ACU0208
Mycetophilidae|[20022]|JSAUG1505-11|Mycetophilidae|658|BOLD:ABV1179
Mycetophilidae|[20023]|CNPKF612-14|Mycetophilidae|658|BOLD:ACI5160
Mycetophilidae|[20024]|HPPPM325-13|Mycetophilidae|639|BOLD:ABV3012
Megalopelma|[20025]|CNNHE3116-14|Mycetophilidae|594|BOLD:ACR7863
Mycetophilidae|[20026]|CNPRK117-15|Mycetophilidae|555|BOLD:ACT8716
Elephantomyia westwoodi|[20027]|BBTEC112-09|Limoniidae|658|BOLD:AAF9064
Limoniidae|[20028]|BBTIP186-10|Limoniidae|658|BOLD:ABV1768
Forcipomyia|[20029]|BBDEE108-10|Ceratopogonidae|658|BOLD:AAG6530
Megophthalmidia occidentalis|[20030]|BBDCM972-10|Mycetophilidae|658|BOLD:AAP6501
Forcipomyia|[20031]|BBDEE120-10|Ceratopogonidae|658|BOLD:AAG6531
Ceratopogonidae|[20032]|CNKOE990-14|Ceratopogonidae|549|BOLD:ACM2771
Penthetria heteroptera|[20033]|NCCA1000-11|Bibionidae|673|BOLD:AAN5137
Penthetria|[20034]|CNBRH299-14|Bibionidae|658|BOLD:ACM1202
Penthetria|[20035]|SMTPJ5450-14|Bibionidae|600|BOLD:ACP9134
Ceratopogonidae|[20036]|TTDFW126-08|Ceratopogonidae|658|BOLD:AAG6377
Ceratopogonidae|[20037]|CHDIP788-12|Ceratopogonidae|658|BOLD:AAP9060
Ceratopogonidae|[20038]|CNPED305-14|Ceratopogonidae|658|BOLD:ACL4925
Ceratopogonidae|[20039]|CNPED291-14|Ceratopogonidae|658|BOLD:ACL5068
Ceratopogonidae|[20040]|BBDCM592-10|Ceratopogonidae|658|BOLD:ABZ4227
Ceratopogonidae|[20041]|PHDIP827-11|Ceratopogonidae|619|BOLD:ACF3207
Ceratopogonidae|[20042]|CNEIC1211-12|Ceratopogonidae|664|BOLD:ACF3206
Ceratopogonidae|[20043]|CNEIC1203-12|Ceratopogonidae|664|BOLD:ACB2226
Ceratopogonidae|[20044]|BBDCN223-10|Ceratopogonidae|658|BOLD:AAP7573
Palpomyia|[20045]|SSBAC338-12|Ceratopogonidae|673|BOLD:ACC5373
Ceratopogonidae|[20046]|CNPEN199-14|Ceratopogonidae|658|BOLD:AAP9066
Clinohelea|[20047]|BBDEC344-09|Ceratopogonidae|658|BOLD:AAG6451
Ceratopogonidae|[20048]|TTDBW402-09|Ceratopogonidae|658|BOLD:AAG6426
Ceratopogonidae|[20049]|CNLMF661-14|Ceratopogonidae|579|BOLD:ACM8957
Keroplatidae|[20050]|CNWLD689-12|Keroplatidae|646|BOLD:ACC9224
Bezzia|[20051]|CHDIP450-12|Ceratopogonidae|658|BOLD:AAG6990
Ceratopogonidae|[20052]|CHDIP1046-12|Ceratopogonidae|658|BOLD:ABY1167
Ceratopogonidae|[20053]|SSBAF854-13|Ceratopogonidae|661|BOLD:ACI4775
Ceratopogonidae|[20054]|BBDIQ054-10|Ceratopogonidae|658|BOLD:AAL7410
Ceratopogonidae|[20055]|CNROE239-13|Ceratopogonidae|664|BOLD:ACK1689
Bezzia|[20056]|CNGBF677-14|Ceratopogonidae|661|BOLD:ABX0900
Ceratopogonidae|[20057]|SSPAA3765-13|Ceratopogonidae|658|BOLD:ACI6162
Ceratopogonidae|[20058]|SSEIC5521-13|Ceratopogonidae|658|BOLD:ACJ1988
Bezzia|[20059]|CNPED1322-14|Ceratopogonidae|664|BOLD:ACB9270
Ceratopogonidae|[20060]|GMDAP782-12|Ceratopogonidae|661|BOLD:ACH0238
Ceratopogonidae|[20061]|GMDAP727-12|Ceratopogonidae|658|BOLD:ACH0378
Ceratopogonidae|[20062]|GMDAP775-12|Ceratopogonidae|661|BOLD:ACH0656
Ceratopogonidae|[20063]|GMDAP891-12|Ceratopogonidae|658|BOLD:ACH0239
Ceratopogonidae|[20064]|GMDAP843-12|Ceratopogonidae|661|BOLD:ACH0657
Ceratopogonidae|[20065]|GMDAP884-12|Ceratopogonidae|658|BOLD:ACH0492
Ceratopogonidae|[20066]|CNBRD754-14|Ceratopogonidae|661|BOLD:ACH0493
Ceratopogonidae|[20067]|CNPEF537-14|Ceratopogonidae|664|BOLD:ACL5212
Ceratopogonidae|[20068]|CNPEF647-14|Ceratopogonidae|654|BOLD:ACL4275
Bezzia|[20069]|CHDIP1275-12|Ceratopogonidae|658|BOLD:AAG6456
Ceratopogonidae|[20070]|JWDCI301-10|Ceratopogonidae|658|BOLD:AAP9064
Ceratopogonidae|[20071]|NGNAX1234-14|Ceratopogonidae|582|BOLD:ACM6338
Ceratopogonidae|[20072]|SSROA1133-14|Ceratopogonidae|588|BOLD:ACN2066
Ceratopogonidae|[20073]|GMDAP772-12|Ceratopogonidae|660|BOLD:ACH0425
Palpomyia|[20074]|BBDEE418-10|Ceratopogonidae|658|BOLD:AAM6196
Ceratopogonidae|[20075]|GMDAP857-12|Ceratopogonidae|658|BOLD:ACH0451
Ceratopogonidae|[20076]|CNFNG648-14|Ceratopogonidae|661|BOLD:ACL5923
Ceratopogonidae|[20077]|CNSLU226-13|Ceratopogonidae|661|BOLD:ACO1780
Ceratopogonidae|[20078]|CNKJE193-14|Ceratopogonidae|658|BOLD:ACM6170
Palpomyia|[20079]|BBDEC644-09|Ceratopogonidae|658|BOLD:AAG6498
Ceratopogonidae|[20080]|BBDCN607-10|Ceratopogonidae|643|BOLD:AAP6913
Palpomyia|[20081]|BBDCM352-10|Ceratopogonidae|658|BOLD:AAP6914
Ceratopogonidae|[20082]|CNWLE688-12|Ceratopogonidae|658|BOLD:ACC6448
Ceratopogonidae|[20083]|JWDCF344-10|Ceratopogonidae|658|BOLD:AAP8782
Palpomyia|[20084]|CNBAC669-12|Ceratopogonidae|658|BOLD:AAG6427
Palpomyia|[20085]|CNPED888-14|Ceratopogonidae|664|BOLD:ACY6351
Ceratopogonidae|[20086]|SSGBB102-14|Ceratopogonidae|658|BOLD:ACO4274
Ceratopogonidae|[20087]|SSGBB707-14|Ceratopogonidae|546|BOLD:ACP6360
Ceratopogonidae|[20088]|NGNAY668-14|Ceratopogonidae|585|BOLD:ACN0441
Ceratopogonidae|[20089]|BBDEE171-10|Ceratopogonidae|658|BOLD:AAG5533
Ceratopogonidae|[20090]|CNPEP614-14|Ceratopogonidae|661|BOLD:ABA0807
Ceratopogonidae|[20091]|CNPEF208-14|Ceratopogonidae|658|BOLD:ABW1331
Monohelea|[20092]|JSDIR297-11|Ceratopogonidae|658|BOLD:ABA0808
Ceratopogonidae|[20093]|CNBPF231-12|Ceratopogonidae|639|BOLD:ACN5166
Monohelea|[20094]|CNGBG905-14|Ceratopogonidae|664|BOLD:ACG6170
Ceratopogonidae|[20095]|SSBAC240-12|Ceratopogonidae|673|BOLD:ACC5125
Ceratopogonidae|[20096]|JSJUN320-11|Ceratopogonidae|658|BOLD:ABV9270

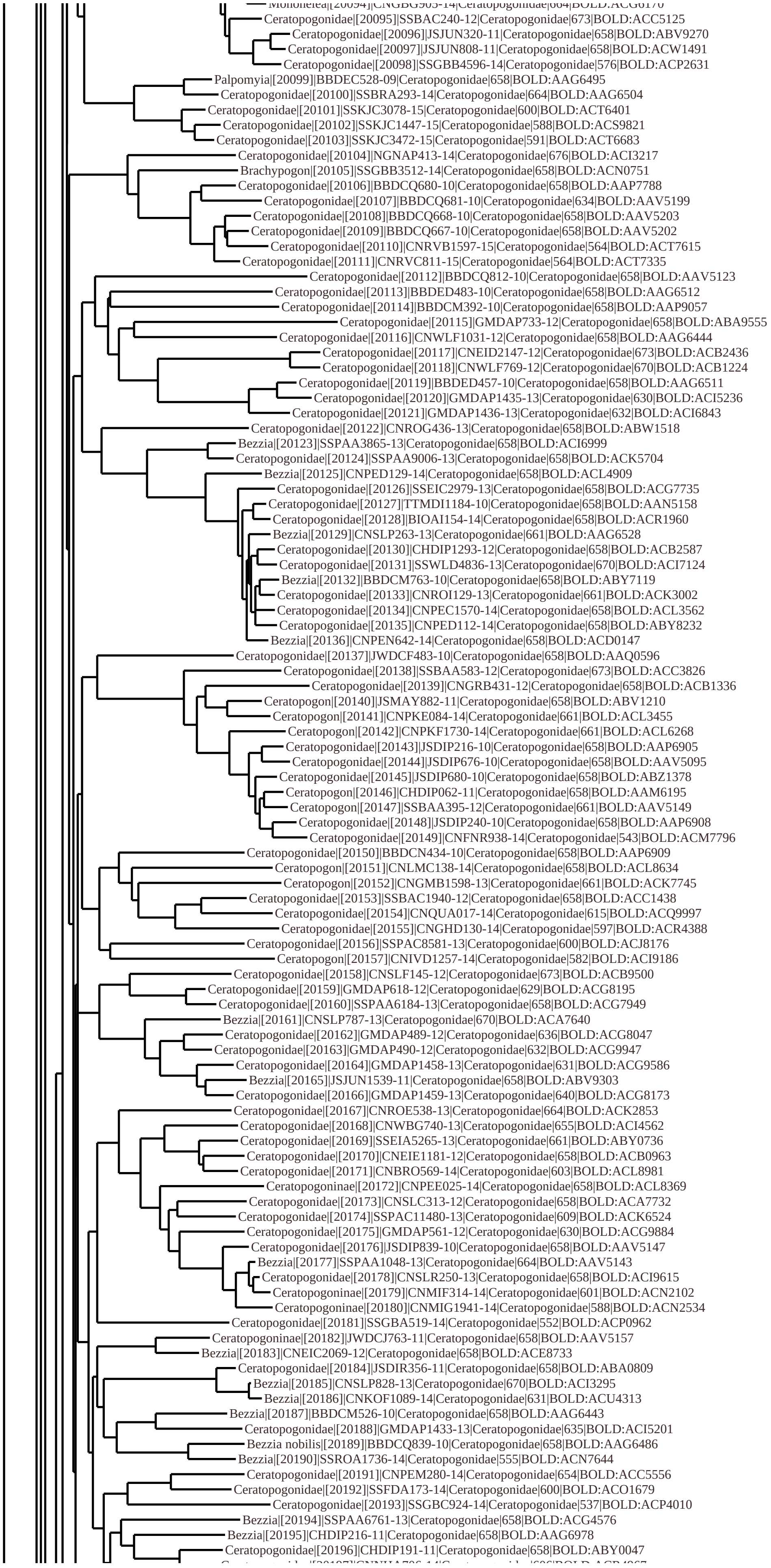

Ceratopogonidae|[20095]|SSBAC240-12|Ceratopogonidae|673|BOLD:ACC5125
Ceratopogonidae|[20096]|JSJUN320-11|Ceratopogonidae|658|BOLD:ABV9270
Ceratopogonidae|[20097]|JSJUN808-11|Ceratopogonidae|658|BOLD:ACW1491
Ceratopogonidae|[20098]|SSGBB4596-14|Ceratopogonidae|576|BOLD:ACP2631
Palpomyia|[20099]|BBDEC528-09|Ceratopogonidae|658|BOLD:AAG6495
Ceratopogonidae|[20100]|SSBRA293-14|Ceratopogonidae|664|BOLD:AAG6504
Ceratopogonidae|[20101]|SSKJC3078-15|Ceratopogonidae|600|BOLD:ACT6401
Ceratopogonidae|[20102]|SSKJC1447-15|Ceratopogonidae|588|BOLD:ACS9821
Ceratopogonidae|[20103]|SSKJC3472-15|Ceratopogonidae|591|BOLD:ACT6683
Ceratopogonidae|[20104]|NGNAP413-14|Ceratopogonidae|676|BOLD:ACI3217
Brachypogon|[20105]|SSGBB3512-14|Ceratopogonidae|658|BOLD:ACN0751
Ceratopogonidae|[20106]|BBDCQ680-10|Ceratopogonidae|658|BOLD:AAP7788
Ceratopogonidae|[20107]|BBDCQ681-10|Ceratopogonidae|634|BOLD:AAV5199
Ceratopogonidae|[20108]|BBDCQ668-10|Ceratopogonidae|658|BOLD:AAV5203
Ceratopogonidae|[20109]|BBDCQ667-10|Ceratopogonidae|658|BOLD:AAV5202
Ceratopogonidae|[20110]|CNRVB1597-15|Ceratopogonidae|564|BOLD:ACT7615
Ceratopogonidae|[20111]|CNRVC811-15|Ceratopogonidae|564|BOLD:ACT7335
Ceratopogonidae|[20112]|BBDCQ812-10|Ceratopogonidae|658|BOLD:AAV5123
Ceratopogonidae|[20113]|BBDED483-10|Ceratopogonidae|658|BOLD:AAG6512
Ceratopogonidae|[20114]|BBDCM392-10|Ceratopogonidae|658|BOLD:AAP9057
Ceratopogonidae|[20115]|GMDAP733-12|Ceratopogonidae|658|BOLD:ABA9555
Ceratopogonidae|[20116]|CNWLF1031-12|Ceratopogonidae|658|BOLD:AAG6444
Ceratopogonidae|[20117]|CNEID2147-12|Ceratopogonidae|673|BOLD:ACB2436
Ceratopogonidae|[20118]|CNWLF769-12|Ceratopogonidae|670|BOLD:ACB1224
Ceratopogonidae|[20119]|BBDED457-10|Ceratopogonidae|658|BOLD:AAG6511
Ceratopogonidae|[20120]|GMDAP1435-13|Ceratopogonidae|630|BOLD:ACI5236
Ceratopogonidae|[20121]|GMDAP1436-13|Ceratopogonidae|632|BOLD:ACI6843
Ceratopogonidae|[20122]|CNROG436-13|Ceratopogonidae|658|BOLD:ABW1518
Bezzia|[20123]|SSPAA3865-13|Ceratopogonidae|658|BOLD:ACI6999
Ceratopogonidae|[20124]|SSPAA9006-13|Ceratopogonidae|658|BOLD:ACK5704
Bezzia|[20125]|CNPED129-14|Ceratopogonidae|658|BOLD:ACL4909
Ceratopogonidae|[20126]|SSEIC2979-13|Ceratopogonidae|658|BOLD:ACG7735
Ceratopogonidae|[20127]|TTMDI1184-10|Ceratopogonidae|658|BOLD:AAN5158
Ceratopogonidae|[20128]|BIOAI154-14|Ceratopogonidae|658|BOLD:ACR1960
Bezzia|[20129]|CNSLP263-13|Ceratopogonidae|661|BOLD:AAG6528
Ceratopogonidae|[20130]|CHDIP1293-12|Ceratopogonidae|658|BOLD:ACB2587
Ceratopogonidae|[20131]|SSWLD4836-13|Ceratopogonidae|670|BOLD:ACI7124
Bezzia|[20132]|BBDCM763-10|Ceratopogonidae|658|BOLD:ABY7119
Ceratopogonidae|[20133]|CNROI129-13|Ceratopogonidae|661|BOLD:ACK3002
Ceratopogonidae|[20134]|CNPEC1570-14|Ceratopogonidae|658|BOLD:ACL3562
Ceratopogonidae|[20135]|CNPED112-14|Ceratopogonidae|658|BOLD:ABY8232
Bezzia|[20136]|CNPEN642-14|Ceratopogonidae|658|BOLD:ACD0147
Ceratopogonidae|[20137]|JWDCF483-10|Ceratopogonidae|658|BOLD:AAQ0596
Ceratopogonidae|[20138]|SSBAA583-12|Ceratopogonidae|673|BOLD:ACC3826
Ceratopogonidae|[20139]|CNGRB431-12|Ceratopogonidae|658|BOLD:ACB1336
Ceratopogon|[20140]|JSMAY882-11|Ceratopogonidae|658|BOLD:ABV1210
Ceratopogon|[20141]|CNPKE084-14|Ceratopogonidae|661|BOLD:ACL3455
Ceratopogon|[20142]|CNPKF1730-14|Ceratopogonidae|661|BOLD:ACL6268
Ceratopogonidae|[20143]|JSDIP216-10|Ceratopogonidae|658|BOLD:AAP6905
Ceratopogonidae|[20144]|JSDIP676-10|Ceratopogonidae|658|BOLD:AAV5095
Ceratopogonidae|[20145]|JSDIP680-10|Ceratopogonidae|658|BOLD:ABZ1378
Ceratopogon|[20146]|CHDIP062-11|Ceratopogonidae|658|BOLD:AAM6195
Ceratopogon|[20147]|SSBAA395-12|Ceratopogonidae|661|BOLD:AAV5149
Ceratopogonidae|[20148]|JSDIP240-10|Ceratopogonidae|658|BOLD:AAP6908
Ceratopogonidae|[20149]|CNFNR938-14|Ceratopogonidae|543|BOLD:ACM7796
Ceratopogonidae|[20150]|BBDCN434-10|Ceratopogonidae|658|BOLD:AAP6909
Ceratopogon|[20151]|CNLMC138-14|Ceratopogonidae|658|BOLD:ACL8634
Ceratopogon|[20152]|CNGMB1598-13|Ceratopogonidae|661|BOLD:ACK7745
Ceratopogonidae|[20153]|SSBAC1940-12|Ceratopogonidae|658|BOLD:ACC1438
Ceratopogonidae|[20154]|CNQUA017-14|Ceratopogonidae|615|BOLD:ACQ9997
Ceratopogonidae|[20155]|CNGHD130-14|Ceratopogonidae|597|BOLD:ACR4388
Ceratopogonidae|[20156]|SSPAC8581-13|Ceratopogonidae|600|BOLD:ACJ8176
Ceratopogon|[20157]|CNIVD1257-14|Ceratopogonidae|582|BOLD:ACI9186
Ceratopogonidae|[20158]|CNSLF145-12|Ceratopogonidae|673|BOLD:ACB9500
Ceratopogonidae|[20159]|GMDAP618-12|Ceratopogonidae|629|BOLD:ACG8195
Ceratopogonidae|[20160]|SSPAA6184-13|Ceratopogonidae|658|BOLD:ACG7949
Bezzia|[20161]|CNSLP787-13|Ceratopogonidae|670|BOLD:ACA7640
Ceratopogonidae|[20162]|GMDAP489-12|Ceratopogonidae|636|BOLD:ACG8047
Ceratopogonidae|[20163]|GMDAP490-12|Ceratopogonidae|632|BOLD:ACG9947
Ceratopogonidae|[20164]|GMDAP1458-13|Ceratopogonidae|631|BOLD:ACG9586
Bezzia|[20165]|JSJUN1539-11|Ceratopogonidae|658|BOLD:ABV9303
Ceratopogonidae|[20166]|GMDAP1459-13|Ceratopogonidae|640|BOLD:ACG8173
Ceratopogonidae|[20167]|CNROE538-13|Ceratopogonidae|664|BOLD:ACK2853
Ceratopogonidae|[20168]|CNWBG740-13|Ceratopogonidae|655|BOLD:ACI4562
Ceratopogonidae|[20169]|SSEIA5265-13|Ceratopogonidae|661|BOLD:ABY0736
Ceratopogonidae|[20170]|CNEIE1181-12|Ceratopogonidae|658|BOLD:ACB0963
Ceratopogonidae|[20171]|CNBRO569-14|Ceratopogonidae|603|BOLD:ACL8981
Ceratopogoninae|[20172]|CNPEE025-14|Ceratopogonidae|658|BOLD:ACL8369
Ceratopogonidae|[20173]|CNSLC313-12|Ceratopogonidae|658|BOLD:ACA7732
Ceratopogonidae|[20174]|SSPAC11480-13|Ceratopogonidae|609|BOLD:ACK6524
Ceratopogonidae|[20175]|GMDAP561-12|Ceratopogonidae|630|BOLD:ACG9884
Ceratopogonidae|[20176]|JSDIP839-10|Ceratopogonidae|658|BOLD:AAV5147
Bezzia|[20177]|SSPAA1048-13|Ceratopogonidae|664|BOLD:AAV5143
Ceratopogonidae|[20178]|CNSLR250-13|Ceratopogonidae|658|BOLD:ACI9615
Ceratopogoninae|[20179]|CNMIF314-14|Ceratopogonidae|601|BOLD:ACN2102
Ceratopogoninae|[20180]|CNMIG1941-14|Ceratopogonidae|588|BOLD:ACN2534
Ceratopogonidae|[20181]|SSGBA519-14|Ceratopogonidae|552|BOLD:ACP0962
Ceratopogoninae|[20182]|JWDCJ763-11|Ceratopogonidae|658|BOLD:AAV5157
Bezzia|[20183]|CNEIC2069-12|Ceratopogonidae|658|BOLD:ACE8733
Ceratopogonidae|[20184]|JSDIR356-11|Ceratopogonidae|658|BOLD:ABA0809
Bezzia|[20185]|CNSLP828-13|Ceratopogonidae|670|BOLD:ACI3295
Bezzia|[20186]|CNKOF1089-14|Ceratopogonidae|631|BOLD:ACU4313
Bezzia|[20187]|BBDCM526-10|Ceratopogonidae|658|BOLD:AAG6443
Ceratopogonidae|[20188]|GMDAP1433-13|Ceratopogonidae|635|BOLD:ACI5201
Bezzia nobilis|[20189]|BBDCQ839-10|Ceratopogonidae|658|BOLD:AAG6486
Bezzia|[20190]|SSROA1736-14|Ceratopogonidae|555|BOLD:ACN7644
Ceratopogonidae|[20191]|CNPEM280-14|Ceratopogonidae|654|BOLD:ACC5556
Ceratopogonidae|[20192]|SSFDA173-14|Ceratopogonidae|600|BOLD:ACO1679
Ceratopogonidae|[20193]|SSGBC924-14|Ceratopogonidae|537|BOLD:ACP4010
Bezzia|[20194]|SSPAA6761-13|Ceratopogonidae|658|BOLD:ACG4576
Bezzia|[20195]|CHDIP216-11|Ceratopogonidae|658|BOLD:AAG6978
Ceratopogonidae|[20196]|CHDIP191-11|Ceratopogonidae|658|BOLD:ABY0047

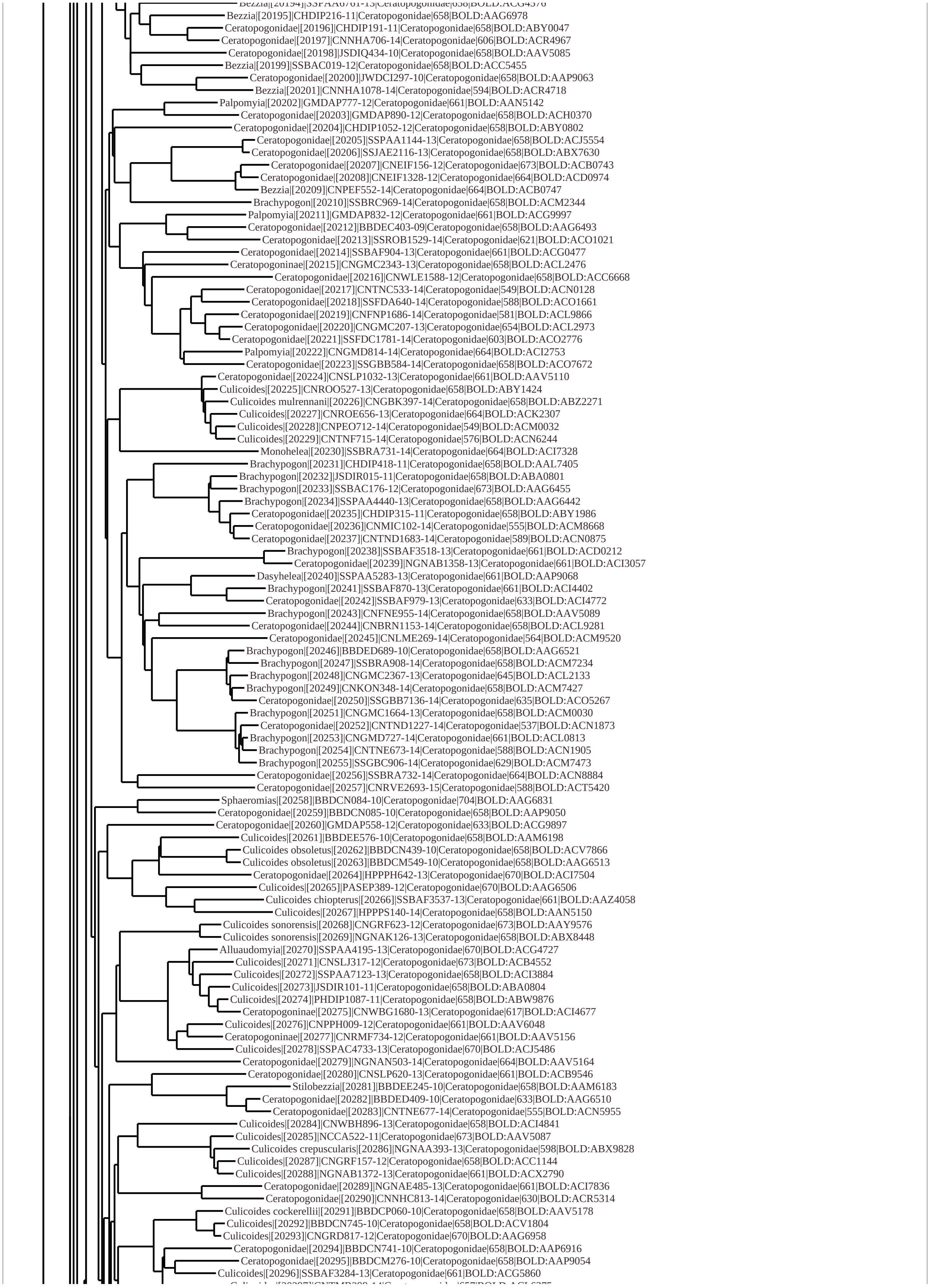

Bezzia|[20195]|CHDIP216-11|Ceratopogonidae|658|BOLD:AAG6978
Ceratopogonidae|[20196]|CHDIP191-11|Ceratopogonidae|658|BOLD:ABY0047
Ceratopogonidae|[20197]|CNNHA706-14|Ceratopogonidae|606|BOLD:ACR4967
Ceratopogonidae|[20198]|JSDIQ434-10|Ceratopogonidae|658|BOLD:AAV5085
Bezzia|[20199]|SSBAC019-12|Ceratopogonidae|658|BOLD:ACC5455
Ceratopogonidae|[20200]|JWDCI297-10|Ceratopogonidae|658|BOLD:AAP9063
Bezzia|[20201]|CNNHA1078-14|Ceratopogonidae|594|BOLD:ACR4718
Palpomyia|[20202]|GMDAP777-12|Ceratopogonidae|661|BOLD:AAN5142
Ceratopogonidae|[20203]|GMDAP890-12|Ceratopogonidae|658|BOLD:ACH0370
Ceratopogonidae|[20204]|CHDIP1052-12|Ceratopogonidae|658|BOLD:ABY0802
Ceratopogonidae|[20205]|SSPAA1144-13|Ceratopogonidae|658|BOLD:ACJ5554
Ceratopogonidae|[20206]|SSJAE2116-13|Ceratopogonidae|658|BOLD:ABX7630
Ceratopogonidae|[20207]|CNEIF156-12|Ceratopogonidae|673|BOLD:ACB0743
Ceratopogonidae|[20208]|CNEIF1328-12|Ceratopogonidae|664|BOLD:ACD0974
Bezzia|[20209]|CNPEF552-14|Ceratopogonidae|664|BOLD:ACB0747
Brachypogon|[20210]|SSBRC969-14|Ceratopogonidae|658|BOLD:ACM2344
Palpomyia|[20211]|GMDAP832-12|Ceratopogonidae|661|BOLD:ACG9997
Ceratopogonidae|[20212]|BBDEC403-09|Ceratopogonidae|658|BOLD:AAG6493
Ceratopogonidae|[20213]|SSROB1529-14|Ceratopogonidae|621|BOLD:ACO1021
Ceratopogonidae|[20214]|SSBAF904-13|Ceratopogonidae|661|BOLD:ACG0477
Ceratopogoninae|[20215]|CNGMC2343-13|Ceratopogonidae|658|BOLD:ACL2476
Ceratopogonidae|[20216]|CNWLE1588-12|Ceratopogonidae|658|BOLD:ACC6668
Ceratopogonidae|[20217]|CNTNC533-14|Ceratopogonidae|549|BOLD:ACN0128
Ceratopogonidae|[20218]|SSFDA640-14|Ceratopogonidae|588|BOLD:ACO1661
Ceratopogonidae|[20219]|CNFNP1686-14|Ceratopogonidae|581|BOLD:ACL9866
Ceratopogonidae|[20220]|CNGMC207-13|Ceratopogonidae|654|BOLD:ACL2973
Ceratopogonidae|[20221]|SSFDC1781-14|Ceratopogonidae|603|BOLD:ACO2776
Palpomyia|[20222]|CNGMD814-14|Ceratopogonidae|664|BOLD:ACI2753
Ceratopogonidae|[20223]|SSGBB584-14|Ceratopogonidae|658|BOLD:ACO7672
Ceratopogonidae|[20224]|CNSLP1032-13|Ceratopogonidae|661|BOLD:AAV5110
Culicoides|[20225]|CNROO527-13|Ceratopogonidae|658|BOLD:ABY1424
Culicoides mulrennani|[20226]|CNGBK397-14|Ceratopogonidae|658|BOLD:ABZ2271
Culicoides|[20227]|CNROE656-13|Ceratopogonidae|664|BOLD:ACK2307
Culicoides|[20228]|CNPEO712-14|Ceratopogonidae|549|BOLD:ACM0032
Culicoides|[20229]|CNTNF715-14|Ceratopogonidae|576|BOLD:ACN6244
Monohelea|[20230]|SSBRA731-14|Ceratopogonidae|664|BOLD:ACI7328
Brachypogon|[20231]|CHDIP418-11|Ceratopogonidae|658|BOLD:AAL7405
Brachypogon|[20232]|JSDIR015-11|Ceratopogonidae|658|BOLD:ABA0801
Brachypogon|[20233]|SSBAC176-12|Ceratopogonidae|673|BOLD:AAG6455
Brachypogon|[20234]|SSPAA4440-13|Ceratopogonidae|658|BOLD:AAG6442
Ceratopogonidae|[20235]|CHDIP315-11|Ceratopogonidae|658|BOLD:ABY1986
Ceratopogonidae|[20236]|CNMIC102-14|Ceratopogonidae|555|BOLD:ACM8668
Ceratopogonidae|[20237]|CNTND1683-14|Ceratopogonidae|589|BOLD:ACN0875
Brachypogon|[20238]|SSBAF3518-13|Ceratopogonidae|661|BOLD:ACD0212
Ceratopogonidae|[20239]|NGNAB1358-13|Ceratopogonidae|661|BOLD:ACI3057
Dasyhelea|[20240]|SSPAA5283-13|Ceratopogonidae|661|BOLD:AAP9068
Brachypogon|[20241]|SSBAF870-13|Ceratopogonidae|661|BOLD:ACI4402
Ceratopogonidae|[20242]|SSBAF979-13|Ceratopogonidae|633|BOLD:ACI4772
Brachypogon|[20243]|CNFNE955-14|Ceratopogonidae|658|BOLD:AAV5089
Ceratopogonidae|[20244]|CNBRN1153-14|Ceratopogonidae|658|BOLD:ACL9281
Ceratopogonidae|[20245]|CNLME269-14|Ceratopogonidae|564|BOLD:ACM9520
Brachypogon|[20246]|BBDED689-10|Ceratopogonidae|658|BOLD:AAG6521
Brachypogon|[20247]|SSBRA908-14|Ceratopogonidae|658|BOLD:ACM7234
Brachypogon|[20248]|CNGMC2367-13|Ceratopogonidae|645|BOLD:ACL2133
Brachypogon|[20249]|CNKON348-14|Ceratopogonidae|658|BOLD:ACM7427
Ceratopogonidae|[20250]|SSGBB7136-14|Ceratopogonidae|635|BOLD:ACO5267
Brachypogon|[20251]|CNGMC1664-13|Ceratopogonidae|658|BOLD:ACM0030
Ceratopogonidae|[20252]|CNTND1227-14|Ceratopogonidae|537|BOLD:ACN1873
Brachypogon|[20253]|CNGMD727-14|Ceratopogonidae|661|BOLD:ACL0813
Brachypogon|[20254]|CNTNE673-14|Ceratopogonidae|588|BOLD:ACN1905
Brachypogon|[20255]|SSGBC906-14|Ceratopogonidae|629|BOLD:ACM7473
Ceratopogonidae|[20256]|SSBRA732-14|Ceratopogonidae|664|BOLD:ACN8884
Ceratopogonidae|[20257]|CNRVE2693-15|Ceratopogonidae|588|BOLD:ACT5420
Sphaeromias|[20258]|BBDCN084-10|Ceratopogonidae|704|BOLD:AAG6831
Ceratopogonidae|[20259]|BBDCN085-10|Ceratopogonidae|658|BOLD:AAP9050
Ceratopogonidae|[20260]|GMDAP558-12|Ceratopogonidae|633|BOLD:ACG9897
Culicoides|[20261]|BBDEE576-10|Ceratopogonidae|658|BOLD:AAM6198
Culicoides obsoletus|[20262]|BBDCN439-10|Ceratopogonidae|658|BOLD:ACV7866
Culicoides obsoletus|[20263]|BBDCM549-10|Ceratopogonidae|658|BOLD:AAG6513
Ceratopogonidae|[20264]|HPPPH642-13|Ceratopogonidae|670|BOLD:ACI7504
Culicoides|[20265]|PASEP389-12|Ceratopogonidae|670|BOLD:AAG6506
Culicoides chiopterus|[20266]|SSBAF3537-13|Ceratopogonidae|661|BOLD:AAZ4058
Culicoides|[20267]|HPPPS140-14|Ceratopogonidae|658|BOLD:AAN5150
Culicoides sonorensis|[20268]|CNGRF623-12|Ceratopogonidae|673|BOLD:AAY9576
Culicoides sonorensis|[20269]|NGNAK126-13|Ceratopogonidae|658|BOLD:ABX8448
Alluaudomyia|[20270]|SSPAA4195-13|Ceratopogonidae|670|BOLD:ACG4727
Culicoides|[20271]|CNSLJ317-12|Ceratopogonidae|673|BOLD:ACB4552
Culicoides|[20272]|SSPAA7123-13|Ceratopogonidae|658|BOLD:ACI3884
Culicoides|[20273]|JSDIR101-11|Ceratopogonidae|658|BOLD:ABA0804
Culicoides|[20274]|PHDIP1087-11|Ceratopogonidae|658|BOLD:ABW9876
Ceratopogoninae|[20275]|CNWBG1680-13|Ceratopogonidae|617|BOLD:ACI4677
Culicoides|[20276]|CNPPH009-12|Ceratopogonidae|661|BOLD:AAV6048
Ceratopogoninae|[20277]|CNRMF734-12|Ceratopogonidae|661|BOLD:AAV5156
Culicoides|[20278]|SSPAC4733-13|Ceratopogonidae|670|BOLD:ACJ5486
Ceratopogonidae|[20279]|NGNAN503-14|Ceratopogonidae|664|BOLD:AAV5164
Ceratopogonidae|[20280]|CNSLP620-13|Ceratopogonidae|661|BOLD:ACB9546
Stilobezzia|[20281]|BBDEE245-10|Ceratopogonidae|658|BOLD:AAM6183
Ceratopogonidae|[20282]|BBDED409-10|Ceratopogonidae|633|BOLD:AAG6510
Ceratopogonidae|[20283]|CNTNE677-14|Ceratopogonidae|555|BOLD:ACN5955
Culicoides|[20284]|CNWBH896-13|Ceratopogonidae|658|BOLD:ACI4841
Culicoides|[20285]|NCCA522-11|Ceratopogonidae|673|BOLD:AAV5087
Culicoides crepuscularis|[20286]|NGNAA393-13|Ceratopogonidae|598|BOLD:ABX9828
Culicoides|[20287]|CNGRF157-12|Ceratopogonidae|658|BOLD:ACC1144
Culicoides|[20288]|NGNAB1372-13|Ceratopogonidae|661|BOLD:ACX2790
Ceratopogonidae|[20289]|NGNAE485-13|Ceratopogonidae|661|BOLD:ACI7836
Ceratopogonidae|[20290]|CNNHC813-14|Ceratopogonidae|630|BOLD:ACR5314
Culicoides cockerellii|[20291]|BBDCP060-10|Ceratopogonidae|658|BOLD:AAV5178
Culicoides|[20292]|BBDCN745-10|Ceratopogonidae|658|BOLD:ACV1804
Culicoides|[20293]|CNGRD817-12|Ceratopogonidae|670|BOLD:AAG6958
Ceratopogonidae|[20294]|BBDCN741-10|Ceratopogonidae|658|BOLD:AAP6916
Ceratopogonidae|[20295]|BBDCM276-10|Ceratopogonidae|658|BOLD:AAP9054
Culicoides|[20296]|SSBAF3284-13|Ceratopogonidae|661|BOLD:ACG5860

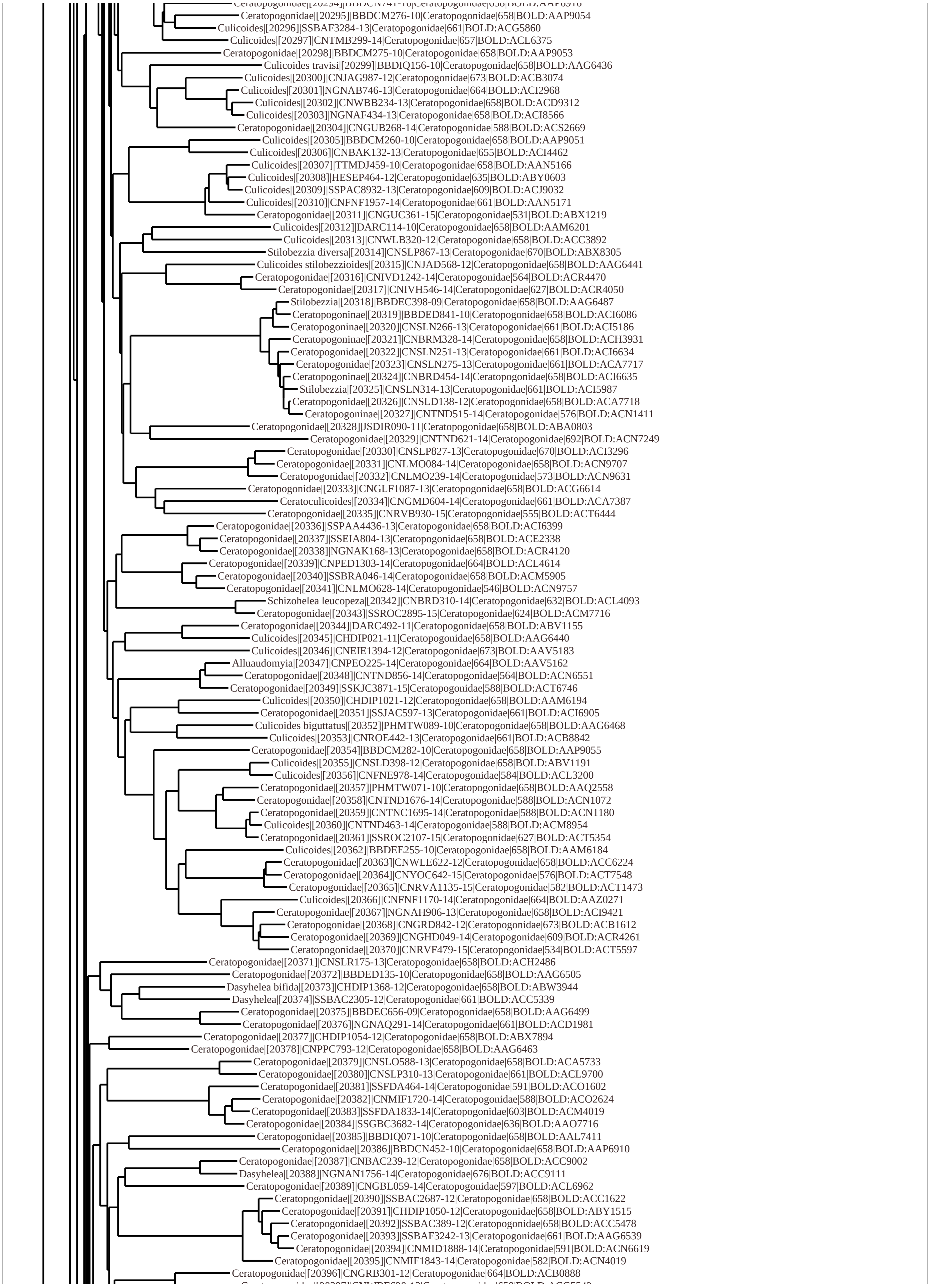
Ceratopogonidae|[20294]|BBDCN741-10|Ceratopogonidae|658|BOLD:AAP6916
Ceratopogonidae|[20295]|BBDCM276-10|Ceratopogonidae|658|BOLD:AAP9054
Culicoides|[20296]|SSBAF3284-13|Ceratopogonidae|661|BOLD:ACG5860
Culicoides|[20297]|CNTMB299-14|Ceratopogonidae|657|BOLD:ACL6375
Ceratopogonidae|[20298]|BBDCM275-10|Ceratopogonidae|658|BOLD:AAP9053
Culicoides travisi|[20299]|BBDIQ156-10|Ceratopogonidae|658|BOLD:AAG6436
Culicoides|[20300]|CNJAG987-12|Ceratopogonidae|673|BOLD:ACB3074
Culicoides|[20301]|NGNAB746-13|Ceratopogonidae|664|BOLD:ACI2968
Culicoides|[20302]|CNWBB234-13|Ceratopogonidae|658|BOLD:ACD9312
Culicoides|[20303]|NGNAF434-13|Ceratopogonidae|658|BOLD:ACI8566
Ceratopogonidae|[20304]|CNGUB268-14|Ceratopogonidae|588|BOLD:ACS2669
Culicoides|[20305]|BBDCM260-10|Ceratopogonidae|658|BOLD:AAP9051
Culicoides|[20306]|CNBAK132-13|Ceratopogonidae|655|BOLD:ACI4462
Culicoides|[20307]|TTMDJ459-10|Ceratopogonidae|658|BOLD:AAN5166
Culicoides|[20308]|HESEP464-12|Ceratopogonidae|635|BOLD:ABY0603
Culicoides|[20309]|SSPAC8932-13|Ceratopogonidae|609|BOLD:ACJ9032
Culicoides|[20310]|CNFNF1957-14|Ceratopogonidae|661|BOLD:AAN5171
Ceratopogonidae|[20311]|CNGUC361-15|Ceratopogonidae|531|BOLD:ABX1219
Culicoides|[20312]|DARC114-10|Ceratopogonidae|658|BOLD:AAM6201
Culicoides|[20313]|CNWLB320-12|Ceratopogonidae|658|BOLD:ACC3892
Stilobezzia diversa|[20314]|CNSLP867-13|Ceratopogonidae|670|BOLD:ABX8305
Culicoides stilobezzioides|[20315]|CNJAD568-12|Ceratopogonidae|658|BOLD:AAG6441
Ceratopogonidae|[20316]|CNIVD1242-14|Ceratopogonidae|564|BOLD:ACR4470
Ceratopogonidae|[20317]|CNIVH546-14|Ceratopogonidae|627|BOLD:ACR4050
Stilobezzia|[20318]|BBDEC398-09|Ceratopogonidae|658|BOLD:AAG6487
Ceratopogoninae|[20319]|BBDED841-10|Ceratopogonidae|658|BOLD:ACI6086
Ceratopogoninae|[20320]|CNSLN266-13|Ceratopogonidae|661|BOLD:ACI5186
Ceratopogoninae|[20321]|CNBRM328-14|Ceratopogonidae|658|BOLD:ACH3931
Ceratopogonidae|[20322]|CNSLN251-13|Ceratopogonidae|661|BOLD:ACI6634
Ceratopogonidae|[20323]|CNSLN275-13|Ceratopogonidae|661|BOLD:ACA7717
Ceratopogoninae|[20324]|CNBRD454-14|Ceratopogonidae|658|BOLD:ACI6635
Stilobezzia|[20325]|CNSLN314-13|Ceratopogonidae|661|BOLD:ACI5987
Ceratopogonidae|[20326]|CNSLD138-12|Ceratopogonidae|658|BOLD:ACA7718
Ceratopogoninae|[20327]|CNTND515-14|Ceratopogonidae|576|BOLD:ACN1411
Ceratopogonidae|[20328]|JSDIR090-11|Ceratopogonidae|658|BOLD:ABA0803
Ceratopogonidae|[20329]|CNTND621-14|Ceratopogonidae|692|BOLD:ACN7249
Ceratopogonidae|[20330]|CNSLP827-13|Ceratopogonidae|670|BOLD:ACI3296
Ceratopogonidae|[20331]|CNLMO084-14|Ceratopogonidae|658|BOLD:ACN9707
Ceratopogonidae|[20332]|CNLMO239-14|Ceratopogonidae|573|BOLD:ACN9631
Ceratopogonidae|[20333]|CNGLF1087-13|Ceratopogonidae|658|BOLD:ACG6614
Ceratoculicoides|[20334]|CNGMD604-14|Ceratopogonidae|661|BOLD:ACA7387
Ceratopogonidae|[20335]|CNRVB930-15|Ceratopogonidae|555|BOLD:ACT6444
Ceratopogonidae|[20336]|SSPAA4436-13|Ceratopogonidae|658|BOLD:ACI6399
Ceratopogonidae|[20337]|SSEIA804-13|Ceratopogonidae|658|BOLD:ACE2338
Ceratopogonidae|[20338]|NGNAK168-13|Ceratopogonidae|658|BOLD:ACR4120
Ceratopogonidae|[20339]|CNPED1303-14|Ceratopogonidae|664|BOLD:ACL4614
Ceratopogonidae|[20340]|SSBRA046-14|Ceratopogonidae|658|BOLD:ACM5905
Ceratopogonidae|[20341]|CNLMO628-14|Ceratopogonidae|546|BOLD:ACN9757
Schizohelea leucopeza|[20342]|CNBRD310-14|Ceratopogonidae|632|BOLD:ACL4093
Ceratopogonidae|[20343]|SSROC2895-15|Ceratopogonidae|624|BOLD:ACM7716
Ceratopogonidae|[20344]|DARC492-11|Ceratopogonidae|658|BOLD:ABV1155
Culicoides|[20345]|CHDIP021-11|Ceratopogonidae|658|BOLD:AAG6440
Culicoides|[20346]|CNEIE1394-12|Ceratopogonidae|673|BOLD:AAV5183
Alluaudomyia|[20347]|CNPEO225-14|Ceratopogonidae|664|BOLD:AAV5162
Ceratopogonidae|[20348]|CNTND856-14|Ceratopogonidae|564|BOLD:ACN6551
Ceratopogonidae|[20349]|SSKJC3871-15|Ceratopogonidae|588|BOLD:ACT6746
Culicoides|[20350]|CHDIP1021-12|Ceratopogonidae|658|BOLD:AAM6194
Ceratopogonidae|[20351]|SSJAC597-13|Ceratopogonidae|661|BOLD:ACI6905
Culicoides biguttatus|[20352]|PHMTW089-10|Ceratopogonidae|658|BOLD:AAG6468
Culicoides|[20353]|CNROE442-13|Ceratopogonidae|661|BOLD:ACB8842
Ceratopogonidae|[20354]|BBDCM282-10|Ceratopogonidae|658|BOLD:AAP9055
Culicoides|[20355]|CNSLD398-12|Ceratopogonidae|658|BOLD:ABV1191
Culicoides|[20356]|CNFNE978-14|Ceratopogonidae|584|BOLD:ACL3200
Ceratopogonidae|[20357]|PHMTW071-10|Ceratopogonidae|658|BOLD:AAQ2558
Ceratopogonidae|[20358]|CNTND1676-14|Ceratopogonidae|588|BOLD:ACN1072
Ceratopogonidae|[20359]|CNTNC1695-14|Ceratopogonidae|588|BOLD:ACN1180
Culicoides|[20360]|CNTND463-14|Ceratopogonidae|588|BOLD:ACM8954
Ceratopogonidae|[20361]|SSROC2107-15|Ceratopogonidae|627|BOLD:ACT5354
Culicoides|[20362]|BBDEE255-10|Ceratopogonidae|658|BOLD:AAM6184
Ceratopogonidae|[20363]|CNWLE622-12|Ceratopogonidae|658|BOLD:ACC6224
Ceratopogonidae|[20364]|CNYOC642-15|Ceratopogonidae|576|BOLD:ACT7548
Ceratopogonidae|[20365]|CNRVA1135-15|Ceratopogonidae|582|BOLD:ACT1473
Culicoides|[20366]|CNFNF1170-14|Ceratopogonidae|664|BOLD:AAZ0271
Ceratopogonidae|[20367]|NGNAH906-13|Ceratopogonidae|658|BOLD:ACI9421
Ceratopogonidae|[20368]|CNGRD842-12|Ceratopogonidae|673|BOLD:ACB1612
Ceratopogonidae|[20369]|CNGHD049-14|Ceratopogonidae|609|BOLD:ACR4261
Ceratopogonidae|[20370]|CNRVF479-15|Ceratopogonidae|534|BOLD:ACT5597
Ceratopogonidae|[20371]|CNSLR175-13|Ceratopogonidae|658|BOLD:ACH2486
Ceratopogonidae|[20372]|BBDED135-10|Ceratopogonidae|658|BOLD:AAG6505
Dasyhelea bifida|[20373]|CHDIP1368-12|Ceratopogonidae|658|BOLD:ABW3944
Dasyhelea|[20374]|SSBAC2305-12|Ceratopogonidae|661|BOLD:ACC5339
Ceratopogonidae|[20375]|BBDEC656-09|Ceratopogonidae|658|BOLD:AAG6499
Ceratopogonidae|[20376]|NGNAQ291-14|Ceratopogonidae|661|BOLD:ACD1981
Ceratopogonidae|[20377]|CHDIP1054-12|Ceratopogonidae|658|BOLD:ABX7894
Ceratopogonidae|[20378]|CNPPC793-12|Ceratopogonidae|658|BOLD:AAG6463
Ceratopogonidae|[20379]|CNSLO588-13|Ceratopogonidae|658|BOLD:ACA5733
Ceratopogonidae|[20380]|CNSLP310-13|Ceratopogonidae|661|BOLD:ACL9700
Ceratopogonidae|[20381]|SSFDA464-14|Ceratopogonidae|591|BOLD:ACO1602
Ceratopogonidae|[20382]|CNMIF1720-14|Ceratopogonidae|588|BOLD:ACO2624
Ceratopogonidae|[20383]|SSFDA1833-14|Ceratopogonidae|603|BOLD:ACM4019
Ceratopogonidae|[20384]|SSGBC3682-14|Ceratopogonidae|636|BOLD:AAO7716
Ceratopogonidae|[20385]|BBDIQ071-10|Ceratopogonidae|658|BOLD:AAL7411
Ceratopogonidae|[20386]|BBDCN452-10|Ceratopogonidae|658|BOLD:AAP6910
Ceratopogonidae|[20387]|CNBAC239-12|Ceratopogonidae|658|BOLD:ACC9002
Dasyhelea|[20388]|NGNAN1756-14|Ceratopogonidae|676|BOLD:ACC9111
Ceratopogonidae|[20389]|CNGBL059-14|Ceratopogonidae|597|BOLD:ACL6962
Ceratopogonidae|[20390]|SSBAC2687-12|Ceratopogonidae|658|BOLD:ACC1622
Ceratopogonidae|[20391]|CHDIP1050-12|Ceratopogonidae|658|BOLD:ABY1515
Ceratopogonidae|[20392]|SSBAC389-12|Ceratopogonidae|658|BOLD:ACC5478
Ceratopogonidae|[20393]|SSBAF3242-13|Ceratopogonidae|661|BOLD:AAG6539
Ceratopogonidae|[20394]|CNMID1888-14|Ceratopogonidae|591|BOLD:ACN6619
Ceratopogonidae|[20395]|CNMIF1843-14|Ceratopogonidae|582|BOLD:ACN4019
Ceratopogonidae|[20396]|CNGRB301-12|Ceratopogonidae|664|BOLD:ACB0888

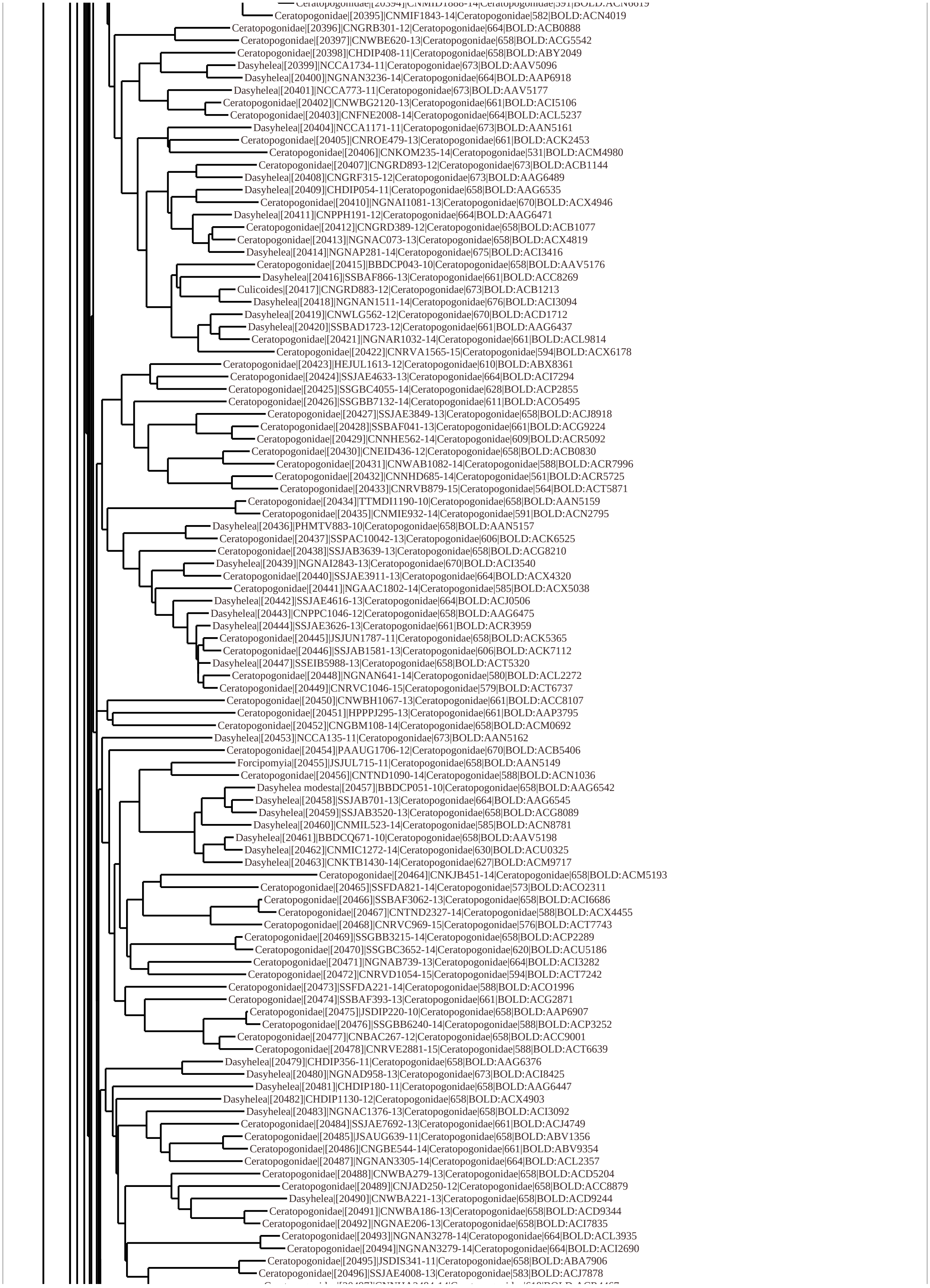

Ceratopogonidae|[20395]|CNMIF1843-14|Ceratopogonidae|582|BOLD:ACN4019
Ceratopogonidae|[20396]|CNGRB301-12|Ceratopogonidae|664|BOLD:ACB0888
Ceratopogonidae|[20397]|CNWBE620-13|Ceratopogonidae|658|BOLD:ACG5542
Ceratopogonidae|[20398]|CHDIP408-11|Ceratopogonidae|658|BOLD:ABY2049
Dasyhelea|[20399]|NCCA1734-11|Ceratopogonidae|673|BOLD:AAV5096
Dasyhelea|[20400]|NGNAN3236-14|Ceratopogonidae|664|BOLD:AAP6918
Dasyhelea|[20401]|NCCA773-11|Ceratopogonidae|673|BOLD:AAV5177
Ceratopogonidae|[20402]|CNWBG2120-13|Ceratopogonidae|661|BOLD:ACI5106
Ceratopogonidae|[20403]|CNFNE2008-14|Ceratopogonidae|664|BOLD:ACL5237
Dasyhelea|[20404]|NCCA1171-11|Ceratopogonidae|673|BOLD:AAN5161
Ceratopogonidae|[20405]|CNROE479-13|Ceratopogonidae|661|BOLD:ACK2453
Ceratopogonidae|[20406]|CNKOM235-14|Ceratopogonidae|531|BOLD:ACM4980
Ceratopogonidae|[20407]|CNGRD893-12|Ceratopogonidae|673|BOLD:ACB1144
Dasyhelea|[20408]|CNGRF315-12|Ceratopogonidae|673|BOLD:AAG6489
Dasyhelea|[20409]|CHDIP054-11|Ceratopogonidae|658|BOLD:AAG6535
Ceratopogonidae|[20410]|NGNAI1081-13|Ceratopogonidae|670|BOLD:ACX4946
Dasyhelea|[20411]|CNPPH191-12|Ceratopogonidae|664|BOLD:AAG6471
Ceratopogonidae|[20412]|CNGRD389-12|Ceratopogonidae|658|BOLD:ACB1077
Ceratopogonidae|[20413]|NGNAC073-13|Ceratopogonidae|658|BOLD:ACX4819
Dasyhelea|[20414]|NGNAP281-14|Ceratopogonidae|675|BOLD:ACI3416
Ceratopogonidae|[20415]|BBDCP043-10|Ceratopogonidae|658|BOLD:AAV5176
Dasyhelea|[20416]|SSBAF866-13|Ceratopogonidae|661|BOLD:ACC8269
Culicoides|[20417]|CNGRD883-12|Ceratopogonidae|673|BOLD:ACB1213
Dasyhelea|[20418]|NGNAN1511-14|Ceratopogonidae|676|BOLD:ACI3094
Dasyhelea|[20419]|CNWLG562-12|Ceratopogonidae|670|BOLD:ACD1712
Dasyhelea|[20420]|SSBAD1723-12|Ceratopogonidae|661|BOLD:AAG6437
Ceratopogonidae|[20421]|NGNAR1032-14|Ceratopogonidae|661|BOLD:ACL9814
Ceratopogonidae|[20422]|CNRVA1565-15|Ceratopogonidae|594|BOLD:ACX6178
Ceratopogonidae|[20423]|HEJUL1613-12|Ceratopogonidae|610|BOLD:ABX8361
Ceratopogonidae|[20424]|SSJAE4633-13|Ceratopogonidae|664|BOLD:ACI7294
Ceratopogonidae|[20425]|SSGBC4055-14|Ceratopogonidae|628|BOLD:ACP2855
Ceratopogonidae|[20426]|SSGBB7132-14|Ceratopogonidae|611|BOLD:ACO5495
Ceratopogonidae|[20427]|SSJAE3849-13|Ceratopogonidae|658|BOLD:ACJ8918
Ceratopogonidae|[20428]|SSBAF041-13|Ceratopogonidae|661|BOLD:ACG9224
Ceratopogonidae|[20429]|CNNHE562-14|Ceratopogonidae|609|BOLD:ACR5092
Ceratopogonidae|[20430]|CNEID436-12|Ceratopogonidae|658|BOLD:ACB0830
Ceratopogonidae|[20431]|CNWAB1082-14|Ceratopogonidae|588|BOLD:ACR7996
Ceratopogonidae|[20432]|CNNHD685-14|Ceratopogonidae|561|BOLD:ACR5725
Ceratopogonidae|[20433]|CNRVB879-15|Ceratopogonidae|564|BOLD:ACT5871
Ceratopogonidae|[20434]|TTMDI1190-10|Ceratopogonidae|658|BOLD:AAN5159
Ceratopogonidae|[20435]|CNMIE932-14|Ceratopogonidae|591|BOLD:ACN2795
Dasyhelea|[20436]|PHMTV883-10|Ceratopogonidae|658|BOLD:AAN5157
Ceratopogonidae|[20437]|SSPAC10042-13|Ceratopogonidae|606|BOLD:ACK6525
Ceratopogonidae|[20438]|SSJAB3639-13|Ceratopogonidae|658|BOLD:ACG8210
Dasyhelea|[20439]|NGNAI2843-13|Ceratopogonidae|670|BOLD:ACI3540
Ceratopogonidae|[20440]|SSJAE3911-13|Ceratopogonidae|664|BOLD:ACX4320
Ceratopogonidae|[20441]|NGAAC1802-14|Ceratopogonidae|585|BOLD:ACX5038
Dasyhelea|[20442]|SSJAE4616-13|Ceratopogonidae|664|BOLD:ACJ0506
Dasyhelea|[20443]|CNPPC1046-12|Ceratopogonidae|658|BOLD:AAG6475
Dasyhelea|[20444]|SSJAE3626-13|Ceratopogonidae|661|BOLD:ACR3959
Ceratopogonidae|[20445]|JSJUN1787-11|Ceratopogonidae|658|BOLD:ACK5365
Ceratopogonidae|[20446]|SSJAB1581-13|Ceratopogonidae|606|BOLD:ACK7112
Dasyhelea|[20447]|SSEIB5988-13|Ceratopogonidae|658|BOLD:ACT5320
Ceratopogonidae|[20448]|NGNAN641-14|Ceratopogonidae|580|BOLD:ACL2272
Ceratopogonidae|[20449]|CNRVC1046-15|Ceratopogonidae|579|BOLD:ACT6737
Ceratopogonidae|[20450]|CNWBH1067-13|Ceratopogonidae|661|BOLD:ACC8107
Ceratopogonidae|[20451]|HPPPJ295-13|Ceratopogonidae|661|BOLD:AAP3795
Ceratopogonidae|[20452]|CNGBM108-14|Ceratopogonidae|658|BOLD:ACM0692
Dasyhelea|[20453]|NCCA135-11|Ceratopogonidae|673|BOLD:AAN5162
Ceratopogonidae|[20454]|PAAUG1706-12|Ceratopogonidae|670|BOLD:ACB5406
Forcipomyia|[20455]|JSJUL715-11|Ceratopogonidae|658|BOLD:AAN5149
Ceratopogonidae|[20456]|CNTND1090-14|Ceratopogonidae|588|BOLD:ACN1036
Dasyhelea modesta|[20457]|BBDCP051-10|Ceratopogonidae|658|BOLD:AAG6542
Dasyhelea|[20458]|SSJAB701-13|Ceratopogonidae|664|BOLD:AAG6545
Dasyhelea|[20459]|SSJAB3520-13|Ceratopogonidae|658|BOLD:ACG8089
Dasyhelea|[20460]|CNMIL523-14|Ceratopogonidae|585|BOLD:ACN8781
Dasyhelea|[20461]|BBDCQ671-10|Ceratopogonidae|658|BOLD:AAV5198
Dasyhelea|[20462]|CNMIC1272-14|Ceratopogonidae|630|BOLD:ACU0325
Dasyhelea|[20463]|CNKTB1430-14|Ceratopogonidae|627|BOLD:ACM9717
Ceratopogonidae|[20464]|CNKJB451-14|Ceratopogonidae|658|BOLD:ACM5193
Ceratopogonidae|[20465]|SSFDA821-14|Ceratopogonidae|573|BOLD:ACO2311
Ceratopogonidae|[20466]|SSBAF3062-13|Ceratopogonidae|658|BOLD:ACI6686
Ceratopogonidae|[20467]|CNTND2327-14|Ceratopogonidae|588|BOLD:ACX4455
Ceratopogonidae|[20468]|CNRVC969-15|Ceratopogonidae|576|BOLD:ACT7743
Ceratopogonidae|[20469]|SSGBB3215-14|Ceratopogonidae|658|BOLD:ACP2289
Ceratopogonidae|[20470]|SSGBC3652-14|Ceratopogonidae|620|BOLD:ACU5186
Ceratopogonidae|[20471]|NGNAB739-13|Ceratopogonidae|664|BOLD:ACI3282
Ceratopogonidae|[20472]|CNRVD1054-15|Ceratopogonidae|594|BOLD:ACT7242
Ceratopogonidae|[20473]|SSFDA221-14|Ceratopogonidae|588|BOLD:ACO1996
Ceratopogonidae|[20474]|SSBAF393-13|Ceratopogonidae|661|BOLD:ACG2871
Ceratopogonidae|[20475]|JSDIP220-10|Ceratopogonidae|658|BOLD:AAP6907
Ceratopogonidae|[20476]|SSGBB6240-14|Ceratopogonidae|588|BOLD:ACP3252
Ceratopogonidae|[20477]|CNBAC267-12|Ceratopogonidae|658|BOLD:ACC9001
Ceratopogonidae|[20478]|CNRVE2881-15|Ceratopogonidae|588|BOLD:ACT6639
Dasyhelea|[20479]|CHDIP356-11|Ceratopogonidae|658|BOLD:AAG6376
Dasyhelea|[20480]|NGNAD958-13|Ceratopogonidae|673|BOLD:ACI8425
Dasyhelea|[20481]|CHDIP180-11|Ceratopogonidae|658|BOLD:AAG6447
Dasyhelea|[20482]|CHDIP1130-12|Ceratopogonidae|658|BOLD:ACX4903
Dasyhelea|[20483]|NGNAC1376-13|Ceratopogonidae|658|BOLD:ACI3092
Ceratopogonidae|[20484]|SSJAE7692-13|Ceratopogonidae|661|BOLD:ACJ4749
Ceratopogonidae|[20485]|JSAUG639-11|Ceratopogonidae|658|BOLD:ABV1356
Ceratopogonidae|[20486]|CNGBE544-14|Ceratopogonidae|661|BOLD:ABV9354
Ceratopogonidae|[20487]|NGNAN3305-14|Ceratopogonidae|664|BOLD:ACL2357
Ceratopogonidae|[20488]|CNWBA279-13|Ceratopogonidae|658|BOLD:ACD5204
Ceratopogonidae|[20489]|CNJAD250-12|Ceratopogonidae|658|BOLD:ACC8879
Dasyhelea|[20490]|CNWBA221-13|Ceratopogonidae|658|BOLD:ACD9244
Ceratopogonidae|[20491]|CNWBA186-13|Ceratopogonidae|658|BOLD:ACD9344
Ceratopogonidae|[20492]|NGNAE206-13|Ceratopogonidae|658|BOLD:ACI7835
Ceratopogonidae|[20493]|NGNAN3278-14|Ceratopogonidae|664|BOLD:ACL3935
Ceratopogonidae|[20494]|NGNAN3279-14|Ceratopogonidae|664|BOLD:ACI2690
Ceratopogonidae|[20495]|JSDIS341-11|Ceratopogonidae|658|BOLD:ABA7906
Ceratopogonidae|[20496]|SSJAE4008-13|Ceratopogonidae|583|BOLD:ACJ7878

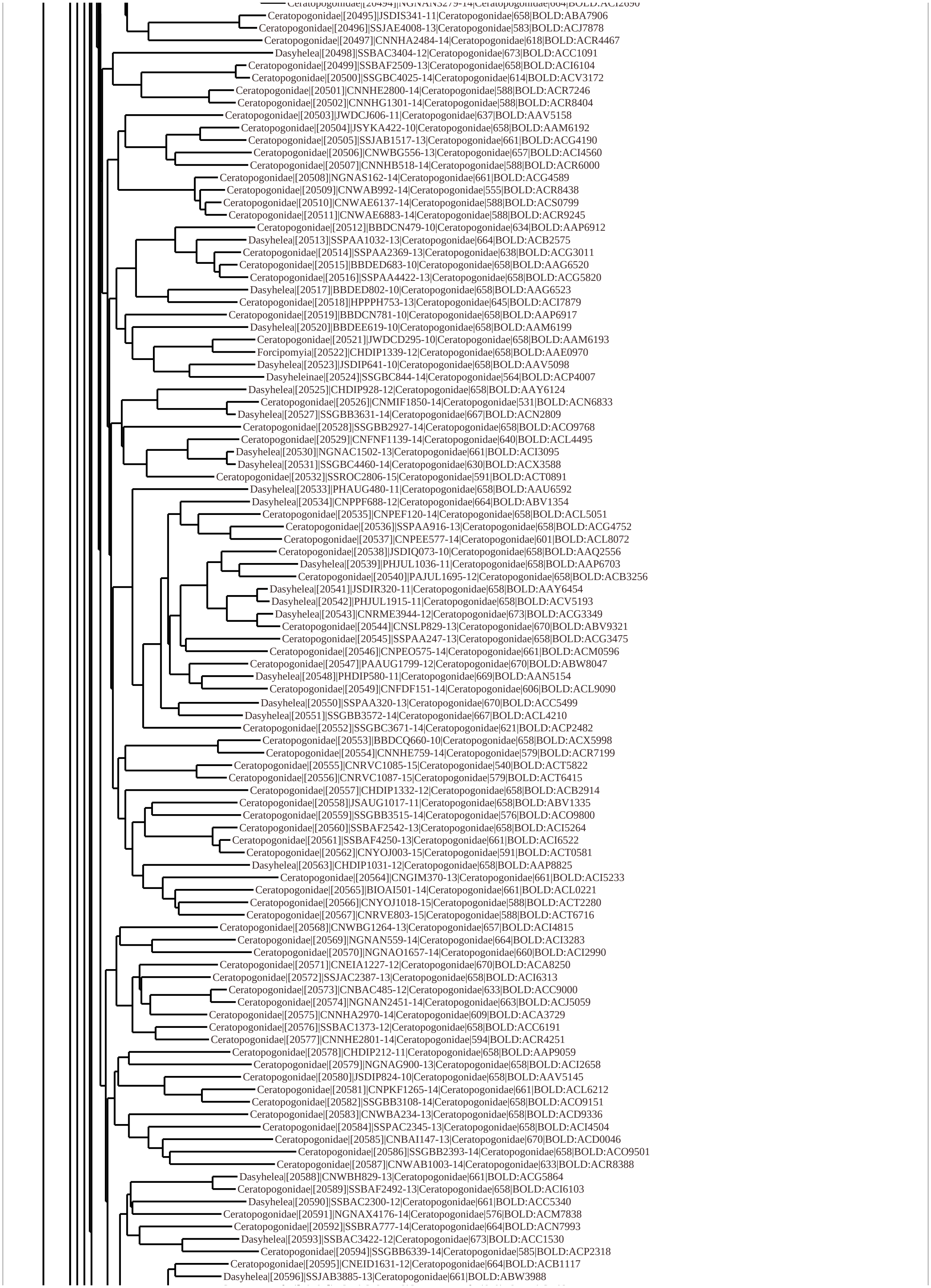
Ceratopogonidae|[20494]|NGNAN3279-14|Ceratopogonidae|664|BOLD:ACI2690
Ceratopogonidae|[20495]|JSDIS341-11|Ceratopogonidae|658|BOLD:ABA7906
Ceratopogonidae|[20496]|SSJAE4008-13|Ceratopogonidae|583|BOLD:ACJ7878
Ceratopogonidae|[20497]|CNNHA2484-14|Ceratopogonidae|618|BOLD:ACR4467
Dasyhelea|[20498]|SSBAC3404-12|Ceratopogonidae|673|BOLD:ACC1091
Ceratopogonidae|[20499]|SSBAF2509-13|Ceratopogonidae|658|BOLD:ACI6104
Ceratopogonidae|[20500]|SSGBC4025-14|Ceratopogonidae|614|BOLD:ACV3172
Ceratopogonidae|[20501]|CNNHE2800-14|Ceratopogonidae|588|BOLD:ACR7246
Ceratopogonidae|[20502]|CNNHG1301-14|Ceratopogonidae|588|BOLD:ACR8404
Ceratopogonidae|[20503]|JWDCJ606-11|Ceratopogonidae|637|BOLD:AAV5158
Ceratopogonidae|[20504]|JSYKA422-10|Ceratopogonidae|658|BOLD:AAM6192
Ceratopogonidae|[20505]|SSJAB1517-13|Ceratopogonidae|661|BOLD:ACG4190
Ceratopogonidae|[20506]|CNWBG556-13|Ceratopogonidae|657|BOLD:ACI4560
Ceratopogonidae|[20507]|CNNHB518-14|Ceratopogonidae|588|BOLD:ACR6000
Ceratopogonidae|[20508]|NGNAS162-14|Ceratopogonidae|661|BOLD:ACG4589
Ceratopogonidae|[20509]|CNWAB992-14|Ceratopogonidae|555|BOLD:ACR8438
Ceratopogonidae|[20510]|CNWAE6137-14|Ceratopogonidae|588|BOLD:ACS0799
Ceratopogonidae|[20511]|CNWAE6883-14|Ceratopogonidae|588|BOLD:ACR9245
Ceratopogonidae|[20512]|BBDCN479-10|Ceratopogonidae|634|BOLD:AAP6912
Dasyhelea|[20513]|SSPAA1032-13|Ceratopogonidae|664|BOLD:ACB2575
Ceratopogonidae|[20514]|SSPAA2369-13|Ceratopogonidae|638|BOLD:ACG3011
Ceratopogonidae|[20515]|BBDED683-10|Ceratopogonidae|658|BOLD:AAG6520
Ceratopogonidae|[20516]|SSPAA4422-13|Ceratopogonidae|658|BOLD:ACG5820
Dasyhelea|[20517]|BBDED802-10|Ceratopogonidae|658|BOLD:AAG6523
Ceratopogonidae|[20518]|HPPPH753-13|Ceratopogonidae|645|BOLD:ACI7879
Ceratopogonidae|[20519]|BBDCN781-10|Ceratopogonidae|658|BOLD:AAP6917
Dasyhelea|[20520]|BBDEE619-10|Ceratopogonidae|658|BOLD:AAM6199
Ceratopogonidae|[20521]|JWDCD295-10|Ceratopogonidae|658|BOLD:AAM6193
Forcipomyia|[20522]|CHDIP1339-12|Ceratopogonidae|658|BOLD:AAE0970
Dasyhelea|[20523]|JSDIP641-10|Ceratopogonidae|658|BOLD:AAV5098
Dasyheleinae|[20524]|SSGBC844-14|Ceratopogonidae|564|BOLD:ACP4007
Dasyhelea|[20525]|CHDIP928-12|Ceratopogonidae|658|BOLD:AAY6124
Ceratopogonidae|[20526]|CNMIF1850-14|Ceratopogonidae|531|BOLD:ACN6833
Dasyhelea|[20527]|SSGBB3631-14|Ceratopogonidae|667|BOLD:ACN2809
Ceratopogonidae|[20528]|SSGBB2927-14|Ceratopogonidae|658|BOLD:ACO9768
Ceratopogonidae|[20529]|CNFNF1139-14|Ceratopogonidae|640|BOLD:ACL4495
Dasyhelea|[20530]|NGNAC1502-13|Ceratopogonidae|661|BOLD:ACI3095
Dasyhelea|[20531]|SSGBC4460-14|Ceratopogonidae|630|BOLD:ACX3588
Ceratopogonidae|[20532]|SSROC2806-15|Ceratopogonidae|591|BOLD:ACT0891
Dasyhelea|[20533]|PHAUG480-11|Ceratopogonidae|658|BOLD:AAU6592
Dasyhelea|[20534]|CNPPF688-12|Ceratopogonidae|664|BOLD:ABV1354
Ceratopogonidae|[20535]|CNPEF120-14|Ceratopogonidae|658|BOLD:ACL5051
Ceratopogonidae|[20536]|SSPAA916-13|Ceratopogonidae|658|BOLD:ACG4752
Ceratopogonidae|[20537]|CNPEE577-14|Ceratopogonidae|601|BOLD:ACL8072
Ceratopogonidae|[20538]|JSDIQ073-10|Ceratopogonidae|658|BOLD:AAQ2556
Dasyhelea|[20539]|PHJUL1036-11|Ceratopogonidae|658|BOLD:AAP6703
Ceratopogonidae|[20540]|PAJUL1695-12|Ceratopogonidae|658|BOLD:ACB3256
Dasyhelea|[20541]|JSDIR320-11|Ceratopogonidae|658|BOLD:AAY6454
Dasyhelea|[20542]|PHJUL1915-11|Ceratopogonidae|658|BOLD:ACV5193
Dasyhelea|[20543]|CNRME3944-12|Ceratopogonidae|673|BOLD:ACG3349
Ceratopogonidae|[20544]|CNSLP829-13|Ceratopogonidae|670|BOLD:ABV9321
Ceratopogonidae|[20545]|SSPAA247-13|Ceratopogonidae|658|BOLD:ACG3475
Ceratopogonidae|[20546]|CNPEO575-14|Ceratopogonidae|661|BOLD:ACM0596
Ceratopogonidae|[20547]|PAAUG1799-12|Ceratopogonidae|670|BOLD:ABW8047
Dasyhelea|[20548]|PHDIP580-11|Ceratopogonidae|669|BOLD:AAN5154
Ceratopogonidae|[20549]|CNFDF151-14|Ceratopogonidae|606|BOLD:ACL9090
Dasyhelea|[20550]|SSPAA320-13|Ceratopogonidae|670|BOLD:ACC5499
Dasyhelea|[20551]|SSGBB3572-14|Ceratopogonidae|667|BOLD:ACL4210
Ceratopogonidae|[20552]|SSGBC3671-14|Ceratopogonidae|621|BOLD:ACP2482
Ceratopogonidae|[20553]|BBDCQ660-10|Ceratopogonidae|658|BOLD:ACX5998
Ceratopogonidae|[20554]|CNNHE759-14|Ceratopogonidae|579|BOLD:ACR7199
Ceratopogonidae|[20555]|CNRVC1085-15|Ceratopogonidae|540|BOLD:ACT5822
Ceratopogonidae|[20556]|CNRVC1087-15|Ceratopogonidae|579|BOLD:ACT6415
Ceratopogonidae|[20557]|CHDIP1332-12|Ceratopogonidae|658|BOLD:ACB2914
Ceratopogonidae|[20558]|JSAUG1017-11|Ceratopogonidae|658|BOLD:ABV1335
Ceratopogonidae|[20559]|SSGBB3515-14|Ceratopogonidae|576|BOLD:ACO9800
Ceratopogonidae|[20560]|SSBAF2542-13|Ceratopogonidae|658|BOLD:ACI5264
Ceratopogonidae|[20561]|SSBAF4250-13|Ceratopogonidae|661|BOLD:ACI6522
Ceratopogonidae|[20562]|CNYOJ003-15|Ceratopogonidae|591|BOLD:ACT0581
Dasyhelea|[20563]|CHDIP1031-12|Ceratopogonidae|658|BOLD:AAP8825
Ceratopogonidae|[20564]|CNGIM370-13|Ceratopogonidae|661|BOLD:ACI5233
Ceratopogonidae|[20565]|BIOAI501-14|Ceratopogonidae|661|BOLD:ACL0221
Ceratopogonidae|[20566]|CNYOJ1018-15|Ceratopogonidae|588|BOLD:ACT2280
Ceratopogonidae|[20567]|CNRVE803-15|Ceratopogonidae|588|BOLD:ACT6716
Ceratopogonidae|[20568]|CNWBG1264-13|Ceratopogonidae|657|BOLD:ACI4815
Ceratopogonidae|[20569]|NGNAN559-14|Ceratopogonidae|664|BOLD:ACI3283
Ceratopogonidae|[20570]|NGNAO1657-14|Ceratopogonidae|660|BOLD:ACI2990
Ceratopogonidae|[20571]|CNEIA1227-12|Ceratopogonidae|670|BOLD:ACA8250
Ceratopogonidae|[20572]|SSJAC2387-13|Ceratopogonidae|658|BOLD:ACI6313
Ceratopogonidae|[20573]|CNBAC485-12|Ceratopogonidae|633|BOLD:ACC9000
Ceratopogonidae|[20574]|NGNAN2451-14|Ceratopogonidae|663|BOLD:ACJ5059
Ceratopogonidae|[20575]|CNNHA2970-14|Ceratopogonidae|609|BOLD:ACA3729
Ceratopogonidae|[20576]|SSBAC1373-12|Ceratopogonidae|658|BOLD:ACC6191
Ceratopogonidae|[20577]|CNNHE2801-14|Ceratopogonidae|594|BOLD:ACR4251
Ceratopogonidae|[20578]|CHDIP212-11|Ceratopogonidae|658|BOLD:AAP9059
Ceratopogonidae|[20579]|NGNAG900-13|Ceratopogonidae|658|BOLD:ACI2658
Ceratopogonidae|[20580]|JSDIP824-10|Ceratopogonidae|658|BOLD:AAV5145
Ceratopogonidae|[20581]|CNPKF1265-14|Ceratopogonidae|661|BOLD:ACL6212
Ceratopogonidae|[20582]|SSGBB3108-14|Ceratopogonidae|658|BOLD:ACO9151
Ceratopogonidae|[20583]|CNWBA234-13|Ceratopogonidae|658|BOLD:ACD9336
Ceratopogonidae|[20584]|SSPAC2345-13|Ceratopogonidae|658|BOLD:ACI4504
Ceratopogonidae|[20585]|CNBAI147-13|Ceratopogonidae|670|BOLD:ACD0046
Ceratopogonidae|[20586]|SSGBB2393-14|Ceratopogonidae|658|BOLD:ACO9501
Ceratopogonidae|[20587]|CNWAB1003-14|Ceratopogonidae|633|BOLD:ACR8388
Dasyhelea|[20588]|CNWBH829-13|Ceratopogonidae|661|BOLD:ACG5864
Ceratopogonidae|[20589]|SSBAF2492-13|Ceratopogonidae|658|BOLD:ACI6103
Dasyhelea|[20590]|SSBAC2300-12|Ceratopogonidae|661|BOLD:ACC5340
Ceratopogonidae|[20591]|NGNAX4176-14|Ceratopogonidae|576|BOLD:ACM7838
Ceratopogonidae|[20592]|SSBRA777-14|Ceratopogonidae|664|BOLD:ACN7993
Dasyhelea|[20593]|SSBAC3422-12|Ceratopogonidae|673|BOLD:ACC1530
Ceratopogonidae|[20594]|SSGBB6339-14|Ceratopogonidae|585|BOLD:ACP2318
Ceratopogonidae|[20595]|CNEID1631-12|Ceratopogonidae|664|BOLD:ACB1117
Dasyhelea|[20596]|SSJAB3885-13|Ceratopogonidae|661|BOLD:ABW3988

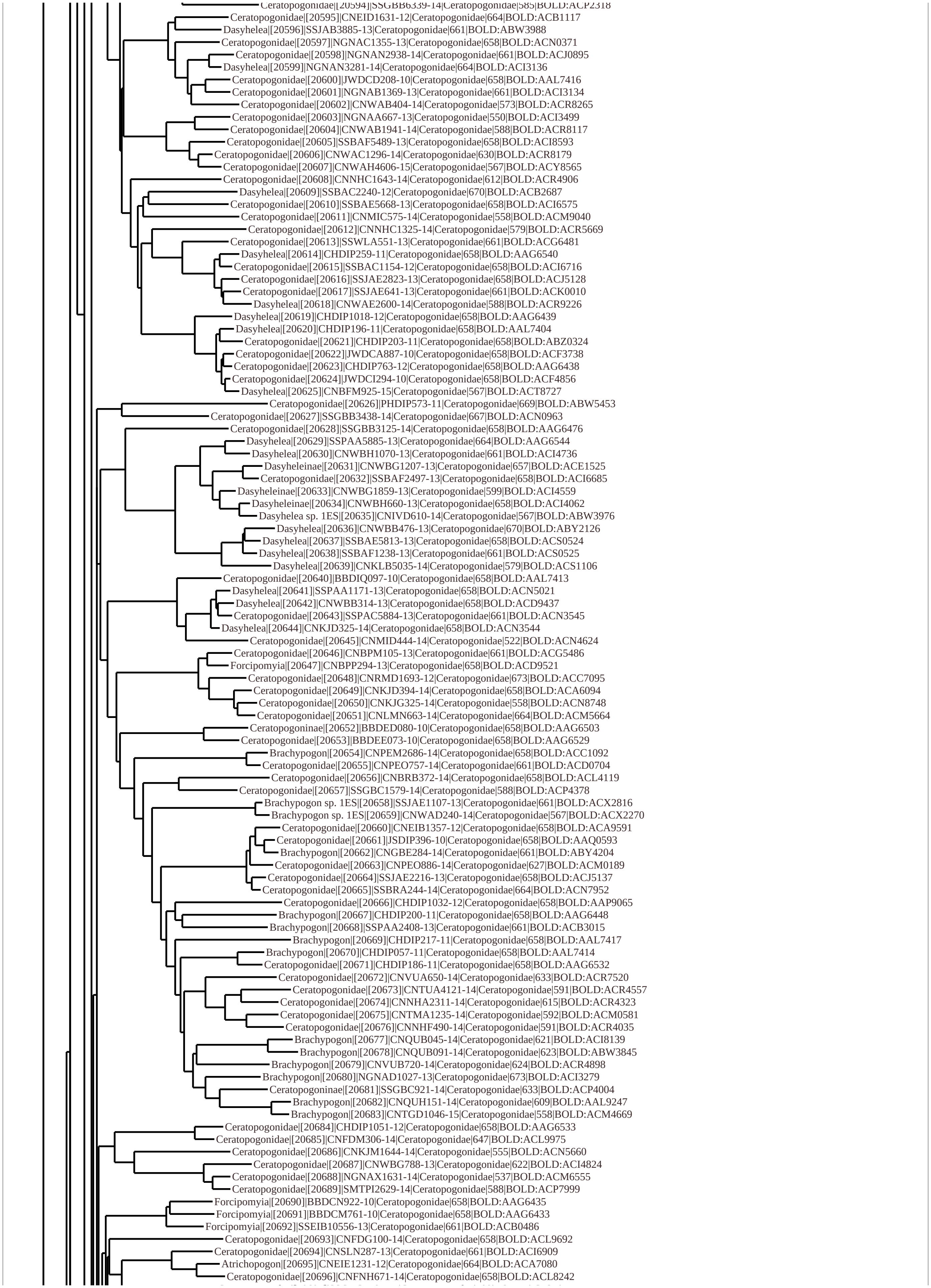

Ceratopogonidae|[20594]|SSGBB6339-14|Ceratopogonidae|585|BOLD:ACP2318
Ceratopogonidae|[20595]|CNEID1631-12|Ceratopogonidae|664|BOLD:ACB1117
Dasyhelea|[20596]|SSJAB3885-13|Ceratopogonidae|661|BOLD:ABW3988
Ceratopogonidae|[20597]|NGNAC1355-13|Ceratopogonidae|658|BOLD:ACN0371
Ceratopogonidae|[20598]|NGNAN2938-14|Ceratopogonidae|661|BOLD:ACJ0895
Dasyhelea|[20599]|NGNAN3281-14|Ceratopogonidae|664|BOLD:ACI3136
Ceratopogonidae|[20600]|JWDCD208-10|Ceratopogonidae|658|BOLD:AAL7416
Ceratopogonidae|[20601]|NGNAB1369-13|Ceratopogonidae|661|BOLD:ACI3134
Ceratopogonidae|[20602]|CNWAB404-14|Ceratopogonidae|573|BOLD:ACR8265
Ceratopogonidae|[20603]|NGNAA667-13|Ceratopogonidae|550|BOLD:ACI3499
Ceratopogonidae|[20604]|CNWAB1941-14|Ceratopogonidae|588|BOLD:ACR8117
Ceratopogonidae|[20605]|SSBAF5489-13|Ceratopogonidae|658|BOLD:ACI8593
Ceratopogonidae|[20606]|CNWAC1296-14|Ceratopogonidae|630|BOLD:ACR8179
Ceratopogonidae|[20607]|CNWAH4606-15|Ceratopogonidae|567|BOLD:ACY8565
Ceratopogonidae|[20608]|CNNHC1643-14|Ceratopogonidae|612|BOLD:ACR4906
Dasyhelea|[20609]|SSBAC2240-12|Ceratopogonidae|670|BOLD:ACB2687
Ceratopogonidae|[20610]|SSBAE5668-13|Ceratopogonidae|658|BOLD:ACI6575
Ceratopogonidae|[20611]|CNMIC575-14|Ceratopogonidae|558|BOLD:ACM9040
Ceratopogonidae|[20612]|CNNHC1325-14|Ceratopogonidae|579|BOLD:ACR5669
Ceratopogonidae|[20613]|SSWLA551-13|Ceratopogonidae|661|BOLD:ACG6481
Dasyhelea|[20614]|CHDIP259-11|Ceratopogonidae|658|BOLD:AAG6540
Ceratopogonidae|[20615]|SSBAC1154-12|Ceratopogonidae|658|BOLD:ACI6716
Ceratopogonidae|[20616]|SSJAE2823-13|Ceratopogonidae|658|BOLD:ACJ5128
Ceratopogonidae|[20617]|SSJAE641-13|Ceratopogonidae|661|BOLD:ACK0010
Dasyhelea|[20618]|CNWAE2600-14|Ceratopogonidae|588|BOLD:ACR9226
Dasyhelea|[20619]|CHDIP1018-12|Ceratopogonidae|658|BOLD:AAG6439
Dasyhelea|[20620]|CHDIP196-11|Ceratopogonidae|658|BOLD:AAL7404
Ceratopogonidae|[20621]|CHDIP203-11|Ceratopogonidae|658|BOLD:ABZ0324
Ceratopogonidae|[20622]|JWDCA887-10|Ceratopogonidae|658|BOLD:ACF3738
Ceratopogonidae|[20623]|CHDIP763-12|Ceratopogonidae|658|BOLD:AAG6438
Ceratopogonidae|[20624]|JWDCI294-10|Ceratopogonidae|658|BOLD:ACF4856
Dasyhelea|[20625]|CNBFM925-15|Ceratopogonidae|567|BOLD:ACT8727
Ceratopogonidae|[20626]|PHDIP573-11|Ceratopogonidae|669|BOLD:ABW5453
Ceratopogonidae|[20627]|SSGBB3438-14|Ceratopogonidae|667|BOLD:ACN0963
Ceratopogonidae|[20628]|SSGBB3125-14|Ceratopogonidae|658|BOLD:AAG6476
Dasyhelea|[20629]|SSPAA5885-13|Ceratopogonidae|664|BOLD:AAG6544
Dasyhelea|[20630]|CNWBH1070-13|Ceratopogonidae|661|BOLD:ACI4736
Dasyheleinae|[20631]|CNWBG1207-13|Ceratopogonidae|657|BOLD:ACE1525
Ceratopogonidae|[20632]|SSBAF2497-13|Ceratopogonidae|658|BOLD:ACI6685
Dasyheleinae|[20633]|CNWBG1859-13|Ceratopogonidae|599|BOLD:ACI4559
Dasyheleinae|[20634]|CNWBH660-13|Ceratopogonidae|658|BOLD:ACI4062
Dasyhelea sp. 1ES|[20635]|CNIVD610-14|Ceratopogonidae|567|BOLD:ABW3976
Dasyhelea|[20636]|CNWBB476-13|Ceratopogonidae|670|BOLD:ABY2126
Dasyhelea|[20637]|SSBAE5813-13|Ceratopogonidae|658|BOLD:ACS0524
Dasyhelea|[20638]|SSBAF1238-13|Ceratopogonidae|661|BOLD:ACS0525
Dasyhelea|[20639]|CNKLB5035-14|Ceratopogonidae|579|BOLD:ACS1106
Ceratopogonidae|[20640]|BBDIQ097-10|Ceratopogonidae|658|BOLD:AAL7413
Dasyhelea|[20641]|SSPAA1171-13|Ceratopogonidae|658|BOLD:ACN5021
Dasyhelea|[20642]|CNWBB314-13|Ceratopogonidae|658|BOLD:ACD9437
Ceratopogonidae|[20643]|SSPAC5884-13|Ceratopogonidae|661|BOLD:ACN3545
Dasyhelea|[20644]|CNKJD325-14|Ceratopogonidae|658|BOLD:ACN3544
Ceratopogonidae|[20645]|CNMID444-14|Ceratopogonidae|522|BOLD:ACN4624
Ceratopogonidae|[20646]|CNBPM105-13|Ceratopogonidae|661|BOLD:ACG5486
Forcipomyia|[20647]|CNBPP294-13|Ceratopogonidae|658|BOLD:ACD9521
Ceratopogonidae|[20648]|CNRMD1693-12|Ceratopogonidae|673|BOLD:ACC7095
Ceratopogonidae|[20649]|CNKJD394-14|Ceratopogonidae|658|BOLD:ACA6094
Ceratopogonidae|[20650]|CNKJG325-14|Ceratopogonidae|558|BOLD:ACN8748
Ceratopogonidae|[20651]|CNLMN663-14|Ceratopogonidae|664|BOLD:ACM5664
Ceratopogoninae|[20652]|BBDED080-10|Ceratopogonidae|658|BOLD:AAG6503
Ceratopogonidae|[20653]|BBDEE073-10|Ceratopogonidae|658|BOLD:AAG6529
Brachypogon|[20654]|CNPEM2686-14|Ceratopogonidae|658|BOLD:ACC1092
Ceratopogonidae|[20655]|CNPEO757-14|Ceratopogonidae|661|BOLD:ACD0704
Ceratopogonidae|[20656]|CNBRB372-14|Ceratopogonidae|658|BOLD:ACL4119
Ceratopogonidae|[20657]|SSGBC1579-14|Ceratopogonidae|588|BOLD:ACP4378
Brachypogon sp. 1ES|[20658]|SSJAE1107-13|Ceratopogonidae|661|BOLD:ACX2816
Brachypogon sp. 1ES|[20659]|CNWAD240-14|Ceratopogonidae|567|BOLD:ACX2270
Ceratopogonidae|[20660]|CNEIB1357-12|Ceratopogonidae|658|BOLD:ACA9591
Ceratopogonidae|[20661]|JSDIP396-10|Ceratopogonidae|658|BOLD:AAQ0593
Brachypogon|[20662]|CNGBE284-14|Ceratopogonidae|661|BOLD:ABY4204
Ceratopogonidae|[20663]|CNPEO886-14|Ceratopogonidae|627|BOLD:ACM0189
Ceratopogonidae|[20664]|SSJAE2216-13|Ceratopogonidae|658|BOLD:ACJ5137
Ceratopogonidae|[20665]|SSBRA244-14|Ceratopogonidae|664|BOLD:ACN7952
Ceratopogonidae|[20666]|CHDIP1032-12|Ceratopogonidae|658|BOLD:AAP9065
Brachypogon|[20667]|CHDIP200-11|Ceratopogonidae|658|BOLD:AAG6448
Brachypogon|[20668]|SSPAA2408-13|Ceratopogonidae|661|BOLD:ACB3015
Brachypogon|[20669]|CHDIP217-11|Ceratopogonidae|658|BOLD:AAL7417
Brachypogon|[20670]|CHDIP057-11|Ceratopogonidae|658|BOLD:AAL7414
Ceratopogonidae|[20671]|CHDIP186-11|Ceratopogonidae|658|BOLD:AAG6532
Ceratopogonidae|[20672]|CNVUA650-14|Ceratopogonidae|633|BOLD:ACR7520
Ceratopogonidae|[20673]|CNTUA4121-14|Ceratopogonidae|591|BOLD:ACR4557
Ceratopogonidae|[20674]|CNNHA2311-14|Ceratopogonidae|615|BOLD:ACR4323
Ceratopogonidae|[20675]|CNTMA1235-14|Ceratopogonidae|592|BOLD:ACM0581
Ceratopogonidae|[20676]|CNNHF490-14|Ceratopogonidae|591|BOLD:ACR4035
Brachypogon|[20677]|CNQUB045-14|Ceratopogonidae|621|BOLD:ACI8139
Brachypogon|[20678]|CNQUB091-14|Ceratopogonidae|623|BOLD:ABW3845
Brachypogon|[20679]|CNVUB720-14|Ceratopogonidae|624|BOLD:ACR4898
Brachypogon|[20680]|NGNAD1027-13|Ceratopogonidae|673|BOLD:ACI3279
Ceratopogoninae|[20681]|SSGBC921-14|Ceratopogonidae|633|BOLD:ACP4004
Brachypogon|[20682]|CNQUH151-14|Ceratopogonidae|609|BOLD:AAL9247
Brachypogon|[20683]|CNTGD1046-15|Ceratopogonidae|558|BOLD:ACM4669
Ceratopogonidae|[20684]|CHDIP1051-12|Ceratopogonidae|658|BOLD:AAG6533
Ceratopogonidae|[20685]|CNFDM306-14|Ceratopogonidae|647|BOLD:ACL9975
Ceratopogonidae|[20686]|CNKJM1644-14|Ceratopogonidae|555|BOLD:ACN5660
Ceratopogonidae|[20687]|CNWBG788-13|Ceratopogonidae|622|BOLD:ACI4824
Ceratopogonidae|[20688]|NGNAX1631-14|Ceratopogonidae|537|BOLD:ACM6555
Ceratopogonidae|[20689]|SMTPI2629-14|Ceratopogonidae|588|BOLD:ACP7999
Forcipomyia|[20690]|BBDCN922-10|Ceratopogonidae|658|BOLD:AAG6435
Forcipomyia|[20691]|BBDCM761-10|Ceratopogonidae|658|BOLD:AAG6433
Forcipomyia|[20692]|SSEIB10556-13|Ceratopogonidae|661|BOLD:ACB0486
Ceratopogonidae|[20693]|CNFDG100-14|Ceratopogonidae|658|BOLD:ACL9692
Ceratopogonidae|[20694]|CNSLN287-13|Ceratopogonidae|661|BOLD:ACI6909
Atrichopogon|[20695]|CNEIE1231-12|Ceratopogonidae|664|BOLD:ACA7080
Ceratopogonidae|[20696]|CNFNH671-14|Ceratopogonidae|658|BOLD:ACL8242

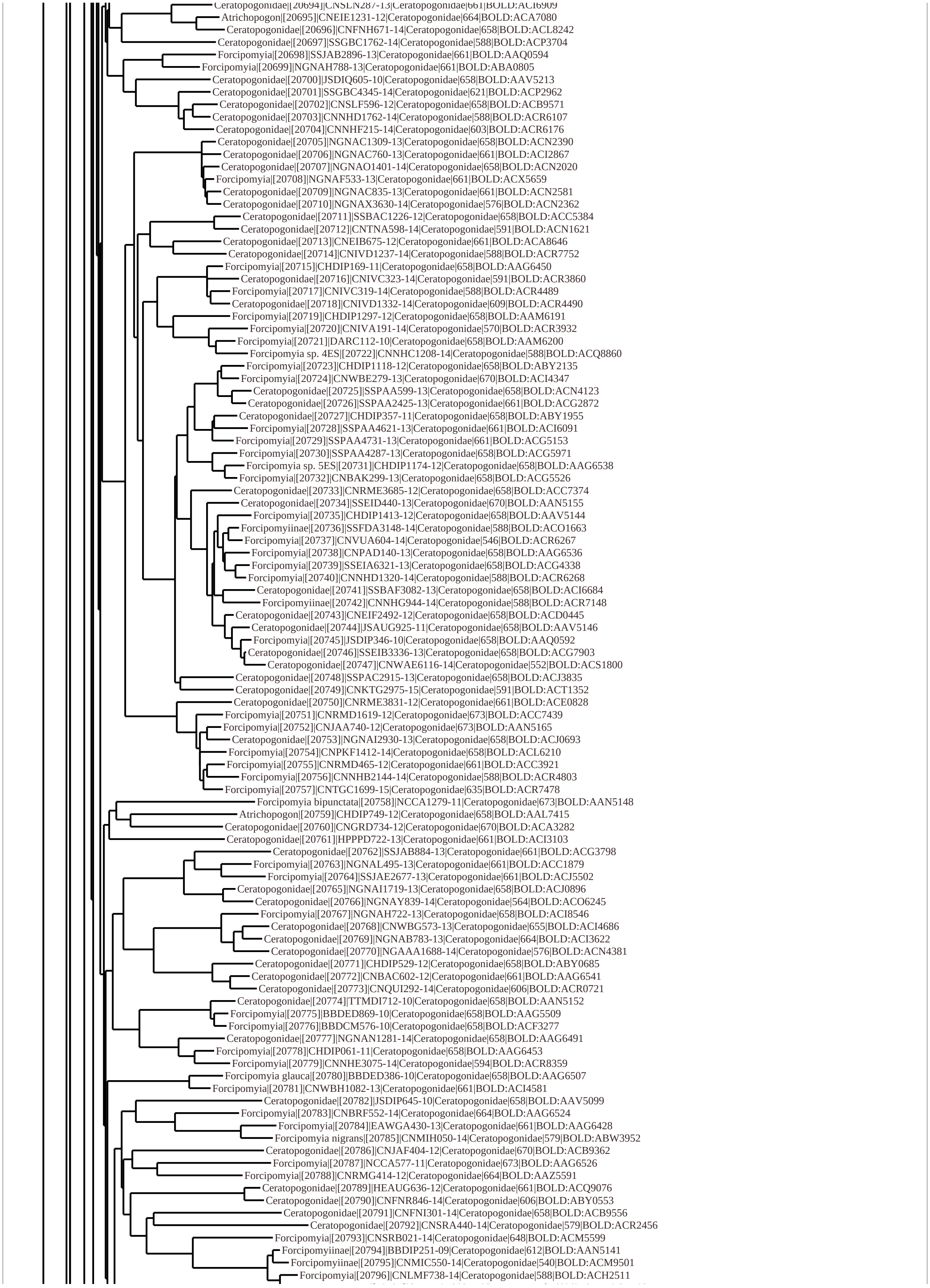
Ceratopogonidae|[20694]|CNSLN287-13|Ceratopogonidae|661|BOLD:ACI6909
Atrichopogon|[20695]|CNEIE1231-12|Ceratopogonidae|664|BOLD:ACA7080
Ceratopogonidae|[20696]|CNFNH671-14|Ceratopogonidae|658|BOLD:ACL8242
Ceratopogonidae|[20697]|SSGBC1762-14|Ceratopogonidae|588|BOLD:ACP3704
Forcipomyia|[20698]|SSJAB2896-13|Ceratopogonidae|661|BOLD:AAQ0594
Forcipomyia|[20699]|NGNAH788-13|Ceratopogonidae|661|BOLD:ABA0805
Ceratopogonidae|[20700]|JSDIQ605-10|Ceratopogonidae|658|BOLD:AAV5213
Ceratopogonidae|[20701]|SSGBC4345-14|Ceratopogonidae|621|BOLD:ACP2962
Ceratopogonidae|[20702]|CNSLF596-12|Ceratopogonidae|658|BOLD:ACB9571
Ceratopogonidae|[20703]|CNNHD1762-14|Ceratopogonidae|588|BOLD:ACR6107
Ceratopogonidae|[20704]|CNNHF215-14|Ceratopogonidae|603|BOLD:ACR6176
Ceratopogonidae|[20705]|NGNAC1309-13|Ceratopogonidae|658|BOLD:ACN2390
Ceratopogonidae|[20706]|NGNAC760-13|Ceratopogonidae|661|BOLD:ACI2867
Ceratopogonidae|[20707]|NGNAO1401-14|Ceratopogonidae|658|BOLD:ACN2020
Forcipomyia|[20708]|NGNAF533-13|Ceratopogonidae|661|BOLD:ACX5659
Ceratopogonidae|[20709]|NGNAC835-13|Ceratopogonidae|661|BOLD:ACN2581
Ceratopogonidae|[20710]|NGNAX3630-14|Ceratopogonidae|576|BOLD:ACN2362
Ceratopogonidae|[20711]|SSBAC1226-12|Ceratopogonidae|658|BOLD:ACC5384
Ceratopogonidae|[20712]|CNTNA598-14|Ceratopogonidae|591|BOLD:ACN1621
Ceratopogonidae|[20713]|CNEIB675-12|Ceratopogonidae|661|BOLD:ACA8646
Ceratopogonidae|[20714]|CNIVD1237-14|Ceratopogonidae|588|BOLD:ACR7752
Forcipomyia|[20715]|CHDIP169-11|Ceratopogonidae|658|BOLD:AAG6450
Ceratopogonidae|[20716]|CNIVC323-14|Ceratopogonidae|591|BOLD:ACR3860
Forcipomyia|[20717]|CNIVC319-14|Ceratopogonidae|588|BOLD:ACR4489
Ceratopogonidae|[20718]|CNIVD1332-14|Ceratopogonidae|609|BOLD:ACR4490
Forcipomyia|[20719]|CHDIP1297-12|Ceratopogonidae|658|BOLD:AAM6191
Forcipomyia|[20720]|CNIVA191-14|Ceratopogonidae|570|BOLD:ACR3932
Forcipomyia|[20721]|DARC112-10|Ceratopogonidae|658|BOLD:AAM6200
Forcipomyia sp. 4ES|[20722]|CNNHC1208-14|Ceratopogonidae|588|BOLD:ACQ8860
Forcipomyia|[20723]|CHDIP1118-12|Ceratopogonidae|658|BOLD:ABY2135
Forcipomyia|[20724]|CNWBE279-13|Ceratopogonidae|670|BOLD:ACI4347
Ceratopogonidae|[20725]|SSPAA599-13|Ceratopogonidae|658|BOLD:ACN4123
Ceratopogonidae|[20726]|SSPAA2425-13|Ceratopogonidae|661|BOLD:ACG2872
Ceratopogonidae|[20727]|CHDIP357-11|Ceratopogonidae|658|BOLD:ABY1955
Forcipomyia|[20728]|SSPAA4621-13|Ceratopogonidae|661|BOLD:ACI6091
Forcipomyia|[20729]|SSPAA4731-13|Ceratopogonidae|661|BOLD:ACG5153
Forcipomyia|[20730]|SSPAA4287-13|Ceratopogonidae|658|BOLD:ACG5971
Forcipomyia sp. 5ES|[20731]|CHDIP1174-12|Ceratopogonidae|658|BOLD:AAG6538
Forcipomyia|[20732]|CNBAK299-13|Ceratopogonidae|658|BOLD:ACG5526
Ceratopogonidae|[20733]|CNRME3685-12|Ceratopogonidae|658|BOLD:ACC7374
Ceratopogonidae|[20734]|SSEID440-13|Ceratopogonidae|670|BOLD:AAN5155
Forcipomyia|[20735]|CHDIP1413-12|Ceratopogonidae|658|BOLD:AAV5144
Forcipomyiinae|[20736]|SSFDA3148-14|Ceratopogonidae|588|BOLD:ACO1663
Forcipomyia|[20737]|CNVUA604-14|Ceratopogonidae|546|BOLD:ACR6267
Forcipomyia|[20738]|CNPAD140-13|Ceratopogonidae|658|BOLD:AAG6536
Forcipomyia|[20739]|SSEIA6321-13|Ceratopogonidae|658|BOLD:ACG4338
Forcipomyia|[20740]|CNNHD1320-14|Ceratopogonidae|588|BOLD:ACR6268
Ceratopogonidae|[20741]|SSBAF3082-13|Ceratopogonidae|658|BOLD:ACI6684
Forcipomyiinae|[20742]|CNNHG944-14|Ceratopogonidae|588|BOLD:ACR7148
Ceratopogonidae|[20743]|CNEIF2492-12|Ceratopogonidae|658|BOLD:ACD0445
Ceratopogonidae|[20744]|JSAUG925-11|Ceratopogonidae|658|BOLD:AAV5146
Forcipomyia|[20745]|JSDIP346-10|Ceratopogonidae|658|BOLD:AAQ0592
Ceratopogonidae|[20746]|SSEIB3336-13|Ceratopogonidae|658|BOLD:ACG7903
Ceratopogonidae|[20747]|CNWAE6116-14|Ceratopogonidae|552|BOLD:ACS1800
Ceratopogonidae|[20748]|SSPAC2915-13|Ceratopogonidae|658|BOLD:ACJ3835
Ceratopogonidae|[20749]|CNKTG2975-15|Ceratopogonidae|591|BOLD:ACT1352
Ceratopogonidae|[20750]|CNRME3831-12|Ceratopogonidae|661|BOLD:ACE0828
Forcipomyia|[20751]|CNRMD1619-12|Ceratopogonidae|673|BOLD:ACC7439
Forcipomyia|[20752]|CNJAA740-12|Ceratopogonidae|673|BOLD:AAN5165
Ceratopogonidae|[20753]|NGNAI2930-13|Ceratopogonidae|658|BOLD:ACJ0693
Forcipomyia|[20754]|CNPKF1412-14|Ceratopogonidae|658|BOLD:ACL6210
Forcipomyia|[20755]|CNRMD465-12|Ceratopogonidae|661|BOLD:ACC3921
Forcipomyia|[20756]|CNNHB2144-14|Ceratopogonidae|588|BOLD:ACR4803
Forcipomyia|[20757]|CNTGC1699-15|Ceratopogonidae|635|BOLD:ACR7478
Forcipomyia bipunctata|[20758]|NCCA1279-11|Ceratopogonidae|673|BOLD:AAN5148
Atrichopogon|[20759]|CHDIP749-12|Ceratopogonidae|658|BOLD:AAL7415
Ceratopogonidae|[20760]|CNGRD734-12|Ceratopogonidae|670|BOLD:ACA3282
Ceratopogonidae|[20761]|HPPPD722-13|Ceratopogonidae|661|BOLD:ACI3103
Ceratopogonidae|[20762]|SSJAB884-13|Ceratopogonidae|661|BOLD:ACG3798
Forcipomyia|[20763]|NGNAL495-13|Ceratopogonidae|661|BOLD:ACC1879
Forcipomyia|[20764]|SSJAE2677-13|Ceratopogonidae|661|BOLD:ACJ5502
Ceratopogonidae|[20765]|NGNAI1719-13|Ceratopogonidae|658|BOLD:ACJ0896
Ceratopogonidae|[20766]|NGNAY839-14|Ceratopogonidae|564|BOLD:ACO6245
Forcipomyia|[20767]|NGNAH722-13|Ceratopogonidae|658|BOLD:ACI8546
Ceratopogonidae|[20768]|CNWBG573-13|Ceratopogonidae|655|BOLD:ACI4686
Ceratopogonidae|[20769]|NGNAB783-13|Ceratopogonidae|664|BOLD:ACI3622
Ceratopogonidae|[20770]|NGAAA1688-14|Ceratopogonidae|576|BOLD:ACN4381
Ceratopogonidae|[20771]|CHDIP529-12|Ceratopogonidae|658|BOLD:ABY0685
Ceratopogonidae|[20772]|CNBAC602-12|Ceratopogonidae|661|BOLD:AAG6541
Ceratopogonidae|[20773]|CNQUI292-14|Ceratopogonidae|606|BOLD:ACR0721
Ceratopogonidae|[20774]|TTMDI712-10|Ceratopogonidae|658|BOLD:AAN5152
Forcipomyia|[20775]|BBDED869-10|Ceratopogonidae|658|BOLD:AAG5509
Forcipomyia|[20776]|BBDCM576-10|Ceratopogonidae|658|BOLD:ACF3277
Ceratopogonidae|[20777]|NGNAN1281-14|Ceratopogonidae|658|BOLD:AAG6491
Forcipomyia|[20778]|CHDIP061-11|Ceratopogonidae|658|BOLD:AAG6453
Forcipomyia|[20779]|CNNHE3075-14|Ceratopogonidae|594|BOLD:ACR8359
Forcipomyia glauca|[20780]|BBDED386-10|Ceratopogonidae|658|BOLD:AAG6507
Forcipomyia|[20781]|CNWBH1082-13|Ceratopogonidae|661|BOLD:ACI4581
Ceratopogonidae|[20782]|JSDIP645-10|Ceratopogonidae|658|BOLD:AAV5099
Forcipomyia|[20783]|CNBRF552-14|Ceratopogonidae|664|BOLD:AAG6524
Forcipomyia|[20784]|EAWGA430-13|Ceratopogonidae|661|BOLD:AAG6428
Forcipomyia nigrans|[20785]|CNMIH050-14|Ceratopogonidae|579|BOLD:ABW3952
Ceratopogonidae|[20786]|CNJAF404-12|Ceratopogonidae|670|BOLD:ACB9362
Forcipomyia|[20787]|NCCA577-11|Ceratopogonidae|673|BOLD:AAG6526
Forcipomyia|[20788]|CNRMG414-12|Ceratopogonidae|664|BOLD:AAZ5591
Ceratopogonidae|[20789]|HEAUG636-12|Ceratopogonidae|661|BOLD:ACQ9076
Ceratopogonidae|[20790]|CNFNR846-14|Ceratopogonidae|606|BOLD:ABY0553
Ceratopogonidae|[20791]|CNFNI301-14|Ceratopogonidae|658|BOLD:ACB9556
Ceratopogonidae|[20792]|CNSRA440-14|Ceratopogonidae|579|BOLD:ACR2456
Forcipomyia|[20793]|CNSRB021-14|Ceratopogonidae|648|BOLD:ACM5599
Forcipomyiinae|[20794]|BBDIP251-09|Ceratopogonidae|612|BOLD:AAN5141
Forcipomyiinae|[20795]|CNMIC550-14|Ceratopogonidae|540|BOLD:ACM9501
Forcipomyia|[20796]|CNLMF738-14|Ceratopogonidae|588|BOLD:ACH2511

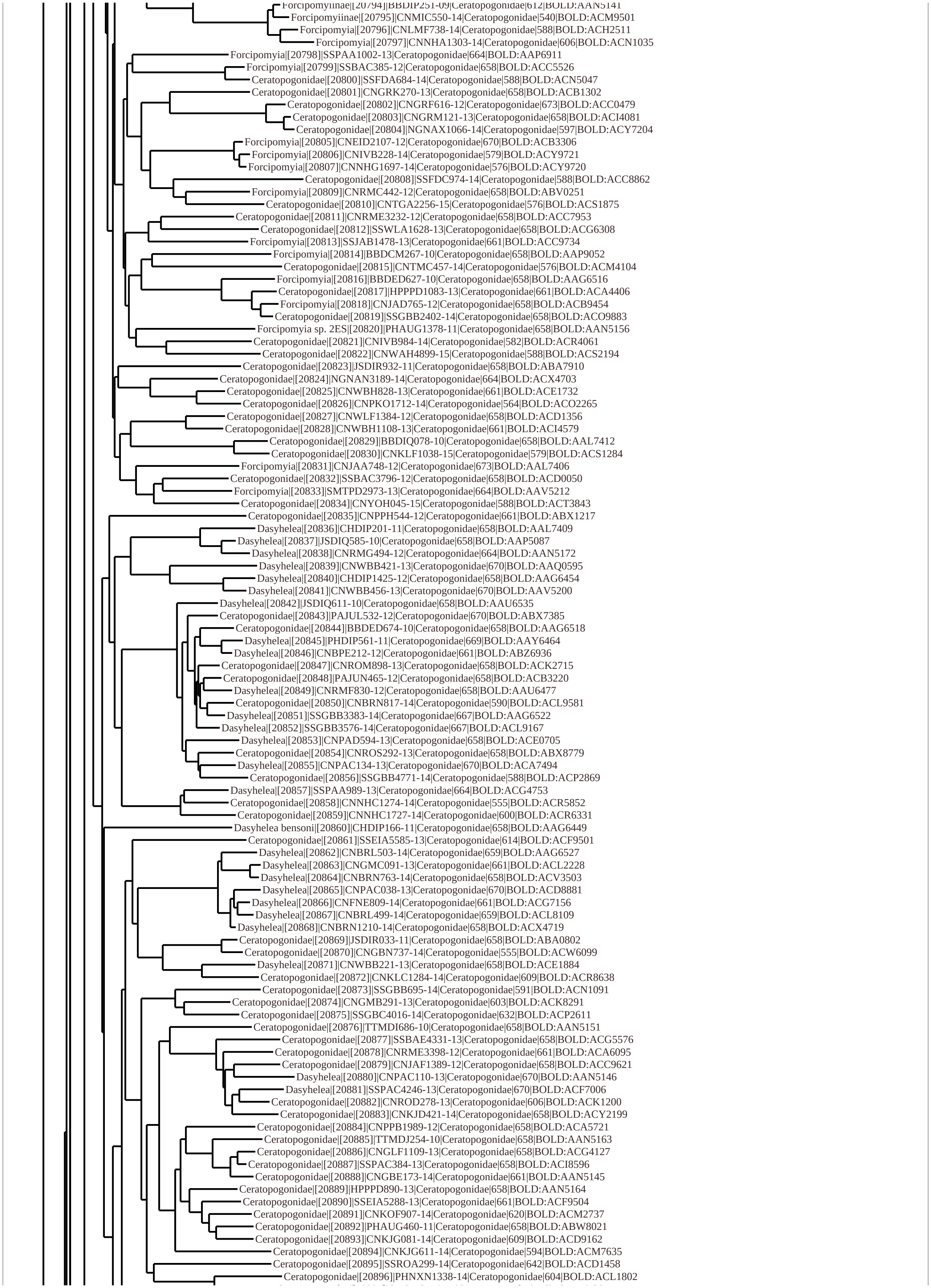
Forcipomyiinae|[20794]|BBDIP251-09|Ceratopogonidae|612|BOLD:AAN5141
Forcipomyiinae|[20795]|CNMIC550-14|Ceratopogonidae|540|BOLD:ACM9501
Forcipomyia|[20796]|CNLMF738-14|Ceratopogonidae|588|BOLD:ACH2511
Forcipomyia|[20797]|CNNHA1303-14|Ceratopogonidae|606|BOLD:ACN1035
Forcipomyia|[20798]|SSPAA1002-13|Ceratopogonidae|664|BOLD:AAP6911
Forcipomyia|[20799]|SSBAC385-12|Ceratopogonidae|658|BOLD:ACC5526
Ceratopogonidae|[20800]|SSFDA684-14|Ceratopogonidae|588|BOLD:ACN5047
Ceratopogonidae|[20801]|CNGRK270-13|Ceratopogonidae|658|BOLD:ACB1302
Ceratopogonidae|[20802]|CNGRF616-12|Ceratopogonidae|673|BOLD:ACC0479
Ceratopogonidae|[20803]|CNGRM121-13|Ceratopogonidae|658|BOLD:ACI4081
Ceratopogonidae|[20804]|NGNAX1066-14|Ceratopogonidae|597|BOLD:ACY7204
Forcipomyia|[20805]|CNEID2107-12|Ceratopogonidae|670|BOLD:ACB3306
Forcipomyia|[20806]|CNIVB228-14|Ceratopogonidae|579|BOLD:ACY9721
Forcipomyia|[20807]|CNNHG1697-14|Ceratopogonidae|576|BOLD:ACY9720
Ceratopogonidae|[20808]|SSFDC974-14|Ceratopogonidae|588|BOLD:ACC8862
Forcipomyia|[20809]|CNRMC442-12|Ceratopogonidae|658|BOLD:ABV0251
Ceratopogonidae|[20810]|CNTGA2256-15|Ceratopogonidae|576|BOLD:ACS1875
Ceratopogonidae|[20811]|CNRME3232-12|Ceratopogonidae|658|BOLD:ACC7953
Ceratopogonidae|[20812]|SSWLA1628-13|Ceratopogonidae|658|BOLD:ACG6308
Forcipomyia|[20813]|SSJAB1478-13|Ceratopogonidae|661|BOLD:ACC9734
Forcipomyia|[20814]|BBDCM267-10|Ceratopogonidae|658|BOLD:AAP9052
Ceratopogonidae|[20815]|CNTMC457-14|Ceratopogonidae|576|BOLD:ACM4104
Forcipomyia|[20816]|BBDED627-10|Ceratopogonidae|658|BOLD:AAG6516
Ceratopogonidae|[20817]|HPPPD1083-13|Ceratopogonidae|661|BOLD:ACA4406
Forcipomyia|[20818]|CNJAD765-12|Ceratopogonidae|658|BOLD:ACB9454
Ceratopogonidae|[20819]|SSGBB2402-14|Ceratopogonidae|658|BOLD:ACO9883
Forcipomyia sp. 2ES|[20820]|PHAUG1378-11|Ceratopogonidae|658|BOLD:AAN5156
Ceratopogonidae|[20821]|CNIVB984-14|Ceratopogonidae|582|BOLD:ACR4061
Ceratopogonidae|[20822]|CNWAH4899-15|Ceratopogonidae|588|BOLD:ACS2194
Ceratopogonidae|[20823]|JSDIR932-11|Ceratopogonidae|658|BOLD:ABA7910
Ceratopogonidae|[20824]|NGNAN3189-14|Ceratopogonidae|664|BOLD:ACX4703
Ceratopogonidae|[20825]|CNWBH828-13|Ceratopogonidae|661|BOLD:ACE1732
Ceratopogonidae|[20826]|CNPKO1712-14|Ceratopogonidae|564|BOLD:ACO2265
Ceratopogonidae|[20827]|CNWLF1384-12|Ceratopogonidae|658|BOLD:ACD1356
Ceratopogonidae|[20828]|CNWBH1108-13|Ceratopogonidae|661|BOLD:ACI4579
Ceratopogonidae|[20829]|BBDIQ078-10|Ceratopogonidae|658|BOLD:AAL7412
Ceratopogonidae|[20830]|CNKLF1038-15|Ceratopogonidae|579|BOLD:ACS1284
Forcipomyia|[20831]|CNJAA748-12|Ceratopogonidae|673|BOLD:AAL7406
Ceratopogonidae|[20832]|SSBAC3796-12|Ceratopogonidae|658|BOLD:ACD0050
Forcipomyia|[20833]|SMTPD2973-13|Ceratopogonidae|664|BOLD:AAV5212
Ceratopogonidae|[20834]|CNYOH045-15|Ceratopogonidae|588|BOLD:ACT3843
Ceratopogonidae|[20835]|CNPPH544-12|Ceratopogonidae|661|BOLD:ABX1217
Dasyhelea|[20836]|CHDIP201-11|Ceratopogonidae|658|BOLD:AAL7409
Dasyhelea|[20837]|JSDIQ585-10|Ceratopogonidae|658|BOLD:AAP5087
Dasyhelea|[20838]|CNRMG494-12|Ceratopogonidae|664|BOLD:AAN5172
Dasyhelea|[20839]|CNWBB421-13|Ceratopogonidae|670|BOLD:AAQ0595
Dasyhelea|[20840]|CHDIP1425-12|Ceratopogonidae|658|BOLD:AAG6454
Dasyhelea|[20841]|CNWBB456-13|Ceratopogonidae|670|BOLD:AAV5200
Dasyhelea|[20842]|JSDIQ611-10|Ceratopogonidae|658|BOLD:AAU6535
Ceratopogonidae|[20843]|PAJUL532-12|Ceratopogonidae|670|BOLD:ABX7385
Ceratopogonidae|[20844]|BBDED674-10|Ceratopogonidae|658|BOLD:AAG6518
Dasyhelea|[20845]|PHDIP561-11|Ceratopogonidae|669|BOLD:AAY6464
Dasyhelea|[20846]|CNBPE212-12|Ceratopogonidae|661|BOLD:ABZ6936
Ceratopogonidae|[20847]|CNROM898-13|Ceratopogonidae|658|BOLD:ACK2715
Ceratopogonidae|[20848]|PAJUN465-12|Ceratopogonidae|658|BOLD:ACB3220
Dasyhelea|[20849]|CNRMF830-12|Ceratopogonidae|658|BOLD:AAU6477
Ceratopogonidae|[20850]|CNBRN817-14|Ceratopogonidae|590|BOLD:ACL9581
Dasyhelea|[20851]|SSGBB3383-14|Ceratopogonidae|667|BOLD:AAG6522
Dasyhelea|[20852]|SSGBB3576-14|Ceratopogonidae|667|BOLD:ACL9167
Dasyhelea|[20853]|CNPAD594-13|Ceratopogonidae|658|BOLD:ACE0705
Ceratopogonidae|[20854]|CNROS292-13|Ceratopogonidae|658|BOLD:ABX8779
Dasyhelea|[20855]|CNPAC134-13|Ceratopogonidae|670|BOLD:ACA7494
Ceratopogonidae|[20856]|SSGBB4771-14|Ceratopogonidae|588|BOLD:ACP2869
Dasyhelea|[20857]|SSPAA989-13|Ceratopogonidae|664|BOLD:ACG4753
Ceratopogonidae|[20858]|CNNHC1274-14|Ceratopogonidae|555|BOLD:ACR5852
Ceratopogonidae|[20859]|CNNHC1727-14|Ceratopogonidae|600|BOLD:ACR6331
Dasyhelea bensoni|[20860]|CHDIP166-11|Ceratopogonidae|658|BOLD:AAG6449
Ceratopogonidae|[20861]|SSEIA5585-13|Ceratopogonidae|614|BOLD:ACF9501
Dasyhelea|[20862]|CNBRL503-14|Ceratopogonidae|659|BOLD:AAG6527
Dasyhelea|[20863]|CNGMC091-13|Ceratopogonidae|661|BOLD:ACL2228
Dasyhelea|[20864]|CNBRN763-14|Ceratopogonidae|658|BOLD:ACV3503
Dasyhelea|[20865]|CNPAC038-13|Ceratopogonidae|670|BOLD:ACD8881
Dasyhelea|[20866]|CNFNE809-14|Ceratopogonidae|661|BOLD:ACG7156
Dasyhelea|[20867]|CNBRL499-14|Ceratopogonidae|659|BOLD:ACL8109
Dasyhelea|[20868]|CNBRN1210-14|Ceratopogonidae|658|BOLD:ACX4719
Ceratopogonidae|[20869]|JSDIR033-11|Ceratopogonidae|658|BOLD:ABA0802
Ceratopogonidae|[20870]|CNGBN737-14|Ceratopogonidae|555|BOLD:ACW6099
Dasyhelea|[20871]|CNWBB221-13|Ceratopogonidae|658|BOLD:ACE1884
Ceratopogonidae|[20872]|CNKLC1284-14|Ceratopogonidae|609|BOLD:ACR8638
Ceratopogonidae|[20873]|SSGBB695-14|Ceratopogonidae|591|BOLD:ACN1091
Ceratopogonidae|[20874]|CNGMB291-13|Ceratopogonidae|603|BOLD:ACK8291
Ceratopogonidae|[20875]|SSGBC4016-14|Ceratopogonidae|632|BOLD:ACP2611
Ceratopogonidae|[20876]|TTMDI686-10|Ceratopogonidae|658|BOLD:AAN5151
Ceratopogonidae|[20877]|SSBAE4331-13|Ceratopogonidae|658|BOLD:ACG5576
Ceratopogonidae|[20878]|CNRME3398-12|Ceratopogonidae|661|BOLD:ACA6095
Ceratopogonidae|[20879]|CNJAF1389-12|Ceratopogonidae|658|BOLD:ACC9621
Dasyhelea|[20880]|CNPAC110-13|Ceratopogonidae|670|BOLD:AAN5146
Dasyhelea|[20881]|SSPAC4246-13|Ceratopogonidae|670|BOLD:ACF7006
Ceratopogonidae|[20882]|CNROD278-13|Ceratopogonidae|606|BOLD:ACK1200
Ceratopogonidae|[20883]|CNKJD421-14|Ceratopogonidae|658|BOLD:ACY2199
Ceratopogonidae|[20884]|CNPPB1989-12|Ceratopogonidae|658|BOLD:ACA5721
Ceratopogonidae|[20885]|TTMDJ254-10|Ceratopogonidae|658|BOLD:AAN5163
Ceratopogonidae|[20886]|CNGLF1109-13|Ceratopogonidae|658|BOLD:ACG4127
Ceratopogonidae|[20887]|SSPAC384-13|Ceratopogonidae|658|BOLD:ACI8596
Ceratopogonidae|[20888]|CNGBE173-14|Ceratopogonidae|661|BOLD:AAN5145
Ceratopogonidae|[20889]|HPPPD890-13|Ceratopogonidae|658|BOLD:AAN5164
Ceratopogonidae|[20890]|SSEIA5288-13|Ceratopogonidae|661|BOLD:ACF9504
Ceratopogonidae|[20891]|CNKOF907-14|Ceratopogonidae|620|BOLD:ACM2737
Ceratopogonidae|[20892]|PHAUG460-11|Ceratopogonidae|658|BOLD:ABW8021
Ceratopogonidae|[20893]|CNKJG081-14|Ceratopogonidae|609|BOLD:ACD9162
Ceratopogonidae|[20894]|CNKJG611-14|Ceratopogonidae|594|BOLD:ACM7635
Ceratopogonidae|[20895]|SSROA299-14|Ceratopogonidae|642|BOLD:ACD1458
Ceratopogonidae|[20896]|PHNXN1338-14|Ceratopogonidae|604|BOLD:ACL1802

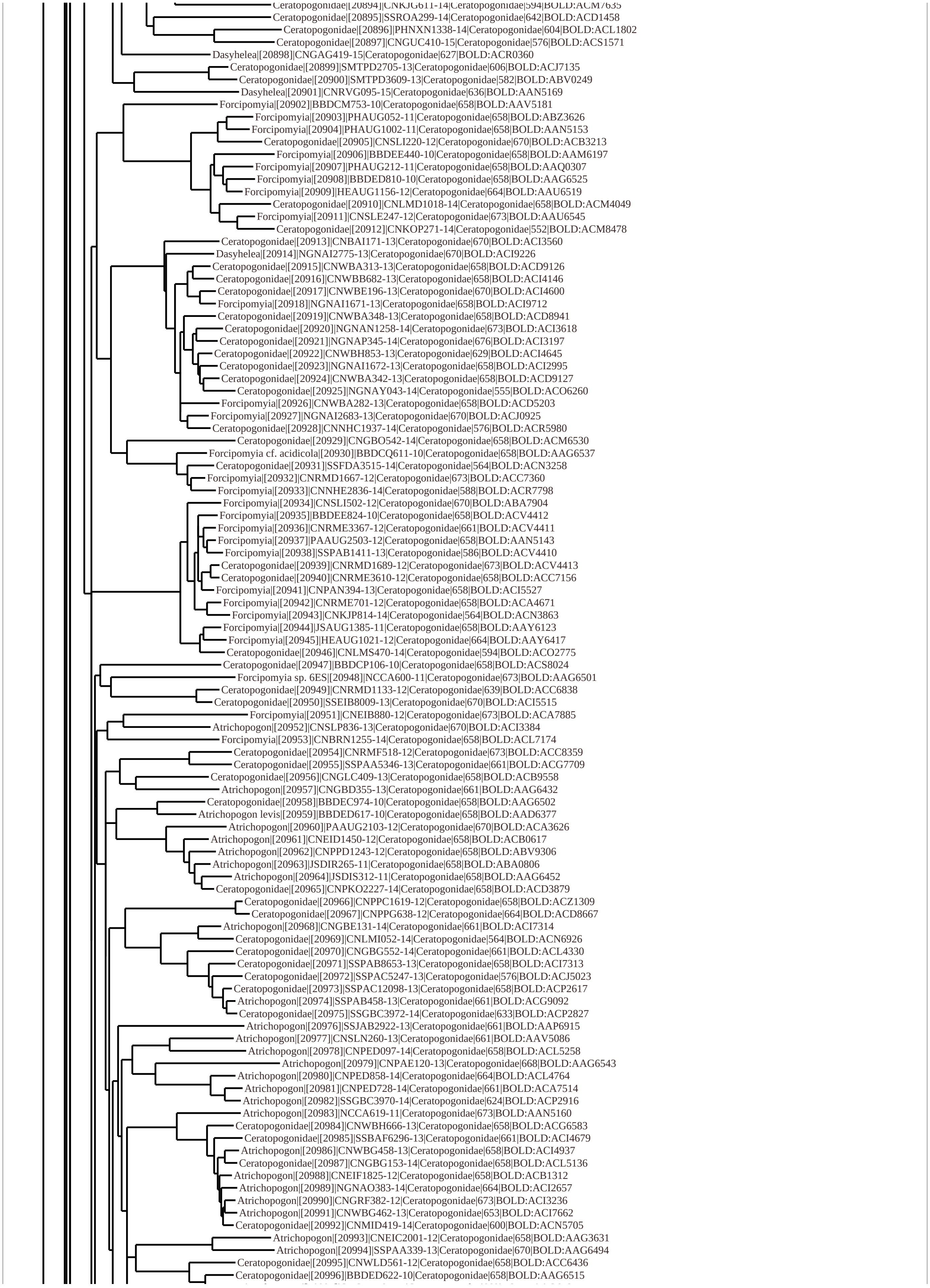
Ceratopogonidae|[20894]|CNKJG611-14|Ceratopogonidae|594|BOLD:ACM7635
Ceratopogonidae|[20895]|SSROA299-14|Ceratopogonidae|642|BOLD:ACD1458
Ceratopogonidae|[20896]|PHNXN1338-14|Ceratopogonidae|604|BOLD:ACL1802
Ceratopogonidae|[20897]|CNGUC410-15|Ceratopogonidae|576|BOLD:ACS1571
Dasyhelea|[20898]|CNGAG419-15|Ceratopogonidae|627|BOLD:ACR0360
Ceratopogonidae|[20899]|SMTPD2705-13|Ceratopogonidae|606|BOLD:ACJ7135
Ceratopogonidae|[20900]|SMTPD3609-13|Ceratopogonidae|582|BOLD:ABV0249
Dasyhelea|[20901]|CNRVG095-15|Ceratopogonidae|636|BOLD:AAN5169
Forcipomyia|[20902]|BBDCM753-10|Ceratopogonidae|658|BOLD:AAV5181
Forcipomyia|[20903]|PHAUG052-11|Ceratopogonidae|658|BOLD:ABZ3626
Forcipomyia|[20904]|PHAUG1002-11|Ceratopogonidae|658|BOLD:AAN5153
Ceratopogonidae|[20905]|CNSLI220-12|Ceratopogonidae|670|BOLD:ACB3213
Forcipomyia|[20906]|BBDEE440-10|Ceratopogonidae|658|BOLD:AAM6197
Forcipomyia|[20907]|PHAUG212-11|Ceratopogonidae|658|BOLD:AAQ0307
Forcipomyia|[20908]|BBDED810-10|Ceratopogonidae|658|BOLD:AAG6525
Forcipomyia|[20909]|HEAUG1156-12|Ceratopogonidae|664|BOLD:AAU6519
Ceratopogonidae|[20910]|CNLMD1018-14|Ceratopogonidae|658|BOLD:ACM4049
Forcipomyia|[20911]|CNSLE247-12|Ceratopogonidae|673|BOLD:AAU6545
Ceratopogonidae|[20912]|CNKOP271-14|Ceratopogonidae|552|BOLD:ACM8478
Ceratopogonidae|[20913]|CNBAI171-13|Ceratopogonidae|670|BOLD:ACI3560
Dasyhelea|[20914]|NGNAI2775-13|Ceratopogonidae|670|BOLD:ACI9226
Ceratopogonidae|[20915]|CNWBA313-13|Ceratopogonidae|658|BOLD:ACD9126
Ceratopogonidae|[20916]|CNWBB682-13|Ceratopogonidae|658|BOLD:ACI4146
Ceratopogonidae|[20917]|CNWBE196-13|Ceratopogonidae|670|BOLD:ACI4600
Forcipomyia|[20918]|NGNAI1671-13|Ceratopogonidae|658|BOLD:ACI9712
Ceratopogonidae|[20919]|CNWBA348-13|Ceratopogonidae|658|BOLD:ACD8941
Ceratopogonidae|[20920]|NGNAN1258-14|Ceratopogonidae|673|BOLD:ACI3618
Ceratopogonidae|[20921]|NGNAP345-14|Ceratopogonidae|676|BOLD:ACI3197
Ceratopogonidae|[20922]|CNWBH853-13|Ceratopogonidae|629|BOLD:ACI4645
Ceratopogonidae|[20923]|NGNAI1672-13|Ceratopogonidae|658|BOLD:ACI2995
Ceratopogonidae|[20924]|CNWBA342-13|Ceratopogonidae|658|BOLD:ACD9127
Ceratopogonidae|[20925]|NGNAY043-14|Ceratopogonidae|555|BOLD:ACO6260
Forcipomyia|[20926]|CNWBA282-13|Ceratopogonidae|658|BOLD:ACD5203
Forcipomyia|[20927]|NGNAI2683-13|Ceratopogonidae|670|BOLD:ACJ0925
Ceratopogonidae|[20928]|CNNHC1937-14|Ceratopogonidae|576|BOLD:ACR5980
Ceratopogonidae|[20929]|CNGBO542-14|Ceratopogonidae|658|BOLD:ACM6530
Forcipomyia cf. acidicola|[20930]|BBDCQ611-10|Ceratopogonidae|658|BOLD:AAG6537
Ceratopogonidae|[20931]|SSFDA3515-14|Ceratopogonidae|564|BOLD:ACN3258
Forcipomyia|[20932]|CNRMD1667-12|Ceratopogonidae|673|BOLD:ACC7360
Forcipomyia|[20933]|CNNHE2836-14|Ceratopogonidae|588|BOLD:ACR7798
Forcipomyia|[20934]|CNSLI502-12|Ceratopogonidae|670|BOLD:ABA7904
Forcipomyia|[20935]|BBDEE824-10|Ceratopogonidae|658|BOLD:ACV4412
Forcipomyia|[20936]|CNRME3367-12|Ceratopogonidae|661|BOLD:ACV4411
Forcipomyia|[20937]|PAAUG2503-12|Ceratopogonidae|658|BOLD:AAN5143
Forcipomyia|[20938]|SSPAB1411-13|Ceratopogonidae|586|BOLD:ACV4410
Ceratopogonidae|[20939]|CNRMD1689-12|Ceratopogonidae|673|BOLD:ACV4413
Ceratopogonidae|[20940]|CNRME3610-12|Ceratopogonidae|658|BOLD:ACC7156
Forcipomyia|[20941]|CNPAN394-13|Ceratopogonidae|658|BOLD:ACI5527
Forcipomyia|[20942]|CNRME701-12|Ceratopogonidae|658|BOLD:ACA4671
Forcipomyia|[20943]|CNKJP814-14|Ceratopogonidae|564|BOLD:ACN3863
Forcipomyia|[20944]|JSAUG1385-11|Ceratopogonidae|658|BOLD:AAY6123
Forcipomyia|[20945]|HEAUG1021-12|Ceratopogonidae|664|BOLD:AAY6417
Ceratopogonidae|[20946]|CNLMS470-14|Ceratopogonidae|594|BOLD:ACO2775
Ceratopogonidae|[20947]|BBDCP106-10|Ceratopogonidae|658|BOLD:ACS8024
Forcipomyia sp. 6ES|[20948]|NCCA600-11|Ceratopogonidae|673|BOLD:AAG6501
Ceratopogonidae|[20949]|CNRMD1133-12|Ceratopogonidae|639|BOLD:ACC6838
Ceratopogonidae|[20950]|SSEIB8009-13|Ceratopogonidae|670|BOLD:ACI5515
Forcipomyia|[20951]|CNEIB880-12|Ceratopogonidae|673|BOLD:ACA7885
Atrichopogon|[20952]|CNSLP836-13|Ceratopogonidae|670|BOLD:ACI3384
Forcipomyia|[20953]|CNBRN1255-14|Ceratopogonidae|658|BOLD:ACL7174
Ceratopogonidae|[20954]|CNRMF518-12|Ceratopogonidae|673|BOLD:ACC8359
Ceratopogonidae|[20955]|SSPAA5346-13|Ceratopogonidae|661|BOLD:ACG7709
Ceratopogonidae|[20956]|CNGLC409-13|Ceratopogonidae|658|BOLD:ACB9558
Atrichopogon|[20957]|CNGBD355-13|Ceratopogonidae|661|BOLD:AAG6432
Ceratopogonidae|[20958]|BBDEC974-10|Ceratopogonidae|658|BOLD:AAG6502
Atrichopogon levis|[20959]|BBDED617-10|Ceratopogonidae|658|BOLD:AAD6377
Atrichopogon|[20960]|PAAUG2103-12|Ceratopogonidae|670|BOLD:ACA3626
Atrichopogon|[20961]|CNEID1450-12|Ceratopogonidae|658|BOLD:ACB0617
Atrichopogon|[20962]|CNPPD1243-12|Ceratopogonidae|658|BOLD:ABV9306
Atrichopogon|[20963]|JSDIR265-11|Ceratopogonidae|658|BOLD:ABA0806
Atrichopogon|[20964]|JSDIS312-11|Ceratopogonidae|658|BOLD:AAG6452
Ceratopogonidae|[20965]|CNPKO2227-14|Ceratopogonidae|658|BOLD:ACD3879
Ceratopogonidae|[20966]|CNPPC1619-12|Ceratopogonidae|658|BOLD:ACZ1309
Ceratopogonidae|[20967]|CNPPG638-12|Ceratopogonidae|664|BOLD:ACD8667
Atrichopogon|[20968]|CNGBE131-14|Ceratopogonidae|661|BOLD:ACI7314
Ceratopogonidae|[20969]|CNLMI052-14|Ceratopogonidae|564|BOLD:ACN6926
Ceratopogonidae|[20970]|CNGBG552-14|Ceratopogonidae|661|BOLD:ACL4330
Ceratopogonidae|[20971]|SSPAB8653-13|Ceratopogonidae|658|BOLD:ACI7313
Ceratopogonidae|[20972]|SSPAC5247-13|Ceratopogonidae|576|BOLD:ACJ5023
Ceratopogonidae|[20973]|SSPAC12098-13|Ceratopogonidae|658|BOLD:ACP2617
Atrichopogon|[20974]|SSPAB458-13|Ceratopogonidae|661|BOLD:ACG9092
Ceratopogonidae|[20975]|SSGBC3972-14|Ceratopogonidae|633|BOLD:ACP2827
Atrichopogon|[20976]|SSJAB2922-13|Ceratopogonidae|661|BOLD:AAP6915
Atrichopogon|[20977]|CNSLN260-13|Ceratopogonidae|661|BOLD:AAV5086
Atrichopogon|[20978]|CNPED097-14|Ceratopogonidae|658|BOLD:ACL5258
Atrichopogon|[20979]|CNPAE120-13|Ceratopogonidae|668|BOLD:AAG6543
Atrichopogon|[20980]|CNPED858-14|Ceratopogonidae|664|BOLD:ACL4764
Atrichopogon|[20981]|CNPED728-14|Ceratopogonidae|661|BOLD:ACA7514
Atrichopogon|[20982]|SSGBC3970-14|Ceratopogonidae|624|BOLD:ACP2916
Atrichopogon|[20983]|NCCA619-11|Ceratopogonidae|673|BOLD:AAN5160
Ceratopogonidae|[20984]|CNWBH666-13|Ceratopogonidae|658|BOLD:ACG6583
Ceratopogonidae|[20985]|SSBAF6296-13|Ceratopogonidae|661|BOLD:ACI4679
Atrichopogon|[20986]|CNWBG458-13|Ceratopogonidae|658|BOLD:ACI4937
Ceratopogonidae|[20987]|CNGBG153-14|Ceratopogonidae|658|BOLD:ACL5136
Atrichopogon|[20988]|CNEIF1825-12|Ceratopogonidae|658|BOLD:ACB1312
Atrichopogon|[20989]|NGNAO383-14|Ceratopogonidae|664|BOLD:ACI2657
Atrichopogon|[20990]|CNGRF382-12|Ceratopogonidae|673|BOLD:ACI3236
Atrichopogon|[20991]|CNWBG462-13|Ceratopogonidae|653|BOLD:ACI7662
Ceratopogonidae|[20992]|CNMID419-14|Ceratopogonidae|600|BOLD:ACN5705
Atrichopogon|[20993]|CNEIC2001-12|Ceratopogonidae|658|BOLD:AAG3631
Atrichopogon|[20994]|SSPAA339-13|Ceratopogonidae|670|BOLD:AAG6494
Ceratopogonidae|[20995]|CNWLD561-12|Ceratopogonidae|658|BOLD:ACC6436
Ceratopogonidae|[20996]|BBDED622-10|Ceratopogonidae|658|BOLD:AAG6515

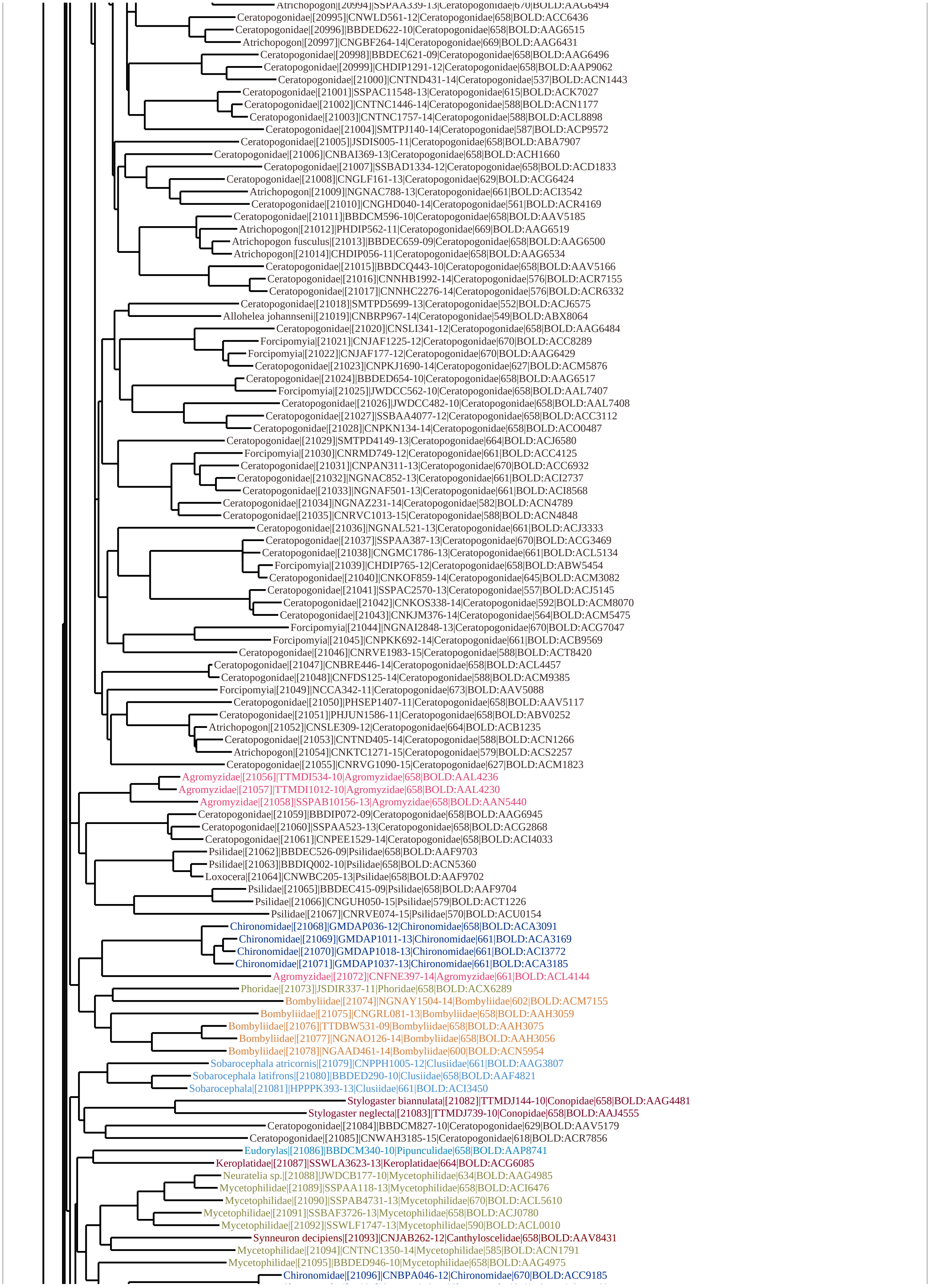
Atrichopogon|[20994]|SSPAA339-13|Ceratopogonidae|670|BOLD:AAG6494
Ceratopogonidae|[20995]|CNWLD561-12|Ceratopogonidae|658|BOLD:ACC6436
Ceratopogonidae|[20996]|BBDED622-10|Ceratopogonidae|658|BOLD:AAG6515
Atrichopogon|[20997]|CNGBF264-14|Ceratopogonidae|669|BOLD:AAG6431
Ceratopogonidae|[20998]|BBDEC621-09|Ceratopogonidae|658|BOLD:AAG6496
Ceratopogonidae|[20999]|CHDIP1291-12|Ceratopogonidae|658|BOLD:AAP9062
Ceratopogonidae|[21000]|CNTND431-14|Ceratopogonidae|537|BOLD:ACN1443
Ceratopogonidae|[21001]|SSPAC11548-13|Ceratopogonidae|615|BOLD:ACK7027
Ceratopogonidae|[21002]|CNTNC1446-14|Ceratopogonidae|588|BOLD:ACN1177
Ceratopogonidae|[21003]|CNTNC1757-14|Ceratopogonidae|588|BOLD:ACL8898
Ceratopogonidae|[21004]|SMTPJ140-14|Ceratopogonidae|587|BOLD:ACP9572
Ceratopogonidae|[21005]|JSDIS005-11|Ceratopogonidae|658|BOLD:ABA7907
Ceratopogonidae|[21006]|CNBAI369-13|Ceratopogonidae|658|BOLD:ACH1660
Ceratopogonidae|[21007]|SSBAD1334-12|Ceratopogonidae|658|BOLD:ACD1833
Ceratopogonidae|[21008]|CNGLF161-13|Ceratopogonidae|629|BOLD:ACG6424
Atrichopogon|[21009]|NGNAC788-13|Ceratopogonidae|661|BOLD:ACI3542
Ceratopogonidae|[21010]|CNGHD040-14|Ceratopogonidae|561|BOLD:ACR4169
Ceratopogonidae|[21011]|BBDCM596-10|Ceratopogonidae|658|BOLD:AAV5185
Atrichopogon|[21012]|PHDIP562-11|Ceratopogonidae|669|BOLD:AAG6519
Atrichopogon fusculus|[21013]|BBDEC659-09|Ceratopogonidae|658|BOLD:AAG6500
Atrichopogon|[21014]|CHDIP056-11|Ceratopogonidae|658|BOLD:AAG6534
Ceratopogonidae|[21015]|BBDCQ443-10|Ceratopogonidae|658|BOLD:AAV5166
Ceratopogonidae|[21016]|CNNHB1992-14|Ceratopogonidae|576|BOLD:ACR7155
Ceratopogonidae|[21017]|CNNHC2276-14|Ceratopogonidae|576|BOLD:ACR6332
Ceratopogonidae|[21018]|SMTPD5699-13|Ceratopogonidae|552|BOLD:ACJ6575
Allohelea johannseni|[21019]|CNBRP967-14|Ceratopogonidae|549|BOLD:ABX8064
Ceratopogonidae|[21020]|CNSLI341-12|Ceratopogonidae|658|BOLD:AAG6484
Forcipomyia|[21021]|CNJAF1225-12|Ceratopogonidae|670|BOLD:ACC8289
Forcipomyia|[21022]|CNJAF177-12|Ceratopogonidae|670|BOLD:AAG6429
Ceratopogonidae|[21023]|CNPKJ1690-14|Ceratopogonidae|627|BOLD:ACM5876
Ceratopogonidae|[21024]|BBDED654-10|Ceratopogonidae|658|BOLD:AAG6517
Forcipomyia|[21025]|JWDCC562-10|Ceratopogonidae|658|BOLD:AAL7407
Ceratopogonidae|[21026]|JWDCC482-10|Ceratopogonidae|658|BOLD:AAL7408
Ceratopogonidae|[21027]|SSBAA4077-12|Ceratopogonidae|658|BOLD:ACC3112
Ceratopogonidae|[21028]|CNPKN134-14|Ceratopogonidae|658|BOLD:ACO0487
Ceratopogonidae|[21029]|SMTPD4149-13|Ceratopogonidae|664|BOLD:ACJ6580
Forcipomyia|[21030]|CNRMD749-12|Ceratopogonidae|661|BOLD:ACC4125
Ceratopogonidae|[21031]|CNPAN311-13|Ceratopogonidae|670|BOLD:ACC6932
Ceratopogonidae|[21032]|NGNAC852-13|Ceratopogonidae|661|BOLD:ACI2737
Ceratopogonidae|[21033]|NGNAF501-13|Ceratopogonidae|661|BOLD:ACI8568
Ceratopogonidae|[21034]|NGNAZ231-14|Ceratopogonidae|582|BOLD:ACN4789
Ceratopogonidae|[21035]|CNRVC1013-15|Ceratopogonidae|588|BOLD:ACN4848
Ceratopogonidae|[21036]|NGNAL521-13|Ceratopogonidae|661|BOLD:ACJ3333
Ceratopogonidae|[21037]|SSPAA387-13|Ceratopogonidae|670|BOLD:ACG3469
Ceratopogonidae|[21038]|CNGMC1786-13|Ceratopogonidae|661|BOLD:ACL5134
Forcipomyia|[21039]|CHDIP765-12|Ceratopogonidae|658|BOLD:ABW5454
Ceratopogonidae|[21040]|CNKOF859-14|Ceratopogonidae|645|BOLD:ACM3082
Ceratopogonidae|[21041]|SSPAC2570-13|Ceratopogonidae|557|BOLD:ACJ5145
Ceratopogonidae|[21042]|CNKOS338-14|Ceratopogonidae|592|BOLD:ACM8070
Ceratopogonidae|[21043]|CNKJM376-14|Ceratopogonidae|564|BOLD:ACM5475
Forcipomyia|[21044]|NGNAI2848-13|Ceratopogonidae|670|BOLD:ACG7047
Forcipomyia|[21045]|CNPKK692-14|Ceratopogonidae|661|BOLD:ACB9569
Ceratopogonidae|[21046]|CNRVE1983-15|Ceratopogonidae|588|BOLD:ACT8420
Ceratopogonidae|[21047]|CNBRE446-14|Ceratopogonidae|658|BOLD:ACL4457
Ceratopogonidae|[21048]|CNFDS125-14|Ceratopogonidae|588|BOLD:ACM9385
Forcipomyia|[21049]|NCCA342-11|Ceratopogonidae|673|BOLD:AAV5088
Ceratopogonidae|[21050]|PHSEP1407-11|Ceratopogonidae|658|BOLD:AAV5117
Ceratopogonidae|[21051]|PHJUN1586-11|Ceratopogonidae|658|BOLD:ABV0252
Atrichopogon|[21052]|CNSLE309-12|Ceratopogonidae|664|BOLD:ACB1235
Ceratopogonidae|[21053]|CNTND405-14|Ceratopogonidae|588|BOLD:ACN1266
Atrichopogon|[21054]|CNKTC1271-15|Ceratopogonidae|579|BOLD:ACS2257
Ceratopogonidae|[21055]|CNRVG1090-15|Ceratopogonidae|627|BOLD:ACM1823
Agromyzidae|[21056]|TTMDI534-10|Agromyzidae|658|BOLD:AAL4236
Agromyzidae|[21057]|TTMDI1012-10|Agromyzidae|658|BOLD:AAL4230
Agromyzidae|[21058]|SSPAB10156-13|Agromyzidae|658|BOLD:AAN5440
Ceratopogonidae|[21059]|BBDIP072-09|Ceratopogonidae|658|BOLD:AAG6945
Ceratopogonidae|[21060]|SSPAA523-13|Ceratopogonidae|658|BOLD:ACG2868
Ceratopogonidae|[21061]|CNPEE1529-14|Ceratopogonidae|658|BOLD:ACI4033
Psilidae|[21062]|BBDEC526-09|Psilidae|658|BOLD:AAF9703
Psilidae|[21063]|BBDIQ002-10|Psilidae|658|BOLD:ACN5360
Loxocera|[21064]|CNWBC205-13|Psilidae|658|BOLD:AAF9702
Psilidae|[21065]|BBDEC415-09|Psilidae|658|BOLD:AAF9704
Psilidae|[21066]|CNGUH050-15|Psilidae|579|BOLD:ACT1226
Psilidae|[21067]|CNRVE074-15|Psilidae|570|BOLD:ACU0154
Chironomidae|[21068]|GMDAP036-12|Chironomidae|658|BOLD:ACA3091
Chironomidae|[21069]|GMDAP1011-13|Chironomidae|661|BOLD:ACA3169
Chironomidae|[21070]|GMDAP1018-13|Chironomidae|661|BOLD:ACI3772
Chironomidae|[21071]|GMDAP1037-13|Chironomidae|661|BOLD:ACA3185
Agromyzidae|[21072]|CNFNE397-14|Agromyzidae|661|BOLD:ACL4144
Phoridae|[21073]|JSDIR337-11|Phoridae|658|BOLD:ACX6289
Bombyliidae|[21074]|NGNAY1504-14|Bombyliidae|602|BOLD:ACM7155
Bombyliidae|[21075]|CNGRL081-13|Bombyliidae|658|BOLD:AAH3059
Bombyliidae|[21076]|TTDBW531-09|Bombyliidae|658|BOLD:AAH3075
Bombyliidae|[21077]|NGNAO126-14|Bombyliidae|658|BOLD:AAH3056
Bombyliidae|[21078]|NGAAD461-14|Bombyliidae|600|BOLD:ACN5954
Sobarocephala atricornis|[21079]|CNPPH1005-12|Clusiidae|661|BOLD:AAG3807
Sobarocephala latifrons|[21080]|BBDED290-10|Clusiidae|658|BOLD:AAF4821
Sobarocephala|[21081]|HPPPK393-13|Clusiidae|661|BOLD:ACI3450
Stylogaster biannulata|[21082]|TTMDJ144-10|Conopidae|658|BOLD:AAG4481
Stylogaster neglecta|[21083]|TTMDJ739-10|Conopidae|658|BOLD:AAJ4555
Ceratopogonidae|[21084]|BBDCM827-10|Ceratopogonidae|629|BOLD:AAV5179
Ceratopogonidae|[21085]|CNWAH3185-15|Ceratopogonidae|618|BOLD:ACR7856
Eudorylas|[21086]|BBDCM340-10|Pipunculidae|658|BOLD:AAP8741
Keroplatidae|[21087]|SSWLA3623-13|Keroplatidae|664|BOLD:ACG6085
Neuratelia sp.|[21088]|JWDCB177-10|Mycetophilidae|634|BOLD:AAG4985
Mycetophilidae|[21089]|SSPAA118-13|Mycetophilidae|658|BOLD:ACI6476
Mycetophilidae|[21090]|SSPAB4731-13|Mycetophilidae|670|BOLD:ACL5610
Mycetophilidae|[21091]|SSBAF3726-13|Mycetophilidae|658|BOLD:ACJ0780
Mycetophilidae|[21092]|SSWLF1747-13|Mycetophilidae|590|BOLD:ACL0010
Synneuron decipiens|[21093]|CNJAB262-12|Canthyloscelidae|658|BOLD:AAV8431
Mycetophilidae|[21094]|CNTNC1350-14|Mycetophilidae|585|BOLD:ACN1791
Mycetophilidae|[21095]|BBDED946-10|Mycetophilidae|658|BOLD:AAG4975
Chironomidae|[21096]|CNBPA046-12|Chironomidae|670|BOLD:ACC9185

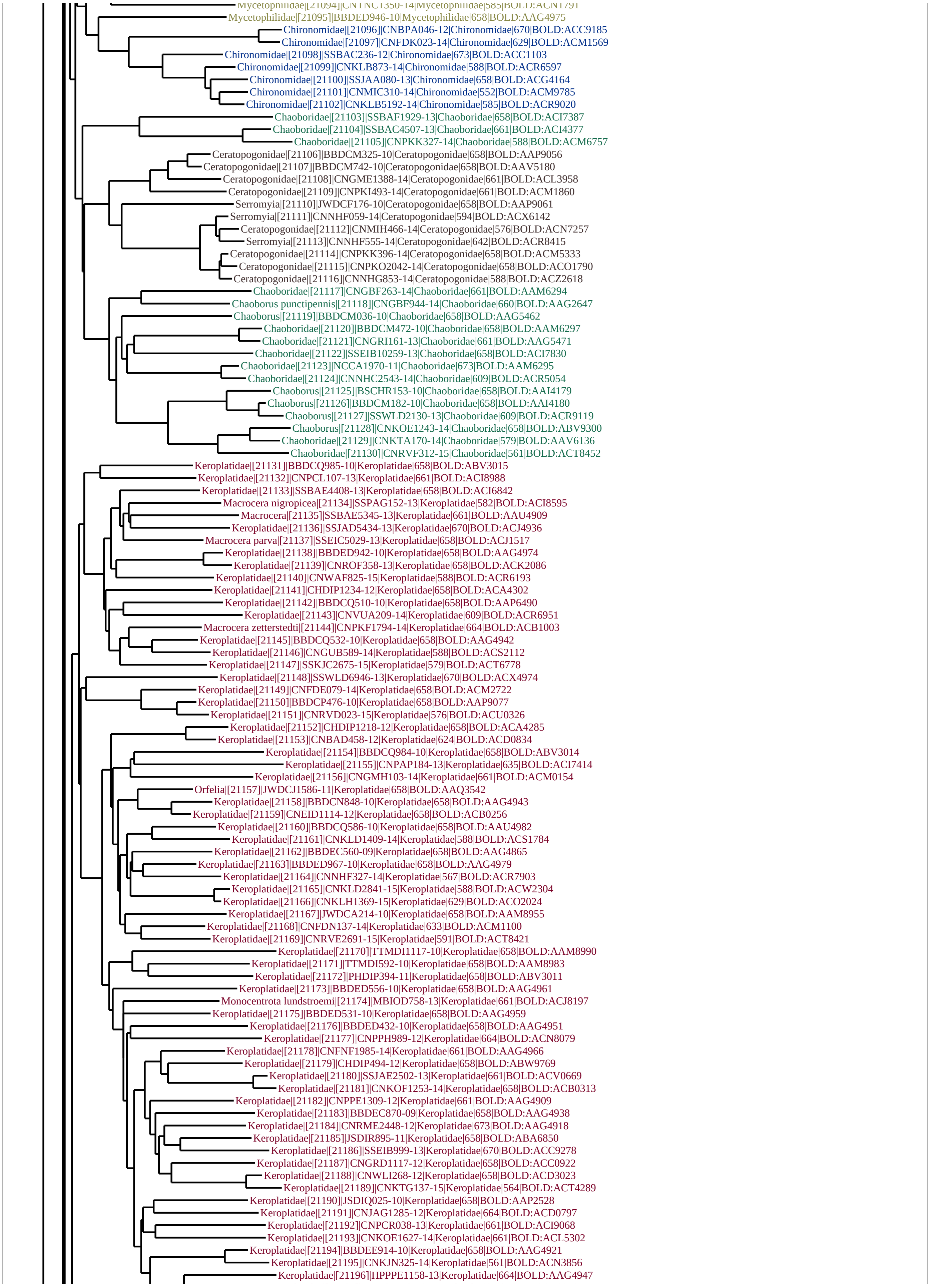
Mycetophilidae|[21094]|CNTNC1350-14|Mycetophilidae|585|BOLD:ACN1791
Mycetophilidae|[21095]|BBDED946-10|Mycetophilidae|658|BOLD:AAG4975
Chironomidae|[21096]|CNBPA046-12|Chironomidae|670|BOLD:ACC9185
Chironomidae|[21097]|CNFDK023-14|Chironomidae|629|BOLD:ACM1569
Chironomidae|[21098]|SSBAC236-12|Chironomidae|673|BOLD:ACC1103
Chironomidae|[21099]|CNKLB873-14|Chironomidae|588|BOLD:ACR6597
Chironomidae|[21100]|SSJAA080-13|Chironomidae|658|BOLD:ACG4164
Chironomidae|[21101]|CNMIC310-14|Chironomidae|552|BOLD:ACM9785
Chironomidae|[21102]|CNKLB5192-14|Chironomidae|585|BOLD:ACR9020
Chaoboridae|[21103]|SSBAF1929-13|Chaoboridae|658|BOLD:ACI7387
Chaoboridae|[21104]|SSBAC4507-13|Chaoboridae|661|BOLD:ACI4377
Chaoboridae|[21105]|CNPKK327-14|Chaoboridae|588|BOLD:ACM6757
Ceratopogonidae|[21106]|BBDCM325-10|Ceratopogonidae|658|BOLD:AAP9056
Ceratopogonidae|[21107]|BBDCM742-10|Ceratopogonidae|658|BOLD:AAV5180
Ceratopogonidae|[21108]|CNGME1388-14|Ceratopogonidae|661|BOLD:ACL3958
Ceratopogonidae|[21109]|CNPKI493-14|Ceratopogonidae|661|BOLD:ACM1860
Serromyia|[21110]|JWDCF176-10|Ceratopogonidae|658|BOLD:AAP9061
Serromyia|[21111]|CNNHF059-14|Ceratopogonidae|594|BOLD:ACX6142
Ceratopogonidae|[21112]|CNMIH466-14|Ceratopogonidae|576|BOLD:ACN7257
Serromyia|[21113]|CNNHF555-14|Ceratopogonidae|642|BOLD:ACR8415
Ceratopogonidae|[21114]|CNPKK396-14|Ceratopogonidae|658|BOLD:ACM5333
Ceratopogonidae|[21115]|CNPKO2042-14|Ceratopogonidae|658|BOLD:ACO1790
Ceratopogonidae|[21116]|CNNHG853-14|Ceratopogonidae|588|BOLD:ACZ2618
Chaoboridae|[21117]|CNGBF263-14|Chaoboridae|661|BOLD:AAM6294
Chaoborus punctipennis|[21118]|CNGBF944-14|Chaoboridae|660|BOLD:AAG2647
Chaoborus|[21119]|BBDCM036-10|Chaoboridae|658|BOLD:AAG5462
Chaoboridae|[21120]|BBDCM472-10|Chaoboridae|658|BOLD:AAM6297
Chaoboridae|[21121]|CNGRI161-13|Chaoboridae|661|BOLD:AAG5471
Chaoboridae|[21122]|SSEIB10259-13|Chaoboridae|658|BOLD:ACI7830
Chaoboridae|[21123]|NCCA1970-11|Chaoboridae|673|BOLD:AAM6295
Chaoboridae|[21124]|CNNHC2543-14|Chaoboridae|609|BOLD:ACR5054
Chaoborus|[21125]|BSCHR153-10|Chaoboridae|658|BOLD:AAI4179
Chaoborus|[21126]|BBDCM182-10|Chaoboridae|658|BOLD:AAI4180
Chaoborus|[21127]|SSWLD2130-13|Chaoboridae|609|BOLD:ACR9119
Chaoborus|[21128]|CNKOE1243-14|Chaoboridae|658|BOLD:ABV9300
Chaoboridae|[21129]|CNKTA170-14|Chaoboridae|579|BOLD:AAV6136
Chaoboridae|[21130]|CNRVF312-15|Chaoboridae|561|BOLD:ACT8452
Keroplatidae|[21131]|BBDCQ985-10|Keroplatidae|658|BOLD:ABV3015
Keroplatidae|[21132]|CNPCL107-13|Keroplatidae|661|BOLD:ACI8988
Keroplatidae|[21133]|SSBAE4408-13|Keroplatidae|658|BOLD:ACI6842
Macrocera nigropicea|[21134]|SSPAG152-13|Keroplatidae|582|BOLD:ACI8595
Macrocera|[21135]|SSBAE5345-13|Keroplatidae|661|BOLD:AAU4909
Keroplatidae|[21136]|SSJAD5434-13|Keroplatidae|670|BOLD:ACJ4936
Macrocera parva|[21137]|SSEIC5029-13|Keroplatidae|658|BOLD:ACJ1517
Keroplatidae|[21138]|BBDED942-10|Keroplatidae|658|BOLD:AAG4974
Keroplatidae|[21139]|CNROF358-13|Keroplatidae|658|BOLD:ACK2086
Keroplatidae|[21140]|CNWAF825-15|Keroplatidae|588|BOLD:ACR6193
Keroplatidae|[21141]|CHDIP1234-12|Keroplatidae|658|BOLD:ACA4302
Keroplatidae|[21142]|BBDCQ510-10|Keroplatidae|658|BOLD:AAP6490
Keroplatidae|[21143]|CNVUA209-14|Keroplatidae|609|BOLD:ACR6951
Macrocera zetterstedti|[21144]|CNPKF1794-14|Keroplatidae|664|BOLD:ACB1003
Keroplatidae|[21145]|BBDCQ532-10|Keroplatidae|658|BOLD:AAG4942
Keroplatidae|[21146]|CNGUB589-14|Keroplatidae|588|BOLD:ACS2112
Keroplatidae|[21147]|SSKJC2675-15|Keroplatidae|579|BOLD:ACT6778
Keroplatidae|[21148]|SSWLD6946-13|Keroplatidae|670|BOLD:ACX4974
Keroplatidae|[21149]|CNFDE079-14|Keroplatidae|658|BOLD:ACM2722
Keroplatidae|[21150]|BBDCP476-10|Keroplatidae|658|BOLD:AAP9077
Keroplatidae|[21151]|CNRVD023-15|Keroplatidae|576|BOLD:ACU0326
Keroplatidae|[21152]|CHDIP1218-12|Keroplatidae|658|BOLD:ACA4285
Keroplatidae|[21153]|CNBAD458-12|Keroplatidae|624|BOLD:ACD0834
Keroplatidae|[21154]|BBDCQ984-10|Keroplatidae|658|BOLD:ABV3014
Keroplatidae|[21155]|CNPAP184-13|Keroplatidae|635|BOLD:ACI7414
Keroplatidae|[21156]|CNGMH103-14|Keroplatidae|661|BOLD:ACM0154
Orfelia|[21157]|JWDCJ1586-11|Keroplatidae|658|BOLD:AAQ3542
Keroplatidae|[21158]|BBDCN848-10|Keroplatidae|658|BOLD:AAG4943
Keroplatidae|[21159]|CNEID1114-12|Keroplatidae|658|BOLD:ACB0256
Keroplatidae|[21160]|BBDCQ586-10|Keroplatidae|658|BOLD:AAU4982
Keroplatidae|[21161]|CNKLD1409-14|Keroplatidae|588|BOLD:ACS1784
Keroplatidae|[21162]|BBDEC560-09|Keroplatidae|658|BOLD:AAG4865
Keroplatidae|[21163]|BBDED967-10|Keroplatidae|658|BOLD:AAG4979
Keroplatidae|[21164]|CNNHF327-14|Keroplatidae|567|BOLD:ACR7903
Keroplatidae|[21165]|CNKLD2841-15|Keroplatidae|588|BOLD:ACW2304
Keroplatidae|[21166]|CNKLH1369-15|Keroplatidae|629|BOLD:ACO2024
Keroplatidae|[21167]|JWDCA214-10|Keroplatidae|658|BOLD:AAM8955
Keroplatidae|[21168]|CNFDN137-14|Keroplatidae|633|BOLD:ACM1100
Keroplatidae|[21169]|CNRVE2691-15|Keroplatidae|591|BOLD:ACT8421
Keroplatidae|[21170]|TTMDI1117-10|Keroplatidae|658|BOLD:AAM8990
Keroplatidae|[21171]|TTMDI592-10|Keroplatidae|658|BOLD:AAM8983
Keroplatidae|[21172]|PHDIP394-11|Keroplatidae|658|BOLD:ABV3011
Keroplatidae|[21173]|BBDED556-10|Keroplatidae|658|BOLD:AAG4961
Monocentrota lundstroemi|[21174]|MBIOD758-13|Keroplatidae|661|BOLD:ACJ8197
Keroplatidae|[21175]|BBDED531-10|Keroplatidae|658|BOLD:AAG4959
Keroplatidae|[21176]|BBDED432-10|Keroplatidae|658|BOLD:AAG4951
Keroplatidae|[21177]|CNPPH989-12|Keroplatidae|664|BOLD:ACN8079
Keroplatidae|[21178]|CNFNF1985-14|Keroplatidae|661|BOLD:AAG4966
Keroplatidae|[21179]|CHDIP494-12|Keroplatidae|658|BOLD:ABW9769
Keroplatidae|[21180]|SSJAE2502-13|Keroplatidae|661|BOLD:ACV0669
Keroplatidae|[21181]|CNKOF1253-14|Keroplatidae|658|BOLD:ACB0313
Keroplatidae|[21182]|CNPPE1309-12|Keroplatidae|661|BOLD:AAG4909
Keroplatidae|[21183]|BBDEC870-09|Keroplatidae|658|BOLD:AAG4938
Keroplatidae|[21184]|CNRME2448-12|Keroplatidae|673|BOLD:AAG4918
Keroplatidae|[21185]|JSDIR895-11|Keroplatidae|658|BOLD:ABA6850
Keroplatidae|[21186]|SSEIB999-13|Keroplatidae|670|BOLD:ACC9278
Keroplatidae|[21187]|CNGRD1117-12|Keroplatidae|658|BOLD:ACC0922
Keroplatidae|[21188]|CNWLI268-12|Keroplatidae|658|BOLD:ACD3023
Keroplatidae|[21189]|CNKTG137-15|Keroplatidae|564|BOLD:ACT4289
Keroplatidae|[21190]|JSDIQ025-10|Keroplatidae|658|BOLD:AAP2528
Keroplatidae|[21191]|CNJAG1285-12|Keroplatidae|664|BOLD:ACD0797
Keroplatidae|[21192]|CNPCR038-13|Keroplatidae|661|BOLD:ACI9068
Keroplatidae|[21193]|CNKOE1627-14|Keroplatidae|661|BOLD:ACL5302
Keroplatidae|[21194]|BBDEE914-10|Keroplatidae|658|BOLD:AAG4921
Keroplatidae|[21195]|CNKJN325-14|Keroplatidae|561|BOLD:ACN3856
Keroplatidae|[21196]|HPPPE1158-13|Keroplatidae|664|BOLD:AAG4947

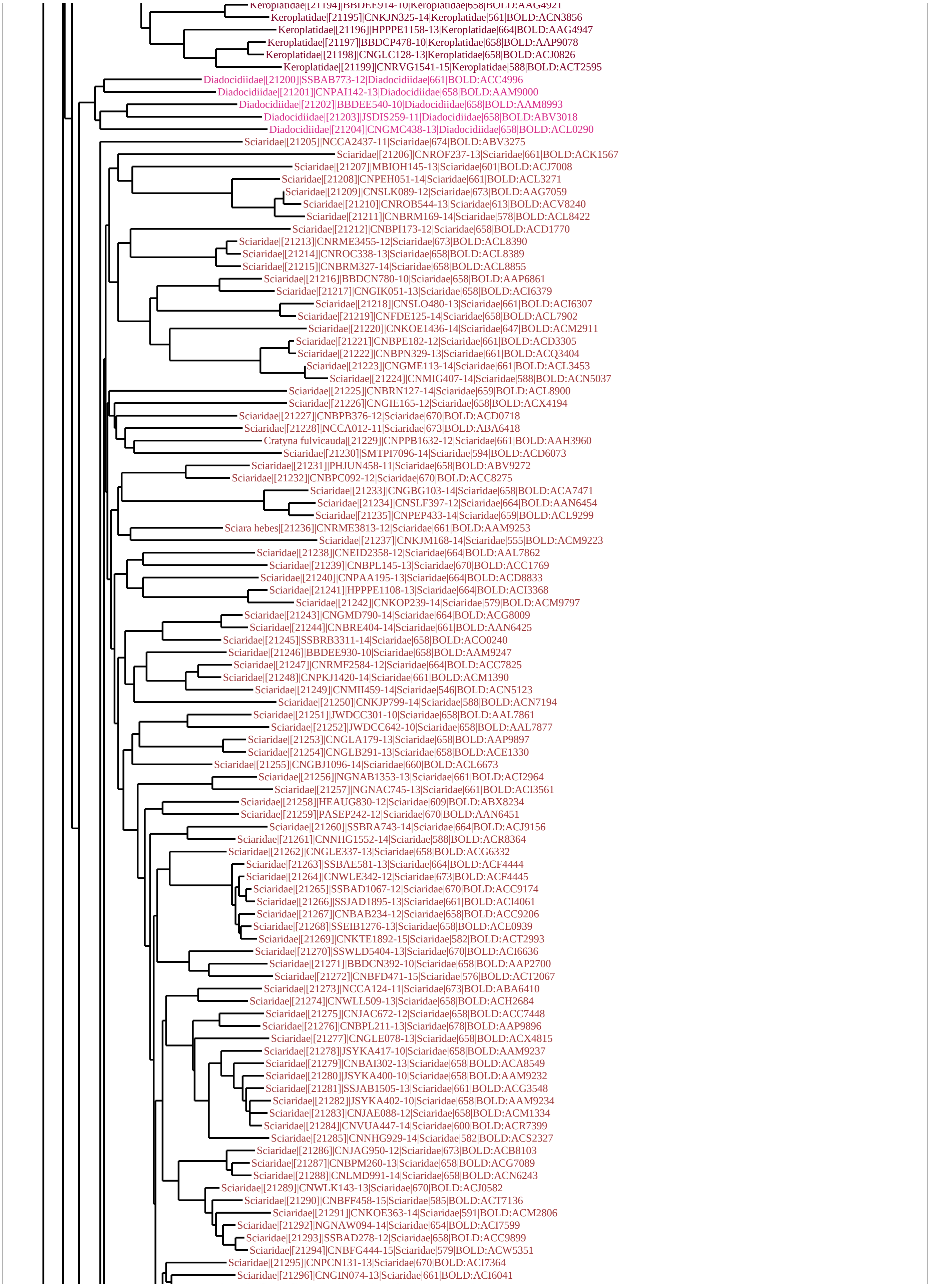
Keroplatidae|[21194]|BBDEE914-10|Keroplatidae|658|BOLD:AAG4921
Keroplatidae|[21195]|CNKJN325-14|Keroplatidae|561|BOLD:ACN3856
Keroplatidae|[21196]|HPPPE1158-13|Keroplatidae|664|BOLD:AAG4947
Keroplatidae|[21197]|BBDCP478-10|Keroplatidae|658|BOLD:AAP9078
Keroplatidae|[21198]|CNGLC128-13|Keroplatidae|658|BOLD:ACJ0826
Keroplatidae|[21199]|CNRVG1541-15|Keroplatidae|588|BOLD:ACT2595
Diadocidiidae|[21200]|SSBAB773-12|Diadocidiidae|661|BOLD:ACC4996
Diadocidiidae|[21201]|CNPAI142-13|Diadocidiidae|658|BOLD:AAM9000
Diadocidiidae|[21202]|BBDEE540-10|Diadocidiidae|658|BOLD:AAM8993
Diadocidiidae|[21203]|JSDIS259-11|Diadocidiidae|658|BOLD:ABV3018
Diadocidiidae|[21204]|CNGMC438-13|Diadocidiidae|658|BOLD:ACL0290
Sciaridae|[21205]|NCCA2437-11|Sciaridae|674|BOLD:ABV3275
Sciaridae|[21206]|CNROF237-13|Sciaridae|661|BOLD:ACK1567
Sciaridae|[21207]|MBIOH145-13|Sciaridae|601|BOLD:ACJ7008
Sciaridae|[21208]|CNPEH051-14|Sciaridae|661|BOLD:ACL3271
Sciaridae|[21209]|CNSLK089-12|Sciaridae|673|BOLD:AAG7059
Sciaridae|[21210]|CNROB544-13|Sciaridae|613|BOLD:ACV8240
Sciaridae|[21211]|CNBRM169-14|Sciaridae|578|BOLD:ACL8422
Sciaridae|[21212]|CNBPI173-12|Sciaridae|658|BOLD:ACD1770
Sciaridae|[21213]|CNRME3455-12|Sciaridae|673|BOLD:ACL8390
Sciaridae|[21214]|CNROC338-13|Sciaridae|658|BOLD:ACL8389
Sciaridae|[21215]|CNBRM327-14|Sciaridae|658|BOLD:ACL8855
Sciaridae|[21216]|BBDCN780-10|Sciaridae|658|BOLD:AAP6861
Sciaridae|[21217]|CNGIK051-13|Sciaridae|658|BOLD:ACL6379
Sciaridae|[21218]|CNSLO480-13|Sciaridae|661|BOLD:ACI6307
Sciaridae|[21219]|CNFDE125-14|Sciaridae|658|BOLD:ACL7902
Sciaridae|[21220]|CNKOE1436-14|Sciaridae|647|BOLD:ACM2911
Sciaridae|[21221]|CNBPE182-12|Sciaridae|661|BOLD:ACD3305
Sciaridae|[21222]|CNBPN329-13|Sciaridae|661|BOLD:ACQ3404
Sciaridae|[21223]|CNGME113-14|Sciaridae|661|BOLD:ACL3453
Sciaridae|[21224]|CNMIG407-14|Sciaridae|588|BOLD:ACN5037
Sciaridae|[21225]|CNBRN127-14|Sciaridae|659|BOLD:ACL8900
Sciaridae|[21226]|CNGIE165-12|Sciaridae|658|BOLD:ACX4194
Sciaridae|[21227]|CNBPB376-12|Sciaridae|670|BOLD:ACD0718
Sciaridae|[21228]|NCCA012-11|Sciaridae|673|BOLD:ABA6418
Cratyna fulvicauda|[21229]|CNPPB1632-12|Sciaridae|661|BOLD:AAH3960
Sciaridae|[21230]|SMTPI7096-14|Sciaridae|594|BOLD:ACD6073
Sciaridae|[21231]|PHJUN458-11|Sciaridae|658|BOLD:ABV9272
Sciaridae|[21232]|CNBPC092-12|Sciaridae|670|BOLD:ACC8275
Sciaridae|[21233]|CNGBG103-14|Sciaridae|658|BOLD:ACA7471
Sciaridae|[21234]|CNSLF397-12|Sciaridae|664|BOLD:AAN6454
Sciaridae|[21235]|CNPEP433-14|Sciaridae|659|BOLD:ACL9299
Sciara hebes|[21236]|CNRME3813-12|Sciaridae|661|BOLD:AAM9253
Sciaridae|[21237]|CNKJM168-14|Sciaridae|555|BOLD:ACM9223
Sciaridae|[21238]|CNEID2358-12|Sciaridae|664|BOLD:AAL7862
Sciaridae|[21239]|CNBPL145-13|Sciaridae|670|BOLD:ACC1769
Sciaridae|[21240]|CNPAA195-13|Sciaridae|664|BOLD:ACD8833
Sciaridae|[21241]|HPPPE1108-13|Sciaridae|664|BOLD:ACI3368
Sciaridae|[21242]|CNKOP239-14|Sciaridae|579|BOLD:ACM9797
Sciaridae|[21243]|CNGMD790-14|Sciaridae|664|BOLD:ACG8009
Sciaridae|[21244]|CNBRE404-14|Sciaridae|661|BOLD:AAN6425
Sciaridae|[21245]|SSBRB3311-14|Sciaridae|658|BOLD:ACO0240
Sciaridae|[21246]|BBDEE930-10|Sciaridae|658|BOLD:AAM9247
Sciaridae|[21247]|CNRMF2584-12|Sciaridae|664|BOLD:ACC7825
Sciaridae|[21248]|CNPKJ1420-14|Sciaridae|661|BOLD:ACM1390
Sciaridae|[21249]|CNMII459-14|Sciaridae|546|BOLD:ACN5123
Sciaridae|[21250]|CNKJP799-14|Sciaridae|588|BOLD:ACN7194
Sciaridae|[21251]|JWDCC301-10|Sciaridae|658|BOLD:AAL7861
Sciaridae|[21252]|JWDCC642-10|Sciaridae|658|BOLD:AAL7877
Sciaridae|[21253]|CNGLA179-13|Sciaridae|658|BOLD:AAP9897
Sciaridae|[21254]|CNGLB291-13|Sciaridae|658|BOLD:ACE1330
Sciaridae|[21255]|CNGBJ1096-14|Sciaridae|660|BOLD:ACL6673
Sciaridae|[21256]|NGNAB1353-13|Sciaridae|661|BOLD:ACI2964
Sciaridae|[21257]|NGNAC745-13|Sciaridae|661|BOLD:ACI3561
Sciaridae|[21258]|HEAUG830-12|Sciaridae|609|BOLD:ABX8234
Sciaridae|[21259]|PASEP242-12|Sciaridae|670|BOLD:AAN6451
Sciaridae|[21260]|SSBRA743-14|Sciaridae|664|BOLD:ACJ9156
Sciaridae|[21261]|CNNHG1552-14|Sciaridae|588|BOLD:ACR8364
Sciaridae|[21262]|CNGLE337-13|Sciaridae|658|BOLD:ACG6332
Sciaridae|[21263]|SSBAE581-13|Sciaridae|664|BOLD:ACF4444
Sciaridae|[21264]|CNWLE342-12|Sciaridae|673|BOLD:ACF4445
Sciaridae|[21265]|SSBAD1067-12|Sciaridae|670|BOLD:ACC9174
Sciaridae|[21266]|SSJAD1895-13|Sciaridae|661|BOLD:ACI4061
Sciaridae|[21267]|CNBAB234-12|Sciaridae|658|BOLD:ACC9206
Sciaridae|[21268]|SSEIB1276-13|Sciaridae|658|BOLD:ACE0939
Sciaridae|[21269]|CNKTE1892-15|Sciaridae|582|BOLD:ACT2993
Sciaridae|[21270]|SSWLD5404-13|Sciaridae|670|BOLD:ACI6636
Sciaridae|[21271]|BBDCN392-10|Sciaridae|658|BOLD:AAP2700
Sciaridae|[21272]|CNBFD471-15|Sciaridae|576|BOLD:ACT2067
Sciaridae|[21273]|NCCA124-11|Sciaridae|673|BOLD:ABA6410
Sciaridae|[21274]|CNWLL509-13|Sciaridae|658|BOLD:ACH2684
Sciaridae|[21275]|CNJAC672-12|Sciaridae|658|BOLD:ACC7448
Sciaridae|[21276]|CNBPL211-13|Sciaridae|678|BOLD:AAP9896
Sciaridae|[21277]|CNGLE078-13|Sciaridae|658|BOLD:ACX4815
Sciaridae|[21278]|JSYKA417-10|Sciaridae|658|BOLD:AAM9237
Sciaridae|[21279]|CNBAI302-13|Sciaridae|658|BOLD:ACA8549
Sciaridae|[21280]|JSYKA400-10|Sciaridae|658|BOLD:AAM9232
Sciaridae|[21281]|SSJAB1505-13|Sciaridae|661|BOLD:ACG3548
Sciaridae|[21282]|JSYKA402-10|Sciaridae|658|BOLD:AAM9234
Sciaridae|[21283]|CNJAE088-12|Sciaridae|658|BOLD:ACM1334
Sciaridae|[21284]|CNVUA447-14|Sciaridae|600|BOLD:ACR7399
Sciaridae|[21285]|CNNHG929-14|Sciaridae|582|BOLD:ACS2327
Sciaridae|[21286]|CNJAG950-12|Sciaridae|673|BOLD:ACB8103
Sciaridae|[21287]|CNBPM260-13|Sciaridae|658|BOLD:ACG7089
Sciaridae|[21288]|CNLMD991-14|Sciaridae|658|BOLD:ACN6243
Sciaridae|[21289]|CNWLK143-13|Sciaridae|670|BOLD:ACJ0582
Sciaridae|[21290]|CNBFF458-15|Sciaridae|585|BOLD:ACT7136
Sciaridae|[21291]|CNKOE363-14|Sciaridae|591|BOLD:ACM2806
Sciaridae|[21292]|NGNAW094-14|Sciaridae|654|BOLD:ACI7599
Sciaridae|[21293]|SSBAD278-12|Sciaridae|658|BOLD:ACC9899
Sciaridae|[21294]|CNBFG444-15|Sciaridae|579|BOLD:ACW5351
Sciaridae|[21295]|CNPCN131-13|Sciaridae|670|BOLD:ACI7364
Sciaridae|[21296]|CNGIN074-13|Sciaridae|661|BOLD:ACI6041

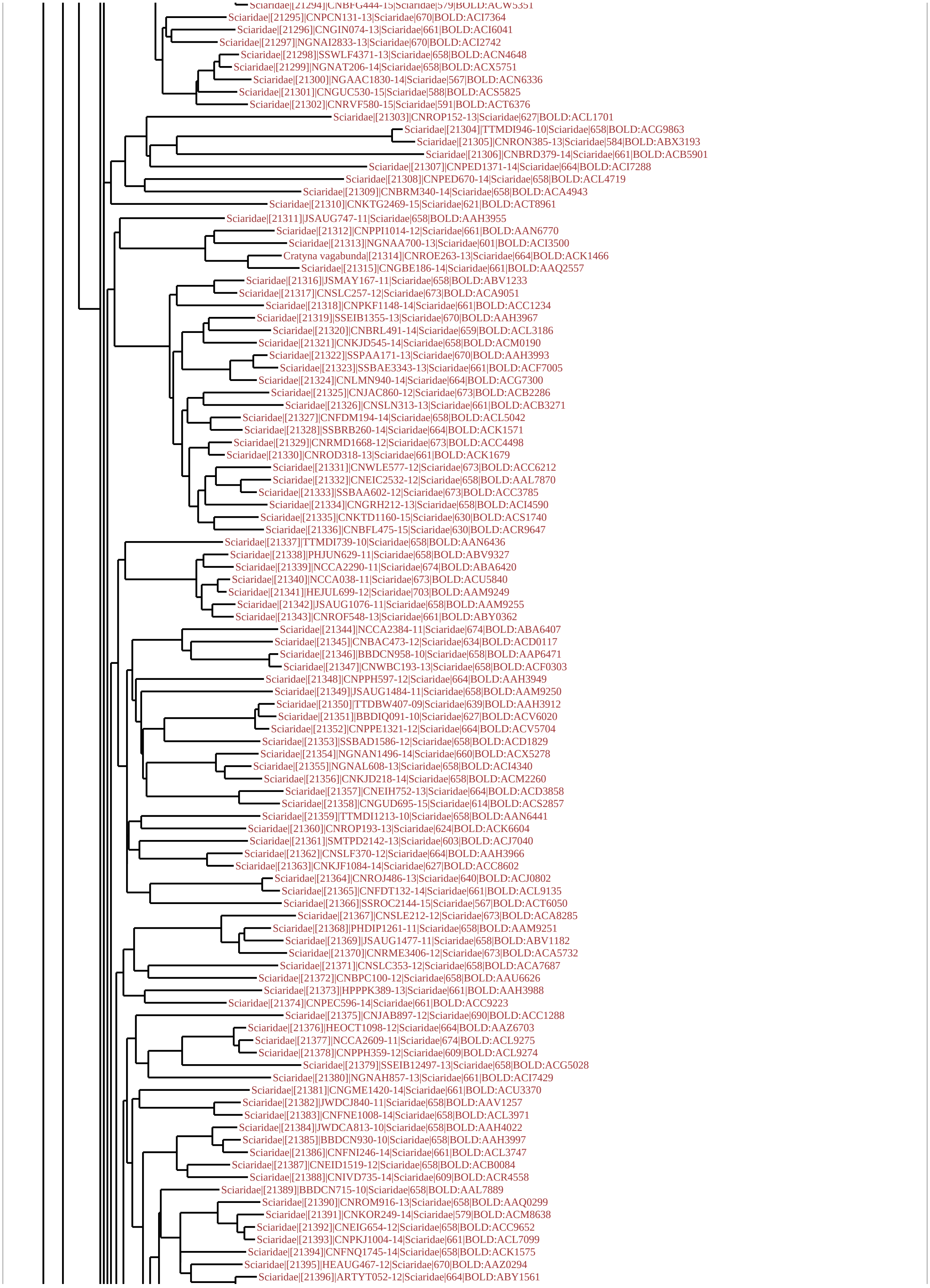

Sciaridae|[21294]|CNBFG444-15|Sciaridae|579|BOLD:ACW5351
Sciaridae|[21295]|CNPCN131-13|Sciaridae|670|BOLD:ACI7364
Sciaridae|[21296]|CNGIN074-13|Sciaridae|661|BOLD:ACI6041
Sciaridae|[21297]|NGNAI2833-13|Sciaridae|670|BOLD:ACI2742
Sciaridae|[21298]|SSWLF4371-13|Sciaridae|658|BOLD:ACN4648
Sciaridae|[21299]|NGNAT206-14|Sciaridae|658|BOLD:ACX5751
Sciaridae|[21300]|NGAAC1830-14|Sciaridae|567|BOLD:ACN6336
Sciaridae|[21301]|CNGUC530-15|Sciaridae|588|BOLD:ACS5825
Sciaridae|[21302]|CNRVF580-15|Sciaridae|591|BOLD:ACT6376
Sciaridae|[21303]|CNROP152-13|Sciaridae|627|BOLD:ACL1701
Sciaridae|[21304]|TTMDI946-10|Sciaridae|658|BOLD:ACG9863
Sciaridae|[21305]|CNRON385-13|Sciaridae|584|BOLD:ABX3193
Sciaridae|[21306]|CNBRD379-14|Sciaridae|661|BOLD:ACB5901
Sciaridae|[21307]|CNPED1371-14|Sciaridae|664|BOLD:ACI7288
Sciaridae|[21308]|CNPED670-14|Sciaridae|658|BOLD:ACL4719
Sciaridae|[21309]|CNBRM340-14|Sciaridae|658|BOLD:ACA4943
Sciaridae|[21310]|CNKTG2469-15|Sciaridae|621|BOLD:ACT8961
Sciaridae|[21311]|JSAUG747-11|Sciaridae|658|BOLD:AAH3955
Sciaridae|[21312]|CNPPI1014-12|Sciaridae|661|BOLD:AAN6770
Sciaridae|[21313]|NGNAA700-13|Sciaridae|601|BOLD:ACI3500
Cratyna vagabunda|[21314]|CNROE263-13|Sciaridae|664|BOLD:ACK1466
Sciaridae|[21315]|CNGBE186-14|Sciaridae|661|BOLD:AAQ2557
Sciaridae|[21316]|JSMAY167-11|Sciaridae|658|BOLD:ABV1233
Sciaridae|[21317]|CNSLC257-12|Sciaridae|673|BOLD:ACA9051
Sciaridae|[21318]|CNPKF1148-14|Sciaridae|661|BOLD:ACC1234
Sciaridae|[21319]|SSEIB1355-13|Sciaridae|670|BOLD:AAH3967
Sciaridae|[21320]|CNBRL491-14|Sciaridae|659|BOLD:ACL3186
Sciaridae|[21321]|CNKJD545-14|Sciaridae|658|BOLD:ACM0190
Sciaridae|[21322]|SSPAA171-13|Sciaridae|670|BOLD:AAH3993
Sciaridae|[21323]|SSBAE3343-13|Sciaridae|661|BOLD:ACF7005
Sciaridae|[21324]|CNLMN940-14|Sciaridae|664|BOLD:ACG7300
Sciaridae|[21325]|CNJAC860-12|Sciaridae|673|BOLD:ACB2286
Sciaridae|[21326]|CNSLN313-13|Sciaridae|661|BOLD:ACB3271
Sciaridae|[21327]|CNFDM194-14|Sciaridae|658|BOLD:ACL5042
Sciaridae|[21328]|SSBRB260-14|Sciaridae|664|BOLD:ACK1571
Sciaridae|[21329]|CNRMD1668-12|Sciaridae|673|BOLD:ACC4498
Sciaridae|[21330]|CNROD318-13|Sciaridae|661|BOLD:ACK1679
Sciaridae|[21331]|CNWLE577-12|Sciaridae|673|BOLD:ACC6212
Sciaridae|[21332]|CNEIC2532-12|Sciaridae|658|BOLD:AAL7870
Sciaridae|[21333]|SSBAA602-12|Sciaridae|673|BOLD:ACC3785
Sciaridae|[21334]|CNGRH212-13|Sciaridae|658|BOLD:ACI4590
Sciaridae|[21335]|CNKTD1160-15|Sciaridae|630|BOLD:ACS1740
Sciaridae|[21336]|CNBFL475-15|Sciaridae|630|BOLD:ACR9647
Sciaridae|[21337]|TTMDI739-10|Sciaridae|658|BOLD:AAN6436
Sciaridae|[21338]|PHJUN629-11|Sciaridae|658|BOLD:ABV9327
Sciaridae|[21339]|NCCA2290-11|Sciaridae|674|BOLD:ABA6420
Sciaridae|[21340]|NCCA038-11|Sciaridae|673|BOLD:ACU5840
Sciaridae|[21341]|HEJUL699-12|Sciaridae|703|BOLD:AAM9249
Sciaridae|[21342]|JSAUG1076-11|Sciaridae|658|BOLD:AAM9255
Sciaridae|[21343]|CNROF548-13|Sciaridae|661|BOLD:ABY0362
Sciaridae|[21344]|NCCA2384-11|Sciaridae|674|BOLD:ABA6407
Sciaridae|[21345]|CNBAC473-12|Sciaridae|634|BOLD:ACD0117
Sciaridae|[21346]|BBDCN958-10|Sciaridae|658|BOLD:AAP6471
Sciaridae|[21347]|CNWBC193-13|Sciaridae|658|BOLD:ACF0303
Sciaridae|[21348]|CNPPH597-12|Sciaridae|664|BOLD:AAH3949
Sciaridae|[21349]|JSAUG1484-11|Sciaridae|658|BOLD:AAM9250
Sciaridae|[21350]|TTDBW407-09|Sciaridae|639|BOLD:AAH3912
Sciaridae|[21351]|BBDIQ091-10|Sciaridae|627|BOLD:ACV6020
Sciaridae|[21352]|CNPPE1321-12|Sciaridae|664|BOLD:ACV5704
Sciaridae|[21353]|SSBAD1586-12|Sciaridae|658|BOLD:ACD1829
Sciaridae|[21354]|NGNAN1496-14|Sciaridae|660|BOLD:ACX5278
Sciaridae|[21355]|NGNAL608-13|Sciaridae|658|BOLD:ACI4340
Sciaridae|[21356]|CNKJD218-14|Sciaridae|658|BOLD:ACM2260
Sciaridae|[21357]|CNEIH752-13|Sciaridae|664|BOLD:ACD3858
Sciaridae|[21358]|CNGUD695-15|Sciaridae|614|BOLD:ACS2857
Sciaridae|[21359]|TTMDI1213-10|Sciaridae|658|BOLD:AAN6441
Sciaridae|[21360]|CNROP193-13|Sciaridae|624|BOLD:ACK6604
Sciaridae|[21361]|SMTPD2142-13|Sciaridae|603|BOLD:ACJ7040
Sciaridae|[21362]|CNSLF370-12|Sciaridae|664|BOLD:AAH3966
Sciaridae|[21363]|CNKJF1084-14|Sciaridae|627|BOLD:ACC8602
Sciaridae|[21364]|CNROJ486-13|Sciaridae|640|BOLD:ACJ0802
Sciaridae|[21365]|CNFDT132-14|Sciaridae|661|BOLD:ACL9135
Sciaridae|[21366]|SSROC2144-15|Sciaridae|567|BOLD:ACT6050
Sciaridae|[21367]|CNSLE212-12|Sciaridae|673|BOLD:ACA8285
Sciaridae|[21368]|PHDIP1261-11|Sciaridae|658|BOLD:AAM9251
Sciaridae|[21369]|JSAUG1477-11|Sciaridae|658|BOLD:ABV1182
Sciaridae|[21370]|CNRME3406-12|Sciaridae|673|BOLD:ACA5732
Sciaridae|[21371]|CNSLC353-12|Sciaridae|658|BOLD:ACA7687
Sciaridae|[21372]|CNBPC100-12|Sciaridae|658|BOLD:AAU6626
Sciaridae|[21373]|HPPPK389-13|Sciaridae|661|BOLD:AAH3988
Sciaridae|[21374]|CNPEC596-14|Sciaridae|661|BOLD:ACC9223
Sciaridae|[21375]|CNJAB897-12|Sciaridae|690|BOLD:ACC1288
Sciaridae|[21376]|HEOCT1098-12|Sciaridae|664|BOLD:AAZ6703
Sciaridae|[21377]|NCCA2609-11|Sciaridae|674|BOLD:ACL9275
Sciaridae|[21378]|CNPPH359-12|Sciaridae|609|BOLD:ACL9274
Sciaridae|[21379]|SSEIB12497-13|Sciaridae|658|BOLD:ACG5028
Sciaridae|[21380]|NGNAH857-13|Sciaridae|661|BOLD:ACI7429
Sciaridae|[21381]|CNGME1420-14|Sciaridae|661|BOLD:ACU3370
Sciaridae|[21382]|JWDCJ840-11|Sciaridae|658|BOLD:AAV1257
Sciaridae|[21383]|CNFNE1008-14|Sciaridae|658|BOLD:ACL3971
Sciaridae|[21384]|JWDCA813-10|Sciaridae|658|BOLD:AAH4022
Sciaridae|[21385]|BBDCN930-10|Sciaridae|658|BOLD:AAH3997
Sciaridae|[21386]|CNFNI246-14|Sciaridae|661|BOLD:ACL3747
Sciaridae|[21387]|CNEID1519-12|Sciaridae|658|BOLD:ACB0084
Sciaridae|[21388]|CNIVD735-14|Sciaridae|609|BOLD:ACR4558
Sciaridae|[21389]|BBDCN715-10|Sciaridae|658|BOLD:AAL7889
Sciaridae|[21390]|CNROM916-13|Sciaridae|658|BOLD:AAQ0299
Sciaridae|[21391]|CNKOR249-14|Sciaridae|579|BOLD:ACM8638
Sciaridae|[21392]|CNEIG654-12|Sciaridae|658|BOLD:ACC9652
Sciaridae|[21393]|CNPKJ1004-14|Sciaridae|661|BOLD:ACL7099
Sciaridae|[21394]|CNFNQ1745-14|Sciaridae|658|BOLD:ACK1575
Sciaridae|[21395]|HEAUG467-12|Sciaridae|670|BOLD:AAZ0294
Sciaridae|[21396]|ARTYT052-12|Sciaridae|664|BOLD:ABY1561

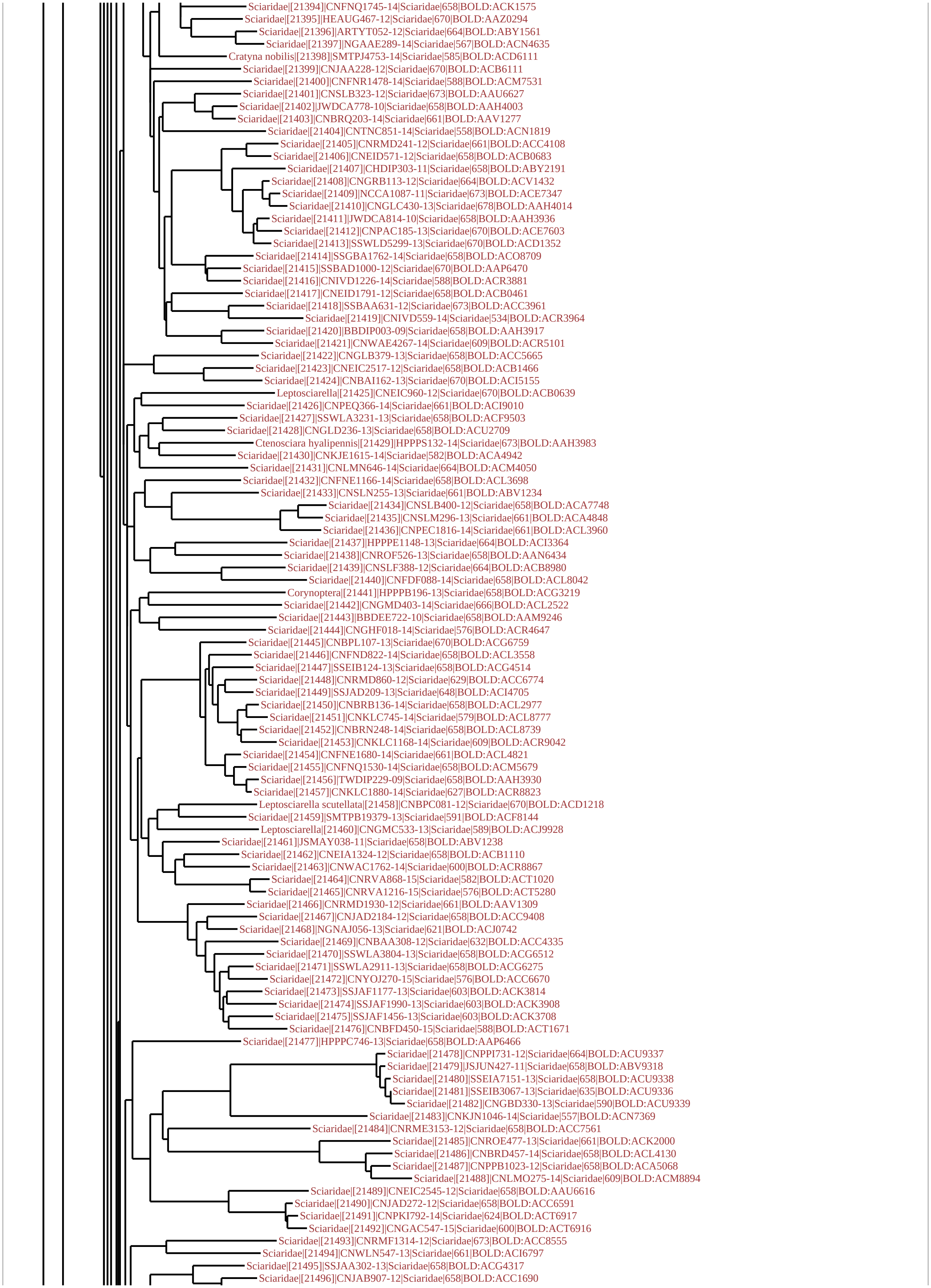
Sciaridae|[21394]|CNFNQ1745-14|Sciaridae|658|BOLD:ACK1575
Sciaridae|[21395]|HEAUG467-12|Sciaridae|670|BOLD:AAZ0294
Sciaridae|[21396]|ARTYT052-12|Sciaridae|664|BOLD:ABY1561
Sciaridae|[21397]|NGAAE289-14|Sciaridae|567|BOLD:ACN4635
Cratyna nobilis|[21398]|SMTPJ4753-14|Sciaridae|585|BOLD:ACD6111
Sciaridae|[21399]|CNJAA228-12|Sciaridae|670|BOLD:ACB6111
Sciaridae|[21400]|CNFNR1478-14|Sciaridae|588|BOLD:ACM7531
Sciaridae|[21401]|CNSLB323-12|Sciaridae|673|BOLD:AAU6627
Sciaridae|[21402]|JWDCA778-10|Sciaridae|658|BOLD:AAH4003
Sciaridae|[21403]|CNBRQ203-14|Sciaridae|661|BOLD:AAV1277
Sciaridae|[21404]|CNTNC851-14|Sciaridae|558|BOLD:ACN1819
Sciaridae|[21405]|CNRMD241-12|Sciaridae|661|BOLD:ACC4108
Sciaridae|[21406]|CNEID571-12|Sciaridae|658|BOLD:ACB0683
Sciaridae|[21407]|CHDIP303-11|Sciaridae|658|BOLD:ABY2191
Sciaridae|[21408]|CNGRB113-12|Sciaridae|664|BOLD:ACV1432
Sciaridae|[21409]|NCCA1087-11|Sciaridae|673|BOLD:ACE7347
Sciaridae|[21410]|CNGLC430-13|Sciaridae|678|BOLD:AAH4014
Sciaridae|[21411]|JWDCA814-10|Sciaridae|658|BOLD:AAH3936
Sciaridae|[21412]|CNPAC185-13|Sciaridae|670|BOLD:ACE7603
Sciaridae|[21413]|SSWLD5299-13|Sciaridae|670|BOLD:ACD1352
Sciaridae|[21414]|SSGBA1762-14|Sciaridae|658|BOLD:ACO8709
Sciaridae|[21415]|SSBAD1000-12|Sciaridae|670|BOLD:AAP6470
Sciaridae|[21416]|CNIVD1226-14|Sciaridae|588|BOLD:ACR3881
Sciaridae|[21417]|CNEID1791-12|Sciaridae|658|BOLD:ACB0461
Sciaridae|[21418]|SSBAA631-12|Sciaridae|673|BOLD:ACC3961
Sciaridae|[21419]|CNIVD559-14|Sciaridae|534|BOLD:ACR3964
Sciaridae|[21420]|BBDIP003-09|Sciaridae|658|BOLD:AAH3917
Sciaridae|[21421]|CNWAE4267-14|Sciaridae|609|BOLD:ACR5101
Sciaridae|[21422]|CNGLB379-13|Sciaridae|658|BOLD:ACC5665
Sciaridae|[21423]|CNEIC2517-12|Sciaridae|658|BOLD:ACB1466
Sciaridae|[21424]|CNBAI162-13|Sciaridae|670|BOLD:ACI5155
Leptosciarella|[21425]|CNEIC960-12|Sciaridae|670|BOLD:ACB0639
Sciaridae|[21426]|CNPEQ366-14|Sciaridae|661|BOLD:ACI9010
Sciaridae|[21427]|SSWLA3231-13|Sciaridae|658|BOLD:ACF9503
Sciaridae|[21428]|CNGLD236-13|Sciaridae|658|BOLD:ACU2709
Ctenosciara hyalipennis|[21429]|HPPPS132-14|Sciaridae|673|BOLD:AAH3983
Sciaridae|[21430]|CNKJE1615-14|Sciaridae|582|BOLD:ACA4942
Sciaridae|[21431]|CNLMN646-14|Sciaridae|664|BOLD:ACM4050
Sciaridae|[21432]|CNFNE1166-14|Sciaridae|658|BOLD:ACL3698
Sciaridae|[21433]|CNSLN255-13|Sciaridae|661|BOLD:ABV1234
Sciaridae|[21434]|CNSLB400-12|Sciaridae|658|BOLD:ACA7748
Sciaridae|[21435]|CNSLM296-13|Sciaridae|661|BOLD:ACA4848
Sciaridae|[21436]|CNPEC1816-14|Sciaridae|661|BOLD:ACL3960
Sciaridae|[21437]|HPPPE1148-13|Sciaridae|664|BOLD:ACI3364
Sciaridae|[21438]|CNROF526-13|Sciaridae|658|BOLD:AAN6434
Sciaridae|[21439]|CNSLF388-12|Sciaridae|664|BOLD:ACB8980
Sciaridae|[21440]|CNFDF088-14|Sciaridae|658|BOLD:ACL8042
Corynoptera|[21441]|HPPPB196-13|Sciaridae|658|BOLD:ACG3219
Sciaridae|[21442]|CNGMD403-14|Sciaridae|666|BOLD:ACL2522
Sciaridae|[21443]|BBDEE722-10|Sciaridae|658|BOLD:AAM9246
Sciaridae|[21444]|CNGHF018-14|Sciaridae|576|BOLD:ACR4647
Sciaridae|[21445]|CNBPL107-13|Sciaridae|670|BOLD:ACG6759
Sciaridae|[21446]|CNFND822-14|Sciaridae|658|BOLD:ACL3558
Sciaridae|[21447]|SSEIB124-13|Sciaridae|658|BOLD:ACG4514
Sciaridae|[21448]|CNRMD860-12|Sciaridae|629|BOLD:ACC6774
Sciaridae|[21449]|SSJAD209-13|Sciaridae|648|BOLD:ACI4705
Sciaridae|[21450]|CNBRB136-14|Sciaridae|658|BOLD:ACL2977
Sciaridae|[21451]|CNKLC745-14|Sciaridae|579|BOLD:ACL8777
Sciaridae|[21452]|CNBRN248-14|Sciaridae|658|BOLD:ACL8739
Sciaridae|[21453]|CNKLC1168-14|Sciaridae|609|BOLD:ACR9042
Sciaridae|[21454]|CNFNE1680-14|Sciaridae|661|BOLD:ACL4821
Sciaridae|[21455]|CNFNQ1530-14|Sciaridae|658|BOLD:ACM5679
Sciaridae|[21456]|TWDIP229-09|Sciaridae|658|BOLD:AAH3930
Sciaridae|[21457]|CNKLC1880-14|Sciaridae|627|BOLD:ACR8823
Leptosciarella scutellata|[21458]|CNBPC081-12|Sciaridae|670|BOLD:ACD1218
Sciaridae|[21459]|SMTPB19379-13|Sciaridae|591|BOLD:ACF8144
Leptosciarella|[21460]|CNGMC533-13|Sciaridae|589|BOLD:ACJ9928
Sciaridae|[21461]|JSMAY038-11|Sciaridae|658|BOLD:ABV1238
Sciaridae|[21462]|CNEIA1324-12|Sciaridae|658|BOLD:ACB1110
Sciaridae|[21463]|CNWAC1762-14|Sciaridae|600|BOLD:ACR8867
Sciaridae|[21464]|CNRVA868-15|Sciaridae|582|BOLD:ACT1020
Sciaridae|[21465]|CNRVA1216-15|Sciaridae|576|BOLD:ACT5280
Sciaridae|[21466]|CNRMD1930-12|Sciaridae|661|BOLD:AAV1309
Sciaridae|[21467]|CNJAD2184-12|Sciaridae|658|BOLD:ACC9408
Sciaridae|[21468]|NGNAJ056-13|Sciaridae|621|BOLD:ACJ0742
Sciaridae|[21469]|CNBAA308-12|Sciaridae|632|BOLD:ACC4335
Sciaridae|[21470]|SSWLA3804-13|Sciaridae|658|BOLD:ACG6512
Sciaridae|[21471]|SSWLA2911-13|Sciaridae|658|BOLD:ACG6275
Sciaridae|[21472]|CNYOJ270-15|Sciaridae|576|BOLD:ACC6670
Sciaridae|[21473]|SSJAF1177-13|Sciaridae|603|BOLD:ACK3814
Sciaridae|[21474]|SSJAF1990-13|Sciaridae|603|BOLD:ACK3908
Sciaridae|[21475]|SSJAF1456-13|Sciaridae|603|BOLD:ACK3708
Sciaridae|[21476]|CNBFD450-15|Sciaridae|588|BOLD:ACT1671
Sciaridae|[21477]|HPPPC746-13|Sciaridae|658|BOLD:AAP6466
Sciaridae|[21478]|CNPPI731-12|Sciaridae|664|BOLD:ACU9337
Sciaridae|[21479]|JSJUN427-11|Sciaridae|658|BOLD:ABV9318
Sciaridae|[21480]|SSEIA7151-13|Sciaridae|658|BOLD:ACU9338
Sciaridae|[21481]|SSEIB3067-13|Sciaridae|635|BOLD:ACU9336
Sciaridae|[21482]|CNGBD330-13|Sciaridae|590|BOLD:ACU9339
Sciaridae|[21483]|CNKJN1046-14|Sciaridae|557|BOLD:ACN7369
Sciaridae|[21484]|CNRME3153-12|Sciaridae|658|BOLD:ACC7561
Sciaridae|[21485]|CNROE477-13|Sciaridae|661|BOLD:ACK2000
Sciaridae|[21486]|CNBRD457-14|Sciaridae|658|BOLD:ACL4130
Sciaridae|[21487]|CNPPB1023-12|Sciaridae|658|BOLD:ACA5068
Sciaridae|[21488]|CNLMO275-14|Sciaridae|609|BOLD:ACM8894
Sciaridae|[21489]|CNEIC2545-12|Sciaridae|658|BOLD:AAU6616
Sciaridae|[21490]|CNJAD272-12|Sciaridae|658|BOLD:ACC6591
Sciaridae|[21491]|CNPKI792-14|Sciaridae|624|BOLD:ACT6917
Sciaridae|[21492]|CNGAC547-15|Sciaridae|600|BOLD:ACT6916
Sciaridae|[21493]|CNRMF1314-12|Sciaridae|673|BOLD:ACC8555
Sciaridae|[21494]|CNWLN547-13|Sciaridae|661|BOLD:ACI6797
Sciaridae|[21495]|SSJAA302-13|Sciaridae|658|BOLD:ACG4317
Sciaridae|[21496]|CNJAB907-12|Sciaridae|658|BOLD:ACC1690

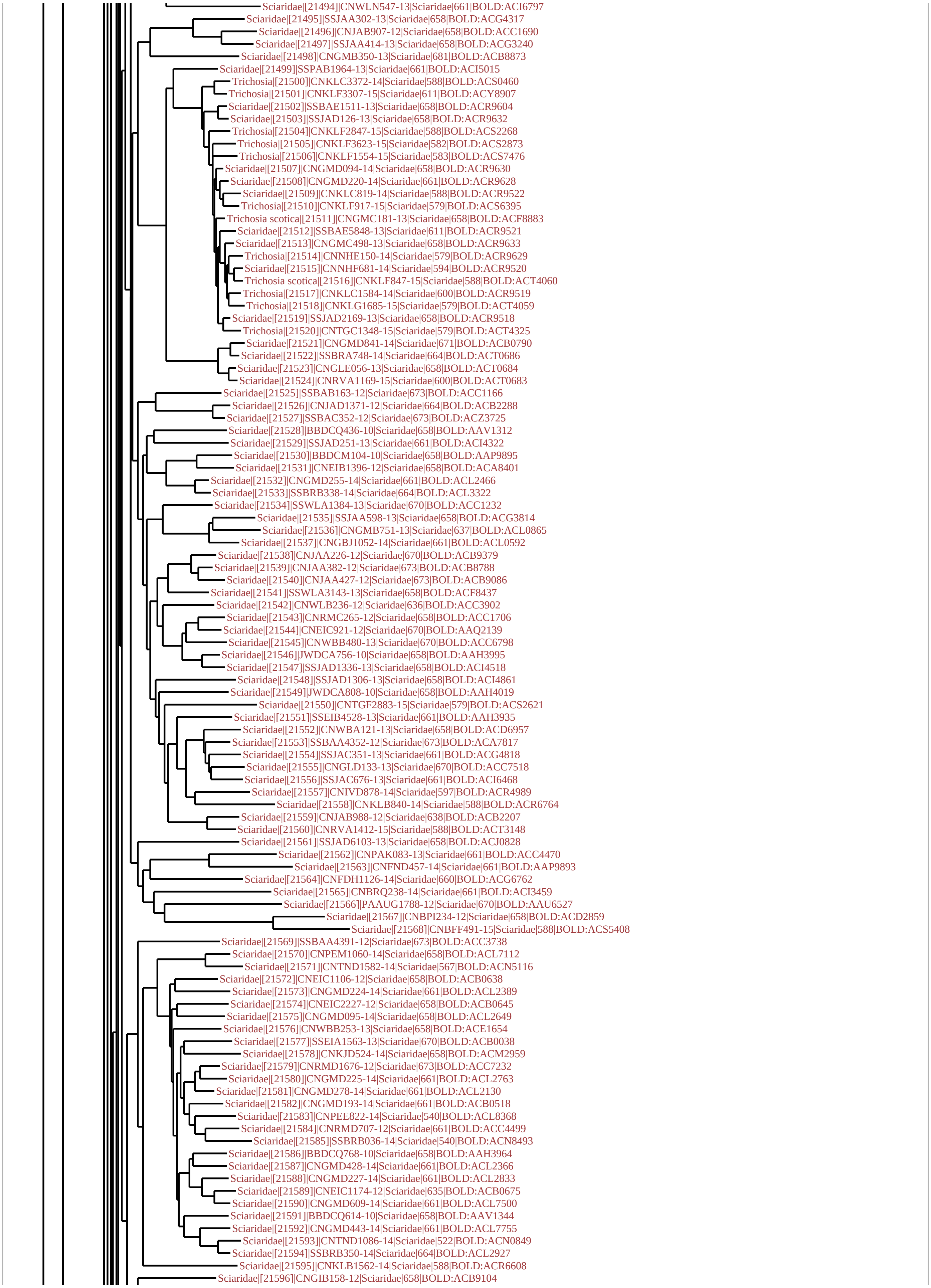
Sciaridae|[21494]|CNWLN547-13|Sciaridae|661|BOLD:ACI6797
Sciaridae|[21495]|SSJAA302-13|Sciaridae|658|BOLD:ACG4317
Sciaridae|[21496]|CNJAB907-12|Sciaridae|658|BOLD:ACC1690
Sciaridae|[21497]|SSJAA414-13|Sciaridae|658|BOLD:ACG3240
Sciaridae|[21498]|CNGMB350-13|Sciaridae|681|BOLD:ACB8873
Sciaridae|[21499]|SSPAB1964-13|Sciaridae|661|BOLD:ACI5015
Trichosia|[21500]|CNKLC3372-14|Sciaridae|588|BOLD:ACS0460
Trichosia|[21501]|CNKLF3307-15|Sciaridae|611|BOLD:ACY8907
Sciaridae|[21502]|SSBAE1511-13|Sciaridae|658|BOLD:ACR9604
Sciaridae|[21503]|SSJAD126-13|Sciaridae|658|BOLD:ACR9632
Trichosia|[21504]|CNKLF2847-15|Sciaridae|588|BOLD:ACS2268
Trichosia|[21505]|CNKLF3623-15|Sciaridae|582|BOLD:ACS2873
Trichosia|[21506]|CNKLF1554-15|Sciaridae|583|BOLD:ACS7476
Sciaridae|[21507]|CNGMD094-14|Sciaridae|658|BOLD:ACR9630
Sciaridae|[21508]|CNGMD220-14|Sciaridae|661|BOLD:ACR9628
Sciaridae|[21509]|CNKLC819-14|Sciaridae|588|BOLD:ACR9522
Trichosia|[21510]|CNKLF917-15|Sciaridae|579|BOLD:ACS6395
Trichosia scotica|[21511]|CNGMC181-13|Sciaridae|658|BOLD:ACF8883
Sciaridae|[21512]|SSBAE5848-13|Sciaridae|611|BOLD:ACR9521
Sciaridae|[21513]|CNGMC498-13|Sciaridae|658|BOLD:ACR9633
Trichosia|[21514]|CNNHE150-14|Sciaridae|579|BOLD:ACR9629
Sciaridae|[21515]|CNNHF681-14|Sciaridae|594|BOLD:ACR9520
Trichosia scotica|[21516]|CNKLF847-15|Sciaridae|588|BOLD:ACT4060
Trichosia|[21517]|CNKLC1584-14|Sciaridae|600|BOLD:ACR9519
Trichosia|[21518]|CNKLG1685-15|Sciaridae|579|BOLD:ACT4059
Sciaridae|[21519]|SSJAD2169-13|Sciaridae|658|BOLD:ACR9518
Trichosia|[21520]|CNTGC1348-15|Sciaridae|579|BOLD:ACT4325
Sciaridae|[21521]|CNGMD841-14|Sciaridae|671|BOLD:ACB0790
Sciaridae|[21522]|SSBRA748-14|Sciaridae|664|BOLD:ACT0686
Sciaridae|[21523]|CNGLE056-13|Sciaridae|658|BOLD:ACT0684
Sciaridae|[21524]|CNRVA1169-15|Sciaridae|600|BOLD:ACT0683
Sciaridae|[21525]|SSBAB163-12|Sciaridae|673|BOLD:ACC1166
Sciaridae|[21526]|CNJAD1371-12|Sciaridae|664|BOLD:ACB2288
Sciaridae|[21527]|SSBAC352-12|Sciaridae|673|BOLD:ACZ3725
Sciaridae|[21528]|BBDCQ436-10|Sciaridae|658|BOLD:AAV1312
Sciaridae|[21529]|SSJAD251-13|Sciaridae|661|BOLD:ACI4322
Sciaridae|[21530]|BBDCM104-10|Sciaridae|658|BOLD:AAP9895
Sciaridae|[21531]|CNEIB1396-12|Sciaridae|658|BOLD:ACA8401
Sciaridae|[21532]|CNGMD255-14|Sciaridae|661|BOLD:ACL2466
Sciaridae|[21533]|SSBRB338-14|Sciaridae|664|BOLD:ACL3322
Sciaridae|[21534]|SSWLA1384-13|Sciaridae|670|BOLD:ACC1232
Sciaridae|[21535]|SSJAA598-13|Sciaridae|658|BOLD:ACG3814
Sciaridae|[21536]|CNGMB751-13|Sciaridae|637|BOLD:ACL0865
Sciaridae|[21537]|CNGBJ1052-14|Sciaridae|661|BOLD:ACL0592
Sciaridae|[21538]|CNJAA226-12|Sciaridae|670|BOLD:ACB9379
Sciaridae|[21539]|CNJAA382-12|Sciaridae|673|BOLD:ACB8788
Sciaridae|[21540]|CNJAA427-12|Sciaridae|673|BOLD:ACB9086
Sciaridae|[21541]|SSWLA3143-13|Sciaridae|658|BOLD:ACF8437
Sciaridae|[21542]|CNWLB236-12|Sciaridae|636|BOLD:ACC3902
Sciaridae|[21543]|CNRMC265-12|Sciaridae|658|BOLD:ACC1706
Sciaridae|[21544]|CNEIC921-12|Sciaridae|670|BOLD:AAQ2139
Sciaridae|[21545]|CNWBB480-13|Sciaridae|670|BOLD:ACC6798
Sciaridae|[21546]|JWDCA756-10|Sciaridae|658|BOLD:AAH3995
Sciaridae|[21547]|SSJAD1336-13|Sciaridae|658|BOLD:ACI4518
Sciaridae|[21548]|SSJAD1306-13|Sciaridae|658|BOLD:ACI4861
Sciaridae|[21549]|JWDCA808-10|Sciaridae|658|BOLD:AAH4019
Sciaridae|[21550]|CNTGF2883-15|Sciaridae|579|BOLD:ACS2621
Sciaridae|[21551]|SSEIB4528-13|Sciaridae|661|BOLD:AAH3935
Sciaridae|[21552]|CNWBA121-13|Sciaridae|658|BOLD:ACD6957
Sciaridae|[21553]|SSBAA4352-12|Sciaridae|673|BOLD:ACA7817
Sciaridae|[21554]|SSJAC351-13|Sciaridae|661|BOLD:ACG4818
Sciaridae|[21555]|CNGLD133-13|Sciaridae|670|BOLD:ACC7518
Sciaridae|[21556]|SSJAC676-13|Sciaridae|661|BOLD:ACI6468
Sciaridae|[21557]|CNIVD878-14|Sciaridae|597|BOLD:ACR4989
Sciaridae|[21558]|CNKLB840-14|Sciaridae|588|BOLD:ACR6764
Sciaridae|[21559]|CNJAB988-12|Sciaridae|638|BOLD:ACB2207
Sciaridae|[21560]|CNRVA1412-15|Sciaridae|588|BOLD:ACT3148
Sciaridae|[21561]|SSJAD6103-13|Sciaridae|658|BOLD:ACJ0828
Sciaridae|[21562]|CNPAK083-13|Sciaridae|661|BOLD:ACC4470
Sciaridae|[21563]|CNFND457-14|Sciaridae|661|BOLD:AAP9893
Sciaridae|[21564]|CNFDH1126-14|Sciaridae|660|BOLD:ACG6762
Sciaridae|[21565]|CNBRQ238-14|Sciaridae|661|BOLD:ACI3459
Sciaridae|[21566]|PAAUG1788-12|Sciaridae|670|BOLD:AAU6527
Sciaridae|[21567]|CNBPI234-12|Sciaridae|658|BOLD:ACD2859
Sciaridae|[21568]|CNBFF491-15|Sciaridae|588|BOLD:ACS5408
Sciaridae|[21569]|SSBAA4391-12|Sciaridae|673|BOLD:ACC3738
Sciaridae|[21570]|CNPEM1060-14|Sciaridae|658|BOLD:ACL7112
Sciaridae|[21571]|CNTND1582-14|Sciaridae|567|BOLD:ACN5116
Sciaridae|[21572]|CNEIC1106-12|Sciaridae|658|BOLD:ACB0638
Sciaridae|[21573]|CNGMD224-14|Sciaridae|661|BOLD:ACL2389
Sciaridae|[21574]|CNEIC2227-12|Sciaridae|658|BOLD:ACB0645
Sciaridae|[21575]|CNGMD095-14|Sciaridae|658|BOLD:ACL2649
Sciaridae|[21576]|CNWBB253-13|Sciaridae|658|BOLD:ACE1654
Sciaridae|[21577]|SSEIA1563-13|Sciaridae|670|BOLD:ACB0038
Sciaridae|[21578]|CNKJD524-14|Sciaridae|658|BOLD:ACM2959
Sciaridae|[21579]|CNRMD1676-12|Sciaridae|673|BOLD:ACC7232
Sciaridae|[21580]|CNGMD225-14|Sciaridae|661|BOLD:ACL2763
Sciaridae|[21581]|CNGMD278-14|Sciaridae|661|BOLD:ACL2130
Sciaridae|[21582]|CNGMD193-14|Sciaridae|661|BOLD:ACB0518
Sciaridae|[21583]|CNPEE822-14|Sciaridae|540|BOLD:ACL8368
Sciaridae|[21584]|CNRMD707-12|Sciaridae|661|BOLD:ACC4499
Sciaridae|[21585]|SSBRB036-14|Sciaridae|540|BOLD:ACN8493
Sciaridae|[21586]|BBDCQ768-10|Sciaridae|658|BOLD:AAH3964
Sciaridae|[21587]|CNGMD428-14|Sciaridae|661|BOLD:ACL2366
Sciaridae|[21588]|CNGMD227-14|Sciaridae|661|BOLD:ACL2833
Sciaridae|[21589]|CNEIC1174-12|Sciaridae|635|BOLD:ACB0675
Sciaridae|[21590]|CNGMD609-14|Sciaridae|661|BOLD:ACL7500
Sciaridae|[21591]|BBDCQ614-10|Sciaridae|658|BOLD:AAV1344
Sciaridae|[21592]|CNGMD443-14|Sciaridae|661|BOLD:ACL7755
Sciaridae|[21593]|CNTND1086-14|Sciaridae|522|BOLD:ACN0849
Sciaridae|[21594]|SSBRB350-14|Sciaridae|664|BOLD:ACL2927
Sciaridae|[21595]|CNKLB1562-14|Sciaridae|588|BOLD:ACR6608
Sciaridae|[21596]|CNGIB158-12|Sciaridae|658|BOLD:ACB9104

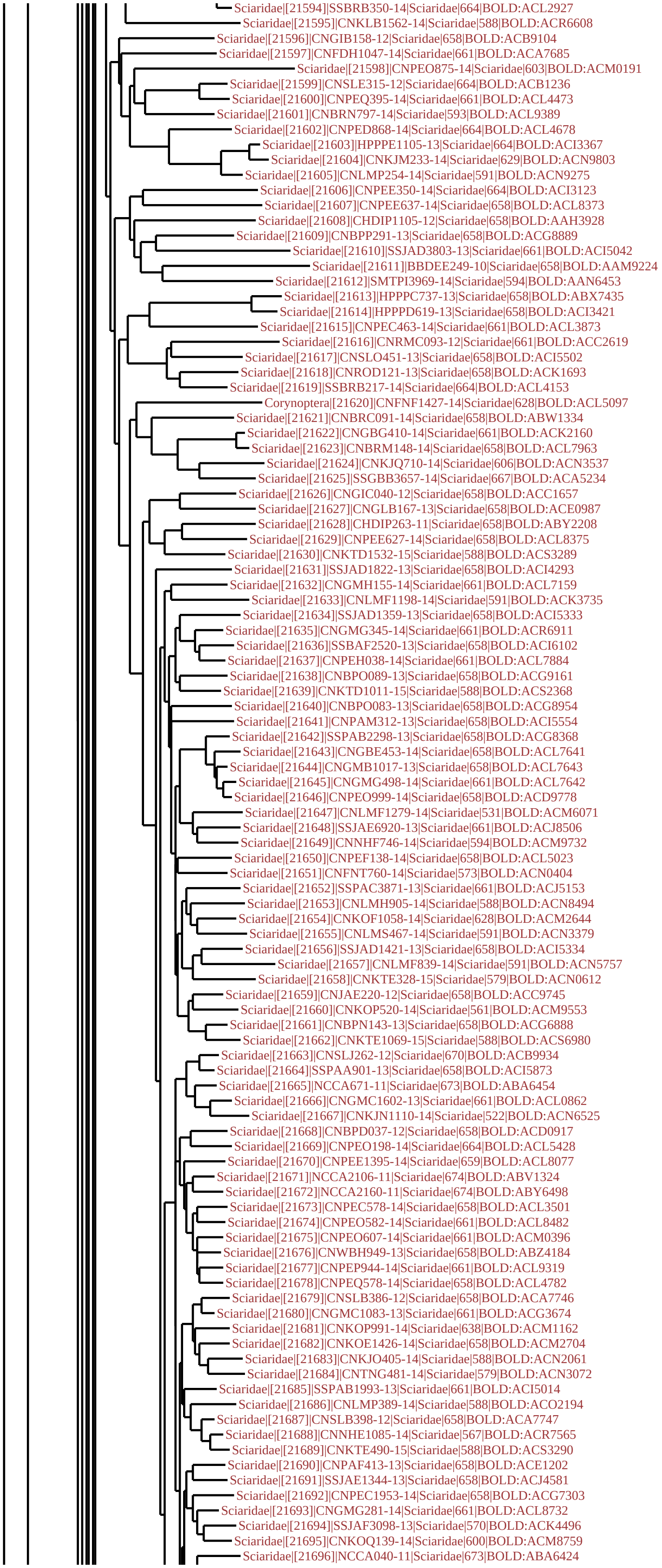
Sciaridae|[21594]|SSBRB350-14|Sciaridae|664|BOLD:ACL2927
Sciaridae|[21595]|CNKLB1562-14|Sciaridae|588|BOLD:ACR6608
Sciaridae|[21596]|CNGIB158-12|Sciaridae|658|BOLD:ACB9104
Sciaridae|[21597]|CNFDH1047-14|Sciaridae|661|BOLD:ACA7685
Sciaridae|[21598]|CNPEO875-14|Sciaridae|603|BOLD:ACM0191
Sciaridae|[21599]|CNSLE315-12|Sciaridae|664|BOLD:ACB1236
Sciaridae|[21600]|CNPEQ395-14|Sciaridae|661|BOLD:ACL4473
Sciaridae|[21601]|CNBRN797-14|Sciaridae|593|BOLD:ACL9389
Sciaridae|[21602]|CNPED868-14|Sciaridae|664|BOLD:ACL4678
Sciaridae|[21603]|HPPPE1105-13|Sciaridae|664|BOLD:ACI3367
Sciaridae|[21604]|CNKJM233-14|Sciaridae|629|BOLD:ACN9803
Sciaridae|[21605]|CNLMP254-14|Sciaridae|591|BOLD:ACN9275
Sciaridae|[21606]|CNPEE350-14|Sciaridae|664|BOLD:ACI3123
Sciaridae|[21607]|CNPEE637-14|Sciaridae|658|BOLD:ACL8373
Sciaridae|[21608]|CHDIP1105-12|Sciaridae|658|BOLD:AAH3928
Sciaridae|[21609]|CNBPP291-13|Sciaridae|658|BOLD:ACG8889
Sciaridae|[21610]|SSJAD3803-13|Sciaridae|661|BOLD:ACI5042
Sciaridae|[21611]|BBDEE249-10|Sciaridae|658|BOLD:AAM9224
Sciaridae|[21612]|SMTPI3969-14|Sciaridae|594|BOLD:AAN6453
Sciaridae|[21613]|HPPPC737-13|Sciaridae|658|BOLD:ABX7435
Sciaridae|[21614]|HPPPD619-13|Sciaridae|658|BOLD:ACI3421
Sciaridae|[21615]|CNPEC463-14|Sciaridae|661|BOLD:ACL3873
Sciaridae|[21616]|CNRMC093-12|Sciaridae|661|BOLD:ACC2619
Sciaridae|[21617]|CNSLO451-13|Sciaridae|658|BOLD:ACI5502
Sciaridae|[21618]|CNROD121-13|Sciaridae|658|BOLD:ACK1693
Sciaridae|[21619]|SSBRB217-14|Sciaridae|664|BOLD:ACL4153
Corynoptera|[21620]|CNFNF1427-14|Sciaridae|628|BOLD:ACL5097
Sciaridae|[21621]|CNBRC091-14|Sciaridae|658|BOLD:ABW1334
Sciaridae|[21622]|CNGBG410-14|Sciaridae|661|BOLD:ACK2160
Sciaridae|[21623]|CNBRM148-14|Sciaridae|658|BOLD:ACL7963
Sciaridae|[21624]|CNKJQ710-14|Sciaridae|606|BOLD:ACN3537
Sciaridae|[21625]|SSGBB3657-14|Sciaridae|667|BOLD:ACA5234
Sciaridae|[21626]|CNGIC040-12|Sciaridae|658|BOLD:ACC1657
Sciaridae|[21627]|CNGLB167-13|Sciaridae|658|BOLD:ACE0987
Sciaridae|[21628]|CHDIP263-11|Sciaridae|658|BOLD:ABY2208
Sciaridae|[21629]|CNPEE627-14|Sciaridae|658|BOLD:ACL8375
Sciaridae|[21630]|CNKTD1532-15|Sciaridae|588|BOLD:ACS3289
Sciaridae|[21631]|SSJAD1822-13|Sciaridae|658|BOLD:ACI4293
Sciaridae|[21632]|CNGMH155-14|Sciaridae|661|BOLD:ACL7159
Sciaridae|[21633]|CNLMF1198-14|Sciaridae|591|BOLD:ACK3735
Sciaridae|[21634]|SSJAD1359-13|Sciaridae|658|BOLD:ACI5333
Sciaridae|[21635]|CNGMG345-14|Sciaridae|661|BOLD:ACR6911
Sciaridae|[21636]|SSBAF2520-13|Sciaridae|658|BOLD:ACI6102
Sciaridae|[21637]|CNPEH038-14|Sciaridae|661|BOLD:ACL7884
Sciaridae|[21638]|CNBPO089-13|Sciaridae|658|BOLD:ACG9161
Sciaridae|[21639]|CNKTD1011-15|Sciaridae|588|BOLD:ACS2368
Sciaridae|[21640]|CNBPO083-13|Sciaridae|658|BOLD:ACG8954
Sciaridae|[21641]|CNPAM312-13|Sciaridae|658|BOLD:ACI5554
Sciaridae|[21642]|SSPAB2298-13|Sciaridae|658|BOLD:ACG8368
Sciaridae|[21643]|CNGBE453-14|Sciaridae|658|BOLD:ACL7641
Sciaridae|[21644]|CNGMB1017-13|Sciaridae|658|BOLD:ACL7643
Sciaridae|[21645]|CNGMG498-14|Sciaridae|661|BOLD:ACL7642
Sciaridae|[21646]|CNPEO999-14|Sciaridae|658|BOLD:ACD9778
Sciaridae|[21647]|CNLMF1279-14|Sciaridae|531|BOLD:ACM6071
Sciaridae|[21648]|SSJAE6920-13|Sciaridae|661|BOLD:ACJ8506
Sciaridae|[21649]|CNNHF746-14|Sciaridae|594|BOLD:ACM9732
Sciaridae|[21650]|CNPEF138-14|Sciaridae|658|BOLD:ACL5023
Sciaridae|[21651]|CNFNT760-14|Sciaridae|573|BOLD:ACN0404
Sciaridae|[21652]|SSPAC3871-13|Sciaridae|661|BOLD:ACJ5153
Sciaridae|[21653]|CNLMH905-14|Sciaridae|588|BOLD:ACN8494
Sciaridae|[21654]|CNKOF1058-14|Sciaridae|628|BOLD:ACM2644
Sciaridae|[21655]|CNLMS467-14|Sciaridae|591|BOLD:ACN3379
Sciaridae|[21656]|SSJAD1421-13|Sciaridae|658|BOLD:ACI5334
Sciaridae|[21657]|CNLMF839-14|Sciaridae|591|BOLD:ACN5757
Sciaridae|[21658]|CNKTE328-15|Sciaridae|579|BOLD:ACN0612
Sciaridae|[21659]|CNJAE220-12|Sciaridae|658|BOLD:ACC9745
Sciaridae|[21660]|CNKOP520-14|Sciaridae|561|BOLD:ACM9553
Sciaridae|[21661]|CNBPN143-13|Sciaridae|658|BOLD:ACG6888
Sciaridae|[21662]|CNKTE1069-15|Sciaridae|588|BOLD:ACS6980
Sciaridae|[21663]|CNSLJ262-12|Sciaridae|670|BOLD:ACB9934
Sciaridae|[21664]|SSPAA901-13|Sciaridae|658|BOLD:ACI5873
Sciaridae|[21665]|NCCA671-11|Sciaridae|673|BOLD:ABA6454
Sciaridae|[21666]|CNGMC1602-13|Sciaridae|661|BOLD:ACL0862
Sciaridae|[21667]|CNKJN1110-14|Sciaridae|522|BOLD:ACN6525
Sciaridae|[21668]|CNBPD037-12|Sciaridae|658|BOLD:ACD0917
Sciaridae|[21669]|CNPEO198-14|Sciaridae|664|BOLD:ACL5428
Sciaridae|[21670]|CNPEE1395-14|Sciaridae|659|BOLD:ACL8077
Sciaridae|[21671]|NCCA2106-11|Sciaridae|674|BOLD:ABV1324
Sciaridae|[21672]|NCCA2160-11|Sciaridae|674|BOLD:ABY6498
Sciaridae|[21673]|CNPEC578-14|Sciaridae|658|BOLD:ACL3501
Sciaridae|[21674]|CNPEO582-14|Sciaridae|661|BOLD:ACL8482
Sciaridae|[21675]|CNPEO607-14|Sciaridae|661|BOLD:ACM0396
Sciaridae|[21676]|CNWBH949-13|Sciaridae|658|BOLD:ABZ4184
Sciaridae|[21677]|CNPEP944-14|Sciaridae|661|BOLD:ACL9319
Sciaridae|[21678]|CNPEQ578-14|Sciaridae|658|BOLD:ACL4782
Sciaridae|[21679]|CNSLB386-12|Sciaridae|658|BOLD:ACA7746
Sciaridae|[21680]|CNGMC1083-13|Sciaridae|661|BOLD:ACG3674
Sciaridae|[21681]|CNKOP991-14|Sciaridae|638|BOLD:ACM1162
Sciaridae|[21682]|CNKOE1426-14|Sciaridae|658|BOLD:ACM2704
Sciaridae|[21683]|CNKJO405-14|Sciaridae|588|BOLD:ACN2061
Sciaridae|[21684]|CNTNG481-14|Sciaridae|579|BOLD:ACN3072
Sciaridae|[21685]|SSPAB1993-13|Sciaridae|661|BOLD:ACI5014
Sciaridae|[21686]|CNLMP389-14|Sciaridae|588|BOLD:ACO2194
Sciaridae|[21687]|CNSLB398-12|Sciaridae|658|BOLD:ACA7747
Sciaridae|[21688]|CNNHE1085-14|Sciaridae|567|BOLD:ACR7565
Sciaridae|[21689]|CNKTE490-15|Sciaridae|588|BOLD:ACS3290
Sciaridae|[21690]|CNPAF413-13|Sciaridae|658|BOLD:ACE1202
Sciaridae|[21691]|SSJAE1344-13|Sciaridae|658|BOLD:ACJ4581
Sciaridae|[21692]|CNPEC1953-14|Sciaridae|658|BOLD:ACG7303
Sciaridae|[21693]|CNGMG281-14|Sciaridae|661|BOLD:ACL8732
Sciaridae|[21694]|SSJAF3098-13|Sciaridae|570|BOLD:ACK4496
Sciaridae|[21695]|CNKOQ139-14|Sciaridae|600|BOLD:ACM8759
Sciaridae|[21696]|NCCA040-11|Sciaridae|673|BOLD:ABA6424

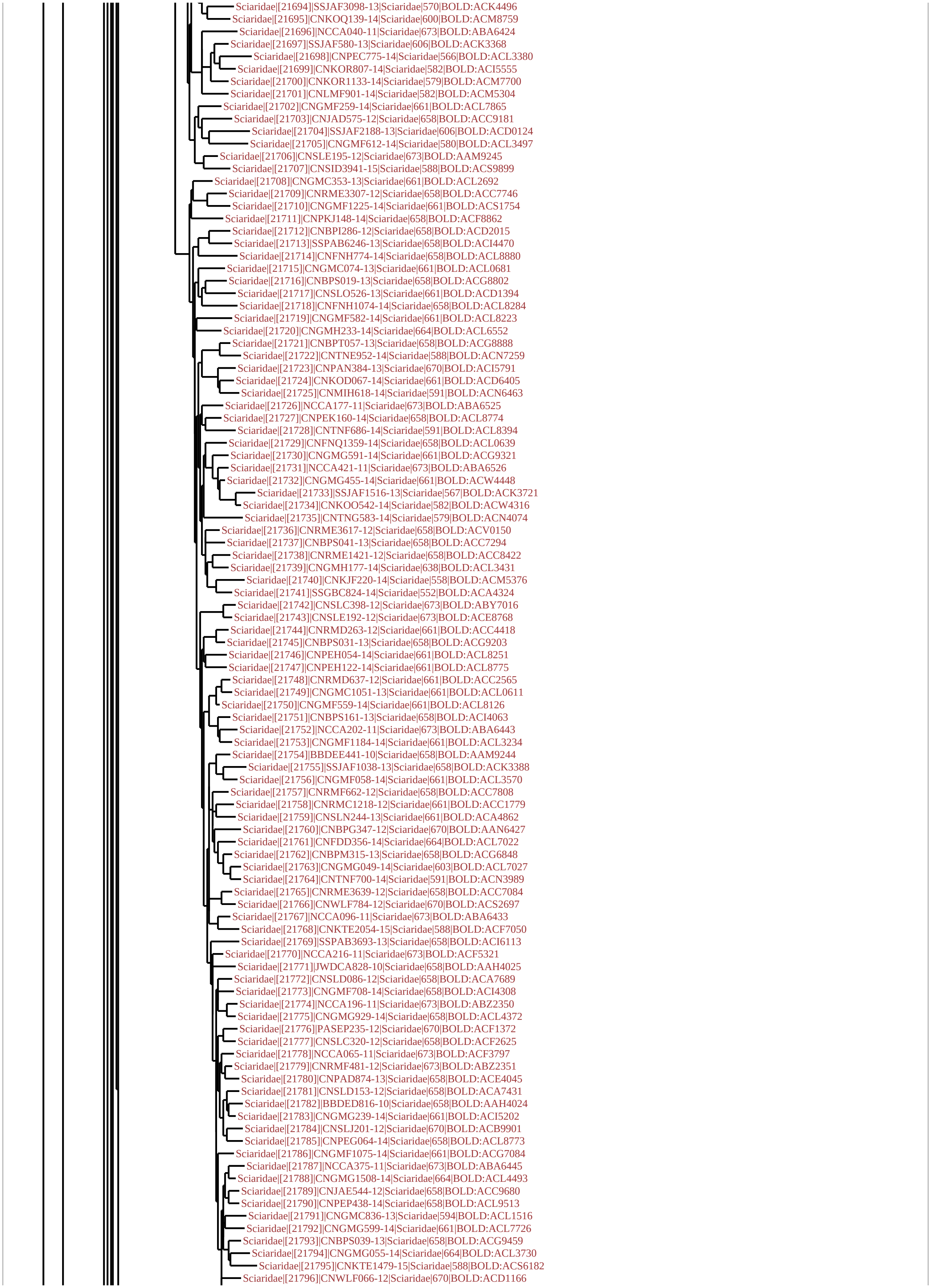
Sciaridae|[21694]|SSJAF3098-13|Sciaridae|570|BOLD:ACK4496
Sciaridae|[21695]|CNKOQ139-14|Sciaridae|600|BOLD:ACM8759
Sciaridae|[21696]|NCCA040-11|Sciaridae|673|BOLD:ABA6424
Sciaridae|[21697]|SSJAF580-13|Sciaridae|606|BOLD:ACK3368
Sciaridae|[21698]|CNPEC775-14|Sciaridae|566|BOLD:ACL3380
Sciaridae|[21699]|CNKOR807-14|Sciaridae|582|BOLD:ACI5555
Sciaridae|[21700]|CNKOR1133-14|Sciaridae|579|BOLD:ACM7700
Sciaridae|[21701]|CNLMF901-14|Sciaridae|582|BOLD:ACM5304
Sciaridae|[21702]|CNGMF259-14|Sciaridae|661|BOLD:ACL7865
Sciaridae|[21703]|CNJAD575-12|Sciaridae|658|BOLD:ACC9181
Sciaridae|[21704]|SSJAF2188-13|Sciaridae|606|BOLD:ACD0124
Sciaridae|[21705]|CNGMF612-14|Sciaridae|580|BOLD:ACL3497
Sciaridae|[21706]|CNSLE195-12|Sciaridae|673|BOLD:AAM9245
Sciaridae|[21707]|CNSID3941-15|Sciaridae|588|BOLD:ACS9899
Sciaridae|[21708]|CNGMC353-13|Sciaridae|661|BOLD:ACL2692
Sciaridae|[21709]|CNRME3307-12|Sciaridae|658|BOLD:ACC7746
Sciaridae|[21710]|CNGMF1225-14|Sciaridae|661|BOLD:ACS1754
Sciaridae|[21711]|CNPKJ148-14|Sciaridae|658|BOLD:ACF8862
Sciaridae|[21712]|CNBPI286-12|Sciaridae|658|BOLD:ACD2015
Sciaridae|[21713]|SSPAB6246-13|Sciaridae|658|BOLD:ACI4470
Sciaridae|[21714]|CNFNH774-14|Sciaridae|658|BOLD:ACL8880
Sciaridae|[21715]|CNGMC074-13|Sciaridae|661|BOLD:ACL0681
Sciaridae|[21716]|CNBPS019-13|Sciaridae|658|BOLD:ACG8802
Sciaridae|[21717]|CNSLO526-13|Sciaridae|661|BOLD:ACD1394
Sciaridae|[21718]|CNFNH1074-14|Sciaridae|658|BOLD:ACL8284
Sciaridae|[21719]|CNGMF582-14|Sciaridae|661|BOLD:ACL8223
Sciaridae|[21720]|CNGMH233-14|Sciaridae|664|BOLD:ACL6552
Sciaridae|[21721]|CNBPT057-13|Sciaridae|658|BOLD:ACG8888
Sciaridae|[21722]|CNTNE952-14|Sciaridae|588|BOLD:ACN7259
Sciaridae|[21723]|CNPAN384-13|Sciaridae|670|BOLD:ACI5791
Sciaridae|[21724]|CNKOD067-14|Sciaridae|661|BOLD:ACD6405
Sciaridae|[21725]|CNMIH618-14|Sciaridae|591|BOLD:ACN6463
Sciaridae|[21726]|NCCA177-11|Sciaridae|673|BOLD:ABA6525
Sciaridae|[21727]|CNPEK160-14|Sciaridae|658|BOLD:ACL8774
Sciaridae|[21728]|CNTNF686-14|Sciaridae|591|BOLD:ACL8394
Sciaridae|[21729]|CNFNQ1359-14|Sciaridae|658|BOLD:ACL0639
Sciaridae|[21730]|CNGMG591-14|Sciaridae|661|BOLD:ACG9321
Sciaridae|[21731]|NCCA421-11|Sciaridae|673|BOLD:ABA6526
Sciaridae|[21732]|CNGMG455-14|Sciaridae|661|BOLD:ACW4448
Sciaridae|[21733]|SSJAF1516-13|Sciaridae|567|BOLD:ACK3721
Sciaridae|[21734]|CNKOO542-14|Sciaridae|582|BOLD:ACW4316
Sciaridae|[21735]|CNTNG583-14|Sciaridae|579|BOLD:ACN4074
Sciaridae|[21736]|CNRME3617-12|Sciaridae|658|BOLD:ACV0150
Sciaridae|[21737]|CNBPS041-13|Sciaridae|658|BOLD:ACC7294
Sciaridae|[21738]|CNRME1421-12|Sciaridae|658|BOLD:ACC8422
Sciaridae|[21739]|CNGMH177-14|Sciaridae|638|BOLD:ACL3431
Sciaridae|[21740]|CNKJF220-14|Sciaridae|558|BOLD:ACM5376
Sciaridae|[21741]|SSGBC824-14|Sciaridae|552|BOLD:ACA4324
Sciaridae|[21742]|CNSLC398-12|Sciaridae|673|BOLD:ABY7016
Sciaridae|[21743]|CNSLE192-12|Sciaridae|673|BOLD:ACE8768
Sciaridae|[21744]|CNRMD263-12|Sciaridae|661|BOLD:ACC4418
Sciaridae|[21745]|CNBPS031-13|Sciaridae|658|BOLD:ACG9203
Sciaridae|[21746]|CNPEH054-14|Sciaridae|661|BOLD:ACL8251
Sciaridae|[21747]|CNPEH122-14|Sciaridae|661|BOLD:ACL8775
Sciaridae|[21748]|CNRMD637-12|Sciaridae|661|BOLD:ACC2565
Sciaridae|[21749]|CNGMC1051-13|Sciaridae|661|BOLD:ACL0611
Sciaridae|[21750]|CNGMF559-14|Sciaridae|661|BOLD:ACL8126
Sciaridae|[21751]|CNBPS161-13|Sciaridae|658|BOLD:ACI4063
Sciaridae|[21752]|NCCA202-11|Sciaridae|673|BOLD:ABA6443
Sciaridae|[21753]|CNGMF1184-14|Sciaridae|661|BOLD:ACL3234
Sciaridae|[21754]|BBDEE441-10|Sciaridae|658|BOLD:AAM9244
Sciaridae|[21755]|SSJAF1038-13|Sciaridae|658|BOLD:ACK3388
Sciaridae|[21756]|CNGMF058-14|Sciaridae|661|BOLD:ACL3570
Sciaridae|[21757]|CNRMF662-12|Sciaridae|658|BOLD:ACC7808
Sciaridae|[21758]|CNRMC1218-12|Sciaridae|661|BOLD:ACC1779
Sciaridae|[21759]|CNSLN244-13|Sciaridae|661|BOLD:ACA4862
Sciaridae|[21760]|CNBPG347-12|Sciaridae|670|BOLD:AAN6427
Sciaridae|[21761]|CNFDD356-14|Sciaridae|664|BOLD:ACL7022
Sciaridae|[21762]|CNBPM315-13|Sciaridae|658|BOLD:ACG6848
Sciaridae|[21763]|CNGMG049-14|Sciaridae|603|BOLD:ACL7027
Sciaridae|[21764]|CNTNF700-14|Sciaridae|591|BOLD:ACN3989
Sciaridae|[21765]|CNRME3639-12|Sciaridae|658|BOLD:ACC7084
Sciaridae|[21766]|CNWLF784-12|Sciaridae|670|BOLD:ACS2697
Sciaridae|[21767]|NCCA096-11|Sciaridae|673|BOLD:ABA6433
Sciaridae|[21768]|CNKTE2054-15|Sciaridae|588|BOLD:ACF7050
Sciaridae|[21769]|SSPAB3693-13|Sciaridae|658|BOLD:ACI6113
Sciaridae|[21770]|NCCA216-11|Sciaridae|673|BOLD:ACF5321
Sciaridae|[21771]|JWDCA828-10|Sciaridae|658|BOLD:AAH4025
Sciaridae|[21772]|CNSLD086-12|Sciaridae|658|BOLD:ACA7689
Sciaridae|[21773]|CNGMF708-14|Sciaridae|658|BOLD:ACI4308
Sciaridae|[21774]|NCCA196-11|Sciaridae|673|BOLD:ABZ2350
Sciaridae|[21775]|CNGMG929-14|Sciaridae|658|BOLD:ACL4372
Sciaridae|[21776]|PASEP235-12|Sciaridae|670|BOLD:ACF1372
Sciaridae|[21777]|CNSLC320-12|Sciaridae|658|BOLD:ACF2625
Sciaridae|[21778]|NCCA065-11|Sciaridae|673|BOLD:ACF3797
Sciaridae|[21779]|CNRMF481-12|Sciaridae|673|BOLD:ABZ2351
Sciaridae|[21780]|CNPAD874-13|Sciaridae|658|BOLD:ACE4045
Sciaridae|[21781]|CNSLD153-12|Sciaridae|658|BOLD:ACA7431
Sciaridae|[21782]|BBDED816-10|Sciaridae|658|BOLD:AAH4024
Sciaridae|[21783]|CNGMG239-14|Sciaridae|661|BOLD:ACI5202
Sciaridae|[21784]|CNSLJ201-12|Sciaridae|670|BOLD:ACB9901
Sciaridae|[21785]|CNPEG064-14|Sciaridae|658|BOLD:ACL8773
Sciaridae|[21786]|CNGMF1075-14|Sciaridae|661|BOLD:ACG7084
Sciaridae|[21787]|NCCA375-11|Sciaridae|673|BOLD:ABA6445
Sciaridae|[21788]|CNGMG1508-14|Sciaridae|664|BOLD:ACL4493
Sciaridae|[21789]|CNJAE544-12|Sciaridae|658|BOLD:ACC9680
Sciaridae|[21790]|CNPEP438-14|Sciaridae|658|BOLD:ACL9513
Sciaridae|[21791]|CNGMC836-13|Sciaridae|594|BOLD:ACL1516
Sciaridae|[21792]|CNGMG599-14|Sciaridae|661|BOLD:ACL7726
Sciaridae|[21793]|CNBPS039-13|Sciaridae|658|BOLD:ACG9459
Sciaridae|[21794]|CNGMG055-14|Sciaridae|664|BOLD:ACL3730
Sciaridae|[21795]|CNKTE1479-15|Sciaridae|588|BOLD:ACS6182
Sciaridae|[21796]|CNWLF066-12|Sciaridae|670|BOLD:ACD1166

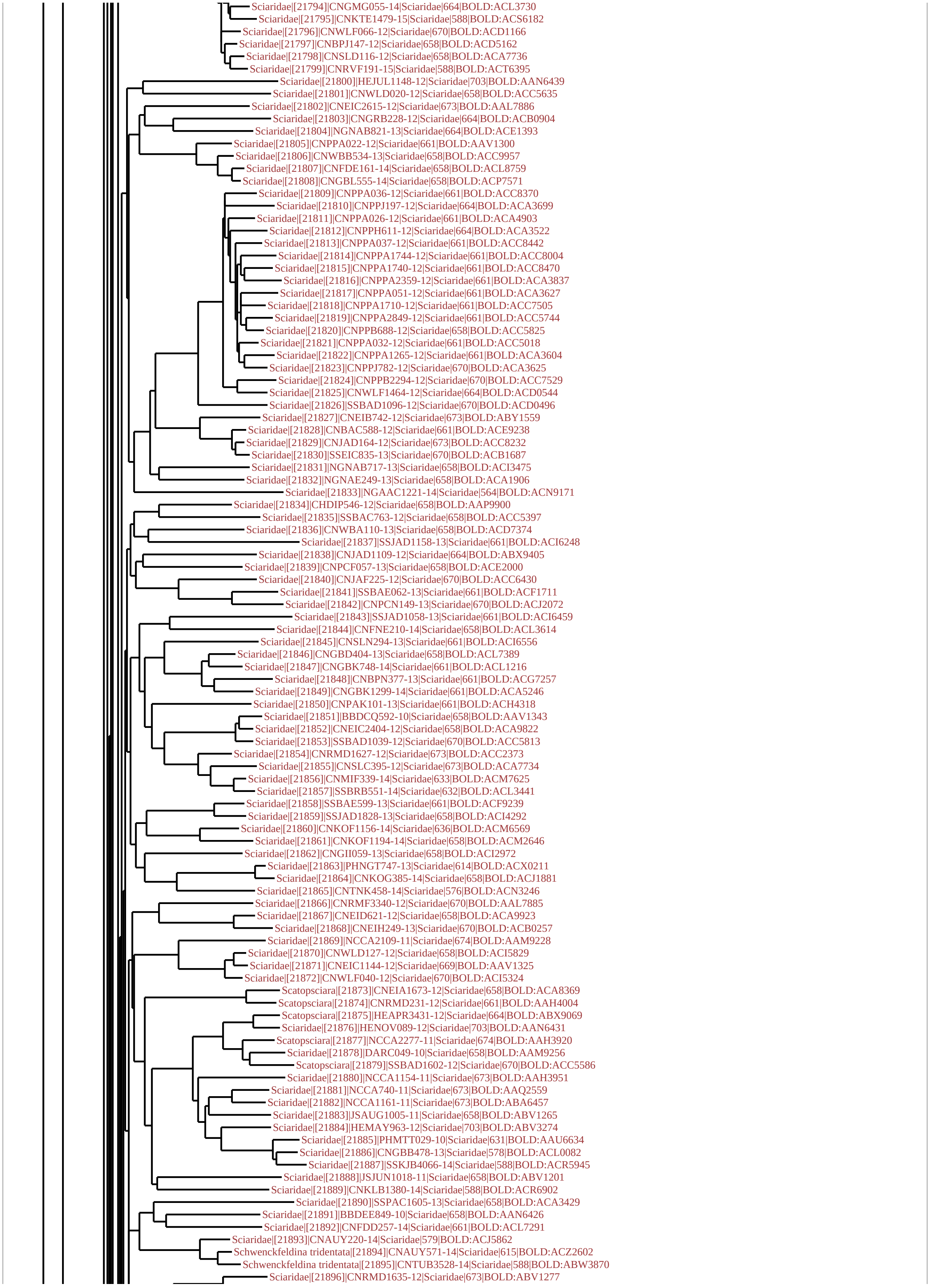

Sciaridae|[21794]|CNGMG055-14|Sciaridae|664|BOLD:ACL3730
Sciaridae|[21795]|CNKTE1479-15|Sciaridae|588|BOLD:ACS6182
Sciaridae|[21796]|CNWLF066-12|Sciaridae|670|BOLD:ACD1166
Sciaridae|[21797]|CNBPJ147-12|Sciaridae|658|BOLD:ACD5162
Sciaridae|[21798]|CNSLD116-12|Sciaridae|658|BOLD:ACA7736
Sciaridae|[21799]|CNRVF191-15|Sciaridae|588|BOLD:ACT6395
Sciaridae|[21800]|HEJUL1148-12|Sciaridae|703|BOLD:AAN6439
Sciaridae|[21801]|CNWLD020-12|Sciaridae|658|BOLD:ACC5635
Sciaridae|[21802]|CNEIC2615-12|Sciaridae|673|BOLD:AAL7886
Sciaridae|[21803]|CNGRB228-12|Sciaridae|664|BOLD:ACB0904
Sciaridae|[21804]|NGNAB821-13|Sciaridae|664|BOLD:ACE1393
Sciaridae|[21805]|CNPPA022-12|Sciaridae|661|BOLD:AAV1300
Sciaridae|[21806]|CNWBB534-13|Sciaridae|658|BOLD:ACC9957
Sciaridae|[21807]|CNFDE161-14|Sciaridae|658|BOLD:ACL8759
Sciaridae|[21808]|CNGBL555-14|Sciaridae|658|BOLD:ACP7571
Sciaridae|[21809]|CNPPA036-12|Sciaridae|661|BOLD:ACC8370
Sciaridae|[21810]|CNPPJ197-12|Sciaridae|664|BOLD:ACA3699
Sciaridae|[21811]|CNPPA026-12|Sciaridae|661|BOLD:ACA4903
Sciaridae|[21812]|CNPPH611-12|Sciaridae|664|BOLD:ACA3522
Sciaridae|[21813]|CNPPA037-12|Sciaridae|661|BOLD:ACC8442
Sciaridae|[21814]|CNPPA1744-12|Sciaridae|661|BOLD:ACC8004
Sciaridae|[21815]|CNPPA1740-12|Sciaridae|661|BOLD:ACC8470
Sciaridae|[21816]|CNPPA2359-12|Sciaridae|661|BOLD:ACA3837
Sciaridae|[21817]|CNPPA051-12|Sciaridae|661|BOLD:ACA3627
Sciaridae|[21818]|CNPPA1710-12|Sciaridae|661|BOLD:ACC7505
Sciaridae|[21819]|CNPPA2849-12|Sciaridae|661|BOLD:ACC5744
Sciaridae|[21820]|CNPPB688-12|Sciaridae|658|BOLD:ACC5825
Sciaridae|[21821]|CNPPA032-12|Sciaridae|661|BOLD:ACC5018
Sciaridae|[21822]|CNPPA1265-12|Sciaridae|661|BOLD:ACA3604
Sciaridae|[21823]|CNPPJ782-12|Sciaridae|670|BOLD:ACA3625
Sciaridae|[21824]|CNPPB2294-12|Sciaridae|670|BOLD:ACC7529
Sciaridae|[21825]|CNWLF1464-12|Sciaridae|664|BOLD:ACD0544
Sciaridae|[21826]|SSBAD1096-12|Sciaridae|670|BOLD:ACD0496
Sciaridae|[21827]|CNEIB742-12|Sciaridae|673|BOLD:ABY1559
Sciaridae|[21828]|CNBAC588-12|Sciaridae|661|BOLD:ACE9238
Sciaridae|[21829]|CNJAD164-12|Sciaridae|673|BOLD:ACC8232
Sciaridae|[21830]|SSEIC835-13|Sciaridae|670|BOLD:ACB1687
Sciaridae|[21831]|NGNAB717-13|Sciaridae|658|BOLD:ACI3475
Sciaridae|[21832]|NGNAE249-13|Sciaridae|658|BOLD:ACA1906
Sciaridae|[21833]|NGAAC1221-14|Sciaridae|564|BOLD:ACN9171
Sciaridae|[21834]|CHDIP546-12|Sciaridae|658|BOLD:AAP9900
Sciaridae|[21835]|SSBAC763-12|Sciaridae|658|BOLD:ACC5397
Sciaridae|[21836]|CNWBA110-13|Sciaridae|658|BOLD:ACD7374
Sciaridae|[21837]|SSJAD1158-13|Sciaridae|661|BOLD:ACI6248
Sciaridae|[21838]|CNJAD1109-12|Sciaridae|664|BOLD:ABX9405
Sciaridae|[21839]|CNPCF057-13|Sciaridae|658|BOLD:ACE2000
Sciaridae|[21840]|CNJAF225-12|Sciaridae|670|BOLD:ACC6430
Sciaridae|[21841]|SSBAE062-13|Sciaridae|661|BOLD:ACF1711
Sciaridae|[21842]|CNPCN149-13|Sciaridae|670|BOLD:ACJ2072
Sciaridae|[21843]|SSJAD1058-13|Sciaridae|661|BOLD:ACI6459
Sciaridae|[21844]|CNFNE210-14|Sciaridae|658|BOLD:ACL3614
Sciaridae|[21845]|CNSLN294-13|Sciaridae|661|BOLD:ACI6556
Sciaridae|[21846]|CNGBD404-13|Sciaridae|658|BOLD:ACL7389
Sciaridae|[21847]|CNGBK748-14|Sciaridae|661|BOLD:ACL1216
Sciaridae|[21848]|CNBPN377-13|Sciaridae|661|BOLD:ACG7257
Sciaridae|[21849]|CNGBK1299-14|Sciaridae|661|BOLD:ACA5246
Sciaridae|[21850]|CNPAK101-13|Sciaridae|661|BOLD:ACH4318
Sciaridae|[21851]|BBDCQ592-10|Sciaridae|658|BOLD:AAV1343
Sciaridae|[21852]|CNEIC2404-12|Sciaridae|658|BOLD:ACA9822
Sciaridae|[21853]|SSBAD1039-12|Sciaridae|670|BOLD:ACC5813
Sciaridae|[21854]|CNRMD1627-12|Sciaridae|673|BOLD:ACC2373
Sciaridae|[21855]|CNSLC395-12|Sciaridae|673|BOLD:ACA7734
Sciaridae|[21856]|CNMIF339-14|Sciaridae|633|BOLD:ACM7625
Sciaridae|[21857]|SSBRB551-14|Sciaridae|632|BOLD:ACL3441
Sciaridae|[21858]|SSBAE599-13|Sciaridae|661|BOLD:ACF9239
Sciaridae|[21859]|SSJAD1828-13|Sciaridae|658|BOLD:ACI4292
Sciaridae|[21860]|CNKOF1156-14|Sciaridae|636|BOLD:ACM6569
Sciaridae|[21861]|CNKOF1194-14|Sciaridae|658|BOLD:ACM2646
Sciaridae|[21862]|CNGII059-13|Sciaridae|658|BOLD:ACI2972
Sciaridae|[21863]|PHNGT747-13|Sciaridae|614|BOLD:ACX0211
Sciaridae|[21864]|CNKOG385-14|Sciaridae|658|BOLD:ACJ1881
Sciaridae|[21865]|CNTNK458-14|Sciaridae|576|BOLD:ACN3246
Sciaridae|[21866]|CNRMF3340-12|Sciaridae|670|BOLD:AAL7885
Sciaridae|[21867]|CNEID621-12|Sciaridae|658|BOLD:ACA9923
Sciaridae|[21868]|CNEIH249-13|Sciaridae|670|BOLD:ACB0257
Sciaridae|[21869]|NCCA2109-11|Sciaridae|674|BOLD:AAM9228
Sciaridae|[21870]|CNWLD127-12|Sciaridae|658|BOLD:ACI5829
Sciaridae|[21871]|CNEIC1144-12|Sciaridae|669|BOLD:AAV1325
Sciaridae|[21872]|CNWLF040-12|Sciaridae|670|BOLD:ACI5324
Scatopsciara|[21873]|CNEIA1673-12|Sciaridae|658|BOLD:ACA8369
Scatopsciara|[21874]|CNRMD231-12|Sciaridae|661|BOLD:AAH4004
Scatopsciara|[21875]|HEAPR3431-12|Sciaridae|664|BOLD:ABX9069
Sciaridae|[21876]|HENOV089-12|Sciaridae|703|BOLD:AAN6431
Scatopsciara|[21877]|NCCA2277-11|Sciaridae|674|BOLD:AAH3920
Sciaridae|[21878]|DARC049-10|Sciaridae|658|BOLD:AAM9256
Scatopsciara|[21879]|SSBAD1602-12|Sciaridae|670|BOLD:ACC5586
Sciaridae|[21880]|NCCA1154-11|Sciaridae|673|BOLD:AAH3951
Sciaridae|[21881]|NCCA740-11|Sciaridae|673|BOLD:AAQ2559
Sciaridae|[21882]|NCCA1161-11|Sciaridae|673|BOLD:ABA6457
Sciaridae|[21883]|JSAUG1005-11|Sciaridae|658|BOLD:ABV1265
Sciaridae|[21884]|HEMAY963-12|Sciaridae|703|BOLD:ABV3274
Sciaridae|[21885]|PHMTT029-10|Sciaridae|631|BOLD:AAU6634
Sciaridae|[21886]|CNGBB478-13|Sciaridae|578|BOLD:ACL0082
Sciaridae|[21887]|SSKJB4066-14|Sciaridae|588|BOLD:ACR5945
Sciaridae|[21888]|JSJUN1018-11|Sciaridae|658|BOLD:ABV1201
Sciaridae|[21889]|CNKLB1380-14|Sciaridae|588|BOLD:ACR6902
Sciaridae|[21890]|SSPAC1605-13|Sciaridae|658|BOLD:ACA3429
Sciaridae|[21891]|BBDEE849-10|Sciaridae|658|BOLD:AAN6426
Sciaridae|[21892]|CNFDD257-14|Sciaridae|661|BOLD:ACL7291
Sciaridae|[21893]|CNAUY220-14|Sciaridae|579|BOLD:ACJ5862
Schwenckfeldina tridentata|[21894]|CNAUY571-14|Sciaridae|615|BOLD:ACZ2602
Schwenckfeldina tridentata|[21895]|CNTUB3528-14|Sciaridae|588|BOLD:ABW3870
Sciaridae|[21896]|CNRMD1635-12|Sciaridae|673|BOLD:ABV1277

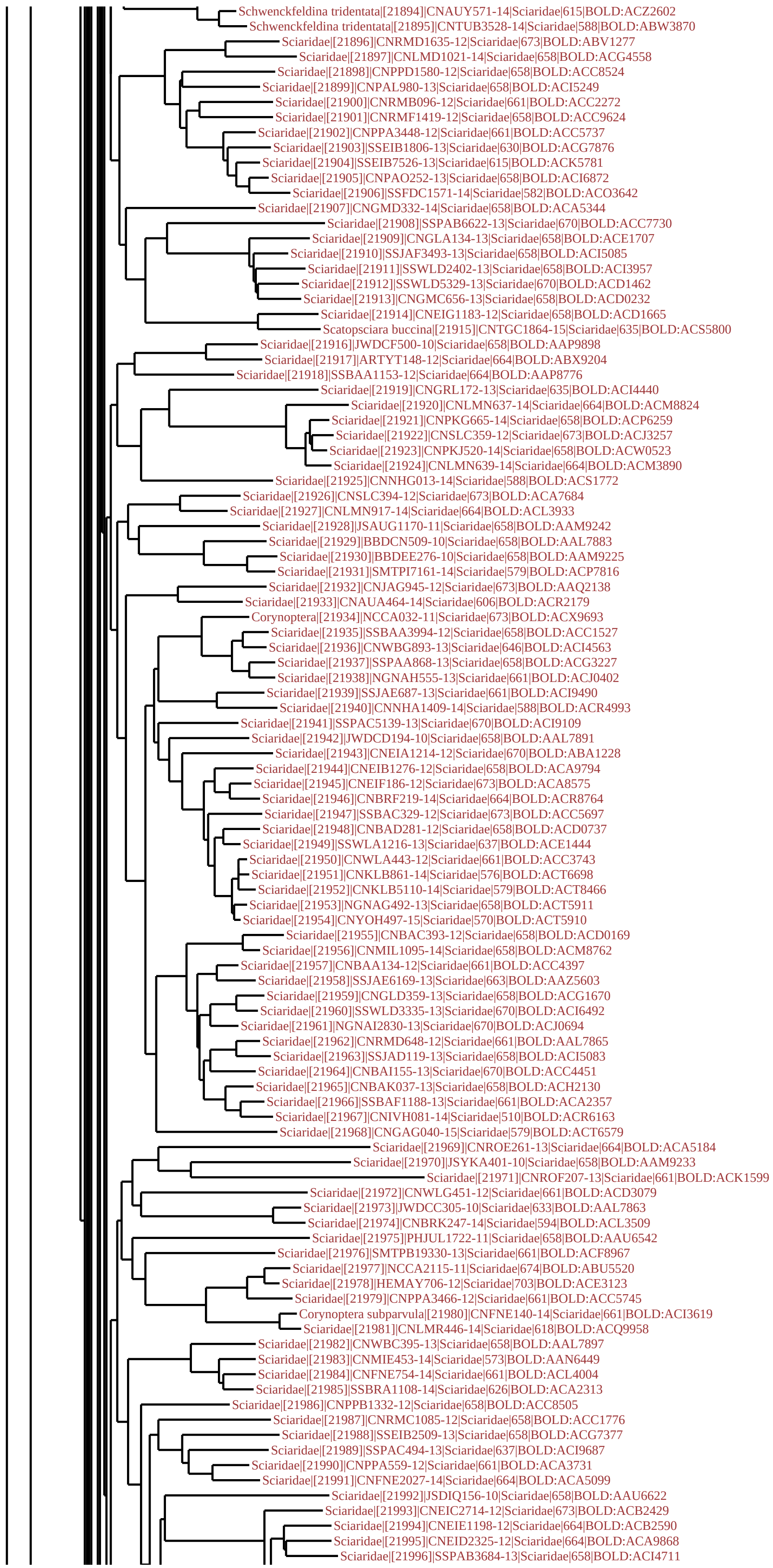

Schwenckfeldina tridentata|[21894]|CNAUY571-14|Sciaridae|615|BOLD:ACZ2602
Schwenckfeldina tridentata|[21895]|CNTUB3528-14|Sciaridae|588|BOLD:ABW3870
Sciaridae|[21896]|CNRMD1635-12|Sciaridae|673|BOLD:ABV1277
Sciaridae|[21897]|CNLMD1021-14|Sciaridae|658|BOLD:ACG4558
Sciaridae|[21898]|CNPPD1580-12|Sciaridae|658|BOLD:ACC8524
Sciaridae|[21899]|CNPAL980-13|Sciaridae|658|BOLD:ACI5249
Sciaridae|[21900]|CNRMB096-12|Sciaridae|661|BOLD:ACC2272
Sciaridae|[21901]|CNRMF1419-12|Sciaridae|658|BOLD:ACC9624
Sciaridae|[21902]|CNPPA3448-12|Sciaridae|661|BOLD:ACC5737
Sciaridae|[21903]|SSEIB1806-13|Sciaridae|630|BOLD:ACG7876
Sciaridae|[21904]|SSEIB7526-13|Sciaridae|615|BOLD:ACK5781
Sciaridae|[21905]|CNPAO252-13|Sciaridae|658|BOLD:ACI6872
Sciaridae|[21906]|SSFDC1571-14|Sciaridae|582|BOLD:ACO3642
Sciaridae|[21907]|CNGMD332-14|Sciaridae|658|BOLD:ACA5344
Sciaridae|[21908]|SSPAB6622-13|Sciaridae|670|BOLD:ACC7730
Sciaridae|[21909]|CNGLA134-13|Sciaridae|658|BOLD:ACE1707
Sciaridae|[21910]|SSJAF3493-13|Sciaridae|658|BOLD:ACI5085
Sciaridae|[21911]|SSWLD2402-13|Sciaridae|658|BOLD:ACI3957
Sciaridae|[21912]|SSWLD5329-13|Sciaridae|670|BOLD:ACD1462
Sciaridae|[21913]|CNGMC656-13|Sciaridae|658|BOLD:ACD0232
Sciaridae|[21914]|CNEIG1183-12|Sciaridae|658|BOLD:ACD1665
Scatopsciara buccina|[21915]|CNTGC1864-15|Sciaridae|635|BOLD:ACS5800
Sciaridae|[21916]|JWDCF500-10|Sciaridae|658|BOLD:AAP9898
Sciaridae|[21917]|ARTYT148-12|Sciaridae|664|BOLD:ABX9204
Sciaridae|[21918]|SSBAA1153-12|Sciaridae|664|BOLD:AAP8776
Sciaridae|[21919]|CNGRL172-13|Sciaridae|635|BOLD:ACI4440
Sciaridae|[21920]|CNLMN637-14|Sciaridae|664|BOLD:ACM8824
Sciaridae|[21921]|CNPKG665-14|Sciaridae|658|BOLD:ACP6259
Sciaridae|[21922]|CNSLC359-12|Sciaridae|673|BOLD:ACJ3257
Sciaridae|[21923]|CNPKJ520-14|Sciaridae|658|BOLD:ACW0523
Sciaridae|[21924]|CNLMN639-14|Sciaridae|664|BOLD:ACM3890
Sciaridae|[21925]|CNNHG013-14|Sciaridae|588|BOLD:ACS1772
Sciaridae|[21926]|CNSLC394-12|Sciaridae|673|BOLD:ACA7684
Sciaridae|[21927]|CNLMN917-14|Sciaridae|664|BOLD:ACL3933
Sciaridae|[21928]|JSAUG1170-11|Sciaridae|658|BOLD:AAM9242
Sciaridae|[21929]|BBDCN509-10|Sciaridae|658|BOLD:AAL7883
Sciaridae|[21930]|BBDEE276-10|Sciaridae|658|BOLD:AAM9225
Sciaridae|[21931]|SMTPI7161-14|Sciaridae|579|BOLD:ACP7816
Sciaridae|[21932]|CNJAG945-12|Sciaridae|673|BOLD:AAQ2138
Sciaridae|[21933]|CNAUA464-14|Sciaridae|606|BOLD:ACR2179
Corynoptera|[21934]|NCCA032-11|Sciaridae|673|BOLD:ACX9693
Sciaridae|[21935]|SSBAA3994-12|Sciaridae|658|BOLD:ACC1527
Sciaridae|[21936]|CNWBG893-13|Sciaridae|646|BOLD:ACI4563
Sciaridae|[21937]|SSPAA868-13|Sciaridae|658|BOLD:ACG3227
Sciaridae|[21938]|NGNAH555-13|Sciaridae|661|BOLD:ACJ0402
Sciaridae|[21939]|SSJAE687-13|Sciaridae|661|BOLD:ACI9490
Sciaridae|[21940]|CNNHA1409-14|Sciaridae|588|BOLD:ACR4993
Sciaridae|[21941]|SSPAC5139-13|Sciaridae|670|BOLD:ACI9109
Sciaridae|[21942]|JWDCD194-10|Sciaridae|658|BOLD:AAL7891
Sciaridae|[21943]|CNEIA1214-12|Sciaridae|670|BOLD:ABA1228
Sciaridae|[21944]|CNEIB1276-12|Sciaridae|658|BOLD:ACA9794
Sciaridae|[21945]|CNEIF186-12|Sciaridae|673|BOLD:ACA8575
Sciaridae|[21946]|CNBRF219-14|Sciaridae|664|BOLD:ACR8764
Sciaridae|[21947]|SSBAC329-12|Sciaridae|673|BOLD:ACC5697
Sciaridae|[21948]|CNBAD281-12|Sciaridae|658|BOLD:ACD0737
Sciaridae|[21949]|SSWLA1216-13|Sciaridae|637|BOLD:ACE1444
Sciaridae|[21950]|CNWLA443-12|Sciaridae|661|BOLD:ACC3743
Sciaridae|[21951]|CNKLB861-14|Sciaridae|576|BOLD:ACT6698
Sciaridae|[21952]|CNKLB5110-14|Sciaridae|579|BOLD:ACT8466
Sciaridae|[21953]|NGNAG492-13|Sciaridae|658|BOLD:ACT5911
Sciaridae|[21954]|CNYOH497-15|Sciaridae|570|BOLD:ACT5910
Sciaridae|[21955]|CNBAC393-12|Sciaridae|658|BOLD:ACD0169
Sciaridae|[21956]|CNMIL1095-14|Sciaridae|658|BOLD:ACM8762
Sciaridae|[21957]|CNBAA134-12|Sciaridae|661|BOLD:ACC4397
Sciaridae|[21958]|SSJAE6169-13|Sciaridae|663|BOLD:AAZ5603
Sciaridae|[21959]|CNGLD359-13|Sciaridae|658|BOLD:ACG1670
Sciaridae|[21960]|SSWLD3335-13|Sciaridae|670|BOLD:ACI6492
Sciaridae|[21961]|NGNAI2830-13|Sciaridae|670|BOLD:ACJ0694
Sciaridae|[21962]|CNRMD648-12|Sciaridae|661|BOLD:AAL7865
Sciaridae|[21963]|SSJAD119-13|Sciaridae|658|BOLD:ACI5083
Sciaridae|[21964]|CNBAI155-13|Sciaridae|670|BOLD:ACC4451
Sciaridae|[21965]|CNBAK037-13|Sciaridae|658|BOLD:ACH2130
Sciaridae|[21966]|SSBAF1188-13|Sciaridae|661|BOLD:ACA2357
Sciaridae|[21967]|CNIVH081-14|Sciaridae|510|BOLD:ACR6163
Sciaridae|[21968]|CNGAG040-15|Sciaridae|579|BOLD:ACT6579
Sciaridae|[21969]|CNROE261-13|Sciaridae|664|BOLD:ACA5184
Sciaridae|[21970]|JSYKA401-10|Sciaridae|658|BOLD:AAM9233
Sciaridae|[21971]|CNROF207-13|Sciaridae|661|BOLD:ACK1599
Sciaridae|[21972]|CNWLG451-12|Sciaridae|661|BOLD:ACD3079
Sciaridae|[21973]|JWDCC305-10|Sciaridae|633|BOLD:AAL7863
Sciaridae|[21974]|CNBRK247-14|Sciaridae|594|BOLD:ACL3509
Sciaridae|[21975]|PHJUL1722-11|Sciaridae|658|BOLD:AAU6542
Sciaridae|[21976]|SMTPB19330-13|Sciaridae|661|BOLD:ACF8967
Sciaridae|[21977]|NCCA2115-11|Sciaridae|674|BOLD:ABU5520
Sciaridae|[21978]|HEMAY706-12|Sciaridae|703|BOLD:ACE3123
Sciaridae|[21979]|CNPPA3466-12|Sciaridae|661|BOLD:ACC5745
Corynoptera subparvula|[21980]|CNFNE140-14|Sciaridae|661|BOLD:ACI3619
Sciaridae|[21981]|CNLMR446-14|Sciaridae|618|BOLD:ACQ9958
Sciaridae|[21982]|CNWBC395-13|Sciaridae|658|BOLD:AAL7897
Sciaridae|[21983]|CNMIE453-14|Sciaridae|573|BOLD:AAN6449
Sciaridae|[21984]|CNFNE754-14|Sciaridae|661|BOLD:ACL4004
Sciaridae|[21985]|SSBRA1108-14|Sciaridae|626|BOLD:ACA2313
Sciaridae|[21986]|CNPPB1332-12|Sciaridae|658|BOLD:ACC8505
Sciaridae|[21987]|CNRMC1085-12|Sciaridae|658|BOLD:ACC1776
Sciaridae|[21988]|SSEIB2509-13|Sciaridae|658|BOLD:ACG7377
Sciaridae|[21989]|SSPAC494-13|Sciaridae|637|BOLD:ACI9687
Sciaridae|[21990]|CNPPA559-12|Sciaridae|661|BOLD:ACA3731
Sciaridae|[21991]|CNFNE2027-14|Sciaridae|664|BOLD:ACA5099
Sciaridae|[21992]|JSDIQ156-10|Sciaridae|658|BOLD:AAU6622
Sciaridae|[21993]|CNEIC2714-12|Sciaridae|673|BOLD:ACB2429
Sciaridae|[21994]|CNEIE1198-12|Sciaridae|664|BOLD:ACB2590
Sciaridae|[21995]|CNEID2325-12|Sciaridae|664|BOLD:ACA9868
Sciaridae|[21996]|SSPAB3684-13|Sciaridae|658|BOLD:ACI4711

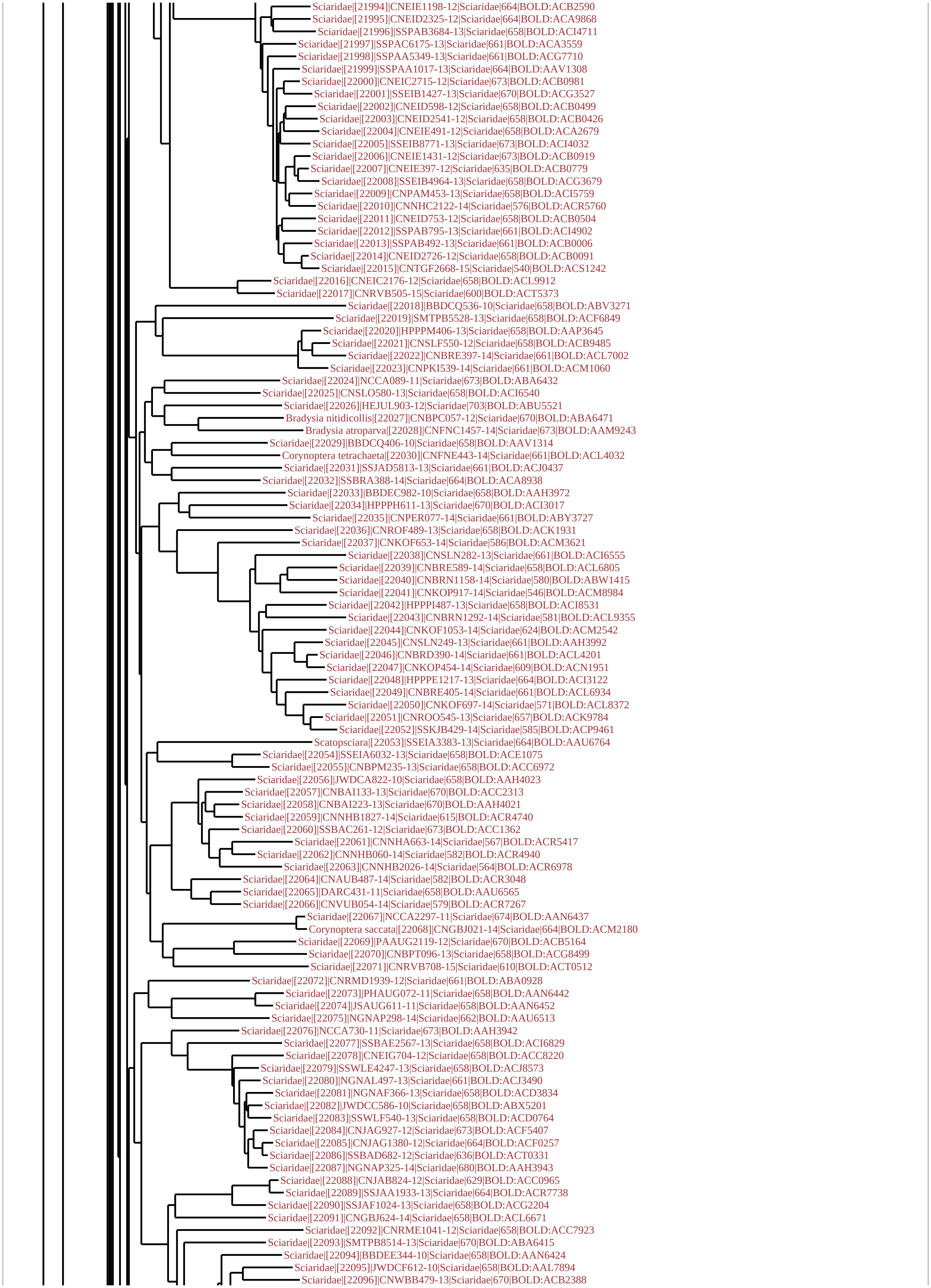
Sciaridae|[21994]|CNEIE1198-12|Sciaridae|664|BOLD:ACB2590
Sciaridae|[21995]|CNEID2325-12|Sciaridae|664|BOLD:ACA9868
Sciaridae|[21996]|SSPAB3684-13|Sciaridae|658|BOLD:ACI4711
Sciaridae|[21997]|SSPAC6175-13|Sciaridae|661|BOLD:ACA3559
Sciaridae|[21998]|SSPAA5349-13|Sciaridae|661|BOLD:ACG7710
Sciaridae|[21999]|SSPAA1017-13|Sciaridae|664|BOLD:AAV1308
Sciaridae|[22000]|CNEIC2715-12|Sciaridae|673|BOLD:ACB0981
Sciaridae|[22001]|SSEIB1427-13|Sciaridae|670|BOLD:ACG3527
Sciaridae|[22002]|CNEID598-12|Sciaridae|658|BOLD:ACB0499
Sciaridae|[22003]|CNEID2541-12|Sciaridae|658|BOLD:ACB0426
Sciaridae|[22004]|CNEIE491-12|Sciaridae|658|BOLD:ACA2679
Sciaridae|[22005]|SSEIB8771-13|Sciaridae|673|BOLD:ACI4032
Sciaridae|[22006]|CNEIE1431-12|Sciaridae|673|BOLD:ACB0919
Sciaridae|[22007]|CNEIE397-12|Sciaridae|635|BOLD:ACB0779
Sciaridae|[22008]|SSEIB4964-13|Sciaridae|658|BOLD:ACG3679
Sciaridae|[22009]|CNPAM453-13|Sciaridae|658|BOLD:ACI5759
Sciaridae|[22010]|CNNHC2122-14|Sciaridae|576|BOLD:ACR5760
Sciaridae|[22011]|CNEID753-12|Sciaridae|658|BOLD:ACB0504
Sciaridae|[22012]|SSPAB795-13|Sciaridae|661|BOLD:ACI4902
Sciaridae|[22013]|SSPAB492-13|Sciaridae|661|BOLD:ACB0006
Sciaridae|[22014]|CNEID2726-12|Sciaridae|658|BOLD:ACB0091
Sciaridae|[22015]|CNTGF2668-15|Sciaridae|540|BOLD:ACS1242
Sciaridae|[22016]|CNEIC2176-12|Sciaridae|658|BOLD:ACL9912
Sciaridae|[22017]|CNRVB505-15|Sciaridae|600|BOLD:ACT5373
Sciaridae|[22018]|BBDCQ536-10|Sciaridae|658|BOLD:ABV3271
Sciaridae|[22019]|SMTPB5528-13|Sciaridae|658|BOLD:ACF6849
Sciaridae|[22020]|HPPPM406-13|Sciaridae|658|BOLD:AAP3645
Sciaridae|[22021]|CNSLF550-12|Sciaridae|658|BOLD:ACB9485
Sciaridae|[22022]|CNBRE397-14|Sciaridae|661|BOLD:ACL7002
Sciaridae|[22023]|CNPKI539-14|Sciaridae|661|BOLD:ACM1060
Sciaridae|[22024]|NCCA089-11|Sciaridae|673|BOLD:ABA6432
Sciaridae|[22025]|CNSLO580-13|Sciaridae|658|BOLD:ACI6540
Sciaridae|[22026]|HEJUL903-12|Sciaridae|703|BOLD:ABU5521
Bradysia nitidicollis|[22027]|CNBPC057-12|Sciaridae|670|BOLD:ABA6471
Bradysia atroparva|[22028]|CNFNC1457-14|Sciaridae|673|BOLD:AAM9243
Sciaridae|[22029]|BBDCQ406-10|Sciaridae|658|BOLD:AAV1314
Corynoptera tetrachaeta|[22030]|CNFNE443-14|Sciaridae|661|BOLD:ACL4032
Sciaridae|[22031]|SSJAD5813-13|Sciaridae|661|BOLD:ACJ0437
Sciaridae|[22032]|SSBRA388-14|Sciaridae|664|BOLD:ACA8938
Sciaridae|[22033]|BBDEC982-10|Sciaridae|658|BOLD:AAH3972
Sciaridae|[22034]|HPPPH611-13|Sciaridae|670|BOLD:ACI3017
Sciaridae|[22035]|CNPER077-14|Sciaridae|661|BOLD:ABY3727
Sciaridae|[22036]|CNROF489-13|Sciaridae|658|BOLD:ACK1931
Sciaridae|[22037]|CNKOF653-14|Sciaridae|586|BOLD:ACM3621
Sciaridae|[22038]|CNSLN282-13|Sciaridae|661|BOLD:ACI6555
Sciaridae|[22039]|CNBRE589-14|Sciaridae|658|BOLD:ACL6805
Sciaridae|[22040]|CNBRN1158-14|Sciaridae|580|BOLD:ABW1415
Sciaridae|[22041]|CNKOP917-14|Sciaridae|546|BOLD:ACM8984
Sciaridae|[22042]|HPPPI487-13|Sciaridae|658|BOLD:ACI8531
Sciaridae|[22043]|CNBRN1292-14|Sciaridae|581|BOLD:ACL9355
Sciaridae|[22044]|CNKOF1053-14|Sciaridae|624|BOLD:ACM2542
Sciaridae|[22045]|CNSLN249-13|Sciaridae|661|BOLD:AAH3992
Sciaridae|[22046]|CNBRD390-14|Sciaridae|661|BOLD:ACL4201
Sciaridae|[22047]|CNKOP454-14|Sciaridae|609|BOLD:ACN1951
Sciaridae|[22048]|HPPPE1217-13|Sciaridae|664|BOLD:ACI3122
Sciaridae|[22049]|CNBRE405-14|Sciaridae|661|BOLD:ACL6934
Sciaridae|[22050]|CNKOF697-14|Sciaridae|571|BOLD:ACL8372
Sciaridae|[22051]|CNROO545-13|Sciaridae|657|BOLD:ACK9784
Sciaridae|[22052]|SSKJB429-14|Sciaridae|585|BOLD:ACP9461
Scatopsciara|[22053]|SSEIA3383-13|Sciaridae|664|BOLD:AAU6764
Sciaridae|[22054]|SSEIA6032-13|Sciaridae|658|BOLD:ACE1075
Sciaridae|[22055]|CNBPM235-13|Sciaridae|658|BOLD:ACC6972
Sciaridae|[22056]|JWDCA822-10|Sciaridae|658|BOLD:AAH4023
Sciaridae|[22057]|CNBAI133-13|Sciaridae|670|BOLD:ACC2313
Sciaridae|[22058]|CNBAI223-13|Sciaridae|670|BOLD:AAH4021
Sciaridae|[22059]|CNNHB1827-14|Sciaridae|615|BOLD:ACR4740
Sciaridae|[22060]|SSBAC261-12|Sciaridae|673|BOLD:ACC1362
Sciaridae|[22061]|CNNHA663-14|Sciaridae|567|BOLD:ACR5417
Sciaridae|[22062]|CNNHB060-14|Sciaridae|582|BOLD:ACR4940
Sciaridae|[22063]|CNNHB2026-14|Sciaridae|564|BOLD:ACR6978
Sciaridae|[22064]|CNAUB487-14|Sciaridae|582|BOLD:ACR3048
Sciaridae|[22065]|DARC431-11|Sciaridae|658|BOLD:AAU6565
Sciaridae|[22066]|CNVUB054-14|Sciaridae|579|BOLD:ACR7267
Sciaridae|[22067]|NCCA2297-11|Sciaridae|674|BOLD:AAN6437
Corynoptera saccata|[22068]|CNGBJ021-14|Sciaridae|664|BOLD:ACM2180
Sciaridae|[22069]|PAAUG2119-12|Sciaridae|670|BOLD:ACB5164
Sciaridae|[22070]|CNBPT096-13|Sciaridae|658|BOLD:ACG8499
Sciaridae|[22071]|CNRVB708-15|Sciaridae|610|BOLD:ACT0512
Sciaridae|[22072]|CNRMD1939-12|Sciaridae|661|BOLD:ABA0928
Sciaridae|[22073]|PHAUG072-11|Sciaridae|658|BOLD:AAN6442
Sciaridae|[22074]|JSAUG611-11|Sciaridae|658|BOLD:AAN6452
Sciaridae|[22075]|NGNAP298-14|Sciaridae|662|BOLD:AAU6513
Sciaridae|[22076]|NCCA730-11|Sciaridae|673|BOLD:AAH3942
Sciaridae|[22077]|SSBAE2567-13|Sciaridae|658|BOLD:ACI6829
Sciaridae|[22078]|CNEIG704-12|Sciaridae|658|BOLD:ACC8220
Sciaridae|[22079]|SSWLE4247-13|Sciaridae|658|BOLD:ACJ8573
Sciaridae|[22080]|NGNAL497-13|Sciaridae|661|BOLD:ACJ3490
Sciaridae|[22081]|NGNAF366-13|Sciaridae|658|BOLD:ACD3834
Sciaridae|[22082]|JWDCC586-10|Sciaridae|658|BOLD:ABX5201
Sciaridae|[22083]|SSWLF540-13|Sciaridae|658|BOLD:ACD0764
Sciaridae|[22084]|CNJAG927-12|Sciaridae|673|BOLD:ACF5407
Sciaridae|[22085]|CNJAG1380-12|Sciaridae|664|BOLD:ACF0257
Sciaridae|[22086]|SSBAD682-12|Sciaridae|636|BOLD:ACT0331
Sciaridae|[22087]|NGNAP325-14|Sciaridae|680|BOLD:AAH3943
Sciaridae|[22088]|CNJAB824-12|Sciaridae|629|BOLD:ACC0965
Sciaridae|[22089]|SSJAA1933-13|Sciaridae|664|BOLD:ACR7738
Sciaridae|[22090]|SSJAF1024-13|Sciaridae|658|BOLD:ACG2204
Sciaridae|[22091]|CNGBJ624-14|Sciaridae|658|BOLD:ACL6671
Sciaridae|[22092]|CNRME1041-12|Sciaridae|658|BOLD:ACC7923
Sciaridae|[22093]|SMTPB8514-13|Sciaridae|670|BOLD:ABA6415
Sciaridae|[22094]|BBDEE344-10|Sciaridae|658|BOLD:AAN6424
Sciaridae|[22095]|JWDCF612-10|Sciaridae|658|BOLD:AAL7894
Sciaridae|[22096]|CNWBB479-13|Sciaridae|670|BOLD:ACB2388

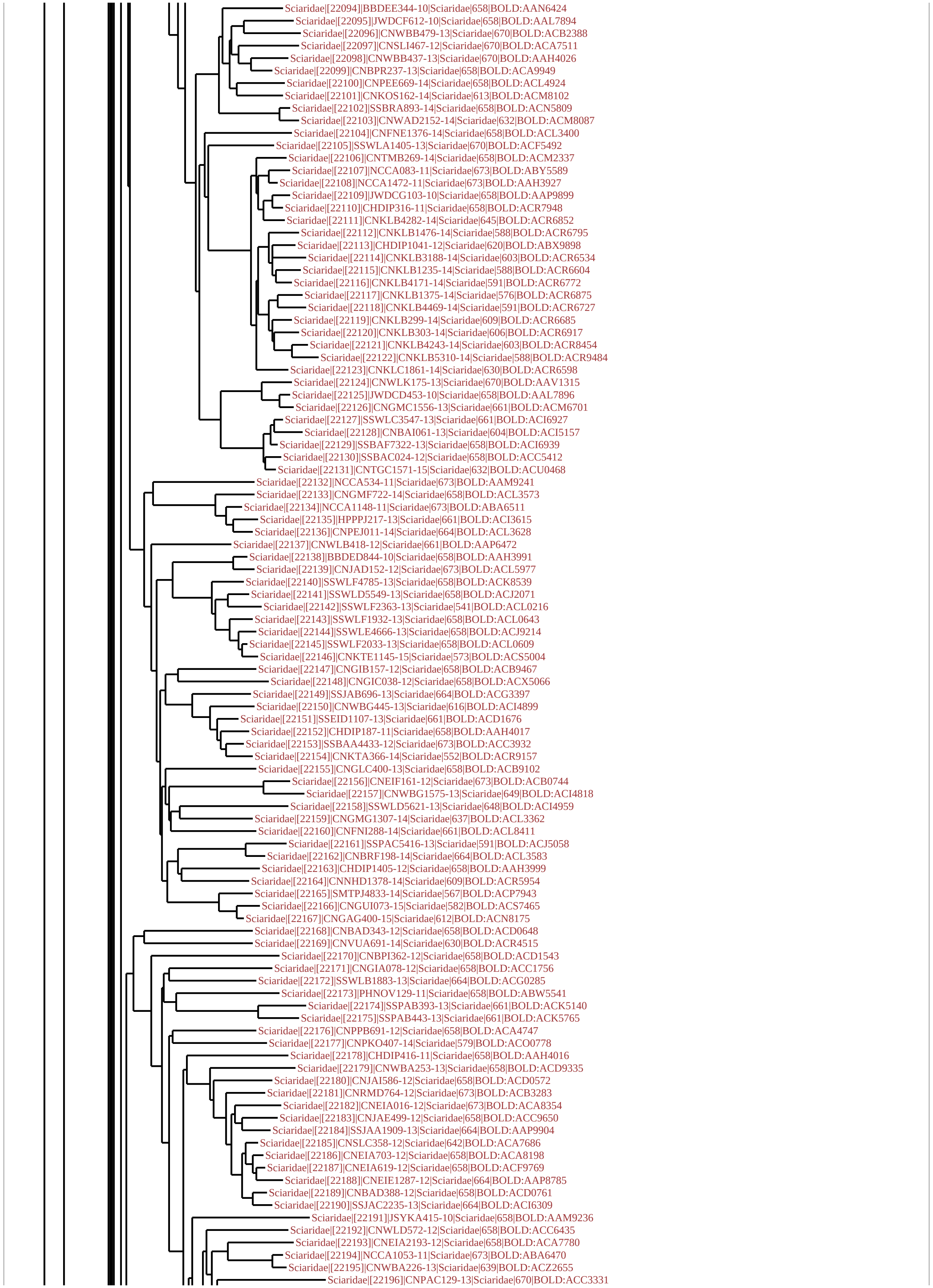
Sciaridae|[22094]|BBDEE344-10|Sciaridae|658|BOLD:AAN6424
Sciaridae|[22095]|JWDCF612-10|Sciaridae|658|BOLD:AAL7894
Sciaridae|[22096]|CNWBB479-13|Sciaridae|670|BOLD:ACB2388
Sciaridae|[22097]|CNSLI467-12|Sciaridae|670|BOLD:ACA7511
Sciaridae|[22098]|CNWBB437-13|Sciaridae|670|BOLD:AAH4026
Sciaridae|[22099]|CNBPR237-13|Sciaridae|658|BOLD:ACA9949
Sciaridae|[22100]|CNPEE669-14|Sciaridae|658|BOLD:ACL4924
Sciaridae|[22101]|CNKOS162-14|Sciaridae|613|BOLD:ACM8102
Sciaridae|[22102]|SSBRA893-14|Sciaridae|658|BOLD:ACN5809
Sciaridae|[22103]|CNWAD2152-14|Sciaridae|632|BOLD:ACM8087
Sciaridae|[22104]|CNFNE1376-14|Sciaridae|658|BOLD:ACL3400
Sciaridae|[22105]|SSWLA1405-13|Sciaridae|670|BOLD:ACF5492
Sciaridae|[22106]|CNTMB269-14|Sciaridae|658|BOLD:ACM2337
Sciaridae|[22107]|NCCA083-11|Sciaridae|673|BOLD:ABY5589
Sciaridae|[22108]|NCCA1472-11|Sciaridae|673|BOLD:AAH3927
Sciaridae|[22109]|JWDCG103-10|Sciaridae|658|BOLD:AAP9899
Sciaridae|[22110]|CHDIP316-11|Sciaridae|658|BOLD:ACR7948
Sciaridae|[22111]|CNKLB4282-14|Sciaridae|645|BOLD:ACR6852
Sciaridae|[22112]|CNKLB1476-14|Sciaridae|588|BOLD:ACR6795
Sciaridae|[22113]|CHDIP1041-12|Sciaridae|620|BOLD:ABX9898
Sciaridae|[22114]|CNKLB3188-14|Sciaridae|603|BOLD:ACR6534
Sciaridae|[22115]|CNKLB1235-14|Sciaridae|588|BOLD:ACR6604
Sciaridae|[22116]|CNKLB4171-14|Sciaridae|591|BOLD:ACR6772
Sciaridae|[22117]|CNKLB1375-14|Sciaridae|576|BOLD:ACR6875
Sciaridae|[22118]|CNKLB4469-14|Sciaridae|591|BOLD:ACR6727
Sciaridae|[22119]|CNKLB299-14|Sciaridae|609|BOLD:ACR6685
Sciaridae|[22120]|CNKLB303-14|Sciaridae|606|BOLD:ACR6917
Sciaridae|[22121]|CNKLB4243-14|Sciaridae|603|BOLD:ACR8454
Sciaridae|[22122]|CNKLB5310-14|Sciaridae|588|BOLD:ACR9484
Sciaridae|[22123]|CNKLC1861-14|Sciaridae|630|BOLD:ACR6598
Sciaridae|[22124]|CNWLK175-13|Sciaridae|670|BOLD:AAV1315
Sciaridae|[22125]|JWDCD453-10|Sciaridae|658|BOLD:AAL7896
Sciaridae|[22126]|CNGMC1556-13|Sciaridae|661|BOLD:ACM6701
Sciaridae|[22127]|SSWLC3547-13|Sciaridae|661|BOLD:ACI6927
Sciaridae|[22128]|CNBAI061-13|Sciaridae|604|BOLD:ACI5157
Sciaridae|[22129]|SSBAF7322-13|Sciaridae|658|BOLD:ACI6939
Sciaridae|[22130]|SSBAC024-12|Sciaridae|658|BOLD:ACC5412
Sciaridae|[22131]|CNTGC1571-15|Sciaridae|632|BOLD:ACU0468
Sciaridae|[22132]|NCCA534-11|Sciaridae|673|BOLD:AAM9241
Sciaridae|[22133]|CNGMF722-14|Sciaridae|658|BOLD:ACL3573
Sciaridae|[22134]|NCCA1148-11|Sciaridae|673|BOLD:ABA6511
Sciaridae|[22135]|HPPPJ217-13|Sciaridae|661|BOLD:ACI3615
Sciaridae|[22136]|CNPEJ011-14|Sciaridae|664|BOLD:ACL3628
Sciaridae|[22137]|CNWLB418-12|Sciaridae|661|BOLD:AAP6472
Sciaridae|[22138]|BBDED844-10|Sciaridae|658|BOLD:AAH3991
Sciaridae|[22139]|CNJAD152-12|Sciaridae|673|BOLD:ACL5977
Sciaridae|[22140]|SSWLF4785-13|Sciaridae|658|BOLD:ACK8539
Sciaridae|[22141]|SSWLD5549-13|Sciaridae|658|BOLD:ACJ2071
Sciaridae|[22142]|SSWLF2363-13|Sciaridae|541|BOLD:ACL0216
Sciaridae|[22143]|SSWLF1932-13|Sciaridae|658|BOLD:ACL0643
Sciaridae|[22144]|SSWLE4666-13|Sciaridae|658|BOLD:ACJ9214
Sciaridae|[22145]|SSWLF2033-13|Sciaridae|658|BOLD:ACL0609
Sciaridae|[22146]|CNKTE1145-15|Sciaridae|573|BOLD:ACS5004
Sciaridae|[22147]|CNGIB157-12|Sciaridae|658|BOLD:ACB9467
Sciaridae|[22148]|CNGIC038-12|Sciaridae|658|BOLD:ACX5066
Sciaridae|[22149]|SSJAB696-13|Sciaridae|664|BOLD:ACG3397
Sciaridae|[22150]|CNWBG445-13|Sciaridae|616|BOLD:ACI4899
Sciaridae|[22151]|SSEID1107-13|Sciaridae|661|BOLD:ACD1676
Sciaridae|[22152]|CHDIP187-11|Sciaridae|658|BOLD:AAH4017
Sciaridae|[22153]|SSBAA4433-12|Sciaridae|673|BOLD:ACC3932
Sciaridae|[22154]|CNKTA366-14|Sciaridae|552|BOLD:ACR9157
Sciaridae|[22155]|CNGLC400-13|Sciaridae|658|BOLD:ACB9102
Sciaridae|[22156]|CNEIF161-12|Sciaridae|673|BOLD:ACB0744
Sciaridae|[22157]|CNWBG1575-13|Sciaridae|649|BOLD:ACI4818
Sciaridae|[22158]|SSWLD5621-13|Sciaridae|648|BOLD:ACI4959
Sciaridae|[22159]|CNGMG1307-14|Sciaridae|637|BOLD:ACL3362
Sciaridae|[22160]|CNFNI288-14|Sciaridae|661|BOLD:ACL8411
Sciaridae|[22161]|SSPAC5416-13|Sciaridae|591|BOLD:ACJ5058
Sciaridae|[22162]|CNBRF198-14|Sciaridae|664|BOLD:ACL3583
Sciaridae|[22163]|CHDIP1405-12|Sciaridae|658|BOLD:AAH3999
Sciaridae|[22164]|CNNHD1378-14|Sciaridae|609|BOLD:ACR5954
Sciaridae|[22165]|SMTPJ4833-14|Sciaridae|567|BOLD:ACP7943
Sciaridae|[22166]|CNGUI073-15|Sciaridae|582|BOLD:ACS7465
Sciaridae|[22167]|CNGAG400-15|Sciaridae|612|BOLD:ACN8175
Sciaridae|[22168]|CNBAD343-12|Sciaridae|658|BOLD:ACD0648
Sciaridae|[22169]|CNVUA691-14|Sciaridae|630|BOLD:ACR4515
Sciaridae|[22170]|CNBPI362-12|Sciaridae|658|BOLD:ACD1543
Sciaridae|[22171]|CNGIA078-12|Sciaridae|658|BOLD:ACC1756
Sciaridae|[22172]|SSWLB1883-13|Sciaridae|664|BOLD:ACG0285
Sciaridae|[22173]|PHNOV129-11|Sciaridae|658|BOLD:ABW5541
Sciaridae|[22174]|SSPAB393-13|Sciaridae|661|BOLD:ACK5140
Sciaridae|[22175]|SSPAB443-13|Sciaridae|661|BOLD:ACK5765
Sciaridae|[22176]|CNPPB691-12|Sciaridae|658|BOLD:ACA4747
Sciaridae|[22177]|CNPKO407-14|Sciaridae|579|BOLD:ACO0778
Sciaridae|[22178]|CHDIP416-11|Sciaridae|658|BOLD:AAH4016
Sciaridae|[22179]|CNWBA253-13|Sciaridae|658|BOLD:ACD9335
Sciaridae|[22180]|CNJAI586-12|Sciaridae|658|BOLD:ACD0572
Sciaridae|[22181]|CNRMD764-12|Sciaridae|673|BOLD:ACB3283
Sciaridae|[22182]|CNEIA016-12|Sciaridae|673|BOLD:ACA8354
Sciaridae|[22183]|CNJAE499-12|Sciaridae|658|BOLD:ACC9650
Sciaridae|[22184]|SSJAA1909-13|Sciaridae|664|BOLD:AAP9904
Sciaridae|[22185]|CNSLC358-12|Sciaridae|642|BOLD:ACA7686
Sciaridae|[22186]|CNEIA703-12|Sciaridae|658|BOLD:ACA8198
Sciaridae|[22187]|CNEIA619-12|Sciaridae|658|BOLD:ACF9769
Sciaridae|[22188]|CNEIE1287-12|Sciaridae|664|BOLD:AAP8785
Sciaridae|[22189]|CNBAD388-12|Sciaridae|658|BOLD:ACD0761
Sciaridae|[22190]|SSJAC2235-13|Sciaridae|664|BOLD:ACI6309
Sciaridae|[22191]|JSYKA415-10|Sciaridae|658|BOLD:AAM9236
Sciaridae|[22192]|CNWLD572-12|Sciaridae|658|BOLD:ACC6435
Sciaridae|[22193]|CNEIA2193-12|Sciaridae|658|BOLD:ACA7780
Sciaridae|[22194]|NCCA1053-11|Sciaridae|673|BOLD:ABA6470
Sciaridae|[22195]|CNWBA226-13|Sciaridae|639|BOLD:ACZ2655
Sciaridae|[22196]|CNPAC129-13|Sciaridae|670|BOLD:ACC3331

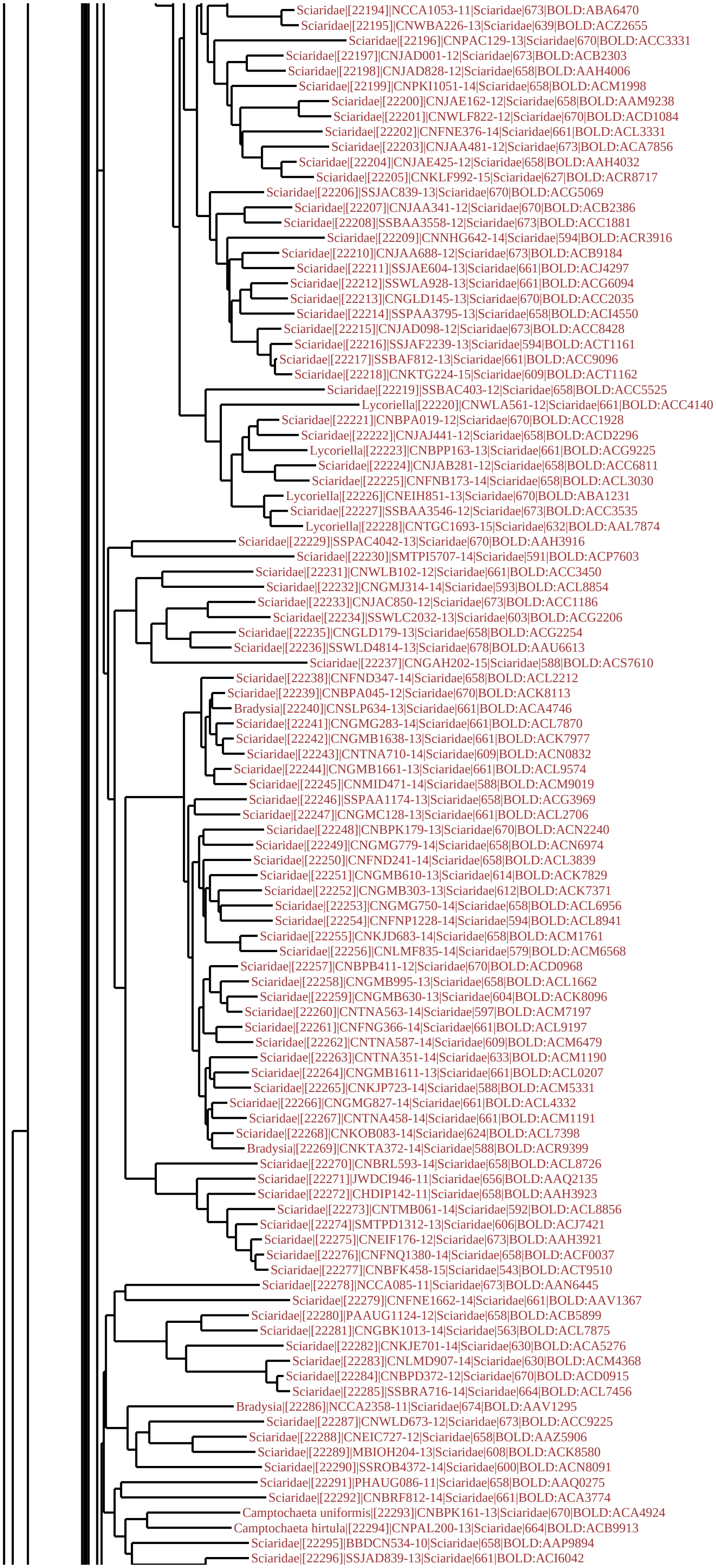
Sciaridae|[22194]|NCCA1053-11|Sciaridae|673|BOLD:ABA6470
Sciaridae|[22195]|CNWBA226-13|Sciaridae|639|BOLD:ACZ2655
Sciaridae|[22196]|CNPAC129-13|Sciaridae|670|BOLD:ACC3331
Sciaridae|[22197]|CNJAD001-12|Sciaridae|673|BOLD:ACB2303
Sciaridae|[22198]|CNJAD828-12|Sciaridae|658|BOLD:AAH4006
Sciaridae|[22199]|CNPKI1051-14|Sciaridae|658|BOLD:ACM1998
Sciaridae|[22200]|CNJAE162-12|Sciaridae|658|BOLD:AAM9238
Sciaridae|[22201]|CNWLF822-12|Sciaridae|670|BOLD:ACD1084
Sciaridae|[22202]|CNFNE376-14|Sciaridae|661|BOLD:ACL3331
Sciaridae|[22203]|CNJAA481-12|Sciaridae|673|BOLD:ACA7856
Sciaridae|[22204]|CNJAE425-12|Sciaridae|658|BOLD:AAH4032
Sciaridae|[22205]|CNKLF992-15|Sciaridae|627|BOLD:ACR8717
Sciaridae|[22206]|SSJAC839-13|Sciaridae|670|BOLD:ACG5069
Sciaridae|[22207]|CNJAA341-12|Sciaridae|670|BOLD:ACB2386
Sciaridae|[22208]|SSBAA3558-12|Sciaridae|673|BOLD:ACC1881
Sciaridae|[22209]|CNNHG642-14|Sciaridae|594|BOLD:ACR3916
Sciaridae|[22210]|CNJAA688-12|Sciaridae|673|BOLD:ACB9184
Sciaridae|[22211]|SSJAE604-13|Sciaridae|661|BOLD:ACJ4297
Sciaridae|[22212]|SSWLA928-13|Sciaridae|661|BOLD:ACG6094
Sciaridae|[22213]|CNGLD145-13|Sciaridae|670|BOLD:ACC2035
Sciaridae|[22214]|SSPAA3795-13|Sciaridae|658|BOLD:ACI4550
Sciaridae|[22215]|CNJAD098-12|Sciaridae|673|BOLD:ACC8428
Sciaridae|[22216]|SSJAF2239-13|Sciaridae|594|BOLD:ACT1161
Sciaridae|[22217]|SSBAF812-13|Sciaridae|661|BOLD:ACC9096
Sciaridae|[22218]|CNKTG224-15|Sciaridae|609|BOLD:ACT1162
Sciaridae|[22219]|SSBAC403-12|Sciaridae|658|BOLD:ACC5525
Lycoriella|[22220]|CNWLA561-12|Sciaridae|661|BOLD:ACC4140
Sciaridae|[22221]|CNBPA019-12|Sciaridae|670|BOLD:ACC1928
Sciaridae|[22222]|CNJAJ441-12|Sciaridae|658|BOLD:ACD2296
Lycoriella|[22223]|CNBPP163-13|Sciaridae|661|BOLD:ACG9225
Sciaridae|[22224]|CNJAB281-12|Sciaridae|658|BOLD:ACC6811
Sciaridae|[22225]|CNFNB173-14|Sciaridae|658|BOLD:ACL3030
Lycoriella|[22226]|CNEIH851-13|Sciaridae|670|BOLD:ABA1231
Sciaridae|[22227]|SSBAA3546-12|Sciaridae|673|BOLD:ACC3535
Lycoriella|[22228]|CNTGC1693-15|Sciaridae|632|BOLD:AAL7874
Sciaridae|[22229]|SSPAC4042-13|Sciaridae|670|BOLD:AAH3916
Sciaridae|[22230]|SMTPI5707-14|Sciaridae|591|BOLD:ACP7603
Sciaridae|[22231]|CNWLB102-12|Sciaridae|661|BOLD:ACC3450
Sciaridae|[22232]|CNGMJ314-14|Sciaridae|593|BOLD:ACL8854
Sciaridae|[22233]|CNJAC850-12|Sciaridae|673|BOLD:ACC1186
Sciaridae|[22234]|SSWLC2032-13|Sciaridae|603|BOLD:ACG2206
Sciaridae|[22235]|CNGLD179-13|Sciaridae|658|BOLD:ACG2254
Sciaridae|[22236]|SSWLD4814-13|Sciaridae|678|BOLD:AAU6613
Sciaridae|[22237]|CNGAH202-15|Sciaridae|588|BOLD:ACS7610
Sciaridae|[22238]|CNFND347-14|Sciaridae|658|BOLD:ACL2212
Sciaridae|[22239]|CNBPA045-12|Sciaridae|670|BOLD:ACK8113
Bradysia|[22240]|CNSLP634-13|Sciaridae|661|BOLD:ACA4746
Sciaridae|[22241]|CNGMG283-14|Sciaridae|661|BOLD:ACL7870
Sciaridae|[22242]|CNGMB1638-13|Sciaridae|661|BOLD:ACK7977
Sciaridae|[22243]|CNTNA710-14|Sciaridae|609|BOLD:ACN0832
Sciaridae|[22244]|CNGMB1661-13|Sciaridae|661|BOLD:ACL9574
Sciaridae|[22245]|CNMID471-14|Sciaridae|588|BOLD:ACM9019
Sciaridae|[22246]|SSPAA1174-13|Sciaridae|658|BOLD:ACG3969
Sciaridae|[22247]|CNGMC128-13|Sciaridae|661|BOLD:ACL2706
Sciaridae|[22248]|CNBPK179-13|Sciaridae|670|BOLD:ACN2240
Sciaridae|[22249]|CNGMG779-14|Sciaridae|658|BOLD:ACN6974
Sciaridae|[22250]|CNFND241-14|Sciaridae|658|BOLD:ACL3839
Sciaridae|[22251]|CNGMB610-13|Sciaridae|614|BOLD:ACK7829
Sciaridae|[22252]|CNGMB303-13|Sciaridae|612|BOLD:ACK7371
Sciaridae|[22253]|CNGMG750-14|Sciaridae|658|BOLD:ACL6956
Sciaridae|[22254]|CNFNP1228-14|Sciaridae|594|BOLD:ACL8941
Sciaridae|[22255]|CNKJD683-14|Sciaridae|658|BOLD:ACM1761
Sciaridae|[22256]|CNLMF835-14|Sciaridae|579|BOLD:ACM6568
Sciaridae|[22257]|CNBPB411-12|Sciaridae|670|BOLD:ACD0968
Sciaridae|[22258]|CNGMB995-13|Sciaridae|658|BOLD:ACL1662
Sciaridae|[22259]|CNGMB630-13|Sciaridae|604|BOLD:ACK8096
Sciaridae|[22260]|CNTNA563-14|Sciaridae|597|BOLD:ACM7197
Sciaridae|[22261]|CNFNG366-14|Sciaridae|661|BOLD:ACL9197
Sciaridae|[22262]|CNTNA587-14|Sciaridae|609|BOLD:ACM6479
Sciaridae|[22263]|CNTNA351-14|Sciaridae|633|BOLD:ACM1190
Sciaridae|[22264]|CNGMB1611-13|Sciaridae|661|BOLD:ACL0207
Sciaridae|[22265]|CNKJP723-14|Sciaridae|588|BOLD:ACM5331
Sciaridae|[22266]|CNGMG827-14|Sciaridae|661|BOLD:ACL4332
Sciaridae|[22267]|CNTNA458-14|Sciaridae|661|BOLD:ACM1191
Sciaridae|[22268]|CNKOB083-14|Sciaridae|624|BOLD:ACL7398
Bradysia|[22269]|CNKTA372-14|Sciaridae|588|BOLD:ACR9399
Sciaridae|[22270]|CNBRL593-14|Sciaridae|658|BOLD:ACL8726
Sciaridae|[22271]|JWDCI946-11|Sciaridae|656|BOLD:AAQ2135
Sciaridae|[22272]|CHDIP142-11|Sciaridae|658|BOLD:AAH3923
Sciaridae|[22273]|CNTMB061-14|Sciaridae|592|BOLD:ACL8856
Sciaridae|[22274]|SMTPD1312-13|Sciaridae|606|BOLD:ACJ7421
Sciaridae|[22275]|CNEIF176-12|Sciaridae|673|BOLD:AAH3921
Sciaridae|[22276]|CNFNQ1380-14|Sciaridae|658|BOLD:ACF0037
Sciaridae|[22277]|CNBFK458-15|Sciaridae|543|BOLD:ACT9510
Sciaridae|[22278]|NCCA085-11|Sciaridae|673|BOLD:AAN6445
Sciaridae|[22279]|CNFNE1662-14|Sciaridae|661|BOLD:AAV1367
Sciaridae|[22280]|PAAUG1124-12|Sciaridae|658|BOLD:ACB5899
Sciaridae|[22281]|CNGBK1013-14|Sciaridae|563|BOLD:ACL7875
Sciaridae|[22282]|CNKJE701-14|Sciaridae|630|BOLD:ACA5276
Sciaridae|[22283]|CNLMD907-14|Sciaridae|630|BOLD:ACM4368
Sciaridae|[22284]|CNBPD372-12|Sciaridae|670|BOLD:ACD0915
Sciaridae|[22285]|SSBRA716-14|Sciaridae|664|BOLD:ACL7456
Bradysia|[22286]|NCCA2358-11|Sciaridae|674|BOLD:AAV1295
Sciaridae|[22287]|CNWLD673-12|Sciaridae|673|BOLD:ACC9225
Sciaridae|[22288]|CNEIC727-12|Sciaridae|658|BOLD:AAZ5906
Sciaridae|[22289]|MBIOH204-13|Sciaridae|608|BOLD:ACK8580
Sciaridae|[22290]|SSROB4372-14|Sciaridae|600|BOLD:ACN8091
Sciaridae|[22291]|PHAUG086-11|Sciaridae|658|BOLD:AAQ0275
Sciaridae|[22292]|CNBRF812-14|Sciaridae|661|BOLD:ACA3774
Camptochaeta uniformis|[22293]|CNBPK161-13|Sciaridae|670|BOLD:ACA4924
Camptochaeta hirtula|[22294]|CNPAL200-13|Sciaridae|664|BOLD:ACB9913
Sciaridae|[22295]|BBDCN534-10|Sciaridae|658|BOLD:AAP9894
Sciaridae|[22296]|SSJAD839-13|Sciaridae|661|BOLD:ACI6042

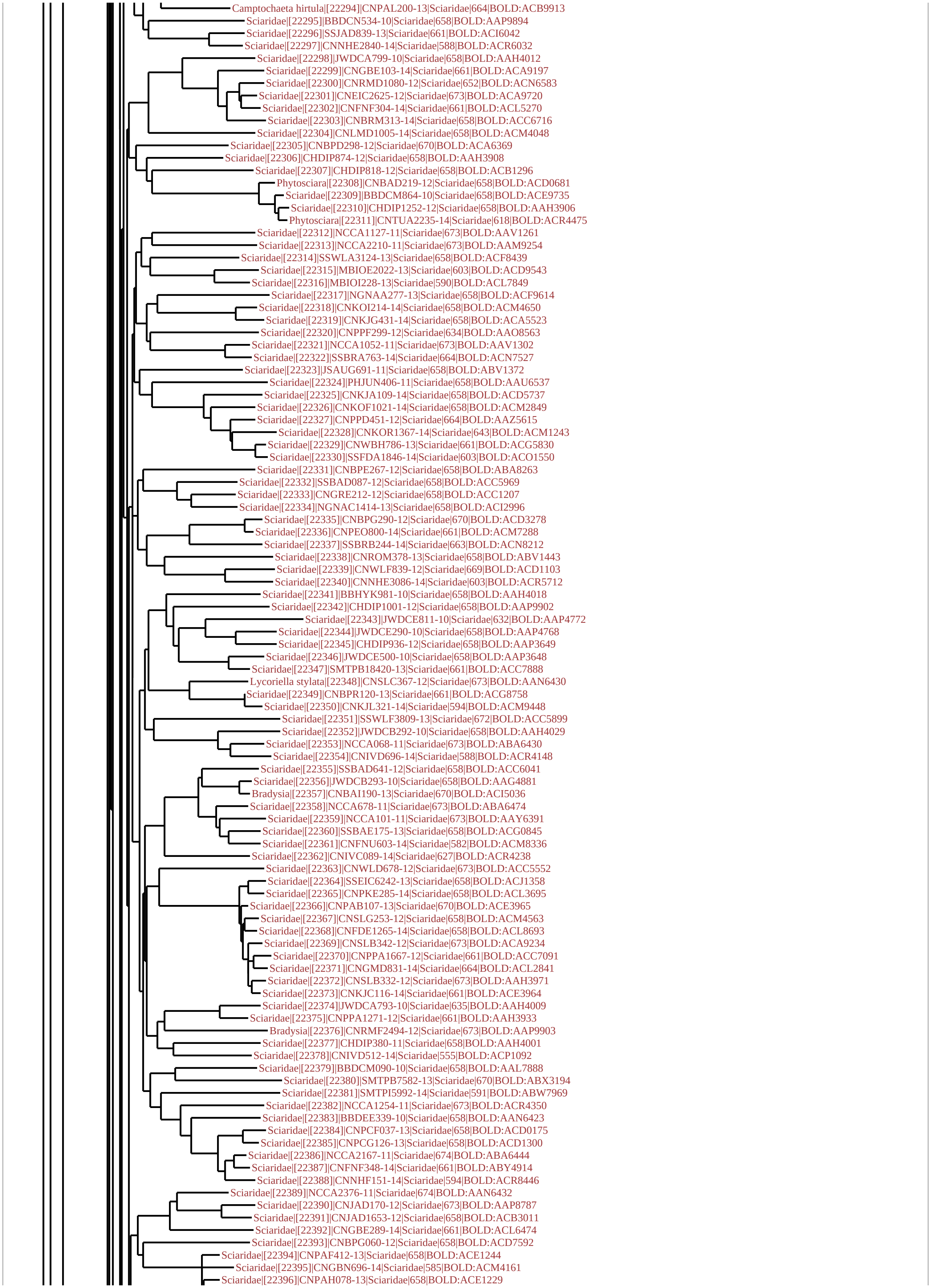
Camptochaeta hirtula|[22294]|CNPAL200-13|Sciaridae|664|BOLD:ACB9913
Sciaridae|[22295]|BBDCN534-10|Sciaridae|658|BOLD:AAP9894
Sciaridae|[22296]|SSJAD839-13|Sciaridae|661|BOLD:ACI6042
Sciaridae|[22297]|CNNHE2840-14|Sciaridae|588|BOLD:ACR6032
Sciaridae|[22298]|JWDCA799-10|Sciaridae|658|BOLD:AAH4012
Sciaridae|[22299]|CNGBE103-14|Sciaridae|661|BOLD:ACA9197
Sciaridae|[22300]|CNRMD1080-12|Sciaridae|652|BOLD:ACN6583
Sciaridae|[22301]|CNEIC2625-12|Sciaridae|673|BOLD:ACA9720
Sciaridae|[22302]|CNFNF304-14|Sciaridae|661|BOLD:ACL5270
Sciaridae|[22303]|CNBRM313-14|Sciaridae|658|BOLD:ACC6716
Sciaridae|[22304]|CNLMD1005-14|Sciaridae|658|BOLD:ACM4048
Sciaridae|[22305]|CNBPD298-12|Sciaridae|670|BOLD:ACA6369
Sciaridae|[22306]|CHDIP874-12|Sciaridae|658|BOLD:AAH3908
Sciaridae|[22307]|CHDIP818-12|Sciaridae|658|BOLD:ACB1296
Phytosciara|[22308]|CNBAD219-12|Sciaridae|658|BOLD:ACD0681
Sciaridae|[22309]|BBDCM864-10|Sciaridae|658|BOLD:ACE9735
Sciaridae|[22310]|CHDIP1252-12|Sciaridae|658|BOLD:AAH3906
Phytosciara|[22311]|CNTUA2235-14|Sciaridae|618|BOLD:ACR4475
Sciaridae|[22312]|NCCA1127-11|Sciaridae|673|BOLD:AAV1261
Sciaridae|[22313]|NCCA2210-11|Sciaridae|673|BOLD:AAM9254
Sciaridae|[22314]|SSWLA3124-13|Sciaridae|658|BOLD:ACF8439
Sciaridae|[22315]|MBIOE2022-13|Sciaridae|603|BOLD:ACD9543
Sciaridae|[22316]|MBIOI228-13|Sciaridae|590|BOLD:ACL7849
Sciaridae|[22317]|NGNAA277-13|Sciaridae|658|BOLD:ACF9614
Sciaridae|[22318]|CNKOI214-14|Sciaridae|658|BOLD:ACM4650
Sciaridae|[22319]|CNKJG431-14|Sciaridae|658|BOLD:ACA5523
Sciaridae|[22320]|CNPPF299-12|Sciaridae|634|BOLD:AAO8563
Sciaridae|[22321]|NCCA1052-11|Sciaridae|673|BOLD:AAV1302
Sciaridae|[22322]|SSBRA763-14|Sciaridae|664|BOLD:ACN7527
Sciaridae|[22323]|JSAUG691-11|Sciaridae|658|BOLD:ABV1372
Sciaridae|[22324]|PHJUN406-11|Sciaridae|658|BOLD:AAU6537
Sciaridae|[22325]|CNKJA109-14|Sciaridae|658|BOLD:ACD5737
Sciaridae|[22326]|CNKOF1021-14|Sciaridae|658|BOLD:ACM2849
Sciaridae|[22327]|CNPPD451-12|Sciaridae|664|BOLD:AAZ5615
Sciaridae|[22328]|CNKOR1367-14|Sciaridae|643|BOLD:ACM1243
Sciaridae|[22329]|CNWBH786-13|Sciaridae|661|BOLD:ACG5830
Sciaridae|[22330]|SSFDA1846-14|Sciaridae|603|BOLD:ACO1550
Sciaridae|[22331]|CNBPE267-12|Sciaridae|658|BOLD:ABA8263
Sciaridae|[22332]|SSBAD087-12|Sciaridae|658|BOLD:ACC5969
Sciaridae|[22333]|CNGRE212-12|Sciaridae|658|BOLD:ACC1207
Sciaridae|[22334]|NGNAC1414-13|Sciaridae|658|BOLD:ACI2996
Sciaridae|[22335]|CNBPG290-12|Sciaridae|670|BOLD:ACD3278
Sciaridae|[22336]|CNPEO800-14|Sciaridae|661|BOLD:ACM7288
Sciaridae|[22337]|SSBRB244-14|Sciaridae|663|BOLD:ACN8212
Sciaridae|[22338]|CNROM378-13|Sciaridae|658|BOLD:ABV1443
Sciaridae|[22339]|CNWLF839-12|Sciaridae|669|BOLD:ACD1103
Sciaridae|[22340]|CNNHE3086-14|Sciaridae|603|BOLD:ACR5712
Sciaridae|[22341]|BBHYK981-10|Sciaridae|658|BOLD:AAH4018
Sciaridae|[22342]|CHDIP1001-12|Sciaridae|658|BOLD:AAP9902
Sciaridae|[22343]|JWDCE811-10|Sciaridae|632|BOLD:AAP4772
Sciaridae|[22344]|JWDCE290-10|Sciaridae|658|BOLD:AAP4768
Sciaridae|[22345]|CHDIP936-12|Sciaridae|658|BOLD:AAP3649
Sciaridae|[22346]|JWDCE500-10|Sciaridae|658|BOLD:AAP3648
Sciaridae|[22347]|SMTPB18420-13|Sciaridae|661|BOLD:ACC7888
Lycoriella stylata|[22348]|CNSLC367-12|Sciaridae|673|BOLD:AAN6430
Sciaridae|[22349]|CNBPR120-13|Sciaridae|661|BOLD:ACG8758
Sciaridae|[22350]|CNKJL321-14|Sciaridae|594|BOLD:ACM9448
Sciaridae|[22351]|SSWLF3809-13|Sciaridae|672|BOLD:ACC5899
Sciaridae|[22352]|JWDCB292-10|Sciaridae|658|BOLD:AAH4029
Sciaridae|[22353]|NCCA068-11|Sciaridae|673|BOLD:ABA6430
Sciaridae|[22354]|CNIVD696-14|Sciaridae|588|BOLD:ACR4148
Sciaridae|[22355]|SSBAD641-12|Sciaridae|658|BOLD:ACC6041
Sciaridae|[22356]|JWDCB293-10|Sciaridae|658|BOLD:AAG4881
Bradysia|[22357]|CNBAI190-13|Sciaridae|670|BOLD:ACI5036
Sciaridae|[22358]|NCCA678-11|Sciaridae|673|BOLD:ABA6474
Sciaridae|[22359]|NCCA101-11|Sciaridae|673|BOLD:AAY6391
Sciaridae|[22360]|SSBAE175-13|Sciaridae|658|BOLD:ACG0845
Sciaridae|[22361]|CNFNU603-14|Sciaridae|582|BOLD:ACM8336
Sciaridae|[22362]|CNIVC089-14|Sciaridae|627|BOLD:ACR4238
Sciaridae|[22363]|CNWLD678-12|Sciaridae|673|BOLD:ACC5552
Sciaridae|[22364]|SSEIC6242-13|Sciaridae|658|BOLD:ACJ1358
Sciaridae|[22365]|CNPKE285-14|Sciaridae|658|BOLD:ACL3695
Sciaridae|[22366]|CNPAB107-13|Sciaridae|670|BOLD:ACE3965
Sciaridae|[22367]|CNSLG253-12|Sciaridae|658|BOLD:ACM4563
Sciaridae|[22368]|CNFDE1265-14|Sciaridae|658|BOLD:ACL8693
Sciaridae|[22369]|CNSLB342-12|Sciaridae|673|BOLD:ACA9234
Sciaridae|[22370]|CNPPA1667-12|Sciaridae|661|BOLD:ACC7091
Sciaridae|[22371]|CNGMD831-14|Sciaridae|664|BOLD:ACL2841
Sciaridae|[22372]|CNSLB332-12|Sciaridae|673|BOLD:AAH3971
Sciaridae|[22373]|CNKJC116-14|Sciaridae|661|BOLD:ACE3964
Sciaridae|[22374]|JWDCA793-10|Sciaridae|635|BOLD:AAH4009
Sciaridae|[22375]|CNPPA1271-12|Sciaridae|661|BOLD:AAH3933
Bradysia|[22376]|CNRMF2494-12|Sciaridae|673|BOLD:AAP9903
Sciaridae|[22377]|CHDIP380-11|Sciaridae|658|BOLD:AAH4001
Sciaridae|[22378]|CNIVD512-14|Sciaridae|555|BOLD:ACP1092
Sciaridae|[22379]|BBDCM090-10|Sciaridae|658|BOLD:AAL7888
Sciaridae|[22380]|SMTPB7582-13|Sciaridae|670|BOLD:ABX3194
Sciaridae|[22381]|SMTPI5992-14|Sciaridae|591|BOLD:ABW7969
Sciaridae|[22382]|NCCA1254-11|Sciaridae|673|BOLD:ACR4350
Sciaridae|[22383]|BBDEE339-10|Sciaridae|658|BOLD:AAN6423
Sciaridae|[22384]|CNPCF037-13|Sciaridae|658|BOLD:ACD0175
Sciaridae|[22385]|CNPCG126-13|Sciaridae|658|BOLD:ACD1300
Sciaridae|[22386]|NCCA2167-11|Sciaridae|674|BOLD:ABA6444
Sciaridae|[22387]|CNFNF348-14|Sciaridae|661|BOLD:ABY4914
Sciaridae|[22388]|CNNHF151-14|Sciaridae|594|BOLD:ACR8446
Sciaridae|[22389]|NCCA2376-11|Sciaridae|674|BOLD:AAN6432
Sciaridae|[22390]|CNJAD170-12|Sciaridae|673|BOLD:AAP8787
Sciaridae|[22391]|CNJAD1653-12|Sciaridae|658|BOLD:ACB3011
Sciaridae|[22392]|CNGBE289-14|Sciaridae|661|BOLD:ACL6474
Sciaridae|[22393]|CNBPG060-12|Sciaridae|658|BOLD:ACD7592
Sciaridae|[22394]|CNPAF412-13|Sciaridae|658|BOLD:ACE1244
Sciaridae|[22395]|CNGBN696-14|Sciaridae|585|BOLD:ACM4161
Sciaridae|[22396]|CNPAH078-13|Sciaridae|658|BOLD:ACE1229

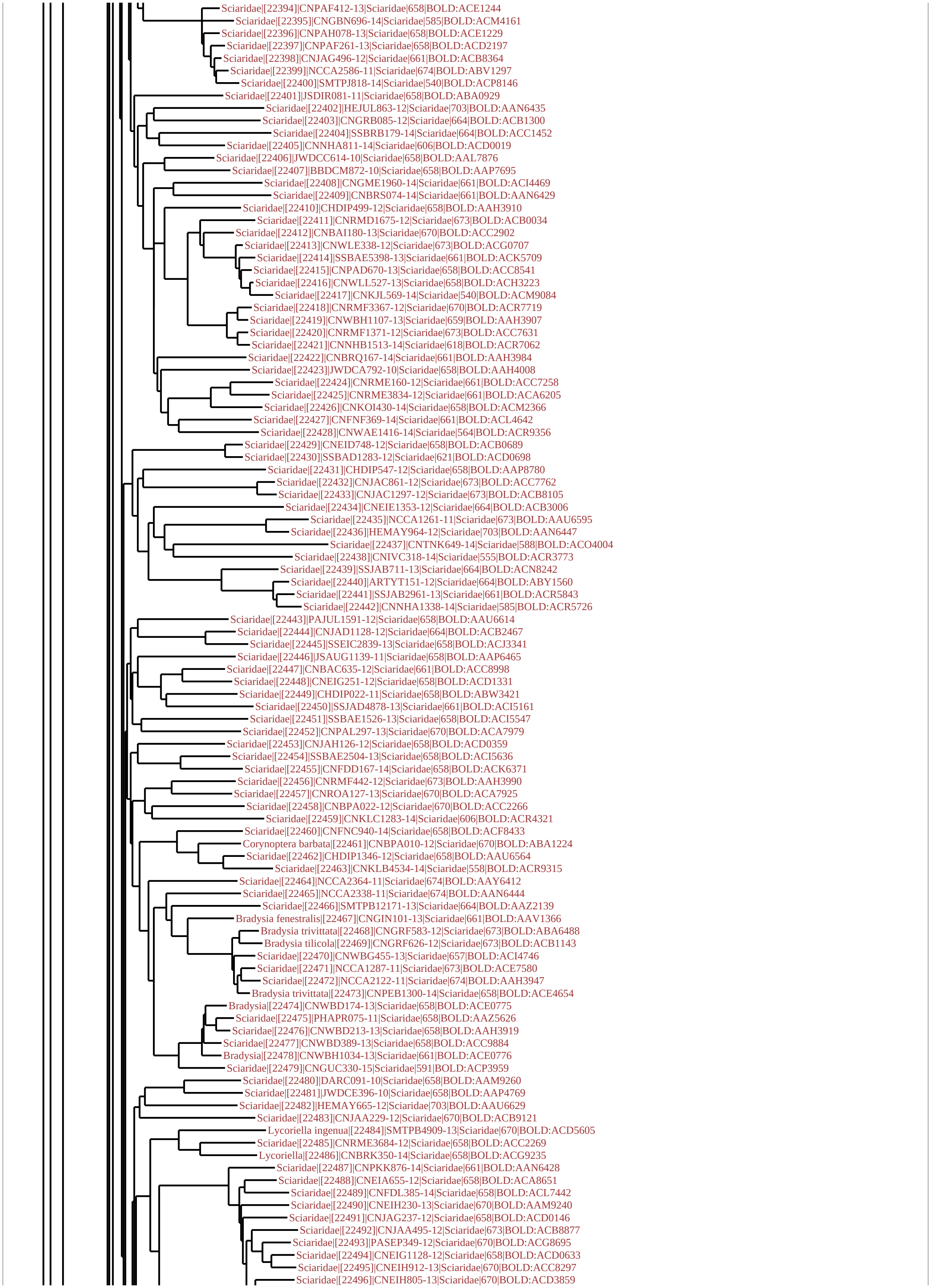
Sciaridae|[22394]|CNPAF412-13|Sciaridae|658|BOLD:ACE1244
Sciaridae|[22395]|CNGBN696-14|Sciaridae|585|BOLD:ACM4161
Sciaridae|[22396]|CNPAH078-13|Sciaridae|658|BOLD:ACE1229
Sciaridae|[22397]|CNPAF261-13|Sciaridae|658|BOLD:ACD2197
Sciaridae|[22398]|CNJAG496-12|Sciaridae|661|BOLD:ACB8364
Sciaridae|[22399]|NCCA2586-11|Sciaridae|674|BOLD:ABV1297
Sciaridae|[22400]|SMTPJ818-14|Sciaridae|540|BOLD:ACP8146
Sciaridae|[22401]|JSDIR081-11|Sciaridae|658|BOLD:ABA0929
Sciaridae|[22402]|HEJUL863-12|Sciaridae|703|BOLD:AAN6435
Sciaridae|[22403]|CNGRB085-12|Sciaridae|664|BOLD:ACB1300
Sciaridae|[22404]|SSBRB179-14|Sciaridae|664|BOLD:ACC1452
Sciaridae|[22405]|CNNHA811-14|Sciaridae|606|BOLD:ACD0019
Sciaridae|[22406]|JWDCC614-10|Sciaridae|658|BOLD:AAL7876
Sciaridae|[22407]|BBDCM872-10|Sciaridae|658|BOLD:AAP7695
Sciaridae|[22408]|CNGME1960-14|Sciaridae|661|BOLD:ACI4469
Sciaridae|[22409]|CNBRS074-14|Sciaridae|661|BOLD:AAN6429
Sciaridae|[22410]|CHDIP499-12|Sciaridae|658|BOLD:AAH3910
Sciaridae|[22411]|CNRMD1675-12|Sciaridae|673|BOLD:ACB0034
Sciaridae|[22412]|CNBAI180-13|Sciaridae|670|BOLD:ACC2902
Sciaridae|[22413]|CNWLE338-12|Sciaridae|673|BOLD:ACG0707
Sciaridae|[22414]|SSBAE5398-13|Sciaridae|661|BOLD:ACK5709
Sciaridae|[22415]|CNPAD670-13|Sciaridae|658|BOLD:ACC8541
Sciaridae|[22416]|CNWLL527-13|Sciaridae|658|BOLD:ACH3223
Sciaridae|[22417]|CNKJL569-14|Sciaridae|540|BOLD:ACM9084
Sciaridae|[22418]|CNRMF3367-12|Sciaridae|670|BOLD:ACR7719
Sciaridae|[22419]|CNWBH1107-13|Sciaridae|659|BOLD:AAH3907
Sciaridae|[22420]|CNRMF1371-12|Sciaridae|673|BOLD:ACC7631
Sciaridae|[22421]|CNNHB1513-14|Sciaridae|618|BOLD:ACR7062
Sciaridae|[22422]|CNBRQ167-14|Sciaridae|661|BOLD:AAH3984
Sciaridae|[22423]|JWDCA792-10|Sciaridae|658|BOLD:AAH4008
Sciaridae|[22424]|CNRME160-12|Sciaridae|661|BOLD:ACC7258
Sciaridae|[22425]|CNRME3834-12|Sciaridae|661|BOLD:ACA6205
Sciaridae|[22426]|CNKOI430-14|Sciaridae|658|BOLD:ACM2366
Sciaridae|[22427]|CNFNF369-14|Sciaridae|661|BOLD:ACL4642
Sciaridae|[22428]|CNWAE1416-14|Sciaridae|564|BOLD:ACR9356
Sciaridae|[22429]|CNEID748-12|Sciaridae|658|BOLD:ACB0689
Sciaridae|[22430]|SSBAD1283-12|Sciaridae|621|BOLD:ACD0698
Sciaridae|[22431]|CHDIP547-12|Sciaridae|658|BOLD:AAP8780
Sciaridae|[22432]|CNJAC861-12|Sciaridae|673|BOLD:ACC7762
Sciaridae|[22433]|CNJAC1297-12|Sciaridae|673|BOLD:ACB8105
Sciaridae|[22434]|CNEIE1353-12|Sciaridae|664|BOLD:ACB3006
Sciaridae|[22435]|NCCA1261-11|Sciaridae|673|BOLD:AAU6595
Sciaridae|[22436]|HEMAY964-12|Sciaridae|703|BOLD:AAN6447
Sciaridae|[22437]|CNTNK649-14|Sciaridae|588|BOLD:ACO4004
Sciaridae|[22438]|CNIVC318-14|Sciaridae|555|BOLD:ACR3773
Sciaridae|[22439]|SSJAB711-13|Sciaridae|664|BOLD:ACN8242
Sciaridae|[22440]|ARTYT151-12|Sciaridae|664|BOLD:ABY1560
Sciaridae|[22441]|SSJAB2961-13|Sciaridae|661|BOLD:ACR5843
Sciaridae|[22442]|CNNHA1338-14|Sciaridae|585|BOLD:ACR5726
Sciaridae|[22443]|PAJUL1591-12|Sciaridae|658|BOLD:AAU6614
Sciaridae|[22444]|CNJAD1128-12|Sciaridae|664|BOLD:ACB2467
Sciaridae|[22445]|SSEIC2839-13|Sciaridae|658|BOLD:ACJ3341
Sciaridae|[22446]|JSAUG1139-11|Sciaridae|658|BOLD:AAP6465
Sciaridae|[22447]|CNBAC635-12|Sciaridae|661|BOLD:ACC8998
Sciaridae|[22448]|CNEIG251-12|Sciaridae|658|BOLD:ACD1331
Sciaridae|[22449]|CHDIP022-11|Sciaridae|658|BOLD:ABW3421
Sciaridae|[22450]|SSJAD4878-13|Sciaridae|661|BOLD:ACI5161
Sciaridae|[22451]|SSBAE1526-13|Sciaridae|658|BOLD:ACI5547
Sciaridae|[22452]|CNPAL297-13|Sciaridae|670|BOLD:ACA7979
Sciaridae|[22453]|CNJAH126-12|Sciaridae|658|BOLD:ACD0359
Sciaridae|[22454]|SSBAE2504-13|Sciaridae|658|BOLD:ACI5636
Sciaridae|[22455]|CNFDD167-14|Sciaridae|658|BOLD:ACK6371
Sciaridae|[22456]|CNRMF442-12|Sciaridae|673|BOLD:AAH3990
Sciaridae|[22457]|CNROA127-13|Sciaridae|670|BOLD:ACA7925
Sciaridae|[22458]|CNBPA022-12|Sciaridae|670|BOLD:ACC2266
Sciaridae|[22459]|CNKLC1283-14|Sciaridae|606|BOLD:ACR4321
Sciaridae|[22460]|CNFNC940-14|Sciaridae|658|BOLD:ACF8433
Corynoptera barbata|[22461]|CNBPA010-12|Sciaridae|670|BOLD:ABA1224
Sciaridae|[22462]|CHDIP1346-12|Sciaridae|658|BOLD:AAU6564
Sciaridae|[22463]|CNKLB4534-14|Sciaridae|558|BOLD:ACR9315
Sciaridae|[22464]|NCCA2364-11|Sciaridae|674|BOLD:AAY6412
Sciaridae|[22465]|NCCA2338-11|Sciaridae|674|BOLD:AAN6444
Sciaridae|[22466]|SMTPB12171-13|Sciaridae|664|BOLD:AAZ2139
Bradysia fenestralis|[22467]|CNGIN101-13|Sciaridae|661|BOLD:AAV1366
Bradysia trivittata|[22468]|CNGRF583-12|Sciaridae|673|BOLD:ABA6488
Bradysia tilicola|[22469]|CNGRF626-12|Sciaridae|673|BOLD:ACB1143
Sciaridae|[22470]|CNWBG455-13|Sciaridae|657|BOLD:ACI4746
Sciaridae|[22471]|NCCA1287-11|Sciaridae|673|BOLD:ACE7580
Sciaridae|[22472]|NCCA2122-11|Sciaridae|674|BOLD:AAH3947
Bradysia trivittata|[22473]|CNPEB1300-14|Sciaridae|658|BOLD:ACE4654
Bradysia|[22474]|CNWBD174-13|Sciaridae|658|BOLD:ACE0775
Sciaridae|[22475]|PHAPR075-11|Sciaridae|658|BOLD:AAZ5626
Sciaridae|[22476]|CNWBD213-13|Sciaridae|658|BOLD:AAH3919
Sciaridae|[22477]|CNWBD389-13|Sciaridae|658|BOLD:ACC9884
Bradysia|[22478]|CNWBH1034-13|Sciaridae|661|BOLD:ACE0776
Sciaridae|[22479]|CNGUC330-15|Sciaridae|591|BOLD:ACP3959
Sciaridae|[22480]|DARC091-10|Sciaridae|658|BOLD:AAM9260
Sciaridae|[22481]|JWDCE396-10|Sciaridae|658|BOLD:AAP4769
Sciaridae|[22482]|HEMAY665-12|Sciaridae|703|BOLD:AAU6629
Sciaridae|[22483]|CNJAA229-12|Sciaridae|670|BOLD:ACB9121
Lycoriella ingenua|[22484]|SMTPB4909-13|Sciaridae|670|BOLD:ACD5605
Sciaridae|[22485]|CNRME3684-12|Sciaridae|658|BOLD:ACC2269
Lycoriella|[22486]|CNBRK350-14|Sciaridae|658|BOLD:ACG9235
Sciaridae|[22487]|CNPKK876-14|Sciaridae|661|BOLD:AAN6428
Sciaridae|[22488]|CNEIA655-12|Sciaridae|658|BOLD:ACA8651
Sciaridae|[22489]|CNFDL385-14|Sciaridae|658|BOLD:ACL7442
Sciaridae|[22490]|CNEIH230-13|Sciaridae|670|BOLD:AAM9240
Sciaridae|[22491]|CNJAG237-12|Sciaridae|658|BOLD:ACD0146
Sciaridae|[22492]|CNJAA495-12|Sciaridae|673|BOLD:ACB8877
Sciaridae|[22493]|PASEP349-12|Sciaridae|670|BOLD:ACG8695
Sciaridae|[22494]|CNEIG1128-12|Sciaridae|658|BOLD:ACD0633
Sciaridae|[22495]|CNEIH912-13|Sciaridae|670|BOLD:ACC8297
Sciaridae|[22496]|CNEIH805-13|Sciaridae|670|BOLD:ACD3859

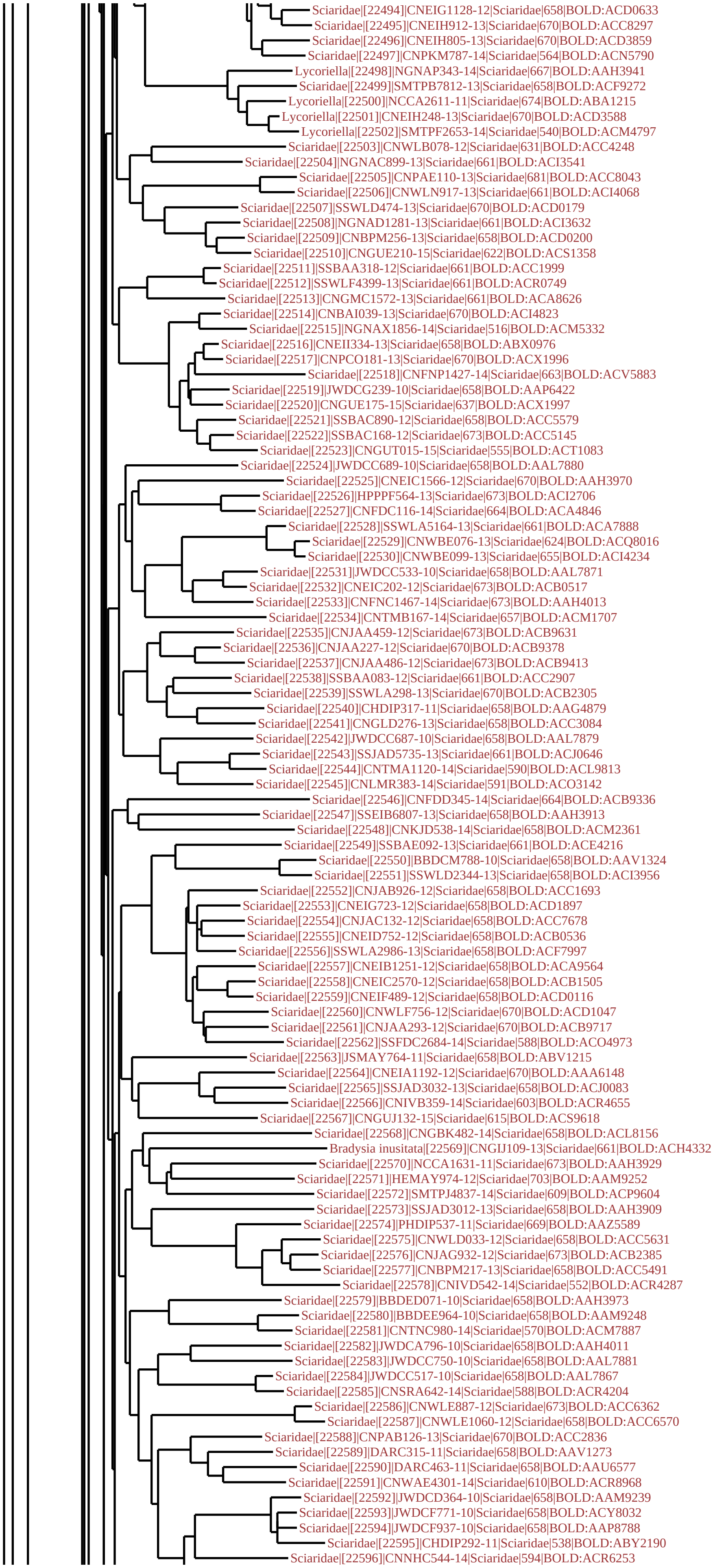

Sciaridae|[22494]|CNEIG1128-12|Sciaridae|658|BOLD:ACD0633
Sciaridae|[22495]|CNEIH912-13|Sciaridae|670|BOLD:ACC8297
Sciaridae|[22496]|CNEIH805-13|Sciaridae|670|BOLD:ACD3859
Sciaridae|[22497]|CNPKM787-14|Sciaridae|564|BOLD:ACN5790
Lycoriella|[22498]|NGNAP343-14|Sciaridae|667|BOLD:AAH3941
Sciaridae|[22499]|SMTPB7812-13|Sciaridae|658|BOLD:ACF9272
Lycoriella|[22500]|NCCA2611-11|Sciaridae|674|BOLD:ABA1215
Lycoriella|[22501]|CNEIH248-13|Sciaridae|670|BOLD:ACD3588
Lycoriella|[22502]|SMTPF2653-14|Sciaridae|540|BOLD:ACM4797
Sciaridae|[22503]|CNWLB078-12|Sciaridae|631|BOLD:ACC4248
Sciaridae|[22504]|NGNAC899-13|Sciaridae|661|BOLD:ACI3541
Sciaridae|[22505]|CNPAE110-13|Sciaridae|681|BOLD:ACC8043
Sciaridae|[22506]|CNWLN917-13|Sciaridae|661|BOLD:ACI4068
Sciaridae|[22507]|SSWLD474-13|Sciaridae|670|BOLD:ACD0179
Sciaridae|[22508]|NGNAD1281-13|Sciaridae|661|BOLD:ACI3632
Sciaridae|[22509]|CNBPM256-13|Sciaridae|658|BOLD:ACD0200
Sciaridae|[22510]|CNGUE210-15|Sciaridae|622|BOLD:ACS1358
Sciaridae|[22511]|SSBAA318-12|Sciaridae|661|BOLD:ACC1999
Sciaridae|[22512]|SSWLF4399-13|Sciaridae|661|BOLD:ACR0749
Sciaridae|[22513]|CNGMC1572-13|Sciaridae|661|BOLD:ACA8626
Sciaridae|[22514]|CNBAI039-13|Sciaridae|670|BOLD:ACI4823
Sciaridae|[22515]|NGNAX1856-14|Sciaridae|516|BOLD:ACM5332
Sciaridae|[22516]|CNEII334-13|Sciaridae|658|BOLD:ABX0976
Sciaridae|[22517]|CNPCO181-13|Sciaridae|670|BOLD:ACX1996
Sciaridae|[22518]|CNFNP1427-14|Sciaridae|663|BOLD:ACV5883
Sciaridae|[22519]|JWDCG239-10|Sciaridae|658|BOLD:AAP6422
Sciaridae|[22520]|CNGUE175-15|Sciaridae|637|BOLD:ACX1997
Sciaridae|[22521]|SSBAC890-12|Sciaridae|658|BOLD:ACC5579
Sciaridae|[22522]|SSBAC168-12|Sciaridae|673|BOLD:ACC5145
Sciaridae|[22523]|CNGUT015-15|Sciaridae|555|BOLD:ACT1083
Sciaridae|[22524]|JWDCC689-10|Sciaridae|658|BOLD:AAL7880
Sciaridae|[22525]|CNEIC1566-12|Sciaridae|670|BOLD:AAH3970
Sciaridae|[22526]|HPPPF564-13|Sciaridae|673|BOLD:ACI2706
Sciaridae|[22527]|CNFDC116-14|Sciaridae|664|BOLD:ACA4846
Sciaridae|[22528]|SSWLA5164-13|Sciaridae|661|BOLD:ACA7888
Sciaridae|[22529]|CNWBE076-13|Sciaridae|624|BOLD:ACQ8016
Sciaridae|[22530]|CNWBE099-13|Sciaridae|655|BOLD:ACI4234
Sciaridae|[22531]|JWDCC533-10|Sciaridae|658|BOLD:AAL7871
Sciaridae|[22532]|CNEIC202-12|Sciaridae|673|BOLD:ACB0517
Sciaridae|[22533]|CNFNC1467-14|Sciaridae|673|BOLD:AAH4013
Sciaridae|[22534]|CNTMB167-14|Sciaridae|657|BOLD:ACM1707
Sciaridae|[22535]|CNJAA459-12|Sciaridae|673|BOLD:ACB9631
Sciaridae|[22536]|CNJAA227-12|Sciaridae|670|BOLD:ACB9378
Sciaridae|[22537]|CNJAA486-12|Sciaridae|673|BOLD:ACB9413
Sciaridae|[22538]|SSBAA083-12|Sciaridae|661|BOLD:ACC2907
Sciaridae|[22539]|SSWLA298-13|Sciaridae|670|BOLD:ACB2305
Sciaridae|[22540]|CHDIP317-11|Sciaridae|658|BOLD:AAG4879
Sciaridae|[22541]|CNGLD276-13|Sciaridae|658|BOLD:ACC3084
Sciaridae|[22542]|JWDCC687-10|Sciaridae|658|BOLD:AAL7879
Sciaridae|[22543]|SSJAD5735-13|Sciaridae|661|BOLD:ACJ0646
Sciaridae|[22544]|CNTMA1120-14|Sciaridae|590|BOLD:ACL9813
Sciaridae|[22545]|CNLMR383-14|Sciaridae|591|BOLD:ACO3142
Sciaridae|[22546]|CNFDD345-14|Sciaridae|664|BOLD:ACB9336
Sciaridae|[22547]|SSEIB6807-13|Sciaridae|658|BOLD:AAH3913
Sciaridae|[22548]|CNKJD538-14|Sciaridae|658|BOLD:ACM2361
Sciaridae|[22549]|SSBAE092-13|Sciaridae|661|BOLD:ACE4216
Sciaridae|[22550]|BBDCM788-10|Sciaridae|658|BOLD:AAV1324
Sciaridae|[22551]|SSWLD2344-13|Sciaridae|658|BOLD:ACI3956
Sciaridae|[22552]|CNJAB926-12|Sciaridae|658|BOLD:ACC1693
Sciaridae|[22553]|CNEIG723-12|Sciaridae|658|BOLD:ACD1897
Sciaridae|[22554]|CNJAC132-12|Sciaridae|658|BOLD:ACC7678
Sciaridae|[22555]|CNEID752-12|Sciaridae|658|BOLD:ACB0536
Sciaridae|[22556]|SSWLA2986-13|Sciaridae|658|BOLD:ACF7997
Sciaridae|[22557]|CNEIB1251-12|Sciaridae|658|BOLD:ACA9564
Sciaridae|[22558]|CNEIC2570-12|Sciaridae|658|BOLD:ACB1505
Sciaridae|[22559]|CNEIF489-12|Sciaridae|658|BOLD:ACD0116
Sciaridae|[22560]|CNWLF756-12|Sciaridae|670|BOLD:ACD1047
Sciaridae|[22561]|CNJAA293-12|Sciaridae|670|BOLD:ACB9717
Sciaridae|[22562]|SSFDC2684-14|Sciaridae|588|BOLD:ACO4973
Sciaridae|[22563]|JSMAY764-11|Sciaridae|658|BOLD:ABV1215
Sciaridae|[22564]|CNEIA1192-12|Sciaridae|670|BOLD:AAA6148
Sciaridae|[22565]|SSJAD3032-13|Sciaridae|658|BOLD:ACJ0083
Sciaridae|[22566]|CNIVB359-14|Sciaridae|603|BOLD:ACR4655
Sciaridae|[22567]|CNGUJ132-15|Sciaridae|615|BOLD:ACS9618
Sciaridae|[22568]|CNGBK482-14|Sciaridae|658|BOLD:ACL8156
Bradysia inusitata|[22569]|CNGIJ109-13|Sciaridae|661|BOLD:ACH4332
Sciaridae|[22570]|NCCA1631-11|Sciaridae|673|BOLD:AAH3929
Sciaridae|[22571]|HEMAY974-12|Sciaridae|703|BOLD:AAM9252
Sciaridae|[22572]|SMTPJ4837-14|Sciaridae|609|BOLD:ACP9604
Sciaridae|[22573]|SSJAD3012-13|Sciaridae|658|BOLD:AAH3909
Sciaridae|[22574]|PHDIP537-11|Sciaridae|669|BOLD:AAZ5589
Sciaridae|[22575]|CNWLD033-12|Sciaridae|658|BOLD:ACC5631
Sciaridae|[22576]|CNJAG932-12|Sciaridae|673|BOLD:ACB2385
Sciaridae|[22577]|CNBPM217-13|Sciaridae|658|BOLD:ACC5491
Sciaridae|[22578]|CNIVD542-14|Sciaridae|552|BOLD:ACR4287
Sciaridae|[22579]|BBDED071-10|Sciaridae|658|BOLD:AAH3973
Sciaridae|[22580]|BBDEE964-10|Sciaridae|658|BOLD:AAM9248
Sciaridae|[22581]|CNTNC980-14|Sciaridae|570|BOLD:ACM7887
Sciaridae|[22582]|JWDCA796-10|Sciaridae|658|BOLD:AAH4011
Sciaridae|[22583]|JWDCC750-10|Sciaridae|658|BOLD:AAL7881
Sciaridae|[22584]|JWDCC517-10|Sciaridae|658|BOLD:AAL7867
Sciaridae|[22585]|CNSRA642-14|Sciaridae|588|BOLD:ACR4204
Sciaridae|[22586]|CNWLE887-12|Sciaridae|673|BOLD:ACC6362
Sciaridae|[22587]|CNWLE1060-12|Sciaridae|658|BOLD:ACC6570
Sciaridae|[22588]|CNPAB126-13|Sciaridae|670|BOLD:ACC2836
Sciaridae|[22589]|DARC315-11|Sciaridae|658|BOLD:AAV1273
Sciaridae|[22590]|DARC463-11|Sciaridae|658|BOLD:AAU6577
Sciaridae|[22591]|CNWAE4301-14|Sciaridae|610|BOLD:ACR8968
Sciaridae|[22592]|JWDCD364-10|Sciaridae|658|BOLD:AAM9239
Sciaridae|[22593]|JWDCF771-10|Sciaridae|658|BOLD:ACY8032
Sciaridae|[22594]|JWDCF937-10|Sciaridae|658|BOLD:AAP8788
Sciaridae|[22595]|CHDIP292-11|Sciaridae|538|BOLD:ABY2190
Sciaridae|[22596]|CNNHC544-14|Sciaridae|594|BOLD:ACR6253

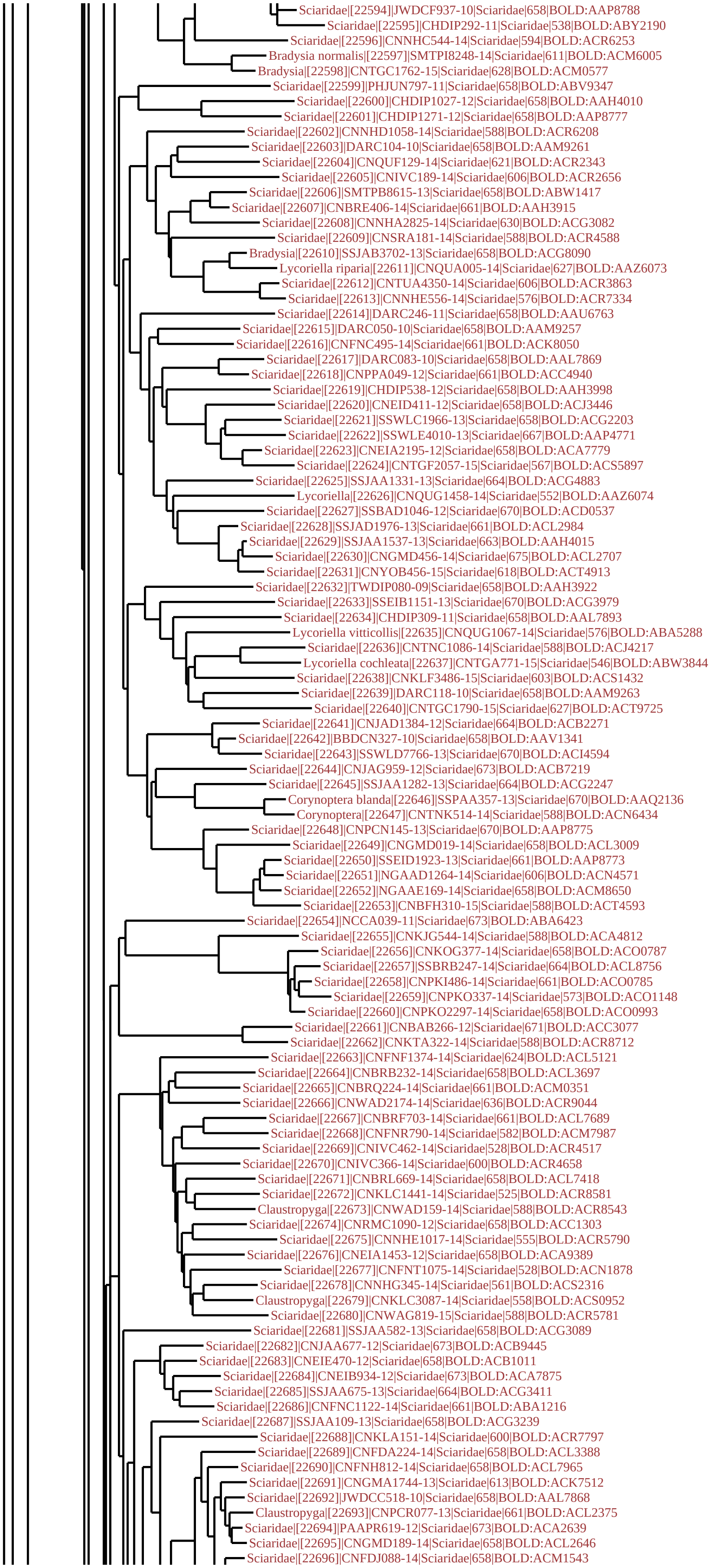

Sciaridae|[22594]|JWDCF937-10|Sciaridae|658|BOLD:AAP8788
Sciaridae|[22595]|CHDIP292-11|Sciaridae|538|BOLD:ABY2190
Sciaridae|[22596]|CNNHC544-14|Sciaridae|594|BOLD:ACR6253
Bradysia normalis|[22597]|SMTPI8248-14|Sciaridae|611|BOLD:ACM6005
Bradysia|[22598]|CNTGC1762-15|Sciaridae|628|BOLD:ACM0577
Sciaridae|[22599]|PHJUN797-11|Sciaridae|658|BOLD:ABV9347
Sciaridae|[22600]|CHDIP1027-12|Sciaridae|658|BOLD:AAH4010
Sciaridae|[22601]|CHDIP1271-12|Sciaridae|658|BOLD:AAP8777
Sciaridae|[22602]|CNNHD1058-14|Sciaridae|588|BOLD:ACR6208
Sciaridae|[22603]|DARC104-10|Sciaridae|658|BOLD:AAM9261
Sciaridae|[22604]|CNQUF129-14|Sciaridae|621|BOLD:ACR2343
Sciaridae|[22605]|CNIVC189-14|Sciaridae|606|BOLD:ACR2656
Sciaridae|[22606]|SMTPB8615-13|Sciaridae|658|BOLD:ABW1417
Sciaridae|[22607]|CNBRE406-14|Sciaridae|661|BOLD:AAH3915
Sciaridae|[22608]|CNNHA2825-14|Sciaridae|630|BOLD:ACG3082
Sciaridae|[22609]|CNSRA181-14|Sciaridae|588|BOLD:ACR4588
Bradysia|[22610]|SSJAB3702-13|Sciaridae|658|BOLD:ACG8090
Lycoriella riparia|[22611]|CNQUA005-14|Sciaridae|627|BOLD:AAZ6073
Sciaridae|[22612]|CNTUA4350-14|Sciaridae|606|BOLD:ACR3863
Sciaridae|[22613]|CNNHE556-14|Sciaridae|576|BOLD:ACR7334
Sciaridae|[22614]|DARC246-11|Sciaridae|658|BOLD:AAU6763
Sciaridae|[22615]|DARC050-10|Sciaridae|658|BOLD:AAM9257
Sciaridae|[22616]|CNFNC495-14|Sciaridae|661|BOLD:ACK8050
Sciaridae|[22617]|DARC083-10|Sciaridae|658|BOLD:AAL7869
Sciaridae|[22618]|CNPPA049-12|Sciaridae|661|BOLD:ACC4940
Sciaridae|[22619]|CHDIP538-12|Sciaridae|658|BOLD:AAH3998
Sciaridae|[22620]|CNEID411-12|Sciaridae|658|BOLD:ACJ3446
Sciaridae|[22621]|SSWLC1966-13|Sciaridae|658|BOLD:ACG2203
Sciaridae|[22622]|SSWLE4010-13|Sciaridae|667|BOLD:AAP4771
Sciaridae|[22623]|CNEIA2195-12|Sciaridae|658|BOLD:ACA7779
Sciaridae|[22624]|CNTGF2057-15|Sciaridae|567|BOLD:ACS5897
Sciaridae|[22625]|SSJAA1331-13|Sciaridae|664|BOLD:ACG4883
Lycoriella|[22626]|CNQUG1458-14|Sciaridae|552|BOLD:AAZ6074
Sciaridae|[22627]|SSBAD1046-12|Sciaridae|670|BOLD:ACD0537
Sciaridae|[22628]|SSJAD1976-13|Sciaridae|661|BOLD:ACL2984
Sciaridae|[22629]|SSJAA1537-13|Sciaridae|663|BOLD:AAH4015
Sciaridae|[22630]|CNGMD456-14|Sciaridae|675|BOLD:ACL2707
Sciaridae|[22631]|CNYOB456-15|Sciaridae|618|BOLD:ACT4913
Sciaridae|[22632]|TWDIP080-09|Sciaridae|658|BOLD:AAH3922
Sciaridae|[22633]|SSEIB1151-13|Sciaridae|670|BOLD:ACG3979
Sciaridae|[22634]|CHDIP309-11|Sciaridae|658|BOLD:AAL7893
Lycoriella vitticollis|[22635]|CNQUG1067-14|Sciaridae|576|BOLD:ABA5288
Sciaridae|[22636]|CNTNC1086-14|Sciaridae|588|BOLD:ACJ4217
Lycoriella cochleata|[22637]|CNTGA771-15|Sciaridae|546|BOLD:ABW3844
Sciaridae|[22638]|CNKLF3486-15|Sciaridae|603|BOLD:ACS1432
Sciaridae|[22639]|DARC118-10|Sciaridae|658|BOLD:AAM9263
Sciaridae|[22640]|CNTGC1790-15|Sciaridae|627|BOLD:ACT9725
Sciaridae|[22641]|CNJAD1384-12|Sciaridae|664|BOLD:ACB2271
Sciaridae|[22642]|BBDCN327-10|Sciaridae|658|BOLD:AAV1341
Sciaridae|[22643]|SSWLD7766-13|Sciaridae|670|BOLD:ACI4594
Sciaridae|[22644]|CNJAG959-12|Sciaridae|673|BOLD:ACB7219
Sciaridae|[22645]|SSJAA1282-13|Sciaridae|664|BOLD:ACG2247
Corynoptera blanda|[22646]|SSPAA357-13|Sciaridae|670|BOLD:AAQ2136
Corynoptera|[22647]|CNTNK514-14|Sciaridae|588|BOLD:ACN6434
Sciaridae|[22648]|CNPCN145-13|Sciaridae|670|BOLD:AAP8775
Sciaridae|[22649]|CNGMD019-14|Sciaridae|658|BOLD:ACL3009
Sciaridae|[22650]|SSEID1923-13|Sciaridae|661|BOLD:AAP8773
Sciaridae|[22651]|NGAAD1264-14|Sciaridae|606|BOLD:ACN4571
Sciaridae|[22652]|NGAAE169-14|Sciaridae|658|BOLD:ACM8650
Sciaridae|[22653]|CNBFH310-15|Sciaridae|588|BOLD:ACT4593
Sciaridae|[22654]|NCCA039-11|Sciaridae|673|BOLD:ABA6423
Sciaridae|[22655]|CNKJG544-14|Sciaridae|588|BOLD:ACA4812
Sciaridae|[22656]|CNKOG377-14|Sciaridae|658|BOLD:ACO0787
Sciaridae|[22657]|SSBRB247-14|Sciaridae|664|BOLD:ACL8756
Sciaridae|[22658]|CNPKI486-14|Sciaridae|661|BOLD:ACO0785
Sciaridae|[22659]|CNPKO337-14|Sciaridae|573|BOLD:ACO1148
Sciaridae|[22660]|CNPKO2297-14|Sciaridae|658|BOLD:ACO0993
Sciaridae|[22661]|CNBAB266-12|Sciaridae|671|BOLD:ACC3077
Sciaridae|[22662]|CNKTA322-14|Sciaridae|588|BOLD:ACR8712
Sciaridae|[22663]|CNFNF1374-14|Sciaridae|624|BOLD:ACL5121
Sciaridae|[22664]|CNBRB232-14|Sciaridae|658|BOLD:ACL3697
Sciaridae|[22665]|CNBRQ224-14|Sciaridae|661|BOLD:ACM0351
Sciaridae|[22666]|CNWAD2174-14|Sciaridae|636|BOLD:ACR9044
Sciaridae|[22667]|CNBRF703-14|Sciaridae|661|BOLD:ACL7689
Sciaridae|[22668]|CNFNR790-14|Sciaridae|582|BOLD:ACM7987
Sciaridae|[22669]|CNIVC462-14|Sciaridae|528|BOLD:ACR4517
Sciaridae|[22670]|CNIVC366-14|Sciaridae|600|BOLD:ACR4658
Sciaridae|[22671]|CNBRL669-14|Sciaridae|658|BOLD:ACL7418
Sciaridae|[22672]|CNKLC1441-14|Sciaridae|525|BOLD:ACR8581
Claustropyga|[22673]|CNWAD159-14|Sciaridae|588|BOLD:ACR8543
Sciaridae|[22674]|CNRMC1090-12|Sciaridae|658|BOLD:ACC1303
Sciaridae|[22675]|CNNHE1017-14|Sciaridae|555|BOLD:ACR5790
Sciaridae|[22676]|CNEIA1453-12|Sciaridae|658|BOLD:ACA9389
Sciaridae|[22677]|CNFNT1075-14|Sciaridae|528|BOLD:ACN1878
Sciaridae|[22678]|CNNHG345-14|Sciaridae|561|BOLD:ACS2316
Claustropyga|[22679]|CNKLC3087-14|Sciaridae|558|BOLD:ACS0952
Sciaridae|[22680]|CNWAG819-15|Sciaridae|588|BOLD:ACR5781
Sciaridae|[22681]|SSJAA582-13|Sciaridae|658|BOLD:ACG3089
Sciaridae|[22682]|CNJAA677-12|Sciaridae|673|BOLD:ACB9445
Sciaridae|[22683]|CNEIE470-12|Sciaridae|658|BOLD:ACB1011
Sciaridae|[22684]|CNEIB934-12|Sciaridae|673|BOLD:ACA7875
Sciaridae|[22685]|SSJAA675-13|Sciaridae|664|BOLD:ACG3411
Sciaridae|[22686]|CNFNC1122-14|Sciaridae|661|BOLD:ABA1216
Sciaridae|[22687]|SSJAA109-13|Sciaridae|658|BOLD:ACG3239
Sciaridae|[22688]|CNKLA151-14|Sciaridae|600|BOLD:ACR7797
Sciaridae|[22689]|CNFDA224-14|Sciaridae|658|BOLD:ACL3388
Sciaridae|[22690]|CNFNH812-14|Sciaridae|658|BOLD:ACL7965
Sciaridae|[22691]|CNGMA1744-13|Sciaridae|613|BOLD:ACK7512
Sciaridae|[22692]|JWDCC518-10|Sciaridae|658|BOLD:AAL7868
Claustropyga|[22693]|CNPCR077-13|Sciaridae|661|BOLD:ACL2375
Sciaridae|[22694]|PAAPR619-12|Sciaridae|673|BOLD:ACA2639
Sciaridae|[22695]|CNGMD189-14|Sciaridae|658|BOLD:ACL2646
Sciaridae|[22696]|CNFDJ088-14|Sciaridae|658|BOLD:ACM1543

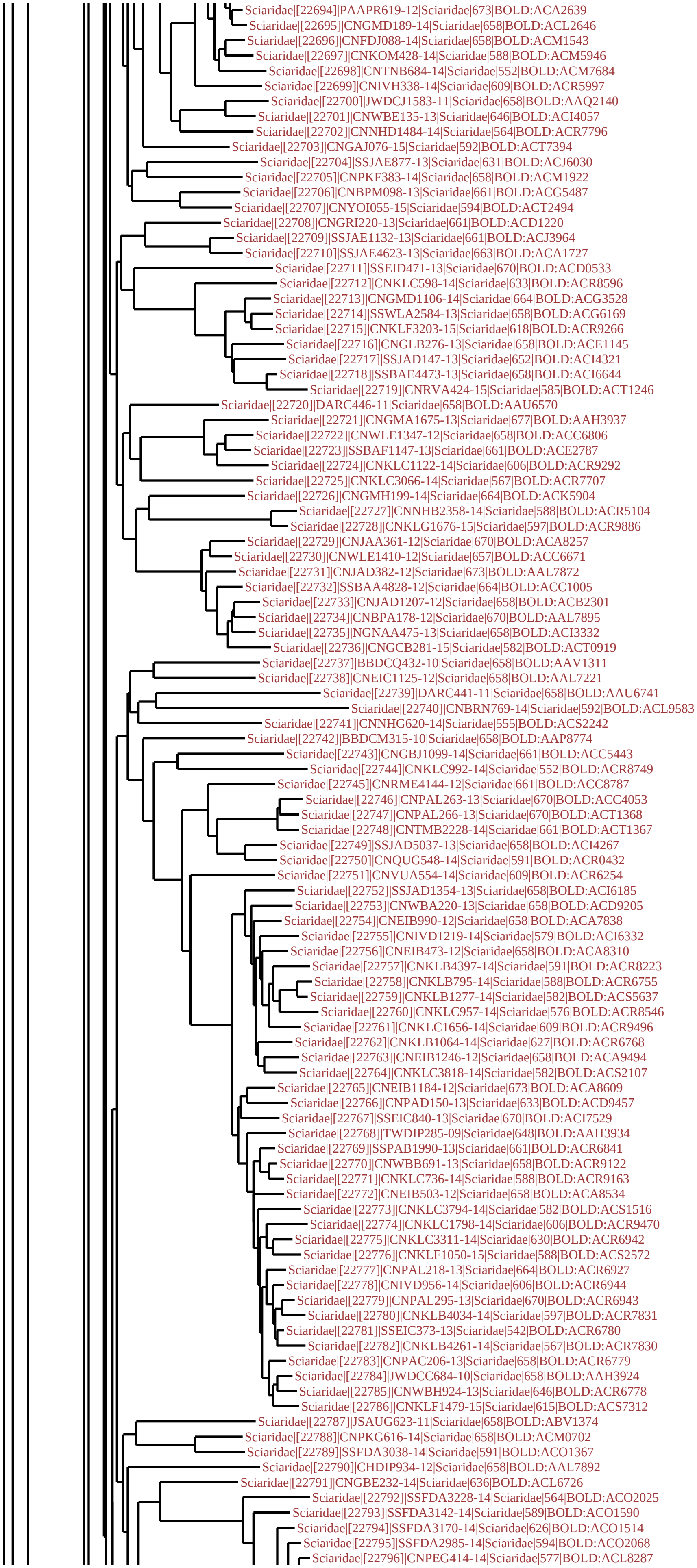
Sciaridae|[22694]|PAAPR619-12|Sciaridae|673|BOLD:ACA2639
Sciaridae|[22695]|CNGMD189-14|Sciaridae|658|BOLD:ACL2646
Sciaridae|[22696]|CNFDJ088-14|Sciaridae|658|BOLD:ACM1543
Sciaridae|[22697]|CNKOM428-14|Sciaridae|588|BOLD:ACM5946
Sciaridae|[22698]|CNTNB684-14|Sciaridae|552|BOLD:ACM7684
Sciaridae|[22699]|CNIVH338-14|Sciaridae|609|BOLD:ACR5997
Sciaridae|[22700]|JWDCJ1583-11|Sciaridae|658|BOLD:AAQ2140
Sciaridae|[22701]|CNWBE135-13|Sciaridae|646|BOLD:ACI4057
Sciaridae|[22702]|CNNHD1484-14|Sciaridae|564|BOLD:ACR7796
Sciaridae|[22703]|CNGAJ076-15|Sciaridae|592|BOLD:ACT7394
Sciaridae|[22704]|SSJAE877-13|Sciaridae|631|BOLD:ACJ6030
Sciaridae|[22705]|CNPKF383-14|Sciaridae|658|BOLD:ACM1922
Sciaridae|[22706]|CNBPM098-13|Sciaridae|661|BOLD:ACG5487
Sciaridae|[22707]|CNYOI055-15|Sciaridae|594|BOLD:ACT2494
Sciaridae|[22708]|CNGRI220-13|Sciaridae|661|BOLD:ACD1220
Sciaridae|[22709]|SSJAE1132-13|Sciaridae|661|BOLD:ACJ3964
Sciaridae|[22710]|SSJAE4623-13|Sciaridae|663|BOLD:ACA1727
Sciaridae|[22711]|SSEID471-13|Sciaridae|670|BOLD:ACD0533
Sciaridae|[22712]|CNKLC598-14|Sciaridae|633|BOLD:ACR8596
Sciaridae|[22713]|CNGMD1106-14|Sciaridae|664|BOLD:ACG3528
Sciaridae|[22714]|SSWLA2584-13|Sciaridae|658|BOLD:ACG6169
Sciaridae|[22715]|CNKLF3203-15|Sciaridae|618|BOLD:ACR9266
Sciaridae|[22716]|CNGLB276-13|Sciaridae|658|BOLD:ACE1145
Sciaridae|[22717]|SSJAD147-13|Sciaridae|652|BOLD:ACI4321
Sciaridae|[22718]|SSBAE4473-13|Sciaridae|658|BOLD:ACI6644
Sciaridae|[22719]|CNRVA424-15|Sciaridae|585|BOLD:ACT1246
Sciaridae|[22720]|DARC446-11|Sciaridae|658|BOLD:AAU6570
Sciaridae|[22721]|CNGMA1675-13|Sciaridae|677|BOLD:AAH3937
Sciaridae|[22722]|CNWLE1347-12|Sciaridae|658|BOLD:ACC6806
Sciaridae|[22723]|SSBAF1147-13|Sciaridae|661|BOLD:ACE2787
Sciaridae|[22724]|CNKLC1122-14|Sciaridae|606|BOLD:ACR9292
Sciaridae|[22725]|CNKLC3066-14|Sciaridae|567|BOLD:ACR7707
Sciaridae|[22726]|CNGMH199-14|Sciaridae|664|BOLD:ACK5904
Sciaridae|[22727]|CNNHB2358-14|Sciaridae|588|BOLD:ACR5104
Sciaridae|[22728]|CNKLG1676-15|Sciaridae|597|BOLD:ACR9886
Sciaridae|[22729]|CNJAA361-12|Sciaridae|670|BOLD:ACA8257
Sciaridae|[22730]|CNWLE1410-12|Sciaridae|657|BOLD:ACC6671
Sciaridae|[22731]|CNJAD382-12|Sciaridae|673|BOLD:AAL7872
Sciaridae|[22732]|SSBAA4828-12|Sciaridae|664|BOLD:ACC1005
Sciaridae|[22733]|CNJAD1207-12|Sciaridae|658|BOLD:ACB2301
Sciaridae|[22734]|CNBPA178-12|Sciaridae|670|BOLD:AAL7895
Sciaridae|[22735]|NGNAA475-13|Sciaridae|658|BOLD:ACI3332
Sciaridae|[22736]|CNGCB281-15|Sciaridae|582|BOLD:ACT0919
Sciaridae|[22737]|BBDCQ432-10|Sciaridae|658|BOLD:AAV1311
Sciaridae|[22738]|CNEIC1125-12|Sciaridae|658|BOLD:AAL7221
Sciaridae|[22739]|DARC441-11|Sciaridae|658|BOLD:AAU6741
Sciaridae|[22740]|CNBRN769-14|Sciaridae|592|BOLD:ACL9583
Sciaridae|[22741]|CNNHG620-14|Sciaridae|555|BOLD:ACS2242
Sciaridae|[22742]|BBDCM315-10|Sciaridae|658|BOLD:AAP8774
Sciaridae|[22743]|CNGBJ1099-14|Sciaridae|661|BOLD:ACC5443
Sciaridae|[22744]|CNKLC992-14|Sciaridae|552|BOLD:ACR8749
Sciaridae|[22745]|CNRME4144-12|Sciaridae|661|BOLD:ACC8787
Sciaridae|[22746]|CNPAL263-13|Sciaridae|670|BOLD:ACC4053
Sciaridae|[22747]|CNPAL266-13|Sciaridae|670|BOLD:ACT1368
Sciaridae|[22748]|CNTMB2228-14|Sciaridae|661|BOLD:ACT1367
Sciaridae|[22749]|SSJAD5037-13|Sciaridae|658|BOLD:ACI4267
Sciaridae|[22750]|CNQUG548-14|Sciaridae|591|BOLD:ACR0432
Sciaridae|[22751]|CNVUA554-14|Sciaridae|609|BOLD:ACR6254
Sciaridae|[22752]|SSJAD1354-13|Sciaridae|658|BOLD:ACI6185
Sciaridae|[22753]|CNWBA220-13|Sciaridae|658|BOLD:ACD9205
Sciaridae|[22754]|CNEIB990-12|Sciaridae|658|BOLD:ACA7838
Sciaridae|[22755]|CNIVD1219-14|Sciaridae|579|BOLD:ACI6332
Sciaridae|[22756]|CNEIB473-12|Sciaridae|658|BOLD:ACA8310
Sciaridae|[22757]|CNKLB4397-14|Sciaridae|591|BOLD:ACR8223
Sciaridae|[22758]|CNKLB795-14|Sciaridae|588|BOLD:ACR6755
Sciaridae|[22759]|CNKLB1277-14|Sciaridae|582|BOLD:ACS5637
Sciaridae|[22760]|CNKLC957-14|Sciaridae|576|BOLD:ACR8546
Sciaridae|[22761]|CNKLC1656-14|Sciaridae|609|BOLD:ACR9496
Sciaridae|[22762]|CNKLB1064-14|Sciaridae|627|BOLD:ACR6768
Sciaridae|[22763]|CNEIB1246-12|Sciaridae|658|BOLD:ACA9494
Sciaridae|[22764]|CNKLC3818-14|Sciaridae|582|BOLD:ACS2107
Sciaridae|[22765]|CNEIB1184-12|Sciaridae|673|BOLD:ACA8609
Sciaridae|[22766]|CNPAD150-13|Sciaridae|633|BOLD:ACD9457
Sciaridae|[22767]|SSEIC840-13|Sciaridae|670|BOLD:ACI7529
Sciaridae|[22768]|TWDIP285-09|Sciaridae|648|BOLD:AAH3934
Sciaridae|[22769]|SSPAB1990-13|Sciaridae|661|BOLD:ACR6841
Sciaridae|[22770]|CNWBB691-13|Sciaridae|658|BOLD:ACR9122
Sciaridae|[22771]|CNKLC736-14|Sciaridae|588|BOLD:ACR9163
Sciaridae|[22772]|CNEIB503-12|Sciaridae|658|BOLD:ACA8534
Sciaridae|[22773]|CNKLC3794-14|Sciaridae|582|BOLD:ACS1516
Sciaridae|[22774]|CNKLC1798-14|Sciaridae|606|BOLD:ACR9470
Sciaridae|[22775]|CNKLC3311-14|Sciaridae|630|BOLD:ACR6942
Sciaridae|[22776]|CNKLF1050-15|Sciaridae|588|BOLD:ACS2572
Sciaridae|[22777]|CNPAL218-13|Sciaridae|664|BOLD:ACR6927
Sciaridae|[22778]|CNIVD956-14|Sciaridae|606|BOLD:ACR6944
Sciaridae|[22779]|CNPAL295-13|Sciaridae|670|BOLD:ACR6943
Sciaridae|[22780]|CNKLB4034-14|Sciaridae|597|BOLD:ACR7831
Sciaridae|[22781]|SSEIC373-13|Sciaridae|542|BOLD:ACR6780
Sciaridae|[22782]|CNKLB4261-14|Sciaridae|567|BOLD:ACR7830
Sciaridae|[22783]|CNPAC206-13|Sciaridae|658|BOLD:ACR6779
Sciaridae|[22784]|JWDCC684-10|Sciaridae|658|BOLD:AAH3924
Sciaridae|[22785]|CNWBH924-13|Sciaridae|646|BOLD:ACR6778
Sciaridae|[22786]|CNKLF1479-15|Sciaridae|615|BOLD:ACS7312
Sciaridae|[22787]|JSAUG623-11|Sciaridae|658|BOLD:ABV1374
Sciaridae|[22788]|CNPKG616-14|Sciaridae|658|BOLD:ACM0702
Sciaridae|[22789]|SSFDA3038-14|Sciaridae|591|BOLD:ACO1367
Sciaridae|[22790]|CHDIP934-12|Sciaridae|658|BOLD:AAL7892
Sciaridae|[22791]|CNGBE232-14|Sciaridae|636|BOLD:ACL6726
Sciaridae|[22792]|SSFDA3228-14|Sciaridae|564|BOLD:ACO2025
Sciaridae|[22793]|SSFDA3142-14|Sciaridae|589|BOLD:ACO1590
Sciaridae|[22794]|SSFDA3170-14|Sciaridae|626|BOLD:ACO1514
Sciaridae|[22795]|SSFDA2985-14|Sciaridae|594|BOLD:ACO2068
Sciaridae|[22796]|CNPEG414-14|Sciaridae|577|BOLD:ACL8287

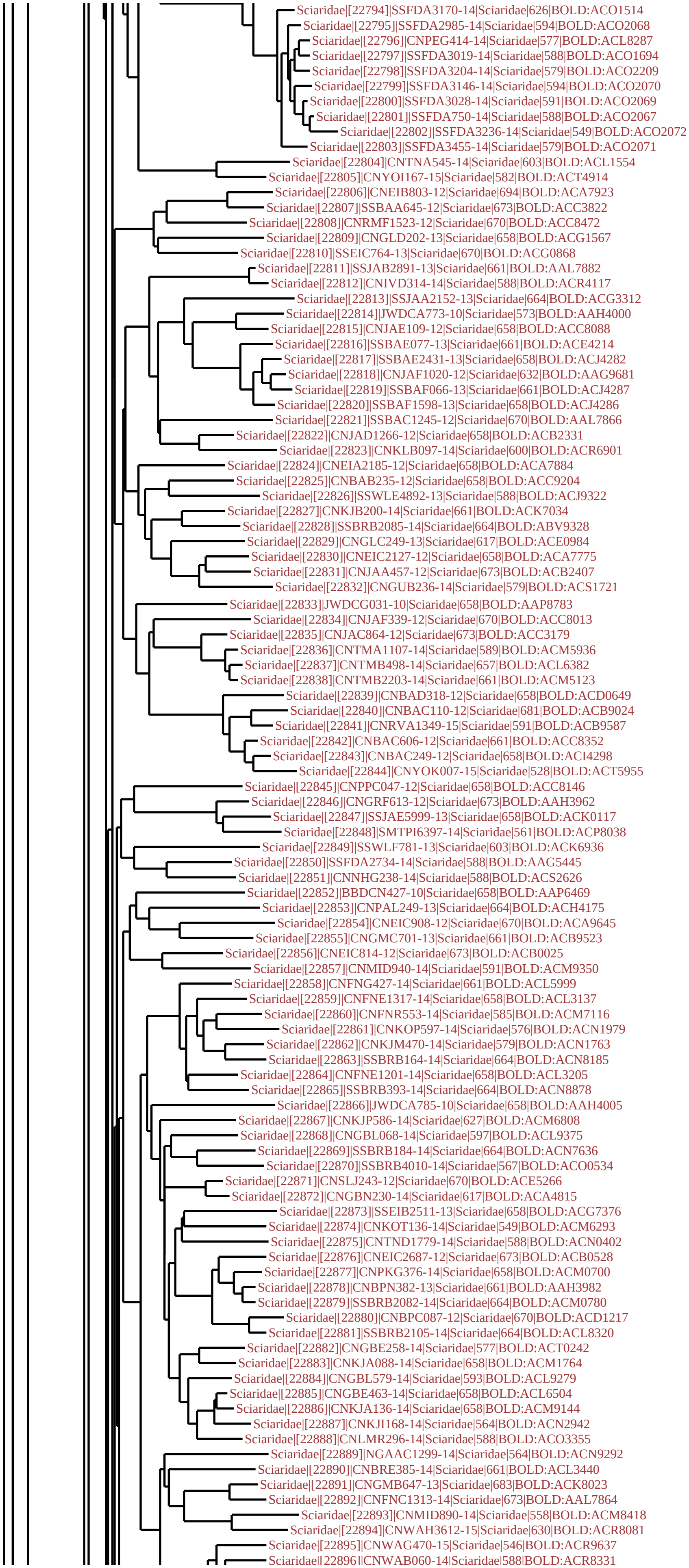
Sciaridae|[22794]|SSFDA3170-14|Sciaridae|626|BOLD:ACO1514
Sciaridae|[22795]|SSFDA2985-14|Sciaridae|594|BOLD:ACO2068
Sciaridae|[22796]|CNPEG414-14|Sciaridae|577|BOLD:ACL8287
Sciaridae|[22797]|SSFDA3019-14|Sciaridae|588|BOLD:ACO1694
Sciaridae|[22798]|SSFDA3204-14|Sciaridae|579|BOLD:ACO2209
Sciaridae|[22799]|SSFDA3146-14|Sciaridae|594|BOLD:ACO2070
Sciaridae|[22800]|SSFDA3028-14|Sciaridae|591|BOLD:ACO2069
Sciaridae|[22801]|SSFDA750-14|Sciaridae|588|BOLD:ACO2067
Sciaridae|[22802]|SSFDA3236-14|Sciaridae|549|BOLD:ACO2072
Sciaridae|[22803]|SSFDA3455-14|Sciaridae|579|BOLD:ACO2071
Sciaridae|[22804]|CNTNA545-14|Sciaridae|603|BOLD:ACL1554
Sciaridae|[22805]|CNYOI167-15|Sciaridae|582|BOLD:ACT4914
Sciaridae|[22806]|CNEIB803-12|Sciaridae|694|BOLD:ACA7923
Sciaridae|[22807]|SSBAA645-12|Sciaridae|673|BOLD:ACC3822
Sciaridae|[22808]|CNRMF1523-12|Sciaridae|670|BOLD:ACC8472
Sciaridae|[22809]|CNGLD202-13|Sciaridae|658|BOLD:ACG1567
Sciaridae|[22810]|SSEIC764-13|Sciaridae|670|BOLD:ACG0868
Sciaridae|[22811]|SSJAB2891-13|Sciaridae|661|BOLD:AAL7882
Sciaridae|[22812]|CNIVD314-14|Sciaridae|588|BOLD:ACR4117
Sciaridae|[22813]|SSJAA2152-13|Sciaridae|664|BOLD:ACG3312
Sciaridae|[22814]|JWDCA773-10|Sciaridae|573|BOLD:AAH4000
Sciaridae|[22815]|CNJAE109-12|Sciaridae|658|BOLD:ACC8088
Sciaridae|[22816]|SSBAE077-13|Sciaridae|661|BOLD:ACE4214
Sciaridae|[22817]|SSBAE2431-13|Sciaridae|658|BOLD:ACJ4282
Sciaridae|[22818]|CNJAF1020-12|Sciaridae|632|BOLD:AAG9681
Sciaridae|[22819]|SSBAF066-13|Sciaridae|661|BOLD:ACJ4287
Sciaridae|[22820]|SSBAF1598-13|Sciaridae|658|BOLD:ACJ4286
Sciaridae|[22821]|SSBAC1245-12|Sciaridae|670|BOLD:AAL7866
Sciaridae|[22822]|CNJAD1266-12|Sciaridae|658|BOLD:ACB2331
Sciaridae|[22823]|CNKLB097-14|Sciaridae|600|BOLD:ACR6901
Sciaridae|[22824]|CNEIA2185-12|Sciaridae|658|BOLD:ACA7884
Sciaridae|[22825]|CNBAB235-12|Sciaridae|658|BOLD:ACC9204
Sciaridae|[22826]|SSWLE4892-13|Sciaridae|588|BOLD:ACJ9322
Sciaridae|[22827]|CNKJB200-14|Sciaridae|661|BOLD:ACK7034
Sciaridae|[22828]|SSBRB2085-14|Sciaridae|664|BOLD:ABV9328
Sciaridae|[22829]|CNGLC249-13|Sciaridae|617|BOLD:ACE0984
Sciaridae|[22830]|CNEIC2127-12|Sciaridae|658|BOLD:ACA7775
Sciaridae|[22831]|CNJAA457-12|Sciaridae|673|BOLD:ACB2407
Sciaridae|[22832]|CNGUB236-14|Sciaridae|579|BOLD:ACS1721
Sciaridae|[22833]|JWDCG031-10|Sciaridae|658|BOLD:AAP8783
Sciaridae|[22834]|CNJAF339-12|Sciaridae|670|BOLD:ACC8013
Sciaridae|[22835]|CNJAC864-12|Sciaridae|673|BOLD:ACC3179
Sciaridae|[22836]|CNTMA1107-14|Sciaridae|589|BOLD:ACM5936
Sciaridae|[22837]|CNTMB498-14|Sciaridae|657|BOLD:ACL6382
Sciaridae|[22838]|CNTMB2203-14|Sciaridae|661|BOLD:ACM5123
Sciaridae|[22839]|CNBAD318-12|Sciaridae|658|BOLD:ACD0649
Sciaridae|[22840]|CNBAC110-12|Sciaridae|681|BOLD:ACB9024
Sciaridae|[22841]|CNRVA1349-15|Sciaridae|591|BOLD:ACB9587
Sciaridae|[22842]|CNBAC606-12|Sciaridae|661|BOLD:ACC8352
Sciaridae|[22843]|CNBAC249-12|Sciaridae|658|BOLD:ACI4298
Sciaridae|[22844]|CNYOK007-15|Sciaridae|528|BOLD:ACT5955
Sciaridae|[22845]|CNPPC047-12|Sciaridae|658|BOLD:ACC8146
Sciaridae|[22846]|CNGRF613-12|Sciaridae|673|BOLD:AAH3962
Sciaridae|[22847]|SSJAE5999-13|Sciaridae|658|BOLD:ACK0117
Sciaridae|[22848]|SMTPI6397-14|Sciaridae|561|BOLD:ACP8038
Sciaridae|[22849]|SSWLF781-13|Sciaridae|603|BOLD:ACK6936
Sciaridae|[22850]|SSFDA2734-14|Sciaridae|588|BOLD:AAG5445
Sciaridae|[22851]|CNNHG238-14|Sciaridae|588|BOLD:ACS2626
Sciaridae|[22852]|BBDCN427-10|Sciaridae|658|BOLD:AAP6469
Sciaridae|[22853]|CNPAL249-13|Sciaridae|664|BOLD:ACH4175
Sciaridae|[22854]|CNEIC908-12|Sciaridae|670|BOLD:ACA9645
Sciaridae|[22855]|CNGMC701-13|Sciaridae|661|BOLD:ACB9523
Sciaridae|[22856]|CNEIC814-12|Sciaridae|673|BOLD:ACB0025
Sciaridae|[22857]|CNMID940-14|Sciaridae|591|BOLD:ACM9350
Sciaridae|[22858]|CNFNG427-14|Sciaridae|661|BOLD:ACL5999
Sciaridae|[22859]|CNFNE1317-14|Sciaridae|658|BOLD:ACL3137
Sciaridae|[22860]|CNFNR553-14|Sciaridae|585|BOLD:ACM7116
Sciaridae|[22861]|CNKOP597-14|Sciaridae|576|BOLD:ACN1979
Sciaridae|[22862]|CNKJM470-14|Sciaridae|579|BOLD:ACN1763
Sciaridae|[22863]|SSBRB164-14|Sciaridae|664|BOLD:ACN8185
Sciaridae|[22864]|CNFNE1201-14|Sciaridae|658|BOLD:ACL3205
Sciaridae|[22865]|SSBRB393-14|Sciaridae|664|BOLD:ACN8878
Sciaridae|[22866]|JWDCA785-10|Sciaridae|658|BOLD:AAH4005
Sciaridae|[22867]|CNKJP586-14|Sciaridae|627|BOLD:ACM6808
Sciaridae|[22868]|CNGBL068-14|Sciaridae|597|BOLD:ACL9375
Sciaridae|[22869]|SSBRB184-14|Sciaridae|664|BOLD:ACN7636
Sciaridae|[22870]|SSBRB4010-14|Sciaridae|567|BOLD:ACO0534
Sciaridae|[22871]|CNSLJ243-12|Sciaridae|670|BOLD:ACE5266
Sciaridae|[22872]|CNGBN230-14|Sciaridae|617|BOLD:ACA4815
Sciaridae|[22873]|SSEIB2511-13|Sciaridae|658|BOLD:ACG7376
Sciaridae|[22874]|CNKOT136-14|Sciaridae|549|BOLD:ACM6293
Sciaridae|[22875]|CNTND1779-14|Sciaridae|588|BOLD:ACN0402
Sciaridae|[22876]|CNEIC2687-12|Sciaridae|673|BOLD:ACB0528
Sciaridae|[22877]|CNPKG376-14|Sciaridae|658|BOLD:ACM0700
Sciaridae|[22878]|CNBPN382-13|Sciaridae|661|BOLD:AAH3982
Sciaridae|[22879]|SSBRB2082-14|Sciaridae|664|BOLD:ACM0780
Sciaridae|[22880]|CNBPC087-12|Sciaridae|670|BOLD:ACD1217
Sciaridae|[22881]|SSBRB2105-14|Sciaridae|664|BOLD:ACL8320
Sciaridae|[22882]|CNGBE258-14|Sciaridae|577|BOLD:ACT0242
Sciaridae|[22883]|CNKJA088-14|Sciaridae|658|BOLD:ACM1764
Sciaridae|[22884]|CNGBL579-14|Sciaridae|593|BOLD:ACL9279
Sciaridae|[22885]|CNGBE463-14|Sciaridae|658|BOLD:ACL6504
Sciaridae|[22886]|CNKJA136-14|Sciaridae|658|BOLD:ACM9144
Sciaridae|[22887]|CNKJI168-14|Sciaridae|564|BOLD:ACN2942
Sciaridae|[22888]|CNLMR296-14|Sciaridae|588|BOLD:ACO3355
Sciaridae|[22889]|NGAAC1299-14|Sciaridae|564|BOLD:ACN9292
Sciaridae|[22890]|CNBRE385-14|Sciaridae|661|BOLD:ACL3440
Sciaridae|[22891]|CNGMB647-13|Sciaridae|683|BOLD:ACK8023
Sciaridae|[22892]|CNFNC1313-14|Sciaridae|673|BOLD:AAL7864
Sciaridae|[22893]|CNMID890-14|Sciaridae|558|BOLD:ACM8418
Sciaridae|[22894]|CNWAH3612-15|Sciaridae|630|BOLD:ACR8081
Sciaridae|[22895]|CNWAG470-15|Sciaridae|546|BOLD:ACR9637
Sciaridae|[22896]|CNWAB060-14|Sciaridae|588|BOLD:ACR8331

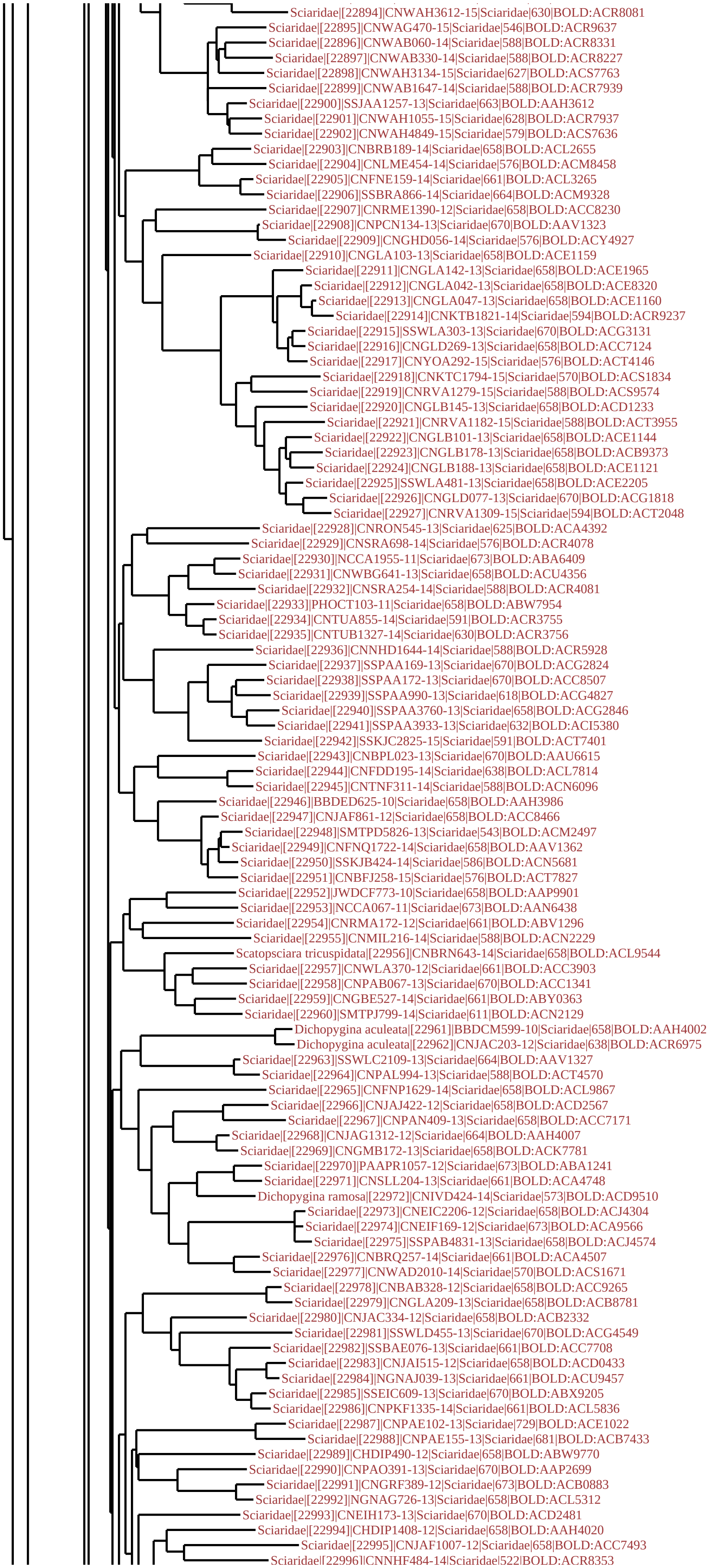

Sciaridae|[22894]|CNWAH3612-15|Sciaridae|630|BOLD:ACR8081
Sciaridae|[22895]|CNWAG470-15|Sciaridae|546|BOLD:ACR9637
Sciaridae|[22896]|CNWAB060-14|Sciaridae|588|BOLD:ACR8331
Sciaridae|[22897]|CNWAB330-14|Sciaridae|588|BOLD:ACR8227
Sciaridae|[22898]|CNWAH3134-15|Sciaridae|627|BOLD:ACS7763
Sciaridae|[22899]|CNWAB1647-14|Sciaridae|588|BOLD:ACR7939
Sciaridae|[22900]|SSJAA1257-13|Sciaridae|663|BOLD:AAH3612
Sciaridae|[22901]|CNWAH1055-15|Sciaridae|628|BOLD:ACR7937
Sciaridae|[22902]|CNWAH4849-15|Sciaridae|579|BOLD:ACS7636
Sciaridae|[22903]|CNBRB189-14|Sciaridae|658|BOLD:ACL2655
Sciaridae|[22904]|CNLME454-14|Sciaridae|576|BOLD:ACM8458
Sciaridae|[22905]|CNFNE159-14|Sciaridae|661|BOLD:ACL3265
Sciaridae|[22906]|SSBRA866-14|Sciaridae|664|BOLD:ACM9328
Sciaridae|[22907]|CNRME1390-12|Sciaridae|658|BOLD:ACC8230
Sciaridae|[22908]|CNPCN134-13|Sciaridae|670|BOLD:AAV1323
Sciaridae|[22909]|CNGHD056-14|Sciaridae|576|BOLD:ACY4927
Sciaridae|[22910]|CNGLA103-13|Sciaridae|658|BOLD:ACE1159
Sciaridae|[22911]|CNGLA142-13|Sciaridae|658|BOLD:ACE1965
Sciaridae|[22912]|CNGLA042-13|Sciaridae|658|BOLD:ACE8320
Sciaridae|[22913]|CNGLA047-13|Sciaridae|658|BOLD:ACE1160
Sciaridae|[22914]|CNKTB1821-14|Sciaridae|594|BOLD:ACR9237
Sciaridae|[22915]|SSWLA303-13|Sciaridae|670|BOLD:ACG3131
Sciaridae|[22916]|CNGLD269-13|Sciaridae|658|BOLD:ACC7124
Sciaridae|[22917]|CNYOA292-15|Sciaridae|576|BOLD:ACT4146
Sciaridae|[22918]|CNKTC1794-15|Sciaridae|570|BOLD:ACS1834
Sciaridae|[22919]|CNRVA1279-15|Sciaridae|588|BOLD:ACS9574
Sciaridae|[22920]|CNGLB145-13|Sciaridae|658|BOLD:ACD1233
Sciaridae|[22921]|CNRVA1182-15|Sciaridae|588|BOLD:ACT3955
Sciaridae|[22922]|CNGLB101-13|Sciaridae|658|BOLD:ACE1144
Sciaridae|[22923]|CNGLB178-13|Sciaridae|658|BOLD:ACB9373
Sciaridae|[22924]|CNGLB188-13|Sciaridae|658|BOLD:ACE1121
Sciaridae|[22925]|SSWLA481-13|Sciaridae|658|BOLD:ACE2205
Sciaridae|[22926]|CNGLD077-13|Sciaridae|670|BOLD:ACG1818
Sciaridae|[22927]|CNRVA1309-15|Sciaridae|594|BOLD:ACT2048
Sciaridae|[22928]|CNRON545-13|Sciaridae|625|BOLD:ACA4392
Sciaridae|[22929]|CNSRA698-14|Sciaridae|576|BOLD:ACR4078
Sciaridae|[22930]|NCCA1955-11|Sciaridae|673|BOLD:ABA6409
Sciaridae|[22931]|CNWBG641-13|Sciaridae|658|BOLD:ACU4356
Sciaridae|[22932]|CNSRA254-14|Sciaridae|588|BOLD:ACR4081
Sciaridae|[22933]|PHOCT103-11|Sciaridae|658|BOLD:ABW7954
Sciaridae|[22934]|CNTUA855-14|Sciaridae|591|BOLD:ACR3755
Sciaridae|[22935]|CNTUB1327-14|Sciaridae|630|BOLD:ACR3756
Sciaridae|[22936]|CNNHD1644-14|Sciaridae|588|BOLD:ACR5928
Sciaridae|[22937]|SSPAA169-13|Sciaridae|670|BOLD:ACG2824
Sciaridae|[22938]|SSPAA172-13|Sciaridae|670|BOLD:ACC8507
Sciaridae|[22939]|SSPAA990-13|Sciaridae|618|BOLD:ACG4827
Sciaridae|[22940]|SSPAA3760-13|Sciaridae|658|BOLD:ACG2846
Sciaridae|[22941]|SSPAA3933-13|Sciaridae|632|BOLD:ACI5380
Sciaridae|[22942]|SSKJC2825-15|Sciaridae|591|BOLD:ACT7401
Sciaridae|[22943]|CNBPL023-13|Sciaridae|670|BOLD:AAU6615
Sciaridae|[22944]|CNFDD195-14|Sciaridae|638|BOLD:ACL7814
Sciaridae|[22945]|CNTNF311-14|Sciaridae|588|BOLD:ACN6096
Sciaridae|[22946]|BBDED625-10|Sciaridae|658|BOLD:AAH3986
Sciaridae|[22947]|CNJAF861-12|Sciaridae|658|BOLD:ACC8466
Sciaridae|[22948]|SMTPD5826-13|Sciaridae|543|BOLD:ACM2497
Sciaridae|[22949]|CNFNQ1722-14|Sciaridae|658|BOLD:AAV1362
Sciaridae|[22950]|SSKJB424-14|Sciaridae|586|BOLD:ACN5681
Sciaridae|[22951]|CNBFJ258-15|Sciaridae|576|BOLD:ACT7827
Sciaridae|[22952]|JWDCF773-10|Sciaridae|658|BOLD:AAP9901
Sciaridae|[22953]|NCCA067-11|Sciaridae|673|BOLD:AAN6438
Sciaridae|[22954]|CNRMA172-12|Sciaridae|661|BOLD:ABV1296
Sciaridae|[22955]|CNMIL216-14|Sciaridae|588|BOLD:ACN2229
Scatopsciara tricuspidata|[22956]|CNBRN643-14|Sciaridae|658|BOLD:ACL9544
Sciaridae|[22957]|CNWLA370-12|Sciaridae|661|BOLD:ACC3903
Sciaridae|[22958]|CNPAB067-13|Sciaridae|670|BOLD:ACC1341
Sciaridae|[22959]|CNGBE527-14|Sciaridae|661|BOLD:ABY0363
Sciaridae|[22960]|SMTPJ799-14|Sciaridae|611|BOLD:ACN2129
Dichopygina aculeata|[22961]|BBDCM599-10|Sciaridae|658|BOLD:AAH4002
Dichopygina aculeata|[22962]|CNJAC203-12|Sciaridae|638|BOLD:ACR6975
Sciaridae|[22963]|SSWLC2109-13|Sciaridae|664|BOLD:AAV1327
Sciaridae|[22964]|CNPAL994-13|Sciaridae|588|BOLD:ACT4570
Sciaridae|[22965]|CNFNP1629-14|Sciaridae|658|BOLD:ACL9867
Sciaridae|[22966]|CNJAJ422-12|Sciaridae|658|BOLD:ACD2567
Sciaridae|[22967]|CNPAN409-13|Sciaridae|658|BOLD:ACC7171
Sciaridae|[22968]|CNJAG1312-12|Sciaridae|664|BOLD:AAH4007
Sciaridae|[22969]|CNGMB172-13|Sciaridae|658|BOLD:ACK7781
Sciaridae|[22970]|PAAPR1057-12|Sciaridae|673|BOLD:ABA1241
Sciaridae|[22971]|CNSLL204-13|Sciaridae|661|BOLD:ACA4748
Dichopygina ramosa|[22972]|CNIVD424-14|Sciaridae|573|BOLD:ACD9510
Sciaridae|[22973]|CNEIC2206-12|Sciaridae|658|BOLD:ACJ4304
Sciaridae|[22974]|CNEIF169-12|Sciaridae|673|BOLD:ACA9566
Sciaridae|[22975]|SSPAB4831-13|Sciaridae|658|BOLD:ACJ4574
Sciaridae|[22976]|CNBRQ257-14|Sciaridae|661|BOLD:ACA4507
Sciaridae|[22977]|CNWAD2010-14|Sciaridae|570|BOLD:ACS1671
Sciaridae|[22978]|CNBAB328-12|Sciaridae|658|BOLD:ACC9265
Sciaridae|[22979]|CNGLA209-13|Sciaridae|658|BOLD:ACB8781
Sciaridae|[22980]|CNJAC334-12|Sciaridae|658|BOLD:ACB2332
Sciaridae|[22981]|SSWLD455-13|Sciaridae|670|BOLD:ACG4549
Sciaridae|[22982]|SSBAE076-13|Sciaridae|661|BOLD:ACC7708
Sciaridae|[22983]|CNJAI515-12|Sciaridae|658|BOLD:ACD0433
Sciaridae|[22984]|NGNAJ039-13|Sciaridae|661|BOLD:ACU9457
Sciaridae|[22985]|SSEIC609-13|Sciaridae|670|BOLD:ABX9205
Sciaridae|[22986]|CNPKF1335-14|Sciaridae|661|BOLD:ACL5836
Sciaridae|[22987]|CNPAE102-13|Sciaridae|729|BOLD:ACE1022
Sciaridae|[22988]|CNPAE155-13|Sciaridae|681|BOLD:ACB7433
Sciaridae|[22989]|CHDIP490-12|Sciaridae|658|BOLD:ABW9770
Sciaridae|[22990]|CNPAO391-13|Sciaridae|670|BOLD:AAP2699
Sciaridae|[22991]|CNGRF389-12|Sciaridae|673|BOLD:ACB0883
Sciaridae|[22992]|NGNAG726-13|Sciaridae|658|BOLD:ACL5312
Sciaridae|[22993]|CNEIH173-13|Sciaridae|670|BOLD:ACD2481
Sciaridae|[22994]|CHDIP1408-12|Sciaridae|658|BOLD:AAH4020
Sciaridae|[22995]|CNJAF1007-12|Sciaridae|658|BOLD:ACC7493
Sciaridae|[22996]|CNNHF484-14|Sciaridae|522|BOLD:ACR8353

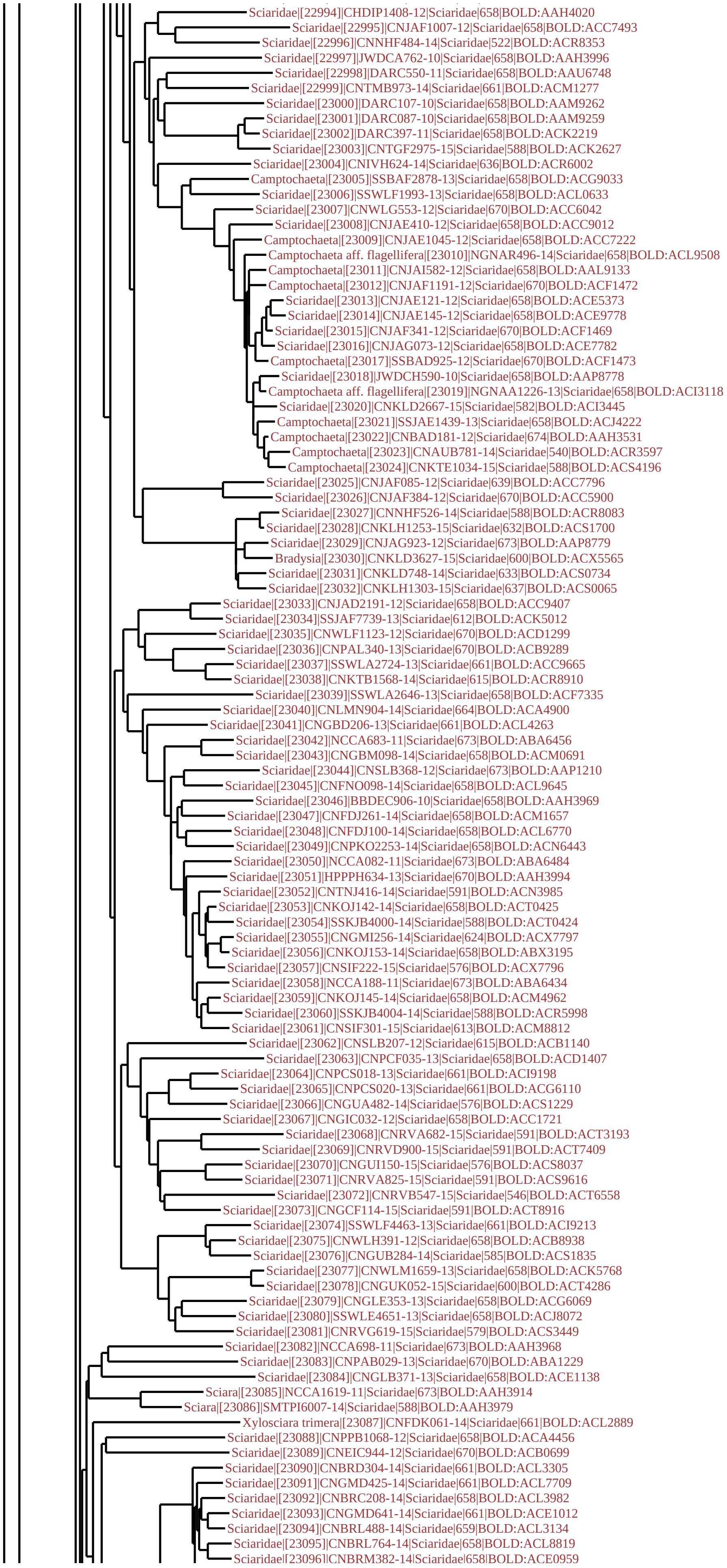

Sciaridae|[22994]|CHDIP1408-12|Sciaridae|658|BOLD:AAH4020
Sciaridae|[22995]|CNJAF1007-12|Sciaridae|658|BOLD:ACC7493
Sciaridae|[22996]|CNNHF484-14|Sciaridae|522|BOLD:ACR8353
Sciaridae|[22997]|JWDCA762-10|Sciaridae|658|BOLD:AAH3996
Sciaridae|[22998]|DARC550-11|Sciaridae|658|BOLD:AAU6748
Sciaridae|[22999]|CNTMB973-14|Sciaridae|661|BOLD:ACM1277
Sciaridae|[23000]|DARC107-10|Sciaridae|658|BOLD:AAM9262
Sciaridae|[23001]|DARC087-10|Sciaridae|658|BOLD:AAM9259
Sciaridae|[23002]|DARC397-11|Sciaridae|658|BOLD:ACK2219
Sciaridae|[23003]|CNTGF2975-15|Sciaridae|588|BOLD:ACK2627
Sciaridae|[23004]|CNIVH624-14|Sciaridae|636|BOLD:ACR6002
Camptochaeta|[23005]|SSBAF2878-13|Sciaridae|658|BOLD:ACG9033
Sciaridae|[23006]|SSWLF1993-13|Sciaridae|658|BOLD:ACL0633
Sciaridae|[23007]|CNWLG553-12|Sciaridae|670|BOLD:ACC6042
Sciaridae|[23008]|CNJAE410-12|Sciaridae|658|BOLD:ACC9012
Camptochaeta|[23009]|CNJAE1045-12|Sciaridae|658|BOLD:ACC7222
Camptochaeta aff. flagellifera|[23010]|NGNAR496-14|Sciaridae|658|BOLD:ACL9508
Camptochaeta|[23011]|CNJAI582-12|Sciaridae|658|BOLD:AAL9133
Camptochaeta|[23012]|CNJAF1191-12|Sciaridae|670|BOLD:ACF1472
Sciaridae|[23013]|CNJAE121-12|Sciaridae|658|BOLD:ACE5373
Sciaridae|[23014]|CNJAE145-12|Sciaridae|658|BOLD:ACE9778
Sciaridae|[23015]|CNJAF341-12|Sciaridae|670|BOLD:ACF1469
Sciaridae|[23016]|CNJAG073-12|Sciaridae|658|BOLD:ACE7782
Camptochaeta|[23017]|SSBAD925-12|Sciaridae|670|BOLD:ACF1473
Sciaridae|[23018]|JWDCH590-10|Sciaridae|658|BOLD:AAP8778
Camptochaeta aff. flagellifera|[23019]|NGNAA1226-13|Sciaridae|658|BOLD:ACI3118
Sciaridae|[23020]|CNKLD2667-15|Sciaridae|582|BOLD:ACI3445
Camptochaeta|[23021]|SSJAE1439-13|Sciaridae|658|BOLD:ACJ4222
Camptochaeta|[23022]|CNBAD181-12|Sciaridae|674|BOLD:AAH3531
Camptochaeta|[23023]|CNAUB781-14|Sciaridae|540|BOLD:ACR3597
Camptochaeta|[23024]|CNKTE1034-15|Sciaridae|588|BOLD:ACS4196
Sciaridae|[23025]|CNJAF085-12|Sciaridae|639|BOLD:ACC7796
Sciaridae|[23026]|CNJAF384-12|Sciaridae|670|BOLD:ACC5900
Sciaridae|[23027]|CNNHF526-14|Sciaridae|588|BOLD:ACR8083
Sciaridae|[23028]|CNKLH1253-15|Sciaridae|632|BOLD:ACS1700
Sciaridae|[23029]|CNJAG923-12|Sciaridae|673|BOLD:AAP8779
Bradysia|[23030]|CNKLD3627-15|Sciaridae|600|BOLD:ACX5565
Sciaridae|[23031]|CNKLD748-14|Sciaridae|633|BOLD:ACS0734
Sciaridae|[23032]|CNKLH1303-15|Sciaridae|637|BOLD:ACS0065
Sciaridae|[23033]|CNJAD2191-12|Sciaridae|658|BOLD:ACC9407
Sciaridae|[23034]|SSJAF7739-13|Sciaridae|612|BOLD:ACK5012
Sciaridae|[23035]|CNWLF1123-12|Sciaridae|670|BOLD:ACD1299
Sciaridae|[23036]|CNPAL340-13|Sciaridae|670|BOLD:ACB9289
Sciaridae|[23037]|SSWLA2724-13|Sciaridae|661|BOLD:ACC9665
Sciaridae|[23038]|CNKTB1568-14|Sciaridae|615|BOLD:ACR8910
Sciaridae|[23039]|SSWLA2646-13|Sciaridae|658|BOLD:ACF7335
Sciaridae|[23040]|CNLMN904-14|Sciaridae|664|BOLD:ACA4900
Sciaridae|[23041]|CNGBD206-13|Sciaridae|661|BOLD:ACL4263
Sciaridae|[23042]|NCCA683-11|Sciaridae|673|BOLD:ABA6456
Sciaridae|[23043]|CNGBM098-14|Sciaridae|658|BOLD:ACM0691
Sciaridae|[23044]|CNSLB368-12|Sciaridae|673|BOLD:AAP1210
Sciaridae|[23045]|CNFNO098-14|Sciaridae|658|BOLD:ACL9645
Sciaridae|[23046]|BBDEC906-10|Sciaridae|658|BOLD:AAH3969
Sciaridae|[23047]|CNFDJ261-14|Sciaridae|658|BOLD:ACM1657
Sciaridae|[23048]|CNFDJ100-14|Sciaridae|658|BOLD:ACL6770
Sciaridae|[23049]|CNPKO2253-14|Sciaridae|658|BOLD:ACN6443
Sciaridae|[23050]|NCCA082-11|Sciaridae|673|BOLD:ABA6484
Sciaridae|[23051]|HPPPH634-13|Sciaridae|670|BOLD:AAH3994
Sciaridae|[23052]|CNTNJ416-14|Sciaridae|591|BOLD:ACN3985
Sciaridae|[23053]|CNKOJ142-14|Sciaridae|658|BOLD:ACT0425
Sciaridae|[23054]|SSKJB4000-14|Sciaridae|588|BOLD:ACT0424
Sciaridae|[23055]|CNGMI256-14|Sciaridae|624|BOLD:ACX7797
Sciaridae|[23056]|CNKOJ153-14|Sciaridae|658|BOLD:ABX3195
Sciaridae|[23057]|CNSIF222-15|Sciaridae|576|BOLD:ACX7796
Sciaridae|[23058]|NCCA188-11|Sciaridae|673|BOLD:ABA6434
Sciaridae|[23059]|CNKOJ145-14|Sciaridae|658|BOLD:ACM4962
Sciaridae|[23060]|SSKJB4004-14|Sciaridae|588|BOLD:ACR5998
Sciaridae|[23061]|CNSIF301-15|Sciaridae|613|BOLD:ACM8812
Sciaridae|[23062]|CNSLB207-12|Sciaridae|615|BOLD:ACB1140
Sciaridae|[23063]|CNPCF035-13|Sciaridae|658|BOLD:ACD1407
Sciaridae|[23064]|CNPCS018-13|Sciaridae|661|BOLD:ACI9198
Sciaridae|[23065]|CNPCS020-13|Sciaridae|661|BOLD:ACG6110
Sciaridae|[23066]|CNGUA482-14|Sciaridae|576|BOLD:ACS1229
Sciaridae|[23067]|CNGIC032-12|Sciaridae|658|BOLD:ACC1721
Sciaridae|[23068]|CNRVA682-15|Sciaridae|591|BOLD:ACT3193
Sciaridae|[23069]|CNRVD900-15|Sciaridae|591|BOLD:ACT7409
Sciaridae|[23070]|CNGUI150-15|Sciaridae|576|BOLD:ACS8037
Sciaridae|[23071]|CNRVA825-15|Sciaridae|591|BOLD:ACS9616
Sciaridae|[23072]|CNRVB547-15|Sciaridae|546|BOLD:ACT6558
Sciaridae|[23073]|CNGCF114-15|Sciaridae|591|BOLD:ACT8916
Sciaridae|[23074]|SSWLF4463-13|Sciaridae|661|BOLD:ACI9213
Sciaridae|[23075]|CNWLH391-12|Sciaridae|658|BOLD:ACB8938
Sciaridae|[23076]|CNGUB284-14|Sciaridae|585|BOLD:ACS1835
Sciaridae|[23077]|CNWLM1659-13|Sciaridae|658|BOLD:ACK5768
Sciaridae|[23078]|CNGUK052-15|Sciaridae|600|BOLD:ACT4286
Sciaridae|[23079]|CNGLE353-13|Sciaridae|658|BOLD:ACG6069
Sciaridae|[23080]|SSWLE4651-13|Sciaridae|658|BOLD:ACJ8072
Sciaridae|[23081]|CNRVG619-15|Sciaridae|579|BOLD:ACS3449
Sciaridae|[23082]|NCCA698-11|Sciaridae|673|BOLD:AAH3968
Sciaridae|[23083]|CNPAB029-13|Sciaridae|670|BOLD:ABA1229
Sciaridae|[23084]|CNGLB371-13|Sciaridae|658|BOLD:ACE1138
Sciara|[23085]|NCCA1619-11|Sciaridae|673|BOLD:AAH3914
Sciara|[23086]|SMTPI6007-14|Sciaridae|588|BOLD:AAH3979
Xylosciara trimera|[23087]|CNFDK061-14|Sciaridae|661|BOLD:ACL2889
Sciaridae|[23088]|CNPPB1068-12|Sciaridae|658|BOLD:ACA4456
Sciaridae|[23089]|CNEIC944-12|Sciaridae|670|BOLD:ACB0699
Sciaridae|[23090]|CNBRD304-14|Sciaridae|661|BOLD:ACL3305
Sciaridae|[23091]|CNGMD425-14|Sciaridae|661|BOLD:ACL7709
Sciaridae|[23092]|CNBRC208-14|Sciaridae|658|BOLD:ACL3982
Sciaridae|[23093]|CNGMD641-14|Sciaridae|661|BOLD:ACE1012
Sciaridae|[23094]|CNBRL488-14|Sciaridae|659|BOLD:ACL3134
Sciaridae|[23095]|CNBRL764-14|Sciaridae|658|BOLD:ACL8819
Sciaridae|[23096]|CNBRM382-14|Sciaridae|658|BOLD:ACE0959

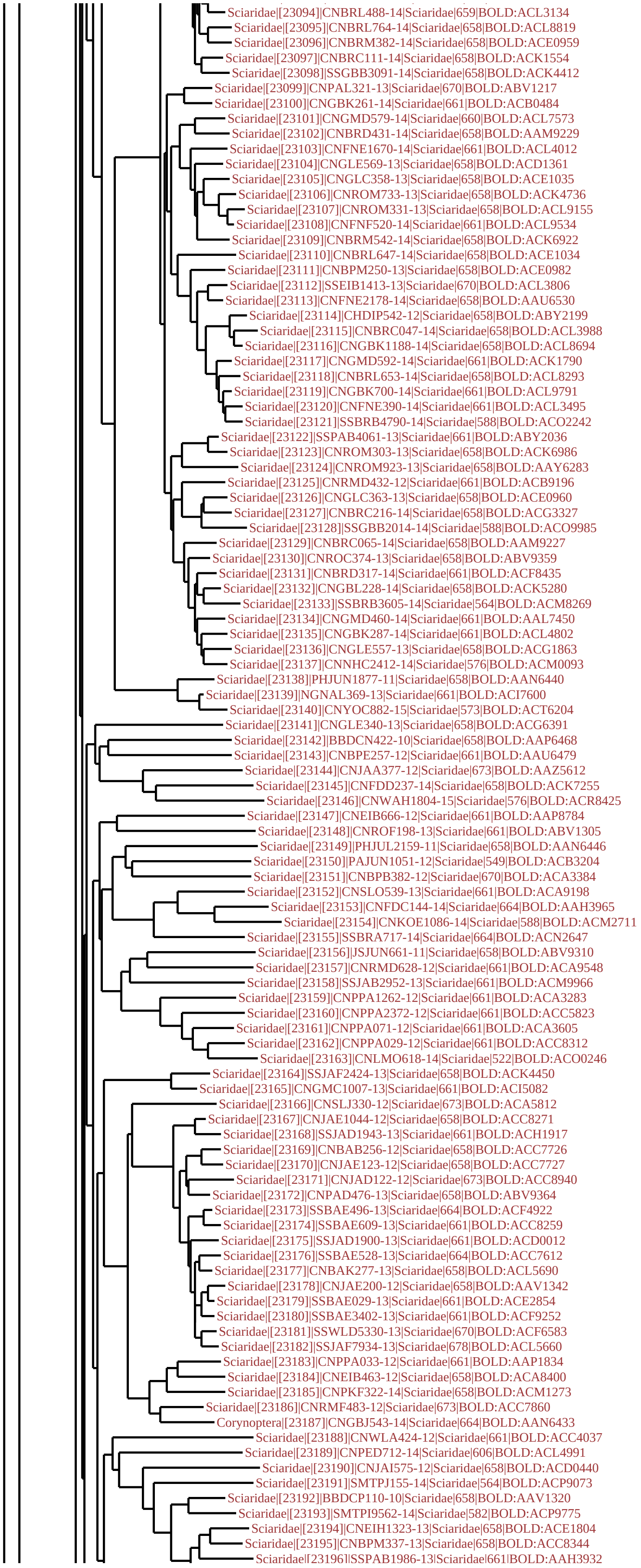

Sciaridae|[23094]|CNBRL488-14|Sciaridae|659|BOLD:ACL3134
Sciaridae|[23095]|CNBRL764-14|Sciaridae|658|BOLD:ACL8819
Sciaridae|[23096]|CNBRM382-14|Sciaridae|658|BOLD:ACE0959
Sciaridae|[23097]|CNBRC111-14|Sciaridae|658|BOLD:ACK1554
Sciaridae|[23098]|SSGBB3091-14|Sciaridae|658|BOLD:ACK4412
Sciaridae|[23099]|CNPAL321-13|Sciaridae|670|BOLD:ABV1217
Sciaridae|[23100]|CNGBK261-14|Sciaridae|661|BOLD:ACB0484
Sciaridae|[23101]|CNGMD579-14|Sciaridae|660|BOLD:ACL7573
Sciaridae|[23102]|CNBRD431-14|Sciaridae|658|BOLD:AAM9229
Sciaridae|[23103]|CNFNE1670-14|Sciaridae|661|BOLD:ACL4012
Sciaridae|[23104]|CNGLE569-13|Sciaridae|658|BOLD:ACD1361
Sciaridae|[23105]|CNGLC358-13|Sciaridae|658|BOLD:ACE1035
Sciaridae|[23106]|CNROM733-13|Sciaridae|658|BOLD:ACK4736
Sciaridae|[23107]|CNROM331-13|Sciaridae|658|BOLD:ACL9155
Sciaridae|[23108]|CNFNF520-14|Sciaridae|661|BOLD:ACL9534
Sciaridae|[23109]|CNBRM542-14|Sciaridae|658|BOLD:ACK6922
Sciaridae|[23110]|CNBRL647-14|Sciaridae|658|BOLD:ACE1034
Sciaridae|[23111]|CNBPM250-13|Sciaridae|658|BOLD:ACE0982
Sciaridae|[23112]|SSEIB1413-13|Sciaridae|670|BOLD:ACL3806
Sciaridae|[23113]|CNFNE2178-14|Sciaridae|658|BOLD:AAU6530
Sciaridae|[23114]|CHDIP542-12|Sciaridae|658|BOLD:ABY2199
Sciaridae|[23115]|CNBRC047-14|Sciaridae|658|BOLD:ACL3988
Sciaridae|[23116]|CNGBK1188-14|Sciaridae|658|BOLD:ACL8694
Sciaridae|[23117]|CNGMD592-14|Sciaridae|661|BOLD:ACK1790
Sciaridae|[23118]|CNBRL653-14|Sciaridae|658|BOLD:ACL8293
Sciaridae|[23119]|CNGBK700-14|Sciaridae|661|BOLD:ACL9791
Sciaridae|[23120]|CNFNE390-14|Sciaridae|661|BOLD:ACL3495
Sciaridae|[23121]|SSBRB4790-14|Sciaridae|588|BOLD:ACO2242
Sciaridae|[23122]|SSPAB4061-13|Sciaridae|661|BOLD:ABY2036
Sciaridae|[23123]|CNROM303-13|Sciaridae|658|BOLD:ACK6986
Sciaridae|[23124]|CNROM923-13|Sciaridae|658|BOLD:AAY6283
Sciaridae|[23125]|CNRMD432-12|Sciaridae|661|BOLD:ACB9196
Sciaridae|[23126]|CNGLC363-13|Sciaridae|658|BOLD:ACE0960
Sciaridae|[23127]|CNBRC216-14|Sciaridae|658|BOLD:ACG3327
Sciaridae|[23128]|SSGBB2014-14|Sciaridae|588|BOLD:ACO9985
Sciaridae|[23129]|CNBRC065-14|Sciaridae|658|BOLD:AAM9227
Sciaridae|[23130]|CNROC374-13|Sciaridae|658|BOLD:ABV9359
Sciaridae|[23131]|CNBRD317-14|Sciaridae|661|BOLD:ACF8435
Sciaridae|[23132]|CNGBL228-14|Sciaridae|658|BOLD:ACK5280
Sciaridae|[23133]|SSBRB3605-14|Sciaridae|564|BOLD:ACM8269
Sciaridae|[23134]|CNGMD460-14|Sciaridae|661|BOLD:AAL7450
Sciaridae|[23135]|CNGBK287-14|Sciaridae|661|BOLD:ACL4802
Sciaridae|[23136]|CNGLE557-13|Sciaridae|658|BOLD:ACG1863
Sciaridae|[23137]|CNNHC2412-14|Sciaridae|576|BOLD:ACM0093
Sciaridae|[23138]|PHJUN1877-11|Sciaridae|658|BOLD:AAN6440
Sciaridae|[23139]|NGNAL369-13|Sciaridae|661|BOLD:ACI7600
Sciaridae|[23140]|CNYOC882-15|Sciaridae|573|BOLD:ACT6204
Sciaridae|[23141]|CNGLE340-13|Sciaridae|658|BOLD:ACG6391
Sciaridae|[23142]|BBDCN422-10|Sciaridae|658|BOLD:AAP6468
Sciaridae|[23143]|CNBPE257-12|Sciaridae|661|BOLD:AAU6479
Sciaridae|[23144]|CNJAA377-12|Sciaridae|673|BOLD:AAZ5612
Sciaridae|[23145]|CNFDD237-14|Sciaridae|658|BOLD:ACK7255
Sciaridae|[23146]|CNWAH1804-15|Sciaridae|576|BOLD:ACR8425
Sciaridae|[23147]|CNEIB666-12|Sciaridae|661|BOLD:AAP8784
Sciaridae|[23148]|CNROF198-13|Sciaridae|661|BOLD:ABV1305
Sciaridae|[23149]|PHJUL2159-11|Sciaridae|658|BOLD:AAN6446
Sciaridae|[23150]|PAJUN1051-12|Sciaridae|549|BOLD:ACB3204
Sciaridae|[23151]|CNBPB382-12|Sciaridae|670|BOLD:ACA3384
Sciaridae|[23152]|CNSLO539-13|Sciaridae|661|BOLD:ACA9198
Sciaridae|[23153]|CNFDC144-14|Sciaridae|664|BOLD:AAH3965
Sciaridae|[23154]|CNKOE1086-14|Sciaridae|588|BOLD:ACM2711
Sciaridae|[23155]|SSBRA717-14|Sciaridae|664|BOLD:ACN2647
Sciaridae|[23156]|JSJUN661-11|Sciaridae|658|BOLD:ABV9310
Sciaridae|[23157]|CNRMD628-12|Sciaridae|661|BOLD:ACA9548
Sciaridae|[23158]|SSJAB2952-13|Sciaridae|661|BOLD:ACM9966
Sciaridae|[23159]|CNPPA1262-12|Sciaridae|661|BOLD:ACA3283
Sciaridae|[23160]|CNPPA2372-12|Sciaridae|661|BOLD:ACC5823
Sciaridae|[23161]|CNPPA071-12|Sciaridae|661|BOLD:ACA3605
Sciaridae|[23162]|CNPPA029-12|Sciaridae|661|BOLD:ACC8312
Sciaridae|[23163]|CNLMO618-14|Sciaridae|522|BOLD:ACO0246
Sciaridae|[23164]|SSJAF2424-13|Sciaridae|658|BOLD:ACK4450
Sciaridae|[23165]|CNGMC1007-13|Sciaridae|661|BOLD:ACI5082
Sciaridae|[23166]|CNSLJ330-12|Sciaridae|673|BOLD:ACA5812
Sciaridae|[23167]|CNJAE1044-12|Sciaridae|658|BOLD:ACC8271
Sciaridae|[23168]|SSJAD1943-13|Sciaridae|661|BOLD:ACH1917
Sciaridae|[23169]|CNBAB256-12|Sciaridae|658|BOLD:ACC7726
Sciaridae|[23170]|CNJAE123-12|Sciaridae|658|BOLD:ACC7727
Sciaridae|[23171]|CNJAD122-12|Sciaridae|673|BOLD:ACC8940
Sciaridae|[23172]|CNPAD476-13|Sciaridae|658|BOLD:ABV9364
Sciaridae|[23173]|SSBAE496-13|Sciaridae|664|BOLD:ACF4922
Sciaridae|[23174]|SSBAE609-13|Sciaridae|661|BOLD:ACC8259
Sciaridae|[23175]|SSJAD1900-13|Sciaridae|661|BOLD:ACD0012
Sciaridae|[23176]|SSBAE528-13|Sciaridae|664|BOLD:ACC7612
Sciaridae|[23177]|CNBAK277-13|Sciaridae|658|BOLD:ACL5690
Sciaridae|[23178]|CNJAE200-12|Sciaridae|658|BOLD:AAV1342
Sciaridae|[23179]|SSBAE029-13|Sciaridae|661|BOLD:ACE2854
Sciaridae|[23180]|SSBAE3402-13|Sciaridae|661|BOLD:ACF9252
Sciaridae|[23181]|SSWLD5330-13|Sciaridae|670|BOLD:ACF6583
Sciaridae|[23182]|SSJAF7934-13|Sciaridae|678|BOLD:ACL5660
Sciaridae|[23183]|CNPPA033-12|Sciaridae|661|BOLD:AAP1834
Sciaridae|[23184]|CNEIB463-12|Sciaridae|658|BOLD:ACA8400
Sciaridae|[23185]|CNPKF322-14|Sciaridae|658|BOLD:ACM1273
Sciaridae|[23186]|CNRMF483-12|Sciaridae|673|BOLD:ACC7860
Corynoptera|[23187]|CNGBJ543-14|Sciaridae|664|BOLD:AAN6433
Sciaridae|[23188]|CNWLA424-12|Sciaridae|661|BOLD:ACC4037
Sciaridae|[23189]|CNPED712-14|Sciaridae|606|BOLD:ACL4991
Sciaridae|[23190]|CNJAI575-12|Sciaridae|658|BOLD:ACD0440
Sciaridae|[23191]|SMTPJ155-14|Sciaridae|564|BOLD:ACP9073
Sciaridae|[23192]|BBDCP110-10|Sciaridae|658|BOLD:AAV1320
Sciaridae|[23193]|SMTPI9562-14|Sciaridae|582|BOLD:ACP9775
Sciaridae|[23194]|CNEIH1323-13|Sciaridae|658|BOLD:ACE1804
Sciaridae|[23195]|CNBPM337-13|Sciaridae|658|BOLD:ACC8344
Sciaridae|[23196]|SSPAB1986-13|Sciaridae|661|BOLD:AAH3932

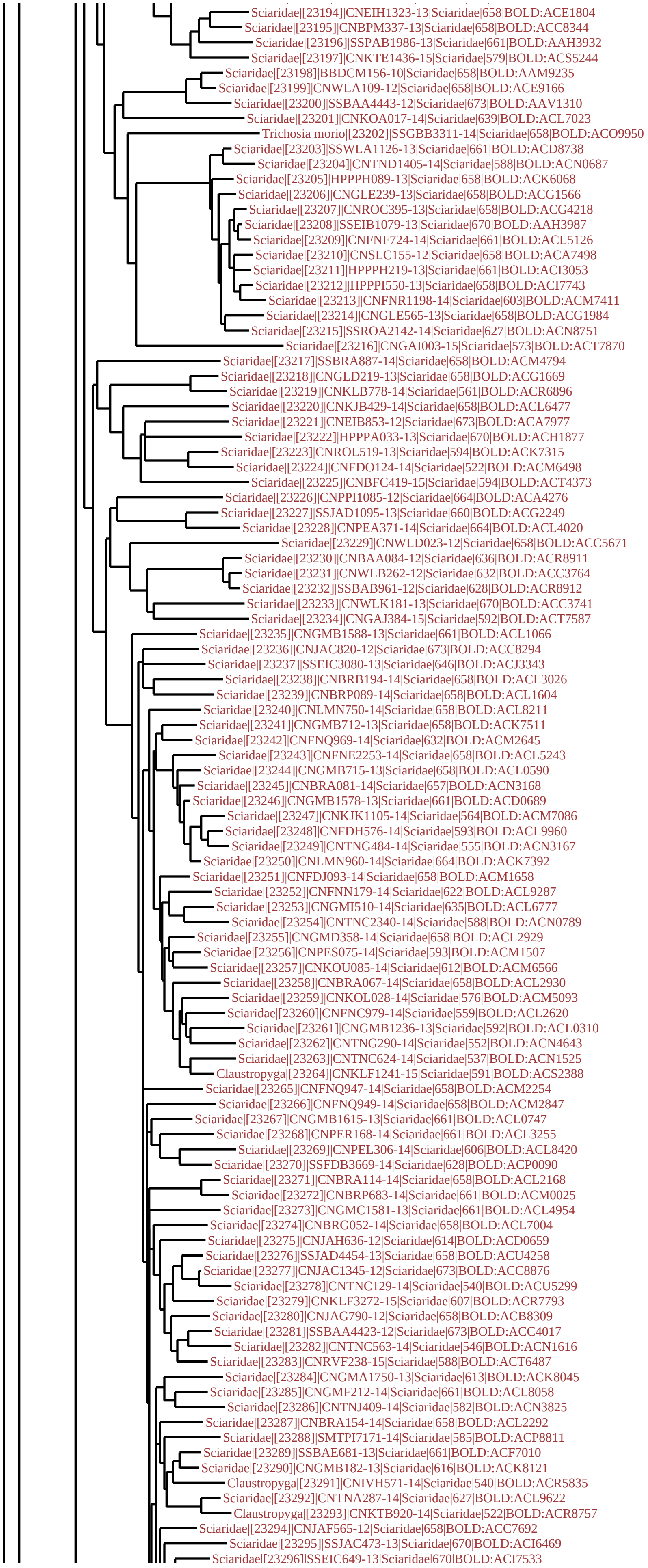
Sciaridae|[23194]|CNEIH1323-13|Sciaridae|658|BOLD:ACE1804
Sciaridae|[23195]|CNBPM337-13|Sciaridae|658|BOLD:ACC8344
Sciaridae|[23196]|SSPAB1986-13|Sciaridae|661|BOLD:AAH3932
Sciaridae|[23197]|CNKTE1436-15|Sciaridae|579|BOLD:ACS5244
Sciaridae|[23198]|BBDCM156-10|Sciaridae|658|BOLD:AAM9235
Sciaridae|[23199]|CNWLA109-12|Sciaridae|658|BOLD:ACE9166
Sciaridae|[23200]|SSBAA4443-12|Sciaridae|673|BOLD:AAV1310
Sciaridae|[23201]|CNKOA017-14|Sciaridae|639|BOLD:ACL7023
Trichosia morio|[23202]|SSGBB3311-14|Sciaridae|658|BOLD:ACO9950
Sciaridae|[23203]|SSWLA1126-13|Sciaridae|661|BOLD:ACD8738
Sciaridae|[23204]|CNTND1405-14|Sciaridae|588|BOLD:ACN0687
Sciaridae|[23205]|HPPPH089-13|Sciaridae|658|BOLD:ACK6068
Sciaridae|[23206]|CNGLE239-13|Sciaridae|658|BOLD:ACG1566
Sciaridae|[23207]|CNROC395-13|Sciaridae|658|BOLD:ACG4218
Sciaridae|[23208]|SSEIB1079-13|Sciaridae|670|BOLD:AAH3987
Sciaridae|[23209]|CNFNF724-14|Sciaridae|661|BOLD:ACL5126
Sciaridae|[23210]|CNSLC155-12|Sciaridae|658|BOLD:ACA7498
Sciaridae|[23211]|HPPPH219-13|Sciaridae|661|BOLD:ACI3053
Sciaridae|[23212]|HPPPI550-13|Sciaridae|658|BOLD:ACI7743
Sciaridae|[23213]|CNFNR1198-14|Sciaridae|603|BOLD:ACM7411
Sciaridae|[23214]|CNGLE565-13|Sciaridae|658|BOLD:ACG1984
Sciaridae|[23215]|SSROA2142-14|Sciaridae|627|BOLD:ACN8751
Sciaridae|[23216]|CNGAI003-15|Sciaridae|573|BOLD:ACT7870
Sciaridae|[23217]|SSBRA887-14|Sciaridae|658|BOLD:ACM4794
Sciaridae|[23218]|CNGLD219-13|Sciaridae|658|BOLD:ACG1669
Sciaridae|[23219]|CNKLB778-14|Sciaridae|561|BOLD:ACR6896
Sciaridae|[23220]|CNKJB429-14|Sciaridae|658|BOLD:ACL6477
Sciaridae|[23221]|CNEIB853-12|Sciaridae|673|BOLD:ACA7977
Sciaridae|[23222]|HPPPA033-13|Sciaridae|670|BOLD:ACH1877
Sciaridae|[23223]|CNROL519-13|Sciaridae|594|BOLD:ACK7315
Sciaridae|[23224]|CNFDO124-14|Sciaridae|522|BOLD:ACM6498
Sciaridae|[23225]|CNBFC419-15|Sciaridae|594|BOLD:ACT4373
Sciaridae|[23226]|CNPPI1085-12|Sciaridae|664|BOLD:ACA4276
Sciaridae|[23227]|SSJAD1095-13|Sciaridae|660|BOLD:ACG2249
Sciaridae|[23228]|CNPEA371-14|Sciaridae|664|BOLD:ACL4020
Sciaridae|[23229]|CNWLD023-12|Sciaridae|658|BOLD:ACC5671
Sciaridae|[23230]|CNBAA084-12|Sciaridae|636|BOLD:ACR8911
Sciaridae|[23231]|CNWLB262-12|Sciaridae|632|BOLD:ACC3764
Sciaridae|[23232]|SSBAB961-12|Sciaridae|628|BOLD:ACR8912
Sciaridae|[23233]|CNWLK181-13|Sciaridae|670|BOLD:ACC3741
Sciaridae|[23234]|CNGAJ384-15|Sciaridae|592|BOLD:ACT7587
Sciaridae|[23235]|CNGMB1588-13|Sciaridae|661|BOLD:ACL1066
Sciaridae|[23236]|CNJAC820-12|Sciaridae|673|BOLD:ACC8294
Sciaridae|[23237]|SSEIC3080-13|Sciaridae|646|BOLD:ACJ3343
Sciaridae|[23238]|CNBRB194-14|Sciaridae|658|BOLD:ACL3026
Sciaridae|[23239]|CNBRP089-14|Sciaridae|658|BOLD:ACL1604
Sciaridae|[23240]|CNLMN750-14|Sciaridae|658|BOLD:ACL8211
Sciaridae|[23241]|CNGMB712-13|Sciaridae|658|BOLD:ACK7511
Sciaridae|[23242]|CNFNQ969-14|Sciaridae|632|BOLD:ACM2645
Sciaridae|[23243]|CNFNE2253-14|Sciaridae|658|BOLD:ACL5243
Sciaridae|[23244]|CNGMB715-13|Sciaridae|658|BOLD:ACL0590
Sciaridae|[23245]|CNBRA081-14|Sciaridae|657|BOLD:ACN3168
Sciaridae|[23246]|CNGMB1578-13|Sciaridae|661|BOLD:ACD0689
Sciaridae|[23247]|CNKJK1105-14|Sciaridae|564|BOLD:ACM7086
Sciaridae|[23248]|CNFDH576-14|Sciaridae|593|BOLD:ACL9960
Sciaridae|[23249]|CNTNG484-14|Sciaridae|555|BOLD:ACN3167
Sciaridae|[23250]|CNLMN960-14|Sciaridae|664|BOLD:ACK7392
Sciaridae|[23251]|CNFDJ093-14|Sciaridae|658|BOLD:ACM1658
Sciaridae|[23252]|CNFNN179-14|Sciaridae|622|BOLD:ACL9287
Sciaridae|[23253]|CNGMI510-14|Sciaridae|635|BOLD:ACL6777
Sciaridae|[23254]|CNTNC2340-14|Sciaridae|588|BOLD:ACN0789
Sciaridae|[23255]|CNGMD358-14|Sciaridae|658|BOLD:ACL2929
Sciaridae|[23256]|CNPES075-14|Sciaridae|593|BOLD:ACM1507
Sciaridae|[23257]|CNKOU085-14|Sciaridae|612|BOLD:ACM6566
Sciaridae|[23258]|CNBRA067-14|Sciaridae|658|BOLD:ACL2930
Sciaridae|[23259]|CNKOL028-14|Sciaridae|576|BOLD:ACM5093
Sciaridae|[23260]|CNFNC979-14|Sciaridae|559|BOLD:ACL2620
Sciaridae|[23261]|CNGMB1236-13|Sciaridae|592|BOLD:ACL0310
Sciaridae|[23262]|CNTNG290-14|Sciaridae|552|BOLD:ACN4643
Sciaridae|[23263]|CNTNC624-14|Sciaridae|537|BOLD:ACN1525
Claustropyga|[23264]|CNKLF1241-15|Sciaridae|591|BOLD:ACS2388
Sciaridae|[23265]|CNFNQ947-14|Sciaridae|658|BOLD:ACM2254
Sciaridae|[23266]|CNFNQ949-14|Sciaridae|658|BOLD:ACM2847
Sciaridae|[23267]|CNGMB1615-13|Sciaridae|661|BOLD:ACL0747
Sciaridae|[23268]|CNPER168-14|Sciaridae|661|BOLD:ACL3255
Sciaridae|[23269]|CNPEL306-14|Sciaridae|606|BOLD:ACL8420
Sciaridae|[23270]|SSFDB3669-14|Sciaridae|628|BOLD:ACP0090
Sciaridae|[23271]|CNBRA114-14|Sciaridae|658|BOLD:ACL2168
Sciaridae|[23272]|CNBRP683-14|Sciaridae|661|BOLD:ACM0025
Sciaridae|[23273]|CNGMC1581-13|Sciaridae|661|BOLD:ACL4954
Sciaridae|[23274]|CNBRG052-14|Sciaridae|658|BOLD:ACL7004
Sciaridae|[23275]|CNJAH636-12|Sciaridae|614|BOLD:ACD0659
Sciaridae|[23276]|SSJAD4454-13|Sciaridae|658|BOLD:ACU4258
Sciaridae|[23277]|CNJAC1345-12|Sciaridae|673|BOLD:ACC8876
Sciaridae|[23278]|CNTNC129-14|Sciaridae|540|BOLD:ACU5299
Sciaridae|[23279]|CNKLF3272-15|Sciaridae|607|BOLD:ACR7793
Sciaridae|[23280]|CNJAG790-12|Sciaridae|658|BOLD:ACB8309
Sciaridae|[23281]|SSBAA4423-12|Sciaridae|673|BOLD:ACC4017
Sciaridae|[23282]|CNTNC563-14|Sciaridae|546|BOLD:ACN1616
Sciaridae|[23283]|CNRVF238-15|Sciaridae|588|BOLD:ACT6487
Sciaridae|[23284]|CNGMA1750-13|Sciaridae|613|BOLD:ACK8045
Sciaridae|[23285]|CNGMF212-14|Sciaridae|661|BOLD:ACL8058
Sciaridae|[23286]|CNTNJ409-14|Sciaridae|582|BOLD:ACN3825
Sciaridae|[23287]|CNBRA154-14|Sciaridae|658|BOLD:ACL2292
Sciaridae|[23288]|SMTPI7171-14|Sciaridae|585|BOLD:ACP8811
Sciaridae|[23289]|SSBAE681-13|Sciaridae|661|BOLD:ACF7010
Sciaridae|[23290]|CNGMB182-13|Sciaridae|616|BOLD:ACK8121
Claustropyga|[23291]|CNIVH571-14|Sciaridae|540|BOLD:ACR5835
Sciaridae|[23292]|CNTNA287-14|Sciaridae|627|BOLD:ACL9622
Claustropyga|[23293]|CNKTB920-14|Sciaridae|522|BOLD:ACR8757
Sciaridae|[23294]|CNJAF565-12|Sciaridae|658|BOLD:ACC7692
Sciaridae|[23295]|SSJAC473-13|Sciaridae|670|BOLD:ACI6469
Sciaridae|[23296]|SSEIC649-13|Sciaridae|670|BOLD:ACI7533

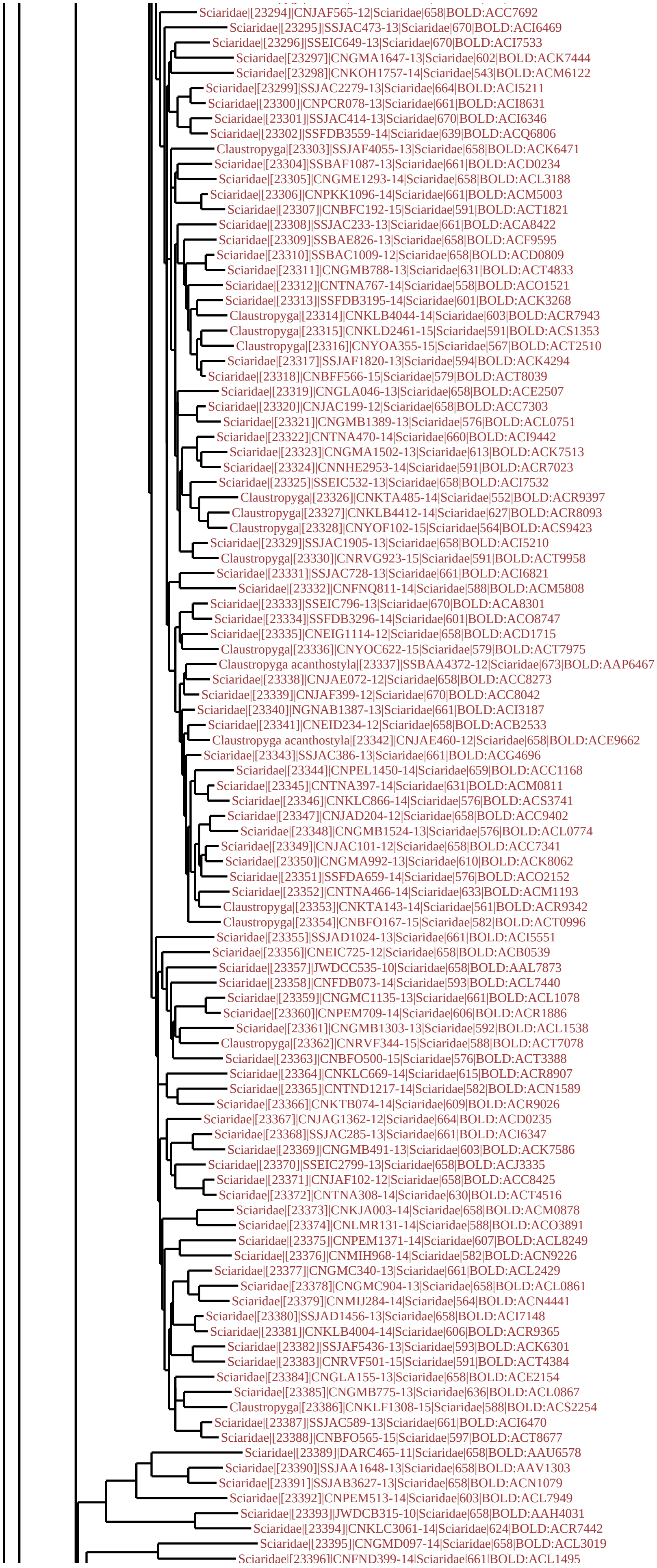
Sciaridae|[23294]|CNJAF565-12|Sciaridae|658|BOLD:ACC7692
Sciaridae|[23295]|SSJAC473-13|Sciaridae|670|BOLD:ACI6469
Sciaridae|[23296]|SSEIC649-13|Sciaridae|670|BOLD:ACI7533
Sciaridae|[23297]|CNGMA1647-13|Sciaridae|602|BOLD:ACK7444
Sciaridae|[23298]|CNKOH1757-14|Sciaridae|543|BOLD:ACM6122
Sciaridae|[23299]|SSJAC2279-13|Sciaridae|664|BOLD:ACI5211
Sciaridae|[23300]|CNPCR078-13|Sciaridae|661|BOLD:ACI8631
Sciaridae|[23301]|SSJAC414-13|Sciaridae|670|BOLD:ACI6346
Sciaridae|[23302]|SSFDB3559-14|Sciaridae|639|BOLD:ACQ6806
Claustropyga|[23303]|SSJAF4055-13|Sciaridae|658|BOLD:ACK6471
Sciaridae|[23304]|SSBAF1087-13|Sciaridae|661|BOLD:ACD0234
Sciaridae|[23305]|CNGME1293-14|Sciaridae|658|BOLD:ACL3188
Sciaridae|[23306]|CNPKK1096-14|Sciaridae|661|BOLD:ACM5003
Sciaridae|[23307]|CNBFC192-15|Sciaridae|591|BOLD:ACT1821
Sciaridae|[23308]|SSJAC233-13|Sciaridae|661|BOLD:ACA8422
Sciaridae|[23309]|SSBAE826-13|Sciaridae|658|BOLD:ACF9595
Sciaridae|[23310]|SSBAC1009-12|Sciaridae|658|BOLD:ACD0809
Sciaridae|[23311]|CNGMB788-13|Sciaridae|631|BOLD:ACT4833
Sciaridae|[23312]|CNTNA767-14|Sciaridae|558|BOLD:ACO1521
Sciaridae|[23313]|SSFDB3195-14|Sciaridae|601|BOLD:ACK3268
Claustropyga|[23314]|CNKLB4044-14|Sciaridae|603|BOLD:ACR7943
Claustropyga|[23315]|CNKLD2461-15|Sciaridae|591|BOLD:ACS1353
Claustropyga|[23316]|CNYOA355-15|Sciaridae|567|BOLD:ACT2510
Sciaridae|[23317]|SSJAF1820-13|Sciaridae|594|BOLD:ACK4294
Sciaridae|[23318]|CNBFF566-15|Sciaridae|579|BOLD:ACT8039
Sciaridae|[23319]|CNGLA046-13|Sciaridae|658|BOLD:ACE2507
Sciaridae|[23320]|CNJAC199-12|Sciaridae|658|BOLD:ACC7303
Sciaridae|[23321]|CNGMB1389-13|Sciaridae|576|BOLD:ACL0751
Sciaridae|[23322]|CNTNA470-14|Sciaridae|660|BOLD:ACI9442
Sciaridae|[23323]|CNGMA1502-13|Sciaridae|613|BOLD:ACK7513
Sciaridae|[23324]|CNNHE2953-14|Sciaridae|591|BOLD:ACR7023
Sciaridae|[23325]|SSEIC532-13|Sciaridae|658|BOLD:ACI7532
Claustropyga|[23326]|CNKTA485-14|Sciaridae|552|BOLD:ACR9397
Claustropyga|[23327]|CNKLB4412-14|Sciaridae|627|BOLD:ACR8093
Claustropyga|[23328]|CNYOF102-15|Sciaridae|564|BOLD:ACS9423
Sciaridae|[23329]|SSJAC1905-13|Sciaridae|658|BOLD:ACI5210
Claustropyga|[23330]|CNRVG923-15|Sciaridae|591|BOLD:ACT9958
Sciaridae|[23331]|SSJAC728-13|Sciaridae|661|BOLD:ACI6821
Sciaridae|[23332]|CNFNQ811-14|Sciaridae|588|BOLD:ACM5808
Sciaridae|[23333]|SSEIC796-13|Sciaridae|670|BOLD:ACA8301
Sciaridae|[23334]|SSFDB3296-14|Sciaridae|601|BOLD:ACO8747
Sciaridae|[23335]|CNEIG1114-12|Sciaridae|658|BOLD:ACD1715
Claustropyga|[23336]|CNYOC622-15|Sciaridae|579|BOLD:ACT7975
Claustropyga acanthostyla|[23337]|SSBAA4372-12|Sciaridae|673|BOLD:AAP6467
Sciaridae|[23338]|CNJAE072-12|Sciaridae|658|BOLD:ACC8273
Sciaridae|[23339]|CNJAF399-12|Sciaridae|670|BOLD:ACC8042
Sciaridae|[23340]|NGNAB1387-13|Sciaridae|661|BOLD:ACI3187
Sciaridae|[23341]|CNEID234-12|Sciaridae|658|BOLD:ACB2533
Claustropyga acanthostyla|[23342]|CNJAE460-12|Sciaridae|658|BOLD:ACE9662
Sciaridae|[23343]|SSJAC386-13|Sciaridae|661|BOLD:ACG4696
Sciaridae|[23344]|CNPEL1450-14|Sciaridae|659|BOLD:ACC1168
Sciaridae|[23345]|CNTNA397-14|Sciaridae|631|BOLD:ACM0811
Sciaridae|[23346]|CNKLC866-14|Sciaridae|576|BOLD:ACS3741
Sciaridae|[23347]|CNJAD204-12|Sciaridae|658|BOLD:ACC9402
Sciaridae|[23348]|CNGMB1524-13|Sciaridae|576|BOLD:ACL0774
Sciaridae|[23349]|CNJAC101-12|Sciaridae|658|BOLD:ACC7341
Sciaridae|[23350]|CNGMA992-13|Sciaridae|610|BOLD:ACK8062
Sciaridae|[23351]|SSFDA659-14|Sciaridae|576|BOLD:ACO2152
Sciaridae|[23352]|CNTNA466-14|Sciaridae|633|BOLD:ACM1193
Claustropyga|[23353]|CNKTA143-14|Sciaridae|561|BOLD:ACR9342
Claustropyga|[23354]|CNBFO167-15|Sciaridae|582|BOLD:ACT0996
Sciaridae|[23355]|SSJAD1024-13|Sciaridae|661|BOLD:ACI5551
Sciaridae|[23356]|CNEIC725-12|Sciaridae|658|BOLD:ACB0539
Sciaridae|[23357]|JWDCC535-10|Sciaridae|658|BOLD:AAL7873
Sciaridae|[23358]|CNFDB073-14|Sciaridae|593|BOLD:ACL7440
Sciaridae|[23359]|CNGMC1135-13|Sciaridae|661|BOLD:ACL1078
Sciaridae|[23360]|CNPEM709-14|Sciaridae|606|BOLD:ACR1886
Sciaridae|[23361]|CNGMB1303-13|Sciaridae|592|BOLD:ACL1538
Claustropyga|[23362]|CNRVF344-15|Sciaridae|588|BOLD:ACT7078
Sciaridae|[23363]|CNBFO500-15|Sciaridae|576|BOLD:ACT3388
Sciaridae|[23364]|CNKLC669-14|Sciaridae|615|BOLD:ACR8907
Sciaridae|[23365]|CNTND1217-14|Sciaridae|582|BOLD:ACN1589
Sciaridae|[23366]|CNKTB074-14|Sciaridae|609|BOLD:ACR9026
Sciaridae|[23367]|CNJAG1362-12|Sciaridae|664|BOLD:ACD0235
Sciaridae|[23368]|SSJAC285-13|Sciaridae|661|BOLD:ACI6347
Sciaridae|[23369]|CNGMB491-13|Sciaridae|603|BOLD:ACK7586
Sciaridae|[23370]|SSEIC2799-13|Sciaridae|658|BOLD:ACJ3335
Sciaridae|[23371]|CNJAF102-12|Sciaridae|658|BOLD:ACC8425
Sciaridae|[23372]|CNTNA308-14|Sciaridae|630|BOLD:ACT4516
Sciaridae|[23373]|CNKJA003-14|Sciaridae|658|BOLD:ACM0878
Sciaridae|[23374]|CNLMR131-14|Sciaridae|588|BOLD:ACO3891
Sciaridae|[23375]|CNPEM1371-14|Sciaridae|607|BOLD:ACL8249
Sciaridae|[23376]|CNMIH968-14|Sciaridae|582|BOLD:ACN9226
Sciaridae|[23377]|CNGMC340-13|Sciaridae|661|BOLD:ACL2429
Sciaridae|[23378]|CNGMC904-13|Sciaridae|658|BOLD:ACL0861
Sciaridae|[23379]|CNMIJ284-14|Sciaridae|564|BOLD:ACN4441
Sciaridae|[23380]|SSJAD1456-13|Sciaridae|658|BOLD:ACI7148
Sciaridae|[23381]|CNKLB4004-14|Sciaridae|606|BOLD:ACR9365
Sciaridae|[23382]|SSJAF5436-13|Sciaridae|593|BOLD:ACK6301
Sciaridae|[23383]|CNRVF501-15|Sciaridae|591|BOLD:ACT4384
Sciaridae|[23384]|CNGLA155-13|Sciaridae|658|BOLD:ACE2154
Sciaridae|[23385]|CNGMB775-13|Sciaridae|636|BOLD:ACL0867
Claustropyga|[23386]|CNKLF1308-15|Sciaridae|588|BOLD:ACS2254
Sciaridae|[23387]|SSJAC589-13|Sciaridae|661|BOLD:ACI6470
Sciaridae|[23388]|CNBFO565-15|Sciaridae|597|BOLD:ACT8677
Sciaridae|[23389]|DARC465-11|Sciaridae|658|BOLD:AAU6578
Sciaridae|[23390]|SSJAA1648-13|Sciaridae|658|BOLD:AAV1303
Sciaridae|[23391]|SSJAB3627-13|Sciaridae|658|BOLD:ACN1079
Sciaridae|[23392]|CNPEM513-14|Sciaridae|603|BOLD:ACL7949
Sciaridae|[23393]|JWDCB315-10|Sciaridae|658|BOLD:AAH4031
Sciaridae|[23394]|CNKLC3061-14|Sciaridae|624|BOLD:ACR7442
Sciaridae|[23395]|CNGMD097-14|Sciaridae|658|BOLD:ACL3019
Sciaridae|[23396]|CNFND399-14|Sciaridae|661|BOLD:ACL1495

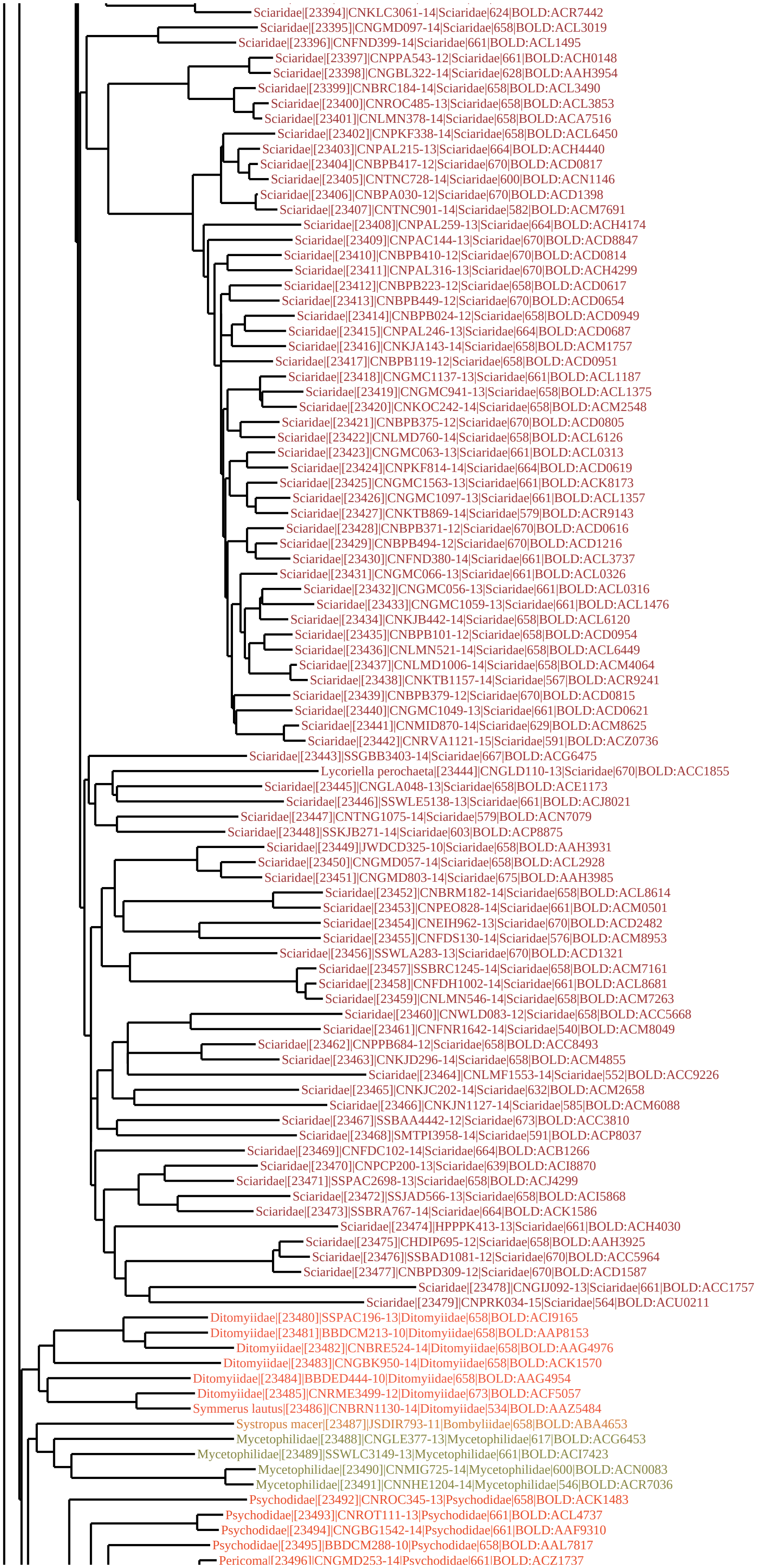

Sciaridae|[23394]|CNKLC3061-14|Sciaridae|624|BOLD:ACR7442
Sciaridae|[23395]|CNGMD097-14|Sciaridae|658|BOLD:ACL3019
Sciaridae|[23396]|CNFND399-14|Sciaridae|661|BOLD:ACL1495
Sciaridae|[23397]|CNPPA543-12|Sciaridae|661|BOLD:ACH0148
Sciaridae|[23398]|CNGBL322-14|Sciaridae|628|BOLD:AAH3954
Sciaridae|[23399]|CNBRC184-14|Sciaridae|658|BOLD:ACL3490
Sciaridae|[23400]|CNROC485-13|Sciaridae|658|BOLD:ACL3853
Sciaridae|[23401]|CNLMN378-14|Sciaridae|658|BOLD:ACA7516
Sciaridae|[23402]|CNPKF338-14|Sciaridae|658|BOLD:ACL6450
Sciaridae|[23403]|CNPAL215-13|Sciaridae|664|BOLD:ACH4440
Sciaridae|[23404]|CNBPB417-12|Sciaridae|670|BOLD:ACD0817
Sciaridae|[23405]|CNTNC728-14|Sciaridae|600|BOLD:ACN1146
Sciaridae|[23406]|CNBPA030-12|Sciaridae|670|BOLD:ACD1398
Sciaridae|[23407]|CNTNC901-14|Sciaridae|582|BOLD:ACM7691
Sciaridae|[23408]|CNPAL259-13|Sciaridae|664|BOLD:ACH4174
Sciaridae|[23409]|CNPAC144-13|Sciaridae|670|BOLD:ACD8847
Sciaridae|[23410]|CNBPB410-12|Sciaridae|670|BOLD:ACD0814
Sciaridae|[23411]|CNPAL316-13|Sciaridae|670|BOLD:ACH4299
Sciaridae|[23412]|CNBPB223-12|Sciaridae|658|BOLD:ACD0617
Sciaridae|[23413]|CNBPB449-12|Sciaridae|670|BOLD:ACD0654
Sciaridae|[23414]|CNBPB024-12|Sciaridae|658|BOLD:ACD0949
Sciaridae|[23415]|CNPAL246-13|Sciaridae|664|BOLD:ACD0687
Sciaridae|[23416]|CNKJA143-14|Sciaridae|658|BOLD:ACM1757
Sciaridae|[23417]|CNBPB119-12|Sciaridae|658|BOLD:ACD0951
Sciaridae|[23418]|CNGMC1137-13|Sciaridae|661|BOLD:ACL1187
Sciaridae|[23419]|CNGMC941-13|Sciaridae|658|BOLD:ACL1375
Sciaridae|[23420]|CNKOC242-14|Sciaridae|658|BOLD:ACM2548
Sciaridae|[23421]|CNBPB375-12|Sciaridae|670|BOLD:ACD0805
Sciaridae|[23422]|CNLMD760-14|Sciaridae|658|BOLD:ACL6126
Sciaridae|[23423]|CNGMC063-13|Sciaridae|661|BOLD:ACL0313
Sciaridae|[23424]|CNPKF814-14|Sciaridae|664|BOLD:ACD0619
Sciaridae|[23425]|CNGMC1563-13|Sciaridae|661|BOLD:ACK8173
Sciaridae|[23426]|CNGMC1097-13|Sciaridae|661|BOLD:ACL1357
Sciaridae|[23427]|CNKTB869-14|Sciaridae|579|BOLD:ACR9143
Sciaridae|[23428]|CNBPB371-12|Sciaridae|670|BOLD:ACD0616
Sciaridae|[23429]|CNBPB494-12|Sciaridae|670|BOLD:ACD1216
Sciaridae|[23430]|CNFND380-14|Sciaridae|661|BOLD:ACL3737
Sciaridae|[23431]|CNGMC066-13|Sciaridae|661|BOLD:ACL0326
Sciaridae|[23432]|CNGMC056-13|Sciaridae|661|BOLD:ACL0316
Sciaridae|[23433]|CNGMC1059-13|Sciaridae|661|BOLD:ACL1476
Sciaridae|[23434]|CNKJB442-14|Sciaridae|658|BOLD:ACL6120
Sciaridae|[23435]|CNBPB101-12|Sciaridae|658|BOLD:ACD0954
Sciaridae|[23436]|CNLMN521-14|Sciaridae|658|BOLD:ACL6449
Sciaridae|[23437]|CNLMD1006-14|Sciaridae|658|BOLD:ACM4064
Sciaridae|[23438]|CNKTB1157-14|Sciaridae|567|BOLD:ACR9241
Sciaridae|[23439]|CNBPB379-12|Sciaridae|670|BOLD:ACD0815
Sciaridae|[23440]|CNGMC1049-13|Sciaridae|661|BOLD:ACD0621
Sciaridae|[23441]|CNMID870-14|Sciaridae|629|BOLD:ACM8625
Sciaridae|[23442]|CNRVA1121-15|Sciaridae|591|BOLD:ACZ0736
Sciaridae|[23443]|SSGBB3403-14|Sciaridae|667|BOLD:ACG6475
Lycoriella perochaeta|[23444]|CNGLD110-13|Sciaridae|670|BOLD:ACC1855
Sciaridae|[23445]|CNGLA048-13|Sciaridae|658|BOLD:ACE1173
Sciaridae|[23446]|SSWLE5138-13|Sciaridae|661|BOLD:ACJ8021
Sciaridae|[23447]|CNTNG1075-14|Sciaridae|579|BOLD:ACN7079
Sciaridae|[23448]|SSKJB271-14|Sciaridae|603|BOLD:ACP8875
Sciaridae|[23449]|JWDCD325-10|Sciaridae|658|BOLD:AAH3931
Sciaridae|[23450]|CNGMD057-14|Sciaridae|658|BOLD:ACL2928
Sciaridae|[23451]|CNGMD803-14|Sciaridae|675|BOLD:AAH3985
Sciaridae|[23452]|CNBRM182-14|Sciaridae|658|BOLD:ACL8614
Sciaridae|[23453]|CNPEO828-14|Sciaridae|661|BOLD:ACM0501
Sciaridae|[23454]|CNEIH962-13|Sciaridae|670|BOLD:ACD2482
Sciaridae|[23455]|CNFDS130-14|Sciaridae|576|BOLD:ACM8953
Sciaridae|[23456]|SSWLA283-13|Sciaridae|670|BOLD:ACD1321
Sciaridae|[23457]|SSBRC1245-14|Sciaridae|658|BOLD:ACM7161
Sciaridae|[23458]|CNFDH1002-14|Sciaridae|661|BOLD:ACL8681
Sciaridae|[23459]|CNLMN546-14|Sciaridae|658|BOLD:ACM7263
Sciaridae|[23460]|CNWLD083-12|Sciaridae|658|BOLD:ACC5668
Sciaridae|[23461]|CNFNR1642-14|Sciaridae|540|BOLD:ACM8049
Sciaridae|[23462]|CNPPB684-12|Sciaridae|658|BOLD:ACC8493
Sciaridae|[23463]|CNKJD296-14|Sciaridae|658|BOLD:ACM4855
Sciaridae|[23464]|CNLMF1553-14|Sciaridae|552|BOLD:ACC9226
Sciaridae|[23465]|CNKJC202-14|Sciaridae|632|BOLD:ACM2658
Sciaridae|[23466]|CNKJN1127-14|Sciaridae|585|BOLD:ACM6088
Sciaridae|[23467]|SSBAA4442-12|Sciaridae|673|BOLD:ACC3810
Sciaridae|[23468]|SMTPI3958-14|Sciaridae|591|BOLD:ACP8037
Sciaridae|[23469]|CNFDC102-14|Sciaridae|664|BOLD:ACB1266
Sciaridae|[23470]|CNPCP200-13|Sciaridae|639|BOLD:ACI8870
Sciaridae|[23471]|SSPAC2698-13|Sciaridae|658|BOLD:ACJ4299
Sciaridae|[23472]|SSJAD566-13|Sciaridae|658|BOLD:ACI5868
Sciaridae|[23473]|SSBRA767-14|Sciaridae|664|BOLD:ACK1586
Sciaridae|[23474]|HPPPK413-13|Sciaridae|661|BOLD:ACH4030
Sciaridae|[23475]|CHDIP695-12|Sciaridae|658|BOLD:AAH3925
Sciaridae|[23476]|SSBAD1081-12|Sciaridae|670|BOLD:ACC5964
Sciaridae|[23477]|CNBPD309-12|Sciaridae|670|BOLD:ACD1587
Sciaridae|[23478]|CNGIJ092-13|Sciaridae|661|BOLD:ACC1757
Sciaridae|[23479]|CNPRK034-15|Sciaridae|564|BOLD:ACU0211
Ditomyiidae|[23480]|SSPAC196-13|Ditomyiidae|658|BOLD:ACI9165
Ditomyiidae|[23481]|BBDCM213-10|Ditomyiidae|658|BOLD:AAP8153
Ditomyiidae|[23482]|CNBRE524-14|Ditomyiidae|658|BOLD:AAG4976
Ditomyiidae|[23483]|CNGBK950-14|Ditomyiidae|658|BOLD:ACK1570
Ditomyiidae|[23484]|BBDED444-10|Ditomyiidae|658|BOLD:AAG4954
Ditomyiidae|[23485]|CNRME3499-12|Ditomyiidae|673|BOLD:ACF5057
Symmerus lautus|[23486]|CNBRN1130-14|Ditomyiidae|534|BOLD:AAZ5484
Systropus macer|[23487]|JSDIR793-11|Bombyliidae|658|BOLD:ABA4653
Mycetophilidae|[23488]|CNGLE377-13|Mycetophilidae|617|BOLD:ACG6453
Mycetophilidae|[23489]|SSWLC3149-13|Mycetophilidae|661|BOLD:ACI7423
Mycetophilidae|[23490]|CNMIG725-14|Mycetophilidae|600|BOLD:ACN0083
Mycetophilidae|[23491]|CNNHE1204-14|Mycetophilidae|546|BOLD:ACR7036
Psychodidae|[23492]|CNROC345-13|Psychodidae|658|BOLD:ACK1483
Psychodidae|[23493]|CNROT111-13|Psychodidae|661|BOLD:ACL4737
Psychodidae|[23494]|CNGBG1542-14|Psychodidae|661|BOLD:AAF9310
Psychodidae|[23495]|BBDCM288-10|Psychodidae|658|BOLD:AAL7817
Pericoma|[23496]|CNGMD253-14|Psychodidae|661|BOLD:ACZ1737

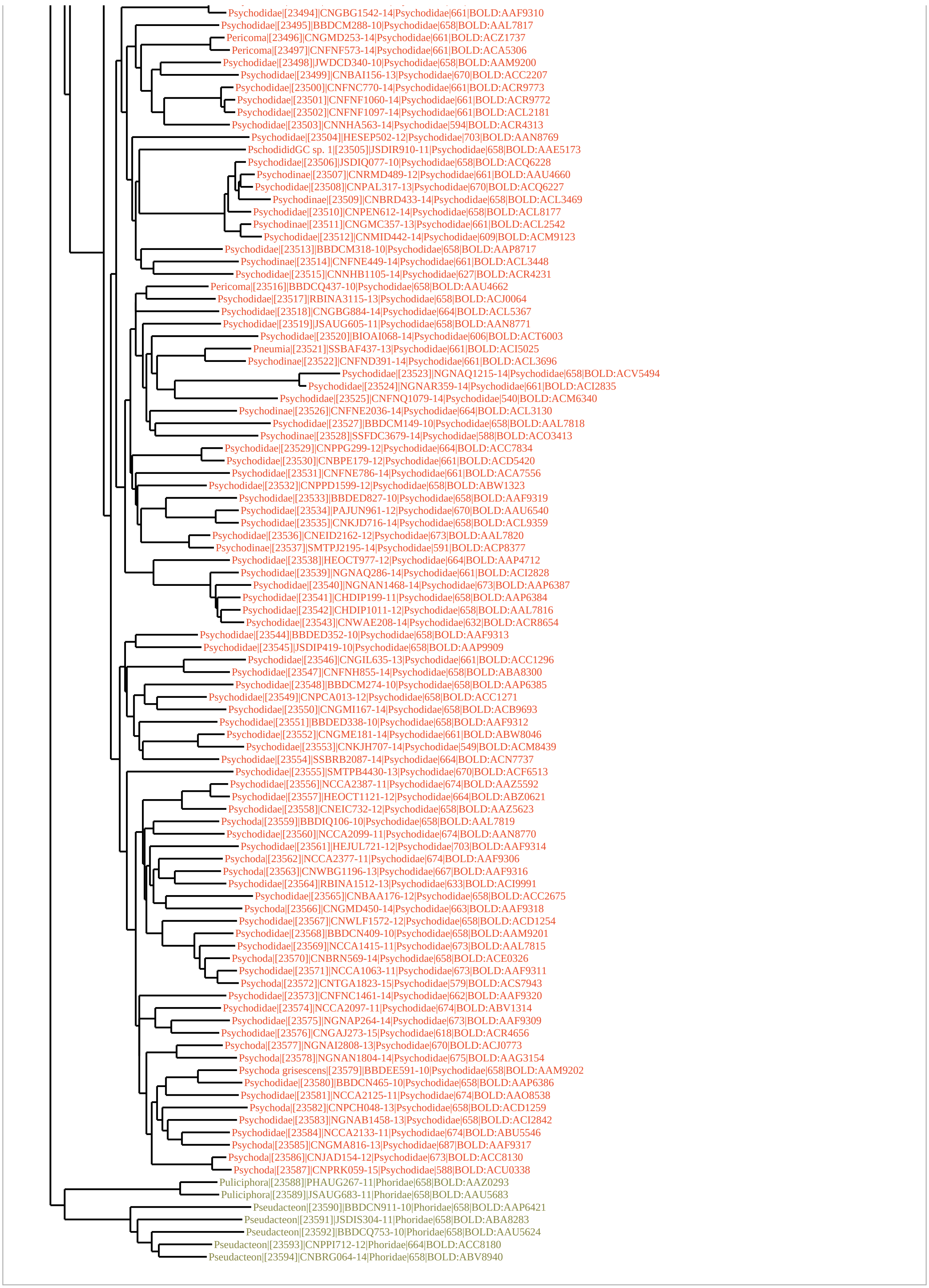
Psychodidae|[23494]|CNGBG1542-14|Psychodidae|661|BOLD:AAF9310
Psychodidae|[23495]|BBDCM288-10|Psychodidae|658|BOLD:AAL7817
Pericoma|[23496]|CNGMD253-14|Psychodidae|661|BOLD:ACZ1737
Pericoma|[23497]|CNFNF573-14|Psychodidae|661|BOLD:ACA5306
Psychodidae|[23498]|JWDCD340-10|Psychodidae|658|BOLD:AAM9200
Psychodidae|[23499]|CNBAI156-13|Psychodidae|670|BOLD:ACC2207
Psychodidae|[23500]|CNFNC770-14|Psychodidae|661|BOLD:ACR9773
Psychodidae|[23501]|CNFNF1060-14|Psychodidae|661|BOLD:ACR9772
Psychodidae|[23502]|CNFNF1097-14|Psychodidae|661|BOLD:ACL2181
Psychodidae|[23503]|CNNHA563-14|Psychodidae|594|BOLD:ACR4313
Psychodidae|[23504]|HESEP502-12|Psychodidae|703|BOLD:AAN8769
PschodididGC sp. 1|[23505]|JSDIR910-11|Psychodidae|658|BOLD:AAE5173
Psychodidae|[23506]|JSDIQ077-10|Psychodidae|658|BOLD:ACQ6228
Psychodinae|[23507]|CNRMD489-12|Psychodidae|661|BOLD:AAU4660
Psychodidae|[23508]|CNPAL317-13|Psychodidae|670|BOLD:ACQ6227
Psychodinae|[23509]|CNBRD433-14|Psychodidae|658|BOLD:ACL3469
Psychodidae|[23510]|CNPEN612-14|Psychodidae|658|BOLD:ACL8177
Psychodinae|[23511]|CNGMC357-13|Psychodidae|661|BOLD:ACL2542
Psychodidae|[23512]|CNMID442-14|Psychodidae|609|BOLD:ACM9123
Psychodidae|[23513]|BBDCM318-10|Psychodidae|658|BOLD:AAP8717
Psychodinae|[23514]|CNFNE449-14|Psychodidae|661|BOLD:ACL3448
Psychodidae|[23515]|CNNHB1105-14|Psychodidae|627|BOLD:ACR4231
Pericoma|[23516]|BBDCQ437-10|Psychodidae|658|BOLD:AAU4662
Psychodidae|[23517]|RBINA3115-13|Psychodidae|658|BOLD:ACJ0064
Psychodidae|[23518]|CNGBG884-14|Psychodidae|664|BOLD:ACL5367
Psychodidae|[23519]|JSAUG605-11|Psychodidae|658|BOLD:AAN8771
Psychodidae|[23520]|BIOAI068-14|Psychodidae|606|BOLD:ACT6003
Pneumia|[23521]|SSBAF437-13|Psychodidae|661|BOLD:ACI5025
Psychodinae|[23522]|CNFND391-14|Psychodidae|661|BOLD:ACL3696
Psychodidae|[23523]|NGNAQ1215-14|Psychodidae|658|BOLD:ACV5494
Psychodidae|[23524]|NGNAR359-14|Psychodidae|661|BOLD:ACI2835
Psychodidae|[23525]|CNFNQ1079-14|Psychodidae|540|BOLD:ACM6340
Psychodinae|[23526]|CNFNE2036-14|Psychodidae|664|BOLD:ACL3130
Psychodidae|[23527]|BBDCM149-10|Psychodidae|658|BOLD:AAL7818
Psychodinae|[23528]|SSFDC3679-14|Psychodidae|588|BOLD:ACO3413
Psychodidae|[23529]|CNPPG299-12|Psychodidae|664|BOLD:ACC7834
Psychodidae|[23530]|CNBPE179-12|Psychodidae|661|BOLD:ACD5420
Psychodidae|[23531]|CNFNE786-14|Psychodidae|661|BOLD:ACA7556
Psychodidae|[23532]|CNPPD1599-12|Psychodidae|658|BOLD:ABW1323
Psychodidae|[23533]|BBDED827-10|Psychodidae|658|BOLD:AAF9319
Psychodidae|[23534]|PAJUN961-12|Psychodidae|670|BOLD:AAU6540
Psychodidae|[23535]|CNKJD716-14|Psychodidae|658|BOLD:ACL9359
Psychodidae|[23536]|CNEID2162-12|Psychodidae|673|BOLD:AAL7820
Psychodinae|[23537]|SMTPJ2195-14|Psychodidae|591|BOLD:ACP8377
Psychodidae|[23538]|HEOCT977-12|Psychodidae|664|BOLD:AAP4712
Psychodidae|[23539]|NGNAQ286-14|Psychodidae|661|BOLD:ACI2828
Psychodidae|[23540]|NGNAN1468-14|Psychodidae|673|BOLD:AAP6387
Psychodidae|[23541]|CHDIP199-11|Psychodidae|658|BOLD:AAP6384
Psychodidae|[23542]|CHDIP1011-12|Psychodidae|658|BOLD:AAL7816
Psychodidae|[23543]|CNWAE208-14|Psychodidae|632|BOLD:ACR8654
Psychodidae|[23544]|BBDED352-10|Psychodidae|658|BOLD:AAF9313
Psychodidae|[23545]|JSDIP419-10|Psychodidae|658|BOLD:AAP9909
Psychodidae|[23546]|CNGIL635-13|Psychodidae|661|BOLD:ACC1296
Psychodidae|[23547]|CNFNH855-14|Psychodidae|658|BOLD:ABA8300
Psychodidae|[23548]|BBDCM274-10|Psychodidae|658|BOLD:AAP6385
Psychodidae|[23549]|CNPCA013-12|Psychodidae|658|BOLD:ACC1271
Psychodidae|[23550]|CNGMI167-14|Psychodidae|658|BOLD:ACB9693
Psychodidae|[23551]|BBDED338-10|Psychodidae|658|BOLD:AAF9312
Psychodidae|[23552]|CNGME181-14|Psychodidae|661|BOLD:ABW8046
Psychodidae|[23553]|CNKJH707-14|Psychodidae|549|BOLD:ACM8439
Psychodidae|[23554]|SSBRB2087-14|Psychodidae|664|BOLD:ACN7737
Psychodidae|[23555]|SMTPB4430-13|Psychodidae|670|BOLD:ACF6513
Psychodidae|[23556]|NCCA2387-11|Psychodidae|674|BOLD:AAZ5592
Psychodidae|[23557]|HEOCT1121-12|Psychodidae|664|BOLD:ABZ0621
Psychodidae|[23558]|CNEIC732-12|Psychodidae|658|BOLD:AAZ5623
Psychoda|[23559]|BBDIQ106-10|Psychodidae|658|BOLD:AAL7819
Psychodidae|[23560]|NCCA2099-11|Psychodidae|674|BOLD:AAN8770
Psychodidae|[23561]|HEJUL721-12|Psychodidae|703|BOLD:AAF9314
Psychoda|[23562]|NCCA2377-11|Psychodidae|674|BOLD:AAF9306
Psychoda|[23563]|CNWBG1196-13|Psychodidae|667|BOLD:AAF9316
Psychodidae|[23564]|RBINA1512-13|Psychodidae|633|BOLD:ACI9991
Psychodidae|[23565]|CNBAA176-12|Psychodidae|658|BOLD:ACC2675
Psychoda|[23566]|CNGMD450-14|Psychodidae|663|BOLD:AAF9318
Psychodidae|[23567]|CNWLF1572-12|Psychodidae|658|BOLD:ACD1254
Psychodidae|[23568]|BBDCN409-10|Psychodidae|658|BOLD:AAM9201
Psychodidae|[23569]|NCCA1415-11|Psychodidae|673|BOLD:AAL7815
Psychoda|[23570]|CNBRN569-14|Psychodidae|658|BOLD:ACE0326
Psychodidae|[23571]|NCCA1063-11|Psychodidae|673|BOLD:AAF9311
Psychoda|[23572]|CNTGA1823-15|Psychodidae|579|BOLD:ACS7943
Psychodidae|[23573]|CNFNC1461-14|Psychodidae|662|BOLD:AAF9320
Psychodidae|[23574]|NCCA2097-11|Psychodidae|674|BOLD:ABV1314
Psychodidae|[23575]|NGNAP264-14|Psychodidae|673|BOLD:AAF9309
Psychodidae|[23576]|CNGAJ273-15|Psychodidae|618|BOLD:ACR4656
Psychoda|[23577]|NGNAI2808-13|Psychodidae|670|BOLD:ACJ0773
Psychoda|[23578]|NGNAN1804-14|Psychodidae|675|BOLD:AAG3154
Psychoda grisescens|[23579]|BBDEE591-10|Psychodidae|658|BOLD:AAM9202
Psychodidae|[23580]|BBDCN465-10|Psychodidae|658|BOLD:AAP6386
Psychodidae|[23581]|NCCA2125-11|Psychodidae|674|BOLD:AAO8538
Psychoda|[23582]|CNPCH048-13|Psychodidae|658|BOLD:ACD1259
Psychodidae|[23583]|NGNAB1458-13|Psychodidae|658|BOLD:ACI2842
Psychodidae|[23584]|NCCA2133-11|Psychodidae|674|BOLD:ABU5546
Psychoda|[23585]|CNGMA816-13|Psychodidae|687|BOLD:AAF9317
Psychoda|[23586]|CNJAD154-12|Psychodidae|673|BOLD:ACC8130
Psychoda|[23587]|CNPRK059-15|Psychodidae|588|BOLD:ACU0338
Puliciphora|[23588]|PHAUG267-11|Phoridae|658|BOLD:AAZ0293
Puliciphora|[23589]|JSAUG683-11|Phoridae|658|BOLD:AAU5683
Pseudacteon|[23590]|BBDCN911-10|Phoridae|658|BOLD:AAP6421
Pseudacteon|[23591]|JSDIS304-11|Phoridae|658|BOLD:ABA8283
Pseudacteon|[23592]|BBDCQ753-10|Phoridae|658|BOLD:AAU5624
Pseudacteon|[23593]|CNPPI712-12|Phoridae|664|BOLD:ACC8180
Pseudacteon|[23594]|CNBRG064-14|Phoridae|658|BOLD:ABV8940

Electronic Supplementary Material for:
Counting Species with DNA Barcodes: Canadian Insects. 2016. PDN Hebert, S Ratnasingham, EV Zakharov, AC Telfer, V Levesque-Beaudin, MA Milton, S Pedersen, P Jannetta, JR deWaard. Philosophical Transactions of the Royal Society B. doi: 10.1098/rstb.2015.0333.

Supplementary Figure S2: Neighbor-Joining tree based on sequence divergences (K2P) at COI for the 23,346 BINs of non-dipteran insects from Canada. Entries in the tree are colorized by family.

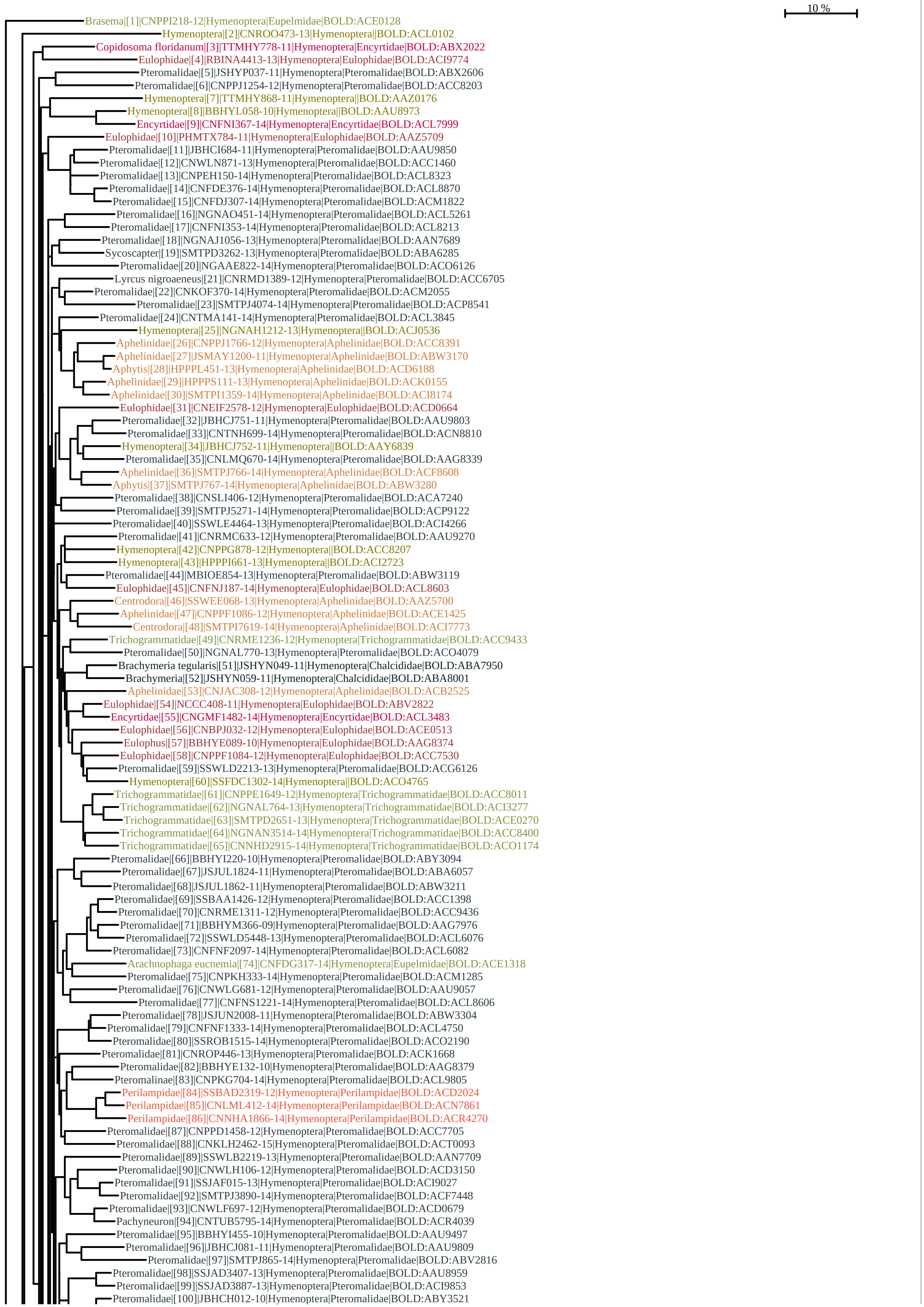

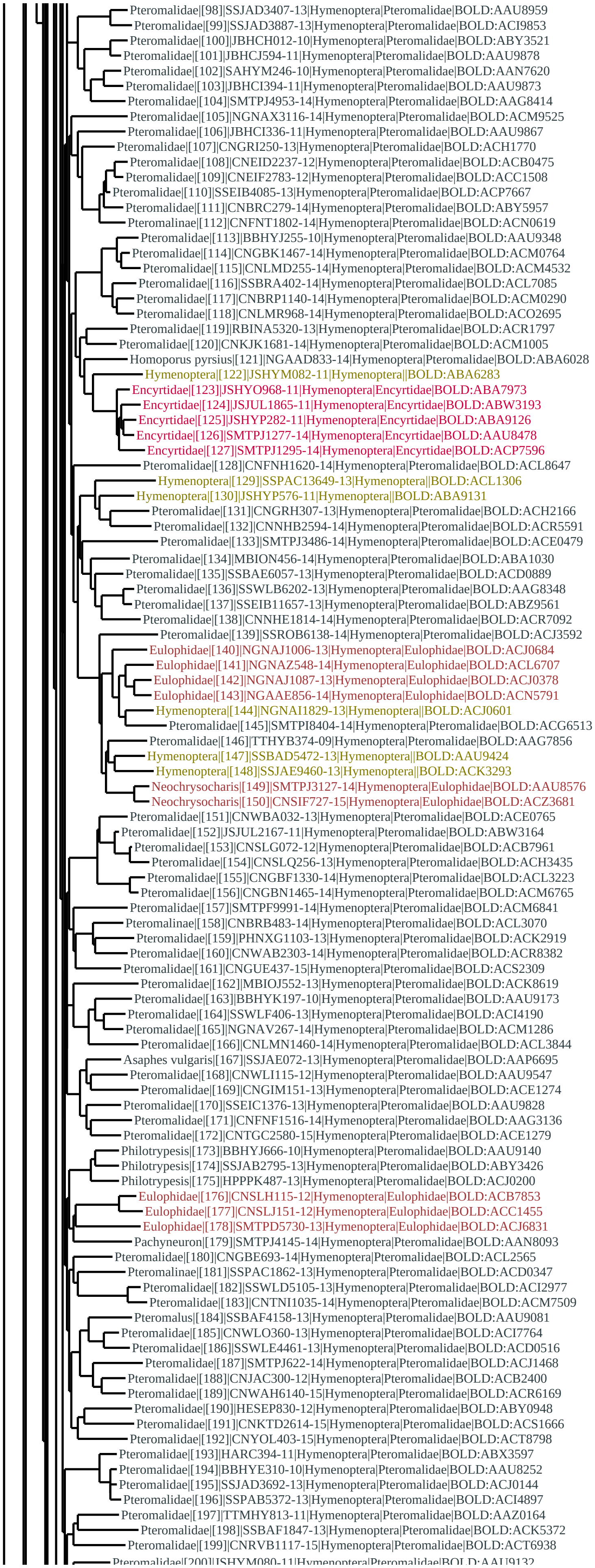

Pteromalidae|[98]|SSJAD3407-13|Hymenoptera|Pteromalidae|BOLD:AAU8959
Pteromalidae|[99]|SSJAD3887-13|Hymenoptera|Pteromalidae|BOLD:ACI9853
Pteromalidae|[100]|JBHCH012-10|Hymenoptera|Pteromalidae|BOLD:ABY3521
Pteromalidae|[101]|JBHCJ594-11|Hymenoptera|Pteromalidae|BOLD:AAU9878
Pteromalidae|[102]|SAHYM246-10|Hymenoptera|Pteromalidae|BOLD:AAN7620
Pteromalidae|[103]|JBHCI394-11|Hymenoptera|Pteromalidae|BOLD:AAU9873
Pteromalidae|[104]|SMTPJ4953-14|Hymenoptera|Pteromalidae|BOLD:AAG8414
Pteromalidae|[105]|NGNAX3116-14|Hymenoptera|Pteromalidae|BOLD:ACM9525
Pteromalidae|[106]|JBHCI336-11|Hymenoptera|Pteromalidae|BOLD:AAU9867
Pteromalidae|[107]|CNGRI250-13|Hymenoptera|Pteromalidae|BOLD:ACH1770
Pteromalidae|[108]|CNEID2237-12|Hymenoptera|Pteromalidae|BOLD:ACB0475
Pteromalidae|[109]|CNEIF2783-12|Hymenoptera|Pteromalidae|BOLD:ACC1508
Pteromalidae|[110]|SSEIB4085-13|Hymenoptera|Pteromalidae|BOLD:ACP7667
Pteromalidae|[111]|CNBRC279-14|Hymenoptera|Pteromalidae|BOLD:ABY5957
Pteromalinae|[112]|CNFNT1802-14|Hymenoptera|Pteromalidae|BOLD:ACN0619
Pteromalidae|[113]|BBHYJ255-10|Hymenoptera|Pteromalidae|BOLD:AAU9348
Pteromalidae|[114]|CNGBK1467-14|Hymenoptera|Pteromalidae|BOLD:ACM0764
Pteromalidae|[115]|CNLMD255-14|Hymenoptera|Pteromalidae|BOLD:ACM4532
Pteromalidae|[116]|SSBRA402-14|Hymenoptera|Pteromalidae|BOLD:ACL7085
Pteromalidae|[117]|CNBRP1140-14|Hymenoptera|Pteromalidae|BOLD:ACM0290
Pteromalidae|[118]|CNLMR968-14|Hymenoptera|Pteromalidae|BOLD:ACO2695
Pteromalidae|[119]|RBINA5320-13|Hymenoptera|Pteromalidae|BOLD:ACR1797
Pteromalidae|[120]|CNKJK1681-14|Hymenoptera|Pteromalidae|BOLD:ACM1005
Homoporus pyrsius|[121]|NGAAD833-14|Hymenoptera|Pteromalidae|BOLD:ABA6028
Hymenoptera|[122]|JSHYM082-11|Hymenoptera||BOLD:ABA6283
Encyrtidae|[123]|JSHYO968-11|Hymenoptera|Encyrtidae|BOLD:ABA7973
Encyrtidae|[124]|JSJUL1865-11|Hymenoptera|Encyrtidae|BOLD:ABW3193
Encyrtidae|[125]|JSHYP282-11|Hymenoptera|Encyrtidae|BOLD:ABA9126
Encyrtidae|[126]|SMTPJ1277-14|Hymenoptera|Encyrtidae|BOLD:AAU8478
Encyrtidae|[127]|SMTPJ1295-14|Hymenoptera|Encyrtidae|BOLD:ACP7596
Pteromalidae|[128]|CNFNH1620-14|Hymenoptera|Pteromalidae|BOLD:ACL8647
Hymenoptera|[129]|SSPAC13649-13|Hymenoptera||BOLD:ACL1306
Hymenoptera|[130]|JSHYP576-11|Hymenoptera||BOLD:ABA9131
Pteromalidae|[131]|CNGRH307-13|Hymenoptera|Pteromalidae|BOLD:ACH2166
Pteromalidae|[132]|CNNHB2594-14|Hymenoptera|Pteromalidae|BOLD:ACR5591
Pteromalidae|[133]|SMTPJ3486-14|Hymenoptera|Pteromalidae|BOLD:ACE0479
Pteromalidae|[134]|MBION456-14|Hymenoptera|Pteromalidae|BOLD:ABA1030
Pteromalidae|[135]|SSBAE6057-13|Hymenoptera|Pteromalidae|BOLD:ACD0889
Pteromalidae|[136]|SSWLB6202-13|Hymenoptera|Pteromalidae|BOLD:AAG8348
Pteromalidae|[137]|SSEIB11657-13|Hymenoptera|Pteromalidae|BOLD:ABZ9561
Pteromalidae|[138]|CNNHE1814-14|Hymenoptera|Pteromalidae|BOLD:ACR7092
Pteromalidae|[139]|SSROB6138-14|Hymenoptera|Pteromalidae|BOLD:ACJ3592
Eulophidae|[140]|NGNAJ1006-13|Hymenoptera|Eulophidae|BOLD:ACJ0684
Eulophidae|[141]|NGNAZ548-14|Hymenoptera|Eulophidae|BOLD:ACL6707
Eulophidae|[142]|NGNAJ1087-13|Hymenoptera|Eulophidae|BOLD:ACJ0378
Eulophidae|[143]|NGAAE856-14|Hymenoptera|Eulophidae|BOLD:ACN5791
Hymenoptera|[144]|NGNAI1829-13|Hymenoptera||BOLD:ACJ0601
Pteromalidae|[145]|SMTPI8404-14|Hymenoptera|Pteromalidae|BOLD:ACG6513
Pteromalidae|[146]|TTHYB374-09|Hymenoptera|Pteromalidae|BOLD:AAG7856
Hymenoptera|[147]|SSBAD5472-13|Hymenoptera||BOLD:AAU9424
Hymenoptera|[148]|SSJAE9460-13|Hymenoptera||BOLD:ACK3293
Neochrysocharis|[149]|SMTPJ3127-14|Hymenoptera|Eulophidae|BOLD:AAU8576
Neochrysocharis|[150]|CNSIF727-15|Hymenoptera|Eulophidae|BOLD:ACZ3681
Pteromalidae|[151]|CNWBA032-13|Hymenoptera|Pteromalidae|BOLD:ACE0765
Pteromalidae|[152]|JSJUL2167-11|Hymenoptera|Pteromalidae|BOLD:ABW3164
Pteromalidae|[153]|CNSLG072-12|Hymenoptera|Pteromalidae|BOLD:ACB7961
Pteromalidae|[154]|CNSLQ256-13|Hymenoptera|Pteromalidae|BOLD:ACH3435
Pteromalidae|[155]|CNGBF1330-14|Hymenoptera|Pteromalidae|BOLD:ACL3223
Pteromalidae|[156]|CNGBN1465-14|Hymenoptera|Pteromalidae|BOLD:ACM6765
Pteromalidae|[157]|SMTPF9991-14|Hymenoptera|Pteromalidae|BOLD:ACM6841
Pteromalinae|[158]|CNBRB483-14|Hymenoptera|Pteromalidae|BOLD:ACL3070
Pteromalidae|[159]|PHNXG1103-13|Hymenoptera|Pteromalidae|BOLD:ACK2919
Pteromalidae|[160]|CNWAB2303-14|Hymenoptera|Pteromalidae|BOLD:ACR8382
Pteromalidae|[161]|CNGUE437-15|Hymenoptera|Pteromalidae|BOLD:ACS2309
Pteromalidae|[162]|MBIOJ552-13|Hymenoptera|Pteromalidae|BOLD:ACK8619
Pteromalidae|[163]|BBHYK197-10|Hymenoptera|Pteromalidae|BOLD:AAU9173
Pteromalidae|[164]|SSWLF406-13|Hymenoptera|Pteromalidae|BOLD:ACI4190
Pteromalidae|[165]|NGNAV267-14|Hymenoptera|Pteromalidae|BOLD:ACM1286
Pteromalidae|[166]|CNLMN1460-14|Hymenoptera|Pteromalidae|BOLD:ACL3844
Asaphes vulgaris|[167]|SSJAE072-13|Hymenoptera|Pteromalidae|BOLD:AAP6695
Pteromalidae|[168]|CNWLI115-12|Hymenoptera|Pteromalidae|BOLD:AAU9547
Pteromalidae|[169]|CNGIM151-13|Hymenoptera|Pteromalidae|BOLD:ACE1274
Pteromalidae|[170]|SSEIC1376-13|Hymenoptera|Pteromalidae|BOLD:AAU9828
Pteromalidae|[171]|CNFNF1516-14|Hymenoptera|Pteromalidae|BOLD:AAG3136
Pteromalidae|[172]|CNTGC2580-15|Hymenoptera|Pteromalidae|BOLD:ACE1279
Philotrypesis|[173]|BBHYJ666-10|Hymenoptera|Pteromalidae|BOLD:AAU9140
Philotrypesis|[174]|SSJAB2795-13|Hymenoptera|Pteromalidae|BOLD:ABY3426
Philotrypesis|[175]|HPPPK487-13|Hymenoptera|Pteromalidae|BOLD:ACJ0200
Eulophidae|[176]|CNSLH115-12|Hymenoptera|Eulophidae|BOLD:ACB7853
Eulophidae|[177]|CNSLJ151-12|Hymenoptera|Eulophidae|BOLD:ACC1455
Eulophidae|[178]|SMTPD5730-13|Hymenoptera|Eulophidae|BOLD:ACJ6831
Pachyneuron|[179]|SMTPJ4145-14|Hymenoptera|Pteromalidae|BOLD:AAN8093
Pteromalidae|[180]|CNGBE693-14|Hymenoptera|Pteromalidae|BOLD:ACL2565
Pteromalinae|[181]|SSPAC1862-13|Hymenoptera|Pteromalidae|BOLD:ACD0347
Pteromalidae|[182]|SSWLD5105-13|Hymenoptera|Pteromalidae|BOLD:ACI2977
Pteromalidae|[183]|CNTNI1035-14|Hymenoptera|Pteromalidae|BOLD:ACM7509
Pteromalus|[184]|SSBAF4158-13|Hymenoptera|Pteromalidae|BOLD:AAU9081
Pteromalidae|[185]|CNWLO360-13|Hymenoptera|Pteromalidae|BOLD:ACI7764
Pteromalidae|[186]|SSWLE4461-13|Hymenoptera|Pteromalidae|BOLD:ACD0516
Pteromalidae|[187]|SMTPJ622-14|Hymenoptera|Pteromalidae|BOLD:ACJ1468
Pteromalidae|[188]|CNJAC300-12|Hymenoptera|Pteromalidae|BOLD:ACB2400
Pteromalidae|[189]|CNWAH6140-15|Hymenoptera|Pteromalidae|BOLD:ACR6169
Pteromalidae|[190]|HESEP830-12|Hymenoptera|Pteromalidae|BOLD:ABY0948
Pteromalidae|[191]|CNKTD2614-15|Hymenoptera|Pteromalidae|BOLD:ACS1666
Pteromalidae|[192]|CNYOL403-15|Hymenoptera|Pteromalidae|BOLD:ACT8798
Pteromalidae|[193]|HARC394-11|Hymenoptera|Pteromalidae|BOLD:ABX3597
Pteromalidae|[194]|BBHYE310-10|Hymenoptera|Pteromalidae|BOLD:AAU8252
Pteromalidae|[195]|SSJAD3692-13|Hymenoptera|Pteromalidae|BOLD:ACJ0144
Pteromalidae|[196]|SSPAB5372-13|Hymenoptera|Pteromalidae|BOLD:ACI4897
Pteromalidae|[197]|TTMHY813-11|Hymenoptera|Pteromalidae|BOLD:AAZ0164
Pteromalidae|[198]|SSBAF1847-13|Hymenoptera|Pteromalidae|BOLD:ACK5372
Pteromalidae|[199]|CNRVB1117-15|Hymenoptera|Pteromalidae|BOLD:ACT6938
Pteromalidae|[200]|JSHYM080-11|Hymenoptera|Pteromalidae|BOLD:AAU9132

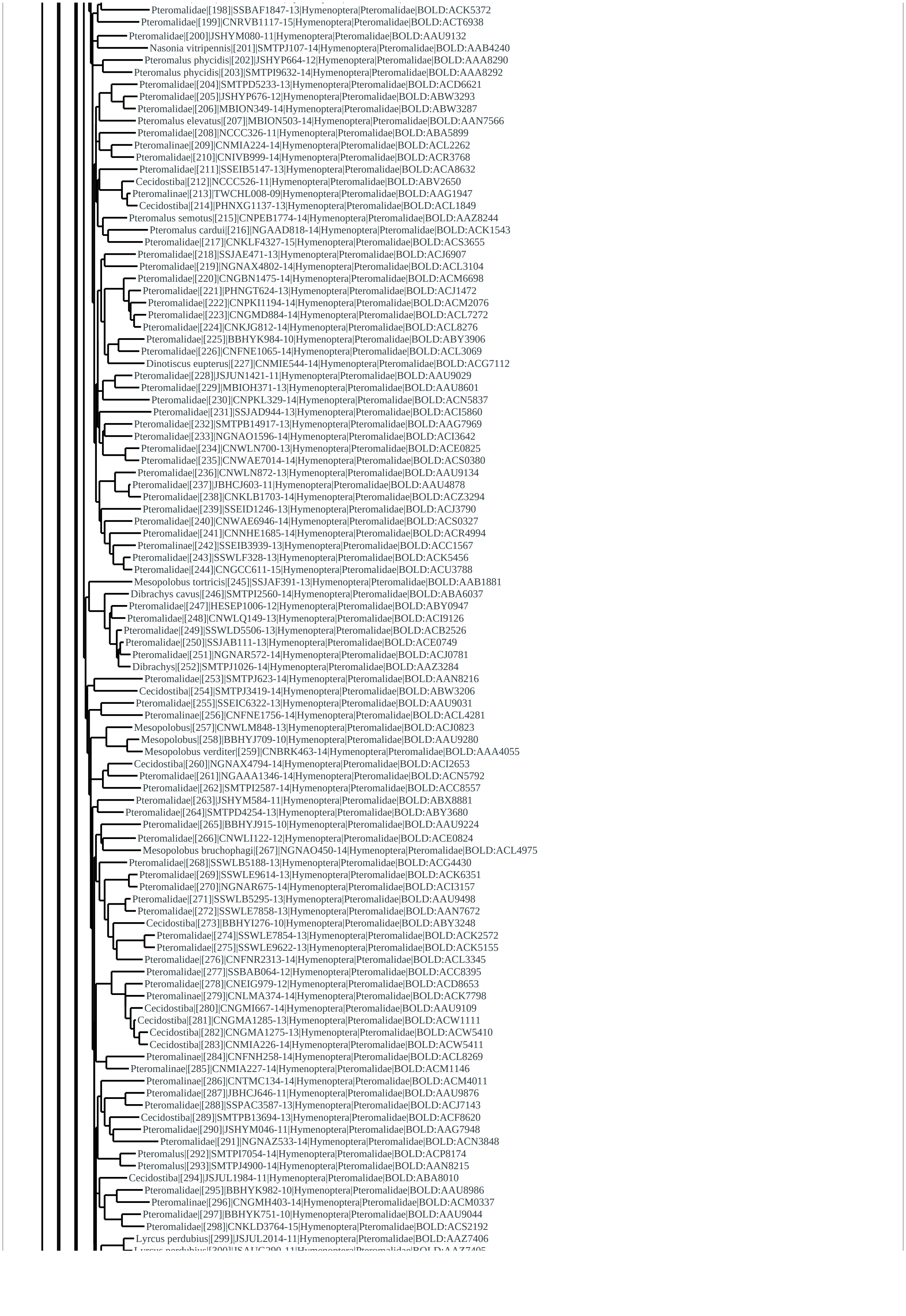
Pteromalidae|[198]|SSBAF1847-13|Hymenoptera|Pteromalidae|BOLD:ACK5372
Pteromalidae|[199]|CNRVB1117-15|Hymenoptera|Pteromalidae|BOLD:ACT6938
Pteromalidae|[200]|JSHYM080-11|Hymenoptera|Pteromalidae|BOLD:AAU9132
Nasonia vitripennis|[201]|SMTPJ107-14|Hymenoptera|Pteromalidae|BOLD:AAB4240
Pteromalus phycidis|[202]|JSHYP664-12|Hymenoptera|Pteromalidae|BOLD:AAA8290
Pteromalus phycidis|[203]|SMTPI9632-14|Hymenoptera|Pteromalidae|BOLD:AAA8292
Pteromalidae|[204]|SMTPD5233-13|Hymenoptera|Pteromalidae|BOLD:ACD6621
Pteromalidae|[205]|JSHYP676-12|Hymenoptera|Pteromalidae|BOLD:ABW3293
Pteromalidae|[206]|MBION349-14|Hymenoptera|Pteromalidae|BOLD:ABW3287
Pteromalus elevatus|[207]|MBION503-14|Hymenoptera|Pteromalidae|BOLD:AAN7566
Pteromalidae|[208]|NCCC326-11|Hymenoptera|Pteromalidae|BOLD:ABA5899
Pteromalinae|[209]|CNMIA224-14|Hymenoptera|Pteromalidae|BOLD:ACL2262
Pteromalidae|[210]|CNIVB999-14|Hymenoptera|Pteromalidae|BOLD:ACR3768
Pteromalidae|[211]|SSEIB5147-13|Hymenoptera|Pteromalidae|BOLD:ACA8632
Cecidostiba|[212]|NCCC526-11|Hymenoptera|Pteromalidae|BOLD:ABV2650
Pteromalinae|[213]|TWCHL008-09|Hymenoptera|Pteromalidae|BOLD:AAG1947
Cecidostiba|[214]|PHNXG1137-13|Hymenoptera|Pteromalidae|BOLD:ACL1849
Pteromalus semotus|[215]|CNPEB1774-14|Hymenoptera|Pteromalidae|BOLD:AAZ8244
Pteromalus cardui|[216]|NGAAD818-14|Hymenoptera|Pteromalidae|BOLD:ACK1543
Pteromalidae|[217]|CNKLF4327-15|Hymenoptera|Pteromalidae|BOLD:ACS3655
Pteromalidae|[218]|SSJAE471-13|Hymenoptera|Pteromalidae|BOLD:ACJ6907
Pteromalidae|[219]|NGNAX4802-14|Hymenoptera|Pteromalidae|BOLD:ACL3104
Pteromalidae|[220]|CNGBN1475-14|Hymenoptera|Pteromalidae|BOLD:ACM6698
Pteromalidae|[221]|PHNGT624-13|Hymenoptera|Pteromalidae|BOLD:ACJ1472
Pteromalidae|[222]|CNPKI1194-14|Hymenoptera|Pteromalidae|BOLD:ACM2076
Pteromalidae|[223]|CNGMD884-14|Hymenoptera|Pteromalidae|BOLD:ACL7272
Pteromalidae|[224]|CNKJG812-14|Hymenoptera|Pteromalidae|BOLD:ACL8276
Pteromalidae|[225]|BBHYK984-10|Hymenoptera|Pteromalidae|BOLD:ABY3906
Pteromalidae|[226]|CNFNE1065-14|Hymenoptera|Pteromalidae|BOLD:ACL3069
Dinotiscus eupterus|[227]|CNMIE544-14|Hymenoptera|Pteromalidae|BOLD:ACG7112
Pteromalidae|[228]|JSJUN1421-11|Hymenoptera|Pteromalidae|BOLD:AAU9029
Pteromalidae|[229]|MBIOH371-13|Hymenoptera|Pteromalidae|BOLD:AAU8601
Pteromalidae|[230]|CNPKL329-14|Hymenoptera|Pteromalidae|BOLD:ACN5837
Pteromalidae|[231]|SSJAD944-13|Hymenoptera|Pteromalidae|BOLD:ACI5860
Pteromalidae|[232]|SMTPB14917-13|Hymenoptera|Pteromalidae|BOLD:AAG7969
Pteromalidae|[233]|NGNAO1596-14|Hymenoptera|Pteromalidae|BOLD:ACI3642
Pteromalidae|[234]|CNWLN700-13|Hymenoptera|Pteromalidae|BOLD:ACE0825
Pteromalidae|[235]|CNWAE7014-14|Hymenoptera|Pteromalidae|BOLD:ACS0380
Pteromalidae|[236]|CNWLN872-13|Hymenoptera|Pteromalidae|BOLD:AAU9134
Pteromalidae|[237]|JBHCJ603-11|Hymenoptera|Pteromalidae|BOLD:AAU4878
Pteromalidae|[238]|CNKLB1703-14|Hymenoptera|Pteromalidae|BOLD:ACZ3294
Pteromalidae|[239]|SSEID1246-13|Hymenoptera|Pteromalidae|BOLD:ACJ3790
Pteromalidae|[240]|CNWAE6946-14|Hymenoptera|Pteromalidae|BOLD:ACS0327
Pteromalidae|[241]|CNNHE1685-14|Hymenoptera|Pteromalidae|BOLD:ACR4994
Pteromalinae|[242]|SSEIB3939-13|Hymenoptera|Pteromalidae|BOLD:ACC1567
Pteromalidae|[243]|SSWLF328-13|Hymenoptera|Pteromalidae|BOLD:ACK5456
Pteromalidae|[244]|CNGCC611-15|Hymenoptera|Pteromalidae|BOLD:ACU3788
Mesopolobus tortricis|[245]|SSJAF391-13|Hymenoptera|Pteromalidae|BOLD:AAB1881
Dibrachys cavus|[246]|SMTPI2560-14|Hymenoptera|Pteromalidae|BOLD:ABA6037
Pteromalidae|[247]|HESEP1006-12|Hymenoptera|Pteromalidae|BOLD:ABY0947
Pteromalidae|[248]|CNWLQ149-13|Hymenoptera|Pteromalidae|BOLD:ACI9126
Pteromalidae|[249]|SSWLD5506-13|Hymenoptera|Pteromalidae|BOLD:ACB2526
Pteromalidae|[250]|SSJAB111-13|Hymenoptera|Pteromalidae|BOLD:ACE0749
Pteromalidae|[251]|NGNAR572-14|Hymenoptera|Pteromalidae|BOLD:ACJ0781
Dibrachys|[252]|SMTPJ1026-14|Hymenoptera|Pteromalidae|BOLD:AAZ3284
Pteromalidae|[253]|SMTPJ623-14|Hymenoptera|Pteromalidae|BOLD:AAN8216
Cecidostiba|[254]|SMTPJ3419-14|Hymenoptera|Pteromalidae|BOLD:ABW3206
Pteromalidae|[255]|SSEIC6322-13|Hymenoptera|Pteromalidae|BOLD:AAU9031
Pteromalinae|[256]|CNFNE1756-14|Hymenoptera|Pteromalidae|BOLD:ACL4281
Mesopolobus|[257]|CNWLM848-13|Hymenoptera|Pteromalidae|BOLD:ACJ0823
Mesopolobus|[258]|BBHYJ709-10|Hymenoptera|Pteromalidae|BOLD:AAU9280
Mesopolobus verditer|[259]|CNBRK463-14|Hymenoptera|Pteromalidae|BOLD:AAA4055
Cecidostiba|[260]|NGNAX4794-14|Hymenoptera|Pteromalidae|BOLD:ACI2653
Pteromalidae|[261]|NGAAA1346-14|Hymenoptera|Pteromalidae|BOLD:ACN5792
Pteromalidae|[262]|SMTPI2587-14|Hymenoptera|Pteromalidae|BOLD:ACC8557
Pteromalidae|[263]|JSHYM584-11|Hymenoptera|Pteromalidae|BOLD:ABX8881
Pteromalidae|[264]|SMTPD4254-13|Hymenoptera|Pteromalidae|BOLD:ABY3680
Pteromalidae|[265]|BBHYJ915-10|Hymenoptera|Pteromalidae|BOLD:AAU9224
Pteromalidae|[266]|CNWLI122-12|Hymenoptera|Pteromalidae|BOLD:ACE0824
Mesopolobus bruchophagi|[267]|NGNAO450-14|Hymenoptera|Pteromalidae|BOLD:ACL4975
Pteromalidae|[268]|SSWLB5188-13|Hymenoptera|Pteromalidae|BOLD:ACG4430
Pteromalidae|[269]|SSWLE9614-13|Hymenoptera|Pteromalidae|BOLD:ACK6351
Pteromalidae|[270]|NGNAR675-14|Hymenoptera|Pteromalidae|BOLD:ACI3157
Pteromalidae|[271]|SSWLB5295-13|Hymenoptera|Pteromalidae|BOLD:AAU9498
Pteromalidae|[272]|SSWLE7858-13|Hymenoptera|Pteromalidae|BOLD:AAN7672
Cecidostiba|[273]|BBHYI276-10|Hymenoptera|Pteromalidae|BOLD:ABY3248
Pteromalidae|[274]|SSWLE7854-13|Hymenoptera|Pteromalidae|BOLD:ACK2572
Pteromalidae|[275]|SSWLE9622-13|Hymenoptera|Pteromalidae|BOLD:ACK5155
Pteromalidae|[276]|CNFNR2313-14|Hymenoptera|Pteromalidae|BOLD:ACL3345
Pteromalidae|[277]|SSBAB064-12|Hymenoptera|Pteromalidae|BOLD:ACC8395
Pteromalidae|[278]|CNEIG979-12|Hymenoptera|Pteromalidae|BOLD:ACD8653
Pteromalinae|[279]|CNLMA374-14|Hymenoptera|Pteromalidae|BOLD:ACK7798
Cecidostiba|[280]|CNGMI667-14|Hymenoptera|Pteromalidae|BOLD:AAU9109
Cecidostiba|[281]|CNGMA1285-13|Hymenoptera|Pteromalidae|BOLD:ACW1111
Cecidostiba|[282]|CNGMA1275-13|Hymenoptera|Pteromalidae|BOLD:ACW5410
Cecidostiba|[283]|CNMIA226-14|Hymenoptera|Pteromalidae|BOLD:ACW5411
Pteromalinae|[284]|CNFNH258-14|Hymenoptera|Pteromalidae|BOLD:ACL8269
Pteromalinae|[285]|CNMIA227-14|Hymenoptera|Pteromalidae|BOLD:ACM1146
Pteromalinae|[286]|CNTMC134-14|Hymenoptera|Pteromalidae|BOLD:ACM4011
Pteromalidae|[287]|JBHCJ646-11|Hymenoptera|Pteromalidae|BOLD:AAU9876
Pteromalidae|[288]|SSPAC3587-13|Hymenoptera|Pteromalidae|BOLD:ACJ7143
Cecidostiba|[289]|SMTPB13694-13|Hymenoptera|Pteromalidae|BOLD:ACF8620
Pteromalidae|[290]|JSHYM046-11|Hymenoptera|Pteromalidae|BOLD:AAG7948
Pteromalidae|[291]|NGNAZ533-14|Hymenoptera|Pteromalidae|BOLD:ACN3848
Pteromalus|[292]|SMTPI7054-14|Hymenoptera|Pteromalidae|BOLD:ACP8174
Pteromalus|[293]|SMTPJ4900-14|Hymenoptera|Pteromalidae|BOLD:AAN8215
Cecidostiba|[294]|JSJUL1984-11|Hymenoptera|Pteromalidae|BOLD:ABA8010
Pteromalidae|[295]|BBHYK982-10|Hymenoptera|Pteromalidae|BOLD:AAU8986
Pteromalinae|[296]|CNGMH403-14|Hymenoptera|Pteromalidae|BOLD:ACM0337
Pteromalidae|[297]|BBHYK751-10|Hymenoptera|Pteromalidae|BOLD:AAU9044
Pteromalidae|[298]|CNKLD3764-15|Hymenoptera|Pteromalidae|BOLD:ACS2192
Lyrcus perdubius|[299]|JSJUL2014-11|Hymenoptera|Pteromalidae|BOLD:AAZ7406

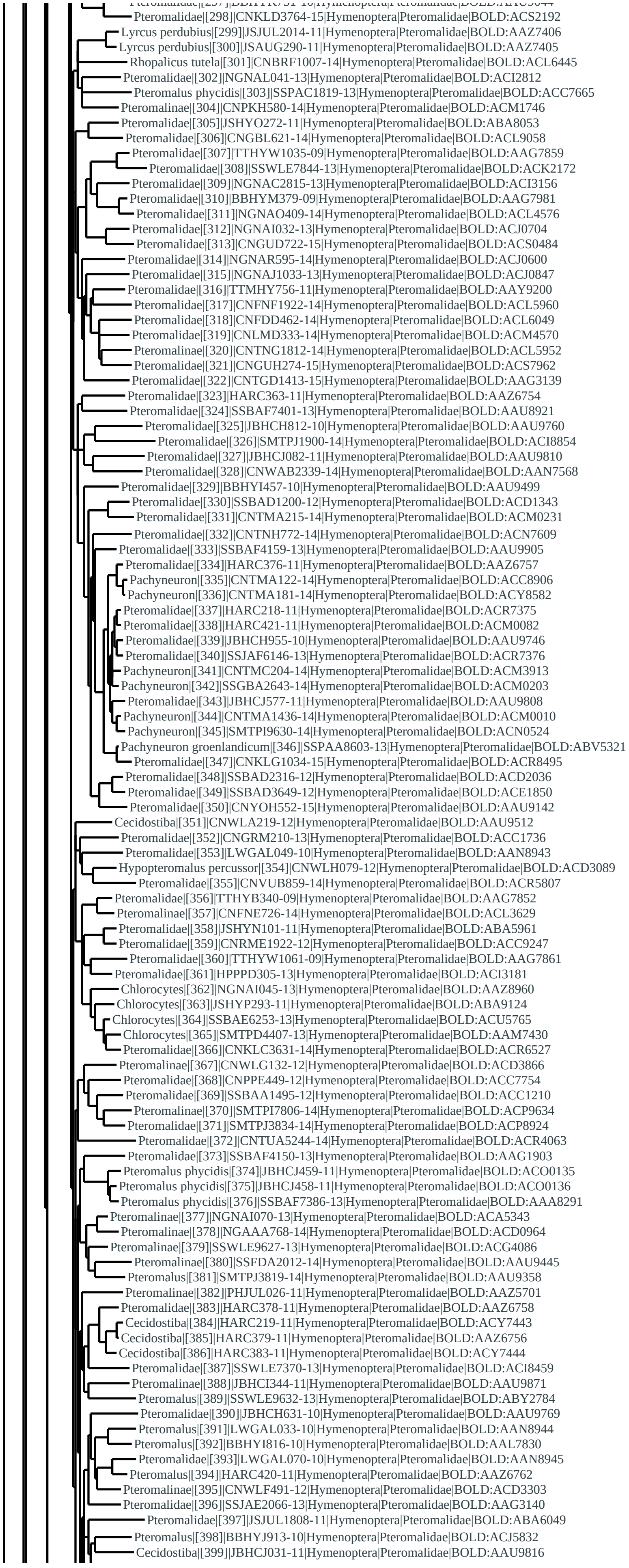
Pteromalidae|[298]|CNKLD3764-15|Hymenoptera|Pteromalidae|BOLD:ACS2192
Lyrcus perdubius|[299]|JSJUL2014-11|Hymenoptera|Pteromalidae|BOLD:AAZ7406
Lyrcus perdubius|[300]|JSAUG290-11|Hymenoptera|Pteromalidae|BOLD:AAZ7405
Rhopalicus tutela|[301]|CNBRF1007-14|Hymenoptera|Pteromalidae|BOLD:ACL6445
Pteromalidae|[302]|NGNAL041-13|Hymenoptera|Pteromalidae|BOLD:ACI2812
Pteromalus phycidis|[303]|SSPAC1819-13|Hymenoptera|Pteromalidae|BOLD:ACC7665
Pteromalinae|[304]|CNPKH580-14|Hymenoptera|Pteromalidae|BOLD:ACM1746
Pteromalidae|[305]|JSHYO272-11|Hymenoptera|Pteromalidae|BOLD:ABA8053
Pteromalidae|[306]|CNGBL621-14|Hymenoptera|Pteromalidae|BOLD:ACL9058
Pteromalidae|[307]|TTHYW1035-09|Hymenoptera|Pteromalidae|BOLD:AAG7859
Pteromalidae|[308]|SSWLE7844-13|Hymenoptera|Pteromalidae|BOLD:ACK2172
Pteromalidae|[309]|NGNAC2815-13|Hymenoptera|Pteromalidae|BOLD:ACI3156
Pteromalidae|[310]|BBHYM379-09|Hymenoptera|Pteromalidae|BOLD:AAG7981
Pteromalidae|[311]|NGNAO409-14|Hymenoptera|Pteromalidae|BOLD:ACL4576
Pteromalidae|[312]|NGNAI032-13|Hymenoptera|Pteromalidae|BOLD:ACJ0704
Pteromalidae|[313]|CNGUD722-15|Hymenoptera|Pteromalidae|BOLD:ACS0484
Pteromalidae|[314]|NGNAR595-14|Hymenoptera|Pteromalidae|BOLD:ACJ0600
Pteromalidae|[315]|NGNAJ1033-13|Hymenoptera|Pteromalidae|BOLD:ACJ0847
Pteromalidae|[316]|TTMHY756-11|Hymenoptera|Pteromalidae|BOLD:AAY9200
Pteromalidae|[317]|CNFNF1922-14|Hymenoptera|Pteromalidae|BOLD:ACL5960
Pteromalidae|[318]|CNFDD462-14|Hymenoptera|Pteromalidae|BOLD:ACL6049
Pteromalidae|[319]|CNLMD333-14|Hymenoptera|Pteromalidae|BOLD:ACM4570
Pteromalinae|[320]|CNTNG1812-14|Hymenoptera|Pteromalidae|BOLD:ACL5952
Pteromalidae|[321]|CNGUH274-15|Hymenoptera|Pteromalidae|BOLD:ACS7962
Pteromalidae|[322]|CNTGD1413-15|Hymenoptera|Pteromalidae|BOLD:AAG3139
Pteromalidae|[323]|HARC363-11|Hymenoptera|Pteromalidae|BOLD:AAZ6754
Pteromalidae|[324]|SSBAF7401-13|Hymenoptera|Pteromalidae|BOLD:AAU8921
Pteromalidae|[325]|JBHCH812-10|Hymenoptera|Pteromalidae|BOLD:AAU9760
Pteromalidae|[326]|SMTPJ1900-14|Hymenoptera|Pteromalidae|BOLD:ACI8854
Pteromalidae|[327]|JBHCJ082-11|Hymenoptera|Pteromalidae|BOLD:AAU9810
Pteromalidae|[328]|CNWAB2339-14|Hymenoptera|Pteromalidae|BOLD:AAN7568
Pteromalidae|[329]|BBHYI457-10|Hymenoptera|Pteromalidae|BOLD:AAU9499
Pteromalidae|[330]|SSBAD1200-12|Hymenoptera|Pteromalidae|BOLD:ACD1343
Pteromalidae|[331]|CNTMA215-14|Hymenoptera|Pteromalidae|BOLD:ACM0231
Pteromalidae|[332]|CNTNH772-14|Hymenoptera|Pteromalidae|BOLD:ACN7609
Pteromalidae|[333]|SSBAF4159-13|Hymenoptera|Pteromalidae|BOLD:AAU9905
Pteromalidae|[334]|HARC376-11|Hymenoptera|Pteromalidae|BOLD:AAZ6757
Pachyneuron|[335]|CNTMA122-14|Hymenoptera|Pteromalidae|BOLD:ACC8906
Pachyneuron|[336]|CNTMA181-14|Hymenoptera|Pteromalidae|BOLD:ACY8582
Pteromalidae|[337]|HARC218-11|Hymenoptera|Pteromalidae|BOLD:ACR7375
Pteromalidae|[338]|HARC421-11|Hymenoptera|Pteromalidae|BOLD:ACM0082
Pteromalidae|[339]|JBHCH955-10|Hymenoptera|Pteromalidae|BOLD:AAU9746
Pteromalidae|[340]|SSJAF6146-13|Hymenoptera|Pteromalidae|BOLD:ACR7376
Pachyneuron|[341]|CNTMC204-14|Hymenoptera|Pteromalidae|BOLD:ACM3913
Pachyneuron|[342]|SSGBA2643-14|Hymenoptera|Pteromalidae|BOLD:ACM0203
Pteromalidae|[343]|JBHCJ577-11|Hymenoptera|Pteromalidae|BOLD:AAU9808
Pachyneuron|[344]|CNTMA1436-14|Hymenoptera|Pteromalidae|BOLD:ACM0010
Pachyneuron|[345]|SMTPI9630-14|Hymenoptera|Pteromalidae|BOLD:ACN0524
Pachyneuron groenlandicum|[346]|SSPAA8603-13|Hymenoptera|Pteromalidae|BOLD:ABV5321
Pteromalidae|[347]|CNKLG1034-15|Hymenoptera|Pteromalidae|BOLD:ACR8495
Pteromalidae|[348]|SSBAD2316-12|Hymenoptera|Pteromalidae|BOLD:ACD2036
Pteromalidae|[349]|SSBAD3649-12|Hymenoptera|Pteromalidae|BOLD:ACE1850
Pteromalidae|[350]|CNYOH552-15|Hymenoptera|Pteromalidae|BOLD:AAU9142
Cecidostiba|[351]|CNWLA219-12|Hymenoptera|Pteromalidae|BOLD:AAU9512
Pteromalidae|[352]|CNGRM210-13|Hymenoptera|Pteromalidae|BOLD:ACC1736
Pteromalidae|[353]|LWGAL049-10|Hymenoptera|Pteromalidae|BOLD:AAN8943
Hypopteromalus percussor|[354]|CNWLH079-12|Hymenoptera|Pteromalidae|BOLD:ACD3089
Pteromalidae|[355]|CNVUB859-14|Hymenoptera|Pteromalidae|BOLD:ACR5807
Pteromalidae|[356]|TTHYB340-09|Hymenoptera|Pteromalidae|BOLD:AAG7852
Pteromalinae|[357]|CNFNE726-14|Hymenoptera|Pteromalidae|BOLD:ACL3629
Pteromalidae|[358]|JSHYN101-11|Hymenoptera|Pteromalidae|BOLD:ABA5961
Pteromalidae|[359]|CNRME1922-12|Hymenoptera|Pteromalidae|BOLD:ACC9247
Pteromalidae|[360]|TTHYW1061-09|Hymenoptera|Pteromalidae|BOLD:AAG7861
Pteromalidae|[361]|HPPPD305-13|Hymenoptera|Pteromalidae|BOLD:ACI3181
Chlorocytes|[362]|NGNAI045-13|Hymenoptera|Pteromalidae|BOLD:AAZ8960
Chlorocytes|[363]|JSHYP293-11|Hymenoptera|Pteromalidae|BOLD:ABA9124
Chlorocytes|[364]|SSBAE6253-13|Hymenoptera|Pteromalidae|BOLD:ACU5765
Chlorocytes|[365]|SMTPD4407-13|Hymenoptera|Pteromalidae|BOLD:AAM7430
Pteromalidae|[366]|CNKLC3631-14|Hymenoptera|Pteromalidae|BOLD:ACR6527
Pteromalinae|[367]|CNWLG132-12|Hymenoptera|Pteromalidae|BOLD:ACD3866
Pteromalidae|[368]|CNPPE449-12|Hymenoptera|Pteromalidae|BOLD:ACC7754
Pteromalidae|[369]|SSBAA1495-12|Hymenoptera|Pteromalidae|BOLD:ACC1210
Pteromalinae|[370]|SMTPI7806-14|Hymenoptera|Pteromalidae|BOLD:ACP9634
Pteromalidae|[371]|SMTPJ3834-14|Hymenoptera|Pteromalidae|BOLD:ACP8924
Pteromalidae|[372]|CNTUA5244-14|Hymenoptera|Pteromalidae|BOLD:ACR4063
Pteromalidae|[373]|SSBAF4150-13|Hymenoptera|Pteromalidae|BOLD:AAG1903
Pteromalus phycidis|[374]|JBHCJ459-11|Hymenoptera|Pteromalidae|BOLD:ACO0135
Pteromalus phycidis|[375]|JBHCJ458-11|Hymenoptera|Pteromalidae|BOLD:ACO0136
Pteromalus phycidis|[376]|SSBAF7386-13|Hymenoptera|Pteromalidae|BOLD:AAA8291
Pteromalinae|[377]|NGNAI070-13|Hymenoptera|Pteromalidae|BOLD:ACA5343
Pteromalinae|[378]|NGAAA768-14|Hymenoptera|Pteromalidae|BOLD:ACD0964
Pteromalinae|[379]|SSWLE9627-13|Hymenoptera|Pteromalidae|BOLD:ACG4086
Pteromalinae|[380]|SSFDA2012-14|Hymenoptera|Pteromalidae|BOLD:AAU9445
Pteromalus|[381]|SMTPJ3819-14|Hymenoptera|Pteromalidae|BOLD:AAU9358
Pteromalinae|[382]|PHJUL026-11|Hymenoptera|Pteromalidae|BOLD:AAZ5701
Pteromalidae|[383]|HARC378-11|Hymenoptera|Pteromalidae|BOLD:AAZ6758
Cecidostiba|[384]|HARC219-11|Hymenoptera|Pteromalidae|BOLD:ACY7443
Cecidostiba|[385]|HARC379-11|Hymenoptera|Pteromalidae|BOLD:AAZ6756
Cecidostiba|[386]|HARC383-11|Hymenoptera|Pteromalidae|BOLD:ACY7444
Pteromalidae|[387]|SSWLE7370-13|Hymenoptera|Pteromalidae|BOLD:ACI8459
Pteromalinae|[388]|JBHCI344-11|Hymenoptera|Pteromalidae|BOLD:AAU9871
Pteromalus|[389]|SSWLE9632-13|Hymenoptera|Pteromalidae|BOLD:ABY2784
Pteromalidae|[390]|JBHCH631-10|Hymenoptera|Pteromalidae|BOLD:AAU9769
Pteromalus|[391]|LWGAL033-10|Hymenoptera|Pteromalidae|BOLD:AAN8944
Pteromalus|[392]|BBHYI816-10|Hymenoptera|Pteromalidae|BOLD:AAL7830
Pteromalidae|[393]|LWGAL070-10|Hymenoptera|Pteromalidae|BOLD:AAN8945
Pteromalus|[394]|HARC420-11|Hymenoptera|Pteromalidae|BOLD:AAZ6762
Pteromalinae|[395]|CNWLF491-12|Hymenoptera|Pteromalidae|BOLD:ACD3303
Pteromalidae|[396]|SSJAE2066-13|Hymenoptera|Pteromalidae|BOLD:AAG3140
Pteromalidae|[397]|JSJUL1808-11|Hymenoptera|Pteromalidae|BOLD:ABA6049
Pteromalus|[398]|BBHYJ913-10|Hymenoptera|Pteromalidae|BOLD:ACJ5832
Cecidostiba|[399]|JBHCJ031-11|Hymenoptera|Pteromalidae|BOLD:AAU9816

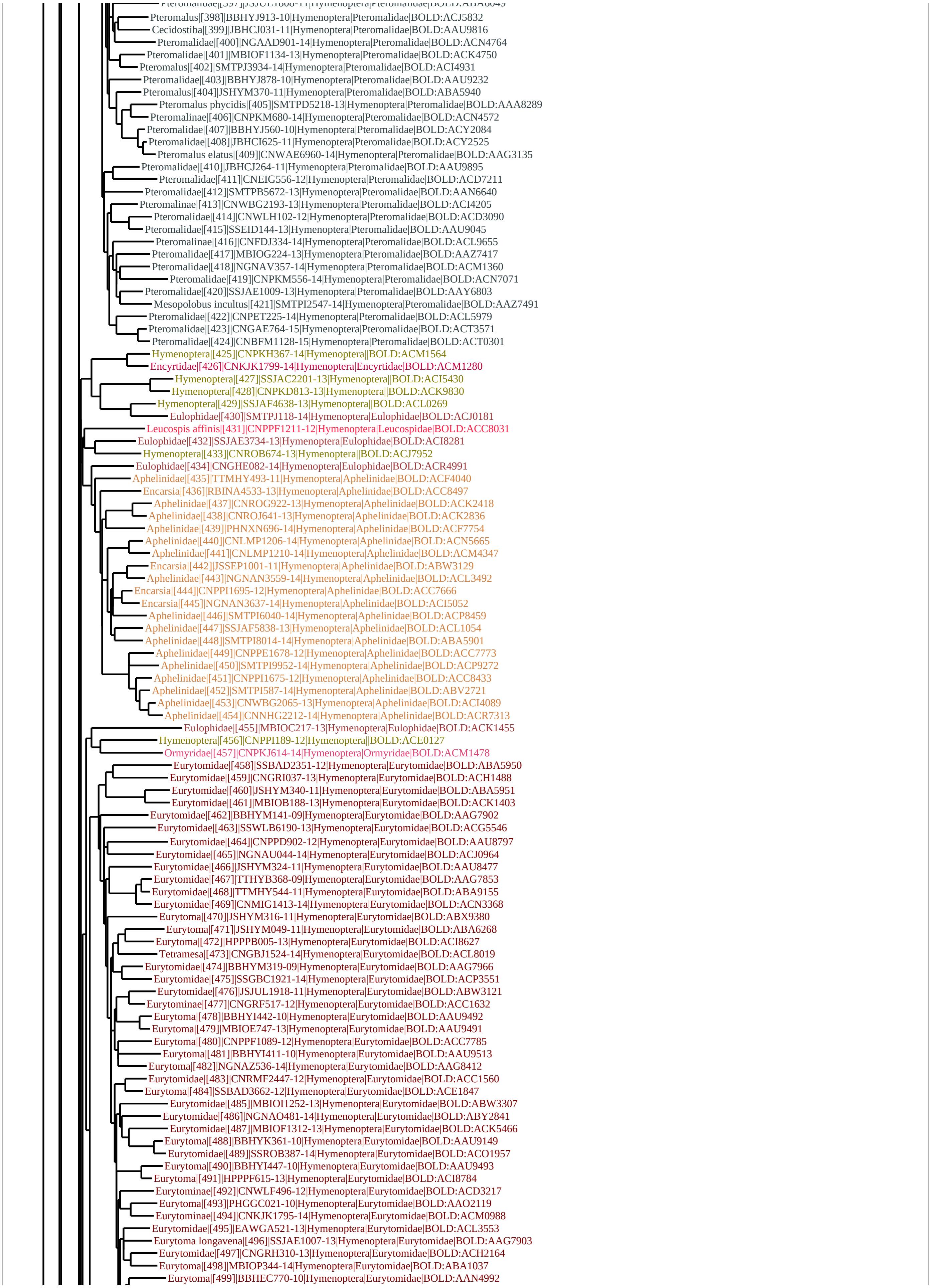
Pteromalidae|[397]|JSJUL1808-11|Hymenoptera|Pteromalidae|BOLD:ABA6049
Pteromalus|[398]|BBHYJ913-10|Hymenoptera|Pteromalidae|BOLD:ACJ5832
Cecidostiba|[399]|JBHCJ031-11|Hymenoptera|Pteromalidae|BOLD:AAU9816
Pteromalidae|[400]|NGAAD901-14|Hymenoptera|Pteromalidae|BOLD:ACN4764
Pteromalidae|[401]|MBIOF1134-13|Hymenoptera|Pteromalidae|BOLD:ACK4750
Pteromalus|[402]|SMTPJ3934-14|Hymenoptera|Pteromalidae|BOLD:ACI4931
Pteromalidae|[403]|BBHYJ878-10|Hymenoptera|Pteromalidae|BOLD:AAU9232
Pteromalus|[404]|JSHYM370-11|Hymenoptera|Pteromalidae|BOLD:ABA5940
Pteromalus phycidis|[405]|SMTPD5218-13|Hymenoptera|Pteromalidae|BOLD:AAA8289
Pteromalinae|[406]|CNPKM680-14|Hymenoptera|Pteromalidae|BOLD:ACN4572
Pteromalidae|[407]|BBHYJ560-10|Hymenoptera|Pteromalidae|BOLD:ACY2084
Pteromalidae|[408]|JBHCI625-11|Hymenoptera|Pteromalidae|BOLD:ACY2525
Pteromalus elatus|[409]|CNWAE6960-14|Hymenoptera|Pteromalidae|BOLD:AAG3135
Pteromalidae|[410]|JBHCJ264-11|Hymenoptera|Pteromalidae|BOLD:AAU9895
Pteromalidae|[411]|CNEIG556-12|Hymenoptera|Pteromalidae|BOLD:ACD7211
Pteromalidae|[412]|SMTPB5672-13|Hymenoptera|Pteromalidae|BOLD:AAN6640
Pteromalinae|[413]|CNWBG2193-13|Hymenoptera|Pteromalidae|BOLD:ACI4205
Pteromalidae|[414]|CNWLH102-12|Hymenoptera|Pteromalidae|BOLD:ACD3090
Pteromalidae|[415]|SSEID144-13|Hymenoptera|Pteromalidae|BOLD:AAU9045
Pteromalinae|[416]|CNFDJ334-14|Hymenoptera|Pteromalidae|BOLD:ACL9655
Pteromalidae|[417]|MBIOG224-13|Hymenoptera|Pteromalidae|BOLD:AAZ7417
Pteromalidae|[418]|NGNAV357-14|Hymenoptera|Pteromalidae|BOLD:ACM1360
Pteromalidae|[419]|CNPKM556-14|Hymenoptera|Pteromalidae|BOLD:ACN7071
Pteromalidae|[420]|SSJAE1009-13|Hymenoptera|Pteromalidae|BOLD:AAY6803
Mesopolobus incultus|[421]|SMTPI2547-14|Hymenoptera|Pteromalidae|BOLD:AAZ7491
Pteromalidae|[422]|CNPET225-14|Hymenoptera|Pteromalidae|BOLD:ACL5979
Pteromalidae|[423]|CNGAE764-15|Hymenoptera|Pteromalidae|BOLD:ACT3571
Pteromalidae|[424]|CNBFM1128-15|Hymenoptera|Pteromalidae|BOLD:ACT0301
Hymenoptera|[425]|CNPKH367-14|Hymenoptera||BOLD:ACM1564
Encyrtidae|[426]|CNKJK1799-14|Hymenoptera|Encyrtidae|BOLD:ACM1280
Hymenoptera|[427]|SSJAC2201-13|Hymenoptera||BOLD:ACI5430
Hymenoptera|[428]|CNPKD813-13|Hymenoptera||BOLD:ACK9830
Hymenoptera|[429]|SSJAF4638-13|Hymenoptera||BOLD:ACL0269
Eulophidae|[430]|SMTPJ118-14|Hymenoptera|Eulophidae|BOLD:ACJ0181
Leucospis affinis|[431]|CNPPF1211-12|Hymenoptera|Leucospidae|BOLD:ACC8031
Eulophidae|[432]|SSJAE3734-13|Hymenoptera|Eulophidae|BOLD:ACI8281
Hymenoptera|[433]|CNROB674-13|Hymenoptera||BOLD:ACJ7952
Eulophidae|[434]|CNGHE082-14|Hymenoptera|Eulophidae|BOLD:ACR4991
Aphelinidae|[435]|TTMHY493-11|Hymenoptera|Aphelinidae|BOLD:ACF4040
Encarsia|[436]|RBINA4533-13|Hymenoptera|Aphelinidae|BOLD:ACC8497
Aphelinidae|[437]|CNROG922-13|Hymenoptera|Aphelinidae|BOLD:ACK2418
Aphelinidae|[438]|CNROJ641-13|Hymenoptera|Aphelinidae|BOLD:ACK2836
Aphelinidae|[439]|PHNXN696-14|Hymenoptera|Aphelinidae|BOLD:ACF7754
Aphelinidae|[440]|CNLMP1206-14|Hymenoptera|Aphelinidae|BOLD:ACN5665
Aphelinidae|[441]|CNLMP1210-14|Hymenoptera|Aphelinidae|BOLD:ACM4347
Encarsia|[442]|JSSEP1001-11|Hymenoptera|Aphelinidae|BOLD:ABW3129
Aphelinidae|[443]|NGNAN3559-14|Hymenoptera|Aphelinidae|BOLD:ACL3492
Encarsia|[444]|CNPPI1695-12|Hymenoptera|Aphelinidae|BOLD:ACC7666
Encarsia|[445]|NGNAN3637-14|Hymenoptera|Aphelinidae|BOLD:ACI5052
Aphelinidae|[446]|SMTPI6040-14|Hymenoptera|Aphelinidae|BOLD:ACP8459
Aphelinidae|[447]|SSJAF5838-13|Hymenoptera|Aphelinidae|BOLD:ACL1054
Aphelinidae|[448]|SMTPI8014-14|Hymenoptera|Aphelinidae|BOLD:ABA5901
Aphelinidae|[449]|CNPPE1678-12|Hymenoptera|Aphelinidae|BOLD:ACC7773
Aphelinidae|[450]|SMTPI9952-14|Hymenoptera|Aphelinidae|BOLD:ACP9272
Aphelinidae|[451]|CNPPI1675-12|Hymenoptera|Aphelinidae|BOLD:ACC8433
Aphelinidae|[452]|SMTPI587-14|Hymenoptera|Aphelinidae|BOLD:ABV2721
Aphelinidae|[453]|CNWBG2065-13|Hymenoptera|Aphelinidae|BOLD:ACI4089
Aphelinidae|[454]|CNNHG2212-14|Hymenoptera|Aphelinidae|BOLD:ACR7313
Eulophidae|[455]|MBIOC217-13|Hymenoptera|Eulophidae|BOLD:ACK1455
Hymenoptera|[456]|CNPPI189-12|Hymenoptera||BOLD:ACE0127
Ormyridae|[457]|CNPKJ614-14|Hymenoptera|Ormyridae|BOLD:ACM1478
Eurytomidae|[458]|SSBAD2351-12|Hymenoptera|Eurytomidae|BOLD:ABA5950
Eurytomidae|[459]|CNGRI037-13|Hymenoptera|Eurytomidae|BOLD:ACH1488
Eurytomidae|[460]|JSHYM340-11|Hymenoptera|Eurytomidae|BOLD:ABA5951
Eurytomidae|[461]|MBIOB188-13|Hymenoptera|Eurytomidae|BOLD:ACK1403
Eurytomidae|[462]|BBHYM141-09|Hymenoptera|Eurytomidae|BOLD:AAG7902
Eurytomidae|[463]|SSWLB6190-13|Hymenoptera|Eurytomidae|BOLD:ACG5546
Eurytomidae|[464]|CNPPD902-12|Hymenoptera|Eurytomidae|BOLD:AAU8797
Eurytomidae|[465]|NGNAU044-14|Hymenoptera|Eurytomidae|BOLD:ACJ0964
Eurytomidae|[466]|JSHYM324-11|Hymenoptera|Eurytomidae|BOLD:AAU8477
Eurytomidae|[467]|TTHYB368-09|Hymenoptera|Eurytomidae|BOLD:AAG7853
Eurytomidae|[468]|TTMHY544-11|Hymenoptera|Eurytomidae|BOLD:ABA9155
Eurytomidae|[469]|CNMIG1413-14|Hymenoptera|Eurytomidae|BOLD:ACN3368
Eurytoma|[470]|JSHYM316-11|Hymenoptera|Eurytomidae|BOLD:ABX9380
Eurytoma|[471]|JSHYM049-11|Hymenoptera|Eurytomidae|BOLD:ABA6268
Eurytoma|[472]|HPPPB005-13|Hymenoptera|Eurytomidae|BOLD:ACI8627
Tetramesa|[473]|CNGBJ1524-14|Hymenoptera|Eurytomidae|BOLD:ACL8019
Eurytomidae|[474]|BBHYM319-09|Hymenoptera|Eurytomidae|BOLD:AAG7966
Eurytomidae|[475]|SSGBC1921-14|Hymenoptera|Eurytomidae|BOLD:ACP3551
Eurytomidae|[476]|JSJUL1918-11|Hymenoptera|Eurytomidae|BOLD:ABW3121
Eurytominae|[477]|CNGRF517-12|Hymenoptera|Eurytomidae|BOLD:ACC1632
Eurytoma|[478]|BBHYI442-10|Hymenoptera|Eurytomidae|BOLD:AAU9492
Eurytoma|[479]|MBIOE747-13|Hymenoptera|Eurytomidae|BOLD:AAU9491
Eurytoma|[480]|CNPPF1089-12|Hymenoptera|Eurytomidae|BOLD:ACC7785
Eurytoma|[481]|BBHYI411-10|Hymenoptera|Eurytomidae|BOLD:AAU9513
Eurytoma|[482]|NGNAZ536-14|Hymenoptera|Eurytomidae|BOLD:AAG8412
Eurytomidae|[483]|CNRMF2447-12|Hymenoptera|Eurytomidae|BOLD:ACC1560
Eurytoma|[484]|SSBAD3662-12|Hymenoptera|Eurytomidae|BOLD:ACE1847
Eurytomidae|[485]|MBIOI1252-13|Hymenoptera|Eurytomidae|BOLD:ABW3307
Eurytomidae|[486]|NGNAO481-14|Hymenoptera|Eurytomidae|BOLD:ABY2841
Eurytomidae|[487]|MBIOF1312-13|Hymenoptera|Eurytomidae|BOLD:ACK5466
Eurytoma|[488]|BBHYK361-10|Hymenoptera|Eurytomidae|BOLD:AAU9149
Eurytomidae|[489]|SSROB387-14|Hymenoptera|Eurytomidae|BOLD:ACO1957
Eurytoma|[490]|BBHYI447-10|Hymenoptera|Eurytomidae|BOLD:AAU9493
Eurytoma|[491]|HPPPF615-13|Hymenoptera|Eurytomidae|BOLD:ACI8784
Eurytominae|[492]|CNWLF496-12|Hymenoptera|Eurytomidae|BOLD:ACD3217
Eurytoma|[493]|PHGGC021-10|Hymenoptera|Eurytomidae|BOLD:AAO2119
Eurytominae|[494]|CNKJK1795-14|Hymenoptera|Eurytomidae|BOLD:ACM0988
Eurytomidae|[495]|EAWGA521-13|Hymenoptera|Eurytomidae|BOLD:ACL3553
Eurytoma longavena|[496]|SSJAE1007-13|Hymenoptera|Eurytomidae|BOLD:AAG7903
Eurytomidae|[497]|CNGRH310-13|Hymenoptera|Eurytomidae|BOLD:ACH2164
Eurytoma|[498]|MBIOP344-14|Hymenoptera|Eurytomidae|BOLD:ABA1037
Eurytoma|[499]|BBHEC770-10|Hymenoptera|Eurytomidae|BOLD:AAN4992

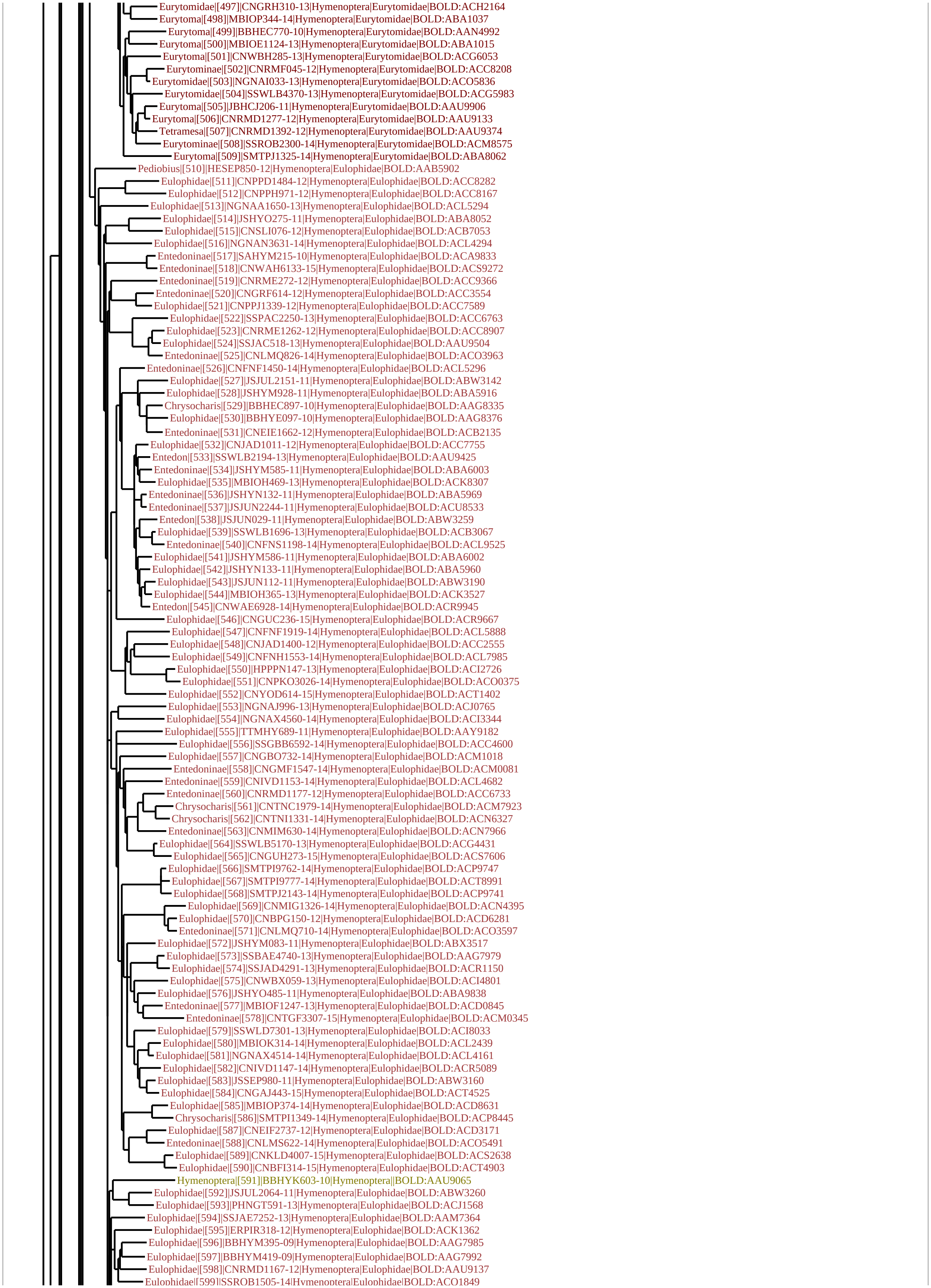

Eurytomidae|[497]|CNGRH310-13|Hymenoptera|Eurytomidae|BOLD:ACH2164
Eurytoma|[498]|MBIOP344-14|Hymenoptera|Eurytomidae|BOLD:ABA1037
Eurytoma|[499]|BBHEC770-10|Hymenoptera|Eurytomidae|BOLD:AAN4992
Eurytoma|[500]|MBIOE1124-13|Hymenoptera|Eurytomidae|BOLD:ABA1015
Eurytoma|[501]|CNWBH285-13|Hymenoptera|Eurytomidae|BOLD:ACG6053
Eurytominae|[502]|CNRMF045-12|Hymenoptera|Eurytomidae|BOLD:ACC8208
Eurytomidae|[503]|NGNAI033-13|Hymenoptera|Eurytomidae|BOLD:ACO5836
Eurytomidae|[504]|SSWLB4370-13|Hymenoptera|Eurytomidae|BOLD:ACG5983
Eurytoma|[505]|JBHCJ206-11|Hymenoptera|Eurytomidae|BOLD:AAU9906
Eurytoma|[506]|CNRMD1277-12|Hymenoptera|Eurytomidae|BOLD:AAU9133
Tetramesa|[507]|CNRMD1392-12|Hymenoptera|Eurytomidae|BOLD:AAU9374
Eurytominae|[508]|SSROB2300-14|Hymenoptera|Eurytomidae|BOLD:ACM8575
Eurytoma|[509]|SMTPJ1325-14|Hymenoptera|Eurytomidae|BOLD:ABA8062
Pediobius|[510]|HESEP850-12|Hymenoptera|Eulophidae|BOLD:AAB5902
Eulophidae|[511]|CNPPD1484-12|Hymenoptera|Eulophidae|BOLD:ACC8282
Eulophidae|[512]|CNPPH971-12|Hymenoptera|Eulophidae|BOLD:ACC8167
Eulophidae|[513]|NGNAA1650-13|Hymenoptera|Eulophidae|BOLD:ACL5294
Eulophidae|[514]|JSHYO275-11|Hymenoptera|Eulophidae|BOLD:ABA8052
Eulophidae|[515]|CNSLI076-12|Hymenoptera|Eulophidae|BOLD:ACB7053
Eulophidae|[516]|NGNAN3631-14|Hymenoptera|Eulophidae|BOLD:ACL4294
Entedoninae|[517]|SAHYM215-10|Hymenoptera|Eulophidae|BOLD:ACA9833
Entedoninae|[518]|CNWAH6133-15|Hymenoptera|Eulophidae|BOLD:ACS9272
Entedoninae|[519]|CNRME272-12|Hymenoptera|Eulophidae|BOLD:ACC9366
Entedoninae|[520]|CNGRF614-12|Hymenoptera|Eulophidae|BOLD:ACC3554
Eulophidae|[521]|CNPPJ1339-12|Hymenoptera|Eulophidae|BOLD:ACC7589
Eulophidae|[522]|SSPAC2250-13|Hymenoptera|Eulophidae|BOLD:ACC6763
Eulophidae|[523]|CNRME1262-12|Hymenoptera|Eulophidae|BOLD:ACC8907
Eulophidae|[524]|SSJAC518-13|Hymenoptera|Eulophidae|BOLD:AAU9504
Entedoninae|[525]|CNLMQ826-14|Hymenoptera|Eulophidae|BOLD:ACO3963
Entedoninae|[526]|CNFNF1450-14|Hymenoptera|Eulophidae|BOLD:ACL5296
Eulophidae|[527]|JSJUL2151-11|Hymenoptera|Eulophidae|BOLD:ABW3142
Eulophidae|[528]|JSHYM928-11|Hymenoptera|Eulophidae|BOLD:ABA5916
Chrysocharis|[529]|BBHEC897-10|Hymenoptera|Eulophidae|BOLD:AAG8335
Eulophidae|[530]|BBHYE097-10|Hymenoptera|Eulophidae|BOLD:AAG8376
Entedoninae|[531]|CNEIE1662-12|Hymenoptera|Eulophidae|BOLD:ACB2135
Eulophidae|[532]|CNJAD1011-12|Hymenoptera|Eulophidae|BOLD:ACC7755
Entedon|[533]|SSWLB2194-13|Hymenoptera|Eulophidae|BOLD:AAU9425
Entedoninae|[534]|JSHYM585-11|Hymenoptera|Eulophidae|BOLD:ABA6003
Eulophidae|[535]|MBIOH469-13|Hymenoptera|Eulophidae|BOLD:ACK8307
Entedoninae|[536]|JSHYN132-11|Hymenoptera|Eulophidae|BOLD:ABA5969
Entedoninae|[537]|JSJUN2244-11|Hymenoptera|Eulophidae|BOLD:ACU8533
Entedon|[538]|JSJUN029-11|Hymenoptera|Eulophidae|BOLD:ABW3259
Eulophidae|[539]|SSWLB1696-13|Hymenoptera|Eulophidae|BOLD:ACB3067
Entedoninae|[540]|CNFNS1198-14|Hymenoptera|Eulophidae|BOLD:ACL9525
Eulophidae|[541]|JSHYM586-11|Hymenoptera|Eulophidae|BOLD:ABA6002
Eulophidae|[542]|JSHYN133-11|Hymenoptera|Eulophidae|BOLD:ABA5960
Eulophidae|[543]|JSJUN112-11|Hymenoptera|Eulophidae|BOLD:ABW3190
Eulophidae|[544]|MBIOH365-13|Hymenoptera|Eulophidae|BOLD:ACK3527
Entedon|[545]|CNWAE6928-14|Hymenoptera|Eulophidae|BOLD:ACR9945
Eulophidae|[546]|CNGUC236-15|Hymenoptera|Eulophidae|BOLD:ACR9667
Eulophidae|[547]|CNFNF1919-14|Hymenoptera|Eulophidae|BOLD:ACL5888
Eulophidae|[548]|CNJAD1400-12|Hymenoptera|Eulophidae|BOLD:ACC2555
Eulophidae|[549]|CNFNH1553-14|Hymenoptera|Eulophidae|BOLD:ACL7985
Eulophidae|[550]|HPPPN147-13|Hymenoptera|Eulophidae|BOLD:ACI2726
Eulophidae|[551]|CNPKO3026-14|Hymenoptera|Eulophidae|BOLD:ACO0375
Eulophidae|[552]|CNYOD614-15|Hymenoptera|Eulophidae|BOLD:ACT1402
Eulophidae|[553]|NGNAJ996-13|Hymenoptera|Eulophidae|BOLD:ACJ0765
Eulophidae|[554]|NGNAX4560-14|Hymenoptera|Eulophidae|BOLD:ACI3344
Eulophidae|[555]|TTMHY689-11|Hymenoptera|Eulophidae|BOLD:AAY9182
Eulophidae|[556]|SSGBB6592-14|Hymenoptera|Eulophidae|BOLD:ACC4600
Eulophidae|[557]|CNGBO732-14|Hymenoptera|Eulophidae|BOLD:ACM1018
Entedoninae|[558]|CNGMF1547-14|Hymenoptera|Eulophidae|BOLD:ACM0081
Entedoninae|[559]|CNIVD1153-14|Hymenoptera|Eulophidae|BOLD:ACL4682
Entedoninae|[560]|CNRMD1177-12|Hymenoptera|Eulophidae|BOLD:ACC6733
Chrysocharis|[561]|CNTNC1979-14|Hymenoptera|Eulophidae|BOLD:ACM7923
Chrysocharis|[562]|CNTNI1331-14|Hymenoptera|Eulophidae|BOLD:ACN6327
Entedoninae|[563]|CNMIM630-14|Hymenoptera|Eulophidae|BOLD:ACN7966
Eulophidae|[564]|SSWLB5170-13|Hymenoptera|Eulophidae|BOLD:ACG4431
Eulophidae|[565]|CNGUH273-15|Hymenoptera|Eulophidae|BOLD:ACS7606
Eulophidae|[566]|SMTPI9762-14|Hymenoptera|Eulophidae|BOLD:ACP9747
Eulophidae|[567]|SMTPI9777-14|Hymenoptera|Eulophidae|BOLD:ACT8991
Eulophidae|[568]|SMTPJ2143-14|Hymenoptera|Eulophidae|BOLD:ACP9741
Eulophidae|[569]|CNMIG1326-14|Hymenoptera|Eulophidae|BOLD:ACN4395
Eulophidae|[570]|CNBPG150-12|Hymenoptera|Eulophidae|BOLD:ACD6281
Entedoninae|[571]|CNLMQ710-14|Hymenoptera|Eulophidae|BOLD:ACO3597
Eulophidae|[572]|JSHYM083-11|Hymenoptera|Eulophidae|BOLD:ABX3517
Eulophidae|[573]|SSBAE4740-13|Hymenoptera|Eulophidae|BOLD:AAG7979
Eulophidae|[574]|SSJAD4291-13|Hymenoptera|Eulophidae|BOLD:ACR1150
Eulophidae|[575]|CNWBX059-13|Hymenoptera|Eulophidae|BOLD:ACI4801
Eulophidae|[576]|JSHYO485-11|Hymenoptera|Eulophidae|BOLD:ABA9838
Entedoninae|[577]|MBIOF1247-13|Hymenoptera|Eulophidae|BOLD:ACD0845
Entedoninae|[578]|CNTGF3307-15|Hymenoptera|Eulophidae|BOLD:ACM0345
Eulophidae|[579]|SSWLD7301-13|Hymenoptera|Eulophidae|BOLD:ACI8033
Eulophidae|[580]|MBIOK314-14|Hymenoptera|Eulophidae|BOLD:ACL2439
Eulophidae|[581]|NGNAX4514-14|Hymenoptera|Eulophidae|BOLD:ACL4161
Eulophidae|[582]|CNIVD1147-14|Hymenoptera|Eulophidae|BOLD:ACR5089
Eulophidae|[583]|JSSEP980-11|Hymenoptera|Eulophidae|BOLD:ABW3160
Eulophidae|[584]|CNGAJ443-15|Hymenoptera|Eulophidae|BOLD:ACT4525
Eulophidae|[585]|MBIOP374-14|Hymenoptera|Eulophidae|BOLD:ACD8631
Chrysocharis|[586]|SMTPI1349-14|Hymenoptera|Eulophidae|BOLD:ACP8445
Eulophidae|[587]|CNEIF2737-12|Hymenoptera|Eulophidae|BOLD:ACD3171
Entedoninae|[588]|CNLMS622-14|Hymenoptera|Eulophidae|BOLD:ACO5491
Eulophidae|[589]|CNKLD4007-15|Hymenoptera|Eulophidae|BOLD:ACS2638
Eulophidae|[590]|CNBFI314-15|Hymenoptera|Eulophidae|BOLD:ACT4903
Hymenoptera|[591]|BBHYK603-10|Hymenoptera||BOLD:AAU9065
Eulophidae|[592]|JSJUL2064-11|Hymenoptera|Eulophidae|BOLD:ABW3260
Eulophidae|[593]|PHNGT591-13|Hymenoptera|Eulophidae|BOLD:ACJ1568
Eulophidae|[594]|SSJAE7252-13|Hymenoptera|Eulophidae|BOLD:AAM7364
Eulophidae|[595]|ERPIR318-12|Hymenoptera|Eulophidae|BOLD:ACK1362
Eulophidae|[596]|BBHYM395-09|Hymenoptera|Eulophidae|BOLD:AAG7985
Eulophidae|[597]|BBHYM419-09|Hymenoptera|Eulophidae|BOLD:AAG7992
Eulophidae|[598]|CNRMD1167-12|Hymenoptera|Eulophidae|BOLD:AAU9137
Eulophidae|[599]|SSROB1505-14|Hymenoptera|Eulophidae|BOLD:ACO1849

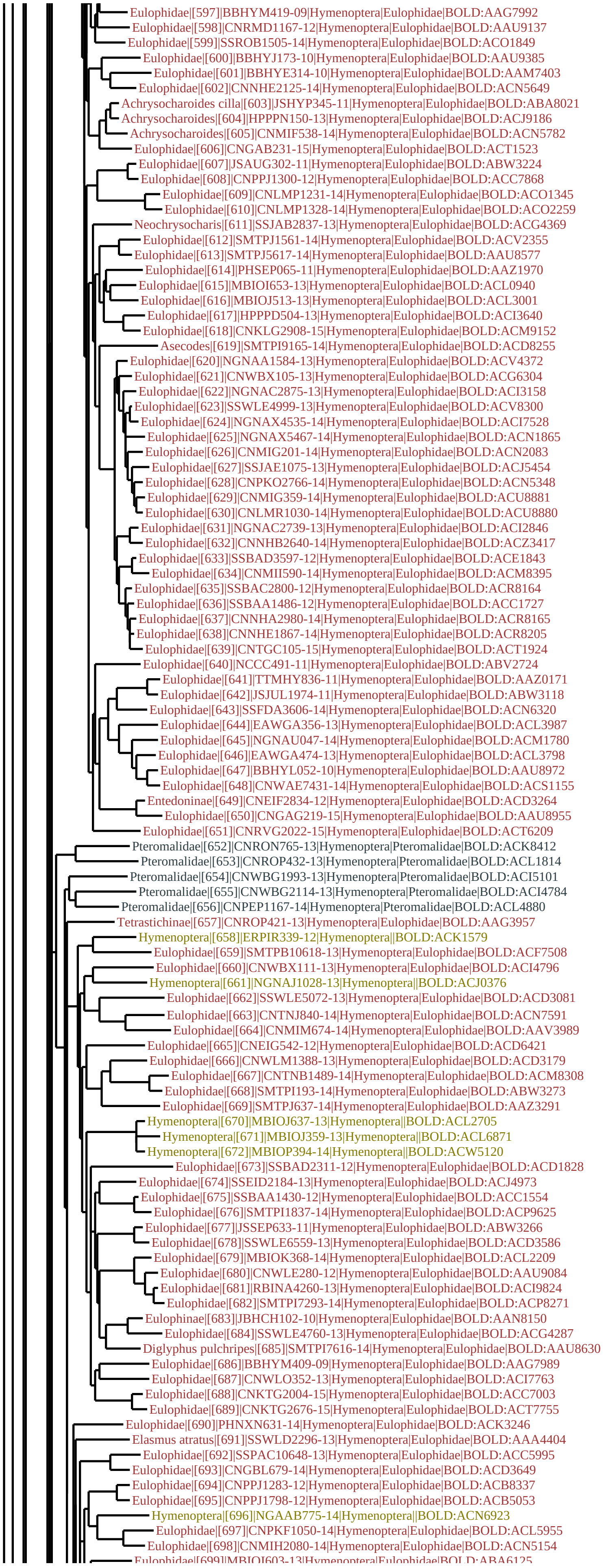

Eulophidae|[597]|BBHYM419-09|Hymenoptera|Eulophidae|BOLD:AAG7992
Eulophidae|[598]|CNRMD1167-12|Hymenoptera|Eulophidae|BOLD:AAU9137
Eulophidae|[599]|SSROB1505-14|Hymenoptera|Eulophidae|BOLD:ACO1849
Eulophidae|[600]|BBHYJ173-10|Hymenoptera|Eulophidae|BOLD:AAU9385
Eulophidae|[601]|BBHYE314-10|Hymenoptera|Eulophidae|BOLD:AAM7403
Eulophidae|[602]|CNNHE2125-14|Hymenoptera|Eulophidae|BOLD:ACN5649
Achrysocharoides cilla|[603]|JSHYP345-11|Hymenoptera|Eulophidae|BOLD:ABA8021
Achrysocharoides|[604]|HPPPN150-13|Hymenoptera|Eulophidae|BOLD:ACJ9186
Achrysocharoides|[605]|CNMIF538-14|Hymenoptera|Eulophidae|BOLD:ACN5782
Eulophidae|[606]|CNGAB231-15|Hymenoptera|Eulophidae|BOLD:ACT1523
Eulophidae|[607]|JSAUG302-11|Hymenoptera|Eulophidae|BOLD:ABW3224
Eulophidae|[608]|CNPPJ1300-12|Hymenoptera|Eulophidae|BOLD:ACC7868
Eulophidae|[609]|CNLMP1231-14|Hymenoptera|Eulophidae|BOLD:ACO1345
Eulophidae|[610]|CNLMP1328-14|Hymenoptera|Eulophidae|BOLD:ACO2259
Neochrysocharis|[611]|SSJAB2837-13|Hymenoptera|Eulophidae|BOLD:ACG4369
Eulophidae|[612]|SMTPJ1561-14|Hymenoptera|Eulophidae|BOLD:ACV2355
Eulophidae|[613]|SMTPJ5617-14|Hymenoptera|Eulophidae|BOLD:AAU8577
Eulophidae|[614]|PHSEP065-11|Hymenoptera|Eulophidae|BOLD:AAZ1970
Eulophidae|[615]|MBIOI653-13|Hymenoptera|Eulophidae|BOLD:ACL0940
Eulophidae|[616]|MBIOJ513-13|Hymenoptera|Eulophidae|BOLD:ACL3001
Eulophidae|[617]|HPPPD504-13|Hymenoptera|Eulophidae|BOLD:ACI3640
Eulophidae|[618]|CNKLG2908-15|Hymenoptera|Eulophidae|BOLD:ACM9152
Asecodes|[619]|SMTPI9165-14|Hymenoptera|Eulophidae|BOLD:ACD8255
Eulophidae|[620]|NGNAA1584-13|Hymenoptera|Eulophidae|BOLD:ACV4372
Eulophidae|[621]|CNWBX105-13|Hymenoptera|Eulophidae|BOLD:ACG6304
Eulophidae|[622]|NGNAC2875-13|Hymenoptera|Eulophidae|BOLD:ACI3158
Eulophidae|[623]|SSWLE4999-13|Hymenoptera|Eulophidae|BOLD:ACV8300
Eulophidae|[624]|NGNAX4535-14|Hymenoptera|Eulophidae|BOLD:ACI7528
Eulophidae|[625]|NGNAX5467-14|Hymenoptera|Eulophidae|BOLD:ACN1865
Eulophidae|[626]|CNMIG201-14|Hymenoptera|Eulophidae|BOLD:ACN2083
Eulophidae|[627]|SSJAE1075-13|Hymenoptera|Eulophidae|BOLD:ACJ5454
Eulophidae|[628]|CNPKO2766-14|Hymenoptera|Eulophidae|BOLD:ACN5348
Eulophidae|[629]|CNMIG359-14|Hymenoptera|Eulophidae|BOLD:ACU8881
Eulophidae|[630]|CNLMR1030-14|Hymenoptera|Eulophidae|BOLD:ACU8880
Eulophidae|[631]|NGNAC2739-13|Hymenoptera|Eulophidae|BOLD:ACI2846
Eulophidae|[632]|CNNHB2640-14|Hymenoptera|Eulophidae|BOLD:ACZ3417
Eulophidae|[633]|SSBAD3597-12|Hymenoptera|Eulophidae|BOLD:ACE1843
Eulophidae|[634]|CNMII590-14|Hymenoptera|Eulophidae|BOLD:ACM8395
Eulophidae|[635]|SSBAC2800-12|Hymenoptera|Eulophidae|BOLD:ACR8164
Eulophidae|[636]|SSBAA1486-12|Hymenoptera|Eulophidae|BOLD:ACC1727
Eulophidae|[637]|CNNHA2980-14|Hymenoptera|Eulophidae|BOLD:ACR8165
Eulophidae|[638]|CNNHE1867-14|Hymenoptera|Eulophidae|BOLD:ACR8205
Eulophidae|[639]|CNTGC105-15|Hymenoptera|Eulophidae|BOLD:ACT1924
Eulophidae|[640]|NCCC491-11|Hymenoptera|Eulophidae|BOLD:ABV2724
Eulophidae|[641]|TTMHY836-11|Hymenoptera|Eulophidae|BOLD:AAZ0171
Eulophidae|[642]|JSJUL1974-11|Hymenoptera|Eulophidae|BOLD:ABW3118
Eulophidae|[643]|SSFDA3606-14|Hymenoptera|Eulophidae|BOLD:ACN6320
Eulophidae|[644]|EAWGA356-13|Hymenoptera|Eulophidae|BOLD:ACL3987
Eulophidae|[645]|NGNAU047-14|Hymenoptera|Eulophidae|BOLD:ACM1780
Eulophidae|[646]|EAWGA474-13|Hymenoptera|Eulophidae|BOLD:ACL3798
Eulophidae|[647]|BBHYL052-10|Hymenoptera|Eulophidae|BOLD:AAU8972
Eulophidae|[648]|CNWAE7431-14|Hymenoptera|Eulophidae|BOLD:ACS1155
Entedoninae|[649]|CNEIF2834-12|Hymenoptera|Eulophidae|BOLD:ACD3264
Eulophidae|[650]|CNGAG219-15|Hymenoptera|Eulophidae|BOLD:AAU8955
Eulophidae|[651]|CNRVG2022-15|Hymenoptera|Eulophidae|BOLD:ACT6209
Pteromalidae|[652]|CNRON765-13|Hymenoptera|Pteromalidae|BOLD:ACK8412
Pteromalidae|[653]|CNROP432-13|Hymenoptera|Pteromalidae|BOLD:ACL1814
Pteromalidae|[654]|CNWBG1993-13|Hymenoptera|Pteromalidae|BOLD:ACI5101
Pteromalidae|[655]|CNWBG2114-13|Hymenoptera|Pteromalidae|BOLD:ACI4784
Pteromalidae|[656]|CNPEP1167-14|Hymenoptera|Pteromalidae|BOLD:ACL4880
Tetrastichinae|[657]|CNROP421-13|Hymenoptera|Eulophidae|BOLD:AAG3957
Hymenoptera|[658]|ERPIR339-12|Hymenoptera||BOLD:ACK1579
Eulophidae|[659]|SMTPB10618-13|Hymenoptera|Eulophidae|BOLD:ACF7508
Eulophidae|[660]|CNWBX111-13|Hymenoptera|Eulophidae|BOLD:ACI4796
Hymenoptera|[661]|NGNAJ1028-13|Hymenoptera||BOLD:ACJ0376
Eulophidae|[662]|SSWLE5072-13|Hymenoptera|Eulophidae|BOLD:ACD3081
Eulophidae|[663]|CNTNJ840-14|Hymenoptera|Eulophidae|BOLD:ACN7591
Eulophidae|[664]|CNMIM674-14|Hymenoptera|Eulophidae|BOLD:AAV3989
Eulophidae|[665]|CNEIG542-12|Hymenoptera|Eulophidae|BOLD:ACD6421
Eulophidae|[666]|CNWLM1388-13|Hymenoptera|Eulophidae|BOLD:ACD3179
Eulophidae|[667]|CNTNB1489-14|Hymenoptera|Eulophidae|BOLD:ACM8308
Eulophidae|[668]|SMTPI193-14|Hymenoptera|Eulophidae|BOLD:ABW3273
Eulophidae|[669]|SMTPJ637-14|Hymenoptera|Eulophidae|BOLD:AAZ3291
Hymenoptera|[670]|MBIOJ637-13|Hymenoptera||BOLD:ACL2705
Hymenoptera|[671]|MBIOJ359-13|Hymenoptera||BOLD:ACL6871
Hymenoptera|[672]|MBIOP394-14|Hymenoptera||BOLD:ACW5120
Eulophidae|[673]|SSBAD2311-12|Hymenoptera|Eulophidae|BOLD:ACD1828
Eulophidae|[674]|SSEID2184-13|Hymenoptera|Eulophidae|BOLD:ACJ4973
Eulophidae|[675]|SSBAA1430-12|Hymenoptera|Eulophidae|BOLD:ACC1554
Eulophidae|[676]|SMTPI1837-14|Hymenoptera|Eulophidae|BOLD:ACP9625
Eulophidae|[677]|JSSEP633-11|Hymenoptera|Eulophidae|BOLD:ABW3266
Eulophidae|[678]|SSWLE6559-13|Hymenoptera|Eulophidae|BOLD:ACD3586
Eulophidae|[679]|MBIOK368-14|Hymenoptera|Eulophidae|BOLD:ACL2209
Eulophidae|[680]|CNWLE280-12|Hymenoptera|Eulophidae|BOLD:AAU9084
Eulophidae|[681]|RBINA4260-13|Hymenoptera|Eulophidae|BOLD:ACI9824
Eulophidae|[682]|SMTPI7293-14|Hymenoptera|Eulophidae|BOLD:ACP8271
Eulophinae|[683]|JBHCH102-10|Hymenoptera|Eulophidae|BOLD:AAN8150
Eulophidae|[684]|SSWLE4760-13|Hymenoptera|Eulophidae|BOLD:ACG4287
Diglyphus pulchripes|[685]|SMTPI7616-14|Hymenoptera|Eulophidae|BOLD:AAU8630
Eulophidae|[686]|BBHYM409-09|Hymenoptera|Eulophidae|BOLD:AAG7989
Eulophidae|[687]|CNWLO352-13|Hymenoptera|Eulophidae|BOLD:ACI7763
Eulophidae|[688]|CNKTG2004-15|Hymenoptera|Eulophidae|BOLD:ACC7003
Eulophidae|[689]|CNKTG2676-15|Hymenoptera|Eulophidae|BOLD:ACT7755
Eulophidae|[690]|PHXN631-14|Hymenoptera|Eulophidae|BOLD:ACK3246
Elasmus atratus|[691]|SSWLD2296-13|Hymenoptera|Eulophidae|BOLD:AAA4404
Eulophidae|[692]|SSPAC10648-13|Hymenoptera|Eulophidae|BOLD:ACC5995
Eulophidae|[693]|CNGBL679-14|Hymenoptera|Eulophidae|BOLD:ACD3649
Eulophidae|[694]|CNPPJ1283-12|Hymenoptera|Eulophidae|BOLD:ACB8337
Eulophidae|[695]|CNPPJ1798-12|Hymenoptera|Eulophidae|BOLD:ACB5053
Hymenoptera|[696]|NGAAB775-14|Hymenoptera||BOLD:ACN6923
Eulophidae|[697]|CNPKF1050-14|Hymenoptera|Eulophidae|BOLD:ACL5955
Eulophidae|[698]|CNMIH2080-14|Hymenoptera|Eulophidae|BOLD:ACN5154
Eulophidae|[699]|MBIOI603-13|Hymenoptera|Eulophidae|BOLD:ABA6125

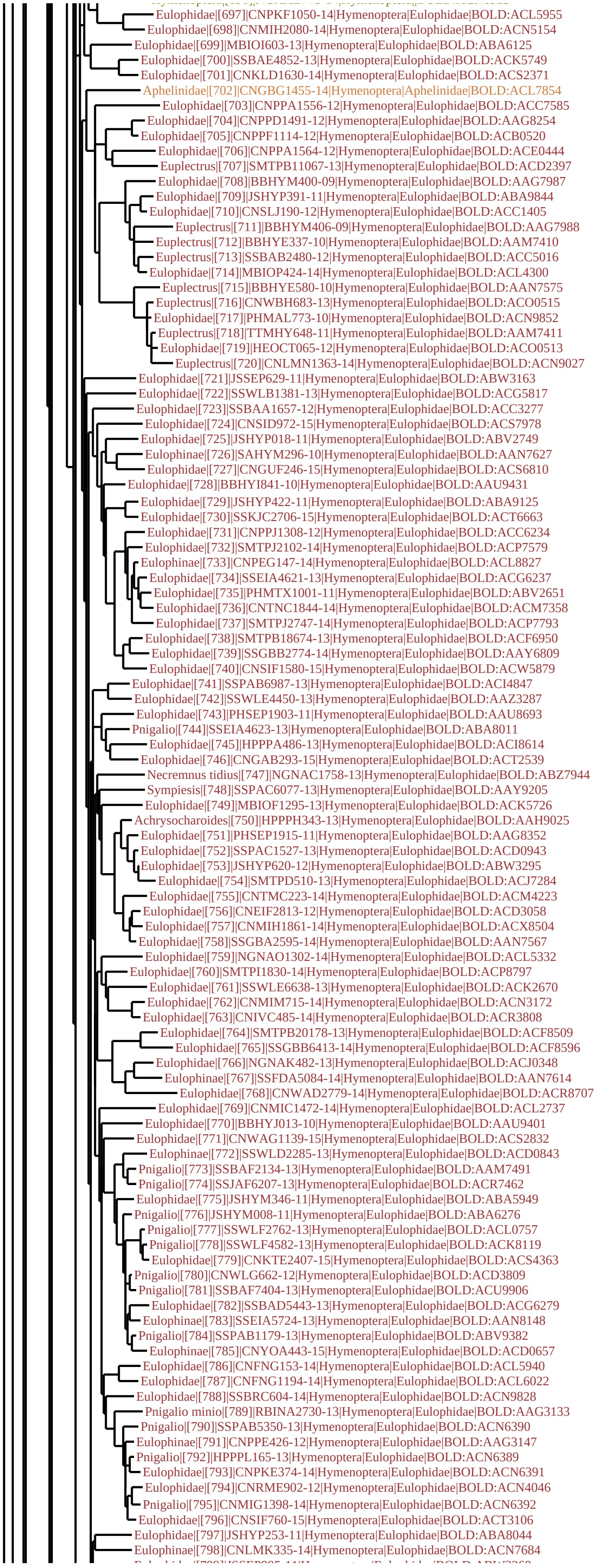
Eulophidae|[697]|CNPKF1050-14|Hymenoptera|Eulophidae|BOLD:ACL5955
Eulophidae|[698]|CNMIH2080-14|Hymenoptera|Eulophidae|BOLD:ACN5154
Eulophidae|[699]|MBIOI603-13|Hymenoptera|Eulophidae|BOLD:ABA6125
Eulophidae|[700]|SSBAE4852-13|Hymenoptera|Eulophidae|BOLD:ACK5749
Eulophidae|[701]|CNKLD1630-14|Hymenoptera|Eulophidae|BOLD:ACS2371
Aphelinidae|[702]|CNGBG1455-14|Hymenoptera|Aphelinidae|BOLD:ACL7854
Eulophidae|[703]|CNPPA1556-12|Hymenoptera|Eulophidae|BOLD:ACC7585
Eulophidae|[704]|CNPPD1491-12|Hymenoptera|Eulophidae|BOLD:AAG8254
Eulophidae|[705]|CNPPF1114-12|Hymenoptera|Eulophidae|BOLD:ACB0520
Eulophidae|[706]|CNPPA1564-12|Hymenoptera|Eulophidae|BOLD:ACE0444
Euplectrus|[707]|SMTPB11067-13|Hymenoptera|Eulophidae|BOLD:ACD2397
Eulophidae|[708]|BBHYM400-09|Hymenoptera|Eulophidae|BOLD:AAG7987
Eulophidae|[709]|JSHYP391-11|Hymenoptera|Eulophidae|BOLD:ABA9844
Eulophidae|[710]|CNSLJ190-12|Hymenoptera|Eulophidae|BOLD:ACC1405
Euplectrus|[711]|BBHYM406-09|Hymenoptera|Eulophidae|BOLD:AAG7988
Euplectrus|[712]|BBHYE337-10|Hymenoptera|Eulophidae|BOLD:AAM7410
Euplectrus|[713]|SSBAB2480-12|Hymenoptera|Eulophidae|BOLD:ACC5016
Eulophidae|[714]|MBIOP424-14|Hymenoptera|Eulophidae|BOLD:ACL4300
Euplectrus|[715]|BBHYE580-10|Hymenoptera|Eulophidae|BOLD:AAN7575
Euplectrus|[716]|CNWBH683-13|Hymenoptera|Eulophidae|BOLD:ACO0515
Eulophidae|[717]|PHMAL773-10|Hymenoptera|Eulophidae|BOLD:ACN9852
Euplectrus|[718]|TTMHY648-11|Hymenoptera|Eulophidae|BOLD:AAM7411
Eulophidae|[719]|HEOCT065-12|Hymenoptera|Eulophidae|BOLD:ACO0513
Euplectrus|[720]|CNLMN1363-14|Hymenoptera|Eulophidae|BOLD:ACN9027
Eulophidae|[721]|JSSEP629-11|Hymenoptera|Eulophidae|BOLD:ABW3163
Eulophidae|[722]|SSWLB1381-13|Hymenoptera|Eulophidae|BOLD:ACG5817
Eulophidae|[723]|SSBAA1657-12|Hymenoptera|Eulophidae|BOLD:ACC3277
Eulophidae|[724]|CNSID972-15|Hymenoptera|Eulophidae|BOLD:ACS7978
Eulophidae|[725]|JSHYP018-11|Hymenoptera|Eulophidae|BOLD:ABV2749
Eulophinae|[726]|SAHYM296-10|Hymenoptera|Eulophidae|BOLD:AAN7627
Eulophidae|[727]|CNGUF246-15|Hymenoptera|Eulophidae|BOLD:ACS6810
Eulophidae|[728]|BBHYI841-10|Hymenoptera|Eulophidae|BOLD:AAU9431
Eulophidae|[729]|JSHYP422-11|Hymenoptera|Eulophidae|BOLD:ABA9125
Eulophidae|[730]|SSKJC2706-15|Hymenoptera|Eulophidae|BOLD:ACT6663
Eulophidae|[731]|CNPPJ1308-12|Hymenoptera|Eulophidae|BOLD:ACC6234
Eulophidae|[732]|SMTPJ2102-14|Hymenoptera|Eulophidae|BOLD:ACP7579
Eulophinae|[733]|CNPEG147-14|Hymenoptera|Eulophidae|BOLD:ACL8827
Eulophidae|[734]|SSEIA4621-13|Hymenoptera|Eulophidae|BOLD:ACG6237
Eulophidae|[735]|PHMTX1001-11|Hymenoptera|Eulophidae|BOLD:ABV2651
Eulophidae|[736]|CNTNC1844-14|Hymenoptera|Eulophidae|BOLD:ACM7358
Eulophidae|[737]|SMTPJ2747-14|Hymenoptera|Eulophidae|BOLD:ACP7793
Eulophidae|[738]|SMTPB18674-13|Hymenoptera|Eulophidae|BOLD:ACF6950
Eulophidae|[739]|SSGBB2774-14|Hymenoptera|Eulophidae|BOLD:AAY6809
Eulophidae|[740]|CNSIF1580-15|Hymenoptera|Eulophidae|BOLD:ACW5879
Eulophidae|[741]|SSPAB6987-13|Hymenoptera|Eulophidae|BOLD:ACI4847
Eulophidae|[742]|SSWLE4450-13|Hymenoptera|Eulophidae|BOLD:AAZ3287
Eulophidae|[743]|PHSEP1903-11|Hymenoptera|Eulophidae|BOLD:AAU8693
Pnigalio|[744]|SSEIA4623-13|Hymenoptera|Eulophidae|BOLD:ABA8011
Eulophidae|[745]|HPPPA486-13|Hymenoptera|Eulophidae|BOLD:ACI8614
Eulophidae|[746]|CNGAB293-15|Hymenoptera|Eulophidae|BOLD:ACT2539
Necremnus tidius|[747]|NGNAC1758-13|Hymenoptera|Eulophidae|BOLD:ABZ7944
Sympiesis|[748]|SSPAC6077-13|Hymenoptera|Eulophidae|BOLD:AAY9205
Eulophidae|[749]|MBIOF1295-13|Hymenoptera|Eulophidae|BOLD:ACK5726
Achrysocharoides|[750]|HPPPH343-13|Hymenoptera|Eulophidae|BOLD:AAH9025
Eulophidae|[751]|PHSEP1915-11|Hymenoptera|Eulophidae|BOLD:AAG8352
Eulophidae|[752]|SSPAC1527-13|Hymenoptera|Eulophidae|BOLD:ACD0943
Eulophidae|[753]|JSHYP620-12|Hymenoptera|Eulophidae|BOLD:ABW3295
Eulophidae|[754]|SMTPD510-13|Hymenoptera|Eulophidae|BOLD:ACJ7284
Eulophidae|[755]|CNTMC223-14|Hymenoptera|Eulophidae|BOLD:ACM4223
Eulophidae|[756]|CNEIF2813-12|Hymenoptera|Eulophidae|BOLD:ACD3058
Eulophidae|[757]|CNMIH1861-14|Hymenoptera|Eulophidae|BOLD:ACX8504
Eulophidae|[758]|SSGBA2595-14|Hymenoptera|Eulophidae|BOLD:AAN7567
Eulophidae|[759]|NGNAO1302-14|Hymenoptera|Eulophidae|BOLD:ACL5332
Eulophidae|[760]|SMTPI1830-14|Hymenoptera|Eulophidae|BOLD:ACP8797
Eulophidae|[761]|SSWLE6638-13|Hymenoptera|Eulophidae|BOLD:ACK2670
Eulophidae|[762]|CNMIM715-14|Hymenoptera|Eulophidae|BOLD:ACN3172
Eulophidae|[763]|CNIVC485-14|Hymenoptera|Eulophidae|BOLD:ACR3808
Eulophidae|[764]|SMTPB20178-13|Hymenoptera|Eulophidae|BOLD:ACF8509
Eulophidae|[765]|SSGBB6413-14|Hymenoptera|Eulophidae|BOLD:ACF8596
Eulophidae|[766]|NGNAK482-13|Hymenoptera|Eulophidae|BOLD:ACJ0348
Eulophinae|[767]|SSFDA5084-14|Hymenoptera|Eulophidae|BOLD:AAN7614
Eulophidae|[768]|CNWAD2779-14|Hymenoptera|Eulophidae|BOLD:ACR8707
Eulophidae|[769]|CNMIC1472-14|Hymenoptera|Eulophidae|BOLD:ACL2737
Eulophidae|[770]|BBHYJ013-10|Hymenoptera|Eulophidae|BOLD:AAU9401
Eulophidae|[771]|CNWAG1139-15|Hymenoptera|Eulophidae|BOLD:ACS2832
Eulophinae|[772]|SSWLD2285-13|Hymenoptera|Eulophidae|BOLD:ACD0843
Pnigalio|[773]|SSBAF2134-13|Hymenoptera|Eulophidae|BOLD:AAM7491
Pnigalio|[774]|SSJAF6207-13|Hymenoptera|Eulophidae|BOLD:ACR7462
Eulophidae|[775]|JSHYM346-11|Hymenoptera|Eulophidae|BOLD:ABA5949
Pnigalio|[776]|JSHYM008-11|Hymenoptera|Eulophidae|BOLD:ABA6276
Pnigalio|[777]|SSWLF2762-13|Hymenoptera|Eulophidae|BOLD:ACL0757
Pnigalio|[778]|SSWLF4582-13|Hymenoptera|Eulophidae|BOLD:ACK8119
Eulophidae|[779]|CNKTE2407-15|Hymenoptera|Eulophidae|BOLD:ACS4363
Pnigalio|[780]|CNWLG662-12|Hymenoptera|Eulophidae|BOLD:ACD3809
Pnigalio|[781]|SSBAF7404-13|Hymenoptera|Eulophidae|BOLD:ACU9906
Eulophidae|[782]|SSBAD5443-13|Hymenoptera|Eulophidae|BOLD:ACG6279
Eulophinae|[783]|SSEIA5724-13|Hymenoptera|Eulophidae|BOLD:AAN8148
Pnigalio|[784]|SSPAB1179-13|Hymenoptera|Eulophidae|BOLD:ABV9382
Eulophinae|[785]|CNYOA443-15|Hymenoptera|Eulophidae|BOLD:ACD0657
Eulophidae|[786]|CNFNG153-14|Hymenoptera|Eulophidae|BOLD:ACL5940
Eulophidae|[787]|CNFNG1194-14|Hymenoptera|Eulophidae|BOLD:ACL6022
Eulophidae|[788]|SSBRC604-14|Hymenoptera|Eulophidae|BOLD:ACN9828
Pnigalio minio|[789]|RBINA2730-13|Hymenoptera|Eulophidae|BOLD:AAG3133
Pnigalio|[790]|SSPAB5350-13|Hymenoptera|Eulophidae|BOLD:ACN6390
Eulophinae|[791]|CNPPE426-12|Hymenoptera|Eulophidae|BOLD:AAG3147
Pnigalio|[792]|HPPPL165-13|Hymenoptera|Eulophidae|BOLD:ACN6389
Eulophidae|[793]|CNPKE374-14|Hymenoptera|Eulophidae|BOLD:ACN6391
Eulophidae|[794]|CNRME902-12|Hymenoptera|Eulophidae|BOLD:ACN4046
Pnigalio|[795]|CNMIG1398-14|Hymenoptera|Eulophidae|BOLD:ACN6392
Eulophidae|[796]|CNSIF760-15|Hymenoptera|Eulophidae|BOLD:ACT3106
Eulophidae|[797]|JSHYP253-11|Hymenoptera|Eulophidae|BOLD:ABA8044
Eulophinae|[798]|CNLMK335-14|Hymenoptera|Eulophidae|BOLD:ACN7684

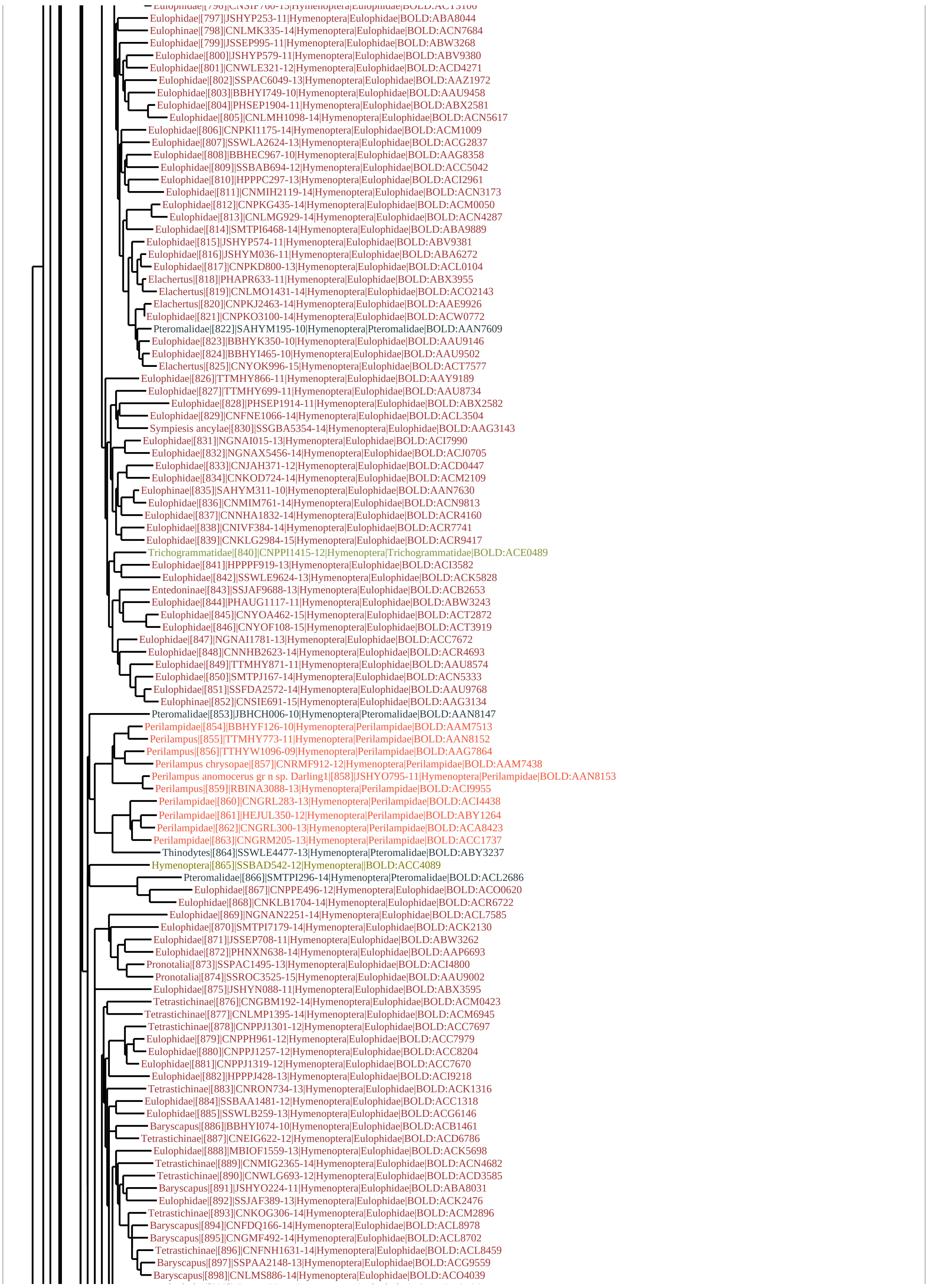

Eulophidae|[797]|JSHYP253-11|Hymenoptera|Eulophidae|BOLD:ABA8044
Eulophinae|[798]|CNLMK335-14|Hymenoptera|Eulophidae|BOLD:ACN7684
Eulophidae|[799]|JSSEP995-11|Hymenoptera|Eulophidae|BOLD:ABW3268
Eulophidae|[800]|JSHYP579-11|Hymenoptera|Eulophidae|BOLD:ABV9380
Eulophidae|[801]|CNWLE321-12|Hymenoptera|Eulophidae|BOLD:ACD4271
Eulophidae|[802]|SSPAC6049-13|Hymenoptera|Eulophidae|BOLD:AAZ1972
Eulophidae|[803]|BBHYI749-10|Hymenoptera|Eulophidae|BOLD:AAU9458
Eulophidae|[804]|PHSEP1904-11|Hymenoptera|Eulophidae|BOLD:ABX2581
Eulophidae|[805]|CNLMH1098-14|Hymenoptera|Eulophidae|BOLD:ACN5617
Eulophidae|[806]|CNPKI1175-14|Hymenoptera|Eulophidae|BOLD:ACM1009
Eulophidae|[807]|SSWLA2624-13|Hymenoptera|Eulophidae|BOLD:ACG2837
Eulophidae|[808]|BBHEC967-10|Hymenoptera|Eulophidae|BOLD:AAG8358
Eulophidae|[809]|SSBAB694-12|Hymenoptera|Eulophidae|BOLD:ACC5042
Eulophidae|[810]|HPPPC297-13|Hymenoptera|Eulophidae|BOLD:ACI2961
Eulophidae|[811]|CNMIH2119-14|Hymenoptera|Eulophidae|BOLD:ACN3173
Eulophidae|[812]|CNPKG435-14|Hymenoptera|Eulophidae|BOLD:ACM0050
Eulophidae|[813]|CNLMG929-14|Hymenoptera|Eulophidae|BOLD:ACN4287
Eulophidae|[814]|SMTPI6468-14|Hymenoptera|Eulophidae|BOLD:ABA9889
Eulophidae|[815]|JSHYP574-11|Hymenoptera|Eulophidae|BOLD:ABV9381
Eulophidae|[816]|JSHYM036-11|Hymenoptera|Eulophidae|BOLD:ABA6272
Eulophidae|[817]|CNPKD800-13|Hymenoptera|Eulophidae|BOLD:ACL0104
Elachertus|[818]|PHAPR633-11|Hymenoptera|Eulophidae|BOLD:ABX3955
Elachertus|[819]|CNLMO1431-14|Hymenoptera|Eulophidae|BOLD:ACO2143
Elachertus|[820]|CNPKJ2463-14|Hymenoptera|Eulophidae|BOLD:AAE9926
Eulophidae|[821]|CNPKO3100-14|Hymenoptera|Eulophidae|BOLD:ACW0772
Pteromalidae|[822]|SAHYM195-10|Hymenoptera|Pteromalidae|BOLD:AAN7609
Eulophidae|[823]|BBHYK350-10|Hymenoptera|Eulophidae|BOLD:AAU9146
Eulophidae|[824]|BBHYI465-10|Hymenoptera|Eulophidae|BOLD:AAU9502
Elachertus|[825]|CNYOK996-15|Hymenoptera|Eulophidae|BOLD:ACT7577
Eulophidae|[826]|TTMHY866-11|Hymenoptera|Eulophidae|BOLD:AAY9189
Eulophidae|[827]|TTMHY699-11|Hymenoptera|Eulophidae|BOLD:AAU8734
Eulophidae|[828]|PHSEP1914-11|Hymenoptera|Eulophidae|BOLD:ABX2582
Eulophidae|[829]|CNFNE1066-14|Hymenoptera|Eulophidae|BOLD:ACL3504
Sympiesis ancylae|[830]|SSGBA5354-14|Hymenoptera|Eulophidae|BOLD:AAG3143
Eulophidae|[831]|NGNAI015-13|Hymenoptera|Eulophidae|BOLD:ACI7990
Eulophidae|[832]|NGNAX5456-14|Hymenoptera|Eulophidae|BOLD:ACJ0705
Eulophidae|[833]|CNJAH371-12|Hymenoptera|Eulophidae|BOLD:ACD0447
Eulophidae|[834]|CNKOD724-14|Hymenoptera|Eulophidae|BOLD:ACM2109
Eulophinae|[835]|SAHYM311-10|Hymenoptera|Eulophidae|BOLD:AAN7630
Eulophidae|[836]|CNMIM761-14|Hymenoptera|Eulophidae|BOLD:ACN9813
Eulophidae|[837]|CNNHA1832-14|Hymenoptera|Eulophidae|BOLD:ACR4160
Eulophidae|[838]|CNIVF384-14|Hymenoptera|Eulophidae|BOLD:ACR7741
Eulophidae|[839]|CNKLG2984-15|Hymenoptera|Eulophidae|BOLD:ACR9417
Trichogrammatidae|[840]|CNPPI1415-12|Hymenoptera|Trichogrammatidae|BOLD:ACE0489
Eulophidae|[841]|HPPPF919-13|Hymenoptera|Eulophidae|BOLD:ACI3582
Eulophidae|[842]|SSWLE9624-13|Hymenoptera|Eulophidae|BOLD:ACK5828
Entedoninae|[843]|SSJAF9688-13|Hymenoptera|Eulophidae|BOLD:ACB2653
Eulophidae|[844]|PHAUG1117-11|Hymenoptera|Eulophidae|BOLD:ABW3243
Eulophidae|[845]|CNYOA462-15|Hymenoptera|Eulophidae|BOLD:ACT2872
Eulophidae|[846]|CNYOF108-15|Hymenoptera|Eulophidae|BOLD:ACT3919
Eulophidae|[847]|NGNAI1781-13|Hymenoptera|Eulophidae|BOLD:ACC7672
Eulophidae|[848]|CNNHB2623-14|Hymenoptera|Eulophidae|BOLD:ACR4693
Eulophidae|[849]|TTMHY871-11|Hymenoptera|Eulophidae|BOLD:AAU8574
Eulophidae|[850]|SMTPJ167-14|Hymenoptera|Eulophidae|BOLD:ACN5333
Eulophidae|[851]|SSFDA2572-14|Hymenoptera|Eulophidae|BOLD:AAU9768
Eulophinae|[852]|CNSIE691-15|Hymenoptera|Eulophidae|BOLD:AAG3134
Pteromalidae|[853]|JBHCH006-10|Hymenoptera|Pteromalidae|BOLD:AAN8147
Perilampidae|[854]|BBHYF126-10|Hymenoptera|Perilampidae|BOLD:AAM7513
Perilampus|[855]|TTMHY773-11|Hymenoptera|Perilampidae|BOLD:AAN8152
Perilampus|[856]|TTHYW1096-09|Hymenoptera|Perilampidae|BOLD:AAG7864
Perilampus chrysopae|[857]|CNRMF912-12|Hymenoptera|Perilampidae|BOLD:AAM7438
Perilampus anomocerus gr n sp. Darling1|[858]|JSHYO795-11|Hymenoptera|Perilampidae|BOLD:AAN8153
Perilampus|[859]|RBINA3088-13|Hymenoptera|Perilampidae|BOLD:ACI9955
Perilampidae|[860]|CNGRL283-13|Hymenoptera|Perilampidae|BOLD:ACI4438
Perilampidae|[861]|HEJUL350-12|Hymenoptera|Perilampidae|BOLD:ABY1264
Perilampidae|[862]|CNGRL300-13|Hymenoptera|Perilampidae|BOLD:ACA8423
Perilampidae|[863]|CNGRM205-13|Hymenoptera|Perilampidae|BOLD:ACC1737
Thinodytes|[864]|SSWLE4477-13|Hymenoptera|Pteromalidae|BOLD:ABY3237
Hymenoptera|[865]|SSBAD542-12|Hymenoptera||BOLD:ACC4089
Pteromalidae|[866]|SMTPI296-14|Hymenoptera|Pteromalidae|BOLD:ACL2686
Eulophidae|[867]|CNPPE496-12|Hymenoptera|Eulophidae|BOLD:ACO0620
Eulophidae|[868]|CNKLB1704-14|Hymenoptera|Eulophidae|BOLD:ACR6722
Eulophidae|[869]|NGNAN2251-14|Hymenoptera|Eulophidae|BOLD:ACL7585
Eulophidae|[870]|SMTPI7179-14|Hymenoptera|Eulophidae|BOLD:ACK2130
Eulophidae|[871]|JSSEP708-11|Hymenoptera|Eulophidae|BOLD:ABW3262
Eulophidae|[872]|PHNXN638-14|Hymenoptera|Eulophidae|BOLD:AAP6693
Pronotalia|[873]|SSPAC1495-13|Hymenoptera|Eulophidae|BOLD:ACI4800
Pronotalia|[874]|SSROC3525-15|Hymenoptera|Eulophidae|BOLD:AAU9002
Eulophidae|[875]|JSHYN088-11|Hymenoptera|Eulophidae|BOLD:ABX3595
Tetrastichinae|[876]|CNGBM192-14|Hymenoptera|Eulophidae|BOLD:ACM0423
Tetrastichinae|[877]|CNLMP1395-14|Hymenoptera|Eulophidae|BOLD:ACM6945
Tetrastichinae|[878]|CNPPJ1301-12|Hymenoptera|Eulophidae|BOLD:ACC7697
Eulophidae|[879]|CNPPH961-12|Hymenoptera|Eulophidae|BOLD:ACC7979
Eulophidae|[880]|CNPPJ1257-12|Hymenoptera|Eulophidae|BOLD:ACC8204
Eulophidae|[881]|CNPPJ1319-12|Hymenoptera|Eulophidae|BOLD:ACC7670
Eulophidae|[882]|HPPPJ428-13|Hymenoptera|Eulophidae|BOLD:ACI9218
Tetrastichinae|[883]|CNRON734-13|Hymenoptera|Eulophidae|BOLD:ACK1316
Eulophidae|[884]|SSBAA1481-12|Hymenoptera|Eulophidae|BOLD:ACC1318
Eulophidae|[885]|SSWLB259-13|Hymenoptera|Eulophidae|BOLD:ACG6146
Baryscapus|[886]|BBHYI074-10|Hymenoptera|Eulophidae|BOLD:ACB1461
Tetrastichinae|[887]|CNEIG622-12|Hymenoptera|Eulophidae|BOLD:ACD6786
Eulophidae|[888]|MBIOF1559-13|Hymenoptera|Eulophidae|BOLD:ACK5698
Tetrastichinae|[889]|CNMIG2365-14|Hymenoptera|Eulophidae|BOLD:ACN4682
Tetrastichinae|[890]|CNWLG693-12|Hymenoptera|Eulophidae|BOLD:ACD3585
Baryscapus|[891]|JSHYO224-11|Hymenoptera|Eulophidae|BOLD:ABA8031
Eulophidae|[892]|SSJAF389-13|Hymenoptera|Eulophidae|BOLD:ACK2476
Tetrastichinae|[893]|CNKOG306-14|Hymenoptera|Eulophidae|BOLD:ACM2896
Baryscapus|[894]|CNFDQ166-14|Hymenoptera|Eulophidae|BOLD:ACL8978
Baryscapus|[895]|CNGMF492-14|Hymenoptera|Eulophidae|BOLD:ACL8702
Tetrastichinae|[896]|CNFNH1631-14|Hymenoptera|Eulophidae|BOLD:ACL8459
Baryscapus|[897]|SSPAA2148-13|Hymenoptera|Eulophidae|BOLD:ACG9559
Baryscapus|[898]|CNLMS886-14|Hymenoptera|Eulophidae|BOLD:ACO4039

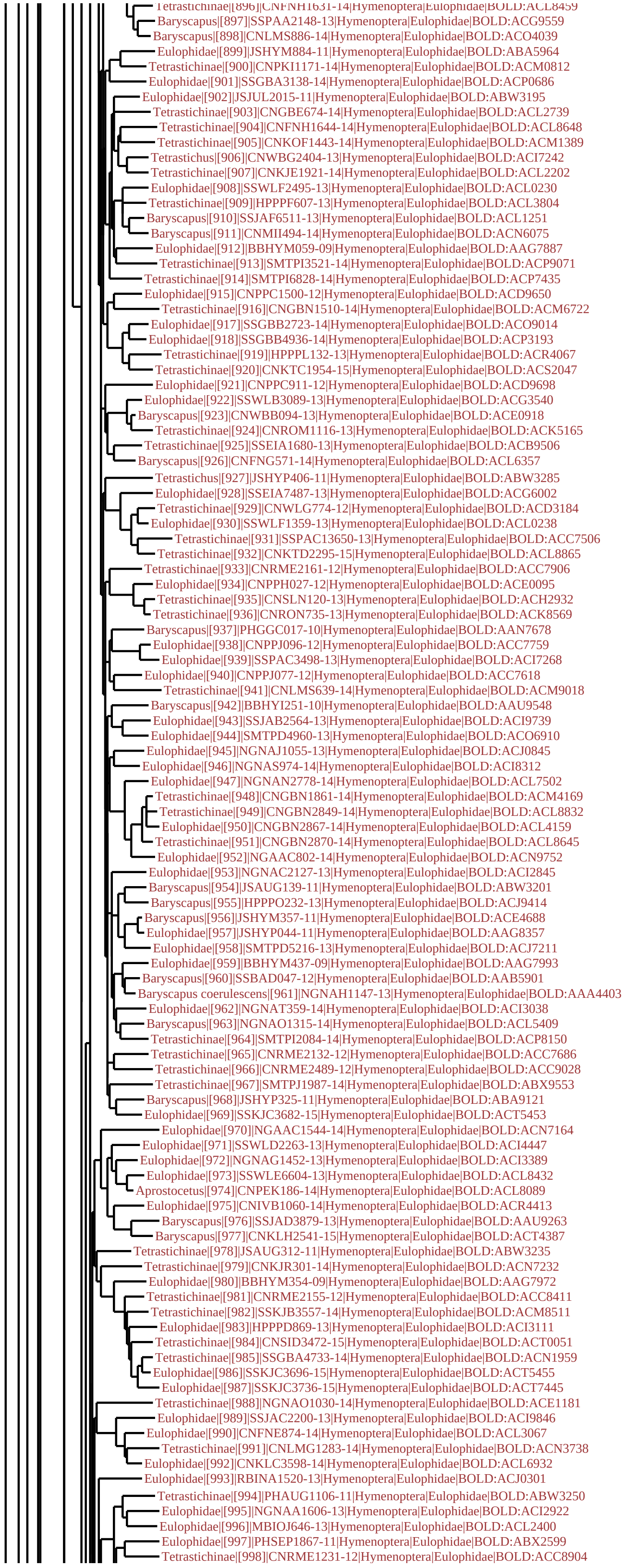

Tetrastichinae|[896]|CNFNH1631-14|Hymenoptera|Eulophidae|BOLD:ACL8459
Baryscapus|[897]|SSPAA2148-13|Hymenoptera|Eulophidae|BOLD:ACG9559
Baryscapus|[898]|CNLMS886-14|Hymenoptera|Eulophidae|BOLD:ACO4039
Eulophidae|[899]|JSHYM884-11|Hymenoptera|Eulophidae|BOLD:ABA5964
Tetrastichinae|[900]|CNPKI1171-14|Hymenoptera|Eulophidae|BOLD:ACM0812
Eulophidae|[901]|SSGBA3138-14|Hymenoptera|Eulophidae|BOLD:ACP0686
Eulophidae|[902]|JSJUL2015-11|Hymenoptera|Eulophidae|BOLD:ABW3195
Tetrastichinae|[903]|CNGBE674-14|Hymenoptera|Eulophidae|BOLD:ACL2739
Tetrastichinae|[904]|CNFNH1644-14|Hymenoptera|Eulophidae|BOLD:ACL8648
Tetrastichinae|[905]|CNKOF1443-14|Hymenoptera|Eulophidae|BOLD:ACM1389
Tetrastichus|[906]|CNWBG2404-13|Hymenoptera|Eulophidae|BOLD:ACI7242
Tetrastichinae|[907]|CNKJE1921-14|Hymenoptera|Eulophidae|BOLD:ACL2202
Eulophidae|[908]|SSWLF2495-13|Hymenoptera|Eulophidae|BOLD:ACL0230
Tetrastichinae|[909]|HPPPF607-13|Hymenoptera|Eulophidae|BOLD:ACL3804
Baryscapus|[910]|SSJAF6511-13|Hymenoptera|Eulophidae|BOLD:ACL1251
Baryscapus|[911]|CNMII494-14|Hymenoptera|Eulophidae|BOLD:ACN6075
Eulophidae|[912]|BBHYM059-09|Hymenoptera|Eulophidae|BOLD:AAG7887
Tetrastichinae|[913]|SMTPI3521-14|Hymenoptera|Eulophidae|BOLD:ACP9071
Tetrastichinae|[914]|SMTPI6828-14|Hymenoptera|Eulophidae|BOLD:ACP7435
Eulophidae|[915]|CNPPC1500-12|Hymenoptera|Eulophidae|BOLD:ACD9650
Tetrastichinae|[916]|CNGBN1510-14|Hymenoptera|Eulophidae|BOLD:ACM6722
Eulophidae|[917]|SSGBB2723-14|Hymenoptera|Eulophidae|BOLD:ACO9014
Eulophidae|[918]|SSGBB4936-14|Hymenoptera|Eulophidae|BOLD:ACP3193
Tetrastichinae|[919]|HPPPL132-13|Hymenoptera|Eulophidae|BOLD:ACR4067
Tetrastichinae|[920]|CNKTC1954-15|Hymenoptera|Eulophidae|BOLD:ACS2047
Eulophidae|[921]|CNPPC911-12|Hymenoptera|Eulophidae|BOLD:ACD9698
Eulophidae|[922]|SSWLB3089-13|Hymenoptera|Eulophidae|BOLD:ACG3540
Baryscapus|[923]|CNWBB094-13|Hymenoptera|Eulophidae|BOLD:ACE0918
Tetrastichinae|[924]|CNROM1116-13|Hymenoptera|Eulophidae|BOLD:ACK5165
Tetrastichinae|[925]|SSEIA1680-13|Hymenoptera|Eulophidae|BOLD:ACB9506
Baryscapus|[926]|CNFNG571-14|Hymenoptera|Eulophidae|BOLD:ACL6357
Tetrastichus|[927]|JSHYP406-11|Hymenoptera|Eulophidae|BOLD:ABW3285
Eulophidae|[928]|SSEIA7487-13|Hymenoptera|Eulophidae|BOLD:ACG6002
Tetrastichinae|[929]|CNWLG774-12|Hymenoptera|Eulophidae|BOLD:ACD3184
Eulophidae|[930]|SSWLF1359-13|Hymenoptera|Eulophidae|BOLD:ACL0238
Tetrastichinae|[931]|SSPAC13650-13|Hymenoptera|Eulophidae|BOLD:ACC7506
Tetrastichinae|[932]|CNKTD2295-15|Hymenoptera|Eulophidae|BOLD:ACL8865
Tetrastichinae|[933]|CNRME2161-12|Hymenoptera|Eulophidae|BOLD:ACC7906
Eulophidae|[934]|CNPPH027-12|Hymenoptera|Eulophidae|BOLD:ACE0095
Tetrastichinae|[935]|CNSLN120-13|Hymenoptera|Eulophidae|BOLD:ACH2932
Tetrastichinae|[936]|CNRON735-13|Hymenoptera|Eulophidae|BOLD:ACK8569
Baryscapus|[937]|PHGGC017-10|Hymenoptera|Eulophidae|BOLD:AAN7678
Eulophidae|[938]|CNPPJ096-12|Hymenoptera|Eulophidae|BOLD:ACC7759
Eulophidae|[939]|SSPAC3498-13|Hymenoptera|Eulophidae|BOLD:ACI7268
Eulophidae|[940]|CNPPJ077-12|Hymenoptera|Eulophidae|BOLD:ACC7618
Tetrastichinae|[941]|CNLMS639-14|Hymenoptera|Eulophidae|BOLD:ACM9018
Baryscapus|[942]|BBHYI251-10|Hymenoptera|Eulophidae|BOLD:AAU9548
Eulophidae|[943]|SSJAB2564-13|Hymenoptera|Eulophidae|BOLD:ACI9739
Eulophidae|[944]|SMTPD4960-13|Hymenoptera|Eulophidae|BOLD:ACO6910
Eulophidae|[945]|NGNAJ1055-13|Hymenoptera|Eulophidae|BOLD:ACJ0845
Eulophidae|[946]|NGNAS974-14|Hymenoptera|Eulophidae|BOLD:ACI8312
Eulophidae|[947]|NGNAN2778-14|Hymenoptera|Eulophidae|BOLD:ACL7502
Tetrastichinae|[948]|CNGBN1861-14|Hymenoptera|Eulophidae|BOLD:ACM4169
Tetrastichinae|[949]|CNGBN2849-14|Hymenoptera|Eulophidae|BOLD:ACL8832
Eulophidae|[950]|CNGBN2867-14|Hymenoptera|Eulophidae|BOLD:ACL4159
Tetrastichinae|[951]|CNGBN2870-14|Hymenoptera|Eulophidae|BOLD:ACL8645
Eulophidae|[952]|NGAAC802-14|Hymenoptera|Eulophidae|BOLD:ACN9752
Eulophidae|[953]|NGNAC2127-13|Hymenoptera|Eulophidae|BOLD:ACI2845
Baryscapus|[954]|JSAUG139-11|Hymenoptera|Eulophidae|BOLD:ABW3201
Baryscapus|[955]|HPPPO232-13|Hymenoptera|Eulophidae|BOLD:ACJ9414
Baryscapus|[956]|JSHYM357-11|Hymenoptera|Eulophidae|BOLD:ACE4688
Eulophidae|[957]|JSHYP044-11|Hymenoptera|Eulophidae|BOLD:AAG8357
Eulophidae|[958]|SMTPD5216-13|Hymenoptera|Eulophidae|BOLD:ACJ7211
Eulophidae|[959]|BBHYM437-09|Hymenoptera|Eulophidae|BOLD:AAG7993
Baryscapus|[960]|SSBAD047-12|Hymenoptera|Eulophidae|BOLD:AAB5901
Baryscapus coerulescens|[961]|NGNAH1147-13|Hymenoptera|Eulophidae|BOLD:AAA4403
Eulophidae|[962]|NGNAT359-14|Hymenoptera|Eulophidae|BOLD:ACI3038
Baryscapus|[963]|NGNAO1315-14|Hymenoptera|Eulophidae|BOLD:ACL5409
Tetrastichinae|[964]|SMTPI2084-14|Hymenoptera|Eulophidae|BOLD:ACP8150
Tetrastichinae|[965]|CNRME2132-12|Hymenoptera|Eulophidae|BOLD:ACC7686
Tetrastichinae|[966]|CNRME2489-12|Hymenoptera|Eulophidae|BOLD:ACC9028
Tetrastichinae|[967]|SMTPJ1987-14|Hymenoptera|Eulophidae|BOLD:ABX9553
Baryscapus|[968]|JSHYP325-11|Hymenoptera|Eulophidae|BOLD:ABA9121
Eulophidae|[969]|SSKJC3682-15|Hymenoptera|Eulophidae|BOLD:ACT5453
Eulophidae|[970]|NGAAC1544-14|Hymenoptera|Eulophidae|BOLD:ACN7164
Eulophidae|[971]|SSWLD2263-13|Hymenoptera|Eulophidae|BOLD:ACI4447
Eulophidae|[972]|NGNAG1452-13|Hymenoptera|Eulophidae|BOLD:ACI3389
Eulophidae|[973]|SSWLE6604-13|Hymenoptera|Eulophidae|BOLD:ACL8432
Aprostocetus|[974]|CNPEK186-14|Hymenoptera|Eulophidae|BOLD:ACL8089
Eulophidae|[975]|CNIVB1060-14|Hymenoptera|Eulophidae|BOLD:ACR4413
Baryscapus|[976]|SSJAD3879-13|Hymenoptera|Eulophidae|BOLD:AAU9263
Baryscapus|[977]|CNKLH2541-15|Hymenoptera|Eulophidae|BOLD:ACT4387
Tetrastichinae|[978]|JSAUG312-11|Hymenoptera|Eulophidae|BOLD:ABW3235
Tetrastichinae|[979]|CNKJR301-14|Hymenoptera|Eulophidae|BOLD:ACN7232
Eulophidae|[980]|BBHYM354-09|Hymenoptera|Eulophidae|BOLD:AAG7972
Tetrastichinae|[981]|CNRME2155-12|Hymenoptera|Eulophidae|BOLD:ACC8411
Tetrastichinae|[982]|SSKJB3557-14|Hymenoptera|Eulophidae|BOLD:ACM8511
Eulophidae|[983]|HPPPD869-13|Hymenoptera|Eulophidae|BOLD:ACI3111
Tetrastichinae|[984]|CNSID3472-15|Hymenoptera|Eulophidae|BOLD:ACT0051
Tetrastichinae|[985]|SSGBA4733-14|Hymenoptera|Eulophidae|BOLD:ACN1959
Eulophidae|[986]|SSKJC3696-15|Hymenoptera|Eulophidae|BOLD:ACT5455
Eulophidae|[987]|SSKJC3736-15|Hymenoptera|Eulophidae|BOLD:ACT7445
Tetrastichinae|[988]|NGNAO1030-14|Hymenoptera|Eulophidae|BOLD:ACE1181
Eulophidae|[989]|SSJAC2200-13|Hymenoptera|Eulophidae|BOLD:ACI9846
Eulophidae|[990]|CNFNE874-14|Hymenoptera|Eulophidae|BOLD:ACL3067
Tetrastichinae|[991]|CNLMG1283-14|Hymenoptera|Eulophidae|BOLD:ACN3738
Eulophidae|[992]|CNKLC3598-14|Hymenoptera|Eulophidae|BOLD:ACL6932
Eulophidae|[993]|RBINA1520-13|Hymenoptera|Eulophidae|BOLD:ACJ0301
Tetrastichinae|[994]|PHAUG1106-11|Hymenoptera|Eulophidae|BOLD:ABW3250
Eulophidae|[995]|NGNAA1606-13|Hymenoptera|Eulophidae|BOLD:ACI2922
Eulophidae|[996]|MBIOJ646-13|Hymenoptera|Eulophidae|BOLD:ACL2400
Eulophidae|[997]|PHSEP1867-11|Hymenoptera|Eulophidae|BOLD:ABX2599
Tetrastichinae|[998]|CNRME1231-12|Hymenoptera|Eulophidae|BOLD:ACC8904

Eulophidae|[996]|MBIOJ646-13|Hymenoptera|Eulophidae|BOLD:ACL2400
Eulophidae|[997]|PHSEP1867-11|Hymenoptera|Eulophidae|BOLD:ABX2599
Tetrastichinae|[998]|CNRME1231-12|Hymenoptera|Eulophidae|BOLD:ACC8904
Eulophidae|[999]|JSSEP593-11|Hymenoptera|Eulophidae|BOLD:ABW3191
Eulophidae|[1000]|MBION403-14|Hymenoptera|Eulophidae|BOLD:ACK8832
Tetrastichinae|[1001]|MBION554-14|Hymenoptera|Eulophidae|BOLD:ACJ9647
Tetrastichinae|[1002]|CNFNS1230-14|Hymenoptera|Eulophidae|BOLD:ACM7746
Tetrastichinae|[1003]|SMTPI3531-14|Hymenoptera|Eulophidae|BOLD:ACP8361
Aprostocetus|[1004]|CNPPJ123-12|Hymenoptera|Eulophidae|BOLD:ACC8010
Aprostocetus|[1005]|BBHYK793-10|Hymenoptera|Eulophidae|BOLD:AAU9030
Aprostocetus meltoftei|[1006]|HARC092-10|Hymenoptera|Eulophidae|BOLD:ABY8710
Aprostocetus|[1007]|CNTMA249-14|Hymenoptera|Eulophidae|BOLD:ACL9878
Aprostocetus|[1008]|SSBAF4176-13|Hymenoptera|Eulophidae|BOLD:ACL1489
Tetrastichinae|[1009]|SSWLF4588-13|Hymenoptera|Eulophidae|BOLD:ACP8959
Aprostocetus|[1010]|CNRMF263-12|Hymenoptera|Eulophidae|BOLD:ACC9349
Aprostocetus|[1011]|NGNAV372-14|Hymenoptera|Eulophidae|BOLD:ACM1428
Aprostocetus|[1012]|SSJAE2083-13|Hymenoptera|Eulophidae|BOLD:ACJ4888
Tetrastichinae|[1013]|HARC023-10|Hymenoptera|Eulophidae|BOLD:ACL1490
Aprostocetus|[1014]|SSJAE076-13|Hymenoptera|Eulophidae|BOLD:ACJ5526
Aprostocetus|[1015]|SSWLF442-13|Hymenoptera|Eulophidae|BOLD:AAM7535
Tetrastichinae|[1016]|SMTPJ5240-14|Hymenoptera|Eulophidae|BOLD:ACL1491
Aprostocetus|[1017]|CNIVD1089-14|Hymenoptera|Eulophidae|BOLD:ACR5461
Eulophidae|[1018]|JBHCJ416-11|Hymenoptera|Eulophidae|BOLD:AAU9885
Eulophidae|[1019]|JSHYN131-11|Hymenoptera|Eulophidae|BOLD:ABA7948
Minotetrastichus SS0231|[1020]|JSHYM635-11|Hymenoptera|Eulophidae|BOLD:ABA5998
Aprostocetus|[1021]|JSHYN074-11|Hymenoptera|Eulophidae|BOLD:ABZ5271
Minotetrastichus SS0231|[1022]|JSHYM879-11|Hymenoptera|Eulophidae|BOLD:ABX2957
Eulophidae|[1023]|JSHYN562-11|Hymenoptera|Eulophidae|BOLD:ABA6058
Tetrastichinae|[1024]|SSWLE3426-13|Hymenoptera|Eulophidae|BOLD:AAG7983
Tetrastichinae|[1025]|CNPKF730-14|Hymenoptera|Eulophidae|BOLD:ACL6376
Eulophidae|[1026]|NGNAA1653-13|Hymenoptera|Eulophidae|BOLD:ACI3190
Eulophidae|[1027]|NGNAN1973-14|Hymenoptera|Eulophidae|BOLD:ACI3200
Eulophidae|[1028]|NGNAN2693-14|Hymenoptera|Eulophidae|BOLD:ACI3039
Tetrastichinae|[1029]|BBHYE130-10|Hymenoptera|Eulophidae|BOLD:AAG8378
Tetrastichinae|[1030]|SSEIB8573-13|Hymenoptera|Eulophidae|BOLD:AAG8381
Tetrastichinae|[1031]|CNBAK405-13|Hymenoptera|Eulophidae|BOLD:ACH2027
Tetrastichinae|[1032]|NGNAZ557-14|Hymenoptera|Eulophidae|BOLD:ACN3175
Aprostocetus|[1033]|JBHCJ575-11|Hymenoptera|Eulophidae|BOLD:AAU9881
Aprostocetus SS0232|[1034]|SMTPD2937-13|Hymenoptera|Eulophidae|BOLD:ACR0898
Aprostocetus|[1035]|PHSEP1891-11|Hymenoptera|Eulophidae|BOLD:ABX2579
Eulophidae|[1036]|JSJUL1850-11|Hymenoptera|Eulophidae|BOLD:ABW3126
Aprostocetus|[1037]|SSWLF2316-13|Hymenoptera|Eulophidae|BOLD:ACI7434
Eulophidae|[1038]|SSWLF4620-13|Hymenoptera|Eulophidae|BOLD:ACJ0504
Tetrastichinae|[1039]|CNGBO696-14|Hymenoptera|Eulophidae|BOLD:ACL6359
Aprostocetus|[1040]|NGNAZ551-14|Hymenoptera|Eulophidae|BOLD:ACM0108
Tetrastichinae|[1041]|CNJAD683-12|Hymenoptera|Eulophidae|BOLD:ACC3765
Tetrastichinae|[1042]|CNGUB193-14|Hymenoptera|Eulophidae|BOLD:ACS2367
Aprostocetus|[1043]|SSBAD3591-12|Hymenoptera|Eulophidae|BOLD:ACE1844
Eulophidae|[1044]|MBIOF1552-13|Hymenoptera|Eulophidae|BOLD:ACK6128
Eulophidae|[1045]|SSKJC3735-15|Hymenoptera|Eulophidae|BOLD:ACT6227
Tetrastichinae|[1046]|CNBAC506-12|Hymenoptera|Eulophidae|BOLD:ACE0082
Eulophidae|[1047]|CNPPJ100-12|Hymenoptera|Eulophidae|BOLD:ACC7865
Eulophidae|[1048]|CNPPJ084-12|Hymenoptera|Eulophidae|BOLD:ACC8205
Eulophidae|[1049]|SSPAA7042-13|Hymenoptera|Eulophidae|BOLD:ACK5568
Eulophidae|[1050]|BBHYE578-10|Hymenoptera|Eulophidae|BOLD:AAN5638
Tetrastichinae|[1051]|CNROJ631-13|Hymenoptera|Eulophidae|BOLD:ACK2298
Eulophidae|[1052]|JSSEP606-11|Hymenoptera|Eulophidae|BOLD:ABW3181
Eulophidae|[1053]|JSSEP648-11|Hymenoptera|Eulophidae|BOLD:ABW3267
Tetrastichinae|[1054]|CNEIF2689-12|Hymenoptera|Eulophidae|BOLD:ACD0842
Tetrastichinae|[1055]|CNKJC338-14|Hymenoptera|Eulophidae|BOLD:AAG8359
Tetrastichinae|[1056]|NCCC546-11|Hymenoptera|Eulophidae|BOLD:ABA2708
Eulophidae|[1057]|NGNAT452-14|Hymenoptera|Eulophidae|BOLD:AAU8811
Eulophidae|[1058]|MBION537-14|Hymenoptera|Eulophidae|BOLD:ABA9129
Tetrastichinae|[1059]|CNLMR891-14|Hymenoptera|Eulophidae|BOLD:ACO2424
Eulophidae|[1060]|HPPPK879-13|Hymenoptera|Eulophidae|BOLD:ACI9880
Eulophidae|[1061]|BBHYJ200-10|Hymenoptera|Eulophidae|BOLD:AAU9363
Eulophidae|[1062]|SSBAF5460-13|Hymenoptera|Eulophidae|BOLD:ACJ0606
Eulophidae|[1063]|CNMIH1571-14|Hymenoptera|Eulophidae|BOLD:ACN5982
Eulophidae|[1064]|SSBAD492-12|Hymenoptera|Eulophidae|BOLD:ACC4091
Eulophidae|[1065]|SSBRA404-14|Hymenoptera|Eulophidae|BOLD:ACN8563
Tetrastichinae|[1066]|PHSEP1907-11|Hymenoptera|Eulophidae|BOLD:ABX2584
Eulophidae|[1067]|CNPPJ089-12|Hymenoptera|Eulophidae|BOLD:ACC8375
Baryscapus|[1068]|SSBAF4143-13|Hymenoptera|Eulophidae|BOLD:ACD3330
Tetrastichinae|[1069]|CNRME881-12|Hymenoptera|Eulophidae|BOLD:ACC8982
Tetrastichinae|[1070]|HPPPJ418-13|Hymenoptera|Eulophidae|BOLD:ACI9217
Eulophidae|[1071]|PHSEP1918-11|Hymenoptera|Eulophidae|BOLD:ABX2586
Eulophidae|[1072]|JSHYP529-11|Hymenoptera|Eulophidae|BOLD:ABV9383
Baryscapus|[1073]|HPPPM203-13|Hymenoptera|Eulophidae|BOLD:AAU8338
Tetrastichinae|[1074]|SMTPI9168-14|Hymenoptera|Eulophidae|BOLD:ACP8918
Eulophidae|[1075]|SMTPD4047-13|Hymenoptera|Eulophidae|BOLD:ACJ6698
Baryscapus|[1076]|SMTPI3543-14|Hymenoptera|Eulophidae|BOLD:AAU8565
Eulophidae|[1077]|CNMIG1403-14|Hymenoptera|Eulophidae|BOLD:ACN3532
Tetrastichinae|[1078]|SMTPJ424-14|Hymenoptera|Eulophidae|BOLD:ACP9481
Eulophidae|[1079]|SSPAA7036-13|Hymenoptera|Eulophidae|BOLD:ACK5851
Eulophidae|[1080]|SSPAA5217-13|Hymenoptera|Eulophidae|BOLD:ACG5213
Tetrastichinae|[1081]|SSBAF2125-13|Hymenoptera|Eulophidae|BOLD:AAG3132
Eulophidae|[1082]|SSEID1208-13|Hymenoptera|Eulophidae|BOLD:ACJ3867
Tetrastichinae|[1083]|CNFNS1105-14|Hymenoptera|Eulophidae|BOLD:ACL5880
Eulophidae|[1084]|CNPPF1135-12|Hymenoptera|Eulophidae|BOLD:ACA7523
Tetrastichinae|[1085]|CNFNT1747-14|Hymenoptera|Eulophidae|BOLD:ACN0795
Baryscapus|[1086]|SMTPI6217-14|Hymenoptera|Eulophidae|BOLD:AAM7511
Eulophidae|[1087]|JSAUG1426-11|Hymenoptera|Eulophidae|BOLD:ABW3180
Tetrastichinae|[1088]|SMTPJ3242-14|Hymenoptera|Eulophidae|BOLD:ACP8769
Aprostocetus|[1089]|BBHYN129-10|Hymenoptera|Eulophidae|BOLD:AAU8806
Tetrastichinae|[1090]|CNBRP1136-14|Hymenoptera|Eulophidae|BOLD:ACL9722
Eulophidae|[1091]|HARC414-11|Hymenoptera|Eulophidae|BOLD:ABX3598
Tetrastichinae|[1092]|CNRMD1178-12|Hymenoptera|Eulophidae|BOLD:ACC6739
Tetrastichinae|[1093]|SSPAA5211-13|Hymenoptera|Eulophidae|BOLD:ACG5211
Tetrastichinae|[1094]|BBHYM321-09|Hymenoptera|Eulophidae|BOLD:AAG7967
Tetrastichinae|[1095]|CNWBG2123-13|Hymenoptera|Eulophidae|BOLD:ACC9351
Eulophidae|[1096]|HARC404-11|Hymenoptera|Eulophidae|BOLD:AAZ6763
Eulophidae|[1097]|HPPPR124-13|Hymenoptera|Eulophidae|BOLD:ACI9985
Eulophidae|[1098]|CNSID906-15|Hymenoptera|Eulophidae|BOLD:ACT7664

Eulophidae|[1096]|HARC404-11|Hymenoptera|Eulophidae|BOLD:AAZ6763
Eulophidae|[1097]|HPPPR124-13|Hymenoptera|Eulophidae|BOLD:ACI9985
Eulophidae|[1098]|CNSID906-15|Hymenoptera|Eulophidae|BOLD:ACT7664
Eulophidae|[1099]|SMTPD2045-13|Hymenoptera|Eulophidae|BOLD:ACJ7416
Eulophidae|[1100]|SSJAD036-13|Hymenoptera|Eulophidae|BOLD:ACG5844
Eulophidae|[1101]|CNSIF725-15|Hymenoptera|Eulophidae|BOLD:AAN8129
Eulophidae|[1102]|JSAUG355-11|Hymenoptera|Eulophidae|BOLD:ABW3232
Tetrastichinae|[1103]|JSHYP367-11|Hymenoptera|Eulophidae|BOLD:ACX6577
Eulophidae|[1104]|JSJUL2166-11|Hymenoptera|Eulophidae|BOLD:ABW3205
Eulophidae|[1105]|MBIOG216-13|Hymenoptera|Eulophidae|BOLD:ACX6360
Tetrastichinae|[1106]|TTMHY757-11|Hymenoptera|Eulophidae|BOLD:AAY9193
Eulophidae|[1107]|JSHYP311-11|Hymenoptera|Eulophidae|BOLD:ACX6359
Eulophidae|[1108]|SMTPB16387-13|Hymenoptera|Eulophidae|BOLD:ACN9593
Tetrastichinae|[1109]|CNGBG1345-14|Hymenoptera|Eulophidae|BOLD:ACN9050
Eulophidae|[1110]|CNRME870-12|Hymenoptera|Eulophidae|BOLD:ACC8917
Eulophidae|[1111]|CNSLI392-12|Hymenoptera|Eulophidae|BOLD:AAN5639
Eulophidae|[1112]|CNJAD721-12|Hymenoptera|Eulophidae|BOLD:ACC4055
Tetrastichinae|[1113]|TTMHY695-11|Hymenoptera|Eulophidae|BOLD:AAY9185
Tetrastichinae|[1114]|SSWLB2174-13|Hymenoptera|Eulophidae|BOLD:ACN2420
Tetrastichinae|[1115]|SSPAC925-13|Hymenoptera|Eulophidae|BOLD:ACI8911
Eulophidae|[1116]|RBINA4673-13|Hymenoptera|Eulophidae|BOLD:ACI9825
Eulophidae|[1117]|CNWBG2175-13|Hymenoptera|Eulophidae|BOLD:ACI4203
Tetrastichinae|[1118]|CNFNT1705-14|Hymenoptera|Eulophidae|BOLD:ACL8026
Aprostocetus|[1119]|TTMHY806-11|Hymenoptera|Eulophidae|BOLD:AAY9179
Aprostocetus|[1120]|CNROH499-13|Hymenoptera|Eulophidae|BOLD:ACK2571
Aprostocetus|[1121]|NGAAC1550-14|Hymenoptera|Eulophidae|BOLD:ACN3563
Tetrastichinae|[1122]|SMTPI6035-14|Hymenoptera|Eulophidae|BOLD:ACP9240
Eulophidae|[1123]|CNRME259-12|Hymenoptera|Eulophidae|BOLD:ACC9391
Aprostocetus|[1124]|CNGAE234-15|Hymenoptera|Eulophidae|BOLD:ACT6901
Tetrastichinae|[1125]|SMTPJ4141-14|Hymenoptera|Eulophidae|BOLD:ACP9558
Eulophidae|[1126]|SSBAE6243-13|Hymenoptera|Eulophidae|BOLD:ACI7318
Eulophidae|[1127]|ERPIR368-12|Hymenoptera|Eulophidae|BOLD:ACK1840
Eulophidae|[1128]|SSBAA1682-12|Hymenoptera|Eulophidae|BOLD:ACC3278
Eulophidae|[1129]|CNRMF264-12|Hymenoptera|Eulophidae|BOLD:ACC9421
Eulophidae|[1130]|SSJAF5864-13|Hymenoptera|Eulophidae|BOLD:ACL0300
Tetrastichinae|[1131]|CNTMC283-14|Hymenoptera|Eulophidae|BOLD:ACM4679
Tetrastichinae|[1132]|CNPKH083-14|Hymenoptera|Eulophidae|BOLD:ACM1679
Tetrastichinae|[1133]|CNFDJ409-14|Hymenoptera|Eulophidae|BOLD:ACL9175
Tetrastichinae|[1134]|CNLMI609-14|Hymenoptera|Eulophidae|BOLD:ACM1112
Eulophidae|[1135]|JSHYM610-11|Hymenoptera|Eulophidae|BOLD:ABA6001
Eulophidae|[1136]|HPPPO223-13|Hymenoptera|Eulophidae|BOLD:ACJ0494
Tetrastichinae|[1137]|CNMIF625-14|Hymenoptera|Eulophidae|BOLD:ACL4787
Eulophidae|[1138]|SSFDA5100-14|Hymenoptera|Eulophidae|BOLD:ACN6600
Eulophidae|[1139]|NGNAJ1043-13|Hymenoptera|Eulophidae|BOLD:AAU9279
Tetrastichinae|[1140]|CNMIE1343-14|Hymenoptera|Eulophidae|BOLD:ACN2344
Tetrastichinae|[1141]|SMTPI7330-14|Hymenoptera|Eulophidae|BOLD:ACP8540
Tetrastichinae|[1142]|CNGBL632-14|Hymenoptera|Eulophidae|BOLD:ACL9057
Eulophidae|[1143]|CNTNG1417-14|Hymenoptera|Eulophidae|BOLD:ACN6726
Tetrastichinae|[1144]|CNFNR2249-14|Hymenoptera|Eulophidae|BOLD:ACL6086
Eulophidae|[1145]|CNTNH854-14|Hymenoptera|Eulophidae|BOLD:ACN7589
Eulophidae|[1146]|SSROA2929-14|Hymenoptera|Eulophidae|BOLD:ACA7698
Tetrastichinae|[1147]|CNLMN1539-14|Hymenoptera|Eulophidae|BOLD:ACL5065
Eulophidae|[1148]|CNNHG1844-14|Hymenoptera|Eulophidae|BOLD:ACR7901
Eulophidae|[1149]|NCCC710-11|Hymenoptera|Eulophidae|BOLD:ABV2722
Eulophidae|[1150]|SSWLF307-13|Hymenoptera|Eulophidae|BOLD:ACD3126
Eulophidae|[1151]|SMTPI6239-14|Hymenoptera|Eulophidae|BOLD:ACB7962
Eulophidae|[1152]|NCCC689-11|Hymenoptera|Eulophidae|BOLD:ABV2723
Eulophidae|[1153]|CNPPI1418-12|Hymenoptera|Eulophidae|BOLD:ACC7698
Tetrastichinae|[1154]|SMTPJ3686-14|Hymenoptera|Eulophidae|BOLD:ACP8539
Eulophidae|[1155]|SSJAB3282-13|Hymenoptera|Eulophidae|BOLD:ACG3340
Eulophidae|[1156]|SSJAB3287-13|Hymenoptera|Eulophidae|BOLD:ACN5667
Eulophidae|[1157]|CNGUC060-15|Hymenoptera|Eulophidae|BOLD:ACS1471
Eulophidae|[1158]|NGNAN1918-14|Hymenoptera|Eulophidae|BOLD:ACL3252
Eulophidae|[1159]|CNSID939-15|Hymenoptera|Eulophidae|BOLD:ACT0209
Eulophidae|[1160]|SSWLD2243-13|Hymenoptera|Eulophidae|BOLD:ABA6122
Tetrastichinae|[1161]|CNRMF309-12|Hymenoptera|Eulophidae|BOLD:ACC8021
Eulophidae|[1162]|NGNAY961-14|Hymenoptera|Eulophidae|BOLD:ACN0381
Eulophidae|[1163]|BBHYE511-10|Hymenoptera|Eulophidae|BOLD:AAM7441
Eulophidae|[1164]|JSJUN2269-11|Hymenoptera|Eulophidae|BOLD:ABW3308
Tetrastichinae|[1165]|CNSLP668-13|Hymenoptera|Eulophidae|BOLD:ACK9747
Eulophidae|[1166]|CNPPJ1279-12|Hymenoptera|Eulophidae|BOLD:ACC8378
Eulophidae|[1167]|JSJUL1807-11|Hymenoptera|Eulophidae|BOLD:ABW3185
Tetrastichinae|[1168]|CNFNT1321-14|Hymenoptera|Eulophidae|BOLD:ACN1599
Tetrastichinae|[1169]|MBIOD940-13|Hymenoptera|Eulophidae|BOLD:ACK1224
Aprostocetus forsteri|[1170]|NGNAW162-14|Hymenoptera|Eulophidae|BOLD:ABU5683
Eulophidae|[1171]|BBHYM026-09|Hymenoptera|Eulophidae|BOLD:AAG7881
Tetrastichinae|[1172]|NGNAY989-14|Hymenoptera|Eulophidae|BOLD:AAG7984
Aprostocetus|[1173]|EAWGA387-13|Hymenoptera|Eulophidae|BOLD:ACL3769
Aprostocetus|[1174]|CNPKE381-14|Hymenoptera|Eulophidae|BOLD:ACL3683
Aprostocetus|[1175]|CNTMA266-14|Hymenoptera|Eulophidae|BOLD:ACS7295
Tetrastichinae|[1176]|SSJAC1847-13|Hymenoptera|Eulophidae|BOLD:ACW4394
Aprostocetus|[1177]|SSBAF7407-13|Hymenoptera|Eulophidae|BOLD:ACW3982
Aprostocetus|[1178]|CNWLH093-12|Hymenoptera|Eulophidae|BOLD:ACW3975
Aprostocetus|[1179]|CNTMA261-14|Hymenoptera|Eulophidae|BOLD:AAG3138
Aprostocetus|[1180]|CNMIH1932-14|Hymenoptera|Eulophidae|BOLD:ACW3983
Aprostocetus|[1181]|CNTNH1024-14|Hymenoptera|Eulophidae|BOLD:ACW3641
Aprostocetus|[1182]|CNWLA592-12|Hymenoptera|Eulophidae|BOLD:ACW4392
Aprostocetus|[1183]|SSEID2217-13|Hymenoptera|Eulophidae|BOLD:ACW3984
Aprostocetus|[1184]|SSJAC1853-13|Hymenoptera|Eulophidae|BOLD:ACW3640
Aprostocetus|[1185]|SSKJA078-14|Hymenoptera|Eulophidae|BOLD:ACW3981
Tetrastichinae|[1186]|SMTPJ3423-14|Hymenoptera|Eulophidae|BOLD:AAU9041
Eulophidae|[1187]|NGNAQ572-14|Hymenoptera|Eulophidae|BOLD:ACL9639
Tetrastichinae|[1188]|SMTPI3269-14|Hymenoptera|Eulophidae|BOLD:ACP8458
Aprostocetus|[1189]|SMTPJ1274-14|Hymenoptera|Eulophidae|BOLD:ABA2854
Aprostocetus|[1190]|CNPPE420-12|Hymenoptera|Eulophidae|BOLD:ACE0313
Aprostocetus|[1191]|BBHYM389-09|Hymenoptera|Eulophidae|BOLD:AAG7950
Aprostocetus|[1192]|JSJUL1817-11|Hymenoptera|Eulophidae|BOLD:ABW3135
Aprostocetus|[1193]|CNGUC074-15|Hymenoptera|Eulophidae|BOLD:ACW6592
Tetrastichinae|[1194]|CNWLF506-12|Hymenoptera|Eulophidae|BOLD:ACD3219
Aprostocetus|[1195]|CNRME3026-12|Hymenoptera|Eulophidae|BOLD:AAG7954
Eulophidae|[1196]|CNGUD530-15|Hymenoptera|Eulophidae|BOLD:ACR9555
Eulophidae|[1197]|NCCC316-11|Hymenoptera|Eulophidae|BOLD:ABA5897
Tetrastichinae|[1198]|SSJAB2521-13|Hymenoptera|Eulophidae|BOLD:AAU9507

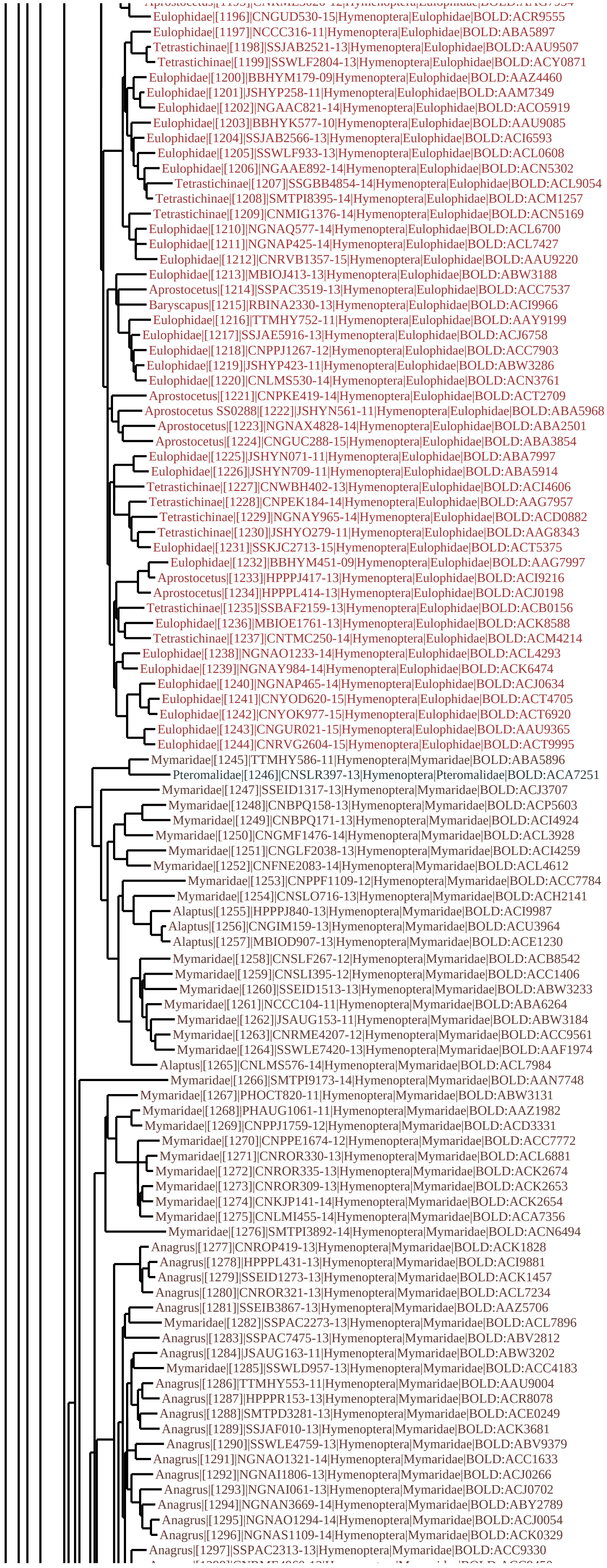
Eulophidae|[1196]|CNGUD530-15|Hymenoptera|Eulophidae|BOLD:ACR9555
Eulophidae|[1197]|NCCC316-11|Hymenoptera|Eulophidae|BOLD:ABA5897
Tetrastichinae|[1198]|SSJAB2521-13|Hymenoptera|Eulophidae|BOLD:AAU9507
Tetrastichinae|[1199]|SSWLF2804-13|Hymenoptera|Eulophidae|BOLD:ACY0871
Eulophidae|[1200]|BBHYM179-09|Hymenoptera|Eulophidae|BOLD:AAZ4460
Eulophidae|[1201]|JSHYP258-11|Hymenoptera|Eulophidae|BOLD:AAM7349
Eulophidae|[1202]|NGAAC821-14|Hymenoptera|Eulophidae|BOLD:ACO5919
Eulophidae|[1203]|BBHYK577-10|Hymenoptera|Eulophidae|BOLD:AAU9085
Eulophidae|[1204]|SSJAB2566-13|Hymenoptera|Eulophidae|BOLD:ACI6593
Eulophidae|[1205]|SSWLF933-13|Hymenoptera|Eulophidae|BOLD:ACL0608
Eulophidae|[1206]|NGAAE892-14|Hymenoptera|Eulophidae|BOLD:ACN5302
Tetrastichinae|[1207]|SSGBB4854-14|Hymenoptera|Eulophidae|BOLD:ACL9054
Tetrastichinae|[1208]|SMTPI8395-14|Hymenoptera|Eulophidae|BOLD:ACM1257
Tetrastichinae|[1209]|CNMIG1376-14|Hymenoptera|Eulophidae|BOLD:ACN5169
Eulophidae|[1210]|NGNAQ577-14|Hymenoptera|Eulophidae|BOLD:ACL6700
Eulophidae|[1211]|NGNAP425-14|Hymenoptera|Eulophidae|BOLD:ACL7427
Eulophidae|[1212]|CNRVB1357-15|Hymenoptera|Eulophidae|BOLD:AAU9220
Eulophidae|[1213]|MBIOJ413-13|Hymenoptera|Eulophidae|BOLD:ABW3188
Aprostocetus|[1214]|SSPAC3519-13|Hymenoptera|Eulophidae|BOLD:ACC7537
Baryscapus|[1215]|RBINA2330-13|Hymenoptera|Eulophidae|BOLD:ACI9966
Eulophidae|[1216]|TTMHY752-11|Hymenoptera|Eulophidae|BOLD:AAY9199
Eulophidae|[1217]|SSJAE5916-13|Hymenoptera|Eulophidae|BOLD:ACJ6758
Eulophidae|[1218]|CNPPJ1267-12|Hymenoptera|Eulophidae|BOLD:ACC7903
Eulophidae|[1219]|JSHYP423-11|Hymenoptera|Eulophidae|BOLD:ABW3286
Eulophidae|[1220]|CNLMS530-14|Hymenoptera|Eulophidae|BOLD:ACN3761
Aprostocetus|[1221]|CNPKE419-14|Hymenoptera|Eulophidae|BOLD:ACT2709
Aprostocetus SS0288|[1222]|JSHYN561-11|Hymenoptera|Eulophidae|BOLD:ABA5968
Aprostocetus|[1223]|NGNAX4828-14|Hymenoptera|Eulophidae|BOLD:ABA2501
Aprostocetus|[1224]|CNGUC288-15|Hymenoptera|Eulophidae|BOLD:ABA3854
Eulophidae|[1225]|JSHYN071-11|Hymenoptera|Eulophidae|BOLD:ABA7997
Eulophidae|[1226]|JSHYN709-11|Hymenoptera|Eulophidae|BOLD:ABA5914
Tetrastichinae|[1227]|CNWBH402-13|Hymenoptera|Eulophidae|BOLD:ACI4606
Tetrastichinae|[1228]|CNPEK184-14|Hymenoptera|Eulophidae|BOLD:AAG7957
Tetrastichinae|[1229]|NGNAY965-14|Hymenoptera|Eulophidae|BOLD:ACD0882
Tetrastichinae|[1230]|JSHYO279-11|Hymenoptera|Eulophidae|BOLD:AAG8343
Eulophidae|[1231]|SSKJC2713-15|Hymenoptera|Eulophidae|BOLD:ACT5375
Eulophidae|[1232]|BBHYM451-09|Hymenoptera|Eulophidae|BOLD:AAG7997
Aprostocetus|[1233]|HPPPJ417-13|Hymenoptera|Eulophidae|BOLD:ACI9216
Aprostocetus|[1234]|HPPPL414-13|Hymenoptera|Eulophidae|BOLD:ACJ0198
Tetrastichinae|[1235]|SSBAF2159-13|Hymenoptera|Eulophidae|BOLD:ACB0156
Eulophidae|[1236]|MBIOE1761-13|Hymenoptera|Eulophidae|BOLD:ACK8588
Tetrastichinae|[1237]|CNTMC250-14|Hymenoptera|Eulophidae|BOLD:ACM4214
Eulophidae|[1238]|NGNAO1233-14|Hymenoptera|Eulophidae|BOLD:ACL4293
Eulophidae|[1239]|NGNAY984-14|Hymenoptera|Eulophidae|BOLD:ACK6474
Eulophidae|[1240]|NGNAP465-14|Hymenoptera|Eulophidae|BOLD:ACJ0634
Eulophidae|[1241]|CNYOD620-15|Hymenoptera|Eulophidae|BOLD:ACT4705
Eulophidae|[1242]|CNYOK977-15|Hymenoptera|Eulophidae|BOLD:ACT6920
Eulophidae|[1243]|CNGUR021-15|Hymenoptera|Eulophidae|BOLD:AAU9365
Eulophidae|[1244]|CNRVG2604-15|Hymenoptera|Eulophidae|BOLD:ACT9995
Mymaridae|[1245]|TTMHY586-11|Hymenoptera|Mymaridae|BOLD:ABA5896
Pteromalidae|[1246]|CNSLR397-13|Hymenoptera|Pteromalidae|BOLD:ACA7251
Mymaridae|[1247]|SSEID1317-13|Hymenoptera|Mymaridae|BOLD:ACJ3707
Mymaridae|[1248]|CNBPQ158-13|Hymenoptera|Mymaridae|BOLD:ACP5603
Mymaridae|[1249]|CNBPQ171-13|Hymenoptera|Mymaridae|BOLD:ACI4924
Mymaridae|[1250]|CNGMF1476-14|Hymenoptera|Mymaridae|BOLD:ACL3928
Mymaridae|[1251]|CNGLF2038-13|Hymenoptera|Mymaridae|BOLD:ACI4259
Mymaridae|[1252]|CNFNE2083-14|Hymenoptera|Mymaridae|BOLD:ACL4612
Mymaridae|[1253]|CNPPF1109-12|Hymenoptera|Mymaridae|BOLD:ACC7784
Mymaridae|[1254]|CNSLO716-13|Hymenoptera|Mymaridae|BOLD:ACH2141
Alaptus|[1255]|HPPPJ840-13|Hymenoptera|Mymaridae|BOLD:ACI9987
Alaptus|[1256]|CNGIM159-13|Hymenoptera|Mymaridae|BOLD:ACU3964
Alaptus|[1257]|MBIOD907-13|Hymenoptera|Mymaridae|BOLD:ACE1230
Mymaridae|[1258]|CNSLF267-12|Hymenoptera|Mymaridae|BOLD:ACB8542
Mymaridae|[1259]|CNSLI395-12|Hymenoptera|Mymaridae|BOLD:ACC1406
Mymaridae|[1260]|SSEID1513-13|Hymenoptera|Mymaridae|BOLD:ABW3233
Mymaridae|[1261]|NCCC104-11|Hymenoptera|Mymaridae|BOLD:ABA6264
Mymaridae|[1262]|JSAUG153-11|Hymenoptera|Mymaridae|BOLD:ABW3184
Mymaridae|[1263]|CNRME4207-12|Hymenoptera|Mymaridae|BOLD:ACC9561
Mymaridae|[1264]|SSWLE7420-13|Hymenoptera|Mymaridae|BOLD:AAF1974
Alaptus|[1265]|CNLMS576-14|Hymenoptera|Mymaridae|BOLD:ACL7984
Mymaridae|[1266]|SMTPI9173-14|Hymenoptera|Mymaridae|BOLD:AAN7748
Mymaridae|[1267]|PHOCT820-11|Hymenoptera|Mymaridae|BOLD:ABW3131
Mymaridae|[1268]|PHAUG1061-11|Hymenoptera|Mymaridae|BOLD:AAZ1982
Mymaridae|[1269]|CNPPJ1759-12|Hymenoptera|Mymaridae|BOLD:ACD3331
Mymaridae|[1270]|CNPPE1674-12|Hymenoptera|Mymaridae|BOLD:ACC7772
Mymaridae|[1271]|CNROR330-13|Hymenoptera|Mymaridae|BOLD:ACL6881
Mymaridae|[1272]|CNROR335-13|Hymenoptera|Mymaridae|BOLD:ACK2674
Mymaridae|[1273]|CNROR309-13|Hymenoptera|Mymaridae|BOLD:ACK2653
Mymaridae|[1274]|CNKJP141-14|Hymenoptera|Mymaridae|BOLD:ACK2654
Mymaridae|[1275]|CNLMI455-14|Hymenoptera|Mymaridae|BOLD:ACA7356
Mymaridae|[1276]|SMTPI3892-14|Hymenoptera|Mymaridae|BOLD:ACN6494
Anagrus|[1277]|CNROP419-13|Hymenoptera|Mymaridae|BOLD:ACK1828
Anagrus|[1278]|HPPPL431-13|Hymenoptera|Mymaridae|BOLD:ACI9881
Anagrus|[1279]|SSEID1273-13|Hymenoptera|Mymaridae|BOLD:ACK1457
Anagrus|[1280]|CNROR321-13|Hymenoptera|Mymaridae|BOLD:ACL7234
Anagrus|[1281]|SSEIB3867-13|Hymenoptera|Mymaridae|BOLD:AAZ5706
Mymaridae|[1282]|SSPAC2273-13|Hymenoptera|Mymaridae|BOLD:ACL7896
Anagrus|[1283]|SSPAC7475-13|Hymenoptera|Mymaridae|BOLD:ABV2812
Anagrus|[1284]|JSAUG163-11|Hymenoptera|Mymaridae|BOLD:ABW3202
Mymaridae|[1285]|SSWLD957-13|Hymenoptera|Mymaridae|BOLD:ACC4183
Anagrus|[1286]|TTMHY553-11|Hymenoptera|Mymaridae|BOLD:AAU9004
Anagrus|[1287]|HPPPR153-13|Hymenoptera|Mymaridae|BOLD:ACR8078
Anagrus|[1288]|SMTPD3281-13|Hymenoptera|Mymaridae|BOLD:ACE0249
Anagrus|[1289]|SSJAF010-13|Hymenoptera|Mymaridae|BOLD:ACK3681
Anagrus|[1290]|SSWLE4759-13|Hymenoptera|Mymaridae|BOLD:ABV9379
Anagrus|[1291]|NGNAO1321-14|Hymenoptera|Mymaridae|BOLD:ACC1633
Anagrus|[1292]|NGNAI1806-13|Hymenoptera|Mymaridae|BOLD:ACJ0266
Anagrus|[1293]|NGNAI061-13|Hymenoptera|Mymaridae|BOLD:ACJ0702
Anagrus|[1294]|NGNAN3669-14|Hymenoptera|Mymaridae|BOLD:ABY2789
Anagrus|[1295]|NGNAO1294-14|Hymenoptera|Mymaridae|BOLD:ACJ0054
Anagrus|[1296]|NGNAS1109-14|Hymenoptera|Mymaridae|BOLD:ACK0329
Anagrus|[1297]|SSPAC2313-13|Hymenoptera|Mymaridae|BOLD:ACC9330

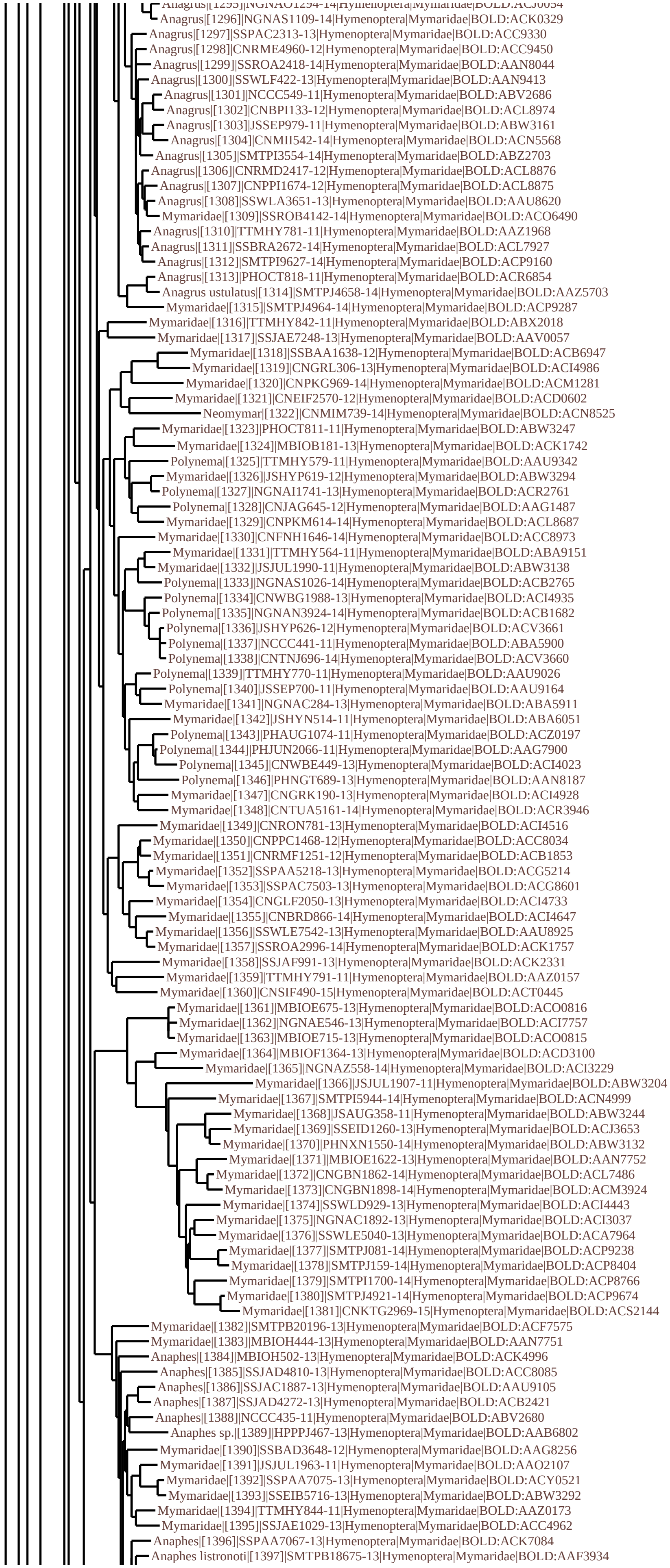

Anagrus|[1295]|NGNAU1294-14|Hymenoptera|Mymaridae|BOLD:ACJ0034
Anagrus|[1296]|NGNAS1109-14|Hymenoptera|Mymaridae|BOLD:ACK0329
Anagrus|[1297]|SSPAC2313-13|Hymenoptera|Mymaridae|BOLD:ACC9330
Anagrus|[1298]|CNRME4960-12|Hymenoptera|Mymaridae|BOLD:ACC9450
Anagrus|[1299]|SSROA2418-14|Hymenoptera|Mymaridae|BOLD:AAN8044
Anagrus|[1300]|SSWLF422-13|Hymenoptera|Mymaridae|BOLD:AAN9413
Anagrus|[1301]|NCCC549-11|Hymenoptera|Mymaridae|BOLD:ABV2686
Anagrus|[1302]|CNBPI133-12|Hymenoptera|Mymaridae|BOLD:ACL8974
Anagrus|[1303]|JSSEP979-11|Hymenoptera|Mymaridae|BOLD:ABW3161
Anagrus|[1304]|CNMII542-14|Hymenoptera|Mymaridae|BOLD:ACN5568
Anagrus|[1305]|SMTPI3554-14|Hymenoptera|Mymaridae|BOLD:ABZ2703
Anagrus|[1306]|CNRMD2417-12|Hymenoptera|Mymaridae|BOLD:ACL8876
Anagrus|[1307]|CNPPI1674-12|Hymenoptera|Mymaridae|BOLD:ACL8875
Anagrus|[1308]|SSWLA3651-13|Hymenoptera|Mymaridae|BOLD:AAU8620
Mymaridae|[1309]|SSROB4142-14|Hymenoptera|Mymaridae|BOLD:ACO6490
Anagrus|[1310]|TTMHY781-11|Hymenoptera|Mymaridae|BOLD:AAZ1968
Anagrus|[1311]|SSBRA2672-14|Hymenoptera|Mymaridae|BOLD:ACL7927
Anagrus|[1312]|SMTPI9627-14|Hymenoptera|Mymaridae|BOLD:ACP9160
Anagrus|[1313]|PHOCT818-11|Hymenoptera|Mymaridae|BOLD:ACR6854
Anagrus ustulatus|[1314]|SMTPJ4658-14|Hymenoptera|Mymaridae|BOLD:AAZ5703
Mymaridae|[1315]|SMTPJ4964-14|Hymenoptera|Mymaridae|BOLD:ACP9287
Mymaridae|[1316]|TTMHY842-11|Hymenoptera|Mymaridae|BOLD:ABX2018
Mymaridae|[1317]|SSJAE7248-13|Hymenoptera|Mymaridae|BOLD:AAV0057
Mymaridae|[1318]|SSBAA1638-12|Hymenoptera|Mymaridae|BOLD:ACB6947
Mymaridae|[1319]|CNGRL306-13|Hymenoptera|Mymaridae|BOLD:ACI4986
Mymaridae|[1320]|CNPKG969-14|Hymenoptera|Mymaridae|BOLD:ACM1281
Mymaridae|[1321]|CNEIF2570-12|Hymenoptera|Mymaridae|BOLD:ACD0602
Neomymar|[1322]|CNMIM739-14|Hymenoptera|Mymaridae|BOLD:ACN8525
Mymaridae|[1323]|PHOCT811-11|Hymenoptera|Mymaridae|BOLD:ABW3247
Mymaridae|[1324]|MBIOB181-13|Hymenoptera|Mymaridae|BOLD:ACK1742
Polynema|[1325]|TTMHY579-11|Hymenoptera|Mymaridae|BOLD:AAU9342
Mymaridae|[1326]|JSHYP619-12|Hymenoptera|Mymaridae|BOLD:ABW3294
Polynema|[1327]|NGNAI1741-13|Hymenoptera|Mymaridae|BOLD:ACR2761
Polynema|[1328]|CNJAG645-12|Hymenoptera|Mymaridae|BOLD:AAG1487
Mymaridae|[1329]|CNPKM614-14|Hymenoptera|Mymaridae|BOLD:ACL8687
Mymaridae|[1330]|CNFNH1646-14|Hymenoptera|Mymaridae|BOLD:ACC8973
Mymaridae|[1331]|TTMHY564-11|Hymenoptera|Mymaridae|BOLD:ABA9151
Mymaridae|[1332]|JSJUL1990-11|Hymenoptera|Mymaridae|BOLD:ABW3138
Polynema|[1333]|NGNAS1026-14|Hymenoptera|Mymaridae|BOLD:ACB2765
Polynema|[1334]|CNWBG1988-13|Hymenoptera|Mymaridae|BOLD:ACI4935
Polynema|[1335]|NGNAN3924-14|Hymenoptera|Mymaridae|BOLD:ACB1682
Polynema|[1336]|JSHYP626-12|Hymenoptera|Mymaridae|BOLD:ACV3661
Polynema|[1337]|NCCC441-11|Hymenoptera|Mymaridae|BOLD:ABA5900
Polynema|[1338]|CNTNJ696-14|Hymenoptera|Mymaridae|BOLD:ACV3660
Polynema|[1339]|TTMHY770-11|Hymenoptera|Mymaridae|BOLD:AAU9026
Polynema|[1340]|JSSEP700-11|Hymenoptera|Mymaridae|BOLD:AAU9164
Mymaridae|[1341]|NGNAC284-13|Hymenoptera|Mymaridae|BOLD:ABA5911
Mymaridae|[1342]|JSHYN514-11|Hymenoptera|Mymaridae|BOLD:ABA6051
Polynema|[1343]|PHAUG1074-11|Hymenoptera|Mymaridae|BOLD:ACZ0197
Polynema|[1344]|PHJUN2066-11|Hymenoptera|Mymaridae|BOLD:AAG7900
Polynema|[1345]|CNWBE449-13|Hymenoptera|Mymaridae|BOLD:ACI4023
Polynema|[1346]|PHNGT689-13|Hymenoptera|Mymaridae|BOLD:AAN8187
Mymaridae|[1347]|CNGRK190-13|Hymenoptera|Mymaridae|BOLD:ACI4928
Mymaridae|[1348]|CNTUA5161-14|Hymenoptera|Mymaridae|BOLD:ACR3946
Mymaridae|[1349]|CNRON781-13|Hymenoptera|Mymaridae|BOLD:ACI4516
Mymaridae|[1350]|CNPPC1468-12|Hymenoptera|Mymaridae|BOLD:ACC8034
Mymaridae|[1351]|CNRMF1251-12|Hymenoptera|Mymaridae|BOLD:ACB1853
Mymaridae|[1352]|SSPAA5218-13|Hymenoptera|Mymaridae|BOLD:ACG5214
Mymaridae|[1353]|SSPAC7503-13|Hymenoptera|Mymaridae|BOLD:ACG8601
Mymaridae|[1354]|CNGLF2050-13|Hymenoptera|Mymaridae|BOLD:ACI4733
Mymaridae|[1355]|CNBRD866-14|Hymenoptera|Mymaridae|BOLD:ACI4647
Mymaridae|[1356]|SSWLE7542-13|Hymenoptera|Mymaridae|BOLD:AAU8925
Mymaridae|[1357]|SSROA2996-14|Hymenoptera|Mymaridae|BOLD:ACK1757
Mymaridae|[1358]|SSJAF991-13|Hymenoptera|Mymaridae|BOLD:ACK2331
Mymaridae|[1359]|TTMHY791-11|Hymenoptera|Mymaridae|BOLD:AAZ0157
Mymaridae|[1360]|CNSIF490-15|Hymenoptera|Mymaridae|BOLD:ACT0445
Mymaridae|[1361]|MBIOE675-13|Hymenoptera|Mymaridae|BOLD:ACO0816
Mymaridae|[1362]|NGNAE546-13|Hymenoptera|Mymaridae|BOLD:ACI7757
Mymaridae|[1363]|MBIOE715-13|Hymenoptera|Mymaridae|BOLD:ACO0815
Mymaridae|[1364]|MBIOF1364-13|Hymenoptera|Mymaridae|BOLD:ACD3100
Mymaridae|[1365]|NGNAZ558-14|Hymenoptera|Mymaridae|BOLD:ACI3229
Mymaridae|[1366]|JSJUL1907-11|Hymenoptera|Mymaridae|BOLD:ABW3204
Mymaridae|[1367]|SMTPI5944-14|Hymenoptera|Mymaridae|BOLD:ACN4999
Mymaridae|[1368]|JSAUG358-11|Hymenoptera|Mymaridae|BOLD:ABW3244
Mymaridae|[1369]|SSEID1260-13|Hymenoptera|Mymaridae|BOLD:ACJ3653
Mymaridae|[1370]|PHNXN1550-14|Hymenoptera|Mymaridae|BOLD:ABW3132
Mymaridae|[1371]|MBIOE1622-13|Hymenoptera|Mymaridae|BOLD:AAN7752
Mymaridae|[1372]|CNGBN1862-14|Hymenoptera|Mymaridae|BOLD:ACL7486
Mymaridae|[1373]|CNGBN1898-14|Hymenoptera|Mymaridae|BOLD:ACM3924
Mymaridae|[1374]|SSWLD929-13|Hymenoptera|Mymaridae|BOLD:ACI4443
Mymaridae|[1375]|NGNAC1892-13|Hymenoptera|Mymaridae|BOLD:ACI3037
Mymaridae|[1376]|SSWLE5040-13|Hymenoptera|Mymaridae|BOLD:ACA7964
Mymaridae|[1377]|SMTPJ081-14|Hymenoptera|Mymaridae|BOLD:ACP9238
Mymaridae|[1378]|SMTPJ159-14|Hymenoptera|Mymaridae|BOLD:ACP8404
Mymaridae|[1379]|SMTPI1700-14|Hymenoptera|Mymaridae|BOLD:ACP8766
Mymaridae|[1380]|SMTPJ4921-14|Hymenoptera|Mymaridae|BOLD:ACP9674
Mymaridae|[1381]|CNKTG2969-15|Hymenoptera|Mymaridae|BOLD:ACS2144
Mymaridae|[1382]|SMTPB20196-13|Hymenoptera|Mymaridae|BOLD:ACF7575
Mymaridae|[1383]|MBIOH444-13|Hymenoptera|Mymaridae|BOLD:AAN7751
Anaphes|[1384]|MBIOH502-13|Hymenoptera|Mymaridae|BOLD:ACK4996
Anaphes|[1385]|SSJAD4810-13|Hymenoptera|Mymaridae|BOLD:ACC8085
Anaphes|[1386]|SSJAC1887-13|Hymenoptera|Mymaridae|BOLD:AAU9105
Anaphes|[1387]|SSJAD4272-13|Hymenoptera|Mymaridae|BOLD:ACB2421
Anaphes|[1388]|NCCC435-11|Hymenoptera|Mymaridae|BOLD:ABV2680
Anaphes sp.|[1389]|HPPPJ467-13|Hymenoptera|Mymaridae|BOLD:AAB6802
Mymaridae|[1390]|SSBAD3648-12|Hymenoptera|Mymaridae|BOLD:AAG8256
Mymaridae|[1391]|JSJUL1963-11|Hymenoptera|Mymaridae|BOLD:AAO2107
Mymaridae|[1392]|SSPAA7075-13|Hymenoptera|Mymaridae|BOLD:ACY0521
Mymaridae|[1393]|SSEIB5716-13|Hymenoptera|Mymaridae|BOLD:ABW3292
Mymaridae|[1394]|TTMHY844-11|Hymenoptera|Mymaridae|BOLD:AAZ0173
Mymaridae|[1395]|SSJAE1029-13|Hymenoptera|Mymaridae|BOLD:ACC4962
Anaphes|[1396]|SSPAA7067-13|Hymenoptera|Mymaridae|BOLD:ACK7084
Anaphes listronoti|[1397]|SMTPB18675-13|Hymenoptera|Mymaridae|BOLD:AAF3934

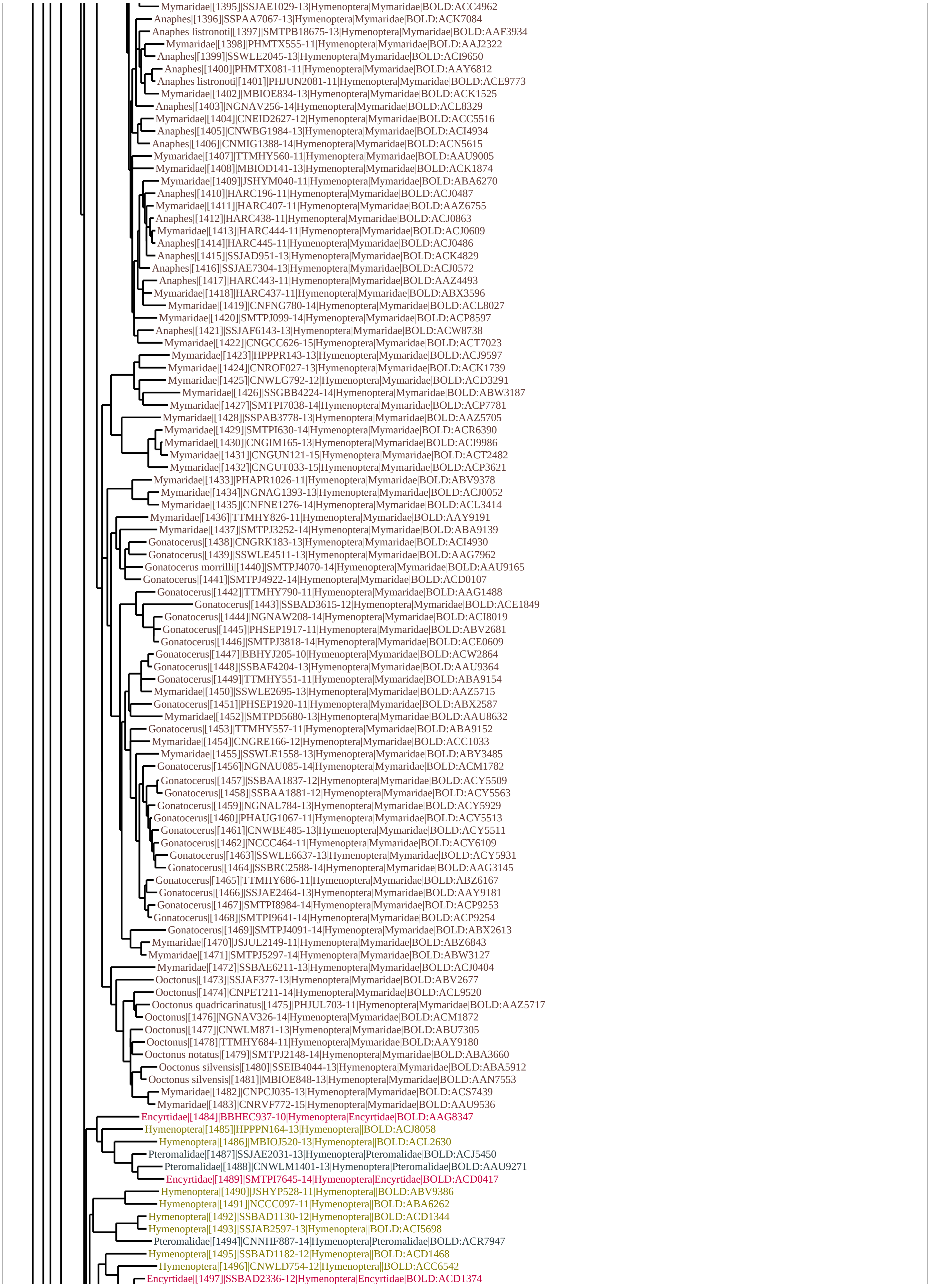
Mymaridae|[1395]|SSJAE1029-13|Hymenoptera|Mymaridae|BOLD:ACC4962
Anaphes|[1396]|SSPAA7067-13|Hymenoptera|Mymaridae|BOLD:ACK7084
Anaphes listronoti|[1397]|SMTPB18675-13|Hymenoptera|Mymaridae|BOLD:AAF3934
Mymaridae|[1398]|PHMTX555-11|Hymenoptera|Mymaridae|BOLD:AAJ2322
Anaphes|[1399]|SSWLE2045-13|Hymenoptera|Mymaridae|BOLD:ACI9650
Anaphes|[1400]|PHMTX081-11|Hymenoptera|Mymaridae|BOLD:AAY6812
Anaphes listronoti|[1401]|PHJUN2081-11|Hymenoptera|Mymaridae|BOLD:ACE9773
Mymaridae|[1402]|MBIOE834-13|Hymenoptera|Mymaridae|BOLD:ACK1525
Anaphes|[1403]|NGNAV256-14|Hymenoptera|Mymaridae|BOLD:ACL8329
Mymaridae|[1404]|CNEID2627-12|Hymenoptera|Mymaridae|BOLD:ACC5516
Anaphes|[1405]|CNWBG1984-13|Hymenoptera|Mymaridae|BOLD:ACI4934
Anaphes|[1406]|CNMIG1388-14|Hymenoptera|Mymaridae|BOLD:ACN5615
Mymaridae|[1407]|TTMHY560-11|Hymenoptera|Mymaridae|BOLD:AAU9005
Mymaridae|[1408]|MBIOD141-13|Hymenoptera|Mymaridae|BOLD:ACK1874
Mymaridae|[1409]|JSHYM040-11|Hymenoptera|Mymaridae|BOLD:ABA6270
Anaphes|[1410]|HARC196-11|Hymenoptera|Mymaridae|BOLD:ACJ0487
Mymaridae|[1411]|HARC407-11|Hymenoptera|Mymaridae|BOLD:AAZ6755
Anaphes|[1412]|HARC438-11|Hymenoptera|Mymaridae|BOLD:ACJ0863
Mymaridae|[1413]|HARC444-11|Hymenoptera|Mymaridae|BOLD:ACJ0609
Anaphes|[1414]|HARC445-11|Hymenoptera|Mymaridae|BOLD:ACJ0486
Anaphes|[1415]|SSJAD951-13|Hymenoptera|Mymaridae|BOLD:ACK4829
Anaphes|[1416]|SSJAE7304-13|Hymenoptera|Mymaridae|BOLD:ACJ0572
Anaphes|[1417]|HARC443-11|Hymenoptera|Mymaridae|BOLD:AAZ4493
Mymaridae|[1418]|HARC437-11|Hymenoptera|Mymaridae|BOLD:ABX3596
Mymaridae|[1419]|CNFNG780-14|Hymenoptera|Mymaridae|BOLD:ACL8027
Mymaridae|[1420]|SMTPJ099-14|Hymenoptera|Mymaridae|BOLD:ACP8597
Anaphes|[1421]|SSJAF6143-13|Hymenoptera|Mymaridae|BOLD:ACW8738
Mymaridae|[1422]|CNGCC626-15|Hymenoptera|Mymaridae|BOLD:ACT7023
Mymaridae|[1423]|HPPPR143-13|Hymenoptera|Mymaridae|BOLD:ACJ9597
Mymaridae|[1424]|CNROF027-13|Hymenoptera|Mymaridae|BOLD:ACK1739
Mymaridae|[1425]|CNWLG792-12|Hymenoptera|Mymaridae|BOLD:ACD3291
Mymaridae|[1426]|SSGBB4224-14|Hymenoptera|Mymaridae|BOLD:ABW3187
Mymaridae|[1427]|SMTPI7038-14|Hymenoptera|Mymaridae|BOLD:ACP7781
Mymaridae|[1428]|SSPAB3778-13|Hymenoptera|Mymaridae|BOLD:AAZ5705
Mymaridae|[1429]|SMTPI630-14|Hymenoptera|Mymaridae|BOLD:ACR6390
Mymaridae|[1430]|CNGIM165-13|Hymenoptera|Mymaridae|BOLD:ACI9986
Mymaridae|[1431]|CNGUN121-15|Hymenoptera|Mymaridae|BOLD:ACT2482
Mymaridae|[1432]|CNGUT033-15|Hymenoptera|Mymaridae|BOLD:ACP3621
Mymaridae|[1433]|PHAPR1026-11|Hymenoptera|Mymaridae|BOLD:ABV9378
Mymaridae|[1434]|NGNAG1393-13|Hymenoptera|Mymaridae|BOLD:ACJ0052
Mymaridae|[1435]|CNFNE1276-14|Hymenoptera|Mymaridae|BOLD:ACL3414
Mymaridae|[1436]|TTMHY826-11|Hymenoptera|Mymaridae|BOLD:AAY9191
Mymaridae|[1437]|SMTPJ3252-14|Hymenoptera|Mymaridae|BOLD:ABA9139
Gonatocerus|[1438]|CNGRK183-13|Hymenoptera|Mymaridae|BOLD:ACI4930
Gonatocerus|[1439]|SSWLE4511-13|Hymenoptera|Mymaridae|BOLD:AAG7962
Gonatocerus morrilli|[1440]|SMTPJ4070-14|Hymenoptera|Mymaridae|BOLD:AAU9165
Gonatocerus|[1441]|SMTPJ4922-14|Hymenoptera|Mymaridae|BOLD:ACD0107
Gonatocerus|[1442]|TTMHY790-11|Hymenoptera|Mymaridae|BOLD:AAG1488
Gonatocerus|[1443]|SSBAD3615-12|Hymenoptera|Mymaridae|BOLD:ACE1849
Gonatocerus|[1444]|NGNAW208-14|Hymenoptera|Mymaridae|BOLD:ACI8019
Gonatocerus|[1445]|PHSEP1917-11|Hymenoptera|Mymaridae|BOLD:ABV2681
Gonatocerus|[1446]|SMTPJ3818-14|Hymenoptera|Mymaridae|BOLD:ACE0609
Gonatocerus|[1447]|BBHYJ205-10|Hymenoptera|Mymaridae|BOLD:ACW2864
Gonatocerus|[1448]|SSBAF4204-13|Hymenoptera|Mymaridae|BOLD:AAU9364
Gonatocerus|[1449]|TTMHY551-11|Hymenoptera|Mymaridae|BOLD:ABA9154
Mymaridae|[1450]|SSWLE2695-13|Hymenoptera|Mymaridae|BOLD:AAZ5715
Gonatocerus|[1451]|PHSEP1920-11|Hymenoptera|Mymaridae|BOLD:ABX2587
Mymaridae|[1452]|SMTPD5680-13|Hymenoptera|Mymaridae|BOLD:AAU8632
Gonatocerus|[1453]|TTMHY557-11|Hymenoptera|Mymaridae|BOLD:ABA9152
Mymaridae|[1454]|CNGRE166-12|Hymenoptera|Mymaridae|BOLD:ACC1033
Mymaridae|[1455]|SSWLE1558-13|Hymenoptera|Mymaridae|BOLD:ABY3485
Gonatocerus|[1456]|NGNAU085-14|Hymenoptera|Mymaridae|BOLD:ACM1782
Gonatocerus|[1457]|SSBAA1837-12|Hymenoptera|Mymaridae|BOLD:ACY5509
Gonatocerus|[1458]|SSBAA1881-12|Hymenoptera|Mymaridae|BOLD:ACY5563
Gonatocerus|[1459]|NGNAL784-13|Hymenoptera|Mymaridae|BOLD:ACY5929
Gonatocerus|[1460]|PHAUG1067-11|Hymenoptera|Mymaridae|BOLD:ACY5513
Gonatocerus|[1461]|CNWBE485-13|Hymenoptera|Mymaridae|BOLD:ACY5511
Gonatocerus|[1462]|NCCC464-11|Hymenoptera|Mymaridae|BOLD:ACY6109
Gonatocerus|[1463]|SSWLE6637-13|Hymenoptera|Mymaridae|BOLD:ACY5931
Gonatocerus|[1464]|SSBRC2588-14|Hymenoptera|Mymaridae|BOLD:AAG3145
Gonatocerus|[1465]|TTMHY686-11|Hymenoptera|Mymaridae|BOLD:ABZ6167
Gonatocerus|[1466]|SSJAE2464-13|Hymenoptera|Mymaridae|BOLD:AAY9181
Gonatocerus|[1467]|SMTPI8984-14|Hymenoptera|Mymaridae|BOLD:ACP9253
Gonatocerus|[1468]|SMTPI9641-14|Hymenoptera|Mymaridae|BOLD:ACP9254
Gonatocerus|[1469]|SMTPJ4091-14|Hymenoptera|Mymaridae|BOLD:ABX2613
Mymaridae|[1470]|JSJUL2149-11|Hymenoptera|Mymaridae|BOLD:ABZ6843
Mymaridae|[1471]|SMTPJ5297-14|Hymenoptera|Mymaridae|BOLD:ABW3127
Mymaridae|[1472]|SSBAE6211-13|Hymenoptera|Mymaridae|BOLD:ACJ0404
Ooctonus|[1473]|SSJAF377-13|Hymenoptera|Mymaridae|BOLD:ABV2677
Ooctonus|[1474]|CNPET211-14|Hymenoptera|Mymaridae|BOLD:ACL9520
Ooctonus quadricarinatus|[1475]|PHJUL703-11|Hymenoptera|Mymaridae|BOLD:AAZ5717
Ooctonus|[1476]|NGNAV326-14|Hymenoptera|Mymaridae|BOLD:ACM1872
Ooctonus|[1477]|CNWLM871-13|Hymenoptera|Mymaridae|BOLD:ABU7305
Ooctonus|[1478]|TTMHY684-11|Hymenoptera|Mymaridae|BOLD:AAY9180
Ooctonus notatus|[1479]|SMTPJ2148-14|Hymenoptera|Mymaridae|BOLD:ABA3660
Ooctonus silvensis|[1480]|SSEIB4044-13|Hymenoptera|Mymaridae|BOLD:ABA5912
Ooctonus silvensis|[1481]|MBIOE848-13|Hymenoptera|Mymaridae|BOLD:AAN7553
Mymaridae|[1482]|CNPCJ035-13|Hymenoptera|Mymaridae|BOLD:ACS7439
Mymaridae|[1483]|CNRVF772-15|Hymenoptera|Mymaridae|BOLD:AAU9536
Encyrtidae|[1484]|BBHEC937-10|Hymenoptera|Encyrtidae|BOLD:AAG8347
Hymenoptera|[1485]|HPPPN164-13|Hymenoptera||BOLD:ACJ8058
Hymenoptera|[1486]|MBIOJ520-13|Hymenoptera||BOLD:ACL2630
Pteromalidae|[1487]|SSJAE2031-13|Hymenoptera|Pteromalidae|BOLD:ACJ5450
Pteromalidae|[1488]|CNWLM1401-13|Hymenoptera|Pteromalidae|BOLD:AAU9271
Encyrtidae|[1489]|SMTPI7645-14|Hymenoptera|Encyrtidae|BOLD:ACD0417
Hymenoptera|[1490]|JSHYP528-11|Hymenoptera||BOLD:ABV9386
Hymenoptera|[1491]|NCCC097-11|Hymenoptera||BOLD:ABA6262
Hymenoptera|[1492]|SSBAD1130-12|Hymenoptera||BOLD:ACD1344
Hymenoptera|[1493]|SSJAB2597-13|Hymenoptera||BOLD:ACI5698
Pteromalidae|[1494]|CNNHF887-14|Hymenoptera|Pteromalidae|BOLD:ACR7947
Hymenoptera|[1495]|SSBAD1182-12|Hymenoptera||BOLD:ACD1468
Hymenoptera|[1496]|CNWLD754-12|Hymenoptera||BOLD:ACC6542
Encyrtidae|[1497]|SSBAD2336-12|Hymenoptera|Encyrtidae|BOLD:ACD1374

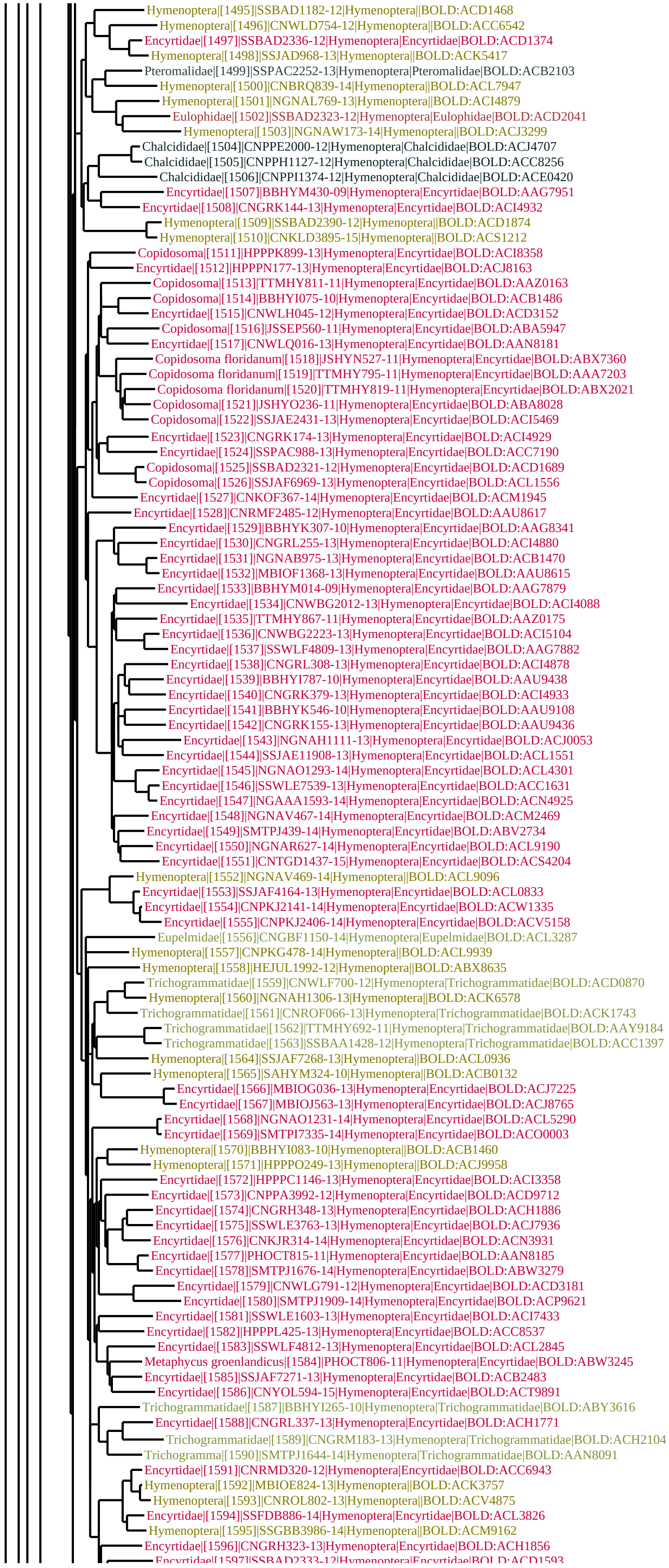

Hymenoptera|[1495]|SSBAD1182-12|Hymenoptera||BOLD:ACD1468
Hymenoptera|[1496]|CNWLD754-12|Hymenoptera||BOLD:ACC6542
Encyrtidae|[1497]|SSBAD2336-12|Hymenoptera|Encyrtidae|BOLD:ACD1374
Hymenoptera|[1498]|SSJAD968-13|Hymenoptera||BOLD:ACK5417
Pteromalidae|[1499]|SSPAC2252-13|Hymenoptera|Pteromalidae|BOLD:ACB2103
Hymenoptera|[1500]|CNBRQ839-14|Hymenoptera||BOLD:ACL7947
Hymenoptera|[1501]|NGNAL769-13|Hymenoptera||BOLD:ACI4879
Eulophidae|[1502]|SSBAD2323-12|Hymenoptera|Eulophidae|BOLD:ACD2041
Hymenoptera|[1503]|NGNAW173-14|Hymenoptera||BOLD:ACJ3299
Chalcididae|[1504]|CNPPE2000-12|Hymenoptera|Chalcididae|BOLD:ACJ4707
Chalcididae|[1505]|CNPPH1127-12|Hymenoptera|Chalcididae|BOLD:ACC8256
Chalcididae|[1506]|CNPPI1374-12|Hymenoptera|Chalcididae|BOLD:ACE0420
Encyrtidae|[1507]|BBHYM430-09|Hymenoptera|Encyrtidae|BOLD:AAG7951
Encyrtidae|[1508]|CNGRK144-13|Hymenoptera|Encyrtidae|BOLD:ACI4932
Hymenoptera|[1509]|SSBAD2390-12|Hymenoptera||BOLD:ACD1874
Hymenoptera|[1510]|CNKLD3895-15|Hymenoptera||BOLD:ACS1212
Copidosoma|[1511]|HPPPK899-13|Hymenoptera|Encyrtidae|BOLD:ACI8358
Encyrtidae|[1512]|HPPPN177-13|Hymenoptera|Encyrtidae|BOLD:ACJ8163
Copidosoma|[1513]|TTMHY811-11|Hymenoptera|Encyrtidae|BOLD:AAZ0163
Copidosoma|[1514]|BBHYI075-10|Hymenoptera|Encyrtidae|BOLD:ACB1486
Encyrtidae|[1515]|CNWLH045-12|Hymenoptera|Encyrtidae|BOLD:ACD3152
Copidosoma|[1516]|JSSEP560-11|Hymenoptera|Encyrtidae|BOLD:ABA5947
Encyrtidae|[1517]|CNWLQ016-13|Hymenoptera|Encyrtidae|BOLD:AAN8181
Copidosoma floridanum|[1518]|JSHYN527-11|Hymenoptera|Encyrtidae|BOLD:ABX7360
Copidosoma floridanum|[1519]|TTMHY795-11|Hymenoptera|Encyrtidae|BOLD:AAA7203
Copidosoma floridanum|[1520]|TTMHY819-11|Hymenoptera|Encyrtidae|BOLD:ABX2021
Copidosoma|[1521]|JSHYO236-11|Hymenoptera|Encyrtidae|BOLD:ABA8028
Copidosoma|[1522]|SSJAE2431-13|Hymenoptera|Encyrtidae|BOLD:ACI5469
Encyrtidae|[1523]|CNGRK174-13|Hymenoptera|Encyrtidae|BOLD:ACI4929
Encyrtidae|[1524]|SSPAC988-13|Hymenoptera|Encyrtidae|BOLD:ACC7190
Copidosoma|[1525]|SSBAD2321-12|Hymenoptera|Encyrtidae|BOLD:ACD1689
Copidosoma|[1526]|SSJAF6969-13|Hymenoptera|Encyrtidae|BOLD:ACL1556
Encyrtidae|[1527]|CNKOF367-14|Hymenoptera|Encyrtidae|BOLD:ACM1945
Encyrtidae|[1528]|CNRMF2485-12|Hymenoptera|Encyrtidae|BOLD:AAU8617
Encyrtidae|[1529]|BBHYK307-10|Hymenoptera|Encyrtidae|BOLD:AAG8341
Encyrtidae|[1530]|CNGRL255-13|Hymenoptera|Encyrtidae|BOLD:ACI4880
Encyrtidae|[1531]|NGNAB975-13|Hymenoptera|Encyrtidae|BOLD:ACB1470
Encyrtidae|[1532]|MBIOF1368-13|Hymenoptera|Encyrtidae|BOLD:AAU8615
Encyrtidae|[1533]|BBHYM014-09|Hymenoptera|Encyrtidae|BOLD:AAG7879
Encyrtidae|[1534]|CNWBG2012-13|Hymenoptera|Encyrtidae|BOLD:ACI4088
Encyrtidae|[1535]|TTMHY867-11|Hymenoptera|Encyrtidae|BOLD:AAZ0175
Encyrtidae|[1536]|CNWBG2223-13|Hymenoptera|Encyrtidae|BOLD:ACI5104
Encyrtidae|[1537]|SSWLF4809-13|Hymenoptera|Encyrtidae|BOLD:AAG7882
Encyrtidae|[1538]|CNGRL308-13|Hymenoptera|Encyrtidae|BOLD:ACI4878
Encyrtidae|[1539]|BBHYI787-10|Hymenoptera|Encyrtidae|BOLD:AAU9438
Encyrtidae|[1540]|CNGRK379-13|Hymenoptera|Encyrtidae|BOLD:ACI4933
Encyrtidae|[1541]|BBHYK546-10|Hymenoptera|Encyrtidae|BOLD:AAU9108
Encyrtidae|[1542]|CNGRK155-13|Hymenoptera|Encyrtidae|BOLD:AAU9436
Encyrtidae|[1543]|NGNAH1111-13|Hymenoptera|Encyrtidae|BOLD:ACJ0053
Encyrtidae|[1544]|SSJAE11908-13|Hymenoptera|Encyrtidae|BOLD:ACL1551
Encyrtidae|[1545]|NGNAO1293-14|Hymenoptera|Encyrtidae|BOLD:ACL4301
Encyrtidae|[1546]|SSWLE7539-13|Hymenoptera|Encyrtidae|BOLD:ACC1631
Encyrtidae|[1547]|NGAAA1593-14|Hymenoptera|Encyrtidae|BOLD:ACN4925
Encyrtidae|[1548]|NGNAV467-14|Hymenoptera|Encyrtidae|BOLD:ACM2469
Encyrtidae|[1549]|SMTPJ439-14|Hymenoptera|Encyrtidae|BOLD:ABV2734
Encyrtidae|[1550]|NGNAR627-14|Hymenoptera|Encyrtidae|BOLD:ACL9190
Encyrtidae|[1551]|CNTGD1437-15|Hymenoptera|Encyrtidae|BOLD:ACS4204
Hymenoptera|[1552]|NGNAV469-14|Hymenoptera||BOLD:ACL9096
Encyrtidae|[1553]|SSJAF4164-13|Hymenoptera|Encyrtidae|BOLD:ACL0833
Encyrtidae|[1554]|CNPKJ2141-14|Hymenoptera|Encyrtidae|BOLD:ACW1335
Encyrtidae|[1555]|CNPKJ2406-14|Hymenoptera|Encyrtidae|BOLD:ACV5158
Eupelmidae|[1556]|CNGBF1150-14|Hymenoptera|Eupelmidae|BOLD:ACL3287
Hymenoptera|[1557]|CNPKG478-14|Hymenoptera||BOLD:ACL9939
Hymenoptera|[1558]|HEJUL1992-12|Hymenoptera||BOLD:ABX8635
Trichogrammatidae|[1559]|CNWLF700-12|Hymenoptera|Trichogrammatidae|BOLD:ACD0870
Hymenoptera|[1560]|NGNAH1306-13|Hymenoptera||BOLD:ACK6578
Trichogrammatidae|[1561]|CNROF066-13|Hymenoptera|Trichogrammatidae|BOLD:ACK1743
Trichogrammatidae|[1562]|TTMHY692-11|Hymenoptera|Trichogrammatidae|BOLD:AAY9184
Trichogrammatidae|[1563]|SSBAA1428-12|Hymenoptera|Trichogrammatidae|BOLD:ACC1397
Hymenoptera|[1564]|SSJAF7268-13|Hymenoptera||BOLD:ACL0936
Hymenoptera|[1565]|SAHYM324-10|Hymenoptera||BOLD:ACB0132
Encyrtidae|[1566]|MBIOG036-13|Hymenoptera|Encyrtidae|BOLD:ACJ7225
Encyrtidae|[1567]|MBIOJ563-13|Hymenoptera|Encyrtidae|BOLD:ACJ8765
Encyrtidae|[1568]|NGNAO1231-14|Hymenoptera|Encyrtidae|BOLD:ACL5290
Encyrtidae|[1569]|SMTPI7335-14|Hymenoptera|Encyrtidae|BOLD:ACO0003
Hymenoptera|[1570]|BBHYI083-10|Hymenoptera||BOLD:ACB1460
Hymenoptera|[1571]|HPPPO249-13|Hymenoptera||BOLD:ACJ9958
Encyrtidae|[1572]|HPPPC1146-13|Hymenoptera|Encyrtidae|BOLD:ACI3358
Encyrtidae|[1573]|CNPPA3992-12|Hymenoptera|Encyrtidae|BOLD:ACD9712
Encyrtidae|[1574]|CNGRH348-13|Hymenoptera|Encyrtidae|BOLD:ACH1886
Encyrtidae|[1575]|SSWLE3763-13|Hymenoptera|Encyrtidae|BOLD:ACJ7936
Encyrtidae|[1576]|CNKJR314-14|Hymenoptera|Encyrtidae|BOLD:ACN3931
Encyrtidae|[1577]|PHOCT815-11|Hymenoptera|Encyrtidae|BOLD:AAN8185
Encyrtidae|[1578]|SMTPJ1676-14|Hymenoptera|Encyrtidae|BOLD:ABW3279
Encyrtidae|[1579]|CNWLG791-12|Hymenoptera|Encyrtidae|BOLD:ACD3181
Encyrtidae|[1580]|SMTPJ1909-14|Hymenoptera|Encyrtidae|BOLD:ACP9621
Encyrtidae|[1581]|SSWLE1603-13|Hymenoptera|Encyrtidae|BOLD:ACI7433
Encyrtidae|[1582]|HPPPL425-13|Hymenoptera|Encyrtidae|BOLD:ACC8537
Encyrtidae|[1583]|SSWLF4812-13|Hymenoptera|Encyrtidae|BOLD:ACL2845
Metaphycus groenlandicus|[1584]|PHOCT806-11|Hymenoptera|Encyrtidae|BOLD:ABW3245
Encyrtidae|[1585]|SSJAF7271-13|Hymenoptera|Encyrtidae|BOLD:ACB2483
Encyrtidae|[1586]|CNYOL594-15|Hymenoptera|Encyrtidae|BOLD:ACT9891
Trichogrammatidae|[1587]|BBHYI265-10|Hymenoptera|Trichogrammatidae|BOLD:ABY3616
Encyrtidae|[1588]|CNGRL337-13|Hymenoptera|Encyrtidae|BOLD:ACH1771
Trichogrammatidae|[1589]|CNGRM183-13|Hymenoptera|Trichogrammatidae|BOLD:ACH2104
Trichogramma|[1590]|SMTPJ1644-14|Hymenoptera|Trichogrammatidae|BOLD:AAN8091
Encyrtidae|[1591]|CNRMD320-12|Hymenoptera|Encyrtidae|BOLD:ACC6943
Hymenoptera|[1592]|MBIOE824-13|Hymenoptera||BOLD:ACK3757
Hymenoptera|[1593]|CNROL802-13|Hymenoptera||BOLD:ACV4875
Encyrtidae|[1594]|SSFDB886-14|Hymenoptera|Encyrtidae|BOLD:ACL3826
Hymenoptera|[1595]|SSGBB3986-14|Hymenoptera||BOLD:ACM9162
Encyrtidae|[1596]|CNGRH323-13|Hymenoptera|Encyrtidae|BOLD:ACH1856
Encyrtidae|[1597]|SSBAD2333-12|Hymenoptera|Encyrtidae|BOLD:ACD1593

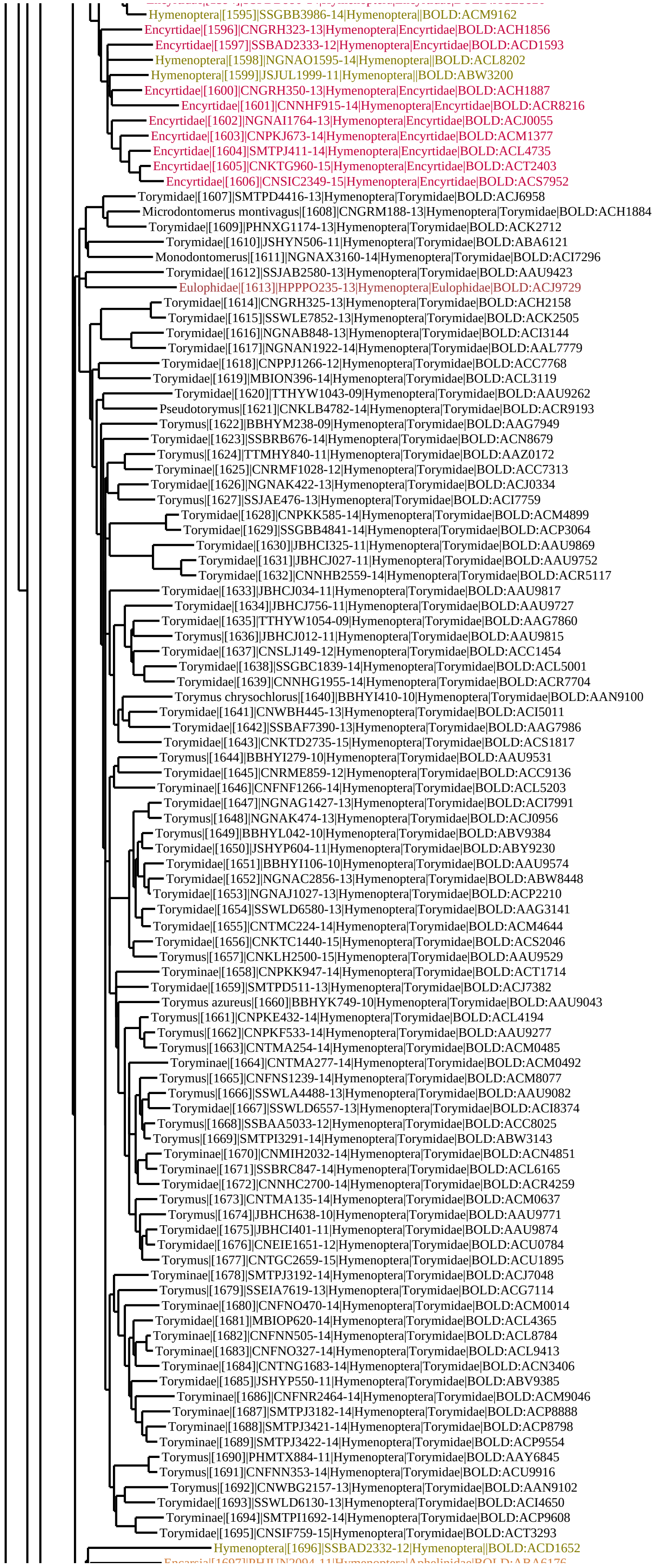

Hymenoptera||[1595]|SSGBB3986-14|Hymenoptera||BOLD:ACM9162
Encyrtidae|[1596]|CNGRH323-13|Hymenoptera|Encyrtidae|BOLD:ACH1856
Encyrtidae|[1597]|SSBAD2333-12|Hymenoptera|Encyrtidae|BOLD:ACD1593
Hymenoptera||[1598]|NGNAO1595-14|Hymenoptera||BOLD:ACL8202
Hymenoptera||[1599]|JSJUL1999-11|Hymenoptera||BOLD:ABW3200
Encyrtidae|[1600]|CNGRH350-13|Hymenoptera|Encyrtidae|BOLD:ACH1887
Encyrtidae|[1601]|CNNHF915-14|Hymenoptera|Encyrtidae|BOLD:ACR8216
Encyrtidae|[1602]|NGNAI1764-13|Hymenoptera|Encyrtidae|BOLD:ACJ0055
Encyrtidae|[1603]|CNPKJ673-14|Hymenoptera|Encyrtidae|BOLD:ACM1377
Encyrtidae|[1604]|SMTPJ411-14|Hymenoptera|Encyrtidae|BOLD:ACL4735
Encyrtidae|[1605]|CNKTG960-15|Hymenoptera|Encyrtidae|BOLD:ACT2403
Encyrtidae|[1606]|CNSIC2349-15|Hymenoptera|Encyrtidae|BOLD:ACS7952
Torymidae|[1607]|SMTPD4416-13|Hymenoptera|Torymidae|BOLD:ACJ6958
Microdontomerus montivagus|[1608]|CNGRM188-13|Hymenoptera|Torymidae|BOLD:ACH1884
Torymidae|[1609]|PHNXG1174-13|Hymenoptera|Torymidae|BOLD:ACK2712
Torymidae|[1610]|JSHYN506-11|Hymenoptera|Torymidae|BOLD:ABA6121
Monodontomerus|[1611]|NGNAX3160-14|Hymenoptera|Torymidae|BOLD:ACI7296
Torymidae|[1612]|SSJAB2580-13|Hymenoptera|Torymidae|BOLD:AAU9423
Eulophidae|[1613]|HPPPO235-13|Hymenoptera|Eulophidae|BOLD:ACJ9729
Torymidae|[1614]|CNGRH325-13|Hymenoptera|Torymidae|BOLD:ACH2158
Torymidae|[1615]|SSWLE7852-13|Hymenoptera|Torymidae|BOLD:ACK2505
Torymidae|[1616]|NGNAB848-13|Hymenoptera|Torymidae|BOLD:ACI3144
Torymidae|[1617]|NGNAN1922-14|Hymenoptera|Torymidae|BOLD:AAL7779
Torymidae|[1618]|CNPPJ1266-12|Hymenoptera|Torymidae|BOLD:ACC7768
Torymidae|[1619]|MBION396-14|Hymenoptera|Torymidae|BOLD:ACL3119
Torymidae|[1620]|TTHYW1043-09|Hymenoptera|Torymidae|BOLD:AAU9262
Pseudotorymus|[1621]|CNKLB4782-14|Hymenoptera|Torymidae|BOLD:ACR9193
Torymus|[1622]|BBHYM238-09|Hymenoptera|Torymidae|BOLD:AAG7949
Torymidae|[1623]|SSBRB676-14|Hymenoptera|Torymidae|BOLD:ACN8679
Torymus|[1624]|TTMHY840-11|Hymenoptera|Torymidae|BOLD:AAZ0172
Toryminae|[1625]|CNRMF1028-12|Hymenoptera|Torymidae|BOLD:ACC7313
Torymidae|[1626]|NGNAK422-13|Hymenoptera|Torymidae|BOLD:ACJ0334
Torymus|[1627]|SSJAE476-13|Hymenoptera|Torymidae|BOLD:ACI7759
Torymidae|[1628]|CNPKK585-14|Hymenoptera|Torymidae|BOLD:ACM4899
Torymidae|[1629]|SSGBB4841-14|Hymenoptera|Torymidae|BOLD:ACP3064
Torymidae|[1630]|JBHCI325-11|Hymenoptera|Torymidae|BOLD:AAU9869
Torymidae|[1631]|JBHCJ027-11|Hymenoptera|Torymidae|BOLD:AAU9752
Torymidae|[1632]|CNNHB2559-14|Hymenoptera|Torymidae|BOLD:ACR5117
Torymidae|[1633]|JBHCJ034-11|Hymenoptera|Torymidae|BOLD:AAU9817
Torymidae|[1634]|JBHCJ756-11|Hymenoptera|Torymidae|BOLD:AAU9727
Torymidae|[1635]|TTHYW1054-09|Hymenoptera|Torymidae|BOLD:AAG7860
Torymus|[1636]|JBHCJ012-11|Hymenoptera|Torymidae|BOLD:AAU9815
Torymidae|[1637]|CNSLJ149-12|Hymenoptera|Torymidae|BOLD:ACC1454
Torymidae|[1638]|SSGBC1839-14|Hymenoptera|Torymidae|BOLD:ACL5001
Torymidae|[1639]|CNNHG1955-14|Hymenoptera|Torymidae|BOLD:ACR7704
Torymus chrysochlorus|[1640]|BBHYI410-10|Hymenoptera|Torymidae|BOLD:AAN9100
Torymidae|[1641]|CNWBH445-13|Hymenoptera|Torymidae|BOLD:ACI5011
Torymidae|[1642]|SSBAF7390-13|Hymenoptera|Torymidae|BOLD:AAG7986
Torymidae|[1643]|CNKTD2735-15|Hymenoptera|Torymidae|BOLD:ACS1817
Torymus|[1644]|BBHYI279-10|Hymenoptera|Torymidae|BOLD:AAU9531
Torymidae|[1645]|CNRME859-12|Hymenoptera|Torymidae|BOLD:ACC9136
Toryminae|[1646]|CNFNF1266-14|Hymenoptera|Torymidae|BOLD:ACL5203
Torymidae|[1647]|NGNAG1427-13|Hymenoptera|Torymidae|BOLD:ACI7991
Torymus|[1648]|NGNAK474-13|Hymenoptera|Torymidae|BOLD:ACJ0956
Torymus|[1649]|BBHYL042-10|Hymenoptera|Torymidae|BOLD:ABV9384
Torymidae|[1650]|JSHYP604-11|Hymenoptera|Torymidae|BOLD:ABY9230
Torymidae|[1651]|BBHYI106-10|Hymenoptera|Torymidae|BOLD:AAU9574
Torymidae|[1652]|NGNAC2856-13|Hymenoptera|Torymidae|BOLD:ABW8448
Torymidae|[1653]|NGNAJ1027-13|Hymenoptera|Torymidae|BOLD:ACP2210
Torymidae|[1654]|SSWLD6580-13|Hymenoptera|Torymidae|BOLD:AAG3141
Torymidae|[1655]|CNTMC224-14|Hymenoptera|Torymidae|BOLD:ACM4644
Torymidae|[1656]|CNKTC1440-15|Hymenoptera|Torymidae|BOLD:ACS2046
Torymus|[1657]|CNKLH2500-15|Hymenoptera|Torymidae|BOLD:AAU9529
Toryminae|[1658]|CNPKK947-14|Hymenoptera|Torymidae|BOLD:ACT1714
Torymidae|[1659]|SMTPD511-13|Hymenoptera|Torymidae|BOLD:ACJ7382
Torymus azureus|[1660]|BBHYK749-10|Hymenoptera|Torymidae|BOLD:AAU9043
Torymus|[1661]|CNPKE432-14|Hymenoptera|Torymidae|BOLD:ACL4194
Torymus|[1662]|CNPKF533-14|Hymenoptera|Torymidae|BOLD:AAU9277
Torymus|[1663]|CNTMA254-14|Hymenoptera|Torymidae|BOLD:ACM0485
Toryminae|[1664]|CNTMA277-14|Hymenoptera|Torymidae|BOLD:ACM0492
Torymus|[1665]|CNFNS1239-14|Hymenoptera|Torymidae|BOLD:ACM8077
Torymus|[1666]|SSWLA4488-13|Hymenoptera|Torymidae|BOLD:AAU9082
Torymidae|[1667]|SSWLD6557-13|Hymenoptera|Torymidae|BOLD:ACI8374
Torymus|[1668]|SSBAA5033-12|Hymenoptera|Torymidae|BOLD:ACC8025
Torymus|[1669]|SMTPI3291-14|Hymenoptera|Torymidae|BOLD:ABW3143
Toryminae|[1670]|CNMIH2032-14|Hymenoptera|Torymidae|BOLD:ACN4851
Toryminae|[1671]|SSBRC847-14|Hymenoptera|Torymidae|BOLD:ACL6165
Torymidae|[1672]|CNNHC2700-14|Hymenoptera|Torymidae|BOLD:ACR4259
Torymus|[1673]|CNTMA135-14|Hymenoptera|Torymidae|BOLD:ACM0637
Torymus|[1674]|JBHCH638-10|Hymenoptera|Torymidae|BOLD:AAU9771
Torymidae|[1675]|JBHCI401-11|Hymenoptera|Torymidae|BOLD:AAU9874
Torymidae|[1676]|CNEIE1651-12|Hymenoptera|Torymidae|BOLD:ACU0784
Torymus|[1677]|CNTGC2659-15|Hymenoptera|Torymidae|BOLD:ACU1895
Toryminae|[1678]|SMTPJ3192-14|Hymenoptera|Torymidae|BOLD:ACJ7048
Torymus|[1679]|SSEIA7619-13|Hymenoptera|Torymidae|BOLD:ACG7114
Toryminae|[1680]|CNFNO470-14|Hymenoptera|Torymidae|BOLD:ACM0014
Torymidae|[1681]|MBIOP620-14|Hymenoptera|Torymidae|BOLD:ACL4365
Toryminae|[1682]|CNFNN505-14|Hymenoptera|Torymidae|BOLD:ACL8784
Toryminae|[1683]|CNFNO327-14|Hymenoptera|Torymidae|BOLD:ACL9413
Toryminae|[1684]|CNTNG1683-14|Hymenoptera|Torymidae|BOLD:ACN3406
Torymidae|[1685]|JSHYP550-11|Hymenoptera|Torymidae|BOLD:ABV9385
Toryminae|[1686]|CNFNR2464-14|Hymenoptera|Torymidae|BOLD:ACM9046
Toryminae|[1687]|SMTPJ3182-14|Hymenoptera|Torymidae|BOLD:ACP8888
Toryminae|[1688]|SMTPJ3421-14|Hymenoptera|Torymidae|BOLD:ACP8798
Toryminae|[1689]|SMTPJ3422-14|Hymenoptera|Torymidae|BOLD:ACP9554
Torymus|[1690]|PHMTX884-11|Hymenoptera|Torymidae|BOLD:AAY6845
Torymus|[1691]|CNFNN353-14|Hymenoptera|Torymidae|BOLD:ACU9916
Torymus|[1692]|CNWBG2157-13|Hymenoptera|Torymidae|BOLD:AAN9102
Torymidae|[1693]|SSWLD6130-13|Hymenoptera|Torymidae|BOLD:ACI4650
Toryminae|[1694]|SMTPI1692-14|Hymenoptera|Torymidae|BOLD:ACP9608
Torymidae|[1695]|CNSIF759-15|Hymenoptera|Torymidae|BOLD:ACT3293
Hymenoptera||[1696]|SSBAD2332-12|Hymenoptera||BOLD:ACD1652

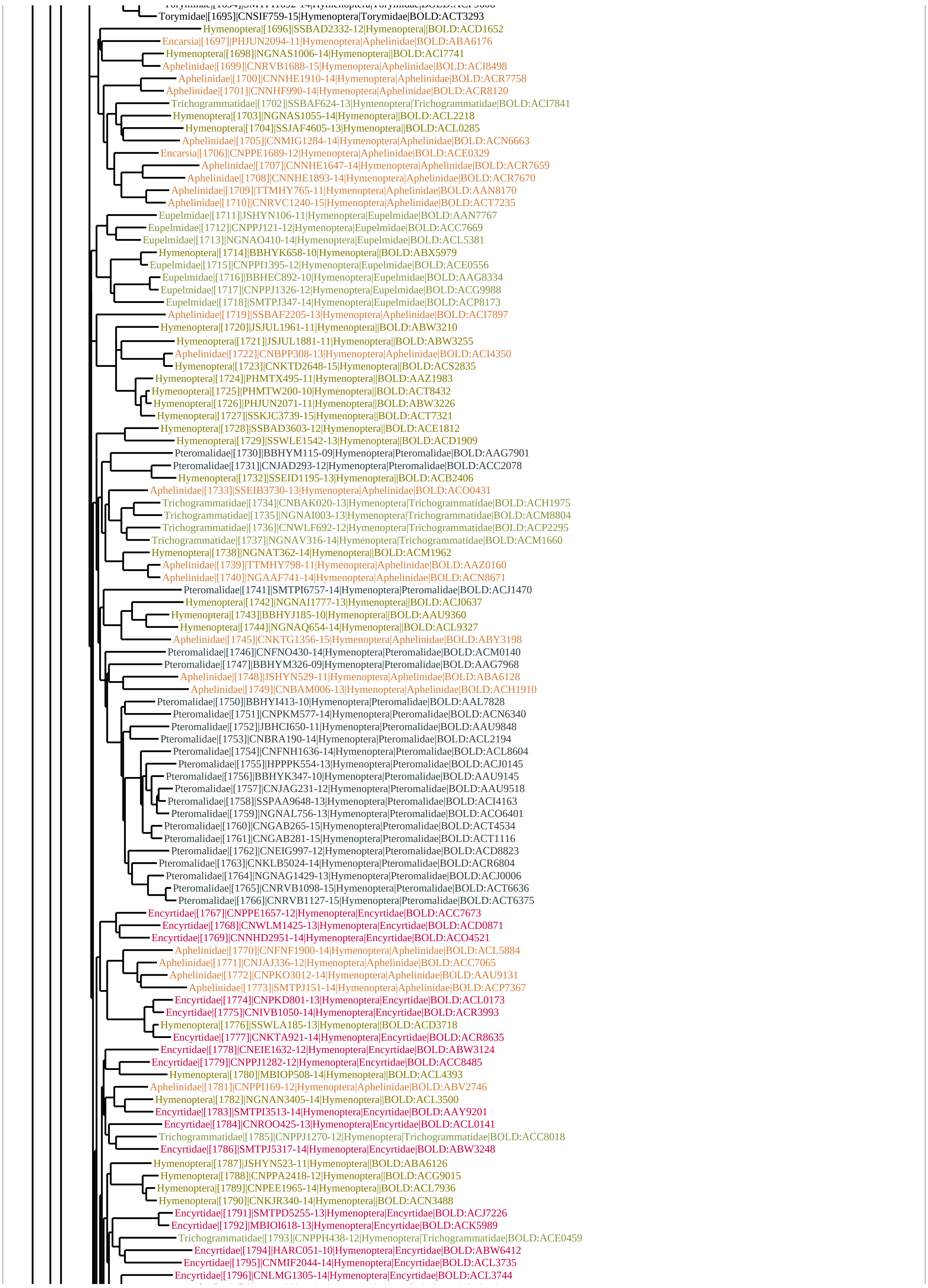

Torymidae|[1695]|CNSIF759-15|Hymenoptera|Torymidae|BOLD:ACT3293
Hymenoptera|[1696]|SSBAD2332-12|Hymenoptera||BOLD:ACD1652
Encarsia|[1697]|PHJUN2094-11|Hymenoptera|Aphelinidae|BOLD:ABA6176
Hymenoptera|[1698]|NGNAS1006-14|Hymenoptera||BOLD:ACI7741
Aphelinidae|[1699]|CNRVB1688-15|Hymenoptera|Aphelinidae|BOLD:ACI8498
Aphelinidae|[1700]|CNNHE1910-14|Hymenoptera|Aphelinidae|BOLD:ACR7758
Aphelinidae|[1701]|CNNHF990-14|Hymenoptera|Aphelinidae|BOLD:ACR8120
Trichogrammatidae|[1702]|SSBAF624-13|Hymenoptera|Trichogrammatidae|BOLD:ACI7841
Hymenoptera|[1703]|NGNAS1055-14|Hymenoptera||BOLD:ACL2218
Hymenoptera|[1704]|SSJAF4605-13|Hymenoptera||BOLD:ACL0285
Aphelinidae|[1705]|CNMIG1284-14|Hymenoptera|Aphelinidae|BOLD:ACN6663
Encarsia|[1706]|CNPPE1689-12|Hymenoptera|Aphelinidae|BOLD:ACE0329
Aphelinidae|[1707]|CNNHE1647-14|Hymenoptera|Aphelinidae|BOLD:ACR7659
Aphelinidae|[1708]|CNNHE1893-14|Hymenoptera|Aphelinidae|BOLD:ACR7670
Aphelinidae|[1709]|TTMHY765-11|Hymenoptera|Aphelinidae|BOLD:AAN8170
Aphelinidae|[1710]|CNRVC1240-15|Hymenoptera|Aphelinidae|BOLD:ACT7235
Eupelmidae|[1711]|JSHYN106-11|Hymenoptera|Eupelmidae|BOLD:AAN7767
Eupelmidae|[1712]|CNPPJ121-12|Hymenoptera|Eupelmidae|BOLD:ACC7669
Eupelmidae|[1713]|NGNAO410-14|Hymenoptera|Eupelmidae|BOLD:ACL5381
Hymenoptera|[1714]|BBHYK658-10|Hymenoptera||BOLD:ABX5979
Eupelmidae|[1715]|CNPPI1395-12|Hymenoptera|Eupelmidae|BOLD:ACE0556
Eupelmidae|[1716]|BBHEC892-10|Hymenoptera|Eupelmidae|BOLD:AAG8334
Eupelmidae|[1717]|CNPPJ1326-12|Hymenoptera|Eupelmidae|BOLD:ACG9988
Eupelmidae|[1718]|SMTPJ347-14|Hymenoptera|Eupelmidae|BOLD:ACP8173
Aphelinidae|[1719]|SSBAF2205-13|Hymenoptera|Aphelinidae|BOLD:ACI7897
Hymenoptera|[1720]|JSJUL1961-11|Hymenoptera||BOLD:ABW3210
Hymenoptera|[1721]|JSJUL1881-11|Hymenoptera||BOLD:ABW3255
Aphelinidae|[1722]|CNBPP308-13|Hymenoptera|Aphelinidae|BOLD:ACI4350
Hymenoptera|[1723]|CNKTD2648-15|Hymenoptera||BOLD:ACS2835
Hymenoptera|[1724]|PHMTX495-11|Hymenoptera||BOLD:AAZ1983
Hymenoptera|[1725]|PHMTW200-10|Hymenoptera||BOLD:ACT8432
Hymenoptera|[1726]|PHJUN2071-11|Hymenoptera||BOLD:ABW3226
Hymenoptera|[1727]|SSKJC3739-15|Hymenoptera||BOLD:ACT7321
Hymenoptera|[1728]|SSBAD3603-12|Hymenoptera||BOLD:ACE1812
Hymenoptera|[1729]|SSWLE1542-13|Hymenoptera||BOLD:ACD1909
Pteromalidae|[1730]|BBHYM115-09|Hymenoptera|Pteromalidae|BOLD:AAG7901
Pteromalidae|[1731]|CNJAD293-12|Hymenoptera|Pteromalidae|BOLD:ACC2078
Hymenoptera|[1732]|SSEID1195-13|Hymenoptera||BOLD:ACB2406
Aphelinidae|[1733]|SSEIB3730-13|Hymenoptera|Aphelinidae|BOLD:ACO0431
Trichogrammatidae|[1734]|CNBAK020-13|Hymenoptera|Trichogrammatidae|BOLD:ACH1975
Trichogrammatidae|[1735]|NGNAI003-13|Hymenoptera|Trichogrammatidae|BOLD:ACM8804
Trichogrammatidae|[1736]|CNWLF692-12|Hymenoptera|Trichogrammatidae|BOLD:ACP2295
Trichogrammatidae|[1737]|NGNAV316-14|Hymenoptera|Trichogrammatidae|BOLD:ACM1660
Hymenoptera|[1738]|NGNAT362-14|Hymenoptera||BOLD:ACM1962
Aphelinidae|[1739]|TTMHY798-11|Hymenoptera|Aphelinidae|BOLD:AAZ0160
Aphelinidae|[1740]|NGAAF741-14|Hymenoptera|Aphelinidae|BOLD:ACN8671
Pteromalidae|[1741]|SMTPI6757-14|Hymenoptera|Pteromalidae|BOLD:ACJ1470
Hymenoptera|[1742]|NGNAI1777-13|Hymenoptera||BOLD:ACJ0637
Hymenoptera|[1743]|BBHYJ185-10|Hymenoptera||BOLD:AAU9360
Hymenoptera|[1744]|NGNAQ654-14|Hymenoptera||BOLD:ACL9327
Aphelinidae|[1745]|CNKTG1356-15|Hymenoptera|Aphelinidae|BOLD:ABY3198
Pteromalidae|[1746]|CNFNO430-14|Hymenoptera|Pteromalidae|BOLD:ACM0140
Pteromalidae|[1747]|BBHYM326-09|Hymenoptera|Pteromalidae|BOLD:AAG7968
Aphelinidae|[1748]|JSHYN529-11|Hymenoptera|Aphelinidae|BOLD:ABA6128
Aphelinidae|[1749]|CNBAM006-13|Hymenoptera|Aphelinidae|BOLD:ACH1910
Pteromalidae|[1750]|BBHYI413-10|Hymenoptera|Pteromalidae|BOLD:AAL7828
Pteromalidae|[1751]|CNPKM577-14|Hymenoptera|Pteromalidae|BOLD:ACN6340
Pteromalidae|[1752]|JBHCI650-11|Hymenoptera|Pteromalidae|BOLD:AAU9848
Pteromalidae|[1753]|CNBRA190-14|Hymenoptera|Pteromalidae|BOLD:ACL2194
Pteromalidae|[1754]|CNFNH1636-14|Hymenoptera|Pteromalidae|BOLD:ACL8604
Pteromalidae|[1755]|HPPPK554-13|Hymenoptera|Pteromalidae|BOLD:ACJ0145
Pteromalidae|[1756]|BBHYK347-10|Hymenoptera|Pteromalidae|BOLD:AAU9145
Pteromalidae|[1757]|CNJAG231-12|Hymenoptera|Pteromalidae|BOLD:AAU9518
Pteromalidae|[1758]|SSPAA9648-13|Hymenoptera|Pteromalidae|BOLD:ACI4163
Pteromalidae|[1759]|NGNAL756-13|Hymenoptera|Pteromalidae|BOLD:ACO6401
Pteromalidae|[1760]|CNGAB265-15|Hymenoptera|Pteromalidae|BOLD:ACT4534
Pteromalidae|[1761]|CNGAB281-15|Hymenoptera|Pteromalidae|BOLD:ACT1116
Pteromalidae|[1762]|CNEIG997-12|Hymenoptera|Pteromalidae|BOLD:ACD8823
Pteromalidae|[1763]|CNKLB5024-14|Hymenoptera|Pteromalidae|BOLD:ACR6804
Pteromalidae|[1764]|NGNAG1429-13|Hymenoptera|Pteromalidae|BOLD:ACJ0006
Pteromalidae|[1765]|CNRVB1098-15|Hymenoptera|Pteromalidae|BOLD:ACT6636
Pteromalidae|[1766]|CNRVB1127-15|Hymenoptera|Pteromalidae|BOLD:ACT6375
Encyrtidae|[1767]|CNPPE1657-12|Hymenoptera|Encyrtidae|BOLD:ACC7673
Encyrtidae|[1768]|CNWLM1425-13|Hymenoptera|Encyrtidae|BOLD:ACD0871
Encyrtidae|[1769]|CNNHD2951-14|Hymenoptera|Encyrtidae|BOLD:ACO4521
Aphelinidae|[1770]|CNFNF1900-14|Hymenoptera|Aphelinidae|BOLD:ACL5884
Aphelinidae|[1771]|CNJAJ336-12|Hymenoptera|Aphelinidae|BOLD:ACC7065
Aphelinidae|[1772]|CNPKO3012-14|Hymenoptera|Aphelinidae|BOLD:AAU9131
Aphelinidae|[1773]|SMTPJ151-14|Hymenoptera|Aphelinidae|BOLD:ACP7367
Encyrtidae|[1774]|CNPKD801-13|Hymenoptera|Encyrtidae|BOLD:ACL0173
Encyrtidae|[1775]|CNIVB1050-14|Hymenoptera|Encyrtidae|BOLD:ACR3993
Hymenoptera|[1776]|SSWLA185-13|Hymenoptera||BOLD:ACD3718
Encyrtidae|[1777]|CNKTA921-14|Hymenoptera|Encyrtidae|BOLD:ACR8635
Encyrtidae|[1778]|CNEIE1632-12|Hymenoptera|Encyrtidae|BOLD:ABW3124
Encyrtidae|[1779]|CNPPJ1282-12|Hymenoptera|Encyrtidae|BOLD:ACC8485
Hymenoptera|[1780]|MBIOP508-14|Hymenoptera||BOLD:ACL4393
Aphelinidae|[1781]|CNPPI169-12|Hymenoptera|Aphelinidae|BOLD:ABV2746
Hymenoptera|[1782]|NGNAN3405-14|Hymenoptera||BOLD:ACL3500
Encyrtidae|[1783]|SMTPI3513-14|Hymenoptera|Encyrtidae|BOLD:AAY9201
Encyrtidae|[1784]|CNROO425-13|Hymenoptera|Encyrtidae|BOLD:ACL0141
Trichogrammatidae|[1785]|CNPPJ1270-12|Hymenoptera|Trichogrammatidae|BOLD:ACC8018
Encyrtidae|[1786]|SMTPJ5317-14|Hymenoptera|Encyrtidae|BOLD:ABW3248
Hymenoptera|[1787]|JSHYN523-11|Hymenoptera||BOLD:ABA6126
Hymenoptera|[1788]|CNPPA2418-12|Hymenoptera||BOLD:ACG9015
Hymenoptera|[1789]|CNPEE1965-14|Hymenoptera||BOLD:ACL7936
Hymenoptera|[1790]|CNKJR340-14|Hymenoptera||BOLD:ACN3488
Encyrtidae|[1791]|SMTPD5255-13|Hymenoptera|Encyrtidae|BOLD:ACJ7226
Encyrtidae|[1792]|MBIOI618-13|Hymenoptera|Encyrtidae|BOLD:ACK5989
Trichogrammatidae|[1793]|CNPPH438-12|Hymenoptera|Trichogrammatidae|BOLD:ACE0459
Encyrtidae|[1794]|HARC051-10|Hymenoptera|Encyrtidae|BOLD:ABW6412
Encyrtidae|[1795]|CNMIF2044-14|Hymenoptera|Encyrtidae|BOLD:ACL3735
Encyrtidae|[1796]|CNLMG1305-14|Hymenoptera|Encyrtidae|BOLD:ACL3744

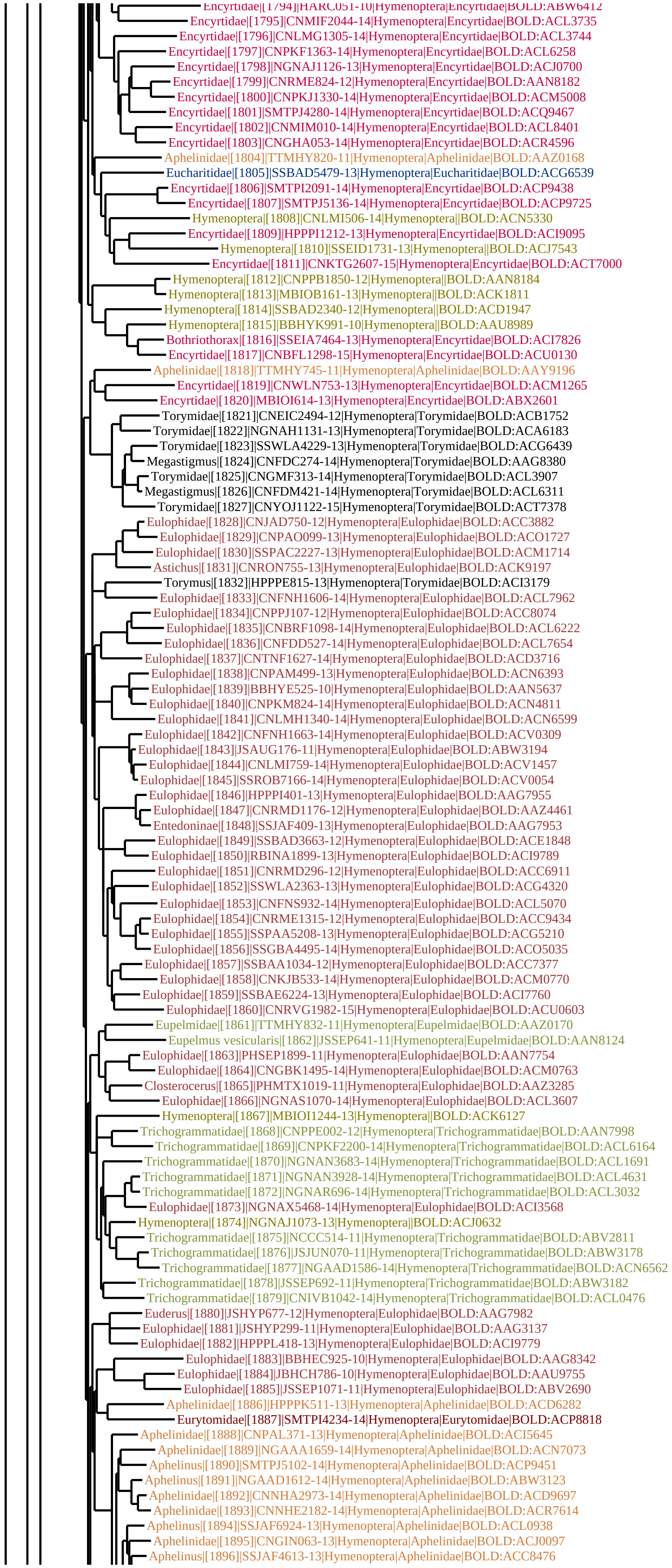
Encyrtidae|[1794]|HARC051-10|Hymenoptera|Encyrtidae|BOLD:ABW6412
Encyrtidae|[1795]|CNMIF2044-14|Hymenoptera|Encyrtidae|BOLD:ACL3735
Encyrtidae|[1796]|CNLMG1305-14|Hymenoptera|Encyrtidae|BOLD:ACL3744
Encyrtidae|[1797]|CNPKF1363-14|Hymenoptera|Encyrtidae|BOLD:ACL6258
Encyrtidae|[1798]|NGNAJ1126-13|Hymenoptera|Encyrtidae|BOLD:ACJ0700
Encyrtidae|[1799]|CNRME824-12|Hymenoptera|Encyrtidae|BOLD:AAN8182
Encyrtidae|[1800]|CNPKJ1330-14|Hymenoptera|Encyrtidae|BOLD:ACM5008
Encyrtidae|[1801]|SMTPJ4280-14|Hymenoptera|Encyrtidae|BOLD:ACQ9467
Encyrtidae|[1802]|CNMIM010-14|Hymenoptera|Encyrtidae|BOLD:ACL8401
Encyrtidae|[1803]|CNGHA053-14|Hymenoptera|Encyrtidae|BOLD:ACR4596
Aphelinidae|[1804]|TTMHY820-11|Hymenoptera|Aphelinidae|BOLD:AAZ0168
Eucharitidae|[1805]|SSBAD5479-13|Hymenoptera|Eucharitidae|BOLD:ACG6539
Encyrtidae|[1806]|SMTPI2091-14|Hymenoptera|Encyrtidae|BOLD:ACP9438
Encyrtidae|[1807]|SMTPJ5136-14|Hymenoptera|Encyrtidae|BOLD:ACP9725
Hymenoptera|[1808]|CNLMI506-14|Hymenoptera||BOLD:ACN5330
Encyrtidae|[1809]|HPPPI1212-13|Hymenoptera|Encyrtidae|BOLD:ACI9095
Hymenoptera|[1810]|SSEID1731-13|Hymenoptera||BOLD:ACJ7543
Encyrtidae|[1811]|CNKTG2607-15|Hymenoptera|Encyrtidae|BOLD:ACT7000
Hymenoptera|[1812]|CNPPB1850-12|Hymenoptera||BOLD:AAN8184
Hymenoptera|[1813]|MBIOB161-13|Hymenoptera||BOLD:ACK1811
Hymenoptera|[1814]|SSBAD2340-12|Hymenoptera||BOLD:ACD1947
Hymenoptera|[1815]|BBHYK991-10|Hymenoptera||BOLD:AAU8989
Bothriothorax|[1816]|SSEIA7464-13|Hymenoptera|Encyrtidae|BOLD:ACI7826
Encyrtidae|[1817]|CNBFL1298-15|Hymenoptera|Encyrtidae|BOLD:ACU0130
Aphelinidae|[1818]|TTMHY745-11|Hymenoptera|Aphelinidae|BOLD:AAY9196
Encyrtidae|[1819]|CNWLN753-13|Hymenoptera|Encyrtidae|BOLD:ACM1265
Encyrtidae|[1820]|MBIOI614-13|Hymenoptera|Encyrtidae|BOLD:ABX2601
Torymidae|[1821]|CNEIC2494-12|Hymenoptera|Torymidae|BOLD:ACB1752
Torymidae|[1822]|NGNAH1131-13|Hymenoptera|Torymidae|BOLD:ACA6183
Torymidae|[1823]|SSWLA4229-13|Hymenoptera|Torymidae|BOLD:ACG6439
Megastigmus|[1824]|CNFDC274-14|Hymenoptera|Torymidae|BOLD:AAG8380
Torymidae|[1825]|CNGMF313-14|Hymenoptera|Torymidae|BOLD:ACL3907
Megastigmus|[1826]|CNFDM421-14|Hymenoptera|Torymidae|BOLD:ACL6311
Torymidae|[1827]|CNYOJ1122-15|Hymenoptera|Torymidae|BOLD:ACT7378
Eulophidae|[1828]|CNJAD750-12|Hymenoptera|Eulophidae|BOLD:ACC3882
Eulophidae|[1829]|CNPAO099-13|Hymenoptera|Eulophidae|BOLD:ACO1727
Eulophidae|[1830]|SSPAC2227-13|Hymenoptera|Eulophidae|BOLD:ACM1714
Astichus|[1831]|CNRON755-13|Hymenoptera|Eulophidae|BOLD:ACK9197
Torymus|[1832]|HPPPE815-13|Hymenoptera|Torymidae|BOLD:ACI3179
Eulophidae|[1833]|CNFNH1606-14|Hymenoptera|Eulophidae|BOLD:ACL7962
Eulophidae|[1834]|CNPPJ107-12|Hymenoptera|Eulophidae|BOLD:ACC8074
Eulophidae|[1835]|CNBRF1098-14|Hymenoptera|Eulophidae|BOLD:ACL6222
Eulophidae|[1836]|CNFDD527-14|Hymenoptera|Eulophidae|BOLD:ACL7654
Eulophidae|[1837]|CNTNF1627-14|Hymenoptera|Eulophidae|BOLD:ACD3716
Eulophidae|[1838]|CNPAM499-13|Hymenoptera|Eulophidae|BOLD:ACN6393
Eulophidae|[1839]|BBHYE525-10|Hymenoptera|Eulophidae|BOLD:AAN5637
Eulophidae|[1840]|CNPKM824-14|Hymenoptera|Eulophidae|BOLD:ACN4811
Eulophidae|[1841]|CNLMH1340-14|Hymenoptera|Eulophidae|BOLD:ACN6599
Eulophidae|[1842]|CNFNH1663-14|Hymenoptera|Eulophidae|BOLD:ACV0309
Eulophidae|[1843]|JSAUG176-11|Hymenoptera|Eulophidae|BOLD:ABW3194
Eulophidae|[1844]|CNLMI759-14|Hymenoptera|Eulophidae|BOLD:ACV1457
Eulophidae|[1845]|SSROB7166-14|Hymenoptera|Eulophidae|BOLD:ACV0054
Eulophidae|[1846]|HPPPI401-13|Hymenoptera|Eulophidae|BOLD:AAG7955
Eulophidae|[1847]|CNRMD1176-12|Hymenoptera|Eulophidae|BOLD:AAZ4461
Entedoninae|[1848]|SSJAF409-13|Hymenoptera|Eulophidae|BOLD:AAG7953
Eulophidae|[1849]|SSBAD3663-12|Hymenoptera|Eulophidae|BOLD:ACE1848
Eulophidae|[1850]|RBINA1899-13|Hymenoptera|Eulophidae|BOLD:ACI9789
Eulophidae|[1851]|CNRMD296-12|Hymenoptera|Eulophidae|BOLD:ACC6911
Eulophidae|[1852]|SSWLA2363-13|Hymenoptera|Eulophidae|BOLD:ACG4320
Eulophidae|[1853]|CNFNS932-14|Hymenoptera|Eulophidae|BOLD:ACL5070
Eulophidae|[1854]|CNRME1315-12|Hymenoptera|Eulophidae|BOLD:ACC9434
Eulophidae|[1855]|SSPAA5208-13|Hymenoptera|Eulophidae|BOLD:ACG5210
Eulophidae|[1856]|SSGBA4495-14|Hymenoptera|Eulophidae|BOLD:ACO5035
Eulophidae|[1857]|SSBAA1034-12|Hymenoptera|Eulophidae|BOLD:ACC7377
Eulophidae|[1858]|CNKJB533-14|Hymenoptera|Eulophidae|BOLD:ACM0770
Eulophidae|[1859]|SSBAE6224-13|Hymenoptera|Eulophidae|BOLD:ACI7760
Eulophidae|[1860]|CNRVG1982-15|Hymenoptera|Eulophidae|BOLD:ACU0603
Eupelmidae|[1861]|TTMHY832-11|Hymenoptera|Eupelmidae|BOLD:AAZ0170
Eupelmus vesicularis|[1862]|JSSEP641-11|Hymenoptera|Eupelmidae|BOLD:AAN8124
Eulophidae|[1863]|PHSEP1899-11|Hymenoptera|Eulophidae|BOLD:AAN7754
Eulophidae|[1864]|CNGBK1495-14|Hymenoptera|Eulophidae|BOLD:ACM0763
Closterocerus|[1865]|PHMTX1019-11|Hymenoptera|Eulophidae|BOLD:AAZ3285
Eulophidae|[1866]|NGNAS1070-14|Hymenoptera|Eulophidae|BOLD:ACL3607
Hymenoptera|[1867]|MBIOI1244-13|Hymenoptera||BOLD:ACK6127
Trichogrammatidae|[1868]|CNPPE002-12|Hymenoptera|Trichogrammatidae|BOLD:AAN7998
Trichogrammatidae|[1869]|CNPKF2200-14|Hymenoptera|Trichogrammatidae|BOLD:ACL6164
Trichogrammatidae|[1870]|NGNAN3683-14|Hymenoptera|Trichogrammatidae|BOLD:ACL1691
Trichogrammatidae|[1871]|NGNAN3928-14|Hymenoptera|Trichogrammatidae|BOLD:ACL4631
Trichogrammatidae|[1872]|NGNAR696-14|Hymenoptera|Trichogrammatidae|BOLD:ACL3032
Eulophidae|[1873]|NGNAX5468-14|Hymenoptera|Eulophidae|BOLD:ACI3568
Hymenoptera|[1874]|NGNAJ1073-13|Hymenoptera||BOLD:ACJ0632
Trichogrammatidae|[1875]|NCCC514-11|Hymenoptera|Trichogrammatidae|BOLD:ABV2811
Trichogrammatidae|[1876]|JSJUN070-11|Hymenoptera|Trichogrammatidae|BOLD:ABW3178
Trichogrammatidae|[1877]|NGAAD1586-14|Hymenoptera|Trichogrammatidae|BOLD:ACN6562
Trichogrammatidae|[1878]|JSSEP692-11|Hymenoptera|Trichogrammatidae|BOLD:ABW3182
Trichogrammatidae|[1879]|CNIVB1042-14|Hymenoptera|Trichogrammatidae|BOLD:ACL0476
Euderus|[1880]|JSHYP677-12|Hymenoptera|Eulophidae|BOLD:AAG7982
Eulophidae|[1881]|JSHYP299-11|Hymenoptera|Eulophidae|BOLD:AAG3137
Eulophidae|[1882]|HPPPL418-13|Hymenoptera|Eulophidae|BOLD:ACI9779
Eulophidae|[1883]|BBHEC925-10|Hymenoptera|Eulophidae|BOLD:AAG8342
Eulophidae|[1884]|JBHCH786-10|Hymenoptera|Eulophidae|BOLD:AAU9755
Eulophidae|[1885]|JSSEP1071-11|Hymenoptera|Eulophidae|BOLD:ABV2690
Aphelinidae|[1886]|HPPPK511-13|Hymenoptera|Aphelinidae|BOLD:ACD6282
Eurytomidae|[1887]|SMTPI4234-14|Hymenoptera|Eurytomidae|BOLD:ACP8818
Aphelinidae|[1888]|CNPAL371-13|Hymenoptera|Aphelinidae|BOLD:ACI5645
Aphelinidae|[1889]|NGAAA1659-14|Hymenoptera|Aphelinidae|BOLD:ACN7073
Aphelinus|[1890]|SMTPJ5102-14|Hymenoptera|Aphelinidae|BOLD:ACP9451
Aphelinus|[1891]|NGAAD1612-14|Hymenoptera|Aphelinidae|BOLD:ABW3123
Aphelinidae|[1892]|CNNHA2973-14|Hymenoptera|Aphelinidae|BOLD:ACD9697
Aphelinidae|[1893]|CNNHE2182-14|Hymenoptera|Aphelinidae|BOLD:ACR7614
Aphelinus|[1894]|SSJAF6924-13|Hymenoptera|Aphelinidae|BOLD:ACL0938
Aphelinidae|[1895]|CNGIN063-13|Hymenoptera|Aphelinidae|BOLD:ACJ0097
Aphelinus|[1896]|SSJAF4613-13|Hymenoptera|Aphelinidae|BOLD:ACC8476

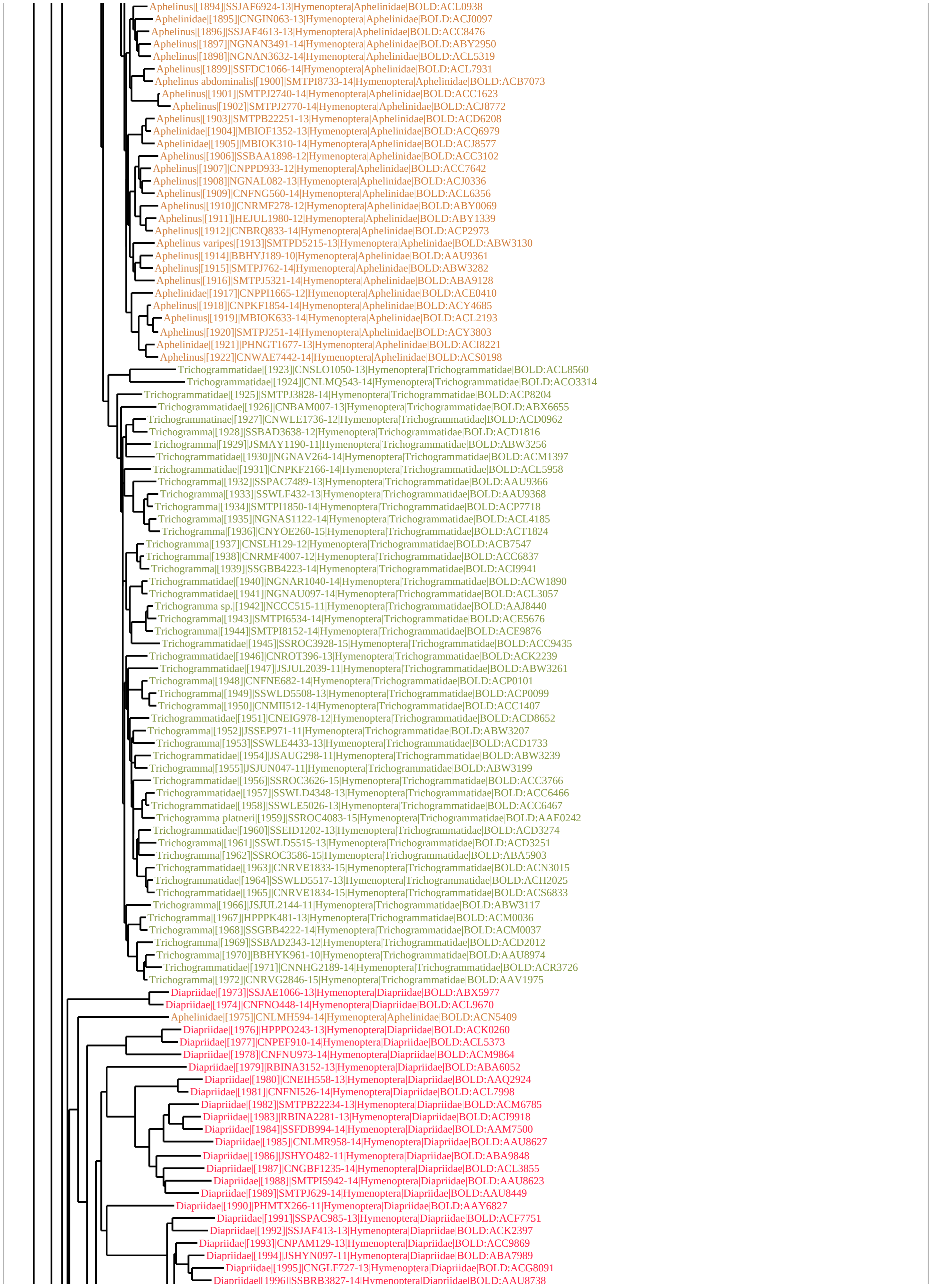

Aphelinus|[1894]|SSJAF6924-13|Hymenoptera|Aphelinidae|BOLD:ACL0938
Aphelinidae|[1895]|CNGIN063-13|Hymenoptera|Aphelinidae|BOLD:ACJ0097
Aphelinus|[1896]|SSJAF4613-13|Hymenoptera|Aphelinidae|BOLD:ACC8476
Aphelinus|[1897]|NGNAN3491-14|Hymenoptera|Aphelinidae|BOLD:ABY2950
Aphelinus|[1898]|NGNAN3632-14|Hymenoptera|Aphelinidae|BOLD:ACL5319
Aphelinus|[1899]|SSFDC1066-14|Hymenoptera|Aphelinidae|BOLD:ACL7931
Aphelinus abdominalis|[1900]|SMTPI8733-14|Hymenoptera|Aphelinidae|BOLD:ACB7073
Aphelinus|[1901]|SMTPJ2740-14|Hymenoptera|Aphelinidae|BOLD:ACC1623
Aphelinus|[1902]|SMTPJ2770-14|Hymenoptera|Aphelinidae|BOLD:ACJ8772
Aphelinus|[1903]|SMTPB22251-13|Hymenoptera|Aphelinidae|BOLD:ACD6208
Aphelinidae|[1904]|MBIOF1352-13|Hymenoptera|Aphelinidae|BOLD:ACQ6979
Aphelinidae|[1905]|MBIOK310-14|Hymenoptera|Aphelinidae|BOLD:ACJ8577
Aphelinus|[1906]|SSBAA1898-12|Hymenoptera|Aphelinidae|BOLD:ACC3102
Aphelinus|[1907]|CNPPD933-12|Hymenoptera|Aphelinidae|BOLD:ACC7642
Aphelinus|[1908]|NGNAL082-13|Hymenoptera|Aphelinidae|BOLD:ACJ0336
Aphelinus|[1909]|CNFNG560-14|Hymenoptera|Aphelinidae|BOLD:ACL6356
Aphelinus|[1910]|CNRMF278-12|Hymenoptera|Aphelinidae|BOLD:ABY0069
Aphelinus|[1911]|HEJUL1980-12|Hymenoptera|Aphelinidae|BOLD:ABY1339
Aphelinus|[1912]|CNBRQ833-14|Hymenoptera|Aphelinidae|BOLD:ACP2973
Aphelinus varipes|[1913]|SMTPD5215-13|Hymenoptera|Aphelinidae|BOLD:ABW3130
Aphelinus|[1914]|BBHYJ189-10|Hymenoptera|Aphelinidae|BOLD:AAU9361
Aphelinus|[1915]|SMTPJ762-14|Hymenoptera|Aphelinidae|BOLD:ABW3282
Aphelinus|[1916]|SMTPJ5321-14|Hymenoptera|Aphelinidae|BOLD:ABA9128
Aphelinidae|[1917]|CNPPI1665-12|Hymenoptera|Aphelinidae|BOLD:ACE0410
Aphelinus|[1918]|CNPKF1854-14|Hymenoptera|Aphelinidae|BOLD:ACY4685
Aphelinus|[1919]|MBIOK633-14|Hymenoptera|Aphelinidae|BOLD:ACL2193
Aphelinus|[1920]|SMTPJ251-14|Hymenoptera|Aphelinidae|BOLD:ACY3803
Aphelinidae|[1921]|PHNGT1677-13|Hymenoptera|Aphelinidae|BOLD:ACI8221
Aphelinus|[1922]|CNWAE7442-14|Hymenoptera|Aphelinidae|BOLD:ACS0198
Trichogrammatidae|[1923]|CNSLO1050-13|Hymenoptera|Trichogrammatidae|BOLD:ACL8560
Trichogrammatidae|[1924]|CNLMQ543-14|Hymenoptera|Trichogrammatidae|BOLD:ACO3314
Trichogrammatidae|[1925]|SMTPJ3828-14|Hymenoptera|Trichogrammatidae|BOLD:ACP8204
Trichogrammatidae|[1926]|CNBAM007-13|Hymenoptera|Trichogrammatidae|BOLD:ABX6655
Trichogrammatinae|[1927]|CNWLE1736-12|Hymenoptera|Trichogrammatidae|BOLD:ACD0962
Trichogramma|[1928]|SSBAD3638-12|Hymenoptera|Trichogrammatidae|BOLD:ACD1816
Trichogramma|[1929]|JSMAY1190-11|Hymenoptera|Trichogrammatidae|BOLD:ABW3256
Trichogrammatidae|[1930]|NGNAV264-14|Hymenoptera|Trichogrammatidae|BOLD:ACM1397
Trichogrammatidae|[1931]|CNPKF2166-14|Hymenoptera|Trichogrammatidae|BOLD:ACL5958
Trichogramma|[1932]|SSPAC7489-13|Hymenoptera|Trichogrammatidae|BOLD:AAU9366
Trichogramma|[1933]|SSWLF432-13|Hymenoptera|Trichogrammatidae|BOLD:AAU9368
Trichogramma|[1934]|SMTPI1850-14|Hymenoptera|Trichogrammatidae|BOLD:ACP7718
Trichogramma|[1935]|NGNAS1122-14|Hymenoptera|Trichogrammatidae|BOLD:ACL4185
Trichogramma|[1936]|CNYOE260-15|Hymenoptera|Trichogrammatidae|BOLD:ACT1824
Trichogramma|[1937]|CNSLH129-12|Hymenoptera|Trichogrammatidae|BOLD:ACB7547
Trichogramma|[1938]|CNRMF4007-12|Hymenoptera|Trichogrammatidae|BOLD:ACC6837
Trichogramma|[1939]|SSGBB4223-14|Hymenoptera|Trichogrammatidae|BOLD:ACI9941
Trichogrammatidae|[1940]|NGNAR1040-14|Hymenoptera|Trichogrammatidae|BOLD:ACW1890
Trichogrammatidae|[1941]|NGNAU097-14|Hymenoptera|Trichogrammatidae|BOLD:ACL3057
Trichogramma sp.|[1942]|NCCC515-11|Hymenoptera|Trichogrammatidae|BOLD:AAJ8440
Trichogramma|[1943]|SMTPI6534-14|Hymenoptera|Trichogrammatidae|BOLD:ACE5676
Trichogramma|[1944]|SMTPI8152-14|Hymenoptera|Trichogrammatidae|BOLD:ACE9876
Trichogrammatidae|[1945]|SSROC3928-15|Hymenoptera|Trichogrammatidae|BOLD:ACC9435
Trichogrammatidae|[1946]|CNROT396-13|Hymenoptera|Trichogrammatidae|BOLD:ACK2239
Trichogrammatidae|[1947]|JSJUL2039-11|Hymenoptera|Trichogrammatidae|BOLD:ABW3261
Trichogramma|[1948]|CNFNE682-14|Hymenoptera|Trichogrammatidae|BOLD:ACP0101
Trichogramma|[1949]|SSWLD5508-13|Hymenoptera|Trichogrammatidae|BOLD:ACP0099
Trichogramma|[1950]|CNMII512-14|Hymenoptera|Trichogrammatidae|BOLD:ACC1407
Trichogrammatidae|[1951]|CNEIG978-12|Hymenoptera|Trichogrammatidae|BOLD:ACD8652
Trichogramma|[1952]|JSSEP971-11|Hymenoptera|Trichogrammatidae|BOLD:ABW3207
Trichogramma|[1953]|SSWLE4433-13|Hymenoptera|Trichogrammatidae|BOLD:ACD1733
Trichogrammatidae|[1954]|JSAUG298-11|Hymenoptera|Trichogrammatidae|BOLD:ABW3239
Trichogramma|[1955]|JSJUN047-11|Hymenoptera|Trichogrammatidae|BOLD:ABW3199
Trichogrammatidae|[1956]|SSROC3626-15|Hymenoptera|Trichogrammatidae|BOLD:ACC3766
Trichogrammatidae|[1957]|SSWLD4348-13|Hymenoptera|Trichogrammatidae|BOLD:ACC6466
Trichogrammatidae|[1958]|SSWLE5026-13|Hymenoptera|Trichogrammatidae|BOLD:ACC6467
Trichogramma platneri|[1959]|SSROC4083-15|Hymenoptera|Trichogrammatidae|BOLD:AAE0242
Trichogrammatidae|[1960]|SSEID1202-13|Hymenoptera|Trichogrammatidae|BOLD:ACD3274
Trichogramma|[1961]|SSWLD5515-13|Hymenoptera|Trichogrammatidae|BOLD:ACD3251
Trichogramma|[1962]|SSROC3586-15|Hymenoptera|Trichogrammatidae|BOLD:ABA5903
Trichogrammatidae|[1963]|CNRVE1833-15|Hymenoptera|Trichogrammatidae|BOLD:ACN3015
Trichogrammatidae|[1964]|SSWLD5517-13|Hymenoptera|Trichogrammatidae|BOLD:ACH2025
Trichogrammatidae|[1965]|CNRVE1834-15|Hymenoptera|Trichogrammatidae|BOLD:ACS6833
Trichogramma|[1966]|JSJUL2144-11|Hymenoptera|Trichogrammatidae|BOLD:ABW3117
Trichogramma|[1967]|HPPPK481-13|Hymenoptera|Trichogrammatidae|BOLD:ACM0036
Trichogramma|[1968]|SSGBB4222-14|Hymenoptera|Trichogrammatidae|BOLD:ACM0037
Trichogramma|[1969]|SSBAD2343-12|Hymenoptera|Trichogrammatidae|BOLD:ACD2012
Trichogramma|[1970]|BBHYK961-10|Hymenoptera|Trichogrammatidae|BOLD:AAU8974
Trichogrammatidae|[1971]|CNNHG2189-14|Hymenoptera|Trichogrammatidae|BOLD:ACR3726
Trichogramma|[1972]|CNRVG2846-15|Hymenoptera|Trichogrammatidae|BOLD:AAV1975
Diapriidae|[1973]|SSJAE1066-13|Hymenoptera|Diapriidae|BOLD:ABX5977
Diapriidae|[1974]|CNFNO448-14|Hymenoptera|Diapriidae|BOLD:ACL9670
Aphelinidae|[1975]|CNLMH594-14|Hymenoptera|Aphelinidae|BOLD:ACN5409
Diapriidae|[1976]|HPPPO243-13|Hymenoptera|Diapriidae|BOLD:ACK0260
Diapriidae|[1977]|CNPEF910-14|Hymenoptera|Diapriidae|BOLD:ACL5373
Diapriidae|[1978]|CNFNU973-14|Hymenoptera|Diapriidae|BOLD:ACM9864
Diapriidae|[1979]|RBINA3152-13|Hymenoptera|Diapriidae|BOLD:ABA6052
Diapriidae|[1980]|CNEIH558-13|Hymenoptera|Diapriidae|BOLD:AAQ2924
Diapriidae|[1981]|CNFNI526-14|Hymenoptera|Diapriidae|BOLD:ACL7998
Diapriidae|[1982]|SMTPB22234-13|Hymenoptera|Diapriidae|BOLD:ACM6785
Diapriidae|[1983]|RBINA2281-13|Hymenoptera|Diapriidae|BOLD:ACI9918
Diapriidae|[1984]|SSFDB994-14|Hymenoptera|Diapriidae|BOLD:AAM7500
Diapriidae|[1985]|CNLMR958-14|Hymenoptera|Diapriidae|BOLD:AAU8627
Diapriidae|[1986]|JSHYO482-11|Hymenoptera|Diapriidae|BOLD:ABA9848
Diapriidae|[1987]|CNGBF1235-14|Hymenoptera|Diapriidae|BOLD:ACL3855
Diapriidae|[1988]|SMTPI5942-14|Hymenoptera|Diapriidae|BOLD:AAU8623
Diapriidae|[1989]|SMTPJ629-14|Hymenoptera|Diapriidae|BOLD:AAU8449
Diapriidae|[1990]|PHMTX266-11|Hymenoptera|Diapriidae|BOLD:AAY6827
Diapriidae|[1991]|SSPAC985-13|Hymenoptera|Diapriidae|BOLD:ACF7751
Diapriidae|[1992]|SSJAF413-13|Hymenoptera|Diapriidae|BOLD:ACK2397
Diapriidae|[1993]|CNPAM129-13|Hymenoptera|Diapriidae|BOLD:ACC9869
Diapriidae|[1994]|JSHYN097-11|Hymenoptera|Diapriidae|BOLD:ABA7989
Diapriidae|[1995]|CNGLF727-13|Hymenoptera|Diapriidae|BOLD:ACG8091
Diapriidae|[1996]|SSBRB3827-14|Hymenoptera|Diapriidae|BOLD:AAU8738

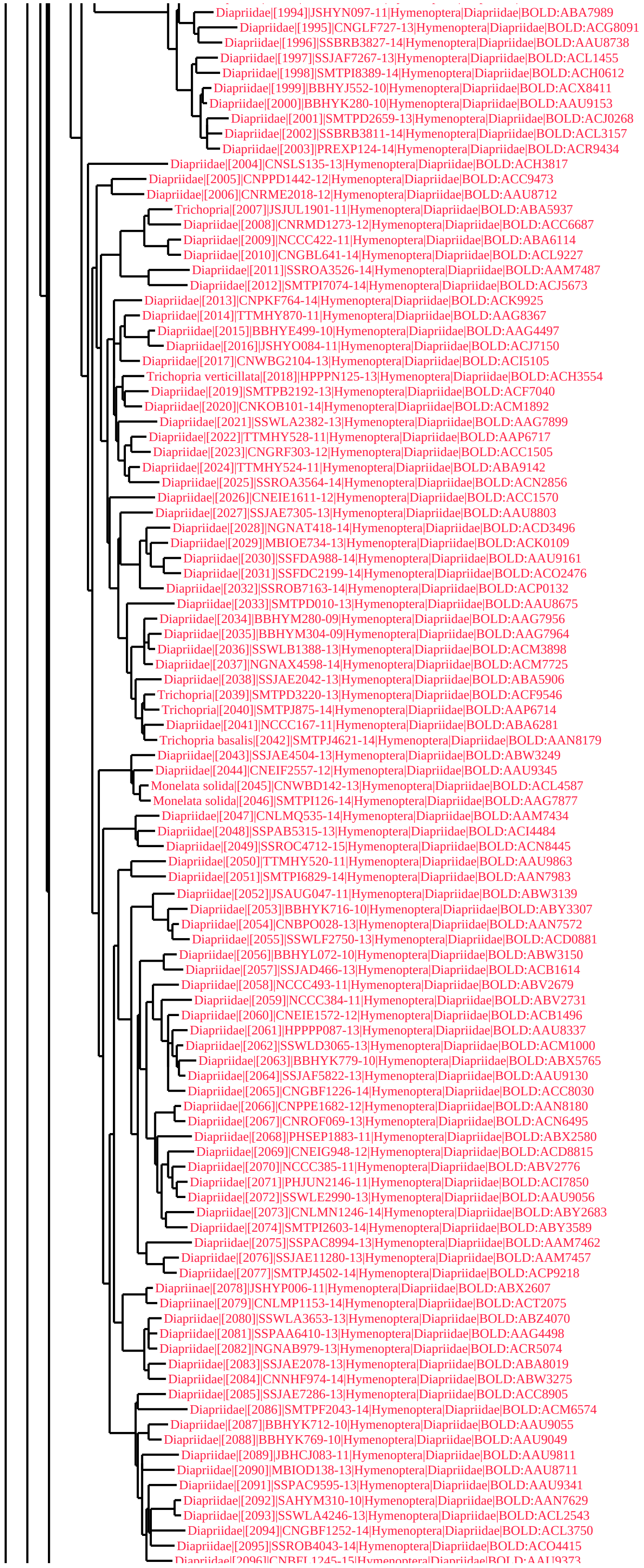
Diapriidae|[1994]|JSHYN097-11|Hymenoptera|Diapriidae|BOLD:ABA7989
Diapriidae|[1995]|CNGLF727-13|Hymenoptera|Diapriidae|BOLD:ACG8091
Diapriidae|[1996]|SSBRB3827-14|Hymenoptera|Diapriidae|BOLD:AAU8738
Diapriidae|[1997]|SSJAF7267-13|Hymenoptera|Diapriidae|BOLD:ACL1455
Diapriidae|[1998]|SMTPI8389-14|Hymenoptera|Diapriidae|BOLD:ACH0612
Diapriidae|[1999]|BBHYJ552-10|Hymenoptera|Diapriidae|BOLD:ACX8411
Diapriidae|[2000]|BBHYK280-10|Hymenoptera|Diapriidae|BOLD:AAU9153
Diapriidae|[2001]|SMTPD2659-13|Hymenoptera|Diapriidae|BOLD:ACJ0268
Diapriidae|[2002]|SSBRB3811-14|Hymenoptera|Diapriidae|BOLD:ACL3157
Diapriidae|[2003]|PREXP124-14|Hymenoptera|Diapriidae|BOLD:ACR9434
Diapriidae|[2004]|CNSLS135-13|Hymenoptera|Diapriidae|BOLD:ACH3817
Diapriidae|[2005]|CNPPD1442-12|Hymenoptera|Diapriidae|BOLD:ACC9473
Diapriidae|[2006]|CNRME2018-12|Hymenoptera|Diapriidae|BOLD:AAU8712
Trichopria|[2007]|JSJUL1901-11|Hymenoptera|Diapriidae|BOLD:ABA5937
Diapriidae|[2008]|CNRMD1273-12|Hymenoptera|Diapriidae|BOLD:ACC6687
Diapriidae|[2009]|NCCC422-11|Hymenoptera|Diapriidae|BOLD:ABA6114
Diapriidae|[2010]|CNGBL641-14|Hymenoptera|Diapriidae|BOLD:ACL9227
Diapriidae|[2011]|SSROA3526-14|Hymenoptera|Diapriidae|BOLD:AAM7487
Diapriidae|[2012]|SMTPI7074-14|Hymenoptera|Diapriidae|BOLD:ACJ5673
Diapriidae|[2013]|CNPKF764-14|Hymenoptera|Diapriidae|BOLD:ACK9925
Diapriidae|[2014]|TTMHY870-11|Hymenoptera|Diapriidae|BOLD:AAG8367
Diapriidae|[2015]|BBHYE499-10|Hymenoptera|Diapriidae|BOLD:AAG4497
Diapriidae|[2016]|JSHYO084-11|Hymenoptera|Diapriidae|BOLD:ACJ7150
Diapriidae|[2017]|CNWBG2104-13|Hymenoptera|Diapriidae|BOLD:ACI5105
Trichopria verticillata|[2018]|HPPPN125-13|Hymenoptera|Diapriidae|BOLD:ACH3554
Diapriidae|[2019]|SMTPB2192-13|Hymenoptera|Diapriidae|BOLD:ACF7040
Diapriidae|[2020]|CNKOB101-14|Hymenoptera|Diapriidae|BOLD:ACM1892
Diapriidae|[2021]|SSWLA2382-13|Hymenoptera|Diapriidae|BOLD:AAG7899
Diapriidae|[2022]|TTMHY528-11|Hymenoptera|Diapriidae|BOLD:AAP6717
Diapriidae|[2023]|CNGRF303-12|Hymenoptera|Diapriidae|BOLD:ACC1505
Diapriidae|[2024]|TTMHY524-11|Hymenoptera|Diapriidae|BOLD:ABA9142
Diapriidae|[2025]|SSROA3564-14|Hymenoptera|Diapriidae|BOLD:ACN2856
Diapriidae|[2026]|CNEIE1611-12|Hymenoptera|Diapriidae|BOLD:ACC1570
Diapriidae|[2027]|SSJAE7305-13|Hymenoptera|Diapriidae|BOLD:AAU8803
Diapriidae|[2028]|NGNAT418-14|Hymenoptera|Diapriidae|BOLD:ACD3496
Diapriidae|[2029]|MBIOE734-13|Hymenoptera|Diapriidae|BOLD:ACK0109
Diapriidae|[2030]|SSFDA988-14|Hymenoptera|Diapriidae|BOLD:AAU9161
Diapriidae|[2031]|SSFDC2199-14|Hymenoptera|Diapriidae|BOLD:ACO2476
Diapriidae|[2032]|SSROB7163-14|Hymenoptera|Diapriidae|BOLD:ACP0132
Diapriidae|[2033]|SMTPD010-13|Hymenoptera|Diapriidae|BOLD:AAU8675
Diapriidae|[2034]|BBHYM280-09|Hymenoptera|Diapriidae|BOLD:AAG7956
Diapriidae|[2035]|BBHYM304-09|Hymenoptera|Diapriidae|BOLD:AAG7964
Diapriidae|[2036]|SSWLB1388-13|Hymenoptera|Diapriidae|BOLD:ACM3898
Diapriidae|[2037]|NGNAX4598-14|Hymenoptera|Diapriidae|BOLD:ACM7725
Diapriidae|[2038]|SSJAE2042-13|Hymenoptera|Diapriidae|BOLD:ABA5906
Trichopria|[2039]|SMTPD3220-13|Hymenoptera|Diapriidae|BOLD:ACF9546
Trichopria|[2040]|SMTPJ875-14|Hymenoptera|Diapriidae|BOLD:AAP6714
Diapriidae|[2041]|NCCC167-11|Hymenoptera|Diapriidae|BOLD:ABA6281
Trichopria basalis|[2042]|SMTPJ4621-14|Hymenoptera|Diapriidae|BOLD:AAN8179
Diapriidae|[2043]|SSJAE4504-13|Hymenoptera|Diapriidae|BOLD:ABW3249
Diapriidae|[2044]|CNEIF2557-12|Hymenoptera|Diapriidae|BOLD:AAU9345
Monelata solida|[2045]|CNWBD142-13|Hymenoptera|Diapriidae|BOLD:ACL4587
Monelata solida|[2046]|SMTPI126-14|Hymenoptera|Diapriidae|BOLD:AAG7877
Diapriidae|[2047]|CNLMQ535-14|Hymenoptera|Diapriidae|BOLD:AAM7434
Diapriidae|[2048]|SSPAB5315-13|Hymenoptera|Diapriidae|BOLD:ACI4484
Diapriidae|[2049]|SSROC4712-15|Hymenoptera|Diapriidae|BOLD:ACN8445
Diapriidae|[2050]|TTMHY520-11|Hymenoptera|Diapriidae|BOLD:AAU9863
Diapriidae|[2051]|SMTPI6829-14|Hymenoptera|Diapriidae|BOLD:AAN7983
Diapriidae|[2052]|JSAUG047-11|Hymenoptera|Diapriidae|BOLD:ABW3139
Diapriidae|[2053]|BBHYK716-10|Hymenoptera|Diapriidae|BOLD:ABY3307
Diapriidae|[2054]|CNBPO028-13|Hymenoptera|Diapriidae|BOLD:AAN7572
Diapriidae|[2055]|SSWLF2750-13|Hymenoptera|Diapriidae|BOLD:ACD0881
Diapriidae|[2056]|BBHYL072-10|Hymenoptera|Diapriidae|BOLD:ABW3150
Diapriidae|[2057]|SSJAD466-13|Hymenoptera|Diapriidae|BOLD:ACB1614
Diapriidae|[2058]|NCCC493-11|Hymenoptera|Diapriidae|BOLD:ABV2679
Diapriidae|[2059]|NCCC384-11|Hymenoptera|Diapriidae|BOLD:ABV2731
Diapriidae|[2060]|CNEIE1572-12|Hymenoptera|Diapriidae|BOLD:ACB1496
Diapriidae|[2061]|HPPPP087-13|Hymenoptera|Diapriidae|BOLD:AAU8337
Diapriidae|[2062]|SSWLD3065-13|Hymenoptera|Diapriidae|BOLD:ACM1000
Diapriidae|[2063]|BBHYK779-10|Hymenoptera|Diapriidae|BOLD:ABX5765
Diapriidae|[2064]|SSJAF5822-13|Hymenoptera|Diapriidae|BOLD:AAU9130
Diapriidae|[2065]|CNGBF1226-14|Hymenoptera|Diapriidae|BOLD:ACC8030
Diapriidae|[2066]|CNPPE1682-12|Hymenoptera|Diapriidae|BOLD:AAN8180
Diapriidae|[2067]|CNROF069-13|Hymenoptera|Diapriidae|BOLD:ACN6495
Diapriidae|[2068]|PHSEP1883-11|Hymenoptera|Diapriidae|BOLD:ABX2580
Diapriidae|[2069]|CNEIG948-12|Hymenoptera|Diapriidae|BOLD:ACD8815
Diapriidae|[2070]|NCCC385-11|Hymenoptera|Diapriidae|BOLD:ABV2776
Diapriidae|[2071]|PHJUN2146-11|Hymenoptera|Diapriidae|BOLD:ACI7850
Diapriidae|[2072]|SSWLE2990-13|Hymenoptera|Diapriidae|BOLD:AAU9056
Diapriidae|[2073]|CNLMN1246-14|Hymenoptera|Diapriidae|BOLD:ABY2683
Diapriidae|[2074]|SMTPI2603-14|Hymenoptera|Diapriidae|BOLD:ABY3589
Diapriidae|[2075]|SSPAC8994-13|Hymenoptera|Diapriidae|BOLD:AAM7462
Diapriidae|[2076]|SSJAE11280-13|Hymenoptera|Diapriidae|BOLD:AAM7457
Diapriidae|[2077]|SMTPJ4502-14|Hymenoptera|Diapriidae|BOLD:ACP9218
Diapriinae|[2078]|JSHYP006-11|Hymenoptera|Diapriidae|BOLD:ABX2607
Diapriinae|[2079]|CNLMP1153-14|Hymenoptera|Diapriidae|BOLD:ACT2075
Diapriidae|[2080]|SSWLA3653-13|Hymenoptera|Diapriidae|BOLD:ABZ4070
Diapriidae|[2081]|SSPAA6410-13|Hymenoptera|Diapriidae|BOLD:AAG4498
Diapriidae|[2082]|NGNAB979-13|Hymenoptera|Diapriidae|BOLD:ACR5074
Diapriidae|[2083]|SSJAE2078-13|Hymenoptera|Diapriidae|BOLD:ABA8019
Diapriidae|[2084]|CNNHF974-14|Hymenoptera|Diapriidae|BOLD:ABW3275
Diapriidae|[2085]|SSJAE7286-13|Hymenoptera|Diapriidae|BOLD:ACC8905
Diapriidae|[2086]|SMTPF2043-14|Hymenoptera|Diapriidae|BOLD:ACM6574
Diapriidae|[2087]|BBHYK712-10|Hymenoptera|Diapriidae|BOLD:AAU9055
Diapriidae|[2088]|BBHYK769-10|Hymenoptera|Diapriidae|BOLD:AAU9049
Diapriidae|[2089]|JBHCJ083-11|Hymenoptera|Diapriidae|BOLD:AAU9811
Diapriidae|[2090]|MBIOD138-13|Hymenoptera|Diapriidae|BOLD:AAU8711
Diapriidae|[2091]|SSPAC9595-13|Hymenoptera|Diapriidae|BOLD:AAU9341
Diapriidae|[2092]|SAHYM310-10|Hymenoptera|Diapriidae|BOLD:AAN7629
Diapriidae|[2093]|SSWLA4246-13|Hymenoptera|Diapriidae|BOLD:ACL2543
Diapriidae|[2094]|CNGBF1252-14|Hymenoptera|Diapriidae|BOLD:ACL3750
Diapriidae|[2095]|SSROB4043-14|Hymenoptera|Diapriidae|BOLD:ACO4415
Diapriidae|[2096]|CNBFL1245-15|Hymenoptera|Diapriidae|BOLD:AAU9373

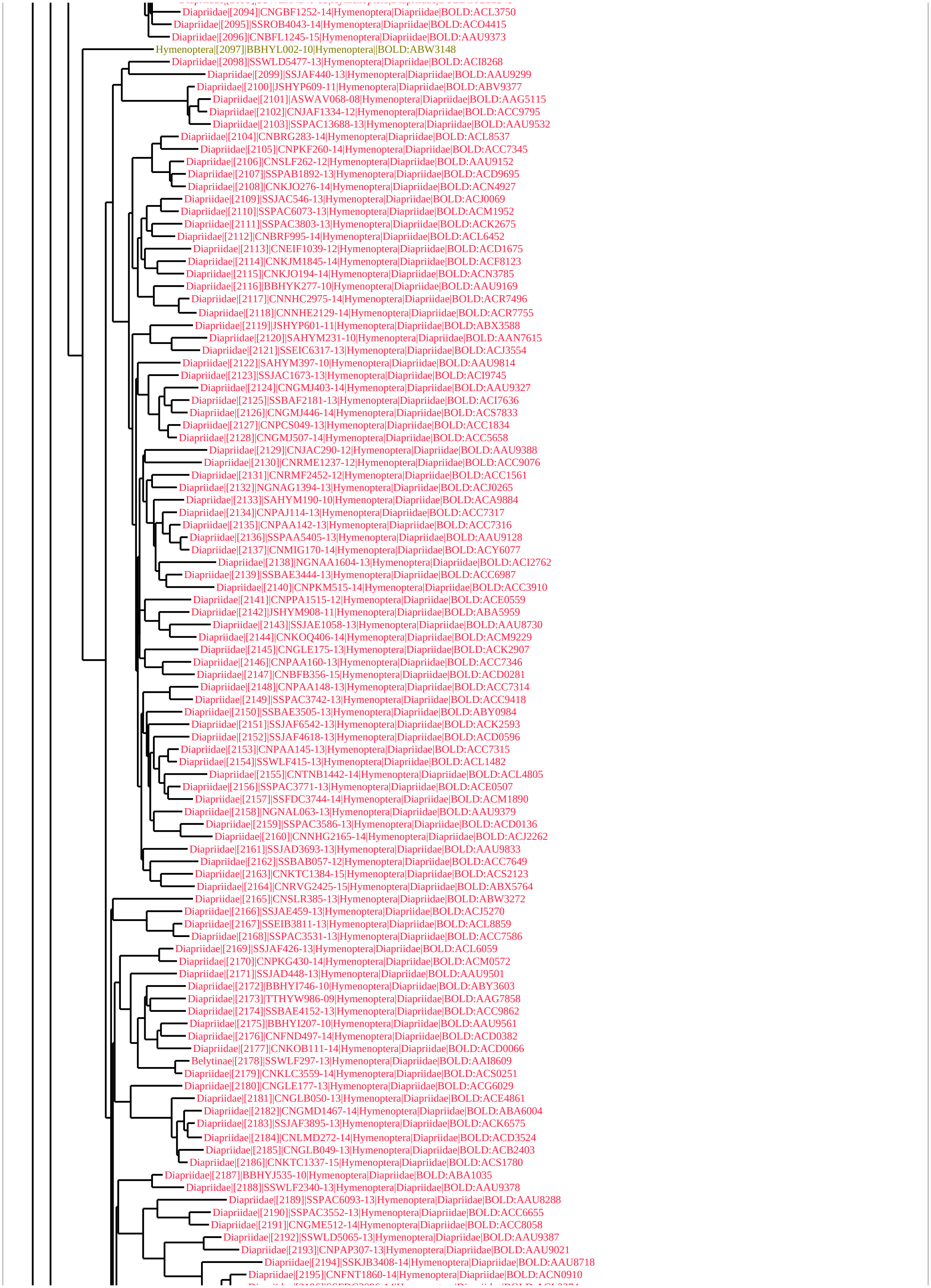
Diapriidae|[2094]|CNGBF1252-14|Hymenoptera|Diapriidae|BOLD:ACL3750
Diapriidae|[2095]|SSROB4043-14|Hymenoptera|Diapriidae|BOLD:ACO4415
Diapriidae|[2096]|CNBFL1245-15|Hymenoptera|Diapriidae|BOLD:AAU9373
Hymenoptera|[2097]|BBHYL002-10|Hymenoptera||BOLD:ABW3148
Diapriidae|[2098]|SSWLD5477-13|Hymenoptera|Diapriidae|BOLD:ACI8268
Diapriidae|[2099]|SSJAF440-13|Hymenoptera|Diapriidae|BOLD:AAU9299
Diapriidae|[2100]|JSHYP609-11|Hymenoptera|Diapriidae|BOLD:ABV9377
Diapriidae|[2101]|ASWAV068-08|Hymenoptera|Diapriidae|BOLD:AAG5115
Diapriidae|[2102]|CNJAF1334-12|Hymenoptera|Diapriidae|BOLD:ACC9795
Diapriidae|[2103]|SSPAC13688-13|Hymenoptera|Diapriidae|BOLD:AAU9532
Diapriidae|[2104]|CNBRG283-14|Hymenoptera|Diapriidae|BOLD:ACL8537
Diapriidae|[2105]|CNPKF260-14|Hymenoptera|Diapriidae|BOLD:ACC7345
Diapriidae|[2106]|CNSLF262-12|Hymenoptera|Diapriidae|BOLD:AAU9152
Diapriidae|[2107]|SSPAB1892-13|Hymenoptera|Diapriidae|BOLD:ACD9695
Diapriidae|[2108]|CNKJO276-14|Hymenoptera|Diapriidae|BOLD:ACN4927
Diapriidae|[2109]|SSJAC546-13|Hymenoptera|Diapriidae|BOLD:ACJ0069
Diapriidae|[2110]|SSPAC6073-13|Hymenoptera|Diapriidae|BOLD:ACM1952
Diapriidae|[2111]|SSPAC3803-13|Hymenoptera|Diapriidae|BOLD:ACK2675
Diapriidae|[2112]|CNBRF995-14|Hymenoptera|Diapriidae|BOLD:ACL6452
Diapriidae|[2113]|CNEIF1039-12|Hymenoptera|Diapriidae|BOLD:ACD1675
Diapriidae|[2114]|CNKJM1845-14|Hymenoptera|Diapriidae|BOLD:ACF8123
Diapriidae|[2115]|CNKJO194-14|Hymenoptera|Diapriidae|BOLD:ACN3785
Diapriidae|[2116]|BBHYK277-10|Hymenoptera|Diapriidae|BOLD:AAU9169
Diapriidae|[2117]|CNNHC2975-14|Hymenoptera|Diapriidae|BOLD:ACR7496
Diapriidae|[2118]|CNNHE2129-14|Hymenoptera|Diapriidae|BOLD:ACR7755
Diapriidae|[2119]|JSHYP601-11|Hymenoptera|Diapriidae|BOLD:ABX3588
Diapriidae|[2120]|SAHYM231-10|Hymenoptera|Diapriidae|BOLD:AAN7615
Diapriidae|[2121]|SSEIC6317-13|Hymenoptera|Diapriidae|BOLD:ACJ3554
Diapriidae|[2122]|SAHYM397-10|Hymenoptera|Diapriidae|BOLD:AAU9814
Diapriidae|[2123]|SSJAC1673-13|Hymenoptera|Diapriidae|BOLD:ACI9745
Diapriidae|[2124]|CNGMJ403-14|Hymenoptera|Diapriidae|BOLD:AAU9327
Diapriidae|[2125]|SSBAF2181-13|Hymenoptera|Diapriidae|BOLD:ACI7636
Diapriidae|[2126]|CNGMJ446-14|Hymenoptera|Diapriidae|BOLD:ACS7833
Diapriidae|[2127]|CNPCS049-13|Hymenoptera|Diapriidae|BOLD:ACC1834
Diapriidae|[2128]|CNGMJ507-14|Hymenoptera|Diapriidae|BOLD:ACC5658
Diapriidae|[2129]|CNJAC290-12|Hymenoptera|Diapriidae|BOLD:AAU9388
Diapriidae|[2130]|CNRME1237-12|Hymenoptera|Diapriidae|BOLD:ACC9076
Diapriidae|[2131]|CNRMF2452-12|Hymenoptera|Diapriidae|BOLD:ACC1561
Diapriidae|[2132]|NGNAG1394-13|Hymenoptera|Diapriidae|BOLD:ACJ0265
Diapriidae|[2133]|SAHYM190-10|Hymenoptera|Diapriidae|BOLD:ACA9884
Diapriidae|[2134]|CNPAJ114-13|Hymenoptera|Diapriidae|BOLD:ACC7317
Diapriidae|[2135]|CNPAA142-13|Hymenoptera|Diapriidae|BOLD:ACC7316
Diapriidae|[2136]|SSPAA5405-13|Hymenoptera|Diapriidae|BOLD:AAU9128
Diapriidae|[2137]|CNMIG170-14|Hymenoptera|Diapriidae|BOLD:ACY6077
Diapriidae|[2138]|NGNAA1604-13|Hymenoptera|Diapriidae|BOLD:ACI2762
Diapriidae|[2139]|SSBAE3444-13|Hymenoptera|Diapriidae|BOLD:ACC6987
Diapriidae|[2140]|CNPKM515-14|Hymenoptera|Diapriidae|BOLD:ACC3910
Diapriidae|[2141]|CNPPA1515-12|Hymenoptera|Diapriidae|BOLD:ACE0559
Diapriidae|[2142]|JSHYM908-11|Hymenoptera|Diapriidae|BOLD:ABA5959
Diapriidae|[2143]|SSJAE1058-13|Hymenoptera|Diapriidae|BOLD:AAU8730
Diapriidae|[2144]|CNKOQ406-14|Hymenoptera|Diapriidae|BOLD:ACM9229
Diapriidae|[2145]|CNGLE175-13|Hymenoptera|Diapriidae|BOLD:ACK2907
Diapriidae|[2146]|CNPAA160-13|Hymenoptera|Diapriidae|BOLD:ACC7346
Diapriidae|[2147]|CNBFB356-15|Hymenoptera|Diapriidae|BOLD:ACD0281
Diapriidae|[2148]|CNPAA148-13|Hymenoptera|Diapriidae|BOLD:ACC7314
Diapriidae|[2149]|SSPAC3742-13|Hymenoptera|Diapriidae|BOLD:ACC9418
Diapriidae|[2150]|SSBAE3505-13|Hymenoptera|Diapriidae|BOLD:ABY0984
Diapriidae|[2151]|SSJAF6542-13|Hymenoptera|Diapriidae|BOLD:ACK2593
Diapriidae|[2152]|SSJAF4618-13|Hymenoptera|Diapriidae|BOLD:ACD0596
Diapriidae|[2153]|CNPAA145-13|Hymenoptera|Diapriidae|BOLD:ACC7315
Diapriidae|[2154]|SSWLF415-13|Hymenoptera|Diapriidae|BOLD:ACL1482
Diapriidae|[2155]|CNTNB1442-14|Hymenoptera|Diapriidae|BOLD:ACL4805
Diapriidae|[2156]|SSPAC3771-13|Hymenoptera|Diapriidae|BOLD:ACE0507
Diapriidae|[2157]|SSFDC3744-14|Hymenoptera|Diapriidae|BOLD:ACM1890
Diapriidae|[2158]|NGNAL063-13|Hymenoptera|Diapriidae|BOLD:AAU9379
Diapriidae|[2159]|SSPAC3586-13|Hymenoptera|Diapriidae|BOLD:ACD0136
Diapriidae|[2160]|CNNHG2165-14|Hymenoptera|Diapriidae|BOLD:ACJ2262
Diapriidae|[2161]|SSJAD3693-13|Hymenoptera|Diapriidae|BOLD:AAU9833
Diapriidae|[2162]|SSBAB057-12|Hymenoptera|Diapriidae|BOLD:ACC7649
Diapriidae|[2163]|CNKTC1384-15|Hymenoptera|Diapriidae|BOLD:ACS2123
Diapriidae|[2164]|CNRVG2425-15|Hymenoptera|Diapriidae|BOLD:ABX5764
Diapriidae|[2165]|CNSLR385-13|Hymenoptera|Diapriidae|BOLD:ABW3272
Diapriidae|[2166]|SSJAE459-13|Hymenoptera|Diapriidae|BOLD:ACJ5270
Diapriidae|[2167]|SSEIB3811-13|Hymenoptera|Diapriidae|BOLD:ACL8859
Diapriidae|[2168]|SSPAC3531-13|Hymenoptera|Diapriidae|BOLD:ACC7586
Diapriidae|[2169]|SSJAF426-13|Hymenoptera|Diapriidae|BOLD:ACL6059
Diapriidae|[2170]|CNPKG430-14|Hymenoptera|Diapriidae|BOLD:ACM0572
Diapriidae|[2171]|SSJAD448-13|Hymenoptera|Diapriidae|BOLD:AAU9501
Diapriidae|[2172]|BBHYI746-10|Hymenoptera|Diapriidae|BOLD:ABY3603
Diapriidae|[2173]|TTHYW986-09|Hymenoptera|Diapriidae|BOLD:AAG7858
Diapriidae|[2174]|SSBAE4152-13|Hymenoptera|Diapriidae|BOLD:ACC9862
Diapriidae|[2175]|BBHYI207-10|Hymenoptera|Diapriidae|BOLD:AAU9561
Diapriidae|[2176]|CNFND497-14|Hymenoptera|Diapriidae|BOLD:ACD0382
Diapriidae|[2177]|CNKOB111-14|Hymenoptera|Diapriidae|BOLD:ACD0066
Belytinae|[2178]|SSWLF297-13|Hymenoptera|Diapriidae|BOLD:AAI8609
Diapriidae|[2179]|CNKLC3559-14|Hymenoptera|Diapriidae|BOLD:ACS0251
Diapriidae|[2180]|CNGLE177-13|Hymenoptera|Diapriidae|BOLD:ACG6029
Diapriidae|[2181]|CNGLB050-13|Hymenoptera|Diapriidae|BOLD:ACE4861
Diapriidae|[2182]|CNGMD1467-14|Hymenoptera|Diapriidae|BOLD:ABA6004
Diapriidae|[2183]|SSJAF3895-13|Hymenoptera|Diapriidae|BOLD:ACK6575
Diapriidae|[2184]|CNLMD272-14|Hymenoptera|Diapriidae|BOLD:ACD3524
Diapriidae|[2185]|CNGLB049-13|Hymenoptera|Diapriidae|BOLD:ACB2403
Diapriidae|[2186]|CNKTC1337-15|Hymenoptera|Diapriidae|BOLD:ACS1780
Diapriidae|[2187]|BBHYJ535-10|Hymenoptera|Diapriidae|BOLD:ABA1035
Diapriidae|[2188]|SSWLF2340-13|Hymenoptera|Diapriidae|BOLD:AAU9378
Diapriidae|[2189]|SSPAC6093-13|Hymenoptera|Diapriidae|BOLD:AAU8288
Diapriidae|[2190]|SSPAC3552-13|Hymenoptera|Diapriidae|BOLD:ACC6655
Diapriidae|[2191]|CNGME512-14|Hymenoptera|Diapriidae|BOLD:ACC8058
Diapriidae|[2192]|SSWLD5065-13|Hymenoptera|Diapriidae|BOLD:AAU9387
Diapriidae|[2193]|CNPAP307-13|Hymenoptera|Diapriidae|BOLD:AAU9021
Diapriidae|[2194]|SSKJB3408-14|Hymenoptera|Diapriidae|BOLD:AAU8718
Diapriidae|[2195]|CNFNT1860-14|Hymenoptera|Diapriidae|BOLD:ACN0910

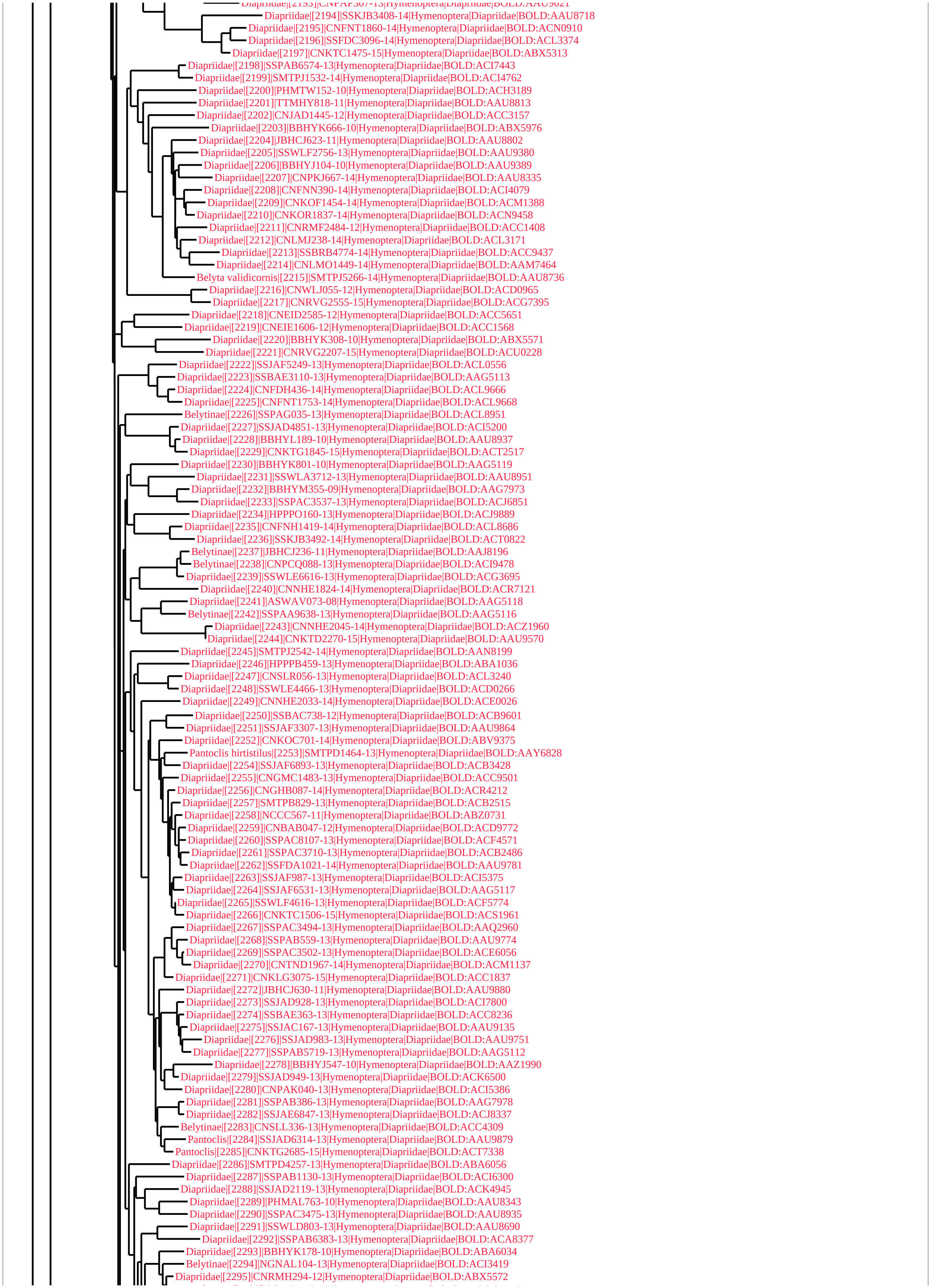

Diapriidae|[2194]|SSKJB3408-14|Hymenoptera|Diapriidae|BOLD:AAU8718
Diapriidae|[2195]|CNFNT1860-14|Hymenoptera|Diapriidae|BOLD:ACN0910
Diapriidae|[2196]|SSFDC3096-14|Hymenoptera|Diapriidae|BOLD:ACL3374
Diapriidae|[2197]|CNKTC1475-15|Hymenoptera|Diapriidae|BOLD:ABX5313
Diapriidae|[2198]|SSPAB6574-13|Hymenoptera|Diapriidae|BOLD:ACI7443
Diapriidae|[2199]|SMTPJ1532-14|Hymenoptera|Diapriidae|BOLD:ACI4762
Diapriidae|[2200]|PHMTW152-10|Hymenoptera|Diapriidae|BOLD:ACH3189
Diapriidae|[2201]|TTMHY818-11|Hymenoptera|Diapriidae|BOLD:AAU8813
Diapriidae|[2202]|CNJAD1445-12|Hymenoptera|Diapriidae|BOLD:ACC3157
Diapriidae|[2203]|BBHYK666-10|Hymenoptera|Diapriidae|BOLD:ABX5976
Diapriidae|[2204]|JBHCJ623-11|Hymenoptera|Diapriidae|BOLD:AAU8802
Diapriidae|[2205]|SSWLF2756-13|Hymenoptera|Diapriidae|BOLD:AAU9380
Diapriidae|[2206]|BBHYJ104-10|Hymenoptera|Diapriidae|BOLD:AAU9389
Diapriidae|[2207]|CNPKJ667-14|Hymenoptera|Diapriidae|BOLD:AAU8335
Diapriidae|[2208]|CNFNN390-14|Hymenoptera|Diapriidae|BOLD:ACI4079
Diapriidae|[2209]|CNKOF1454-14|Hymenoptera|Diapriidae|BOLD:ACM1388
Diapriidae|[2210]|CNKOR1837-14|Hymenoptera|Diapriidae|BOLD:ACN9458
Diapriidae|[2211]|CNRMF2484-12|Hymenoptera|Diapriidae|BOLD:ACC1408
Diapriidae|[2212]|CNLMJ238-14|Hymenoptera|Diapriidae|BOLD:ACL3171
Diapriidae|[2213]|SSBRB4774-14|Hymenoptera|Diapriidae|BOLD:ACC9437
Diapriidae|[2214]|CNLMO1449-14|Hymenoptera|Diapriidae|BOLD:AAM7464
Belyta validicornis|[2215]|SMTPJ5266-14|Hymenoptera|Diapriidae|BOLD:AAU8736
Diapriidae|[2216]|CNWLJ055-12|Hymenoptera|Diapriidae|BOLD:ACD0965
Diapriidae|[2217]|CNRVG2555-15|Hymenoptera|Diapriidae|BOLD:ACG7395
Diapriidae|[2218]|CNEID2585-12|Hymenoptera|Diapriidae|BOLD:ACC5651
Diapriidae|[2219]|CNEIE1606-12|Hymenoptera|Diapriidae|BOLD:ACC1568
Diapriidae|[2220]|BBHYK308-10|Hymenoptera|Diapriidae|BOLD:ABX5571
Diapriidae|[2221]|CNRVG2207-15|Hymenoptera|Diapriidae|BOLD:ACU0228
Diapriidae|[2222]|SSJAF5249-13|Hymenoptera|Diapriidae|BOLD:ACL0556
Diapriidae|[2223]|SSBAE3110-13|Hymenoptera|Diapriidae|BOLD:AAG5113
Diapriidae|[2224]|CNFDH436-14|Hymenoptera|Diapriidae|BOLD:ACL9666
Diapriidae|[2225]|CNFNT1753-14|Hymenoptera|Diapriidae|BOLD:ACL9668
Belytinae|[2226]|SSPAG035-13|Hymenoptera|Diapriidae|BOLD:ACL8951
Diapriidae|[2227]|SSJAD4851-13|Hymenoptera|Diapriidae|BOLD:ACI5200
Diapriidae|[2228]|BBHYL189-10|Hymenoptera|Diapriidae|BOLD:AAU8937
Diapriidae|[2229]|CNKTG1845-15|Hymenoptera|Diapriidae|BOLD:ACT2517
Diapriidae|[2230]|BBHYK801-10|Hymenoptera|Diapriidae|BOLD:AAG5119
Diapriidae|[2231]|SSWLA3712-13|Hymenoptera|Diapriidae|BOLD:AAU8951
Diapriidae|[2232]|BBHYM355-09|Hymenoptera|Diapriidae|BOLD:AAG7973
Diapriidae|[2233]|SSPAC3537-13|Hymenoptera|Diapriidae|BOLD:ACJ6851
Diapriidae|[2234]|HPPPO160-13|Hymenoptera|Diapriidae|BOLD:ACJ9889
Diapriidae|[2235]|CNFNH1419-14|Hymenoptera|Diapriidae|BOLD:ACL8686
Diapriidae|[2236]|SSKJB3492-14|Hymenoptera|Diapriidae|BOLD:ACT0822
Belytinae|[2237]|JBHCJ236-11|Hymenoptera|Diapriidae|BOLD:AAJ8196
Belytinae|[2238]|CNPCQ088-13|Hymenoptera|Diapriidae|BOLD:ACI9478
Diapriidae|[2239]|SSWLE6616-13|Hymenoptera|Diapriidae|BOLD:ACG3695
Diapriidae|[2240]|CNNHE1824-14|Hymenoptera|Diapriidae|BOLD:ACR7121
Diapriidae|[2241]|ASWAV073-08|Hymenoptera|Diapriidae|BOLD:AAG5118
Belytinae|[2242]|SSPAA9638-13|Hymenoptera|Diapriidae|BOLD:AAG5116
Diapriidae|[2243]|CNNHE2045-14|Hymenoptera|Diapriidae|BOLD:ACZ1960
Diapriidae|[2244]|CNKTD2270-15|Hymenoptera|Diapriidae|BOLD:AAU9570
Diapriidae|[2245]|SMTPJ2542-14|Hymenoptera|Diapriidae|BOLD:AAN8199
Diapriidae|[2246]|HPPPB459-13|Hymenoptera|Diapriidae|BOLD:ABA1036
Diapriidae|[2247]|CNSLR056-13|Hymenoptera|Diapriidae|BOLD:ACL3240
Diapriidae|[2248]|SSWLE4466-13|Hymenoptera|Diapriidae|BOLD:ACD0266
Diapriidae|[2249]|CNNHE2033-14|Hymenoptera|Diapriidae|BOLD:ACE0026
Diapriidae|[2250]|SSBAC738-12|Hymenoptera|Diapriidae|BOLD:ACB9601
Diapriidae|[2251]|SSJAF3307-13|Hymenoptera|Diapriidae|BOLD:AAU9864
Diapriidae|[2252]|CNKOC701-14|Hymenoptera|Diapriidae|BOLD:ABV9375
Pantoclis hirtistilus|[2253]|SMTPD1464-13|Hymenoptera|Diapriidae|BOLD:AAY6828
Diapriidae|[2254]|SSJAF6893-13|Hymenoptera|Diapriidae|BOLD:ACB3428
Diapriidae|[2255]|CNGMC1483-13|Hymenoptera|Diapriidae|BOLD:ACC9501
Diapriidae|[2256]|CNGHB087-14|Hymenoptera|Diapriidae|BOLD:ACR4212
Diapriidae|[2257]|SMTPB829-13|Hymenoptera|Diapriidae|BOLD:ACB2515
Diapriidae|[2258]|NCCC567-11|Hymenoptera|Diapriidae|BOLD:ABZ0731
Diapriidae|[2259]|CNBAB047-12|Hymenoptera|Diapriidae|BOLD:ACD9772
Diapriidae|[2260]|SSPAC8107-13|Hymenoptera|Diapriidae|BOLD:ACF4571
Diapriidae|[2261]|SSPAC3710-13|Hymenoptera|Diapriidae|BOLD:ACB2486
Diapriidae|[2262]|SSFDA1021-14|Hymenoptera|Diapriidae|BOLD:AAU9781
Diapriidae|[2263]|SSJAF987-13|Hymenoptera|Diapriidae|BOLD:ACI5375
Diapriidae|[2264]|SSJAF6531-13|Hymenoptera|Diapriidae|BOLD:AAG5117
Diapriidae|[2265]|SSWLF4616-13|Hymenoptera|Diapriidae|BOLD:ACF5774
Diapriidae|[2266]|CNKTC1506-15|Hymenoptera|Diapriidae|BOLD:ACS1961
Diapriidae|[2267]|SSPAC3494-13|Hymenoptera|Diapriidae|BOLD:AAQ2960
Diapriidae|[2268]|SSPAB559-13|Hymenoptera|Diapriidae|BOLD:AAU9774
Diapriidae|[2269]|SSPAC3502-13|Hymenoptera|Diapriidae|BOLD:ACE6056
Diapriidae|[2270]|CNTND1967-14|Hymenoptera|Diapriidae|BOLD:ACM1137
Diapriidae|[2271]|CNKLG3075-15|Hymenoptera|Diapriidae|BOLD:ACC1837
Diapriidae|[2272]|JBHCJ630-11|Hymenoptera|Diapriidae|BOLD:AAU9880
Diapriidae|[2273]|SSJAD928-13|Hymenoptera|Diapriidae|BOLD:ACI7800
Diapriidae|[2274]|SSBAE363-13|Hymenoptera|Diapriidae|BOLD:ACC8236
Diapriidae|[2275]|SSJAC167-13|Hymenoptera|Diapriidae|BOLD:AAU9135
Diapriidae|[2276]|SSJAD983-13|Hymenoptera|Diapriidae|BOLD:AAU9751
Diapriidae|[2277]|SSPAB5719-13|Hymenoptera|Diapriidae|BOLD:AAG5112
Diapriidae|[2278]|BBHYJ547-10|Hymenoptera|Diapriidae|BOLD:AAZ1990
Diapriidae|[2279]|SSJAD949-13|Hymenoptera|Diapriidae|BOLD:ACK6500
Diapriidae|[2280]|CNPAK040-13|Hymenoptera|Diapriidae|BOLD:ACI5386
Diapriidae|[2281]|SSPAB386-13|Hymenoptera|Diapriidae|BOLD:AAG7978
Diapriidae|[2282]|SSJAE6847-13|Hymenoptera|Diapriidae|BOLD:ACJ8337
Belytinae|[2283]|CNSLL336-13|Hymenoptera|Diapriidae|BOLD:ACC4309
Pantoclis|[2284]|SSJAD6314-13|Hymenoptera|Diapriidae|BOLD:AAU9879
Pantoclis|[2285]|CNKTG2685-15|Hymenoptera|Diapriidae|BOLD:ACT7338
Diapriidae|[2286]|SMTPD4257-13|Hymenoptera|Diapriidae|BOLD:ABA6056
Diapriidae|[2287]|SSPAB1130-13|Hymenoptera|Diapriidae|BOLD:ACI6300
Diapriidae|[2288]|SSJAD2119-13|Hymenoptera|Diapriidae|BOLD:ACK4945
Diapriidae|[2289]|PHMAL763-10|Hymenoptera|Diapriidae|BOLD:AAU8343
Diapriidae|[2290]|SSPAC3475-13|Hymenoptera|Diapriidae|BOLD:AAU8935
Diapriidae|[2291]|SSWLD803-13|Hymenoptera|Diapriidae|BOLD:AAU8690
Diapriidae|[2292]|SSPAB6383-13|Hymenoptera|Diapriidae|BOLD:ACA8377
Diapriidae|[2293]|BBHYK178-10|Hymenoptera|Diapriidae|BOLD:ABA6034
Belytinae|[2294]|NGNAL104-13|Hymenoptera|Diapriidae|BOLD:ACI3419
Diapriidae|[2295]|CNRMH294-12|Hymenoptera|Diapriidae|BOLD:ABX5572

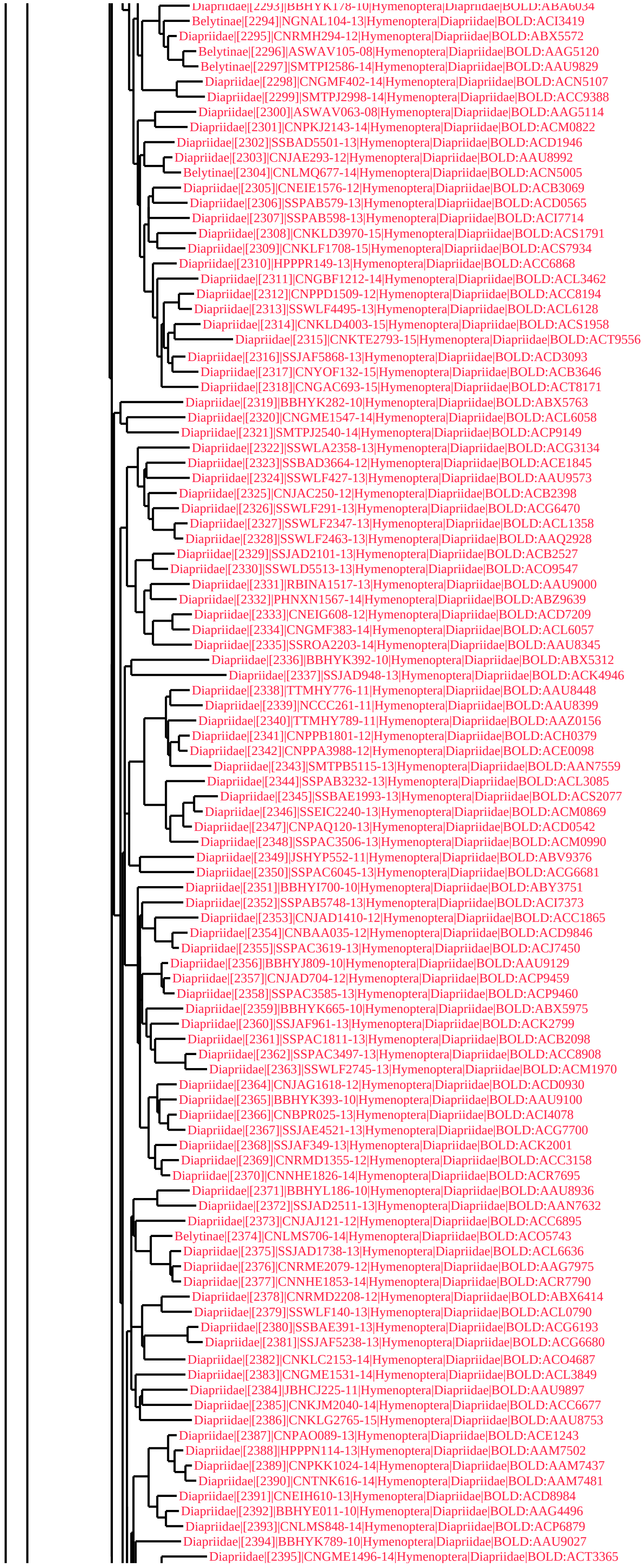
Diapriidae|[2293]|BBHYK178-10|Hymenoptera|Diapriidae|BOLD:ABA6034
Belytinae|[2294]|NGNAL104-13|Hymenoptera|Diapriidae|BOLD:ACI3419
Diapriidae|[2295]|CNRMH294-12|Hymenoptera|Diapriidae|BOLD:ABX5572
Belytinae|[2296]|ASWAV105-08|Hymenoptera|Diapriidae|BOLD:AAG5120
Belytinae|[2297]|SMTPI2586-14|Hymenoptera|Diapriidae|BOLD:AAU9829
Diapriidae|[2298]|CNGMF402-14|Hymenoptera|Diapriidae|BOLD:ACN5107
Diapriidae|[2299]|SMTPJ2998-14|Hymenoptera|Diapriidae|BOLD:ACC9388
Diapriidae|[2300]|ASWAV063-08|Hymenoptera|Diapriidae|BOLD:AAG5114
Diapriidae|[2301]|CNPKJ2143-14|Hymenoptera|Diapriidae|BOLD:ACM0822
Diapriidae|[2302]|SSBAD5501-13|Hymenoptera|Diapriidae|BOLD:ACD1946
Diapriidae|[2303]|CNJAE293-12|Hymenoptera|Diapriidae|BOLD:AAU8992
Belytinae|[2304]|CNLMQ677-14|Hymenoptera|Diapriidae|BOLD:ACN5005
Diapriidae|[2305]|CNEIE1576-12|Hymenoptera|Diapriidae|BOLD:ACB3069
Diapriidae|[2306]|SSPAB579-13|Hymenoptera|Diapriidae|BOLD:ACD0565
Diapriidae|[2307]|SSPAB598-13|Hymenoptera|Diapriidae|BOLD:ACI7714
Diapriidae|[2308]|CNKLD3970-15|Hymenoptera|Diapriidae|BOLD:ACS1791
Diapriidae|[2309]|CNKLF1708-15|Hymenoptera|Diapriidae|BOLD:ACS7934
Diapriidae|[2310]|HPPPR149-13|Hymenoptera|Diapriidae|BOLD:ACC6868
Diapriidae|[2311]|CNGBF1212-14|Hymenoptera|Diapriidae|BOLD:ACL3462
Diapriidae|[2312]|CNPPD1509-12|Hymenoptera|Diapriidae|BOLD:ACC8194
Diapriidae|[2313]|SSWLF4495-13|Hymenoptera|Diapriidae|BOLD:ACL6128
Diapriidae|[2314]|CNKLD4003-15|Hymenoptera|Diapriidae|BOLD:ACS1958
Diapriidae|[2315]|CNKTE2793-15|Hymenoptera|Diapriidae|BOLD:ACT9556
Diapriidae|[2316]|SSJAF5868-13|Hymenoptera|Diapriidae|BOLD:ACD3093
Diapriidae|[2317]|CNYOF132-15|Hymenoptera|Diapriidae|BOLD:ACB3646
Diapriidae|[2318]|CNGAC693-15|Hymenoptera|Diapriidae|BOLD:ACT8171
Diapriidae|[2319]|BBHYK282-10|Hymenoptera|Diapriidae|BOLD:ABX5763
Diapriidae|[2320]|CNGME1547-14|Hymenoptera|Diapriidae|BOLD:ACL6058
Diapriidae|[2321]|SMTPJ2540-14|Hymenoptera|Diapriidae|BOLD:ACP9149
Diapriidae|[2322]|SSWLA2358-13|Hymenoptera|Diapriidae|BOLD:ACG3134
Diapriidae|[2323]|SSBAD3664-12|Hymenoptera|Diapriidae|BOLD:ACE1845
Diapriidae|[2324]|SSWLF427-13|Hymenoptera|Diapriidae|BOLD:AAU9573
Diapriidae|[2325]|CNJAC250-12|Hymenoptera|Diapriidae|BOLD:ACB2398
Diapriidae|[2326]|SSWLF291-13|Hymenoptera|Diapriidae|BOLD:ACG6470
Diapriidae|[2327]|SSWLF2347-13|Hymenoptera|Diapriidae|BOLD:ACL1358
Diapriidae|[2328]|SSWLF2463-13|Hymenoptera|Diapriidae|BOLD:AAQ2928
Diapriidae|[2329]|SSJAD2101-13|Hymenoptera|Diapriidae|BOLD:ACB2527
Diapriidae|[2330]|SSWLD5513-13|Hymenoptera|Diapriidae|BOLD:ACO9547
Diapriidae|[2331]|RBINA1517-13|Hymenoptera|Diapriidae|BOLD:AAU9000
Diapriidae|[2332]|PHNXN1567-14|Hymenoptera|Diapriidae|BOLD:ABZ9639
Diapriidae|[2333]|CNEIG608-12|Hymenoptera|Diapriidae|BOLD:ACD7209
Diapriidae|[2334]|CNGMF383-14|Hymenoptera|Diapriidae|BOLD:ACL6057
Diapriidae|[2335]|SSROA2203-14|Hymenoptera|Diapriidae|BOLD:AAU8345
Diapriidae|[2336]|BBHYK392-10|Hymenoptera|Diapriidae|BOLD:ABX5312
Diapriidae|[2337]|SSJAD948-13|Hymenoptera|Diapriidae|BOLD:ACK4946
Diapriidae|[2338]|TTMHY776-11|Hymenoptera|Diapriidae|BOLD:AAU8448
Diapriidae|[2339]|NCCC261-11|Hymenoptera|Diapriidae|BOLD:AAU8399
Diapriidae|[2340]|TTMHY789-11|Hymenoptera|Diapriidae|BOLD:AAZ0156
Diapriidae|[2341]|CNPPB1801-12|Hymenoptera|Diapriidae|BOLD:ACH0379
Diapriidae|[2342]|CNPPA3988-12|Hymenoptera|Diapriidae|BOLD:ACE0098
Diapriidae|[2343]|SMTPB5115-13|Hymenoptera|Diapriidae|BOLD:AAN7559
Diapriidae|[2344]|SSPAB3232-13|Hymenoptera|Diapriidae|BOLD:ACL3085
Diapriidae|[2345]|SSBAE1993-13|Hymenoptera|Diapriidae|BOLD:ACS2077
Diapriidae|[2346]|SSEIC2240-13|Hymenoptera|Diapriidae|BOLD:ACM0869
Diapriidae|[2347]|CNPAQ120-13|Hymenoptera|Diapriidae|BOLD:ACD0542
Diapriidae|[2348]|SSPAC3506-13|Hymenoptera|Diapriidae|BOLD:ACM0990
Diapriidae|[2349]|JSHYP552-11|Hymenoptera|Diapriidae|BOLD:ABV9376
Diapriidae|[2350]|SSPAC6045-13|Hymenoptera|Diapriidae|BOLD:ACG6681
Diapriidae|[2351]|BBHYI700-10|Hymenoptera|Diapriidae|BOLD:ABY3751
Diapriidae|[2352]|SSPAB5748-13|Hymenoptera|Diapriidae|BOLD:ACI7373
Diapriidae|[2353]|CNJAD1410-12|Hymenoptera|Diapriidae|BOLD:ACC1865
Diapriidae|[2354]|CNBAA035-12|Hymenoptera|Diapriidae|BOLD:ACD9846
Diapriidae|[2355]|SSPAC3619-13|Hymenoptera|Diapriidae|BOLD:ACJ7450
Diapriidae|[2356]|BBHYJ809-10|Hymenoptera|Diapriidae|BOLD:AAU9129
Diapriidae|[2357]|CNJAD704-12|Hymenoptera|Diapriidae|BOLD:ACP9459
Diapriidae|[2358]|SSPAC3585-13|Hymenoptera|Diapriidae|BOLD:ACP9460
Diapriidae|[2359]|BBHYK665-10|Hymenoptera|Diapriidae|BOLD:ABX5975
Diapriidae|[2360]|SSJAF961-13|Hymenoptera|Diapriidae|BOLD:ACK2799
Diapriidae|[2361]|SSPAC1811-13|Hymenoptera|Diapriidae|BOLD:ACB2098
Diapriidae|[2362]|SSPAC3497-13|Hymenoptera|Diapriidae|BOLD:ACC8908
Diapriidae|[2363]|SSWLF2745-13|Hymenoptera|Diapriidae|BOLD:ACM1970
Diapriidae|[2364]|CNJAG1618-12|Hymenoptera|Diapriidae|BOLD:ACD0930
Diapriidae|[2365]|BBHYK393-10|Hymenoptera|Diapriidae|BOLD:AAU9100
Diapriidae|[2366]|CNBPR025-13|Hymenoptera|Diapriidae|BOLD:ACI4078
Diapriidae|[2367]|SSJAE4521-13|Hymenoptera|Diapriidae|BOLD:ACG7700
Diapriidae|[2368]|SSJAF349-13|Hymenoptera|Diapriidae|BOLD:ACK2001
Diapriidae|[2369]|CNRMD1355-12|Hymenoptera|Diapriidae|BOLD:ACC3158
Diapriidae|[2370]|CNNHE1826-14|Hymenoptera|Diapriidae|BOLD:ACR7695
Diapriidae|[2371]|BBHYL186-10|Hymenoptera|Diapriidae|BOLD:AAU8936
Diapriidae|[2372]|SSJAD2511-13|Hymenoptera|Diapriidae|BOLD:AAN7632
Diapriidae|[2373]|CNJAJ121-12|Hymenoptera|Diapriidae|BOLD:ACC6895
Belytinae|[2374]|CNLMS706-14|Hymenoptera|Diapriidae|BOLD:ACO5743
Diapriidae|[2375]|SSJAD1738-13|Hymenoptera|Diapriidae|BOLD:ACL6636
Diapriidae|[2376]|CNRME2079-12|Hymenoptera|Diapriidae|BOLD:AAG7975
Diapriidae|[2377]|CNNHE1853-14|Hymenoptera|Diapriidae|BOLD:ACR7790
Diapriidae|[2378]|CNRMD2208-12|Hymenoptera|Diapriidae|BOLD:ABX6414
Diapriidae|[2379]|SSWLF140-13|Hymenoptera|Diapriidae|BOLD:ACL0790
Diapriidae|[2380]|SSBAE391-13|Hymenoptera|Diapriidae|BOLD:ACG6193
Diapriidae|[2381]|SSJAF5238-13|Hymenoptera|Diapriidae|BOLD:ACG6680
Diapriidae|[2382]|CNKLC2153-14|Hymenoptera|Diapriidae|BOLD:ACO4687
Diapriidae|[2383]|CNGME1531-14|Hymenoptera|Diapriidae|BOLD:ACL3849
Diapriidae|[2384]|JBHCJ225-11|Hymenoptera|Diapriidae|BOLD:AAU9897
Diapriidae|[2385]|CNKJM2040-14|Hymenoptera|Diapriidae|BOLD:ACC6677
Diapriidae|[2386]|CNKLG2765-15|Hymenoptera|Diapriidae|BOLD:AAU8753
Diapriidae|[2387]|CNPAO089-13|Hymenoptera|Diapriidae|BOLD:ACE1243
Diapriidae|[2388]|HPPPN114-13|Hymenoptera|Diapriidae|BOLD:AAM7502
Diapriidae|[2389]|CNPKK1024-14|Hymenoptera|Diapriidae|BOLD:AAM7437
Diapriidae|[2390]|CNTNK616-14|Hymenoptera|Diapriidae|BOLD:AAM7481
Diapriidae|[2391]|CNEIH610-13|Hymenoptera|Diapriidae|BOLD:ACD8984
Diapriidae|[2392]|BBHYE011-10|Hymenoptera|Diapriidae|BOLD:AAG4496
Diapriidae|[2393]|CNLMS848-14|Hymenoptera|Diapriidae|BOLD:ACP6879
Diapriidae|[2394]|BBHYK789-10|Hymenoptera|Diapriidae|BOLD:AAU9027
Diapriidae|[2395]|CNGME1496-14|Hymenoptera|Diapriidae|BOLD:ACT3365

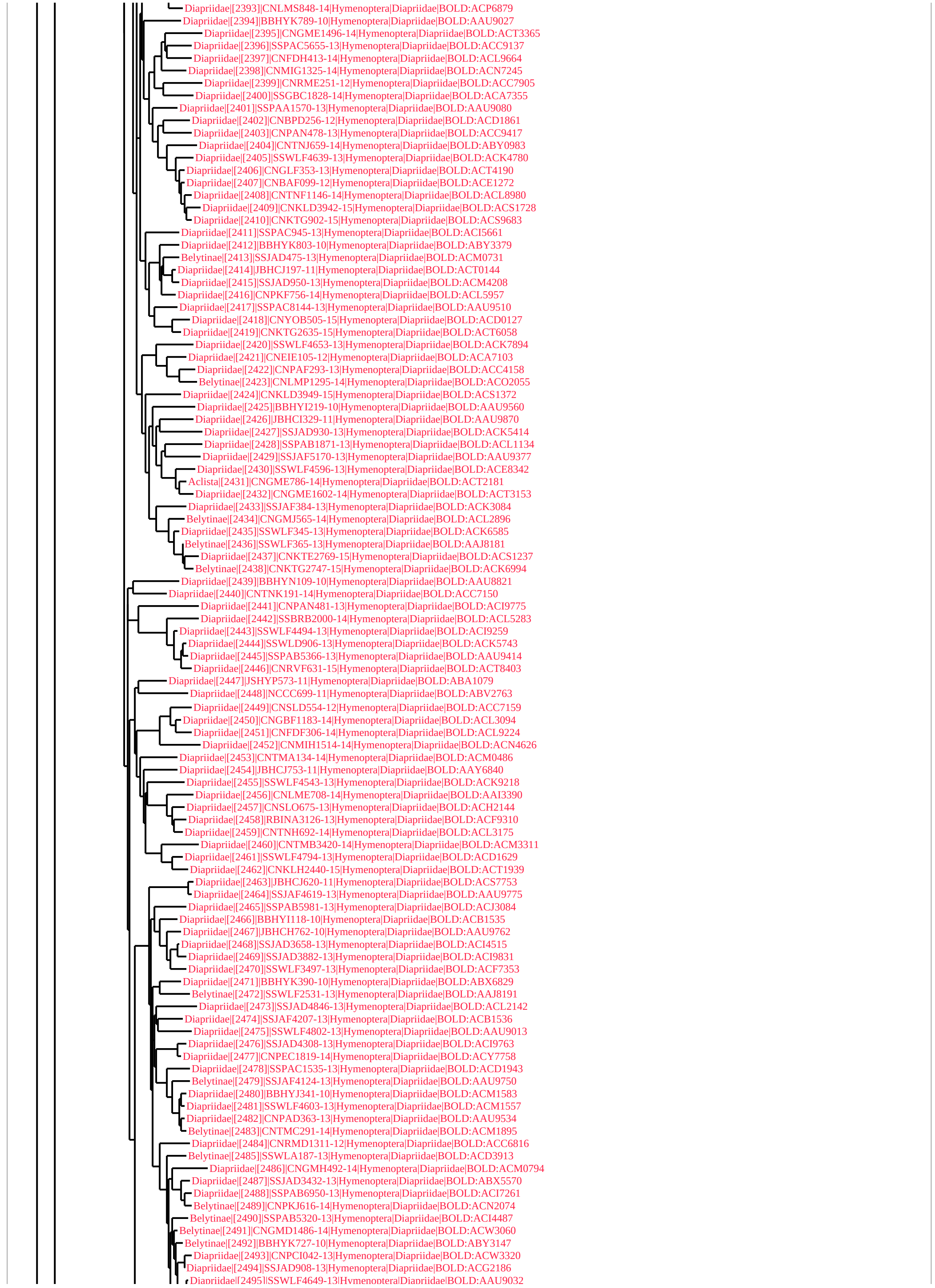
Diapriidae|[2393]|CNLMS848-14|Hymenoptera|Diapriidae|BOLD:ACP6879
Diapriidae|[2394]|BBHYK789-10|Hymenoptera|Diapriidae|BOLD:AAU9027
Diapriidae|[2395]|CNGME1496-14|Hymenoptera|Diapriidae|BOLD:ACT3365
Diapriidae|[2396]|SSPAC5655-13|Hymenoptera|Diapriidae|BOLD:ACC9137
Diapriidae|[2397]|CNFDH413-14|Hymenoptera|Diapriidae|BOLD:ACL9664
Diapriidae|[2398]|CNMIG1325-14|Hymenoptera|Diapriidae|BOLD:ACN7245
Diapriidae|[2399]|CNRME251-12|Hymenoptera|Diapriidae|BOLD:ACC7905
Diapriidae|[2400]|SSGBC1828-14|Hymenoptera|Diapriidae|BOLD:ACA7355
Diapriidae|[2401]|SSPAA1570-13|Hymenoptera|Diapriidae|BOLD:AAU9080
Diapriidae|[2402]|CNBPD256-12|Hymenoptera|Diapriidae|BOLD:ACD1861
Diapriidae|[2403]|CNPAN478-13|Hymenoptera|Diapriidae|BOLD:ACC9417
Diapriidae|[2404]|CNTNJ659-14|Hymenoptera|Diapriidae|BOLD:ABY0983
Diapriidae|[2405]|SSWLF4639-13|Hymenoptera|Diapriidae|BOLD:ACK4780
Diapriidae|[2406]|CNGLF353-13|Hymenoptera|Diapriidae|BOLD:ACT4190
Diapriidae|[2407]|CNBAF099-12|Hymenoptera|Diapriidae|BOLD:ACE1272
Diapriidae|[2408]|CNTNF1146-14|Hymenoptera|Diapriidae|BOLD:ACL8980
Diapriidae|[2409]|CNKLD3942-15|Hymenoptera|Diapriidae|BOLD:ACS1728
Diapriidae|[2410]|CNKTG902-15|Hymenoptera|Diapriidae|BOLD:ACS9683
Diapriidae|[2411]|SSPAC945-13|Hymenoptera|Diapriidae|BOLD:ACI5661
Diapriidae|[2412]|BBHYK803-10|Hymenoptera|Diapriidae|BOLD:ABY3379
Belytinae|[2413]|SSJAD475-13|Hymenoptera|Diapriidae|BOLD:ACM0731
Diapriidae|[2414]|JBHCJ197-11|Hymenoptera|Diapriidae|BOLD:ACT0144
Diapriidae|[2415]|SSJAD950-13|Hymenoptera|Diapriidae|BOLD:ACM4208
Diapriidae|[2416]|CNPKF756-14|Hymenoptera|Diapriidae|BOLD:ACL5957
Diapriidae|[2417]|SSPAC8144-13|Hymenoptera|Diapriidae|BOLD:AAU9510
Diapriidae|[2418]|CNYOB505-15|Hymenoptera|Diapriidae|BOLD:ACD0127
Diapriidae|[2419]|CNKTG2635-15|Hymenoptera|Diapriidae|BOLD:ACT6058
Diapriidae|[2420]|SSWLF4653-13|Hymenoptera|Diapriidae|BOLD:ACK7894
Diapriidae|[2421]|CNEIE105-12|Hymenoptera|Diapriidae|BOLD:ACA7103
Diapriidae|[2422]|CNPAF293-13|Hymenoptera|Diapriidae|BOLD:ACC4158
Belytinae|[2423]|CNLMP1295-14|Hymenoptera|Diapriidae|BOLD:ACO2055
Diapriidae|[2424]|CNKLD3949-15|Hymenoptera|Diapriidae|BOLD:ACS1372
Diapriidae|[2425]|BBHYI219-10|Hymenoptera|Diapriidae|BOLD:AAU9560
Diapriidae|[2426]|JBHCI329-11|Hymenoptera|Diapriidae|BOLD:AAU9870
Diapriidae|[2427]|SSJAD930-13|Hymenoptera|Diapriidae|BOLD:ACK5414
Diapriidae|[2428]|SSPAB1871-13|Hymenoptera|Diapriidae|BOLD:ACL1134
Diapriidae|[2429]|SSJAF5170-13|Hymenoptera|Diapriidae|BOLD:AAU9377
Diapriidae|[2430]|SSWLF4596-13|Hymenoptera|Diapriidae|BOLD:ACE8342
Aclista|[2431]|CNGME786-14|Hymenoptera|Diapriidae|BOLD:ACT2181
Diapriidae|[2432]|CNGME1602-14|Hymenoptera|Diapriidae|BOLD:ACT3153
Diapriidae|[2433]|SSJAF384-13|Hymenoptera|Diapriidae|BOLD:ACK3084
Belytinae|[2434]|CNGMJ565-14|Hymenoptera|Diapriidae|BOLD:ACL2896
Diapriidae|[2435]|SSWLF345-13|Hymenoptera|Diapriidae|BOLD:ACK6585
Belytinae|[2436]|SSWLF365-13|Hymenoptera|Diapriidae|BOLD:AAJ8181
Diapriidae|[2437]|CNKTE2769-15|Hymenoptera|Diapriidae|BOLD:ACS1237
Belytinae|[2438]|CNKTG2747-15|Hymenoptera|Diapriidae|BOLD:ACK6994
Diapriidae|[2439]|BBHYN109-10|Hymenoptera|Diapriidae|BOLD:AAU8821
Diapriidae|[2440]|CNTNK191-14|Hymenoptera|Diapriidae|BOLD:ACC7150
Diapriidae|[2441]|CNPAN481-13|Hymenoptera|Diapriidae|BOLD:ACI9775
Diapriidae|[2442]|SSBRB2000-14|Hymenoptera|Diapriidae|BOLD:ACL5283
Diapriidae|[2443]|SSWLF4494-13|Hymenoptera|Diapriidae|BOLD:ACI9259
Diapriidae|[2444]|SSWLD906-13|Hymenoptera|Diapriidae|BOLD:ACK5743
Diapriidae|[2445]|SSPAB5366-13|Hymenoptera|Diapriidae|BOLD:AAU9414
Diapriidae|[2446]|CNRVF631-15|Hymenoptera|Diapriidae|BOLD:ACT8403
Diapriidae|[2447]|JSHYP573-11|Hymenoptera|Diapriidae|BOLD:ABA1079
Diapriidae|[2448]|NCCC699-11|Hymenoptera|Diapriidae|BOLD:ABV2763
Diapriidae|[2449]|CNSLD554-12|Hymenoptera|Diapriidae|BOLD:ACC7159
Diapriidae|[2450]|CNGBF1183-14|Hymenoptera|Diapriidae|BOLD:ACL3094
Diapriidae|[2451]|CNFDF306-14|Hymenoptera|Diapriidae|BOLD:ACL9224
Diapriidae|[2452]|CNMIH1514-14|Hymenoptera|Diapriidae|BOLD:ACN4626
Diapriidae|[2453]|CNTMA134-14|Hymenoptera|Diapriidae|BOLD:ACM0486
Diapriidae|[2454]|JBHCJ753-11|Hymenoptera|Diapriidae|BOLD:AAY6840
Diapriidae|[2455]|SSWLF4543-13|Hymenoptera|Diapriidae|BOLD:ACK9218
Diapriidae|[2456]|CNLME708-14|Hymenoptera|Diapriidae|BOLD:AAI3390
Diapriidae|[2457]|CNSLO675-13|Hymenoptera|Diapriidae|BOLD:ACH2144
Diapriidae|[2458]|RBINA3126-13|Hymenoptera|Diapriidae|BOLD:ACF9310
Diapriidae|[2459]|CNTNH692-14|Hymenoptera|Diapriidae|BOLD:ACL3175
Diapriidae|[2460]|CNTMB3420-14|Hymenoptera|Diapriidae|BOLD:ACM3311
Diapriidae|[2461]|SSWLF4794-13|Hymenoptera|Diapriidae|BOLD:ACD1629
Diapriidae|[2462]|CNKLH2440-15|Hymenoptera|Diapriidae|BOLD:ACT1939
Diapriidae|[2463]|JBHCJ620-11|Hymenoptera|Diapriidae|BOLD:ACS7753
Diapriidae|[2464]|SSJAF4619-13|Hymenoptera|Diapriidae|BOLD:AAU9775
Diapriidae|[2465]|SSPAB5981-13|Hymenoptera|Diapriidae|BOLD:ACJ3084
Diapriidae|[2466]|BBHYI118-10|Hymenoptera|Diapriidae|BOLD:ACB1535
Diapriidae|[2467]|JBHCH762-10|Hymenoptera|Diapriidae|BOLD:AAU9762
Diapriidae|[2468]|SSJAD3658-13|Hymenoptera|Diapriidae|BOLD:ACI4515
Diapriidae|[2469]|SSJAD3882-13|Hymenoptera|Diapriidae|BOLD:ACI9831
Diapriidae|[2470]|SSWLF3497-13|Hymenoptera|Diapriidae|BOLD:ACF7353
Diapriidae|[2471]|BBHYK390-10|Hymenoptera|Diapriidae|BOLD:ABX6829
Belytinae|[2472]|SSWLF2531-13|Hymenoptera|Diapriidae|BOLD:AAJ8191
Diapriidae|[2473]|SSJAD4846-13|Hymenoptera|Diapriidae|BOLD:ACL2142
Diapriidae|[2474]|SSJAF4207-13|Hymenoptera|Diapriidae|BOLD:ACB1536
Diapriidae|[2475]|SSWLF4802-13|Hymenoptera|Diapriidae|BOLD:AAU9013
Diapriidae|[2476]|SSJAD4308-13|Hymenoptera|Diapriidae|BOLD:ACI9763
Diapriidae|[2477]|CNPEC1819-14|Hymenoptera|Diapriidae|BOLD:ACY7758
Diapriidae|[2478]|SSPAC1535-13|Hymenoptera|Diapriidae|BOLD:ACD1943
Belytinae|[2479]|SSJAF4124-13|Hymenoptera|Diapriidae|BOLD:AAU9750
Diapriidae|[2480]|BBHYJ341-10|Hymenoptera|Diapriidae|BOLD:ACM1583
Diapriidae|[2481]|SSWLF4603-13|Hymenoptera|Diapriidae|BOLD:ACM1557
Diapriidae|[2482]|CNPAD363-13|Hymenoptera|Diapriidae|BOLD:AAU9534
Belytinae|[2483]|CNTMC291-14|Hymenoptera|Diapriidae|BOLD:ACM1895
Diapriidae|[2484]|CNRMD1311-12|Hymenoptera|Diapriidae|BOLD:ACC6816
Belytinae|[2485]|SSWLA187-13|Hymenoptera|Diapriidae|BOLD:ACD3913
Diapriidae|[2486]|CNGMH492-14|Hymenoptera|Diapriidae|BOLD:ACM0794
Diapriidae|[2487]|SSJAD3432-13|Hymenoptera|Diapriidae|BOLD:ABX5570
Diapriidae|[2488]|SSPAB6950-13|Hymenoptera|Diapriidae|BOLD:ACI7261
Belytinae|[2489]|CNPKJ616-14|Hymenoptera|Diapriidae|BOLD:ACN2074
Belytinae|[2490]|SSPAB5320-13|Hymenoptera|Diapriidae|BOLD:ACI4487
Belytinae|[2491]|CNGMD1486-14|Hymenoptera|Diapriidae|BOLD:ACW3060
Belytinae|[2492]|BBHYK727-10|Hymenoptera|Diapriidae|BOLD:ABY3147
Diapriidae|[2493]|CNPCI042-13|Hymenoptera|Diapriidae|BOLD:ACW3320
Diapriidae|[2494]|SSJAD908-13|Hymenoptera|Diapriidae|BOLD:ACG2186
Diapriidae|[2495]|SSWLF4649-13|Hymenoptera|Diapriidae|BOLD:AAU9032

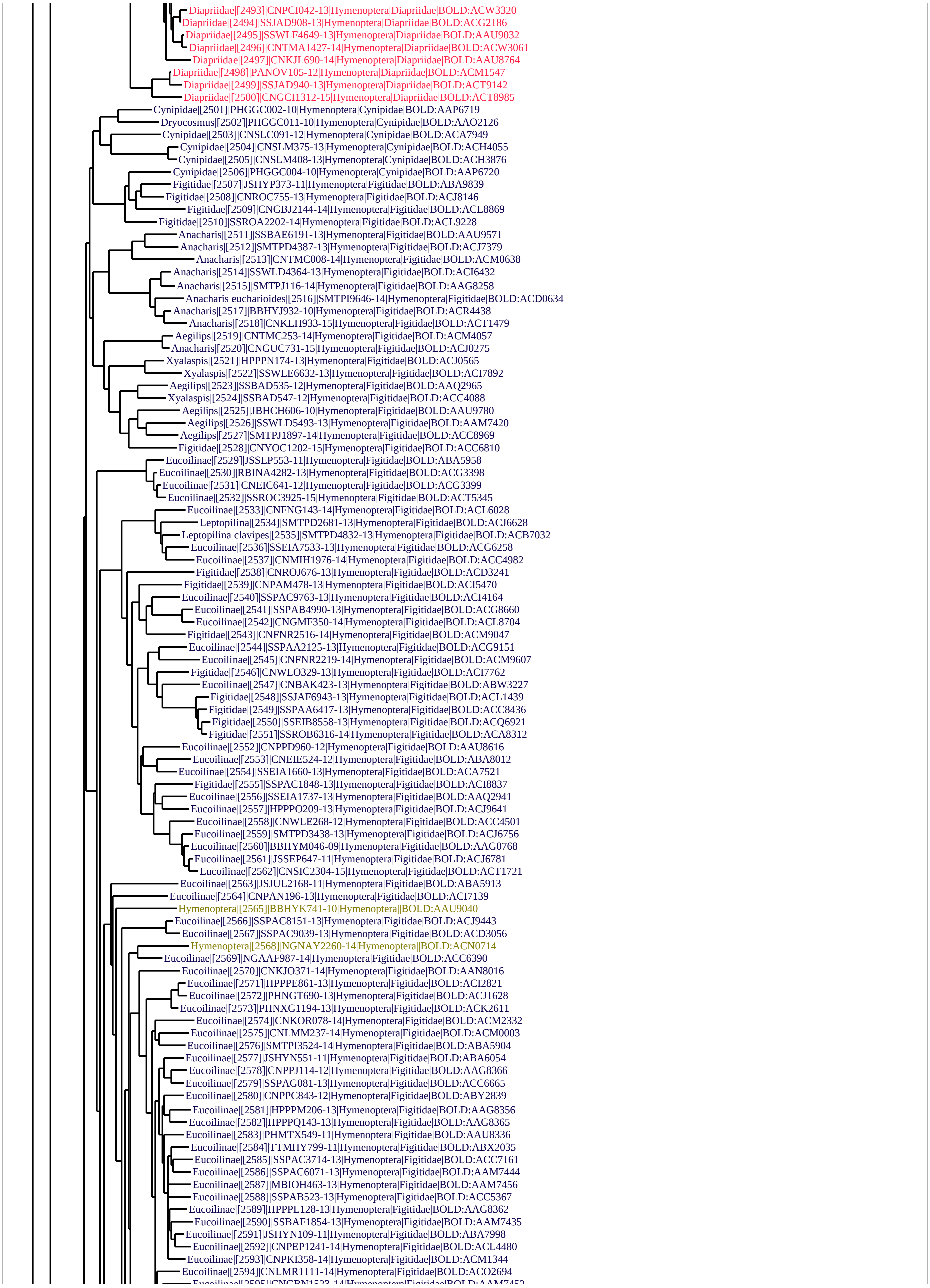

Diapriidae|[2493]|CNPCI042-13|Hymenoptera|Diapriidae|BOLD:ACW3320
Diapriidae|[2494]|SSJAD908-13|Hymenoptera|Diapriidae|BOLD:ACG2186
Diapriidae|[2495]|SSWLF4649-13|Hymenoptera|Diapriidae|BOLD:AAU9032
Diapriidae|[2496]|CNTMA1427-14|Hymenoptera|Diapriidae|BOLD:ACW3061
Diapriidae|[2497]|CNKJL690-14|Hymenoptera|Diapriidae|BOLD:AAU8764
Diapriidae|[2498]|PANOV105-12|Hymenoptera|Diapriidae|BOLD:ACM1547
Diapriidae|[2499]|SSJAD940-13|Hymenoptera|Diapriidae|BOLD:ACT9142
Diapriidae|[2500]|CNGCI1312-15|Hymenoptera|Diapriidae|BOLD:ACT8985
Cynipidae|[2501]|PHGGC002-10|Hymenoptera|Cynipidae|BOLD:AAP6719
Dryocosmus|[2502]|PHGGC011-10|Hymenoptera|Cynipidae|BOLD:AAO2126
Cynipidae|[2503]|CNSLC091-12|Hymenoptera|Cynipidae|BOLD:ACA7949
Cynipidae|[2504]|CNSLM375-13|Hymenoptera|Cynipidae|BOLD:ACH4055
Cynipidae|[2505]|CNSLM408-13|Hymenoptera|Cynipidae|BOLD:ACH3876
Cynipidae|[2506]|PHGGC004-10|Hymenoptera|Cynipidae|BOLD:AAP6720
Figitidae|[2507]|JSHYP373-11|Hymenoptera|Figitidae|BOLD:ABA9839
Figitidae|[2508]|CNROC755-13|Hymenoptera|Figitidae|BOLD:ACJ8146
Figitidae|[2509]|CNGBJ2144-14|Hymenoptera|Figitidae|BOLD:ACL8869
Figitidae|[2510]|SSROA2202-14|Hymenoptera|Figitidae|BOLD:ACL9228
Anacharis|[2511]|SSBAE6191-13|Hymenoptera|Figitidae|BOLD:AAU9571
Anacharis|[2512]|SMTPD4387-13|Hymenoptera|Figitidae|BOLD:ACJ7379
Anacharis|[2513]|CNTMC008-14|Hymenoptera|Figitidae|BOLD:ACM0638
Anacharis|[2514]|SSWLD4364-13|Hymenoptera|Figitidae|BOLD:ACI6432
Anacharis|[2515]|SMTPJ116-14|Hymenoptera|Figitidae|BOLD:AAG8258
Anacharis eucharioides|[2516]|SMTPI9646-14|Hymenoptera|Figitidae|BOLD:ACD0634
Anacharis|[2517]|BBHYJ932-10|Hymenoptera|Figitidae|BOLD:ACR4438
Anacharis|[2518]|CNKLH933-15|Hymenoptera|Figitidae|BOLD:ACT1479
Aegilips|[2519]|CNTMC253-14|Hymenoptera|Figitidae|BOLD:ACM4057
Anacharis|[2520]|CNGUC731-15|Hymenoptera|Figitidae|BOLD:ACJ0275
Xyalaspis|[2521]|HPPPN174-13|Hymenoptera|Figitidae|BOLD:ACJ0565
Xyalaspis|[2522]|SSWLE6632-13|Hymenoptera|Figitidae|BOLD:ACI7892
Aegilips|[2523]|SSBAD535-12|Hymenoptera|Figitidae|BOLD:AAQ2965
Xyalaspis|[2524]|SSBAD547-12|Hymenoptera|Figitidae|BOLD:ACC4088
Aegilips|[2525]|JBHCH606-10|Hymenoptera|Figitidae|BOLD:AAU9780
Aegilips|[2526]|SSWLD5493-13|Hymenoptera|Figitidae|BOLD:AAM7420
Aegilips|[2527]|SMTPJ1897-14|Hymenoptera|Figitidae|BOLD:ACC8969
Figitidae|[2528]|CNYOC1202-15|Hymenoptera|Figitidae|BOLD:ACC6810
Eucoilinae|[2529]|JSSEP553-11|Hymenoptera|Figitidae|BOLD:ABA5958
Eucoilinae|[2530]|RBINA4282-13|Hymenoptera|Figitidae|BOLD:ACG3398
Eucoilinae|[2531]|CNEIC641-12|Hymenoptera|Figitidae|BOLD:ACG3399
Eucoilinae|[2532]|SSROC3925-15|Hymenoptera|Figitidae|BOLD:ACT5345
Eucoilinae|[2533]|CNFNG143-14|Hymenoptera|Figitidae|BOLD:ACL6028
Leptopilina|[2534]|SMTPD2681-13|Hymenoptera|Figitidae|BOLD:ACJ6628
Leptopilina clavipes|[2535]|SMTPD4832-13|Hymenoptera|Figitidae|BOLD:ACB7032
Eucoilinae|[2536]|SSEIA7533-13|Hymenoptera|Figitidae|BOLD:ACG6258
Eucoilinae|[2537]|CNMIH1976-14|Hymenoptera|Figitidae|BOLD:ACC4982
Figitidae|[2538]|CNROJ676-13|Hymenoptera|Figitidae|BOLD:ACD3241
Figitidae|[2539]|CNPAM478-13|Hymenoptera|Figitidae|BOLD:ACI5470
Eucoilinae|[2540]|SSPAC9763-13|Hymenoptera|Figitidae|BOLD:ACI4164
Eucoilinae|[2541]|SSPAB4990-13|Hymenoptera|Figitidae|BOLD:ACG8660
Eucoilinae|[2542]|CNGMF350-14|Hymenoptera|Figitidae|BOLD:ACL8704
Figitidae|[2543]|CNFNR2516-14|Hymenoptera|Figitidae|BOLD:ACM9047
Eucoilinae|[2544]|SSPAA2125-13|Hymenoptera|Figitidae|BOLD:ACG9151
Eucoilinae|[2545]|CNFNR2219-14|Hymenoptera|Figitidae|BOLD:ACM9607
Figitidae|[2546]|CNWLO329-13|Hymenoptera|Figitidae|BOLD:ACI7762
Eucoilinae|[2547]|CNBAK423-13|Hymenoptera|Figitidae|BOLD:ABW3227
Figitidae|[2548]|SSJAF6943-13|Hymenoptera|Figitidae|BOLD:ACL1439
Figitidae|[2549]|SSPAA6417-13|Hymenoptera|Figitidae|BOLD:ACC8436
Figitidae|[2550]|SSEIB8558-13|Hymenoptera|Figitidae|BOLD:ACQ6921
Figitidae|[2551]|SSROB6316-14|Hymenoptera|Figitidae|BOLD:ACA8312
Eucoilinae|[2552]|CNPPD960-12|Hymenoptera|Figitidae|BOLD:AAU8616
Eucoilinae|[2553]|CNEIE524-12|Hymenoptera|Figitidae|BOLD:ABA8012
Eucoilinae|[2554]|SSEIA1660-13|Hymenoptera|Figitidae|BOLD:ACA7521
Figitidae|[2555]|SSPAC1848-13|Hymenoptera|Figitidae|BOLD:ACI8837
Eucoilinae|[2556]|SSEIA1737-13|Hymenoptera|Figitidae|BOLD:AAQ2941
Eucoilinae|[2557]|HPPPO209-13|Hymenoptera|Figitidae|BOLD:ACJ9641
Eucoilinae|[2558]|CNWLE268-12|Hymenoptera|Figitidae|BOLD:ACC4501
Eucoilinae|[2559]|SMTPD3438-13|Hymenoptera|Figitidae|BOLD:ACJ6756
Eucoilinae|[2560]|BBHYM046-09|Hymenoptera|Figitidae|BOLD:AAG0768
Eucoilinae|[2561]|JSSEP647-11|Hymenoptera|Figitidae|BOLD:ACJ6781
Eucoilinae|[2562]|CNSIC2304-15|Hymenoptera|Figitidae|BOLD:ACT1721
Eucoilinae|[2563]|JSJUL2168-11|Hymenoptera|Figitidae|BOLD:ABA5913
Eucoilinae|[2564]|CNPAN196-13|Hymenoptera|Figitidae|BOLD:ACI7139
Hymenoptera|[2565]|BBHYK741-10|Hymenoptera||BOLD:AAU9040
Eucoilinae|[2566]|SSPAC8151-13|Hymenoptera|Figitidae|BOLD:ACJ9443
Eucoilinae|[2567]|SSPAC9039-13|Hymenoptera|Figitidae|BOLD:ACD3056
Hymenoptera|[2568]|NGNAY2260-14|Hymenoptera||BOLD:ACN0714
Eucoilinae|[2569]|NGAAF987-14|Hymenoptera|Figitidae|BOLD:ACC6390
Eucoilinae|[2570]|CNKJO371-14|Hymenoptera|Figitidae|BOLD:AAN8016
Eucoilinae|[2571]|HPPPE861-13|Hymenoptera|Figitidae|BOLD:ACI2821
Eucoilinae|[2572]|PHNGT690-13|Hymenoptera|Figitidae|BOLD:ACJ1628
Eucoilinae|[2573]|PHNXG1194-13|Hymenoptera|Figitidae|BOLD:ACK2611
Eucoilinae|[2574]|CNKOR078-14|Hymenoptera|Figitidae|BOLD:ACM2332
Eucoilinae|[2575]|CNLMM237-14|Hymenoptera|Figitidae|BOLD:ACM0003
Eucoilinae|[2576]|SMTPI3524-14|Hymenoptera|Figitidae|BOLD:ABA5904
Eucoilinae|[2577]|JSHYN551-11|Hymenoptera|Figitidae|BOLD:ABA6054
Eucoilinae|[2578]|CNPPJ114-12|Hymenoptera|Figitidae|BOLD:AAG8366
Eucoilinae|[2579]|SSPAG081-13|Hymenoptera|Figitidae|BOLD:ACC6665
Eucoilinae|[2580]|CNPPC843-12|Hymenoptera|Figitidae|BOLD:ABY2839
Eucoilinae|[2581]|HPPPM206-13|Hymenoptera|Figitidae|BOLD:AAG8356
Eucoilinae|[2582]|HPPPQ143-13|Hymenoptera|Figitidae|BOLD:AAG8365
Eucoilinae|[2583]|PHMTX549-11|Hymenoptera|Figitidae|BOLD:AAU8336
Eucoilinae|[2584]|TTMHY799-11|Hymenoptera|Figitidae|BOLD:ABX2035
Eucoilinae|[2585]|SSPAC3714-13|Hymenoptera|Figitidae|BOLD:ACC7161
Eucoilinae|[2586]|SSPAC6071-13|Hymenoptera|Figitidae|BOLD:AAM7444
Eucoilinae|[2587]|MBIOH463-13|Hymenoptera|Figitidae|BOLD:AAM7456
Eucoilinae|[2588]|SSPAB523-13|Hymenoptera|Figitidae|BOLD:ACC5367
Eucoilinae|[2589]|HPPPL128-13|Hymenoptera|Figitidae|BOLD:AAG8362
Eucoilinae|[2590]|SSBAF1854-13|Hymenoptera|Figitidae|BOLD:AAM7435
Eucoilinae|[2591]|JSHYN109-11|Hymenoptera|Figitidae|BOLD:ABA7998
Eucoilinae|[2592]|CNPEP1241-14|Hymenoptera|Figitidae|BOLD:ACL4480
Eucoilinae|[2593]|CNPKI358-14|Hymenoptera|Figitidae|BOLD:ACM1344
Eucoilinae|[2594]|CNLMR1111-14|Hymenoptera|Figitidae|BOLD:ACO2694

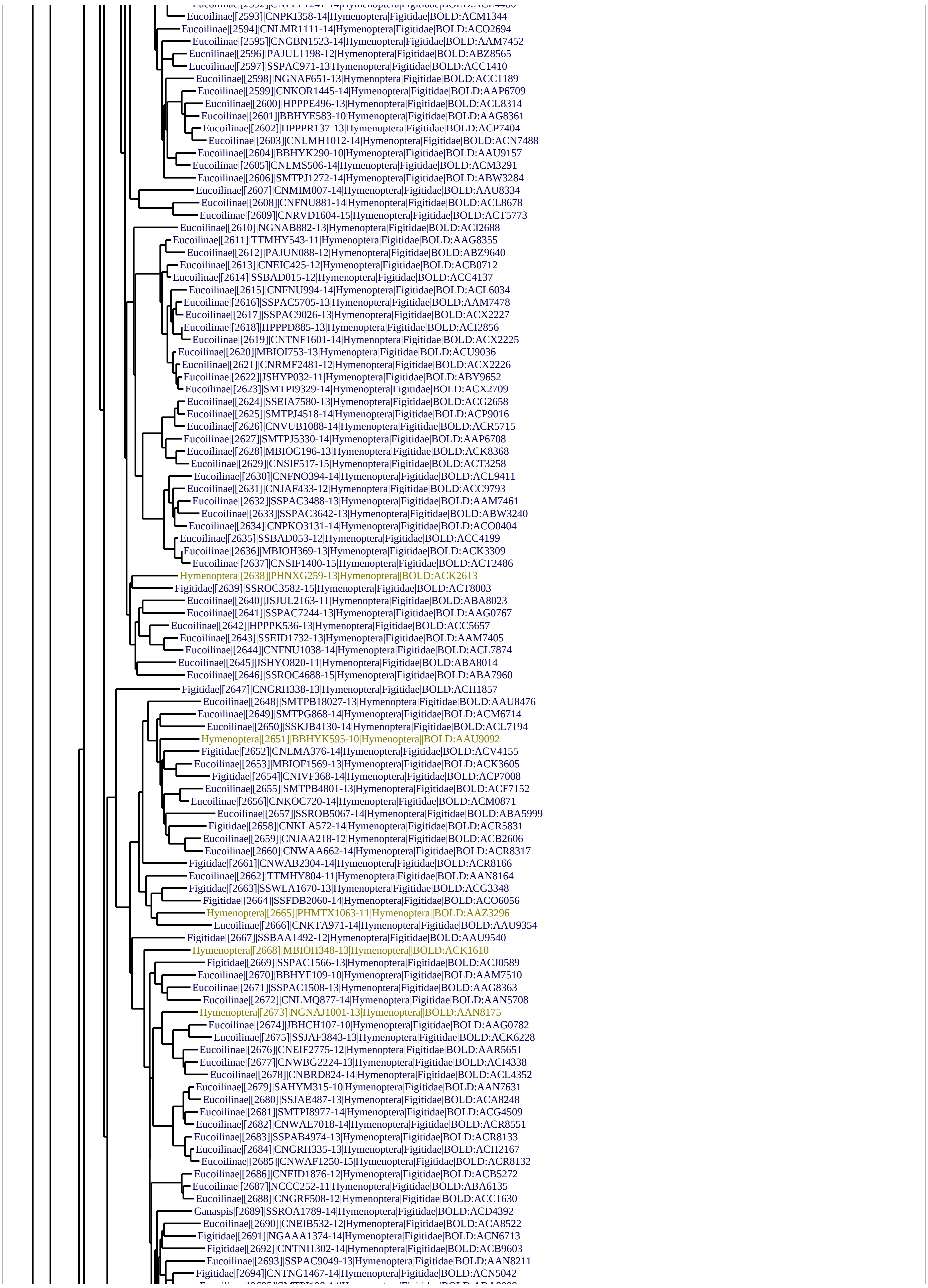

Eucoilinae|[2593]|CNPKI358-14|Hymenoptera|Figitidae|BOLD:ACM1344
Eucoilinae|[2594]|CNLMR1111-14|Hymenoptera|Figitidae|BOLD:ACO2694
Eucoilinae|[2595]|CNGBN1523-14|Hymenoptera|Figitidae|BOLD:AAM7452
Eucoilinae|[2596]|PAJUL1198-12|Hymenoptera|Figitidae|BOLD:ABZ8565
Eucoilinae|[2597]|SSPAC971-13|Hymenoptera|Figitidae|BOLD:ACC1410
Eucoilinae|[2598]|NGNAF651-13|Hymenoptera|Figitidae|BOLD:ACC1189
Eucoilinae|[2599]|CNKOR1445-14|Hymenoptera|Figitidae|BOLD:AAP6709
Eucoilinae|[2600]|HPPPE496-13|Hymenoptera|Figitidae|BOLD:ACL8314
Eucoilinae|[2601]|BBHYE583-10|Hymenoptera|Figitidae|BOLD:AAG8361
Eucoilinae|[2602]|HPPPR137-13|Hymenoptera|Figitidae|BOLD:ACP7404
Eucoilinae|[2603]|CNLMH1012-14|Hymenoptera|Figitidae|BOLD:ACN7488
Eucoilinae|[2604]|BBHYK290-10|Hymenoptera|Figitidae|BOLD:AAU9157
Eucoilinae|[2605]|CNLMS506-14|Hymenoptera|Figitidae|BOLD:ACM3291
Eucoilinae|[2606]|SMTPJ1272-14|Hymenoptera|Figitidae|BOLD:ABW3284
Eucoilinae|[2607]|CNMIM007-14|Hymenoptera|Figitidae|BOLD:AAU8334
Eucoilinae|[2608]|CNFNU881-14|Hymenoptera|Figitidae|BOLD:ACL8678
Eucoilinae|[2609]|CNRVD1604-15|Hymenoptera|Figitidae|BOLD:ACT5773
Eucoilinae|[2610]|NGNAB882-13|Hymenoptera|Figitidae|BOLD:ACI2688
Eucoilinae|[2611]|TTMHY543-11|Hymenoptera|Figitidae|BOLD:AAG8355
Eucoilinae|[2612]|PAJUN088-12|Hymenoptera|Figitidae|BOLD:ABZ9640
Eucoilinae|[2613]|CNEIC425-12|Hymenoptera|Figitidae|BOLD:ACB0712
Eucoilinae|[2614]|SSBAD015-12|Hymenoptera|Figitidae|BOLD:ACC4137
Eucoilinae|[2615]|CNFNU994-14|Hymenoptera|Figitidae|BOLD:ACL6034
Eucoilinae|[2616]|SSPAC5705-13|Hymenoptera|Figitidae|BOLD:AAM7478
Eucoilinae|[2617]|SSPAC9026-13|Hymenoptera|Figitidae|BOLD:ACX2227
Eucoilinae|[2618]|HPPPD885-13|Hymenoptera|Figitidae|BOLD:ACI2856
Eucoilinae|[2619]|CNTNF1601-14|Hymenoptera|Figitidae|BOLD:ACX2225
Eucoilinae|[2620]|MBIOI753-13|Hymenoptera|Figitidae|BOLD:ACU9036
Eucoilinae|[2621]|CNRMF2481-12|Hymenoptera|Figitidae|BOLD:ACX2226
Eucoilinae|[2622]|JSHYP032-11|Hymenoptera|Figitidae|BOLD:ABY9652
Eucoilinae|[2623]|SMTPI9329-14|Hymenoptera|Figitidae|BOLD:ACX2709
Eucoilinae|[2624]|SSEIA7580-13|Hymenoptera|Figitidae|BOLD:ACG2658
Eucoilinae|[2625]|SMTPJ4518-14|Hymenoptera|Figitidae|BOLD:ACP9016
Eucoilinae|[2626]|CNVUB1088-14|Hymenoptera|Figitidae|BOLD:ACR5715
Eucoilinae|[2627]|SMTPJ5330-14|Hymenoptera|Figitidae|BOLD:AAP6708
Eucoilinae|[2628]|MBIOG196-13|Hymenoptera|Figitidae|BOLD:ACK8368
Eucoilinae|[2629]|CNSIF517-15|Hymenoptera|Figitidae|BOLD:ACT3258
Eucoilinae|[2630]|CNFNO394-14|Hymenoptera|Figitidae|BOLD:ACL9411
Eucoilinae|[2631]|CNJAF433-12|Hymenoptera|Figitidae|BOLD:ACC9793
Eucoilinae|[2632]|SSPAC3488-13|Hymenoptera|Figitidae|BOLD:AAM7461
Eucoilinae|[2633]|SSPAC3642-13|Hymenoptera|Figitidae|BOLD:ABW3240
Eucoilinae|[2634]|CNPKO3131-14|Hymenoptera|Figitidae|BOLD:ACO0404
Eucoilinae|[2635]|SSBAD053-12|Hymenoptera|Figitidae|BOLD:ACC4199
Eucoilinae|[2636]|MBIOH369-13|Hymenoptera|Figitidae|BOLD:ACK3309
Eucoilinae|[2637]|CNSIF1400-15|Hymenoptera|Figitidae|BOLD:ACT2486
Hymenoptera|[2638]|PHNXG259-13|Hymenoptera||BOLD:ACK2613
Figitidae|[2639]|SSROC3582-15|Hymenoptera|Figitidae|BOLD:ACT8003
Eucoilinae|[2640]|JSJUL2163-11|Hymenoptera|Figitidae|BOLD:ABA8023
Eucoilinae|[2641]|SSPAC7244-13|Hymenoptera|Figitidae|BOLD:AAG0767
Eucoilinae|[2642]|HPPPK536-13|Hymenoptera|Figitidae|BOLD:ACC5657
Eucoilinae|[2643]|SSEID1732-13|Hymenoptera|Figitidae|BOLD:AAM7405
Eucoilinae|[2644]|CNFNU1038-14|Hymenoptera|Figitidae|BOLD:ACL7874
Eucoilinae|[2645]|JSHYO820-11|Hymenoptera|Figitidae|BOLD:ABA8014
Eucoilinae|[2646]|SSROC4688-15|Hymenoptera|Figitidae|BOLD:ABA7960
Figitidae|[2647]|CNGRH338-13|Hymenoptera|Figitidae|BOLD:ACH1857
Eucoilinae|[2648]|SMTPB18027-13|Hymenoptera|Figitidae|BOLD:AAU8476
Eucoilinae|[2649]|SMTPG868-14|Hymenoptera|Figitidae|BOLD:ACM6714
Eucoilinae|[2650]|SSKJB4130-14|Hymenoptera|Figitidae|BOLD:ACL7194
Hymenoptera|[2651]|BBHYK595-10|Hymenoptera||BOLD:AAU9092
Figitidae|[2652]|CNLMA376-14|Hymenoptera|Figitidae|BOLD:ACV4155
Eucoilinae|[2653]|MBIOF1569-13|Hymenoptera|Figitidae|BOLD:ACK3605
Figitidae|[2654]|CNIVF368-14|Hymenoptera|Figitidae|BOLD:ACP7008
Eucoilinae|[2655]|SMTPB4801-13|Hymenoptera|Figitidae|BOLD:ACF7152
Eucoilinae|[2656]|CNKOC720-14|Hymenoptera|Figitidae|BOLD:ACM0871
Eucoilinae|[2657]|SSROB5067-14|Hymenoptera|Figitidae|BOLD:ABA5999
Figitidae|[2658]|CNKLA572-14|Hymenoptera|Figitidae|BOLD:ACR5831
Eucoilinae|[2659]|CNJAA218-12|Hymenoptera|Figitidae|BOLD:ACB2606
Eucoilinae|[2660]|CNWAA662-14|Hymenoptera|Figitidae|BOLD:ACR8317
Figitidae|[2661]|CNWAB2304-14|Hymenoptera|Figitidae|BOLD:ACR8166
Eucoilinae|[2662]|TTMHY804-11|Hymenoptera|Figitidae|BOLD:AAN8164
Figitidae|[2663]|SSWLA1670-13|Hymenoptera|Figitidae|BOLD:ACG3348
Figitidae|[2664]|SSFDB2060-14|Hymenoptera|Figitidae|BOLD:ACO6056
Hymenoptera|[2665]|PHMTX1063-11|Hymenoptera||BOLD:AAZ3296
Eucoilinae|[2666]|CNKTA971-14|Hymenoptera|Figitidae|BOLD:AAU9354
Figitidae|[2667]|SSBAA1492-12|Hymenoptera|Figitidae|BOLD:AAU9540
Hymenoptera|[2668]|MBIOH348-13|Hymenoptera||BOLD:ACK1610
Figitidae|[2669]|SSPAC1566-13|Hymenoptera|Figitidae|BOLD:ACJ0589
Eucoilinae|[2670]|BBHYF109-10|Hymenoptera|Figitidae|BOLD:AAM7510
Eucoilinae|[2671]|SSPAC1508-13|Hymenoptera|Figitidae|BOLD:AAG8363
Eucoilinae|[2672]|CNLMQ877-14|Hymenoptera|Figitidae|BOLD:AAN5708
Hymenoptera|[2673]|NGNAJ1001-13|Hymenoptera||BOLD:AAN8175
Eucoilinae|[2674]|JBHCH107-10|Hymenoptera|Figitidae|BOLD:AAG0782
Eucoilinae|[2675]|SSJAF3843-13|Hymenoptera|Figitidae|BOLD:ACK6228
Eucoilinae|[2676]|CNEIF2775-12|Hymenoptera|Figitidae|BOLD:AAR5651
Eucoilinae|[2677]|CNWBG2224-13|Hymenoptera|Figitidae|BOLD:ACI4338
Eucoilinae|[2678]|CNBRD824-14|Hymenoptera|Figitidae|BOLD:ACL4352
Eucoilinae|[2679]|SAHYM315-10|Hymenoptera|Figitidae|BOLD:AAN7631
Eucoilinae|[2680]|SSJAE487-13|Hymenoptera|Figitidae|BOLD:ACA8248
Eucoilinae|[2681]|SMTPI8977-14|Hymenoptera|Figitidae|BOLD:ACG4509
Eucoilinae|[2682]|CNWAE7018-14|Hymenoptera|Figitidae|BOLD:ACR8551
Eucoilinae|[2683]|SSPAB4974-13|Hymenoptera|Figitidae|BOLD:ACR8133
Eucoilinae|[2684]|CNGRH335-13|Hymenoptera|Figitidae|BOLD:ACH2167
Eucoilinae|[2685]|CNWAF1250-15|Hymenoptera|Figitidae|BOLD:ACR8132
Eucoilinae|[2686]|CNEID1876-12|Hymenoptera|Figitidae|BOLD:ACB5272
Eucoilinae|[2687]|NCCC252-11|Hymenoptera|Figitidae|BOLD:ABA6135
Eucoilinae|[2688]|CNGRF508-12|Hymenoptera|Figitidae|BOLD:ACC1630
Ganaspis|[2689]|SSROA1789-14|Hymenoptera|Figitidae|BOLD:ACD4392
Eucoilinae|[2690]|CNEIB532-12|Hymenoptera|Figitidae|BOLD:ACA8522
Figitidae|[2691]|NGAAA1374-14|Hymenoptera|Figitidae|BOLD:ACN6713
Figitidae|[2692]|CNTNI1302-14|Hymenoptera|Figitidae|BOLD:ACB9603
Eucoilinae|[2693]|SSPAC9049-13|Hymenoptera|Figitidae|BOLD:AAN8211
Figitidae|[2694]|CNTNG1467-14|Hymenoptera|Figitidae|BOLD:ACN5042

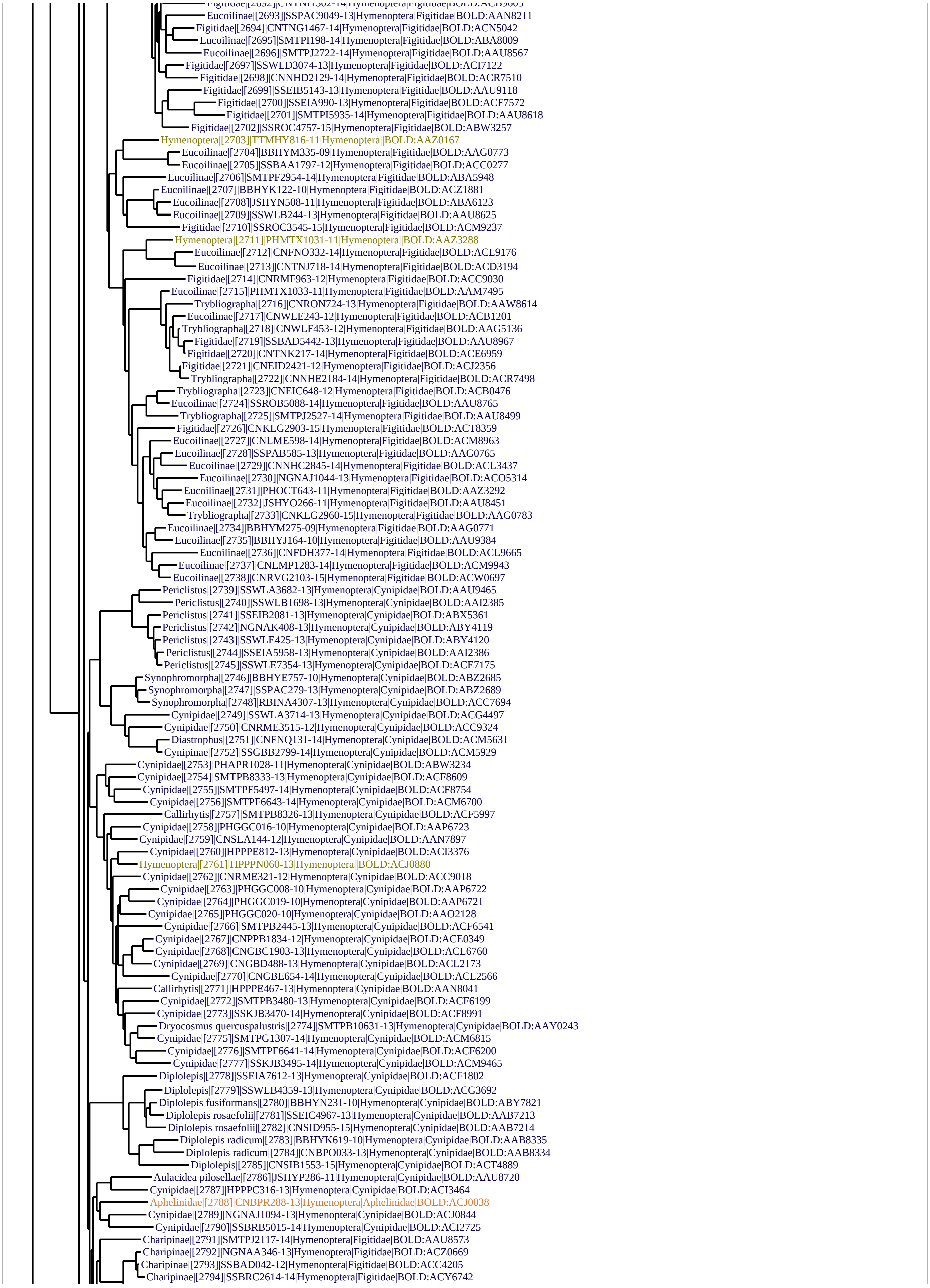
Eucoilinae|[2693]|SSPAC9049-13|Hymenoptera|Figitidae|BOLD:AAN8211
Figitidae|[2694]|CNTNG1467-14|Hymenoptera|Figitidae|BOLD:ACN5042
Eucoilinae|[2695]|SMTPI198-14|Hymenoptera|Figitidae|BOLD:ABA8009
Eucoilinae|[2696]|SMTPJ2722-14|Hymenoptera|Figitidae|BOLD:AAU8567
Figitidae|[2697]|SSWLD3074-13|Hymenoptera|Figitidae|BOLD:ACI7122
Figitidae|[2698]|CNNHD2129-14|Hymenoptera|Figitidae|BOLD:ACR7510
Figitidae|[2699]|SSEIB5143-13|Hymenoptera|Figitidae|BOLD:AAU9118
Figitidae|[2700]|SSEIA990-13|Hymenoptera|Figitidae|BOLD:ACF7572
Figitidae|[2701]|SMTPI5935-14|Hymenoptera|Figitidae|BOLD:AAU8618
Figitidae|[2702]|SSROC4757-15|Hymenoptera|Figitidae|BOLD:ABW3257
Hymenoptera|[2703]|TTMHY816-11|Hymenoptera||BOLD:AAZ0167
Eucoilinae|[2704]|BBHYM335-09|Hymenoptera|Figitidae|BOLD:AAG0773
Eucoilinae|[2705]|SSBAA1797-12|Hymenoptera|Figitidae|BOLD:ACC0277
Eucoilinae|[2706]|SMTPF2954-14|Hymenoptera|Figitidae|BOLD:ABA5948
Eucoilinae|[2707]|BBHYK122-10|Hymenoptera|Figitidae|BOLD:ACZ1881
Eucoilinae|[2708]|JSHYN508-11|Hymenoptera|Figitidae|BOLD:ABA6123
Eucoilinae|[2709]|SSWLB244-13|Hymenoptera|Figitidae|BOLD:AAU8625
Figitidae|[2710]|SSROC3545-15|Hymenoptera|Figitidae|BOLD:ACM9237
Hymenoptera|[2711]|PHMTX1031-11|Hymenoptera||BOLD:AAZ3288
Eucoilinae|[2712]|CNFNO332-14|Hymenoptera|Figitidae|BOLD:ACL9176
Eucoilinae|[2713]|CNTNJ718-14|Hymenoptera|Figitidae|BOLD:ACD3194
Figitidae|[2714]|CNRMF963-12|Hymenoptera|Figitidae|BOLD:ACC9030
Eucoilinae|[2715]|PHMTX1033-11|Hymenoptera|Figitidae|BOLD:AAM7495
Trybliographa|[2716]|CNRON724-13|Hymenoptera|Figitidae|BOLD:AAW8614
Eucoilinae|[2717]|CNWLE243-12|Hymenoptera|Figitidae|BOLD:ACB1201
Trybliographa|[2718]|CNWLF453-12|Hymenoptera|Figitidae|BOLD:AAG5136
Figitidae|[2719]|SSBAD5442-13|Hymenoptera|Figitidae|BOLD:AAU8967
Figitidae|[2720]|CNTNK217-14|Hymenoptera|Figitidae|BOLD:ACE6959
Figitidae|[2721]|CNEID2421-12|Hymenoptera|Figitidae|BOLD:ACJ2356
Trybliographa|[2722]|CNNHE2184-14|Hymenoptera|Figitidae|BOLD:ACR7498
Trybliographa|[2723]|CNEIC648-12|Hymenoptera|Figitidae|BOLD:ACB0476
Eucoilinae|[2724]|SSROB5088-14|Hymenoptera|Figitidae|BOLD:AAU8765
Trybliographa|[2725]|SMTPJ2527-14|Hymenoptera|Figitidae|BOLD:AAU8499
Figitidae|[2726]|CNKLG2903-15|Hymenoptera|Figitidae|BOLD:ACT8359
Eucoilinae|[2727]|CNLME598-14|Hymenoptera|Figitidae|BOLD:ACM8963
Eucoilinae|[2728]|SSPAB585-13|Hymenoptera|Figitidae|BOLD:AAG0765
Eucoilinae|[2729]|CNNHC2845-14|Hymenoptera|Figitidae|BOLD:ACL3437
Eucoilinae|[2730]|NGNAJ1044-13|Hymenoptera|Figitidae|BOLD:ACO5314
Eucoilinae|[2731]|PHOCT643-11|Hymenoptera|Figitidae|BOLD:AAZ3292
Eucoilinae|[2732]|JSHYO266-11|Hymenoptera|Figitidae|BOLD:AAU8451
Trybliographa|[2733]|CNKLG2960-15|Hymenoptera|Figitidae|BOLD:AAG0783
Eucoilinae|[2734]|BBHYM275-09|Hymenoptera|Figitidae|BOLD:AAG0771
Eucoilinae|[2735]|BBHYJ164-10|Hymenoptera|Figitidae|BOLD:AAU9384
Eucoilinae|[2736]|CNFDH377-14|Hymenoptera|Figitidae|BOLD:ACL9665
Eucoilinae|[2737]|CNLMP1283-14|Hymenoptera|Figitidae|BOLD:ACM9943
Eucoilinae|[2738]|CNRVG2103-15|Hymenoptera|Figitidae|BOLD:ACW0697
Periclistus|[2739]|SSWLA3682-13|Hymenoptera|Cynipidae|BOLD:AAU9465
Periclistus|[2740]|SSWLB1698-13|Hymenoptera|Cynipidae|BOLD:AAI2385
Periclistus|[2741]|SSEIB2081-13|Hymenoptera|Cynipidae|BOLD:ABX5361
Periclistus|[2742]|NGNAK408-13|Hymenoptera|Cynipidae|BOLD:ABY4119
Periclistus|[2743]|SSWLE425-13|Hymenoptera|Cynipidae|BOLD:ABY4120
Periclistus|[2744]|SSEIA5958-13|Hymenoptera|Cynipidae|BOLD:AAI2386
Periclistus|[2745]|SSWLE7354-13|Hymenoptera|Cynipidae|BOLD:ACE7175
Synophromorpha|[2746]|BBHYE757-10|Hymenoptera|Cynipidae|BOLD:ABZ2685
Synophromorpha|[2747]|SSPAC279-13|Hymenoptera|Cynipidae|BOLD:ABZ2689
Synophromorpha|[2748]|RBINA4307-13|Hymenoptera|Cynipidae|BOLD:ACC7694
Cynipidae|[2749]|SSWLA3714-13|Hymenoptera|Cynipidae|BOLD:ACG4497
Cynipidae|[2750]|CNRME3515-12|Hymenoptera|Cynipidae|BOLD:ACC9324
Diastrophus|[2751]|CNFNQ131-14|Hymenoptera|Cynipidae|BOLD:ACM5631
Cynipinae|[2752]|SSGBB2799-14|Hymenoptera|Cynipidae|BOLD:ACM5929
Cynipidae|[2753]|PHAPR1028-11|Hymenoptera|Cynipidae|BOLD:ABW3234
Cynipidae|[2754]|SMTPB8333-13|Hymenoptera|Cynipidae|BOLD:ACF8609
Cynipidae|[2755]|SMTPF5497-14|Hymenoptera|Cynipidae|BOLD:ACF8754
Cynipidae|[2756]|SMTPF6643-14|Hymenoptera|Cynipidae|BOLD:ACM6700
Callirhytis|[2757]|SMTPB8326-13|Hymenoptera|Cynipidae|BOLD:ACF5997
Cynipidae|[2758]|PHGGC016-10|Hymenoptera|Cynipidae|BOLD:AAP6723
Cynipidae|[2759]|CNSLA144-12|Hymenoptera|Cynipidae|BOLD:AAN7897
Cynipidae|[2760]|HPPPE812-13|Hymenoptera|Cynipidae|BOLD:ACI3376
Hymenoptera|[2761]|HPPPN060-13|Hymenoptera||BOLD:ACJ0880
Cynipidae|[2762]|CNRME321-12|Hymenoptera|Cynipidae|BOLD:ACC9018
Cynipidae|[2763]|PHGGC008-10|Hymenoptera|Cynipidae|BOLD:AAP6722
Cynipidae|[2764]|PHGGC019-10|Hymenoptera|Cynipidae|BOLD:AAP6721
Cynipidae|[2765]|PHGGC020-10|Hymenoptera|Cynipidae|BOLD:AAO2128
Cynipidae|[2766]|SMTPB2445-13|Hymenoptera|Cynipidae|BOLD:ACF6541
Cynipidae|[2767]|CNPPB1834-12|Hymenoptera|Cynipidae|BOLD:ACE0349
Cynipidae|[2768]|CNGBC1903-13|Hymenoptera|Cynipidae|BOLD:ACL6760
Cynipidae|[2769]|CNGBD488-13|Hymenoptera|Cynipidae|BOLD:ACL2173
Cynipidae|[2770]|CNGBE654-14|Hymenoptera|Cynipidae|BOLD:ACL2566
Callirhytis|[2771]|HPPPE467-13|Hymenoptera|Cynipidae|BOLD:AAN8041
Cynipidae|[2772]|SMTPB3480-13|Hymenoptera|Cynipidae|BOLD:ACF6199
Cynipidae|[2773]|SSKJB3470-14|Hymenoptera|Cynipidae|BOLD:ACF8991
Dryocosmus quercuspalustris|[2774]|SMTPB10631-13|Hymenoptera|Cynipidae|BOLD:AAY0243
Cynipidae|[2775]|SMTPG1307-14|Hymenoptera|Cynipidae|BOLD:ACM6815
Cynipidae|[2776]|SMTPF6641-14|Hymenoptera|Cynipidae|BOLD:ACF6200
Cynipidae|[2777]|SSKJB3495-14|Hymenoptera|Cynipidae|BOLD:ACM9465
Diplolepis|[2778]|SSEIA7612-13|Hymenoptera|Cynipidae|BOLD:ACF1802
Diplolepis|[2779]|SSWLB4359-13|Hymenoptera|Cynipidae|BOLD:ACG3692
Diplolepis fusiformans|[2780]|BBHYN231-10|Hymenoptera|Cynipidae|BOLD:ABY7821
Diplolepis rosaefolii|[2781]|SSEIC4967-13|Hymenoptera|Cynipidae|BOLD:AAB7213
Diplolepis rosaefolii|[2782]|CNSID955-15|Hymenoptera|Cynipidae|BOLD:AAB7214
Diplolepis radicum|[2783]|BBHYK619-10|Hymenoptera|Cynipidae|BOLD:AAB8335
Diplolepis radicum|[2784]|CNBPO033-13|Hymenoptera|Cynipidae|BOLD:AAB8334
Diplolepis|[2785]|CNSIB1553-15|Hymenoptera|Cynipidae|BOLD:ACT4889
Aulacidea pilosellae|[2786]|JSHYP286-11|Hymenoptera|Cynipidae|BOLD:AAU8720
Cynipidae|[2787]|HPPPC316-13|Hymenoptera|Cynipidae|BOLD:ACI3464
Aphelinidae|[2788]|CNBPR288-13|Hymenoptera|Aphelinidae|BOLD:ACJ0038
Cynipidae|[2789]|NGNAJ1094-13|Hymenoptera|Cynipidae|BOLD:ACJ0844
Cynipidae|[2790]|SSBRB5015-14|Hymenoptera|Cynipidae|BOLD:ACI2725
Charipinae|[2791]|SMTPJ2117-14|Hymenoptera|Figitidae|BOLD:AAU8573
Charipinae|[2792]|NGNAA346-13|Hymenoptera|Figitidae|BOLD:ACZ0669
Charipinae|[2793]|SSBAD042-12|Hymenoptera|Figitidae|BOLD:ACC4205
Charipinae|[2794]|SSBRC2614-14|Hymenoptera|Figitidae|BOLD:ACY6742

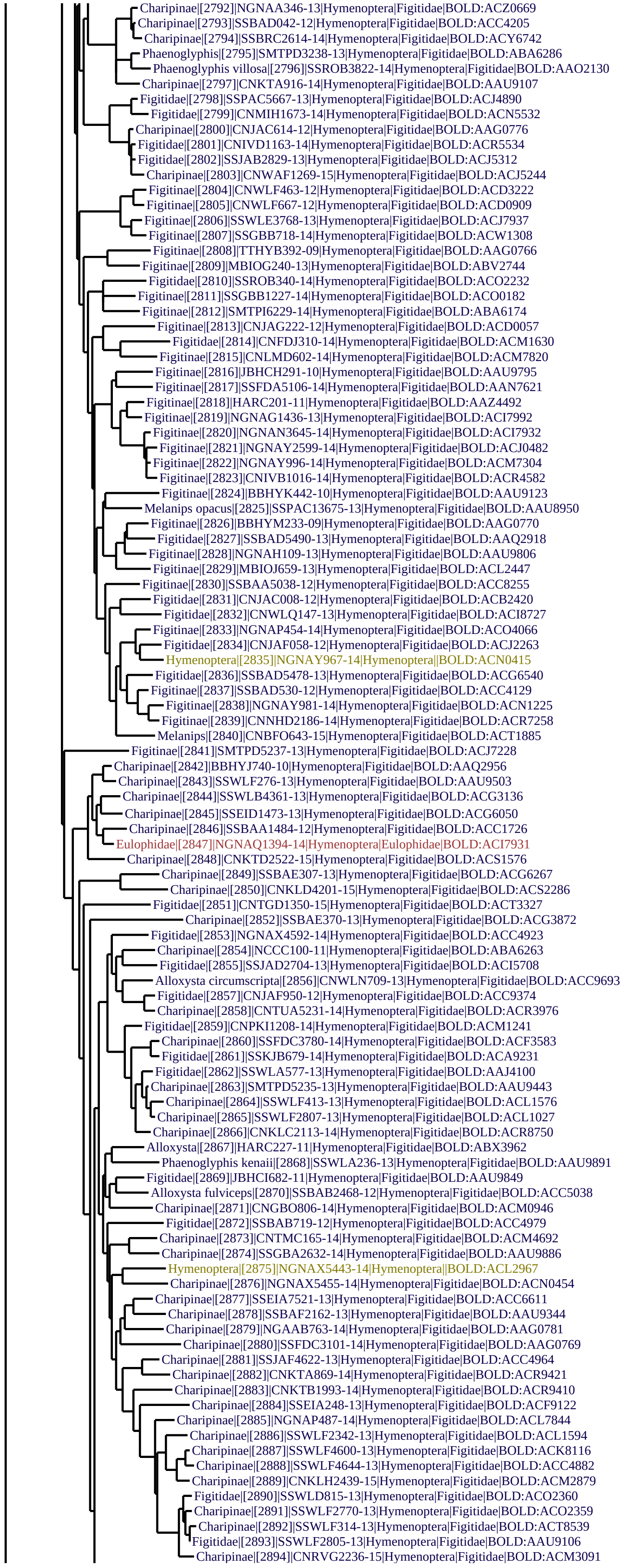
Charipinae|[2792]|NGNAA346-13|Hymenoptera|Figitidae|BOLD:ACZ0669
Charipinae|[2793]|SSBAD042-12|Hymenoptera|Figitidae|BOLD:ACC4205
Charipinae|[2794]|SSBRC2614-14|Hymenoptera|Figitidae|BOLD:ACY6742
Phaenoglyphis|[2795]|SMTPD3238-13|Hymenoptera|Figitidae|BOLD:ABA6286
Phaenoglyphis villosa|[2796]|SSROB3822-14|Hymenoptera|Figitidae|BOLD:AAO2130
Charipinae|[2797]|CNKTA916-14|Hymenoptera|Figitidae|BOLD:AAU9107
Figitidae|[2798]|SSPAC5667-13|Hymenoptera|Figitidae|BOLD:ACJ4890
Figitidae|[2799]|CNMIH1673-14|Hymenoptera|Figitidae|BOLD:ACN5532
Charipinae|[2800]|CNJAC614-12|Hymenoptera|Figitidae|BOLD:AAG0776
Figitidae|[2801]|CNIVD1163-14|Hymenoptera|Figitidae|BOLD:ACR5534
Figitidae|[2802]|SSJAB2829-13|Hymenoptera|Figitidae|BOLD:ACJ5312
Charipinae|[2803]|CNWAF1269-15|Hymenoptera|Figitidae|BOLD:ACJ5244
Figitinae|[2804]|CNWLF463-12|Hymenoptera|Figitidae|BOLD:ACD3222
Figitinae|[2805]|CNWLF667-12|Hymenoptera|Figitidae|BOLD:ACD0909
Figitinae|[2806]|SSWLE3768-13|Hymenoptera|Figitidae|BOLD:ACJ7937
Figitinae|[2807]|SSGBB718-14|Hymenoptera|Figitidae|BOLD:ACW1308
Figitinae|[2808]|TTHYB392-09|Hymenoptera|Figitidae|BOLD:AAG0766
Figitinae|[2809]|MBIOG240-13|Hymenoptera|Figitidae|BOLD:ABV2744
Figitidae|[2810]|SSROB340-14|Hymenoptera|Figitidae|BOLD:ACO2232
Figitinae|[2811]|SSGBB1227-14|Hymenoptera|Figitidae|BOLD:ACO0182
Figitinae|[2812]|SMTPI6229-14|Hymenoptera|Figitidae|BOLD:ABA6174
Figitinae|[2813]|CNJAG222-12|Hymenoptera|Figitidae|BOLD:ACD0057
Figitidae|[2814]|CNFDJ310-14|Hymenoptera|Figitidae|BOLD:ACM1630
Figitinae|[2815]|CNLMD602-14|Hymenoptera|Figitidae|BOLD:ACM7820
Figitinae|[2816]|JBHCH291-10|Hymenoptera|Figitidae|BOLD:AAU9795
Figitinae|[2817]|SSFDA5106-14|Hymenoptera|Figitidae|BOLD:AAN7621
Figitinae|[2818]|HARC201-11|Hymenoptera|Figitidae|BOLD:AAZ4492
Figitinae|[2819]|NGNAG1436-13|Hymenoptera|Figitidae|BOLD:ACI7992
Figitinae|[2820]|NGNAN3645-14|Hymenoptera|Figitidae|BOLD:ACI7932
Figitinae|[2821]|NGNAY2599-14|Hymenoptera|Figitidae|BOLD:ACJ0482
Figitinae|[2822]|NGNAY996-14|Hymenoptera|Figitidae|BOLD:ACM7304
Figitinae|[2823]|CNIVB1016-14|Hymenoptera|Figitidae|BOLD:ACR4582
Figitinae|[2824]|BBHYK442-10|Hymenoptera|Figitidae|BOLD:AAU9123
Melanips opacus|[2825]|SSPAC13675-13|Hymenoptera|Figitidae|BOLD:AAU8950
Figitinae|[2826]|BBHYM233-09|Hymenoptera|Figitidae|BOLD:AAG0770
Figitidae|[2827]|SSBAD5490-13|Hymenoptera|Figitidae|BOLD:AAQ2918
Figitinae|[2828]|NGNAH109-13|Hymenoptera|Figitidae|BOLD:AAU9806
Figitinae|[2829]|MBIOJ659-13|Hymenoptera|Figitidae|BOLD:ACL2447
Figitinae|[2830]|SSBAA5038-12|Hymenoptera|Figitidae|BOLD:ACC8255
Figitidae|[2831]|CNJAC008-12|Hymenoptera|Figitidae|BOLD:ACB2420
Figitidae|[2832]|CNWLQ147-13|Hymenoptera|Figitidae|BOLD:ACI8727
Figitinae|[2833]|NGNAP454-14|Hymenoptera|Figitidae|BOLD:ACO4066
Figitidae|[2834]|CNJAF058-12|Hymenoptera|Figitidae|BOLD:ACJ2263
Hymenoptera|[2835]|NGNAY967-14|Hymenoptera||BOLD:ACN0415
Figitidae|[2836]|SSBAD5478-13|Hymenoptera|Figitidae|BOLD:ACG6540
Figitinae|[2837]|SSBAD530-12|Hymenoptera|Figitidae|BOLD:ACC4129
Figitinae|[2838]|NGNAY981-14|Hymenoptera|Figitidae|BOLD:ACN1225
Figitinae|[2839]|CNNHD2186-14|Hymenoptera|Figitidae|BOLD:ACR7258
Melanips|[2840]|CNBFO643-15|Hymenoptera|Figitidae|BOLD:ACT1885
Figitinae|[2841]|SMTPD5237-13|Hymenoptera|Figitidae|BOLD:ACJ7228
Charipinae|[2842]|BBHYJ740-10|Hymenoptera|Figitidae|BOLD:AAQ2956
Charipinae|[2843]|SSWLF276-13|Hymenoptera|Figitidae|BOLD:AAU9503
Charipinae|[2844]|SSWLB4361-13|Hymenoptera|Figitidae|BOLD:ACG3136
Charipinae|[2845]|SSEID1473-13|Hymenoptera|Figitidae|BOLD:ACG6050
Charipinae|[2846]|SSBAA1484-12|Hymenoptera|Figitidae|BOLD:ACC1726
Eulophidae|[2847]|NGNAQ1394-14|Hymenoptera|Eulophidae|BOLD:ACI7931
Charipinae|[2848]|CNKTD2522-15|Hymenoptera|Figitidae|BOLD:ACS1576
Charipinae|[2849]|SSBAE307-13|Hymenoptera|Figitidae|BOLD:ACG6267
Charipinae|[2850]|CNKLD4201-15|Hymenoptera|Figitidae|BOLD:ACS2286
Figitidae|[2851]|CNTGD1350-15|Hymenoptera|Figitidae|BOLD:ACT3327
Charipinae|[2852]|SSBAE370-13|Hymenoptera|Figitidae|BOLD:ACG3872
Figitidae|[2853]|NGNAX4592-14|Hymenoptera|Figitidae|BOLD:ACC4923
Charipinae|[2854]|NCCC100-11|Hymenoptera|Figitidae|BOLD:ABA6263
Figitidae|[2855]|SSJAD2704-13|Hymenoptera|Figitidae|BOLD:ACI5708
Alloxysta circumscripta|[2856]|CNWLN709-13|Hymenoptera|Figitidae|BOLD:ACC9693
Figitidae|[2857]|CNJAF950-12|Hymenoptera|Figitidae|BOLD:ACC9374
Charipinae|[2858]|CNTUA5231-14|Hymenoptera|Figitidae|BOLD:ACR3976
Figitidae|[2859]|CNPKI1208-14|Hymenoptera|Figitidae|BOLD:ACM1241
Charipinae|[2860]|SSFDC3780-14|Hymenoptera|Figitidae|BOLD:ACF3583
Figitidae|[2861]|SSKJB679-14|Hymenoptera|Figitidae|BOLD:ACA9231
Figitidae|[2862]|SSWLA577-13|Hymenoptera|Figitidae|BOLD:AAJ4100
Charipinae|[2863]|SMTPD5235-13|Hymenoptera|Figitidae|BOLD:AAU9443
Charipinae|[2864]|SSWLF413-13|Hymenoptera|Figitidae|BOLD:ACL1576
Charipinae|[2865]|SSWLF2807-13|Hymenoptera|Figitidae|BOLD:ACL1027
Charipinae|[2866]|CNKLC2113-14|Hymenoptera|Figitidae|BOLD:ACR8750
Alloxysta|[2867]|HARC227-11|Hymenoptera|Figitidae|BOLD:ABX3962
Phaenoglyphis kenaii|[2868]|SSWLA236-13|Hymenoptera|Figitidae|BOLD:AAU9891
Figitidae|[2869]|JBHCI682-11|Hymenoptera|Figitidae|BOLD:AAU9849
Alloxysta fulviceps|[2870]|SSBAB2468-12|Hymenoptera|Figitidae|BOLD:ACC5038
Charipinae|[2871]|CNGBO806-14|Hymenoptera|Figitidae|BOLD:ACM0946
Figitidae|[2872]|SSBAB719-12|Hymenoptera|Figitidae|BOLD:ACC4979
Charipinae|[2873]|CNTMC165-14|Hymenoptera|Figitidae|BOLD:ACM4692
Charipinae|[2874]|SSGBA2632-14|Hymenoptera|Figitidae|BOLD:AAU9886
Hymenoptera|[2875]|NGNAX5443-14|Hymenoptera||BOLD:ACL2967
Charipinae|[2876]|NGNAX5455-14|Hymenoptera|Figitidae|BOLD:ACN0454
Charipinae|[2877]|SSEIA7521-13|Hymenoptera|Figitidae|BOLD:ACC6611
Charipinae|[2878]|SSBAF2162-13|Hymenoptera|Figitidae|BOLD:AAU9344
Charipinae|[2879]|NGAAB763-14|Hymenoptera|Figitidae|BOLD:AAG0781
Charipinae|[2880]|SSFDC3101-14|Hymenoptera|Figitidae|BOLD:AAG0769
Charipinae|[2881]|SSJAF4622-13|Hymenoptera|Figitidae|BOLD:ACC4964
Charipinae|[2882]|CNKTA869-14|Hymenoptera|Figitidae|BOLD:ACR9421
Charipinae|[2883]|CNKTB1993-14|Hymenoptera|Figitidae|BOLD:ACR9410
Charipinae|[2884]|SSEIA248-13|Hymenoptera|Figitidae|BOLD:ACF9122
Charipinae|[2885]|NGNAP487-14|Hymenoptera|Figitidae|BOLD:ACL7844
Charipinae|[2886]|SSWLF2342-13|Hymenoptera|Figitidae|BOLD:ACL1594
Charipinae|[2887]|SSWLF4600-13|Hymenoptera|Figitidae|BOLD:ACK8116
Charipinae|[2888]|SSWLF4644-13|Hymenoptera|Figitidae|BOLD:ACC4882
Charipinae|[2889]|CNKLH2439-15|Hymenoptera|Figitidae|BOLD:ACM2879
Figitidae|[2890]|SSWLD815-13|Hymenoptera|Figitidae|BOLD:ACO2360
Charipinae|[2891]|SSWLF2770-13|Hymenoptera|Figitidae|BOLD:ACO2359
Charipinae|[2892]|SSWLF314-13|Hymenoptera|Figitidae|BOLD:ACT8539
Figitidae|[2893]|SSWLF2805-13|Hymenoptera|Figitidae|BOLD:AAU9106
Charipinae|[2894]|CNRVG2236-15|Hymenoptera|Figitidae|BOLD:ACM3091

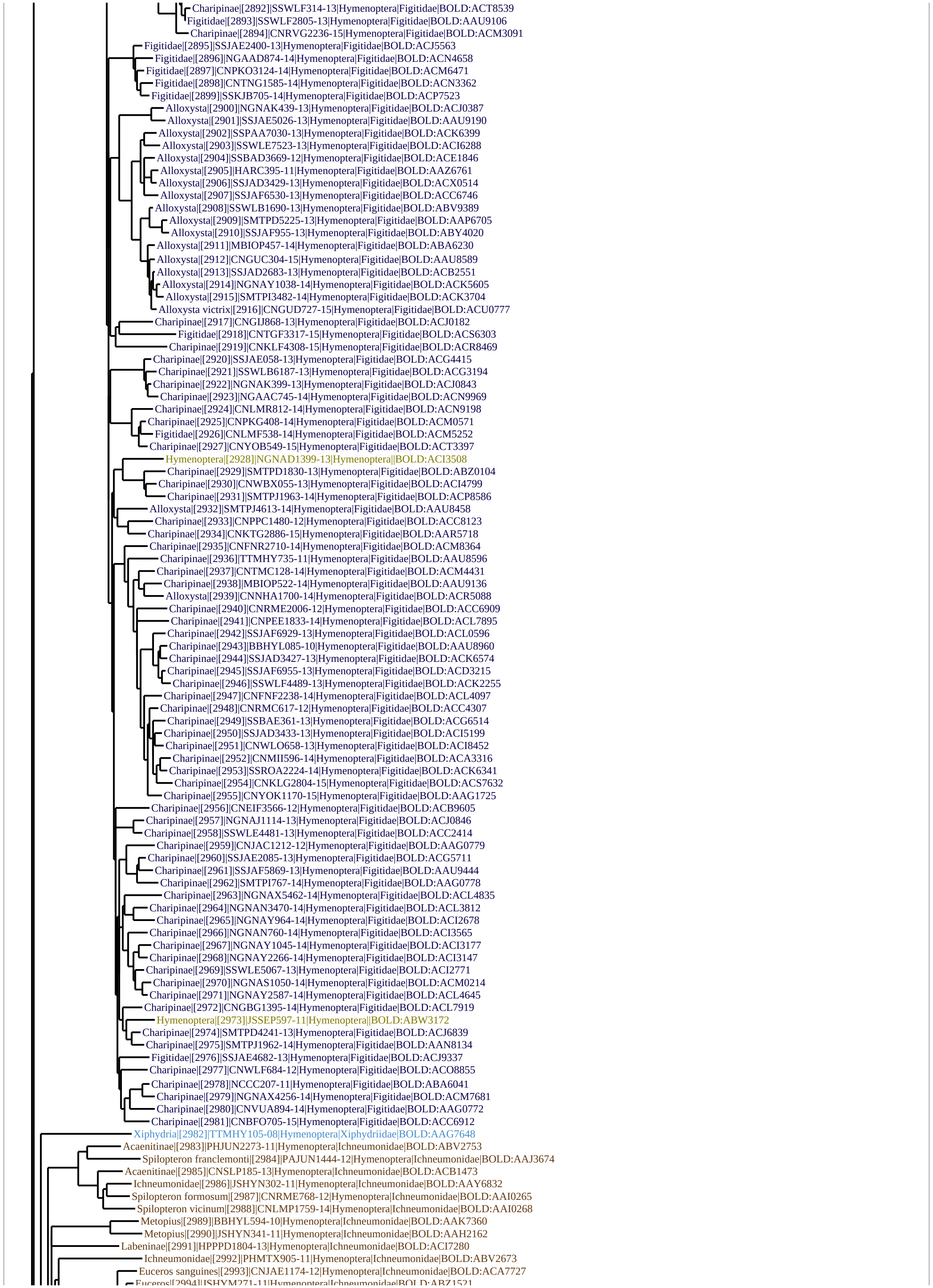
Charipinae|[2892]|SSWLF314-13|Hymenoptera|Figitidae|BOLD:ACT8539
Figitidae|[2893]|SSWLF2805-13|Hymenoptera|Figitidae|BOLD:AAU9106
Charipinae|[2894]|CNRVG2236-15|Hymenoptera|Figitidae|BOLD:ACM3091
Figitidae|[2895]|SSJAE2400-13|Hymenoptera|Figitidae|BOLD:ACJ5563
Figitidae|[2896]|NGAAD874-14|Hymenoptera|Figitidae|BOLD:ACN4658
Figitidae|[2897]|CNPKO3124-14|Hymenoptera|Figitidae|BOLD:ACM6471
Figitidae|[2898]|CNTNG1585-14|Hymenoptera|Figitidae|BOLD:ACN3362
Figitidae|[2899]|SSKJB705-14|Hymenoptera|Figitidae|BOLD:ACP7523
Alloxysta|[2900]|NGNAK439-13|Hymenoptera|Figitidae|BOLD:ACJ0387
Alloxysta|[2901]|SSJAE5026-13|Hymenoptera|Figitidae|BOLD:AAU9190
Alloxysta|[2902]|SSPAA7030-13|Hymenoptera|Figitidae|BOLD:ACK6399
Alloxysta|[2903]|SSWLE7523-13|Hymenoptera|Figitidae|BOLD:ACI6288
Alloxysta|[2904]|SSBAD3669-12|Hymenoptera|Figitidae|BOLD:ACE1846
Alloxysta|[2905]|HARC395-11|Hymenoptera|Figitidae|BOLD:AAZ6761
Alloxysta|[2906]|SSJAD3429-13|Hymenoptera|Figitidae|BOLD:ACX0514
Alloxysta|[2907]|SSJAF6530-13|Hymenoptera|Figitidae|BOLD:ACC6746
Alloxysta|[2908]|SSWLB1690-13|Hymenoptera|Figitidae|BOLD:ABV9389
Alloxysta|[2909]|SMTPD5225-13|Hymenoptera|Figitidae|BOLD:AAP6705
Alloxysta|[2910]|SSJAF955-13|Hymenoptera|Figitidae|BOLD:ABY4020
Alloxysta|[2911]|MBIOP457-14|Hymenoptera|Figitidae|BOLD:ABA6230
Alloxysta|[2912]|CNGUC304-15|Hymenoptera|Figitidae|BOLD:AAU8589
Alloxysta|[2913]|SSJAD2683-13|Hymenoptera|Figitidae|BOLD:ACB2551
Alloxysta|[2914]|NGNAY1038-14|Hymenoptera|Figitidae|BOLD:ACK5605
Alloxysta|[2915]|SMTPI3482-14|Hymenoptera|Figitidae|BOLD:ACK3704
Alloxysta victrix|[2916]|CNGUD727-15|Hymenoptera|Figitidae|BOLD:ACU0777
Charipinae|[2917]|CNGIJ868-13|Hymenoptera|Figitidae|BOLD:ACJ0182
Figitidae|[2918]|CNTGF3317-15|Hymenoptera|Figitidae|BOLD:ACS6303
Charipinae|[2919]|CNKLF4308-15|Hymenoptera|Figitidae|BOLD:ACR8469
Charipinae|[2920]|SSJAE058-13|Hymenoptera|Figitidae|BOLD:ACG4415
Charipinae|[2921]|SSWLB6187-13|Hymenoptera|Figitidae|BOLD:ACG3194
Charipinae|[2922]|NGNAK399-13|Hymenoptera|Figitidae|BOLD:ACJ0843
Charipinae|[2923]|NGAAC745-14|Hymenoptera|Figitidae|BOLD:ACN9969
Charipinae|[2924]|CNLMR812-14|Hymenoptera|Figitidae|BOLD:ACN9198
Charipinae|[2925]|CNPKG408-14|Hymenoptera|Figitidae|BOLD:ACM0571
Figitidae|[2926]|CNLMF538-14|Hymenoptera|Figitidae|BOLD:ACM5252
Charipinae|[2927]|CNYOB549-15|Hymenoptera|Figitidae|BOLD:ACT3397
Hymenoptera|[2928]|NGNAD1399-13|Hymenoptera||BOLD:ACI3508
Charipinae|[2929]|SMTPD1830-13|Hymenoptera|Figitidae|BOLD:ABZ0104
Charipinae|[2930]|CNWBX055-13|Hymenoptera|Figitidae|BOLD:ACI4799
Charipinae|[2931]|SMTPJ1963-14|Hymenoptera|Figitidae|BOLD:ACP8586
Alloxysta|[2932]|SMTPJ4613-14|Hymenoptera|Figitidae|BOLD:AAU8458
Charipinae|[2933]|CNPPC1480-12|Hymenoptera|Figitidae|BOLD:ACC8123
Charipinae|[2934]|CNKTG2886-15|Hymenoptera|Figitidae|BOLD:AAR5718
Charipinae|[2935]|CNFNR2710-14|Hymenoptera|Figitidae|BOLD:ACM8364
Charipinae|[2936]|TTMHY735-11|Hymenoptera|Figitidae|BOLD:AAU8596
Charipinae|[2937]|CNTMC128-14|Hymenoptera|Figitidae|BOLD:ACM4431
Charipinae|[2938]|MBIOP522-14|Hymenoptera|Figitidae|BOLD:AAU9136
Alloxysta|[2939]|CNNHA1700-14|Hymenoptera|Figitidae|BOLD:ACR5088
Charipinae|[2940]|CNRME2006-12|Hymenoptera|Figitidae|BOLD:ACC6909
Charipinae|[2941]|CNPEE1833-14|Hymenoptera|Figitidae|BOLD:ACL7895
Charipinae|[2942]|SSJAF6929-13|Hymenoptera|Figitidae|BOLD:ACL0596
Charipinae|[2943]|BBHYL085-10|Hymenoptera|Figitidae|BOLD:AAU8960
Charipinae|[2944]|SSJAD3427-13|Hymenoptera|Figitidae|BOLD:ACK6574
Charipinae|[2945]|SSJAF6955-13|Hymenoptera|Figitidae|BOLD:ACD3215
Charipinae|[2946]|SSWLF4489-13|Hymenoptera|Figitidae|BOLD:ACK2255
Charipinae|[2947]|CNFNF2238-14|Hymenoptera|Figitidae|BOLD:ACL4097
Charipinae|[2948]|CNRMC617-12|Hymenoptera|Figitidae|BOLD:ACC4307
Charipinae|[2949]|SSBAE361-13|Hymenoptera|Figitidae|BOLD:ACG6514
Charipinae|[2950]|SSJAD3433-13|Hymenoptera|Figitidae|BOLD:ACI5199
Charipinae|[2951]|CNWLO658-13|Hymenoptera|Figitidae|BOLD:ACI8452
Charipinae|[2952]|CNMII596-14|Hymenoptera|Figitidae|BOLD:ACA3316
Charipinae|[2953]|SSROA2224-14|Hymenoptera|Figitidae|BOLD:ACK6341
Charipinae|[2954]|CNKLG2804-15|Hymenoptera|Figitidae|BOLD:ACS7632
Charipinae|[2955]|CNYOK1170-15|Hymenoptera|Figitidae|BOLD:AAG1725
Charipinae|[2956]|CNEIF3566-12|Hymenoptera|Figitidae|BOLD:ACB9605
Charipinae|[2957]|NGNAJ1114-13|Hymenoptera|Figitidae|BOLD:ACJ0846
Charipinae|[2958]|SSWLE4481-13|Hymenoptera|Figitidae|BOLD:ACC2414
Charipinae|[2959]|CNJAC1212-12|Hymenoptera|Figitidae|BOLD:AAG0779
Charipinae|[2960]|SSJAE2085-13|Hymenoptera|Figitidae|BOLD:ACG5711
Charipinae|[2961]|SSJAF5869-13|Hymenoptera|Figitidae|BOLD:AAU9444
Charipinae|[2962]|SMTPI767-14|Hymenoptera|Figitidae|BOLD:AAG0778
Charipinae|[2963]|NGNAX5462-14|Hymenoptera|Figitidae|BOLD:ACL4835
Charipinae|[2964]|NGNAN3470-14|Hymenoptera|Figitidae|BOLD:ACL3812
Charipinae|[2965]|NGNAY964-14|Hymenoptera|Figitidae|BOLD:ACI2678
Charipinae|[2966]|NGNAN760-14|Hymenoptera|Figitidae|BOLD:ACI3565
Charipinae|[2967]|NGNAY1045-14|Hymenoptera|Figitidae|BOLD:ACI3177
Charipinae|[2968]|NGNAY2266-14|Hymenoptera|Figitidae|BOLD:ACI3147
Charipinae|[2969]|SSWLE5067-13|Hymenoptera|Figitidae|BOLD:ACI2771
Charipinae|[2970]|NGNAS1050-14|Hymenoptera|Figitidae|BOLD:ACM0214
Charipinae|[2971]|NGNAY2587-14|Hymenoptera|Figitidae|BOLD:ACL4645
Charipinae|[2972]|CNGBG1395-14|Hymenoptera|Figitidae|BOLD:ACL7919
Hymenoptera|[2973]|JSSEP597-11|Hymenoptera||BOLD:ABW3172
Charipinae|[2974]|SMTPD4241-13|Hymenoptera|Figitidae|BOLD:ACJ6839
Charipinae|[2975]|SMTPJ1962-14|Hymenoptera|Figitidae|BOLD:AAN8134
Figitidae|[2976]|SSJAE4682-13|Hymenoptera|Figitidae|BOLD:ACJ9337
Charipinae|[2977]|CNWLF684-12|Hymenoptera|Figitidae|BOLD:ACO8855
Charipinae|[2978]|NCCC207-11|Hymenoptera|Figitidae|BOLD:ABA6041
Charipinae|[2979]|NGNAX4256-14|Hymenoptera|Figitidae|BOLD:ACM7681
Charipinae|[2980]|CNVUA894-14|Hymenoptera|Figitidae|BOLD:AAG0772
Charipinae|[2981]|CNBFO705-15|Hymenoptera|Figitidae|BOLD:ACC6912
Xiphydria|[2982]|TTMHY105-08|Hymenoptera|Xiphydriidae|BOLD:AAG7648
Acaenitinae|[2983]|PHJUN2273-11|Hymenoptera|Ichneumonidae|BOLD:ABV2753
Spilopteron franclemonti|[2984]|PAJUN1444-12|Hymenoptera|Ichneumonidae|BOLD:AAJ3674
Acaenitinae|[2985]|CNSLP185-13|Hymenoptera|Ichneumonidae|BOLD:ACB1473
Ichneumonidae|[2986]|JSHYN302-11|Hymenoptera|Ichneumonidae|BOLD:AAY6832
Spilopteron formosum|[2987]|CNRME768-12|Hymenoptera|Ichneumonidae|BOLD:AAI0265
Spilopteron vicinum|[2988]|CNLMP1759-14|Hymenoptera|Ichneumonidae|BOLD:AAI0268
Metopius|[2989]|BBHYL594-10|Hymenoptera|Ichneumonidae|BOLD:AAK7360
Metopius|[2990]|JSHYN341-11|Hymenoptera|Ichneumonidae|BOLD:AAH2162
Labeninae|[2991]|HPPPD1804-13|Hymenoptera|Ichneumonidae|BOLD:ACI7280
Ichneumonidae|[2992]|PHMTX905-11|Hymenoptera|Ichneumonidae|BOLD:ABV2673
Euceros sanguines|[2993]|CNJAE1174-12|Hymenoptera|Ichneumonidae|BOLD:ACA7727
Euceros|[2994]|JSHYM271-11|Hymenoptera|Ichneumonidae|BOLD:ABZ1521

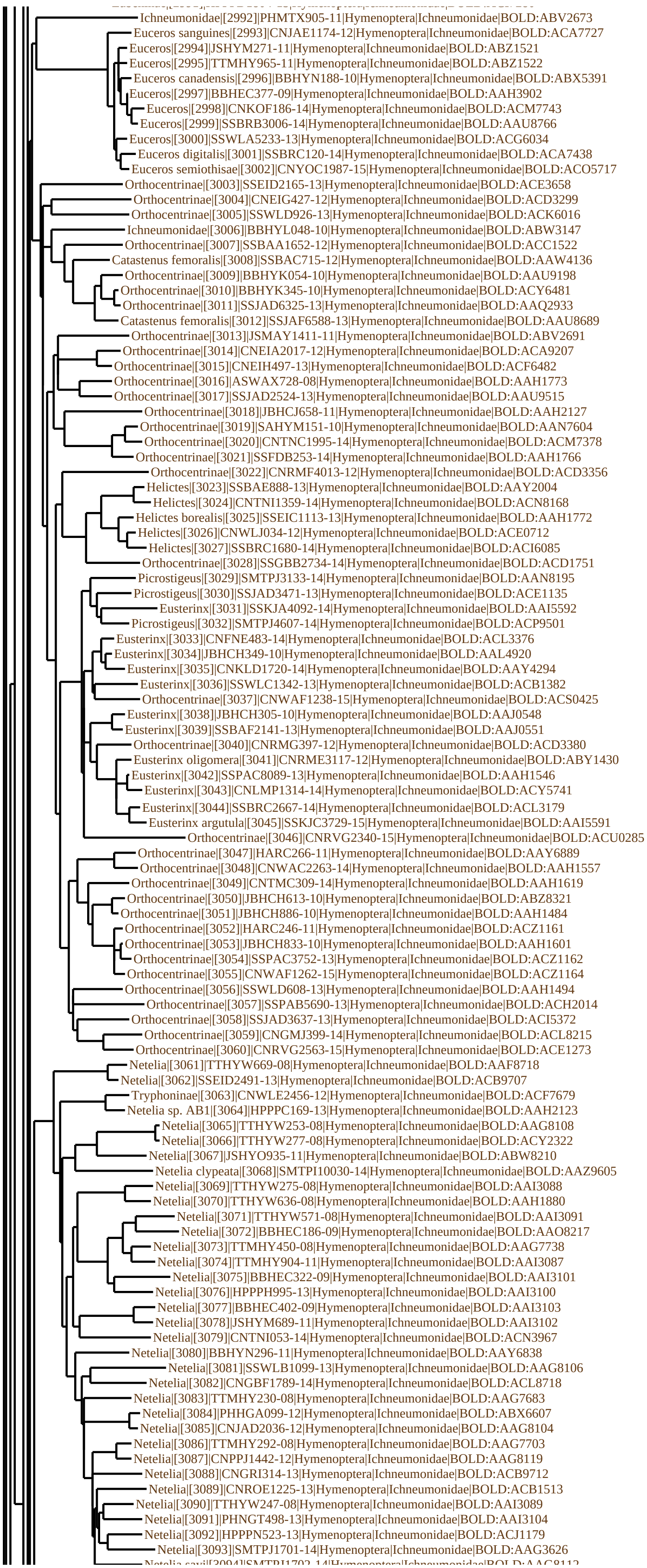
Ichneumonidae|[2992]|PHMTX905-11|Hymenoptera|Ichneumonidae|BOLD:ABV2673
Euceros sanguines|[2993]|CNJAE1174-12|Hymenoptera|Ichneumonidae|BOLD:ACA7727
Euceros|[2994]|JSHYM271-11|Hymenoptera|Ichneumonidae|BOLD:ABZ1521
Euceros|[2995]|TTMHY965-11|Hymenoptera|Ichneumonidae|BOLD:ABZ1522
Euceros canadensis|[2996]|BBHYN188-10|Hymenoptera|Ichneumonidae|BOLD:ABX5391
Euceros|[2997]|BBHEC377-09|Hymenoptera|Ichneumonidae|BOLD:AAH3902
Euceros|[2998]|CNKOF186-14|Hymenoptera|Ichneumonidae|BOLD:ACM7743
Euceros|[2999]|SSBRB3006-14|Hymenoptera|Ichneumonidae|BOLD:AAU8766
Euceros|[3000]|SSWLA5233-13|Hymenoptera|Ichneumonidae|BOLD:ACG6034
Euceros digitalis|[3001]|SSBRC120-14|Hymenoptera|Ichneumonidae|BOLD:ACA7438
Euceros semiothisae|[3002]|CNYOC1987-15|Hymenoptera|Ichneumonidae|BOLD:ACO5717
Orthocentrinae|[3003]|SSEID2165-13|Hymenoptera|Ichneumonidae|BOLD:ACE3658
Orthocentrinae|[3004]|CNEIG427-12|Hymenoptera|Ichneumonidae|BOLD:ACD3299
Orthocentrinae|[3005]|SSWLD926-13|Hymenoptera|Ichneumonidae|BOLD:ACK6016
Ichneumonidae|[3006]|BBHYL048-10|Hymenoptera|Ichneumonidae|BOLD:ABW3147
Orthocentrinae|[3007]|SSBAA1652-12|Hymenoptera|Ichneumonidae|BOLD:ACC1522
Catastenus femoralis|[3008]|SSBAC715-12|Hymenoptera|Ichneumonidae|BOLD:AAW4136
Orthocentrinae|[3009]|BBHYK054-10|Hymenoptera|Ichneumonidae|BOLD:AAU9198
Orthocentrinae|[3010]|BBHYK345-10|Hymenoptera|Ichneumonidae|BOLD:ACY6481
Orthocentrinae|[3011]|SSJAD6325-13|Hymenoptera|Ichneumonidae|BOLD:AAQ2933
Catastenus femoralis|[3012]|SSJAF6588-13|Hymenoptera|Ichneumonidae|BOLD:AAU8689
Orthocentrinae|[3013]|JSMAY1411-11|Hymenoptera|Ichneumonidae|BOLD:ABV2691
Orthocentrinae|[3014]|CNEIA2017-12|Hymenoptera|Ichneumonidae|BOLD:ACA9207
Orthocentrinae|[3015]|CNEIH497-13|Hymenoptera|Ichneumonidae|BOLD:ACF6482
Orthocentrinae|[3016]|ASWAX728-08|Hymenoptera|Ichneumonidae|BOLD:AAH1773
Orthocentrinae|[3017]|SSJAD2524-13|Hymenoptera|Ichneumonidae|BOLD:AAU9515
Orthocentrinae|[3018]|JBHCJ658-11|Hymenoptera|Ichneumonidae|BOLD:AAH2127
Orthocentrinae|[3019]|SAHYM151-10|Hymenoptera|Ichneumonidae|BOLD:AAN7604
Orthocentrinae|[3020]|CNTNC1995-14|Hymenoptera|Ichneumonidae|BOLD:ACM7378
Orthocentrinae|[3021]|SSFDB253-14|Hymenoptera|Ichneumonidae|BOLD:AAH1766
Orthocentrinae|[3022]|CNRMF4013-12|Hymenoptera|Ichneumonidae|BOLD:ACD3356
Helictes|[3023]|SSBAE888-13|Hymenoptera|Ichneumonidae|BOLD:AAY2004
Helictes|[3024]|CNTNI1359-14|Hymenoptera|Ichneumonidae|BOLD:ACN8168
Helictes borealis|[3025]|SSEIC1113-13|Hymenoptera|Ichneumonidae|BOLD:AAH1772
Helictes|[3026]|CNWLJ034-12|Hymenoptera|Ichneumonidae|BOLD:ACE0712
Helictes|[3027]|SSBRC1680-14|Hymenoptera|Ichneumonidae|BOLD:ACI6085
Orthocentrinae|[3028]|SSGBB2734-14|Hymenoptera|Ichneumonidae|BOLD:ACD1751
Picrostigeus|[3029]|SMTPJ3133-14|Hymenoptera|Ichneumonidae|BOLD:AAN8195
Picrostigeus|[3030]|SSJAD3471-13|Hymenoptera|Ichneumonidae|BOLD:ACE1135
Eusterinx|[3031]|SSKJA4092-14|Hymenoptera|Ichneumonidae|BOLD:AAI5592
Picrostigeus|[3032]|SMTPJ4607-14|Hymenoptera|Ichneumonidae|BOLD:ACP9501
Eusterinx|[3033]|CNFNE483-14|Hymenoptera|Ichneumonidae|BOLD:ACL3376
Eusterinx|[3034]|JBHCH349-10|Hymenoptera|Ichneumonidae|BOLD:AAL4920
Eusterinx|[3035]|CNKLD1720-14|Hymenoptera|Ichneumonidae|BOLD:AAY4294
Eusterinx|[3036]|SSWLC1342-13|Hymenoptera|Ichneumonidae|BOLD:ACB1382
Orthocentrinae|[3037]|CNWAF1238-15|Hymenoptera|Ichneumonidae|BOLD:ACS0425
Eusterinx|[3038]|JBHCH305-10|Hymenoptera|Ichneumonidae|BOLD:AAJ0548
Eusterinx|[3039]|SSBAF2141-13|Hymenoptera|Ichneumonidae|BOLD:AAJ0551
Orthocentrinae|[3040]|CNRMG397-12|Hymenoptera|Ichneumonidae|BOLD:ACD3380
Eusterinx oligomera|[3041]|CNRME3117-12|Hymenoptera|Ichneumonidae|BOLD:ABY1430
Eusterinx|[3042]|SSPAC8089-13|Hymenoptera|Ichneumonidae|BOLD:AAH1546
Eusterinx|[3043]|CNLMP1314-14|Hymenoptera|Ichneumonidae|BOLD:ACY5741
Eusterinx|[3044]|SSBRC2667-14|Hymenoptera|Ichneumonidae|BOLD:ACL3179
Eusterinx argutula|[3045]|SSKJC3729-15|Hymenoptera|Ichneumonidae|BOLD:AAI5591
Orthocentrinae|[3046]|CNRVG2340-15|Hymenoptera|Ichneumonidae|BOLD:ACU0285
Orthocentrinae|[3047]|HARC266-11|Hymenoptera|Ichneumonidae|BOLD:AAY6889
Orthocentrinae|[3048]|CNWAC2263-14|Hymenoptera|Ichneumonidae|BOLD:AAH1557
Orthocentrinae|[3049]|CNTMC309-14|Hymenoptera|Ichneumonidae|BOLD:AAH1619
Orthocentrinae|[3050]|JBHCH613-10|Hymenoptera|Ichneumonidae|BOLD:ABZ8321
Orthocentrinae|[3051]|JBHCH886-10|Hymenoptera|Ichneumonidae|BOLD:AAH1484
Orthocentrinae|[3052]|HARC246-11|Hymenoptera|Ichneumonidae|BOLD:ACZ1161
Orthocentrinae|[3053]|JBHCH833-10|Hymenoptera|Ichneumonidae|BOLD:AAH1601
Orthocentrinae|[3054]|SSPAC3752-13|Hymenoptera|Ichneumonidae|BOLD:ACZ1162
Orthocentrinae|[3055]|CNWAF1262-15|Hymenoptera|Ichneumonidae|BOLD:ACZ1164
Orthocentrinae|[3056]|SSWLD608-13|Hymenoptera|Ichneumonidae|BOLD:AAH1494
Orthocentrinae|[3057]|SSPAB5690-13|Hymenoptera|Ichneumonidae|BOLD:ACH2014
Orthocentrinae|[3058]|SSJAD3637-13|Hymenoptera|Ichneumonidae|BOLD:ACI5372
Orthocentrinae|[3059]|CNGMJ399-14|Hymenoptera|Ichneumonidae|BOLD:ACL8215
Orthocentrinae|[3060]|CNRVG2563-15|Hymenoptera|Ichneumonidae|BOLD:ACE1273
Netelia|[3061]|TTHYW669-08|Hymenoptera|Ichneumonidae|BOLD:AAF8718
Netelia|[3062]|SSEID2491-13|Hymenoptera|Ichneumonidae|BOLD:ACB9707
Tryphoninae|[3063]|CNWLE2456-12|Hymenoptera|Ichneumonidae|BOLD:ACF7679
Netelia sp. AB1|[3064]|HPPPC169-13|Hymenoptera|Ichneumonidae|BOLD:AAH2123
Netelia|[3065]|TTHYW253-08|Hymenoptera|Ichneumonidae|BOLD:AAG8108
Netelia|[3066]|TTHYW277-08|Hymenoptera|Ichneumonidae|BOLD:ACY2322
Netelia|[3067]|JSHYO935-11|Hymenoptera|Ichneumonidae|BOLD:ABW8210
Netelia clypeata|[3068]|SMTPI10030-14|Hymenoptera|Ichneumonidae|BOLD:AAZ9605
Netelia|[3069]|TTHYW275-08|Hymenoptera|Ichneumonidae|BOLD:AAI3088
Netelia|[3070]|TTHYW636-08|Hymenoptera|Ichneumonidae|BOLD:AAH1880
Netelia|[3071]|TTHYW571-08|Hymenoptera|Ichneumonidae|BOLD:AAI3091
Netelia|[3072]|BBHEC186-09|Hymenoptera|Ichneumonidae|BOLD:AAO8217
Netelia|[3073]|TTMHY450-08|Hymenoptera|Ichneumonidae|BOLD:AAG7738
Netelia|[3074]|TTMHY904-11|Hymenoptera|Ichneumonidae|BOLD:AAI3087
Netelia|[3075]|BBHEC322-09|Hymenoptera|Ichneumonidae|BOLD:AAI3101
Netelia|[3076]|HPPPH995-13|Hymenoptera|Ichneumonidae|BOLD:AAI3100
Netelia|[3077]|BBHEC402-09|Hymenoptera|Ichneumonidae|BOLD:AAI3103
Netelia|[3078]|JSHYM689-11|Hymenoptera|Ichneumonidae|BOLD:AAI3102
Netelia|[3079]|CNTNI053-14|Hymenoptera|Ichneumonidae|BOLD:ACN3967
Netelia|[3080]|BBHYN296-11|Hymenoptera|Ichneumonidae|BOLD:AAY6838
Netelia|[3081]|SSWLB1099-13|Hymenoptera|Ichneumonidae|BOLD:AAG8106
Netelia|[3082]|CNGBF1789-14|Hymenoptera|Ichneumonidae|BOLD:ACL8718
Netelia|[3083]|TTMHY230-08|Hymenoptera|Ichneumonidae|BOLD:AAG7683
Netelia|[3084]|PHHGA099-12|Hymenoptera|Ichneumonidae|BOLD:ABX6607
Netelia|[3085]|CNJAD2036-12|Hymenoptera|Ichneumonidae|BOLD:AAG8104
Netelia|[3086]|TTMHY292-08|Hymenoptera|Ichneumonidae|BOLD:AAG7703
Netelia|[3087]|CNPPJ1442-12|Hymenoptera|Ichneumonidae|BOLD:AAG8119
Netelia|[3088]|CNGRI314-13|Hymenoptera|Ichneumonidae|BOLD:ACB9712
Netelia|[3089]|CNROE1225-13|Hymenoptera|Ichneumonidae|BOLD:ACB1513
Netelia|[3090]|TTHYW247-08|Hymenoptera|Ichneumonidae|BOLD:AAI3089
Netelia|[3091]|PHNGT498-13|Hymenoptera|Ichneumonidae|BOLD:AAI3104
Netelia|[3092]|HPPPN523-13|Hymenoptera|Ichneumonidae|BOLD:ACJ1179
Netelia|[3093]|SMTPJ1701-14|Hymenoptera|Ichneumonidae|BOLD:AAG3626

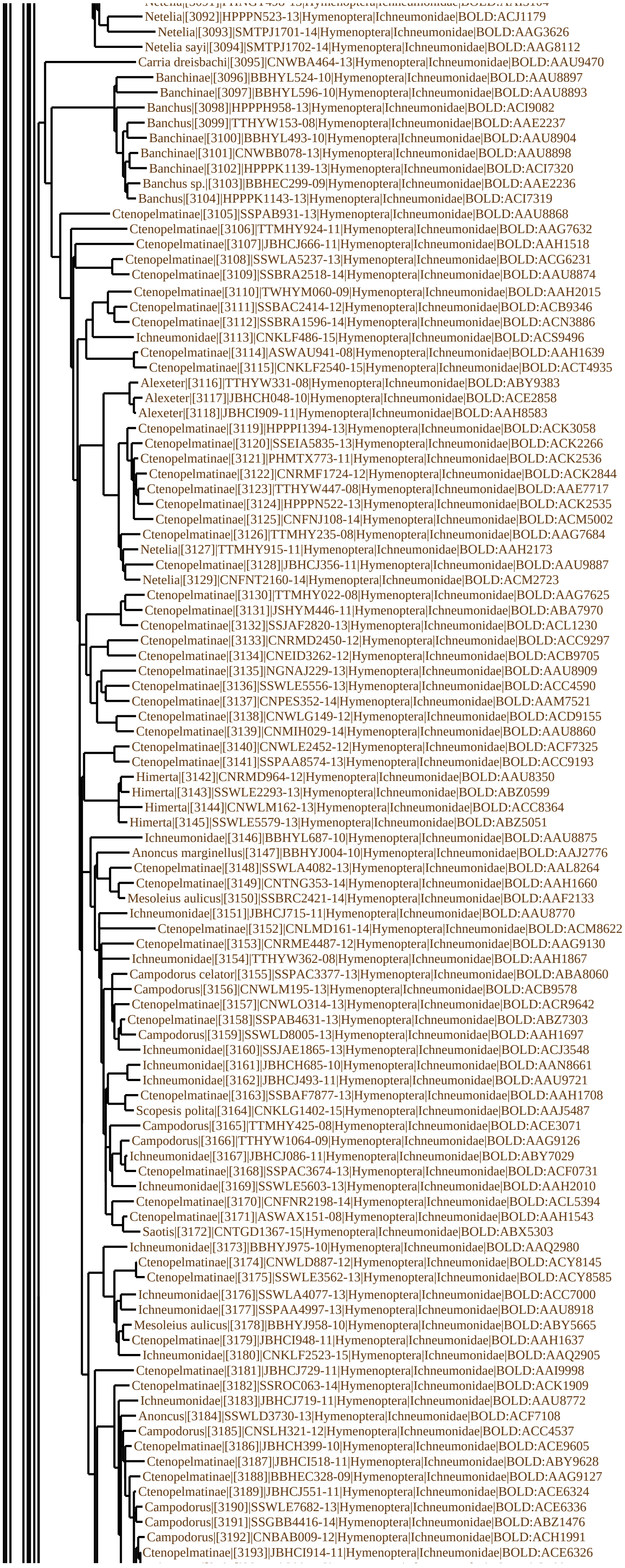
Netelia|[3092]|HPPPN523-13|Hymenoptera|Ichneumonidae|BOLD:ACJ1179
Netelia|[3093]|SMTPJ1701-14|Hymenoptera|Ichneumonidae|BOLD:AAG3626
Netelia sayi|[3094]|SMTPJ1702-14|Hymenoptera|Ichneumonidae|BOLD:AAG8112
Carria dreisbachi|[3095]|CNWBA464-13|Hymenoptera|Ichneumonidae|BOLD:AAU9470
Banchinae|[3096]|BBHYL524-10|Hymenoptera|Ichneumonidae|BOLD:AAU8897
Banchinae|[3097]|BBHYL596-10|Hymenoptera|Ichneumonidae|BOLD:AAU8893
Banchus|[3098]|HPPPH958-13|Hymenoptera|Ichneumonidae|BOLD:ACI9082
Banchus|[3099]|TTHYW153-08|Hymenoptera|Ichneumonidae|BOLD:AAE2237
Banchinae|[3100]|BBHYL493-10|Hymenoptera|Ichneumonidae|BOLD:AAU8904
Banchinae|[3101]|CNWBB078-13|Hymenoptera|Ichneumonidae|BOLD:AAU8898
Banchinae|[3102]|HPPPK1139-13|Hymenoptera|Ichneumonidae|BOLD:ACI7320
Banchus sp.|[3103]|BBHEC299-09|Hymenoptera|Ichneumonidae|BOLD:AAE2236
Banchus|[3104]|HPPPK1143-13|Hymenoptera|Ichneumonidae|BOLD:ACI7319
Ctenopelmatinae|[3105]|SSPAB931-13|Hymenoptera|Ichneumonidae|BOLD:AAU8868
Ctenopelmatinae|[3106]|TTMHY924-11|Hymenoptera|Ichneumonidae|BOLD:AAG7632
Ctenopelmatinae|[3107]|JBHCJ666-11|Hymenoptera|Ichneumonidae|BOLD:AAH1518
Ctenopelmatinae|[3108]|SSWLA5237-13|Hymenoptera|Ichneumonidae|BOLD:ACG6231
Ctenopelmatinae|[3109]|SSBRA2518-14|Hymenoptera|Ichneumonidae|BOLD:AAU8874
Ctenopelmatinae|[3110]|TWHYM060-09|Hymenoptera|Ichneumonidae|BOLD:AAH2015
Ctenopelmatinae|[3111]|SSBAC2414-12|Hymenoptera|Ichneumonidae|BOLD:ACB9346
Ctenopelmatinae|[3112]|SSBRA1596-14|Hymenoptera|Ichneumonidae|BOLD:ACN3886
Ichneumonidae|[3113]|CNKLF486-15|Hymenoptera|Ichneumonidae|BOLD:ACS9496
Ctenopelmatinae|[3114]|ASWAU941-08|Hymenoptera|Ichneumonidae|BOLD:AAH1639
Ctenopelmatinae|[3115]|CNKLF2540-15|Hymenoptera|Ichneumonidae|BOLD:ACT4935
Alexeter|[3116]|TTHYW331-08|Hymenoptera|Ichneumonidae|BOLD:ABY9383
Alexeter|[3117]|JBHCH048-10|Hymenoptera|Ichneumonidae|BOLD:ACE2858
Alexeter|[3118]|JBHCI909-11|Hymenoptera|Ichneumonidae|BOLD:AAH8583
Ctenopelmatinae|[3119]|HPPPI1394-13|Hymenoptera|Ichneumonidae|BOLD:ACK3058
Ctenopelmatinae|[3120]|SSEIA5835-13|Hymenoptera|Ichneumonidae|BOLD:ACK2266
Ctenopelmatinae|[3121]|PHMTX773-11|Hymenoptera|Ichneumonidae|BOLD:ACK2536
Ctenopelmatinae|[3122]|CNRMF1724-12|Hymenoptera|Ichneumonidae|BOLD:ACK2844
Ctenopelmatinae|[3123]|TTHYW447-08|Hymenoptera|Ichneumonidae|BOLD:AAE7717
Ctenopelmatinae|[3124]|HPPPN522-13|Hymenoptera|Ichneumonidae|BOLD:ACK2535
Ctenopelmatinae|[3125]|CNFNJ108-14|Hymenoptera|Ichneumonidae|BOLD:ACM5002
Ctenopelmatinae|[3126]|TTMHY235-08|Hymenoptera|Ichneumonidae|BOLD:AAG7684
Netelia|[3127]|TTMHY915-11|Hymenoptera|Ichneumonidae|BOLD:AAH2173
Ctenopelmatinae|[3128]|JBHCJ356-11|Hymenoptera|Ichneumonidae|BOLD:AAU9887
Netelia|[3129]|CNFNT2160-14|Hymenoptera|Ichneumonidae|BOLD:ACM2723
Ctenopelmatinae|[3130]|TTMHY022-08|Hymenoptera|Ichneumonidae|BOLD:AAG7625
Ctenopelmatinae|[3131]|JSHYM446-11|Hymenoptera|Ichneumonidae|BOLD:ABA7970
Ctenopelmatinae|[3132]|SSJAF2820-13|Hymenoptera|Ichneumonidae|BOLD:ACL1230
Ctenopelmatinae|[3133]|CNRMD2450-12|Hymenoptera|Ichneumonidae|BOLD:ACC9297
Ctenopelmatinae|[3134]|CNEID3262-12|Hymenoptera|Ichneumonidae|BOLD:ACB9705
Ctenopelmatinae|[3135]|NGNAJ229-13|Hymenoptera|Ichneumonidae|BOLD:AAU8909
Ctenopelmatinae|[3136]|SSWLE5556-13|Hymenoptera|Ichneumonidae|BOLD:ACC4590
Ctenopelmatinae|[3137]|CNPES352-14|Hymenoptera|Ichneumonidae|BOLD:AAM7521
Ctenopelmatinae|[3138]|CNWLG149-12|Hymenoptera|Ichneumonidae|BOLD:ACD9155
Ctenopelmatinae|[3139]|CNMIH029-14|Hymenoptera|Ichneumonidae|BOLD:AAU8860
Ctenopelmatinae|[3140]|CNWLE2452-12|Hymenoptera|Ichneumonidae|BOLD:ACF7325
Ctenopelmatinae|[3141]|SSPAA8574-13|Hymenoptera|Ichneumonidae|BOLD:ACC9193
Himerta|[3142]|CNRMD964-12|Hymenoptera|Ichneumonidae|BOLD:AAU8350
Himerta|[3143]|SSWLE2293-13|Hymenoptera|Ichneumonidae|BOLD:ABZ0599
Himerta|[3144]|CNWLM162-13|Hymenoptera|Ichneumonidae|BOLD:ACC8364
Himerta|[3145]|SSWLE5579-13|Hymenoptera|Ichneumonidae|BOLD:ABZ5051
Ichneumonidae|[3146]|BBHYL687-10|Hymenoptera|Ichneumonidae|BOLD:AAU8875
Anoncus marginellus|[3147]|BBHYJ004-10|Hymenoptera|Ichneumonidae|BOLD:AAJ2776
Ctenopelmatinae|[3148]|SSWLA4082-13|Hymenoptera|Ichneumonidae|BOLD:AAL8264
Ctenopelmatinae|[3149]|CNTNG353-14|Hymenoptera|Ichneumonidae|BOLD:AAH1660
Mesoleius aulicus|[3150]|SSBRC2421-14|Hymenoptera|Ichneumonidae|BOLD:AAF2133
Ichneumonidae|[3151]|JBHCJ715-11|Hymenoptera|Ichneumonidae|BOLD:AAU8770
Ctenopelmatinae|[3152]|CNLMD161-14|Hymenoptera|Ichneumonidae|BOLD:ACM8622
Ctenopelmatinae|[3153]|CNRME4487-12|Hymenoptera|Ichneumonidae|BOLD:AAG9130
Ichneumonidae|[3154]|TTHYW362-08|Hymenoptera|Ichneumonidae|BOLD:AAH1867
Campodorus celator|[3155]|SSPAC3377-13|Hymenoptera|Ichneumonidae|BOLD:ABA8060
Campodorus|[3156]|CNWLM195-13|Hymenoptera|Ichneumonidae|BOLD:ACB9578
Ctenopelmatinae|[3157]|CNWLO314-13|Hymenoptera|Ichneumonidae|BOLD:ACR9642
Ctenopelmatinae|[3158]|SSPAB4631-13|Hymenoptera|Ichneumonidae|BOLD:ABZ7303
Campodorus|[3159]|SSWLD8005-13|Hymenoptera|Ichneumonidae|BOLD:AAH1697
Ichneumonidae|[3160]|SSJAE1865-13|Hymenoptera|Ichneumonidae|BOLD:ACJ3548
Ichneumonidae|[3161]|JBHCH685-10|Hymenoptera|Ichneumonidae|BOLD:AAN8661
Ichneumonidae|[3162]|JBHCJ493-11|Hymenoptera|Ichneumonidae|BOLD:AAU9721
Ctenopelmatinae|[3163]|SSBAF7877-13|Hymenoptera|Ichneumonidae|BOLD:AAH1708
Scopesis polita|[3164]|CNKLG1402-15|Hymenoptera|Ichneumonidae|BOLD:AAJ5487
Campodorus|[3165]|TTMHY425-08|Hymenoptera|Ichneumonidae|BOLD:ACE3071
Campodorus|[3166]|TTHYW1064-09|Hymenoptera|Ichneumonidae|BOLD:AAG9126
Ichneumonidae|[3167]|JBHCJ086-11|Hymenoptera|Ichneumonidae|BOLD:ABY7029
Ctenopelmatinae|[3168]|SSPAC3674-13|Hymenoptera|Ichneumonidae|BOLD:ACF0731
Ichneumonidae|[3169]|SSWLE5603-13|Hymenoptera|Ichneumonidae|BOLD:AAH2010
Ctenopelmatinae|[3170]|CNFNR2198-14|Hymenoptera|Ichneumonidae|BOLD:ACL5394
Ctenopelmatinae|[3171]|ASWAX151-08|Hymenoptera|Ichneumonidae|BOLD:AAH1543
Saotis|[3172]|CNTGD1367-15|Hymenoptera|Ichneumonidae|BOLD:ABX5303
Ichneumonidae|[3173]|BBHYJ975-10|Hymenoptera|Ichneumonidae|BOLD:AAQ2980
Ctenopelmatinae|[3174]|CNWLD887-12|Hymenoptera|Ichneumonidae|BOLD:ACY8145
Ctenopelmatinae|[3175]|SSWLE3562-13|Hymenoptera|Ichneumonidae|BOLD:ACY8585
Ichneumonidae|[3176]|SSWLA4077-13|Hymenoptera|Ichneumonidae|BOLD:ACC7000
Ichneumonidae|[3177]|SSPAA4997-13|Hymenoptera|Ichneumonidae|BOLD:AAU8918
Mesoleius aulicus|[3178]|BBHYJ958-10|Hymenoptera|Ichneumonidae|BOLD:ABY5665
Ctenopelmatinae|[3179]|JBHCI948-11|Hymenoptera|Ichneumonidae|BOLD:AAH1637
Ichneumonidae|[3180]|CNKLF2523-15|Hymenoptera|Ichneumonidae|BOLD:AAQ2905
Ctenopelmatinae|[3181]|JBHCJ729-11|Hymenoptera|Ichneumonidae|BOLD:AAI9998
Ctenopelmatinae|[3182]|SSROC063-14|Hymenoptera|Ichneumonidae|BOLD:ACK1909
Ichneumonidae|[3183]|JBHCJ719-11|Hymenoptera|Ichneumonidae|BOLD:AAU8772
Anoncus|[3184]|SSWLD3730-13|Hymenoptera|Ichneumonidae|BOLD:ACF7108
Campodorus|[3185]|CNSLH321-12|Hymenoptera|Ichneumonidae|BOLD:ACC4537
Ctenopelmatinae|[3186]|JBHCH399-10|Hymenoptera|Ichneumonidae|BOLD:ACE9605
Ctenopelmatinae|[3187]|JBHCI518-11|Hymenoptera|Ichneumonidae|BOLD:ABY9628
Ctenopelmatinae|[3188]|BBHEC328-09|Hymenoptera|Ichneumonidae|BOLD:AAG9127
Ctenopelmatinae|[3189]|JBHCJ551-11|Hymenoptera|Ichneumonidae|BOLD:ACE6324
Campodorus|[3190]|SSWLE7682-13|Hymenoptera|Ichneumonidae|BOLD:ACE6336
Campodorus|[3191]|SSGBB4416-14|Hymenoptera|Ichneumonidae|BOLD:ABZ1476
Campodorus|[3192]|CNBAB009-12|Hymenoptera|Ichneumonidae|BOLD:ACH1991
Ctenopelmatinae|[3193]|JBHCI914-11|Hymenoptera|Ichneumonidae|BOLD:ACE6326

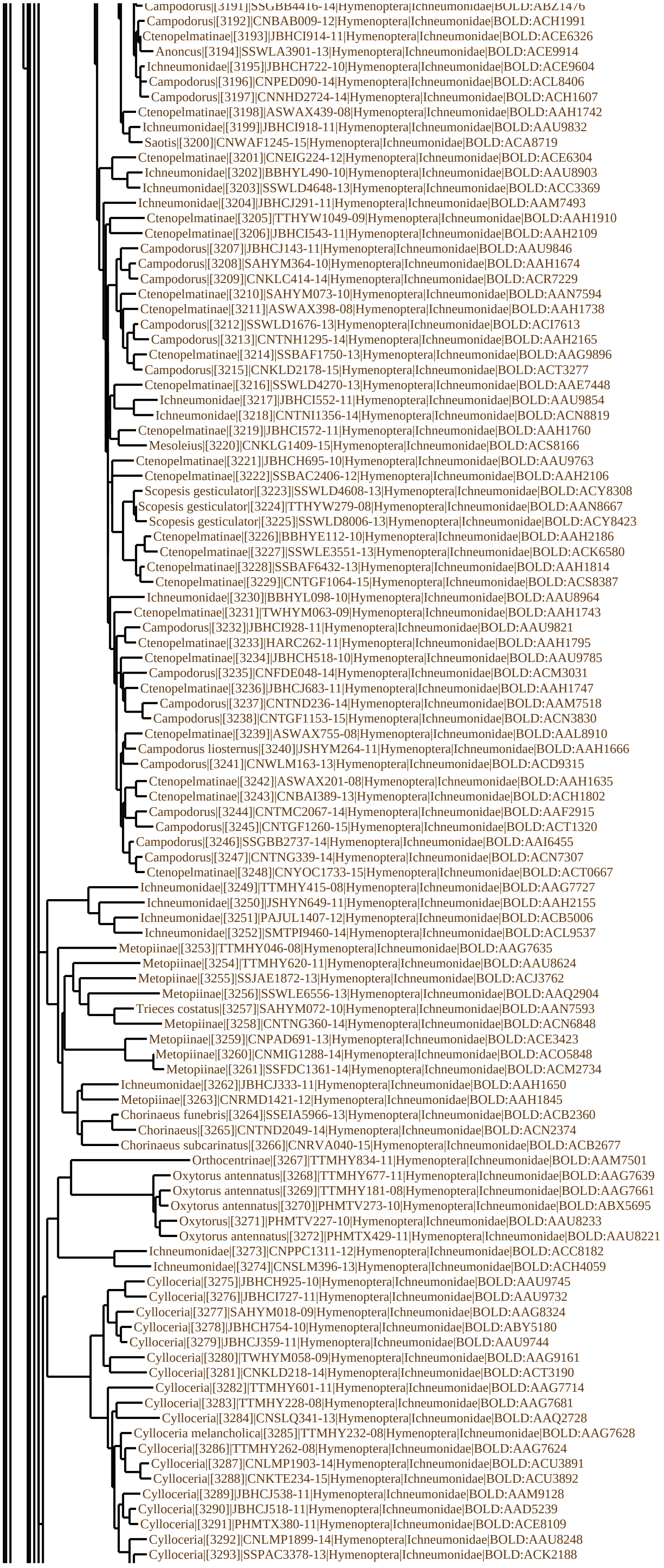
Campodorus|[3191]|SSGBB4416-14|Hymenoptera|Ichneumonidae|BOLD:ABZ1476
Campodorus|[3192]|CNBAB009-12|Hymenoptera|Ichneumonidae|BOLD:ACH1991
Ctenopelmatinae|[3193]|JBHCI914-11|Hymenoptera|Ichneumonidae|BOLD:ACE6326
Anoncus|[3194]|SSWLA3901-13|Hymenoptera|Ichneumonidae|BOLD:ACE9914
Ichneumonidae|[3195]|JBHCH722-10|Hymenoptera|Ichneumonidae|BOLD:ACE9604
Campodorus|[3196]|CNPED090-14|Hymenoptera|Ichneumonidae|BOLD:ACL8406
Campodorus|[3197]|CNNHD2724-14|Hymenoptera|Ichneumonidae|BOLD:ACH1607
Ctenopelmatinae|[3198]|ASWAX439-08|Hymenoptera|Ichneumonidae|BOLD:AAH1742
Ichneumonidae|[3199]|JBHCI918-11|Hymenoptera|Ichneumonidae|BOLD:AAU9832
Saotis|[3200]|CNWAF1245-15|Hymenoptera|Ichneumonidae|BOLD:ACA8719
Ctenopelmatinae|[3201]|CNEIG224-12|Hymenoptera|Ichneumonidae|BOLD:ACE6304
Ichneumonidae|[3202]|BBHYL490-10|Hymenoptera|Ichneumonidae|BOLD:AAU8903
Ichneumonidae|[3203]|SSWLD4648-13|Hymenoptera|Ichneumonidae|BOLD:ACC3369
Ichneumonidae|[3204]|JBHCJ291-11|Hymenoptera|Ichneumonidae|BOLD:AAM7493
Ctenopelmatinae|[3205]|TTHYW1049-09|Hymenoptera|Ichneumonidae|BOLD:AAH1910
Ctenopelmatinae|[3206]|JBHCI543-11|Hymenoptera|Ichneumonidae|BOLD:AAH2109
Campodorus|[3207]|JBHCJ143-11|Hymenoptera|Ichneumonidae|BOLD:AAU9846
Campodorus|[3208]|SAHYM364-10|Hymenoptera|Ichneumonidae|BOLD:AAH1674
Campodorus|[3209]|CNKLC414-14|Hymenoptera|Ichneumonidae|BOLD:ACR7229
Ctenopelmatinae|[3210]|SAHYM073-10|Hymenoptera|Ichneumonidae|BOLD:AAN7594
Ctenopelmatinae|[3211]|ASWAX398-08|Hymenoptera|Ichneumonidae|BOLD:AAH1738
Campodorus|[3212]|SSWLD1676-13|Hymenoptera|Ichneumonidae|BOLD:ACI7613
Campodorus|[3213]|CNTNH1295-14|Hymenoptera|Ichneumonidae|BOLD:AAH2165
Ctenopelmatinae|[3214]|SSBAF1750-13|Hymenoptera|Ichneumonidae|BOLD:AAG9896
Campodorus|[3215]|CNKLD2178-15|Hymenoptera|Ichneumonidae|BOLD:ACT3277
Ctenopelmatinae|[3216]|SSWLD4270-13|Hymenoptera|Ichneumonidae|BOLD:AAE7448
Ichneumonidae|[3217]|JBHCI552-11|Hymenoptera|Ichneumonidae|BOLD:AAU9854
Ichneumonidae|[3218]|CNTNI1356-14|Hymenoptera|Ichneumonidae|BOLD:ACN8819
Ctenopelmatinae|[3219]|JBHCI572-11|Hymenoptera|Ichneumonidae|BOLD:AAH1760
Mesoleius|[3220]|CNKLG1409-15|Hymenoptera|Ichneumonidae|BOLD:ACS8166
Ctenopelmatinae|[3221]|JBHCH695-10|Hymenoptera|Ichneumonidae|BOLD:AAU9763
Ctenopelmatinae|[3222]|SSBAC2406-12|Hymenoptera|Ichneumonidae|BOLD:AAH2106
Scopesis gesticulator|[3223]|SSWLD4608-13|Hymenoptera|Ichneumonidae|BOLD:ACY8308
Scopesis gesticulator|[3224]|TTHYW279-08|Hymenoptera|Ichneumonidae|BOLD:AAN8667
Scopesis gesticulator|[3225]|SSWLD8006-13|Hymenoptera|Ichneumonidae|BOLD:ACY8423
Ctenopelmatinae|[3226]|BBHYE112-10|Hymenoptera|Ichneumonidae|BOLD:AAH2186
Ctenopelmatinae|[3227]|SSWLE3551-13|Hymenoptera|Ichneumonidae|BOLD:ACK6580
Ctenopelmatinae|[3228]|SSBAF6432-13|Hymenoptera|Ichneumonidae|BOLD:AAH1814
Ctenopelmatinae|[3229]|CNTGF1064-15|Hymenoptera|Ichneumonidae|BOLD:ACS8387
Ichneumonidae|[3230]|BBHYL098-10|Hymenoptera|Ichneumonidae|BOLD:AAU8964
Ctenopelmatinae|[3231]|TWHYM063-09|Hymenoptera|Ichneumonidae|BOLD:AAH1743
Campodorus|[3232]|JBHCI928-11|Hymenoptera|Ichneumonidae|BOLD:AAU9821
Ctenopelmatinae|[3233]|HARC262-11|Hymenoptera|Ichneumonidae|BOLD:AAH1795
Ctenopelmatinae|[3234]|JBHCH518-10|Hymenoptera|Ichneumonidae|BOLD:AAU9785
Campodorus|[3235]|CNFDE048-14|Hymenoptera|Ichneumonidae|BOLD:ACM3031
Ctenopelmatinae|[3236]|JBHCJ683-11|Hymenoptera|Ichneumonidae|BOLD:AAH1747
Campodorus|[3237]|CNTND236-14|Hymenoptera|Ichneumonidae|BOLD:AAM7518
Campodorus|[3238]|CNTGF1153-15|Hymenoptera|Ichneumonidae|BOLD:ACN3830
Ctenopelmatinae|[3239]|ASWAX755-08|Hymenoptera|Ichneumonidae|BOLD:AAL8910
Campodorus liosternus|[3240]|JSHYM264-11|Hymenoptera|Ichneumonidae|BOLD:AAH1666
Campodorus|[3241]|CNWLM163-13|Hymenoptera|Ichneumonidae|BOLD:ACD9315
Ctenopelmatinae|[3242]|ASWAX201-08|Hymenoptera|Ichneumonidae|BOLD:AAH1635
Ctenopelmatinae|[3243]|CNBAI389-13|Hymenoptera|Ichneumonidae|BOLD:ACH1802
Campodorus|[3244]|CNTMC2067-14|Hymenoptera|Ichneumonidae|BOLD:AAF2915
Campodorus|[3245]|CNTGF1260-15|Hymenoptera|Ichneumonidae|BOLD:ACT1320
Campodorus|[3246]|SSGBB2737-14|Hymenoptera|Ichneumonidae|BOLD:AAI6455
Campodorus|[3247]|CNTNG339-14|Hymenoptera|Ichneumonidae|BOLD:ACN7307
Ctenopelmatinae|[3248]|CNYOC1733-15|Hymenoptera|Ichneumonidae|BOLD:ACT0667
Ichneumonidae|[3249]|TTMHY415-08|Hymenoptera|Ichneumonidae|BOLD:AAG7727
Ichneumonidae|[3250]|JSHYN649-11|Hymenoptera|Ichneumonidae|BOLD:AAH2155
Ichneumonidae|[3251]|PAJUL1407-12|Hymenoptera|Ichneumonidae|BOLD:ACB5006
Ichneumonidae|[3252]|SMTPI9460-14|Hymenoptera|Ichneumonidae|BOLD:ACL9537
Metopiinae|[3253]|TTMHY046-08|Hymenoptera|Ichneumonidae|BOLD:AAG7635
Metopiinae|[3254]|TTMHY620-11|Hymenoptera|Ichneumonidae|BOLD:AAU8624
Metopiinae|[3255]|SSJAE1872-13|Hymenoptera|Ichneumonidae|BOLD:ACJ3762
Metopiinae|[3256]|SSWLE6556-13|Hymenoptera|Ichneumonidae|BOLD:AAQ2904
Trieces costatus|[3257]|SAHYM072-10|Hymenoptera|Ichneumonidae|BOLD:AAN7593
Metopiinae|[3258]|CNTNG360-14|Hymenoptera|Ichneumonidae|BOLD:ACN6848
Metopiinae|[3259]|CNPAD691-13|Hymenoptera|Ichneumonidae|BOLD:ACE3423
Metopiinae|[3260]|CNMIG1288-14|Hymenoptera|Ichneumonidae|BOLD:ACO5848
Metopiinae|[3261]|SSFDC1361-14|Hymenoptera|Ichneumonidae|BOLD:ACM2734
Ichneumonidae|[3262]|JBHCJ333-11|Hymenoptera|Ichneumonidae|BOLD:AAH1650
Metopiinae|[3263]|CNRMD1421-12|Hymenoptera|Ichneumonidae|BOLD:AAH1845
Chorinaeus funebris|[3264]|SSEIA5966-13|Hymenoptera|Ichneumonidae|BOLD:ACB2360
Chorinaeus|[3265]|CNTND2049-14|Hymenoptera|Ichneumonidae|BOLD:ACN2374
Chorinaeus subcarinatus|[3266]|CNRVA040-15|Hymenoptera|Ichneumonidae|BOLD:ACB2677
Orthocentrinae|[3267]|TTMHY834-11|Hymenoptera|Ichneumonidae|BOLD:AAM7501
Oxytorus antennatus|[3268]|TTMHY677-11|Hymenoptera|Ichneumonidae|BOLD:AAG7639
Oxytorus antennatus|[3269]|TTMHY181-08|Hymenoptera|Ichneumonidae|BOLD:AAG7661
Oxytorus antennatus|[3270]|PHMTV273-10|Hymenoptera|Ichneumonidae|BOLD:ABX5695
Oxytorus|[3271]|PHMTV227-10|Hymenoptera|Ichneumonidae|BOLD:AAU8233
Oxytorus antennatus|[3272]|PHMTX429-11|Hymenoptera|Ichneumonidae|BOLD:AAU8221
Ichneumonidae|[3273]|CNPPC1311-12|Hymenoptera|Ichneumonidae|BOLD:ACC8182
Ichneumonidae|[3274]|CNSLM396-13|Hymenoptera|Ichneumonidae|BOLD:ACH4059
Cylloceria|[3275]|JBHCH925-10|Hymenoptera|Ichneumonidae|BOLD:AAU9745
Cylloceria|[3276]|JBHCI727-11|Hymenoptera|Ichneumonidae|BOLD:AAU9732
Cylloceria|[3277]|SAHYM018-09|Hymenoptera|Ichneumonidae|BOLD:AAG8324
Cylloceria|[3278]|JBHCH754-10|Hymenoptera|Ichneumonidae|BOLD:ABY5180
Cylloceria|[3279]|JBHCJ359-11|Hymenoptera|Ichneumonidae|BOLD:AAU9744
Cylloceria|[3280]|TWHYM058-09|Hymenoptera|Ichneumonidae|BOLD:AAG9161
Cylloceria|[3281]|CNKLD218-14|Hymenoptera|Ichneumonidae|BOLD:ACT3190
Cylloceria|[3282]|TTMHY601-11|Hymenoptera|Ichneumonidae|BOLD:AAG7714
Cylloceria|[3283]|TTMHY228-08|Hymenoptera|Ichneumonidae|BOLD:AAG7681
Cylloceria|[3284]|CNSLQ341-13|Hymenoptera|Ichneumonidae|BOLD:AAQ2728
Cylloceria melancholica|[3285]|TTMHY232-08|Hymenoptera|Ichneumonidae|BOLD:AAG7628
Cylloceria|[3286]|TTMHY262-08|Hymenoptera|Ichneumonidae|BOLD:AAG7624
Cylloceria|[3287]|CNLMP1903-14|Hymenoptera|Ichneumonidae|BOLD:ACU3891
Cylloceria|[3288]|CNKTE234-15|Hymenoptera|Ichneumonidae|BOLD:ACU3892
Cylloceria|[3289]|JBHCJ538-11|Hymenoptera|Ichneumonidae|BOLD:AAM9128
Cylloceria|[3290]|JBHCJ518-11|Hymenoptera|Ichneumonidae|BOLD:AAD5239
Cylloceria|[3291]|PHMTX380-11|Hymenoptera|Ichneumonidae|BOLD:ACE8109
Cylloceria|[3292]|CNLMP1899-14|Hymenoptera|Ichneumonidae|BOLD:AAU8248
Cylloceria|[3293]|SSPAC3378-13|Hymenoptera|Ichneumonidae|BOLD:ACK2188

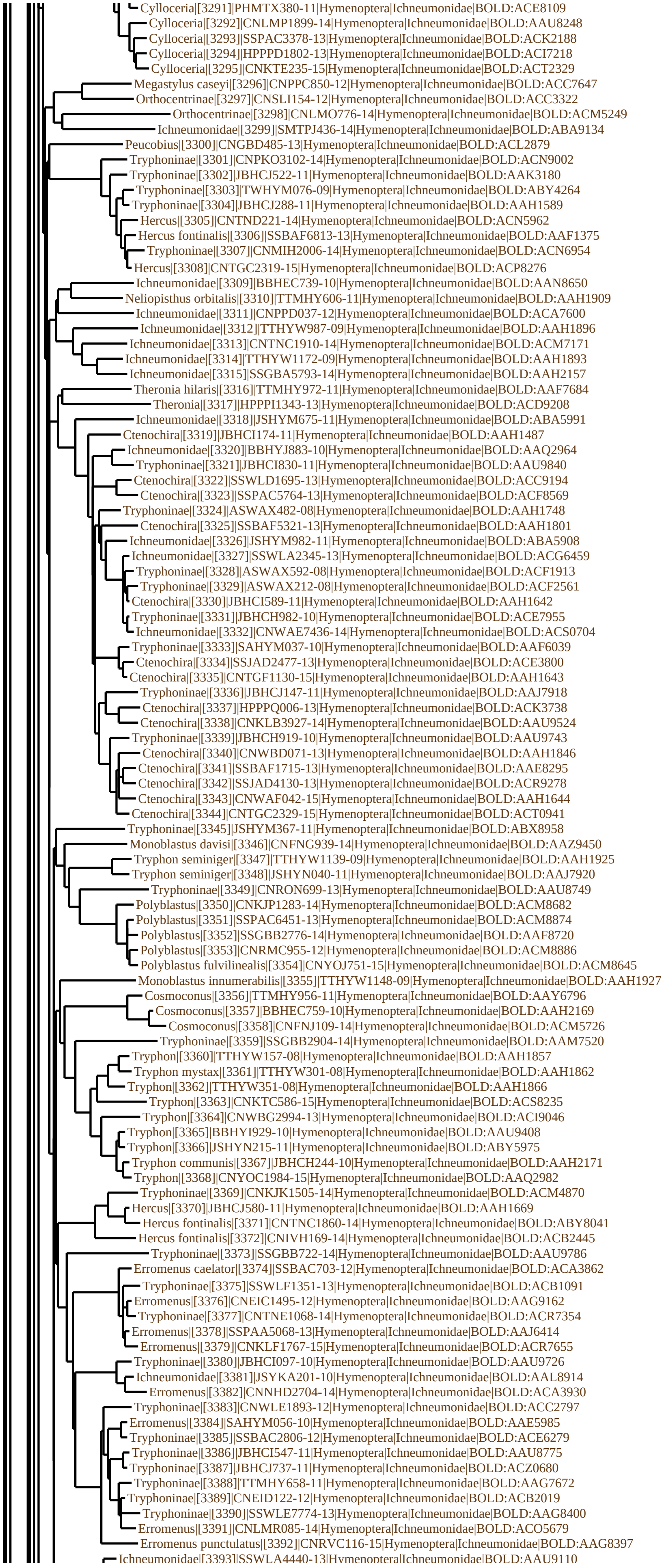
Cylloceria|[3291]|PHMTX380-11|Hymenoptera|Ichneumonidae|BOLD:ACE8109
Cylloceria|[3292]|CNLMP1899-14|Hymenoptera|Ichneumonidae|BOLD:AAU8248
Cylloceria|[3293]|SSPAC3378-13|Hymenoptera|Ichneumonidae|BOLD:ACK2188
Cylloceria|[3294]|HPPPD1802-13|Hymenoptera|Ichneumonidae|BOLD:ACI7218
Cylloceria|[3295]|CNKTE235-15|Hymenoptera|Ichneumonidae|BOLD:ACT2329
Megastylus caseyi|[3296]|CNPPC850-12|Hymenoptera|Ichneumonidae|BOLD:ACC7647
Orthocentrinae|[3297]|CNSLI154-12|Hymenoptera|Ichneumonidae|BOLD:ACC3322
Orthocentrinae|[3298]|CNLMO776-14|Hymenoptera|Ichneumonidae|BOLD:ACM5249
Ichneumonidae|[3299]|SMTPJ436-14|Hymenoptera|Ichneumonidae|BOLD:ABA9134
Peucobius|[3300]|CNGBD485-13|Hymenoptera|Ichneumonidae|BOLD:ACL2879
Tryphoninae|[3301]|CNPKO3102-14|Hymenoptera|Ichneumonidae|BOLD:ACN9002
Tryphoninae|[3302]|JBHCJ522-11|Hymenoptera|Ichneumonidae|BOLD:AAK3180
Tryphoninae|[3303]|TWHYM076-09|Hymenoptera|Ichneumonidae|BOLD:ABY4264
Tryphoninae|[3304]|JBHCJ288-11|Hymenoptera|Ichneumonidae|BOLD:AAH1589
Hercus|[3305]|CNTND221-14|Hymenoptera|Ichneumonidae|BOLD:ACN5962
Hercus fontinalis|[3306]|SSBAF6813-13|Hymenoptera|Ichneumonidae|BOLD:AAF1375
Tryphoninae|[3307]|CNMIH2006-14|Hymenoptera|Ichneumonidae|BOLD:ACN6954
Hercus|[3308]|CNTGC2319-15|Hymenoptera|Ichneumonidae|BOLD:ACP8276
Ichneumonidae|[3309]|BBHEC739-10|Hymenoptera|Ichneumonidae|BOLD:AAN8650
Neliopisthus orbitalis|[3310]|TTMHY606-11|Hymenoptera|Ichneumonidae|BOLD:AAH1909
Ichneumonidae|[3311]|CNPPD037-12|Hymenoptera|Ichneumonidae|BOLD:ACA7600
Ichneumonidae|[3312]|TTHYW987-09|Hymenoptera|Ichneumonidae|BOLD:AAH1896
Ichneumonidae|[3313]|CNTNC1910-14|Hymenoptera|Ichneumonidae|BOLD:ACM7171
Ichneumonidae|[3314]|TTHYW1172-09|Hymenoptera|Ichneumonidae|BOLD:AAH1893
Ichneumonidae|[3315]|SSGBA5793-14|Hymenoptera|Ichneumonidae|BOLD:AAH2157
Theronia hilaris|[3316]|TTMHY972-11|Hymenoptera|Ichneumonidae|BOLD:AAF7684
Theronia|[3317]|HPPPI1343-13|Hymenoptera|Ichneumonidae|BOLD:ACD9208
Ichneumonidae|[3318]|JSHYM675-11|Hymenoptera|Ichneumonidae|BOLD:ABA5991
Ctenochira|[3319]|JBHCI174-11|Hymenoptera|Ichneumonidae|BOLD:AAH1487
Ichneumonidae|[3320]|BBHYJ883-10|Hymenoptera|Ichneumonidae|BOLD:AAQ2964
Tryphoninae|[3321]|JBHCI830-11|Hymenoptera|Ichneumonidae|BOLD:AAU9840
Ctenochira|[3322]|SSWLD1695-13|Hymenoptera|Ichneumonidae|BOLD:ACC9194
Ctenochira|[3323]|SSPAC5764-13|Hymenoptera|Ichneumonidae|BOLD:ACF8569
Tryphoninae|[3324]|ASWAX482-08|Hymenoptera|Ichneumonidae|BOLD:AAH1748
Ctenochira|[3325]|SSBAF5321-13|Hymenoptera|Ichneumonidae|BOLD:AAH1801
Ichneumonidae|[3326]|JSHYM982-11|Hymenoptera|Ichneumonidae|BOLD:ABA5908
Ichneumonidae|[3327]|SSWLA2345-13|Hymenoptera|Ichneumonidae|BOLD:ACG6459
Tryphoninae|[3328]|ASWAX592-08|Hymenoptera|Ichneumonidae|BOLD:ACF1913
Tryphoninae|[3329]|ASWAX212-08|Hymenoptera|Ichneumonidae|BOLD:ACF2561
Ctenochira|[3330]|JBHCI589-11|Hymenoptera|Ichneumonidae|BOLD:AAH1642
Tryphoninae|[3331]|JBHCH982-10|Hymenoptera|Ichneumonidae|BOLD:ACE7955
Ichneumonidae|[3332]|CNWAE7436-14|Hymenoptera|Ichneumonidae|BOLD:ACS0704
Tryphoninae|[3333]|SAHYM037-10|Hymenoptera|Ichneumonidae|BOLD:AAF6039
Ctenochira|[3334]|SSJAD2477-13|Hymenoptera|Ichneumonidae|BOLD:ACE3800
Ctenochira|[3335]|CNTGF1130-15|Hymenoptera|Ichneumonidae|BOLD:AAH1643
Tryphoninae|[3336]|JBHCJ147-11|Hymenoptera|Ichneumonidae|BOLD:AAJ7918
Ctenochira|[3337]|HPPPQ006-13|Hymenoptera|Ichneumonidae|BOLD:ACK3738
Ctenochira|[3338]|CNKLB3927-14|Hymenoptera|Ichneumonidae|BOLD:AAU9524
Tryphoninae|[3339]|JBHCH919-10|Hymenoptera|Ichneumonidae|BOLD:AAU9743
Ctenochira|[3340]|CNWBD071-13|Hymenoptera|Ichneumonidae|BOLD:AAH1846
Ctenochira|[3341]|SSBAF1715-13|Hymenoptera|Ichneumonidae|BOLD:AAE8295
Ctenochira|[3342]|SSJAD4130-13|Hymenoptera|Ichneumonidae|BOLD:ACR9278
Ctenochira|[3343]|CNWAF042-15|Hymenoptera|Ichneumonidae|BOLD:AAH1644
Ctenochira|[3344]|CNTGC2329-15|Hymenoptera|Ichneumonidae|BOLD:ACT0941
Tryphoninae|[3345]|JSHYM367-11|Hymenoptera|Ichneumonidae|BOLD:ABX8958
Monoblastus davisi|[3346]|CNFNG939-14|Hymenoptera|Ichneumonidae|BOLD:AAZ9450
Tryphon seminiger|[3347]|TTHYW1139-09|Hymenoptera|Ichneumonidae|BOLD:AAH1925
Tryphon seminiger|[3348]|JSHYN040-11|Hymenoptera|Ichneumonidae|BOLD:AAJ7920
Tryphoninae|[3349]|CNRON699-13|Hymenoptera|Ichneumonidae|BOLD:AAU8749
Polyblastus|[3350]|CNKJP1283-14|Hymenoptera|Ichneumonidae|BOLD:ACM8682
Polyblastus|[3351]|SSPAC6451-13|Hymenoptera|Ichneumonidae|BOLD:ACM8874
Polyblastus|[3352]|SSGBB2776-14|Hymenoptera|Ichneumonidae|BOLD:AAF8720
Polyblastus|[3353]|CNRMC955-12|Hymenoptera|Ichneumonidae|BOLD:ACM8886
Polyblastus fulvilinealis|[3354]|CNYOJ751-15|Hymenoptera|Ichneumonidae|BOLD:ACM8645
Monoblastus innumerabilis|[3355]|TTHYW1148-09|Hymenoptera|Ichneumonidae|BOLD:AAH1927
Cosmoconus|[3356]|TTMHY956-11|Hymenoptera|Ichneumonidae|BOLD:AAY6796
Cosmoconus|[3357]|BBHEC759-10|Hymenoptera|Ichneumonidae|BOLD:AAH2169
Cosmoconus|[3358]|CNFNJ109-14|Hymenoptera|Ichneumonidae|BOLD:ACM5726
Tryphoninae|[3359]|SSGBB2904-14|Hymenoptera|Ichneumonidae|BOLD:AAM7520
Tryphon|[3360]|TTHYW157-08|Hymenoptera|Ichneumonidae|BOLD:AAH1857
Tryphon mystax|[3361]|TTHYW301-08|Hymenoptera|Ichneumonidae|BOLD:AAH1862
Tryphon|[3362]|TTHYW351-08|Hymenoptera|Ichneumonidae|BOLD:AAH1866
Tryphon|[3363]|CNKTC586-15|Hymenoptera|Ichneumonidae|BOLD:ACS8235
Tryphon|[3364]|CNWBG2994-13|Hymenoptera|Ichneumonidae|BOLD:ACI9046
Tryphon|[3365]|BBHYI929-10|Hymenoptera|Ichneumonidae|BOLD:AAU9408
Tryphon|[3366]|JSHYN215-11|Hymenoptera|Ichneumonidae|BOLD:ABY5975
Tryphon communis|[3367]|JBHCH244-10|Hymenoptera|Ichneumonidae|BOLD:AAH2171
Tryphon|[3368]|CNYOC1984-15|Hymenoptera|Ichneumonidae|BOLD:AAQ2982
Tryphoninae|[3369]|CNKJK1505-14|Hymenoptera|Ichneumonidae|BOLD:ACM4870
Hercus|[3370]|JBHCJ580-11|Hymenoptera|Ichneumonidae|BOLD:AAH1669
Hercus fontinalis|[3371]|CNTNC1860-14|Hymenoptera|Ichneumonidae|BOLD:ABY8041
Hercus fontinalis|[3372]|CNIVH169-14|Hymenoptera|Ichneumonidae|BOLD:ACB2445
Tryphoninae|[3373]|SSGBB722-14|Hymenoptera|Ichneumonidae|BOLD:AAU9786
Erromenus caelator|[3374]|SSBAC703-12|Hymenoptera|Ichneumonidae|BOLD:ACA3862
Tryphoninae|[3375]|SSWLF1351-13|Hymenoptera|Ichneumonidae|BOLD:ACB1091
Erromenus|[3376]|CNEIC1495-12|Hymenoptera|Ichneumonidae|BOLD:AAG9162
Tryphoninae|[3377]|CNTNE1068-14|Hymenoptera|Ichneumonidae|BOLD:ACR7354
Erromenus|[3378]|SSPAA5068-13|Hymenoptera|Ichneumonidae|BOLD:AAJ6414
Erromenus|[3379]|CNKLF1767-15|Hymenoptera|Ichneumonidae|BOLD:ACR7655
Tryphoninae|[3380]|JBHCI097-10|Hymenoptera|Ichneumonidae|BOLD:AAU9726
Ichneumonidae|[3381]|JSYKA201-10|Hymenoptera|Ichneumonidae|BOLD:AAL8914
Erromenus|[3382]|CNNHD2704-14|Hymenoptera|Ichneumonidae|BOLD:ACA3930
Tryphoninae|[3383]|CNWLE1893-12|Hymenoptera|Ichneumonidae|BOLD:ACC2797
Erromenus|[3384]|SAHYM056-10|Hymenoptera|Ichneumonidae|BOLD:AAE5985
Tryphoninae|[3385]|SSBAC2806-12|Hymenoptera|Ichneumonidae|BOLD:ACE6279
Tryphoninae|[3386]|JBHCI547-11|Hymenoptera|Ichneumonidae|BOLD:AAU8775
Tryphoninae|[3387]|JBHCJ737-11|Hymenoptera|Ichneumonidae|BOLD:ACZ0680
Tryphoninae|[3388]|TTMHY658-11|Hymenoptera|Ichneumonidae|BOLD:AAG7672
Tryphoninae|[3389]|CNEID122-12|Hymenoptera|Ichneumonidae|BOLD:ACB2019
Tryphoninae|[3390]|SSWLE7774-13|Hymenoptera|Ichneumonidae|BOLD:AAG8400
Erromenus|[3391]|CNLMR085-14|Hymenoptera|Ichneumonidae|BOLD:ACO5679
Erromenus punctulatus|[3392]|CNRVC116-15|Hymenoptera|Ichneumonidae|BOLD:AAG8397
Ichneumonidae|[3393]|SSWLA4440-13|Hymenoptera|Ichneumonidae|BOLD:AAU9110

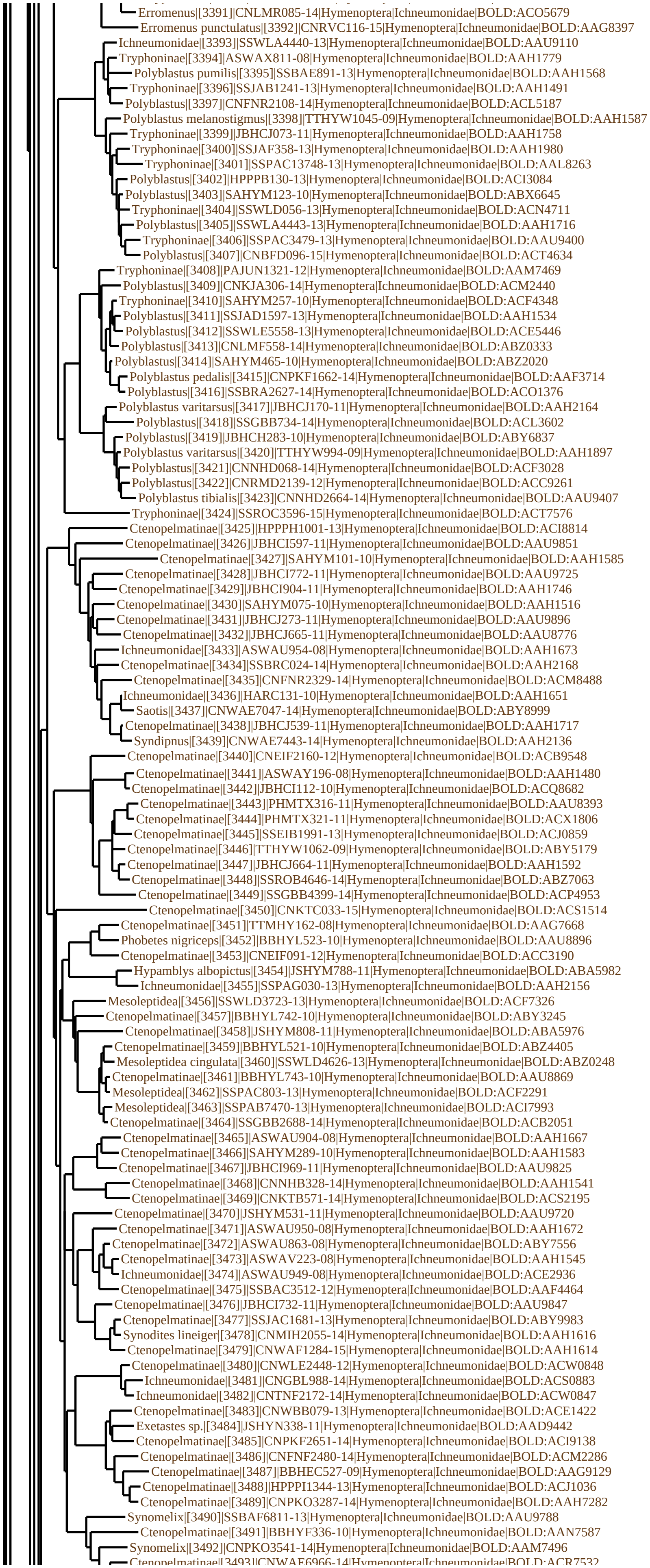
Erromenus|[3391]|CNLMR085-14|Hymenoptera|Ichneumonidae|BOLD:ACO5679
Erromenus punctulatus|[3392]|CNRVC116-15|Hymenoptera|Ichneumonidae|BOLD:AAG8397
Ichneumonidae|[3393]|SSWLA4440-13|Hymenoptera|Ichneumonidae|BOLD:AAU9110
Tryphoninae|[3394]|ASWAX811-08|Hymenoptera|Ichneumonidae|BOLD:AAH1779
Polyblastus pumilis|[3395]|SSBAE891-13|Hymenoptera|Ichneumonidae|BOLD:AAH1568
Tryphoninae|[3396]|SSJAB1241-13|Hymenoptera|Ichneumonidae|BOLD:AAH1491
Polyblastus|[3397]|CNFNR2108-14|Hymenoptera|Ichneumonidae|BOLD:ACL5187
Polyblastus melanostigmus|[3398]|TTHYW1045-09|Hymenoptera|Ichneumonidae|BOLD:AAH1587
Tryphoninae|[3399]|JBHCJ073-11|Hymenoptera|Ichneumonidae|BOLD:AAH1758
Tryphoninae|[3400]|SSJAF358-13|Hymenoptera|Ichneumonidae|BOLD:AAH1980
Tryphoninae|[3401]|SSPAC13748-13|Hymenoptera|Ichneumonidae|BOLD:AAL8263
Polyblastus|[3402]|HPPPB130-13|Hymenoptera|Ichneumonidae|BOLD:ACI3084
Polyblastus|[3403]|SAHYM123-10|Hymenoptera|Ichneumonidae|BOLD:ABX6645
Tryphoninae|[3404]|SSWLD056-13|Hymenoptera|Ichneumonidae|BOLD:ACN4711
Polyblastus|[3405]|SSWLA4443-13|Hymenoptera|Ichneumonidae|BOLD:AAH1716
Tryphoninae|[3406]|SSPAC3479-13|Hymenoptera|Ichneumonidae|BOLD:AAU9400
Polyblastus|[3407]|CNBFD096-15|Hymenoptera|Ichneumonidae|BOLD:ACT4634
Tryphoninae|[3408]|PAJUN1321-12|Hymenoptera|Ichneumonidae|BOLD:AAM7469
Polyblastus|[3409]|CNKJA306-14|Hymenoptera|Ichneumonidae|BOLD:ACM2440
Tryphoninae|[3410]|SAHYM257-10|Hymenoptera|Ichneumonidae|BOLD:ACF4348
Polyblastus|[3411]|SSJAD1597-13|Hymenoptera|Ichneumonidae|BOLD:AAH1534
Polyblastus|[3412]|SSWLE5558-13|Hymenoptera|Ichneumonidae|BOLD:ACE5446
Polyblastus|[3413]|CNLMF558-14|Hymenoptera|Ichneumonidae|BOLD:ABZ0333
Polyblastus|[3414]|SAHYM465-10|Hymenoptera|Ichneumonidae|BOLD:ABZ2020
Polyblastus pedalis|[3415]|CNPKF1662-14|Hymenoptera|Ichneumonidae|BOLD:AAF3714
Polyblastus|[3416]|SSBRA2627-14|Hymenoptera|Ichneumonidae|BOLD:ACO1376
Polyblastus varitarsus|[3417]|JBHCJ170-11|Hymenoptera|Ichneumonidae|BOLD:AAH2164
Polyblastus|[3418]|SSGBB734-14|Hymenoptera|Ichneumonidae|BOLD:ACL3602
Polyblastus|[3419]|JBHCH283-10|Hymenoptera|Ichneumonidae|BOLD:ABY6837
Polyblastus varitarsus|[3420]|TTHYW994-09|Hymenoptera|Ichneumonidae|BOLD:AAH1897
Polyblastus|[3421]|CNNHD068-14|Hymenoptera|Ichneumonidae|BOLD:ACF3028
Polyblastus|[3422]|CNRMD2139-12|Hymenoptera|Ichneumonidae|BOLD:ACC9261
Polyblastus tibialis|[3423]|CNNHD2664-14|Hymenoptera|Ichneumonidae|BOLD:AAU9407
Tryphoninae|[3424]|SSROC3596-15|Hymenoptera|Ichneumonidae|BOLD:ACT7576
Ctenopelmatinae|[3425]|HPPPH1001-13|Hymenoptera|Ichneumonidae|BOLD:ACI8814
Ctenopelmatinae|[3426]|JBHCI597-11|Hymenoptera|Ichneumonidae|BOLD:AAU9851
Ctenopelmatinae|[3427]|SAHYM101-10|Hymenoptera|Ichneumonidae|BOLD:AAH1585
Ctenopelmatinae|[3428]|JBHCI772-11|Hymenoptera|Ichneumonidae|BOLD:AAU9725
Ctenopelmatinae|[3429]|JBHCI904-11|Hymenoptera|Ichneumonidae|BOLD:AAH1746
Ctenopelmatinae|[3430]|SAHYM075-10|Hymenoptera|Ichneumonidae|BOLD:AAH1516
Ctenopelmatinae|[3431]|JBHCJ273-11|Hymenoptera|Ichneumonidae|BOLD:AAU9896
Ctenopelmatinae|[3432]|JBHCJ665-11|Hymenoptera|Ichneumonidae|BOLD:AAU8776
Ichneumonidae|[3433]|ASWAU954-08|Hymenoptera|Ichneumonidae|BOLD:AAH1673
Ctenopelmatinae|[3434]|SSBRC024-14|Hymenoptera|Ichneumonidae|BOLD:AAH2168
Ctenopelmatinae|[3435]|CNFNR2329-14|Hymenoptera|Ichneumonidae|BOLD:ACM8488
Ichneumonidae|[3436]|HARC131-10|Hymenoptera|Ichneumonidae|BOLD:AAH1651
Saotis|[3437]|CNWAE7047-14|Hymenoptera|Ichneumonidae|BOLD:ABY8999
Ctenopelmatinae|[3438]|JBHCJ539-11|Hymenoptera|Ichneumonidae|BOLD:AAH1717
Syndipnus|[3439]|CNWAE7443-14|Hymenoptera|Ichneumonidae|BOLD:AAH2136
Ctenopelmatinae|[3440]|CNEIF2160-12|Hymenoptera|Ichneumonidae|BOLD:ACB9548
Ctenopelmatinae|[3441]|ASWAY196-08|Hymenoptera|Ichneumonidae|BOLD:AAH1480
Ctenopelmatinae|[3442]|JBHCI112-10|Hymenoptera|Ichneumonidae|BOLD:ACQ8682
Ctenopelmatinae|[3443]|PHMTX316-11|Hymenoptera|Ichneumonidae|BOLD:AAU8393
Ctenopelmatinae|[3444]|PHMTX321-11|Hymenoptera|Ichneumonidae|BOLD:ACX1806
Ctenopelmatinae|[3445]|SSEIB1991-13|Hymenoptera|Ichneumonidae|BOLD:ACJ0859
Ctenopelmatinae|[3446]|TTHYW1062-09|Hymenoptera|Ichneumonidae|BOLD:ABY5179
Ctenopelmatinae|[3447]|JBHCJ664-11|Hymenoptera|Ichneumonidae|BOLD:AAH1592
Ctenopelmatinae|[3448]|SSROB4646-14|Hymenoptera|Ichneumonidae|BOLD:ABZ7063
Ctenopelmatinae|[3449]|SSGBB4399-14|Hymenoptera|Ichneumonidae|BOLD:ACP4953
Ctenopelmatinae|[3450]|CNKTC033-15|Hymenoptera|Ichneumonidae|BOLD:ACS1514
Ctenopelmatinae|[3451]|TTMHY162-08|Hymenoptera|Ichneumonidae|BOLD:AAG7668
Phobetes nigriceps|[3452]|BBHYL523-10|Hymenoptera|Ichneumonidae|BOLD:AAU8896
Ctenopelmatinae|[3453]|CNEIF091-12|Hymenoptera|Ichneumonidae|BOLD:ACC3190
Hypamblys albopictus|[3454]|JSHYM788-11|Hymenoptera|Ichneumonidae|BOLD:ABA5982
Ichneumonidae|[3455]|SSPAG030-13|Hymenoptera|Ichneumonidae|BOLD:AAH2156
Mesoleptidea|[3456]|SSWLD3723-13|Hymenoptera|Ichneumonidae|BOLD:ACF7326
Ctenopelmatinae|[3457]|BBHYL742-10|Hymenoptera|Ichneumonidae|BOLD:ABY3245
Ctenopelmatinae|[3458]|JSHYM808-11|Hymenoptera|Ichneumonidae|BOLD:ABA5976
Ctenopelmatinae|[3459]|BBHYL521-10|Hymenoptera|Ichneumonidae|BOLD:ABZ4405
Mesoleptidea cingulata|[3460]|SSWLD4626-13|Hymenoptera|Ichneumonidae|BOLD:ABZ0248
Ctenopelmatinae|[3461]|BBHYL743-10|Hymenoptera|Ichneumonidae|BOLD:AAU8869
Mesoleptidea|[3462]|SSPAC803-13|Hymenoptera|Ichneumonidae|BOLD:ACF2291
Mesoleptidea|[3463]|SSPAB7470-13|Hymenoptera|Ichneumonidae|BOLD:ACI7993
Ctenopelmatinae|[3464]|SSGBB2688-14|Hymenoptera|Ichneumonidae|BOLD:ACB2051
Ctenopelmatinae|[3465]|ASWAU904-08|Hymenoptera|Ichneumonidae|BOLD:AAH1667
Ctenopelmatinae|[3466]|SAHYM289-10|Hymenoptera|Ichneumonidae|BOLD:AAH1583
Ctenopelmatinae|[3467]|JBHCI969-11|Hymenoptera|Ichneumonidae|BOLD:AAU9825
Ctenopelmatinae|[3468]|CNNHB328-14|Hymenoptera|Ichneumonidae|BOLD:AAH1541
Ctenopelmatinae|[3469]|CNKTB571-14|Hymenoptera|Ichneumonidae|BOLD:ACS2195
Ctenopelmatinae|[3470]|JSHYM531-11|Hymenoptera|Ichneumonidae|BOLD:AAU9720
Ctenopelmatinae|[3471]|ASWAU950-08|Hymenoptera|Ichneumonidae|BOLD:AAH1672
Ctenopelmatinae|[3472]|ASWAU863-08|Hymenoptera|Ichneumonidae|BOLD:ABY7556
Ctenopelmatinae|[3473]|ASWAV223-08|Hymenoptera|Ichneumonidae|BOLD:AAH1545
Ichneumonidae|[3474]|ASWAU949-08|Hymenoptera|Ichneumonidae|BOLD:ACE2936
Ctenopelmatinae|[3475]|SSBAC3512-12|Hymenoptera|Ichneumonidae|BOLD:AAF4464
Ctenopelmatinae|[3476]|JBHCI732-11|Hymenoptera|Ichneumonidae|BOLD:AAU9847
Ctenopelmatinae|[3477]|SSJAC1681-13|Hymenoptera|Ichneumonidae|BOLD:ABY9983
Synodites lineiger|[3478]|CNMIH2055-14|Hymenoptera|Ichneumonidae|BOLD:AAH1616
Ctenopelmatinae|[3479]|CNWAF1284-15|Hymenoptera|Ichneumonidae|BOLD:AAH1614
Ctenopelmatinae|[3480]|CNWLE2448-12|Hymenoptera|Ichneumonidae|BOLD:ACW0848
Ichneumonidae|[3481]|CNGBL988-14|Hymenoptera|Ichneumonidae|BOLD:ACS0883
Ichneumonidae|[3482]|CNTNF2172-14|Hymenoptera|Ichneumonidae|BOLD:ACW0847
Ctenopelmatinae|[3483]|CNWBB079-13|Hymenoptera|Ichneumonidae|BOLD:ACE1422
Exetastes sp.|[3484]|JSHYN338-11|Hymenoptera|Ichneumonidae|BOLD:AAD9442
Ctenopelmatinae|[3485]|CNPKF2651-14|Hymenoptera|Ichneumonidae|BOLD:ACI9138
Ctenopelmatinae|[3486]|CNFNF2480-14|Hymenoptera|Ichneumonidae|BOLD:ACM2286
Ctenopelmatinae|[3487]|BBHEC527-09|Hymenoptera|Ichneumonidae|BOLD:AAG9129
Ctenopelmatinae|[3488]|HPPPI1344-13|Hymenoptera|Ichneumonidae|BOLD:ACJ1036
Ctenopelmatinae|[3489]|CNPKO3287-14|Hymenoptera|Ichneumonidae|BOLD:AAH7282
Synomelix|[3490]|SSBAF6811-13|Hymenoptera|Ichneumonidae|BOLD:AAU9788
Ctenopelmatinae|[3491]|BBHYF336-10|Hymenoptera|Ichneumonidae|BOLD:AAN7587
Synomelix|[3492]|CNPKO3541-14|Hymenoptera|Ichneumonidae|BOLD:AAM7496
Ctenopelmatinae|[3493]|CNWAF6966-14|Hymenoptera|Ichneumonidae|BOLD:ACR7532

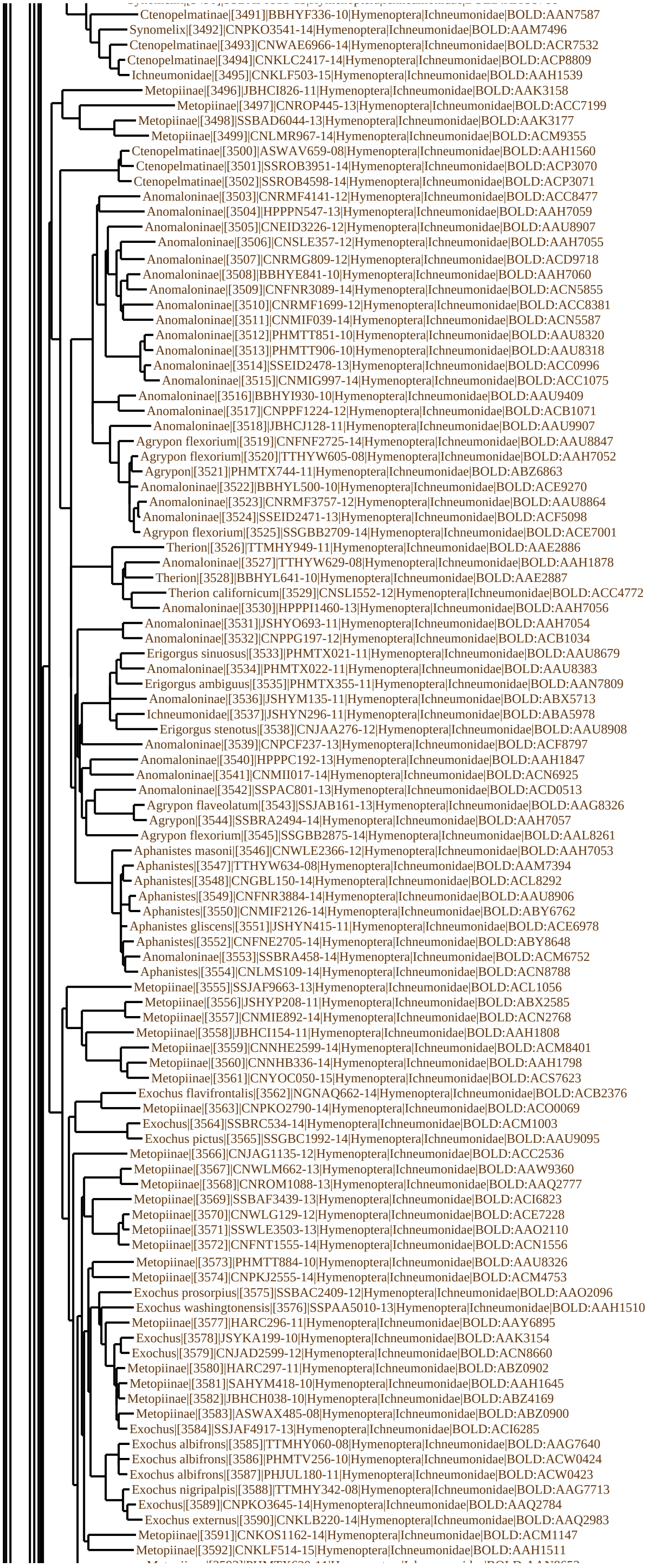
Ctenopelmatinae|[3491]|BBHYF336-10|Hymenoptera|Ichneumonidae|BOLD:AAN7587
Synomelix|[3492]|CNPKO3541-14|Hymenoptera|Ichneumonidae|BOLD:AAM7496
Ctenopelmatinae|[3493]|CNWAE6966-14|Hymenoptera|Ichneumonidae|BOLD:ACR7532
Ctenopelmatinae|[3494]|CNKLC2417-14|Hymenoptera|Ichneumonidae|BOLD:ACP8809
Ichneumonidae|[3495]|CNKLF503-15|Hymenoptera|Ichneumonidae|BOLD:AAH1539
Metopiinae|[3496]|JBHCI826-11|Hymenoptera|Ichneumonidae|BOLD:AAK3158
Metopiinae|[3497]|CNROP445-13|Hymenoptera|Ichneumonidae|BOLD:ACC7199
Metopiinae|[3498]|SSBAD6044-13|Hymenoptera|Ichneumonidae|BOLD:AAK3177
Metopiinae|[3499]|CNLMR967-14|Hymenoptera|Ichneumonidae|BOLD:ACM9355
Ctenopelmatinae|[3500]|ASWAV659-08|Hymenoptera|Ichneumonidae|BOLD:AAH1560
Ctenopelmatinae|[3501]|SSROB3951-14|Hymenoptera|Ichneumonidae|BOLD:ACP3070
Ctenopelmatinae|[3502]|SSROB4598-14|Hymenoptera|Ichneumonidae|BOLD:ACP3071
Anomaloninae|[3503]|CNRMF4141-12|Hymenoptera|Ichneumonidae|BOLD:ACC8477
Anomaloninae|[3504]|HPPPN547-13|Hymenoptera|Ichneumonidae|BOLD:AAH7059
Anomaloninae|[3505]|CNEID3226-12|Hymenoptera|Ichneumonidae|BOLD:AAU8907
Anomaloninae|[3506]|CNSLE357-12|Hymenoptera|Ichneumonidae|BOLD:AAH7055
Anomaloninae|[3507]|CNRMG809-12|Hymenoptera|Ichneumonidae|BOLD:ACD9718
Anomaloninae|[3508]|BBHYE841-10|Hymenoptera|Ichneumonidae|BOLD:AAH7060
Anomaloninae|[3509]|CNFNR3089-14|Hymenoptera|Ichneumonidae|BOLD:ACN5855
Anomaloninae|[3510]|CNRMF1699-12|Hymenoptera|Ichneumonidae|BOLD:ACC8381
Anomaloninae|[3511]|CNMIF039-14|Hymenoptera|Ichneumonidae|BOLD:ACN5587
Anomaloninae|[3512]|PHMTT851-10|Hymenoptera|Ichneumonidae|BOLD:AAU8320
Anomaloninae|[3513]|PHMTT906-10|Hymenoptera|Ichneumonidae|BOLD:AAU8318
Anomaloninae|[3514]|SSEID2478-13|Hymenoptera|Ichneumonidae|BOLD:ACC0996
Anomaloninae|[3515]|CNMIG997-14|Hymenoptera|Ichneumonidae|BOLD:ACC1075
Anomaloninae|[3516]|BBHYI930-10|Hymenoptera|Ichneumonidae|BOLD:AAU9409
Anomaloninae|[3517]|CNPPF1224-12|Hymenoptera|Ichneumonidae|BOLD:ACB1071
Anomaloninae|[3518]|JBHCJ128-11|Hymenoptera|Ichneumonidae|BOLD:AAU9907
Agrypon flexorium|[3519]|CNFNF2725-14|Hymenoptera|Ichneumonidae|BOLD:AAU8847
Agrypon flexorium|[3520]|TTHYW605-08|Hymenoptera|Ichneumonidae|BOLD:AAH7052
Agrypon|[3521]|PHMTX744-11|Hymenoptera|Ichneumonidae|BOLD:ABZ6863
Anomaloninae|[3522]|BBHYL500-10|Hymenoptera|Ichneumonidae|BOLD:ACE9270
Anomaloninae|[3523]|CNRMF3757-12|Hymenoptera|Ichneumonidae|BOLD:AAU8864
Anomaloninae|[3524]|SSEID2471-13|Hymenoptera|Ichneumonidae|BOLD:ACF5098
Agrypon flexorium|[3525]|SSGBB2709-14|Hymenoptera|Ichneumonidae|BOLD:ACE7001
Therion|[3526]|TTMHY949-11|Hymenoptera|Ichneumonidae|BOLD:AAE2886
Anomaloninae|[3527]|TTHYW629-08|Hymenoptera|Ichneumonidae|BOLD:AAH1878
Therion|[3528]|BBHYL641-10|Hymenoptera|Ichneumonidae|BOLD:AAE2887
Therion californicum|[3529]|CNSLI552-12|Hymenoptera|Ichneumonidae|BOLD:ACC4772
Anomaloninae|[3530]|HPPPI1460-13|Hymenoptera|Ichneumonidae|BOLD:AAH7056
Anomaloninae|[3531]|JSHYO693-11|Hymenoptera|Ichneumonidae|BOLD:AAH7054
Anomaloninae|[3532]|CNPPG197-12|Hymenoptera|Ichneumonidae|BOLD:ACB1034
Erigorgus sinuosus|[3533]|PHMTX021-11|Hymenoptera|Ichneumonidae|BOLD:AAU8679
Anomaloninae|[3534]|PHMTX022-11|Hymenoptera|Ichneumonidae|BOLD:AAU8383
Erigorgus ambiguus|[3535]|PHMTX355-11|Hymenoptera|Ichneumonidae|BOLD:AAN7809
Anomaloninae|[3536]|JSHYM135-11|Hymenoptera|Ichneumonidae|BOLD:ABX5713
Ichneumonidae|[3537]|JSHYN296-11|Hymenoptera|Ichneumonidae|BOLD:ABA5978
Erigorgus stenotus|[3538]|CNJAA276-12|Hymenoptera|Ichneumonidae|BOLD:AAU8908
Anomaloninae|[3539]|CNPCF237-13|Hymenoptera|Ichneumonidae|BOLD:ACF8797
Anomaloninae|[3540]|HPPPC192-13|Hymenoptera|Ichneumonidae|BOLD:AAH1847
Anomaloninae|[3541]|CNMII017-14|Hymenoptera|Ichneumonidae|BOLD:ACN6925
Anomaloninae|[3542]|SSPAC801-13|Hymenoptera|Ichneumonidae|BOLD:ACD0513
Agrypon flaveolatum|[3543]|SSJAB161-13|Hymenoptera|Ichneumonidae|BOLD:AAG8326
Agrypon|[3544]|SSBRA2494-14|Hymenoptera|Ichneumonidae|BOLD:AAH7057
Agrypon flexorium|[3545]|SSGBB2875-14|Hymenoptera|Ichneumonidae|BOLD:AAL8261
Aphanistes masoni|[3546]|CNWLE2366-12|Hymenoptera|Ichneumonidae|BOLD:AAH7053
Aphanistes|[3547]|TTHYW634-08|Hymenoptera|Ichneumonidae|BOLD:AAM7394
Aphanistes|[3548]|CNGBL150-14|Hymenoptera|Ichneumonidae|BOLD:ACL8292
Aphanistes|[3549]|CNFNR3884-14|Hymenoptera|Ichneumonidae|BOLD:AAU8906
Aphanistes|[3550]|CNMIF2126-14|Hymenoptera|Ichneumonidae|BOLD:ABY6762
Aphanistes gliscens|[3551]|JSHYN415-11|Hymenoptera|Ichneumonidae|BOLD:ACE6978
Aphanistes|[3552]|CNFNE2705-14|Hymenoptera|Ichneumonidae|BOLD:ABY8648
Anomaloninae|[3553]|SSBRA458-14|Hymenoptera|Ichneumonidae|BOLD:ACM6752
Aphanistes|[3554]|CNLMS109-14|Hymenoptera|Ichneumonidae|BOLD:ACN8788
Metopiinae|[3555]|SSJAF9663-13|Hymenoptera|Ichneumonidae|BOLD:ACL1056
Metopiinae|[3556]|JSHYP208-11|Hymenoptera|Ichneumonidae|BOLD:ABX2585
Metopiinae|[3557]|CNMIE892-14|Hymenoptera|Ichneumonidae|BOLD:ACN2768
Metopiinae|[3558]|JBHCI154-11|Hymenoptera|Ichneumonidae|BOLD:AAH1808
Metopiinae|[3559]|CNNHE2599-14|Hymenoptera|Ichneumonidae|BOLD:ACM8401
Metopiinae|[3560]|CNNHB336-14|Hymenoptera|Ichneumonidae|BOLD:AAH1798
Metopiinae|[3561]|CNYOC050-15|Hymenoptera|Ichneumonidae|BOLD:ACS7623
Exochus flavifrontalis|[3562]|NGNAQ662-14|Hymenoptera|Ichneumonidae|BOLD:ACB2376
Metopiinae|[3563]|CNPKO2790-14|Hymenoptera|Ichneumonidae|BOLD:ACO0069
Exochus|[3564]|SSBRC534-14|Hymenoptera|Ichneumonidae|BOLD:ACM1003
Exochus pictus|[3565]|SSGBC1992-14|Hymenoptera|Ichneumonidae|BOLD:AAU9095
Metopiinae|[3566]|CNJAG1135-12|Hymenoptera|Ichneumonidae|BOLD:ACC2536
Metopiinae|[3567]|CNWLM662-13|Hymenoptera|Ichneumonidae|BOLD:AAW9360
Metopiinae|[3568]|CNROM1088-13|Hymenoptera|Ichneumonidae|BOLD:AAQ2777
Metopiinae|[3569]|SSBAF3439-13|Hymenoptera|Ichneumonidae|BOLD:ACI6823
Metopiinae|[3570]|CNWLG129-12|Hymenoptera|Ichneumonidae|BOLD:ACE7228
Metopiinae|[3571]|SSWLE3503-13|Hymenoptera|Ichneumonidae|BOLD:AAO2110
Metopiinae|[3572]|CNFNT1555-14|Hymenoptera|Ichneumonidae|BOLD:ACN1556
Metopiinae|[3573]|PHMTT884-10|Hymenoptera|Ichneumonidae|BOLD:AAU8326
Metopiinae|[3574]|CNPKJ2555-14|Hymenoptera|Ichneumonidae|BOLD:ACM4753
Exochus prosorpius|[3575]|SSBAC2409-12|Hymenoptera|Ichneumonidae|BOLD:AAO2096
Exochus washingtonensis|[3576]|SSPAA5010-13|Hymenoptera|Ichneumonidae|BOLD:AAH1510
Metopiinae|[3577]|HARC296-11|Hymenoptera|Ichneumonidae|BOLD:AAY6895
Exochus|[3578]|JSYKA199-10|Hymenoptera|Ichneumonidae|BOLD:AAK3154
Exochus|[3579]|CNJAD2599-12|Hymenoptera|Ichneumonidae|BOLD:ACN8660
Metopiinae|[3580]|HARC297-11|Hymenoptera|Ichneumonidae|BOLD:ABZ0902
Metopiinae|[3581]|SAHYM418-10|Hymenoptera|Ichneumonidae|BOLD:AAH1645
Metopiinae|[3582]|JBHCH038-10|Hymenoptera|Ichneumonidae|BOLD:ABZ4169
Metopiinae|[3583]|ASWAX485-08|Hymenoptera|Ichneumonidae|BOLD:ABZ0900
Exochus|[3584]|SSJAF4917-13|Hymenoptera|Ichneumonidae|BOLD:ACI6285
Exochus albifrons|[3585]|TTMHY060-08|Hymenoptera|Ichneumonidae|BOLD:AAG7640
Exochus albifrons|[3586]|PHMTV256-10|Hymenoptera|Ichneumonidae|BOLD:ACW0424
Exochus albifrons|[3587]|PHJUL180-11|Hymenoptera|Ichneumonidae|BOLD:ACW0423
Exochus nigripalpis|[3588]|TTMHY342-08|Hymenoptera|Ichneumonidae|BOLD:AAG7713
Exochus|[3589]|CNPKO3645-14|Hymenoptera|Ichneumonidae|BOLD:AAQ2784
Exochus externus|[3590]|CNKLB220-14|Hymenoptera|Ichneumonidae|BOLD:AAQ2983
Metopiinae|[3591]|CNKOS1162-14|Hymenoptera|Ichneumonidae|BOLD:ACM1147
Metopiinae|[3592]|CNKLF514-15|Hymenoptera|Ichneumonidae|BOLD:AAH1511

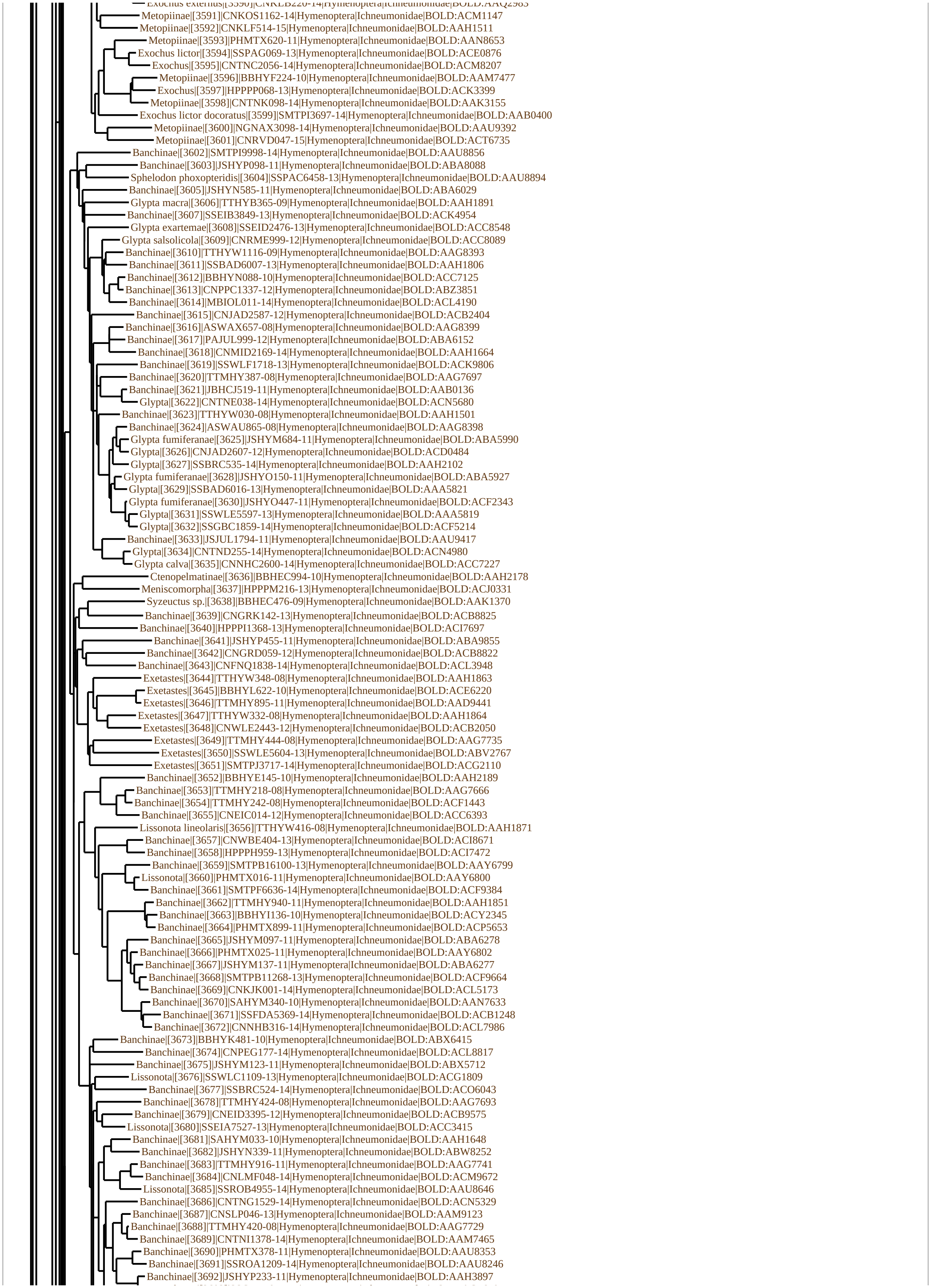
Exochus externus|[3590]|CNKLB220-14|Hymenoptera|Ichneumonidae|BOLD:AAQ2983
Metopiinae|[3591]|CNKOS1162-14|Hymenoptera|Ichneumonidae|BOLD:ACM1147
Metopiinae|[3592]|CNKLF514-15|Hymenoptera|Ichneumonidae|BOLD:AAH1511
Metopiinae|[3593]|PHMTX620-11|Hymenoptera|Ichneumonidae|BOLD:AAN8653
Exochus lictor|[3594]|SSPAG069-13|Hymenoptera|Ichneumonidae|BOLD:ACE0876
Exochus|[3595]|CNTNC2056-14|Hymenoptera|Ichneumonidae|BOLD:ACM8207
Metopiinae|[3596]|BBHYF224-10|Hymenoptera|Ichneumonidae|BOLD:AAM7477
Exochus|[3597]|HPPPP068-13|Hymenoptera|Ichneumonidae|BOLD:ACK3399
Metopiinae|[3598]|CNTNK098-14|Hymenoptera|Ichneumonidae|BOLD:AAK3155
Exochus lictor docoratus|[3599]|SMTPI3697-14|Hymenoptera|Ichneumonidae|BOLD:AAB0400
Metopiinae|[3600]|NGNAX3098-14|Hymenoptera|Ichneumonidae|BOLD:AAU9392
Metopiinae|[3601]|CNRVD047-15|Hymenoptera|Ichneumonidae|BOLD:ACT6735
Banchinae|[3602]|SMTPI9998-14|Hymenoptera|Ichneumonidae|BOLD:AAU8856
Banchinae|[3603]|JSHYP098-11|Hymenoptera|Ichneumonidae|BOLD:ABA8088
Sphelodon phoxopteridis|[3604]|SSPAC6458-13|Hymenoptera|Ichneumonidae|BOLD:AAU8894
Banchinae|[3605]|JSHYN585-11|Hymenoptera|Ichneumonidae|BOLD:ABA6029
Glypta macra|[3606]|TTHYB365-09|Hymenoptera|Ichneumonidae|BOLD:AAH1891
Banchinae|[3607]|SSEIB3849-13|Hymenoptera|Ichneumonidae|BOLD:ACK4954
Glypta exartemae|[3608]|SSEID2476-13|Hymenoptera|Ichneumonidae|BOLD:ACC8548
Glypta salsolicola|[3609]|CNRME999-12|Hymenoptera|Ichneumonidae|BOLD:ACC8089
Banchinae|[3610]|TTHYW1116-09|Hymenoptera|Ichneumonidae|BOLD:AAG8393
Banchinae|[3611]|SSBAD6007-13|Hymenoptera|Ichneumonidae|BOLD:AAH1806
Banchinae|[3612]|BBHYN088-10|Hymenoptera|Ichneumonidae|BOLD:ACC7125
Banchinae|[3613]|CNPPC1337-12|Hymenoptera|Ichneumonidae|BOLD:ABZ3851
Banchinae|[3614]|MBIOL011-14|Hymenoptera|Ichneumonidae|BOLD:ACL4190
Banchinae|[3615]|CNJAD2587-12|Hymenoptera|Ichneumonidae|BOLD:ACB2404
Banchinae|[3616]|ASWAX657-08|Hymenoptera|Ichneumonidae|BOLD:AAG8399
Banchinae|[3617]|PAJUL999-12|Hymenoptera|Ichneumonidae|BOLD:ABA6152
Banchinae|[3618]|CNMID2169-14|Hymenoptera|Ichneumonidae|BOLD:AAH1664
Banchinae|[3619]|SSWLF1718-13|Hymenoptera|Ichneumonidae|BOLD:ACK9806
Banchinae|[3620]|TTMHY387-08|Hymenoptera|Ichneumonidae|BOLD:AAG7697
Banchinae|[3621]|JBHCJ519-11|Hymenoptera|Ichneumonidae|BOLD:AAB0136
Glypta|[3622]|CNTNE038-14|Hymenoptera|Ichneumonidae|BOLD:ACN5680
Banchinae|[3623]|TTHYW030-08|Hymenoptera|Ichneumonidae|BOLD:AAH1501
Banchinae|[3624]|ASWAU865-08|Hymenoptera|Ichneumonidae|BOLD:AAG8398
Glypta fumiferanae|[3625]|JSHYM684-11|Hymenoptera|Ichneumonidae|BOLD:ABA5990
Glypta|[3626]|CNJAD2607-12|Hymenoptera|Ichneumonidae|BOLD:ACD0484
Glypta|[3627]|SSBRC535-14|Hymenoptera|Ichneumonidae|BOLD:AAH2102
Glypta fumiferanae|[3628]|JSHYO150-11|Hymenoptera|Ichneumonidae|BOLD:ABA5927
Glypta|[3629]|SSBAD6016-13|Hymenoptera|Ichneumonidae|BOLD:AAA5821
Glypta fumiferanae|[3630]|JSHYO447-11|Hymenoptera|Ichneumonidae|BOLD:ACF2343
Glypta|[3631]|SSWLE5597-13|Hymenoptera|Ichneumonidae|BOLD:AAA5819
Glypta|[3632]|SSGBC1859-14|Hymenoptera|Ichneumonidae|BOLD:ACF5214
Banchinae|[3633]|JSJUL1794-11|Hymenoptera|Ichneumonidae|BOLD:AAU9417
Glypta|[3634]|CNTND255-14|Hymenoptera|Ichneumonidae|BOLD:ACN4980
Glypta calva|[3635]|CNNHC2600-14|Hymenoptera|Ichneumonidae|BOLD:ACC7227
Ctenopelmatinae|[3636]|BBHEC994-10|Hymenoptera|Ichneumonidae|BOLD:AAH2178
Meniscomorpha|[3637]|HPPPM216-13|Hymenoptera|Ichneumonidae|BOLD:ACJ0331
Syzeuctus sp.|[3638]|BBHEC476-09|Hymenoptera|Ichneumonidae|BOLD:AAK1370
Banchinae|[3639]|CNGRK142-13|Hymenoptera|Ichneumonidae|BOLD:ACB8825
Banchinae|[3640]|HPPPI1368-13|Hymenoptera|Ichneumonidae|BOLD:ACI7697
Banchinae|[3641]|JSHYP455-11|Hymenoptera|Ichneumonidae|BOLD:ABA9855
Banchinae|[3642]|CNGRD059-12|Hymenoptera|Ichneumonidae|BOLD:ACB8822
Banchinae|[3643]|CNFNQ1838-14|Hymenoptera|Ichneumonidae|BOLD:ACL3948
Exetastes|[3644]|TTHYW348-08|Hymenoptera|Ichneumonidae|BOLD:AAH1863
Exetastes|[3645]|BBHYL622-10|Hymenoptera|Ichneumonidae|BOLD:ACE6220
Exetastes|[3646]|TTMHY895-11|Hymenoptera|Ichneumonidae|BOLD:AAD9441
Exetastes|[3647]|TTHYW332-08|Hymenoptera|Ichneumonidae|BOLD:AAH1864
Exetastes|[3648]|CNWLE2443-12|Hymenoptera|Ichneumonidae|BOLD:ACB2050
Exetastes|[3649]|TTMHY444-08|Hymenoptera|Ichneumonidae|BOLD:AAG7735
Exetastes|[3650]|SSWLE5604-13|Hymenoptera|Ichneumonidae|BOLD:ABV2767
Exetastes|[3651]|SMTPJ3717-14|Hymenoptera|Ichneumonidae|BOLD:ACG2110
Banchinae|[3652]|BBHYE145-10|Hymenoptera|Ichneumonidae|BOLD:AAH2189
Banchinae|[3653]|TTMHY218-08|Hymenoptera|Ichneumonidae|BOLD:AAG7666
Banchinae|[3654]|TTMHY242-08|Hymenoptera|Ichneumonidae|BOLD:ACF1443
Banchinae|[3655]|CNEIC014-12|Hymenoptera|Ichneumonidae|BOLD:ACC6393
Lissonota lineolaris|[3656]|TTHYW416-08|Hymenoptera|Ichneumonidae|BOLD:AAH1871
Banchinae|[3657]|CNWBE404-13|Hymenoptera|Ichneumonidae|BOLD:ACI8671
Banchinae|[3658]|HPPPH959-13|Hymenoptera|Ichneumonidae|BOLD:ACI7472
Banchinae|[3659]|SMTPB16100-13|Hymenoptera|Ichneumonidae|BOLD:AAY6799
Lissonota|[3660]|PHMTX016-11|Hymenoptera|Ichneumonidae|BOLD:AAY6800
Banchinae|[3661]|SMTPF6636-14|Hymenoptera|Ichneumonidae|BOLD:ACF9384
Banchinae|[3662]|TTMHY940-11|Hymenoptera|Ichneumonidae|BOLD:AAH1851
Banchinae|[3663]|BBHYI136-10|Hymenoptera|Ichneumonidae|BOLD:ACY2345
Banchinae|[3664]|PHMTX899-11|Hymenoptera|Ichneumonidae|BOLD:ACP5653
Banchinae|[3665]|JSHYM097-11|Hymenoptera|Ichneumonidae|BOLD:ABA6278
Banchinae|[3666]|PHMTX025-11|Hymenoptera|Ichneumonidae|BOLD:AAY6802
Banchinae|[3667]|JSHYM137-11|Hymenoptera|Ichneumonidae|BOLD:ABA6277
Banchinae|[3668]|SMTPB11268-13|Hymenoptera|Ichneumonidae|BOLD:ACF9664
Banchinae|[3669]|CNKJK001-14|Hymenoptera|Ichneumonidae|BOLD:ACL5173
Banchinae|[3670]|SAHYM340-10|Hymenoptera|Ichneumonidae|BOLD:AAN7633
Banchinae|[3671]|SSFDA5369-14|Hymenoptera|Ichneumonidae|BOLD:ACB1248
Banchinae|[3672]|CNNHB316-14|Hymenoptera|Ichneumonidae|BOLD:ACL7986
Banchinae|[3673]|BBHYK481-10|Hymenoptera|Ichneumonidae|BOLD:ABX6415
Banchinae|[3674]|CNPEG177-14|Hymenoptera|Ichneumonidae|BOLD:ACL8817
Banchinae|[3675]|JSHYM123-11|Hymenoptera|Ichneumonidae|BOLD:ABX5712
Lissonota|[3676]|SSWLC1109-13|Hymenoptera|Ichneumonidae|BOLD:ACG1809
Banchinae|[3677]|SSBRC524-14|Hymenoptera|Ichneumonidae|BOLD:ACO6043
Banchinae|[3678]|TTMHY424-08|Hymenoptera|Ichneumonidae|BOLD:AAG7693
Banchinae|[3679]|CNEID3395-12|Hymenoptera|Ichneumonidae|BOLD:ACB9575
Lissonota|[3680]|SSEIA7527-13|Hymenoptera|Ichneumonidae|BOLD:ACC3415
Banchinae|[3681]|SAHYM033-10|Hymenoptera|Ichneumonidae|BOLD:AAH1648
Banchinae|[3682]|JSHYN339-11|Hymenoptera|Ichneumonidae|BOLD:ABW8252
Banchinae|[3683]|TTMHY916-11|Hymenoptera|Ichneumonidae|BOLD:AAG7741
Banchinae|[3684]|CNLMF048-14|Hymenoptera|Ichneumonidae|BOLD:ACM9672
Lissonota|[3685]|SSROB4955-14|Hymenoptera|Ichneumonidae|BOLD:AAU8646
Banchinae|[3686]|CNTNG1529-14|Hymenoptera|Ichneumonidae|BOLD:ACN5329
Banchinae|[3687]|CNSLP046-13|Hymenoptera|Ichneumonidae|BOLD:AAM9123
Banchinae|[3688]|TTMHY420-08|Hymenoptera|Ichneumonidae|BOLD:AAG7729
Banchinae|[3689]|CNTNI1378-14|Hymenoptera|Ichneumonidae|BOLD:AAM7465
Banchinae|[3690]|PHMTX378-11|Hymenoptera|Ichneumonidae|BOLD:AAU8353
Banchinae|[3691]|SSROA1209-14|Hymenoptera|Ichneumonidae|BOLD:AAU8246
Banchinae|[3692]|JSHYP233-11|Hymenoptera|Ichneumonidae|BOLD:AAH3897

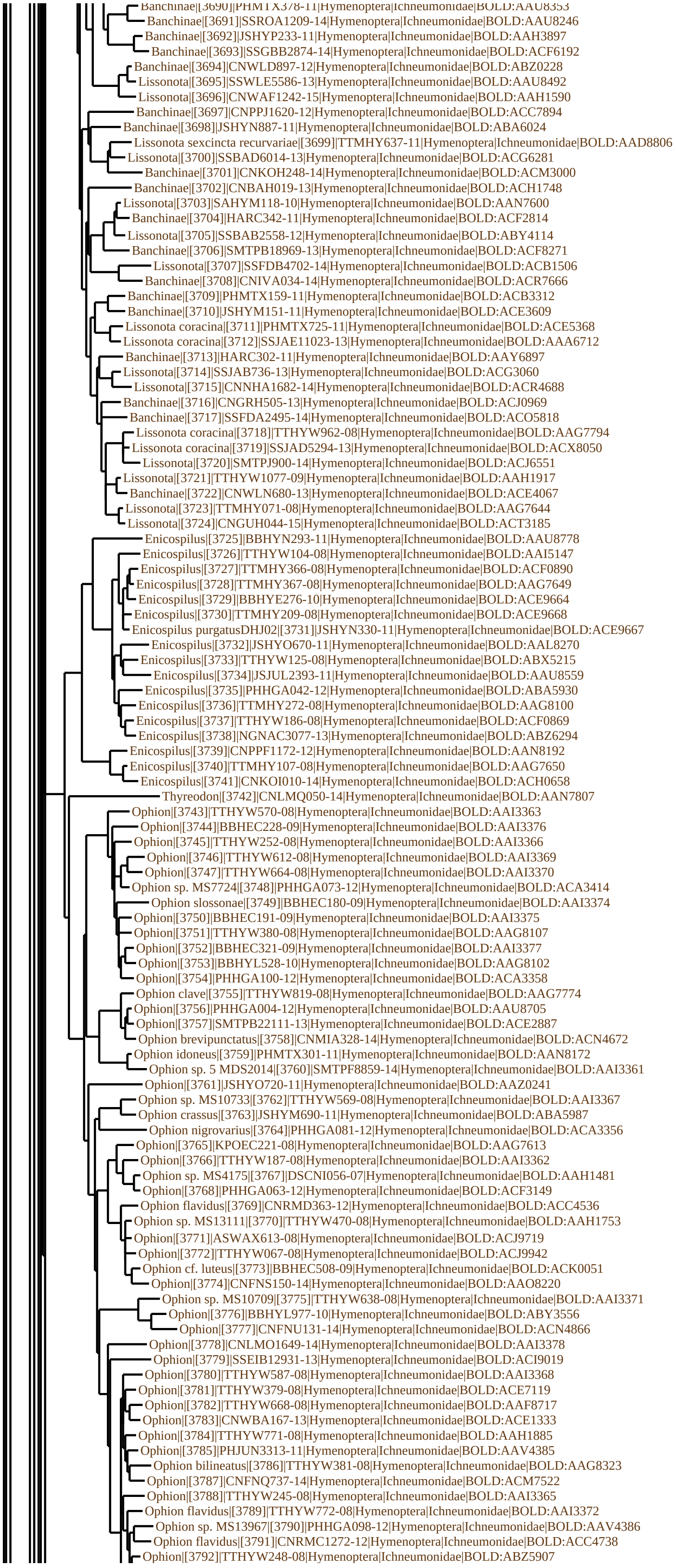

Banchinae|[3690]|PHMTX378-11|Hymenoptera|Ichneumonidae|BOLD:AAU8353
Banchinae|[3691]|SSROA1209-14|Hymenoptera|Ichneumonidae|BOLD:AAU8246
Banchinae|[3692]|JSHYP233-11|Hymenoptera|Ichneumonidae|BOLD:AAH3897
Banchinae|[3693]|SSGBB2874-14|Hymenoptera|Ichneumonidae|BOLD:ACF6192
Banchinae|[3694]|CNWLD897-12|Hymenoptera|Ichneumonidae|BOLD:ABZ0228
Lissonota|[3695]|SSWLE5586-13|Hymenoptera|Ichneumonidae|BOLD:AAU8492
Lissonota|[3696]|CNWAF1242-15|Hymenoptera|Ichneumonidae|BOLD:AAH1590
Banchinae|[3697]|CNPPJ1620-12|Hymenoptera|Ichneumonidae|BOLD:ACC7894
Banchinae|[3698]|JSHYN887-11|Hymenoptera|Ichneumonidae|BOLD:ABA6024
Lissonota sexcincta recurvariae|[3699]|TTMHY637-11|Hymenoptera|Ichneumonidae|BOLD:AAD8806
Lissonota|[3700]|SSBAD6014-13|Hymenoptera|Ichneumonidae|BOLD:ACG6281
Banchinae|[3701]|CNKOH248-14|Hymenoptera|Ichneumonidae|BOLD:ACM3000
Banchinae|[3702]|CNBAH019-13|Hymenoptera|Ichneumonidae|BOLD:ACH1748
Lissonota|[3703]|SAHYM118-10|Hymenoptera|Ichneumonidae|BOLD:AAN7600
Banchinae|[3704]|HARC342-11|Hymenoptera|Ichneumonidae|BOLD:ACF2814
Lissonota|[3705]|SSBAB2558-12|Hymenoptera|Ichneumonidae|BOLD:ABY4114
Banchinae|[3706]|SMTPB18969-13|Hymenoptera|Ichneumonidae|BOLD:ACF8271
Lissonota|[3707]|SSFDB4702-14|Hymenoptera|Ichneumonidae|BOLD:ACB1506
Banchinae|[3708]|CNIVA034-14|Hymenoptera|Ichneumonidae|BOLD:ACR7666
Banchinae|[3709]|PHMTX159-11|Hymenoptera|Ichneumonidae|BOLD:ACB3312
Banchinae|[3710]|JSHYM151-11|Hymenoptera|Ichneumonidae|BOLD:ACE3609
Lissonota coracina|[3711]|PHMTX725-11|Hymenoptera|Ichneumonidae|BOLD:ACE5368
Lissonota coracina|[3712]|SSJAE11023-13|Hymenoptera|Ichneumonidae|BOLD:AAA6712
Banchinae|[3713]|HARC302-11|Hymenoptera|Ichneumonidae|BOLD:AAY6897
Lissonota|[3714]|SSJAB736-13|Hymenoptera|Ichneumonidae|BOLD:ACG3060
Lissonota|[3715]|CNNHA1682-14|Hymenoptera|Ichneumonidae|BOLD:ACR4688
Banchinae|[3716]|CNGRH505-13|Hymenoptera|Ichneumonidae|BOLD:ACJ0969
Banchinae|[3717]|SSFDA2495-14|Hymenoptera|Ichneumonidae|BOLD:ACO5818
Lissonota coracina|[3718]|TTHYW962-08|Hymenoptera|Ichneumonidae|BOLD:AAG7794
Lissonota coracina|[3719]|SSJAD5294-13|Hymenoptera|Ichneumonidae|BOLD:ACX8050
Lissonota|[3720]|SMTPJ900-14|Hymenoptera|Ichneumonidae|BOLD:ACJ6551
Lissonota|[3721]|TTHYW1077-09|Hymenoptera|Ichneumonidae|BOLD:AAH1917
Banchinae|[3722]|CNWLN680-13|Hymenoptera|Ichneumonidae|BOLD:ACE4067
Lissonota|[3723]|TTMHY071-08|Hymenoptera|Ichneumonidae|BOLD:AAG7644
Lissonota|[3724]|CNGUH044-15|Hymenoptera|Ichneumonidae|BOLD:ACT3185
Enicospilus|[3725]|BBHYN293-11|Hymenoptera|Ichneumonidae|BOLD:AAU8778
Enicospilus|[3726]|TTHYW104-08|Hymenoptera|Ichneumonidae|BOLD:AAI5147
Enicospilus|[3727]|TTMHY366-08|Hymenoptera|Ichneumonidae|BOLD:ACF0890
Enicospilus|[3728]|TTMHY367-08|Hymenoptera|Ichneumonidae|BOLD:AAG7649
Enicospilus|[3729]|BBHYE276-10|Hymenoptera|Ichneumonidae|BOLD:ACE9664
Enicospilus|[3730]|TTMHY209-08|Hymenoptera|Ichneumonidae|BOLD:ACE9668
Enicospilus purgatusDHJ02|[3731]|JSHYN330-11|Hymenoptera|Ichneumonidae|BOLD:ACE9667
Enicospilus|[3732]|JSHYO670-11|Hymenoptera|Ichneumonidae|BOLD:AAL8270
Enicospilus|[3733]|TTHYW125-08|Hymenoptera|Ichneumonidae|BOLD:ABX5215
Enicospilus|[3734]|JSJUL2393-11|Hymenoptera|Ichneumonidae|BOLD:AAU8559
Enicospilus|[3735]|PHHGA042-12|Hymenoptera|Ichneumonidae|BOLD:ABA5930
Enicospilus|[3736]|TTMHY272-08|Hymenoptera|Ichneumonidae|BOLD:AAG8100
Enicospilus|[3737]|TTHYW186-08|Hymenoptera|Ichneumonidae|BOLD:ACF0869
Enicospilus|[3738]|NGNAC3077-13|Hymenoptera|Ichneumonidae|BOLD:ABZ6294
Enicospilus|[3739]|CNPPF1172-12|Hymenoptera|Ichneumonidae|BOLD:AAN8192
Enicospilus|[3740]|TTMHY107-08|Hymenoptera|Ichneumonidae|BOLD:AAG7650
Enicospilus|[3741]|CNKOI010-14|Hymenoptera|Ichneumonidae|BOLD:ACH0658
Thyreodon|[3742]|CNLMQ050-14|Hymenoptera|Ichneumonidae|BOLD:AAN7807
Ophion|[3743]|TTHYW570-08|Hymenoptera|Ichneumonidae|BOLD:AAI3363
Ophion|[3744]|BBHEC228-09|Hymenoptera|Ichneumonidae|BOLD:AAI3376
Ophion|[3745]|TTHYW252-08|Hymenoptera|Ichneumonidae|BOLD:AAI3366
Ophion|[3746]|TTHYW612-08|Hymenoptera|Ichneumonidae|BOLD:AAI3369
Ophion|[3747]|TTHYW664-08|Hymenoptera|Ichneumonidae|BOLD:AAI3370
Ophion sp. MS7724|[3748]|PHHGA073-12|Hymenoptera|Ichneumonidae|BOLD:ACA3414
Ophion slossonae|[3749]|BBHEC180-09|Hymenoptera|Ichneumonidae|BOLD:AAI3374
Ophion|[3750]|BBHEC191-09|Hymenoptera|Ichneumonidae|BOLD:AAI3375
Ophion|[3751]|TTHYW380-08|Hymenoptera|Ichneumonidae|BOLD:AAG8107
Ophion|[3752]|BBHEC321-09|Hymenoptera|Ichneumonidae|BOLD:AAI3377
Ophion|[3753]|BBHYL528-10|Hymenoptera|Ichneumonidae|BOLD:AAG8102
Ophion|[3754]|PHHGA100-12|Hymenoptera|Ichneumonidae|BOLD:ACA3358
Ophion clave|[3755]|TTHYW819-08|Hymenoptera|Ichneumonidae|BOLD:AAG7774
Ophion|[3756]|PHHGA004-12|Hymenoptera|Ichneumonidae|BOLD:AAU8705
Ophion|[3757]|SMTPB22111-13|Hymenoptera|Ichneumonidae|BOLD:ACE2887
Ophion brevipunctatus|[3758]|CNMIA328-14|Hymenoptera|Ichneumonidae|BOLD:ACN4672
Ophion idoneus|[3759]|PHMTX301-11|Hymenoptera|Ichneumonidae|BOLD:AAN8172
Ophion sp. 5 MDS2014|[3760]|SMTPF8859-14|Hymenoptera|Ichneumonidae|BOLD:AAI3361
Ophion|[3761]|JSHYO720-11|Hymenoptera|Ichneumonidae|BOLD:AAZ0241
Ophion sp. MS10733|[3762]|TTHYW569-08|Hymenoptera|Ichneumonidae|BOLD:AAI3367
Ophion crassus|[3763]|JSHYM690-11|Hymenoptera|Ichneumonidae|BOLD:ABA5987
Ophion nigrovarius|[3764]|PHHGA081-12|Hymenoptera|Ichneumonidae|BOLD:ACA3356
Ophion|[3765]|KPOEC221-08|Hymenoptera|Ichneumonidae|BOLD:AAG7613
Ophion|[3766]|TTHYW187-08|Hymenoptera|Ichneumonidae|BOLD:AAI3362
Ophion sp. MS4175|[3767]|DSCNI056-07|Hymenoptera|Ichneumonidae|BOLD:AAH1481
Ophion|[3768]|PHHGA063-12|Hymenoptera|Ichneumonidae|BOLD:ACF3149
Ophion flavidus|[3769]|CNRMD363-12|Hymenoptera|Ichneumonidae|BOLD:ACC4536
Ophion sp. MS13111|[3770]|TTHYW470-08|Hymenoptera|Ichneumonidae|BOLD:AAH1753
Ophion|[3771]|ASWAX613-08|Hymenoptera|Ichneumonidae|BOLD:ACJ9719
Ophion|[3772]|TTHYW067-08|Hymenoptera|Ichneumonidae|BOLD:ACJ9942
Ophion cf. luteus|[3773]|BBHEC508-09|Hymenoptera|Ichneumonidae|BOLD:ACK0051
Ophion|[3774]|CNFNS150-14|Hymenoptera|Ichneumonidae|BOLD:AAO8220
Ophion sp. MS10709|[3775]|TTHYW638-08|Hymenoptera|Ichneumonidae|BOLD:AAI3371
Ophion|[3776]|BBHYL977-10|Hymenoptera|Ichneumonidae|BOLD:ABY3556
Ophion|[3777]|CNFNU131-14|Hymenoptera|Ichneumonidae|BOLD:ACN4866
Ophion|[3778]|CNLMO1649-14|Hymenoptera|Ichneumonidae|BOLD:AAI3378
Ophion|[3779]|SSEIB12931-13|Hymenoptera|Ichneumonidae|BOLD:ACI9019
Ophion|[3780]|TTHYW587-08|Hymenoptera|Ichneumonidae|BOLD:AAI3368
Ophion|[3781]|TTHYW379-08|Hymenoptera|Ichneumonidae|BOLD:ACE7119
Ophion|[3782]|TTHYW668-08|Hymenoptera|Ichneumonidae|BOLD:AAF8717
Ophion|[3783]|CNWBA167-13|Hymenoptera|Ichneumonidae|BOLD:ACE1333
Ophion|[3784]|TTHYW771-08|Hymenoptera|Ichneumonidae|BOLD:AAH1885
Ophion|[3785]|PHJUN3313-11|Hymenoptera|Ichneumonidae|BOLD:AAV4385
Ophion bilineatus|[3786]|TTHYW381-08|Hymenoptera|Ichneumonidae|BOLD:AAG8323
Ophion|[3787]|CNFNQ737-14|Hymenoptera|Ichneumonidae|BOLD:ACM7522
Ophion|[3788]|TTHYW245-08|Hymenoptera|Ichneumonidae|BOLD:AAI3365
Ophion flavidus|[3789]|TTHYW772-08|Hymenoptera|Ichneumonidae|BOLD:AAI3372
Ophion sp. MS13967|[3790]|PHHGA098-12|Hymenoptera|Ichneumonidae|BOLD:AAV4386
Ophion flavidus|[3791]|CNRMC1272-12|Hymenoptera|Ichneumonidae|BOLD:ACC4738
Ophion|[3792]|TTHYW248-08|Hymenoptera|Ichneumonidae|BOLD:ABZ5907

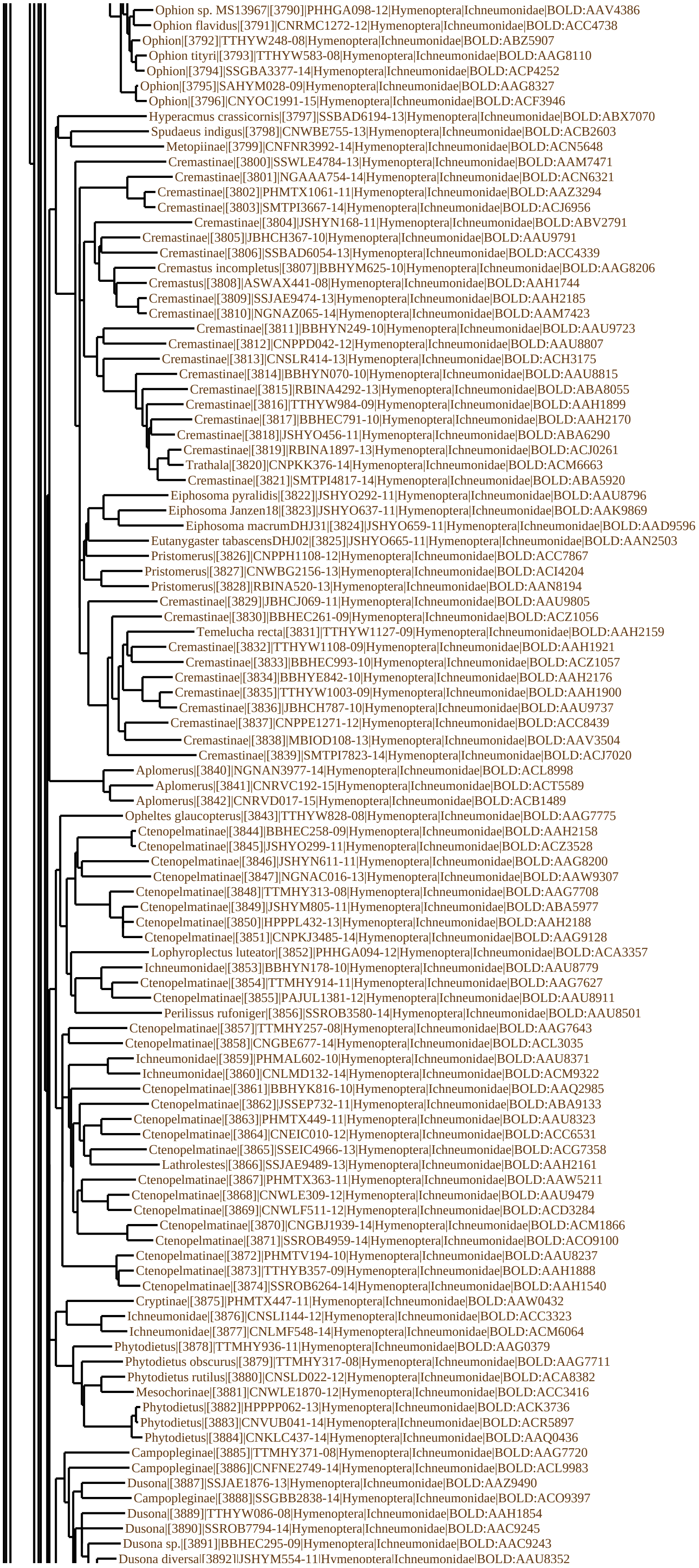

Ophion sp. MS13967|[3790]|PHHGA098-12|Hymenoptera|Ichneumonidae|BOLD:AAV4386
Ophion flavidus|[3791]|CNRMC1272-12|Hymenoptera|Ichneumonidae|BOLD:ACC4738
Ophion|[3792]|TTHYW248-08|Hymenoptera|Ichneumonidae|BOLD:ABZ5907
Ophion tityri|[3793]|TTHYW583-08|Hymenoptera|Ichneumonidae|BOLD:AAG8110
Ophion|[3794]|SSGBA3377-14|Hymenoptera|Ichneumonidae|BOLD:ACP4252
Ophion|[3795]|SAHYM028-09|Hymenoptera|Ichneumonidae|BOLD:AAG8327
Ophion|[3796]|CNYOC1991-15|Hymenoptera|Ichneumonidae|BOLD:ACF3946
Hyperacmus crassicornis|[3797]|SSBAD6194-13|Hymenoptera|Ichneumonidae|BOLD:ABX7070
Spudaeus indigus|[3798]|CNWBE755-13|Hymenoptera|Ichneumonidae|BOLD:ACB2603
Metopiinae|[3799]|CNFNR3992-14|Hymenoptera|Ichneumonidae|BOLD:ACN5648
Cremastinae|[3800]|SSWLE4784-13|Hymenoptera|Ichneumonidae|BOLD:AAM7471
Cremastinae|[3801]|NGAAA754-14|Hymenoptera|Ichneumonidae|BOLD:ACN6321
Cremastinae|[3802]|PHMTX1061-11|Hymenoptera|Ichneumonidae|BOLD:AAZ3294
Cremastinae|[3803]|SMTPI3667-14|Hymenoptera|Ichneumonidae|BOLD:ACJ6956
Cremastinae|[3804]|JSHYN168-11|Hymenoptera|Ichneumonidae|BOLD:ABV2791
Cremastinae|[3805]|JBHCH367-10|Hymenoptera|Ichneumonidae|BOLD:AAU9791
Cremastinae|[3806]|SSBAD6054-13|Hymenoptera|Ichneumonidae|BOLD:ACC4339
Cremastus incompletus|[3807]|BBHYM625-10|Hymenoptera|Ichneumonidae|BOLD:AAG8206
Cremastus|[3808]|ASWAX441-08|Hymenoptera|Ichneumonidae|BOLD:AAH1744
Cremastinae|[3809]|SSJAE9474-13|Hymenoptera|Ichneumonidae|BOLD:AAH2185
Cremastinae|[3810]|NGNAZ065-14|Hymenoptera|Ichneumonidae|BOLD:AAM7423
Cremastinae|[3811]|BBHYN249-10|Hymenoptera|Ichneumonidae|BOLD:AAU9723
Cremastinae|[3812]|CNPPD042-12|Hymenoptera|Ichneumonidae|BOLD:AAU8807
Cremastinae|[3813]|CNSLR414-13|Hymenoptera|Ichneumonidae|BOLD:ACH3175
Cremastinae|[3814]|BBHYN070-10|Hymenoptera|Ichneumonidae|BOLD:AAU8815
Cremastinae|[3815]|RBINA4292-13|Hymenoptera|Ichneumonidae|BOLD:ABA8055
Cremastinae|[3816]|TTHYW984-09|Hymenoptera|Ichneumonidae|BOLD:AAH1899
Cremastinae|[3817]|BBHEC791-10|Hymenoptera|Ichneumonidae|BOLD:AAH2170
Cremastinae|[3818]|JSHYO456-11|Hymenoptera|Ichneumonidae|BOLD:ABA6290
Cremastinae|[3819]|RBINA1897-13|Hymenoptera|Ichneumonidae|BOLD:ACJ0261
Trathala|[3820]|CNPKK376-14|Hymenoptera|Ichneumonidae|BOLD:ACM6663
Cremastinae|[3821]|SMTPI4817-14|Hymenoptera|Ichneumonidae|BOLD:ABA5920
Eiphosoma pyralidis|[3822]|JSHYO292-11|Hymenoptera|Ichneumonidae|BOLD:AAU8796
Eiphosoma Janzen18|[3823]|JSHYO637-11|Hymenoptera|Ichneumonidae|BOLD:AAK9869
Eiphosoma macrumDHJ31|[3824]|JSHYO659-11|Hymenoptera|Ichneumonidae|BOLD:AAD9596
Eutanygaster tabascensDHJ02|[3825]|JSHYO665-11|Hymenoptera|Ichneumonidae|BOLD:AAN2503
Pristomerus|[3826]|CNPPH1108-12|Hymenoptera|Ichneumonidae|BOLD:ACC7867
Pristomerus|[3827]|CNWBG2156-13|Hymenoptera|Ichneumonidae|BOLD:ACI4204
Pristomerus|[3828]|RBINA520-13|Hymenoptera|Ichneumonidae|BOLD:AAN8194
Cremastinae|[3829]|JBHCJ069-11|Hymenoptera|Ichneumonidae|BOLD:AAU9805
Cremastinae|[3830]|BBHEC261-09|Hymenoptera|Ichneumonidae|BOLD:ACZ1056
Temelucha recta|[3831]|TTHYW1127-09|Hymenoptera|Ichneumonidae|BOLD:AAH2159
Cremastinae|[3832]|TTHYW1108-09|Hymenoptera|Ichneumonidae|BOLD:AAH1921
Cremastinae|[3833]|BBHEC993-10|Hymenoptera|Ichneumonidae|BOLD:ACZ1057
Cremastinae|[3834]|BBHYE842-10|Hymenoptera|Ichneumonidae|BOLD:AAH2176
Cremastinae|[3835]|TTHYW1003-09|Hymenoptera|Ichneumonidae|BOLD:AAH1900
Cremastinae|[3836]|JBHCH787-10|Hymenoptera|Ichneumonidae|BOLD:AAU9737
Cremastinae|[3837]|CNPPE1271-12|Hymenoptera|Ichneumonidae|BOLD:ACC8439
Cremastinae|[3838]|MBIOD108-13|Hymenoptera|Ichneumonidae|BOLD:AAV3504
Cremastinae|[3839]|SMTPI7823-14|Hymenoptera|Ichneumonidae|BOLD:ACJ7020
Aplomerus|[3840]|NGNAN3977-14|Hymenoptera|Ichneumonidae|BOLD:ACL8998
Aplomerus|[3841]|CNRVC192-15|Hymenoptera|Ichneumonidae|BOLD:ACT5589
Aplomerus|[3842]|CNRVD017-15|Hymenoptera|Ichneumonidae|BOLD:ACB1489
Opheltes glaucopterus|[3843]|TTHYW828-08|Hymenoptera|Ichneumonidae|BOLD:AAG7775
Ctenopelmatinae|[3844]|BBHEC258-09|Hymenoptera|Ichneumonidae|BOLD:AAH2158
Ctenopelmatinae|[3845]|JSHYO299-11|Hymenoptera|Ichneumonidae|BOLD:ACZ3528
Ctenopelmatinae|[3846]|JSHYN611-11|Hymenoptera|Ichneumonidae|BOLD:AAG8200
Ctenopelmatinae|[3847]|NGNAC016-13|Hymenoptera|Ichneumonidae|BOLD:AAW9307
Ctenopelmatinae|[3848]|TTMHY313-08|Hymenoptera|Ichneumonidae|BOLD:AAG7708
Ctenopelmatinae|[3849]|JSHYM805-11|Hymenoptera|Ichneumonidae|BOLD:ABA5977
Ctenopelmatinae|[3850]|HPPPL432-13|Hymenoptera|Ichneumonidae|BOLD:AAH2188
Ctenopelmatinae|[3851]|CNPKJ3485-14|Hymenoptera|Ichneumonidae|BOLD:AAG9128
Lophyroplectus luteator|[3852]|PHHGA094-12|Hymenoptera|Ichneumonidae|BOLD:ACA3357
Ichneumonidae|[3853]|BBHYN178-10|Hymenoptera|Ichneumonidae|BOLD:AAU8779
Ctenopelmatinae|[3854]|TTMHY914-11|Hymenoptera|Ichneumonidae|BOLD:AAG7627
Ctenopelmatinae|[3855]|PAJUL1381-12|Hymenoptera|Ichneumonidae|BOLD:AAU8911
Perilissus rufoniger|[3856]|SSROB3580-14|Hymenoptera|Ichneumonidae|BOLD:AAU8501
Ctenopelmatinae|[3857]|TTMHY257-08|Hymenoptera|Ichneumonidae|BOLD:AAG7643
Ctenopelmatinae|[3858]|CNGBE677-14|Hymenoptera|Ichneumonidae|BOLD:ACL3035
Ichneumonidae|[3859]|PHMAL602-10|Hymenoptera|Ichneumonidae|BOLD:AAU8371
Ichneumonidae|[3860]|CNLMD132-14|Hymenoptera|Ichneumonidae|BOLD:ACM9322
Ctenopelmatinae|[3861]|BBHYK816-10|Hymenoptera|Ichneumonidae|BOLD:AAQ2985
Ctenopelmatinae|[3862]|JSSEP732-11|Hymenoptera|Ichneumonidae|BOLD:ABA9133
Ctenopelmatinae|[3863]|PHMTX449-11|Hymenoptera|Ichneumonidae|BOLD:AAU8323
Ctenopelmatinae|[3864]|CNEIC010-12|Hymenoptera|Ichneumonidae|BOLD:ACC6531
Ctenopelmatinae|[3865]|SSEIC4966-13|Hymenoptera|Ichneumonidae|BOLD:ACG7358
Lathrolestes|[3866]|SSJAE9489-13|Hymenoptera|Ichneumonidae|BOLD:AAH2161
Ctenopelmatinae|[3867]|PHMTX363-11|Hymenoptera|Ichneumonidae|BOLD:AAW5211
Ctenopelmatinae|[3868]|CNWLE309-12|Hymenoptera|Ichneumonidae|BOLD:AAU9479
Ctenopelmatinae|[3869]|CNWLF511-12|Hymenoptera|Ichneumonidae|BOLD:ACD3284
Ctenopelmatinae|[3870]|CNGBJ1939-14|Hymenoptera|Ichneumonidae|BOLD:ACM1866
Ctenopelmatinae|[3871]|SSROB4959-14|Hymenoptera|Ichneumonidae|BOLD:ACO9100
Ctenopelmatinae|[3872]|PHMTV194-10|Hymenoptera|Ichneumonidae|BOLD:AAU8237
Ctenopelmatinae|[3873]|TTHYB357-09|Hymenoptera|Ichneumonidae|BOLD:AAH1888
Ctenopelmatinae|[3874]|SSROB6264-14|Hymenoptera|Ichneumonidae|BOLD:AAH1540
Cryptinae|[3875]|PHMTX447-11|Hymenoptera|Ichneumonidae|BOLD:AAW0432
Ichneumonidae|[3876]|CNSLI144-12|Hymenoptera|Ichneumonidae|BOLD:ACC3323
Ichneumonidae|[3877]|CNLMF548-14|Hymenoptera|Ichneumonidae|BOLD:ACM6064
Phytodietus|[3878]|TTMHY936-11|Hymenoptera|Ichneumonidae|BOLD:AAG0379
Phytodietus obscurus|[3879]|TTMHY317-08|Hymenoptera|Ichneumonidae|BOLD:AAG7711
Phytodietus rutilus|[3880]|CNSLD022-12|Hymenoptera|Ichneumonidae|BOLD:ACA8382
Mesochorinae|[3881]|CNWLE1870-12|Hymenoptera|Ichneumonidae|BOLD:ACC3416
Phytodietus|[3882]|HPPPP062-13|Hymenoptera|Ichneumonidae|BOLD:ACK3736
Phytodietus|[3883]|CNVUB041-14|Hymenoptera|Ichneumonidae|BOLD:ACR5897
Phytodietus|[3884]|CNKLC437-14|Hymenoptera|Ichneumonidae|BOLD:AAQ0436
Campopleginae|[3885]|TTMHY371-08|Hymenoptera|Ichneumonidae|BOLD:AAG7720
Campopleginae|[3886]|CNFNE2749-14|Hymenoptera|Ichneumonidae|BOLD:ACL9983
Dusona|[3887]|SSJAE1876-13|Hymenoptera|Ichneumonidae|BOLD:AAZ9490
Campopleginae|[3888]|SSGBB2838-14|Hymenoptera|Ichneumonidae|BOLD:ACO9397
Dusona|[3889]|TTHYW086-08|Hymenoptera|Ichneumonidae|BOLD:AAH1854
Dusona|[3890]|SSROB7794-14|Hymenoptera|Ichneumonidae|BOLD:AAC9245
Dusona sp.|[3891]|BBHEC295-09|Hymenoptera|Ichneumonidae|BOLD:AAC9243
Dusona diversa|[3892]|JSHYM554-11|Hymenoptera|Ichneumonidae|BOLD:AAU8352

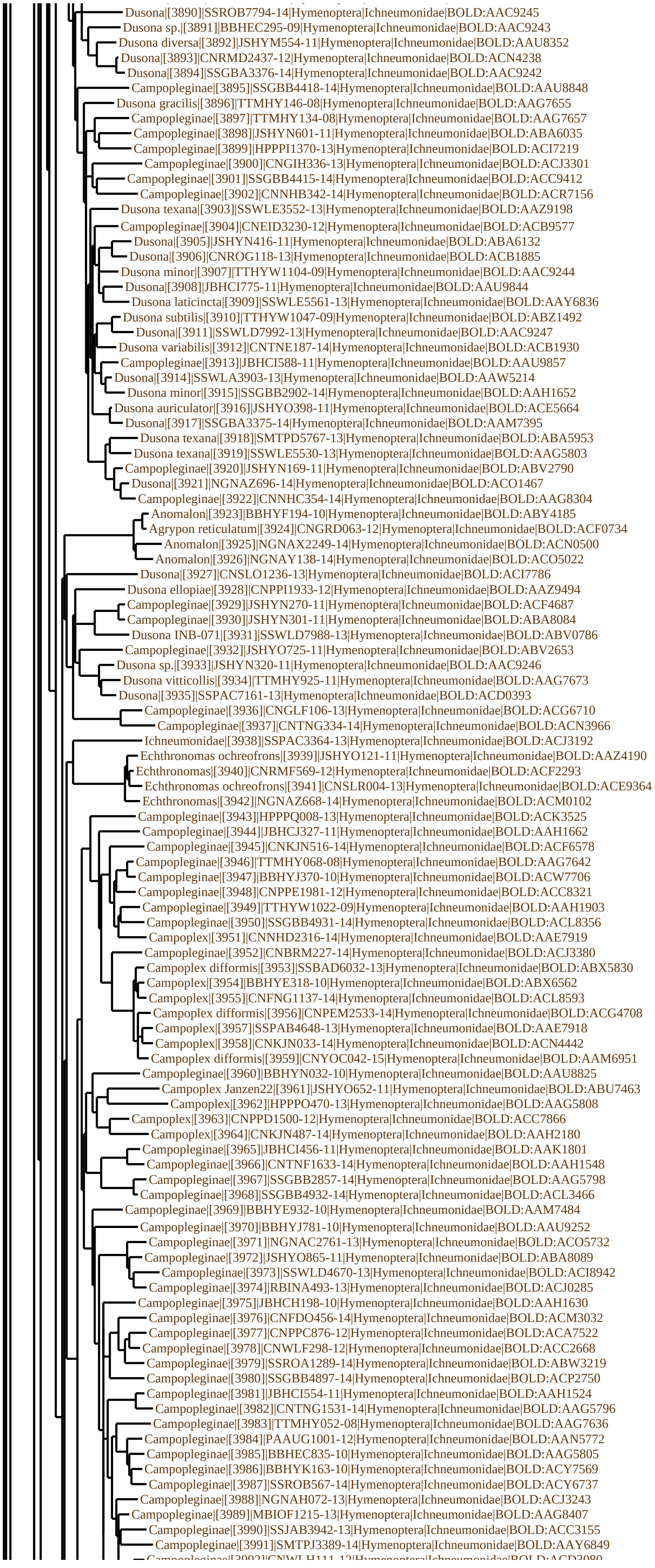

Dusona|[3890]|SSROB7794-14|Hymenoptera|Ichneumonidae|BOLD:AAC9245
Dusona sp.|[3891]|BBHEC295-09|Hymenoptera|Ichneumonidae|BOLD:AAC9243
Dusona diversa|[3892]|JSHYM554-11|Hymenoptera|Ichneumonidae|BOLD:AAU8352
Dusona|[3893]|CNRMD2437-12|Hymenoptera|Ichneumonidae|BOLD:ACN4238
Dusona|[3894]|SSGBA3376-14|Hymenoptera|Ichneumonidae|BOLD:AAC9242
Campopleginae|[3895]|SSGBB4418-14|Hymenoptera|Ichneumonidae|BOLD:AAU8848
Dusona gracilis|[3896]|TTMHY146-08|Hymenoptera|Ichneumonidae|BOLD:AAG7655
Campopleginae|[3897]|TTMHY134-08|Hymenoptera|Ichneumonidae|BOLD:AAG7657
Campopleginae|[3898]|JSHYN601-11|Hymenoptera|Ichneumonidae|BOLD:ABA6035
Campopleginae|[3899]|HPPPI1370-13|Hymenoptera|Ichneumonidae|BOLD:ACI7219
Campopleginae|[3900]|CNGIH336-13|Hymenoptera|Ichneumonidae|BOLD:ACJ3301
Campopleginae|[3901]|SSGBB4415-14|Hymenoptera|Ichneumonidae|BOLD:ACC9412
Campopleginae|[3902]|CNNHB342-14|Hymenoptera|Ichneumonidae|BOLD:ACR7156
Dusona texana|[3903]|SSWLE3552-13|Hymenoptera|Ichneumonidae|BOLD:AAZ9198
Campopleginae|[3904]|CNEID3230-12|Hymenoptera|Ichneumonidae|BOLD:ACB9577
Dusona|[3905]|JSHYN416-11|Hymenoptera|Ichneumonidae|BOLD:ABA6132
Dusona|[3906]|CNROG118-13|Hymenoptera|Ichneumonidae|BOLD:ACB1885
Dusona minor|[3907]|TTHYW1104-09|Hymenoptera|Ichneumonidae|BOLD:AAC9244
Dusona|[3908]|JBHCI775-11|Hymenoptera|Ichneumonidae|BOLD:AAU9844
Dusona laticincta|[3909]|SSWLE5561-13|Hymenoptera|Ichneumonidae|BOLD:AAY6836
Dusona subtilis|[3910]|TTHYW1047-09|Hymenoptera|Ichneumonidae|BOLD:ABZ1492
Dusona|[3911]|SSWLD7992-13|Hymenoptera|Ichneumonidae|BOLD:AAC9247
Dusona variabilis|[3912]|CNTNE187-14|Hymenoptera|Ichneumonidae|BOLD:ACB1930
Campopleginae|[3913]|JBHCI588-11|Hymenoptera|Ichneumonidae|BOLD:AAU9857
Dusona|[3914]|SSWLA3903-13|Hymenoptera|Ichneumonidae|BOLD:AAW5214
Dusona minor|[3915]|SSGBB2902-14|Hymenoptera|Ichneumonidae|BOLD:AAH1652
Dusona auriculator|[3916]|JSHYO398-11|Hymenoptera|Ichneumonidae|BOLD:ACE5664
Dusona|[3917]|SSGBA3375-14|Hymenoptera|Ichneumonidae|BOLD:AAM7395
Dusona texana|[3918]|SMTPD5767-13|Hymenoptera|Ichneumonidae|BOLD:ABA5953
Dusona texana|[3919]|SSWLE5530-13|Hymenoptera|Ichneumonidae|BOLD:AAG5803
Campopleginae|[3920]|JSHYN169-11|Hymenoptera|Ichneumonidae|BOLD:ABV2790
Dusona|[3921]|NGNAZ696-14|Hymenoptera|Ichneumonidae|BOLD:ACO1467
Campopleginae|[3922]|CNNHC354-14|Hymenoptera|Ichneumonidae|BOLD:AAG8304
Anomalon|[3923]|BBHYF194-10|Hymenoptera|Ichneumonidae|BOLD:ABY4185
Agrypon reticulatum|[3924]|CNGRD063-12|Hymenoptera|Ichneumonidae|BOLD:ACF0734
Anomalon|[3925]|NGNAX2249-14|Hymenoptera|Ichneumonidae|BOLD:ACN0500
Anomalon|[3926]|NGNAY138-14|Hymenoptera|Ichneumonidae|BOLD:ACO5022
Dusona|[3927]|CNSLO1236-13|Hymenoptera|Ichneumonidae|BOLD:ACI7786
Dusona ellopiae|[3928]|CNPPI1933-12|Hymenoptera|Ichneumonidae|BOLD:AAZ9494
Campopleginae|[3929]|JSHYN270-11|Hymenoptera|Ichneumonidae|BOLD:ACF4687
Campopleginae|[3930]|JSHYN301-11|Hymenoptera|Ichneumonidae|BOLD:ABA8084
Dusona INB-071|[3931]|SSWLD7988-13|Hymenoptera|Ichneumonidae|BOLD:ABV0786
Campopleginae|[3932]|JSHYO725-11|Hymenoptera|Ichneumonidae|BOLD:ABV2653
Dusona sp.|[3933]|JSHYN320-11|Hymenoptera|Ichneumonidae|BOLD:AAC9246
Dusona vitticollis|[3934]|TTMHY925-11|Hymenoptera|Ichneumonidae|BOLD:AAG7673
Dusona|[3935]|SSPAC7161-13|Hymenoptera|Ichneumonidae|BOLD:ACD0393
Campopleginae|[3936]|CNGLF106-13|Hymenoptera|Ichneumonidae|BOLD:ACG6710
Campopleginae|[3937]|CNTNG334-14|Hymenoptera|Ichneumonidae|BOLD:ACN3966
Ichneumonidae|[3938]|SSPAC3364-13|Hymenoptera|Ichneumonidae|BOLD:ACJ3192
Echthronomas ochreofrons|[3939]|JSHYO121-11|Hymenoptera|Ichneumonidae|BOLD:AAZ4190
Echthronomas|[3940]|CNRMF569-12|Hymenoptera|Ichneumonidae|BOLD:ACF2293
Echthronomas ochreofrons|[3941]|CNSLR004-13|Hymenoptera|Ichneumonidae|BOLD:ACE9364
Echthronomas|[3942]|NGNAZ668-14|Hymenoptera|Ichneumonidae|BOLD:ACM0102
Campopleginae|[3943]|HPPPQ008-13|Hymenoptera|Ichneumonidae|BOLD:ACK3525
Campopleginae|[3944]|JBHCJ327-11|Hymenoptera|Ichneumonidae|BOLD:AAH1662
Campopleginae|[3945]|CNKJN516-14|Hymenoptera|Ichneumonidae|BOLD:ACF6578
Campopleginae|[3946]|TTMHY068-08|Hymenoptera|Ichneumonidae|BOLD:AAG7642
Campopleginae|[3947]|BBHYJ370-10|Hymenoptera|Ichneumonidae|BOLD:ACW7706
Campopleginae|[3948]|CNPPE1981-12|Hymenoptera|Ichneumonidae|BOLD:ACC8321
Campopleginae|[3949]|TTHYW1022-09|Hymenoptera|Ichneumonidae|BOLD:AAH1903
Campopleginae|[3950]|SSGBB4931-14|Hymenoptera|Ichneumonidae|BOLD:ACL8356
Campoplex|[3951]|CNNHD2316-14|Hymenoptera|Ichneumonidae|BOLD:AAE7919
Campopleginae|[3952]|CNBRM227-14|Hymenoptera|Ichneumonidae|BOLD:ACJ3380
Campoplex difformis|[3953]|SSBAD6032-13|Hymenoptera|Ichneumonidae|BOLD:ABX5830
Campoplex|[3954]|BBHYE318-10|Hymenoptera|Ichneumonidae|BOLD:ABX6562
Campoplex|[3955]|CNFNG1137-14|Hymenoptera|Ichneumonidae|BOLD:ACL8593
Campoplex difformis|[3956]|CNPEM2533-14|Hymenoptera|Ichneumonidae|BOLD:ACG4708
Campoplex|[3957]|SSPAB4648-13|Hymenoptera|Ichneumonidae|BOLD:AAE7918
Campoplex|[3958]|CNKJN033-14|Hymenoptera|Ichneumonidae|BOLD:ACN4442
Campoplex difformis|[3959]|CNYOC042-15|Hymenoptera|Ichneumonidae|BOLD:AAM6951
Campopleginae|[3960]|BBHYN032-10|Hymenoptera|Ichneumonidae|BOLD:AAU8825
Campoplex Janzen22|[3961]|JSHYO652-11|Hymenoptera|Ichneumonidae|BOLD:ABU7463
Campoplex|[3962]|HPPPO470-13|Hymenoptera|Ichneumonidae|BOLD:AAG5808
Campoplex|[3963]|CNPPD1500-12|Hymenoptera|Ichneumonidae|BOLD:ACC7866
Campoplex|[3964]|CNKJN487-14|Hymenoptera|Ichneumonidae|BOLD:AAH2180
Campopleginae|[3965]|JBHCI456-11|Hymenoptera|Ichneumonidae|BOLD:AAK1801
Campopleginae|[3966]|CNTNF1633-14|Hymenoptera|Ichneumonidae|BOLD:AAH1548
Campopleginae|[3967]|SSGBB2857-14|Hymenoptera|Ichneumonidae|BOLD:AAG5798
Campopleginae|[3968]|SSGBB4932-14|Hymenoptera|Ichneumonidae|BOLD:ACL3466
Campopleginae|[3969]|BBHYE932-10|Hymenoptera|Ichneumonidae|BOLD:AAM7484
Campopleginae|[3970]|BBHYJ781-10|Hymenoptera|Ichneumonidae|BOLD:AAU9252
Campopleginae|[3971]|NGNAC2761-13|Hymenoptera|Ichneumonidae|BOLD:ACO5732
Campopleginae|[3972]|JSHYO865-11|Hymenoptera|Ichneumonidae|BOLD:ABA8089
Campopleginae|[3973]|SSWLD4670-13|Hymenoptera|Ichneumonidae|BOLD:ACI8942
Campopleginae|[3974]|RBINA493-13|Hymenoptera|Ichneumonidae|BOLD:ACJ0285
Campopleginae|[3975]|JBHCH198-10|Hymenoptera|Ichneumonidae|BOLD:AAH1630
Campopleginae|[3976]|CNFDO456-14|Hymenoptera|Ichneumonidae|BOLD:ACM3032
Campopleginae|[3977]|CNPPC876-12|Hymenoptera|Ichneumonidae|BOLD:ACA7522
Campopleginae|[3978]|CNWLF298-12|Hymenoptera|Ichneumonidae|BOLD:ACC2668
Campopleginae|[3979]|SSROA1289-14|Hymenoptera|Ichneumonidae|BOLD:ABW3219
Campopleginae|[3980]|SSGBB4897-14|Hymenoptera|Ichneumonidae|BOLD:ACP2750
Campopleginae|[3981]|JBHCI554-11|Hymenoptera|Ichneumonidae|BOLD:AAH1524
Campopleginae|[3982]|CNTNG1531-14|Hymenoptera|Ichneumonidae|BOLD:AAG5796
Campopleginae|[3983]|TTMHY052-08|Hymenoptera|Ichneumonidae|BOLD:AAG7636
Campopleginae|[3984]|PAAUG1001-12|Hymenoptera|Ichneumonidae|BOLD:AAN5772
Campopleginae|[3985]|BBHEC835-10|Hymenoptera|Ichneumonidae|BOLD:AAG5805
Campopleginae|[3986]|BBHYK163-10|Hymenoptera|Ichneumonidae|BOLD:ACY7569
Campopleginae|[3987]|SSROB567-14|Hymenoptera|Ichneumonidae|BOLD:ACY6737
Campopleginae|[3988]|NGNAH072-13|Hymenoptera|Ichneumonidae|BOLD:ACJ3243
Campopleginae|[3989]|MBIOF1215-13|Hymenoptera|Ichneumonidae|BOLD:AAG8407
Campopleginae|[3990]|SSJAB3942-13|Hymenoptera|Ichneumonidae|BOLD:ACC3155
Campopleginae|[3991]|SMTPJ3389-14|Hymenoptera|Ichneumonidae|BOLD:AAY6849
Campopleginae|[3992]|CNWLH111-12|Hymenoptera|Ichneumonidae|BOLD:ACD3080

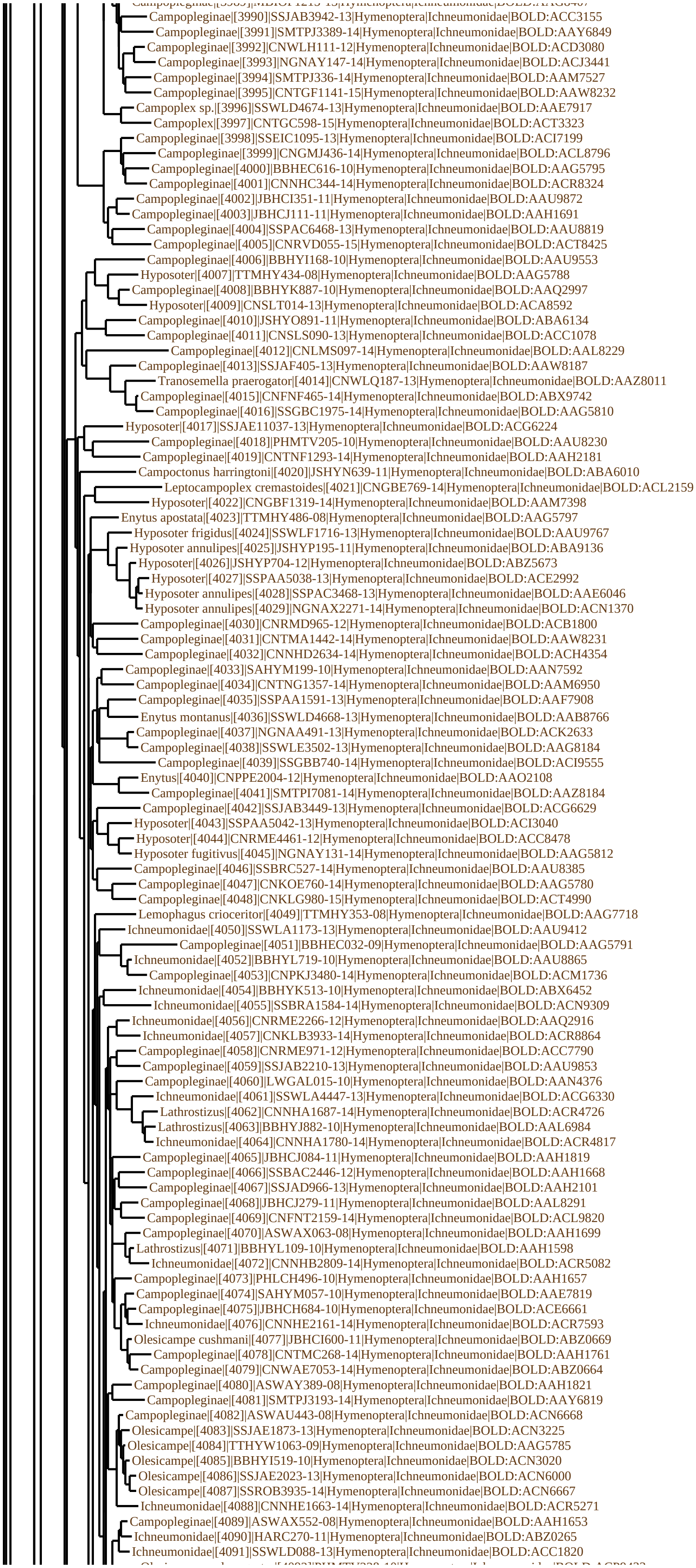

Campopleginae|[3990]|SSJAB3942-13|Hymenoptera|Ichneumonidae|BOLD:ACC3155
Campopleginae|[3991]|SMTPJ3389-14|Hymenoptera|Ichneumonidae|BOLD:AAY6849
Campopleginae|[3992]|CNWLH111-12|Hymenoptera|Ichneumonidae|BOLD:ACD3080
Campopleginae|[3993]|NGNAY147-14|Hymenoptera|Ichneumonidae|BOLD:ACJ3441
Campopleginae|[3994]|SMTPJ336-14|Hymenoptera|Ichneumonidae|BOLD:AAM7527
Campopleginae|[3995]|CNTGF1141-15|Hymenoptera|Ichneumonidae|BOLD:AAW8232
Campoplex sp.|[3996]|SSWLD4674-13|Hymenoptera|Ichneumonidae|BOLD:AAE7917
Campoplex|[3997]|CNTGC598-15|Hymenoptera|Ichneumonidae|BOLD:ACT3323
Campopleginae|[3998]|SSEIC1095-13|Hymenoptera|Ichneumonidae|BOLD:ACI7199
Campopleginae|[3999]|CNGMJ436-14|Hymenoptera|Ichneumonidae|BOLD:ACL8796
Campopleginae|[4000]|BBHEC616-10|Hymenoptera|Ichneumonidae|BOLD:AAG5795
Campopleginae|[4001]|CNNHC344-14|Hymenoptera|Ichneumonidae|BOLD:ACR8324
Campopleginae|[4002]|JBHCI351-11|Hymenoptera|Ichneumonidae|BOLD:AAU9872
Campopleginae|[4003]|JBHCJ111-11|Hymenoptera|Ichneumonidae|BOLD:AAH1691
Campopleginae|[4004]|SSPAC6468-13|Hymenoptera|Ichneumonidae|BOLD:AAU8819
Campopleginae|[4005]|CNRVD055-15|Hymenoptera|Ichneumonidae|BOLD:ACT8425
Campopleginae|[4006]|BBHYI168-10|Hymenoptera|Ichneumonidae|BOLD:AAU9553
Hyposoter|[4007]|TTMHY434-08|Hymenoptera|Ichneumonidae|BOLD:AAG5788
Campopleginae|[4008]|BBHYK887-10|Hymenoptera|Ichneumonidae|BOLD:AAQ2997
Hyposoter|[4009]|CNSLT014-13|Hymenoptera|Ichneumonidae|BOLD:ACA8592
Campopleginae|[4010]|JSHYO891-11|Hymenoptera|Ichneumonidae|BOLD:ABA6134
Campopleginae|[4011]|CNSLS090-13|Hymenoptera|Ichneumonidae|BOLD:ACC1078
Campopleginae|[4012]|CNLMS097-14|Hymenoptera|Ichneumonidae|BOLD:AAL8229
Campopleginae|[4013]|SSJAF405-13|Hymenoptera|Ichneumonidae|BOLD:AAW8187
Tranosemella praerogator|[4014]|CNWLQ187-13|Hymenoptera|Ichneumonidae|BOLD:AAZ8011
Campopleginae|[4015]|CNFNF465-14|Hymenoptera|Ichneumonidae|BOLD:ABX9742
Campopleginae|[4016]|SSGBC1975-14|Hymenoptera|Ichneumonidae|BOLD:AAG5810
Hyposoter|[4017]|SSJAE11037-13|Hymenoptera|Ichneumonidae|BOLD:ACG6224
Campopleginae|[4018]|PHMTV205-10|Hymenoptera|Ichneumonidae|BOLD:AAU8230
Campopleginae|[4019]|CNTNF1293-14|Hymenoptera|Ichneumonidae|BOLD:AAH2181
Campoctonus harringtoni|[4020]|JSHYN639-11|Hymenoptera|Ichneumonidae|BOLD:ABA6010
Leptocampoplex cremastoides|[4021]|CNGBE769-14|Hymenoptera|Ichneumonidae|BOLD:ACL2159
Hyposoter|[4022]|CNGBF1319-14|Hymenoptera|Ichneumonidae|BOLD:AAM7398
Enytus apostata|[4023]|TTMHY486-08|Hymenoptera|Ichneumonidae|BOLD:AAG5797
Hyposoter frigidus|[4024]|SSWLF1716-13|Hymenoptera|Ichneumonidae|BOLD:AAU9767
Hyposoter annulipes|[4025]|JSHYP195-11|Hymenoptera|Ichneumonidae|BOLD:ABA9136
Hyposoter|[4026]|JSHYP704-12|Hymenoptera|Ichneumonidae|BOLD:ABZ5673
Hyposoter|[4027]|SSPAA5038-13|Hymenoptera|Ichneumonidae|BOLD:ACE2992
Hyposoter annulipes|[4028]|SSPAC3468-13|Hymenoptera|Ichneumonidae|BOLD:AAE6046
Hyposoter annulipes|[4029]|NGNAX2271-14|Hymenoptera|Ichneumonidae|BOLD:ACN1370
Campopleginae|[4030]|CNRMD965-12|Hymenoptera|Ichneumonidae|BOLD:ACB1800
Campopleginae|[4031]|CNTMA1442-14|Hymenoptera|Ichneumonidae|BOLD:AAW8231
Campopleginae|[4032]|CNNHD2634-14|Hymenoptera|Ichneumonidae|BOLD:ACH4354
Campopleginae|[4033]|SAHYM199-10|Hymenoptera|Ichneumonidae|BOLD:AAN7592
Campopleginae|[4034]|CNTNG1357-14|Hymenoptera|Ichneumonidae|BOLD:AAM6950
Campopleginae|[4035]|SSPAA1591-13|Hymenoptera|Ichneumonidae|BOLD:AAF7908
Enytus montanus|[4036]|SSWLD4668-13|Hymenoptera|Ichneumonidae|BOLD:AAB8766
Campopleginae|[4037]|NGNAA491-13|Hymenoptera|Ichneumonidae|BOLD:ACK2633
Campopleginae|[4038]|SSWLE3502-13|Hymenoptera|Ichneumonidae|BOLD:AAG8184
Campopleginae|[4039]|SSGBB740-14|Hymenoptera|Ichneumonidae|BOLD:ACI9555
Enytus|[4040]|CNPPE2004-12|Hymenoptera|Ichneumonidae|BOLD:AAO2108
Campopleginae|[4041]|SMTPI7081-14|Hymenoptera|Ichneumonidae|BOLD:AAZ8184
Campopleginae|[4042]|SSJAB3449-13|Hymenoptera|Ichneumonidae|BOLD:ACG6629
Hyposoter|[4043]|SSPAA5042-13|Hymenoptera|Ichneumonidae|BOLD:ACI3040
Hyposoter|[4044]|CNRME4461-12|Hymenoptera|Ichneumonidae|BOLD:ACC8478
Hyposoter fugitivus|[4045]|NGNAY131-14|Hymenoptera|Ichneumonidae|BOLD:AAG5812
Campopleginae|[4046]|SSBRC527-14|Hymenoptera|Ichneumonidae|BOLD:AAU8385
Campopleginae|[4047]|CNKOE760-14|Hymenoptera|Ichneumonidae|BOLD:AAG5780
Campopleginae|[4048]|CNKLG980-15|Hymenoptera|Ichneumonidae|BOLD:ACT4990
Lemophagus crioceritor|[4049]|TTMHY353-08|Hymenoptera|Ichneumonidae|BOLD:AAG7718
Ichneumonidae|[4050]|SSWLA1173-13|Hymenoptera|Ichneumonidae|BOLD:AAU9412
Campopleginae|[4051]|BBHEC032-09|Hymenoptera|Ichneumonidae|BOLD:AAG5791
Ichneumonidae|[4052]|BBHYL719-10|Hymenoptera|Ichneumonidae|BOLD:AAU8865
Campopleginae|[4053]|CNPKJ3480-14|Hymenoptera|Ichneumonidae|BOLD:ACM1736
Ichneumonidae|[4054]|BBHYK513-10|Hymenoptera|Ichneumonidae|BOLD:ABX6452
Ichneumonidae|[4055]|SSBRA1584-14|Hymenoptera|Ichneumonidae|BOLD:ACN9309
Ichneumonidae|[4056]|CNRME2266-12|Hymenoptera|Ichneumonidae|BOLD:AAQ2916
Ichneumonidae|[4057]|CNKLB3933-14|Hymenoptera|Ichneumonidae|BOLD:ACR8864
Campopleginae|[4058]|CNRME971-12|Hymenoptera|Ichneumonidae|BOLD:ACC7790
Campopleginae|[4059]|SSJAB2210-13|Hymenoptera|Ichneumonidae|BOLD:AAU9853
Campopleginae|[4060]|LWGAL015-10|Hymenoptera|Ichneumonidae|BOLD:AAN4376
Ichneumonidae|[4061]|SSWLA4447-13|Hymenoptera|Ichneumonidae|BOLD:ACG6330
Lathrostizus|[4062]|CNNHA1687-14|Hymenoptera|Ichneumonidae|BOLD:ACR4726
Lathrostizus|[4063]|BBHYJ882-10|Hymenoptera|Ichneumonidae|BOLD:AAL6984
Ichneumonidae|[4064]|CNNHA1780-14|Hymenoptera|Ichneumonidae|BOLD:ACR4817
Campopleginae|[4065]|JBHCJ084-11|Hymenoptera|Ichneumonidae|BOLD:AAH1819
Campopleginae|[4066]|SSBAC2446-12|Hymenoptera|Ichneumonidae|BOLD:AAH1668
Campopleginae|[4067]|SSJAD966-13|Hymenoptera|Ichneumonidae|BOLD:AAH2101
Campopleginae|[4068]|JBHCJ279-11|Hymenoptera|Ichneumonidae|BOLD:AAL8291
Campopleginae|[4069]|CNFNT2159-14|Hymenoptera|Ichneumonidae|BOLD:ACL9820
Campopleginae|[4070]|ASWAX063-08|Hymenoptera|Ichneumonidae|BOLD:AAH1699
Lathrostizus|[4071]|BBHYL109-10|Hymenoptera|Ichneumonidae|BOLD:AAH1598
Ichneumonidae|[4072]|CNNHB2809-14|Hymenoptera|Ichneumonidae|BOLD:ACR5082
Campopleginae|[4073]|PHLCH496-10|Hymenoptera|Ichneumonidae|BOLD:AAH1657
Campopleginae|[4074]|SAHYM057-10|Hymenoptera|Ichneumonidae|BOLD:AAE7819
Campopleginae|[4075]|JBHCH684-10|Hymenoptera|Ichneumonidae|BOLD:ACE6661
Ichneumonidae|[4076]|CNNHE2161-14|Hymenoptera|Ichneumonidae|BOLD:ACR7593
Olesicampe cushmani|[4077]|JBHCI600-11|Hymenoptera|Ichneumonidae|BOLD:ABZ0669
Campopleginae|[4078]|CNTMC268-14|Hymenoptera|Ichneumonidae|BOLD:AAH1761
Campopleginae|[4079]|CNWAE7053-14|Hymenoptera|Ichneumonidae|BOLD:ABZ0664
Campopleginae|[4080]|ASWAY389-08|Hymenoptera|Ichneumonidae|BOLD:AAH1821
Campopleginae|[4081]|SMTPJ3193-14|Hymenoptera|Ichneumonidae|BOLD:AAY6819
Campopleginae|[4082]|ASWAU443-08|Hymenoptera|Ichneumonidae|BOLD:ACN6668
Olesicampe|[4083]|SSJAE1873-13|Hymenoptera|Ichneumonidae|BOLD:ACN3225
Olesicampe|[4084]|TTHYW1063-09|Hymenoptera|Ichneumonidae|BOLD:AAG5785
Olesicampe|[4085]|BBHYI519-10|Hymenoptera|Ichneumonidae|BOLD:ACN3020
Olesicampe|[4086]|SSJAE2023-13|Hymenoptera|Ichneumonidae|BOLD:ACN6000
Olesicampe|[4087]|SSROB3935-14|Hymenoptera|Ichneumonidae|BOLD:ACN6667
Ichneumonidae|[4088]|CNNHE1663-14|Hymenoptera|Ichneumonidae|BOLD:ACR5271
Campopleginae|[4089]|ASWAX552-08|Hymenoptera|Ichneumonidae|BOLD:AAH1653
Ichneumonidae|[4090]|HARC270-11|Hymenoptera|Ichneumonidae|BOLD:ABZ0265
Ichneumonidae|[4091]|SSWLD088-13|Hymenoptera|Ichneumonidae|BOLD:ACC1820

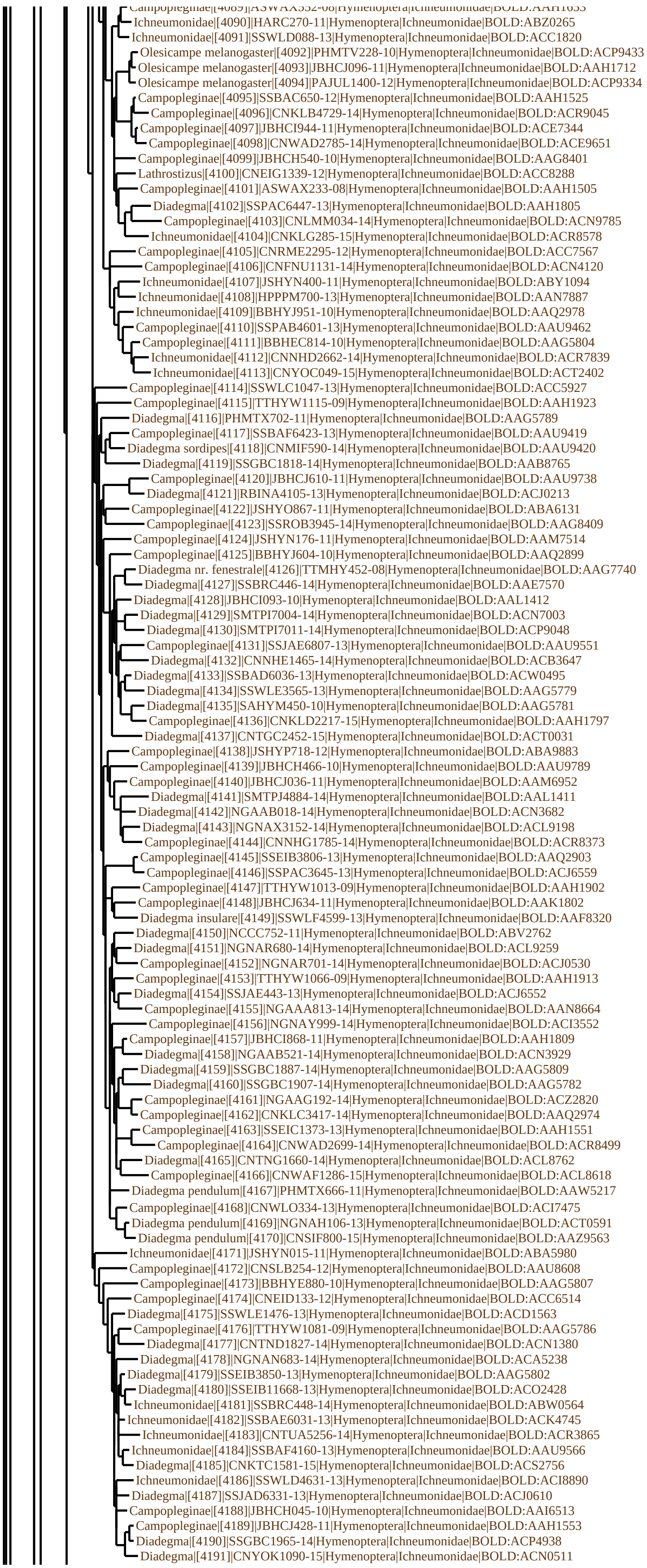
Campopleginae|[4089]|ASWAX332-08|Hymenoptera|Ichneumonidae|BOLD:AAH1633
Ichneumonidae|[4090]|HARC270-11|Hymenoptera|Ichneumonidae|BOLD:ABZ0265
Ichneumonidae|[4091]|SSWLD088-13|Hymenoptera|Ichneumonidae|BOLD:ACC1820
Olesicampe melanogaster|[4092]|PHMTV228-10|Hymenoptera|Ichneumonidae|BOLD:ACP9433
Olesicampe melanogaster|[4093]|JBHCJ096-11|Hymenoptera|Ichneumonidae|BOLD:AAH1712
Olesicampe melanogaster|[4094]|PAJUL1400-12|Hymenoptera|Ichneumonidae|BOLD:ACP9334
Campopleginae|[4095]|SSBAC650-12|Hymenoptera|Ichneumonidae|BOLD:AAH1525
Campopleginae|[4096]|CNKLB4729-14|Hymenoptera|Ichneumonidae|BOLD:ACR9045
Campopleginae|[4097]|JBHCI944-11|Hymenoptera|Ichneumonidae|BOLD:ACE7344
Campopleginae|[4098]|CNWAD2785-14|Hymenoptera|Ichneumonidae|BOLD:ACE9651
Campopleginae|[4099]|JBHCH540-10|Hymenoptera|Ichneumonidae|BOLD:AAG8401
Lathrostizus|[4100]|CNEIG1339-12|Hymenoptera|Ichneumonidae|BOLD:ACC8288
Campopleginae|[4101]|ASWAX233-08|Hymenoptera|Ichneumonidae|BOLD:AAH1505
Diadegma|[4102]|SSPAC6447-13|Hymenoptera|Ichneumonidae|BOLD:AAH1805
Campopleginae|[4103]|CNLMM034-14|Hymenoptera|Ichneumonidae|BOLD:ACN9785
Ichneumonidae|[4104]|CNKLG285-15|Hymenoptera|Ichneumonidae|BOLD:ACR8578
Campopleginae|[4105]|CNRME2295-12|Hymenoptera|Ichneumonidae|BOLD:ACC7567
Campopleginae|[4106]|CNFNU1131-14|Hymenoptera|Ichneumonidae|BOLD:ACN4120
Ichneumonidae|[4107]|JSHYN400-11|Hymenoptera|Ichneumonidae|BOLD:ABY1094
Ichneumonidae|[4108]|HPPPM700-13|Hymenoptera|Ichneumonidae|BOLD:AAN7887
Ichneumonidae|[4109]|BBHYJ951-10|Hymenoptera|Ichneumonidae|BOLD:AAQ2978
Campopleginae|[4110]|SSPAB4601-13|Hymenoptera|Ichneumonidae|BOLD:AAU9462
Campopleginae|[4111]|BBHEC814-10|Hymenoptera|Ichneumonidae|BOLD:AAG5804
Ichneumonidae|[4112]|CNNHD2662-14|Hymenoptera|Ichneumonidae|BOLD:ACR7839
Ichneumonidae|[4113]|CNYOC049-15|Hymenoptera|Ichneumonidae|BOLD:ACT2402
Campopleginae|[4114]|SSWLC1047-13|Hymenoptera|Ichneumonidae|BOLD:ACC5927
Campopleginae|[4115]|TTHYW1115-09|Hymenoptera|Ichneumonidae|BOLD:AAH1923
Diadegma|[4116]|PHMTX702-11|Hymenoptera|Ichneumonidae|BOLD:AAG5789
Campopleginae|[4117]|SSBAF6423-13|Hymenoptera|Ichneumonidae|BOLD:AAU9419
Diadegma sordipes|[4118]|CNMIF590-14|Hymenoptera|Ichneumonidae|BOLD:AAU9420
Diadegma|[4119]|SSGBC1818-14|Hymenoptera|Ichneumonidae|BOLD:AAB8765
Campopleginae|[4120]|JBHCJ610-11|Hymenoptera|Ichneumonidae|BOLD:AAU9738
Diadegma|[4121]|RBINA4105-13|Hymenoptera|Ichneumonidae|BOLD:ACJ0213
Campopleginae|[4122]|JSHYO867-11|Hymenoptera|Ichneumonidae|BOLD:ABA6131
Campopleginae|[4123]|SSROB3945-14|Hymenoptera|Ichneumonidae|BOLD:AAG8409
Campopleginae|[4124]|JSHYN176-11|Hymenoptera|Ichneumonidae|BOLD:AAM7514
Campopleginae|[4125]|BBHYJ604-10|Hymenoptera|Ichneumonidae|BOLD:AAQ2899
Diadegma nr. fenestrale|[4126]|TTMHY452-08|Hymenoptera|Ichneumonidae|BOLD:AAG7740
Diadegma|[4127]|SSBRC446-14|Hymenoptera|Ichneumonidae|BOLD:AAE7570
Diadegma|[4128]|JBHCI093-10|Hymenoptera|Ichneumonidae|BOLD:AAL1412
Diadegma|[4129]|SMTPI7004-14|Hymenoptera|Ichneumonidae|BOLD:ACN7003
Diadegma|[4130]|SMTPI7011-14|Hymenoptera|Ichneumonidae|BOLD:ACP9048
Campopleginae|[4131]|SSJAE6807-13|Hymenoptera|Ichneumonidae|BOLD:AAU9551
Diadegma|[4132]|CNNHE1465-14|Hymenoptera|Ichneumonidae|BOLD:ACB3647
Diadegma|[4133]|SSBAD6036-13|Hymenoptera|Ichneumonidae|BOLD:ACW0495
Diadegma|[4134]|SSWLE3565-13|Hymenoptera|Ichneumonidae|BOLD:AAG5779
Diadegma|[4135]|SAHYM450-10|Hymenoptera|Ichneumonidae|BOLD:AAG5781
Campopleginae|[4136]|CNKLD2217-15|Hymenoptera|Ichneumonidae|BOLD:AAH1797
Diadegma|[4137]|CNTGC2452-15|Hymenoptera|Ichneumonidae|BOLD:ACT0031
Campopleginae|[4138]|JSHYP718-12|Hymenoptera|Ichneumonidae|BOLD:ABA9883
Campopleginae|[4139]|JBHCH466-10|Hymenoptera|Ichneumonidae|BOLD:AAU9789
Campopleginae|[4140]|JBHCJ036-11|Hymenoptera|Ichneumonidae|BOLD:AAM6952
Diadegma|[4141]|SMTPJ4884-14|Hymenoptera|Ichneumonidae|BOLD:AAL1411
Diadegma|[4142]|NGAAB018-14|Hymenoptera|Ichneumonidae|BOLD:ACN3682
Diadegma|[4143]|NGNAX3152-14|Hymenoptera|Ichneumonidae|BOLD:ACL9198
Campopleginae|[4144]|CNNHG1785-14|Hymenoptera|Ichneumonidae|BOLD:ACR8373
Campopleginae|[4145]|SSEIB3806-13|Hymenoptera|Ichneumonidae|BOLD:AAQ2903
Campopleginae|[4146]|SSPAC3645-13|Hymenoptera|Ichneumonidae|BOLD:ACJ6559
Campopleginae|[4147]|TTHYW1013-09|Hymenoptera|Ichneumonidae|BOLD:AAH1902
Campopleginae|[4148]|JBHCJ634-11|Hymenoptera|Ichneumonidae|BOLD:AAK1802
Diadegma insulare|[4149]|SSWLF4599-13|Hymenoptera|Ichneumonidae|BOLD:AAF8320
Diadegma|[4150]|NCCC752-11|Hymenoptera|Ichneumonidae|BOLD:ABV2762
Diadegma|[4151]|NGNAR680-14|Hymenoptera|Ichneumonidae|BOLD:ACL9259
Campopleginae|[4152]|NGNAR701-14|Hymenoptera|Ichneumonidae|BOLD:ACJ0530
Campopleginae|[4153]|TTHYW1066-09|Hymenoptera|Ichneumonidae|BOLD:AAH1913
Diadegma|[4154]|SSJAE443-13|Hymenoptera|Ichneumonidae|BOLD:ACJ6552
Campopleginae|[4155]|NGAAA813-14|Hymenoptera|Ichneumonidae|BOLD:AAN8664
Campopleginae|[4156]|NGNAY999-14|Hymenoptera|Ichneumonidae|BOLD:ACI3552
Campopleginae|[4157]|JBHCI868-11|Hymenoptera|Ichneumonidae|BOLD:AAH1809
Diadegma|[4158]|NGAAB521-14|Hymenoptera|Ichneumonidae|BOLD:ACN3929
Diadegma|[4159]|SSGBC1887-14|Hymenoptera|Ichneumonidae|BOLD:AAG5809
Diadegma|[4160]|SSGBC1907-14|Hymenoptera|Ichneumonidae|BOLD:AAG5782
Campopleginae|[4161]|NGAAG192-14|Hymenoptera|Ichneumonidae|BOLD:ACZ2820
Campopleginae|[4162]|CNKLC3417-14|Hymenoptera|Ichneumonidae|BOLD:AAQ2974
Campopleginae|[4163]|SSEIC1373-13|Hymenoptera|Ichneumonidae|BOLD:AAH1551
Campopleginae|[4164]|CNWAD2699-14|Hymenoptera|Ichneumonidae|BOLD:ACR8499
Diadegma|[4165]|CNTNG1660-14|Hymenoptera|Ichneumonidae|BOLD:ACL8762
Campopleginae|[4166]|CNWAF1286-15|Hymenoptera|Ichneumonidae|BOLD:ACL8618
Diadegma pendulum|[4167]|PHMTX666-11|Hymenoptera|Ichneumonidae|BOLD:AAW5217
Campopleginae|[4168]|CNWLO334-13|Hymenoptera|Ichneumonidae|BOLD:ACI7475
Diadegma pendulum|[4169]|NGNAH106-13|Hymenoptera|Ichneumonidae|BOLD:ACT0591
Diadegma pendulum|[4170]|CNSIF800-15|Hymenoptera|Ichneumonidae|BOLD:AAZ9563
Ichneumonidae|[4171]|JSHYN015-11|Hymenoptera|Ichneumonidae|BOLD:ABA5980
Campopleginae|[4172]|CNSLB254-12|Hymenoptera|Ichneumonidae|BOLD:AAU8608
Campopleginae|[4173]|BBHYE880-10|Hymenoptera|Ichneumonidae|BOLD:AAG5807
Campopleginae|[4174]|CNEID133-12|Hymenoptera|Ichneumonidae|BOLD:ACC6514
Diadegma|[4175]|SSWLE1476-13|Hymenoptera|Ichneumonidae|BOLD:ACD1563
Campopleginae|[4176]|TTHYW1081-09|Hymenoptera|Ichneumonidae|BOLD:AAG5786
Diadegma|[4177]|CNTND1827-14|Hymenoptera|Ichneumonidae|BOLD:ACN1380
Diadegma|[4178]|NGNAN683-14|Hymenoptera|Ichneumonidae|BOLD:ACA5238
Diadegma|[4179]|SSEIB3850-13|Hymenoptera|Ichneumonidae|BOLD:AAG5802
Diadegma|[4180]|SSEIB11668-13|Hymenoptera|Ichneumonidae|BOLD:ACO2428
Ichneumonidae|[4181]|SSBRC448-14|Hymenoptera|Ichneumonidae|BOLD:ABW0564
Ichneumonidae|[4182]|SSBAE6031-13|Hymenoptera|Ichneumonidae|BOLD:ACK4745
Ichneumonidae|[4183]|CNTUA5256-14|Hymenoptera|Ichneumonidae|BOLD:ACR3865
Ichneumonidae|[4184]|SSBAF4160-13|Hymenoptera|Ichneumonidae|BOLD:AAU9566
Diadegma|[4185]|CNKTC1581-15|Hymenoptera|Ichneumonidae|BOLD:ACS2756
Ichneumonidae|[4186]|SSWLD4631-13|Hymenoptera|Ichneumonidae|BOLD:ACI8890
Diadegma|[4187]|SSJAD6331-13|Hymenoptera|Ichneumonidae|BOLD:ACJ0610
Campopleginae|[4188]|JBHCH045-10|Hymenoptera|Ichneumonidae|BOLD:AAI6513
Campopleginae|[4189]|JBHCJ428-11|Hymenoptera|Ichneumonidae|BOLD:AAH1553
Diadegma|[4190]|SSGBC1965-14|Hymenoptera|Ichneumonidae|BOLD:ACP4938
Diadegma|[4191]|CNYOK1090-15|Hymenoptera|Ichneumonidae|BOLD:ACN0511

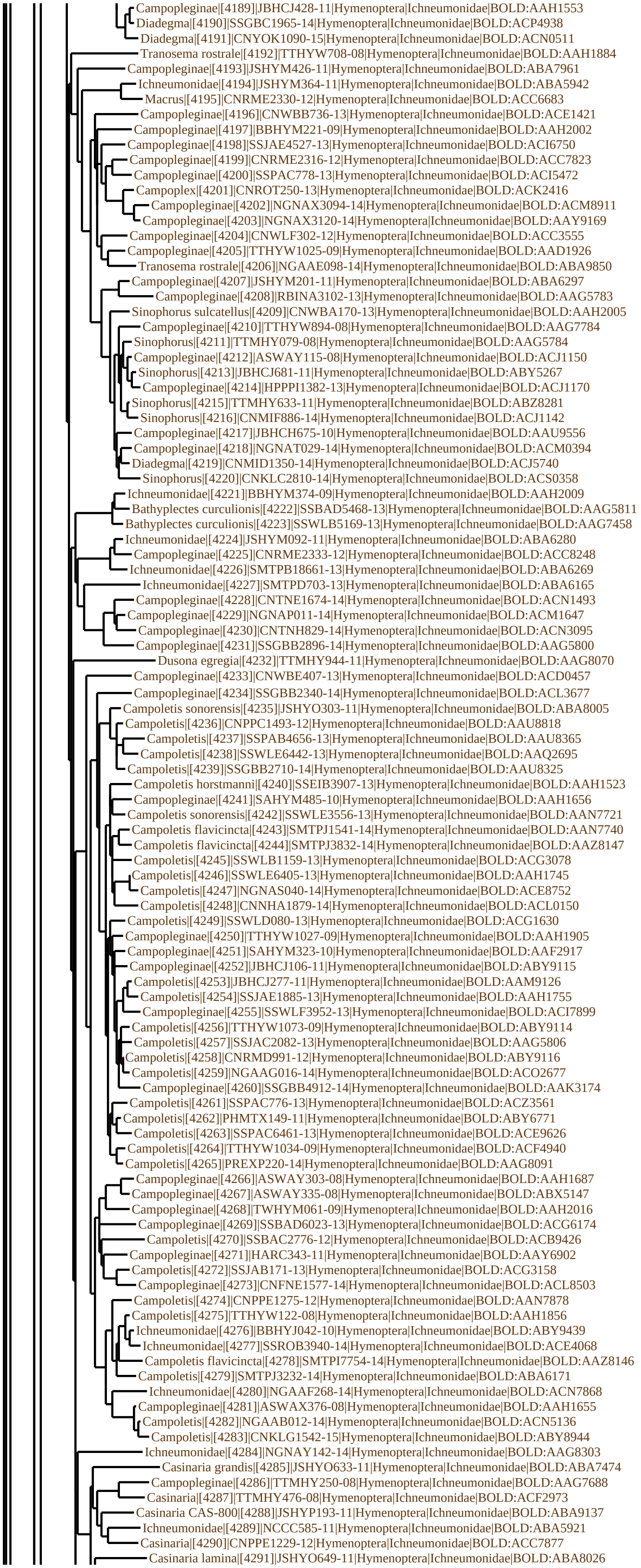

Campopleginae|[4189]|JBHCJ428-11|Hymenoptera|Ichneumonidae|BOLD:AAH1553
Diadegma|[4190]|SSGBC1965-14|Hymenoptera|Ichneumonidae|BOLD:ACP4938
Diadegma|[4191]|CNYOK1090-15|Hymenoptera|Ichneumonidae|BOLD:ACN0511
Tranosema rostrale|[4192]|TTHYW708-08|Hymenoptera|Ichneumonidae|BOLD:AAH1884
Campopleginae|[4193]|JSHYM426-11|Hymenoptera|Ichneumonidae|BOLD:ABA7961
Ichneumonidae|[4194]|JSHYM364-11|Hymenoptera|Ichneumonidae|BOLD:ABA5942
Macrus|[4195]|CNRME2330-12|Hymenoptera|Ichneumonidae|BOLD:ACC6683
Campopleginae|[4196]|CNWBB736-13|Hymenoptera|Ichneumonidae|BOLD:ACE1421
Campopleginae|[4197]|BBHYM221-09|Hymenoptera|Ichneumonidae|BOLD:AAH2002
Campopleginae|[4198]|SSJAE4527-13|Hymenoptera|Ichneumonidae|BOLD:ACI6750
Campopleginae|[4199]|CNRME2316-12|Hymenoptera|Ichneumonidae|BOLD:ACC7823
Campopleginae|[4200]|SSPAC778-13|Hymenoptera|Ichneumonidae|BOLD:ACI5472
Campoplex|[4201]|CNROT250-13|Hymenoptera|Ichneumonidae|BOLD:ACK2416
Campopleginae|[4202]|NGNAX3094-14|Hymenoptera|Ichneumonidae|BOLD:ACM8911
Campopleginae|[4203]|NGNAX3120-14|Hymenoptera|Ichneumonidae|BOLD:AAY9169
Campopleginae|[4204]|CNWLF302-12|Hymenoptera|Ichneumonidae|BOLD:ACC3555
Campopleginae|[4205]|TTHYW1025-09|Hymenoptera|Ichneumonidae|BOLD:AAD1926
Tranosema rostrale|[4206]|NGAAE098-14|Hymenoptera|Ichneumonidae|BOLD:ABA9850
Campopleginae|[4207]|JSHYM201-11|Hymenoptera|Ichneumonidae|BOLD:ABA6297
Campopleginae|[4208]|RBINA3102-13|Hymenoptera|Ichneumonidae|BOLD:AAG5783
Sinophorus sulcatellus|[4209]|CNWBA170-13|Hymenoptera|Ichneumonidae|BOLD:AAH2005
Campopleginae|[4210]|TTHYW894-08|Hymenoptera|Ichneumonidae|BOLD:AAG7784
Sinophorus|[4211]|TTMHY079-08|Hymenoptera|Ichneumonidae|BOLD:AAG5784
Campopleginae|[4212]|ASWAY115-08|Hymenoptera|Ichneumonidae|BOLD:ACJ1150
Sinophorus|[4213]|JBHCJ681-11|Hymenoptera|Ichneumonidae|BOLD:ABY5267
Campopleginae|[4214]|HPPPI1382-13|Hymenoptera|Ichneumonidae|BOLD:ACJ1170
Sinophorus|[4215]|TTMHY633-11|Hymenoptera|Ichneumonidae|BOLD:ABZ8281
Sinophorus|[4216]|CNMIF886-14|Hymenoptera|Ichneumonidae|BOLD:ACJ1142
Campopleginae|[4217]|JBHCH675-10|Hymenoptera|Ichneumonidae|BOLD:AAU9556
Campopleginae|[4218]|NGNAT029-14|Hymenoptera|Ichneumonidae|BOLD:ACM0394
Diadegma|[4219]|CNMID1350-14|Hymenoptera|Ichneumonidae|BOLD:ACJ5740
Sinophorus|[4220]|CNKLC2810-14|Hymenoptera|Ichneumonidae|BOLD:ACS0358
Ichneumonidae|[4221]|BBHYM374-09|Hymenoptera|Ichneumonidae|BOLD:AAH2009
Bathyplectes curculionis|[4222]|SSBAD5468-13|Hymenoptera|Ichneumonidae|BOLD:AAG5811
Bathyplectes curculionis|[4223]|SSWLB5169-13|Hymenoptera|Ichneumonidae|BOLD:AAG7458
Ichneumonidae|[4224]|JSHYM092-11|Hymenoptera|Ichneumonidae|BOLD:ABA6280
Campopleginae|[4225]|CNRME2333-12|Hymenoptera|Ichneumonidae|BOLD:ACC8248
Ichneumonidae|[4226]|SMTPB18661-13|Hymenoptera|Ichneumonidae|BOLD:ABA6269
Ichneumonidae|[4227]|SMTPD703-13|Hymenoptera|Ichneumonidae|BOLD:ABA6165
Campopleginae|[4228]|CNTNE1674-14|Hymenoptera|Ichneumonidae|BOLD:ACN1493
Campopleginae|[4229]|NGNAP011-14|Hymenoptera|Ichneumonidae|BOLD:ACM1647
Campopleginae|[4230]|CNTNH829-14|Hymenoptera|Ichneumonidae|BOLD:ACN3095
Campopleginae|[4231]|SSGBB2896-14|Hymenoptera|Ichneumonidae|BOLD:AAG5800
Dusona egregia|[4232]|TTMHY944-11|Hymenoptera|Ichneumonidae|BOLD:AAG8070
Campopleginae|[4233]|CNWBE407-13|Hymenoptera|Ichneumonidae|BOLD:ACD0457
Campopleginae|[4234]|SSGBB2340-14|Hymenoptera|Ichneumonidae|BOLD:ACL3677
Campoletis sonorensis|[4235]|JSHYO303-11|Hymenoptera|Ichneumonidae|BOLD:ABA8005
Campoletis|[4236]|CNPPC1493-12|Hymenoptera|Ichneumonidae|BOLD:AAU8818
Campoletis|[4237]|SSPAB4656-13|Hymenoptera|Ichneumonidae|BOLD:AAU8365
Campoletis|[4238]|SSWLE6442-13|Hymenoptera|Ichneumonidae|BOLD:AAQ2695
Campoletis|[4239]|SSGBB2710-14|Hymenoptera|Ichneumonidae|BOLD:AAU8325
Campoletis horstmanni|[4240]|SSEIB3907-13|Hymenoptera|Ichneumonidae|BOLD:AAH1523
Campopleginae|[4241]|SAHYM485-10|Hymenoptera|Ichneumonidae|BOLD:AAH1656
Campoletis sonorensis|[4242]|SSWLE3556-13|Hymenoptera|Ichneumonidae|BOLD:AAN7721
Campoletis flavicincta|[4243]|SMTPJ1541-14|Hymenoptera|Ichneumonidae|BOLD:AAN7740
Campoletis flavicincta|[4244]|SMTPJ3832-14|Hymenoptera|Ichneumonidae|BOLD:AAZ8147
Campoletis|[4245]|SSWLB1159-13|Hymenoptera|Ichneumonidae|BOLD:ACG3078
Campoletis|[4246]|SSWLE6405-13|Hymenoptera|Ichneumonidae|BOLD:AAH1745
Campoletis|[4247]|NGNAS040-14|Hymenoptera|Ichneumonidae|BOLD:ACE8752
Campoletis|[4248]|CNNHA1879-14|Hymenoptera|Ichneumonidae|BOLD:ACL0150
Campoletis|[4249]|SSWLD080-13|Hymenoptera|Ichneumonidae|BOLD:ACG1630
Campopleginae|[4250]|TTHYW1027-09|Hymenoptera|Ichneumonidae|BOLD:AAH1905
Campopleginae|[4251]|SAHYM323-10|Hymenoptera|Ichneumonidae|BOLD:AAF2917
Campopleginae|[4252]|JBHCJ106-11|Hymenoptera|Ichneumonidae|BOLD:ABY9115
Campoletis|[4253]|JBHCJ277-11|Hymenoptera|Ichneumonidae|BOLD:AAM9126
Campoletis|[4254]|SSJAE1885-13|Hymenoptera|Ichneumonidae|BOLD:AAH1755
Campopleginae|[4255]|SSWLF3952-13|Hymenoptera|Ichneumonidae|BOLD:ACI7899
Campoletis|[4256]|TTHYW1073-09|Hymenoptera|Ichneumonidae|BOLD:ABY9114
Campoletis|[4257]|SSJAC2082-13|Hymenoptera|Ichneumonidae|BOLD:AAG5806
Campoletis|[4258]|CNRMD991-12|Hymenoptera|Ichneumonidae|BOLD:ABY9116
Campoletis|[4259]|NGAAG016-14|Hymenoptera|Ichneumonidae|BOLD:ACO2677
Campopleginae|[4260]|SSGBB4912-14|Hymenoptera|Ichneumonidae|BOLD:AAK3174
Campoletis|[4261]|SSPAC776-13|Hymenoptera|Ichneumonidae|BOLD:ACZ3561
Campoletis|[4262]|PHMTX149-11|Hymenoptera|Ichneumonidae|BOLD:ABY6771
Campoletis|[4263]|SSPAC6461-13|Hymenoptera|Ichneumonidae|BOLD:ACE9626
Campoletis|[4264]|TTHYW1034-09|Hymenoptera|Ichneumonidae|BOLD:ACF4940
Campoletis|[4265]|PREXP220-14|Hymenoptera|Ichneumonidae|BOLD:AAG8091
Campopleginae|[4266]|ASWAY303-08|Hymenoptera|Ichneumonidae|BOLD:AAH1687
Campopleginae|[4267]|ASWAY335-08|Hymenoptera|Ichneumonidae|BOLD:ABX5147
Campopleginae|[4268]|TWHYM061-09|Hymenoptera|Ichneumonidae|BOLD:AAH2016
Campopleginae|[4269]|SSBAD6023-13|Hymenoptera|Ichneumonidae|BOLD:ACG6174
Campoletis|[4270]|SSBAC2776-12|Hymenoptera|Ichneumonidae|BOLD:ACB9426
Campopleginae|[4271]|HARC343-11|Hymenoptera|Ichneumonidae|BOLD:AAY6902
Campoletis|[4272]|SSJAB171-13|Hymenoptera|Ichneumonidae|BOLD:ACG3158
Campopleginae|[4273]|CNFNE1577-14|Hymenoptera|Ichneumonidae|BOLD:ACL8503
Campoletis|[4274]|CNPPE1275-12|Hymenoptera|Ichneumonidae|BOLD:AAN7878
Campoletis|[4275]|TTHYW122-08|Hymenoptera|Ichneumonidae|BOLD:AAH1856
Ichneumonidae|[4276]|BBHYJ042-10|Hymenoptera|Ichneumonidae|BOLD:ABY9439
Ichneumonidae|[4277]|SSROB3940-14|Hymenoptera|Ichneumonidae|BOLD:ACE4068
Campoletis flavicincta|[4278]|SMTPI7754-14|Hymenoptera|Ichneumonidae|BOLD:AAZ8146
Campoletis|[4279]|SMTPJ3232-14|Hymenoptera|Ichneumonidae|BOLD:ABA6171
Ichneumonidae|[4280]|NGAAF268-14|Hymenoptera|Ichneumonidae|BOLD:ACN7868
Campopleginae|[4281]|ASWAX376-08|Hymenoptera|Ichneumonidae|BOLD:AAH1655
Campoletis|[4282]|NGAAB012-14|Hymenoptera|Ichneumonidae|BOLD:ACN5136
Campoletis|[4283]|CNKLG1542-15|Hymenoptera|Ichneumonidae|BOLD:ABY8944
Ichneumonidae|[4284]|NGNAY142-14|Hymenoptera|Ichneumonidae|BOLD:AAG8303
Casinaria grandis|[4285]|JSHYO633-11|Hymenoptera|Ichneumonidae|BOLD:ABA7474
Campopleginae|[4286]|TTMHY250-08|Hymenoptera|Ichneumonidae|BOLD:AAG7688
Casinaria|[4287]|TTMHY476-08|Hymenoptera|Ichneumonidae|BOLD:ACF2973
Casinaria CAS-800|[4288]|JSHYP193-11|Hymenoptera|Ichneumonidae|BOLD:ABA9137
Ichneumonidae|[4289]|NCCC585-11|Hymenoptera|Ichneumonidae|BOLD:ABA5921
Casinaria|[4290]|CNPPE1229-12|Hymenoptera|Ichneumonidae|BOLD:ACC7877
Casinaria lamina|[4291]|JSHYO649-11|Hymenoptera|Ichneumonidae|BOLD:ABA8026

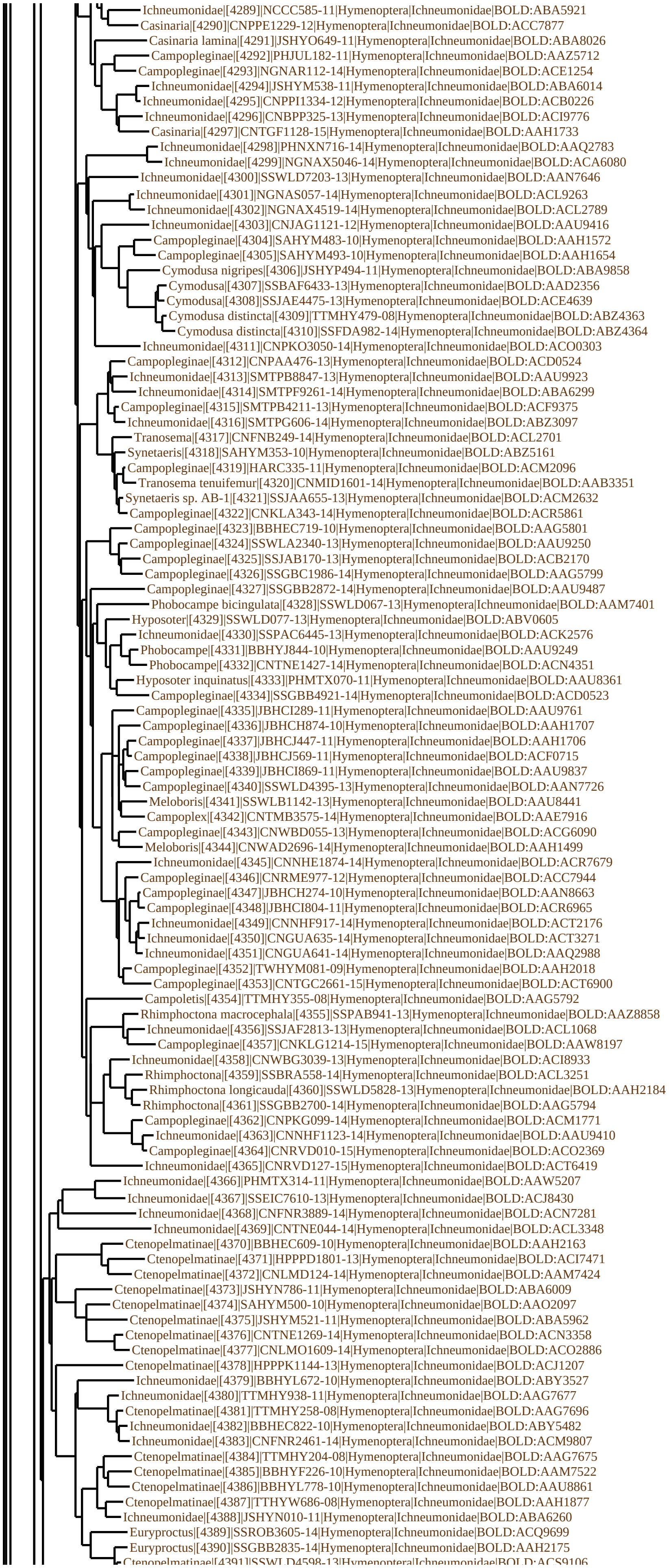
Ichneumonidae|[4289]|NCCC585-11|Hymenoptera|Ichneumonidae|BOLD:ABA5921
Casinaria|[4290]|CNPPE1229-12|Hymenoptera|Ichneumonidae|BOLD:ACC7877
Casinaria lamina|[4291]|JSHYO649-11|Hymenoptera|Ichneumonidae|BOLD:ABA8026
Campopleginae|[4292]|PHJUL182-11|Hymenoptera|Ichneumonidae|BOLD:AAZ5712
Campopleginae|[4293]|NGNAR112-14|Hymenoptera|Ichneumonidae|BOLD:ACE1254
Ichneumonidae|[4294]|JSHYM538-11|Hymenoptera|Ichneumonidae|BOLD:ABA6014
Ichneumonidae|[4295]|CNPPI1334-12|Hymenoptera|Ichneumonidae|BOLD:ACB0226
Ichneumonidae|[4296]|CNBPP325-13|Hymenoptera|Ichneumonidae|BOLD:ACI9776
Casinaria|[4297]|CNTGF1128-15|Hymenoptera|Ichneumonidae|BOLD:AAH1733
Ichneumonidae|[4298]|PHNXN716-14|Hymenoptera|Ichneumonidae|BOLD:AAQ2783
Ichneumonidae|[4299]|NGNAX5046-14|Hymenoptera|Ichneumonidae|BOLD:ACA6080
Ichneumonidae|[4300]|SSWLD7203-13|Hymenoptera|Ichneumonidae|BOLD:AAN7646
Ichneumonidae|[4301]|NGNAS057-14|Hymenoptera|Ichneumonidae|BOLD:ACL9263
Ichneumonidae|[4302]|NGNAX4519-14|Hymenoptera|Ichneumonidae|BOLD:ACL2789
Ichneumonidae|[4303]|CNJAG1121-12|Hymenoptera|Ichneumonidae|BOLD:AAU9416
Campopleginae|[4304]|SAHYM483-10|Hymenoptera|Ichneumonidae|BOLD:AAH1572
Campopleginae|[4305]|SAHYM493-10|Hymenoptera|Ichneumonidae|BOLD:AAH1654
Cymodusa nigripes|[4306]|JSHYP494-11|Hymenoptera|Ichneumonidae|BOLD:ABA9858
Cymodusa|[4307]|SSBAF6433-13|Hymenoptera|Ichneumonidae|BOLD:AAD2356
Cymodusa|[4308]|SSJAE4475-13|Hymenoptera|Ichneumonidae|BOLD:ACE4639
Cymodusa distincta|[4309]|TTMHY479-08|Hymenoptera|Ichneumonidae|BOLD:ABZ4363
Cymodusa distincta|[4310]|SSFDA982-14|Hymenoptera|Ichneumonidae|BOLD:ABZ4364
Ichneumonidae|[4311]|CNPKO3050-14|Hymenoptera|Ichneumonidae|BOLD:ACO0303
Campopleginae|[4312]|CNPAA476-13|Hymenoptera|Ichneumonidae|BOLD:ACD0524
Ichneumonidae|[4313]|SMTPB8847-13|Hymenoptera|Ichneumonidae|BOLD:AAU9923
Ichneumonidae|[4314]|SMTPF9261-14|Hymenoptera|Ichneumonidae|BOLD:ABA6299
Campopleginae|[4315]|SMTPB4211-13|Hymenoptera|Ichneumonidae|BOLD:ACF9375
Ichneumonidae|[4316]|SMTPG606-14|Hymenoptera|Ichneumonidae|BOLD:ABZ3097
Tranosema|[4317]|CNFNB249-14|Hymenoptera|Ichneumonidae|BOLD:ACL2701
Synetaeris|[4318]|SAHYM353-10|Hymenoptera|Ichneumonidae|BOLD:ABZ5161
Campopleginae|[4319]|HARC335-11|Hymenoptera|Ichneumonidae|BOLD:ACM2096
Tranosema tenuifemur|[4320]|CNMID1601-14|Hymenoptera|Ichneumonidae|BOLD:AAB3351
Synetaeris sp. AB-1|[4321]|SSJAA655-13|Hymenoptera|Ichneumonidae|BOLD:ACM2632
Campopleginae|[4322]|CNKLA343-14|Hymenoptera|Ichneumonidae|BOLD:ACR5861
Campopleginae|[4323]|BBHEC719-10|Hymenoptera|Ichneumonidae|BOLD:AAG5801
Campopleginae|[4324]|SSWLA2340-13|Hymenoptera|Ichneumonidae|BOLD:AAU9250
Campopleginae|[4325]|SSJAB170-13|Hymenoptera|Ichneumonidae|BOLD:ACB2170
Campopleginae|[4326]|SSGBC1986-14|Hymenoptera|Ichneumonidae|BOLD:AAG5799
Campopleginae|[4327]|SSGBB2872-14|Hymenoptera|Ichneumonidae|BOLD:AAU9487
Phobocampe bicingulata|[4328]|SSWLD067-13|Hymenoptera|Ichneumonidae|BOLD:AAM7401
Hyposoter|[4329]|SSWLD077-13|Hymenoptera|Ichneumonidae|BOLD:ABV0605
Ichneumonidae|[4330]|SSPAC6445-13|Hymenoptera|Ichneumonidae|BOLD:ACK2576
Phobocampe|[4331]|BBHYJ844-10|Hymenoptera|Ichneumonidae|BOLD:AAU9249
Phobocampe|[4332]|CNTNE1427-14|Hymenoptera|Ichneumonidae|BOLD:ACN4351
Hyposoter inquinatus|[4333]|PHMTX070-11|Hymenoptera|Ichneumonidae|BOLD:AAU8361
Campopleginae|[4334]|SSGBB4921-14|Hymenoptera|Ichneumonidae|BOLD:ACD0523
Campopleginae|[4335]|JBHCI289-11|Hymenoptera|Ichneumonidae|BOLD:AAU9761
Campopleginae|[4336]|JBHCH874-10|Hymenoptera|Ichneumonidae|BOLD:AAH1707
Campopleginae|[4337]|JBHCJ447-11|Hymenoptera|Ichneumonidae|BOLD:AAH1706
Campopleginae|[4338]|JBHCJ569-11|Hymenoptera|Ichneumonidae|BOLD:ACF0715
Campopleginae|[4339]|JBHCI869-11|Hymenoptera|Ichneumonidae|BOLD:AAU9837
Campopleginae|[4340]|SSWLD4395-13|Hymenoptera|Ichneumonidae|BOLD:AAN7726
Meloboris|[4341]|SSWLB1142-13|Hymenoptera|Ichneumonidae|BOLD:AAU8441
Campoplex|[4342]|CNTMB3575-14|Hymenoptera|Ichneumonidae|BOLD:AAE7916
Campopleginae|[4343]|CNWBD055-13|Hymenoptera|Ichneumonidae|BOLD:ACG6090
Meloboris|[4344]|CNWAD2696-14|Hymenoptera|Ichneumonidae|BOLD:AAH1499
Ichneumonidae|[4345]|CNNHE1874-14|Hymenoptera|Ichneumonidae|BOLD:ACR7679
Campopleginae|[4346]|CNRME977-12|Hymenoptera|Ichneumonidae|BOLD:ACC7944
Campopleginae|[4347]|JBHCH274-10|Hymenoptera|Ichneumonidae|BOLD:AAN8663
Campopleginae|[4348]|JBHCI804-11|Hymenoptera|Ichneumonidae|BOLD:ACR6965
Ichneumonidae|[4349]|CNNHF917-14|Hymenoptera|Ichneumonidae|BOLD:ACT2176
Ichneumonidae|[4350]|CNGUA635-14|Hymenoptera|Ichneumonidae|BOLD:ACT3271
Ichneumonidae|[4351]|CNGUA641-14|Hymenoptera|Ichneumonidae|BOLD:AAQ2988
Campopleginae|[4352]|TWHYM081-09|Hymenoptera|Ichneumonidae|BOLD:AAH2018
Campopleginae|[4353]|CNTGC2661-15|Hymenoptera|Ichneumonidae|BOLD:ACT6900
Campoletis|[4354]|TTMHY355-08|Hymenoptera|Ichneumonidae|BOLD:AAG5792
Rhimphoctona macrocephala|[4355]|SSPAB941-13|Hymenoptera|Ichneumonidae|BOLD:AAZ8858
Ichneumonidae|[4356]|SSJAF2813-13|Hymenoptera|Ichneumonidae|BOLD:ACL1068
Campopleginae|[4357]|CNKLG1214-15|Hymenoptera|Ichneumonidae|BOLD:AAW8197
Ichneumonidae|[4358]|CNWBG3039-13|Hymenoptera|Ichneumonidae|BOLD:ACI8933
Rhimphoctona|[4359]|SSBRA558-14|Hymenoptera|Ichneumonidae|BOLD:ACL3251
Rhimphoctona longicauda|[4360]|SSWLD5828-13|Hymenoptera|Ichneumonidae|BOLD:AAH2184
Rhimphoctona|[4361]|SSGBB2700-14|Hymenoptera|Ichneumonidae|BOLD:AAG5794
Campopleginae|[4362]|CNPKG099-14|Hymenoptera|Ichneumonidae|BOLD:ACM1771
Ichneumonidae|[4363]|CNNHF1123-14|Hymenoptera|Ichneumonidae|BOLD:AAU9410
Campopleginae|[4364]|CNRVD010-15|Hymenoptera|Ichneumonidae|BOLD:ACO2369
Ichneumonidae|[4365]|CNRVD127-15|Hymenoptera|Ichneumonidae|BOLD:ACT6419
Ichneumonidae|[4366]|PHMTX314-11|Hymenoptera|Ichneumonidae|BOLD:AAW5207
Ichneumonidae|[4367]|SSEIC7610-13|Hymenoptera|Ichneumonidae|BOLD:ACJ8430
Ichneumonidae|[4368]|CNFNR3889-14|Hymenoptera|Ichneumonidae|BOLD:ACN7281
Ichneumonidae|[4369]|CNTNE044-14|Hymenoptera|Ichneumonidae|BOLD:ACL3348
Ctenopelmatinae|[4370]|BBHEC609-10|Hymenoptera|Ichneumonidae|BOLD:AAH2163
Ctenopelmatinae|[4371]|HPPPD1801-13|Hymenoptera|Ichneumonidae|BOLD:ACI7471
Ctenopelmatinae|[4372]|CNLMD124-14|Hymenoptera|Ichneumonidae|BOLD:AAM7424
Ctenopelmatinae|[4373]|JSHYN786-11|Hymenoptera|Ichneumonidae|BOLD:ABA6009
Ctenopelmatinae|[4374]|SAHYM500-10|Hymenoptera|Ichneumonidae|BOLD:AAO2097
Ctenopelmatinae|[4375]|JSHYM521-11|Hymenoptera|Ichneumonidae|BOLD:ABA5962
Ctenopelmatinae|[4376]|CNTNE1269-14|Hymenoptera|Ichneumonidae|BOLD:ACN3358
Ctenopelmatinae|[4377]|CNLMO1609-14|Hymenoptera|Ichneumonidae|BOLD:ACO2886
Ctenopelmatinae|[4378]|HPPPK1144-13|Hymenoptera|Ichneumonidae|BOLD:ACJ1207
Ichneumonidae|[4379]|BBHYL672-10|Hymenoptera|Ichneumonidae|BOLD:ABY3527
Ichneumonidae|[4380]|TTMHY938-11|Hymenoptera|Ichneumonidae|BOLD:AAG7677
Ctenopelmatinae|[4381]|TTMHY258-08|Hymenoptera|Ichneumonidae|BOLD:AAG7696
Ichneumonidae|[4382]|BBHEC822-10|Hymenoptera|Ichneumonidae|BOLD:ABY5482
Ichneumonidae|[4383]|CNFNR2461-14|Hymenoptera|Ichneumonidae|BOLD:ACM9807
Ctenopelmatinae|[4384]|TTMHY204-08|Hymenoptera|Ichneumonidae|BOLD:AAG7675
Ctenopelmatinae|[4385]|BBHYF226-10|Hymenoptera|Ichneumonidae|BOLD:AAM7522
Ctenopelmatinae|[4386]|BBHYL778-10|Hymenoptera|Ichneumonidae|BOLD:AAU8861
Ctenopelmatinae|[4387]|TTHYW686-08|Hymenoptera|Ichneumonidae|BOLD:AAH1877
Ichneumonidae|[4388]|JSHYN010-11|Hymenoptera|Ichneumonidae|BOLD:ABA6260
Euryproctus|[4389]|SSROB3605-14|Hymenoptera|Ichneumonidae|BOLD:ACQ9699
Euryproctus|[4390]|SSGBB2835-14|Hymenoptera|Ichneumonidae|BOLD:AAH2175
Ctenopelmatinae|[4391]|SSWLD4598-13|Hymenoptera|Ichneumonidae|BOLD:ACS9106

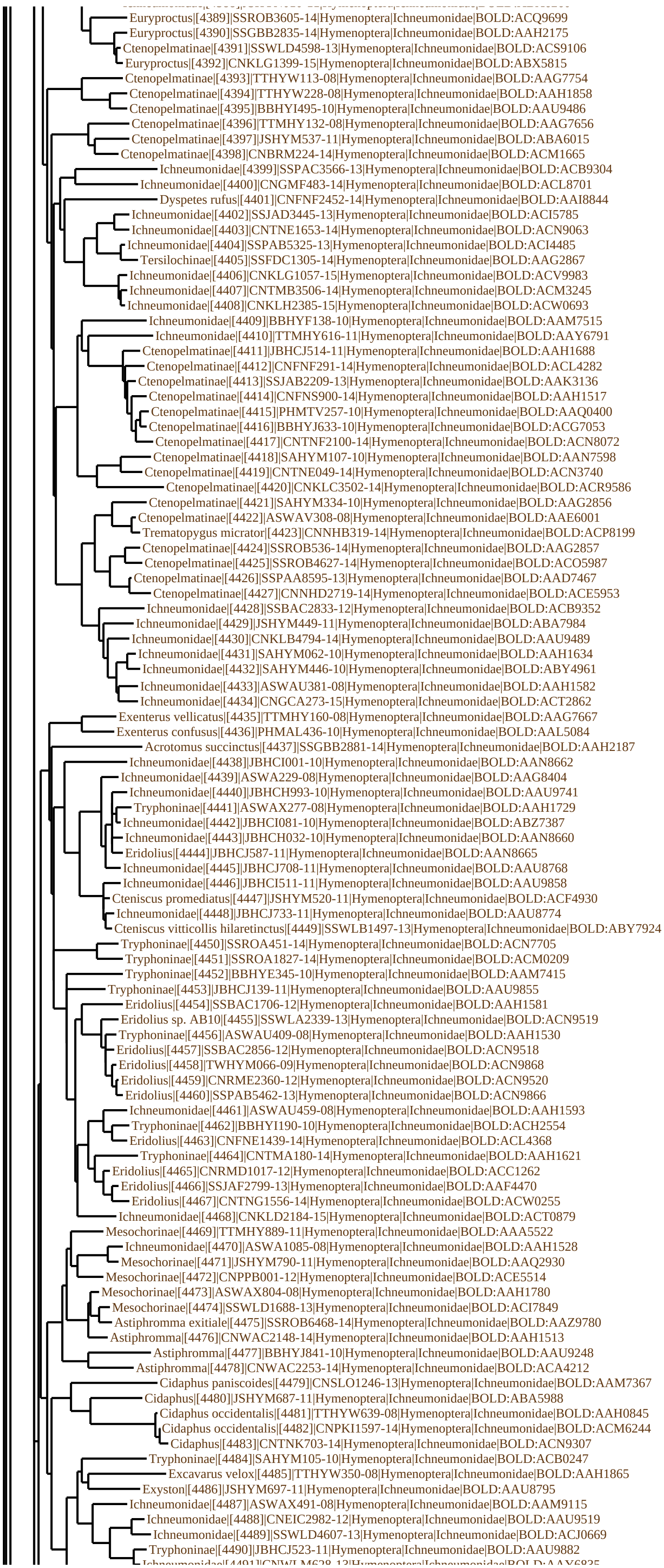

Euryproctus|[4389]|SSROB3605-14|Hymenoptera|Ichneumonidae|BOLD:ACQ9699
Euryproctus|[4390]|SSGBB2835-14|Hymenoptera|Ichneumonidae|BOLD:AAH2175
Ctenopelmatinae|[4391]|SSWLD4598-13|Hymenoptera|Ichneumonidae|BOLD:ACS9106
Euryproctus|[4392]|CNKLG1399-15|Hymenoptera|Ichneumonidae|BOLD:ABX5815
Ctenopelmatinae|[4393]|TTHYW113-08|Hymenoptera|Ichneumonidae|BOLD:AAG7754
Ctenopelmatinae|[4394]|TTHYW228-08|Hymenoptera|Ichneumonidae|BOLD:AAH1858
Ctenopelmatinae|[4395]|BBHYI495-10|Hymenoptera|Ichneumonidae|BOLD:AAU9486
Ctenopelmatinae|[4396]|TTMHY132-08|Hymenoptera|Ichneumonidae|BOLD:AAG7656
Ctenopelmatinae|[4397]|JSHYM537-11|Hymenoptera|Ichneumonidae|BOLD:ABA6015
Ctenopelmatinae|[4398]|CNBRM224-14|Hymenoptera|Ichneumonidae|BOLD:ACM1665
Ichneumonidae|[4399]|SSPAC3566-13|Hymenoptera|Ichneumonidae|BOLD:ACB9304
Ichneumonidae|[4400]|CNGMF483-14|Hymenoptera|Ichneumonidae|BOLD:ACL8701
Dyspetes rufus|[4401]|CNFNF2452-14|Hymenoptera|Ichneumonidae|BOLD:AAI8844
Ichneumonidae|[4402]|SSJAD3445-13|Hymenoptera|Ichneumonidae|BOLD:ACI5785
Ichneumonidae|[4403]|CNTNE1653-14|Hymenoptera|Ichneumonidae|BOLD:ACN9063
Ichneumonidae|[4404]|SSPAB5325-13|Hymenoptera|Ichneumonidae|BOLD:ACI4485
Tersilochinae|[4405]|SSFDC1305-14|Hymenoptera|Ichneumonidae|BOLD:AAG2867
Ichneumonidae|[4406]|CNKLG1057-15|Hymenoptera|Ichneumonidae|BOLD:ACV9983
Ichneumonidae|[4407]|CNTMB3506-14|Hymenoptera|Ichneumonidae|BOLD:ACM3245
Ichneumonidae|[4408]|CNKLH2385-15|Hymenoptera|Ichneumonidae|BOLD:ACW0693
Ichneumonidae|[4409]|BBHYF138-10|Hymenoptera|Ichneumonidae|BOLD:AAM7515
Ichneumonidae|[4410]|TTMHY616-11|Hymenoptera|Ichneumonidae|BOLD:AAY6791
Ctenopelmatinae|[4411]|JBHCJ514-11|Hymenoptera|Ichneumonidae|BOLD:AAH1688
Ctenopelmatinae|[4412]|CNFNF291-14|Hymenoptera|Ichneumonidae|BOLD:ACL4282
Ctenopelmatinae|[4413]|SSJAB2209-13|Hymenoptera|Ichneumonidae|BOLD:AAK3136
Ctenopelmatinae|[4414]|CNFNS900-14|Hymenoptera|Ichneumonidae|BOLD:AAH1517
Ctenopelmatinae|[4415]|PHMTV257-10|Hymenoptera|Ichneumonidae|BOLD:AAQ0400
Ctenopelmatinae|[4416]|BBHYJ633-10|Hymenoptera|Ichneumonidae|BOLD:ACG7053
Ctenopelmatinae|[4417]|CNTNF2100-14|Hymenoptera|Ichneumonidae|BOLD:ACN8072
Ctenopelmatinae|[4418]|SAHYM107-10|Hymenoptera|Ichneumonidae|BOLD:AAN7598
Ctenopelmatinae|[4419]|CNTNE049-14|Hymenoptera|Ichneumonidae|BOLD:ACN3740
Ctenopelmatinae|[4420]|CNKLC3502-14|Hymenoptera|Ichneumonidae|BOLD:ACR9586
Ctenopelmatinae|[4421]|SAHYM334-10|Hymenoptera|Ichneumonidae|BOLD:AAG2856
Ctenopelmatinae|[4422]|ASWAV308-08|Hymenoptera|Ichneumonidae|BOLD:AAE6001
Trematopygus micrator|[4423]|CNNHB319-14|Hymenoptera|Ichneumonidae|BOLD:ACP8199
Ctenopelmatinae|[4424]|SSROB536-14|Hymenoptera|Ichneumonidae|BOLD:AAG2857
Ctenopelmatinae|[4425]|SSROB4627-14|Hymenoptera|Ichneumonidae|BOLD:ACO5987
Ctenopelmatinae|[4426]|SSPAA8595-13|Hymenoptera|Ichneumonidae|BOLD:AAD7467
Ctenopelmatinae|[4427]|CNNHD2719-14|Hymenoptera|Ichneumonidae|BOLD:ACE5953
Ichneumonidae|[4428]|SSBAC2833-12|Hymenoptera|Ichneumonidae|BOLD:ACB9352
Ichneumonidae|[4429]|JSHYM449-11|Hymenoptera|Ichneumonidae|BOLD:ABA7984
Ichneumonidae|[4430]|CNKLB4794-14|Hymenoptera|Ichneumonidae|BOLD:AAU9489
Ichneumonidae|[4431]|SAHYM062-10|Hymenoptera|Ichneumonidae|BOLD:AAH1634
Ichneumonidae|[4432]|SAHYM446-10|Hymenoptera|Ichneumonidae|BOLD:ABY4961
Ichneumonidae|[4433]|ASWAU381-08|Hymenoptera|Ichneumonidae|BOLD:AAH1582
Ichneumonidae|[4434]|CNGCA273-15|Hymenoptera|Ichneumonidae|BOLD:ACT2862
Exenterus vellicatus|[4435]|TTMHY160-08|Hymenoptera|Ichneumonidae|BOLD:AAG7667
Exenterus confusus|[4436]|PHMAL436-10|Hymenoptera|Ichneumonidae|BOLD:AAL5084
Acrotomus succinctus|[4437]|SSGBB2881-14|Hymenoptera|Ichneumonidae|BOLD:AAH2187
Ichneumonidae|[4438]|JBHCI001-10|Hymenoptera|Ichneumonidae|BOLD:AAN8662
Ichneumonidae|[4439]|ASWA229-08|Hymenoptera|Ichneumonidae|BOLD:AAG8404
Ichneumonidae|[4440]|JBHCH993-10|Hymenoptera|Ichneumonidae|BOLD:AAU9741
Tryphoninae|[4441]|ASWAX277-08|Hymenoptera|Ichneumonidae|BOLD:AAH1729
Ichneumonidae|[4442]|JBHCI081-10|Hymenoptera|Ichneumonidae|BOLD:ABZ7387
Ichneumonidae|[4443]|JBHCH032-10|Hymenoptera|Ichneumonidae|BOLD:AAN8660
Eridolius|[4444]|JBHCJ587-11|Hymenoptera|Ichneumonidae|BOLD:AAN8665
Ichneumonidae|[4445]|JBHCJ708-11|Hymenoptera|Ichneumonidae|BOLD:AAU8768
Ichneumonidae|[4446]|JBHCI511-11|Hymenoptera|Ichneumonidae|BOLD:AAU9858
Cteniscus promediatus|[4447]|JSHYM520-11|Hymenoptera|Ichneumonidae|BOLD:ACF4930
Ichneumonidae|[4448]|JBHCJ733-11|Hymenoptera|Ichneumonidae|BOLD:AAU8774
Cteniscus vitticollis hilaretinctus|[4449]|SSWLB1497-13|Hymenoptera|Ichneumonidae|BOLD:ABY7924
Tryphoninae|[4450]|SSROA451-14|Hymenoptera|Ichneumonidae|BOLD:ACN7705
Tryphoninae|[4451]|SSROA1827-14|Hymenoptera|Ichneumonidae|BOLD:ACM0209
Tryphoninae|[4452]|BBHYE345-10|Hymenoptera|Ichneumonidae|BOLD:AAM7415
Tryphoninae|[4453]|JBHCJ139-11|Hymenoptera|Ichneumonidae|BOLD:AAU9855
Eridolius|[4454]|SSBAC1706-12|Hymenoptera|Ichneumonidae|BOLD:AAH1581
Eridolius sp. AB10|[4455]|SSWLA2339-13|Hymenoptera|Ichneumonidae|BOLD:ACN9519
Tryphoninae|[4456]|ASWAU409-08|Hymenoptera|Ichneumonidae|BOLD:AAH1530
Eridolius|[4457]|SSBAC2856-12|Hymenoptera|Ichneumonidae|BOLD:ACN9518
Eridolius|[4458]|TWHYM066-09|Hymenoptera|Ichneumonidae|BOLD:ACN9868
Eridolius|[4459]|CNRME2360-12|Hymenoptera|Ichneumonidae|BOLD:ACN9520
Eridolius|[4460]|SSPAB5462-13|Hymenoptera|Ichneumonidae|BOLD:ACN9866
Ichneumonidae|[4461]|ASWAU459-08|Hymenoptera|Ichneumonidae|BOLD:AAH1593
Tryphoninae|[4462]|BBHYI190-10|Hymenoptera|Ichneumonidae|BOLD:ACH2554
Eridolius|[4463]|CNFNE1439-14|Hymenoptera|Ichneumonidae|BOLD:ACL4368
Tryphoninae|[4464]|CNTMA180-14|Hymenoptera|Ichneumonidae|BOLD:AAH1621
Eridolius|[4465]|CNRMD1017-12|Hymenoptera|Ichneumonidae|BOLD:ACC1262
Eridolius|[4466]|SSJAF2799-13|Hymenoptera|Ichneumonidae|BOLD:AAF4470
Eridolius|[4467]|CNTNG1556-14|Hymenoptera|Ichneumonidae|BOLD:ACW0255
Ichneumonidae|[4468]|CNKLD2184-15|Hymenoptera|Ichneumonidae|BOLD:ACT0879
Mesochorinae|[4469]|TTMHY889-11|Hymenoptera|Ichneumonidae|BOLD:AAA5522
Ichneumonidae|[4470]|ASWA1085-08|Hymenoptera|Ichneumonidae|BOLD:AAH1528
Mesochorinae|[4471]|JSHYM790-11|Hymenoptera|Ichneumonidae|BOLD:AAQ2930
Mesochorinae|[4472]|CNPPB001-12|Hymenoptera|Ichneumonidae|BOLD:ACE5514
Mesochorinae|[4473]|ASWAX804-08|Hymenoptera|Ichneumonidae|BOLD:AAH1780
Mesochorinae|[4474]|SSWLD1688-13|Hymenoptera|Ichneumonidae|BOLD:ACI7849
Astiphromma exitiale|[4475]|SSROB6468-14|Hymenoptera|Ichneumonidae|BOLD:AAZ9780
Astiphromma|[4476]|CNWAC2148-14|Hymenoptera|Ichneumonidae|BOLD:AAH1513
Astiphromma|[4477]|BBHYJ841-10|Hymenoptera|Ichneumonidae|BOLD:AAU9248
Astiphromma|[4478]|CNWAC2253-14|Hymenoptera|Ichneumonidae|BOLD:ACA4212
Cidaphus paniscoides|[4479]|CNSLO1246-13|Hymenoptera|Ichneumonidae|BOLD:AAM7367
Cidaphus|[4480]|JSHYM687-11|Hymenoptera|Ichneumonidae|BOLD:ABA5988
Cidaphus occidentalis|[4481]|TTHYW639-08|Hymenoptera|Ichneumonidae|BOLD:AAH0845
Cidaphus occidentalis|[4482]|CNPKI1597-14|Hymenoptera|Ichneumonidae|BOLD:ACM6244
Cidaphus|[4483]|CNTNK703-14|Hymenoptera|Ichneumonidae|BOLD:ACN9307
Tryphoninae|[4484]|SAHYM105-10|Hymenoptera|Ichneumonidae|BOLD:ACB0247
Excavarus velox|[4485]|TTHYW350-08|Hymenoptera|Ichneumonidae|BOLD:AAH1865
Exyston|[4486]|JSHYM697-11|Hymenoptera|Ichneumonidae|BOLD:AAU8795
Ichneumonidae|[4487]|ASWAX491-08|Hymenoptera|Ichneumonidae|BOLD:AAM9115
Ichneumonidae|[4488]|CNEIC2982-12|Hymenoptera|Ichneumonidae|BOLD:AAU9519
Ichneumonidae|[4489]|SSWLD4607-13|Hymenoptera|Ichneumonidae|BOLD:ACJ0669
Tryphoninae|[4490]|JBHCJ523-11|Hymenoptera|Ichneumonidae|BOLD:AAU9882

[illegible]
Ichneumonidae|[4489]|SSWLD4607-13|Hymenoptera|Ichneumonidae|BOLD:ACJ0669
Tryphoninae|[4490]|JBHCJ523-11|Hymenoptera|Ichneumonidae|BOLD:AAU9882
Ichneumonidae|[4491]|CNWLM628-13|Hymenoptera|Ichneumonidae|BOLD:AAY6835
Ichneumonidae|[4492]|ASWAV019-08|Hymenoptera|Ichneumonidae|BOLD:AAH1544
Smicroplectrus|[4493]|BBHYI490-10|Hymenoptera|Ichneumonidae|BOLD:AAU9485
Ichneumonidae|[4494]|ASWAX182-08|Hymenoptera|Ichneumonidae|BOLD:AAH1661
Smicroplectrus jucundus|[4495]|CNTGC2422-15|Hymenoptera|Ichneumonidae|BOLD:AAH1665
Mesochorinae|[4496]|CNPPD878-12|Hymenoptera|Ichneumonidae|BOLD:ACA6038
Mesochorinae|[4497]|CNPPD044-12|Hymenoptera|Ichneumonidae|BOLD:ACC7575
Mesochorinae|[4498]|SSJAE2429-13|Hymenoptera|Ichneumonidae|BOLD:ACJ4870
Mesochorinae|[4499]|CNKJP129-14|Hymenoptera|Ichneumonidae|BOLD:ACN3908
Stictopisthus|[4500]|BBHYE032-10|Hymenoptera|Ichneumonidae|BOLD:AAG9567
Mesochorinae|[4501]|TTMHY664-11|Hymenoptera|Ichneumonidae|BOLD:AAA5524
Mesochorinae|[4502]|CNTNG1603-14|Hymenoptera|Ichneumonidae|BOLD:ACK3032
Mesochorinae|[4503]|JBHCI881-11|Hymenoptera|Ichneumonidae|BOLD:AAU9826
Mesochorus americanus|[4504]|TTMHY634-11|Hymenoptera|Ichneumonidae|BOLD:AAY6795
Mesochorus americanus|[4505]|SMTPI2544-14|Hymenoptera|Ichneumonidae|BOLD:ACP8673
Mesochorus|[4506]|PHMTW159-10|Hymenoptera|Ichneumonidae|BOLD:AAU8208
Mesochorinae|[4507]|CNWBG2258-13|Hymenoptera|Ichneumonidae|BOLD:AAA5518
Mesochorinae|[4508]|CNWLO646-13|Hymenoptera|Ichneumonidae|BOLD:ABW0562
Mesochorinae|[4509]|CNMIH1913-14|Hymenoptera|Ichneumonidae|BOLD:ACN4221
Mesochorinae|[4510]|CNTNH655-14|Hymenoptera|Ichneumonidae|BOLD:ACN5745
Stictopisthus lanceolatus|[4511]|JSHYO481-11|Hymenoptera|Ichneumonidae|BOLD:AAA5520
Stictopisthus|[4512]|SSPAA1574-13|Hymenoptera|Ichneumonidae|BOLD:AAA5521
Stictopisthus|[4513]|CNTNK603-14|Hymenoptera|Ichneumonidae|BOLD:AAA5525
Mesochorinae|[4514]|HPPPN118-13|Hymenoptera|Ichneumonidae|BOLD:ACJ0813
Mesochorus discitergus|[4515]|CNRVG2370-15|Hymenoptera|Ichneumonidae|BOLD:ACE0759
Mesochorinae|[4516]|JSHYP412-11|Hymenoptera|Ichneumonidae|BOLD:ABA9846
Mesochorinae|[4517]|CNRVD690-15|Hymenoptera|Ichneumonidae|BOLD:ACB2480
Mesochorus unicarinatus|[4518]|TTHYB418-09|Hymenoptera|Ichneumonidae|BOLD:AAG3627
Mesochorinae|[4519]|BBHYF369-10|Hymenoptera|Ichneumonidae|BOLD:AAN7590
Mesochorinae|[4520]|CNBRQ724-14|Hymenoptera|Ichneumonidae|BOLD:ACL8440
Mesochorinae|[4521]|CNRMD1142-12|Hymenoptera|Ichneumonidae|BOLD:ACC6749
Mesochorinae|[4522]|SSWLD6556-13|Hymenoptera|Ichneumonidae|BOLD:AAA5519
Mesochorinae|[4523]|BBHYF296-10|Hymenoptera|Ichneumonidae|BOLD:AAN7583
Mesochorinae|[4524]|HARC284-11|Hymenoptera|Ichneumonidae|BOLD:AAY6893
Mesochorus|[4525]|SSWLA2355-13|Hymenoptera|Ichneumonidae|BOLD:ACG6326
Mesochorus|[4526]|SSPAC797-13|Hymenoptera|Ichneumonidae|BOLD:ACJ0390
Mesochorinae|[4527]|HPPPK533-13|Hymenoptera|Ichneumonidae|BOLD:ACI8500
Mesochorus|[4528]|CNPPD024-12|Hymenoptera|Ichneumonidae|BOLD:ACC7956
Mesochorus|[4529]|SSPAC6463-13|Hymenoptera|Ichneumonidae|BOLD:ACK2014
Mesochorinae|[4530]|TTMHY649-11|Hymenoptera|Ichneumonidae|BOLD:AAG7746
Mesochorus|[4531]|JBHCH998-10|Hymenoptera|Ichneumonidae|BOLD:AAU9742
Mesochorus|[4532]|SSJAF6951-13|Hymenoptera|Ichneumonidae|BOLD:ABZ3487
Mesochorus|[4533]|JSJUL1776-11|Hymenoptera|Ichneumonidae|BOLD:ABZ3488
Mesochorus|[4534]|SSJAB3972-13|Hymenoptera|Ichneumonidae|BOLD:ACG5789
Mesochorus|[4535]|SSBRA2668-14|Hymenoptera|Ichneumonidae|BOLD:AAU9405
Mesochorinae|[4536]|BBHYF154-10|Hymenoptera|Ichneumonidae|BOLD:AAM7517
Mesochorus curvulus|[4537]|SSPAC3453-13|Hymenoptera|Ichneumonidae|BOLD:AAY6820
Mesochorus|[4538]|CNLMP1278-14|Hymenoptera|Ichneumonidae|BOLD:ACM6098
Mesochorus intonsus|[4539]|JSHYM789-11|Hymenoptera|Ichneumonidae|BOLD:ABA5981
Mesochorus dimidiator|[4540]|PHMTT876-10|Hymenoptera|Ichneumonidae|BOLD:AAU8324
Mesochorinae|[4541]|CNFDF057-14|Hymenoptera|Ichneumonidae|BOLD:ACM2098
Mesochorus|[4542]|SSWLF1866-13|Hymenoptera|Ichneumonidae|BOLD:AAF0483
Stictopisthus|[4543]|SSBRB2888-14|Hymenoptera|Ichneumonidae|BOLD:AAA5523
Mesochorus nuncupator|[4544]|SSGBB6443-14|Hymenoptera|Ichneumonidae|BOLD:ACP0120
Mesochorinae|[4545]|CNSLO653-13|Hymenoptera|Ichneumonidae|BOLD:AAU8742
Mesochorinae|[4546]|NGNAY149-14|Hymenoptera|Ichneumonidae|BOLD:ACD3153
Mesochorinae|[4547]|CNWLN677-13|Hymenoptera|Ichneumonidae|BOLD:ACC2966
Mesochorus|[4548]|SSPAB5388-13|Hymenoptera|Ichneumonidae|BOLD:ACA9291
Mesochorus|[4549]|JBHCJ506-11|Hymenoptera|Ichneumonidae|BOLD:AAH0844
Mesochorinae|[4550]|CNTNI1401-14|Hymenoptera|Ichneumonidae|BOLD:ACN8499
Mesochorinae|[4551]|JSHYP056-11|Hymenoptera|Ichneumonidae|BOLD:AAU8651
Mesochorus|[4552]|SSJAE1884-13|Hymenoptera|Ichneumonidae|BOLD:AAG9511
Mesochorus|[4553]|TTHYW1156-09|Hymenoptera|Ichneumonidae|BOLD:AAH1928
Mesochorus|[4554]|CNGMI715-14|Hymenoptera|Ichneumonidae|BOLD:ACM0804
Mesochorus|[4555]|CNTMA082-14|Hymenoptera|Ichneumonidae|BOLD:ACM1599
Mesochorus|[4556]|SSJAE2405-13|Hymenoptera|Ichneumonidae|BOLD:AAE9332
Mesochorus suomiensis|[4557]|SSWLD5830-13|Hymenoptera|Ichneumonidae|BOLD:AAZ1979
Mesochorus|[4558]|CNTNH1291-14|Hymenoptera|Ichneumonidae|BOLD:ACL8591
Mesochorus|[4559]|NGAAF261-14|Hymenoptera|Ichneumonidae|BOLD:ACA1803
Mesochorus zygaenae|[4560]|NGAAG025-14|Hymenoptera|Ichneumonidae|BOLD:ACG0345
Mesochorus|[4561]|CNTNH1311-14|Hymenoptera|Ichneumonidae|BOLD:AAH0848
Mesochorus prolatus|[4562]|SSGBB728-14|Hymenoptera|Ichneumonidae|BOLD:ACE4725
Mesochorus|[4563]|SMTPI8741-14|Hymenoptera|Ichneumonidae|BOLD:AAZ0182
Mesochorus|[4564]|SSBAF4381-13|Hymenoptera|Ichneumonidae|BOLD:AAH1804
Mesochorinae|[4565]|JSHYM638-11|Hymenoptera|Ichneumonidae|BOLD:ABA5996
Mesochorinae|[4566]|JBHCI365-11|Hymenoptera|Ichneumonidae|BOLD:ABZ3561
Mesochorinae|[4567]|JBHCJ720-11|Hymenoptera|Ichneumonidae|BOLD:AAU8773
Mesochorus|[4568]|SSBAF4166-13|Hymenoptera|Ichneumonidae|BOLD:ACI6452
Mesochorinae|[4569]|TTHYW667-08|Hymenoptera|Ichneumonidae|BOLD:AAH0846
Mesochorinae|[4570]|SSWLD3726-13|Hymenoptera|Ichneumonidae|BOLD:ACT3572
Mesochorinae|[4571]|CNJAC1034-12|Hymenoptera|Ichneumonidae|BOLD:ACB3786
Mesochorus|[4572]|SSWLD7389-13|Hymenoptera|Ichneumonidae|BOLD:AAI1444
Mesochorus|[4573]|CNNHF1027-14|Hymenoptera|Ichneumonidae|BOLD:ACR8182
Mesochorus ottawaensis|[4574]|SSBRC1818-14|Hymenoptera|Ichneumonidae|BOLD:AAH0849
Mesochorus|[4575]|SSROB4071-14|Hymenoptera|Ichneumonidae|BOLD:AAM7412
Mesochorinae|[4576]|SSKJB1525-14|Hymenoptera|Ichneumonidae|BOLD:AAA5517
Mesochorinae|[4577]|SSBAF4673-13|Hymenoptera|Ichneumonidae|BOLD:AAH1799
Mesochorinae|[4578]|CNBAK410-13|Hymenoptera|Ichneumonidae|BOLD:ACE0079
Mesochorus testaceus|[4579]|CNNHD2652-14|Hymenoptera|Ichneumonidae|BOLD:ACE0839
Mesochorinae|[4580]|CNNHE1438-14|Hymenoptera|Ichneumonidae|BOLD:ACR7515
Mesochorinae|[4581]|CNNHG1945-14|Hymenoptera|Ichneumonidae|BOLD:ACR7898
Mesochorinae|[4582]|JBHCI855-11|Hymenoptera|Ichneumonidae|BOLD:AAU9836
Mesochorinae|[4583]|SSWLD4673-13|Hymenoptera|Ichneumonidae|BOLD:ACI9215
Mesochorinae|[4584]|CNMIG1364-14|Hymenoptera|Ichneumonidae|BOLD:ACN5929
Mesochorus|[4585]|SAHYM084-10|Hymenoptera|Ichneumonidae|BOLD:AAN7595
Mesochorinae|[4586]|JBHCJ177-11|Hymenoptera|Ichneumonidae|BOLD:AAU9902
Mesochorus|[4587]|ASWAY343-08|Hymenoptera|Ichneumonidae|BOLD:AAG9390
Mesochorus|[4588]|SSJAE11041-13|Hymenoptera|Ichneumonidae|BOLD:AAK7580
Mesochorus|[4589]|CNLMS033-14|Hymenoptera|Ichneumonidae|BOLD:AAM7404
Mesochorus|[4590]|TTHYW278-08|Hymenoptera|Ichneumonidae|BOLD:AAI1431
[illegible]

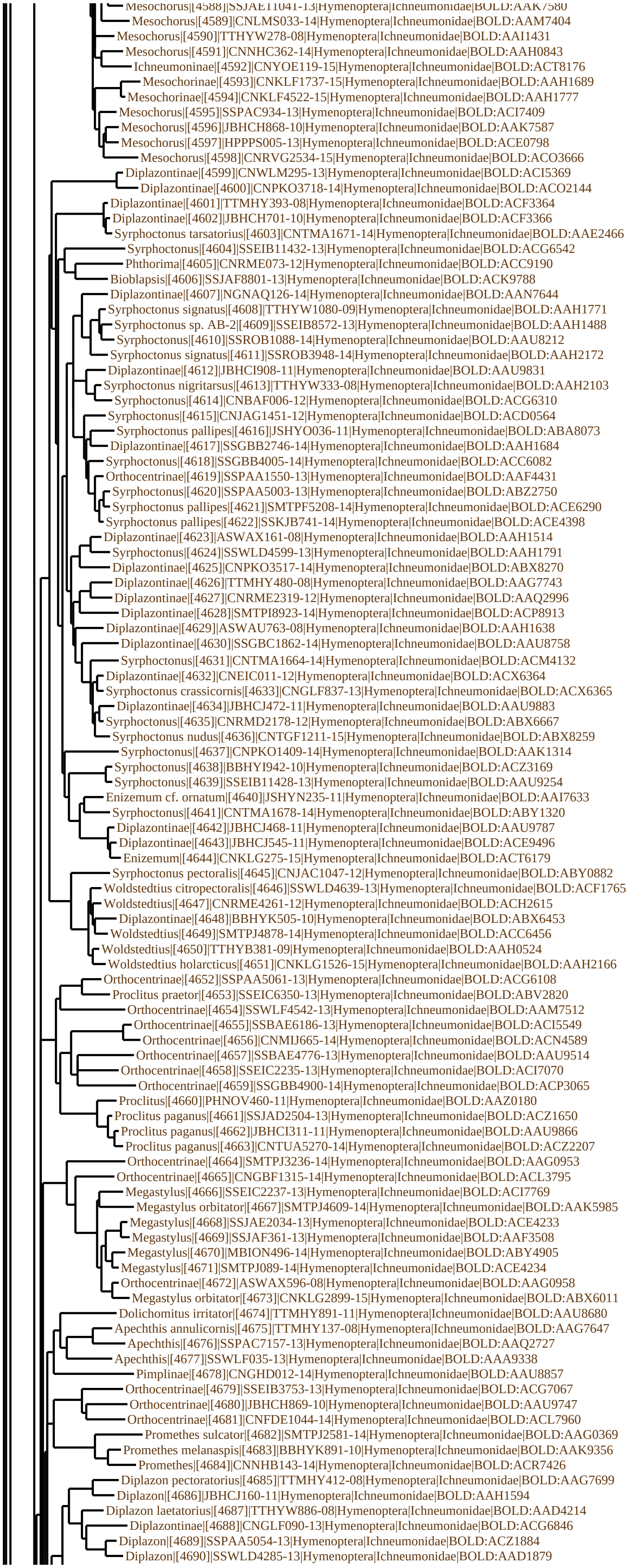
Mesochorus|[4588]|SSJAE11041-13|Hymenoptera|Ichneumonidae|BOLD:AAK7580
Mesochorus|[4589]|CNLMS033-14|Hymenoptera|Ichneumonidae|BOLD:AAM7404
Mesochorus|[4590]|TTHYW278-08|Hymenoptera|Ichneumonidae|BOLD:AAI1431
Mesochorus|[4591]|CNNHC362-14|Hymenoptera|Ichneumonidae|BOLD:AAH0843
Ichneumoninae|[4592]|CNYOE119-15|Hymenoptera|Ichneumonidae|BOLD:ACT8176
Mesochorinae|[4593]|CNKLF1737-15|Hymenoptera|Ichneumonidae|BOLD:AAH1689
Mesochorinae|[4594]|CNKLF4522-15|Hymenoptera|Ichneumonidae|BOLD:AAH1777
Mesochorus|[4595]|SSPAC934-13|Hymenoptera|Ichneumonidae|BOLD:ACI7409
Mesochorus|[4596]|JBHCH868-10|Hymenoptera|Ichneumonidae|BOLD:AAK7587
Mesochorus|[4597]|HPPPS005-13|Hymenoptera|Ichneumonidae|BOLD:ACE0798
Mesochorus|[4598]|CNRVG2534-15|Hymenoptera|Ichneumonidae|BOLD:ACO3666
Diplazontinae|[4599]|CNWLM295-13|Hymenoptera|Ichneumonidae|BOLD:ACI5369
Diplazontinae|[4600]|CNPKO3718-14|Hymenoptera|Ichneumonidae|BOLD:ACO2144
Diplazontinae|[4601]|TTMHY393-08|Hymenoptera|Ichneumonidae|BOLD:ACF3364
Diplazontinae|[4602]|JBHCH701-10|Hymenoptera|Ichneumonidae|BOLD:ACF3366
Syrphoctonus tarsatorius|[4603]|CNTMA1671-14|Hymenoptera|Ichneumonidae|BOLD:AAE2466
Syrphoctonus|[4604]|SSEIB11432-13|Hymenoptera|Ichneumonidae|BOLD:ACG6542
Phthorima|[4605]|CNRME073-12|Hymenoptera|Ichneumonidae|BOLD:ACC9190
Bioblapsis|[4606]|SSJAF8801-13|Hymenoptera|Ichneumonidae|BOLD:ACK9788
Diplazontinae|[4607]|NGNAQ126-14|Hymenoptera|Ichneumonidae|BOLD:AAN7644
Syrphoctonus signatus|[4608]|TTHYW1080-09|Hymenoptera|Ichneumonidae|BOLD:AAH1771
Syrphoctonus sp. AB-2|[4609]|SSEIB8572-13|Hymenoptera|Ichneumonidae|BOLD:AAH1488
Syrphoctonus|[4610]|SSROB1088-14|Hymenoptera|Ichneumonidae|BOLD:AAU8212
Syrphoctonus signatus|[4611]|SSROB3948-14|Hymenoptera|Ichneumonidae|BOLD:AAH2172
Diplazontinae|[4612]|JBHCI908-11|Hymenoptera|Ichneumonidae|BOLD:AAU9831
Syrphoctonus nigritarsus|[4613]|TTHYW333-08|Hymenoptera|Ichneumonidae|BOLD:AAH2103
Syrphoctonus|[4614]|CNBAF006-12|Hymenoptera|Ichneumonidae|BOLD:ACG6310
Syrphoctonus|[4615]|CNJAG1451-12|Hymenoptera|Ichneumonidae|BOLD:ACD0564
Syrphoctonus pallipes|[4616]|JSHYO036-11|Hymenoptera|Ichneumonidae|BOLD:ABA8073
Diplazontinae|[4617]|SSGBB2746-14|Hymenoptera|Ichneumonidae|BOLD:AAH1684
Syrphoctonus|[4618]|SSGBB4005-14|Hymenoptera|Ichneumonidae|BOLD:ACC6082
Orthocentrinae|[4619]|SSPAA1550-13|Hymenoptera|Ichneumonidae|BOLD:AAF4431
Syrphoctonus|[4620]|SSPAA5003-13|Hymenoptera|Ichneumonidae|BOLD:ABZ2750
Syrphoctonus pallipes|[4621]|SMTPF5208-14|Hymenoptera|Ichneumonidae|BOLD:ACE6290
Syrphoctonus pallipes|[4622]|SSKJB741-14|Hymenoptera|Ichneumonidae|BOLD:ACE4398
Diplazontinae|[4623]|ASWAX161-08|Hymenoptera|Ichneumonidae|BOLD:AAH1514
Syrphoctonus|[4624]|SSWLD4599-13|Hymenoptera|Ichneumonidae|BOLD:AAH1791
Diplazontinae|[4625]|CNPKO3517-14|Hymenoptera|Ichneumonidae|BOLD:ABX8270
Diplazontinae|[4626]|TTMHY480-08|Hymenoptera|Ichneumonidae|BOLD:AAG7743
Diplazontinae|[4627]|CNRME2319-12|Hymenoptera|Ichneumonidae|BOLD:AAQ2996
Diplazontinae|[4628]|SMTPI8923-14|Hymenoptera|Ichneumonidae|BOLD:ACP8913
Diplazontinae|[4629]|ASWAU763-08|Hymenoptera|Ichneumonidae|BOLD:AAH1638
Diplazontinae|[4630]|SSGBC1862-14|Hymenoptera|Ichneumonidae|BOLD:AAU8758
Syrphoctonus|[4631]|CNTMA1664-14|Hymenoptera|Ichneumonidae|BOLD:ACM4132
Diplazontinae|[4632]|CNEIC011-12|Hymenoptera|Ichneumonidae|BOLD:ACX6364
Syrphoctonus crassicornis|[4633]|CNGLF837-13|Hymenoptera|Ichneumonidae|BOLD:ACX6365
Diplazontinae|[4634]|JBHCJ472-11|Hymenoptera|Ichneumonidae|BOLD:AAU9883
Syrphoctonus|[4635]|CNRMD2178-12|Hymenoptera|Ichneumonidae|BOLD:ABX6667
Syrphoctonus nudus|[4636]|CNTGF1211-15|Hymenoptera|Ichneumonidae|BOLD:ABX8259
Syrphoctonus|[4637]|CNPKO1409-14|Hymenoptera|Ichneumonidae|BOLD:AAK1314
Syrphoctonus|[4638]|BBHYI942-10|Hymenoptera|Ichneumonidae|BOLD:ACZ3169
Syrphoctonus|[4639]|SSEIB11428-13|Hymenoptera|Ichneumonidae|BOLD:AAU9254
Enizemum cf. ornatum|[4640]|JSHYN235-11|Hymenoptera|Ichneumonidae|BOLD:AAI7633
Syrphoctonus|[4641]|CNTMA1678-14|Hymenoptera|Ichneumonidae|BOLD:ABY1320
Diplazontinae|[4642]|JBHCJ468-11|Hymenoptera|Ichneumonidae|BOLD:AAU9787
Diplazontinae|[4643]|JBHCJ545-11|Hymenoptera|Ichneumonidae|BOLD:ACE9496
Enizemum|[4644]|CNKLG275-15|Hymenoptera|Ichneumonidae|BOLD:ACT6179
Syrphoctonus pectoralis|[4645]|CNJAC1047-12|Hymenoptera|Ichneumonidae|BOLD:ABY0882
Woldstedtius citropectoralis|[4646]|SSWLD4639-13|Hymenoptera|Ichneumonidae|BOLD:ACF1765
Woldstedtius|[4647]|CNRME4261-12|Hymenoptera|Ichneumonidae|BOLD:ACH2615
Diplazontinae|[4648]|BBHYK505-10|Hymenoptera|Ichneumonidae|BOLD:ABX6453
Woldstedtius|[4649]|SMTPJ4878-14|Hymenoptera|Ichneumonidae|BOLD:ACC6456
Woldstedtius|[4650]|TTHYB381-09|Hymenoptera|Ichneumonidae|BOLD:AAH0524
Woldstedtius holarcticus|[4651]|CNKLG1526-15|Hymenoptera|Ichneumonidae|BOLD:AAH2166
Orthocentrinae|[4652]|SSPAA5061-13|Hymenoptera|Ichneumonidae|BOLD:ACG6108
Proclitus praetor|[4653]|SSEIC6350-13|Hymenoptera|Ichneumonidae|BOLD:ABV2820
Orthocentrinae|[4654]|SSWLF4542-13|Hymenoptera|Ichneumonidae|BOLD:AAM7512
Orthocentrinae|[4655]|SSBAE6186-13|Hymenoptera|Ichneumonidae|BOLD:ACI5549
Orthocentrinae|[4656]|CNMIJ665-14|Hymenoptera|Ichneumonidae|BOLD:ACN4589
Orthocentrinae|[4657]|SSBAE4776-13|Hymenoptera|Ichneumonidae|BOLD:AAU9514
Orthocentrinae|[4658]|SSEIC2235-13|Hymenoptera|Ichneumonidae|BOLD:ACI7070
Orthocentrinae|[4659]|SSGBB4900-14|Hymenoptera|Ichneumonidae|BOLD:ACP3065
Proclitus|[4660]|PHNOV460-11|Hymenoptera|Ichneumonidae|BOLD:AAZ0180
Proclitus paganus|[4661]|SSJAD2504-13|Hymenoptera|Ichneumonidae|BOLD:ACZ1650
Proclitus paganus|[4662]|JBHCI311-11|Hymenoptera|Ichneumonidae|BOLD:AAU9866
Proclitus paganus|[4663]|CNTUA5270-14|Hymenoptera|Ichneumonidae|BOLD:ACZ2207
Orthocentrinae|[4664]|SMTPJ3236-14|Hymenoptera|Ichneumonidae|BOLD:AAG0953
Orthocentrinae|[4665]|CNGBF1315-14|Hymenoptera|Ichneumonidae|BOLD:ACL3795
Megastylus|[4666]|SSEIC2237-13|Hymenoptera|Ichneumonidae|BOLD:ACI7769
Megastylus orbitator|[4667]|SMTPJ4609-14|Hymenoptera|Ichneumonidae|BOLD:AAK5985
Megastylus|[4668]|SSJAE2034-13|Hymenoptera|Ichneumonidae|BOLD:ACE4233
Megastylus|[4669]|SSJAF361-13|Hymenoptera|Ichneumonidae|BOLD:AAF3508
Megastylus|[4670]|MBION496-14|Hymenoptera|Ichneumonidae|BOLD:ABY4905
Megastylus|[4671]|SMTPJ089-14|Hymenoptera|Ichneumonidae|BOLD:ACE4234
Orthocentrinae|[4672]|ASWAX596-08|Hymenoptera|Ichneumonidae|BOLD:AAG0958
Megastylus orbitator|[4673]|CNKLG2899-15|Hymenoptera|Ichneumonidae|BOLD:ABX6011
Dolichomitus irritator|[4674]|TTMHY891-11|Hymenoptera|Ichneumonidae|BOLD:AAU8680
Apechthis annulicornis|[4675]|TTMHY137-08|Hymenoptera|Ichneumonidae|BOLD:AAG7647
Apechthis|[4676]|SSPAC7157-13|Hymenoptera|Ichneumonidae|BOLD:AAQ2727
Apechthis|[4677]|SSWLF035-13|Hymenoptera|Ichneumonidae|BOLD:AAA9338
Pimplinae|[4678]|CNGHD012-14|Hymenoptera|Ichneumonidae|BOLD:AAU8857
Orthocentrinae|[4679]|SSEIB3753-13|Hymenoptera|Ichneumonidae|BOLD:ACG7067
Orthocentrinae|[4680]|JBHCH869-10|Hymenoptera|Ichneumonidae|BOLD:AAU9747
Orthocentrinae|[4681]|CNFDE1044-14|Hymenoptera|Ichneumonidae|BOLD:ACL7960
Promethes sulcator|[4682]|SMTPJ2581-14|Hymenoptera|Ichneumonidae|BOLD:AAG0369
Promethes melanaspis|[4683]|BBHYK891-10|Hymenoptera|Ichneumonidae|BOLD:AAK9356
Promethes|[4684]|CNNHB143-14|Hymenoptera|Ichneumonidae|BOLD:ACR7426
Diplazon pectoratorius|[4685]|TTMHY412-08|Hymenoptera|Ichneumonidae|BOLD:AAG7699
Diplazon|[4686]|JBHCJ160-11|Hymenoptera|Ichneumonidae|BOLD:AAH1594
Diplazon laetatorius|[4687]|TTHYW886-08|Hymenoptera|Ichneumonidae|BOLD:AAD4214
Diplazontinae|[4688]|CNGLF090-13|Hymenoptera|Ichneumonidae|BOLD:ACG6846
Diplazon|[4689]|SSPAA5054-13|Hymenoptera|Ichneumonidae|BOLD:ACZ1884
Diplazon|[4690]|SSWLD4285-13|Hymenoptera|Ichneumonidae|BOLD:AAD1879

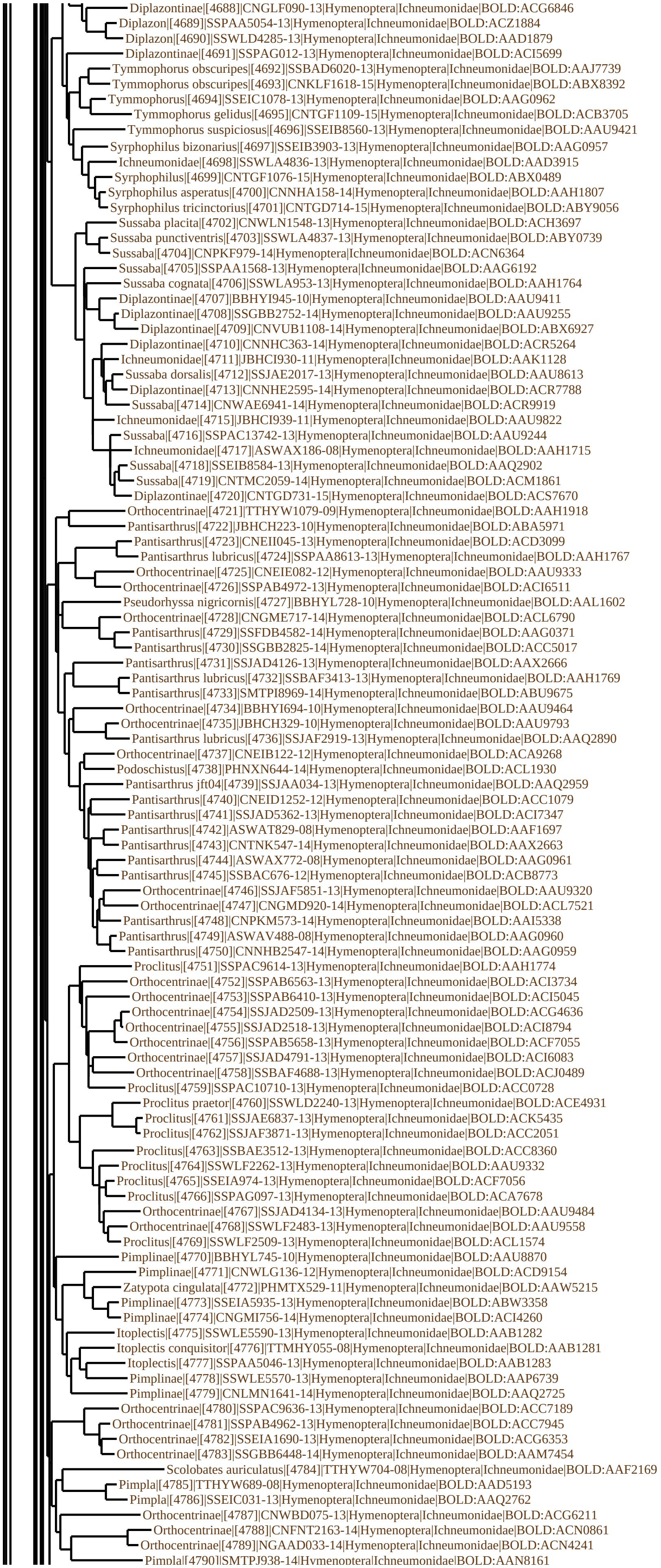
Diplazontinae|[4688]|CNGLF090-13|Hymenoptera|Ichneumonidae|BOLD:ACG6846
Diplazon|[4689]|SSPAA5054-13|Hymenoptera|Ichneumonidae|BOLD:ACZ1884
Diplazon|[4690]|SSWLD4285-13|Hymenoptera|Ichneumonidae|BOLD:AAD1879
Diplazontinae|[4691]|SSPAG012-13|Hymenoptera|Ichneumonidae|BOLD:ACI5699
Tymmophorus obscuripes|[4692]|SSBAD6020-13|Hymenoptera|Ichneumonidae|BOLD:AAJ7739
Tymmophorus obscuripes|[4693]|CNKLF1618-15|Hymenoptera|Ichneumonidae|BOLD:ABX8392
Tymmophorus|[4694]|SSEIC1078-13|Hymenoptera|Ichneumonidae|BOLD:AAG0962
Tymmophorus gelidus|[4695]|CNTGF1109-15|Hymenoptera|Ichneumonidae|BOLD:ACB3705
Tymmophorus suspiciosus|[4696]|SSEIB8560-13|Hymenoptera|Ichneumonidae|BOLD:AAU9421
Syrphophilus bizonarius|[4697]|SSEIB3903-13|Hymenoptera|Ichneumonidae|BOLD:AAG0957
Ichneumonidae|[4698]|SSWLA4836-13|Hymenoptera|Ichneumonidae|BOLD:AAD3915
Syrphophilus|[4699]|CNTGF1076-15|Hymenoptera|Ichneumonidae|BOLD:ABX0489
Syrphophilus asperatus|[4700]|CNNHA158-14|Hymenoptera|Ichneumonidae|BOLD:AAH1807
Syrphophilus tricinctorius|[4701]|CNTGD714-15|Hymenoptera|Ichneumonidae|BOLD:ABY9056
Sussaba placita|[4702]|CNWLN1548-13|Hymenoptera|Ichneumonidae|BOLD:ACH3697
Sussaba punctiventris|[4703]|SSWLA4837-13|Hymenoptera|Ichneumonidae|BOLD:ABY0739
Sussaba|[4704]|CNPKF979-14|Hymenoptera|Ichneumonidae|BOLD:ACN6364
Sussaba|[4705]|SSPAA1568-13|Hymenoptera|Ichneumonidae|BOLD:AAG6192
Sussaba cognata|[4706]|SSWLA953-13|Hymenoptera|Ichneumonidae|BOLD:AAH1764
Diplazontinae|[4707]|BBHYI945-10|Hymenoptera|Ichneumonidae|BOLD:AAU9411
Diplazontinae|[4708]|SSGBB2752-14|Hymenoptera|Ichneumonidae|BOLD:AAU9255
Diplazontinae|[4709]|CNVUB1108-14|Hymenoptera|Ichneumonidae|BOLD:ABX6927
Diplazontinae|[4710]|CNNHC363-14|Hymenoptera|Ichneumonidae|BOLD:ACR5264
Ichneumonidae|[4711]|JBHCI930-11|Hymenoptera|Ichneumonidae|BOLD:AAK1128
Sussaba dorsalis|[4712]|SSJAE2017-13|Hymenoptera|Ichneumonidae|BOLD:AAU8613
Diplazontinae|[4713]|CNNHE2595-14|Hymenoptera|Ichneumonidae|BOLD:ACR7788
Sussaba|[4714]|CNWAE6941-14|Hymenoptera|Ichneumonidae|BOLD:ACR9919
Ichneumonidae|[4715]|JBHCI939-11|Hymenoptera|Ichneumonidae|BOLD:AAU9822
Sussaba|[4716]|SSPAC13742-13|Hymenoptera|Ichneumonidae|BOLD:AAU9244
Ichneumonidae|[4717]|ASWAX186-08|Hymenoptera|Ichneumonidae|BOLD:AAH1715
Sussaba|[4718]|SSEIB8584-13|Hymenoptera|Ichneumonidae|BOLD:AAQ2902
Sussaba|[4719]|CNTMC2059-14|Hymenoptera|Ichneumonidae|BOLD:ACM1861
Diplazontinae|[4720]|CNTGD731-15|Hymenoptera|Ichneumonidae|BOLD:ACS7670
Orthocentrinae|[4721]|TTHYW1079-09|Hymenoptera|Ichneumonidae|BOLD:AAH1918
Pantisarthrus|[4722]|JBHCH223-10|Hymenoptera|Ichneumonidae|BOLD:ABA5971
Pantisarthrus|[4723]|CNEII045-13|Hymenoptera|Ichneumonidae|BOLD:ACD3099
Pantisarthrus lubricus|[4724]|SSPAA8613-13|Hymenoptera|Ichneumonidae|BOLD:AAH1767
Orthocentrinae|[4725]|CNEIE082-12|Hymenoptera|Ichneumonidae|BOLD:AAU9333
Orthocentrinae|[4726]|SSPAB4972-13|Hymenoptera|Ichneumonidae|BOLD:ACI6511
Pseudorhyssa nigricornis|[4727]|BBHYL728-10|Hymenoptera|Ichneumonidae|BOLD:AAL1602
Orthocentrinae|[4728]|CNGME717-14|Hymenoptera|Ichneumonidae|BOLD:ACL6790
Pantisarthrus|[4729]|SSFDB4582-14|Hymenoptera|Ichneumonidae|BOLD:AAG0371
Pantisarthrus|[4730]|SSGBB2825-14|Hymenoptera|Ichneumonidae|BOLD:ACC5017
Pantisarthrus|[4731]|SSJAD4126-13|Hymenoptera|Ichneumonidae|BOLD:AAX2666
Pantisarthrus lubricus|[4732]|SSBAF3413-13|Hymenoptera|Ichneumonidae|BOLD:AAH1769
Pantisarthrus|[4733]|SMTPI8969-14|Hymenoptera|Ichneumonidae|BOLD:ABU9675
Orthocentrinae|[4734]|BBHYI694-10|Hymenoptera|Ichneumonidae|BOLD:AAU9464
Orthocentrinae|[4735]|JBHCH329-10|Hymenoptera|Ichneumonidae|BOLD:AAU9793
Pantisarthrus lubricus|[4736]|SSJAF2919-13|Hymenoptera|Ichneumonidae|BOLD:AAQ2890
Orthocentrinae|[4737]|CNEIB122-12|Hymenoptera|Ichneumonidae|BOLD:ACA9268
Podoschistus|[4738]|PHNXN644-14|Hymenoptera|Ichneumonidae|BOLD:ACL1930
Pantisarthrus jft04|[4739]|SSJAA034-13|Hymenoptera|Ichneumonidae|BOLD:AAQ2959
Pantisarthrus|[4740]|CNEID1252-12|Hymenoptera|Ichneumonidae|BOLD:ACC1079
Pantisarthrus|[4741]|SSJAD5362-13|Hymenoptera|Ichneumonidae|BOLD:ACI7347
Pantisarthrus|[4742]|ASWAT829-08|Hymenoptera|Ichneumonidae|BOLD:AAF1697
Pantisarthrus|[4743]|CNTNK547-14|Hymenoptera|Ichneumonidae|BOLD:AAX2663
Pantisarthrus|[4744]|ASWAX772-08|Hymenoptera|Ichneumonidae|BOLD:AAG0961
Pantisarthrus|[4745]|SSBAC676-12|Hymenoptera|Ichneumonidae|BOLD:ACB8773
Orthocentrinae|[4746]|SSJAF5851-13|Hymenoptera|Ichneumonidae|BOLD:AAU9320
Orthocentrinae|[4747]|CNGMD920-14|Hymenoptera|Ichneumonidae|BOLD:ACL7521
Pantisarthrus|[4748]|CNPKM573-14|Hymenoptera|Ichneumonidae|BOLD:AAI5338
Pantisarthrus|[4749]|ASWAV488-08|Hymenoptera|Ichneumonidae|BOLD:AAG0960
Pantisarthrus|[4750]|CNNHB2547-14|Hymenoptera|Ichneumonidae|BOLD:AAG0959
Proclitus|[4751]|SSPAC9614-13|Hymenoptera|Ichneumonidae|BOLD:AAH1774
Orthocentrinae|[4752]|SSPAB6563-13|Hymenoptera|Ichneumonidae|BOLD:ACI3734
Orthocentrinae|[4753]|SSPAB6410-13|Hymenoptera|Ichneumonidae|BOLD:ACI5045
Orthocentrinae|[4754]|SSJAD2509-13|Hymenoptera|Ichneumonidae|BOLD:ACG4636
Orthocentrinae|[4755]|SSJAD2518-13|Hymenoptera|Ichneumonidae|BOLD:ACI8794
Orthocentrinae|[4756]|SSPAB5658-13|Hymenoptera|Ichneumonidae|BOLD:ACF7055
Orthocentrinae|[4757]|SSJAD4791-13|Hymenoptera|Ichneumonidae|BOLD:ACI6083
Orthocentrinae|[4758]|SSBAF4688-13|Hymenoptera|Ichneumonidae|BOLD:ACJ0489
Proclitus|[4759]|SSPAC10710-13|Hymenoptera|Ichneumonidae|BOLD:ACC0728
Proclitus praetor|[4760]|SSWLD2240-13|Hymenoptera|Ichneumonidae|BOLD:ACE4931
Proclitus|[4761]|SSJAE6837-13|Hymenoptera|Ichneumonidae|BOLD:ACK5435
Proclitus|[4762]|SSJAF3871-13|Hymenoptera|Ichneumonidae|BOLD:ACC2051
Proclitus|[4763]|SSBAE3512-13|Hymenoptera|Ichneumonidae|BOLD:ACC8360
Proclitus|[4764]|SSWLF2262-13|Hymenoptera|Ichneumonidae|BOLD:AAU9332
Proclitus|[4765]|SSEIA974-13|Hymenoptera|Ichneumonidae|BOLD:ACF7056
Proclitus|[4766]|SSPAG097-13|Hymenoptera|Ichneumonidae|BOLD:ACA7678
Orthocentrinae|[4767]|SSJAD4134-13|Hymenoptera|Ichneumonidae|BOLD:AAU9484
Orthocentrinae|[4768]|SSWLF2483-13|Hymenoptera|Ichneumonidae|BOLD:AAU9558
Proclitus|[4769]|SSWLF2509-13|Hymenoptera|Ichneumonidae|BOLD:ACL1574
Pimplinae|[4770]|BBHYL745-10|Hymenoptera|Ichneumonidae|BOLD:AAU8870
Pimplinae|[4771]|CNWLG136-12|Hymenoptera|Ichneumonidae|BOLD:ACD9154
Zatypota cingulata|[4772]|PHMTX529-11|Hymenoptera|Ichneumonidae|BOLD:AAW5215
Pimplinae|[4773]|SSEIA5935-13|Hymenoptera|Ichneumonidae|BOLD:ABW3358
Pimplinae|[4774]|CNGMI756-14|Hymenoptera|Ichneumonidae|BOLD:ACI4260
Itoplectis|[4775]|SSWLE5590-13|Hymenoptera|Ichneumonidae|BOLD:AAB1282
Itoplectis conquisitor|[4776]|TTMHY055-08|Hymenoptera|Ichneumonidae|BOLD:AAB1281
Itoplectis|[4777]|SSPAA5046-13|Hymenoptera|Ichneumonidae|BOLD:AAB1283
Pimplinae|[4778]|SSWLE5570-13|Hymenoptera|Ichneumonidae|BOLD:AAP6739
Pimplinae|[4779]|CNLMN1641-14|Hymenoptera|Ichneumonidae|BOLD:AAQ2725
Orthocentrinae|[4780]|SSPAC9636-13|Hymenoptera|Ichneumonidae|BOLD:ACC7189
Orthocentrinae|[4781]|SSPAB4962-13|Hymenoptera|Ichneumonidae|BOLD:ACC7945
Orthocentrinae|[4782]|SSEIA1690-13|Hymenoptera|Ichneumonidae|BOLD:ACG6353
Orthocentrinae|[4783]|SSGBB6448-14|Hymenoptera|Ichneumonidae|BOLD:AAM7454
Scolobates auriculatus|[4784]|TTHYW704-08|Hymenoptera|Ichneumonidae|BOLD:AAF2169
Pimpla|[4785]|TTHYW689-08|Hymenoptera|Ichneumonidae|BOLD:AAD5193
Pimpla|[4786]|SSEIC031-13|Hymenoptera|Ichneumonidae|BOLD:AAQ2762
Orthocentrinae|[4787]|CNWBD075-13|Hymenoptera|Ichneumonidae|BOLD:ACG6211
Orthocentrinae|[4788]|CNFNT2163-14|Hymenoptera|Ichneumonidae|BOLD:ACN0861
Orthocentrinae|[4789]|NGAAD033-14|Hymenoptera|Ichneumonidae|BOLD:ACN4241
Pimpla|[4790]|SMTPJ938-14|Hymenoptera|Ichneumonidae|BOLD:AAN8161

[illegible]
Orthocentrinae|[4788]|CNFNT2163-14|Hymenoptera|Ichneumonidae|BOLD:ACN0861
Orthocentrinae|[4789]|NGAAD033-14|Hymenoptera|Ichneumonidae|BOLD:ACN4241
Pimpla|[4790]|SMTPJ938-14|Hymenoptera|Ichneumonidae|BOLD:AAN8161
Pimpla|[4791]|TTMHY930-11|Hymenoptera|Ichneumonidae|BOLD:AAD5194
Pimpla sodalis|[4792]|SAHYM497-10|Hymenoptera|Ichneumonidae|BOLD:AAH1503
Pimpla|[4793]|TTMHY478-08|Hymenoptera|Ichneumonidae|BOLD:AAG0387
Pimpla stricklandi|[4794]|SAHYM115-10|Hymenoptera|Ichneumonidae|BOLD:AAN7599
Pimplinae|[4795]|SSWLE6547-13|Hymenoptera|Ichneumonidae|BOLD:ACB1611
Pimpla nuda|[4796]|JSHYM527-11|Hymenoptera|Ichneumonidae|BOLD:ABY8397
Pimpla stricklandi|[4797]|SSWLE7692-13|Hymenoptera|Ichneumonidae|BOLD:AAG0385
Pimpla|[4798]|SSGBC1984-14|Hymenoptera|Ichneumonidae|BOLD:AAG0383
Pimpla|[4799]|TTMHY929-11|Hymenoptera|Ichneumonidae|BOLD:AAG7721
Pimpla aequalis|[4800]|TTMHY934-11|Hymenoptera|Ichneumonidae|BOLD:AAG7634
Pimpla aequalis|[4801]|SMTPI7752-14|Hymenoptera|Ichneumonidae|BOLD:ACP8990
Pimplinae|[4802]|CNRME2314-12|Hymenoptera|Ichneumonidae|BOLD:ACC8257
Orthocentrinae|[4803]|SSJAD958-13|Hymenoptera|Ichneumonidae|BOLD:AAK5983
Gnathochorisis|[4804]|ASWAX415-08|Hymenoptera|Ichneumonidae|BOLD:AAH1741
Orthocentrinae|[4805]|SSJAD979-13|Hymenoptera|Ichneumonidae|BOLD:AAH1754
Orthocentrinae|[4806]|SSWLE6404-13|Hymenoptera|Ichneumonidae|BOLD:AAU9478
Orthocentrinae|[4807]|SSWLE6407-13|Hymenoptera|Ichneumonidae|BOLD:ACI7775
Orthocentrinae|[4808]|SSJAF1009-13|Hymenoptera|Ichneumonidae|BOLD:ACB1308
Pimplinae|[4809]|SSJAF411-13|Hymenoptera|Ichneumonidae|BOLD:AAU8217
Gnathochorisis crassulus|[4810]|SSJAF5215-13|Hymenoptera|Ichneumonidae|BOLD:AAF0574
Orthocentrinae|[4811]|TTMHY254-08|Hymenoptera|Ichneumonidae|BOLD:AAG7694
Orthocentrinae|[4812]|CNFNR3125-14|Hymenoptera|Ichneumonidae|BOLD:ACM2323
Perithous divinator|[4813]|JSHYM965-11|Hymenoptera|Ichneumonidae|BOLD:AAY2114
Pimplinae|[4814]|SSGBB4417-14|Hymenoptera|Ichneumonidae|BOLD:AAU8373
Rhyssa howdenorum|[4815]|TTMHY877-11|Hymenoptera|Ichneumonidae|BOLD:AAJ3938
Rhyssa crevieri|[4816]|SSBRA2526-14|Hymenoptera|Ichneumonidae|BOLD:ABV2646
Rhyssa|[4817]|CNYOB876-15|Hymenoptera|Ichneumonidae|BOLD:ACT0586
Pimplinae|[4818]|NGNAF607-13|Hymenoptera|Ichneumonidae|BOLD:ACB3725
Zatypota|[4819]|SSWLE9132-13|Hymenoptera|Ichneumonidae|BOLD:ACK5829
Orthocentrinae|[4820]|JBHCH714-10|Hymenoptera|Ichneumonidae|BOLD:AAU9766
Orthocentrinae|[4821]|JSHYM637-11|Hymenoptera|Ichneumonidae|BOLD:ABA5997
Acrotaphus wiltii tounes|[4822]|CNLME061-14|Hymenoptera|Ichneumonidae|BOLD:AAG8064
Pimpla pedalis|[4823]|TTMHY899-11|Hymenoptera|Ichneumonidae|BOLD:AAD5192
Pimplinae|[4824]|JSHYO243-11|Hymenoptera|Ichneumonidae|BOLD:ABA8061
Ichneumonidae|[4825]|CNPAG321-13|Hymenoptera|Ichneumonidae|BOLD:ACO2520
Pimplinae|[4826]|CNWLM785-13|Hymenoptera|Ichneumonidae|BOLD:ACD3195
Pimplinae|[4827]|BBHYI320-10|Hymenoptera|Ichneumonidae|BOLD:AAK3258
Pimplinae|[4828]|JBHCI119-10|Hymenoptera|Ichneumonidae|BOLD:AAU9719
Schizopyga|[4829]|SMTPD1268-13|Hymenoptera|Ichneumonidae|BOLD:ACJ6635
Schizopyga|[4830]|SSROB1011-14|Hymenoptera|Ichneumonidae|BOLD:AAH2006
Dreisbachia slossonae|[4831]|SSJAF8776-13|Hymenoptera|Ichneumonidae|BOLD:ACN6024
Dreisbachia|[4832]|SSBRC583-14|Hymenoptera|Ichneumonidae|BOLD:ACO0124
Dreisbachia slossonae|[4833]|SSJAF2937-13|Hymenoptera|Ichneumonidae|BOLD:ACN6941
Dreisbachia|[4834]|CNMIG1281-14|Hymenoptera|Ichneumonidae|BOLD:ACN3656
Dreisbachia slossonae|[4835]|SSROB6262-14|Hymenoptera|Ichneumonidae|BOLD:AAN8649
Pimplinae|[4836]|TTHYW985-09|Hymenoptera|Ichneumonidae|BOLD:AAG0384
Iseropus|[4837]|PHMTX728-11|Hymenoptera|Ichneumonidae|BOLD:AAW0437
Iseropus stercorator|[4838]|SSPAC3380-13|Hymenoptera|Ichneumonidae|BOLD:AAO2094
Pimplinae|[4839]|ASWAU945-08|Hymenoptera|Ichneumonidae|BOLD:AAH1671
Ephialtes manifestator|[4840]|TTMHY975-11|Hymenoptera|Ichneumonidae|BOLD:AAU8667
Pimplinae|[4841]|JSHYP492-11|Hymenoptera|Ichneumonidae|BOLD:ABA9857
Perithous|[4842]|SSGBB055-14|Hymenoptera|Ichneumonidae|BOLD:ACO3734
Pimplinae|[4843]|CNLMO1674-14|Hymenoptera|Ichneumonidae|BOLD:ACM9065
Perithous|[4844]|SMTPI3042-14|Hymenoptera|Ichneumonidae|BOLD:ACP6162
Dolichomitus tuberculatus|[4845]|SMTPJ4584-14|Hymenoptera|Ichneumonidae|BOLD:AAZ9575
Dolichomitus messor|[4846]|BBHYJ833-10|Hymenoptera|Ichneumonidae|BOLD:AAU9247
Dolichomitus|[4847]|CNWLM159-13|Hymenoptera|Ichneumonidae|BOLD:ACJ0555
Dolichomitus|[4848]|TTHYW160-08|Hymenoptera|Ichneumonidae|BOLD:AAH1682
Dolichomitus foxleei|[4849]|SSJAF2797-13|Hymenoptera|Ichneumonidae|BOLD:ACA3842
Dolichomitus|[4850]|CNVUB092-14|Hymenoptera|Ichneumonidae|BOLD:AAH2122
Pimplinae|[4851]|CNNHE2565-14|Hymenoptera|Ichneumonidae|BOLD:ACR8278
Dolichomitus|[4852]|SSBRA2497-14|Hymenoptera|Ichneumonidae|BOLD:AAW5210
Dolichomitus terebrans|[4853]|SSEIC027-13|Hymenoptera|Ichneumonidae|BOLD:ACA4061
Pimplinae|[4854]|BBHYL754-10|Hymenoptera|Ichneumonidae|BOLD:AAU8855
Dolichomitus|[4855]|CNLMD134-14|Hymenoptera|Ichneumonidae|BOLD:ACN3213
Dolichomitus imperator|[4856]|TTHYW194-08|Hymenoptera|Ichneumonidae|BOLD:AAG7815
Dolichomitus|[4857]|SSGBB4402-14|Hymenoptera|Ichneumonidae|BOLD:ACX1370
Dolichomitus|[4858]|PHMTT744-10|Hymenoptera|Ichneumonidae|BOLD:AAU8333
Pimplinae|[4859]|CNKTG1940-15|Hymenoptera|Ichneumonidae|BOLD:ACS8611
Pimplinae|[4860]|CNBRQ733-14|Hymenoptera|Ichneumonidae|BOLD:ACL7087
Zatypota percontatoria|[4861]|SSPAA8598-13|Hymenoptera|Ichneumonidae|BOLD:AAH1695
Zaglyptus varipes|[4862]|TTHYW1094-09|Hymenoptera|Ichneumonidae|BOLD:AAG0382
Zaglyptus|[4863]|CNNHE2007-14|Hymenoptera|Ichneumonidae|BOLD:ACR7542
Pimplinae|[4864]|CNPPB2246-12|Hymenoptera|Ichneumonidae|BOLD:ACA7508
Tromatobia variabilis|[4865]|CNPPE1278-12|Hymenoptera|Ichneumonidae|BOLD:AAY4010
Tromatobia variabilis|[4866]|JBHCJ643-11|Hymenoptera|Ichneumonidae|BOLD:ACI7676
Tromatobia|[4867]|SSPAB4661-13|Hymenoptera|Ichneumonidae|BOLD:AAG0372
Tromatobia|[4868]|TTHYW298-08|Hymenoptera|Ichneumonidae|BOLD:AAG0381
Tromatobia ovivora|[4869]|PHMTX973-11|Hymenoptera|Ichneumonidae|BOLD:AAU8354
Pimplinae|[4870]|JSHYO418-11|Hymenoptera|Ichneumonidae|BOLD:ABA5923
Pimplinae|[4871]|NGNAW016-14|Hymenoptera|Ichneumonidae|BOLD:AAN7850
Tromatobia notator|[4872]|SMTPJ2490-14|Hymenoptera|Ichneumonidae|BOLD:AAC5504
Acrodactyla quadrisculpta|[4873]|SSPAG063-13|Hymenoptera|Ichneumonidae|BOLD:AAD7584
Pimplinae|[4874]|CNTMA060-14|Hymenoptera|Ichneumonidae|BOLD:ABW3360
Acrodactyla degener|[4875]|SSBAE6060-13|Hymenoptera|Ichneumonidae|BOLD:ABW3362
Pimplinae|[4876]|CNJAC1417-12|Hymenoptera|Ichneumonidae|BOLD:AAG0386
Pimplinae|[4877]|JBHCI361-11|Hymenoptera|Ichneumonidae|BOLD:AAU9861
Pimplinae|[4878]|SSEIA7454-13|Hymenoptera|Ichneumonidae|BOLD:AAG0374
Pimplinae|[4879]|CNTND238-14|Hymenoptera|Ichneumonidae|BOLD:ACM1730
Eruga|[4880]|CNPKI1164-14|Hymenoptera|Ichneumonidae|BOLD:ACM1346
Eruga|[4881]|SSPAC284-13|Hymenoptera|Ichneumonidae|BOLD:AAU9121
Eruga|[4882]|SMTPJ4902-14|Hymenoptera|Ichneumonidae|BOLD:ACX3532
Pimplinae|[4883]|JSJUL1752-11|Hymenoptera|Ichneumonidae|BOLD:ABA5919
Clistopyga canadensis|[4884]|SSJAE2026-13|Hymenoptera|Ichneumonidae|BOLD:AAB3568
Polysphincta|[4885]|SSJAE1882-13|Hymenoptera|Ichneumonidae|BOLD:AAH1739
Polysphincta jft03|[4886]|TTHYW232-08|Hymenoptera|Ichneumonidae|BOLD:AAG2937
Polysphincta tuberosa|[4887]|CNBRQ002-14|Hymenoptera|Ichneumonidae|BOLD:AAU9253
Pimplinae|[4888]|SMTPJ4955-14|Hymenoptera|Ichneumonidae|BOLD:AAQ2915
Scambus|[4889]|SSJAF2814-13|Hymenoptera|Ichneumonidae|BOLD:AAB0894
Scambus|[4890]|SSGBB2848-14|Hymenoptera|Ichneumonidae|BOLD:ACP9166

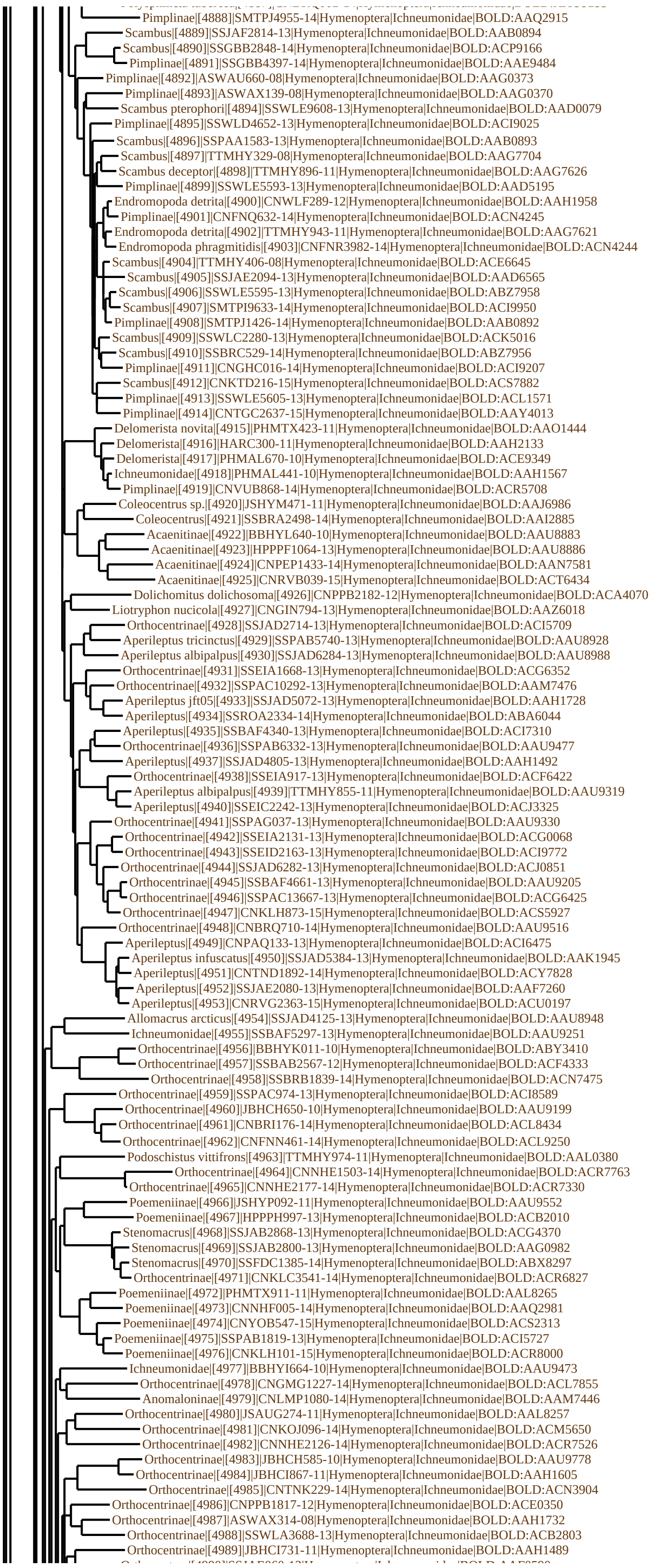

Pimplinae|[4888]|SMTPJ4955-14|Hymenoptera|Ichneumonidae|BOLD:AAQ2915
Scambus|[4889]|SSJAF2814-13|Hymenoptera|Ichneumonidae|BOLD:AAB0894
Scambus|[4890]|SSGBB2848-14|Hymenoptera|Ichneumonidae|BOLD:ACP9166
Pimplinae|[4891]|SSGBB4397-14|Hymenoptera|Ichneumonidae|BOLD:AAE9484
Pimplinae|[4892]|ASWAU660-08|Hymenoptera|Ichneumonidae|BOLD:AAG0373
Pimplinae|[4893]|ASWAX139-08|Hymenoptera|Ichneumonidae|BOLD:AAG0370
Scambus pterophori|[4894]|SSWLE9608-13|Hymenoptera|Ichneumonidae|BOLD:AAD0079
Pimplinae|[4895]|SSWLD4652-13|Hymenoptera|Ichneumonidae|BOLD:ACI9025
Scambus|[4896]|SSPAA1583-13|Hymenoptera|Ichneumonidae|BOLD:AAB0893
Scambus|[4897]|TTMHY329-08|Hymenoptera|Ichneumonidae|BOLD:AAG7704
Scambus deceptor|[4898]|TTMHY896-11|Hymenoptera|Ichneumonidae|BOLD:AAG7626
Pimplinae|[4899]|SSWLE5593-13|Hymenoptera|Ichneumonidae|BOLD:AAD5195
Endromopoda detrita|[4900]|CNWLF289-12|Hymenoptera|Ichneumonidae|BOLD:AAH1958
Pimplinae|[4901]|CNFNQ632-14|Hymenoptera|Ichneumonidae|BOLD:ACN4245
Endromopoda detrita|[4902]|TTMHY943-11|Hymenoptera|Ichneumonidae|BOLD:AAG7621
Endromopoda phragmitidis|[4903]|CNFNR3982-14|Hymenoptera|Ichneumonidae|BOLD:ACN4244
Scambus|[4904]|TTMHY406-08|Hymenoptera|Ichneumonidae|BOLD:ACE6645
Scambus|[4905]|SSJAE2094-13|Hymenoptera|Ichneumonidae|BOLD:AAD6565
Scambus|[4906]|SSWLE5595-13|Hymenoptera|Ichneumonidae|BOLD:ABZ7958
Scambus|[4907]|SMTPI9633-14|Hymenoptera|Ichneumonidae|BOLD:ACI9950
Pimplinae|[4908]|SMTPJ1426-14|Hymenoptera|Ichneumonidae|BOLD:AAB0892
Scambus|[4909]|SSWLC2280-13|Hymenoptera|Ichneumonidae|BOLD:ACK5016
Scambus|[4910]|SSBRC529-14|Hymenoptera|Ichneumonidae|BOLD:ABZ7956
Pimplinae|[4911]|CNGHC016-14|Hymenoptera|Ichneumonidae|BOLD:ACI9207
Scambus|[4912]|CNKTD216-15|Hymenoptera|Ichneumonidae|BOLD:ACS7882
Pimplinae|[4913]|SSWLE5605-13|Hymenoptera|Ichneumonidae|BOLD:ACL1571
Pimplinae|[4914]|CNTGC2637-15|Hymenoptera|Ichneumonidae|BOLD:AAY4013
Delomerista novita|[4915]|PHMTX423-11|Hymenoptera|Ichneumonidae|BOLD:AAO1444
Delomerista|[4916]|HARC300-11|Hymenoptera|Ichneumonidae|BOLD:AAH2133
Delomerista|[4917]|PHMAL670-10|Hymenoptera|Ichneumonidae|BOLD:ACE9349
Ichneumonidae|[4918]|PHMAL441-10|Hymenoptera|Ichneumonidae|BOLD:AAH1567
Pimplinae|[4919]|CNVUB868-14|Hymenoptera|Ichneumonidae|BOLD:ACR5708
Coleocentrus sp.|[4920]|JSHYM471-11|Hymenoptera|Ichneumonidae|BOLD:AAJ6986
Coleocentrus|[4921]|SSBRA2498-14|Hymenoptera|Ichneumonidae|BOLD:AAI2885
Acaenitinae|[4922]|BBHYL640-10|Hymenoptera|Ichneumonidae|BOLD:AAU8883
Acaenitinae|[4923]|HPPPF1064-13|Hymenoptera|Ichneumonidae|BOLD:AAU8886
Acaenitinae|[4924]|CNPEP1433-14|Hymenoptera|Ichneumonidae|BOLD:AAN7581
Acaenitinae|[4925]|CNRVB039-15|Hymenoptera|Ichneumonidae|BOLD:ACT6434
Dolichomitus dolichosoma|[4926]|CNPPB2182-12|Hymenoptera|Ichneumonidae|BOLD:ACA4070
Liotryphon nucicola|[4927]|CNGIN794-13|Hymenoptera|Ichneumonidae|BOLD:AAZ6018
Orthocentrinae|[4928]|SSJAD2714-13|Hymenoptera|Ichneumonidae|BOLD:ACI5709
Aperileptus tricinctus|[4929]|SSPAB5740-13|Hymenoptera|Ichneumonidae|BOLD:AAU8928
Aperileptus albipalpus|[4930]|SSJAD6284-13|Hymenoptera|Ichneumonidae|BOLD:AAU8988
Orthocentrinae|[4931]|SSEIA1668-13|Hymenoptera|Ichneumonidae|BOLD:ACG6352
Orthocentrinae|[4932]|SSPAC10292-13|Hymenoptera|Ichneumonidae|BOLD:AAM7476
Aperileptus jft05|[4933]|SSJAD5072-13|Hymenoptera|Ichneumonidae|BOLD:AAH1728
Aperileptus|[4934]|SSROA2334-14|Hymenoptera|Ichneumonidae|BOLD:ABA6044
Aperileptus|[4935]|SSBAF4340-13|Hymenoptera|Ichneumonidae|BOLD:ACI7310
Orthocentrinae|[4936]|SSPAB6332-13|Hymenoptera|Ichneumonidae|BOLD:AAU9477
Aperileptus|[4937]|SSJAD4805-13|Hymenoptera|Ichneumonidae|BOLD:AAH1492
Orthocentrinae|[4938]|SSEIA917-13|Hymenoptera|Ichneumonidae|BOLD:ACF6422
Aperileptus albipalpus|[4939]|TTMHY855-11|Hymenoptera|Ichneumonidae|BOLD:AAU9319
Aperileptus|[4940]|SSEIC2242-13|Hymenoptera|Ichneumonidae|BOLD:ACJ3325
Orthocentrinae|[4941]|SSPAG037-13|Hymenoptera|Ichneumonidae|BOLD:AAU9330
Orthocentrinae|[4942]|SSEIA2131-13|Hymenoptera|Ichneumonidae|BOLD:ACG0068
Orthocentrinae|[4943]|SSEID2163-13|Hymenoptera|Ichneumonidae|BOLD:ACI9772
Orthocentrinae|[4944]|SSJAD6282-13|Hymenoptera|Ichneumonidae|BOLD:ACJ0851
Orthocentrinae|[4945]|SSBAF4661-13|Hymenoptera|Ichneumonidae|BOLD:AAU9205
Orthocentrinae|[4946]|SSPAC13667-13|Hymenoptera|Ichneumonidae|BOLD:ACG6425
Orthocentrinae|[4947]|CNKLH873-15|Hymenoptera|Ichneumonidae|BOLD:ACS5927
Orthocentrinae|[4948]|CNBRQ710-14|Hymenoptera|Ichneumonidae|BOLD:AAU9516
Aperileptus|[4949]|CNPAQ133-13|Hymenoptera|Ichneumonidae|BOLD:ACI6475
Aperileptus infuscatus|[4950]|SSJAD5384-13|Hymenoptera|Ichneumonidae|BOLD:AAK1945
Aperileptus|[4951]|CNTND1892-14|Hymenoptera|Ichneumonidae|BOLD:ACY7828
Aperileptus|[4952]|SSJAE2080-13|Hymenoptera|Ichneumonidae|BOLD:AAF7260
Aperileptus|[4953]|CNRVG2363-15|Hymenoptera|Ichneumonidae|BOLD:ACU0197
Allomacrus arcticus|[4954]|SSJAD4125-13|Hymenoptera|Ichneumonidae|BOLD:AAU8948
Ichneumonidae|[4955]|SSBAF5297-13|Hymenoptera|Ichneumonidae|BOLD:AAU9251
Orthocentrinae|[4956]|BBHYK011-10|Hymenoptera|Ichneumonidae|BOLD:ABY3410
Orthocentrinae|[4957]|SSBAB2567-12|Hymenoptera|Ichneumonidae|BOLD:ACF4333
Orthocentrinae|[4958]|SSBRB1839-14|Hymenoptera|Ichneumonidae|BOLD:ACN7475
Orthocentrinae|[4959]|SSPAC974-13|Hymenoptera|Ichneumonidae|BOLD:ACI8589
Orthocentrinae|[4960]|JBHCH650-10|Hymenoptera|Ichneumonidae|BOLD:AAU9199
Orthocentrinae|[4961]|CNBRI176-14|Hymenoptera|Ichneumonidae|BOLD:ACL8434
Orthocentrinae|[4962]|CNFNN461-14|Hymenoptera|Ichneumonidae|BOLD:ACL9250
Podoschistus vittifrons|[4963]|TTMHY974-11|Hymenoptera|Ichneumonidae|BOLD:AAL0380
Orthocentrinae|[4964]|CNNHE1503-14|Hymenoptera|Ichneumonidae|BOLD:ACR7763
Orthocentrinae|[4965]|CNNHE2177-14|Hymenoptera|Ichneumonidae|BOLD:ACR7330
Poemeniinae|[4966]|JSHYP092-11|Hymenoptera|Ichneumonidae|BOLD:AAU9552
Poemeniinae|[4967]|HPPPH997-13|Hymenoptera|Ichneumonidae|BOLD:ACB2010
Stenomacrus|[4968]|SSJAB2868-13|Hymenoptera|Ichneumonidae|BOLD:ACG4370
Stenomacrus|[4969]|SSJAB2800-13|Hymenoptera|Ichneumonidae|BOLD:AAG0982
Stenomacrus|[4970]|SSFDC1385-14|Hymenoptera|Ichneumonidae|BOLD:ABX8297
Orthocentrinae|[4971]|CNKLC3541-14|Hymenoptera|Ichneumonidae|BOLD:ACR6827
Poemeniinae|[4972]|PHMTX911-11|Hymenoptera|Ichneumonidae|BOLD:AAL8265
Poemeniinae|[4973]|CNNHF005-14|Hymenoptera|Ichneumonidae|BOLD:AAQ2981
Poemeniinae|[4974]|CNYOB547-15|Hymenoptera|Ichneumonidae|BOLD:ACS2313
Poemeniinae|[4975]|SSPAB1819-13|Hymenoptera|Ichneumonidae|BOLD:ACI5727
Poemeniinae|[4976]|CNKLH101-15|Hymenoptera|Ichneumonidae|BOLD:ACR8000
Ichneumonidae|[4977]|BBHYI664-10|Hymenoptera|Ichneumonidae|BOLD:AAU9473
Orthocentrinae|[4978]|CNGMG1227-14|Hymenoptera|Ichneumonidae|BOLD:ACL7855
Anomaloninae|[4979]|CNLMP1080-14|Hymenoptera|Ichneumonidae|BOLD:AAM7446
Orthocentrinae|[4980]|JSAUG274-11|Hymenoptera|Ichneumonidae|BOLD:AAL8257
Orthocentrinae|[4981]|CNKOJ096-14|Hymenoptera|Ichneumonidae|BOLD:ACM5650
Orthocentrinae|[4982]|CNNHE2126-14|Hymenoptera|Ichneumonidae|BOLD:ACR7526
Orthocentrinae|[4983]|JBHCH585-10|Hymenoptera|Ichneumonidae|BOLD:AAU9778
Orthocentrinae|[4984]|JBHCI867-11|Hymenoptera|Ichneumonidae|BOLD:AAH1605
Orthocentrinae|[4985]|CNTNK229-14|Hymenoptera|Ichneumonidae|BOLD:ACN3904
Orthocentrinae|[4986]|CNPPB1817-12|Hymenoptera|Ichneumonidae|BOLD:ACE0350
Orthocentrinae|[4987]|ASWAX314-08|Hymenoptera|Ichneumonidae|BOLD:AAH1732
Orthocentrinae|[4988]|SSWLA3688-13|Hymenoptera|Ichneumonidae|BOLD:ACB2803
Orthocentrinae|[4989]|JBHCI731-11|Hymenoptera|Ichneumonidae|BOLD:AAH1489

Orthocentrinae|[4987]|ASWAX314-08|Hymenoptera|Ichneumonidae|BOLD:AAH1732
Orthocentrinae|[4988]|SSWLA3688-13|Hymenoptera|Ichneumonidae|BOLD:ACB2803
Orthocentrinae|[4989]|JBHCI731-11|Hymenoptera|Ichneumonidae|BOLD:AAH1489
Orthocentrus|[4990]|SSJAE060-13|Hymenoptera|Ichneumonidae|BOLD:AAF0590
Neurateles|[4991]|CNWBX039-13|Hymenoptera|Ichneumonidae|BOLD:AAK8152
Orthocentrinae|[4992]|CNTND2149-14|Hymenoptera|Ichneumonidae|BOLD:ACL3521
Orthocentrinae|[4993]|SSBRA1177-14|Hymenoptera|Ichneumonidae|BOLD:AAL8282
Orthocentrinae|[4994]|SSJAF5833-13|Hymenoptera|Ichneumonidae|BOLD:ABY0125
Orthocentrinae|[4995]|SSKJB3434-14|Hymenoptera|Ichneumonidae|BOLD:AAG0973
Neurateles|[4996]|SAHYM314-10|Hymenoptera|Ichneumonidae|BOLD:AAH1559
Neurateles|[4997]|HARC041-10|Hymenoptera|Ichneumonidae|BOLD:ACE9541
Neurateles|[4998]|SSJAD981-13|Hymenoptera|Ichneumonidae|BOLD:ABY5384
Orthocentrinae|[4999]|SSGBB2690-14|Hymenoptera|Ichneumonidae|BOLD:ACL7987
Orthocentrinae|[5000]|CNGLE225-13|Hymenoptera|Ichneumonidae|BOLD:ACD3807
Orthocentrinae|[5001]|ASWAV225-08|Hymenoptera|Ichneumonidae|BOLD:AAH1561
Orthocentrinae|[5002]|SSJAD6294-13|Hymenoptera|Ichneumonidae|BOLD:ACS5770
Orthocentrinae|[5003]|CNWAH6113-15|Hymenoptera|Ichneumonidae|BOLD:AAH1570
Orthocentrinae|[5004]|SMTPJ1286-14|Hymenoptera|Ichneumonidae|BOLD:AAH1826
Orthocentrinae|[5005]|CNTGF3143-15|Hymenoptera|Ichneumonidae|BOLD:ACP7298
Orthocentrinae|[5006]|BBHYJ075-10|Hymenoptera|Ichneumonidae|BOLD:AAQ2779
Orthocentrinae|[5007]|CNWLM852-13|Hymenoptera|Ichneumonidae|BOLD:ACI5368
Orthocentrinae|[5008]|CNTNH633-14|Hymenoptera|Ichneumonidae|BOLD:ACK9829
Orthocentrus|[5009]|SSPAC8718-13|Hymenoptera|Ichneumonidae|BOLD:AAE4989
Orthocentrus|[5010]|SMTPI4235-14|Hymenoptera|Ichneumonidae|BOLD:AAU8604
Orthocentrinae|[5011]|BBHYK268-10|Hymenoptera|Ichneumonidae|BOLD:AAU8612
Orthocentrinae|[5012]|CNKOD744-14|Hymenoptera|Ichneumonidae|BOLD:ACD8997
Orthocentrus|[5013]|PHNOV476-11|Hymenoptera|Ichneumonidae|BOLD:AAF0572
Orthocentrinae|[5014]|SMTPJ5498-14|Hymenoptera|Ichneumonidae|BOLD:AAH2142
Orthocentrus|[5015]|SSPAC8095-13|Hymenoptera|Ichneumonidae|BOLD:AAU9466
Orthocentrus|[5016]|TTHYB414-09|Hymenoptera|Ichneumonidae|BOLD:AAI3459
Orthocentrus|[5017]|PHJUN2126-11|Hymenoptera|Ichneumonidae|BOLD:ACM2706
Orthocentrus|[5018]|CNPPA1500-12|Hymenoptera|Ichneumonidae|BOLD:ACN3597
Orthocentrus|[5019]|SMTPJ4916-14|Hymenoptera|Ichneumonidae|BOLD:AAU8218
Orthocentrinae|[5020]|SMTPJ2317-14|Hymenoptera|Ichneumonidae|BOLD:AAY6848
Orthocentrinae|[5021]|CNKLF4356-15|Hymenoptera|Ichneumonidae|BOLD:ACS4538
Orthocentrus|[5022]|JBHCI786-11|Hymenoptera|Ichneumonidae|BOLD:AAU9756
Orthocentrus|[5023]|JBHCJ721-11|Hymenoptera|Ichneumonidae|BOLD:AAK5989
Orthocentrinae|[5024]|ASWA1115-08|Hymenoptera|Ichneumonidae|BOLD:AAG0955
Orthocentrinae|[5025]|SSWLD5489-13|Hymenoptera|Ichneumonidae|BOLD:ACY3886
Orthocentrus|[5026]|SAHYM321-10|Hymenoptera|Ichneumonidae|BOLD:ACF2213
Orthocentrus|[5027]|SSJAF4199-13|Hymenoptera|Ichneumonidae|BOLD:AAE1614
Orthocentrus spurius|[5028]|SSJAF5209-13|Hymenoptera|Ichneumonidae|BOLD:AAC3876
Orthocentrus|[5029]|HARC410-11|Hymenoptera|Ichneumonidae|BOLD:AAY6894
Orthocentrinae|[5030]|JBHCI884-11|Hymenoptera|Ichneumonidae|BOLD:AAU9827
Orthocentrinae|[5031]|SSPAB5338-13|Hymenoptera|Ichneumonidae|BOLD:AAV4407
Orthocentrus fulvipes|[5032]|SSJAF4659-13|Hymenoptera|Ichneumonidae|BOLD:AAM9125
Stenomacrus|[5033]|TTMHY828-11|Hymenoptera|Ichneumonidae|BOLD:ACN3086
Stenomacrus jft19|[5034]|SSEIC1048-13|Hymenoptera|Ichneumonidae|BOLD:AAF3664
Orthocentrinae|[5035]|SSBAF5375-13|Hymenoptera|Ichneumonidae|BOLD:AAU8991
Orthocentrinae|[5036]|SSJAF2117-13|Hymenoptera|Ichneumonidae|BOLD:AAU8582
Orthocentrinae|[5037]|CNEIG1266-12|Hymenoptera|Ichneumonidae|BOLD:ACD9088
Plectiscus|[5038]|SSBAE3135-13|Hymenoptera|Ichneumonidae|BOLD:ABU9109
Plectiscus callidulus|[5039]|SSJAF4131-13|Hymenoptera|Ichneumonidae|BOLD:ACC8246
Orthocentrinae|[5040]|SSJAE2441-13|Hymenoptera|Ichneumonidae|BOLD:AAU9483
Leipaulus|[5041]|JBHCJ749-11|Hymenoptera|Ichneumonidae|BOLD:AAC8798
Orthocentrinae|[5042]|SSJAF2943-13|Hymenoptera|Ichneumonidae|BOLD:AAD8974
Orthocentrinae|[5043]|SSGBB4844-14|Hymenoptera|Ichneumonidae|BOLD:AAG0983
Orthocentrus jft06|[5044]|SSEIC6348-13|Hymenoptera|Ichneumonidae|BOLD:AAW9549
Orthocentrus|[5045]|SSJAD473-13|Hymenoptera|Ichneumonidae|BOLD:ACG5893
Orthocentrus|[5046]|SSWLF416-13|Hymenoptera|Ichneumonidae|BOLD:AAB1188
Orthocentrus|[5047]|SSWLF1350-13|Hymenoptera|Ichneumonidae|BOLD:ACO2477
Neurateles|[5048]|SSWLF4643-13|Hymenoptera|Ichneumonidae|BOLD:ACC7897
Stenomacrus jft12|[5049]|ASWA175-08|Hymenoptera|Ichneumonidae|BOLD:AAH1500
Batakomacrus caudatus|[5050]|SSWLF925-13|Hymenoptera|Ichneumonidae|BOLD:ACC2831
Orthocentrinae|[5051]|CNTNF1154-14|Hymenoptera|Ichneumonidae|BOLD:ACL8703
Orthocentrinae|[5052]|CNPPI1373-12|Hymenoptera|Ichneumonidae|BOLD:ACB2448
Orthocentrinae|[5053]|SSGBB2775-14|Hymenoptera|Ichneumonidae|BOLD:ACB2423
Orthocentrus|[5054]|JBHCJ481-11|Hymenoptera|Ichneumonidae|BOLD:AAI2361
Stenomacrus|[5055]|SSPAC5750-13|Hymenoptera|Ichneumonidae|BOLD:AAG0952
Orthocentrinae|[5056]|SMTPJ3191-14|Hymenoptera|Ichneumonidae|BOLD:AAG8257
Orthocentrinae|[5057]|ASWA231-08|Hymenoptera|Ichneumonidae|BOLD:AAH1507
Stenomacrus|[5058]|JBHCJ635-11|Hymenoptera|Ichneumonidae|BOLD:AAE2018
Orthocentrinae|[5059]|SSWLF336-13|Hymenoptera|Ichneumonidae|BOLD:AAG0975
Orthocentrinae|[5060]|SSPAC3667-13|Hymenoptera|Ichneumonidae|BOLD:ACC6470
Orthocentrinae|[5061]|CNKOE679-14|Hymenoptera|Ichneumonidae|BOLD:ACM2381
Stenomacrus nemoralis|[5062]|TTMHY726-11|Hymenoptera|Ichneumonidae|BOLD:AAM7494
Orthocentrinae|[5063]|JBHCI299-11|Hymenoptera|Ichneumonidae|BOLD:AAU9772
Stenomacrus|[5064]|JBHCJ746-11|Hymenoptera|Ichneumonidae|BOLD:AAC3396
Stenomacrus|[5065]|SSBAC2788-12|Hymenoptera|Ichneumonidae|BOLD:AAC1404
Orthocentrinae|[5066]|JBHCJ230-11|Hymenoptera|Ichneumonidae|BOLD:AAU9898
Orthocentrinae|[5067]|SSPAB385-13|Hymenoptera|Ichneumonidae|BOLD:AAH1785
Stenomacrus|[5068]|SMTPJ1316-14|Hymenoptera|Ichneumonidae|BOLD:AAJ5372
Stenomacrus|[5069]|CNKLC2035-14|Hymenoptera|Ichneumonidae|BOLD:ACD1949
Stenomacrus jft16|[5070]|ASWAY392-08|Hymenoptera|Ichneumonidae|BOLD:AAB4997
Stenomacrus|[5071]|JBHCI323-11|Hymenoptera|Ichneumonidae|BOLD:AAH1610
Orthocentrinae|[5072]|CNPEC1838-14|Hymenoptera|Ichneumonidae|BOLD:ACL4531
Stenomacrus|[5073]|SAHYM360-10|Hymenoptera|Ichneumonidae|BOLD:AAG0980
Stenomacrus exserens|[5074]|SSWLA1662-13|Hymenoptera|Ichneumonidae|BOLD:AAH1611
Orthocentrinae|[5075]|SSPAB4942-13|Hymenoptera|Ichneumonidae|BOLD:ACI5221
Orthocentrinae|[5076]|CNRMF053-12|Hymenoptera|Ichneumonidae|BOLD:ACC8109
Stenomacrus|[5077]|SMTPJ1984-14|Hymenoptera|Ichneumonidae|BOLD:AAH1571
Stenomacrus|[5078]|SSPAB1864-13|Hymenoptera|Ichneumonidae|BOLD:AAP6689
Orthocentrinae|[5079]|SSEIC3704-13|Hymenoptera|Ichneumonidae|BOLD:AAG0974
Stenomacrus groenlandicus|[5080]|SSWLF2500-13|Hymenoptera|Ichneumonidae|BOLD:ACA4473
Stenomacrus|[5081]|CNKLF4527-15|Hymenoptera|Ichneumonidae|BOLD:ACS5323
Orthocentrinae|[5082]|CNPCK025-13|Hymenoptera|Ichneumonidae|BOLD:ACI7226
Stenomacrus|[5083]|SSPAC5708-13|Hymenoptera|Ichneumonidae|BOLD:AAU8210
Stenomacrus|[5084]|SSEIA7632-13|Hymenoptera|Ichneumonidae|BOLD:ACG7128
Orthocentrinae|[5085]|CNEIH623-13|Hymenoptera|Ichneumonidae|BOLD:ACB1307
Orthocentrinae|[5086]|CNWLO963-13|Hymenoptera|Ichneumonidae|BOLD:ACC8950
Stenomacrus|[5087]|SSWLD944-13|Hymenoptera|Ichneumonidae|BOLD:AAH1823
Stenomacrus|[5088]|SSPAB6327-13|Hymenoptera|Ichneumonidae|BOLD:ACC0639
Orthocentrinae|[5089]|SSPAC3725-13|Hymenoptera|Ichneumonidae|BOLD:AAD0245
[illegible]

Stenomacrus|[5087]|SSWLD944-13|Hymenoptera|Ichneumonidae|BOLD:AAH1823
Stenomacrus|[5088]|SSPAB6327-13|Hymenoptera|Ichneumonidae|BOLD:ACC0639
Orthocentrinae|[5089]|SSPAC3725-13|Hymenoptera|Ichneumonidae|BOLD:AAD0245
Leipaulus|[5090]|CNKLG2734-15|Hymenoptera|Ichneumonidae|BOLD:AAF0476
Orthocentrinae|[5091]|CNGLF912-13|Hymenoptera|Ichneumonidae|BOLD:ACN4080
Orthocentrinae|[5092]|NGAAE908-14|Hymenoptera|Ichneumonidae|BOLD:AAH1830
Orthocentrinae|[5093]|SMTPI8960-14|Hymenoptera|Ichneumonidae|BOLD:ACP7701
Orthocentrinae|[5094]|CNPKK950-14|Hymenoptera|Ichneumonidae|BOLD:AAG0972
Stenomacrus|[5095]|ASWAU692-08|Hymenoptera|Ichneumonidae|BOLD:AAH1493
Stenomacrus|[5096]|SSPAC8104-13|Hymenoptera|Ichneumonidae|BOLD:AAD0179
Orthocentrinae|[5097]|SSWLC992-13|Hymenoptera|Ichneumonidae|BOLD:ACE5147
Stenomacrus|[5098]|SSWLF4798-13|Hymenoptera|Ichneumonidae|BOLD:AAH9934
Stenomacrus|[5099]|CNKLB4967-14|Hymenoptera|Ichneumonidae|BOLD:AAE9001
Orthocentrinae|[5100]|JBHCH584-10|Hymenoptera|Ichneumonidae|BOLD:AAU9777
Stenomacrus|[5101]|PHMTX1083-11|Hymenoptera|Ichneumonidae|BOLD:AAZ0181
Orthocentrinae|[5102]|CNTND2044-14|Hymenoptera|Ichneumonidae|BOLD:ACN2370
Ichneumonidae|[5103]|HARC094-10|Hymenoptera|Ichneumonidae|BOLD:AAO8223
Orthocentrinae|[5104]|HARC419-11|Hymenoptera|Ichneumonidae|BOLD:ACE2974
Stenomacrus sp. 2ZERO|[5105]|HARC015-10|Hymenoptera|Ichneumonidae|BOLD:AAM7533
Orthocentrinae|[5106]|CNSRA974-14|Hymenoptera|Ichneumonidae|BOLD:ACR4925
Orthocentrinae|[5107]|JBHCJ597-11|Hymenoptera|Ichneumonidae|BOLD:AAH1737
Stenomacrus|[5108]|JBHCI871-11|Hymenoptera|Ichneumonidae|BOLD:AAH1623
Orthocentrinae|[5109]|SSPAA1600-13|Hymenoptera|Ichneumonidae|BOLD:AAH1632
Stenomacrus|[5110]|CNWBG2165-13|Hymenoptera|Ichneumonidae|BOLD:ACG2500
Orthocentrinae|[5111]|SMTPI6460-14|Hymenoptera|Ichneumonidae|BOLD:ACP8963
Orthocentrinae|[5112]|JBHCI360-11|Hymenoptera|Ichneumonidae|BOLD:AAU9773
Stenomacrus|[5113]|SSBAF4225-13|Hymenoptera|Ichneumonidae|BOLD:AAF3467
Stenomacrus|[5114]|SSWLE1606-13|Hymenoptera|Ichneumonidae|BOLD:ACE4848
Stenomacrus|[5115]|SSJAF983-13|Hymenoptera|Ichneumonidae|BOLD:AAD0219
Stenomacrus|[5116]|JBHCJ638-11|Hymenoptera|Ichneumonidae|BOLD:AAD8052
Stenomacrus|[5117]|SMTPI8396-14|Hymenoptera|Ichneumonidae|BOLD:AAF3476
Stenomacrus|[5118]|JBHCH621-10|Hymenoptera|Ichneumonidae|BOLD:AAE9002
Stenomacrus|[5119]|SMTPI1374-14|Hymenoptera|Ichneumonidae|BOLD:AAN8654
Stenomacrus|[5120]|SSBAE721-13|Hymenoptera|Ichneumonidae|BOLD:AAH1490
Orthocentrinae|[5121]|SMTPJ3955-14|Hymenoptera|Ichneumonidae|BOLD:ACP7752
Stenomacrus|[5122]|CNWLM1427-13|Hymenoptera|Ichneumonidae|BOLD:AAH1508
Stenomacrus groenlandicus|[5123]|SSJAD904-13|Hymenoptera|Ichneumonidae|BOLD:ACA4472
Orthocentrinae|[5124]|SSBAF4341-13|Hymenoptera|Ichneumonidae|BOLD:AAG0965
Stenomacrus|[5125]|SSJAB734-13|Hymenoptera|Ichneumonidae|BOLD:ACG3877
Orthocentrinae|[5126]|SSFDA5094-14|Hymenoptera|Ichneumonidae|BOLD:ACN5554
Orthocentrinae|[5127]|JBHCI321-11|Hymenoptera|Ichneumonidae|BOLD:AAU9868
Stenomacrus|[5128]|CNTUA5243-14|Hymenoptera|Ichneumonidae|BOLD:AAH1506
Orthocentrinae|[5129]|CNKLF1623-15|Hymenoptera|Ichneumonidae|BOLD:ACS1595
Stenomacrus|[5130]|JBHCJ257-11|Hymenoptera|Ichneumonidae|BOLD:AAH1782
Stenomacrus|[5131]|SSBAC1684-12|Hymenoptera|Ichneumonidae|BOLD:AAH1609
Stenomacrus|[5132]|SSPAA2142-13|Hymenoptera|Ichneumonidae|BOLD:ACM2072
Orthocentrinae|[5133]|CNPEP1206-14|Hymenoptera|Ichneumonidae|BOLD:ACL9781
Stenomacrus|[5134]|SSPAC1513-13|Hymenoptera|Ichneumonidae|BOLD:ACC7160
Orthocentrinae|[5135]|SSEIB3674-13|Hymenoptera|Ichneumonidae|BOLD:ACC6632
Orthocentrinae|[5136]|SSWLA3729-13|Hymenoptera|Ichneumonidae|BOLD:AAG0976
Orthocentrinae|[5137]|CNWAF1276-15|Hymenoptera|Ichneumonidae|BOLD:AAG0966
Picrostigeus debilis|[5138]|SMTPI6110-14|Hymenoptera|Ichneumonidae|BOLD:AAU8205
Orthocentrinae|[5139]|SSBAE756-13|Hymenoptera|Ichneumonidae|BOLD:ACC8952
Orthocentrinae|[5140]|CNGLF909-13|Hymenoptera|Ichneumonidae|BOLD:AAU9114
Orthocentrinae|[5141]|CNFNG1205-14|Hymenoptera|Ichneumonidae|BOLD:AAH1787
Orthocentrinae|[5142]|CNFNF1302-14|Hymenoptera|Ichneumonidae|BOLD:ACL5316
Orthocentrinae|[5143]|SSWLA4868-13|Hymenoptera|Ichneumonidae|BOLD:ACA7963
Orthocentrinae|[5144]|SSGBC1840-14|Hymenoptera|Ichneumonidae|BOLD:AAG0981
Orthocentrinae|[5145]|CNJAF1305-12|Hymenoptera|Ichneumonidae|BOLD:AAH1509
Orthocentrinae|[5146]|SSKJB684-14|Hymenoptera|Ichneumonidae|BOLD:AAU8746
Orthocentrinae|[5147]|CNKLH2452-15|Hymenoptera|Ichneumonidae|BOLD:ACW8771
Orthocentrinae|[5148]|ASWAT935-08|Hymenoptera|Ichneumonidae|BOLD:AAH1828
Orthocentrinae|[5149]|CNPAE387-13|Hymenoptera|Ichneumonidae|BOLD:ACG6508
Orthocentrus winnertzii|[5150]|SSJAD974-13|Hymenoptera|Ichneumonidae|BOLD:ACG5294
Stenomacrus jft08|[5151]|SSJAD2508-13|Hymenoptera|Ichneumonidae|BOLD:AAH1710
Orthocentrus winnertzii|[5152]|SSJAD957-13|Hymenoptera|Ichneumonidae|BOLD:ACM5848
Orthocentrus winnertzii|[5153]|SSJAD6315-13|Hymenoptera|Ichneumonidae|BOLD:AAU8611
Orthocentrus|[5154]|CNWAD2679-14|Hymenoptera|Ichneumonidae|BOLD:AAH2131
Orthocentrus winnertzii|[5155]|CNKTB2065-14|Hymenoptera|Ichneumonidae|BOLD:AAU9494
Orthocentrus asper|[5156]|SMTPI4243-14|Hymenoptera|Ichneumonidae|BOLD:AAG0956
Orthocentrinae|[5157]|SSBRA1655-14|Hymenoptera|Ichneumonidae|BOLD:ACO0573
Orthocentrinae|[5158]|CNNHG1825-14|Hymenoptera|Ichneumonidae|BOLD:ACR8188
Orthocentrinae|[5159]|CNFDS371-14|Hymenoptera|Ichneumonidae|BOLD:ACM1889
Orthocentrinae|[5160]|SSWLD3088-13|Hymenoptera|Ichneumonidae|BOLD:ACC2805
Orthocentrinae|[5161]|SMTPI8987-14|Hymenoptera|Ichneumonidae|BOLD:ACJ6556
Orthocentrinae|[5162]|SMTPD4055-13|Hymenoptera|Ichneumonidae|BOLD:ACJ6707
Orthocentrinae|[5163]|CNYOK1123-15|Hymenoptera|Ichneumonidae|BOLD:ACT8014
Orthocentrinae|[5164]|CNWLI058-12|Hymenoptera|Ichneumonidae|BOLD:ACE1260
Orthocentrinae|[5165]|SSJAE6829-13|Hymenoptera|Ichneumonidae|BOLD:ACJ8806
Orthocentrinae|[5166]|SSWLF940-13|Hymenoptera|Ichneumonidae|BOLD:ACI5707
Orthocentrinae|[5167]|CNEIH504-13|Hymenoptera|Ichneumonidae|BOLD:ACD0601
Plectiscidea jft03|[5168]|ASWA029-08|Hymenoptera|Ichneumonidae|BOLD:AAF4323
Plectiscidea jft03|[5169]|SSJAD3650-13|Hymenoptera|Ichneumonidae|BOLD:ACN5142
Plectiscidea|[5170]|SSBAE1844-13|Hymenoptera|Ichneumonidae|BOLD:ACC6916
Plectiscidea|[5171]|SSBAE3180-13|Hymenoptera|Ichneumonidae|BOLD:ACL8476
Orthocentrinae|[5172]|SSJAD6327-13|Hymenoptera|Ichneumonidae|BOLD:AAH1588
Orthocentrinae|[5173]|SSPAC1871-13|Hymenoptera|Ichneumonidae|BOLD:AAM7466
Orthocentrinae|[5174]|SSJAF5835-13|Hymenoptera|Ichneumonidae|BOLD:AAG0380
Orthocentrinae|[5175]|JBHCH430-10|Hymenoptera|Ichneumonidae|BOLD:AAM7407
Orthocentrinae|[5176]|SSEIB3837-13|Hymenoptera|Ichneumonidae|BOLD:ACC8520
Dialipsis|[5177]|SSWLF922-13|Hymenoptera|Ichneumonidae|BOLD:AAD6970
Dialipsis|[5178]|JBHCI646-11|Hymenoptera|Ichneumonidae|BOLD:AAC7200
Dialipsis|[5179]|SSWLF1343-13|Hymenoptera|Ichneumonidae|BOLD:ACI6847
Orthocentrinae|[5180]|SSBAE1847-13|Hymenoptera|Ichneumonidae|BOLD:ACI6849
Plectiscidea|[5181]|SSPAB4958-13|Hymenoptera|Ichneumonidae|BOLD:AAH1832
Plectiscidea|[5182]|SSBAC747-12|Hymenoptera|Ichneumonidae|BOLD:ACB9714
Plectiscidea venusta|[5183]|SSPAA9639-13|Hymenoptera|Ichneumonidae|BOLD:ABX9075
Orthocentrinae|[5184]|ASWAY441-08|Hymenoptera|Ichneumonidae|BOLD:AAH1822
Plectiscidea|[5185]|SSBAE2034-13|Hymenoptera|Ichneumonidae|BOLD:ACV6901
Orthocentrinae|[5186]|SSPAG050-13|Hymenoptera|Ichneumonidae|BOLD:AAU9318
Plectiscidea jft04|[5187]|SSJAD2681-13|Hymenoptera|Ichneumonidae|BOLD:AAH9172
Plectiscidea|[5188]|SSJAD2501-13|Hymenoptera|Ichneumonidae|BOLD:ACG6440
Plectiscidea|[5189]|SSJAD5296-13|Hymenoptera|Ichneumonidae|BOLD:ACI5550

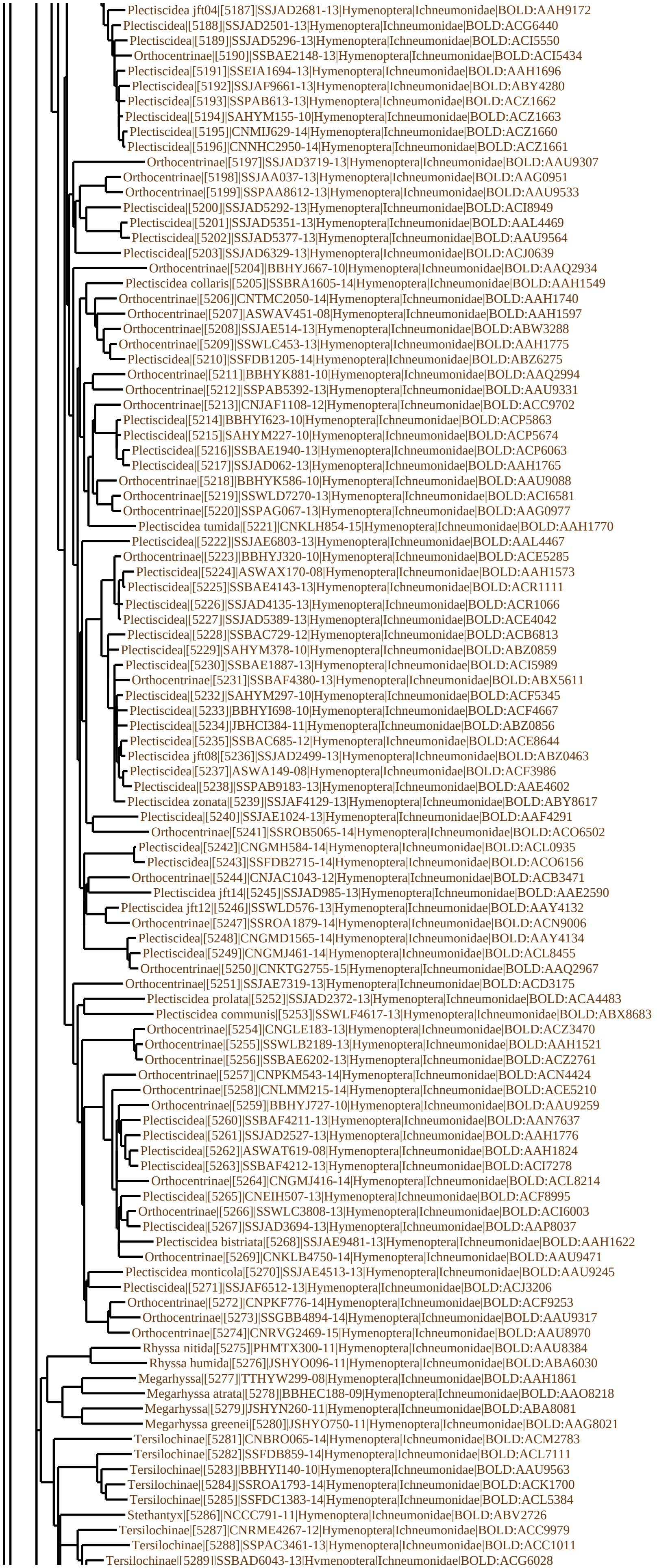
Plectiscidea jft04|[5187]|SSJAD2681-13|Hymenoptera|Ichneumonidae|BOLD:AAH9172
Plectiscidea|[5188]|SSJAD2501-13|Hymenoptera|Ichneumonidae|BOLD:ACG6440
Plectiscidea|[5189]|SSJAD5296-13|Hymenoptera|Ichneumonidae|BOLD:ACI5550
Orthocentrinae|[5190]|SSBAE2148-13|Hymenoptera|Ichneumonidae|BOLD:ACI5434
Plectiscidea|[5191]|SSEIA1694-13|Hymenoptera|Ichneumonidae|BOLD:AAH1696
Plectiscidea|[5192]|SSJAF9661-13|Hymenoptera|Ichneumonidae|BOLD:ABY4280
Plectiscidea|[5193]|SSPAB613-13|Hymenoptera|Ichneumonidae|BOLD:ACZ1662
Plectiscidea|[5194]|SAHYM155-10|Hymenoptera|Ichneumonidae|BOLD:ACZ1663
Plectiscidea|[5195]|CNMIJ629-14|Hymenoptera|Ichneumonidae|BOLD:ACZ1660
Plectiscidea|[5196]|CNNHC2950-14|Hymenoptera|Ichneumonidae|BOLD:ACZ1661
Orthocentrinae|[5197]|SSJAD3719-13|Hymenoptera|Ichneumonidae|BOLD:AAU9307
Orthocentrinae|[5198]|SSJAA037-13|Hymenoptera|Ichneumonidae|BOLD:AAG0951
Orthocentrinae|[5199]|SSPAA8612-13|Hymenoptera|Ichneumonidae|BOLD:AAU9533
Plectiscidea|[5200]|SSJAD5292-13|Hymenoptera|Ichneumonidae|BOLD:ACI8949
Plectiscidea|[5201]|SSJAD5351-13|Hymenoptera|Ichneumonidae|BOLD:AAL4469
Plectiscidea|[5202]|SSJAD5377-13|Hymenoptera|Ichneumonidae|BOLD:AAU9564
Plectiscidea|[5203]|SSJAD6329-13|Hymenoptera|Ichneumonidae|BOLD:ACJ0639
Orthocentrinae|[5204]|BBHYJ667-10|Hymenoptera|Ichneumonidae|BOLD:AAQ2934
Plectiscidea collaris|[5205]|SSBRA1605-14|Hymenoptera|Ichneumonidae|BOLD:AAH1549
Orthocentrinae|[5206]|CNTMC2050-14|Hymenoptera|Ichneumonidae|BOLD:AAH1740
Orthocentrinae|[5207]|ASWAV451-08|Hymenoptera|Ichneumonidae|BOLD:AAH1597
Orthocentrinae|[5208]|SSJAE514-13|Hymenoptera|Ichneumonidae|BOLD:ABW3288
Orthocentrinae|[5209]|SSWLC453-13|Hymenoptera|Ichneumonidae|BOLD:AAH1775
Plectiscidea|[5210]|SSFDB1205-14|Hymenoptera|Ichneumonidae|BOLD:ABZ6275
Orthocentrinae|[5211]|BBHYK881-10|Hymenoptera|Ichneumonidae|BOLD:AAQ2994
Orthocentrinae|[5212]|SSPAB5392-13|Hymenoptera|Ichneumonidae|BOLD:AAU9331
Orthocentrinae|[5213]|CNJAF1108-12|Hymenoptera|Ichneumonidae|BOLD:ACC9702
Plectiscidea|[5214]|BBHYI623-10|Hymenoptera|Ichneumonidae|BOLD:ACP5863
Plectiscidea|[5215]|SAHYM227-10|Hymenoptera|Ichneumonidae|BOLD:ACP5674
Plectiscidea|[5216]|SSBAE1940-13|Hymenoptera|Ichneumonidae|BOLD:ACP6063
Plectiscidea|[5217]|SSJAD062-13|Hymenoptera|Ichneumonidae|BOLD:AAH1765
Orthocentrinae|[5218]|BBHYK586-10|Hymenoptera|Ichneumonidae|BOLD:AAU9088
Orthocentrinae|[5219]|SSWLD7270-13|Hymenoptera|Ichneumonidae|BOLD:ACI6581
Orthocentrinae|[5220]|SSPAG067-13|Hymenoptera|Ichneumonidae|BOLD:AAG0977
Plectiscidea tumida|[5221]|CNKLH854-15|Hymenoptera|Ichneumonidae|BOLD:AAH1770
Plectiscidea|[5222]|SSJAE6803-13|Hymenoptera|Ichneumonidae|BOLD:AAL4467
Orthocentrinae|[5223]|BBHYJ320-10|Hymenoptera|Ichneumonidae|BOLD:ACE5285
Plectiscidea|[5224]|ASWAX170-08|Hymenoptera|Ichneumonidae|BOLD:AAH1573
Plectiscidea|[5225]|SSBAE4143-13|Hymenoptera|Ichneumonidae|BOLD:ACR1111
Plectiscidea|[5226]|SSJAD4135-13|Hymenoptera|Ichneumonidae|BOLD:ACR1066
Plectiscidea|[5227]|SSJAD5389-13|Hymenoptera|Ichneumonidae|BOLD:ACE4042
Plectiscidea|[5228]|SSBAC729-12|Hymenoptera|Ichneumonidae|BOLD:ACB6813
Plectiscidea|[5229]|SAHYM378-10|Hymenoptera|Ichneumonidae|BOLD:ABZ0859
Plectiscidea|[5230]|SSBAE1887-13|Hymenoptera|Ichneumonidae|BOLD:ACI5989
Orthocentrinae|[5231]|SSBAF4380-13|Hymenoptera|Ichneumonidae|BOLD:ABX5611
Plectiscidea|[5232]|SAHYM297-10|Hymenoptera|Ichneumonidae|BOLD:ACF5345
Plectiscidea|[5233]|BBHYI698-10|Hymenoptera|Ichneumonidae|BOLD:ACF4667
Plectiscidea|[5234]|JBHCI384-11|Hymenoptera|Ichneumonidae|BOLD:ABZ0856
Plectiscidea|[5235]|SSBAC685-12|Hymenoptera|Ichneumonidae|BOLD:ACE8644
Plectiscidea jft08|[5236]|SSJAD2499-13|Hymenoptera|Ichneumonidae|BOLD:ABZ0463
Plectiscidea|[5237]|ASWA149-08|Hymenoptera|Ichneumonidae|BOLD:ACF3986
Plectiscidea|[5238]|SSPAB9183-13|Hymenoptera|Ichneumonidae|BOLD:AAE4602
Plectiscidea zonata|[5239]|SSJAF4129-13|Hymenoptera|Ichneumonidae|BOLD:ABY8617
Plectiscidea|[5240]|SSJAE1024-13|Hymenoptera|Ichneumonidae|BOLD:AAF4291
Orthocentrinae|[5241]|SSROB5065-14|Hymenoptera|Ichneumonidae|BOLD:ACO6502
Plectiscidea|[5242]|CNGMH584-14|Hymenoptera|Ichneumonidae|BOLD:ACL0935
Plectiscidea|[5243]|SSFDB2715-14|Hymenoptera|Ichneumonidae|BOLD:ACO6156
Orthocentrinae|[5244]|CNJAC1043-12|Hymenoptera|Ichneumonidae|BOLD:ACB3471
Plectiscidea jft14|[5245]|SSJAD985-13|Hymenoptera|Ichneumonidae|BOLD:AAE2590
Plectiscidea jft12|[5246]|SSWLD576-13|Hymenoptera|Ichneumonidae|BOLD:AAY4132
Orthocentrinae|[5247]|SSROA1879-14|Hymenoptera|Ichneumonidae|BOLD:ACN9006
Plectiscidea|[5248]|CNGMD1565-14|Hymenoptera|Ichneumonidae|BOLD:AAY4134
Plectiscidea|[5249]|CNGMJ461-14|Hymenoptera|Ichneumonidae|BOLD:ACL8455
Orthocentrinae|[5250]|CNKTG2755-15|Hymenoptera|Ichneumonidae|BOLD:AAQ2967
Orthocentrinae|[5251]|SSJAE7319-13|Hymenoptera|Ichneumonidae|BOLD:ACD3175
Plectiscidea prolata|[5252]|SSJAD2372-13|Hymenoptera|Ichneumonidae|BOLD:ACA4483
Plectiscidea communis|[5253]|SSWLF4617-13|Hymenoptera|Ichneumonidae|BOLD:ABX8683
Orthocentrinae|[5254]|CNGLE183-13|Hymenoptera|Ichneumonidae|BOLD:ACZ3470
Orthocentrinae|[5255]|SSWLB2189-13|Hymenoptera|Ichneumonidae|BOLD:AAH1521
Orthocentrinae|[5256]|SSBAE6202-13|Hymenoptera|Ichneumonidae|BOLD:ACZ2761
Orthocentrinae|[5257]|CNPKM543-14|Hymenoptera|Ichneumonidae|BOLD:ACN4424
Orthocentrinae|[5258]|CNLMM215-14|Hymenoptera|Ichneumonidae|BOLD:ACE5210
Orthocentrinae|[5259]|BBHYJ727-10|Hymenoptera|Ichneumonidae|BOLD:AAU9259
Plectiscidea|[5260]|SSBAF4211-13|Hymenoptera|Ichneumonidae|BOLD:AAN7637
Plectiscidea|[5261]|SSJAD2527-13|Hymenoptera|Ichneumonidae|BOLD:AAH1776
Plectiscidea|[5262]|ASWAT619-08|Hymenoptera|Ichneumonidae|BOLD:AAH1824
Plectiscidea|[5263]|SSBAF4212-13|Hymenoptera|Ichneumonidae|BOLD:ACI7278
Orthocentrinae|[5264]|CNGMJ416-14|Hymenoptera|Ichneumonidae|BOLD:ACL8214
Plectiscidea|[5265]|CNEIH507-13|Hymenoptera|Ichneumonidae|BOLD:ACF8995
Orthocentrinae|[5266]|SSWLC3808-13|Hymenoptera|Ichneumonidae|BOLD:ACI6003
Plectiscidea|[5267]|SSJAD3694-13|Hymenoptera|Ichneumonidae|BOLD:AAP8037
Plectiscidea bistriata|[5268]|SSJAE9481-13|Hymenoptera|Ichneumonidae|BOLD:AAH1622
Orthocentrinae|[5269]|CNKLB4750-14|Hymenoptera|Ichneumonidae|BOLD:AAU9471
Plectiscidea monticola|[5270]|SSJAE4513-13|Hymenoptera|Ichneumonidae|BOLD:AAU9245
Plectiscidea|[5271]|SSJAF6512-13|Hymenoptera|Ichneumonidae|BOLD:ACJ3206
Orthocentrinae|[5272]|CNPKF776-14|Hymenoptera|Ichneumonidae|BOLD:ACF9253
Orthocentrinae|[5273]|SSGBB4894-14|Hymenoptera|Ichneumonidae|BOLD:AAU9317
Orthocentrinae|[5274]|CNRVG2469-15|Hymenoptera|Ichneumonidae|BOLD:AAU8970
Rhyssa nitida|[5275]|PHMTX300-11|Hymenoptera|Ichneumonidae|BOLD:AAU8384
Rhyssa humida|[5276]|JSHYO096-11|Hymenoptera|Ichneumonidae|BOLD:ABA6030
Megarhyssa|[5277]|TTHYW299-08|Hymenoptera|Ichneumonidae|BOLD:AAH1861
Megarhyssa atrata|[5278]|BBHEC188-09|Hymenoptera|Ichneumonidae|BOLD:AAO8218
Megarhyssa|[5279]|JSHYN260-11|Hymenoptera|Ichneumonidae|BOLD:ABA8081
Megarhyssa greenei|[5280]|JSHYO750-11|Hymenoptera|Ichneumonidae|BOLD:AAG8021
Tersilochinae|[5281]|CNBRO065-14|Hymenoptera|Ichneumonidae|BOLD:ACM2783
Tersilochinae|[5282]|SSFDB859-14|Hymenoptera|Ichneumonidae|BOLD:ACL7111
Tersilochinae|[5283]|BBHYI140-10|Hymenoptera|Ichneumonidae|BOLD:AAU9563
Tersilochinae|[5284]|SSROA1793-14|Hymenoptera|Ichneumonidae|BOLD:ACK1700
Tersilochinae|[5285]|SSFDC1383-14|Hymenoptera|Ichneumonidae|BOLD:ACL5384
Stethantyx|[5286]|NCCC791-11|Hymenoptera|Ichneumonidae|BOLD:ABV2726
Tersilochinae|[5287]|CNRME4267-12|Hymenoptera|Ichneumonidae|BOLD:ACC9979
Tersilochinae|[5288]|SSPAC3461-13|Hymenoptera|Ichneumonidae|BOLD:ACC1011
Tersilochinae|[5289]|SSBAD6043-13|Hymenoptera|Ichneumonidae|BOLD:ACG6028

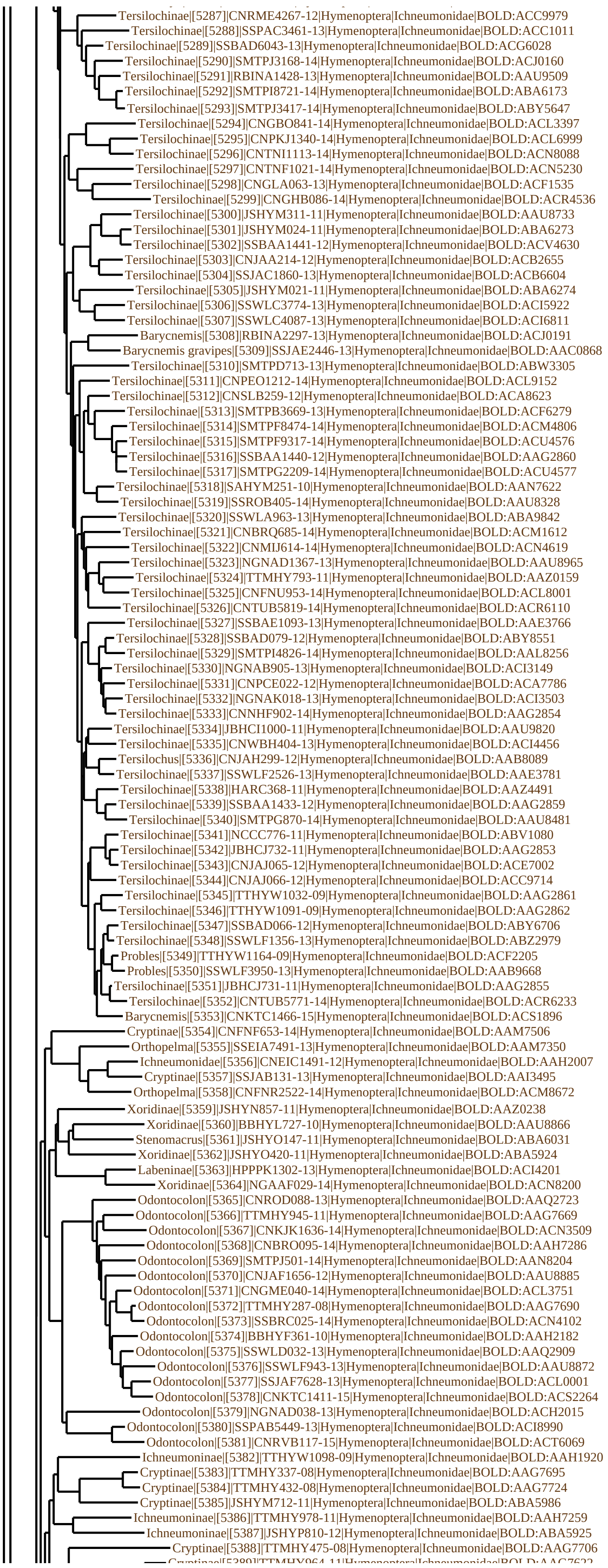
Tersilochinae|[5287]|CNRME4267-12|Hymenoptera|Ichneumonidae|BOLD:ACC9979
Tersilochinae|[5288]|SSPAC3461-13|Hymenoptera|Ichneumonidae|BOLD:ACC1011
Tersilochinae|[5289]|SSBAD6043-13|Hymenoptera|Ichneumonidae|BOLD:ACG6028
Tersilochinae|[5290]|SMTPJ3168-14|Hymenoptera|Ichneumonidae|BOLD:ACJ0160
Tersilochinae|[5291]|RBINA1428-13|Hymenoptera|Ichneumonidae|BOLD:AAU9509
Tersilochinae|[5292]|SMTPI8721-14|Hymenoptera|Ichneumonidae|BOLD:ABA6173
Tersilochinae|[5293]|SMTPJ3417-14|Hymenoptera|Ichneumonidae|BOLD:ABY5647
Tersilochinae|[5294]|CNGBO841-14|Hymenoptera|Ichneumonidae|BOLD:ACL3397
Tersilochinae|[5295]|CNPKJ1340-14|Hymenoptera|Ichneumonidae|BOLD:ACL6999
Tersilochinae|[5296]|CNTNI1113-14|Hymenoptera|Ichneumonidae|BOLD:ACN8088
Tersilochinae|[5297]|CNTNF1021-14|Hymenoptera|Ichneumonidae|BOLD:ACN5230
Tersilochinae|[5298]|CNGLA063-13|Hymenoptera|Ichneumonidae|BOLD:ACF1535
Tersilochinae|[5299]|CNGHB086-14|Hymenoptera|Ichneumonidae|BOLD:ACR4536
Tersilochinae|[5300]|JSHYM311-11|Hymenoptera|Ichneumonidae|BOLD:AAU8733
Tersilochinae|[5301]|JSHYM024-11|Hymenoptera|Ichneumonidae|BOLD:ABA6273
Tersilochinae|[5302]|SSBAA1441-12|Hymenoptera|Ichneumonidae|BOLD:ACV4630
Tersilochinae|[5303]|CNJAA214-12|Hymenoptera|Ichneumonidae|BOLD:ACB2655
Tersilochinae|[5304]|SSJAC1860-13|Hymenoptera|Ichneumonidae|BOLD:ACB6604
Tersilochinae|[5305]|JSHYM021-11|Hymenoptera|Ichneumonidae|BOLD:ABA6274
Tersilochinae|[5306]|SSWLC3774-13|Hymenoptera|Ichneumonidae|BOLD:ACI5922
Tersilochinae|[5307]|SSWLC4087-13|Hymenoptera|Ichneumonidae|BOLD:ACI6811
Barycnemis|[5308]|RBINA2297-13|Hymenoptera|Ichneumonidae|BOLD:ACJ0191
Barycnemis gravipes|[5309]|SSJAE2446-13|Hymenoptera|Ichneumonidae|BOLD:AAC0868
Tersilochinae|[5310]|SMTPD713-13|Hymenoptera|Ichneumonidae|BOLD:ABW3305
Tersilochinae|[5311]|CNPEO1212-14|Hymenoptera|Ichneumonidae|BOLD:ACL9152
Tersilochinae|[5312]|CNSLB259-12|Hymenoptera|Ichneumonidae|BOLD:ACA8623
Tersilochinae|[5313]|SMTPB3669-13|Hymenoptera|Ichneumonidae|BOLD:ACF6279
Tersilochinae|[5314]|SMTPF8474-14|Hymenoptera|Ichneumonidae|BOLD:ACM4806
Tersilochinae|[5315]|SMTPF9317-14|Hymenoptera|Ichneumonidae|BOLD:ACU4576
Tersilochinae|[5316]|SSBAA1440-12|Hymenoptera|Ichneumonidae|BOLD:AAG2860
Tersilochinae|[5317]|SMTPG2209-14|Hymenoptera|Ichneumonidae|BOLD:ACU4577
Tersilochinae|[5318]|SAHYM251-10|Hymenoptera|Ichneumonidae|BOLD:AAN7622
Tersilochinae|[5319]|SSROB405-14|Hymenoptera|Ichneumonidae|BOLD:AAU8328
Tersilochinae|[5320]|SSWLA963-13|Hymenoptera|Ichneumonidae|BOLD:ABA9842
Tersilochinae|[5321]|CNBRQ685-14|Hymenoptera|Ichneumonidae|BOLD:ACM1612
Tersilochinae|[5322]|CNMIJ614-14|Hymenoptera|Ichneumonidae|BOLD:ACN4619
Tersilochinae|[5323]|NGNAD1367-13|Hymenoptera|Ichneumonidae|BOLD:AAU8965
Tersilochinae|[5324]|TTMHY793-11|Hymenoptera|Ichneumonidae|BOLD:AAZ0159
Tersilochinae|[5325]|CNFNU953-14|Hymenoptera|Ichneumonidae|BOLD:ACL8001
Tersilochinae|[5326]|CNTUB5819-14|Hymenoptera|Ichneumonidae|BOLD:ACR6110
Tersilochinae|[5327]|SSBAE1093-13|Hymenoptera|Ichneumonidae|BOLD:AAE3766
Tersilochinae|[5328]|SSBAD079-12|Hymenoptera|Ichneumonidae|BOLD:ABY8551
Tersilochinae|[5329]|SMTPI4826-14|Hymenoptera|Ichneumonidae|BOLD:AAL8256
Tersilochinae|[5330]|NGNAB905-13|Hymenoptera|Ichneumonidae|BOLD:ACI3149
Tersilochinae|[5331]|CNPCE022-12|Hymenoptera|Ichneumonidae|BOLD:ACA7786
Tersilochinae|[5332]|NGNAK018-13|Hymenoptera|Ichneumonidae|BOLD:ACI3503
Tersilochinae|[5333]|CNNHF902-14|Hymenoptera|Ichneumonidae|BOLD:AAG2854
Tersilochinae|[5334]|JBHCI1000-11|Hymenoptera|Ichneumonidae|BOLD:AAU9820
Tersilochinae|[5335]|CNWBH404-13|Hymenoptera|Ichneumonidae|BOLD:ACI4456
Tersilochus|[5336]|CNJAH299-12|Hymenoptera|Ichneumonidae|BOLD:AAB8089
Tersilochinae|[5337]|SSWLF2526-13|Hymenoptera|Ichneumonidae|BOLD:AAE3781
Tersilochinae|[5338]|HARC368-11|Hymenoptera|Ichneumonidae|BOLD:AAZ4491
Tersilochinae|[5339]|SSBAA1433-12|Hymenoptera|Ichneumonidae|BOLD:AAG2859
Tersilochinae|[5340]|SMTPG870-14|Hymenoptera|Ichneumonidae|BOLD:AAU8481
Tersilochinae|[5341]|NCCC776-11|Hymenoptera|Ichneumonidae|BOLD:ABV1080
Tersilochinae|[5342]|JBHCJ732-11|Hymenoptera|Ichneumonidae|BOLD:AAG2853
Tersilochinae|[5343]|CNJAJ065-12|Hymenoptera|Ichneumonidae|BOLD:ACE7002
Tersilochinae|[5344]|CNJAJ066-12|Hymenoptera|Ichneumonidae|BOLD:ACC9714
Tersilochinae|[5345]|TTHYW1032-09|Hymenoptera|Ichneumonidae|BOLD:AAG2861
Tersilochinae|[5346]|TTHYW1091-09|Hymenoptera|Ichneumonidae|BOLD:AAG2862
Tersilochinae|[5347]|SSBAD066-12|Hymenoptera|Ichneumonidae|BOLD:ABY6706
Tersilochinae|[5348]|SSWLF1356-13|Hymenoptera|Ichneumonidae|BOLD:ABZ2979
Probles|[5349]|TTHYW1164-09|Hymenoptera|Ichneumonidae|BOLD:ACF2205
Probles|[5350]|SSWLF3950-13|Hymenoptera|Ichneumonidae|BOLD:AAB9668
Tersilochinae|[5351]|JBHCJ731-11|Hymenoptera|Ichneumonidae|BOLD:AAG2855
Tersilochinae|[5352]|CNTUB5771-14|Hymenoptera|Ichneumonidae|BOLD:ACR6233
Barycnemis|[5353]|CNKTC1466-15|Hymenoptera|Ichneumonidae|BOLD:ACS1896
Cryptinae|[5354]|CNFNF653-14|Hymenoptera|Ichneumonidae|BOLD:AAM7506
Orthopelma|[5355]|SSEIA7491-13|Hymenoptera|Ichneumonidae|BOLD:AAM7350
Ichneumonidae|[5356]|CNEIC1491-12|Hymenoptera|Ichneumonidae|BOLD:AAH2007
Cryptinae|[5357]|SSJAB131-13|Hymenoptera|Ichneumonidae|BOLD:AAI3495
Orthopelma|[5358]|CNFNR2522-14|Hymenoptera|Ichneumonidae|BOLD:ACM8672
Xoridinae|[5359]|JSHYN857-11|Hymenoptera|Ichneumonidae|BOLD:AAZ0238
Xoridinae|[5360]|BBHYL727-10|Hymenoptera|Ichneumonidae|BOLD:AAU8866
Stenomacrus|[5361]|JSHYO147-11|Hymenoptera|Ichneumonidae|BOLD:ABA6031
Xoridinae|[5362]|JSHYO420-11|Hymenoptera|Ichneumonidae|BOLD:ABA5924
Labeninae|[5363]|HPPPK1302-13|Hymenoptera|Ichneumonidae|BOLD:ACI4201
Xoridinae|[5364]|NGAAF029-14|Hymenoptera|Ichneumonidae|BOLD:ACN8200
Odontocolon|[5365]|CNROD088-13|Hymenoptera|Ichneumonidae|BOLD:AAQ2723
Odontocolon|[5366]|TTMHY945-11|Hymenoptera|Ichneumonidae|BOLD:AAG7669
Odontocolon|[5367]|CNKJK1636-14|Hymenoptera|Ichneumonidae|BOLD:ACN3509
Odontocolon|[5368]|CNBRO095-14|Hymenoptera|Ichneumonidae|BOLD:AAH7286
Odontocolon|[5369]|SMTPJ501-14|Hymenoptera|Ichneumonidae|BOLD:AAN8204
Odontocolon|[5370]|CNJAF1656-12|Hymenoptera|Ichneumonidae|BOLD:AAU8885
Odontocolon|[5371]|CNGME040-14|Hymenoptera|Ichneumonidae|BOLD:ACL3751
Odontocolon|[5372]|TTMHY287-08|Hymenoptera|Ichneumonidae|BOLD:AAG7690
Odontocolon|[5373]|SSBRC025-14|Hymenoptera|Ichneumonidae|BOLD:ACN4102
Odontocolon|[5374]|BBHYF361-10|Hymenoptera|Ichneumonidae|BOLD:AAH2182
Odontocolon|[5375]|SSWLD032-13|Hymenoptera|Ichneumonidae|BOLD:AAQ2909
Odontocolon|[5376]|SSWLF943-13|Hymenoptera|Ichneumonidae|BOLD:AAU8872
Odontocolon|[5377]|SSJAF7628-13|Hymenoptera|Ichneumonidae|BOLD:ACL0001
Odontocolon|[5378]|CNKTC1411-15|Hymenoptera|Ichneumonidae|BOLD:ACS2264
Odontocolon|[5379]|NGNAD038-13|Hymenoptera|Ichneumonidae|BOLD:ACH2015
Odontocolon|[5380]|SSPAB5449-13|Hymenoptera|Ichneumonidae|BOLD:ACI8990
Odontocolon|[5381]|CNRVB117-15|Hymenoptera|Ichneumonidae|BOLD:ACT6069
Ichneumoninae|[5382]|TTHYW1098-09|Hymenoptera|Ichneumonidae|BOLD:AAH1920
Cryptinae|[5383]|TTMHY337-08|Hymenoptera|Ichneumonidae|BOLD:AAG7695
Cryptinae|[5384]|TTMHY432-08|Hymenoptera|Ichneumonidae|BOLD:AAG7724
Cryptinae|[5385]|JSHYM712-11|Hymenoptera|Ichneumonidae|BOLD:ABA5986
Ichneumoninae|[5386]|TTMHY978-11|Hymenoptera|Ichneumonidae|BOLD:AAH7259
Ichneumoninae|[5387]|JSHYP810-12|Hymenoptera|Ichneumonidae|BOLD:ABA5925
Cryptinae|[5388]|TTMHY475-08|Hymenoptera|Ichneumonidae|BOLD:AAG7706

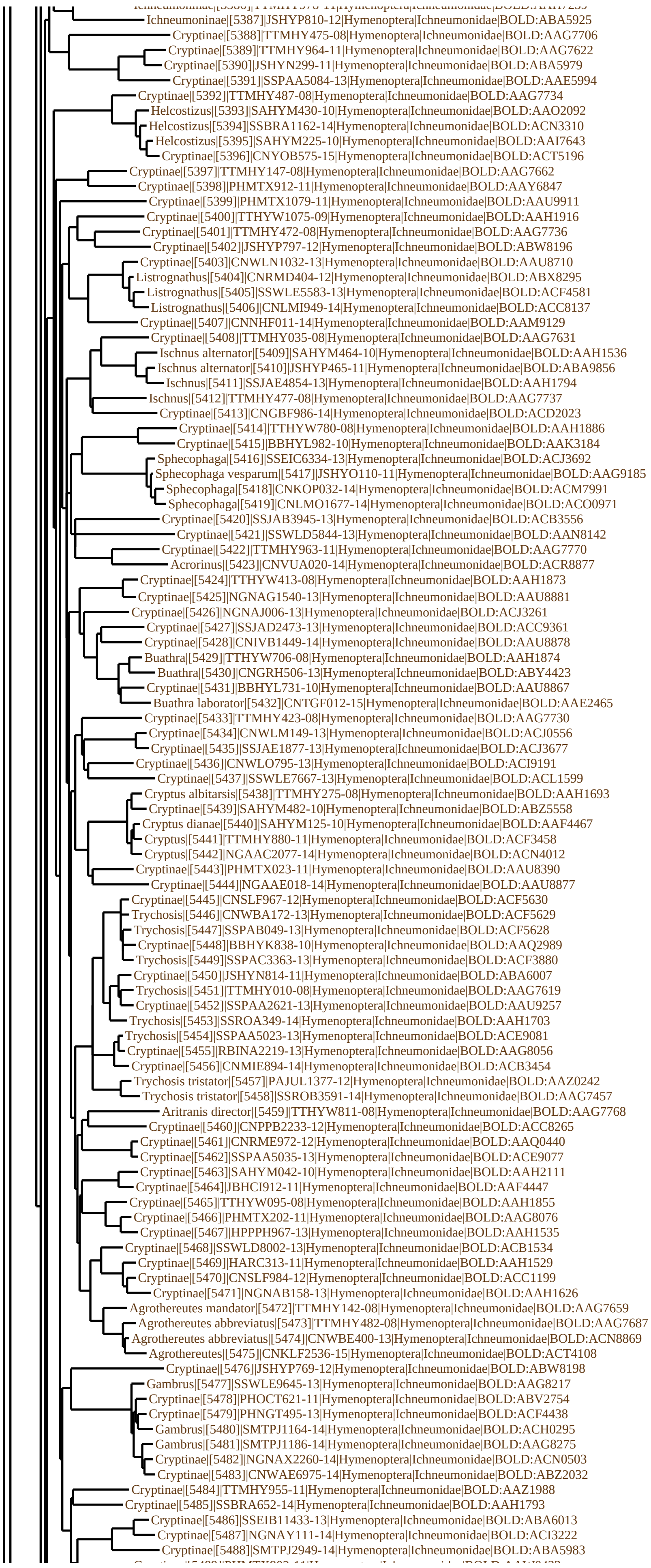

Ichneumoninae|[5387]|JSHYP810-12|Hymenoptera|Ichneumonidae|BOLD:ABA5925
Cryptinae|[5388]|TTMHY475-08|Hymenoptera|Ichneumonidae|BOLD:AAG7706
Cryptinae|[5389]|TTMHY964-11|Hymenoptera|Ichneumonidae|BOLD:AAG7622
Cryptinae|[5390]|JSHYN299-11|Hymenoptera|Ichneumonidae|BOLD:ABA5979
Cryptinae|[5391]|SSPAA5084-13|Hymenoptera|Ichneumonidae|BOLD:AAE5994
Cryptinae|[5392]|TTMHY487-08|Hymenoptera|Ichneumonidae|BOLD:AAG7734
Helcostizus|[5393]|SAHYM430-10|Hymenoptera|Ichneumonidae|BOLD:AAO2092
Helcostizus|[5394]|SSBRA1162-14|Hymenoptera|Ichneumonidae|BOLD:ACN3310
Helcostizus|[5395]|SAHYM225-10|Hymenoptera|Ichneumonidae|BOLD:AAI7643
Cryptinae|[5396]|CNYOB575-15|Hymenoptera|Ichneumonidae|BOLD:ACT5196
Cryptinae|[5397]|TTMHY147-08|Hymenoptera|Ichneumonidae|BOLD:AAG7662
Cryptinae|[5398]|PHMTX912-11|Hymenoptera|Ichneumonidae|BOLD:AAY6847
Cryptinae|[5399]|PHMTX1079-11|Hymenoptera|Ichneumonidae|BOLD:AAU9911
Cryptinae|[5400]|TTHYW1075-09|Hymenoptera|Ichneumonidae|BOLD:AAH1916
Cryptinae|[5401]|TTMHY472-08|Hymenoptera|Ichneumonidae|BOLD:AAG7736
Cryptinae|[5402]|JSHYP797-12|Hymenoptera|Ichneumonidae|BOLD:ABW8196
Cryptinae|[5403]|CNWLN1032-13|Hymenoptera|Ichneumonidae|BOLD:AAU8710
Listrognathus|[5404]|CNRMD404-12|Hymenoptera|Ichneumonidae|BOLD:ABX8295
Listrognathus|[5405]|SSWLE5583-13|Hymenoptera|Ichneumonidae|BOLD:ACF4581
Listrognathus|[5406]|CNLMI949-14|Hymenoptera|Ichneumonidae|BOLD:ACC8137
Cryptinae|[5407]|CNNHF011-14|Hymenoptera|Ichneumonidae|BOLD:AAM9129
Cryptinae|[5408]|TTMHY035-08|Hymenoptera|Ichneumonidae|BOLD:AAG7631
Ischnus alternator|[5409]|SAHYM464-10|Hymenoptera|Ichneumonidae|BOLD:AAH1536
Ischnus alternator|[5410]|JSHYP465-11|Hymenoptera|Ichneumonidae|BOLD:ABA9856
Ischnus|[5411]|SSJAE4854-13|Hymenoptera|Ichneumonidae|BOLD:AAH1794
Ischnus|[5412]|TTMHY477-08|Hymenoptera|Ichneumonidae|BOLD:AAG7737
Cryptinae|[5413]|CNGBF986-14|Hymenoptera|Ichneumonidae|BOLD:ACD2023
Cryptinae|[5414]|TTHYW780-08|Hymenoptera|Ichneumonidae|BOLD:AAH1886
Cryptinae|[5415]|BBHYL982-10|Hymenoptera|Ichneumonidae|BOLD:AAK3184
Sphecophaga|[5416]|SSEIC6334-13|Hymenoptera|Ichneumonidae|BOLD:ACJ3692
Sphecophaga vesparum|[5417]|JSHYO110-11|Hymenoptera|Ichneumonidae|BOLD:AAG9185
Sphecophaga|[5418]|CNKOP032-14|Hymenoptera|Ichneumonidae|BOLD:ACM7991
Sphecophaga|[5419]|CNLMO1677-14|Hymenoptera|Ichneumonidae|BOLD:ACO0971
Cryptinae|[5420]|SSJAB3945-13|Hymenoptera|Ichneumonidae|BOLD:ACB3556
Cryptinae|[5421]|SSWLD5844-13|Hymenoptera|Ichneumonidae|BOLD:AAN8142
Cryptinae|[5422]|TTMHY963-11|Hymenoptera|Ichneumonidae|BOLD:AAG7770
Acrorinus|[5423]|CNVUA020-14|Hymenoptera|Ichneumonidae|BOLD:ACR8877
Cryptinae|[5424]|TTHYW413-08|Hymenoptera|Ichneumonidae|BOLD:AAH1873
Cryptinae|[5425]|NGNAG1540-13|Hymenoptera|Ichneumonidae|BOLD:AAU8881
Cryptinae|[5426]|NGNAJ006-13|Hymenoptera|Ichneumonidae|BOLD:ACJ3261
Cryptinae|[5427]|SSJAD2473-13|Hymenoptera|Ichneumonidae|BOLD:ACC9361
Cryptinae|[5428]|CNIVB1449-14|Hymenoptera|Ichneumonidae|BOLD:AAU8878
Buathra|[5429]|TTHYW706-08|Hymenoptera|Ichneumonidae|BOLD:AAH1874
Buathra|[5430]|CNGRH506-13|Hymenoptera|Ichneumonidae|BOLD:ABY4423
Cryptinae|[5431]|BBHYL731-10|Hymenoptera|Ichneumonidae|BOLD:AAU8867
Buathra laborator|[5432]|CNTGF012-15|Hymenoptera|Ichneumonidae|BOLD:AAE2465
Cryptinae|[5433]|TTMHY423-08|Hymenoptera|Ichneumonidae|BOLD:AAG7730
Cryptinae|[5434]|CNWLM149-13|Hymenoptera|Ichneumonidae|BOLD:ACJ0556
Cryptinae|[5435]|SSJAE1877-13|Hymenoptera|Ichneumonidae|BOLD:ACJ3677
Cryptinae|[5436]|CNWLO795-13|Hymenoptera|Ichneumonidae|BOLD:ACI9191
Cryptinae|[5437]|SSWLE7667-13|Hymenoptera|Ichneumonidae|BOLD:ACL1599
Cryptus albitarsis|[5438]|TTMHY275-08|Hymenoptera|Ichneumonidae|BOLD:AAH1693
Cryptinae|[5439]|SAHYM482-10|Hymenoptera|Ichneumonidae|BOLD:ABZ5558
Cryptus dianae|[5440]|SAHYM125-10|Hymenoptera|Ichneumonidae|BOLD:AAF4467
Cryptus|[5441]|TTMHY880-11|Hymenoptera|Ichneumonidae|BOLD:ACF3458
Cryptus|[5442]|NGAAC2077-14|Hymenoptera|Ichneumonidae|BOLD:ACN4012
Cryptinae|[5443]|PHMTX023-11|Hymenoptera|Ichneumonidae|BOLD:AAU8390
Cryptinae|[5444]|NGAAE018-14|Hymenoptera|Ichneumonidae|BOLD:AAU8877
Cryptinae|[5445]|CNSLF967-12|Hymenoptera|Ichneumonidae|BOLD:ACF5630
Trychosis|[5446]|CNWBA172-13|Hymenoptera|Ichneumonidae|BOLD:ACF5629
Trychosis|[5447]|SSPAB049-13|Hymenoptera|Ichneumonidae|BOLD:ACF5628
Cryptinae|[5448]|BBHYK838-10|Hymenoptera|Ichneumonidae|BOLD:AAQ2989
Trychosis|[5449]|SSPAC3363-13|Hymenoptera|Ichneumonidae|BOLD:ACF3880
Cryptinae|[5450]|JSHYN814-11|Hymenoptera|Ichneumonidae|BOLD:ABA6007
Trychosis|[5451]|TTMHY010-08|Hymenoptera|Ichneumonidae|BOLD:AAG7619
Cryptinae|[5452]|SSPAA2621-13|Hymenoptera|Ichneumonidae|BOLD:AAU9257
Trychosis|[5453]|SSROA349-14|Hymenoptera|Ichneumonidae|BOLD:AAH1703
Trychosis|[5454]|SSPAA5023-13|Hymenoptera|Ichneumonidae|BOLD:ACE9081
Cryptinae|[5455]|RBINA2219-13|Hymenoptera|Ichneumonidae|BOLD:AAG8056
Cryptinae|[5456]|CNMIE894-14|Hymenoptera|Ichneumonidae|BOLD:ACB3454
Trychosis tristator|[5457]|PAJUL1377-12|Hymenoptera|Ichneumonidae|BOLD:AAZ0242
Trychosis tristator|[5458]|SSROB3591-14|Hymenoptera|Ichneumonidae|BOLD:AAG7457
Aritranis director|[5459]|TTHYW811-08|Hymenoptera|Ichneumonidae|BOLD:AAG7768
Cryptinae|[5460]|CNPPB2233-12|Hymenoptera|Ichneumonidae|BOLD:ACC8265
Cryptinae|[5461]|CNRME972-12|Hymenoptera|Ichneumonidae|BOLD:AAQ0440
Cryptinae|[5462]|SSPAA5035-13|Hymenoptera|Ichneumonidae|BOLD:ACE9077
Cryptinae|[5463]|SAHYM042-10|Hymenoptera|Ichneumonidae|BOLD:AAH2111
Cryptinae|[5464]|JBHCI912-11|Hymenoptera|Ichneumonidae|BOLD:AAF4447
Cryptinae|[5465]|TTHYW095-08|Hymenoptera|Ichneumonidae|BOLD:AAH1855
Cryptinae|[5466]|PHMTX202-11|Hymenoptera|Ichneumonidae|BOLD:AAG8076
Cryptinae|[5467]|HPPPH967-13|Hymenoptera|Ichneumonidae|BOLD:AAH1535
Cryptinae|[5468]|SSWLD8002-13|Hymenoptera|Ichneumonidae|BOLD:ACB1534
Cryptinae|[5469]|HARC313-11|Hymenoptera|Ichneumonidae|BOLD:AAH1529
Cryptinae|[5470]|CNSLF984-12|Hymenoptera|Ichneumonidae|BOLD:ACC1199
Cryptinae|[5471]|NGNAB158-13|Hymenoptera|Ichneumonidae|BOLD:AAH1626
Agrothereutes mandator|[5472]|TTMHY142-08|Hymenoptera|Ichneumonidae|BOLD:AAG7659
Agrothereutes abbreviatus|[5473]|TTMHY482-08|Hymenoptera|Ichneumonidae|BOLD:AAG7687
Agrothereutes abbreviatus|[5474]|CNWBE400-13|Hymenoptera|Ichneumonidae|BOLD:ACN8869
Agrothereutes|[5475]|CNKLF2536-15|Hymenoptera|Ichneumonidae|BOLD:ACT4108
Cryptinae|[5476]|JSHYP769-12|Hymenoptera|Ichneumonidae|BOLD:ABW8198
Gambrus|[5477]|SSWLE9645-13|Hymenoptera|Ichneumonidae|BOLD:AAG8217
Cryptinae|[5478]|PHOCT621-11|Hymenoptera|Ichneumonidae|BOLD:ABV2754
Cryptinae|[5479]|PHNGT495-13|Hymenoptera|Ichneumonidae|BOLD:ACF4438
Gambrus|[5480]|SMTPJ1164-14|Hymenoptera|Ichneumonidae|BOLD:ACH0295
Gambrus|[5481]|SMTPJ1186-14|Hymenoptera|Ichneumonidae|BOLD:AAG8275
Cryptinae|[5482]|NGNAX2260-14|Hymenoptera|Ichneumonidae|BOLD:ACN0503
Cryptinae|[5483]|CNWAE6975-14|Hymenoptera|Ichneumonidae|BOLD:ABZ2032
Cryptinae|[5484]|TTMHY955-11|Hymenoptera|Ichneumonidae|BOLD:AAZ1988
Cryptinae|[5485]|SSBRA652-14|Hymenoptera|Ichneumonidae|BOLD:AAH1793
Cryptinae|[5486]|SSEIB11433-13|Hymenoptera|Ichneumonidae|BOLD:ABA6013
Cryptinae|[5487]|NGNAY111-14|Hymenoptera|Ichneumonidae|BOLD:ACI3222
Cryptinae|[5488]|SMTPJ2949-14|Hymenoptera|Ichneumonidae|BOLD:ABA5983

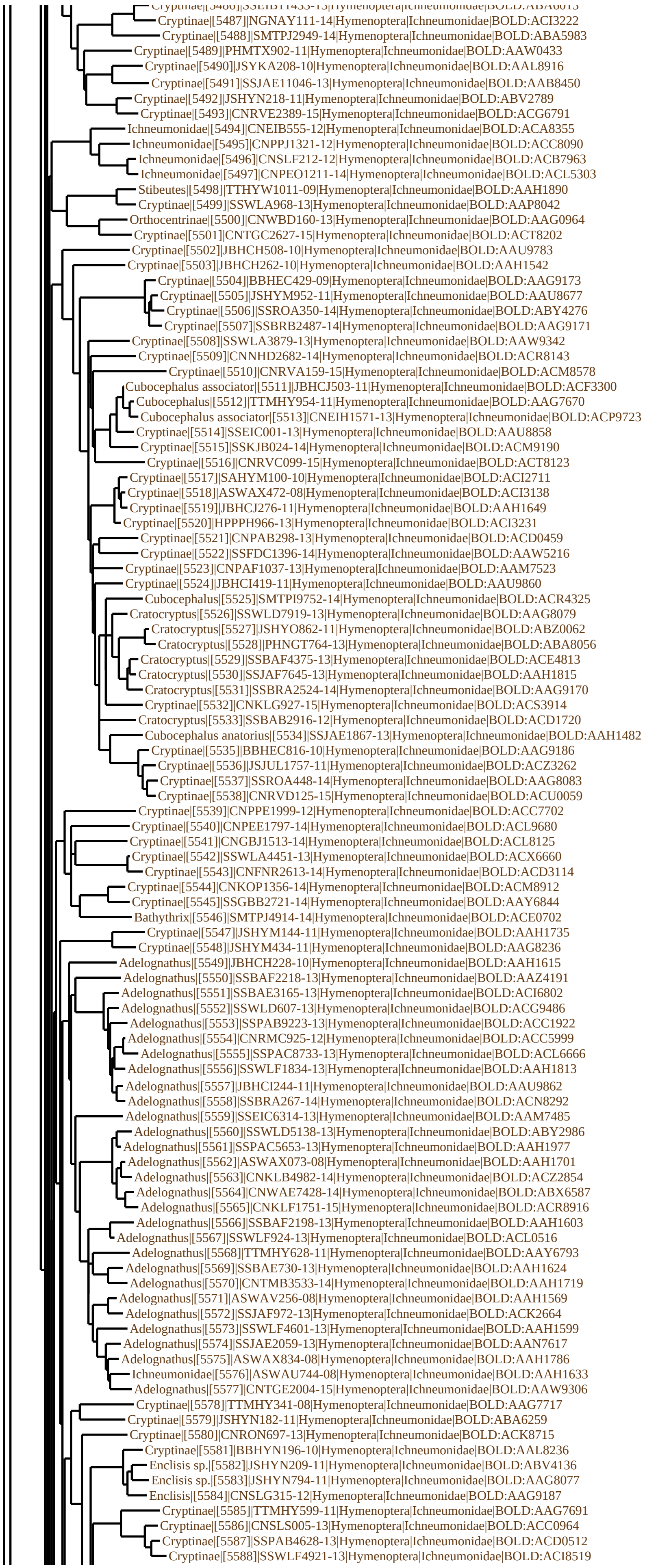

Cryptinae|[5486]|SSEIB11433-13|Hymenoptera|Ichneumonidae|BOLD:ABA6013
Cryptinae|[5487]|NGNAY111-14|Hymenoptera|Ichneumonidae|BOLD:ACI3222
Cryptinae|[5488]|SMTPJ2949-14|Hymenoptera|Ichneumonidae|BOLD:ABA5983
Cryptinae|[5489]|PHMTX902-11|Hymenoptera|Ichneumonidae|BOLD:AAW0433
Cryptinae|[5490]|JSYKA208-10|Hymenoptera|Ichneumonidae|BOLD:AAL8916
Cryptinae|[5491]|SSJAE11046-13|Hymenoptera|Ichneumonidae|BOLD:AAB8450
Cryptinae|[5492]|JSHYN218-11|Hymenoptera|Ichneumonidae|BOLD:ABV2789
Cryptinae|[5493]|CNRVE2389-15|Hymenoptera|Ichneumonidae|BOLD:ACG6791
Ichneumonidae|[5494]|CNEIB555-12|Hymenoptera|Ichneumonidae|BOLD:ACA8355
Ichneumonidae|[5495]|CNPPJ1321-12|Hymenoptera|Ichneumonidae|BOLD:ACC8090
Ichneumonidae|[5496]|CNSLF212-12|Hymenoptera|Ichneumonidae|BOLD:ACB7963
Ichneumonidae|[5497]|CNPEO1211-14|Hymenoptera|Ichneumonidae|BOLD:ACL5303
Stibeutes|[5498]|TTHYW1011-09|Hymenoptera|Ichneumonidae|BOLD:AAH1890
Cryptinae|[5499]|SSWLA968-13|Hymenoptera|Ichneumonidae|BOLD:AAP8042
Orthocentrinae|[5500]|CNWBD160-13|Hymenoptera|Ichneumonidae|BOLD:AAG0964
Cryptinae|[5501]|CNTGC2627-15|Hymenoptera|Ichneumonidae|BOLD:ACT8202
Cryptinae|[5502]|JBHCH508-10|Hymenoptera|Ichneumonidae|BOLD:AAU9783
Cryptinae|[5503]|JBHCH262-10|Hymenoptera|Ichneumonidae|BOLD:AAH1542
Cryptinae|[5504]|BBHEC429-09|Hymenoptera|Ichneumonidae|BOLD:AAG9173
Cryptinae|[5505]|JSHYM952-11|Hymenoptera|Ichneumonidae|BOLD:AAU8677
Cryptinae|[5506]|SSROA350-14|Hymenoptera|Ichneumonidae|BOLD:ABY4276
Cryptinae|[5507]|SSBRB2487-14|Hymenoptera|Ichneumonidae|BOLD:AAG9171
Cryptinae|[5508]|SSWLA3879-13|Hymenoptera|Ichneumonidae|BOLD:AAW9342
Cryptinae|[5509]|CNNHD2682-14|Hymenoptera|Ichneumonidae|BOLD:ACR8143
Cryptinae|[5510]|CNRVA159-15|Hymenoptera|Ichneumonidae|BOLD:ACM8578
Cubocephalus associator|[5511]|JBHCJ503-11|Hymenoptera|Ichneumonidae|BOLD:ACF3300
Cubocephalus|[5512]|TTMHY954-11|Hymenoptera|Ichneumonidae|BOLD:AAG7670
Cubocephalus associator|[5513]|CNEIH1571-13|Hymenoptera|Ichneumonidae|BOLD:ACP9723
Cryptinae|[5514]|SSEIC001-13|Hymenoptera|Ichneumonidae|BOLD:AAU8858
Cryptinae|[5515]|SSKJB024-14|Hymenoptera|Ichneumonidae|BOLD:ACM9190
Cryptinae|[5516]|CNRVC099-15|Hymenoptera|Ichneumonidae|BOLD:ACT8123
Cryptinae|[5517]|SAHYM100-10|Hymenoptera|Ichneumonidae|BOLD:ACI2711
Cryptinae|[5518]|ASWAX472-08|Hymenoptera|Ichneumonidae|BOLD:ACI3138
Cryptinae|[5519]|JBHCJ276-11|Hymenoptera|Ichneumonidae|BOLD:AAH1649
Cryptinae|[5520]|HPPPH966-13|Hymenoptera|Ichneumonidae|BOLD:ACI3231
Cryptinae|[5521]|CNPAB298-13|Hymenoptera|Ichneumonidae|BOLD:ACD0459
Cryptinae|[5522]|SSFDC1396-14|Hymenoptera|Ichneumonidae|BOLD:AAW5216
Cryptinae|[5523]|CNPAF1037-13|Hymenoptera|Ichneumonidae|BOLD:AAM7523
Cryptinae|[5524]|JBHCI419-11|Hymenoptera|Ichneumonidae|BOLD:AAU9860
Cubocephalus|[5525]|SMTPI9752-14|Hymenoptera|Ichneumonidae|BOLD:ACR4325
Cratocryptus|[5526]|SSWLD7919-13|Hymenoptera|Ichneumonidae|BOLD:AAG8079
Cratocryptus|[5527]|JSHYO862-11|Hymenoptera|Ichneumonidae|BOLD:ABZ0062
Cratocryptus|[5528]|PHNGT764-13|Hymenoptera|Ichneumonidae|BOLD:ABA8056
Cratocryptus|[5529]|SSBAF4375-13|Hymenoptera|Ichneumonidae|BOLD:ACE4813
Cratocryptus|[5530]|SSJAF7645-13|Hymenoptera|Ichneumonidae|BOLD:AAH1815
Cratocryptus|[5531]|SSBRA2524-14|Hymenoptera|Ichneumonidae|BOLD:AAG9170
Cryptinae|[5532]|CNKLG927-15|Hymenoptera|Ichneumonidae|BOLD:ACS3914
Cratocryptus|[5533]|SSBAB2916-12|Hymenoptera|Ichneumonidae|BOLD:ACD1720
Cubocephalus anatorius|[5534]|SSJAE1867-13|Hymenoptera|Ichneumonidae|BOLD:AAH1482
Cryptinae|[5535]|BBHEC816-10|Hymenoptera|Ichneumonidae|BOLD:AAG9186
Cryptinae|[5536]|JSJUL1757-11|Hymenoptera|Ichneumonidae|BOLD:ACZ3262
Cryptinae|[5537]|SSROA448-14|Hymenoptera|Ichneumonidae|BOLD:AAG8083
Cryptinae|[5538]|CNRVD125-15|Hymenoptera|Ichneumonidae|BOLD:ACU0059
Cryptinae|[5539]|CNPPE1999-12|Hymenoptera|Ichneumonidae|BOLD:ACC7702
Cryptinae|[5540]|CNPEE1797-14|Hymenoptera|Ichneumonidae|BOLD:ACL9680
Cryptinae|[5541]|CNGBJ1513-14|Hymenoptera|Ichneumonidae|BOLD:ACL8125
Cryptinae|[5542]|SSWLA4451-13|Hymenoptera|Ichneumonidae|BOLD:ACX6660
Cryptinae|[5543]|CNFNR2613-14|Hymenoptera|Ichneumonidae|BOLD:ACD3114
Cryptinae|[5544]|CNKOP1356-14|Hymenoptera|Ichneumonidae|BOLD:ACM8912
Cryptinae|[5545]|SSGBB2721-14|Hymenoptera|Ichneumonidae|BOLD:AAY6844
Bathythrix|[5546]|SMTPJ4914-14|Hymenoptera|Ichneumonidae|BOLD:ACE0702
Cryptinae|[5547]|JSHYM144-11|Hymenoptera|Ichneumonidae|BOLD:AAH1735
Cryptinae|[5548]|JSHYM434-11|Hymenoptera|Ichneumonidae|BOLD:AAG8236
Adelognathus|[5549]|JBHCH228-10|Hymenoptera|Ichneumonidae|BOLD:AAH1615
Adelognathus|[5550]|SSBAF2218-13|Hymenoptera|Ichneumonidae|BOLD:AAZ4191
Adelognathus|[5551]|SSBAE3165-13|Hymenoptera|Ichneumonidae|BOLD:ACI6802
Adelognathus|[5552]|SSWLD607-13|Hymenoptera|Ichneumonidae|BOLD:ACG9486
Adelognathus|[5553]|SSPAB9223-13|Hymenoptera|Ichneumonidae|BOLD:ACC1922
Adelognathus|[5554]|CNRMC925-12|Hymenoptera|Ichneumonidae|BOLD:ACC5999
Adelognathus|[5555]|SSPAC8733-13|Hymenoptera|Ichneumonidae|BOLD:ACL6666
Adelognathus|[5556]|SSWLF1834-13|Hymenoptera|Ichneumonidae|BOLD:AAH1813
Adelognathus|[5557]|JBHCI244-11|Hymenoptera|Ichneumonidae|BOLD:AAU9862
Adelognathus|[5558]|SSBRA267-14|Hymenoptera|Ichneumonidae|BOLD:ACN8292
Adelognathus|[5559]|SSEIC6314-13|Hymenoptera|Ichneumonidae|BOLD:AAM7485
Adelognathus|[5560]|SSWLD5138-13|Hymenoptera|Ichneumonidae|BOLD:ABY2986
Adelognathus|[5561]|SSPAC5653-13|Hymenoptera|Ichneumonidae|BOLD:AAH1977
Adelognathus|[5562]|ASWAX073-08|Hymenoptera|Ichneumonidae|BOLD:AAH1701
Adelognathus|[5563]|CNKLB4982-14|Hymenoptera|Ichneumonidae|BOLD:ACZ2854
Adelognathus|[5564]|CNWAE7428-14|Hymenoptera|Ichneumonidae|BOLD:ABX6587
Adelognathus|[5565]|CNKLF1751-15|Hymenoptera|Ichneumonidae|BOLD:ACR8916
Adelognathus|[5566]|SSBAF2198-13|Hymenoptera|Ichneumonidae|BOLD:AAH1603
Adelognathus|[5567]|SSWLF924-13|Hymenoptera|Ichneumonidae|BOLD:ACL0516
Adelognathus|[5568]|TTMHY628-11|Hymenoptera|Ichneumonidae|BOLD:AAY6793
Adelognathus|[5569]|SSBAE730-13|Hymenoptera|Ichneumonidae|BOLD:AAH1624
Adelognathus|[5570]|CNTMB3533-14|Hymenoptera|Ichneumonidae|BOLD:AAH1719
Adelognathus|[5571]|ASWAV256-08|Hymenoptera|Ichneumonidae|BOLD:AAH1569
Adelognathus|[5572]|SSJAF972-13|Hymenoptera|Ichneumonidae|BOLD:ACK2664
Adelognathus|[5573]|SSWLF4601-13|Hymenoptera|Ichneumonidae|BOLD:AAH1599
Adelognathus|[5574]|SSJAE2059-13|Hymenoptera|Ichneumonidae|BOLD:AAN7617
Adelognathus|[5575]|ASWAX834-08|Hymenoptera|Ichneumonidae|BOLD:AAH1786
Ichneumonidae|[5576]|ASWAU744-08|Hymenoptera|Ichneumonidae|BOLD:AAH1633
Adelognathus|[5577]|CNTGE2004-15|Hymenoptera|Ichneumonidae|BOLD:AAW9306
Cryptinae|[5578]|TTMHY341-08|Hymenoptera|Ichneumonidae|BOLD:AAG7717
Cryptinae|[5579]|JSHYN182-11|Hymenoptera|Ichneumonidae|BOLD:ABA6259
Cryptinae|[5580]|CNRON697-13|Hymenoptera|Ichneumonidae|BOLD:ACK8715
Cryptinae|[5581]|BBHYN196-10|Hymenoptera|Ichneumonidae|BOLD:AAL8236
Enclisis sp.|[5582]|JSHYN209-11|Hymenoptera|Ichneumonidae|BOLD:ABV4136
Enclisis sp.|[5583]|JSHYN794-11|Hymenoptera|Ichneumonidae|BOLD:AAG8077
Enclisis|[5584]|CNSLG315-12|Hymenoptera|Ichneumonidae|BOLD:AAG9187
Cryptinae|[5585]|TTMHY599-11|Hymenoptera|Ichneumonidae|BOLD:AAG7691
Cryptinae|[5586]|CNSLS005-13|Hymenoptera|Ichneumonidae|BOLD:ACC0964
Cryptinae|[5587]|SSPAB4628-13|Hymenoptera|Ichneumonidae|BOLD:ACD0512
Cryptinae|[5588]|SSWLF4921-13|Hymenoptera|Ichneumonidae|BOLD:ACI8519

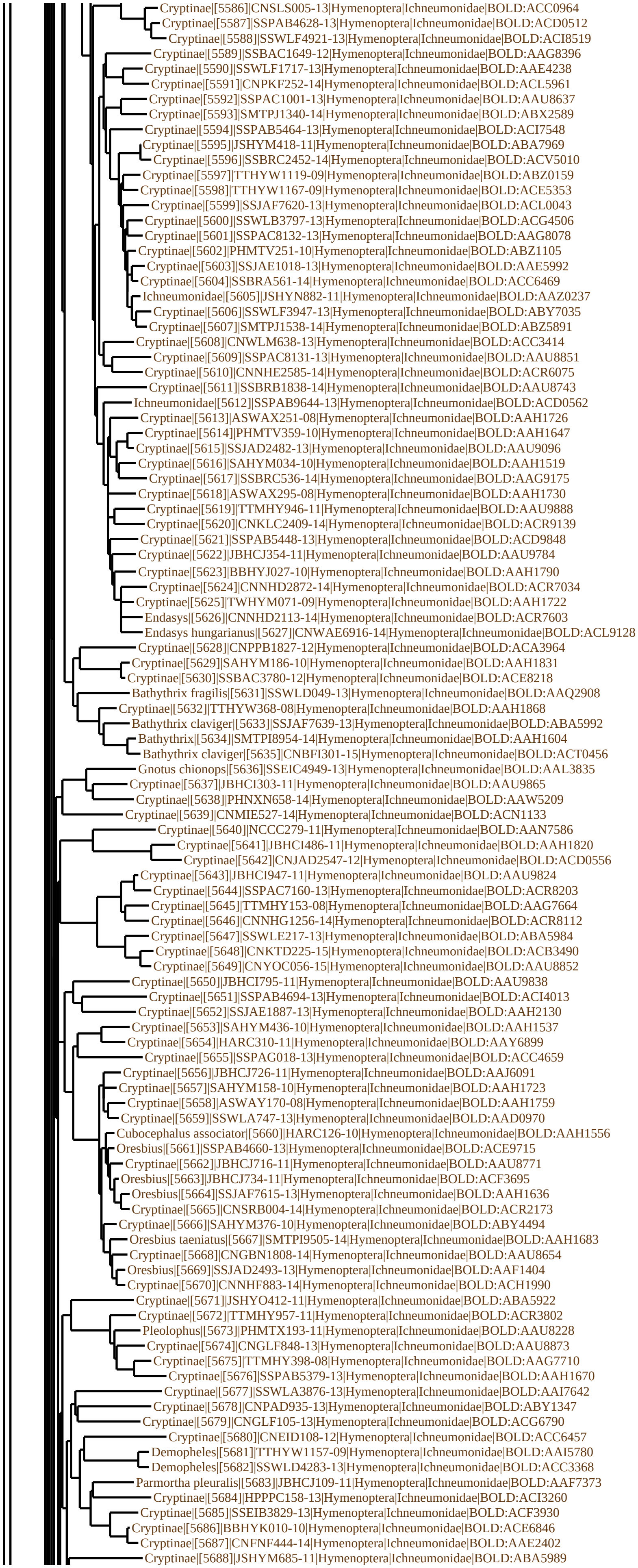
Cryptinae|[5586]|CNSLS005-13|Hymenoptera|Ichneumonidae|BOLD:ACC0964
Cryptinae|[5587]|SSPAB4628-13|Hymenoptera|Ichneumonidae|BOLD:ACD0512
Cryptinae|[5588]|SSWLF4921-13|Hymenoptera|Ichneumonidae|BOLD:ACI8519
Cryptinae|[5589]|SSBAC1649-12|Hymenoptera|Ichneumonidae|BOLD:AAG8396
Cryptinae|[5590]|SSWLF1717-13|Hymenoptera|Ichneumonidae|BOLD:AAE4238
Cryptinae|[5591]|CNPKF252-14|Hymenoptera|Ichneumonidae|BOLD:ACL5961
Cryptinae|[5592]|SSPAC1001-13|Hymenoptera|Ichneumonidae|BOLD:AAU8637
Cryptinae|[5593]|SMTPJ1340-14|Hymenoptera|Ichneumonidae|BOLD:ABX2589
Cryptinae|[5594]|SSPAB5464-13|Hymenoptera|Ichneumonidae|BOLD:ACI7548
Cryptinae|[5595]|JSHYM418-11|Hymenoptera|Ichneumonidae|BOLD:ABA7969
Cryptinae|[5596]|SSBRC2452-14|Hymenoptera|Ichneumonidae|BOLD:ACV5010
Cryptinae|[5597]|TTHYW1119-09|Hymenoptera|Ichneumonidae|BOLD:ABZ0159
Cryptinae|[5598]|TTHYW1167-09|Hymenoptera|Ichneumonidae|BOLD:ACE5353
Cryptinae|[5599]|SSJAF7620-13|Hymenoptera|Ichneumonidae|BOLD:ACL0043
Cryptinae|[5600]|SSWLB3797-13|Hymenoptera|Ichneumonidae|BOLD:ACG4506
Cryptinae|[5601]|SSPAC8132-13|Hymenoptera|Ichneumonidae|BOLD:AAG8078
Cryptinae|[5602]|PHMTV251-10|Hymenoptera|Ichneumonidae|BOLD:ABZ1105
Cryptinae|[5603]|SSJAE1018-13|Hymenoptera|Ichneumonidae|BOLD:AAE5992
Cryptinae|[5604]|SSBRA561-14|Hymenoptera|Ichneumonidae|BOLD:ACC6469
Ichneumonidae|[5605]|JSHYN882-11|Hymenoptera|Ichneumonidae|BOLD:AAZ0237
Cryptinae|[5606]|SSWLF3947-13|Hymenoptera|Ichneumonidae|BOLD:ABY7035
Cryptinae|[5607]|SMTPJ1538-14|Hymenoptera|Ichneumonidae|BOLD:ABZ5891
Cryptinae|[5608]|CNWLM638-13|Hymenoptera|Ichneumonidae|BOLD:ACC3414
Cryptinae|[5609]|SSPAC8131-13|Hymenoptera|Ichneumonidae|BOLD:AAU8851
Cryptinae|[5610]|CNNHE2585-14|Hymenoptera|Ichneumonidae|BOLD:ACR6075
Cryptinae|[5611]|SSBRB1838-14|Hymenoptera|Ichneumonidae|BOLD:AAU8743
Ichneumonidae|[5612]|SSPAB9644-13|Hymenoptera|Ichneumonidae|BOLD:ACD0562
Cryptinae|[5613]|ASWAX251-08|Hymenoptera|Ichneumonidae|BOLD:AAH1726
Cryptinae|[5614]|PHMTV359-10|Hymenoptera|Ichneumonidae|BOLD:AAH1647
Cryptinae|[5615]|SSJAD2482-13|Hymenoptera|Ichneumonidae|BOLD:AAU9096
Cryptinae|[5616]|SAHYM034-10|Hymenoptera|Ichneumonidae|BOLD:AAH1519
Cryptinae|[5617]|SSBRC536-14|Hymenoptera|Ichneumonidae|BOLD:AAG9175
Cryptinae|[5618]|ASWAX295-08|Hymenoptera|Ichneumonidae|BOLD:AAH1730
Cryptinae|[5619]|TTMHY946-11|Hymenoptera|Ichneumonidae|BOLD:AAU9888
Cryptinae|[5620]|CNKLC2409-14|Hymenoptera|Ichneumonidae|BOLD:ACR9139
Cryptinae|[5621]|SSPAB5448-13|Hymenoptera|Ichneumonidae|BOLD:ACD9848
Cryptinae|[5622]|JBHCJ354-11|Hymenoptera|Ichneumonidae|BOLD:AAU9784
Cryptinae|[5623]|BBHYJ027-10|Hymenoptera|Ichneumonidae|BOLD:AAH1790
Cryptinae|[5624]|CNNHD2872-14|Hymenoptera|Ichneumonidae|BOLD:ACR7034
Cryptinae|[5625]|TWHYM071-09|Hymenoptera|Ichneumonidae|BOLD:AAH1722
Endasys|[5626]|CNNHD2113-14|Hymenoptera|Ichneumonidae|BOLD:ACR7603
Endasys hungarianus|[5627]|CNWAE6916-14|Hymenoptera|Ichneumonidae|BOLD:ACL9128
Cryptinae|[5628]|CNPPB1827-12|Hymenoptera|Ichneumonidae|BOLD:ACA3964
Cryptinae|[5629]|SAHYM186-10|Hymenoptera|Ichneumonidae|BOLD:AAH1831
Cryptinae|[5630]|SSBAC3780-12|Hymenoptera|Ichneumonidae|BOLD:ACE8218
Bathythrix fragilis|[5631]|SSWLD049-13|Hymenoptera|Ichneumonidae|BOLD:AAQ2908
Cryptinae|[5632]|TTHYW368-08|Hymenoptera|Ichneumonidae|BOLD:AAH1868
Bathythrix claviger|[5633]|SSJAF7639-13|Hymenoptera|Ichneumonidae|BOLD:ABA5992
Bathythrix|[5634]|SMTPI8954-14|Hymenoptera|Ichneumonidae|BOLD:AAH1604
Bathythrix claviger|[5635]|CNBFI301-15|Hymenoptera|Ichneumonidae|BOLD:ACT0456
Gnotus chionops|[5636]|SSEIC4949-13|Hymenoptera|Ichneumonidae|BOLD:AAL3835
Cryptinae|[5637]|JBHCI303-11|Hymenoptera|Ichneumonidae|BOLD:AAU9865
Cryptinae|[5638]|PHNXN658-14|Hymenoptera|Ichneumonidae|BOLD:AAW5209
Cryptinae|[5639]|CNMIE527-14|Hymenoptera|Ichneumonidae|BOLD:ACN1133
Cryptinae|[5640]|NCCC279-11|Hymenoptera|Ichneumonidae|BOLD:AAN7586
Cryptinae|[5641]|JBHCI486-11|Hymenoptera|Ichneumonidae|BOLD:AAH1820
Cryptinae|[5642]|CNJAD2547-12|Hymenoptera|Ichneumonidae|BOLD:ACD0556
Cryptinae|[5643]|JBHCI947-11|Hymenoptera|Ichneumonidae|BOLD:AAU9824
Cryptinae|[5644]|SSPAC7160-13|Hymenoptera|Ichneumonidae|BOLD:ACR8203
Cryptinae|[5645]|TTMHY153-08|Hymenoptera|Ichneumonidae|BOLD:AAG7664
Cryptinae|[5646]|CNNHG1256-14|Hymenoptera|Ichneumonidae|BOLD:ACR8112
Cryptinae|[5647]|SSWLE217-13|Hymenoptera|Ichneumonidae|BOLD:ABA5984
Cryptinae|[5648]|CNKTD225-15|Hymenoptera|Ichneumonidae|BOLD:ACB3490
Cryptinae|[5649]|CNYOC056-15|Hymenoptera|Ichneumonidae|BOLD:AAU8852
Cryptinae|[5650]|JBHCI795-11|Hymenoptera|Ichneumonidae|BOLD:AAU9838
Cryptinae|[5651]|SSPAB4694-13|Hymenoptera|Ichneumonidae|BOLD:ACI4013
Cryptinae|[5652]|SSJAE1887-13|Hymenoptera|Ichneumonidae|BOLD:AAH2130
Cryptinae|[5653]|SAHYM436-10|Hymenoptera|Ichneumonidae|BOLD:AAH1537
Cryptinae|[5654]|HARC310-11|Hymenoptera|Ichneumonidae|BOLD:AAY6899
Cryptinae|[5655]|SSPAG018-13|Hymenoptera|Ichneumonidae|BOLD:ACC4659
Cryptinae|[5656]|JBHCJ726-11|Hymenoptera|Ichneumonidae|BOLD:AAJ6091
Cryptinae|[5657]|SAHYM158-10|Hymenoptera|Ichneumonidae|BOLD:AAH1723
Cryptinae|[5658]|ASWAY170-08|Hymenoptera|Ichneumonidae|BOLD:AAH1759
Cryptinae|[5659]|SSWLA747-13|Hymenoptera|Ichneumonidae|BOLD:AAD0970
Cubocephalus associator|[5660]|HARC126-10|Hymenoptera|Ichneumonidae|BOLD:AAH1556
Oresbius|[5661]|SSPAB4660-13|Hymenoptera|Ichneumonidae|BOLD:ACE9715
Cryptinae|[5662]|JBHCJ716-11|Hymenoptera|Ichneumonidae|BOLD:AAU8771
Oresbius|[5663]|JBHCJ734-11|Hymenoptera|Ichneumonidae|BOLD:ACF3695
Oresbius|[5664]|SSJAF7615-13|Hymenoptera|Ichneumonidae|BOLD:AAH1636
Cryptinae|[5665]|CNSRB004-14|Hymenoptera|Ichneumonidae|BOLD:ACR2173
Cryptinae|[5666]|SAHYM376-10|Hymenoptera|Ichneumonidae|BOLD:ABY4494
Oresbius taeniatus|[5667]|SMTPI9505-14|Hymenoptera|Ichneumonidae|BOLD:AAH1683
Cryptinae|[5668]|CNGBN1808-14|Hymenoptera|Ichneumonidae|BOLD:AAU8654
Oresbius|[5669]|SSJAD2493-13|Hymenoptera|Ichneumonidae|BOLD:AAF1404
Cryptinae|[5670]|CNNHF883-14|Hymenoptera|Ichneumonidae|BOLD:ACH1990
Cryptinae|[5671]|JSHYO412-11|Hymenoptera|Ichneumonidae|BOLD:ABA5922
Cryptinae|[5672]|TTMHY957-11|Hymenoptera|Ichneumonidae|BOLD:ACR3802
Pleolophus|[5673]|PHMTX193-11|Hymenoptera|Ichneumonidae|BOLD:AAU8228
Cryptinae|[5674]|CNGLF848-13|Hymenoptera|Ichneumonidae|BOLD:AAU8873
Cryptinae|[5675]|TTMHY398-08|Hymenoptera|Ichneumonidae|BOLD:AAG7710
Cryptinae|[5676]|SSPAB5379-13|Hymenoptera|Ichneumonidae|BOLD:AAH1670
Cryptinae|[5677]|SSWLA3876-13|Hymenoptera|Ichneumonidae|BOLD:AAI7642
Cryptinae|[5678]|CNPAD935-13|Hymenoptera|Ichneumonidae|BOLD:ABY1347
Cryptinae|[5679]|CNGLF105-13|Hymenoptera|Ichneumonidae|BOLD:ACG6790
Cryptinae|[5680]|CNEID108-12|Hymenoptera|Ichneumonidae|BOLD:ACC6457
Demopheles|[5681]|TTHYW1157-09|Hymenoptera|Ichneumonidae|BOLD:AAI5780
Demopheles|[5682]|SSWLD4283-13|Hymenoptera|Ichneumonidae|BOLD:ACC3368
Parmortha pleuralis|[5683]|JBHCJ109-11|Hymenoptera|Ichneumonidae|BOLD:AAF7373
Cryptinae|[5684]|HPPPC158-13|Hymenoptera|Ichneumonidae|BOLD:ACI3260
Cryptinae|[5685]|SSEIB3829-13|Hymenoptera|Ichneumonidae|BOLD:ACF3930
Cryptinae|[5686]|BBHYK010-10|Hymenoptera|Ichneumonidae|BOLD:ACE6846
Cryptinae|[5687]|CNFNF444-14|Hymenoptera|Ichneumonidae|BOLD:AAE2402
Cryptinae|[5688]|JSHYM685-11|Hymenoptera|Ichneumonidae|BOLD:ABA5989

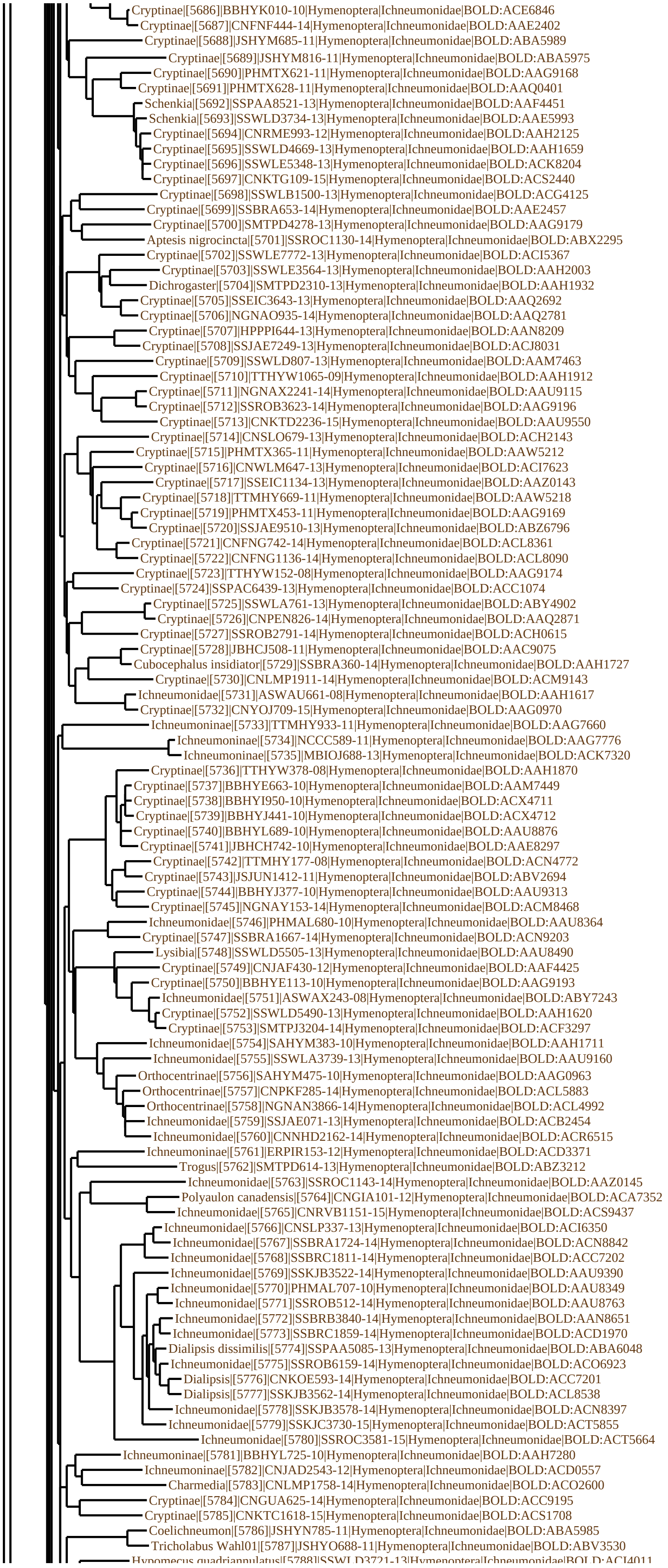
Cryptinae|[5686]|BBHYK010-10|Hymenoptera|Ichneumonidae|BOLD:ACE6846
Cryptinae|[5687]|CNFNF444-14|Hymenoptera|Ichneumonidae|BOLD:AAE2402
Cryptinae|[5688]|JSHYM685-11|Hymenoptera|Ichneumonidae|BOLD:ABA5989
Cryptinae|[5689]|JSHYM816-11|Hymenoptera|Ichneumonidae|BOLD:ABA5975
Cryptinae|[5690]|PHMTX621-11|Hymenoptera|Ichneumonidae|BOLD:AAG9168
Cryptinae|[5691]|PHMTX628-11|Hymenoptera|Ichneumonidae|BOLD:AAQ0401
Schenkia|[5692]|SSPAA8521-13|Hymenoptera|Ichneumonidae|BOLD:AAF4451
Schenkia|[5693]|SSWLD3734-13|Hymenoptera|Ichneumonidae|BOLD:AAE5993
Cryptinae|[5694]|CNRME993-12|Hymenoptera|Ichneumonidae|BOLD:AAH2125
Cryptinae|[5695]|SSWLD4669-13|Hymenoptera|Ichneumonidae|BOLD:AAH1659
Cryptinae|[5696]|SSWLE5348-13|Hymenoptera|Ichneumonidae|BOLD:ACK8204
Cryptinae|[5697]|CNKTG109-15|Hymenoptera|Ichneumonidae|BOLD:ACS2440
Cryptinae|[5698]|SSWLB1500-13|Hymenoptera|Ichneumonidae|BOLD:ACG4125
Cryptinae|[5699]|SSBRA653-14|Hymenoptera|Ichneumonidae|BOLD:AAE2457
Cryptinae|[5700]|SMTPD4278-13|Hymenoptera|Ichneumonidae|BOLD:AAG9179
Aptesis nigrocincta|[5701]|SSROC1130-14|Hymenoptera|Ichneumonidae|BOLD:ABX2295
Cryptinae|[5702]|SSWLE7772-13|Hymenoptera|Ichneumonidae|BOLD:ACI5367
Cryptinae|[5703]|SSWLE3564-13|Hymenoptera|Ichneumonidae|BOLD:AAH2003
Dichrogaster|[5704]|SMTPD2310-13|Hymenoptera|Ichneumonidae|BOLD:AAH1932
Cryptinae|[5705]|SSEIC3643-13|Hymenoptera|Ichneumonidae|BOLD:AAQ2692
Cryptinae|[5706]|NGNAO935-14|Hymenoptera|Ichneumonidae|BOLD:AAQ2781
Cryptinae|[5707]|HPPPI644-13|Hymenoptera|Ichneumonidae|BOLD:AAN8209
Cryptinae|[5708]|SSJAE7249-13|Hymenoptera|Ichneumonidae|BOLD:ACJ8031
Cryptinae|[5709]|SSWLD807-13|Hymenoptera|Ichneumonidae|BOLD:AAM7463
Cryptinae|[5710]|TTHYW1065-09|Hymenoptera|Ichneumonidae|BOLD:AAH1912
Cryptinae|[5711]|NGNAX2241-14|Hymenoptera|Ichneumonidae|BOLD:AAU9115
Cryptinae|[5712]|SSROB3623-14|Hymenoptera|Ichneumonidae|BOLD:AAG9196
Cryptinae|[5713]|CNKTD2236-15|Hymenoptera|Ichneumonidae|BOLD:AAU9550
Cryptinae|[5714]|CNSLO679-13|Hymenoptera|Ichneumonidae|BOLD:ACH2143
Cryptinae|[5715]|PHMTX365-11|Hymenoptera|Ichneumonidae|BOLD:AAW5212
Cryptinae|[5716]|CNWLM647-13|Hymenoptera|Ichneumonidae|BOLD:ACI7623
Cryptinae|[5717]|SSEIC1134-13|Hymenoptera|Ichneumonidae|BOLD:AAZ0143
Cryptinae|[5718]|TTMHY669-11|Hymenoptera|Ichneumonidae|BOLD:AAW5218
Cryptinae|[5719]|PHMTX453-11|Hymenoptera|Ichneumonidae|BOLD:AAG9169
Cryptinae|[5720]|SSJAE9510-13|Hymenoptera|Ichneumonidae|BOLD:ABZ6796
Cryptinae|[5721]|CNFNG742-14|Hymenoptera|Ichneumonidae|BOLD:ACL8361
Cryptinae|[5722]|CNFNG1136-14|Hymenoptera|Ichneumonidae|BOLD:ACL8090
Cryptinae|[5723]|TTHYW152-08|Hymenoptera|Ichneumonidae|BOLD:AAG9174
Cryptinae|[5724]|SSPAC6439-13|Hymenoptera|Ichneumonidae|BOLD:ACC1074
Cryptinae|[5725]|SSWLA761-13|Hymenoptera|Ichneumonidae|BOLD:ABY4902
Cryptinae|[5726]|CNPEN826-14|Hymenoptera|Ichneumonidae|BOLD:AAQ2871
Cryptinae|[5727]|SSROB2791-14|Hymenoptera|Ichneumonidae|BOLD:ACH0615
Cryptinae|[5728]|JBHCJ508-11|Hymenoptera|Ichneumonidae|BOLD:AAC9075
Cubocephalus insidiator|[5729]|SSBRA360-14|Hymenoptera|Ichneumonidae|BOLD:AAH1727
Cryptinae|[5730]|CNLMP1911-14|Hymenoptera|Ichneumonidae|BOLD:ACM9143
Ichneumonidae|[5731]|ASWAU661-08|Hymenoptera|Ichneumonidae|BOLD:AAH1617
Cryptinae|[5732]|CNYOJ709-15|Hymenoptera|Ichneumonidae|BOLD:AAG0970
Ichneumoninae|[5733]|TTMHY933-11|Hymenoptera|Ichneumonidae|BOLD:AAG7660
Ichneumoninae|[5734]|NCCC589-11|Hymenoptera|Ichneumonidae|BOLD:AAG7776
Ichneumoninae|[5735]|MBIOJ688-13|Hymenoptera|Ichneumonidae|BOLD:ACK7320
Cryptinae|[5736]|TTHYW378-08|Hymenoptera|Ichneumonidae|BOLD:AAH1870
Cryptinae|[5737]|BBHYE663-10|Hymenoptera|Ichneumonidae|BOLD:AAM7449
Cryptinae|[5738]|BBHYI950-10|Hymenoptera|Ichneumonidae|BOLD:ACX4711
Cryptinae|[5739]|BBHYJ441-10|Hymenoptera|Ichneumonidae|BOLD:ACX4712
Cryptinae|[5740]|BBHYL689-10|Hymenoptera|Ichneumonidae|BOLD:AAU8876
Cryptinae|[5741]|JBHCH742-10|Hymenoptera|Ichneumonidae|BOLD:AAE8297
Cryptinae|[5742]|TTMHY177-08|Hymenoptera|Ichneumonidae|BOLD:ACN4772
Cryptinae|[5743]|JSJUN1412-11|Hymenoptera|Ichneumonidae|BOLD:ABV2694
Cryptinae|[5744]|BBHYJ377-10|Hymenoptera|Ichneumonidae|BOLD:AAU9313
Cryptinae|[5745]|NGNAY153-14|Hymenoptera|Ichneumonidae|BOLD:ACM8468
Ichneumonidae|[5746]|PHMAL680-10|Hymenoptera|Ichneumonidae|BOLD:AAU8364
Cryptinae|[5747]|SSBRA1667-14|Hymenoptera|Ichneumonidae|BOLD:ACN9203
Lysibia|[5748]|SSWLD5505-13|Hymenoptera|Ichneumonidae|BOLD:AAU8490
Cryptinae|[5749]|CNJAF430-12|Hymenoptera|Ichneumonidae|BOLD:AAF4425
Cryptinae|[5750]|BBHYE113-10|Hymenoptera|Ichneumonidae|BOLD:AAG9193
Ichneumonidae|[5751]|ASWAX243-08|Hymenoptera|Ichneumonidae|BOLD:ABY7243
Cryptinae|[5752]|SSWLD5490-13|Hymenoptera|Ichneumonidae|BOLD:AAH1620
Cryptinae|[5753]|SMTPJ3204-14|Hymenoptera|Ichneumonidae|BOLD:ACF3297
Ichneumonidae|[5754]|SAHYM383-10|Hymenoptera|Ichneumonidae|BOLD:AAH1711
Ichneumonidae|[5755]|SSWLA3739-13|Hymenoptera|Ichneumonidae|BOLD:AAU9160
Orthocentrinae|[5756]|SAHYM475-10|Hymenoptera|Ichneumonidae|BOLD:AAG0963
Orthocentrinae|[5757]|CNPKF285-14|Hymenoptera|Ichneumonidae|BOLD:ACL5883
Orthocentrinae|[5758]|NGNAN3866-14|Hymenoptera|Ichneumonidae|BOLD:ACL4992
Ichneumonidae|[5759]|SSJAE071-13|Hymenoptera|Ichneumonidae|BOLD:ACB2454
Ichneumonidae|[5760]|CNNHD2162-14|Hymenoptera|Ichneumonidae|BOLD:ACR6515
Ichneumoninae|[5761]|ERPIR153-12|Hymenoptera|Ichneumonidae|BOLD:ACD3371
Trogus|[5762]|SMTPD614-13|Hymenoptera|Ichneumonidae|BOLD:ABZ3212
Ichneumonidae|[5763]|SSROC1143-14|Hymenoptera|Ichneumonidae|BOLD:AAZ0145
Polyaulon canadensis|[5764]|CNGIA101-12|Hymenoptera|Ichneumonidae|BOLD:ACA7352
Ichneumonidae|[5765]|CNRVB1151-15|Hymenoptera|Ichneumonidae|BOLD:ACS9437
Ichneumonidae|[5766]|CNSLP337-13|Hymenoptera|Ichneumonidae|BOLD:ACI6350
Ichneumonidae|[5767]|SSBRA1724-14|Hymenoptera|Ichneumonidae|BOLD:ACN8842
Ichneumonidae|[5768]|SSBRC1811-14|Hymenoptera|Ichneumonidae|BOLD:ACC7202
Ichneumonidae|[5769]|SSKJB3522-14|Hymenoptera|Ichneumonidae|BOLD:AAU9390
Ichneumonidae|[5770]|PHMAL707-10|Hymenoptera|Ichneumonidae|BOLD:AAU8349
Ichneumonidae|[5771]|SSROB512-14|Hymenoptera|Ichneumonidae|BOLD:AAU8763
Ichneumonidae|[5772]|SSBRB3840-14|Hymenoptera|Ichneumonidae|BOLD:AAN8651
Ichneumonidae|[5773]|SSBRC1859-14|Hymenoptera|Ichneumonidae|BOLD:ACD1970
Dialipsis dissimilis|[5774]|SSPAA5085-13|Hymenoptera|Ichneumonidae|BOLD:ABA6048
Ichneumonidae|[5775]|SSROB6159-14|Hymenoptera|Ichneumonidae|BOLD:ACO6923
Dialipsis|[5776]|CNKOE593-14|Hymenoptera|Ichneumonidae|BOLD:ACC7201
Dialipsis|[5777]|SSKJB3562-14|Hymenoptera|Ichneumonidae|BOLD:ACL8538
Ichneumonidae|[5778]|SSKJB3578-14|Hymenoptera|Ichneumonidae|BOLD:ACN8397
Ichneumonidae|[5779]|SSKJC3730-15|Hymenoptera|Ichneumonidae|BOLD:ACT5855
Ichneumonidae|[5780]|SSROC3581-15|Hymenoptera|Ichneumonidae|BOLD:ACT5664
Ichneumoninae|[5781]|BBHYL725-10|Hymenoptera|Ichneumonidae|BOLD:AAH7280
Ichneumoninae|[5782]|CNJAD2543-12|Hymenoptera|Ichneumonidae|BOLD:ACD0557
Charmedia|[5783]|CNLMP1758-14|Hymenoptera|Ichneumonidae|BOLD:ACO2600
Cryptinae|[5784]|CNGUA625-14|Hymenoptera|Ichneumonidae|BOLD:ACC9195
Cryptinae|[5785]|CNKTC1618-15|Hymenoptera|Ichneumonidae|BOLD:ACS1708
Coelichneumon|[5786]|JSHYN785-11|Hymenoptera|Ichneumonidae|BOLD:ABA5985
Tricholabus Wahl01|[5787]|JSHYO688-11|Hymenoptera|Ichneumonidae|BOLD:ABV3530

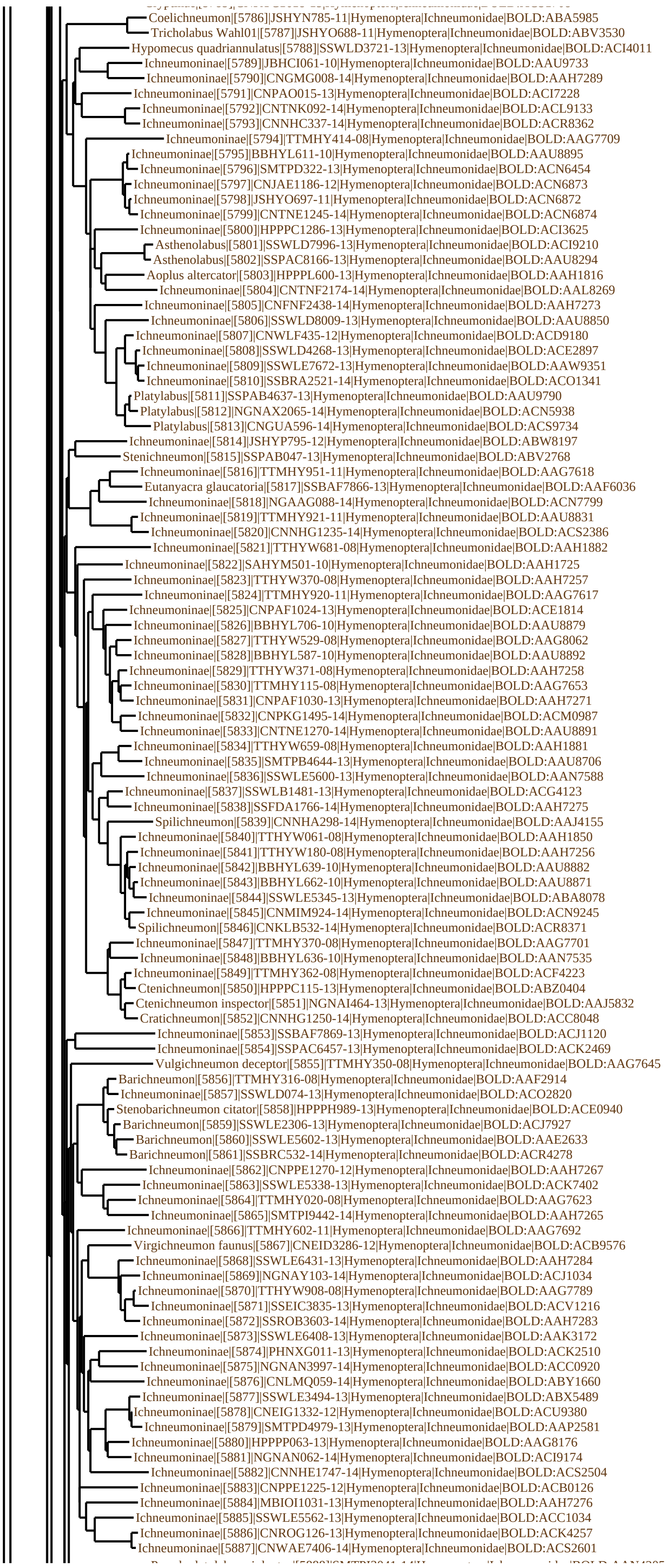

Coelichneumon|[5786]|JSHYN785-11|Hymenoptera|Ichneumonidae|BOLD:ABA5985
Tricholabus Wahl01|[5787]|JSHYO688-11|Hymenoptera|Ichneumonidae|BOLD:ABV3530
Hypomecus quadriannulatus|[5788]|SSWLD3721-13|Hymenoptera|Ichneumonidae|BOLD:ACI4011
Ichneumoninae|[5789]|JBHCI061-10|Hymenoptera|Ichneumonidae|BOLD:AAU9733
Ichneumoninae|[5790]|CNGMG008-14|Hymenoptera|Ichneumonidae|BOLD:AAH7289
Ichneumoninae|[5791]|CNPAO015-13|Hymenoptera|Ichneumonidae|BOLD:ACI7228
Ichneumoninae|[5792]|CNTNK092-14|Hymenoptera|Ichneumonidae|BOLD:ACL9133
Ichneumoninae|[5793]|CNNHC337-14|Hymenoptera|Ichneumonidae|BOLD:ACR8362
Ichneumoninae|[5794]|TTMHY414-08|Hymenoptera|Ichneumonidae|BOLD:AAG7709
Ichneumoninae|[5795]|BBHYL611-10|Hymenoptera|Ichneumonidae|BOLD:AAU8895
Ichneumoninae|[5796]|SMTPD322-13|Hymenoptera|Ichneumonidae|BOLD:ACN6454
Ichneumoninae|[5797]|CNJAE1186-12|Hymenoptera|Ichneumonidae|BOLD:ACN6873
Ichneumoninae|[5798]|JSHYO697-11|Hymenoptera|Ichneumonidae|BOLD:ACN6872
Ichneumoninae|[5799]|CNTNE1245-14|Hymenoptera|Ichneumonidae|BOLD:ACN6874
Ichneumoninae|[5800]|HPPPC1286-13|Hymenoptera|Ichneumonidae|BOLD:ACI3625
Asthenolabus|[5801]|SSWLD7996-13|Hymenoptera|Ichneumonidae|BOLD:ACI9210
Asthenolabus|[5802]|SSPAC8166-13|Hymenoptera|Ichneumonidae|BOLD:AAU8294
Aoplus altercator|[5803]|HPPPL600-13|Hymenoptera|Ichneumonidae|BOLD:AAH1816
Ichneumoninae|[5804]|CNTNF2174-14|Hymenoptera|Ichneumonidae|BOLD:AAL8269
Ichneumoninae|[5805]|CNFNF2438-14|Hymenoptera|Ichneumonidae|BOLD:AAH7273
Ichneumoninae|[5806]|SSWLD8009-13|Hymenoptera|Ichneumonidae|BOLD:AAU8850
Ichneumoninae|[5807]|CNWLF435-12|Hymenoptera|Ichneumonidae|BOLD:ACD9180
Ichneumoninae|[5808]|SSWLD4268-13|Hymenoptera|Ichneumonidae|BOLD:ACE2897
Ichneumoninae|[5809]|SSWLE7672-13|Hymenoptera|Ichneumonidae|BOLD:AAW9351
Ichneumoninae|[5810]|SSBRA2521-14|Hymenoptera|Ichneumonidae|BOLD:ACO1341
Platylabus|[5811]|SSPAB4637-13|Hymenoptera|Ichneumonidae|BOLD:AAU9790
Platylabus|[5812]|NGNAX2065-14|Hymenoptera|Ichneumonidae|BOLD:ACN5938
Platylabus|[5813]|CNGUA596-14|Hymenoptera|Ichneumonidae|BOLD:ACS9734
Ichneumoninae|[5814]|JSHYP795-12|Hymenoptera|Ichneumonidae|BOLD:ABW8197
Stenichneumon|[5815]|SSPAB047-13|Hymenoptera|Ichneumonidae|BOLD:ABV2768
Ichneumoninae|[5816]|TTMHY951-11|Hymenoptera|Ichneumonidae|BOLD:AAG7618
Eutanyacra glaucatoria|[5817]|SSBAF7866-13|Hymenoptera|Ichneumonidae|BOLD:AAF6036
Ichneumoninae|[5818]|NGAAG088-14|Hymenoptera|Ichneumonidae|BOLD:ACN7799
Ichneumoninae|[5819]|TTMHY921-11|Hymenoptera|Ichneumonidae|BOLD:AAU8831
Ichneumoninae|[5820]|CNNHG1235-14|Hymenoptera|Ichneumonidae|BOLD:ACS2386
Ichneumoninae|[5821]|TTHYW681-08|Hymenoptera|Ichneumonidae|BOLD:AAH1882
Ichneumoninae|[5822]|SAHYM501-10|Hymenoptera|Ichneumonidae|BOLD:AAH1725
Ichneumoninae|[5823]|TTHYW370-08|Hymenoptera|Ichneumonidae|BOLD:AAH7257
Ichneumoninae|[5824]|TTMHY920-11|Hymenoptera|Ichneumonidae|BOLD:AAG7617
Ichneumoninae|[5825]|CNPAF1024-13|Hymenoptera|Ichneumonidae|BOLD:ACE1814
Ichneumoninae|[5826]|BBHYL706-10|Hymenoptera|Ichneumonidae|BOLD:AAU8879
Ichneumoninae|[5827]|TTHYW529-08|Hymenoptera|Ichneumonidae|BOLD:AAG8062
Ichneumoninae|[5828]|BBHYL587-10|Hymenoptera|Ichneumonidae|BOLD:AAU8892
Ichneumoninae|[5829]|TTHYW371-08|Hymenoptera|Ichneumonidae|BOLD:AAH7258
Ichneumoninae|[5830]|TTMHY115-08|Hymenoptera|Ichneumonidae|BOLD:AAG7653
Ichneumoninae|[5831]|CNPAF1030-13|Hymenoptera|Ichneumonidae|BOLD:AAH7271
Ichneumoninae|[5832]|CNPKG1495-14|Hymenoptera|Ichneumonidae|BOLD:ACM0987
Ichneumoninae|[5833]|CNTNE1270-14|Hymenoptera|Ichneumonidae|BOLD:AAU8891
Ichneumoninae|[5834]|TTHYW659-08|Hymenoptera|Ichneumonidae|BOLD:AAH1881
Ichneumoninae|[5835]|SMTPB4644-13|Hymenoptera|Ichneumonidae|BOLD:AAU8706
Ichneumoninae|[5836]|SSWLE5600-13|Hymenoptera|Ichneumonidae|BOLD:AAN7588
Ichneumoninae|[5837]|SSWLB1481-13|Hymenoptera|Ichneumonidae|BOLD:ACG4123
Ichneumoninae|[5838]|SSFDA1766-14|Hymenoptera|Ichneumonidae|BOLD:AAH7275
Spilichneumon|[5839]|CNNHA298-14|Hymenoptera|Ichneumonidae|BOLD:AAJ4155
Ichneumoninae|[5840]|TTHYW061-08|Hymenoptera|Ichneumonidae|BOLD:AAH1850
Ichneumoninae|[5841]|TTHYW180-08|Hymenoptera|Ichneumonidae|BOLD:AAH7256
Ichneumoninae|[5842]|BBHYL639-10|Hymenoptera|Ichneumonidae|BOLD:AAU8882
Ichneumoninae|[5843]|BBHYL662-10|Hymenoptera|Ichneumonidae|BOLD:AAU8871
Ichneumoninae|[5844]|SSWLE5345-13|Hymenoptera|Ichneumonidae|BOLD:ABA8078
Ichneumoninae|[5845]|CNMIM924-14|Hymenoptera|Ichneumonidae|BOLD:ACN9245
Spilichneumon|[5846]|CNKLB532-14|Hymenoptera|Ichneumonidae|BOLD:ACR8371
Ichneumoninae|[5847]|TTMHY370-08|Hymenoptera|Ichneumonidae|BOLD:AAG7701
Ichneumoninae|[5848]|BBHYL636-10|Hymenoptera|Ichneumonidae|BOLD:AAN7535
Ichneumoninae|[5849]|TTMHY362-08|Hymenoptera|Ichneumonidae|BOLD:ACF4223
Ctenichneumon|[5850]|HPPPC115-13|Hymenoptera|Ichneumonidae|BOLD:ABZ0404
Ctenichneumon inspector|[5851]|NGNAI464-13|Hymenoptera|Ichneumonidae|BOLD:AAJ5832
Cratichneumon|[5852]|CNNHG1250-14|Hymenoptera|Ichneumonidae|BOLD:ACC8048
Ichneumoninae|[5853]|SSBAF7869-13|Hymenoptera|Ichneumonidae|BOLD:ACJ1120
Ichneumoninae|[5854]|SSPAC6457-13|Hymenoptera|Ichneumonidae|BOLD:ACK2469
Vulgichneumon deceptor|[5855]|TTMHY350-08|Hymenoptera|Ichneumonidae|BOLD:AAG7645
Barichneumon|[5856]|TTMHY316-08|Hymenoptera|Ichneumonidae|BOLD:AAF2914
Ichneumoninae|[5857]|SSWLD074-13|Hymenoptera|Ichneumonidae|BOLD:ACO2820
Stenobarichneumon citator|[5858]|HPPPH989-13|Hymenoptera|Ichneumonidae|BOLD:ACE0940
Barichneumon|[5859]|SSWLE2306-13|Hymenoptera|Ichneumonidae|BOLD:ACJ7927
Barichneumon|[5860]|SSWLE5602-13|Hymenoptera|Ichneumonidae|BOLD:AAE2633
Barichneumon|[5861]|SSBRC532-14|Hymenoptera|Ichneumonidae|BOLD:ACR4278
Ichneumoninae|[5862]|CNPPE1270-12|Hymenoptera|Ichneumonidae|BOLD:AAH7267
Ichneumoninae|[5863]|SSWLE5338-13|Hymenoptera|Ichneumonidae|BOLD:ACK7402
Ichneumoninae|[5864]|TTMHY020-08|Hymenoptera|Ichneumonidae|BOLD:AAG7623
Ichneumoninae|[5865]|SMTPI9442-14|Hymenoptera|Ichneumonidae|BOLD:AAH7265
Ichneumoninae|[5866]|TTMHY602-11|Hymenoptera|Ichneumonidae|BOLD:AAG7692
Virgichneumon faunus|[5867]|CNEID3286-12|Hymenoptera|Ichneumonidae|BOLD:ACB9576
Ichneumoninae|[5868]|SSWLE6431-13|Hymenoptera|Ichneumonidae|BOLD:AAH7284
Ichneumoninae|[5869]|NGNAY103-14|Hymenoptera|Ichneumonidae|BOLD:ACJ1034
Ichneumoninae|[5870]|TTHYW908-08|Hymenoptera|Ichneumonidae|BOLD:AAG7789
Ichneumoninae|[5871]|SSEIC3835-13|Hymenoptera|Ichneumonidae|BOLD:ACV1216
Ichneumoninae|[5872]|SSROB3603-14|Hymenoptera|Ichneumonidae|BOLD:AAH7283
Ichneumoninae|[5873]|SSWLE6408-13|Hymenoptera|Ichneumonidae|BOLD:AAK3172
Ichneumoninae|[5874]|PHNXG011-13|Hymenoptera|Ichneumonidae|BOLD:ACK2510
Ichneumoninae|[5875]|NGNAN3997-14|Hymenoptera|Ichneumonidae|BOLD:ACC0920
Ichneumoninae|[5876]|CNLMQ059-14|Hymenoptera|Ichneumonidae|BOLD:ABY1660
Ichneumoninae|[5877]|SSWLE3494-13|Hymenoptera|Ichneumonidae|BOLD:ABX5489
Ichneumoninae|[5878]|CNEIG1332-12|Hymenoptera|Ichneumonidae|BOLD:ACU9380
Ichneumoninae|[5879]|SMTPD4979-13|Hymenoptera|Ichneumonidae|BOLD:AAP2581
Ichneumoninae|[5880]|HPPPP063-13|Hymenoptera|Ichneumonidae|BOLD:AAG8176
Ichneumoninae|[5881]|NGNAN062-14|Hymenoptera|Ichneumonidae|BOLD:ACI9174
Ichneumoninae|[5882]|CNNHE1747-14|Hymenoptera|Ichneumonidae|BOLD:ACS2504
Ichneumoninae|[5883]|CNPPE1225-12|Hymenoptera|Ichneumonidae|BOLD:ACB0126
Ichneumoninae|[5884]|MBIOI1031-13|Hymenoptera|Ichneumonidae|BOLD:AAH7276
Ichneumoninae|[5885]|SSWLE5562-13|Hymenoptera|Ichneumonidae|BOLD:ACC1034
Ichneumoninae|[5886]|CNROG126-13|Hymenoptera|Ichneumonidae|BOLD:ACK4257
Ichneumoninae|[5887]|CNWAE7406-14|Hymenoptera|Ichneumonidae|BOLD:ACS2601

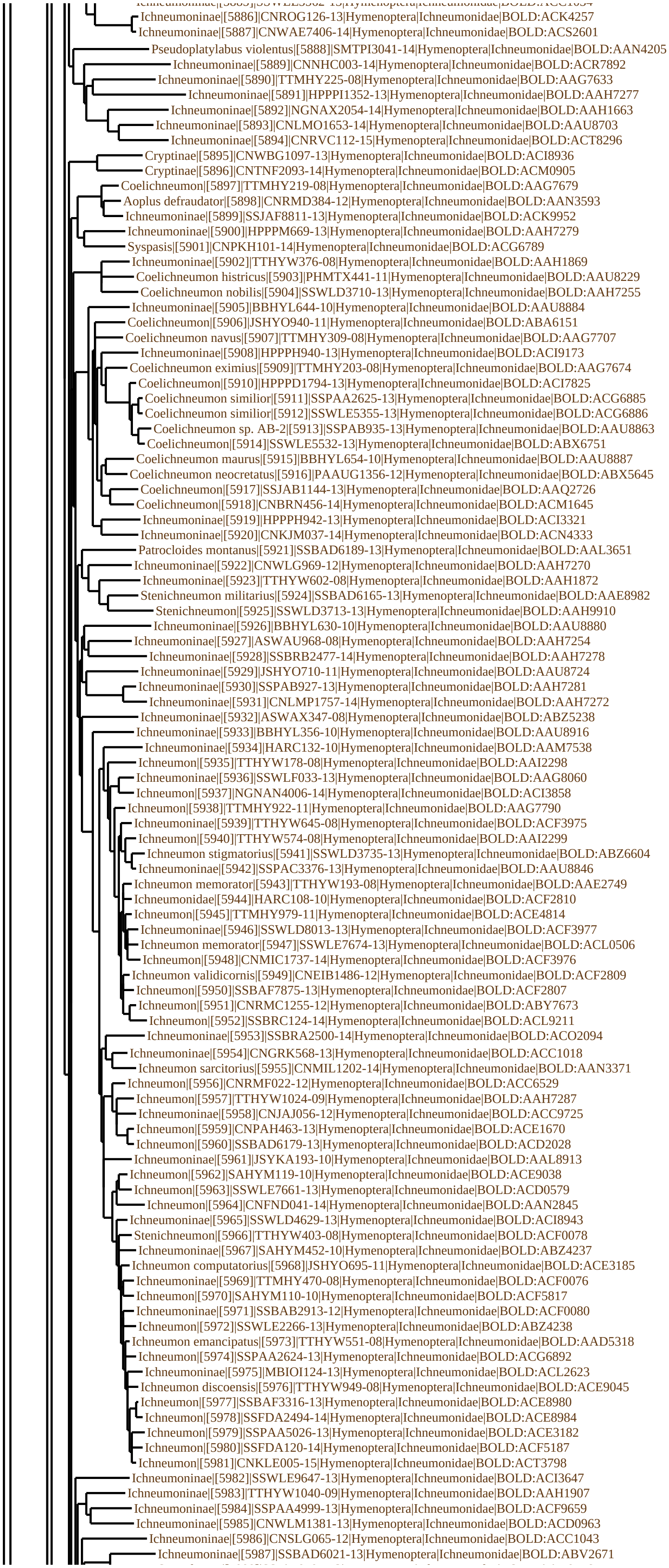
Ichneumoninae|[5886]|CNROG126-13|Hymenoptera|Ichneumonidae|BOLD:ACK4257
Ichneumoninae|[5887]|CNWAE7406-14|Hymenoptera|Ichneumonidae|BOLD:ACS2601
Pseudoplatylabus violentus|[5888]|SMTPI3041-14|Hymenoptera|Ichneumonidae|BOLD:AAN4205
Ichneumoninae|[5889]|CNNHC003-14|Hymenoptera|Ichneumonidae|BOLD:ACR7892
Ichneumoninae|[5890]|TTMHY225-08|Hymenoptera|Ichneumonidae|BOLD:AAG7633
Ichneumoninae|[5891]|HPPPI1352-13|Hymenoptera|Ichneumonidae|BOLD:AAH7277
Ichneumoninae|[5892]|NGNAX2054-14|Hymenoptera|Ichneumonidae|BOLD:AAH1663
Ichneumoninae|[5893]|CNLMO1653-14|Hymenoptera|Ichneumonidae|BOLD:AAU8703
Ichneumoninae|[5894]|CNRVC112-15|Hymenoptera|Ichneumonidae|BOLD:ACT8296
Cryptinae|[5895]|CNWBG1097-13|Hymenoptera|Ichneumonidae|BOLD:ACI8936
Cryptinae|[5896]|CNTNF2093-14|Hymenoptera|Ichneumonidae|BOLD:ACM0905
Coelichneumon|[5897]|TTMHY219-08|Hymenoptera|Ichneumonidae|BOLD:AAG7679
Aoplus defraudator|[5898]|CNRMD384-12|Hymenoptera|Ichneumonidae|BOLD:AAN3593
Ichneumoninae|[5899]|SSJAF8811-13|Hymenoptera|Ichneumonidae|BOLD:ACK9952
Ichneumoninae|[5900]|HPPPM669-13|Hymenoptera|Ichneumonidae|BOLD:AAH7279
Syspasis|[5901]|CNPKH101-14|Hymenoptera|Ichneumonidae|BOLD:ACG6789
Ichneumoninae|[5902]|TTHYW376-08|Hymenoptera|Ichneumonidae|BOLD:AAH1869
Coelichneumon histricus|[5903]|PHMTX441-11|Hymenoptera|Ichneumonidae|BOLD:AAU8229
Coelichneumon nobilis|[5904]|SSWLD3710-13|Hymenoptera|Ichneumonidae|BOLD:AAH7255
Ichneumoninae|[5905]|BBHYL644-10|Hymenoptera|Ichneumonidae|BOLD:AAU8884
Coelichneumon|[5906]|JSHYO940-11|Hymenoptera|Ichneumonidae|BOLD:ABA6151
Coelichneumon navus|[5907]|TTMHY309-08|Hymenoptera|Ichneumonidae|BOLD:AAG7707
Ichneumoninae|[5908]|HPPPH940-13|Hymenoptera|Ichneumonidae|BOLD:ACI9173
Coelichneumon eximius|[5909]|TTMHY203-08|Hymenoptera|Ichneumonidae|BOLD:AAG7674
Coelichneumon|[5910]|HPPPD1794-13|Hymenoptera|Ichneumonidae|BOLD:ACI7825
Coelichneumon similior|[5911]|SSPAA2625-13|Hymenoptera|Ichneumonidae|BOLD:ACG6885
Coelichneumon similior|[5912]|SSWLE5355-13|Hymenoptera|Ichneumonidae|BOLD:ACG6886
Coelichneumon sp. AB-2|[5913]|SSPAB935-13|Hymenoptera|Ichneumonidae|BOLD:AAU8863
Coelichneumon|[5914]|SSWLE5532-13|Hymenoptera|Ichneumonidae|BOLD:ABX6751
Coelichneumon maurus|[5915]|BBHYL654-10|Hymenoptera|Ichneumonidae|BOLD:AAU8887
Coelichneumon neocretatus|[5916]|PAAUG1356-12|Hymenoptera|Ichneumonidae|BOLD:ABX5645
Coelichneumon|[5917]|SSJAB1144-13|Hymenoptera|Ichneumonidae|BOLD:AAQ2726
Coelichneumon|[5918]|CNBRN456-14|Hymenoptera|Ichneumonidae|BOLD:ACM1645
Ichneumoninae|[5919]|HPPPH942-13|Hymenoptera|Ichneumonidae|BOLD:ACI3321
Ichneumoninae|[5920]|CNKJM037-14|Hymenoptera|Ichneumonidae|BOLD:ACN4333
Patrocloides montanus|[5921]|SSBAD6189-13|Hymenoptera|Ichneumonidae|BOLD:AAL3651
Ichneumoninae|[5922]|CNWLG969-12|Hymenoptera|Ichneumonidae|BOLD:AAH7270
Ichneumoninae|[5923]|TTHYW602-08|Hymenoptera|Ichneumonidae|BOLD:AAH1872
Stenichneumon militarius|[5924]|SSBAD6165-13|Hymenoptera|Ichneumonidae|BOLD:AAE8982
Stenichneumon|[5925]|SSWLD3713-13|Hymenoptera|Ichneumonidae|BOLD:AAH9910
Ichneumoninae|[5926]|BBHYL630-10|Hymenoptera|Ichneumonidae|BOLD:AAU8880
Ichneumoninae|[5927]|ASWAU968-08|Hymenoptera|Ichneumonidae|BOLD:AAH7254
Ichneumoninae|[5928]|SSBRB2477-14|Hymenoptera|Ichneumonidae|BOLD:AAH7278
Ichneumoninae|[5929]|JSHYO710-11|Hymenoptera|Ichneumonidae|BOLD:AAU8724
Ichneumoninae|[5930]|SSPAB927-13|Hymenoptera|Ichneumonidae|BOLD:AAH7281
Ichneumoninae|[5931]|CNLMP1757-14|Hymenoptera|Ichneumonidae|BOLD:AAH7272
Ichneumoninae|[5932]|ASWAX347-08|Hymenoptera|Ichneumonidae|BOLD:ABZ5238
Ichneumoninae|[5933]|BBHYL356-10|Hymenoptera|Ichneumonidae|BOLD:AAU8916
Ichneumoninae|[5934]|HARC132-10|Hymenoptera|Ichneumonidae|BOLD:AAM7538
Ichneumon|[5935]|TTHYW178-08|Hymenoptera|Ichneumonidae|BOLD:AAI2298
Ichneumoninae|[5936]|SSWLF033-13|Hymenoptera|Ichneumonidae|BOLD:AAG8060
Ichneumon|[5937]|NGNAN4006-14|Hymenoptera|Ichneumonidae|BOLD:ACI3858
Ichneumon|[5938]|TTMHY922-11|Hymenoptera|Ichneumonidae|BOLD:AAG7790
Ichneumoninae|[5939]|TTHYW645-08|Hymenoptera|Ichneumonidae|BOLD:ACF3975
Ichneumon|[5940]|TTHYW574-08|Hymenoptera|Ichneumonidae|BOLD:AAI2299
Ichneumon stigmatorius|[5941]|SSWLD3735-13|Hymenoptera|Ichneumonidae|BOLD:ABZ6604
Ichneumoninae|[5942]|SSPAC3376-13|Hymenoptera|Ichneumonidae|BOLD:AAU8846
Ichneumon memorator|[5943]|TTHYW193-08|Hymenoptera|Ichneumonidae|BOLD:AAE2749
Ichneumonidae|[5944]|HARC108-10|Hymenoptera|Ichneumonidae|BOLD:ACF2810
Ichneumon|[5945]|TTMHY979-11|Hymenoptera|Ichneumonidae|BOLD:ACE4814
Ichneumoninae|[5946]|SSWLD8013-13|Hymenoptera|Ichneumonidae|BOLD:ACF3977
Ichneumon memorator|[5947]|SSWLE7674-13|Hymenoptera|Ichneumonidae|BOLD:ACL0506
Ichneumon|[5948]|CNMIC1737-14|Hymenoptera|Ichneumonidae|BOLD:ACF3976
Ichneumon validicornis|[5949]|CNEIB1486-12|Hymenoptera|Ichneumonidae|BOLD:ACF2809
Ichneumon|[5950]|SSBAF7875-13|Hymenoptera|Ichneumonidae|BOLD:ACF2807
Ichneumon|[5951]|CNRMC1255-12|Hymenoptera|Ichneumonidae|BOLD:ABY7673
Ichneumon|[5952]|SSBRC124-14|Hymenoptera|Ichneumonidae|BOLD:ACL9211
Ichneumoninae|[5953]|SSBRA2500-14|Hymenoptera|Ichneumonidae|BOLD:ACO2094
Ichneumoninae|[5954]|CNGRK568-13|Hymenoptera|Ichneumonidae|BOLD:ACC1018
Ichneumon sarcitorius|[5955]|CNMIL1202-14|Hymenoptera|Ichneumonidae|BOLD:AAN3371
Ichneumon|[5956]|CNRMF022-12|Hymenoptera|Ichneumonidae|BOLD:ACC6529
Ichneumon|[5957]|TTHYW1024-09|Hymenoptera|Ichneumonidae|BOLD:AAH7287
Ichneumoninae|[5958]|CNJAJ056-12|Hymenoptera|Ichneumonidae|BOLD:ACC9725
Ichneumon|[5959]|CNPAH463-13|Hymenoptera|Ichneumonidae|BOLD:ACE1670
Ichneumon|[5960]|SSBAD6179-13|Hymenoptera|Ichneumonidae|BOLD:ACD2028
Ichneumoninae|[5961]|JSYKA193-10|Hymenoptera|Ichneumonidae|BOLD:AAL8913
Ichneumon|[5962]|SAHYM119-10|Hymenoptera|Ichneumonidae|BOLD:ACE9038
Ichneumon|[5963]|SSWLE7661-13|Hymenoptera|Ichneumonidae|BOLD:ACD0579
Ichneumon|[5964]|CNFND041-14|Hymenoptera|Ichneumonidae|BOLD:AAN2845
Ichneumoninae|[5965]|SSWLD4629-13|Hymenoptera|Ichneumonidae|BOLD:ACI8943
Stenichneumon|[5966]|TTHYW403-08|Hymenoptera|Ichneumonidae|BOLD:ACF0078
Ichneumoninae|[5967]|SAHYM452-10|Hymenoptera|Ichneumonidae|BOLD:ABZ4237
Ichneumon computatorius|[5968]|JSHYO695-11|Hymenoptera|Ichneumonidae|BOLD:ACE3185
Ichneumoninae|[5969]|TTMHY470-08|Hymenoptera|Ichneumonidae|BOLD:ACF0076
Ichneumon|[5970]|SAHYM110-10|Hymenoptera|Ichneumonidae|BOLD:ACF5817
Ichneumoninae|[5971]|SSBAB2913-12|Hymenoptera|Ichneumonidae|BOLD:ACF0080
Ichneumon|[5972]|SSWLE2266-13|Hymenoptera|Ichneumonidae|BOLD:ABZ4238
Ichneumon emancipatus|[5973]|TTHYW551-08|Hymenoptera|Ichneumonidae|BOLD:AAD5318
Ichneumon|[5974]|SSPAA2624-13|Hymenoptera|Ichneumonidae|BOLD:ACG6892
Ichneumoninae|[5975]|MBIOI124-13|Hymenoptera|Ichneumonidae|BOLD:ACL2623
Ichneumon discoensis|[5976]|TTHYW949-08|Hymenoptera|Ichneumonidae|BOLD:ACE9045
Ichneumon|[5977]|SSBAF3316-13|Hymenoptera|Ichneumonidae|BOLD:ACE8980
Ichneumon|[5978]|SSFDA2494-14|Hymenoptera|Ichneumonidae|BOLD:ACE8984
Ichneumon|[5979]|SSPAA5026-13|Hymenoptera|Ichneumonidae|BOLD:ACE3182
Ichneumon|[5980]|SSFDA120-14|Hymenoptera|Ichneumonidae|BOLD:ACF5187
Ichneumon|[5981]|CNKLE005-15|Hymenoptera|Ichneumonidae|BOLD:ACT3798
Ichneumoninae|[5982]|SSWLE9647-13|Hymenoptera|Ichneumonidae|BOLD:ACI3647
Ichneumoninae|[5983]|TTHYW1040-09|Hymenoptera|Ichneumonidae|BOLD:AAH1907
Ichneumoninae|[5984]|SSPAA4999-13|Hymenoptera|Ichneumonidae|BOLD:ACF9659
Ichneumoninae|[5985]|CNWLM1381-13|Hymenoptera|Ichneumonidae|BOLD:ACD0963
Ichneumoninae|[5986]|CNSLG065-12|Hymenoptera|Ichneumonidae|BOLD:ACC1043
Ichneumoninae|[5987]|SSBAD6021-13|Hymenoptera|Ichneumonidae|BOLD:ABV2671

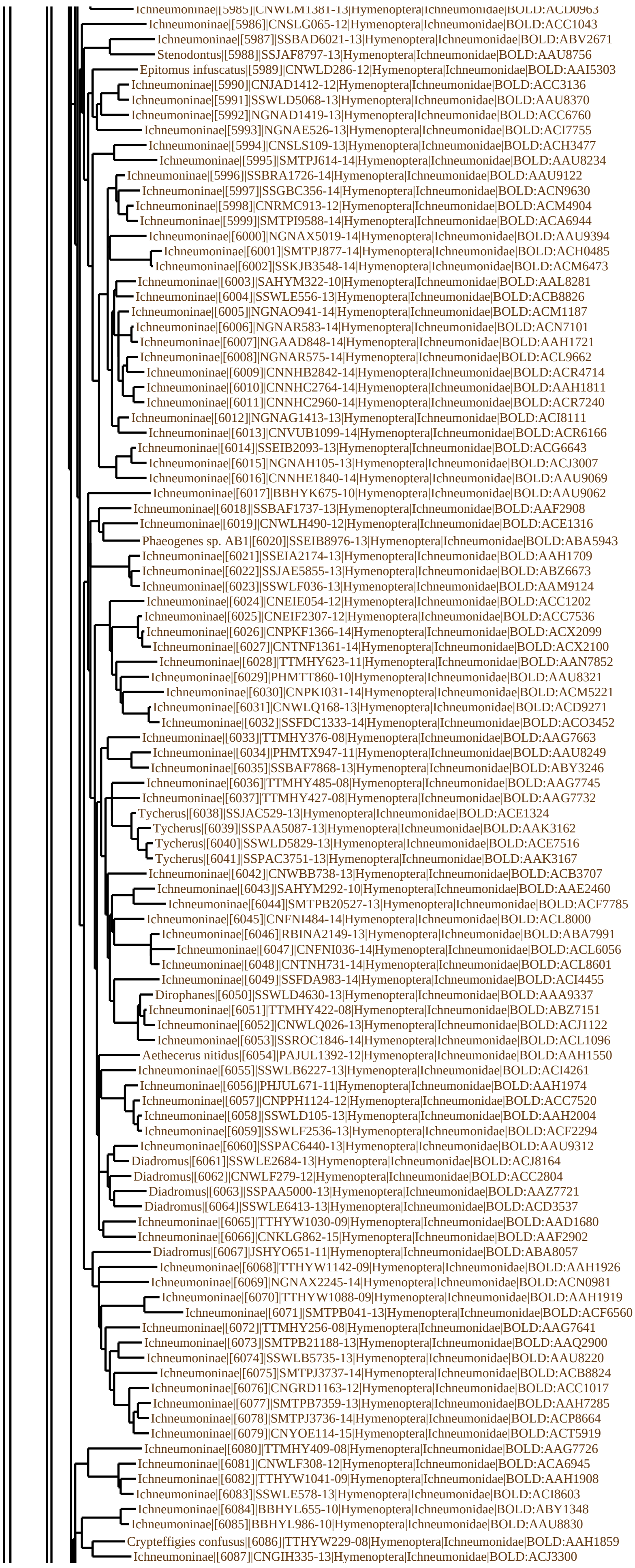

Ichneumoninae|[5985]|CNWLM1381-13|Hymenoptera|Ichneumonidae|BOLD:ACD0963
Ichneumoninae|[5986]|CNSLG065-12|Hymenoptera|Ichneumonidae|BOLD:ACC1043
Ichneumoninae|[5987]|SSBAD6021-13|Hymenoptera|Ichneumonidae|BOLD:ABV2671
Stenodontus|[5988]|SSJAF8797-13|Hymenoptera|Ichneumonidae|BOLD:AAU8756
Epitomus infuscatus|[5989]|CNWLD286-12|Hymenoptera|Ichneumonidae|BOLD:AAI5303
Ichneumoninae|[5990]|CNJAD1412-12|Hymenoptera|Ichneumonidae|BOLD:ACC3136
Ichneumoninae|[5991]|SSWLD5068-13|Hymenoptera|Ichneumonidae|BOLD:AAU8370
Ichneumoninae|[5992]|NGNAD1419-13|Hymenoptera|Ichneumonidae|BOLD:ACC6760
Ichneumoninae|[5993]|NGNAE526-13|Hymenoptera|Ichneumonidae|BOLD:ACI7755
Ichneumoninae|[5994]|CNSLS109-13|Hymenoptera|Ichneumonidae|BOLD:ACH3477
Ichneumoninae|[5995]|SMTPJ614-14|Hymenoptera|Ichneumonidae|BOLD:AAU8234
Ichneumoninae|[5996]|SSBRA1726-14|Hymenoptera|Ichneumonidae|BOLD:AAU9122
Ichneumoninae|[5997]|SSGBC356-14|Hymenoptera|Ichneumonidae|BOLD:ACN9630
Ichneumoninae|[5998]|CNRMC913-12|Hymenoptera|Ichneumonidae|BOLD:ACM4904
Ichneumoninae|[5999]|SMTPI9588-14|Hymenoptera|Ichneumonidae|BOLD:ACA6944
Ichneumoninae|[6000]|NGNAX5019-14|Hymenoptera|Ichneumonidae|BOLD:AAU9394
Ichneumoninae|[6001]|SMTPJ877-14|Hymenoptera|Ichneumonidae|BOLD:ACH0485
Ichneumoninae|[6002]|SSKJB3548-14|Hymenoptera|Ichneumonidae|BOLD:ACM6473
Ichneumoninae|[6003]|SAHYM322-10|Hymenoptera|Ichneumonidae|BOLD:AAL8281
Ichneumoninae|[6004]|SSWLE556-13|Hymenoptera|Ichneumonidae|BOLD:ACB8826
Ichneumoninae|[6005]|NGNAO941-14|Hymenoptera|Ichneumonidae|BOLD:ACM1187
Ichneumoninae|[6006]|NGNAR583-14|Hymenoptera|Ichneumonidae|BOLD:ACN7101
Ichneumoninae|[6007]|NGAAD848-14|Hymenoptera|Ichneumonidae|BOLD:AAH1721
Ichneumoninae|[6008]|NGNAR575-14|Hymenoptera|Ichneumonidae|BOLD:ACL9662
Ichneumoninae|[6009]|CNNHB2842-14|Hymenoptera|Ichneumonidae|BOLD:ACR4714
Ichneumoninae|[6010]|CNNHC2764-14|Hymenoptera|Ichneumonidae|BOLD:AAH1811
Ichneumoninae|[6011]|CNNHC2960-14|Hymenoptera|Ichneumonidae|BOLD:ACR7240
Ichneumoninae|[6012]|NGNAG1413-13|Hymenoptera|Ichneumonidae|BOLD:ACI8111
Ichneumoninae|[6013]|CNVUB1099-14|Hymenoptera|Ichneumonidae|BOLD:ACR6166
Ichneumoninae|[6014]|SSEIB2093-13|Hymenoptera|Ichneumonidae|BOLD:ACG6643
Ichneumoninae|[6015]|NGNAH105-13|Hymenoptera|Ichneumonidae|BOLD:ACJ3007
Ichneumoninae|[6016]|CNNHE1840-14|Hymenoptera|Ichneumonidae|BOLD:AAU9069
Ichneumoninae|[6017]|BBHYK675-10|Hymenoptera|Ichneumonidae|BOLD:AAU9062
Ichneumoninae|[6018]|SSBAF1737-13|Hymenoptera|Ichneumonidae|BOLD:AAF2908
Ichneumoninae|[6019]|CNWLH490-12|Hymenoptera|Ichneumonidae|BOLD:ACE1316
Phaeogenes sp. AB1|[6020]|SSEIB8976-13|Hymenoptera|Ichneumonidae|BOLD:ABA5943
Ichneumoninae|[6021]|SSEIA2174-13|Hymenoptera|Ichneumonidae|BOLD:AAH1709
Ichneumoninae|[6022]|SSJAE5855-13|Hymenoptera|Ichneumonidae|BOLD:ABZ6673
Ichneumoninae|[6023]|SSWLF036-13|Hymenoptera|Ichneumonidae|BOLD:AAM9124
Ichneumoninae|[6024]|CNEIE054-12|Hymenoptera|Ichneumonidae|BOLD:ACC1202
Ichneumoninae|[6025]|CNEIF2307-12|Hymenoptera|Ichneumonidae|BOLD:ACC7536
Ichneumoninae|[6026]|CNPKF1366-14|Hymenoptera|Ichneumonidae|BOLD:ACX2099
Ichneumoninae|[6027]|CNTNF1361-14|Hymenoptera|Ichneumonidae|BOLD:ACX2100
Ichneumoninae|[6028]|TTMHY623-11|Hymenoptera|Ichneumonidae|BOLD:AAN7852
Ichneumoninae|[6029]|PHMTT860-10|Hymenoptera|Ichneumonidae|BOLD:AAU8321
Ichneumoninae|[6030]|CNPKI031-14|Hymenoptera|Ichneumonidae|BOLD:ACM5221
Ichneumoninae|[6031]|CNWLQ168-13|Hymenoptera|Ichneumonidae|BOLD:ACD9271
Ichneumoninae|[6032]|SSFDC1333-14|Hymenoptera|Ichneumonidae|BOLD:ACO3452
Ichneumoninae|[6033]|TTMHY376-08|Hymenoptera|Ichneumonidae|BOLD:AAG7663
Ichneumoninae|[6034]|PHMTX947-11|Hymenoptera|Ichneumonidae|BOLD:AAU8249
Ichneumoninae|[6035]|SSBAF7868-13|Hymenoptera|Ichneumonidae|BOLD:ABY3246
Ichneumoninae|[6036]|TTMHY485-08|Hymenoptera|Ichneumonidae|BOLD:AAG7745
Ichneumoninae|[6037]|TTMHY427-08|Hymenoptera|Ichneumonidae|BOLD:AAG7732
Tycherus|[6038]|SSJAC529-13|Hymenoptera|Ichneumonidae|BOLD:ACE1324
Tycherus|[6039]|SSPAA5087-13|Hymenoptera|Ichneumonidae|BOLD:AAK3162
Tycherus|[6040]|SSWLD5829-13|Hymenoptera|Ichneumonidae|BOLD:ACE7516
Tycherus|[6041]|SSPAC3751-13|Hymenoptera|Ichneumonidae|BOLD:AAK3167
Ichneumoninae|[6042]|CNWBB738-13|Hymenoptera|Ichneumonidae|BOLD:ACB3707
Ichneumoninae|[6043]|SAHYM292-10|Hymenoptera|Ichneumonidae|BOLD:AAE2460
Ichneumoninae|[6044]|SMTPB20527-13|Hymenoptera|Ichneumonidae|BOLD:ACF7785
Ichneumoninae|[6045]|CNFNI484-14|Hymenoptera|Ichneumonidae|BOLD:ACL8000
Ichneumoninae|[6046]|RBINA2149-13|Hymenoptera|Ichneumonidae|BOLD:ABA7991
Ichneumoninae|[6047]|CNFNI036-14|Hymenoptera|Ichneumonidae|BOLD:ACL6056
Ichneumoninae|[6048]|CNTNH731-14|Hymenoptera|Ichneumonidae|BOLD:ACL8601
Ichneumoninae|[6049]|SSFDA983-14|Hymenoptera|Ichneumonidae|BOLD:ACI4455
Dirophanes|[6050]|SSWLD4630-13|Hymenoptera|Ichneumonidae|BOLD:AAA9337
Ichneumoninae|[6051]|TTMHY422-08|Hymenoptera|Ichneumonidae|BOLD:ABZ7151
Ichneumoninae|[6052]|CNWLQ026-13|Hymenoptera|Ichneumonidae|BOLD:ACJ1122
Ichneumoninae|[6053]|SSROC1846-14|Hymenoptera|Ichneumonidae|BOLD:ACL1096
Aethecerus nitidus|[6054]|PAJUL1392-12|Hymenoptera|Ichneumonidae|BOLD:AAH1550
Ichneumoninae|[6055]|SSWLB6227-13|Hymenoptera|Ichneumonidae|BOLD:ACI4261
Ichneumoninae|[6056]|PHJUL671-11|Hymenoptera|Ichneumonidae|BOLD:AAH1974
Ichneumoninae|[6057]|CNPPH1124-12|Hymenoptera|Ichneumonidae|BOLD:ACC7520
Ichneumoninae|[6058]|SSWLD105-13|Hymenoptera|Ichneumonidae|BOLD:AAH2004
Ichneumoninae|[6059]|SSWLF2536-13|Hymenoptera|Ichneumonidae|BOLD:ACF2294
Ichneumoninae|[6060]|SSPAC6440-13|Hymenoptera|Ichneumonidae|BOLD:AAU9312
Diadromus|[6061]|SSWLE2684-13|Hymenoptera|Ichneumonidae|BOLD:ACJ8164
Diadromus|[6062]|CNWLF279-12|Hymenoptera|Ichneumonidae|BOLD:ACC2804
Diadromus|[6063]|SSPAA5000-13|Hymenoptera|Ichneumonidae|BOLD:AAZ7721
Diadromus|[6064]|SSWLE6413-13|Hymenoptera|Ichneumonidae|BOLD:ACD3537
Ichneumoninae|[6065]|TTHYW1030-09|Hymenoptera|Ichneumonidae|BOLD:AAD1680
Ichneumoninae|[6066]|CNKLG862-15|Hymenoptera|Ichneumonidae|BOLD:AAF2902
Diadromus|[6067]|JSHYO651-11|Hymenoptera|Ichneumonidae|BOLD:ABA8057
Ichneumoninae|[6068]|TTHYW1142-09|Hymenoptera|Ichneumonidae|BOLD:AAH1926
Ichneumoninae|[6069]|NGNAX2245-14|Hymenoptera|Ichneumonidae|BOLD:ACN0981
Ichneumoninae|[6070]|TTHYW1088-09|Hymenoptera|Ichneumonidae|BOLD:AAH1919
Ichneumoninae|[6071]|SMTPB041-13|Hymenoptera|Ichneumonidae|BOLD:ACF6560
Ichneumoninae|[6072]|TTMHY256-08|Hymenoptera|Ichneumonidae|BOLD:AAG7641
Ichneumoninae|[6073]|SMTPB21188-13|Hymenoptera|Ichneumonidae|BOLD:AAQ2900
Ichneumoninae|[6074]|SSWLB5735-13|Hymenoptera|Ichneumonidae|BOLD:AAU8220
Ichneumoninae|[6075]|SMTPJ3737-14|Hymenoptera|Ichneumonidae|BOLD:ACB8824
[truncated: 1,318,094 more chars]
